# Molecular organization and comparative analysis of chromosome 5B of the wild wheat ancestor *Triticum dicoccoides*

**DOI:** 10.1038/srep10763

**Published:** 2015-06-18

**Authors:** Bala Ani Akpinar, Meral Yuce, Stuart Lucas, Jan Vrána, Veronika Burešová, Jaroslav Doležel, Hikmet Budak

**Affiliations:** 1 Sabanci University Nanotechnology Research and Application Centre (SUNUM), Sabanci University, Orhanlı, 34956 Tuzla, Istanbul, Turkey; 2 Institute of Experimental Botany, Centre of the Region Haná for Biotechnological and Agricultural Research, Slechtitelu 31, CZ-78371 Olomouc, Czech Republic; 3 Faculty of Engineering and Natural Sciences, Molecular Biology, Genetics and Bioengineering, Sabanci University, Orhanlı, 34956 Tuzla, Istanbul, Turkey

## Abstract

Wild emmer wheat, *Triticum turgidum* ssp. *dicoccoides* is the wild relative of *Triticum turgidum*, the progenitor of durum and bread wheat, and maintains a rich allelic diversity among its wild populations. The lack of adequate genetic and genomic resources, however, restricts its exploitation in wheat improvement. Here, we report next-generation sequencing of the flow-sorted chromosome 5B of *T. dicoccoides* to shed light into its genome structure, function and organization by exploring the repetitive elements, protein-encoding genes and putative microRNA and tRNA coding sequences. Comparative analyses with its counterparts in modern and wild wheats suggest clues into the B-genome evolution. Syntenic relationships of chromosome 5B with the model grasses can facilitate further efforts for fine-mapping of traits of interest. Mapping of 5B sequences onto the root transcriptomes of two additional *T. dicoccoides* genotypes, with contrasting drought tolerances, revealed several thousands of single nucleotide polymorphisms, of which 584 shared polymorphisms on 228 transcripts were specific to the drought-tolerant genotype. To our knowledge, this study presents the largest genomics resource currently available for *T. dicoccoides*, which, we believe, will encourage the exploitation of its genetic and genomic potential for wheat improvement to meet the increasing demand to feed the world.

With an annual global production of more than 700 million tons across over 200 million hectares, wheat is the most widely grown crop worldwide (http://faostat.fao.org/). While the allohexaploid bread wheat (*Triticum aestivum*, 2n = 6x= 42, AABBDD genome) and the allotetraploid durum wheat (*Triticum turgidum* ssp. *durum*, 2n = 4x = 28, AABB genome) account for almost all global production, wild diploid and tetraploid wheat species and their relatives are still grown around the Fertile Crescent where they originated. For decades, wild species have been attractive sources of genetic diversity to be introduced into the narrow gene pool of modern cultivated wheats^1^. Introgression of genes and alleles from wild relatives is gaining increasing attention due to the urgent need to increase global wheat production^2^,^3^.

Recent research indicates that the evolution of bread wheat involved three hybridization events^4^. Following the divergence of the *Triticum* and *Aegilops* lineages from a common ancestor ~6.5 million years ago, the first of these events is thought to involve A and B genome lineages which eventually gave rise to the diploid wheat D-genome progenitor, *Aegilops tauschii* (2n = 2x = 14, DD genome). The second event is dated back to a few hundred thousand years ago and resulted in the formation of the tetraploid AABB genome of *Triticum turgidum*, through the hybridization between *Triticum urartu* (A genome) and a close relative of *Aegilops speltoides* (B genome), followed by whole genome duplication, probably via the production of non-reduced gametes^5^. Although several *T. turgidum* subspecies cultivated for thousands of years have lost their importance along the agricultural history, durum wheat, *T. turgidum* ssp. *durum* remains an important crop^1^. Finally, the most recent hybridization, dating back to ~10.000 years, involved *T. turgidum* and *Ae. tauschii*, and resulted in the emergence of the allohexaploid bread wheat, *Triticum aestivum*^1^,^4^. Domestication and, more recently, intensive breeding programs for better agricultural gain have considerably depleted the genetic diversity in today’s elite cultivars. Fortunately, this diversity is still maintained in wild populations, which are adapted to a range of environmental conditions^2^,^6^.

Wild emmer wheat, *Triticum turgidum* ssp. *dicoccoides* (2n = 4x = 28, AABB genome), is the wild relative of durum wheat and is capable of producing fertile offspring with both tetraploid and hexaploid wheat cultivars^2^,^7^. Some wild emmer genotypes exhibit remarkable tolerance against drought, the major abiotic stress factor responsible for severe yield losses worldwide^6^. One such genotype, TR39477, exhibits a strong and consistent tolerance against shock and prolonged drought stress, sharply contrasting with another genotype, TTD-22, highly sensitive to drought^8^,^9^. Such genetic diversity found within the natural populations of wild emmer wheat might provide clues into the key players of the drought response which may be targeted for introgression into the elite cultivars^7^,^10^.

Wild emmer wheat genotypes are also recognized for high grain micronutrient content, tolerance against herbicides and resistance genes against biotic stresses, particularly against powdery mildew^2^,^7^. While the great potential that *T. dicoccoides* holds for wheat improvement has been recognized for decades, this potential remains largely unexploited to date. The rich gene pool and direct ancestry of *T. dicoccoides* enable the transfer of beneficial traits into elite cultivars relatively easily; however, ‘linkage drag’, caused by the co-transfer of chromosomal segments with negative effects on crop performance, complicates the introgression of such traits. If possible at all, the elimination of these undesirable segments, thereby minimizing the linkage drag, may take years of back-crosses^1^,^2^. While extensive genetic and genomic resources can largely circumvent these difficulties through marker-assisted selection or transgenic approaches^2^,^6^, such resources are currently very limited for *T. dicoccoides*.

Advances in chromosome genomics, in particular, flow-cytometric isolation of individual chromosomes or chromosome arms enabling the construction of chromosome-specific Bacterial Artificial Chromosome (BAC) libraries or shotgun sequencing of isolated chromosomes by Next Generation Sequencing (NGS) methods have been pivotal in wheat genomics research^11^,^12^,^13^. Recently, draft sequences of all 21 bread wheat chromosomes have been published^14^. This important advance followed the publication of the draft genome sequences of A and D genome progenitors, *T. urartu*^15^ and *Ae. tauschii*^16^, altogether providing valuable insights into the genome organization and evolution of wheat. These sequencing efforts are likely to extend into the wild relatives of wheat, not only to complement and further broaden the comparative evolutionary genomics studies, but also to explore and exploit these rich sources for the benefit of the humankind.

In this study, we report the flow-cytometric sorting and sequencing of chromosome 5B of *Triticum turgidum* ssp. *dicoccoides*, which is known to harbor genes encoding resistance against powdery mildew disease, as well as quantitative trait loci for grain protein and mineral content^7^. As the first genomics study carried out on wild emmer wheat, the large-scale sequence information on chromosome 5B should enable the development of molecular markers linked to beneficial traits and facilitate gene transfer to support bread and durum wheat improvement.

## Results

### Flow-sorting, sequencing and assembly of Tdic5B

**Figure 1:**
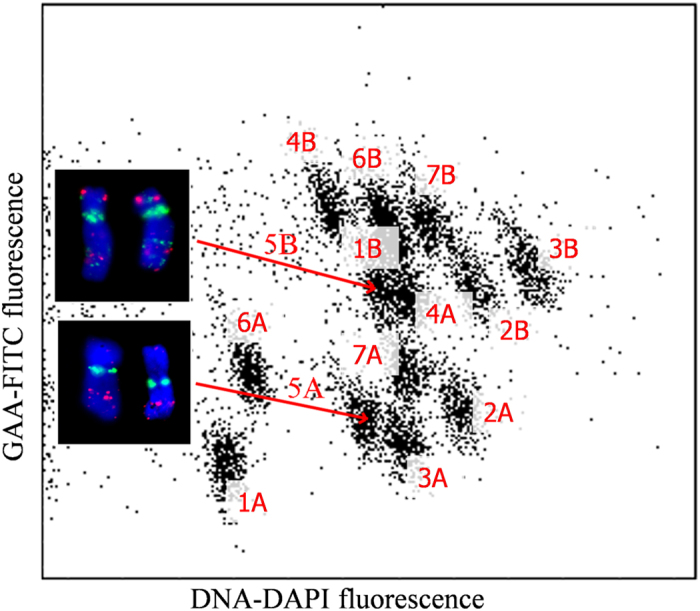
Biparametric flow karyotype of chromosomes isolated from T. dicoccoides. Prior to the analysis, GAA microsatellites were labeled by FITC and chromosomal DNA was stained by DAPI. FITC fluorescence was acquired at logarithmic scale, while DAPI fluorescence was measured at linear scale. This approach permitted separation from other chromosomes in the karyotype, including its homoeolog 5A. Insets: Images of flow-sorted chromosomes 5A and 5B. The chromosomes were identified after FISH with probes for GAA microsatellites (yellow-green) and for *Afa* family repeats (red).

Flow cytometric analysis of fluorescence of DAPI-stained chromosomes alone did not permit the discrimination of chromosome 5B from other chromosomes of wild emmer wheat, *Triticum dicoccoides* variety 26676. Thus, biparametric analysis of GAA microsatellite content and DAPI fluorescence intensity was employed. This approach enabled the discrimination of all wild emmer wheat chromosomes and permitted sorting of chromosome 5B (Fig. 1). Fluorescence *in situ* hybridization (FISH) with probes for GAA microsatellites and *Afa* repeat family indicated an average purity of 95.24% from three independent samples. As obtaining sufficient amounts of DNA for direct sequencing by flow-cytometry is prohibitively resource-intensive, three flow-sorted 5B chromosome fractions were amplified by three independent rounds of Multiple Displacement Amplification (MDA) that yielded a total of 12.56 μg of *T. dicoccoides* 5B chromosome (Tdic5B hereafter) DNA.

Three sequencing runs on GS FLX Titanium platform were performed on two Tdic5B libraries, giving a total of 1.57 Gb of good-quality sequence data (Table 1). Assuming that the size of Tdic5B is similar to its modern counterpart, the 840 Mbp-long *T. durum* 5B chromosome^17^, the sequence data obtained in this study represent a coverage of 1.87x, with the probability of any given position being represented as least once in this dataset being 0.799.

Repetitive elements comprise a notable fraction of *Triticeae* genomes^18^ and interfere with the accurate assembly of genomic sequences. Hence, reads identified as repetitive elements, together with the reads exhibiting significant similarities to ribosomal RNA and chloroplast/mitochondrial DNA, deemed to have derived from contaminants of sorted chromosome fractions, were excluded. The remaining sequence reads were assembled using gsAssembler tool of Newbler 2.6 software. This assembly, referred to as Low-Copy Number (LCN) assembly hereafter, is comprised of 26,225 contigs and 256,685 singletons (Table 1), which is expected to cover majority of the non-repetitive regions of Tdic5B. The contigs of the LCN assembly had a peak depth of 2.1, close to the sequencing depth, indicating the accuracy of the contig construction. The cumulative length of the assembly was 100.9 Mb, shorter than the estimated 127 Mb non-repetitive loci, based on the repetitive fraction of the chromosome described below.

### Repetitive content of Tdic5B

**Figure 2:**
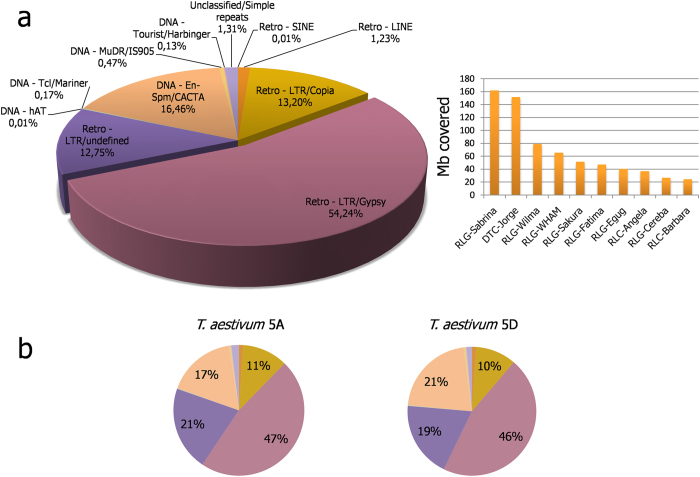
Repetitive element composition of Tdic5B. **a.** Repeat fractions by superfamily (left) and the cumulative sizes covered by the most abundant repeat families (right) of Tdic5B. **b.** Repeat fractions of *T. aestivum* 5A and 5D chromosomes by superfamily as in (**a**). DTC = DNA transposon, CACTA; RLG = retroelement, LTR, Gypsy; RLC = retroelement, LTR, Copia.

Repeat masking of Tdic5B sequences against known *Poaceae* repeat elements revealed that 84.9% of all Tdic5B sequences were repetitive, largely dominated by Long Terminal Repeat (LTR) retroelements (67.8% of all sequences). Within the LTR retroelements, Gypsy superfamily repeats had a marked abundance, accounting for over half of LTR elements, while the second most abundant Copia superfamily comprised 13% of all repeat elements (Fig. 2a). DNA transposons were mainly represented by En-Spm/CACTA repeats, which made up 17% of all repeats. Despite the predominance of LTR retroelements among Tdic5B repeats, the DTC-Jorge family of En-Spm/CACTA superfamily DNA transposons had a notable coverage of the chromosome (Fig. 2a).

In order to compare the repeat content and distribution of Tdic5B with its modern and wild counterparts, raw sequences from the 5D chromosomes of *T. aestivum*^19^ and *Ae. tauschii*^20^ and the 5A chromosome of *T. aestivum*^13^, obtained with the same NGS platform, were retrieved and masked against the same *Poaceae* repeat element database. The repeat content of Tdic5B was comparable to that of 5D chromosomes of *T. aestivum* and *Ae. tauschii* (82% and 81.1%, respectively)^19^,^20^, while *T. aestivum* 5A chromosome contained fewer repetitive elements (72.8%)^13^. This is highly intriguing as the recently published chromosome-based draft sequences of bread wheat suggested repeat contents 5A > 5B > 5D^14^. However, the reference sequencing of chromosome 3B^21^, the only bread wheat chromosome sequenced to this quality so far, reported a much higher repeat content than assessed by its draft sequence^14^. These inconsistencies may result from either different sequencing platforms being prone to different kinds of errors or amplification biases caused by MDA, both of which may have profound effects on the interpretation of low coverage NGS data. Therefore, a firm comparison of the repeat contents between group 5 chromosomes of the wheat ancestry may await reference sequencing of these chromosomes.

Despite the inconsistencies on the overall repeat content estimates, the chromosome-based draft genome sequence of bread wheat revealed higher abundance for class I retroelements for the A subgenome compared to B and D subgenomes (A > B > D), and an opposite trend for the class II DNA transposons (D > B > A)^14^, in accordance with our observations for *T. aestivum* chromosomes 5A and 5D which were applied the same procedure as Tdic5B (Fig. 2b). As the undefined LTR elements, presumably representing older repeats, were the scarcest in the B genome, Mayer and his colleagues hypothesized that the modern B genome had undergone extensive transposon activity following polyploidization, giving rise to a higher retrotransposon content representing more recent proliferations^14^. In fact, this would be consistent with the repeat element distribution of Tdic5B, where undefined LTRs make up only 13% of all repeat annotations. It is tempting to speculate that, following tetraploidization, certain LTR families, in particular, those belonging to the Gypsy superfamily might have been proliferated in Tdic5B (Fig. 2a). Indeed, the repeat distribution of *Ae. tauschii* 5D chromosome suggests that the modern wheat D genome has undergone an expansion of the specific LTR retroelements coupled with the reduction of the relative contribution of DNA transposons compared to its progenitor^20^.Since transposable elements are known to have family-specific and species-specific evolutionary trajectories^22^, which repeat families might have expanded in Tdic5B remains elusive at the time. All repeat annotations with regard to repeat families are given in Supplementary Table 1.

### Gene content and conservation

**Figure 3:**
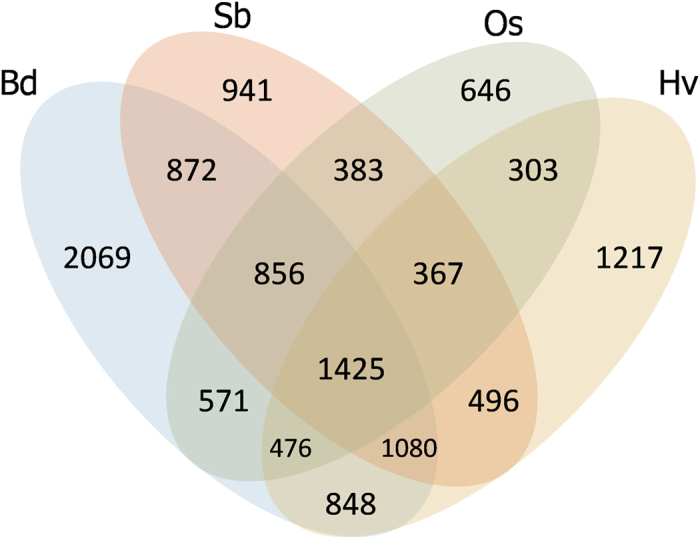
Venn diagram exhibiting Tdic5B sequence reads matching *Brachypodium* (Bd: *Brachypodium distachyon*), sorghum (Sb: *Sorghum bicolor*), rice (Os: *Oryza sativa*), and barley (Hv: *Hordeum vulgare*) proteins.

To explore the gene content and conservation of Tdic5B, the LCN assembly was compared against the fully annotated proteomes of model grasses *Brachypodium distachyon*^23^, rice^24^ and sorghum^25^, in addition to the high-confidence proteins of its close relative, barley^26^, and wheat UniGene and UniProt sequences. A total of 19,669 sequences from the LCN assembly (5,635 contigs and 14,034 singletons) were deemed as gene-associated, as suggested by significant matches to related grass proteins and UniGene/UniProt sequences (Supplementary Table 2). Over half of these sequences, 3,161 contigs and 9,389 singletons, retrieved matches from at least one related grass proteome, indicating ‘conserved’ genes among grasses (Fig. 3). Among these, 2,555 contigs and 4,850 singletons were also supported by matches from wheat UniGene and UniProt sequences. A total of 1,425 sequences of the LCN assembly retrieved matches from all four proteomes, which possibly correspond to highly conserved genes, suggestive of central cellular processes, or, of a shared ancient origin (Fig. 3). Considering the fully annotated proteomes of model grasses, LCN assembly sequences matching *Brachypodium* proteins (8,197) outnumbered that of rice (5,027) and sorghum (6,420), as would be expected from the evolutionary distances, although the high number of matches with sorghum proteins is intriguing. In addition to these ‘conserved’ gene-associated sequences, 2,474 contigs and 4,645 singletons were found to have significant matches to only wheat UniGene or UniProt sequences indicating a collection of gene fragments, pseudogenes and a number of putatively *Triticum*-specific genes; for simplicity, these are collectively referred as ‘non-conserved’ gene-associated sequences. Due to the prevalence of pseudogenes in polyploid wheat genomes^27^, several of these non-conserved gene-associated sequences are suspected to represent non-functional gene copies which might have undergone extensive rearrangements or accumulated too many mutations through the wheat genme evolution. To estimate the total genic content of Tdic5B and interpolating the estimate to the entire genome, *Brachypodium*, rice, sorghum and barley proteins exhibiting significant similarities to the LCN assembly were used as references onto which masked Tdic5B sequences were mapped. This approach merged non-overlapping sequences of the LCN assembly that matched the same query protein, and resulted in the construction of 4,818 ‘conserved gene models’ for the Tdic5B (Supplementary File 1). Assuming an average coding sequence length of 2000 bases^13^ and a chromosome length of 840 Mbp^17^, the genic fraction (~9.63 Mb estimated coding length) of Tdic5B equals to 1.15%, similar to that of *Triticum aestivum* 5A (~1.23%)^13^ and 5D (~1.15%)^19^ chromosomes, but considerably lower than *Aegilops tauschii* 5D chromosome (2.1-2.9%)^20^. At a size of approximately 12 Gbp, this genic fraction corresponds to a total estimate of over 68,800 genes for the entire genome of *T. dicoccoides*. At the whole genome level, this estimate is consistent with both diploid wheat progenitors *Ae. tauschii*^16^ and *T. urartu*^15^, for which ~35,000 protein-coding loci were predicted, while considerably lower than the sum of high-confidence gene loci reported for the A and B genomes of *T. aestivum* (40,253 for the A genome and 44,523 for the B genome)^14^. The actual number of genes may be slightly higher than estimated for *T. dicoccoides*, as a fraction of the non-conserved gene associated sequences that did not match any of the four related grass proteomes likely represents genuine *Triticum*-specific genes. Additionally, the cumulative length of the LCN assembly being shorter than the coding length estimated by repeat annotations (100.9 Mb vs. 127 Mb) suggest that some paralogous loci might have been collapsed into single contigs in the LCN assembly, causing a slight underestimate of the coding fraction of the chromosome. All conserved gene models for Tdic5B are given in Supplementary File 1.

**Figure 4:**
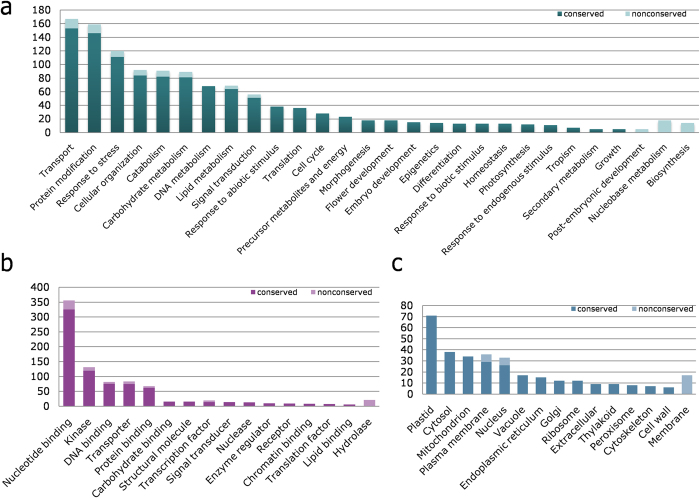
Gene-Ontology annotations of Tdic5B conserved and non-conserved genes in terms of, **a.** Biological Process, **b.** Molecular Function, **c.** Cellular Component.

To gain insight into the functional gene space of Tdic5B, the LCN assembly contigs and singletons corresponding to the 7,612 putative conserved and 4,011 putative non-conserved gene associated sequences were annotated based on *Viridiplantae* proteins. Gene Ontology (GO) annotation of these sequences with regard to Biological Process (BP), Molecular Function (MF) and Cellular Component (CC) suggested a variety of GO terms (Fig. 4). Among BP terms, ‘transport’ and ‘protein modification’ processes were the most prominent, with a significant share of ‘response to stress’, for which wild progenitors are generally attributed (Fig. 4a). In terms of MF, ‘nucleotide binding’ and ‘kinase’ activities together, essential to all central pathways, accounted for more than half of all annotations (Fig. 4b). ‘Transporter’ function was also evident among MF terms, possibly in connection to the ‘transport’ process in BP terms. Although the LCN assembly was filtered against cpDNA and mtDNA sequences, ‘plastid’ terms alone took up almost a quarter of all CC annotations, (Fig. 4c). Similarly, mitochondrion-related sequences were also abundant among CC terms. Since energy or photosynthesis-related processes or functions were not among top terms for BP and MF, these abundances in CC terms were not expected. Interestingly, more than 72% of GO annotations related to either plastid or mitochondrion were observed to be hypothetical or predicted proteins, suggesting that the unusual abundance of these CC terms may be due to mis-annotations. Despite a number of leading terms in each classification, Tdic5B annotations revealed an array of processes, functions and components in general. This observation is, in fact, in accordance with the transcriptional autonomy of wheat sub-genomes^14^, such that Tdic5B appears to encode a variety of genes capable to carrying out diverse functions.

### Syntenic relationships

Conserved genes between Tdic5B and model grasses *Brachypodium*, rice and sorghum were observed to be organized into large-scale syntenic blocks on *Brachypodium* chromosomes 1 and 4 (Bd1 & Bd4), rice chromosomes 3, 9 and 12 (Os3, Os9 & Os12), and sorghum chromosomes 1 and 2 (Sb1 & Sb2) (Supplementary Fig. 1, 2). These syntenic blocks defined three groups of syntenic relationships between the model grass genomes, in accordance with the previous findings^23^(Supplementary Fig. 2, ribbons). The first syntenic group involved proximal ends of Bd1 and Sb1 and the distal end of Os3 and, conversely, involved distal ends of Bd1 and Sb1 and the proximal end of Os3. The second syntenic group connected the distal ends of Bd4, Os9 and Sb2. Finally, the third group involved only *Brachypodium* and rice, in which the proximal end of the Bd4 was connected to the distal end of Os12. Syntenic genes conserved within these blocks are likely to be found in syntenic blocks along Tdic5B. As indicated by the dark red histograms in Supplementary Fig. 2, conserved genes of Tdic5B were usually found at the telomeric regions of model grass chromosomes, in accordance with the overall gene density trends along these chromosomes (light blue and light gray histograms flanking chromosomes for genes on ‘+’ and ‘−’ strands, respectively). Furthermore, these conserved genes were widely supported by barley homologues (Supplementary Fig. 2, light red histograms), implying that these are indeed functional genes.

Among the non-syntenic Tdic5B sequences (matching *Brachypodium*, rice or sorghum genes on non-orthologous chromosomes), 69 contigs and 206 singletons were found to match genes that were syntenic within *Brachypodium*, rice or sorghum genomes. Considering the evolutionary relationships between *Brachypodium*, rice or sorghum, a gene that is found on a non- colinear position in *Brachypodium*, but on colinear positions in rice and sorghum, is deemed as ‘moved’ (i.e. rearranged) specifically in the *Brachypodium* genome^28^. Similarly, non-syntenic Tdic5B sequences matching *Brachypodium*, rice and sorghum genes that are syntenic with each other indicate genes that are rearranged in the wheat lineage. Of such sequences (69 contigs and 206 singletons), 64 contigs and 191 singletons could be annotated based on *Viridiplantae* proteins, although 113 of these were hypothetical/predicted proteins (Supplementary Table 2). Intriguingly, 20 of these sequences did not match any known *Viridiplantae* proteins, a subset of which may actually correspond to pseudogenes or gene fragments that have lost their functionality through extensive rearrangements.

### Putative tRNA and miRNA repertoire of Tdic5B

The analysis of Tdic5B sequences for putative tRNA genes revealed that the LCN assembly and the unmasked reads encode up to 78 and 875 tRNA genes, respectively, with a marked abundance for tRNA^Lys^ species among unmasked reads (Supplementary Fig. 3a). This marked abundance was also reported for the unmasked low coverage sequences from *T. aestivum* 6B^29^ and 5D^19^ chromosomes, as well as *Ae. tauschii* 5D chromosome^20^, and, is generally attributed to a Transposable Element (TE)-driven capture and subsequent co-proliferation. Targeted insertion of transposable elements into high copy small RNA genes have been observed previously, and, implicated as a potential tool for gene delivery^30^. Consistent with these observations, repetitive sequences predicted to contain putative tRNA^Lys^ genes belonged almost exclusively to the LTR/Gypsy superfamily. Conversely, putative tRNA genes encoded by the non-repetitive LCN assembly were slightly less than that of *T. aestivum* and *Ae. tauschii* 5D chromosomes, as well as, much smaller orthologous *Brachypodium* chromosomes 1 & 4, indicating that tRNA genes are not likely expanded in *T. dicoccoides* (Supplementary Fig. 3b).

**Figure 5:**
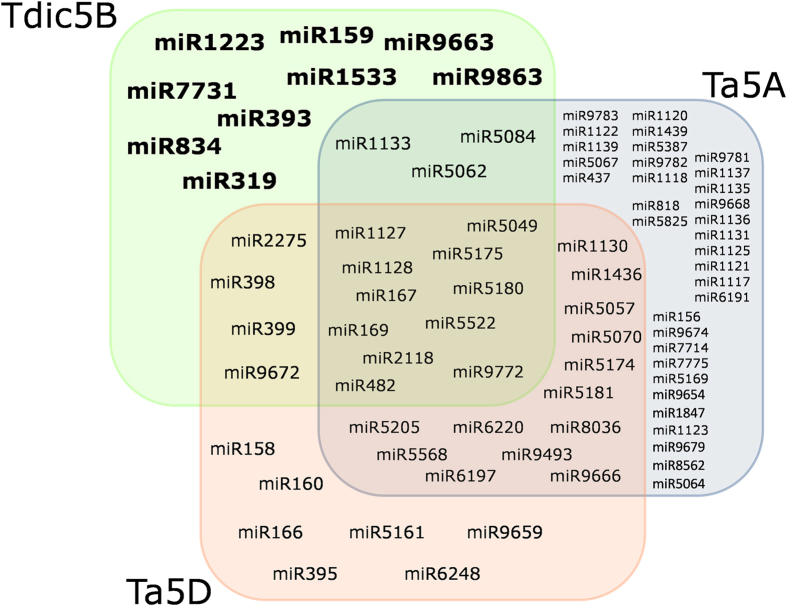
Predicted miRNA repertoires of *T. aestivum* 5A & 5D (Ta5A & Ta5D) and Tdic5B.

MicroRNAs (miRNAs) are an important subclass of small RNAs and carry out crucial functions in growth, development and stress responses by regulating gene expression^31^. The LCN assembly of Tdic5B identified 217 genomic loci for 64 miRNAs, based on sequence homology to known *Viridiplantae* miRNAs (miRBase, Release 21) and secondary structure preservation (Supplementary Table 3). The minimal folding free-energy index (MFEI) of miRNA precursors is generally higher than other types of RNAs, such as tRNAs (0.64), rRNAs (0.59), and mRNAs (0.62 − 0.66), and, thus, is utilized in computational miRNA prediction approaches^32^. Accordingly, MFEI values of miRNA precursors predicted from Tdic5B assembly were 0.95 ± 0.13. Among the predicted miRNAs, over half (54.8%) belonged to the miR2118 family. Three other miRNA families with well-established roles in plants, miR167, miR169 and miR399, were also prominent (10.1%, 6.9% and 7.8%, respectively) among miRNAs putatively encoded by Tdic5B. Computational prediction of miRNAs from the LCN assemblies constructed from raw 454 sequences of *T. aestivum* chromosomes 5A^13^ and 5D^19^, using the same procedure as Tdic5B, suggested that 9 miRNA families detected from Tdic5B are not present in these chromosomes (Fig. 5), although experimental validation is required for a firm conclusion.

To explore the functional networks regulated by the miRNAs predicted from Tdic5B sequences, miRNA-targeted genes were predicted from the transcriptome sequences assembled from RNA-Sequencing (RNA-Seq) of five wheat tissues (http://wheat-urgi.versailles.inra.fr/Seq-Repository/RNA-Seq)^14^,^33^. The wheat transcriptome assembly provided a comprehensive source for target genes, as reflected by the one-third of transcripts that could not be annotated based on known plant proteins (Supplementary Table 3). These, along with the hypothetical and predicted proteins, together comprising over two-thirds of all targets, suggest that our knowledge on miRNA-target interactions is going to evolve as more wheat miRNAs and proteins are annotated and characterized. Disease resistance-associated proteins alone comprised over 10% of all annotations, emphasizing the abundance of biotic stress related loci on Tdic5B. The remaining annotations revealed proteins involved in a variety of biological pathways; multiple targets regulated by the same miRNA, or, conversely, common targets of a number of different miRNAs point out to a complex and intermingled network of miRNA-regulated gene expression.

### Single Nucleotide Polymorphisms on Tdic5B

Despite the rich allelic diversity maintained among wild wheat populations, saturated genetic maps to exploit this diversity are scarce. Therefore, Tdic5B sequences were mapped against the transcriptome sequences of two different *T. dicoccoides* varieties, TR39477 and TTD-22, assembled from RNA-Seq data obtained recently (Budak *et al.*, in review), to reveal Single Nucleotide Polymorphisms (SNPs), following the pipeline proposed by You *et al.*^34^. In the absence of a reference-quality genome sequence, You *et al.*^34^ recently suggested a methodology to discover potential SNPs, by mapping short reads generated by NGS technologies on relatively longer reads or sequence assemblies, such as full length cDNA sequences or transcriptome assemblies. Using this approach, RNA-Seq sequences from drought-treated and control root tissues of TR39477 and TTD-22 varieties were assembled to generate longer transcriptome sequences to be used as reference. To minimize false alignments with transcripts from elsewhere in the genome, 5B-related transcripts were retrieved by blast searches against Tdic5B sequences. Despite the high stringency used to filter out 5B related sequences, it should be noted that a small number of highly similar homoeologous sequences from Tidc5A chromosome or paralogous loci from elsewhere may not be excluded and remain among the filtered transcripts. Unmasked Tdic5B reads were then mapped onto these 5B-related transcripts and sequence variations within positive alignments were filtered against depth and SNP proximity^34^. Consequently, a total of 9275, 10034, 8913 and 9242 SNPs in 1827, 1879, 2064 and 2137 5B-related transcripts from drought-treated TR39477, control TR39477, drought-treated TTD-22, and control TTD-22 samples, were identified, respectively (Supplementary Table 4). These corresponded to the average SNP frequencies of 1,043.4 bases/SNP (1,047.8 for drought, 1,038.9 for control) for TR39477, and, 1,368.3 bases/SNP (1,370.6 for drought, 1,365.9 for control) for TTD-22 varieties, considering the total length of all respective 5B-related transcripts.

The two *T. dicoccoides* varieties used to discover potential SNPs exhibited contrasting levels of drought tolerance, consistent across different drought exposures. TR39477 is characterized by its high tolerance against drought, compared to highly sensitive TTD-22^8^,^9^. Transcripts from the drought-treated TR39477 roots were further examined, as SNPs within these transcripts may be utilized in breeding programmes if linked to drought stress tolerance. Of the 1,827 SNP-containing transcripts from the drought-treated TR39477 transcriptome, 507 exhibited significant similarities to transcripts from control TR39477, drought-treated TTD-22, and control TTD-22 transcriptomes. On these 507 transcripts, positions corresponding to SNPs identified in TR39477 samples were examined across other samples through pair-wise alignments and only those that are covered by transcripts from both control and drought-treated samples and that are consistent (having the same nucleotide) in control and drought-treated samples of the same variety were recorded. A total of 584 SNPs in 228 transcripts identified in TR39477 had the same nucleotide in TTD-22 transcripts as in Tdic5B sequences (for instance, C in TR39477 but T in TTD-22 and Tdic5B; “Group 1” in Supplementary Table 5). Conversely, 1,092 SNPs in 290 transcripts had the same nucleotide in TTD-22 and TR39477, but differed in Tdic5B (“Group 2” in Supplementary File 5). Interestingly, 3 SNPs on 3 transcripts identified in TR39477 had a different nucleotide in each of the three varieties. For instance, the transcript c23780_g2_i1 from TR39477 drought sample had the base ‘Thymine’ at position 967 (as well as the corresponding transcript from TR39477 control sample). However, the corresponding position in corresponding transcripts from TTD-22 control and drought samples had ‘Guanine’ instead, while the Tdic5B sequences mapping to this position had ‘Cytosine’ (“Group 3” in Supplementary Table 5). As these transcripts can be readily differentiated based on SNPs in all three genotypes, phenotypic traits conferred by these transcripts can also be readily screened using linked molecular markers. However, functional annotations of these transcripts through the comparison against known *Viridiplantae* proteins revealed sequence similarities to only hypothetical proteins with currently unknown functions. Functional characterization of these transcripts and physiological characterization of *T. dicoccoides* 26676 variety used in this study, particularly against drought stress conditions, may provide candidate genes for wheat improvement, for which SNP-based molecular markers for gene cloning and transfer can then be designed and implemented in breeding programs.

## Discussion

Domestication and breeding for modern agriculture have narrowed gene pools within crop populations for improved yield, rendering crops susceptible to stress factors. Wild germplasms adapted to a range of environments maintain a rich genetic diversity and are a promising source for crop breeding programmes. Wild emmer wheat, *Triticum turgidum* ssp. *dicoccoides* is the wild relative of the tetraploid durum wheat progenitor, *Triticum turgidum*. The potential that *T. dicoccoides* holds for wheat improvement has been recognized for almost a century; accordingly, a number of genes associated with abiotic and biotic stress tolerance, grain protein and micronutrient content have been mapped to several wild emmer chromosomes. A subset of these genes have also been introgressed into modern wheat cultivars^7^. A majority of genes introgressed from *T. dicoccoides* into modern cultivars comprised disease-resistance genes, particularly against powdery mildew and rust. Fine mapping and characterization of additional resistance genes, including powdery mildew resistance, continue as pathogen evolution necessitates the identification of novel alleles against novel pathogen strains^35^,^36^,^37^,^38^,^39^. A few loci controlling important agronomic traits, such as grain protein and micronutrient content have also been mapped to 5B chromosome *T. dicoccoides*^7^,^35^,^36^,^40^. In addition, *T. dicoccoides* exhibits allelic variation for the *Ph1* locus located on the long arm of 5B chromosome. This locus is responsible for the suppression of homoeologous chromosome pairing during meiosis, extending the utility of studying this chromosome beyond agronomically relevant traits^41^,^42^,^43^.

Despite its rich genetic diversity and direct ancestry to durum and bread wheat, genomic resources are highly limited for *T. dicoccoides*, restricting its exploitation in wheat improvement. In this study, we present the next-generation sequencing of flow-sorted *T. dicoccoides* 5B chromosome to 1.87x coverage, enabling us to explore its repeat content and composition, conserved protein-coding and tRNA-encoding genes, miRNA repertoire and nucleotide variations with two related genotypes with contrasting levels of drought tolerance. To our knowledge, the sequence information generated in this study is currently the largest genomics resource available for *T. dicoccoides*, providing an in-depth view into its genome structure and organization.

Comparison of Tdic5B sequences against the known *Poaceae* repeats revealed that repetitive sequences make up 84.9% of the chromosome, consistent with the highly repetitive nature of *Triticeae* genomes. Recently, low-coverage 454 sequencing of *T. aestivum* 5B chromosome has been reported^44^. Despite representing only 7% of the chromosome (61 Mb of sequence data, thus, not included in the main comparative analyses), *T. aestivum* 5B sequences, which were applied the same repeat-masking procedure, suggested a repeat content of 83.7%, similar to Tdic5B (Supplementary Fig. 4). Repeat superfamily distribution of Tdic5B suggested recent amplification of the Gypsy superfamily, as suggested by the comparative analysis of the recent draft chromosome sequences of bread wheat^14^. Repetitive element distributions revealed from the limited 454 sequencing data, from the *T. aestivum* 5B chromosome support this view (Supplementary Fig. 4). Differential expansion of high-copy and low-copy elements following polyploidization and diploidization is a known phenomenon^45^; however, due to the highly dynamic profileration profiles of repetitive elements in different backgrounds, which family members of the Gypsy superfamily might have expanded in Tdic5B could not be determined with the present data on its counterparts. The genic fraction of Tdic5B (1.15%) assessed from a total of 4,818 conserved gene models was comparable to that of *T. aestivum* 5A and 5D chromosomes. Recently, over 5,500 functional gene or gene-models were reported for the reference sequence of the 3B chromosome including the unanchored scaffolds^21^. The estimated gene content is considerably lower for Tdic5B, which is largely attributable to the differences in chromosome sizes (1 Gb for 3B vs. estimated 840 Mb for Tdic5B), and to a lesser extent, can be explained by the exclusion of non-conserved gene associated sequences in Tdic5B gene content estimation. The reference sequence of 3B chromosome revealed ~27% pseudogenic loci among all identified coding loci^21^. As distinguishing pseudogenic loci from genuine genes at this level of coverage would be impractical, these non-conserved gene associated sequences were excluded from gene estimation. Accordingly, the actual gene content of Tdic5B is expected to slightly exceed 4,818 gene models constructed in this study. The LCN assembly of Tdic5B matched 7,612 conserved genes from model grasses, *Brachypodium*, rice and sorghum, and revealed 3 syntenic blocks, involving, (1) Bd1-Os3-Sb1, (2) Bd4-Os9-Sb2, (3) Bd4-Os12 chromosomes (Supplementary Fig. 2), consistent with the previous observations^23^. The presence of large syntenic blocks and colinearity within these blocks is crucial, especially for species with limited genetic mapping data. Indeed, fine mapping of a number of traits in *T. dicoccoides* relied heavily on the syntenic relationships and colinearity^35^,^38^,^39^,^46^. In addition to protein-coding loci, Tdic5B was observed to contain slightly fewer putative tRNA genes and miRNAs, compared to its modern counterparts *T. aestivum* chromosomes 5A and 5D, for which raw sequence data obtained with the same NGS platform were retrieved and processed using the same procedures as Tdic5B. While NGS data for *T. aestivum* 5B chromosome is available from two sources^14^,^44^, these could not be used for direct comparisons due to either limited data size^44^ or different sequencing technology^14^.

Homology-based miRNA prediction identified 64 unique miRNAs putatively encoded by Tdic5B. Among the predictions, miR2118 family were the most abundant, representing over half of the putative miRNA-coding genomic loci. Additionally, Tdic5B was found to encode 11 members of miR167 family, 10 members of miR169 family and 6 members of miR399 family. Remarkably, the precursors of miR2118 and miR169 have been experimentally verified to be specific to the 5D chromosome of modern bread wheat^47^. miR2118 family was also reported to be represented by 42 family members in *Ae. tauschii* draft genome^16^. It is tempting to speculate that the coding regions for miR2118 and miR169 on ancient B-genome might have been lost through wheat genome evolution due to functional redundancy in homoeologous genomes, while these regions are still retained in the B-genomes of tetraploid wild populations. miR169 has been identified as an abiotic stress-responsive miRNA family in plants, specifically targeting NF-YA subunit of Nuclear Transcription Factor Y (NF-Y)^48^. Consistently, target annotations of wheat transcriptome sequences identified several NF-Y subunits exclusively targeted by miR169 and miR2118 (Supplementary Table 3). miR2118 has also been implicated to target NBS-LRR disease resistance genes^49^, as reflected in the target annotations of putative Tdic5B miR2118 family. Intriguingly, these observations indicate that several putative miR2118-targeted wheat transcriptome sequences assembled from RNA-Seq of five different wheat tissues^14^,^33^ that lacked an annotation or annotated as hypothetical proteins may actually correspond to biotic or abiotic stress-related genes. The lack of an apparent sequence similarity to known *Viridiplantae* proteins implies that these transcripts may code for novel or highly diverged proteins and their further characterization may reveal new candidates for wheat improvement.

Mapping of Tdic5B reads onto 5B-related transcriptome sequences of two *T. dicoccoides* varieties, TR39477 and TTD-22, revealed one SNP in every 1,043.4 and 1,368.3 bases on average, respectively. It should be noted that, however, some of these SNPs may arise from highly similar homoeologous Tdic5A sequences or, to a lesser extent, highly similar paralogous loci elsewhere in the genome, which could not be differentiated from 5B-related transcripts computationally, despite the highly stringent filtering criteria. Recently, Brenchley and her colleagues could differentiate homoeologous sequences with high precision for 66% of gene assemblies obtained from 5X coverage sequences of the entire bread wheat genome^50^. Similarly, among approximately 30% of the transcriptome assemblies of TR39477 and TTD-22 that are probably highly similar, transcripts that differ by 2% of less by sequence composition on the homoeologous 5A chromosome are likely to be retained among the 5B-related transcripts used for SNP analyses. Thus, it is important to implement SNPs reported in this study cautiously for functional studies, until they are verified experimentally. The SNP frequencies observed in this study imply that coding regions carry more sequence divergence between 26676 and TR39477 genotypes, which may be utilized to design SNP-based markers, particularly for traits linked to the remarkable drought tolerance of the TR39477 genotype. The contrasting drought tolerances of TR39477 and TTD-22 potentiates the use of the SNPs for novel molecular marker design to aid in genetic and physical mapping of genomic drought-resistance loci. Through effective genotyping of wild populations these SNPs could be useful for gene discovery and mapping, as demonstrated by the SNP-based genome-wide association mapping of stripe rust resistance reported recently^51^. NGS mediated discovery of SNPs was previously utilized for the fine mapping of a grain protein content locus in durum wheat^52^. Although the SNP frequencies reported here are relatively lower than the study of Sela *et al.*^51^, and, another study reporting SNP discovery via NGS in two *Ae. tauschii* accessions^34^, the accumulation of high-throughput NGS data is likely to play pivotal role in gene discovery and mapping in wild emmer wheat that can further be implemented into wheat improvement.

## Methods

### Flow-sorting, sequencing and assembly of Tdic5B

Seeds of *Triticum dicoccoides* accession 26676 were kindly provided by Dr. Etienne Paux (INRA, France). The seeds were germinated and their primary roots used for preparation of aqueous suspensions of intact mitotic metaphase chromosomes^11^. GAA microsatellites of chromosomes in suspension were labeled by FITC^53^, chromosomal DNA was stained by DAPI at 2 μg/ml and the samples were analyzed using FACSAria SORP (BD Biosciences, San José, USA) at rate of 1,500 chromosomes/sec. Blue laser (488 nm, 100 mW) was used to excite FITC fluorescence of GAA microsatellites, while UV laser (355 nm, 100 mW) was used for DAPI excitation. Biparametric flow karyotypes of FITC fluorescence (logarithmic scale) and DAPI fluorescence (linear scale) were obtained after analyzing 20,000 chromosomes. In order to sort chromosome 5B, sort window was set up on the dot plot and the chromosome was sorted at rate of 20 chromosomes/sec. In order to assess contamination of the sorted fraction by other chromosomes, 2,000 chromosomes were sorted into a drop of P5 buffer^54^and air-dried. FISH with probes for GAA microsatellites and *Afa* repeat family was used to facilitate identification of chromosomes, which were counterstained by DAPI and observed by fluorescence microscopy. Three independent samples were prepared and average purity of sorted fraction was determined. To produce the required amounts of chromosomal DNA for sequencing, 30,000 chromosomes (equivalent to 50 ng DNA) were sorted into PCR tube filled with 40 μl deionized water in three batches, and their DNA was amplified by isothermal multiple displacement amplification (MDA)^55^.

Sequencing Tdic5B DNA was carried out on GS FLX Titanium platform (Roche 454 Life Sciences, Branford, CT, USA), following manufacturer’s instructions. Two shotgun libraries were prepared from 0.5 μg of amplified Tdic5B and sequenced in three rounds. Raw reads are submitted to the EBI Sequence Read Archive under the primary accession number PRJEB8079.

All sequence reads were compared against MIPS Repeat Element Database v9.3 p for *Poaceae* (ftp://ftpmips.helmholtz-muenchen.de/plants/REdat/)^56^, using RepeatMasker v.3.3.0 software (http://www.repeatmasker.org/) to identify repetitive elements. Organellar genome and rRNA associated reads were identified through BLAST searches against *Triticum turgidum* ssp. *dicoccoides* TA0073 (GenBank: KJ614400.1), TA0060 (GenBank: KJ614401.1), TA1133 (GenBank: KJ614402.1) chloroplast, complete genome (1E-15, -dust “no”); *Triticum aestivum* mitochondrion, complete genome (NC_007579.1, 1E-15, -dust “no”); all *Triticum* rRNA sequences (419 sequences on 08.09.14) deposited in NCBI Nucleotide database (1E-05, -dust “no”). Sequence reads identified as repetitive or organellar genome/rRNA-associated were excluded from the sequence assembly. The remaining sequences were used to construct a Low Copy-Number (LCN) assembly using gsAssembler software (Newbler 2.6, Roche 454 Life Sciences, Branford, CT, USA) with the “Large and complex genome”, “Heterozygotic genome”, “Extend low-depth overlaps” options and a minimum overlap identity of 98%^20^. Sequencing and assembly metrics are given in Table 1.

For comparative analyses, raw sequences for *T. aestivum* 5A^13^, 5B^44^ and 5D^19^ chromosomes, and, *Ae. tauschii* 5D^20^ chromosome, all of which were obtained with GS FLX Titanium as Tdic5B, were retrieved, and the same procedures and criteria were applied using the same databases as Tdic5B.

### Identification of protein-coding genes, putative tRNAs and miRNAs

Protein-coding gene-associated reads of the LCN assembly were identified using BLAST searches against the fully annotated *Brachypodium distachyon* (v1.2, http://mips.helmholtz-muenchen.de/plant/brachypodium)^23^, *Oryza sativa (*assembly IRGSP-1.0, http://rapdb.dna.affrc.go.jp/download/irgsp1.html)^24^, *Sorghum bicolor* (v1.4, http://mips.helmholtz-muenchen.de/plant/sorghum/)^25^ proteins (1E-6, -length 30, -ppos 75); high-confidence *Hordeum vulgare* proteins (http://mips.helmholtz-muenchen.de/plant/barley/)^26^ (1E-6, -length 30, -ppos 90); *Triticum aestivum* UniGenes (Build#63, ftp://ftp.ncbi.nih.gov/repository/UniGene/Triticum_aestivum/, 1E-30, -length 90, -pident 98) and *Triticum* UniProt sequences (14,4397 entries, http://www.uniprot.org/, (1E-6, -length 30, -ppos 100). The blast parameters were essentially adopted from previous studies to ensure consistency^13^,^19^,^20^ and similarity/identity cutoffs were increased for the close relatives, barley and wheat species. To increase stringency, ‘Best Reciprocal Hit’ approach was applied for protein queries, where BLAST searches were performed as blastx and tblastn, and only reciprocal best hits were retained. For all BLAST searches, redundant LCN assembly singletons covering the exact same portion of a protein or gene query were eliminated to avoid amplification bias deriving from MDA. BLAST+ stand-alone toolkit, version 2.2.25^57^ were used for all BLAST searches. Gene models were constructed by mapping masked Tdic5B reads onto the coding sequences of *Brachypodium*, rice, sorghum and barley proteins that exhibited significant similarities to the LCN assembly through BLAST searches. If an LCN contig or singleton is associated with multiple hits from the grass proteomes through BLAST searches the reference sequence is picked by this precedence: *Brachypodium*, rice, sorghum and barley. Mapping was performed using gsMapper software (Newbler 2.6, Roche 454 Life Sciences, Branford, CT, USA) with default settings, except for All Contig Threshold=40. Mapping results were processed with an in-house Perl script which merged non-overlapping sequences mapping to the different sections of the same reference sequence and filled the gaps (where no Tdic5B sequence was mapped) by strings of ‘n’.

Circle plots and heatmaps demonstrating gene conservation and syntenic relationships were visualized using Circos software^58^ and MATLAB R2010b, respectively. Ribbons in Circos image were generated with >100 members along 1 Mb intervals. Gene densities were counted on 500 kb intervals (light blue & light grey). Heatmaps were drawn with a sliding window approach of 50 kb step size and the genomic positions of annotated proteins were retrieved from MIPS database of plants (http://mips.helmholtz-muenchen.de/plant/genomes.jsp). All functional annotations were performed on BLAST2GO^59^ using locally run BLAST results against *Viridiplantae* proteins (1E-6, -outfmt 5, -max_target_seq 1).

The tRNAscan-SE 1.21 program^60^ was run locally with the default parameters for eukaryotic genomes to predict putative tRNA genes. Pseudogenes and other undetermined annotations were not evaluated.

Prediction of putative miRNAs was performed using two in-house Perl scripts, SUmirFind and SUmirFold. Mature miRNA sequences for *Viridiplantae* were retrieved from miRBase Release 21 (http://mirbase.org/) and used as query for homology searches. Hairpin structures were evaluated for miRNA characteristics as previously reported^47^. Potential miRNA targets were predicted online using psRNATarget (http://plantgrn.noble.org/psRNATarget/) among transcriptome assemblies from RNA-Seq data of five *T. aestivum* tissues (http://wheat-urgi.versailles.inra.fr/Seq-Repository/RNA-Seq)^14^,^33^.

### Discovery of Single Nucleotide Polymorphisms

Single Nucleotide Polymorphisms (SNPs) were investigated essentially following You *et al.*^34^. RNA-Sequencing data from drought-treated and control roots of *T. dicoccoides* varieties TR39477 and TTD-22 were assembled using Trinity pipeline (http://trinityrnaseq.sourceforge.net/). The assembled transcriptome sequences were blasted against Tdic5B reads to identify 5B-related transcripts (1E-30, -pident 98). The 5B-related transcripts sequences were then separately used as reference onto which Tdic5B unmasked reads were mapped using gsMapper software (Newbler 2.6, Roche 454 Life Sciences, Branford, CT, USA) with default settings. Nucleotide variations on single positions were retained and filtered for mapped read depth (3 ≤ depth ≤ 10) and SNP proximity (>3 bp between SNPs). To identify shared SNP positions, drought-treated TR39477 transcripts were blasted against remaining three sets of transcriptome sequences (1E-30, -pident 98) and positions corresponding to SNPs in drought-treated TR39477 transcripts were manually evaluated through pairwise sequence alignments on NCBI Blast (http://blast.ncbi.nlm.nih.gov/BlastAlign.cgi).

## Additional Information

**How to cite this article**: Ani Akpinar, B. *et al*. Molecular organization and comparative analysis of chromosome 5B of the wild wheat ancestor *Triticum dicoccoides*. *Sci. Rep.* **5**, 10763; doi: 10.1038/srep10763 (2015).

## Supplementary Material

Supplementary Information

## Figures and Tables

**Table 1:** Sequencing and assembly metrics for Tdic5B.

Sequencing library	No.of reads N	Mean read length L (bp)	Total read length (Mb)	Sequencing Coverage^a^	Probability^b^
Tdic5B-1	953,680	294.8	281.2		
Tdic5B-2	1,694,938	357.2	605.4		
Tdic5B-2	1,640,921	419.2	687.9		
Combined	4,289,539	357.1	1,574.4	1.87	0.799

Assembly statistics	No. of reads/contigs	Mean length (bp)	Total length (Mb)	Length (% of chromosome)	N50 contig size (bp)
Filtered reads	501,177	357	100.8	12	
LCN assembly:					
Large contigs	14,302	1045	14.9	1.77	1117
All contigs	26,225	697	18.3	2.18	
Singletons	256,685	322	82.6	9.83	

^a^Sequencing coverage was calculated using a chromosome size estimate of 840 Mbp^17^

^b^The probability of representation of any position in the dataset was calculated as follows: P = [1 – (1 − L/S)^N x Purity^ ], where S is the chromosome size and L & N are as listed in the table.
