# Supplementary material for: Molecular organization and comparative analysis of chromosome 5B of the wild wheat ancestor Triticum dicoccoides
Source: Sci Rep. 2015 Jun 18;5:10763. doi: 10.1038/srep10763 (PMC4471722; doi:10.1038/srep10763)
Supplement: Supplementary Information [file srep10763-s1.pdf]

# **Molecular organization and comparative analysis of chromosome 5B of the wild wheat ancestor *Triticum dicoccoides***

Bala Ani Akpinar[1], Meral Yuce[1], Stuart Lucas[1], Jan Vrána[2], Veronika Burešová[2], Jaroslav Doležel[2], Hikmet Budak[1,3]*

## Supplementary Information

**Supplementary Figure 1.** Detailed heatmaps showing gene conservation between Tdic5B and the model grasses, *Brachypodium*, rice and sorghum. Orthologous chromosomes are the focus, however, entire genomes of the model grasses are also given as minimized objects.

**Supplementary Figure 2.** Circular representation of Tdic5B syntenic relationships with the three model grass genomes (Bd: *Brachypodium distachyon*, Sb: *Sorghum bicolor*, Os: *Oryza sativa*). Heatmaps are given below the histograms represent conserved genes along each respective chromosome. Ribbons indicate regions with ≥50 genes counted along 1Mb intervals. Dark red bars correspond to histograms of the links; light red bars indicate the presence of a barley ortholog. Light blue and light grey bars surrounding chromosomes indicate gene densities, where dark blue and dark grey bars indicate a gene density of >50 genes/Mb.

**Supplementary Figure 3.** Putative tRNA genes of Tdic5B **a.** A comparison of the unmasked Tdic5B reads containing repetitive elements and the LCN assembly. **b.** Comparison of putative tRNA gene contents of *T. aestivum* 5D (Ta5D), *Ae. tauschii* 5D (Aeg5D), Tdic5B and orthologous chromosomes 1 & 4 of the model grasses *B. distachyon* (Bd1 & Bd4).**Supplementary Figure 4.** Repeat fractions of Tdic5B and *T. aestivum* 5B (Ta5B).

**Supplementary Table 1.** Complete list of repeat annotations for Tdic5B

**Supplementary Table 2.** Gene-associated Tdic5B contigs and singletons of the LCN assembly

**Supplementary Table 3.** Predicted Tdic5B miRNAs and their respective targets

**Supplementary Table 4.** Putative SNPs between Tdic5B and the root transcriptomes of TR39477 and TTD-22

**Supplementary Table 5.** Shared SNPs between Tdic5B, TR39477 and TTD-22

**Supplementary File 1.** All conserved gene models for Tdic5B and their sequences in FASTA format.

**Supplementary Figure 1.**

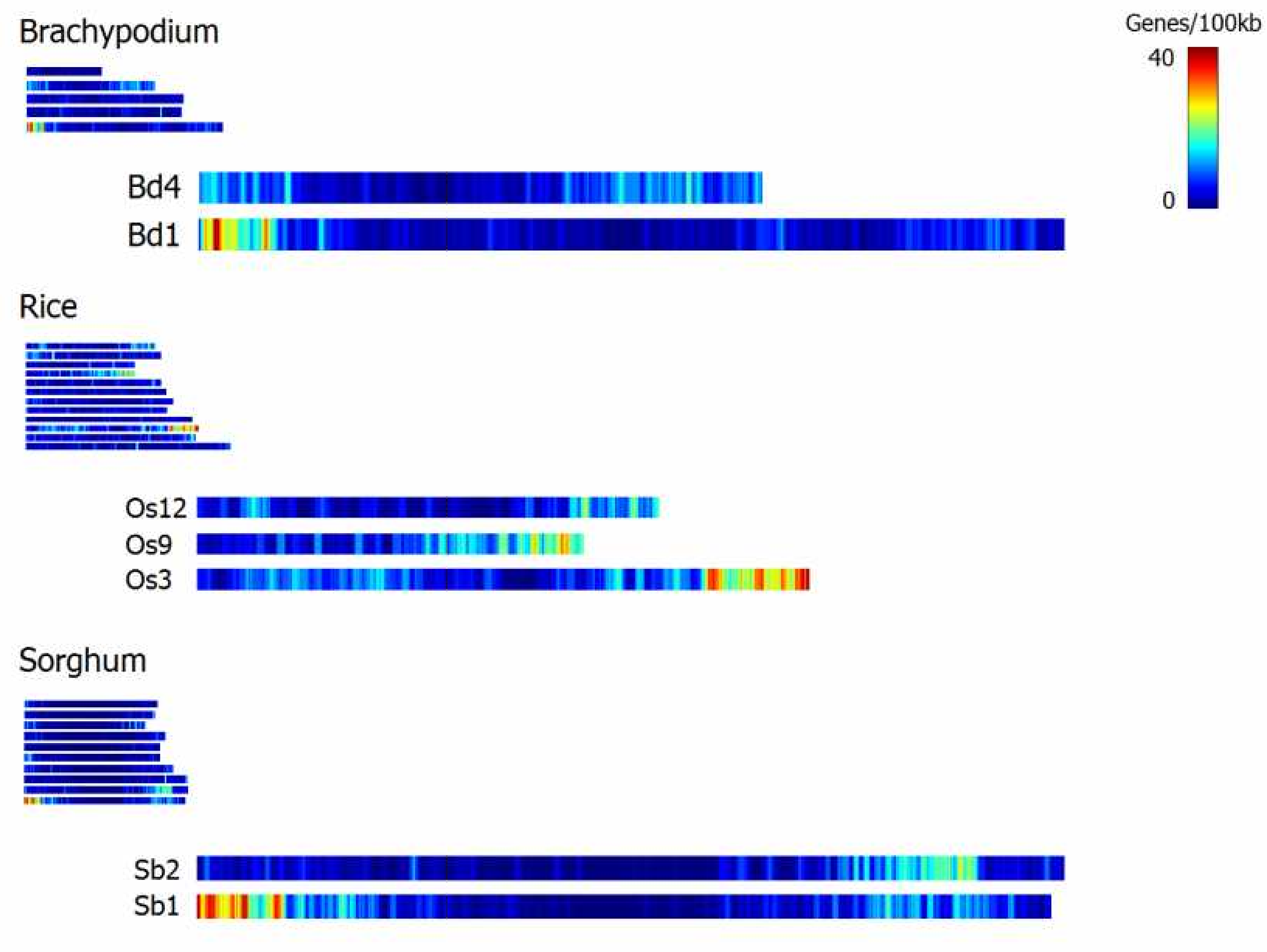

**Supplementary Figure 2.**

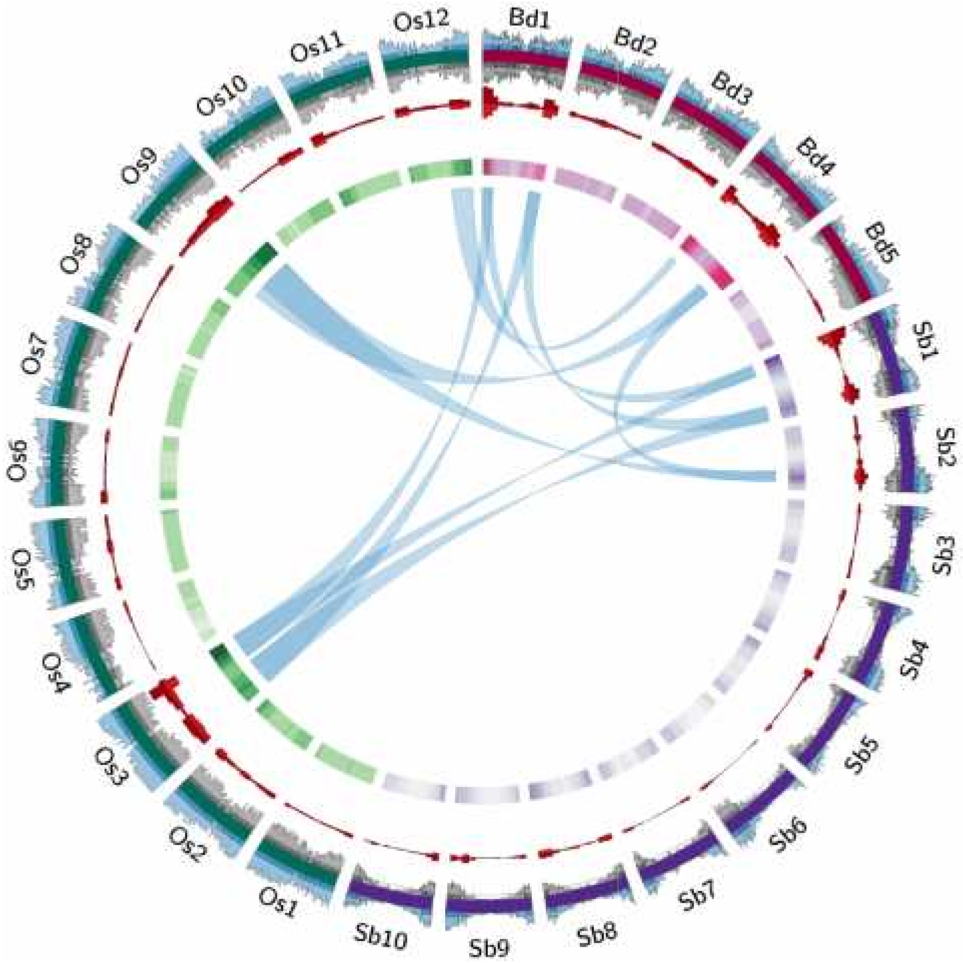

**Supplementary Figure 3.**

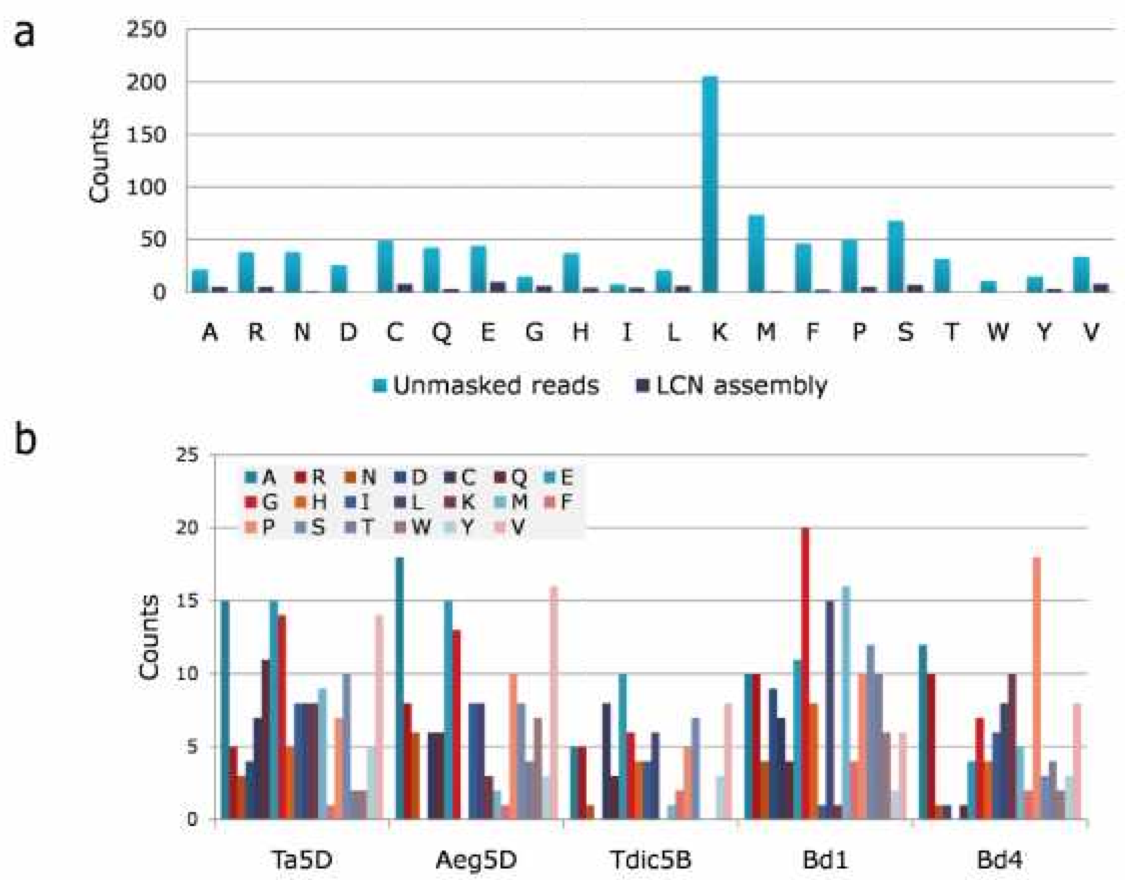

**Supplementary Figure 4.**

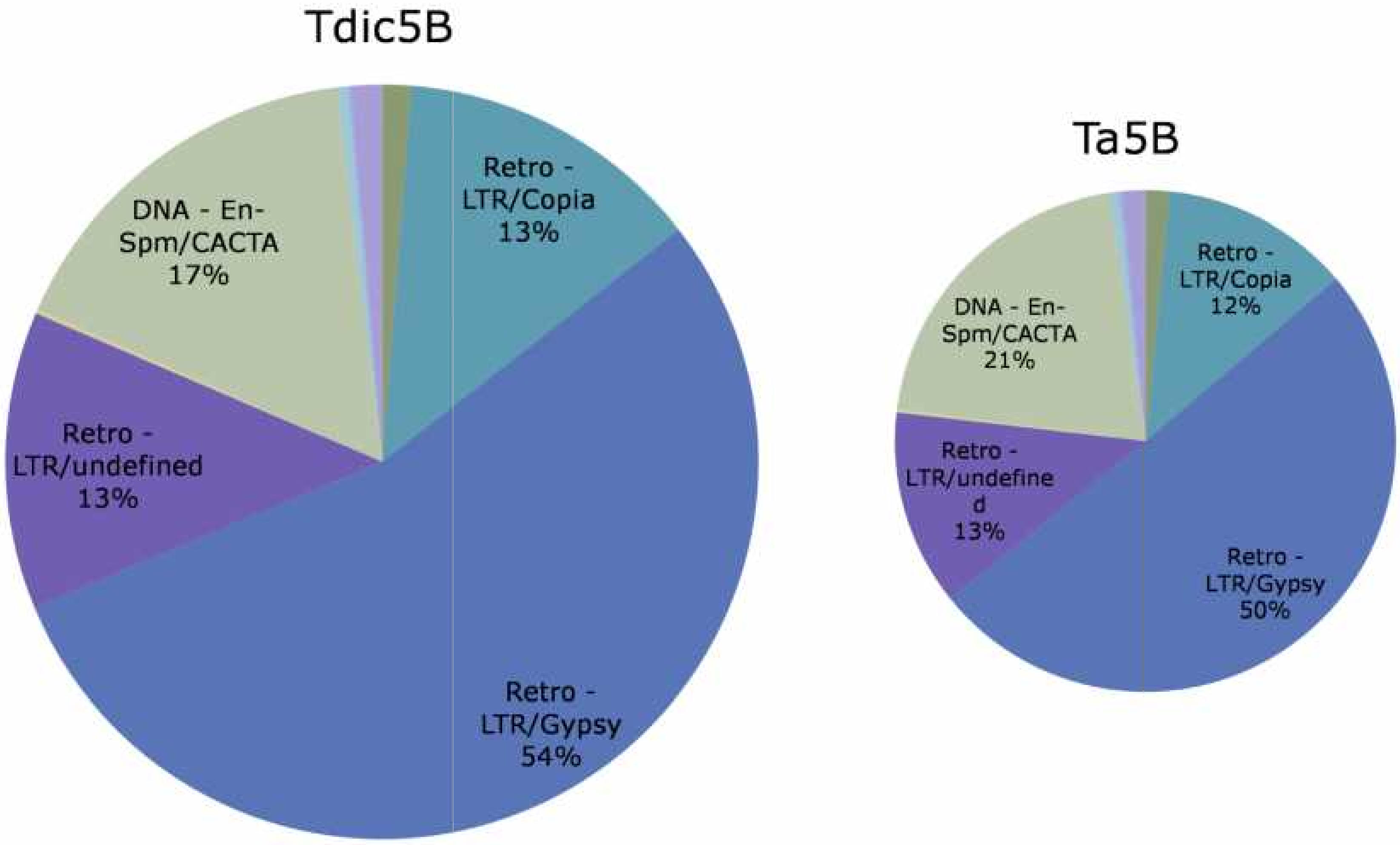

**Molecula**

Bala Ani Ak

**Supplemen**

>Bradi1g00
tgcatgatgtg
>Bradi1g00
agcatgcatga
>Bradi1g00
cattagccgcg
>Bradi1g00
ggcctcctcga
>Bradi1g00
GGACTGG
>Bradi1g00
caggatgacc
>Bradi1g00
TTCTAGGT
>Bradi1g00
AGAATAcc
>Bradi1g00
agcagctaca
>Bradi1g00
acaaagaaca
>Bradi1g00
aacagcatgt
>Bradi1g00
ggtgacatcca
>Bradi1g00
TTGCTAGC
>Bradi1g00
tattaacagga
>Bradi1g00
ggaattacaa
>Bradi1g00
atgggcatctc
>Bradi1g00
caggtgggcg
>Bradi1g00
ggtttgccggc
>Bradi1g00
agcaggTcgc
>Bradi1g00
atggcggcga
>Bradi1g00
aggtggtcaga
>Bradi1g01
AGAGAAAG
>Bradi1g01

gctcgctggcc
>Bradi1g01
gagaaccgttg
>Bradi1g01
atggcatccgt
>Bradi1g01
GTgGGacGt
>Bradi1g01
gggaaaaacc
>Bradi1g01
ttcaaatgaaa
>Bradi1g01
AAGATCGC
>Bradi1g01
cagaggatga
>Bradi1g01
GAGGTTAA
>Bradi1g01
ctgaccaacac
>Bradi1g01
tgctccttcgcc
>Bradi1g01
ctgTTGatCt
>Bradi1g01
tgctcgtccac
>Bradi1g01
CGGCGgCG
>Bradi1g01
CTTATATGT
>Bradi1g01
acacactattc
>Bradi1g01
gggcaatgctg
>Bradi1g01
AGTACCTG
>Bradi1g01
ATGAAGGA
>Bradi1g01
TAAGGGAG
>Bradi1g01
ccggctcgcag
>Bradi1g01
AGGTaGTT
>Bradi1g01
aggggctgctg
>Bradi1g01
ACCTGCTG
>Bradi1g02
atagacattgt
>Bradi1g02

GCGGCACG
>Bradi1g02
taaGggTTG
>Bradi1g02
atggagagcc
>Bradi1g02
ggccggaggt
>Bradi1g02
gccgggAGC
>Bradi1g02
aatacaatgc
>Bradi1g02
CAGCAACT
>Bradi1g02
gagagtcagt
>Bradi1g02
attgcggacct
>Bradi1g02
gggcacgaaa
>Bradi1g02
tttgagggcac
>Bradi1g02
agttgaggttg
>Bradi1g02
CCAGATGA
>Bradi1g02
aagaagaaac
>Bradi1g02
ccctccgccat
>Bradi1g02
aggtatcgat
>Bradi1g02
gactcattgct
>Bradi1g02
gcaggcgacg
>Bradi1g02
ctttttagtac
>Bradi1g02
aggccttgca
>Bradi1g02
TACCAGAT
>Bradi1g02
agggtttgac
>Bradi1g02
cagattcctgg
>Bradi1g02
TGACTCTT
>Bradi1g02
tgcaggaaat
>Bradi1g02

catcttgatgg
>Bradi1g02
CTCTTCCtC
>Bradi1g02
CAGAGGA
>Bradi1g02
CAGcCATC
>Bradi1g03
tgttatccagc
>Bradi1g03
aggtaatggg
>Bradi1g03
cgcccgtcgc
>Bradi1g03
gacgagctca
>Bradi1g03
ctctaggtgct
>Bradi1g03
atggattggg
>Bradi1g03
ttcctgcagat
>Bradi1g03
AGgaTGGT
>Bradi1g03
ccgccgcgac
>Bradi1g03
ctacaatgttt
>Bradi1g03
GACTAACA
>Bradi1g03
atgggggttc
>Bradi1g03
ggtatggcag
>Bradi1g03
ATTAATCTT
>Bradi1g03
tctcggtagac
>Bradi1g03
TGTTAAaAT
>Bradi1g03
gtggaatgtg
>Bradi1g03
aaaattacca
>Bradi1g03
atggcgccga
>Bradi1g03
tgatgctgtca
>Bradi1g03
gacgactactc
>Bradi1g03

atgaggcaaa
>Bradi1g03
CAGGAGAA
>Bradi1g04
aagtttgtgct
>Bradi1g04
agggtgttca
>Bradi1g04
aagggaaact
>Bradi1g04
ATGGGGCT
>Bradi1g04
tggctcccgC
>Bradi1g04
gcattctgga
>Bradi1g04
gatgcgcacg
>Bradi1g04
AAGTCCTT
>Bradi1g04
TATGATTG
>Bradi1g04
gataaagaat
>Bradi1g04
ttcagccgtgt
>Bradi1g04
ttaatcattctt
>Bradi1g05
gactgatgca
>Bradi1g05
atgagggcgt
>Bradi1g05
TGGGAGTC
>Bradi1g05
aggtatgcaa
>Bradi1g05
gactccgggg
>Bradi1g05
CCTCGTCC
>Bradi1g05
ggtgttgttat
>Bradi1g05
TGTTCGAA
>Bradi1g05
ACGGCGAC
>Bradi1g05
atgctcgcggt
>Bradi1g05
TTCACTAG
>Bradi1g05

tgtatgtccag
>Bradi1g05
gttcagaaat
>Bradi1g05
CAGTTGCT
>Bradi1g05
ccagctggta
>Bradi1g05
agggttctAT
>Bradi1g05
gaagataact
>Bradi1g05
gccttgtcttg
>Bradi1g05
atgatgcgca
>Bradi1g06
atggtgaagc
>Bradi1g06
agaacctgat
>Bradi1g06
tccggcgggtt
>Bradi1g06
aggtgaatgc
>Bradi1g06
agcttctaacc
>Bradi1g06
tgcaggttgat
>Bradi1g06
ACAGGAAT
>Bradi1g06
gccttcctcgc
>Bradi1g06
agagcgaggc
>Bradi1g07
GCAGGGA
>Bradi1g07
ATGGGCGA
>Bradi1g07
gataaccttca
>Bradi1g07
gctggcccga
>Bradi1g07
AGAGGAC
>Bradi1g07
agaatgtggc
>Bradi1g07
cctccccgccc
>Bradi1g07
gctccagccg
>Bradi1g07

gaagaccaat
>Bradi1g07
ctgcaggcatt
>Bradi1g07
ATGGAGCG
>Bradi1g07
agtctcagttc
>Bradi1g07
caaaagattc
>Bradi1g07
attttccctgg
>Bradi1g07
aaggggctaa
>Bradi1g07
agctatctgg
>Bradi1g07
tcccctcgctg
>Bradi1g07
attggacaga
>Bradi1g07
tcccgagcgg
>Bradi1g07
gcatggccga
>Bradi1g07
cagagcgtgg
>Bradi1g07
ggatctatgg
>Bradi1g08
ggaaggacga
>Bradi1g08
attgcaggtg
>Bradi1g08
TTtACCCCT
>Bradi1g08
ATGGTGAA
>Bradi1g08
ATGGcccGC
>Bradi1g08
cagggataca
>Bradi1g08
CAATTCTAC
>Bradi1g08
CTCCTccTG
>Bradi1g08
aaagttgttgg
>Bradi1g08
tgcctccggga
>Bradi1g08
ATGGGTCG
>Bradi1g08

gaagaggagg
>Bradi1g08
gagatgttccg
>Bradi1g08
gaataacaga
>Bradi1g08
ggggtcgctgc
>Bradi1g08
GCTCCTAGA
>Bradi1g08
ttctattcgaag
>Bradi1g08
aggaattgaag
>Bradi1g08
atgcagcaact
>Bradi1g08
ATGCGCCG
>Bradi1g08
CAGGTTTTC
>Bradi1g08
tatattgctcgt
>Bradi1g08
ACaGGATTC
>Bradi1g08
caagtggggg
>Bradi1g08
ggtctatgttgc
>Bradi1g08
agatcgaggca
>Bradi1g09
tcaggtggctg
>Bradi1g09
ATGGAAGa
>Bradi1g09
gtctgcaagtt
>Bradi1g09
TTTGgCGGC
>Bradi1g09
ttcgagaaccc
>Bradi1g09
ACcAGGATC
>Bradi1g09
cggcgaggga
>Bradi1g09
ccgagcaagta
>Bradi1g09
caagattgagg
>Bradi1g09
cagtattctga
>Bradi1g09

GCCGTTCG
>Bradi1g09
aggttaagttg
>Bradi1g09
attctagggg
>Bradi1g09
acggagctct
>Bradi1g09
ggggtattca
>Bradi1g10
ctgtatcagg
>Bradi1g10
agtggcgaag
>Bradi1g10
aagaacagtc
>Bradi1g10
agatcaagga
>Bradi1g10
GCAGAGTT
>Bradi1g10
ctggagctcg
>Bradi1g10
aggttacatat
>Bradi1g11
aagttccgct
>Bradi1g11
tgacggagct
>Bradi1g11
gctcaatcata
>Bradi1g11
tatgcgttttg
>Bradi1g11
ggggtttttct
>Bradi1g11
gatacttctgt
>Bradi1g12
actcaggacc
>Bradi1g12
ttatgcataag
>Bradi1g12
atgaaggtta
>Bradi1g12
TTcTTCTCT
>Bradi1g12
caggttatatt
>Bradi1g12
gctagtgaga
>Bradi1g12
GATCtcGG
>Bradi1g12

GGGTGATC
>Bradi1g13
gccgaccaac
>Bradi1g13
caacAGCG
>Bradi1g13
tgcttctacag
>Bradi1g13
TGGGCGCA
>Bradi1g13
ggatgggacg
>Bradi1g13
agagtggtgg
>Bradi1g13
gcagatattc
>Bradi1g13
CTCAACTG
>Bradi1g13
ATGTCAGG
>Bradi1g13
GGCAGAA
>Bradi1g13
gattgcgtac
>Bradi1g13
agtatgtgag
>Bradi1g13
CAGACTGG
>Bradi1g13
aaaaagctct
>Bradi1g14
gattgagctat
>Bradi1g14
gatgcacacc
>Bradi1g14
gtgggatcca
>Bradi1g14
tgcaggggat
>Bradi1g14
GTGaACGC
>Bradi1g14
agatgggtga
>Bradi1g14
ttggcgacgc
>Bradi1g14
cacgtcgcgct
>Bradi1g14
cgtcagaggt
>Bradi1g14
tcgggcatgtt
>Bradi1g14

CAGAAGTG
>Bradi1g15
acttgattaag
>Bradi1g15
tgaaacttgag
>Bradi1g15
caggtactgcg
>Bradi1g15
GGATACAT
>Bradi1g15
CATTCTTAT
>Bradi1g15
gaggggCAC
>Bradi1g15
ggatgtggtga
>Bradi1g15
aggatgagag
>Bradi1g15
gacctgccttt
>Bradi1g15
TATTGGAa
>Bradi1g16
AGGGTGAG
>Bradi1g16
ggaggacgaa
>Bradi1g17
gctgtggtggt
>Bradi1g17
agatggacat
>Bradi1g19
gaaaaggtat
>Bradi1g20
GAGGCCGG
>Bradi1g20
atggagaata
>Bradi1g21
AAGGTGTA
>Bradi1g21
aaggcctcttg
>Bradi1g22
gcaggcttggt
>Bradi1g22
cgagcagcgg
>Bradi1g22
AggCAGAA
>Bradi1g22
GGCGAGCA
>Bradi1g23
ggaGCCTAC
>Bradi1g23

ggacgccggg
>Bradi1g23
ttcgggtgaga
>Bradi1g24
cctgctagagt
>Bradi1g24
GTGGACAC
>Bradi1g25
cagccgctgc
>Bradi1g25
ttcgtcggagg
>Bradi1g25
gctgcagcca
>Bradi1g26
atggaggagg
>Bradi1g26
tgtgtccatca
>Bradi1g26
gttaccTTAC
>Bradi1g27
ctgtggaaac
>Bradi1g27
GCGAGGG
>Bradi1g27
cgcgccgccg
>Bradi1g28
aatcttatcta
>Bradi1g28
TTGCGACG
>Bradi1g28
TGcAGGTG
>Bradi1g28
ggcgaatgac
>Bradi1g29
ATGGCGGA
>Bradi1g29
gctgaaggca
>Bradi1g29
atacttggtag
>Bradi1g29
tgAGTCCAT
>Bradi1g29
CAaTGCTG
>Bradi1g29
attatgccatc
>Bradi1g30
gggttaatca
>Bradi1g30
caattcatgca
>Bradi1g30

GTTTTATG
>Bradi1g30
ctcaagcctg
>Bradi1g30
ttatttcattag
>Bradi1g30
aaaattgaca
>Bradi1g30
aagattttcn
>Bradi1g31
tgggaccacc
>Bradi1g31
ctgccctccc
>Bradi1g31
accttcactG
>Bradi1g32
gacaattATT
>Bradi1g32
tataggttgtt
>Bradi1g33
agaagattgt
>Bradi1g33
tgatttgatgg
>Bradi1g33
tgtgaatctga
>Bradi1g33
GGtTCTGT
>Bradi1g33
gaacgtaaat
>Bradi1g33
ataatgtcctc
>Bradi1g33
attaaaatatg
>Bradi1g34
aacgagaag
>Bradi1g34
gaggagaag
>Bradi1g34
ggtatcaGgC
>Bradi1g35
tctaacaggt
>Bradi1g35
GGAAGTG
>Bradi1g35
tgacaagctc
>Bradi1g36
ggctgaacaa
>Bradi1g36
gtagtgatCA
>Bradi1g36

aaggttgattt

>Bradi1g36

atatggattgc

>Bradi1g37

tcgacgatctc

>Bradi1g37

gggacatcaa

>Bradi1g38

gccggcctgtg

>Bradi1g41

AGCTAACC

>Bradi1g41

tttgcAGTTT

>Bradi1g42

ATGTGGAG

>Bradi1g42

cgttcccttctc

>Bradi1g43

TAGCAATG

>Bradi1g44

atcgagaggg

>Bradi1g44

ttcaatttaga

>Bradi1g44

GAGCTATC

>Bradi1g44

tgaccatcgtt

>Bradi1g45

GCATAAAA

>Bradi1g45

gttgcgttcct

>Bradi1g45

aatggtcagta

>Bradi1g47

gctatcgagg

>Bradi1g47

atggatatcat

>Bradi1g48

gaagggttta

>Bradi1g48

ATGTATGT

>Bradi1g48

CACAGTGG

>Bradi1g48

ccagctttgct

>Bradi1g49

ACGAGGTC

>Bradi1g49

cctccatcaT

>Bradi1g49

tggtggcgttc
>Bradi1g49
gatcaaagca
>Bradi1g49
tactatataaa
>Bradi1g49
atcctttgccg
>Bradi1g49
CGAgCCAG
>Bradi1g50
GTTGTttGT
>Bradi1g50
atggaccaat
>Bradi1g50
ggagatctact
>Bradi1g50
tacctcagga
>Bradi1g50
aggcagtgaa
>Bradi1g50
TCATATGCT
>Bradi1g50
gaaaaatctg
>Bradi1g51
cagaaagaat
>Bradi1g51
CCAAGGGC
>Bradi1g51
gcaggaatgt
>Bradi1g51
gcagctccgtg
>Bradi1g51
tgcaggaaaa
>Bradi1g51
GCAGGAGC
>Bradi1g51
ggtgaggact
>Bradi1g51
gttacagtggt
>Bradi1g51
CAGCTCTTt
>Bradi1g51
cacaatggttc
>Bradi1g51
gatcctctttg
>Bradi1g52
atgaCGACG
>Bradi1g52
AAGGACTG
>Bradi1g52

TTTcaaaTC
>Bradi1g52
cgctccgccg
>Bradi1g52
ggcGatcAA
>Bradi1g52
GCATGTGA
>Bradi1g52
gaggggagc
>Bradi1g52
cagggagtag
>Bradi1g52
tgttgatgatg
>Bradi1g52
agggggtgga
>Bradi1g53
AGTTTATG
>Bradi1g53
tccGCCGCg
>Bradi1g53
tcagtgacgtt
>Bradi1g53
AGGATGG
>Bradi1g54
cagagcgagt
>Bradi1g54
taggttattat
>Bradi1g54
gccccgccgc
>Bradi1g54
cagaaaatta
>Bradi1g55
ggttgatcatc
>Bradi1g55
ATGAAGCA
>Bradi1g56
atggggacag
>Bradi1g56
acttctcagcg
>Bradi1g56
gaatctgtgtg
>Bradi1g56
gcagacaaat
>Bradi1g56
tccagttgaca
>Bradi1g57
actccttcaaa
>Bradi1g57
CagATTGG
>Bradi1g58

tcctcacgtcg
>Bradi1g58
CCACTCATT
>Bradi1g59
ctaccagttcc
>Bradi1g59
tctctccgtga
>Bradi1g59
ctatggttggc
>Bradi1g60
acattgccagt
>Bradi1g60
AACGGCGg
>Bradi1g60
gagaataaat
>Bradi1g61
AGATGCCA
>Bradi1g61
GCTCTCGG
>Bradi1g61
atggaggaag
>Bradi1g61
GACCATGC
>Bradi1g62
cagagcgggc
>Bradi1g62
gatccagggt
>Bradi1g62
gatgccataa
>Bradi1g62
aagtctctact
>Bradi1g62
GGTACGGG
>Bradi1g62
agcgacgccc
>Bradi1g62
tgttacaatgt
>Bradi1g62
ccatgcataa
>Bradi1g62
CTCCTAAA
>Bradi1g62
gtgctggagct
>Bradi1g63
aagggcaagt
>Bradi1g63
ctctttcaggtt
>Bradi1g63
aggtaccattt
>Bradi1g63

accacgttcag
>Bradi1g64
cagtgtcaag
>Bradi1g64
cagctttgtat
>Bradi1g64
aaacacatac
>Bradi1g64
atggcgtcgt
>Bradi1g64
AAGAgATG
>Bradi1g64
tacaccgccg
>Bradi1g65
TTGAAAGA
>Bradi1g66
agtcattcctt
>Bradi1g66
ctaccttgcat
>Bradi1g66
atctgggttca
>Bradi1g66
gaggagaaca
>Bradi1g66
atggggtcca
>Bradi1g67
aaatcaagtg
>Bradi1g67
TCAGGGTT
>Bradi1g67
CTGCTTCCa
>Bradi1g67
GATGGACa
>Bradi1g67
aattcatgca
>Bradi1g67
atagttcaaat
>Bradi1g68
AGGATATC
>Bradi1g68
AGGTTGAA
>Bradi1g68
aggaagatgg
>Bradi1g68
GGTCcATG
>Bradi1g69
ataggataat
>Bradi1g69
actccagtatt
>Bradi1g69

GAGTTTTAT
>Bradi1g69
aaagcctggg
>Bradi1g69
tcaacactact
>Bradi1g70
ccttcaaatga
>Bradi1g70
gtttgtgctcc
>Bradi1g70
caggcgaattc
>Bradi1g70
ttgcagagatt
>Bradi1g70
acaccgaggA
>Bradi1g70
ATGgCtaGT
>Bradi1g71
CTCtAGAAT
>Bradi1g71
cgaaagcaga
>Bradi1g71
atggctgccat
>Bradi1g71
agatatgcaa
>Bradi1g71
atgaagccag
>Bradi1g71
gtcaatatcct
>Bradi1g72
atactggagg
>Bradi1g72
cagtgtactat
>Bradi1g72
AGACTCGT
>Bradi1g72
cagtggcacg
>Bradi1g72
GAAAATGG
>Bradi1g73
aagaaggaca
>Bradi1g74
atatcttgtga
>Bradi1g74
aagcattaaa
>Bradi1g74
GCgGATGC
>Bradi1g74
TGaCaAACA
>Bradi1g74

atggcgacat
>Bradi1g74
atgaagaac
>Bradi1g74
ggcgcGTGc
>Bradi1g74
ccaccacca
>Bradi1g74
agtgtaagaa
>Bradi1g74
TGcAGGTC
>Bradi1g74
gtgcagcatc
>Bradi1g75
gtatgttttca
>Bradi1g75
agcttgttcta
>Bradi1g75
tagtttatgat
>Bradi1g75
tagttggactc
>Bradi1g75
aagatagtgt
>Bradi1g75
atcctgagag
>Bradi1g75
tctcgggtgct
>Bradi1g76
caggagaagc
>Bradi1g77
atcagaatgg
>Bradi1g77
CTGCTCCA
>Bradi1g78
GAGGGTA
>Bradi2g00
CGTATtaCA
>Bradi2g00
cattggatatg
>Bradi2g00
ccatggtgac
>Bradi2g00
agatgtcaac
>Bradi2g00
tacttaatgag
>Bradi2g01
CGtGATCG
>Bradi2g02
ttggagggat
>Bradi2g03

AGGAGGA
>Bradi2g03
TCCAAGGC
>Bradi2g03
GATATGTT
>Bradi2g03
tgcggcggcg
>Bradi2g04
GTtgTAGTg
>Bradi2g04
ctgggaggtg
>Bradi2g04
agagagtgca
>Bradi2g04
TCATGTGC
>Bradi2g05
atgataagca
>Bradi2g05
caggagaaac
>Bradi2g05
cagcggcatc
>Bradi2g06
AAATGCTA
>Bradi2g06
tacttcttacct
>Bradi2g07
GATACTCT
>Bradi2g08
taatcaacatt
>Bradi2g08
TGGAGGA
>Bradi2g08
GTGCCGTA
>Bradi2g08
tctttatttgga
>Bradi2g09
aggttatactg
>Bradi2g09
GCCCAGTT
>Bradi2g09
AAATTGTT
>Bradi2g10
gggactttata
>Bradi2g10
accccgcact
>Bradi2g10
tgtgccaagga
>Bradi2g10
tgactcatatg
>Bradi2g11

CACTTTTTg
>Bradi2g11
gatgtggatta
>Bradi2g12
ccatttgaag
>Bradi2g12
ACCGATAT
>Bradi2g12
caggggtcac
>Bradi2g12
ATGGAgCa
>Bradi2g13
ggttctgactg
>Bradi2g13
ACACGCCA
>Bradi2g14
gcgctccgac
>Bradi2g14
GTCTTCAc
>Bradi2g14
tcaccgaagc
>Bradi2g15
ttaaacacttt
>Bradi2g15
ggatcgcttta
>Bradi2g16
TCGTcGGC
>Bradi2g16
tggccaagttg
>Bradi2g16
ggaaaagctg
>Bradi2g17
gtggtacatct
>Bradi2g17
tgatatatttg
>Bradi2g17
cactttgtgat
>Bradi2g17
GGATCCGG
>Bradi2g18
aggtggtgctc
>Bradi2g18
ATGACtAC
>Bradi2g18
aaaacaccag
>Bradi2g19
acaggctacg
>Bradi2g19
gctcagacctt
>Bradi2g19

caggttacatg
>Bradi2g19
atagctctcag
>Bradi2g19
agcagtgtttg
>Bradi2g20
AATGTATAT
>Bradi2g20
AGGAAAAC
>Bradi2g20
cggcggagca
>Bradi2g20
tactgtgaaga
>Bradi2g21
tggagtgctca
>Bradi2g21
gaacTACCT
>Bradi2g21
ggtatacggag
>Bradi2g22
tcttgttttcca
>Bradi2g22
ctgccaagatt
>Bradi2g22
cgccgctccgc
>Bradi2g22
GCTCTCAG
>Bradi2g23
gtcggggctgc
>Bradi2g24
agaaataattg
>Bradi2g24
agtttggggt
>Bradi2g25
ggtcgcttcta
>Bradi2g25
ggccggggcg
>Bradi2g25
GCCAGGTT
>Bradi2g25
tccatctgtaca
>Bradi2g25
gtgacaaactt
>Bradi2g26
ggctaaaactg
>Bradi2g26
aggttttggat
>Bradi2g26
TCTGCACCt
>Bradi2g26

aagaaagagg
>Bradi2g26
caggtcccagg
>Bradi2g27
gcagtcgatgg
>Bradi2g27
AGGAGTCT
>Bradi2g27
AGAGTGAT
>Bradi2g28
ACAgATTA
>Bradi2g28
AAGTATGC
>Bradi2g29
cagaagaggc
>Bradi2g29
gcatgtctggc
>Bradi2g31
tggttcttatgc
>Bradi2g31
taccatgagga
>Bradi2g31
TCAAGCTC
>Bradi2g32
agatggttacc
>Bradi2g34
ggcggcaccc
>Bradi2g34
ctcgcgatgga
>Bradi2g34
gcagaaaaac
>Bradi2g34
cttgttgcttca
>Bradi2g35
cagattcacac
>Bradi2g35
CCGTGACT
>Bradi2g36
ctgaagttgag
>Bradi2g36
ccaagttcaaa
>Bradi2g36
gagactatccg
>Bradi2g36
gttcgatagtg
>Bradi2g36
ATGGCtGT
>Bradi2g37
AAAGCTAT
>Bradi2g37

gaattCTAC
>Bradi2g37
ggcaaggag
>Bradi2g37
atgaggtgct
>Bradi2g38
ccggtgttccg
>Bradi2g38
gcaaggaact
>Bradi2g38
TGgAGCAA
>Bradi2g38
ggcgtcgagc
>Bradi2g38
atcttGCCAC
>Bradi2g38
GTAcCAGG
>Bradi2g39
AACTCaTC
>Bradi2g39
tatctggaatg
>Bradi2g39
tgattacaggt
>Bradi2g39
tggagcgctta
>Bradi2g39
caaagccaag
>Bradi2g39
cacttctctaa
>Bradi2g40
cgagttccga
>Bradi2g40
aacattatcgt
>Bradi2g40
aaactttgatt
>Bradi2g41
ggaaaatcaa
>Bradi2g41
cagattgcatt
>Bradi2g41
AGTTTGTT
>Bradi2g41
aaactctcatt
>Bradi2g42
GATCCTGA
>Bradi2g42
ctcgtgataag
>Bradi2g42
cggcatcgaC
>Bradi2g42

tacatagtgga
>Bradi2g42
gctctccaaca
>Bradi2g43
gaatgttatga
>Bradi2g44
ctcccatccct
>Bradi2g44
ttggaccggtg
>Bradi2g44
ATGGCTgTc
>Bradi2g46
acaattcttga
>Bradi2g48
gtacttatcca
>Bradi2g48
gtggtggtaat
>Bradi2g49
cctcggttgca
>Bradi2g49
atgaagcaga
>Bradi2g51
ACTGCCCA
>Bradi2g51
ttctagaaggc
>Bradi2g54
gatgcttggac
>Bradi2g55
gatcggtttct
>Bradi2g57
caccaagatc
>Bradi2g57
aaagtcaggc
>Bradi2g57
ATCCCAGCA
>Bradi2g57
atggcgactct
>Bradi2g58
atgatagggtg
>Bradi2g59
ttgtcataaat
>Bradi2g59
AGTAGAGT
>Bradi2g60
ATGGTGCA
>Bradi2g60
GAACTTGC
>Bradi2g60
cctgatgactg
>Bradi2g60

CAGGTGGG
>Bradi2g60
TCATTAACT
>Bradi2g60
CCATTTCTG
>Bradi2g61
AGAGGGAC
>Bradi2g61
TCTGCCCAC
>Bradi2g61
GGCAGGAC
>Bradi2g62
acgctGCTCT
>Bradi2g62
AAATCCGG
>Bradi3g00
TCTTGCTTA
>Bradi3g00
CACCAAGG
>Bradi3g00
aggagtttgag
>Bradi3g00
AGGGTTCA
>Bradi3g00
aaaggcgatta
>Bradi3g00
TCTCCGCG
>Bradi3g00
GAGACTAT
>Bradi3g01
agacgaaggg
>Bradi3g02
tgtcaggcttc
>Bradi3g02
cagtgctgctg
>Bradi3g02
CTtACaGGT
>Bradi3g03
ttgccagtaac
>Bradi3g03
AGGTTCTtT
>Bradi3g03
caggataagc
>Bradi3g04
ctggacgaga
>Bradi3g05
actgctgccag
>Bradi3g06
attatacctAa
>Bradi3g06

acaggaccgc
>Bradi3g07
aagCCgGaa
>Bradi3g07
gatgtttctgtg
>Bradi3g07
AAGGCgTC
>Bradi3g07
tcaaataatat
>Bradi3g08
agaaggtcga
>Bradi3g08
ttctcctttgac
>Bradi3g08
ggttggtgtta
>Bradi3g08
cggcggcgac
>Bradi3g08
GGGTgACA
>Bradi3g09
ATGATGTA
>Bradi3g09
GTGTTCAA
>Bradi3g09
agcttttctca
>Bradi3g10
AGCAAGGA
>Bradi3g10
ggcttcgatat
>Bradi3g10
tagaaggctt
>Bradi3g10
acggagccgc
>Bradi3g10
atactcaacct
>Bradi3g10
ggctcttggcc
>Bradi3g10
catcctgtaca
>Bradi3g12
ATGGAccTt
>Bradi3g12
CAGTCTGA
>Bradi3g13
cgcatccccaa
>Bradi3g14
caacgcacctg
>Bradi3g15
ccgttgaccct
>Bradi3g15

ATGGACGC
>Bradi3g15
acgaggggtt
>Bradi3g16
CCATCATA
>Bradi3g16
cggagtaaac
>Bradi3g17
ATGACTTC
>Bradi3g17
CAgtCTCGG
>Bradi3g17
AGATAACT
>Bradi3g18
TTTGCTtGC
>Bradi3g18
ttcacgggaa
>Bradi3g18
tggctaacaa
>Bradi3g19
GAAGtGTT
>Bradi3g20
agaggacatg
>Bradi3g20
acgatgcaga
>Bradi3g20
tggatgctttt
>Bradi3g21
gtggatttacc
>Bradi3g21
AAAATATT
>Bradi3g22
tatgtttgctgt
>Bradi3g22
ggtttatgtAG
>Bradi3g22
ATTCAGGC
>Bradi3g22
tcgcagcttgg
>Bradi3g22
ttcttccaagg
>Bradi3g22
cactgaagaa
>Bradi3g23
ggggaatgag
>Bradi3g23
caggttatgtg
>Bradi3g24
aggtacacaa
>Bradi3g26

ATTTTcGCA
>Bradi3g27
ggggcagatt
>Bradi3g27
tgcttttgtttc
>Bradi3g28
ttgacagaga
>Bradi3g28
gggaaaagtc
>Bradi3g29
ggcaaatgaa
>Bradi3g29
gtggattgata
>Bradi3g29
gttacatacca
>Bradi3g29
tctagaagac
>Bradi3g30
ttactccctct
>Bradi3g31
cagattcttga
>Bradi3g32
tttctggatct
>Bradi3g32
ccaacAGGT
>Bradi3g32
taggttggcg
>Bradi3g33
aggttttatct
>Bradi3g33
aggaatgttc
>Bradi3g34
atctttatctgt
>Bradi3g34
atgattgcaa
>Bradi3g35
tgccccggcct
>Bradi3g35
AGGGAGG
>Bradi3g35
GAGCTGAA
>Bradi3g36
ATGTCAGG
>Bradi3g36
tggaagaaga
>Bradi3g36
GCAGtTGA
>Bradi3g37
ccggcgaggt
>Bradi3g37

GTTTcTGTA
>Bradi3g37
gtatcccacgc
>Bradi3g38
gtgctgcacga
>Bradi3g38
cgccgccggg
>Bradi3g39
caagctccgga
>Bradi3g39
tatattcccaca
>Bradi3g39
gccgatgtgtc
>Bradi3g40
cagcatgttgt
>Bradi3g40
AGGCTGAT
>Bradi3g41
ccagtatgctg
>Bradi3g41
TCCCTGAG
>Bradi3g41
TGTAAAAT
>Bradi3g41
TCAGCGgA
>Bradi3g41
agatacttggt
>Bradi3g42
ctgaaggagc
>Bradi3g42
ACGAcGgTg
>Bradi3g43
gcacttaaagt
>Bradi3g43
GGAGGCGC
>Bradi3g43
gttattgctaa
>Bradi3g44
caggatggttc
>Bradi3g44
CTTTGTGA
>Bradi3g44
cagtgtattgg
>Bradi3g44
TTgATTCAA
>Bradi3g44
ACTCAAAG
>Bradi3g44
aggttgattag
>Bradi3g45

gagctccgacg
>Bradi3g45
GCCTCGTG
>Bradi3g45
ctaggaaccg
>Bradi3g45
CTTTGTGCa
>Bradi3g46
ggtggaggcg
>Bradi3g46
acagctctaT
>Bradi3g47
GGTTCAAA
>Bradi3g47
gtcaacttttg
>Bradi3g48
GATGATggT
>Bradi3g48
gttgtctgcag
>Bradi3g48
ggtccatccat
>Bradi3g49
gagcttggtcg
>Bradi3g50
acgttgcatcc
>Bradi3g50
ggaggaggag
>Bradi3g50
GTGGGTTC
>Bradi3g51
atggaaggaa
>Bradi3g51
ccgcaggtgcg
>Bradi3g52
caggtcagctt
>Bradi3g53
atggaagagg
>Bradi3g53
ttgcaggggC
>Bradi3g53
ggactccaaca
>Bradi3g55
aatggttgcat
>Bradi3g55
ttgttgcctga
>Bradi3g56
cagtgggggcg
>Bradi3g56
GGtGtAGG
>Bradi3g56

gtgaactaga
>Bradi3g57
gatgcaggag
>Bradi3g57
gttgcactagc
>Bradi3g59
gctgacctcgc
>Bradi3g59
GGTGAGCa
>Bradi3g60
AAGAAGAA
>Bradi3g60
tccagtgtcgc
>Bradi3g60
cgccatgctgc
>Bradi3g60
ggcggcgcgc
>Bradi3g60
cttgctttgat
>Bradi3g60
ttcccgccctt
>Bradi3g60
ggttgCaagg
>Bradi3g61
gcgatgctttg
>Bradi4g00
gttcAcATCA
>Bradi4g00
agggttttctg
>Bradi4g00
CCgAGGAT
>Bradi4g00
tgaatgccttg
>Bradi4g00
tctgaaatagt
>Bradi4g00
CCgATAGA
>Bradi4g00
gaggaggag
>Bradi4g01
AAAATAAA
>Bradi4g01
ACTTTGGC
>Bradi4g01
ggcacacagc
>Bradi4g01
gcaggccttg
>Bradi4g01
tgcAGCgcc
>Bradi4g01

gttgtggtagc
>Bradi4g01
CGGTTCCT
>Bradi4g01
CAAATCAA
>Bradi4g01
tggcattgtgg
>Bradi4g01
aacataCCA
>Bradi4g01
gggaggtgct
>Bradi4g01
AAGGAGAT
>Bradi4g01
agttagaaat
>Bradi4g02
gccgccgcca
>Bradi4g02
ccgccgccgc
>Bradi4g02
ctggtcttcat
>Bradi4g02
accgaggtgt
>Bradi4g02
GGAAACAG
>Bradi4g02
GGAAATCT
>Bradi4g02
GGAGcGAa
>Bradi4g02
aggactcccc
>Bradi4g02
GGTGACTT
>Bradi4g02
AGACTGTT
>Bradi4g02
ctgccgcggt
>Bradi4g02
agcatggtgc
>Bradi4g02
AGGATACA
>Bradi4g02
attccttgcag
>Bradi4g02
TGCTCGCC
>Bradi4g02
AGGGAGCC
>Bradi4g02
gcaggtgaG
>Bradi4g02

AAGGcGAT
>Bradi4g03
atcctcagaa
>Bradi4g03
caggaagtg
>Bradi4g03
cagggtatcc
>Bradi4g03
ccgacggcac
>Bradi4g03
aaactgacag
>Bradi4g03
ttCATTTTC
>Bradi4g03
atgctgcggt
>Bradi4g03
ctcacatgtgg
>Bradi4g03
tctgctaaTG
>Bradi4g03
AGGAAGG
>Bradi4g03
TGCAGATG
>Bradi4g03
tgccgaggct
>Bradi4g04
ggcggaactg
>Bradi4g04
ttgaggggga
>Bradi4g04
atgtcatcga
>Bradi4g04
GCCACAAT
>Bradi4g04
aggctgcagc
>Bradi4g04
gatcttcctga
>Bradi4g04
tcacgtttgtg
>Bradi4g04
taggaaaact
>Bradi4g04
ggagtacgca
>Bradi4g04
atggccgccg
>Bradi4g04
ctaagacggt
>Bradi4g04
ATGATATT
>Bradi4g05

ctccgtgaacg
>Bradi4g05
gaggagccaa
>Bradi4g05
gaggagctga
>Bradi4g05
cccctcctacg
>Bradi4g05
ggaaatattag
>Bradi4g05
ccatgtttggg
>Bradi4g05
GGTGACCC
>Bradi4g05
cagtgggtaa
>Bradi4g05
cctatactcaa
>Bradi4g05
aagAATCTT
>Bradi4g06
gcacttcattt
>Bradi4g06
AGAATATT
>Bradi4g06
ATGGAGGA
>Bradi4g06
TCTTTTCAG
>Bradi4g06
cggtgcacca
>Bradi4g06
CAgACTACT
>Bradi4g06
ATGGCTTC
>Bradi4g06
AGGAGCCT
>Bradi4g06
TCTGAaTTA
>Bradi4g07
atggagctcga
>Bradi4g07
ggaacctagca
>Bradi4g07
tcaggtatcaa
>Bradi4g07
AGACTGAG
>Bradi4g07
atggcagtctg
>Bradi4g07
ctccttgcggt
>Bradi4g07

gccgcgcctg
>Bradi4g07
CAGTCACA
>Bradi4g07
ATGaGCAG
>Bradi4g07
AAAgAGTC
>Bradi4g07
CACTTATC
>Bradi4g07
CATTTCATT
>Bradi4g07
CCgTGAGA
>Bradi4g07
GGTACCAC
>Bradi4g08
tcggcaggag
>Bradi4g08
aatcttttgg
>Bradi4g08
aagtggcttc
>Bradi4g08
ATATGCAG
>Bradi4g08
ggctcTCgct
>Bradi4g08
agatgtgaag
>Bradi4g08
aacaggtcat
>Bradi4g08
CTGTTACA
>Bradi4g08
aattttgggA
>Bradi4g08
tctgggagca
>Bradi4g08
ttagttggaac
>Bradi4g08
TCAGGTCA
>Bradi4g08
gttcgaccttt
>Bradi4g08
AGGATTGA
>Bradi4g08
GTACGAGC
>Bradi4g08
ATACAGGT
>Bradi4g09
cctatgaaatt
>Bradi4g09

TGCAAAGA
>Bradi4g09
TtGAGCAA
>Bradi4g09
tgaagggttG
>Bradi4g09
tctatgctatg
>Bradi4g09
tctgtaattgg
>Bradi4g09
gcagatttgtc
>Bradi4g10
tgcggcagca
>Bradi4g10
cagagtcctc
>Bradi4g11
ACaCAACG
>Bradi4g11
agaagAGAT
>Bradi4g11
aggcaggtga
>Bradi4g11
gtgagaggat
>Bradi4g12
ggagggga
>Bradi4g12
AGGATGTG
>Bradi4g12
agagcttgag
>Bradi4g12
aggtacttact
>Bradi4g12
agcaccagat
>Bradi4g13
AGATTTTG
>Bradi4g13
ctacaaagga
>Bradi4g13
tataaGCCC
>Bradi4g13
aggatggaca
>Bradi4g13
ttcctagcgag
>Bradi4g14
GTTCCACC
>Bradi4g15
TCTCTGatG
>Bradi4g15
gacatctgcct
>Bradi4g16

ATGGGGAG
>Bradi4g16
GGcGGAGT
>Bradi4g16
ttaaaagatgg
>Bradi4g16
aggaagcagc
>Bradi4g17
acctccggcgt
>Bradi4g17
ccatatcatgt
>Bradi4g18
gtctatgctttc
>Bradi4g18
tgaatcacctg
>Bradi4g19
ccgcgggtccc
>Bradi4g19
cgtaatccttg
>Bradi4g19
ttctgaaggaa
>Bradi4g20
GCCCTCACA
>Bradi4g20
tttgccataca
>Bradi4g21
aagggtgtgtt
>Bradi4g21
atgaagataca
>Bradi4g21
ttgcagaggaa
>Bradi4g21
GAgCCGGA
>Bradi4g21
aattggagcac
>Bradi4g21
aatcaCAGA
>Bradi4g21
ccaatagcaTC
>Bradi4g22
acaaactaccc
>Bradi4g22
agcttagagcc
>Bradi4g22
cctgatcccac
>Bradi4g23
agccaggatga
>Bradi4g23
tgggccgtgaa
>Bradi4g23

gcggtgttgtg
>Bradi4g23
agagggaTG
>Bradi4g23
cagctcatcg
>Bradi4g23
gggtcatgga
>Bradi4g23
TCCGgCAt
>Bradi4g24
tccagctggT
>Bradi4g24
ATGTCATT
>Bradi4g24
cagctaaaag
>Bradi4g24
AGGATACC
>Bradi4g24
ccaacaaaag
>Bradi4g24
atgggaagg
>Bradi4g24
aagctcaggg
>Bradi4g25
AAAGATGA
>Bradi4g25
agaaaacaa
>Bradi4g25
tatctaggcta
>Bradi4g26
ttcaggtgcca
>Bradi4g26
atgggtcgca
>Bradi4g26
gccgcgggat
>Bradi4g26
tctgtacttga
>Bradi4g26
ATGGCAGA
>Bradi4g26
gtcatctgtttt
>Bradi4g26
CCCTCGCC
>Bradi4g26
tgtgatccag
>Bradi4g26
AGGTCTCA
>Bradi4g27
gcagaatcgc
>Bradi4g27

gtgcaggttga
>Bradi4g27
cttggggttta
>Bradi4g27
gagacaattg
>Bradi4g27
CCTGGATA
>Bradi4g27
tcaatgacaga
>Bradi4g27
gagctcaagg
>Bradi4g27
atctcatgttca
>Bradi4g27
GTCTATAG
>Bradi4g28
cgcggcagca
>Bradi4g28
tggcgttcggt
>Bradi4g28
cgtccctcccg
>Bradi4g28
tggattcagtg
>Bradi4g28
CAGGCTTC
>Bradi4g28
tggcgacaga
>Bradi4g28
aatctctgtaa
>Bradi4g28
tggaggtcgtc
>Bradi4g28
ggtttgtggag
>Bradi4g28
AGGTTCgA
>Bradi4g28
aatactatcta
>Bradi4g28
aggtaattcct
>Bradi4g28
gatccatcagc
>Bradi4g29
aaccttgcagc
>Bradi4g29
TGAAATAC
>Bradi4g29
TTCCTGGT
>Bradi4g29
GTTtGTCAA
>Bradi4g29

aggattgcatc
>Bradi4g29
agggggtggg
>Bradi4g29
acggcgtcgcg
>Bradi4g29
ttgactgattg
>Bradi4g29
gtggctcagcc
>Bradi4g29
TTGcagggTC
>Bradi4g29
atggtgaagcc
>Bradi4g29
ATTATATTT
>Bradi4g29
aaaggaggaa
>Bradi4g29
gggcgcaaag
>Bradi4g29
tgtggatcaaa
>Bradi4g30
gggaccgccg
>Bradi4g30
CTCTGTCTA
>Bradi4g30
TGAAGGAT
>Bradi4g30
gcggacgtcga
>Bradi4g30
AGGCgAGg
>Bradi4g30
tctcgaagaga
>Bradi4g30
ATGGAGTT
>Bradi4g30
aggtacatgag
>Bradi4g30
cctgccccagt
>Bradi4g30
agcaggaact
>Bradi4g30
aggaatggtac
>Bradi4g30
agaaGCACA
>Bradi4g30
gacccgccgca
>Bradi4g30
aactccatact
>Bradi4g30

atcatcgtgat
>Bradi4g31
atgtcgtacaa
>Bradi4g31
aggaactcttt
>Bradi4g31
caggttacaga
>Bradi4g31
tgcccattatg
>Bradi4g31
ggcggcggCC
>Bradi4g31
acaagcctcc
>Bradi4g31
cggagagcga
>Bradi4g31
aggttgctaga
>Bradi4g31
ggcggaggcg
>Bradi4g31
gaggtcggca
>Bradi4g31
GTTGGCAA
>Bradi4g31
AAGGGTGG
>Bradi4g31
gttgaggacg
>Bradi4g31
GgCTTATAT
>Bradi4g32
gcggctgggt
>Bradi4g32
atgcgtcgcc
>Bradi4g32
ATGGAGCT
>Bradi4g32
cgtaacatAG
>Bradi4g32
gcgctgcccg
>Bradi4g32
GCAGATTA
>Bradi4g32
atcccaagag
>Bradi4g32
ccggccagcg
>Bradi4g32
tcgccatcatg
>Bradi4g32
TGCAATTT
>Bradi4g32

gtgtcaagcc
>Bradi4g32
ctcgccgccct
>Bradi4g33
AGGGACG
>Bradi4g33
gggatactgc
>Bradi4g33
gagacgcttC
>Bradi4g33
GGCGAGC
>Bradi4g33
gtagacatgc
>Bradi4g33
CAGCGTAG
>Bradi4g33
ggtggtcttc
>Bradi4g33
atgctgtggat
>Bradi4g33
actagattcg
>Bradi4g33
GCGCATGC
>Bradi4g33
tatggaagta
>Bradi4g34
atggagaggg
>Bradi4g34
gagcggtcgg
>Bradi4g34
tagtcaagga
>Bradi4g34
tgtgccTTCC
>Bradi4g34
ATAAGtGG
>Bradi4g34
gtacggtccct
>Bradi4g34
GGGGcTaC
>Bradi4g34
CAGGCAG
>Bradi4g34
ggcatgattg
>Bradi4g34
tgatttgagtg
>Bradi4g34
gatgttcgac
>Bradi4g34
ggagctgtca
>Bradi4g35

acatgaaaaa
>Bradi4g35
ctaggatcgt
>Bradi4g35
tcatcccttctt
>Bradi4g35
acttttggggc
>Bradi4g35
aagttaacatt
>Bradi4g35
ggctcaagac
>Bradi4g35
atgccttccat
>Bradi4g35
gtatgccaac
>Bradi4g35
gcagcagcag
>Bradi4g35
tatctaacgct
>Bradi4g36
aagaagcaa
>Bradi4g36
CGgCcAtG
>Bradi4g36
ggcgtgggca
>Bradi4g36
aggcggttgc
>Bradi4g36
GTGAGCGa
>Bradi4g36
gagttcaggt
>Bradi4g36
tgctttaggag
>Bradi4g36
tggacatcac
>Bradi4g36
tgtttgtaGA
>Bradi4g36
tggttggggc
>Bradi4g36
ATGGCAG
>Bradi4g36
acaggcaaag
>Bradi4g36
ATGGTtaG
>Bradi4g36
ATGGCCag
>Bradi4g37
ATGATCCA
>Bradi4g37

tccaggctgat
>Bradi4g37
GGAGGGG
>Bradi4g37
atttcatttctt
>Bradi4g37
catttcctggc
>Bradi4g37
CAAAGGG
>Bradi4g37
gcaaggggtt
>Bradi4g37
cacctGAtcG
>Bradi4g37
ACTCCAGC
>Bradi4g37
gccccatgca
>Bradi4g37
TCCTGGAA
>Bradi4g37
gccgaccccc
>Bradi4g37
ccgtGATGA
>Bradi4g37
atgcactcctt
>Bradi4g37
ctggagtcctc
>Bradi4g37
ACAGTtCCt
>Bradi4g37
ttcaaaagttg
>Bradi4g37
cctcgacccct
>Bradi4g37
gtcgaccggt
>Bradi4g37
ataggttaagt
>Bradi4g37
gttataagag
>Bradi4g38
tccagcctgg
>Bradi4g38
AGACCTAC
>Bradi4g38
TGaCAggTC
>Bradi4g38
ATCAACTA
>Bradi4g38
ttaggaagac
>Bradi4g38

cggcggcgga
>Bradi4g38
ctggatgatgt
>Bradi4g38
atggaggtgg
>Bradi4g38
aaaagcctgg
>Bradi4g38
aggttccaagt
>Bradi4g38
ACACCATG
>Bradi4g38
gaccatgccg
>Bradi4g38
GTGATCGA
>Bradi4g38
AAGTCCAT
>Bradi4g38
attgtgcagat
>Bradi4g38
gcgttcgagg
>Bradi4g39
cttctccaaag
>Bradi4g39
ccattgatga
>Bradi4g39
aggcaaggat
>Bradi4g39
AGCTTGAG
>Bradi4g39
gcccccgcgg
>Bradi4g40
gcacctgagg
>Bradi4g41
gaggcgtaca
>Bradi4g41
cggcggccgc
>Bradi4g41
acaccttccca
>Bradi4g41
AGGATGAA
>Bradi4g41
agctgcgtgtg
>Bradi4g41
gaaagcaatt
>Bradi4g41
CAACAGAA
>Bradi4g41
AGCTCCTG
>Bradi4g41

tgaacgagga
>Bradi4g42
ttggaaacaa
>Bradi4g42
gacatacaca
>Bradi4g42
catccttgatg
>Bradi4g42
tgtacaacctg
>Bradi4g42
ggacaggtta
>Bradi4g42
CAGACTAc
>Bradi4g42
gtcggaaaga
>Bradi4g42
cagtgcaggg
>Bradi4g42
agtgattgcg
>Bradi4g43
aaGGACTA
>Bradi4g43
ATTGGATG
>Bradi4g43
gcagcccaag
>Bradi4g43
aggtcctcca
>Bradi4g43
gatggcacctt
>Bradi4g43
tcaaaggagt
>Bradi4g43
gtgttcaaaa
>Bradi4g44
cttgacaacg
>Bradi4g44
tttgctgccgg
>Bradi4g44
ATGGTCAA
>Bradi4g44
tctggtgtgtt
>Bradi4g45
AGGACAA
>Bradi4g45
ggggataccg
>Bradi4g45
TCCACGCC
>Bradi4g45
AGCTGCCA
>Bradi4g45

GCAAaCAC
>Bradi4g45
ttgaatcccc
>Bradi4g45
atgttgcaga
>Bradi4g45
gagaaatgaa
>Bradi4g45
gtcgacgccg
>Bradi5g00
acaaggcgtc
>Bradi5g00
gcgctcaggt
>Bradi5g00
tggaccttcca
>Bradi5g01
ctcctaatgag
>Bradi5g01
agagctggtt
>Bradi5g01
AGCTCAAA
>Bradi5g01
gccggtgtag
>Bradi5g02
tgcaatggat
>Bradi5g02
CCTCTTCG
>Bradi5g02
ctgataatga
>Bradi5g02
ggcgccggga
>Bradi5g03
gacgatgaac
>Bradi5g03
tggcagggat
>Bradi5g03
CTCAgAGA
>Bradi5g04
gagagtgacc
>Bradi5g04
agagtggcgt
>Bradi5g05
TTCAGCAC
>Bradi5g07
ttagtattcat
>Bradi5g09
ccgacaccac
>Bradi5g09
ATGGGTTT
>Bradi5g10

ATGcAGGG
>Bradi5g12
gcaaggtcGC
>Bradi5g13
tgggaaccAT
>Bradi5g13
gacctcaagt
>Bradi5g14
atagttcatga
>Bradi5g15
agaattatgg
>Bradi5g15
ccgggtgctg
>Bradi5g16
CAGCGCCA
>Bradi5g17
attttctggaa
>Bradi5g20
ggagttgact
>Bradi5g20
agcctgtattc
>Bradi5g20
aatgtcaagc
>Bradi5g20
gacttggcca
>Bradi5g20
aagattgccc
>Bradi5g21
agaagtatgg
>Bradi5g22
gattttagaaa
>Bradi5g22
gtttggaattc
>Bradi5g22
ttgcaggagtt
>Bradi5g22
ATGCCATT
>Bradi5g22
gaggtggact
>Bradi5g22
agattgttgat
>Bradi5g22
gtcgCTCAg
>Bradi5g24
ATgCCTGA
>Bradi5g24
CAGCTTGg
>Bradi5g24
GGAGCATA
>Bradi5g25

caaattaCCT
>Bradi5g25
gacaagtgcg
>Bradi5g25
TACAAGGA
>Bradi5g25
gatcaatatg
>Bradi5g26
gcccgctcgct
>Bradi5g26
tcctttgggca
>Bradi5g27
AGGTCGTT
>Bradi5g27
TTCaGttCT
>Bradi5g27
cctgcgccgc
>Bradi0023
TGCCcAGA
>Sb01g000
aggttctttcg
>Sb01g000
caagtacttgt
>Sb01g000
gtggccaaca
>Sb01g000
gccgtcgcgct
>Sb01g000
gaaataatttg
>Sb01g000
CTTCTTTCC
>Sb01g000
aggatggcaa
>Sb01g001
tggtgtggag
>Sb01g001
GGCTTCCT
>Sb01g001
ggcggggTT
>Sb01g001
gaacgccgtg
>Sb01g001
atgcgcgccc
>Sb01g001
TGCCTgtAG
>Sb01g001
ATGAgTGG
>Sb01g001
cgcaccactg
>Sb01g001

ggcgttaactt
>Sb01g001
cttttgggggc
>Sb01g001
GGTGCTCT
>Sb01g001
GGaCTCCT
>Sb01g001
catctacgatg
>Sb01g001
ctgcccgggg
>Sb01g002
ggggcccact
>Sb01g002
aggcagtaga
>Sb01g002
ctttaaattca
>Sb01g002
AGCAGCCA
>Sb01g002
cctgcagaga
>Sb01g002
gctccaacttt
>Sb01g003
gaagttaatta
>Sb01g003
cgaccacggt
>Sb01g003
gccacggcgt
>Sb01g003
CGGCGAGg
>Sb01g004
TGGCTTGA
>Sb01g004
actatcctgat
>Sb01g004
ttggccttata
>Sb01g004
taccaaaagg
>Sb01g004
TTTCCCGA
>Sb01g004
gaaaaactct
>Sb01g005
CTCAACAA
>Sb01g005
GAGAGGC
>Sb01g005
AGACaGCG
>Sb01g005

catcatctacg
>Sb01g005
tcatggaaca
>Sb01g005
TATgaTTTc
>Sb01g005
ttgtcgacca
>Sb01g005
gaggcaaacg
>Sb01g006
gcatgcgctg
>Sb01g006
gattctatccc
>Sb01g006
atcaattgctt
>Sb01g006
tgtcatacga
>Sb01g006
GCTTCTGG
>Sb01g007
agatcaagat
>Sb01g007
ccgtcgccgt
>Sb01g007
tgtgctctaga
>Sb01g007
gcgtggttcga
>Sb01g007
cagttcatcgg
>Sb01g007
gcggatcgag
>Sb01g007
ttgtccaccca
>Sb01g007
cataggatgg
>Sb01g008
taaagcagga
>Sb01g008
aaacaTGGC
>Sb01g008
gcaaggaaa
>Sb01g008
gaaacctgaa
>Sb01g008
TTATCTGC
>Sb01g008
cgcaggccac
>Sb01g008
tttcgtccata
>Sb01g008

GGTGGATG
>Sb01g009
gaatacgtcta
>Sb01g009
cgtgaccgaca
>Sb01g009
atgggcgtgg
>Sb01g009
aatgggctaa
>Sb01g010
cacccctggg
>Sb01g010
agcgtcttgct
>Sb01g010
gagatgggct
>Sb01g011
cctacctccaa
>Sb01g011
tcggagataga
>Sb01g011
gacgggcagg
>Sb01g011
aatcagtttca
>Sb01g012
tggaagaaga
>Sb01g012
gccggAGGT
>Sb01g012
agcaccgtgg
>Sb01g012
ggtatgtactg
>Sb01g013
aggtctacga
>Sb01g013
gctgacggA
>Sb01g013
atgattcagca
>Sb01g013
atgggaaagc
>Sb01g013
gacgtgccgg
>Sb01g013
gctgctcctg
>Sb01g013
cttgatgcag
>Sb01g014
aatgtgcccc
>Sb01g014
tgccattcgtg
>Sb01g014

ggtcgAGgga
>Sb01g014
tgagaatttga
>Sb01g015
GGTAAGAC
>Sb01g015
ggcgagacgt
>Sb01g016
TGATCACA
>Sb01g016
atgcattagca
>Sb01g016
gctacctggat
>Sb01g016
ttaaagcttgc
>Sb01g016
AAGTATTC
>Sb01g017
ttgtgctgacc
>Sb01g017
cgaggccggg
>Sb01g017
gcggtgctggt
>Sb01g017
gctcaatctca
>Sb01g018
ggtggaatag
>Sb01g018
tcctgacgctc
>Sb01g018
gatctgtttca
>Sb01g019
gagctccgcga
>Sb01g019
agactgtgata
>Sb01g020
aggtgactgtt
>Sb01g020
ctcccgcccaa
>Sb01g020
tgcattcgtga
>Sb01g021
TACTACTA
>Sb01g022
agctcttggag
>Sb01g022
ggtagagtcc
>Sb01g026
aaaccatcag
>Sb01g026

GAACAACT
>Sb01g026
ATTGCTGA
>Sb01g027
ggggcaacta
>Sb01g027
ttcccgaaggt
>Sb01g028
gggcaagtgc
>Sb01g028
atatgtgaatt
>Sb01g028
gcacacatgc
>Sb01g029
ATTTAAGA
>Sb01g031
catatagaac
>Sb01g033
caagaaacca
>Sb01g034
tctggggcctc
>Sb01g034
GGCGGAA
>Sb01g034
agaatctcaa
>Sb01g034
aaccggcata
>Sb01g034
ggacttggat
>Sb01g035
ctagggtggg
>Sb01g036
ccctgatgag
>Sb01g036
aatgctttatt
>Sb01g036
GATGATGT
>Sb01g036
CATTGTGa
>Sb01g036
ATGTATCA
>Sb01g037
aatggtgcatt
>Sb01g037
accccggagg
>Sb01g037
ccggccgcac
>Sb01g038
agccagaatg
>Sb01g038

cagtgactgca
>Sb01g039
TGAaGCTC
>Sb01g039
ctcaccagcga
>Sb01g041
AAGCAGGT
>Sb01g041
ggagctgactt
>Sb01g041
cggctcgacct
>Sb01g042
gttgttgctgta
>Sb01g042
agtttctggtg
>Sb01g042
AACAAGGC
>Sb01g042
caaagaaagt
>Sb01g043
gtagccatgaa
>Sb01g043
tggacagaaa
>Sb01g043
aaaaggctgg
>Sb01g043
gtctatggaag
>Sb01g043
CGGACCAG
>Sb01g043
ttgctaggaag
>Sb01g043
ctcctacgcca
>Sb01g044
ccacggagga
>Sb01g044
accattcctaa
>Sb01g044
CAAACAAG
>Sb01g044
gctgtggggcg
>Sb01g044
cagcaagctcg
>Sb01g045
GGCcGATG
>Sb01g045
AAGTTCAT
>Sb01g046
ggggacatgtg
>Sb01g047

TGAACATT
>Sb01g048
TCAAGAAC
>Sb01g049
aagccagaga
>Sb02g000
AACCAATT
>Sb02g001
atttgaagat
>Sb02g001
ATAATTTG
>Sb02g002
gagtcacagt
>Sb02g002
GCAGGACT
>Sb02g003
atccccagag
>Sb02g003
atggagaaag
>Sb02g004
ctgcatactat
>Sb02g004
ctgtttAaCT
>Sb02g005
tgtggacgttc
>Sb02g005
ggagatagaa
>Sb02g005
aacacattgct
>Sb02g006
TATGATGG
>Sb02g006
cagcgggacc
>Sb02g006
aatagtcact
>Sb02g006
acgtgggcgc
>Sb02g007
CGCCGGaC
>Sb02g008
CGAAAaGC
>Sb02g008
atggacccag
>Sb02g008
GGGCTAGG
>Sb02g008
gatgaaaaaa
>Sb02g008
CTAaTAAca
>Sb02g009

atggatgtcaa
>Sb02g009
ggaggagatg
>Sb02g010
tagcaacgga
>Sb02g011
aagcatggaa
>Sb02g011
AGGATGG
>Sb02g013
AGCATCGA
>Sb02g015
GTCAAGTA
>Sb02g018
gacccagaca
>Sb02g018
GTCTAAGA
>Sb02g020
atactaccaa
>Sb02g020
cagtcctgtat
>Sb02g020
tacagcggct
>Sb02g020
attatgcatat
>Sb02g021
GCTAAAGG
>Sb02g021
GACTTCCc
>Sb02g021
GAATTtAG
>Sb02g022
CCATcTCcG
>Sb02g022
AGAGAATG
>Sb02g022
tccgctcattt
>Sb02g022
cgcgcgtgct
>Sb02g022
gcagccGCG
>Sb02g022
ATATATAC
>Sb02g022
tctccagcaga
>Sb02g023
TTTTAGCC
>Sb02g023
cgctctacgcc
>Sb02g023

gcagcttttag
>Sb02g023
tatactaggca
>Sb02g024
TAATGGTT
>Sb02g024
aaaaagaaa
>Sb02g024
atcctcccgcg
>Sb02g024
gtgctccatct
>Sb02g025
tataggttgtc
>Sb02g025
AAATTAAA
>Sb02g025
ctgaaatgtcg
>Sb02g025
tgggtgttcgt
>Sb02g025
caggagatat
>Sb02g025
CAGAGTGA
>Sb02g025
tttgatttagct
>Sb02g025
gtcgtcgtcga
>Sb02g025
GGTTCCaT
>Sb02g026
gtcgccgcgg
>Sb02g026
ATGCaCTat
>Sb02g026
tactttaattg
>Sb02g026
tatgggctaca
>Sb02g026
cacgtcgctg
>Sb02g026
atttttgtcctg
>Sb02g026
agaaacttga
>Sb02g026
cgAGGAGt
>Sb02g027
ggttaatcaa
>Sb02g027
TAGGGGTG
>Sb02g027

ttgagaaaga
>Sb02g027
ATGGCTTC
>Sb02g027
CTCAAGgT
>Sb02g028
aatgaagata
>Sb02g028
CCTCCaTC
>Sb02g028
GGGCTTTC
>Sb02g029
CGAGGAG
>Sb02g029
aagtcttggt
>Sb02g029
GAtGATaG
>Sb02g029
tttgcaaggtc
>Sb02g029
ggcgctccgc
>Sb02g029
atgctgggcg
>Sb02g029
ttttgctgttgc
>Sb02g029
gccatcgaca
>Sb02g029
gctgccggcg
>Sb02g029
acatcagaga
>Sb02g029
aaccctcaga
>Sb02g029
catgggacac
>Sb02g029
TGACAAGA
>Sb02g029
ttggtgcccgg
>Sb02g030
tcatacttgta
>Sb02g030
atgacataga
>Sb02g030
ctcgtcaccgt
>Sb02g030
caccactgaa
>Sb02g031
gcaccgccatt
>Sb02g031

gtggcaggtc
>Sb02g031
gcaggtcgga
>Sb02g031
ccaagaacct
>Sb02g031
gatagttgatg
>Sb02g032
GCCgACGT
>Sb02g032
CGGCGGTG
>Sb02g032
TATCCCCG
>Sb02g032
gctgttggtac
>Sb02g032
ggccacgccc
>Sb02g032
GTCGGAGT
>Sb02g032
acttcatccac
>Sb02g032
ttcagagttgg
>Sb02g032
gataggcaga
>Sb02g033
GATTGCTG
>Sb02g034
AGGACTTC
>Sb02g035
tgaggcggac
>Sb02g036
atcaccattga
>Sb02g036
AAGCTACA
>Sb02g036
TCAAATaT
>Sb02g037
tcgtcgttcct
>Sb02g039
agaggctccg
>Sb02g039
tgtttcagcaa
>Sb02g039
aggttatggtg
>Sb02g039
ttggatcccat
>Sb02g041
gagggcgagt
>Sb02g043

acagatgatg
>Sb03g001
tacactgctcc
>Sb03g001
atcattgtgag
>Sb03g003
AATATGGG
>Sb03g005
tctgcaggctg
>Sb03g005
TCTGTcCGt
>Sb03g008
tttggagggtt
>Sb03g011
GACACAAG
>Sb03g011
tttggccttag
>Sb03g011
tgaccaagctt
>Sb03g013
ATTGATGA
>Sb03g014
gtgccggccg
>Sb03g015
aattttacagg
>Sb03g017
gttgagatatg
>Sb03g018
aagtatgtatg
>Sb03g020
TTATCCGC
>Sb03g022
gagaagattc
>Sb03g023
tgcagtgtttg
>Sb03g025
aagctcgcaa
>Sb03g025
taccacgtctt
>Sb03g027
gatatctcaaa
>Sb03g027
CTTGATCT
>Sb03g027
gacctgccca
>Sb03g028
caaGGAAT
>Sb03g029
ctcgtgctccc
>Sb03g029

ATGGCGTT
>Sb03g036
ATTTTCTGT
>Sb03g039
gaaaggtacc
>Sb03g042
tctacaagaa
>Sb03g044
GATAATGT
>Sb03g046
tttctgacttc
>Sb03g047
gattatggcc
>Sb04g000
tcgacaaggc
>Sb04g000
cgaggagttt
>Sb04g001
GACCAAGA
>Sb04g001
AGCTATGG
>Sb04g002
gtggaggtga
>Sb04g003
cacaagaata
>Sb04g003
CTGTACAC
>Sb04g003
cagtatgtga
>Sb04g003
tatattcctac
>Sb04g004
tttgttcagta
>Sb04g004
gtacccaaa
>Sb04g005
cggctgcagg
>Sb04g005
ggaggaagg
>Sb04g006
AATGAGTA
>Sb04g008
GTTCCACC
>Sb04g009
ttcggcgattc
>Sb04g010
atagggaatg
>Sb04g010
tcttggtattg
>Sb04g012

tttattgtaaa
>Sb04g019
gtaacatctcg
>Sb04g021
aagaataaga
>Sb04g021
gacccagtgg
>Sb04g022
ggcgacccgg
>Sb04g025
GAGATAGA
>Sb04g026
aaccttgttgg
>Sb04g027
CATCTGCC
>Sb04g028
ggctgcacgg
>Sb04g028
tgcgatctgag
>Sb04g030
aggggatatg
>Sb04g030
atccgcagttg
>Sb04g031
tgcattggcca
>Sb04g032
cttgatgtggc
>Sb04g033
gaacaataG
>Sb04g034
ggtccagggg
>Sb04g035
ttgatgatgtt
>Sb04g035
tgtttgcagttt
>Sb04g036
ccttcctctca
>Sb04g038
acttgagaat
>Sb04g038
agcggctgcc
>Sb04g038
TTCTACGC
>Sb05g000
cttaccagtgt
>Sb05g000
cctgggaatg
>Sb05g000
gaagcagacc
>Sb05g001

taccatctcac
>Sb05g001
gtcgatgtgta
>Sb05g001
atctcctggac
>Sb05g001
GAAGCCTT
>Sb05g001
atggcggaga
>Sb05g001
agctacaatc
>Sb05g001
gtgcatcttgg
>Sb05g001
GAACCTCC
>Sb05g001
ggagacgacg
>Sb05g002
tctaggattca
>Sb05g002
TGCCTCTA
>Sb05g002
gagcctccaa
>Sb05g002
agacttgcttg
>Sb05g002
ttaagctttat
>Sb05g003
cagattatgtg
>Sb05g003
gcagtgcgtc
>Sb05g004
AAGAACTT
>Sb05g004
GAtAGCAT
>Sb05g004
AATGATgC
>Sb05g004
CAGGTGGT
>Sb05g005
gtctgatgaa
>Sb05g007
gccttcatttc
>Sb05g008
ctgggacaga
>Sb05g009
CATCCTTC
>Sb05g009
gcgacgattta
>Sb05g010

gagcagcggc
>Sb05g018
agATGGCC
>Sb05g021
GCGTGAAG
>Sb05g021
CAGTGGGG
>Sb05g022
gtaatcgatgg
>Sb05g023
attgataagac
>Sb05g024
GGCTACCT
>Sb05g025
agcagatgaa
>Sb05g025
atgtggatttt
>Sb05g025
cgttttccagc
>Sb05g025
ggtgctggac
>Sb05g026
caagCTGTT
>Sb05g026
cttccttttgag
>Sb05g026
TGCTTAGA
>Sb05g026
gtgttggaaga
>Sb05g027
actgcggacc
>Sb06g000
ATgCTGCA
>Sb06g000
TAGTCCAC
>Sb06g000
acgccacgca
>Sb06g001
agactggggc
>Sb06g001
ccagttcgtgt
>Sb06g001
cggcgaggcg
>Sb06g002
gggcaagcag
>Sb06g004
gcatagaaga
>Sb06g015
gcgtcccccga
>Sb06g016

gctaacatgct
>Sb06g016
GGAAACAA
>Sb06g019
tcttgttcaat
>Sb06g019
TATGCGAT
>Sb06g019
ccaaggtctt
>Sb06g020
aaagagaag
>Sb06g021
atgtccctctt
>Sb06g021
ggagtttgatg
>Sb06g024
GcGTGTTC
>Sb06g027
GGGAGAG
>Sb06g027
gacttggcca
>Sb06g028
ttcaggtggtg
>Sb06g029
catgacctact
>Sb06g029
aaagttggga
>Sb06g029
TATCTTCAT
>Sb06g030
gacaTGATC
>Sb06g030
gctagcaagc
>Sb06g031
ggactgggtct
>Sb07g000
TTGGTTCC
>Sb07g001
CTGGTTTC
>Sb07g002
cagaactccc
>Sb07g003
TGCTGCAA
>Sb07g003
accTCTGGA
>Sb07g005
GAGTTACC
>Sb07g006
gggacttgtg
>Sb07g006

gcggctgcat
>Sb07g009
caccttcccg
>Sb07g020
ctatattgcct
>Sb07g021
GTGGGATA
>Sb07g022
cctgttgggga
>Sb07g022
cgtggcgacg
>Sb07g023
CAGGATGC
>Sb07g025
TCCCgGAA
>Sb07g025
aagcagatga
>Sb07g025
ctaaagaaat
>Sb07g025
TGGTGGCG
>Sb07g027
tcattgtttgg
>Sb07g027
agaaacccct
>Sb07g028
ccgatggacc
>Sb07g029
tgtgtgctgct
>Sb08g001
ATGTGCGG
>Sb08g001
aaaattgctg
>Sb08g001
AGGGCACT
>Sb08g002
ccgtcgccgg
>Sb08g002
taaaaagtca
>Sb08g002
atACCATG
>Sb08g003
ctaaaaagac
>Sb08g003
ggtgaacgac
>Sb08g003
tccctgttcat
>Sb08g003
tacgacatcct
>Sb08g003

tgtatgagctg
>Sb08g004
AGCTCCAA
>Sb08g004
ggcaaacccg
>Sb08g008
GACCCGCC
>Sb08g008
GAAAGCCA
>Sb08g011
GAGCTGCT
>Sb08g014
gatggacGC
>Sb08g015
tgaagccaa
>Sb08g015
tcagtcctggg
>Sb08g015
AGCTTATA
>Sb08g015
tggagcgcag
>Sb08g016
AGAAGCAC
>Sb08g016
tctatatgaag
>Sb08g017
TGACTCGT
>Sb08g018
aaggaggtgg
>Sb08g018
CATAGATT
>Sb08g018
tgttctccgcc
>Sb08g019
atggatcgcg
>Sb08g019
tagagaagga
>Sb08g019
TATTTATCA
>Sb08g020
cccgccaggg
>Sb08g020
cctgcttctgc
>Sb08g020
tggccaattta
>Sb08g021
acagacacct
>Sb08g021
ctccattcaat
>Sb08g021

cagaggaagg
>Sb08g021
tgaagattgct
>Sb08g021
GGtACGGC
>Sb08g022
GAGTACGC
>Sb08g022
ttttatgttcat
>Sb08g023
attcttgtttgg
>Sb09g000
AACAAGTT
>Sb09g001
atggtaccat
>Sb09g001
gcagtgaaaa
>Sb09g003
cactgaagca
>Sb09g004
ttggtccggtg
>Sb09g006
aaagcTTTT
>Sb09g017
cttgctgcggg
>Sb09g021
CAATGGAG
>Sb09g023
gtggaaaacc
>Sb09g024
aaaaaatgaa
>Sb09g024
accacatatat
>Sb09g025
acagtggtcg
>Sb09g025
aaataagatc
>Sb09g026
gtccaagtac
>Sb09g026
acgccaccac
>Sb09g027
agctgtcagg
>Sb09g030
gggcgttttga
>Sb10g000
catctagttct
>Sb10g000
caaaattcaa
>Sb10g000

ctgaccttcca
>Sb10g000
TGGAcATA
>Sb10g000
gaagcagcca
>Sb10g001
aagaaattcc
>Sb10g001
caggatcttt
>Sb10g001
gctacgaata
>Sb10g001
gccaacgcgc
>Sb10g002
TATGTcGA
>Sb10g002
tgccaattgtc
>Sb10g004
CAcTGCTG
>Sb10g005
ctcctcctatg
>Sb10g006
ccagtcgtatg
>Sb10g008
atagatccag
>Sb10g010
aagccttgaa
>Sb10g023
GCTTGtAG
>Sb10g024
tcttgactgca
>Sb10g025
gccacccGg
>Sb10g025
aaatggctca
>Sb10g029
TGTGATAT
>Sb10g030
atggcggact
>Sb10g030
gctgtcttgtt
>Sb0011s0
gagattggag
>Os01t015
gacgtcctgg
>Os01t015
aaggcctctt
>Os01t015
GACACCAA
>Os01t017

atgacgctga
>Os01t018
ttataggtga
>Os01t018
AGGCTATG
>Os01t018
cccaaacacc
>Os01t020
gggggggctg
>Os01t027
aggggaagg
>Os01t027
ggtgtcagta
>Os01t028
CTCCgCGA
>Os01t035
ttttcttgtact
>Os01t036
aagggaaag
>Os01t036
gcagcgggC
>Os01t037
tcgatcaacgt
>Os01t037
ATGCGCGA
>Os01t051
agctaaggat
>Os01t051
TCCTATGA
>Os01t052
atgatctgcct
>Os01t053
ggaaccgagg
>Os01t056
cagattgcatt
>Os01t056
ggcggcgtga
>Os01t057
aacttggtga
>Os01t059
aggcactaga
>Os01t060
aaatacaaaa
>Os01t060
CTcGATGG
>Os01t060
TGTGGAgG
>Os01t060
gactgctgcc
>Os01t062

gccacgaggg
>Os01t070
AGGTCAGT
>Os01t076
TCGGtCTA
>Os01t077
agattaatga
>Os01t078
tgaatttgatt
>Os01t078
ttggtgagctg
>Os01t084
AGGAAGAT
>Os01t085
aaagctgaca
>Os01t085
cggaagcgag
>Os01t089
TGCATCAT
>Os01t089
taccagttgtg
>Os01t093
TTGAAAAG
>Os01t093
TTCTAATC
>Os01t094
agttgAtcAC
>Os01t096
cctgTTGGT
>Os01t096
catccaatatg
>Os01t097
gatttagctca
>Os01t097
TTTGATGA
>Os02t010
tcttgcttaca
>Os02t010
AGTGCATG
>Os02t010
CGgTCACA
>Os02t010
gcaagaagct
>Os02t010
gcagcgcacc
>Os02t010
ttgaaaatgga
>Os02t011
ttgcaccagga
>Os02t013

CGGTGCAC
>Os02t013
tgttgcatatg
>Os02t016
gtgagacgtg
>Os02t016
AACTTGAC
>Os02t017
atggcctacgt
>Os02t018
CCACTGTC
>Os02t019
ggtaaacgtc
>Os02t020
gttaaagttct
>Os02t023
aagttcgacg
>Os02t026
ttacggcgatt
>Os02t027
ggttcatcctg
>Os02t027
AATCATTG
>Os02t028
CCGCGGTT
>Os02t028
ggaatcgggg
>Os02t028
aaacagctgg
>Os02t029
cgggtcgttcc
>Os02t032
agatcgtaca
>Os02t045
GCCCTGCA
>Os02t046
ggacgcggag
>Os02t049
ctgctagaag
>Os02t051
GAAGGAG
>Os02t052
GtcaGTGG
>Os02t053
ggtcgccagtt
>Os02t057
ccgatgatga
>Os02t058
ttgcccttctct
>Os02t061

AAAAGACC
>Os02t061
CAGGCCAA
>Os02t061
gtccATCTA
>Os02t065
GAATTGGT
>Os02t067
aagtatcgctt
>Os02t068
ctcattgcggt
>Os02t068
ttgcagactgt
>Os02t069
agtgttcgaat
>Os02t071
agataaatttg
>Os02t071
cgccgacgga
>Os02t072
TGGTTCAC
>Os02t079
gtctataatgg
>Os02t079
AATGGCTC
>Os02t080
acttcaacaga
>Os02t083
ccatattttac
>Os03t010
aagaatttcat
>Os03t010
gttcacaaga
>Os03t011
agcccatgta
>Os03t011
tctgatcggat
>Os03t011
cgactccgtca
>Os03t013
aacATACTT
>Os03t014
gagattgttga
>Os03t015
tttggggtatg
>Os03t015
ctcgtcgtcga
>Os03t017
gacacctaata
>Os03t017

tttcgtgttgg
>Os03t018
GACTCAGA
>Os03t018
agggaagcc
>Os03t019
atggaacga
>Os03t019
gaggggtcgg
>Os03t020
ttgctgggaa
>Os03t020
gtgagcagga
>Os03t021
TTGAAGCT
>Os03t021
CAAACAGC
>Os03t022
tctctcaaag
>Os03t022
gccttgtcgca
>Os03t022
gtgaagcagt
>Os03t023
gggaaaatgc
>Os03t023
ggtgactcca
>Os03t024
ggtctggaaa
>Os03t025
atacagttca
>Os03t027
ctgtactgcga
>Os03t028
ggatcctcca
>Os03t030
agtacgaggt
>Os03t030
atgggaaagt
>Os03t030
cgactatccca
>Os03t031
tctaggttcta
>Os03t031
agcggatgcg
>Os03t033
TGAAACAA
>Os03t034
gtacgtgagc
>Os03t034

ggcattgatct
>Os03t035
atttgatatgg
>Os03t035
gcacaccttct
>Os03t040
aggtcggtga
>Os03t041
ggaggaagtg
>Os03t043
AGTCTTGT
>Os03t043
TGAAgAcT
>Os03t057
gtgtccaagtt
>Os03t057
GCTCGCCA
>Os03t061
ccgcgcgtcg
>Os03t061
GAAATTAT
>Os03t061
gagtccaggc
>Os03t062
atcctgctata
>Os03t062
aggaccaaca
>Os03t063
atgcagagcc
>Os03t064
agaccgccgc
>Os03t064
gttctacaaa
>Os03t064
cccgtcctcct
>Os03t065
CATGGCTC
>Os03t065
gcggtcatcc
>Os03t065
tatgctcttat
>Os03t066
cagatgatcg
>Os03t067
agatggacaa
>Os03t068
gaaaaagatg
>Os03t068
aaccaacttg
>Os03t069

gagattattgt
>Os03t070
ccgcGGtGG
>Os03t071
gtggagagat
>Os03t071
acttgctatat
>Os03t071
gataTCATT
>Os03t072
cggccgtggc
>Os03t073
GCTGTTGC
>Os03t073
aggaagtaca
>Os03t073
caagtacagc
>Os03t074
aactgaggtc
>Os03t074
ccctttttgtta
>Os03t074
GAGGGGG
>Os03t074
GTCCAAAA
>Os03t074
GGGGcGG
>Os03t074
gatcatccact
>Os03t075
gcgctccacc
>Os03t075
AGGTTAAT
>Os03t075
atgattccgca
>Os03t075
aggtaggaag
>Os03t075
AAACTTTT
>Os03t075
agagtgggcg
>Os03t076
CAGTTCCA
>Os03t076
AGGCTCTC
>Os03t076
GcGGACCc
>Os03t077
cagctgccgg
>Os03t077

gccagggtgc
>Os03t077
cagtatcaaa
>Os03t078
gcgagaccat
>Os03t078
tttgctcagga
>Os03t079
gatttcaaga
>Os03t079
gcacgccgaa
>Os03t079
tcttCTTTTA
>Os03t079
GCaGGTGC
>Os03t080
aggtgataga
>Os03t080
gctataaaaa
>Os03t080
tggatgatga
>Os03t080
ttgatgacta
>Os03t080
actatcctga
>Os03t081
cgggagataa
>Os03t081
CAGAAGCt
>Os03t081
AGAACaTC
>Os03t081
TTCCGGTC
>Os03t082
agcagaagca
>Os03t082
GGATGCCA
>Os03t082
AGCAGCCA
>Os03t083
GTGAAaCT
>Os03t083
tggcaggtca
>Os03t083
gggatggttc
>Os03t083
gaaatgtatg
>Os03t084
aactgtgagtt
>Os03t084

acgtgctccc
>Os03t084
cttcttccagg
>Os03t084
gagctcctcg
>Os03t085
tgagggccca
>Os03t085
gttaaagttg
>Os03t085
ggtacagcag
>Os03t085
acaacgaatt
>Os04t013
gtgccctaca
>Os04t016
AGCTGAAG
>Os04t017
TGGGCAAA
>Os04t020
atcgtctcccg
>Os04t021
cttggtagagt
>Os04t030
ATGGGGA
>Os04t035
actAAATaa
>Os04t036
caagtccgtgt
>Os04t037
gtcaagtcca
>Os04t037
aaagggatca
>Os04t045
CTCGGTGA
>Os04t046
atggcagttg
>Os04t048
GATATCTT
>Os04t050
AACTGGGA
>Os04t057
CTTCCTCCT
>Os04t059
gcgaagagct
>Os04t061
agaaagaag
>Os04t065
gaagatcctg
>Os04t069

cacgtgttgtt
>Os05t010
cggttcagctt
>Os05t010
agatcctcttc
>Os05t011
caaattttcga
>Os05t012
tggaagattat
>Os05t013
gaagcaagtt
>Os05t013
gctctgacgc
>Os05t014
gtcacctttgc
>Os05t015
ATAGAGCA
>Os05t018
agccaatact
>Os05t020
ggcggcggct
>Os05t021
ccaagttccct
>Os05t022
TACAAGGG
>Os05t025
ATGCATGA
>Os05t027
CCTCCGCC
>Os05t029
AATGATGA
>Os05t030
agattcacaa
>Os05t031
ttagagaaga
>Os05t035
atgatatgga
>Os05t035
tatctgttcga
>Os05t037
GGACtCTG
>Os05t039
gagtgcagtc
>Os05t040
aaagttaaac
>Os05t041
gatcggtttgg
>Os05t041
ccgccgccgc
>Os05t044

atgagaagat
>Os05t045
gagcgggcca
>Os05t050
acaggctcgt
>Os05t052
acattcagaa
>Os05t052
gactttgtgca
>Os05t053
tctgtATGTG
>Os05t057
CTtCAAGTg
>Os05t058
acctaggatg
>Os06t010
caggttctga
>Os06t010
gcctgtggcg
>Os06t011
ggaaagagca
>Os06t011
gtGAAGGT
>Os06t012
cacaattatct
>Os06t013
gttgttgccct
>Os06t013
aggtaaagca
>Os06t014
aggcttgaga
>Os06t014
ggtctaggca
>Os06t015
catggaatta
>Os06t020
AATCTTTC
>Os06t021
ccggaggagc
>Os06t022
gtggtcaagc
>Os06t025
caagtggagg
>Os06t025
tctacgtcggc
>Os06t026
tttgtagggtt
>Os06t027
cTGGTGGA
>Os06t028

ttaccctagct
>Os06t047
cggggctaga
>Os06t048
ttcgacccag
>Os06t053
tgtttggagct
>Os06t054
cctccgctccg
>Os06t061
AGATTGAA
>Os06t062
ggagctggag
>Os06t062
GCCTCCAA
>Os06t066
tggtgcttttg
>Os06t070
GTTACTGg
>Os07t010
ccggccccctg
>Os07t016
atactggata
>Os07t018
gggaaatcca
>Os07t018
tttggagttgg
>Os07t019
caggagatga
>Os07t019
gatgtgtggtc
>Os07t024
tactacccgtt
>Os07t026
ctcctcgcgga
>Os07t027
tctcaggtgct
>Os07t027
gccacatctat
>Os07t027
AAAATACG
>Os07t042
ggcatcccgtg
>Os07t042
CTcTCcgaC
>Os07t047
cagacagagg
>Os07t047
gttgatggaa
>Os07t051

gaggtcttgg
>Os07t051
AAGATCTT
>Os07t053
atactcgtat
>Os07t054
tgaagatagt
>Os07t055
gacctcctcg
>Os07t056
gacacgttcc
>Os07t064
tacttGCAA
>Os07t065
tactttggga
>Os07t068
TATCTGAT
>Os08t011
cacccggtgc
>Os08t011
tgctgcacgct
>Os08t013
gacgaggtct
>Os08t014
ctgaacaaga
>Os08t015
cacgccggtg
>Os08t017
gcagatggag
>Os08t020
tatggggagg
>Os08t022
TTCAAGGC
>Os08t024
ctcgccgccc
>Os08t026
GTATGGGT
>Os08t039
CATTaTCTT
>Os08t040
CGAGATCC
>Os08t040
gctcggctcc
>Os08t041
gctgaaaatg
>Os08t042
caggttttgat
>Os08t043
ttcgaatgcca
>Os08t045

acggtgctgct
>Os08t047
cagttgttgaa
>Os08t048
aaccatgggg
>Os08t048
caaagatgcg
>Os08t049
tttacatcagg
>Os08t050
gacgaggagc
>Os08t053
gtggagctcg
>Os08t053
AGATGACT
>Os08t053
gttgggtgacc
>Os08t053
agagccaatg
>Os08t054
ccaatttatca
>Os08t054
tccatcattga
>Os08t054
tggatgaagg
>Os08t054
tgtaaaatgc
>Os09t012
CTGCAGGT
>Os09t024
ATaCAGGT
>Os09t024
TTGTTGAG
>Os09t025
atggcgatga
>Os09t026
gacaccctcc
>Os09t026
cttcggcgaat
>Os09t026
atggggttcgt
>Os09t027
gcccaaggcc
>Os09t028
ttggttcggaa
>Os09t028
tgcaggctttt
>Os09t029
atgaaagagt
>Os09t029

ggttgccagc
>Os09t030
gatactccaa
>Os09t030
gcattctacga
>Os09t031
atcattccact
>Os09t032
GTTGATtgA
>Os09t032
tgcgacggcg
>Os09t032
CCTGGatTA
>Os09t033
ttgaacatgca
>Os09t039
GAGAAGG
>Os09t039
ttctcccccac
>Os09t039
GATATTGT
>Os09t040
gacggagagg
>Os09t040
gcTCTaCAT
>Os09t042
agtttccccttc
>Os09t042
gtacgacggc
>Os09t042
aagcattgca
>Os09t042
aagtcaggtc
>Os09t043
ggacggcggg
>Os09t043
ccccggntctc
>Os09t043
ctaatgtcaa
>Os09t044
ttaaTGGCA
>Os09t045
gttcaggagtt
>Os09t045
TCTTGATGT
>Os09t045
cagccccatcg
>Os09t045
ccaccggcga
>Os09t046

aagaaacttg
>Os09t046
ctgcagcgca
>Os09t046
gcggccgtca
>Os09t046
gcaatggagg
>Os09t047
TTATTTTAT
>Os09t047
gggggccggc
>Os09t047
gtcaacatat
>Os09t047
tttgcttgtact
>Os09t048
CAGGCGC
>Os09t049
cttgccaaga
>Os09t049
cagcaccagc
>Os09t049
tccccgacaa
>Os09t050
ggaaggGGg
>Os09t050
gcggcgccgg
>Os09t050
ataggaatgg
>Os09t051
GATGGAA
>Os09t051
ATGTCACG
>Os09t051
TCAAGATA
>Os09t051
ttgcttcacag
>Os09t052
gtggccgtcc
>Os09t052
ccccgtcggc
>Os09t052
ggcaaggttg
>Os09t052
GAGAAGG
>Os09t053
cgctggtggt
>Os09t053
aaccatgacc
>Os09t054

TACCTCGT
>Os09t054
agtactagat
>Os09t054
tgggcgttcc
>Os09t054
aggttataga
>Os09t054
ATGGCAGG
>Os09t054
GGcAaGAT
>Os09t054
AGTGCCaG
>Os09t054
gccgagctcc
>Os09t054
GAcGGTCA
>Os09t055
ttcaccataa
>Os09t055
atcaggacac
>Os09t056
aaactttttgg
>Os09t056
accgcctaca
>Os09t056
CTTGATCA
>Os09t056
agctattggcg
>Os09t056
gtaaactgtgt
>Os09t056
cctcccccgc
>Os09t056
gaacatcac
>Os09t056
tgctatctgct
>Os09t056
AAGTACCA
>Os10t013
ccatggtgaa
>Os10t013
AAGCTACA
>Os10t014
gacagtcaag
>Os10t014
tctgttgagcg
>Os10t015
tatatgcactc
>Os10t018

tgtcgccaac
>Os10t019
tggttggcttg
>Os10t019
ggtctcctcct
>Os10t020
CTTATATT
>Os10t034
aggggagtgg
>Os10t035
tttggtaggat
>Os10t037
caagtattga
>Os10t037
cggtgaacct
>Os10t039
CTcGCTGT
>Os10t040
tggagtttggc
>Os10t043
AGCCCAGa
>Os10t044
ctcctGGAC
>Os10t044
AGCATTGC
>Os10t045
gagttcataa
>Os10t045
tgccagggag
>Os10t047
ggttcaggag
>Os10t047
gataaaagtc
>Os10t048
AGTACCTG
>Os10t049
CGgAACGG
>Os10t051
ggacagtttt
>Os11t010
gaaattgaag
>Os11t010
GGGGTGG
>Os11t012
tcatacgccat
>Os11t012
caagaatgct
>Os11t013
aggaaaaaa
>Os11t013

tctctgtgatg
>Os11t013
gcttcttttan
>Os11t014
cagtggtgca
>Os11t014
gagcctcgcc
>Os11t015
ccttcaactg
>Os11t017
cctcaggtga
>Os11t020
ataattctttc
>Os11t021
TTGAgtGA
>Os11t021
tgatgctgCA
>Os11t021
gagtcaattg
>Os11t021
aTCCTTCca
>Os11t022
ccgcgtcgtg
>Os11t026
gtgaagcag
>Os11t027
atgggcgcgg
>Os11t029
aggtgaaagt
>Os11t043
tgtctcgcgg
>Os11t043
ggccttttcct
>Os11t044
ttgactctttc
>Os11t046
GAGCAGcA
>Os11t047
gtggaatggg
>Os11t048
GCAGCCTT
>Os11t055
attttacggaa
>Os11t055
tgaagcactt
>Os11t057
GCAGGCcC
>Os11t058
CCATCcCCg
>Os11t059

tatcaaGGT
>Os11t060
AGGGAGT
>Os11t061
AGGTGATT
>Os11t061
ggaggggagt
>Os11t063
agaagagac
>Os11t064
GAGAAcAT
>Os11t065
TTttAGCTA
>Os11t067
tattactatga
>Os11t067
cctaaaagtg
>Os11t067
agagtacttgt
>Os11t068
tttccagaatc
>Os11t068
cttcggaagc
>Os11t069
gtggggaaat
>Os11t070
AGGAGTG
>Os12t013
ATGCCTAG
>Os12t015
CTGCTCAA
>Os12t015
TTTAAGTC
>Os12t016
caataaccgt
>Os12t016
GTTTGGgC
>Os12t016
gctcgaggtgt
>Os12t017
TGCCAAAG
>Os12t017
TCTCAAGC
>Os12t017
caggttgtact
>Os12t017
CAGTGATT
>Os12t019
cagtgctatg
>Os12t019

tacctgcagg
>Os12t020
gttcatcctgt
>Os12t020
cagatttggat
>Os12t021
CCGCCGgC
>Os12t023
aggcgggga
>Os12t023
ATGCCACA
>Os12t027
ttcattggttct
>Os12t027
ctgctttctttc
>Os12t027
gaaaataaca
>Os12t028
ctgttgtttcg
>Os12t029
AAAGCtcaa
>Os12t042
gtttctcgccg
>Os12t042
gccagaatct
>Os12t043
gtcagttgtca
>Os12t043
atgggggaga
>Os12t044
tacaatgagtt
>Os12t048
caatttctctta
>Os12t049
tcctcgccgcc
>Os12t050
ACAAAAGG
>Os12t050
gtcaaatttct
>Os12t051
caaactgaat
>Os12t051
gccggtggcg
>Os12t053
cccggcgtcgt
>Os12t053
tgggctttcaa
>Os12t054
agtcacagtct
>Os12t055

CTTGGGCA
>Os12t055
gctgccaaag
>Os12t055
ttgaacaaG
>Os12t056
gattttgaatt
>Os12t056
gcgcgctcgt
>Os12t056
gtcctgaaca
>Os12t056
tgaagataag
>Os12t057
tgcactgaac
>Os12t058
TGCTGCGG
>Os12t058
ttggaacttga
>Os12t059
AATTCCGC
>Os12t060
ggaggccgg
>Os12t061
aggttgacta
>Os12t061
gagggccaga
>Os12t061
cgggcccttct
>Os12t061
ggtcatggaa
>Os12t061
acggtggctct
>Os12t061
gacctgccca
>Os12t061
ttcagcaaat
>Os12t061
gcaaatgtgc
>Os12t062
aaaatatcca
>Os12t063
GGTGCGTG
>Os12t063
aagtgcttgtt
>Os12t064
gctaatgcctg
>Os12t064
ttttatatccat
>MLOC_91

atggttggta
>MLOC_61
ATGGAAAT
>MLOC_61
GCCTCCGC
>MLOC_63
GTAGCTTT
>MLOC_65
aggaagcaag
>MLOC_82
atgtcgtcgt
>MLOC_82
tcaacaagtt
>MLOC_88
aggaggagg
>MLOC_93
GGAAGGT
>MLOC_10
ATGACGCG
>MLOC_10
atgatgggaa
>MLOC_11
gcagtttaag
>MLOC_13
ttttccatctg
>MLOC_14
gcgatctgat
>MLOC_14
AGACTTAT
>MLOC_14
ggcggagacg
>MLOC_14
atgtttgactg
>MLOC_15
ggtggaacag
>MLOC_16
atggagatca
>MLOC_21
gtggagatgt
>MLOC_23
ATGCAGAG
>MLOC_24
tcctacgccg
>MLOC_24
agatcaagag
>MLOC_25
atggagccgg
>MLOC_26
tttacggcgtg
>MLOC_26

GGTcGTGG
>MLOC_29
ccaaggccgg
>MLOC_30
ggggacgagt
>MLOC_30
atgaatctgtt
>MLOC_30
ATGCCCctG
>MLOC_30
tgcaaatatc
>MLOC_31
gacatcgccg
>MLOC_31
ttgtaggatga
>MLOC_32
ATGGCGAT
>MLOC_33
tctccagggg
>MLOC_33
ATGGACCT
>MLOC_34
accaggtcca
>MLOC_34
ctgtagaattc
>MLOC_35
ATGGCGAC
>MLOC_35
TTCCGGAC
>MLOC_35
gaaggctact
>MLOC_37
actggcgcgc
>MLOC_38
ggtttgacaa
>MLOC_38
TTGCAGAT
>MLOC_39
CAGAGATC
>MLOC_39
aggcgatcgc
>MLOC_39
ctacatgtgat
>MLOC_39
tcaggaaaag
>MLOC_40
gaccagactg
>MLOC_40
ATGGAAGA
>MLOC_41

AGGAATGA
>MLOC_41
atggccactc
>MLOC_42
caggggcagc
>MLOC_42
atggcggacc
>MLOC_42
tttcaggtgg
>MLOC_42
tctttttgaact
>MLOC_43
CTAGGTGT
>MLOC_43
tggcagagttc
>MLOC_43
gggtggtgtg
>MLOC_43
tcccttcgtcg
>MLOC_44
aggaagatga
>MLOC_44
ATGTTGGC
>MLOC_44
AGAAGCTG
>MLOC_44
ttcatacaggt
>MLOC_44
ATGATCAA
>MLOC_44
cctgatgatgt
>MLOC_45
ATGTTGGA
>MLOC_45
ATGAGCTC
>MLOC_46
ccgcccaggc
>MLOC_46
agaatttcgg
>MLOC_46
ATGTTGCC
>MLOC_46
agaatctttca
>MLOC_46
ttctcaagaca
>MLOC_47
atgaagatca
>MLOC_47
ATGGCTAT
>MLOC_47

atgcgtcgcca
>MLOC_48
atggctcttAA
>MLOC_48
gccccggcgc
>MLOC_48
AGGGAGAT
>MLOC_49
atgaagggcc
>MLOC_49
aTTCTCCAA
>MLOC_49
ggcgccggtc
>MLOC_49
atgaatttcat
>MLOC_49
gtttttgactg
>MLOC_49
agaaaggttg
>MLOC_49
gcaaatctcca
>MLOC_49
gcagcagtaa
>MLOC_50
atggggtcgtc
>MLOC_51
agaatattttc
>MLOC_51
aggctatctgc
>MLOC_52
gccagcctgc
>MLOC_52
gcaggcatatt
>MLOC_53
ccttgaaggg
>MLOC_53
agtgggatcg
>MLOC_54
atggctactta
>MLOC_54
GGGCACCC
>MLOC_54
ATGGCCGA
>MLOC_54
cacacgatCc
>MLOC_55
ATGTGCAA
>MLOC_56
tgcagttatgt
>MLOC_56

attcttgccat
>MLOC_56
atggaggcca
>MLOC_56
ttgttcagctg
>MLOC_57
ggaaagcatc
>MLOC_57
GGTACCTC
>MLOC_57
gttcaggccat
>MLOC_57
agatctttGG
>MLOC_57
atctccgcaa
>MLOC_58
cttaatgtagt
>MLOC_58
ATGTCCTC
>MLOC_58
aggatgtattt
>MLOC_58
ccccctccgcc
>MLOC_59
accaggggg
>MLOC_59
tgtccgtgctg
>MLOC_59
gtggaacttg
>MLOC_60
ATGTCTGA
>MLOC_60
ggcttaggga
>MLOC_60
atgcaccctgt
>MLOC_60
gatgacgatg
>MLOC_60
attagtgaga
>MLOC_61
gggaaacccg
>MLOC_61
caggcaaacc
>MLOC_61
ccacggccat
>MLOC_61
tggnggaaac
>MLOC_61
actgtaggag
>MLOC_61

cagagagaa
>MLOC_61
GGTAGATG
>MLOC_62
caggcagaga
>MLOC_62
cccgaaatgtt
>MLOC_62
ctgcatcgac
>MLOC_63
ccgttttgagc
>MLOC_63
agaaagtgaa
>MLOC_63
gtccattagtt
>MLOC_64
taccggaagg
>MLOC_64
ggtggatcaa
>MLOC_64
atggcgtcgtt
>MLOC_64
tgaaggagct
>MLOC_64
gaagcgggcg
>MLOC_65
ccaaacagat
>MLOC_65
ATGGTGCA
>MLOC_65
tccgcagctac
>MLOC_65
CAGGTAGG
>MLOC_67
atGCCGCTT
>MLOC_67
ATGTCTCC
>MLOC_67
GGGTTCAA
>MLOC_67
atggccatgg
>MLOC_67
ATGGCtCC
>MLOC_67
ATGGGCAA
>MLOC_68
ctgcTCcAA
>MLOC_68
cactcattctc
>MLOC_68

atggacagcg
>MLOC_68
atggaggcac
>MLOC_68
atgaagtcga
>MLOC_68
aggggaggaa
>MLOC_69
ccaaaggatg
>MLOC_69
AGGTGCTC
>MLOC_69
ATGAAGCC
>MLOC_70
aggccacgcc
>MLOC_70
ataaggatgc
>MLOC_70
ctgcacatcga
>MLOC_72
ATGGCtGA
>MLOC_72
ATGTGaTG
>MLOC_72
ATgCAaTG
>MLOC_74
ATGtCTCA
>MLOC_75
tttcctccata
>MLOC_76
CtCCTCCTC
>MLOC_77
ctgatgaacc
>MLOC_77
CTCATTTG
>MLOC_77
aggtttctgat
>MLOC_78
atggccaccg
>MLOC_81
agctggttttg
>MLOC_81
AGATtAAG
>MLOC_81
atggctgcga
>MLOC_82
ATGGCAGC
>MLOC_89
atgatttaagt
>MLOC_92

atattattctgt
>MLOC_97
gctctctatag
>MLOC_97
ccggggcagc
>MLOC_98
AGATGAGT
>MLOC_98
atgggctcctt
>MLOC_98
cagcaaggga
>MLOC_98
actggttgatg
>MLOC_99
aggttgttgaa
>MLOC_99
AAGGTCAA
>MLOC_99
ggctcggcgg
>MLOC_99
ctaccaatctc
>MLOC_99
ATGCATCT
>MLOC_99
atgagtcacc
>MLOC_99
gctgttgctaa
>MLOC_10
gcgaaaagg
>MLOC_10
atgtcgtcgtt
>MLOC_10
ggggagtgcg
>MLOC_10
GCCGAgGA
>MLOC_10
cgtcacccac
>MLOC_10
AGAATCTG
>MLOC_10
ATGGACCA
>MLOC_10
gcaaatgaag
>MLOC_10
aggtggatgg
>MLOC_10
ATGACTGA
>MLOC_10
aaatatggca
>MLOC_10

ATGGtGGC
>MLOC_10
ggtggggtgg
>MLOC_10
ggagctcctg
>MLOC_10
gtgcggagaa
>MLOC_10
ttcaaggtttt
>MLOC_10
ATGTCTAA
>MLOC_10
tggtgTTATC
>MLOC_10
atgggtgagg
>MLOC_10
cagctcctGG
>MLOC_10
ctctgattgac
>MLOC_10
AGAGCCCC
>MLOC_10
CAGAGAAA
>MLOC_10
atggagatcg
>MLOC_10
cgTGACCCC
>MLOC_10
aggtaaatgc
>MLOC_10
GggcAGGa
>MLOC_10
GATCTGTC
>MLOC_10
tgttcgaggcc
>MLOC_10
atgcactttca
>MLOC_10
tcgagcaggc
>MLOC_10
tctACCAGT
>MLOC_10
cagccattga
>MLOC_10
atggatgtga
>MLOC_10
aggggcaaga
>MLOC_10
AGTGggAG
>MLOC_10

ATGGAAGT
>MLOC_10
ATGTCTGA
>MLOC_10
tcctcctcgcc
>MLOC_10
ACAGCAAA
>MLOC_10
CCTTGCAT
>MLOC_10
ATGcGTCC
>MLOC_10
gttgtagcaa
>MLOC_10
GTGTaGCG
>MLOC_10
aatccacttg
>MLOC_10
gtatgatgttg
>MLOC_10
GGATCCAA
>MLOC_10
GCCTGGCA
>MLOC_10
agctacaagg
>MLOC_10
aaggaatatt
>MLOC_10
caatacatcg
>MLOC_10
ggagatgcca
>MLOC_10
TGCCCGTT
>MLOC_11
gtcctcgacg
>MLOC_11
caccatgtcg
>MLOC_11
gtggctgggt
>MLOC_11
AGACTGAG
>MLOC_11
ttacataggtt
>MLOC_11
atgcccttatc
>MLOC_11
gttagagaaa
>MLOC_11
agaaatggca
>MLOC_11

GCAGTTAT
>MLOC_11
atggttttcctt
>MLOC_11
aggtttgtggc
>MLOC_11
ATTTCTCCT
>MLOC_11
AGAGTgCA
>MLOC_11
ttcaatagcct
>MLOC_11
ATGTCAAA
>MLOC_11
tcctcctgggg
>MLOC_11
AGGGGACT
>MLOC_11
agtgaggatg
>MLOC_11
atgtcggcac
>MLOC_11
gttgccagcc
>MLOC_11
atggcggccg
>MLOC_11
ATGGCGGC
>MLOC_11
aggTTTCAG
>MLOC_11
tggcaatagg
>MLOC_11
ATGCAGGG
>MLOC_11
ATGCGCGA
>MLOC_11
atgtccaccgc
>MLOC_11
ggaagcgtca
>MLOC_11
atggatgagg
>MLOC_11
ggtcattggat
>MLOC_11
atggggggcg
>MLOC_11
atggacagga
>MLOC_11
cttgtggagtg
>MLOC_11

ataggaagga
>MLOC_12
TAGTCAGG
>MLOC_12
agttgCTTgg
>MLOC_12
atgcagtctca
>MLOC_12
atggcatcgc
>MLOC_12
atggagaggg
>MLOC_12
ccgtctccctc
>MLOC_12
CCAGGAGG
>MLOC_12
tcgcgcgctg
>MLOC_12
GTATTCCT
>MLOC_12
ctgaagcgct
>MLOC_12
TCACTGTC
>MLOC_12
aggtcatgaa
>MLOC_12
ATGGTTGC
>MLOC_12
agcctgaagc
>MLOC_12
ATGGTATA
>MLOC_12
agattgcttcT
>MLOC_12
TTCAGGGT
>MLOC_12
AGACTCAG
>MLOC_12
AcTGCAGG
>MLOC_12
tccggaggct
>MLOC_12
GGTATCCC
>MLOC_12
gatgaaaaac
>MLOC_12
ggaaaacgca
>MLOC_12
aggctgagca
>MLOC_12

ctcctccttgc
>MLOC_12
agattatccat
>MLOC_12
atggtgagcg
>MLOC_12
ctagggatcat
>MLOC_12
ATGTATCT
>MLOC_12
attggaaactt
>MLOC_12
ATGCTTCC
>MLOC_12
ggtgcagata
>MLOC_12
ggcaagaag
>MLOC_12
accgcaagca
>MLOC_12
gaacagattc
>MLOC_12
AGGTtGAG
>MLOC_13
tcccattcctc
>MLOC_13
cgtacaagga
>MLOC_13
GCaGATGC
>MLOC_13
ctacAGACg
>MLOC_13
gagtcactca
>MLOC_13
caggttactg
>MLOC_13
ATTtCAaGC
>MLOC_13
ttgggtatatc
>MLOC_13
ATTTCAGA
>MLOC_13
gagccaagag
>MLOC_13
cACTTACCT
>MLOC_13
ATGGTgTT
>MLOC_13
atgctaccctt
>MLOC_13

agccgaagct
>MLOC_13
gatccatcttg
>MLOC_13
atggATGAG
>MLOC_13
AGACGACC
>MLOC_13
catactgctgg
>MLOC_13
cccatctctctt
>MLOC_13
ATGGGtAT
>MLOC_13
aggcttgggg
>MLOC_13
caggtggctgt
>MLOC_13
aggttgatgtt
>MLOC_13
TGtGAgTaG
>MLOC_13
tcagtaaagc
>MLOC_13
atactgctcct
>MLOC_14
cagattggag
>MLOC_14
AGATACCA
>MLOC_14
ACAGAAAa
>MLOC_14
cagctgccag
>MLOC_14
atgctttgcct
>MLOC_14
atGGcCGTC
>MLOC_14
ATGTGCTG
>MLOC_14
gtctccgcctc
>MLOC_14
tactgggccct
>MLOC_14
gaaacttatcc
>MLOC_14
ctctcactatc
>MLOC_14
ATGGGCAT
>MLOC_14

ATGCAAAT
>MLOC_14
ggccacctcct
>MLOC_14
atgtggatttA
>MLOC_14
ATGCGTCa
>MLOC_14
ccaGATACA
>MLOC_14
caactcctgca
>MLOC_15
ggatggcatg
>MLOC_15
atggcgaagc
>MLOC_15
GATCGTCa
>MLOC_15
ggcatacatg
>MLOC_15
ggcgctctccg
>MLOC_15
atggcgacgg
>MLOC_15
ATGGCCAT
>MLOC_15
TTcGATGAT
>MLOC_15
ccatgcagac
>MLOC_15
AGGTGTGA
>MLOC_15
gacctagcct
>MLOC_15
tcatgcgctct
>MLOC_15
caggggttga
>MLOC_15
atcctcaacga
>MLOC_15
aatacctgatc
>MLOC_15
atgctcggtgt
>MLOC_15
catgcaattga
>MLOC_15
tacgccgagg
>MLOC_15
ATGACCGC
>MLOC_15

ttgactttgag
>MLOC_15
ATGGAtGG
>MLOC_15
gagcttcgag
>MLOC_15
gacaaagaat
>MLOC_15
atggggttca
>MLOC_15
tcgccgccgc
>MLOC_16
TGCTTTCT
>MLOC_16
GGGAGGT
>MLOC_16
atgttcaaga
>MLOC_16
ATGTCAAG
>MLOC_16
tgtggacaga
>MLOC_16
atggcggcg
>MLOC_16
atggcggggg
>MLOC_16
atggagaata
>MLOC_16
atggccatcc
>MLOC_16
cagcgcttctc
>MLOC_16
tcAGCGTG
>MLOC_16
agatgggagc
>MLOC_16
ATGGCCTC
>MLOC_16
aactctggag
>MLOC_16
ATGCTCGA
>MLOC_17
gtccctgcgcg
>MLOC_17
tcgttgcagat
>MLOC_17
ATGAGTGG
>MLOC_17
gtatcaatgc
>MLOC_17

cctcaaggag
>MLOC_17
ATGGACCC
>MLOC_17
cagataaata
>MLOC_17
cgatgataaa
>MLOC_17
gatgaaccag
>MLOC_17
tcaggttttc
>MLOC_17
gtacaaggtg
>MLOC_17
acaacttctct
>MLOC_17
atggtgttcgg
>MLOC_17
ATGGATTG
>MLOC_17
atggaccggt
>MLOC_18
ctttcaaccaa
>MLOC_18
gatgctccaca
>MLOC_18
gatgatcacg
>MLOC_18
atggcgggtc
>MLOC_18
ctgggtggcgt
>MLOC_18
atgtctccgat
>MLOC_18
tgatgcatcct
>MLOC_18
cgttgaaaga
>MLOC_18
ccaagaaaac
>MLOC_18
aagctggatg
>MLOC_18
cggcgcatcg
>MLOC_18
ATGGCGAA
>MLOC_18
atggcgactc
>MLOC_18
ATGAACcA
>MLOC_18

ATGGgCAG
>MLOC_18
acggggTGA
>MLOC_18
TCAGAAGA
>MLOC_18
ttggcagacg
>MLOC_18
ttcagaccccg
>MLOC_18
tgcgatcatgg
>MLOC_19
gctccacaac
>MLOC_19
agaaggtgca
>MLOC_19
gatgaaaatg
>MLOC_19
ATGGAAAa
>MLOC_19
gtgatcaagg
>MLOC_19
atgggaaaaa
>MLOC_19
ATGGCCAA
>MLOC_19
atggtcgccc
>MLOC_19
ATGTACGC
>MLOC_19
ttattttagcc
>MLOC_19
AAggAGGg
>MLOC_19
gatatcacgtt
>MLOC_19
atcacGGAT
>MLOC_19
cacctcaagg
>MLOC_19
atgccaagcc
>MLOC_19
agaaaccagg
>MLOC_19
agggccagct
>MLOC_20
cctggccggc
>MLOC_20
GCACAATA
>MLOC_20

ttcctggcgtt
>MLOC_20
atggacaaat
>MLOC_20
tccaggaaaa
>MLOC_20
TATGCTCC
>MLOC_20
atggcgttgtt
>MLOC_20
ATGGATAA
>MLOC_20
atgcggtatg
>MLOC_20
caggtaccttc
>MLOC_20
aggattcagt
>MLOC_21
cagaatggaa
>MLOC_21
GCCGGAGT
>MLOC_21
ATGGACAT
>MLOC_21
atgggcctgct
>MLOC_21
ATGCttAAA
>MLOC_21
ggcgtaattg
>MLOC_21
AGGCCGAG
>MLOC_21
GTTATTGG
>MLOC_21
tggtatccttg
>MLOC_21
ATGTgcATC
>MLOC_21
ATGGgAAG
>MLOC_21
atggaccgca
>MLOC_22
gtgtccacgg
>MLOC_22
GAGAAGG
>MLOC_22
tctagttgagg
>MLOC_22
atggtgggca
>MLOC_22

ATGGCGGG
>MLOC_22
ccagcggcaa
>MLOC_22
atggtctgatg
>MLOC_22
atgtccatgaa
>MLOC_22
atgggaccctc
>MLOC_23
gaggcgtgcca
>MLOC_23
atgcagccttc
>MLOC_24
CcGGCCGG
>MLOC_24
ATGCTTTG
>MLOC_24
ctgatacttga
>MLOC_24
cgganccgga
>MLOC_24
tcaaaatCGA
>MLOC_25
gctgacaaat
>MLOC_25
ATGGgGAA
>MLOC_25
GCAAcTTCA
>MLOC_25
ATGtATCAG
>MLOC_25
gcaaaggttca
>MLOC_26
TTGAAGgTt
>MLOC_26
TTtgaGGTG
>MLOC_26
GTATTGGT
>MLOC_26
agaagcccga
>MLOC_26
atgagcgtgg
>MLOC_26
gtcctatggta
>MLOC_26
ATGGTGGT
>MLOC_27
CAGATTCG
>MLOC_28

ttgcttgggtc
>MLOC_28
AGAGTGAA
>MLOC_28
ATGAGGCC
>MLOC_28
AGACCAGC
>MLOC_28
ggttcgaagc
>MLOC_28
ATGATTGA
>MLOC_29
agctttaccag
>MLOC_29
cagacgccgc
>MLOC_29
aatgataaca
>MLOC_29
atgtcaaattg
>MLOC_29
gcagggacgg
>MLOC_30
ATGACAGA
>MLOC_30
gatgatatgat
>MLOC_30
ATGGAcGG
>MLOC_30
atggcggana
>MLOC_31
aagcagtggt
>MLOC_31
GTGAAGTC
>MLOC_31
ATGCcGAT
>MLOC_31
ATGGCGAT
>MLOC_32
ATGCTTTG
>MLOC_32
tcctccagctt
>MLOC_32
actcttcgagt
>MLOC_32
aaaggtggtg
>MLOC_32
attgagatgct
>MLOC_33
gaaggtgaat
>MLOC_33

atggcggcgg
>MLOC_33
CAGGCCCG
>MLOC_33
AAcAAAGA
>MLOC_33
caggatgcaa
>MLOC_33
ctcaccgaga
>MLOC_34
caacggcggt
>MLOC_34
ATGACCcc
>MLOC_34
cacagggttg
>MLOC_34
ATGGAGG
>MLOC_34
ATGCGTCC
>MLOC_34
ATGGACGA
>MLOC_34
aaagggagta
>MLOC_34
agaatcttgct
>MLOC_34
ATGCAGCG
>MLOC_34
caagtcaatg
>MLOC_34
TACTCATTT
>MLOC_34
tGTAGTCT
>MLOC_34
caggtagctc
>MLOC_34
ATGTCCTc
>MLOC_34
gtctcggcgg
>MLOC_34
agctctaatat
>MLOC_34
atggccggcg
>MLOC_34
atgggccggt
>MLOC_34
ATGTCCAG
>MLOC_34
ccaccgccgg
>MLOC_35

atggggtggg
>MLOC_35
aaaacttcca
>MLOC_36
agcccttgatg
>MLOC_36
agccttgcca
>MLOC_36
tgcttgcgtca
>MLOC_36
ATGAAATT
>MLOC_36
ATGGACGA
>MLOC_36
ATGGCAgC
>MLOC_36
gatactattg
>MLOC_36
ttcttcaggT
>MLOC_36
ATGGTTCT
>MLOC_36
GATAcTgC
>MLOC_36
tccgcacgct
>MLOC_36
attatttgca
>MLOC_36
cactgtatga
>MLOC_36
gatgcagatc
>MLOC_36
aattctatca
>MLOC_36
ggaggcgacg
>MLOC_36
ATCCACCc
>MLOC_36
ATGgCGGC
>MLOC_36
agttctaggtg
>MLOC_36
gcctgctttc
>MLOC_36
GAGCTGGT
>MLOC_36
ctggtaatcta
>MLOC_36
CAGCTTAT
>MLOC_36

tgcaagctga
>MLOC_36
ATGATTGA
>MLOC_36
aggcgtatgc
>MLOC_36
CCAAtATCT
>MLOC_36
ATGTCTGA
>MLOC_36
atggcggcca
>MLOC_36
ATGGTTCA
>MLOC_37
agtttggctgc
>MLOC_37
atgacatcca
>MLOC_37
atcttggttga
>MLOC_37
ctgcaggactt
>MLOC_37
GCTTGTCT
>MLOC_37
atggcaagta
>MLOC_37
tttccattttt
>MLOC_37
ggcatcTTCT
>MLOC_37
atgagacccc
>MLOC_37
aggggttgaa
>MLOC_37
ATGGCAAA
>MLOC_37
atgtatactga
>MLOC_37
cagagggaag
>MLOC_37
CAGgAGTT
>MLOC_37
ttgcttcaatc
>MLOC_37
ggacgaagtc
>MLOC_37
cagtcaaaga
>MLOC_37
AGAAATCA
>MLOC_37

atgaagatta
>MLOC_37
ggatacacat
>MLOC_37
gtggagacag
>MLOC_37
TTTGcaGG
>MLOC_37
ATGCAcTT
>MLOC_37
atggcgatgg
>MLOC_37
ATGAGCAA
>MLOC_37
gggctccaca
>MLOC_37
ATGGCaCC
>MLOC_37
GCTCTCCC
>MLOC_37
ctacgctcgtt
>MLOC_38
GAATTCaT
>MLOC_38
acttactttac
>MLOC_38
agggtcagct
>MLOC_38
AGTCATAT
>MLOC_38
atgaatgcag
>MLOC_38
atggcggggt
>MLOC_38
AGACCACC
>MLOC_38
ATGTCTGg
>MLOC_38
atgattttcaa
>MLOC_38
ATGGCCGC
>MLOC_38
atgaacagcc
>MLOC_38
ATGGCAGA
>MLOC_38
atggcgacca
>MLOC_38
atggctgcatc
>MLOC_38

ctctcATGTC
>MLOC_38
agaGgAATC
>MLOC_38
ATGGATAG
>MLOC_38
ttgtatgcaga
>MLOC_38
agtATGATG
>MLOC_38
atgagcggcg
>MLOC_38
ATGGAAGC
>MLOC_38
AGGTACAA
>MLOC_38
gcacgCAGG
>MLOC_38
ttcagagttcc
>MLOC_39
ccgCggAGT
>MLOC_39
atggaccctg
>MLOC_39
acaaaagcgt
>MLOC_39
AGCTCCAG
>MLOC_39
aggctgcagt
>MLOC_39
agatcctcag
>MLOC_39
ATGGCGCA
>MLOC_39
aagctcacca
>MLOC_39
aaggaggacc
>MLOC_39
tatgggatttg
>MLOC_39
caggcagcag
>MLOC_39
ATGCAATG
>MLOC_39
aggcctgtaa
>MLOC_40
atgagcactg
>MLOC_40
atggccgctc
>MLOC_40

ggtttggatcg
>MLOC_40
aatcaaacTC
>MLOC_43
ccctgtccttg
>MLOC_43
TGCTCTTA
>MLOC_43
atgggaaaga
>MLOC_43
gaatgtgctc
>MLOC_43
tacatggtgtt
>MLOC_43
ATGCAGTG
>MLOC_43
ATGGATCG
>MLOC_43
ACTTGAGA
>MLOC_43
aaaatgatgc
>MLOC_43
AGGTGATG
>MLOC_44
atattgacag
>MLOC_44
gtcgccatctc
>MLOC_44
ctagtccatct
>MLOC_44
gttcgggatgt
>MLOC_44
ctatagttcac
>MLOC_44
tgtttttcagg
>MLOC_44
caggactgcc
>MLOC_44
ggctccggcg
>MLOC_44
gcaccatggg
>MLOC_44
agctggacgt
>MLOC_44
agggaaagg
>MLOC_44
aacaccATC
>MLOC_44
tcatagatga
>MLOC_44

actttcAAGT
>MLOC_44
cgtcgtCACA
>MLOC_44
caggcgaata
>MLOC_44
acgaccatgg
>MLOC_44
aagcagttga
>MLOC_44
agccgacaag
>MLOC_44
GGAGTGGA
>MLOC_44
ATGGACCC
>MLOC_44
GATGAAGT
>MLOC_44
ATGACTAT
>MLOC_45
atgggccgctt
>MLOC_45
atgtcggtcag
>MLOC_45
ATGATGAG
>MLOC_45
gcccactgtct
>MLOC_46
AaaCATTGT
>MLOC_46
agtatttttata
>MLOC_46
tagatgcggg
>MLOC_46
ATGgCcGG
>MLOC_46
ccaTGTTTG
>MLOC_47
AATAgcACt
>MLOC_47
cagagttctca
>MLOC_47
ggttgcactga
>MLOC_48
ACCCcGGg
>MLOC_48
ATGAAGGA
>MLOC_48
gttcctaatgg
>MLOC_48

gccgatctcca
>MLOC_48
gccgccgctct
>MLOC_49
GAAGCTTC
>MLOC_49
gtaggcttggc
>MLOC_49
ggttacgtgga
>MLOC_49
aggaggggct
>MLOC_50
ATGGCTTA
>MLOC_50
ctcgtaaatga
>MLOC_50
GCCATGTC
>MLOC_50
atgctgcgga
>MLOC_50
cataaggttct
>MLOC_50
atgccgacaa
>MLOC_50
atgggcttctt
>MLOC_51
ATGGACAT
>MLOC_51
gatgattctgg
>MLOC_51
gatattgaaa
>MLOC_51
cactcacagca
>MLOC_51
catGCAGG
>MLOC_51
tcttggactac
>MLOC_51
CCGCcGCC
>MLOC_51
ctccagatgga
>MLOC_51
tgtgttgctga
>MLOC_51
atcaaggtat
>MLOC_51
ATGGTGGA
>MLOC_51
gtcaagggag
>MLOC_51

gtatgctcaa
>MLOC_51
CTaCCgCaG
>MLOC_51
agttccccctg
>MLOC_51
GAtATCGT
>MLOC_51
ATGAAAaa
>MLOC_51
tccccacgag
>MLOC_51
cagctaggta
>MLOC_51
ctaCtgtAGA
>MLOC_51
cagaactggc
>MLOC_51
cctccaccatc
>MLOC_51
agagggagg
>MLOC_51
ATGTCGAG
>MLOC_52
atgatgactgt
>MLOC_52
ATGGCaAA
>MLOC_52
TTGAGGCA
>MLOC_52
ATGCCCAtt
>MLOC_52
atggccaccg
>MLOC_52
ATGAGCAG
>MLOC_52
agtccatcgc
>MLOC_52
atgcctcctcg
>MLOC_52
ATAAGCAC
>MLOC_52
agatttggca
>MLOC_52
tgaaggacaa
>MLOC_52
AGGTGAAG
>MLOC_52
CAGtAaCAC
>MLOC_52

TCTgCTGA
>MLOC_52
tctccccgcg
>MLOC_52
gcaatcatga
>MLOC_52
atggagttat
>MLOC_52
acaggcaggg
>MLOC_52
gcaacatcca
>MLOC_52
ATGGTGAC
>MLOC_52
aaccacctct
>MLOC_52
ccgccggctct
>MLOC_52
atgccgatgct
>MLOC_52
gggggtctt
>MLOC_52
aggtttatatc
>MLOC_52
CACCTAAT
>MLOC_52
GGCTATTG
>MLOC_52
atggcggaca
>MLOC_52
GAAGCCTG
>MLOC_52
ATGGCAGC
>MLOC_52
aaagaagga
>MLOC_52
TTTGACAT
>MLOC_52
ggccatggcc
>MLOC_52
CGCTtGcTT
>MLOC_52
ATGACCAG
>MLOC_53
tttgcatggcc
>MLOC_53
cggcgatgac
>MLOC_53
atggccgcca
>MLOC_53

TGCTACTT
>MLOC_53
ATGGGACC
>MLOC_53
aaggccttcgt
>MLOC_53
attgttggtgg
>MLOC_53
cagaagaaca
>MLOC_53
aaaggtcaag
>MLOC_53
ATGGAGAC
>MLOC_53
atgtctaatta
>MLOC_53
ATGAACCC
>MLOC_53
agtgagtttca
>MLOC_53
gttcccaagtt
>MLOC_53
gggcgggtga
>MLOC_53
atggcgatgg
>MLOC_53
ATGcAGAA
>MLOC_53
ggacgccgac
>MLOC_53
ATGCAAGA
>MLOC_53
tctctggcgc
>MLOC_53
atgggacgcc
>MLOC_53
ATGGCGCC
>MLOC_53
ATGTCTCA
>MLOC_53
AGGAAATC
>MLOC_53
catagggtgg
>MLOC_53
ctttgtctctg
>MLOC_53
aaaccaatat
>MLOC_53
agatagtattg
>MLOC_53

ttgtttggcag
>MLOC_53
tgcaggaggg
>MLOC_53
cagactttatt
>MLOC_53
gtcacagctg
>MLOC_53
cagacctgga
>MLOC_53
gcgaatgtca
>MLOC_54
taacttgagca
>MLOC_54
ttgaccctagc
>MLOC_54
ggattgtgtca
>MLOC_54
atggcgccG
>MLOC_54
gtggtggtggt
>MLOC_54
gatgttgatct
>MLOC_54
ATGGCCGC
>MLOC_54
tccgctccagg
>MLOC_54
cagcgcctttt
>MLOC_54
GGGTAATC
>MLOC_54
AGCAAAAT
>MLOC_54
atggcttacgg
>MLOC_54
ATGGATCa
>MLOC_54
agacgtacca
>MLOC_54
gtagaccagg
>MLOC_54
cccggagctct
>MLOC_54
gcgtcctggag
>MLOC_54
agaagctttat
>MLOC_54
ATGGCAGC
>MLOC_54

AGGGAAG
>MLOC_54
catcgccgcc
>MLOC_54
GGcGAGG
>MLOC_54
GggAAGAA
>MLOC_54
acgcctcgcc
>MLOC_54
TATTAAAC
>MLOC_54
CTCGCCTG
>MLOC_54
ttcgaatcagt
>MLOC_54
tatgagacaa
>MLOC_54
ctttctatgtt
>MLOC_54
ggaatgcccc
>MLOC_54
atggggtaca
>MLOC_54
CTGCAGGC
>MLOC_54
ctggacctggt
>MLOC_54
ATGGCGG
>MLOC_54
atccacatcc
>MLOC_54
ATGGAGAC
>MLOC_54
atggctcaaa
>MLOC_54
ttggagaagg
>MLOC_54
TTCTCAGA
>MLOC_54
ATGCTTTG
>MLOC_54
tagatgaaga
>MLOC_54
ggcactgcgc
>MLOC_54
aggcgctcgt
>MLOC_54
tgatgaggac
>MLOC_54

ATGGCAgT
>MLOC_54
acccagggaa
>MLOC_54
cgcagacatg
>MLOC_54
ATGGGTGA
>MLOC_54
aacataagaa
>MLOC_55
ATGGCGCC
>MLOC_55
atgcgtcccct
>MLOC_55
tgcaggacgc
>MLOC_55
attgcagttgt
>MLOC_55
ttcttgggttc
>MLOC_55
taccacttcag
>MLOC_55
gattggcctgg
>MLOC_55
tgcagggcgg
>MLOC_55
agtccattgat
>MLOC_55
acgtacggtt
>MLOC_55
gaagaacggc
>MLOC_55
GAGCTTCA
>MLOC_55
ccaaggggag
>MLOC_55
tgaggagatc
>MLOC_55
gggtttttcaa
>MLOC_55
atggcgccgg
>MLOC_55
ATGACCAC
>MLOC_55
TCAGGAAG
>MLOC_55
atgaagcctt
>MLOC_55
GATGATGC
>MLOC_55

ATGGgAAA
>MLOC_55
gaaatggatg
>MLOC_55
gcttgagaca
>MLOC_55
tcatatatctg
>MLOC_55
aggagaaca
>MLOC_55
atgcgggagg
>MLOC_55
atggacgagc
>MLOC_55
cgaagcagaa
>MLOC_55
ggagcacccc
>MLOC_55
catctcagtcc
>MLOC_55
ATGGCTGC
>MLOC_55
agtccacgcc
>MLOC_55
atggacgcgc
>MLOC_55
gatgacgatg
>MLOC_55
AGCTCGAC
>MLOC_55
cagggacatc
>MLOC_55
attcgcacttg
>MLOC_55
caaatcaatg
>MLOC_55
actctagcac
>MLOC_55
cagggtgccg
>MLOC_56
atggcccgcg
>MLOC_56
tgaaagcgca
>MLOC_56
ggccgataaa
>MLOC_56
atgggttcca
>MLOC_56
ATGGAGGg
>MLOC_56

ctcggcgtcg
>MLOC_56
atgtacttccc
>MLOC_56
gttgatggctg
>MLOC_56
acaggctaaa
>MLOC_56
gatccgccgc
>MLOC_56
gtcagtgCTA
>MLOC_56
ATGGATCG
>MLOC_56
tttgcagctgg
>MLOC_56
cagcgcctga
>MLOC_56
TATTGCAT
>MLOC_56
ggttaaagaa
>MLOC_56
atgggcattc
>MLOC_56
cttgacgccca
>MLOC_56
atgatttacta
>MLOC_56
gccgccggcg
>MLOC_56
TTcAtAGCT
>MLOC_56
atggctgcac
>MLOC_56
ATGGCGCT
>MLOC_56
ATGGATCA
>MLOC_56
cttcaggtcgc
>MLOC_56
ATGGCGCT
>MLOC_56
atggggagcg
>MLOC_56
AGATACCT
>MLOC_56
ATGGAgTt
>MLOC_56
ATGGGGG
>MLOC_56

GCGGAcTT
>MLOC_57
aggcaggaat
>MLOC_57
ggggaggga
>MLOC_57
tgagcaaca
>MLOC_57
agggaatatt
>MLOC_57
ACCCGGCA
>MLOC_57
TGCAGATT
>MLOC_57
tggtttccta
>MLOC_57
GATTTGGA
>MLOC_57
tgcttcaact
>MLOC_57
cgcgcgatg
>MLOC_57
ATCCTTGa
>MLOC_57
tgaagctgg
>MLOC_57
ggtgaaacc
>MLOC_57
ATGGGGA
>MLOC_57
CAAGGGC
>MLOC_57
ggtggccgc
>MLOC_57
acatccaac
>MLOC_57
ATGGCCTT
>MLOC_57
GAACGAC
>MLOC_57
ATGGACC
>MLOC_57
atggagagc
>MLOC_57
AGTTTCcT
>MLOC_57
gacggcatc
>MLOC_57
gatttctaag
>MLOC_57

gcggtggcgg
>MLOC_57
agcttcacggc
>MLOC_57
ATGACGTC
>MLOC_57
gctgcctgtat
>MLOC_57
ATGTCTGA
>MLOC_57
caggtggaga
>MLOC_57
ATGGCGGC
>MLOC_57
caaccatggtt
>MLOC_57
tcccggtatgt
>MLOC_57
atggacagca
>MLOC_57
gtcaaggggg
>MLOC_57
ATTGCAGA
>MLOC_57
ccactacTAA
>MLOC_57
gcagaaatgc
>MLOC_57
catctgttgcc
>MLOC_57
atggcctcgaa
>MLOC_57
cagattaaag
>MLOC_57
tttgtgattcag
>MLOC_57
ATGCGTGA
>MLOC_57
cactttgcagt
>MLOC_57
atgggcatcgt
>MLOC_57
atgcggatccg
>MLOC_58
ATGGCCGG
>MLOC_58
CTTTTGGG
>MLOC_58
tccccgagcag
>MLOC_58

atgacgccgg
>MLOC_58
AGCTTCTG
>MLOC_58
aataaaagtg
>MLOC_58
ggtcgtgaag
>MLOC_58
atcaacctac
>MLOC_58
tagctcgtcaa
>MLOC_58
gctgcccgcg
>MLOC_58
tcaaacagttt
>MLOC_58
ATGGCGTC
>MLOC_58
CAGGTTGG
>MLOC_58
ccatatttcta
>MLOC_58
aggatccggg
>MLOC_58
gctcggcctc
>MLOC_58
gcagatttgca
>MLOC_58
GACTGTAA
>MLOC_58
gccgaggctg
>MLOC_58
acatttgcaa
>MLOC_58
ATGGCTGG
>MLOC_58
AGATTAAT
>MLOC_58
cagatttgccg
>MLOC_58
ATGGCGAG
>MLOC_58
ctttcggatca
>MLOC_58
TTATCTGA
>MLOC_58
ATGATTCA
>MLOC_58
ATGATgcAT
>MLOC_58

tagtgcataca
>MLOC_58
ggtatggttgc
>MLOC_58
agatttgcgag
>MLOC_58
CAGGGAGA
>MLOC_58
atggagaaca
>MLOC_58
gcaggtcgtga
>MLOC_58
ctctcactcctg
>MLOC_58
GTTGATCCg
>MLOC_58
ATGGCTCT
>MLOC_58
caaaatctaca
>MLOC_58
ATGGCCTG
>MLOC_58
atgtcggcaga
>MLOC_58
agcctACTG
>MLOC_58
ccgccgatctc
>MLOC_59
tcgcggcggc
>MLOC_59
GGTCTTTC
>MLOC_59
GTTTTcCAG
>MLOC_59
TGcAGGTA
>MLOC_59
GATTTATCT
>MLOC_59
gttcaggactc
>MLOC_59
atggccgcca
>MLOC_59
ATGGGAGG
>MLOC_59
AGATGTCT
>MLOC_59
atgatcttaca
>MLOC_59
TGCAGcGG
>MLOC_59

cagagaatga
>MLOC_594
AGGCGCAC
>MLOC_594
caagaaggag
>MLOC_594
tcacagacag
>MLOC_594
atgagtgtgca
>MLOC_594
GCCCAcACc
>MLOC_59
ATGGGCGA
>MLOC_59
tctcatttatac
>MLOC_59
ATGGAGCT
>MLOC_59
ATGGAGTG
>MLOC_596
tttgctgttgct
>MLOC_596
ccaacggcctt
>MLOC_596
GGCTTTTG
>MLOC_596
ggtgcttggat
>MLOC_596
atgctacatgg
>MLOC_597
ctgcgcacaag
>MLOC_597
ttaattttattа
>MLOC_597
ctgcaggatct
>MLOC_597
ttcctgatggc
>MLOC_598
GTACCAGA
>MLOC_598
CtAGGACAT
>MLOC_598
ATGATCGG
>MLOC_598
atgtggtgcct
>MLOC_599
ATGCAGCC
>MLOC_599
aaaaggattc
>MLOC_600

atggatatggt
>MLOC_60
ATGGAATC
>MLOC_60
atggctccgg
>MLOC_60
atggttggga
>MLOC_60
AGGCAGAT
>MLOC_60
aatggtctaa
>MLOC_60
cttcattctgt
>MLOC_60
aatccgaccg
>MLOC_60
ATgaCTAA
>MLOC_60
GTTTTGGG
>MLOC_60
atgggggaca
>MLOC_60
agttaagttag
>MLOC_60
cggaagctcg
>MLOC_60
ATGAAACA
>MLOC_60
gataagttga
>MLOC_60
aggagtttca
>MLOC_60
ggctggtagc
>MLOC_60
atggatgggt
>MLOC_60
aagatggcgg
>MLOC_60
taccgccaaa
>MLOC_60
gcttcggcat
>MLOC_60
ttgatagaac
>MLOC_60
atgggcatgg
>MLOC_60
atgggcatgg
>MLOC_60
atggtgggag
>MLOC_60

atgaggtggc
>MLOC_60
cctcccaaaca
>MLOC_60
tcatGATCG
>MLOC_60
atgggcaggc
>MLOC_60
TCATGTGA
>MLOC_60
acaagcagct
>MLOC_60
GCAGACTC
>MLOC_60
ATGGGGAg
>MLOC_60
tgccttgaggc
>MLOC_60
ctctggactac
>MLOC_61
atgatgaggg
>MLOC_61
GCAGAaGA
>MLOC_61
tcctttgccatc
>MLOC_61
ATGGGTTT
>MLOC_61
ATGggAAC
>MLOC_61
atggaggcgg
>MLOC_61
tggagcaggt
>MLOC_61
atggcggcttc
>MLOC_61
CAGGCTAT
>MLOC_61
atgaggttcca
>MLOC_61
CAGAATGC
>MLOC_61
aggtaaagaa
>MLOC_61
gcaggtccag
>MLOC_61
gcacccagcc
>MLOC_61
ATGGCTAG
>MLOC_61

caagtgcgag
>MLOC_61
atggctgccg
>MLOC_61
tgccacagtc
>MLOC_61
ATGGAGGT
>MLOC_61
agaaattcta
>MLOC_61
tggcaggtcg
>MLOC_61
aacccaccgt
>MLOC_61
atgggtcgcg
>MLOC_61
acatcagcta
>MLOC_61
acagagaag
>MLOC_61
ATGGATAG
>MLOC_61
caggtgatcc
>MLOC_61
gcagttgatg
>MLOC_61
acatgcAGG
>MLOC_62
atgaatgtgg
>MLOC_62
ttaaaggacc
>MLOC_62
agaaacTGG
>MLOC_62
atactttctcg
>MLOC_62
tccccgagga
>MLOC_62
ggcagagtca
>MLOC_62
atggagaggc
>MLOC_62
atggcttccac
>MLOC_62
agctgttcctc
>MLOC_62
GTGTCACT
>MLOC_62
TTTGCAGA
>MLOC_62

ctcccctcccc
>MLOC_62
tgcgccttcct
>MLOC_62
gggtcagggc
>MLOC_62
agcagattgc
>MLOC_62
ATGTTCAT
>MLOC_62
aggacgagg
>MLOC_62
gatgtggatt
>MLOC_62
atgacccagg
>MLOC_62
GCAGGCTT
>MLOC_62
aaggatccat
>MLOC_62
tggcgctgca
>MLOC_62
tgatgtcatc
>MLOC_62
GGCGTGG
>MLOC_62
aggatctccct
>MLOC_62
TCAAATGA
>MLOC_62
aggtgatgtg
>MLOC_62
gaaaggcaat
>MLOC_62
gtctgattgCT
>MLOC_62
AGATGAG
>MLOC_62
aggctggggt
>MLOC_62
gatatcttgtt
>MLOC_62
aggtgtttagt
>MLOC_62
TGAATGCA
>MLOC_62
caggagagg
>MLOC_63
tagacaaaat
>MLOC_63

atggatgtgg
>MLOC_63
gtgaatggag
>MLOC_63
AGGTGGG
>MLOC_63
ctaggaaccg
>MLOC_63
gcctcggcct
>MLOC_63
acttatcacc
>MLOC_63
ggagatgtta
>MLOC_63
aggtaccgta
>MLOC_63
gcagaagaca
>MLOC_63
ccttcttggcg
>MLOC_63
cagggcagaa
>MLOC_63
agtgtgctgta
>MLOC_63
agggtgcatt
>MLOC_63
agattgaaga
>MLOC_63
caggctagtt
>MLOC_63
ATGGGGG
>MLOC_63
tcttgcactgg
>MLOC_63
gcggACAA
>MLOC_63
ttcgatggtgc
>MLOC_63
gtcaacatcta
>MLOC_63
cagagaaatg
>MLOC_63
atggctcctgc
>MLOC_63
atggaggaga
>MLOC_63
ttcctattgta
>MLOC_63
ATGAGGG
>MLOC_63

tattataggtg
>MLOC_63
CTGCTCAG
>MLOC_63
GCTGCTcC
>MLOC_63
ctacgcattcg
>MLOC_63
GAGCCTCT
>MLOC_63
gagtgcacgg
>MLOC_63
ttggcatggg
>MLOC_64
tagatggata
>MLOC_64
ATGGCGGC
>MLOC_64
ATGCAGTC
>MLOC_64
agaaatgcca
>MLOC_64
atggcatacc
>MLOC_64
AGCAGATC
>MLOC_64
atgcatcccg
>MLOC_64
gtctcgctcga
>MLOC_64
ATGGCGAT
>MLOC_64
atggaaaacc
>MLOC_64
GTGACTGC
>MLOC_64
atggacctcg
>MLOC_64
atgcccacac
>MLOC_64
gacatgggat
>MLOC_64
ggtaactctat
>MLOC_64
ctaggttgata
>MLOC_64
atgttcggaT
>MLOC_64
TCACAGGG
>MLOC_64

gtacttgaaag
>MLOC_64
cagacgaagg
>MLOC_64
gcagggacag
>MLOC_64
cggcgctgctg
>MLOC_64
AGGGAaTC
>MLOC_64
gggcgacggt
>MLOC_64
gtaatcgatgg
>MLOC_64
ttcttcaagga
>MLOC_64
gactggggag
>MLOC_64
atgctcctcct
>MLOC_64
atgggtaact
>MLOC_64
GGTGCATA
>MLOC_64
GCAGGGA
>MLOC_64
acaagtccgc
>MLOC_64
tgctcttctctg
>MLOC_64
atggattggA
>MLOC_64
atgcctttcttt
>MLOC_64
gacaataaga
>MLOC_65
AGGATATT
>MLOC_65
cgaagcccgc
>MLOC_65
cgccggcatc
>MLOC_65
cccactcggcg
>MLOC_65
ATGCATGA
>MLOC_65
TGATTGTT
>MLOC_65
ATGGACGC
>MLOC_65

gctggctggat
>MLOC_65
ATGCCAGG
>MLOC_65
aggtgtcgttg
>MLOC_65
ggcgacggcg
>MLOC_65
atgattgcga
>MLOC_65
atgcagatgc
>MLOC_65
TTCAATAG
>MLOC_65
atgataagtC
>MLOC_65
tctggagagg
>MLOC_65
GCAGCAAA
>MLOC_65
cgacggcggc
>MLOC_65
AGTCAGAT
>MLOC_65
ttcacagaaa
>MLOC_65
acaatttttaa
>MLOC_65
GTGAAGTT
>MLOC_65
AGTTGATT
>MLOC_65
caatcttctca
>MLOC_65
atggcttccaa
>MLOC_65
ATGGCGAC
>MLOC_65
cctccaacag
>MLOC_65
cagttagctga
>MLOC_65
tgtgagtccag
>MLOC_65
tgcgagggcg
>MLOC_65
cgtgccaccc
>MLOC_65
ggcgcgTCT
>MLOC_65

taggtgaata
>MLOC_65
cagctgcgaa
>MLOC_66
gaactagatg
>MLOC_66
ttggataaag
>MLOC_66
GGAGAaGG
>MLOC_66
ATGGGCGG
>MLOC_66
ATGTCGTC
>MLOC_66
tctctatatag
>MLOC_66
ATGTCTTCT
>MLOC_66
ggagatgaac
>MLOC_66
tccccaGTG
>MLOC_66
acacagagct
>MLOC_66
atggacagtt
>MLOC_66
atgaaggtgc
>MLOC_66
aggtccctcat
>MLOC_66
ggatgacatc
>MLOC_66
CCtCgCCGG
>MLOC_66
atGGAGAA
>MLOC_66
CCATATTTT
>MLOC_66
cagtgtcgag
>MLOC_66
cgcgctcctgc
>MLOC_66
agcgaagaga
>MLOC_66
GCGAGAAG
>MLOC_67
TGAATGCc
>MLOC_67
atgcccccga
>MLOC_67

CAGATAAA
>MLOC_67
cttgtgcctcc
>MLOC_67
gatggttctaa
>MLOC_67
ATGGCCAT
>MLOC_67
ATGAaaCC
>MLOC_67
AGGTTGCA
>MLOC_67
aggtaagaga
>MLOC_67
cggcggctgg
>MLOC_67
atggagccgc
>MLOC_67
GAAACTGA
>MLOC_67
atgtgcatgca
>MLOC_67
cagacatgaa
>MLOC_67
aggtatctaaa
>MLOC_67
gggatgccta
>MLOC_67
tcgATGCCG
>MLOC_67
GTTGCCAC
>MLOC_67
ttttgcaaaG
>MLOC_67
AGATGACA
>MLOC_67
ggaggcgcag
>MLOC_67
CCATGCGC
>MLOC_67
acaatttctcg
>MLOC_68
tgatgtgctcc
>MLOC_68
gtatggggat
>MLOC_68
agctgcggaa
>MLOC_68
ATGACCGG
>MLOC_68

atggagaaac
>MLOC_68
atgttacgaag
>MLOC_68
ttcggactgac
>MLOC_68
TtGAAGGA
>MLOC_68
gggacggcca
>MLOC_68
caggaattact
>MLOC_68
gcctacatcta
>MLOC_68
atggaggcgg
>MLOC_68
GCaGGAAC
>MLOC_68
gtcaacaacg
>MLOC_68
ctaacccgcg
>MLOC_68
ATGCCACT
>MLOC_68
atggaggcca
>MLOC_68
ATGGCCGG
>MLOC_68
gcctttgagaa
>MLOC_68
acccccaaatt
>MLOC_68
CcGCACGC
>MLOC_68
taggtctccta
>MLOC_68
acagcggcgc
>MLOC_68
tggagttgaag
>MLOC_68
cccacccgcc
>MLOC_69
atggacgccg
>MLOC_69
atcgctgagcg
>MLOC_69
cctaaagctcg
>MLOC_69
agagagccaa
>MLOC_69

ttgttgccact
>MLOC_69
aggctcagat
>MLOC_69
GTAGATGT
>MLOC_69
agaagcgcga
>MLOC_69
atcctctacgt
>MLOC_69
TCAAATCT
>MLOC_69
cccggccacc
>MLOC_69
gtagagtattg
>MLOC_69
agatgggtgc
>MLOC_69
tgtattttgtt
>MLOC_69
ttgccttatta
>MLOC_69
tcactcgatct
>MLOC_69
atggctagac
>MLOC_69
tcattctacct
>MLOC_69
GTGCCATG
>MLOC_69
ggaaTGacT
>MLOC_69
cagggagaca
>MLOC_69
CCTCCTCtG
>MLOC_69
gctggaggtc
>MLOC_69
attcaaggtgt
>MLOC_69
ATGGCGG
>MLOC_69
atgtccgcgtc
>MLOC_69
atggatgatct
>MLOC_70
atgaggaggc
>MLOC_70
atggctctcaa
>MLOC_70

tttagtcaagg
>MLOC_70
agtgcttactt
>MLOC_70
tgactggataa
>MLOC_70
ATGCTGAG
>MLOC_70
gtggctgtttg
>MLOC_70
ctggaaaaaa
>MLOC_70
CTAGATAA
>MLOC_70
ATGGGAGA
>MLOC_70
tgcaacattgt
>MLOC_70
ccaagcgagc
>MLOC_70
gatatggcac
>MLOC_70
ttgcggcgcat
>MLOC_70
TTgCAGGA
>MLOC_70
tgaggctggc
>MLOC_70
CAGGAaaG
>MLOC_70
CGtCTCAA
>MLOC_70
CAGCtCCT
>MLOC_70
atGGAtGTT
>MLOC_70
caggagccac
>MLOC_70
ctccgatgac
>MLOC_71
atgtcaagtg
>MLOC_71
CCtGCAGT
>MLOC_71
ctggaggatg
>MLOC_71
cagcttccac
>MLOC_71
tcagtgttgca
>MLOC_71

ATcAATTG
>MLOC_71
ATGGATGA
>MLOC_71
CAAaTAAA
>MLOC_71
ATGAATCA
>MLOC_71
AAaaTTCC
>MLOC_71
ATGGGTTC
>MLOC_71
atgccggcctt
>MLOC_71
aattaggcaa
>MLOC_71
atggagtggt
>MLOC_71
atgccaaatg
>MLOC_71
atggaaacat
>MLOC_71
atggcggcgg
>MLOC_71
atgaccggag
>MLOC_71
gccagacatg
>MLOC_71
tctggattcat
>MLOC_71
AATAACTC
>MLOC_72
cgggctccttc
>MLOC_72
gccttctcgtg
>MLOC_72
GTAGAGAA
>MLOC_72
AGGCaTCC
>MLOC_72
ttttcaacgag
>MLOC_72
GGTTGTtC
>MLOC_72
cggtccttcgc
>MLOC_72
gcaagaatca
>MLOC_72
atggagcata
>MLOC_72

CAGGTTGT
>MLOC_72
ctcttgtaca
>MLOC_72
cggaaaggtt
>MLOC_72
tacctttggtc
>MLOC_72
ccttcccgctc
>MLOC_72
ATGGGTGC
>MLOC_72
TATCAcaTT
>MLOC_72
ATGGCGTC
>MLOC_72
gacccgcacg
>MLOC_72
atggcggaga
>MLOC_72
gcgccggagc
>MLOC_72
cgttggagcc
>MLOC_73
catAAAAaa
>MLOC_73
atggagctgt
>MLOC_73
ttgctgctcat
>MLOC_73
ggttcttaatg
>MLOC_73
tatcctccgcg
>MLOC_73
gcccgcgcag
>MLOC_73
atgtccgggtt
>MLOC_73
atggagcagg
>MLOC_73
gtatatgaca
>MLOC_73
CCaGACAT
>MLOC_73
cagagtcaca
>MLOC_73
GTAAAATA
>MLOC_73
aacaggatca
>MLOC_73

gctgccatcgg
>MLOC_73
tggccccgga
>MLOC_73
agtttcaagtt
>MLOC_73
atgctgttgctg
>MLOC_73
gtccgcaccg
>MLOC_73
gtatggtgatc
>MLOC_73
cttttgtagctg
>MLOC_74
caggccaatcg
>MLOC_74
aagttctgctc
>MLOC_74
ATGGCGAC
>MLOC_74
agcgtttgcac
>MLOC_74
tacacatcaa
>MLOC_74
atggtgagcg
>MLOC_74
tggaccgtgct
>MLOC_74
ATGGCCTT
>MLOC_74
TGCAGATA
>MLOC_74
gactttttgcta
>MLOC_74
AGACaCCA
>MLOC_74
ATGGCCAC
>MLOC_74
cagaccccga
>MLOC_74
atgcaggtAG
>MLOC_74
ATGGATCT
>MLOC_74
tgcttattcata
>MLOC_74
ggtcgtagtag
>MLOC_74
tttgtgcagaa
>MLOC_74

gtttgccaggt
>MLOC_74
atggctacaa
>MLOC_74
agatagtgca
>MLOC_74
ATGTCaTT
>MLOC_74
agggccTGA
>MLOC_74
aaatacagaa
>MLOC_74
atgctcttcgc
>MLOC_74
aacttgggat
>MLOC_75
taagtggtcat
>MLOC_75
ATGGCGCT
>MLOC_75
gcgagcaaga
>MLOC_75
caaggggcgg
>MLOC_75
ggctccctgcg
>MLOC_75
ATGGGttG
>MLOC_75
tccaagggtg
>MLOC_75
atgctccgcg
>MLOC_75
gatgcatgtag
>MLOC_75
gagagtctcG
>MLOC_75
atgtcgcacct
>MLOC_75
ATGGCACC
>MLOC_75
ATGGATAT
>MLOC_75
AGATTGAC
>MLOC_75
cttgtcaggag
>MLOC_75
CAAGCAAG
>MLOC_75
gttgctggtttt
>MLOC_75

tttctaggccg
>MLOC_75
gagggtaaat
>MLOC_76
atggaaacat
>MLOC_76
aggaaacccc
>MLOC_76
aacatcgtcg
>MLOC_76
cagattcaag
>MLOC_76
gatctcatga
>MLOC_76
ATGGAGG
>MLOC_76
aatagactct
>MLOC_76
tccaaggcttc
>MLOC_76
ctctacctccc
>MLOC_76
agaaatctac
>MLOC_76
ggagatcacg
>MLOC_76
cattgggTTC
>MLOC_77
catcagaaaa
>MLOC_77
ATGGCGAA
>MLOC_77
AGGTTGAT
>MLOC_77
gttgacagac
>MLOC_77
aatgttcaaa
>MLOC_77
ATGGGGA
>MLOC_77
ATGAGGGT
>MLOC_77
tgcaggcaaa
>MLOC_77
aggtacctga
>MLOC_77
gtccccgtctt
>MLOC_77
atggcggaga
>MLOC_77

agtccatcca
>MLOC_77
cagtttgatcc
>MLOC_78
ctggccgccc
>MLOC_78
gtgccttgag
>MLOC_78
caatcgaaca
>MLOC_78
gctgcagttt
>MLOC_78
ttcaggtgcca
>MLOC_78
gaaagagatg
>MLOC_78
tcagggaaga
>MLOC_79
ACaGAATT
>MLOC_79
ATGGTGAA
>MLOC_79
agctgctcgtg
>MLOC_79
ggagggggA
>MLOC_79
caggtacttca
>MLOC_80
cccaagagcg
>MLOC_80
aacaaggacc
>MLOC_80
cggccatcgg
>MLOC_80
TAtggCAGA
>MLOC_80
GCgGATTT
>MLOC_80
aaaaggatga
>MLOC_80
ccttgtcactt
>MLOC_81
caggaagcta
>MLOC_81
gagtgcggga
>MLOC_81
atgagcttcct
>MLOC_81
atggaacagg
>MLOC_81

atgcactgcca
>MLOC_81
ggggtagctga
>MLOC_81
catcgataTC
>MLOC_81
aggattagaa
>MLOC_81
ctgcaaaaca
>MLOC_81
caggggattct
>MLOC_81
ggggaagagg
>MLOC_81
AGGAGGT
>MLOC_81
atggccttcgc
>MLOC_81
TAtTATATt
>MLOC_81
CATTTTTAT
>MLOC_82
gatcgtttgct
>AK375316
ATGGATTG
>AK252846
ACATCGAA
>AK361872
atggccatctt
>AK354390
ccctccccgac
>AK355206
tggcaggtttt
>AK375460
ATGGTTCC
>MLOC_39
aacagttagct
>AK361568
caGCaGcTC
>AK365828
atgaaaggaa
>AK357819
gcatcagCA
>AK358200
atgcgctgctc
>AK354917
aatggagcaa
>AK249956
ATGCTCAa
>AK354860

gagagcctga
>AK251637
accacgccgc
>AK359113
aggtgtcttta
>MLOC_15
ATgCTGGG
>AK366062
TTaCACTGA
>MLOC_66
tgtctaggtac
>MLOC_62
tatcttcaaga
>AK373109
agtcgggagg
>AK368229
gactcgcctcc
>MLOC_74
ATGGCTTC
>AK375893
aggttgtatgt
>MLOC_64
ccggccgcgg
>AK371801
tgtctatgcat
>AK248573
gcgccgcggc
>AK363695
cttgccccccт
>AK366806
gagggcgcca
>AK375629
cctttactggg
>AK248596
ctgctcgcctt
>AK369673
aggagtacttt
>AK365368
gacctcagatt
>AK369768
aaccaggaga
>AK361988
tgctgcttcttg
>AK359587
ATGGCATC
>AK251051
cgatgagaag
>AK370654
ATGGCAAa
>AK354830

ATGGGCaC
>AK359885
cgccagctcga
>AK361251
ctcaagaacg
>AK370620
ccagcgccga
>AK358014
ggttccgaac
>AK376057
tttaaaagtaa
>AK251848
caggtggcga
>MLOC_10
ATGGCAAC
>AK370509
atggaatcca
>AK365734
ggcacgatgg
>AK367957
agtcgctatg
>AK358358
ATGGGggT
>AK357711
cggcggcggc
>AK356433
AGGATTTG
>AK357267
tcgcgggaac
>AK360179
AGTCCTGG
>AK375542
ggcaagtact
>AK248434
ATGGCGCG
>AK250553
cttaggaagc
>AK367106
ttcgcgggcc
>MLOC_59
TCaTCCaG
>MLOC_67
atgagcaaga
>AK364882
acaatgagca
>AK367623
caggtttccga
>AK372481
atgtgcggcg
>AK371754

aggacgagtc
>AK375510
gcagcagccg
>AK365855
atgaagtttgg
>AK354110
GGATGACT
>MLOC_62
ccaagcacca
>AK252578
atggcgacga
>AK377122
tccgcctccaa
>AK373633
CCTCTTATA
>AK372882
cagacaccac
>AK354651
aggtacaaag
>AK248246
AGGTCAAC
>AK371586
TCAGGTGT
>AK374885
gacggcagcg
>AK353868
aggagctctg
>AK370183
AGGTGCAT
>AK372516
tgactatagtt
>AK354347
ATGGCCCG
>AK365364
ggtgcgagttg
>AK252546
cgtcgagccc
>AK373795
gggcggctgg
>AK362848
cggggaggag
>AK364313
GACCCATA
>AK360305
GAGTGGG
>AK375855
GGAGGAG
>MLOC_65
aggtgagtgg
>AK357949

atggcccccgc
>MLOC_61
atgccgccgca
>AK251443
cacggaggag
>AK369747
AGATCGAT
>AK372381
ggaggacccc
>AK359220
atggacaaCt
>AK355874
caggagacga
>AK248880
attacaactgt
>AK371533
ggaagaaatg
>AK376689
tggccctacgg
>AK250598
atgttcggccg
>AK367046
cgggctgctcc
>AK355948
gcTgCAGCA
>AK252801
gcaacatgag
>AK371454
GTATACATA
>AK369426
GTGATGGT
>AK371672
ggccaacctcT
>AK252642
tgCATCAGC
>AK368749
tgttgtacaag
>AK365783
ggcggagacc
>AK364675
tgaggacttcc
>MLOC_34
ttgctaacatg
>AK371791
gggctgccatc
>AK362344
atggtgctgcg
>AK357977
GGTTTCAAT
>AK360950

acttgttatct
>MLOC_15
ATGCGgCG
>AK368346
atgaaggata
>AK251570
agatgacagt
>AK249099
gcttgcagca
>MLOC_59
gtgcAGGAA
>AK355004
ATGGCGAT
>AK370601
CAGAACAG
>MLOC_30
AACTACGT
>AK248678
CAGGAATA
>MLOC_54
atgtcgtccg
>AK252532
tctacaggttg
>MLOC_17
GCAGATGT
>AK368497
gtgcgtagttc
>AK360042
agattgtctca
>AK370751
ataattaatgg
>AK374936
gcagacagtc
>AK252400
TGGGAGG
>AK358569
atgacggaca
>AK355790
aggtggtgttc
>AK374386
ATGGAGG
>MLOC_68
agctgctgat
>AK373499
gccgggagag
>MLOC_52
ATGGCGG
>AK369963
gcgcgacgtg
>AK365960

GGTGTGCC
>AK357861
AGGAAAAT
>AK368335
GGGTGTGC
>AK368910
acgcgttggtg
>AK364131
AGGCGGTC
>AK248864
ctcaagtcagt
>AK360494
tatgctaggtg
>AK355525
tcggcgccaa
>MLOC_70
CAGcATTG
>AK366952
CAGGTATT
>AK372747
atggggaaga
>AK252937
caagctccag
>AK355680
aggtacataa
>AK373955
GGTTTTCG
>MLOC_36
gtctgggatgt
>AK370995
tgtacaagaa
>AK376249
gcagattctgg
>AK251535
atGTATTGT
>AK374742
GAtATATCT
>AK375493
cagggctaca
>AK355534
AGGTGCAG
>MLOC_19
tcgcggacag
>AK360859
gctgctttggg
>AK373775
GGTGCAGg
>AK369809
atggcgcgaa
>AK353827

ATGGGCAA
>AK364743
GCAGtTGA
>AK372643
GCGttCAA
>AK373279
gtaatggggg
>AK362522
cacagctgttg
>MLOC_56
gggctagtga
>AK360154
cgtcagcctca
>AK377103
atggaggcca
>AK359447
cctgcagaga
>AK249476
CTGcAGTG
>AK374624
AGTTCTAC
>AK363158
attcaaatggt
>AK366262
GGCGGcgt
>AK248140
AGGTAGCA
>AK362557
ATGGGCCA
>AK365226
gtttgatgatg
>AK373528
atgatggcca
>AK373937
ATGTCTTC
>AK252445
atggcgctctc
>AK371929
ctggggcggc
>AK249972
CTAAcGAA
>AK363680
tcatgcagcta
>AK360635
ATGGCGCT
>AK366216
gctgctggcg
>AK373945
ATGTATCT
>AK363200

gccgagttgtt
>MLOC_60
GTTCCTCG
>AK362627
atggatgggc
>AK369445
CGGCCGCG
>AK369869
ggaagcgggt
>AK249699
ATGAATTC
>AK367504
CTCACCCG
>AK248976
atgaacagca
>AK368548
gctcaagaag
>AK373703
tggaggtgca
>AK358428
agatctttgac
>AK371972
taccctccaac
>AK377038
ATGGCGGC
>AK361159
ggcacgatga
>AK370417
atgatggtgtc
>AK376759
ggaaggtgta
>AK369723
ttcaggTTTG
>AK374298
agtggcgagg
>AK376623
ggggctacaa
>AK374328
TTACTGGA
>AK372875
agaagaagaa
>AK373095
atagaccaag
>AK370542
AGGAAGCA
>AK360105
ctggcggtgcg
>AK374953
ATGGCGTC
>AK354786

CCACGGCC
>AK363509
aggcacatat
>AK249109
ccattgagtca
>MLOC_53
gctaattgtga
>AK363437
agggtgaaga
>AK367778
ccgccaccac
>AK364029
gtcctatcaat
>AK373346
ccagcagcag
>AK355785
agaaagagat
>AK252929
AGGTTGAA
>AK367303
tgtgcaggtta
>AK249378
atgaggaaga
>MLOC_58
tttctatcacta
>MLOC_76
tttattaaggt
>AK364560
ggtgacgtgc
>AK366468
ctttgccgtga
>AK359906
atggaggacg
>AK250022
ATGGCGAC
>AK356321
agagcaagcc
>AK369637
atgaatcctta
>AK356799
gagcccggtc
>AK252312
ATGAaGCT
>AK251444
gaggccatcg
>AK361961
ATGCCCGC
>AK250552
ctaacagcga
>AK361404

cctcagtggag
>AK376614
CAGTtCATT
>AK250731
tgacagatgct
>AK373293
gccaaacagg
>MLOC_63
cgcgcgccgc
>AK375935
AGTGGGTC
>AK366111
acgccgacct
>AK362468
GCaGGTTG
>AK248724
agcactttcac
>AK377052
ATGATGGG
>MLOC_57
atgtcgtcctg
>AK362794
aacattgggg
>AK372954
agtgcagtac
>AK370689
gaatcattttg
>AK252367
AGCTAGGC
>AK369762
aagtttactgt
>AK368217
gctgctgttcg
>AK373246
aggtcagctg
>AK249382
atgataattga
>AK356191
AGGCTTtG
>AK375087
atgtacgccat
>AK361560
tcagaacccc
>AK371998
gacgactcct
>AK358485
atggaaggta
>AK365210
GACATGGA
>AK373567

gtgcccgattg
>AK375183
tcccccgccgc
>AK367874
tccaaggactg
>MLOC_54
caggaactatg
>AK360603
GGCCttCTG
>AK250045
cccatggattg
>AK250992
aaaaTaTtCa
>AK249691
GGtCTTaCA
>AK372604
GGaTCTTC
>AK374103
ATGGATtG
>AK353905
CGCAGGCA
>AK359713
AGGTCAAA
>AK362230
AGGAAGCA
>AK363414
tagatgagatt
>AK369503
ctcaaaactgc
>AK368023
ATGGGCTC
>AK250178
acaccgggatt
>AK355134
taatGGTgA
>AK249324
atggggtgctc
>AK251043
CGtTCCGCa
>AK362277
caggggagctg
>AK358224
taaaaaTaCA
>AK373149
ATGGAGgg
>AK353724
cgacgacgac
>AK361070
ATGGTCAG
>AK365206

ataggttcttg
>AK365747
attgaaaTGC
>AK360806
TGtAGGTG
>AK366127
tttgagcaac
>AK367129
aacaggttag
>AK354076
catgttgtttt
>AK362376
gagaatgacc
>MLOC_74
ATGGGTaC
>AK354583
tatggcaggtt
>AK358997
tgtagggata
>AK366097
CCAGATTG
>AK359188
gttcatcaag
>AK361972
atgatggcgt
>AK371412
cagcaacaac
>AK370249
ATGGACCG
>MLOC_15
tatggtggttg
>AK372711
atacAAGCG
>AK368463
gagggcgagc
>AK365524
ttccagatgac
>AK250638
GCTGCCGC
>MLOC_81
tctctctggtc
>AK354857
gttaatggatg
>AK252044
atgagctccg
>MLOC_66
gaccccttcg
>AK356364
tggcaattccc
>AK367148

atctccattgc
>AK361816
tcgagcagaa
>AK371781
atggtggtgcg
>AK359410
atctccaacc
>AK358246
gccacattgC
>AK252538
atggccacgg
>MLOC_52
atggccgccg
>AK374259
atcctcaagtc
>AK250343
ATTTcTCGC
>MLOC_54
gtcaggaacc
>AK360968
cgggctgggg
>AK252025
TTCACTTCT
>AK370634
gttttCAtGT
>MLOC_45
caggaactgg
>MLOC_71
ggcatgggtG
>AK364926
gcaggtgtgg
>AK252960
aggattcgaa
>AK366562
ATGGGAAG
>AK251055
gccaagttcga
>AK370611
ATGGGGAG
>AK374713
TGATtTTAT
>AK371991
tccgggcaatg
>MLOC_78
accatGATG
>MLOC_65
atggcggcttc
>AK353909
tgccttctgga
>AK370298

gtctcgctgcc
>AK357778
caagtcatgca
>AK249523
TCAGGCTCT
>AK364984
AGTCTGCAT
>MLOC_13
AGTATGGT
>AK362143
GGCTGATG
>MLOC_72
acatctccccc
>MLOC_14
cgtagtccccc
>AK365251
ATGAGGTC
>AK375490
aggttgaatgg
>AK370152
tgganaTTTG
>AK377032
ccgcgcgcctc
>AK373820
acattgttcac
>AK366848
ccagcaactgg
>AK364318
AGGTGAGC
>AK373755
gtgtttttcaga
>AK362688
gagttctacta
>MLOC_60
ATGGCCGG
>AK251992
CGtGCCGAC
>AK367480
gtggcactggc
>AK372460
gcagagttggc
>AK373030
atggtcgaccg
>AK354681
aatCGTTGG
>AK372303
TTGTCAATT
>AK374134
atgaggctctc
>AK354787

caggttagcc
>AK249926
atgggaatgg
>MLOC_43
ttttCAGGA
>AK248344
tctgatggcct
>AK353826
ggccgtagcg
>AK370672
ATGTTGCC
>AK359497
aaattcctcct
>MLOC_57
gcgacgacca
>AK375616
gtaatctcctt
>AK250388
gtcgccagtg
>AK373739
ccaagactca
>AK355248
ATGcGGCA
>AK356239
aagtccaagg
>AK251930
cganacgatg
>AK368629
atggcggcg
>AK370330
ATGGCACC
>AK249847
GGTTGGGT
>AK372211
CCTACGAG
>AK248429
tgccgtcgac
>AK365854
atggcaaagg
>AK372273
cactgTTCA
>MLOC_18
TATAATAG
>AK372591
ATGGAGCA
>AK363456
tttagtttgcc
>AK354845
agaatggtga
>AK373768

ccgcgtcctcc
>MLOC_11
GGTAGAGG
>AK373661
atgaagggcg
>AK359692
ggagggaaag
>AK248775
ccaagggagg
>AK353650
tcctttcagca
>AK249402
tcaaataagtt
>MLOC_61
catctccagac
>AK366261
agATGAGC
>AK360753
ACACCAGT
>MLOC_70
ATGGCGCC
>AK365033
atgtccgggct
>AK358941
GTATAAGT
>AK370645
ctgaagaagg
>MLOC_12
ATGCcGCC
>AK354358
acagcagagt
>AK364034
ggtacaatgg
>AK248453
tctcagcatgc
>AK369248
GGCGTAcG
>MLOC_53
ggagatcgac
>AK356612
gcagagtaga
>AK368525
ttcttctcatta
>MLOC_11
gagaagttctt
>AK251945
aggtgcactct
>AK251471
ggagtttacta
>AK356672

TGTGCAGC
>AK368403
gatcacatcg
>AK355570
AATCAAGT
>AK371709
cttgcagggg
>AK252569
atgtcggtgc
>AK371721
CAGGAAGA
>AK252610
CTCTTTCAG
>AK248785
ATGGAGAa
>AK363081
gacatgtcgg
>AK368868
gaatgggttgt
>AK362311
gctatgtatgg
>AK353969
atggggccat
>AK369808
attccaacatc
>AK249769
GCTTGTTG
>AK371603
gtgtagcgttt
>AK365736
AGaGAAAa
>AK250340
ggtgcagaaa
>MLOC_10
atggccgccg
>AK252911
atggtggagg
>MLOC_29
ggaaaaaatg
>AK252411
aggagggcag
>MLOC_65
ccaaatctctc
>AK368609
AGGCAACA
>AK372289
aggtgagggc
>AK356347
ctccatgcgct
>AK369373

ATGGCCGG
>AK370908
gtgggcgccg
>AK374423
ATGGATCC
>AK361476
agatctcttcc
>MLOC_20
gaactcccccc
>AK370665
CAGGTcAA
>MLOC_44
agctatgagg
>AK370127
ggcgcaccgg
>AK367927
cagatcatcgt
>AK372025
ggagtcctgta
>AK251358
TAgTttATT
>AK373558
TGcAGCAA
>AK357469
atcagtcctac
>AK363819
CAGGAAAT
>AK361882
ggttcaagttg
>MLOC_55
agcatcacca
>AK361316
ACGACTTA
>AK355627
ctagtatctgt
>AK252293
aggttgcatct
>AK363464
GgTTATGC
>AK371182
aagatggcac
>AK365230
atgctcccctt
>AK369309
caggtgtatg
>AK370998
atgatggagg
>AK248279
ATGTTACC
>AK373596

GTGGCCAG
>AK248308
atggaggtga
>AK362409
aggaggagct
>AK366557
aggaggtcat
>AK357647
cgtgtccgcca
>AK363693
atggcaggac
>AK250529
ATGGACTG
>AK365785
ccagccaggc
>AK370682
ttcctcctcaa
>AK252561
gaacaaaggc
>AK366149
atgctgaaga
>MLOC_57
gtggaatcga
>AK374248
ggtgagggtt
>AK361745
aaccctggtca
>AK363387
aaggataaag
>AK370619
GGATACTT
>AK360530
caggtgaatc
>AK371555
gccaacatct
>AK358827
cccgcagggc
>AK366137
aagcacacaa
>AK359073
agaccggcgg
>MLOC_65
AGGAAGG
>AK370873
atgacgcagg
>AK248684
AGGATCCT
>MLOC_10
GGTTCAAG
>AK358219

tttcatctcta
>AK363507
atgcaggaaa
>AK365240
aaccagtctg
>AK377004
ctctccaagct
>AK364622
ctggtgtgga
>AK362988
tgagcagctg
>MLOC_64
GTcACGGG
>AK248980
tatgttgtagg
>AK372511
ggaggacgac
>AK368276
tagccgcacc
>AK369117
cagtcttggca
>AK366549
atgggggccc
>AK250029
ATGAAGGT
>AK370108
GGTTCTTG
>AK375335
cctcttcctgc
>AK251756
atgaattcca
>AK362384
ATGATAaa
>AK363884
gcggcggcga
>AK365190
gaaagctccc
>AK367104
gtgtttggca
>AK355185
AGGAGGG
>MLOC_16
gagaaagtga
>AK365802
atgtaggaga
>AK369779
aggtctattca
>AK358982
atggccagct
>AK249200

AGGTGACa
>AK249626
atgtttacttca
>AK248477
gaaatttttat
>AK363790
GATTCaAA
>AK371706
ggaactcaag
>AK353972
ATGTCTCT
>AK354753
ttctcagcaga
>AK355795
CTGAAGGC
>AK375835
aggagcgttt
>AK366742
ATGGAGCC
>AK356305
ctcttgcggtg
>AK374520
atggcggccg
>AK373752
aagggatatg
>AK252749
tccagagtgtt
>AK376822
atgtctgtgcg
>AK361911
tctccggtggc
>AK364772
AGGGATGA
>AK358630
CAGGCCAA
>AK367195
ACAGGAGC
>AK362698
gatcggcacc
>AK358556
ccatccccGG
>AK363359
AGGATTAC
>MLOC_36
cgttgatgcgt
>AK367438
ATGGAGGC
>AK375706
aggatgacat
>AK360311

ttcaggctgca
>AK364520
GTTTCCGAT
>AK354434
AGGACTCC
>AK358964
ATGGCCtCC
>AK357293
atggcgtcga
>AK249250
ATGGCGGG
>MLOC_48
CTTTGtCAG
>AK353660
ggaccttcagt
>AK248532
gttggacaga
>AK251236
gcatagtgga
>AK373812
atgcgtgatga
>MLOC_10
atgTTTACAT
>AK365985
acAGAAATT
>AK249984
gatgaaggcta
>AK367116
ATGCCTCCC
>AK364175
ggtgactggca
>AK371091
cctgtcctgaa
>AK357244
ccgctcctccc
>MLOC_51
ATGGCaAAT
>AK362285
tcagcggatcc
>AK368296
aggtgtatgca
>AK367972
tgcagagcca
>AK251832
AATGCTGG
>AK374504
AGATTCTG
>AK365078
aggcagaaat
>AK364665

taatttaggaa
>AK370272
gcggtggcgg
>AK364297
cctgtgtattt
>AK371401
caaGGCTCT
>AK377105
caaaggggt
>AK364570
aaagcttcta
>AK364505
tcctggcgtct
>AK375754
gccggCCttg
>AK356315
ggcgaggccg
>AK359028
gtccggtggc
>AK355834
gttgntcccc
>MLOC_60
gttttactgtc
>AK252670
agttcccagg
>MLOC_75
atggggttact
>AK353866
agactgtccta
>AK354532
tgcagcattg
>AK374311
actgatgcaa
>AK358163
atgcagattgt
>AK375623
agcagtacga
>AK356300
ATGTACTA
>AK371492
ATGGCGGC
>AK370019
ATGcggAA
>AK250156
AGGCAATG
>AK373215
AGGTTGCA
>AK358153
GCCAGCAC
>AK373258

TCCTGGTC
>AK367025
ACAGGTCC
>AK374671
AGAAAGAC
>MLOC_67
ATGGtCAT
>MLOC_14
caagaggacc
>AK357591
ccgccctccg
>AK249459
CAGATTcA
>AK366132
caacctccta
>AK355342
gttggagatg
>AK373392
ctgcaggaca
>AK373675
attggatagtt
>AK354972
GGCCTcGA
>AK368828
TCAAGGGA
>AK363711
ATGGACCC
>AK365058
ctctgcgagg
>AK367324
ataggaggcg
>AK376142
ATGGATGA
>AK368262
gaagagacac
>AK374818
TCAGCTCa
>AK248364
gcccgcaccc
>AK359869
cgtgggaggg
>AK372775
agtactatgca
>AK373093
ggctgcggcg
>AK249011
tgggaggatg
>MLOC_55
actacgccttc
>AK358846

cgtcatcgag[illegible]
>AK376040
AAAaCcAA[illegible]
>MLOC_64[illegible]
gcagcgaaag
>AK358106
TTTcTAACT
>AK369833
TGTACAGT[illegible]
>AK249161
gctttgtgcag
>MLOC_14[illegible]
ATGGCACT[illegible]
>AK371866
tacctgacag[illegible]
>AK360995
tcggcgaggg[illegible]
>AK354633
agCTTGTTT
>AK361278
gggctgtgtcc
>AK365926
ttttgttgggc[illegible]
>AK251691
cctgccgcgg[illegible]
>AK251905
ATaCACAG[illegible]
>AK364936
atgagccggg[illegible]
>AK364816
gcttgcaattg
>AK374173
ATGCCGAA
>AK376547
ggacgagggg
>AK368907
ATGGCCGA
>AK364444
ATGGCTGA
>AK371325
ctgaaattggt
>AK375886
gctgctgcccc
>AK354521
atggatagaa[illegible]
>AK368962
caagtgggat[illegible]
>AK368885
atgaacaaag
>AK355408

cttcctcctccg
>AK370794
agtattgggat
>AK374115
gtcgtacgtgg
>AK361767
cccgctcaggc
>AK362381
AGACTGCA
>AK368680
ccgcaggatct
>AK359280
GGCGCGCA
>AK369711
tgaagaggAG
>AK376438
gatattgacca
>AK355001
atggcgtcccc
>AK358124
TCgAAcAGA
>AK360808
atgacggagg
>AK369305
agattttgcca
>AK363868
CAGGCTGC
>AK375511
ggggacggag
>AK366345
caggatcttat
>AK355984
ctgaaggtctt
>AK357034
gggggctctgc
>MLOC_69
AGGACAAG
>AK363800
cagggatatta
>AK368065
ggacggaccc
>AK249522
atgggctcctt
>MLOC_63
ctgtggcccaa
>AK363908
AGGACAAC
>AK370761
AGAGCTGG
>AK353915

atgtcgcaaat
>AK369923
atggAGGTC
>AK355417
ATGCTTGTt
>AK363211
gtctgctgcac
>AK374709
ggtgaaagttc
>AK364215
ccacacaggt
>MLOC_57
cattgtttggg
>AK369180
ATGGCTTC
>AK356427
atggctgtccg
>AK362473
tcggactGA
>AK374032
tgctctccact
>MLOC_73
atggagagct
>AK371711
ATGGCCTT
>AK359773
gttgatggatt
>AK367079
GTTGTGGA
>AK354495
GAGATCAG
>AK252415
ggtttcgcgaa
>AK370517
aggtgaaaga
>AK364599
atggacttcgc
>AK250586
caggggcttc
>AK251773
atgggggcgc
>AK366232
atgggttatgg
>AK355646
caggatgcaa
>AK366489
ATGGCGAC
>AK355291
GGCGTGCA
>AK361994

ATGGCCTC
>AK363897
atggtcacag
>AK370749
AGCTCCGC
>AK353886
CGTtCgCG
>AK376220
atgcggAGC
>AK357513
aaagtgccgc
>AK365509
ATGGGcAA
>AK377101
accaatggat
>AK251104
GGTTAGtG
>MLOC_21
gtggtggcgg
>AK364487
TAcAGAGA
>AK376201
atgtcgccgta
>AK361970
atgtccaggg
>AK355067
CAGGATAT
>AK367105
gcccagcagc
>AK374778
gctacagaga
>AK357471
tacatcctcta
>AK356991
agaaaatccg
>AK357261
atggatcacc
>AK375138
TCcAGGAC
>MLOC_81
actggggagt
>AK356819
ATGCGGGg
>MLOC_52
ATGGGGAg
>AK376014
AGGACGAT
>MLOC_53
gcccgtccac
>AK370312

accatctgatt
>AK358368
CCAGATCT
>AK368079
aaggaggagg
>AK369392
AGATTAGC
>AK374530
gcgccgccttc
>AK354479
atggaagagg
>AK376394
CAGATGAt(
>AK252722
cagatgggtga
>AK365959
AaGAGATtc
>MLOC_14
ttgaccgtgat
>AK251984
aaggcaacag
>AK354470
aggttaaaga
>AK251150
ATGGCTAT
>AK365436
agaaaatcttg
>AK366160
tctttgcagtg
>AK360693
atggggatgtg
>MLOC_41
AAAGGTGT
>AK376987
ATGTtCGTT
>AK249921
ggcttattcta
>AK364168
cagtccacacc
>AK365922
cctcgcctcTG
>AK365963
ggcgagatcc
>AK370319
ATGGAAGC
>AK370781
TGGCAGTG
>AK354375
CAGGATAT
>AK369109

ggcactacttc
>AK375730
ACTGGAGG
>AK368140
TGCAGATT
>AK249518
ccgacagatg
>AK364849
cgcggcggag
>AK375108
tcccttcggcg
>AK376028
agggtaccag
>MLOC_11
gcaagcgtga
>AK364839
ggcggcagct
>AK250482
acatgtcaaa
>AK367062
ggacggcaat
>AK363003
gaaaaaaata
>AK359827
gcccgtgctgt
>AK377003
TCCaGGAA
>AK371221
ATCCTCTTC
>AK371597
ggccgcgacg
>AK376038
aggaggagca
>AK367045
agataacatca
>MLOC_15
ctgcgccagta
>AK365103
agctacgatgt
>AK249319
ATGAGGAA
>MLOC_65
atgtcgacaat
>AK376852
GATGATGA
>AK363561
atgtatttccta
>AK359097
atgatggggct
>MLOC_53

AGGTAtCT
>AK375481
ATGAtGAT
>AK371245
ctatgggatg
>AK376864
tgaaaatagg
>AK373447
cggcgtcaaa
>AK358687
actgggacga
>AK369212
AGGTCCAt
>AK366243
acaaggtgac
>MLOC_60
atgccatgctt
>AK369767
GACTCAGT
>AK365503
aggcgctcga
>AK367064
CTCTCATC
>AK252129
AGGACAA
>AK364458
agcctggttg
>MLOC_37
acccggcggc
>AK369576
cattactcgc
>AK361521
atgatgaga
>AK248486
ttcttcacata
>AK356188
CTGCtTGC
>AK369964
atggggtcttg
>AK365017
gtcagaagct
>MLOC_64
acgattctcgg
>AK370211
cgctacatcc
>AK371916
agtcagcgac
>AK354986
atgctgcgga
>AK376270

gcagcaatgg
>AK248772
atgatcccgct
>AK249886
aggtgcacaa
>AK373811
ggcggacgag
>AK362199
tGCaGTGC
>AK366349
tttattgtgca
>AK356045
atggcctccct
>AK362483
atgcatctgag
>AK373354
agtaccaggc
>AK358948
ccgcgtgaag
>AK354084
atcaggcgag
>AK366333
GGTAACTT
>AK366571
GGGtGCAG
>AK248269
GACTTTGA
>MLOC_55
gatatgcaga
>AK359191
AGAAATTT
>AK252280
TTTGTAGG
>AK367098
cgcatgataga
>MLOC_61
AAGGGATG
>AK364488
ggcttggagg
>AK354365
accagagggg
>AK376401
ggaactcaat
>AK366523
attccatgtca
>AK369105
agcataagct
>MLOC_38
atggagaggg
>AK361228

CGCGCTTC
>AK376471
TGGcTTTCg
>AK249059
tttatgtttgtg
>AK372407
gtattggtgtt
>AK358331
atataacata
>AK362963
ttcatctgcat
>AK364742
ATGGGTTC
>AB447465
ATGTCTAG
>AK373959
ggcggcgtgc
>AK368628
atggacaaaa
>AK375918
gaatgcagaa
>AK371804
atgttgtcgag
>AK252460
GGGCAAG
>AK362622
AGGCTGTG
>AK373944
ATGGCGGA
>AK371707
tggtgggcga
>AK369379
atggcccgca
>AK370260
gcttcctggtt
>AK363157
ATGGCTCC
>AK364634
agtatgacag
>AK353941
atgaatgcttt
>AK248352
ATGGTACC
>AK364381
attttcaggga
>AK363932
AGATGCAA
>AK366863
tccgtgctctc
>AK371992

atggcggagc
>AK375914
atgttccagct
>AK370762
tgcagagtgc
>AK376429
atgactgtgtt
>AK252584
ataatgctgg
>AK369111
cggcggcccg
>MLOC_58
catgggagga
>AK370148
atggcgcgaa
>AK357959
atggaggtctc
>AK374037
TCTTgtCtC
>AK354908
ATGGCAGT
>AK369519
atgaacttca
>AK353756
ATTGTGAT
>AK366898
gctacaactgt
>AK355901
aaaataataa
>AK371413
ccagacaatt
>MLOC_68
atggccgtgg
>AK248835
ggagtggagg
>AK367590
ctgacgatgc
>AK359376
AGAGCTAT
>AK370395
ccaagcttaat
>AK364579
CTGTTCGA
>AK363730
GCGAATGG
>AK355259
gattgactgc
>AK373015
gggagaaca
>MLOC_61

atgctttgtgtt
>AK365917
ctgagttcact
>AK368037
GGATCCAA
>AK250899
ATGGCAGC
>AK365357
ATGACGAC
>AK248234
TAGTACTT
>AK366381
ggcggcggcg
>AK358227
atggcgccgt
>AK372734
gtccgtctccg
>AK373995
atgtggccctc
>MLOC_69
atgtcagatcc
>AK373335
agcatgggca
>AK361277
atggaggtca
>AK249839
agtccaacat
>AK354730
atggatcgcg
>AK368758
actcccccga
>AK358057
CTCTTCAG
>AK362216
ATGACTCA
>AK357041
catggtttcaa
>AK248999
CTTCTTcTG
>AK362566
AGGAGCCA
>AK251844
attggactcct
>AK359823
tttgccaggaa
>AK353840
gaactttgAT
>AK357695
atgtctcgcac
>AK369530

atggagatgct
>AK375054
atgatgatgcc
>AK250130
aggttttatag
>AK248871
atgctatttgtt
>AK362524
aacaggtgaa
>MLOC_12
caccatgaga
>AK365864
ATGTCTTaC
>AK360162
tacatatgctc
>AK369262
gctgctaataa
>AK250233
cttcatagggc
>AK252321
caggctcacc
>AK354697
caggacctgcc
>MLOC_54
gaaaatAaa
>MLOC_16
gccgccgcca
>AK364106
tgtttctttgat
>AK364395
gtagacttctg
>AK369331
ATGGCTTC
>AK368575
cgaaccatgtc
>AK370769
cggCAGTCG
>MLOC_81
CCTGCAGA
>AK377006
cgacggtggc
>AK362554
atggcgactga
>AK369954
tgcaggtgcct
>AK362289
ggaacctatg
>AK249030
ccaggtgttcc
>MLOC_59

ccggccaccg
>AK376476
CAGCACTT
>MLOC_17
ATGAGAGA
>AK374452
ATGGCGAA
>AK365907
atgccactat
>AK376083
atgctgccgct
>AK353726
atggcgacca
>AK359881
cggccgtggt
>AK252511
atggcgacag
>AK368582
agactgcagt
>AK356629
gtactacggc
>AK252323
gccaggaatt
>AK365745
gagagaggc
>AK365473
ggcaatgttg
>AK373952
tgcagtgactt
>AK354222
gtacagctcc
>MLOC_69
ttctctcccttg
>AK359711
tggcaagctt
>AK365822
CTCaGggC
>AK354330
aggggtgaca
>AK370512
ctttcgactct

# Molecular organization and comparative analysis of chromosome 5B of the wild wheat

Bala Ani Akpinar[1], Meral Yuce[1], Stuart Lucas[1], Jan Vrána[2], Veronika Burešová[2], Jaroslav Doležel[2], Hikmet Buda

**Supplementary Table 1. Complete list of repeat annotations for Tdic5B**

| Repeat name | Repeat class | Reads matched | Bases covered |
|---|---|---|---|
| (A)n | Simple_repeat | 2549 | 271680 |
| (AGGGGG)n | Simple_repeat | 214 | 16849 |
| (CA)n | Simple_repeat | 8113 | 405471 |
| (CAA)n | Simple_repeat | 2629 | 177990 |
| (CAAA)n | Simple_repeat | 147 | 5252 |
| (CAAAA)n | Simple_repeat | 339 | 32383 |
| (CAAAAA)n | Simple_repeat | 792 | 76295 |
| (CAAAC)n | Simple_repeat | 32 | 963 |
| (CAAAG)n | Simple_repeat | 11 | 339 |
| (CAAAT)n | Simple_repeat | 40 | 1301 |
| (CAACC)n | Simple_repeat | 8 | 203 |
| (CAACT)n | Simple_repeat | 1 | 32 |
| (CAAG)n | Simple_repeat | 33 | 1044 |
| (CAAGA)n | Simple_repeat | 6 | 319 |
| (CAAGC)n | Simple_repeat | 1 | 36 |
| (CAAGG)n | Simple_repeat | 2 | 43 |
| (CAAT)n | Simple_repeat | 122 | 4468 |
| (CAATA)n | Simple_repeat | 11 | 276 |
| (CAATC)n | Simple_repeat | 48 | 2091 |
| (CAATG)n | Simple_repeat | 25 | 528 |
| (CAATT)n | Simple_repeat | 9 | 443 |
| (CACAA)n | Simple_repeat | 25 | 767 |
| (CACAC)n | Simple_repeat | 27 | 904 |
| (CACAG)n | Simple_repeat | 50 | 2938 |
| (CACAT)n | Simple_repeat | 6 | 250 |
| (CACCAT)n | Simple_repeat | 321 | 10335 |
| (CACCC)n | Simple_repeat | 45 | 1657 |
| (CACCG)n | Simple_repeat | 5 | 442 |
| (CACCT)n | Simple_repeat | 13 | 318 |
| (CACG)n | Simple_repeat | 156 | 6885 |
| (CACGA)n | Simple_repeat | 54 | 1390 |
| (CACGC)n | Simple_repeat | 20 | 666 |
| (CACTA)n | Simple_repeat | 3 | 125 |
| (CACTC)n | Simple_repeat | 16 | 515 |
| (CACTG)n | Simple_repeat | 9 | 230 |
| (CACTT)n | Simple_repeat | 5 | 137 |
| (CAG)n | Simple_repeat | 313 | 14465 |
| (CAGA)n | Simple_repeat | 15 | 392 |
| (CAGAA)n | Simple_repeat | 5 | 153 |
| (CAGAC)n | Simple_repeat | 4 | 108 |

| | | | |
|---|---|---|---|
| (CAGAG)n | Simple_repeat | 30 | 893 |
| (CAGAGA)n | Simple_repeat | 52 | 2479 |
| (CAGAT)n | Simple_repeat | 6 | 161 |
| (CAGC)n | Simple_repeat | 20 | 577 |
| (CAGCC)n | Simple_repeat | 17 | 549 |
| (CAGCG)n | Simple_repeat | 1 | 76 |
| (CAGCT)n | Simple_repeat | 2 | 54 |
| (CAGG)n | Simple_repeat | 16 | 395 |
| (CAGGC)n | Simple_repeat | 8 | 225 |
| (CAGGG)n | Simple_repeat | 8 | 348 |
| (CAGGT)n | Simple_repeat | 3 | 62 |
| (CAGT)n | Simple_repeat | 12 | 338 |
| (CAGTA)n | Simple_repeat | 2 | 99 |
| (CAGTC)n | Simple_repeat | 9 | 203 |
| (CAGTT)n | Simple_repeat | 20 | 1463 |
| (CAT)n | Simple_repeat | 1736 | 65212 |
| (CATA)n | Simple_repeat | 258 | 12644 |
| (CATAA)n | Simple_repeat | 1 | 111 |
| (CATAC)n | Simple_repeat | 16 | 467 |
| (CATAG)n | Simple_repeat | 8 | 285 |
| (CATAT)n | Simple_repeat | 8 | 178 |
| (CATATA)n | Simple_repeat | 67 | 2654 |
| (CATCC)n | Simple_repeat | 13 | 415 |
| (CATCG)n | Simple_repeat | 3 | 90 |
| (CATCT)n | Simple_repeat | 15 | 448 |
| (CATG)n | Simple_repeat | 285 | 8515 |
| (CATGC)n | Simple_repeat | 14 | 319 |
| (CATGT)n | Simple_repeat | 34 | 1141 |
| (CATTA)n | Simple_repeat | 23 | 627 |
| (CATTC)n | Simple_repeat | 11 | 260 |
| (CATTT)n | Simple_repeat | 26 | 1271 |
| (CCAA)n | Simple_repeat | 18 | 697 |
| (CCCA)n | Simple_repeat | 26 | 1054 |
| (CCCCAA)n | Simple_repeat | 14 | 714 |
| (CCCCAG)n | Simple_repeat | 9 | 442 |
| (CCCCG)n | Simple_repeat | 94 | 10601 |
| (CCCGA)n | Simple_repeat | 10 | 427 |
| (CCCGAA)n | Simple_repeat | 5 | 464 |
| (CCCTAA)n | Simple_repeat | 157 | 10456 |
| (CCCTAG)n | Simple_repeat | 1 | 23 |
| (CCGAA)n | Simple_repeat | 8 | 268 |
| (CCGAG)n | Simple_repeat | 16 | 385 |
| (CCGCG)n | Simple_repeat | 29 | 2722 |
| (CCGG)n | Simple_repeat | 4 | 141 |
| (CCGGA)n | Simple_repeat | 10 | 258 |
| (CCGGG)n | Simple_repeat | 78 | 3108 |
| (CCGTA)n | Simple_repeat | 5 | 292 |

| | | | |
|---|---|---|---|
| (CCTAA)n | Simple_repeat | 15 | 635 |
| (CCTAT)n | Simple_repeat | 6 | 239 |
| (CG)n | Simple_repeat | 51 | 1519 |
| (CGA)n | Simple_repeat | 161 | 7252 |
| (CGAA)n | Simple_repeat | 23 | 910 |
| (CGAAA)n | Simple_repeat | 5 | 470 |
| (CGAAT)n | Simple_repeat | 11 | 452 |
| (CGACG)n | Simple_repeat | 5 | 111 |
| (CGAG)n | Simple_repeat | 59 | 3495 |
| (CGAGA)n | Simple_repeat | 2 | 43 |
| (CGAGG)n | Simple_repeat | 62 | 3122 |
| (CGAGT)n | Simple_repeat | 2 | 58 |
| (CGAT)n | Simple_repeat | 4 | 104 |
| (CGG)n | Simple_repeat | 805 | 58811 |
| (CGGA)n | Simple_repeat | 27 | 727 |
| (CGGAA)n | Simple_repeat | 2 | 52 |
| (CGGAG)n | Simple_repeat | 4 | 150 |
| (CGGG)n | Simple_repeat | 35 | 2145 |
| (CGGGA)n | Simple_repeat | 5 | 332 |
| (CGGGGG)n | Simple_repeat | 144 | 14138 |
| (CGGT)n | Simple_repeat | 6 | 247 |
| (CGTAA)n | Simple_repeat | 13 | 956 |
| (CTAA)n | Simple_repeat | 17 | 688 |
| (CTAAA)n | Simple_repeat | 57 | 4312 |
| (CTAAG)n | Simple_repeat | 4 | 174 |
| (CTAAT)n | Simple_repeat | 19 | 1404 |
| (CTACT)n | Simple_repeat | 5 | 143 |
| (CTAG)n | Simple_repeat | 40 | 1277 |
| (CTAGG)n | Simple_repeat | 2 | 57 |
| (CTAGT)n | Simple_repeat | 15 | 367 |
| (CTATA)n | Simple_repeat | 2 | 72 |
| (CTATT)n | Simple_repeat | 32 | 1060 |
| (CTTAT)n | Simple_repeat | 1 | 44 |
| (CTTTA)n | Simple_repeat | 17 | 706 |
| (G)n | Simple_repeat | 438 | 12359 |
| (GA)n | Simple_repeat | 12174 | 417180 |
| (GAA)n | Simple_repeat | 2003 | 97234 |
| (GAAA)n | Simple_repeat | 1184 | 109897 |
| (GAAAA)n | Simple_repeat | 3988 | 361088 |
| (GAGAA)n | Simple_repeat | 166 | 9107 |
| (GGA)n | Simple_repeat | 1067 | 63347 |
| (GGAA)n | Simple_repeat | 58 | 2334 |
| (GGAAA)n | Simple_repeat | 67 | 4341 |
| (GGAGA)n | Simple_repeat | 174 | 8558 |
| (GGAGAA)n | Simple_repeat | 202 | 13990 |
| (GGGA)n | Simple_repeat | 236 | 13162 |
| (GGGAA)n | Simple_repeat | 30 | 1664 |

| | | | |
|---|---|---|---|
| (GGGAGA)n | Simple_repeat | 403 | 21252 |
| (GGGGA)n | Simple_repeat | 169 | 12458 |
| (TA)n | Simple_repeat | 6740 | 233643 |
| (TAA)n | Simple_repeat | 1092 | 56704 |
| (TAAA)n | Simple_repeat | 749 | 23943 |
| (TAAAA)n | Simple_repeat | 597 | 35257 |
| (TAAAAA)n | Simple_repeat | 1390 | 126807 |
| (TAAG)n | Simple_repeat | 1 | 28 |
| (TAG)n | Simple_repeat | 432 | 18597 |
| (TAGA)n | Simple_repeat | 372 | 17323 |
| (TAGG)n | Simple_repeat | 11 | 366 |
| (TAGGG)n | Simple_repeat | 7 | 203 |
| (TATAA)n | Simple_repeat | 50 | 1796 |
| (TGAA)n | Simple_repeat | 25 | 633 |
| (TGAG)n | Simple_repeat | 37 | 1458 |
| (TGG)n | Simple_repeat | 530 | 39830 |
| (TGGA)n | Simple_repeat | 145 | 5308 |
| (TGGGGG)n | Simple_repeat | 107 | 5111 |
| (TTAA)n | Simple_repeat | 118 | 5595 |
| (TTAAA)n | Simple_repeat | 10 | 970 |
| (TTAAG)n | Simple_repeat | 32 | 1962 |
| (TTAGGC)n | Simple_repeat | 8 | 620 |
| AT_rich | Low_complexity | 16620 | 620870 |
| BAGY-2-LTR-1 | LTR | 8 | 1527 |
| BAGY-2-LTR-1.1 | LTR | 17 | 1735 |
| BAGY-2-LTR-1.2 | LTR | 20 | 2976 |
| BAGY-2-LTR-1.3 | LTR | 5 | 808 |
| BAGY-2-LTR-2 | LTR | 16 | 1400 |
| BARE-1-LTR-5 | LTR/Copia | 1 | 82 |
| Bd1-RLC | LTR/Copia | 2204 | 440636 |
| Bd1-RLG | LTR/Gypsy | 1724 | 279794 |
| Bd1-RLX | LTR | 1578 | 313285 |
| Bd2-RLC | LTR/Copia | 866 | 157290 |
| Bd2-RLG | LTR/Gypsy | 1733 | 302392 |
| Bd2-RLX | LTR | 524 | 79194 |
| Bd3-RLC | LTR/Copia | 1913 | 495938 |
| Bd3-RLG | LTR/Gypsy | 1914 | 348384 |
| Bd3-RLX | LTR | 206 | 31458 |
| Bd4-RLC | LTR/Copia | 1760 | 290353 |
| Bd4-RLG | LTR/Gypsy | 1581 | 299197 |
| Bd4-RLX | LTR | 1004 | 132389 |
| Bd5-RLC | LTR/Copia | 435 | 101937 |
| Bd5-RLG | LTR/Gypsy | 1318 | 247226 |
| Bd5-RLX | LTR | 321 | 52951 |
| DHH-Helios | RC/Helitron | 403 | 124558 |
| DHH-unnamedfam1 | RC/Helitron | 163 | 35080 |
| DHH-unnamedfam2 | RC/Helitron | 91 | 16977 |

| | | | |
|---|---|---|---|
| DTA-George | DNA/hAT | 158 | 31495 |
| DTC-Balduin | DNA/En-Spm | 5942 | 1554009 |
| DTC-Boris | DNA/En-Spm | 7126 | 1831327 |
| DTC-Byron | DNA/En-Spm | 2644 | 607436 |
| DTC-Caspar | DNA/En-Spm | 15742 | 3835250 |
| DTC-Clifford | DNA/En-Spm | 19428 | 5363948 |
| DTC-Conan | DNA/En-Spm | 4180 | 1211624 |
| DTC-Dagobert | DNA/En-Spm | 1 | 39 |
| DTC-Donald | DNA/En-Spm | 2292 | 541451 |
| DTC-Enac | DNA/En-Spm | 284 | 23577 |
| DTC-Fergat | DNA/En-Spm | 1693 | 419902 |
| DTC-Hamlet | DNA/En-Spm | 2319 | 477843 |
| DTC-Heyjude | DNA/En-Spm | 837 | 197118 |
| DTC-Isaac | DNA/En-Spm | 3089 | 710512 |
| DTC-Jorge | DNA/En-Spm | 549548 | 151452739 |
| DTC-Jude | DNA/En-Spm | 559 | 126766 |
| DTC-Mandrake | DNA/En-Spm | 4622 | 1098996 |
| DTC-Pavel | DNA/En-Spm | 15403 | 3873540 |
| DTC-Sherlock | DNA/En-Spm | 1195 | 251271 |
| DTC-TAT1 | DNA/En-Spm | 9697 | 2525327 |
| DTC-TAT2 | DNA/En-Spm | 47 | 4428 |
| DTC-Vincent | DNA/En-Spm | 12720 | 3487691 |
| DTC-unnamedfam1 | DNA/En-Spm | 41 | 4762 |
| DTC-unnamedfam10 | DNA/En-Spm | 16 | 1137 |
| DTC-unnamedfam12 | DNA/En-Spm | 599 | 121967 |
| DTC-unnamedfam13 | DNA/En-Spm | 384 | 100563 |
| DTC-unnamedfam14 | DNA/En-Spm | 382 | 58313 |
| DTC-unnamedfam15 | DNA/En-Spm | 179 | 43568 |
| DTC-unnamedfam16 | DNA/En-Spm | 237 | 68948 |
| DTC-unnamedfam17 | DNA/En-Spm | 4 | 524 |
| DTC-unnamedfam18 | DNA/En-Spm | 227 | 54161 |
| DTC-unnamedfam19 | DNA/En-Spm | 31 | 4718 |
| DTC-unnamedfam2 | DNA/En-Spm | 9436 | 2569364 |
| DTC-unnamedfam20 | DNA/En-Spm | 306 | 47810 |
| DTC-unnamedfam21 | DNA/En-Spm | 40 | 5548 |
| DTC-unnamedfam22 | DNA/En-Spm | 22 | 3176 |
| DTC-unnamedfam23 | DNA/En-Spm | 113 | 10781 |
| DTC-unnamedfam24 | DNA/En-Spm | 1884 | 487971 |
| DTC-unnamedfam25 | DNA/En-Spm | 177 | 33535 |
| DTC-unnamedfam26 | DNA/En-Spm | 153 | 30383 |
| DTC-unnamedfam27 | DNA/En-Spm | 808 | 109164 |
| DTC-unnamedfam28 | DNA/En-Spm | 6 | 552 |
| DTC-unnamedfam29 | DNA/En-Spm | 279 | 49027 |
| DTC-unnamedfam3 | DNA/En-Spm | 761 | 182927 |
| DTC-unnamedfam30 | DNA/En-Spm | 324 | 56626 |
| DTC-unnamedfam31 | DNA/En-Spm | 79 | 8971 |
| DTC-unnamedfam32 | DNA/En-Spm | 1 | 81 |

| | | | |
|---|---|---|---|
| DTC-unnamedfam33 | DNA/En-Spm | 388 | 89376 |
| DTC-unnamedfam34 | DNA/En-Spm | 50 | 7235 |
| DTC-unnamedfam35 | DNA/En-Spm | 76 | 20515 |
| DTC-unnamedfam36 | DNA/En-Spm | 228 | 47393 |
| DTC-unnamedfam37 | DNA/En-Spm | 166 | 30711 |
| DTC-unnamedfam38 | DNA/En-Spm | 98 | 21642 |
| DTC-unnamedfam39 | DNA/En-Spm | 292 | 47365 |
| DTC-unnamedfam4 | DNA/En-Spm | 353 | 57885 |
| DTC-unnamedfam40 | DNA/En-Spm | 111 | 19094 |
| DTC-unnamedfam41 | DNA/En-Spm | 1105 | 284730 |
| DTC-unnamedfam42 | DNA/En-Spm | 297 | 53330 |
| DTC-unnamedfam43 | DNA/En-Spm | 98 | 15357 |
| DTC-unnamedfam44 | DNA/En-Spm | 751 | 139269 |
| DTC-unnamedfam45 | DNA/En-Spm | 1981 | 579023 |
| DTC-unnamedfam46 | DNA/En-Spm | 605 | 101730 |
| DTC-unnamedfam47 | DNA/En-Spm | 53 | 8889 |
| DTC-unnamedfam48 | DNA/En-Spm | 11393 | 2570610 |
| DTC-unnamedfam49 | DNA/En-Spm | 2713 | 701390 |
| DTC-unnamedfam5 | DNA/En-Spm | 868 | 184976 |
| DTC-unnamedfam50 | DNA/En-Spm | 1461 | 355805 |
| DTC-unnamedfam52 | DNA/En-Spm | 95 | 24270 |
| DTC-unnamedfam53 | DNA/En-Spm | 827 | 170830 |
| DTC-unnamedfam54 | DNA/En-Spm | 2661 | 739392 |
| DTC-unnamedfam55 | DNA/En-Spm | 1053 | 276204 |
| DTC-unnamedfam56 | DNA/En-Spm | 104 | 10590 |
| DTC-unnamedfam57 | DNA/En-Spm | 126 | 25359 |
| DTC-unnamedfam58 | DNA/En-Spm | 14 | 2193 |
| DTC-unnamedfam59 | DNA/En-Spm | 1 | 138 |
| DTC-unnamedfam6 | DNA/En-Spm | 3 | 450 |
| DTC-unnamedfam60 | DNA/En-Spm | 90 | 8766 |
| DTC-unnamedfam61 | DNA/En-Spm | 1351 | 241829 |
| DTC-unnamedfam62 | DNA/En-Spm | 648 | 121354 |
| DTC-unnamedfam63 | DNA/En-Spm | 473 | 65411 |
| DTC-unnamedfam64 | DNA/En-Spm | 3232 | 781046 |
| DTC-unnamedfam65 | DNA/En-Spm | 171 | 37302 |
| DTC-unnamedfam66 | DNA/En-Spm | 1158 | 277406 |
| DTC-unnamedfam67 | DNA/En-Spm | 22 | 2363 |
| DTC-unnamedfam68 | DNA/En-Spm | 996 | 263550 |
| DTC-unnamedfam69 | DNA/En-Spm | 207 | 39988 |
| DTC-unnamedfam7 | DNA/En-Spm | 10782 | 2778081 |
| DTC-unnamedfam70 | DNA/En-Spm | 164 | 29968 |
| DTC-unnamedfam71 | DNA/En-Spm | 1444 | 377143 |
| DTC-unnamedfam72 | DNA/En-Spm | 39 | 4427 |
| DTC-unnamedfam73 | DNA/En-Spm | 153 | 15698 |
| DTC-unnamedfam74 | DNA/En-Spm | 4 | 641 |
| DTC-unnamedfam75 | DNA/En-Spm | 714 | 218565 |
| DTC-unnamedfam76 | DNA/En-Spm | 113 | 24782 |

| | | | |
|---|---|---|---|
| DTC-unnamedfam8 | DNA/En-Spm | 21 | 2931 |
| DTC-unnamedfam9 | DNA/En-Spm | 2895 | 597968 |
| DTH-Coeus | DNA/Harbinger | 310 | 29159 |
| DTH-Islay | DNA/Harbinger | 135 | 28387 |
| DTH-Kerberos | DNA/Harbinger | 384 | 52026 |
| DTH-Kong | DNA/Harbinger | 488 | 97448 |
| DTH-Orpheus | DNA/Harbinger | 137 | 15985 |
| DTH-Rong | DNA/Harbinger | 1084 | 234491 |
| DTH-Victor | DNA/Harbinger | 29 | 4566 |
| DTH-Xenon | DNA/Harbinger | 70 | 7250 |
| DTH-unnamedfam1 | DNA/Harbinger | 7 | 660 |
| DTH-unnamedfam2 | DNA/Harbinger | 87 | 5562 |
| DTH-unnamedfam3 | DNA/Harbinger | 81 | 6303 |
| DTH-unnamedfam4 | DNA/Harbinger | 13 | 1026 |
| DTH-unnamedfam5 | DNA/Harbinger | 516 | 124735 |
| DTH-unnamedfam6 | DNA/Harbinger | 674 | 85709 |
| DTH-unnamedfam7 | DNA/Harbinger | 497 | 115647 |
| DTH-unnamedfam8 | DNA/Harbinger | 9 | 1404 |
| DTH-unnamedfam9 | DNA/Harbinger | 120 | 28683 |
| DTM-Argus | DNA/MuDR | 85 | 10600 |
| DTM-Deimos | DNA/MuDR | 5538 | 1386151 |
| DTM-Gerald | DNA/MuDR | 7 | 960 |
| DTM-Hermes | DNA/MuDR | 297 | 52548 |
| DTM-Remus | DNA/MuDR | 101 | 12334 |
| DTM-Spring | DNA/MuDR | 30 | 2281 |
| DTM-Vacuna | DNA/MuDR | 11 | 1768 |
| DTM-unnamedfam1 | DNA/MuDR | 26 | 2046 |
| DTM-unnamedfam10 | DNA/MuDR | 433 | 76419 |
| DTM-unnamedfam11 | DNA/MuDR | 47 | 5541 |
| DTM-unnamedfam12 | DNA/MuDR | 85 | 10157 |
| DTM-unnamedfam13 | DNA/MuDR | 420 | 121096 |
| DTM-unnamedfam14 | DNA/MuDR | 162 | 35888 |
| DTM-unnamedfam15 | DNA/MuDR | 382 | 71994 |
| DTM-unnamedfam16 | DNA/MuDR | 71 | 12650 |
| DTM-unnamedfam17 | DNA/MuDR | 26 | 3360 |
| DTM-unnamedfam18 | DNA/MuDR | 104 | 10369 |
| DTM-unnamedfam19 | DNA/MuDR | 64 | 9991 |
| DTM-unnamedfam2 | DNA/MuDR | 143 | 16944 |
| DTM-unnamedfam20 | DNA/MuDR | 461 | 63531 |
| DTM-unnamedfam21 | DNA/MuDR | 84 | 11062 |
| DTM-unnamedfam22 | DNA/MuDR | 113 | 15309 |
| DTM-unnamedfam23 | DNA/MuDR | 16 | 1533 |
| DTM-unnamedfam24 | DNA/MuDR | 9 | 1108 |
| DTM-unnamedfam25 | DNA/MuDR | 3435 | 785277 |
| DTM-unnamedfam26 | DNA/MuDR | 84 | 15006 |
| DTM-unnamedfam27 | DNA/MuDR | 584 | 105855 |
| DTM-unnamedfam28 | DNA/MuDR | 63 | 6444 |

| | | | |
|---|---|---|---|
| DTM-unnamedfam29 | DNA/MuDR | 37 | 5268 |
| DTM-unnamedfam30 | DNA/MuDR | 912 | 175731 |
| DTM-unnamedfam31 | DNA/MuDR | 327 | 47447 |
| DTM-unnamedfam32 | DNA/MuDR | 246 | 38733 |
| DTM-unnamedfam33 | DNA/MuDR | 424 | 59719 |
| DTM-unnamedfam34 | DNA/MuDR | 38 | 4397 |
| DTM-unnamedfam35 | DNA/MuDR | 131 | 19278 |
| DTM-unnamedfam36 | DNA/MuDR | 255 | 57271 |
| DTM-unnamedfam37 | DNA/MuDR | 84 | 12380 |
| DTM-unnamedfam38 | DNA/MuDR | 505 | 119211 |
| DTM-unnamedfam39 | DNA/MuDR | 796 | 132980 |
| DTM-unnamedfam4 | DNA/MuDR | 1 | 63 |
| DTM-unnamedfam40 | DNA/MuDR | 106 | 24560 |
| DTM-unnamedfam41 | DNA/MuDR | 145 | 17712 |
| DTM-unnamedfam42 | DNA/MuDR | 34 | 2991 |
| DTM-unnamedfam43 | DNA/MuDR | 10 | 1045 |
| DTM-unnamedfam44 | DNA/MuDR | 242 | 32162 |
| DTM-unnamedfam45 | DNA/MuDR | 355 | 55225 |
| DTM-unnamedfam46 | DNA/MuDR | 6 | 872 |
| DTM-unnamedfam47 | DNA/MuDR | 3 | 222 |
| DTM-unnamedfam5 | DNA/MuDR | 26 | 3849 |
| DTM-unnamedfam6 | DNA/MuDR | 3 | 204 |
| DTM-unnamedfam7 | DNA/MuDR | 142 | 38777 |
| DTM-unnamedfam8 | DNA/MuDR | 69 | 8417 |
| DTM-unnamedfam9 | DNA/MuDR | 112 | 28284 |
| DTT-Athos | DNA/TcMar | 1458 | 87212 |
| DTT-Fortuna | DNA/TcMar | 454 | 59188 |
| DTT-Hades | DNA/TcMar | 937 | 66346 |
| DTT-Icarus | DNA/TcMar | 2806 | 263041 |
| DTT-Oleus | DNA/TcMar | 879 | 92319 |
| DTT-Pan | DNA/TcMar | 1036 | 100889 |
| DTT-Phoebus | DNA/TcMar | 112 | 20683 |
| DTT-Polyphemus | DNA/TcMar | 2713 | 393647 |
| DTT-Stolos | DNA/TcMar | 735 | 77086 |
| DTT-Thalos | DNA/TcMar | 3284 | 363456 |
| DTT-unnamedfam1 | DNA/TcMar | 12 | 943 |
| DTT-unnamedfam2 | DNA/TcMar | 1 | 96 |
| DTT-unnamedfam3 | DNA/TcMar | 17 | 1102 |
| DTT-unnamedfam4 | DNA/TcMar | 41 | 5603 |
| DTT-unnamedfam5 | DNA/TcMar | 18 | 1829 |
| DTT-unnamedfam6 | DNA/TcMar | 33 | 4518 |
| DTT-unnamedfam7 | DNA/TcMar | 31 | 3317 |
| DTT-unnamedfam8 | DNA/TcMar | 17 | 1236 |
| DTT-unnamedfam9 | DNA/TcMar | 2 | 141 |
| DTX-Nisos | DNA | 2 | 107 |
| DTX-unnamedfam1 | DNA | 552 | 76766 |
| DTX-unnamedfam10 | DNA | 796 | 107035 |

| | | | |
|---|---|---|---|
| DTX-unnamedfam100 | DNA | 77 | 16888 |
| DTX-unnamedfam101 | DNA | 117 | 22230 |
| DTX-unnamedfam102 | DNA | 1 | 66 |
| DTX-unnamedfam103 | DNA | 9 | 1042 |
| DTX-unnamedfam104 | DNA | 20 | 2441 |
| DTX-unnamedfam105 | DNA | 5 | 604 |
| DTX-unnamedfam106 | DNA | 8 | 611 |
| DTX-unnamedfam107 | DNA | 14 | 1172 |
| DTX-unnamedfam108 | DNA | 37 | 2143 |
| DTX-unnamedfam109 | DNA | 212 | 30520 |
| DTX-unnamedfam11 | DNA | 101 | 8683 |
| DTX-unnamedfam110 | DNA | 36 | 5006 |
| DTX-unnamedfam111 | DNA | 18 | 1098 |
| DTX-unnamedfam112 | DNA | 8 | 890 |
| DTX-unnamedfam113 | DNA | 146 | 15432 |
| DTX-unnamedfam12 | DNA | 116 | 7555 |
| DTX-unnamedfam13 | DNA | 29 | 2089 |
| DTX-unnamedfam14 | DNA | 10 | 747 |
| DTX-unnamedfam15 | DNA | 8 | 620 |
| DTX-unnamedfam16 | DNA | 10 | 1072 |
| DTX-unnamedfam17 | DNA | 210 | 27075 |
| DTX-unnamedfam18 | DNA | 105 | 14753 |
| DTX-unnamedfam19 | DNA | 216 | 12568 |
| DTX-unnamedfam2 | DNA | 66 | 5595 |
| DTX-unnamedfam20 | DNA | 2 | 106 |
| DTX-unnamedfam21 | DNA | 9 | 1006 |
| DTX-unnamedfam22 | DNA | 1 | 136 |
| DTX-unnamedfam23 | DNA | 38 | 3264 |
| DTX-unnamedfam24 | DNA | 2 | 236 |
| DTX-unnamedfam25 | DNA | 9 | 541 |
| DTX-unnamedfam27 | DNA | 83 | 11363 |
| DTX-unnamedfam28 | DNA | 3 | 227 |
| DTX-unnamedfam29 | DNA | 12 | 819 |
| DTX-unnamedfam3 | DNA | 348 | 29476 |
| DTX-unnamedfam30 | DNA | 7 | 351 |
| DTX-unnamedfam31 | DNA | 62 | 9279 |
| DTX-unnamedfam32 | DNA | 36 | 2528 |
| DTX-unnamedfam33 | DNA | 1 | 47 |
| DTX-unnamedfam34 | DNA | 17 | 871 |
| DTX-unnamedfam35 | DNA | 3 | 199 |
| DTX-unnamedfam36 | DNA | 1 | 54 |
| DTX-unnamedfam37 | DNA | 44 | 7146 |
| DTX-unnamedfam38 | DNA | 6 | 482 |
| DTX-unnamedfam4 | DNA | 299 | 39179 |
| DTX-unnamedfam40 | DNA | 41 | 4151 |
| DTX-unnamedfam41 | DNA | 3 | 144 |
| DTX-unnamedfam42 | DNA | 129 | 7811 |

| | | | |
|---|---|---|---|
| DTX-unnamedfam43 | DNA | 2 | 116 |
| DTX-unnamedfam44 | DNA | 45 | 3755 |
| DTX-unnamedfam45 | DNA | 4 | 266 |
| DTX-unnamedfam46 | DNA | 12 | 1476 |
| DTX-unnamedfam47 | DNA | 263 | 31330 |
| DTX-unnamedfam49 | DNA | 6 | 740 |
| DTX-unnamedfam5 | DNA | 460 | 71553 |
| DTX-unnamedfam50 | DNA | 3 | 390 |
| DTX-unnamedfam51 | DNA | 39 | 2678 |
| DTX-unnamedfam54 | DNA | 34 | 1967 |
| DTX-unnamedfam55 | DNA | 1 | 70 |
| DTX-unnamedfam56 | DNA | 2 | 109 |
| DTX-unnamedfam57 | DNA | 40 | 2428 |
| DTX-unnamedfam58 | DNA | 52 | 8002 |
| DTX-unnamedfam6 | DNA | 27 | 3506 |
| DTX-unnamedfam60 | DNA | 4 | 233 |
| DTX-unnamedfam61 | DNA | 4 | 367 |
| DTX-unnamedfam62 | DNA | 177 | 8856 |
| DTX-unnamedfam63 | DNA | 23 | 3047 |
| DTX-unnamedfam64 | DNA | 93 | 13076 |
| DTX-unnamedfam65 | DNA | 4 | 215 |
| DTX-unnamedfam66 | DNA | 1 | 133 |
| DTX-unnamedfam67 | DNA | 22 | 2767 |
| DTX-unnamedfam68 | DNA | 103 | 7090 |
| DTX-unnamedfam69 | DNA | 12 | 525 |
| DTX-unnamedfam7 | DNA | 56 | 4784 |
| DTX-unnamedfam70 | DNA | 325 | 51823 |
| DTX-unnamedfam71 | DNA | 86 | 24549 |
| DTX-unnamedfam72 | DNA | 2383 | 349485 |
| DTX-unnamedfam73 | DNA | 10 | 1010 |
| DTX-unnamedfam74 | DNA | 118 | 14323 |
| DTX-unnamedfam76 | DNA/TcMar | 134 | 10372 |
| DTX-unnamedfam77 | DNA | 5 | 367 |
| DTX-unnamedfam78 | DNA | 4 | 210 |
| DTX-unnamedfam79 | DNA | 7 | 374 |
| DTX-unnamedfam8 | DNA | 148 | 10478 |
| DTX-unnamedfam80 | DNA | 17 | 1196 |
| DTX-unnamedfam81 | DNA | 14 | 1161 |
| DTX-unnamedfam82 | DNA | 985 | 158369 |
| DTX-unnamedfam83 | DNA | 121 | 19146 |
| DTX-unnamedfam84 | DNA | 18 | 2217 |
| DTX-unnamedfam85 | DNA | 19 | 3601 |
| DTX-unnamedfam86 | DNA | 1 | 74 |
| DTX-unnamedfam87 | DNA | 4 | 332 |
| DTX-unnamedfam88 | DNA | 20 | 1225 |
| DTX-unnamedfam89 | DNA | 36 | 4822 |
| DTX-unnamedfam9 | DNA | 108 | 15420 |

| | | | |
|---|---|---|---|
| DTX-unnamedfam90 | DNA | 3 | 131 |
| DTX-unnamedfam91 | DNA | 55 | 4899 |
| DTX-unnamedfam92 | DNA | 11 | 1021 |
| DTX-unnamedfam93 | DNA | 40 | 6158 |
| DTX-unnamedfam94 | DNA | 24 | 3104 |
| DTX-unnamedfam95 | DNA | 16 | 785 |
| DTX-unnamedfam96 | DNA | 42 | 4388 |
| DTX-unnamedfam97 | DNA | 201 | 33027 |
| DTX-unnamedfam99 | DNA | 510 | 74549 |
| DXX-Misenos | DNA | 382 | 49517 |
| GC_rich | Low_complexity | 1820 | 59237 |
| GENBANK-AB061327.1 | LTR/Copia | 26 | 3427 |
| GENBANK-AF538614.1 | LTR | 23 | 3033 |
| GENBANK-AF538615.1 | LTR | 16 | 2453 |
| GENBANK-AF538617.1 | LTR | 8 | 1509 |
| GENBANK-AY271960.1 | LTR | 8 | 1081 |
| GENBANK-AY359471.1 | LTR | 604 | 130808 |
| HRSiCMCMOOT00003 | Other | 2 | 322 |
| HRSiCMCMOOT00004 | Other | 471 | 62237 |
| HRSiCMCMOOT00005 | Retroelement | 30 | 4281 |
| HRSiCMCMOOT00006 | Other | 62 | 7352 |
| HRSiRGRR00000031 | rRNA | 2 | 256 |
| HRSiRGRR00500001 | rRNA | 4 | 321 |
| HRSiRGRR00500002 | rRNA | 1 | 79 |
| HRSiRGRR00500004 | rRNA | 1 | 98 |
| HRSiRGRR00500013 | rRNA | 1 | 50 |
| HRSiRGRR00500015 | rRNA | 1 | 45 |
| HRSiRGRR00500017 | rRNA | 1 | 143 |
| HRSiRGRR00500018 | rRNA | 4 | 234 |
| HRSiRGRR00500020 | rRNA | 1 | 59 |
| HRSiRGRR00500022 | rRNA | 2 | 179 |
| HRSiRGRR00500024 | rRNA | 3 | 298 |
| HRSiRGRR00500030 | rRNA | 11 | 1150 |
| HRSiRGRR00500032 | rRNA | 1 | 68 |
| HRSiRGRR00500033 | rRNA | 3 | 268 |
| HRSiRGRR00500035 | rRNA | 3 | 214 |
| HRSiRGRR00500041 | rRNA | 4 | 190 |
| HRSiRGRR00500042 | rRNA | 28 | 1529 |
| HRSiRGRR00500043 | rRNA | 1 | 61 |
| HRSiRGRR00500044 | rRNA | 1 | 79 |
| HRSiRGRR00500046 | rRNA | 1 | 33 |
| HRSiRGRR00500047 | rRNA | 2 | 164 |
| HRSiRGRR00500052 | rRNA | 2 | 420 |
| HRSiRGRR00500057 | rRNA | 1 | 135 |
| HRSiRGRR00500062 | rRNA | 1 | 75 |
| HRSiRGRR00500064 | rRNA | 1 | 56 |
| HRSiRGRR00500066 | rRNA | 1 | 128 |

| | | | |
|---|---|---|---|
| HRSiRGRR00500068 | rRNA | 2 | 151 |
| HRSiRGRR00500070 | rRNA | 2 | 55 |
| HRSiRGRR00500074 | rRNA | 1 | 116 |
| HRSiRGRR00500075 | rRNA | 10 | 750 |
| HRSiRGRR00500077 | rRNA | 2 | 293 |
| HRSiRGRR00500080 | rRNA | 1 | 189 |
| HRSiRGRR00500085 | rRNA | 4 | 328 |
| HRSiRGRR00500090 | rRNA | 1 | 43 |
| HRSiRGRR00500096 | rRNA | 2 | 255 |
| HRSiRGRR00500101 | rRNA | 2 | 91 |
| HRSiRGRR00500102 | rRNA | 3 | 139 |
| HRSiRGRR00500111 | rRNA | 6 | 388 |
| HRSiRGRR00500113 | rRNA | 5 | 487 |
| HRSiRGRR00500116 | rRNA | 4 | 216 |
| HRSiRGRR00500120 | rRNA | 60 | 4018 |
| HRSiRGRR00500123 | rRNA | 2 | 62 |
| HRSiRGRR00500126 | rRNA | 1 | 32 |
| HRSiRGRR00500129 | rRNA | 8 | 1017 |
| HRSiRGRR00500131 | rRNA | 1 | 41 |
| HRSiRGRR00500132 | rRNA | 2 | 167 |
| HRSiRGRR00500135 | rRNA | 4 | 261 |
| HRSiRGRR00500137 | rRNA | 6 | 525 |
| HRSiRGRR00500140 | rRNA | 1 | 95 |
| HRSiRGRR00500141 | rRNA | 2 | 154 |
| HRSiRGRR00500150 | rRNA | 7 | 721 |
| HRSiRGRR00500152 | rRNA | 1 | 119 |
| HRSiRGRR00500154 | rRNA | 1 | 36 |
| HRSiRGRR00500157 | rRNA | 4 | 793 |
| HRSiRGRR00500161 | rRNA | 13 | 1227 |
| HRSiRGRR00500167 | rRNA | 9 | 624 |
| HRSiRGRR00500170 | rRNA | 7 | 660 |
| HRSiRGRR00500174 | rRNA | 2 | 272 |
| HRSiRGRR00500176 | rRNA | 1 | 296 |
| HRSiRGRR00500177 | rRNA | 1 | 67 |
| HRSiRGRR00500180 | rRNA | 3 | 393 |
| HRSiRGRR00500184 | rRNA | 2 | 144 |
| HRSiRGRR00500185 | rRNA | 3 | 160 |
| HRSiRGRR00500193 | rRNA | 2 | 279 |
| HRSiRGRR00500195 | rRNA | 2 | 80 |
| HRSiRGRR00500201 | rRNA | 3 | 203 |
| HRSiRGRR00500202 | rRNA | 2 | 149 |
| HRSiRGRR00500203 | rRNA | 3 | 313 |
| HRSiRGRR00500207 | rRNA | 3 | 480 |
| HRSiRGRR00500208 | rRNA | 1 | 111 |
| HRSiRGRR00500212 | rRNA | 4 | 108 |
| HRSiRGRR00500216 | rRNA | 1 | 90 |
| HRSiRGRR00500222 | rRNA | 1 | 40 |

| | | | |
|---|---|---|---|
| HRSiRGRR00500225 | rRNA | 11 | 964 |
| HRSiRGRR00500229 | rRNA | 1 | 64 |
| HRSiRGRR00500237 | rRNA | 1 | 69 |
| HRSiRGRR00500238 | rRNA | 2 | 160 |
| HRSiRGRR00500239 | rRNA | 2 | 134 |
| HRSiRGRR00500244 | rRNA | 2 | 121 |
| HRSiRGRR00500247 | rRNA | 4 | 530 |
| HRSiRGRR00500248 | rRNA | 12 | 1250 |
| HRSiRGRR00500251 | rRNA | 3 | 259 |
| HRSiRGRR00500252 | rRNA | 5 | 821 |
| HRSiRGRR00500260 | rRNA | 3 | 393 |
| HRSiRGRR00500263 | rRNA | 199 | 82508 |
| HRSiRGRR00500270 | rRNA | 2 | 162 |
| HRSiRGRR00500278 | rRNA | 2 | 178 |
| HRSiRGRR00500280 | rRNA | 2 | 117 |
| HRSiRGRR00500282 | rRNA | 3 | 141 |
| HRSiRGRR00500286 | rRNA | 1 | 245 |
| HRSiRGRR00500289 | rRNA | 3 | 362 |
| HRSiRGRR00500292 | rRNA | 3 | 443 |
| HRSiRGRR00500297 | rRNA | 1 | 133 |
| HRSiRGRR00500298 | rRNA | 4 | 209 |
| HRSiRGRR00500301 | rRNA | 4 | 306 |
| HRSiRGRR00500303 | rRNA | 1 | 99 |
| HRSiRGRR00500304 | rRNA | 2 | 172 |
| HRSiRGRR00500308 | rRNA | 2 | 149 |
| HRSiRGRR00500314 | rRNA | 1 | 105 |
| HRSiRGRR00500316 | rRNA | 2 | 192 |
| HRSiRGRR00500319 | rRNA | 3 | 122 |
| HRSiTEMT06000002 | DNA/Mite | 3 | 251 |
| HRSiTERT00100001 | LTR/Copia | 6 | 930 |
| HRSiTERT00100002 | LTR/Copia | 1 | 86 |
| HRSiTERT00100004 | LTR/Copia | 6 | 1745 |
| HRSiTERT00100008 | LTR/Copia | 2 | 147 |
| HRSiTERT00100011 | LTR/Copia | 5 | 513 |
| HRSiTERT00100013 | LTR/Copia | 10 | 904 |
| HRSiTERT00100014 | LTR/Copia | 1 | 152 |
| HRSiTERT00100015 | LTR/Copia | 2 | 408 |
| HRSiTERT00100019 | LTR/Copia | 34 | 4632 |
| HRSiTERT00100021 | LTR/Copia | 1 | 126 |
| HRSiTERT00100039 | LTR/Copia | 4 | 403 |
| HRSiTERT00100042 | LTR/Copia | 1 | 104 |
| HRSiTERT00100045 | LTR/Copia | 1 | 45 |
| HRSiTERT00100058 | LTR/Copia | 4 | 441 |
| HRSiTERT00100070 | LTR/Copia | 3 | 522 |
| HRSiTERT00100073 | LTR/Copia | 37 | 2899 |
| HRSiTERT00100078 | LTR/Copia | 4 | 930 |
| HRSiTERT00100091 | LTR/Copia | 4 | 547 |

| | | | |
|---|---|---|---|
| HRSiTERT00100095 | LTR/Copia | 3 | 190 |
| HRSiTERT00100096 | LTR/Copia | 9 | 1286 |
| HRSiTERT00100103 | LTR/Copia | 4 | 292 |
| HRSiTERT00100104 | LTR/Copia | 5 | 510 |
| HRSiTERT00100108 | LTR/Copia | 13 | 2115 |
| HRSiTERT00100119 | LTR/Copia | 6 | 857 |
| HRSiTERT00100128 | LTR/Copia | 3 | 542 |
| HRSiTERT00100135 | LTR/Copia | 1 | 171 |
| HRSiTERT00100141 | LTR/Copia | 2 | 229 |
| HRSiTERT00100143 | LTR/Copia | 12 | 1192 |
| HRSiTERT00100150 | LTR/Copia | 1 | 125 |
| HRSiTERT00100151 | LTR/Copia | 7 | 1134 |
| HRSiTERT00100155 | LTR/Copia | 1 | 139 |
| HRSiTERT00100157 | LTR/Copia | 1 | 59 |
| HRSiTERT00100163 | LTR/Copia | 3 | 252 |
| HRSiTERT00200005 | LTR/Gypsy | 3 | 679 |
| HRSiTERT00200006 | LTR/Gypsy | 1 | 86 |
| HRSiTERT00200009 | LTR/Gypsy | 6 | 566 |
| HRSiTERT00200010 | LTR/Copia | 2 | 250 |
| HRSiTERT00200014 | LTR/Gypsy | 32 | 5438 |
| HRSiTERT00200015 | LTR/Gypsy | 175 | 35780 |
| HRSiTERT00200025 | LTR/Gypsy | 5 | 967 |
| HRSiTERT00200026 | LTR/Gypsy | 4 | 387 |
| HRSiTERT00200027 | LTR/Gypsy | 5 | 893 |
| HRSiTERT00200028 | LTR/Gypsy | 5 | 895 |
| HRSiTERT00200029 | LTR/Gypsy | 2 | 301 |
| HRSiTERT00200030 | LTR/Gypsy | 2 | 246 |
| HRSiTERT00200031 | LTR/Gypsy | 4 | 685 |
| HRSiTERT00200033 | LTR/Gypsy | 7 | 509 |
| HRSiTERT00200034 | LTR/Gypsy | 4 | 824 |
| HRSiTERT00200035 | LTR/Gypsy | 10 | 2512 |
| HRSiTERT00200039 | LTR/Gypsy | 1 | 68 |
| HRSiTERT00200040 | LTR/Gypsy | 4 | 834 |
| HRSiTERT00200041 | LTR/Gypsy | 5 | 1115 |
| HRSiTERT00200042 | LTR/Gypsy | 5 | 588 |
| HRSiTERTOOT00001 | LTR/Gypsy | 500 | 58398 |
| HRSiTERTOOT00002 | LTR | 193 | 28070 |
| HRSiTERTOOT00003 | LTR | 150 | 8720 |
| HRSiTERTOOT00004 | LTR | 83 | 3591 |
| HRSiTERTOOT00005 | LTR | 153 | 7890 |
| HRSiTERTOOT00006 | LTR | 3 | 480 |
| HRSiTERTOOT00007 | LTR | 6 | 625 |
| HRSiTERTOOT00019 | LTR | 16 | 2022 |
| HRSiTERTOOT00020 | LTR/Gypsy | 22 | 3531 |
| HRSiTERTOOT00021 | LTR/Gypsy | 37 | 7763 |
| HRSiTERTOOT00022 | LTR | 9 | 914 |
| HRSiTERTOOT00023 | LTR/Copia | 36 | 8523 |

| | | | |
|---|---|---|---|
| HRSiTERTOOT00024 | LTR/Copia | 52 | 10604 |
| HRSiTERTOOT00025 | LTR/Gypsy | 30 | 6234 |
| HRSiTERTOOT00026 | LTR | 11 | 1382 |
| HRSiTERTOOT00027 | LTR/Gypsy | 71 | 7489 |
| HRSiTERTOOT00029 | LTR/Gypsy | 93 | 17363 |
| HRSiTERTOOT00031 | LTR/Gypsy | 36 | 6914 |
| HRSiTERTOOT00032 | LTR/Gypsy | 18 | 2896 |
| HRSiTERTOOT00033 | LTR/Gypsy | 55 | 12363 |
| HRSiTERTOOT00034 | LTR/Gypsy | 51 | 8549 |
| HRSiTERTOOT00035 | LTR/Gypsy | 46 | 9057 |
| HRSiTERTOOT00041 | LTR/Gypsy | 6 | 976 |
| HRSiTETN00400002 | DNA/TcMar | 19 | 4000 |
| HRSiTETN00400003 | DNA/TcMar | 2 | 461 |
| HRSiTETN00400007 | DNA/TcMar | 7 | 2160 |
| HSATII | Satellite | 14 | 1704 |
| LTR-102N04 | LTR | 2169 | 690931 |
| LTR-109K13 | LTR | 5293 | 1390292 |
| LTR-213F23 | LTR/Copia | 350 | 91283 |
| LTR-28F09 | LTR | 142 | 29856 |
| LTR-5B23 | LTR | 8765 | 2576919 |
| LTR-79D10 | LTR/Copia | 115 | 31194 |
| LTR-AB | LTR/Copia | 2130 | 670516 |
| LTR-AB298185.1 | LTR/Copia | 1071 | 375630 |
| LTR-AC177814.2 | LTR/Gypsy | 1 | 40 |
| LTR-AC177815.1 | LTR/Gypsy | 5 | 1058 |
| LTR-AC177820.3 | LTR/Gypsy | 3 | 521 |
| LTR-AC177824.1 | LTR/Gypsy | 1 | 259 |
| LTR-AC177826.3 | LTR | 1 | 78 |
| LTR-AC177828.3 | LTR | 3 | 393 |
| LTR-AC177830.2 | LTR/Copia | 9 | 1157 |
| LTR-AC177831.2 | LTR | 16 | 2618 |
| LTR-AC177833.1 | LTR | 1 | 43 |
| LTR-AC177834.3 | LTR | 20 | 1449 |
| LTR-AC177839.3 | LTR | 1 | 128 |
| LTR-AC177840.2 | LTR | 6 | 1371 |
| LTR-AC177841.1 | LTR | 1 | 54 |
| LTR-AC177847.1 | LTR | 6 | 625 |
| LTR-AC177851.4 | LTR/Copia | 9 | 1556 |
| LTR-AC177852.2 | LTR/Gypsy | 12 | 1115 |
| LTR-AC177857.2 | LTR/Gypsy | 2 | 151 |
| LTR-AC177858.4 | LTR | 1 | 135 |
| LTR-AC177859.4 | LTR/Copia | 1 | 440 |
| LTR-AC177863.2 | LTR | 13 | 2073 |
| LTR-AC177864.4 | LTR/Copia | 1 | 310 |
| LTR-AC177866.1 | LTR | 1 | 183 |
| LTR-AC177868.2 | LTR | 9 | 689 |
| LTR-AC177870.3 | LTR/Copia | 18 | 2185 |

| | | | |
|---|---|---|---|
| LTR-AC177872.1 | LTR | 3 | 164 |
| LTR-AC177873.3 | LTR | 1 | 98 |
| LTR-AC177874.2 | LTR/Gypsy | 3 | 218 |
| LTR-AC177878.4 | LTR | 3 | 401 |
| LTR-AC177879.1 | LTR/Gypsy | 2 | 84 |
| LTR-AC177881.1 | LTR | 1 | 33 |
| LTR-AC177882.3 | LTR/Copia | 3 | 801 |
| LTR-AC177887.2 | LTR/Copia | 1 | 381 |
| LTR-AC177888.2 | LTR/Gypsy | 2 | 193 |
| LTR-AC177889.2 | LTR/Copia | 1 | 187 |
| LTR-AC177891.2 | LTR | 2 | 122 |
| LTR-AC177895.2 | LTR/Gypsy | 2 | 139 |
| LTR-AC177896.4 | LTR/Copia | 6 | 700 |
| LTR-AC177897.1 | LTR | 21 | 2132 |
| LTR-AC177898.2 | LTR/Gypsy | 2 | 258 |
| LTR-AC177899.1 | LTR/Gypsy | 2 | 154 |
| LTR-AC177900.3 | LTR/Copia | 1 | 207 |
| LTR-AC177902.2 | LTR/Copia | 2 | 239 |
| LTR-AC177903.4 | LTR | 3 | 268 |
| LTR-AC177904.2 | LTR | 15 | 2057 |
| LTR-AC177905.2 | LTR/Gypsy | 22 | 3396 |
| LTR-AC177908.3 | LTR | 10 | 1991 |
| LTR-AC177909.3 | LTR/Gypsy | 1 | 39 |
| LTR-AC177910.2 | LTR/Gypsy | 1 | 175 |
| LTR-AC177911.4 | LTR | 2 | 146 |
| LTR-AC177912.1 | LTR | 1 | 89 |
| LTR-AC177916.3 | LTR/Copia | 10 | 976 |
| LTR-AC177917.1 | LTR | 1 | 44 |
| LTR-AC177918.3 | LTR/Gypsy | 3 | 249 |
| LTR-AC177919.2 | LTR | 1 | 136 |
| LTR-AC177921.1 | LTR/Gypsy | 5 | 527 |
| LTR-AC177927.3 | LTR/Gypsy | 3 | 335 |
| LTR-AC177929.2 | LTR | 34 | 4934 |
| LTR-AC177931.2 | LTR/Gypsy | 29 | 3844 |
| LTR-AC177932.2 | LTR/Gypsy | 8 | 658 |
| LTR-AC177934.1 | LTR/Gypsy | 4 | 510 |
| LTR-AC177937.4 | LTR/Gypsy | 1 | 194 |
| LTR-AC177941.3 | LTR/Gypsy | 1 | 42 |
| LTR-AC177942.3 | LTR | 1 | 60 |
| LTR-AC177944.1 | LTR | 1 | 135 |
| LTR-AC177945.3 | LTR/Gypsy | 16 | 1090 |
| LTR-AC177946.2 | LTR/Gypsy | 8 | 979 |
| LTR-AC177947.2 | LTR/Copia | 2 | 356 |
| LTR-AC182104.4 | LTR/Copia | 2 | 672 |
| LTR-AC182105.3 | LTR/Copia | 1 | 55 |
| LTR-AC182112.4 | LTR/Gypsy | 4 | 784 |
| LTR-AC182413.4 | LTR | 1 | 90 |

| | | | |
|---|---|---|---|
| LTR-AC182415.3 | LTR | 2 | 153 |
| LTR-AC182416.2 | LTR | 1 | 161 |
| LTR-AC182419.4 | LTR | 14 | 3145 |
| LTR-AC182421.3 | LTR/Gypsy | 7 | 608 |
| LTR-AC182422.3 | LTR/Gypsy | 9 | 1314 |
| LTR-AC182423.2 | LTR | 4 | 573 |
| LTR-AC182439.3 | LTR | 1 | 71 |
| LTR-AC182440.4 | LTR/Copia | 25 | 1640 |
| LTR-AC182441.5 | LTR | 6 | 722 |
| LTR-AC182481.3 | LTR | 2 | 113 |
| LTR-AC182482.3 | LTR/Copia | 1 | 153 |
| LTR-AC182602.5 | LTR | 4 | 648 |
| LTR-AC182603.4 | LTR/Gypsy | 2 | 107 |
| LTR-AC182605.3 | LTR/Gypsy | 2 | 88 |
| LTR-AC182611.4 | LTR | 4 | 456 |
| LTR-AC182613.4 | LTR | 1 | 55 |
| LTR-AC182615.3 | LTR/Copia | 13 | 862 |
| LTR-AC182618.3 | LTR/Gypsy | 4 | 355 |
| LTR-AC182622.2 | LTR/Gypsy | 1 | 69 |
| LTR-AC182623.2 | LTR | 2 | 121 |
| LTR-AC182624.2 | LTR | 4 | 238 |
| LTR-AC182625.4 | LTR | 2 | 198 |
| LTR-AC182626.3 | LTR/Gypsy | 1 | 66 |
| LTR-AC182627.2 | LTR/Copia | 1 | 54 |
| LTR-AC182818.2 | LTR/Copia | 3 | 183 |
| LTR-AC182819.3 | LTR/Copia | 1 | 81 |
| LTR-AC182822.3 | LTR/Copia | 10 | 651 |
| LTR-AC182826.4 | LTR/Copia | 1 | 39 |
| LTR-AC182828.4 | LTR/Copia | 12 | 660 |
| LTR-AC182829.2 | LTR | 1 | 51 |
| LTR-AC182831.2 | LTR | 12 | 1144 |
| LTR-AC182834.2 | LTR | 10 | 1108 |
| LTR-AC182835.3 | LTR/Gypsy | 3 | 416 |
| LTR-AC182838.2 | LTR/Copia | 1 | 154 |
| LTR-AC183266.3 | LTR | 6 | 549 |
| LTR-AC183311.2 | LTR | 1 | 38 |
| LTR-AC183312.4 | LTR/Copia | 1 | 137 |
| LTR-AC183313.1 | LTR | 1 | 67 |
| LTR-AC183316.3 | LTR/Gypsy | 2 | 370 |
| LTR-AC183318.2 | LTR/Gypsy | 3 | 123 |
| LTR-AC183372.3 | LTR/Copia | 6 | 694 |
| LTR-AC183412.5 | LTR | 19 | 1493 |
| LTR-AC183414.3 | LTR/Gypsy | 7 | 708 |
| LTR-AC183437.2 | LTR/Copia | 1 | 389 |
| LTR-AC183500.2 | LTR/Copia | 1 | 85 |
| LTR-AC183503.2 | LTR/Gypsy | 6 | 630 |
| LTR-AC183504.3 | LTR/Copia | 4 | 648 |

| | | | |
|---|---|---|---|
| LTR-AC183505.3 | LTR/Copia | 4 | 543 |
| LTR-AC183506.3 | LTR/Gypsy | 5 | 294 |
| LTR-AC183508.3 | LTR | 7 | 869 |
| LTR-AC183511.3 | LTR/Gypsy | 1 | 67 |
| LTR-AC183514.3 | LTR/Copia | 1 | 87 |
| LTR-AC183516.2 | LTR/Gypsy | 1 | 73 |
| LTR-AC183518.1 | LTR/Copia | 1 | 48 |
| LTR-AC183590.3 | LTR | 5 | 560 |
| LTR-AC183591.3 | LTR | 2 | 238 |
| LTR-AC183654.2 | LTR | 4 | 244 |
| LTR-AC183655.2 | LTR | 3 | 383 |
| LTR-AC183656.1 | LTR | 2 | 129 |
| LTR-AC183657.2 | LTR | 12 | 1254 |
| LTR-AC183661.2 | LTR/Copia | 479 | 76020 |
| LTR-AC183754.2 | LTR | 4 | 293 |
| LTR-AC183755.2 | LTR/Copia | 4 | 226 |
| LTR-AC183757.3 | LTR/Gypsy | 1 | 161 |
| LTR-AC183758.4 | LTR/Gypsy | 2 | 113 |
| LTR-AC183778.3 | LTR/Gypsy | 7 | 852 |
| LTR-AC183780.2 | LTR | 1 | 72 |
| LTR-AC183783.3 | LTR/Copia | 1 | 43 |
| LTR-AC183784.2 | LTR/Gypsy | 3 | 243 |
| LTR-AC183811.2 | LTR/Gypsy | 1 | 115 |
| LTR-AC183814.2 | LTR | 4 | 489 |
| LTR-AC183816.2 | LTR | 4 | 351 |
| LTR-AC183821.2 | LTR/Gypsy | 1 | 85 |
| LTR-AC183825.3 | LTR | 3 | 285 |
| LTR-AC183891.3 | LTR/Gypsy | 4 | 533 |
| LTR-AC183893.2 | LTR | 1 | 187 |
| LTR-AC183894.2 | LTR/Gypsy | 1 | 51 |
| LTR-AC183895.2 | LTR/Copia | 4 | 519 |
| LTR-AC183896.2 | LTR/Gypsy | 1 | 51 |
| LTR-AC183899.2 | LTR | 8 | 1041 |
| LTR-AC183901.2 | LTR/Gypsy | 1 | 108 |
| LTR-AC183907.2 | LTR/Copia | 1 | 86 |
| LTR-AC183908.2 | LTR/Copia | 2 | 172 |
| LTR-AC183912.2 | LTR/Copia | 2 | 64 |
| LTR-AC183913.3 | LTR/Copia | 2 | 134 |
| LTR-AC183915.4 | LTR | 2 | 188 |
| LTR-AC183916.4 | LTR | 3 | 591 |
| LTR-AC183917.3 | LTR | 4 | 398 |
| LTR-AC183924.4 | LTR/Copia | 3 | 222 |
| LTR-AC183930.3 | LTR/Gypsy | 2 | 194 |
| LTR-AC183932.3 | LTR | 5 | 370 |
| LTR-AC183936.2 | LTR | 2 | 458 |
| LTR-AC183939.2 | LTR | 4 | 265 |
| LTR-AC183942.3 | LTR/Gypsy | 44 | 10064 |

| | | | |
|---|---|---|---|
| LTR-AC183943.3 | LTR | 1 | 103 |
| LTR-AC183944.3 | LTR/Copia | 2 | 360 |
| LTR-AC183945.2 | LTR | 1 | 165 |
| LTR-AC183947.3 | LTR | 4 | 632 |
| LTR-AC183954.3 | LTR | 29 | 1649 |
| LTR-AC184114.3 | LTR/Copia | 1 | 104 |
| LTR-AC184116.3 | LTR/Gypsy | 1 | 127 |
| LTR-AC184118.2 | LTR/Gypsy | 28 | 3325 |
| LTR-AC184128.3 | LTR/Copia | 2 | 124 |
| LTR-AC184129.2 | LTR/Gypsy | 2 | 185 |
| LTR-AC184130.3 | LTR | 1 | 101 |
| LTR-AC184132.3 | LTR/Copia | 1 | 94 |
| LTR-AC184136.3 | LTR | 1 | 88 |
| LTR-AC184138.3 | LTR/Copia | 1 | 168 |
| LTR-AC184139.3 | LTR/Gypsy | 3 | 630 |
| LTR-AC184140.2 | LTR/Gypsy | 7 | 1772 |
| LTR-AC184141.4 | LTR/Copia | 7 | 806 |
| LTR-AC184142.2 | LTR/Copia | 7 | 830 |
| LTR-AC184146.3 | LTR | 5 | 550 |
| LTR-AC184164.2 | LTR/Gypsy | 3 | 442 |
| LTR-AC184166.2 | LTR | 3 | 139 |
| LTR-AC184167.4 | LTR | 2 | 211 |
| LTR-AC184169.2 | LTR | 33 | 4102 |
| LTR-AC184170.3 | LTR | 1 | 61 |
| LTR-AC184171.3 | LTR/Copia | 1 | 140 |
| LTR-AC184692.3 | LTR | 1 | 75 |
| LTR-AC184697.5 | LTR/Gypsy | 4 | 479 |
| LTR-AC184699.2 | LTR/Copia | 10 | 926 |
| LTR-AC184700.3 | LTR | 7 | 399 |
| LTR-AC184701.2 | LTR/Gypsy | 2 | 227 |
| LTR-AC184702.3 | LTR | 4 | 335 |
| LTR-AC184703.4 | LTR | 3 | 468 |
| LTR-AC184706.2 | LTR/Gypsy | 19 | 3107 |
| LTR-AC184765.4 | LTR/Copia | 2 | 383 |
| LTR-AC184768.3 | LTR | 5 | 391 |
| LTR-AC184769.2 | LTR/Gypsy | 3 | 194 |
| LTR-AC184770.3 | LTR/Gypsy | 2 | 233 |
| LTR-AC184772.2 | LTR | 6 | 354 |
| LTR-AC184773.3 | LTR/Gypsy | 1 | 393 |
| LTR-AC184774.4 | LTR/Copia | 44 | 7802 |
| LTR-AC184775.2 | LTR | 4 | 336 |
| LTR-AC184778.2 | LTR/Copia | 1 | 151 |
| LTR-AC184781.3 | LTR/Gypsy | 5 | 465 |
| LTR-AC184784.2 | LTR/Gypsy | 1 | 242 |
| LTR-AC184788.2 | LTR | 3 | 233 |
| LTR-AC184791.3 | LTR/Gypsy | 1 | 66 |
| LTR-AC184792.2 | LTR/Gypsy | 1 | 72 |

| | | | |
|---|---|---|---|
| LTR-AC184794.2 | LTR/Gypsy | 2 | 154 |
| LTR-AC184795.2 | LTR | 1 | 91 |
| LTR-AC184831.3 | LTR/Gypsy | 6 | 836 |
| LTR-AC184832.2 | LTR | 10 | 1257 |
| LTR-AC184833.2 | LTR | 5 | 440 |
| LTR-AC184835.2 | LTR/Copia | 10 | 1662 |
| LTR-AC184836.2 | LTR/Gypsy | 2 | 124 |
| LTR-AC184837.3 | LTR/Gypsy | 1 | 51 |
| LTR-AC184839.3 | LTR/Copia | 1 | 214 |
| LTR-AC184840.3 | LTR/Copia | 1 | 182 |
| LTR-AC184841.2 | LTR/Gypsy | 1 | 170 |
| LTR-AC184843.3 | LTR/Gypsy | 4 | 683 |
| LTR-AC184846.4 | LTR | 1 | 64 |
| LTR-AC184851.3 | LTR/Gypsy | 8 | 1256 |
| LTR-AC184855.3 | LTR | 2 | 83 |
| LTR-AC184858.3 | LTR/Copia | 14 | 1014 |
| LTR-AC184859.3 | LTR/Gypsy | 2 | 78 |
| LTR-AC184860.3 | LTR/Copia | 1 | 67 |
| LTR-AC184862.3 | LTR/Copia | 1 | 137 |
| LTR-AC184865.4 | LTR | 2 | 155 |
| LTR-AC184866.3 | LTR | 9 | 1140 |
| LTR-AC184867.1 | LTR | 6 | 1075 |
| LTR-AC184869.4 | LTR | 3 | 190 |
| LTR-AC184871.2 | LTR/Gypsy | 1 | 199 |
| LTR-AC185109.2 | LTR | 2 | 136 |
| LTR-AC185111.2 | LTR | 7 | 620 |
| LTR-AC185112.2 | LTR/Copia | 3 | 677 |
| LTR-AC185113.2 | LTR/Gypsy | 4 | 325 |
| LTR-AC185114.3 | LTR | 2 | 95 |
| LTR-AC185115.2 | LTR | 2 | 124 |
| LTR-AC185118.2 | LTR | 6 | 797 |
| LTR-AC185119.2 | LTR/Gypsy | 31 | 4078 |
| LTR-AC185120.2 | LTR | 1 | 43 |
| LTR-AC185121.2 | LTR/Gypsy | 8 | 947 |
| LTR-AC185123.1 | LTR/Gypsy | 3 | 514 |
| LTR-AC185126.3 | LTR/Gypsy | 1 | 150 |
| LTR-AC185130.2 | LTR | 9 | 1205 |
| LTR-AC185211.3 | LTR/Gypsy | 6 | 612 |
| LTR-AC185214.3 | LTR | 4 | 238 |
| LTR-AC185215.1 | LTR | 3 | 245 |
| LTR-AC185219.3 | LTR/Gypsy | 36 | 2573 |
| LTR-AC185221.2 | LTR/Copia | 1 | 87 |
| LTR-AC185225.2 | LTR | 1 | 158 |
| LTR-AC185227.2 | LTR | 5 | 738 |
| LTR-AC185228.4 | LTR | 1 | 289 |
| LTR-AC185229.2 | LTR/Gypsy | 9 | 1319 |
| LTR-AC185230.3 | LTR/Copia | 5 | 235 |

| | | | |
|---|---|---|---|
| LTR-AC185231.3 | LTR/Gypsy | 2 | 188 |
| LTR-AC185236.2 | LTR | 1 | 49 |
| LTR-AC185238.3 | LTR | 1 | 104 |
| LTR-AC185250.2 | LTR | 2 | 264 |
| LTR-AC185251.3 | LTR | 35 | 7569 |
| LTR-AC185252.3 | LTR | 1 | 101 |
| LTR-AC185253.3 | LTR/Copia | 2 | 280 |
| LTR-AC185254.3 | LTR | 5 | 305 |
| LTR-AC185256.2 | LTR/Copia | 1 | 64 |
| LTR-AC185260.3 | LTR | 9 | 1383 |
| LTR-AC185261.3 | LTR | 1 | 208 |
| LTR-AC185263.2 | LTR | 43 | 5863 |
| LTR-AC185265.2 | LTR/Gypsy | 3 | 515 |
| LTR-AC185266.2 | LTR | 1 | 46 |
| LTR-AC185268.4 | LTR | 1 | 53 |
| LTR-AC185269.3 | LTR | 2 | 149 |
| LTR-AC185276.2 | LTR | 3 | 262 |
| LTR-AC185277.2 | LTR | 4 | 335 |
| LTR-AC185280.3 | LTR | 3 | 266 |
| LTR-AC185281.3 | LTR/Gypsy | 6 | 277 |
| LTR-AC185284.3 | LTR/Copia | 3 | 225 |
| LTR-AC185287.3 | LTR/Gypsy | 4 | 530 |
| LTR-AC185296.2 | LTR | 1 | 79 |
| LTR-AC185297.2 | LTR/Copia | 1 | 74 |
| LTR-AC185299.3 | LTR | 1 | 146 |
| LTR-AC185300.3 | LTR/Copia | 4 | 575 |
| LTR-AC185302.4 | LTR/Gypsy | 3 | 425 |
| LTR-AC185303.3 | LTR/Gypsy | 2 | 262 |
| LTR-AC185304.3 | LTR/Gypsy | 13 | 653 |
| LTR-AC185305.3 | LTR | 1 | 42 |
| LTR-AC185308.2 | LTR | 253 | 59215 |
| LTR-AC185312.5 | LTR/Gypsy | 13 | 3857 |
| LTR-AC185313.5 | LTR/Gypsy | 1 | 157 |
| LTR-AC185314.3 | LTR/Gypsy | 1 | 50 |
| LTR-AC185315.2 | LTR/Gypsy | 2 | 114 |
| LTR-AC185316.2 | LTR/Gypsy | 1 | 133 |
| LTR-AC185319.3 | LTR/Gypsy | 5 | 573 |
| LTR-AC185321.2 | LTR | 2 | 147 |
| LTR-AC185323.3 | LTR/Gypsy | 2 | 70 |
| LTR-AC185416.2 | LTR | 48 | 8086 |
| LTR-AC185418.2 | LTR/Gypsy | 2 | 205 |
| LTR-AC185419.2 | LTR/Copia | 1 | 226 |
| LTR-AC185420.3 | LTR/Copia | 1 | 119 |
| LTR-AC185422.3 | LTR | 1 | 53 |
| LTR-AC185426.2 | LTR/Gypsy | 3 | 471 |
| LTR-AC185427.5 | LTR/Gypsy | 5 | 307 |
| LTR-AC185428.3 | LTR | 2 | 111 |

| | | | |
|---|---|---|---|
| LTR-AC185430.2 | LTR | 15 | 1925 |
| LTR-AC185431.3 | LTR/Gypsy | 1 | 88 |
| LTR-AC185432.2 | LTR/Gypsy | 5 | 471 |
| LTR-AC185433.2 | LTR/Gypsy | 1 | 52 |
| LTR-AC185434.4 | LTR | 8 | 1620 |
| LTR-AC185435.3 | LTR/Copia | 1 | 97 |
| LTR-AC185436.3 | LTR | 3 | 127 |
| LTR-AC185438.2 | LTR/Copia | 1 | 112 |
| LTR-AC185439.4 | LTR | 1 | 95 |
| LTR-AC185444.2 | LTR | 1 | 419 |
| LTR-AC185445.2 | LTR | 20 | 2685 |
| LTR-AC185446.5 | LTR | 11 | 2371 |
| LTR-AC185452.2 | LTR/Gypsy | 3 | 305 |
| LTR-AC185453.2 | LTR/Gypsy | 14 | 1055 |
| LTR-AC185457.2 | LTR | 1 | 67 |
| LTR-AC185458.3 | LTR | 2 | 294 |
| LTR-AC185462.3 | LTR | 1 | 68 |
| LTR-AC185464.3 | LTR/Gypsy | 2 | 108 |
| LTR-AC185467.2 | LTR | 2 | 97 |
| LTR-AC185470.2 | LTR | 2 | 252 |
| LTR-AC185473.2 | LTR | 24 | 6304 |
| LTR-AC185478.2 | LTR/Copia | 1 | 70 |
| LTR-AC185479.2 | LTR/Gypsy | 19 | 2224 |
| LTR-AC185480.4 | LTR/Copia | 1 | 95 |
| LTR-AC185481.5 | LTR | 2 | 161 |
| LTR-AC185485.2 | LTR/Copia | 2 | 658 |
| LTR-AC185486.2 | LTR/Copia | 4 | 585 |
| LTR-AC185487.4 | LTR | 2 | 226 |
| LTR-AC185488.2 | LTR | 7 | 747 |
| LTR-AC185490.3 | LTR/Gypsy | 12 | 1547 |
| LTR-AC185492.2 | LTR/Copia | 1 | 76 |
| LTR-AC185493.3 | LTR/Copia | 2 | 295 |
| LTR-AC185497.2 | LTR | 1 | 103 |
| LTR-AC185498.3 | LTR/Gypsy | 1 | 128 |
| LTR-AC185500.2 | LTR | 11 | 1383 |
| LTR-AC185501.3 | LTR | 2 | 108 |
| LTR-AC185502.3 | LTR | 3 | 483 |
| LTR-AC185503.2 | LTR | 3 | 370 |
| LTR-AC185505.2 | LTR | 1 | 264 |
| LTR-AC185506.4 | LTR | 2 | 145 |
| LTR-AC185508.3 | LTR/Copia | 8 | 720 |
| LTR-AC185512.2 | LTR | 9 | 1111 |
| LTR-AC185514.2 | LTR/Copia | 1 | 129 |
| LTR-AC185517.2 | LTR | 1 | 48 |
| LTR-AC185519.2 | LTR/Copia | 5 | 588 |
| LTR-AC185521.2 | LTR/Gypsy | 4 | 932 |
| LTR-AC185522.2 | LTR | 1 | 46 |

| | | | |
|---|---|---|---|
| LTR-AC185526.2 | LTR | 2 | 155 |
| LTR-AC185529.2 | LTR/Copia | 19 | 2184 |
| LTR-AC185530.1 | LTR/Gypsy | 1 | 40 |
| LTR-AC185532.3 | LTR/Gypsy | 7 | 1892 |
| LTR-AC185592.4 | LTR/Copia | 2 | 92 |
| LTR-AC185593.3 | LTR/Copia | 1 | 290 |
| LTR-AC185595.2 | LTR/Gypsy | 1 | 220 |
| LTR-AC185596.3 | LTR/Copia | 1 | 201 |
| LTR-AC185597.2 | LTR/Copia | 2 | 439 |
| LTR-AC185598.3 | LTR/Gypsy | 8 | 683 |
| LTR-AC185601.2 | LTR | 1 | 105 |
| LTR-AC185603.4 | LTR | 2 | 145 |
| LTR-AC185605.2 | LTR | 5 | 1301 |
| LTR-AC185608.2 | LTR/Gypsy | 9 | 848 |
| LTR-AC185610.3 | LTR | 1 | 57 |
| LTR-AC185612.2 | LTR/Copia | 48 | 6460 |
| LTR-AC185613.4 | LTR/Gypsy | 1 | 77 |
| LTR-AC185618.2 | LTR/Gypsy | 2 | 447 |
| LTR-AC185620.2 | LTR/Copia | 1 | 50 |
| LTR-AC185625.4 | LTR | 3 | 388 |
| LTR-AC185626.2 | LTR/Gypsy | 1 | 327 |
| LTR-AC185628.2 | LTR/Gypsy | 14 | 1521 |
| LTR-AC185633.2 | LTR | 1 | 87 |
| LTR-AC185635.2 | LTR/Gypsy | 2 | 129 |
| LTR-AC185639.3 | LTR/Gypsy | 4 | 581 |
| LTR-AC185640.3 | LTR | 1 | 53 |
| LTR-AC185642.4 | LTR/Gypsy | 1 | 247 |
| LTR-AC185644.4 | LTR | 8 | 1379 |
| LTR-AC185648.2 | LTR/Copia | 2 | 172 |
| LTR-AC185650.3 | LTR/Copia | 1 | 345 |
| LTR-AC185651.2 | LTR/Copia | 9 | 1100 |
| LTR-AC185653.3 | LTR | 5 | 795 |
| LTR-AC185654.2 | LTR | 46 | 5797 |
| LTR-AC185655.2 | LTR | 1 | 49 |
| LTR-AC185662.2 | LTR/Gypsy | 6 | 599 |
| LTR-AC185664.2 | LTR | 2 | 115 |
| LTR-AC185666.2 | LTR/Gypsy | 2 | 365 |
| LTR-AC185667.2 | LTR/Copia | 1 | 89 |
| LTR-AC185668.2 | LTR | 1 | 40 |
| LTR-AC185669.3 | LTR | 1 | 44 |
| LTR-AC186008.3 | LTR | 2 | 220 |
| LTR-AC186011.2 | LTR/Copia | 1 | 317 |
| LTR-AC186013.3 | LTR/Gypsy | 2 | 106 |
| LTR-AC186015.3 | LTR | 1 | 49 |
| LTR-AC186020.3 | LTR/Gypsy | 2 | 94 |
| LTR-AC186023.2 | LTR/Copia | 6 | 776 |
| LTR-AC186025.3 | LTR | 2 | 384 |

| | | | |
|---|---|---|---|
| LTR-AC186027.2 | LTR/Copia | 5 | 426 |
| LTR-AC186028.2 | LTR/Gypsy | 2 | 308 |
| LTR-AC186030.2 | LTR/Copia | 1 | 267 |
| LTR-AC186139.2 | LTR/Gypsy | 15 | 2773 |
| LTR-AC186142.3 | LTR/Gypsy | 1 | 275 |
| LTR-AC186145.2 | LTR/Gypsy | 4 | 575 |
| LTR-AC186151.2 | LTR | 1 | 48 |
| LTR-AC186152.3 | LTR | 5 | 240 |
| LTR-AC186158.1 | LTR | 3 | 415 |
| LTR-AC186161.3 | LTR/Gypsy | 1 | 245 |
| LTR-AC186162.4 | LTR/Copia | 1 | 77 |
| LTR-AC186163.3 | LTR/Copia | 12 | 1500 |
| LTR-AC186164.2 | LTR | 2 | 329 |
| LTR-AC186167.2 | LTR/Copia | 1 | 175 |
| LTR-AC186187.3 | LTR/Gypsy | 1 | 50 |
| LTR-AC186189.3 | LTR | 5 | 524 |
| LTR-AC186190.4 | LTR | 9 | 1572 |
| LTR-AC186191.3 | LTR/Gypsy | 1 | 51 |
| LTR-AC186220.3 | LTR/Gypsy | 10 | 1764 |
| LTR-AC186221.4 | LTR/Copia | 1 | 54 |
| LTR-AC186224.3 | LTR | 1 | 63 |
| LTR-AC186225.3 | LTR | 93 | 20099 |
| LTR-AC186226.5 | LTR/Copia | 1 | 288 |
| LTR-AC186235.2 | LTR | 3 | 139 |
| LTR-AC186282.3 | LTR/Copia | 1 | 128 |
| LTR-AC186283.4 | LTR/Copia | 2 | 246 |
| LTR-AC186284.2 | LTR | 1 | 450 |
| LTR-AC186285.3 | LTR/Gypsy | 3 | 90 |
| LTR-AC186286.2 | LTR | 2 | 161 |
| LTR-AC186288.3 | LTR/Gypsy | 2 | 98 |
| LTR-AC186302.3 | LTR/Copia | 1 | 39 |
| LTR-AC186306.2 | LTR/Gypsy | 1 | 54 |
| LTR-AC186307.3 | LTR/Gypsy | 2 | 136 |
| LTR-AC186309.2 | LTR | 12 | 2076 |
| LTR-AC186310.3 | LTR/Gypsy | 2 | 205 |
| LTR-AC186311.2 | LTR | 1 | 197 |
| LTR-AC186317.3 | LTR/Gypsy | 57 | 6671 |
| LTR-AC186319.3 | LTR/Gypsy | 7 | 835 |
| LTR-AC186321.5 | LTR/Gypsy | 6 | 615 |
| LTR-AC186322.3 | LTR | 7 | 1803 |
| LTR-AC186326.4 | LTR | 1 | 80 |
| LTR-AC186328.2 | LTR | 15 | 1247 |
| LTR-AC186330.4 | LTR/Gypsy | 2 | 316 |
| LTR-AC186334.4 | LTR/Gypsy | 8 | 810 |
| LTR-AC186335.3 | LTR/Gypsy | 8 | 750 |
| LTR-AC186338.2 | LTR | 1 | 56 |
| LTR-AC186339.2 | LTR/Gypsy | 8 | 943 |

| | | | |
|---|---|---|---|
| LTR-AC186340.4 | LTR/Gypsy | 7 | 741 |
| LTR-AC186341.3 | LTR/Gypsy | 3 | 487 |
| LTR-AC186370.1 | LTR/Copia | 1 | 193 |
| LTR-AC186371.2 | LTR/Copia | 4 | 183 |
| LTR-AC186377.2 | LTR/Gypsy | 3 | 654 |
| LTR-AC186400.5 | LTR/Copia | 1 | 53 |
| LTR-AC186404.2 | LTR | 9 | 645 |
| LTR-AC186406.3 | LTR/Gypsy | 5 | 694 |
| LTR-AC186412.4 | LTR/Gypsy | 1 | 37 |
| LTR-AC186415.2 | LTR | 1 | 272 |
| LTR-AC186416.2 | LTR/Gypsy | 1 | 47 |
| LTR-AC186421.4 | LTR | 54 | 3273 |
| LTR-AC186422.2 | LTR/Gypsy | 1 | 37 |
| LTR-AC186423.2 | LTR/Copia | 1 | 57 |
| LTR-AC186425.4 | LTR/Copia | 1 | 90 |
| LTR-AC186426.3 | LTR/Gypsy | 6 | 333 |
| LTR-AC186430.3 | LTR/Gypsy | 1 | 164 |
| LTR-AC186494.4 | LTR | 3 | 209 |
| LTR-AC186495.2 | LTR/Gypsy | 3 | 152 |
| LTR-AC186498.2 | LTR/Gypsy | 1 | 96 |
| LTR-AC186500.2 | LTR/Gypsy | 1 | 261 |
| LTR-AC186503.2 | LTR/Gypsy | 16 | 1309 |
| LTR-AC186507.3 | LTR/Gypsy | 1 | 45 |
| LTR-AC186508.3 | LTR/Gypsy | 9 | 1386 |
| LTR-AC186509.2 | LTR/Copia | 2 | 251 |
| LTR-AC186510.2 | LTR/Copia | 2 | 118 |
| LTR-AC186513.3 | LTR | 1 | 36 |
| LTR-AC186515.2 | LTR/Copia | 91 | 11005 |
| LTR-AC186518.2 | LTR | 5 | 498 |
| LTR-AC186521.2 | LTR/Copia | 1 | 268 |
| LTR-AC186523.3 | LTR | 3 | 329 |
| LTR-AC186524.2 | LTR | 1 | 45 |
| LTR-AC186526.4 | LTR/Gypsy | 8 | 670 |
| LTR-AC186527.2 | LTR | 5 | 566 |
| LTR-AC186528.2 | LTR/Copia | 3 | 196 |
| LTR-AC186531.5 | LTR | 2 | 128 |
| LTR-AC186563.3 | LTR/Copia | 3 | 212 |
| LTR-AC186573.3 | LTR/Gypsy | 2 | 132 |
| LTR-AC186580.4 | LTR/Copia | 10 | 876 |
| LTR-AC186583.2 | LTR | 10 | 1097 |
| LTR-AC186586.3 | LTR | 6 | 1799 |
| LTR-AC186587.2 | LTR | 2 | 177 |
| LTR-AC186588.2 | LTR/Gypsy | 2 | 367 |
| LTR-AC186590.2 | LTR | 3 | 335 |
| LTR-AC186593.3 | LTR | 2 | 82 |
| LTR-AC186596.2 | LTR/Copia | 1 | 51 |
| LTR-AC186600.2 | LTR/Gypsy | 1 | 148 |

| | | | |
|---|---|---|---|
| LTR-AC186601.3 | LTR/Copia | 1 | 312 |
| LTR-AC186603.3 | LTR/Copia | 2 | 160 |
| LTR-AC186604.3 | LTR | 10 | 1652 |
| LTR-AC186605.2 | LTR | 1 | 38 |
| LTR-AC186606.3 | LTR | 1 | 74 |
| LTR-AC186608.5 | LTR/Gypsy | 3 | 157 |
| LTR-AC186610.3 | LTR/Gypsy | 5 | 404 |
| LTR-AC186611.3 | LTR | 1 | 53 |
| LTR-AC186615.2 | LTR/Gypsy | 2 | 151 |
| LTR-AC186619.2 | LTR/Copia | 3 | 198 |
| LTR-AC186621.3 | LTR/Gypsy | 6 | 460 |
| LTR-AC186622.3 | LTR/Gypsy | 2 | 223 |
| LTR-AC186623.3 | LTR | 1 | 86 |
| LTR-AC186625.2 | LTR/Gypsy | 3 | 327 |
| LTR-AC186626.4 | LTR/Gypsy | 4 | 404 |
| LTR-AC186644.3 | LTR/Gypsy | 2 | 222 |
| LTR-AC186645.3 | LTR/Copia | 3 | 218 |
| LTR-AC186646.3 | LTR | 5 | 375 |
| LTR-AC186647.2 | LTR/Gypsy | 2 | 141 |
| LTR-AC186648.2 | LTR/Copia | 5 | 612 |
| LTR-AC186649.3 | LTR | 19 | 3686 |
| LTR-AC186652.2 | LTR | 2 | 82 |
| LTR-AC186653.3 | LTR/Gypsy | 1 | 70 |
| LTR-AC186654.4 | LTR | 4 | 430 |
| LTR-AC186655.3 | LTR | 1 | 38 |
| LTR-AC186658.2 | LTR/Gypsy | 4 | 279 |
| LTR-AC186659.3 | LTR | 2 | 164 |
| LTR-AC186662.2 | LTR | 15 | 1496 |
| LTR-AC186663.2 | LTR/Gypsy | 2 | 161 |
| LTR-AC186664.3 | LTR | 1 | 102 |
| LTR-AC186669.3 | LTR/Gypsy | 6 | 238 |
| LTR-AC186681.2 | LTR | 2 | 141 |
| LTR-AC186684.3 | LTR/Gypsy | 12 | 993 |
| LTR-AC186687.2 | LTR/Copia | 1 | 52 |
| LTR-AC186689.3 | LTR/Copia | 2 | 364 |
| LTR-AC186691.3 | LTR/Copia | 8 | 445 |
| LTR-AC186741.3 | LTR/Gypsy | 3 | 219 |
| LTR-AC186745.3 | LTR/Gypsy | 1 | 309 |
| LTR-AC186784.3 | LTR/Copia | 2 | 413 |
| LTR-AC186785.3 | LTR/Gypsy | 2 | 234 |
| LTR-AC186788.3 | LTR | 6 | 442 |
| LTR-AC186790.4 | LTR/Copia | 2 | 426 |
| LTR-AC186792.2 | LTR/Gypsy | 5 | 452 |
| LTR-AC186793.2 | LTR/Copia | 2 | 508 |
| LTR-AC186794.3 | LTR | 20 | 3367 |
| LTR-AC186797.2 | LTR | 20 | 1197 |
| LTR-AC186801.4 | LTR | 34 | 7114 |

| | | | |
|---|---|---|---|
| LTR-AC186802.4 | LTR/Copia | 4 | 318 |
| LTR-AC186807.4 | LTR | 1 | 123 |
| LTR-AC186810.3 | LTR/Gypsy | 1 | 32 |
| LTR-AC186818.2 | LTR | 2 | 88 |
| LTR-AC186819.3 | LTR | 1 | 64 |
| LTR-AC186828.2 | LTR/Gypsy | 5 | 567 |
| LTR-AC186862.2 | LTR/Gypsy | 1 | 213 |
| LTR-AC186863.3 | LTR/Gypsy | 3 | 180 |
| LTR-AC186864.2 | LTR | 4 | 384 |
| LTR-AC186866.4 | LTR | 1 | 50 |
| LTR-AC186883.3 | LTR/Gypsy | 1 | 44 |
| LTR-AC186885.3 | LTR/Copia | 2 | 512 |
| LTR-AC186888.2 | LTR | 1 | 63 |
| LTR-AC186890.2 | LTR | 1 | 426 |
| LTR-AC186891.2 | LTR/Gypsy | 2 | 186 |
| LTR-AC186896.4 | LTR | 14 | 2343 |
| LTR-AC186897.3 | LTR | 1 | 280 |
| LTR-AC186898.4 | LTR/Gypsy | 2 | 176 |
| LTR-AC186905.2 | LTR/Gypsy | 2 | 468 |
| LTR-AC187032.5 | LTR/Copia | 13 | 2631 |
| LTR-AC187033.2 | LTR | 1 | 58 |
| LTR-AC187036.2 | LTR | 4 | 165 |
| LTR-AC187037.3 | LTR/Copia | 4 | 661 |
| LTR-AC187047.4 | LTR | 24 | 2044 |
| LTR-AC187049.2 | LTR/Gypsy | 11 | 728 |
| LTR-AC187051.4 | LTR/Gypsy | 1 | 53 |
| LTR-AC187053.3 | LTR | 5 | 1152 |
| LTR-AC187054.3 | LTR/Copia | 1 | 45 |
| LTR-AC187056.3 | LTR | 13 | 2265 |
| LTR-AC187059.2 | LTR/Gypsy | 8 | 699 |
| LTR-AC187060.6 | LTR/Copia | 10 | 662 |
| LTR-AC187063.3 | LTR/Gypsy | 4 | 644 |
| LTR-AC187067.4 | LTR/Gypsy | 1 | 337 |
| LTR-AC187071.4 | LTR | 3 | 250 |
| LTR-AC187072.3 | LTR/Gypsy | 2 | 100 |
| LTR-AC187075.3 | LTR | 1 | 325 |
| LTR-AC187076.3 | LTR | 9 | 938 |
| LTR-AC187078.3 | LTR | 4 | 370 |
| LTR-AC187080.3 | LTR/Gypsy | 1 | 35 |
| LTR-AC187082.2 | LTR | 3 | 491 |
| LTR-AC187084.2 | LTR | 1 | 48 |
| LTR-AC187085.2 | LTR/Gypsy | 19 | 2254 |
| LTR-AC187087.3 | LTR/Gypsy | 3 | 425 |
| LTR-AC187088.2 | LTR/Copia | 11 | 920 |
| LTR-AC187090.3 | LTR/Copia | 1 | 61 |
| LTR-AC187092.5 | LTR | 1 | 63 |
| LTR-AC187093.3 | LTR/Copia | 3 | 265 |

| | | | |
|---|---|---|---|
| LTR-AC187095.3 | LTR/Copia | 1 | 365 |
| LTR-AC187096.4 | LTR | 6 | 402 |
| LTR-AC187097.2 | LTR/Gypsy | 1 | 344 |
| LTR-AC187099.4 | LTR | 3 | 461 |
| LTR-AC187134.3 | LTR | 1 | 266 |
| LTR-AC187135.5 | LTR/Gypsy | 2 | 231 |
| LTR-AC187140.3 | LTR | 5 | 445 |
| LTR-AC187143.3 | LTR/Copia | 1 | 232 |
| LTR-AC187144.3 | LTR/Gypsy | 1 | 120 |
| LTR-AC187150.3 | LTR | 5 | 414 |
| LTR-AC187152.3 | LTR | 1 | 44 |
| LTR-AC187157.3 | LTR/Copia | 1 | 172 |
| LTR-AC187158.4 | LTR/Copia | 6 | 808 |
| LTR-AC187159.2 | LTR | 2 | 250 |
| LTR-AC187162.2 | LTR/Gypsy | 28 | 4337 |
| LTR-AC187203.2 | LTR/Gypsy | 3 | 182 |
| LTR-AC187204.4 | LTR | 3 | 227 |
| LTR-AC187205.3 | LTR | 1 | 52 |
| LTR-AC187212.3 | LTR/Copia | 1 | 45 |
| LTR-AC187217.4 | LTR/Copia | 32 | 9035 |
| LTR-AC187224.3 | LTR | 1 | 89 |
| LTR-AC187226.3 | LTR/Gypsy | 2 | 186 |
| LTR-AC187227.2 | LTR | 1 | 229 |
| LTR-AC187233.3 | LTR/Copia | 2 | 314 |
| LTR-AC187234.2 | LTR/Gypsy | 3 | 197 |
| LTR-AC187235.2 | LTR/Gypsy | 3 | 170 |
| LTR-AC187236.5 | LTR/Gypsy | 2 | 626 |
| LTR-AC187237.3 | LTR/Gypsy | 4 | 508 |
| LTR-AC187239.2 | LTR/Gypsy | 2 | 145 |
| LTR-AC187240.3 | LTR | 2 | 149 |
| LTR-AC187244.4 | LTR/Gypsy | 5 | 788 |
| LTR-AC187248.5 | LTR/Copia | 1 | 63 |
| LTR-AC187251.4 | LTR | 1 | 149 |
| LTR-AC187252.5 | LTR/Copia | 4 | 678 |
| LTR-AC187255.2 | LTR | 13 | 1946 |
| LTR-AC187256.2 | LTR/Gypsy | 12 | 1062 |
| LTR-AC187260.3 | LTR/Copia | 2 | 409 |
| LTR-AC187261.2 | LTR/Copia | 3 | 356 |
| LTR-AC187263.4 | LTR/Copia | 1 | 146 |
| LTR-AC187264.2 | LTR/Gypsy | 8 | 1159 |
| LTR-AC187265.2 | LTR/Gypsy | 2 | 150 |
| LTR-AC187280.3 | LTR/Gypsy | 3 | 220 |
| LTR-AC187283.3 | LTR | 158 | 33791 |
| LTR-AC187284.4 | LTR/Gypsy | 12 | 2399 |
| LTR-AC187285.3 | LTR/Gypsy | 1 | 77 |
| LTR-AC187287.3 | LTR/Gypsy | 4 | 226 |
| LTR-AC187289.2 | LTR/Gypsy | 13 | 2752 |

| | | | |
|---|---|---|---|
| LTR-AC187291.4 | LTR | 10 | 545 |
| LTR-AC187338.2 | LTR/Copia | 1 | 28 |
| LTR-AC187343.3 | LTR | 4 | 424 |
| LTR-AC187384.3 | LTR/Copia | 1 | 183 |
| LTR-AC187386.3 | LTR/Gypsy | 1 | 73 |
| LTR-AC187387.3 | LTR/Copia | 1 | 95 |
| LTR-AC187388.4 | LTR | 2 | 230 |
| LTR-AC187393.2 | LTR | 29 | 3379 |
| LTR-AC187394.3 | LTR | 3 | 183 |
| LTR-AC187399.2 | LTR | 4 | 453 |
| LTR-AC187401.2 | LTR/Gypsy | 3 | 165 |
| LTR-AC187402.4 | LTR | 1 | 49 |
| LTR-AC187403.3 | LTR/Copia | 1 | 181 |
| LTR-AC187404.2 | LTR/Gypsy | 1 | 209 |
| LTR-AC187409.3 | LTR | 4 | 200 |
| LTR-AC187411.4 | LTR/Copia | 22 | 3878 |
| LTR-AC187468.2 | LTR | 4 | 481 |
| LTR-AC187475.3 | LTR | 2 | 137 |
| LTR-AC187476.2 | LTR/Copia | 1 | 75 |
| LTR-AC187480.3 | LTR | 49 | 9438 |
| LTR-AC187481.4 | LTR | 1 | 87 |
| LTR-AC187484.3 | LTR/Gypsy | 1 | 116 |
| LTR-AC187485.3 | LTR/Gypsy | 3 | 502 |
| LTR-AC187551.2 | LTR/Gypsy | 1 | 56 |
| LTR-AC187552.6 | LTR/Gypsy | 1 | 92 |
| LTR-AC187558.4 | LTR/Copia | 11 | 1029 |
| LTR-AC187559.1 | LTR | 3 | 696 |
| LTR-AC187560.4 | LTR/Gypsy | 3 | 293 |
| LTR-AC187562.5 | LTR/Copia | 1 | 109 |
| LTR-AC187564.4 | LTR/Gypsy | 2 | 446 |
| LTR-AC187565.3 | LTR/Gypsy | 2 | 600 |
| LTR-AC187566.4 | LTR | 2 | 133 |
| LTR-AC187580.3 | LTR/Gypsy | 10 | 774 |
| LTR-AC187581.4 | LTR/Gypsy | 3 | 80 |
| LTR-AC187582.4 | LTR | 1 | 86 |
| LTR-AC187658.4 | LTR/Gypsy | 3 | 486 |
| LTR-AC187775.2 | LTR | 2 | 290 |
| LTR-AC187776.5 | LTR | 1 | 71 |
| LTR-AC187777.3 | LTR/Copia | 9 | 1400 |
| LTR-AC187780.3 | LTR/Copia | 1 | 201 |
| LTR-AC187783.2 | LTR/Copia | 1 | 162 |
| LTR-AC187785.2 | LTR/Copia | 1 | 52 |
| LTR-AC187786.3 | LTR/Copia | 5 | 272 |
| LTR-AC187787.2 | LTR/Gypsy | 10 | 1382 |
| LTR-AC187790.2 | LTR | 2 | 100 |
| LTR-AC187791.3 | LTR/Gypsy | 3 | 273 |
| LTR-AC187792.2 | LTR/Copia | 1 | 114 |

| | | | |
|---|---|---|---|
| LTR-AC187797.3 | LTR | 1 | 175 |
| LTR-AC187798.3 | LTR | 17 | 2731 |
| LTR-AC187807.3 | LTR/Gypsy | 1 | 84 |
| LTR-AC187814.3 | LTR/Gypsy | 1 | 125 |
| LTR-AC187819.3 | LTR | 1 | 48 |
| LTR-AC187824.3 | LTR/Gypsy | 2 | 138 |
| LTR-AC187826.2 | LTR/Gypsy | 2 | 166 |
| LTR-AC187832.2 | LTR/Gypsy | 7 | 538 |
| LTR-AC187834.2 | LTR/Gypsy | 2 | 233 |
| LTR-AC187835.4 | LTR | 3 | 306 |
| LTR-AC187839.3 | LTR/Gypsy | 8 | 1300 |
| LTR-AC187840.2 | LTR/Gypsy | 8 | 1167 |
| LTR-AC187844.5 | LTR/Gypsy | 1 | 206 |
| LTR-AC187845.3 | LTR | 9 | 479 |
| LTR-AC187873.2 | LTR/Copia | 1 | 58 |
| LTR-AC187875.1 | LTR | 5 | 276 |
| LTR-AC187878.2 | LTR | 5 | 346 |
| LTR-AC187879.3 | LTR/Gypsy | 3 | 181 |
| LTR-AC187885.2 | LTR | 2 | 186 |
| LTR-AC187886.3 | LTR/Gypsy | 4 | 313 |
| LTR-AC187889.2 | LTR/Gypsy | 5 | 610 |
| LTR-AC187890.4 | LTR | 1 | 237 |
| LTR-AC187891.3 | LTR/Copia | 3 | 408 |
| LTR-AC187893.2 | LTR/Gypsy | 3 | 429 |
| LTR-AC187894.2 | LTR/Gypsy | 1 | 155 |
| LTR-AC187895.2 | LTR/Gypsy | 2 | 147 |
| LTR-AC187898.4 | LTR | 2 | 141 |
| LTR-AC187902.2 | LTR/Copia | 1 | 196 |
| LTR-AC187907.3 | LTR/Gypsy | 13 | 1330 |
| LTR-AC187908.2 | LTR | 18 | 2025 |
| LTR-AC187911.3 | LTR | 2 | 263 |
| LTR-AC187916.2 | LTR/Gypsy | 21 | 3574 |
| LTR-AC187917.2 | LTR | 5 | 680 |
| LTR-AC187918.3 | LTR | 4 | 571 |
| LTR-AC188003.2 | LTR/Gypsy | 1 | 30 |
| LTR-AC188004.2 | LTR/Gypsy | 1 | 47 |
| LTR-AC188006.2 | LTR | 3 | 211 |
| LTR-AC188007.3 | LTR/Gypsy | 7 | 334 |
| LTR-AC188008.3 | LTR | 3 | 407 |
| LTR-AC188009.2 | LTR/Gypsy | 175 | 37370 |
| LTR-AC188010.2 | LTR | 6 | 739 |
| LTR-AC188019.2 | LTR | 11 | 1775 |
| LTR-AC188020.2 | LTR | 1 | 81 |
| LTR-AC188022.4 | LTR | 1 | 69 |
| LTR-AC188023.2 | LTR/Gypsy | 3 | 638 |
| LTR-AC188024.3 | LTR/Gypsy | 5 | 576 |
| LTR-AC188025.2 | LTR/Gypsy | 1 | 54 |

| | | | |
|---|---|---|---|
| LTR-AC188031.4 | LTR/Gypsy | 2 | 133 |
| LTR-AC188032.2 | LTR/Copia | 9 | 624 |
| LTR-AC188033.2 | LTR/Copia | 11 | 1669 |
| LTR-AC188034.4 | LTR/Gypsy | 1 | 210 |
| LTR-AC188124.3 | LTR | 2 | 259 |
| LTR-AC188125.2 | LTR | 23 | 4030 |
| LTR-AC188126.2 | LTR/Gypsy | 10 | 2357 |
| LTR-AC188129.3 | LTR | 2 | 84 |
| LTR-AC188130.2 | LTR/Gypsy | 2 | 251 |
| LTR-AC188145.3 | LTR/Gypsy | 3 | 558 |
| LTR-AC188194.3 | LTR/Copia | 2 | 140 |
| LTR-AC188195.2 | LTR | 1 | 37 |
| LTR-AC188197.2 | LTR/Gypsy | 5 | 304 |
| LTR-AC188377.2 | LTR/Gypsy | 3 | 527 |
| LTR-AC188379.4 | LTR | 1 | 105 |
| LTR-AC188395.3 | LTR | 2 | 101 |
| LTR-AC188396.4 | LTR/Gypsy | 65 | 8046 |
| LTR-AC188582.2 | LTR | 2 | 289 |
| LTR-AC188583.4 | LTR/Copia | 3 | 407 |
| LTR-AC188584.2 | LTR/Gypsy | 1 | 112 |
| LTR-AC188612.2 | LTR/Copia | 1 | 87 |
| LTR-AC188613.4 | LTR | 11 | 1224 |
| LTR-AC188709.2 | LTR | 1 | 358 |
| LTR-AC188710.3 | LTR/Copia | 9 | 2073 |
| LTR-AC188711.2 | LTR/Copia | 50 | 4978 |
| LTR-AC188712.2 | LTR/Gypsy | 6 | 906 |
| LTR-AC188713.2 | LTR | 1 | 29 |
| LTR-AC188715.2 | LTR/Gypsy | 1 | 67 |
| LTR-AC188723.3 | LTR/Gypsy | 4 | 566 |
| LTR-AC188724.3 | LTR | 1 | 128 |
| LTR-AC188725.2 | LTR/Gypsy | 6 | 424 |
| LTR-AC188729.2 | LTR | 2 | 187 |
| LTR-AC188732.3 | LTR/Gypsy | 1 | 116 |
| LTR-AC188733.2 | LTR | 1 | 318 |
| LTR-AC188734.2 | LTR/Gypsy | 79 | 12185 |
| LTR-AC188739.2 | LTR/Copia | 2 | 123 |
| LTR-AC188740.3 | LTR/Gypsy | 2 | 346 |
| LTR-AC188741.3 | LTR | 4 | 392 |
| LTR-AC188742.2 | LTR/Gypsy | 5 | 789 |
| LTR-AC188744.3 | LTR/Gypsy | 17 | 1218 |
| LTR-AC188745.2 | LTR | 13 | 1629 |
| LTR-AC188746.2 | LTR/Gypsy | 1 | 184 |
| LTR-AC188749.4 | LTR | 7 | 657 |
| LTR-AC188751.3 | LTR/Gypsy | 7 | 618 |
| LTR-AC188753.2 | LTR/Gypsy | 3 | 466 |
| LTR-AC188757.4 | LTR/Gypsy | 1 | 149 |
| LTR-AC188758.2 | LTR | 3 | 231 |

| | | | |
|---|---|---|---|
| LTR-AC188773.3 | LTR/Copia | 1 | 114 |
| LTR-AC188777.3 | LTR/Gypsy | 8 | 605 |
| LTR-AC188823.3 | LTR | 9 | 1137 |
| LTR-AC188824.1 | LTR | 7 | 831 |
| LTR-AC188825.2 | LTR/Gypsy | 22 | 2821 |
| LTR-AC188826.3 | LTR/Gypsy | 5 | 1141 |
| LTR-AC188828.2 | LTR | 1 | 68 |
| LTR-AC188829.2 | LTR/Copia | 2 | 283 |
| LTR-AC188833.2 | LTR/Gypsy | 8 | 1577 |
| LTR-AC188835.2 | LTR/Copia | 1 | 179 |
| LTR-AC188836.2 | LTR/Gypsy | 1 | 59 |
| LTR-AC188837.3 | LTR/Gypsy | 3 | 186 |
| LTR-AC188839.3 | LTR/Copia | 1 | 37 |
| LTR-AC188843.2 | LTR/Copia | 3 | 159 |
| LTR-AC188960.3 | LTR/Gypsy | 32 | 4620 |
| LTR-AC188965.2 | LTR/Gypsy | 2 | 226 |
| LTR-AC188967.2 | LTR | 3 | 350 |
| LTR-AC188968.2 | LTR/Gypsy | 3 | 249 |
| LTR-AC188970.3 | LTR/Copia | 14 | 1419 |
| LTR-AC188973.2 | LTR/Copia | 1 | 258 |
| LTR-AC188974.4 | LTR/Copia | 10 | 1593 |
| LTR-AC188975.2 | LTR/Gypsy | 1 | 141 |
| LTR-AC188977.2 | LTR | 2 | 109 |
| LTR-AC188980.2 | LTR/Gypsy | 4 | 240 |
| LTR-AC188981.2 | LTR/Copia | 2 | 125 |
| LTR-AC188985.2 | LTR/Gypsy | 37 | 2954 |
| LTR-AC188987.2 | LTR/Gypsy | 3 | 317 |
| LTR-AC189040.2 | LTR/Copia | 4 | 490 |
| LTR-AC189042.2 | LTR/Gypsy | 6 | 1046 |
| LTR-AC189044.2 | LTR/Gypsy | 1 | 143 |
| LTR-AC189046.2 | LTR/Copia | 3 | 268 |
| LTR-AC189054.2 | LTR/Copia | 1 | 283 |
| LTR-AC189055.3 | LTR/Copia | 1 | 60 |
| LTR-AC189056.2 | LTR | 2 | 119 |
| LTR-AC189057.3 | LTR/Gypsy | 1 | 52 |
| LTR-AC189059.5 | LTR | 10 | 1412 |
| LTR-AC189060.3 | LTR/Gypsy | 1 | 78 |
| LTR-AC189063.2 | LTR | 1 | 54 |
| LTR-AC189065.2 | LTR/Gypsy | 9 | 1242 |
| LTR-AC189067.4 | LTR/Gypsy | 15 | 1833 |
| LTR-AC189069.3 | LTR/Gypsy | 2 | 397 |
| LTR-AC189073.2 | LTR/Copia | 1 | 252 |
| LTR-AC189074.2 | LTR | 1 | 101 |
| LTR-AC189076.2 | LTR/Copia | 1 | 54 |
| LTR-AC189077.2 | LTR/Copia | 3 | 431 |
| LTR-AC189079.2 | LTR | 16 | 1094 |
| LTR-AC189080.2 | LTR | 1 | 49 |

| | | | |
|---|---|---|---|
| LTR-AC189083.3 | LTR | 1 | 100 |
| LTR-AC189091.1 | LTR/Copia | 1 | 57 |
| LTR-AC189092.2 | LTR/Copia | 2 | 262 |
| LTR-AC189093.2 | LTR/Gypsy | 34 | 3534 |
| LTR-AC189099.2 | LTR | 1 | 60 |
| LTR-AC189281.4 | LTR/Copia | 4 | 213 |
| LTR-AC189283.2 | LTR/Gypsy | 59 | 8445 |
| LTR-AC189284.3 | LTR/Gypsy | 4 | 316 |
| LTR-AC189674.3 | LTR | 8 | 866 |
| LTR-AC189695.2 | LTR | 3 | 193 |
| LTR-AC189741.5 | LTR/Gypsy | 1 | 74 |
| LTR-AC189746.2 | LTR/Gypsy | 3 | 193 |
| LTR-AC189753.2 | LTR/Gypsy | 1 | 70 |
| LTR-AC189758.3 | LTR/Gypsy | 6 | 1051 |
| LTR-AC189760.3 | LTR | 4 | 288 |
| LTR-AC189762.3 | LTR/Copia | 2 | 319 |
| LTR-AC189764.2 | LTR | 1 | 70 |
| LTR-AC189766.2 | LTR/Gypsy | 1 | 34 |
| LTR-AC189768.2 | LTR | 1 | 43 |
| LTR-AC189770.3 | LTR/Gypsy | 4 | 236 |
| LTR-AC189771.3 | LTR | 3 | 179 |
| LTR-AC189774.3 | LTR/Copia | 8 | 1421 |
| LTR-AC189776.3 | LTR | 24 | 3066 |
| LTR-AC189780.2 | LTR/Copia | 1 | 151 |
| LTR-AC189784.3 | LTR/Gypsy | 1 | 189 |
| LTR-AC189785.2 | LTR/Copia | 2 | 83 |
| LTR-AC189789.3 | LTR/Gypsy | 9 | 739 |
| LTR-AC189791.2 | LTR | 1 | 39 |
| LTR-AC189792.3 | LTR | 1 | 108 |
| LTR-AC189796.3 | LTR/Gypsy | 16 | 2738 |
| LTR-AC189798.3 | LTR/Gypsy | 1 | 61 |
| LTR-AC189799.3 | LTR/Copia | 34 | 2545 |
| LTR-AC189800.3 | LTR | 6 | 476 |
| LTR-AC189876.3 | LTR/Gypsy | 1 | 233 |
| LTR-AC189877.3 | LTR/Gypsy | 1 | 69 |
| LTR-AC189879.2 | LTR | 4 | 597 |
| LTR-AC189881.3 | LTR | 1 | 69 |
| LTR-AC189882.2 | LTR | 3 | 160 |
| LTR-AC189932.1 | LTR | 2 | 84 |
| LTR-AC189933.1 | LTR/Gypsy | 3 | 209 |
| LTR-AC190037.1 | LTR/Copia | 6 | 881 |
| LTR-AC190483.1 | LTR/Gypsy | 1 | 197 |
| LTR-AC190484.1 | LTR/Gypsy | 17 | 2601 |
| LTR-AC190485.1 | LTR | 8 | 1642 |
| LTR-AC190486.1 | LTR/Gypsy | 3 | 170 |
| LTR-AC190489.1 | LTR | 4 | 270 |
| LTR-AC190490.1 | LTR/Copia | 7 | 980 |

| | | | |
|---|---|---|---|
| LTR-AC190491.1 | LTR | 1 | 100 |
| LTR-AC190492.1 | LTR | 4 | 469 |
| LTR-AC190495.1 | LTR/Copia | 7 | 543 |
| LTR-AC190497.1 | LTR/Gypsy | 3 | 163 |
| LTR-AC190498.1 | LTR | 6 | 500 |
| LTR-AC190500.1 | LTR/Gypsy | 7 | 909 |
| LTR-AC190501.1 | LTR/Gypsy | 2 | 169 |
| LTR-AC190502.1 | LTR | 7 | 534 |
| LTR-AC190503.1 | LTR/Gypsy | 4 | 274 |
| LTR-AC190504.1 | LTR/Gypsy | 2 | 379 |
| LTR-AC190505.1 | LTR | 1 | 101 |
| LTR-AC190506.1 | LTR | 7 | 1269 |
| LTR-AC190507.1 | LTR | 72 | 7852 |
| LTR-AC190509.1 | LTR | 1 | 85 |
| LTR-AC190510.1 | LTR/Gypsy | 40 | 7369 |
| LTR-AC190511.1 | LTR | 3 | 194 |
| LTR-AC190512.1 | LTR/Copia | 2 | 495 |
| LTR-AC190515.1 | LTR | 3 | 177 |
| LTR-AC190516.1 | LTR/Gypsy | 4 | 522 |
| LTR-AC190518.1 | LTR/Gypsy | 2 | 263 |
| LTR-AC190519.1 | LTR/Gypsy | 8 | 585 |
| LTR-AC190521.1 | LTR/Gypsy | 1 | 126 |
| LTR-AC190522.1 | LTR/Copia | 7 | 428 |
| LTR-AC190523.1 | LTR | 43 | 4946 |
| LTR-AC190528.2 | LTR/Copia | 1 | 93 |
| LTR-AC190529.3 | LTR | 17 | 1219 |
| LTR-AC190531.2 | LTR | 1 | 43 |
| LTR-AC190535.3 | LTR | 1 | 208 |
| LTR-AC190536.2 | LTR | 1 | 161 |
| LTR-AC190537.2 | LTR | 3 | 153 |
| LTR-AC190538.1 | LTR | 1 | 169 |
| LTR-AC190540.3 | LTR/Gypsy | 1 | 98 |
| LTR-AC190544.1 | LTR/Copia | 1 | 105 |
| LTR-AC190545.2 | LTR/Gypsy | 10 | 1790 |
| LTR-AC190548.2 | LTR/Gypsy | 2 | 227 |
| LTR-AC190550.2 | LTR/Gypsy | 16 | 1230 |
| LTR-AC190553.2 | LTR | 1 | 56 |
| LTR-AC190555.1 | LTR | 1 | 154 |
| LTR-AC190557.3 | LTR/Gypsy | 1 | 37 |
| LTR-AC190558.2 | LTR | 1 | 176 |
| LTR-AC190560.2 | LTR/Copia | 1 | 193 |
| LTR-AC190563.2 | LTR/Copia | 1 | 146 |
| LTR-AC190565.1 | LTR/Gypsy | 12 | 799 |
| LTR-AC190568.2 | LTR/Gypsy | 5 | 219 |
| LTR-AC190572.4 | LTR | 16 | 1226 |
| LTR-AC190575.2 | LTR/Copia | 1 | 40 |
| LTR-AC190576.2 | LTR/Gypsy | 1 | 129 |

| | | | |
|---|---|---|---|
| LTR-AC190579.3 | LTR/Gypsy | 1 | 53 |
| LTR-AC190580.2 | LTR | 10 | 1050 |
| LTR-AC190582.3 | LTR | 4 | 297 |
| LTR-AC190586.3 | LTR/Copia | 4 | 243 |
| LTR-AC190587.2 | LTR/Copia | 2 | 368 |
| LTR-AC190589.2 | LTR/Copia | 2 | 334 |
| LTR-AC190591.2 | LTR | 31 | 2158 |
| LTR-AC190594.3 | LTR/Gypsy | 1 | 159 |
| LTR-AC190595.3 | LTR/Gypsy | 17 | 2725 |
| LTR-AC190600.1 | LTR/Copia | 125 | 17573 |
| LTR-AC190602.3 | LTR/Copia | 1 | 81 |
| LTR-AC190603.2 | LTR | 1 | 41 |
| LTR-AC190609.2 | LTR | 5 | 270 |
| LTR-AC190610.2 | LTR | 7 | 428 |
| LTR-AC190611.1 | LTR/Gypsy | 1 | 117 |
| LTR-AC190612.2 | LTR | 6 | 723 |
| LTR-AC190617.3 | LTR/Gypsy | 5 | 343 |
| LTR-AC190618.3 | LTR/Gypsy | 5 | 354 |
| LTR-AC190620.3 | LTR | 96 | 17128 |
| LTR-AC190625.2 | LTR | 5 | 595 |
| LTR-AC190629.2 | LTR/Gypsy | 2 | 109 |
| LTR-AC190631.2 | LTR/Copia | 7 | 616 |
| LTR-AC190632.1 | LTR/Gypsy | 1 | 214 |
| LTR-AC190633.1 | LTR/Gypsy | 9 | 507 |
| LTR-AC190634.1 | LTR/Gypsy | 2 | 151 |
| LTR-AC190635.3 | LTR/Gypsy | 2 | 229 |
| LTR-AC190636.2 | LTR/Copia | 1 | 143 |
| LTR-AC190637.2 | LTR/Gypsy | 1 | 115 |
| LTR-AC190639.3 | LTR/Gypsy | 7 | 722 |
| LTR-AC190641.2 | LTR/Gypsy | 1 | 43 |
| LTR-AC190642.1 | LTR/Gypsy | 5 | 309 |
| LTR-AC190644.3 | LTR | 1 | 39 |
| LTR-AC190646.3 | LTR/Gypsy | 2 | 238 |
| LTR-AC190650.3 | LTR/Gypsy | 14 | 1255 |
| LTR-AC190651.2 | LTR/Copia | 1 | 120 |
| LTR-AC190652.3 | LTR/Gypsy | 5 | 489 |
| LTR-AC190655.1 | LTR | 1 | 133 |
| LTR-AC190671.3 | LTR | 14 | 1512 |
| LTR-AC190674.3 | LTR/Copia | 3 | 714 |
| LTR-AC190676.1 | LTR/Copia | 3 | 132 |
| LTR-AC190677.3 | LTR/Gypsy | 6 | 617 |
| LTR-AC190679.3 | LTR/Copia | 3 | 402 |
| LTR-AC190685.3 | LTR | 1 | 240 |
| LTR-AC190687.3 | LTR | 1 | 107 |
| LTR-AC190688.2 | LTR/Gypsy | 17 | 2236 |
| LTR-AC190691.2 | LTR/Gypsy | 8 | 701 |
| LTR-AC190694.2 | LTR/Copia | 2 | 116 |

| | | | |
|---|---|---|---|
| LTR-AC190695.2 | LTR/Gypsy | 1 | 30 |
| LTR-AC190699.1 | LTR | 1 | 74 |
| LTR-AC190700.2 | LTR/Gypsy | 5 | 618 |
| LTR-AC190701.3 | LTR/Gypsy | 3 | 260 |
| LTR-AC190702.3 | LTR | 4 | 700 |
| LTR-AC190708.2 | LTR/Gypsy | 13 | 1263 |
| LTR-AC190709.1 | LTR/Gypsy | 1 | 48 |
| LTR-AC190713.2 | LTR/Gypsy | 5 | 272 |
| LTR-AC190715.2 | LTR/Copia | 8 | 1112 |
| LTR-AC190716.3 | LTR/Copia | 6 | 924 |
| LTR-AC190718.2 | LTR/Gypsy | 3 | 436 |
| LTR-AC190719.1 | LTR/Gypsy | 9 | 712 |
| LTR-AC190723.2 | LTR/Gypsy | 1 | 83 |
| LTR-AC190724.3 | LTR | 12 | 851 |
| LTR-AC190725.3 | LTR/Copia | 5 | 692 |
| LTR-AC190727.1 | LTR/Copia | 2 | 112 |
| LTR-AC190728.3 | LTR/Gypsy | 4 | 703 |
| LTR-AC190730.2 | LTR/Gypsy | 2 | 338 |
| LTR-AC190732.2 | LTR/Copia | 7 | 1125 |
| LTR-AC190733.1 | LTR/Copia | 1 | 70 |
| LTR-AC190734.1 | LTR/Gypsy | 1 | 55 |
| LTR-AC190736.2 | LTR | 7 | 553 |
| LTR-AC190738.2 | LTR/Copia | 19 | 1273 |
| LTR-AC190741.4 | LTR/Gypsy | 10 | 1001 |
| LTR-AC190743.3 | LTR | 1 | 55 |
| LTR-AC190747.2 | LTR/Gypsy | 2 | 333 |
| LTR-AC190751.3 | LTR/Copia | 1 | 163 |
| LTR-AC190756.4 | LTR/Copia | 1 | 475 |
| LTR-AC190762.2 | LTR/Copia | 6 | 421 |
| LTR-AC190763.2 | LTR/Gypsy | 3 | 156 |
| LTR-AC190764.3 | LTR/Gypsy | 7 | 560 |
| LTR-AC190766.1 | LTR/Gypsy | 1 | 222 |
| LTR-AC190768.2 | LTR/Gypsy | 1 | 70 |
| LTR-AC190769.2 | LTR | 3 | 159 |
| LTR-AC190770.2 | LTR/Gypsy | 1 | 105 |
| LTR-AC190771.2 | LTR/Copia | 11 | 728 |
| LTR-AC190774.2 | LTR/Gypsy | 12 | 1019 |
| LTR-AC190780.4 | LTR/Copia | 1 | 70 |
| LTR-AC190785.3 | LTR/Copia | 1 | 80 |
| LTR-AC190786.3 | LTR | 1 | 95 |
| LTR-AC190787.3 | LTR | 6 | 1105 |
| LTR-AC190789.2 | LTR/Copia | 1 | 50 |
| LTR-AC190791.1 | LTR | 1 | 57 |
| LTR-AC190797.3 | LTR/Copia | 1 | 50 |
| LTR-AC190798.2 | LTR/Gypsy | 10 | 2051 |
| LTR-AC190799.1 | LTR | 9 | 1406 |
| LTR-AC190802.3 | LTR | 2 | 151 |

| | | | |
|---|---|---|---|
| LTR-AC190804.3 | LTR/Gypsy | 2 | 291 |
| LTR-AC190806.3 | LTR/Copia | 14 | 1770 |
| LTR-AC190809.1 | LTR | 15 | 1216 |
| LTR-AC190816.3 | LTR | 13 | 1915 |
| LTR-AC190818.2 | LTR/Gypsy | 6 | 1037 |
| LTR-AC190822.1 | LTR/Gypsy | 2 | 162 |
| LTR-AC190823.2 | LTR/Gypsy | 4 | 331 |
| LTR-AC190829.3 | LTR | 21 | 1556 |
| LTR-AC190830.2 | LTR/Gypsy | 5 | 636 |
| LTR-AC190835.3 | LTR/Gypsy | 3 | 175 |
| LTR-AC190839.2 | LTR/Gypsy | 6 | 331 |
| LTR-AC190843.2 | LTR | 1 | 42 |
| LTR-AC190845.2 | LTR/Copia | 1 | 173 |
| LTR-AC190848.2 | LTR | 1 | 57 |
| LTR-AC190849.1 | LTR | 1 | 70 |
| LTR-AC190851.2 | LTR | 10 | 1121 |
| LTR-AC190854.3 | LTR/Gypsy | 4 | 637 |
| LTR-AC190858.3 | LTR/Copia | 2 | 120 |
| LTR-AC190860.1 | LTR/Gypsy | 2 | 272 |
| LTR-AC190861.1 | LTR | 9 | 931 |
| LTR-AC190862.2 | LTR | 2 | 365 |
| LTR-AC190864.4 | LTR/Gypsy | 2 | 80 |
| LTR-AC190865.1 | LTR/Gypsy | 7 | 878 |
| LTR-AC190866.2 | LTR | 1 | 30 |
| LTR-AC190872.1 | LTR/Gypsy | 1 | 160 |
| LTR-AC190874.1 | LTR | 3 | 541 |
| LTR-AC190875.2 | LTR/Copia | 2 | 165 |
| LTR-AC190877.3 | LTR | 21 | 3338 |
| LTR-AC190880.2 | LTR/Copia | 13 | 802 |
| LTR-AC190883.2 | LTR/Copia | 7 | 738 |
| LTR-AC190884.3 | LTR/Copia | 8 | 560 |
| LTR-AC190885.3 | LTR | 2 | 275 |
| LTR-AC190887.3 | LTR/Gypsy | 7 | 1099 |
| LTR-AC190889.2 | LTR/Gypsy | 3 | 238 |
| LTR-AC190895.3 | LTR | 2 | 163 |
| LTR-AC190897.3 | LTR/Gypsy | 17 | 3270 |
| LTR-AC190898.4 | LTR/Copia | 2 | 187 |
| LTR-AC190900.2 | LTR/Copia | 3 | 283 |
| LTR-AC190902.1 | LTR/Copia | 1 | 121 |
| LTR-AC190903.3 | LTR/Gypsy | 1 | 57 |
| LTR-AC190904.1 | LTR | 3 | 743 |
| LTR-AC190909.2 | LTR/Gypsy | 3 | 234 |
| LTR-AC190911.3 | LTR/Gypsy | 2 | 255 |
| LTR-AC190912.2 | LTR/Copia | 10 | 864 |
| LTR-AC190914.1 | LTR | 3 | 522 |
| LTR-AC190917.2 | LTR/Copia | 1 | 169 |
| LTR-AC190919.5 | LTR/Gypsy | 1 | 39 |

| | | | |
|---|---|---|---|
| LTR-AC190923.2 | LTR/Gypsy | 1 | 175 |
| LTR-AC190928.1 | LTR | 11 | 471 |
| LTR-AC190933.3 | LTR/Gypsy | 4 | 389 |
| LTR-AC190935.1 | LTR | 4 | 1208 |
| LTR-AC190937.3 | LTR | 19 | 1259 |
| LTR-AC190938.1 | LTR/Copia | 1 | 68 |
| LTR-AC190939.2 | LTR/Gypsy | 9 | 869 |
| LTR-AC190942.2 | LTR/Gypsy | 3 | 241 |
| LTR-AC190943.2 | LTR/Copia | 2 | 128 |
| LTR-AC190944.2 | LTR/Gypsy | 16 | 4056 |
| LTR-AC190947.2 | LTR | 7 | 343 |
| LTR-AC190949.3 | LTR | 17 | 884 |
| LTR-AC190950.1 | LTR/Gypsy | 8 | 819 |
| LTR-AC190951.2 | LTR/Gypsy | 1 | 56 |
| LTR-AC190953.2 | LTR/Gypsy | 16 | 2389 |
| LTR-AC190956.2 | LTR/Copia | 5 | 805 |
| LTR-AC190958.3 | LTR/Copia | 1 | 92 |
| LTR-AC190959.3 | LTR/Gypsy | 6 | 338 |
| LTR-AC190961.2 | LTR/Gypsy | 1 | 80 |
| LTR-AC190966.2 | LTR/Copia | 1 | 44 |
| LTR-AC190967.2 | LTR/Gypsy | 1 | 53 |
| LTR-AC190969.3 | LTR/Gypsy | 2 | 109 |
| LTR-AC190970.1 | LTR/Copia | 2 | 139 |
| LTR-AC190971.2 | LTR/Copia | 4 | 429 |
| LTR-AC190974.3 | LTR | 1 | 117 |
| LTR-AC190975.3 | LTR/Gypsy | 362 | 52175 |
| LTR-AC190977.2 | LTR | 2 | 236 |
| LTR-AC190980.4 | LTR/Gypsy | 3 | 217 |
| LTR-AC190982.2 | LTR/Copia | 1 | 43 |
| LTR-AC190983.2 | LTR/Copia | 6 | 393 |
| LTR-AC190984.2 | LTR | 5 | 363 |
| LTR-AC190986.2 | LTR/Copia | 7 | 615 |
| LTR-AC190989.2 | LTR/Gypsy | 1 | 170 |
| LTR-AC190992.2 | LTR/Copia | 3 | 477 |
| LTR-AC190995.2 | LTR | 24 | 2161 |
| LTR-AC190996.4 | LTR/Copia | 3 | 155 |
| LTR-AC190997.3 | LTR/Copia | 28 | 4193 |
| LTR-AC190998.3 | LTR | 1 | 71 |
| LTR-AC191002.3 | LTR | 4 | 206 |
| LTR-AC191006.2 | LTR/Gypsy | 8 | 474 |
| LTR-AC191007.2 | LTR | 1 | 43 |
| LTR-AC191009.2 | LTR | 1 | 36 |
| LTR-AC191011.2 | LTR | 1 | 119 |
| LTR-AC191012.1 | LTR/Copia | 3 | 212 |
| LTR-AC191016.3 | LTR | 1611 | 112391 |
| LTR-AC191017.2 | LTR | 6 | 281 |
| LTR-AC191018.4 | LTR | 2 | 100 |

| | | | |
|---|---|---|---|
| LTR-AC191019.1 | LTR | 7 | 601 |
| LTR-AC191020.1 | LTR/Copia | 3 | 303 |
| LTR-AC191023.1 | LTR/Gypsy | 1 | 59 |
| LTR-AC191024.2 | LTR/Gypsy | 1 | 62 |
| LTR-AC191027.2 | LTR/Gypsy | 8 | 595 |
| LTR-AC191030.2 | LTR/Copia | 1 | 52 |
| LTR-AC191033.2 | LTR/Copia | 2 | 108 |
| LTR-AC191035.2 | LTR | 2 | 169 |
| LTR-AC191038.3 | LTR/Gypsy | 2 | 569 |
| LTR-AC191044.2 | LTR/Copia | 2 | 76 |
| LTR-AC191045.2 | LTR/Copia | 1 | 194 |
| LTR-AC191048.1 | LTR | 4 | 410 |
| LTR-AC191049.2 | LTR/Gypsy | 13 | 1330 |
| LTR-AC191051.3 | LTR/Gypsy | 12 | 2424 |
| LTR-AC191053.3 | LTR | 5 | 417 |
| LTR-AC191055.2 | LTR | 1 | 115 |
| LTR-AC191059.2 | LTR | 2 | 119 |
| LTR-AC191061.2 | LTR | 3 | 224 |
| LTR-AC191063.1 | LTR/Copia | 2 | 327 |
| LTR-AC191064.2 | LTR | 2 | 171 |
| LTR-AC191066.1 | LTR/Copia | 4 | 411 |
| LTR-AC191067.3 | LTR | 1 | 47 |
| LTR-AC191068.1 | LTR/Gypsy | 1 | 122 |
| LTR-AC191069.2 | LTR/Gypsy | 1 | 54 |
| LTR-AC191070.2 | LTR | 7 | 764 |
| LTR-AC191071.2 | LTR/Gypsy | 1 | 148 |
| LTR-AC191076.1 | LTR/Copia | 2 | 405 |
| LTR-AC191077.1 | LTR/Copia | 2 | 453 |
| LTR-AC191079.1 | LTR | 14 | 2021 |
| LTR-AC191080.1 | LTR | 10 | 933 |
| LTR-AC191083.2 | LTR | 2 | 236 |
| LTR-AC191084.3 | LTR/Gypsy | 4 | 316 |
| LTR-AC191087.3 | LTR | 1 | 158 |
| LTR-AC191089.3 | LTR/Copia | 8 | 1059 |
| LTR-AC191090.2 | LTR/Gypsy | 7 | 739 |
| LTR-AC191094.2 | LTR | 1 | 133 |
| LTR-AC191097.2 | LTR/Gypsy | 5 | 468 |
| LTR-AC191098.2 | LTR/Copia | 6 | 1960 |
| LTR-AC191099.1 | LTR/Gypsy | 9 | 1248 |
| LTR-AC191101.4 | LTR/Gypsy | 2 | 121 |
| LTR-AC191102.2 | LTR | 4 | 259 |
| LTR-AC191105.1 | LTR/Copia | 8 | 1008 |
| LTR-AC191106.2 | LTR | 8 | 791 |
| LTR-AC191107.3 | LTR/Gypsy | 1 | 47 |
| LTR-AC191111.3 | LTR | 7 | 1040 |
| LTR-AC191112.2 | LTR/Copia | 5 | 421 |
| LTR-AC191113.1 | LTR/Copia | 1 | 237 |

| | | | |
|---|---|---|---|
| LTR-AC191116.2 | LTR/Gypsy | 2 | 578 |
| LTR-AC191117.2 | LTR | 2 | 110 |
| LTR-AC191118.2 | LTR/Gypsy | 5 | 342 |
| LTR-AC191119.1 | LTR/Gypsy | 2 | 72 |
| LTR-AC191121.2 | LTR | 59 | 5654 |
| LTR-AC191122.3 | LTR | 7 | 1486 |
| LTR-AC191123.3 | LTR/Gypsy | 10 | 849 |
| LTR-AC191124.2 | LTR | 2 | 118 |
| LTR-AC191125.3 | LTR/Gypsy | 6 | 853 |
| LTR-AC191127.4 | LTR | 88 | 6366 |
| LTR-AC191134.1 | LTR/Copia | 2 | 174 |
| LTR-AC191138.2 | LTR/Gypsy | 3 | 329 |
| LTR-AC191139.2 | LTR/Gypsy | 29 | 6041 |
| LTR-AC191141.3 | LTR | 23 | 3552 |
| LTR-AC191146.2 | LTR/Copia | 2 | 282 |
| LTR-AC191147.2 | LTR | 7 | 350 |
| LTR-AC191176.2 | LTR/Gypsy | 2 | 275 |
| LTR-AC191177.4 | LTR/Gypsy | 33 | 5132 |
| LTR-AC191242.1 | LTR | 1 | 58 |
| LTR-AC191246.3 | LTR/Copia | 1 | 210 |
| LTR-AC191249.1 | LTR | 3 | 137 |
| LTR-AC191253.2 | LTR/Gypsy | 1 | 153 |
| LTR-AC191257.2 | LTR/Gypsy | 4 | 765 |
| LTR-AC191260.2 | LTR/Gypsy | 1 | 155 |
| LTR-AC191261.2 | LTR | 3 | 281 |
| LTR-AC191262.2 | LTR | 3 | 297 |
| LTR-AC191263.3 | LTR/Gypsy | 1 | 85 |
| LTR-AC191265.3 | LTR/Gypsy | 10 | 1114 |
| LTR-AC191267.2 | LTR/Copia | 1 | 61 |
| LTR-AC191268.2 | LTR/Copia | 4 | 456 |
| LTR-AC191269.2 | LTR/Gypsy | 10 | 817 |
| LTR-AC191270.3 | LTR/Gypsy | 12 | 2006 |
| LTR-AC191273.3 | LTR/Copia | 8 | 475 |
| LTR-AC191276.1 | LTR/Gypsy | 3 | 249 |
| LTR-AC191278.2 | LTR/Copia | 1 | 33 |
| LTR-AC191280.3 | LTR/Copia | 4 | 571 |
| LTR-AC191282.1 | LTR | 3 | 576 |
| LTR-AC191287.1 | LTR | 1 | 157 |
| LTR-AC191291.2 | LTR | 1 | 43 |
| LTR-AC191292.2 | LTR | 1 | 49 |
| LTR-AC191294.2 | LTR/Gypsy | 12 | 2180 |
| LTR-AC191295.1 | LTR | 2 | 170 |
| LTR-AC191305.2 | LTR | 1 | 35 |
| LTR-AC191306.2 | LTR | 2 | 126 |
| LTR-AC191307.2 | LTR | 3 | 118 |
| LTR-AC191310.2 | LTR | 3 | 591 |
| LTR-AC191314.2 | LTR | 2 | 347 |

| | | | |
|---|---|---|---|
| LTR-AC191315.1 | LTR | 4 | 433 |
| LTR-AC191319.3 | LTR | 14 | 1125 |
| LTR-AC191320.2 | LTR | 1 | 50 |
| LTR-AC191321.2 | LTR/Gypsy | 8 | 512 |
| LTR-AC191322.1 | LTR | 1 | 131 |
| LTR-AC191323.2 | LTR/Gypsy | 3 | 689 |
| LTR-AC191324.2 | LTR/Gypsy | 24 | 1149 |
| LTR-AC191325.2 | LTR/Copia | 1 | 81 |
| LTR-AC191327.1 | LTR | 1 | 47 |
| LTR-AC191328.2 | LTR/Copia | 1 | 112 |
| LTR-AC191330.2 | LTR/Copia | 2 | 141 |
| LTR-AC191331.2 | LTR/Gypsy | 21 | 2038 |
| LTR-AC191332.2 | LTR/Gypsy | 3 | 421 |
| LTR-AC191333.2 | LTR/Gypsy | 16 | 1532 |
| LTR-AC191334.1 | LTR/Copia | 5 | 455 |
| LTR-AC191335.2 | LTR/Gypsy | 1 | 116 |
| LTR-AC191337.2 | LTR | 2 | 116 |
| LTR-AC191338.2 | LTR/Gypsy | 8 | 1015 |
| LTR-AC191341.2 | LTR/Gypsy | 1 | 157 |
| LTR-AC191343.2 | LTR | 1 | 157 |
| LTR-AC191345.3 | LTR/Copia | 135 | 12259 |
| LTR-AC191349.3 | LTR | 1 | 65 |
| LTR-AC191350.2 | LTR/Copia | 18 | 1880 |
| LTR-AC191353.2 | LTR/Copia | 5 | 1332 |
| LTR-AC191356.2 | LTR/Gypsy | 3 | 132 |
| LTR-AC191358.2 | LTR | 3 | 252 |
| LTR-AC191360.3 | LTR/Gypsy | 6 | 633 |
| LTR-AC191361.2 | LTR/Copia | 1 | 155 |
| LTR-AC191362.2 | LTR/Gypsy | 48 | 6176 |
| LTR-AC191363.2 | LTR | 40 | 4311 |
| LTR-AC191367.2 | LTR | 3 | 160 |
| LTR-AC191375.2 | LTR/Copia | 5 | 388 |
| LTR-AC191376.3 | LTR | 1 | 160 |
| LTR-AC191377.2 | LTR | 1 | 61 |
| LTR-AC191379.2 | LTR | 1 | 60 |
| LTR-AC191380.3 | LTR | 2 | 117 |
| LTR-AC191382.3 | LTR/Copia | 1 | 132 |
| LTR-AC191384.1 | LTR/Gypsy | 20 | 2922 |
| LTR-AC191386.2 | LTR/Gypsy | 3 | 272 |
| LTR-AC191389.2 | LTR | 2 | 145 |
| LTR-AC191391.2 | LTR/Copia | 4 | 368 |
| LTR-AC191393.3 | LTR | 2 | 70 |
| LTR-AC191397.2 | LTR | 3 | 141 |
| LTR-AC191398.1 | LTR/Gypsy | 2 | 142 |
| LTR-AC191399.2 | LTR | 1 | 43 |
| LTR-AC191407.2 | LTR | 2 | 331 |
| LTR-AC191408.2 | LTR/Gypsy | 3 | 698 |

| | | | |
|---|---|---|---|
| LTR-AC191409.2 | LTR/Copia | 1 | 136 |
| LTR-AC191412.2 | LTR | 126 | 30980 |
| LTR-AC191414.2 | LTR/Gypsy | 30 | 5399 |
| LTR-AC191416.2 | LTR | 1 | 68 |
| LTR-AC191423.2 | LTR/Copia | 1 | 43 |
| LTR-AC191425.4 | LTR/Copia | 3 | 201 |
| LTR-AC191426.2 | LTR | 6 | 443 |
| LTR-AC191427.3 | LTR | 6 | 483 |
| LTR-AC191428.2 | LTR | 1 | 37 |
| LTR-AC191430.2 | LTR/Copia | 5 | 331 |
| LTR-AC191433.3 | LTR/Gypsy | 5 | 640 |
| LTR-AC191455.2 | LTR/Gypsy | 3 | 332 |
| LTR-AC191456.2 | LTR | 6 | 583 |
| LTR-AC191513.3 | LTR/Copia | 4 | 337 |
| LTR-AC191514.3 | LTR/Copia | 1 | 270 |
| LTR-AC191516.3 | LTR/Copia | 7 | 1038 |
| LTR-AC191518.2 | LTR/Copia | 3 | 598 |
| LTR-AC191522.2 | LTR | 4 | 362 |
| LTR-AC191523.2 | LTR/Copia | 1 | 67 |
| LTR-AC191534.2 | LTR | 47 | 5608 |
| LTR-AC191540.2 | LTR/Gypsy | 12 | 943 |
| LTR-AC191541.3 | LTR | 1 | 68 |
| LTR-AC191545.3 | LTR | 1 | 69 |
| LTR-AC191553.3 | LTR/Copia | 1 | 216 |
| LTR-AC191556.2 | LTR | 2 | 184 |
| LTR-AC191558.2 | LTR/Gypsy | 10 | 1159 |
| LTR-AC191561.2 | LTR/Gypsy | 1 | 145 |
| LTR-AC191564.3 | LTR/Copia | 2 | 252 |
| LTR-AC191565.2 | LTR | 14 | 1675 |
| LTR-AC191566.2 | LTR/Copia | 2 | 86 |
| LTR-AC191569.2 | LTR | 20 | 1424 |
| LTR-AC191573.2 | LTR/Gypsy | 1 | 36 |
| LTR-AC191575.2 | LTR/Copia | 1 | 94 |
| LTR-AC191576.2 | LTR/Gypsy | 44 | 10620 |
| LTR-AC191577.2 | LTR | 3 | 390 |
| LTR-AC191583.2 | LTR/Copia | 1 | 54 |
| LTR-AC191584.2 | LTR/Gypsy | 1 | 62 |
| LTR-AC191585.2 | LTR/Copia | 3 | 554 |
| LTR-AC191587.2 | LTR/Gypsy | 6 | 765 |
| LTR-AC191592.2 | LTR | 11 | 1193 |
| LTR-AC191598.2 | LTR | 8 | 1107 |
| LTR-AC191601.1 | LTR/Copia | 23 | 5441 |
| LTR-AC191608.2 | LTR/Gypsy | 2 | 440 |
| LTR-AC191609.2 | LTR/Gypsy | 4 | 449 |
| LTR-AC191610.2 | LTR/Gypsy | 2 | 139 |
| LTR-AC191611.3 | LTR | 1 | 65 |
| LTR-AC191613.2 | LTR | 1 | 52 |

| | | | |
|---|---|---|---|
| LTR-AC191614.2 | LTR/Gypsy | 1 | 125 |
| LTR-AC191616.2 | LTR | 4 | 191 |
| LTR-AC191619.2 | LTR/Gypsy | 31 | 9175 |
| LTR-AC191620.2 | LTR | 1 | 97 |
| LTR-AC191621.2 | LTR | 17 | 2591 |
| LTR-AC191623.3 | LTR | 2 | 462 |
| LTR-AC191624.2 | LTR/Gypsy | 2 | 104 |
| LTR-AC191631.3 | LTR/Gypsy | 3 | 430 |
| LTR-AC191632.3 | LTR/Copia | 4 | 624 |
| LTR-AC191633.2 | LTR | 21 | 3742 |
| LTR-AC191636.2 | LTR | 2 | 253 |
| LTR-AC191639.2 | LTR/Gypsy | 17 | 2647 |
| LTR-AC191643.3 | LTR | 32 | 4841 |
| LTR-AC191649.3 | LTR/Gypsy | 1 | 80 |
| LTR-AC191650.2 | LTR/Gypsy | 3 | 143 |
| LTR-AC191653.2 | LTR/Gypsy | 3 | 280 |
| LTR-AC191654.2 | LTR | 2 | 112 |
| LTR-AC191658.2 | LTR/Gypsy | 1 | 45 |
| LTR-AC191661.2 | LTR/Gypsy | 3 | 222 |
| LTR-AC191662.3 | LTR/Copia | 3 | 121 |
| LTR-AC191663.2 | LTR | 3 | 193 |
| LTR-AC191664.2 | LTR/Copia | 2 | 331 |
| LTR-AC191665.2 | LTR/Gypsy | 3 | 324 |
| LTR-AC191668.2 | LTR/Copia | 2 | 492 |
| LTR-AC191672.2 | LTR | 7 | 653 |
| LTR-AC191674.2 | LTR/Copia | 2 | 252 |
| LTR-AC191675.2 | LTR/Gypsy | 4 | 534 |
| LTR-AC191677.2 | LTR | 18 | 1654 |
| LTR-AC191678.3 | LTR/Gypsy | 1 | 94 |
| LTR-AC191682.3 | LTR/Gypsy | 2 | 525 |
| LTR-AC191683.2 | LTR/Copia | 1 | 58 |
| LTR-AC191685.3 | LTR/Gypsy | 1 | 131 |
| LTR-AC191686.3 | LTR | 8 | 487 |
| LTR-AC191687.2 | LTR/Gypsy | 1 | 40 |
| LTR-AC191690.2 | LTR | 6 | 606 |
| LTR-AC191693.2 | LTR/Copia | 2 | 476 |
| LTR-AC191697.2 | LTR | 31 | 4554 |
| LTR-AC191704.2 | LTR | 2 | 124 |
| LTR-AC191708.2 | LTR | 3 | 227 |
| LTR-AC191714.2 | LTR/Copia | 1 | 118 |
| LTR-AC191718.2 | LTR | 8 | 489 |
| LTR-AC191719.2 | LTR/Copia | 1 | 51 |
| LTR-AC191723.2 | LTR | 1 | 188 |
| LTR-AC191724.2 | LTR/Copia | 1 | 67 |
| LTR-AC191727.2 | LTR/Gypsy | 6 | 692 |
| LTR-AC191732.3 | LTR/Copia | 2 | 68 |
| LTR-AC191736.1 | LTR/Copia | 1 | 158 |

| | | | |
|---|---|---|---|
| LTR-AC191740.3 | LTR | 1 | 113 |
| LTR-AC191741.3 | LTR | 2 | 86 |
| LTR-AC191744.2 | LTR/Gypsy | 1 | 92 |
| LTR-AC191747.2 | LTR/Copia | 5 | 982 |
| LTR-AC191756.2 | LTR | 1 | 31 |
| LTR-AC191759.2 | LTR/Copia | 7 | 838 |
| LTR-AC191764.3 | LTR/Gypsy | 1 | 178 |
| LTR-AC191769.3 | LTR/Gypsy | 16 | 2165 |
| LTR-AC191772.2 | LTR | 3 | 152 |
| LTR-AC191775.3 | LTR/Gypsy | 1 | 33 |
| LTR-AC191779.3 | LTR | 1 | 86 |
| LTR-AC191780.2 | LTR/Gypsy | 1 | 96 |
| LTR-AC191783.3 | LTR/Gypsy | 14 | 566 |
| LTR-AC191786.2 | LTR | 1 | 31 |
| LTR-AC191791.2 | LTR/Gypsy | 1 | 66 |
| LTR-AC191797.2 | LTR/Copia | 80 | 5445 |
| LTR-AC191798.2 | LTR/Copia | 1 | 49 |
| LTR-AC191800.3 | LTR | 4 | 220 |
| LTR-AC191802.2 | LTR | 63 | 5749 |
| LTR-AC191806.2 | LTR/Gypsy | 2 | 228 |
| LTR-AC191808.2 | LTR/Gypsy | 26 | 4026 |
| LTR-AC191809.4 | LTR | 6 | 297 |
| LTR-AC191925.2 | LTR/Gypsy | 1 | 63 |
| LTR-AC191926.2 | LTR/Gypsy | 30 | 4423 |
| LTR-AC191928.3 | LTR | 3 | 189 |
| LTR-AC191931.3 | LTR/Copia | 38 | 3018 |
| LTR-AC191932.2 | LTR/Gypsy | 1 | 92 |
| LTR-AC191950.3 | LTR/Gypsy | 15 | 2355 |
| LTR-AC191952.2 | LTR | 4 | 336 |
| LTR-AC191996.1 | LTR | 4 | 808 |
| LTR-AC191999.1 | LTR/Gypsy | 6 | 513 |
| LTR-AC192096.2 | LTR | 1 | 138 |
| LTR-AC192170.2 | LTR | 2 | 112 |
| LTR-AC192176.2 | LTR/Copia | 3 | 443 |
| LTR-AC192198.3 | LTR/Gypsy | 2 | 226 |
| LTR-AC192201.2 | LTR | 1 | 127 |
| LTR-AC192211.2 | LTR/Copia | 2 | 325 |
| LTR-AC192214.3 | LTR | 54 | 12247 |
| LTR-AC192220.2 | LTR/Gypsy | 1 | 65 |
| LTR-AC192226.3 | LTR/Copia | 1 | 114 |
| LTR-AC192230.2 | LTR | 2 | 179 |
| LTR-AC192231.2 | LTR/Gypsy | 22 | 2169 |
| LTR-AC192232.3 | LTR/Gypsy | 1 | 66 |
| LTR-AC192233.2 | LTR | 3 | 493 |
| LTR-AC192236.2 | LTR | 40 | 5189 |
| LTR-AC192238.2 | LTR | 3 | 494 |
| LTR-AC192247.2 | LTR | 2 | 173 |

| | | | |
|---|---|---|---|
| LTR-AC192249.2 | LTR/Gypsy | 8 | 749 |
| LTR-AC192250.2 | LTR | 17 | 2524 |
| LTR-AC192251.2 | LTR | 13 | 1468 |
| LTR-AC192252.2 | LTR/Copia | 1 | 66 |
| LTR-AC192260.3 | LTR/Gypsy | 1 | 45 |
| LTR-AC192261.2 | LTR | 2 | 144 |
| LTR-AC192263.2 | LTR/Gypsy | 94 | 11042 |
| LTR-AC192264.2 | LTR | 1 | 176 |
| LTR-AC192267.2 | LTR/Gypsy | 8 | 480 |
| LTR-AC192270.2 | LTR | 1 | 62 |
| LTR-AC192282.2 | LTR/Gypsy | 1 | 51 |
| LTR-AC192329.2 | LTR | 1 | 55 |
| LTR-AC192353.2 | LTR/Gypsy | 4 | 823 |
| LTR-AC192354.2 | LTR/Gypsy | 14 | 1300 |
| LTR-AC192355.2 | LTR | 2 | 107 |
| LTR-AC192357.3 | LTR | 2 | 131 |
| LTR-AC192358.3 | LTR/Gypsy | 1 | 58 |
| LTR-AC192359.2 | LTR | 1 | 112 |
| LTR-AC192360.2 | LTR/Copia | 4 | 1005 |
| LTR-AC192362.2 | LTR | 1 | 56 |
| LTR-AC192364.3 | LTR/Copia | 3 | 1005 |
| LTR-AC192365.2 | LTR/Gypsy | 11 | 1706 |
| LTR-AC192366.2 | LTR/Copia | 6 | 785 |
| LTR-AC192369.3 | LTR/Gypsy | 4 | 563 |
| LTR-AC192370.2 | LTR | 1 | 48 |
| LTR-AC192371.3 | LTR/Copia | 1 | 31 |
| LTR-AC192372.2 | LTR | 1 | 54 |
| LTR-AC192373.2 | LTR/Gypsy | 2 | 319 |
| LTR-AC192375.4 | LTR/Gypsy | 1 | 168 |
| LTR-AC192377.3 | LTR/Copia | 6 | 545 |
| LTR-AC192379.2 | LTR | 2 | 258 |
| LTR-AC192382.2 | LTR/Copia | 3 | 538 |
| LTR-AC192383.3 | LTR/Gypsy | 1 | 156 |
| LTR-AC192401.2 | LTR/Gypsy | 2 | 111 |
| LTR-AC192403.2 | LTR | 5 | 548 |
| LTR-AC192405.2 | LTR/Gypsy | 3 | 582 |
| LTR-AC192406.3 | LTR/Gypsy | 8 | 814 |
| LTR-AC192449.3 | LTR | 1 | 56 |
| LTR-AC192450.2 | LTR/Copia | 2 | 371 |
| LTR-AC192452.2 | LTR | 2 | 100 |
| LTR-AC192453.2 | LTR/Gypsy | 1 | 88 |
| LTR-AC192454.2 | LTR/Copia | 2 | 191 |
| LTR-AC192456.2 | LTR/Gypsy | 3 | 205 |
| LTR-AC192457.2 | LTR | 3 | 209 |
| LTR-AC192460.2 | LTR/Gypsy | 2 | 263 |
| LTR-AC192559.2 | LTR/Copia | 1 | 45 |
| LTR-AC192560.3 | LTR | 5 | 370 |

| | | | |
|---|---|---|---|
| LTR-AC192561.2 | LTR/Copia | 1 | 193 |
| LTR-AC192572.3 | LTR | 2 | 121 |
| LTR-AC192589.2 | LTR | 10 | 668 |
| LTR-AC192596.2 | LTR/Copia | 1 | 231 |
| LTR-AC192600.3 | LTR/Gypsy | 13 | 1992 |
| LTR-AC192602.2 | LTR/Gypsy | 3 | 283 |
| LTR-AC193309.2 | LTR/Gypsy | 34 | 5428 |
| LTR-AC193311.2 | LTR/Gypsy | 1 | 93 |
| LTR-AC193313.2 | LTR | 1 | 76 |
| LTR-AC193315.2 | LTR/Copia | 1 | 379 |
| LTR-AC193320.2 | LTR | 1 | 63 |
| LTR-AC193328.2 | LTR/Copia | 1 | 134 |
| LTR-AC193340.3 | LTR | 1 | 110 |
| LTR-AC193341.2 | LTR | 2 | 201 |
| LTR-AC193342.3 | LTR/Copia | 1 | 31 |
| LTR-AC193344.3 | LTR/Gypsy | 2 | 84 |
| LTR-AC193348.2 | LTR | 3 | 259 |
| LTR-AC193356.2 | LTR | 1 | 105 |
| LTR-AC193359.3 | LTR/Gypsy | 9 | 797 |
| LTR-AC193360.2 | LTR/Gypsy | 2 | 251 |
| LTR-AC193363.3 | LTR | 1 | 40 |
| LTR-AC193364.2 | LTR | 1 | 40 |
| LTR-AC193368.2 | LTR/Gypsy | 2 | 138 |
| LTR-AC193369.2 | LTR | 1 | 325 |
| LTR-AC193370.2 | LTR/Copia | 3 | 419 |
| LTR-AC193375.2 | LTR | 2 | 106 |
| LTR-AC193378.3 | LTR/Copia | 5 | 676 |
| LTR-AC193379.2 | LTR/Copia | 1 | 166 |
| LTR-AC193384.3 | LTR/Gypsy | 10 | 1936 |
| LTR-AC193385.2 | LTR | 1 | 198 |
| LTR-AC193391.3 | LTR/Gypsy | 1 | 151 |
| LTR-AC193392.2 | LTR/Copia | 3 | 785 |
| LTR-AC193394.2 | LTR/Gypsy | 2 | 97 |
| LTR-AC193395.3 | LTR/Gypsy | 1 | 85 |
| LTR-AC193396.2 | LTR/Gypsy | 2 | 333 |
| LTR-AC193399.2 | LTR/Gypsy | 2 | 113 |
| LTR-AC193401.3 | LTR/Gypsy | 15 | 1673 |
| LTR-AC193404.2 | LTR | 7 | 622 |
| LTR-AC193406.2 | LTR | 15 | 1174 |
| LTR-AC193409.3 | LTR/Copia | 1 | 33 |
| LTR-AC193412.2 | LTR | 3 | 180 |
| LTR-AC193415.2 | LTR | 1 | 28 |
| LTR-AC193416.2 | LTR/Gypsy | 10 | 928 |
| LTR-AC193419.3 | LTR | 7 | 604 |
| LTR-AC193420.2 | LTR | 10 | 1508 |
| LTR-AC193424.2 | LTR | 1 | 453 |
| LTR-AC193429.2 | LTR/Gypsy | 18 | 2733 |

| | | | |
|---|---|---|---|
| LTR-AC193433.2 | LTR | 2 | 147 |
| LTR-AC193436.3 | LTR/Copia | 2 | 122 |
| LTR-AC193438.3 | LTR | 1 | 89 |
| LTR-AC193440.2 | LTR | 2 | 135 |
| LTR-AC193443.2 | LTR | 2 | 93 |
| LTR-AC193444.2 | LTR | 1 | 72 |
| LTR-AC193445.2 | LTR/Gypsy | 1 | 119 |
| LTR-AC193447.2 | LTR | 5 | 407 |
| LTR-AC193448.3 | LTR | 1 | 54 |
| LTR-AC193449.2 | LTR/Gypsy | 2 | 159 |
| LTR-AC193450.2 | LTR/Copia | 18 | 1239 |
| LTR-AC193454.2 | LTR/Copia | 1 | 38 |
| LTR-AC193455.2 | LTR/Gypsy | 1 | 79 |
| LTR-AC193457.2 | LTR/Gypsy | 1 | 93 |
| LTR-AC193459.2 | LTR/Copia | 1 | 239 |
| LTR-AC193463.2 | LTR/Gypsy | 4 | 330 |
| LTR-AC193466.2 | LTR | 4 | 249 |
| LTR-AC193470.2 | LTR/Copia | 14 | 581 |
| LTR-AC193474.2 | LTR/Gypsy | 4 | 471 |
| LTR-AC193479.2 | LTR | 1 | 112 |
| LTR-AC193484.2 | LTR/Copia | 1 | 83 |
| LTR-AC193485.3 | LTR/Gypsy | 1 | 318 |
| LTR-AC193487.2 | LTR/Copia | 4 | 180 |
| LTR-AC193488.2 | LTR | 1 | 411 |
| LTR-AC193490.3 | LTR | 1 | 119 |
| LTR-AC193497.3 | LTR/Gypsy | 2 | 206 |
| LTR-AC193500.2 | LTR/Gypsy | 7 | 389 |
| LTR-AC193502.2 | LTR/Copia | 19 | 1237 |
| LTR-AC193503.2 | LTR | 4 | 603 |
| LTR-AC193504.3 | LTR/Gypsy | 1 | 302 |
| LTR-AC193519.3 | LTR/Gypsy | 1 | 177 |
| LTR-AC193528.2 | LTR | 12 | 1649 |
| LTR-AC193529.3 | LTR | 6 | 497 |
| LTR-AC193573.3 | LTR | 1 | 89 |
| LTR-AC193578.2 | LTR/Gypsy | 4 | 584 |
| LTR-AC193581.2 | LTR | 1 | 124 |
| LTR-AC193583.2 | LTR | 5 | 510 |
| LTR-AC193584.2 | LTR/Copia | 1 | 194 |
| LTR-AC193587.3 | LTR/Gypsy | 2 | 277 |
| LTR-AC193588.2 | LTR/Copia | 1 | 371 |
| LTR-AC193589.2 | LTR/Gypsy | 6 | 1006 |
| LTR-AC193592.2 | LTR/Gypsy | 1 | 251 |
| LTR-AC193599.3 | LTR/Gypsy | 4 | 403 |
| LTR-AC193615.2 | LTR/Gypsy | 19 | 1275 |
| LTR-AC193626.2 | LTR | 8 | 656 |
| LTR-AC193628.2 | LTR/Gypsy | 2 | 577 |
| LTR-AC193629.3 | LTR/Gypsy | 4 | 180 |

| | | | |
|---|---|---|---|
| LTR-AC193630.2 | LTR/Gypsy | 1 | 64 |
| LTR-AC193647.2 | LTR/Copia | 2 | 149 |
| LTR-AC193656.2 | LTR | 2 | 78 |
| LTR-AC193658.2 | LTR | 7 | 507 |
| LTR-AC193659.3 | LTR | 3 | 180 |
| LTR-AC193661.2 | LTR | 3 | 259 |
| LTR-AC193664.2 | LTR/Gypsy | 2 | 190 |
| LTR-AC193667.3 | LTR | 6 | 443 |
| LTR-AC193668.3 | LTR | 2 | 120 |
| LTR-AC193670.2 | LTR/Gypsy | 1 | 59 |
| LTR-AC193745.2 | LTR/Gypsy | 8 | 1322 |
| LTR-AC193746.2 | LTR | 1 | 140 |
| LTR-AC193747.3 | LTR/Gypsy | 3 | 292 |
| LTR-AC193749.2 | LTR/Copia | 2 | 154 |
| LTR-AC193752.2 | LTR | 1 | 52 |
| LTR-AC193754.2 | LTR/Gypsy | 4 | 224 |
| LTR-AC193755.2 | LTR/Copia | 2 | 342 |
| LTR-AC193756.2 | LTR/Gypsy | 3 | 642 |
| LTR-AC193767.3 | LTR/Gypsy | 1 | 61 |
| LTR-AC193772.2 | LTR/Gypsy | 1 | 65 |
| LTR-AC193773.1 | LTR/Gypsy | 20 | 1374 |
| LTR-AC193782.2 | LTR | 2 | 244 |
| LTR-AC193784.2 | LTR | 2 | 449 |
| LTR-AC193785.2 | LTR/Gypsy | 6 | 1085 |
| LTR-AC193786.2 | LTR/Gypsy | 1 | 109 |
| LTR-AC193788.2 | LTR/Gypsy | 3 | 265 |
| LTR-AC193789.2 | LTR | 1 | 90 |
| LTR-AC193790.2 | LTR | 1 | 281 |
| LTR-AC193791.2 | LTR/Gypsy | 1 | 58 |
| LTR-AC193800.2 | LTR/Gypsy | 1 | 78 |
| LTR-AC193937.2 | LTR | 3 | 213 |
| LTR-AC193939.2 | LTR/Copia | 1 | 302 |
| LTR-AC193943.2 | LTR | 1 | 154 |
| LTR-AC193944.2 | LTR/Gypsy | 5 | 1016 |
| LTR-AC193946.2 | LTR/Copia | 117 | 20196 |
| LTR-AC193947.2 | LTR | 7 | 767 |
| LTR-AC193948.2 | LTR/Gypsy | 2 | 286 |
| LTR-AC193949.2 | LTR | 1 | 141 |
| LTR-AC193951.2 | LTR/Gypsy | 2 | 164 |
| LTR-AC193953.3 | LTR/Gypsy | 9 | 1626 |
| LTR-AC193958.2 | LTR/Gypsy | 3 | 859 |
| LTR-AC193960.2 | LTR/Copia | 3 | 135 |
| LTR-AC193966.2 | LTR/Gypsy | 6 | 621 |
| LTR-AC193967.2 | LTR/Copia | 3 | 513 |
| LTR-AC193972.3 | LTR/Gypsy | 2 | 145 |
| LTR-AC193974.2 | LTR/Copia | 2 | 100 |
| LTR-AC193984.2 | LTR/Copia | 4 | 207 |

| | | | |
|---|---|---|---|
| LTR-AC193996.2 | LTR/Gypsy | 2 | 193 |
| LTR-AC193997.2 | LTR | 2 | 146 |
| LTR-AC194000.3 | LTR/Gypsy | 13 | 1940 |
| LTR-AC194002.2 | LTR/Gypsy | 7 | 1159 |
| LTR-AC194004.2 | LTR/Gypsy | 1 | 90 |
| LTR-AC194005.2 | LTR | 1 | 47 |
| LTR-AC194009.2 | LTR | 2 | 88 |
| LTR-AC194016.2 | LTR | 31 | 2727 |
| LTR-AC194022.2 | LTR | 1 | 128 |
| LTR-AC194025.2 | LTR/Gypsy | 1 | 74 |
| LTR-AC194040.2 | LTR/Copia | 5 | 1180 |
| LTR-AC194041.2 | LTR/Copia | 1 | 144 |
| LTR-AC194050.2 | LTR/Copia | 2 | 217 |
| LTR-AC194061.2 | LTR/Copia | 3 | 336 |
| LTR-AC194062.2 | LTR | 2 | 371 |
| LTR-AC194063.2 | LTR/Gypsy | 2 | 248 |
| LTR-AC194064.2 | LTR/Gypsy | 3 | 333 |
| LTR-AC194068.2 | LTR/Gypsy | 4 | 562 |
| LTR-AC194072.2 | LTR/Copia | 5 | 207 |
| LTR-AC194076.2 | LTR/Copia | 45 | 2472 |
| LTR-AC194077.2 | LTR/Copia | 8 | 621 |
| LTR-AC194084.2 | LTR/Gypsy | 2 | 72 |
| LTR-AC194085.2 | LTR | 3 | 466 |
| LTR-AC194088.2 | LTR/Gypsy | 3 | 201 |
| LTR-AC194089.2 | LTR/Gypsy | 1 | 223 |
| LTR-AC194091.2 | LTR/Copia | 2 | 140 |
| LTR-AC194092.2 | LTR | 18 | 1721 |
| LTR-AC194093.2 | LTR | 1 | 26 |
| LTR-AC194098.2 | LTR | 2 | 349 |
| LTR-AC194105.2 | LTR/Gypsy | 1 | 157 |
| LTR-AC194106.2 | LTR/Gypsy | 17 | 4029 |
| LTR-AC194112.2 | LTR/Copia | 1 | 65 |
| LTR-AC194116.2 | LTR/Gypsy | 3 | 220 |
| LTR-AC194119.3 | LTR | 3 | 344 |
| LTR-AC194120.2 | LTR/Copia | 3 | 243 |
| LTR-AC194122.2 | LTR/Gypsy | 1 | 48 |
| LTR-AC194123.2 | LTR | 9 | 1251 |
| LTR-AC194130.2 | LTR/Gypsy | 3 | 321 |
| LTR-AC194133.2 | LTR/Gypsy | 6 | 496 |
| LTR-AC194138.2 | LTR/Gypsy | 5 | 448 |
| LTR-AC194139.2 | LTR | 1 | 46 |
| LTR-AC194142.2 | LTR | 3 | 574 |
| LTR-AC194143.2 | LTR/Copia | 1 | 45 |
| LTR-AC194147.2 | LTR | 2 | 173 |
| LTR-AC194149.2 | LTR | 1 | 86 |
| LTR-AC194152.2 | LTR/Gypsy | 6 | 352 |
| LTR-AC194154.2 | LTR/Copia | 2 | 190 |

| | | | |
|---|---|---|---|
| LTR-AC194162.2 | LTR | 9 | 755 |
| LTR-AC194163.3 | LTR | 4 | 378 |
| LTR-AC194166.2 | LTR | 1 | 127 |
| LTR-AC194168.2 | LTR | 3 | 466 |
| LTR-AC194172.2 | LTR | 1 | 105 |
| LTR-AC194174.2 | LTR/Copia | 1 | 110 |
| LTR-AC194176.2 | LTR/Gypsy | 1 | 32 |
| LTR-AC194185.2 | LTR/Copia | 1 | 76 |
| LTR-AC194187.3 | LTR | 97 | 15677 |
| LTR-AC194189.2 | LTR/Gypsy | 3 | 323 |
| LTR-AC194192.2 | LTR/Copia | 6 | 408 |
| LTR-AC194204.2 | LTR/Gypsy | 13 | 1585 |
| LTR-AC194205.2 | LTR | 4 | 525 |
| LTR-AC194206.2 | LTR/Gypsy | 1 | 182 |
| LTR-AC194210.2 | LTR | 9 | 1209 |
| LTR-AC194211.2 | LTR/Gypsy | 10 | 742 |
| LTR-AC194213.2 | LTR | 6 | 901 |
| LTR-AC194215.2 | LTR/Gypsy | 1 | 131 |
| LTR-AC194218.2 | LTR | 2 | 148 |
| LTR-AC194223.2 | LTR | 3 | 172 |
| LTR-AC194227.2 | LTR/Gypsy | 11 | 1956 |
| LTR-AC194228.2 | LTR/Gypsy | 1 | 74 |
| LTR-AC194229.3 | LTR/Gypsy | 2 | 135 |
| LTR-AC194230.2 | LTR | 109 | 16414 |
| LTR-AC194231.2 | LTR/Gypsy | 4 | 756 |
| LTR-AC194234.2 | LTR | 1 | 62 |
| LTR-AC194238.2 | LTR | 1 | 72 |
| LTR-AC194240.2 | LTR | 1 | 44 |
| LTR-AC194242.2 | LTR | 1 | 40 |
| LTR-AC194243.2 | LTR | 1 | 192 |
| LTR-AC194244.1 | LTR | 14 | 3346 |
| LTR-AC194245.2 | LTR/Copia | 2 | 416 |
| LTR-AC194246.2 | LTR/Gypsy | 6 | 233 |
| LTR-AC194249.3 | LTR | 1 | 67 |
| LTR-AC194250.2 | LTR/Copia | 2 | 200 |
| LTR-AC194253.2 | LTR | 1 | 70 |
| LTR-AC194254.2 | LTR/Gypsy | 3 | 125 |
| LTR-AC194258.2 | LTR | 4 | 323 |
| LTR-AC194259.2 | LTR/Copia | 1 | 79 |
| LTR-AC194260.2 | LTR/Gypsy | 3 | 1016 |
| LTR-AC194263.2 | LTR | 2 | 192 |
| LTR-AC194266.2 | LTR/Copia | 2 | 85 |
| LTR-AC194268.2 | LTR/Gypsy | 20 | 3885 |
| LTR-AC194272.2 | LTR | 1 | 55 |
| LTR-AC194273.2 | LTR/Copia | 2 | 163 |
| LTR-AC194274.2 | LTR/Gypsy | 1 | 71 |
| LTR-AC194275.2 | LTR | 1 | 77 |

| | | | |
|---|---|---|---|
| LTR-AC194281.2 | LTR | 3 | 510 |
| LTR-AC194282.2 | LTR | 1 | 77 |
| LTR-AC194285.2 | LTR/Gypsy | 4 | 949 |
| LTR-AC194286.2 | LTR/Gypsy | 5 | 259 |
| LTR-AC194289.2 | LTR | 3 | 391 |
| LTR-AC194291.2 | LTR/Gypsy | 8 | 1050 |
| LTR-AC194292.2 | LTR/Gypsy | 9 | 1003 |
| LTR-AC194293.2 | LTR/Gypsy | 7 | 1079 |
| LTR-AC194295.3 | LTR/Gypsy | 1 | 143 |
| LTR-AC194296.2 | LTR/Copia | 1 | 95 |
| LTR-AC194298.2 | LTR/Copia | 1 | 279 |
| LTR-AC194301.2 | LTR | 13 | 2265 |
| LTR-AC194303.2 | LTR | 9 | 864 |
| LTR-AC194308.2 | LTR | 7 | 697 |
| LTR-AC194312.2 | LTR/Copia | 6 | 865 |
| LTR-AC194320.2 | LTR | 3 | 237 |
| LTR-AC194321.2 | LTR/Gypsy | 8 | 1125 |
| LTR-AC194322.2 | LTR/Gypsy | 1 | 154 |
| LTR-AC194324.2 | LTR | 5 | 511 |
| LTR-AC194325.2 | LTR | 3 | 222 |
| LTR-AC194327.2 | LTR/Copia | 1 | 125 |
| LTR-AC194329.2 | LTR/Gypsy | 6 | 466 |
| LTR-AC194331.2 | LTR | 2 | 260 |
| LTR-AC194346.2 | LTR/Gypsy | 6 | 1069 |
| LTR-AC194347.2 | LTR | 1 | 76 |
| LTR-AC194351.2 | LTR | 6 | 435 |
| LTR-AC194352.2 | LTR | 2 | 91 |
| LTR-AC194360.2 | LTR/Copia | 5 | 431 |
| LTR-AC194367.2 | LTR/Gypsy | 1 | 54 |
| LTR-AC194371.2 | LTR/Copia | 10 | 1374 |
| LTR-AC194373.3 | LTR/Copia | 2 | 157 |
| LTR-AC194375.2 | LTR/Copia | 5 | 573 |
| LTR-AC194379.2 | LTR/Gypsy | 5 | 455 |
| LTR-AC194381.2 | LTR/Copia | 3 | 698 |
| LTR-AC194385.2 | LTR/Gypsy | 3 | 179 |
| LTR-AC194386.2 | LTR | 20 | 1175 |
| LTR-AC194399.2 | LTR | 1 | 78 |
| LTR-AC194404.2 | LTR/Gypsy | 11 | 2330 |
| LTR-AC194405.2 | LTR/Gypsy | 10 | 2256 |
| LTR-AC194407.3 | LTR | 1 | 37 |
| LTR-AC194410.3 | LTR/Gypsy | 1 | 38 |
| LTR-AC194414.2 | LTR | 1 | 37 |
| LTR-AC194416.2 | LTR | 1 | 132 |
| LTR-AC194419.2 | LTR/Gypsy | 4 | 273 |
| LTR-AC194420.2 | LTR/Gypsy | 2 | 225 |
| LTR-AC194421.2 | LTR | 2 | 142 |
| LTR-AC194423.2 | LTR/Gypsy | 1 | 36 |

| | | | |
|---|---|---|---|
| LTR-AC194425.2 | LTR/Gypsy | 1 | 68 |
| LTR-AC194428.2 | LTR/Copia | 2 | 321 |
| LTR-AC194431.2 | LTR/Gypsy | 13 | 3310 |
| LTR-AC194433.2 | LTR/Copia | 2 | 192 |
| LTR-AC194438.2 | LTR/Copia | 13 | 687 |
| LTR-AC194441.2 | LTR/Gypsy | 1 | 69 |
| LTR-AC194442.2 | LTR | 2 | 187 |
| LTR-AC194443.2 | LTR | 1 | 349 |
| LTR-AC194444.2 | LTR/Gypsy | 8 | 826 |
| LTR-AC194446.2 | LTR | 1 | 33 |
| LTR-AC194447.2 | LTR/Gypsy | 7 | 540 |
| LTR-AC194449.2 | LTR/Gypsy | 1 | 125 |
| LTR-AC194451.2 | LTR/Gypsy | 2 | 430 |
| LTR-AC194454.2 | LTR | 5 | 743 |
| LTR-AC194458.2 | LTR/Copia | 1 | 41 |
| LTR-AC194459.2 | LTR/Copia | 1 | 125 |
| LTR-AC194461.2 | LTR/Copia | 4 | 461 |
| LTR-AC194462.2 | LTR/Copia | 1 | 273 |
| LTR-AC194463.2 | LTR/Copia | 13 | 1408 |
| LTR-AC194464.2 | LTR/Gypsy | 1 | 184 |
| LTR-AC194467.2 | LTR/Gypsy | 1 | 167 |
| LTR-AC194473.2 | LTR/Gypsy | 2 | 271 |
| LTR-AC194474.2 | LTR/Copia | 10 | 1538 |
| LTR-AC194477.2 | LTR | 47 | 4133 |
| LTR-AC194479.2 | LTR/Copia | 1 | 107 |
| LTR-AC194481.2 | LTR/Gypsy | 3 | 181 |
| LTR-AC194482.2 | LTR/Copia | 1 | 134 |
| LTR-AC194483.2 | LTR/Gypsy | 2 | 482 |
| LTR-AC194588.2 | LTR/Gypsy | 1 | 182 |
| LTR-AC194589.1 | LTR/Gypsy | 5 | 478 |
| LTR-AC194590.1 | LTR/Gypsy | 44 | 6828 |
| LTR-AC194594.1 | LTR/Gypsy | 5 | 509 |
| LTR-AC194600.1 | LTR/Copia | 3 | 651 |
| LTR-AC194601.1 | LTR/Gypsy | 2 | 116 |
| LTR-AC194602.1 | LTR | 3 | 392 |
| LTR-AC194603.1 | LTR/Copia | 13 | 2701 |
| LTR-AC194604.2 | LTR | 2 | 181 |
| LTR-AC194605.1 | LTR/Copia | 1 | 37 |
| LTR-AC194607.1 | LTR | 1 | 211 |
| LTR-AC194608.1 | LTR | 1 | 121 |
| LTR-AC194610.1 | LTR/Copia | 5 | 625 |
| LTR-AC194612.1 | LTR | 2 | 153 |
| LTR-AC194614.2 | LTR | 1 | 58 |
| LTR-AC194616.1 | LTR/Copia | 1 | 46 |
| LTR-AC194617.1 | LTR | 1 | 53 |
| LTR-AC194630.2 | LTR/Gypsy | 14 | 2660 |
| LTR-AC194632.1 | LTR | 2 | 106 |

| | | | |
|---|---|---|---|
| LTR-AC194633.2 | LTR/Copia | 1 | 118 |
| LTR-AC194637.2 | LTR | 1 | 99 |
| LTR-AC194641.1 | LTR/Copia | 2 | 258 |
| LTR-AC194642.1 | LTR/Gypsy | 5 | 1032 |
| LTR-AC194648.2 | LTR/Gypsy | 18 | 2337 |
| LTR-AC194650.1 | LTR | 3 | 224 |
| LTR-AC194653.1 | LTR/Gypsy | 4 | 835 |
| LTR-AC194654.2 | LTR | 1 | 134 |
| LTR-AC194659.1 | LTR/Gypsy | 2 | 138 |
| LTR-AC194664.2 | LTR/Gypsy | 3 | 376 |
| LTR-AC194674.1 | LTR | 2 | 106 |
| LTR-AC194676.2 | LTR | 31 | 6007 |
| LTR-AC194695.2 | LTR/Copia | 5 | 1143 |
| LTR-AC194696.2 | LTR | 4 | 294 |
| LTR-AC194698.2 | LTR/Gypsy | 1 | 33 |
| LTR-AC194699.2 | LTR | 2 | 110 |
| LTR-AC194700.2 | LTR | 2 | 203 |
| LTR-AC194702.2 | LTR/Gypsy | 1 | 40 |
| LTR-AC194704.2 | LTR/Gypsy | 1 | 51 |
| LTR-AC194706.2 | LTR/Copia | 3 | 152 |
| LTR-AC194709.2 | LTR/Gypsy | 6 | 357 |
| LTR-AC194720.3 | LTR | 31 | 6044 |
| LTR-AC194724.3 | LTR/Gypsy | 15 | 1290 |
| LTR-AC194728.2 | LTR/Gypsy | 1 | 141 |
| LTR-AC194731.2 | LTR/Copia | 12 | 1450 |
| LTR-AC194734.2 | LTR | 53 | 3540 |
| LTR-AC194736.1 | LTR/Copia | 1 | 217 |
| LTR-AC194737.2 | LTR/Copia | 5 | 1021 |
| LTR-AC194738.2 | LTR | 3 | 556 |
| LTR-AC194739.2 | LTR/Copia | 3 | 372 |
| LTR-AC194741.2 | LTR/Gypsy | 19 | 2801 |
| LTR-AC194745.2 | LTR/Gypsy | 5 | 825 |
| LTR-AC194818.2 | LTR/Copia | 1 | 77 |
| LTR-AC194822.2 | LTR/Gypsy | 6 | 427 |
| LTR-AC194825.2 | LTR/Copia | 1 | 70 |
| LTR-AC194829.2 | LTR | 1 | 45 |
| LTR-AC194831.2 | LTR/Gypsy | 28 | 2160 |
| LTR-AC194834.2 | LTR | 1 | 416 |
| LTR-AC194835.2 | LTR/Copia | 10 | 850 |
| LTR-AC194837.2 | LTR | 11 | 944 |
| LTR-AC194847.2 | LTR/Gypsy | 3 | 93 |
| LTR-AC194848.2 | LTR | 1 | 45 |
| LTR-AC194852.2 | LTR/Copia | 1 | 43 |
| LTR-AC194855.2 | LTR | 2 | 307 |
| LTR-AC194857.2 | LTR | 3 | 329 |
| LTR-AC194858.2 | LTR | 1 | 103 |
| LTR-AC194860.2 | LTR/Gypsy | 3 | 316 |

| | | | |
|---|---|---|---|
| LTR-AC194861.2 | LTR | 2 | 248 |
| LTR-AC194864.2 | LTR/Gypsy | 3 | 132 |
| LTR-AC194869.2 | LTR | 2 | 289 |
| LTR-AC194872.2 | LTR/Gypsy | 14 | 1714 |
| LTR-AC194874.2 | LTR/Gypsy | 8 | 638 |
| LTR-AC194877.2 | LTR | 1 | 466 |
| LTR-AC194879.2 | LTR | 1 | 134 |
| LTR-AC194882.2 | LTR/Copia | 8 | 546 |
| LTR-AC194884.2 | LTR | 3 | 183 |
| LTR-AC194886.2 | LTR/Gypsy | 2 | 171 |
| LTR-AC194888.2 | LTR/Gypsy | 1 | 63 |
| LTR-AC194890.2 | LTR | 1 | 109 |
| LTR-AC194891.2 | LTR/Gypsy | 21 | 5281 |
| LTR-AC194893.2 | LTR | 1 | 82 |
| LTR-AC194894.2 | LTR | 2 | 145 |
| LTR-AC194895.2 | LTR/Copia | 2 | 212 |
| LTR-AC194899.2 | LTR | 1 | 73 |
| LTR-AC194900.2 | LTR/Gypsy | 7 | 554 |
| LTR-AC194901.2 | LTR | 14 | 857 |
| LTR-AC194904.2 | LTR/Gypsy | 2 | 117 |
| LTR-AC194905.2 | LTR/Copia | 13 | 685 |
| LTR-AC194908.2 | LTR/Copia | 9 | 924 |
| LTR-AC194909.2 | LTR/Copia | 1 | 168 |
| LTR-AC194910.2 | LTR | 2 | 127 |
| LTR-AC194913.2 | LTR/Gypsy | 4 | 589 |
| LTR-AC194916.2 | LTR/Gypsy | 1 | 81 |
| LTR-AC194922.1 | LTR/Gypsy | 2 | 169 |
| LTR-AC194923.2 | LTR/Gypsy | 1 | 101 |
| LTR-AC194925.2 | LTR/Copia | 2 | 274 |
| LTR-AC194926.2 | LTR/Copia | 2 | 90 |
| LTR-AC194930.2 | LTR/Gypsy | 3 | 424 |
| LTR-AC194931.2 | LTR | 10 | 1157 |
| LTR-AC194932.1 | LTR | 8 | 1092 |
| LTR-AC194933.2 | LTR | 6 | 378 |
| LTR-AC194935.2 | LTR | 3 | 297 |
| LTR-AC194936.2 | LTR/Copia | 1 | 100 |
| LTR-AC194937.2 | LTR/Gypsy | 7 | 346 |
| LTR-AC194939.2 | LTR | 2 | 80 |
| LTR-AC194940.2 | LTR/Copia | 1 | 171 |
| LTR-AC194942.2 | LTR | 8 | 1792 |
| LTR-AC194946.2 | LTR/Gypsy | 1 | 110 |
| LTR-AC194947.2 | LTR | 5 | 798 |
| LTR-AC194949.2 | LTR | 12 | 1005 |
| LTR-AC194953.2 | LTR/Gypsy | 2 | 390 |
| LTR-AC194956.2 | LTR | 1 | 53 |
| LTR-AC194957.2 | LTR | 1 | 58 |
| LTR-AC194958.2 | LTR | 7 | 891 |

| | | | |
|---|---|---|---|
| LTR-AC194959.2 | LTR/Copia | 2 | 172 |
| LTR-AC194962.2 | LTR/Gypsy | 1 | 114 |
| LTR-AC194963.2 | LTR/Copia | 1 | 75 |
| LTR-AC194964.3 | LTR | 14 | 1963 |
| LTR-AC194965.2 | LTR/Copia | 2 | 383 |
| LTR-AC194966.2 | LTR | 1 | 59 |
| LTR-AC194967.2 | LTR/Gypsy | 9 | 1046 |
| LTR-AC194968.2 | LTR | 14 | 1119 |
| LTR-AC194974.2 | LTR | 5 | 721 |
| LTR-AC194978.2 | LTR/Gypsy | 2 | 230 |
| LTR-AC194979.2 | LTR | 4 | 460 |
| LTR-AC195058.2 | LTR/Copia | 1 | 138 |
| LTR-AC195059.2 | LTR/Gypsy | 3 | 526 |
| LTR-AC195060.2 | LTR/Gypsy | 5 | 260 |
| LTR-AC195120.2 | LTR | 7 | 921 |
| LTR-AC195121.1 | LTR/Gypsy | 1 | 203 |
| LTR-AC195124.2 | LTR | 2 | 237 |
| LTR-AC195127.2 | LTR/Gypsy | 1 | 41 |
| LTR-AC195132.2 | LTR | 1 | 86 |
| LTR-AC195133.1 | LTR/Copia | 1 | 354 |
| LTR-AC195134.2 | LTR | 1 | 56 |
| LTR-AC195135.2 | LTR | 1 | 115 |
| LTR-AC195137.1 | LTR | 2 | 329 |
| LTR-AC195138.2 | LTR/Gypsy | 21 | 3535 |
| LTR-AC195139.2 | LTR/Gypsy | 2 | 135 |
| LTR-AC195140.1 | LTR | 1 | 313 |
| LTR-AC195142.2 | LTR/Copia | 2 | 665 |
| LTR-AC195144.1 | LTR/Gypsy | 19 | 2023 |
| LTR-AC195146.1 | LTR | 4 | 172 |
| LTR-AC195153.2 | LTR | 3 | 274 |
| LTR-AC195155.2 | LTR/Copia | 3 | 407 |
| LTR-AC195158.2 | LTR | 7 | 418 |
| LTR-AC195160.2 | LTR/Copia | 5 | 311 |
| LTR-AC195164.2 | LTR/Gypsy | 2 | 126 |
| LTR-AC195168.1 | LTR | 1 | 47 |
| LTR-AC195174.1 | LTR/Gypsy | 20 | 3837 |
| LTR-AC195175.1 | LTR | 1 | 83 |
| LTR-AC195177.2 | LTR/Copia | 1 | 150 |
| LTR-AC195178.2 | LTR/Gypsy | 2 | 130 |
| LTR-AC195184.2 | LTR/Gypsy | 3 | 184 |
| LTR-AC195185.2 | LTR/Gypsy | 1 | 43 |
| LTR-AC195186.1 | LTR/Gypsy | 3 | 375 |
| LTR-AC195189.2 | LTR | 2 | 100 |
| LTR-AC195191.2 | LTR | 1 | 95 |
| LTR-AC195192.2 | LTR/Copia | 2 | 209 |
| LTR-AC195197.1 | LTR/Gypsy | 2 | 376 |
| LTR-AC195198.2 | LTR | 1 | 37 |

| | | | |
|---|---|---|---|
| LTR-AC195199.2 | LTR/Gypsy | 11 | 693 |
| LTR-AC195200.2 | LTR/Gypsy | 1 | 69 |
| LTR-AC195201.2 | LTR | 10 | 596 |
| LTR-AC195205.2 | LTR | 4 | 160 |
| LTR-AC195206.2 | LTR/Gypsy | 5 | 543 |
| LTR-AC195207.1 | LTR/Copia | 2 | 239 |
| LTR-AC195208.2 | LTR/Gypsy | 23 | 1966 |
| LTR-AC195211.2 | LTR | 1 | 74 |
| LTR-AC195212.2 | LTR | 8 | 465 |
| LTR-AC195213.2 | LTR | 10 | 633 |
| LTR-AC195215.2 | LTR | 1 | 87 |
| LTR-AC195223.2 | LTR/Copia | 6 | 585 |
| LTR-AC195226.1 | LTR | 11 | 1050 |
| LTR-AC195227.2 | LTR/Gypsy | 4 | 685 |
| LTR-AC195229.2 | LTR | 2 | 125 |
| LTR-AC195233.2 | LTR | 1 | 56 |
| LTR-AC195237.3 | LTR/Gypsy | 4 | 364 |
| LTR-AC195239.2 | LTR | 166 | 35380 |
| LTR-AC195243.1 | LTR/Gypsy | 1 | 56 |
| LTR-AC195244.2 | LTR/Gypsy | 8 | 588 |
| LTR-AC195245.2 | LTR/Gypsy | 3 | 369 |
| LTR-AC195247.2 | LTR/Gypsy | 1 | 149 |
| LTR-AC195248.2 | LTR/Gypsy | 9 | 396 |
| LTR-AC195256.1 | LTR/Copia | 3 | 162 |
| LTR-AC195260.1 | LTR/Copia | 2 | 171 |
| LTR-AC195269.2 | LTR/Copia | 2 | 239 |
| LTR-AC195274.2 | LTR/Gypsy | 2 | 261 |
| LTR-AC195300.2 | LTR/Gypsy | 1 | 99 |
| LTR-AC195301.1 | LTR/Gypsy | 30 | 4499 |
| LTR-AC195302.2 | LTR/Copia | 1 | 41 |
| LTR-AC195306.2 | LTR/Copia | 6 | 360 |
| LTR-AC195309.2 | LTR/Gypsy | 5 | 369 |
| LTR-AC195310.2 | LTR | 3 | 332 |
| LTR-AC195311.2 | LTR | 1 | 93 |
| LTR-AC195314.2 | LTR/Gypsy | 28 | 3369 |
| LTR-AC195316.2 | LTR | 8 | 1085 |
| LTR-AC195318.2 | LTR/Gypsy | 2 | 139 |
| LTR-AC195321.2 | LTR/Gypsy | 4 | 304 |
| LTR-AC195322.2 | LTR/Gypsy | 3 | 277 |
| LTR-AC195325.2 | LTR/Gypsy | 11 | 1069 |
| LTR-AC195327.3 | LTR/Gypsy | 29 | 2682 |
| LTR-AC195329.2 | LTR | 2 | 140 |
| LTR-AC195331.2 | LTR | 1 | 180 |
| LTR-AC195335.2 | LTR/Copia | 2 | 287 |
| LTR-AC195337.2 | LTR | 1 | 60 |
| LTR-AC195339.2 | LTR | 1 | 84 |
| LTR-AC195342.2 | LTR | 9 | 1500 |

| | | | |
|---|---|---|---|
| LTR-AC195343.2 | LTR | 1 | 113 |
| LTR-AC195346.2 | LTR/Copia | 2 | 139 |
| LTR-AC195347.2 | LTR | 62 | 10017 |
| LTR-AC195348.2 | LTR/Gypsy | 1 | 83 |
| LTR-AC195352.2 | LTR/Gypsy | 1 | 77 |
| LTR-AC195355.2 | LTR/Gypsy | 3 | 195 |
| LTR-AC195359.2 | LTR | 1 | 95 |
| LTR-AC195360.2 | LTR/Gypsy | 2 | 194 |
| LTR-AC195364.2 | LTR | 36 | 5431 |
| LTR-AC195372.2 | LTR/Gypsy | 3 | 266 |
| LTR-AC195373.2 | LTR/Gypsy | 1 | 49 |
| LTR-AC195378.2 | LTR/Gypsy | 7 | 835 |
| LTR-AC195383.2 | LTR | 1 | 50 |
| LTR-AC195390.2 | LTR/Gypsy | 2 | 962 |
| LTR-AC195391.2 | LTR | 4 | 460 |
| LTR-AC195394.2 | LTR/Gypsy | 1 | 214 |
| LTR-AC195395.2 | LTR/Gypsy | 1 | 87 |
| LTR-AC195398.2 | LTR/Gypsy | 1 | 285 |
| LTR-AC195402.3 | LTR/Gypsy | 2 | 308 |
| LTR-AC195411.2 | LTR | 1 | 74 |
| LTR-AC195457.2 | LTR/Copia | 3 | 118 |
| LTR-AC195463.2 | LTR/Gypsy | 4 | 405 |
| LTR-AC195466.2 | LTR | 2 | 72 |
| LTR-AC195478.2 | LTR/Gypsy | 8 | 862 |
| LTR-AC195482.2 | LTR | 2 | 130 |
| LTR-AC195534.2 | LTR | 11 | 1794 |
| LTR-AC195536.2 | LTR/Copia | 4 | 164 |
| LTR-AC195540.2 | LTR | 1 | 95 |
| LTR-AC195541.2 | LTR/Copia | 1 | 291 |
| LTR-AC195542.2 | LTR/Gypsy | 4 | 500 |
| LTR-AC195543.2 | LTR/Copia | 8 | 1110 |
| LTR-AC195547.2 | LTR | 7 | 991 |
| LTR-AC195551.2 | LTR | 4 | 261 |
| LTR-AC195573.2 | LTR | 2 | 186 |
| LTR-AC195576.2 | LTR/Gypsy | 2 | 139 |
| LTR-AC195581.3 | LTR/Gypsy | 8 | 543 |
| LTR-AC195587.2 | LTR/Copia | 1 | 51 |
| LTR-AC195588.2 | LTR/Copia | 1 | 43 |
| LTR-AC195589.2 | LTR/Gypsy | 2 | 253 |
| LTR-AC195590.1 | LTR | 1 | 77 |
| LTR-AC195593.2 | LTR/Copia | 1 | 71 |
| LTR-AC195595.2 | LTR | 4 | 377 |
| LTR-AC195596.2 | LTR/Gypsy | 12 | 1905 |
| LTR-AC195602.2 | LTR | 127 | 6876 |
| LTR-AC195604.2 | LTR | 4 | 337 |
| LTR-AC195606.2 | LTR/Copia | 8 | 450 |
| LTR-AC195672.2 | LTR | 1 | 181 |

| | | | |
|---|---|---|---|
| LTR-AC195674.2 | LTR | 27 | 2321 |
| LTR-AC195675.2 | LTR/Gypsy | 2 | 92 |
| LTR-AC195676.2 | LTR/Copia | 3 | 471 |
| LTR-AC195688.2 | LTR/Gypsy | 12 | 1276 |
| LTR-AC195782.1 | LTR | 2 | 146 |
| LTR-AC195784.2 | LTR | 2 | 147 |
| LTR-AC195785.2 | LTR | 1 | 50 |
| LTR-AC195787.2 | LTR | 1 | 69 |
| LTR-AC195789.2 | LTR | 5 | 775 |
| LTR-AC195790.2 | LTR | 21 | 1810 |
| LTR-AC195791.2 | LTR/Gypsy | 12 | 831 |
| LTR-AC195795.2 | LTR/Gypsy | 15 | 2590 |
| LTR-AC195797.1 | LTR | 1 | 123 |
| LTR-AC195798.2 | LTR | 2 | 77 |
| LTR-AC195800.2 | LTR | 1 | 84 |
| LTR-AC195802.2 | LTR/Copia | 1 | 48 |
| LTR-AC195804.2 | LTR | 1 | 56 |
| LTR-AC195805.1 | LTR | 1 | 54 |
| LTR-AC195806.2 | LTR | 2 | 94 |
| LTR-AC195807.2 | LTR/Copia | 13 | 1933 |
| LTR-AC195809.2 | LTR/Gypsy | 2 | 516 |
| LTR-AC195811.2 | LTR/Copia | 6 | 460 |
| LTR-AC195814.1 | LTR | 21 | 2097 |
| LTR-AC195815.2 | LTR | 3 | 283 |
| LTR-AC195818.1 | LTR/Gypsy | 1 | 42 |
| LTR-AC195820.2 | LTR | 1 | 39 |
| LTR-AC195822.2 | LTR | 3 | 102 |
| LTR-AC195823.2 | LTR | 23 | 2700 |
| LTR-AC195824.2 | LTR/Gypsy | 2 | 145 |
| LTR-AC195830.2 | LTR/Gypsy | 1 | 174 |
| LTR-AC195832.1 | LTR/Gypsy | 2 | 114 |
| LTR-AC195836.2 | LTR | 2 | 105 |
| LTR-AC195838.2 | LTR/Copia | 1 | 60 |
| LTR-AC195840.2 | LTR/Gypsy | 1 | 109 |
| LTR-AC195841.2 | LTR | 2 | 162 |
| LTR-AC195842.2 | LTR/Gypsy | 1 | 99 |
| LTR-AC195845.2 | LTR | 2 | 516 |
| LTR-AC195846.2 | LTR | 15 | 1748 |
| LTR-AC195848.2 | LTR | 2 | 401 |
| LTR-AC195849.2 | LTR | 1 | 104 |
| LTR-AC195854.1 | LTR | 2 | 112 |
| LTR-AC195855.2 | LTR | 1 | 107 |
| LTR-AC195859.2 | LTR | 9 | 765 |
| LTR-AC195860.2 | LTR | 1 | 95 |
| LTR-AC195861.2 | LTR/Gypsy | 2 | 374 |
| LTR-AC195865.2 | LTR | 4 | 296 |
| LTR-AC195866.3 | LTR | 22 | 2743 |

| | | | |
|---|---|---|---|
| LTR-AC195871.2 | LTR/Gypsy | 1 | 160 |
| LTR-AC195874.1 | LTR | 1 | 109 |
| LTR-AC195877.2 | LTR | 1 | 363 |
| LTR-AC195878.2 | LTR | 2 | 290 |
| LTR-AC195888.2 | LTR/Gypsy | 5 | 654 |
| LTR-AC195890.2 | LTR/Copia | 1 | 75 |
| LTR-AC195891.2 | LTR | 5 | 1132 |
| LTR-AC195893.1 | LTR/Copia | 2 | 229 |
| LTR-AC195895.2 | LTR | 10 | 1504 |
| LTR-AC195897.2 | LTR | 2 | 230 |
| LTR-AC195898.2 | LTR/Gypsy | 2 | 448 |
| LTR-AC195901.2 | LTR | 8 | 895 |
| LTR-AC195905.2 | LTR/Gypsy | 6 | 897 |
| LTR-AC195906.2 | LTR/Copia | 10 | 1622 |
| LTR-AC195907.2 | LTR/Gypsy | 5 | 445 |
| LTR-AC195911.2 | LTR/Gypsy | 7 | 556 |
| LTR-AC195914.1 | LTR | 6 | 452 |
| LTR-AC195915.2 | LTR/Gypsy | 17 | 680 |
| LTR-AC195919.3 | LTR/Gypsy | 3 | 216 |
| LTR-AC195923.2 | LTR/Copia | 3 | 753 |
| LTR-AC195925.3 | LTR/Gypsy | 3 | 367 |
| LTR-AC195926.1 | LTR | 1 | 60 |
| LTR-AC195927.2 | LTR/Gypsy | 2 | 122 |
| LTR-AC195929.1 | LTR | 12 | 1626 |
| LTR-AC195932.2 | LTR | 3 | 289 |
| LTR-AC195933.2 | LTR/Gypsy | 2 | 156 |
| LTR-AC195934.2 | LTR | 14 | 1573 |
| LTR-AC195935.1 | LTR | 4 | 498 |
| LTR-AC195936.1 | LTR | 1 | 98 |
| LTR-AC195937.3 | LTR | 18 | 1643 |
| LTR-AC195938.1 | LTR | 1 | 105 |
| LTR-AC195940.1 | LTR/Gypsy | 3 | 380 |
| LTR-AC195943.2 | LTR/Gypsy | 2 | 130 |
| LTR-AC195945.2 | LTR/Copia | 2 | 323 |
| LTR-AC195946.2 | LTR | 5 | 309 |
| LTR-AC195950.2 | LTR | 3 | 184 |
| LTR-AC195951.1 | LTR/Gypsy | 2 | 177 |
| LTR-AC195952.2 | LTR/Gypsy | 10 | 1356 |
| LTR-AC195954.3 | LTR/Gypsy | 9 | 1546 |
| LTR-AC195957.2 | LTR/Copia | 2 | 280 |
| LTR-AC195959.2 | LTR | 1 | 42 |
| LTR-AC195961.2 | LTR/Copia | 3 | 133 |
| LTR-AC195967.2 | LTR | 1 | 150 |
| LTR-AC195969.1 | LTR/Copia | 1 | 51 |
| LTR-AC195973.2 | LTR | 1 | 48 |
| LTR-AC195975.3 | LTR/Gypsy | 1 | 59 |
| LTR-AC195976.2 | LTR/Gypsy | 3 | 169 |

| | | | |
|---|---|---|---|
| LTR-AC195977.2 | LTR/Gypsy | 2 | 114 |
| LTR-AC195978.1 | LTR | 1 | 132 |
| LTR-AC195980.1 | LTR | 2 | 168 |
| LTR-AC195985.2 | LTR/Gypsy | 5 | 232 |
| LTR-AC195987.2 | LTR/Gypsy | 2 | 216 |
| LTR-AC195991.2 | LTR/Gypsy | 3 | 177 |
| LTR-AC195994.2 | LTR/Copia | 1 | 96 |
| LTR-AC195995.2 | LTR/Gypsy | 8 | 553 |
| LTR-AC195997.2 | LTR/Gypsy | 13 | 1729 |
| LTR-AC196000.1 | LTR/Gypsy | 13 | 1309 |
| LTR-AC196003.2 | LTR/Gypsy | 2 | 273 |
| LTR-AC196004.2 | LTR/Gypsy | 1 | 159 |
| LTR-AC196005.2 | LTR/Gypsy | 5 | 275 |
| LTR-AC196010.3 | LTR | 6 | 448 |
| LTR-AC196013.2 | LTR/Gypsy | 1 | 101 |
| LTR-AC196016.2 | LTR/Gypsy | 5 | 720 |
| LTR-AC196019.3 | LTR/Copia | 1 | 53 |
| LTR-AC196022.2 | LTR | 46 | 5423 |
| LTR-AC196025.2 | LTR/Copia | 2 | 90 |
| LTR-AC196027.2 | LTR/Gypsy | 2 | 168 |
| LTR-AC196028.1 | LTR/Copia | 13 | 1271 |
| LTR-AC196029.2 | LTR/Copia | 1 | 34 |
| LTR-AC196032.2 | LTR/Gypsy | 1 | 108 |
| LTR-AC196034.3 | LTR/Gypsy | 1 | 116 |
| LTR-AC196035.3 | LTR | 4 | 342 |
| LTR-AC196036.2 | LTR | 1 | 41 |
| LTR-AC196040.2 | LTR/Gypsy | 2 | 113 |
| LTR-AC196045.2 | LTR/Copia | 1 | 124 |
| LTR-AC196046.3 | LTR/Copia | 5 | 619 |
| LTR-AC196047.2 | LTR/Gypsy | 5 | 311 |
| LTR-AC196048.2 | LTR/Copia | 11 | 1891 |
| LTR-AC196053.2 | LTR/Gypsy | 2 | 176 |
| LTR-AC196054.2 | LTR | 2 | 114 |
| LTR-AC196055.1 | LTR | 2 | 225 |
| LTR-AC196057.3 | LTR/Copia | 11 | 1373 |
| LTR-AC196058.2 | LTR | 4 | 251 |
| LTR-AC196059.2 | LTR | 3 | 172 |
| LTR-AC196061.1 | LTR | 121 | 14812 |
| LTR-AC196064.2 | LTR/Copia | 3 | 262 |
| LTR-AC196066.2 | LTR | 1 | 57 |
| LTR-AC196067.2 | LTR/Gypsy | 4 | 326 |
| LTR-AC196068.2 | LTR | 5 | 920 |
| LTR-AC196069.2 | LTR | 1 | 60 |
| LTR-AC196072.2 | LTR/Copia | 4 | 379 |
| LTR-AC196073.2 | LTR | 2 | 123 |
| LTR-AC196076.2 | LTR/Copia | 3 | 468 |
| LTR-AC196078.3 | LTR/Gypsy | 7 | 629 |

| | | | |
|---|---|---|---|
| LTR-AC196079.2 | LTR | 9 | 1328 |
| LTR-AC196080.2 | LTR/Copia | 1 | 170 |
| LTR-AC196086.2 | LTR | 3 | 144 |
| LTR-AC196087.2 | LTR/Gypsy | 4 | 454 |
| LTR-AC196089.2 | LTR/Gypsy | 2 | 326 |
| LTR-AC196090.2 | LTR/Gypsy | 2 | 176 |
| LTR-AC196092.2 | LTR | 1 | 40 |
| LTR-AC196099.3 | LTR | 29 | 1648 |
| LTR-AC196101.1 | LTR | 51 | 5863 |
| LTR-AC196103.1 | LTR | 5 | 552 |
| LTR-AC196109.2 | LTR/Gypsy | 9 | 952 |
| LTR-AC196110.2 | LTR/Gypsy | 1 | 45 |
| LTR-AC196112.3 | LTR | 2 | 311 |
| LTR-AC196113.1 | LTR | 19 | 1397 |
| LTR-AC196115.1 | LTR | 2 | 111 |
| LTR-AC196116.3 | LTR/Gypsy | 7 | 1374 |
| LTR-AC196117.2 | LTR/Gypsy | 1 | 39 |
| LTR-AC196125.2 | LTR/Copia | 12 | 1648 |
| LTR-AC196127.2 | LTR/Copia | 1 | 98 |
| LTR-AC196132.2 | LTR/Gypsy | 3 | 437 |
| LTR-AC196140.2 | LTR/Gypsy | 2 | 308 |
| LTR-AC196141.3 | LTR/Copia | 2 | 175 |
| LTR-AC196148.2 | LTR | 2 | 253 |
| LTR-AC196151.2 | LTR | 6 | 740 |
| LTR-AC196155.2 | LTR | 5 | 220 |
| LTR-AC196156.2 | LTR | 2 | 263 |
| LTR-AC196163.2 | LTR/Gypsy | 61 | 7749 |
| LTR-AC196166.2 | LTR/Copia | 11 | 1422 |
| LTR-AC196172.2 | LTR/Gypsy | 10 | 1457 |
| LTR-AC196173.2 | LTR | 2 | 105 |
| LTR-AC196175.2 | LTR/Copia | 1 | 60 |
| LTR-AC196176.2 | LTR/Gypsy | 6 | 634 |
| LTR-AC196180.2 | LTR | 1 | 30 |
| LTR-AC196181.1 | LTR | 3 | 180 |
| LTR-AC196182.2 | LTR | 1 | 38 |
| LTR-AC196183.2 | LTR/Copia | 1 | 71 |
| LTR-AC196184.2 | LTR/Copia | 3 | 256 |
| LTR-AC196186.2 | LTR/Gypsy | 3 | 324 |
| LTR-AC196188.2 | LTR | 13 | 2111 |
| LTR-AC196193.2 | LTR | 6 | 471 |
| LTR-AC196196.2 | LTR/Copia | 8 | 1421 |
| LTR-AC196199.2 | LTR/Gypsy | 17 | 1219 |
| LTR-AC196202.2 | LTR/Gypsy | 3 | 395 |
| LTR-AC196203.2 | LTR | 62 | 3610 |
| LTR-AC196205.2 | LTR/Gypsy | 1 | 51 |
| LTR-AC196207.2 | LTR | 1 | 44 |
| LTR-AC196209.2 | LTR | 1 | 46 |

| | | | |
|---|---|---|---|
| LTR-AC196212.2 | LTR | 5 | 210 |
| LTR-AC196213.2 | LTR/Copia | 5 | 378 |
| LTR-AC196214.2 | LTR/Gypsy | 1 | 122 |
| LTR-AC196260.2 | LTR | 3 | 163 |
| LTR-AC196261.2 | LTR/Gypsy | 6 | 839 |
| LTR-AC196262.2 | LTR | 5 | 484 |
| LTR-AC196264.3 | LTR/Gypsy | 4 | 157 |
| LTR-AC196268.2 | LTR | 204 | 42695 |
| LTR-AC196270.2 | LTR | 2 | 329 |
| LTR-AC196272.2 | LTR/Gypsy | 1 | 71 |
| LTR-AC196273.2 | LTR | 28 | 5236 |
| LTR-AC196278.2 | LTR | 1 | 71 |
| LTR-AC196279.2 | LTR | 1 | 44 |
| LTR-AC196288.2 | LTR | 1 | 71 |
| LTR-AC196291.2 | LTR/Gypsy | 3 | 114 |
| LTR-AC196292.2 | LTR | 2 | 76 |
| LTR-AC196294.2 | LTR/Copia | 1 | 175 |
| LTR-AC196296.2 | LTR/Gypsy | 2 | 182 |
| LTR-AC196297.2 | LTR/Copia | 7 | 306 |
| LTR-AC196366.2 | LTR/Gypsy | 3 | 346 |
| LTR-AC196368.2 | LTR | 15 | 1118 |
| LTR-AC196369.2 | LTR/Gypsy | 7 | 568 |
| LTR-AC196387.2 | LTR/Gypsy | 2 | 302 |
| LTR-AC196389.2 | LTR | 21 | 2622 |
| LTR-AC196395.2 | LTR/Copia | 1 | 49 |
| LTR-AC196399.2 | LTR/Copia | 1 | 181 |
| LTR-AC196403.2 | LTR/Copia | 6 | 1613 |
| LTR-AC196405.2 | LTR/Copia | 1 | 62 |
| LTR-AC196406.2 | LTR | 6 | 413 |
| LTR-AC196409.2 | LTR | 4 | 464 |
| LTR-AC196413.2 | LTR/Copia | 3 | 394 |
| LTR-AC196415.2 | LTR | 1 | 75 |
| LTR-AC196420.2 | LTR | 14 | 2284 |
| LTR-AC196421.2 | LTR/Gypsy | 7 | 1487 |
| LTR-AC196422.2 | LTR/Gypsy | 2 | 104 |
| LTR-AC196423.2 | LTR | 2 | 196 |
| LTR-AC196426.2 | LTR/Copia | 2 | 155 |
| LTR-AC196433.2 | LTR/Copia | 18 | 1454 |
| LTR-AC196435.2 | LTR/Copia | 1 | 288 |
| LTR-AC196438.2 | LTR/Gypsy | 3 | 688 |
| LTR-AC196439.2 | LTR/Gypsy | 8 | 575 |
| LTR-AC196459.2 | LTR/Gypsy | 78 | 10459 |
| LTR-AC196464.2 | LTR | 7 | 1557 |
| LTR-AC196465.2 | LTR/Copia | 1 | 102 |
| LTR-AC196466.2 | LTR/Gypsy | 2 | 401 |
| LTR-AC196468.3 | LTR | 1 | 44 |
| LTR-AC196470.2 | LTR | 1 | 43 |

| | | | |
|---|---|---|---|
| LTR-AC196473.2 | LTR/Gypsy | 6 | 640 |
| LTR-AC196476.2 | LTR/Gypsy | 1 | 48 |
| LTR-AC196479.2 | LTR | 13 | 2094 |
| LTR-AC196484.2 | LTR/Gypsy | 1 | 248 |
| LTR-AC196485.2 | LTR/Copia | 9 | 836 |
| LTR-AC196487.2 | LTR | 14 | 3224 |
| LTR-AC196489.2 | LTR | 4 | 318 |
| LTR-AC196491.2 | LTR | 1 | 35 |
| LTR-AC196492.2 | LTR | 1 | 58 |
| LTR-AC196575.2 | LTR/Copia | 3 | 262 |
| LTR-AC196576.2 | LTR/Gypsy | 12 | 799 |
| LTR-AC196579.2 | LTR/Gypsy | 5 | 299 |
| LTR-AC196637.1 | LTR/Gypsy | 1 | 210 |
| LTR-AC196638.2 | LTR/Gypsy | 1 | 56 |
| LTR-AC196642.3 | LTR | 1 | 95 |
| LTR-AC196643.2 | LTR | 5 | 298 |
| LTR-AC196646.2 | LTR/Gypsy | 4 | 424 |
| LTR-AC196678.2 | LTR | 1 | 57 |
| LTR-AC196679.3 | LTR/Copia | 1 | 414 |
| LTR-AC196680.2 | LTR | 2 | 438 |
| LTR-AC196681.2 | LTR/Copia | 2 | 237 |
| LTR-AC196682.2 | LTR | 1 | 88 |
| LTR-AC196683.2 | LTR | 1 | 94 |
| LTR-AC196686.2 | LTR | 1 | 48 |
| LTR-AC196708.2 | LTR/Gypsy | 3 | 242 |
| LTR-AC196711.2 | LTR | 4 | 140 |
| LTR-AC196713.2 | LTR/Gypsy | 2 | 197 |
| LTR-AC196714.2 | LTR/Gypsy | 3 | 529 |
| LTR-AC196715.2 | LTR | 1 | 335 |
| LTR-AC196717.2 | LTR | 9 | 1200 |
| LTR-AC196718.2 | LTR | 3 | 289 |
| LTR-AC196719.2 | LTR/Gypsy | 2 | 715 |
| LTR-AC196720.2 | LTR | 3 | 249 |
| LTR-AC196725.3 | LTR | 1 | 45 |
| LTR-AC196729.2 | LTR/Gypsy | 5 | 448 |
| LTR-AC196766.2 | LTR/Gypsy | 1 | 75 |
| LTR-AC196767.2 | LTR | 2 | 105 |
| LTR-AC196768.2 | LTR/Gypsy | 2 | 337 |
| LTR-AC196771.2 | LTR | 3 | 556 |
| LTR-AC196779.2 | LTR/Copia | 1 | 76 |
| LTR-AC196780.2 | LTR | 14 | 977 |
| LTR-AC196782.2 | LTR/Copia | 1 | 51 |
| LTR-AC196789.2 | LTR | 6 | 609 |
| LTR-AC196790.2 | LTR/Gypsy | 3 | 493 |
| LTR-AC196791.2 | LTR/Gypsy | 1 | 40 |
| LTR-AC196792.2 | LTR | 2 | 138 |
| LTR-AC196795.3 | LTR | 3 | 302 |

| | | | |
|---|---|---|---|
| LTR-AC196796.3 | LTR | 1 | 424 |
| LTR-AC196799.2 | LTR | 1 | 42 |
| LTR-AC196800.2 | LTR | 1 | 312 |
| LTR-AC196953.2 | LTR | 4 | 185 |
| LTR-AC196955.2 | LTR/Copia | 4 | 273 |
| LTR-AC196956.2 | LTR/Gypsy | 10 | 1529 |
| LTR-AC196960.3 | LTR/Gypsy | 5 | 492 |
| LTR-AC196962.2 | LTR/Gypsy | 3 | 276 |
| LTR-AC196963.2 | LTR | 1 | 46 |
| LTR-AC196965.2 | LTR | 2 | 193 |
| LTR-AC196969.3 | LTR/Gypsy | 1 | 90 |
| LTR-AC196970.2 | LTR/Gypsy | 3 | 251 |
| LTR-AC196972.2 | LTR | 1 | 369 |
| LTR-AC196974.2 | LTR/Gypsy | 3 | 149 |
| LTR-AC196981.1 | LTR | 6 | 842 |
| LTR-AC196984.2 | LTR/Gypsy | 2 | 172 |
| LTR-AC196985.2 | LTR | 4 | 220 |
| LTR-AC196986.2 | LTR | 2 | 332 |
| LTR-AC196988.2 | LTR | 1 | 55 |
| LTR-AC196989.2 | LTR | 1 | 132 |
| LTR-AC196991.3 | LTR/Copia | 3 | 477 |
| LTR-AC196992.2 | LTR/Copia | 1 | 148 |
| LTR-AC196993.3 | LTR | 3 | 185 |
| LTR-AC196997.2 | LTR | 1 | 66 |
| LTR-AC196998.3 | LTR | 1 | 87 |
| LTR-AC197004.2 | LTR/Copia | 2 | 221 |
| LTR-AC197007.2 | LTR/Gypsy | 2 | 267 |
| LTR-AC197008.2 | LTR/Gypsy | 2 | 216 |
| LTR-AC197009.2 | LTR/Gypsy | 1 | 270 |
| LTR-AC197011.2 | LTR/Copia | 1 | 88 |
| LTR-AC197013.2 | LTR/Gypsy | 1 | 59 |
| LTR-AC197014.2 | LTR/Gypsy | 7 | 723 |
| LTR-AC197016.3 | LTR/Copia | 1 | 68 |
| LTR-AC197017.2 | LTR/Gypsy | 14 | 1292 |
| LTR-AC197018.2 | LTR | 1 | 43 |
| LTR-AC197020.2 | LTR/Gypsy | 1 | 75 |
| LTR-AC197023.2 | LTR | 1 | 89 |
| LTR-AC197028.2 | LTR/Gypsy | 3 | 421 |
| LTR-AC197029.2 | LTR/Copia | 1 | 59 |
| LTR-AC197034.2 | LTR/Copia | 2 | 153 |
| LTR-AC197040.2 | LTR/Copia | 1 | 43 |
| LTR-AC197042.2 | LTR | 2 | 316 |
| LTR-AC197043.2 | LTR/Copia | 1 | 53 |
| LTR-AC197045.2 | LTR/Gypsy | 8 | 1314 |
| LTR-AC197048.2 | LTR | 2 | 135 |
| LTR-AC197049.2 | LTR/Copia | 4 | 250 |
| LTR-AC197054.2 | LTR/Gypsy | 5 | 371 |

| | | | |
|---|---|---|---|
| LTR-AC197055.2 | LTR/Copia | 1 | 205 |
| LTR-AC197061.1 | LTR/Gypsy | 2 | 149 |
| LTR-AC197062.2 | LTR | 1 | 87 |
| LTR-AC197063.2 | LTR/Gypsy | 1 | 59 |
| LTR-AC197067.2 | LTR/Gypsy | 2 | 94 |
| LTR-AC197068.2 | LTR/Copia | 1 | 102 |
| LTR-AC197069.2 | LTR/Copia | 6 | 754 |
| LTR-AC197070.2 | LTR/Gypsy | 1 | 52 |
| LTR-AC197071.2 | LTR | 1 | 133 |
| LTR-AC197074.2 | LTR | 17 | 1725 |
| LTR-AC197075.2 | LTR/Gypsy | 3 | 487 |
| LTR-AC197080.2 | LTR | 3 | 505 |
| LTR-AC197084.2 | LTR | 29 | 3351 |
| LTR-AC197086.2 | LTR | 1 | 109 |
| LTR-AC197087.2 | LTR | 12 | 696 |
| LTR-AC197089.2 | LTR/Gypsy | 15 | 2514 |
| LTR-AC197091.2 | LTR | 6 | 1525 |
| LTR-AC197094.2 | LTR | 1 | 57 |
| LTR-AC197103.2 | LTR | 3 | 201 |
| LTR-AC197105.2 | LTR/Gypsy | 3 | 920 |
| LTR-AC197111.2 | LTR/Gypsy | 9 | 577 |
| LTR-AC197114.2 | LTR | 1 | 41 |
| LTR-AC197119.2 | LTR/Gypsy | 3 | 188 |
| LTR-AC197122.2 | LTR/Copia | 13 | 1080 |
| LTR-AC197123.2 | LTR/Copia | 2 | 188 |
| LTR-AC197124.2 | LTR/Copia | 3 | 390 |
| LTR-AC197130.2 | LTR | 2 | 90 |
| LTR-AC197132.2 | LTR | 2 | 155 |
| LTR-AC197133.2 | LTR/Copia | 1 | 242 |
| LTR-AC197134.3 | LTR/Gypsy | 1 | 184 |
| LTR-AC197136.2 | LTR | 1 | 59 |
| LTR-AC197137.2 | LTR/Gypsy | 2 | 108 |
| LTR-AC197139.2 | LTR | 13 | 1121 |
| LTR-AC197141.2 | LTR/Copia | 1 | 41 |
| LTR-AC197145.2 | LTR | 23 | 1690 |
| LTR-AC197147.2 | LTR | 20 | 1537 |
| LTR-AC197148.2 | LTR/Gypsy | 4 | 1313 |
| LTR-AC197150.2 | LTR/Copia | 2 | 124 |
| LTR-AC197151.2 | LTR/Copia | 5 | 400 |
| LTR-AC197163.2 | LTR/Copia | 1 | 83 |
| LTR-AC197169.2 | LTR | 1 | 86 |
| LTR-AC197170.1 | LTR | 66 | 9945 |
| LTR-AC197171.2 | LTR/Copia | 4 | 431 |
| LTR-AC197185.2 | LTR/Gypsy | 80 | 5092 |
| LTR-AC197188.2 | LTR | 34 | 3888 |
| LTR-AC197195.2 | LTR/Copia | 1 | 49 |
| LTR-AC197196.2 | LTR | 2 | 162 |

| | | | |
|---|---|---|---|
| LTR-AC197201.2 | LTR/Copia | 6 | 712 |
| LTR-AC197218.2 | LTR/Gypsy | 5 | 235 |
| LTR-AC197220.2 | LTR | 1 | 63 |
| LTR-AC197222.2 | LTR | 1 | 68 |
| LTR-AC197226.2 | LTR/Copia | 4 | 553 |
| LTR-AC197228.2 | LTR/Gypsy | 1 | 98 |
| LTR-AC197229.2 | LTR | 22 | 3237 |
| LTR-AC197235.2 | LTR | 4 | 369 |
| LTR-AC197236.1 | LTR/Gypsy | 7 | 1360 |
| LTR-AC197238.1 | LTR/Gypsy | 1 | 52 |
| LTR-AC197243.2 | LTR/Gypsy | 4 | 273 |
| LTR-AC197248.2 | LTR/Gypsy | 147 | 32281 |
| LTR-AC197250.2 | LTR | 5 | 308 |
| LTR-AC197251.2 | LTR | 2 | 107 |
| LTR-AC197254.2 | LTR | 1 | 271 |
| LTR-AC197256.2 | LTR/Gypsy | 4 | 684 |
| LTR-AC197261.2 | LTR | 9 | 616 |
| LTR-AC197263.2 | LTR/Copia | 1 | 78 |
| LTR-AC197264.2 | LTR | 1 | 45 |
| LTR-AC197267.2 | LTR/Gypsy | 1 | 274 |
| LTR-AC197270.2 | LTR | 1 | 65 |
| LTR-AC197271.1 | LTR/Gypsy | 6 | 409 |
| LTR-AC197273.2 | LTR | 6 | 1066 |
| LTR-AC197274.2 | LTR/Gypsy | 3 | 253 |
| LTR-AC197326.2 | LTR | 83 | 11013 |
| LTR-AC197328.2 | LTR/Gypsy | 2 | 368 |
| LTR-AC197332.2 | LTR/Gypsy | 1 | 186 |
| LTR-AC197333.2 | LTR | 4 | 297 |
| LTR-AC197336.2 | LTR/Gypsy | 1 | 193 |
| LTR-AC197342.2 | LTR/Copia | 3 | 172 |
| LTR-AC197352.2 | LTR/Gypsy | 2 | 202 |
| LTR-AC197353.2 | LTR/Gypsy | 2 | 311 |
| LTR-AC197355.2 | LTR/Gypsy | 6 | 755 |
| LTR-AC197356.2 | LTR/Copia | 3 | 1237 |
| LTR-AC197357.2 | LTR/Copia | 6 | 727 |
| LTR-AC197359.2 | LTR/Gypsy | 15 | 1851 |
| LTR-AC197360.2 | LTR | 1 | 100 |
| LTR-AC197365.2 | LTR/Gypsy | 2 | 111 |
| LTR-AC197368.2 | LTR | 9 | 1311 |
| LTR-AC197370.2 | LTR/Copia | 1 | 138 |
| LTR-AC197371.2 | LTR | 1 | 36 |
| LTR-AC197373.2 | LTR/Copia | 2 | 444 |
| LTR-AC197374.2 | LTR | 31 | 3178 |
| LTR-AC197376.2 | LTR | 1 | 44 |
| LTR-AC197380.2 | LTR/Gypsy | 1 | 124 |
| LTR-AC197382.2 | LTR/Copia | 1 | 118 |
| LTR-AC197384.2 | LTR/Gypsy | 1 | 86 |

| | | | |
|---|---|---|---|
| LTR-AC197387.2 | LTR | 6 | 509 |
| LTR-AC197425.2 | LTR | 1 | 43 |
| LTR-AC197426.2 | LTR/Copia | 1 | 81 |
| LTR-AC197429.2 | LTR/Copia | 1 | 106 |
| LTR-AC197431.2 | LTR/Gypsy | 1 | 108 |
| LTR-AC197434.2 | LTR | 4 | 589 |
| LTR-AC197437.2 | LTR | 10 | 420 |
| LTR-AC197438.2 | LTR | 1 | 44 |
| LTR-AC197443.2 | LTR | 1 | 87 |
| LTR-AC197444.1 | LTR/Gypsy | 3 | 312 |
| LTR-AC197473.2 | LTR | 11 | 852 |
| LTR-AC197474.1 | LTR/Gypsy | 1 | 405 |
| LTR-AC197479.2 | LTR/Copia | 7 | 315 |
| LTR-AC197480.2 | LTR/Copia | 2 | 110 |
| LTR-AC197482.2 | LTR/Gypsy | 1 | 142 |
| LTR-AC197483.2 | LTR | 7 | 677 |
| LTR-AC197484.2 | LTR | 3 | 281 |
| LTR-AC197485.2 | LTR | 3 | 108 |
| LTR-AC197486.1 | LTR/Copia | 2 | 191 |
| LTR-AC197487.1 | LTR | 1 | 342 |
| LTR-AC197489.2 | LTR | 2 | 296 |
| LTR-AC197493.2 | LTR/Copia | 1 | 137 |
| LTR-AC197498.2 | LTR | 1 | 91 |
| LTR-AC197522.2 | LTR | 3 | 300 |
| LTR-AC197525.2 | LTR | 27 | 2403 |
| LTR-AC197530.2 | LTR/Gypsy | 5 | 1160 |
| LTR-AC197531.2 | LTR | 94 | 9295 |
| LTR-AC197532.2 | LTR | 2 | 300 |
| LTR-AC197535.2 | LTR/Gypsy | 1 | 34 |
| LTR-AC197536.2 | LTR/Gypsy | 1 | 138 |
| LTR-AC197538.2 | LTR/Gypsy | 3 | 283 |
| LTR-AC197541.2 | LTR/Gypsy | 2 | 286 |
| LTR-AC197547.2 | LTR/Copia | 2 | 212 |
| LTR-AC197549.2 | LTR/Gypsy | 2 | 256 |
| LTR-AC197552.2 | LTR/Gypsy | 1 | 123 |
| LTR-AC197560.1 | LTR/Gypsy | 3 | 338 |
| LTR-AC197561.2 | LTR/Copia | 3 | 186 |
| LTR-AC197562.2 | LTR/Gypsy | 2 | 238 |
| LTR-AC197563.2 | LTR | 2 | 147 |
| LTR-AC197566.2 | LTR/Copia | 1 | 123 |
| LTR-AC197569.2 | LTR | 1 | 94 |
| LTR-AC197570.2 | LTR/Gypsy | 1 | 141 |
| LTR-AC197572.2 | LTR/Gypsy | 3 | 245 |
| LTR-AC197573.2 | LTR/Copia | 1 | 192 |
| LTR-AC197574.2 | LTR | 2 | 68 |
| LTR-AC197578.2 | LTR/Copia | 1 | 126 |
| LTR-AC197581.2 | LTR | 1 | 49 |

| | | | |
|---|---|---|---|
| LTR-AC197582.2 | LTR | 1 | 80 |
| LTR-AC197583.1 | LTR/Gypsy | 2 | 163 |
| LTR-AC197585.2 | LTR/Gypsy | 4 | 731 |
| LTR-AC197601.2 | LTR/Gypsy | 3 | 214 |
| LTR-AC197603.2 | LTR | 1 | 92 |
| LTR-AC197669.2 | LTR | 1 | 118 |
| LTR-AC197670.2 | LTR/Copia | 4 | 457 |
| LTR-AC197673.2 | LTR | 2 | 80 |
| LTR-AC197674.2 | LTR | 1 | 42 |
| LTR-AC197677.2 | LTR/Copia | 3 | 192 |
| LTR-AC197678.2 | LTR/Gypsy | 9 | 1881 |
| LTR-AC197680.2 | LTR | 1 | 142 |
| LTR-AC197683.2 | LTR/Gypsy | 9 | 1931 |
| LTR-AC197684.2 | LTR/Gypsy | 2 | 394 |
| LTR-AC197686.2 | LTR/Gypsy | 23 | 5193 |
| LTR-AC197687.2 | LTR | 1 | 72 |
| LTR-AC197689.2 | LTR | 1 | 57 |
| LTR-AC197690.2 | LTR/Gypsy | 3 | 252 |
| LTR-AC197695.2 | LTR | 1 | 225 |
| LTR-AC197696.2 | LTR/Gypsy | 1 | 47 |
| LTR-AC197697.2 | LTR/Copia | 3 | 225 |
| LTR-AC197700.2 | LTR/Gypsy | 8 | 877 |
| LTR-AC197701.2 | LTR/Gypsy | 1 | 212 |
| LTR-AC197710.2 | LTR/Gypsy | 2 | 138 |
| LTR-AC197712.2 | LTR | 8 | 590 |
| LTR-AC197715.2 | LTR/Copia | 1 | 57 |
| LTR-AC197720.2 | LTR/Gypsy | 2 | 228 |
| LTR-AC197721.2 | LTR | 4 | 402 |
| LTR-AC197722.2 | LTR/Gypsy | 2 | 119 |
| LTR-AC197724.2 | LTR | 13 | 1473 |
| LTR-AC197726.2 | LTR/Copia | 2 | 194 |
| LTR-AC197727.2 | LTR | 1 | 94 |
| LTR-AC197728.2 | LTR/Gypsy | 1 | 151 |
| LTR-AC197729.2 | LTR | 1 | 59 |
| LTR-AC197733.2 | LTR | 2 | 285 |
| LTR-AC197735.2 | LTR/Gypsy | 2 | 136 |
| LTR-AC197741.2 | LTR | 23 | 2651 |
| LTR-AC197757.2 | LTR | 1 | 63 |
| LTR-AC197760.2 | LTR/Copia | 1 | 79 |
| LTR-AC197764.1 | LTR/Gypsy | 2 | 214 |
| LTR-AC197765.2 | LTR/Copia | 1 | 52 |
| LTR-AC197767.2 | LTR | 38 | 3540 |
| LTR-AC197769.2 | LTR | 3 | 225 |
| LTR-AC197770.2 | LTR/Gypsy | 9 | 665 |
| LTR-AC197775.2 | LTR | 5 | 457 |
| LTR-AC197782.2 | LTR/Gypsy | 1 | 89 |
| LTR-AC197783.2 | LTR/Gypsy | 1 | 222 |

| | | | |
|---|---|---|---|
| LTR-AC197785.2 | LTR/Gypsy | 1 | 75 |
| LTR-AC197830.2 | LTR/Gypsy | 10 | 1137 |
| LTR-AC197832.2 | LTR/Gypsy | 1 | 235 |
| LTR-AC197833.2 | LTR | 1 | 162 |
| LTR-AC197836.2 | LTR/Copia | 3 | 864 |
| LTR-AC197838.2 | LTR | 16 | 1506 |
| LTR-AC197841.2 | LTR/Gypsy | 16 | 2180 |
| LTR-AC197843.2 | LTR | 10 | 554 |
| LTR-AC197897.2 | LTR | 1 | 37 |
| LTR-AC197900.2 | LTR/Copia | 1 | 87 |
| LTR-AC197901.2 | LTR/Gypsy | 2 | 582 |
| LTR-AC197906.2 | LTR | 5 | 535 |
| LTR-AC197907.2 | LTR/Gypsy | 7 | 1095 |
| LTR-AC197908.2 | LTR/Copia | 3 | 531 |
| LTR-AC197909.2 | LTR | 2 | 132 |
| LTR-AC197910.2 | LTR/Copia | 10 | 820 |
| LTR-AC197920.2 | LTR | 7 | 665 |
| LTR-AC197924.2 | LTR | 4 | 426 |
| LTR-AC198012.2 | LTR/Gypsy | 1 | 109 |
| LTR-AC198013.2 | LTR/Copia | 12 | 1226 |
| LTR-AC198014.2 | LTR/Gypsy | 5 | 578 |
| LTR-AC198016.2 | LTR/Gypsy | 4 | 220 |
| LTR-AC198017.2 | LTR/Gypsy | 19 | 2616 |
| LTR-AC198019.2 | LTR/Copia | 1 | 56 |
| LTR-AC198021.2 | LTR/Copia | 2 | 122 |
| LTR-AC198022.2 | LTR/Gypsy | 1 | 203 |
| LTR-AC198028.2 | LTR/Gypsy | 1 | 144 |
| LTR-AC198029.2 | LTR | 2 | 206 |
| LTR-AC198033.2 | LTR/Gypsy | 1 | 49 |
| LTR-AC198056.2 | LTR | 5 | 362 |
| LTR-AC198075.2 | LTR/Gypsy | 3 | 251 |
| LTR-AC198076.2 | LTR/Gypsy | 1 | 53 |
| LTR-AC198080.2 | LTR/Gypsy | 2 | 64 |
| LTR-AC198083.1 | LTR/Gypsy | 101 | 14611 |
| LTR-AC198111.2 | LTR/Copia | 1 | 129 |
| LTR-AC198113.1 | LTR/Gypsy | 7 | 740 |
| LTR-AC198115.2 | LTR/Gypsy | 5 | 497 |
| LTR-AC198118.2 | LTR/Gypsy | 1 | 86 |
| LTR-AC198120.2 | LTR/Gypsy | 1 | 42 |
| LTR-AC198122.2 | LTR/Gypsy | 2 | 127 |
| LTR-AC198126.2 | LTR | 1 | 44 |
| LTR-AC198127.2 | LTR/Gypsy | 18 | 3104 |
| LTR-AC198128.2 | LTR/Gypsy | 49 | 6671 |
| LTR-AC198129.2 | LTR | 1 | 83 |
| LTR-AC198131.1 | LTR/Gypsy | 3 | 457 |
| LTR-AC198133.2 | LTR/Gypsy | 1 | 85 |
| LTR-AC198135.2 | LTR | 1 | 156 |

| | | | |
|---|---|---|---|
| LTR-AC198136.2 | LTR/Copia | 2 | 366 |
| LTR-AC198140.2 | LTR/Gypsy | 1 | 285 |
| LTR-AC198175.2 | LTR/Copia | 3 | 524 |
| LTR-AC198176.2 | LTR/Copia | 2 | 264 |
| LTR-AC198178.1 | LTR | 1 | 126 |
| LTR-AC198179.2 | LTR/Copia | 1 | 83 |
| LTR-AC198180.2 | LTR | 2 | 145 |
| LTR-AC198198.2 | LTR | 3 | 193 |
| LTR-AC198201.2 | LTR | 2 | 361 |
| LTR-AC198214.2 | LTR | 47 | 11414 |
| LTR-AC198215.1 | LTR/Copia | 8 | 1644 |
| LTR-AC198220.1 | LTR | 1 | 83 |
| LTR-AC198221.1 | LTR/Gypsy | 4 | 456 |
| LTR-AC198224.2 | LTR/Gypsy | 6 | 485 |
| LTR-AC198226.1 | LTR | 27 | 3408 |
| LTR-AC198227.2 | LTR/Gypsy | 3 | 93 |
| LTR-AC198234.2 | LTR | 27 | 1213 |
| LTR-AC198289.2 | LTR/Gypsy | 1 | 108 |
| LTR-AC198290.2 | LTR | 9 | 1816 |
| LTR-AC198291.2 | LTR/Gypsy | 2 | 293 |
| LTR-AC198320.2 | LTR/Gypsy | 3 | 284 |
| LTR-AC198322.2 | LTR | 14 | 1251 |
| LTR-AC198323.2 | LTR/Gypsy | 5 | 352 |
| LTR-AC198344.2 | LTR/Gypsy | 1 | 48 |
| LTR-AC198350.2 | LTR | 1 | 76 |
| LTR-AC198353.2 | LTR/Gypsy | 1 | 84 |
| LTR-AC198356.1 | LTR | 1 | 63 |
| LTR-AC198358.2 | LTR/Gypsy | 2 | 396 |
| LTR-AC198362.2 | LTR/Gypsy | 18 | 2826 |
| LTR-AC198365.2 | LTR | 2 | 42 |
| LTR-AC198369.2 | LTR | 2 | 192 |
| LTR-AC198374.2 | LTR | 13 | 1862 |
| LTR-AC198375.2 | LTR/Gypsy | 6 | 590 |
| LTR-AC198378.2 | LTR/Gypsy | 1 | 73 |
| LTR-AC198379.2 | LTR | 1 | 45 |
| LTR-AC198380.2 | LTR/Copia | 9 | 1759 |
| LTR-AC198382.2 | LTR | 3 | 591 |
| LTR-AC198385.2 | LTR | 8 | 544 |
| LTR-AC198386.2 | LTR/Gypsy | 48 | 7899 |
| LTR-AC198390.2 | LTR | 9 | 546 |
| LTR-AC198391.2 | LTR/Copia | 1 | 53 |
| LTR-AC198396.2 | LTR | 1 | 187 |
| LTR-AC198399.2 | LTR | 147 | 7942 |
| LTR-AC198403.2 | LTR/Copia | 1 | 206 |
| LTR-AC198413.2 | LTR/Gypsy | 15 | 3243 |
| LTR-AC198416.2 | LTR | 1 | 71 |
| LTR-AC198418.2 | LTR | 1 | 82 |

| | | | |
|---|---|---|---|
| LTR-AC198419.2 | LTR | 1 | 130 |
| LTR-AC198422.2 | LTR/Copia | 1 | 195 |
| LTR-AC198425.2 | LTR | 30 | 4243 |
| LTR-AC198426.2 | LTR/Copia | 2 | 225 |
| LTR-AC198428.2 | LTR/Gypsy | 4 | 426 |
| LTR-AC198475.2 | LTR/Copia | 4 | 448 |
| LTR-AC198478.2 | LTR | 16 | 1674 |
| LTR-AC198481.2 | LTR | 118 | 13775 |
| LTR-AC198482.2 | LTR | 1 | 31 |
| LTR-AC198483.2 | LTR | 1 | 42 |
| LTR-AC198491.2 | LTR/Gypsy | 2 | 94 |
| LTR-AC198492.2 | LTR | 2 | 331 |
| LTR-AC198494.2 | LTR/Copia | 3 | 314 |
| LTR-AC198497.2 | LTR/Gypsy | 2 | 301 |
| LTR-AC198502.2 | LTR | 1 | 67 |
| LTR-AC198504.2 | LTR/Copia | 2 | 66 |
| LTR-AC198508.2 | LTR | 1 | 60 |
| LTR-AC198512.2 | LTR | 17 | 2556 |
| LTR-AC198514.2 | LTR | 6 | 899 |
| LTR-AC198587.2 | LTR/Copia | 3 | 253 |
| LTR-AC198589.2 | LTR/Gypsy | 47 | 4911 |
| LTR-AC198593.2 | LTR/Copia | 1 | 354 |
| LTR-AC198595.2 | LTR/Gypsy | 10 | 1041 |
| LTR-AC198597.2 | LTR/Gypsy | 1 | 168 |
| LTR-AC198600.2 | LTR | 2 | 309 |
| LTR-AC198602.2 | LTR | 5 | 284 |
| LTR-AC198606.2 | LTR/Gypsy | 3 | 345 |
| LTR-AC198608.2 | LTR/Copia | 1 | 196 |
| LTR-AC198610.2 | LTR/Gypsy | 1 | 68 |
| LTR-AC198647.2 | LTR/Copia | 2 | 198 |
| LTR-AC198649.1 | LTR | 2 | 120 |
| LTR-AC198651.2 | LTR/Gypsy | 1 | 288 |
| LTR-AC198652.1 | LTR | 1 | 50 |
| LTR-AC198654.1 | LTR/Copia | 1 | 58 |
| LTR-AC198655.2 | LTR/Gypsy | 5 | 596 |
| LTR-AC198657.1 | LTR | 1 | 68 |
| LTR-AC198658.2 | LTR/Copia | 1 | 47 |
| LTR-AC198661.1 | LTR | 1 | 53 |
| LTR-AC198662.2 | LTR/Copia | 1 | 102 |
| LTR-AC198667.1 | LTR/Copia | 1 | 228 |
| LTR-AC198689.2 | LTR | 1 | 167 |
| LTR-AC198691.2 | LTR | 10 | 1222 |
| LTR-AC198692.2 | LTR | 2 | 92 |
| LTR-AC198693.2 | LTR/Gypsy | 2 | 128 |
| LTR-AC198694.2 | LTR | 1 | 60 |
| LTR-AC198698.2 | LTR/Gypsy | 6 | 602 |
| LTR-AC198700.2 | LTR/Copia | 2 | 143 |

| | | | |
|---|---|---|---|
| LTR-AC198701.2 | LTR | 4 | 232 |
| LTR-AC198704.2 | LTR/Gypsy | 2 | 107 |
| LTR-AC198726.2 | LTR/Copia | 1 | 43 |
| LTR-AC198728.2 | LTR/Gypsy | 2 | 278 |
| LTR-AC198731.2 | LTR/Gypsy | 5 | 891 |
| LTR-AC198732.2 | LTR/Gypsy | 6 | 769 |
| LTR-AC198735.2 | LTR | 3 | 246 |
| LTR-AC198736.2 | LTR | 1 | 61 |
| LTR-AC198738.2 | LTR/Gypsy | 4 | 432 |
| LTR-AC198739.2 | LTR | 6 | 911 |
| LTR-AC198743.2 | LTR | 75 | 5220 |
| LTR-AC198745.1 | LTR | 2 | 234 |
| LTR-AC198746.2 | LTR | 5 | 237 |
| LTR-AC198747.2 | LTR/Gypsy | 1 | 63 |
| LTR-AC198751.2 | LTR | 1 | 83 |
| LTR-AC198753.2 | LTR/Gypsy | 4 | 575 |
| LTR-AC198754.2 | LTR | 2 | 112 |
| LTR-AC198756.2 | LTR/Gypsy | 7 | 703 |
| LTR-AC198758.2 | LTR/Gypsy | 3 | 558 |
| LTR-AC198759.2 | LTR/Gypsy | 15 | 1703 |
| LTR-AC198760.2 | LTR | 1 | 84 |
| LTR-AC198762.2 | LTR | 1 | 295 |
| LTR-AC198765.2 | LTR/Copia | 2 | 88 |
| LTR-AC198773.2 | LTR | 9 | 682 |
| LTR-AC198775.2 | LTR | 37 | 5375 |
| LTR-AC198776.2 | LTR | 3 | 229 |
| LTR-AC198777.2 | LTR/Copia | 1 | 143 |
| LTR-AC198778.1 | LTR | 1 | 37 |
| LTR-AC198779.2 | LTR/Gypsy | 22 | 3156 |
| LTR-AC198780.2 | LTR | 1 | 64 |
| LTR-AC198782.2 | LTR | 1 | 67 |
| LTR-AC198787.2 | LTR/Copia | 1 | 62 |
| LTR-AC198859.2 | LTR/Copia | 1 | 290 |
| LTR-AC198860.2 | LTR/Copia | 5 | 523 |
| LTR-AC198861.2 | LTR/Copia | 1 | 148 |
| LTR-AC198863.2 | LTR | 2 | 138 |
| LTR-AC198864.2 | LTR/Gypsy | 1 | 194 |
| LTR-AC198868.2 | LTR | 1 | 293 |
| LTR-AC198869.2 | LTR | 13 | 2155 |
| LTR-AC198872.2 | LTR | 2 | 186 |
| LTR-AC198873.2 | LTR | 15 | 2644 |
| LTR-AC198874.2 | LTR | 2 | 297 |
| LTR-AC198888.2 | LTR | 1 | 105 |
| LTR-AC198891.2 | LTR | 24 | 2437 |
| LTR-AC198894.2 | LTR/Copia | 2 | 390 |
| LTR-AC198895.2 | LTR/Copia | 1 | 152 |
| LTR-AC198898.2 | LTR/Gypsy | 1 | 133 |

| | | | |
|---|---|---|---|
| LTR-AC198903.2 | LTR/Gypsy | 1 | 76 |
| LTR-AC198923.2 | LTR/Gypsy | 1 | 55 |
| LTR-AC198924.2 | LTR/Gypsy | 1 | 110 |
| LTR-AC198925.2 | LTR | 1 | 84 |
| LTR-AC198927.2 | LTR/Copia | 2 | 183 |
| LTR-AC198928.2 | LTR/Gypsy | 1 | 72 |
| LTR-AC198931.2 | LTR/Gypsy | 2 | 296 |
| LTR-AC198932.2 | LTR/Gypsy | 2 | 256 |
| LTR-AC198934.2 | LTR/Gypsy | 1 | 251 |
| LTR-AC198935.2 | LTR/Copia | 13 | 2910 |
| LTR-AC198936.2 | LTR/Copia | 4 | 514 |
| LTR-AC198939.2 | LTR/Gypsy | 2 | 129 |
| LTR-AC198940.2 | LTR/Copia | 1 | 91 |
| LTR-AC198963.2 | LTR | 1 | 47 |
| LTR-AC198966.2 | LTR/Gypsy | 15 | 3441 |
| LTR-AC198967.2 | LTR/Gypsy | 3 | 558 |
| LTR-AC198968.1 | LTR/Gypsy | 3 | 146 |
| LTR-AC198969.2 | LTR/Gypsy | 1 | 81 |
| LTR-AC198970.2 | LTR/Copia | 2 | 476 |
| LTR-AC198972.2 | LTR/Copia | 1 | 154 |
| LTR-AC198973.2 | LTR | 1 | 60 |
| LTR-AC198978.2 | LTR | 1 | 208 |
| LTR-AC198980.2 | LTR | 5 | 402 |
| LTR-AC198987.2 | LTR/Gypsy | 2 | 410 |
| LTR-AC198989.2 | LTR/Gypsy | 28 | 5402 |
| LTR-AC198991.2 | LTR | 8 | 783 |
| LTR-AC198992.2 | LTR | 11 | 716 |
| LTR-AC198995.2 | LTR/Gypsy | 4 | 385 |
| LTR-AC199001.2 | LTR/Copia | 14 | 1294 |
| LTR-AC199004.2 | LTR/Gypsy | 1 | 113 |
| LTR-AC199008.2 | LTR/Gypsy | 2 | 320 |
| LTR-AC199009.2 | LTR | 2 | 133 |
| LTR-AC199020.2 | LTR/Copia | 1 | 83 |
| LTR-AC199037.2 | LTR/Copia | 3 | 217 |
| LTR-AC199043.2 | LTR/Gypsy | 6 | 780 |
| LTR-AC199044.2 | LTR/Gypsy | 8 | 1626 |
| LTR-AC199045.2 | LTR/Gypsy | 18 | 2910 |
| LTR-AC199047.2 | LTR/Gypsy | 1 | 80 |
| LTR-AC199048.2 | LTR/Gypsy | 1 | 45 |
| LTR-AC199051.2 | LTR/Gypsy | 1 | 69 |
| LTR-AC199053.2 | LTR | 2 | 150 |
| LTR-AC199054.2 | LTR | 1 | 138 |
| LTR-AC199055.2 | LTR/Gypsy | 2 | 212 |
| LTR-AC199056.2 | LTR/Gypsy | 1 | 88 |
| LTR-AC199061.2 | LTR | 1 | 53 |
| LTR-AC199062.2 | LTR | 1 | 62 |
| LTR-AC199063.2 | LTR | 3 | 168 |

| | | | |
|---|---|---|---|
| LTR-AC199064.2 | LTR | 1 | 73 |
| LTR-AC199065.2 | LTR | 1 | 68 |
| LTR-AC199066.2 | LTR/Gypsy | 5 | 492 |
| LTR-AC199067.2 | LTR | 26 | 2463 |
| LTR-AC199069.2 | LTR | 40 | 8269 |
| LTR-AC199070.2 | LTR/Copia | 2 | 157 |
| LTR-AC199071.2 | LTR/Copia | 2 | 561 |
| LTR-AC199072.2 | LTR/Gypsy | 1 | 38 |
| LTR-AC199077.2 | LTR/Gypsy | 11 | 721 |
| LTR-AC199078.2 | LTR/Copia | 2 | 121 |
| LTR-AC199080.2 | LTR | 1 | 59 |
| LTR-AC199084.2 | LTR/Copia | 1 | 71 |
| LTR-AC199089.2 | LTR/Gypsy | 3 | 540 |
| LTR-AC199114.2 | LTR/Gypsy | 4 | 216 |
| LTR-AC199115.2 | LTR/Gypsy | 2 | 113 |
| LTR-AC199117.2 | LTR/Gypsy | 1 | 139 |
| LTR-AC199118.1 | LTR | 4 | 212 |
| LTR-AC199148.2 | LTR/Gypsy | 1 | 60 |
| LTR-AC199154.2 | LTR | 1 | 60 |
| LTR-AC199155.2 | LTR | 4 | 551 |
| LTR-AC199157.2 | LTR/Copia | 14 | 846 |
| LTR-AC199173.1 | LTR/Gypsy | 2 | 139 |
| LTR-AC199177.2 | LTR | 1 | 112 |
| LTR-AC199178.2 | LTR | 2 | 88 |
| LTR-AC199179.2 | LTR | 4 | 250 |
| LTR-AC199181.2 | LTR/Gypsy | 2 | 140 |
| LTR-AC199182.2 | LTR/Gypsy | 6 | 517 |
| LTR-AC199183.2 | LTR/Copia | 5 | 907 |
| LTR-AC199184.2 | LTR/Copia | 7 | 1126 |
| LTR-AC199188.2 | LTR | 3 | 195 |
| LTR-AC199193.2 | LTR | 3 | 218 |
| LTR-AC199194.2 | LTR | 1 | 38 |
| LTR-AC199198.2 | LTR/Gypsy | 1 | 79 |
| LTR-AC199199.2 | LTR/Copia | 1 | 40 |
| LTR-AC199201.2 | LTR/Gypsy | 1 | 40 |
| LTR-AC199210.2 | LTR | 29 | 1576 |
| LTR-AC199214.2 | LTR/Gypsy | 1 | 133 |
| LTR-AC199215.2 | LTR/Gypsy | 11 | 1523 |
| LTR-AC199216.2 | LTR | 2 | 93 |
| LTR-AC199217.2 | LTR | 1 | 50 |
| LTR-AC199218.2 | LTR | 1 | 41 |
| LTR-AC199219.2 | LTR/Gypsy | 8 | 974 |
| LTR-AC199221.2 | LTR | 90 | 15352 |
| LTR-AC199223.2 | LTR | 97 | 11376 |
| LTR-AC199224.2 | LTR | 1 | 110 |
| LTR-AC199315.2 | LTR/Copia | 1 | 61 |
| LTR-AC199317.2 | LTR/Copia | 2 | 347 |

| | | | |
|---|---|---|---|
| LTR-AC199319.2 | LTR/Gypsy | 30 | 4528 |
| LTR-AC199322.2 | LTR/Gypsy | 2 | 618 |
| LTR-AC199323.2 | LTR/Gypsy | 1 | 222 |
| LTR-AC199325.2 | LTR/Copia | 6 | 1169 |
| LTR-AC199326.1 | LTR/Gypsy | 1 | 119 |
| LTR-AC199328.2 | LTR | 1 | 44 |
| LTR-AC199329.2 | LTR | 9 | 572 |
| LTR-AC199332.2 | LTR/Copia | 1 | 83 |
| LTR-AC199334.2 | LTR/Gypsy | 8 | 1520 |
| LTR-AC199335.2 | LTR | 2 | 136 |
| LTR-AC199339.2 | LTR/Gypsy | 2 | 111 |
| LTR-AC199341.1 | LTR | 3 | 391 |
| LTR-AC199343.2 | LTR/Copia | 13 | 886 |
| LTR-AC199346.2 | LTR/Copia | 1 | 92 |
| LTR-AC199347.2 | LTR/Gypsy | 2 | 70 |
| LTR-AC199348.2 | LTR/Copia | 1 | 44 |
| LTR-AC199352.2 | LTR | 34 | 3488 |
| LTR-AC199356.2 | LTR/Gypsy | 8 | 941 |
| LTR-AC199357.2 | LTR/Copia | 12 | 1477 |
| LTR-AC199359.2 | LTR | 6 | 558 |
| LTR-AC199364.2 | LTR/Copia | 1 | 134 |
| LTR-AC199367.2 | LTR | 1 | 57 |
| LTR-AC199368.2 | LTR | 1 | 94 |
| LTR-AC199372.2 | LTR/Gypsy | 3 | 320 |
| LTR-AC199375.2 | LTR/Gypsy | 1 | 279 |
| LTR-AC199376.2 | LTR | 4 | 650 |
| LTR-AC199377.2 | LTR/Gypsy | 3 | 211 |
| LTR-AC199381.1 | LTR/Gypsy | 5 | 366 |
| LTR-AC199383.2 | LTR/Copia | 4 | 240 |
| LTR-AC199384.2 | LTR | 23 | 3781 |
| LTR-AC199387.2 | LTR | 3 | 638 |
| LTR-AC199389.2 | LTR | 1 | 56 |
| LTR-AC199391.2 | LTR/Gypsy | 4 | 234 |
| LTR-AC199394.2 | LTR | 1 | 125 |
| LTR-AC199396.2 | LTR/Copia | 27 | 4642 |
| LTR-AC199401.2 | LTR | 1 | 66 |
| LTR-AC199402.2 | LTR/Copia | 2 | 336 |
| LTR-AC199403.2 | LTR/Copia | 1 | 170 |
| LTR-AC199404.2 | LTR/Copia | 2 | 389 |
| LTR-AC199405.2 | LTR | 16 | 2036 |
| LTR-AC199407.2 | LTR | 4 | 294 |
| LTR-AC199409.2 | LTR/Gypsy | 2 | 96 |
| LTR-AC199411.2 | LTR/Copia | 1 | 74 |
| LTR-AC199412.1 | LTR/Gypsy | 2 | 152 |
| LTR-AC199414.2 | LTR/Copia | 2 | 134 |
| LTR-AC199418.2 | LTR | 2 | 215 |
| LTR-AC199420.2 | LTR | 15 | 1073 |

| | | | |
|---|---|---|---|
| LTR-AC199437.2 | LTR | 34 | 4226 |
| LTR-AC199442.2 | LTR | 1 | 123 |
| LTR-AC199443.2 | LTR | 1 | 41 |
| LTR-AC199471.2 | LTR | 1 | 68 |
| LTR-AC199472.2 | LTR/Gypsy | 1 | 58 |
| LTR-AC199475.2 | LTR | 2 | 168 |
| LTR-AC199477.2 | LTR | 16 | 1689 |
| LTR-AC199478.2 | LTR/Copia | 2 | 88 |
| LTR-AC199483.2 | LTR/Gypsy | 7 | 472 |
| LTR-AC199486.2 | LTR | 8 | 606 |
| LTR-AC199496.2 | LTR | 1 | 93 |
| LTR-AC199507.2 | LTR/Copia | 1 | 177 |
| LTR-AC199508.2 | LTR/Gypsy | 2 | 233 |
| LTR-AC199510.2 | LTR/Gypsy | 17 | 3723 |
| LTR-AC199514.2 | LTR/Gypsy | 6 | 324 |
| LTR-AC199515.2 | LTR | 62 | 7315 |
| LTR-AC199516.2 | LTR | 4 | 689 |
| LTR-AC199519.1 | LTR/Gypsy | 1 | 42 |
| LTR-AC199521.2 | LTR/Gypsy | 2 | 202 |
| LTR-AC199525.2 | LTR/Copia | 10 | 675 |
| LTR-AC199528.2 | LTR | 23 | 2878 |
| LTR-AC199529.2 | LTR/Copia | 1 | 110 |
| LTR-AC199530.2 | LTR | 1 | 36 |
| LTR-AC199531.2 | LTR | 2 | 215 |
| LTR-AC199537.2 | LTR/Copia | 1 | 47 |
| LTR-AC199538.2 | LTR | 9 | 808 |
| LTR-AC199541.2 | LTR | 1 | 324 |
| LTR-AC199545.2 | LTR | 2 | 126 |
| LTR-AC199546.2 | LTR/Copia | 1 | 81 |
| LTR-AC199548.2 | LTR | 1 | 61 |
| LTR-AC199549.2 | LTR | 23 | 2028 |
| LTR-AC199552.2 | LTR | 1 | 91 |
| LTR-AC199553.1 | LTR/Gypsy | 4 | 336 |
| LTR-AC199554.1 | LTR/Copia | 1 | 69 |
| LTR-AC199556.2 | LTR | 1 | 114 |
| LTR-AC199564.1 | LTR/Copia | 1 | 230 |
| LTR-AC199567.2 | LTR/Copia | 2 | 186 |
| LTR-AC199598.2 | LTR | 2 | 194 |
| LTR-AC199619.2 | LTR/Gypsy | 4 | 177 |
| LTR-AC199621.1 | LTR/Gypsy | 2 | 148 |
| LTR-AC199624.1 | LTR | 3 | 188 |
| LTR-AC199625.2 | LTR/Copia | 2 | 252 |
| LTR-AC199637.2 | LTR/Gypsy | 1 | 359 |
| LTR-AC199700.1 | LTR | 1 | 42 |
| LTR-AC199702.1 | LTR | 2 | 198 |
| LTR-AC199703.2 | LTR | 15 | 2976 |
| LTR-AC199704.1 | LTR | 6 | 394 |

| | | | |
|---|---|---|---|
| LTR-AC199707.2 | LTR/Gypsy | 8 | 751 |
| LTR-AC199709.2 | LTR/Copia | 3 | 386 |
| LTR-AC199727.2 | LTR/Gypsy | 1 | 118 |
| LTR-AC199728.2 | LTR/Copia | 1 | 85 |
| LTR-AC199764.2 | LTR/Gypsy | 1 | 397 |
| LTR-AC199767.2 | LTR | 3 | 737 |
| LTR-AC199768.2 | LTR | 1 | 78 |
| LTR-AC199769.2 | LTR | 3 | 327 |
| LTR-AC199771.1 | LTR/Gypsy | 1 | 95 |
| LTR-AC199773.1 | LTR | 39 | 3385 |
| LTR-AC199775.1 | LTR | 1 | 45 |
| LTR-AC199786.1 | LTR/Copia | 1 | 196 |
| LTR-AC199789.2 | LTR/Gypsy | 5 | 667 |
| LTR-AC199790.2 | LTR | 1 | 100 |
| LTR-AC199791.1 | LTR/Gypsy | 1 | 75 |
| LTR-AC199800.1 | LTR/Gypsy | 3 | 305 |
| LTR-AC199804.1 | LTR/Copia | 1 | 74 |
| LTR-AC199807.1 | LTR | 2 | 294 |
| LTR-AC199812.2 | LTR/Copia | 18 | 1576 |
| LTR-AC199814.2 | LTR/Gypsy | 7 | 908 |
| LTR-AC199818.1 | LTR/Gypsy | 3 | 198 |
| LTR-AC199821.1 | LTR | 1 | 212 |
| LTR-AC199824.1 | LTR/Gypsy | 21 | 3052 |
| LTR-AC199827.2 | LTR | 8 | 447 |
| LTR-AC199828.1 | LTR | 4 | 208 |
| LTR-AC199841.2 | LTR | 2 | 139 |
| LTR-AC199844.1 | LTR | 1 | 36 |
| LTR-AC199852.2 | LTR/Gypsy | 2 | 144 |
| LTR-AC199855.2 | LTR/Gypsy | 3 | 150 |
| LTR-AC199857.2 | LTR/Gypsy | 2 | 96 |
| LTR-AC199876.1 | LTR | 2 | 78 |
| LTR-AC199882.1 | LTR | 1 | 83 |
| LTR-AC199883.1 | LTR | 2 | 100 |
| LTR-AC199887.1 | LTR | 7 | 831 |
| LTR-AC199896.1 | LTR/Gypsy | 6 | 1025 |
| LTR-AC199902.1 | LTR/Copia | 2 | 81 |
| LTR-AC199904.1 | LTR/Copia | 1 | 43 |
| LTR-AC199906.1 | LTR | 1 | 59 |
| LTR-AC199916.1 | LTR/Gypsy | 3 | 562 |
| LTR-AC199918.1 | LTR | 1 | 80 |
| LTR-AC199922.1 | LTR | 2 | 299 |
| LTR-AC199926.1 | LTR/Gypsy | 78 | 11778 |
| LTR-AC199933.1 | LTR | 1 | 53 |
| LTR-AC199934.1 | LTR | 7 | 547 |
| LTR-AC199944.1 | LTR/Gypsy | 1 | 46 |
| LTR-AC199957.1 | LTR | 2 | 120 |
| LTR-AC199958.1 | LTR/Copia | 2 | 717 |

| | | | |
|---|---|---|---|
| LTR-AC199961.1 | LTR | 1 | 93 |
| LTR-AC200033.1 | LTR | 10 | 979 |
| LTR-AC200034.1 | LTR/Copia | 6 | 433 |
| LTR-AC200041.1 | LTR | 1 | 51 |
| LTR-AC200045.1 | LTR/Gypsy | 11 | 1514 |
| LTR-AC200047.1 | LTR/Gypsy | 1 | 114 |
| LTR-AC200048.1 | LTR | 7 | 701 |
| LTR-AC200053.1 | LTR/Gypsy | 2 | 250 |
| LTR-AC200058.1 | LTR/Gypsy | 1 | 179 |
| LTR-AC200059.1 | LTR/Gypsy | 4 | 220 |
| LTR-AC200060.1 | LTR | 1 | 46 |
| LTR-AC200061.1 | LTR | 1 | 113 |
| LTR-AC200062.1 | LTR/Gypsy | 1 | 54 |
| LTR-AC200063.1 | LTR | 5 | 833 |
| LTR-AC200064.1 | LTR | 2 | 109 |
| LTR-AC200067.1 | LTR/Gypsy | 1 | 168 |
| LTR-AC200092.1 | LTR | 2 | 291 |
| LTR-AC200094.1 | LTR/Gypsy | 21 | 2130 |
| LTR-AC200095.1 | LTR/Gypsy | 1 | 182 |
| LTR-AC200103.1 | LTR | 4 | 264 |
| LTR-AC200107.1 | LTR | 1 | 52 |
| LTR-AC200112.1 | LTR/Gypsy | 1 | 79 |
| LTR-AC200116.1 | LTR/Gypsy | 7 | 1439 |
| LTR-AC200120.1 | LTR | 1 | 33 |
| LTR-AC200121.1 | LTR | 4 | 656 |
| LTR-AC200128.1 | LTR/Copia | 4 | 675 |
| LTR-AC200133.1 | LTR/Gypsy | 3 | 195 |
| LTR-AC200134.1 | LTR/Gypsy | 5 | 415 |
| LTR-AC200135.1 | LTR/Copia | 1 | 155 |
| LTR-AC200147.1 | LTR/Copia | 1 | 95 |
| LTR-AC200171.1 | LTR/Copia | 6 | 536 |
| LTR-AC200174.1 | LTR/Gypsy | 3 | 264 |
| LTR-AC200175.1 | LTR/Gypsy | 3 | 710 |
| LTR-AC200177.1 | LTR/Gypsy | 1 | 282 |
| LTR-AC200179.1 | LTR/Gypsy | 2 | 160 |
| LTR-AC200180.2 | LTR/Copia | 2 | 122 |
| LTR-AC200183.1 | LTR/Copia | 1 | 129 |
| LTR-AC200187.1 | LTR/Copia | 1 | 188 |
| LTR-AC200189.1 | LTR | 4 | 233 |
| LTR-AC200190.1 | LTR/Copia | 6 | 1000 |
| LTR-AC200195.1 | LTR/Copia | 1 | 30 |
| LTR-AC200196.1 | LTR | 4 | 686 |
| LTR-AC200200.1 | LTR | 6 | 355 |
| LTR-AC200201.1 | LTR/Gypsy | 11 | 1287 |
| LTR-AC200203.1 | LTR | 2 | 230 |
| LTR-AC200208.1 | LTR | 1 | 151 |
| LTR-AC200209.1 | LTR/Gypsy | 7 | 905 |

| | | | |
|---|---|---|---|
| LTR-AC200212.1 | LTR/Gypsy | 4 | 654 |
| LTR-AC200213.1 | LTR/Gypsy | 3 | 318 |
| LTR-AC200234.1 | LTR | 4 | 902 |
| LTR-AC200248.1 | LTR | 8 | 934 |
| LTR-AC200252.1 | LTR/Gypsy | 1 | 46 |
| LTR-AC200254.1 | LTR/Gypsy | 16 | 3643 |
| LTR-AC200257.1 | LTR | 1 | 88 |
| LTR-AC200259.1 | LTR | 4 | 631 |
| LTR-AC200260.1 | LTR | 17 | 724 |
| LTR-AC200261.1 | LTR | 1 | 49 |
| LTR-AC200263.1 | LTR | 1 | 34 |
| LTR-AC200281.1 | LTR | 1 | 143 |
| LTR-AC200285.1 | LTR/Copia | 2 | 504 |
| LTR-AC200286.1 | LTR/Copia | 1 | 43 |
| LTR-AC200290.1 | LTR/Gypsy | 2 | 242 |
| LTR-AC200297.1 | LTR/Gypsy | 1 | 137 |
| LTR-AC200299.1 | LTR | 2 | 108 |
| LTR-AC200310.1 | LTR | 2 | 212 |
| LTR-AC200320.1 | LTR | 4 | 544 |
| LTR-AC200323.1 | LTR | 7 | 196 |
| LTR-AC200401.1 | LTR/Gypsy | 5 | 1554 |
| LTR-AC200408.1 | LTR/Copia | 1 | 72 |
| LTR-AC200420.1 | LTR/Gypsy | 3 | 604 |
| LTR-AC200468.1 | LTR/Gypsy | 1 | 115 |
| LTR-AC200477.1 | LTR/Gypsy | 12 | 823 |
| LTR-AC200481.1 | LTR/Gypsy | 4 | 200 |
| LTR-AC200484.1 | LTR/Gypsy | 4 | 684 |
| LTR-AC200488.1 | LTR | 1 | 176 |
| LTR-AC200490.1 | LTR/Copia | 28 | 4114 |
| LTR-AC200496.1 | LTR | 7 | 914 |
| LTR-AC200497.1 | LTR | 67 | 9770 |
| LTR-AC200500.1 | LTR/Copia | 1 | 275 |
| LTR-AC200511.1 | LTR/Gypsy | 1 | 59 |
| LTR-AC200540.1 | LTR | 17 | 2022 |
| LTR-AC200543.1 | LTR | 3 | 601 |
| LTR-AC200544.1 | LTR/Gypsy | 149 | 34133 |
| LTR-AC200553.1 | LTR | 2 | 155 |
| LTR-AC200556.1 | LTR/Copia | 1 | 72 |
| LTR-AC200562.1 | LTR/Copia | 3 | 563 |
| LTR-AC200563.1 | LTR/Gypsy | 1 | 83 |
| LTR-AC200609.1 | LTR | 11 | 1026 |
| LTR-AC200617.1 | LTR | 2 | 366 |
| LTR-AC200618.1 | LTR/Gypsy | 1 | 58 |
| LTR-AC200619.1 | LTR | 18 | 1542 |
| LTR-AC200655.1 | LTR | 2 | 135 |
| LTR-AC200656.1 | LTR | 2 | 348 |
| LTR-AC200683.1 | LTR | 3 | 132 |

| | | | |
|---|---|---|---|
| LTR-AC200689.1 | LTR | 1 | 61 |
| LTR-AC200725.1 | LTR/Copia | 1 | 307 |
| LTR-AC200727.1 | LTR/Gypsy | 2 | 191 |
| LTR-AC200739.1 | LTR/Gypsy | 5 | 312 |
| LTR-AC200746.1 | LTR | 2 | 612 |
| LTR-AC200756.1 | LTR/Gypsy | 12 | 1344 |
| LTR-AC200765.1 | LTR | 1509 | 425508 |
| LTR-AC200767.1 | LTR/Gypsy | 2278 | 507235 |
| LTR-AC200768.1 | LTR | 2653 | 719641 |
| LTR-AC200769.1 | LTR | 263 | 79421 |
| LTR-AC200770.1 | LTR | 3764 | 1103778 |
| LTR-AC200774.1 | LTR/Copia | 593 | 198870 |
| LTR-AC200775.1 | LTR/Copia | 1338 | 191208 |
| LTR-AC200776.1 | LTR | 9320 | 2399486 |
| LTR-AC200777.1 | LTR | 1498 | 482479 |
| LTR-AC200778.1 | LTR | 3788 | 1103387 |
| LTR-AC200779.1 | LTR | 487 | 160124 |
| LTR-AC200780.1 | LTR | 2293 | 664947 |
| LTR-AC200781.1 | LTR | 2110 | 662405 |
| LTR-AC200783.1 | LTR | 1219 | 326937 |
| LTR-AC200784.1 | LTR/Gypsy | 700 | 220550 |
| LTR-AC200785.1 | LTR | 1184 | 258020 |
| LTR-AC200788.1 | LTR/Copia | 5021 | 1278036 |
| LTR-AC200789.1 | LTR | 2019 | 587702 |
| LTR-AC200792.1 | LTR | 375 | 88779 |
| LTR-AC200793.1 | LTR | 626 | 182824 |
| LTR-AC200794.1 | LTR | 2584 | 704560 |
| LTR-AC200795.1 | LTR | 3727 | 1127810 |
| LTR-AC200797.1 | LTR | 2607 | 724333 |
| LTR-AC200798.1 | LTR | 5105 | 1491935 |
| LTR-AC200799.1 | LTR | 5257 | 1424553 |
| LTR-AC200800.1 | LTR | 107 | 21314 |
| LTR-AC200802.1 | LTR | 169 | 42453 |
| LTR-AC200805.1 | LTR | 1225 | 331733 |
| LTR-AC200806.1 | LTR/Gypsy | 2663 | 806002 |
| LTR-AC200807.1 | LTR | 7048 | 2119006 |
| LTR-AC200808.1 | LTR | 464 | 140900 |
| LTR-AC200812.1 | LTR/Gypsy | 430 | 130578 |
| LTR-AC200813.1 | LTR | 165 | 50237 |
| LTR-AC200815.1 | LTR | 444 | 129199 |
| LTR-AC200816.1 | LTR/Gypsy | 1591 | 468230 |
| LTR-AC200819.1 | LTR | 9928 | 3196919 |
| LTR-AC200820.1 | LTR | 319 | 91343 |
| LTR-AC200821.1 | LTR/Gypsy | 3103 | 969407 |
| LTR-AC200822.1 | LTR/Gypsy | 2829 | 699915 |
| LTR-AC200824.1 | LTR/Gypsy | 1395 | 449966 |
| LTR-AC200825.1 | LTR/Gypsy | 526 | 149317 |

| | | | |
|---|---|---|---|
| LTR-AC200826.1 | LTR | 5287 | 1426685 |
| LTR-AC200827.1 | LTR | 3697 | 931866 |
| LTR-AC200828.1 | LTR | 1359 | 373887 |
| LTR-AC200829.1 | LTR | 7141 | 1929271 |
| LTR-AC200831.1 | LTR | 2858 | 833701 |
| LTR-AC200832.1 | LTR | 1112 | 289048 |
| LTR-AC200833.1 | LTR | 1494 | 387033 |
| LTR-AC200834.1 | LTR | 4833 | 1222023 |
| LTR-AC200835.1 | LTR/Copia | 20 | 5675 |
| LTR-AC200838.1 | LTR | 800 | 241059 |
| LTR-AC200841.1 | LTR | 564 | 172976 |
| LTR-AC200842.1 | LTR/Copia | 4078 | 1315582 |
| LTR-AC200843.1 | LTR | 819 | 235593 |
| LTR-AC200846.1 | LTR/Copia | 733 | 252336 |
| LTR-AC200847.1 | LTR/Copia | 3257 | 953035 |
| LTR-AC200848.1 | LTR | 496 | 142483 |
| LTR-AC200850.1 | LTR/Copia | 258 | 47522 |
| LTR-AC200851.1 | LTR/Copia | 1937 | 547502 |
| LTR-AC200853.1 | LTR | 2 | 114 |
| LTR-AC200869.1 | LTR | 2 | 143 |
| LTR-AC200874.1 | LTR | 1 | 40 |
| LTR-AC200876.1 | LTR/Copia | 1 | 76 |
| LTR-AC200878.1 | LTR/Copia | 2 | 119 |
| LTR-AC200879.1 | LTR | 1 | 44 |
| LTR-AC200904.1 | LTR/Copia | 1 | 44 |
| LTR-AC201742.1 | LTR | 1 | 41 |
| LTR-AC201746.1 | LTR | 3 | 373 |
| LTR-AC201750.1 | LTR/Gypsy | 3 | 290 |
| LTR-AC201754.1 | LTR/Gypsy | 1 | 126 |
| LTR-AC201759.1 | LTR | 91 | 7685 |
| LTR-AC201760.1 | LTR/Gypsy | 3 | 172 |
| LTR-AC201761.1 | LTR | 3 | 644 |
| LTR-AC201773.1 | LTR | 4 | 503 |
| LTR-AC201775.1 | LTR | 2 | 345 |
| LTR-AC201777.1 | LTR/Gypsy | 12 | 1417 |
| LTR-AC201778.1 | LTR | 4 | 232 |
| LTR-AC201781.1 | LTR/Gypsy | 1 | 73 |
| LTR-AC201785.1 | LTR | 1 | 95 |
| LTR-AC201786.1 | LTR/Gypsy | 1 | 101 |
| LTR-AC201787.1 | LTR/Copia | 1 | 107 |
| LTR-AC201795.1 | LTR/Gypsy | 1 | 213 |
| LTR-AC201800.1 | LTR/Gypsy | 29 | 2039 |
| LTR-AC201805.1 | LTR | 1 | 80 |
| LTR-AC201806.1 | LTR | 1 | 60 |
| LTR-AC201810.1 | LTR/Copia | 1 | 134 |
| LTR-AC201811.1 | LTR | 1 | 51 |
| LTR-AC201812.1 | LTR | 1 | 55 |

| | | | |
|---|---|---|---|
| LTR-AC201821.1 | LTR | 2 | 216 |
| LTR-AC201830.1 | LTR/Copia | 1 | 74 |
| LTR-AC201831.1 | LTR/Gypsy | 1 | 34 |
| LTR-AC201834.1 | LTR | 6 | 828 |
| LTR-AC201878.1 | LTR/Gypsy | 2 | 126 |
| LTR-AC201884.1 | LTR/Gypsy | 1 | 47 |
| LTR-AC201889.1 | LTR/Gypsy | 2 | 123 |
| LTR-AC201891.1 | LTR | 57 | 14197 |
| LTR-AC201895.1 | LTR/Gypsy | 12 | 816 |
| LTR-AC201898.1 | LTR/Copia | 1 | 121 |
| LTR-AC201902.1 | LTR | 1 | 379 |
| LTR-AC201906.1 | LTR/Copia | 1 | 47 |
| LTR-AC201917.1 | LTR/Copia | 7 | 1178 |
| LTR-AC201919.1 | LTR/Gypsy | 2 | 232 |
| LTR-AC201920.1 | LTR | 10 | 451 |
| LTR-AC201957.1 | LTR/Gypsy | 10 | 2136 |
| LTR-AC201968.1 | LTR | 5 | 913 |
| LTR-AC201979.1 | LTR | 1 | 82 |
| LTR-AC201980.1 | LTR | 2 | 155 |
| LTR-AC201992.1 | LTR/Copia | 1 | 89 |
| LTR-AC201994.1 | LTR | 5 | 403 |
| LTR-AC202002.1 | LTR | 2 | 311 |
| LTR-AC202005.1 | LTR/Gypsy | 1 | 85 |
| LTR-AC202006.1 | LTR | 24 | 1950 |
| LTR-AC202012.1 | LTR/Gypsy | 2 | 171 |
| LTR-AC202017.1 | LTR/Gypsy | 2 | 92 |
| LTR-AC202018.1 | LTR | 2 | 129 |
| LTR-AC202035.1 | LTR/Copia | 4 | 375 |
| LTR-AC202036.1 | LTR | 311 | 20964 |
| LTR-AC202041.1 | LTR/Copia | 2 | 455 |
| LTR-AC202059.1 | LTR/Gypsy | 4 | 283 |
| LTR-AC202061.1 | LTR | 2 | 153 |
| LTR-AC202063.1 | LTR/Gypsy | 3 | 289 |
| LTR-AC202064.1 | LTR/Copia | 60 | 7031 |
| LTR-AC202065.1 | LTR/Gypsy | 27 | 3178 |
| LTR-AC202068.1 | LTR | 1 | 300 |
| LTR-AC202069.1 | LTR | 2 | 191 |
| LTR-AC202072.1 | LTR/Gypsy | 2 | 125 |
| LTR-AC202073.1 | LTR/Gypsy | 2 | 157 |
| LTR-AC202075.1 | LTR/Gypsy | 1 | 160 |
| LTR-AC202082.1 | LTR | 4 | 463 |
| LTR-AC202092.1 | LTR/Copia | 2 | 110 |
| LTR-AC202095.1 | LTR/Copia | 1 | 89 |
| LTR-AC202102.1 | LTR | 1 | 78 |
| LTR-AC202109.1 | LTR/Copia | 2 | 330 |
| LTR-AC202116.1 | LTR/Copia | 1 | 96 |
| LTR-AC202121.1 | LTR | 1 | 73 |

| | | | |
|---|---|---|---|
| LTR-AC202122.1 | LTR/Gypsy | 1 | 399 |
| LTR-AC202131.1 | LTR | 1 | 68 |
| LTR-AC202137.1 | LTR/Copia | 2 | 142 |
| LTR-AC202146.1 | LTR | 10 | 758 |
| LTR-AC202148.1 | LTR/Gypsy | 24 | 3850 |
| LTR-AC202155.1 | LTR/Gypsy | 9 | 934 |
| LTR-AC202156.1 | LTR/Gypsy | 1 | 34 |
| LTR-AC202159.1 | LTR | 1 | 165 |
| LTR-AC202160.1 | LTR/Gypsy | 1 | 92 |
| LTR-AC202162.1 | LTR | 1 | 53 |
| LTR-AC202164.1 | LTR/Gypsy | 1 | 64 |
| LTR-AC202167.1 | LTR/Copia | 1 | 88 |
| LTR-AC202174.1 | LTR | 7 | 753 |
| LTR-AC202178.1 | LTR/Copia | 1 | 76 |
| LTR-AC202179.1 | LTR/Copia | 1 | 72 |
| LTR-AC202183.1 | LTR | 4 | 377 |
| LTR-AC202184.1 | LTR/Gypsy | 1 | 97 |
| LTR-AC202190.1 | LTR/Gypsy | 4 | 926 |
| LTR-AC202191.1 | LTR/Gypsy | 10 | 845 |
| LTR-AC202192.1 | LTR/Copia | 3 | 179 |
| LTR-AC202383.1 | LTR/Gypsy | 10 | 768 |
| LTR-AC202385.1 | LTR/Gypsy | 6 | 348 |
| LTR-AC202390.1 | LTR | 1 | 68 |
| LTR-AC202399.1 | LTR/Gypsy | 1 | 51 |
| LTR-AC202430.1 | LTR | 1 | 103 |
| LTR-AC202432.1 | LTR | 1 | 78 |
| LTR-AC202434.1 | LTR | 2 | 181 |
| LTR-AC202437.1 | LTR | 1 | 53 |
| LTR-AC202443.1 | LTR | 1 | 54 |
| LTR-AC202449.1 | LTR/Gypsy | 3 | 263 |
| LTR-AC202450.1 | LTR/Gypsy | 8 | 1073 |
| LTR-AC202527.1 | LTR/Copia | 5 | 277 |
| LTR-AC202530.1 | LTR/Gypsy | 1 | 150 |
| LTR-AC202531.1 | LTR/Gypsy | 1 | 35 |
| LTR-AC202536.1 | LTR/Copia | 5 | 1250 |
| LTR-AC202538.1 | LTR/Gypsy | 2 | 193 |
| LTR-AC202539.1 | LTR | 6 | 345 |
| LTR-AC202540.1 | LTR/Copia | 2 | 150 |
| LTR-AC202541.1 | LTR/Copia | 1 | 99 |
| LTR-AC202562.1 | LTR/Gypsy | 5 | 375 |
| LTR-AC202563.1 | LTR | 1 | 41 |
| LTR-AC202703.1 | LTR/Gypsy | 9 | 513 |
| LTR-AC202705.1 | LTR/Gypsy | 1 | 84 |
| LTR-AC202708.1 | LTR/Copia | 1 | 394 |
| LTR-AC202818.1 | LTR/Copia | 1 | 246 |
| LTR-AC202855.1 | LTR | 9 | 828 |
| LTR-AC202858.1 | LTR | 1 | 269 |

| | | | |
|---|---|---|---|
| LTR-AC202860.1 | LTR/Gypsy | 6 | 741 |
| LTR-AC202861.1 | LTR | 41 | 3067 |
| LTR-AC202865.1 | LTR/Gypsy | 1 | 59 |
| LTR-AC202867.1 | LTR/Gypsy | 2 | 97 |
| LTR-AC202869.1 | LTR/Gypsy | 1 | 65 |
| LTR-AC202875.1 | LTR | 3 | 252 |
| LTR-AC202877.1 | LTR | 18 | 3422 |
| LTR-AC202878.1 | LTR | 1 | 83 |
| LTR-AC202885.1 | LTR/Gypsy | 6 | 725 |
| LTR-AC202886.1 | LTR | 6 | 231 |
| LTR-AC202889.1 | LTR/Gypsy | 5 | 324 |
| LTR-AC202892.1 | LTR/Gypsy | 1 | 178 |
| LTR-AC202896.1 | LTR/Gypsy | 1 | 56 |
| LTR-AC202908.1 | LTR | 26 | 6685 |
| LTR-AC202910.1 | LTR/Gypsy | 7 | 384 |
| LTR-AC202917.1 | LTR/Gypsy | 9 | 1007 |
| LTR-AC202924.1 | LTR/Gypsy | 3 | 353 |
| LTR-AC202929.1 | LTR/Gypsy | 3 | 195 |
| LTR-AC202930.1 | LTR/Gypsy | 3 | 150 |
| LTR-AC202934.1 | LTR | 4 | 328 |
| LTR-AC202943.1 | LTR/Gypsy | 3 | 269 |
| LTR-AC202951.1 | LTR | 1 | 106 |
| LTR-AC202972.1 | LTR | 1 | 67 |
| LTR-AC202974.1 | LTR | 1 | 103 |
| LTR-AC202976.1 | LTR/Gypsy | 2 | 236 |
| LTR-AC202978.1 | LTR/Copia | 1 | 56 |
| LTR-AC202980.1 | LTR | 13 | 2149 |
| LTR-AC202981.1 | LTR | 3 | 140 |
| LTR-AC202987.1 | LTR | 11 | 1283 |
| LTR-AC202988.1 | LTR | 1 | 65 |
| LTR-AC202999.1 | LTR/Copia | 6 | 567 |
| LTR-AC203001.1 | LTR | 1 | 95 |
| LTR-AC203002.1 | LTR/Gypsy | 5 | 287 |
| LTR-AC203008.1 | LTR | 8 | 947 |
| LTR-AC203009.1 | LTR | 1 | 227 |
| LTR-AC203023.1 | LTR | 12 | 2041 |
| LTR-AC203034.1 | LTR | 2 | 262 |
| LTR-AC203036.1 | LTR/Gypsy | 1 | 29 |
| LTR-AC203052.1 | LTR/Copia | 8 | 1422 |
| LTR-AC203056.1 | LTR/Copia | 3 | 177 |
| LTR-AC203062.1 | LTR/Gypsy | 2 | 487 |
| LTR-AC203065.1 | LTR | 3 | 304 |
| LTR-AC203069.1 | LTR | 1 | 51 |
| LTR-AC203099.1 | LTR/Gypsy | 1 | 67 |
| LTR-AC203153.1 | LTR/Gypsy | 8 | 488 |
| LTR-AC203158.1 | LTR | 8 | 847 |
| LTR-AC203160.1 | LTR/Copia | 64 | 6916 |

| | | | |
|---|---|---|---|
| LTR-AC203164.1 | LTR/Gypsy | 1 | 171 |
| LTR-AC203166.1 | LTR/Gypsy | 3 | 225 |
| LTR-AC203175.1 | LTR | 58 | 12233 |
| LTR-AC203176.1 | LTR/Gypsy | 14 | 1237 |
| LTR-AC203179.1 | LTR/Gypsy | 9 | 1018 |
| LTR-AC203210.1 | LTR | 10 | 1475 |
| LTR-AC203212.1 | LTR | 5 | 772 |
| LTR-AC203213.1 | LTR | 2 | 105 |
| LTR-AC207902.1 | LTR | 637 | 215973 |
| LTR-AC207903.1 | LTR | 414 | 101923 |
| LTR-AC207906.1 | LTR/Copia | 99 | 28465 |
| LTR-AC207907.1 | LTR | 1922 | 577922 |
| LTR-AC207908.1 | LTR/Copia | 3503 | 1127254 |
| LTR-AC207909.1 | LTR | 99 | 22712 |
| LTR-AC207910.1 | LTR/Gypsy | 51 | 9631 |
| LTR-AC207912.1 | LTR | 3277 | 891599 |
| LTR-AC207914.1 | LTR | 18790 | 5313305 |
| LTR-AC207915.1 | LTR/Gypsy | 931 | 288125 |
| LTR-AC207916.1 | LTR/Gypsy | 1202 | 286091 |
| LTR-AC207917.1 | LTR | 3344 | 948662 |
| LTR-AC207921.1 | LTR | 1911 | 531053 |
| LTR-AC207923.1 | LTR | 3116 | 880432 |
| LTR-AC207927.1 | LTR/Copia | 163 | 40883 |
| LTR-AC207928.1 | LTR/Gypsy | 1561 | 438285 |
| LTR-AC207931.1 | LTR | 1708 | 522950 |
| LTR-AC207934.1 | LTR/Copia | 6640 | 2251943 |
| LTR-AC207935.1 | LTR | 698 | 239345 |
| LTR-AC207936.1 | LTR | 2654 | 801201 |
| LTR-AC207937.1 | LTR/Gypsy | 4146 | 1250795 |
| LTR-AC207938.1 | LTR | 2743 | 614266 |
| LTR-AC207939.1 | LTR/Gypsy | 4338 | 1479398 |
| LTR-AC207940.1 | LTR | 4915 | 1148973 |
| LTR-AC207941.1 | LTR | 574 | 181768 |
| LTR-AC207943.1 | LTR/Copia | 1234 | 359106 |
| LTR-AC207944.1 | LTR | 5062 | 1412988 |
| LTR-AC207945.1 | LTR/Copia | 883 | 278053 |
| LTR-AC207946.1 | LTR/Gypsy | 296 | 82112 |
| LTR-AC207947.1 | LTR | 837 | 233298 |
| LTR-AC207948.1 | LTR | 1473 | 440149 |
| LTR-AC207949.1 | LTR | 1203 | 377471 |
| LTR-AC207950.1 | LTR | 8771 | 2785036 |
| LTR-AC207951.1 | LTR | 1419 | 430215 |
| LTR-AC207952.1 | LTR | 1955 | 514612 |
| LTR-AC207953.1 | LTR | 4371 | 1269054 |
| LTR-AC207954.1 | LTR | 748 | 185038 |
| LTR-AC207955.1 | LTR | 5920 | 1823440 |
| LTR-AC207956.1 | LTR | 13557 | 4300552 |

| | | | |
|---|---|---|---|
| LTR-AC207957.1 | LTR | 1528 | 433431 |
| LTR-AC207958.1 | LTR | 5038 | 1489051 |
| LTR-AC207959.1 | LTR/Gypsy | 388 | 84123 |
| LTR-AC216550.1 | LTR | 5100 | 1465576 |
| LTR-AC216552.1 | LTR/Gypsy | 147 | 40154 |
| LTR-AC216553.1 | LTR | 7120 | 1819718 |
| LTR-AC216554.1 | LTR | 3882 | 1111547 |
| LTR-AC216555.1 | LTR/Gypsy | 786 | 232292 |
| LTR-AC216556.1 | LTR | 718 | 186005 |
| LTR-AC216561.1 | LTR | 24 | 3458 |
| LTR-AC216562.1 | LTR | 288 | 75917 |
| LTR-AC216564.1 | LTR/Gypsy | 4343 | 1212266 |
| LTR-AC216566.1 | LTR | 4113 | 1206355 |
| LTR-AC216567.1 | LTR/Gypsy | 268 | 77735 |
| LTR-AC216568.1 | LTR/Gypsy | 495 | 135676 |
| LTR-AC216570.1 | LTR/Gypsy | 283 | 75373 |
| LTR-AC216571.1 | LTR/Gypsy | 4149 | 1248637 |
| LTR-AC216573.1 | LTR | 12237 | 3589323 |
| LTR-AC216577.1 | LTR/Copia | 203 | 47069 |
| LTR-AC216579.1 | LTR | 4038 | 1075708 |
| LTR-AC216582.1 | LTR | 5087 | 1491205 |
| LTR-AC216583.1 | LTR/Copia | 1078 | 345160 |
| LTR-AC216584.1 | LTR/Copia | 2515 | 761500 |
| LTR-AC216585.1 | LTR/Gypsy | 863 | 270732 |
| LTR-AD | LTR/Copia | 1392 | 379886 |
| LTR-AF | LTR | 4391 | 1306564 |
| LTR-AF488415.1 | LTR/Gypsy | 133 | 36766 |
| LTR-AK | LTR | 3607 | 918592 |
| LTR-AM | LTR | 1198 | 340758 |
| LTR-AM050668.1 | LTR | 842 | 280509 |
| LTR-AM050676.1 | LTR/Copia | 5859 | 1425388 |
| LTR-AM050678.1 | LTR | 2522 | 854225 |
| LTR-AM050685.1 | LTR | 1385 | 444381 |
| LTR-AM050694.1 | LTR | 396 | 112420 |
| LTR-AM050695.1 | LTR | 880 | 214217 |
| LTR-AM087556.1 | LTR | 543 | 171235 |
| LTR-AM932680.1 | LTR/Gypsy | 600 | 159538 |
| LTR-AM932681.1 | LTR/Copia | 1743 | 554152 |
| LTR-AM932682.1 | LTR | 19139 | 5628835 |
| LTR-AM932683.1 | LTR/Copia | 7541 | 2304991 |
| LTR-AM932684.1 | LTR/Gypsy | 4121 | 1313797 |
| LTR-AM932685.1 | LTR | 8740 | 2549141 |
| LTR-AM932686.1 | LTR | 2362 | 727156 |
| LTR-AM932689.1 | LTR | 2246 | 629553 |
| LTR-AP | LTR | 4721 | 1259374 |
| LTR-AR | LTR | 5422 | 1401326 |
| LTR-AT | LTR | 836 | 222072 |

| | | | |
|---|---|---|---|
| LTR-AVB | LTR | 17467 | 5248756 |
| LTR-AY146588.1 | LTR | 269 | 36208 |
| LTR-AY188332.1 | LTR/Copia | 1167 | 273802 |
| LTR-AY188333.1 | LTR | 1773 | 504423 |
| LTR-AY368673.1 | LTR/Gypsy | 1268 | 300832 |
| LTR-AY485644.1 | LTR/Copia | 2327 | 521152 |
| LTR-AY494981.1 | LTR | 3129 | 976184 |
| LTR-AY663391.1 | LTR | 597 | 128316 |
| LTR-AY663392.1 | LTR | 2999 | 908372 |
| LTR-AY772733.1 | LTR/Copia | 316 | 74331 |
| LTR-AY914081.1 | LTR/Copia | 853 | 243064 |
| LTR-AY914082.1 | LTR/Copia | 1083 | 307489 |
| LTR-AY914083.1 | LTR/Copia | 3156 | 832423 |
| LTR-AY914084.1 | LTR | 163 | 41111 |
| LTR-AY914085.1 | LTR/Copia | 207 | 58715 |
| LTR-AY914086.1 | LTR/Gypsy | 7944 | 2523273 |
| LTR-AY951945.1 | LTR/Copia | 75 | 15920 |
| LTR-BA | LTR | 6374 | 1690829 |
| LTR-BB | LTR/Copia | 1325 | 400429 |
| LTR-BC | LTR | 19735 | 6092809 |
| LTR-BD | LTR | 5006 | 1338804 |
| LTR-BE | LTR | 307 | 79806 |
| LTR-BF | LTR | 9776 | 2956414 |
| LTR-BG | LTR | 840 | 256669 |
| LTR-BH | LTR | 1948 | 612447 |
| LTR-BI | LTR/Copia | 2499 | 764251 |
| LTR-BK | LTR | 9811 | 2917854 |
| LTR-CD | LTR/Gypsy | 4309 | 1272079 |
| LTR-CE | LTR/Copia | 1180 | 415211 |
| LTR-CF | LTR | 3632 | 967133 |
| LTR-CR626934.1 | LTR | 1682 | 409994 |
| LTR-CT009585.1 | LTR/Copia | 535 | 157419 |
| LTR-CT009587.1 | LTR | 737 | 180350 |
| LTR-CT009735.1 | LTR/Copia | 1227 | 371960 |
| LTR-DJ344414.1 | LTR/Copia | 92 | 21272 |
| LTR-DQ157836.1 | LTR/Gypsy | 716 | 235275 |
| LTR-DQ157837.1 | LTR | 307 | 92649 |
| LTR-DQ157841.1 | LTR/Copia | 128 | 35410 |
| LTR-DQ267105.1 | LTR | 5292 | 1477090 |
| LTR-DQ267106.1 | LTR | 1491 | 414263 |
| LTR-DQ537335.1 | LTR | 6989 | 2049773 |
| LTR-DQ537336.1 | LTR/Gypsy | 1805 | 590875 |
| LTR-DQ537337.1 | LTR | 21849 | 6688405 |
| LTR-DQ767610.1 | LTR/Gypsy | 2559 | 830089 |
| LTR-DQ767611.1 | LTR/Gypsy | 750 | 247529 |
| LTR-DQ767612.1 | LTR | 720 | 227643 |
| LTR-DQ767613.1 | LTR | 1855 | 559626 |

| | | | |
|---|---|---|---|
| LTR-DQ767614.1 | LTR/Copia | 127 | 37752 |
| LTR-DQ767620.1 | LTR | 1489 | 488675 |
| LTR-DQ767621.1 | LTR/Gypsy | 2313 | 761693 |
| LTR-DQ767623.1 | LTR/Copia | 1255 | 407225 |
| LTR-DQ767625.1 | LTR | 429 | 115867 |
| LTR-DQ767626.1 | LTR | 591 | 169557 |
| LTR-DQ767627.1 | LTR/Gypsy | 1302 | 398127 |
| LTR-DQ767628.1 | LTR/Copia | 1885 | 581065 |
| LTR-DQ767630.1 | LTR | 537 | 146443 |
| LTR-DQ871219.1 | LTR/Gypsy | 2820 | 795177 |
| LTR-EF081025.1 | LTR | 172 | 47868 |
| LTR-EF081026.1 | LTR | 5228 | 1521312 |
| LTR-EF081027.1 | LTR/Copia | 9447 | 2971036 |
| LTR-EF081029.1 | LTR | 3223 | 1053987 |
| LTR-EF081030.1 | LTR | 6916 | 2114053 |
| LTR-EF426565.1 | LTR | 1617 | 518129 |
| LTR-EU660892.1 | LTR | 5933 | 1852001 |
| LTR-EU660893.1 | LTR/Copia | 3855 | 1225579 |
| LTR-EU660894.1 | LTR/Copia | 4047 | 1004133 |
| LTR-EU660895.1 | LTR/Gypsy | 3567 | 1120452 |
| LTR-EU660896.1 | LTR/Gypsy | 997 | 340337 |
| LTR-EU660898.1 | LTR/Copia | 1354 | 313322 |
| LTR-EU660899.1 | LTR | 12705 | 3709972 |
| LTR-EU660900.1 | LTR/Copia | 370 | 108906 |
| LTR-EU660901.1 | LTR/Gypsy | 6782 | 2108115 |
| LTR-EU660903.1 | LTR/Copia | 1524 | 433648 |
| LTR-HVVMRX83KhA0001B23 | LTR | 46 | 7863 |
| LTR-HVVMRX83KhA0001I13 | LTR | 54 | 8140 |
| LTR-HVVMRX83KhA0001K06 | LTR/Copia | 39 | 4818 |
| LTR-HVVMRX83KhA0001N08 | LTR | 127 | 20032 |
| LTR-HVVMRX83KhA0004B15 | LTR | 1091 | 222271 |
| LTR-HVVMRX83KhA0004E13 | LTR/Gypsy | 43 | 6450 |
| LTR-HVVMRX83KhA0004I17 | LTR/Copia | 28 | 5668 |
| LTR-HVVMRX83KhA0004K03 | LTR | 49 | 8664 |
| LTR-HVVMRX83KhA0006E16 | LTR/Gypsy | 74 | 10612 |
| LTR-HVVMRX83KhA0006F04 | LTR | 104 | 17623 |
| LTR-HVVMRX83KhA0007C15 | LTR/Gypsy | 2 | 557 |
| LTR-HVVMRX83KhA0007F10 | LTR | 38 | 5487 |
| LTR-HVVMRX83KhA0007H24 | LTR | 34 | 5274 |
| LTR-HVVMRX83KhA0007M11 | LTR | 253 | 35261 |
| LTR-HVVMRX83KhA0008J17 | LTR/Gypsy | 70 | 12852 |
| LTR-HVVMRX83KhA0009D19 | LTR | 67 | 10556 |
| LTR-HVVMRX83KhA0009E10 | LTR/Gypsy | 171 | 27116 |
| LTR-HVVMRX83KhA0009M03 | LTR | 138 | 21387 |
| LTR-HVVMRX83KhA0009N04 | LTR | 155 | 27117 |
| LTR-HVVMRX83KhA0010A07 | LTR | 32 | 4892 |
| LTR-HVVMRX83KhA0010A10 | LTR/Gypsy | 200 | 42046 |

| | | | |
|---|---|---|---|
| LTR-HVVMRX83KhA0010F02 | LTR | 86 | 14740 |
| LTR-HVVMRX83KhA0011E17 | LTR | 793 | 94371 |
| LTR-HVVMRX83KhA0012C20 | LTR | 43 | 6588 |
| LTR-HVVMRX83KhA0012D03 | LTR | 570 | 116216 |
| LTR-HVVMRX83KhA0012N09 | LTR | 138 | 16542 |
| LTR-HVVMRX83KhA0013I23 | LTR/Copia | 3 | 587 |
| LTR-HVVMRX83KhA0013N10 | LTR | 137 | 22848 |
| LTR-HVVMRX83KhA0014D14 | LTR/Copia | 102 | 14533 |
| LTR-HVVMRX83KhA0014M23 | LTR | 111 | 17024 |
| LTR-HVVMRX83KhA0015L14 | LTR/Copia | 2 | 167 |
| LTR-HVVMRX83KhA0015M24 | LTR | 231 | 47535 |
| LTR-HVVMRX83KhA0016B16 | LTR | 67 | 9263 |
| LTR-HVVMRX83KhA0016H19 | LTR | 131 | 22421 |
| LTR-HVVMRX83KhA0017E24 | LTR | 919 | 133465 |
| LTR-HVVMRX83KhA0017H09 | LTR | 78 | 10457 |
| LTR-HVVMRX83KhA0017N24 | LTR | 66 | 8706 |
| LTR-HVVMRX83KhA0018L20 | LTR/Copia | 7 | 1217 |
| LTR-HVVMRX83KhA0019F09 | LTR/Copia | 39 | 4144 |
| LTR-HVVMRX83KhA0019F13 | LTR | 201 | 30903 |
| LTR-HVVMRX83KhA0019I19 | LTR | 130 | 34853 |
| LTR-HVVMRX83KhA0019K18 | LTR/Copia | 1 | 182 |
| LTR-HVVMRX83KhA0020F07 | LTR/Copia | 20 | 2173 |
| LTR-HVVMRX83KhA0020P14 | LTR | 32 | 4722 |
| LTR-HVVMRX83KhA0022C01 | LTR/Copia | 6 | 730 |
| LTR-HVVMRX83KhA0022N16 | LTR/Gypsy | 111 | 18631 |
| LTR-HVVMRX83KhA0024E19 | LTR | 592 | 94865 |
| LTR-HVVMRX83KhA0024I15 | LTR | 214 | 30668 |
| LTR-HVVMRX83KhA0026G08 | LTR/Copia | 6 | 220 |
| LTR-HVVMRX83KhA0029I17 | LTR | 17 | 4371 |
| LTR-HVVMRX83KhA0031B10 | LTR/Copia | 4 | 233 |
| LTR-HVVMRX83KhA0032J08 | LTR | 112 | 17766 |
| LTR-HVVMRX83KhA0032L01 | LTR/Copia | 86 | 12323 |
| LTR-HVVMRX83KhA0032L12 | LTR/Gypsy | 40 | 5199 |
| LTR-HVVMRX83KhA0033A19 | LTR | 35 | 5426 |
| LTR-HVVMRX83KhA0033M12 | LTR | 221 | 43724 |
| LTR-HVVMRX83KhA0034O13 | LTR/Gypsy | 7 | 947 |
| LTR-HVVMRX83KhA0038O15 | LTR/Copia | 70 | 10573 |
| LTR-HVVMRX83KhA0039A18 | LTR | 119 | 20840 |
| LTR-HVVMRX83KhA0039D21 | LTR/Gypsy | 235 | 33511 |
| LTR-HVVMRX83KhA0041A17 | LTR/Copia | 2 | 177 |
| LTR-HVVMRX83KhA0042B12 | LTR/Gypsy | 12 | 2182 |
| LTR-HVVMRX83KhA0043C24 | LTR | 29 | 4131 |
| LTR-HVVMRX83KhA0043H20 | LTR/Copia | 43 | 5389 |
| LTR-HVVMRX83KhA0044A01 | LTR | 163 | 18220 |
| LTR-HVVMRX83KhA0044A15 | LTR/Copia | 111 | 16239 |
| LTR-HVVMRX83KhA0045K13 | LTR/Copia | 4 | 657 |
| LTR-HVVMRX83KhA0045N16 | LTR/Copia | 14 | 3037 |

| | | | |
|---|---|---|---|
| LTR-HVVMRX83KhA0047G15 | LTR/Gypsy | 47 | 6105 |
| LTR-HVVMRX83KhA0048D11 | LTR | 199 | 33668 |
| LTR-HVVMRX83KhA0048M17 | LTR | 145 | 35344 |
| LTR-HVVMRX83KhA0049F12 | LTR/Copia | 3 | 247 |
| LTR-HVVMRX83KhA0051K04 | LTR/Copia | 19 | 3595 |
| LTR-HVVMRX83KhA0051L21 | LTR/Gypsy | 120 | 14769 |
| LTR-HVVMRX83KhA0051O05 | LTR | 449 | 74489 |
| LTR-HVVMRX83KhA0052A02 | LTR | 54 | 6742 |
| LTR-HVVMRX83KhA0052F09 | LTR | 176 | 31160 |
| LTR-HVVMRX83KhA0052L16 | LTR | 158 | 23725 |
| LTR-HVVMRX83KhA0053O18 | LTR | 10 | 1043 |
| LTR-HVVMRX83KhA0054B11 | LTR | 1015 | 113939 |
| LTR-HVVMRX83KhA0054D11 | LTR/Copia | 25 | 5607 |
| LTR-HVVMRX83KhA0054O16 | LTR | 5 | 501 |
| LTR-HVVMRX83KhA0055L18 | LTR/Gypsy | 44 | 6914 |
| LTR-HVVMRX83KhA0056F10 | LTR | 623 | 162674 |
| LTR-HVVMRX83KhA0057B02 | LTR/Gypsy | 601 | 75567 |
| LTR-HVVMRX83KhA0057D20 | LTR/Copia | 653 | 66882 |
| LTR-HVVMRX83KhA0057F16 | LTR | 74 | 11062 |
| LTR-HVVMRX83KhA0057G03 | LTR | 3 | 527 |
| LTR-HVVMRX83KhA0057H07 | LTR | 537 | 76413 |
| LTR-HVVMRX83KhA0058C08 | LTR | 76 | 12571 |
| LTR-HVVMRX83KhA0058D01 | LTR/Copia | 61 | 13989 |
| LTR-HVVMRX83KhA0058F02 | LTR/Gypsy | 861 | 129138 |
| LTR-HVVMRX83KhA0058H22 | LTR/Gypsy | 17 | 2928 |
| LTR-HVVMRX83KhA0058O10 | LTR | 249 | 35866 |
| LTR-HVVMRX83KhA0058P17 | LTR | 62 | 10657 |
| LTR-HVVMRX83KhA0059A15 | LTR/Gypsy | 13 | 2687 |
| LTR-HVVMRX83KhA0059D03 | LTR | 84 | 13150 |
| LTR-HVVMRX83KhA0059E09 | LTR/Gypsy | 6 | 680 |
| LTR-HVVMRX83KhA0059H23 | LTR | 166 | 23446 |
| LTR-HVVMRX83KhA0059P16 | LTR | 194 | 34429 |
| LTR-HVVMRX83KhA0060C11 | LTR | 364 | 43445 |
| LTR-HVVMRX83KhA0060F03 | LTR | 168 | 26202 |
| LTR-HVVMRX83KhA0060F14 | LTR | 244 | 40047 |
| LTR-HVVMRX83KhA0060H03 | LTR/Copia | 61 | 15614 |
| LTR-HVVMRX83KhA0060N03 | LTR | 116 | 18909 |
| LTR-HVVMRX83KhA0060N20 | LTR | 143 | 24144 |
| LTR-HVVMRX83KhA0060O03 | LTR/Copia | 9 | 1328 |
| LTR-HVVMRX83KhA0061M19 | LTR/Gypsy | 18 | 2842 |
| LTR-HVVMRX83KhA0061N11 | LTR/Gypsy | 13 | 2969 |
| LTR-HVVMRX83KhA0062C09 | LTR/Gypsy | 20 | 2466 |
| LTR-HVVMRX83KhA0062D04 | LTR | 7 | 914 |
| LTR-HVVMRX83KhA0062L15 | LTR | 105 | 15801 |
| LTR-HVVMRX83KhA0062M20 | LTR | 42 | 5371 |
| LTR-HVVMRX83KhA0063A05 | LTR | 1 | 100 |
| LTR-HVVMRX83KhA0063C17 | LTR/Gypsy | 110 | 13956 |

| | | | |
|---|---|---|---|
| LTR-HVVMRX83KhA0063I02 | LTR/Copia | 38 | 5275 |
| LTR-HVVMRX83KhA0064F07 | LTR | 278 | 33141 |
| LTR-HVVMRX83KhA0064M04 | LTR/Gypsy | 113 | 15638 |
| LTR-HVVMRX83KhA0064P17 | LTR/Copia | 4 | 476 |
| LTR-HVVMRX83KhA0065K02 | LTR/Copia | 1 | 367 |
| LTR-HVVMRX83KhA0066I12 | LTR | 69 | 9830 |
| LTR-HVVMRX83KhA0067M04 | LTR/Copia | 21 | 6608 |
| LTR-HVVMRX83KhA0068F14 | LTR | 15 | 3160 |
| LTR-HVVMRX83KhA0068K01 | LTR/Copia | 251 | 61734 |
| LTR-HVVMRX83KhA0068O17 | LTR/Copia | 1 | 76 |
| LTR-HVVMRX83KhA0069B02 | LTR/Copia | 2 | 271 |
| LTR-HVVMRX83KhA0069H02 | LTR | 3 | 351 |
| LTR-HVVMRX83KhA0069M05 | LTR | 1161 | 167546 |
| LTR-HVVMRX83KhA0070G11 | LTR | 50 | 15292 |
| LTR-HVVMRX83KhA0070H13 | LTR/Gypsy | 45 | 7368 |
| LTR-HVVMRX83KhA0070O12 | LTR | 62 | 12259 |
| LTR-HVVMRX83KhA0071A09 | LTR | 119 | 14964 |
| LTR-HVVMRX83KhA0071C16 | LTR/Copia | 3 | 423 |
| LTR-HVVMRX83KhA0071E05 | LTR/Gypsy | 6 | 906 |
| LTR-HVVMRX83KhA0071K21 | LTR/Gypsy | 10 | 1386 |
| LTR-HVVMRX83KhA0072L07 | LTR | 281 | 40372 |
| LTR-HVVMRX83KhA0074A03 | LTR | 13 | 1596 |
| LTR-HVVMRX83KhA0075C14 | LTR | 8 | 2005 |
| LTR-HVVMRX83KhA0075F03 | LTR/Gypsy | 58 | 9373 |
| LTR-HVVMRX83KhA0075I20 | LTR | 250 | 45589 |
| LTR-HVVMRX83KhA0075O17 | LTR | 12 | 1377 |
| LTR-HVVMRX83KhA0078D14 | LTR/Copia | 13 | 2890 |
| LTR-HVVMRX83KhA0078D21 | LTR | 58 | 9607 |
| LTR-HVVMRX83KhA0078L23 | LTR/Copia | 7 | 664 |
| LTR-HVVMRX83KhA0079B20 | LTR | 532 | 72614 |
| LTR-HVVMRX83KhA0081H16 | LTR | 7 | 858 |
| LTR-HVVMRX83KhA0082F18 | LTR | 134 | 19536 |
| LTR-HVVMRX83KhA0083J17 | LTR/Copia | 31 | 4129 |
| LTR-HVVMRX83KhA0085I11 | LTR/Gypsy | 29 | 4529 |
| LTR-HVVMRX83KhA0085P08 | LTR/Gypsy | 5 | 1147 |
| LTR-HVVMRX83KhA0087D17 | LTR | 40 | 5195 |
| LTR-HVVMRX83KhA0088B16 | LTR | 1466 | 161122 |
| LTR-HVVMRX83KhA0088E23 | LTR | 97 | 15216 |
| LTR-HVVMRX83KhA0088P15 | LTR/Copia | 18 | 2644 |
| LTR-HVVMRX83KhA0089L20 | LTR | 76 | 13059 |
| LTR-HVVMRX83KhA0089L24 | LTR/Gypsy | 15 | 2270 |
| LTR-HVVMRX83KhA0090F07 | LTR | 50 | 6776 |
| LTR-HVVMRX83KhA0091D03 | LTR/Gypsy | 6 | 657 |
| LTR-HVVMRX83KhA0091D06 | LTR/Gypsy | 18 | 2422 |
| LTR-HVVMRX83KhA0091I11 | LTR/Copia | 7 | 1210 |
| LTR-HVVMRX83KhA0092B12 | LTR | 101 | 20061 |
| LTR-HVVMRX83KhA0092C20 | LTR | 53 | 9147 |

| | | | |
|---|---|---|---|
| LTR-HVVMRX83KhA0092I19 | LTR/Copia | 382 | 100808 |
| LTR-HVVMRX83KhA0093P06 | LTR/Copia | 323 | 95830 |
| LTR-HVVMRX83KhA0094C22 | LTR | 12 | 1714 |
| LTR-HVVMRX83KhA0094J01 | LTR/Copia | 25 | 4780 |
| LTR-HVVMRX83KhA0094K20 | LTR | 42 | 6774 |
| LTR-HVVMRX83KhA0095N05 | LTR/Copia | 96 | 16183 |
| LTR-HVVMRX83KhA0096A09 | LTR | 221 | 56684 |
| LTR-HVVMRX83KhA0096G13 | LTR/Copia | 1 | 148 |
| LTR-HVVMRX83KhA0096I23 | LTR | 143 | 27890 |
| LTR-HVVMRX83KhA0097O15 | LTR/Copia | 19 | 3155 |
| LTR-HVVMRX83KhA0098D16 | LTR/Gypsy | 12 | 1166 |
| LTR-HVVMRX83KhA0098H22 | LTR/Copia | 2 | 137 |
| LTR-HVVMRX83KhA0098K23 | LTR | 58 | 8145 |
| LTR-HVVMRX83KhA0098O19 | LTR | 65 | 15899 |
| LTR-HVVMRX83KhA0098P07 | LTR/Gypsy | 7 | 1737 |
| LTR-HVVMRX83KhA0099B15 | LTR | 99 | 16587 |
| LTR-HVVMRX83KhA0099F21 | LTR/Copia | 5 | 1098 |
| LTR-HVVMRX83KhA0099H09 | LTR/Copia | 16 | 2464 |
| LTR-HVVMRX83KhA0099K19 | LTR | 339 | 43964 |
| LTR-HVVMRX83KhA0101B16 | LTR/Copia | 14 | 1895 |
| LTR-HVVMRX83KhA0102D03 | LTR/Gypsy | 16 | 2052 |
| LTR-HVVMRX83KhA0102D09 | LTR | 9 | 1514 |
| LTR-HVVMRX83KhA0102D12 | LTR/Gypsy | 82 | 10545 |
| LTR-HVVMRX83KhA0102E06 | LTR/Copia | 1 | 111 |
| LTR-HVVMRX83KhA0102M04 | LTR/Copia | 8 | 1404 |
| LTR-HVVMRX83KhA0102N15 | LTR | 98 | 16359 |
| LTR-HVVMRX83KhA0103I01 | LTR | 28 | 4073 |
| LTR-HVVMRX83KhA0104L05 | LTR/Gypsy | 45 | 6182 |
| LTR-HVVMRX83KhA0104N15 | LTR/Copia | 103 | 15116 |
| LTR-HVVMRX83KhA0104O18 | LTR | 10 | 1386 |
| LTR-HVVMRX83KhA0105F16 | LTR/Copia | 6 | 942 |
| LTR-HVVMRX83KhA0105L22 | LTR | 40 | 7528 |
| LTR-HVVMRX83KhA0106J08 | LTR | 1723 | 239350 |
| LTR-HVVMRX83KhA0106K07 | LTR/Copia | 5 | 565 |
| LTR-HVVMRX83KhA0107H17 | LTR/Copia | 248 | 67891 |
| LTR-HVVMRX83KhA0109B08 | LTR | 57 | 8699 |
| LTR-HVVMRX83KhA0110B01 | LTR | 11 | 1291 |
| LTR-HVVMRX83KhA0110D14 | LTR | 86 | 14535 |
| LTR-HVVMRX83KhA0110E04 | LTR | 56 | 9922 |
| LTR-HVVMRX83KhA0111E19 | LTR/Copia | 8 | 754 |
| LTR-HVVMRX83KhA0112B11 | LTR/Copia | 11 | 2494 |
| LTR-HVVMRX83KhA0113E09 | LTR | 22 | 4040 |
| LTR-HVVMRX83KhA0113P06 | LTR | 4 | 541 |
| LTR-HVVMRX83KhA0114G23 | LTR/Copia | 16 | 2588 |
| LTR-HVVMRX83KhA0115B14 | LTR/Gypsy | 524 | 103149 |
| LTR-HVVMRX83KhA0115J03 | LTR/Gypsy | 48 | 7514 |
| LTR-HVVMRX83KhA0116B20 | LTR/Gypsy | 131 | 18112 |

| | | | |
|---|---|---|---|
| LTR-HVVMRX83KhA0117B17 | LTR/Gypsy | 1 | 59 |
| LTR-HVVMRX83KhA0117E19 | LTR | 111 | 14393 |
| LTR-HVVMRX83KhA0117I14 | LTR/Copia | 27 | 3233 |
| LTR-HVVMRX83KhA0117K05 | LTR | 148 | 20854 |
| LTR-HVVMRX83KhA0117N23 | LTR/Copia | 6 | 666 |
| LTR-HVVMRX83KhA0118C17 | LTR | 32 | 5408 |
| LTR-HVVMRX83KhA0118E04 | LTR/Copia | 5 | 1059 |
| LTR-HVVMRX83KhA0118L02 | LTR | 74 | 13134 |
| LTR-HVVMRX83KhA0119B03 | LTR/Copia | 30 | 6515 |
| LTR-HVVMRX83KhA0119B12 | LTR | 111 | 19790 |
| LTR-HVVMRX83KhA0119F12 | LTR | 26 | 3219 |
| LTR-HVVMRX83KhA0119L05 | LTR/Gypsy | 476 | 54121 |
| LTR-HVVMRX83KhA0119M05 | LTR/Copia | 104 | 15598 |
| LTR-HVVMRX83KhA0120L18 | LTR/Gypsy | 19 | 2945 |
| LTR-HVVMRX83KhA0120P07 | LTR/Copia | 24 | 3930 |
| LTR-HVVMRX83KhA0124C05 | LTR | 313 | 54277 |
| LTR-HVVMRX83KhA0124C21 | LTR/Copia | 1 | 131 |
| LTR-HVVMRX83KhA0124D20 | LTR/Copia | 5 | 919 |
| LTR-HVVMRX83KhA0124E10 | LTR | 279 | 39123 |
| LTR-HVVMRX83KhA0124F14 | LTR | 10 | 1169 |
| LTR-HVVMRX83KhA0124G21 | LTR/Gypsy | 15 | 2741 |
| LTR-HVVMRX83KhA0125A09 | LTR/Copia | 30 | 5287 |
| LTR-HVVMRX83KhA0125A14 | LTR/Copia | 18 | 2613 |
| LTR-HVVMRX83KhA0125F24 | LTR | 57 | 9065 |
| LTR-HVVMRX83KhA0126B14 | LTR/Gypsy | 247 | 35498 |
| LTR-HVVMRX83KhA0126E05 | LTR/Gypsy | 170 | 27596 |
| LTR-HVVMRX83KhA0126O09 | LTR/Gypsy | 12 | 2772 |
| LTR-HVVMRX83KhA0127B11 | LTR | 210 | 47823 |
| LTR-HVVMRX83KhA0127B17 | LTR/Gypsy | 4 | 588 |
| LTR-HVVMRX83KhA0127E04 | LTR/Copia | 11 | 2215 |
| LTR-HVVMRX83KhA0127F15 | LTR/Gypsy | 128 | 24505 |
| LTR-HVVMRX83KhA0128O21 | LTR/Copia | 26 | 3562 |
| LTR-HVVMRX83KhA0129C13 | LTR/Gypsy | 5 | 831 |
| LTR-HVVMRX83KhA0129G12 | LTR/Gypsy | 19 | 3087 |
| LTR-HVVMRX83KhA0129I21 | LTR/Copia | 2 | 280 |
| LTR-HVVMRX83KhA0129P08 | LTR | 67 | 14671 |
| LTR-HVVMRX83KhA0130C21 | LTR | 400 | 56670 |
| LTR-HVVMRX83KhA0130F01 | LTR | 88 | 13047 |
| LTR-HVVMRX83KhA0130F11 | LTR | 8 | 957 |
| LTR-HVVMRX83KhA0130F22 | LTR | 191 | 26885 |
| LTR-HVVMRX83KhA0131B16 | LTR/Copia | 2 | 144 |
| LTR-HVVMRX83KhA0131O09 | LTR/Copia | 2 | 311 |
| LTR-HVVMRX83KhA0132K06 | LTR/Copia | 1 | 219 |
| LTR-HVVMRX83KhA0140D05 | LTR | 40 | 6224 |
| LTR-HVVMRX83KhA0140J05 | LTR/Copia | 6 | 695 |
| LTR-HVVMRX83KhA0140M03 | LTR/Gypsy | 12 | 2684 |
| LTR-HVVMRX83KhA0146O22 | LTR/Copia | 5 | 646 |

| | | | |
|---|---|---|---|
| LTR-HVVMRX83KhA0148I02 | LTR | 599 | 85382 |
| LTR-HVVMRX83KhA0148L11 | LTR/Copia | 40 | 4682 |
| LTR-HVVMRX83KhA0148O16 | LTR | 27 | 4727 |
| LTR-HVVMRX83KhA0149C01 | LTR | 27 | 3815 |
| LTR-HVVMRX83KhA0149E13 | LTR | 88 | 11767 |
| LTR-HVVMRX83KhA0149H18 | LTR | 35 | 4969 |
| LTR-HVVMRX83KhA0149M03 | LTR/Copia | 3 | 503 |
| LTR-HVVMRX83KhA0149P16 | LTR | 175 | 21801 |
| LTR-HVVMRX83KhA0149P18 | LTR | 158 | 28745 |
| LTR-HVVMRX83KhA0150J13 | LTR/Copia | 1 | 60 |
| LTR-HVVMRX83KhA0150L18 | LTR | 49 | 7412 |
| LTR-HVVMRX83KhA0151O14 | LTR/Gypsy | 16 | 2721 |
| LTR-HVVMRX83KhA0155D17 | LTR | 237 | 52320 |
| LTR-HVVMRX83KhA0155E10 | LTR/Copia | 5 | 862 |
| LTR-HVVMRX83KhA0155F13 | LTR/Copia | 9 | 981 |
| LTR-HVVMRX83KhA0155L10 | LTR/Gypsy | 21 | 3247 |
| LTR-HVVMRX83KhA0156I21 | LTR/Copia | 30 | 4596 |
| LTR-HVVMRX83KhA0157F19 | LTR/Gypsy | 113 | 14878 |
| LTR-HVVMRX83KhA0157N21 | LTR | 221 | 36866 |
| LTR-HVVMRX83KhA0158D06 | LTR/Copia | 5 | 401 |
| LTR-HVVMRX83KhA0158F08 | LTR/Copia | 340 | 54145 |
| LTR-HVVMRX83KhA0158G04 | LTR/Gypsy | 12 | 1585 |
| LTR-HVVMRX83KhA0158M21 | LTR/Copia | 3 | 620 |
| LTR-HVVMRX83KhA0158O22 | LTR | 275 | 51269 |
| LTR-HVVMRX83KhA0159A16 | LTR/Copia | 2 | 100 |
| LTR-HVVMRX83KhA0159G07 | LTR | 256 | 37887 |
| LTR-HVVMRX83KhA0159K08 | LTR/Copia | 4 | 647 |
| LTR-HVVMRX83KhA0161I02 | LTR/Copia | 18 | 2368 |
| LTR-HVVMRX83KhA0162E17 | LTR | 100 | 15802 |
| LTR-HVVMRX83KhA0162I23 | LTR/Copia | 2 | 557 |
| LTR-HVVMRX83KhA0163E19 | LTR | 20 | 3432 |
| LTR-HVVMRX83KhA0163L21 | LTR/Copia | 46 | 7954 |
| LTR-HVVMRX83KhA0163O10 | LTR/Gypsy | 13 | 2048 |
| LTR-HVVMRX83KhA0164F12 | LTR | 202 | 31269 |
| LTR-HVVMRX83KhA0164I21 | LTR/Gypsy | 8 | 1436 |
| LTR-HVVMRX83KhA0164O10 | LTR | 10 | 1513 |
| LTR-HVVMRX83KhA0164O11 | LTR/Gypsy | 165 | 22835 |
| LTR-HVVMRX83KhA0165C08 | LTR | 9 | 1170 |
| LTR-HVVMRX83KhA0165F17 | LTR | 1045 | 179304 |
| LTR-HVVMRX83KhA0166B18 | LTR/Gypsy | 255 | 49409 |
| LTR-HVVMRX83KhA0166L12 | LTR/Copia | 269 | 72889 |
| LTR-HVVMRX83KhA0166P08 | LTR/Copia | 90 | 10985 |
| LTR-HVVMRX83KhA0167G01 | LTR/Copia | 5 | 1477 |
| LTR-HVVMRX83KhA0167L13 | LTR | 12 | 1450 |
| LTR-HVVMRX83KhA0167L14 | LTR/Copia | 4 | 551 |
| LTR-HVVMRX83KhA0167O16 | LTR | 8 | 851 |
| LTR-HVVMRX83KhA0168O15 | LTR | 545 | 126456 |

| | | | |
|---|---|---|---|
| LTR-HVVMRX83KhA0168P17 | LTR | 109 | 14954 |
| LTR-HVVMRX83KhA0169C05 | LTR/Copia | 1 | 139 |
| LTR-HVVMRX83KhA0169O15 | LTR/Gypsy | 4 | 345 |
| LTR-HVVMRX83KhA0170B07 | LTR/Gypsy | 2 | 175 |
| LTR-HVVMRX83KhA0170B09 | LTR | 7 | 988 |
| LTR-HVVMRX83KhA0170B15 | LTR | 692 | 141431 |
| LTR-HVVMRX83KhA0170C07 | LTR/Copia | 22 | 3058 |
| LTR-HVVMRX83KhA0170K04 | LTR/Copia | 14 | 1598 |
| LTR-HVVMRX83KhA0170L14 | LTR/Copia | 200 | 54967 |
| LTR-HVVMRX83KhA0170P17 | LTR | 51 | 7328 |
| LTR-HVVMRX83KhA0171H02 | LTR | 214 | 30308 |
| LTR-HVVMRX83KhA0171H04 | LTR | 24 | 4538 |
| LTR-HVVMRX83KhA0171I05 | LTR | 352 | 54737 |
| LTR-HVVMRX83KhA0171L17 | LTR/Gypsy | 264 | 45558 |
| LTR-HVVMRX83KhA0172E11 | LTR/Copia | 3 | 319 |
| LTR-HVVMRX83KhA0172K23 | LTR | 55 | 7187 |
| LTR-HVVMRX83KhA0173D19 | LTR | 248 | 34501 |
| LTR-HVVMRX83KhA0173N06 | LTR/Copia | 573 | 73415 |
| LTR-HVVMRX83KhA0174E07 | LTR | 63 | 9864 |
| LTR-HVVMRX83KhA0174I06 | LTR/Copia | 2 | 488 |
| LTR-HVVMRX83KhA0175F14 | LTR/Copia | 1 | 73 |
| LTR-HVVMRX83KhA0177J07 | LTR/Copia | 3 | 654 |
| LTR-HVVMRX83KhA0177J12 | LTR | 89 | 15639 |
| LTR-HVVMRX83KhA0178B13 | LTR/Gypsy | 12 | 1808 |
| LTR-HVVMRX83KhA0178E07 | LTR/Copia | 11 | 1456 |
| LTR-HVVMRX83KhA0178F16 | LTR | 36 | 6774 |
| LTR-HVVMRX83KhA0178K02 | LTR | 183 | 35044 |
| LTR-HVVMRX83KhA0179C14 | LTR/Copia | 2 | 186 |
| LTR-HVVMRX83KhA0181D24 | LTR | 346 | 53102 |
| LTR-HVVMRX83KhA0181L03 | LTR/Copia | 3 | 838 |
| LTR-HVVMRX83KhA0185E19 | LTR/Gypsy | 35 | 5080 |
| LTR-HVVMRX83KhA0185L23 | LTR/Gypsy | 53 | 7450 |
| LTR-HVVMRX83KhA0186D10 | LTR/Gypsy | 66 | 11243 |
| LTR-HVVMRX83KhA0186K07 | LTR | 150 | 26292 |
| LTR-HVVMRX83KhA0187J20 | LTR | 1 | 38 |
| LTR-HVVMRX83KhA0188G05 | LTR | 137 | 24332 |
| LTR-HVVMRX83KhA0188J23 | LTR | 403 | 60390 |
| LTR-HVVMRX83KhA0188O19 | LTR | 132 | 34163 |
| LTR-HVVMRX83KhA0191M21 | LTR/Gypsy | 16 | 2732 |
| LTR-HVVMRX83KhA0192D04 | LTR/Gypsy | 223 | 26645 |
| LTR-HVVMRX83KhA0193P05 | LTR | 44 | 7266 |
| LTR-HVVMRX83KhA0195E12 | LTR/Gypsy | 52 | 7973 |
| LTR-HVVMRX83KhA0195G10 | LTR | 186 | 29048 |
| LTR-HVVMRX83KhA0195L11 | LTR | 9 | 1660 |
| LTR-HVVMRX83KhA0195O11 | LTR/Copia | 128 | 20963 |
| LTR-HVVMRX83KhA0196A15 | LTR | 105 | 17798 |
| LTR-HVVMRX83KhA0196D14 | LTR | 6 | 798 |

| | | | |
|---|---|---|---|
| LTR-HVVMRX83KhA0196D24 | LTR/Gypsy | 103 | 15814 |
| LTR-HVVMRX83KhA0196G18 | LTR | 36 | 5779 |
| LTR-HVVMRX83KhA0197E08 | LTR | 268 | 41916 |
| LTR-HVVMRX83KhA0197G10 | LTR | 49 | 9031 |
| LTR-HVVMRX83KhA0197H15 | LTR | 433 | 46802 |
| LTR-HVVMRX83KhA0197K07 | LTR/Gypsy | 32 | 4553 |
| LTR-HVVMRX83KhA0197M10 | LTR | 48 | 7565 |
| LTR-HVVMRX83KhA0197N04 | LTR/Copia | 5 | 701 |
| LTR-HVVMRX83KhA0198B01 | LTR/Gypsy | 15 | 2094 |
| LTR-HVVMRX83KhA0198F13 | LTR/Copia | 101 | 13954 |
| LTR-HVVMRX83KhA0198F17 | LTR | 387 | 61014 |
| LTR-HVVMRX83KhA0199B01 | LTR | 640 | 131214 |
| LTR-HVVMRX83KhA0201C07 | LTR | 49 | 9326 |
| LTR-HVVMRX83KhA0201J04 | LTR | 3 | 510 |
| LTR-HVVMRX83KhA0201K09 | LTR/Gypsy | 55 | 10400 |
| LTR-HVVMRX83KhA0201P15 | LTR/Copia | 47 | 10153 |
| LTR-HVVMRX83KhA0202I12 | LTR/Gypsy | 86 | 12443 |
| LTR-HVVMRX83KhA0203B24 | LTR | 339 | 49769 |
| LTR-HVVMRX83KhA0203N15 | LTR/Copia | 4 | 676 |
| LTR-HVVMRX83KhA0204E15 | LTR/Copia | 893 | 183160 |
| LTR-HVVMRX83KhA0204J18 | LTR | 270 | 39279 |
| LTR-HVVMRX83KhA0204O04 | LTR/Copia | 2 | 363 |
| LTR-HVVMRX83KhA0205N14 | LTR/Gypsy | 51 | 7664 |
| LTR-HVVMRX83KhA0206E12 | LTR/Copia | 6 | 915 |
| LTR-HVVMRX83KhA0206K06 | LTR/Gypsy | 5 | 733 |
| LTR-HVVMRX83KhA0207B21 | LTR/Gypsy | 23 | 2346 |
| LTR-HVVMRX83KhA0207D14 | LTR/Gypsy | 85 | 15135 |
| LTR-HVVMRX83KhA0208G16 | LTR/Gypsy | 11 | 1346 |
| LTR-HVVMRX83KhA0208L20 | LTR | 59 | 9484 |
| LTR-HVVMRX83KhA0209J24 | LTR/Copia | 16 | 2123 |
| LTR-HVVMRX83KhA0209K21 | LTR | 342 | 46652 |
| LTR-HVVMRX83KhA0209N07 | LTR/Copia | 10 | 1449 |
| LTR-HVVMRX83KhA0210F20 | LTR/Gypsy | 10 | 1659 |
| LTR-HVVMRX83KhA0211J24 | LTR | 37 | 6941 |
| LTR-HVVMRX83KhA0213B22 | LTR | 109 | 18242 |
| LTR-HVVMRX83KhA0213C09 | LTR | 135 | 22685 |
| LTR-HVVMRX83KhA0213H13 | LTR/Copia | 6 | 1785 |
| LTR-HVVMRX83KhA0214A24 | LTR/Copia | 55 | 10323 |
| LTR-HVVMRX83KhA0214M01 | LTR/Gypsy | 62 | 7372 |
| LTR-HVVMRX83KhA0214M18 | LTR/Gypsy | 9 | 1324 |
| LTR-HVVMRX83KhA0218N24 | LTR | 253 | 32278 |
| LTR-HVVMRX83KhA0218O06 | LTR | 26 | 4151 |
| LTR-HVVMRX83KhA0219C24 | LTR/Copia | 21 | 2518 |
| LTR-HVVMRX83khA0003H08 | LTR | 188 | 31880 |
| LTR-HVVMRX83khA0006J12 | LTR/Copia | 49 | 10607 |
| LTR-HVVMRX83khA0007N02 | LTR | 281 | 48790 |
| LTR-HVVMRX83khA0008F05 | LTR | 433 | 66852 |

| | | | |
|---|---|---|---|
| LTR-HVVMRX83khA0009H10 | LTR | 183 | 26288 |
| LTR-HVVMRX83khA0010L08 | LTR/Copia | 509 | 84041 |
| LTR-HVVMRX83khA0011O12 | LTR/Gypsy | 13 | 1892 |
| LTR-HVVMRX83khA0014A07 | LTR | 60 | 9614 |
| LTR-HVVMRX83khA0014H19 | LTR | 128 | 20454 |
| LTR-HVVMRX83khA0016L10 | LTR/Copia | 3 | 159 |
| LTR-HVVMRX83khA0017D09 | LTR | 14 | 2162 |
| LTR-HVVMRX83khA0017I01 | LTR/Copia | 5 | 1104 |
| LTR-HVVMRX83khA0018A05 | LTR/Copia | 38 | 5262 |
| LTR-HVVMRX83khA0019E20 | LTR/Gypsy | 32 | 5529 |
| LTR-HVVMRX83khA0019O04 | LTR/Copia | 21 | 2962 |
| LTR-HVVMRX83khA0020G23 | LTR/Copia | 3 | 523 |
| LTR-HVVMRX83khA0020I01 | LTR | 30 | 4212 |
| LTR-HVVMRX83khA0020K17 | LTR | 109 | 16923 |
| LTR-HVVMRX83khA0021C02 | LTR | 740 | 100895 |
| LTR-HVVMRX83khA0021E18 | LTR | 9 | 1333 |
| LTR-HVVMRX83khA0022M04 | LTR/Copia | 5 | 521 |
| LTR-HVVMRX83khA0023L23 | LTR | 147 | 23442 |
| LTR-HVVMRX83khA0023N07 | LTR | 1 | 122 |
| LTR-HVVMRX83khA0023N13 | LTR | 71 | 10078 |
| LTR-HVVMRX83khA0023P07 | LTR/Copia | 54 | 9979 |
| LTR-HVVMRX83khA0024I07 | LTR/Gypsy | 4 | 441 |
| LTR-HVVMRX83khA0026I14 | LTR | 210 | 51591 |
| LTR-HVVMRX83khA0026N10 | LTR | 47 | 7092 |
| LTR-HVVMRX83khA0030I09 | LTR/Gypsy | 9 | 1569 |
| LTR-HVVMRX83khA0031C08 | LTR | 490 | 101948 |
| LTR-HVVMRX83khA0035I17 | LTR/Gypsy | 95 | 12638 |
| LTR-HVVMRX83khA0036I22 | LTR | 104 | 16824 |
| LTR-HVVMRX83khA0037L24 | LTR/Gypsy | 10 | 732 |
| LTR-HVVMRX83khA0040C03 | LTR | 182 | 45705 |
| LTR-HVVMRX83khA0040L02 | LTR/Copia | 9 | 1379 |
| LTR-HVVMRX83khA0041E21 | LTR/Gypsy | 95 | 14392 |
| LTR-HVVMRX83khA0042N03 | LTR | 111 | 17885 |
| LTR-HVVMRX83khA0043B02 | LTR/Gypsy | 74 | 20695 |
| LTR-HVVMRX83khA0043B12 | LTR | 7 | 1769 |
| LTR-HVVMRX83khA0043G03 | LTR/Copia | 3 | 396 |
| LTR-HVVMRX83khA0043H08 | LTR/Copia | 29 | 4068 |
| LTR-HVVMRX83khA0043J10 | LTR | 12 | 3638 |
| LTR-HVVMRX83khA0043K21 | LTR | 12 | 1603 |
| LTR-HVVMRX83khA0044A09 | LTR/Copia | 11 | 2451 |
| LTR-HVVMRX83khA0044I02 | LTR | 117 | 17009 |
| LTR-HVVMRX83khA0047O19 | LTR/Gypsy | 299 | 40979 |
| LTR-HVVMRX83khA0050D08 | LTR | 104 | 16766 |
| LTR-HVVMRX83khA0050E11 | LTR | 113 | 18150 |
| LTR-HVVMRX83khA0051D23 | LTR | 3 | 258 |
| LTR-HVVMRX83khA0052B03 | LTR | 74 | 10879 |
| LTR-HVVMRX83khA0052C09 | LTR | 47 | 7322 |

| | | | |
|---|---|---|---|
| LTR-HVVMRX83khA0052D10 | LTR | 6 | 800 |
| LTR-HVVMRX83khA0052K10 | LTR | 166 | 20615 |
| LTR-HVVMRX83khA0052L04 | LTR/Gypsy | 210 | 29864 |
| LTR-HVVMRX83khA0053C17 | LTR/Copia | 2 | 406 |
| LTR-HVVMRX83khA0054M24 | LTR | 406 | 27574 |
| LTR-HVVMRX83khA0055E02 | LTR | 87 | 13786 |
| LTR-HVVMRX83khA0056O07 | LTR/Copia | 10 | 2188 |
| LTR-HVVMRX83khA0057A09 | LTR/Copia | 4 | 948 |
| LTR-HVVMRX83khA0057C14 | LTR | 346 | 51346 |
| LTR-HVVMRX83khA0057L07 | LTR/Copia | 10 | 1078 |
| LTR-HVVMRX83khA0057M11 | LTR/Gypsy | 44 | 6114 |
| LTR-HVVMRX83khA0057N04 | LTR | 365 | 56908 |
| LTR-HVVMRX83khA0057O09 | LTR | 281 | 41272 |
| LTR-HVVMRX83khA0058D07 | LTR | 152 | 24599 |
| LTR-HVVMRX83khA0059F02 | LTR | 40 | 5681 |
| LTR-HVVMRX83khA0060G15 | LTR/Gypsy | 412 | 101911 |
| LTR-HVVMRX83khA0060H02 | LTR | 92 | 11527 |
| LTR-HVVMRX83khA0060K21 | LTR/Copia | 2 | 237 |
| LTR-HVVMRX83khA0061E11 | LTR | 85 | 13961 |
| LTR-HVVMRX83khA0061J07 | LTR | 215 | 28874 |
| LTR-HVVMRX83khA0061L24 | LTR/Copia | 52 | 8575 |
| LTR-HVVMRX83khA0063C16 | LTR/Copia | 36 | 5751 |
| LTR-HVVMRX83khA0063E12 | LTR/Gypsy | 19 | 3023 |
| LTR-HVVMRX83khA0064D23 | LTR/Copia | 3 | 712 |
| LTR-HVVMRX83khA0064G22 | LTR | 67 | 8804 |
| LTR-HVVMRX83khA0064M22 | LTR | 225 | 60217 |
| LTR-HVVMRX83khA0064P13 | LTR/Gypsy | 5 | 584 |
| LTR-HVVMRX83khA0065E13 | LTR/Copia | 4 | 676 |
| LTR-HVVMRX83khA0065K08 | LTR | 6 | 1102 |
| LTR-HVVMRX83khA0065L11 | LTR/Copia | 6 | 783 |
| LTR-HVVMRX83khA0065M16 | LTR | 254 | 34810 |
| LTR-HVVMRX83khA0066C17 | LTR | 247 | 39385 |
| LTR-HVVMRX83khA0066C19 | LTR/Copia | 18 | 4784 |
| LTR-HVVMRX83khA0066F13 | LTR | 35 | 7032 |
| LTR-HVVMRX83khA0066I07 | LTR | 26 | 3918 |
| LTR-HVVMRX83khA0067D20 | LTR/Copia | 11 | 1605 |
| LTR-HVVMRX83khA0067H21 | LTR/Copia | 9 | 1938 |
| LTR-HVVMRX83khA0068H05 | LTR/Gypsy | 17 | 2421 |
| LTR-HVVMRX83khA0068O09 | LTR/Gypsy | 153 | 22424 |
| LTR-HVVMRX83khA0069A23 | LTR | 20 | 3210 |
| LTR-HVVMRX83khA0069M21 | LTR | 236 | 32526 |
| LTR-HVVMRX83khA0069N04 | LTR/Gypsy | 12 | 2499 |
| LTR-HVVMRX83khA0070D21 | LTR/Copia | 3 | 393 |
| LTR-HVVMRX83khA0070N09 | LTR | 107 | 19576 |
| LTR-HVVMRX83khA0070P07 | LTR | 51 | 7032 |
| LTR-HVVMRX83khA0071G03 | LTR/Copia | 12 | 1208 |
| LTR-HVVMRX83khA0071M01 | LTR | 12 | 1962 |

| | | | |
|---|---|---|---|
| LTR-HVVMRX83khA0071P08 | LTR | 234 | 33907 |
| LTR-HVVMRX83khA0072L15 | LTR | 39 | 5898 |
| LTR-HVVMRX83khA0073E06 | LTR | 220 | 32881 |
| LTR-HVVMRX83khA0073H02 | LTR | 22 | 4549 |
| LTR-HVVMRX83khA0073H16 | LTR | 79 | 12110 |
| LTR-HVVMRX83khA0074H09 | LTR/Gypsy | 21 | 3036 |
| LTR-HVVMRX83khA0074K22 | LTR | 36 | 5024 |
| LTR-HVVMRX83khA0079A19 | LTR/Gypsy | 210 | 47135 |
| LTR-HVVMRX83khA0079D08 | LTR | 70 | 8615 |
| LTR-HVVMRX83khA0079M11 | LTR | 12 | 2205 |
| LTR-HVVMRX83khA0080E20 | LTR/Copia | 3 | 505 |
| LTR-HVVMRX83khA0080G24 | LTR | 30 | 4275 |
| LTR-HVVMRX83khA0081E20 | LTR/Gypsy | 45 | 8661 |
| LTR-HVVMRX83khA0082H11 | LTR | 92 | 14013 |
| LTR-HVVMRX83khA0084J07 | LTR | 12 | 2001 |
| LTR-HVVMRX83khA0085G23 | LTR/Copia | 15 | 1995 |
| LTR-HVVMRX83khA0086N18 | LTR/Gypsy | 3 | 628 |
| LTR-HVVMRX83khA0087F05 | LTR | 215 | 36359 |
| LTR-HVVMRX83khA0087H15 | LTR | 66 | 9140 |
| LTR-HVVMRX83khA0088D15 | LTR/Gypsy | 19 | 2310 |
| LTR-HVVMRX83khA0088J17 | LTR/Gypsy | 64 | 9315 |
| LTR-HVVMRX83khA0088P21 | LTR/Gypsy | 6 | 785 |
| LTR-HVVMRX83khA0090A14 | LTR/Copia | 3 | 265 |
| LTR-HVVMRX83khA0090P15 | LTR | 381 | 50374 |
| LTR-HVVMRX83khA0092H23 | LTR | 206 | 24659 |
| LTR-HVVMRX83khA0093G12 | LTR/Gypsy | 137 | 16060 |
| LTR-HVVMRX83khA0093I06 | LTR/Copia | 71 | 12757 |
| LTR-HVVMRX83khA0095M03 | LTR/Copia | 5 | 929 |
| LTR-HVVMRX83khA0097F03 | LTR | 568 | 80757 |
| LTR-HVVMRX83khA0097K18 | LTR | 274 | 36239 |
| LTR-HVVMRX83khA0098D04 | LTR | 673 | 130593 |
| LTR-HVVMRX83khA0098J08 | LTR | 61 | 10919 |
| LTR-HVVMRX83khA0098J11 | LTR/Copia | 239 | 62514 |
| LTR-HVVMRX83khA0099E12 | LTR/Gypsy | 48 | 10036 |
| LTR-HVVMRX83khA0099I02 | LTR/Copia | 8 | 790 |
| LTR-HVVMRX83khA0100N02 | LTR | 311 | 49063 |
| LTR-HVVMRX83khA0101P12 | LTR | 84 | 11394 |
| LTR-HVVMRX83khA0102B10 | LTR | 1968 | 504641 |
| LTR-HVVMRX83khA0102K03 | LTR/Gypsy | 6 | 758 |
| LTR-HVVMRX83khA0104J10 | LTR | 89 | 13840 |
| LTR-HVVMRX83khA0108H10 | LTR/Copia | 328 | 47528 |
| LTR-HVVMRX83khA0108K03 | LTR | 46 | 7930 |
| LTR-HVVMRX83khA0113L21 | LTR/Copia | 6 | 1821 |
| LTR-HVVMRX83khA0114C14 | LTR | 42 | 5077 |
| LTR-HVVMRX83khA0114G03 | LTR/Copia | 3 | 442 |
| LTR-HVVMRX83khA0116G11 | LTR/Gypsy | 40 | 5071 |
| LTR-HVVMRX83khA0116J03 | LTR | 8 | 1461 |

| | | | |
|---|---|---|---|
| LTR-HVVMRX83khA0116J04 | LTR | 44 | 5859 |
| LTR-HVVMRX83khA0116L17 | LTR/Gypsy | 32 | 4569 |
| LTR-HVVMRX83khA0117I01 | LTR/Gypsy | 191 | 30538 |
| LTR-HVVMRX83khA0118H10 | LTR | 10 | 1751 |
| LTR-HVVMRX83khA0118H15 | LTR/Gypsy | 7 | 735 |
| LTR-HVVMRX83khA0119J05 | LTR/Gypsy | 327 | 38751 |
| LTR-HVVMRX83khA0120D23 | LTR/Copia | 21 | 2941 |
| LTR-HVVMRX83khA0120I14 | LTR | 146 | 39723 |
| LTR-HVVMRX83khA0120K04 | LTR | 210 | 15769 |
| LTR-HVVMRX83khA0124K15 | LTR/Gypsy | 31 | 5489 |
| LTR-HVVMRX83khA0125I22 | LTR | 1971 | 244913 |
| LTR-HVVMRX83khA0125P15 | LTR | 27 | 3729 |
| LTR-HVVMRX83khA0126B13 | LTR | 21 | 2101 |
| LTR-HVVMRX83khA0126G14 | LTR/Copia | 3 | 705 |
| LTR-HVVMRX83khA0127A13 | LTR | 33 | 6706 |
| LTR-HVVMRX83khA0129C03 | LTR | 63 | 16164 |
| LTR-HVVMRX83khA0130K01 | LTR | 198 | 27374 |
| LTR-HVVMRX83khA0131C09 | LTR | 101 | 16548 |
| LTR-HVVMRX83khA0131E21 | LTR | 346 | 35855 |
| LTR-HVVMRX83khA0131F18 | LTR/Copia | 306 | 33565 |
| LTR-HVVMRX83khA0131I09 | LTR | 36 | 3988 |
| LTR-HVVMRX83khA0131P08 | LTR | 225 | 39848 |
| LTR-HVVMRX83khA0139I16 | LTR/Gypsy | 64 | 11600 |
| LTR-HVVMRX83khA0139K10 | LTR/Gypsy | 190 | 23901 |
| LTR-HVVMRX83khA0140C02 | LTR | 371 | 39556 |
| LTR-HVVMRX83khA0140E03 | LTR/Copia | 233 | 34115 |
| LTR-HVVMRX83khA0146E04 | LTR | 75 | 10633 |
| LTR-HVVMRX83khA0147D21 | LTR | 5 | 524 |
| LTR-HVVMRX83khA0149G11 | LTR | 62 | 10859 |
| LTR-HVVMRX83khA0149G19 | LTR | 100 | 12529 |
| LTR-HVVMRX83khA0155B07 | LTR/Gypsy | 9 | 1299 |
| LTR-HVVMRX83khA0156I18 | LTR/Gypsy | 51 | 6797 |
| LTR-HVVMRX83khA0156M07 | LTR | 130 | 20707 |
| LTR-HVVMRX83khA0157E13 | LTR | 102 | 13825 |
| LTR-HVVMRX83khA0158A23 | LTR | 195 | 42956 |
| LTR-HVVMRX83khA0158D22 | LTR/Gypsy | 39 | 4718 |
| LTR-HVVMRX83khA0158E01 | LTR/Copia | 8 | 954 |
| LTR-HVVMRX83khA0158E06 | LTR | 16 | 2466 |
| LTR-HVVMRX83khA0158O16 | LTR/Gypsy | 14 | 3247 |
| LTR-HVVMRX83khA0159D17 | LTR/Gypsy | 46 | 7546 |
| LTR-HVVMRX83khA0159G08 | LTR | 118 | 13175 |
| LTR-HVVMRX83khA0159G11 | LTR/Gypsy | 19 | 2505 |
| LTR-HVVMRX83khA0159L01 | LTR/Gypsy | 238 | 39533 |
| LTR-HVVMRX83khA0160K07 | LTR | 38 | 6606 |
| LTR-HVVMRX83khA0161F16 | LTR | 9 | 2775 |
| LTR-HVVMRX83khA0162F16 | LTR | 600 | 102145 |
| LTR-HVVMRX83khA0162K19 | LTR/Copia | 1 | 31 |

| | | | |
|---|---|---|---|
| LTR-HVVMRX83khA0163C08 | LTR/Copia | 7 | 1186 |
| LTR-HVVMRX83khA0163D12 | LTR | 27 | 5015 |
| LTR-HVVMRX83khA0164A08 | LTR | 47 | 6483 |
| LTR-HVVMRX83khA0164N06 | LTR/Copia | 127 | 18706 |
| LTR-HVVMRX83khA0165K21 | LTR/Gypsy | 10 | 1455 |
| LTR-HVVMRX83khA0165O07 | LTR/Copia | 1 | 117 |
| LTR-HVVMRX83khA0165O15 | LTR | 96 | 12495 |
| LTR-HVVMRX83khA0167H08 | LTR/Gypsy | 152 | 24821 |
| LTR-HVVMRX83khA0167H11 | LTR/Gypsy | 352 | 37566 |
| LTR-HVVMRX83khA0167I17 | LTR | 228 | 24792 |
| LTR-HVVMRX83khA0167J13 | LTR/Copia | 2 | 405 |
| LTR-HVVMRX83khA0167N13 | LTR/Gypsy | 56 | 13963 |
| LTR-HVVMRX83khA0168B20 | LTR | 237 | 34557 |
| LTR-HVVMRX83khA0168H05 | LTR/Copia | 7 | 1903 |
| LTR-HVVMRX83khA0168K03 | LTR/Gypsy | 44 | 6238 |
| LTR-HVVMRX83khA0169C01 | LTR | 141 | 17214 |
| LTR-HVVMRX83khA0169J15 | LTR | 33 | 5345 |
| LTR-HVVMRX83khA0169P16 | LTR/Copia | 41 | 4643 |
| LTR-HVVMRX83khA0170D09 | LTR/Copia | 26 | 2547 |
| LTR-HVVMRX83khA0170L15 | LTR | 327 | 36133 |
| LTR-HVVMRX83khA0170P04 | LTR | 178 | 26066 |
| LTR-HVVMRX83khA0172A15 | LTR/Gypsy | 17 | 2427 |
| LTR-HVVMRX83khA0172C04 | LTR/Gypsy | 18 | 2308 |
| LTR-HVVMRX83khA0172H11 | LTR/Gypsy | 290 | 33056 |
| LTR-HVVMRX83khA0173A10 | LTR | 337 | 33620 |
| LTR-HVVMRX83khA0174N21 | LTR/Copia | 51 | 7911 |
| LTR-HVVMRX83khA0175A03 | LTR/Copia | 3 | 492 |
| LTR-HVVMRX83khA0176P12 | LTR/Copia | 7 | 1167 |
| LTR-HVVMRX83khA0177G11 | LTR/Gypsy | 39 | 5351 |
| LTR-HVVMRX83khA0177J18 | LTR | 60 | 10697 |
| LTR-HVVMRX83khA0178G20 | LTR/Gypsy | 233 | 27225 |
| LTR-HVVMRX83khA0179G19 | LTR | 183 | 31511 |
| LTR-HVVMRX83khA0179N08 | LTR | 45 | 6661 |
| LTR-HVVMRX83khA0180G01 | LTR | 366 | 61152 |
| LTR-HVVMRX83khA0180J15 | LTR/Gypsy | 473 | 85567 |
| LTR-HVVMRX83khA0180J17 | LTR/Copia | 117 | 27864 |
| LTR-HVVMRX83khA0180K02 | LTR | 8 | 1615 |
| LTR-HVVMRX83khA0180M07 | LTR/Copia | 35 | 4730 |
| LTR-HVVMRX83khA0180O15 | LTR | 7 | 1071 |
| LTR-HVVMRX83khA0181G04 | LTR | 33 | 3063 |
| LTR-HVVMRX83khA0182D14 | LTR | 201 | 39859 |
| LTR-HVVMRX83khA0182O06 | LTR/Copia | 75 | 11660 |
| LTR-HVVMRX83khA0185K02 | LTR | 2 | 180 |
| LTR-HVVMRX83khA0186G15 | LTR | 32 | 4557 |
| LTR-HVVMRX83khA0186K15 | LTR/Gypsy | 55 | 7146 |
| LTR-HVVMRX83khA0186O03 | LTR | 163 | 24268 |
| LTR-HVVMRX83khA0187E03 | LTR/Copia | 4 | 544 |

| | | | |
|---|---|---|---|
| LTR-HVVMRX83khA0187K09 | LTR/Copia | 20 | 2736 |
| LTR-HVVMRX83khA0187L08 | LTR/Gypsy | 114 | 15273 |
| LTR-HVVMRX83khA0187P22 | LTR | 20 | 3599 |
| LTR-HVVMRX83khA0188E14 | LTR/Copia | 46 | 7813 |
| LTR-HVVMRX83khA0189A17 | LTR/Gypsy | 55 | 8961 |
| LTR-HVVMRX83khA0189E03 | LTR/Copia | 3 | 644 |
| LTR-HVVMRX83khA0189F10 | LTR/Gypsy | 743 | 97924 |
| LTR-HVVMRX83khA0189G13 | LTR | 155 | 29909 |
| LTR-HVVMRX83khA0189O18 | LTR/Gypsy | 545 | 77902 |
| LTR-HVVMRX83khA0191P18 | LTR | 346 | 50066 |
| LTR-HVVMRX83khA0195O22 | LTR/Copia | 171 | 23260 |
| LTR-HVVMRX83khA0195P19 | LTR | 21 | 3115 |
| LTR-HVVMRX83khA0196A21 | LTR/Gypsy | 83 | 21344 |
| LTR-HVVMRX83khA0196B21 | LTR/Gypsy | 49 | 6097 |
| LTR-HVVMRX83khA0196J12 | LTR | 126 | 35325 |
| LTR-HVVMRX83khA0196P08 | LTR/Gypsy | 401 | 62026 |
| LTR-HVVMRX83khA0197A10 | LTR/Copia | 759 | 109969 |
| LTR-HVVMRX83khA0197F20 | LTR | 118 | 20551 |
| LTR-HVVMRX83khA0197F21 | LTR | 7 | 1363 |
| LTR-HVVMRX83khA0197P11 | LTR | 58 | 9226 |
| LTR-HVVMRX83khA0198E01 | LTR/Copia | 9 | 1664 |
| LTR-HVVMRX83khA0199G18 | LTR/Copia | 2 | 268 |
| LTR-HVVMRX83khA0200E03 | LTR | 61 | 7916 |
| LTR-HVVMRX83khA0202A14 | LTR/Copia | 390 | 103040 |
| LTR-HVVMRX83khA0202C12 | LTR | 937 | 104562 |
| LTR-HVVMRX83khA0204B21 | LTR | 112 | 15407 |
| LTR-HVVMRX83khA0204K18 | LTR | 1435 | 185079 |
| LTR-HVVMRX83khA0205B20 | LTR/Copia | 66 | 15356 |
| LTR-HVVMRX83khA0205C16 | LTR | 50 | 7612 |
| LTR-HVVMRX83khA0205K10 | LTR | 41 | 6709 |
| LTR-HVVMRX83khA0206B15 | LTR/Gypsy | 6 | 735 |
| LTR-HVVMRX83khA0206K01 | LTR | 216 | 47962 |
| LTR-HVVMRX83khA0207M01 | LTR/Copia | 61 | 8819 |
| LTR-HVVMRX83khA0209G21 | LTR | 523 | 83491 |
| LTR-HVVMRX83khA0209K10 | LTR | 192 | 41368 |
| LTR-HVVMRX83khA0210D23 | LTR | 265 | 38407 |
| LTR-HVVMRX83khA0212A22 | LTR | 72 | 12771 |
| LTR-HVVMRX83khA0212I10 | LTR | 15 | 2357 |
| LTR-HVVMRX83khA0213F23 | LTR/Gypsy | 44 | 5508 |
| LTR-HVVMRX83khA0213K02 | LTR/Gypsy | 47 | 6771 |
| LTR-HVVMRX83khA0213M19 | LTR | 56 | 9680 |
| LTR-HVVMRX83khA0213O09 | LTR/Copia | 2 | 156 |
| LTR-HVVMRX83khA0214I20 | LTR/Copia | 4 | 915 |
| LTR-HVVMRX83khA0215L15 | LTR/Gypsy | 24 | 6153 |
| LTR-HVVMRX83khA0215M21 | LTR/Copia | 8 | 1358 |
| LTR-HVVMRX83khA0216F15 | LTR | 541 | 158779 |
| LTR-HVVMRX83khA0217D04 | LTR | 141 | 20971 |

| | | | |
|---|---|---|---|
| LTR-HVVMRX83khA0217D17 | LTR/Copia | 28 | 3358 |
| LTR-HVVMRX83khA0217I11 | LTR/Copia | 4 | 659 |
| LTR-HVVMRX83khA0217J15 | LTR | 157 | 23871 |
| LTR-HVVMRX83khA0218E24 | LTR | 27 | 3915 |
| LTR-HVVMRX83khA0218J14 | LTR/Gypsy | 10 | 1709 |
| LTR-HVVMRX83khA0218N11 | LTR/Copia | 2 | 188 |
| LTR-HVVMRX83khA0219I03 | LTR | 176 | 29110 |
| LTR-HVVMRXALLeA0006B24 | LTR | 87 | 13252 |
| LTR-HVVMRXALLeA0006N02 | LTR | 4 | 888 |
| LTR-HVVMRXALLeA0006P03 | LTR | 181 | 28813 |
| LTR-HVVMRXALLeA0007L11 | LTR/Gypsy | 25 | 4863 |
| LTR-HVVMRXALLeA0008A01 | LTR/Gypsy | 66 | 10547 |
| LTR-HVVMRXALLeA0008E01 | LTR | 748 | 127923 |
| LTR-HVVMRXALLeA0008J21 | LTR | 23 | 3456 |
| LTR-HVVMRXALLeA0008N19 | LTR | 1 | 109 |
| LTR-HVVMRXALLeA0008O01 | LTR/Gypsy | 42 | 6271 |
| LTR-HVVMRXALLeA0008P07 | LTR/Gypsy | 31 | 4373 |
| LTR-HVVMRXALLeA0009A13 | LTR | 63 | 9872 |
| LTR-HVVMRXALLeA0009P21 | LTR/Gypsy | 99 | 19093 |
| LTR-HVVMRXALLeA0011K14 | LTR | 47 | 8358 |
| LTR-HVVMRXALLeA0011L11 | LTR | 57 | 8507 |
| LTR-HVVMRXALLeA0012A04 | LTR/Copia | 4 | 529 |
| LTR-HVVMRXALLeA0012K02 | LTR/Gypsy | 87 | 12385 |
| LTR-HVVMRXALLeA0012K09 | LTR/Copia | 17 | 2226 |
| LTR-HVVMRXALLeA0012N09 | LTR/Copia | 39 | 6671 |
| LTR-HVVMRXALLeA0013B10 | LTR | 122 | 19829 |
| LTR-HVVMRXALLeA0014E01 | LTR | 376 | 57039 |
| LTR-HVVMRXALLeA0014E10 | LTR/Copia | 7 | 1575 |
| LTR-HVVMRXALLeA0015H17 | LTR/Gypsy | 10 | 1927 |
| LTR-HVVMRXALLeA0016G01 | LTR | 1 | 61 |
| LTR-HVVMRXALLeA0016I01 | LTR | 33 | 5479 |
| LTR-HVVMRXALLeA0018P12 | LTR/Gypsy | 400 | 49263 |
| LTR-HVVMRXALLeA0019F01 | LTR/Copia | 13 | 1423 |
| LTR-HVVMRXALLeA0019J06 | LTR/Gypsy | 81 | 12027 |
| LTR-HVVMRXALLeA0019N06 | LTR/Copia | 287 | 45751 |
| LTR-HVVMRXALLeA0020K05 | LTR | 5 | 617 |
| LTR-HVVMRXALLeA0020N20 | LTR/Copia | 1 | 256 |
| LTR-HVVMRXALLeA0023J19 | LTR/Copia | 176 | 26126 |
| LTR-HVVMRXALLeA0026O07 | LTR/Gypsy | 19 | 2878 |
| LTR-HVVMRXALLeA0027J10 | LTR/Gypsy | 36 | 5093 |
| LTR-HVVMRXALLeA0028F21 | LTR | 19 | 2711 |
| LTR-HVVMRXALLeA0028J19 | LTR/Copia | 2 | 462 |
| LTR-HVVMRXALLeA0030J05 | LTR | 298 | 48297 |
| LTR-HVVMRXALLeA0030L15 | LTR/Copia | 5 | 657 |
| LTR-HVVMRXALLeA0032E01 | LTR | 55 | 7106 |
| LTR-HVVMRXALLeA0035C14 | LTR/Gypsy | 19 | 3124 |
| LTR-HVVMRXALLeA0038D14 | LTR | 989 | 131279 |

| | | | |
|---|---|---|---|
| LTR-HVVMRXALLeA0038D17 | LTR | 9 | 1218 |
| LTR-HVVMRXALLeA0038M01 | LTR | 95 | 12760 |
| LTR-HVVMRXALLeA0040C01 | LTR/Copia | 13 | 1735 |
| LTR-HVVMRXALLeA0040I01 | LTR/Copia | 172 | 27005 |
| LTR-HVVMRXALLeA0040I10 | LTR | 70 | 9329 |
| LTR-HVVMRXALLeA0046N06 | LTR | 94 | 21885 |
| LTR-HVVMRXALLeA0047C14 | LTR/Copia | 3 | 253 |
| LTR-HVVMRXALLeA0048E01 | LTR | 146 | 18218 |
| LTR-HVVMRXALLeA0049A05 | LTR/Gypsy | 25 | 3265 |
| LTR-HVVMRXALLeA0049G10 | LTR | 40 | 5848 |
| LTR-HVVMRXALLeA0049I02 | LTR/Copia | 3 | 401 |
| LTR-HVVMRXALLeA0049N11 | LTR | 2 | 300 |
| LTR-HVVMRXALLeA0050G03 | LTR/Gypsy | 319 | 34260 |
| LTR-HVVMRXALLeA0052D08 | LTR/Copia | 25 | 2586 |
| LTR-HVVMRXALLeA0054A09 | LTR | 49 | 7664 |
| LTR-HVVMRXALLeA0054P14 | LTR | 114 | 20324 |
| LTR-HVVMRXALLeA0055P16 | LTR | 74 | 15722 |
| LTR-HVVMRXALLeA0056M01 | LTR | 163 | 24110 |
| LTR-HVVMRXALLeA0058K22 | LTR/Copia | 11 | 1240 |
| LTR-HVVMRXALLeA0060M19 | LTR/Gypsy | 1 | 186 |
| LTR-HVVMRXALLeA0063P10 | LTR | 55 | 8498 |
| LTR-HVVMRXALLeA0064C09 | LTR | 262 | 37712 |
| LTR-HVVMRXALLeA0064G06 | LTR | 64 | 10439 |
| LTR-HVVMRXALLeA0064K01 | LTR/Gypsy | 15 | 1921 |
| LTR-HVVMRXALLeA0065M03 | LTR/Copia | 2 | 150 |
| LTR-HVVMRXALLeA0066C04 | LTR/Gypsy | 6 | 1304 |
| LTR-HVVMRXALLeA0066K09 | LTR | 71 | 10222 |
| LTR-HVVMRXALLeA0066N18 | LTR | 96 | 18738 |
| LTR-HVVMRXALLeA0067C09 | LTR | 1258 | 203289 |
| LTR-HVVMRXALLeA0067F18 | LTR | 109 | 14708 |
| LTR-HVVMRXALLeA0067O06 | LTR/Gypsy | 81 | 16235 |
| LTR-HVVMRXALLeA0067P03 | LTR | 711 | 82318 |
| LTR-HVVMRXALLeA0068F06 | LTR | 7 | 1104 |
| LTR-HVVMRXALLeA0069B18 | LTR/Copia | 3 | 702 |
| LTR-HVVMRXALLeA0069E14 | LTR | 44 | 7731 |
| LTR-HVVMRXALLeA0069G13 | LTR/Copia | 4 | 390 |
| LTR-HVVMRXALLeA0070P21 | LTR/Gypsy | 134 | 17063 |
| LTR-HVVMRXALLeA0071L01 | LTR/Gypsy | 16 | 1709 |
| LTR-HVVMRXALLeA0072A01 | LTR | 345 | 43988 |
| LTR-HVVMRXALLeA0072I01 | LTR/Copia | 34 | 3552 |
| LTR-HVVMRXALLeA0075F23 | LTR/Copia | 50 | 8197 |
| LTR-HVVMRXALLeA0076C02 | LTR/Copia | 33 | 4787 |
| LTR-HVVMRXALLeA0076L06 | LTR | 8 | 1076 |
| LTR-HVVMRXALLeA0077F08 | LTR | 55 | 7672 |
| LTR-HVVMRXALLeA0078C24 | LTR | 31 | 3889 |
| LTR-HVVMRXALLeA0078E21 | LTR | 131 | 16714 |
| LTR-HVVMRXALLeA0080M01 | LTR/Copia | 7 | 1004 |

| | | | |
|---|---|---|---|
| LTR-HVVMRXALLeA0080P03 | LTR/Gypsy | 95 | 15257 |
| LTR-HVVMRXALLeA0081K17 | LTR | 85 | 12344 |
| LTR-HVVMRXALLeA0082H12 | LTR/Gypsy | 12 | 2959 |
| LTR-HVVMRXALLeA0082I13 | LTR/Copia | 6 | 1874 |
| LTR-HVVMRXALLeA0082L22 | LTR/Copia | 6 | 972 |
| LTR-HVVMRXALLeA0083D22 | LTR/Copia | 4 | 528 |
| LTR-HVVMRXALLeA0086C18 | LTR | 499 | 82171 |
| LTR-HVVMRXALLeA0086E19 | LTR | 95 | 14177 |
| LTR-HVVMRXALLeA0088B20 | LTR/Copia | 16 | 1904 |
| LTR-HVVMRXALLeA0088C01 | LTR/Copia | 2 | 237 |
| LTR-HVVMRXALLeA0088C17 | LTR/Copia | 8 | 1643 |
| LTR-HVVMRXALLeA0088K01 | LTR/Copia | 15 | 1899 |
| LTR-HVVMRXALLeA0089D06 | LTR | 47 | 8096 |
| LTR-HVVMRXALLeA0089D12 | LTR/Copia | 2 | 154 |
| LTR-HVVMRXALLeA0090F17 | LTR/Copia | 90 | 23981 |
| LTR-HVVMRXALLeA0090P12 | LTR | 52 | 7761 |
| LTR-HVVMRXALLeA0092P05 | LTR/Gypsy | 48 | 8167 |
| LTR-HVVMRXALLeA0094I17 | LTR/Gypsy | 47 | 7076 |
| LTR-HVVMRXALLeA0094K17 | LTR | 5 | 784 |
| LTR-HVVMRXALLeA0095C24 | LTR/Copia | 498 | 64434 |
| LTR-HVVMRXALLeA0096A01 | LTR | 61 | 7041 |
| LTR-HVVMRXALLeA0097H01 | LTR/Gypsy | 3 | 719 |
| LTR-HVVMRXALLeA0099J18 | LTR/Gypsy | 24 | 3759 |
| LTR-HVVMRXALLeA0100P04 | LTR/Copia | 98 | 18479 |
| LTR-HVVMRXALLeA0100P16 | LTR/Copia | 102 | 10809 |
| LTR-HVVMRXALLeA0101F06 | LTR/Copia | 10 | 1254 |
| LTR-HVVMRXALLeA0104E01 | LTR | 7 | 759 |
| LTR-HVVMRXALLeA0104K01 | LTR/Copia | 6 | 644 |
| LTR-HVVMRXALLeA0105P06 | LTR/Copia | 18 | 4474 |
| LTR-HVVMRXALLeA0106G18 | LTR | 71 | 14050 |
| LTR-HVVMRXALLeA0108K14 | LTR | 92 | 13937 |
| LTR-HVVMRXALLeA0109E18 | LTR | 46 | 6275 |
| LTR-HVVMRXALLeA0109J02 | LTR | 19 | 2562 |
| LTR-HVVMRXALLeA0110O06 | LTR/Gypsy | 8 | 1601 |
| LTR-HVVMRXALLeA0111K13 | LTR | 365 | 70373 |
| LTR-HVVMRXALLeA0112O12 | LTR | 390 | 44200 |
| LTR-HVVMRXALLeA0113J08 | LTR | 42 | 7610 |
| LTR-HVVMRXALLeA0113K22 | LTR/Copia | 36 | 5221 |
| LTR-HVVMRXALLeA0113N21 | LTR/Copia | 3 | 214 |
| LTR-HVVMRXALLeA0114B22 | LTR/Gypsy | 30 | 4097 |
| LTR-HVVMRXALLeA0115O18 | LTR | 7 | 660 |
| LTR-HVVMRXALLeA0116A14 | LTR/Gypsy | 33 | 5378 |
| LTR-HVVMRXALLeA0118N07 | LTR | 57 | 9539 |
| LTR-HVVMRXALLeA0119D04 | LTR/Copia | 126 | 33842 |
| LTR-HVVMRXALLeA0119K17 | LTR | 93 | 14267 |
| LTR-HVVMRXALLeA0119O21 | LTR | 143 | 32717 |
| LTR-HVVMRXALLeA0120A01 | LTR | 54 | 8227 |

| | | | |
|---|---|---|---|
| LTR-HVVMRXALLeA0120B11 | LTR | 712 | 72213 |
| LTR-HVVMRXALLeA0120E01 | LTR/Copia | 2 | 160 |
| LTR-HVVMRXALLeA0120E05 | LTR | 142 | 19414 |
| LTR-HVVMRXALLeA0120I02 | LTR/Gypsy | 74 | 10991 |
| LTR-HVVMRXALLeA0120M01 | LTR/Copia | 12 | 3172 |
| LTR-HVVMRXALLeA0121L17 | LTR | 501 | 91872 |
| LTR-HVVMRXALLeA0123D11 | LTR | 52 | 7638 |
| LTR-HVVMRXALLeA0123M03 | LTR/Copia | 30 | 6063 |
| LTR-HVVMRXALLeA0124E21 | LTR | 45 | 7995 |
| LTR-HVVMRXALLeA0124I06 | LTR | 75 | 11688 |
| LTR-HVVMRXALLeA0124M19 | LTR/Copia | 23 | 2800 |
| LTR-HVVMRXALLeA0125N09 | LTR/Gypsy | 4 | 819 |
| LTR-HVVMRXALLeA0127P17 | LTR/Copia | 1 | 162 |
| LTR-HVVMRXALLeA0128G01 | LTR | 810 | 98832 |
| LTR-HVVMRXALLeA0128O01 | LTR | 128 | 19174 |
| LTR-HVVMRXALLeA0130I23 | LTR | 698 | 84399 |
| LTR-HVVMRXALLeA0130L01 | LTR | 46 | 5835 |
| LTR-HVVMRXALLeA0130P06 | LTR/Gypsy | 104 | 14121 |
| LTR-HVVMRXALLeA0131P08 | LTR | 1705 | 240350 |
| LTR-HVVMRXALLeA0132D03 | LTR | 145 | 20948 |
| LTR-HVVMRXALLeA0132N09 | LTR/Copia | 34 | 4706 |
| LTR-HVVMRXALLeA0133J21 | LTR | 69 | 11363 |
| LTR-HVVMRXALLeA0133P15 | LTR | 209 | 34358 |
| LTR-HVVMRXALLeA0135C06 | LTR | 84 | 19929 |
| LTR-HVVMRXALLeA0135G06 | LTR | 307 | 46773 |
| LTR-HVVMRXALLeA0135J20 | LTR/Gypsy | 76 | 12709 |
| LTR-HVVMRXALLeA0135M05 | LTR/Gypsy | 795 | 150090 |
| LTR-HVVMRXALLeA0135N15 | LTR/Gypsy | 53 | 8399 |
| LTR-HVVMRXALLeA0136C09 | LTR/Copia | 1 | 58 |
| LTR-HVVMRXALLeA0136G01 | LTR | 32 | 4164 |
| LTR-HVVMRXALLeA0136K01 | LTR | 271 | 56405 |
| LTR-HVVMRXALLeA0136K06 | LTR | 576 | 88336 |
| LTR-HVVMRXALLeA0137E17 | LTR | 502 | 70969 |
| LTR-HVVMRXALLeA0137G17 | LTR | 40 | 6312 |
| LTR-HVVMRXALLeA0138I04 | LTR | 220 | 34581 |
| LTR-HVVMRXALLeA0139D12 | LTR/Gypsy | 142 | 23482 |
| LTR-HVVMRXALLeA0139G02 | LTR | 14 | 3000 |
| LTR-HVVMRXALLeA0139K11 | LTR | 76 | 10219 |
| LTR-HVVMRXALLeA0140N22 | LTR | 115 | 18191 |
| LTR-HVVMRXALLeA0141K18 | LTR | 35 | 7406 |
| LTR-HVVMRXALLeA0141L15 | LTR/Copia | 10 | 2303 |
| LTR-HVVMRXALLeA0142P13 | LTR/Copia | 2 | 505 |
| LTR-HVVMRXALLeA0143O20 | LTR/Copia | 5 | 594 |
| LTR-HVVMRXALLeA0144E01 | LTR/Copia | 40 | 7107 |
| LTR-HVVMRXALLeA0144M01 | LTR/Gypsy | 23 | 2517 |
| LTR-HVVMRXALLeA0145H16 | LTR | 5 | 624 |
| LTR-HVVMRXALLeA0146G15 | LTR/Copia | 2 | 89 |

| | | | |
|---|---|---|---|
| LTR-HVVMRXALLeA0146P16 | LTR/Copia | 1 | 72 |
| LTR-HVVMRXALLeA0147E17 | LTR | 70 | 11651 |
| LTR-HVVMRXALLeA0147I05 | LTR/Copia | 3 | 200 |
| LTR-HVVMRXALLeA0147O14 | LTR/Copia | 15 | 1531 |
| LTR-HVVMRXALLeA0147P20 | LTR | 180 | 24185 |
| LTR-HVVMRXALLeA0148A18 | LTR | 62 | 10854 |
| LTR-HVVMRXALLeA0148H12 | LTR/Copia | 16 | 2981 |
| LTR-HVVMRXALLeA0149I13 | LTR/Copia | 4 | 703 |
| LTR-HVVMRXALLeA0150D01 | LTR | 392 | 53184 |
| LTR-HVVMRXALLeA0150H15 | LTR/Gypsy | 35 | 5566 |
| LTR-HVVMRXALLeA0151J13 | LTR/Copia | 4 | 417 |
| LTR-HVVMRXALLeA0152A01 | LTR | 164 | 26281 |
| LTR-HVVMRXALLeA0152E10 | LTR | 53 | 7220 |
| LTR-HVVMRXALLeA0152I01 | LTR | 52 | 8096 |
| LTR-HVVMRXALLeA0152L08 | LTR/Gypsy | 10 | 1992 |
| LTR-HVVMRXALLeA0152M21 | LTR/Copia | 7 | 989 |
| LTR-HVVMRXALLeA0152O01 | LTR/Gypsy | 41 | 6019 |
| LTR-HVVMRXALLeA0157I17 | LTR | 137 | 20345 |
| LTR-HVVMRXALLeA0159P14 | LTR/Gypsy | 8 | 947 |
| LTR-HVVMRXALLeA0160B07 | LTR | 117 | 31937 |
| LTR-HVVMRXALLeA0160L19 | LTR | 461 | 62242 |
| LTR-HVVMRXALLeA0161F09 | LTR | 22 | 3424 |
| LTR-HVVMRXALLeA0162F11 | LTR | 109 | 14384 |
| LTR-HVVMRXALLeA0162F17 | LTR/Gypsy | 87 | 13314 |
| LTR-HVVMRXALLeA0163P21 | LTR/Copia | 8 | 1486 |
| LTR-HVVMRXALLeA0164J18 | LTR | 25 | 5562 |
| LTR-HVVMRXALLeA0164L03 | LTR | 155 | 18025 |
| LTR-HVVMRXALLeA0168D20 | LTR | 1494 | 167971 |
| LTR-HVVMRXALLeA0168E01 | LTR/Gypsy | 20 | 1923 |
| LTR-HVVMRXALLeA0168I01 | LTR/Copia | 12 | 1568 |
| LTR-HVVMRXALLeA0169B20 | LTR/Copia | 17 | 4261 |
| LTR-HVVMRXALLeA0169D18 | LTR/Copia | 13 | 2065 |
| LTR-HVVMRXALLeA0174C03 | LTR/Copia | 10 | 1337 |
| LTR-HVVMRXALLeA0174F16 | LTR | 16 | 2105 |
| LTR-HVVMRXALLeA0175F10 | LTR | 4 | 546 |
| LTR-HVVMRXALLeA0175H06 | LTR/Gypsy | 36 | 4411 |
| LTR-HVVMRXALLeA0176M01 | LTR/Gypsy | 10 | 1546 |
| LTR-HVVMRXALLeA0178A01 | LTR | 442 | 60319 |
| LTR-HVVMRXALLeA0178B14 | LTR/Gypsy | 67 | 9358 |
| LTR-HVVMRXALLeA0178D14 | LTR/Copia | 357 | 102594 |
| LTR-HVVMRXALLeA0178E11 | LTR | 319 | 47287 |
| LTR-HVVMRXALLeA0180O06 | LTR | 46 | 7469 |
| LTR-HVVMRXALLeA0181P16 | LTR | 749 | 113950 |
| LTR-HVVMRXALLeA0184I01 | LTR | 44 | 9159 |
| LTR-HVVMRXALLeA0184I12 | LTR | 85 | 11093 |
| LTR-HVVMRXALLeA0186D08 | LTR | 17 | 2780 |
| LTR-HVVMRXALLeA0186K04 | LTR/Copia | 2 | 72 |

| | | | |
|---|---|---|---|
| LTR-HVVMRXALLeA0187F06 | LTR/Gypsy | 15 | 1793 |
| LTR-HVVMRXALLeA0187H08 | LTR | 229 | 28777 |
| LTR-HVVMRXALLeA0190E23 | LTR | 64 | 11132 |
| LTR-HVVMRXALLeA0191K22 | LTR | 78 | 13656 |
| LTR-HVVMRXALLeA0192C19 | LTR/Gypsy | 12 | 2385 |
| LTR-HVVMRXALLeA0192E01 | LTR/Gypsy | 9 | 1335 |
| LTR-HVVMRXALLeA0192E20 | LTR | 697 | 95069 |
| LTR-HVVMRXALLeA0192M01 | LTR | 36 | 8731 |
| LTR-HVVMRXALLeA0192O08 | LTR/Copia | 95 | 19717 |
| LTR-HVVMRXALLeA0194H14 | LTR | 64 | 15288 |
| LTR-HVVMRXALLeA0196L03 | LTR/Copia | 124 | 17349 |
| LTR-HVVMRXALLeA0197C12 | LTR/Gypsy | 10 | 2351 |
| LTR-HVVMRXALLeA0197F10 | LTR/Gypsy | 160 | 25352 |
| LTR-HVVMRXALLeA0197F14 | LTR | 43 | 5962 |
| LTR-HVVMRXALLeA0197L17 | LTR | 1 | 136 |
| LTR-HVVMRXALLeA0197N08 | LTR/Copia | 72 | 8819 |
| LTR-HVVMRXALLeA0198K13 | LTR | 60 | 9611 |
| LTR-HVVMRXALLeA0200G01 | LTR/Copia | 9 | 1357 |
| LTR-HVVMRXALLeA0200K01 | LTR | 110 | 14420 |
| LTR-HVVMRXALLeA0201B02 | LTR | 71 | 8848 |
| LTR-HVVMRXALLeA0202O05 | LTR | 341 | 61914 |
| LTR-HVVMRXALLeA0207K08 | LTR | 31 | 6098 |
| LTR-HVVMRXALLeA0208A15 | LTR/Gypsy | 168 | 34024 |
| LTR-HVVMRXALLeA0208F01 | LTR/Copia | 3 | 389 |
| LTR-HVVMRXALLeA0208M01 | LTR/Gypsy | 22 | 3120 |
| LTR-HVVMRXALLeA0210K07 | LTR/Copia | 2 | 289 |
| LTR-HVVMRXALLeA0211G14 | LTR/Gypsy | 65 | 8869 |
| LTR-HVVMRXALLeA0211H24 | LTR | 12 | 1506 |
| LTR-HVVMRXALLeA0211L18 | LTR | 63 | 9574 |
| LTR-HVVMRXALLeA0212B05 | LTR | 417 | 56077 |
| LTR-HVVMRXALLeA0212G15 | LTR/Copia | 135 | 25290 |
| LTR-HVVMRXALLeA0213G20 | LTR | 108 | 18510 |
| LTR-HVVMRXALLeA0213O03 | LTR/Copia | 2 | 493 |
| LTR-HVVMRXALLeA0216A01 | LTR | 80 | 13388 |
| LTR-HVVMRXALLeA0216E01 | LTR/Copia | 67 | 6947 |
| LTR-HVVMRXALLeA0216O01 | LTR/Gypsy | 105 | 14542 |
| LTR-HVVMRXALLeA0218B17 | LTR/Gypsy | 117 | 14396 |
| LTR-HVVMRXALLeA0219G06 | LTR/Gypsy | 7 | 806 |
| LTR-HVVMRXALLeA0221F19 | LTR | 31 | 4481 |
| LTR-HVVMRXALLeA0221F22 | LTR | 91 | 14008 |
| LTR-HVVMRXALLeA0221F23 | LTR | 55 | 8161 |
| LTR-HVVMRXALLeA0222A01 | LTR/Gypsy | 221 | 32157 |
| LTR-HVVMRXALLeA0222D17 | LTR | 49 | 6271 |
| LTR-HVVMRXALLeA0222L16 | LTR | 96 | 10242 |
| LTR-HVVMRXALLeA0222M09 | LTR | 11 | 3038 |
| LTR-HVVMRXALLeA0222M24 | LTR/Copia | 21 | 2534 |
| LTR-HVVMRXALLeA0224A01 | LTR/Copia | 1 | 350 |

| | | | |
|---|---|---|---|
| LTR-HVVMRXALLeA0225F17 | LTR | 68 | 9524 |
| LTR-HVVMRXALLeA0230L01 | LTR/Copia | 10 | 1498 |
| LTR-HVVMRXALLeA0231L05 | LTR | 14 | 2436 |
| LTR-HVVMRXALLeA0231N14 | LTR/Copia | 5 | 592 |
| LTR-HVVMRXALLeA0232C01 | LTR/Copia | 17 | 2997 |
| LTR-HVVMRXALLeA0232G01 | LTR/Gypsy | 2 | 329 |
| LTR-HVVMRXALLeA0236K01 | LTR | 52 | 7469 |
| LTR-HVVMRXALLeA0239A18 | LTR/Copia | 19 | 5684 |
| LTR-HVVMRXALLeA0239E04 | LTR | 130 | 23542 |
| LTR-HVVMRXALLeA0240G01 | LTR/Gypsy | 302 | 51437 |
| LTR-HVVMRXALLeA0240K01 | LTR | 914 | 157189 |
| LTR-HVVMRXALLeA0240M15 | LTR/Copia | 5 | 884 |
| LTR-HVVMRXALLeA0241F12 | LTR | 143 | 15644 |
| LTR-HVVMRXALLeA0243B14 | LTR/Copia | 8 | 935 |
| LTR-HVVMRXALLeA0246M24 | LTR/Copia | 11 | 1939 |
| LTR-HVVMRXALLeA0247J20 | LTR | 279 | 38168 |
| LTR-HVVMRXALLeA0249P22 | LTR | 21 | 1987 |
| LTR-HVVMRXALLeA0251B15 | LTR | 200 | 26912 |
| LTR-HVVMRXALLeA0253F17 | LTR | 156 | 19939 |
| LTR-HVVMRXALLeA0256C01 | LTR | 474 | 56536 |
| LTR-HVVMRXALLeA0256E01 | LTR/Copia | 3 | 297 |
| LTR-HVVMRXALLeA0256J17 | LTR | 5 | 544 |
| LTR-HVVMRXALLeA0256M01 | LTR | 90 | 13134 |
| LTR-HVVMRXALLeA0260H11 | LTR | 36 | 5378 |
| LTR-HVVMRXALLeA0263J01 | LTR | 23 | 4424 |
| LTR-HVVMRXALLeA0264C01 | LTR/Gypsy | 171 | 24586 |
| LTR-HVVMRXALLeA0267E10 | LTR/Copia | 10 | 1359 |
| LTR-HVVMRXALLeA0268G20 | LTR/Copia | 82 | 9233 |
| LTR-HVVMRXALLeA0269E08 | LTR/Copia | 273 | 35122 |
| LTR-HVVMRXALLeA0270J23 | LTR | 77 | 10441 |
| LTR-HVVMRXALLeA0270M10 | LTR | 7 | 1708 |
| LTR-HVVMRXALLeA0272A01 | LTR/Copia | 8 | 1493 |
| LTR-HVVMRXALLeA0272G01 | LTR/Copia | 147 | 28476 |
| LTR-HVVMRXALLeA0272K01 | LTR/Gypsy | 64 | 8795 |
| LTR-HVVMRXALLeA0273B16 | LTR/Gypsy | 54 | 9260 |
| LTR-HVVMRXALLeA0273F01 | LTR/Copia | 11 | 2517 |
| LTR-HVVMRXALLeA0273I21 | LTR | 42 | 5658 |
| LTR-HVVMRXALLeA0274B17 | LTR/Gypsy | 19 | 2312 |
| LTR-HVVMRXALLeA0275O19 | LTR | 57 | 8391 |
| LTR-HVVMRXALLeA0280C24 | LTR | 29 | 5662 |
| LTR-HVVMRXALLeA0280G01 | LTR/Gypsy | 42 | 7142 |
| LTR-HVVMRXALLeA0282G01 | LTR/Copia | 2 | 208 |
| LTR-HVVMRXALLeA0282M17 | LTR/Copia | 16 | 2550 |
| LTR-HVVMRXALLeA0285O08 | LTR/Gypsy | 21 | 2724 |
| LTR-HVVMRXALLeA0288C01 | LTR/Gypsy | 398 | 52670 |
| LTR-HVVMRXALLeA0289L21 | LTR/Gypsy | 22 | 2562 |
| LTR-HVVMRXALLeA0289M17 | LTR | 77 | 11756 |

| | | | |
|---|---|---|---|
| LTR-HVVMRXALLeA0291F05 | LTR | 93 | 15680 |
| LTR-HVVMRXALLeA0292A23 | LTR/Gypsy | 75 | 10938 |
| LTR-HVVMRXALLeA0292H02 | LTR/Copia | 56 | 10233 |
| LTR-HVVMRXALLeA0293A01 | LTR | 9 | 778 |
| LTR-HVVMRXALLeA0293E13 | LTR/Copia | 5 | 606 |
| LTR-HVVMRXALLeA0296C01 | LTR/Copia | 2 | 471 |
| LTR-HVVMRXALLeA0296G01 | LTR/Gypsy | 42 | 6573 |
| LTR-HVVMRXALLeA0296M01 | LTR | 37 | 6657 |
| LTR-HVVMRXALLeA0296O01 | LTR/Gypsy | 9 | 963 |
| LTR-HVVMRXALLeA0297L06 | LTR | 79 | 12535 |
| LTR-HVVMRXALLeA0299L17 | LTR | 22 | 3598 |
| LTR-HVVMRXALLeA0312A01 | LTR | 54 | 8060 |
| LTR-HVVMRXALLeA0315D15 | LTR/Copia | 109 | 18843 |
| LTR-HVVMRXALLeA0315F15 | LTR | 83 | 13108 |
| LTR-HVVMRXALLeA0318G04 | LTR | 30 | 4143 |
| LTR-HVVMRXALLeA0320E06 | LTR/Gypsy | 139 | 21176 |
| LTR-HVVMRXALLeA0320E22 | LTR/Gypsy | 9 | 2529 |
| LTR-HVVMRXALLeA0320G01 | LTR/Gypsy | 13 | 2173 |
| LTR-HVVMRXALLeA0320K01 | LTR | 241 | 42696 |
| LTR-HVVMRXALLeA0322A20 | LTR | 292 | 32180 |
| LTR-HVVMRXALLeA0322D01 | LTR/Gypsy | 2 | 354 |
| LTR-HVVMRXALLeA0328A01 | LTR | 94 | 14046 |
| LTR-HVVMRXALLeA0328C01 | LTR/Gypsy | 99 | 14657 |
| LTR-HVVMRXALLeA0328E01 | LTR/Gypsy | 261 | 40011 |
| LTR-HVVMRXALLeA0328O01 | LTR | 210 | 31264 |
| LTR-HVVMRXALLeA0330H06 | LTR | 390 | 61776 |
| LTR-HVVMRXALLeA0332G10 | LTR/Gypsy | 50 | 7963 |
| LTR-HVVMRXALLeA0333N24 | LTR/Copia | 4 | 951 |
| LTR-HVVMRXALLeA0336G01 | LTR | 68 | 9644 |
| LTR-HVVMRXALLeA0336M01 | LTR/Gypsy | 16 | 2102 |
| LTR-HVVMRXALLeA0338I02 | LTR/Copia | 23 | 3243 |
| LTR-HVVMRXALLeA0342B05 | LTR | 82 | 15465 |
| LTR-HVVMRXALLeA0342F04 | LTR | 195 | 42634 |
| LTR-HVVMRXALLeA0342N20 | LTR/Gypsy | 26 | 2365 |
| LTR-HVVMRXALLeA0343C08 | LTR | 23 | 3946 |
| LTR-HVVMRXALLeA0344O01 | LTR/Copia | 24 | 3631 |
| LTR-HVVMRXALLeA0346J10 | LTR | 186 | 28650 |
| LTR-HVVMRXALLeA0349H05 | LTR | 51 | 7931 |
| LTR-HVVMRXALLeA0349N17 | LTR | 5 | 765 |
| LTR-HVVMRXALLeA0352K01 | LTR | 423 | 53674 |
| LTR-HVVMRXALLeA0355O03 | LTR | 197 | 26844 |
| LTR-HVVMRXALLeA0358A08 | LTR/Copia | 2 | 177 |
| LTR-HVVMRXALLeA0358C08 | LTR/Copia | 93 | 28369 |
| LTR-HVVMRXALLeA0358L18 | LTR | 98 | 16462 |
| LTR-HVVMRXALLeA0359I09 | LTR | 149 | 22776 |
| LTR-HVVMRXALLeA0361C21 | LTR/Copia | 7 | 1058 |
| LTR-HVVMRXALLeA0362L12 | LTR/Gypsy | 82 | 12590 |

| | | | |
|---|---|---|---|
| LTR-HVVMRXALLeA0364O13 | LTR/Copia | 21 | 2706 |
| LTR-HVVMRXALLeA0373C01 | LTR/Copia | 56 | 9754 |
| LTR-HVVMRXALLeA0378E13 | LTR/Copia | 3 | 552 |
| LTR-HVVMRXALLeA0379G22 | LTR | 523 | 62627 |
| LTR-HVVMRXALLeA0382A06 | LTR | 32 | 4791 |
| LTR-HVVMRXALLeA0382I21 | LTR | 76 | 10710 |
| LTR-HVVMRXALLeA0384G08 | LTR/Gypsy | 21 | 7905 |
| LTR-HVVMRXALLhA0001C16 | LTR | 26 | 4693 |
| LTR-HVVMRXALLhA0001E21 | LTR/Copia | 2 | 131 |
| LTR-HVVMRXALLhA0001G06 | LTR/Gypsy | 50 | 6332 |
| LTR-HVVMRXALLhA0004B16 | LTR | 466 | 65654 |
| LTR-HVVMRXALLhA0004J20 | LTR | 168 | 26261 |
| LTR-HVVMRXALLhA0006C12 | LTR/Copia | 22 | 2560 |
| LTR-HVVMRXALLhA0006E21 | LTR/Copia | 85 | 11049 |
| LTR-HVVMRXALLhA0006H18 | LTR | 6 | 1455 |
| LTR-HVVMRXALLhA0008G07 | LTR/Copia | 15 | 2424 |
| LTR-HVVMRXALLhA0009M04 | LTR/Gypsy | 144 | 19145 |
| LTR-HVVMRXALLhA0010L22 | LTR/Copia | 13 | 1737 |
| LTR-HVVMRXALLhA0010P16 | LTR/Copia | 6 | 639 |
| LTR-HVVMRXALLhA0012E19 | LTR/Copia | 29 | 5914 |
| LTR-HVVMRXALLhA0016L08 | LTR | 12 | 2324 |
| LTR-HVVMRXALLhA0022J09 | LTR | 81 | 12114 |
| LTR-HVVMRXALLhA0025N11 | LTR/Copia | 13 | 1857 |
| LTR-HVVMRXALLhA0026J15 | LTR/Copia | 67 | 10146 |
| LTR-HVVMRXALLhA0028G15 | LTR | 114 | 30774 |
| LTR-HVVMRXALLhA0029I02 | LTR/Copia | 21 | 2839 |
| LTR-HVVMRXALLhA0030P07 | LTR/Copia | 10 | 1342 |
| LTR-HVVMRXALLhA0031L21 | LTR | 155 | 29595 |
| LTR-HVVMRXALLhA0032F19 | LTR/Copia | 2 | 199 |
| LTR-HVVMRXALLhA0033D11 | LTR | 1101 | 134403 |
| LTR-HVVMRXALLhA0034L04 | LTR | 27 | 3858 |
| LTR-HVVMRXALLhA0036K08 | LTR/Copia | 32 | 4182 |
| LTR-HVVMRXALLhA0037F21 | LTR/Copia | 22 | 3171 |
| LTR-HVVMRXALLhA0040F06 | LTR | 39 | 4970 |
| LTR-HVVMRXALLhA0042K01 | LTR | 134 | 37960 |
| LTR-HVVMRXALLhA0043P04 | LTR/Copia | 15 | 2083 |
| LTR-HVVMRXALLhA0044B14 | LTR | 80 | 9045 |
| LTR-HVVMRXALLhA0044F04 | LTR/Copia | 8 | 1190 |
| LTR-HVVMRXALLhA0045G09 | LTR/Gypsy | 30 | 3976 |
| LTR-HVVMRXALLhA0045L09 | LTR | 256 | 25393 |
| LTR-HVVMRXALLhA0046E23 | LTR/Gypsy | 68 | 9197 |
| LTR-HVVMRXALLhA0046H04 | LTR/Copia | 6 | 984 |
| LTR-HVVMRXALLhA0046K06 | LTR | 304 | 41546 |
| LTR-HVVMRXALLhA0048C16 | LTR/Copia | 3 | 420 |
| LTR-HVVMRXALLhA0048D02 | LTR | 85 | 10371 |
| LTR-HVVMRXALLhA0049F03 | LTR | 44 | 6862 |
| LTR-HVVMRXALLhA0049H17 | LTR | 108 | 14251 |

| | | | |
|---|---|---|---|
| LTR-HVVMRXALLhA0049K10 | LTR/Gypsy | 292 | 50320 |
| LTR-HVVMRXALLhA0049K16 | LTR/Copia | 144 | 37690 |
| LTR-HVVMRXALLhA0049O17 | LTR/Copia | 19 | 1942 |
| LTR-HVVMRXALLhA0050I22 | LTR | 121 | 18384 |
| LTR-HVVMRXALLhA0051G21 | LTR/Copia | 3 | 743 |
| LTR-HVVMRXALLhA0052P22 | LTR | 4 | 625 |
| LTR-HVVMRXALLhA0053C09 | LTR/Copia | 92 | 15339 |
| LTR-HVVMRXALLhA0053M06 | LTR/Copia | 6 | 982 |
| LTR-HVVMRXALLhA0054E18 | LTR | 258 | 43540 |
| LTR-HVVMRXALLhA0055O11 | LTR | 43 | 8669 |
| LTR-HVVMRXALLhA0056B13 | LTR/Copia | 1 | 269 |
| LTR-HVVMRXALLhA0056L11 | LTR/Copia | 8 | 1142 |
| LTR-HVVMRXALLhA0057A13 | LTR/Copia | 6 | 649 |
| LTR-HVVMRXALLhA0057M08 | LTR/Copia | 2 | 228 |
| LTR-HVVMRXALLhA0058J13 | LTR | 67 | 9284 |
| LTR-HVVMRXALLhA0058K05 | LTR/Copia | 1 | 137 |
| LTR-HVVMRXALLhA0059A15 | LTR | 59 | 7074 |
| LTR-HVVMRXALLhA0060I16 | LTR | 105 | 15029 |
| LTR-HVVMRXALLhA0062B20 | LTR | 47 | 8189 |
| LTR-HVVMRXALLhA0066A11 | LTR/Copia | 3 | 263 |
| LTR-HVVMRXALLhA0067D13 | LTR/Gypsy | 52 | 9411 |
| LTR-HVVMRXALLhA0068A17 | LTR/Gypsy | 135 | 38166 |
| LTR-HVVMRXALLhA0069E04 | LTR | 247 | 39716 |
| LTR-HVVMRXALLhA0070C16 | LTR/Copia | 16 | 2710 |
| LTR-HVVMRXALLhA0070M16 | LTR/Copia | 1 | 109 |
| LTR-HVVMRXALLhA0072B09 | LTR | 65 | 10326 |
| LTR-HVVMRXALLhA0074D06 | LTR | 84 | 17757 |
| LTR-HVVMRXALLhA0075H17 | LTR | 79 | 8665 |
| LTR-HVVMRXALLhA0075I10 | LTR | 129 | 22935 |
| LTR-HVVMRXALLhA0075J11 | LTR | 58 | 7418 |
| LTR-HVVMRXALLhA0076J07 | LTR/Gypsy | 74 | 12349 |
| LTR-HVVMRXALLhA0076M06 | LTR/Gypsy | 146 | 21582 |
| LTR-HVVMRXALLhA0077G20 | LTR | 24 | 3470 |
| LTR-HVVMRXALLhA0077M08 | LTR/Copia | 33 | 5787 |
| LTR-HVVMRXALLhA0078K03 | LTR/Gypsy | 7 | 1276 |
| LTR-HVVMRXALLhA0079C23 | LTR | 245 | 73209 |
| LTR-HVVMRXALLhA0079L17 | LTR | 234 | 48004 |
| LTR-HVVMRXALLhA0080H05 | LTR/Gypsy | 8 | 1454 |
| LTR-HVVMRXALLhA0081D16 | LTR/Copia | 2 | 555 |
| LTR-HVVMRXALLhA0081F11 | LTR/Copia | 3 | 1418 |
| LTR-HVVMRXALLhA0085C18 | LTR | 15 | 2351 |
| LTR-HVVMRXALLhA0085O13 | LTR/Copia | 6 | 498 |
| LTR-HVVMRXALLhA0086N02 | LTR | 1 | 132 |
| LTR-HVVMRXALLhA0088M06 | LTR | 33 | 4535 |
| LTR-HVVMRXALLhA0090F09 | LTR/Copia | 12 | 1250 |
| LTR-HVVMRXALLhA0090I21 | LTR/Copia | 2 | 142 |
| LTR-HVVMRXALLhA0092F20 | LTR | 73 | 12517 |

| | | | |
|---|---|---|---|
| LTR-HVVMRXALLhA0093L13 | LTR | 90 | 12781 |
| LTR-HVVMRXALLhA0093P13 | LTR | 100 | 15291 |
| LTR-HVVMRXALLhA0094H12 | LTR | 36 | 4096 |
| LTR-HVVMRXALLhA0094K08 | LTR | 62 | 12233 |
| LTR-HVVMRXALLhA0095F14 | LTR/Gypsy | 56 | 8926 |
| LTR-HVVMRXALLhA0097I02 | LTR | 131 | 18974 |
| LTR-HVVMRXALLhA0099G23 | LTR/Copia | 3 | 451 |
| LTR-HVVMRXALLhA0100G24 | LTR/Gypsy | 5 | 960 |
| LTR-HVVMRXALLhA0100P08 | LTR/Copia | 1 | 112 |
| LTR-HVVMRXALLhA0107B18 | LTR/Gypsy | 5 | 683 |
| LTR-HVVMRXALLhA0107I03 | LTR | 446 | 89022 |
| LTR-HVVMRXALLhA0110P04 | LTR/Gypsy | 110 | 17514 |
| LTR-HVVMRXALLhA0112C01 | LTR | 3 | 319 |
| LTR-HVVMRXALLhA0113O23 | LTR | 23 | 6804 |
| LTR-HVVMRXALLhA0113P23 | LTR | 158 | 19815 |
| LTR-HVVMRXALLhA0114I01 | LTR | 23 | 3304 |
| LTR-HVVMRXALLhA0119G03 | LTR/Gypsy | 37 | 5103 |
| LTR-HVVMRXALLhA0119I01 | LTR/Gypsy | 23 | 2478 |
| LTR-HVVMRXALLhA0123E09 | LTR | 38 | 5747 |
| LTR-HVVMRXALLhA0125H11 | LTR/Copia | 3 | 601 |
| LTR-HVVMRXALLhA0125P05 | LTR | 1026 | 151490 |
| LTR-HVVMRXALLhA0127H11 | LTR/Copia | 8 | 1176 |
| LTR-HVVMRXALLhA0128C22 | LTR | 79 | 12345 |
| LTR-HVVMRXALLhA0130C07 | LTR/Copia | 3 | 463 |
| LTR-HVVMRXALLhA0131C23 | LTR/Gypsy | 54 | 10159 |
| LTR-HVVMRXALLhA0131J02 | LTR/Copia | 46 | 5153 |
| LTR-HVVMRXALLhA0133A21 | LTR | 66 | 9603 |
| LTR-HVVMRXALLhA0135B11 | LTR | 49 | 7343 |
| LTR-HVVMRXALLhA0135D02 | LTR | 112 | 13932 |
| LTR-HVVMRXALLhA0135I04 | LTR | 473 | 69895 |
| LTR-HVVMRXALLhA0136D16 | LTR | 7 | 1266 |
| LTR-HVVMRXALLhA0136J06 | LTR/Copia | 8 | 1514 |
| LTR-HVVMRXALLhA0137K14 | LTR | 442 | 61421 |
| LTR-HVVMRXALLhA0138C03 | LTR/Gypsy | 3 | 788 |
| LTR-HVVMRXALLhA0138D15 | LTR/Copia | 29 | 4802 |
| LTR-HVVMRXALLhA0138L06 | LTR/Gypsy | 489 | 127381 |
| LTR-HVVMRXALLhA0141I09 | LTR | 3 | 560 |
| LTR-HVVMRXALLhA0141K03 | LTR | 749 | 148578 |
| LTR-HVVMRXALLhA0142A22 | LTR | 9 | 1830 |
| LTR-HVVMRXALLhA0142F09 | LTR/Gypsy | 10 | 1608 |
| LTR-HVVMRXALLhA0142M19 | LTR | 68 | 8803 |
| LTR-HVVMRXALLhA0143D02 | LTR/Copia | 6 | 746 |
| LTR-HVVMRXALLhA0144D15 | LTR | 1054 | 148997 |
| LTR-HVVMRXALLhA0144J02 | LTR | 183 | 24895 |
| LTR-HVVMRXALLhA0145N01 | LTR | 44 | 8960 |
| LTR-HVVMRXALLhA0146L24 | LTR | 1145 | 141228 |
| LTR-HVVMRXALLhA0147A04 | LTR/Gypsy | 13 | 1742 |

| | | | |
|---|---|---|---|
| LTR-HVVMRXALLhA0147D21 | LTR | 244 | 45092 |
| LTR-HVVMRXALLhA0148E17 | LTR | 24 | 5246 |
| LTR-HVVMRXALLhA0149F13 | LTR | 604 | 98191 |
| LTR-HVVMRXALLhA0151K05 | LTR | 48 | 11417 |
| LTR-HVVMRXALLhA0156D07 | LTR/Gypsy | 13 | 2346 |
| LTR-HVVMRXALLhA0157G20 | LTR | 57 | 11179 |
| LTR-HVVMRXALLhA0160F22 | LTR | 179 | 26492 |
| LTR-HVVMRXALLhA0160M05 | LTR | 25 | 3376 |
| LTR-HVVMRXALLhA0161J01 | LTR/Copia | 6 | 751 |
| LTR-HVVMRXALLhA0162L20 | LTR/Copia | 13 | 1788 |
| LTR-HVVMRXALLhA0163D24 | LTR | 21 | 2465 |
| LTR-HVVMRXALLhA0163E15 | LTR/Copia | 1 | 471 |
| LTR-HVVMRXALLhA0163F12 | LTR | 282 | 32384 |
| LTR-HVVMRXALLhA0164G23 | LTR/Gypsy | 16 | 2016 |
| LTR-HVVMRXALLhA0165G19 | LTR/Copia | 544 | 67091 |
| LTR-HVVMRXALLhA0167K14 | LTR | 281 | 45518 |
| LTR-HVVMRXALLhA0168L19 | LTR | 63 | 16976 |
| LTR-HVVMRXALLhA0174B19 | LTR | 376 | 53232 |
| LTR-HVVMRXALLhA0174C10 | LTR | 38 | 6155 |
| LTR-HVVMRXALLhA0175F21 | LTR/Copia | 122 | 23001 |
| LTR-HVVMRXALLhA0176N06 | LTR/Copia | 29 | 4220 |
| LTR-HVVMRXALLhA0178I23 | LTR | 84 | 11533 |
| LTR-HVVMRXALLhA0178J06 | LTR | 62 | 8813 |
| LTR-HVVMRXALLhA0183I03 | LTR/Copia | 31 | 7826 |
| LTR-HVVMRXALLhA0183N14 | LTR/Copia | 3 | 1071 |
| LTR-HVVMRXALLhA0184G09 | LTR/Copia | 232 | 54687 |
| LTR-HVVMRXALLhA0184H13 | LTR/Copia | 6 | 645 |
| LTR-HVVMRXALLhA0184I13 | LTR/Copia | 9 | 1182 |
| LTR-HVVMRXALLhA0184J07 | LTR/Gypsy | 507 | 76313 |
| LTR-HVVMRXALLhA0185D04 | LTR/Gypsy | 28 | 4142 |
| LTR-HVVMRXALLhA0186C02 | LTR/Copia | 1 | 149 |
| LTR-HVVMRXALLhA0187I24 | LTR/Copia | 70 | 13512 |
| LTR-HVVMRXALLhA0187N01 | LTR/Copia | 5 | 1327 |
| LTR-HVVMRXALLhA0187P12 | LTR/Gypsy | 149 | 20796 |
| LTR-HVVMRXALLhA0188M04 | LTR | 706 | 118459 |
| LTR-HVVMRXALLhA0188N02 | LTR | 590 | 74633 |
| LTR-HVVMRXALLhA0190B08 | LTR/Gypsy | 148 | 23114 |
| LTR-HVVMRXALLhA0190L20 | LTR/Copia | 1 | 36 |
| LTR-HVVMRXALLhA0191E18 | LTR/Gypsy | 96 | 14290 |
| LTR-HVVMRXALLhA0194O15 | LTR/Copia | 10 | 1123 |
| LTR-HVVMRXALLhA0195N02 | LTR/Copia | 104 | 24944 |
| LTR-HVVMRXALLhA0196I10 | LTR | 38 | 4980 |
| LTR-HVVMRXALLhA0198M14 | LTR | 39 | 8215 |
| LTR-HVVMRXALLhA0204M14 | LTR | 212 | 26444 |
| LTR-HVVMRXALLhA0205M01 | LTR | 775 | 105119 |
| LTR-HVVMRXALLhA0205O11 | LTR/Copia | 3 | 309 |
| LTR-HVVMRXALLhA0206N16 | LTR/Gypsy | 101 | 14299 |

| | | | |
|---|---|---|---|
| LTR-HVVMRXALLhA0207K08 | LTR/Gypsy | 181 | 27630 |
| LTR-HVVMRXALLhA0208P16 | LTR | 4 | 728 |
| LTR-HVVMRXALLhA0210H06 | LTR/Copia | 23 | 2757 |
| LTR-HVVMRXALLhA0213G22 | LTR/Gypsy | 8 | 671 |
| LTR-HVVMRXALLhA0213K12 | LTR | 40 | 4954 |
| LTR-HVVMRXALLhA0215B11 | LTR/Gypsy | 20 | 2466 |
| LTR-HVVMRXALLhA0215G19 | LTR/Gypsy | 124 | 19127 |
| LTR-HVVMRXALLhA0217B18 | LTR | 1256 | 178206 |
| LTR-HVVMRXALLhA0217B21 | LTR | 26 | 4431 |
| LTR-HVVMRXALLhA0221J01 | LTR/Copia | 3 | 669 |
| LTR-HVVMRXALLhA0221P08 | LTR/Gypsy | 43 | 7269 |
| LTR-HVVMRXALLhA0222G02 | LTR/Copia | 5 | 1313 |
| LTR-HVVMRXALLhA0222O19 | LTR | 25 | 4778 |
| LTR-HVVMRXALLhA0225C23 | LTR | 233 | 45595 |
| LTR-HVVMRXALLhA0228P05 | LTR | 48 | 6960 |
| LTR-HVVMRXALLhA0231A14 | LTR | 372 | 51540 |
| LTR-HVVMRXALLhA0232C02 | LTR | 63 | 13561 |
| LTR-HVVMRXALLhA0232C09 | LTR/Copia | 4 | 1154 |
| LTR-HVVMRXALLhA0233A09 | LTR/Copia | 3 | 602 |
| LTR-HVVMRXALLhA0233B04 | LTR | 91 | 13395 |
| LTR-HVVMRXALLhA0234B19 | LTR | 115 | 16967 |
| LTR-HVVMRXALLhA0234L21 | LTR/Copia | 6 | 454 |
| LTR-HVVMRXALLhA0235B04 | LTR/Copia | 35 | 7146 |
| LTR-HVVMRXALLhA0236H15 | LTR | 38 | 5757 |
| LTR-HVVMRXALLhA0236I13 | LTR/Copia | 73 | 19843 |
| LTR-HVVMRXALLhA0236P01 | LTR/Gypsy | 70 | 10813 |
| LTR-HVVMRXALLhA0237E09 | LTR | 16 | 3544 |
| LTR-HVVMRXALLhA0238A04 | LTR/Gypsy | 54 | 7253 |
| LTR-HVVMRXALLhA0238O03 | LTR/Copia | 129 | 22522 |
| LTR-HVVMRXALLhA0239A20 | LTR | 74 | 11758 |
| LTR-HVVMRXALLhA0241D11 | LTR/Copia | 33 | 4182 |
| LTR-HVVMRXALLhA0241M03 | LTR/Copia | 12 | 1476 |
| LTR-HVVMRXALLhA0242A23 | LTR/Gypsy | 88 | 11016 |
| LTR-HVVMRXALLhA0242H05 | LTR/Gypsy | 70 | 11373 |
| LTR-HVVMRXALLhA0242L08 | LTR/Copia | 22 | 5187 |
| LTR-HVVMRXALLhA0242L10 | LTR | 39 | 6623 |
| LTR-HVVMRXALLhA0244G12 | LTR/Copia | 166 | 23578 |
| LTR-HVVMRXALLhA0246O12 | LTR/Gypsy | 7 | 1492 |
| LTR-HVVMRXALLhA0248B24 | LTR/Copia | 34 | 6922 |
| LTR-HVVMRXALLhA0249D06 | LTR/Copia | 5 | 615 |
| LTR-HVVMRXALLhA0251I08 | LTR/Copia | 14 | 1374 |
| LTR-HVVMRXALLhA0252H16 | LTR | 57 | 8234 |
| LTR-HVVMRXALLhA0252J04 | LTR/Copia | 4 | 943 |
| LTR-HVVMRXALLhA0253E22 | LTR/Gypsy | 146 | 21447 |
| LTR-HVVMRXALLhA0254C19 | LTR | 141 | 24383 |
| LTR-HVVMRXALLhA0258C12 | LTR | 30 | 5349 |
| LTR-HVVMRXALLhA0258H12 | LTR/Copia | 2 | 403 |

| | | | |
|---|---|---|---|
| LTR-HVVMRXALLhA0259H19 | LTR | 105 | 19102 |
| LTR-HVVMRXALLhA0260M22 | LTR/Gypsy | 45 | 7550 |
| LTR-HVVMRXALLhA0260O24 | LTR | 92 | 11752 |
| LTR-HVVMRXALLhA0260P20 | LTR | 426 | 72645 |
| LTR-HVVMRXALLhA0261D13 | LTR | 22 | 4414 |
| LTR-HVVMRXALLhA0261M10 | LTR | 94 | 15826 |
| LTR-HVVMRXALLhA0262H03 | LTR/Copia | 55 | 10864 |
| LTR-HVVMRXALLhA0262I17 | LTR/Copia | 10 | 1141 |
| LTR-HVVMRXALLhA0264C01 | LTR/Copia | 555 | 122973 |
| LTR-HVVMRXALLhA0264P11 | LTR | 1368 | 261405 |
| LTR-HVVMRXALLhA0265N11 | LTR/Copia | 27 | 4450 |
| LTR-HVVMRXALLhA0266G02 | LTR/Copia | 99 | 30119 |
| LTR-HVVMRXALLhA0266M10 | LTR | 808 | 132782 |
| LTR-HVVMRXALLhA0268E23 | LTR/Copia | 11 | 2721 |
| LTR-HVVMRXALLhA0270D18 | LTR/Gypsy | 3227 | 490965 |
| LTR-HVVMRXALLhA0270N14 | LTR/Copia | 47 | 5932 |
| LTR-HVVMRXALLhA0271J23 | LTR | 25 | 4712 |
| LTR-HVVMRXALLhA0272H15 | LTR | 956 | 140958 |
| LTR-HVVMRXALLhA0272H22 | LTR | 62 | 8981 |
| LTR-HVVMRXALLhA0273B11 | LTR | 25 | 3164 |
| LTR-HVVMRXALLhA0274E14 | LTR | 12 | 2410 |
| LTR-HVVMRXALLhA0276C19 | LTR/Copia | 91 | 19683 |
| LTR-HVVMRXALLhA0277D24 | LTR/Gypsy | 559 | 60968 |
| LTR-HVVMRXALLhA0277J13 | LTR | 144 | 21612 |
| LTR-HVVMRXALLhA0277K16 | LTR | 28 | 4820 |
| LTR-HVVMRXALLhA0277O06 | LTR/Copia | 2 | 698 |
| LTR-HVVMRXALLhA0278H23 | LTR | 15 | 1567 |
| LTR-HVVMRXALLhA0279E13 | LTR | 7 | 1044 |
| LTR-HVVMRXALLhA0280G12 | LTR | 53 | 9078 |
| LTR-HVVMRXALLhA0280L05 | LTR | 8 | 1044 |
| LTR-HVVMRXALLhA0281J18 | LTR | 185 | 21348 |
| LTR-HVVMRXALLhA0282C14 | LTR | 12 | 1960 |
| LTR-HVVMRXALLhA0282N24 | LTR/Gypsy | 52 | 6831 |
| LTR-HVVMRXALLhA0283C03 | LTR | 16 | 3011 |
| LTR-HVVMRXALLhA0284I17 | LTR | 198 | 38586 |
| LTR-HVVMRXALLhA0286J08 | LTR/Copia | 30 | 7203 |
| LTR-HVVMRXALLhA0287J10 | LTR | 201 | 42429 |
| LTR-HVVMRXALLhA0288A11 | LTR/Copia | 4 | 582 |
| LTR-HVVMRXALLhA0288B17 | LTR/Gypsy | 58 | 9331 |
| LTR-HVVMRXALLhA0288N04 | LTR/Copia | 4 | 798 |
| LTR-HVVMRXALLhA0292C12 | LTR/Copia | 33 | 4714 |
| LTR-HVVMRXALLhA0292K18 | LTR/Copia | 221 | 30128 |
| LTR-HVVMRXALLhA0293B08 | LTR | 41 | 5452 |
| LTR-HVVMRXALLhA0293C17 | LTR | 453 | 78954 |
| LTR-HVVMRXALLhA0294A16 | LTR/Copia | 38 | 4959 |
| LTR-HVVMRXALLhA0294D24 | LTR/Copia | 47 | 10434 |
| LTR-HVVMRXALLhA0294E23 | LTR/Copia | 3 | 721 |

| | | | |
|---|---|---|---|
| LTR-HVVMRXALLhA0294I09 | LTR/Copia | 1 | 58 |
| LTR-HVVMRXALLhA0294J14 | LTR | 75 | 11569 |
| LTR-HVVMRXALLhA0295J13 | LTR/Copia | 3 | 687 |
| LTR-HVVMRXALLhA0295N13 | LTR | 151 | 22165 |
| LTR-HVVMRXALLhA0296A10 | LTR | 69 | 9443 |
| LTR-HVVMRXALLhA0296C08 | LTR/Gypsy | 63 | 9554 |
| LTR-HVVMRXALLhA0299G07 | LTR | 169 | 32822 |
| LTR-HVVMRXALLhA0299J10 | LTR | 183 | 31519 |
| LTR-HVVMRXALLhA0300K24 | LTR/Copia | 2 | 298 |
| LTR-HVVMRXALLhA0300O21 | LTR | 5 | 588 |
| LTR-HVVMRXALLhA0301D09 | LTR/Copia | 39 | 10907 |
| LTR-HVVMRXALLhA0302M05 | LTR | 522 | 76860 |
| LTR-HVVMRXALLhA0303B04 | LTR/Gypsy | 79 | 13537 |
| LTR-HVVMRXALLhA0305B18 | LTR | 152 | 20548 |
| LTR-HVVMRXALLhA0305G07 | LTR | 1216 | 166987 |
| LTR-HVVMRXALLhA0306E02 | LTR | 38 | 8641 |
| LTR-HVVMRXALLhA0306G21 | LTR/Copia | 1 | 86 |
| LTR-HVVMRXALLhA0306N10 | LTR/Gypsy | 13 | 2093 |
| LTR-HVVMRXALLhA0307G15 | LTR/Gypsy | 8 | 1758 |
| LTR-HVVMRXALLhA0308F17 | LTR/Gypsy | 30 | 4463 |
| LTR-HVVMRXALLhA0309K16 | LTR/Gypsy | 9 | 1082 |
| LTR-HVVMRXALLhA0311A16 | LTR/Copia | 11 | 3502 |
| LTR-HVVMRXALLhA0311J16 | LTR | 2091 | 319255 |
| LTR-HVVMRXALLhA0311K12 | LTR | 430 | 57001 |
| LTR-HVVMRXALLhA0311P23 | LTR | 27 | 3289 |
| LTR-HVVMRXALLhA0312H11 | LTR | 43 | 6069 |
| LTR-HVVMRXALLhA0312K12 | LTR/Gypsy | 66 | 15700 |
| LTR-HVVMRXALLhA0314G16 | LTR/Copia | 32 | 4624 |
| LTR-HVVMRXALLhA0315A01 | LTR/Gypsy | 3 | 329 |
| LTR-HVVMRXALLhA0317P07 | LTR/Copia | 9 | 3552 |
| LTR-HVVMRXALLhA0318G23 | LTR/Gypsy | 149 | 33545 |
| LTR-HVVMRXALLhA0319O19 | LTR | 50 | 6528 |
| LTR-HVVMRXALLhA0320J11 | LTR | 55 | 9309 |
| LTR-HVVMRXALLhA0321D08 | LTR/Copia | 16 | 4173 |
| LTR-HVVMRXALLhA0321I14 | LTR | 133 | 20377 |
| LTR-HVVMRXALLhA0322B05 | LTR | 101 | 13182 |
| LTR-HVVMRXALLhA0322F15 | LTR/Copia | 2 | 583 |
| LTR-HVVMRXALLhA0323F22 | LTR | 13 | 2080 |
| LTR-HVVMRXALLhA0325M13 | LTR | 2 | 275 |
| LTR-HVVMRXALLhA0325N20 | LTR | 155 | 25171 |
| LTR-HVVMRXALLhA0326J12 | LTR/Gypsy | 288 | 35275 |
| LTR-HVVMRXALLhA0328A10 | LTR/Copia | 4 | 657 |
| LTR-HVVMRXALLhA0328E19 | LTR/Gypsy | 122 | 17276 |
| LTR-HVVMRXALLhA0328I07 | LTR/Gypsy | 15 | 3004 |
| LTR-HVVMRXALLhA0328L03 | LTR/Gypsy | 13 | 1372 |
| LTR-HVVMRXALLhA0329D17 | LTR/Copia | 30 | 5808 |
| LTR-HVVMRXALLhA0329E13 | LTR | 954 | 144126 |

| | | | |
|---|---|---|---|
| LTR-HVVMRXALLhA0329F14 | LTR | 37 | 5579 |
| LTR-HVVMRXALLhA0330H14 | LTR | 1 | 86 |
| LTR-HVVMRXALLhA0332A16 | LTR/Gypsy | 9 | 945 |
| LTR-HVVMRXALLhA0332M12 | LTR | 14 | 1700 |
| LTR-HVVMRXALLhA0333D13 | LTR/Gypsy | 10 | 1787 |
| LTR-HVVMRXALLhA0333E20 | LTR/Copia | 3 | 324 |
| LTR-HVVMRXALLhA0334B21 | LTR/Copia | 30 | 6687 |
| LTR-HVVMRXALLhA0334I01 | LTR | 56 | 10332 |
| LTR-HVVMRXALLhA0335G24 | LTR | 15 | 1824 |
| LTR-HVVMRXALLhA0335I03 | LTR | 58 | 8411 |
| LTR-HVVMRXALLhA0335I17 | LTR | 92 | 17454 |
| LTR-HVVMRXALLhA0335M19 | LTR | 32 | 5454 |
| LTR-HVVMRXALLhA0336A03 | LTR/Copia | 2 | 346 |
| LTR-HVVMRXALLhA0336I01 | LTR/Copia | 4 | 545 |
| LTR-HVVMRXALLhA0336I24 | LTR | 83 | 13324 |
| LTR-HVVMRXALLhA0337E06 | LTR/Gypsy | 43 | 5584 |
| LTR-HVVMRXALLhA0337F14 | LTR | 350 | 56392 |
| LTR-HVVMRXALLhA0337J01 | LTR | 491 | 76443 |
| LTR-HVVMRXALLhA0338C15 | LTR | 129 | 21047 |
| LTR-HVVMRXALLhA0340A23 | LTR/Copia | 2 | 326 |
| LTR-HVVMRXALLhA0341G04 | LTR/Copia | 10 | 1786 |
| LTR-HVVMRXALLhA0341G11 | LTR | 1084 | 137568 |
| LTR-HVVMRXALLhA0341I06 | LTR/Copia | 1 | 55 |
| LTR-HVVMRXALLhA0341N04 | LTR/Copia | 1 | 103 |
| LTR-HVVMRXALLhA0342P10 | LTR/Copia | 5 | 679 |
| LTR-HVVMRXALLhA0343C05 | LTR | 14 | 2658 |
| LTR-HVVMRXALLhA0346F01 | LTR | 70 | 10326 |
| LTR-HVVMRXALLhA0346K08 | LTR/Copia | 3 | 379 |
| LTR-HVVMRXALLhA0346N18 | LTR/Gypsy | 7 | 773 |
| LTR-HVVMRXALLhA0347F16 | LTR | 8 | 985 |
| LTR-HVVMRXALLhA0348A09 | LTR/Copia | 1 | 163 |
| LTR-HVVMRXALLhA0348M23 | LTR/Copia | 19 | 2451 |
| LTR-HVVMRXALLhA0349F15 | LTR | 233 | 58444 |
| LTR-HVVMRXALLhA0349H04 | LTR/Copia | 3 | 651 |
| LTR-HVVMRXALLhA0351L23 | LTR | 46 | 5169 |
| LTR-HVVMRXALLhA0351O20 | LTR | 166 | 24895 |
| LTR-HVVMRXALLhA0352I19 | LTR | 1240 | 174282 |
| LTR-HVVMRXALLhA0353D24 | LTR | 186 | 26668 |
| LTR-HVVMRXALLhA0353M18 | LTR/Gypsy | 26 | 4382 |
| LTR-HVVMRXALLhA0354F03 | LTR/Gypsy | 53 | 7611 |
| LTR-HVVMRXALLhA0355K12 | LTR | 236 | 29329 |
| LTR-HVVMRXALLhA0356F14 | LTR/Gypsy | 22 | 3736 |
| LTR-HVVMRXALLhA0357B07 | LTR/Gypsy | 24 | 5043 |
| LTR-HVVMRXALLhA0357G07 | LTR | 63 | 8395 |
| LTR-HVVMRXALLhA0357M01 | LTR/Gypsy | 59 | 8435 |
| LTR-HVVMRXALLhA0357M23 | LTR | 42 | 6321 |
| LTR-HVVMRXALLhA0357N15 | LTR | 18 | 3255 |

| | | | |
|---|---|---|---|
| LTR-HVVMRXALLhA0359A01 | LTR | 395 | 97559 |
| LTR-HVVMRXALLhA0360O22 | LTR | 173 | 24217 |
| LTR-HVVMRXALLhA0361E15 | LTR | 8 | 1068 |
| LTR-HVVMRXALLhA0361M02 | LTR | 101 | 15801 |
| LTR-HVVMRXALLhA0362K14 | LTR/Gypsy | 6 | 1207 |
| LTR-HVVMRXALLhA0362M12 | LTR/Gypsy | 581 | 55860 |
| LTR-HVVMRXALLhA0363N16 | LTR/Copia | 15 | 1359 |
| LTR-HVVMRXALLhA0364C07 | LTR | 37 | 6019 |
| LTR-HVVMRXALLhA0365M22 | LTR/Gypsy | 84 | 18634 |
| LTR-HVVMRXALLhA0366C06 | LTR/Copia | 5 | 2051 |
| LTR-HVVMRXALLhA0366H14 | LTR/Copia | 9 | 1447 |
| LTR-HVVMRXALLhA0366I07 | LTR/Gypsy | 9 | 1584 |
| LTR-HVVMRXALLhA0367M09 | LTR | 52 | 8883 |
| LTR-HVVMRXALLhA0367P10 | LTR/Copia | 9 | 1080 |
| LTR-HVVMRXALLhA0368B18 | LTR/Gypsy | 39 | 5822 |
| LTR-HVVMRXALLhA0368H23 | LTR | 26 | 5299 |
| LTR-HVVMRXALLhA0368P10 | LTR/Copia | 3 | 499 |
| LTR-HVVMRXALLhA0369B03 | LTR/Copia | 2 | 380 |
| LTR-HVVMRXALLhA0369B05 | LTR/Copia | 20 | 3463 |
| LTR-HVVMRXALLhA0369I04 | LTR/Copia | 13 | 2462 |
| LTR-HVVMRXALLhA0371B04 | LTR/Copia | 2 | 426 |
| LTR-HVVMRXALLhA0371F13 | LTR | 58 | 9184 |
| LTR-HVVMRXALLhA0372F17 | LTR/Copia | 6 | 787 |
| LTR-HVVMRXALLhA0373H12 | LTR/Copia | 3 | 514 |
| LTR-HVVMRXALLhA0373P07 | LTR | 68 | 9027 |
| LTR-HVVMRXALLhA0374E20 | LTR | 56 | 8627 |
| LTR-HVVMRXALLhA0374O19 | LTR/Copia | 9 | 1197 |
| LTR-HVVMRXALLhA0376B03 | LTR | 51 | 6478 |
| LTR-HVVMRXALLhA0376E12 | LTR | 931 | 114163 |
| LTR-HVVMRXALLhA0376H03 | LTR/Gypsy | 5 | 669 |
| LTR-HVVMRXALLhA0376O20 | LTR | 155 | 20058 |
| LTR-HVVMRXALLhA0377G09 | LTR/Copia | 21 | 3048 |
| LTR-HVVMRXALLhA0378E10 | LTR/Gypsy | 1 | 155 |
| LTR-HVVMRXALLhA0379G06 | LTR/Copia | 34 | 5861 |
| LTR-HVVMRXALLhA0380C18 | LTR | 10 | 1471 |
| LTR-HVVMRXALLhA0380G06 | LTR | 2 | 304 |
| LTR-HVVMRXALLhA0380P17 | LTR | 6 | 666 |
| LTR-HVVMRXALLhA0381B07 | LTR | 36 | 5037 |
| LTR-HVVMRXALLhA0383F01 | LTR/Copia | 24 | 3620 |
| LTR-HVVMRXALLhA0384D01 | LTR | 131 | 17357 |
| LTR-HVVMRXALLhA0384J03 | LTR | 11 | 1800 |
| LTR-HVVMRXALLhA0384L13 | LTR/Copia | 595 | 111837 |
| LTR-HVVMRXALLhA0384L20 | LTR | 4 | 684 |
| LTR-HVVMRXALLhA0384P11 | LTR | 3 | 184 |
| LTR-HVVMRXALLhA0385H13 | LTR | 28 | 4155 |
| LTR-HVVMRXALLhA0385H21 | LTR | 85 | 12265 |
| LTR-HVVMRXALLhA0387A11 | LTR/Copia | 1 | 294 |

| | | | |
|---|---|---|---|
| LTR-HVVMRXALLhA0389H01 | LTR/Copia | 2 | 233 |
| LTR-HVVMRXALLhA0390H12 | LTR | 111 | 16572 |
| LTR-HVVMRXALLhA0390L10 | LTR/Copia | 26 | 5765 |
| LTR-HVVMRXALLhA0391B15 | LTR/Copia | 47 | 7025 |
| LTR-HVVMRXALLhA0391B23 | LTR | 37 | 4722 |
| LTR-HVVMRXALLhA0391F17 | LTR/Gypsy | 114 | 17463 |
| LTR-HVVMRXALLhA0391L17 | LTR | 204 | 25809 |
| LTR-HVVMRXALLhA0393E04 | LTR/Copia | 41 | 5698 |
| LTR-HVVMRXALLhA0394F21 | LTR/Copia | 12 | 479 |
| LTR-HVVMRXALLhA0394G18 | LTR/Gypsy | 177 | 24874 |
| LTR-HVVMRXALLhA0395I13 | LTR/Copia | 11 | 3615 |
| LTR-HVVMRXALLhA0395K20 | LTR | 154 | 19335 |
| LTR-HVVMRXALLhA0397D22 | LTR/Copia | 11 | 1105 |
| LTR-HVVMRXALLhA0397J24 | LTR/Gypsy | 54 | 7803 |
| LTR-HVVMRXALLhA0398K23 | LTR/Copia | 31 | 4666 |
| LTR-HVVMRXALLhA0402E14 | LTR/Gypsy | 360 | 63485 |
| LTR-HVVMRXALLhA0403G01 | LTR/Copia | 73 | 18889 |
| LTR-HVVMRXALLhA0406E01 | LTR | 9 | 1298 |
| LTR-HVVMRXALLhA0406M11 | LTR/Gypsy | 18 | 2442 |
| LTR-HVVMRXALLhA0408H14 | LTR | 106 | 14932 |
| LTR-HVVMRXALLhA0409M08 | LTR | 3 | 523 |
| LTR-HVVMRXALLhA0410G06 | LTR/Gypsy | 136 | 22352 |
| LTR-HVVMRXALLhA0411E05 | LTR | 48 | 8683 |
| LTR-HVVMRXALLhA0412K18 | LTR | 89 | 12981 |
| LTR-HVVMRXALLhA0413N13 | LTR | 17 | 3609 |
| LTR-HVVMRXALLhA0414M21 | LTR | 30 | 4901 |
| LTR-HVVMRXALLhA0415E05 | LTR/Copia | 112 | 16200 |
| LTR-HVVMRXALLhA0416J09 | LTR | 479 | 77402 |
| LTR-HVVMRXALLhA0416L06 | LTR/Gypsy | 8 | 1186 |
| LTR-HVVMRXALLhA0418F22 | LTR | 43 | 7155 |
| LTR-HVVMRXALLhA0418H24 | LTR | 26 | 4046 |
| LTR-HVVMRXALLhA0418J08 | LTR/Gypsy | 69 | 10686 |
| LTR-HVVMRXALLhA0420J19 | LTR/Gypsy | 133 | 21851 |
| LTR-HVVMRXALLhA0422J04 | LTR/Copia | 36 | 8025 |
| LTR-HVVMRXALLhA0424G19 | LTR/Copia | 1 | 113 |
| LTR-HVVMRXALLhA0424O24 | LTR/Copia | 24 | 3938 |
| LTR-HVVMRXALLhA0425O23 | LTR | 172 | 16846 |
| LTR-HVVMRXALLhA0426B23 | LTR | 14 | 2714 |
| LTR-HVVMRXALLhA0426C16 | LTR | 42 | 7554 |
| LTR-HVVMRXALLhA0426I24 | LTR/Copia | 8 | 1347 |
| LTR-HVVMRXALLhA0426O05 | LTR | 111 | 18339 |
| LTR-HVVMRXALLhA0427B06 | LTR | 273 | 44011 |
| LTR-HVVMRXALLhA0429C22 | LTR/Gypsy | 8 | 735 |
| LTR-HVVMRXALLhA0430D14 | LTR | 9 | 912 |
| LTR-HVVMRXALLhA0431A06 | LTR | 25 | 5170 |
| LTR-HVVMRXALLhA0431D12 | LTR/Gypsy | 2 | 425 |
| LTR-HVVMRXALLhA0432B18 | LTR | 44 | 7304 |

| | | | |
|---|---|---|---|
| LTR-HVVMRXALLhA0434A01 | LTR/Copia | 2 | 397 |
| LTR-HVVMRXALLhA0435A22 | LTR | 9 | 980 |
| LTR-HVVMRXALLhA0435B24 | LTR/Copia | 11 | 1127 |
| LTR-HVVMRXALLhA0437K03 | LTR/Copia | 90 | 14623 |
| LTR-HVVMRXALLhA0438K11 | LTR/Copia | 6 | 1547 |
| LTR-HVVMRXALLhA0438O22 | LTR | 6 | 998 |
| LTR-HVVMRXALLhA0439C02 | LTR/Gypsy | 31 | 5052 |
| LTR-HVVMRXALLhA0439K19 | LTR | 71 | 20879 |
| LTR-HVVMRXALLhA0441E09 | LTR/Gypsy | 50 | 6911 |
| LTR-HVVMRXALLhA0442B15 | LTR/Copia | 56 | 8436 |
| LTR-HVVMRXALLhA0443A20 | LTR | 58 | 8676 |
| LTR-HVVMRXALLhA0444N07 | LTR | 7 | 778 |
| LTR-HVVMRXALLhA0448M06 | LTR/Gypsy | 11 | 1565 |
| LTR-HVVMRXALLhA0450B16 | LTR | 54 | 9715 |
| LTR-HVVMRXALLhA0451K12 | LTR/Copia | 8 | 1265 |
| LTR-HVVMRXALLhA0452H02 | LTR | 60 | 5677 |
| LTR-HVVMRXALLhA0453C17 | LTR | 39 | 5735 |
| LTR-HVVMRXALLhA0454B23 | LTR/Copia | 4 | 576 |
| LTR-HVVMRXALLhA0454G20 | LTR/Gypsy | 28 | 3700 |
| LTR-HVVMRXALLhA0454H20 | LTR/Copia | 5 | 849 |
| LTR-HVVMRXALLhA0457M07 | LTR | 56 | 6642 |
| LTR-HVVMRXALLhA0457N20 | LTR | 578 | 88512 |
| LTR-HVVMRXALLhA0460K05 | LTR/Copia | 6 | 571 |
| LTR-HVVMRXALLhA0463P18 | LTR | 40 | 8560 |
| LTR-HVVMRXALLhA0465H17 | LTR | 222 | 29232 |
| LTR-HVVMRXALLhA0466L17 | LTR | 8 | 1005 |
| LTR-HVVMRXALLhA0466N14 | LTR/Gypsy | 15 | 3230 |
| LTR-HVVMRXALLhA0467D01 | LTR | 296 | 34561 |
| LTR-HVVMRXALLhA0467M15 | LTR/Copia | 20 | 2897 |
| LTR-HVVMRXALLhA0467O05 | LTR | 4326 | 1223230 |
| LTR-HVVMRXALLhA0468D16 | LTR/Copia | 25 | 3216 |
| LTR-HVVMRXALLhA0468F21 | LTR/Gypsy | 508 | 78524 |
| LTR-HVVMRXALLhA0469I10 | LTR | 174 | 34794 |
| LTR-HVVMRXALLhA0470B14 | LTR | 83 | 12506 |
| LTR-HVVMRXALLhA0471I12 | LTR | 38 | 6468 |
| LTR-HVVMRXALLhA0472C01 | LTR/Copia | 72 | 16973 |
| LTR-HVVMRXALLhA0474N23 | LTR/Copia | 9 | 1220 |
| LTR-HVVMRXALLhA0474O16 | LTR | 597 | 78758 |
| LTR-HVVMRXALLhA0476B18 | LTR | 66 | 9151 |
| LTR-HVVMRXALLhA0476O11 | LTR/Gypsy | 149 | 37328 |
| LTR-HVVMRXALLhA0477K13 | LTR/Copia | 4 | 347 |
| LTR-HVVMRXALLhA0478M10 | LTR | 2 | 130 |
| LTR-HVVMRXALLhA0479G13 | LTR/Copia | 3 | 313 |
| LTR-HVVMRXALLhA0479N07 | LTR | 459 | 59302 |
| LTR-HVVMRXALLhA0480E02 | LTR/Copia | 4 | 793 |
| LTR-HVVMRXALLhA0480K23 | LTR/Gypsy | 15 | 4724 |
| LTR-HVVMRXALLhA0481A06 | LTR | 952 | 111881 |

| | | | |
|---|---|---|---|
| LTR-HVVMRXALLhA0481A11 | LTR | 42 | 7753 |
| LTR-HVVMRXALLhA0483A05 | LTR | 5 | 543 |
| LTR-HVVMRXALLhA0487O17 | LTR/Copia | 3 | 564 |
| LTR-HVVMRXALLhA0489D17 | LTR/Gypsy | 242 | 44063 |
| LTR-HVVMRXALLhA0489E01 | LTR | 9 | 2322 |
| LTR-HVVMRXALLhA0489F09 | LTR | 22 | 3753 |
| LTR-HVVMRXALLhA0490K16 | LTR | 72 | 9439 |
| LTR-HVVMRXALLhA0491C07 | LTR | 94 | 20045 |
| LTR-HVVMRXALLhA0491G10 | LTR | 78 | 11228 |
| LTR-HVVMRXALLhA0492H15 | LTR/Copia | 6 | 855 |
| LTR-HVVMRXALLhA0492J24 | LTR/Copia | 44 | 5185 |
| LTR-HVVMRXALLhA0494J04 | LTR | 85 | 12713 |
| LTR-HVVMRXALLhA0495J02 | LTR | 40 | 8687 |
| LTR-HVVMRXALLhA0495M13 | LTR/Copia | 32 | 5666 |
| LTR-HVVMRXALLhA0496E12 | LTR/Copia | 3 | 202 |
| LTR-HVVMRXALLhA0498E18 | LTR | 62 | 10536 |
| LTR-HVVMRXALLhA0498N09 | LTR | 34 | 5865 |
| LTR-HVVMRXALLhA0500F19 | LTR/Gypsy | 594 | 86827 |
| LTR-HVVMRXALLhA0500J15 | LTR | 86 | 16015 |
| LTR-HVVMRXALLhA0503H11 | LTR/Gypsy | 135 | 20257 |
| LTR-HVVMRXALLhA0504M13 | LTR/Copia | 139 | 21272 |
| LTR-HVVMRXALLhA0507G08 | LTR/Copia | 17 | 4238 |
| LTR-HVVMRXALLhA0508J20 | LTR/Copia | 1 | 132 |
| LTR-HVVMRXALLhA0508L16 | LTR | 25 | 3996 |
| LTR-HVVMRXALLhA0510D08 | LTR/Copia | 3 | 492 |
| LTR-HVVMRXALLhA0510H08 | LTR | 141 | 21987 |
| LTR-HVVMRXALLhA0510I03 | LTR/Copia | 1 | 139 |
| LTR-HVVMRXALLhA0510L12 | LTR/Copia | 418 | 72632 |
| LTR-HVVMRXALLhA0511K09 | LTR/Copia | 5 | 466 |
| LTR-HVVMRXALLhA0512D19 | LTR | 48 | 8092 |
| LTR-HVVMRXALLhA0516J19 | LTR | 50 | 6023 |
| LTR-HVVMRXALLhA0519I09 | LTR | 26 | 3946 |
| LTR-HVVMRXALLhA0522D08 | LTR/Copia | 495 | 148738 |
| LTR-HVVMRXALLhA0522J06 | LTR | 33 | 6871 |
| LTR-HVVMRXALLhA0522O07 | LTR/Copia | 1 | 198 |
| LTR-HVVMRXALLhA0522P15 | LTR/Copia | 31 | 8645 |
| LTR-HVVMRXALLhA0523P01 | LTR | 198 | 26333 |
| LTR-HVVMRXALLhA0524G20 | LTR | 13 | 2693 |
| LTR-HVVMRXALLhA0528E01 | LTR | 250 | 35755 |
| LTR-HVVMRXALLhA0535K16 | LTR/Gypsy | 17 | 1897 |
| LTR-HVVMRXALLhA0543J14 | LTR | 333 | 69509 |
| LTR-HVVMRXALLhA0545G08 | LTR | 1 | 251 |
| LTR-HVVMRXALLhA0545K07 | LTR/Gypsy | 55 | 8988 |
| LTR-HVVMRXALLhA0545N14 | LTR | 38 | 6289 |
| LTR-HVVMRXALLhA0545O15 | LTR | 6 | 1102 |
| LTR-HVVMRXALLhA0548N22 | LTR/Copia | 10 | 1527 |
| LTR-HVVMRXALLhA0549E10 | LTR/Copia | 17 | 3056 |

| | | | |
|---|---|---|---|
| LTR-HVVMRXALLhA0549F07 | LTR/Gypsy | 15 | 3454 |
| LTR-HVVMRXALLhA0549K14 | LTR | 58 | 9738 |
| LTR-HVVMRXALLhA0552B19 | LTR | 86 | 15803 |
| LTR-HVVMRXALLhA0552H13 | LTR/Copia | 2 | 854 |
| LTR-HVVMRXALLhA0553K12 | LTR/Copia | 35 | 6888 |
| LTR-HVVMRXALLhA0556D08 | LTR/Gypsy | 6 | 560 |
| LTR-HVVMRXALLhA0558H09 | LTR/Copia | 1 | 43 |
| LTR-HVVMRXALLhA0558J15 | LTR/Copia | 87 | 23243 |
| LTR-HVVMRXALLhA0560E07 | LTR | 10 | 1284 |
| LTR-HVVMRXALLhA0560L12 | LTR | 84 | 13520 |
| LTR-HVVMRXALLhA0560N23 | LTR | 54 | 7649 |
| LTR-HVVMRXALLhA0560O12 | LTR/Copia | 3 | 473 |
| LTR-HVVMRXALLhA0564O07 | LTR/Gypsy | 5 | 894 |
| LTR-HVVMRXALLhA0565E21 | LTR/Gypsy | 15 | 1965 |
| LTR-HVVMRXALLhA0565F08 | LTR/Gypsy | 5 | 771 |
| LTR-HVVMRXALLhA0566P05 | LTR/Gypsy | 83 | 16844 |
| LTR-HVVMRXALLhA0568K12 | LTR/Copia | 10 | 1499 |
| LTR-HVVMRXALLhA0573L13 | LTR/Copia | 1 | 294 |
| LTR-HVVMRXALLhA0575D21 | LTR/Gypsy | 153 | 30214 |
| LTR-HVVMRXALLhA0575J02 | LTR | 47 | 6684 |
| LTR-HVVMRXALLhA0577H01 | LTR | 27 | 4365 |
| LTR-HVVMRXALLhA0578K13 | LTR/Gypsy | 10 | 781 |
| LTR-HVVMRXALLhA0581E02 | LTR | 37 | 7602 |
| LTR-HVVMRXALLhA0582D13 | LTR | 59 | 8612 |
| LTR-HVVMRXALLhA0585F19 | LTR/Copia | 3 | 706 |
| LTR-HVVMRXALLhA0585I20 | LTR | 128 | 18425 |
| LTR-HVVMRXALLhA0588H14 | LTR | 6 | 664 |
| LTR-HVVMRXALLhA0588J09 | LTR/Copia | 4 | 784 |
| LTR-HVVMRXALLhA0591E22 | LTR | 109 | 14225 |
| LTR-HVVMRXALLhA0591F23 | LTR | 24 | 4101 |
| LTR-HVVMRXALLhA0591I11 | LTR | 68 | 7996 |
| LTR-HVVMRXALLhA0591J24 | LTR | 37 | 4837 |
| LTR-HVVMRXALLhA0594O06 | LTR/Copia | 4 | 953 |
| LTR-HVVMRXALLhA0595G17 | LTR | 21 | 2123 |
| LTR-HVVMRXALLhA0595N20 | LTR | 103 | 12860 |
| LTR-HVVMRXALLhA0597D19 | LTR/Gypsy | 26 | 3446 |
| LTR-HVVMRXALLhA0597O22 | LTR/Copia | 555 | 87107 |
| LTR-HVVMRXALLhA0598A09 | LTR | 11 | 1450 |
| LTR-HVVMRXALLhA0600D10 | LTR | 99 | 14979 |
| LTR-HVVMRXALLhA0600H23 | LTR | 75 | 14380 |
| LTR-HVVMRXALLhA0601B11 | LTR/Gypsy | 297 | 53001 |
| LTR-HVVMRXALLhA0601C20 | LTR | 100 | 17062 |
| LTR-HVVMRXALLhA0601E22 | LTR | 4 | 517 |
| LTR-HVVMRXALLhA0602G23 | LTR | 23 | 4119 |
| LTR-HVVMRXALLhA0602K15 | LTR | 192 | 26805 |
| LTR-HVVMRXALLhA0602N15 | LTR/Copia | 23 | 3661 |
| LTR-HVVMRXALLhA0602P13 | LTR | 66 | 9980 |

| | | | |
|---|---|---|---|
| LTR-HVVMRXALLhA0605A17 | LTR/Copia | 5 | 585 |
| LTR-HVVMRXALLhA0605B18 | LTR/Copia | 26 | 3833 |
| LTR-HVVMRXALLhA0606I19 | LTR/Gypsy | 142 | 42828 |
| LTR-HVVMRXALLhA0607G10 | LTR/Copia | 339 | 49378 |
| LTR-HVVMRXALLhA0609I08 | LTR/Copia | 18 | 2261 |
| LTR-HVVMRXALLhA0611E01 | LTR/Copia | 16 | 3122 |
| LTR-HVVMRXALLhA0611P01 | LTR | 3 | 531 |
| LTR-HVVMRXALLhA0613B22 | LTR | 74 | 9994 |
| LTR-HVVMRXALLhA0614L10 | LTR | 73 | 10729 |
| LTR-HVVMRXALLhA0615P02 | LTR/Copia | 12 | 1740 |
| LTR-HVVMRXALLhA0617C14 | LTR | 4 | 398 |
| LTR-HVVMRXALLhA0617N08 | LTR/Gypsy | 272 | 48122 |
| LTR-HVVMRXALLhA0618P23 | LTR/Copia | 20 | 3764 |
| LTR-HVVMRXALLhA0620G10 | LTR | 2 | 300 |
| LTR-HVVMRXALLhA0620I09 | LTR | 1 | 59 |
| LTR-HVVMRXALLhA0620I16 | LTR/Copia | 3 | 204 |
| LTR-HVVMRXALLhA0620N04 | LTR/Copia | 9 | 704 |
| LTR-HVVMRXALLhA0620P19 | LTR/Copia | 1 | 45 |
| LTR-HVVMRXALLhA0622N18 | LTR/Copia | 7 | 1102 |
| LTR-HVVMRXALLhA0623B12 | LTR | 311 | 59899 |
| LTR-HVVMRXALLhA0623E08 | LTR | 194 | 36034 |
| LTR-HVVMRXALLhA0623O07 | LTR | 38 | 5499 |
| LTR-HVVMRXALLhA0623O20 | LTR/Gypsy | 21 | 2675 |
| LTR-HVVMRXALLhA0624D13 | LTR/Copia | 99 | 12319 |
| LTR-HVVMRXALLhA0626L01 | LTR/Copia | 20 | 2824 |
| LTR-HVVMRXALLhA0627O23 | LTR/Copia | 3 | 178 |
| LTR-HVVMRXALLhA0628M10 | LTR/Copia | 24 | 3400 |
| LTR-HVVMRXALLhA0630P18 | LTR/Copia | 23 | 2899 |
| LTR-HVVMRXALLhA0631P08 | LTR/Gypsy | 63 | 8609 |
| LTR-HVVMRXALLhA0632I20 | LTR/Copia | 9 | 2961 |
| LTR-HVVMRXALLhA0633G20 | LTR | 126 | 18032 |
| LTR-HVVMRXALLhA0634B05 | LTR | 266 | 38922 |
| LTR-HVVMRXALLhA0634J12 | LTR/Copia | 4 | 481 |
| LTR-HVVMRXALLhA0635A06 | LTR/Copia | 2 | 139 |
| LTR-HVVMRXALLhA0635I20 | LTR/Copia | 1967 | 253548 |
| LTR-HVVMRXALLhA0635J11 | LTR/Copia | 9 | 2719 |
| LTR-HVVMRXALLhA0638L13 | LTR | 28 | 3806 |
| LTR-HVVMRXALLhA0639B21 | LTR | 487 | 122159 |
| LTR-HVVMRXALLhA0640L03 | LTR/Copia | 331 | 98100 |
| LTR-HVVMRXALLhA0644K20 | LTR/Copia | 28 | 5449 |
| LTR-HVVMRXALLhA0645N18 | LTR/Copia | 5 | 776 |
| LTR-HVVMRXALLhA0645P03 | LTR/Copia | 37 | 3373 |
| LTR-HVVMRXALLhA0646I21 | LTR/Copia | 6 | 1027 |
| LTR-HVVMRXALLhA0647K01 | LTR/Copia | 116 | 16459 |
| LTR-HVVMRXALLhA0647N06 | LTR | 113 | 18031 |
| LTR-HVVMRXALLhA0648A23 | LTR | 17 | 1321 |
| LTR-HVVMRXALLhA0648H19 | LTR/Gypsy | 75 | 10990 |

| | | | |
|---|---|---|---|
| LTR-HVVMRXALLhA0650E01 | LTR | 111 | 15339 |
| LTR-HVVMRXALLhA0650M05 | LTR | 55 | 7283 |
| LTR-HVVMRXALLhA0652D10 | LTR | 158 | 22571 |
| LTR-HVVMRXALLhA0653G03 | LTR | 648 | 94019 |
| LTR-HVVMRXALLhA0653M14 | LTR | 44 | 14419 |
| LTR-HVVMRXALLhA0654C20 | LTR/Copia | 23 | 2887 |
| LTR-HVVMRXALLhA0654J06 | LTR/Copia | 20 | 4284 |
| LTR-HVVMRXALLhA0654M19 | LTR/Copia | 3 | 251 |
| LTR-HVVMRXALLhA0655M18 | LTR/Copia | 5 | 1023 |
| LTR-HVVMRXALLhA0657C22 | LTR | 67 | 8794 |
| LTR-HVVMRXALLhA0657P24 | LTR | 62 | 14360 |
| LTR-HVVMRXALLhA0660J20 | LTR | 8 | 902 |
| LTR-HVVMRXALLhA0661J21 | LTR/Copia | 102 | 16885 |
| LTR-HVVMRXALLhA0664F10 | LTR/Copia | 5 | 1002 |
| LTR-HVVMRXALLhA0664F23 | LTR/Gypsy | 42 | 6419 |
| LTR-HVVMRXALLhA0665A05 | LTR | 24 | 2181 |
| LTR-HVVMRXALLhA0665P23 | LTR/Gypsy | 14 | 2457 |
| LTR-HVVMRXALLhA0669P06 | LTR | 1 | 129 |
| LTR-HVVMRXALLhA0672F21 | LTR | 223 | 30509 |
| LTR-HVVMRXALLhA0674B08 | LTR | 63 | 10814 |
| LTR-HVVMRXALLhA0674O16 | LTR | 7 | 1724 |
| LTR-HVVMRXALLhA0675O12 | LTR | 655 | 86993 |
| LTR-HVVMRXALLhA0678H04 | LTR | 81 | 10712 |
| LTR-HVVMRXALLhA0679G23 | LTR/Copia | 3 | 433 |
| LTR-HVVMRXALLhA0680I06 | LTR | 1280 | 197846 |
| LTR-HVVMRXALLhA0683C01 | LTR/Copia | 23 | 3381 |
| LTR-HVVMRXALLhA0683L06 | LTR | 10 | 958 |
| LTR-HVVMRXALLhA0686F03 | LTR | 16 | 2718 |
| LTR-HVVMRXALLhA0686J13 | LTR/Copia | 45 | 11854 |
| LTR-HVVMRXALLhA0686N23 | LTR | 733 | 93848 |
| LTR-HVVMRXALLhA0687B24 | LTR | 285 | 37194 |
| LTR-HVVMRXALLhA0687I07 | LTR | 135 | 26002 |
| LTR-HVVMRXALLhA0688E13 | LTR/Copia | 15 | 3268 |
| LTR-HVVMRXALLhA0697K01 | LTR | 22 | 2788 |
| LTR-HVVMRXALLhA0697M23 | LTR | 8 | 2629 |
| LTR-HVVMRXALLhA0698B24 | LTR/Copia | 5 | 642 |
| LTR-HVVMRXALLhA0699D14 | LTR/Copia | 24 | 6454 |
| LTR-HVVMRXALLhA0699G20 | LTR | 87 | 13205 |
| LTR-HVVMRXALLhA0699N18 | LTR | 271 | 43858 |
| LTR-HVVMRXALLhA0700A13 | LTR/Gypsy | 266 | 41417 |
| LTR-HVVMRXALLhA0700H04 | LTR | 2 | 253 |
| LTR-HVVMRXALLhA0702L07 | LTR/Copia | 27 | 4742 |
| LTR-HVVMRXALLhA0706G13 | LTR | 12 | 1422 |
| LTR-HVVMRXALLhA0707E16 | LTR/Copia | 2 | 164 |
| LTR-HVVMRXALLhA0707J23 | LTR/Copia | 21 | 3451 |
| LTR-HVVMRXALLhA0711H01 | LTR/Gypsy | 12 | 1674 |
| LTR-HVVMRXALLhA0712B05 | LTR | 78 | 13445 |

| | | | |
|---|---|---|---|
| LTR-HVVMRXALLhA0712E11 | LTR/Gypsy | 355 | 41307 |
| LTR-HVVMRXALLhA0712G04 | LTR/Copia | 20 | 2886 |
| LTR-HVVMRXALLhA0712M07 | LTR | 25 | 5224 |
| LTR-HVVMRXALLhA0713G12 | LTR/Copia | 1 | 45 |
| LTR-HVVMRXALLhA0713J13 | LTR/Copia | 40 | 9698 |
| LTR-HVVMRXALLhA0713O21 | LTR | 76 | 10359 |
| LTR-HVVMRXALLhA0713P24 | LTR | 76 | 6376 |
| LTR-HVVMRXALLhA0715E12 | LTR | 51 | 13595 |
| LTR-HVVMRXALLhA0715F10 | LTR/Copia | 53 | 11531 |
| LTR-HVVMRXALLhA0715J24 | LTR | 605 | 102456 |
| LTR-HVVMRXALLhA0715N09 | LTR/Gypsy | 14 | 3035 |
| LTR-HVVMRXALLhA0717J16 | LTR/Gypsy | 60 | 7166 |
| LTR-HVVMRXALLhA0718B18 | LTR/Copia | 10 | 1165 |
| LTR-HVVMRXALLhA0718N07 | LTR | 7 | 1101 |
| LTR-HVVMRXALLhA0720L21 | LTR/Copia | 7 | 1336 |
| LTR-HVVMRXALLhA0720P06 | LTR/Copia | 3 | 489 |
| LTR-HVVMRXALLhA0721H07 | LTR/Copia | 19 | 4476 |
| LTR-HVVMRXALLhA0724C08 | LTR | 348 | 37826 |
| LTR-HVVMRXALLhA0725M02 | LTR | 98 | 13647 |
| LTR-HVVMRXALLhA0727F08 | LTR | 1 | 142 |
| LTR-HVVMRXALLhA0728K02 | LTR/Gypsy | 28 | 5216 |
| LTR-HVVMRXALLhA0730M08 | LTR/Copia | 1 | 348 |
| LTR-HVVMRXALLhA0731H15 | LTR/Copia | 1 | 86 |
| LTR-HVVMRXALLhA0731O24 | LTR/Copia | 17 | 2736 |
| LTR-HVVMRXALLhA0733L19 | LTR | 533 | 78016 |
| LTR-HVVMRXALLhA0734L12 | LTR | 286 | 37071 |
| LTR-HVVMRXALLhA0736C18 | LTR/Copia | 2 | 235 |
| LTR-HVVMRXALLhA0736D18 | LTR/Copia | 36 | 7428 |
| LTR-HVVMRXALLhA0737B04 | LTR | 42 | 6293 |
| LTR-HVVMRXALLhA0737H21 | LTR/Copia | 1 | 171 |
| LTR-HVVMRXALLhA0737M15 | LTR | 86 | 20245 |
| LTR-HVVMRXALLhA0739E08 | LTR | 151 | 25474 |
| LTR-HVVMRXALLhA0739E22 | LTR/Gypsy | 26 | 3245 |
| LTR-HVVMRXALLhA0740F09 | LTR | 24 | 2993 |
| LTR-HVVMRXALLhA0741M11 | LTR | 11 | 2215 |
| LTR-HVVMRXALLhA0743G10 | LTR/Gypsy | 123 | 15558 |
| LTR-HVVMRXALLhA0743J09 | LTR/Copia | 3 | 402 |
| LTR-HVVMRXALLhA0744H17 | LTR | 4 | 449 |
| LTR-HVVMRXALLhA0745C22 | LTR | 5 | 873 |
| LTR-HVVMRXALLhA0746I17 | LTR | 379 | 57367 |
| LTR-HVVMRXALLhA0747A23 | LTR/Copia | 2 | 228 |
| LTR-HVVMRXALLhA0750I20 | LTR | 51 | 8896 |
| LTR-HVVMRXALLhA0751B20 | LTR/Gypsy | 7 | 805 |
| LTR-HVVMRXALLhA0751D21 | LTR/Gypsy | 40 | 5212 |
| LTR-HVVMRXALLhA0751F04 | LTR/Gypsy | 32 | 4230 |
| LTR-HVVMRXALLhA0751P17 | LTR/Gypsy | 151 | 21562 |
| LTR-HVVMRXALLhA0752A11 | LTR/Gypsy | 16 | 2527 |

| | | | |
|---|---|---|---|
| LTR-HVVMRXALLhA0752P07 | LTR | 17 | 2165 |
| LTR-HVVMRXALLhA0753M17 | LTR | 67 | 8390 |
| LTR-HVVMRXALLhA0754K24 | LTR/Copia | 9 | 1647 |
| LTR-HVVMRXALLhA0754P10 | LTR/Copia | 3 | 832 |
| LTR-HVVMRXALLhA0755A13 | LTR | 236 | 52259 |
| LTR-HVVMRXALLhA0756E06 | LTR/Gypsy | 54 | 8096 |
| LTR-HVVMRXALLhA0757C05 | LTR/Gypsy | 103 | 32060 |
| LTR-HVVMRXALLhA0758B08 | LTR/Copia | 13 | 4300 |
| LTR-HVVMRXALLhA0759H10 | LTR | 196 | 28128 |
| LTR-HVVMRXALLhA0760M16 | LTR/Gypsy | 15 | 2176 |
| LTR-HVVMRXALLhA0762D04 | LTR/Copia | 5 | 783 |
| LTR-HVVMRXALLhA0764E23 | LTR/Copia | 21 | 3970 |
| LTR-HVVMRXALLhA0771H01 | LTR/Copia | 36 | 4418 |
| LTR-HVVMRXALLhA0772E15 | LTR/Gypsy | 2 | 209 |
| LTR-HVVMRXALLhA0772G18 | LTR | 49 | 6903 |
| LTR-HVVMRXALLhA0773O07 | LTR/Copia | 1 | 85 |
| LTR-HVVMRXALLhA0774H16 | LTR/Copia | 1 | 338 |
| LTR-HVVMRXALLhA0778H16 | LTR | 2391 | 535814 |
| LTR-HVVMRXALLhA0779H22 | LTR/Gypsy | 4 | 653 |
| LTR-HVVMRXALLhA0779M15 | LTR | 272 | 47363 |
| LTR-HVVMRXALLhA0780I16 | LTR/Copia | 8 | 709 |
| LTR-HVVMRXALLhA0783J21 | LTR | 187 | 28894 |
| LTR-HVVMRXALLhA0783K02 | LTR/Copia | 19 | 3422 |
| LTR-HVVMRXALLhA0786K02 | LTR/Gypsy | 22 | 3220 |
| LTR-HVVMRXALLhA0788C12 | LTR | 16 | 3138 |
| LTR-HVVMRXALLhA0788K01 | LTR | 51 | 6296 |
| LTR-HVVMRXALLhA0789G01 | LTR/Gypsy | 80 | 13550 |
| LTR-HVVMRXALLhA0790N05 | LTR/Copia | 28 | 4230 |
| LTR-HVVMRXALLhA0790O18 | LTR | 117 | 16221 |
| LTR-HVVMRXALLhA0791F17 | LTR | 135 | 22230 |
| LTR-HVVMRXALLhA0792E04 | LTR/Copia | 1 | 449 |
| LTR-HVVMRXALLhA0792J20 | LTR | 109 | 19165 |
| LTR-HVVMRXALLhA0793C12 | LTR/Gypsy | 95 | 10423 |
| LTR-HVVMRXALLhA0793I12 | LTR | 81 | 14358 |
| LTR-HVVMRXALLhA0793L03 | LTR/Copia | 41 | 10080 |
| LTR-HVVMRXALLhA0794L18 | LTR | 3 | 397 |
| LTR-HVVMRXALLhA0795C17 | LTR/Gypsy | 6 | 984 |
| LTR-HVVMRXALLhA0798A06 | LTR | 154 | 29724 |
| LTR-HVVMRXALLhA0798N18 | LTR | 719 | 151340 |
| LTR-HVVMRXALLhA0799C08 | LTR/Copia | 1 | 307 |
| LTR-HVVMRXALLhA0799G08 | LTR/Copia | 8 | 691 |
| LTR-HVVMRXALLhA0800C22 | LTR | 9 | 2280 |
| LTR-HVVMRXALLhA0802H13 | LTR | 262 | 37137 |
| LTR-HVVMRXALLhA0806F23 | LTR | 18 | 3140 |
| LTR-HVVMRXALLhA0808C02 | LTR/Copia | 27 | 6483 |
| LTR-HVVMRXALLhA0808M17 | LTR/Copia | 12 | 1613 |
| LTR-HVVMRXALLhA0809B08 | LTR | 27 | 4024 |

| | | | |
|---|---|---|---|
| LTR-HVVMRXALLhA0809K04 | LTR | 89 | 13133 |
| LTR-HVVMRXALLhA0810E04 | LTR/Copia | 17 | 3051 |
| LTR-HVVMRXALLhA0810G20 | LTR | 11 | 1325 |
| LTR-HVVMRXALLhA0811B06 | LTR/Gypsy | 4 | 333 |
| LTR-HVVMRXALLhA0811D23 | LTR | 208 | 24876 |
| LTR-HVVMRXALLhA0812K02 | LTR/Copia | 9 | 1970 |
| LTR-HVVMRXALLhA0812K11 | LTR/Copia | 2 | 161 |
| LTR-HVVMRXALLhA0814K22 | LTR | 130 | 19640 |
| LTR-HVVMRXALLhA0815B17 | LTR/Gypsy | 54 | 9383 |
| LTR-HVVMRXALLhA0816C23 | LTR | 63 | 8480 |
| LTR-HVVMRXALLhB0030B01 | LTR/Copia | 3 | 344 |
| LTR-HVVMRXALLhB0030D01 | LTR | 325 | 50036 |
| LTR-HVVMRXALLhB0030G01 | LTR/Gypsy | 73 | 12396 |
| LTR-HVVMRXALLhB0040B01 | LTR | 14 | 1945 |
| LTR-HVVMRXALLhB0040C24 | LTR/Gypsy | 13 | 1832 |
| LTR-HVVMRXALLhB0040E01 | LTR/Gypsy | 33 | 4814 |
| LTR-HVVMRXALLhB0040G24 | LTR | 1007 | 217599 |
| LTR-HVVMRXALLhB0040H01 | LTR | 120 | 19167 |
| LTR-HVVMRXALLhB0050A01 | LTR | 24 | 3896 |
| LTR-HVVMRXALLhB0050F01 | LTR | 72 | 10113 |
| LTR-HVVMRXALLhB0058D03 | LTR | 6 | 631 |
| LTR-HVVMRXALLhB0060E01 | LTR | 200 | 32355 |
| LTR-HVVMRXALLhB0060H01 | LTR/Gypsy | 9 | 1020 |
| LTR-HVVMRXALLhB0070A01 | LTR | 156 | 21880 |
| LTR-HVVMRXALLhB0070C01 | LTR | 98 | 13534 |
| LTR-HVVMRXALLhB0070C24 | LTR/Copia | 100 | 9797 |
| LTR-HVVMRXALLhB0072A15 | LTR/Gypsy | 186 | 38342 |
| LTR-HVVMRXALLhB0074D08 | LTR | 893 | 119185 |
| LTR-HVVMRXALLhB0080F01 | LTR | 94 | 12613 |
| LTR-HVVMRXALLhB0080N11 | LTR/Copia | 3 | 390 |
| LTR-HVVMRXALLhB0089G08 | LTR/Copia | 3 | 286 |
| LTR-HVVMRXALLhB0090D01 | LTR | 4 | 398 |
| LTR-HVVMRXALLhB0090H01 | LTR/Gypsy | 31 | 4232 |
| LTR-HVVMRXALLhB0090O20 | LTR | 15 | 2324 |
| LTR-HVVMRXALLhB0092F24 | LTR/Copia | 34 | 8343 |
| LTR-HVVMRXALLhB0093D02 | LTR | 40 | 5897 |
| LTR-HVVMRXALLhB0093N03 | LTR/Copia | 2 | 226 |
| LTR-HVVMRXALLhB0098M14 | LTR | 135 | 26584 |
| LTR-HVVMRXALLhB0099E04 | LTR | 68 | 10240 |
| LTR-HVVMRXALLhB0100A01 | LTR | 292 | 44416 |
| LTR-HVVMRXALLhB0100A24 | LTR | 8 | 1251 |
| LTR-HVVMRXALLhB0100E01 | LTR | 26 | 5854 |
| LTR-HVVMRXALLhB0100G01 | LTR | 3 | 286 |
| LTR-HVVMRXALLhB0100G24 | LTR | 5 | 759 |
| LTR-HVVMRXALLhB0102D01 | LTR/Copia | 90 | 13220 |
| LTR-HVVMRXALLhB0105N13 | LTR/Gypsy | 2 | 471 |
| LTR-HVVMRXALLhB0110A24 | LTR | 4 | 580 |

| | | | |
|---|---|---|---|
| LTR-HVVMRXALLhB0110B24 | LTR/Copia | 2 | 287 |
| LTR-HVVMRXALLhB0110C01 | LTR | 94 | 13202 |
| LTR-HVVMRXALLhB0110E24 | LTR/Copia | 44 | 6174 |
| LTR-HVVMRXALLhB0110H24 | LTR/Gypsy | 22 | 3722 |
| LTR-HVVMRXALLhB0110O19 | LTR | 133 | 23421 |
| LTR-HVVMRXALLhB0114I05 | LTR | 77 | 16884 |
| LTR-HVVMRXALLhB0115D15 | LTR/Copia | 6 | 553 |
| LTR-HVVMRXALLhB0115K08 | LTR | 200 | 35194 |
| LTR-HVVMRXALLhB0118C08 | LTR | 59 | 7121 |
| LTR-HVVMRXALLhB0118L11 | LTR | 8 | 1190 |
| LTR-HVVMRXALLhB0120C24 | LTR/Gypsy | 19 | 2891 |
| LTR-HVVMRXALLhB0120G01 | LTR | 88 | 11272 |
| LTR-HVVMRXALLhB0120H01 | LTR | 779 | 136719 |
| LTR-HVVMRXALLhB0130A01 | LTR/Gypsy | 118 | 19721 |
| LTR-HVVMRXALLhB0130A24 | LTR/Copia | 6 | 1321 |
| LTR-HVVMRXALLhB0130F01 | LTR/Gypsy | 695 | 119004 |
| LTR-HVVMRXALLhB0133M10 | LTR/Copia | 8 | 1197 |
| LTR-HVVMRXALLhB0133M20 | LTR/Gypsy | 193 | 25989 |
| LTR-HVVMRXALLhB0133M21 | LTR | 142 | 19312 |
| LTR-HVVMRXALLhB0134C01 | LTR | 18 | 3983 |
| LTR-HVVMRXALLhB0134F24 | LTR | 241 | 38066 |
| LTR-HVVMRXALLhB0137A03 | LTR/Copia | 1 | 333 |
| LTR-HVVMRXALLhB0139L12 | LTR/Gypsy | 108 | 23270 |
| LTR-HVVMRXALLhB0149H18 | LTR/Gypsy | 7 | 1784 |
| LTR-HVVMRXALLhB0149M05 | LTR | 116 | 18679 |
| LTR-HVVMRXALLhB0175P10 | LTR | 169 | 33980 |
| LTR-HVVMRXALLhC0020A01 | LTR | 203 | 38344 |
| LTR-HVVMRXALLhC0020I01 | LTR/Gypsy | 2 | 345 |
| LTR-HVVMRXALLhC0020M01 | LTR/Copia | 8 | 833 |
| LTR-HVVMRXALLhC0030M01 | LTR/Gypsy | 484 | 135725 |
| LTR-HVVMRXALLhC0040A01 | LTR/Copia | 64 | 7969 |
| LTR-HVVMRXALLhC0050K01 | LTR/Copia | 336 | 42507 |
| LTR-HVVMRXALLhC0060A01 | LTR/Gypsy | 5 | 348 |
| LTR-HVVMRXALLhC0070C01 | LTR | 215 | 26764 |
| LTR-HVVMRXALLhC0070O01 | LTR/Gypsy | 676 | 88930 |
| LTR-HVVMRXALLhC0080E01 | LTR/Gypsy | 18 | 3162 |
| LTR-HVVMRXALLhC0080K01 | LTR/Copia | 162 | 24933 |
| LTR-HVVMRXALLhC0090A01 | LTR | 756 | 137174 |
| LTR-HVVMRXALLhC0090C01 | LTR/Copia | 170 | 20803 |
| LTR-HVVMRXALLhC0118D08 | LTR | 246 | 54530 |
| LTR-HVVMRXALLhC0120E01 | LTR | 115 | 15224 |
| LTR-HVVMRXALLhC0140M01 | LTR | 243 | 37824 |
| LTR-HVVMRXALLhC0160A01 | LTR/Copia | 3 | 341 |
| LTR-HVVMRXALLhC0160K01 | LTR/Copia | 26 | 3450 |
| LTR-HVVMRXALLhC0170A01 | LTR | 22 | 3289 |
| LTR-HVVMRXALLhC0170O01 | LTR/Gypsy | 49 | 6947 |
| LTR-HVVMRXALLhC0185C01 | LTR/Copia | 43 | 6133 |

| | | | |
|---|---|---|---|
| LTR-HVVMRXALLhC0185O01 | LTR | 655 | 74258 |
| LTR-HVVMRXALLhC0190I01 | LTR | 54 | 6985 |
| LTR-HVVMRXALLhC0195A01 | LTR/Copia | 74 | 14186 |
| LTR-HVVMRXALLhC0200K01 | LTR/Gypsy | 306 | 38534 |
| LTR-HVVMRXALLhC0205A01 | LTR | 62 | 9126 |
| LTR-HVVMRXALLhC0210G01 | LTR | 53 | 10758 |
| LTR-HVVMRXALLhC0219J10 | LTR | 26 | 3737 |
| LTR-HVVMRXALLhC0240M01 | LTR | 87 | 14300 |
| LTR-HVVMRXALLhC0255O01 | LTR | 776 | 107953 |
| LTR-HVVMRXALLhC0270C01 | LTR/Copia | 7 | 857 |
| LTR-HVVMRXALLhC0280M01 | LTR | 183 | 27859 |
| LTR-HVVMRXALLhC0290A01 | LTR/Copia | 14 | 1333 |
| LTR-HVVMRXALLmA0002F15 | LTR | 5 | 735 |
| LTR-HVVMRXALLmA0004M22 | LTR | 58 | 8315 |
| LTR-HVVMRXALLmA0005H05 | LTR/Copia | 320 | 44715 |
| LTR-HVVMRXALLmA0005M05 | LTR | 245 | 39225 |
| LTR-HVVMRXALLmA0007E08 | LTR | 69 | 12639 |
| LTR-HVVMRXALLmA0008E01 | LTR/Gypsy | 8 | 1126 |
| LTR-HVVMRXALLmA0008M01 | LTR | 11 | 1253 |
| LTR-HVVMRXALLmA0016O01 | LTR/Copia | 35 | 4301 |
| LTR-HVVMRXALLmA0018K20 | LTR/Copia | 84 | 19102 |
| LTR-HVVMRXALLmA0018O09 | LTR/Gypsy | 58 | 7052 |
| LTR-HVVMRXALLmA0024C01 | LTR | 529 | 75666 |
| LTR-HVVMRXALLmA0028G23 | LTR | 775 | 87799 |
| LTR-HVVMRXALLmA0028H18 | LTR | 161 | 16519 |
| LTR-HVVMRXALLmA0028J12 | LTR | 2 | 211 |
| LTR-HVVMRXALLmA0029J16 | LTR | 50 | 6084 |
| LTR-HVVMRXALLmA0032K01 | LTR/Gypsy | 13 | 2553 |
| LTR-HVVMRXALLmA0033A15 | LTR | 143 | 33005 |
| LTR-HVVMRXALLmA0040E01 | LTR | 139 | 19315 |
| LTR-HVVMRXALLmA0040I01 | LTR | 54 | 11081 |
| LTR-HVVMRXALLmA0041C02 | LTR/Gypsy | 406 | 47800 |
| LTR-HVVMRXALLmA0041M10 | LTR | 117 | 21338 |
| LTR-HVVMRXALLmA0053H10 | LTR | 2220 | 527938 |
| LTR-HVVMRXALLmA0054C16 | LTR | 26 | 4361 |
| LTR-HVVMRXALLmA0056A01 | LTR | 87 | 11945 |
| LTR-HVVMRXALLmA0056G01 | LTR/Copia | 1 | 115 |
| LTR-HVVMRXALLmA0057M07 | LTR | 9 | 1334 |
| LTR-HVVMRXALLmA0064C01 | LTR | 30 | 3662 |
| LTR-HVVMRXALLmA0064I01 | LTR | 46 | 7402 |
| LTR-HVVMRXALLmA0064K01 | LTR/Copia | 1 | 373 |
| LTR-HVVMRXALLmA0069O21 | LTR/Gypsy | 46 | 6666 |
| LTR-HVVMRXALLmA0071N18 | LTR | 561 | 57746 |
| LTR-HVVMRXALLmA0071P20 | LTR/Gypsy | 42 | 4334 |
| LTR-HVVMRXALLmA0072E01 | LTR/Copia | 7 | 681 |
| LTR-HVVMRXALLmA0072N11 | LTR/Gypsy | 108 | 16357 |
| LTR-HVVMRXALLmA0072O01 | LTR/Gypsy | 103 | 13260 |

| | | | |
|---|---|---|---|
| LTR-HVVMRXALLmA0077A22 | LTR/Copia | 3 | 505 |
| LTR-HVVMRXALLmA0077C02 | LTR | 17 | 2624 |
| LTR-HVVMRXALLmA0077N05 | LTR | 297 | 38569 |
| LTR-HVVMRXALLmA0080A01 | LTR | 50 | 7748 |
| LTR-HVVMRXALLmA0080K01 | LTR | 191 | 28357 |
| LTR-HVVMRXALLmA0083O10 | LTR | 54 | 7848 |
| LTR-HVVMRXALLmA0088M01 | LTR | 260 | 42888 |
| LTR-HVVMRXALLmA0088O01 | LTR/Copia | 34 | 5500 |
| LTR-HVVMRXALLmA0092D17 | LTR | 1101 | 165970 |
| LTR-HVVMRXALLmA0092N12 | LTR/Copia | 2 | 433 |
| LTR-HVVMRXALLmA0092O20 | LTR/Gypsy | 17 | 3710 |
| LTR-HVVMRXALLmA0095I08 | LTR/Copia | 34 | 3996 |
| LTR-HVVMRXALLmA0095I10 | LTR/Gypsy | 22 | 3193 |
| LTR-HVVMRXALLmA0096K01 | LTR/Gypsy | 87 | 11490 |
| LTR-HVVMRXALLmA0104A01 | LTR | 3 | 507 |
| LTR-HVVMRXALLmA0104E01 | LTR/Copia | 12 | 1874 |
| LTR-HVVMRXALLmA0104M01 | LTR/Gypsy | 11 | 1561 |
| LTR-HVVMRXALLmA0107P12 | LTR | 57 | 10231 |
| LTR-HVVMRXALLmA0108I10 | LTR/Copia | 1 | 175 |
| LTR-HVVMRXALLmA0112I01 | LTR | 420 | 64436 |
| LTR-HVVMRXALLmA0120B03 | LTR/Copia | 161 | 17321 |
| LTR-HVVMRXALLmA0120B11 | LTR | 335 | 41637 |
| LTR-HVVMRXALLmA0120B18 | LTR/Gypsy | 27 | 3612 |
| LTR-HVVMRXALLmA0120C02 | LTR/Copia | 114 | 18187 |
| LTR-HVVMRXALLmA0120E01 | LTR | 75 | 9764 |
| LTR-HVVMRXALLmA0120I12 | LTR/Gypsy | 140 | 19399 |
| LTR-HVVMRXALLmA0120P11 | LTR/Gypsy | 15 | 2433 |
| LTR-HVVMRXALLmA0136I01 | LTR/Copia | 10 | 1325 |
| LTR-HVVMRXALLmA0142I01 | LTR/Copia | 15 | 1518 |
| LTR-HVVMRXALLmA0142K01 | LTR | 286 | 39170 |
| LTR-HVVMRXALLmA0146L05 | LTR | 35 | 6461 |
| LTR-HVVMRXALLmA0146M18 | LTR | 24 | 4162 |
| LTR-HVVMRXALLmA0150A01 | LTR/Gypsy | 24 | 2424 |
| LTR-HVVMRXALLmA0150C01 | LTR/Gypsy | 8 | 922 |
| LTR-HVVMRXALLmA0150G01 | LTR | 7 | 1343 |
| LTR-HVVMRXALLmA0156H19 | LTR | 43 | 7883 |
| LTR-HVVMRXALLmA0158K01 | LTR | 1200 | 229167 |
| LTR-HVVMRXALLmA0158O01 | LTR/Copia | 51 | 7152 |
| LTR-HVVMRXALLmA0161H17 | LTR/Copia | 4 | 800 |
| LTR-HVVMRXALLmA0166M01 | LTR | 136 | 19225 |
| LTR-HVVMRXALLmA0172I01 | LTR | 96 | 14755 |
| LTR-HVVMRXALLmA0176P03 | LTR | 100 | 12388 |
| LTR-HVVMRXALLmA0180A01 | LTR/Gypsy | 81 | 11203 |
| LTR-HVVMRXALLmA0180K01 | LTR/Gypsy | 219 | 36658 |
| LTR-HVVMRXALLmA0188I01 | LTR | 6 | 833 |
| LTR-HVVMRXALLmA0188O01 | LTR | 14 | 1987 |
| LTR-HVVMRXALLmA0196C01 | LTR | 75 | 10605 |

| | | | |
|---|---|---|---|
| LTR-HVVMRXALLmA0196G01 | LTR | 59 | 9523 |
| LTR-HVVMRXALLmA0196G20 | LTR | 18 | 2419 |
| LTR-HVVMRXALLmA0196K01 | LTR/Copia | 42 | 5782 |
| LTR-HVVMRXALLmA0197D09 | LTR/Copia | 7 | 968 |
| LTR-HVVMRXALLmA0197I24 | LTR | 187 | 31632 |
| LTR-HVVMRXALLmA0204A01 | LTR | 58 | 8557 |
| LTR-HVVMRXALLmA0209C05 | LTR/Gypsy | 73 | 9985 |
| LTR-HVVMRXALLmA0212E01 | LTR/Copia | 3 | 370 |
| LTR-HVVMRXALLmA0212I01 | LTR/Gypsy | 117 | 18086 |
| LTR-HVVMRXALLmA0214B06 | LTR | 8 | 1687 |
| LTR-HVVMRXALLmA0214M15 | LTR/Gypsy | 67 | 11594 |
| LTR-HVVMRXALLmA0217M04 | LTR/Gypsy | 10 | 1478 |
| LTR-HVVMRXALLmA0220C01 | LTR/Copia | 12 | 1415 |
| LTR-HVVMRXALLmA0220O01 | LTR/Gypsy | 44 | 7457 |
| LTR-HVVMRXALLmA0223G02 | LTR/Gypsy | 73 | 12196 |
| LTR-HVVMRXALLmA0225G09 | LTR/Copia | 8 | 1746 |
| LTR-HVVMRXALLmA0225G22 | LTR | 473 | 60140 |
| LTR-HVVMRXALLmA0225G23 | LTR | 41 | 5269 |
| LTR-HVVMRXALLmA0226B11 | LTR | 8 | 1187 |
| LTR-HVVMRXALLmA0228E01 | LTR/Copia | 5 | 961 |
| LTR-HVVMRXALLmA0231D22 | LTR/Copia | 7 | 1161 |
| LTR-HVVMRXALLmA0234E07 | LTR/Copia | 11 | 1846 |
| LTR-HVVMRXALLmA0236M01 | LTR/Gypsy | 11 | 1724 |
| LTR-HVVMRXALLmA0244A01 | LTR | 60 | 8937 |
| LTR-HVVMRXALLmA0244I01 | LTR/Gypsy | 82 | 12618 |
| LTR-HVVMRXALLmA0244K07 | LTR/Copia | 63 | 17345 |
| LTR-HVVMRXALLmA0249H03 | LTR/Gypsy | 10 | 1007 |
| LTR-HVVMRXALLmA0252I01 | LTR | 121 | 16059 |
| LTR-HVVMRXALLmA0252O01 | LTR/Gypsy | 39 | 6469 |
| LTR-HVVMRXALLmA0260K01 | LTR/Gypsy | 5 | 603 |
| LTR-HVVMRXALLmA0263M22 | LTR/Copia | 25 | 4126 |
| LTR-HVVMRXALLmA0268A01 | LTR/Gypsy | 97 | 13569 |
| LTR-HVVMRXALLmA0269A04 | LTR | 80 | 11436 |
| LTR-HVVMRXALLmA0269K02 | LTR/Gypsy | 7 | 808 |
| LTR-HVVMRXALLmA0270H23 | LTR/Copia | 10 | 1819 |
| LTR-HVVMRXALLmA0270M16 | LTR | 50 | 7374 |
| LTR-HVVMRXALLmA0271E20 | LTR | 362 | 44031 |
| LTR-HVVMRXALLmA0275A09 | LTR/Copia | 46 | 8261 |
| LTR-HVVMRXALLmA0275F13 | LTR | 59 | 6853 |
| LTR-HVVMRXALLmA0275O04 | LTR | 148 | 25291 |
| LTR-HVVMRXALLmA0276I01 | LTR | 10 | 2287 |
| LTR-HVVMRXALLmA0276M01 | LTR | 27 | 3765 |
| LTR-HVVMRXALLmA0279D01 | LTR/Copia | 249 | 32374 |
| LTR-HVVMRXALLmA0280D14 | LTR | 335 | 57901 |
| LTR-HVVMRXALLmA0284G01 | LTR | 2 | 462 |
| LTR-HVVMRXALLmA0284O01 | LTR | 201 | 30262 |
| LTR-HVVMRXALLmA0288C10 | LTR | 300 | 78392 |

| | | | |
|---|---|---|---|
| LTR-HVVMRXALLmA0288D22 | LTR | 69 | 12113 |
| LTR-HVVMRXALLmA0292G01 | LTR | 263 | 33400 |
| LTR-HVVMRXALLmA0294L17 | LTR | 159 | 22117 |
| LTR-HVVMRXALLmA0294N12 | LTR | 89 | 13810 |
| LTR-HVVMRXALLmA0295A01 | LTR/Copia | 3 | 580 |
| LTR-HVVMRXALLmA0300K01 | LTR | 421 | 67785 |
| LTR-HVVMRXALLmA0301B12 | LTR | 204 | 30343 |
| LTR-HVVMRXALLmA0301F08 | LTR | 539 | 85245 |
| LTR-HVVMRXALLmA0302J18 | LTR | 126 | 18836 |
| LTR-HVVMRXALLmA0305B16 | LTR/Gypsy | 62 | 8252 |
| LTR-HVVMRXALLmA0306A20 | LTR | 50 | 6749 |
| LTR-HVVMRXALLmA0307L06 | LTR | 500 | 69550 |
| LTR-HVVMRXALLmA0308C01 | LTR/Gypsy | 43 | 7251 |
| LTR-HVVMRXALLmA0316J11 | LTR | 138 | 18532 |
| LTR-HVVMRXALLmA0316O05 | LTR | 47 | 6659 |
| LTR-HVVMRXALLmA0317H08 | LTR | 204 | 24469 |
| LTR-HVVMRXALLmA0319K22 | LTR | 407 | 63041 |
| LTR-HVVMRXALLmA0319P18 | LTR | 8 | 1276 |
| LTR-HVVMRXALLmA0321J24 | LTR | 747 | 124035 |
| LTR-HVVMRXALLmA0322A10 | LTR | 30 | 5047 |
| LTR-HVVMRXALLmA0322H14 | LTR/Copia | 157 | 16438 |
| LTR-HVVMRXALLmA0324A01 | LTR | 61 | 10318 |
| LTR-HVVMRXALLmA0324I01 | LTR/Gypsy | 74 | 18996 |
| LTR-HVVMRXALLmA0325G09 | LTR/Copia | 111 | 15942 |
| LTR-HVVMRXALLmA0325K06 | LTR | 92 | 12576 |
| LTR-HVVMRXALLmA0325L06 | LTR/Gypsy | 102 | 14178 |
| LTR-HVVMRXALLmA0329D13 | LTR | 15 | 2404 |
| LTR-HVVMRXALLmA0329F19 | LTR | 81 | 12334 |
| LTR-HVVMRXALLmA0332G01 | LTR | 190 | 31805 |
| LTR-HVVMRXALLmA0334F08 | LTR/Copia | 7 | 803 |
| LTR-HVVMRXALLmA0340A01 | LTR/Copia | 2 | 298 |
| LTR-HVVMRXALLmA0340H04 | LTR | 71 | 10931 |
| LTR-HVVMRXALLmA0340I01 | LTR/Copia | 26 | 3931 |
| LTR-HVVMRXALLmA0348C01 | LTR/Gypsy | 11 | 1887 |
| LTR-HVVMRXALLmA0348M01 | LTR | 235 | 32189 |
| LTR-HVVMRXALLmA0354N08 | LTR/Gypsy | 66 | 8751 |
| LTR-HVVMRXALLmA0356G01 | LTR/Gypsy | 32 | 4144 |
| LTR-HVVMRXALLmA0356N05 | LTR | 37 | 5012 |
| LTR-HVVMRXALLmA0359A13 | LTR | 146 | 20395 |
| LTR-HVVMRXALLmA0359B02 | LTR/Gypsy | 140 | 23720 |
| LTR-HVVMRXALLmA0370K10 | LTR | 2 | 541 |
| LTR-HVVMRXALLmA0373I10 | LTR/Copia | 1 | 61 |
| LTR-HVVMRXALLmA0378G04 | LTR | 76 | 10629 |
| LTR-HVVMRXALLmA0382I24 | LTR | 67 | 9031 |
| LTR-HVVMRXALLmA0382J20 | LTR/Gypsy | 5 | 669 |
| LTR-HVVMRXALLmA0382M24 | LTR | 145 | 20649 |
| LTR-HVVMRXALLmA0383C02 | LTR | 30 | 4138 |

| | | | |
|---|---|---|---|
| LTR-HVVMRXALLmA0383J02 | LTR/Copia | 1 | 229 |
| LTR-HVVMRXALLmA0383P03 | LTR/Copia | 7 | 771 |
| LTR-HVVMRXALLmA0385E08 | LTR | 99 | 18808 |
| LTR-HVVMRXALLmA0390G03 | LTR | 51 | 7816 |
| LTR-HVVMRXALLmA0392K10 | LTR/Gypsy | 62 | 9833 |
| LTR-HVVMRXALLmA0402F19 | LTR | 156 | 19525 |
| LTR-HVVMRXALLmA0402H01 | LTR | 2104 | 473896 |
| LTR-HVVMRXALLmA0402P06 | LTR | 100 | 13876 |
| LTR-HVVMRXALLmA0409L10 | LTR/Gypsy | 702 | 97724 |
| LTR-HVVMRXALLmA0410F12 | LTR | 97 | 15554 |
| LTR-HVVMRXALLmA0410J05 | LTR | 8 | 1070 |
| LTR-HVVMRXALLmA0410J20 | LTR | 76 | 13267 |
| LTR-HVVMRXALLmA0411I08 | LTR | 243 | 31320 |
| LTR-HVVMRXALLmA0415B13 | LTR | 76 | 9552 |
| LTR-HVVMRXALLmA0417P20 | LTR | 141 | 22608 |
| LTR-HVVMRXALLmA0420F08 | LTR/Gypsy | 246 | 34362 |
| LTR-HVVMRXALLmA0422J12 | LTR/Gypsy | 9 | 960 |
| LTR-HVVMRXALLmA0423F17 | LTR | 83 | 15346 |
| LTR-HVVMRXALLmA0429F07 | LTR | 139 | 20532 |
| LTR-HVVMRXALLmA0431J18 | LTR | 312 | 41838 |
| LTR-HVVMRXALLmA0431L24 | LTR/Copia | 6 | 1097 |
| LTR-HVVMRXALLmA0432H21 | LTR/Gypsy | 86 | 9025 |
| LTR-HVVMRXALLmA0432J16 | LTR | 276 | 47961 |
| LTR-HVVMRXALLmA0432L21 | LTR | 73 | 11620 |
| LTR-HVVMRXALLmA0433M21 | LTR | 148 | 18511 |
| LTR-HVVMRXALLmA0445J06 | LTR | 79 | 13611 |
| LTR-HVVMRXALLmA0445M05 | LTR | 83 | 13413 |
| LTR-HVVMRXALLmA0448A02 | LTR/Gypsy | 55 | 8572 |
| LTR-HVVMRXALLmA0448K21 | LTR/Gypsy | 81 | 11116 |
| LTR-HVVMRXALLmA0460D10 | LTR | 35 | 5073 |
| LTR-HVVMRXALLmA0460I23 | LTR/Copia | 1 | 393 |
| LTR-HVVMRXALLmA0475E08 | LTR/Copia | 1 | 182 |
| LTR-HVVMRXALLmA0478M01 | LTR | 367 | 43269 |
| LTR-HVVMRXALLmA0486J02 | LTR/Gypsy | 141 | 36910 |
| LTR-HVVMRXALLmA0488J11 | LTR | 69 | 9064 |
| LTR-HVVMRXALLmA0492P10 | LTR | 178 | 31809 |
| LTR-HVVMRXALLmA0496H01 | LTR | 100 | 28120 |
| LTR-HVVMRXALLmA0499H17 | LTR/Copia | 64 | 10976 |
| LTR-HVVMRXALLmA0505N20 | LTR | 127 | 20057 |
| LTR-HVVMRXALLmA0506M19 | LTR/Gypsy | 78 | 13041 |
| LTR-HVVMRXALLmA0506N10 | LTR/Copia | 1 | 174 |
| LTR-HVVMRXALLmA0514G20 | LTR | 257 | 38388 |
| LTR-HVVMRXALLmA0514M13 | LTR | 70 | 10630 |
| LTR-HVVMRXALLmA0514N09 | LTR/Copia | 1 | 48 |
| LTR-HVVMRXALLmA0514N12 | LTR/Gypsy | 51 | 7260 |
| LTR-HVVMRXALLmA0519F20 | LTR/Gypsy | 248 | 44643 |
| LTR-HVVMRXALLmA0520F23 | LTR/Gypsy | 189 | 25967 |

| | | | |
|---|---|---|---|
| LTR-HVVMRXALLrA0007C09 | LTR | 182 | 24437 |
| LTR-HVVMRXALLrA0007J13 | LTR/Gypsy | 1151 | 159429 |
| LTR-HVVMRXALLrA0007J19 | LTR/Gypsy | 18 | 2328 |
| LTR-HVVMRXALLrA0007K05 | LTR | 189 | 32305 |
| LTR-HVVMRXALLrA0015M01 | LTR | 51 | 8370 |
| LTR-HVVMRXALLrA0015O01 | LTR | 5 | 274 |
| LTR-HVVMRXALLrA0021H17 | LTR | 41 | 6220 |
| LTR-HVVMRXALLrA0021P12 | LTR/Gypsy | 137 | 29985 |
| LTR-HVVMRXALLrA0025G01 | LTR | 107 | 16997 |
| LTR-HVVMRXALLrA0030K01 | LTR | 138 | 17946 |
| LTR-HVVMRXALLrA0037I21 | LTR/Gypsy | 26 | 4069 |
| LTR-HVVMRXALLrA0037K09 | LTR | 26 | 4858 |
| LTR-HVVMRXALLrA0037O22 | LTR/Gypsy | 59 | 7517 |
| LTR-HVVMRXALLrA0038P08 | LTR/Gypsy | 18 | 2096 |
| LTR-HVVMRXALLrA0039N11 | LTR | 107 | 21634 |
| LTR-HVVMRXALLrA0045N13 | LTR/Copia | 6 | 1835 |
| LTR-HVVMRXALLrA0050A01 | LTR | 53 | 6509 |
| LTR-HVVMRXALLrA0050K01 | LTR | 46 | 6348 |
| LTR-HVVMRXALLrA0052G16 | LTR/Gypsy | 8 | 1119 |
| LTR-HVVMRXALLrA0058F23 | LTR | 98 | 17043 |
| LTR-HVVMRXALLrA0060C01 | LTR/Gypsy | 47 | 8480 |
| LTR-HVVMRXALLrA0060I01 | LTR | 97 | 16247 |
| LTR-HVVMRXALLrA0064H07 | LTR | 12 | 1204 |
| LTR-HVVMRXALLrA0074G08 | LTR | 82 | 11310 |
| LTR-HVVMRXALLrA0074K19 | LTR/Copia | 27 | 3617 |
| LTR-HVVMRXALLrA0075F06 | LTR | 66 | 8886 |
| LTR-HVVMRXALLrA0075M24 | LTR | 2 | 255 |
| LTR-HVVMRXALLrA0083L19 | LTR | 108 | 10957 |
| LTR-HVVMRXALLrA0084C10 | LTR | 298 | 44223 |
| LTR-HVVMRXALLrA0084J11 | LTR/Gypsy | 9 | 1557 |
| LTR-HVVMRXALLrA0088H17 | LTR | 3 | 256 |
| LTR-HVVMRXALLrA0089F22 | LTR | 207 | 31533 |
| LTR-HVVMRXALLrA0090C01 | LTR | 506 | 58396 |
| LTR-HVVMRXALLrA0090E01 | LTR/Gypsy | 87 | 14051 |
| LTR-HVVMRXALLrA0093C09 | LTR/Gypsy | 32 | 5165 |
| LTR-HVVMRXALLrA0095L10 | LTR | 43 | 5688 |
| LTR-HVVMRXALLrA0095N06 | LTR/Copia | 1 | 127 |
| LTR-HVVMRXALLrA0107P12 | LTR | 75 | 11990 |
| LTR-HVVMRXALLrA0109F15 | LTR/Gypsy | 47 | 5424 |
| LTR-HVVMRXALLrA0109P09 | LTR | 161 | 23561 |
| LTR-HVVMRXALLrA0110C01 | LTR/Copia | 1 | 55 |
| LTR-HVVMRXALLrA0110G01 | LTR/Copia | 31 | 5166 |
| LTR-HVVMRXALLrA0112K01 | LTR | 86 | 14841 |
| LTR-HVVMRXALLrA0112P05 | LTR/Copia | 1 | 72 |
| LTR-HVVMRXALLrA0113E08 | LTR/Copia | 293 | 31604 |
| LTR-HVVMRXALLrA0113F18 | LTR/Copia | 47 | 6818 |
| LTR-HVVMRXALLrA0113P08 | LTR | 47 | 7550 |

| | | | |
|---|---|---|---|
| LTR-HVVMRXALLrA0117C22 | LTR | 434 | 67801 |
| LTR-HVVMRXALLrA0120A01 | LTR/Copia | 24 | 2711 |
| LTR-HVVMRXALLrA0128A08 | LTR | 128 | 17696 |
| LTR-HVVMRXALLrA0128O06 | LTR | 78 | 11894 |
| LTR-HVVMRXALLrA0130C03 | LTR/Gypsy | 3 | 376 |
| LTR-HVVMRXALLrA0132F12 | LTR | 266 | 41891 |
| LTR-HVVMRXALLrA0150E01 | LTR | 1052 | 145038 |
| LTR-HVVMRXALLrA0150I01 | LTR | 44 | 6721 |
| LTR-HVVMRXALLrA0156N01 | LTR/Copia | 5 | 701 |
| LTR-HVVMRXALLrA0158D13 | LTR | 69 | 9753 |
| LTR-HVVMRXALLrA0159M08 | LTR | 100 | 14713 |
| LTR-HVVMRXALLrA0169E04 | LTR/Gypsy | 6 | 1136 |
| LTR-HVVMRXALLrA0170A01 | LTR/Copia | 3 | 449 |
| LTR-HVVMRXALLrA0170B10 | LTR | 363 | 44832 |
| LTR-HVVMRXALLrA0170D03 | LTR | 243 | 44847 |
| LTR-HVVMRXALLrA0170E04 | LTR | 68 | 10766 |
| LTR-HVVMRXALLrA0170N05 | LTR | 590 | 66915 |
| LTR-HVVMRXALLrA0170N09 | LTR/Gypsy | 163 | 25878 |
| LTR-HVVMRXALLrA0172M23 | LTR/Copia | 12 | 2180 |
| LTR-HVVMRXALLrA0176M01 | LTR/Gypsy | 7 | 925 |
| LTR-HVVMRXALLrA0180G01 | LTR | 7 | 898 |
| LTR-HVVMRXALLrA0187C04 | LTR | 125 | 17306 |
| LTR-HVVMRXALLrA0187J16 | LTR/Copia | 89 | 15122 |
| LTR-HVVMRXALLrA0188G09 | LTR/Gypsy | 110 | 17703 |
| LTR-HVVMRXALLrA0188P06 | LTR/Gypsy | 13 | 1859 |
| LTR-HVVMRXALLrA0193B03 | LTR | 7 | 936 |
| LTR-HVVMRXALLrA0193F01 | LTR | 133 | 21146 |
| LTR-HVVMRXALLrA0200C01 | LTR/Gypsy | 46 | 6310 |
| LTR-HVVMRXALLrA0200M01 | LTR/Gypsy | 4 | 921 |
| LTR-HVVMRXALLrA0200M05 | LTR | 68 | 8894 |
| LTR-HVVMRXALLrA0204I14 | LTR | 91 | 14671 |
| LTR-HVVMRXALLrA0207F09 | LTR | 90 | 14974 |
| LTR-HVVMRXALLrA0207J19 | LTR/Copia | 9 | 1260 |
| LTR-HVVMRXALLrA0210A01 | LTR/Gypsy | 27 | 3785 |
| LTR-HVVMRXALLrA0210I01 | LTR | 77 | 10006 |
| LTR-HVVMRXALLrA0217D06 | LTR | 2277 | 309277 |
| LTR-HVVMRXALLrA0217H07 | LTR/Copia | 46 | 5057 |
| LTR-HVVMRXALLrA0217P07 | LTR | 57 | 7439 |
| LTR-HVVMRXALLrA0224H08 | LTR/Gypsy | 721 | 122885 |
| LTR-HVVMRXALLrA0224L09 | LTR | 81 | 10656 |
| LTR-HVVMRXALLrA0227E17 | LTR/Copia | 7 | 859 |
| LTR-HVVMRXALLrA0229I08 | LTR | 40 | 6192 |
| LTR-HVVMRXALLrA0229L19 | LTR | 623 | 115822 |
| LTR-HVVMRXALLrA0230I01 | LTR | 1035 | 84285 |
| LTR-HVVMRXALLrA0236F03 | LTR/Copia | 4 | 742 |
| LTR-HVVMRXALLrA0236O20 | LTR/Gypsy | 9 | 1109 |
| LTR-HVVMRXALLrA0240C01 | LTR | 17 | 2428 |

| | | | |
|---|---|---|---|
| LTR-HVVMRXALLrA0241P12 | LTR | 201 | 41179 |
| LTR-HVVMRXALLrA0260A01 | LTR | 263 | 40457 |
| LTR-HVVMRXALLrA0260C01 | LTR/Gypsy | 95 | 12333 |
| LTR-HVVMRXALLrA0260K01 | LTR | 163 | 25883 |
| LTR-HVVMRXALLrA0261J14 | LTR/Gypsy | 323 | 41391 |
| LTR-HVVMRXALLrA0265H04 | LTR/Gypsy | 90 | 11271 |
| LTR-Os | LTR/Gypsy | 11799 | 1681783 |
| LTR-Sb | LTR/Gypsy | 21819 | 2977568 |
| LTR-Sb1 | LTR | 2137 | 271684 |
| MITE-barke | DNA/Mite | 14952 | 2055117 |
| MITE-morex | DNA/Mite | 14786 | 1949592 |
| Machiavelli | LTR/Copia | 6 | 710 |
| Nikita-LTR-1.1 | LTR | 21 | 2851 |
| Nikita-LTR-1.2 | LTR | 34 | 4527 |
| ORSiCMCM00100001 | Other | 1 | 48 |
| ORSiCMCM00100002 | Retroelement | 1 | 210 |
| ORSiCMCM00100003 | Other | 1 | 231 |
| ORSiCMCM00100005 | Other | 3 | 647 |
| ORSiCMCM00100008 | LTR/Copia | 6 | 877 |
| ORSiCMCM00100009 | Other | 7 | 991 |
| ORSiCMCMOOT00002 | Other | 2 | 431 |
| ORSiOTOT00000031 | Other | 2 | 187 |
| ORSiOTOT00000033 | Other | 107 | 10551 |
| ORSiRGRR00000005 | rRNA | 1 | 52 |
| ORSiRGRR00000006 | rRNA | 20 | 4695 |
| ORSiRGRR00000008 | rRNA | 58 | 19665 |
| ORSiRGRR00000010 | rRNA | 1 | 87 |
| ORSiRGRR00000011 | rRNA | 20 | 4650 |
| ORSiRGRR00000015 | rRNA | 3 | 323 |
| ORSiRGRR00000019 | rRNA | 2 | 105 |
| ORSiRGRR00000081 | rRNA | 1 | 212 |
| ORSiRGRR00000932 | rRNA | 3 | 719 |
| ORSiRGRR00500002 | rRNA | 3 | 111 |
| ORSiRGRR00500003 | rRNA | 2 | 196 |
| ORSiRGRR00500004 | rRNA | 2 | 178 |
| ORSiTEMT00100005 | DNA/Tourist | 1 | 187 |
| ORSiTEMT00100009 | DNA/Tourist | 1 | 169 |
| ORSiTEMT00600001 | DNA/Mite | 27 | 4459 |
| ORSiTEMT00700003 | DNA/Mite | 8 | 1077 |
| ORSiTEMT00900001 | DNA/Mite | 2 | 56 |
| ORSiTEMT00900002 | DNA/Mite | 1 | 142 |
| ORSiTEMT00900004 | DNA/Mite | 1 | 77 |
| ORSiTEMT00900005 | DNA/Mite | 2 | 200 |
| ORSiTEMT00900006 | DNA/Mite | 1 | 207 |
| ORSiTEMT00900007 | DNA/Mite | 19 | 827 |
| ORSiTEMT00900008 | DNA/Mite | 5 | 330 |
| ORSiTEMT01000001 | DNA/Tourist | 2 | 92 |

| | | | |
|---|---|---|---|
| ORSiTEMT01100003 | SINE | 1 | 46 |
| ORSiTEMT01100006 | SINE | 1 | 62 |
| ORSiTEMT01100008 | SINE | 1 | 67 |
| ORSiTEMT01100015 | SINE | 2 | 241 |
| ORSiTEMT01100018 | SINE | 51 | 4032 |
| ORSiTEMT01100028 | SINE | 2 | 104 |
| ORSiTEMT01400001 | DNA/Mite | 1 | 68 |
| ORSiTEMT01400002 | DNA/Mite | 4 | 518 |
| ORSiTEMT01500001 | DNA/Mite | 1 | 200 |
| ORSiTEMT01500003 | DNA/Mite | 1 | 54 |
| ORSiTEMT01500004 | DNA/Mite | 4 | 604 |
| ORSiTEMT01500005 | DNA/Mite | 1 | 66 |
| ORSiTEMT01700001 | DNA/Mite | 1 | 118 |
| ORSiTEMT01800001 | DNA/Mite | 4 | 657 |
| ORSiTEMT01900003 | DNA/Mite | 1 | 37 |
| ORSiTEMT02500003 | DNA/Mite | 15 | 1975 |
| ORSiTEMT03400001 | DNA/hAT | 2 | 143 |
| ORSiTEMT05300001 | DNA/Mite | 19 | 1676 |
| ORSiTEMT05300003 | DNA/Mite | 4 | 327 |
| ORSiTEMT05300006 | DNA/Mite | 5 | 168 |
| ORSiTEMT06000002 | DNA/Mite | 5 | 975 |
| ORSiTEMT06000005 | DNA/Mite | 7 | 1530 |
| ORSiTEMT06000009 | DNA/Mite | 5 | 771 |
| ORSiTEMT06000906 | DNA/Mite | 5 | 290 |
| ORSiTEMT06100001 | DNA/Mite | 12 | 1251 |
| ORSiTERT00100019 | LTR/Copia | 1 | 53 |
| ORSiTERT00100028 | LTR/Copia | 1 | 86 |
| ORSiTERT00100041 | LTR/Copia | 1 | 123 |
| ORSiTERT00100052 | LTR/Copia | 1 | 96 |
| ORSiTERT00100059 | LTR/Copia | 3 | 155 |
| ORSiTERT00100060 | LTR/Copia | 10 | 2962 |
| ORSiTERT00100062 | LTR/Copia | 5 | 220 |
| ORSiTERT00100063 | LTR/Copia | 5 | 657 |
| ORSiTERT00100067 | LTR/Copia | 1 | 132 |
| ORSiTERT00100068 | LTR/Copia | 1 | 327 |
| ORSiTERT00200002 | LTR/Gypsy | 7 | 945 |
| ORSiTERT00200005 | LTR/Gypsy | 6 | 599 |
| ORSiTERT00200030 | LTR/Gypsy | 2 | 113 |
| ORSiTERT00200062 | LTR/Gypsy | 1 | 81 |
| ORSiTERT00200072 | LTR/Gypsy | 1 | 90 |
| ORSiTERT00200076 | LTR/Gypsy | 2 | 320 |
| ORSiTERT00200915 | LTR/Gypsy | 2 | 238 |
| ORSiTERT00200916 | LTR/Gypsy | 2 | 331 |
| ORSiTERT00200925 | LTR/Gypsy | 1 | 193 |
| ORSiTERT00200936 | LTR/Gypsy | 2 | 353 |
| ORSiTERT00200938 | LTR/Gypsy | 1 | 151 |
| ORSiTERT00200944 | LTR/Gypsy | 1 | 297 |

| | | | |
|---|---|---|---|
| ORSiTERT00200947 | LTR/Gypsy | 1 | 73 |
| ORSiTERT00200948 | LTR/Gypsy | 1 | 123 |
| ORSiTERT00200962 | LTR/Gypsy | 1 | 171 |
| ORSiTERT00300001 | LINE | 10 | 916 |
| ORSiTERT00300004 | LINE | 6 | 976 |
| ORSiTERT00300006 | LINE | 4 | 361 |
| ORSiTERT00300008 | LINE | 18 | 2120 |
| ORSiTERT00300009 | LINE | 41 | 8226 |
| ORSiTERT00300012 | LINE | 18 | 2707 |
| ORSiTERT00400002 | SINE | 1 | 44 |
| ORSiTERTOOT00005 | LINE | 93 | 10185 |
| ORSiTERTOOT00006 | LTR | 1 | 95 |
| ORSiTERTOOT00007 | LTR | 1 | 109 |
| ORSiTERTOOT00008 | LTR | 2 | 274 |
| ORSiTERTOOT00010 | LTR | 1 | 171 |
| ORSiTERTOOT00012 | LINE | 34 | 3297 |
| ORSiTERTOOT00014 | LTR/Copia | 7 | 582 |
| ORSiTERTOOT00015 | LTR/Gypsy | 11 | 2697 |
| ORSiTERTOOT00016 | LTR | 2 | 127 |
| ORSiTERTOOT00017 | LTR/Gypsy | 9 | 1273 |
| ORSiTERTOOT00018 | LTR/Gypsy | 17 | 2759 |
| ORSiTERTOOT00904 | LTR | 2 | 167 |
| ORSiTETN00100005 | DNA/hAT-Ac | 1 | 100 |
| ORSiTETN00100014 | DNA/hAT-Ac | 3 | 482 |
| ORSiTETN00100901 | DNA/hAT-Ac | 1 | 104 |
| ORSiTETN00200002 | DNA/En-Spm | 1 | 56 |
| ORSiTETN00200004 | DNA/En-Spm | 12 | 1408 |
| ORSiTETN00200006 | DNA/En-Spm | 30 | 8310 |
| ORSiTETN00200007 | DNA/En-Spm | 2 | 167 |
| ORSiTETN00200009 | DNA/En-Spm | 104 | 21764 |
| ORSiTETN00200010 | DNA/En-Spm | 24 | 3453 |
| ORSiTETN00200011 | DNA/En-Spm | 26 | 3595 |
| ORSiTETN00200012 | DNA/En-Spm | 72 | 13629 |
| ORSiTETN00200013 | DNA/En-Spm | 24 | 3194 |
| ORSiTETN00300006 | DNA/MuDR | 3 | 258 |
| ORSiTETN00400001 | DNA/TcMar | 3 | 515 |
| ORSiTETN00400007 | DNA/TcMar | 1 | 304 |
| ORSiTETN00500001 | DNA | 7 | 1151 |
| ORSiTETNOOT00005 | DNA | 29 | 4498 |
| ORSiTETNOOT00012 | DNA | 1 | 40 |
| ORSiTETNOOT00013 | DNA | 3 | 368 |
| ORSiTETNOOT00017 | DNA | 3 | 454 |
| ORSiTETNOOT00021 | DNA | 1 | 79 |
| ORSiTETNOOT00022 | DNA/hAT | 9 | 771 |
| ORSiTETNOOT00039 | DNA | 2 | 413 |
| ORSiTETNOOT00061 | DNA | 4 | 473 |
| ORSiTETNOOT00064 | DNA | 1 | 94 |

| | | | |
|---|---|---|---|
| ORSiTETNOOT00067 | DNA | 1 | 65 |
| ORSiTETNOOT00075 | DNA/hAT | 1 | 90 |
| ORSiTETNOOT00084 | DNA | 2 | 130 |
| ORSiTETNOOT00086 | DNA | 1 | 169 |
| ORSiTETNOOT00087 | LTR/Gypsy | 3 | 279 |
| ORSiTETNOOT00088 | DNA | 1 | 130 |
| ORSiTETNOOT00089 | DNA | 8 | 1154 |
| ORSiTETNOOT00090 | DNA | 2 | 263 |
| ORSiTETNOOT00091 | DNA | 1 | 106 |
| ORSiTETNOOT00092 | DNA | 8 | 639 |
| ORSiTETNOOT00095 | DNA | 1 | 79 |
| ORSiTETNOOT00102 | DNA | 9 | 1083 |
| ORSiTETNOOT00103 | DNA | 5 | 606 |
| ORSiTETNOOT00104 | DNA | 4 | 354 |
| ORSiTETNOOT00105 | DNA | 1 | 192 |
| ORSiTETNOOT00106 | DNA | 9 | 827 |
| ORSiTETNOOT00107 | DNA | 1 | 121 |
| ORSiTETNOOT00108 | DNA | 2 | 107 |
| ORSiTETNOOT00109 | DNA | 1 | 120 |
| ORSiTETNOOT00110 | DNA | 3 | 257 |
| ORSiTETNOOT00111 | DNA | 1 | 74 |
| ORSiTETNOOT00112 | DNA | 7 | 507 |
| ORSiTETNOOT00114 | DNA | 3 | 349 |
| ORSiTETNOOT00115 | DNA | 1 | 94 |
| ORSiTETNOOT00116 | DNA | 44 | 7762 |
| ORSiTETNOOT00908 | DNA | 4 | 329 |
| ORSiTETNOOT00999 | DNA | 8 | 861 |
| Os1-19 | Retroelement | 2 | 225 |
| Os11-01 | LTR | 4 | 317 |
| Os11-09 | LTR/Copia | 9 | 1480 |
| Os11-13 | LTR | 2 | 199 |
| Os2-10 | LTR/Copia | 25 | 3975 |
| Os3S-11L | LTR/Gypsy | 21 | 3441 |
| Os3S-12L | LTR/Gypsy | 2 | 479 |
| Os3S-25L | LTR/Gypsy | 1 | 241 |
| Os3S-9L | LTR/Gypsy | 2 | 109 |
| Os4-05 | LTR | 1281 | 253140 |
| Os4-18 | LTR/Gypsy | 4 | 423 |
| Os5-04 | LTR/Copia | 14 | 3009 |
| Os5-05 | LTR | 12 | 1374 |
| Os5-13 | Retroelement | 4 | 270 |
| Os6-12 | LTR/Gypsy | 4 | 390 |
| Osr09-AP000969-0 | LTR/Copia | 1 | 64 |
| Osr10-AC069324-0 | Other | 1 | 72 |
| Osr16-AP002845-0 | Other | 5 | 227 |
| Osr19-AC069300-0 | LTR/Copia | 6 | 631 |
| Osr23-AP002843-0 | LTR/Copia | 17 | 3184 |

| | | | |
|---|---|---|---|
| Osr24-AC016781-0 | LTR | 12 | 1572 |
| Osr25-AP001278-0 | LTR | 1 | 51 |
| Osr26-AP001111-0 | LTR/Gypsy | 1 | 252 |
| Osr28-AP002539-0 | LTR/Gypsy | 4 | 1434 |
| Osr31-AP003054-0 | Other | 30 | 3260 |
| Osr37-AC068654-0 | LTR | 9 | 821 |
| RIX-Karin | LINE | 1958 | 490700 |
| RIX-Morpheus | LINE | 168 | 13172 |
| RIX-Nicole | LINE | 369 | 30927 |
| RIX-Paula | LINE | 13 | 751 |
| RIX-Stasy | LINE | 3121 | 740385 |
| RIX-unnamedfam1 | LINE | 540 | 148073 |
| RIX-unnamedfam10 | LINE | 620 | 66502 |
| RIX-unnamedfam100 | LINE | 3 | 452 |
| RIX-unnamedfam101 | LINE | 205 | 50876 |
| RIX-unnamedfam102 | LINE | 71 | 13874 |
| RIX-unnamedfam103 | LINE | 31 | 6005 |
| RIX-unnamedfam104 | LINE | 267 | 46574 |
| RIX-unnamedfam105 | LINE | 492 | 131489 |
| RIX-unnamedfam106 | LINE | 284 | 65519 |
| RIX-unnamedfam107 | LINE | 1319 | 353729 |
| RIX-unnamedfam108 | LINE | 130 | 36058 |
| RIX-unnamedfam109 | LINE | 56 | 8503 |
| RIX-unnamedfam11 | LINE | 358 | 95949 |
| RIX-unnamedfam110 | LINE | 298 | 76659 |
| RIX-unnamedfam111 | LINE | 802 | 184746 |
| RIX-unnamedfam112 | LINE | 123 | 27797 |
| RIX-unnamedfam113 | LINE | 244 | 32491 |
| RIX-unnamedfam114 | LINE | 236 | 41499 |
| RIX-unnamedfam115 | LINE | 322 | 21284 |
| RIX-unnamedfam117 | LINE | 7 | 1449 |
| RIX-unnamedfam118 | LINE | 20 | 4802 |
| RIX-unnamedfam119 | LINE | 2 | 280 |
| RIX-unnamedfam12 | LINE | 36 | 8834 |
| RIX-unnamedfam120 | LINE | 5 | 586 |
| RIX-unnamedfam121 | LINE | 562 | 124147 |
| RIX-unnamedfam124 | LINE | 256 | 55771 |
| RIX-unnamedfam125 | LINE | 98 | 14093 |
| RIX-unnamedfam126 | LINE | 728 | 168586 |
| RIX-unnamedfam127 | LINE | 46 | 8017 |
| RIX-unnamedfam129 | LINE | 6 | 1954 |
| RIX-unnamedfam13 | LINE | 239 | 67295 |
| RIX-unnamedfam14 | LINE | 736 | 204216 |
| RIX-unnamedfam15 | LINE | 49 | 11024 |
| RIX-unnamedfam16 | LINE | 99 | 25396 |
| RIX-unnamedfam17 | LINE | 623 | 169223 |
| RIX-unnamedfam18 | LINE | 853 | 242179 |

| | | | |
|---|---|---|---|
| RIX-unnamedfam19 | LINE | 205 | 50247 |
| RIX-unnamedfam2 | LINE | 173 | 32599 |
| RIX-unnamedfam20 | LINE | 264 | 44852 |
| RIX-unnamedfam21 | LINE | 1000 | 317300 |
| RIX-unnamedfam22 | LINE | 1006 | 288724 |
| RIX-unnamedfam23 | LINE | 59 | 9528 |
| RIX-unnamedfam24 | LINE | 2 | 231 |
| RIX-unnamedfam25 | LINE | 271 | 39907 |
| RIX-unnamedfam26 | LINE | 756 | 178702 |
| RIX-unnamedfam27 | LINE | 480 | 115540 |
| RIX-unnamedfam28 | LINE | 438 | 128733 |
| RIX-unnamedfam29 | LINE | 309 | 66426 |
| RIX-unnamedfam3 | LINE | 194 | 39785 |
| RIX-unnamedfam30 | LINE | 61 | 10073 |
| RIX-unnamedfam31 | LINE | 957 | 283927 |
| RIX-unnamedfam32 | LINE | 28 | 2660 |
| RIX-unnamedfam33 | LINE | 61 | 13382 |
| RIX-unnamedfam34 | LINE | 112 | 17126 |
| RIX-unnamedfam35 | LINE | 340 | 40631 |
| RIX-unnamedfam36 | LINE | 211 | 53748 |
| RIX-unnamedfam37 | LINE | 224 | 60752 |
| RIX-unnamedfam38 | LINE | 76 | 5885 |
| RIX-unnamedfam39 | LINE | 345 | 94552 |
| RIX-unnamedfam4 | LINE | 848 | 210136 |
| RIX-unnamedfam40 | LINE | 9 | 585 |
| RIX-unnamedfam41 | LINE | 42 | 10626 |
| RIX-unnamedfam42 | LINE | 44 | 9022 |
| RIX-unnamedfam43 | LINE | 2 | 218 |
| RIX-unnamedfam44 | LINE | 284 | 63363 |
| RIX-unnamedfam45 | LINE | 196 | 18893 |
| RIX-unnamedfam46 | LINE | 94 | 24808 |
| RIX-unnamedfam47 | LINE | 12 | 1259 |
| RIX-unnamedfam48 | LINE | 45 | 4251 |
| RIX-unnamedfam49 | LINE | 28 | 2926 |
| RIX-unnamedfam5 | LINE | 141 | 24580 |
| RIX-unnamedfam50 | LINE | 16 | 4440 |
| RIX-unnamedfam51 | LINE | 1 | 95 |
| RIX-unnamedfam52 | LINE | 14 | 2236 |
| RIX-unnamedfam53 | LINE | 7 | 1680 |
| RIX-unnamedfam54 | LINE | 163 | 40550 |
| RIX-unnamedfam55 | LINE | 12 | 1314 |
| RIX-unnamedfam56 | LINE | 21 | 2507 |
| RIX-unnamedfam57 | LINE | 192 | 51011 |
| RIX-unnamedfam58 | LINE | 829 | 230510 |
| RIX-unnamedfam59 | LINE | 60 | 12155 |
| RIX-unnamedfam6 | LINE | 958 | 253772 |
| RIX-unnamedfam60 | LINE | 134 | 38404 |

| | | | |
|---|---|---|---|
| RIX-unnamedfam61 | LINE | 8 | 780 |
| RIX-unnamedfam62 | LINE | 207 | 25290 |
| RIX-unnamedfam63 | LINE | 162 | 41790 |
| RIX-unnamedfam64 | LINE | 198 | 51759 |
| RIX-unnamedfam65 | LINE | 86 | 18783 |
| RIX-unnamedfam66 | LINE | 207 | 32434 |
| RIX-unnamedfam67 | LINE | 38 | 4410 |
| RIX-unnamedfam68 | LINE | 424 | 118363 |
| RIX-unnamedfam69 | LINE | 18 | 4117 |
| RIX-unnamedfam7 | LINE | 23 | 1420 |
| RIX-unnamedfam70 | LINE | 28 | 5902 |
| RIX-unnamedfam71 | LINE | 6 | 829 |
| RIX-unnamedfam72 | LINE | 46 | 7031 |
| RIX-unnamedfam73 | LINE | 1307 | 374259 |
| RIX-unnamedfam74 | LINE | 1202 | 311954 |
| RIX-unnamedfam75 | LINE | 4801 | 1458992 |
| RIX-unnamedfam76 | LINE | 2081 | 608337 |
| RIX-unnamedfam77 | LINE | 383 | 102395 |
| RIX-unnamedfam78 | LINE | 949 | 259737 |
| RIX-unnamedfam79 | LINE | 103 | 24524 |
| RIX-unnamedfam8 | LINE | 108 | 10674 |
| RIX-unnamedfam80 | LINE | 1144 | 338237 |
| RIX-unnamedfam81 | LINE | 422 | 106172 |
| RIX-unnamedfam82 | LINE | 199 | 44480 |
| RIX-unnamedfam83 | LINE | 234 | 49192 |
| RIX-unnamedfam84 | LINE | 836 | 230529 |
| RIX-unnamedfam85 | LINE | 920 | 235331 |
| RIX-unnamedfam86 | LINE | 552 | 163100 |
| RIX-unnamedfam87 | LINE | 295 | 66108 |
| RIX-unnamedfam88 | LINE | 605 | 161016 |
| RIX-unnamedfam89 | LINE | 363 | 98420 |
| RIX-unnamedfam9 | LINE | 5 | 334 |
| RIX-unnamedfam90 | LINE | 167 | 36867 |
| RIX-unnamedfam91 | LINE | 614 | 138585 |
| RIX-unnamedfam92 | LINE | 16 | 2025 |
| RIX-unnamedfam93 | LINE | 162 | 42521 |
| RIX-unnamedfam94 | LINE | 58 | 14229 |
| RIX-unnamedfam95 | LINE | 603 | 156952 |
| RIX-unnamedfam96 | LINE | 218 | 51401 |
| RIX-unnamedfam97 | LINE | 1160 | 312534 |
| RIX-unnamedfam98 | LINE | 37 | 6070 |
| RIX-unnamedfam99 | LINE | 2050 | 420908 |
| RLC-Ale | LTR/Copia | 191 | 48120 |
| RLC-Angela | LTR/Copia | 124706 | 36773835 |
| RLC-BARE1 | LTR/Copia | 759 | 234125 |
| RLC-Barbara | LTR/Copia | 80784 | 24413827 |
| RLC-Claudia | LTR/Copia | 24976 | 7653616 |

| | | | |
|---|---|---|---|
| RLC-Eugene | LTR/Copia | 16059 | 4830559 |
| RLC-Ikya | LTR/Copia | 183 | 46603 |
| RLC-Inga | LTR/Copia | 23205 | 7051453 |
| RLC-Jorge | LTR/Copia | 2685 | 831125 |
| RLC-Leojyg | LTR/Copia | 92 | 15998 |
| RLC-Maximus | LTR/Copia | 52102 | 16089031 |
| RLC-Oref | LTR/Copia | 147 | 38571 |
| RLC-Rada | LTR/Copia | 4 | 407 |
| RLC-Sasanda | LTR/Copia | 8 | 1342 |
| RLC-TAR2 | LTR/Copia | 2946 | 905534 |
| RLC-TAR3 | LTR/Copia | 445 | 114538 |
| RLC-Usier | LTR/Copia | 3910 | 1156757 |
| RLC-Valerie | LTR/Copia | 77 | 15110 |
| RLC-WIS | LTR/Copia | 45196 | 14192057 |
| RLC-unnamedfam1 | LTR/Copia | 383 | 100632 |
| RLC-unnamedfam10 | LTR/Copia | 3 | 352 |
| RLC-unnamedfam11 | LTR/Copia | 9 | 1254 |
| RLC-unnamedfam12 | LTR/Copia | 255 | 52004 |
| RLC-unnamedfam13 | LTR/Copia | 81 | 23562 |
| RLC-unnamedfam14 | LTR/Copia | 240 | 66949 |
| RLC-unnamedfam15 | LTR/Copia | 23 | 5061 |
| RLC-unnamedfam16 | LTR/Copia | 171 | 44165 |
| RLC-unnamedfam17 | LTR/Copia | 160 | 42278 |
| RLC-unnamedfam18 | LTR/Copia | 71 | 13786 |
| RLC-unnamedfam19 | LTR/Copia | 29 | 5158 |
| RLC-unnamedfam2 | LTR/Copia | 124 | 29548 |
| RLC-unnamedfam20 | LTR/Copia | 71 | 13306 |
| RLC-unnamedfam21 | LTR/Copia | 54 | 13321 |
| RLC-unnamedfam22 | LTR/Copia | 23 | 2994 |
| RLC-unnamedfam23 | LTR/Copia | 84 | 11465 |
| RLC-unnamedfam24 | LTR/Copia | 9 | 1315 |
| RLC-unnamedfam25 | LTR/Copia | 1091 | 296422 |
| RLC-unnamedfam26 | LTR/Copia | 29 | 4806 |
| RLC-unnamedfam27 | LTR/Copia | 28 | 5550 |
| RLC-unnamedfam28 | LTR/Copia | 22 | 3379 |
| RLC-unnamedfam29 | LTR/Copia | 39 | 6782 |
| RLC-unnamedfam3 | LTR/Copia | 1 | 43 |
| RLC-unnamedfam30 | LTR/Copia | 52 | 9098 |
| RLC-unnamedfam31 | LTR/Copia | 83 | 12124 |
| RLC-unnamedfam32 | LTR/Copia | 96 | 22869 |
| RLC-unnamedfam33 | LTR/Copia | 275 | 31700 |
| RLC-unnamedfam34 | LTR/Copia | 176 | 52894 |
| RLC-unnamedfam35 | LTR/Copia | 231 | 64095 |
| RLC-unnamedfam36 | LTR/Copia | 48 | 12763 |
| RLC-unnamedfam37 | LTR/Copia | 1068 | 291928 |
| RLC-unnamedfam38 | LTR/Copia | 270 | 41076 |
| RLC-unnamedfam39 | LTR/Copia | 2278 | 607092 |

| | | | |
|---|---|---|---|
| RLC-unnamedfam4 | LTR/Copia | 34 | 6974 |
| RLC-unnamedfam40 | LTR/Copia | 3463 | 961275 |
| RLC-unnamedfam41 | LTR/Copia | 139 | 35829 |
| RLC-unnamedfam42 | LTR/Copia | 615 | 146026 |
| RLC-unnamedfam43 | LTR/Copia | 605 | 157775 |
| RLC-unnamedfam44 | LTR/Copia | 502 | 97658 |
| RLC-unnamedfam45 | LTR/Copia | 1470 | 295413 |
| RLC-unnamedfam46 | LTR/Copia | 33 | 8854 |
| RLC-unnamedfam47 | LTR/Copia | 112 | 25011 |
| RLC-unnamedfam48 | LTR/Copia | 99 | 22360 |
| RLC-unnamedfam49 | LTR/Copia | 33 | 5773 |
| RLC-unnamedfam5 | LTR/Copia | 1560 | 459486 |
| RLC-unnamedfam51 | LTR/Copia | 250 | 56189 |
| RLC-unnamedfam52 | LTR/Copia | 83 | 14479 |
| RLC-unnamedfam53 | LTR/Copia | 93 | 22396 |
| RLC-unnamedfam54 | LTR/Copia | 55 | 8390 |
| RLC-unnamedfam55 | LTR/Copia | 24 | 5328 |
| RLC-unnamedfam56 | LTR/Copia | 49 | 7851 |
| RLC-unnamedfam58 | LTR/Copia | 38 | 7001 |
| RLC-unnamedfam6 | LTR/Copia | 7508 | 2066765 |
| RLC-unnamedfam7 | LTR/Copia | 615 | 139039 |
| RLC-unnamedfam8 | LTR/Copia | 119 | 31534 |
| RLC-unnamedfam9 | LTR/Copia | 48 | 4910 |
| RLG-Carmilla | LTR/Gypsy | 6398 | 2159749 |
| RLG-Cereba | LTR/Gypsy | 80916 | 26327710 |
| RLG-Danae | LTR/Gypsy | 3472 | 918463 |
| RLG-Daniela | LTR/Gypsy | 2024 | 592329 |
| RLG-Derami | LTR/Gypsy | 76499 | 21621969 |
| RLG-Egug | LTR/Gypsy | 138511 | 40309662 |
| RLG-Erika | LTR/Gypsy | 7214 | 2214239 |
| RLG-Fatima | LTR/Gypsy | 149865 | 46907119 |
| RLG-HORGY | LTR/Gypsy | 45 | 12852 |
| RLG-Hawi | LTR/Gypsy | 875 | 225871 |
| RLG-Ifis | LTR/Gypsy | 45726 | 14502309 |
| RLG-Jeli | LTR/Gypsy | 24501 | 7334543 |
| RLG-Latidu | LTR/Gypsy | 7427 | 2079922 |
| RLG-Laura | LTR/Gypsy | 30638 | 9539135 |
| RLG-Lila | LTR/Gypsy | 3173 | 901544 |
| RLG-Lisa | LTR/Gypsy | 791 | 206790 |
| RLG-Nusif | LTR/Gypsy | 13627 | 3713320 |
| RLG-Olivia | LTR/Gypsy | 142 | 30964 |
| RLG-Quinta | LTR/Gypsy | 13161 | 3941317 |
| RLG-Romana | LTR/Gypsy | 1004 | 247955 |
| RLG-Romani | LTR/Gypsy | 21747 | 6431662 |
| RLG-Sabrina | LTR/Gypsy | 549395 | 161872316 |
| RLG-Sakura | LTR/Gypsy | 175746 | 51538969 |
| RLG-Sumana | LTR/Gypsy | 42916 | 12370278 |

| | | | |
|---|---|---|---|
| RLG-Sumaya | LTR/Gypsy | 5465 | 1437701 |
| RLG-WHAM | LTR/Gypsy | 211526 | 65445213 |
| RLG-Wilma | LTR/Gypsy | 257020 | 78748989 |
| RLG-unnamedfam1 | LTR/Gypsy | 210 | 48212 |
| RLG-unnamedfam10 | LTR/Gypsy | 957 | 292711 |
| RLG-unnamedfam100 | LTR/Gypsy | 319 | 81310 |
| RLG-unnamedfam101 | LTR/Gypsy | 560 | 160986 |
| RLG-unnamedfam102 | LTR/Gypsy | 1743 | 560104 |
| RLG-unnamedfam103 | LTR/Gypsy | 7 | 1198 |
| RLG-unnamedfam104 | LTR/Gypsy | 536 | 123722 |
| RLG-unnamedfam105 | LTR/Gypsy | 138 | 35393 |
| RLG-unnamedfam106 | LTR/Gypsy | 328 | 73710 |
| RLG-unnamedfam107 | LTR/Gypsy | 3 | 647 |
| RLG-unnamedfam108 | LTR/Gypsy | 989 | 298294 |
| RLG-unnamedfam109 | LTR/Gypsy | 167 | 50150 |
| RLG-unnamedfam11 | LTR/Gypsy | 8 | 2536 |
| RLG-unnamedfam110 | LTR/Gypsy | 326 | 75459 |
| RLG-unnamedfam111 | LTR/Gypsy | 189 | 54121 |
| RLG-unnamedfam112 | LTR/Gypsy | 541 | 133356 |
| RLG-unnamedfam113 | LTR/Gypsy | 376 | 90811 |
| RLG-unnamedfam114 | LTR/Gypsy | 651 | 192704 |
| RLG-unnamedfam115 | LTR/Gypsy | 218 | 65732 |
| RLG-unnamedfam116 | LTR/Gypsy | 11 | 1578 |
| RLG-unnamedfam117 | LTR/Gypsy | 138 | 24986 |
| RLG-unnamedfam118 | LTR/Gypsy | 141 | 24353 |
| RLG-unnamedfam119 | LTR/Gypsy | 52 | 10922 |
| RLG-unnamedfam12 | LTR/Gypsy | 223 | 32230 |
| RLG-unnamedfam120 | LTR/Gypsy | 207 | 39072 |
| RLG-unnamedfam121 | LTR/Gypsy | 17 | 2927 |
| RLG-unnamedfam122 | LTR/Gypsy | 33 | 3760 |
| RLG-unnamedfam123 | LTR/Gypsy | 94 | 21315 |
| RLG-unnamedfam13 | LTR/Gypsy | 7687 | 2274154 |
| RLG-unnamedfam14 | LTR/Gypsy | 2488 | 689295 |
| RLG-unnamedfam15 | LTR/Gypsy | 271 | 68801 |
| RLG-unnamedfam16 | LTR/Gypsy | 1597 | 338955 |
| RLG-unnamedfam17 | LTR/Gypsy | 37 | 6996 |
| RLG-unnamedfam18 | LTR/Gypsy | 40 | 11803 |
| RLG-unnamedfam19 | LTR/Gypsy | 13 | 2464 |
| RLG-unnamedfam2 | LTR/Gypsy | 131 | 23855 |
| RLG-unnamedfam20 | LTR/Gypsy | 116 | 23251 |
| RLG-unnamedfam21 | LTR/Gypsy | 181 | 41183 |
| RLG-unnamedfam22 | LTR/Gypsy | 189 | 41705 |
| RLG-unnamedfam23 | LTR/Gypsy | 18 | 3124 |
| RLG-unnamedfam24 | LTR/Gypsy | 438 | 108430 |
| RLG-unnamedfam25 | LTR/Gypsy | 1189 | 249421 |
| RLG-unnamedfam26 | LTR/Gypsy | 244 | 43937 |
| RLG-unnamedfam27 | LTR/Gypsy | 502 | 122147 |

| | | | |
|---|---|---|---|
| RLG-unnamedfam28 | LTR/Gypsy | 22 | 7055 |
| RLG-unnamedfam29 | LTR/Gypsy | 178 | 35678 |
| RLG-unnamedfam3 | LTR/Gypsy | 146 | 26536 |
| RLG-unnamedfam30 | LTR/Gypsy | 1306 | 337659 |
| RLG-unnamedfam31 | LTR/Gypsy | 55 | 15992 |
| RLG-unnamedfam32 | LTR/Gypsy | 102 | 21470 |
| RLG-unnamedfam33 | LTR/Gypsy | 369 | 87149 |
| RLG-unnamedfam34 | LTR/Gypsy | 51 | 12588 |
| RLG-unnamedfam35 | LTR/Gypsy | 80 | 19185 |
| RLG-unnamedfam36 | LTR/Gypsy | 452 | 124244 |
| RLG-unnamedfam37 | LTR/Gypsy | 1002 | 195617 |
| RLG-unnamedfam38 | LTR/Gypsy | 128 | 34163 |
| RLG-unnamedfam4 | LTR/Gypsy | 514 | 146405 |
| RLG-unnamedfam40 | LTR/Gypsy | 26 | 5032 |
| RLG-unnamedfam41 | LTR/Gypsy | 285 | 39754 |
| RLG-unnamedfam42 | LTR/Gypsy | 120 | 24421 |
| RLG-unnamedfam43 | LTR/Gypsy | 88 | 19978 |
| RLG-unnamedfam44 | LTR/Gypsy | 592 | 129204 |
| RLG-unnamedfam45 | LTR/Gypsy | 668 | 182924 |
| RLG-unnamedfam46 | LTR/Gypsy | 94 | 15986 |
| RLG-unnamedfam47 | LTR/Gypsy | 44 | 11529 |
| RLG-unnamedfam48 | LTR/Gypsy | 587 | 185582 |
| RLG-unnamedfam49 | LTR/Gypsy | 345 | 50399 |
| RLG-unnamedfam5 | LTR/Gypsy | 33 | 7413 |
| RLG-unnamedfam50 | LTR/Gypsy | 825 | 175835 |
| RLG-unnamedfam51 | LTR/Gypsy | 12 | 2341 |
| RLG-unnamedfam53 | LTR/Gypsy | 69 | 19481 |
| RLG-unnamedfam54 | LTR/Gypsy | 2445 | 736379 |
| RLG-unnamedfam55 | LTR/Gypsy | 39 | 10668 |
| RLG-unnamedfam56 | LTR/Gypsy | 101 | 21710 |
| RLG-unnamedfam57 | LTR/Gypsy | 57 | 11958 |
| RLG-unnamedfam58 | LTR/Gypsy | 939 | 246802 |
| RLG-unnamedfam59 | LTR/Gypsy | 168 | 35052 |
| RLG-unnamedfam6 | LTR/Gypsy | 196 | 45997 |
| RLG-unnamedfam61 | LTR/Gypsy | 445 | 114176 |
| RLG-unnamedfam62 | LTR/Gypsy | 123 | 22055 |
| RLG-unnamedfam63 | LTR/Gypsy | 111 | 27575 |
| RLG-unnamedfam64 | LTR/Gypsy | 1239 | 338153 |
| RLG-unnamedfam65 | LTR/Gypsy | 256 | 69436 |
| RLG-unnamedfam66 | LTR/Gypsy | 381 | 106468 |
| RLG-unnamedfam67 | LTR/Gypsy | 32 | 7381 |
| RLG-unnamedfam68 | LTR/Gypsy | 495 | 145793 |
| RLG-unnamedfam69 | LTR/Gypsy | 21 | 3063 |
| RLG-unnamedfam7 | LTR/Gypsy | 466 | 84325 |
| RLG-unnamedfam70 | LTR/Gypsy | 155 | 35914 |
| RLG-unnamedfam71 | LTR/Gypsy | 88 | 24830 |
| RLG-unnamedfam72 | LTR/Gypsy | 1305 | 358060 |

| | | | |
|---|---|---|---|
| RLG-unnamedfam73 | LTR/Gypsy | 188 | 40287 |
| RLG-unnamedfam74 | LTR/Gypsy | 209 | 46418 |
| RLG-unnamedfam75 | LTR/Gypsy | 11 | 1373 |
| RLG-unnamedfam76 | LTR/Gypsy | 474 | 104112 |
| RLG-unnamedfam77 | LTR/Gypsy | 1 | 159 |
| RLG-unnamedfam78 | LTR/Gypsy | 472 | 126065 |
| RLG-unnamedfam79 | LTR/Gypsy | 1619 | 290210 |
| RLG-unnamedfam8 | LTR/Gypsy | 258 | 62698 |
| RLG-unnamedfam80 | LTR/Gypsy | 962 | 252412 |
| RLG-unnamedfam81 | LTR/Gypsy | 430 | 116583 |
| RLG-unnamedfam82 | LTR/Gypsy | 2789 | 875538 |
| RLG-unnamedfam83 | LTR/Gypsy | 720 | 191944 |
| RLG-unnamedfam84 | LTR/Gypsy | 880 | 241106 |
| RLG-unnamedfam85 | LTR/Gypsy | 1476 | 432669 |
| RLG-unnamedfam86 | LTR/Gypsy | 622 | 172890 |
| RLG-unnamedfam87 | LTR/Gypsy | 2780 | 853380 |
| RLG-unnamedfam88 | LTR/Gypsy | 207 | 54997 |
| RLG-unnamedfam89 | LTR/Gypsy | 691 | 157833 |
| RLG-unnamedfam9 | LTR/Gypsy | 233 | 57653 |
| RLG-unnamedfam90 | LTR/Gypsy | 122 | 29079 |
| RLG-unnamedfam91 | LTR/Gypsy | 20 | 2950 |
| RLG-unnamedfam92 | LTR/Gypsy | 158 | 40335 |
| RLG-unnamedfam93 | LTR/Gypsy | 904 | 258944 |
| RLG-unnamedfam94 | LTR/Gypsy | 1087 | 299289 |
| RLG-unnamedfam95 | LTR/Gypsy | 246 | 40033 |
| RLG-unnamedfam96 | LTR/Gypsy | 61 | 11160 |
| RLG-unnamedfam97 | LTR/Gypsy | 386 | 89393 |
| RLG-unnamedfam98 | LTR/Gypsy | 59 | 7111 |
| RLG-unnamedfam99 | LTR/Gypsy | 65 | 12661 |
| RLX-Artem | LTR | 169 | 31079 |
| RLX-Cassandra | LTR | 76 | 7421 |
| RLX-Eway | LTR | 122 | 25040 |
| RLX-Ginger | LTR | 6919 | 1862683 |
| RLX-Gujog | LTR | 7343 | 2091428 |
| RLX-Varna | LTR | 136 | 30753 |
| RLX-Veju | LTR | 439 | 104587 |
| RLX-Xalax | LTR | 5494 | 1648074 |
| RLX-unnamedfam1 | LTR | 156 | 30067 |
| RLX-unnamedfam10 | LTR | 82 | 13684 |
| RLX-unnamedfam11 | LTR | 42 | 7259 |
| RLX-unnamedfam12 | LTR | 166 | 42929 |
| RLX-unnamedfam13 | LTR | 46 | 6761 |
| RLX-unnamedfam14 | LTR | 1138 | 366377 |
| RLX-unnamedfam15 | LTR | 9 | 2472 |
| RLX-unnamedfam16 | LTR/Gypsy | 1065 | 255371 |
| RLX-unnamedfam17 | LTR | 2524 | 736188 |
| RLX-unnamedfam18 | LTR | 140 | 27698 |

| | | | |
|---|---|---|---|
| RLX-unnamedfam19 | LTR | 212 | 46785 |
| RLX-unnamedfam2 | LTR | 359 | 106199 |
| RLX-unnamedfam21 | LTR | 177 | 36831 |
| RLX-unnamedfam22 | LTR | 553 | 156619 |
| RLX-unnamedfam23 | LTR | 81 | 13596 |
| RLX-unnamedfam24 | LTR | 106 | 29419 |
| RLX-unnamedfam26 | LTR | 51 | 14364 |
| RLX-unnamedfam27 | LTR | 191 | 49540 |
| RLX-unnamedfam28 | LTR | 640 | 113410 |
| RLX-unnamedfam29 | LTR | 6 | 1552 |
| RLX-unnamedfam3 | LTR | 179 | 49784 |
| RLX-unnamedfam30 | LTR | 110 | 23380 |
| RLX-unnamedfam31 | LTR | 1627 | 413161 |
| RLX-unnamedfam32 | LTR | 145 | 37018 |
| RLX-unnamedfam33 | LTR | 793 | 230620 |
| RLX-unnamedfam34 | LTR | 189 | 41366 |
| RLX-unnamedfam35 | LTR | 498 | 103576 |
| RLX-unnamedfam36 | LTR | 806 | 94157 |
| RLX-unnamedfam37 | LTR | 127 | 33582 |
| RLX-unnamedfam38 | LTR | 22 | 4577 |
| RLX-unnamedfam39 | LTR | 79 | 22624 |
| RLX-unnamedfam4 | LTR | 1124 | 355010 |
| RLX-unnamedfam5 | LTR | 261 | 74290 |
| RLX-unnamedfam7 | LTR | 305 | 84475 |
| RLX-unnamedfam8 | LTR | 180 | 49606 |
| RLX-unnamedfam9 | LTR | 71 | 10633 |
| SIX-Dido | SINE | 106 | 14329 |
| SIX-unnamedfam1 | SINE | 380 | 56992 |
| SIX-unnamedfam2 | SINE | 10 | 958 |
| SRSiCMCM00100001 | LTR/Copia | 4 | 629 |
| SRSiCMCMOOT00001 | Retroelement | 1 | 108 |
| SRSiOTOT00000004 | Other | 1 | 59 |
| SRSiOTOT00000014 | Other | 3 | 658 |
| SRSiOTOT00000015 | LTR/Gypsy | 4 | 701 |
| SRSiRGRR00000001 | rRNA | 4 | 610 |
| SRSiRGRR00000035 | rRNA | 7 | 273 |
| SRSiRGRR00000058 | rRNA | 1 | 30 |
| SRSiRGRR00000063 | rRNA | 1 | 77 |
| SRSiRGRR00000069 | rRNA | 1 | 100 |
| SRSiRGRR00500003 | rRNA | 4 | 114 |
| SRSiTEMT06000001 | DNA/Mite | 2 | 292 |
| SRSiTEMT06000002 | DNA/Mite | 1 | 123 |
| SRSiTEMT06000004 | DNA/Mite | 5 | 309 |
| SRSiTEMTOOT00003 | DNA/Mite | 1 | 37 |
| SRSiTERT00200003 | LTR/Gypsy | 1 | 193 |
| SRSiTERTOOT00028 | LTR | 7 | 489 |
| SRSiTERTOOT00050 | LTR | 1 | 83 |

| | | | |
|---|---|---|---|
| SRSiTERTOOT00058 | nonLTR | 2 | 226 |
| SRSiTERTOOT00059 | nonLTR | 23 | 3257 |
| SRSiTERTOOT00060 | nonLTR | 66 | 11835 |
| SRSiTERTOOT00061 | nonLTR | 6 | 918 |
| SRSiTERTOOT00069 | nonLTR | 30 | 5454 |
| SRSiTERTOOT00070 | nonLTR | 2 | 129 |
| SRSiTERTOOT00071 | LTR | 1 | 317 |
| SRSiTERTOOT00072 | LTR | 9 | 421 |
| SRSiTERTOOT00074 | nonLTR | 7 | 511 |
| SRSiTERTOOT00077 | nonLTR | 136 | 32931 |
| SUBTEL_sat | Satellite | 1 | 65 |
| Sabrina-LTR-1.2 | LTR | 6 | 1063 |
| Sabrina-LTR-2.1 | LTR | 8 | 1065 |
| Sabrina-LTR-3 | LTR | 4 | 730 |
| Sc-Revolver | DNA | 683 | 103555 |
| Sc-Superior | DNA | 19 | 2288 |
| Sukkula-LTR-2.2 | LTR/Gypsy | 5 | 665 |
| TIGR-ORSgTERT00100053 | LTR/Copia | 4 | 570 |
| TIGR-ORSgTERT00100127 | LTR/Copia | 1 | 48 |
| TIGR-ORSgTERT00100133 | LTR/Copia | 4 | 1376 |
| TIGR-ORSgTERT00100240 | LTR/Copia | 2 | 443 |
| TIGR-ORSgTERT00100363 | LTR/Copia | 6 | 953 |
| TIGR-ORSgTERT00100416 | LTR/Copia | 4 | 710 |
| TIGR-ORSgTERT00100558 | LTR/Copia | 1 | 227 |
| TIGR-ORSgTERT00200888 | LTR/Gypsy | 2 | 84 |
| TIGR-ORSgTERT00201131 | LTR/Gypsy | 3 | 462 |
| TIGR-ORSgTERT00201155 | LTR/Gypsy | 1 | 80 |
| TIGR-ORSgTERT00201289 | LTR/Gypsy | 2 | 150 |
| TIGR-ORSgTERT00201382 | LTR/Gypsy | 1 | 238 |
| TIGR-TRSiTERTOOT00060 | LTR | 27 | 2746 |
| TIGR-ZRSgTERTOOT00064 | LTR/Copia | 2 | 194 |
| TIGR-ZRSiTERTOOT00348 | LTR | 1 | 125 |
| TIGR-ZRSiTERTOOT00349 | LTR | 1 | 299 |
| TRSiCMCMOOT00001 | LTR | 38 | 3824 |
| TRSiOTOT00000001 | LINE | 265 | 50169 |
| TRSiOTOT00000003 | Other | 340 | 57611 |
| TRSiOTOT00000004 | DNA/hAT | 6 | 657 |
| TRSiOTOT00000005 | DNA/hAT | 4 | 455 |
| TRSiOTOT00000006 | Other | 750 | 156614 |
| TRSiOTOT00000008 | Other | 4 | 442 |
| TRSiOTOT00000009 | Other | 3 | 178 |
| TRSiOTOT00000010 | Other | 9 | 761 |
| TRSiOTOT00000019 | rRNA | 108 | 23091 |
| TRSiRGRR00000002 | rRNA | 3 | 714 |
| TRSiRGRR00000006 | rRNA | 4 | 497 |
| TRSiRGRR00000009 | rRNA | 10 | 2074 |
| TRSiRGRR00000012 | rRNA | 1 | 49 |

| | | | |
|---|---|---|---|
| TRSiRGRR00000013 | rRNA | 2 | 170 |
| TRSiRGRR00000014 | rRNA | 3 | 358 |
| TRSiRGRR00000015 | rRNA | 1 | 63 |
| TRSiRGRR00000016 | rRNA | 129 | 34234 |
| TRSiRGRR00000017 | rRNA | 1 | 44 |
| TRSiRGRR00000024 | rRNA | 22 | 3636 |
| TRSiRGRR00000029 | rRNA | 1 | 112 |
| TRSiRGRR00000051 | rRNA | 7 | 2044 |
| TRSiRGRR00000062 | rRNA | 84 | 28676 |
| TRSiRGRR00500006 | rRNA | 3 | 737 |
| TRSiRGRR00500039 | rRNA | 29 | 7299 |
| TRSiRGRR00500055 | rRNA | 2 | 78 |
| TRSiRGRR00500062 | rRNA | 2 | 289 |
| TRSiRGRR00500068 | rRNA | 373 | 97368 |
| TRSiRGRR00500078 | rRNA | 1 | 57 |
| TRSiRGRR00500081 | rRNA | 5 | 501 |
| TRSiRGRR00500101 | rRNA | 455 | 66725 |
| TRSiRGRR00500106 | rRNA | 13 | 1384 |
| TRSiRGRR00500120 | rRNA | 2 | 157 |
| TRSiRGRR00500121 | rRNA | 2 | 271 |
| TRSiRGRR00500123 | rRNA | 2 | 132 |
| TRSiRGRR00500141 | rRNA | 8 | 830 |
| TRSiRGRR00500176 | rRNA | 26 | 5637 |
| TRSiRGRR00500182 | rRNA | 48 | 8064 |
| TRSiRGRR00500189 | rRNA | 172 | 32298 |
| TRSiRGRR00500190 | rRNA | 134 | 48474 |
| TRSiRGRR00500209 | rRNA | 4 | 490 |
| TRSiRGRR00500218 | rRNA | 10 | 1120 |
| TRSiRGRR00500226 | rRNA | 9 | 967 |
| TRSiRGRR00500227 | rRNA | 57 | 12220 |
| TRSiRGRR00500243 | rRNA | 5 | 357 |
| TRSiRGRR00500254 | rRNA | 4 | 865 |
| TRSiRGRR00500260 | rRNA | 5 | 415 |
| TRSiRGRR00500266 | rRNA | 82 | 22545 |
| TRSiRGRR00500274 | rRNA | 3 | 250 |
| TRSiRGRR00500281 | rRNA | 29 | 3103 |
| TRSiRGRR00500286 | rRNA | 3 | 547 |
| TRSiRGRR00500288 | rRNA | 20 | 2931 |
| TRSiRGRR00500289 | rRNA | 24 | 6194 |
| TRSiRGRR00500290 | rRNA | 12 | 1777 |
| TRSiRGRR00500291 | rRNA | 2 | 338 |
| TRSiRGRR00500292 | rRNA | 2 | 193 |
| TRSiRGRR00500293 | rRNA | 3 | 311 |
| TRSiRGRR00500320 | rRNA | 6 | 731 |
| TRSiRGRR00500329 | rRNA | 9 | 1341 |
| TRSiRGRR00500330 | rRNA | 7 | 1000 |
| TRSiRGRR00500332 | rRNA | 1 | 310 |

| | | | |
|---|---|---|---|
| TRSiRGRR00500334 | rRNA | 20 | 2706 |
| TRSiTEMT06000002 | DNA/Mite | 37 | 5391 |
| TRSiTEMT06000003 | DNA/Mite | 5 | 358 |
| TRSiTERT00100004 | LTR/Copia | 4 | 749 |
| TRSiTERT00100022 | LTR/Copia | 3 | 358 |
| TRSiTERT00100024 | LTR/Copia | 1 | 54 |
| TRSiTERT00100025 | LTR/Copia | 2 | 145 |
| TRSiTERT00100032 | LTR/Copia | 8 | 1028 |
| TRSiTERT00100036 | LTR/Copia | 4 | 427 |
| TRSiTERT00100037 | LTR/Copia | 1 | 71 |
| TRSiTERT00100051 | LTR/Copia | 3 | 249 |
| TRSiTERT00100054 | LTR/Copia | 2 | 115 |
| TRSiTERT00100055 | LTR/Copia | 8 | 989 |
| TRSiTERT00100056 | LTR/Copia | 15 | 2104 |
| TRSiTERT00100061 | LTR/Copia | 2 | 224 |
| TRSiTERT00100062 | LTR/Copia | 1 | 80 |
| TRSiTERT00100069 | LTR/Copia | 2 | 396 |
| TRSiTERT00100070 | LTR/Copia | 1 | 151 |
| TRSiTERT00100073 | LTR/Copia | 2 | 296 |
| TRSiTERT00100074 | LTR/Copia | 3 | 291 |
| TRSiTERT00100077 | LTR/Copia | 3 | 195 |
| TRSiTERT00100078 | LTR/Copia | 2 | 209 |
| TRSiTERT00100081 | LTR/Copia | 1 | 111 |
| TRSiTERT00100082 | LTR/Copia | 4 | 371 |
| TRSiTERT00100083 | LTR/Copia | 1 | 57 |
| TRSiTERT00100084 | Retroelement | 4 | 491 |
| TRSiTERT00100085 | LTR/Copia | 2 | 361 |
| TRSiTERT00100087 | LTR/Copia | 3 | 681 |
| TRSiTERT00100088 | LTR/Copia | 28 | 2820 |
| TRSiTERT00100090 | LTR/Copia | 3 | 563 |
| TRSiTERT00100091 | LTR/Copia | 1 | 91 |
| TRSiTERT00100092 | LTR/Copia | 2 | 100 |
| TRSiTERT00100093 | LTR/Copia | 1 | 228 |
| TRSiTERT00100094 | LTR/Copia | 1 | 179 |
| TRSiTERT00100095 | LTR/Copia | 47 | 10599 |
| TRSiTERT00100097 | LTR/Copia | 34 | 6533 |
| TRSiTERT00200001 | LTR/Gypsy | 240 | 69431 |
| TRSiTERT00200002 | LTR/Gypsy | 97 | 20507 |
| TRSiTERT00200003 | LTR/Gypsy | 22 | 3192 |
| TRSiTERT00200004 | LTR/Gypsy | 20 | 5411 |
| TRSiTERT00200005 | LTR/Gypsy | 18 | 4414 |
| TRSiTERT00200006 | LTR/Gypsy | 7 | 1102 |
| TRSiTERT00200008 | LTR/Gypsy | 1583 | 501394 |
| TRSiTERT00200009 | LTR/Gypsy | 215 | 50795 |
| TRSiTERT00200010 | LTR/Gypsy | 330 | 87311 |
| TRSiTERT00200011 | LTR/Gypsy | 215 | 63072 |
| TRSiTERT00200013 | LTR/Gypsy | 366 | 96415 |

| | | | |
|---|---|---|---|
| TRSiTERT00200014 | LTR/Gypsy | 480 | 153224 |
| TRSiTERT00200017 | LTR/Gypsy | 100 | 28807 |
| TRSiTERT00200018 | LTR/Gypsy | 345 | 90025 |
| TRSiTERTOOT00003 | LTR | 33 | 4837 |
| TRSiTERTOOT00004 | Retroelement | 5 | 826 |
| TRSiTERTOOT00005 | LTR | 4 | 263 |
| TRSiTERTOOT00006 | LTR | 12 | 700 |
| TRSiTERTOOT00007 | LTR | 1 | 60 |
| TRSiTERTOOT00008 | LTR | 21 | 5340 |
| TRSiTERTOOT00009 | LTR | 13 | 1849 |
| TRSiTERTOOT00010 | LTR | 49 | 12191 |
| TRSiTERTOOT00011 | LTR | 8 | 857 |
| TRSiTERTOOT00012 | LTR | 24 | 3700 |
| TRSiTERTOOT00014 | Retroelement | 17 | 2561 |
| TRSiTERTOOT00016 | Retroelement | 34 | 2627 |
| TRSiTERTOOT00020 | LTR | 41 | 2816 |
| TRSiTERTOOT00021 | LTR | 33 | 2062 |
| TRSiTETN00200002 | DNA/En-Spm | 86 | 19032 |
| TRSiTETN00400001 | DNA/TcMar | 1 | 82 |
| TRSiTETN00400002 | DNA/TcMar | 3 | 688 |
| TRSiTETN00400003 | DNA/TcMar | 3 | 397 |
| TRSiTETN00400005 | DNA/TcMar | 7 | 1655 |
| XXX-XC | Other | 598 | 112792 |
| XXX-Xabor | Other | 3150 | 629509 |
| XXX-Xanti | Other | 70 | 8934 |
| XXX-Xenos | Other | 63 | 6609 |
| XXX-unnamedfam1 | Other | 1742 | 375796 |
| XXX-unnamedfam10 | Other | 8 | 446 |
| XXX-unnamedfam100 | Other | 1045 | 148617 |
| XXX-unnamedfam101 | Other | 409 | 72943 |
| XXX-unnamedfam102 | Other | 30 | 4300 |
| XXX-unnamedfam103 | Other | 4 | 373 |
| XXX-unnamedfam104 | Other | 5 | 563 |
| XXX-unnamedfam105 | Other | 88 | 20098 |
| XXX-unnamedfam106 | Other | 232 | 31932 |
| XXX-unnamedfam107 | Other | 12 | 1001 |
| XXX-unnamedfam108 | Other | 9 | 1318 |
| XXX-unnamedfam109 | Other | 48 | 5902 |
| XXX-unnamedfam11 | Other | 9 | 637 |
| XXX-unnamedfam110 | Other | 59 | 12809 |
| XXX-unnamedfam111 | Other | 217 | 36110 |
| XXX-unnamedfam112 | Other | 55 | 6736 |
| XXX-unnamedfam113 | Other | 43 | 10869 |
| XXX-unnamedfam114 | Other | 1 | 109 |
| XXX-unnamedfam115 | Other | 40 | 6355 |
| XXX-unnamedfam116 | Other | 1563 | 396997 |
| XXX-unnamedfam117 | Other | 874 | 177550 |

| | | | |
|---|---|---|---|
| XXX-unnamedfam118 | Other | 19 | 2822 |
| XXX-unnamedfam119 | Other | 5 | 525 |
| XXX-unnamedfam12 | Other | 129 | 18304 |
| XXX-unnamedfam120 | Other | 40 | 6379 |
| XXX-unnamedfam121 | Other | 239 | 37993 |
| XXX-unnamedfam122 | Other | 196 | 50995 |
| XXX-unnamedfam123 | Other | 1 | 125 |
| XXX-unnamedfam124 | Other | 171 | 35679 |
| XXX-unnamedfam125 | Other | 162 | 44530 |
| XXX-unnamedfam126 | Other | 77 | 11079 |
| XXX-unnamedfam127 | Other | 22 | 2656 |
| XXX-unnamedfam128 | Other | 1 | 74 |
| XXX-unnamedfam129 | Other | 41 | 6026 |
| XXX-unnamedfam13 | Other | 147 | 9880 |
| XXX-unnamedfam130 | Other | 84 | 10719 |
| XXX-unnamedfam131 | Other | 35 | 7966 |
| XXX-unnamedfam132 | Other | 16 | 2096 |
| XXX-unnamedfam133 | Other | 2 | 123 |
| XXX-unnamedfam134 | Other | 499 | 141540 |
| XXX-unnamedfam135 | Other | 23 | 5993 |
| XXX-unnamedfam136 | Other | 22 | 6051 |
| XXX-unnamedfam137 | Other | 141 | 17710 |
| XXX-unnamedfam138 | Other | 10 | 801 |
| XXX-unnamedfam139 | Other | 397 | 68803 |
| XXX-unnamedfam14 | Other | 558 | 88775 |
| XXX-unnamedfam140 | Other | 8 | 1649 |
| XXX-unnamedfam141 | Other | 1198 | 351462 |
| XXX-unnamedfam142 | Other | 150 | 32833 |
| XXX-unnamedfam143 | Other | 263 | 56328 |
| XXX-unnamedfam144 | Other | 343 | 69224 |
| XXX-unnamedfam145 | Other | 21 | 6048 |
| XXX-unnamedfam147 | Other | 904 | 281225 |
| XXX-unnamedfam149 | Other | 1660 | 424001 |
| XXX-unnamedfam15 | Other | 1536 | 260291 |
| XXX-unnamedfam150 | Other | 222 | 28794 |
| XXX-unnamedfam151 | Other | 42 | 5965 |
| XXX-unnamedfam152 | Other | 388 | 80349 |
| XXX-unnamedfam153 | Other | 4 | 164 |
| XXX-unnamedfam154 | Other | 26 | 2483 |
| XXX-unnamedfam155 | Other | 3144 | 782544 |
| XXX-unnamedfam156 | Other | 401 | 80741 |
| XXX-unnamedfam157 | Other | 62 | 10688 |
| XXX-unnamedfam158 | Other | 150 | 27216 |
| XXX-unnamedfam159 | Other | 448 | 62417 |
| XXX-unnamedfam16 | Other | 63 | 14518 |
| XXX-unnamedfam160 | Other | 29 | 4841 |
| XXX-unnamedfam161 | Other | 6 | 744 |

| | | | |
|---|---|---|---|
| XXX-unnamedfam162 | Other | 141 | 39240 |
| XXX-unnamedfam163 | Other | 70 | 16608 |
| XXX-unnamedfam164 | Other | 1312 | 397729 |
| XXX-unnamedfam165 | Other | 1461 | 380149 |
| XXX-unnamedfam166 | Other | 577 | 145672 |
| XXX-unnamedfam167 | Other | 24 | 1343 |
| XXX-unnamedfam168 | Other | 8 | 695 |
| XXX-unnamedfam169 | Other | 178 | 33075 |
| XXX-unnamedfam17 | Other | 7 | 1010 |
| XXX-unnamedfam170 | Other | 445 | 89360 |
| XXX-unnamedfam171 | Other | 11 | 1253 |
| XXX-unnamedfam172 | Other | 47 | 9651 |
| XXX-unnamedfam173 | Other | 817 | 167341 |
| XXX-unnamedfam174 | Other | 2 | 261 |
| XXX-unnamedfam175 | Other | 11 | 1921 |
| XXX-unnamedfam176 | Other | 10 | 2810 |
| XXX-unnamedfam177 | Other | 1092 | 191957 |
| XXX-unnamedfam178 | Other | 2103 | 426949 |
| XXX-unnamedfam179 | Other | 109 | 9817 |
| XXX-unnamedfam18 | Other | 185 | 22796 |
| XXX-unnamedfam180 | Other | 6 | 1225 |
| XXX-unnamedfam181 | Other | 831 | 165040 |
| XXX-unnamedfam182 | Other | 119 | 26275 |
| XXX-unnamedfam183 | Other | 314 | 83875 |
| XXX-unnamedfam184 | Other | 52 | 4230 |
| XXX-unnamedfam185 | Other | 545 | 76062 |
| XXX-unnamedfam186 | Other | 291 | 63905 |
| XXX-unnamedfam187 | Other | 713 | 68721 |
| XXX-unnamedfam188 | Other | 388 | 57917 |
| XXX-unnamedfam189 | Other | 11 | 801 |
| XXX-unnamedfam19 | Other | 805 | 131824 |
| XXX-unnamedfam191 | Other | 1088 | 222801 |
| XXX-unnamedfam192 | Other | 1 | 187 |
| XXX-unnamedfam193 | Other | 127 | 34786 |
| XXX-unnamedfam195 | Other | 187 | 31205 |
| XXX-unnamedfam196 | Other | 250 | 54364 |
| XXX-unnamedfam197 | Other | 53 | 12433 |
| XXX-unnamedfam198 | Other | 64 | 7898 |
| XXX-unnamedfam199 | Other | 1226 | 213341 |
| XXX-unnamedfam2 | Other | 605 | 103833 |
| XXX-unnamedfam20 | Other | 394 | 73362 |
| XXX-unnamedfam200 | Other | 358 | 87580 |
| XXX-unnamedfam201 | Other | 591 | 115087 |
| XXX-unnamedfam202 | Other | 169 | 38054 |
| XXX-unnamedfam22 | Other | 27 | 5864 |
| XXX-unnamedfam23 | Other | 99 | 11152 |
| XXX-unnamedfam24 | Other | 22 | 5865 |

| | | | |
|---|---|---|---|
| XXX-unnamedfam25 | Other | 26 | 3763 |
| XXX-unnamedfam26 | Other | 11 | 1386 |
| XXX-unnamedfam27 | Other | 72 | 8016 |
| XXX-unnamedfam28 | Other | 18 | 2475 |
| XXX-unnamedfam29 | Other | 31 | 8046 |
| XXX-unnamedfam3 | Other | 876 | 74554 |
| XXX-unnamedfam30 | Other | 34 | 6331 |
| XXX-unnamedfam31 | Other | 100 | 16040 |
| XXX-unnamedfam32 | Other | 218 | 30948 |
| XXX-unnamedfam33 | Other | 106 | 26547 |
| XXX-unnamedfam35 | Other | 70 | 10795 |
| XXX-unnamedfam36 | Other | 10 | 2280 |
| XXX-unnamedfam37 | Other | 408 | 62715 |
| XXX-unnamedfam38 | Other | 321 | 64002 |
| XXX-unnamedfam39 | Other | 1 | 169 |
| XXX-unnamedfam4 | Other | 287 | 22021 |
| XXX-unnamedfam40 | Other | 22 | 5182 |
| XXX-unnamedfam41 | Other | 31 | 8107 |
| XXX-unnamedfam42 | Other | 300 | 31470 |
| XXX-unnamedfam43 | Other | 20 | 1429 |
| XXX-unnamedfam44 | Other | 466 | 121730 |
| XXX-unnamedfam45 | Other | 66 | 19468 |
| XXX-unnamedfam46 | Other | 27 | 6099 |
| XXX-unnamedfam47 | Other | 11 | 2459 |
| XXX-unnamedfam48 | Other | 30 | 6208 |
| XXX-unnamedfam49 | Other | 81 | 19730 |
| XXX-unnamedfam5 | Other | 167 | 23757 |
| XXX-unnamedfam51 | Other | 212 | 23294 |
| XXX-unnamedfam52 | Other | 94 | 21683 |
| XXX-unnamedfam53 | Other | 1028 | 217098 |
| XXX-unnamedfam54 | Other | 1105 | 201837 |
| XXX-unnamedfam55 | Other | 264 | 61776 |
| XXX-unnamedfam56 | Other | 107 | 21975 |
| XXX-unnamedfam57 | Other | 2334 | 510127 |
| XXX-unnamedfam58 | Other | 127 | 30217 |
| XXX-unnamedfam59 | Other | 235 | 50417 |
| XXX-unnamedfam6 | Other | 80 | 12981 |
| XXX-unnamedfam60 | Other | 67 | 18241 |
| XXX-unnamedfam61 | Other | 35 | 5324 |
| XXX-unnamedfam63 | Other | 1034 | 271876 |
| XXX-unnamedfam65 | Other | 8 | 1285 |
| XXX-unnamedfam66 | Other | 53 | 11335 |
| XXX-unnamedfam67 | Other | 120 | 14607 |
| XXX-unnamedfam68 | Other | 61 | 9899 |
| XXX-unnamedfam69 | Other | 28 | 3701 |
| XXX-unnamedfam7 | Other | 170 | 29593 |
| XXX-unnamedfam70 | Other | 3 | 580 |

| | | | |
|---|---|---|---|
| XXX-unnamedfam71 | Other | 246 | 35759 |
| XXX-unnamedfam72 | Other | 648 | 98702 |
| XXX-unnamedfam73 | Other | 16 | 3789 |
| XXX-unnamedfam74 | Other | 59 | 13792 |
| XXX-unnamedfam75 | Other | 554 | 85285 |
| XXX-unnamedfam76 | Other | 54 | 6851 |
| XXX-unnamedfam77 | Other | 61 | 6229 |
| XXX-unnamedfam78 | Other | 136 | 26270 |
| XXX-unnamedfam79 | Other | 92 | 22797 |
| XXX-unnamedfam8 | Other | 141 | 13854 |
| XXX-unnamedfam80 | Other | 165 | 36711 |
| XXX-unnamedfam81 | Other | 697 | 154452 |
| XXX-unnamedfam83 | Other | 46 | 8893 |
| XXX-unnamedfam84 | Other | 58 | 12449 |
| XXX-unnamedfam85 | Other | 134 | 24816 |
| XXX-unnamedfam86 | Other | 6 | 781 |
| XXX-unnamedfam87 | Other | 331 | 61247 |
| XXX-unnamedfam88 | Other | 107 | 21505 |
| XXX-unnamedfam89 | Other | 7 | 929 |
| XXX-unnamedfam9 | Other | 363 | 61343 |
| XXX-unnamedfam90 | Other | 4 | 264 |
| XXX-unnamedfam91 | Other | 3 | 339 |
| XXX-unnamedfam92 | Other | 20 | 3859 |
| XXX-unnamedfam93 | Other | 8 | 948 |
| XXX-unnamedfam94 | Other | 204 | 23166 |
| XXX-unnamedfam95 | Other | 2 | 207 |
| XXX-unnamedfam96 | Other | 8 | 643 |
| XXX-unnamedfam97 | Other | 287 | 66908 |
| XXX-unnamedfam98 | Other | 21 | 2130 |
| XXX-unnamedfam99 | Other | 35 | 3719 |
| ZRSiCMCMOOT00011 | Other | 1 | 138 |
| ZRSiCMCMOOT00014 | Other | 9 | 1054 |
| ZRSiCMCMOOT00016 | Other | 1 | 60 |
| ZRSiCMCMOOT00020 | Other | 1 | 54 |
| ZRSiCMCMOOT00033 | Other | 41 | 7338 |
| ZRSiOTOT00000008 | Other | 40 | 5034 |
| ZRSiOTOT00000009 | Other | 8 | 560 |
| ZRSiOTOT00000010 | Other | 119 | 20373 |
| ZRSiOTOT00000011 | Other | 1 | 49 |
| ZRSiOTOT00000015 | Other | 66 | 8262 |
| ZRSiOTOT00000018 | Other | 46 | 6153 |
| ZRSiOTOT00000019 | DNA/hAT | 20 | 1562 |
| ZRSiOTOT00000020 | DNA/hAT | 26 | 2309 |
| ZRSiOTOT00000021 | DNA/hAT | 38 | 4428 |
| ZRSiOTOT00000022 | DNA/hAT | 52 | 5102 |
| ZRSiOTOT00000023 | DNA/hAT | 21 | 1772 |
| ZRSiOTOT00000043 | DNA | 1 | 59 |

| | | | |
|---|---|---|---|
| ZRSiOTOT00000045 | Other | 2 | 90 |
| ZRSiRGRR00000004 | rRNA | 13 | 3901 |
| ZRSiRGRR00000019 | rRNA | 2 | 233 |
| ZRSiRGRR00000035 | rRNA | 832 | 119883 |
| ZRSiRGRR00000037 | rRNA | 1 | 91 |
| ZRSiRGRR00000041 | rRNA | 566 | 103124 |
| ZRSiRGRR00000042 | rRNA | 347 | 65613 |
| ZRSiRGRR00500007 | rRNA | 89 | 11270 |
| ZRSiRGRR00500008 | rRNA | 35 | 12404 |
| ZRSiRGRR00500009 | rRNA | 138 | 24765 |
| ZRSiTEMT00600001 | DNA/Mite | 2 | 186 |
| ZRSiTEMT05500008 | DNA/Mite | 1 | 177 |
| ZRSiTEMT05500014 | DNA/Mite | 9 | 852 |
| ZRSiTEMT05500017 | DNA/Mite | 13 | 840 |
| ZRSiTEMT05500030 | DNA/Mite | 2 | 204 |
| ZRSiTEMT05500042 | DNA/Mite | 2 | 290 |
| ZRSiTEMT05900032 | DNA/Harbinger | 17 | 1725 |
| ZRSiTEMT05900033 | DNA/Harbinger | 52 | 6316 |
| ZRSiTEMT05900034 | DNA/Harbinger | 19 | 1677 |
| ZRSiTERT00100007 | LTR/Copia | 1 | 188 |
| ZRSiTERT00100023 | LTR/Copia | 1 | 78 |
| ZRSiTERT00100057 | LTR/Copia | 1 | 45 |
| ZRSiTERT00100066 | LTR/Copia | 1 | 203 |
| ZRSiTERT00100076 | DNA | 11 | 2316 |
| ZRSiTERT00100077 | LTR/Copia | 1 | 85 |
| ZRSiTERT00100079 | LTR/Copia | 7 | 762 |
| ZRSiTERT00200057 | LTR/Gypsy | 3 | 144 |
| ZRSiTERT00300001 | LINE | 4 | 522 |
| ZRSiTERT00300003 | LINE | 1 | 37 |
| ZRSiTERT00300004 | LINE | 19 | 1811 |
| ZRSiTERT00300005 | LINE | 50 | 7427 |
| ZRSiTERT00300006 | LINE | 50 | 4327 |
| ZRSiTERTOOT00001 | LTR/Gypsy | 1 | 76 |
| ZRSiTERTOOT00032 | LTR/Gypsy | 4 | 156 |
| ZRSiTERTOOT00090 | LTR | 24 | 3761 |
| ZRSiTERTOOT00106 | LTR/Gypsy | 7 | 444 |
| ZRSiTERTOOT00126 | LTR/Gypsy | 4 | 268 |
| ZRSiTERTOOT00130 | DNA | 12 | 2866 |
| ZRSiTERTOOT00132 | LTR | 73 | 9061 |
| ZRSiTERTOOT00133 | LTR/Gypsy | 41 | 7519 |
| ZRSiTERTOOT00326 | LTR | 7 | 1036 |
| ZRSiTETN00100001 | DNA/hAT | 16 | 2197 |
| ZRSiTETN00100007 | DNA/hAT-Ac | 1 | 178 |
| ZRSiTETN00300001 | DNA/MuDR | 3 | 460 |
| ZRSiTETN00300003 | DNA/MuDR | 1 | 162 |
| ZRSiTETN00300006 | DNA/MuDR | 25 | 5227 |
| ZRSiTETN00300007 | DNA/MuDR | 5 | 1868 |

| | | | |
|---|---|---|---|
| ZRSiTETN00300009 | DNA/MuDR | 2 | 216 |
| ZRSiTETN00300014 | DNA/MuDR | 33 | 7514 |
| ZRSiTETN00300037 | DNA/MuDR | 13 | 759 |
| ZRSiTETN00400001 | DNA/TcMar | 1 | 91 |
| ZRSiTETN00400004 | DNA/TcMar | 1 | 143 |
| ZRSiTETN00400012 | DNA/TcMar | 1 | 355 |
| ZRSiTETNOOT00005 | DNA/En-Spm | 4 | 581 |
| ZRSiTETNOOT00008 | DNA/Harbinger | 1 | 79 |
| ZRSiTETNOOT00011 | Other | 77 | 13646 |
| ZRSiTETNOOT00016 | DNA/Harbinger | 49 | 10977 |
| ZRSiTETNOOT00018 | DNA | 2 | 72 |
| Zm-AC145225.7 | LTR/Copia | 1 | 193 |
| Zm-AC145226.4 | LTR | 145 | 20648 |
| Zm-AC145227.7 | LTR/Copia | 2 | 358 |
| Zm-AC145228.9 | LTR/Gypsy | 11 | 1901 |
| Zm-AC145261.8 | LTR/Gypsy | 11 | 1652 |
| Zm-AC145262.7 | LTR | 15 | 1487 |
| Zm-AC145263.8 | Retroelement | 6 | 876 |
| Zm-AC145726.6 | LTR/Copia | 5 | 460 |
| Zm-AC145729.6 | LTR/Gypsy | 94 | 22800 |
| Zm-AC146349.4 | LTR/Copia | 8 | 1298 |
| Zm-AC146713.7 | LTR/Gypsy | 2 | 285 |
| Zm-AC146762.2 | LTR | 1 | 145 |
| Zm-AC146763.2 | LTR/Gypsy | 3 | 317 |
| Zm-AC146796.5 | LTR/Gypsy | 9 | 823 |
| Zm-AC146809.3 | LTR/Copia | 1 | 49 |
| Zm-AC146811.6 | LTR | 8 | 1031 |
| Zm-AC146812.6 | LTR/Gypsy | 2 | 98 |
| Zm-AC146813.3 | LTR | 167 | 38972 |
| Zm-AC146814.6 | LTR | 2 | 137 |
| Zm-AC146974.4 | LTR/Copia | 41 | 10674 |
| Zm-AC147015.8 | Retroelement | 9 | 922 |
| Zm-AC147016.3 | LTR/Gypsy | 2 | 191 |
| Zm-AC147814.3 | LTR/Copia | 1 | 86 |
| Zm-AC148080.4 | LTR/Gypsy | 3 | 280 |
| Zm-AC148081.5 | LTR/Gypsy | 2 | 199 |
| Zm-AC148083.6 | LTR/Gypsy | 1 | 136 |
| Zm-AC148084.5 | LTR/Copia | 1 | 115 |
| Zm-AC148093.6 | LTR/Gypsy | 2 | 223 |
| Zm-AC148099.5 | LTR/Gypsy | 34 | 6485 |
| Zm-AC148100.2 | LTR | 3 | 243 |
| Zm-AC148111.4 | LTR/Gypsy | 6 | 593 |
| Zm-AC148112.7 | LTR | 15 | 1316 |
| Zm-AC148152.3 | LTR/Copia | 10 | 1503 |
| Zm-AC148159.5 | Retroelement | 1 | 61 |
| Zm-AC148160.3 | LTR/Copia | 1 | 36 |
| Zm-AC148161.3 | LTR/Copia | 2 | 206 |

| | | | |
|---|---|---|---|
| Zm-AC148163.6 | LTR | 4 | 500 |
| Zm-AC148164.3 | LTR | 2 | 123 |
| Zm-AC148167.6 | LTR/Gypsy | 1 | 35 |
| Zm-AC148169.2 | LTR/Copia | 4 | 878 |
| Zm-AC148173.2 | LTR | 15 | 2417 |
| Zm-AC148243.4 | LTR/Gypsy | 2 | 149 |
| Zm-AC148350.3 | LTR | 2 | 205 |
| Zm-AC148394.3 | Retroelement | 2 | 294 |
| Zm-AC148479.2 | LTR | 6 | 1053 |
| Zm-AC148480.2 | LTR | 23 | 5700 |
| Zm-AC149477.2 | LTR/Gypsy | 23 | 3287 |
| Zm-AC149833.2 | LTR | 2 | 84 |
| Zm-AC149834.1 | LTR/Gypsy | 3 | 223 |
| Zm-AC149835.2 | LTR/Copia | 2 | 217 |
| Zm1S-101L | LTR/Copia | 101 | 7415 |
| Zm1S-102L | LTR/Copia | 1 | 171 |
| Zm1S-106L | LTR/Copia | 4 | 232 |
| Zm1S-107L | LTR/Copia | 1 | 52 |
| Zm1S-108L | LTR/Gypsy | 56 | 8308 |
| Zm1S-110L | LTR/Copia | 1 | 221 |
| Zm1S-114L | LTR/Copia | 9 | 734 |
| Zm1S-117L | LTR/Gypsy | 1 | 64 |
| Zm1S-121L | LTR/Copia | 1 | 93 |
| Zm1S-125L | LTR/Gypsy | 2 | 179 |
| Zm1S-126L | LTR | 2 | 188 |
| Zm1S-130L | LTR/Gypsy | 1 | 237 |
| Zm1S-133L | LTR | 1 | 55 |
| Zm1S-134L | LTR/Copia | 1 | 110 |
| Zm1S-135L | LTR | 1 | 72 |
| Zm1S-136L | LTR/Copia | 31 | 4718 |
| Zm1S-139L | LTR | 3 | 781 |
| Zm1S-13L | LTR/Copia | 2 | 200 |
| Zm1S-144L | LTR/Copia | 1 | 85 |
| Zm1S-145L | LTR/Gypsy | 2 | 218 |
| Zm1S-19L | LTR/Gypsy | 1 | 103 |
| Zm1S-1L | LTR/Gypsy | 4 | 891 |
| Zm1S-23L | LTR/Gypsy | 12 | 912 |
| Zm1S-24L | LTR/Copia | 1 | 275 |
| Zm1S-25L | LTR | 1 | 141 |
| Zm1S-29L | LTR/Copia | 1 | 457 |
| Zm1S-2L | LTR | 1 | 28 |
| Zm1S-31L | LTR | 1 | 47 |
| Zm1S-32L | LTR/Copia | 3 | 387 |
| Zm1S-3L | LTR/Copia | 1 | 256 |
| Zm1S-42L | LTR | 2 | 151 |
| Zm1S-43L | LTR/Gypsy | 6 | 353 |
| Zm1S-4L | LTR/Gypsy | 1 | 59 |

| | | | |
|---|---|---|---|
| Zm1S-50L | LTR/Gypsy | 4 | 321 |
| Zm1S-53L | LTR/Gypsy | 7 | 744 |
| Zm1S-56L | LTR | 4 | 317 |
| Zm1S-57L | LTR/Gypsy | 3 | 235 |
| Zm1S-58L | LTR | 1 | 129 |
| Zm1S-60L | Retroelement | 133 | 28345 |
| Zm1S-62L | LTR/Copia | 9 | 548 |
| Zm1S-69L | LTR/Copia | 5 | 384 |
| Zm1S-6L | LTR/Gypsy | 1 | 43 |
| Zm1S-72L | LTR/Copia | 7 | 675 |
| Zm1S-74L | LTR | 2 | 557 |
| Zm1S-80L | LTR | 1 | 61 |
| Zm1S-83L | LTR | 8 | 394 |
| Zm1S-85L | Retroelement | 3 | 282 |
| Zm1S-88L | LTR | 6 | 339 |
| Zm1S-89L | LTR | 1 | 79 |
| Zm1S-90B | LTR | 2 | 346 |
| Zm1S-93L | LTR/Gypsy | 3 | 478 |
| Zm1S-98L | LTR | 1 | 58 |
| Zm1S-9L | LTR | 4 | 457 |
| Zm9L-100L | LTR/Copia | 14 | 910 |
| Zm9L-102L | LTR/Gypsy | 1 | 43 |
| Zm9L-108L | LTR/Copia | 4 | 232 |
| Zm9L-112L | LTR/Copia | 2 | 96 |
| Zm9L-115L | LTR | 2 | 150 |
| Zm9L-11L | LTR/Copia | 2 | 153 |
| Zm9L-13L | Retroelement | 1 | 186 |
| Zm9L-15L | LTR/Copia | 4 | 649 |
| Zm9L-16L | LTR/Gypsy | 1 | 71 |
| Zm9L-18L | LTR/Copia | 4 | 1479 |
| Zm9L-25L | LTR/Copia | 1 | 316 |
| Zm9L-29L | LTR/Copia | 2 | 106 |
| Zm9L-2L | LTR | 2 | 202 |
| Zm9L-32L | LTR | 4 | 409 |
| Zm9L-34L | LTR | 2 | 181 |
| Zm9L-37L | LTR/Copia | 14 | 1609 |
| Zm9L-39L | LTR/Copia | 1 | 94 |
| Zm9L-3L | LTR/Copia | 1 | 324 |
| Zm9L-41L | LTR | 1 | 78 |
| Zm9L-49L | LTR/Copia | 3 | 436 |
| Zm9L-50L | LTR/Copia | 1 | 89 |
| Zm9L-53L | LTR | 1 | 78 |
| Zm9L-54L | LTR/Gypsy | 1 | 172 |
| Zm9L-56L | LTR | 1 | 88 |
| Zm9L-58L | LTR/Gypsy | 2 | 425 |
| Zm9L-59L | LTR/Copia | 2 | 101 |
| Zm9L-61L | LTR/Gypsy | 1 | 150 |

| | | | |
|---|---|---|---|
| Zm9L-67L | LTR/Gypsy | 3 | 175 |
| Zm9L-69L | LTR | 123 | 16075 |
| Zm9L-70L | LTR | 2 | 664 |
| Zm9L-71L | LTR/Gypsy | 1 | 40 |
| Zm9L-72L | LTR/Copia | 1 | 107 |
| Zm9L-78L | LTR | 3 | 293 |
| Zm9L-80L | LTR | 7 | 1327 |
| Zm9L-98L | LTR/Gypsy | 1 | 80 |
| adyrog-311G2-1 | LTR | 2 | 68 |
| adyrog-311G2-2 | Retroelement | 7 | 365 |
| alari-405L8-1 | LTR/Copia | 442 | 112654 |
| alejandra-354M17-1 | LTR | 498 | 139361 |
| angela-206C17-1 | LTR/Copia | 530 | 148793 |
| angela-206C17-2 | LTR/Copia | 274 | 48800 |
| angela-258C22-1 | LTR/Copia | 183 | 42293 |
| angela-258C22-2solo | LTR/Copia | 70 | 23326 |
| angela-258C22-3 | LTR/Copia | 703 | 161840 |
| angela-258C22-4solohdnm | LTR/Copia | 66 | 14100 |
| angela-258C22-5part | LTR/Copia | 324 | 85637 |
| angela-258C22-6part | LTR/Copia | 412 | 93960 |
| angela-354M17-1 | LTR/Copia | 347 | 78733 |
| angela-354M17-3 | LTR/Copia | 339 | 79836 |
| angela-354M17-4 | LTR/Copia | 282 | 57629 |
| angela-354M17-5part | LTR/Copia | 659 | 225471 |
| angela-405L8-1 | LTR/Copia | 181 | 29893 |
| angela-5K14-1 | LTR/Copia | 148 | 34233 |
| angela-5K14-2 | LTR/Copia | 565 | 109352 |
| angela-5K14-3part | LTR/Copia | 217 | 59550 |
| angela-5K14-4part | LTR/Copia | 7 | 733 |
| angela-5K14-5part | LTR/Copia | 517 | 157666 |
| angela-5K14-6part | DNA/En-Spm | 6 | 1249 |
| angela-5K14-7part | LTR/Copia | 24 | 6024 |
| angela-af326781-4 | LTR/Copia | 192 | 33802 |
| angela-af326781-8solo | LTR/Copia | 19 | 2261 |
| angela-af326781-9 | LTR/Copia | 49 | 16588 |
| angela-af326781-a | LTR/Copia | 217 | 48947 |
| angela-af326781-bsolo | LTR/Copia | 12 | 2021 |
| antechinus-osj-retroryza | LTR | 6 | 873 |
| bare-259I16-2 | LTR/Copia | 3 | 388 |
| bare-635P2-2 | LTR/Copia | 3 | 638 |
| bsr1 | LTR | 3 | 201 |
| cinful-2 | LTR/Gypsy | 1 | 59 |
| cinful-573F08-2 | LTR/Gypsy | 2 | 108 |
| cinful-ac146814-1 | LTR/Gypsy | 1 | 150 |
| cinful-ac148161-1 | LTR/Gypsy | 1 | 39 |
| copia | LTR/Copia | 1 | 56 |
| dagaf-576C20-1 | LTR | 2 | 148 |

| | | | |
|---|---|---|---|
| dagaf-af391808-1solopart | LTR | 1 | 148 |
| deiho-35P03-1 | LTR/Gypsy | 2 | 402 |
| dendrobat-osj | LTR | 2 | 312 |
| doerum-ac079037-1 | LTR | 1 | 283 |
| donuil-106M24-1 | LTR/Copia | 1 | 60 |
| ebeuk-311G2-1 | LTR | 1 | 52 |
| echidne-osj | LTR | 21 | 3823 |
| ekasi-35P03-1 | LTR/Copia | 36 | 6418 |
| erika-326E2-1part | LTR/Gypsy | 1209 | 369315 |
| etut-573F08-1 | Other | 19 | 4591 |
| eugene | LTR/Gypsy | 1 | 383 |
| evum-134O12-2 | LTR/Gypsy | 1 | 141 |
| fasu-333J11-1part | LTR | 8 | 1513 |
| fatima-258C22-1part | LTR/Gypsy | 177 | 50534 |
| fatima-326E2-1 | LTR/Gypsy | 367 | 109634 |
| fatima-af326781-1 | LTR/Gypsy | 737 | 243638 |
| fourf | LTR/Copia | 2 | 482 |
| fourf-138B04-1 | LTR/Copia | 1 | 165 |
| fourf-ac146814-1 | LTR/Copia | 2 | 303 |
| giepum-333J11-1hdnm | LTR/Copia | 3 | 289 |
| giepum-438D03-1 | LTR/Copia | 1 | 51 |
| giepum-ac116033-1 | LTR/Copia | 1 | 80 |
| giepum-ac145226-1 | LTR/Copia | 2 | 183 |
| giepum-ac145452-1 | LTR/Copia | 9 | 709 |
| giepum-ac146811-1 | LTR/Copia | 2 | 147 |
| giepum-ac147518-1 | LTR/Copia | 1 | 123 |
| giepum-ac148100-1 | LTR/Copia | 8 | 692 |
| giepum-ac148350-1 | LTR/Copia | 3 | 159 |
| giepum-ac149034-1 | LTR/Copia | 2 | 238 |
| grande-013I05-1 | LTR/Gypsy | 3 | 265 |
| grande-092E12-1part | LTR/Gypsy | 2 | 199 |
| grande-464F01-1part | LTR/Gypsy | 4 | 192 |
| grande-ac145228-1 | LTR/Gypsy | 1 | 81 |
| grande-af090447-1 | LTR/Gypsy | 5 | 324 |
| grande-af448416-1 | LTR/Gypsy | 2 | 120 |
| grande-af546188-1 | LTR/Gypsy | 1 | 209 |
| grande3 | LTR/Gypsy | 1 | 40 |
| gyma-438D03-1 | LTR | 2 | 521 |
| gypsy | LTR/Gypsy | 1 | 63 |
| hobe-311G2-1 | Other | 13 | 1269 |
| hopi-retroryza | LTR | 1 | 176 |
| huck-138B04-1part | LTR/Gypsy | 17 | 1189 |
| huck-333J11-1 | LTR/Gypsy | 1 | 138 |
| huck-438D03-1 | LTR/Gypsy | 1 | 125 |
| huck-438D03-2 | LTR/Gypsy | 2 | 185 |
| huck-573F08-1 | LTR/Gypsy | 1 | 31 |
| huck-573F08-2part | LTR/Gypsy | 1 | 72 |

| | | | |
|---|---|---|---|
| huck-573L14-2 | LTR/Gypsy | 1 | 109 |
| huck-5BL-1 | LTR/Gypsy | 8 | 260 |
| huck-9C20-1 | LTR/Gypsy | 4 | 448 |
| huck-ac144718-2 | LTR/Gypsy | 2 | 217 |
| huck-ac146813-1hdnm | LTR/Gypsy | 2 | 104 |
| huck-ac148082-1hdnm | LTR/Gypsy | 1 | 56 |
| huck-af391808-2 | LTR/Gypsy | 1 | 72 |
| huck-af391808-4hdnm | LTR/Gypsy | 1 | 51 |
| huck-at1 | LTR/Gypsy | 1 | 75 |
| ifisi-84L17-1 | LTR/Gypsy | 4 | 404 |
| ifisi-AP003348-1 | LTR/Gypsy | 1 | 64 |
| ifisi-ac079037-1 | LTR/Gypsy | 1 | 81 |
| ikeros-635P2-1 | LTR/Copia | 76 | 11155 |
| ilaw-464F01-1 | LTR/Copia | 5 | 865 |
| jaku-354M17-1 | LTR/Copia | 53 | 15704 |
| jaku-5K14-1 | LTR/Copia | 108 | 31323 |
| jeli-206C17-2solo | LTR | 56 | 11785 |
| ji-092E12-1 | LTR/Copia | 1 | 113 |
| ji-238E11-2part | LTR/Copia | 1 | 32 |
| ji-276N13-2 | LTR/Copia | 4 | 272 |
| ji-276N13-3 | LTR/Copia | 4 | 307 |
| ji-333J11-2 | LTR/Copia | 1 | 95 |
| ji-333J11-5hdnm | LTR/Copia | 1 | 75 |
| ji-334B07-3 | LTR/Copia | 1 | 249 |
| ji-573L14-2hdnm | LTR/Copia | 1 | 97 |
| ji-573L14-3part | LTR/Copia | 3 | 299 |
| ji-5BL-1 | LTR/Copia | 13 | 1189 |
| ji-9C20-3 | LTR/Copia | 3 | 255 |
| ji-ZMMBBc0007A01-1 | LTR/Copia | 3 | 215 |
| ji-ac145227-1 | LTR/Copia | 2 | 294 |
| ji-ac145227-2 | LTR/Copia | 1 | 109 |
| ji-ac145261-1 | LTR/Copia | 1 | 68 |
| ji-ac145727-1 | LTR/Copia | 1 | 68 |
| ji-ac146795-1 | LTR/Copia | 1 | 59 |
| ji-ac147016-1 | LTR/Copia | 1 | 46 |
| ji-ac147776-1hdnm | LTR/Copia | 3 | 526 |
| ji-ac147808-1hdnm | LTR/Copia | 1 | 53 |
| ji-ac148109-1 | LTR/Copia | 1 | 51 |
| ji-ac148163-2 | LTR/Copia | 1 | 46 |
| ji-ac148234-1 | LTR/Copia | 1 | 194 |
| ji-af090447-2 | LTR/Copia | 3 | 149 |
| ji-af391808-1 | LTR/Copia | 2 | 573 |
| josephine-af326781-1part | LTR | 2865 | 558654 |
| juriah-311G2-1 | LTR/Gypsy | 5 | 907 |
| k-16 | SINE | 2 | 104 |
| k-20 | SINE | 6 | 401 |
| k-27 | SINE | 2 | 173 |

| | | | |
|---|---|---|---|
| k-28 | SINE | 1 | 45 |
| k-29 | SINE | 2 | 295 |
| k-3 | DNA | 2 | 174 |
| k-57 | DNA | 1 | 93 |
| k-9 | DNA | 4 | 1205 |
| kake-334B07-1 | LTR | 1 | 479 |
| kangourou-osj | LTR | 5 | 794 |
| latidu-5K14-1 | LTR/Gypsy | 420 | 108968 |
| leviathan-98N8-1 | LTR/Gypsy | 1 | 52 |
| line-615K1-1 | LINE | 231 | 25140 |
| lolium-LTR | LTR | 593 | 93505 |
| machiavelli-ac145224-1 | LTR/Copia | 5 | 592 |
| machiavelli-ac148165-1 | LTR/Copia | 3 | 450 |
| magellan-cone | LTR/Gypsy | 46 | 4246 |
| milt-013I05-1 | LTR/Gypsy | 1 | 68 |
| milt-573L14-1 | LTR/Gypsy | 5 | 336 |
| misfit-5BL-1 | DNA/En-Spm | 1 | 118 |
| misfit-af391808-1 | DNA/En-Spm | 10 | 1013 |
| niwywi-354M17-1hdnm | LTR | 144 | 28404 |
| ofovin-311G2-1 | LTR/Copia | 5 | 441 |
| ofovin-311G2-3part | LTR/Copia | 1 | 117 |
| onap-SB45I19-1 | LTR/Gypsy | 1 | 213 |
| opie-092E12-1 | LTR/Copia | 1 | 125 |
| opie-573F08-3solo | LTR/Copia | 15 | 1692 |
| opie-ZMMBBc0007A01-1 | LTR/Copia | 4 | 337 |
| opie-ac145227-1 | LTR/Copia | 2 | 435 |
| opie-ac145261-1 | LTR/Copia | 1 | 50 |
| opie-ac146974-2 | LTR/Copia | 1 | 128 |
| opie-ac147776-1 | LTR/Copia | 1 | 54 |
| opie-ac148163-1 | LTR/Copia | 1 | 79 |
| opie-af090447-1 | LTR/Copia | 1 | 97 |
| opie-af090447-2 | LTR/Copia | 4 | 230 |
| opie-af090447-4 | LTR/Copia | 1 | 206 |
| opie-af448416-1part | LTR/Copia | 1 | 56 |
| opil-98N8-1part | LTR/Copia | 2 | 535 |
| ornithorynque-osj | LTR | 14 | 886 |
| osr01-AP005250-1 | LTR/Copia | 1 | 79 |
| osr12-retroryza | LTR/Copia | 7 | 1754 |
| osr14-retroryza | LTR | 14 | 3543 |
| osr15-retroryza | LTR/Copia | 4 | 414 |
| osr2-retroryza | LTR/Copia | 6 | 490 |
| osr20-retroryza | LTR/Copia | 1 | 127 |
| osr25-AP005618-2 | LTR | 2 | 287 |
| osr25-retroryza | LTR | 1 | 55 |
| osr28-retroryza | LTR | 51 | 9011 |
| osr29-retroryza | LTR/Gypsy | 4 | 615 |
| osr30-AP005618-1hdnm | LTR/Gypsy | 2 | 205 |

| | | | |
|---|---|---|---|
| osr30-retroryza | LTR/Gypsy | 3 | 303 |
| osr34-retroryza | LTR | 7 | 636 |
| osr36-retroryza | LTR/Gypsy | 15 | 1681 |
| osr37-AP005618-1hdnm | LTR | 23 | 2378 |
| osr37-retroryza | LTR | 18 | 3153 |
| osr40-AP005618-1 | LTR/Gypsy | 2 | 251 |
| osr40-retroryza | LTR/Gypsy | 1 | 37 |
| osr5-retroryza | LTR/Copia | 3 | 182 |
| osr6-retroryza | LTR | 5 | 269 |
| osr7-retroryza | LTR/Copia | 11 | 636 |
| osr9-retroryza | LTR | 51 | 7536 |
| park-24K23-1hdnm | LTR/Gypsy | 4 | 576 |
| pifi-SB95A23-1hdnm | LTR/Copia | 11 | 1969 |
| prem-1 | LTR/Gypsy | 1 | 126 |
| prem1-276N13-1 | LTR/Gypsy | 6 | 648 |
| prem1-276N13-2 | LTR/Gypsy | 2 | 235 |
| prem1-276N13-3 | LTR/Gypsy | 1 | 32 |
| prem1-276N13-4 | LTR/Gypsy | 2 | 145 |
| prem1-573F08-1 | LTR/Gypsy | 4 | 338 |
| prem1-Z195D10-1 | LTR/Gypsy | 1 | 47 |
| prem1-af391808-1 | LTR/Gypsy | 1 | 105 |
| psau3a9 | Other | 1 | 51 |
| pyrubu-170F08-1 | LTR/Copia | 1 | 157 |
| rafalski-4F | LTR/Copia | 5 | 1171 |
| rcs1 | Other | 44 | 5493 |
| retro-138B04-1 | LTR/Gypsy | 1 | 73 |
| retro-Z195D10-1 | LTR/Gypsy | 1 | 49 |
| retrosat1-retroryza | LTR/Gypsy | 3 | 534 |
| retrosor1 | LTR/Gypsy | 1 | 81 |
| rire1-ac148152-1 | LTR/Copia | 3 | 370 |
| rire1-af391808-1 | LTR/Copia | 7 | 1435 |
| rire2-84L17-1part | LTR/Gypsy | 1 | 140 |
| rire3 | LTR/Gypsy | 1 | 218 |
| rire3-ac022352-0 | LTR/Gypsy | 1 | 146 |
| rire3-retroryza | LTR | 1 | 121 |
| rire4-ltr | LTR/Gypsy | 2 | 570 |
| rire5-retroryza | LTR | 4 | 718 |
| rire7 | LTR/Copia | 3 | 292 |
| rire7-retroryza | LTR | 17 | 1998 |
| rire8-retroryza | LTR | 18 | 1628 |
| rire9-ltr | LTR/Gypsy | 10 | 972 |
| rn-103-58 | LTR/Gypsy | 57 | 13739 |
| rn-106-362 | LTR | 3 | 319 |
| rn-107-61 | LTR/Copia | 4 | 325 |
| rn-11-2 | LTR | 10 | 2012 |
| rn-110-271 | LTR/Copia | 2 | 170 |
| rn-111-158 | LTR/Copia | 1 | 41 |

| | | | |
|---|---|---|---|
| rn-116-67 | LTR | 10 | 1589 |
| rn-118-68 | LTR | 26 | 2433 |
| rn-119-116 | LTR | 11 | 2122 |
| rn-12-3 | LTR/Gypsy | 3 | 340 |
| rn-120-70 | LTR/Copia | 1 | 35 |
| rn-126-72 | LTR/Copia | 6 | 552 |
| rn-128-73 | LTR | 6 | 1198 |
| rn-129-74 | LTR | 42 | 8958 |
| rn-14-5 | LTR | 1 | 48 |
| rn-15-6 | LTR | 139 | 38379 |
| rn-150-84 | LTR | 40 | 3892 |
| rn-151-85 | LTR | 3 | 818 |
| rn-153-87 | LTR | 22 | 4259 |
| rn-154-162 | LTR | 36 | 2909 |
| rn-155-90 | LTR | 2 | 620 |
| rn-157-332 | LTR/Copia | 5 | 891 |
| rn-16-305 | LTR | 46 | 11430 |
| rn-161-163 | LTR/Gypsy | 27 | 3874 |
| rn-163-96 | LTR | 12 | 2420 |
| rn-171-247 | LTR/Gypsy | 6 | 835 |
| rn-172-100 | LTR | 65 | 8045 |
| rn-176-103 | LTR | 4 | 533 |
| rn-179-105 | LTR | 17 | 2385 |
| rn-18-313 | LTR | 3 | 200 |
| rn-180-106 | LTR | 164 | 35297 |
| rn-189-107 | LTR | 11 | 2152 |
| rn-2-350 | LTR/Copia | 23 | 6557 |
| rn-20-10 | LTR/Gypsy | 20 | 3213 |
| rn-200-117 | LTR | 9 | 2255 |
| rn-201-118 | LTR/Copia | 1 | 54 |
| rn-202-119 | LTR | 18 | 2787 |
| rn-206-352 | LTR | 3 | 362 |
| rn-207-122 | LTR/Copia | 14 | 3884 |
| rn-21-12 | LTR/Copia | 2 | 133 |
| rn-211-124 | LTR | 6 | 1173 |
| rn-215-125 | LTR | 4 | 347 |
| rn-216-127 | LTR | 19 | 1208 |
| rn-219-129 | LTR | 12 | 2978 |
| rn-22-13 | LTR | 21 | 4228 |
| rn-222-131 | LTR | 5 | 578 |
| rn-223-132 | LTR | 13 | 2619 |
| rn-226-134 | LTR | 1 | 342 |
| rn-228-214 | LTR | 2 | 588 |
| rn-24-14 | LTR | 29 | 5241 |
| rn-25-15 | LTR | 6 | 1410 |
| rn-250-144 | LTR/Gypsy | 23 | 4390 |
| rn-251-145 | LTR/Gypsy | 23 | 4727 |

| | | | |
|---|---|---|---|
| rn-252-146 | LTR | 1 | 340 |
| rn-253-147 | LTR | 2 | 298 |
| rn-255-148 | LTR | 8 | 963 |
| rn-26-390 | LTR/Gypsy | 31 | 5526 |
| rn-262-149 | LTR | 14 | 3374 |
| rn-265-150 | LTR | 23 | 5487 |
| rn-267-152 | LTR/Gypsy | 36 | 7647 |
| rn-27-17 | LTR | 4 | 794 |
| rn-28-18 | LTR | 1 | 80 |
| rn-3-19 | LTR | 21 | 3547 |
| rn-302-177 | LTR/Gypsy | 15 | 2929 |
| rn-303-178 | LTR/Gypsy | 54 | 11388 |
| rn-304-179 | LTR | 3 | 420 |
| rn-305-180 | LTR | 12 | 1179 |
| rn-306-181 | LTR | 15 | 3159 |
| rn-307-182 | LTR | 14 | 2994 |
| rn-308-183 | LTR | 1 | 229 |
| rn-312-184 | LTR | 3 | 930 |
| rn-313-186 | LTR | 9 | 964 |
| rn-314-187 | LTR | 9 | 1200 |
| rn-315-188 | LTR | 93 | 23104 |
| rn-351-194 | LTR/Copia | 2 | 444 |
| rn-352-195 | LTR/Gypsy | 34 | 6401 |
| rn-353-366 | LTR | 15 | 2521 |
| rn-356-197 | LTR | 2 | 254 |
| rn-359-198 | LTR | 13 | 1070 |
| rn-360-199 | LTR | 28 | 5796 |
| rn-363-200 | LTR | 106 | 13947 |
| rn-364-201 | LTR | 6 | 510 |
| rn-366-202 | LTR | 5 | 876 |
| rn-367-203 | LTR | 5 | 1345 |
| rn-369-204 | LTR/Gypsy | 27 | 5177 |
| rn-374-205 | LTR | 4 | 1218 |
| rn-375-206 | LTR | 22 | 2265 |
| rn-376-207 | LTR | 22 | 4188 |
| rn-377-208 | LTR | 24 | 5616 |
| rn-4-189 | LTR | 2 | 144 |
| rn-40-22 | LTR/Copia | 38 | 8752 |
| rn-402-75 | LTR | 2 | 206 |
| rn-403-215 | LTR/Gypsy | 20 | 2880 |
| rn-405-384 | LTR | 6 | 1293 |
| rn-406-217 | LTR | 8 | 1688 |
| rn-408-218 | LTR | 6 | 625 |
| rn-409-219 | LTR | 15 | 2379 |
| rn-410-220 | LTR | 11 | 2586 |
| rn-411-154 | LTR | 3 | 247 |
| rn-413-222 | LTR/Gypsy | 13 | 1762 |

| | | | |
|---|---|---|---|
| rn-414-223 | LTR/Copia | 10 | 1464 |
| rn-415-224 | LTR | 7 | 507 |
| rn-416-225 | LTR/Copia | 10 | 1885 |
| rn-417-136 | LTR | 58 | 11603 |
| rn-42-23 | LTR | 21 | 5627 |
| rn-43-24 | LTR | 6 | 878 |
| rn-44-26 | LTR | 165 | 32363 |
| rn-450-233 | LTR | 3 | 322 |
| rn-453-234 | LTR | 11 | 2593 |
| rn-455-235 | LTR/Gypsy | 6 | 971 |
| rn-456-236 | LTR | 2 | 376 |
| rn-457-190 | LTR | 3 | 171 |
| rn-458-237 | LTR | 17 | 4232 |
| rn-460-239 | LTR/Gypsy | 44 | 8547 |
| rn-462-240 | LTR | 2 | 155 |
| rn-463-241 | LTR/Gypsy | 29 | 3660 |
| rn-5-28 | LTR/Gypsy | 21 | 3400 |
| rn-50-33 | LTR | 13 | 2404 |
| rn-502-253 | LTR | 29 | 4691 |
| rn-503-254 | LTR | 5 | 411 |
| rn-504-255 | LTR | 11 | 1800 |
| rn-505-256 | LTR | 26 | 6185 |
| rn-507-257 | LTR | 6 | 528 |
| rn-51-338 | LTR | 20 | 5301 |
| rn-510-259 | LTR | 41 | 11384 |
| rn-511-260 | LTR/Gypsy | 22 | 3369 |
| rn-512-261 | LTR | 3 | 278 |
| rn-513-262 | LTR | 10 | 2049 |
| rn-517-264 | LTR | 11 | 3110 |
| rn-518-265 | LTR/Gypsy | 27 | 5330 |
| rn-52-35 | LTR | 8 | 1365 |
| rn-521-266 | LTR/Gypsy | 8 | 822 |
| rn-523-268 | LTR | 14 | 4084 |
| rn-525-269 | LTR | 8 | 1575 |
| rn-54-36 | LTR | 86 | 10882 |
| rn-55-37 | LTR/Gypsy | 4 | 308 |
| rn-550-280 | LTR | 1 | 335 |
| rn-551-281 | LTR | 15 | 2233 |
| rn-552-282 | LTR | 16 | 2688 |
| rn-56-38 | LTR | 3 | 185 |
| rn-566-287 | LTR | 23 | 4199 |
| rn-567-288 | LTR | 4 | 322 |
| rn-568-289 | LTR | 8 | 1305 |
| rn-569-290 | LTR | 1 | 36 |
| rn-57-39 | LTR/Gypsy | 28 | 5665 |
| rn-570-291 | LTR/Gypsy | 27 | 4456 |
| rn-573-292 | LTR | 10 | 1277 |

| | | | |
|---|---|---|---|
| rn-574-293 | LTR | 1 | 115 |
| rn-575-294 | LTR | 16 | 2433 |
| rn-60-156 | LTR | 26 | 4706 |
| rn-62-43 | LTR | 9 | 1394 |
| rn-65-44 | LTR | 12 | 2063 |
| rn-66-340 | LTR/Copia | 6 | 1187 |
| rn-68-46 | LTR/Copia | 7 | 2100 |
| rn-7-29 | LTR | 6 | 889 |
| rn-71-47 | LTR | 1 | 51 |
| rn-72-48 | LTR | 21 | 3095 |
| rn-73-138 | LTR | 46 | 6117 |
| rn-74-211 | LTR | 3 | 349 |
| rn-77-52 | LTR | 14 | 2859 |
| rn-81-157 | LTR | 3 | 374 |
| rn-83-55 | LTR | 407 | 21524 |
| rn-85-323 | LTR | 2 | 183 |
| ryba-311G2-1 | LTR/Copia | 6 | 1008 |
| sabrina-115G1-1 | LTR/Gypsy | 3532 | 996532 |
| sabrina-115G1-2 | LTR | 5164 | 1506400 |
| sabrina-116F2-2 | LTR | 3444 | 885406 |
| sabrina-5K14-1solo | LTR | 344 | 73449 |
| sabrina-5K14-2part | LTR | 1349 | 378910 |
| sabrina-745C13-1 | LTR/Gypsy | 61 | 8701 |
| sabrina-745C13-2hdnm | LTR/Gypsy | 453 | 73447 |
| sb-mite | DNA/Mite | 381 | 48483 |
| scaffold-25 | LTR | 3 | 301 |
| scaffold-78 | LTR/Copia | 34 | 9896 |
| sukkula-259I16-1solo | LTR/Gypsy | 19 | 2878 |
| taz-retroryza | LTR | 1 | 73 |
| tekay | LTR/Gypsy | 3 | 213 |
| tekay-af448416-1 | LTR/Gypsy | 2 | 326 |
| tos17-retroryza | LTR | 6 | 569 |
| trep10 | LTR/Copia | 183 | 40605 |
| trep100 | LTR/Copia | 6 | 670 |
| trep1000 | DNA/Stowaway | 4 | 238 |
| trep1004 | DNA/Stowaway | 8 | 422 |
| trep1009 | DNA/Stowaway | 7 | 426 |
| trep1016 | DNA/Stowaway | 96 | 7468 |
| trep1018 | DNA/Stowaway | 3 | 192 |
| trep1019 | DNA/Stowaway | 4 | 303 |
| trep102 | LTR/Gypsy | 11 | 1255 |
| trep1020 | DNA/Stowaway | 9 | 485 |
| trep1025 | DNA/Stowaway | 2 | 111 |
| trep1026 | DNA/Stowaway | 24 | 1682 |
| trep1027 | DNA/Stowaway | 14 | 666 |
| trep1029 | DNA/Stowaway | 2 | 139 |
| trep1030 | DNA/Stowaway | 7 | 405 |

| | | | |
|---|---|---|---|
| trep1031 | DNA/Stowaway | 2 | 134 |
| trep1034 | DNA/Stowaway | 1 | 39 |
| trep1035 | DNA/Stowaway | 14 | 735 |
| trep1036 | DNA/Stowaway | 1 | 47 |
| trep1037 | DNA/Stowaway | 3 | 249 |
| trep1038 | DNA/Stowaway | 7 | 485 |
| trep1041 | DNA/Stowaway | 8 | 637 |
| trep1042 | DNA/Stowaway | 5 | 491 |
| trep1045 | DNA/Stowaway | 1 | 58 |
| trep1047 | DNA/Stowaway | 12 | 916 |
| trep1048 | DNA/Stowaway | 2 | 112 |
| trep1049 | DNA/Stowaway | 19 | 1711 |
| trep105 | LTR/Copia | 1 | 141 |
| trep1050 | DNA/Stowaway | 41 | 3008 |
| trep1051 | DNA/Stowaway | 44 | 3284 |
| trep1054 | DNA/Stowaway | 50 | 4036 |
| trep1056 | DNA/Stowaway | 56 | 5347 |
| trep1066 | DNA/Stowaway | 29 | 2898 |
| trep1067 | DNA/Stowaway | 16 | 1031 |
| trep1068 | DNA/Stowaway | 106 | 10089 |
| trep1074 | DNA/Stowaway | 62 | 6264 |
| trep1076 | DNA/Stowaway | 9 | 721 |
| trep1077 | DNA/Stowaway | 29 | 2566 |
| trep1079 | DNA/Stowaway | 127 | 12567 |
| trep108 | LTR/Gypsy | 2 | 175 |
| trep1080 | DNA/Stowaway | 1 | 86 |
| trep1082 | DNA/Stowaway | 2 | 127 |
| trep1083 | DNA/Stowaway | 5 | 324 |
| trep1088 | DNA/Stowaway | 6 | 452 |
| trep1089 | DNA/Stowaway | 16 | 1127 |
| trep109 | LTR/Gypsy | 14 | 1086 |
| trep1091 | DNA/Stowaway | 3 | 185 |
| trep1092 | DNA/Stowaway | 33 | 3447 |
| trep1093 | DNA/Stowaway | 6 | 424 |
| trep1094 | DNA/Stowaway | 3 | 269 |
| trep1095 | DNA/Stowaway | 8 | 417 |
| trep1096 | DNA/Stowaway | 30 | 2337 |
| trep1097 | DNA/Stowaway | 31 | 2096 |
| trep1098 | DNA/Stowaway | 6 | 478 |
| trep1099 | DNA/Stowaway | 12 | 788 |
| trep110 | LTR/Copia | 6 | 639 |
| trep1100 | DNA/Stowaway | 29 | 2644 |
| trep1103 | DNA/Stowaway | 11 | 1009 |
| trep1104 | DNA/Stowaway | 11 | 804 |
| trep1105 | DNA/Stowaway | 2 | 184 |
| trep1107 | DNA/Stowaway | 25 | 2069 |
| trep1109 | DNA/Stowaway | 76 | 8104 |

| | | | |
|---|---|---|---|
| trep111 | LTR/Copia | 448 | 125255 |
| trep1110 | DNA/Stowaway | 16 | 1474 |
| trep1111 | DNA/Stowaway | 7 | 565 |
| trep1118 | DNA/Stowaway | 7 | 575 |
| trep112 | LTR/Copia | 297 | 74250 |
| trep1121 | DNA/Stowaway | 18 | 1186 |
| trep1123 | DNA/Stowaway | 11 | 1144 |
| trep1124 | DNA/Stowaway | 5 | 393 |
| trep1129 | DNA/Stowaway | 8 | 491 |
| trep1130 | DNA/Stowaway | 13 | 1130 |
| trep1131 | DNA/Stowaway | 4 | 323 |
| trep1132 | DNA/Stowaway | 5 | 266 |
| trep1133 | DNA/Stowaway | 5 | 423 |
| trep1138 | DNA/Stowaway | 2 | 114 |
| trep1139 | DNA/Stowaway | 88 | 10144 |
| trep114 | LTR/Copia | 136 | 28413 |
| trep1140 | DNA/Stowaway | 3 | 276 |
| trep1141 | DNA/Stowaway | 19 | 1603 |
| trep1142 | DNA/Stowaway | 7 | 812 |
| trep1143 | DNA/Stowaway | 6 | 473 |
| trep1145 | DNA/Stowaway | 62 | 5812 |
| trep1146 | DNA/Stowaway | 253 | 34071 |
| trep1148 | DNA/Stowaway | 4 | 377 |
| trep1149 | DNA/Stowaway | 8 | 674 |
| trep115 | LTR/Copia | 92 | 20024 |
| trep1150 | DNA/Stowaway | 33 | 5014 |
| trep1151 | DNA/Stowaway | 1 | 141 |
| trep1154 | DNA/Stowaway | 5 | 276 |
| trep1155 | DNA/Stowaway | 15 | 1506 |
| trep1156 | DNA/Stowaway | 7 | 802 |
| trep1157 | DNA/Stowaway | 7 | 971 |
| trep1159 | DNA/Stowaway | 4 | 267 |
| trep1160 | DNA/Stowaway | 8 | 811 |
| trep1161 | LTR/Copia | 52 | 9678 |
| trep1162 | LTR/Copia | 8 | 321 |
| trep1163 | Other | 1 | 163 |
| trep1164 | DNA/Mite | 19 | 2205 |
| trep1165 | Other | 2 | 106 |
| trep1167 | Other | 1 | 147 |
| trep1168 | Other | 4 | 500 |
| trep1169 | DNA | 338 | 57986 |
| trep1171 | DNA/MuDR | 201 | 34763 |
| trep119 | DNA/Stowaway | 2 | 84 |
| trep120 | DNA/Stowaway | 13 | 445 |
| trep1200 | DNA/Stowaway | 36 | 2872 |
| trep1202 | DNA/Stowaway | 25 | 2152 |
| trep1204 | DNA/En-Spm | 1 | 121 |

| | | | |
|---|---|---|---|
| trep1212 | DNA/En-Spm | 6 | 440 |
| trep1213 | LTR/Gypsy | 112 | 20209 |
| trep1214 | LTR/Gypsy | 139 | 20426 |
| trep1215 | LTR/Gypsy | 126 | 19736 |
| trep1217 | LTR/Gypsy | 40 | 7909 |
| trep1219 | LTR/Copia | 165 | 37513 |
| trep1220 | LTR/Copia | 256 | 77324 |
| trep1221 | LTR/Copia | 885 | 245088 |
| trep1223 | LTR/Gypsy | 143 | 36383 |
| trep1224 | LTR/Copia | 539 | 147063 |
| trep1225 | DNA/En-Spm | 81 | 13585 |
| trep1226 | LTR/Gypsy | 409 | 118153 |
| trep1228 | LTR/Gypsy | 158 | 32407 |
| trep123 | DNA/Stowaway | 3 | 154 |
| trep1230 | LTR/Gypsy | 87 | 10485 |
| trep1231 | LTR/Gypsy | 561 | 162624 |
| trep1234 | DNA/En-Spm | 345 | 84227 |
| trep1235 | LTR/Gypsy | 114 | 30981 |
| trep1236 | LTR/Gypsy | 28 | 9691 |
| trep1237 | LTR/Gypsy | 134 | 42506 |
| trep1238 | LTR/Gypsy | 1005 | 329198 |
| trep124 | DNA/En-Spm | 37 | 6259 |
| trep1240 | LTR/Gypsy | 1020 | 296847 |
| trep1241 | DNA/En-Spm | 665 | 144859 |
| trep1242 | LTR/Gypsy | 1041 | 277552 |
| trep1243 | LTR/Gypsy | 4212 | 1332526 |
| trep1244 | LTR/Gypsy | 3541 | 996672 |
| trep1245 | DNA/En-Spm | 4 | 739 |
| trep1246 | DNA/Stowaway | 79 | 15895 |
| trep1247 | DNA/MuDR | 71 | 11329 |
| trep1248 | DNA/En-Spm | 92 | 20744 |
| trep126 | DNA/En-Spm | 468 | 78475 |
| trep127 | LTR/Copia | 2849 | 846526 |
| trep128 | LTR/Copia | 192 | 36696 |
| trep129 | LTR/Copia | 239 | 63545 |
| trep1302 | DNA/En-Spm | 261 | 58374 |
| trep1303 | DNA/En-Spm | 446 | 119477 |
| trep1304 | DNA/En-Spm | 927 | 245294 |
| trep1305 | LTR/Gypsy | 263 | 66294 |
| trep1306 | LTR/Gypsy | 294 | 78768 |
| trep1307 | LTR/Copia | 197 | 57983 |
| trep1308 | LTR/Gypsy | 180 | 34132 |
| trep1309 | LTR/Gypsy | 246 | 66197 |
| trep1310 | LINE | 862 | 248825 |
| trep1311 | LTR/Gypsy | 430 | 118046 |
| trep1312 | LTR/Gypsy | 1233 | 361320 |
| trep1314 | LTR/Gypsy | 813 | 253232 |

| | | | |
|---|---|---|---|
| trep1316 | LTR/Copia | 624 | 164463 |
| trep1317 | LTR/Copia | 49 | 12027 |
| trep1318 | DNA/En-Spm | 1200 | 314543 |
| trep1320 | LTR/Gypsy | 3141 | 926632 |
| trep1321 | LTR/Gypsy | 4216 | 1321862 |
| trep1322 | DNA/En-Spm | 1448 | 422267 |
| trep1323 | LTR/Gypsy | 29 | 5980 |
| trep1325 | LTR/Copia | 146 | 47019 |
| trep1326 | LTR/Copia | 29 | 8413 |
| trep1401 | LTR/Copia | 5023 | 1423197 |
| trep1402 | LTR/Copia | 519 | 119268 |
| trep1403 | LTR/Copia | 3 | 588 |
| trep1405 | LTR/Copia | 6 | 1125 |
| trep1407 | LTR/Copia | 32 | 9120 |
| trep1408 | LTR/Gypsy | 172 | 38327 |
| trep1412 | LTR/Gypsy | 2623 | 847983 |
| trep1414 | LTR/Gypsy | 607 | 165136 |
| trep1415 | LTR/Gypsy | 967 | 283028 |
| trep1416 | LTR | 421 | 101545 |
| trep1417 | LTR/Gypsy | 413 | 111329 |
| trep1418 | LTR/Gypsy | 1502 | 449871 |
| trep1419 | LTR/Gypsy | 596 | 159180 |
| trep1420 | LTR/Gypsy | 261 | 62736 |
| trep1421 | LTR/Gypsy | 333 | 93422 |
| trep1422 | LTR/Gypsy | 1066 | 326517 |
| trep1423 | LTR/Copia | 5 | 1071 |
| trep1424 | LTR/Copia | 78 | 15118 |
| trep1425 | LTR/Gypsy | 2221 | 650430 |
| trep1430 | LTR/Gypsy | 1840 | 414249 |
| trep1435 | LTR/Copia | 60 | 7817 |
| trep1437 | LTR/Gypsy | 29 | 3589 |
| trep1438 | LTR/Gypsy | 6652 | 1993473 |
| trep1439 | LTR/Copia | 437 | 118889 |
| trep1440 | LTR/Copia | 1165 | 378159 |
| trep1441 | LTR/Copia | 720 | 212105 |
| trep1442 | LTR/Copia | 367 | 113228 |
| trep1448 | LTR/Copia | 5 | 1269 |
| trep1450 | LTR/Copia | 9 | 1392 |
| trep1471 | LTR/Gypsy | 863 | 210248 |
| trep1473 | LTR/Gypsy | 1077 | 189482 |
| trep1477 | DNA/En-Spm | 195 | 34031 |
| trep1478 | DNA/En-Spm | 1234 | 268390 |
| trep1501 | LTR/Copia | 3 | 366 |
| trep1503 | LTR/Copia | 16 | 2694 |
| trep1504 | LTR/Copia | 1 | 45 |
| trep1506 | LTR/Copia | 3 | 807 |
| trep1509 | DNA/En-Spm | 2 | 213 |

| | | | |
|---|---|---|---|
| trep1510 | DNA/En-Spm | 20 | 2323 |
| trep1511 | DNA/En-Spm | 43 | 5047 |
| trep1512 | DNA/En-Spm | 27 | 3553 |
| trep1513 | DNA/En-Spm | 28 | 3049 |
| trep1514 | DNA/En-Spm | 43 | 4983 |
| trep1516 | DNA/En-Spm | 205 | 45891 |
| trep1517 | DNA/En-Spm | 67 | 7667 |
| trep1518 | DNA/En-Spm | 105 | 20332 |
| trep1519 | DNA/En-Spm | 47 | 6530 |
| trep1521 | LTR/Copia | 2 | 355 |
| trep1523 | DNA/MuDR | 292 | 69318 |
| trep1524 | LTR/Copia | 7 | 1332 |
| trep1525 | DNA/Stowaway | 37 | 2437 |
| trep1527 | LTR/Copia | 66 | 7424 |
| trep1528 | LTR/Gypsy | 70 | 11080 |
| trep1529 | LTR/Gypsy | 29 | 3154 |
| trep1530 | LTR/Gypsy | 3 | 774 |
| trep1531 | LTR/Gypsy | 12 | 1828 |
| trep1533 | LTR/Gypsy | 11 | 1843 |
| trep1534 | LTR/Gypsy | 8 | 992 |
| trep1536 | LTR/Copia | 10 | 1138 |
| trep1538 | LTR/Copia | 4 | 1027 |
| trep1539 | LTR/Copia | 3 | 292 |
| trep1540 | DNA/En-Spm | 3 | 174 |
| trep1541 | DNA/En-Spm | 62 | 11093 |
| trep1542 | DNA/En-Spm | 364 | 41051 |
| trep1543 | DNA/En-Spm | 84 | 11541 |
| trep1544 | DNA/En-Spm | 148 | 16331 |
| trep1545 | DNA/En-Spm | 3 | 551 |
| trep1546 | DNA/En-Spm | 4 | 1189 |
| trep1550 | LTR/Gypsy | 18 | 3595 |
| trep1551 | LTR/Gypsy | 14 | 1509 |
| trep1552 | LTR/Gypsy | 7 | 937 |
| trep1553 | LTR/Gypsy | 17 | 2326 |
| trep1554 | LTR/Gypsy | 29 | 5136 |
| trep1555 | LTR/Gypsy | 14 | 1494 |
| trep1556 | LTR/Gypsy | 14 | 1940 |
| trep1557 | DNA/Stowaway | 16 | 1596 |
| trep1558 | DNA/Stowaway | 9 | 958 |
| trep1559 | DNA/Stowaway | 4 | 434 |
| trep1560 | DNA/Stowaway | 2 | 106 |
| trep1561 | LTR/Copia | 42 | 8750 |
| trep1562 | LTR/Copia | 239 | 65894 |
| trep1563 | LTR/Copia | 26 | 3187 |
| trep1564 | LTR/Copia | 25 | 3157 |
| trep1565 | DNA/En-Spm | 281 | 40218 |
| trep1568 | DNA/En-Spm | 129 | 18844 |

| | | | |
|---|---|---|---|
| trep1569 | DNA/En-Spm | 89 | 21955 |
| trep1570 | DNA/Stowaway | 59 | 7872 |
| trep1571 | LTR | 58 | 12767 |
| trep1574 | LTR | 10 | 1592 |
| trep1575 | LTR | 9 | 989 |
| trep1576 | Other | 24 | 4425 |
| trep1578 | Other | 66 | 5912 |
| trep1579 | Other | 24 | 3019 |
| trep1580 | DNA/Stowaway | 5 | 420 |
| trep1600 | DNA/Stowaway | 4 | 162 |
| trep1601 | DNA/Stowaway | 37 | 3480 |
| trep1602 | LTR/Copia | 763 | 231765 |
| trep1605 | LTR/Gypsy | 299 | 87328 |
| trep1606 | LTR/Copia | 445 | 113824 |
| trep1607 | DNA/Harbinger | 9 | 1342 |
| trep1608 | LINE | 907 | 223483 |
| trep1609 | DNA/Stowaway | 12 | 708 |
| trep1610 | LTR/Copia | 54 | 14816 |
| trep1612 | DNA/Stowaway | 31 | 4981 |
| trep1620 | LTR/Gypsy | 5 | 896 |
| trep1621 | LTR/Gypsy | 7 | 1701 |
| trep1622 | LTR/Gypsy | 74 | 9160 |
| trep1625 | LTR/Copia | 2 | 199 |
| trep1626 | LTR/Copia | 6 | 1151 |
| trep1628 | LTR/Copia | 10 | 861 |
| trep1629 | LTR/Copia | 5 | 237 |
| trep1630 | LTR/Copia | 8 | 846 |
| trep1634 | LINE | 281 | 42269 |
| trep1635 | DNA/En-Spm | 44 | 5885 |
| trep1636 | DNA/En-Spm | 28 | 3868 |
| trep1637 | LTR/Gypsy | 22 | 3488 |
| trep1638 | LTR/Gypsy | 12 | 1359 |
| trep1639 | LTR/Copia | 5 | 1062 |
| trep1640 | LTR/Copia | 5 | 589 |
| trep1641 | Other | 114 | 31481 |
| trep1642 | LTR/Gypsy | 56 | 8238 |
| trep1643 | LINE | 667 | 144932 |
| trep1644 | LTR/Gypsy | 5 | 985 |
| trep1645 | LTR/Copia | 41 | 8032 |
| trep1648 | LTR/Gypsy | 16 | 2699 |
| trep1649 | LTR/Gypsy | 8 | 1753 |
| trep1650 | LTR | 18 | 1397 |
| trep1652 | DNA/MuDR | 94 | 10859 |
| trep1653 | DNA/Stowaway | 12 | 1201 |
| trep1655 | DNA/Stowaway | 6 | 722 |
| trep1657 | DNA/Stowaway | 1 | 75 |
| trep1658 | DNA/Stowaway | 4 | 249 |

| | | | |
|---|---|---|---|
| trep1659 | DNA/Stowaway | 8 | 587 |
| trep1660 | DNA/Tourist | 9 | 1249 |
| trep1661 | DNA/Tourist | 56 | 4202 |
| trep1662 | DNA/Tourist | 2 | 195 |
| trep1664 | Other | 60 | 8325 |
| trep1665 | Other | 1 | 99 |
| trep1670 | DNA/Tourist | 1 | 97 |
| trep1672 | DNA/Tourist | 27 | 2651 |
| trep1673 | DNA/Tourist | 117 | 17949 |
| trep1674 | DNA/MuDR | 117 | 15689 |
| trep17 | Other | 10 | 1494 |
| trep1700 | LTR/Copia | 110 | 34102 |
| trep1701 | LTR/Copia | 79 | 18231 |
| trep1702 | LTR/Copia | 234 | 56304 |
| trep1704 | DNA/En-Spm | 276 | 53419 |
| trep1710 | DNA/En-Spm | 641 | 167306 |
| trep1711 | LTR/Gypsy | 784 | 218286 |
| trep1712 | LINE | 801 | 203248 |
| trep1713 | LINE | 307 | 92358 |
| trep1714 | LTR/Gypsy | 353 | 106464 |
| trep1715 | LTR/Gypsy | 372 | 125820 |
| trep1716 | LTR/Gypsy | 343 | 116261 |
| trep1717 | DNA/En-Spm | 131 | 23706 |
| trep1718 | DNA | 73 | 6263 |
| trep1719 | DNA | 79 | 9295 |
| trep1720 | LTR/Copia | 59 | 10961 |
| trep1722 | LTR/Gypsy | 3199 | 862810 |
| trep1723 | LTR/Copia | 277 | 87370 |
| trep1724 | LTR/Copia | 643 | 211965 |
| trep1725 | DNA | 69 | 14986 |
| trep1726 | DNA | 129 | 37083 |
| trep18 | LTR/Copia | 6 | 584 |
| trep1800 | LTR/Copia | 890 | 307087 |
| trep1801 | DNA/En-Spm | 762 | 204002 |
| trep1802 | DNA/En-Spm | 516 | 111461 |
| trep1803 | DNA/En-Spm | 248 | 72125 |
| trep1804 | LTR/Gypsy | 236 | 65721 |
| trep1805 | DNA/Stowaway | 90 | 6951 |
| trep1806 | LTR/Copia | 13 | 1266 |
| trep1807 | DNA/Stowaway | 86 | 7672 |
| trep1808 | DNA/Stowaway | 2 | 86 |
| trep1809 | DNA/Stowaway | 168 | 16402 |
| trep1810 | DNA/Stowaway | 1 | 76 |
| trep1811 | DNA/En-Spm | 61 | 13055 |
| trep1812 | DNA/En-Spm | 453 | 133462 |
| trep1813 | DNA/En-Spm | 895 | 252784 |
| trep1815 | LINE | 459 | 120828 |

| | | | |
|---|---|---|---|
| trep1816 | Other | 52 | 15001 |
| trep1817 | DNA | 2 | 160 |
| trep1818 | LINE | 60 | 4666 |
| trep1819 | DNA/Tourist | 7 | 696 |
| trep1821 | DNA/Stowaway | 5 | 373 |
| trep1822 | LTR | 25 | 3433 |
| trep1823 | LTR/Copia | 242 | 64570 |
| trep1824 | LTR/Copia | 356 | 94758 |
| trep1825 | LTR/Copia | 234 | 57194 |
| trep1826 | LTR/Copia | 313 | 88058 |
| trep19 | LTR/Copia | 8 | 1466 |
| trep20 | LINE | 364 | 88861 |
| trep200 | LTR/Gypsy | 14 | 1765 |
| trep2000 | DNA/Stowaway | 3 | 129 |
| trep2002 | DNA/Stowaway | 1 | 39 |
| trep2003 | LTR/Gypsy | 42 | 5982 |
| trep2004 | DNA/En-Spm | 5 | 273 |
| trep2005 | DNA/En-Spm | 168 | 29079 |
| trep2006 | DNA/En-Spm | 13 | 1339 |
| trep2007 | DNA/En-Spm | 4 | 344 |
| trep2009 | DNA/En-Spm | 28 | 4345 |
| trep2010 | DNA/En-Spm | 10 | 1408 |
| trep2011 | DNA/En-Spm | 54 | 5614 |
| trep2012 | LTR/Copia | 77 | 21574 |
| trep2013 | Other | 210 | 31398 |
| trep2015 | DNA/Stowaway | 5 | 472 |
| trep2016 | LTR/Copia | 10 | 764 |
| trep2017 | LTR/Gypsy | 17 | 2661 |
| trep2018 | DNA/En-Spm | 49 | 5478 |
| trep2019 | DNA/En-Spm | 57 | 11187 |
| trep2022 | Other | 229 | 50329 |
| trep2023 | DNA/En-Spm | 355 | 74199 |
| trep2024 | DNA/MuDR | 903 | 208807 |
| trep2026 | DNA/Stowaway | 3 | 256 |
| trep2027 | DNA/Stowaway | 2 | 131 |
| trep2029 | LTR/Gypsy | 13 | 1941 |
| trep2030 | LTR/Gypsy | 8 | 1504 |
| trep2032 | LINE | 14 | 2584 |
| trep2033 | DNA/Stowaway | 3 | 150 |
| trep2034 | DNA/Stowaway | 146 | 21470 |
| trep2035 | DNA/Stowaway | 2 | 98 |
| trep2036 | DNA/Stowaway | 12 | 1780 |
| trep2038 | LTR/Gypsy | 17 | 2544 |
| trep2039 | LTR/Copia | 24 | 3063 |
| trep2040 | LTR/Copia | 59 | 6069 |
| trep2041 | DNA/En-Spm | 118 | 15954 |
| trep2042 | DNA/Stowaway | 4 | 231 |

| | | | |
|---|---|---|---|
| trep2043 | DNA/Stowaway | 5 | 291 |
| trep2044 | DNA/Stowaway | 13 | 995 |
| trep2045 | DNA/Stowaway | 5 | 371 |
| trep2047 | LTR | 1 | 72 |
| trep2048 | LTR/Gypsy | 22 | 2649 |
| trep2049 | LTR/Gypsy | 32 | 3687 |
| trep2050 | SINE | 198 | 29578 |
| trep2051 | Other | 149 | 21164 |
| trep2052 | LTR/Copia | 78 | 10024 |
| trep2053 | Other | 72 | 17093 |
| trep2054 | Other | 381 | 49800 |
| trep2055 | DNA/En-Spm | 20 | 1552 |
| trep2056 | LINE | 197 | 59490 |
| trep2057 | LTR/Copia | 65 | 14990 |
| trep2058 | LTR/Gypsy | 25 | 3664 |
| trep2059 | LTR/Copia | 18 | 1767 |
| trep206 | LTR/Gypsy | 23 | 3159 |
| trep2060 | LTR/Copia | 2 | 174 |
| trep2061 | LTR/Copia | 1 | 111 |
| trep2062 | LTR/Copia | 9 | 1147 |
| trep2063 | DNA/En-Spm | 209 | 38548 |
| trep2064 | DNA/En-Spm | 658 | 157135 |
| trep2065 | DNA/En-Spm | 1924 | 315110 |
| trep2066 | DNA/En-Spm | 45 | 10586 |
| trep2067 | DNA/En-Spm | 23 | 3179 |
| trep2068 | DNA/En-Spm | 90 | 16839 |
| trep2069 | DNA/En-Spm | 167 | 20648 |
| trep2070 | LTR/Copia | 5 | 1404 |
| trep2071 | LTR/Copia | 32 | 7669 |
| trep2072 | DNA/MuDR | 353 | 89812 |
| trep2079 | LTR/Copia | 31 | 8057 |
| trep208 | LTR/Gypsy | 5 | 1143 |
| trep2080 | LTR/Copia | 4 | 585 |
| trep2081 | DNA/Tourist | 2 | 111 |
| trep2084 | LINE | 156 | 44788 |
| trep2085 | LTR/Copia | 8 | 2090 |
| trep2088 | LTR/Gypsy | 54 | 8983 |
| trep2089 | DNA/En-Spm | 1 | 71 |
| trep209 | LTR/Gypsy | 8 | 649 |
| trep2090 | DNA/Stowaway | 18 | 1379 |
| trep2091 | DNA/Stowaway | 2 | 279 |
| trep2092 | LTR/Copia | 72 | 15950 |
| trep2093 | LTR/Copia | 16 | 1394 |
| trep2095 | DNA/Stowaway | 1 | 92 |
| trep2096 | DNA/Stowaway | 7 | 546 |
| trep2097 | DNA/Stowaway | 1 | 58 |
| trep2099 | DNA/Stowaway | 3 | 220 |

| | | | |
|---|---|---|---|
| trep21 | LTR/Copia | 79 | 13187 |
| trep2100 | DNA/Stowaway | 1 | 59 |
| trep2101 | DNA/Stowaway | 3 | 259 |
| trep2103 | DNA/Stowaway | 5 | 637 |
| trep2104 | DNA/Harbinger | 41 | 5744 |
| trep2105 | LTR/Gypsy | 28 | 3036 |
| trep2106 | Other | 5 | 1327 |
| trep2107 | Other | 137 | 13982 |
| trep2108 | Other | 1 | 135 |
| trep2109 | Other | 47 | 4450 |
| trep2110 | Other | 10 | 2160 |
| trep212 | LINE | 27 | 6142 |
| trep213 | DNA/Tourist | 2 | 102 |
| trep214 | LINE | 264 | 68069 |
| trep216 | DNA/Stowaway | 7 | 586 |
| trep217 | DNA/Stowaway | 146 | 8594 |
| trep218 | DNA/Stowaway | 60 | 5802 |
| trep22 | LTR/Copia | 10 | 1573 |
| trep2200 | LTR/Copia | 18 | 2732 |
| trep2201 | LTR/Copia | 1308 | 377288 |
| trep2202 | DNA/MuDR | 137 | 28788 |
| trep2203 | LTR/Copia | 581 | 181377 |
| trep2204 | DNA/En-Spm | 30 | 4846 |
| trep2205 | DNA/En-Spm | 375 | 92652 |
| trep2206 | DNA/En-Spm | 2 | 88 |
| trep2207 | DNA/En-Spm | 35 | 5171 |
| trep2208 | LTR/Gypsy | 33 | 4657 |
| trep2209 | LTR/Gypsy | 388 | 112116 |
| trep2210 | LTR/Gypsy | 9 | 1170 |
| trep2211 | DNA/Stowaway | 4 | 207 |
| trep2212 | LINE | 1092 | 325173 |
| trep2213 | LTR/Gypsy | 374 | 115704 |
| trep2214 | DNA/Stowaway | 20 | 1964 |
| trep2215 | DNA/Stowaway | 120 | 18521 |
| trep2216 | DNA/Stowaway | 31 | 3765 |
| trep2217 | DNA/MuDR | 214 | 32049 |
| trep2218 | LTR/Gypsy | 4168 | 1153580 |
| trep2219 | LTR/Gypsy | 1553 | 503726 |
| trep2220 | LTR/Gypsy | 424 | 121842 |
| trep2221 | DNA/En-Spm | 376 | 79655 |
| trep2222 | DNA/En-Spm | 158 | 39687 |
| trep2224 | LTR/Gypsy | 8356 | 2615481 |
| trep2225 | Other | 406 | 82594 |
| trep2226 | DNA/Tourist | 55 | 7194 |
| trep2227 | Other | 83 | 18914 |
| trep2228 | LTR/Copia | 107 | 28265 |
| trep2229 | LTR/Copia | 257 | 88606 |

| | | | |
|---|---|---|---|
| trep2230 | LTR/Copia | 217 | 49360 |
| trep2231 | LTR/Copia | 1 | 63 |
| trep2232 | LTR/Copia | 1 | 176 |
| trep2235 | LTR/Copia | 7 | 1135 |
| trep2236 | LTR/Copia | 16 | 1716 |
| trep2237 | LTR/Copia | 42 | 5597 |
| trep2238 | LTR/Copia | 69 | 20509 |
| trep2241 | LTR/Copia | 6 | 312 |
| trep2242 | DNA/En-Spm | 31 | 4815 |
| trep2243 | DNA/En-Spm | 52 | 9683 |
| trep2244 | DNA/En-Spm | 91 | 22375 |
| trep2245 | DNA/En-Spm | 69 | 16333 |
| trep2246 | DNA/En-Spm | 52 | 6046 |
| trep2247 | DNA/En-Spm | 4 | 343 |
| trep2248 | DNA/En-Spm | 215 | 61832 |
| trep2249 | DNA/En-Spm | 1148 | 281962 |
| trep2250 | DNA/En-Spm | 35 | 6459 |
| trep2252 | LTR/Copia | 72 | 15577 |
| trep2253 | LTR/Copia | 442 | 126742 |
| trep2254 | LTR/Copia | 17 | 2684 |
| trep2255 | DNA/Stowaway | 16 | 1719 |
| trep2257 | LTR/Gypsy | 394 | 84243 |
| trep2258 | LTR/Gypsy | 369 | 97514 |
| trep2259 | Other | 921 | 190550 |
| trep226 | DNA/Stowaway | 25 | 1579 |
| trep2260 | DNA/Stowaway | 1 | 37 |
| trep2261 | LTR/Gypsy | 4358 | 1290106 |
| trep2262 | LTR/Gypsy | 377 | 105705 |
| trep2263 | LTR/Copia | 72 | 17586 |
| trep2264 | LTR/Copia | 75 | 15099 |
| trep2265 | LTR/Copia | 922 | 202545 |
| trep2266 | LTR/Gypsy | 695 | 232187 |
| trep2267 | LTR/Gypsy | 187 | 48314 |
| trep2268 | LTR/Gypsy | 75 | 19232 |
| trep2269 | LTR/Gypsy | 291 | 78561 |
| trep227 | LTR/Copia | 375 | 56944 |
| trep2271 | DNA/MuDR | 65 | 9742 |
| trep2274 | LTR | 523 | 151944 |
| trep2275 | LTR/Gypsy | 3 | 645 |
| trep2278 | DNA/Stowaway | 6 | 301 |
| trep2279 | DNA/Stowaway | 6 | 387 |
| trep228 | LTR/Copia | 929 | 288155 |
| trep2280 | DNA/Stowaway | 2 | 104 |
| trep2281 | DNA/Stowaway | 3 | 149 |
| trep2282 | LTR/Gypsy | 18 | 1799 |
| trep2283 | LTR/Gypsy | 30 | 8715 |
| trep2284 | Other | 235 | 38560 |

| | | | |
|---|---|---|---|
| trep2285 | DNA/Stowaway | 7 | 330 |
| trep2287 | LTR/Gypsy | 66 | 12824 |
| trep2288 | LTR/Copia | 4 | 629 |
| trep2289 | LTR/Copia | 24 | 4879 |
| trep229 | DNA | 2 | 92 |
| trep2292 | DNA/TcMar | 196 | 24451 |
| trep2293 | LTR/Gypsy | 9 | 1152 |
| trep2294 | LTR/Gypsy | 10 | 1081 |
| trep2295 | LTR/Gypsy | 5 | 1488 |
| trep2296 | LINE | 335 | 72162 |
| trep2297 | LTR/Copia | 4 | 618 |
| trep2298 | LTR/Copia | 14 | 2105 |
| trep2299 | LTR/Copia | 117 | 14951 |
| trep23 | LTR/Copia | 539 | 91045 |
| trep230 | DNA/MuDR | 113 | 17637 |
| trep2300 | LTR/Gypsy | 2 | 161 |
| trep2301 | LTR/Gypsy | 4 | 439 |
| trep2302 | LTR/Gypsy | 665 | 217687 |
| trep2303 | LINE | 11 | 1628 |
| trep2304 | LINE | 18 | 3505 |
| trep2305 | LTR/Gypsy | 11 | 1998 |
| trep2306 | LTR | 48 | 6612 |
| trep2307 | LTR | 19 | 1969 |
| trep2308 | DNA/En-Spm | 458 | 121109 |
| trep2309 | DNA/En-Spm | 135 | 27578 |
| trep231 | LTR/Gypsy | 136 | 33731 |
| trep2310 | LTR/Copia | 31 | 5086 |
| trep2311 | LTR/Copia | 24 | 3878 |
| trep2312 | LTR/Copia | 23 | 3576 |
| trep2313 | LTR/Copia | 3 | 321 |
| trep2315 | LINE | 114 | 23286 |
| trep2316 | LTR/Gypsy | 129 | 31757 |
| trep2317 | LTR/Gypsy | 293 | 80086 |
| trep2318 | LTR/Gypsy | 261 | 72732 |
| trep232 | DNA/MuDR | 2383 | 632785 |
| trep2321 | LINE | 50 | 7246 |
| trep2323 | DNA/Stowaway | 7 | 590 |
| trep2326 | DNA/Harbinger | 229 | 60841 |
| trep2327 | LTR/Copia | 58 | 12424 |
| trep2328 | LTR/Gypsy | 986 | 309933 |
| trep2329 | LTR/Gypsy | 18 | 3518 |
| trep233 | DNA/Stowaway | 445 | 81388 |
| trep2332 | LTR/Gypsy | 38 | 8890 |
| trep2337 | LTR/Gypsy | 7 | 863 |
| trep2338 | LTR/Gypsy | 211 | 35195 |
| trep2339 | LTR/Gypsy | 1 | 344 |
| trep234 | LTR/Gypsy | 204 | 41821 |

| | | | |
|---|---|---|---|
| trep2341 | LTR/Gypsy | 2430 | 690409 |
| trep2343 | LTR/Gypsy | 5684 | 1685162 |
| trep2344 | LTR/Gypsy | 155 | 20978 |
| trep2345 | Other | 67 | 9234 |
| trep2346 | LTR/Gypsy | 45 | 5820 |
| trep2347 | LTR/Gypsy | 105 | 16356 |
| trep2348 | LTR/Gypsy | 1361 | 403692 |
| trep2349 | LTR/Gypsy | 1635 | 511464 |
| trep235 | LTR/Gypsy | 108 | 35590 |
| trep2350 | DNA/En-Spm | 72 | 13819 |
| trep2352 | DNA/Stowaway | 10 | 684 |
| trep2353 | DNA/Stowaway | 10 | 899 |
| trep2356 | LTR | 1 | 46 |
| trep2357 | LTR/Gypsy | 22 | 4108 |
| trep2358 | DNA/En-Spm | 69 | 9615 |
| trep2359 | LTR/Gypsy | 82 | 14703 |
| trep236 | DNA/Stowaway | 49 | 6111 |
| trep2361 | LTR/Gypsy | 227 | 43154 |
| trep2362 | LTR/Gypsy | 13 | 1602 |
| trep2363 | LTR/Gypsy | 102 | 16441 |
| trep2364 | LTR/Gypsy | 608 | 152493 |
| trep2365 | LTR/Gypsy | 2658 | 759245 |
| trep2366 | LTR/Gypsy | 68 | 11154 |
| trep2367 | LTR/Gypsy | 226 | 22655 |
| trep2368 | LTR/Gypsy | 252 | 33888 |
| trep2369 | LTR/Gypsy | 177 | 49569 |
| trep237 | DNA | 1 | 65 |
| trep2370 | LTR/Copia | 58 | 19546 |
| trep2371 | DNA/Tourist | 170 | 25439 |
| trep2372 | Other | 47 | 9265 |
| trep2373 | Other | 82 | 13578 |
| trep2374 | Other | 234 | 35576 |
| trep2375 | LTR/Gypsy | 169 | 32885 |
| trep2376 | LTR/Copia | 108 | 22864 |
| trep2377 | LTR/Copia | 17 | 4071 |
| trep238 | LINE | 1974 | 498076 |
| trep239 | Other | 100 | 15458 |
| trep24 | LINE | 523 | 136413 |
| trep240 | Other | 132 | 18437 |
| trep242 | DNA/Stowaway | 69 | 8226 |
| trep243 | LINE | 1187 | 310780 |
| trep244 | DNA | 18 | 1242 |
| trep245 | Other | 7 | 716 |
| trep246 | LTR/Copia | 252 | 57058 |
| trep247 | LTR/Copia | 518 | 136847 |
| trep248 | LTR/Copia | 305 | 85186 |
| trep249 | LTR/Copia | 67 | 13044 |

| | | | |
|---|---|---|---|
| trep25 | LTR/Copia | 2064 | 648368 |
| trep250 | LTR/Copia | 1764 | 490101 |
| trep251 | LTR | 91 | 19688 |
| trep252 | LTR/Gypsy | 348 | 96892 |
| trep253 | LTR/Gypsy | 319 | 80526 |
| trep254 | LTR/Gypsy | 1365 | 201356 |
| trep255 | LTR | 191 | 39270 |
| trep256 | LINE | 2015 | 592754 |
| trep257 | LTR/Gypsy | 57 | 9188 |
| trep258 | LTR/Gypsy | 1352 | 371778 |
| trep26 | LTR/Copia | 2256 | 673318 |
| trep261 | LTR/Gypsy | 4534 | 1279320 |
| trep262 | LTR/Copia | 313 | 87347 |
| trep263 | LTR/Gypsy | 2317 | 682192 |
| trep264 | LTR/Gypsy | 967 | 322765 |
| trep265 | LTR/Gypsy | 2620 | 789981 |
| trep27 | LTR/Copia | 253 | 54411 |
| trep28 | DNA/En-Spm | 329 | 68325 |
| trep29 | LTR/Copia | 919 | 269601 |
| trep30 | DNA/Stowaway | 6 | 533 |
| trep3000 | RC/Helitron | 260 | 65316 |
| trep3001 | RC/Helitron | 1100 | 240745 |
| trep3002 | DNA/hAT | 434 | 90808 |
| trep3003 | DNA/hAT | 139 | 33027 |
| trep3004 | DNA/En-Spm | 16 | 2565 |
| trep3005 | DNA/En-Spm | 1220 | 365392 |
| trep3006 | DNA/En-Spm | 737 | 112678 |
| trep3008 | DNA/En-Spm | 200 | 41335 |
| trep3009 | DNA/En-Spm | 2430 | 762246 |
| trep3010 | DNA/En-Spm | 12 | 2652 |
| trep3012 | DNA/En-Spm | 384 | 97938 |
| trep3013 | DNA/En-Spm | 86 | 23810 |
| trep3014 | DNA/En-Spm | 737 | 200018 |
| trep3015 | DNA/En-Spm | 472 | 133586 |
| trep3016 | DNA/En-Spm | 32 | 8338 |
| trep3017 | DNA/En-Spm | 49 | 8682 |
| trep3018 | DNA/En-Spm | 522 | 129671 |
| trep3019 | DNA/En-Spm | 31 | 5569 |
| trep3020 | DNA/En-Spm | 101 | 21267 |
| trep3021 | DNA/En-Spm | 221 | 52473 |
| trep3022 | DNA/En-Spm | 12 | 1345 |
| trep3023 | DNA/En-Spm | 75 | 20603 |
| trep3024 | DNA/En-Spm | 8 | 1187 |
| trep3025 | DNA/En-Spm | 1564 | 439058 |
| trep3026 | DNA/En-Spm | 4148 | 1001832 |
| trep3027 | DNA/En-Spm | 354 | 122248 |
| trep3028 | DNA/En-Spm | 394 | 130486 |

| | | | |
|---|---|---|---|
| trep3029 | DNA/En-Spm | 64 | 11361 |
| trep3030 | DNA/En-Spm | 228 | 49671 |
| trep3031 | DNA/En-Spm | 7 | 1731 |
| trep3032 | DNA/En-Spm | 15 | 3464 |
| trep3033 | DNA/En-Spm | 68 | 15655 |
| trep3034 | DNA/Harbinger | 28 | 5400 |
| trep3035 | DNA/Harbinger | 17 | 2966 |
| trep3036 | DNA/Harbinger | 30 | 6500 |
| trep3037 | DNA/Harbinger | 67 | 19256 |
| trep3038 | DNA/Harbinger | 70 | 18931 |
| trep3039 | DNA/Harbinger | 58 | 12334 |
| trep3040 | DNA/Harbinger | 330 | 89251 |
| trep3041 | DNA/Harbinger | 45 | 11006 |
| trep3042 | DNA/Harbinger | 188 | 52635 |
| trep3043 | DNA/Harbinger | 34 | 8994 |
| trep3044 | DNA/Harbinger | 14 | 2203 |
| trep3045 | DNA/Harbinger | 17 | 4164 |
| trep3046 | DNA/Harbinger | 12 | 2746 |
| trep3047 | DNA/Harbinger | 15 | 1580 |
| trep3048 | DNA/Harbinger | 19 | 4040 |
| trep3049 | DNA/Harbinger | 11 | 2085 |
| trep3050 | DNA/Harbinger | 25 | 3264 |
| trep3052 | DNA/Harbinger | 151 | 39196 |
| trep3053 | DNA/Tourist | 59 | 8528 |
| trep3054 | DNA/Harbinger | 116 | 18818 |
| trep3055 | DNA/MuDR | 77 | 18993 |
| trep3056 | DNA/MuDR | 57 | 6898 |
| trep3057 | DNA/MuDR | 158 | 16748 |
| trep3058 | DNA/MuDR | 196 | 25992 |
| trep3059 | DNA/MuDR | 21 | 2758 |
| trep3060 | DNA/MuDR | 68 | 8428 |
| trep3061 | DNA/MuDR | 162 | 21521 |
| trep3062 | DNA/MuDR | 227 | 38031 |
| trep3064 | DNA/MuDR | 148 | 17368 |
| trep3065 | DNA/MuDR | 441 | 53120 |
| trep3067 | DNA/MuDR | 798 | 178720 |
| trep3068 | DNA/MuDR | 273 | 43146 |
| trep3069 | DNA/TcMar | 2 | 233 |
| trep3070 | DNA/TcMar | 20 | 1591 |
| trep3072 | DNA/TcMar | 114 | 17319 |
| trep3075 | DNA/TcMar | 55 | 4043 |
| trep3076 | DNA/TcMar | 479 | 36612 |
| trep3078 | DNA/TcMar | 89 | 6228 |
| trep3079 | DNA/TcMar | 12 | 844 |
| trep3082 | DNA/TcMar | 41 | 2595 |
| trep3083 | DNA/TcMar | 322 | 25983 |
| trep3085 | DNA/TcMar | 294 | 25059 |

| | | | |
|---|---|---|---|
| trep3086 | DNA/TcMar | 3 | 186 |
| trep3087 | DNA/TcMar | 1 | 54 |
| trep3088 | DNA/TcMar | 231 | 20301 |
| trep3089 | DNA/TcMar | 21 | 1743 |
| trep3090 | DNA/TcMar | 7 | 613 |
| trep3091 | DNA/TcMar | 25 | 1946 |
| trep3092 | DNA/Stowaway | 8 | 728 |
| trep3093 | DNA/TcMar | 12 | 931 |
| trep3094 | DNA/TcMar | 6 | 484 |
| trep3095 | DNA/TcMar | 209 | 30410 |
| trep3097 | DNA/TcMar | 37 | 3738 |
| trep3098 | DNA/Stowaway | 6 | 392 |
| trep3099 | DNA/TcMar | 2 | 202 |
| trep31 | DNA/Stowaway | 11 | 827 |
| trep3100 | DNA/TcMar | 19 | 2655 |
| trep3101 | DNA/TcMar | 156 | 21533 |
| trep3102 | DNA/TcMar | 14 | 1259 |
| trep3103 | DNA/TcMar | 5 | 542 |
| trep3104 | DNA/TcMar | 121 | 16855 |
| trep3105 | DNA/TcMar | 37 | 5869 |
| trep3106 | DNA/TcMar | 6 | 807 |
| trep3107 | DNA/Stowaway | 282 | 34399 |
| trep3108 | DNA/TcMar | 10 | 748 |
| trep3109 | DNA/TcMar | 6 | 582 |
| trep3110 | DNA/TcMar | 4 | 190 |
| trep3111 | DNA/TcMar | 17 | 1733 |
| trep3112 | DNA | 1 | 52 |
| trep3115 | LINE | 449 | 111927 |
| trep3116 | LINE | 179 | 34860 |
| trep3117 | LINE | 216 | 55315 |
| trep3118 | LINE | 106 | 21345 |
| trep3119 | LINE | 298 | 73429 |
| trep3120 | LINE | 2 | 281 |
| trep3124 | LINE | 6 | 1278 |
| trep3125 | LTR/Copia | 470 | 99729 |
| trep3127 | LTR/Copia | 1339 | 425894 |
| trep3128 | LTR/Copia | 1074 | 324094 |
| trep3129 | LTR/Copia | 230 | 47716 |
| trep3130 | LTR/Copia | 1468 | 400965 |
| trep3131 | LTR/Copia | 1642 | 527076 |
| trep3132 | LTR/Copia | 4 | 480 |
| trep3134 | LTR/Copia | 5 | 1192 |
| trep3135 | LTR/Copia | 5 | 1097 |
| trep3136 | LTR/Copia | 2 | 196 |
| trep3138 | LTR/Copia | 140 | 22463 |
| trep3139 | LTR/Copia | 5 | 641 |
| trep3140 | LTR/Copia | 3 | 316 |

| | | | |
|---|---|---|---|
| trep3143 | LTR/Copia | 17 | 3435 |
| trep3144 | LTR/Copia | 1313 | 461267 |
| trep3147 | LTR/Copia | 2551 | 679873 |
| trep3148 | LTR/Copia | 115 | 28467 |
| trep3149 | LTR/Copia | 28 | 5591 |
| trep3150 | LTR/Copia | 103 | 33605 |
| trep3151 | LTR/Copia | 385 | 40800 |
| trep3152 | LTR/Copia | 1223 | 358072 |
| trep3153 | LTR/Copia | 34 | 6954 |
| trep3154 | LTR/Copia | 41 | 14394 |
| trep3155 | LTR/Copia | 543 | 153153 |
| trep3156 | LTR/Copia | 376 | 112878 |
| trep3157 | LTR/Copia | 777 | 234178 |
| trep3159 | LTR/Copia | 738 | 241074 |
| trep3160 | LTR/Copia | 3305 | 1220292 |
| trep3161 | LTR/Copia | 1298 | 461251 |
| trep3162 | LTR/Gypsy | 85 | 17469 |
| trep3163 | LTR/Gypsy | 66 | 9211 |
| trep3164 | LTR/Gypsy | 95 | 13141 |
| trep3165 | LTR/Gypsy | 51 | 7525 |
| trep3166 | LTR/Gypsy | 9 | 1232 |
| trep3167 | LTR/Gypsy | 81 | 11155 |
| trep3168 | LTR/Gypsy | 1403 | 464958 |
| trep3170 | LTR/Gypsy | 622 | 126531 |
| trep3171 | LTR/Gypsy | 339 | 93153 |
| trep3174 | LTR/Gypsy | 955 | 190386 |
| trep3175 | LTR/Gypsy | 120 | 25312 |
| trep3176 | LTR/Gypsy | 622 | 114775 |
| trep3177 | LTR/Gypsy | 5714 | 1685709 |
| trep3178 | LTR/Gypsy | 1452 | 448102 |
| trep3179 | LTR/Gypsy | 436 | 128972 |
| trep3180 | LTR/Gypsy | 1213 | 438646 |
| trep3181 | LTR/Gypsy | 465 | 137796 |
| trep3182 | LTR/Gypsy | 261 | 74968 |
| trep3185 | LTR/Gypsy | 393 | 126062 |
| trep3187 | LTR/Gypsy | 2839 | 951044 |
| trep3188 | LTR/Gypsy | 1988 | 655083 |
| trep3189 | LTR/Gypsy | 3089 | 1102679 |
| trep3191 | LTR/Gypsy | 598 | 155657 |
| trep3192 | LTR/Gypsy | 529 | 147915 |
| trep3193 | LTR/Gypsy | 980 | 303229 |
| trep3195 | LTR/Gypsy | 120 | 33569 |
| trep3196 | LTR/Gypsy | 295 | 85186 |
| trep3197 | LTR/Gypsy | 1114 | 324893 |
| trep3198 | LTR/Gypsy | 5789 | 1864959 |
| trep3199 | LTR/Gypsy | 78 | 26097 |
| trep32 | DNA | 60 | 9042 |

| | | | |
|---|---|---|---|
| trep3200 | LTR/Gypsy | 219 | 45944 |
| trep3202 | LTR/Gypsy | 746 | 222763 |
| trep3203 | LTR/Gypsy | 422 | 127325 |
| trep3204 | LTR/Gypsy | 1147 | 298601 |
| trep3205 | LTR/Gypsy | 328 | 108653 |
| trep3206 | LTR/Gypsy | 163 | 40595 |
| trep3207 | LTR/Gypsy | 290 | 88231 |
| trep3208 | LTR/Gypsy | 394 | 123088 |
| trep3209 | LTR/Gypsy | 433 | 118890 |
| trep3210 | LTR/Gypsy | 714 | 209018 |
| trep3211 | LTR/Gypsy | 663 | 220944 |
| trep3214 | LTR/Gypsy | 100 | 27521 |
| trep3215 | LTR/Gypsy | 69 | 17151 |
| trep3216 | LTR/Gypsy | 54 | 14734 |
| trep3217 | LTR/Gypsy | 93 | 28119 |
| trep3218 | LTR/Gypsy | 20 | 4599 |
| trep3219 | LTR/Gypsy | 229 | 64062 |
| trep3220 | LTR/Gypsy | 1 | 56 |
| trep3221 | LTR/Gypsy | 864 | 283761 |
| trep3222 | LTR/Gypsy | 387 | 122346 |
| trep3223 | LTR/Gypsy | 636 | 188406 |
| trep3224 | LTR/Gypsy | 2642 | 757238 |
| trep3226 | LTR/Gypsy | 15 | 2447 |
| trep3227 | LTR/Gypsy | 87 | 11514 |
| trep3228 | LTR/Gypsy | 62 | 9103 |
| trep3229 | LTR/Gypsy | 7054 | 2114080 |
| trep3231 | LTR/Gypsy | 7314 | 2220567 |
| trep3232 | LTR/Gypsy | 7920 | 2308915 |
| trep3235 | LTR/Gypsy | 66 | 11218 |
| trep3236 | LTR/Gypsy | 3231 | 913975 |
| trep3237 | LTR/Gypsy | 112 | 16956 |
| trep3238 | LTR/Gypsy | 62 | 7467 |
| trep3239 | LTR/Gypsy | 353 | 61045 |
| trep3240 | LTR/Gypsy | 51 | 7241 |
| trep3241 | LTR/Gypsy | 43 | 6972 |
| trep3242 | LTR/Gypsy | 85 | 11529 |
| trep3243 | LTR/Gypsy | 63 | 10117 |
| trep3244 | LTR/Gypsy | 4177 | 1144374 |
| trep3245 | LTR/Gypsy | 5729 | 1495155 |
| trep3249 | LTR/Gypsy | 150 | 26453 |
| trep3253 | LTR/Gypsy | 910 | 189214 |
| trep3254 | LTR/Gypsy | 6770 | 2123426 |
| trep3257 | LTR/Gypsy | 1310 | 372363 |
| trep3258 | LTR/Gypsy | 1 | 203 |
| trep3259 | LTR/Gypsy | 43 | 7314 |
| trep3260 | LTR/Gypsy | 59 | 8956 |
| trep3261 | LTR/Gypsy | 15 | 1538 |

| | | | |
|---|---|---|---|
| trep3262 | LTR/Gypsy | 289 | 47287 |
| trep3263 | LTR/Gypsy | 5276 | 1703812 |
| trep3264 | LTR/Gypsy | 6018 | 1893618 |
| trep3265 | LTR/Gypsy | 5925 | 1915495 |
| trep3266 | LTR/Gypsy | 3905 | 1221481 |
| trep3267 | LTR/Gypsy | 3811 | 1198783 |
| trep3268 | LTR/Gypsy | 6377 | 2143948 |
| trep3269 | LTR/Gypsy | 20588 | 6978527 |
| trep3271 | LTR | 171 | 40485 |
| trep3272 | LTR | 17 | 4643 |
| trep3273 | LTR | 133 | 30155 |
| trep3274 | LTR | 592 | 143026 |
| trep3275 | LTR | 155 | 35351 |
| trep3276 | LTR | 364 | 116185 |
| trep3277 | LTR | 521 | 132988 |
| trep3278 | LTR | 56 | 7758 |
| trep3280 | LTR | 96 | 16116 |
| trep3282 | Other | 357 | 26648 |
| trep3286 | Other | 81 | 16867 |
| trep3287 | Other | 22 | 3385 |
| trep3288 | Other | 29 | 5132 |
| trep3289 | Other | 6 | 1075 |
| trep3290 | Other | 20 | 4231 |
| trep3292 | Other | 65 | 17599 |
| trep3293 | Other | 12 | 2682 |
| trep3294 | Other | 23 | 6266 |
| trep3295 | Other | 58 | 10801 |
| trep3300 | RC/Helitron | 352 | 70459 |
| trep3301 | RC/Helitron | 27 | 5905 |
| trep3302 | RC/Helitron | 12 | 2652 |
| trep3303 | RC/Helitron | 7 | 998 |
| trep3304 | RC/Helitron | 13 | 3025 |
| trep3305 | RC/Helitron | 18 | 4000 |
| trep3306 | RC/Helitron | 14 | 3463 |
| trep3307 | RC/Helitron | 30 | 5370 |
| trep3308 | RC/Helitron | 13 | 2638 |
| trep3309 | DNA/En-Spm | 1597 | 286577 |
| trep3310 | DNA/En-Spm | 67 | 10873 |
| trep3311 | DNA/Harbinger | 14 | 2133 |
| trep3312 | DNA/Harbinger | 2 | 151 |
| trep3313 | DNA/MuDR | 35 | 6489 |
| trep3315 | DNA | 71 | 9991 |
| trep3316 | DNA | 201 | 50575 |
| trep3317 | LINE | 53 | 8327 |
| trep3318 | LINE | 5 | 981 |
| trep3319 | LINE | 2 | 495 |
| trep3321 | LINE | 6 | 1087 |

| | | | |
|---|---|---|---|
| trep3322 | LINE | 714 | 201977 |
| trep3323 | LINE | 949 | 230114 |
| trep3324 | LINE | 17 | 3930 |
| trep3325 | LTR/Copia | 17 | 4786 |
| trep3326 | LTR/Copia | 728 | 209061 |
| trep3327 | LTR/Copia | 16 | 1890 |
| trep3328 | LTR/Copia | 93 | 25239 |
| trep3329 | LTR/Gypsy | 198 | 55875 |
| trep3330 | LTR/Copia | 497 | 130764 |
| trep3331 | LTR/Copia | 5 | 1058 |
| trep3333 | LTR/Copia | 1 | 160 |
| trep3334 | LTR/Copia | 1 | 46 |
| trep3335 | LTR/Gypsy | 414 | 122197 |
| trep3336 | LTR/Gypsy | 33 | 7417 |
| trep3337 | LTR/Gypsy | 61 | 12374 |
| trep3338 | LTR/Gypsy | 1186 | 310698 |
| trep3339 | LTR/Gypsy | 2090 | 676203 |
| trep3340 | LTR/Gypsy | 182 | 41747 |
| trep3341 | LTR/Gypsy | 3108 | 964155 |
| trep3342 | LTR | 101 | 25076 |
| trep3343 | LTR | 431 | 79310 |
| trep3344 | LTR | 107 | 27982 |
| trep3345 | LTR | 605 | 190186 |
| trep3347 | Other | 3 | 232 |
| trep3348 | Other | 343 | 43348 |
| trep3351 | Other | 1251 | 275081 |
| trep3352 | Other | 8 | 1207 |
| trep3353 | SINE | 5 | 814 |
| trep3354 | Other | 131 | 14200 |
| trep3355 | Other | 30 | 2869 |
| trep3356 | Other | 1 | 266 |
| trep34 | LTR/Copia | 762 | 234586 |
| trep3403 | DNA/En-Spm | 5 | 1210 |
| trep3404 | DNA/En-Spm | 2 | 66 |
| trep3405 | DNA/En-Spm | 1 | 57 |
| trep3407 | DNA/En-Spm | 1 | 50 |
| trep3410 | DNA/En-Spm | 7 | 519 |
| trep3411 | DNA/En-Spm | 1 | 107 |
| trep3412 | DNA/En-Spm | 506 | 122333 |
| trep3413 | DNA/En-Spm | 75 | 10970 |
| trep3414 | DNA/En-Spm | 1298 | 316205 |
| trep3415 | DNA/En-Spm | 3168 | 940281 |
| trep3416 | DNA/En-Spm | 419 | 104488 |
| trep3417 | DNA/En-Spm | 705 | 191214 |
| trep3418 | DNA/En-Spm | 29 | 5482 |
| trep3419 | DNA/En-Spm | 26 | 3717 |
| trep3420 | DNA/En-Spm | 119 | 26400 |

| | | | |
|---|---|---|---|
| trep3422 | DNA/En-Spm | 16 | 2212 |
| trep3423 | DNA/En-Spm | 216 | 43516 |
| trep3424 | DNA/En-Spm | 12 | 2500 |
| trep3425 | DNA/En-Spm | 12 | 1679 |
| trep3426 | DNA/En-Spm | 39 | 8197 |
| trep3427 | DNA/En-Spm | 13 | 1004 |
| trep3428 | DNA/En-Spm | 1 | 107 |
| trep3429 | DNA/En-Spm | 285 | 67747 |
| trep3430 | DNA/En-Spm | 17 | 2677 |
| trep3431 | DNA/En-Spm | 17 | 1625 |
| trep3432 | DNA/En-Spm | 19 | 2056 |
| trep3433 | DNA/En-Spm | 55 | 11697 |
| trep3434 | DNA/En-Spm | 73 | 16939 |
| trep3435 | DNA/En-Spm | 181 | 23275 |
| trep3436 | DNA/En-Spm | 4 | 259 |
| trep3438 | DNA/En-Spm | 2 | 195 |
| trep3439 | DNA/En-Spm | 144 | 21249 |
| trep3440 | DNA/En-Spm | 30 | 5998 |
| trep3441 | DNA/En-Spm | 21 | 2681 |
| trep3442 | DNA/En-Spm | 53 | 3519 |
| trep3443 | DNA/En-Spm | 39 | 2854 |
| trep3444 | DNA/En-Spm | 34 | 3453 |
| trep3446 | DNA/Harbinger | 70 | 8994 |
| trep3447 | DNA/Tourist | 415 | 68889 |
| trep3448 | DNA/Harbinger | 34 | 6512 |
| trep3449 | DNA/MuDR | 147 | 32524 |
| trep3450 | DNA/Stowaway | 34 | 3109 |
| trep3451 | DNA/TcMar | 264 | 24380 |
| trep3452 | DNA/TcMar | 30 | 3532 |
| trep3453 | DNA/TcMar | 27 | 2486 |
| trep3454 | LINE | 498 | 101847 |
| trep3455 | LTR/Copia | 60 | 15245 |
| trep3456 | LTR/Copia | 7280 | 2448783 |
| trep3457 | LTR/Gypsy | 1308 | 412973 |
| trep3458 | LTR/Gypsy | 343 | 78311 |
| trep3459 | LTR/Gypsy | 1608 | 420721 |
| trep35 | LTR/Copia | 379 | 110571 |
| trep3500 | DNA/MuDR | 187 | 38841 |
| trep3501 | DNA/MuDR | 20 | 3337 |
| trep3502 | DNA/MuDR | 35 | 5471 |
| trep3503 | DNA/MuDR | 8 | 1566 |
| trep3504 | DNA/MuDR | 33 | 3552 |
| trep3505 | DNA/MuDR | 21 | 2325 |
| trep3510 | DNA/En-Spm | 15 | 1451 |
| trep3511 | DNA/Harbinger | 43 | 5930 |
| trep3512 | DNA/MuDR | 161 | 29418 |
| trep3513 | DNA/TcMar | 247 | 30645 |

| | | | |
|---|---|---|---|
| trep3514 | DNA/TcMar | 43 | 2266 |
| trep3515 | LINE | 1073 | 284935 |
| trep3516 | LTR/Copia | 1120 | 349400 |
| trep3521 | LTR | 1 | 80 |
| trep3522 | LTR | 8 | 1412 |
| trep3523 | LTR | 16 | 3435 |
| trep3525 | LTR | 16 | 2258 |
| trep3528 | DNA/Stowaway | 76 | 4961 |
| trep3530 | LTR/Copia | 3854 | 1354427 |
| trep36 | LTR/Copia | 256 | 55221 |
| trep40 | LTR/Gypsy | 39 | 6468 |
| trep4000 | LTR/Gypsy | 16180 | 4929955 |
| trep4001 | LTR/Gypsy | 7140 | 1951360 |
| trep4002 | LTR/Gypsy | 5457 | 1488937 |
| trep4003 | LTR/Gypsy | 55 | 10005 |
| trep4004 | LTR/Gypsy | 3904 | 1198002 |
| trep4005 | LTR/Gypsy | 758 | 208114 |
| trep4006 | LTR/Gypsy | 4 | 360 |
| trep4008 | LTR/Gypsy | 2 | 473 |
| trep4013 | LTR/Gypsy | 139 | 21207 |
| trep4015 | LTR/Gypsy | 232 | 35963 |
| trep4017 | LTR/Gypsy | 2 | 273 |
| trep4018 | LTR/Gypsy | 405 | 64857 |
| trep4019 | LTR/Gypsy | 54 | 8629 |
| trep4021 | LTR/Gypsy | 97 | 13684 |
| trep4022 | LTR/Copia | 2 | 743 |
| trep4023 | LTR/Copia | 60 | 11300 |
| trep4026 | LTR/Copia | 1 | 229 |
| trep4027 | LTR/Copia | 8 | 1136 |
| trep4032 | LTR/Copia | 6 | 940 |
| trep4033 | LTR/Copia | 2 | 170 |
| trep4034 | LTR/Copia | 21 | 2327 |
| trep4035 | LTR/Copia | 7 | 984 |
| trep4037 | LTR/Copia | 2 | 489 |
| trep4041 | LTR/Copia | 3 | 333 |
| trep4042 | LTR/Copia | 14 | 2706 |
| trep4043 | LTR/Copia | 13 | 1438 |
| trep4044 | LTR/Copia | 60 | 7808 |
| trep4045 | LTR/Copia | 71 | 11260 |
| trep4046 | LTR/Copia | 12 | 1879 |
| trep4056 | LTR/Copia | 397 | 68622 |
| trep4057 | LTR/Copia | 101 | 21078 |
| trep4058 | LTR/Copia | 674 | 213004 |
| trep4059 | LTR/Copia | 868 | 206623 |
| trep4060 | LTR/Copia | 1554 | 573249 |
| trep4061 | LTR/Copia | 1050 | 304337 |
| trep4062 | LTR/Copia | 273 | 69426 |

| | | | |
|---|---|---|---|
| trep4065 | DNA/En-Spm | 130 | 17565 |
| trep4066 | DNA/En-Spm | 120 | 17496 |
| trep4067 | DNA/En-Spm | 114 | 18305 |
| trep4068 | DNA/En-Spm | 173 | 36116 |
| trep4069 | DNA/En-Spm | 199 | 33363 |
| trep4070 | DNA/En-Spm | 293 | 30183 |
| trep4071 | DNA/En-Spm | 540 | 93502 |
| trep4072 | DNA/En-Spm | 157 | 25627 |
| trep4073 | DNA/En-Spm | 160 | 22269 |
| trep4074 | DNA/En-Spm | 11 | 1291 |
| trep4075 | DNA/En-Spm | 1541 | 387669 |
| trep4076 | DNA/En-Spm | 1816 | 473395 |
| trep4077 | DNA/En-Spm | 55 | 5944 |
| trep4078 | DNA/En-Spm | 413 | 112719 |
| trep4079 | DNA/En-Spm | 436 | 74495 |
| trep4080 | DNA/En-Spm | 1930 | 359252 |
| trep4081 | DNA/En-Spm | 152 | 32074 |
| trep4082 | DNA/En-Spm | 63 | 19079 |
| trep4083 | DNA/En-Spm | 402 | 103112 |
| trep4084 | DNA/En-Spm | 99 | 16800 |
| trep4085 | DNA/En-Spm | 750 | 177430 |
| trep4086 | DNA/En-Spm | 4453 | 1093732 |
| trep4087 | DNA/En-Spm | 247 | 49370 |
| trep4088 | DNA/En-Spm | 1814 | 509719 |
| trep4089 | DNA/En-Spm | 235 | 49751 |
| trep4090 | DNA/En-Spm | 4 | 320 |
| trep4091 | DNA/hAT | 105 | 14649 |
| trep4092 | LTR/Gypsy | 60 | 8740 |
| trep4093 | LTR/Gypsy | 41 | 5686 |
| trep4095 | LTR/Gypsy | 15 | 2323 |
| trep4096 | LTR/Gypsy | 395 | 42283 |
| trep4097 | LTR/Gypsy | 14 | 1612 |
| trep4098 | LTR/Gypsy | 124 | 26677 |
| trep4099 | LTR/Gypsy | 534 | 135534 |
| trep41 | LTR/Copia | 15 | 1834 |
| trep4100 | LTR/Gypsy | 1928 | 417113 |
| trep4101 | LTR/Gypsy | 1906 | 570200 |
| trep4102 | LTR/Gypsy | 102 | 15414 |
| trep4103 | LTR/Gypsy | 678 | 190677 |
| trep4104 | LTR/Gypsy | 84 | 15096 |
| trep4105 | LTR/Gypsy | 22 | 4514 |
| trep4106 | LTR/Gypsy | 126 | 19402 |
| trep4107 | LTR/Gypsy | 30 | 4516 |
| trep4108 | LTR/Gypsy | 14 | 1592 |
| trep4109 | LTR/Gypsy | 128 | 20144 |
| trep4110 | LTR/Gypsy | 31 | 4945 |
| trep4111 | LTR/Gypsy | 67 | 14845 |

| | | | |
|---|---|---|---|
| trep4112 | LTR/Gypsy | 5 | 1455 |
| trep4113 | LTR/Gypsy | 36 | 6828 |
| trep4114 | LTR/Gypsy | 4 | 405 |
| trep4115 | LTR/Gypsy | 4121 | 1046714 |
| trep4116 | LTR/Gypsy | 72 | 9903 |
| trep4117 | LTR/Gypsy | 25 | 1886 |
| trep4118 | LTR/Gypsy | 153 | 25323 |
| trep4120 | DNA/MuDR | 288 | 59311 |
| trep4121 | DNA/MuDR | 353 | 63385 |
| trep4124 | DNA/MuDR | 720 | 207619 |
| trep4125 | DNA/MuDR | 28 | 4356 |
| trep4126 | DNA/MuDR | 2 | 282 |
| trep4127 | DNA/MuDR | 111 | 14462 |
| trep4128 | LTR/Copia | 44 | 8906 |
| trep4129 | LTR/Copia | 57 | 9900 |
| trep4130 | LTR/Copia | 932 | 245938 |
| trep4131 | LTR/Copia | 3 | 702 |
| trep4132 | LTR/Copia | 77 | 14299 |
| trep4133 | LTR/Copia | 1 | 41 |
| trep4134 | LTR/Copia | 510 | 139511 |
| trep4135 | LTR/Copia | 456 | 113955 |
| trep4136 | LTR/Copia | 767 | 196007 |
| trep4138 | LTR/Copia | 212 | 57546 |
| trep4139 | LTR/Copia | 197 | 51307 |
| trep4140 | LTR/Copia | 91 | 25068 |
| trep4141 | LINE | 40 | 9646 |
| trep4142 | LINE | 34 | 8696 |
| trep4143 | LINE | 122 | 31169 |
| trep4144 | LINE | 1 | 28 |
| trep4145 | LINE | 91 | 18015 |
| trep4146 | LINE | 62 | 12335 |
| trep4147 | LINE | 108 | 28195 |
| trep4148 | Other | 467 | 50600 |
| trep4149 | Other | 11 | 1601 |
| trep4150 | Other | 14 | 3832 |
| trep4151 | Other | 731 | 85062 |
| trep4152 | Other | 437 | 70057 |
| trep4153 | Other | 121 | 25547 |
| trep4154 | Other | 1577 | 366822 |
| trep4156 | Other | 89 | 14251 |
| trep4157 | Other | 20 | 2466 |
| trep4158 | Other | 11 | 939 |
| trep4159 | Other | 1181 | 274044 |
| trep4160 | Other | 444 | 52793 |
| trep4161 | Other | 202 | 48091 |
| trep4162 | Other | 23 | 5985 |
| trep4163 | Other | 5 | 561 |

| | | | |
|---|---|---|---|
| trep4165 | Other | 1 | 178 |
| trep4167 | LTR | 52 | 5543 |
| trep4168 | LTR | 42 | 6636 |
| trep4169 | LTR | 74 | 12806 |
| trep4171 | SINE | 271 | 56466 |
| trep4172 | RC/Helitron | 59 | 10345 |
| trep4173 | DNA/hAT | 9 | 1301 |
| trep4174 | DNA/Harbinger | 278 | 53796 |
| trep4175 | DNA/Harbinger | 5 | 504 |
| trep4176 | DNA/Harbinger | 15 | 2196 |
| trep4177 | DNA/Harbinger | 92 | 17930 |
| trep4178 | DNA/Harbinger | 164 | 24304 |
| trep4179 | DNA/TcMar | 2 | 186 |
| trep4181 | DNA/TcMar | 1 | 78 |
| trep4182 | DNA/TcMar | 75 | 4910 |
| trep4183 | DNA/En-Spm | 1965 | 503337 |
| trep4184 | DNA/En-Spm | 398 | 90747 |
| trep4185 | DNA/En-Spm | 331 | 74368 |
| trep4186 | DNA/En-Spm | 102 | 17560 |
| trep4187 | DNA/En-Spm | 13612 | 3800482 |
| trep4188 | DNA/En-Spm | 3960 | 989009 |
| trep4189 | DNA/En-Spm | 709 | 195588 |
| trep4190 | DNA/En-Spm | 50 | 7088 |
| trep4191 | DNA/En-Spm | 18 | 2167 |
| trep4192 | DNA/En-Spm | 104 | 18581 |
| trep4193 | DNA/En-Spm | 511 | 126220 |
| trep4194 | DNA/En-Spm | 10510 | 2800015 |
| trep4195 | DNA/En-Spm | 2729 | 819807 |
| trep4196 | DNA/En-Spm | 215 | 35211 |
| trep4197 | DNA/MuDR | 199 | 36999 |
| trep4198 | DNA/MuDR | 947 | 179195 |
| trep4199 | DNA/MuDR | 105 | 15588 |
| trep42 | LTR/Gypsy | 59 | 4598 |
| trep4200 | DNA/MuDR | 19 | 3780 |
| trep4201 | DNA/Harbinger | 230 | 37931 |
| trep4202 | DNA/Harbinger | 232 | 31398 |
| trep4205 | DNA | 173 | 54253 |
| trep4206 | LINE | 101 | 8662 |
| trep4207 | LINE | 130 | 23748 |
| trep4209 | LTR/Copia | 136 | 33937 |
| trep4210 | LTR/Copia | 14684 | 4770781 |
| trep4211 | LTR/Copia | 15 | 5412 |
| trep4213 | LTR/Copia | 3476 | 1141831 |
| trep4214 | LTR/Copia | 3 | 387 |
| trep4215 | LTR/Copia | 27 | 7883 |
| trep4216 | LTR/Gypsy | 34612 | 10002660 |
| trep4217 | LTR/Gypsy | 837 | 282126 |

| | | | |
|---|---|---|---|
| trep4218 | LTR/Gypsy | 4690 | 1195147 |
| trep4219 | LTR/Gypsy | 11136 | 3518216 |
| trep4220 | LTR/Gypsy | 48 | 9048 |
| trep4221 | LTR/Gypsy | 2888 | 950484 |
| trep4222 | LTR/Gypsy | 15 | 2610 |
| trep4223 | LTR/Gypsy | 726 | 202302 |
| trep4224 | LTR/Gypsy | 4084 | 1331061 |
| trep4225 | LTR/Gypsy | 535 | 99806 |
| trep4226 | LTR/Gypsy | 393 | 121486 |
| trep4227 | LTR/Gypsy | 447 | 135939 |
| trep4228 | LTR/Gypsy | 351 | 91013 |
| trep4229 | LTR/Gypsy | 816 | 236772 |
| trep4230 | LTR/Gypsy | 3350 | 792378 |
| trep4231 | LTR/Gypsy | 856 | 252405 |
| trep4232 | LTR/Gypsy | 6849 | 1697174 |
| trep4233 | LTR/Gypsy | 21 | 2874 |
| trep4234 | LTR/Gypsy | 3638 | 1098701 |
| trep4235 | LTR/Gypsy | 97 | 19118 |
| trep4236 | LTR/Gypsy | 13602 | 4476677 |
| trep4237 | LTR | 3801 | 1160073 |
| trep4238 | LTR | 1834 | 563943 |
| trep4239 | LTR | 437 | 107008 |
| trep4240 | LTR | 60 | 14396 |
| trep4241 | LTR | 1135 | 340964 |
| trep4242 | Other | 565 | 165870 |
| trep4243 | Other | 77 | 18238 |
| trep4244 | Other | 186 | 26146 |
| trep4245 | Other | 1781 | 390061 |
| trep4246 | Other | 77 | 16652 |
| trep4247 | DNA/En-Spm | 3415 | 839043 |
| trep4248 | DNA/En-Spm | 2502 | 623751 |
| trep4249 | DNA/Harbinger | 639 | 109470 |
| trep4250 | DNA/TcMar | 265 | 49428 |
| trep4251 | DNA/TcMar | 10 | 820 |
| trep4252 | LTR/Gypsy | 798 | 205215 |
| trep4253 | LTR/Gypsy | 2914 | 906347 |
| trep4254 | LTR/Gypsy | 769 | 236446 |
| trep4256 | LTR/Gypsy | 2366 | 510112 |
| trep4257 | LTR | 358 | 106563 |
| trep4258 | LTR | 348 | 99952 |
| trep4259 | LTR | 11 | 2573 |
| trep4260 | LTR | 715 | 224116 |
| trep4261 | Other | 2 | 171 |
| trep4262 | Other | 47 | 6755 |
| trep4263 | Other | 89 | 12619 |
| trep4264 | Other | 163 | 17620 |
| trep4265 | Other | 51 | 4199 |

| | | | |
|---|---|---|---|
| trep4266 | Other | 378 | 55372 |
| trep4267 | DNA | 168 | 27576 |
| trep4268 | DNA | 509 | 88219 |
| trep4269 | DNA | 668 | 87369 |
| trep4270 | DNA | 162 | 14047 |
| trep4271 | Other | 32 | 3604 |
| trep4272 | LTR/Gypsy | 926 | 207428 |
| trep4273 | LTR/Gypsy | 738 | 176528 |
| trep4274 | LTR/Gypsy | 558 | 146536 |
| trep4276 | LTR/Gypsy | 848 | 200886 |
| trep4277 | LTR/Gypsy | 1061 | 281411 |
| trep4278 | LTR/Gypsy | 769 | 167520 |
| trep4279 | LTR/Gypsy | 1028 | 229098 |
| trep4280 | LTR/Gypsy | 861 | 212632 |
| trep4281 | DNA/En-Spm | 5272 | 1331367 |
| trep4282 | DNA/En-Spm | 1139 | 226697 |
| trep4283 | DNA/En-Spm | 1842 | 495413 |
| trep4284 | DNA/En-Spm | 4548 | 1166424 |
| trep4285 | DNA/En-Spm | 2795 | 742590 |
| trep4286 | DNA/En-Spm | 1738 | 440036 |
| trep4287 | DNA/En-Spm | 103 | 23140 |
| trep4288 | DNA/En-Spm | 187 | 50008 |
| trep4289 | DNA/En-Spm | 1470 | 345809 |
| trep4290 | DNA/En-Spm | 1046 | 216824 |
| trep4291 | DNA/Harbinger | 48 | 5815 |
| trep4292 | LTR/Copia | 1130 | 286202 |
| trep4293 | LTR/Gypsy | 2330 | 610616 |
| trep4294 | LTR/Gypsy | 1194 | 306406 |
| trep4295 | LTR/Gypsy | 1260 | 347619 |
| trep4296 | LTR/Gypsy | 2005 | 552531 |
| trep4297 | LTR/Gypsy | 4060 | 1193112 |
| trep4298 | LTR/Gypsy | 3829 | 1036110 |
| trep4299 | LTR/Gypsy | 4973 | 1575712 |
| trep4300 | LTR/Gypsy | 412 | 117146 |
| trep4301 | LTR/Gypsy | 463 | 134394 |
| trep4302 | LTR/Gypsy | 427 | 118820 |
| trep4303 | LTR/Gypsy | 862 | 277251 |
| trep4304 | LTR/Gypsy | 496 | 149688 |
| trep4305 | LTR/Gypsy | 1509 | 463385 |
| trep4306 | LTR/Gypsy | 1617 | 533917 |
| trep4307 | LTR/Gypsy | 1851 | 470373 |
| trep4308 | LTR/Gypsy | 162 | 46462 |
| trep4309 | LTR/Gypsy | 740 | 170587 |
| trep4310 | LTR | 3600 | 1159271 |
| trep4311 | LTR | 1611 | 473997 |
| trep4312 | LTR | 672 | 200942 |
| trep4313 | LTR | 1545 | 490505 |

| | | | |
|---|---|---|---|
| trep4314 | Other | 294 | 46129 |
| trep4315 | DNA/TcMar | 151 | 19775 |
| trep4317 | DNA/TcMar | 2 | 192 |
| trep4318 | LINE | 234 | 47389 |
| trep4319 | LTR/Copia | 549 | 168559 |
| trep4320 | LTR/Gypsy | 2270 | 720750 |
| trep4321 | LTR/Gypsy | 2257 | 682308 |
| trep4322 | LTR/Gypsy | 504 | 128503 |
| trep4323 | DNA | 14 | 1462 |
| trep4324 | DNA | 142 | 16349 |
| trep4325 | Other | 579 | 97278 |
| trep4326 | LINE | 3 | 520 |
| trep4327 | Other | 29 | 1187 |
| trep4328 | Other | 132 | 15539 |
| trep4329 | Other | 32 | 3670 |
| trep4330 | DNA | 6 | 305 |
| trep4331 | DNA | 38 | 3014 |
| trep4332 | LTR/Copia | 6 | 1306 |
| trep4333 | LTR/Copia | 263 | 51212 |
| trep4339 | LTR/Copia | 12 | 2244 |
| trep4340 | LTR/Copia | 5 | 903 |
| trep4342 | LTR/Copia | 9 | 1852 |
| trep4344 | LTR/Copia | 1 | 202 |
| trep4345 | LTR/Copia | 24 | 5498 |
| trep4346 | LTR/Copia | 5 | 1291 |
| trep4347 | LTR/Copia | 6 | 896 |
| trep4349 | LTR/Copia | 3 | 243 |
| trep4350 | LTR/Copia | 5 | 1624 |
| trep4352 | LTR/Copia | 14 | 3252 |
| trep4353 | LTR/Gypsy | 10 | 834 |
| trep4354 | LTR/Gypsy | 6 | 776 |
| trep4355 | LTR/Gypsy | 3 | 714 |
| trep4356 | LTR/Gypsy | 4 | 457 |
| trep4357 | LTR/Gypsy | 17 | 3190 |
| trep4358 | LTR/Gypsy | 15 | 1698 |
| trep4359 | LTR/Gypsy | 65 | 4040 |
| trep4360 | LTR/Gypsy | 10 | 2081 |
| trep4362 | LTR/Gypsy | 10 | 1594 |
| trep4363 | LTR/Gypsy | 9 | 1301 |
| trep4364 | LTR/Gypsy | 100 | 27445 |
| trep4365 | LTR/Gypsy | 15 | 5328 |
| trep4366 | LTR/Gypsy | 10 | 788 |
| trep4367 | LTR/Gypsy | 6 | 754 |
| trep4368 | LTR/Gypsy | 3 | 374 |
| trep4369 | LTR/Gypsy | 87 | 15200 |
| trep4370 | LTR/Gypsy | 9 | 1586 |
| trep4371 | LTR | 8 | 888 |

| | | | |
|---|---|---|---|
| trep4372 | LTR | 4 | 498 |
| trep4373 | LTR | 10 | 911 |
| trep4374 | LTR | 2 | 372 |
| trep4375 | LTR | 58 | 12773 |
| trep4376 | DNA/TcMar | 3 | 231 |
| trep4378 | DNA/TcMar | 4 | 346 |
| trep4379 | DNA/TcMar | 9 | 1056 |
| trep4380 | DNA/TcMar | 6 | 356 |
| trep4381 | DNA/TcMar | 112 | 8902 |
| trep4382 | DNA/TcMar | 27 | 3099 |
| trep4383 | DNA/TcMar | 23 | 1787 |
| trep4384 | DNA/TcMar | 1 | 83 |
| trep4386 | DNA/TcMar | 109 | 13022 |
| trep4387 | DNA/TcMar | 2 | 138 |
| trep4389 | DNA/TcMar | 24 | 3352 |
| trep4390 | DNA/TcMar | 24 | 3547 |
| trep4391 | DNA/TcMar | 2 | 144 |
| trep4392 | DNA/TcMar | 52 | 3701 |
| trep4393 | DNA/TcMar | 35 | 2048 |
| trep4394 | DNA/TcMar | 16 | 1072 |
| trep4395 | DNA/TcMar | 14 | 993 |
| trep4396 | DNA/TcMar | 20 | 2810 |
| trep4397 | DNA/En-Spm | 35 | 5839 |
| trep4398 | DNA/En-Spm | 10 | 656 |
| trep4399 | DNA/En-Spm | 55 | 12658 |
| trep4400 | DNA/En-Spm | 78 | 6746 |
| trep4401 | DNA/En-Spm | 158 | 19237 |
| trep4402 | DNA/En-Spm | 42 | 9187 |
| trep4403 | DNA/En-Spm | 107 | 16332 |
| trep4404 | DNA/En-Spm | 66 | 15587 |
| trep4405 | DNA/En-Spm | 28 | 4179 |
| trep4406 | DNA/En-Spm | 224 | 41650 |
| trep4408 | DNA/En-Spm | 13 | 1573 |
| trep4409 | DNA/En-Spm | 136 | 23488 |
| trep4410 | DNA/TcMar | 32 | 5769 |
| trep4411 | DNA/TcMar | 20 | 3664 |
| trep4412 | DNA/TcMar | 16 | 4298 |
| trep4413 | DNA/TcMar | 64 | 13757 |
| trep4414 | DNA/TcMar | 26 | 5837 |
| trep4415 | DNA/TcMar | 21 | 4863 |
| trep4417 | DNA/TcMar | 12 | 2855 |
| trep4418 | DNA/TcMar | 12 | 1375 |
| trep4419 | DNA/TcMar | 4 | 390 |
| trep4420 | DNA/TcMar | 240 | 26061 |
| trep4421 | DNA/TcMar | 41 | 9173 |
| trep4422 | DNA/TcMar | 11 | 3964 |
| trep4423 | DNA/TcMar | 12 | 3075 |

| | | | |
|---|---|---|---|
| trep4424 | DNA/TcMar | 20 | 3427 |
| trep4425 | DNA/TcMar | 13 | 3707 |
| trep4426 | DNA/TcMar | 18 | 3966 |
| trep4427 | DNA/TcMar | 2 | 589 |
| trep4428 | DNA/TcMar | 4 | 755 |
| trep4429 | DNA/TcMar | 11 | 2150 |
| trep4430 | DNA/TcMar | 3 | 615 |
| trep4432 | DNA/TcMar | 4 | 216 |
| trep4433 | DNA/TcMar | 3 | 245 |
| trep4435 | DNA/TcMar | 1 | 45 |
| trep4436 | DNA/Harbinger | 82 | 13974 |
| trep4437 | DNA/Harbinger | 51 | 10624 |
| trep4438 | DNA/Harbinger | 116 | 28176 |
| trep4439 | DNA/Harbinger | 25 | 4316 |
| trep4440 | DNA/Harbinger | 91 | 17641 |
| trep4441 | DNA/Harbinger | 49 | 6629 |
| trep4442 | DNA/Harbinger | 307 | 78151 |
| trep4444 | DNA/Harbinger | 43 | 7841 |
| trep4445 | DNA/Harbinger | 1 | 62 |
| trep4446 | DNA/Harbinger | 17 | 2725 |
| trep4447 | DNA/Harbinger | 36 | 7306 |
| trep4448 | DNA/Harbinger | 40 | 8713 |
| trep4450 | DNA/Harbinger | 15 | 2638 |
| trep4451 | DNA/Harbinger | 8 | 854 |
| trep4452 | DNA/Harbinger | 77 | 12840 |
| trep4453 | DNA/Harbinger | 11 | 2405 |
| trep4454 | DNA/Harbinger | 56 | 7285 |
| trep4455 | DNA/Harbinger | 59 | 8860 |
| trep4456 | DNA/Harbinger | 2 | 754 |
| trep4457 | DNA/Harbinger | 14 | 2366 |
| trep4458 | DNA/Harbinger | 13 | 3770 |
| trep4459 | DNA/Harbinger | 21 | 3416 |
| trep4460 | DNA/Harbinger | 22 | 3475 |
| trep4461 | DNA/Harbinger | 126 | 34803 |
| trep4462 | DNA/Harbinger | 44 | 5702 |
| trep4464 | DNA/Harbinger | 15 | 1449 |
| trep4465 | DNA/Harbinger | 2 | 74 |
| trep4466 | DNA/Harbinger | 37 | 4678 |
| trep4470 | DNA/Harbinger | 20 | 1496 |
| trep4472 | DNA/Harbinger | 2 | 185 |
| trep4473 | DNA/Harbinger | 24 | 2483 |
| trep4474 | DNA/Harbinger | 31 | 2776 |
| trep4475 | DNA/Harbinger | 46 | 6140 |
| trep4476 | DNA/Harbinger | 27 | 3024 |
| trep4477 | DNA/Harbinger | 7 | 773 |
| trep4478 | DNA/Harbinger | 1 | 89 |
| trep4479 | DNA/Harbinger | 3 | 534 |

| | | | |
|---|---|---|---|
| trep4480 | DNA/Harbinger | 94 | 8957 |
| trep4481 | DNA/MuDR | 8 | 713 |
| trep4483 | DNA/MuDR | 81 | 9035 |
| trep4485 | DNA/MuDR | 2 | 147 |
| trep4486 | DNA/MuDR | 12 | 1169 |
| trep4487 | DNA/MuDR | 8 | 1237 |
| trep4488 | DNA/MuDR | 1 | 40 |
| trep4489 | DNA/MuDR | 12 | 1251 |
| trep4491 | DNA/MuDR | 398 | 80400 |
| trep4493 | DNA/MuDR | 44 | 4384 |
| trep4494 | DNA/MuDR | 2 | 201 |
| trep4499 | DNA/MuDR | 3 | 472 |
| trep4501 | DNA/MuDR | 64 | 7039 |
| trep4502 | DNA/MuDR | 48 | 5285 |
| trep4503 | DNA/MuDR | 17 | 1707 |
| trep4504 | DNA/MuDR | 3 | 401 |
| trep4506 | DNA/MuDR | 16 | 1667 |
| trep4507 | DNA/MuDR | 9 | 886 |
| trep4508 | DNA/MuDR | 3 | 318 |
| trep4509 | DNA/MuDR | 15 | 2192 |
| trep4510 | DNA/MuDR | 23 | 2566 |
| trep4511 | DNA/MuDR | 46 | 12687 |
| trep4512 | DNA/MuDR | 27 | 1663 |
| trep4513 | DNA/MuDR | 1059 | 285406 |
| trep4514 | DNA/MuDR | 6 | 520 |
| trep4516 | DNA/MuDR | 16 | 1706 |
| trep4517 | DNA/MuDR | 21 | 4682 |
| trep4518 | DNA/MuDR | 3 | 311 |
| trep4519 | DNA/MuDR | 137 | 26063 |
| trep4520 | DNA/MuDR | 150 | 42398 |
| trep4522 | DNA/MuDR | 52 | 11691 |
| trep4523 | DNA/MuDR | 146 | 29179 |
| trep4524 | DNA/MuDR | 469 | 128641 |
| trep4525 | DNA/MuDR | 223 | 52064 |
| trep4526 | DNA/MuDR | 35 | 7619 |
| trep4528 | DNA/MuDR | 21 | 1642 |
| trep4529 | DNA/MuDR | 2 | 101 |
| trep4530 | DNA/MuDR | 1 | 140 |
| trep4531 | DNA/MuDR | 240 | 59933 |
| trep4532 | DNA/MuDR | 308 | 65293 |
| trep4533 | DNA/MuDR | 87 | 23110 |
| trep4535 | DNA/MuDR | 58 | 5903 |
| trep4536 | DNA/MuDR | 166 | 28398 |
| trep4537 | DNA/MuDR | 2 | 153 |
| trep4538 | DNA/MuDR | 11 | 970 |
| trep4539 | DNA/MuDR | 1 | 112 |
| trep4540 | DNA/MuDR | 15 | 1823 |

| | | | |
|---|---|---|---|
| trep4541 | DNA/MuDR | 23 | 2610 |
| trep4542 | DNA/MuDR | 2 | 418 |
| trep48 | DNA | 1 | 80 |
| trep49 | LTR/Gypsy | 5 | 656 |
| trep50 | LTR/Gypsy | 23 | 1252 |
| trep51 | LINE | 34 | 8728 |
| trep52 | LINE | 162 | 34131 |
| trep53 | LINE | 4 | 1087 |
| trep54 | LTR/Gypsy | 3 | 426 |
| trep56 | LTR/Gypsy | 1 | 99 |
| trep57 | LTR/Gypsy | 2 | 136 |
| trep58 | LINE | 43 | 6835 |
| trep614 | LTR/Copia | 15 | 2974 |
| trep616 | LTR/Gypsy | 25 | 2406 |
| trep617 | LTR/Gypsy | 229 | 20158 |
| trep618 | LTR/Copia | 40 | 8519 |
| trep619 | LTR/Gypsy | 377 | 45273 |
| trep620 | LTR/Gypsy | 1141 | 246659 |
| trep621 | LTR/Gypsy | 146 | 14293 |
| trep622 | LTR/Gypsy | 175 | 17690 |
| trep625 | Other | 1 | 59 |
| trep627 | Other | 108 | 18165 |
| trep702 | LTR/Copia | 14 | 1780 |
| trep703 | LTR/Copia | 3 | 467 |
| trep704 | LTR/Copia | 36 | 6567 |
| trep705 | LTR/Copia | 2 | 238 |
| trep706 | LTR/Copia | 18 | 2127 |
| trep707 | LTR/Copia | 12 | 1524 |
| trep713 | LTR/Gypsy | 402 | 81617 |
| trep714 | LTR/Gypsy | 23 | 3433 |
| trep715 | LTR/Gypsy | 4 | 760 |
| trep716 | LTR/Gypsy | 1091 | 232533 |
| trep717 | LTR/Gypsy | 6 | 1473 |
| trep718 | DNA/hAT | 23 | 5333 |
| trep719 | LINE | 233 | 30981 |
| trep720 | LTR/Gypsy | 39 | 5117 |
| trep722 | LTR/Copia | 7 | 644 |
| trep723 | LTR/Copia | 3 | 548 |
| trep724 | LTR/Copia | 3 | 636 |
| trep725 | LTR/Copia | 3 | 335 |
| trep727 | LTR/Copia | 1 | 142 |
| trep728 | LTR/Gypsy | 4 | 521 |
| trep729 | DNA/MuDR | 46 | 10614 |
| trep731 | DNA/MuDR | 147 | 41938 |
| trep733 | DNA | 116 | 16467 |
| trep734 | DNA | 69 | 8322 |
| trep735 | DNA/Stowaway | 4 | 172 |

| | | | |
|---|---|---|---|
| trep738 | DNA/Stowaway | 5 | 378 |
| trep740 | LTR/Gypsy | 5 | 974 |
| trep741 | DNA/Stowaway | 26 | 2752 |
| trep743 | DNA/Stowaway | 9 | 592 |
| trep744 | DNA/Stowaway | 1 | 58 |
| trep745 | DNA/Stowaway | 11 | 1086 |
| trep746 | DNA/En-Spm | 429 | 78244 |
| trep747 | DNA/En-Spm | 628 | 119245 |
| trep748 | DNA/En-Spm | 81 | 10280 |
| trep749 | DNA/En-Spm | 905 | 85850 |
| trep750 | DNA/En-Spm | 48 | 5625 |
| trep751 | DNA/En-Spm | 33 | 2847 |
| trep752 | DNA/En-Spm | 83 | 18456 |
| trep753 | DNA/En-Spm | 4 | 343 |
| trep754 | DNA/En-Spm | 28 | 4801 |
| trep756 | DNA/En-Spm | 1 | 171 |
| trep757 | DNA/En-Spm | 206 | 37022 |
| trep758 | DNA/En-Spm | 13 | 1715 |
| trep759 | DNA/En-Spm | 43 | 5604 |
| trep760 | DNA/En-Spm | 37 | 3601 |
| trep761 | DNA/En-Spm | 25 | 3442 |
| trep762 | DNA/En-Spm | 33 | 4835 |
| trep763 | DNA/En-Spm | 59 | 6400 |
| trep764 | DNA/En-Spm | 243 | 66867 |
| trep765 | DNA/En-Spm | 16815 | 4706772 |
| trep766 | DNA/En-Spm | 3333 | 943231 |
| trep767 | DNA/En-Spm | 621 | 129926 |
| trep768 | DNA/En-Spm | 391 | 51091 |
| trep769 | DNA/En-Spm | 5 | 507 |
| trep770 | DNA/En-Spm | 981 | 242125 |
| trep771 | DNA/En-Spm | 266 | 70442 |
| trep772 | DNA/En-Spm | 287 | 46756 |
| trep773 | DNA/En-Spm | 2635 | 742884 |
| trep774 | DNA/En-Spm | 455 | 119390 |
| trep775 | DNA/En-Spm | 241 | 41522 |
| trep776 | DNA/En-Spm | 199 | 38949 |
| trep777 | DNA/En-Spm | 1388 | 262761 |
| trep778 | DNA/En-Spm | 1021 | 120066 |
| trep779 | DNA/En-Spm | 27 | 3665 |
| trep780 | DNA/En-Spm | 294 | 72707 |
| trep781 | DNA/En-Spm | 579 | 104545 |
| trep782 | DNA/En-Spm | 91 | 16287 |
| trep783 | DNA/En-Spm | 23 | 3702 |
| trep784 | DNA/En-Spm | 75 | 10687 |
| trep785 | DNA/En-Spm | 33 | 5881 |
| trep786 | DNA/En-Spm | 527 | 119630 |
| trep787 | DNA/En-Spm | 192 | 38245 |

| | | | |
|---|---|---|---|
| trep788 | DNA/En-Spm | 41 | 6242 |
| trep789 | LTR/Copia | 11 | 1436 |
| trep790 | LTR/Copia | 114 | 23052 |
| trep792 | LTR/Copia | 240 | 49062 |
| trep793 | LTR/Copia | 171 | 31959 |
| trep794 | LTR/Copia | 110 | 28505 |
| trep795 | DNA/MuDR | 122 | 22038 |
| trep796 | LTR/Gypsy | 61 | 7808 |
| trep797 | DNA/En-Spm | 656 | 162873 |
| trep798 | LTR/Gypsy | 1012 | 253216 |
| trep80 | LINE | 73 | 12523 |
| trep800 | LINE | 170 | 56020 |
| trep801 | LINE | 86 | 17597 |
| trep802 | LINE | 269 | 72421 |
| trep803 | LINE | 7 | 836 |
| trep804 | LINE | 554 | 126127 |
| trep805 | DNA/MuDR | 318 | 60451 |
| trep807 | LTR/Gypsy | 1138 | 284755 |
| trep809 | LTR/Gypsy | 1534 | 328063 |
| trep810 | LTR/Gypsy | 3051 | 728142 |
| trep811 | LTR/Gypsy | 1660 | 468417 |
| trep812 | LTR/Gypsy | 3575 | 889154 |
| trep814 | DNA/Stowaway | 7 | 629 |
| trep815 | LTR/Gypsy | 825 | 255704 |
| trep817 | LTR/Gypsy | 1053 | 223553 |
| trep818 | LTR/Copia | 81 | 13718 |
| trep819 | LTR/Copia | 38 | 10613 |
| trep820 | LTR/Gypsy | 124 | 14119 |
| trep821 | LTR/Gypsy | 4712 | 1286588 |
| trep822 | LTR/Gypsy | 218 | 38860 |
| trep823 | Other | 258 | 71576 |
| trep824 | LINE | 833 | 193226 |
| trep825 | DNA/MuDR | 74 | 15056 |
| trep826 | LTR/Copia | 204 | 44515 |
| trep827 | LTR/Gypsy | 104 | 23584 |
| trep828 | LTR/Gypsy | 1612 | 343221 |
| trep829 | LTR/Copia | 58 | 10207 |
| trep831 | DNA/MuDR | 358 | 78557 |
| trep832 | LINE | 304 | 51842 |
| trep833 | DNA/Tourist | 44 | 5822 |
| trep834 | DNA/Tourist | 45 | 5896 |
| trep835 | LTR/Gypsy | 2073 | 590294 |
| trep836 | LTR/Gypsy | 5660 | 1739173 |
| trep837 | LTR/Gypsy | 9852 | 3200860 |
| trep838 | LTR/Gypsy | 1539 | 432998 |
| trep839 | LTR/Copia | 334 | 97082 |
| trep84 | Other | 17 | 1529 |

| | | | |
|---|---|---|---|
| trep840 | LTR/Copia | 208 | 53093 |
| trep841 | LTR/Copia | 40 | 11915 |
| trep842 | LTR/Gypsy | 1240 | 373999 |
| trep850 | DNA/Stowaway | 11 | 1108 |
| trep862 | DNA/Stowaway | 1 | 34 |
| trep866 | DNA/Stowaway | 7 | 331 |
| trep868 | DNA/Stowaway | 2 | 98 |
| trep870 | DNA/Stowaway | 46 | 2326 |
| trep871 | DNA/Stowaway | 8 | 509 |
| trep875 | DNA/Stowaway | 15 | 860 |
| trep876 | DNA/Stowaway | 1 | 63 |
| trep878 | DNA/Stowaway | 15 | 872 |
| trep880 | DNA/Stowaway | 1 | 39 |
| trep881 | DNA/Stowaway | 77 | 4331 |
| trep884 | DNA/Stowaway | 22 | 1631 |
| trep886 | DNA/Stowaway | 3 | 177 |
| trep889 | DNA/Stowaway | 14 | 763 |
| trep890 | DNA/Stowaway | 43 | 2725 |
| trep893 | DNA/Stowaway | 34 | 1660 |
| trep894 | DNA/Stowaway | 10 | 496 |
| trep896 | DNA/Stowaway | 7 | 371 |
| trep898 | DNA/Stowaway | 24 | 1519 |
| trep899 | DNA/Stowaway | 25 | 1388 |
| trep900 | DNA/Stowaway | 24 | 1459 |
| trep903 | DNA/Stowaway | 90 | 5713 |
| trep905 | DNA/Stowaway | 3 | 162 |
| trep907 | DNA/Stowaway | 19 | 803 |
| trep908 | DNA/Stowaway | 10 | 687 |
| trep913 | DNA/Stowaway | 45 | 2646 |
| trep914 | DNA/Stowaway | 11 | 532 |
| trep922 | DNA/Stowaway | 4 | 264 |
| trep923 | DNA/Stowaway | 16 | 725 |
| trep924 | DNA/Stowaway | 5 | 273 |
| trep925 | DNA/Stowaway | 2 | 113 |
| trep926 | DNA/Stowaway | 2 | 130 |
| trep929 | DNA/Stowaway | 5 | 314 |
| trep934 | DNA/Stowaway | 2 | 109 |
| trep935 | DNA/Stowaway | 2 | 115 |
| trep936 | DNA/Stowaway | 1 | 67 |
| trep939 | DNA/Stowaway | 3 | 133 |
| trep941 | DNA/Stowaway | 9 | 462 |
| trep942 | DNA/Stowaway | 45 | 2480 |
| trep944 | DNA/Stowaway | 9 | 496 |
| trep946 | DNA/Stowaway | 16 | 1119 |
| trep947 | DNA/Stowaway | 35 | 1900 |
| trep948 | DNA/Stowaway | 18 | 1186 |
| trep949 | DNA/Stowaway | 7 | 408 |

| | | | |
|---|---|---|---|
| trep953 | DNA/Stowaway | 2 | 244 |
| trep955 | DNA/Stowaway | 17 | 1031 |
| trep958 | DNA/Stowaway | 14 | 1304 |
| trep96 | LTR/Copia | 128 | 32339 |
| trep961 | DNA/Stowaway | 2 | 196 |
| trep962 | DNA/Stowaway | 1 | 142 |
| trep967 | DNA/Stowaway | 168 | 17511 |
| trep97 | LTR/Copia | 32 | 13089 |
| trep971 | DNA/Stowaway | 25 | 2115 |
| trep972 | DNA/Stowaway | 12 | 1197 |
| trep973 | DNA/Stowaway | 3 | 245 |
| trep974 | DNA/Stowaway | 3 | 178 |
| trep975 | DNA/Stowaway | 2 | 198 |
| trep98 | LTR/Gypsy | 827 | 72303 |
| trep980 | DNA/Stowaway | 7 | 719 |
| trep987 | DNA/Stowaway | 9 | 648 |
| trep988 | DNA/Stowaway | 15 | 856 |
| trep99 | LTR/Gypsy | 141 | 28143 |
| trep991 | DNA/Stowaway | 19 | 1102 |
| trep992 | DNA/Stowaway | 12 | 660 |
| trep993 | DNA/Stowaway | 5 | 287 |
| trep994 | DNA/Stowaway | 12 | 770 |
| trep995 | DNA/Stowaway | 10 | 471 |
| trep996 | DNA/Stowaway | 2 | 141 |
| trep997 | DNA/Stowaway | 9 | 554 |
| trep998 | DNA/Stowaway | 4 | 188 |
| trep999 | DNA/Stowaway | 36 | 2095 |
| ty3 | LTR/Gypsy | 2 | 127 |
| upat-311G2-1 | Retroelement | 1 | 94 |
| uwum-573F08-1 | Other | 1 | 53 |
| veju-206C17-1part | LTR | 66 | 12158 |
| victim-334B07-1 | LTR/Copia | 1 | 436 |
| wham-115G1-2 | LTR/Gypsy | 4692 | 1245611 |
| wis | LTR/Copia | 124 | 35585 |
| wis-354M17-2 | LTR/Copia | 266 | 76436 |
| wis-60B2-1 | Other | 214 | 38867 |
| wombat-retroryza | LTR | 19 | 5719 |
| xilon-333J11-1 | LTR/Gypsy | 1 | 142 |
| xilon-333J11-2hdnm | LTR/Gypsy | 2 | 210 |
| xilon-af448416-2hdnm | LTR/Gypsy | 2 | 214 |
| yemi-013I05-1hdnm | LTR | 20 | 1709 |
| yote-438D03-1 | DNA/En-Spm | 10 | 1097 |
| yote-af090447-1 | DNA/En-Spm | 1 | 139 |
| zebulon-retroryza | LTR | 1 | 76 |
| zeon-Z178A11-1 | LTR/Gypsy | 3 | 165 |
| zeon-ac148112-1hdnm | LTR/Gypsy | 1 | 51 |

| | | | |
|---|---|---|---|
| zeon-ac148169-1 | LTR/Gypsy | 2 | 166 |

**ancestor *Triticum***

ak[1,3]*

## Molecular organization and comparative analysis of chromosome 5B of the wild

Bala Ani Akpinar[1], Meral Yuce[1], Stuart Lucas[1], Jan Vrána[2], Veronika Burešová[2], Jaroslav Doležel[2], Hikm

**Supplementary Table 2. Gene-associated Tdic5B contigs/singletons**

| Contig/read name | Brachypodium | Rice | Sorghum | Barley |
|---|---|---|---|---|
| IV9RU5F01BCDPJ | Bradi5g27650.1 | - | - | - |
| IV9RU5F01BF1MW | Bradi5g27080.1 | - | - | - |
| IV9RU5F01DLXUL | Bradi5g27080.1 | - | - | - |
| IV9RU5F01A9LMK | Bradi5g26790.1 | - | - | - |
| IV9RU5F02HTW53 | Bradi5g26790.1 | - | - | - |
| IVLNWEC01AQQFF | Bradi5g26790.1 | - | - | - |
| IV9RU5F02JUSYP | Bradi5g25180.1 | - | - | - |
| IV9RU5F02JXOXH | Bradi5g24890.1 | - | - | - |
| IUIVLUD02GXNX0 | Bradi5g24095.1 | - | - | - |
| IV9RU5F01AJ6B1 | Bradi5g24095.1 | - | - | - |
| IV9RU5F01C3HTN | Bradi5g24095.1 | - | - | - |
| IV9RU5F02FS633 | Bradi5g24095.1 | - | - | - |
| IV9RU5F02HQU9B | Bradi5g24095.1 | - | - | - |
| IVLNWEC02IMNBK | Bradi5g24095.1 | - | - | - |
| IUIVLUD02IWHLT | Bradi5g22870.1 | - | - | - |
| IV9RU5F01BN4RO | Bradi5g22870.1 | - | - | - |
| IVLNWEC02FWR69 | Bradi5g22547.1 | - | - | - |
| IUIVLUD01BQYMP | Bradi5g22187.1 | - | - | - |
| IVLNWEC02GNSXD | Bradi5g22187.1 | - | - | - |
| IV9RU5F01BUMXZ | Bradi5g20587.1 | - | - | - |
| IV9RU5F01AYRUG | Bradi5g20077.1 | - | - | - |
| IV9RU5F01AOYQZ | Bradi5g16900.1 | - | - | - |
| IUIVLUD02JPNL4 | Bradi5g15797.1 | - | - | - |
| IV9RU5F02H6TC4 | Bradi5g15797.1 | - | - | - |
| IV9RU5F02HOKRS | Bradi5g15797.1 | - | - | - |
| IVLNWEC02GUDQS | Bradi5g15797.1 | - | - | - |
| IUIVLUD02HFJUN | Bradi5g15240.1 | - | - | - |
| IV9RU5F02IXHTG | Bradi5g15240.1 | - | - | - |
| IVLNWEC02GZD7R | Bradi5g15240.1 | - | - | - |
| IV9RU5F01EOP1H | Bradi5g14930.1 | - | - | - |
| IVLNWEC01C2IDX | Bradi5g14930.1 | - | - | - |
| IV9RU5F01BN40G | Bradi5g14110.1 | - | - | - |
| IUIVLUD01BR7TE | Bradi5g13180.1 | - | - | - |
| IUIVLUD01EIUSA | Bradi5g13130.1 | - | - | - |
| IV9RU5F01CB5LW | Bradi5g13130.1 | - | - | - |
| IV9RU5F02GWWGM | Bradi5g13130.1 | - | - | - |
| IV9RU5F02GWZZJ | Bradi5g13130.1 | - | - | - |
| IVLNWEC01AFWT3 | Bradi5g13130.1 | - | - | - |
| IVLNWEC01B3DBM | Bradi5g13130.1 | - | - | - |
| IVLNWEC02FNSNL | Bradi5g13130.1 | - | - | - |

| | | | | |
|---|---|---|---|---|
| IVLNWEC02IPOCC | Bradi5g12976.1 | - | - | - |
| IV9RU5F01EPX6F | Bradi5g12900.1 | - | - | - |
| IVLNWEC01CMZXM | Bradi5g12900.1 | - | - | - |
| IVLNWEC01CQ6ZA | Bradi5g12900.1 | - | - | - |
| IV9RU5F01A5LTL | Bradi5g12090.1 | - | - | - |
| IVLNWEC02G643E | Bradi5g12090.1 | - | - | - |
| IV9RU5F02GZBGV | Bradi5g10940.1 | - | - | - |
| IVLNWEC01BLD1W | Bradi5g09350.1 | - | - | - |
| IVLNWEC01C492A | Bradi5g09350.1 | - | - | - |
| IV9RU5F02G9EZI | Bradi5g07160.1 | - | - | - |
| IUIVLUD02F3X9X | Bradi5g05900.1 | - | - | - |
| IUIVLUD02GGR8H | Bradi5g05900.1 | - | - | - |
| IV9RU5F02FUOQJ | Bradi5g04000.1 | - | - | - |
| IVLNWEC01D3CFY | Bradi5g03890.1 | - | - | - |
| IUIVLUD02HNPGF | Bradi5g03840.1 | - | - | - |
| IVLNWEC01C353J | Bradi5g03760.1 | - | - | - |
| IVLNWEC02F9NG5 | Bradi5g03760.1 | - | - | - |
| IV9RU5F01DHGHQ | Bradi5g03627.1 | - | - | - |
| IVLNWEC01B6SXP | Bradi5g03627.1 | - | - | - |
| IVLNWEC01CBDIH | Bradi5g03627.1 | - | - | - |
| IVLNWEC02HZXH0 | Bradi5g03627.1 | - | - | - |
| IVLNWEC02IBT70 | Bradi5g03627.1 | - | - | - |
| IV9RU5F01AEO4T | Bradi5g03570.1 | - | - | - |
| IVLNWEC01A7R8Y | Bradi5g03570.1 | - | - | - |
| IVLNWEC01AZLMF | Bradi5g03570.1 | - | - | - |
| IVLNWEC02FX5AC | Bradi5g03570.1 | - | - | - |
| IUIVLUD02HCTN0 | Bradi5g03200.1 | - | - | - |
| IUIVLUD02IBBU4 | Bradi5g03200.1 | - | - | - |
| IV9RU5F01DSIWP | Bradi5g03180.1 | - | - | - |
| IVLNWEC01DFS08 | Bradi5g03180.1 | - | - | - |
| IV9RU5F01D2YG3 | Bradi5g02430.1 | - | - | - |
| IVLNWEC01AN57A | Bradi5g02430.1 | - | - | - |
| IUIVLUD01DCWTW | Bradi5g02420.1 | - | - | - |
| IVLNWEC02GI6QG | Bradi5g02420.1 | - | - | - |
| IVLNWEC02HJED5 | Bradi5g02420.1 | - | - | - |
| IV9RU5F02FYN2C | Bradi5g02240.1 | - | - | - |
| IV9RU5F02IXAWH | Bradi5g02240.1 | - | - | - |
| IVLNWEC01BRJZP | Bradi5g02240.1 | - | - | - |
| IUIVLUD01AO3EG | Bradi5g01462.1 | - | - | - |
| IV9RU5F02F3ALQ | Bradi5g01462.1 | - | - | - |
| IV9RU5F02FUG41 | Bradi5g01230.1 | - | - | - |
| IUIVLUD02IC2ZW | Bradi5g01167.1 | - | - | - |
| IV9RU5F02HTUP2 | Bradi5g01167.1 | - | - | - |
| IV9RU5F02IPVD0 | Bradi5g01167.1 | - | - | - |
| IVLNWEC01DEZYL | Bradi5g01167.1 | - | - | - |
| IV9RU5F02HF2P3 | Bradi5g01070.1 | - | - | - |
| IV9RU5F02IE36J | Bradi5g00990.1 | - | - | - |

| | | | | |
|---|---|---|---|---|
| IV9RU5F01B32G3 | Bradi5g00970.1 | - | - | - |
| IVLNWEC02I92XQ | Bradi5g00590.1 | - | - | - |
| IUIVLUD02HAQ2C | Bradi5g00440.1 | - | - | - |
| IVLNWEC01CX860 | Bradi5g00440.1 | - | - | - |
| IUIVLUD02GN2TJ | Bradi4g45397.1 | - | - | - |
| IUIVLUD02H7XA1 | Bradi4g45160.1 | - | - | - |
| IV9RU5F02HGCA6 | Bradi4g45160.1 | - | - | - |
| IV9RU5F02HLAUT | Bradi4g45160.1 | - | - | - |
| IVLNWEC02FOHEW | Bradi4g45160.1 | - | - | - |
| IVLNWEC02HHAOM | Bradi4g45160.1 | - | - | - |
| IV9RU5F02F161V | Bradi4g43877.1 | - | - | - |
| IV9RU5F02HGZE7 | Bradi4g43870.1 | - | - | - |
| IV9RU5F01AMIW8 | Bradi4g43680.2 | - | - | - |
| IV9RU5F02IKZEM | Bradi4g42836.1 | - | - | - |
| IVLNWEC02IQBU4 | Bradi4g42830.1 | - | - | - |
| IV9RU5F01DTEFI | Bradi4g42780.1 | - | - | - |
| IVLNWEC02JT153 | Bradi4g42780.1 | - | - | - |
| IVLNWEC02ISQR5 | Bradi4g42060.1 | - | - | - |
| IUIVLUD02GWTXP | Bradi4g42020.1 | - | - | - |
| IVLNWEC02GBSK4 | Bradi4g42020.1 | - | - | - |
| IVLNWEC02GF6RL | Bradi4g41830.1 | - | - | - |
| IV9RU5F02GV0ZO | Bradi4g41470.1 | - | - | - |
| IV9RU5F02FOGKE | Bradi4g40450.2 | - | - | - |
| IV9RU5F01B1A80 | Bradi4g39840.1 | - | - | - |
| IV9RU5F01CCNW7 | Bradi4g39380.1 | - | - | - |
| IVLNWEC02GSQZ2 | Bradi4g39380.1 | - | - | - |
| IV9RU5F01D3GMJ | Bradi4g38700.1 | - | - | - |
| IVLNWEC02GS1ZE | Bradi4g38700.1 | - | - | - |
| IUIVLUD01AYNST | Bradi4g38560.1 | - | - | - |
| IV9RU5F01EULKU | Bradi4g38560.1 | - | - | - |
| IV9RU5F02JG8LD | Bradi4g38560.1 | - | - | - |
| IVLNWEC01DUTG2 | Bradi4g38560.1 | - | - | - |
| IV9RU5F02GL4MX | Bradi4g38357.1 | - | - | - |
| IV9RU5F02H7RBJ | Bradi4g38170.1 | - | - | - |
| IVLNWEC02IQB8N | Bradi4g38170.1 | - | - | - |
| IV9RU5F01CWG71 | Bradi4g38140.1 | - | - | - |
| IUIVLUD02GLJXC | Bradi4g38110.1 | - | - | - |
| IV9RU5F02GNZS4 | Bradi4g38110.1 | - | - | - |
| IUIVLUD01ET67X | Bradi4g38010.1 | - | - | - |
| IV9RU5F01CQ6N9 | Bradi4g38010.1 | - | - | - |
| IV9RU5F01D06TT | Bradi4g37260.1 | - | - | - |
| IV9RU5F01A43DY | Bradi4g37190.1 | - | - | - |
| IV9RU5F02GKQ94 | Bradi4g37190.1 | - | - | - |
| IVLNWEC01D5EMF | Bradi4g37190.1 | - | - | - |
| IVLNWEC01DTPXF | Bradi4g37190.1 | - | - | - |
| IVLNWEC02FSW8P | Bradi4g37190.1 | - | - | - |
| IVLNWEC02HOKEE | Bradi4g37190.1 | - | - | - |

| | | | | |
|---|---|---|---|---|
| IUIVLUD02INR1Z | Bradi4g37170.2 | - | - | - |
| IV9RU5F01DZSM2 | Bradi4g37120.1 | - | - | - |
| IV9RU5F01BIOZA | Bradi4g36990.1 | - | - | - |
| IVLNWEC01A0P1B | Bradi4g36990.1 | - | - | - |
| IVLNWEC01EBAFB | Bradi4g36976.1 | - | - | - |
| IV9RU5F01EIX5D | Bradi4g36850.1 | - | - | - |
| IUIVLUD01DARE2 | Bradi4g36680.1 | - | - | - |
| IV9RU5F01BVSCE | Bradi4g36220.3 | - | - | - |
| IV9RU5F01DAG6F | Bradi4g36160.2 | - | - | - |
| IVLNWEC01EY5YL | Bradi4g36045.1 | - | - | - |
| IVLNWEC02HMAB2 | Bradi4g36045.1 | - | - | - |
| IUIVLUD02G6XB7 | Bradi4g36020.1 | - | - | - |
| IV9RU5F02H21YG | Bradi4g35440.1 | - | - | - |
| IV9RU5F01EHIUT | Bradi4g35257.1 | - | - | - |
| IVLNWEC01BVM89 | Bradi4g34927.1 | - | - | - |
| IVLNWEC01D71X6 | Bradi4g34927.1 | - | - | - |
| IVLNWEC01C9V76 | Bradi4g34760.1 | - | - | - |
| IVLNWEC01BJDEY | Bradi4g34470.1 | - | - | - |
| IVLNWEC01D2HPA | Bradi4g34470.1 | - | - | - |
| IV9RU5F02GVGXS | Bradi4g34207.1 | - | - | - |
| IVLNWEC02HE9LS | Bradi4g34207.1 | - | - | - |
| IVLNWEC02HGC10 | Bradi4g34207.1 | - | - | - |
| IVLNWEC02HRNZR | Bradi4g34207.1 | - | - | - |
| IV9RU5F01B92C8 | Bradi4g33990.1 | - | - | - |
| IV9RU5F02JK8IX | Bradi4g33990.1 | - | - | - |
| IV9RU5F01EL3A4 | Bradi4g33920.2 | - | - | - |
| IUIVLUD02G4TLX | Bradi4g33630.1 | - | - | - |
| IVLNWEC02I0TJP | Bradi4g33630.1 | - | - | - |
| IVLNWEC02H0Y01 | Bradi4g33520.1 | - | - | - |
| IV9RU5F01B8MW2 | Bradi4g33480.1 | - | - | - |
| IVLNWEC01ASGXJ | Bradi4g33480.1 | - | - | - |
| IVLNWEC01BOPMQ | Bradi4g33480.1 | - | - | - |
| IVLNWEC01CWID5 | Bradi4g33480.1 | - | - | - |
| IVLNWEC02HMLPU | Bradi4g33480.1 | - | - | - |
| IVLNWEC02HNQBW | Bradi4g33480.1 | - | - | - |
| IVLNWEC02I6SRZ | Bradi4g33480.1 | - | - | - |
| IV9RU5F01D0ZQG | Bradi4g33310.1 | - | - | - |
| IVLNWEC01CXY52 | Bradi4g33310.1 | - | - | - |
| IVLNWEC01AZ4SC | Bradi4g33180.1 | - | - | - |
| IVLNWEC02GMI0L | Bradi4g33180.1 | - | - | - |
| IUIVLUD01DTQS2 | Bradi4g32830.1 | - | - | - |
| IVLNWEC02H2UUY | Bradi4g32830.1 | - | - | - |
| IV9RU5F01AMVB0 | Bradi4g32620.1 | - | - | - |
| IV9RU5F01B99AL | Bradi4g32620.1 | - | - | - |
| IV9RU5F01ARARW | Bradi4g32530.1 | - | - | - |
| IV9RU5F02H2D1N | Bradi4g32530.1 | - | - | - |
| IVLNWEC02JDKRJ | Bradi4g32390.1 | - | - | - |

| | | | | |
|---|---|---|---|---|
| IVLNWEC01BH9ZF | Bradi4g32140.1 | - | - | - |
| IVLNWEC01DEUO1 | Bradi4g32140.1 | - | - | - |
| IV9RU5F01CPMIY | Bradi4g31790.1 | - | - | - |
| IV9RU5F02FXAE0 | Bradi4g31790.1 | - | - | - |
| IV9RU5F02JZFZ9 | Bradi4g31400.1 | - | - | - |
| IVLNWEC02HHTC9 | Bradi4g31400.1 | - | - | - |
| IUIVLUD02GY0S8 | Bradi4g31090.1 | - | - | - |
| IV9RU5F01BBCI2 | Bradi4g31090.1 | - | - | - |
| IUIVLUD02JRKWL | Bradi4g30850.1 | - | - | - |
| IV9RU5F02JYU1H | Bradi4g30850.1 | - | - | - |
| IVLNWEC02I3T7N | Bradi4g30850.1 | - | - | - |
| IV9RU5F02JO742 | Bradi4g30840.1 | - | - | - |
| IVLNWEC02I1K54 | Bradi4g30810.1 | - | - | - |
| IV9RU5F01A51FH | Bradi4g30520.1 | - | - | - |
| IVLNWEC02FO4BP | Bradi4g30520.1 | - | - | - |
| IUIVLUD01EMFLJ | Bradi4g30370.1 | - | - | - |
| IV9RU5F01EXC6H | Bradi4g30370.1 | - | - | - |
| IV9RU5F02HNSAZ | Bradi4g30370.1 | - | - | - |
| IV9RU5F02IF2WR | Bradi4g30370.1 | - | - | - |
| IV9RU5F02JU5TM | Bradi4g30370.1 | - | - | - |
| IVLNWEC01C1F90 | Bradi4g30370.1 | - | - | - |
| IUIVLUD01DUD3I | Bradi4g30190.1 | - | - | - |
| IUIVLUD01BE91M | Bradi4g29947.1 | - | - | - |
| IUIVLUD02GI3VV | Bradi4g29760.1 | - | - | - |
| IVLNWEC02F4S64 | Bradi4g29720.1 | - | - | - |
| IUIVLUD01CA27J | Bradi4g29390.1 | - | - | - |
| IVLNWEC01EX2V7 | Bradi4g29390.1 | - | - | - |
| IVLNWEC01ETIMR | Bradi4g29190.2 | - | - | - |
| IV9RU5F02IU3PT | Bradi4g28960.1 | - | - | - |
| IV9RU5F02JRYYV | Bradi4g28900.1 | - | - | - |
| IV9RU5F01E3B5M | Bradi4g28750.1 | - | - | - |
| IV9RU5F02HEHD2 | Bradi4g28750.1 | - | - | - |
| IVLNWEC02IH1HE | Bradi4g28750.1 | - | - | - |
| IUIVLUD02FKS0J | Bradi4g28107.1 | - | - | - |
| IVLNWEC02HCT20 | Bradi4g28070.1 | - | - | - |
| IVLNWEC01CH5KG | Bradi4g27417.1 | - | - | - |
| IV9RU5F01D7D1Q | Bradi4g27090.1 | - | - | - |
| IVLNWEC02IDV87 | Bradi4g27090.1 | - | - | - |
| IV9RU5F01DM2EQ | Bradi4g26990.1 | - | - | - |
| IVLNWEC01C6KQL | Bradi4g26990.1 | - | - | - |
| IVLNWEC01AI568 | Bradi4g26877.1 | - | - | - |
| IUIVLUD02GZUGU | Bradi4g26580.1 | - | - | - |
| IV9RU5F01DKQ4W | Bradi4g26390.1 | - | - | - |
| IVLNWEC01A80BE | Bradi4g26342.1 | - | - | - |
| IV9RU5F02GHJIO | Bradi4g24660.1 | - | - | - |
| IVLNWEC01C24C0 | Bradi4g24340.1 | - | - | - |
| IV9RU5F02G7EI6 | Bradi4g24280.1 | - | - | - |

| | | | | |
|---|---|---|---|---|
| IV9RU5F02I4EJE | Bradi4g23810.1 | - | - | - |
| IV9RU5F01CTQYM | Bradi4g23730.1 | - | - | - |
| IVLNWEC02IG9BV | Bradi4g23695.1 | - | - | - |
| IV9RU5F02H39VW | Bradi4g23650.1 | - | - | - |
| IV9RU5F02JJYY8 | Bradi4g23650.1 | - | - | - |
| IUIVLUD01AG1X1 | Bradi4g23510.1 | - | - | - |
| IVLNWEC02HIYUE | Bradi4g23510.1 | - | - | - |
| IV9RU5F02I4GT8 | Bradi4g23480.1 | - | - | - |
| IVLNWEC01ESW6B | Bradi4g23480.1 | - | - | - |
| IV9RU5F02GLWY8 | Bradi4g23417.1 | - | - | - |
| IV9RU5F02F38NJ | Bradi4g23080.1 | - | - | - |
| IVLNWEC01BB8NJ | Bradi4g23080.1 | - | - | - |
| IVLNWEC02JFG8S | Bradi4g23080.1 | - | - | - |
| IUIVLUD02JSIV6 | Bradi4g21980.1 | - | - | - |
| IUIVLUD01CJDUT | Bradi4g21860.1 | - | - | - |
| IV9RU5F02GBK2E | Bradi4g21860.1 | - | - | - |
| IV9RU5F02H3IKW | Bradi4g21860.1 | - | - | - |
| IV9RU5F02H5ISH | Bradi4g21860.1 | - | - | - |
| IUIVLUD01DMX17 | Bradi4g21730.1 | - | - | - |
| IV9RU5F01BXI6V | Bradi4g21730.1 | - | - | - |
| IVLNWEC01BFY09 | Bradi4g21730.1 | - | - | - |
| IV9RU5F02G7W54 | Bradi4g21670.1 | - | - | - |
| IV9RU5F02ID5TW | Bradi4g21670.1 | - | - | - |
| IVLNWEC01BNL1R | Bradi4g21670.1 | - | - | - |
| IV9RU5F02IK7F3 | Bradi4g21510.1 | - | - | - |
| IV9RU5F01DJ4KX | Bradi4g21363.1 | - | - | - |
| IV9RU5F01ECVM0 | Bradi4g21363.1 | - | - | - |
| IV9RU5F02H3H4K | Bradi4g21363.1 | - | - | - |
| IVLNWEC01AO0RS | Bradi4g21363.1 | - | - | - |
| IVLNWEC01D5VL7 | Bradi4g21363.1 | - | - | - |
| IVLNWEC01EO5KZ | Bradi4g21363.1 | - | - | - |
| IVLNWEC02GVI2M | Bradi4g21363.1 | - | - | - |
| IVLNWEC02IHDQ7 | Bradi4g21363.1 | - | - | - |
| IV9RU5F01DELX2 | Bradi4g21250.1 | - | - | - |
| IVLNWEC02I7BTC | Bradi4g18220.1 | - | - | - |
| IV9RU5F01CZNWR | Bradi4g17190.1 | - | - | - |
| IV9RU5F01DUJ3V | Bradi4g17190.1 | - | - | - |
| IVLNWEC02JS4US | Bradi4g17190.1 | - | - | - |
| IUIVLUD01BRRQP | Bradi4g16492.1 | - | - | - |
| IVLNWEC01BRFIN | Bradi4g16492.1 | - | - | - |
| IVLNWEC02GIMO9 | Bradi4g16492.1 | - | - | - |
| IV9RU5F01C838Q | Bradi4g16377.1 | - | - | - |
| IVLNWEC02FZRCP | Bradi4g16377.1 | - | - | - |
| IUIVLUD02F4Q15 | Bradi4g16290.1 | - | - | - |
| IV9RU5F02GTL9V | Bradi4g15000.1 | - | - | - |
| IUIVLUD02HW55L | Bradi4g14750.1 | - | - | - |
| IUIVLUD02JIX6N | Bradi4g14180.1 | - | - | - |

| | | | | |
|---|---|---|---|---|
| IV9RU5F01BCCIJ | Bradi4g14180.1 | - | - | - |
| IVLNWEC01AEY45 | Bradi4g14180.1 | - | - | - |
| IV9RU5F01CDFT2 | Bradi4g14080.1 | - | - | - |
| IV9RU5F02IXPMX | Bradi4g14070.1 | - | - | - |
| IV9RU5F02JI6I9 | Bradi4g14070.1 | - | - | - |
| IV9RU5F01BVHPB | Bradi4g13990.1 | - | - | - |
| IV9RU5F02F3C0H | Bradi4g13990.1 | - | - | - |
| IV9RU5F02G4RF4 | Bradi4g13990.1 | - | - | - |
| IV9RU5F02I5ABG | Bradi4g13990.1 | - | - | - |
| IVLNWEC02G4G1H | Bradi4g13990.1 | - | - | - |
| IVLNWEC02GJ8LJ | Bradi4g13990.1 | - | - | - |
| IV9RU5F02IBLHQ | Bradi4g13975.1 | - | - | - |
| IVLNWEC02HXAWV | Bradi4g13975.1 | - | - | - |
| IVLNWEC02I6UV5 | Bradi4g13720.1 | - | - | - |
| IV9RU5F02GGYWX | Bradi4g13710.1 | - | - | - |
| IVLNWEC02JVV59 | Bradi4g13710.1 | - | - | - |
| IV9RU5F02HFD3K | Bradi4g12680.1 | - | - | - |
| IV9RU5F02J5MK3 | Bradi4g12680.1 | - | - | - |
| IV9RU5F01B3NY9 | Bradi4g12227.1 | - | - | - |
| IV9RU5F02FPNU5 | Bradi4g12227.1 | - | - | - |
| IV9RU5F02GNA9C | Bradi4g11740.1 | - | - | - |
| IV9RU5F01BNT75 | Bradi4g11635.1 | - | - | - |
| IVLNWEC01EQ9BW | Bradi4g11635.1 | - | - | - |
| IUIVLUD01C53QW | Bradi4g11120.1 | - | - | - |
| IV9RU5F01D8USO | Bradi4g11120.1 | - | - | - |
| IV9RU5F01EHWX0 | Bradi4g11120.1 | - | - | - |
| IV9RU5F02FT5RA | Bradi4g11120.1 | - | - | - |
| IV9RU5F02IRTWA | Bradi4g11120.1 | - | - | - |
| IV9RU5F02JKWFR | Bradi4g11120.1 | - | - | - |
| IVLNWEC01DRGC0 | Bradi4g11120.1 | - | - | - |
| IVLNWEC01EH64G | Bradi4g11120.1 | - | - | - |
| IV9RU5F01DWDVP | Bradi4g10700.1 | - | - | - |
| IUIVLUD01DCQPR | Bradi4g10670.1 | - | - | - |
| IUIVLUD02H1LTG | Bradi4g10670.1 | - | - | - |
| IV9RU5F01CNSF1 | Bradi4g10670.1 | - | - | - |
| IV9RU5F02JEHHB | Bradi4g10670.1 | - | - | - |
| IUIVLUD02F7QPF | Bradi4g10250.1 | - | - | - |
| IV9RU5F01BYGPS | Bradi4g09957.1 | - | - | - |
| IV9RU5F01CEBOM | Bradi4g09870.1 | - | - | - |
| IV9RU5F01BQT47 | Bradi4g09640.1 | - | - | - |
| IV9RU5F01DBUM5 | Bradi4g09640.1 | - | - | - |
| IV9RU5F02GSZ44 | Bradi4g09640.1 | - | - | - |
| IUIVLUD02HZE5J | Bradi4g09597.1 | - | - | - |
| IV9RU5F01C7QYP | Bradi4g09285.1 | - | - | - |
| IV9RU5F01CLIW7 | Bradi4g09285.1 | - | - | - |
| IV9RU5F01BCVWL | Bradi4g09247.1 | - | - | - |
| IV9RU5F01CLSY8 | Bradi4g09247.1 | - | - | - |

| | | | | |
|---|---|---|---|---|
| IV9RU5F02IDN8B | Bradi4g09247.1 | - | - | - |
| IV9RU5F02JP2FN | Bradi4g09247.1 | - | - | - |
| IV9RU5F02JS5IH | Bradi4g09247.1 | - | - | - |
| IVLNWEC02H33FA | Bradi4g09247.1 | - | - | - |
| IVLNWEC02IBCSF | Bradi4g09247.1 | - | - | - |
| IV9RU5F02IJC7Y | Bradi4g08870.1 | - | - | - |
| IVLNWEC01BGGZH | Bradi4g08640.1 | - | - | - |
| IV9RU5F02GH6G3 | Bradi4g08567.1 | - | - | - |
| IVLNWEC01BO8C3 | Bradi4g08567.1 | - | - | - |
| IV9RU5F02G4VPG | Bradi4g08152.1 | - | - | - |
| IV9RU5F02IEHS0 | Bradi4g07760.1 | - | - | - |
| IUIVLUD02I2HZP | Bradi4g07717.1 | - | - | - |
| IV9RU5F01CVC44 | Bradi4g07610.1 | - | - | - |
| IVLNWEC01DDCVL | Bradi4g07460.1 | - | - | - |
| IVLNWEC02HWSTS | Bradi4g07460.1 | - | - | - |
| IVLNWEC02JT9B7 | Bradi4g07430.1 | - | - | - |
| IVLNWEC01ASHU8 | Bradi4g07060.1 | - | - | - |
| IVLNWEC01BPPUW | Bradi4g07060.1 | - | - | - |
| IUIVLUD01CUQJ7 | Bradi4g05870.1 | - | - | - |
| IUIVLUD02ICZXT | Bradi4g05870.1 | - | - | - |
| IVLNWEC01DL3YH | Bradi4g05870.1 | - | - | - |
| IUIVLUD02JBE1P | Bradi4g05817.1 | - | - | - |
| IV9RU5F02ID8GQ | Bradi4g05817.1 | - | - | - |
| IV9RU5F02GBNTE | Bradi4g05370.1 | - | - | - |
| IVLNWEC01BE2K0 | Bradi4g05370.1 | - | - | - |
| IV9RU5F02I44ER | Bradi4g05010.1 | - | - | - |
| IVLNWEC01EXDT9 | Bradi4g04930.1 | - | - | - |
| IV9RU5F01C6INM | Bradi4g04400.1 | - | - | - |
| IV9RU5F01A4JTD | Bradi4g03890.1 | - | - | - |
| IUIVLUD02JTDOO | Bradi4g03720.1 | - | - | - |
| IVLNWEC01DHRYB | Bradi4g03690.1 | - | - | - |
| IVLNWEC01B14DR | Bradi4g03215.1 | - | - | - |
| IVLNWEC01B1GAF | Bradi4g03215.1 | - | - | - |
| IV9RU5F02GPJSS | Bradi4g03107.1 | - | - | - |
| IV9RU5F02HLLSN | Bradi4g03107.1 | - | - | - |
| IUIVLUD01CS1ZO | Bradi4g03040.1 | - | - | - |
| IV9RU5F01B5DDQ | Bradi4g03040.1 | - | - | - |
| IV9RU5F02INU68 | Bradi4g03040.1 | - | - | - |
| IVLNWEC01CNK4Y | Bradi4g03040.1 | - | - | - |
| IVLNWEC01DKCBS | Bradi4g03040.1 | - | - | - |
| IVLNWEC01EGB81 | Bradi4g03040.1 | - | - | - |
| IVLNWEC01ESWPB | Bradi4g03040.1 | - | - | - |
| IVLNWEC02I8PSM | Bradi4g03040.1 | - | - | - |
| IVLNWEC02JNIOE | Bradi4g03040.1 | - | - | - |
| IUIVLUD01A6FXZ | Bradi4g02990.1 | - | - | - |
| IVLNWEC01EHSMG | Bradi4g02990.1 | - | - | - |
| IVLNWEC02G7P6Z | Bradi4g02990.1 | - | - | - |

| | | | | |
|---|---|---|---|---|
| IV9RU5F02GGJ1G | Bradi4g02950.1 | - | - | - |
| IV9RU5F02GY390 | Bradi4g02950.1 | - | - | - |
| IVLNWEC02GQ202 | Bradi4g02950.1 | - | - | - |
| IVLNWEC02HUA2Z | Bradi4g02950.1 | - | - | - |
| IUIVLUD01EDSS1 | Bradi4g02786.1 | - | - | - |
| IV9RU5F01BK61R | Bradi4g02786.1 | - | - | - |
| IUIVLUD01BBWAW | Bradi4g02710.1 | - | - | - |
| IV9RU5F02FV3HZ | Bradi4g02660.1 | - | - | - |
| IV9RU5F01CQ60L | Bradi4g02570.1 | - | - | - |
| IVLNWEC01CTMLL | Bradi4g02420.1 | - | - | - |
| IVLNWEC02GEBT2 | Bradi4g02420.1 | - | - | - |
| IUIVLUD01DMCJE | Bradi4g02160.2 | - | - | - |
| IV9RU5F01D8W88 | Bradi4g01660.1 | - | - | - |
| IV9RU5F02JZH7Z | Bradi4g01660.1 | - | - | - |
| IV9RU5F02JOEI1 | Bradi4g01610.1 | - | - | - |
| IVLNWEC01DJWXS | Bradi4g01410.1 | - | - | - |
| IV9RU5F01EZPCK | Bradi4g01320.1 | - | - | - |
| IVLNWEC01CGVH4 | Bradi4g01230.2 | - | - | - |
| IVLNWEC01EY8E5 | Bradi4g00910.3 | - | - | - |
| IV9RU5F01CZO2O | Bradi4g00747.1 | - | - | - |
| IVLNWEC01D3Z65 | Bradi4g00497.1 | - | - | - |
| IVLNWEC02IUDFF | Bradi4g00367.1 | - | - | - |
| IUIVLUD01AOA3Z | Bradi3g61040.1 | - | - | - |
| IVLNWEC02FMNXY | Bradi3g61040.1 | - | - | - |
| IVLNWEC02FTLZR | Bradi3g61040.1 | - | - | - |
| IVLNWEC01B2JVE | Bradi3g60720.1 | - | - | - |
| IVLNWEC01B5H6P | Bradi3g60550.1 | - | - | - |
| IV9RU5F01CTV1V | Bradi3g60337.1 | - | - | - |
| IV9RU5F01D3Y35 | Bradi3g60337.1 | - | - | - |
| IV9RU5F02H7JX2 | Bradi3g60337.1 | - | - | - |
| IVLNWEC01B3JBB | Bradi3g60337.1 | - | - | - |
| IVLNWEC01DGEG4 | Bradi3g60337.1 | - | - | - |
| IV9RU5F02JP839 | Bradi3g60250.1 | - | - | - |
| IVLNWEC02JNA0K | Bradi3g60250.1 | - | - | - |
| IVLNWEC02JXJ3E | Bradi3g60160.1 | - | - | - |
| IVLNWEC01BD5NE | Bradi3g59726.1 | - | - | - |
| IVLNWEC01BNUZ7 | Bradi3g57600.1 | - | - | - |
| IUIVLUD01EWYK8 | Bradi3g57130.1 | - | - | - |
| IV9RU5F01AGOJD | Bradi3g57130.1 | - | - | - |
| IVLNWEC01DRPNG | Bradi3g57130.1 | - | - | - |
| IV9RU5F02HUCQP | Bradi3g56980.1 | - | - | - |
| IVLNWEC02HEPFW | Bradi3g56087.1 | - | - | - |
| IVLNWEC01CTCNB | Bradi3g55640.1 | - | - | - |
| IVLNWEC02JKWOJ | Bradi3g55640.1 | - | - | - |
| IVLNWEC02G7QQF | Bradi3g54950.1 | - | - | - |
| IVLNWEC01C385P | Bradi3g53887.1 | - | - | - |
| IV9RU5F02ILV62 | Bradi3g53370.1 | - | - | - |

| | | | | |
|---|---|---|---|---|
| IUIVLUD02J1E3A | Bradi3g52630.1 | - | - | - |
| IVLNWEC02H044N | Bradi3g52630.1 | - | - | - |
| IVLNWEC02I5CYJ | Bradi3g52630.1 | - | - | - |
| IVLNWEC02JIMV9 | Bradi3g52630.1 | - | - | - |
| IV9RU5F01ETHTS | Bradi3g52470.2 | - | - | - |
| IUIVLUD01BBIZB | Bradi3g50300.1 | - | - | - |
| IUIVLUD01BLGUU | Bradi3g50300.1 | - | - | - |
| IUIVLUD01DXYRV | Bradi3g50300.1 | - | - | - |
| IUIVLUD01ERMXS | Bradi3g50300.1 | - | - | - |
| IV9RU5F01CUXXI | Bradi3g50300.1 | - | - | - |
| IV9RU5F02HDS56 | Bradi3g49840.1 | - | - | - |
| IV9RU5F01BQSXU | Bradi3g49640.1 | - | - | - |
| IV9RU5F01DS0QP | Bradi3g48257.1 | - | - | - |
| IV9RU5F01COUSP | Bradi3g46500.1 | - | - | - |
| IV9RU5F02HAKI6 | Bradi3g46500.1 | - | - | - |
| IUIVLUD01BE51M | Bradi3g45660.1 | - | - | - |
| IV9RU5F02HNETV | Bradi3g45580.1 | - | - | - |
| IVLNWEC01CTJAH | Bradi3g45580.1 | - | - | - |
| IVLNWEC02F7HJL | Bradi3g45107.1 | - | - | - |
| IUIVLUD01A73TB | Bradi3g44940.1 | - | - | - |
| IUIVLUD02H0ZG6 | Bradi3g44740.1 | - | - | - |
| IUIVLUD01B75MX | Bradi3g44270.1 | - | - | - |
| IV9RU5F01AN2IL | Bradi3g44270.1 | - | - | - |
| IVLNWEC01A5TTX | Bradi3g44270.1 | - | - | - |
| IUIVLUD02IAYFU | Bradi3g41987.1 | - | - | - |
| IV9RU5F01DL3PU | Bradi3g41987.1 | - | - | - |
| IVLNWEC02HDTGZ | Bradi3g41987.1 | - | - | - |
| IVLNWEC01D6B2X | Bradi3g41960.1 | - | - | - |
| IVLNWEC02G6ZGL | Bradi3g41960.1 | - | - | - |
| IVLNWEC02GG6DA | Bradi3g41870.1 | - | - | - |
| IVLNWEC02HWJZL | Bradi3g41870.1 | - | - | - |
| IV9RU5F02GLVQJ | Bradi3g41810.1 | - | - | - |
| IV9RU5F02IO4R1 | Bradi3g41810.1 | - | - | - |
| IUIVLUD02H0LNY | Bradi3g41720.2 | - | - | - |
| IVLNWEC02GN5LC | Bradi3g41520.1 | - | - | - |
| IV9RU5F02IJ781 | Bradi3g38400.1 | - | - | - |
| IUIVLUD02J3Z1W | Bradi3g37780.1 | - | - | - |
| IV9RU5F01EWX97 | Bradi3g37780.1 | - | - | - |
| IVLNWEC02IRMQ6 | Bradi3g37780.1 | - | - | - |
| IVLNWEC02IIA9C | Bradi3g37010.1 | - | - | - |
| IV9RU5F02FSSTZ | Bradi3g36850.1 | - | - | - |
| IVLNWEC02F2CYU | Bradi3g36850.1 | - | - | - |
| IVLNWEC02HJPJF | Bradi3g36850.1 | - | - | - |
| IUIVLUD02INIDK | Bradi3g36100.1 | - | - | - |
| IUIVLUD02JODJK | Bradi3g36100.1 | - | - | - |
| IV9RU5F01DQSW6 | Bradi3g36100.1 | - | - | - |
| IVLNWEC01DNB3S | Bradi3g35910.1 | - | - | - |

| | | | | |
|---|---|---|---|---|
| IUIVLUD02F9PNZ | Bradi3g35460.1 | - | - | - |
| IV9RU5F02JUDO6 | Bradi3g35460.1 | - | - | - |
| IVLNWEC01ANH29 | Bradi3g35460.1 | - | - | - |
| IVLNWEC01APEN1 | Bradi3g35460.1 | - | - | - |
| IVLNWEC01CLSA3 | Bradi3g35460.1 | - | - | - |
| IUIVLUD01DT1FR | Bradi3g35300.1 | - | - | - |
| IVLNWEC02I11U0 | Bradi3g35300.1 | - | - | - |
| IUIVLUD02HF5X1 | Bradi3g32440.1 | - | - | - |
| IUIVLUD02JMUTM | Bradi3g32440.1 | - | - | - |
| IV9RU5F02FPKMR | Bradi3g32440.1 | - | - | - |
| IVLNWEC02F61KM | Bradi3g32440.1 | - | - | - |
| IVLNWEC02IMGNG | Bradi3g32440.1 | - | - | - |
| IUIVLUD01AZ1VL | Bradi3g32276.1 | - | - | - |
| IV9RU5F02IY9UU | Bradi3g30810.3 | - | - | - |
| IUIVLUD02JU9OA | Bradi3g28390.1 | - | - | - |
| IV9RU5F02H8YOF | Bradi3g28390.1 | - | - | - |
| IUIVLUD01EUL7V | Bradi3g28380.1 | - | - | - |
| IVLNWEC02GRVQA | Bradi3g28380.1 | - | - | - |
| IVLNWEC02JSG2C | Bradi3g27560.1 | - | - | - |
| IVLNWEC01EBP48 | Bradi3g27210.2 | - | - | - |
| IUIVLUD01A2QXP | Bradi3g27160.1 | - | - | - |
| IV9RU5F01ALHRF | Bradi3g27160.1 | - | - | - |
| IV9RU5F02G5MCO | Bradi3g27074.1 | - | - | - |
| IV9RU5F02JH6GK | Bradi3g27074.1 | - | - | - |
| IVLNWEC01BN4B3 | Bradi3g25900.1 | - | - | - |
| IUIVLUD02IH05Z | Bradi3g24417.1 | - | - | - |
| IVLNWEC02HX7L3 | Bradi3g24417.1 | - | - | - |
| IV9RU5F02GW4IB | Bradi3g21590.1 | - | - | - |
| IUIVLUD01AZSR8 | Bradi3g21350.2 | - | - | - |
| IVLNWEC02I5QAX | Bradi3g21350.2 | - | - | - |
| IVLNWEC01AHI7D | Bradi3g20570.1 | - | - | - |
| IVLNWEC02H274V | Bradi3g20570.1 | - | - | - |
| IUIVLUD01BRRQD | Bradi3g19890.1 | - | - | - |
| IVLNWEC02I1YL5 | Bradi3g19890.1 | - | - | - |
| IVLNWEC01EULTC | Bradi3g18390.1 | - | - | - |
| IVLNWEC02IA819 | Bradi3g17440.1 | - | - | - |
| IVLNWEC02JUVNY | Bradi3g17440.1 | - | - | - |
| IUIVLUD01C9JKJ | Bradi3g17240.1 | - | - | - |
| IUIVLUD02F2OAY | Bradi3g17240.1 | - | - | - |
| IUIVLUD02FR6WR | Bradi3g17240.1 | - | - | - |
| IUIVLUD02HIDDO | Bradi3g17240.1 | - | - | - |
| IUIVLUD02IY5EW | Bradi3g17240.1 | - | - | - |
| IUIVLUD02JDQYS | Bradi3g17240.1 | - | - | - |
| IV9RU5F01DATIA | Bradi3g17240.1 | - | - | - |
| IV9RU5F01DKYW3 | Bradi3g17240.1 | - | - | - |
| IV9RU5F01DWPMK | Bradi3g17240.1 | - | - | - |
| IV9RU5F01DWRWR | Bradi3g17240.1 | - | - | - |

| | | | | |
|---|---|---|---|---|
| IV9RU5F02GGA0P | Bradi3g17240.1 | - | - | - |
| IV9RU5F02GPN36 | Bradi3g17240.1 | - | - | - |
| IV9RU5F02IRBAX | Bradi3g17240.1 | - | - | - |
| IV9RU5F02J22Q1 | Bradi3g17240.1 | - | - | - |
| IV9RU5F02JIIF5 | Bradi3g17240.1 | - | - | - |
| IVLNWEC01CJVDG | Bradi3g17240.1 | - | - | - |
| IVLNWEC01EVTZX | Bradi3g17240.1 | - | - | - |
| IVLNWEC02F01I5 | Bradi3g17240.1 | - | - | - |
| IVLNWEC02FV10M | Bradi3g17240.1 | - | - | - |
| IVLNWEC02GRX8P | Bradi3g17240.1 | - | - | - |
| IVLNWEC02GUB5I | Bradi3g17240.1 | - | - | - |
| IVLNWEC02HBEFG | Bradi3g17240.1 | - | - | - |
| IVLNWEC02I86ZQ | Bradi3g17240.1 | - | - | - |
| IVLNWEC02IFNHT | Bradi3g17240.1 | - | - | - |
| IVLNWEC02IHI3Q | Bradi3g17240.1 | - | - | - |
| IVLNWEC02JBQAN | Bradi3g17240.1 | - | - | - |
| IV9RU5F01C7NNB | Bradi3g16515.1 | - | - | - |
| IVLNWEC01AM4EB | Bradi3g16515.1 | - | - | - |
| IVLNWEC01BGTQK | Bradi3g16250.1 | - | - | - |
| IV9RU5F01A9XOA | Bradi3g16110.1 | - | - | - |
| IVLNWEC01CMDOY | Bradi3g16110.1 | - | - | - |
| IUIVLUD02I2MUE | Bradi3g15935.1 | - | - | - |
| IV9RU5F01DGF2G | Bradi3g15277.1 | - | - | - |
| IV9RU5F02IM13S | Bradi3g15277.1 | - | - | - |
| IVLNWEC01AW6WN | Bradi3g15277.1 | - | - | - |
| IUIVLUD02IS0YX | Bradi3g15270.1 | - | - | - |
| IV9RU5F01AQSU1 | Bradi3g15270.1 | - | - | - |
| IVLNWEC01AM584 | Bradi3g15270.1 | - | - | - |
| IVLNWEC02GT14W | Bradi3g15270.1 | - | - | - |
| IVLNWEC02ICOOQ | Bradi3g15270.1 | - | - | - |
| IUIVLUD02FU98Y | Bradi3g14460.1 | - | - | - |
| IV9RU5F01AJX6P | Bradi3g14460.1 | - | - | - |
| IV9RU5F01BIFVQ | Bradi3g14030.1 | - | - | - |
| IVLNWEC02HQI5Y | Bradi3g14030.1 | - | - | - |
| IUIVLUD01DPZY9 | Bradi3g13600.1 | - | - | - |
| IV9RU5F01D343Z | Bradi3g11810.1 | - | - | - |
| IVLNWEC02FV1BB | Bradi3g11810.1 | - | - | - |
| IVLNWEC02JJ7ZS | Bradi3g11810.1 | - | - | - |
| IV9RU5F01AZX5B | Bradi3g10480.1 | - | - | - |
| IV9RU5F01AY3NH | Bradi3g10416.1 | - | - | - |
| IVLNWEC01ETRX8 | Bradi3g10416.1 | - | - | - |
| IUIVLUD02JW4C7 | Bradi3g10370.1 | - | - | - |
| IV9RU5F02H85RF | Bradi3g10370.1 | - | - | - |
| IV9RU5F02HAVZI | Bradi3g10370.1 | - | - | - |
| IVLNWEC02F8S2I | Bradi3g09770.1 | - | - | - |
| IUIVLUD02HCLY9 | Bradi3g09535.1 | - | - | - |
| IUIVLUD01AI0VV | Bradi3g08490.1 | - | - | - |

| | | | | |
|---|---|---|---|---|
| IV9RU5F01CWIRV | Bradi3g08490.1 | - | - | - |
| IV9RU5F02I98AP | Bradi3g08490.1 | - | - | - |
| IVLNWEC02H6ZIA | Bradi3g08110.1 | - | - | - |
| IV9RU5F02HXYUX | Bradi3g07790.1 | - | - | - |
| IUIVLUD01CRBI7 | Bradi3g06727.1 | - | - | - |
| IV9RU5F02J2YG6 | Bradi3g05292.1 | - | - | - |
| IV9RU5F02IWKLN | Bradi3g04980.1 | - | - | - |
| IVLNWEC02JKAE7 | Bradi3g04980.1 | - | - | - |
| IUIVLUD01BGR5X | Bradi3g02870.1 | - | - | - |
| IV9RU5F01BYUIN | Bradi3g02606.1 | - | - | - |
| IV9RU5F02HAOR8 | Bradi3g02606.1 | - | - | - |
| IUIVLUD02HIPS9 | Bradi3g02380.1 | - | - | - |
| IVLNWEC01DTE7G | Bradi3g02380.1 | - | - | - |
| IVLNWEC02HGOMI | Bradi3g02380.1 | - | - | - |
| IVLNWEC02HQDYI | Bradi3g02380.1 | - | - | - |
| IVLNWEC01BL8Y0 | Bradi3g02247.1 | - | - | - |
| IVLNWEC01CHY2P | Bradi3g02247.1 | - | - | - |
| IUIVLUD02GIGN7 | Bradi3g01190.1 | - | - | - |
| IV9RU5F02GU3MW | Bradi3g01190.1 | - | - | - |
| IV9RU5F01AIZOX | Bradi3g01150.1 | - | - | - |
| IV9RU5F01BED23 | Bradi3g01150.1 | - | - | - |
| IVLNWEC01CVZI8 | Bradi3g01150.1 | - | - | - |
| IVLNWEC01BVLGI | Bradi3g00670.1 | - | - | - |
| IUIVLUD01AYBQQ | Bradi3g00340.1 | - | - | - |
| IV9RU5F02HANY4 | Bradi3g00340.1 | - | - | - |
| IVLNWEC01D8CGA | Bradi3g00340.1 | - | - | - |
| IV9RU5F01B5JEF | Bradi2g62560.1 | - | - | - |
| IV9RU5F02JYNNQ | Bradi2g60350.1 | - | - | - |
| IV9RU5F01A6TRS | Bradi2g55330.1 | - | - | - |
| IV9RU5F01E0ROQ | Bradi2g55330.1 | - | - | - |
| IVLNWEC02J3A43 | Bradi2g52830.1 | - | - | - |
| IV9RU5F01EA3B9 | Bradi2g52010.1 | - | - | - |
| IVLNWEC01ALC04 | Bradi2g52010.1 | - | - | - |
| IVLNWEC02GC6WK | Bradi2g52010.1 | - | - | - |
| IUIVLUD01BEP56 | Bradi2g51807.1 | - | - | - |
| IUIVLUD01DBKUW | Bradi2g51807.1 | - | - | - |
| IUIVLUD01EFY3P | Bradi2g51807.1 | - | - | - |
| IUIVLUD02GCF69 | Bradi2g51807.1 | - | - | - |
| IV9RU5F01A46OM | Bradi2g51807.1 | - | - | - |
| IV9RU5F01AS75S | Bradi2g51807.1 | - | - | - |
| IV9RU5F01DYALP | Bradi2g51807.1 | - | - | - |
| IV9RU5F01E0YNG | Bradi2g51807.1 | - | - | - |
| IV9RU5F01EH2A5 | Bradi2g51807.1 | - | - | - |
| IV9RU5F02FWDYB | Bradi2g51807.1 | - | - | - |
| IV9RU5F02GW13U | Bradi2g51807.1 | - | - | - |
| IV9RU5F02GY6PH | Bradi2g51807.1 | - | - | - |
| IV9RU5F02I8FTQ | Bradi2g51807.1 | - | - | - |

| | | | | |
|---|---|---|---|---|
| IVLNWEC01C6Z4C | Bradi2g51807.1 | - | - | - |
| IVLNWEC01CEDMG | Bradi2g51807.1 | - | - | - |
| IVLNWEC01EI0UD | Bradi2g51807.1 | - | - | - |
| IVLNWEC02F4WSS | Bradi2g51807.1 | - | - | - |
| IVLNWEC02GMLWZ | Bradi2g51807.1 | - | - | - |
| IVLNWEC02HAHNW | Bradi2g51807.1 | - | - | - |
| IVLNWEC02HOONM | Bradi2g51807.1 | - | - | - |
| IVLNWEC02JU9M3 | Bradi2g51807.1 | - | - | - |
| IUIVLUD02GLRKA | Bradi2g51100.1 | - | - | - |
| IV9RU5F02ILBM2 | Bradi2g51100.1 | - | - | - |
| IVLNWEC02JPMHM | Bradi2g49067.1 | - | - | - |
| IV9RU5F01AGX0Z | Bradi2g46150.1 | - | - | - |
| IV9RU5F02GCEDZ | Bradi2g44785.1 | - | - | - |
| IUIVLUD02GMIQX | Bradi2g44310.1 | - | - | - |
| IVLNWEC01CEH2K | Bradi2g44310.1 | - | - | - |
| IVLNWEC02FZU6U | Bradi2g44310.1 | - | - | - |
| IVLNWEC02GL0T8 | Bradi2g44300.1 | - | - | - |
| IV9RU5F01EUKUD | Bradi2g43557.1 | - | - | - |
| IVLNWEC01DBLXM | Bradi2g42820.1 | - | - | - |
| IVLNWEC02HG1ZY | Bradi2g42820.1 | - | - | - |
| IVLNWEC02I6B1C | Bradi2g42820.1 | - | - | - |
| IV9RU5F02FWS8U | Bradi2g42607.1 | - | - | - |
| IUIVLUD01B7DQN | Bradi2g42200.1 | - | - | - |
| IVLNWEC02F32AH | Bradi2g42200.1 | - | - | - |
| IUIVLUD02FTQZ2 | Bradi2g42000.1 | - | - | - |
| IV9RU5F01DE882 | Bradi2g42000.1 | - | - | - |
| IVLNWEC01AEB3W | Bradi2g42000.1 | - | - | - |
| IUIVLUD01A5U64 | Bradi2g41920.1 | - | - | - |
| IVLNWEC02HG7GP | Bradi2g41920.1 | - | - | - |
| IV9RU5F01DX4FB | Bradi2g41080.1 | - | - | - |
| IV9RU5F02H2OLQ | Bradi2g41080.1 | - | - | - |
| IVLNWEC01AHOK2 | Bradi2g40170.2 | - | - | - |
| IUIVLUD01AK35P | Bradi2g40170.1 | - | - | - |
| IV9RU5F02JX8H5 | Bradi2g39470.1 | - | - | - |
| IV9RU5F02FUBRT | Bradi2g39091.1 | - | - | - |
| IV9RU5F02H2JWM | Bradi2g39091.1 | - | - | - |
| IVLNWEC02HRT6I | Bradi2g39091.1 | - | - | - |
| IVLNWEC02J2VFZ | Bradi2g39091.1 | - | - | - |
| IUIVLUD01AHV4G | Bradi2g38930.1 | - | - | - |
| IUIVLUD02H7CMC | Bradi2g38930.1 | - | - | - |
| IUIVLUD01APX6X | Bradi2g38810.1 | - | - | - |
| IVLNWEC01AYR7A | Bradi2g38810.1 | - | - | - |
| IVLNWEC01BTLVE | Bradi2g38577.1 | - | - | - |
| IUIVLUD02GCJQI | Bradi2g38510.1 | - | - | - |
| IUIVLUD01CSORT | Bradi2g37980.2 | - | - | - |
| IVLNWEC01EFFZN | Bradi2g37795.1 | - | - | - |
| IVLNWEC01ENRGK | Bradi2g37795.1 | - | - | - |

| | | | | |
|---|---|---|---|---|
| IVLNWEC02G0NJM | Bradi2g37795.1 | - | - | - |
| IVLNWEC02HY112 | Bradi2g37795.1 | - | - | - |
| IVLNWEC02J3EVS | Bradi2g37795.1 | - | - | - |
| IV9RU5F02FTDKH | Bradi2g37580.1 | - | - | - |
| IV9RU5F02IQY9A | Bradi2g37580.1 | - | - | - |
| IVLNWEC01DA5VV | Bradi2g37570.1 | - | - | - |
| IVLNWEC02F73GY | Bradi2g37387.1 | - | - | - |
| IVLNWEC01A4J7J | Bradi2g36810.1 | - | - | - |
| IVLNWEC01BGO9Q | Bradi2g36810.1 | - | - | - |
| IV9RU5F01AXZZO | Bradi2g36525.1 | - | - | - |
| IVLNWEC02HFXEB | Bradi2g36525.1 | - | - | - |
| IUIVLUD02GLNIV | Bradi2g36210.1 | - | - | - |
| IVLNWEC01CRSGD | Bradi2g36210.1 | - | - | - |
| IV9RU5F01D8DL4 | Bradi2g36180.1 | - | - | - |
| IV9RU5F02JD8LM | Bradi2g36180.1 | - | - | - |
| IVLNWEC02GMHJO | Bradi2g36180.1 | - | - | - |
| IV9RU5F02J2TQ0 | Bradi2g36170.1 | - | - | - |
| IV9RU5F02JE3DY | Bradi2g36170.1 | - | - | - |
| IVLNWEC02GIKNE | Bradi2g36150.1 | - | - | - |
| IV9RU5F02G6RKT | Bradi2g31690.1 | - | - | - |
| IV9RU5F02H25XF | Bradi2g31530.1 | - | - | - |
| IV9RU5F02HXZTO | Bradi2g30425.1 | - | - | - |
| IUIVLUD02GZRU7 | Bradi2g28410.1 | - | - | - |
| IV9RU5F02I4PUF | Bradi2g28410.1 | - | - | - |
| IUIVLUD01A5NSQ | Bradi2g27230.1 | - | - | - |
| IVLNWEC01AQNZX | Bradi2g26180.1 | - | - | - |
| IV9RU5F01CQ48D | Bradi2g25745.1 | - | - | - |
| IUIVLUD01AITYU | Bradi2g24255.1 | - | - | - |
| IV9RU5F01APY4S | Bradi2g24255.1 | - | - | - |
| IVLNWEC01AXT42 | Bradi2g24255.1 | - | - | - |
| IUIVLUD02H12FP | Bradi2g23620.1 | - | - | - |
| IVLNWEC02FYYIZ | Bradi2g23620.1 | - | - | - |
| IVLNWEC02JU1XK | Bradi2g23620.1 | - | - | - |
| IUIVLUD01E46D9 | Bradi2g23410.1 | - | - | - |
| IUIVLUD01EREKU | Bradi2g23410.1 | - | - | - |
| IUIVLUD02HTASX | Bradi2g23410.1 | - | - | - |
| IUIVLUD02GY8NB | Bradi2g22710.1 | - | - | - |
| IV9RU5F02JGBDJ | Bradi2g22630.1 | - | - | - |
| IV9RU5F02JRACQ | Bradi2g22550.1 | - | - | - |
| IVLNWEC01BQW39 | Bradi2g21510.1 | - | - | - |
| IV9RU5F02GXVP7 | Bradi2g19830.1 | - | - | - |
| IV9RU5F01B95JX | Bradi2g19250.1 | - | - | - |
| IV9RU5F02H2U3X | Bradi2g18550.1 | - | - | - |
| IVLNWEC02G488O | Bradi2g18270.1 | - | - | - |
| IUIVLUD02HWMAR | Bradi2g17926.1 | - | - | - |
| IUIVLUD01C5LAE | Bradi2g17187.1 | - | - | - |
| IUIVLUD02ILHSM | Bradi2g17187.1 | - | - | - |

| | | | | |
|---|---|---|---|---|
| IVLNWEC01AG5G2 | Bradi2g17187.1 | - | - | - |
| IVLNWEC02GLO3F | Bradi2g17187.1 | - | - | - |
| IVLNWEC02IVYA6 | Bradi2g17187.1 | - | - | - |
| IVLNWEC02JLMIZ | Bradi2g17187.1 | - | - | - |
| IUIVLUD01DCM24 | Bradi2g16060.1 | - | - | - |
| IVLNWEC01EFH4R | Bradi2g16060.1 | - | - | - |
| IVLNWEC02GOUSR | Bradi2g16060.1 | - | - | - |
| IUIVLUD01AZZK1 | Bradi2g16040.1 | - | - | - |
| IV9RU5F01AYPPY | Bradi2g16040.1 | - | - | - |
| IV9RU5F02JH0FE | Bradi2g16040.1 | - | - | - |
| IVLNWEC02G7KUC | Bradi2g15560.1 | - | - | - |
| IVLNWEC02IEZ5G | Bradi2g15560.1 | - | - | - |
| IV9RU5F01EOFM6 | Bradi2g14620.1 | - | - | - |
| IUIVLUD01CQQOM | Bradi2g14450.1 | - | - | - |
| IUIVLUD01D6VGH | Bradi2g14450.1 | - | - | - |
| IUIVLUD02HPKVB | Bradi2g14450.1 | - | - | - |
| IV9RU5F02GI9NN | Bradi2g14450.1 | - | - | - |
| IVLNWEC01BZAOZ | Bradi2g14450.1 | - | - | - |
| IVLNWEC02HJ9OM | Bradi2g14450.1 | - | - | - |
| IVLNWEC02JKMI1 | Bradi2g14450.1 | - | - | - |
| IVLNWEC02IHWRO | Bradi2g14340.2 | - | - | - |
| IUIVLUD02GLY17 | Bradi2g13330.1 | - | - | - |
| IVLNWEC01AXVKA | Bradi2g13330.1 | - | - | - |
| IVLNWEC01DNM27 | Bradi2g13330.1 | - | - | - |
| IVLNWEC02HOE1S | Bradi2g13330.1 | - | - | - |
| IVLNWEC02GLE66 | Bradi2g12850.1 | - | - | - |
| IVLNWEC02ISKJE | Bradi2g12850.1 | - | - | - |
| IUIVLUD02HCWS9 | Bradi2g11590.1 | - | - | - |
| IV9RU5F01BUBQF | Bradi2g11590.1 | - | - | - |
| IV9RU5F01BXVMU | Bradi2g11590.1 | - | - | - |
| IV9RU5F02HLG3D | Bradi2g11590.1 | - | - | - |
| IVLNWEC01B0OQD | Bradi2g11590.1 | - | - | - |
| IVLNWEC02HQF9M | Bradi2g11590.1 | - | - | - |
| IVLNWEC02I9052 | Bradi2g11590.1 | - | - | - |
| IUIVLUD02FXFCS | Bradi2g10950.1 | - | - | - |
| IUIVLUD01DHCWH | Bradi2g10800.2 | - | - | - |
| IUIVLUD01AH2FR | Bradi2g09434.1 | - | - | - |
| IUIVLUD01BFU2G | Bradi2g09434.1 | - | - | - |
| IV9RU5F01DS02A | Bradi2g09434.1 | - | - | - |
| IV9RU5F02H9CIS | Bradi2g09434.1 | - | - | - |
| IUIVLUD01A18V0 | Bradi2g09427.1 | - | - | - |
| IV9RU5F02HZ85E | Bradi2g09427.1 | - | - | - |
| IV9RU5F02JIX8W | Bradi2g09427.1 | - | - | - |
| IV9RU5F01DKN4J | Bradi2g08750.1 | - | - | - |
| IV9RU5F02HBWV9 | Bradi2g08750.1 | - | - | - |
| IVLNWEC01C9JZQ | Bradi2g08750.1 | - | - | - |
| IUIVLUD02HAO5Q | Bradi2g08630.1 | - | - | - |

| | | | | |
|---|---|---|---|---|
| IUIVLUD02JNQD9 | Bradi2g08390.1 | - | - | - |
| IV9RU5F01A5OLO | Bradi2g08390.1 | - | - | - |
| IV9RU5F02FIWA7 | Bradi2g08390.1 | - | - | - |
| IVLNWEC02IJQ8Z | Bradi2g08390.1 | - | - | - |
| IV9RU5F01A3V1M | Bradi2g07910.1 | - | - | - |
| IV9RU5F02IWWXD | Bradi2g07910.1 | - | - | - |
| IVLNWEC01AQHDM | Bradi2g07910.1 | - | - | - |
| IVLNWEC02I85UA | Bradi2g07910.1 | - | - | - |
| IVLNWEC02J3REJ | Bradi2g07910.1 | - | - | - |
| IVLNWEC02HSZ7Q | Bradi2g07310.1 | - | - | - |
| IV9RU5F02JCEIA | Bradi2g06780.1 | - | - | - |
| IVLNWEC01A1YYG | Bradi2g06780.1 | - | - | - |
| IVLNWEC01C3308 | Bradi2g06780.1 | - | - | - |
| IV9RU5F01BJP1M | Bradi2g06320.1 | - | - | - |
| IV9RU5F02GX96F | Bradi2g06320.1 | - | - | - |
| IV9RU5F02IQ9Z8 | Bradi2g06320.1 | - | - | - |
| IVLNWEC02F4KWZ | Bradi2g06320.1 | - | - | - |
| IVLNWEC02HD1K8 | Bradi2g06320.1 | - | - | - |
| IVLNWEC02I8ZTT | Bradi2g06320.1 | - | - | - |
| IV9RU5F01BPQZV | Bradi2g05890.1 | - | - | - |
| IVLNWEC01BE43X | Bradi2g05890.1 | - | - | - |
| IUIVLUD01AZ7UB | Bradi2g05120.1 | - | - | - |
| IUIVLUD01BI9XR | Bradi2g05120.1 | - | - | - |
| IV9RU5F01BLV89 | Bradi2g05120.1 | - | - | - |
| IV9RU5F01A567Y | Bradi2g03530.1 | - | - | - |
| IV9RU5F01D4ZJY | Bradi2g02510.1 | - | - | - |
| IVLNWEC02JC3R6 | Bradi2g02510.1 | - | - | - |
| IVLNWEC01BVSN4 | Bradi2g01550.1 | - | - | - |
| IUIVLUD01C2CGC | Bradi2g00650.1 | - | - | - |
| IV9RU5F02FX31W | Bradi2g00500.1 | - | - | - |
| IV9RU5F01CLGMU | Bradi2g00417.1 | - | - | - |
| IUIVLUD02HB83M | Bradi2g00400.1 | - | - | - |
| IVLNWEC02G3Y7H | Bradi2g00400.1 | - | - | - |
| IV9RU5F02G46YV | Bradi1g77537.1 | - | - | - |
| IV9RU5F02HNIJ3 | Bradi1g77537.1 | - | - | - |
| IV9RU5F01DAM8F | Bradi1g77310.1 | - | - | - |
| IV9RU5F02F5HPJ | Bradi1g77310.1 | - | - | - |
| IVLNWEC01CSE3T | Bradi1g76970.3 | - | - | - |
| IVLNWEC02J1DQ0 | Bradi1g75540.1 | - | - | - |
| IV9RU5F02FK28Y | Bradi1g75530.1 | - | - | - |
| IV9RU5F02IHMXF | Bradi1g75330.1 | - | - | - |
| IVLNWEC01BRD8X | Bradi1g75320.1 | - | - | - |
| IVLNWEC01DM15D | Bradi1g75320.1 | - | - | - |
| IVLNWEC02H2QC3 | Bradi1g75320.1 | - | - | - |
| IVLNWEC01AJNH9 | Bradi1g75250.1 | - | - | - |
| IVLNWEC01B7JMK | Bradi1g75250.1 | - | - | - |
| IUIVLUD02G8L38 | Bradi1g75165.1 | - | - | - |

| | | | | |
|---|---|---|---|---|
| IV9RU5F02HY18F | Bradi1g75140.1 | - | - | - |
| IV9RU5F02JP2S5 | Bradi1g75140.1 | - | - | - |
| IVLNWEC01CU8YM | Bradi1g74997.1 | - | - | - |
| IV9RU5F02JNL5V | Bradi1g74827.1 | - | - | - |
| IVLNWEC02IHZCL | Bradi1g74450.1 | - | - | - |
| IV9RU5F01DF3J9 | Bradi1g74430.1 | - | - | - |
| IVLNWEC02F01PL | Bradi1g74430.1 | - | - | - |
| IUIVLUD01DCFZN | Bradi1g74287.1 | - | - | - |
| IVLNWEC02JB3TZ | Bradi1g74140.1 | - | - | - |
| IVLNWEC02HJZSU | Bradi1g74070.1 | - | - | - |
| IVLNWEC02I31BB | Bradi1g73810.1 | - | - | - |
| IUIVLUD01DVRIT | Bradi1g73150.1 | - | - | - |
| IV9RU5F01CF75H | Bradi1g72490.1 | - | - | - |
| IVLNWEC01BHZFM | Bradi1g72490.1 | - | - | - |
| IUIVLUD02JTXUZ | Bradi1g70980.1 | - | - | - |
| IV9RU5F01DHETB | Bradi1g70980.1 | - | - | - |
| IVLNWEC01B48JI | Bradi1g70980.1 | - | - | - |
| IVLNWEC01CWQBZ | Bradi1g70980.1 | - | - | - |
| IVLNWEC01D0ZFW | Bradi1g70980.1 | - | - | - |
| IVLNWEC01DEYT4 | Bradi1g70980.1 | - | - | - |
| IVLNWEC02G4YK3 | Bradi1g70980.1 | - | - | - |
| IVLNWEC02HWUXF | Bradi1g70980.1 | - | - | - |
| IVLNWEC02JC57U | Bradi1g70980.1 | - | - | - |
| IVLNWEC02JLVSI | Bradi1g70980.1 | - | - | - |
| IVLNWEC02JQLD4 | Bradi1g70980.1 | - | - | - |
| IV9RU5F01C3DF4 | Bradi1g70970.1 | - | - | - |
| IV9RU5F01DRSWQ | Bradi1g70970.1 | - | - | - |
| IVLNWEC02F6GAR | Bradi1g70970.1 | - | - | - |
| IVLNWEC02GOO4F | Bradi1g70970.1 | - | - | - |
| IVLNWEC02GZ88X | Bradi1g70970.1 | - | - | - |
| IVLNWEC01CAF5S | Bradi1g70460.1 | - | - | - |
| IUIVLUD01BQ5CS | Bradi1g69617.2 | - | - | - |
| IV9RU5F02GJ691 | Bradi1g69617.2 | - | - | - |
| IV9RU5F02FM4UA | Bradi1g69350.1 | - | - | - |
| IUIVLUD01DESCG | Bradi1g69050.1 | - | - | - |
| IV9RU5F02I16DP | Bradi1g69050.1 | - | - | - |
| IVLNWEC02JIF99 | Bradi1g69050.1 | - | - | - |
| IVLNWEC01A1SH3 | Bradi1g68980.1 | - | - | - |
| IV9RU5F01E3IG2 | Bradi1g68957.1 | - | - | - |
| IVLNWEC01B61I7 | Bradi1g68697.1 | - | - | - |
| IUIVLUD02HY23N | Bradi1g68220.1 | - | - | - |
| IV9RU5F01E4W9I | Bradi1g68220.1 | - | - | - |
| IUIVLUD01D1NHS | Bradi1g67610.1 | - | - | - |
| IV9RU5F02JOIDX | Bradi1g67610.1 | - | - | - |
| IV9RU5F02FYGDB | Bradi1g67210.1 | - | - | - |
| IV9RU5F02G50DB | Bradi1g67210.1 | - | - | - |
| IV9RU5F02GSBEU | Bradi1g67210.1 | - | - | - |

| | | | | |
|---|---|---|---|---|
| IUIVLUD02GYJPW | Bradi1g64687.1 | - | - | - |
| IVLNWEC02GGZ1M | Bradi1g64210.1 | - | - | - |
| IUIVLUD02I660Y | Bradi1g63880.2 | - | - | - |
| IVLNWEC01DLF5B | Bradi1g63870.1 | - | - | - |
| IUIVLUD01ASYSR | Bradi1g63510.1 | - | - | - |
| IV9RU5F01EN6Q7 | Bradi1g62650.1 | - | - | - |
| IVLNWEC01CRIKK | Bradi1g62650.1 | - | - | - |
| IVLNWEC02JPUOF | Bradi1g62650.1 | - | - | - |
| IUIVLUD02F0HRB | Bradi1g62640.1 | - | - | - |
| IUIVLUD02JJJ58 | Bradi1g62600.1 | - | - | - |
| IVLNWEC02IQZKE | Bradi1g62277.1 | - | - | - |
| IV9RU5F01BUKI2 | Bradi1g62100.1 | - | - | - |
| IVLNWEC02H6YMM | Bradi1g62100.1 | - | - | - |
| IVLNWEC02JIIU5 | Bradi1g62007.1 | - | - | - |
| IV9RU5F01C7DVI | Bradi1g61310.1 | - | - | - |
| IVLNWEC01CUUJZ | Bradi1g61310.1 | - | - | - |
| IVLNWEC02HT2A1 | Bradi1g61310.1 | - | - | - |
| IUIVLUD01DGKWQ | Bradi1g61140.1 | - | - | - |
| IUIVLUD02FPZ6R | Bradi1g61140.1 | - | - | - |
| IUIVLUD02FZC07 | Bradi1g61140.1 | - | - | - |
| IV9RU5F02G9JOE | Bradi1g61140.1 | - | - | - |
| IV9RU5F02IMIX9 | Bradi1g61140.1 | - | - | - |
| IV9RU5F02F7A9O | Bradi1g60380.1 | - | - | - |
| IVLNWEC02F11Z7 | Bradi1g60380.1 | - | - | - |
| IV9RU5F01AITRS | Bradi1g59960.1 | - | - | - |
| IVLNWEC02GZSJB | Bradi1g59960.1 | - | - | - |
| IVLNWEC01BXOZQ | Bradi1g59950.1 | - | - | - |
| IVLNWEC01DP4VC | Bradi1g59950.1 | - | - | - |
| IVLNWEC02HNEE5 | Bradi1g59950.1 | - | - | - |
| IVLNWEC01BF52P | Bradi1g59370.1 | - | - | - |
| IV9RU5F02JM2DJ | Bradi1g59360.1 | - | - | - |
| IVLNWEC02GTN9O | Bradi1g59360.1 | - | - | - |
| IV9RU5F01CEOKW | Bradi1g59317.1 | - | - | - |
| IV9RU5F02IHXVA | Bradi1g59317.1 | - | - | - |
| IV9RU5F01BH3UY | Bradi1g58930.1 | - | - | - |
| IVLNWEC01BPNDQ | Bradi1g58930.1 | - | - | - |
| IVLNWEC02GJ1KY | Bradi1g58030.1 | - | - | - |
| IV9RU5F01BSUAH | Bradi1g57975.1 | - | - | - |
| IV9RU5F02FVL83 | Bradi1g57975.1 | - | - | - |
| IVLNWEC01AH3T7 | Bradi1g57975.1 | - | - | - |
| IV9RU5F01D18CP | Bradi1g57840.1 | - | - | - |
| IV9RU5F01DI6L7 | Bradi1g57840.1 | - | - | - |
| IVLNWEC01DEW3S | Bradi1g57840.1 | - | - | - |
| IVLNWEC01DNGST | Bradi1g57840.1 | - | - | - |
| IV9RU5F01ALT80 | Bradi1g56107.1 | - | - | - |
| IV9RU5F01B151N | Bradi1g56107.1 | - | - | - |
| IV9RU5F01EXTRU | Bradi1g56107.1 | - | - | - |

| | | | | |
|---|---|---|---|---|
| IV9RU5F01BERJS | Bradi1g55250.1 | - | - | - |
| IV9RU5F01BI0MU | Bradi1g54590.1 | - | - | - |
| IVLNWEC01DLZWU | Bradi1g54590.1 | - | - | - |
| IVLNWEC02IOLX9 | Bradi1g54590.1 | - | - | - |
| IV9RU5F02I34VM | Bradi1g54510.1 | - | - | - |
| IVLNWEC01E3198 | Bradi1g54510.1 | - | - | - |
| IV9RU5F02HGDWU | Bradi1g53280.1 | - | - | - |
| IUIVLUD01BLH3D | Bradi1g53027.1 | - | - | - |
| IV9RU5F01CPMFI | Bradi1g53027.1 | - | - | - |
| IV9RU5F02FMUO5 | Bradi1g52280.1 | - | - | - |
| IV9RU5F02JJEJY | Bradi1g52280.1 | - | - | - |
| IVLNWEC02JFUEQ | Bradi1g52280.1 | - | - | - |
| IV9RU5F02F787U | Bradi1g52220.1 | - | - | - |
| IVLNWEC02JUTG8 | Bradi1g52220.1 | - | - | - |
| IVLNWEC01AJRXF | Bradi1g52150.3 | - | - | - |
| IVLNWEC01AT0C7 | Bradi1g52150.3 | - | - | - |
| IUIVLUD01E21EI | Bradi1g51960.1 | - | - | - |
| IV9RU5F02IWXM8 | Bradi1g51950.1 | - | - | - |
| IUIVLUD02FZ4KP | Bradi1g51807.1 | - | - | - |
| IV9RU5F02GXX64 | Bradi1g51807.1 | - | - | - |
| IVLNWEC01BKFEE | Bradi1g51807.1 | - | - | - |
| IV9RU5F01B334R | Bradi1g51757.1 | - | - | - |
| IV9RU5F02FL9UA | Bradi1g51650.1 | - | - | - |
| IVLNWEC01CD46Y | Bradi1g51650.1 | - | - | - |
| IUIVLUD01BJ1O5 | Bradi1g51320.1 | - | - | - |
| IV9RU5F02JGYM2 | Bradi1g51300.1 | - | - | - |
| IVLNWEC02F14LJ | Bradi1g51300.1 | - | - | - |
| IUIVLUD01D09NR | Bradi1g51150.1 | - | - | - |
| IV9RU5F01EBT3X | Bradi1g51150.1 | - | - | - |
| IVLNWEC01BQDHE | Bradi1g51150.1 | - | - | - |
| IV9RU5F02JGQJ6 | Bradi1g51097.1 | - | - | - |
| IV9RU5F02JTJ54 | Bradi1g50890.1 | - | - | - |
| IV9RU5F02JSIRW | Bradi1g50530.1 | - | - | - |
| IV9RU5F01EJS60 | Bradi1g50420.1 | - | - | - |
| IV9RU5F01CWUJ0 | Bradi1g50360.1 | - | - | - |
| IV9RU5F02FWA03 | Bradi1g50360.1 | - | - | - |
| IVLNWEC02H2I1P | Bradi1g50360.1 | - | - | - |
| IUIVLUD02H6EC1 | Bradi1g50260.1 | - | - | - |
| IVLNWEC01CUMMO | Bradi1g50260.1 | - | - | - |
| IVLNWEC01DKCHD | Bradi1g50260.1 | - | - | - |
| IV9RU5F01DZWK3 | Bradi1g49930.1 | - | - | - |
| IVLNWEC02HDCYM | Bradi1g49840.1 | - | - | - |
| IV9RU5F01EFCK0 | Bradi1g49790.1 | - | - | - |
| IV9RU5F02GXOCI | Bradi1g49790.1 | - | - | - |
| IVLNWEC02GR66Z | Bradi1g49790.1 | - | - | - |
| IV9RU5F01AMVVI | Bradi1g49450.1 | - | - | - |
| IVLNWEC02JEMRU | Bradi1g49030.1 | - | - | - |

| | | | | |
|---|---|---|---|---|
| IV9RU5F01AGVU1 | Bradi1g48680.1 | - | - | - |
| IVLNWEC02HPX6G | Bradi1g48680.1 | - | - | - |
| IV9RU5F02FNWZ4 | Bradi1g48410.1 | - | - | - |
| IVLNWEC02GLSYA | Bradi1g48410.1 | - | - | - |
| IUIVLUD01D6J36 | Bradi1g48170.1 | - | - | - |
| IUIVLUD01B77UH | Bradi1g47920.1 | - | - | - |
| IV9RU5F02F7PUN | Bradi1g47880.1 | - | - | - |
| IV9RU5F02JBO0H | Bradi1g47880.1 | - | - | - |
| IUIVLUD02HM6RA | Bradi1g46220.1 | - | - | - |
| IVLNWEC01C42GP | Bradi1g45860.1 | - | - | - |
| IUIVLUD01C749F | Bradi1g45720.1 | - | - | - |
| IVLNWEC02ISG45 | Bradi1g45620.1 | - | - | - |
| IUIVLUD01CL12G | Bradi1g44620.1 | - | - | - |
| IV9RU5F01EAS64 | Bradi1g44590.1 | - | - | - |
| IUIVLUD02GQ88Q | Bradi1g44450.1 | - | - | - |
| IV9RU5F02I29ZG | Bradi1g44450.1 | - | - | - |
| IVLNWEC01DWPY7 | Bradi1g44450.1 | - | - | - |
| IVLNWEC02GN97O | Bradi1g44450.1 | - | - | - |
| IVLNWEC01D9YQT | Bradi1g44090.1 | - | - | - |
| IVLNWEC02IYHKI | Bradi1g44090.1 | - | - | - |
| IUIVLUD01BI33N | Bradi1g43906.1 | - | - | - |
| IVLNWEC02I8DDW | Bradi1g43906.1 | - | - | - |
| IV9RU5F02FURG0 | Bradi1g43880.1 | - | - | - |
| IVLNWEC01AXUDI | Bradi1g43880.1 | - | - | - |
| IUIVLUD01AUSLS | Bradi1g43500.1 | - | - | - |
| IUIVLUD01BJD8F | Bradi1g43500.1 | - | - | - |
| IV9RU5F01DLW96 | Bradi1g43500.1 | - | - | - |
| IV9RU5F01ERLX5 | Bradi1g43500.1 | - | - | - |
| IV9RU5F02I2HHH | Bradi1g43500.1 | - | - | - |
| IVLNWEC01EVNR6 | Bradi1g43500.1 | - | - | - |
| IVLNWEC02IW9NP | Bradi1g42690.1 | - | - | - |
| IUIVLUD02JPUN6 | Bradi1g42000.1 | - | - | - |
| IV9RU5F01DBLTW | Bradi1g42000.1 | - | - | - |
| IV9RU5F01DLP7V | Bradi1g42000.1 | - | - | - |
| IVLNWEC02I1HR6 | Bradi1g42000.1 | - | - | - |
| IUIVLUD02HPNF0 | Bradi1g41860.1 | - | - | - |
| IV9RU5F01A1IIB | Bradi1g41860.1 | - | - | - |
| IUIVLUD01DAKY5 | Bradi1g38240.1 | - | - | - |
| IVLNWEC02HEHNL | Bradi1g38240.1 | - | - | - |
| IUIVLUD02HUXY7 | Bradi1g38220.1 | - | - | - |
| IVLNWEC01ERHGO | Bradi1g38220.1 | - | - | - |
| IV9RU5F01ESNGG | Bradi1g37800.1 | - | - | - |
| IVLNWEC01AQP9A | Bradi1g37800.1 | - | - | - |
| IV9RU5F02GV3X7 | Bradi1g36387.1 | - | - | - |
| IV9RU5F02HMUFD | Bradi1g35960.1 | - | - | - |
| IVLNWEC01BN28Q | Bradi1g35470.1 | - | - | - |
| IV9RU5F02HI1MF | Bradi1g34407.1 | - | - | - |

| | | | | |
|---|---|---|---|---|
| IVLNWEC02FSM1S | Bradi1g34407.1 | - | - | - |
| IUIVLUD01EPS6G | Bradi1g34370.1 | - | - | - |
| IVLNWEC01AVLRE | Bradi1g34030.1 | - | - | - |
| IVLNWEC02HEZQ6 | Bradi1g34030.1 | - | - | - |
| IVLNWEC01BCCC6 | Bradi1g33457.2 | - | - | - |
| IV9RU5F01CHBZ7 | Bradi1g33010.1 | - | - | - |
| IUIVLUD02FYF6Z | Bradi1g32950.1 | - | - | - |
| IV9RU5F01A51YA | Bradi1g32950.1 | - | - | - |
| IV9RU5F02GFVL8 | Bradi1g32950.1 | - | - | - |
| IVLNWEC01BO9O6 | Bradi1g32950.1 | - | - | - |
| IVLNWEC01DELR9 | Bradi1g32950.1 | - | - | - |
| IV9RU5F01DGF6D | Bradi1g32520.1 | - | - | - |
| IVLNWEC01EL8F6 | Bradi1g32310.2 | - | - | - |
| IV9RU5F02GRV9U | Bradi1g31970.1 | - | - | - |
| IV9RU5F02I7QJD | Bradi1g31970.1 | - | - | - |
| IV9RU5F02JV3KY | Bradi1g31970.1 | - | - | - |
| IV9RU5F01CQT9J | Bradi1g31350.1 | - | - | - |
| IV9RU5F01D8320 | Bradi1g31350.1 | - | - | - |
| IV9RU5F02G2TAV | Bradi1g31350.1 | - | - | - |
| IUIVLUD02HWGZ4 | Bradi1g31130.1 | - | - | - |
| IVLNWEC01ASCWR | Bradi1g31130.1 | - | - | - |
| IVLNWEC02IPC57 | Bradi1g31130.1 | - | - | - |
| IUIVLUD02FR63N | Bradi1g30672.1 | - | - | - |
| IV9RU5F01A2D3R | Bradi1g30672.1 | - | - | - |
| IV9RU5F01CCUCT | Bradi1g30672.1 | - | - | - |
| IV9RU5F01ED5UC | Bradi1g30672.1 | - | - | - |
| IV9RU5F02HCAPI | Bradi1g30672.1 | - | - | - |
| IVLNWEC01A44F9 | Bradi1g30672.1 | - | - | - |
| IVLNWEC01CA71M | Bradi1g30672.1 | - | - | - |
| IVLNWEC01DKCED | Bradi1g30672.1 | - | - | - |
| IVLNWEC01DM8P9 | Bradi1g30648.1 | - | - | - |
| IUIVLUD01E04XS | Bradi1g30610.1 | - | - | - |
| IUIVLUD02GYHOE | Bradi1g29810.1 | - | - | - |
| IUIVLUD02HACKP | Bradi1g29810.1 | - | - | - |
| IV9RU5F01AR9QB | Bradi1g29810.1 | - | - | - |
| IV9RU5F01BFFVB | Bradi1g29810.1 | - | - | - |
| IV9RU5F02IQWJ5 | Bradi1g29810.1 | - | - | - |
| IV9RU5F02IT5FY | Bradi1g29810.1 | - | - | - |
| IV9RU5F02JK1Q3 | Bradi1g29810.1 | - | - | - |
| IVLNWEC01B4P2E | Bradi1g29810.1 | - | - | - |
| IVLNWEC01DMLIV | Bradi1g29810.1 | - | - | - |
| IVLNWEC01ENH3T | Bradi1g29810.1 | - | - | - |
| IVLNWEC02FX0IF | Bradi1g29810.1 | - | - | - |
| IVLNWEC02G81IM | Bradi1g29810.1 | - | - | - |
| IVLNWEC02GWKP7 | Bradi1g29810.1 | - | - | - |
| IUIVLUD02J3KZC | Bradi1g29658.2 | - | - | - |
| IV9RU5F01ARIB0 | Bradi1g29658.2 | - | - | - |

| | | | | |
|---|---|---|---|---|
| IV9RU5F02I14BM | Bradi1g29622.1 | - | - | - |
| IV9RU5F02H6QLC | Bradi1g29427.1 | - | - | - |
| IV9RU5F02H0AKA | Bradi1g29360.1 | - | - | - |
| IV9RU5F02HNLWH | Bradi1g29360.1 | - | - | - |
| IVLNWEC01A83BR | Bradi1g29360.1 | - | - | - |
| IVLNWEC02GHUWU | Bradi1g29360.1 | - | - | - |
| IV9RU5F02I6ESL | Bradi1g29267.1 | - | - | - |
| IVLNWEC02GP5U9 | Bradi1g29267.1 | - | - | - |
| IUIVLUD02GVR9P | Bradi1g29247.1 | - | - | - |
| IUIVLUD01BGE9N | Bradi1g27940.1 | - | - | - |
| IUIVLUD01ETNC9 | Bradi1g27940.1 | - | - | - |
| IV9RU5F01C4FAP | Bradi1g27890.1 | - | - | - |
| IV9RU5F02ITDNB | Bradi1g27700.1 | - | - | - |
| IVLNWEC01EWU6Z | Bradi1g27700.1 | - | - | - |
| IUIVLUD01C0ILM | Bradi1g27020.1 | - | - | - |
| IV9RU5F02I83OP | Bradi1g27020.1 | - | - | - |
| IUIVLUD01D744K | Bradi1g26480.1 | - | - | - |
| IVLNWEC01AYJIC | Bradi1g26480.1 | - | - | - |
| IUIVLUD01A2EOU | Bradi1g26270.1 | - | - | - |
| IVLNWEC01AHUHG | Bradi1g26180.1 | - | - | - |
| IVLNWEC01DZL9A | Bradi1g25497.1 | - | - | - |
| IUIVLUD01EF098 | Bradi1g24780.1 | - | - | - |
| IV9RU5F01BWJUC | Bradi1g24525.1 | - | - | - |
| IV9RU5F02JIYOJ | Bradi1g24525.1 | - | - | - |
| IVLNWEC01ANER7 | Bradi1g23840.1 | - | - | - |
| IVLNWEC01DU6GJ | Bradi1g23840.1 | - | - | - |
| IVLNWEC02HIZKC | Bradi1g23840.1 | - | - | - |
| IUIVLUD02JG8KA | Bradi1g23360.1 | - | - | - |
| IVLNWEC02ISPKF | Bradi1g23360.1 | - | - | - |
| IV9RU5F01D4VPW | Bradi1g23350.1 | - | - | - |
| IV9RU5F01DUQRX | Bradi1g23350.1 | - | - | - |
| IVLNWEC01B13PJ | Bradi1g23350.1 | - | - | - |
| IV9RU5F01A955C | Bradi1g22780.1 | - | - | - |
| IV9RU5F01ANO4H | Bradi1g22780.1 | - | - | - |
| IV9RU5F01BC37H | Bradi1g22780.1 | - | - | - |
| IV9RU5F01BUMCG | Bradi1g22780.1 | - | - | - |
| IV9RU5F02G3096 | Bradi1g22780.1 | - | - | - |
| IV9RU5F02HEYRF | Bradi1g22780.1 | - | - | - |
| IV9RU5F02HZ96S | Bradi1g22780.1 | - | - | - |
| IVLNWEC01ELDX7 | Bradi1g22780.1 | - | - | - |
| IVLNWEC02HHTBS | Bradi1g22780.1 | - | - | - |
| IVLNWEC02I8U63 | Bradi1g22780.1 | - | - | - |
| IVLNWEC02JVVO0 | Bradi1g22780.1 | - | - | - |
| IV9RU5F01AYT2N | Bradi1g22640.1 | - | - | - |
| IV9RU5F01BOMBH | Bradi1g22640.1 | - | - | - |
| IVLNWEC01BC6KL | Bradi1g22640.1 | - | - | - |
| IVLNWEC01CU6QC | Bradi1g22640.1 | - | - | - |

| | | | | |
|---|---|---|---|---|
| IVLNWEC02F168O | Bradi1g22640.1 | - | - | - |
| IVLNWEC02HCNSK | Bradi1g22480.1 | - | - | - |
| IVLNWEC02HGCBQ | Bradi1g22480.1 | - | - | - |
| IV9RU5F02HUQLC | Bradi1g22340.1 | - | - | - |
| IVLNWEC02JWV5W | Bradi1g22340.1 | - | - | - |
| IVLNWEC02F5A2L | Bradi1g21280.1 | - | - | - |
| IV9RU5F02GBOE0 | Bradi1g21130.1 | - | - | - |
| IVLNWEC02GNVT8 | Bradi1g21130.1 | - | - | - |
| IVLNWEC02F2EF1 | Bradi1g18910.1 | - | - | - |
| IVLNWEC01B6C57 | Bradi1g17060.1 | - | - | - |
| IV9RU5F01A0E91 | Bradi1g16560.1 | - | - | - |
| IV9RU5F02F2O2D | Bradi1g16250.1 | - | - | - |
| IV9RU5F02FTP3J | Bradi1g16250.1 | - | - | - |
| IV9RU5F02G28QT | Bradi1g16250.1 | - | - | - |
| IVLNWEC01AZ07K | Bradi1g16250.1 | - | - | - |
| IVLNWEC02HS5BU | Bradi1g16250.1 | - | - | - |
| IUIVLUD02HAJ8O | Bradi1g16130.1 | - | - | - |
| IVLNWEC01BGV79 | Bradi1g16130.1 | - | - | - |
| IVLNWEC01CFMFG | Bradi1g16130.1 | - | - | - |
| IV9RU5F01CO2X7 | Bradi1g15870.1 | - | - | - |
| IVLNWEC01BA5IO | Bradi1g15870.1 | - | - | - |
| IV9RU5F02GTFGX | Bradi1g15680.1 | - | - | - |
| IVLNWEC01APKZL | Bradi1g15680.1 | - | - | - |
| IUIVLUD02G9WT9 | Bradi1g15123.1 | - | - | - |
| IV9RU5F02JZEV7 | Bradi1g15123.1 | - | - | - |
| IV9RU5F01AW249 | Bradi1g14500.1 | - | - | - |
| IV9RU5F01CTYYI | Bradi1g14500.1 | - | - | - |
| IV9RU5F01EE6BJ | Bradi1g14500.1 | - | - | - |
| IV9RU5F02IZSH8 | Bradi1g14500.1 | - | - | - |
| IV9RU5F02J2JPF | Bradi1g14500.1 | - | - | - |
| IUIVLUD02FK8Q6 | Bradi1g14450.1 | - | - | - |
| IUIVLUD01ECFKR | Bradi1g14410.1 | - | - | - |
| IV9RU5F02HZVZ1 | Bradi1g14350.1 | - | - | - |
| IV9RU5F01EJKYL | Bradi1g14190.1 | - | - | - |
| IVLNWEC01A21LY | Bradi1g14190.1 | - | - | - |
| IV9RU5F01A67RN | Bradi1g13787.1 | - | - | - |
| IV9RU5F01BGDYF | Bradi1g13580.1 | - | - | - |
| IUIVLUD01CAJMZ | Bradi1g12530.1 | - | - | - |
| IV9RU5F01C2CZH | Bradi1g11330.1 | - | - | - |
| IVLNWEC02GGP66 | Bradi1g11330.1 | - | - | - |
| IV9RU5F01B6EYM | Bradi1g10750.1 | - | - | - |
| IVLNWEC01DMA3L | Bradi1g10750.1 | - | - | - |
| IVLNWEC01DVP4Y | Bradi1g10750.1 | - | - | - |
| IV9RU5F02I3EBL | Bradi1g10447.1 | - | - | - |
| IV9RU5F02FUXPD | Bradi1g10360.1 | - | - | - |
| IUIVLUD01B9NYE | Bradi1g10120.1 | - | - | - |
| IV9RU5F02GSC36 | Bradi1g10120.1 | - | - | - |

| | | | | |
|---|---|---|---|---|
| IUIVLUD01AY2WC | Bradi1g09260.1 | - | - | - |
| IVLNWEC02IXYDM | Bradi1g08840.1 | - | - | - |
| IV9RU5F01AVOYR | Bradi1g08727.1 | - | - | - |
| IVLNWEC02H25QB | Bradi1g08301.1 | - | - | - |
| IVLNWEC02HJVGA | Bradi1g08287.1 | - | - | - |
| IV9RU5F02GH744 | Bradi1g08170.1 | - | - | - |
| IVLNWEC02G73I5 | Bradi1g08170.1 | - | - | - |
| IVLNWEC02HNP3N | Bradi1g08157.1 | - | - | - |
| IVLNWEC01B84LY | Bradi1g08140.1 | - | - | - |
| IV9RU5F01A7K3B | Bradi1g08120.1 | - | - | - |
| IVLNWEC01CN4MB | Bradi1g08120.1 | - | - | - |
| IV9RU5F02JT7I8 | Bradi1g08060.1 | - | - | - |
| IVLNWEC01B73GJ | Bradi1g07960.1 | - | - | - |
| IVLNWEC02HU9QT | Bradi1g07755.1 | - | - | - |
| IUIVLUD01CS2XH | Bradi1g07730.1 | - | - | - |
| IV9RU5F01C9HVT | Bradi1g07730.1 | - | - | - |
| IV9RU5F01CFGIH | Bradi1g07730.1 | - | - | - |
| IVLNWEC01BL03Y | Bradi1g07730.1 | - | - | - |
| IVLNWEC01BS630 | Bradi1g07730.1 | - | - | - |
| IVLNWEC02IG147 | Bradi1g07730.1 | - | - | - |
| IVLNWEC02FYMUJ | Bradi1g07717.1 | - | - | - |
| IV9RU5F01DUU9G | Bradi1g07650.1 | - | - | - |
| IV9RU5F01AQBMH | Bradi1g07590.1 | - | - | - |
| IVLNWEC02IUKE2 | Bradi1g07570.2 | - | - | - |
| IV9RU5F01EMZ9B | Bradi1g07360.1 | - | - | - |
| IV9RU5F01DIXWU | Bradi1g07340.1 | - | - | - |
| IUIVLUD01B8YPL | Bradi1g07020.1 | - | - | - |
| IUIVLUD01ETXKL | Bradi1g07020.1 | - | - | - |
| IV9RU5F02HJ6PK | Bradi1g06880.1 | - | - | - |
| IVLNWEC01DMMFO | Bradi1g06880.1 | - | - | - |
| IVLNWEC01A65M8 | Bradi1g06470.1 | - | - | - |
| IUIVLUD02F9XX1 | Bradi1g06250.1 | - | - | - |
| IVLNWEC01AUB0N | Bradi1g06110.1 | - | - | - |
| IVLNWEC02HDRIV | Bradi1g05960.1 | - | - | - |
| IVLNWEC02I85EU | Bradi1g05960.1 | - | - | - |
| IV9RU5F01BZW9Y | Bradi1g05950.1 | - | - | - |
| IUIVLUD01D9QPW | Bradi1g05890.1 | - | - | - |
| IUIVLUD02G6UCZ | Bradi1g05890.1 | - | - | - |
| IUIVLUD02I7K23 | Bradi1g05890.1 | - | - | - |
| IVLNWEC01BQSR0 | Bradi1g05890.1 | - | - | - |
| IVLNWEC01C398A | Bradi1g05890.1 | - | - | - |
| IUIVLUD01CM1ZS | Bradi1g05540.1 | - | - | - |
| IVLNWEC02JYXQZ | Bradi1g05540.1 | - | - | - |
| IVLNWEC02GCBTM | Bradi1g05500.1 | - | - | - |
| IUIVLUD02GT5GZ | Bradi1g05480.1 | - | - | - |
| IVLNWEC02IF3YA | Bradi1g05390.1 | - | - | - |
| IV9RU5F02FWEBK | Bradi1g05380.1 | - | - | - |

| | | | | |
|---|---|---|---|---|
| IVLNWEC01CUPUI | Bradi1g05380.1 | - | - | - |
| IVLNWEC02JTXIP | Bradi1g05380.1 | - | - | - |
| IUIVLUD01AQ0HK | Bradi1g05190.1 | - | - | - |
| IV9RU5F02IU6X1 | Bradi1g04980.1 | - | - | - |
| IV9RU5F01CEFYM | Bradi1g04720.1 | - | - | - |
| IUIVLUD01CK33R | Bradi1g04677.1 | - | - | - |
| IVLNWEC01C4DB5 | Bradi1g04610.1 | - | - | - |
| IV9RU5F01DQRLX | Bradi1g04477.1 | - | - | - |
| IUIVLUD01CHKM6 | Bradi1g04000.1 | - | - | - |
| IUIVLUD02HTP6R | Bradi1g04000.1 | - | - | - |
| IV9RU5F02GXY81 | Bradi1g04000.1 | - | - | - |
| IVLNWEC01AVD7I | Bradi1g04000.1 | - | - | - |
| IVLNWEC01CH0YX | Bradi1g04000.1 | - | - | - |
| IVLNWEC02GRWFS | Bradi1g03827.1 | - | - | - |
| IV9RU5F02JFT97 | Bradi1g03720.1 | - | - | - |
| IVLNWEC01BW9IS | Bradi1g03720.1 | - | - | - |
| IVLNWEC01B5GY3 | Bradi1g03680.1 | - | - | - |
| IVLNWEC01DQA0A | Bradi1g03680.1 | - | - | - |
| IVLNWEC01DXQLM | Bradi1g03680.1 | - | - | - |
| IVLNWEC02FSGDQ | Bradi1g03650.1 | - | - | - |
| IUIVLUD02J3GJB | Bradi1g03307.1 | - | - | - |
| IV9RU5F01CR2RN | Bradi1g03307.1 | - | - | - |
| IV9RU5F01DYDNM | Bradi1g03307.1 | - | - | - |
| IVLNWEC02HUUUE | Bradi1g03307.1 | - | - | - |
| IV9RU5F02GKAG5 | Bradi1g03277.1 | - | - | - |
| IVLNWEC01DX5SS | Bradi1g03277.1 | - | - | - |
| IV9RU5F01AXAMY | Bradi1g03136.1 | - | - | - |
| IV9RU5F01EYNEA | Bradi1g03090.1 | - | - | - |
| IVLNWEC01AME9Q | Bradi1g03090.1 | - | - | - |
| IV9RU5F02F2BCP | Bradi1g02980.1 | - | - | - |
| IV9RU5F01BLAVB | Bradi1g02830.1 | - | - | - |
| IV9RU5F01CQJGF | Bradi1g02830.1 | - | - | - |
| IV9RU5F02JDF24 | Bradi1g02830.1 | - | - | - |
| IVLNWEC02IFDJW | Bradi1g02505.1 | - | - | - |
| IUIVLUD01DEZFQ | Bradi1g02360.1 | - | - | - |
| IV9RU5F01BS1Q1 | Bradi1g02360.1 | - | - | - |
| IV9RU5F02I2GS9 | Bradi1g02360.1 | - | - | - |
| IV9RU5F02IDYLW | Bradi1g02360.1 | - | - | - |
| IVLNWEC01A9MEQ | Bradi1g02360.1 | - | - | - |
| IVLNWEC01C099I | Bradi1g02360.1 | - | - | - |
| IVLNWEC02IR2XV | Bradi1g02360.1 | - | - | - |
| IUIVLUD01D5PLN | Bradi1g02290.1 | - | - | - |
| IUIVLUD02HWNH7 | Bradi1g02290.1 | - | - | - |
| IV9RU5F01AQPPN | Bradi1g02290.1 | - | - | - |
| IV9RU5F01DQ8VX | Bradi1g02290.1 | - | - | - |
| IV9RU5F01DPMXK | Bradi1g02247.1 | - | - | - |
| IUIVLUD02F667H | Bradi1g02240.1 | - | - | - |

| | | | | |
|---|---|---|---|---|
| IV9RU5F01BUD1U | Bradi1g02240.1 | - | - | - |
| IVLNWEC01D4RQV | Bradi1g02240.1 | - | - | - |
| IVLNWEC01EDJVQ | Bradi1g02240.1 | - | - | - |
| IVLNWEC02HCU2W | Bradi1g02240.1 | - | - | - |
| IVLNWEC02JAXDI | Bradi1g02240.1 | - | - | - |
| IV9RU5F01C141H | Bradi1g02190.1 | - | - | - |
| IV9RU5F02HMXXS | Bradi1g02190.1 | - | - | - |
| IVLNWEC01DJTFE | Bradi1g02190.1 | - | - | - |
| IVLNWEC01EFPS3 | Bradi1g02190.1 | - | - | - |
| IUIVLUD01DQ302 | Bradi1g02180.1 | - | - | - |
| IUIVLUD02HMY34 | Bradi1g02180.1 | - | - | - |
| IUIVLUD02HRPVA | Bradi1g02180.1 | - | - | - |
| IV9RU5F01ATX2R | Bradi1g02180.1 | - | - | - |
| IV9RU5F01DJLI1 | Bradi1g02180.1 | - | - | - |
| IV9RU5F01EFY07 | Bradi1g02180.1 | - | - | - |
| IV9RU5F01EGXIK | Bradi1g02180.1 | - | - | - |
| IVLNWEC01A079E | Bradi1g02180.1 | - | - | - |
| IVLNWEC02HH7XY | Bradi1g02180.1 | - | - | - |
| IVLNWEC02IB16M | Bradi1g02180.1 | - | - | - |
| IVLNWEC02IGYOU | Bradi1g02180.1 | - | - | - |
| IVLNWEC01A5INN | Bradi1g02160.1 | - | - | - |
| IVLNWEC02F0NYA | Bradi1g02160.1 | - | - | - |
| IV9RU5F01EV9O4 | Bradi1g02080.1 | - | - | - |
| IUIVLUD02F9R4K | Bradi1g01936.1 | - | - | - |
| IV9RU5F01D245R | Bradi1g01530.1 | - | - | - |
| IUIVLUD01ARUTQ | Bradi1g01377.1 | - | - | - |
| IVLNWEC01BWIYR | Bradi1g01377.1 | - | - | - |
| IV9RU5F01AKPBX | Bradi1g01257.1 | - | - | - |
| IVLNWEC02GF5KQ | Bradi1g01257.1 | - | - | - |
| IVLNWEC02J0LJL | Bradi1g01257.1 | - | - | - |
| IV9RU5F01AXYTC | Bradi1g01250.1 | - | - | - |
| IV9RU5F01DGLQ5 | Bradi1g01250.1 | - | - | - |
| IVLNWEC01CBXH1 | Bradi1g01250.1 | - | - | - |
| IVLNWEC02I282G | Bradi1g01250.1 | - | - | - |
| IV9RU5F01DMKFA | Bradi1g01180.1 | - | - | - |
| IVLNWEC02GC72V | Bradi1g01070.1 | - | - | - |
| IUIVLUD02I8L9O | Bradi1g01040.1 | - | - | - |
| IV9RU5F01AQ91P | Bradi1g01040.1 | - | - | - |
| IVLNWEC01B9JHX | Bradi1g01040.1 | - | - | - |
| IVLNWEC01B2ORH | Bradi1g00710.1 | - | - | - |
| IVLNWEC02IE9VJ | Bradi1g00690.1 | - | - | - |
| IUIVLUD01EKTN5 | Bradi1g00440.1 | - | - | - |
| IUIVLUD02F6VD3 | Bradi1g00440.1 | - | - | - |
| IVLNWEC02ISUF6 | Bradi1g00440.1 | - | - | - |
| IUIVLUD01A4YRR | Bradi1g00370.1 | - | - | - |
| IV9RU5F02FZWAU | Bradi1g00370.1 | - | - | - |
| IV9RU5F02G48K2 | Bradi1g00370.1 | - | - | - |

| | | | | |
|---|---|---|---|---|
| IUIVLUD02G6PKH | Bradi1g00237.1 | - | - | - |
| IV9RU5F02J1EKP | Bradi1g00237.1 | - | - | - |
| IVLNWEC01DXRV2 | Bradi1g00237.1 | - | - | - |
| IVLNWEC02HRHPA | Bradi1g00237.1 | - | - | - |
| IUIVLUD01BSTH8 | Bradi1g00227.1 | - | - | - |
| IUIVLUD02H65XM | Bradi1g00227.1 | - | - | - |
| IUIVLUD02JO9SM | Bradi1g00227.1 | - | - | - |
| IV9RU5F01D4WR5 | Bradi1g00227.1 | - | - | - |
| IV9RU5F01DOXGM | Bradi1g00227.1 | - | - | - |
| IVLNWEC01BUI76 | Bradi1g00227.1 | - | - | - |
| IVLNWEC01BW7VL | Bradi1g00227.1 | - | - | - |
| IVLNWEC01DPOA9 | Bradi1g00227.1 | - | - | - |
| IVLNWEC02GRRVZ | Bradi1g51097.1 | - | - | - |
| IUIVLUD01EUJ40 | Bradi3g01190.1 | - | - | - |
| IV9RU5F02H232W | Bradi1g10390.1 | - | - | - |
| IV9RU5F02FZUWC | Bradi4g08727.1 | - | - | - |
| IV9RU5F02HOEXS | Bradi4g08727.1 | - | - | - |
| IVLNWEC02JSIDB | Bradi4g08727.1 | - | - | - |
| IV9RU5F02JVJO7 | Bradi1g43906.1 | - | - | - |
| IV9RU5F02IO8JM | Bradi5g10940.1 | - | - | - |
| IVLNWEC01C2TJF | Bradi4g21980.1 | - | - | - |
| IV9RU5F01AXURJ | Bradi3g15600.1 | - | - | - |
| IV9RU5F02IRNTU | Bradi3g15600.1 | - | - | - |
| IVLNWEC02IP5QA | Bradi1g01377.1 | - | - | - |
| IVLNWEC02JZTRD | Bradi1g03307.1 | - | - | - |
| IVLNWEC02IX512 | Bradi1g25920.1 | - | - | - |
| IVLNWEC02FXZE4 | Bradi1g65860.1 | - | - | - |
| IV9RU5F02JB3LE | Bradi4g27334.1 | - | - | - |
| IV9RU5F01BFL1I | Bradi1g33010.1 | - | - | - |
| IVLNWEC01EQR5L | Bradi1g07870.1 | - | - | - |
| IV9RU5F02HBV9B | Bradi2g19660.1 | - | - | - |
| IV9RU5F02FX9US | Bradi4g31003.1 | - | - | - |
| IV9RU5F02GADDL | Bradi4g31003.1 | - | - | - |
| IV9RU5F02GBWL5 | Bradi4g26970.1 | - | - | - |
| IV9RU5F02JBCWX | Bradi4g26970.1 | - | - | - |
| IVLNWEC01B3HWT | Bradi4g26970.1 | - | - | - |
| IVLNWEC02HS8K1 | Bradi4g26970.1 | - | - | - |
| IVLNWEC02IBX39 | Bradi4g26970.1 | - | - | - |
| IVLNWEC02IJ6IG | Bradi4g24010.1 | - | - | - |
| IV9RU5F02GYYPN | Bradi2g55660.1 | - | - | - |
| IVLNWEC02JFG01 | Bradi2g55660.1 | - | - | - |
| IV9RU5F02GM542 | Bradi4g16885.1 | - | - | - |
| IVLNWEC01AOPM7 | Bradi2g04240.1 | - | - | - |
| IVLNWEC01BNEKA | Bradi1g14190.1 | - | - | - |
| IVLNWEC01AV1B9 | Bradi4g26627.1 | - | - | - |
| IUIVLUD01DZ9DW | Bradi2g04240.1 | - | - | - |
| IVLNWEC02H3B79 | Bradi1g75320.1 | - | - | - |

| | | | | |
|---|---|---|---|---|
| IVLNWEC02INASK | Bradi1g75320.1 | - | - | - |
| IUIVLUD01DSQR7 | Bradi3g56087.1 | - | - | - |
| IV9RU5F02GYGBA | Bradi1g67750.1 | - | - | - |
| IVLNWEC02H2991 | Bradi1g06430.1 | - | - | - |
| IUIVLUD01AF71U | Bradi4g10670.1 | - | - | - |
| IUIVLUD02FNL2Q | Bradi4g37170.2 | - | - | - |
| IVLNWEC01BVSVW | Bradi1g51790.1 | - | - | - |
| IV9RU5F01DSNAD | Bradi3g15277.1 | - | - | - |
| IUIVLUD01DO35W | Bradi1g52090.1 | - | - | - |
| IVLNWEC01CJWM5 | Bradi4g34927.1 | - | - | - |
| IVLNWEC02FKY93 | Bradi4g34927.1 | - | - | - |
| IVLNWEC02HLBWR | Bradi3g15277.1 | - | - | - |
| IVLNWEC02G4GKN | Bradi2g03850.1 | - | - | - |
| IVLNWEC01CIFON | Bradi4g03000.1 | - | - | - |
| IV9RU5F01B9MFD | Bradi1g04980.1 | - | - | - |
| IV9RU5F02H71QX | Bradi1g34370.1 | - | - | - |
| IV9RU5F01AXG5G | Bradi1g58930.1 | - | - | - |
| IVLNWEC01EK1ED | Bradi1g11650.1 | - | - | - |
| IUIVLUD01DCJDY | Bradi5g17947.1 | - | - | - |
| IV9RU5F02G6E1S | Bradi1g06270.1 | - | - | - |
| IV9RU5F01CQKZF | Bradi4g02950.1 | - | - | - |
| IV9RU5F02I1VJF | Bradi3g03467.1 | - | - | - |
| IUIVLUD01E2EIM | Bradi1g42000.1 | - | - | - |
| IUIVLUD02IPPTA | Bradi1g42000.1 | - | - | - |
| IV9RU5F01D2XH5 | Bradi1g42000.1 | - | - | - |
| IVLNWEC01BLTTN | Bradi4g31070.2 | - | - | - |
| IV9RU5F01B7COJ | Bradi2g26180.1 | - | - | - |
| IVLNWEC02J3UN5 | Bradi4g39210.1 | - | - | - |
| IV9RU5F02GSFMY | Bradi1g03827.1 | - | - | - |
| IVLNWEC02I25EJ | Bradi1g09810.1 | - | - | - |
| IVLNWEC02GLLM4 | Bradi4g09247.1 | - | - | - |
| IUIVLUD02IKD60 | Bradi4g05610.1 | - | - | - |
| IUIVLUD02HAA57 | Bradi1g01250.1 | - | - | - |
| IUIVLUD01AP4YC | Bradi1g04980.1 | - | - | - |
| IV9RU5F02HYS05 | Bradi4g45397.1 | - | - | - |
| IV9RU5F02H5WVT | Bradi1g78750.1 | - | - | - |
| IV9RU5F01BZURL | Bradi2g19610.3 | - | - | - |
| IV9RU5F01AI03S | Bradi4g32150.1 | - | - | - |
| IV9RU5F02HHT74 | Bradi4g32150.1 | - | - | - |
| IV9RU5F02GIH98 | Bradi1g14500.1 | - | - | - |
| IVLNWEC02I2VF5 | Bradi1g74170.1 | - | - | - |
| IUIVLUD02GNBX3 | Bradi4g20717.1 | - | - | - |
| IV9RU5F02HMDD7 | Bradi4g29820.1 | - | - | - |
| IV9RU5F02GZKCQ | Bradi1g74450.1 | - | - | - |
| IVLNWEC01C5J5E | Bradi1g66167.1 | - | - | - |
| IVLNWEC01E5I2S | Bradi4g35150.1 | - | - | - |
| IV9RU5F02ISPHE | Bradi1g76970.3 | - | - | - |

| | | | | |
|---|---|---|---|---|
| IVLNWEC02GI76Q | Bradi1g04000.1 | - | - | - |
| IVLNWEC02JNCPS | Bradi1g04000.1 | - | - | - |
| IVLNWEC01EXSR4 | Bradi3g43340.1 | - | - | - |
| IV9RU5F01BENKH | Bradi1g58410.1 | - | - | - |
| IVLNWEC02GN6US | Bradi1g38380.1 | - | - | - |
| IVLNWEC02H6AU8 | Bradi1g13710.1 | - | - | - |
| IUIVLUD01CV3RV | Bradi4g07110.1 | - | - | - |
| IVLNWEC01AZ0Y6 | Bradi3g52470.2 | - | - | - |
| IV9RU5F02JCZI6 | Bradi1g48264.1 | - | - | - |
| IV9RU5F01BW0KD | Bradi1g57017.1 | - | - | - |
| IV9RU5F01D32JL | Bradi1g57017.1 | - | - | - |
| IUIVLUD02G6XQQ | Bradi3g34450.2 | - | - | - |
| IVLNWEC01BB0BX | Bradi1g31130.1 | - | - | - |
| IVLNWEC01EM8UG | Bradi1g31130.1 | - | - | - |
| IUIVLUD01BV32I | Bradi1g02510.1 | - | - | - |
| IV9RU5F01B4J0T | Bradi1g62000.1 | - | - | - |
| IV9RU5F01D9N0T | Bradi1g62000.1 | - | - | - |
| IVLNWEC01EAD7R | Bradi2g39470.1 | - | - | - |
| IVLNWEC02IPK24 | Bradi1g02240.1 | - | - | - |
| IV9RU5F02JA4C1 | Bradi2g07910.1 | - | - | - |
| IUIVLUD02JYOSQ | Bradi1g23840.1 | - | - | - |
| IV9RU5F01AM52J | Bradi1g71730.2 | - | - | - |
| IV9RU5F02IBU32 | Bradi5g24095.1 | - | - | - |
| IUIVLUD02JF9BV | Bradi1g02240.1 | - | - | - |
| IVLNWEC01CGE2Y | Bradi2g42890.1 | - | - | - |
| IV9RU5F01BFI2X | Bradi1g01040.1 | - | - | - |
| IV9RU5F02G2C40 | Bradi1g74800.1 | - | - | - |
| IUIVLUD02IK97O | Bradi1g03136.1 | - | - | - |
| IVLNWEC01DFGLY | Bradi5g02170.1 | - | - | - |
| IV9RU5F02H2SJU | Bradi2g42890.1 | - | - | - |
| IVLNWEC02HX1BM | Bradi4g30810.1 | - | - | - |
| IV9RU5F02JPM5T | Bradi2g19010.1 | - | - | - |
| IVLNWEC02I72P7 | Bradi3g22460.1 | - | - | - |
| IUIVLUD02FVMOV | Bradi4g36020.1 | - | - | - |
| IV9RU5F01AJ602 | Bradi4g33886.1 | - | - | - |
| IV9RU5F01C86US | Bradi4g01513.1 | - | - | - |
| IVLNWEC01CNKSA | Bradi3g41720.2 | - | - | - |
| IV9RU5F01CJOAZ | Bradi4g30580.1 | - | - | - |
| IV9RU5F02IJME2 | Bradi3g07790.1 | - | - | - |
| IV9RU5F02JHTNC | Bradi1g66250.1 | - | - | - |
| IV9RU5F02IWM04 | Bradi4g45210.1 | - | - | - |
| IVLNWEC02ITBU5 | Bradi2g21510.1 | - | - | - |
| IUIVLUD01BT4KA | Bradi1g12760.1 | - | - | - |
| IVLNWEC02FUM7A | Bradi1g67510.1 | - | - | - |
| IV9RU5F02GX9WF | Bradi4g43950.1 | - | - | - |
| IVLNWEC01CVMMS | Bradi4g43950.1 | - | - | - |
| IV9RU5F01ARK2G | Bradi1g23350.1 | - | - | - |

| | | | | |
|---|---|---|---|---|
| IV9RU5F02H4BZ1 | Bradi1g23350.1 | - | - | - |
| IV9RU5F02IFA0V | Bradi1g14270.1 | - | - | - |
| IUIVLUD01ENCSV | Bradi1g14000.2 | - | - | - |
| IVLNWEC01EAT7S | Bradi1g14000.2 | - | - | - |
| IV9RU5F01CDZNW | Bradi1g05890.1 | - | - | - |
| IV9RU5F01B2CUG | Bradi2g23610.1 | - | - | - |
| IUIVLUD01EMZGF | Bradi4g37197.1 | - | - | - |
| IVLNWEC01DJJXM | Bradi3g24740.1 | - | - | - |
| IV9RU5F01AXX4N | Bradi1g01560.2 | - | - | - |
| IVLNWEC02G0WWB | Bradi1g14410.1 | - | - | - |
| IV9RU5F02H3UH8 | Bradi4g09957.1 | - | - | - |
| IV9RU5F02INHHZ | Bradi4g06827.1 | - | - | - |
| IV9RU5F01DDDAX | Bradi1g64220.1 | - | - | - |
| IV9RU5F01EGSED | Bradi4g07060.1 | - | - | - |
| IVLNWEC01A8GLC | Bradi1g28540.1 | - | - | - |
| IV9RU5F01CUC31 | Bradi2g37980.2 | - | - | - |
| IVLNWEC01A8EJF | Bradi1g62600.1 | - | - | - |
| IVLNWEC02JIHPL | Bradi4g08380.1 | - | - | - |
| IV9RU5F02JM18B | Bradi1g02247.1 | - | - | - |
| IVLNWEC01DCNTG | Bradi2g39240.1 | - | - | - |
| IVLNWEC01AH7WY | Bradi3g37010.1 | - | - | - |
| IVLNWEC02I9UET | Bradi2g16237.1 | - | - | - |
| IV9RU5F01BW6M4 | Bradi4g07110.1 | - | - | - |
| IV9RU5F02GBCPS | Bradi3g10180.2 | - | - | - |
| IUIVLUD02F1D1H | Bradi2g36810.1 | - | - | - |
| IVLNWEC02ILEJW | Bradi4g09997.1 | - | - | - |
| IUIVLUD02GJXVP | Bradi1g05920.1 | - | - | - |
| IV9RU5F01EGB6U | Bradi1g51807.1 | - | - | - |
| IV9RU5F02ION4E | Bradi1g51807.1 | - | - | - |
| IV9RU5F02IOQT1 | Bradi1g65290.1 | - | - | - |
| IV9RU5F01CLR78 | Bradi1g67387.1 | - | - | - |
| IVLNWEC02FOES1 | Bradi4g25047.1 | - | - | - |
| IV9RU5F02G4QCA | Bradi1g04980.1 | - | - | - |
| IVLNWEC02IAX8N | Bradi3g43260.1 | - | - | - |
| IUIVLUD02GXD0Q | Bradi2g09434.1 | - | - | - |
| IV9RU5F01BFXUZ | Bradi2g09434.1 | - | - | - |
| IUIVLUD02G84W8 | Bradi1g05890.1 | - | - | - |
| IV9RU5F01DUGPE | Bradi1g05890.1 | - | - | - |
| IUIVLUD01CNQTR | Bradi3g01150.1 | - | - | - |
| IV9RU5F01DRFQM | Bradi4g35440.1 | - | - | - |
| IV9RU5F01C8WDQ | Bradi1g08120.1 | - | - | - |
| IV9RU5F01BFJF5 | Bradi4g09120.2 | - | - | - |
| IV9RU5F01A2HKI | Bradi4g02010.1 | - | - | - |
| IV9RU5F01B72QU | Bradi4g02010.1 | - | - | - |
| IVLNWEC01APYSW | Bradi4g31310.1 | - | - | - |
| IVLNWEC02HAD3R | Bradi1g01257.1 | - | - | - |
| IVLNWEC02HLDQF | Bradi4g41970.2 | - | - | - |

| | | | | |
|---|---|---|---|---|
| IV9RU5F01DQSE2 | Bradi2g36210.1 | - | - | - |
| IV9RU5F01A6L1X | Bradi4g28400.1 | - | - | - |
| IV9RU5F02IFXAE | Bradi5g22790.1 | - | - | - |
| IV9RU5F02JN4LQ | Bradi1g57017.1 | - | - | - |
| IVLNWEC02JFR9O | Bradi4g39550.1 | - | - | - |
| IVLNWEC02GZGQV | Bradi1g15857.1 | - | - | - |
| IV9RU5F01C3NHD | Bradi4g31090.1 | - | - | - |
| IV9RU5F02H1ZOS | Bradi3g22720.1 | - | - | - |
| IV9RU5F01BURCH | Bradi1g30610.1 | - | - | - |
| IVLNWEC01DH8QN | Bradi2g37990.1 | - | - | - |
| IV9RU5F02FPYJU | Bradi2g21080.1 | - | - | - |
| IVLNWEC01D8M2E | Bradi1g71530.1 | - | - | - |
| IV9RU5F02GTPDS | Bradi4g09640.1 | - | - | - |
| IUIVLUD02HDJLH | Bradi3g00360.2 | - | - | - |
| IV9RU5F02GGDVZ | Bradi4g03000.1 | - | - | - |
| IVLNWEC01DAX24 | Bradi1g04677.1 | - | - | - |
| IVLNWEC01D2KHZ | Bradi3g43260.1 | - | - | - |
| IVLNWEC02HBHXF | Bradi1g14550.1 | - | - | - |
| IVLNWEC02GPHGM | Bradi1g06060.1 | - | - | - |
| IVLNWEC02H6NGJ | Bradi3g27970.1 | - | - | - |
| IVLNWEC01EBAA4 | Bradi1g52150.3 | - | - | - |
| IVLNWEC01DIWWY | Bradi4g03000.1 | - | - | - |
| IUIVLUD01AU8A3 | Bradi1g07755.1 | - | - | - |
| IVLNWEC01BJR3V | Bradi1g03307.1 | - | - | - |
| IVLNWEC02HR2QN | Bradi5g20077.1 | - | - | - |
| IVLNWEC01C5DRK | Bradi1g53085.1 | - | - | - |
| IV9RU5F01A1T9B | Bradi3g20710.1 | - | - | - |
| IVLNWEC01E0470 | Bradi2g24255.1 | - | - | - |
| IVLNWEC02JONCO | Bradi1g06230.1 | - | - | - |
| IV9RU5F01C56G5 | Bradi2g19660.1 | - | - | - |
| IVLNWEC01DC1QD | Bradi5g02240.1 | - | - | - |
| IV9RU5F01ECNU0 | Bradi4g12770.1 | - | - | - |
| IV9RU5F01EPU2V | Bradi3g14710.1 | - | - | - |
| IVLNWEC01E2BAF | Bradi1g11650.1 | - | - | - |
| IV9RU5F02ID20Q | Bradi1g04980.1 | - | - | - |
| IV9RU5F02FZNWC | Bradi5g22547.1 | - | - | - |
| IVLNWEC02GEHTC | Bradi3g18390.1 | - | - | - |
| IV9RU5F01BM2G1 | Bradi1g00237.1 | - | - | - |
| IVLNWEC02IWK30 | Bradi5g03760.1 | - | - | - |
| IUIVLUD01CDUR0 | Bradi1g68550.1 | - | - | - |
| IVLNWEC01DVEMC | Bradi4g42830.1 | - | - | - |
| IVLNWEC02ISHJF | Bradi3g27912.1 | - | - | - |
| IVLNWEC01EEDQT | Bradi1g61140.1 | - | - | - |
| IV9RU5F02H2K1B | Bradi4g12940.1 | - | - | - |
| IV9RU5F01CTG3C | Bradi4g00730.1 | - | - | - |
| IV9RU5F02H1UXD | Bradi1g64380.1 | - | - | - |
| IVLNWEC02FOBNA | Bradi1g64380.1 | - | - | - |

| | | | | |
|---|---|---|---|---|
| IUIVLUD02IQ9HO | Bradi5g03890.1 | - | - | - |
| IUIVLUD01APJTU | Bradi1g62600.1 | - | - | - |
| IVLNWEC02FXZHP | Bradi1g12530.1 | - | - | - |
| IVLNWEC02FRX92 | Bradi1g03307.1 | - | - | - |
| IUIVLUD02JAZU9 | Bradi3g56980.1 | - | - | - |
| IVLNWEC01E4GYV | Bradi1g07717.1 | - | - | - |
| IV9RU5F01DRABG | Bradi1g75210.1 | - | - | - |
| IV9RU5F02FY4R8 | Bradi2g39320.1 | - | - | - |
| IUIVLUD02I5PPG | Bradi2g26180.1 | - | - | - |
| IV9RU5F01BQ3Q0 | Bradi2g08750.1 | - | - | - |
| IVLNWEC02G23TX | Bradi5g02770.1 | - | - | - |
| IVLNWEC01EXRBS | Bradi1g02180.1 | - | - | - |
| IV9RU5F02J3VRI | Bradi4g32140.1 | - | - | - |
| IVLNWEC02FNDZU | Bradi1g52090.1 | - | - | - |
| IV9RU5F02HE7YT | Bradi1g09260.1 | - | - | - |
| IUIVLUD02GIIWU | Bradi1g53280.1 | - | - | - |
| IV9RU5F02GNQVD | Bradi1g70980.1 | - | - | - |
| IVLNWEC02FM9LS | Bradi1g70980.1 | - | - | - |
| IVLNWEC02F9KIJ | Bradi4g07717.1 | - | - | - |
| IV9RU5F02F8Z1V | Bradi1g14550.1 | - | - | - |
| IUIVLUD01CN9BC | Bradi5g01070.1 | - | - | - |
| IV9RU5F01DXKYF | Bradi1g53020.1 | - | - | - |
| IVLNWEC01BDSBW | Bradi1g53020.1 | - | - | - |
| IVLNWEC01A03BN | Bradi4g24660.1 | - | - | - |
| IUIVLUD01BYBS3 | Bradi3g30000.1 | - | - | - |
| IV9RU5F02HF5RN | Bradi1g75240.1 | - | - | - |
| IV9RU5F02JSSNP | Bradi2g19290.1 | - | - | - |
| IV9RU5F01DCME7 | Bradi4g09247.1 | - | - | - |
| IUIVLUD02F7A01 | Bradi2g39470.1 | - | - | - |
| IV9RU5F02H1JIB | Bradi3g21350.2 | - | - | - |
| IUIVLUD01DWKX8 | Bradi1g57975.1 | - | - | - |
| IUIVLUD01BMSJP | Bradi2g14750.1 | - | - | - |
| IV9RU5F02JXLL8 | Bradi2g11590.1 | - | - | - |
| IVLNWEC01AQ0BD | Bradi1g09210.1 | - | - | - |
| IV9RU5F01A8NDQ | Bradi1g75460.1 | - | - | - |
| IV9RU5F01ER8AV | Bradi1g22700.1 | - | - | - |
| IV9RU5F02GNYI9 | Bradi1g22700.1 | - | - | - |
| IV9RU5F01BMAFJ | Bradi4g09640.1 | - | - | - |
| IVLNWEC01A4PZN | Bradi1g02290.1 | - | - | - |
| IV9RU5F01A9YBK | Bradi4g24410.1 | - | - | - |
| IV9RU5F02IYHXI | Bradi2g09434.1 | - | - | - |
| IVLNWEC01D33K4 | Bradi2g61830.1 | - | - | - |
| IVLNWEC01AYCEW | Bradi1g51960.1 | - | - | - |
| IVLNWEC02JKZYW | Bradi1g12117.1 | - | - | - |
| IUIVLUD01EHLFA | Bradi1g68550.1 | - | - | - |
| IUIVLUD01C2LJ5 | Bradi2g37795.1 | - | - | - |
| IUIVLUD02HDWK3 | Bradi1g04980.1 | - | - | - |

| | | | | |
|---|---|---|---|---|
| IUIVLUD01COI6M | Bradi4g03000.1 | - | - | - |
| IVLNWEC01AV9V2 | Bradi1g02290.1 | - | - | - |
| IV9RU5F02HNLK3 | Bradi5g25030.1 | - | - | - |
| IVLNWEC02HJO8E | Bradi5g25030.1 | - | - | - |
| IVLNWEC01D7IKA | Bradi4g37260.1 | - | - | - |
| IVLNWEC02HSSN3 | Bradi4g37260.1 | - | - | - |
| IVLNWEC02H1USV | Bradi1g42690.1 | - | - | - |
| IVLNWEC01BFOM2 | Bradi4g32120.1 | - | - | - |
| IV9RU5F01BYAH3 | Bradi1g08190.1 | - | - | - |
| IV9RU5F01EQR6C | Bradi1g08190.1 | - | - | - |
| IV9RU5F01ESWV9 | Bradi4g45190.1 | - | - | - |
| IV9RU5F02F8KDM | Bradi1g08145.1 | - | - | - |
| IVLNWEC02I30M6 | Bradi4g33467.1 | - | - | - |
| IV9RU5F02HFMI3 | Bradi4g35940.1 | - | - | - |
| IV9RU5F01B55KP | Bradi1g08157.1 | - | - | - |
| IUIVLUD01C3YOL | Bradi1g01040.1 | - | - | - |
| IVLNWEC01BYO41 | Bradi1g02180.1 | - | - | - |
| IUIVLUD01B52I0 | Bradi4g01513.1 | - | - | - |
| IUIVLUD01CHP0O | Bradi1g00960.1 | - | - | - |
| IV9RU5F02HBL74 | Bradi3g54950.1 | - | - | - |
| IV9RU5F02GTCJX | Bradi4g26990.1 | - | - | - |
| IUIVLUD01AOR2V | Bradi3g10030.1 | - | - | - |
| IVLNWEC02ICN6X | Bradi4g06970.1 | - | - | - |
| IVLNWEC01BQHKH | Bradi1g07420.1 | - | - | - |
| IVLNWEC02FF2DJ | Bradi2g07310.1 | - | - | - |
| IVLNWEC01A2BJI | Bradi2g34230.1 | - | - | - |
| IVLNWEC02HJHLT | Bradi3g43260.1 | - | - | - |
| IV9RU5F02JCBSI | Bradi1g74710.1 | - | - | - |
| IV9RU5F01BZVPN | Bradi1g09260.1 | - | - | - |
| IUIVLUD02ISHJX | Bradi1g31970.1 | - | - | - |
| IVLNWEC02IVH7D | Bradi1g06450.1 | - | - | - |
| IUIVLUD01CGLC7 | Bradi4g09247.1 | - | - | - |
| IV9RU5F01CE4B7 | Bradi4g09247.1 | - | - | - |
| IV9RU5F02GUO2S | Bradi4g09247.1 | - | - | - |
| IVLNWEC01DQPS9 | Bradi1g04980.1 | - | - | - |
| IVLNWEC01ATG31 | Bradi3g10370.1 | - | - | - |
| IVLNWEC02IL09R | Bradi1g00960.1 | - | - | - |
| IV9RU5F02JC511 | Bradi1g05890.1 | - | - | - |
| IV9RU5F01B2DG5 | Bradi4g36680.1 | - | - | - |
| IUIVLUD01BWU2T | Bradi4g24887.1 | - | - | - |
| IUIVLUD01EQ6G4 | Bradi4g35150.1 | - | - | - |
| IUIVLUD01CX77Q | Bradi4g31257.1 | - | - | - |
| IV9RU5F02JC4OI | Bradi4g42130.1 | - | - | - |
| IVLNWEC01D3CUS | Bradi2g20430.1 | - | - | - |
| IV9RU5F01DPOPW | Bradi4g34022.1 | - | - | - |
| IV9RU5F02FY024 | Bradi3g10480.1 | - | - | - |
| IVLNWEC02H1CK6 | Bradi3g00340.1 | - | - | - |

| | | | | |
|---|---|---|---|---|
| IV9RU5F02HC8I8 | Bradi4g00790.1 | - | - | - |
| IUIVLUD01D3U1F | Bradi4g02030.1 | - | - | - |
| IUIVLUD02JD0XN | Bradi4g05817.1 | - | - | - |
| IV9RU5F02IS9WJ | Bradi2g17926.1 | - | - | - |
| IUIVLUD02I93EZ | Bradi3g49640.1 | - | - | - |
| IVLNWEC01DRAKK | Bradi4g37780.1 | - | - | - |
| IVLNWEC02F306X | Bradi4g38700.1 | - | - | - |
| IUIVLUD01BEZWM | Bradi2g10800.2 | - | - | - |
| IUIVLUD01APTWW | Bradi1g70980.1 | - | - | - |
| IV9RU5F01EIVVG | Bradi4g02660.1 | - | - | - |
| IV9RU5F02IMQS6 | Bradi4g21860.1 | - | - | - |
| IVLNWEC02HV1LE | Bradi2g58917.1 | - | - | - |
| IVLNWEC02I9UCZ | Bradi2g00500.1 | - | - | - |
| IV9RU5F01A943M | Bradi1g68980.1 | - | - | - |
| IV9RU5F01D8B2N | Bradi1g44440.1 | - | - | - |
| IVLNWEC02FZ59T | Bradi2g24255.1 | - | - | - |
| IUIVLUD02JKLTG | Bradi1g00237.1 | - | - | - |
| IVLNWEC02GYL0G | Bradi4g38240.1 | - | - | - |
| IVLNWEC01DY369 | Bradi1g00227.1 | - | - | - |
| IVLNWEC02HC51E | Bradi1g51097.1 | - | - | - |
| IV9RU5F01CRH3D | Bradi1g03720.1 | - | - | - |
| IVLNWEC02HIGGB | Bradi1g32950.1 | - | - | - |
| IUIVLUD01ALA18 | Bradi4g30840.1 | - | - | - |
| IVLNWEC01A7K2R | Bradi2g07310.1 | - | - | - |
| IVLNWEC02F2H1O | Bradi2g22710.1 | - | - | - |
| IV9RU5F02HS38J | Bradi4g06370.1 | - | - | - |
| IUIVLUD01CMOQO | Bradi4g03000.1 | - | - | - |
| IVLNWEC02JYDO4 | Bradi2g51807.1 | - | - | - |
| IVLNWEC02IF9ED | Bradi4g21250.1 | - | - | - |
| IV9RU5F01CZ0AC | Bradi2g10800.2 | - | - | - |
| IVLNWEC01AOP02 | Bradi4g44277.1 | - | - | - |
| IUIVLUD01B6SCR | Bradi1g66167.1 | - | - | - |
| IV9RU5F01D77VD | Bradi2g19610.3 | - | - | - |
| IVLNWEC02GMKXN | Bradi3g52470.2 | - | - | - |
| IVLNWEC01B1KGG | Bradi1g48264.1 | - | - | - |
| IVLNWEC02ITL7Z | Bradi4g24887.1 | - | - | - |
| IVLNWEC01CAQHE | Bradi1g51097.1 | - | - | - |
| IVLNWEC01DPSO9 | Bradi4g42060.1 | - | - | - |
| IVLNWEC01ECXF4 | Bradi3g55470.1 | - | - | - |
| IV9RU5F01BDLO7 | Bradi4g08470.1 | - | - | - |
| IUIVLUD02JKP9K | Bradi4g00747.1 | - | - | - |
| IV9RU5F02IW9Y7 | Bradi4g45210.1 | - | - | - |
| IVLNWEC02GKS61 | Bradi2g04680.1 | - | - | - |
| IVLNWEC01DLSIJ | Bradi1g06945.1 | - | - | - |
| IV9RU5F01AVLI0 | Bradi1g08301.1 | - | - | - |
| IUIVLUD02IQ90K | Bradi4g34220.3 | - | - | - |
| IUIVLUD01BTG66 | Bradi3g24740.1 | - | - | - |

| | | | | |
|---|---|---|---|---|
| IVLNWEC01B5GNP | Bradi3g24740.1 | - | - | - |
| IVLNWEC01BBP20 | Bradi3g27160.1 | - | - | - |
| IUIVLUD01CK7OS | Bradi4g09247.1 | - | - | - |
| IVLNWEC02JM6JK | Bradi4g00730.1 | - | - | - |
| IV9RU5F01EAVPM | Bradi4g35977.1 | - | - | - |
| IVLNWEC02HA9IN | Bradi3g28787.1 | - | - | - |
| IV9RU5F01BV075 | Bradi3g34000.1 | - | - | - |
| IV9RU5F02F4NYC | Bradi2g49050.1 | - | - | - |
| IV9RU5F02FT5V8 | Bradi1g70000.1 | - | - | - |
| IV9RU5F01ESE1S | Bradi4g07760.1 | - | - | - |
| IV9RU5F01AON6Z | Bradi1g26270.1 | - | - | - |
| IVLNWEC01AIXBG | Bradi4g40450.2 | - | - | - |
| IVLNWEC02HHXO9 | Bradi1g12117.1 | - | - | - |
| IVLNWEC01C3ZWX | Bradi1g44440.1 | - | - | - |
| IV9RU5F01ANIOP | Bradi2g60170.1 | - | - | - |
| IVLNWEC01AH5HF | Bradi2g60170.1 | - | - | - |
| IV9RU5F02JM0TR | Bradi5g15337.2 | - | - | - |
| IVLNWEC01C5CF3 | Bradi4g43670.1 | - | - | - |
| IVLNWEC01COHUJ | Bradi1g02117.1 | - | - | - |
| IV9RU5F01BS4LK | Bradi3g10370.1 | - | - | - |
| IVLNWEC01AKERR | Bradi3g18390.1 | - | - | - |
| IV9RU5F01D65VZ | Bradi1g51757.1 | - | - | - |
| IVLNWEC02I0IHD | Bradi5g01462.1 | - | - | - |
| IV9RU5F01ASNKO | Bradi1g68550.1 | - | - | - |
| IVLNWEC02HEW2T | Bradi1g04860.2 | - | - | AK248344.1 |
| IV9RU5F01C3ZGO | Bradi1g49470.1 | - | - | AK248429.1 |
| IV9RU5F01DT31C | Bradi1g00750.1 | - | - | AK251756.1 |
| IVLNWEC02H5YQ8 | Bradi4g26342.1 | - | - | AK252280.1 |
| IVLNWEC02HE955 | Bradi4g26342.1 | - | - | AK252280.1 |
| IV9RU5F02HSBT1 | Bradi5g00990.1 | - | - | AK252561.1 |
| IVLNWEC02HYRWY | Bradi4g07110.1 | - | - | AK252801.1 |
| IV9RU5F02JTL6Y | Bradi1g52280.1 | - | - | AK353650 |
| IVLNWEC01BDDTA | Bradi4g07957.1 | - | - | AK353660 |
| IVLNWEC01BRWSZ | Bradi4g32967.1 | - | - | AK353724 |
| IV9RU5F01BAR9N | Bradi1g71800.2 | - | - | AK353969 |
| IV9RU5F02H2Y90 | Bradi1g71800.2 | - | - | AK353969 |
| IVLNWEC02JKGZW | Bradi4g00640.1 | - | - | AK354479 |
| IV9RU5F02HTKRJ | Bradi3g02870.1 | - | - | AK354730 |
| IV9RU5F01DT7A6 | Bradi1g30830.1 | - | - | AK354860 |
| IUIVLUD02JWG4Y | Bradi1g01130.2 | - | - | AK354917 |
| IV9RU5F02GAPZX | Bradi2g44785.1 | - | - | AK356427 |
| IVLNWEC01EXEAA | Bradi4g07350.1 | - | - | AK356629 |
| IVLNWEC01CGUM6 | Bradi1g05610.1 | - | - | AK357244 |
| IUIVLUD01AY2CM | Bradi5g01230.1 | - | - | AK358163 |
| IV9RU5F02HCG7N | Bradi4g24887.1 | - | - | AK358948 |
| IVLNWEC02ICI2T | Bradi2g39700.1 | - | - | AK359191 |
| IV9RU5F01DH426 | Bradi1g36067.1 | - | - | AK359410 |

| | | | | |
|---|---|---|---|---|
| IV9RU5F02ISAHH | Bradi4g14750.1 | - | - | AK359881 |
| IV9RU5F02HEN7K | Bradi1g04440.1 | - | - | AK360154 |
| IUIVLUD02JIPV2 | Bradi1g35585.1 | - | - | AK360693 |
| IVLNWEC01BMSTW | Bradi4g23695.1 | - | - | AK361159 |
| IV9RU5F01A9RWN | Bradi4g05570.1 | - | - | AK361994 |
| IV9RU5F02IFJOB | Bradi4g35250.2 | - | - | AK362289 |
| IV9RU5F02IP61T | Bradi1g06050.1 | - | - | AK363211 |
| IVLNWEC01DZET9 | Bradi3g34387.1 | - | - | AK364034 |
| IUIVLUD01C4VCS | Bradi1g20240.1 | - | - | AK364622 |
| IVLNWEC02G8HI6 | Bradi1g28847.1 | - | - | AK365190 |
| IVLNWEC01ELTB4 | Bradi3g43260.1 | - | - | AK365473 |
| IUIVLUD01EWPG6 | Bradi4g26990.1 | - | - | AK365802 |
| IV9RU5F01AG0YG | Bradi4g26990.1 | - | - | AK365802 |
| IV9RU5F01BMM9B | Bradi4g07267.1 | - | - | AK365985 |
| IV9RU5F01DN6MH | Bradi4g07267.1 | - | - | AK365985 |
| IUIVLUD02G5HZW | Bradi4g08130.1 | - | - | AK366062 |
| IVLNWEC01C6E2W | Bradi4g08490.1 | - | - | AK366742 |
| IV9RU5F01ENRDJ | Bradi5g01990.1 | - | - | AK366848 |
| IV9RU5F02G3FV6 | Bradi1g09340.3 | - | - | AK367045 |
| IV9RU5F01EF86T | Bradi2g26930.1 | - | - | AK367623 |
| IV9RU5F01BGS2E | Bradi1g00980.2 | - | - | AK367778 |
| IVLNWEC02HH22R | Bradi1g08060.1 | - | - | AK367927 |
| IV9RU5F01C8N6L | Bradi1g03277.1 | - | - | AK368276 |
| IVLNWEC02I4ZMB | Bradi4g36976.1 | - | - | AK368548 |
| IV9RU5F01EWVGI | Bradi4g23380.1 | - | - | AK368758 |
| IUIVLUD01A80F9 | Bradi4g33467.1 | - | - | AK368907 |
| IV9RU5F01C9JBC | Bradi4g33467.1 | - | - | AK368907 |
| IV9RU5F01D3YD7 | Bradi4g33467.1 | - | - | AK368907 |
| IUIVLUD01CIJKN | Bradi1g02170.1 | - | - | AK369519 |
| IV9RU5F01A3YA1 | Bradi2g09434.1 | - | - | AK369963 |
| IVLNWEC01CMQ9R | Bradi1g43260.1 | - | - | AK370018 |
| IVLNWEC01EIQLU | Bradi1g02800.1 | - | - | AK370148 |
| IV9RU5F01EQUWO | Bradi1g72086.1 | - | - | AK370665 |
| IV9RU5F02JACJP | Bradi1g37304.1 | - | - | AK370749 |
| IV9RU5F01BFX2G | Bradi2g11337.1 | - | - | AK370761 |
| IUIVLUD02IP1W9 | Bradi1g31350.1 | - | - | AK371221 |
| IV9RU5F02HU7LD | Bradi1g31350.1 | - | - | AK371221 |
| IV9RU5F01ETP52 | Bradi4g38980.1 | - | - | AK371791 |
| IVLNWEC01DP0YW | Bradi4g38980.1 | - | - | AK371791 |
| IVLNWEC01DJ72R | Bradi5g00440.1 | - | - | AK371992 |
| IUIVLUD02IFI9G | Bradi1g03010.1 | - | - | AK371998 |
| IVLNWEC01A03T1 | Bradi1g03010.1 | - | - | AK371998 |
| IVLNWEC02JAA4T | Bradi1g03010.1 | - | - | AK371998 |
| IVLNWEC01D4X6O | Bradi1g59970.1 | - | - | AK372747 |
| IV9RU5F01EB06E | Bradi1g29247.1 | - | - | AK373567 |
| IUIVLUD01DZ8YM | Bradi1g76520.1 | - | - | AK373775 |
| IUIVLUD01C4KN1 | Bradi2g49067.1 | - | - | AK373811 |

| | | | | |
|---|---|---|---|---|
| IVLNWEC02JNGQR | Bradi2g27097.1 | - | - | AK374248 |
| IVLNWEC02G8I14 | Bradi1g67210.1 | - | - | AK374520 |
| IV9RU5F02H3UFY | Bradi1g07027.1 | - | - | AK374530 |
| IV9RU5F01DXWSI | Bradi1g08727.1 | - | - | AK374936 |
| IVLNWEC02IUHTU | Bradi4g42957.1 | - | - | AK375623 |
| IUIVLUD01D8ISS | Bradi1g04527.1 | - | - | AK376547 |
| IUIVLUD01CXCLA | Bradi4g08597.1 | - | - | AK376689 |
| IUIVLUD02HJSZG | Bradi5g03840.1 | - | - | AK377006 |
| IVLNWEC02JUVOC | Bradi1g68510.2 | - | - | MLOC_10030.4 |
| IUIVLUD02H5W7H | Bradi4g28960.1 | - | - | MLOC_10144.1 |
| IV9RU5F01EFF43 | Bradi3g18300.1 | - | - | MLOC_10180.1 |
| IUIVLUD01AJJOJ | Bradi1g05200.1 | - | - | MLOC_10339.1 |
| IV9RU5F02JJS6G | Bradi4g02630.1 | - | - | MLOC_10438.1 |
| IVLNWEC01DZX30 | Bradi4g37120.1 | - | - | MLOC_10503.1 |
| IVLNWEC02I167D | Bradi3g27970.1 | - | - | MLOC_10621.1 |
| IUIVLUD01B8FR4 | Bradi2g51807.1 | - | - | MLOC_10643.2 |
| IUIVLUD02H0DW2 | Bradi2g51807.1 | - | - | MLOC_10643.2 |
| IV9RU5F02FJNVH | Bradi2g51807.1 | - | - | MLOC_10643.2 |
| IV9RU5F01A26GH | Bradi1g13960.1 | - | - | MLOC_10667.2 |
| IVLNWEC02GULKQ | Bradi4g11740.1 | - | - | MLOC_10793.6 |
| IVLNWEC01CBASB | Bradi1g63500.1 | - | - | MLOC_10915.1 |
| IUIVLUD01C3C8G | Bradi1g42967.1 | - | - | MLOC_10971.1 |
| IUIVLUD02J19U0 | Bradi1g42967.1 | - | - | MLOC_10971.1 |
| IV9RU5F01EVF8N | Bradi1g42967.1 | - | - | MLOC_10971.1 |
| IV9RU5F02JZUNQ | Bradi1g42967.1 | - | - | MLOC_10971.1 |
| IV9RU5F01ATIQQ | Bradi1g05380.1 | - | - | MLOC_10988.2 |
| IVLNWEC01DC3WT | Bradi4g08870.1 | - | - | MLOC_11707.1 |
| IVLNWEC01DDJ29 | Bradi4g08870.1 | - | - | MLOC_11707.1 |
| IV9RU5F01ASB4D | Bradi1g53020.1 | - | - | MLOC_11868.1 |
| IVLNWEC01AS1D2 | Bradi1g64420.1 | - | - | MLOC_12000.2 |
| IUIVLUD02IEY2M | Bradi4g26510.1 | - | - | MLOC_12394.1 |
| IV9RU5F01DW6FN | Bradi4g26510.1 | - | - | MLOC_12394.1 |
| IVLNWEC01BJP6H | Bradi1g68957.1 | - | - | MLOC_12936.1 |
| IVLNWEC02IVZJT | Bradi1g68957.1 | - | - | MLOC_12936.1 |
| IV9RU5F01CWFS9 | Bradi4g36680.1 | - | - | MLOC_13649.1 |
| IUIVLUD01ANLEC | Bradi1g05500.1 | - | - | MLOC_14311.2 |
| IVLNWEC02IBGDM | Bradi2g35680.1 | - | - | MLOC_14915.2 |
| IV9RU5F02J2GIP | Bradi2g51807.1 | - | - | MLOC_18121.2 |
| IVLNWEC01EYP6W | Bradi2g51807.1 | - | - | MLOC_18121.2 |
| IV9RU5F01BDTRW | Bradi1g29267.1 | - | - | MLOC_18226.2 |
| IV9RU5F02GJ15M | Bradi1g35960.1 | - | - | MLOC_18265.2 |
| IVLNWEC02F8LR3 | Bradi4g31090.1 | - | - | MLOC_25077.2 |
| IV9RU5F02GP5VX | Bradi3g10416.1 | - | - | MLOC_26193.1 |
| IV9RU5F01BYRAJ | Bradi1g10750.1 | - | - | MLOC_28634.1 |
| IV9RU5F01DCW0P | Bradi1g00227.1 | - | - | MLOC_29057.1 |
| IVLNWEC02IOMJ6 | Bradi3g37780.1 | - | - | MLOC_30125.1 |
| IV9RU5F01BZISB | Bradi4g17230.1 | - | - | MLOC_3110.1 |

| | | | | |
|---|---|---|---|---|
| IUIVLUD02G22N3 | Bradi1g59950.1 | - | - | MLOC_31206.1 |
| IUIVLUD01DZOZE | Bradi1g03827.1 | - | - | MLOC_34058.1 |
| IVLNWEC01CITH3 | Bradi4g43780.1 | - | - | MLOC_34755.2 |
| IVLNWEC01EQI1Z | Bradi1g13710.1 | - | - | MLOC_37399.1 |
| IUIVLUD02H2QEE | Bradi4g24410.1 | - | - | MLOC_37499.1 |
| IVLNWEC02JV1VI | Bradi4g24410.1 | - | - | MLOC_37499.1 |
| IV9RU5F02J2E9Z | Bradi1g04980.1 | - | - | MLOC_37586.1 |
| IVLNWEC02HW7BP | Bradi1g01800.1 | - | - | MLOC_38862.3 |
| IV9RU5F01EV0R9 | Bradi1g03600.1 | - | - | MLOC_38902.3 |
| IV9RU5F02H0N9I | Bradi1g04677.1 | - | - | MLOC_39053.2 |
| IVLNWEC01EKOZ7 | Bradi1g53690.1 | - | - | MLOC_39183.1 |
| IV9RU5F02FYS30 | Bradi1g70057.1 | - | - | MLOC_4101.1 |
| IUIVLUD02HTN2Q | Bradi1g43906.1 | - | - | MLOC_43923.3 |
| IUIVLUD02H0LP1 | Bradi1g02020.1 | - | - | MLOC_44001.3 |
| IVLNWEC01D8BGN | Bradi1g02020.1 | - | - | MLOC_44001.3 |
| IV9RU5F01CSCKB | Bradi3g32440.1 | - | - | MLOC_44099.1 |
| IV9RU5F01BGJGA | Bradi1g32520.1 | - | - | MLOC_44207.4 |
| IV9RU5F01BKW7A | Bradi1g67750.1 | - | - | MLOC_4458.1 |
| IV9RU5F02HEV54 | Bradi1g67750.1 | - | - | MLOC_4458.1 |
| IVLNWEC02HE7RG | Bradi4g33920.2 | - | - | MLOC_44877.1 |
| IUIVLUD02IVVMO | Bradi1g03670.1 | - | - | MLOC_4490.1 |
| IUIVLUD01DFUWA | Bradi4g13710.1 | - | - | MLOC_4685.6 |
| IVLNWEC02GVZIR | Bradi4g13710.1 | - | - | MLOC_4685.6 |
| IUIVLUD02G16UN | Bradi1g70980.1 | - | - | MLOC_47578.1 |
| IV9RU5F02FRXYQ | Bradi1g70980.1 | - | - | MLOC_47578.1 |
| IV9RU5F02JRYLL | Bradi1g70980.1 | - | - | MLOC_47578.1 |
| IVLNWEC01D54BI | Bradi1g47920.1 | - | - | MLOC_47634.1 |
| IVLNWEC01CP7EI | Bradi3g38310.1 | - | - | MLOC_4917.1 |
| IVLNWEC01D0QJ0 | Bradi3g38310.1 | - | - | MLOC_4917.1 |
| IVLNWEC02IV91E | Bradi3g38310.1 | - | - | MLOC_4917.1 |
| IUIVLUD01BCTYQ | Bradi1g00490.1 | - | - | MLOC_49327.6 |
| IVLNWEC01B8LQB | Bradi1g06960.1 | - | - | MLOC_4953.1 |
| IV9RU5F01CS2UH | Bradi4g02710.1 | - | - | MLOC_49625.2 |
| IUIVLUD01CM30L | Bradi1g01730.3 | - | - | MLOC_4983.1 |
| IV9RU5F01AFN4N | Bradi1g51000.2 | - | - | MLOC_5024.1 |
| IVLNWEC02JU610 | Bradi4g43690.1 | - | - | MLOC_50612.1 |
| IVLNWEC02I14X9 | Bradi3g18400.1 | - | - | MLOC_50726.1 |
| IVLNWEC02JTNTT | Bradi3g18400.1 | - | - | MLOC_50726.1 |
| IV9RU5F01DG34O | Bradi3g15410.1 | - | - | MLOC_51413.1 |
| IV9RU5F02IZQNG | Bradi1g09210.1 | - | - | MLOC_51599.3 |
| IV9RU5F01C8UN6 | Bradi1g13530.1 | - | - | MLOC_51763.4 |
| IUIVLUD01EMNY7 | Bradi4g02420.1 | - | - | MLOC_51985.1 |
| IV9RU5F02G5YIY | Bradi4g02420.1 | - | - | MLOC_51985.1 |
| IVLNWEC01DP3RJ | Bradi1g66040.1 | - | - | MLOC_52259.1 |
| IVLNWEC02F94GQ | Bradi3g22460.1 | - | - | MLOC_54069.1 |
| IUIVLUD01EJ731 | Bradi4g32850.1 | - | - | MLOC_54243.2 |
| IV9RU5F02GCT95 | Bradi4g04400.1 | - | - | MLOC_5444.3 |

| | | | | |
|---|---|---|---|---|
| IVLNWEC02GPRG4 | Bradi4g04400.1 | - | - | MLOC_5444.3 |
| IUIVLUD02HYKTE | Bradi1g02290.1 | - | - | MLOC_54841.1 |
| IUIVLUD01EQDFX | Bradi1g08430.1 | - | - | MLOC_54983.5 |
| IV9RU5F01BNJ2N | Bradi5g02420.1 | - | - | MLOC_54986.1 |
| IVLNWEC01D6ZWE | Bradi1g51507.1 | - | - | MLOC_55033.1 |
| IUIVLUD02GEHW7 | Bradi3g24417.1 | - | - | MLOC_55367.1 |
| IUIVLUD02II4OW | Bradi3g24417.1 | - | - | MLOC_55367.1 |
| IV9RU5F02HHM47 | Bradi3g24417.1 | - | - | MLOC_55367.1 |
| IV9RU5F02JFY5B | Bradi3g24417.1 | - | - | MLOC_55367.1 |
| IV9RU5F02JQU3P | Bradi3g24417.1 | - | - | MLOC_55367.1 |
| IV9RU5F01DXTE8 | Bradi1g00510.1 | - | - | MLOC_55857.2 |
| IVLNWEC02FL18P | Bradi4g45240.1 | - | - | MLOC_55862.2 |
| IVLNWEC02HLRKN | Bradi4g45240.1 | - | - | MLOC_55862.2 |
| IUIVLUD01CFBVF | Bradi3g06847.1 | - | - | MLOC_55923.3 |
| IVLNWEC02FOQ7Y | Bradi1g43570.1 | - | - | MLOC_55934.1 |
| IV9RU5F01EHJKP | Bradi3g21720.1 | - | - | MLOC_56410.1 |
| IV9RU5F01DQDE6 | Bradi1g14270.1 | - | - | MLOC_56602.1 |
| IVLNWEC01ATW4R | Bradi3g18250.1 | - | - | MLOC_56876.1 |
| IV9RU5F02FF5GX | Bradi1g64380.1 | - | - | MLOC_57538.2 |
| IVLNWEC02I6BYY | Bradi1g64380.1 | - | - | MLOC_57538.2 |
| IV9RU5F02FVDSK | Bradi3g00670.1 | - | - | MLOC_57715.1 |
| IV9RU5F02JHOR5 | Bradi1g10550.1 | - | - | MLOC_57872.1 |
| IUIVLUD02GM4ZG | Bradi5g10940.1 | - | - | MLOC_58269.1 |
| IVLNWEC01B5WKX | Bradi1g08510.1 | - | - | MLOC_59424.2 |
| IUIVLUD01BKHTG | Bradi3g14710.1 | - | - | MLOC_59480.2 |
| IV9RU5F01D4P9W | Bradi1g56695.1 | - | - | MLOC_59980.1 |
| IVLNWEC01AV29Y | Bradi1g02180.1 | - | - | MLOC_60056.5 |
| IV9RU5F02GR1NC | Bradi1g63330.1 | - | - | MLOC_60118.1 |
| IV9RU5F01CWZ2Y | Bradi2g37387.1 | - | - | MLOC_60460.3 |
| IV9RU5F01A283X | Bradi1g02240.1 | - | - | MLOC_60551.2 |
| IV9RU5F01EL48X | Bradi1g02240.1 | - | - | MLOC_60551.2 |
| IVLNWEC02GCN5J | Bradi4g34760.1 | - | - | MLOC_60694.3 |
| IVLNWEC01BFRW6 | Bradi4g34760.1 | - | - | MLOC_60695.1 |
| IUIVLUD02HJYR2 | Bradi4g42530.1 | - | - | MLOC_60742.2 |
| IVLNWEC01AXW8C | Bradi4g42530.1 | - | - | MLOC_60742.2 |
| IVLNWEC02HBFHN | Bradi1g09390.1 | - | - | MLOC_61341.1 |
| IUIVLUD01EGVTC | Bradi2g58930.1 | - | - | MLOC_61347.1 |
| IV9RU5F01EG7HV | Bradi2g58930.1 | - | - | MLOC_61347.1 |
| IUIVLUD01CYRY3 | Bradi4g31336.1 | - | - | MLOC_61654.1 |
| IVLNWEC01ECPBJ | Bradi4g31336.1 | - | - | MLOC_61654.1 |
| IVLNWEC01CB0JP | Bradi1g01620.1 | - | - | MLOC_61965.1 |
| IUIVLUD01C7S2B | Bradi1g53920.1 | - | - | MLOC_62284.1 |
| IVLNWEC01BSARC | Bradi1g53920.1 | - | - | MLOC_62284.1 |
| IVLNWEC01AV1R9 | Bradi2g23220.1 | - | - | MLOC_62417.3 |
| IV9RU5F01EFZ3L | Bradi3g34967.1 | - | - | MLOC_6270.1 |
| IVLNWEC01EASHV | Bradi3g34967.1 | - | - | MLOC_6270.1 |
| IV9RU5F02FR4VE | Bradi2g51807.1 | - | - | MLOC_62897.1 |

| | | | | |
|---|---|---|---|---|
| IVLNWEC02ILO7W | Bradi3g44197.1 | - | - | MLOC_63002.1 |
| IUIVLUD01BLFAH | Bradi1g38250.1 | - | - | MLOC_63550.2 |
| IV9RU5F02I2ZYF | Bradi3g20530.1 | - | - | MLOC_63968.2 |
| IUIVLUD01B41Y9 | Bradi1g08190.1 | - | - | MLOC_64192.1 |
| IUIVLUD02GAZ6J | Bradi2g09434.1 | - | - | MLOC_64708.1 |
| IV9RU5F02FTTI5 | Bradi1g19050.1 | - | - | MLOC_64812.2 |
| IVLNWEC01EK4A0 | Bradi1g32310.2 | - | - | MLOC_64997.1 |
| IVLNWEC01BF9MQ | Bradi4g33540.1 | - | - | MLOC_65370.3 |
| IV9RU5F02JM5OX | Bradi4g05370.1 | - | - | MLOC_65526.2 |
| IV9RU5F01DWODW | Bradi2g39320.1 | - | - | MLOC_65684.2 |
| IVLNWEC01A0PVK | Bradi4g04440.1 | - | - | MLOC_657.1 |
| IUIVLUD01EZMZA | Bradi3g10600.1 | - | - | MLOC_65804.3 |
| IVLNWEC01CEMHW | Bradi1g23350.1 | - | - | MLOC_65838.1 |
| IVLNWEC01D3G52 | Bradi1g23350.1 | - | - | MLOC_65838.1 |
| IVLNWEC01EQT80 | Bradi4g22180.1 | - | - | MLOC_65936.3 |
| IV9RU5F02G54P3 | Bradi4g38170.1 | - | - | MLOC_66453.1 |
| IV9RU5F02GLBEI | Bradi2g39330.1 | - | - | MLOC_66457.1 |
| IV9RU5F02HS1LE | Bradi3g44740.1 | - | - | MLOC_66803.2 |
| IVLNWEC01B87H3 | Bradi1g08847.1 | - | - | MLOC_66914.1 |
| IV9RU5F01DLSFL | Bradi4g28070.1 | - | - | MLOC_66998.1 |
| IV9RU5F01EWWBQ | Bradi4g28070.1 | - | - | MLOC_66998.1 |
| IUIVLUD01BHT3E | Bradi4g32420.1 | - | - | MLOC_67231.2 |
| IUIVLUD02IY1QG | Bradi1g38250.1 | - | - | MLOC_67318.1 |
| IV9RU5F01EI95U | Bradi3g27912.1 | - | - | MLOC_68408.1 |
| IUIVLUD01CTBBR | Bradi4g07110.1 | - | - | MLOC_68734.1 |
| IUIVLUD01EHAK8 | Bradi4g07110.1 | - | - | MLOC_68734.1 |
| IV9RU5F02FWIR1 | Bradi3g15935.1 | - | - | MLOC_68738.1 |
| IV9RU5F01EJPL2 | Bradi4g05950.1 | - | - | MLOC_6879.1 |
| IVLNWEC02GTF5D | Bradi1g01250.1 | - | - | MLOC_69418.1 |
| IV9RU5F01CZ4SS | Bradi2g37990.1 | - | - | MLOC_69663.1 |
| IVLNWEC01A09JV | Bradi1g09260.1 | - | - | MLOC_70664.2 |
| IVLNWEC02G5HGG | Bradi1g74170.1 | - | - | MLOC_7075.1 |
| IV9RU5F01DW1GR | Bradi1g08070.1 | - | - | MLOC_71287.2 |
| IVLNWEC01DGQM4 | Bradi4g42240.1 | - | - | MLOC_71746.1 |
| IUIVLUD01DE4LM | Bradi1g03170.1 | - | - | MLOC_71913.1 |
| IV9RU5F01AQIBW | Bradi1g07717.1 | - | - | MLOC_72276.1 |
| IVLNWEC01AER7R | Bradi4g13975.1 | - | - | MLOC_72572.1 |
| IVLNWEC01CL721 | Bradi4g09247.1 | - | - | MLOC_72805.2 |
| IVLNWEC01DH67L | Bradi4g08567.1 | - | - | MLOC_72899.1 |
| IV9RU5F01AGZBW | Bradi1g50890.1 | - | - | MLOC_73860.2 |
| IV9RU5F02IDX19 | Bradi1g50890.1 | - | - | MLOC_73860.2 |
| IV9RU5F01ALVQT | Bradi1g04080.1 | - | - | MLOC_73874.1 |
| IVLNWEC02JO9CV | Bradi1g51570.1 | - | - | MLOC_74285.1 |
| IVLNWEC01EJXKY | Bradi4g08380.1 | - | - | MLOC_74303.1 |
| IVLNWEC01CEIFX | Bradi4g32200.1 | - | - | MLOC_75452.1 |
| IV9RU5F01AI5QK | Bradi1g03330.1 | - | - | MLOC_75924.1 |
| IVLNWEC02F97UO | Bradi4g03070.1 | - | - | MLOC_76486.1 |

| | | | | |
|---|---|---|---|---|
| IVLNWEC01BVPVD | Bradi2g09434.1 | - | - | MLOC_76493.1 |
| IUIVLUD02IVN6O | Bradi1g08170.1 | - | - | MLOC_77757.1 |
| IVLNWEC01DY1VT | Bradi5g01167.1 | - | - | MLOC_77773.1 |
| IUIVLUD02IQ2RO | Bradi2g37990.1 | - | - | MLOC_77918.2 |
| IV9RU5F01CDXZH | Bradi4g02570.1 | - | - | MLOC_81003.8 |
| IVLNWEC01AH02R | Bradi1g74710.1 | - | - | MLOC_8296.1 |
| IUIVLUD01C3TNC | Bradi4g37260.1 | - | - | MLOC_9823.2 |
| IV9RU5F01CS85Y | Bradi3g01150.1 | - | - | MLOC_9988.2 |
| IVLNWEC01CTNKJ | Bradi3g01150.1 | - | - | MLOC_9988.2 |
| IV9RU5F02INXHI | Bradi4g09120.2 | - | - | MLOC_70817.1 |
| IVLNWEC02IQX1G | Bradi1g35960.1 | - | - | MLOC_18265.2 |
| IV9RU5F02F1W2K | Bradi1g53690.1 | - | - | MLOC_39183.1 |
| IV9RU5F01EK52R | Bradi1g42810.1 | - | - | AK373246 |
| IV9RU5F01BZ1JJ | Bradi4g42100.1 | - | - | MLOC_21993.1 |
| IV9RU5F01CIDO2 | Bradi1g31350.1 | - | - | AK371221 |
| IV9RU5F02G2L4F | Bradi1g28760.2 | - | - | MLOC_56278.2 |
| IV9RU5F02JDL7B | Bradi1g75330.1 | - | - | MLOC_12131.1 |
| IVLNWEC01AOKAQ | Bradi4g43680.2 | - | - | MLOC_73193.3 |
| IV9RU5F01D3YUT | Bradi1g08070.1 | - | - | MLOC_71287.2 |
| IVLNWEC02HUQUH | Bradi1g08070.1 | - | - | MLOC_71287.2 |
| IVLNWEC01AZI2K | Bradi4g07861.1 | - | - | AK371603 |
| IVLNWEC01DYFCT | Bradi4g11510.3 | - | - | AK369248 |
| IV9RU5F01EU9TN | Bradi4g21740.1 | - | - | MLOC_65122.3 |
| IVLNWEC01DJWYO | Bradi4g05817.1 | - | - | MLOC_50356.2 |
| IVLNWEC01C1BZY | Bradi3g47930.1 | - | - | MLOC_58168.1 |
| IVLNWEC02GIPKS | Bradi3g47930.1 | - | - | MLOC_58168.1 |
| IV9RU5F01ASTTM | Bradi1g68957.1 | - | - | MLOC_12936.1 |
| IV9RU5F02JHH39 | Bradi1g68957.1 | - | - | MLOC_12936.1 |
| IV9RU5F02JDSW2 | Bradi1g04080.1 | - | - | MLOC_73874.1 |
| IVLNWEC02GG5JP | Bradi1g05950.1 | - | - | AK361882 |
| IV9RU5F02F1C9L | Bradi3g19100.1 | - | - | AK252538.1 |
| IUIVLUD01CTWMB | Bradi1g67760.1 | - | - | MLOC_56418.3 |
| IVLNWEC02GD21M | Bradi1g09210.1 | - | - | MLOC_51599.3 |
| IVLNWEC01BCS6A | Bradi1g67750.1 | - | - | MLOC_4458.1 |
| IVLNWEC01B7YSE | Bradi1g31350.1 | - | - | AK371221 |
| IV9RU5F01A8KXQ | Bradi1g58410.1 | - | - | MLOC_62747.4 |
| IVLNWEC02JDRVD | Bradi1g51000.2 | - | - | MLOC_5024.1 |
| IV9RU5F01BPAEL | Bradi2g16960.1 | - | - | MLOC_53410.2 |
| IV9RU5F02HB185 | Bradi2g16960.1 | - | - | MLOC_53410.2 |
| IUIVLUD01CWH90 | Bradi1g61230.2 | - | - | MLOC_70283.1 |
| IV9RU5F02I1W5Q | Bradi1g61230.2 | - | - | MLOC_70283.1 |
| IUIVLUD01BBK7Z | Bradi4g04390.1 | - | - | MLOC_17197.3 |
| IV9RU5F01E1P4E | Bradi4g04390.1 | - | - | MLOC_17197.3 |
| IVLNWEC01EOC9I | Bradi1g00750.1 | - | - | AK251756.1 |
| IVLNWEC02HU9C0 | Bradi4g02570.1 | - | - | MLOC_81003.8 |
| IUIVLUD02IPTBA | Bradi4g23700.1 | - | - | AK362688 |
| IV9RU5F01B8MJ9 | Bradi4g02950.1 | - | - | MLOC_54060.1 |

| | | | | |
|---|---|---|---|---|
| IVLNWEC02F8TCI | Bradi1g02180.1 | - | - | MLOC_60056.5 |
| IV9RU5F01EUOVQ | Bradi4g38140.1 | - | - | MLOC_21732.1 |
| IV9RU5F01CWINP | Bradi1g72160.1 | - | - | MLOC_65664.1 |
| IVLNWEC02JZEFE | Bradi1g72160.1 | - | - | MLOC_65664.1 |
| IUIVLUD01D8ZC2 | Bradi1g74997.1 | - | - | MLOC_57109.1 |
| IVLNWEC01D8PNN | Bradi4g02420.1 | - | - | MLOC_51985.1 |
| IV9RU5F01D14RS | Bradi4g45227.1 | - | - | MLOC_48965.1 |
| IVLNWEC02H7E2V | Bradi1g13450.1 | - | - | AK251104.1 |
| IVLNWEC01C165K | Bradi1g08400.1 | - | - | MLOC_824.2 |
| IV9RU5F02IALGK | Bradi4g22180.1 | - | - | MLOC_65936.3 |
| IUIVLUD02JF141 | Bradi4g03720.1 | - | - | MLOC_54490.8 |
| IVLNWEC02G9CD3 | Bradi1g74480.1 | - | - | MLOC_59190.1 |
| IV9RU5F02I35CJ | Bradi1g66167.1 | - | - | MLOC_59780.1 |
| IV9RU5F01A1HPQ | Bradi4g38387.1 | - | - | MLOC_81592.1 |
| IVLNWEC01A1HOS | Bradi1g62640.1 | - | - | MLOC_66033.2 |
| IV9RU5F02HHD25 | Bradi1g68510.2 | - | - | MLOC_10030.4 |
| IVLNWEC01EBFSC | Bradi1g68510.2 | - | - | MLOC_10030.4 |
| IV9RU5F01DMV3N | Bradi4g02725.1 | - | - | AK358630 |
| IUIVLUD02HTUY0 | Bradi2g14340.2 | - | - | MLOC_37459.1 |
| IV9RU5F02IMXG5 | Bradi1g15110.1 | - | - | MLOC_55271.1 |
| IVLNWEC01A165S | Bradi4g03690.1 | - | - | MLOC_59257.1 |
| IVLNWEC01DQY17 | Bradi1g13710.1 | - | - | MLOC_37399.1 |
| IV9RU5F01A6IE2 | Bradi3g24417.1 | - | - | MLOC_55367.1 |
| IVLNWEC01BGERA | Bradi4g04280.1 | - | - | MLOC_10781.1 |
| IVLNWEC02H0OCS | Bradi2g22927.1 | - | - | MLOC_69831.1 |
| IUIVLUD02GTKR1 | Bradi1g78470.1 | - | - | MLOC_36655.1 |
| IVLNWEC01AQC13 | Bradi1g78470.1 | - | - | MLOC_36655.1 |
| IVLNWEC01DOCID | Bradi1g62000.1 | - | - | MLOC_63567.1 |
| IUIVLUD02GLU4X | Bradi3g24417.1 | - | - | MLOC_55367.1 |
| IV9RU5F01EQJP6 | Bradi4g35162.1 | - | - | MLOC_19573.5 |
| IV9RU5F01DN9ZS | Bradi4g43490.5 | - | - | AK251443.1 |
| IV9RU5F01DCQPO | Bradi2g14620.1 | - | - | MLOC_62097.2 |
| IUIVLUD01B001R | Bradi5g20420.1 | - | - | MLOC_55154.1 |
| IUIVLUD01CLWM3 | Bradi5g20420.1 | - | - | MLOC_55154.1 |
| IVLNWEC01CSRDC | Bradi5g20420.1 | - | - | MLOC_55154.1 |
| IV9RU5F01EGJTK | Bradi1g03160.1 | - | - | AK365917 |
| IUIVLUD02GETVT | Bradi4g07267.1 | - | - | AK365985 |
| IV9RU5F01APFBX | Bradi4g07267.1 | - | - | AK365985 |
| IV9RU5F02G6EHY | Bradi4g33467.1 | - | - | AK368907 |
| IV9RU5F01EPKKJ | Bradi3g48257.1 | - | - | MLOC_9802.1 |
| IV9RU5F02JZ1CY | Bradi4g01230.2 | - | - | MLOC_70613.1 |
| IV9RU5F01B5GP8 | Bradi5g02170.1 | - | - | AK249518.1 |
| IV9RU5F02JV0Q1 | Bradi1g03600.1 | - | - | MLOC_38902.3 |
| IVLNWEC02G7M3G | Bradi2g19010.1 | - | - | MLOC_72837.2 |
| IUIVLUD01ALD3R | Bradi4g35557.1 | - | - | MLOC_69413.1 |
| IVLNWEC02IT2BT | Bradi4g26870.1 | - | - | AK355004 |
| IVLNWEC02IVR13 | Bradi4g26870.1 | - | - | AK355004 |

| | | | | |
|---|---|---|---|---|
| IVLNWEC01DLEJJ | Bradi4g30580.1 | - | - | MLOC_63433.1 |
| IUIVLUD01A5J7P | Bradi1g01007.1 | - | - | MLOC_49689.2 |
| IV9RU5F02I6HQJ | Bradi4g04560.1 | - | - | MLOC_68577.1 |
| IVLNWEC01C5822 | Bradi1g03010.2 | - | - | AK371998 |
| IVLNWEC01A88DL | Bradi4g42337.1 | - | - | MLOC_5857.3 |
| IVLNWEC01BI9SV | Bradi2g26680.1 | - | - | MLOC_10169.2 |
| IV9RU5F02JE0SE | Bradi4g26210.3 | - | - | MLOC_57851.1 |
| IVLNWEC02H6SAV | Bradi1g03170.1 | - | - | MLOC_71913.1 |
| IVLNWEC01DLQQF | Bradi4g16885.1 | - | - | AK362199 |
| IVLNWEC02ICFYG | Bradi1g74437.1 | - | - | MLOC_59792.1 |
| IVLNWEC01CBA6T | Bradi3g30010.1 | - | - | MLOC_45140.1 |
| IV9RU5F02FPQ6F | Bradi2g25260.1 | - | - | MLOC_57607.1 |
| IV9RU5F02GZY2G | Bradi4g44320.1 | - | - | MLOC_65070.2 |
| IV9RU5F02GGO37 | Bradi1g01560.2 | - | - | AK252411.1 |
| IV9RU5F02IV3I7 | Bradi4g06000.2 | - | - | MLOC_80410.1 |
| IUIVLUD02H4VER | Bradi1g05480.1 | - | - | AK360603 |
| IV9RU5F01C3QVZ | Bradi4g34650.1 | - | - | MLOC_12052.2 |
| IUIVLUD01ALF5Q | Bradi4g04700.1 | - | - | MLOC_74300.1 |
| IVLNWEC01CW55I | Bradi4g04700.1 | - | - | MLOC_74300.1 |
| IV9RU5F02HZX1U | Bradi2g27400.1 | - | - | MLOC_57041.2 |
| IV9RU5F01CUK09 | Bradi1g07960.1 | - | - | MLOC_72552.2 |
| IV9RU5F02GA31T | Bradi2g08080.1 | - | - | AK354365 |
| IVLNWEC01DXY30 | Bradi2g08080.1 | - | - | AK354365 |
| IUIVLUD01A74P1 | Bradi2g16237.1 | - | - | AK354470 |
| IVLNWEC02IB8EY | Bradi3g23290.1 | - | - | AK252293.1 |
| IV9RU5F01B64E3 | Bradi1g01847.1 | - | - | MLOC_4674.2 |
| IVLNWEC02JMDZG | Bradi2g09434.1 | - | - | MLOC_76493.1 |
| IV9RU5F01CUMR1 | Bradi5g00430.1 | - | - | MLOC_57244.2 |
| IUIVLUD02H4DFS | Bradi1g02800.1 | - | - | AK370148 |
| IVLNWEC01D42LD | Bradi1g02800.1 | - | - | AK370148 |
| IV9RU5F01EYTI8 | Bradi4g29617.1 | - | - | AK373293 |
| IV9RU5F01C8TMZ | Bradi1g72092.1 | - | - | MLOC_60439.1 |
| IVLNWEC01A36I1 | Bradi1g72092.1 | - | - | MLOC_60439.1 |
| IVLNWEC01BSBUD | Bradi4g07430.1 | - | - | MLOC_5095.1 |
| IV9RU5F02GMSE5 | Bradi1g67387.1 | - | - | MLOC_13606.1 |
| IVLNWEC02HTP99 | Bradi1g35630.1 | - | - | MLOC_74748.1 |
| IVLNWEC02ICMIR | Bradi1g35630.1 | - | - | MLOC_74748.1 |
| IVLNWEC01ENQY1 | Bradi4g25047.1 | - | - | AK371325 |
| IUIVLUD02J2Q4W | Bradi2g25745.1 | - | - | AK361560 |
| IV9RU5F01A3U1M | Bradi2g25745.1 | - | - | AK361560 |
| IV9RU5F02JJ39R | Bradi2g25745.1 | - | - | AK361560 |
| IVLNWEC01ESSUQ | Bradi2g25745.1 | - | - | AK361560 |
| IUIVLUD02HBFQM | Bradi1g63160.1 | - | - | AK364634 |
| IV9RU5F01EH9T2 | Bradi1g23350.1 | - | - | MLOC_65838.1 |
| IVLNWEC02G2TUZ | Bradi1g07717.1 | - | - | MLOC_19181.1 |
| IV9RU5F02I8JQR | Bradi4g29947.1 | - | - | MLOC_68788.2 |
| IUIVLUD02GRL7O | Bradi4g04400.1 | - | - | MLOC_5444.3 |

| | | | | |
|---|---|---|---|---|
| IV9RU5F02HEMFC | Bradi4g04400.1 | - | - | MLOC_5444.3 |
| IVLNWEC02JBBT8 | Bradi1g10447.1 | - | - | MLOC_13554.5 |
| IVLNWEC01EGGVN | Bradi4g08870.1 | - | - | MLOC_11707.1 |
| IV9RU5F02HAMMQ | Bradi1g06766.1 | - | - | MLOC_51163.2 |
| IV9RU5F02F90OF | Bradi1g06945.1 | - | - | MLOC_17956.6 |
| IV9RU5F02JKHMR | Bradi3g01150.1 | - | - | MLOC_9988.2 |
| IV9RU5F01C9OK8 | Bradi3g43630.1 | - | - | MLOC_66893.1 |
| IVLNWEC01CPVIN | Bradi3g43630.1 | - | - | MLOC_66893.1 |
| IVLNWEC01DR2RB | Bradi3g18970.1 | - | - | AK376028 |
| IV9RU5F01CNXAA | Bradi1g02610.1 | - | - | AK374311 |
| IV9RU5F01BQGJA | Bradi4g26627.1 | - | - | MLOC_81426.2 |
| IVLNWEC01BHRKA | Bradi4g06087.1 | - | - | AK354495 |
| IVLNWEC01CUUR9 | Bradi1g13770.1 | - | - | AK356347 |
| IVLNWEC01AY9HY | Bradi4g07760.1 | - | - | AK361911 |
| IV9RU5F02FN3WA | Bradi1g03420.1 | - | - | AK359113 |
| IVLNWEC01BIM1X | Bradi2g54390.1 | - | - | MLOC_26628.1 |
| IUIVLUD01CCAQK | Bradi4g06390.1 | - | - | MLOC_73857.1 |
| IV9RU5F01AZXQ2 | Bradi1g64380.1 | - | - | MLOC_57538.2 |
| IV9RU5F02HHDLM | Bradi1g64380.1 | - | - | MLOC_57538.2 |
| IV9RU5F01AK5FX | Bradi1g55250.1 | - | - | MLOC_62977.1 |
| IV9RU5F01B8O7K | Bradi4g41970.2 | - | - | MLOC_63606.1 |
| IV9RU5F02GK2W1 | Bradi4g41970.2 | - | - | MLOC_63606.1 |
| IVLNWEC02FTOZ4 | Bradi4g31680.1 | - | - | MLOC_12614.1 |
| IV9RU5F02J2GSC | Bradi1g67050.2 | - | - | MLOC_4483.1 |
| IV9RU5F02HHRAY | Bradi4g40450.2 | - | - | MLOC_21002.1 |
| IVLNWEC02JHECD | Bradi4g40450.2 | - | - | MLOC_21002.1 |
| IVLNWEC02FY13V | Bradi4g02440.1 | - | - | MLOC_56719.2 |
| IVLNWEC01D8LHW | Bradi5g22790.1 | - | - | MLOC_52352.2 |
| IV9RU5F02FSTZ9 | Bradi1g13910.1 | - | - | AK364215 |
| IV9RU5F01ERSXT | Bradi2g01550.1 | - | - | MLOC_59709.1 |
| IUIVLUD01BU3QF | Bradi1g06310.1 | - | - | MLOC_55774.1 |
| IV9RU5F02I7NPX | Bradi1g06310.1 | - | - | MLOC_55774.1 |
| IV9RU5F01E262K | Bradi2g62760.1 | - | - | MLOC_55536.1 |
| IVLNWEC01EPJKL | Bradi3g58300.1 | - | - | MLOC_52021.2 |
| IV9RU5F02ILPRE | Bradi1g13710.1 | - | - | AK375108 |
| IVLNWEC02HG0GG | Bradi1g13710.1 | - | - | AK375108 |
| IUIVLUD01CEO87 | Bradi4g32530.1 | - | - | MLOC_1497.2 |
| IV9RU5F01C85UM | Bradi1g75250.1 | - | - | MLOC_75859.1 |
| IV9RU5F02IN2FT | Bradi1g75250.1 | - | - | MLOC_75859.1 |
| IVLNWEC01CWB6B | Bradi2g28900.1 | - | - | MLOC_1154.2 |
| IV9RU5F01A56MQ | Bradi1g74430.1 | - | - | MLOC_44284.2 |
| IV9RU5F02HQVZE | Bradi1g15800.1 | - | - | AK360494 |
| IVLNWEC01B8GLU | Bradi1g15800.1 | - | - | AK360494 |
| IVLNWEC01A8RWY | Bradi1g51757.1 | - | - | MLOC_54721.2 |
| IVLNWEC01BTPYP | Bradi3g31487.1 | - | - | MLOC_32790.2 |
| IVLNWEC01DCW81 | Bradi3g31487.1 | - | - | MLOC_32790.2 |
| IVLNWEC02HMDYK | Bradi4g33467.1 | - | - | AK368907 |

| | | | | |
|---|---|---|---|---|
| IV9RU5F01DC5SI | Bradi4g37780.1 | - | - | AK374953 |
| IVLNWEC02HR1LZ | Bradi4g36680.1 | - | - | MLOC_13649.1 |
| IVLNWEC01EKYKU | Bradi1g06060.1 | - | - | AK358687 |
| IVLNWEC02HVT82 | Bradi1g36067.1 | - | - | MLOC_65351.2 |
| IV9RU5F01C9WDT | Bradi3g45107.1 | - | - | MLOC_78400.1 |
| IV9RU5F02JHES1 | Bradi4g24410.1 | - | - | MLOC_37499.1 |
| IV9RU5F01AW3BC | Bradi4g43137.1 | - | - | AK373675 |
| IV9RU5F02G91VM | Bradi4g05570.1 | - | - | AK361994 |
| IUIVLUD02GM8O8 | Bradi4g12200.1 | - | - | MLOC_74552.2 |
| IVLNWEC01DYY8O | Bradi4g12200.1 | - | - | MLOC_74552.2 |
| IUIVLUD01BZQG5 | Bradi4g23520.1 | - | - | MLOC_54710.1 |
| IVLNWEC01A2SLD | Bradi1g53085.1 | - | - | MLOC_79808.1 |
| IVLNWEC02G55XT | Bradi1g01970.1 | - | - | AK357261 |
| IV9RU5F01BS65G | Bradi3g27912.1 | - | - | MLOC_68408.1 |
| IVLNWEC02FY3RL | Bradi4g35250.2 | - | - | AK362289 |
| IVLNWEC02HLBX3 | Bradi4g35250.2 | - | - | AK362289 |
| IUIVLUD01C2IPV | Bradi1g05200.1 | - | - | MLOC_10339.1 |
| IVLNWEC01D0QKF | Bradi1g62320.1 | - | - | MLOC_52203.1 |
| IVLNWEC02I2W6R | Bradi1g09390.1 | - | - | MLOC_61341.1 |
| IV9RU5F02J4A4I | Bradi1g00760.1 | - | - | MLOC_69405.3 |
| IVLNWEC02II31M | Bradi1g66130.1 | - | - | MLOC_33094.1 |
| IVLNWEC02IAP8G | Bradi3g43260.1 | - | - | AK365473 |
| IV9RU5F01EJAGJ | Bradi3g33190.1 | - | - | MLOC_65819.2 |
| IV9RU5F01BTFQZ | Bradi1g64210.1 | - | - | MLOC_53997.2 |
| IUIVLUD01EX3GD | Bradi3g34967.1 | - | - | MLOC_6270.1 |
| IUIVLUD02IT7WD | Bradi1g74800.1 | - | - | MLOC_51140.2 |
| IV9RU5F01AK7C0 | Bradi2g39330.1 | - | - | MLOC_66457.1 |
| IVLNWEC02JJAEP | Bradi4g17960.1 | - | - | MLOC_3482.3 |
| IV9RU5F01EWCQK | Bradi4g17230.1 | - | - | MLOC_3110.1 |
| IVLNWEC01DLJK6 | Bradi1g09260.1 | - | - | MLOC_70664.2 |
| IVLNWEC01DP6AX | Bradi1g09260.1 | - | - | MLOC_70664.2 |
| IVLNWEC01EZYON | Bradi4g33467.1 | - | - | AK368907 |
| IVLNWEC01BK8IJ | Bradi1g49790.1 | - | - | MLOC_69412.1 |
| IVLNWEC01EIJ34 | Bradi2g34450.1 | - | - | MLOC_10762.1 |
| IVLNWEC02FR944 | Bradi2g10180.1 | - | - | MLOC_6843.2 |
| IVLNWEC01BK0C1 | Bradi2g51807.1 | - | - | MLOC_18121.2 |
| IVLNWEC01DJM2X | Bradi2g23610.1 | - | - | MLOC_65120.2 |
| IVLNWEC01B7PJ5 | Bradi1g56695.1 | - | - | MLOC_59980.1 |
| IV9RU5F02G9R07 | Bradi3g27906.1 | - | - | MLOC_37545.1 |
| IUIVLUD01EUQ0Y | Bradi2g36210.1 | - | - | MLOC_61578.2 |
| IUIVLUD01BDFLX | Bradi2g42890.1 | - | - | AK248596.1 |
| IVLNWEC02HU59Q | Bradi1g38227.1 | - | - | MLOC_10482.5 |
| IV9RU5F02JTR5Z | Bradi1g70980.1 | - | - | MLOC_47578.1 |
| IUIVLUD01BMB5Z | Bradi1g14970.1 | - | - | MLOC_63226.1 |
| IVLNWEC02J22LM | Bradi1g09390.1 | - | - | MLOC_61341.1 |
| IV9RU5F01ENUFW | Bradi4g24190.1 | - | - | AK251570.1 |
| IUIVLUD01EADYJ | Bradi2g51807.1 | - | - | MLOC_6393.2 |

| | | | | |
|---|---|---|---|---|
| IUIVLUD01EQB8R | Bradi2g62760.1 | - | - | MLOC_55536.1 |
| IUIVLUD02GA3VQ | Bradi4g12940.1 | - | - | MLOC_5462.2 |
| IUIVLUD02IZDUT | Bradi1g51097.1 | - | - | AK252642.1 |
| IVLNWEC02I6MCR | Bradi1g13060.1 | - | - | MLOC_39318.1 |
| IUIVLUD01CA2HR | Bradi1g02240.1 | - | - | MLOC_60551.2 |
| IUIVLUD01C21VO | Bradi1g09530.1 | - | - | AK353866 |
| IUIVLUD02GYF4N | Bradi4g13710.1 | - | - | MLOC_4685.6 |
| IVLNWEC02HQ2RI | Bradi4g13710.1 | - | - | MLOC_4685.6 |
| IV9RU5F01EUK09 | Bradi2g51807.1 | - | - | MLOC_10643.2 |
| IVLNWEC01C9NSN | Bradi2g51807.1 | - | - | MLOC_10643.2 |
| IVLNWEC02HIW8Y | Bradi1g00607.1 | - | - | MLOC_52235.1 |
| IV9RU5F01DAPR6 | Bradi1g00607.1 | - | - | MLOC_72134.2 |
| IVLNWEC02F98CD | Bradi1g00607.1 | - | - | MLOC_72134.2 |
| IVLNWEC02JKSG9 | Bradi1g63870.1 | - | - | MLOC_30704.1 |
| IUIVLUD02GHLEG | Bradi1g65010.1 | - | - | MLOC_58282.1 |
| IV9RU5F01D1GEG | Bradi1g05610.1 | - | - | AK357244 |
| IV9RU5F01BA3Z7 | Bradi1g09260.1 | - | - | MLOC_70664.2 |
| IUIVLUD01C9UBG | Bradi3g21720.1 | - | - | MLOC_56410.1 |
| IVLNWEC02HPZXG | Bradi3g35840.1 | - | - | AK372604 |
| IUIVLUD02HW44Y | Bradi1g13770.1 | - | - | AK356347 |
| IUIVLUD02I1LNV | Bradi1g02800.1 | - | - | AK370148 |
| IV9RU5F01AT2AH | Bradi4g13690.1 | - | - | AK354110 |
| IV9RU5F01B7FGH | Bradi4g29310.3 | - | - | MLOC_67515.1 |
| IV9RU5F02GGU5H | Bradi4g37260.1 | - | - | MLOC_9823.2 |
| IVLNWEC01EOPHX | Bradi1g43500.1 | - | - | MLOC_58047.2 |
| IVLNWEC01D52UZ | Bradi4g31450.1 | - | - | AK249378.1 |
| IVLNWEC02HRCHT | Bradi1g06880.1 | - | - | AK249378.1 |
| IUIVLUD02FZBLI | Bradi1g08145.1 | - | - | MLOC_57500.1 |
| IVLNWEC02GNBNE | Bradi3g00670.1 | - | - | MLOC_57715.1 |
| IVLNWEC02IPAV8 | Bradi4g29070.1 | - | - | MLOC_15248.2 |
| IUIVLUD01BR63H | Bradi5g25870.1 | - | - | MLOC_69382.6 |
| IUIVLUD01BXSHO | Bradi4g31400.1 | - | - | MLOC_13783.2 |
| IV9RU5F01DJU1A | Bradi4g36077.1 | - | - | MLOC_57021.1 |
| IV9RU5F02GMQVF | Bradi4g31750.1 | - | - | MLOC_13048.8 |
| IV9RU5F02GYIT9 | Bradi4g31750.1 | - | - | MLOC_13048.8 |
| IV9RU5F02ISP8J | Bradi1g20240.1 | - | - | MLOC_55008.1 |
| IVLNWEC02HJAOP | Bradi5g00430.1 | - | - | MLOC_57244.2 |
| IVLNWEC01DYX2E | Bradi4g26990.1 | - | - | AK365802 |
| IV9RU5F02IGFGM | Bradi4g33780.1 | - | - | MLOC_60855.1 |
| IUIVLUD01CX9HZ | Bradi4g12770.1 | - | - | MLOC_64296.4 |
| IV9RU5F02I8DFG | Bradi4g36810.1 | - | - | MLOC_60174.1 |
| IV9RU5F02HCKK7 | Bradi2g62760.1 | - | - | MLOC_55536.1 |
| IUIVLUD02GC4QF | Bradi3g38310.1 | - | - | MLOC_4917.1 |
| IVLNWEC01DESKB | Bradi3g38310.1 | - | - | MLOC_4917.1 |
| IVLNWEC01EAV8F | Bradi3g38310.1 | - | - | MLOC_4917.1 |
| IUIVLUD01EOYDH | Bradi5g12976.1 | - | - | MLOC_54883.1 |
| IV9RU5F01DBVB5 | Bradi3g18250.1 | - | - | MLOC_56876.1 |

| | | | | |
|---|---|---|---|---|
| IV9RU5F01CPTN5 | Bradi4g44890.1 | - | - | AK368828 |
| IV9RU5F01D2BMZ | Bradi1g02980.1 | - | - | MLOC_63062.2 |
| IVLNWEC02HXJB3 | Bradi4g35977.1 | - | - | MLOC_4008.2 |
| IVLNWEC02ILF2A | Bradi4g31990.1 | - | - | MLOC_9952.1 |
| IVLNWEC02H5KZZ | Bradi4g43130.1 | - | - | MLOC_69804.2 |
| IVLNWEC01DVOE7 | Bradi1g08190.1 | - | - | MLOC_64192.1 |
| IV9RU5F02GX391 | Bradi1g06945.1 | - | - | MLOC_17956.6 |
| IUIVLUD01C30YH | Bradi2g61830.1 | - | - | AK356300 |
| IVLNWEC02HUOGQ | Bradi1g05960.1 | - | - | MLOC_14677.1 |
| IVLNWEC02G55BV | Bradi1g30860.1 | - | - | MLOC_14578.3 |
| IVLNWEC02GGIWI | Bradi1g30860.1 | - | - | MLOC_14578.3 |
| IVLNWEC01C092Q | Bradi4g21740.1 | - | - | MLOC_65122.3 |
| IV9RU5F02ITB0L | Bradi4g34022.1 | - | - | MLOC_11138.1 |
| IVLNWEC02GEW3J | Bradi4g28900.1 | - | - | AK371492 |
| IV9RU5F01ANWUM | Bradi4g06400.1 | - | - | MLOC_18166.1 |
| IV9RU5F02F9CQU | Bradi4g06400.1 | - | - | MLOC_18166.1 |
| IVLNWEC02HUB38 | Bradi4g03070.1 | - | - | MLOC_76486.1 |
| IV9RU5F02JEVFB | Bradi4g02117.1 | - | - | MLOC_52525.1 |
| IV9RU5F02JZCLL | Bradi1g21860.1 | - | - | MLOC_63937.1 |
| IV9RU5F02IDRV4 | Bradi4g03050.1 | - | - | MLOC_75843.3 |
| IVLNWEC02F8YRR | Bradi4g03050.1 | - | - | MLOC_75843.3 |
| IV9RU5F02IT9T9 | Bradi4g03690.1 | - | - | MLOC_59257.1 |
| IVLNWEC02HJ9CG | Bradi4g03390.1 | - | - | AK369809 |
| IVLNWEC02GR0FP | Bradi4g07610.1 | - | - | AK370873 |
| IVLNWEC02HU52U | Bradi1g07930.1 | - | - | MLOC_67514.1 |
| IUIVLUD02JAJPK | Bradi4g06137.1 | - | - | AK361476 |
| IV9RU5F02GM3QV | Bradi1g08140.1 | - | - | MLOC_63805.2 |
| IVLNWEC02G3KCO | Bradi1g08140.1 | - | - | MLOC_63805.2 |
| IV9RU5F02HRUVI | Bradi3g24417.1 | - | - | MLOC_55367.1 |
| IUIVLUD01CUFQM | Bradi1g78750.1 | - | - | MLOC_37495.1 |
| IV9RU5F02GALVU | Bradi1g51950.1 | - | - | MLOC_4344.2 |
| IV9RU5F01A1SAI | Bradi2g20250.1 | - | - | MLOC_22624.2 |
| IV9RU5F02G3M2G | Bradi4g34760.1 | - | - | MLOC_60694.3 |
| IUIVLUD01AHJN6 | Bradi4g33780.1 | - | - | MLOC_60855.1 |
| IV9RU5F01ENY7U | Bradi4g35730.1 | - | - | MLOC_6781.1 |
| IVLNWEC01D9H1B | Bradi1g43570.1 | - | - | MLOC_55934.1 |
| IV9RU5F01BM35A | Bradi4g05817.1 | - | - | MLOC_50356.2 |
| IUIVLUD01EYURP | Bradi4g42880.1 | - | - | AK356045 |
| IV9RU5F01EVQ82 | Bradi4g08700.1 | - | - | MLOC_58205.1 |
| IVLNWEC01EFKMX | Bradi1g68957.1 | - | - | MLOC_12936.1 |
| IV9RU5F02JSTSY | Bradi4g30200.2 | - | - | MLOC_5225.1 |
| IVLNWEC01CYJ49 | Bradi1g46360.1 | - | - | AK370995 |
| IVLNWEC01CF3CT | Bradi3g10416.1 | - | - | MLOC_58126.1 |
| IV9RU5F01BO4Y4 | Bradi1g01936.1 | - | - | AK373596 |
| IV9RU5F02HXMD5 | Bradi4g13690.1 | - | - | AK354110 |
| IUIVLUD01EUNR9 | Bradi1g05200.1 | - | - | MLOC_10339.1 |
| IV9RU5F02HRZU7 | Bradi2g09427.1 | - | - | MLOC_64709.2 |

| | | | | |
|---|---|---|---|---|
| IVLNWEC02I2OVB | Bradi4g28960.1 | - | - | MLOC_10144.1 |
| IUIVLUD02GKC8S | Bradi3g27750.1 | - | - | MLOC_10199.2 |
| IV9RU5F01ALMI0 | Bradi1g04540.1 | - | - | MLOC_62782.5 |
| IV9RU5F01C6R5F | Bradi1g62770.1 | - | - | AK368525 |
| IUIVLUD01C7PUK | Bradi1g72160.1 | - | - | MLOC_65664.1 |
| IV9RU5F01B1Y5D | Bradi4g20717.1 | - | - | MLOC_13610.1 |
| IVLNWEC02H6RO7 | Bradi4g04470.1 | - | - | AK370509 |
| IVLNWEC01BZKS0 | Bradi1g72160.1 | - | - | MLOC_65664.1 |
| IV9RU5F01CF5VK | Bradi3g33190.1 | - | - | MLOC_65819.2 |
| IVLNWEC02HZF5X | Bradi1g58030.1 | - | - | MLOC_57767.2 |
| IVLNWEC02GE71H | Bradi1g69300.1 | - | - | AK248532.1 |
| IUIVLUD01B0JW7 | Bradi1g74480.1 | - | - | MLOC_59190.1 |
| IUIVLUD02GFDBT | Bradi4g02020.1 | - | - | MLOC_54218.3 |
| IVLNWEC01CC1ET | Bradi4g02020.1 | - | - | MLOC_54218.3 |
| IVLNWEC01AZKSW | Bradi4g20717.1 | - | - | MLOC_13610.1 |
| IV9RU5F02JK60N | Bradi4g12940.1 | - | - | MLOC_5462.2 |
| IV9RU5F01BPN6V | Bradi4g43670.1 | - | - | MLOC_74044.2 |
| IV9RU5F02HUZJM | Bradi4g43670.1 | - | - | MLOC_74044.2 |
| IUIVLUD02I168A | Bradi1g62277.1 | - | - | MLOC_60035.1 |
| IVLNWEC01EIVYL | Bradi1g68510.2 | - | - | MLOC_10030.4 |
| IV9RU5F01C9GLV | Bradi1g51807.1 | - | - | MLOC_64607.5 |
| IVLNWEC01CJHHJ | Bradi3g43340.1 | - | - | MLOC_8921.1 |
| IUIVLUD02HW9FX | Bradi3g33240.1 | - | - | MLOC_51141.6 |
| IVLNWEC01CGMBI | Bradi3g33240.1 | - | - | MLOC_51141.6 |
| IVLNWEC02HL4TI | Bradi3g33240.1 | - | - | MLOC_51141.6 |
| IVLNWEC01CV4BY | Bradi1g08977.1 | - | - | MLOC_4611.1 |
| IVLNWEC02H9SKO | Bradi4g07717.1 | - | - | MLOC_19401.1 |
| IV9RU5F02H7KPW | Bradi4g16492.1 | - | - | MLOC_6032.2 |
| IV9RU5F02IF60L | Bradi4g16492.1 | - | - | MLOC_6032.2 |
| IV9RU5F02IVVJD | Bradi4g16492.1 | - | - | MLOC_6032.2 |
| IVLNWEC01DOX89 | Bradi4g16492.1 | - | - | MLOC_6032.2 |
| IVLNWEC01EVQL7 | Bradi4g16492.1 | - | - | MLOC_6032.2 |
| IVLNWEC01DYYE6 | Bradi1g37304.1 | - | - | AK370749 |
| IV9RU5F02HCJ16 | Bradi4g26366.1 | - | - | AK249921.1 |
| IV9RU5F02IQQ1L | Bradi3g48157.1 | - | - | AK363695 |
| IVLNWEC01DNNQW | Bradi1g50890.1 | - | - | MLOC_73860.2 |
| IV9RU5F01A13UT | Bradi5g27270.1 | - | - | MLOC_6266.2 |
| IV9RU5F01CPKOE | Bradi4g38387.1 | - | - | MLOC_81592.1 |
| IV9RU5F02GOL3V | Bradi3g13600.1 | - | - | AK375754 |
| IVLNWEC02HS2FF | Bradi1g01630.1 | - | - | AK250529.1 |
| IUIVLUD01DG5JF | Bradi4g42337.1 | - | - | MLOC_5857.3 |
| IV9RU5F02FUA9B | Bradi4g37197.1 | - | - | AK376014 |
| IVLNWEC02GJYG8 | Bradi1g02800.1 | - | - | AK370148 |
| IV9RU5F02IYZR1 | Bradi4g29190.2 | - | - | AK372711 |
| IVLNWEC01EEIB1 | Bradi2g39420.1 | - | - | MLOC_63684.1 |
| IVLNWEC01C83EX | Bradi4g08380.1 | - | - | MLOC_74303.1 |
| IVLNWEC01DVTD4 | Bradi4g08380.1 | - | - | MLOC_74303.1 |

| | | | | |
|---|---|---|---|---|
| IV9RU5F01EF8RX | Bradi4g04440.1 | - | - | MLOC_657.1 |
| IVLNWEC01CKKQZ | Bradi4g04440.1 | - | - | MLOC_657.1 |
| IVLNWEC01DB5OB | Bradi1g72092.1 | - | - | MLOC_60439.1 |
| IV9RU5F02JNP3B | Bradi3g48647.1 | - | - | MLOC_73368.2 |
| IUIVLUD01BCH8K | Bradi1g12340.2 | - | - | MLOC_12803.1 |
| IV9RU5F02JH9MQ | Bradi1g74430.1 | - | - | MLOC_17519.2 |
| IVLNWEC02F8JZO | Bradi4g25047.1 | - | - | AK371325 |
| IVLNWEC02JQX4Q | Bradi1g23350.1 | - | - | MLOC_65838.1 |
| IVLNWEC02GBL6B | Bradi3g59627.1 | - | - | MLOC_54546.1 |
| IV9RU5F02FTKRT | Bradi4g08870.1 | - | - | MLOC_11707.1 |
| IVLNWEC01E3LBV | Bradi4g08870.1 | - | - | MLOC_11707.1 |
| IUIVLUD02H98SQ | Bradi4g24480.1 | - | - | MLOC_60365.2 |
| IUIVLUD02JQ557 | Bradi4g07760.1 | - | - | AK361911 |
| IV9RU5F01D326L | Bradi4g07760.1 | - | - | AK361911 |
| IVLNWEC02GHJN4 | Bradi4g06390.1 | - | - | MLOC_73857.1 |
| IVLNWEC02GA9RI | Bradi4g40450.2 | - | - | MLOC_21002.1 |
| IV9RU5F01ENSLC | Bradi5g22790.1 | - | - | MLOC_52352.2 |
| IVLNWEC02F1OMT | Bradi4g07110.1 | - | - | MLOC_68734.1 |
| IV9RU5F02GEERR | Bradi1g75250.1 | - | - | MLOC_47627.2 |
| IUIVLUD02F7ALD | Bradi1g60357.1 | - | - | AK369262 |
| IUIVLUD02GUUJL | Bradi2g37990.1 | - | - | MLOC_69663.1 |
| IV9RU5F02H2GXB | Bradi4g35162.1 | - | - | MLOC_19573.5 |
| IV9RU5F02GL7G9 | Bradi4g03000.1 | - | - | MLOC_74632.1 |
| IVLNWEC01BKU5T | Bradi2g43557.1 | - | - | AK363414 |
| IVLNWEC01CDWEI | Bradi4g31830.1 | - | - | MLOC_34487.1 |
| IVLNWEC01CXXBN | Bradi3g56980.1 | - | - | AK373279 |
| IV9RU5F02HB8MU | Bradi4g37147.1 | - | - | MLOC_9982.2 |
| IVLNWEC02IXLRK | Bradi4g12770.1 | - | - | MLOC_64296.4 |
| IV9RU5F02HUPU6 | Bradi1g01970.1 | - | - | AK357261 |
| IUIVLUD01C6O4F | Bradi1g07027.1 | - | - | AK374530 |
| IUIVLUD01CD70G | Bradi3g27912.1 | - | - | MLOC_68408.1 |
| IVLNWEC02FQWRT | Bradi4g19650.1 | - | Sb0014s003010.1 | - |
| IUIVLUD02HAAO7 | Bradi3g35840.1 | - | Sb0183s002030.1 | - |
| IV9RU5F02HQQ8Q | Bradi1g00460.1 | - | Sb01g000220.1 | - |
| IV9RU5F02IR1JD | Bradi1g00460.1 | - | Sb01g000220.1 | - |
| IVLNWEC01D7ZKL | Bradi1g00460.1 | - | Sb01g000220.1 | - |
| IUIVLUD02GXXFB | Bradi1g00490.1 | - | Sb01g000255.1 | - |
| IUIVLUD02I9HE6 | Bradi1g00607.1 | - | Sb01g000330.1 | - |
| IVLNWEC02H0XTH | Bradi1g00690.1 | - | Sb01g000357.1 | - |
| IVLNWEC02HRG6H | Bradi1g00790.1 | - | Sb01g000430.1 | - |
| IVLNWEC02H9772 | Bradi1g00950.1 | - | Sb01g000580.1 | - |
| IUIVLUD01A5M8T | Bradi1g00990.1 | - | Sb01g000600.1 | - |
| IUIVLUD02JFS6P | Bradi1g00990.1 | - | Sb01g000600.1 | - |
| IVLNWEC01BQMDZ | Bradi1g00990.1 | - | Sb01g000600.1 | - |
| IV9RU5F01A907Y | Bradi1g01070.1 | - | Sb01g000790.1 | - |
| IVLNWEC01CABBU | Bradi1g01460.1 | - | Sb01g000970.1 | - |
| IVLNWEC01DOWN4 | Bradi1g01630.1 | - | Sb01g001120.1 | - |

| | | | | |
|---|---|---|---|---|
| IV9RU5F02IO9ET | Bradi1g22340.1 | - | Sb01g001180.1 | - |
| IVLNWEC02HF9PV | Bradi1g22340.1 | - | Sb01g001180.1 | - |
| IVLNWEC02IPCJQ | Bradi4g39650.1 | - | Sb01g001200.1 | - |
| IV9RU5F01EJS02 | Bradi1g01750.1 | - | Sb01g001260.1 | - |
| IV9RU5F02GZJ2Q | Bradi1g01710.1 | - | Sb01g001300.1 | - |
| IVLNWEC02GBOPG | Bradi1g01710.1 | - | Sb01g001300.1 | - |
| IVLNWEC02IFKVZ | Bradi1g01710.1 | - | Sb01g001300.1 | - |
| IVLNWEC01CS50T | Bradi1g01810.1 | - | Sb01g001360.1 | - |
| IVLNWEC01EAEFW | Bradi1g01810.1 | - | Sb01g001360.1 | - |
| IVLNWEC02H5YE4 | Bradi1g01810.1 | - | Sb01g001360.1 | - |
| IUIVLUD01A8KZG | Bradi1g02160.1 | - | Sb01g001760.1 | - |
| IUIVLUD01DE1U0 | Bradi1g02160.1 | - | Sb01g001760.1 | - |
| IUIVLUD02I2H64 | Bradi1g02160.1 | - | Sb01g001760.1 | - |
| IV9RU5F02GHLGC | Bradi1g02160.1 | - | Sb01g001760.1 | - |
| IVLNWEC02IH9VI | Bradi1g02170.1 | - | Sb01g001770.1 | - |
| IVLNWEC02FS58S | Bradi1g02440.1 | - | Sb01g001980.1 | - |
| IUIVLUD01BBSDF | Bradi1g02450.2 | - | Sb01g001990.1 | - |
| IVLNWEC01AZKU1 | Bradi1g03470.1 | - | Sb01g002950.1 | - |
| IV9RU5F01AUIT2 | Bradi1g03570.1 | - | Sb01g003040.1 | - |
| IUIVLUD01DBZRZ | Bradi1g03650.1 | - | Sb01g003130.1 | - |
| IUIVLUD01B8TR6 | Bradi1g03827.1 | - | Sb01g003320.1 | - |
| IUIVLUD02G7V6E | Bradi1g04140.1 | - | Sb01g003700.1 | - |
| IV9RU5F01BZ30S | Bradi1g04140.1 | - | Sb01g003700.1 | - |
| IV9RU5F02HTNKX | Bradi1g04140.1 | - | Sb01g003700.1 | - |
| IVLNWEC01CG94D | Bradi1g04140.1 | - | Sb01g003700.1 | - |
| IV9RU5F01E3YKM | Bradi1g04300.2 | - | Sb01g003860.1 | - |
| IVLNWEC01CGGJ6 | Bradi1g04310.1 | - | Sb01g003870.1 | - |
| IV9RU5F02JFQ2Y | Bradi1g04720.1 | - | Sb01g004360.1 | - |
| IVLNWEC02H0AQG | Bradi1g04720.1 | - | Sb01g004360.1 | - |
| IVLNWEC01EAHZF | Bradi1g04760.1 | - | Sb01g004400.1 | - |
| IV9RU5F01E1XQB | Bradi1g04980.1 | - | Sb01g004640.1 | - |
| IV9RU5F02HY6R9 | Bradi1g05156.1 | - | Sb01g004870.1 | - |
| IVLNWEC02HENGF | Bradi1g05162.1 | - | Sb01g004920.1 | - |
| IV9RU5F02FJE6R | Bradi1g05180.1 | - | Sb01g004940.1 | - |
| IUIVLUD01A6YZN | Bradi1g05380.1 | - | Sb01g005084.1 | - |
| IUIVLUD01CQRMP | Bradi1g05380.1 | - | Sb01g005084.1 | - |
| IV9RU5F01BOBZM | Bradi1g05380.1 | - | Sb01g005084.1 | - |
| IV9RU5F01CPBSA | Bradi1g05380.1 | - | Sb01g005084.1 | - |
| IVLNWEC01AE2A6 | Bradi1g05380.1 | - | Sb01g005084.1 | - |
| IVLNWEC01CT0NK | Bradi1g05380.1 | - | Sb01g005084.1 | - |
| IV9RU5F01COSCY | Bradi1g05560.1 | - | Sb01g005260.1 | - |
| IUIVLUD02HO24L | Bradi1g05970.2 | - | Sb01g005500.1 | - |
| IV9RU5F02IGXPF | Bradi1g05970.2 | - | Sb01g005500.1 | - |
| IV9RU5F01C6W8A | Bradi1g06110.1 | - | Sb01g005550.1 | - |
| IUIVLUD01CRB03 | Bradi3g35460.1 | - | Sb01g005660.1 | - |
| IUIVLUD01BIATF | Bradi2g11590.1 | - | Sb01g005660.1 | - |
| IVLNWEC01CHUUJ | Bradi1g06230.1 | - | Sb01g005710.1 | - |

| | | | | |
|---|---|---|---|---|
| IV9RU5F02GQLIZ | Bradi1g06250.1 | - | Sb01g005730.1 | - |
| IV9RU5F02HSQ5L | Bradi1g06270.1 | - | Sb01g005750.1 | - |
| IUIVLUD01BA5TR | Bradi1g06540.1 | - | Sb01g006100.1 | - |
| IVLNWEC02GQZ3T | Bradi1g06540.1 | - | Sb01g006100.1 | - |
| IVLNWEC02GO2BY | Bradi1g07390.1 | - | Sb01g006930.1 | - |
| IVLNWEC02G1C3O | Bradi1g07577.1 | - | Sb01g007090.1 | - |
| IVLNWEC02GEQN7 | Bradi1g07755.1 | - | Sb01g007195.1 | - |
| IUIVLUD01BJGMD | Bradi1g07930.1 | - | Sb01g007400.1 | - |
| IV9RU5F01E2TRI | Bradi1g08060.1 | - | Sb01g007550.1 | - |
| IUIVLUD01C9AE9 | Bradi1g08240.1 | - | Sb01g007680.1 | - |
| IUIVLUD01DXJ07 | Bradi1g08280.1 | - | Sb01g007710.1 | - |
| IVLNWEC02JH6JI | Bradi1g08400.1 | - | Sb01g007850.1 | - |
| IUIVLUD02JT3CB | Bradi1g08470.1 | - | Sb01g007950.1 | - |
| IUIVLUD02JLD3D | Bradi1g08540.1 | - | Sb01g008020.1 | - |
| IUIVLUD02HEB4Y | Bradi1g08607.1 | - | Sb01g008080.1 | - |
| IUIVLUD02FW1CO | Bradi1g08660.2 | - | Sb01g008130.1 | - |
| IVLNWEC01DHTML | Bradi1g08847.1 | - | Sb01g008310.1 | - |
| IV9RU5F02GAO44 | Bradi1g09200.1 | - | Sb01g008600.1 | - |
| IV9RU5F02ITQNI | Bradi1g09210.1 | - | Sb01g008630.1 | - |
| IVLNWEC01ECT2A | Bradi1g09210.1 | - | Sb01g008630.1 | - |
| IV9RU5F02GYJMQ | Bradi1g09390.1 | - | Sb01g008790.1 | - |
| IUIVLUD02GQSH8 | Bradi1g09810.1 | - | Sb01g009225.1 | - |
| IUIVLUD01DC75U | Bradi1g22780.1 | - | Sb01g009615.1 | - |
| IV9RU5F02JCGT8 | Bradi1g22780.1 | - | Sb01g009615.1 | - |
| IVLNWEC01DIIBH | Bradi1g22780.1 | - | Sb01g009615.1 | - |
| IV9RU5F01A4OHL | Bradi1g10210.1 | - | Sb01g009630.1 | - |
| IUIVLUD02JA2I1 | Bradi1g10470.1 | - | Sb01g009870.1 | - |
| IUIVLUD01A684C | Bradi1g11520.2 | - | Sb01g010210.1 | - |
| IV9RU5F01D3ZLN | Bradi1g12370.1 | - | Sb01g011860.1 | - |
| IV9RU5F01BO17N | Bradi1g13910.1 | - | Sb01g013710.1 | - |
| IUIVLUD01EW76L | Bradi2g37795.1 | - | Sb01g014270.1 | - |
| IUIVLUD02FQYQT | Bradi2g37795.1 | - | Sb01g014270.1 | - |
| IUIVLUD02GJILM | Bradi2g37795.1 | - | Sb01g014270.1 | - |
| IUIVLUD02GPUMC | Bradi2g37795.1 | - | Sb01g014270.1 | - |
| IUIVLUD02IKYJS | Bradi2g37795.1 | - | Sb01g014270.1 | - |
| IV9RU5F01CZHE6 | Bradi2g37795.1 | - | Sb01g014270.1 | - |
| IV9RU5F01DS6UA | Bradi2g37795.1 | - | Sb01g014270.1 | - |
| IV9RU5F01E28KH | Bradi2g37795.1 | - | Sb01g014270.1 | - |
| IVLNWEC01CHIJN | Bradi2g37795.1 | - | Sb01g014270.1 | - |
| IVLNWEC01C88EH | Bradi4g23730.1 | - | Sb01g015810.1 | - |
| IV9RU5F02GNQNV | Bradi1g06070.1 | - | Sb01g015810.1 | - |
| IVLNWEC01BC6Y1 | Bradi2g37795.1 | - | Sb01g016055.1 | - |
| IV9RU5F01AL5DH | Bradi3g30220.1 | - | Sb01g017800.1 | - |
| IUIVLUD02HUJGX | Bradi2g17187.1 | - | Sb01g017870.1 | - |
| IUIVLUD01C41N3 | Bradi3g29690.1 | - | Sb01g018510.1 | - |
| IV9RU5F02GQTB6 | Bradi3g29690.1 | - | Sb01g018510.1 | - |
| IVLNWEC02HN7VQ | Bradi1g27750.1 | - | Sb01g019620.1 | - |

| | | | | |
|---|---|---|---|---|
| IV9RU5F01CJG5A | Bradi3g28390.1 | - | Sb01g019680.1 | - |
| IVLNWEC02HGV82 | Bradi3g28390.1 | - | Sb01g019680.1 | - |
| IV9RU5F02FKSTE | Bradi1g56107.1 | - | Sb01g021220.1 | - |
| IVLNWEC02G9ZMD | Bradi1g74430.1 | - | Sb01g021680.1 | - |
| IVLNWEC02G5G87 | Bradi3g26697.1 | - | Sb01g022040.1 | - |
| IUIVLUD02F600V | Bradi1g26180.1 | - | Sb01g022280.1 | - |
| IUIVLUD02HXKGF | Bradi1g73150.1 | - | Sb01g022460.1 | - |
| IUIVLUD02GWE4C | Bradi2g31690.1 | - | Sb01g024390.1 | - |
| IV9RU5F02IJ3ZN | Bradi2g31690.1 | - | Sb01g024390.1 | - |
| IV9RU5F01BN40R | Bradi2g17187.1 | - | Sb01g025305.1 | - |
| IVLNWEC01AP7AW | Bradi4g05870.1 | - | Sb01g026690.1 | - |
| IVLNWEC01BZQBJ | Bradi1g24780.1 | - | Sb01g027310.1 | - |
| IV9RU5F02JUNCK | Bradi4g24340.1 | - | Sb01g031070.1 | - |
| IVLNWEC02JDBNS | Bradi4g24340.1 | - | Sb01g031070.1 | - |
| IV9RU5F02IBRUG | Bradi1g60890.1 | - | Sb01g033760.1 | - |
| IVLNWEC02GXKKN | Bradi4g36680.1 | - | Sb01g034300.1 | - |
| IUIVLUD02HMBXZ | Bradi3g36100.1 | - | Sb01g034805.1 | - |
| IV9RU5F02HQ8U4 | Bradi1g62007.1 | - | Sb01g034960.1 | - |
| IV9RU5F01A50XD | Bradi1g62600.1 | - | Sb01g035520.1 | - |
| IUIVLUD01ASNIK | Bradi1g17670.1 | - | Sb01g036170.1 | - |
| IV9RU5F02JMGKM | Bradi1g63880.2 | - | Sb01g036840.1 | - |
| IV9RU5F02HB0HR | Bradi1g64687.1 | - | Sb01g037670.1 | - |
| IVLNWEC02JNBGM | Bradi3g53020.1 | - | Sb01g037910.1 | - |
| IVLNWEC01DF4WY | Bradi1g66040.1 | - | Sb01g038920.1 | - |
| IVLNWEC02G9B86 | Bradi1g66130.1 | - | Sb01g038980.1 | - |
| IV9RU5F01A1FST | Bradi2g59280.1 | - | Sb01g039150.1 | - |
| IVLNWEC02JZ80Y | Bradi1g67210.1 | - | Sb01g040155.1 | - |
| IUIVLUD01AMCD9 | Bradi1g67800.1 | - | Sb01g040800.1 | - |
| IV9RU5F02H74FX | Bradi1g68180.1 | - | Sb01g041140.1 | - |
| IUIVLUD02FWH9R | Bradi1g68697.1 | - | Sb01g041600.1 | - |
| IV9RU5F01AGT2D | Bradi1g68697.1 | - | Sb01g041600.1 | - |
| IUIVLUD02JQPUX | Bradi1g69880.1 | - | Sb01g042830.1 | - |
| IVLNWEC01BS5C2 | Bradi4g24340.1 | - | Sb01g042855.1 | - |
| IUIVLUD01CHBCV | Bradi1g70570.1 | - | Sb01g043330.1 | - |
| IV9RU5F02IVI3C | Bradi1g71530.1 | - | Sb01g044130.1 | - |
| IV9RU5F01ET8NI | Bradi2g40170.2 | - | Sb01g045820.1 | - |
| IV9RU5F02F2EPK | Bradi1g74940.1 | - | Sb01g046850.1 | - |
| IVLNWEC01C8RB4 | Bradi1g74940.1 | - | Sb01g046850.1 | - |
| IV9RU5F01A5S4D | Bradi1g75390.1 | - | Sb01g047200.1 | - |
| IVLNWEC02F9BSJ | Bradi1g76520.1 | - | Sb01g048520.1 | - |
| IV9RU5F01EJDC5 | Bradi1g51757.1 | - | Sb01g050480.1 | - |
| IUIVLUD02ISW2F | Bradi1g03560.1 | - | Sb02g001010.1 | - |
| IV9RU5F02HQD4K | Bradi1g50420.1 | - | Sb02g001150.1 | - |
| IVLNWEC01EUSIS | Bradi5g15797.1 | - | Sb02g002097.1 | - |
| IVLNWEC02IDVXK | Bradi4g32830.1 | - | Sb02g002110.1 | - |
| IVLNWEC01C0Y0E | Bradi2g36810.1 | - | Sb02g002703.1 | - |
| IV9RU5F01E34RX | Bradi1g56720.3 | - | Sb02g003700.1 | - |

| | | | | |
|---|---|---|---|---|
| IUIVLUD01EB29N | Bradi1g05380.1 | - | Sb02g004680.1 | - |
| IV9RU5F01EF9MM | Bradi1g05380.1 | - | Sb02g004680.1 | - |
| IV9RU5F02HIJQ1 | Bradi1g05380.1 | - | Sb02g004680.1 | - |
| IVLNWEC01CFDYM | Bradi1g49930.1 | - | Sb02g004980.1 | - |
| IV9RU5F01AW5WZ | Bradi1g01250.1 | - | Sb02g005230.1 | - |
| IUIVLUD02HHDVB | Bradi1g01250.1 | - | Sb02g005235.1 | - |
| IV9RU5F01EBKTV | Bradi1g01250.1 | - | Sb02g005235.1 | - |
| IVLNWEC02JCWXG | Bradi1g01250.1 | - | Sb02g005235.1 | - |
| IV9RU5F02I08VM | Bradi1g04990.1 | - | Sb02g005780.1 | - |
| IUIVLUD01D4TT0 | Bradi1g54327.1 | - | Sb02g006140.1 | - |
| IUIVLUD02GIUNS | Bradi1g53840.1 | - | Sb02g006620.1 | - |
| IV9RU5F01BVJDA | Bradi1g02710.1 | - | Sb02g007460.1 | - |
| IVLNWEC01DNOMR | Bradi3g36100.1 | - | Sb02g008045.1 | - |
| IUIVLUD01CGQ0W | Bradi2g11590.1 | - | Sb02g008710.1 | - |
| IVLNWEC02IER0C | Bradi1g00607.1 | - | Sb02g010040.1 | - |
| IV9RU5F02JXY45 | Bradi1g28790.5 | - | Sb02g010840.1 | - |
| IUIVLUD01BXBP8 | Bradi1g46220.1 | - | Sb02g013070.1 | - |
| IUIVLUD01C496S | Bradi1g46220.1 | - | Sb02g013070.1 | - |
| IVLNWEC01D1BO0 | Bradi4g08420.1 | - | Sb02g013180.1 | - |
| IUIVLUD01BMGR7 | Bradi4g08350.1 | - | Sb02g014460.1 | - |
| IUIVLUD02JCN5N | Bradi4g27470.1 | - | Sb02g019400.1 | - |
| IV9RU5F02I4HOK | Bradi2g14450.1 | - | Sb02g021133.1 | - |
| IVLNWEC01CZ1SO | Bradi2g14450.1 | - | Sb02g021133.1 | - |
| IUIVLUD02FXYUR | Bradi4g27977.1 | - | Sb02g021490.1 | - |
| IVLNWEC02FP1EW | Bradi4g27977.1 | - | Sb02g021490.1 | - |
| IV9RU5F01AR2CG | Bradi2g42000.1 | - | Sb02g023900.1 | - |
| IUIVLUD02JNX97 | Bradi3g36180.1 | - | Sb02g024010.1 | - |
| IV9RU5F02FR31M | Bradi4g30210.1 | - | Sb02g024630.1 | - |
| IV9RU5F01BE2MA | Bradi4g31227.1 | - | Sb02g025710.1 | - |
| IV9RU5F01DL8EW | Bradi1g06880.1 | - | Sb02g025930.2 | - |
| IV9RU5F01BMYX0 | Bradi4g31727.1 | - | Sb02g026170.1 | - |
| IVLNWEC01DZXMX | Bradi4g31727.1 | - | Sb02g026170.1 | - |
| IVLNWEC01DEFKY | Bradi1g07570.2 | - | Sb02g026400.1 | - |
| IVLNWEC01AVGTG | Bradi4g32250.1 | - | Sb02g026720.1 | - |
| IUIVLUD02FV1UA | Bradi4g32420.1 | - | Sb02g026840.1 | - |
| IV9RU5F02GOM17 | Bradi4g32530.1 | - | Sb02g027000.1 | - |
| IV9RU5F01DSBIY | Bradi4g34290.1 | - | Sb02g029000.1 | - |
| IV9RU5F01D6VFC | Bradi2g58917.1 | - | Sb02g029210.1 | - |
| IVLNWEC02HIIJX | Bradi2g58917.1 | - | Sb02g029210.1 | - |
| IV9RU5F02GU9FT | Bradi4g34846.1 | - | Sb02g029500.1 | - |
| IVLNWEC02GNEMC | Bradi4g34846.1 | - | Sb02g029500.1 | - |
| IV9RU5F02G8JB7 | Bradi4g35557.1 | - | Sb02g030290.1 | - |
| IVLNWEC02I6EMP | Bradi3g42080.1 | - | Sb02g030430.1 | - |
| IVLNWEC01BL6UB | Bradi4g36160.2 | - | Sb02g030960.1 | - |
| IVLNWEC01DH8WI | Bradi4g36160.2 | - | Sb02g030960.1 | - |
| IV9RU5F02I65AB | Bradi4g36477.1 | - | Sb02g031210.1 | - |
| IVLNWEC02FXL9J | Bradi4g37110.1 | - | Sb02g031940.1 | - |

| | | | | |
|---|---|---|---|---|
| IUIVLUD01EJ4NI | Bradi4g37197.1 | - | Sb02g032020.1 | - |
| IUIVLUD01AU9IC | Bradi4g37190.1 | - | Sb02g032130.1 | - |
| IV9RU5F01EH78X | Bradi4g37190.1 | - | Sb02g032135.1 | - |
| IV9RU5F02HDGU5 | Bradi4g37260.1 | - | Sb02g032150.1 | - |
| IV9RU5F02H2S7J | Bradi1g28000.1 | - | Sb02g033170.1 | - |
| IVLNWEC01AQ16O | Bradi1g25497.1 | - | Sb02g035420.1 | - |
| IVLNWEC01AMRY5 | Bradi3g56550.1 | - | Sb02g035430.1 | - |
| IV9RU5F02I9SKR | Bradi1g25090.1 | - | Sb02g036040.1 | - |
| IVLNWEC02IV6EM | Bradi1g25090.1 | - | Sb02g036040.1 | - |
| IV9RU5F01DO9M8 | Bradi3g18250.1 | - | Sb02g036820.1 | - |
| IV9RU5F02GUAEH | Bradi1g03277.1 | - | Sb02g039465.1 | - |
| IVLNWEC02JAA3A | Bradi1g02180.1 | - | Sb02g041520.1 | - |
| IV9RU5F02GYY2D | Bradi1g18910.1 | - | Sb02g041910.1 | - |
| IVLNWEC02INYZ2 | Bradi1g18910.1 | - | Sb02g041910.1 | - |
| IUIVLUD01C84XG | Bradi4g08380.1 | - | Sb03g001440.1 | - |
| IV9RU5F02INJMN | Bradi2g01550.1 | - | Sb03g003250.1 | - |
| IUIVLUD01BP9NO | Bradi2g05460.1 | - | Sb03g003420.1 | - |
| IVLNWEC01DKOI5 | Bradi3g09535.1 | - | Sb03g005350.1 | - |
| IVLNWEC02GU4EF | Bradi3g09535.1 | - | Sb03g005350.1 | - |
| IVLNWEC01BZ6Y1 | Bradi1g43500.1 | - | Sb03g007345.1 | - |
| IVLNWEC01EDQTC | Bradi2g10800.2 | - | Sb03g011360.1 | - |
| IV9RU5F01BZM2A | Bradi2g12850.1 | - | Sb03g014690.1 | - |
| IV9RU5F01BT4TZ | Bradi2g40430.1 | - | Sb03g023490.1 | - |
| IVLNWEC01C56VC | Bradi1g51757.1 | - | Sb03g023520.1 | - |
| IUIVLUD01APMHW | Bradi4g33310.1 | - | Sb03g029110.1 | - |
| IVLNWEC02F9DQ7 | Bradi5g01990.1 | - | Sb03g030490.1 | - |
| IUIVLUD01ELIUW | Bradi2g46550.1 | - | Sb03g031160.1 | - |
| IVLNWEC02F84Z8 | Bradi2g46550.1 | - | Sb03g031160.1 | - |
| IV9RU5F02JSZJF | Bradi2g11590.1 | - | Sb03g032665.1 | - |
| IV9RU5F01EC9BT | Bradi2g51630.1 | - | Sb03g036250.2 | - |
| IUIVLUD02FN1S5 | Bradi2g51807.1 | - | Sb03g036450.1 | - |
| IUIVLUD02G0O2K | Bradi2g51807.1 | - | Sb03g036450.1 | - |
| IUIVLUD02I39SW | Bradi2g51807.1 | - | Sb03g036450.1 | - |
| IV9RU5F01CDRE4 | Bradi2g51807.1 | - | Sb03g036450.1 | - |
| IV9RU5F01DEOGO | Bradi2g51807.1 | - | Sb03g036450.1 | - |
| IV9RU5F02JNZZE | Bradi2g51807.1 | - | Sb03g036450.1 | - |
| IVLNWEC01A7IDZ | Bradi2g51807.1 | - | Sb03g036450.1 | - |
| IVLNWEC01BGMTZ | Bradi2g51807.1 | - | Sb03g036450.1 | - |
| IVLNWEC01BQNJ7 | Bradi2g51807.1 | - | Sb03g036450.1 | - |
| IVLNWEC01BZOPQ | Bradi2g51807.1 | - | Sb03g036450.1 | - |
| IVLNWEC01ENAVS | Bradi2g51807.1 | - | Sb03g036450.1 | - |
| IVLNWEC02IUJHO | Bradi2g51807.1 | - | Sb03g036450.1 | - |
| IUIVLUD02FQL5Q | Bradi3g37780.1 | - | Sb03g036763.1 | - |
| IV9RU5F01DMIHV | Bradi2g54390.1 | - | Sb03g039200.1 | - |
| IV9RU5F01DNWZH | Bradi2g23610.1 | - | Sb03g039940.1 | - |
| IV9RU5F01C8YN7 | Bradi5g20997.1 | - | Sb03g040220.1 | - |
| IVLNWEC02GP6F6 | Bradi5g27650.1 | - | Sb03g042560.1 | - |

| | | | | |
|---|---|---|---|---|
| IVLNWEC01BNH8B | Bradi2g58967.1 | - | Sb03g044000.1 | - |
| IVLNWEC02G13FL | Bradi2g58967.1 | - | Sb03g044000.1 | - |
| IV9RU5F01CWI64 | Bradi1g17060.1 | - | Sb03g045640.1 | - |
| IUIVLUD01DR1SM | Bradi2g61830.1 | - | Sb03g047060.1 | - |
| IV9RU5F02HRDC1 | Bradi2g61830.1 | - | Sb03g047060.1 | - |
| IVLNWEC01DJBDT | Bradi2g61830.1 | - | Sb03g047060.1 | - |
| IVLNWEC01EZARF | Bradi2g61830.1 | - | Sb03g047060.1 | - |
| IVLNWEC02HIJZN | Bradi2g61830.1 | - | Sb03g047060.1 | - |
| IV9RU5F01CZIUJ | Bradi3g00340.1 | - | Sb04g000330.1 | - |
| IV9RU5F02GBBYC | Bradi1g52210.1 | - | Sb04g000620.1 | - |
| IUIVLUD01BQNL3 | Bradi3g10370.1 | - | Sb04g001410.1 | - |
| IUIVLUD02HG176 | Bradi3g32276.1 | - | Sb04g001610.1 | - |
| IV9RU5F01ESQ9E | Bradi3g32276.1 | - | Sb04g001610.1 | - |
| IVLNWEC01A0KLB | Bradi3g02560.1 | - | Sb04g002180.1 | - |
| IVLNWEC01E0XT1 | Bradi3g03317.1 | - | Sb04g003000.1 | - |
| IUIVLUD02I9ERZ | Bradi1g58930.1 | - | Sb04g003073.1 | - |
| IUIVLUD02I2LA7 | Bradi3g49640.1 | - | Sb04g004040.1 | - |
| IV9RU5F01DAYIN | Bradi3g07457.1 | - | Sb04g006980.1 | - |
| IUIVLUD01APCEH | Bradi1g02840.1 | - | Sb04g009720.1 | - |
| IVLNWEC01ANKHR | Bradi3g10030.1 | - | Sb04g010330.1 | - |
| IUIVLUD02GHUYE | Bradi4g30370.1 | - | Sb04g016965.1 | - |
| IV9RU5F01C3X0W | Bradi4g30370.1 | - | Sb04g016965.1 | - |
| IV9RU5F02GM1YI | Bradi3g44197.1 | - | Sb04g020730.1 | - |
| IVLNWEC01APEA8 | Bradi1g52280.1 | - | Sb04g021130.1 | - |
| IUIVLUD01BB3F1 | Bradi3g44960.1 | - | Sb04g021790.1 | - |
| IVLNWEC01B4PS6 | Bradi2g11590.1 | - | Sb04g023610.1 | - |
| IV9RU5F01D1F1E | Bradi3g46500.1 | - | Sb04g023620.1 | - |
| IVLNWEC02HJCVV | Bradi3g37780.1 | - | Sb04g027285.1 | - |
| IVLNWEC01BYM85 | Bradi3g59910.1 | - | Sb04g028630.1 | - |
| IVLNWEC02G9BY9 | Bradi1g69880.1 | - | Sb04g029070.1 | - |
| IV9RU5F02HOLPN | Bradi3g52470.2 | - | Sb04g030880.1 | - |
| IVLNWEC01BN4FI | Bradi3g52470.2 | - | Sb04g030880.1 | - |
| IV9RU5F02H3T9Q | Bradi5g10450.1 | - | Sb04g031810.1 | - |
| IVLNWEC02GRNQF | Bradi3g50300.1 | - | Sb04g033300.1 | - |
| IVLNWEC02J2D1T | Bradi3g50300.1 | - | Sb04g033300.1 | - |
| IUIVLUD02H5J3Y | Bradi3g58300.1 | - | Sb04g033870.1 | - |
| IV9RU5F01AZNF1 | Bradi4g37560.1 | - | Sb04g037070.1 | - |
| IVLNWEC02IEW91 | Bradi4g37560.1 | - | Sb04g037070.1 | - |
| IUIVLUD01B9L9O | Bradi1g56107.1 | - | Sb05g001950.1 | - |
| IVLNWEC02HHI1T | Bradi2g62760.1 | - | Sb05g002400.1 | - |
| IV9RU5F01DGX6L | Bradi4g43230.1 | - | Sb05g002450.1 | - |
| IVLNWEC02H5S1A | Bradi4g24960.2 | - | Sb05g003650.1 | - |
| IUIVLUD01ARFI4 | Bradi4g21860.1 | - | Sb05g005345.1 | - |
| IUIVLUD01DEMMC | Bradi4g21860.1 | - | Sb05g005345.1 | - |
| IVLNWEC01AYQQX | Bradi4g23695.1 | - | Sb05g005360.1 | - |
| IVLNWEC02I182C | Bradi2g38510.1 | - | Sb05g008053.1 | - |
| IV9RU5F02GP3GT | Bradi3g22840.2 | - | Sb05g011430.1 | - |

| | | | | |
|---|---|---|---|---|
| IUIVLUD01CVJLG | Bradi5g20587.1 | - | Sb05g018835.1 | - |
| IVLNWEC02JPJ6W | Bradi2g31530.1 | - | Sb05g019526.1 | - |
| IVLNWEC01EQAG6 | Bradi4g16290.1 | - | Sb05g021820.1 | - |
| IV9RU5F01BXPYR | Bradi3g18300.1 | - | Sb05g022140.1 | - |
| IUIVLUD02H6YJ8 | Bradi1g75540.1 | - | Sb05g022270.1 | - |
| IVLNWEC01EMJM4 | Bradi4g33630.1 | - | Sb05g022920.1 | - |
| IUIVLUD01E1BAS | Bradi4g11687.1 | - | Sb05g025620.1 | - |
| IV9RU5F02GC0XC | Bradi4g11687.1 | - | Sb05g025620.1 | - |
| IV9RU5F01EOP9M | Bradi4g08130.1 | - | Sb05g027420.1 | - |
| IV9RU5F02F74OJ | Bradi4g08130.1 | - | Sb05g027420.1 | - |
| IVLNWEC01BST9Q | Bradi1g49450.1 | - | Sb05g027506.1 | - |
| IVLNWEC01EQSVG | Bradi4g45160.1 | - | Sb05g027773.1 | - |
| IV9RU5F01CAZ1R | Bradi4g12680.1 | - | Sb06g000340.1 | - |
| IV9RU5F02JAK7J | Bradi4g23810.1 | - | Sb06g000740.1 | - |
| IVLNWEC01BEB3K | Bradi5g00590.1 | - | Sb06g001580.1 | - |
| IVLNWEC01D1DDI | Bradi5g02770.1 | - | Sb06g002540.1 | - |
| IVLNWEC01A1QSL | Bradi5g00970.1 | - | Sb06g002820.1 | - |
| IVLNWEC01EUEXD | Bradi3g25900.1 | - | Sb06g003220.1 | - |
| IV9RU5F01D1G3C | Bradi2g17187.1 | - | Sb06g004070.1 | - |
| IV9RU5F02JFY1O | Bradi2g17187.1 | - | Sb06g004070.1 | - |
| IVLNWEC01D9FUT | Bradi2g17187.1 | - | Sb06g004070.1 | - |
| IV9RU5F01ASKUN | Bradi2g17187.1 | - | Sb06g005665.1 | - |
| IVLNWEC01CGYLJ | Bradi5g03840.1 | - | Sb06g006100.1 | - |
| IVLNWEC02HK7MJ | Bradi5g07160.1 | - | Sb06g010096.1 | - |
| IVLNWEC01BGKDP | Bradi2g31530.1 | - | Sb06g016240.1 | - |
| IUIVLUD02IG03B | Bradi1g00440.1 | - | Sb06g016240.1 | - |
| IUIVLUD01D0VW1 | Bradi1g01180.1 | - | Sb06g016690.1 | - |
| IV9RU5F01B1FJF | Bradi1g01180.1 | - | Sb06g016690.1 | - |
| IV9RU5F02GI2X1 | Bradi3g59910.1 | - | Sb06g019330.1 | - |
| IUIVLUD01D8YKC | Bradi5g13180.1 | - | Sb06g019760.1 | - |
| IVLNWEC01EJ06I | Bradi5g13180.1 | - | Sb06g019760.1 | - |
| IV9RU5F02HO8CZ | Bradi1g03680.1 | - | Sb06g021060.1 | - |
| IUIVLUD02JWKO4 | Bradi5g15797.1 | - | Sb06g022700.1 | - |
| IV9RU5F02IR43Z | Bradi5g15797.1 | - | Sb06g022700.1 | - |
| IV9RU5F02F9MEC | Bradi5g22077.1 | - | Sb06g028880.1 | - |
| IVLNWEC02GPN4Z | Bradi5g22077.1 | - | Sb06g028880.1 | - |
| IVLNWEC01AIU11 | Bradi5g24890.1 | - | Sb06g031650.1 | - |
| IUIVLUD01EH7Y8 | Bradi4g42430.2 | - | Sb07g001477.1 | - |
| IV9RU5F01DPAGJ | Bradi1g43500.1 | - | Sb07g001797.1 | - |
| IVLNWEC02HULJW | Bradi1g75540.1 | - | Sb07g003260.1 | - |
| IVLNWEC01AR18Z | Bradi2g04960.1 | - | Sb07g004420.1 | - |
| IVLNWEC02FP2ZN | Bradi3g14710.1 | - | Sb07g005130.1 | - |
| IV9RU5F02GUL6K | Bradi2g36170.1 | - | Sb07g005720.1 | - |
| IVLNWEC01DPRU5 | Bradi3g18250.1 | - | Sb07g006450.1 | - |
| IVLNWEC01ECRS5 | Bradi2g03850.1 | - | Sb07g009340.1 | - |
| IVLNWEC01CA1CJ | Bradi4g11120.1 | - | Sb07g016021.1 | - |
| IUIVLUD01E1CXU | Bradi1g30656.1 | - | Sb07g020130.1 | - |

| | | | | |
|---|---|---|---|---|
| IUIVLUD02JGCLW | Bradi3g38310.1 | - | Sb07g023220.1 | - |
| IUIVLUD01DZCON | Bradi3g41810.1 | - | Sb07g025500.1 | - |
| IUIVLUD02GRQI6 | Bradi3g41810.1 | - | Sb07g025500.1 | - |
| IV9RU5F01C6NZK | Bradi2g39517.1 | - | Sb07g025650.1 | - |
| IUIVLUD01COUHJ | Bradi4g38170.1 | - | Sb07g025850.1 | - |
| IVLNWEC02G0FW5 | Bradi1g48670.1 | - | Sb07g027770.1 | - |
| IVLNWEC02JBOZR | Bradi4g32840.1 | - | Sb07g029085.1 | - |
| IVLNWEC01APE8L | Bradi4g26430.1 | - | Sb08g001480.1 | - |
| IVLNWEC01EFQB5 | Bradi4g26430.1 | - | Sb08g001480.1 | - |
| IV9RU5F01A7IMK | Bradi1g78750.1 | - | Sb08g002910.1 | - |
| IV9RU5F01CQQWT | Bradi2g04240.1 | - | Sb08g005370.1 | - |
| IV9RU5F02HRFX1 | Bradi2g51807.1 | - | Sb08g005460.1 | - |
| IVLNWEC02JFOWG | Bradi3g22840.2 | - | Sb08g007250.1 | - |
| IVLNWEC01C8TGC | Bradi4g39550.1 | - | Sb08g008650.1 | - |
| IVLNWEC02FYHS4 | Bradi4g05870.1 | - | Sb08g015400.1 | - |
| IUIVLUD02GL12N | Bradi3g24417.1 | - | Sb08g016820.1 | - |
| IV9RU5F01CPGGZ | Bradi3g24417.1 | - | Sb08g016820.1 | - |
| IV9RU5F02GR67N | Bradi3g24417.1 | - | Sb08g016820.1 | - |
| IVLNWEC01BJP39 | Bradi3g24417.1 | - | Sb08g016820.1 | - |
| IVLNWEC02F633M | Bradi3g24417.1 | - | Sb08g016820.1 | - |
| IVLNWEC02GBO0W | Bradi3g24417.1 | - | Sb08g016820.1 | - |
| IVLNWEC01A9HYK | Bradi4g05840.1 | - | Sb08g017010.1 | - |
| IVLNWEC02HC1OG | Bradi4g05370.1 | - | Sb08g017550.1 | - |
| IVLNWEC02FMX9H | Bradi4g05310.1 | - | Sb08g017590.1 | - |
| IV9RU5F01B2IW4 | Bradi4g01215.1 | - | Sb08g017730.1 | - |
| IVLNWEC02ICKJY | Bradi4g04620.1 | - | Sb08g018360.1 | - |
| IUIVLUD01CRWOC | Bradi4g03150.1 | - | Sb08g019730.1 | - |
| IV9RU5F01B1BRC | Bradi2g38810.1 | - | Sb08g019990.1 | - |
| IV9RU5F02JPFYZ | Bradi4g02910.1 | - | Sb08g020025.1 | - |
| IVLNWEC01A8UZY | Bradi4g02810.1 | - | Sb08g020140.1 | - |
| IV9RU5F02JEZ1C | Bradi4g06000.2 | - | Sb08g020420.1 | - |
| IUIVLUD02G937L | Bradi4g02420.1 | - | Sb08g020780.1 | - |
| IVLNWEC02JBNNU | Bradi4g22740.1 | - | Sb08g021280.1 | - |
| IUIVLUD01DGGPQ | Bradi4g01930.1 | - | Sb08g021320.1 | - |
| IUIVLUD01B8NFK | Bradi1g02290.1 | - | Sb08g021540.1 | - |
| IUIVLUD01D67FN | Bradi1g02290.1 | - | Sb08g021540.1 | - |
| IV9RU5F02GY9LX | Bradi1g02290.1 | - | Sb08g021540.1 | - |
| IUIVLUD01BW4AB | Bradi2g09434.1 | - | Sb08g021870.1 | - |
| IVLNWEC01BYKXY | Bradi2g14450.1 | - | Sb08g022140.1 | - |
| IV9RU5F02JZJY8 | Bradi4g01100.1 | - | Sb08g022310.1 | - |
| IUIVLUD02IZLWH | Bradi4g00747.1 | - | Sb08g022950.1 | - |
| IVLNWEC02IYDVX | Bradi4g00480.1 | - | Sb08g023130.1 | - |
| IUIVLUD01EMQHF | Bradi2g39900.1 | - | Sb09g000380.1 | - |
| IVLNWEC02ICR1J | Bradi2g39240.1 | - | Sb09g001090.1 | - |
| IV9RU5F01EZT30 | Bradi1g49030.1 | - | Sb09g001485.1 | - |
| IV9RU5F02G2KQ3 | Bradi2g37570.1 | - | Sb09g001850.1 | - |
| IUIVLUD02FUECG | Bradi2g38490.1 | - | Sb09g002640.2 | - |

| | | | | |
|---|---|---|---|---|
| IUIVLUD02I3BDN | Bradi2g38490.1 | - | Sb09g002640.2 | - |
| IVLNWEC02JWKWK | Bradi3g61040.1 | - | Sb09g003480.1 | - |
| IUIVLUD02JRV95 | Bradi3g61040.1 | - | Sb09g015250.1 | - |
| IVLNWEC01AMXOP | Bradi5g03627.1 | - | Sb09g017563.1 | - |
| IUIVLUD01CR19V | Bradi2g28007.1 | - | Sb09g017790.1 | - |
| IV9RU5F02FKZMR | Bradi2g28007.1 | - | Sb09g017790.1 | - |
| IV9RU5F02G6W40 | Bradi2g28007.1 | - | Sb09g017790.1 | - |
| IVLNWEC02F6BV5 | Bradi2g21420.1 | - | Sb09g024060.1 | - |
| IVLNWEC02G2622 | Bradi2g21420.1 | - | Sb09g024060.1 | - |
| IUIVLUD01CRK1P | Bradi2g20310.1 | - | Sb09g025080.1 | - |
| IV9RU5F02JJQV5 | Bradi3g01150.1 | - | Sb09g025250.1 | - |
| IVLNWEC01EB5SJ | Bradi3g01150.1 | - | Sb09g025250.1 | - |
| IUIVLUD02H2FGI | Bradi2g19640.1 | - | Sb09g025790.1 | - |
| IUIVLUD02HDE2J | Bradi2g46150.1 | - | Sb09g026140.1 | - |
| IVLNWEC02FQWFU | Bradi2g19290.1 | - | Sb09g026160.1 | - |
| IV9RU5F02IH5SG | Bradi2g19250.1 | - | Sb09g026180.1 | - |
| IV9RU5F02GQZL7 | Bradi2g17000.1 | - | Sb09g028070.1 | - |
| IVLNWEC01EGV7G | Bradi2g16940.1 | - | Sb09g028090.1 | - |
| IUIVLUD02I0TV1 | Bradi2g06780.1 | - | Sb09g028670.1 | - |
| IUIVLUD02F5OX7 | Bradi2g14450.1 | - | Sb09g030570.1 | - |
| IUIVLUD02HOC4U | Bradi2g14450.1 | - | Sb09g030570.1 | - |
| IV9RU5F01AV214 | Bradi2g14450.1 | - | Sb09g030570.1 | - |
| IV9RU5F02H2MPR | Bradi2g14450.1 | - | Sb09g030570.1 | - |
| IVLNWEC01C4EMF | Bradi2g14450.1 | - | Sb09g030570.1 | - |
| IVLNWEC01CAE2N | Bradi2g14450.1 | - | Sb09g030570.1 | - |
| IVLNWEC01DVH6D | Bradi2g14450.1 | - | Sb09g030570.1 | - |
| IV9RU5F01E1K1N | Bradi2g14340.2 | - | Sb09g030650.1 | - |
| IVLNWEC01CX9TR | Bradi1g02240.1 | - | Sb09g030660.1 | - |
| IVLNWEC01BLURH | Bradi1g52320.1 | - | Sb10g000390.1 | - |
| IVLNWEC01DKEIC | Bradi1g52330.1 | - | Sb10g000400.1 | - |
| IV9RU5F02IBIUT | Bradi5g02430.1 | - | Sb10g000860.1 | - |
| IV9RU5F02IXZ3B | Bradi1g49450.1 | - | Sb10g001470.1 | - |
| IVLNWEC02F2SMW | Bradi1g49790.1 | - | Sb10g001840.1 | - |
| IVLNWEC02FKJV7 | Bradi1g49790.1 | - | Sb10g001840.1 | - |
| IVLNWEC02F6Q7W | Bradi1g49840.1 | - | Sb10g001880.1 | - |
| IV9RU5F02HJH9D | Bradi1g50890.1 | - | Sb10g003430.1 | - |
| IVLNWEC01C4VSS | Bradi1g48264.1 | - | Sb10g004682.1 | - |
| IVLNWEC01EJDME | Bradi1g48264.1 | - | Sb10g004682.1 | - |
| IVLNWEC02FPUFI | Bradi1g48170.1 | - | Sb10g004780.1 | - |
| IUIVLUD01AZRUD | Bradi1g27750.1 | - | Sb10g006030.1 | - |
| IV9RU5F02JWYNT | Bradi1g46360.1 | - | Sb10g006740.1 | - |
| IVLNWEC01BEZON | Bradi1g46360.1 | - | Sb10g006740.1 | - |
| IV9RU5F01BYYDN | Bradi1g44530.1 | - | Sb10g008780.1 | - |
| IVLNWEC01DE7T4 | Bradi1g44530.1 | - | Sb10g008780.1 | - |
| IVLNWEC02IMEPX | Bradi1g75240.1 | - | Sb10g011270.1 | - |
| IV9RU5F02FWT49 | Bradi1g02840.1 | - | Sb10g020060.1 | - |
| IVLNWEC02GN9QT | Bradi1g51320.1 | - | Sb10g021850.1 | - |

| | | | | |
|---|---|---|---|---|
| IV9RU5F01DSGKM | Bradi1g36387.1 | - | Sb10g023780.1 | - |
| IVLNWEC02ITED5 | Bradi1g36387.1 | - | Sb10g023780.1 | - |
| IV9RU5F02HGXCU | Bradi1g35960.1 | - | Sb10g024200.1 | - |
| IV9RU5F01ANGYY | Bradi1g30648.1 | - | Sb10g025390.1 | - |
| IVLNWEC01AJ60Z | Bradi1g30656.1 | - | Sb10g025400.1 | - |
| IV9RU5F01AMHYQ | Bradi1g32420.1 | - | Sb10g027000.1 | - |
| IV9RU5F01C56WU | Bradi1g30672.1 | - | Sb1736s002010.1 | - |
| IV9RU5F01C9MSM | Bradi1g30672.1 | - | Sb1736s002010.1 | - |
| IV9RU5F01BUFN1 | Bradi2g61830.1 | - | Sb03g047060.1 | - |
| IV9RU5F02IHTMK | Bradi2g61830.1 | - | Sb03g047060.1 | - |
| IUIVLUD01AIHG9 | Bradi1g28210.1 | - | Sb01g026670.1 | - |
| IV9RU5F02JIAOW | Bradi4g39650.1 | - | Sb01g001200.1 | - |
| IVLNWEC01EPTOO | Bradi1g25090.1 | - | Sb02g036040.1 | - |
| IV9RU5F02IB4YT | Bradi4g36160.2 | - | Sb02g030960.1 | - |
| IVLNWEC01CMT63 | Bradi4g36160.2 | - | Sb02g030960.1 | - |
| IVLNWEC02JLQU2 | Bradi4g36160.2 | - | Sb02g030960.1 | - |
| IVLNWEC01BU4AK | Bradi1g44530.1 | - | Sb10g008780.1 | - |
| IUIVLUD02I3M7F | Bradi1g08607.1 | - | Sb01g008080.1 | - |
| IV9RU5F01BK69A | Bradi1g08607.1 | - | Sb01g008080.1 | - |
| IVLNWEC01CT5B8 | Bradi1g08607.1 | - | Sb01g008080.1 | - |
| IVLNWEC01CGNLF | Bradi1g06430.1 | - | Sb01g005960.1 | - |
| IVLNWEC02HTS3F | Bradi1g06430.1 | - | Sb01g005960.1 | - |
| IVLNWEC02G227I | Bradi4g29840.1 | - | Sb02g024280.1 | - |
| IUIVLUD02GHKB7 | Bradi1g01800.1 | - | Sb01g001140.1 | - |
| IVLNWEC01A9OIV | Bradi1g69780.1 | - | Sb01g042750.1 | - |
| IVLNWEC01A4LFL | Bradi4g04620.1 | - | Sb08g018360.1 | - |
| IVLNWEC01C86UO | Bradi4g02990.1 | - | Sb08g019930.1 | - |
| IV9RU5F01BOD5D | Bradi1g08900.1 | - | Sb01g008390.1 | - |
| IVLNWEC02I4K7H | Bradi1g03920.1 | - | Sb01g003490.1 | - |
| IVLNWEC02G8LW4 | Bradi1g62007.1 | - | Sb01g034960.1 | - |
| IV9RU5F01BHIA1 | Bradi1g14360.1 | - | Sb01g014220.1 | - |
| IVLNWEC02G0V1Y | Bradi1g03560.1 | - | Sb02g001010.1 | - |
| IV9RU5F01A8HJT | Bradi4g06370.1 | - | Sb08g016560.1 | - |
| IUIVLUD02I53BA | Bradi1g00460.1 | - | Sb01g000220.1 | - |
| IV9RU5F01C8THQ | Bradi1g01810.1 | - | Sb01g001360.1 | - |
| IUIVLUD02GKR00 | Bradi2g28007.1 | - | Sb09g017790.1 | - |
| IV9RU5F01AY6IN | Bradi1g27020.1 | - | Sb01g046340.1 | - |
| IV9RU5F02IRMYG | Bradi4g31630.1 | - | Sb02g026110.1 | - |
| IV9RU5F01EROEZ | Bradi3g01150.1 | - | Sb09g025250.1 | - |
| IVLNWEC02J3KTH | Bradi2g49010.1 | - | Sb03g033770.1 | - |
| IV9RU5F02F8RFK | Bradi1g00460.1 | - | Sb01g000220.1 | - |
| IV9RU5F02GD702 | Bradi1g02420.1 | - | Sb01g001970.1 | - |
| IVLNWEC01BVLH3 | Bradi4g30570.1 | - | Sb07g007540.1 | - |
| IV9RU5F02JJQ1O | Bradi1g33410.1 | - | Sb07g004330.1 | - |
| IUIVLUD02I25T1 | Bradi4g37190.1 | - | Sb02g032145.1 | - |
| IV9RU5F01BFHRQ | Bradi2g60230.1 | - | Sb03g045290.1 | - |
| IV9RU5F02JIDRF | Bradi4g32830.1 | - | Sb02g002110.1 | - |

| | | | | |
|---|---|---|---|---|
| IUIVLUD02J1UGJ | Bradi1g03650.1 | - | Sb01g003130.1 | - |
| IV9RU5F01EJBL3 | Bradi1g03650.1 | - | Sb01g003130.1 | - |
| IVLNWEC01CNZER | Bradi1g09810.1 | - | Sb01g009225.1 | - |
| IVLNWEC02JB1E9 | Bradi4g37420.1 | - | Sb02g032310.1 | - |
| IV9RU5F02JGWMK | Bradi1g04830.1 | - | Sb01g004510.1 | - |
| IV9RU5F01BB8KK | Bradi1g03550.1 | - | Sb02g001020.1 | - |
| IVLNWEC01A01KP | Bradi1g11960.1 | - | Sb01g011410.1 | - |
| IV9RU5F02HMJ1D | Bradi1g74170.1 | - | Sb01g032760.1 | - |
| IVLNWEC02GKJWP | Bradi4g22690.1 | - | Sb05g009420.1 | - |
| IUIVLUD02JCNBZ | Bradi1g00580.1 | - | Sb01g000310.1 | - |
| IVLNWEC02JQUL1 | Bradi1g00580.1 | - | Sb01g000310.1 | - |
| IV9RU5F01DX57Z | Bradi1g10210.1 | - | Sb01g009630.1 | - |
| IV9RU5F02FR6AJ | Bradi3g05950.1 | - | Sb02g041940.1 | - |
| IV9RU5F01BL4H1 | Bradi1g48264.1 | - | Sb10g004682.1 | - |
| IVLNWEC02GGOYA | Bradi1g67960.1 | - | Sb01g040970.1 | - |
| IVLNWEC02HTG4B | Bradi4g43490.5 | - | Sb05g002140.1 | - |
| IUIVLUD02HEGRW | Bradi5g22187.1 | - | Sb06g028930.1 | - |
| IV9RU5F02IKV1K | Bradi3g57690.1 | - | Sb04g007853.1 | - |
| IUIVLUD01D5L53 | Bradi4g33960.1 | - | Sb07g013780.1 | - |
| IUIVLUD01AJJ27 | Bradi4g26430.1 | - | Sb08g001480.1 | - |
| IVLNWEC02F9G11 | Bradi1g71650.1 | - | Sb07g021830.1 | - |
| IUIVLUD02GDYXV | Bradi3g54430.1 | - | Sb04g036270.1 | - |
| IVLNWEC01BIT2A | Bradi1g07590.1 | - | Sb01g007100.1 | - |
| IUIVLUD02JJ2ZX | Bradi1g03330.1 | - | Sb01g002785.1 | - |
| IUIVLUD02IUNS4 | Bradi3g05370.1 | - | Sb08g000310.1 | - |
| IV9RU5F01C0PF7 | Bradi1g01980.1 | - | Sb01g001560.1 | - |
| IVLNWEC01C1BX0 | Bradi1g03650.1 | - | Sb01g003130.1 | - |
| IV9RU5F02GRJ17 | Bradi1g15330.1 | - | Sb01g015490.1 | - |
| IVLNWEC01D44EE | Bradi4g01513.1 | - | Sb02g027700.1 | - |
| IUIVLUD01CAK8Z | Bradi1g70330.1 | - | Sb01g043190.1 | - |
| IVLNWEC02J16ZP | Bradi1g70330.1 | - | Sb01g043190.1 | - |
| IVLNWEC01CH2OQ | Bradi1g55840.1 | - | Sb02g007040.1 | - |
| IVLNWEC02GCT0H | Bradi4g33886.1 | - | Sb02g028600.1 | - |
| IVLNWEC01DOE5H | Bradi1g62640.1 | - | Sb01g035540.1 | - |
| IVLNWEC02JAK7W | Bradi3g44960.1 | - | Sb04g021790.1 | - |
| IVLNWEC02G4AEA | Bradi1g50950.1 | - | Sb06g001320.1 | - |
| IVLNWEC01AZRTF | Bradi4g16885.1 | - | Sb05g020820.2 | - |
| IVLNWEC02GAQDN | Bradi4g16885.1 | - | Sb05g020820.2 | - |
| IV9RU5F01BKUWN | Bradi1g08301.1 | - | Sb01g007740.1 | - |
| IV9RU5F01A1HQF | Bradi1g73860.1 | - | Sb01g022240.1 | - |
| IV9RU5F02G9P1L | Bradi1g12530.1 | - | Sb04g019750.1 | - |
| IUIVLUD02F4XFU | Bradi4g03150.1 | - | Sb08g019730.1 | - |
| IV9RU5F01BMP03 | Bradi4g03150.1 | - | Sb08g019730.1 | - |
| IVLNWEC02GZIWR | Bradi1g02247.1 | - | Sb03g025820.1 | - |
| IVLNWEC02GK2CI | Bradi4g35301.1 | - | Sb02g029920.1 | - |
| IUIVLUD01AJAL3 | Bradi2g20360.2 | - | Sb09g025030.1 | - |
| IV9RU5F01DKR3K | Bradi4g02587.1 | - | Sb02g036590.1 | - |

| | | | | |
|---|---|---|---|---|
| IV9RU5F01DNRPF | Bradi4g02587.1 | - | Sb02g036590.1 | - |
| IVLNWEC02JE6L7 | Bradi4g02587.1 | - | Sb02g036590.1 | - |
| IV9RU5F01BTNHX | Bradi4g41717.1 | - | Sb08g004180.1 | - |
| IV9RU5F02F9A29 | Bradi2g59226.1 | - | Sb03g044310.1 | - |
| IVLNWEC01ENXD5 | Bradi1g63240.1 | - | Sb01g036190.1 | - |
| IVLNWEC01EPNGW | Bradi2g25527.1 | - | Sb09g020000.1 | - |
| IVLNWEC01CF4F1 | Bradi1g63210.1 | - | Sb01g036170.1 | - |
| IVLNWEC01DROFQ | Bradi1g17670.1 | - | Sb01g036170.1 | - |
| IV9RU5F01CPZDD | Bradi3g10180.2 | - | Sb04g010370.1 | - |
| IVLNWEC02IHDSW | Bradi3g48860.1 | - | Sb04g026110.1 | - |
| IV9RU5F01BAGKQ | Bradi1g64770.1 | - | Sb01g037790.1 | - |
| IUIVLUD02JDSM4 | Bradi4g44780.1 | - | Sb08g000780.1 | - |
| IUIVLUD01AUE9Z | Bradi3g15520.1 | - | Sb07g004940.1 | - |
| IV9RU5F01DX2FY | Bradi4g24170.1 | - | Sb03g003740.1 | - |
| IVLNWEC02F6IER | Bradi1g04980.1 | - | Sb01g004640.1 | - |
| IVLNWEC01E1MP3 | Bradi1g07717.1 | - | Sb08g014960.1 | - |
| IUIVLUD02F5H9L | Bradi1g45620.1 | - | Sb06g029476.1 | - |
| IVLNWEC02JR83C | Bradi1g13720.1 | - | Sb01g013470.1 | - |
| IV9RU5F01C0MDC | Bradi1g08500.1 | - | Sb01g007970.1 | - |
| IV9RU5F01D8QNI | Bradi4g26877.1 | - | Sb05g000450.1 | - |
| IV9RU5F02GOGSE | Bradi1g10447.1 | - | Sb01g009860.1 | - |
| IVLNWEC02HUBCP | Bradi4g05830.1 | - | Sb08g017020.1 | - |
| IV9RU5F01ATMH1 | Bradi3g01150.1 | - | Sb09g025250.1 | - |
| IV9RU5F01APMO5 | Bradi2g49050.1 | - | Sb02g026560.1 | - |
| IV9RU5F02IG784 | Bradi1g68237.1 | - | Sb01g041380.1 | - |
| IV9RU5F02ISEJI | Bradi1g68237.1 | - | Sb01g041380.1 | - |
| IV9RU5F02IGH0J | Bradi1g66687.1 | - | Sb01g039660.1 | - |
| IVLNWEC02G81XS | Bradi1g03420.1 | - | Sb01g002850.1 | - |
| IVLNWEC02H4YM4 | Bradi2g54390.1 | - | Sb03g039200.1 | - |
| IVLNWEC01A33PI | Bradi1g37960.1 | - | Sb10g022060.1 | - |
| IUIVLUD01ERBOE | Bradi4g28400.1 | - | Sb02g022580.1 | - |
| IV9RU5F02HLCPM | Bradi1g07755.1 | - | Sb01g007195.1 | - |
| IVLNWEC01DX1HX | Bradi1g07755.1 | - | Sb01g007195.1 | - |
| IVLNWEC01B60MR | Bradi4g02100.1 | - | Sb08g020980.1 | - |
| IV9RU5F02FQZAB | Bradi1g13910.1 | - | Sb01g013710.1 | - |
| IVLNWEC02F5ALY | Bradi2g01550.1 | - | Sb03g007840.1 | - |
| IVLNWEC01EPNM2 | Bradi2g31550.1 | - | Sb01g044380.1 | - |
| IUIVLUD01B9B02 | Bradi3g03720.1 | - | Sb04g003410.1 | - |
| IUIVLUD01ELI13 | Bradi3g58300.1 | - | Sb04g033870.1 | - |
| IV9RU5F01BXCGR | Bradi3g58300.1 | - | Sb04g033870.1 | - |
| IUIVLUD01B3RQZ | Bradi2g08980.1 | - | Sb06g020700.1 | - |
| IV9RU5F02GRQJB | Bradi1g12140.1 | - | Sb01g011620.1 | - |
| IV9RU5F01DZX72 | Bradi1g75470.2 | - | Sb01g047340.1 | - |
| IVLNWEC02IVNXQ | Bradi1g75470.2 | - | Sb01g047340.1 | - |
| IVLNWEC02F0JLW | Bradi2g34820.1 | - | Sb09g004530.1 | - |
| IUIVLUD01DHIE6 | Bradi3g06847.1 | - | Sb04g006380.1 | - |
| IV9RU5F02HQQEC | Bradi2g37990.1 | - | Sb05g005853.1 | - |

| | | | | |
|---|---|---|---|---|
| IUIVLUD01CSJWN | Bradi4g21057.1 | - | Sb03g036320.1 | - |
| IUIVLUD02J2KPT | Bradi4g21057.1 | - | Sb03g036320.1 | - |
| IV9RU5F01CF48W | Bradi4g21057.1 | - | Sb03g036320.1 | - |
| IVLNWEC01B5SBT | Bradi1g11317.2 | - | Sb01g010410.1 | - |
| IVLNWEC01BS31O | Bradi1g11317.2 | - | Sb01g010410.1 | - |
| IV9RU5F02HHW7J | Bradi2g40170.1 | - | Sb01g045820.1 | - |
| IV9RU5F01D0XB2 | Bradi1g28210.1 | - | Sb01g026670.1 | - |
| IUIVLUD02GTUKX | Bradi4g00480.1 | - | Sb08g023130.1 | - |
| IVLNWEC02JGVN8 | Bradi4g00480.1 | - | Sb08g023130.1 | - |
| IVLNWEC02JL8P3 | Bradi1g08420.1 | - | Sb01g007880.1 | - |
| IV9RU5F02GPUSK | Bradi1g53160.1 | - | Sb02g007870.1 | - |
| IV9RU5F02GMAWR | Bradi4g30980.1 | - | Sb02g025460.1 | - |
| IVLNWEC02JSYK6 | Bradi1g05560.1 | - | Sb01g005260.1 | - |
| IV9RU5F01CGAFT | Bradi1g42650.1 | - | Sb04g030010.1 | - |
| IVLNWEC02IP6FS | Bradi1g10130.1 | - | Sb01g009540.1 | - |
| IVLNWEC01C0NIY | Bradi1g04830.1 | - | Sb01g004510.1 | - |
| IV9RU5F02JA79F | Bradi1g54590.1 | - | Sb08g003880.1 | - |
| IV9RU5F02JWOW8 | Bradi1g48170.1 | - | Sb10g004780.1 | - |
| IVLNWEC01EM7TM | Bradi1g48170.1 | - | Sb10g004780.1 | - |
| IVLNWEC01BMNCZ | Bradi1g68550.1 | - | Sb01g041470.1 | - |
| IUIVLUD01D9HIA | Bradi1g45720.1 | - | Sb05g008515.1 | - |
| IV9RU5F01BU1P1 | Bradi4g30980.1 | - | Sb02g025460.1 | - |
| IUIVLUD01D9LD4 | Bradi4g05950.1 | - | Sb08g016860.1 | - |
| IVLNWEC01CW7DT | Bradi1g49470.1 | - | Sb10g001530.1 | - |
| IVLNWEC02FY67T | Bradi1g74940.1 | - | Sb01g046850.1 | - |
| IUIVLUD02JL79R | Bradi4g03890.1 | - | Sb08g018890.1 | - |
| IV9RU5F02IS5X4 | Bradi1g07390.1 | - | Sb01g006930.1 | - |
| IV9RU5F02FSSRD | Bradi3g39900.2 | - | Sb07g027850.1 | - |
| IVLNWEC02HLX4C | Bradi1g58930.1 | - | Sb04g003073.1 | - |
| IUIVLUD01C7TY4 | Bradi5g22017.1 | - | Sb03g005130.1 | - |
| IVLNWEC02IRWLV | Bradi3g57690.1 | - | Sb04g007853.1 | - |
| IUIVLUD02INXOO | Bradi1g06230.1 | - | Sb01g005710.1 | - |
| IUIVLUD01AGKBJ | Bradi4g41650.1 | - | Sb08g004240.1 | - |
| IV9RU5F01ECD8Z | Bradi4g11120.1 | - | Sb10g016950.1 | - |
| IUIVLUD02JBT99 | Bradi4g32390.1 | - | Sb02g026840.1 | - |
| IVLNWEC01EQ27X | Bradi4g27417.1 | - | Sb01g015210.1 | - |
| IUIVLUD02I01JK | Bradi3g34387.1 | - | Sb01g028140.1 | - |
| IVLNWEC01CY0AR | Bradi2g14440.1 | - | Sb09g030580.1 | - |
| IV9RU5F02G4VI3 | Bradi2g31550.1 | - | Sb01g044380.1 | - |
| IUIVLUD02IXSTR | Bradi2g38510.1 | - | Sb09g000630.1 | - |
| IV9RU5F02H5ACL | Bradi3g18390.1 | - | Sb07g006470.1 | - |
| IVLNWEC01CAKTB | Bradi1g37304.1 | - | Sb09g004960.1 | - |
| IV9RU5F02I5W9O | Bradi2g14750.1 | - | Sb06g024560.1 | - |
| IV9RU5F01EGXWC | Bradi1g01250.1 | - | Sb02g005235.1 | - |
| IV9RU5F02J4HDG | Bradi1g00607.1 | - | Sb01g000330.1 | - |
| IUIVLUD01DW3R0 | Bradi1g16680.1 | - | Sb02g043880.1 | - |
| IUIVLUD02GAEYO | Bradi1g66130.1 | - | Sb01g038980.1 | - |

| | | | | |
|---|---|---|---|---|
| IV9RU5F02GC99E | Bradi4g31947.1 | - | Sb02g026420.1 | - |
| IV9RU5F02HX5T4 | Bradi4g01100.1 | - | Sb08g022310.1 | - |
| IUIVLUD02F94AN | Bradi4g35376.1 | - | Sb02g030160.1 | - |
| IVLNWEC02HO98Y | Bradi1g01830.1 | - | Sb01g001380.1 | - |
| IV9RU5F01C8KMI | Bradi4g37120.1 | - | Sb06g001960.1 | - |
| IUIVLUD01DTIQ2 | Bradi1g04790.1 | - | Sb01g004420.1 | - |
| IV9RU5F01AWCDD | Bradi4g38080.1 | - | Sb07g024480.1 | - |
| IV9RU5F01ENVT1 | Bradi1g05180.1 | - | Sb01g004940.1 | - |
| IVLNWEC01BWX38 | Bradi3g49640.1 | - | Sb04g032380.1 | - |
| IV9RU5F01BUNET | Bradi4g31820.1 | - | Sb04g034520.1 | - |
| IV9RU5F02HDRYB | Bradi2g08750.1 | - | Sb07g028225.1 | - |
| IV9RU5F01DRHLM | Bradi3g20070.1 | - | Sb04g006250.1 | - |
| IV9RU5F02H0WDQ | Bradi1g04980.1 | - | Sb01g004640.1 | - |
| IV9RU5F02GQDCE | Bradi4g41830.1 | - | Sb08g003990.1 | - |
| IVLNWEC01DGUZW | Bradi4g28400.1 | - | Sb02g022580.1 | - |
| IV9RU5F01C2HU3 | Bradi4g30850.1 | - | Sb02g025340.1 | - |
| IUIVLUD01B36SO | Bradi4g00790.1 | - | Sb01g013840.1 | - |
| IV9RU5F01BARP4 | Bradi4g02030.1 | - | Sb08g021110.1 | - |
| IVLNWEC02JWZLW | Bradi2g08390.1 | - | Sb01g038510.1 | - |
| IVLNWEC01D56FY | Bradi4g32250.1 | - | Sb02g026720.1 | - |
| IV9RU5F02JV5R2 | Bradi2g12960.1 | - | Sb03g014800.1 | - |
| IVLNWEC01BM51N | Bradi3g57690.1 | - | Sb04g007853.1 | - |
| IVLNWEC02HIYSW | Bradi3g10600.1 | - | Sb10g022860.1 | - |
| IVLNWEC02J273Z | Bradi5g00440.1 | - | Sb03g014390.1 | - |
| IVLNWEC01CB6O9 | Bradi5g20420.1 | - | Sb06g027382.1 | - |
| IV9RU5F02H9VTI | Bradi4g36160.2 | - | Sb02g030960.1 | - |
| IVLNWEC02JV8LC | Bradi4g31336.1 | - | Sb02g031620.1 | - |
| IVLNWEC02IUHGB | Bradi2g11860.1 | - | Sb03g012830.1 | - |
| IV9RU5F01A3NPY | Bradi3g20070.1 | - | Sb04g006250.1 | - |
| IV9RU5F02ILDMM | Bradi4g37030.1 | - | Sb02g031850.1 | - |
| IUIVLUD02GBBFG | Bradi1g62007.1 | - | Sb01g034960.1 | - |
| IVLNWEC02JQ8RB | Bradi4g31630.1 | - | Sb02g026110.1 | - |
| IUIVLUD01A6NKH | Bradi4g31070.2 | - | Sb02g025590.1 | - |
| IV9RU5F02JF331 | Bradi1g10360.1 | - | Sb01g009790.1 | - |
| IVLNWEC01EGB3E | Bradi4g31070.2 | - | Sb02g025590.1 | - |
| IUIVLUD01AR92E | Bradi1g01250.1 | - | Sb02g005235.1 | - |
| IV9RU5F02JE60I | Bradi1g66167.1 | - | Sb09g007390.1 | - |
| IVLNWEC01C5FG2 | Bradi1g66167.1 | - | Sb09g007390.1 | - |
| IV9RU5F01DIUG9 | Bradi1g67140.1 | - | Sb01g040090.1 | - |
| IUIVLUD01A32WQ | Bradi2g08390.1 | - | Sb01g038510.1 | - |
| IVLNWEC02G3YI1 | Bradi1g31130.1 | - | Sb06g019540.1 | - |
| IV9RU5F02GFYKS | Bradi1g78470.1 | - | Sb01g010390.1 | - |
| IVLNWEC02JPG8K | Bradi1g75340.1 | - | Sb01g047150.1 | - |
| IVLNWEC02IR9QH | Bradi1g02710.1 | - | Sb02g007460.1 | - |
| IV9RU5F01BQBYE | Bradi1g72967.1 | - | Sb01g045520.1 | - |
| IV9RU5F01AL5KU | Bradi1g63240.1 | - | Sb01g036190.1 | - |
| IV9RU5F01EO6UX | Bradi1g51430.1 | - | Sb10g002570.1 | - |

| | | | | |
|---|---|---|---|---|
| IUIVLUD02F2JRH | Bradi1g17670.1 | - | Sb01g036170.1 | - |
| IUIVLUD01DIUWK | Bradi4g27157.1 | - | Sb02g018910.1 | - |
| IV9RU5F01DZXZX | Bradi1g70570.1 | - | Sb01g043330.1 | - |
| IVLNWEC01BG172 | Bradi2g17000.1 | - | Sb09g028070.1 | - |
| IVLNWEC01D1B1S | Bradi1g07650.1 | - | Sb01g007150.1 | - |
| IVLNWEC01CGX1B | Bradi4g39550.1 | - | Sb08g008650.1 | - |
| IV9RU5F01EU9BZ | Bradi3g03720.1 | - | Sb04g003410.1 | - |
| IV9RU5F02HNP03 | Bradi5g04000.1 | - | Sb06g003140.1 | - |
| IV9RU5F02IX4GI | Bradi1g31970.1 | - | Sb03g027070.1 | - |
| IVLNWEC02IQMC2 | Bradi4g01080.1 | - | Sb05g002340.1 | AK248684.1 |
| IV9RU5F02H4HZ4 | Bradi4g29830.1 | - | Sb02g024270.1 | AK250045.1 |
| IVLNWEC01DS7IA | Bradi1g05560.1 | - | Sb01g005260.1 | AK251691.1 |
| IV9RU5F02H7DBH | Bradi4g08260.3 | - | Sb02g013050.1 | AK252445.1 |
| IVLNWEC02HEUV6 | Bradi4g08260.3 | - | Sb02g013050.1 | AK252445.1 |
| IV9RU5F01DWD29 | Bradi3g19100.1 | - | Sb07g007870.1 | AK252538.1 |
| IV9RU5F01BY43O | Bradi5g00990.1 | - | Sb03g033755.1 | AK252561.1 |
| IVLNWEC01AHK23 | Bradi1g02420.1 | - | Sb01g001970.1 | AK252569.1 |
| IVLNWEC02HAOCC | Bradi1g02420.1 | - | Sb01g001970.1 | AK252569.1 |
| IV9RU5F01DBBHD | Bradi1g52280.1 | - | Sb10g000350.1 | AK353650 |
| IVLNWEC02J1W3T | Bradi4g32967.1 | - | Sb02g027460.1 | AK353724 |
| IUIVLUD01AY52L | Bradi2g08080.1 | - | Sb03g000570.1 | AK354365 |
| IUIVLUD01D0W10 | Bradi1g14590.1 | - | Sb01g014570.1 | AK354697 |
| IV9RU5F02JCLZ8 | Bradi1g12140.1 | - | Sb01g011620.1 | AK354857 |
| IV9RU5F01DKEWV | Bradi1g01130.2 | - | Sb01g000730.1 | AK354917 |
| IV9RU5F02F7XBP | Bradi4g26870.1 | - | Sb09g019780.1 | AK355004 |
| IVLNWEC02H8DMM | Bradi1g04830.1 | - | Sb01g004510.1 | AK355259 |
| IVLNWEC01EC94T | Bradi4g43877.1 | - | Sb05g001790.1 | AK355408 |
| IVLNWEC01CZRVX | Bradi1g63510.1 | - | Sb01g036420.1 | AK355834 |
| IVLNWEC01E21CP | Bradi1g10530.2 | - | Sb01g009940.1 | AK355901 |
| IV9RU5F02F1BWA | Bradi1g06772.1 | - | Sb01g006280.1 | AK356364 |
| IVLNWEC01DOGJ0 | Bradi4g30390.1 | - | Sb07g021660.1 | AK357267 |
| IVLNWEC01E3RMW | Bradi4g30390.1 | - | Sb07g021660.1 | AK357267 |
| IUIVLUD02HIIKS | Bradi1g16210.1 | - | Sb01g032340.1 | AK357977 |
| IVLNWEC01BO5YY | Bradi1g16210.1 | - | Sb01g032340.1 | AK357977 |
| IVLNWEC01CWN57 | Bradi1g05156.1 | - | Sb01g004870.1 | AK358246 |
| IV9RU5F01CVMUW | Bradi4g07040.1 | - | Sb08g015410.1 | AK358846 |
| IV9RU5F01A4OQW | Bradi3g30120.1 | - | Sb01g035200.1 | AK358994 |
| IVLNWEC01D2RC8 | Bradi3g30120.1 | - | Sb01g035200.1 | AK358994 |
| IUIVLUD01AOGVO | Bradi4g31540.1 | - | Sb02g026010.1 | AK359376 |
| IV9RU5F01EFZRM | Bradi4g31540.1 | - | Sb02g026010.1 | AK359376 |
| IVLNWEC01C563Z | Bradi4g31540.1 | - | Sb02g026010.1 | AK359376 |
| IV9RU5F01CGVO3 | Bradi1g24310.1 | - | Sb05g001500.1 | AK359827 |
| IV9RU5F01ALV5F | Bradi1g04440.1 | - | Sb01g004150.1 | AK360154 |
| IV9RU5F02GEZ0B | Bradi1g04440.1 | - | Sb01g004150.1 | AK360154 |
| IVLNWEC01B39NQ | Bradi1g04440.1 | - | Sb01g004150.1 | AK360154 |
| IVLNWEC02JDB34 | Bradi1g04440.1 | - | Sb01g004150.1 | AK360154 |
| IUIVLUD01AMU8V | Bradi1g05480.1 | - | Sb01g005170.1 | AK360603 |

| | | | | |
|---|---|---|---|---|
| IV9RU5F02GNVKR | Bradi1g00790.1 | - | Sb01g000430.1 | AK361568 |
| IV9RU5F01BOPMS | Bradi4g32960.1 | - | Sb02g027450.1 | AK361970 |
| IVLNWEC02HUBXT | Bradi4g32960.1 | - | Sb02g027450.1 | AK361970 |
| IVLNWEC01AOSYD | Bradi3g48860.1 | - | Sb04g026110.1 | AK362230 |
| IVLNWEC02GD5KM | Bradi3g53310.1 | - | Sb04g029950.1 | AK362483 |
| IVLNWEC02G7N1T | Bradi1g06540.1 | - | Sb01g006100.1 | AK362698 |
| IUIVLUD02H0GVP | Bradi1g67140.1 | - | Sb01g040090.1 | AK362963 |
| IV9RU5F01AMJQB | Bradi1g67140.1 | - | Sb01g040090.1 | AK362963 |
| IV9RU5F01ANR66 | Bradi1g67140.1 | - | Sb01g040090.1 | AK362963 |
| IV9RU5F02FNUOH | Bradi1g67140.1 | - | Sb01g040090.1 | AK362963 |
| IV9RU5F02JJXRZ | Bradi1g67140.1 | - | Sb01g040090.1 | AK362963 |
| IVLNWEC01BQ0NB | Bradi1g67140.1 | - | Sb01g040090.1 | AK362963 |
| IVLNWEC02HUJY2 | Bradi1g67140.1 | - | Sb01g040090.1 | AK362963 |
| IUIVLUD01EPJYR | Bradi1g33410.1 | - | Sb07g004330.1 | AK363003 |
| IV9RU5F01D2BLE | Bradi1g33410.1 | - | Sb07g004330.1 | AK363003 |
| IV9RU5F02IKK9L | Bradi1g50067.1 | - | Sb10g002070.1 | AK363081 |
| IVLNWEC02F4N76 | Bradi2g37990.1 | - | Sb08g017060.1 | AK363200 |
| IUIVLUD02GSY1R | Bradi4g42810.1 | - | Sb05g002900.1 | AK363464 |
| IV9RU5F02IC7AO | Bradi3g34387.1 | - | Sb01g028140.1 | AK364034 |
| IV9RU5F02FU2UO | Bradi4g01215.1 | - | Sb08g017730.1 | AK364168 |
| IVLNWEC02JU16E | Bradi4g01215.1 | - | Sb08g017730.1 | AK364168 |
| IVLNWEC02JGEA3 | Bradi1g13910.1 | - | Sb01g013710.1 | AK364215 |
| IVLNWEC01DF9W8 | Bradi1g14450.1 | - | Sb01g014290.1 | AK364857 |
| IV9RU5F02JXORS | Bradi2g46550.1 | - | Sb03g031160.1 | AK365017 |
| IVLNWEC02HISNL | Bradi1g13440.1 | - | Sb01g013100.1 | AK365734 |
| IV9RU5F02HMUUV | Bradi4g38037.1 | - | Sb02g011230.1 | AK365745 |
| IUIVLUD01BR2WU | Bradi2g38030.2 | - | Sb08g000380.1 | AK366345 |
| IV9RU5F01EOJ6K | Bradi2g38030.2 | - | Sb08g000380.1 | AK366345 |
| IUIVLUD02I0BYQ | Bradi1g01460.1 | - | Sb01g000970.1 | AK366381 |
| IVLNWEC01A62HW | Bradi1g02510.1 | - | Sb01g002050.1 | AK366571 |
| IUIVLUD01EBL0O | Bradi5g01990.1 | - | Sb03g030490.1 | AK366848 |
| IV9RU5F01EN569 | Bradi1g20630.1 | - | Sb09g018670.1 | AK368262 |
| IUIVLUD02HL90I | Bradi1g03277.1 | - | Sb02g039465.1 | AK368276 |
| IV9RU5F01BGXVF | Bradi3g60550.1 | - | Sb04g038310.1 | AK368463 |
| IUIVLUD02H7FYD | Bradi4g36976.1 | - | Sb02g021250.1 | AK368548 |
| IUIVLUD02HFJWV | Bradi4g44890.1 | - | Sb08g000535.1 | AK368828 |
| IV9RU5F02JGMLJ | Bradi1g02170.1 | - | Sb01g001770.1 | AK369519 |
| IVLNWEC02IC7EY | Bradi1g15330.1 | - | Sb01g015490.1 | AK369530 |
| IVLNWEC02F28BZ | Bradi1g15547.1 | - | Sb01g015850.1 | AK369964 |
| IUIVLUD01DUDV8 | Bradi1g15547.1 | - | Sb01g015860.1 | AK369964 |
| IV9RU5F01B9QS5 | Bradi4g33520.1 | - | Sb02g028170.1 | AK370417 |
| IV9RU5F01EPI2V | Bradi4g33520.1 | - | Sb02g028170.1 | AK370417 |
| IV9RU5F02GA8F6 | Bradi4g33520.1 | - | Sb02g028170.1 | AK370417 |
| IV9RU5F01DD4MT | Bradi4g21277.1 | - | Sb05g007160.1 | AK370601 |
| IVLNWEC01BNAC0 | Bradi4g21277.1 | - | Sb05g007160.1 | AK370601 |
| IUIVLUD01A31XD | Bradi3g10600.1 | - | Sb06g003140.1 | AK370749 |
| IVLNWEC01D4O7L | Bradi1g37304.1 | - | Sb09g004960.1 | AK370749 |

| | | | | |
|---|---|---|---|---|
| IV9RU5F02GBPI3 | Bradi2g26170.1 | - | Sb09g019480.1 | AK371091 |
| IUIVLUD01DDLYZ | Bradi1g51890.1 | - | Sb01g014880.1 | AK371801 |
| IVLNWEC01E4522 | Bradi1g51890.1 | - | Sb01g014880.1 | AK371801 |
| IUIVLUD02HT2Q6 | Bradi4g04930.1 | - | Sb01g012860.2 | AK372604 |
| IUIVLUD02H4R99 | Bradi4g02380.2 | - | Sb08g020810.1 | AK372643 |
| IVLNWEC01DZKDF | Bradi4g02380.2 | - | Sb08g020810.1 | AK372643 |
| IVLNWEC01DHF7T | Bradi1g59970.1 | - | Sb01g032360.1 | AK372747 |
| IV9RU5F01BR6EL | Bradi1g06430.1 | - | Sb01g005960.1 | AK373354 |
| IV9RU5F01CHMLP | Bradi1g76520.1 | - | Sb01g048520.1 | AK373775 |
| IVLNWEC01C4PLE | Bradi1g76520.1 | - | Sb01g048520.1 | AK373775 |
| IVLNWEC01A66TG | Bradi1g66410.1 | - | Sb01g039310.1 | AK373812 |
| IVLNWEC01EFZ3Z | Bradi1g62340.1 | - | Sb01g035300.1 | AK374778 |
| IV9RU5F01E1PO9 | Bradi4g00367.1 | - | Sb08g023210.1 | AK375316 |
| IUIVLUD02JVBN2 | Bradi4g03150.1 | - | Sb08g019730.1 | AK375460 |
| IUIVLUD01B04IS | Bradi4g01910.1 | - | Sb08g021330.1 | AK375893 |
| IVLNWEC01DNKO1 | Bradi1g04527.1 | - | Sb01g004020.1 | AK376547 |
| IVLNWEC02IZBG6 | Bradi2g40170.1 | - | Sb01g045820.1 | AK377122 |
| IV9RU5F02HI7IW | Bradi4g28107.1 | - | Sb02g022100.1 | MLOC_10243.2 |
| IVLNWEC01DLRUQ | Bradi4g01790.1 | - | Sb08g002530.1 | MLOC_10435.2 |
| IVLNWEC01AJ5XI | Bradi1g03260.1 | - | Sb01g002760.1 | MLOC_10450.1 |
| IVLNWEC01B1CST | Bradi1g03260.1 | - | Sb01g002760.1 | MLOC_10450.1 |
| IUIVLUD02GLKHT | Bradi1g07942.1 | - | Sb01g007450.1 | MLOC_10633.1 |
| IV9RU5F02IO9UJ | Bradi1g07942.1 | - | Sb01g007450.1 | MLOC_10633.1 |
| IVLNWEC01DE79E | Bradi1g07942.1 | - | Sb01g007450.1 | MLOC_10633.1 |
| IVLNWEC01BTWQL | Bradi2g51807.1 | - | Sb03g036450.1 | MLOC_10643.2 |
| IVLNWEC02JMZFG | Bradi1g13960.1 | - | Sb01g013810.1 | MLOC_10667.2 |
| IV9RU5F02GSD1S | Bradi1g05380.1 | - | Sb01g005084.1 | MLOC_10988.2 |
| IVLNWEC02FSKIU | Bradi1g05380.1 | - | Sb01g005084.1 | MLOC_10988.2 |
| IVLNWEC02HF9AU | Bradi1g08710.1 | - | Sb01g008180.1 | MLOC_11351.1 |
| IUIVLUD01AN69N | Bradi2g28900.1 | - | Sb03g045540.1 | MLOC_1154.2 |
| IV9RU5F02FWOW1 | Bradi4g25410.1 | - | Sb08g007640.1 | MLOC_11551.2 |
| IVLNWEC01DCGEL | Bradi1g02000.1 | - | Sb01g001620.1 | MLOC_11570.6 |
| IVLNWEC01EGXA6 | Bradi1g02000.1 | - | Sb01g001620.1 | MLOC_11570.6 |
| IUIVLUD01B4N0U | Bradi4g15000.1 | - | Sb05g022850.1 | MLOC_11883.1 |
| IVLNWEC02GZLRA | Bradi4g34650.1 | - | Sb02g029270.1 | MLOC_12052.2 |
| IVLNWEC02IA1RQ | Bradi4g31680.1 | - | Sb02g026140.1 | MLOC_12614.1 |
| IV9RU5F01BHMA9 | Bradi4g27417.1 | - | Sb01g015210.1 | MLOC_12624.1 |
| IV9RU5F01D2A1L | Bradi2g14440.1 | - | Sb09g030580.1 | MLOC_13176.1 |
| IUIVLUD02GBC92 | Bradi1g67387.1 | - | Sb01g040350.1 | MLOC_13606.1 |
| IUIVLUD01BW1NR | Bradi1g02290.1 | - | Sb08g021540.1 | MLOC_14275.1 |
| IV9RU5F01AG0EU | Bradi1g05500.1 | - | Sb01g005190.1 | MLOC_14311.2 |
| IUIVLUD01CZAGX | Bradi1g11720.1 | - | Sb01g011080.1 | MLOC_15467.1 |
| IVLNWEC02G7WPC | Bradi1g11720.1 | - | Sb01g011080.1 | MLOC_15467.1 |
| IUIVLUD01DFAB2 | Bradi1g62210.1 | - | Sb0598s002020.1 | MLOC_15738.2 |
| IVLNWEC02HLYHG | Bradi3g09770.1 | - | Sb04g009720.1 | MLOC_15887.2 |
| IUIVLUD01CYDOH | Bradi5g22077.1 | - | Sb06g028880.1 | MLOC_15955.1 |
| IV9RU5F02I5QB9 | Bradi5g22077.1 | - | Sb06g028880.1 | MLOC_15955.1 |

| | | | | |
|---|---|---|---|---|
| IVLNWEC01BL7HA | Bradi5g22077.1 | - | Sb06g028880.1 | MLOC_15955.1 |
| IVLNWEC02FTT46 | Bradi1g00950.1 | - | Sb01g000580.1 | MLOC_16059.1 |
| IV9RU5F01ASSBP | Bradi1g29267.1 | - | Sb10g030950.1 | MLOC_18226.2 |
| IV9RU5F01BCHIB | Bradi4g31570.1 | - | Sb02g026050.1 | MLOC_19049.1 |
| IV9RU5F01B8QK0 | Bradi1g10447.1 | - | Sb01g009860.1 | MLOC_19422.5 |
| IV9RU5F01DK4IK | Bradi3g33880.1 | - | Sb01g028540.1 | MLOC_19478.1 |
| IV9RU5F02IU3C4 | Bradi4g23680.1 | - | Sb05g005390.1 | MLOC_20977.1 |
| IVLNWEC01CXA19 | Bradi2g00650.1 | - | Sb03g008650.2 | MLOC_21379.2 |
| IVLNWEC02HAW8J | Bradi1g05170.1 | - | Sb01g004930.1 | MLOC_21567.1 |
| IVLNWEC02JS5JA | Bradi1g05170.1 | - | Sb01g004930.1 | MLOC_21567.1 |
| IUIVLUD01A38O7 | Bradi4g31947.1 | - | Sb02g026420.1 | MLOC_26365.1 |
| IUIVLUD01ASBT1 | Bradi4g31947.1 | - | Sb02g026420.1 | MLOC_26365.1 |
| IV9RU5F02F6GAN | Bradi4g31947.1 | - | Sb02g026420.1 | MLOC_26365.1 |
| IVLNWEC01AQ26I | Bradi4g31947.1 | - | Sb02g026420.1 | MLOC_26365.1 |
| IVLNWEC02I4S3U | Bradi4g31947.1 | - | Sb02g026420.1 | MLOC_26365.1 |
| IV9RU5F01A1N9Y | Bradi1g37370.1 | - | Sb10g022700.1 | MLOC_3122.1 |
| IV9RU5F01ANOLU | Bradi1g13520.1 | - | Sb01g013210.1 | MLOC_33781.1 |
| IV9RU5F02HERM0 | Bradi1g13520.1 | - | Sb01g013210.1 | MLOC_33781.1 |
| IVLNWEC02IELW0 | Bradi1g00540.1 | - | Sb04g020690.1 | MLOC_34972.1 |
| IVLNWEC02I39SK | Bradi1g78470.1 | - | Sb01g010390.1 | MLOC_36655.1 |
| IVLNWEC02JGQLO | Bradi1g33020.1 | - | Sb01g022050.1 | MLOC_36684.1 |
| IV9RU5F01BWVXT | Bradi1g69780.1 | - | Sb01g042750.1 | MLOC_36871.1 |
| IV9RU5F02FMSTW | Bradi4g02910.1 | - | Sb08g020025.1 | MLOC_36925.1 |
| IUIVLUD01AWWO5 | Bradi1g10660.1 | - | Sb01g010070.1 | MLOC_36949.2 |
| IUIVLUD02F9V4Y | Bradi4g08780.1 | - | Sb03g002730.1 | MLOC_36986.1 |
| IV9RU5F01BOUA9 | Bradi4g08780.1 | - | Sb03g002730.1 | MLOC_36986.1 |
| IVLNWEC01B8R37 | Bradi4g08780.1 | - | Sb03g002730.1 | MLOC_36986.1 |
| IVLNWEC01BLY5U | Bradi4g08780.1 | - | Sb03g002730.1 | MLOC_36986.1 |
| IVLNWEC02ILV8Y | Bradi2g09434.1 | - | Sb05g008160.1 | MLOC_37321.1 |
| IV9RU5F01AEJ3E | Bradi2g14340.2 | - | Sb09g030650.1 | MLOC_37459.1 |
| IV9RU5F02GRRU7 | Bradi2g14340.2 | - | Sb09g030650.1 | MLOC_37459.1 |
| IVLNWEC01DK97M | Bradi1g78750.1 | - | Sb08g002900.1 | MLOC_37495.1 |
| IVLNWEC01BDBS5 | Bradi4g08470.1 | - | Sb06g020050.1 | MLOC_3762.1 |
| IUIVLUD01D884E | Bradi3g52470.2 | - | Sb04g030880.1 | MLOC_38181.1 |
| IV9RU5F02GON1F | Bradi1g01800.1 | - | Sb01g001140.1 | MLOC_38862.3 |
| IV9RU5F02G8NYN | Bradi1g70057.1 | - | Sb01g042980.1 | MLOC_4101.1 |
| IV9RU5F01C3HN7 | Bradi1g48264.1 | - | Sb10g004682.1 | MLOC_44147.1 |
| IUIVLUD01EQZ3D | Bradi4g37560.1 | - | Sb04g037070.1 | MLOC_44238.5 |
| IV9RU5F02GMTBA | Bradi4g37560.1 | - | Sb04g037070.1 | MLOC_44238.5 |
| IVLNWEC01AX22U | Bradi1g03670.1 | - | Sb01g003150.1 | MLOC_4490.1 |
| IVLNWEC01DUA6I | Bradi4g45227.1 | - | Sb05g000280.1 | MLOC_48965.1 |
| IVLNWEC01CKG7W | Bradi3g38310.1 | - | Sb07g023230.1 | MLOC_4917.1 |
| IV9RU5F01ADZCD | Bradi4g43690.1 | - | Sb09g008070.1 | MLOC_50612.1 |
| IUIVLUD02FZ10H | Bradi4g02420.1 | - | Sb08g020780.1 | MLOC_51985.1 |
| IV9RU5F01C1KFF | Bradi1g00607.1 | - | Sb02g010040.1 | MLOC_52235.1 |
| IVLNWEC02HNVQL | Bradi1g00607.1 | - | Sb02g010040.1 | MLOC_52235.1 |
| IV9RU5F01CSZ81 | Bradi1g63210.1 | - | Sb05g001160.1 | MLOC_53134.1 |

| | | | | |
|---|---|---|---|---|
| IUIVLUD02HQPO0 | Bradi3g34450.2 | - | Sb01g028220.1 | MLOC_5314.1 |
| IV9RU5F01BM8F0 | Bradi4g31727.1 | - | Sb02g026170.1 | MLOC_53246.1 |
| IUIVLUD01APYSX | Bradi2g16960.1 | - | Sb09g028110.1 | MLOC_53410.2 |
| IVLNWEC01ATBE4 | Bradi2g16960.1 | - | Sb09g028110.1 | MLOC_53410.2 |
| IUIVLUD01BCD01 | Bradi1g69350.1 | - | Sb01g042290.1 | MLOC_53518.1 |
| IVLNWEC01A7L5Q | Bradi4g12940.1 | - | Sb04g021030.1 | MLOC_5462.2 |
| IVLNWEC01AQ5RE | Bradi4g26627.1 | - | Sb05g002930.1 | MLOC_54689.2 |
| IUIVLUD02GKYKH | Bradi2g27230.1 | - | Sb04g002060.1 | MLOC_54934.2 |
| IVLNWEC02F684O | Bradi2g27230.1 | - | Sb04g002060.1 | MLOC_54934.2 |
| IVLNWEC01EX9SF | Bradi2g28435.1 | - | Sb09g016865.1 | MLOC_5527.1 |
| IVLNWEC02JIZLD | Bradi3g24417.1 | - | Sb08g016820.1 | MLOC_55367.1 |
| IUIVLUD01DRX3Q | Bradi2g62760.1 | - | Sb05g002400.1 | MLOC_55536.1 |
| IV9RU5F02G4ZYA | Bradi2g00490.1 | - | Sb02g007200.1 | MLOC_56473.2 |
| IVLNWEC01AMZFA | Bradi5g17947.1 | - | Sb06g024850.1 | MLOC_56535.1 |
| IV9RU5F01C8U73 | Bradi1g13850.1 | - | Sb01g028520.1 | MLOC_56768.1 |
| IVLNWEC01A9U6O | Bradi1g53160.1 | - | Sb02g007870.1 | MLOC_5701.1 |
| IUIVLUD02FW1GS | Bradi1g08240.1 | - | Sb01g007680.1 | MLOC_57253.1 |
| IV9RU5F01CJVAZ | Bradi2g61830.1 | - | Sb03g047060.1 | MLOC_57254.8 |
| IV9RU5F01A3UMA | Bradi4g06880.1 | - | Sb07g001420.1 | MLOC_57831.1 |
| IV9RU5F01BY1EX | Bradi4g06880.1 | - | Sb07g001420.1 | MLOC_57831.1 |
| IVLNWEC01CWO3Q | Bradi4g06880.1 | - | Sb07g001420.1 | MLOC_57831.1 |
| IVLNWEC01E3U02 | Bradi4g26210.3 | - | Sb05g001780.1 | MLOC_57851.1 |
| IVLNWEC01BXXZP | Bradi1g60740.1 | - | Sb01g033580.1 | MLOC_57937.2 |
| IV9RU5F01EP2UJ | Bradi3g05950.1 | - | Sb02g041940.1 | MLOC_58105.1 |
| IVLNWEC01C9Q3X | Bradi3g05950.1 | - | Sb02g041940.1 | MLOC_58105.1 |
| IVLNWEC01CRBLP | Bradi3g28890.1 | - | Sb01g019180.1 | MLOC_58230.2 |
| IUIVLUD02IHJ83 | Bradi1g63330.1 | - | Sb01g036270.1 | MLOC_60118.1 |
| IV9RU5F01AJ2GE | Bradi1g01090.1 | - | Sb01g000770.1 | MLOC_60310.1 |
| IV9RU5F02JVM7V | Bradi1g02240.1 | - | Sb03g025820.1 | MLOC_60551.2 |
| IVLNWEC01EDHL5 | Bradi1g02240.1 | - | Sb03g025820.1 | MLOC_60551.2 |
| IUIVLUD01BY2HG | Bradi1g75210.1 | - | Sb01g047070.1 | MLOC_60571.1 |
| IVLNWEC01C4SM5 | Bradi1g75210.1 | - | Sb01g047070.1 | MLOC_60571.1 |
| IV9RU5F02F511R | Bradi4g27440.1 | - | Sb02g019470.1 | MLOC_60829.1 |
| IV9RU5F02FVFY1 | Bradi4g27440.1 | - | Sb02g019470.1 | MLOC_60829.1 |
| IVLNWEC02GUOEY | Bradi3g39900.2 | - | Sb02g028290.1 | MLOC_6132.2 |
| IVLNWEC02HC7VI | Bradi3g39900.2 | - | Sb02g028290.1 | MLOC_6132.2 |
| IV9RU5F01CM4IC | Bradi1g09390.1 | - | Sb01g008790.1 | MLOC_61341.1 |
| IV9RU5F02HEBHT | Bradi1g64310.1 | - | Sb01g037300.1 | MLOC_61348.1 |
| IV9RU5F01ERTNB | Bradi2g20360.2 | - | Sb09g025030.1 | MLOC_61697.2 |
| IV9RU5F01EP8BJ | Bradi5g10450.1 | - | Sb04g031810.1 | MLOC_62259.1 |
| IV9RU5F01AOHNG | Bradi1g01810.1 | - | Sb01g001360.1 | MLOC_62353.2 |
| IV9RU5F01B89NI | Bradi1g01810.1 | - | Sb01g001360.1 | MLOC_62353.2 |
| IV9RU5F02HU1WR | Bradi1g01810.1 | - | Sb01g001360.1 | MLOC_62353.2 |
| IVLNWEC02GG7FI | Bradi1g01810.1 | - | Sb01g001360.1 | MLOC_62353.2 |
| IVLNWEC02I9QQX | Bradi1g01810.1 | - | Sb01g001360.1 | MLOC_62353.2 |
| IV9RU5F02I4PZH | Bradi1g03550.1 | - | Sb02g001020.1 | MLOC_62464.1 |
| IVLNWEC01EI2Y2 | Bradi1g03550.1 | - | Sb02g001020.1 | MLOC_62464.1 |

| | | | | |
|---|---|---|---|---|
| IVLNWEC02JCYV3 | Bradi1g03550.1 | - | Sb02g001020.1 | MLOC_62464.1 |
| IV9RU5F01C9MNR | Bradi2g51807.1 | - | Sb03g036450.1 | MLOC_62897.1 |
| IUIVLUD01DH35N | Bradi1g14970.1 | - | Sb01g014995.1 | MLOC_63226.1 |
| IV9RU5F02JJGIY | Bradi1g02280.1 | - | Sb01g001890.1 | MLOC_63395.1 |
| IV9RU5F02F4Z92 | Bradi1g67430.2 | - | Sb01g040400.1 | MLOC_639.2 |
| IVLNWEC02GS8HR | Bradi1g14350.1 | - | Sb01g014210.1 | MLOC_63976.1 |
| IVLNWEC01D56SS | Bradi1g04610.1 | - | Sb01g004190.1 | MLOC_6444.1 |
| IVLNWEC01CV570 | Bradi4g23680.1 | - | Sb05g005390.1 | MLOC_66265.2 |
| IVLNWEC02FL16W | Bradi2g51807.1 | - | Sb08g005463.1 | MLOC_67378.2 |
| IUIVLUD02FU2JN | Bradi4g35730.1 | - | Sb02g030440.1 | MLOC_6781.1 |
| IUIVLUD01AU1R1 | Bradi4g05950.1 | - | Sb08g016860.1 | MLOC_6879.1 |
| IVLNWEC01EHHVC | Bradi4g35557.1 | - | Sb02g030290.1 | MLOC_69413.1 |
| IVLNWEC02IE9YI | Bradi1g10530.1 | - | Sb01g009940.1 | MLOC_70277.1 |
| IV9RU5F01BYQJL | Bradi3g53020.1 | - | Sb01g037910.1 | MLOC_73930.1 |
| IV9RU5F02HOD0H | Bradi4g39570.1 | - | Sb05g003020.1 | MLOC_74448.5 |
| IVLNWEC02H3SN9 | Bradi4g39570.1 | - | Sb05g003020.1 | MLOC_74448.5 |
| IUIVLUD01D4CBP | Bradi1g10697.1 | - | Sb01g010130.1 | MLOC_74741.1 |
| IVLNWEC01DBVB7 | Bradi1g10697.1 | - | Sb01g010130.1 | MLOC_74741.1 |
| IUIVLUD02IF6W5 | Bradi1g07830.1 | - | Sb01g007290.1 | MLOC_75007.5 |
| IVLNWEC01BZ7EA | Bradi1g07830.1 | - | Sb01g007290.1 | MLOC_75007.5 |
| IVLNWEC01ESAQO | Bradi1g07830.1 | - | Sb01g007290.1 | MLOC_75007.5 |
| IV9RU5F01DL41O | Bradi1g02230.1 | - | Sb07g025050.1 | MLOC_75184.1 |
| IVLNWEC01CPDGI | Bradi1g02230.1 | - | Sb07g025050.1 | MLOC_75184.1 |
| IVLNWEC01EPRN0 | Bradi1g02230.1 | - | Sb07g025050.1 | MLOC_75184.1 |
| IV9RU5F01CWUVD | Bradi1g72967.1 | - | Sb01g045520.1 | MLOC_75508.1 |
| IV9RU5F02J0KAA | Bradi1g72967.1 | - | Sb01g045520.1 | MLOC_75508.1 |
| IVLNWEC02H4ARI | Bradi1g53085.1 | - | Sb02g008130.1 | MLOC_77526.5 |
| IVLNWEC02JO9SQ | Bradi2g31690.1 | - | Sb01g024390.1 | MLOC_77701.1 |
| IV9RU5F02HX4QM | Bradi4g33310.1 | - | Sb03g029110.1 | MLOC_78764.1 |
| IVLNWEC02HOM15 | Bradi1g06110.1 | - | Sb01g005550.1 | MLOC_79115.1 |
| IVLNWEC02HMY7I | Bradi1g03840.1 | - | Sb01g003330.1 | MLOC_80571.3 |
| IVLNWEC02ITU3S | Bradi1g03840.1 | - | Sb01g003330.1 | MLOC_80571.3 |
| IV9RU5F02FOHRX | Bradi1g08400.1 | - | Sb01g007850.1 | MLOC_824.2 |
| IVLNWEC01D1KWT | Bradi1g08400.1 | - | Sb01g007850.1 | MLOC_824.2 |
| IVLNWEC02FK8PV | Bradi1g08400.1 | - | Sb01g007850.1 | MLOC_824.2 |
| IVLNWEC02IA9J5 | Bradi1g08400.1 | - | Sb01g007850.1 | MLOC_824.2 |
| IV9RU5F01B8Y3B | Bradi1g01630.1 | - | Sb01g001120.1 | MLOC_880.1 |
| IUIVLUD02IIY52 | Bradi3g01150.1 | - | Sb09g025250.1 | MLOC_9988.2 |
| IV9RU5F02HXGVY | Bradi3g01150.1 | - | Sb09g025250.1 | MLOC_9988.2 |
| IVLNWEC02JKPPN | Bradi4g10700.1 | - | Sb05g026170.1 | MLOC_9994.3 |
| IUIVLUD02FUTFG | Bradi1g12860.1 | - | Sb01g012430.1 | MLOC_57969.1 |
| IVLNWEC02GO10J | Bradi4g13990.1 | - | Sb09g003330.1 | AK356321 |
| IV9RU5F01E052Z | Bradi1g05600.1 | - | Sb01g005300.1 | MLOC_2682.1 |
| IVLNWEC02JOFAR | Bradi1g53920.1 | - | Sb02g006500.1 | MLOC_62284.1 |
| IV9RU5F01BAYY0 | Bradi2g61830.1 | - | Sb03g047060.1 | AK356300 |
| IVLNWEC02I049Y | Bradi4g36730.1 | - | Sb02g031470.1 | MLOC_60595.1 |
| IVLNWEC01CAC8O | Bradi1g01630.1 | - | Sb01g001120.1 | MLOC_880.1 |

| | | | | |
|---|---|---|---|---|
| IV9RU5F02I4YNJ | Bradi3g38310.1 | - | Sb07g023230.1 | MLOC_62095.2 |
| IUIVLUD02GD6M9 | Bradi3g49840.1 | - | Sb04g027220.1 | MLOC_8151.2 |
| IV9RU5F02HI6N5 | Bradi1g08430.1 | - | Sb01g007890.1 | MLOC_54983.5 |
| IV9RU5F01EDZN5 | Bradi1g04980.1 | - | Sb01g004640.1 | MLOC_37586.1 |
| IUIVLUD01BYBV8 | Bradi1g08070.1 | - | Sb01g007560.2 | MLOC_71287.2 |
| IVLNWEC02JMQFN | Bradi1g08070.1 | - | Sb01g007560.2 | MLOC_71287.2 |
| IUIVLUD01DTSQQ | Bradi4g07861.1 | - | Sb08g012570.1 | AK371603 |
| IVLNWEC02FVVLN | Bradi1g63330.1 | - | Sb01g036270.1 | MLOC_60118.1 |
| IVLNWEC02H88SZ | Bradi1g63330.1 | - | Sb01g036270.1 | MLOC_60118.1 |
| IV9RU5F02H6LH3 | Bradi1g62590.1 | - | Sb01g035510.1 | AK373558 |
| IV9RU5F01EEA2Z | Bradi4g22230.1 | - | Sb05g008760.1 | AK252584.1 |
| IVLNWEC01CP71Q | Bradi4g22230.1 | - | Sb05g008760.1 | AK252584.1 |
| IV9RU5F01A32KM | Bradi4g31336.1 | - | Sb02g031620.1 | MLOC_61654.1 |
| IUIVLUD02JQQ1S | Bradi1g65860.1 | - | Sb01g038770.1 | AK358331 |
| IV9RU5F02HOH9V | Bradi1g65860.1 | - | Sb01g038770.1 | AK358331 |
| IVLNWEC01CYUGI | Bradi4g02380.2 | - | Sb08g020810.1 | AK372643 |
| IUIVLUD01DFWJN | Bradi4g31947.1 | - | Sb02g026420.1 | MLOC_26365.1 |
| IV9RU5F01AU8JI | Bradi4g29170.1 | - | Sb02g023390.1 | MLOC_4202.1 |
| IV9RU5F02II1OW | Bradi4g29170.1 | - | Sb02g023390.1 | MLOC_4202.1 |
| IVLNWEC02HXJHP | Bradi4g00320.1 | - | Sb08g023310.1 | AK362376 |
| IVLNWEC01D741G | Bradi4g43930.1 | - | Sb05g001750.1 | MLOC_11293.1 |
| IUIVLUD02FP0A3 | Bradi4g37147.1 | - | Sb02g031970.1 | MLOC_9982.2 |
| IVLNWEC02H5ICA | Bradi4g33520.1 | - | Sb02g028170.1 | AK370417 |
| IUIVLUD02INWGU | Bradi1g07870.1 | - | Sb01g007340.1 | AK364849 |
| IV9RU5F02HWA8L | Bradi3g58120.1 | - | Sb04g034030.1 | MLOC_64085.2 |
| IVLNWEC01BWQNH | Bradi4g30190.1 | - | Sb02g024600.1 | MLOC_71161.1 |
| IV9RU5F02GGO7B | Bradi4g41230.1 | - | Sb08g004790.1 | AK362277 |
| IVLNWEC01DLKVG | Bradi1g75460.1 | - | Sb01g047320.1 | MLOC_77263.1 |
| IUIVLUD01D1H8J | Bradi1g00510.1 | - | Sb02g010960.1 | MLOC_55857.2 |
| IUIVLUD01DB2DP | Bradi1g06070.1 | - | Sb01g005590.1 | AK362622 |
| IVLNWEC01DY8WV | Bradi1g53840.1 | - | Sb02g006620.1 | MLOC_63544.1 |
| IUIVLUD02GABAO | Bradi3g20560.1 | - | Sb08g016920.1 | MLOC_6864.1 |
| IVLNWEC01B26ME | Bradi3g20560.1 | - | Sb08g016920.1 | MLOC_6864.1 |
| IV9RU5F01APIBP | Bradi4g28877.1 | - | Sb02g023040.1 | MLOC_54108.2 |
| IVLNWEC01EBTEU | Bradi1g50530.1 | - | Sb04g010020.1 | AK250992.1 |
| IVLNWEC02FXBJC | Bradi1g10360.1 | - | Sb01g009790.1 | MLOC_64588.1 |
| IV9RU5F02GKU0G | Bradi4g24010.1 | - | Sb05g005210.1 | AK369762 |
| IV9RU5F01BU4VG | Bradi1g10697.1 | - | Sb01g010130.1 | MLOC_74741.1 |
| IV9RU5F01EHI1C | Bradi1g10697.1 | - | Sb01g010130.1 | MLOC_74741.1 |
| IV9RU5F02F3EMR | Bradi1g10697.1 | - | Sb01g010130.1 | MLOC_74741.1 |
| IVLNWEC01EF9I3 | Bradi1g10697.1 | - | Sb01g010130.1 | MLOC_74741.1 |
| IV9RU5F01COFQK | Bradi1g51570.1 | - | Sb10g002420.1 | MLOC_74285.1 |
| IUIVLUD01EZYH9 | Bradi4g02390.1 | - | Sb08g020800.1 | AK354434 |
| IV9RU5F01DP4CB | Bradi4g08420.1 | - | Sb02g013180.1 | MLOC_61378.1 |
| IVLNWEC02JPZ5L | Bradi4g28430.1 | - | Sb02g022610.1 | AK369117 |
| IV9RU5F02GHBR0 | Bradi2g35680.1 | - | Sb09g003930.1 | MLOC_14915.2 |
| IUIVLUD01EGDP4 | Bradi4g43130.1 | - | Sb05g002540.1 | MLOC_69804.2 |

| | | | | |
|---|---|---|---|---|
| IVLNWEC01A4PAI | Bradi4g43130.1 | - | Sb05g002540.1 | MLOC_69804.2 |
| IVLNWEC01AP69E | Bradi3g16515.1 | - | Sb07g003870.1 | MLOC_14118.2 |
| IUIVLUD02IKZNZ | Bradi1g63800.1 | - | Sb01g036760.1 | AK252749.1 |
| IV9RU5F01EMZDY | Bradi3g19100.1 | - | Sb07g007870.1 | AK252538.1 |
| IVLNWEC01DAHAR | Bradi2g11107.1 | - | Sb03g011670.1 | AK370908 |
| IV9RU5F02HU7PM | Bradi4g08780.1 | - | Sb03g002730.1 | MLOC_36986.1 |
| IUIVLUD02HR5GJ | Bradi3g47770.1 | - | Sb01g042620.1 | MLOC_58525.1 |
| IVLNWEC02HXSTW | Bradi3g47770.1 | - | Sb01g042620.1 | MLOC_58525.1 |
| IVLNWEC01DR0LX | Bradi1g03480.1 | - | Sb01g002960.1 | MLOC_72726.2 |
| IVLNWEC01DLWO7 | Bradi1g13440.1 | - | Sb01g013100.1 | AK248871.1 |
| IV9RU5F01BXQE8 | Bradi1g08900.1 | - | Sb01g008390.1 | AK366333 |
| IUIVLUD01BHYLC | Bradi4g33920.2 | - | Sb02g028640.1 | MLOC_44877.1 |
| IV9RU5F02JGJKF | Bradi1g03920.1 | - | Sb01g003490.1 | MLOC_37861.2 |
| IV9RU5F01DQZ4P | Bradi1g62007.1 | - | Sb01g034960.1 | AK369109 |
| IV9RU5F02FMCOD | Bradi3g45830.1 | - | Sb04g022730.1 | MLOC_70636.1 |
| IV9RU5F01EL5GT | Bradi2g19640.1 | - | Sb09g025790.1 | MLOC_72532.1 |
| IUIVLUD01D687W | Bradi1g13520.1 | - | Sb01g013210.1 | MLOC_33781.1 |
| IV9RU5F01DB3RR | Bradi4g41940.1 | - | Sb08g003920.1 | MLOC_10892.1 |
| IUIVLUD01EESV8 | Bradi3g10630.2 | - | Sb04g011310.1 | AK248453.1 |
| IVLNWEC02J37R8 | Bradi3g10630.2 | - | Sb04g011310.1 | AK248453.1 |
| IV9RU5F01CM4QF | Bradi1g09100.1 | - | Sb01g008490.1 | AK364599 |
| IV9RU5F02INVYB | Bradi1g09100.1 | - | Sb01g008490.1 | AK364599 |
| IV9RU5F02JU0T7 | Bradi1g09100.1 | - | Sb01g008490.1 | AK364599 |
| IVLNWEC01A3RB7 | Bradi1g09100.1 | - | Sb01g008490.1 | AK364599 |
| IV9RU5F01A36K2 | Bradi4g13690.1 | - | Sb05g024480.1 | AK354110 |
| IVLNWEC02IY4QP | Bradi3g30000.1 | - | Sb01g018120.1 | AK365078 |
| IUIVLUD02I9L56 | Bradi1g06700.1 | - | Sb01g006220.1 | MLOC_6875.2 |
| IVLNWEC02FW6J8 | Bradi4g37560.1 | - | Sb04g037070.1 | MLOC_44238.5 |
| IV9RU5F02F9XVA | Bradi1g07140.1 | - | Sb01g006630.1 | MLOC_39143.3 |
| IV9RU5F02HMIOQ | Bradi4g30570.1 | - | Sb07g007540.1 | MLOC_50938.1 |
| IVLNWEC02HXYDU | Bradi4g36077.1 | - | Sb02g030870.1 | MLOC_57021.1 |
| IV9RU5F01AKUQE | Bradi3g44340.3 | - | Sb06g014590.1 | MLOC_73713.1 |
| IUIVLUD01EXEPX | Bradi1g66290.1 | - | Sb01g039110.1 | MLOC_5907.1 |
| IV9RU5F01BSEVO | Bradi1g01810.1 | - | Sb01g001360.1 | MLOC_62353.2 |
| IUIVLUD01A05W6 | Bradi4g08567.1 | - | Sb07g010440.1 | MLOC_72899.1 |
| IVLNWEC02F8MFD | Bradi4g08567.1 | - | Sb07g010440.1 | MLOC_72899.1 |
| IVLNWEC01CE55F | Bradi2g38510.1 | - | Sb05g008360.1 | MLOC_7020.1 |
| IV9RU5F02G1I73 | Bradi3g45107.1 | - | Sb07g009440.1 | MLOC_78400.1 |
| IVLNWEC02G5QU6 | Bradi4g23680.1 | - | Sb05g005390.1 | MLOC_20977.1 |
| IV9RU5F01A54SR | Bradi1g38227.1 | - | Sb02g006000.1 | MLOC_10482.5 |
| IV9RU5F01DNT2O | Bradi1g09210.1 | - | Sb01g008630.1 | MLOC_51599.3 |
| IVLNWEC01C1DTU | Bradi1g09210.1 | - | Sb01g008630.1 | MLOC_51599.3 |
| IUIVLUD02HFCAG | Bradi1g70057.1 | - | Sb01g042980.1 | MLOC_4101.1 |
| IV9RU5F02GHGH0 | Bradi1g70057.1 | - | Sb01g042980.1 | MLOC_4101.1 |
| IV9RU5F02IFE1U | Bradi1g70057.1 | - | Sb01g042980.1 | MLOC_4101.1 |
| IVLNWEC01DBHAF | Bradi1g06270.1 | - | Sb01g005750.1 | MLOC_6391.1 |
| IV9RU5F02I3AQK | Bradi4g08567.1 | - | Sb07g010440.1 | MLOC_72899.1 |

| | | | | |
|---|---|---|---|---|
| IV9RU5F02II3IX | Bradi4g08567.1 | - | Sb07g010440.1 | MLOC_72899.1 |
| IVLNWEC02HFKOS | Bradi4g08567.1 | - | Sb07g010440.1 | MLOC_72899.1 |
| IVLNWEC02FNSLO | Bradi4g45190.1 | - | Sb05g000330.1 | MLOC_21503.1 |
| IV9RU5F02F6OKJ | Bradi2g23220.1 | - | Sb09g022620.1 | MLOC_62417.3 |
| IV9RU5F01BNLPA | Bradi2g26547.2 | - | Sb09g019370.1 | MLOC_44485.1 |
| IUIVLUD01CVBCO | Bradi1g02000.1 | - | Sb01g001620.1 | MLOC_11570.6 |
| IV9RU5F02FYMBE | Bradi1g02000.1 | - | Sb01g001620.1 | MLOC_11570.6 |
| IVLNWEC01BPFNN | Bradi1g02000.1 | - | Sb01g001620.1 | MLOC_11570.6 |
| IV9RU5F02IEBBE | Bradi1g74997.1 | - | Sb01g046870.1 | MLOC_57109.1 |
| IVLNWEC02H3CIL | Bradi1g06580.1 | - | Sb03g038260.1 | MLOC_64013.1 |
| IVLNWEC02IIBW5 | Bradi1g67430.2 | - | Sb01g040400.1 | MLOC_639.2 |
| IV9RU5F02JFYGW | Bradi1g72160.1 | - | Sb01g044820.1 | MLOC_65664.1 |
| IUIVLUD02H6HE8 | Bradi1g75520.1 | - | Sb01g020010.1 | MLOC_66031.1 |
| IV9RU5F01BLVOB | Bradi1g08400.1 | - | Sb01g007850.1 | MLOC_824.2 |
| IVLNWEC02H42A9 | Bradi1g27400.1 | - | Sb03g008500.1 | MLOC_9787.1 |
| IUIVLUD01B7NPI | Bradi2g28007.1 | - | Sb09g017790.1 | MLOC_13770.2 |
| IVLNWEC01DBMNU | Bradi1g03050.1 | - | Sb01g002300.1 | MLOC_57570.3 |
| IV9RU5F01AIALE | Bradi1g70657.1 | - | Sb01g043410.1 | MLOC_6166.1 |
| IV9RU5F01AIBAJ | Bradi1g74997.1 | - | Sb01g046870.1 | MLOC_57109.1 |
| IV9RU5F01DCV0J | Bradi4g20717.1 | - | Sb06g021370.1 | MLOC_13610.1 |
| IV9RU5F02I9PTK | Bradi4g20717.1 | - | Sb06g021370.1 | MLOC_13610.1 |
| IV9RU5F01CSHYA | Bradi4g12940.1 | - | Sb04g021030.1 | MLOC_5462.2 |
| IV9RU5F01AWK8S | Bradi4g35150.1 | - | Sb02g029830.1 | MLOC_16969.1 |
| IV9RU5F02GKJXD | Bradi1g68510.2 | - | Sb01g041425.1 | MLOC_10030.4 |
| IV9RU5F02GY43C | Bradi1g67140.1 | - | Sb01g040090.1 | AK362963 |
| IVLNWEC02HTDYU | Bradi4g02725.1 | - | Sb08g020210.1 | AK358630 |
| IV9RU5F01CD5IW | Bradi1g11530.1 | - | Sb01g010920.1 | MLOC_51052.3 |
| IV9RU5F01CLL3P | Bradi3g05950.1 | - | Sb02g041940.1 | MLOC_58105.1 |
| IV9RU5F01DA0V4 | Bradi3g05950.1 | - | Sb02g041940.1 | MLOC_58105.1 |
| IV9RU5F02I0ZSO | Bradi3g05950.1 | - | Sb02g041940.1 | MLOC_58105.1 |
| IUIVLUD02GKHMR | Bradi4g10700.1 | - | Sb05g026170.1 | MLOC_9994.3 |
| IV9RU5F02IV562 | Bradi4g10700.1 | - | Sb05g026170.1 | MLOC_9994.3 |
| IVLNWEC01COPG9 | Bradi4g10700.1 | - | Sb05g026170.1 | MLOC_9994.3 |
| IV9RU5F02GQVR9 | Bradi1g38380.1 | - | Sb01g012080.1 | MLOC_70394.1 |
| IVLNWEC02GVOJK | Bradi1g38380.1 | - | Sb01g012080.1 | MLOC_70394.1 |
| IV9RU5F02HD8Z8 | Bradi2g04960.1 | - | Sb07g004420.1 | MLOC_21642.1 |
| IVLNWEC02HYKTD | Bradi4g07970.1 | - | Sb05g016000.1 | MLOC_14105.3 |
| IV9RU5F02I55DS | Bradi2g16820.1 | - | Sb09g028250.1 | AK368296 |
| IUIVLUD02GT2VL | Bradi1g01900.1 | - | Sb01g001520.1 | AK370512 |
| IVLNWEC01BLKDK | Bradi1g48264.1 | - | Sb10g004682.1 | MLOC_44147.1 |
| IVLNWEC01EA96Q | Bradi1g14590.1 | - | Sb01g014570.1 | AK354697 |
| IV9RU5F02IL9RP | Bradi3g34450.2 | - | Sb01g028220.1 | MLOC_36898.1 |
| IUIVLUD01EWO84 | Bradi1g35630.1 | - | Sb08g018670.1 | MLOC_74748.1 |
| IV9RU5F02JF74B | Bradi1g12117.1 | - | Sb01g011610.1 | MLOC_52920.4 |
| IV9RU5F01B2LAY | Bradi4g08527.1 | - | Sb02g019360.1 | AK355795 |
| IV9RU5F02GKJA4 | Bradi1g01830.1 | - | Sb01g001380.1 | AK363387 |
| IV9RU5F02GZ3PE | Bradi1g01830.1 | - | Sb01g001380.1 | AK363387 |

| | | | | |
|---|---|---|---|---|
| IV9RU5F02HRRZP | Bradi1g67960.1 | - | Sb01g040970.1 | MLOC_53996.2 |
| IVLNWEC02FXPTY | Bradi1g08977.1 | - | Sb01g008430.1 | MLOC_4611.1 |
| IVLNWEC02JVHMX | Bradi5g02240.1 | - | Sb06g016947.1 | MLOC_66775.1 |
| IV9RU5F02F0KTR | Bradi4g06030.1 | - | Sb08g016760.1 | AK371413 |
| IVLNWEC01EK3U2 | Bradi4g06030.1 | - | Sb08g016760.1 | AK371413 |
| IV9RU5F02GIPP1 | Bradi1g03160.1 | - | Sb01g002690.1 | AK365917 |
| IV9RU5F02H05ZD | Bradi1g03160.1 | - | Sb01g002690.1 | AK365917 |
| IV9RU5F01B3QYR | Bradi4g04440.1 | - | Sb08g018430.1 | MLOC_657.1 |
| IV9RU5F02IBUXQ | Bradi1g12760.1 | - | Sb01g012250.1 | MLOC_68896.2 |
| IV9RU5F01A4PAI | Bradi4g07040.1 | - | Sb08g015410.1 | AK358846 |
| IVLNWEC01AM3ZX | Bradi2g26930.1 | - | Sb03g046360.1 | AK367623 |
| IVLNWEC02FMX3A | Bradi1g02280.1 | - | Sb01g001890.1 | MLOC_63395.1 |
| IV9RU5F01DCB81 | Bradi1g04440.1 | - | Sb01g004150.1 | AK360154 |
| IVLNWEC02JUL1C | Bradi4g36730.1 | - | Sb02g031470.1 | MLOC_60595.1 |
| IVLNWEC01BBXA6 | Bradi1g04677.1 | - | Sb01g031620.1 | MLOC_39053.2 |
| IV9RU5F02JZPRZ | Bradi1g03050.1 | - | Sb01g002300.1 | MLOC_57570.3 |
| IV9RU5F01DSE1G | Bradi4g04280.1 | - | Sb08g018810.1 | MLOC_10781.1 |
| IV9RU5F01EG2W9 | Bradi3g05370.1 | - | Sb08g000310.1 | MLOC_24495.4 |
| IV9RU5F02ILOY3 | Bradi3g05370.1 | - | Sb08g000310.1 | MLOC_24495.4 |
| IVLNWEC01BQZ8B | Bradi1g03737.1 | - | Sb01g003240.1 | AK363800 |
| IV9RU5F02JZ2XV | Bradi3g48010.1 | - | Sb04g025195.1 | MLOC_59646.3 |
| IVLNWEC01AYM7F | Bradi1g70330.1 | - | Sb01g043190.1 | MLOC_36338.1 |
| IV9RU5F02HDXVP | Bradi4g30580.1 | - | Sb02g025050.1 | MLOC_63433.1 |
| IV9RU5F02I9UKQ | Bradi4g43230.1 | - | Sb05g002450.1 | AK248835.1 |
| IVLNWEC02GZ7GD | Bradi4g42836.1 | - | Sb05g002890.1 | MLOC_20904.1 |
| IVLNWEC01E0S7D | Bradi1g55840.1 | - | Sb02g007040.1 | AK373955 |
| IV9RU5F02IMIG1 | Bradi3g33880.1 | - | Sb01g028540.1 | MLOC_19478.1 |
| IV9RU5F01BJ7NZ | Bradi4g06987.1 | - | Sb08g015430.1 | AK252511.1 |
| IV9RU5F02F4KDN | Bradi4g06987.1 | - | Sb08g015430.1 | AK252511.1 |
| IVLNWEC02JV8O8 | Bradi4g06987.1 | - | Sb08g015430.1 | AK252511.1 |
| IV9RU5F02HUMJJ | Bradi1g05000.1 | - | Sb01g004670.1 | MLOC_55291.2 |
| IUIVLUD02FZBRB | Bradi4g30210.1 | - | Sb02g024630.1 | AK354786 |
| IV9RU5F02FPN05 | Bradi4g08120.1 | - | Sb02g035910.1 | AK368037 |
| IUIVLUD02IIIIY | Bradi1g70657.1 | - | Sb01g043410.1 | MLOC_6166.1 |
| IV9RU5F02IL3NI | Bradi1g70657.1 | - | Sb01g043410.1 | MLOC_6166.1 |
| IV9RU5F02G8FRT | Bradi1g03910.1 | - | Sb01g003470.1 | MLOC_3543.1 |
| IV9RU5F02GUP2C | Bradi1g00567.1 | - | Sb01g000300.1 | AK251043.1 |
| IUIVLUD02JYR9V | Bradi4g07350.1 | - | Sb08g014630.1 | AK356629 |
| IVLNWEC01CVVNN | Bradi1g53747.1 | - | Sb02g006800.1 | MLOC_16846.1 |
| IV9RU5F02GK1DW | Bradi1g03010.2 | - | Sb01g002260.1 | AK371998 |
| IUIVLUD02IWTEZ | Bradi4g26900.1 | - | Sb02g001400.1 | MLOC_53218.1 |
| IVLNWEC01EK4ON | Bradi4g30897.1 | - | Sb02g025370.1 | MLOC_23179.3 |
| IVLNWEC02JSGFP | Bradi4g30897.1 | - | Sb02g025370.1 | MLOC_23179.3 |
| IVLNWEC01DGOWT | Bradi4g27550.1 | - | Sb02g020360.1 | AK367025 |
| IV9RU5F02HD5A3 | Bradi4g32070.1 | - | Sb02g026540.1 | AK357695 |
| IVLNWEC01A6FKR | Bradi4g32070.1 | - | Sb02g026540.1 | AK357695 |
| IUIVLUD01A873U | Bradi4g19560.1 | - | Sb01g003450.1 | MLOC_4066.1 |

| | | | | |
|---|---|---|---|---|
| IVLNWEC01C9VOA | Bradi1g10270.1 | - | Sb01g009680.1 | MLOC_64122.1 |
| IV9RU5F01DMSPJ | Bradi1g06772.1 | - | Sb01g006280.1 | AK356364 |
| IV9RU5F01DWWMI | Bradi4g08350.1 | - | Sb02g014460.1 | AK251055.1 |
| IV9RU5F02IT6M8 | Bradi5g08810.1 | - | Sb06g015125.1 | MLOC_63780.6 |
| IVLNWEC02G76KU | Bradi4g31277.1 | - | Sb02g025760.1 | MLOC_3871.2 |
| IV9RU5F01CMD41 | Bradi1g73860.1 | - | Sb01g022240.1 | MLOC_67589.1 |
| IVLNWEC02FXI1F | Bradi1g75470.2 | - | Sb01g047340.1 | MLOC_14123.2 |
| IV9RU5F02GFMJ8 | Bradi1g03470.1 | - | Sb01g002950.1 | MLOC_56527.2 |
| IUIVLUD01DMQB4 | Bradi3g08910.1 | - | Sb04g008330.1 | MLOC_10622.1 |
| IV9RU5F01EQ20E | Bradi3g60190.1 | - | Sb04g035270.1 | MLOC_68157.5 |
| IV9RU5F01CEP3Y | Bradi1g01090.1 | - | Sb01g000770.1 | MLOC_60310.1 |
| IV9RU5F01B5EOA | Bradi4g06000.2 | - | Sb08g020450.1 | MLOC_80410.1 |
| IVLNWEC02JFG24 | Bradi1g05162.1 | - | Sb01g004920.1 | AK374742 |
| IUIVLUD02G1PB7 | Bradi4g41717.1 | - | Sb08g004180.1 | MLOC_54805.1 |
| IVLNWEC02JFUCU | Bradi1g64597.1 | - | Sb01g037570.1 | MLOC_7062.1 |
| IV9RU5F02JSIRG | Bradi4g00950.2 | - | Sb04g004300.1 | AK368346 |
| IVLNWEC01EV1VV | Bradi4g00950.2 | - | Sb04g004300.1 | AK368346 |
| IUIVLUD01D37XL | Bradi1g64390.1 | - | Sb01g037380.1 | MLOC_13679.4 |
| IVLNWEC01EBM8L | Bradi4g34650.1 | - | Sb02g029270.1 | MLOC_12052.2 |
| IUIVLUD01B63XB | Bradi4g04700.1 | - | Sb08g018200.1 | MLOC_74300.1 |
| IV9RU5F02FZJ0D | Bradi2g27400.1 | - | Sb09g018450.1 | MLOC_57041.2 |
| IVLNWEC02JN8ZC | Bradi1g51430.1 | - | Sb10g002570.1 | MLOC_55782.1 |
| IV9RU5F01A13YL | Bradi1g52210.1 | - | Sb04g000620.1 | MLOC_67531.6 |
| IUIVLUD02HNMDR | Bradi2g16237.1 | - | Sb09g028730.1 | AK354470 |
| IVLNWEC02HDMZX | Bradi3g23290.1 | - | Sb0013s011230.1 | MLOC_13755.2 |
| IUIVLUD01DZMER | Bradi1g09200.1 | - | Sb01g008600.1 | AK369711 |
| IVLNWEC01EIUTY | Bradi4g08490.1 | - | Sb02g013160.1 | AK366742 |
| IVLNWEC02JR5R0 | Bradi3g59910.1 | - | Sb04g028630.1 | MLOC_75294.1 |
| IV9RU5F01BASN7 | Bradi1g75210.1 | - | Sb01g047070.1 | MLOC_60571.1 |
| IUIVLUD01BN3XM | Bradi3g29287.1 | - | Sb01g015670.1 | MLOC_53494.2 |
| IUIVLUD01A1Y2M | Bradi4g07430.1 | - | Sb08g014350.1 | MLOC_5095.1 |
| IVLNWEC01AIM6M | Bradi4g13710.1 | - | Sb05g024490.1 | MLOC_4685.6 |
| IUIVLUD02JFU1X | Bradi2g26600.1 | - | Sb09g019350.1 | MLOC_58956.3 |
| IUIVLUD01BMZNW | Bradi4g44780.1 | - | Sb08g000780.1 | MLOC_45555.1 |
| IVLNWEC01CWPNW | Bradi1g62210.2 | - | Sb0598s002020.1 | MLOC_15738.2 |
| IV9RU5F01ASOO1 | Bradi1g35630.1 | - | Sb08g018670.1 | MLOC_74748.1 |
| IV9RU5F01BMZO5 | Bradi1g51757.1 | - | Sb01g050480.1 | MLOC_54721.2 |
| IUIVLUD02JMW0K | Bradi4g25047.1 | - | Sb05g003760.1 | AK371325 |
| IV9RU5F01C8E8F | Bradi1g05162.1 | - | Sb01g004920.1 | AK374742 |
| IV9RU5F02J2BZ2 | Bradi1g05162.1 | - | Sb01g004920.1 | AK374742 |
| IVLNWEC02F4KAX | Bradi3g32780.1 | - | Sb01g029925.1 | AK359773 |
| IVLNWEC02J1UMD | Bradi3g32780.1 | - | Sb01g029925.1 | AK359773 |
| IV9RU5F01BV7TC | Bradi1g13720.1 | - | Sb01g013470.1 | MLOC_25582.1 |
| IV9RU5F02FVMXJ | Bradi1g13720.1 | - | Sb01g013470.1 | MLOC_25582.1 |
| IV9RU5F02IF50V | Bradi1g13720.1 | - | Sb01g013470.1 | MLOC_25582.1 |
| IUIVLUD01AS9A7 | Bradi1g20630.1 | - | Sb09g018670.1 | AK368262 |
| IUIVLUD02GOBJ8 | Bradi1g20630.1 | - | Sb09g018670.1 | AK368262 |

| | | | | |
|---|---|---|---|---|
| IUIVLUD02HB8AC | Bradi1g20630.1 | - | Sb09g018670.1 | AK368262 |
| IVLNWEC02IVR4C | Bradi3g34000.1 | - | Sb01g028420.1 | MLOC_53285.1 |
| IV9RU5F01CF3XS | Bradi1g62320.1 | - | Sb01g035270.1 | MLOC_52203.1 |
| IV9RU5F02H4BLT | Bradi1g62320.1 | - | Sb01g035270.1 | MLOC_52203.1 |
| IVLNWEC01CI17T | Bradi4g27157.1 | - | Sb02g018910.1 | MLOC_63338.1 |
| IVLNWEC02FTIBA | Bradi1g10447.1 | - | Sb01g009860.1 | AK373633 |
| IVLNWEC01A39CM | Bradi1g10447.1 | - | Sb01g009860.1 | MLOC_19422.5 |
| IVLNWEC01ASU3V | Bradi1g00980.2 | - | Sb01g000590.1 | AK367778 |
| IUIVLUD02GNEB2 | Bradi1g06945.1 | - | Sb01g030350.1 | MLOC_17956.6 |
| IV9RU5F01BWDEP | Bradi1g06945.1 | - | Sb01g030350.1 | MLOC_17956.6 |
| IVLNWEC02GPIHR | Bradi1g06945.1 | - | Sb01g030350.1 | MLOC_17956.6 |
| IV9RU5F01DFGNP | Bradi4g05830.1 | - | Sb08g017020.1 | AK366262 |
| IV9RU5F02HTGY4 | Bradi1g02910.1 | - | Sb01g002470.1 | AK360179 |
| IVLNWEC02JM993 | Bradi1g02910.1 | - | Sb01g002470.1 | AK360179 |
| IUIVLUD02GV7W2 | Bradi3g43630.1 | - | Sb04g019570.1 | MLOC_66893.1 |
| IV9RU5F02FJH6K | Bradi4g19650.1 | - | Sb0014s003010.1 | AK357819 |
| IV9RU5F02GTAL5 | Bradi4g00747.1 | - | Sb08g022950.1 | AK365959 |
| IVLNWEC01AHISC | Bradi1g70170.2 | - | Sb01g043030.1 | MLOC_15694.3 |
| IUIVLUD01ANFLQ | Bradi4g26627.1 | - | Sb08g002920.1 | MLOC_81426.2 |
| IV9RU5F01EIAL7 | Bradi1g70570.1 | - | Sb01g043330.1 | MLOC_15010.1 |
| IV9RU5F02H88SP | Bradi4g12060.1 | - | Sb05g025510.1 | AK371597 |
| IV9RU5F02HHHD7 | Bradi4g12060.1 | - | Sb05g025510.1 | AK371597 |
| IV9RU5F01DKM0K | Bradi2g54390.1 | - | Sb03g039200.1 | MLOC_26628.1 |
| IV9RU5F01C0S70 | Bradi1g12920.1 | - | Sb01g012510.1 | AK364395 |
| IV9RU5F02FU262 | Bradi1g12920.1 | - | Sb01g012510.1 | AK364395 |
| IVLNWEC02IN2S7 | Bradi1g12920.1 | - | Sb01g012510.1 | AK364395 |
| IV9RU5F02H31YQ | Bradi1g00567.1 | - | Sb01g000300.1 | AK251043.1 |
| IVLNWEC02JG2EN | Bradi4g43070.1 | - | Sb05g002630.2 | AK355248 |
| IVLNWEC01EZOK6 | Bradi4g42810.1 | - | Sb05g002900.1 | AK363464 |
| IV9RU5F01BH8LT | Bradi1g13890.1 | - | Sb01g013680.1 | MLOC_69902.1 |
| IUIVLUD02JYK6R | Bradi4g31310.1 | - | Sb02g025790.1 | MLOC_72011.2 |
| IV9RU5F02H6CIE | Bradi1g15330.1 | - | Sb01g015490.1 | AK369530 |
| IUIVLUD02HH39U | Bradi4g02440.1 | - | Sb08g020750.1 | MLOC_56719.2 |
| IUIVLUD02IEKJT | Bradi4g02440.1 | - | Sb08g020750.1 | MLOC_56719.2 |
| IV9RU5F02GN96S | Bradi4g02440.1 | - | Sb08g020750.1 | MLOC_56719.2 |
| IV9RU5F01A0DI6 | Bradi1g59970.1 | - | Sb01g032360.1 | AK372747 |
| IV9RU5F02GGV95 | Bradi1g06540.1 | - | Sb01g006100.1 | AK362698 |
| IV9RU5F02JRPOS | Bradi1g06540.1 | - | Sb01g006100.1 | AK362698 |
| IV9RU5F02IYT64 | Bradi1g15857.1 | - | Sb01g031810.1 | AK355570 |
| IV9RU5F01EOQ0L | Bradi1g69350.1 | - | Sb01g042290.1 | MLOC_53518.1 |
| IV9RU5F02JP2RB | Bradi1g64310.1 | - | Sb01g037300.1 | MLOC_61348.1 |
| IVLNWEC01AXKE0 | Bradi1g12117.1 | - | Sb01g011610.1 | MLOC_52920.4 |
| IV9RU5F01A2S3C | Bradi1g64190.1 | - | Sb01g037170.1 | MLOC_61417.2 |
| IVLNWEC02H7K5Q | Bradi1g65940.2 | - | Sb01g038840.1 | AK372875 |
| IV9RU5F01BSULV | Bradi1g12140.1 | - | Sb01g011620.1 | AK354857 |
| IVLNWEC01CSYK7 | Bradi1g75470.2 | - | Sb01g047340.1 | MLOC_64091.1 |
| IVLNWEC01D4O13 | Bradi1g75470.2 | - | Sb01g047340.1 | MLOC_64091.1 |

| | | | | |
|---|---|---|---|---|
| IVLNWEC01EVCHZ | Bradi1g75470.2 | - | Sb01g047340.1 | MLOC_64091.1 |
| IV9RU5F01CCYG8 | Bradi1g75470.2 | - | Sb01g047340.1 | MLOC_74468.1 |
| IV9RU5F01EWX7Z | Bradi1g75470.2 | - | Sb01g047340.1 | MLOC_74468.1 |
| IV9RU5F02GIDQH | Bradi3g60160.1 | - | Sb04g035220.1 | AK369768 |
| IV9RU5F01DRHYN | Bradi4g32530.1 | - | Sb02g027000.1 | MLOC_1497.2 |
| IVLNWEC02IVXQY | Bradi1g75250.1 | - | Sb01g047100.1 | MLOC_75859.1 |
| IV9RU5F01BESYP | Bradi3g29287.1 | - | Sb01g018890.1 | MLOC_63339.1 |
| IUIVLUD01EHLBE | Bradi3g28760.1 | - | Sb01g019330.1 | MLOC_71676.1 |
| IVLNWEC01B0QDX | Bradi5g15337.2 | - | Sb06g022180.1 | MLOC_12299.1 |
| IVLNWEC02HHC3O | Bradi4g05110.2 | - | Sb08g017700.1 | AK252415.1 |
| IUIVLUD01BWQ1T | Bradi2g21080.1 | - | Sb09g024390.1 | MLOC_12687.1 |
| IUIVLUD02FOIRT | Bradi2g21080.1 | - | Sb09g024390.1 | MLOC_12687.1 |
| IV9RU5F01EJXFO | Bradi1g70057.1 | - | Sb01g042980.1 | MLOC_5747.2 |
| IV9RU5F02HDVSA | Bradi1g70057.1 | - | Sb01g042980.1 | MLOC_5747.2 |
| IVLNWEC02HMTIG | Bradi1g11317.2 | - | Sb01g010410.1 | MLOC_5817.1 |
| IVLNWEC02JHSGK | Bradi1g11317.2 | - | Sb01g010410.1 | MLOC_5817.1 |
| IV9RU5F01AJU2P | Bradi1g68180.1 | - | Sb01g041140.1 | MLOC_37049.1 |
| IUIVLUD02H9NLH | Bradi3g15410.1 | - | Sb07g005020.1 | MLOC_51413.1 |
| IV9RU5F02GHJP3 | Bradi4g44680.1 | - | Sb08g000870.1 | MLOC_15454.1 |
| IV9RU5F01BTSHK | Bradi1g04310.1 | - | Sb01g003870.1 | AK369954 |
| IVLNWEC01E110F | Bradi1g04310.1 | - | Sb01g003870.1 | AK369954 |
| IVLNWEC01C2XLD | Bradi3g20580.1 | - | Sb01g000350.1 | AK374115 |
| IUIVLUD02HW13V | Bradi4g26580.1 | - | Sb08g001060.1 | MLOC_55601.1 |
| IV9RU5F01BIDAF | Bradi4g00480.1 | - | Sb08g023130.1 | MLOC_73145.1 |
| IVLNWEC01DI2UJ | Bradi4g00480.1 | - | Sb08g023130.1 | MLOC_73145.1 |
| IV9RU5F01DTA3G | Bradi1g04310.1 | - | Sb01g003870.1 | AK369954 |
| IUIVLUD01CYHVB | Bradi1g02170.1 | - | Sb01g001770.1 | AK369519 |
| IVLNWEC02ISV8G | Bradi1g02170.1 | - | Sb01g001770.1 | AK369519 |
| IVLNWEC02I06CD | Bradi1g05560.1 | - | Sb01g005260.1 | AK251691.1 |
| IUIVLUD01BPWJR | Bradi5g02740.1 | - | Sb09g020050.1 | MLOC_51122.1 |
| IV9RU5F02JQ1QD | Bradi1g08430.1 | - | Sb01g007890.1 | MLOC_54983.5 |
| IV9RU5F01BWFVF | Bradi1g02280.1 | - | Sb01g001890.1 | MLOC_63395.1 |
| IV9RU5F02IFTT0 | Bradi1g02080.1 | - | Sb01g001680.1 | AK363359 |
| IV9RU5F02FXSI2 | Bradi1g01750.1 | - | Sb01g001260.1 | MLOC_11345.4 |
| IVLNWEC02JGSFE | Bradi1g01750.1 | - | Sb01g001260.1 | MLOC_11345.4 |
| IUIVLUD01CBL28 | Bradi3g20060.1 | - | Sb02g040900.1 | AK358219 |
| IVLNWEC02HNK02 | Bradi3g20060.1 | - | Sb02g040900.1 | AK358219 |
| IUIVLUD01A36V3 | Bradi4g08567.1 | - | Sb07g010440.1 | MLOC_72899.1 |
| IV9RU5F01BO75R | Bradi4g08567.1 | - | Sb07g010440.1 | MLOC_72899.1 |
| IV9RU5F01CNAN9 | Bradi4g08567.1 | - | Sb07g010440.1 | MLOC_72899.1 |
| IV9RU5F01D354T | Bradi4g08567.1 | - | Sb07g010440.1 | MLOC_72899.1 |
| IV9RU5F01EDZ4B | Bradi4g08567.1 | - | Sb07g010440.1 | MLOC_72899.1 |
| IV9RU5F02HRUEP | Bradi4g08567.1 | - | Sb07g010440.1 | MLOC_72899.1 |
| IV9RU5F02I5CHU | Bradi4g08567.1 | - | Sb07g010440.1 | MLOC_72899.1 |
| IVLNWEC01DSVBV | Bradi4g08567.1 | - | Sb07g010440.1 | MLOC_72899.1 |
| IVLNWEC01E0G66 | Bradi4g08567.1 | - | Sb07g010440.1 | MLOC_72899.1 |
| IVLNWEC02FL73E | Bradi4g08567.1 | - | Sb07g010440.1 | MLOC_72899.1 |

| | | | | | |
|---|---|---|---|---|---|
| IVLNWEC02GSXNS | Bradi4g08567.1 | - | | Sb07g010440.1 | MLOC_72899.1 |
| IVLNWEC01CILVK | Bradi3g20710.1 | - | | Sb04g038240.1 | MLOC_57657.1 |
| IVLNWEC02F3Q90 | Bradi4g05570.1 | - | | Sb08g017220.1 | AK361994 |
| IV9RU5F01AMZPC | Bradi1g12860.1 | - | | Sb01g012430.1 | MLOC_57969.1 |
| IUIVLUD01DGEE8 | Bradi3g33450.1 | - | | Sb10g028820.1 | AK375183 |
| IVLNWEC01BO0EO | Bradi1g62277.1 | - | | Sb01g035230.1 | MLOC_3982.4 |
| IUIVLUD02IBWLV | Bradi4g29260.1 | - | | Sb02g023500.1 | MLOC_38178.1 |
| IUIVLUD01DNFWZ | Bradi1g68550.1 | - | | Sb01g041470.1 | MLOC_59553.1 |
| IV9RU5F02H5G2T | Bradi1g68550.1 | - | | Sb01g041470.1 | MLOC_59553.1 |
| IV9RU5F02FKVW8 | Bradi4g30980.1 | - | | Sb02g025460.1 | MLOC_55280.1 |
| IV9RU5F02JD6FI | Bradi2g14340.2 | - | | Sb09g030650.1 | MLOC_37459.1 |
| IVLNWEC02H8JS9 | Bradi1g04440.1 | - | | Sb01g004150.1 | AK360154 |
| IV9RU5F02I0EFY | Bradi1g03550.1 | - | | Sb02g001020.1 | MLOC_62464.1 |
| IV9RU5F02IZ4KO | Bradi1g03550.1 | - | | Sb02g001020.1 | MLOC_62464.1 |
| IV9RU5F01DCS04 | Bradi1g62770.1 | - | | Sb01g035670.1 | AK368525 |
| IUIVLUD01CTP9P | Bradi1g03480.1 | - | | Sb01g002960.1 | MLOC_72726.2 |
| IUIVLUD02I2YFL | Bradi4g21277.1 | - | | Sb05g007160.1 | AK370601 |
| IVLNWEC01A1WDJ | Bradi1g71600.2 | - | | Sb07g024800.1 | MLOC_81613.2 |
| IV9RU5F02H6YET | Bradi4g07970.1 | - | | Sb05g016000.1 | MLOC_14105.3 |
| IV9RU5F02HC5FQ | Bradi1g04980.1 | - | | Sb01g004640.1 | MLOC_37586.1 |
| IVLNWEC02HDIML | Bradi1g08500.1 | - | | Sb01g007970.1 | MLOC_58630.2 |
| IV9RU5F02HWF42 | Bradi1g08270.1 | - | | Sb01g007700.1 | AK372882 |
| IV9RU5F01BNF8O | Bradi1g30610.1 | - | | Sb04g035150.1 | MLOC_4966.5 |
| IVLNWEC02HJW5P | Bradi4g07610.1 | - | | Sb0011s011820.1 | AK370873 |
| IUIVLUD02G6GP9 | Bradi2g26500.1 | - | | Sb09g019410.1 | MLOC_81833.1 |
| IV9RU5F01D32TA | Bradi1g00607.1 | - | | Sb01g000330.1 | MLOC_4461.5 |
| IV9RU5F02J144I | Bradi4g27334.1 | - | | Sb02g019500.2 | MLOC_4504.5 |
| IV9RU5F02JWX8P | Bradi1g62640.1 | - | | Sb01g035540.1 | AK373952 |
| IV9RU5F01D6RG6 | Bradi4g37620.1 | - | | Sb02g032450.1 | AK361872 |
| IUIVLUD01CVD8A | Bradi1g52150.3 | - | | Sb10g000430.1 | AK249926.1 |
| IVLNWEC02GF3JX | Bradi1g65910.1 | - | | Sb01g038820.1 | MLOC_12732.2 |
| IV9RU5F01BRH4J | Bradi1g03840.1 | - | | Sb01g003330.1 | MLOC_80571.3 |
| IUIVLUD01BVQTH | Bradi3g33880.1 | - | | Sb01g028540.1 | MLOC_19478.1 |
| IVLNWEC01EXWUJ | Bradi4g26900.1 | - | | Sb02g001400.1 | MLOC_53218.1 |
| IVLNWEC02FV76X | Bradi4g07970.1 | - | | Sb05g016000.1 | MLOC_14105.3 |
| IUIVLUD01C5JRQ | Bradi1g13890.1 | - | | Sb01g013680.1 | MLOC_69902.1 |
| IV9RU5F01CHALP | Bradi3g28390.1 | - | | Sb01g019680.1 | AK363693 |
| IV9RU5F02IDHT2 | Bradi3g28890.1 | - | | Sb01g019180.1 | MLOC_58230.2 |
| IVLNWEC02HV2RF | Bradi3g28890.1 | - | | Sb01g019180.1 | MLOC_58230.2 |
| IVLNWEC01DUVKO | Bradi2g39230.1 | - | | Sb03g023990.1 | AK250130.1 |
| IVLNWEC01E0CGQ | Bradi2g39230.1 | - | | Sb03g023990.1 | AK250130.1 |
| IV9RU5F01CMOMK | Bradi2g16710.2 | - | | Sb09g028380.1 | AK372954 |
| IV9RU5F02IB85L | Bradi3g30010.1 | - | | Sb01g018110.1 | MLOC_45140.1 |
| IVLNWEC02G3CZI | Bradi1g50890.1 | - | | Sb10g003430.1 | MLOC_73860.2 |
| IV9RU5F02JP0DM | Bradi4g00480.1 | - | | Sb08g023130.1 | MLOC_73145.1 |
| IUIVLUD01BOUWS | Bradi2g61830.1 | - | | Sb03g047060.1 | MLOC_57254.8 |
| IV9RU5F02H4DVT | Bradi5g20997.1 | - | | Sb03g040220.1 | AK362988 |

| | | | | |
|---|---|---|---|---|
| IVLNWEC01DBCL6 | Bradi1g62210.1 | - | Sb0598s002020.1 | MLOC_15738.2 |
| IUIVLUD02G4S5D | Bradi2g51807.1 | - | Sb03g036450.1 | MLOC_10643.2 |
| IV9RU5F02JB6ZO | Bradi1g52320.1 | - | Sb10g000390.1 | AK249030.1 |
| IV9RU5F02GE35A | Bradi1g62920.1 | - | Sb01g035850.1 | MLOC_7704.1 |
| IVLNWEC01CH873 | Bradi1g62920.1 | - | Sb01g035850.1 | MLOC_7704.1 |
| IV9RU5F01EBVN7 | Bradi1g71160.1 | - | Sb01g043810.1 | MLOC_10095.1 |
| IV9RU5F02IPTW4 | Bradi1g64390.1 | - | Sb01g037380.1 | MLOC_13679.4 |
| IV9RU5F02JQ8VJ | Bradi1g71600.2 | - | Sb07g024800.1 | AK375616 |
| IVLNWEC01DNKBM | Bradi4g26430.1 | - | Sb08g001480.1 | MLOC_59721.1 |
| IVLNWEC02F2MTU | Bradi1g12340.2 | - | Sb01g011845.1 | MLOC_12803.1 |
| IUIVLUD01D31DL | Bradi4g31680.1 | - | Sb02g026140.1 | MLOC_12614.1 |
| IVLNWEC02INJ30 | Bradi4g08597.1 | - | Sb08g021590.1 | AK376689 |
| IV9RU5F02G9IIB | Bradi3g33450.1 | - | Sb10g028820.1 | AK375183 |
| IVLNWEC02GRM6V | Bradi4g01580.1 | - | Sb08g021670.1 | MLOC_17022.2 |
| IV9RU5F01EB0IA | Bradi4g00490.1 | - | Sb08g023090.1 | MLOC_61021.2 |
| IVLNWEC02HZ3HJ | Bradi1g51807.1 | - | Sb02g028030.1 | MLOC_25208.2 |
| IVLNWEC01A899L | Bradi1g62007.1 | - | Sb01g034960.1 | AK369109 |
| IVLNWEC01BGX1E | Bradi1g62007.1 | - | Sb01g034960.1 | AK369109 |
| IV9RU5F02IRFZW | Bradi4g30570.1 | - | Sb07g007540.1 | MLOC_50938.1 |
| IV9RU5F02JHEG0 | Bradi4g30570.1 | - | Sb07g007540.1 | MLOC_50938.1 |
| IV9RU5F02IL8RZ | Bradi1g03470.1 | - | Sb01g002950.1 | MLOC_56527.2 |
| IVLNWEC01D146S | Bradi1g05600.1 | - | Sb01g005300.1 | MLOC_2682.1 |
| IV9RU5F02HAIH0 | Bradi3g19100.1 | - | Sb07g007870.1 | AK252538.1 |
| IV9RU5F01CB2AZ | Bradi1g02440.1 | - | Sb01g001980.1 | AK361988 |
| IVLNWEC02I4FBR | Bradi1g02440.1 | - | Sb01g001980.1 | AK361988 |
| IVLNWEC01B0DXQ | Bradi4g29070.1 | - | Sb02g023310.1 | MLOC_15248.2 |
| IVLNWEC02JJ9XS | Bradi4g29070.1 | - | Sb02g023310.1 | MLOC_15248.2 |
| IV9RU5F01CI62T | Bradi1g08140.1 | - | Sb01g007590.1 | MLOC_63806.1 |
| IV9RU5F01DPHL4 | Bradi1g08140.1 | - | Sb01g007590.1 | MLOC_63806.1 |
| IV9RU5F02I1RCX | Bradi1g08470.1 | - | Sb01g007950.1 | MLOC_34938.1 |
| IV9RU5F02IWK4U | Bradi1g06470.1 | - | Sb01g006030.2 | MLOC_55659.1 |
| IV9RU5F02INVW9 | Bradi1g02910.1 | - | Sb01g002470.1 | AK360179 |
| IUIVLUD01C7VIH | Bradi1g02980.1 | - | Sb01g002240.1 | MLOC_63062.2 |
| IVLNWEC02HHIN0 | Bradi1g08430.1 | - | Sb01g007890.1 | MLOC_54983.5 |
| IV9RU5F02INIG7 | Bradi1g13520.1 | - | Sb01g013210.1 | MLOC_33781.1 |
| IVLNWEC02G62BM | Bradi1g06380.1 | - | Sb01g005910.1 | MLOC_14680.1 |
| IV9RU5F02IQRAF | Bradi4g33520.1 | - | Sb02g028165.1 | AK370417 |
| IVLNWEC01DVQKA | Bradi4g33520.1 | - | Sb02g028165.1 | AK370417 |
| IVLNWEC01D86QC | Bradi1g24310.1 | - | Sb05g001500.1 | AK359827 |
| IV9RU5F02GJX85 | Bradi4g38037.1 | - | Sb02g011230.1 | AK365745 |
| IV9RU5F02I4H1A | Bradi4g38037.1 | - | Sb02g011230.1 | AK365745 |
| IVLNWEC02HYI6O | Bradi4g38037.1 | - | Sb02g011230.1 | AK365745 |
| IVLNWEC01DV6SW | Bradi1g13890.1 | - | Sb01g013680.1 | MLOC_69902.1 |
| IVLNWEC02IW54J | Bradi4g29410.1 | - | Sb02g023690.1 | MLOC_13051.1 |
| IV9RU5F01DGMRB | Bradi4g38070.1 | - | Sb02g011240.1 | MLOC_13650.1 |
| IUIVLUD02I6OY8 | Bradi3g19100.1 | - | Sb07g007870.1 | AK252538.1 |
| IVLNWEC01D7HY6 | Bradi1g04310.1 | - | Sb01g003870.1 | AK369954 |

| | | | | |
|---|---|---|---|---|
| IV9RU5F02IXALA | Bradi1g21860.1 | - | Sb04g000470.1 | MLOC_63937.1 |
| IV9RU5F01A1YVJ | Bradi4g01910.1 | - | Sb08g021330.1 | AK375893 |
| IV9RU5F01CTR1A | Bradi1g59970.1 | - | Sb01g032360.1 | AK372747 |
| IUIVLUD02F1DE8 | Bradi1g28790.5 | - | Sb02g010840.1 | AK356672 |
| IV9RU5F01EN5RU | Bradi1g55840.1 | - | Sb02g007040.1 | AK373955 |
| IUIVLUD01APDGE | Bradi3g33450.1 | - | Sb10g028820.1 | AK375183 |
| IVLNWEC02HQ8UE | Bradi4g02630.1 | - | Sb08g020520.1 | MLOC_10438.1 |
| IVLNWEC02I9KB4 | Bradi4g02630.1 | - | Sb08g020520.1 | MLOC_10438.1 |
| IUIVLUD01BXKIE | Bradi4g36127.1 | - | Sb02g030880.1 | MLOC_52297.1 |
| IV9RU5F01CZCDL | Bradi1g45860.1 | - | Sb04g034505.1 | MLOC_58047.2 |
| IUIVLUD01DI9HS | Bradi3g45830.1 | - | Sb04g022730.1 | MLOC_70636.1 |
| IV9RU5F01EK24V | Bradi1g33457.2 | - | Sb10g028240.1 | MLOC_10884.2 |
| IV9RU5F01EDCLT | Bradi5g01990.1 | - | Sb03g030490.1 | AK366848 |
| IV9RU5F02FX9LI | Bradi1g00950.1 | - | Sb01g000580.1 | MLOC_16059.1 |
| IVLNWEC01BDR6E | Bradi1g02420.1 | - | Sb01g001970.1 | AK252569.1 |
| IVLNWEC02H058N | Bradi1g02420.1 | - | Sb01g001970.1 | AK252569.1 |
| IV9RU5F02JJ8E1 | Bradi4g38980.1 | - | Sb02g012880.2 | AK371791 |
| IVLNWEC02GPQ5J | Bradi4g07290.1 | - | Sb08g014900.1 | MLOC_6161.1 |
| IV9RU5F01D5TXU | Bradi4g40440.1 | - | Sb08g006380.1 | MLOC_72040.1 |
| IVLNWEC02FO01Z | Bradi4g07861.1 | - | Sb08g012570.1 | AK371603 |
| IV9RU5F01B63NN | Bradi1g62590.1 | - | Sb01g035510.1 | AK373558 |
| IVLNWEC01CJMPF | Bradi4g22230.1 | - | Sb05g008760.1 | AK252584.1 |
| IVLNWEC02FW6NG | Bradi4g29170.1 | - | Sb02g023390.1 | MLOC_4202.1 |
| IV9RU5F01D5Z3C | Bradi4g00320.1 | - | Sb08g023310.1 | AK362376 |
| IV9RU5F02HO0PC | Bradi4g08597.1 | - | Sb08g021590.1 | AK376689 |
| IV9RU5F01C3F0D | Bradi1g69360.1 | - | Sb01g042300.1 | MLOC_69036.1 |
| IV9RU5F01EIG1J | Bradi1g69360.1 | - | Sb01g042300.1 | MLOC_69036.1 |
| IV9RU5F01AS6TA | Bradi2g27230.1 | - | Sb04g002060.1 | MLOC_54934.2 |
| IUIVLUD02G2M25 | Bradi4g41230.1 | - | Sb08g004790.1 | AK362277 |
| IVLNWEC01DIQMK | Bradi1g75460.1 | - | Sb01g047320.1 | MLOC_77263.1 |
| IUIVLUD02ID66A | Bradi1g11180.1 | - | Sb01g010590.1 | AK369576 |
| IVLNWEC01DL77K | Bradi1g11180.1 | - | Sb01g010590.1 | AK369576 |
| IUIVLUD02G1VN6 | Bradi4g29840.1 | - | Sb02g024280.1 | AK366468 |
| IVLNWEC02HLCH9 | Bradi4g35150.1 | - | Sb02g029830.1 | MLOC_16969.1 |
| IV9RU5F01B8ODL | Bradi1g06070.1 | - | Sb01g005590.1 | AK362622 |
| IUIVLUD01CXTP6 | Bradi1g53840.1 | - | Sb02g006620.1 | MLOC_63544.1 |
| IVLNWEC02I8OJ1 | Bradi4g31300.1 | - | Sb02g025780.1 | AK362794 |
| IV9RU5F01B7NL1 | Bradi4g04460.1 | - | Sb08g018230.1 | MLOC_18312.1 |
| IV9RU5F02HIBRX | Bradi4g43130.1 | - | Sb05g002540.1 | MLOC_69804.2 |
| IVLNWEC02I1KPW | Bradi1g63800.1 | - | Sb01g036760.1 | AK252749.1 |
| IV9RU5F02I5TG2 | Bradi1g03480.1 | - | Sb01g002960.1 | MLOC_72726.2 |
| IVLNWEC01CIUFC | Bradi4g02930.1 | - | Sb08g019970.1 | AK355185 |
| IV9RU5F01CKDHS | Bradi4g02930.1 | - | Sb08g019970.1 | AK355185 |
| IVLNWEC01CVXRD | Bradi4g37030.1 | - | Sb02g031850.1 | AK375054 |
| IV9RU5F02JTSMZ | Bradi1g13520.1 | - | Sb01g013210.1 | MLOC_33781.1 |
| IVLNWEC02IBR6X | Bradi1g65910.1 | - | Sb01g038820.1 | MLOC_12732.2 |
| IV9RU5F02GRIEQ | Bradi1g10640.1 | - | Sb01g010050.2 | MLOC_74226.3 |

| | | | | |
|---|---|---|---|---|
| IUIVLUD01EJCL2 | Bradi1g02420.1 | - | Sb01g001970.1 | AK252569.1 |
| IV9RU5F02I6DVF | Bradi1g02420.1 | - | Sb01g001970.1 | AK252569.1 |
| IVLNWEC01CHXN3 | Bradi4g30570.1 | - | Sb07g007540.1 | MLOC_50938.1 |
| IUIVLUD01DQ3AE | Bradi4g04390.1 | - | Sb05g027180.1 | MLOC_17197.3 |
| IVLNWEC01BALX6 | Bradi2g25527.1 | - | Sb09g020000.1 | MLOC_55035.9 |
| IV9RU5F01EC61G | Bradi1g01810.1 | - | Sb01g001360.1 | MLOC_62353.2 |
| IVLNWEC02HV87V | Bradi4g37147.1 | - | Sb02g031970.1 | MLOC_9982.2 |
| IV9RU5F01B7II1 | Bradi1g70057.1 | - | Sb01g042980.1 | MLOC_4101.1 |
| IV9RU5F01C6E3Y | Bradi1g70057.1 | - | Sb01g042980.1 | MLOC_4101.1 |
| IV9RU5F01BIWGY | Bradi4g08567.1 | - | Sb07g010440.1 | MLOC_72899.1 |
| IVLNWEC01BWL5D | Bradi4g08567.1 | - | Sb07g010440.1 | MLOC_72899.1 |
| IV9RU5F01ENSTL | Bradi4g40440.1 | - | Sb08g006380.1 | MLOC_72040.1 |
| IVLNWEC01DKT7D | Bradi2g23220.1 | - | Sb09g022620.1 | MLOC_62417.3 |
| IV9RU5F02JXA54 | Bradi1g66850.3 | - | Sb09g028275.1 | AK354358 |
| IV9RU5F02JFHNE | Bradi1g59970.1 | - | Sb01g032360.1 | AK372747 |
| IV9RU5F02I80TU | Bradi4g38140.1 | - | Sb02g011280.1 | MLOC_21732.1 |
| IV9RU5F01D0PNF | Bradi1g72160.1 | - | Sb01g044820.1 | MLOC_65664.1 |
| IV9RU5F02FQUD1 | Bradi3g29440.1 | - | Sb01g018770.1 | MLOC_68088.1 |
| IV9RU5F01DUWLR | Bradi2g26547.2 | - | Sb09g019370.1 | MLOC_44485.1 |
| IUIVLUD02IU3WC | Bradi1g08400.1 | - | Sb01g007850.1 | MLOC_824.2 |
| IV9RU5F02F10CE | Bradi1g62640.1 | - | Sb01g035540.1 | MLOC_66033.2 |
| IV9RU5F02HFLEI | Bradi4g33310.1 | - | Sb03g029110.1 | MLOC_78764.1 |
| IV9RU5F01DQRH2 | Bradi1g62277.1 | - | Sb01g035230.1 | MLOC_3982.4 |
| IV9RU5F02HIVFX | Bradi2g16820.1 | - | Sb09g028250.1 | AK368296 |
| IUIVLUD02HSH7E | Bradi3g34450.2 | - | Sb01g028220.1 | MLOC_36898.1 |
| IV9RU5F02JZOE6 | Bradi3g34450.2 | - | Sb01g028220.1 | MLOC_5314.1 |
| IUIVLUD02JGUW6 | Bradi1g35630.1 | - | Sb08g018670.1 | MLOC_74748.1 |
| IV9RU5F01D41H6 | Bradi1g71730.2 | - | Sb01g044400.1 | AK374709 |
| IVLNWEC02JAMRO | Bradi4g36470.1 | - | Sb02g031200.1 | AK373959 |
| IVLNWEC01CB5MB | Bradi3g55470.1 | - | Sb04g037320.1 | MLOC_57016.2 |
| IVLNWEC01BFA6S | Bradi4g02117.1 | - | Sb08g020970.1 | MLOC_52525.1 |
| IVLNWEC01BJ5EM | Bradi4g40140.1 | - | Sb08g006760.1 | MLOC_74333.3 |
| IVLNWEC02HJBBD | Bradi4g28107.1 | - | Sb02g022100.1 | MLOC_10243.2 |
| IV9RU5F02H9D5O | Bradi1g67247.1 | - | Sb01g040190.1 | MLOC_5745.4 |
| IV9RU5F02HJCWI | Bradi4g30580.1 | - | Sb02g025050.1 | MLOC_63433.1 |
| IVLNWEC02IJ4K2 | Bradi4g42836.1 | - | Sb05g002890.1 | MLOC_20904.1 |
| IV9RU5F01CPNME | Bradi5g27270.1 | - | Sb06g033900.1 | MLOC_6266.2 |
| IV9RU5F01ELQBD | Bradi4g06987.1 | - | Sb08g015430.1 | AK252511.1 |
| IV9RU5F02IB7L9 | Bradi1g51230.1 | - | Sb10g003060.1 | AK371533 |
| IVLNWEC02G9OV8 | Bradi1g13060.1 | - | Sb01g012710.1 | MLOC_39318.1 |
| IV9RU5F01B7IUU | Bradi1g02000.1 | - | Sb01g001620.1 | MLOC_11570.6 |
| IUIVLUD01C58C5 | Bradi3g05420.1 | - | Sb04g004880.1 | MLOC_19066.1 |
| IVLNWEC02JQ0GU | Bradi1g03010.2 | - | Sb01g002260.1 | AK371998 |
| IV9RU5F01DPRP7 | Bradi4g41650.1 | - | Sb08g004240.1 | AK367116 |
| IV9RU5F02GOIQO | Bradi4g19560.1 | - | Sb01g003450.1 | MLOC_4066.1 |
| IV9RU5F01D4UVB | Bradi1g06470.1 | - | Sb01g006030.2 | MLOC_55659.1 |
| IV9RU5F01BJS4C | Bradi4g08350.1 | - | Sb02g014460.1 | AK251055.1 |

| | | | | |
|---|---|---|---|---|
| IUIVLUD01ARNEE | Bradi4g31277.1 | - | Sb02g025760.1 | MLOC_3871.2 |
| IV9RU5F01EG9CX | Bradi3g07580.1 | - | Sb04g007100.1 | MLOC_3451.1 |
| IV9RU5F01BSWAX | Bradi4g06000.2 | - | Sb04g019783.1 | MLOC_80410.1 |
| IVLNWEC01D2QO4 | Bradi4g41717.1 | - | Sb08g004180.1 | MLOC_54805.1 |
| IVLNWEC02H2Q2U | Bradi1g64597.1 | - | Sb01g037570.1 | MLOC_7062.1 |
| IV9RU5F01AGK7D | Bradi4g00950.2 | - | Sb04g004300.1 | AK368346 |
| IUIVLUD01CB87I | Bradi4g01580.1 | - | Sb08g021670.1 | MLOC_17022.2 |
| IVLNWEC02IPYZO | Bradi4g05840.1 | - | Sb08g017010.1 | AK365226 |
| IUIVLUD02I2V4M | Bradi1g02597.1 | - | Sb01g002200.1 | AK355984 |
| IV9RU5F02GIWBX | Bradi4g00490.1 | - | Sb08g023090.1 | MLOC_61021.2 |
| IV9RU5F02IKED4 | Bradi1g64770.1 | - | Sb01g037790.1 | MLOC_63204.1 |
| IUIVLUD01AY5NW | Bradi3g48647.1 | - | Sb04g025910.1 | MLOC_73368.2 |
| IV9RU5F02G85RL | Bradi3g48647.1 | - | Sb04g025910.1 | MLOC_73368.2 |
| IUIVLUD02HHO2N | Bradi4g07430.1 | - | Sb08g014350.1 | MLOC_5095.1 |
| IUIVLUD01C9BZW | Bradi1g10660.1 | - | Sb01g010070.1 | MLOC_36949.2 |
| IVLNWEC02H2IZL | Bradi4g44780.1 | - | Sb08g000780.1 | MLOC_45555.1 |
| IV9RU5F02HQ2D4 | Bradi1g51757.1 | - | Sb01g050480.1 | MLOC_54721.2 |
| IV9RU5F02I2KDK | Bradi1g51757.1 | - | Sb01g050480.1 | MLOC_54721.2 |
| IUIVLUD02GJP22 | Bradi4g35977.1 | - | Sb03g034370.1 | MLOC_4008.2 |
| IVLNWEC01C1IGF | Bradi1g62320.1 | - | Sb01g035270.1 | MLOC_52203.1 |
| IV9RU5F01EG1YP | Bradi4g27157.1 | - | Sb02g018910.1 | MLOC_63338.1 |
| IVLNWEC02FSDVP | Bradi4g27157.1 | - | Sb02g018910.1 | MLOC_63338.1 |
| IVLNWEC02GNRWI | Bradi4g27157.1 | - | Sb02g018910.1 | MLOC_63338.1 |
| IVLNWEC02GXHAX | Bradi3g59627.1 | - | Sb04g028320.1 | MLOC_54546.1 |
| IVLNWEC01DJ5VT | Bradi3g59627.1 | - | Sb04g028320.1 | MLOC_54546.1 |
| IV9RU5F02HRFYM | Bradi1g73810.1 | - | Sb01g045978.1 | MLOC_70176.1 |
| IV9RU5F01ANATC | Bradi1g06945.1 | - | Sb01g030350.1 | MLOC_17956.6 |
| IVLNWEC02JKLVT | Bradi1g06945.1 | - | Sb01g030350.1 | MLOC_17956.6 |
| IV9RU5F01DIZI3 | Bradi4g05830.1 | - | Sb08g017020.1 | AK366262 |
| IVLNWEC02GY38T | Bradi1g04270.1 | - | Sb01g003820.1 | MLOC_15858.1 |
| IV9RU5F01DJR3H | Bradi1g04527.1 | - | Sb01g004020.1 | AK376547 |
| IUIVLUD02HD6HK | Bradi4g43070.1 | - | Sb05g002630.2 | AK355248 |
| IVLNWEC01DS5GR | Bradi4g31680.1 | - | Sb02g026140.1 | MLOC_12614.1 |
| IV9RU5F01BY9V8 | Bradi1g67760.1 | - | Sb01g040750.1 | MLOC_56418.3 |
| IVLNWEC01A3QX4 | Bradi1g14877.1 | - | Sb01g014940.1 | MLOC_10859.4 |
| IUIVLUD01B625Q | Bradi1g06540.1 | - | Sb01g006100.1 | AK362698 |
| IVLNWEC02JPMPR | Bradi1g64190.1 | - | Sb01g037170.1 | MLOC_61417.2 |
| IV9RU5F01D2AG9 | Bradi1g73110.1 | - | Sb01g045670.1 | MLOC_57182.2 |
| IV9RU5F02HQ81B | Bradi1g12140.1 | - | Sb01g011620.1 | AK354857 |
| IV9RU5F02GICP5 | Bradi4g06137.1 | - | Sb08g016700.1 | AK361476 |
| IVLNWEC02HKHQQ | Bradi1g75470.2 | - | Sb01g047340.1 | MLOC_14123.2 |
| IV9RU5F01CC3X6 | Bradi1g75470.2 | - | Sb01g047340.1 | MLOC_74468.1 |
| IUIVLUD01B0A6X | Bradi1g75250.1 | - | Sb01g047100.1 | MLOC_75859.1 |
| IV9RU5F02G1SL2 | Bradi2g28900.1 | - | Sb03g045540.1 | MLOC_1154.2 |
| IV9RU5F01D69DK | Bradi4g17960.1 | - | Sb05g019010.1 | MLOC_3482.3 |
| IVLNWEC02HOLLO | Bradi3g15410.1 | - | Sb07g005020.1 | MLOC_51413.1 |
| IVLNWEC02JSFZM | Bradi1g02420.1 | - | Sb01g001970.1 | AK252569.1 |

| | | | | |
|---|---|---|---|---|
| IVLNWEC01DF8CC | Bradi4g04030.1 | - | Sb08g018540.1 | AK367062 |
| IV9RU5F01AG5MP | Bradi1g04760.1 | - | Sb01g004400.1 | AK364675 |
| IV9RU5F01EC8PX | Bradi4g08567.1 | - | Sb07g010440.1 | MLOC_72899.1 |
| IV9RU5F01DY4NP | Bradi1g03207.1 | - | Sb01g002630.1 | MLOC_52484.1 |
| IVLNWEC02HG42E | Bradi3g13460.1 | - | Sb01g044300.1 | MLOC_36653.1 |
| IVLNWEC02H042J | Bradi4g12770.1 | - | Sb02g021226.1 | MLOC_64296.4 |
| IVLNWEC01C1UKE | Bradi3g33450.1 | - | Sb10g028820.1 | AK375183 |
| IVLNWEC02GYIQN | Bradi3g38100.1 | - | Sb07g023060.1 | MLOC_57586.2 |
| IUIVLUD02GI1JL | Bradi1g06050.1 | - | Sb01g005610.1 | MLOC_63574.2 |
| IVLNWEC02FUOL4 | Bradi4g29260.1 | - | Sb02g023500.1 | MLOC_38178.1 |
| IV9RU5F01AWBCF | Bradi1g04980.1 | - | Sb01g004640.1 | MLOC_37586.1 |
| IVLNWEC01DXEW0 | Bradi2g03530.1 | Os01t0103600-01 | - | - |
| IUIVLUD02HRS5L | Bradi2g03530.1 | Os01t0103600-02 | - | - |
| IV9RU5F01EEOLL | Bradi2g00490.1 | Os01t0152000-01 | - | - |
| IVLNWEC02ILXAU | Bradi2g04397.1 | Os01t0170800-01 | - | - |
| IV9RU5F02IZA3U | Bradi2g05870.1 | Os01t0194300-01 | - | - |
| IVLNWEC01A0HHF | Bradi2g10180.1 | Os01t0271200-01 | - | - |
| IVLNWEC02H8UCN | Bradi2g10950.1 | Os01t0281400-00 | - | - |
| IV9RU5F02JZ6JO | Bradi1g51807.1 | Os01t0304100-00 | - | - |
| IVLNWEC01BWJMM | Bradi1g51807.1 | Os01t0304100-00 | - | - |
| IVLNWEC01C3XBK | Bradi1g51807.1 | Os01t0304100-00 | - | - |
| IVLNWEC02HIK8U | Bradi5g01462.1 | Os01t0364100-00 | - | - |
| IUIVLUD02HKMGX | Bradi4g32830.1 | Os01t0364100-00 | - | - |
| IVLNWEC02GJ8VW | Bradi1g00227.1 | Os01t0521600-00 | - | - |
| IUIVLUD01C27YU | Bradi1g02240.1 | Os01t0574400-01 | - | - |
| IV9RU5F01C8422 | Bradi1g02240.1 | Os01t0574400-01 | - | - |
| IVLNWEC01BMF9V | Bradi1g02240.1 | Os01t0574400-01 | - | - |
| IVLNWEC01CIR7Q | Bradi5g25030.1 | Os01t0601675-01 | - | - |
| IV9RU5F01ERTAX | Bradi2g42890.1 | Os01t0601675-01 | - | - |
| IUIVLUD02HPMYC | Bradi1g31970.1 | Os01t0601675-01 | - | - |
| IVLNWEC01B9H3I | Bradi1g31970.1 | Os01t0601675-01 | - | - |
| IUIVLUD01AI491 | Bradi2g44300.1 | Os01t0628700-01 | - | - |
| IV9RU5F01ALMF3 | Bradi2g44856.1 | Os01t0639900-01 | - | - |
| IVLNWEC01EGN80 | Bradi2g51807.1 | Os01t0781200-01 | - | - |
| IVLNWEC02G17P8 | Bradi1g02440.1 | Os01t0890600-00 | - | - |
| IUIVLUD02H9H5P | Bradi1g13960.1 | Os01t0901200-01 | - | - |
| IUIVLUD02FLCVQ | Bradi2g60230.1 | Os01t0937300-01 | - | - |
| IV9RU5F02H7B1C | Bradi2g60230.1 | Os01t0937300-01 | - | - |
| IV9RU5F01ANIWJ | Bradi5g22540.2 | Os01t0946850-00 | - | - |
| IV9RU5F01BX354 | Bradi2g61830.1 | Os01t0966700-02 | - | - |
| IVLNWEC01B053Q | Bradi3g00360.2 | Os02t0101800-01 | - | - |
| IVLNWEC02I40NJ | Bradi1g42000.1 | Os02t0139500-02 | - | - |
| IV9RU5F02F5ERM | Bradi4g07267.1 | Os02t0139600-01 | - | - |
| IV9RU5F01C02XH | Bradi1g03277.1 | Os02t0156000-00 | - | - |
| IV9RU5F02IRFN5 | Bradi3g06727.1 | Os02t0192400-01 | - | - |
| IV9RU5F01AIA2E | Bradi3g08910.1 | Os02t0232400-02 | - | - |
| IVLNWEC02IBMRB | Bradi5g04000.1 | Os02t0277700-01 | - | - |

| | | | | |
|---|---|---|---|---|
| IV9RU5F01ECKW3 | Bradi2g00417.1 | Os02t0288400-00 | - | - |
| IV9RU5F02H1H1A | Bradi4g38240.1 | Os02t0297132-00 | - | - |
| IV9RU5F01E3OEE | Bradi4g23730.1 | Os02t0309200-00 | - | - |
| IV9RU5F02IACJ9 | Bradi3g56710.1 | Os02t0468200-01 | - | - |
| IVLNWEC01A51J5 | Bradi3g43260.1 | Os02t0469200-01 | - | - |
| IVLNWEC01BOSAE | Bradi3g44940.1 | Os02t0533300-01 | - | - |
| IV9RU5F02F1RJG | Bradi1g01180.1 | Os02t0537400-01 | - | - |
| IVLNWEC02JXCEX | Bradi3g46100.1 | Os02t0564000-01 | - | - |
| IVLNWEC01C2RBR | Bradi3g53370.1 | Os02t0716500-01 | - | - |
| IV9RU5F01EKJDT | Bradi3g57600.1 | Os02t0771800-01 | - | - |
| IV9RU5F01CKMQT | Bradi5g25870.1 | Os02t0782200-01 | - | - |
| IV9RU5F01BHLY5 | Bradi3g53740.1 | Os02t0788800-01 | - | - |
| IV9RU5F02HBE5P | Bradi4g43570.1 | Os02t0792500-00 | - | - |
| IUIVLUD01A6ZW0 | Bradi2g39517.1 | Os02t0818450-00 | - | - |
| IVLNWEC02F34XZ | Bradi3g55870.1 | Os02t0822300-00 | - | - |
| IV9RU5F02HGP3L | Bradi1g78420.1 | Os03t0101600-01 | - | - |
| IV9RU5F02GSW6O | Bradi4g05680.1 | Os03t0110800-01 | - | - |
| IVLNWEC02JWZV7 | Bradi4g05680.1 | Os03t0110800-01 | - | - |
| IUIVLUD01BEG9C | Bradi1g51757.1 | Os03t0119500-01 | - | - |
| IVLNWEC01CNBG6 | Bradi1g75340.1 | Os03t0146000-01 | - | - |
| IUIVLUD01EE5V2 | Bradi1g74827.1 | Os03t0152900-01 | - | - |
| IVLNWEC01BOLLM | Bradi2g37795.1 | Os03t0164400-01 | - | - |
| IUIVLUD02GBOZC | Bradi1g72120.1 | Os03t0186800-01 | - | - |
| IVLNWEC02JADIK | Bradi1g72120.1 | Os03t0186800-01 | - | - |
| IVLNWEC02HRPF3 | Bradi1g70570.1 | Os03t0209600-01 | - | - |
| IV9RU5F01CGI8W | Bradi1g70330.1 | Os03t0211600-01 | - | - |
| IV9RU5F02FYY97 | Bradi1g70170.2 | Os03t0212950-00 | - | - |
| IUIVLUD01BYRMD | Bradi1g69617.2 | Os03t0221300-01 | - | - |
| IVLNWEC02HAE2H | Bradi1g69360.1 | Os03t0225500-01 | - | - |
| IVLNWEC02H6N43 | Bradi1g68980.1 | Os03t0226800-01 | - | - |
| IUIVLUD02JRSWK | Bradi1g68250.1 | Os03t0242200-01 | - | - |
| IVLNWEC01BX3JG | Bradi1g68250.1 | Os03t0242200-01 | - | - |
| IVLNWEC02HRGPA | Bradi1g68250.1 | Os03t0242200-01 | - | - |
| IV9RU5F02GXLJQ | Bradi1g51650.1 | Os03t0249400-01 | - | - |
| IV9RU5F01C5P0P | Bradi1g15370.2 | Os03t0586800-02 | - | - |
| IV9RU5F01DALPO | Bradi1g15330.1 | Os03t0587200-01 | - | - |
| IV9RU5F01AM1SA | Bradi1g15317.1 | Os03t0588200-01 | - | - |
| IUIVLUD02IRI40 | Bradi1g14730.1 | Os03t0610650-01 | - | - |
| IV9RU5F02I3TO5 | Bradi1g14200.1 | Os03t0634400-01 | - | - |
| IV9RU5F01C0GVR | Bradi4g02420.1 | Os03t0638000-00 | - | - |
| IVLNWEC02GO197 | Bradi1g13710.1 | Os03t0646900-01 | - | - |
| IV9RU5F01BTGM5 | Bradi4g01230.2 | Os03t0655100-00 | - | - |
| IV9RU5F02GTH77 | Bradi3g35840.1 | Os03t0658000-00 | - | - |
| IV9RU5F01B2BYF | Bradi1g09260.1 | Os03t0700700-03 | - | - |
| IVLNWEC01E1SMN | Bradi1g11580.1 | Os03t0701500-01 | - | - |
| IVLNWEC02JUVLS | Bradi1g11580.1 | Os03t0701500-01 | - | - |
| IV9RU5F02FZZ9K | Bradi1g10447.1 | Os03t0721200-01 | - | - |

| | | | | |
|---|---|---|---|---|
| IUIVLUD02H4TZ4 | Bradi1g10390.1 | Os03t0721900-00 | - | - |
| IV9RU5F01BX7XZ | Bradi1g10190.1 | Os03t0725300-01 | - | - |
| IUIVLUD02GBV3S | Bradi1g09810.1 | Os03t0730800-01 | - | - |
| IV9RU5F02FMK8N | Bradi1g08620.1 | Os03t0748700-01 | - | - |
| IUIVLUD01CY0DI | Bradi1g08301.1 | Os03t0754800-01 | - | - |
| IV9RU5F01E0TD1 | Bradi1g07942.1 | Os03t0759600-00 | - | - |
| IVLNWEC02H0JFL | Bradi1g06380.1 | Os03t0786900-01 | - | - |
| IVLNWEC02IWLSI | Bradi1g05920.1 | Os03t0793600-00 | - | - |
| IUIVLUD01D1T8T | Bradi1g05190.1 | Os03t0799900-01 | - | - |
| IV9RU5F02GW8J8 | Bradi1g05000.1 | Os03t0803500-01 | - | - |
| IV9RU5F01CCSK7 | Bradi1g04610.1 | Os03t0808000-00 | - | - |
| IVLNWEC02JBC3X | Bradi1g04500.1 | Os03t0809100-01 | - | - |
| IVLNWEC01B70F8 | Bradi1g04000.1 | Os03t0816900-01 | - | - |
| IVLNWEC01ETZND | Bradi1g04000.1 | Os03t0816900-01 | - | - |
| IV9RU5F02JPURZ | Bradi1g03910.1 | Os03t0818200-01 | - | - |
| IUIVLUD01DNKM7 | Bradi1g03730.1 | Os03t0820900-01 | - | - |
| IUIVLUD01ECZ1R | Bradi4g33960.1 | Os03t0822100-01 | - | - |
| IV9RU5F02H2QXN | Bradi1g03050.1 | Os03t0830900-01 | - | - |
| IVLNWEC01BEI7D | Bradi1g03030.1 | Os03t0831100-01 | - | - |
| IV9RU5F01CDR7M | Bradi1g02980.1 | Os03t0831700-01 | - | - |
| IVLNWEC02JDE4G | Bradi1g02490.1 | Os03t0837300-02 | - | - |
| IVLNWEC02HW80I | Bradi1g02400.1 | Os03t0838900-01 | - | - |
| IV9RU5F01EWX60 | Bradi1g02380.1 | Os03t0839100-01 | - | - |
| IV9RU5F01ANRXJ | Bradi1g01870.1 | Os03t0845000-01 | - | - |
| IVLNWEC01BMP6E | Bradi1g01870.1 | Os03t0845000-01 | - | - |
| IUIVLUD02GATK0 | Bradi1g01847.1 | Os03t0845500-01 | - | - |
| IVLNWEC01D0KRQ | Bradi1g01847.1 | Os03t0845500-01 | - | - |
| IUIVLUD02GAUZ2 | Bradi1g01620.1 | Os03t0850800-01 | - | - |
| IV9RU5F02HL5QN | Bradi1g75520.1 | Os03t0859600-01 | - | - |
| IUIVLUD01BI4QD | Bradi1g03277.1 | Os04t0109100-01 | - | - |
| IV9RU5F02ICZ5F | Bradi5g01230.1 | Os04t0131900-01 | - | - |
| IVLNWEC01CQGYP | Bradi5g02770.1 | Os04t0186800-00 | - | - |
| IV9RU5F02F9Q7X | Bradi5g14110.1 | Os04t0489800-01 | - | - |
| IVLNWEC01DI2D2 | Bradi3g00507.1 | Os04t0492300-01 | - | - |
| IVLNWEC01EG2DG | Bradi2g39700.1 | Os05t0106000-01 | - | - |
| IVLNWEC02IDP2N | Bradi2g39700.1 | Os05t0106000-01 | - | - |
| IVLNWEC01BQR1Q | Bradi2g38490.1 | Os05t0132100-02 | - | - |
| IUIVLUD01C5BDK | Bradi3g16660.1 | Os05t0151000-00 | - | - |
| IUIVLUD02FXLEN | Bradi3g16660.1 | Os05t0151000-00 | - | - |
| IV9RU5F01ENW7F | Bradi3g16660.1 | Os05t0151000-00 | - | - |
| IV9RU5F01ET1AJ | Bradi3g16660.1 | Os05t0151000-00 | - | - |
| IVLNWEC01B3J5G | Bradi3g16660.1 | Os05t0151000-00 | - | - |
| IVLNWEC01C831Q | Bradi3g16660.1 | Os05t0151000-00 | - | - |
| IUIVLUD02G6GQ7 | Bradi2g34450.1 | Os05t0169000-01 | - | - |
| IV9RU5F01C276T | Bradi2g14600.1 | Os05t0196500-01 | - | - |
| IV9RU5F02GEXPD | Bradi2g14600.1 | Os05t0196500-01 | - | - |
| IUIVLUD02I3WFM | Bradi4g38170.1 | Os05t0216950-00 | - | - |

| | | | | |
|---|---|---|---|---|
| IV9RU5F02GTUEC | Bradi4g33960.1 | Os05t0219300-00 | - | - |
| IV9RU5F01AVQHS | Bradi2g31690.1 | Os05t0244500-01 | - | - |
| IV9RU5F01B1WA5 | Bradi2g31690.1 | Os05t0244500-01 | - | - |
| IV9RU5F02HCFT3 | Bradi2g31690.1 | Os05t0244500-01 | - | - |
| IV9RU5F02HCLFG | Bradi2g31690.1 | Os05t0244500-01 | - | - |
| IVLNWEC01C4MVU | Bradi2g31690.1 | Os05t0244500-01 | - | - |
| IVLNWEC01CKVF0 | Bradi2g31690.1 | Os05t0244500-01 | - | - |
| IV9RU5F02FNR6X | Bradi1g00237.1 | Os05t0250700-00 | - | - |
| IVLNWEC01CMNT0 | Bradi2g31720.1 | Os05t0251900-00 | - | - |
| IV9RU5F01CDJDF | Bradi2g36930.1 | Os05t0277300-02 | - | - |
| IVLNWEC01BCEAQ | Bradi2g36930.1 | Os05t0277300-02 | - | - |
| IV9RU5F02ITHV1 | Bradi1g38380.1 | Os05t0289400-01 | - | - |
| IV9RU5F01AX3HF | Bradi1g30672.1 | Os05t0311600-01 | - | - |
| IVLNWEC01BWHVP | Bradi1g30672.1 | Os05t0311600-01 | - | - |
| IUIVLUD01EO6TK | Bradi2g28435.1 | Os05t0354300-01 | - | - |
| IVLNWEC02F3D4O | Bradi2g51807.1 | Os05t0365300-01 | - | - |
| IVLNWEC01B4Q36 | Bradi2g25930.1 | Os05t0400800-01 | - | - |
| IVLNWEC02I5Z63 | Bradi2g25500.1 | Os05t0409400-01 | - | - |
| IVLNWEC01BM58O | Bradi4g36976.1 | Os05t0479800-01 | - | - |
| IVLNWEC02HJQUQ | Bradi2g19830.1 | Os05t0515500-01 | - | - |
| IUIVLUD02IY5XR | Bradi2g16710.2 | Os05t0561500-01 | - | - |
| IV9RU5F01COJ6X | Bradi5g10450.1 | Os05t0569500-01 | - | - |
| IUIVLUD01D89NH | Bradi4g42430.2 | Os05t0571700-01 | - | - |
| IVLNWEC02JSU0Y | Bradi1g50360.1 | Os06t0111200-01 | - | - |
| IV9RU5F02HWEAV | Bradi1g50010.1 | Os06t0131100-02 | - | - |
| IUIVLUD02GVDM2 | Bradi1g51620.1 | Os06t0134800-01 | - | - |
| IVLNWEC01E2RNJ | Bradi1g50420.1 | Os06t0146100-01 | - | - |
| IV9RU5F02G09IP | Bradi1g51000.2 | Os06t0147800-01 | - | - |
| IVLNWEC01CTYOO | Bradi1g48670.1 | Os06t0158900-00 | - | - |
| IVLNWEC02J4977 | Bradi1g48410.1 | Os06t0164100-02 | - | - |
| IUIVLUD02FP5U0 | Bradi1g48264.1 | Os06t0167000-02 | - | - |
| IVLNWEC02HS8WD | Bradi2g30425.1 | Os06t0226000-01 | - | - |
| IV9RU5F01BBO7Q | Bradi1g44620.1 | Os06t0242000-01 | - | - |
| IVLNWEC02H735D | Bradi3g19890.1 | Os06t0255700-01 | - | - |
| IV9RU5F02JWUQP | Bradi4g09957.1 | Os06t0279900-00 | - | - |
| IVLNWEC02JGZXD | Bradi1g50420.1 | Os06t0286500-01 | - | - |
| IUIVLUD01C5ZFF | Bradi2g60170.1 | Os06t0313440-00 | - | - |
| IVLNWEC01BI0GL | Bradi2g60170.1 | Os06t0313440-00 | - | - |
| IV9RU5F02IUI8A | Bradi2g60170.1 | Os06t0314600-01 | - | - |
| IUIVLUD02JLMMH | Bradi3g22540.1 | Os06t0330100-00 | - | - |
| IVLNWEC01EPMUA | Bradi3g22540.1 | Os06t0330100-00 | - | - |
| IV9RU5F02GHDUM | Bradi3g22520.1 | Os06t0330100-00 | - | - |
| IVLNWEC02IWK50 | Bradi3g22520.1 | Os06t0330100-00 | - | - |
| IV9RU5F02GCZWJ | Bradi3g35460.1 | Os06t0484800-00 | - | - |
| IVLNWEC02HUU62 | Bradi2g11590.1 | Os06t0484800-00 | - | - |
| IV9RU5F02J1K45 | Bradi1g30830.1 | Os06t0647400-02 | - | - |
| IV9RU5F01DJR0Y | Bradi1g34180.1 | Os06t0701200-01 | - | - |

| | | | | |
|---|---|---|---|---|
| IUIVLUD01DARWF | Bradi1g59170.1 | Os07t0111600-01 | - | - |
| IV9RU5F01CKE6U | Bradi1g07020.1 | Os07t0134800-01 | - | - |
| IVLNWEC01CMNT2 | Bradi1g22240.1 | Os07t0164100-01 | - | - |
| IUIVLUD02I3PLU | Bradi1g05630.1 | Os07t0172900-01 | - | - |
| IUIVLUD01EE0H8 | Bradi1g51150.1 | Os07t0180100-00 | - | - |
| IV9RU5F01CWLVJ | Bradi1g54590.1 | Os07t0197000-00 | - | - |
| IV9RU5F01ARA02 | Bradi1g54327.1 | Os07t0205700-01 | - | - |
| IVLNWEC01DJQGL | Bradi3g30220.1 | Os07t0244200-01 | - | - |
| IVLNWEC02JR5UR | Bradi1g75240.1 | Os07t0270800-00 | - | - |
| IV9RU5F02JY57O | Bradi1g59370.1 | Os07t0271700-00 | - | - |
| IV9RU5F01BSJYK | Bradi2g08630.1 | Os07t0515700-01 | - | - |
| IV9RU5F02J2DET | Bradi1g25920.1 | Os07t0525900-00 | - | - |
| IVLNWEC02F06PT | Bradi1g25487.1 | Os07t0541400-01 | - | - |
| IVLNWEC02G89VC | Bradi1g25487.1 | Os07t0541400-01 | - | - |
| IV9RU5F01BWIQO | Bradi1g02180.1 | Os07t0645701-00 | - | - |
| IUIVLUD02FP3PU | Bradi3g06253.1 | Os08t0106900-00 | - | - |
| IVLNWEC02H6VSR | Bradi3g06253.1 | Os08t0106900-00 | - | - |
| IVLNWEC02JA4AQ | Bradi3g18350.1 | Os08t0226000-00 | - | - |
| IVLNWEC02GPPE5 | Bradi3g18350.1 | Os08t0227400-00 | - | - |
| IV9RU5F01CIF4F | Bradi1g29360.1 | Os08t0246300-01 | - | - |
| IVLNWEC01BRG80 | Bradi1g29360.1 | Os08t0246300-01 | - | - |
| IVLNWEC01DANV8 | Bradi1g29267.1 | Os08t0246300-01 | - | - |
| IV9RU5F02IHVY2 | Bradi1g14500.1 | Os08t0324300-01 | - | - |
| IV9RU5F02IYO90 | Bradi1g14500.1 | Os08t0324300-01 | - | - |
| IVLNWEC01D09UW | Bradi1g14500.1 | Os08t0324300-01 | - | - |
| IVLNWEC02IE3XQ | Bradi1g14500.1 | Os08t0324300-01 | - | - |
| IVLNWEC02GZEQY | Bradi1g71650.1 | Os08t0359600-01 | - | - |
| IV9RU5F02JPYON | Bradi3g35910.1 | Os08t0417000-01 | - | - |
| IVLNWEC01EOI5L | Bradi3g37020.1 | Os08t0439000-01 | - | - |
| IUIVLUD01EEUNF | Bradi3g41810.1 | Os08t0541300-01 | - | - |
| IV9RU5F02G1PBE | Bradi3g41810.1 | Os08t0541300-01 | - | - |
| IUIVLUD01EKA0V | Bradi1g50380.1 | Os09t0123300-01 | - | - |
| IV9RU5F02IPAJL | Bradi1g02505.1 | Os09t0266100-01 | - | - |
| IUIVLUD02JWDYS | Bradi1g61140.1 | Os09t0268600-01 | - | - |
| IVLNWEC02FOCY1 | Bradi4g27977.1 | Os09t0321200-00 | - | - |
| IV9RU5F01DY38Z | Bradi5g27320.1 | Os09t0330700-00 | - | - |
| IUIVLUD02GATA8 | Bradi4g45160.1 | Os09t0330700-00 | - | - |
| IV9RU5F02HHUQK | Bradi4g32830.1 | Os09t0373800-00 | - | - |
| IVLNWEC01EFM5P | Bradi4g29760.1 | Os09t0397900-01 | - | - |
| IVLNWEC02JFJJO | Bradi4g29810.1 | Os09t0401100-00 | - | - |
| IVLNWEC01BQS8D | Bradi4g30630.1 | Os09t0424200-01 | - | - |
| IV9RU5F02IJESP | Bradi1g31350.1 | Os09t0429200-00 | - | - |
| IV9RU5F01D5TQK | Bradi4g30840.1 | Os09t0431100-01 | - | - |
| IVLNWEC01CO018 | Bradi4g30850.1 | Os09t0431200-01 | - | - |
| IUIVLUD01EV41L | Bradi4g31450.1 | Os09t0442400-01 | - | - |
| IVLNWEC01BT1NA | Bradi4g31450.1 | Os09t0442400-01 | - | - |
| IV9RU5F01B9YOW | Bradi1g06880.1 | Os09t0442400-01 | - | - |

| | | | | |
|---|---|---|---|---|
| IV9RU5F02GULPR | Bradi1g10360.1 | Os09t0442700-00 | - | - |
| IV9RU5F02H1HKN | Bradi1g46020.1 | Os09t0458100-01 | - | - |
| IUIVLUD01C2HR3 | Bradi4g32620.1 | Os09t0467300-02 | - | - |
| IVLNWEC02HWDF7 | Bradi4g32830.1 | Os09t0471400-01 | - | - |
| IVLNWEC01DHPEX | Bradi2g11337.1 | Os09t0491788-01 | - | - |
| IVLNWEC01BBPZH | Bradi2g62560.1 | Os09t0499400-01 | - | - |
| IV9RU5F02IF939 | Bradi4g37110.1 | Os09t0548400-01 | - | - |
| IV9RU5F01ES5GT | Bradi4g37620.1 | Os09t0556500-01 | - | - |
| IV9RU5F01CI8UA | Bradi1g75240.1 | Os09t0562750-00 | - | - |
| IVLNWEC02JW4B3 | Bradi4g38070.1 | Os09t0563200-01 | - | - |
| IV9RU5F02GO6A5 | Bradi2g03850.1 | Os10t0103140-00 | - | - |
| IV9RU5F02JQXC2 | Bradi5g01462.1 | Os10t0162836-01 | - | - |
| IUIVLUD02JVBHQ | Bradi3g22760.1 | Os10t0182000-01 | - | - |
| IV9RU5F01BGLW2 | Bradi3g22760.1 | Os10t0182000-01 | - | - |
| IVLNWEC01BPVAK | Bradi3g22760.1 | Os10t0182000-01 | - | - |
| IUIVLUD02I2DM7 | Bradi4g09247.1 | Os10t0358200-00 | - | - |
| IV9RU5F01D0TS8 | Bradi4g09247.1 | Os10t0358200-00 | - | - |
| IVLNWEC01EABFF | Bradi2g09434.1 | Os10t0370100-01 | - | - |
| IV9RU5F02GGT7A | Bradi1g75165.1 | Os10t0384600-01 | - | - |
| IUIVLUD02HCYBA | Bradi1g69880.1 | Os10t0400100-01 | - | - |
| IVLNWEC02JL0NU | Bradi3g59847.1 | Os10t0405500-01 | - | - |
| IV9RU5F02JBEYN | Bradi3g27210.2 | Os10t0436800-01 | - | - |
| IVLNWEC02GCGY0 | Bradi3g27740.1 | Os10t0446200-01 | - | - |
| IVLNWEC02I1FS7 | Bradi3g27740.1 | Os10t0446200-01 | - | - |
| IUIVLUD01EL6C4 | Bradi3g28380.1 | Os10t0468500-01 | - | - |
| IVLNWEC02IGIYK | Bradi1g33020.1 | Os10t0469700-01 | - | - |
| IV9RU5F01B635D | Bradi3g29190.1 | Os10t0484400-02 | - | - |
| IVLNWEC01DR4GI | Bradi3g29190.1 | Os10t0484400-02 | - | - |
| IUIVLUD01C2HST | Bradi4g44320.1 | Os11t0118500-00 | - | - |
| IUIVLUD02IZTSE | Bradi4g42957.1 | Os11t0146800-00 | - | - |
| IVLNWEC02G2YZP | Bradi4g26627.1 | Os11t0148700-01 | - | - |
| IV9RU5F02IW5SM | Bradi4g24410.1 | Os11t0171800-01 | - | - |
| IV9RU5F01AGN6C | Bradi4g23695.1 | Os11t0182900-00 | - | - |
| IUIVLUD02GBV1N | Bradi4g23600.2 | Os11t0186400-00 | - | - |
| IUIVLUD02I419H | Bradi4g23600.2 | Os11t0186400-00 | - | - |
| IUIVLUD02GWL2A | Bradi1g01442.1 | Os11t0208900-01 | - | - |
| IVLNWEC01EW2AG | Bradi4g21510.1 | Os11t0242200-00 | - | - |
| IVLNWEC02IPHE4 | Bradi3g03467.1 | Os11t0285000-01 | - | - |
| IV9RU5F02JU2K5 | Bradi1g42000.1 | Os11t0285000-01 | - | - |
| IUIVLUD01CQPGB | Bradi2g09427.1 | Os11t0462500-00 | - | - |
| IV9RU5F02F91GY | Bradi2g09427.1 | Os11t0462500-00 | - | - |
| IV9RU5F02I6X4P | Bradi2g09427.1 | Os11t0462500-00 | - | - |
| IV9RU5F02IZY6Y | Bradi2g09427.1 | Os11t0462500-00 | - | - |
| IV9RU5F02JGO75 | Bradi2g09427.1 | Os11t0462500-00 | - | - |
| IV9RU5F01CQ5QX | Bradi4g16492.1 | Os11t0550100-01 | - | - |
| IV9RU5F01C0BSD | Bradi4g16492.1 | Os11t0550500-01 | - | - |
| IV9RU5F01BXAOE | Bradi2g51807.1 | Os11t0555300-00 | - | - |

| | | | | |
|---|---|---|---|---|
| IV9RU5F02G6GTG | Bradi2g51807.1 | Os11t0555300-00 | - | - |
| IVLNWEC01CD39K | Bradi2g51807.1 | Os11t0555300-00 | - | - |
| IUIVLUD02FTN7C | Bradi4g06880.1 | Os11t0597400-00 | - | - |
| IV9RU5F02GL5AT | Bradi1g58930.1 | Os11t0608300-01 | - | - |
| IV9RU5F02IFKQY | Bradi4g13850.1 | Os11t0619500-00 | - | - |
| IVLNWEC01C1868 | Bradi1g64770.1 | Os11t0672400-00 | - | - |
| IVLNWEC02I2SQR | Bradi1g01442.1 | Os11t0691800-01 | - | - |
| IVLNWEC02JLLCH | Bradi4g39840.1 | Os12t0153700-00 | - | - |
| IUIVLUD01EAN6G | Bradi4g42100.1 | Os12t0157700-00 | - | - |
| IUIVLUD01DGN05 | Bradi4g41470.1 | Os12t0171200-01 | - | - |
| IVLNWEC01EP9PC | Bradi4g41470.1 | Os12t0171200-01 | - | - |
| IVLNWEC01AY2BK | Bradi4g45030.1 | Os12t0175500-01 | - | - |
| IV9RU5F02JIBTY | Bradi4g41137.1 | Os12t0178700-01 | - | - |
| IVLNWEC02HQBP1 | Bradi4g08152.1 | Os12t0291000-00 | - | - |
| IUIVLUD01DVA3H | Bradi4g05010.1 | Os12t0556200-01 | - | - |
| IVLNWEC02HPRZU | Bradi4g02660.1 | Os12t0600100-01 | - | - |
| IUIVLUD01CHZDG | Bradi1g02290.1 | Os12t0614800-00 | - | - |
| IV9RU5F01ESA0S | Bradi4g00910.3 | Os12t0632700-01 | - | - |
| IUIVLUD01BFUBC | Bradi4g00747.1 | Os12t0636100-01 | - | - |
| IVLNWEC02F7N12 | Bradi1g42650.1 | Os02t0714100-01 | - | - |
| IV9RU5F02FY0BF | Bradi4g34070.1 | Os09t0495200-01 | - | - |
| IV9RU5F01AQS6I | Bradi1g09260.1 | Os03t0699700-01 | - | - |
| IUIVLUD02GHHVX | Bradi1g01870.1 | Os03t0845000-01 | - | - |
| IUIVLUD02IZ5T9 | Bradi1g01870.1 | Os03t0845000-01 | - | - |
| IVLNWEC02G8CUS | Bradi1g00540.1 | Os03t0861400-00 | - | - |
| IV9RU5F01AM5BA | Bradi4g26227.1 | Os12t0126800-01 | - | - |
| IVLNWEC01CPNVI | Bradi2g27230.1 | Os01t0355600-01 | - | - |
| IV9RU5F02G3AJ2 | Bradi1g10360.1 | Os09t0442700-00 | - | - |
| IV9RU5F02GZ7RG | Bradi4g02390.1 | Os12t0605500-01 | - | - |
| IVLNWEC02IG2H4 | Bradi1g67740.1 | Os03t0255400-01 | - | - |
| IVLNWEC02GSVEG | Bradi4g03690.1 | Os02t0294600-01 | - | - |
| IVLNWEC01CP4BM | Bradi3g37020.1 | Os08t0439000-01 | - | - |
| IV9RU5F02GU0U7 | Bradi5g22700.1 | Os04t0628600-01 | - | - |
| IVLNWEC01DVM11 | Bradi2g36810.1 | Os11t0681300-00 | - | - |
| IVLNWEC02GHKM0 | Bradi4g04650.1 | Os12t0564600-01 | - | - |
| IVLNWEC01DZDOC | Bradi3g10370.1 | Os02t0281200-01 | - | - |
| IVLNWEC02GOXZV | Bradi3g10370.1 | Os02t0281200-01 | - | - |
| IUIVLUD01EW6OR | Bradi5g27320.1 | Os09t0343200-01 | - | - |
| IV9RU5F02FQP3O | Bradi4g07717.1 | Os06t0102800-01 | - | - |
| IV9RU5F02GDW7F | Bradi3g23150.1 | Os10t0206800-01 | - | - |
| IVLNWEC01ELJQ0 | Bradi4g42110.1 | Os12t0157200-01 | - | - |
| IVLNWEC02GLZHM | Bradi4g41790.1 | Os11t0167500-00 | - | - |
| IVLNWEC02I2YKN | Bradi4g41790.1 | Os11t0167500-00 | - | - |
| IVLNWEC02HOD34 | Bradi4g38357.1 | Os09t0565500-01 | - | - |
| IUIVLUD01EFIG1 | Bradi1g62000.1 | Os03t0356700-01 | - | - |
| IV9RU5F02JLAS6 | Bradi4g34070.1 | Os09t0495200-01 | - | - |
| IVLNWEC01C1252 | Bradi4g43490.4 | Os11t0136800-02 | - | - |

| | | | | |
|---|---|---|---|---|
| IUIVLUD01AHFXB | Bradi3g60720.1 | Os02t0831800-01 | - | - |
| IVLNWEC01E1BZM | Bradi4g02950.1 | Os12t0592300-00 | - | - |
| IUIVLUD01B01IP | Bradi1g12750.1 | Os03t0672300-01 | - | - |
| IVLNWEC01CEAN4 | Bradi4g22080.1 | Os11t0220400-01 | - | - |
| IUIVLUD01C6JIH | Bradi2g42890.1 | Os01t0601675-01 | - | - |
| IV9RU5F02JA9ZC | Bradi1g01900.1 | Os01t0151200-01 | - | - |
| IUIVLUD02GFWLQ | Bradi4g31500.1 | Os09t0443500-01 | - | - |
| IUIVLUD01AIA0T | Bradi1g75530.1 | Os09t0556500-01 | - | - |
| IVLNWEC02HYC8P | Bradi3g59847.1 | Os10t0405500-01 | - | - |
| IV9RU5F02GNX1L | Bradi4g37260.1 | Os09t0551800-00 | - | - |
| IVLNWEC02G0KMQ | Bradi4g03520.1 | Os12t0580900-01 | - | - |
| IV9RU5F02FQ844 | Bradi2g28435.1 | Os05t0354300-01 | - | - |
| IVLNWEC01DCUIW | Bradi1g08301.1 | Os03t0754800-01 | - | - |
| IV9RU5F01EB72O | Bradi4g02710.1 | Os02t0523300-01 | - | - |
| IVLNWEC02I814L | Bradi4g34220.3 | Os09t0498600-01 | - | - |
| IVLNWEC01E4251 | Bradi1g03470.1 | Os03t0436300-00 | - | - |
| IV9RU5F01EJS2B | Bradi1g41860.1 | Os06t0473200-01 | - | - |
| IVLNWEC02HU2M5 | Bradi5g20070.1 | Os04t0593600-01 | - | - |
| IVLNWEC02G5QOS | Bradi3g27740.1 | Os10t0446200-01 | - | - |
| IV9RU5F01DZTP8 | Bradi4g05370.1 | Os12t0547500-01 | - | - |
| IV9RU5F01BQ6P2 | Bradi1g51807.1 | Os01t0304100-00 | - | - |
| IV9RU5F02I4Z3C | Bradi1g51807.1 | Os01t0304100-00 | - | - |
| IVLNWEC01DFRTV | Bradi1g51807.1 | Os01t0304100-00 | - | - |
| IV9RU5F02FLFHS | Bradi1g65290.1 | Os03t0295700-01 | - | - |
| IUIVLUD02GDS7F | Bradi1g13710.1 | Os03t0646900-01 | - | - |
| IVLNWEC01DPNVN | Bradi3g43260.1 | Os02t0469200-01 | - | - |
| IV9RU5F01CJM3T | Bradi1g08500.1 | Os03t0750300-00 | - | - |
| IVLNWEC01DUH2S | Bradi2g16460.1 | Os01t0530500-01 | - | - |
| IVLNWEC02H7Z76 | Bradi1g05890.1 | Os04t0103500-01 | - | - |
| IV9RU5F02H8MOH | Bradi1g09680.1 | Os03t0733800-00 | - | - |
| IVLNWEC02I3CWN | Bradi1g02610.1 | Os03t0835800-01 | - | - |
| IV9RU5F02I3KTW | Bradi4g02010.1 | Os03t0850100-01 | - | - |
| IV9RU5F01D1UW7 | Bradi4g05680.1 | Os03t0110800-01 | - | - |
| IV9RU5F02HL6C2 | Bradi3g55640.1 | Os02t0826000-00 | - | - |
| IVLNWEC02HVCWB | Bradi3g33190.1 | Os10t0556600-00 | - | - |
| IV9RU5F02G42YC | Bradi3g27970.1 | Os10t0457700-01 | - | - |
| IUIVLUD01AOHTU | Bradi1g57017.1 | Os11t0131900-01 | - | - |
| IV9RU5F02IWXX1 | Bradi4g39550.1 | Os12t0238100-01 | - | - |
| IV9RU5F01DFV3W | Bradi3g29870.1 | Os10t0495000-01 | - | - |
| IVLNWEC02ITNQF | Bradi3g29870.1 | Os10t0495000-01 | - | - |
| IV9RU5F01DPM1A | Bradi3g29440.1 | Os10t0488800-01 | - | - |
| IUIVLUD01CXHKF | Bradi4g06137.1 | Os12t0527800-01 | - | - |
| IVLNWEC01A4GK8 | Bradi1g12340.2 | Os03t0683866-00 | - | - |
| IVLNWEC01DK5TN | Bradi1g04990.1 | Os03t0803600-01 | - | - |
| IVLNWEC02IRUQ1 | Bradi4g00270.1 | Os08t0407200-01 | - | - |
| IUIVLUD01CBHU9 | Bradi4g41250.1 | Os12t0176300-01 | - | - |
| IV9RU5F02GM6B9 | Bradi3g20710.1 | Os08t0320200-01 | - | - |

| | | | | |
|---|---|---|---|---|
| IV9RU5F01A44KF | Bradi2g36430.1 | Os11t0433200-02 | - | - |
| IV9RU5F02HMMTU | Bradi2g16460.1 | Os08t0410266-00 | - | - |
| IV9RU5F01BXIDF | Bradi1g61140.1 | Os09t0268600-01 | - | - |
| IV9RU5F01C83DW | Bradi3g16110.1 | Os03t0690500-00 | - | - |
| IV9RU5F01AM9SR | Bradi1g01870.1 | Os03t0845000-01 | - | - |
| IVLNWEC01EJ7FU | Bradi1g60357.1 | Os03t0399532-00 | - | - |
| IVLNWEC02GL8HY | Bradi1g15370.2 | Os03t0586800-02 | - | - |
| IVLNWEC02HMLXQ | Bradi3g12660.1 | Os08t0564800-01 | - | - |
| IVLNWEC01AE8WS | Bradi3g16660.1 | Os05t0151000-00 | - | - |
| IUIVLUD01EYJQ1 | Bradi1g74140.1 | Os03t0164800-00 | - | - |
| IV9RU5F01AML4R | Bradi1g74620.1 | Os03t0157600-00 | - | - |
| IVLNWEC01DWOZ4 | Bradi2g59226.1 | Os01t0924300-01 | - | - |
| IVLNWEC02GEPCZ | Bradi1g49840.1 | Os06t0129900-01 | - | - |
| IV9RU5F01AM9GU | Bradi2g42820.1 | Os01t0601625-00 | - | - |
| IV9RU5F01BO9ZU | Bradi4g38560.1 | Os09t0568700-01 | - | - |
| IUIVLUD01BCGG4 | Bradi1g02180.1 | Os07t0644700-00 | - | - |
| IV9RU5F01A8X6B | Bradi2g10180.1 | Os01t0271200-01 | - | - |
| IVLNWEC02FWNER | Bradi2g18800.1 | Os02t0187400-01 | - | - |
| IV9RU5F01BPZLI | Bradi4g00730.1 | Os05t0353400-01 | - | - |
| IUIVLUD01BKOJB | Bradi3g00360.2 | Os02t0101800-01 | - | - |
| IVLNWEC01DHPHO | Bradi3g53160.1 | Os02t0712000-01 | - | - |
| IUIVLUD01EOXSI | Bradi3g35390.1 | Os08t0398300-01 | - | - |
| IVLNWEC02HID2C | Bradi3g35390.1 | Os08t0398300-01 | - | - |
| IUIVLUD02ISAFD | Bradi1g02290.1 | Os12t0614800-00 | - | - |
| IVLNWEC01BN7U5 | Bradi3g03467.1 | Os11t0285000-01 | - | - |
| IVLNWEC01EQITI | Bradi2g23690.1 | Os05t0451100-01 | - | - |
| IVLNWEC02FWHDM | Bradi2g20430.1 | Os05t0508500-01 | - | - |
| IV9RU5F01EOCYM | Bradi4g34470.1 | Os09t0504900-01 | - | - |
| IVLNWEC02G1K9W | Bradi1g65010.1 | Os03t0298700-01 | - | - |
| IV9RU5F01CG2I6 | Bradi4g01100.1 | Os06t0729000-03 | - | - |
| IVLNWEC01D5WTZ | Bradi1g06350.1 | Os03t0787200-01 | - | - |
| IV9RU5F02JCGJ2 | Bradi3g16660.1 | Os05t0151000-00 | - | - |
| IV9RU5F01DYMBP | Bradi1g31970.1 | Os01t0601675-01 | - | - |
| IVLNWEC01ELMW4 | Bradi1g04270.1 | Os03t0811500-01 | - | - |
| IV9RU5F02HUOK1 | Bradi4g33180.1 | Os09t0479300-01 | - | - |
| IV9RU5F02GQB47 | Bradi4g35940.1 | Os09t0530275-00 | - | - |
| IUIVLUD02H1F2P | Bradi2g60230.1 | Os01t0937300-01 | - | - |
| IV9RU5F02JEDX1 | Bradi1g15680.1 | Os03t0571900-01 | - | - |
| IVLNWEC02HKI9J | Bradi4g34460.1 | Os09t0504800-01 | - | - |
| IV9RU5F01CGFKN | Bradi4g30630.1 | Os09t0424200-01 | - | - |
| IVLNWEC02HMZX3 | Bradi1g06250.1 | Os03t0789200-01 | - | - |
| IV9RU5F01EWCD7 | Bradi1g53027.1 | Os07t0265100-01 | - | - |
| IV9RU5F01DJNGO | Bradi1g01870.1 | Os03t0845000-01 | - | - |
| IVLNWEC01D13M3 | Bradi1g04550.1 | Os03t0809800-01 | - | - |
| IV9RU5F02GRIAU | Bradi4g33930.1 | Os09t0492800-00 | - | - |
| IVLNWEC01C0EM6 | Bradi4g42130.1 | Os12t0156400-01 | - | - |
| IVLNWEC02IY8VZ | Bradi4g02030.1 | Os12t0615800-01 | - | - |

| | | | | |
|---|---|---|---|---|
| IVLNWEC02F1OYG | Bradi2g31690.1 | Os05t0244500-01 | - | - |
| IUIVLUD01ALQ6Y | Bradi2g12960.1 | Os01t0368900-01 | - | - |
| IUIVLUD02HEWSM | Bradi1g15680.1 | Os03t0571900-01 | - | - |
| IUIVLUD02HZNWV | Bradi1g15680.1 | Os03t0571900-01 | - | - |
| IV9RU5F01CD194 | Bradi4g00730.1 | Os05t0353400-01 | - | - |
| IV9RU5F02I4OPR | Bradi2g42820.1 | Os01t0601625-00 | - | - |
| IVLNWEC02GSAT4 | Bradi1g42810.1 | Os06t0308100-01 | - | - |
| IVLNWEC02G2W79 | Bradi1g31350.1 | Os09t0428300-00 | - | - |
| IUIVLUD02ILSC6 | Bradi3g21350.2 | Os04t0176400-00 | - | - |
| IV9RU5F02G97NE | Bradi1g67740.1 | Os03t0255400-01 | - | - |
| IV9RU5F02ITVGL | Bradi3g37020.1 | Os08t0439000-01 | - | - |
| IV9RU5F02F0I9G | Bradi4g11330.1 | Os11t0655800-01 | - | - |
| IV9RU5F02I1PEW | Bradi2g36150.1 | Os11t0213800-01 | - | - |
| IVLNWEC01CANAB | Bradi1g42000.1 | Os02t0140200-01 | - | - |
| IVLNWEC02FO7CA | Bradi1g44590.1 | Os04t0650200-01 | - | - |
| IV9RU5F01CD4EH | Bradi3g16660.1 | Os05t0151000-00 | - | - |
| IVLNWEC01ER8ET | Bradi4g01513.1 | Os09t0479000-01 | - | - |
| IV9RU5F01BFFES | Bradi4g37260.1 | Os09t0551800-00 | - | - |
| IUIVLUD01CI1BI | Bradi5g25180.1 | Os10t0116701-00 | - | - |
| IV9RU5F02GHB2L | Bradi5g25180.1 | Os10t0116701-00 | - | - |
| IV9RU5F01D7FT5 | Bradi4g44750.1 | Os11t0109000-01 | - | - |
| IVLNWEC01BWSOJ | Bradi4g41470.1 | Os12t0171200-01 | - | - |
| IV9RU5F01B0WIO | Bradi4g05010.1 | Os12t0556200-01 | - | - |
| IV9RU5F01EQQG3 | Bradi5g05900.1 | Os04t0211700-00 | - | - |
| IUIVLUD02IHVFN | Bradi1g52230.1 | Os12t0279000-01 | - | - |
| IV9RU5F01DG0UD | Bradi4g41250.1 | Os12t0176300-01 | - | - |
| IVLNWEC02IKGBM | Bradi4g33270.1 | Os10t0444500-00 | - | - |
| IV9RU5F02I2LRB | Bradi4g06970.1 | Os08t0261000-01 | - | - |
| IV9RU5F01EA3RP | Bradi4g05310.1 | Os12t0548200-01 | - | - |
| IVLNWEC02GCQ28 | Bradi1g04860.2 | Os03t0805200-01 | - | AK248344.1 |
| IVLNWEC01ALGVA | Bradi3g54710.1 | Os02t0806000-01 | - | AK248678.1 |
| IVLNWEC01C3FNL | Bradi2g10800.2 | Os01t0279400-01 | - | AK249459.1 |
| IV9RU5F02FV9M2 | Bradi4g26366.1 | Os11t0135900-01 | - | AK249921.1 |
| IUIVLUD01B1P72 | Bradi1g13450.1 | Os03t0652100-01 | - | AK251104.1 |
| IUIVLUD02GN1ZN | Bradi4g24190.1 | Os12t0172500-01 | - | AK251570.1 |
| IV9RU5F01DYEDF | Bradi1g02117.1 | Os03t0842700-02 | - | AK251992.1 |
| IUIVLUD02GDYEP | Bradi1g02117.1 | Os03t0842700-04 | - | AK251992.1 |
| IV9RU5F02I76FA | Bradi3g22760.1 | Os10t0182000-01 | - | AK252546.1 |
| IVLNWEC01ATM7A | Bradi3g22760.1 | Os10t0182000-01 | - | AK252546.1 |
| IVLNWEC01DY9GK | Bradi3g22760.1 | Os10t0182000-01 | - | AK252546.1 |
| IV9RU5F02GL4M8 | Bradi3g02870.1 | Os09t0249600-01 | - | AK354730 |
| IV9RU5F01D0QBZ | Bradi1g28540.1 | Os07t0459200-02 | - | AK357778 |
| IV9RU5F01ARHBP | Bradi5g01230.1 | Os04t0131850-00 | - | AK358163 |
| IUIVLUD02G1O7S | Bradi4g37420.1 | Os09t0554000-01 | - | AK360530 |
| IV9RU5F02GRIUB | Bradi3g35350.1 | Os08t0398700-01 | - | AK360995 |
| IVLNWEC02H97H0 | Bradi3g35350.1 | Os08t0398700-01 | - | AK360995 |
| IV9RU5F01EFZAH | Bradi3g14710.1 | Os08t0139700-01 | - | AK361404 |

| | | | | |
|---|---|---|---|---|
| IVLNWEC02HPPKU | Bradi3g48157.1 | Os02t0606300-00 | - | AK363695 |
| IV9RU5F02IGD3I | Bradi1g05490.1 | Os07t0181700-01 | - | AK364520 |
| IUIVLUD01B7MWA | Bradi3g43260.1 | Os02t0469200-01 | - | AK365473 |
| IV9RU5F01D4DYV | Bradi4g29820.1 | Os09t0401200-01 | - | AK365854 |
| IV9RU5F02HRICX | Bradi4g00747.1 | Os12t0636100-01 | - | AK365959 |
| IVLNWEC01A9WCJ | Bradi4g08490.1 | Os09t0123100-01 | - | AK366742 |
| IUIVLUD01DKDIW | Bradi1g15317.1 | Os03t0588200-01 | - | AK367098 |
| IUIVLUD02JI2IZ | Bradi3g38680.1 | Os08t0484700-01 | - | AK367324 |
| IV9RU5F02HKP3C | Bradi2g15560.1 | Os01t0641000-01 | - | AK369833 |
| IVLNWEC02HE46D | Bradi2g15560.1 | Os01t0641000-01 | - | AK369833 |
| IV9RU5F02FMASH | Bradi4g34960.1 | Os09t0512700-01 | - | AK370272 |
| IVLNWEC01C00F8 | Bradi4g34960.1 | Os09t0512700-01 | - | AK370272 |
| IUIVLUD02F1SZV | Bradi1g08917.1 | Os03t0744675-00 | - | AK370634 |
| IUIVLUD01B5WJB | Bradi3g03730.1 | Os05t0317200-01 | - | AK370654 |
| IVLNWEC02I8BF4 | Bradi3g03730.1 | Os05t0317200-01 | - | AK370654 |
| IVLNWEC01BEGFX | Bradi1g00710.1 | Os03t0859900-01 | - | AK371412 |
| IVLNWEC02G1XEF | Bradi1g68250.1 | Os03t0242200-01 | - | AK371586 |
| IVLNWEC01DA0PJ | Bradi1g25090.1 | Os07t0553633-01 | - | AK371972 |
| IV9RU5F01BL21O | Bradi1g09187.1 | Os03t0740800-01 | - | AK372381 |
| IUIVLUD01AJA6N | Bradi1g08287.1 | Os03t0755000-01 | - | AK372734 |
| IUIVLUD02F2TJU | Bradi2g57987.1 | Os12t0621700-01 | - | AK373015 |
| IV9RU5F01CDHXZ | Bradi1g10447.1 | Os03t0721200-01 | - | AK373633 |
| IUIVLUD02FUOH5 | Bradi1g02610.1 | Os03t0835800-01 | - | AK374311 |
| IV9RU5F02F9EGZ | Bradi1g02610.1 | Os03t0835800-01 | - | AK374311 |
| IVLNWEC01BH6XH | Bradi1g02610.1 | Os03t0835800-01 | - | AK374311 |
| IUIVLUD01DSG1E | Bradi4g03150.1 | Os03t0240900-01 | - | AK375460 |
| IVLNWEC02I8U6Z | Bradi4g32120.1 | Os03t0423300-01 | - | AK375481 |
| IVLNWEC01BEDLK | Bradi4g08640.1 | Os09t0128400-01 | - | AK376270 |
| IV9RU5F02HV916 | Bradi2g31690.1 | Os05t0244500-01 | - | AK376623 |
| IVLNWEC01CUMD7 | Bradi2g31690.1 | Os05t0244500-01 | - | AK376623 |
| IVLNWEC02H0J45 | Bradi1g09970.2 | Os03t0728800-01 | - | AK377003 |
| IVLNWEC01C5LIQ | Bradi3g59870.1 | Os02t0736600-01 | - | MLOC_10268.4 |
| IV9RU5F02FN4VM | Bradi3g27970.1 | Os10t0457700-01 | - | MLOC_10621.1 |
| IV9RU5F02FYBL7 | Bradi2g51807.1 | Os11t0555300-00 | - | MLOC_10643.2 |
| IV9RU5F01CGCE7 | Bradi2g22840.1 | Os12t0552300-00 | - | MLOC_1068.2 |
| IVLNWEC02JT6GB | Bradi2g22840.1 | Os12t0552300-00 | - | MLOC_1068.2 |
| IV9RU5F01D1H00 | Bradi4g11740.1 | Os02t0231700-00 | - | MLOC_10793.6 |
| IV9RU5F01DHXY8 | Bradi4g41940.1 | Os11t0160300-01 | - | MLOC_10892.1 |
| IVLNWEC01BA1Y1 | Bradi4g42050.1 | Os12t0158800-01 | - | MLOC_11415.2 |
| IVLNWEC02I2AXL | Bradi1g06380.1 | Os03t0786900-01 | - | MLOC_12376.1 |
| IVLNWEC02IURP7 | Bradi1g63650.1 | Os01t0277600-00 | - | MLOC_12897.1 |
| IV9RU5F02IB6QU | Bradi4g43570.1 | Os02t0792500-00 | - | MLOC_13245.1 |
| IVLNWEC01D2YAM | Bradi3g50020.1 | Os02t0642600-01 | - | MLOC_13310.1 |
| IVLNWEC02INRKI | Bradi4g44680.1 | Os11t0109800-00 | - | MLOC_15454.1 |
| IVLNWEC01EYN9V | Bradi1g62210.2 | Os03t0355600-01 | - | MLOC_15738.2 |
| IVLNWEC01BPZ9L | Bradi1g00237.1 | Os05t0250700-00 | - | MLOC_16020.1 |
| IV9RU5F02JDP3W | Bradi1g01847.1 | Os03t0845500-01 | - | MLOC_17240.4 |

| | | | | |
|---|---|---|---|---|
| IV9RU5F02GNO9X | Bradi3g16660.1 | Os05t0151000-00 | - | MLOC_17526.1 |
| IV9RU5F02GOE43 | Bradi4g33700.1 | Os09t0488600-00 | - | MLOC_18305.1 |
| IUIVLUD01A2RS7 | Bradi4g07717.1 | Os06t0102800-01 | - | MLOC_19401.1 |
| IV9RU5F01D8CSH | Bradi2g11590.1 | Os06t0484800-00 | - | MLOC_21373.1 |
| IVLNWEC01AW407 | Bradi2g11590.1 | Os06t0484800-00 | - | MLOC_21373.1 |
| IVLNWEC01BCY5D | Bradi2g11590.1 | Os06t0484800-00 | - | MLOC_21373.1 |
| IVLNWEC01DZVSF | Bradi2g11590.1 | Os06t0484800-00 | - | MLOC_21373.1 |
| IVLNWEC02I0QC4 | Bradi2g11590.1 | Os06t0484800-00 | - | MLOC_21373.1 |
| IUIVLUD02GU076 | Bradi1g04610.1 | Os03t0808000-00 | - | MLOC_22135.1 |
| IVLNWEC01ETQIH | Bradi1g00227.1 | Os11t0678400-00 | - | MLOC_29057.1 |
| IV9RU5F01BR66Y | Bradi2g06100.1 | Os08t0496700-00 | - | MLOC_33543.1 |
| IVLNWEC02JG1VO | Bradi2g06100.1 | Os08t0496700-00 | - | MLOC_33543.1 |
| IV9RU5F02IZ4Y8 | Bradi1g00540.1 | Os03t0861400-00 | - | MLOC_34972.1 |
| IUIVLUD01CU18R | Bradi1g29622.1 | Os02t0832400-02 | - | MLOC_36683.1 |
| IVLNWEC01AIKHE | Bradi2g38810.1 | Os08t0539700-01 | - | MLOC_36685.1 |
| IUIVLUD01DISHI | Bradi2g38810.1 | Os12t0559300-00 | - | MLOC_36685.1 |
| IVLNWEC02HY8ZZ | Bradi2g18270.1 | Os09t0241100-01 | - | MLOC_36749.1 |
| IUIVLUD02INC9O | Bradi2g32290.1 | Os05t0212100-01 | - | MLOC_36835.1 |
| IVLNWEC01D1UMR | Bradi2g21470.1 | Os05t0491000-01 | - | MLOC_36992.1 |
| IVLNWEC01BWG1D | Bradi1g52210.1 | Os02t0106100-01 | - | MLOC_37242.1 |
| IVLNWEC01DGZK5 | Bradi4g24410.1 | Os11t0171800-01 | - | MLOC_37499.1 |
| IVLNWEC01DW23I | Bradi3g30360.2 | Os10t0506800-01 | - | MLOC_38557.8 |
| IUIVLUD01A6IIZ | Bradi2g44856.1 | Os01t0639900-01 | - | MLOC_43331.1 |
| IVLNWEC01EYR1W | Bradi4g37560.1 | Os01t0664000-00 | - | MLOC_44238.5 |
| IUIVLUD01EWOEO | Bradi1g00607.1 | Os02t0816900-01 | - | MLOC_4461.5 |
| IV9RU5F01D6P2F | Bradi1g50920.1 | Os08t0559700-00 | - | MLOC_49593.1 |
| IV9RU5F02JJ8T2 | Bradi1g30610.1 | Os06t0645400-01 | - | MLOC_4966.5 |
| IVLNWEC02JNE4X | Bradi1g11530.1 | Os03t0701800-01 | - | MLOC_51052.3 |
| IVLNWEC01D08EZ | Bradi3g33240.1 | Os10t0559600-01 | - | MLOC_51141.6 |
| IVLNWEC02ITQQF | Bradi3g33240.1 | Os10t0559600-01 | - | MLOC_51141.6 |
| IVLNWEC01E3FFV | Bradi4g02420.1 | Os03t0638000-00 | - | MLOC_51985.1 |
| IVLNWEC02IO25K | Bradi4g02420.1 | Os03t0638000-00 | - | MLOC_51985.1 |
| IV9RU5F02JPZHR | Bradi1g65390.1 | Os03t0294600-01 | - | MLOC_53998.2 |
| IVLNWEC01BQ3IM | Bradi2g08527.1 | Os01t0243200-02 | - | MLOC_54047.1 |
| IVLNWEC02FKNQH | Bradi1g04000.1 | Os03t0816900-01 | - | MLOC_54074.2 |
| IV9RU5F01DZVYM | Bradi1g51757.1 | Os06t0113150-00 | - | MLOC_54721.2 |
| IUIVLUD02FLZGK | Bradi1g03380.1 | Os03t0825700-01 | - | MLOC_55007.1 |
| IUIVLUD02F02VP | Bradi2g28435.1 | Os05t0354300-01 | - | MLOC_5527.1 |
| IVLNWEC02HQR6L | Bradi2g28435.1 | Os05t0354300-01 | - | MLOC_5527.1 |
| IV9RU5F01BNY7Y | Bradi4g43570.1 | Os02t0792500-00 | - | MLOC_55397.1 |
| IV9RU5F01EAK3I | Bradi1g13690.1 | Os03t0647500-02 | - | MLOC_55532.1 |
| IV9RU5F02GNET5 | Bradi1g28760.2 | Os02t0666250-00 | - | MLOC_56278.2 |
| IV9RU5F02IK7G8 | Bradi4g42160.1 | Os12t0156900-00 | - | MLOC_56529.1 |
| IVLNWEC01C2S7N | Bradi3g38100.1 | Os08t0474700-01 | - | MLOC_57586.2 |
| IV9RU5F01DFDJO | Bradi4g06950.1 | Os12t0498800-01 | - | MLOC_59107.2 |
| IV9RU5F01E0ACL | Bradi4g36810.1 | Os09t0543400-00 | - | MLOC_60174.1 |
| IUIVLUD01AXEIS | Bradi4g24480.1 | Os11t0175500-01 | - | MLOC_60365.2 |

| | | | | |
|---|---|---|---|---|
| IV9RU5F02H4AV5 | Bradi1g06350.1 | Os03t0787200-01 | - | MLOC_60795.1 |
| IVLNWEC02GC4FE | Bradi4g01090.1 | Os12t0626300-01 | - | MLOC_60893.1 |
| IV9RU5F02H7M5A | Bradi1g09390.1 | Os03t0737200-00 | - | MLOC_61341.1 |
| IVLNWEC02HJ5H3 | Bradi1g09390.1 | Os03t0737200-00 | - | MLOC_61341.1 |
| IV9RU5F02G7KD3 | Bradi3g16660.1 | Os05t0151000-00 | - | MLOC_61462.1 |
| IVLNWEC01C24PG | Bradi4g07290.1 | Os12t0485600-01 | - | MLOC_6161.1 |
| IVLNWEC01EHCI6 | Bradi2g10180.1 | Os01t0271200-01 | - | MLOC_61627.1 |
| IVLNWEC02I8CEM | Bradi2g10180.1 | Os01t0271200-01 | - | MLOC_61627.1 |
| IVLNWEC01CW0YR | Bradi3g41520.1 | Os08t0537600-01 | - | MLOC_6232.1 |
| IV9RU5F02HVGTW | Bradi1g71810.2 | Os03t0191000-01 | - | MLOC_63242.2 |
| IVLNWEC02GST7P | Bradi1g22340.1 | Os12t0512800-01 | - | MLOC_63458.1 |
| IUIVLUD01DDKEE | Bradi4g23600.2 | Os11t0186400-00 | - | MLOC_64602.2 |
| IUIVLUD01DEG0A | Bradi4g23600.2 | Os11t0186400-00 | - | MLOC_64602.2 |
| IVLNWEC02GELKX | Bradi1g51807.1 | Os01t0304100-00 | - | MLOC_64607.5 |
| IV9RU5F02HVCBB | Bradi3g59910.1 | Os02t0736200-01 | - | MLOC_65625.1 |
| IUIVLUD02GS3FO | Bradi4g22180.1 | Os11t0216300-01 | - | MLOC_65936.3 |
| IV9RU5F02IVENS | Bradi1g07360.1 | Os03t0769400-01 | - | MLOC_67384.1 |
| IVLNWEC02I6351 | Bradi4g35730.1 | Os05t0269500-01 | - | MLOC_6781.1 |
| IVLNWEC01DOLG9 | Bradi1g69880.1 | Os10t0400100-01 | - | MLOC_68522.2 |
| IVLNWEC02GW49F | Bradi3g45620.1 | Os02t0552100-01 | - | MLOC_6928.1 |
| IVLNWEC02GAV66 | Bradi3g06727.1 | Os02t0192400-01 | - | MLOC_6946.1 |
| IV9RU5F01DIGUB | Bradi1g30360.2 | Os06t0639500-01 | - | MLOC_70404.1 |
| IVLNWEC01EOY57 | Bradi1g30360.2 | Os06t0639500-01 | - | MLOC_70404.1 |
| IV9RU5F02JA3JE | Bradi3g16660.1 | Os05t0151000-00 | - | MLOC_73160.1 |
| IV9RU5F01BC2NU | Bradi1g00227.1 | Os08t0260800-00 | - | MLOC_74542.1 |
| IV9RU5F01BSNUA | Bradi2g36230.1 | Os05t0270200-01 | - | MLOC_74926.1 |
| IVLNWEC01A2TQ8 | Bradi1g48670.1 | Os06t0158900-00 | - | MLOC_76366.3 |
| IV9RU5F02HOJZ7 | Bradi4g08860.1 | Os09t0251100-00 | - | MLOC_76583.1 |
| IV9RU5F02HWXGS | Bradi2g31690.1 | Os05t0244500-01 | - | MLOC_77701.1 |
| IUIVLUD01A0BOA | Bradi2g00627.1 | Os01t0106700-00 | - | MLOC_9291.1 |
| IV9RU5F02HOC79 | Bradi3g38100.1 | Os08t0474700-01 | - | MLOC_57586.2 |
| IVLNWEC02IJRWT | Bradi3g38100.1 | Os08t0474700-01 | - | MLOC_57586.2 |
| IV9RU5F01DQ2ZL | Bradi1g08190.1 | Os03t0757200-01 | - | MLOC_64192.1 |
| IV9RU5F01BRGBS | Bradi4g08060.1 | Os12t0405200-01 | - | AK359711 |
| IV9RU5F01DYKU0 | Bradi3g54710.1 | Os02t0806000-01 | - | AK248678.1 |
| IV9RU5F02JMVHC | Bradi4g42240.1 | Os11t0153500-01 | - | MLOC_71746.1 |
| IUIVLUD01EKTTM | Bradi4g26227.1 | Os11t0130100-01 | - | AK248724.1 |
| IV9RU5F01CULPG | Bradi2g27097.1 | Os05t0376600-01 | - | AK374248 |
| IVLNWEC01DG9JQ | Bradi4g34960.1 | Os09t0512700-01 | - | AK370272 |
| IVLNWEC02I9594 | Bradi4g34960.1 | Os09t0512700-01 | - | AK370272 |
| IVLNWEC02F3ET4 | Bradi1g64580.1 | Os03t0305700-01 | - | MLOC_54120.2 |
| IVLNWEC02GAGGH | Bradi1g64580.1 | Os03t0305700-01 | - | MLOC_54120.2 |
| IVLNWEC01DL3GI | Bradi4g30190.1 | Os09t0413300-00 | - | MLOC_71161.1 |
| IUIVLUD02H0D4G | Bradi2g27230.1 | Os01t0355600-01 | - | MLOC_54934.2 |
| IUIVLUD02JMI9I | Bradi2g27230.1 | Os01t0355600-01 | - | MLOC_54934.2 |
| IV9RU5F02H7AB6 | Bradi2g27230.1 | Os01t0355600-01 | - | MLOC_54934.2 |
| IVLNWEC01ARYGU | Bradi2g27230.1 | Os01t0355600-01 | - | MLOC_54934.2 |

| | | | | |
|---|---|---|---|---|
| IVLNWEC01B0050 | Bradi2g27230.1 | Os01t0355600-01 | - | MLOC_54934.2 |
| IVLNWEC01D0E3X | Bradi2g27230.1 | Os01t0355600-01 | - | MLOC_54934.2 |
| IUIVLUD02HZMHV | Bradi4g42880.1 | Os11t0148101-00 | - | AK356045 |
| IV9RU5F01CRV8Q | Bradi4g42880.1 | Os11t0148101-00 | - | AK356045 |
| IUIVLUD01D4X3X | Bradi4g26970.1 | Os09t0267500-01 | - | MLOC_1337.1 |
| IV9RU5F01EC9SO | Bradi4g26970.1 | Os09t0267500-01 | - | MLOC_1337.1 |
| IVLNWEC02JLUDP | Bradi4g26970.1 | Os09t0267500-01 | - | MLOC_1337.1 |
| IUIVLUD02GQGI5 | Bradi4g26970.1 | Os09t0267500-01 | - | MLOC_39605.1 |
| IV9RU5F02H66QV | Bradi2g16500.1 | Os03t0619400-01 | - | MLOC_38451.1 |
| IV9RU5F02IAUMK | Bradi4g28877.1 | Os09t0364000-01 | - | MLOC_54108.2 |
| IVLNWEC02I4F0H | Bradi1g69880.1 | Os10t0400100-01 | - | MLOC_68522.2 |
| IV9RU5F01BKW5E | Bradi1g10360.1 | Os09t0442700-00 | - | MLOC_64588.1 |
| IV9RU5F01E2GUT | Bradi4g03050.1 | Os12t0590500-00 | - | MLOC_75843.3 |
| IUIVLUD02FNZP5 | Bradi3g03730.1 | Os05t0317200-01 | - | AK370654 |
| IV9RU5F01A8NLK | Bradi1g06450.1 | Os03t0785900-01 | - | MLOC_69873.1 |
| IV9RU5F01DQPHO | Bradi3g04270.1 | Os01t0507700-01 | - | MLOC_13110.1 |
| IUIVLUD01A4C9V | Bradi4g31040.1 | Os09t0434500-01 | - | AK372481 |
| IVLNWEC01BCTHK | Bradi4g31040.1 | Os09t0434500-01 | - | AK372481 |
| IVLNWEC01D2IZM | Bradi4g31040.1 | Os09t0434500-01 | - | AK372481 |
| IVLNWEC01CR8KN | Bradi4g37917.1 | Os09t0560100-00 | - | MLOC_71830.3 |
| IVLNWEC01DVBNV | Bradi4g37917.1 | Os09t0560100-00 | - | MLOC_71830.3 |
| IV9RU5F01EA9UA | Bradi2g38510.1 | Os11t0212300-00 | - | MLOC_7020.1 |
| IUIVLUD01EODFM | Bradi2g62760.1 | Os01t0977600-02 | - | MLOC_55536.1 |
| IV9RU5F01EVGQ7 | Bradi4g44760.1 | Os12t0108500-01 | - | AK372025 |
| IV9RU5F01B23PS | Bradi1g28847.1 | Os07t0444000-02 | - | MLOC_44210.2 |
| IVLNWEC02HNOTG | Bradi4g45227.1 | Os11t0102900-01 | - | MLOC_48965.1 |
| IV9RU5F01CVEWA | Bradi1g13450.1 | Os03t0652100-01 | - | AK251104.1 |
| IVLNWEC01B60P3 | Bradi4g00270.1 | Os08t0407200-01 | - | MLOC_62326.2 |
| IUIVLUD01A01WI | Bradi3g21117.1 | Os10t0140200-01 | - | AK363456 |
| IVLNWEC01CN7OT | Bradi1g11960.1 | Os05t0220900-01 | - | MLOC_63149.1 |
| IV9RU5F02I9VA2 | Bradi1g15110.1 | Os03t0598200-01 | - | MLOC_55271.1 |
| IV9RU5F01D1EE6 | Bradi4g03690.1 | Os02t0294600-01 | - | MLOC_59257.1 |
| IVLNWEC02F6X6Z | Bradi4g03690.1 | Os02t0294600-01 | - | MLOC_59257.1 |
| IV9RU5F01DLERA | Bradi3g03730.1 | Os05t0317200-01 | - | AK370654 |
| IVLNWEC01BIQEN | Bradi1g14590.1 | Os03t0617900-01 | - | AK354697 |
| IV9RU5F01EJZDE | Bradi1g68957.1 | Os10t0105400-02 | - | MLOC_12936.1 |
| IUIVLUD01AQD3X | Bradi3g33240.1 | Os10t0559600-01 | - | MLOC_51141.6 |
| IUIVLUD02G8NEL | Bradi4g08527.1 | Os09t0121000-01 | - | MLOC_11195.1 |
| IUIVLUD01AN4QN | Bradi1g29622.1 | Os02t0832400-02 | - | MLOC_36683.1 |
| IV9RU5F01CH9W5 | Bradi1g02180.1 | Os07t0644700-00 | - | MLOC_60056.5 |
| IV9RU5F01DBC6Y | Bradi2g25930.1 | Os05t0400800-02 | - | AK357293 |
| IUIVLUD01EO5XI | Bradi2g10800.2 | Os01t0279400-01 | - | AK249459.1 |
| IUIVLUD02JAAYR | Bradi4g31500.1 | Os09t0443500-01 | - | AK362143 |
| IV9RU5F01D7YU1 | Bradi3g44230.1 | Os02t0512300-01 | - | MLOC_44370.1 |
| IUIVLUD02JF58Z | Bradi2g18710.1 | Os07t0603200-01 | - | AK364029 |
| IVLNWEC01AY5OY | Bradi2g18710.1 | Os07t0603200-01 | - | AK364029 |
| IV9RU5F02GN4TX | Bradi4g15150.1 | Os11t0585700-01 | - | AK369305 |

| | | | | |
|---|---|---|---|---|
| IVLNWEC02I7GB9 | Bradi1g10190.1 | Os03t0725300-01 | - | MLOC_12321.3 |
| IV9RU5F02G9KGL | Bradi1g06900.1 | Os03t0778000-01 | - | MLOC_66071.3 |
| IVLNWEC01BP8HT | Bradi4g08180.1 | Os09t0103600-01 | - | AK365783 |
| IV9RU5F02JGAG3 | Bradi4g33700.1 | Os09t0488600-00 | - | MLOC_18305.1 |
| IVLNWEC01D4VQP | Bradi4g45030.1 | Os12t0175500-01 | - | AK252532.1 |
| IVLNWEC02G1Q62 | Bradi3g05420.1 | Os02t0173500-01 | - | MLOC_19066.1 |
| IV9RU5F02GH0QT | Bradi4g42860.1 | Os11t0148200-01 | - | AK367874 |
| IVLNWEC02IMCDH | Bradi4g42860.1 | Os11t0148200-01 | - | AK367874 |
| IV9RU5F02I7M4Q | Bradi3g34040.1 | Os10t0573000-01 | - | AK364381 |
| IVLNWEC01A1B4V | Bradi1g06470.1 | Os03t0785500-01 | - | MLOC_55659.1 |
| IVLNWEC01CQ45M | Bradi4g02710.1 | Os02t0523300-01 | - | MLOC_61177.1 |
| IVLNWEC01A9OYR | Bradi4g30550.1 | Os09t0422600-01 | - | MLOC_10572.1 |
| IVLNWEC02HAARU | Bradi4g30550.1 | Os09t0422600-01 | - | MLOC_10572.1 |
| IUIVLUD01A8RJ3 | Bradi2g25260.1 | Os05t0417100-01 | - | MLOC_57607.1 |
| IVLNWEC02JD16C | Bradi1g70460.1 | Os02t0787500-01 | - | MLOC_75001.1 |
| IUIVLUD01DVRMU | Bradi1g64220.1 | Os03t0313300-01 | - | MLOC_5906.1 |
| IUIVLUD01D4V7P | Bradi1g28540.1 | Os07t0459200-02 | - | AK357778 |
| IV9RU5F01CGHBU | Bradi3g42080.1 | Os08t0546400-01 | - | AK376864 |
| IV9RU5F02HPG8T | Bradi1g65390.1 | Os03t0294600-01 | - | MLOC_53998.2 |
| IVLNWEC02F4BW6 | Bradi1g65390.1 | Os03t0294600-01 | - | MLOC_53998.2 |
| IV9RU5F01EEJZ3 | Bradi1g01007.1 | Os03t0856200-01 | - | MLOC_49689.2 |
| IVLNWEC02IORVN | Bradi4g08527.1 | Os09t0121050-00 | - | AK355795 |
| IUIVLUD01ECSHJ | Bradi1g51507.1 | Os03t0152900-01 | - | MLOC_55033.1 |
| IVLNWEC02FXAGR | Bradi1g51507.1 | Os06t0137100-01 | - | MLOC_55033.1 |
| IV9RU5F02IDLC4 | Bradi3g29917.1 | Os10t0495600-01 | - | AK375542 |
| IVLNWEC02I4EB4 | Bradi3g29917.1 | Os10t0495600-01 | - | AK375542 |
| IVLNWEC01DCAMN | Bradi3g25380.1 | Os10t0346200-01 | - | AK368868 |
| IVLNWEC02G9X7B | Bradi3g25380.1 | Os10t0346200-01 | - | AK368868 |
| IUIVLUD01AHH62 | Bradi3g43630.1 | Os02t0490000-01 | - | MLOC_66893.1 |
| IVLNWEC02HQNMH | Bradi3g43630.1 | Os02t0495400-01 | - | MLOC_66893.1 |
| IVLNWEC01DPIKK | Bradi1g67247.1 | Os03t0264300-00 | - | MLOC_5745.4 |
| IVLNWEC01C5ORC | Bradi2g36230.1 | Os05t0270200-01 | - | MLOC_74926.1 |
| IUIVLUD02I075P | Bradi4g06390.1 | Os12t0514400-01 | - | MLOC_73857.1 |
| IVLNWEC02GITG8 | Bradi3g56035.1 | Os02t0822900-01 | - | AK368403 |
| IV9RU5F01AZ7LN | Bradi1g04860.2 | Os03t0805200-01 | - | AK248344.1 |
| IV9RU5F01BTCHH | Bradi1g04860.2 | Os03t0805200-01 | - | AK248344.1 |
| IV9RU5F02IJHA1 | Bradi3g27970.1 | Os10t0457700-01 | - | MLOC_10620.1 |
| IVLNWEC01BLNPH | Bradi3g27970.1 | Os10t0457700-01 | - | MLOC_10620.1 |
| IVLNWEC02F10VL | Bradi3g27970.1 | Os10t0457700-01 | - | MLOC_10620.1 |
| IVLNWEC02GJH12 | Bradi3g27970.1 | Os10t0457700-01 | - | MLOC_10620.1 |
| IVLNWEC01D9U7J | Bradi4g39550.1 | Os12t0238100-01 | - | MLOC_82054.2 |
| IVLNWEC01CPX9G | Bradi1g14877.1 | Os03t0604600-01 | - | MLOC_10859.4 |
| IVLNWEC01CD389 | Bradi4g07957.1 | Os12t0433200-01 | - | AK353660 |
| IV9RU5F01BL5XI | Bradi3g35350.1 | Os08t0398700-01 | - | AK360995 |
| IUIVLUD02H5WFR | Bradi1g65940.2 | Os01t0835000-01 | - | MLOC_36790.3 |
| IUIVLUD01BFPOE | Bradi1g74430.1 | Os03t0161100-00 | - | MLOC_44284.2 |
| IUIVLUD02IUIA7 | Bradi1g74430.1 | Os03t0161100-00 | - | MLOC_44284.2 |

| | | | | |
|---|---|---|---|---|
| IV9RU5F02JAGXG | Bradi4g36976.1 | Os11t0684700-01 | - | AK368548 |
| IV9RU5F02HLCJO | Bradi4g06650.1 | Os12t0508100-00 | - | MLOC_69555.3 |
| IVLNWEC01CVQQI | Bradi4g00270.1 | Os08t0407200-01 | - | MLOC_62326.2 |
| IVLNWEC01CDFTC | Bradi4g26750.1 | Os12t0104300-01 | - | MLOC_34565.4 |
| IVLNWEC01AZ11U | Bradi4g28177.1 | Os09t0327800-00 | - | MLOC_2429.1 |
| IVLNWEC02JT2UV | Bradi4g02420.1 | Os03t0637900-01 | - | MLOC_51985.1 |
| IUIVLUD01ENT6W | Bradi3g30360.2 | Os10t0506800-01 | - | MLOC_38557.8 |
| IV9RU5F01AO3S0 | Bradi1g74480.1 | Os03t0160100-01 | - | MLOC_59190.1 |
| IV9RU5F02HY895 | Bradi1g09390.1 | Os03t0737200-00 | - | MLOC_61341.1 |
| IVLNWEC01EE0LC | Bradi1g34180.1 | Os06t0701200-01 | - | AK366137 |
| IVLNWEC01BPY3G | Bradi1g04560.1 | Os03t0809900-01 | - | MLOC_64220.1 |
| IVLNWEC02FW48R | Bradi2g26930.1 | Os01t0957000-01 | - | AK367623 |
| IV9RU5F01EM1D5 | Bradi4g32840.1 | Os08t0484400-01 | - | MLOC_63540.1 |
| IV9RU5F01CLK9E | Bradi1g11730.1 | Os03t0699200-01 | - | MLOC_70449.2 |
| IUIVLUD01D3QTU | Bradi2g26680.1 | Os05t0389700-01 | - | MLOC_10169.2 |
| IV9RU5F01CPRWO | Bradi3g27906.1 | Os10t0456900-01 | - | MLOC_37545.1 |
| IV9RU5F01DOEPI | Bradi1g14190.1 | Os04t0351333-00 | - | AK373258 |
| IV9RU5F01BCFHU | Bradi4g25410.1 | Os03t0287600-00 | - | MLOC_11551.2 |
| IUIVLUD02H12JK | Bradi1g50212.1 | Os06t0111600-01 | - | AK372516 |
| IVLNWEC02IV2FM | Bradi1g66240.1 | Os03t0280700-01 | - | AK364570 |
| IV9RU5F01AYDXK | Bradi1g72490.1 | Os03t0182400-01 | - | AK248477.1 |
| IUIVLUD02HZ747 | Bradi4g42530.1 | Os11t0151500-01 | - | MLOC_60742.2 |
| IUIVLUD01AMYXX | Bradi4g30190.1 | Os09t0413300-00 | - | MLOC_71161.1 |
| IV9RU5F01ENJMW | Bradi4g42640.1 | Os11t0149900-00 | - | MLOC_72578.1 |
| IV9RU5F02HLIBD | Bradi4g42640.1 | Os11t0149900-00 | - | MLOC_72578.1 |
| IV9RU5F02FS3Y3 | Bradi2g26910.1 | Os03t0853700-01 | - | MLOC_57217.1 |
| IUIVLUD02GQ395 | Bradi1g13690.1 | Os03t0647500-02 | - | MLOC_55532.1 |
| IVLNWEC01B05SR | Bradi1g13690.1 | Os03t0647500-02 | - | MLOC_55532.1 |
| IV9RU5F02GNKKC | Bradi1g16030.1 | Os12t0129000-01 | - | MLOC_36543.1 |
| IVLNWEC01B268L | Bradi1g16030.1 | Os12t0129000-01 | - | MLOC_36543.1 |
| IV9RU5F02GIV4Z | Bradi1g08145.1 | Os03t0757500-01 | - | MLOC_57500.1 |
| IV9RU5F01AQALA | Bradi2g32290.1 | Os05t0212100-01 | - | MLOC_36835.1 |
| IV9RU5F02GJTG2 | Bradi3g16110.1 | Os03t0860600-01 | - | MLOC_58934.1 |
| IVLNWEC01B801S | Bradi1g13530.1 | Os03t0650400-01 | - | MLOC_51763.4 |
| IV9RU5F01BXFLX | Bradi4g34330.1 | Os09t0501100-00 | - | MLOC_18171.1 |
| IV9RU5F01CFTAF | Bradi2g62760.1 | Os01t0977600-02 | - | MLOC_55536.1 |
| IV9RU5F01D3PER | Bradi2g62760.1 | Os01t0977600-02 | - | MLOC_55536.1 |
| IV9RU5F01BI7XC | Bradi4g42860.1 | Os11t0148200-01 | - | AK367874 |
| IVLNWEC02HJS6D | Bradi1g56800.2 | Os07t0595800-01 | - | MLOC_7288.1 |
| IVLNWEC01A8U53 | Bradi4g35977.1 | Os09t0530500-01 | - | MLOC_4008.2 |
| IV9RU5F02FXDKK | Bradi1g06570.1 | Os03t0784600-01 | - | MLOC_62889.1 |
| IVLNWEC01AQA34 | Bradi1g06570.1 | Os03t0784600-01 | - | MLOC_62889.1 |
| IV9RU5F01A9N79 | Bradi3g41987.1 | Os08t0544400-01 | - | MLOC_11283.1 |
| IV9RU5F01EKUDR | Bradi1g08350.1 | Os03t0752700-01 | - | MLOC_53865.1 |
| IV9RU5F01CYXIY | Bradi4g27600.1 | Os09t0298700-01 | - | AK359869 |
| IV9RU5F01DIGNJ | Bradi4g27600.1 | Os09t0298700-01 | - | AK359869 |
| IV9RU5F02FKDQO | Bradi4g07267.1 | Os02t0139600-02 | - | AK365985 |

| | | | | |
|---|---|---|---|---|
| IV9RU5F01DBI8H | Bradi4g34022.1 | Os09t0493700-01 | - | MLOC_11138.1 |
| IVLNWEC02FNDSE | Bradi1g04310.1 | Os01t0762000-01 | - | AK369954 |
| IV9RU5F01DB5WR | Bradi4g02420.1 | Os03t0638000-00 | - | MLOC_51985.1 |
| IV9RU5F01DQ0ZV | Bradi4g02420.1 | Os12t0605200-01 | - | MLOC_51985.1 |
| IVLNWEC02IO3RP | Bradi4g02420.1 | Os12t0605200-01 | - | MLOC_51985.1 |
| IV9RU5F01EC3EH | Bradi4g01320.1 | Os03t0649000-01 | - | AK363790 |
| IV9RU5F02IZJYG | Bradi4g03050.1 | Os12t0590500-00 | - | MLOC_75843.3 |
| IVLNWEC01EG8ZB | Bradi4g03050.1 | Os12t0590500-00 | - | MLOC_75843.3 |
| IV9RU5F02F49J8 | Bradi2g22840.1 | Os12t0552300-00 | - | MLOC_1068.2 |
| IV9RU5F02G3W5I | Bradi4g06950.1 | Os12t0498800-01 | - | MLOC_59107.2 |
| IVLNWEC01EI2LW | Bradi2g05460.1 | Os01t0185200-01 | - | MLOC_8121.1 |
| IV9RU5F01DL8N9 | Bradi1g08140.1 | Os03t0757600-01 | - | MLOC_63805.2 |
| IUIVLUD02FY7I0 | Bradi1g08810.1 | Os03t0746800-01 | - | AK359906 |
| IUIVLUD01ER1D7 | Bradi4g33310.1 | Os12t0233600-01 | - | MLOC_78764.1 |
| IVLNWEC01BJQ28 | Bradi4g33310.1 | Os12t0233600-01 | - | MLOC_78764.1 |
| IUIVLUD02JE2ZT | Bradi2g11590.1 | Os06t0484800-00 | - | MLOC_21373.1 |
| IVLNWEC01EB6G1 | Bradi1g74620.1 | Os03t0157600-00 | - | MLOC_6726.1 |
| IUIVLUD01CB0L8 | Bradi2g34230.1 | Os10t0489301-00 | - | MLOC_32569.1 |
| IVLNWEC02IUEZN | Bradi1g01870.1 | Os03t0845000-01 | - | MLOC_70257.1 |
| IUIVLUD02H1EWG | Bradi4g43930.1 | Os11t0128700-00 | - | MLOC_11293.1 |
| IVLNWEC01EWC65 | Bradi1g42340.1 | Os06t0489500-01 | - | MLOC_80715.2 |
| IVLNWEC01BNJMY | Bradi1g64580.1 | Os03t0305700-01 | - | MLOC_54120.2 |
| IV9RU5F02HPTH2 | Bradi2g38577.1 | Os05t0133900-01 | - | AK358106 |
| IV9RU5F01EQFWS | Bradi2g27230.1 | Os01t0355600-01 | - | MLOC_54934.2 |
| IV9RU5F02G1DCJ | Bradi2g11860.1 | Os01t0312500-01 | - | MLOC_54267.1 |
| IV9RU5F01AZ1FP | Bradi4g08700.1 | Os09t0258500-01 | - | MLOC_58205.1 |
| IVLNWEC01AJ75V | Bradi1g68957.1 | Os03t0226600-02 | - | MLOC_12936.1 |
| IVLNWEC01D75WK | Bradi4g26970.1 | Os09t0267500-01 | - | MLOC_1337.1 |
| IUIVLUD02ITV84 | Bradi4g07790.1 | Os12t0456100-00 | - | MLOC_65209.1 |
| IV9RU5F02H97PI | Bradi4g42330.1 | Os12t0152600-01 | - | MLOC_5856.1 |
| IVLNWEC01E0TB0 | Bradi4g42330.1 | Os12t0152600-01 | - | MLOC_5856.1 |
| IUIVLUD01CK2JH | Bradi1g10640.1 | Os03t0717900-01 | - | MLOC_74226.3 |
| IVLNWEC02I32PH | Bradi1g04540.1 | Os03t0809700-01 | - | MLOC_62782.5 |
| IV9RU5F01DBYTW | Bradi4g44760.1 | Os12t0108500-01 | - | AK372025 |
| IVLNWEC02GC6KV | Bradi4g22180.1 | Os11t0216300-01 | - | MLOC_65936.3 |
| IUIVLUD01C7FZB | Bradi1g06380.1 | Os03t0786900-01 | - | MLOC_14680.1 |
| IVLNWEC01BUE9F | Bradi3g33240.1 | Os10t0559700-02 | - | MLOC_51141.6 |
| IV9RU5F02JJ7IN | Bradi4g43490.4 | Os11t0136800-01 | - | AK251443.1 |
| IVLNWEC02HSE07 | Bradi1g02180.1 | Os07t0644700-00 | - | MLOC_60056.5 |
| IV9RU5F01CZQ24 | Bradi4g36250.1 | Os09t0534000-01 | - | MLOC_52513.1 |
| IV9RU5F02GEPDL | Bradi4g36250.1 | Os09t0534000-01 | - | MLOC_52513.1 |
| IV9RU5F01C14ME | Bradi1g06900.1 | Os03t0778000-01 | - | MLOC_62114.2 |
| IVLNWEC01AV5R6 | Bradi1g06900.1 | Os03t0778000-01 | - | MLOC_62114.2 |
| IV9RU5F02IPQ5R | Bradi2g18710.1 | Os07t0603200-01 | - | AK364029 |
| IV9RU5F02I4K95 | Bradi4g15150.1 | Os11t0585700-01 | - | AK369305 |
| IV9RU5F01B56MS | Bradi4g42337.1 | Os12t0152100-01 | - | MLOC_5857.3 |
| IVLNWEC01BWBIV | Bradi1g74437.1 | Os03t0160600-01 | - | MLOC_59792.1 |

| | | | | |
|---|---|---|---|---|
| IV9RU5F02FY2SU | Bradi1g16560.1 | Os03t0289300-00 | - | MLOC_58308.2 |
| IVLNWEC02HSTB4 | Bradi4g29410.1 | Os09t0380000-02 | - | MLOC_13051.1 |
| IV9RU5F01BXU17 | Bradi4g44750.1 | Os11t0109000-01 | - | MLOC_75827.1 |
| IV9RU5F01EPTWI | Bradi1g03030.1 | Os03t0831100-01 | - | MLOC_63484.2 |
| IVLNWEC01ALTPK | Bradi1g01847.1 | Os03t0845500-01 | - | MLOC_4674.2 |
| IVLNWEC01DGCXC | Bradi3g27740.1 | Os10t0446200-01 | - | AK369392 |
| IUIVLUD01A73PF | Bradi4g25047.1 | Os11t0159000-01 | - | AK371325 |
| IUIVLUD01D7MYI | Bradi1g08500.1 | Os03t0750300-00 | - | MLOC_58630.2 |
| IV9RU5F01DGHWT | Bradi1g08500.1 | Os03t0750300-00 | - | MLOC_58630.2 |
| IVLNWEC01E3RKT | Bradi1g66850.3 | Os01t0103800-01 | - | AK354358 |
| IVLNWEC02H3JRO | Bradi3g08110.1 | Os10t0378400-01 | - | MLOC_44285.1 |
| IUIVLUD01DZRST | Bradi1g70000.1 | Os03t0213900-02 | - | AK360808 |
| IUIVLUD02I5482 | Bradi1g70000.1 | Os03t0213900-02 | - | AK360808 |
| IVLNWEC01C34NX | Bradi1g42340.1 | Os06t0489500-01 | - | MLOC_80715.2 |
| IVLNWEC02G54RQ | Bradi1g64380.1 | Os03t0308900-01 | - | MLOC_57538.2 |
| IV9RU5F01EDL25 | Bradi3g22720.1 | Os10t0181200-01 | - | AK357959 |
| IUIVLUD01EAYW1 | Bradi3g44230.1 | Os02t0512300-01 | - | MLOC_44370.1 |
| IVLNWEC02JKJAW | Bradi1g30910.1 | Os06t0649700-00 | - | AK367046 |
| IVLNWEC01CONKL | Bradi2g22840.1 | Os12t0552300-00 | - | MLOC_1068.2 |
| IV9RU5F01ESUKH | Bradi4g30665.1 | Os09t0424701-00 | - | MLOC_19512.1 |
| IV9RU5F02J2NIJ | Bradi3g30360.2 | Os10t0506800-01 | - | MLOC_38557.8 |
| IV9RU5F01CREYW | Bradi1g17670.1 | Os10t0346600-00 | Sb0013s004030.1 | - |
| IUIVLUD01EA8FJ | Bradi1g00460.1 | Os07t0522500-01 | Sb01g000220.1 | - |
| IV9RU5F01CPWCN | Bradi1g00460.1 | Os07t0522500-01 | Sb01g000220.1 | - |
| IVLNWEC01ATG8L | Bradi1g00460.1 | Os07t0522500-01 | Sb01g000220.1 | - |
| IVLNWEC02I0IKG | Bradi1g00760.1 | Os03t0859500-01 | Sb01g000400.1 | - |
| IV9RU5F01D8XM0 | Bradi1g01007.1 | Os03t0856200-04 | Sb01g000620.1 | - |
| IUIVLUD01B9NEV | Bradi1g01140.1 | Os03t0852900-01 | Sb01g000850.1 | - |
| IUIVLUD02FVTPD | Bradi1g01140.1 | Os03t0852900-01 | Sb01g000850.1 | - |
| IV9RU5F01AE4JZ | Bradi1g01140.1 | Os03t0852900-01 | Sb01g000850.1 | - |
| IVLNWEC01AHFYK | Bradi1g01140.1 | Os03t0852900-01 | Sb01g000850.1 | - |
| IUIVLUD01AYWQW | Bradi1g01280.1 | Os03t0852000-01 | Sb01g000920.1 | - |
| IV9RU5F02HA28W | Bradi1g01280.1 | Os03t0852000-01 | Sb01g000920.1 | - |
| IV9RU5F01C2CJK | Bradi1g01800.1 | Os03t0850400-01 | Sb01g001140.1 | - |
| IUIVLUD01DR7WN | Bradi1g01847.1 | Os03t0845500-01 | Sb01g001445.1 | - |
| IVLNWEC01CLI7P | Bradi1g01980.1 | Os03t0843900-01 | Sb01g001560.1 | - |
| IUIVLUD02GPKKL | Bradi4g20557.1 | Os11t0265600-00 | Sb01g001720.1 | - |
| IV9RU5F01DDHA1 | Bradi1g02400.1 | Os03t0838900-01 | Sb01g001940.1 | - |
| IVLNWEC01BCFDD | Bradi1g02400.1 | Os03t0838900-01 | Sb01g001940.1 | - |
| IV9RU5F01EWPA5 | Bradi1g02980.1 | Os03t0831700-01 | Sb01g002240.1 | - |
| IV9RU5F01ENQES | Bradi1g03050.1 | Os03t0830900-01 | Sb01g002300.1 | - |
| IUIVLUD01AFXTO | Bradi1g04140.1 | Os03t0815200-01 | Sb01g003700.1 | - |
| IUIVLUD02JJ5CY | Bradi1g04300.2 | Os03t0811100-01 | Sb01g003860.1 | - |
| IVLNWEC01DP9H9 | Bradi1g36810.1 | Os02t0596100-01 | Sb01g003970.1 | - |
| IV9RU5F02GBKNR | Bradi1g04500.1 | Os03t0809100-01 | Sb01g004100.1 | - |
| IV9RU5F02GPRSZ | Bradi4g01740.1 | Os04t0312100-01 | Sb01g004110.1 | - |
| IVLNWEC01ELO6S | Bradi4g01740.1 | Os04t0312100-01 | Sb01g004110.1 | - |

| | | | | |
|---|---|---|---|---|
| IV9RU5F02F34GR | Bradi1g04610.1 | Os03t0808000-00 | Sb01g004190.1 | - |
| IUIVLUD01B01CG | Bradi1g05170.1 | Os03t0800050-00 | Sb01g004930.1 | - |
| IV9RU5F01A1VG7 | Bradi1g05590.1 | Os03t0795400-00 | Sb01g005290.1 | - |
| IVLNWEC01BBSSZ | Bradi1g05630.1 | Os03t0795000-01 | Sb01g005330.1 | - |
| IVLNWEC01DJI9Y | Bradi1g05630.1 | Os03t0795000-01 | Sb01g005330.1 | - |
| IVLNWEC02JGVV7 | Bradi1g05630.1 | Os03t0795000-01 | Sb01g005330.1 | - |
| IVLNWEC01EK312 | Bradi1g06380.1 | Os03t0786900-01 | Sb01g005910.1 | - |
| IVLNWEC02HG29S | Bradi1g06766.1 | Os03t0781100-00 | Sb01g006260.1 | - |
| IV9RU5F01BOILO | Bradi1g06930.1 | Os03t0777500-01 | Sb01g006390.1 | - |
| IV9RU5F01EVCD0 | Bradi1g06930.1 | Os03t0777500-01 | Sb01g006390.1 | - |
| IVLNWEC02IMQGY | Bradi1g06930.1 | Os03t0777500-01 | Sb01g006390.1 | - |
| IUIVLUD01AP0RW | Bradi1g07132.1 | Os03t0774400-01 | Sb01g006620.1 | - |
| IUIVLUD02IQNJ2 | Bradi1g07390.1 | Os03t0769000-00 | Sb01g006930.1 | - |
| IV9RU5F02I5GBS | Bradi1g07420.1 | Os03t0768900-00 | Sb01g006940.1 | - |
| IV9RU5F02ID0AM | Bradi1g07650.1 | Os03t0764300-01 | Sb01g007150.1 | - |
| IV9RU5F01CX51P | Bradi1g08270.1 | Os03t0755800-00 | Sb01g007700.1 | - |
| IVLNWEC01CEY1D | Bradi1g08270.1 | Os03t0755800-00 | Sb01g007700.1 | - |
| IV9RU5F01AJDBF | Bradi1g08280.1 | Os03t0755100-01 | Sb01g007710.1 | - |
| IV9RU5F02HM6EJ | Bradi1g08280.1 | Os03t0755100-01 | Sb01g007710.1 | - |
| IUIVLUD02ITX36 | Bradi1g08540.1 | Os03t0749800-01 | Sb01g008020.1 | - |
| IV9RU5F02IIXCT | Bradi1g08540.1 | Os03t0749800-02 | Sb01g008020.1 | - |
| IVLNWEC01D0PWR | Bradi1g08620.1 | Os03t0748700-01 | Sb01g008090.1 | - |
| IV9RU5F02HIPUM | Bradi1g08840.1 | Os03t0746400-01 | Sb01g008320.1 | - |
| IVLNWEC01DMD4P | Bradi1g09030.1 | Os03t0743900-01 | Sb01g008450.1 | - |
| IVLNWEC01BT8UR | Bradi1g09187.1 | Os03t0740800-01 | Sb01g008590.1 | - |
| IVLNWEC01BQKJP | Bradi1g09250.1 | Os03t0738700-01 | Sb01g008680.1 | - |
| IUIVLUD01EIXXZ | Bradi1g10530.1 | Os03t0719500-02 | Sb01g009940.1 | - |
| IVLNWEC02I0DEX | Bradi1g10550.1 | Os03t0719300-01 | Sb01g009960.1 | - |
| IV9RU5F01CB7PZ | Bradi1g11490.1 | Os03t0713000-00 | Sb01g010250.1 | - |
| IVLNWEC02HCH3Z | Bradi1g11780.1 | Os03t0698350-00 | Sb01g011170.1 | - |
| IVLNWEC01CX4V4 | Bradi1g12370.1 | Os05t0116800-00 | Sb01g011860.1 | - |
| IUIVLUD02G08GQ | Bradi1g12830.1 | Os03t0669100-01 | Sb01g012360.1 | - |
| IV9RU5F02G85A1 | Bradi1g12830.1 | Os03t0669100-01 | Sb01g012360.1 | - |
| IV9RU5F02IXV6T | Bradi1g12830.1 | Os03t0669100-01 | Sb01g012360.1 | - |
| IVLNWEC01BFFIW | Bradi3g35840.1 | Os03t0658000-00 | Sb01g012860.2 | - |
| IVLNWEC01AL1EX | Bradi2g62740.1 | Os01t0977400-01 | Sb01g012973.1 | - |
| IVLNWEC01EAPPF | Bradi1g13450.1 | Os03t0652100-01 | Sb01g013110.1 | - |
| IVLNWEC02GKT0G | Bradi1g13910.1 | Os03t0640800-01 | Sb01g013710.1 | - |
| IVLNWEC01CL6LX | Bradi4g00790.1 | Os12t0634900-01 | Sb01g013840.1 | - |
| IUIVLUD01CCRLI | Bradi1g14730.1 | Os03t0610650-01 | Sb01g014740.1 | - |
| IVLNWEC02G5V6U | Bradi1g14830.1 | Os12t0581700-01 | Sb01g014860.1 | - |
| IVLNWEC01EF7GF | Bradi1g14877.1 | Os03t0604600-01 | Sb01g014940.1 | - |
| IVLNWEC01CDUDN | Bradi2g36180.1 | Os09t0314200-00 | Sb01g015550.1 | - |
| IVLNWEC01CZCOF | Bradi3g29870.1 | Os10t0495000-01 | Sb01g018300.1 | - |
| IV9RU5F01BOCG1 | Bradi1g74430.1 | Os03t0103950-00 | Sb01g021680.1 | - |
| IVLNWEC01ALDZB | Bradi3g26697.1 | Os10t0414700-01 | Sb01g022040.1 | - |
| IVLNWEC01DJ8W3 | Bradi3g26697.1 | Os10t0414700-01 | Sb01g022040.1 | - |

| | | | | |
|---|---|---|---|---|
| IVLNWEC01AQQL7 | Bradi3g20790.1 | Os10t0160000-01 | Sb01g025350.1 | - |
| IVLNWEC01BRYHR | Bradi4g05870.1 | Os10t0131100-00 | Sb01g026690.1 | - |
| IVLNWEC02ILGFJ | Bradi3g22520.1 | Os06t0330100-00 | Sb01g028490.1 | - |
| IUIVLUD01DGI8Z | Bradi3g33190.1 | Os10t0556600-00 | Sb01g029180.1 | - |
| IVLNWEC02IBT3C | Bradi3g32780.1 | Os10t0548200-01 | Sb01g029925.1 | - |
| IV9RU5F01B4O23 | Bradi2g11590.1 | Os06t0484800-00 | Sb01g031070.1 | - |
| IVLNWEC01C38BF | Bradi2g42607.1 | Os04t0208600-01 | Sb01g032350.1 | - |
| IV9RU5F01AGRHB | Bradi1g59910.2 | Os03t0417900-01 | Sb01g032420.1 | - |
| IVLNWEC02H1ZE6 | Bradi1g62007.1 | Os04t0650900-01 | Sb01g034960.1 | - |
| IV9RU5F02HQUOF | Bradi3g30120.1 | Os11t0432600-01 | Sb01g035200.1 | - |
| IV9RU5F02I3FGZ | Bradi1g62330.1 | Os03t0352400-01 | Sb01g035280.1 | - |
| IVLNWEC02I96ZY | Bradi1g62957.1 | Os03t0340500-01 | Sb01g035890.1 | - |
| IVLNWEC01C8BQZ | Bradi1g63320.1 | Os01t0769700-01 | Sb01g036260.1 | - |
| IV9RU5F02H2U4K | Bradi1g63500.1 | Os03t0330300-02 | Sb01g036410.1 | - |
| IUIVLUD01BDGLC | Bradi1g64420.1 | Os03t0308200-01 | Sb01g037420.1 | - |
| IVLNWEC01A6U8D | Bradi1g65290.1 | Os03t0295700-01 | Sb01g038270.1 | - |
| IVLNWEC02FYBD4 | Bradi1g65290.1 | Os03t0295700-01 | Sb01g038270.1 | - |
| IVLNWEC01AYXLE | Bradi1g65310.1 | Os03t0295500-01 | Sb01g038290.1 | - |
| IVLNWEC01BSYGQ | Bradi1g66240.1 | Os03t0280700-01 | Sb01g039070.1 | - |
| IUIVLUD01CF2X5 | Bradi2g59280.1 | Os07t0645900-00 | Sb01g039150.1 | - |
| IV9RU5F02IGBZL | Bradi1g66410.1 | Os03t0278500-01 | Sb01g039310.1 | - |
| IVLNWEC01DLW61 | Bradi1g66687.1 | Os03t0274000-01 | Sb01g039660.1 | - |
| IV9RU5F01EW4KJ | Bradi1g67140.1 | Os03t0265700-01 | Sb01g040090.1 | - |
| IV9RU5F02IA3Y3 | Bradi1g51650.1 | Os03t0249400-01 | Sb01g041040.1 | - |
| IVLNWEC02JKVY6 | Bradi1g69360.1 | Os03t0225500-01 | Sb01g042300.1 | - |
| IV9RU5F02IHH6R | Bradi1g69520.1 | Os03t0223700-01 | Sb01g042440.1 | - |
| IVLNWEC01ES41X | Bradi1g71000.1 | Os03t0202300-01 | Sb01g043670.1 | - |
| IUIVLUD02IWG2K | Bradi3g22990.1 | Os10t0194300-01 | Sb01g044200.1 | - |
| IV9RU5F01ALR3Q | Bradi3g13460.1 | Os02t0301500-01 | Sb01g044300.1 | - |
| IUIVLUD02IBEBA | Bradi1g72100.1 | Os03t0187000-01 | Sb01g044760.1 | - |
| IVLNWEC02GTVWA | Bradi1g72100.1 | Os03t0187000-01 | Sb01g044760.1 | - |
| IUIVLUD02JIRNH | Bradi1g72490.1 | Os03t0182400-01 | Sb01g045110.1 | - |
| IVLNWEC01BX8M6 | Bradi1g72490.1 | Os03t0182400-01 | Sb01g045110.1 | - |
| IVLNWEC02JG0YA | Bradi2g60350.1 | Os05t0333200-00 | Sb01g045320.1 | - |
| IV9RU5F01A4QOG | Bradi1g76520.1 | Os05t0301500-01 | Sb01g048520.1 | - |
| IUIVLUD02IX81O | Bradi3g27560.1 | Os01t0967700-01 | Sb01g048580.1 | - |
| IV9RU5F01ET9RT | Bradi1g77560.1 | Os03t0115000-01 | Sb01g049630.1 | - |
| IUIVLUD01B8C2O | Bradi1g59360.1 | Os07t0107800-01 | Sb02g000750.1 | - |
| IV9RU5F02FX7D2 | Bradi1g59170.1 | Os07t0111600-01 | Sb02g000950.1 | - |
| IV9RU5F01BYGUB | Bradi3g10370.1 | Os02t0281200-01 | Sb02g001410.1 | - |
| IVLNWEC01EHJYW | Bradi1g56720.3 | Os07t0164300-01 | Sb02g003700.1 | - |
| IV9RU5F02I5BFT | Bradi1g54960.1 | Os07t0188266-00 | Sb02g005170.1 | - |
| IUIVLUD02HM4ID | Bradi1g54807.1 | Os07t0191700-01 | Sb02g005590.1 | - |
| IV9RU5F02H1RRY | Bradi1g54807.1 | Os07t0191700-01 | Sb02g005590.1 | - |
| IV9RU5F02IU1FR | Bradi1g54327.1 | Os07t0205700-01 | Sb02g006140.1 | - |
| IVLNWEC02FRKNS | Bradi1g54327.1 | Os07t0205700-01 | Sb02g006140.1 | - |
| IVLNWEC01C917D | Bradi2g36950.1 | Os07t0227800-00 | Sb02g006790.1 | - |

| | | | | |
|---|---|---|---|---|
| IUIVLUD01D9SQ4 | Bradi1g02710.1 | Os03t0833900-02 | Sb02g007460.1 | - |
| IV9RU5F02H87ST | Bradi1g53020.1 | Os07t0271000-01 | Sb02g008810.1 | - |
| IVLNWEC01DZJHD | Bradi1g28820.1 | Os07t0446100-01 | Sb02g010610.1 | - |
| IVLNWEC02JGROG | Bradi4g38080.1 | Os09t0563250-01 | Sb02g011250.1 | - |
| IVLNWEC01DLM6L | Bradi4g38270.1 | Os09t0565400-01 | Sb02g011350.1 | - |
| IVLNWEC02G4PFR | Bradi4g08490.1 | Os09t0123100-01 | Sb02g013160.1 | - |
| IVLNWEC01BQY3W | Bradi4g08520.1 | Os09t0240500-01 | Sb02g013390.1 | - |
| IVLNWEC02IHLUJ | Bradi4g08520.1 | Os09t0240500-01 | Sb02g013390.1 | - |
| IVLNWEC01BR5YN | Bradi4g36976.1 | Os05t0479800-01 | Sb02g021250.1 | - |
| IUIVLUD01DDACT | Bradi4g28400.1 | Os09t0346400-01 | Sb02g022580.1 | - |
| IVLNWEC01EWFMD | Bradi4g35940.1 | Os09t0530275-00 | Sb02g022600.1 | - |
| IVLNWEC02ILJ6Z | Bradi4g28430.1 | Os09t0346700-01 | Sb02g022610.1 | - |
| IUIVLUD01EQKL8 | Bradi4g29520.1 | Os09t0383400-01 | Sb02g023840.1 | - |
| IVLNWEC02HZID1 | Bradi4g29520.1 | Os09t0383400-01 | Sb02g023840.1 | - |
| IV9RU5F01B52LN | Bradi3g36180.1 | Os09t0393200-01 | Sb02g024010.1 | - |
| IV9RU5F01BMVL1 | Bradi4g29630.1 | Os09t0394100-01 | Sb02g024040.1 | - |
| IV9RU5F02F2TQN | Bradi4g29720.1 | Os04t0463400-01 | Sb02g024130.1 | - |
| IVLNWEC02GH211 | Bradi4g30630.1 | Os09t0424200-01 | Sb02g025100.1 | - |
| IVLNWEC02H2P12 | Bradi4g30840.1 | Os09t0431100-01 | Sb02g025330.1 | - |
| IV9RU5F02JPYZJ | Bradi4g30840.1 | Os09t0431100-01 | Sb02g025340.1 | - |
| IUIVLUD02GQ234 | Bradi4g30850.1 | Os09t0431200-01 | Sb02g025340.1 | - |
| IV9RU5F01CCV3X | Bradi4g30850.1 | Os09t0431200-01 | Sb02g025340.1 | - |
| IV9RU5F01CKNG7 | Bradi4g30850.1 | Os09t0431200-01 | Sb02g025340.1 | - |
| IVLNWEC02J3BAE | Bradi4g30850.1 | Os09t0431200-01 | Sb02g025340.1 | - |
| IUIVLUD01D3YI6 | Bradi4g30897.1 | Os09t0431600-00 | Sb02g025370.1 | - |
| IUIVLUD02JV65H | Bradi4g30897.1 | Os09t0431600-00 | Sb02g025370.1 | - |
| IVLNWEC01ENP5E | Bradi4g31990.1 | Os09t0454100-01 | Sb02g026450.1 | - |
| IV9RU5F02IQ5CM | Bradi4g32420.1 | Os09t0463100-01 | Sb02g026860.1 | - |
| IV9RU5F01D8JVI | Bradi3g38400.1 | Os09t0464400-00 | Sb02g026980.1 | - |
| IVLNWEC01AWSGN | Bradi4g32530.1 | Os09t0465600-01 | Sb02g027000.1 | - |
| IVLNWEC02FVPHC | Bradi4g01513.1 | Os09t0479000-01 | Sb02g027700.1 | - |
| IVLNWEC01DYVDE | Bradi4g06970.1 | Os08t0261000-01 | Sb02g027790.1 | - |
| IUIVLUD01C9U1O | Bradi4g33930.1 | Os09t0492800-00 | Sb02g028650.1 | - |
| IVLNWEC02FMZ59 | Bradi4g34022.1 | Os09t0493700-01 | Sb02g028750.1 | - |
| IV9RU5F02GGHUB | Bradi4g34290.1 | Os09t0500600-01 | Sb02g029000.1 | - |
| IUIVLUD02IVP1J | Bradi3g53770.1 | Os09t0504400-01 | Sb02g029140.1 | - |
| IVLNWEC02JHSOE | Bradi4g35382.1 | Os09t0520200-00 | Sb02g030150.1 | - |
| IVLNWEC02GLVJ0 | Bradi4g35490.1 | Os09t0520700-01 | Sb02g030210.1 | - |
| IUIVLUD02HXO3T | Bradi4g35540.2 | Os09t0521500-01 | Sb02g030280.1 | - |
| IVLNWEC02G17QG | Bradi4g36220.3 | Os09t0532700-01 | Sb02g030890.3 | - |
| IVLNWEC02JOOQC | Bradi4g36200.1 | Os09t0533100-01 | Sb02g030920.1 | - |
| IV9RU5F02GZAVJ | Bradi4g36250.1 | Os09t0534000-01 | Sb02g031000.1 | - |
| IUIVLUD02I7CV0 | Bradi4g36380.1 | Os09t0536000-01 | Sb02g031100.1 | - |
| IV9RU5F01ENZAF | Bradi4g36380.1 | Os09t0536000-01 | Sb02g031100.1 | - |
| IVLNWEC01CD4NM | Bradi4g36470.1 | Os09t0538500-01 | Sb02g031200.1 | - |
| IV9RU5F02GQGOU | Bradi1g26380.1 | Os09t0542600-01 | Sb02g031500.1 | - |
| IVLNWEC02IDDFO | Bradi4g36850.1 | Os09t0544000-01 | Sb02g031580.1 | - |

| | | | | |
|---|---|---|---|---|
| IV9RU5F01AHLUM | Bradi4g37110.1 | Os09t0548400-01 | Sb02g031940.1 | - |
| IVLNWEC01B08QM | Bradi4g37170.2 | Os09t0549600-00 | Sb02g032000.1 | - |
| IVLNWEC01B17SM | Bradi4g37260.1 | Os09t0551800-00 | Sb02g032150.1 | - |
| IV9RU5F02GO2AJ | Bradi4g37420.1 | Os09t0554000-01 | Sb02g032310.1 | - |
| IV9RU5F01BPJ13 | Bradi4g37580.1 | Os09t0556000-01 | Sb02g032420.1 | - |
| IVLNWEC01B930G | Bradi4g37580.1 | Os09t0556000-01 | Sb02g032420.1 | - |
| IV9RU5F02HZAZC | Bradi4g37910.1 | Os09t0560000-01 | Sb02g032725.1 | - |
| IVLNWEC02HY1HW | Bradi1g28000.1 | Os07t0479300-01 | Sb02g033170.1 | - |
| IVLNWEC02GB3U6 | Bradi1g26607.1 | Os07t0508000-01 | Sb02g034295.1 | - |
| IVLNWEC02HRDUG | Bradi1g26607.1 | Os07t0508000-01 | Sb02g034295.1 | - |
| IV9RU5F01B3G0V | Bradi1g25780.1 | Os07t0531700-01 | Sb02g035270.1 | - |
| IVLNWEC01CV4KX | Bradi1g25780.1 | Os07t0531700-01 | Sb02g035270.1 | - |
| IVLNWEC02G1750 | Bradi1g25780.1 | Os07t0531700-01 | Sb02g035270.1 | - |
| IVLNWEC02I9D43 | Bradi1g25780.1 | Os07t0531700-01 | Sb02g035270.1 | - |
| IV9RU5F02GHQLP | Bradi1g25090.1 | Os07t0553633-01 | Sb02g036040.1 | - |
| IVLNWEC01BRCXS | Bradi1g20870.1 | Os07t0616500-01 | Sb02g039240.1 | - |
| IUIVLUD01AWZCB | Bradi1g21280.1 | Os07t0621500-01 | Sb02g039580.1 | - |
| IV9RU5F02H0IG4 | Bradi1g21280.1 | Os07t0621500-01 | Sb02g039580.1 | - |
| IVLNWEC01BVF79 | Bradi1g21280.1 | Os07t0621500-01 | Sb02g039580.1 | - |
| IV9RU5F02FTAAG | Bradi1g02180.1 | Os07t0645701-00 | Sb02g041520.1 | - |
| IVLNWEC02JZ9MZ | Bradi1g17950.1 | Os07t0675000-01 | Sb02g042770.1 | - |
| IVLNWEC01B6BZH | Bradi1g17950.1 | Os07t0675000-02 | Sb02g042770.1 | - |
| IVLNWEC02IONCP | Bradi2g01550.1 | Os01t0120800-01 | Sb03g003250.1 | - |
| IVLNWEC01AZ16F | Bradi2g05460.1 | Os01t0185200-03 | Sb03g003420.1 | - |
| IV9RU5F02I6HGD | Bradi2g04397.1 | Os01t0170800-01 | Sb03g004480.1 | - |
| IVLNWEC01DRIEU | Bradi1g27400.1 | Os06t0535300-01 | Sb03g008500.1 | - |
| IVLNWEC02HNPFE | Bradi2g00620.1 | Os12t0638600-01 | Sb03g008700.1 | - |
| IUIVLUD02JWMTT | Bradi2g10302.1 | Os01t0273800-01 | Sb03g010910.1 | - |
| IUIVLUD02HKMN5 | Bradi2g10800.2 | Os01t0279400-01 | Sb03g011240.1 | - |
| IVLNWEC01CYB15 | Bradi2g11107.1 | Os01t0286600-01 | Sb03g011670.1 | - |
| IV9RU5F02J3N61 | Bradi2g40430.1 | Os01t0533000-00 | Sb03g023490.1 | - |
| IVLNWEC01CIX2C | Bradi2g40430.1 | Os01t0533000-00 | Sb03g023490.1 | - |
| IV9RU5F02HKSAV | Bradi3g05292.1 | Os01t0603500-00 | Sb03g025650.1 | - |
| IV9RU5F01DTV3N | Bradi1g02247.1 | Os01t0574400-01 | Sb03g025820.1 | - |
| IUIVLUD01CR972 | Bradi5g25030.1 | Os01t0601675-01 | Sb03g027070.1 | - |
| IV9RU5F02IJ2PZ | Bradi1g31970.1 | Os01t0601675-01 | Sb03g027070.1 | - |
| IVLNWEC02IIW75 | Bradi2g42820.1 | Os01t0604150-00 | Sb03g027080.1 | - |
| IVLNWEC01D1FE5 | Bradi5g25030.1 | Os01t0601675-01 | Sb03g027090.1 | - |
| IV9RU5F01DA6MZ | Bradi2g43110.1 | Os01t0607900-01 | Sb03g027400.1 | - |
| IV9RU5F02JY73H | Bradi4g29760.1 | Os09t0397900-01 | Sb03g028040.1 | - |
| IVLNWEC01CI53K | Bradi4g39580.1 | Os12t0236900-01 | Sb03g029140.1 | - |
| IVLNWEC01DBC6I | Bradi4g39580.1 | Os12t0236900-01 | Sb03g029140.1 | - |
| IVLNWEC01DTOIP | Bradi4g39580.1 | Os12t0236900-01 | Sb03g029140.1 | - |
| IUIVLUD01ED174 | Bradi2g45050.1 | Os01t0644200-01 | Sb03g029390.1 | - |
| IV9RU5F01ECUSE | Bradi2g45050.1 | Os01t0644200-01 | Sb03g029390.1 | - |
| IV9RU5F02JOFGA | Bradi2g45050.1 | Os01t0644200-01 | Sb03g029390.1 | - |
| IUIVLUD02JVPYD | Bradi2g46550.1 | Os01t0679000-02 | Sb03g031160.1 | - |

| | | | | |
|---|---|---|---|---|
| IVLNWEC01CKQYK | Bradi2g49010.1 | Os01t0733600-01 | Sb03g033770.1 | - |
| IV9RU5F01AGQNO | Bradi4g05930.1 | Os12t0533700-01 | Sb03g033850.2 | - |
| IVLNWEC01DHLZT | Bradi2g51630.1 | Os01t0777200-01 | Sb03g036250.2 | - |
| IUIVLUD02G1WSZ | Bradi2g51807.1 | Os11t0555300-00 | Sb03g036450.1 | - |
| IUIVLUD01AI36Q | Bradi1g09260.1 | Os03t0738600-01 | Sb03g042440.1 | - |
| IUIVLUD01EYYT8 | Bradi1g02550.1 | Os01t0938100-01 | Sb03g045320.1 | - |
| IV9RU5F02HD0JF | Bradi1g02550.1 | Os01t0938100-01 | Sb03g045320.1 | - |
| IVLNWEC02FTRC4 | Bradi2g61560.1 | Os01t0962600-01 | Sb03g046750.1 | - |
| IV9RU5F01B7YKX | Bradi2g61830.1 | Os01t0966700-01 | Sb03g047060.1 | - |
| IV9RU5F01EHDUX | Bradi2g61830.1 | Os01t0966700-01 | Sb03g047060.1 | - |
| IV9RU5F02GR9KN | Bradi3g02560.1 | Os02t0125600-00 | Sb04g002180.1 | - |
| IV9RU5F01EY5XH | Bradi3g02900.1 | Os02t0131200-01 | Sb04g002630.2 | - |
| IVLNWEC01BVY0W | Bradi3g02900.1 | Os02t0131200-01 | Sb04g002630.2 | - |
| IVLNWEC02HQJ41 | Bradi4g23600.2 | Os11t0186400-00 | Sb04g004200.1 | - |
| IVLNWEC01EKJJ2 | Bradi1g17710.1 | Os02t0167700-01 | Sb04g004540.1 | - |
| IVLNWEC02G77XQ | Bradi1g26380.1 | Os09t0542600-01 | Sb04g004780.1 | - |
| IUIVLUD01CA2MO | Bradi3g06727.1 | Os02t0192400-01 | Sb04g006240.1 | - |
| IUIVLUD01CDXRL | Bradi3g06727.1 | Os02t0192400-01 | Sb04g006240.1 | - |
| IUIVLUD02IX0AT | Bradi3g06727.1 | Os02t0192400-01 | Sb04g006240.1 | - |
| IV9RU5F01EQCFD | Bradi3g06727.1 | Os02t0192400-01 | Sb04g006240.1 | - |
| IVLNWEC01D4E0H | Bradi3g06727.1 | Os02t0192400-01 | Sb04g006240.1 | - |
| IV9RU5F02F4HPV | Bradi3g07457.1 | Os02t0201900-01 | Sb04g006980.1 | - |
| IVLNWEC01DW9BR | Bradi3g07490.1 | Os02t0202250-01 | Sb04g007000.1 | - |
| IVLNWEC01DH1FI | Bradi3g57690.1 | Os02t0770700-01 | Sb04g007853.1 | - |
| IUIVLUD02F773U | Bradi1g37960.1 | Os02t0227700-01 | Sb04g008110.1 | - |
| IVLNWEC01A02RE | Bradi2g13450.1 | Os01t0528800-01 | Sb04g011550.1 | - |
| IVLNWEC02HFUUV | Bradi4g39430.1 | Os02t0329300-01 | Sb04g014390.1 | - |
| IUIVLUD02GLS5P | Bradi3g44347.2 | Os02t0516600-01 | Sb04g021110.1 | - |
| IVLNWEC02IDLZ0 | Bradi1g30672.1 | Os05t0311600-01 | Sb04g021690.1 | - |
| IVLNWEC02JO22M | Bradi1g30672.1 | Os05t0311600-01 | Sb04g021690.1 | - |
| IV9RU5F02IJUKJ | Bradi3g44940.1 | Os02t0533300-01 | Sb04g021740.1 | - |
| IVLNWEC01AJC7P | Bradi3g46100.1 | Os11t0245100-01 | Sb04g023210.1 | - |
| IV9RU5F02JWEDW | Bradi3g48850.1 | Os02t0617500-01 | Sb04g026080.1 | - |
| IVLNWEC01EECM7 | Bradi3g53160.1 | Os02t0712000-01 | Sb04g030100.1 | - |
| IVLNWEC01BXF97 | Bradi3g50300.1 | Os02t0650800-00 | Sb04g033300.1 | - |
| IV9RU5F02F5PC0 | Bradi2g26500.1 | Os02t0650800-00 | Sb04g033300.1 | - |
| IV9RU5F02II6H0 | Bradi3g58130.1 | Os10t0400800-01 | Sb04g034020.1 | - |
| IVLNWEC01A15FQ | Bradi3g58130.1 | Os10t0400800-01 | Sb04g034020.1 | - |
| IVLNWEC01BPC6J | Bradi3g58130.1 | Os10t0400800-01 | Sb04g034020.1 | - |
| IVLNWEC01BJD3X | Bradi3g60190.1 | Os02t0785900-00 | Sb04g035270.1 | - |
| IVLNWEC02IUQ2Q | Bradi3g53887.1 | Os02t0793300-01 | Sb04g035685.1 | - |
| IUIVLUD02JFRAB | Bradi3g54100.1 | Os02t0796400-00 | Sb04g035970.1 | - |
| IUIVLUD02H0X44 | Bradi3g55920.1 | Os02t0824000-00 | Sb04g037860.1 | - |
| IV9RU5F02JX6K1 | Bradi3g55920.1 | Os02t0824000-00 | Sb04g037860.1 | - |
| IVLNWEC02FQ1AY | Bradi3g55920.1 | Os02t0824000-00 | Sb04g037860.1 | - |
| IV9RU5F01EWRJX | Bradi3g60710.1 | Os02t0831700-01 | Sb04g038430.1 | - |
| IVLNWEC01A26IU | Bradi3g60710.1 | Os02t0831700-01 | Sb04g038430.1 | - |

| | | | | |
|---|---|---|---|---|
| IVLNWEC02I29CH | Bradi3g60710.1 | Os02t0831700-01 | Sb04g038430.1 | - |
| IVLNWEC01BR5YQ | Bradi4g26750.1 | Os12t0104300-01 | Sb05g000365.1 | - |
| IVLNWEC02FYY57 | Bradi4g45030.1 | Os12t0175500-01 | Sb05g000420.1 | - |
| IUIVLUD02JWE4V | Bradi4g26877.1 | Os11t0104900-00 | Sb05g000450.1 | - |
| IV9RU5F01EPIUV | Bradi4g43517.3 | Os11t0137300-01 | Sb05g002190.1 | - |
| IVLNWEC01AMZ7O | Bradi4g01080.1 | Os12t0163200-01 | Sb05g002340.1 | - |
| IUIVLUD02IWPV6 | Bradi2g62760.1 | Os01t0977600-02 | Sb05g002400.1 | - |
| IVLNWEC02I3S4R | Bradi4g42530.1 | Os11t0151500-01 | Sb05g003130.1 | - |
| IVLNWEC02JB76G | Bradi4g24960.2 | Os12t0159700-00 | Sb05g003650.1 | - |
| IVLNWEC02F5R2J | Bradi1g00237.1 | Os11t0479100-00 | Sb05g004300.1 | - |
| IUIVLUD01B4QO8 | Bradi1g00237.1 | Os05t0250700-00 | Sb05g004310.1 | - |
| IV9RU5F01DFBWX | Bradi4g24190.1 | Os11t0176000-01 | Sb05g004850.1 | - |
| IV9RU5F02GM8JH | Bradi4g23450.1 | Os11t0191400-01 | Sb05g005750.1 | - |
| IVLNWEC01DUJWW | Bradi4g23450.1 | Os11t0191400-01 | Sb05g005750.1 | - |
| IVLNWEC02I8L7R | Bradi4g23417.1 | Os11t0191300-02 | Sb05g005751.1 | - |
| IUIVLUD01B0514 | Bradi2g09427.1 | Os11t0462500-00 | Sb05g008150.1 | - |
| IUIVLUD01CHSPS | Bradi2g09427.1 | Os11t0462500-00 | Sb05g008150.1 | - |
| IVLNWEC01AT2OH | Bradi2g09427.1 | Os11t0462500-00 | Sb05g008150.1 | - |
| IVLNWEC02GT42Q | Bradi2g09427.1 | Os11t0462500-00 | Sb05g008150.1 | - |
| IUIVLUD01D8GXP | Bradi4g13720.1 | Os11t0615100-01 | Sb05g024500.1 | - |
| IUIVLUD01D8Z5Y | Bradi4g13720.1 | Os11t0615100-01 | Sb05g024500.1 | - |
| IUIVLUD01EUGXA | Bradi4g13720.1 | Os11t0615100-01 | Sb05g024500.1 | - |
| IVLNWEC01DM8EC | Bradi4g13720.1 | Os11t0615100-01 | Sb05g024500.1 | - |
| IVLNWEC01EGJ4E | Bradi4g09597.1 | Os11t0480000-01 | Sb06g000340.1 | - |
| IVLNWEC02IIP75 | Bradi5g01230.1 | Os04t0131900-01 | Sb06g001270.2 | - |
| IV9RU5F01D1CEM | Bradi2g15526.1 | Os04t0394300-01 | Sb06g014350.1 | - |
| IV9RU5F02JWIIN | Bradi2g15526.1 | Os04t0394300-01 | Sb06g014350.1 | - |
| IV9RU5F01DBLFE | Bradi5g09120.1 | Os04t0407900-01 | Sb06g015320.1 | - |
| IVLNWEC01CEJRZ | Bradi5g09120.1 | Os04t0407900-01 | Sb06g015320.1 | - |
| IV9RU5F02HEAZV | Bradi1g33920.1 | Os04t0465700-01 | Sb06g019330.1 | - |
| IUIVLUD02ITE1G | Bradi2g08980.1 | Os12t0165900-01 | Sb06g020700.1 | - |
| IUIVLUD01A3RKY | Bradi3g00507.1 | Os04t0492300-01 | Sb06g021120.1 | - |
| IVLNWEC01AZ9FI | Bradi1g44090.1 | Os06t0127900-01 | Sb06g021460.1 | - |
| IUIVLUD02J3KNC | Bradi2g31720.1 | Os05t0251900-00 | Sb06g023020.1 | - |
| IV9RU5F01AJJXR | Bradi1g77537.1 | Os04t0625400-00 | Sb06g029130.1 | - |
| IV9RU5F01BIOC1 | Bradi1g29427.1 | Os11t0633500-01 | Sb06g029290.1 | - |
| IVLNWEC01CEEZG | Bradi5g26017.1 | Os04t0675101-00 | Sb06g032770.1 | - |
| IVLNWEC01B8WTP | Bradi3g16660.1 | Os05t0151000-00 | Sb07g003680.1 | - |
| IUIVLUD01EEKIW | Bradi2g04960.1 | Os03t0335600-01 | Sb07g004420.1 | - |
| IVLNWEC02JHQQ8 | Bradi3g15910.1 | Os08t0169800-01 | Sb07g004540.1 | - |
| IVLNWEC01CAVNO | Bradi3g15520.1 | Os08t0178100-01 | Sb07g004940.1 | - |
| IVLNWEC02GHKAJ | Bradi3g15520.1 | Os08t0178100-01 | Sb07g004940.1 | - |
| IVLNWEC01EMPF5 | Bradi3g18390.1 | Os01t0604150-00 | Sb07g006470.1 | - |
| IUIVLUD01CWJ7Z | Bradi3g18390.1 | Os11t0558400-00 | Sb07g006490.1 | - |
| IVLNWEC01BX99C | Bradi4g29810.1 | Os09t0401100-00 | Sb07g009430.1 | - |
| IV9RU5F01CKMJM | Bradi3g10980.1 | Os02t0317800-01 | Sb07g009530.1 | - |
| IV9RU5F01AQKRF | Bradi3g35390.1 | Os08t0398300-01 | Sb07g019540.1 | - |

| | | | | |
|---|---|---|---|---|
| IV9RU5F01AUSLI | Bradi3g35517.1 | Os08t0407000-01 | Sb07g020060.1 | - |
| IV9RU5F02JFG5S | Bradi3g35517.1 | Os08t0407000-01 | Sb07g020060.1 | - |
| IVLNWEC02H6CZ5 | Bradi3g35517.1 | Os08t0407000-01 | Sb07g020060.1 | - |
| IUIVLUD01B7R0U | Bradi2g05890.1 | Os04t0354200-00 | Sb07g020145.1 | - |
| IV9RU5F02I9R7M | Bradi3g37650.1 | Os08t0459600-01 | Sb07g022500.1 | - |
| IV9RU5F01EPNS1 | Bradi3g12660.1 | Os08t0564800-01 | Sb07g023630.1 | - |
| IUIVLUD01D1P7P | Bradi1g50920.1 | Os08t0559700-00 | Sb07g024060.1 | - |
| IUIVLUD02IDGZZ | Bradi3g41810.1 | Os08t0541300-01 | Sb07g025500.1 | - |
| IV9RU5F01A9MRR | Bradi3g41810.1 | Os08t0541300-01 | Sb07g025500.1 | - |
| IVLNWEC02JNRSM | Bradi3g41810.1 | Os08t0541300-01 | Sb07g025500.1 | - |
| IV9RU5F02J5TRW | Bradi3g39360.1 | Os08t0499100-01 | Sb07g028470.1 | - |
| IVLNWEC01D2H11 | Bradi3g39360.1 | Os08t0499100-01 | Sb07g028470.1 | - |
| IVLNWEC01AF4BD | Bradi4g44780.1 | Os07t0540366-01 | Sb08g000780.1 | - |
| IUIVLUD02IEFC8 | Bradi4g26390.1 | Os11t0134500-01 | Sb08g001420.1 | - |
| IVLNWEC02G25A7 | Bradi4g26627.1 | Os11t0148700-01 | Sb08g002920.1 | - |
| IUIVLUD02HNIDS | Bradi4g42160.1 | Os12t0156900-00 | Sb08g003540.1 | - |
| IV9RU5F01CL2IO | Bradi4g13850.1 | Os11t0619500-00 | Sb08g003810.1 | - |
| IUIVLUD01BUFMA | Bradi4g41137.1 | Os12t0178700-01 | Sb08g004890.1 | - |
| IUIVLUD01B719Z | Bradi3g22760.1 | Os10t0182000-01 | Sb08g008230.1 | - |
| IVLNWEC02GH3W8 | Bradi3g22760.1 | Os10t0182000-01 | Sb08g008230.1 | - |
| IVLNWEC02GIMXY | Bradi3g22760.1 | Os10t0182000-01 | Sb08g008230.1 | - |
| IVLNWEC01C2W5O | Bradi4g09870.1 | Os11t0607900-01 | Sb08g015237.1 | - |
| IVLNWEC02FOUJ1 | Bradi4g06950.1 | Os12t0498800-01 | Sb08g015480.1 | - |
| IV9RU5F01BNHEZ | Bradi4g06827.1 | Os12t0502300-01 | Sb08g015590.1 | - |
| IVLNWEC01BTASY | Bradi4g05940.1 | Os06t0168500-01 | Sb08g016910.1 | - |
| IVLNWEC01B4ODF | Bradi4g04650.1 | Os12t0564600-01 | Sb08g018400.1 | - |
| IV9RU5F02FJEZB | Bradi4g04030.1 | Os12t0566900-01 | Sb08g018540.1 | - |
| IVLNWEC02FS391 | Bradi1g35630.1 | Os12t0568800-01 | Sb08g018670.1 | - |
| IVLNWEC02H7RR5 | Bradi4g02910.1 | Os12t0594200-00 | Sb08g020025.1 | - |
| IVLNWEC02I21WM | Bradi4g02160.2 | Os12t0609500-01 | Sb08g020890.1 | - |
| IVLNWEC01DINJG | Bradi4g01930.1 | Os12t0612300-01 | Sb08g021320.1 | - |
| IV9RU5F01BVQBY | Bradi4g01610.1 | Os12t0615700-01 | Sb08g021640.1 | - |
| IVLNWEC02JAXJA | Bradi4g01610.1 | Os12t0615700-01 | Sb08g021640.1 | - |
| IVLNWEC02JZD9U | Bradi4g01610.1 | Os12t0615700-01 | Sb08g021640.1 | - |
| IVLNWEC01A1Q7W | Bradi1g13580.1 | Os03t0649000-01 | Sb08g022010.1 | - |
| IV9RU5F01CC9Y2 | Bradi4g01320.1 | Os12t0620600-01 | Sb08g022010.1 | - |
| IVLNWEC01BTTOW | Bradi2g39790.1 | Os05t0105000-01 | Sb09g000490.1 | - |
| IUIVLUD01EX0U1 | Bradi2g39420.1 | Os05t0107600-01 | Sb09g000670.1 | - |
| IVLNWEC02FQ9GO | Bradi1g00580.1 | Os07t0438500-01 | Sb09g004060.1 | - |
| IV9RU5F01BB7DS | Bradi2g32290.1 | Os05t0212100-01 | Sb09g006640.1 | - |
| IV9RU5F01A5BV4 | Bradi2g31690.1 | Os05t0244500-01 | Sb09g008170.1 | - |
| IV9RU5F01EL5DL | Bradi2g27267.1 | Os05t0373300-01 | Sb09g018600.1 | - |
| IV9RU5F02IKXV0 | Bradi2g27267.1 | Os05t0373300-01 | Sb09g018600.1 | - |
| IVLNWEC02JHJL4 | Bradi2g26930.1 | Os05t0388400-01 | Sb09g019130.1 | - |
| IVLNWEC01EXM7A | Bradi2g26500.1 | Os05t0392400-01 | Sb09g019410.1 | - |
| IV9RU5F01DZ544 | Bradi2g25500.1 | Os05t0409400-01 | Sb09g020180.1 | - |
| IUIVLUD01EXPFT | Bradi2g25410.1 | Os05t0411200-01 | Sb09g020310.1 | - |

| | | | | |
|---|---|---|---|---|
| IV9RU5F02HXXQG | Bradi2g23690.1 | Os05t0451100-01 | Sb09g022090.1 | - |
| IUIVLUD01AI6KQ | Bradi2g22630.1 | Os05t0470800-01 | Sb09g023080.1 | - |
| IV9RU5F02IBQHU | Bradi2g22550.1 | Os05t0472200-01 | Sb09g023140.1 | - |
| IV9RU5F01EY1PF | Bradi2g20290.1 | Os05t0510200-01 | Sb09g025100.1 | - |
| IVLNWEC02JZA4G | Bradi2g20290.1 | Os05t0510200-01 | Sb09g025100.1 | - |
| IV9RU5F02H81EX | Bradi2g19010.1 | Os05t0529700-01 | Sb09g026410.1 | - |
| IUIVLUD02F99S7 | Bradi2g16940.1 | Os05t0557500-01 | Sb09g028090.1 | - |
| IV9RU5F02IADLX | Bradi2g14450.1 | Os05t0592300-01 | Sb09g030570.1 | - |
| IV9RU5F02J3HJL | Bradi2g14450.1 | Os05t0592300-01 | Sb09g030570.1 | - |
| IVLNWEC02HPXQC | Bradi2g14450.1 | Os05t0592300-01 | Sb09g030570.1 | - |
| IUIVLUD02IRAJV | Bradi2g14220.2 | Os05t0595300-01 | Sb09g030830.1 | - |
| IVLNWEC02JJK28 | Bradi2g14220.2 | Os05t0595300-02 | Sb09g030830.1 | - |
| IV9RU5F01DUYMF | Bradi1g52150.3 | Os06t0103800-01 | Sb10g000430.1 | - |
| IV9RU5F01C9V81 | Bradi1g52170.2 | Os06t0104000-01 | Sb10g000445.1 | - |
| IV9RU5F02IERKG | Bradi1g51940.2 | Os06t0109400-01 | Sb10g000870.1 | - |
| IVLNWEC02I8K8U | Bradi1g50212.1 | Os06t0111600-01 | Sb10g001030.1 | - |
| IUIVLUD01BOI2J | Bradi1g49470.1 | Os06t0125400-00 | Sb10g001530.1 | - |
| IV9RU5F01DSLQ4 | Bradi1g49750.1 | Os04t0320200-01 | Sb10g001830.1 | - |
| IVLNWEC02H0ZMW | Bradi1g49750.1 | Os04t0320200-01 | Sb10g001830.1 | - |
| IVLNWEC02I7KJ8 | Bradi1g50010.1 | Os06t0131100-02 | Sb10g002050.1 | - |
| IV9RU5F01DJNIM | Bradi1g51650.1 | Os06t0134000-01 | Sb10g002240.1 | - |
| IVLNWEC01EQGN6 | Bradi1g51620.1 | Os06t0134800-01 | Sb10g002310.1 | - |
| IUIVLUD01A5VPN | Bradi1g51290.1 | Os06t0140100-01 | Sb10g002720.1 | - |
| IV9RU5F02IXCQM | Bradi1g47097.1 | Os06t0186300-00 | Sb10g005860.1 | - |
| IVLNWEC01CGBIQ | Bradi1g47097.1 | Os06t0186300-00 | Sb10g005860.1 | - |
| IV9RU5F01EI0NI | Bradi1g45320.1 | Os06t0225100-01 | Sb10g007930.1 | - |
| IV9RU5F02JZUAA | Bradi1g45320.1 | Os06t0225100-01 | Sb10g007930.1 | - |
| IVLNWEC02JFDUJ | Bradi1g45320.1 | Os06t0225100-01 | Sb10g007930.1 | - |
| IVLNWEC02JQ3KD | Bradi1g45320.1 | Os06t0225100-01 | Sb10g007930.1 | - |
| IVLNWEC01DJ3DJ | Bradi1g43260.1 | Os06t0297400-03 | Sb10g010800.1 | - |
| IVLNWEC02HR5TC | Bradi1g35960.1 | Os05t0370600-01 | Sb10g024200.1 | - |
| IVLNWEC01CWMC5 | Bradi1g35585.1 | Os06t0632700-01 | Sb10g024700.1 | - |
| IVLNWEC02GD36S | Bradi1g35585.1 | Os06t0632700-01 | Sb10g024700.1 | - |
| IVLNWEC01A8M97 | Bradi1g35470.1 | Os06t0634600-01 | Sb10g024840.1 | - |
| IVLNWEC02FNJPZ | Bradi1g30360.2 | Os06t0639500-01 | Sb10g025070.1 | - |
| IUIVLUD02FSYJH | Bradi1g30910.1 | Os06t0649700-00 | Sb10g025630.1 | - |
| IUIVLUD02I72K0 | Bradi1g30910.1 | Os06t0649700-00 | Sb10g025630.1 | - |
| IV9RU5F01B6ZC0 | Bradi1g30910.1 | Os06t0649700-00 | Sb10g025630.1 | - |
| IVLNWEC01C33AO | Bradi1g32420.1 | Os06t0671800-01 | Sb10g027000.1 | - |
| IVLNWEC02FXCVU | Bradi1g29622.1 | Os02t0832400-01 | Sb10g030970.1 | - |
| IVLNWEC02IXAIY | Bradi1g04610.1 | Os03t0808000-00 | Sb01g004190.1 | - |
| IV9RU5F01DYYAO | Bradi1g07910.1 | Os03t0760700-01 | Sb01g007370.1 | - |
| IUIVLUD02IRHRU | Bradi1g65310.1 | Os03t0295500-01 | Sb01g038290.1 | - |
| IV9RU5F01C3N3T | Bradi2g61830.1 | Os01t0966700-01 | Sb03g047060.1 | - |
| IVLNWEC01A4L0I | Bradi1g07190.1 | Os03t0773600-01 | Sb01g006700.1 | - |
| IUIVLUD01EHCHZ | Bradi4g32830.1 | Os09t0471800-01 | Sb02g028550.1 | - |
| IV9RU5F02HZ8KW | Bradi4g36160.2 | Os07t0689300-01 | Sb02g030960.1 | - |

| | | | | |
|---|---|---|---|---|
| IUIVLUD02G0A3U | Bradi1g63330.1 | Os03t0333100-01 | Sb01g036270.1 | - |
| IV9RU5F02JT4FT | Bradi1g63330.1 | Os03t0333100-01 | Sb01g036270.1 | - |
| IVLNWEC01EXHPI | Bradi1g63330.1 | Os03t0333100-01 | Sb01g036270.1 | - |
| IVLNWEC01CIK0F | Bradi4g31336.1 | Os09t0573200-01 | Sb02g031620.1 | - |
| IV9RU5F02FZ798 | Bradi4g36380.1 | Os09t0536000-01 | Sb02g031100.1 | - |
| IVLNWEC02I65CK | Bradi4g11510.3 | Os05t0188500-02 | Sb05g025690.1 | - |
| IV9RU5F01DSPRS | Bradi1g04300.2 | Os03t0811100-01 | Sb01g003860.1 | - |
| IUIVLUD01AUJ7J | Bradi1g08720.1 | Os03t0747700-01 | Sb01g008190.1 | - |
| IVLNWEC02HXFJV | Bradi1g08720.1 | Os03t0747700-01 | Sb01g008190.1 | - |
| IVLNWEC01DCC8Y | Bradi1g69360.1 | Os03t0225500-01 | Sb01g042300.1 | - |
| IV9RU5F02JEWRX | Bradi3g05847.1 | Os03t0403100-01 | Sb10g006995.1 | - |
| IVLNWEC01C3EMP | Bradi3g05847.1 | Os03t0403100-01 | Sb10g006995.1 | - |
| IVLNWEC02F7THL | Bradi2g24670.1 | Os05t0428100-00 | Sb09g021140.1 | - |
| IV9RU5F02GNJLT | Bradi1g07340.1 | Os03t0769600-01 | Sb01g006880.1 | - |
| IUIVLUD02F5OHM | Bradi1g08607.1 | Os03t0748800-01 | Sb01g008080.1 | - |
| IUIVLUD02IE26P | Bradi1g08607.1 | Os03t0748800-01 | Sb01g008080.1 | - |
| IVLNWEC02ILH0Z | Bradi1g02810.1 | Os03t0832600-01 | Sb01g002480.1 | - |
| IV9RU5F01AN2O1 | Bradi4g37040.1 | Os09t0547300-01 | Sb02g031860.1 | - |
| IUIVLUD01B1M6B | Bradi2g16500.1 | Os03t0619400-01 | Sb03g005190.1 | - |
| IV9RU5F01BONBL | Bradi2g16500.1 | Os03t0619400-01 | Sb03g005190.1 | - |
| IV9RU5F02HBV2A | Bradi4g28877.1 | Os09t0364000-01 | Sb02g023040.1 | - |
| IUIVLUD01DHOS4 | Bradi1g30360.2 | Os06t0639500-01 | Sb10g025070.1 | - |
| IV9RU5F01ECC6Y | Bradi1g50530.1 | Os12t0297500-01 | Sb04g010020.1 | - |
| IVLNWEC01C15O2 | Bradi1g50530.1 | Os12t0297500-01 | Sb04g010020.1 | - |
| IVLNWEC01DYL2C | Bradi1g50530.1 | Os12t0297500-01 | Sb04g010020.1 | - |
| IVLNWEC01AXP6W | Bradi4g08880.1 | Os09t0246200-01 | Sb02g039520.1 | - |
| IVLNWEC01CYYKI | Bradi4g08420.1 | Os09t0114500-01 | Sb02g013180.1 | - |
| IV9RU5F01AVJR1 | Bradi1g04330.2 | Os03t0810600-01 | Sb01g003900.2 | - |
| IV9RU5F01B8O3F | Bradi4g36200.1 | Os09t0533100-01 | Sb02g030920.1 | - |
| IV9RU5F02GQWUD | Bradi4g36200.1 | Os09t0533100-01 | Sb02g030920.1 | - |
| IUIVLUD02GJ2I7 | Bradi2g11107.1 | Os01t0286600-01 | Sb03g011670.1 | - |
| IUIVLUD01C6YZI | Bradi1g30580.1 | Os06t0644600-01 | Sb10g025320.1 | - |
| IV9RU5F01DVTBD | Bradi2g14440.1 | Os05t0592400-01 | Sb09g030580.1 | - |
| IV9RU5F01DOSB9 | Bradi1g51940.2 | Os06t0109400-01 | Sb10g000870.1 | - |
| IV9RU5F01CJZZ9 | Bradi1g50212.1 | Os06t0111600-01 | Sb10g001030.1 | - |
| IVLNWEC01CMHOU | Bradi1g01740.1 | Os03t0849600-01 | Sb01g001270.1 | - |
| IUIVLUD01CPG3R | Bradi1g08540.1 | Os03t0749800-02 | Sb01g008020.1 | - |
| IVLNWEC01DD5F4 | Bradi1g13580.1 | Os03t0649000-01 | Sb01g013300.1 | - |
| IVLNWEC01CU8HM | Bradi3g26697.1 | Os10t0414700-01 | Sb01g022040.1 | - |
| IVLNWEC02JE9X8 | Bradi4g01930.1 | Os12t0612300-01 | Sb08g021320.1 | - |
| IV9RU5F02JA0ZA | Bradi4g31630.1 | Os09t0516800-01 | Sb02g026110.1 | - |
| IUIVLUD01BZUWB | Bradi4g33450.1 | Os09t0482720-00 | Sb02g028080.1 | - |
| IV9RU5F01DD533 | Bradi4g33450.1 | Os09t0482720-00 | Sb02g028080.1 | - |
| IVLNWEC02J0L1L | Bradi4g33450.1 | Os09t0482720-00 | Sb02g028080.1 | - |
| IVLNWEC01B62LL | Bradi1g67800.1 | Os03t0254700-01 | Sb01g040800.1 | - |
| IV9RU5F01BN4LA | Bradi1g00830.1 | Os03t0858700-01 | Sb01g000446.1 | - |
| IVLNWEC01ETVYG | Bradi1g00830.1 | Os03t0858700-01 | Sb01g000446.1 | - |

| | | | | |
|---|---|---|---|---|
| IVLNWEC01ARDPH | Bradi1g08620.1 | Os03t0748700-01 | Sb01g008090.1 | - |
| IV9RU5F02HO91Q | Bradi1g72490.1 | Os03t0182400-01 | Sb01g045110.1 | - |
| IVLNWEC01CAJTZ | Bradi3g04270.1 | Os01t0507700-01 | Sb07g000380.1 | - |
| IVLNWEC02GHEKK | Bradi3g33810.3 | Os10t0567000-01 | Sb01g028580.1 | - |
| IVLNWEC01DY25V | Bradi5g25030.1 | Os01t0601675-01 | Sb03g027070.1 | - |
| IVLNWEC02G8NHJ | Bradi1g13760.1 | Os03t0645900-00 | Sb01g013520.1 | - |
| IUIVLUD01A47G3 | Bradi4g37960.1 | Os09t0560450-01 | Sb02g032760.1 | - |
| IV9RU5F01B4XIA | Bradi4g37960.1 | Os09t0560450-01 | Sb02g032760.1 | - |
| IUIVLUD01D6RJA | Bradi1g01250.1 | Os11t0689100-02 | Sb02g005235.1 | - |
| IV9RU5F01AVBN9 | Bradi4g21057.1 | Os12t0209300-00 | Sb03g036320.1 | - |
| IV9RU5F02F6N71 | Bradi1g02680.1 | Os03t0835100-00 | Sb01g002230.1 | - |
| IVLNWEC01D6MQV | Bradi1g08917.1 | Os03t0744650-00 | Sb01g008410.1 | - |
| IVLNWEC02IASR6 | Bradi1g08400.1 | Os03t0752100-01 | Sb01g007850.1 | - |
| IVLNWEC02J2DWX | Bradi4g30160.1 | Os09t0413100-01 | Sb02g024580.1 | - |
| IUIVLUD01D3UCP | Bradi3g20580.1 | Os08t0326700-00 | Sb01g000350.1 | - |
| IVLNWEC01CI9FN | Bradi1g11530.1 | Os03t0701800-01 | Sb01g010920.1 | - |
| IVLNWEC02FXG4R | Bradi3g41520.1 | Os08t0537600-01 | Sb02g029540.1 | - |
| IUIVLUD01C0R1O | Bradi4g01410.1 | Os12t0617900-01 | Sb08g021850.1 | - |
| IUIVLUD01DCVPX | Bradi1g16680.1 | Os03t0294900-01 | Sb01g038300.1 | - |
| IV9RU5F02F0Y02 | Bradi1g16680.1 | Os03t0294900-01 | Sb01g038300.1 | - |
| IVLNWEC02HTPKM | Bradi1g16680.1 | Os03t0294900-01 | Sb01g038300.1 | - |
| IUIVLUD01EFXR0 | Bradi1g16680.1 | Os03t0294900-01 | Sb02g043880.1 | - |
| IV9RU5F01BU8P9 | Bradi4g05110.2 | Os12t0554400-01 | Sb08g017700.1 | - |
| IV9RU5F01DQVEM | Bradi1g35630.1 | Os05t0353400-01 | Sb08g018670.1 | - |
| IVLNWEC02JZACS | Bradi1g35630.1 | Os05t0353400-01 | Sb08g018670.1 | - |
| IV9RU5F01CM6TT | Bradi1g17710.1 | Os02t0167700-01 | Sb04g004540.1 | - |
| IUIVLUD01EUAQ0 | Bradi2g16820.1 | Os05t0559900-01 | Sb09g028250.2 | - |
| IVLNWEC01CPVEU | Bradi2g16820.1 | Os05t0559900-01 | Sb09g028250.2 | - |
| IVLNWEC02JD639 | Bradi1g52330.1 | Os06t0103600-01 | Sb10g000400.1 | - |
| IV9RU5F02GVJ03 | Bradi1g11260.1 | Os03t0710100-02 | Sb01g010480.1 | - |
| IV9RU5F02HQ2EI | Bradi4g41777.1 | Os12t0165000-01 | Sb08g004050.2 | - |
| IV9RU5F02JO3JA | Bradi1g21280.1 | Os07t0621500-01 | Sb02g039580.1 | - |
| IVLNWEC02I48RT | Bradi1g45320.1 | Os06t0225100-01 | Sb10g007930.1 | - |
| IV9RU5F02JMMX2 | Bradi3g43050.1 | Os04t0492300-01 | Sb06g021120.1 | - |
| IUIVLUD02HKO4T | Bradi5g00430.1 | Os09t0101800-01 | Sb02g013000.1 | - |
| IVLNWEC02IU0XJ | Bradi4g00910.3 | Os12t0632700-01 | Sb08g022770.1 | - |
| IVLNWEC02HQINX | Bradi2g09177.1 | Os01t0256800-01 | Sb03g009880.1 | - |
| IUIVLUD02ICLXK | Bradi3g48850.1 | Os02t0617500-01 | Sb04g026080.1 | - |
| IVLNWEC02GIB32 | Bradi4g37917.1 | Os09t0560100-00 | Sb02g032730.1 | - |
| IVLNWEC02HEX9U | Bradi4g31500.1 | Os09t0443500-01 | Sb02g025970.2 | - |
| IV9RU5F01BJD1S | Bradi2g20310.1 | Os01t0772200-02 | Sb09g025080.1 | - |
| IV9RU5F02FKZAE | Bradi4g33010.2 | Os09t0476000-01 | Sb02g027490.1 | - |
| IV9RU5F02GDXAD | Bradi4g26870.1 | Os01t0182200-01 | Sb05g007520.1 | - |
| IUIVLUD01AOFQ1 | Bradi4g37260.1 | Os09t0551800-00 | Sb02g032150.1 | - |
| IVLNWEC01CUH9X | Bradi1g20870.1 | Os07t0616500-01 | Sb02g039240.1 | - |
| IV9RU5F02FLMEZ | Bradi1g09030.1 | Os03t0743900-01 | Sb01g008450.1 | - |
| IV9RU5F01BGZ1X | Bradi3g35517.1 | Os08t0407000-01 | Sb07g020060.1 | - |

| | | | | |
|---|---|---|---|---|
| IVLNWEC02H5GR4 | Bradi4g03520.1 | Os12t0580900-01 | Sb01g007340.1 | - |
| IV9RU5F01C43HH | Bradi1g11780.1 | Os03t0698350-00 | Sb01g011170.1 | - |
| IUIVLUD02IJBEO | Bradi1g53747.1 | Os01t0971600-01 | Sb02g006800.1 | - |
| IVLNWEC02JM5JQ | Bradi4g30600.1 | Os09t0423600-01 | Sb02g025070.1 | - |
| IV9RU5F02HK3VO | Bradi1g51430.1 | Os06t0138000-01 | Sb10g002570.1 | - |
| IVLNWEC02F43SC | Bradi1g50950.1 | Os09t0128600-01 | Sb06g001320.1 | - |
| IVLNWEC01DEUUI | Bradi1g05390.1 | Os03t0798200-01 | Sb01g005090.1 | - |
| IVLNWEC02IIIUV | Bradi4g35257.1 | Os09t0516500-00 | Sb02g029890.1 | - |
| IVLNWEC01DIZ2H | Bradi4g03150.1 | Os03t0240900-01 | Sb08g019730.1 | - |
| IVLNWEC02HWAAK | Bradi4g01090.1 | Os12t0626300-01 | Sb03g010430.1 | - |
| IV9RU5F01BZH5D | Bradi1g63240.1 | Os03t0334800-01 | Sb01g036190.1 | - |
| IVLNWEC02JZCQH | Bradi2g27267.1 | Os05t0373300-01 | Sb09g018600.1 | - |
| IVLNWEC02I1UA0 | Bradi1g03030.1 | Os03t0831100-01 | Sb01g002280.1 | - |
| IVLNWEC02HF0ZI | Bradi4g01090.1 | Os12t0626300-01 | Sb03g010430.1 | - |
| IVLNWEC01AUD9T | Bradi1g11020.1 | Os03t0704400-01 | Sb01g010730.1 | - |
| IUIVLUD01BEZ3X | Bradi4g05610.1 | Os12t0538800-01 | Sb05g025915.1 | - |
| IUIVLUD01AG67C | Bradi1g69520.1 | Os03t0223700-01 | Sb01g042440.1 | - |
| IVLNWEC01EUNVI | Bradi4g29617.1 | Os09t0393200-01 | Sb02g024010.1 | - |
| IV9RU5F01D6FVO | Bradi4g41137.1 | Os12t0178700-01 | Sb08g004890.1 | - |
| IUIVLUD02I6O98 | Bradi3g15520.1 | Os08t0178100-01 | Sb07g004940.1 | - |
| IV9RU5F01EEFKR | Bradi1g67387.1 | Os03t0262300-01 | Sb01g040350.1 | - |
| IVLNWEC02GQPR1 | Bradi3g22760.1 | Os10t0182000-01 | Sb08g008230.1 | - |
| IV9RU5F02IMNZQ | Bradi3g32780.1 | Os10t0548200-01 | Sb01g029925.1 | - |
| IUIVLUD01CDP63 | Bradi1g63160.1 | Os03t0336300-01 | Sb01g036110.1 | - |
| IV9RU5F02H7HXV | Bradi1g62320.1 | Os03t0352450-00 | Sb01g035270.1 | - |
| IV9RU5F02HEQ5T | Bradi4g08490.1 | Os09t0123100-01 | Sb02g013160.1 | - |
| IVLNWEC01ASDMG | Bradi2g03530.1 | Os01t0103600-02 | Sb03g008930.1 | - |
| IVLNWEC01DRFIT | Bradi1g08830.1 | Os03t0746600-01 | Sb01g008300.1 | - |
| IV9RU5F02HOBY5 | Bradi4g24480.1 | Os11t0175500-01 | Sb05g004790.1 | - |
| IUIVLUD02H3WAY | Bradi4g19650.1 | Os11t0414000-01 | Sb0014s003010.1 | - |
| IV9RU5F02JMK2Z | Bradi4g26627.1 | Os11t0148700-01 | Sb05g002930.1 | - |
| IUIVLUD01CM11I | Bradi4g39580.1 | Os12t0236900-01 | Sb03g029140.1 | - |
| IV9RU5F02HRL41 | Bradi4g39580.1 | Os12t0236900-01 | Sb03g029140.1 | - |
| IVLNWEC01DKL07 | Bradi4g39580.1 | Os12t0236900-01 | Sb03g029140.1 | - |
| IUIVLUD02HT9I7 | Bradi2g16500.1 | Os03t0619400-01 | Sb03g005190.1 | - |
| IVLNWEC01DEU82 | Bradi1g66687.1 | Os03t0274000-01 | Sb01g039660.1 | - |
| IVLNWEC02HFBXA | Bradi1g66687.1 | Os03t0274000-01 | Sb01g039660.1 | - |
| IVLNWEC01A4AGU | Bradi1g70570.1 | Os03t0209600-01 | Sb01g043330.1 | - |
| IV9RU5F01DN25D | Bradi1g03420.1 | Os03t0825300-01 | Sb01g002850.1 | - |
| IVLNWEC01BWH47 | Bradi1g00567.1 | Os03t0860900-01 | Sb01g000300.1 | - |
| IVLNWEC01C6O4G | Bradi3g28760.1 | Os10t0477000-01 | Sb01g019330.1 | - |
| IVLNWEC02HSJ79 | Bradi4g41970.2 | Os11t0158500-01 | Sb05g003740.1 | - |
| IV9RU5F02GO6ER | Bradi3g29440.1 | Os10t0488800-01 | Sb01g018770.1 | - |
| IVLNWEC02GZC08 | Bradi3g58300.1 | Os07t0620800-01 | Sb04g033870.1 | - |
| IVLNWEC01B5E9N | Bradi1g13710.1 | Os12t0617900-01 | Sb01g013460.1 | - |
| IV9RU5F01BEXY7 | Bradi1g52530.1 | Os07t0413700-01 | Sb02g009900.1 | - |
| IVLNWEC01CGKL3 | Bradi4g11687.1 | Os11t0643700-01 | Sb05g025620.1 | - |

| | | | | |
|---|---|---|---|---|
| IV9RU5F02INTIH | Bradi4g32530.1 | Os08t0478800-01 | Sb02g027000.1 | - |
| IV9RU5F02GFQVS | Bradi2g34820.1 | Os05t0159000-01 | Sb09g004530.1 | - |
| IVLNWEC01EHDSQ | Bradi1g60357.1 | Os03t0399532-00 | Sb01g033120.1 | - |
| IUIVLUD02H3ZTF | Bradi1g60357.1 | Os08t0428900-01 | Sb01g033120.1 | - |
| IV9RU5F01AZTOG | Bradi4g05110.2 | Os12t0554400-01 | Sb08g017700.1 | - |
| IVLNWEC01DXFJB | Bradi1g60890.1 | Os03t0387000-01 | Sb01g033760.1 | - |
| IVLNWEC02HJE8H | Bradi4g21057.1 | Os12t0209300-00 | Sb03g036320.1 | - |
| IV9RU5F02GDCUB | Bradi4g43517.3 | Os12t0134200-01 | Sb05g002180.1 | - |
| IVLNWEC02IECTE | Bradi4g43517.3 | Os12t0134200-01 | Sb05g002180.1 | - |
| IUIVLUD01DPRJL | Bradi1g68180.1 | Os03t0246800-01 | Sb01g041140.1 | - |
| IVLNWEC02H8OLE | Bradi1g62957.1 | Os03t0340500-01 | Sb01g035890.1 | - |
| IVLNWEC02IV2SE | Bradi4g44660.1 | Os11t0110100-01 | Sb05g000870.1 | - |
| IUIVLUD01BT7E6 | Bradi3g24750.1 | Os10t0407000-01 | Sb01g022290.1 | - |
| IVLNWEC01BNDJ4 | Bradi1g71420.1 | Os03t0196800-01 | Sb01g044040.1 | - |
| IVLNWEC01BGG87 | Bradi4g18220.1 | Os11t0515500-01 | Sb05g018860.1 | - |
| IUIVLUD01A02G8 | Bradi4g31990.1 | Os09t0454100-01 | Sb02g026450.1 | - |
| IUIVLUD02JSHYK | Bradi3g13840.1 | Os08t0117800-01 | Sb07g001700.1 | - |
| IVLNWEC02GB2FG | Bradi4g27370.1 | Os09t0287300-01 | Sb02g019520.1 | - |
| IV9RU5F02FRWVC | Bradi4g30665.1 | Os09t0424701-00 | Sb02g025140.1 | - |
| IVLNWEC01ESIDK | Bradi5g16900.1 | Os04t0535600-01 | Sb06g023760.1 | - |
| IVLNWEC02I95OS | Bradi1g67387.1 | Os03t0262300-01 | Sb01g040350.1 | - |
| IV9RU5F02H9PA8 | Bradi1g00710.1 | Os03t0859900-01 | Sb01g000365.1 | - |
| IVLNWEC01BRB74 | Bradi1g64380.1 | Os03t0308850-00 | Sb01g037370.1 | - |
| IVLNWEC02JMQI7 | Bradi1g07132.1 | Os03t0774400-01 | Sb01g006620.1 | - |
| IV9RU5F01CMSSA | Bradi1g66250.1 | Os02t0221600-00 | Sb01g039080.1 | - |
| IV9RU5F02I4DKY | Bradi1g74070.1 | Os03t0165266-00 | Sb01g046180.1 | - |
| IVLNWEC02GFBEK | Bradi1g12750.1 | Os03t0672300-01 | Sb01g012240.1 | - |
| IVLNWEC01D8Y5A | Bradi1g46020.1 | Os06t0212300-00 | Sb10g007120.1 | - |
| IV9RU5F01BY5B9 | Bradi4g22740.1 | Os11t0429100-00 | Sb07g020500.1 | - |
| IV9RU5F01CPVTY | Bradi4g27550.1 | Os08t0344600-01 | Sb02g020360.1 | - |
| IV9RU5F01C693L | Bradi3g22760.1 | Os10t0182000-01 | Sb08g008230.1 | - |
| IV9RU5F01EXKNU | Bradi1g66410.1 | Os03t0278500-01 | Sb01g039310.1 | - |
| IVLNWEC01CG6DW | Bradi1g66410.1 | Os03t0278500-01 | Sb01g039310.1 | - |
| IVLNWEC01C7CMN | Bradi2g36170.1 | Os11t0212100-00 | Sb07g005720.1 | - |
| IV9RU5F02HFXLP | Bradi3g20790.1 | Os10t0160000-01 | Sb01g025350.1 | - |
| IVLNWEC02HDWQ4 | Bradi2g31690.1 | Os10t0370800-01 | Sb01g024390.1 | - |
| IV9RU5F01BHS82 | Bradi4g21730.1 | Os07t0515900-00 | Sb02g028950.1 | - |
| IV9RU5F01ELC47 | Bradi1g01740.1 | Os03t0849600-01 | Sb01g001270.1 | - |
| IVLNWEC02JZ8DC | Bradi1g01250.1 | Os08t0170200-00 | Sb02g005230.1 | - |
| IV9RU5F01EUJYF | Bradi1g01530.1 | Os02t0234800-01 | Sb01g001040.1 | - |
| IVLNWEC02JMSR2 | Bradi1g31130.1 | Os10t0205500-01 | Sb06g019540.1 | - |
| IVLNWEC02F1XI5 | Bradi2g51807.1 | Os01t0781100-01 | Sb03g036450.1 | - |
| IVLNWEC02IHDPL | Bradi1g63650.1 | Os03t0326000-01 | Sb01g036650.1 | - |
| IV9RU5F01DZEQM | Bradi1g74287.1 | Os11t0708600-02 | Sb01g027020.1 | - |
| IV9RU5F01AY9HH | Bradi1g04560.1 | Os03t0809900-01 | Sb01g004010.1 | - |
| IV9RU5F02HQRG9 | Bradi4g30850.1 | Os09t0431200-01 | Sb02g025340.1 | - |
| IVLNWEC02JS2H1 | Bradi3g16660.1 | Os05t0151000-00 | Sb07g003680.1 | - |

| | | | | |
|---|---|---|---|---|
| IVLNWEC02GOX2P | Bradi1g04790.1 | Os03t0806500-01 | Sb01g004420.1 | - |
| IVLNWEC01A12AY | Bradi1g02117.1 | Os03t0842700-01 | Sb01g001730.1 | - |
| IUIVLUD01EKFTH | Bradi1g09250.1 | Os03t0738700-01 | Sb01g008680.1 | - |
| IV9RU5F02IYWK3 | Bradi4g38000.2 | Os09t0560900-01 | Sb02g032810.1 | - |
| IV9RU5F01CKNBH | Bradi4g06970.1 | Os08t0261000-01 | Sb02g027790.1 | - |
| IVLNWEC02HTB66 | Bradi1g06700.1 | Os03t0781700-01 | Sb01g006220.1 | - |
| IV9RU5F02HB1W7 | Bradi1g19050.1 | Os07t0658700-01 | Sb02g041770.1 | - |
| IVLNWEC02IBSGF | Bradi1g02190.1 | Os07t0645701-00 | Sb02g041520.1 | - |
| IUIVLUD01BCV5V | Bradi1g08847.1 | Os03t0746500-01 | Sb01g008310.1 | - |
| IV9RU5F01BVBFA | Bradi3g37650.1 | Os08t0459600-01 | Sb07g022500.1 | - |
| IVLNWEC01ASHBS | Bradi1g06930.1 | Os03t0777500-01 | Sb01g006390.1 | - |
| IVLNWEC02JG3QL | Bradi1g06930.1 | Os03t0777500-01 | Sb01g006390.1 | - |
| IVLNWEC01EYLZG | Bradi4g23730.1 | Os10t0425900-01 | Sb01g018710.1 | - |
| IVLNWEC01CT6ZL | Bradi3g55920.1 | Os02t0824000-00 | Sb04g037860.1 | - |
| IVLNWEC01DPFR4 | Bradi3g55920.1 | Os02t0824000-00 | Sb04g037860.1 | - |
| IVLNWEC02F7TMR | Bradi3g55920.1 | Os02t0824000-00 | Sb04g037860.1 | - |
| IUIVLUD01AFBT8 | Bradi4g04030.1 | Os12t0566900-01 | Sb08g018540.1 | - |
| IVLNWEC02HF9K5 | Bradi2g05460.1 | Os01t0185200-03 | Sb03g003420.1 | - |
| IUIVLUD01A0N94 | Bradi4g30630.1 | Os09t0424200-01 | Sb02g025100.1 | - |
| IUIVLUD01CRQGT | Bradi1g48670.1 | Os06t0158900-00 | Sb10g004070.1 | - |
| IVLNWEC02HUTDJ | Bradi4g30730.1 | Os07t0124500-01 | Sb02g028310.1 | - |
| IVLNWEC02IR1BV | Bradi4g37960.1 | Os09t0560450-01 | Sb02g032760.1 | - |
| IV9RU5F02HTXVZ | Bradi2g10302.1 | Os01t0273800-01 | Sb03g010910.1 | - |
| IVLNWEC02IKRAC | Bradi1g08070.1 | Os03t0758100-01 | Sb01g007560.2 | - |
| IV9RU5F01C3US3 | Bradi1g63330.1 | Os03t0333100-01 | Sb01g036270.1 | - |
| IVLNWEC02IQ5RR | Bradi2g29750.2 | Os05t0345400-02 | Sb09g015940.1 | - |
| IVLNWEC01CFFZT | Bradi2g03850.1 | Os10t0116600-00 | Sb07g009340.1 | - |
| IUIVLUD01AYGAQ | Bradi2g03850.1 | Os10t0162836-01 | Sb07g021550.1 | - |
| IV9RU5F01EA6TB | Bradi1g13760.1 | Os03t0645900-00 | Sb01g013520.1 | - |
| IV9RU5F02J151W | Bradi1g10130.1 | Os03t0673800-02 | Sb08g022840.1 | - |
| IVLNWEC02GK8PJ | Bradi4g44277.1 | Os11t0119200-01 | Sb05g001320.1 | - |
| IV9RU5F02GDOMT | Bradi4g39430.1 | Os02t0329300-01 | Sb04g014390.1 | - |
| IV9RU5F01A6GU1 | Bradi4g21057.1 | Os12t0209300-00 | Sb03g036320.1 | - |
| IVLNWEC01CP35D | Bradi4g21057.1 | Os12t0209300-00 | Sb03g036320.1 | - |
| IV9RU5F01AIK2H | Bradi1g08917.1 | Os03t0744650-00 | Sb01g008410.1 | - |
| IVLNWEC02HQYJQ | Bradi2g18800.1 | Os02t0187400-01 | Sb10g025000.1 | - |
| IUIVLUD02HSHWG | Bradi1g01140.1 | Os03t0852900-01 | Sb01g000850.1 | - |
| IV9RU5F01A5PN4 | Bradi4g33310.1 | Os12t0233600-01 | Sb03g029110.1 | - |
| IV9RU5F02GZXWG | Bradi4g35382.1 | Os09t0520200-00 | Sb02g030150.1 | - |
| IUIVLUD02JTPTE | Bradi1g16680.1 | Os03t0294900-01 | Sb01g038300.1 | - |
| IV9RU5F02FO9H5 | Bradi4g04280.1 | Os12t0570500-01 | Sb08g018810.1 | - |
| IVLNWEC01D4XPS | Bradi1g31130.1 | Os10t0205500-01 | Sb06g019540.1 | - |
| IV9RU5F01AJHY0 | Bradi3g43050.1 | Os04t0492300-01 | Sb06g021120.1 | - |
| IVLNWEC02HYH9W | Bradi5g22540.2 | Os05t0571700-01 | Sb07g002050.1 | - |
| IUIVLUD01DXPUL | Bradi3g45660.1 | Os11t0678000-01 | Sb05g026530.1 | - |
| IV9RU5F02F3BPQ | Bradi1g04550.1 | Os03t0809800-01 | Sb01g004016.1 | - |
| IV9RU5F01BXH8H | Bradi2g04680.1 | Os01t0189100-01 | Sb01g028256.1 | - |

| | | | | |
|---|---|---|---|---|
| IUIVLUD01B50JZ | Bradi5g20070.1 | Os04t0593600-01 | Sb06g027130.1 | - |
| IV9RU5F02GG43W | Bradi1g68750.1 | Os03t0237000-01 | Sb01g041650.1 | - |
| IV9RU5F01CX1QD | Bradi3g15520.1 | Os08t0178100-01 | Sb07g004940.1 | - |
| IV9RU5F01DJOBK | Bradi3g22760.1 | Os10t0182000-01 | Sb08g008230.1 | - |
| IUIVLUD01EVQS2 | Bradi1g08500.1 | Os12t0590500-00 | Sb01g007970.1 | - |
| IV9RU5F01CV6JI | Bradi5g22017.1 | Os03t0306200-01 | Sb03g005120.1 | - |
| IVLNWEC02HBZCS | Bradi1g09680.1 | Os03t0733800-00 | Sb01g009070.1 | - |
| IV9RU5F02F6YSA | Bradi1g62330.1 | Os03t0352400-01 | Sb01g035280.1 | - |
| IV9RU5F02GXPPL | Bradi1g62330.1 | Os03t0352400-01 | Sb01g035280.1 | - |
| IV9RU5F01EGDNG | Bradi3g56035.1 | Os02t0822900-01 | Sb04g037780.1 | - |
| IVLNWEC01DV461 | Bradi4g29500.1 | Os08t0421800-01 | Sb02g023830.1 | - |
| IVLNWEC02FN8RO | Bradi3g45660.1 | Os11t0678000-01 | Sb05g026530.1 | - |
| IUIVLUD01AL689 | Bradi1g14877.1 | Os03t0604600-01 | Sb01g014940.1 | - |
| IVLNWEC01CLWYB | Bradi3g33510.1 | Os10t0563200-01 | Sb01g028950.1 | - |
| IVLNWEC02IZMRJ | Bradi3g29870.1 | Os10t0495000-01 | Sb01g018300.1 | - |
| IV9RU5F02GB4HB | Bradi4g11687.1 | Os11t0643700-01 | Sb05g025620.1 | - |
| IV9RU5F01BIRZ7 | Bradi3g12240.1 | Os07t0476200-02 | Sb06g025910.1 | - |
| IVLNWEC01D2OAF | Bradi2g00627.1 | Os01t0106700-00 | Sb03g008670.1 | - |
| IVLNWEC01A8JDT | Bradi2g00627.1 | Os01t0106700-00 | Sb03g008680.1 | - |
| IVLNWEC01CH1U0 | Bradi2g00627.1 | Os01t0106700-00 | Sb03g008680.1 | - |
| IV9RU5F02HWDLY | Bradi1g08840.1 | Os03t0746400-01 | Sb01g008320.1 | - |
| IV9RU5F02JRHQQ | Bradi1g71420.1 | Os01t0579900-01 | Sb01g044040.1 | - |
| IUIVLUD02H5S26 | Bradi3g13840.1 | Os08t0117800-01 | Sb07g001700.1 | - |
| IVLNWEC01EJD9H | Bradi1g04830.1 | Os03t0805500-01 | Sb01g004510.1 | - |
| IV9RU5F02F9FWT | Bradi3g49640.1 | Os11t0569500-01 | Sb04g032380.1 | - |
| IVLNWEC02G9XLT | Bradi4g33270.1 | Os10t0444500-00 | Sb02g027830.1 | - |
| IUIVLUD01DHAOB | Bradi3g33980.1 | Os10t0572100-01 | Sb10g026610.1 | - |
| IVLNWEC01DEO4I | Bradi3g33980.1 | Os10t0572100-01 | Sb10g026610.1 | - |
| IVLNWEC02F8666 | Bradi3g12240.1 | Os07t0476200-01 | Sb06g025910.1 | AK248246.1 |
| IV9RU5F02JE547 | Bradi1g07390.1 | Os03t0769000-00 | Sb01g006930.1 | AK248308.1 |
| IVLNWEC01ARC3W | Bradi1g07390.1 | Os03t0769000-00 | Sb01g006930.1 | AK248308.1 |
| IVLNWEC01B5E0L | Bradi1g07390.1 | Os03t0769000-00 | Sb01g006930.1 | AK248308.1 |
| IV9RU5F02IOHTI | Bradi1g75390.1 | Os03t0145500-01 | Sb01g047200.1 | AK248976.1 |
| IUIVLUD01B515L | Bradi1g78420.1 | Os03t0101600-01 | Sb01g050560.1 | AK249011.1 |
| IVLNWEC02F5PL5 | Bradi1g78420.1 | Os03t0101600-01 | Sb01g050560.1 | AK249011.1 |
| IVLNWEC02FY65R | Bradi3g23150.1 | Os10t0206800-01 | Sb07g001800.1 | AK249059.1 |
| IVLNWEC02IZR0D | Bradi1g63050.1 | Os03t0338600-01 | Sb01g035980.1 | AK249109.1 |
| IV9RU5F01C200R | Bradi2g32930.1 | Os11t0525800-02 | Sb05g019630.1 | AK249200.1 |
| IUIVLUD01CCM4H | Bradi1g07190.1 | Os03t0773600-01 | Sb01g006700.1 | AK249476.1 |
| IVLNWEC02HD4DG | Bradi1g07190.1 | Os03t0773600-01 | Sb01g006700.1 | AK249476.1 |
| IV9RU5F02FOMYZ | Bradi4g08080.1 | Os12t0407500-01 | Sb02g000980.1 | AK249633.1 |
| IV9RU5F01EW7S1 | Bradi1g04140.1 | Os03t0815200-01 | Sb01g003700.1 | AK250029.1 |
| IVLNWEC01EN3ER | Bradi4g41137.1 | Os12t0178700-01 | Sb08g004890.1 | AK251832.1 |
| IUIVLUD02JC5VM | Bradi3g08040.1 | Os02t0215000-01 | Sb04g007460.1 | AK251905.1 |
| IV9RU5F02GTHMS | Bradi1g02117.1 | Os03t0842700-03 | Sb01g001730.1 | AK251992.1 |
| IV9RU5F01BQAFN | Bradi2g13450.1 | Os01t0528800-01 | Sb04g011550.1 | AK252044.1 |
| IUIVLUD01EU6HD | Bradi4g26342.1 | Os11t0136400-00 | Sb05g008475.1 | AK252280.1 |

| | | | | |
|---|---|---|---|---|
| IUIVLUD01EHOPC | Bradi4g05110.2 | Os12t0554400-01 | Sb08g017700.1 | AK252415.1 |
| IV9RU5F01E2GGC | Bradi4g05110.2 | Os12t0554400-01 | Sb08g017700.1 | AK252415.1 |
| IV9RU5F02FO7CI | Bradi4g05110.2 | Os12t0554400-01 | Sb08g017700.1 | AK252415.1 |
| IUIVLUD01C7ULQ | Bradi3g22760.1 | Os10t0182000-01 | Sb08g008230.1 | AK252546.1 |
| IV9RU5F02H93BA | Bradi3g22760.1 | Os10t0182000-01 | Sb08g008230.1 | AK252546.1 |
| IUIVLUD01DFQM2 | Bradi4g36580.1 | Os09t0540600-01 | Sb02g031330.1 | AK353826 |
| IUIVLUD02GU0L3 | Bradi3g11150.1 | Os02t0489500-01 | Sb04g002920.1 | AK353886 |
| IV9RU5F01AMBXW | Bradi3g11150.1 | Os02t0489500-01 | Sb04g002920.1 | AK353886 |
| IV9RU5F02JWHG2 | Bradi1g33920.1 | Os04t0465700-01 | Sb06g019330.1 | AK354076 |
| IVLNWEC01CS82J | Bradi4g00640.1 | Os06t0498900-01 | Sb05g001920.1 | AK354479 |
| IVLNWEC02IPYTD | Bradi4g06087.1 | Os11t0642800-01 | Sb08g016750.1 | AK354495 |
| IV9RU5F02HS9FP | Bradi4g25490.1 | Os11t0153600-01 | Sb05g003290.1 | AK354532 |
| IV9RU5F02HGN9K | Bradi4g02930.1 | Os12t0593000-01 | Sb08g019970.1 | AK355185 |
| IV9RU5F02FWHAC | Bradi4g43070.1 | Os11t0143500-01 | Sb05g002630.1 | AK355248 |
| IV9RU5F02JKP8H | Bradi4g43070.1 | Os11t0143500-01 | Sb05g002630.1 | AK355248 |
| IVLNWEC02IP3JL | Bradi1g04300.2 | Os03t0811100-01 | Sb01g003860.1 | AK355646 |
| IUIVLUD01BK0GO | Bradi1g52230.1 | Os12t0279000-01 | Sb10g000500.1 | AK355680 |
| IUIVLUD01BW4B9 | Bradi1g52230.1 | Os12t0279000-01 | Sb10g000500.1 | AK355680 |
| IV9RU5F02HGUI7 | Bradi1g52230.1 | Os12t0279000-01 | Sb10g000500.1 | AK355680 |
| IVLNWEC02HAYY0 | Bradi3g55870.1 | Os02t0822300-00 | Sb04g037690.1 | AK356305 |
| IVLNWEC02HJSGC | Bradi4g19650.1 | Os11t0414000-01 | Sb0014s003010.1 | AK357819 |
| IVLNWEC01DSLTH | Bradi4g02810.1 | Os10t0493800-01 | Sb08g020060.1 | AK357861 |
| IUIVLUD01BM1GQ | Bradi5g01230.1 | Os04t0131850-00 | Sb06g001270.2 | AK358163 |
| IVLNWEC02GT55G | Bradi5g01230.1 | Os04t0131850-00 | Sb06g001270.2 | AK358163 |
| IVLNWEC02I6D8Z | Bradi4g07040.1 | Os12t0497400-01 | Sb08g015410.1 | AK358846 |
| IV9RU5F02G5FLQ | Bradi4g31540.1 | Os09t0444200-01 | Sb02g026010.1 | AK359376 |
| IVLNWEC02I4UMO | Bradi4g31540.1 | Os09t0444200-01 | Sb02g026010.1 | AK359376 |
| IVLNWEC02GOX63 | Bradi4g31820.1 | Os09t0451400-01 | Sb02g026280.1 | AK359587 |
| IV9RU5F01EVCNE | Bradi4g27600.1 | Os09t0298700-01 | Sb02g020460.1 | AK359869 |
| IUIVLUD01A6C8O | Bradi1g08810.1 | Os03t0746800-01 | Sb01g008280.1 | AK359906 |
| IV9RU5F02GHR6N | Bradi1g08810.1 | Os03t0746800-01 | Sb01g008280.1 | AK359906 |
| IVLNWEC02F8WNI | Bradi1g08810.1 | Os03t0746800-01 | Sb01g008280.1 | AK359906 |
| IV9RU5F02H2R8K | Bradi1g03560.1 | Os11t0312340-00 | Sb02g001010.1 | AK360635 |
| IVLNWEC02HMGM8 | Bradi1g03560.1 | Os11t0312340-00 | Sb02g001010.1 | AK360635 |
| IV9RU5F02FPKN1 | Bradi4g36477.1 | Os09t0538700-01 | Sb02g031210.1 | AK361816 |
| IUIVLUD01CEBDZ | Bradi1g69510.1 | Os06t0305200-01 | Sb01g042430.1 | AK361972 |
| IUIVLUD01EB24H | Bradi1g51790.1 | Os06t0110100-01 | Sb09g002540.1 | AK362311 |
| IUIVLUD01ASW20 | Bradi1g02450.2 | Os03t0837900-01 | Sb01g001990.1 | AK362468 |
| IUIVLUD02G4XJO | Bradi1g02450.2 | Os03t0837900-01 | Sb01g001990.1 | AK362468 |
| IV9RU5F01DN2TI | Bradi2g36430.1 | Os11t0433200-01 | Sb06g020770.1 | AK362473 |
| IVLNWEC01ARB5H | Bradi2g36430.1 | Os11t0433200-01 | Sb06g020770.1 | AK362473 |
| IUIVLUD01DA0L0 | Bradi2g36430.1 | Os11t0433200-02 | Sb06g020770.1 | AK362473 |
| IVLNWEC01DP1SY | Bradi4g37960.1 | Os09t0560450-01 | Sb02g032760.1 | AK362524 |
| IUIVLUD01DX0U9 | Bradi1g67140.1 | Os03t0265700-01 | Sb01g040090.1 | AK362963 |
| IV9RU5F02GOQ37 | Bradi1g50067.1 | Os06t0131300-00 | Sb10g002070.1 | AK363081 |
| IUIVLUD01DTQHW | Bradi3g21117.1 | Os10t0140200-01 | Sb01g026390.1 | AK363456 |
| IV9RU5F02ILE63 | Bradi4g42810.1 | Os11t0148500-02 | Sb05g002900.1 | AK363464 |

| | | | | |
|---|---|---|---|---|
| IVLNWEC01E4UG4 | Bradi1g14450.1 | Os11t0276000-01 | Sb01g014290.1 | AK363680 |
| IVLNWEC01B4N8Z | Bradi1g03737.1 | Os03t0820700-01 | Sb01g003240.1 | AK363800 |
| IVLNWEC01AVQDD | Bradi4g35540.2 | Os09t0521500-01 | Sb02g030280.1 | AK363819 |
| IV9RU5F02HF2ZP | Bradi3g34040.1 | Os10t0573000-01 | Sb01g028370.1 | AK364381 |
| IVLNWEC02H2UHF | Bradi3g41660.1 | Os08t0538800-01 | Sb07g025740.1 | AK364458 |
| IVLNWEC02JY8EA | Bradi1g05490.1 | Os07t0181700-01 | Sb01g005180.1 | AK364520 |
| IUIVLUD01ETBX4 | Bradi1g63160.1 | Os03t0336300-01 | Sb01g036110.1 | AK364634 |
| IUIVLUD02H305K | Bradi1g63160.1 | Os03t0336300-01 | Sb01g036110.1 | AK364634 |
| IV9RU5F02JZG4P | Bradi1g63160.1 | Os03t0336300-01 | Sb01g036110.1 | AK364634 |
| IUIVLUD02HS69N | Bradi1g02380.1 | Os03t0839100-01 | Sb01g001905.1 | AK364926 |
| IVLNWEC02JAIJK | Bradi1g13760.1 | Os03t0645900-00 | Sb01g013520.1 | AK365103 |
| IV9RU5F02HH11J | Bradi4g05840.1 | Os12t0535900-01 | Sb08g017010.1 | AK365226 |
| IVLNWEC02F9TQW | Bradi4g30850.1 | Os09t0431200-01 | Sb02g025340.1 | AK365357 |
| IVLNWEC02JIUG8 | Bradi4g08180.1 | Os09t0103600-01 | Sb02g012970.1 | AK365783 |
| IUIVLUD01ASF9P | Bradi4g29590.2 | Os09t0392000-01 | Sb02g024000.1 | AK365922 |
| IVLNWEC01B2QQ7 | Bradi4g29590.2 | Os09t0392000-01 | Sb02g024000.1 | AK365922 |
| IVLNWEC01C1U44 | Bradi4g00747.1 | Os12t0636100-01 | Sb01g013840.1 | AK365959 |
| IVLNWEC02IOI6R | Bradi4g00747.1 | Os12t0636100-01 | Sb08g022950.1 | AK365959 |
| IVLNWEC01B7UCI | Bradi4g29840.1 | Os09t0401600-00 | Sb02g024280.1 | AK366468 |
| IUIVLUD01DBD3H | Bradi4g04030.1 | Os12t0566900-01 | Sb08g018540.1 | AK367062 |
| IV9RU5F02IZN6N | Bradi1g03277.1 | Os02t0156000-00 | Sb02g039465.1 | AK368276 |
| IV9RU5F01EWU5Z | Bradi3g60550.1 | Os02t0830100-01 | Sb04g038310.1 | AK368463 |
| IVLNWEC01BZUC8 | Bradi1g62770.1 | Os05t0516900-01 | Sb01g035670.1 | AK368525 |
| IVLNWEC01DPKD1 | Bradi1g62770.1 | Os05t0516900-01 | Sb01g035670.1 | AK368525 |
| IVLNWEC01CGHPR | Bradi4g36976.1 | Os05t0479800-01 | Sb02g021250.1 | AK368548 |
| IUIVLUD01DKHDV | Bradi1g05630.1 | Os03t0795000-01 | Sb01g005330.1 | AK368582 |
| IVLNWEC02HQAKX | Bradi4g23380.1 | Os11t0195100-00 | Sb05g005960.1 | AK368758 |
| IUIVLUD01B7UUI | Bradi4g15150.1 | Os11t0585700-01 | Sb01g004650.1 | AK369305 |
| IVLNWEC01ATT6K | Bradi1g15330.1 | Os03t0587200-01 | Sb01g015490.1 | AK369530 |
| IVLNWEC02G2T03 | Bradi1g09200.1 | Os03t0740700-01 | Sb01g008600.1 | AK369711 |
| IVLNWEC01D67UQ | Bradi2g15560.1 | Os01t0641000-01 | Sb03g029240.1 | AK369833 |
| IUIVLUD01EPHP8 | Bradi1g02800.1 | Os03t0832900-01 | Sb01g002500.1 | AK370148 |
| IV9RU5F01D28CP | Bradi1g07430.1 | Os03t0767900-01 | Sb01g006950.1 | AK370260 |
| IVLNWEC02I34T1 | Bradi1g07430.1 | Os03t0767900-01 | Sb01g006950.1 | AK370260 |
| IV9RU5F01BZSXH | Bradi4g34960.1 | Os09t0512700-01 | Sb02g029650.1 | AK370272 |
| IUIVLUD02G847S | Bradi1g01740.1 | Os03t0849600-01 | Sb01g001270.1 | AK370312 |
| IV9RU5F01DA2XV | Bradi4g40960.1 | Os12t0182700-01 | Sb03g038950.1 | AK370542 |
| IV9RU5F01C67S1 | Bradi1g72086.1 | Os03t0187300-01 | Sb01g044740.1 | AK370665 |
| IUIVLUD01BD1V5 | Bradi3g48510.1 | Os02t0611400-01 | Sb04g025740.1 | AK370682 |
| IV9RU5F01EOJUE | Bradi3g15520.1 | Os08t0178100-01 | Sb07g004940.1 | AK371182 |
| IV9RU5F01CB5ZG | Bradi4g06030.1 | Os12t0529400-01 | Sb08g016760.1 | AK371413 |
| IVLNWEC02F04OE | Bradi1g08660.2 | Os03t0748400-01 | Sb01g008130.1 | AK371916 |
| IV9RU5F01AJGKF | Bradi3g30860.1 | Os10t0517500-02 | Sb01g017060.1 | AK371929 |
| IV9RU5F01BF78A | Bradi3g30860.1 | Os10t0517500-02 | Sb01g017060.1 | AK371929 |
| IV9RU5F01A0FZQ | Bradi1g08620.1 | Os03t0748700-01 | Sb01g008100.1 | AK372289 |
| IUIVLUD01ESNXX | Bradi2g36937.1 | Os07t0227700-01 | Sb02g005340.1 | AK372407 |
| IVLNWEC02JGRF0 | Bradi4g01810.1 | Os03t0625900-01 | Sb01g013700.1 | AK372511 |

| | | | | |
|---|---|---|---|---|
| IV9RU5F02FY2Q2 | Bradi3g15910.1 | Os08t0169800-01 | Sb07g004540.1 | AK372775 |
| IUIVLUD02I751A | Bradi2g57987.1 | Os12t0621700-01 | Sb08g022070.1 | AK373015 |
| IV9RU5F01CU48K | Bradi1g02680.1 | Os03t0835100-00 | Sb01g002230.1 | AK373768 |
| IV9RU5F02HPSCK | Bradi4g36470.1 | Os09t0538500-01 | Sb02g031200.1 | AK373959 |
| IVLNWEC01CNQ3N | Bradi5g22017.1 | Os03t0306200-01 | Sb03g005120.1 | AK373995 |
| IUIVLUD01B2O9T | Bradi4g33067.1 | Os09t0477900-01 | Sb02g027560.1 | AK374386 |
| IVLNWEC01AXW15 | Bradi4g33067.1 | Os09t0477900-01 | Sb02g027560.1 | AK374386 |
| IVLNWEC01CHNOE | Bradi4g33067.1 | Os09t0477900-01 | Sb02g027560.1 | AK374386 |
| IVLNWEC01D1O5S | Bradi1g12750.1 | Os03t0672300-01 | Sb01g012240.1 | AK375629 |
| IVLNWEC02FV3VS | Bradi4g38750.1 | Os09t0570500-01 | Sb04g037810.1 | AK375835 |
| IVLNWEC01EYZEC | Bradi3g59847.1 | Os10t0405500-01 | Sb04g005260.1 | AK375886 |
| IUIVLUD01EVLN5 | Bradi1g08420.1 | Os03t0751100-01 | Sb01g007880.1 | AK376249 |
| IV9RU5F01AT40G | Bradi1g13580.1 | Os03t0649000-01 | Sb01g013300.1 | AK376438 |
| IUIVLUD01EJCFP | Bradi4g08597.1 | Os09t0132900-01 | Sb08g021590.1 | AK376689 |
| IV9RU5F02GYKGD | Bradi3g29190.1 | Os10t0484400-02 | Sb07g027720.1 | AK376852 |
| IVLNWEC02GJLA9 | Bradi1g09970.2 | Os03t0728800-01 | Sb01g009400.1 | AK377003 |
| IVLNWEC02H8FS6 | Bradi3g27750.1 | Os10t0532100-01 | Sb01g030750.1 | MLOC_10199.2 |
| IVLNWEC01DF5KY | Bradi4g01790.1 | Os12t0613200-01 | Sb08g002530.1 | MLOC_10435.2 |
| IVLNWEC02I9RFE | Bradi4g01790.1 | Os12t0613200-01 | Sb08g002530.1 | MLOC_10435.2 |
| IUIVLUD01B329L | Bradi1g38227.1 | Os07t0203300-01 | Sb02g006000.1 | MLOC_10482.5 |
| IVLNWEC01ESK9R | Bradi1g38227.1 | Os07t0203300-01 | Sb02g006000.1 | MLOC_10482.5 |
| IVLNWEC02IYPJ3 | Bradi1g06810.1 | Os03t0780500-01 | Sb01g006320.1 | MLOC_10567.1 |
| IUIVLUD02FSXVN | Bradi1g07942.1 | Os03t0759600-00 | Sb01g007450.1 | MLOC_10633.1 |
| IUIVLUD01CPRZD | Bradi2g51807.1 | Os01t0781200-01 | Sb03g036450.1 | MLOC_10643.2 |
| IUIVLUD02IWE5W | Bradi2g14600.1 | Os05t0196500-01 | Sb04g036630.1 | MLOC_1083.2 |
| IVLNWEC02JCD70 | Bradi1g05380.1 | Os03t0798300-01 | Sb01g005082.1 | MLOC_10988.2 |
| IVLNWEC01AZ0PE | Bradi1g05380.1 | Os03t0798300-01 | Sb02g004680.1 | MLOC_10988.2 |
| IVLNWEC02JYILX | Bradi1g08710.1 | Os03t0747900-00 | Sb01g023150.1 | MLOC_11351.1 |
| IVLNWEC02GIAS1 | Bradi4g42050.1 | Os12t0158800-01 | Sb08g003670.1 | MLOC_11415.2 |
| IV9RU5F01DDWAB | Bradi1g02000.1 | Os03t0843600-01 | Sb01g001620.1 | MLOC_11570.6 |
| IUIVLUD01ETLE5 | Bradi4g35376.1 | Os09t0520300-00 | Sb02g030160.1 | MLOC_11733.1 |
| IV9RU5F02GQ4N4 | Bradi5g20420.1 | Os04t0599650-00 | Sb06g027382.1 | MLOC_11906.1 |
| IVLNWEC02GR1Q3 | Bradi1g64420.1 | Os03t0308200-01 | Sb01g037420.1 | MLOC_12000.2 |
| IVLNWEC01C2Q84 | Bradi3g31377.1 | Os10t0520700-01 | Sb01g031320.1 | MLOC_12086.1 |
| IV9RU5F01CUTYB | Bradi1g06380.1 | Os03t0786900-01 | Sb01g005910.1 | MLOC_12376.1 |
| IV9RU5F02JT315 | Bradi1g06380.1 | Os03t0786900-01 | Sb01g005910.1 | MLOC_12376.1 |
| IVLNWEC01ENUZL | Bradi1g06380.1 | Os03t0786900-01 | Sb01g005910.1 | MLOC_12376.1 |
| IVLNWEC02JT6OA | Bradi1g06380.1 | Os03t0786900-01 | Sb01g005910.1 | MLOC_12376.1 |
| IV9RU5F01CUP0P | Bradi1g65650.1 | Os03t0288800-01 | Sb01g038570.1 | MLOC_12718.1 |
| IV9RU5F01EU688 | Bradi1g65650.1 | Os03t0288800-01 | Sb01g038570.1 | MLOC_12718.1 |
| IVLNWEC02JPG1V | Bradi1g12340.2 | Os03t0683866-00 | Sb01g011845.1 | MLOC_12803.1 |
| IVLNWEC02GBOWS | Bradi1g51290.1 | Os06t0140100-01 | Sb10g002720.1 | MLOC_13246.1 |
| IUIVLUD01COVHD | Bradi2g28680.1 | Os05t0350500-01 | Sb09g016610.1 | MLOC_13565.1 |
| IVLNWEC02I16R4 | Bradi2g28680.1 | Os05t0350500-01 | Sb09g016610.1 | MLOC_13565.1 |
| IUIVLUD02IBLB9 | Bradi1g67387.1 | Os03t0262300-01 | Sb01g040350.1 | MLOC_13606.1 |
| IVLNWEC02HLD3V | Bradi1g02290.1 | Os12t0614800-00 | Sb08g021540.1 | MLOC_14275.1 |
| IVLNWEC02GZELA | Bradi2g28007.1 | Os05t0235300-01 | Sb09g017790.1 | MLOC_14340.1 |

| | | | | |
|---|---|---|---|---|
| IV9RU5F01DHE6B | Bradi4g04650.1 | Os12t0564600-01 | Sb08g018400.1 | MLOC_1444.1 |
| IVLNWEC02FUBHX | Bradi3g10980.1 | Os02t0317800-01 | Sb07g009530.1 | MLOC_15210.1 |
| IV9RU5F02GCM6L | Bradi4g44720.1 | Os03t0849700-01 | Sb10g002910.1 | MLOC_15553.1 |
| IVLNWEC01DR7FR | Bradi4g44720.1 | Os03t0849700-01 | Sb10g002910.1 | MLOC_15553.1 |
| IVLNWEC02G3Z13 | Bradi1g04270.1 | Os03t0811500-01 | Sb01g003820.1 | MLOC_15858.1 |
| IUIVLUD02G17HO | Bradi1g22240.1 | Os07t0602200-01 | Sb02g038550.1 | MLOC_16422.1 |
| IV9RU5F01D6VLV | Bradi1g68750.1 | Os03t0237000-01 | Sb01g041650.1 | MLOC_16677.1 |
| IV9RU5F01BHNB3 | Bradi1g53590.1 | Os07t0240200-01 | Sb02g007050.1 | MLOC_16913.1 |
| IVLNWEC02F1F83 | Bradi4g29570.1 | Os09t0386600-01 | Sb02g023980.1 | MLOC_16987.2 |
| IVLNWEC02ITXK2 | Bradi4g34330.1 | Os09t0501100-00 | Sb02g029020.1 | MLOC_18171.1 |
| IUIVLUD02HBSMA | Bradi4g31570.1 | Os09t0444800-01 | Sb02g026050.1 | MLOC_19049.1 |
| IV9RU5F01BEX4J | Bradi4g30665.1 | Os09t0424701-00 | Sb02g025140.1 | MLOC_19512.1 |
| IV9RU5F01DN933 | Bradi1g71000.1 | Os03t0202300-01 | Sb01g043670.1 | MLOC_20830.1 |
| IUIVLUD02FTMUF | Bradi4g23680.1 | Os11t0183800-00 | Sb05g005390.1 | MLOC_20977.1 |
| IV9RU5F01DSST9 | Bradi4g45190.1 | Os12t0103000-01 | Sb05g000330.1 | MLOC_21503.1 |
| IV9RU5F02HQSPB | Bradi2g02790.1 | Os01t0595300-01 | Sb03g002010.1 | MLOC_22173.1 |
| IV9RU5F02J087N | Bradi2g02790.1 | Os01t0595300-01 | Sb03g002010.1 | MLOC_22173.1 |
| IVLNWEC01BA70E | Bradi4g30897.1 | Os09t0431600-00 | Sb02g025370.1 | MLOC_23179.3 |
| IV9RU5F01ER6VP | Bradi3g33980.1 | Os10t0572100-01 | Sb10g026610.1 | MLOC_2499.3 |
| IV9RU5F01BVG65 | Bradi4g27820.1 | Os09t0309100-01 | Sb01g001310.1 | MLOC_26274.1 |
| IUIVLUD01CH805 | Bradi1g04000.1 | Os03t0816900-01 | Sb01g003570.1 | MLOC_27721.1 |
| IVLNWEC01A0KVJ | Bradi3g35517.1 | Os08t0407000-01 | Sb07g020060.1 | MLOC_28103.2 |
| IV9RU5F01AL8DP | Bradi1g37370.1 | Os06t0574400-01 | Sb10g022700.1 | MLOC_3122.1 |
| IV9RU5F02FK54J | Bradi5g00430.1 | Os09t0101800-01 | Sb02g013000.1 | MLOC_3301.2 |
| IVLNWEC02F7MFB | Bradi1g03827.1 | Os03t0819700-01 | Sb01g003320.1 | MLOC_34058.1 |
| IV9RU5F02GFQV7 | Bradi4g31830.1 | Os09t0451500-02 | Sb02g026300.1 | MLOC_34487.1 |
| IV9RU5F02JZW8A | Bradi4g31830.1 | Os09t0451500-02 | Sb02g026300.1 | MLOC_34487.1 |
| IVLNWEC01B38AU | Bradi4g43780.1 | Os06t0715000-01 | Sb08g001600.1 | MLOC_34755.2 |
| IUIVLUD02JUBJ3 | Bradi1g03910.1 | Os03t0818200-01 | Sb01g003480.1 | MLOC_3543.1 |
| IUIVLUD01CFX63 | Bradi4g27057.1 | Os09t0279600-01 | Sb02g018660.1 | MLOC_36393.1 |
| IUIVLUD01CY5S2 | Bradi4g27057.1 | Os09t0279600-01 | Sb02g018660.1 | MLOC_36393.1 |
| IV9RU5F01CDFL4 | Bradi3g00507.1 | Os04t0492300-01 | Sb06g021120.1 | MLOC_36681.1 |
| IUIVLUD01CPMIO | Bradi2g18270.1 | Os09t0241100-01 | Sb10g019730.1 | MLOC_36749.1 |
| IVLNWEC02JJ1UK | Bradi1g69780.1 | Os03t0216600-01 | Sb01g042750.1 | MLOC_36871.1 |
| IV9RU5F01DTYJK | Bradi4g02910.1 | Os12t0594200-00 | Sb08g020025.1 | MLOC_36971.7 |
| IVLNWEC02HVADN | Bradi4g02910.1 | Os12t0594200-00 | Sb08g020025.1 | MLOC_36971.7 |
| IV9RU5F02JR4F1 | Bradi4g22690.1 | Os11t0209700-02 | Sb05g009420.1 | MLOC_36974.1 |
| IVLNWEC01CCXLM | Bradi3g57600.1 | Os02t0771800-01 | Sb04g034540.1 | MLOC_36985.5 |
| IUIVLUD01A2CE8 | Bradi2g21470.1 | Os05t0491000-01 | Sb09g024030.1 | MLOC_36992.1 |
| IV9RU5F01BXSE0 | Bradi2g00620.1 | Os12t0638600-01 | Sb03g008700.1 | MLOC_37453.3 |
| IVLNWEC02GPKA8 | Bradi1g03920.1 | Os03t0818100-01 | Sb01g003490.1 | MLOC_37861.2 |
| IV9RU5F02JHI7I | Bradi4g05940.1 | Os06t0168500-01 | Sb08g016910.1 | MLOC_38005.1 |
| IV9RU5F01DET5L | Bradi1g68220.1 | Os05t0571700-01 | Sb05g000850.1 | MLOC_38081.1 |
| IV9RU5F01DWPIO | Bradi3g36960.1 | Os08t0440200-01 | Sb07g021600.1 | MLOC_38205.5 |
| IV9RU5F02GSSAB | Bradi3g36960.1 | Os08t0440200-01 | Sb07g021600.1 | MLOC_38205.5 |
| IVLNWEC01DYXVP | Bradi1g01330.1 | Os03t0852600-01 | Sb01g000870.1 | MLOC_39992.2 |
| IVLNWEC02FUWL8 | Bradi1g01330.1 | Os03t0852600-01 | Sb01g000870.1 | MLOC_39992.2 |

| | | | | |
|---|---|---|---|---|
| IV9RU5F01CAA4R | Bradi2g18550.1 | Os05t0536400-01 | Sb09g026810.1 | MLOC_4273.2 |
| IVLNWEC02HFDGD | Bradi1g08840.1 | Os03t0746400-01 | Sb01g008320.1 | MLOC_43312.1 |
| IUIVLUD01C2RJH | Bradi4g35140.1 | Os09t0514900-01 | Sb02g029820.1 | MLOC_4408.1 |
| IUIVLUD02IUD6S | Bradi4g35140.1 | Os09t0514900-01 | Sb02g029820.1 | MLOC_4408.1 |
| IV9RU5F01AH900 | Bradi4g35140.1 | Os09t0514900-01 | Sb02g029820.1 | MLOC_4408.1 |
| IVLNWEC01BK7OA | Bradi1g15547.1 | Os03t0580200-01 | Sb01g015890.1 | MLOC_44661.1 |
| IV9RU5F01AD889 | Bradi1g15547.1 | Os03t0581400-00 | Sb01g015890.1 | MLOC_44661.1 |
| IV9RU5F02JWV28 | Bradi4g13710.1 | Os05t0462400-01 | Sb05g024490.1 | MLOC_4685.6 |
| IV9RU5F01ENLMO | Bradi1g05590.1 | Os03t0795400-00 | Sb01g005290.1 | MLOC_4861.1 |
| IV9RU5F02GWN2Y | Bradi1g05590.1 | Os03t0795400-00 | Sb01g005290.1 | MLOC_4861.1 |
| IVLNWEC01CQKDW | Bradi4g08907.3 | Os09t0266400-01 | Sb02g015540.1 | MLOC_4863.2 |
| IUIVLUD01DSHQ7 | Bradi3g59726.1 | Os02t0740300-01 | Sb04g028420.1 | MLOC_4872.1 |
| IVLNWEC02F82M6 | Bradi4g45227.1 | Os11t0102900-01 | Sb05g000280.1 | MLOC_48965.1 |
| IVLNWEC01AUCD6 | Bradi1g01730.3 | Os03t0848600-01 | Sb01g001280.2 | MLOC_4983.1 |
| IUIVLUD02GAJHG | Bradi3g33240.1 | Os10t0559600-01 | Sb01g029120.1 | MLOC_51141.6 |
| IV9RU5F01CF7A3 | Bradi1g69590.1 | Os03t0221800-01 | Sb01g042540.1 | MLOC_51331.2 |
| IVLNWEC01ELJEA | Bradi1g69590.1 | Os03t0221800-01 | Sb01g042540.1 | MLOC_51331.2 |
| IUIVLUD01B7IXY | Bradi1g03730.1 | Os03t0820900-01 | Sb01g003230.1 | MLOC_51706.2 |
| IV9RU5F01C3N5C | Bradi1g03730.1 | Os03t0820900-01 | Sb01g003230.1 | MLOC_51706.2 |
| IVLNWEC01A07RP | Bradi1g03730.1 | Os03t0820900-01 | Sb01g003230.1 | MLOC_51706.2 |
| IUIVLUD02FNXND | Bradi1g66980.1 | Os03t0267800-01 | Sb01g039940.1 | MLOC_51938.1 |
| IVLNWEC01ED0OD | Bradi1g66980.1 | Os03t0267800-01 | Sb01g039940.1 | MLOC_51938.1 |
| IUIVLUD01BH0G7 | Bradi4g02420.1 | Os12t0605200-01 | Sb08g020780.1 | MLOC_51985.1 |
| IVLNWEC02G5V41 | Bradi1g11640.1 | Os03t0701000-02 | Sb01g011010.1 | MLOC_52027.1 |
| IVLNWEC01AMQJR | Bradi4g36127.1 | Os09t0532500-01 | Sb02g030880.1 | MLOC_52297.1 |
| IV9RU5F01AKS15 | Bradi4g36250.1 | Os09t0534000-01 | Sb02g031000.1 | MLOC_52513.1 |
| IUIVLUD01AVIXC | Bradi1g03980.1 | Os03t0817200-01 | Sb01g003540.1 | MLOC_53542.2 |
| IV9RU5F01BKUZH | Bradi1g03980.1 | Os03t0817200-01 | Sb01g003540.1 | MLOC_53542.2 |
| IV9RU5F01BOIX7 | Bradi1g03980.1 | Os03t0817200-01 | Sb01g003540.1 | MLOC_53542.2 |
| IV9RU5F02JKAX1 | Bradi1g08350.1 | Os03t0752700-01 | Sb01g007800.1 | MLOC_53865.1 |
| IVLNWEC01CMIP9 | Bradi1g13787.1 | Os03t0645000-00 | Sb01g013560.1 | MLOC_53948.1 |
| IUIVLUD01EPYKS | Bradi1g67960.1 | Os03t0251500-01 | Sb01g040970.1 | MLOC_53996.2 |
| IVLNWEC02JPI78 | Bradi1g64210.1 | Os03t0313600-01 | Sb01g037190.1 | MLOC_53997.2 |
| IVLNWEC02JB4EH | Bradi4g28877.1 | Os09t0364000-01 | Sb02g023040.1 | MLOC_54108.2 |
| IV9RU5F02IUPWH | Bradi1g11260.1 | Os03t0710100-02 | Sb01g010480.1 | MLOC_54143.1 |
| IV9RU5F01B7W7Q | Bradi1g74827.1 | Os03t0152900-01 | Sb01g046750.1 | MLOC_54161.7 |
| IVLNWEC02F7OLX | Bradi4g32850.1 | Os09t0472100-01 | Sb02g027350.1 | MLOC_54243.2 |
| IUIVLUD01B78M3 | Bradi3g59627.1 | Os02t0741400-01 | Sb04g028320.1 | MLOC_54546.1 |
| IV9RU5F02GSSTM | Bradi1g51757.1 | Os06t0113150-00 | Sb01g050480.1 | MLOC_54721.2 |
| IUIVLUD02FYOTY | Bradi4g29760.1 | Os09t0397900-01 | Sb03g028040.1 | MLOC_55056.1 |
| IVLNWEC02IUF6W | Bradi1g13690.1 | Os03t0647500-02 | Sb08g021890.1 | MLOC_55532.1 |
| IVLNWEC02H6OT3 | Bradi2g62760.1 | Os01t0977600-02 | Sb05g002400.1 | MLOC_55536.1 |
| IV9RU5F01E1L7I | Bradi1g04930.1 | Os03t0804200-01 | Sb01g004610.1 | MLOC_56327.1 |
| IUIVLUD01C7A23 | Bradi4g42160.1 | Os12t0156900-00 | Sb08g003540.1 | MLOC_56529.1 |
| IVLNWEC01BPW97 | Bradi4g42160.1 | Os12t0156900-00 | Sb08g003540.1 | MLOC_56529.1 |
| IV9RU5F02F7UT0 | Bradi1g30580.1 | Os06t0644600-01 | Sb10g025320.1 | MLOC_5664.2 |
| IVLNWEC01EWEZF | Bradi1g13850.1 | Os10t0569200-01 | Sb01g028520.1 | MLOC_56768.1 |

| | | | | |
|---|---|---|---|---|
| IVLNWEC02JO7OS | Bradi1g01280.1 | Os03t0852000-01 | Sb01g000920.1 | MLOC_57150.1 |
| IVLNWEC01DM2FH | Bradi1g73110.1 | Os03t0172000-01 | Sb01g045670.1 | MLOC_57182.2 |
| IVLNWEC02I2IRT | Bradi1g73110.1 | Os03t0172000-01 | Sb01g045670.1 | MLOC_57182.2 |
| IV9RU5F01BDOIX | Bradi2g26910.1 | Os03t0853700-01 | Sb01g000820.1 | MLOC_57217.1 |
| IVLNWEC02G03VM | Bradi2g26910.1 | Os03t0853700-01 | Sb01g000820.1 | MLOC_57217.1 |
| IVLNWEC02JXU5F | Bradi1g11020.1 | Os03t0704400-01 | Sb01g010730.1 | MLOC_57909.2 |
| IVLNWEC01EUJ78 | Bradi3g58130.1 | Os10t0400800-01 | Sb04g034020.1 | MLOC_58108.2 |
| IVLNWEC01BHWEH | Bradi3g38400.1 | Os08t0478500-01 | Sb02g026980.1 | MLOC_58115.2 |
| IV9RU5F01CRKXQ | Bradi2g51637.1 | Os01t0777300-00 | Sb03g036255.1 | MLOC_58514.8 |
| IV9RU5F02JAG2A | Bradi2g51637.1 | Os01t0777300-00 | Sb03g036255.1 | MLOC_58514.8 |
| IV9RU5F01EH2DP | Bradi4g06130.1 | Os12t0527900-01 | Sb08g016710.1 | MLOC_58573.1 |
| IVLNWEC02GOIM6 | Bradi2g26600.1 | Os05t0391500-01 | Sb09g019350.1 | MLOC_58956.3 |
| IV9RU5F01EYNM8 | Bradi4g24767.1 | Os11t0162000-01 | Sb05g003870.1 | MLOC_59247.2 |
| IVLNWEC02JMH78 | Bradi3g48010.1 | Os02t0602400-01 | Sb04g025195.1 | MLOC_59646.3 |
| IUIVLUD01D8320 | Bradi1g63330.1 | Os03t0333100-01 | Sb01g036270.1 | MLOC_60118.1 |
| IV9RU5F01EB2BW | Bradi1g72092.1 | Os03t0187100-00 | Sb01g044750.1 | MLOC_60439.1 |
| IUIVLUD01E08Z8 | Bradi4g08880.1 | Os09t0246200-01 | Sb02g039520.1 | MLOC_60620.1 |
| IV9RU5F01BMWDM | Bradi4g01090.1 | Os12t0626300-01 | Sb03g010430.1 | MLOC_60893.1 |
| IVLNWEC01BOKB2 | Bradi4g01090.1 | Os12t0626300-01 | Sb03g010430.1 | MLOC_60893.1 |
| IUIVLUD02G7UG3 | Bradi3g05847.1 | Os03t0403100-01 | Sb10g006995.1 | MLOC_61052.6 |
| IVLNWEC01ENONC | Bradi4g25980.1 | Os01t0897000-01 | Sb08g002240.1 | MLOC_6120.2 |
| IUIVLUD01A6B8E | Bradi4g35301.1 | Os09t0516900-01 | Sb02g029920.1 | MLOC_61380.1 |
| IVLNWEC01BHH2B | Bradi4g36620.1 | Os09t0541100-00 | Sb02g031400.1 | MLOC_62173.1 |
| IVLNWEC02IODS3 | Bradi4g36620.1 | Os09t0541100-00 | Sb02g031400.1 | MLOC_62173.1 |
| IVLNWEC01DPOSO | Bradi4g22080.1 | Os11t0220400-01 | Sb05g008540.1 | MLOC_62275.3 |
| IV9RU5F01DVP6E | Bradi1g21280.1 | Os07t0621500-01 | Sb02g039580.1 | MLOC_62356.1 |
| IUIVLUD01AOVCF | Bradi4g31350.1 | Os09t0573100-01 | Sb02g013170.1 | MLOC_62866.1 |
| IVLNWEC02GHDUK | Bradi4g31350.1 | Os09t0573100-01 | Sb02g013170.1 | MLOC_62866.1 |
| IV9RU5F01CUO7C | Bradi1g06570.1 | Os03t0784500-01 | Sb01g006110.1 | MLOC_62889.1 |
| IVLNWEC02J37D1 | Bradi2g51807.1 | Os01t0781200-01 | Sb03g036450.1 | MLOC_62897.1 |
| IVLNWEC02JJVKO | Bradi1g02980.1 | Os03t0831700-01 | Sb01g002240.1 | MLOC_63062.2 |
| IVLNWEC01BQQDV | Bradi1g08140.1 | Os03t0757600-01 | Sb01g007590.1 | MLOC_63805.2 |
| IV9RU5F02G8BP6 | Bradi1g07577.1 | Os03t0765100-01 | Sb01g007090.1 | MLOC_63880.1 |
| IV9RU5F01D2RZE | Bradi1g21860.1 | Os04t0422300-01 | Sb04g000470.1 | MLOC_63937.1 |
| IVLNWEC01DNIIM | Bradi1g06580.1 | Os03t0784400-01 | Sb03g038260.1 | MLOC_64013.1 |
| IUIVLUD01DE093 | Bradi3g58120.1 | Os01t0598900-00 | Sb04g034030.1 | MLOC_64085.2 |
| IUIVLUD01DELUS | Bradi1g75470.2 | Os03t0143851-01 | Sb01g047340.1 | MLOC_64091.1 |
| IVLNWEC01C7O5W | Bradi1g75470.2 | Os03t0143851-01 | Sb01g047340.1 | MLOC_64091.1 |
| IV9RU5F02F2NFN | Bradi4g42110.1 | Os12t0157200-01 | Sb08g003590.1 | MLOC_64847.1 |
| IUIVLUD01D8WTI | Bradi1g49750.1 | Os04t0320200-01 | Sb10g001830.1 | MLOC_6485.2 |
| IV9RU5F02IMRRV | Bradi1g04590.2 | Os07t0212300-01 | Sb01g003940.1 | MLOC_64862.1 |
| IV9RU5F01EO1QK | Bradi2g05870.1 | Os01t0194300-01 | Sb03g002980.1 | MLOC_64922.1 |
| IV9RU5F01DOV4J | Bradi4g27470.1 | Os09t0296700-01 | Sb02g019400.1 | MLOC_65221.1 |
| IVLNWEC01DDP3J | Bradi4g27470.1 | Os09t0296700-01 | Sb02g019400.1 | MLOC_65221.1 |
| IVLNWEC02HZZ8Y | Bradi4g27470.1 | Os09t0296700-01 | Sb02g019400.1 | MLOC_65221.1 |
| IVLNWEC02IW876 | Bradi1g11780.1 | Os03t0698350-00 | Sb01g011170.1 | MLOC_65929.1 |
| IVLNWEC01CJPGE | Bradi1g00460.1 | Os07t0522500-01 | Sb01g000220.1 | MLOC_66589.2 |

| | | | | |
|---|---|---|---|---|
| IVLNWEC01A39TO | Bradi3g41670.1 | Os08t0539100-01 | Sb07g025730.1 | MLOC_66731.2 |
| IVLNWEC01BK340 | Bradi1g08830.1 | Os03t0746600-01 | Sb01g008300.1 | MLOC_66915.2 |
| IV9RU5F01CGCMC | Bradi2g60350.1 | Os05t0333200-00 | Sb01g045320.1 | MLOC_67224.3 |
| IV9RU5F01DFCV2 | Bradi2g60350.1 | Os05t0333200-00 | Sb01g045320.1 | MLOC_67224.3 |
| IVLNWEC01A3FWG | Bradi2g57720.1 | Os01t0513800-01 | Sb09g024910.1 | MLOC_67376.2 |
| IV9RU5F01C7WO8 | Bradi4g29600.1 | Os09t0392100-01 | Sb02g024030.1 | MLOC_67615.1 |
| IVLNWEC01DCJDT | Bradi4g29600.1 | Os09t0392100-01 | Sb02g024030.1 | MLOC_67615.1 |
| IVLNWEC02JTNY7 | Bradi4g29600.1 | Os09t0392100-01 | Sb02g024030.1 | MLOC_67615.1 |
| IV9RU5F02GBOZP | Bradi4g40560.1 | Os12t0194800-01 | Sb08g005650.1 | MLOC_67682.1 |
| IUIVLUD01CZLP5 | Bradi2g51807.1 | Os01t0781100-01 | Sb03g036450.1 | MLOC_67712.3 |
| IUIVLUD01CQKGI | Bradi1g17710.1 | Os02t0167700-01 | Sb04g004540.1 | MLOC_67779.1 |
| IV9RU5F02G0GM3 | Bradi4g37410.1 | Os09t0553900-01 | Sb02g032300.1 | MLOC_67933.1 |
| IUIVLUD02JQ8Z9 | Bradi4g38200.1 | Os03t0670200-01 | Sb02g011300.1 | MLOC_67950.1 |
| IV9RU5F01DVGKA | Bradi1g64460.4 | Os03t0307800-01 | Sb01g037450.1 | MLOC_69040.3 |
| IV9RU5F02IRKJM | Bradi1g64460.4 | Os03t0307800-01 | Sb01g037450.1 | MLOC_69040.3 |
| IV9RU5F02GR41U | Bradi1g00760.1 | Os03t0859500-01 | Sb01g000400.1 | MLOC_69405.3 |
| IV9RU5F02I47Z2 | Bradi1g00760.1 | Os03t0859500-01 | Sb01g000400.1 | MLOC_69405.3 |
| IVLNWEC02GPWKU | Bradi1g00760.1 | Os03t0859500-01 | Sb01g000400.1 | MLOC_69405.3 |
| IUIVLUD01EZEIZ | Bradi4g01620.1 | Os12t0615600-00 | Sb08g021630.1 | MLOC_69487.3 |
| IVLNWEC01C3AJF | Bradi4g01620.1 | Os12t0615600-00 | Sb08g021630.1 | MLOC_69487.3 |
| IVLNWEC02IZXZK | Bradi4g01620.1 | Os12t0615600-00 | Sb08g021630.1 | MLOC_69487.3 |
| IV9RU5F01CN969 | Bradi3g54700.1 | Os02t0805900-01 | Sb10g028040.1 | MLOC_70182.1 |
| IVLNWEC01B48QJ | Bradi1g10530.2 | Os03t0719500-02 | Sb01g009940.1 | MLOC_70277.1 |
| IV9RU5F01AMJ9R | Bradi1g11730.1 | Os03t0699200-01 | Sb01g011100.1 | MLOC_70449.2 |
| IV9RU5F01EM4D9 | Bradi1g02490.1 | Os03t0837300-02 | Sb01g002020.1 | MLOC_72184.1 |
| IVLNWEC02I5RYM | Bradi1g02490.1 | Os03t0837300-02 | Sb01g002020.1 | MLOC_72184.1 |
| IV9RU5F02HFNQ2 | Bradi4g42640.1 | Os11t0149900-00 | Sb05g003000.1 | MLOC_72578.1 |
| IV9RU5F01CNBXZ | Bradi3g50020.1 | Os02t0642600-01 | Sb04g033640.1 | MLOC_73192.2 |
| IVLNWEC01BTGD2 | Bradi1g07830.1 | Os03t0761700-01 | Sb01g007290.1 | MLOC_75007.5 |
| IV9RU5F02JR820 | Bradi4g08152.1 | Os12t0291000-00 | Sb08g011950.1 | MLOC_75102.1 |
| IV9RU5F01AWLHU | Bradi3g28570.1 | Os01t0511200-01 | Sb04g001080.1 | MLOC_75183.1 |
| IUIVLUD02GQUQP | Bradi4g32200.1 | Os09t0458700-01 | Sb02g026670.1 | MLOC_75452.1 |
| IV9RU5F01C1UYA | Bradi1g72967.1 | Os03t0174600-01 | Sb01g045520.1 | MLOC_75508.1 |
| IV9RU5F02JVHJ9 | Bradi4g03050.1 | Os12t0590500-00 | Sb08g019800.1 | MLOC_75843.3 |
| IV9RU5F02HD4PP | Bradi4g08860.1 | Os09t0251100-00 | Sb03g013430.1 | MLOC_76583.1 |
| IVLNWEC01CEB30 | Bradi1g03660.1 | Os03t0821800-01 | Sb01g003140.1 | MLOC_76878.1 |
| IVLNWEC01EVF0G | Bradi1g72280.1 | Os03t0185500-01 | Sb01g044850.1 | MLOC_80833.1 |
| IVLNWEC02GYCHC | Bradi1g72280.1 | Os03t0185500-01 | Sb01g044850.1 | MLOC_80833.1 |
| IVLNWEC02HCEI3 | Bradi1g72280.1 | Os03t0185500-01 | Sb01g044850.1 | MLOC_80833.1 |
| IV9RU5F02IQUQ6 | Bradi2g05460.1 | Os01t0185200-03 | Sb03g003420.1 | MLOC_8121.1 |
| IV9RU5F02J34OY | Bradi2g05460.1 | Os01t0185200-03 | Sb03g003420.1 | MLOC_8121.1 |
| IVLNWEC02ILRH3 | Bradi2g05460.1 | Os01t0185200-03 | Sb03g003420.1 | MLOC_8121.1 |
| IVLNWEC01B96D7 | Bradi1g00887.1 | Os03t0858100-01 | Sb01g000490.1 | MLOC_81454.2 |
| IV9RU5F02FXGNM | Bradi1g08400.1 | Os03t0752100-01 | Sb01g007850.1 | MLOC_824.2 |
| IVLNWEC01DDDN4 | Bradi1g08400.1 | Os03t0752100-01 | Sb01g007850.1 | MLOC_824.2 |
| IVLNWEC02HJ1N6 | Bradi4g28177.1 | Os09t0327600-01 | Sb02g022180.1 | MLOC_91.1 |
| IV9RU5F02G1847 | Bradi4g15010.1 | Os11t0586300-01 | Sb05g022830.1 | MLOC_9792.1 |

| | | | | |
|---|---|---|---|---|
| IUIVLUD02INEAE | Bradi4g37260.1 | Os09t0551800-00 | Sb02g032150.1 | MLOC_9823.2 |
| IVLNWEC01C1YTK | Bradi1g53590.1 | Os07t0240200-01 | Sb02g007050.1 | MLOC_16913.1 |
| IUIVLUD02IKERD | Bradi1g01980.1 | Os03t0843900-01 | Sb01g001560.1 | AK364505 |
| IUIVLUD01CTRGK | Bradi1g09250.1 | Os03t0738700-01 | Sb01g008680.1 | MLOC_17465.1 |
| IUIVLUD01BI9OA | Bradi4g08520.1 | Os09t0240500-01 | Sb02g013390.1 | AK250156.1 |
| IUIVLUD01DZ9W9 | Bradi1g07910.1 | Os03t0760700-01 | Sb01g007370.1 | AK359713 |
| IV9RU5F02JWQZN | Bradi1g69690.1 | Os03t0219100-01 | Sb01g042670.1 | MLOC_18330.1 |
| IV9RU5F01B4T5H | Bradi1g13770.1 | Os12t0617100-01 | Sb01g013530.1 | AK356347 |
| IVLNWEC01BNR85 | Bradi4g08060.1 | Os12t0405200-01 | Sb08g014610.2 | AK359711 |
| IVLNWEC02HWPTH | Bradi4g08060.1 | Os12t0405200-01 | Sb08g014610.2 | AK359711 |
| IVLNWEC02HLT9K | Bradi1g04930.1 | Os03t0804200-01 | Sb01g004610.1 | MLOC_56327.1 |
| IVLNWEC02GZXV7 | Bradi1g63050.1 | Os03t0338600-01 | Sb01g035980.1 | AK249109.1 |
| IV9RU5F01CDHYS | Bradi3g33510.1 | Os10t0563200-01 | Sb01g028950.1 | AK370762 |
| IV9RU5F02IVITV | Bradi4g32830.1 | Os09t0471800-01 | Sb02g028550.1 | AK252312.1 |
| IV9RU5F02F3CJB | Bradi1g03207.1 | Os03t0828300-01 | Sb01g002630.1 | MLOC_52484.1 |
| IUIVLUD02IUW7L | Bradi4g26227.1 | Os11t0130100-01 | Sb05g001810.1 | AK248724.1 |
| IUIVLUD02JGF9X | Bradi4g36160.2 | Os09t0533400-01 | Sb02g030960.1 | MLOC_71112.1 |
| IVLNWEC02JQMNU | Bradi4g36160.2 | Os09t0533400-01 | Sb02g030960.1 | MLOC_71112.1 |
| IV9RU5F01COBQT | Bradi3g49830.1 | Os02t0639000-01 | Sb04g027200.1 | AK362381 |
| IUIVLUD02FWMV1 | Bradi4g36380.1 | Os09t0536000-01 | Sb02g031100.1 | AK251535.1 |
| IUIVLUD02GKQMI | Bradi4g36380.1 | Os09t0536000-01 | Sb02g031100.1 | AK251535.1 |
| IVLNWEC01ARNET | Bradi4g33067.1 | Os09t0477900-01 | Sb02g027560.1 | AK374386 |
| IV9RU5F02H8F20 | Bradi4g29170.1 | Os09t0371700-01 | Sb02g023390.1 | MLOC_4202.1 |
| IV9RU5F02II14Y | Bradi4g29170.1 | Os09t0371700-01 | Sb02g023390.1 | MLOC_4202.1 |
| IVLNWEC02FZTJQ | Bradi1g04300.2 | Os03t0811100-01 | Sb01g003860.1 | AK355646 |
| IUIVLUD02HPN68 | Bradi1g69510.1 | Os06t0305200-01 | Sb01g042430.1 | AK361972 |
| IVLNWEC02G99M6 | Bradi4g08597.1 | Os09t0132900-01 | Sb08g021590.1 | AK376689 |
| IV9RU5F01B8Z1D | Bradi4g10250.1 | Os11t0672400-00 | Sb05g026290.1 | MLOC_4990.2 |
| IV9RU5F02JASK0 | Bradi4g04800.1 | Os12t0560600-01 | Sb02g037450.1 | AK375918 |
| IV9RU5F01CJRE0 | Bradi3g05847.1 | Os03t0403100-01 | Sb10g006995.1 | MLOC_61052.6 |
| IVLNWEC01BISDE | Bradi4g30190.1 | Os09t0413300-00 | Sb02g024600.1 | MLOC_71161.1 |
| IV9RU5F01CXBH0 | Bradi2g29750.2 | Os05t0345400-02 | Sb09g015940.1 | MLOC_5286.2 |
| IUIVLUD01BI775 | Bradi1g02810.1 | Os03t0832600-01 | Sb01g002480.1 | MLOC_61231.1 |
| IUIVLUD02FU687 | Bradi1g02810.1 | Os03t0832600-01 | Sb01g002480.1 | MLOC_61231.1 |
| IVLNWEC01EQLXM | Bradi1g02810.1 | Os03t0832600-01 | Sb01g002480.1 | MLOC_61231.1 |
| IV9RU5F01AMRCW | Bradi4g37040.1 | Os09t0547300-01 | Sb02g031860.1 | AK353981 |
| IV9RU5F02HQ8OX | Bradi3g53887.1 | Os02t0793300-01 | Sb04g035685.1 | AK354651 |
| IVLNWEC01BIULG | Bradi3g53887.1 | Os02t0793300-01 | Sb04g035685.1 | AK354651 |
| IVLNWEC01CK51S | Bradi3g53887.1 | Os02t0793300-01 | Sb04g035685.1 | AK354651 |
| IV9RU5F02FPSED | Bradi4g14080.1 | Os03t0597200-01 | Sb01g016560.1 | MLOC_81731.1 |
| IVLNWEC01C4BP2 | Bradi4g14080.1 | Os03t0597200-01 | Sb01g016560.1 | MLOC_81731.1 |
| IVLNWEC01DGX7K | Bradi4g14080.1 | Os03t0597200-01 | Sb01g016560.1 | MLOC_81731.1 |
| IV9RU5F02GIRAF | Bradi4g29840.1 | Os09t0401600-00 | Sb02g024280.1 | AK366468 |
| IVLNWEC02HUM9K | Bradi4g42880.1 | Os11t0148101-00 | Sb03g008600.1 | AK356045 |
| IUIVLUD01EUL93 | Bradi4g35150.1 | Os09t0515100-01 | Sb02g029830.1 | MLOC_16969.1 |
| IV9RU5F02JHG84 | Bradi4g35150.1 | Os09t0515100-01 | Sb02g029830.1 | MLOC_16969.1 |
| IVLNWEC02GKAAS | Bradi4g35150.1 | Os09t0515100-02 | Sb02g029830.1 | MLOC_16969.1 |

| | | | | |
|---|---|---|---|---|
| IVLNWEC02F5O9J | Bradi3g38680.1 | Os08t0484700-01 | Sb07g029070.1 | AK367324 |
| IUIVLUD02JPO7Q | Bradi4g28877.1 | Os09t0364000-01 | Sb02g023040.1 | MLOC_54108.2 |
| IV9RU5F01BVLMF | Bradi4g28877.1 | Os09t0364000-01 | Sb02g023040.1 | MLOC_54108.2 |
| IV9RU5F01CIN7G | Bradi4g30200.2 | Os09t0413600-01 | Sb02g024620.1 | MLOC_5225.1 |
| IV9RU5F01CSRZP | Bradi4g30200.2 | Os09t0413600-01 | Sb02g024620.1 | MLOC_5225.1 |
| IUIVLUD02I2Z23 | Bradi1g69780.1 | Os03t0216600-01 | Sb01g042750.1 | MLOC_36871.1 |
| IV9RU5F01EJXSE | Bradi4g04460.1 | Os12t0562300-01 | Sb08g018230.1 | MLOC_18312.1 |
| IVLNWEC01BM2H2 | Bradi1g08287.1 | Os03t0755000-01 | Sb01g007720.1 | AK372734 |
| IV9RU5F01A4G08 | Bradi1g04477.1 | Os07t0475800-01 | Sb02g033090.1 | AK250388.1 |
| IVLNWEC02GRUYF | Bradi4g28430.1 | Os09t0346700-01 | Sb02g022610.1 | AK369117 |
| IV9RU5F01DA9NH | Bradi4g43130.1 | Os11t0142500-00 | Sb05g002540.1 | MLOC_69804.2 |
| IVLNWEC01A9E12 | Bradi1g14830.1 | Os03t0607500-01 | Sb01g014860.1 | MLOC_76706.3 |
| IVLNWEC01BU4L4 | Bradi2g11107.1 | Os01t0286600-01 | Sb03g011670.1 | AK370908 |
| IVLNWEC01CDH0V | Bradi2g11107.1 | Os01t0286600-01 | Sb03g011670.1 | AK370908 |
| IUIVLUD01AJ1U3 | Bradi1g07190.1 | Os03t0773600-01 | Sb01g006700.1 | AK249476.1 |
| IV9RU5F02J2ACJ | Bradi1g07190.1 | Os03t0773600-01 | Sb01g006700.1 | AK249476.1 |
| IV9RU5F02JEOKU | Bradi4g38000.2 | Os09t0560900-01 | Sb02g032810.1 | AK358014 |
| IVLNWEC01DFT55 | Bradi1g09340.3 | Os03t0737800-01 | Sb01g008740.1 | AK367045 |
| IV9RU5F02H139W | Bradi1g77440.1 | Os03t0116500-01 | Sb01g049510.1 | AK367957 |
| IV9RU5F02JDFOM | Bradi4g24237.1 | Os11t0169100-01 | Sb09g030030.1 | MLOC_14088.3 |
| IV9RU5F02I3BAF | Bradi2g37030.1 | Os03t0667400-01 | Sb01g012310.1 | MLOC_54332.1 |
| IUIVLUD02G28AW | Bradi4g06650.1 | Os12t0508100-00 | Sb08g016300.1 | MLOC_69555.3 |
| IV9RU5F01CGX5J | Bradi1g27460.1 | Os07t0485100-01 | Sb02g033430.1 | MLOC_73067.1 |
| IV9RU5F01DICMG | Bradi1g27460.1 | Os07t0485100-01 | Sb02g033430.1 | MLOC_73067.1 |
| IV9RU5F02JSOPU | Bradi1g27460.1 | Os07t0485100-01 | Sb02g033430.1 | MLOC_73067.1 |
| IUIVLUD01A9EVE | Bradi1g03730.1 | Os03t0820900-01 | Sb01g003230.1 | MLOC_51706.2 |
| IV9RU5F02GWX6L | Bradi1g66850.3 | Os01t0103800-01 | Sb09g028270.1 | AK356612 |
| IV9RU5F02IZJRU | Bradi4g20557.1 | Os11t0265600-00 | Sb01g001720.1 | AK252846.1 |
| IVLNWEC02JX5J4 | Bradi3g22990.1 | Os10t0194300-01 | Sb01g044200.1 | AK365028 |
| IV9RU5F02IC6EH | Bradi1g12820.1 | Os03t0669200-01 | Sb01g012370.1 | AK251844.1 |
| IVLNWEC02JFTI9 | Bradi1g12820.1 | Os03t0669200-01 | Sb01g012370.1 | AK251844.1 |
| IV9RU5F01B7LVD | Bradi1g67800.1 | Os03t0254700-01 | Sb01g040800.1 | MLOC_66422.2 |
| IV9RU5F01BTWKJ | Bradi1g07140.1 | Os03t0774300-01 | Sb01g006630.1 | MLOC_39143.3 |
| IVLNWEC01AZDSF | Bradi5g25870.1 | Os02t0782200-01 | Sb04g035110.1 | MLOC_69382.6 |
| IV9RU5F02H04DN | Bradi1g08620.1 | Os03t0748700-01 | Sb01g008100.1 | AK372289 |
| IVLNWEC02HX3F0 | Bradi1g08620.1 | Os03t0748700-01 | Sb01g008100.1 | AK372289 |
| IV9RU5F01BZ86M | Bradi4g04650.1 | Os12t0564600-01 | Sb08g018400.1 | MLOC_1444.1 |
| IVLNWEC02GB36W | Bradi4g40990.1 | Os12t0182300-01 | Sb08g005060.1 | MLOC_60385.1 |
| IVLNWEC02JTAJ7 | Bradi4g40990.1 | Os12t0182300-01 | Sb08g005060.1 | MLOC_60385.1 |
| IUIVLUD02I6RNJ | Bradi1g01530.1 | Os02t0234800-01 | Sb01g001040.1 | AK368609 |
| IV9RU5F02HO68S | Bradi4g38350.1 | Os03t0428800-01 | Sb02g011390.1 | MLOC_69252.1 |
| IVLNWEC01BUDCS | Bradi4g38350.1 | Os03t0428800-01 | Sb02g011390.1 | MLOC_69252.1 |
| IVLNWEC01DJZYC | Bradi4g38350.1 | Os03t0428800-01 | Sb02g011390.1 | MLOC_69252.1 |
| IV9RU5F02FL8ZY | Bradi4g38350.1 | Os03t0428800-01 | Sb02g011390.2 | MLOC_69252.1 |
| IUIVLUD01BA96C | Bradi3g04270.1 | Os01t0507700-01 | Sb07g000380.1 | MLOC_13110.1 |
| IV9RU5F01E1SJ9 | Bradi3g04270.1 | Os01t0507700-01 | Sb07g000380.1 | MLOC_13110.1 |
| IV9RU5F02GCGZQ | Bradi4g13940.1 | Os11t0610900-01 | Sb05g024270.1 | AK369779 |

| | | | | |
|---|---|---|---|---|
| IVLNWEC01EUBXQ | Bradi4g13940.1 | Os11t0610900-01 | Sb05g024270.1 | AK369779 |
| IVLNWEC02H4J9U | Bradi4g37110.1 | Os09t0548400-01 | Sb02g031940.1 | MLOC_11897.1 |
| IUIVLUD01EY9OU | Bradi4g37110.1 | Os09t0548400-01 | Sb02g031940.1 | MLOC_11896.2 |
| IVLNWEC02IDP7S | Bradi3g44340.3 | Os04t0397100-01 | Sb06g014590.1 | MLOC_73713.1 |
| IVLNWEC02GK3R5 | Bradi1g01007.1 | Os03t0856200-03 | Sb01g000620.1 | MLOC_49689.2 |
| IV9RU5F01DZE9J | Bradi1g01007.1 | Os03t0856200-04 | Sb01g000620.1 | MLOC_49689.2 |
| IUIVLUD01DKA84 | Bradi1g66290.1 | Os03t0280000-01 | Sb01g039110.1 | MLOC_5907.1 |
| IV9RU5F01DJLMT | Bradi1g65010.1 | Os03t0298700-01 | Sb01g038070.1 | MLOC_58282.1 |
| IV9RU5F02HT94X | Bradi1g72380.1 | Os06t0314100-01 | Sb01g044940.1 | MLOC_69835.1 |
| IV9RU5F01EA8QH | Bradi4g02810.1 | Os10t0493800-01 | Sb08g020140.1 | AK357861 |
| IVLNWEC01EIQ5O | Bradi4g02810.1 | Os10t0493800-01 | Sb08g020140.1 | AK357861 |
| IVLNWEC02JIOXW | Bradi1g06270.1 | Os03t0789000-00 | Sb01g005750.1 | MLOC_6391.1 |
| IVLNWEC02HKZ6O | Bradi3g20530.1 | Os03t0644200-01 | Sb01g027350.1 | MLOC_63968.2 |
| IVLNWEC02J19KB | Bradi4g08060.1 | Os12t0405200-01 | Sb08g014610.2 | AK359711 |
| IV9RU5F01BI42P | Bradi1g70850.1 | Os03t0205400-00 | Sb01g043560.1 | MLOC_1607.2 |
| IVLNWEC01ERN7H | Bradi4g37380.1 | Os09t0553600-01 | Sb02g032270.1 | AK375490 |
| IVLNWEC02IXOTV | Bradi4g37380.1 | Os09t0553600-01 | Sb02g032270.1 | AK375490 |
| IUIVLUD02ICGJ0 | Bradi4g39210.1 | Os12t0271600-01 | Sb0011s005460.1 | MLOC_74657.2 |
| IVLNWEC02HXP7Q | Bradi2g36937.1 | Os07t0227700-01 | Sb02g005340.1 | AK372407 |
| IVLNWEC01DFFLP | Bradi4g29520.1 | Os09t0383400-01 | Sb02g023840.1 | MLOC_34448.1 |
| IUIVLUD01ESEIP | Bradi1g04500.1 | Os03t0809100-01 | Sb01g004100.1 | MLOC_65434.2 |
| IVLNWEC01D1IF5 | Bradi4g22690.1 | Os11t0209700-02 | Sb05g009420.1 | MLOC_36974.1 |
| IUIVLUD01EJGRK | Bradi1g70937.1 | Os03t0203100-01 | Sb01g043640.1 | AK373755 |
| IUIVLUD01CTMB8 | Bradi1g07100.1 | Os03t0775300-01 | Sb01g006590.1 | AK375493 |
| IV9RU5F01CI27N | Bradi1g07100.1 | Os03t0775300-01 | Sb01g006590.1 | AK375493 |
| IVLNWEC01EFIA4 | Bradi4g24237.1 | Os11t0169100-01 | Sb09g030030.1 | MLOC_14088.3 |
| IV9RU5F02H8BMV | Bradi4g44320.1 | Os11t0118500-00 | Sb08g002310.1 | MLOC_65070.2 |
| IV9RU5F02JMTL3 | Bradi1g69510.1 | Os06t0305200-01 | Sb01g042430.1 | AK361972 |
| IV9RU5F01EN5YB | Bradi3g29247.1 | Os10t0485000-01 | Sb01g018930.1 | AK367129 |
| IVLNWEC02IY6OF | Bradi3g29247.1 | Os10t0485000-01 | Sb01g018930.1 | AK367129 |
| IVLNWEC01ASW8P | Bradi1g06570.1 | Os03t0784500-01 | Sb01g006110.1 | MLOC_62889.1 |
| IV9RU5F02I7JRZ | Bradi3g08040.1 | Os02t0215000-01 | Sb04g007460.1 | AK251905.1 |
| IUIVLUD01DEKQW | Bradi4g38750.1 | Os09t0570500-01 | Sb02g012440.1 | AK375835 |
| IVLNWEC02G4M2C | Bradi4g39430.1 | Os02t0329300-01 | Sb04g014390.1 | MLOC_44349.1 |
| IVLNWEC02IT7QZ | Bradi4g35140.1 | Os09t0514900-01 | Sb02g029820.1 | MLOC_4408.1 |
| IVLNWEC01DR35L | Bradi3g57220.1 | Os02t0778200-01 | Sb04g034870.1 | AK371866 |
| IVLNWEC02HABR9 | Bradi1g08400.1 | Os03t0309200-01 | Sb01g007850.1 | MLOC_824.2 |
| IVLNWEC01ECFGC | Bradi2g28007.1 | Os05t0235300-01 | Sb09g017790.1 | MLOC_13770.2 |
| IVLNWEC01C9XCY | Bradi2g28007.1 | Os05t0235300-01 | Sb09g017790.1 | MLOC_14340.1 |
| IV9RU5F01ENOBQ | Bradi2g28007.1 | Os05t0235300-01 | Sb09g017790.1 | MLOC_77928.2 |
| IV9RU5F02F90OT | Bradi1g58030.1 | Os07t0137900-02 | Sb02g002600.1 | MLOC_57767.2 |
| IVLNWEC02IQDCP | Bradi4g42110.1 | Os12t0157200-01 | Sb08g003590.1 | MLOC_64847.1 |
| IV9RU5F01DHG98 | Bradi2g26910.1 | Os03t0853700-01 | Sb01g000820.1 | MLOC_57217.1 |
| IVLNWEC01DJIW0 | Bradi4g01790.1 | Os12t0613200-02 | Sb08g002530.1 | MLOC_10435.2 |
| IV9RU5F02GIGSF | Bradi4g32150.1 | Os09t0458000-00 | Sb02g026635.1 | MLOC_6152.2 |
| IVLNWEC01ENGGQ | Bradi4g32150.1 | Os09t0458000-00 | Sb02g026635.1 | MLOC_6152.2 |
| IV9RU5F01DW3XP | Bradi2g08527.1 | Os01t0243200-01 | Sb03g003850.1 | MLOC_54047.1 |

| | | | | |
|---|---|---|---|---|
| IV9RU5F01B1PL5 | Bradi2g36950.1 | Os07t0227800-00 | Sb02g006790.1 | MLOC_37418.3 |
| IUIVLUD02G8YBY | Bradi4g29570.1 | Os09t0386600-01 | Sb02g023980.1 | MLOC_11861.2 |
| IUIVLUD01CX078 | Bradi4g29570.1 | Os09t0386600-01 | Sb02g023980.1 | MLOC_16987.2 |
| IV9RU5F02GB0IH | Bradi4g29570.1 | Os09t0386600-01 | Sb02g023980.1 | MLOC_16987.2 |
| IV9RU5F01DPSVL | Bradi3g06727.1 | Os02t0192400-01 | Sb04g006240.1 | MLOC_6946.1 |
| IVLNWEC02F60VP | Bradi4g15150.1 | Os11t0585700-01 | Sb01g004650.1 | AK369305 |
| IVLNWEC02HUKX5 | Bradi1g01140.1 | Os03t0852900-01 | Sb01g000850.1 | MLOC_71240.1 |
| IV9RU5F01CLVIN | Bradi1g28820.1 | Os07t0446100-01 | Sb02g010610.1 | AK363868 |
| IUIVLUD01D705Q | Bradi4g23680.1 | Os11t0183800-00 | Sb05g005390.1 | MLOC_20977.1 |
| IV9RU5F01CU7AA | Bradi4g01430.1 | Os12t0617800-01 | Sb08g021840.1 | AK363884 |
| IVLNWEC02IZIZU | Bradi4g01430.1 | Os12t0617800-01 | Sb08g021840.1 | AK363884 |
| IVLNWEC02JERQC | Bradi4g01430.1 | Os12t0617800-01 | Sb08g021840.1 | AK363884 |
| IV9RU5F01AUOWO | Bradi1g14450.1 | Os11t0276000-01 | Sb01g014290.1 | AK364857 |
| IV9RU5F02IGRWK | Bradi1g67140.1 | Os03t0265700-01 | Sb01g040090.1 | AK362963 |
| IUIVLUD01ALHOW | Bradi4g35382.1 | Os09t0520200-00 | Sb02g030150.1 | MLOC_6428.2 |
| IV9RU5F02IREEW | Bradi4g35382.1 | Os09t0520200-00 | Sb02g030150.1 | MLOC_6428.2 |
| IUIVLUD01EWF9J | Bradi1g50380.1 | Os09t0123300-01 | Sb01g027530.1 | MLOC_64891.2 |
| IUIVLUD01BEWYV | Bradi1g11530.1 | Os03t0701800-01 | Sb01g010920.1 | MLOC_51052.3 |
| IUIVLUD01D3I7B | Bradi2g14440.1 | Os05t0592400-01 | Sb09g030580.1 | MLOC_13176.1 |
| IVLNWEC02GAFQE | Bradi1g59910.2 | Os03t0417900-01 | Sb01g032420.1 | AK361277 |
| IUIVLUD01AW38E | Bradi3g05950.1 | Os12t0541500-02 | Sb02g041940.1 | MLOC_58105.1 |
| IVLNWEC02FPEBL | Bradi1g08727.1 | Os03t0747600-02 | Sb01g008195.1 | AK374936 |
| IVLNWEC01EXBPR | Bradi1g72490.1 | Os03t0182400-01 | Sb01g045110.1 | AK248477.1 |
| IVLNWEC02F7I7Q | Bradi1g38380.1 | Os05t0289400-01 | Sb01g012080.1 | MLOC_70394.1 |
| IV9RU5F01D816D | Bradi1g03380.1 | Os03t0825700-01 | Sb01g002810.1 | MLOC_55007.1 |
| IV9RU5F02IPXOD | Bradi1g71810.2 | Os03t0191000-01 | Sb01g044480.1 | MLOC_63242.2 |
| IV9RU5F01B3AGV | Bradi2g04960.1 | Os03t0694900-01 | Sb07g004420.1 | MLOC_21642.1 |
| IVLNWEC01D2E2Q | Bradi1g48264.1 | Os06t0167000-02 | Sb10g004680.1 | MLOC_44147.1 |
| IUIVLUD02GXEKO | Bradi3g34450.2 | Os10t0575200-01 | Sb01g028220.1 | MLOC_36898.1 |
| IV9RU5F01ENZ5U | Bradi3g34450.2 | Os10t0575200-01 | Sb01g028220.1 | MLOC_36898.1 |
| IV9RU5F02G010O | Bradi3g34450.2 | Os10t0575200-01 | Sb01g028220.1 | MLOC_36898.1 |
| IV9RU5F02GESKT | Bradi4g04280.1 | Os12t0570600-00 | Sb08g018810.1 | MLOC_10781.1 |
| IV9RU5F02G88MT | Bradi4g05110.2 | Os12t0554400-01 | Sb08g017700.1 | AK252415.1 |
| IVLNWEC02GQ80C | Bradi4g05110.2 | Os12t0554400-01 | Sb08g017700.1 | AK252415.1 |
| IV9RU5F02HQH9A | Bradi2g22927.1 | Os05t0463900-01 | Sb09g022820.1 | MLOC_69831.1 |
| IVLNWEC02G8G2V | Bradi1g11520.2 | Os03t0713400-01 | Sb01g010210.1 | AK364772 |
| IVLNWEC02I93R3 | Bradi1g11520.2 | Os03t0713400-01 | Sb01g010210.1 | AK364772 |
| IUIVLUD02GD09V | Bradi1g11640.1 | Os03t0701000-02 | Sb01g011010.1 | MLOC_52027.1 |
| IVLNWEC02GK0S6 | Bradi1g11640.1 | Os07t0575100-00 | Sb02g037270.1 | MLOC_52027.1 |
| IVLNWEC01EJ33G | Bradi1g17710.1 | Os02t0167700-01 | Sb04g004540.1 | MLOC_67779.1 |
| IV9RU5F01D9L6Q | Bradi1g12050.1 | Os02t0523500-01 | Sb04g021280.1 | MLOC_36424.1 |
| IV9RU5F01B1XIC | Bradi1g08977.1 | Os03t0744300-01 | Sb01g008430.1 | MLOC_4611.1 |
| IVLNWEC01BG7GD | Bradi2g39900.1 | Os05t0103500-01 | Sb09g000380.1 | MLOC_57152.1 |
| IV9RU5F02IZRVW | Bradi4g43490.4 | Os11t0136800-02 | Sb05g002140.1 | AK251443.1 |
| IV9RU5F01DGSOK | Bradi2g39790.1 | Os05t0105000-01 | Sb09g000490.1 | AK366216 |
| IV9RU5F01E2KVQ | Bradi4g06030.1 | Os12t0529400-01 | Sb08g016760.1 | AK371413 |
| IV9RU5F02F9R3Y | Bradi1g36810.1 | Os02t0596100-01 | Sb01g003970.1 | AK367972 |

| | | | | |
|---|---|---|---|---|
| IVLNWEC01B7DZS | Bradi4g36250.1 | Os09t0534000-01 | Sb02g031000.1 | MLOC_52513.1 |
| IUIVLUD01ASY1O | Bradi3g51720.1 | Os02t0290900-00 | Sb04g011320.1 | MLOC_10139.2 |
| IV9RU5F02GOKTA | Bradi1g51890.1 | Os09t0407950-01 | Sb01g014880.1 | AK371801 |
| IVLNWEC01CJ9KP | Bradi4g40140.1 | Os12t0211400-00 | Sb08g006760.1 | MLOC_74333.3 |
| IV9RU5F01AMZ4Y | Bradi3g03317.1 | Os02t0137400-01 | Sb04g003000.1 | MLOC_78250.1 |
| IVLNWEC02GSZQW | Bradi4g36200.1 | Os09t0533100-01 | Sb02g030920.1 | AK251984.1 |
| IVLNWEC02JAKGD | Bradi1g09187.1 | Os03t0740800-01 | Sb01g008590.1 | AK372381 |
| IUIVLUD01AKV0L | Bradi4g33010.2 | Os09t0476000-01 | Sb02g027490.1 | MLOC_71276.1 |
| IUIVLUD01EU7P5 | Bradi4g33010.1 | Os09t0476000-01 | Sb02g027490.1 | MLOC_71276.1 |
| IV9RU5F01BTRZU | Bradi1g01330.1 | Os03t0852600-01 | Sb01g000870.1 | MLOC_39992.2 |
| IV9RU5F02HYB0Y | Bradi1g01330.1 | Os03t0852600-01 | Sb01g000870.1 | MLOC_39992.2 |
| IV9RU5F02F6NMZ | Bradi1g60540.1 | Os03t0395000-01 | Sb01g033330.1 | AK249626.1 |
| IV9RU5F02FQ8YM | Bradi1g60540.1 | Os03t0395000-01 | Sb01g033330.1 | AK249626.1 |
| IV9RU5F02JN6FE | Bradi1g27890.1 | Os09t0104300-01 | Sb01g032070.1 | MLOC_6913.1 |
| IUIVLUD01AF66C | Bradi4g05930.1 | Os12t0533700-01 | Sb03g033850.2 | AK353840 |
| IVLNWEC02GLFL7 | Bradi4g05930.1 | Os12t0533700-01 | Sb03g033850.2 | AK353840 |
| IUIVLUD02HPHYQ | Bradi3g57690.1 | Os02t0770700-01 | Sb04g007853.1 | MLOC_54603.1 |
| IV9RU5F01C1FJP | Bradi1g03660.1 | Os03t0821800-01 | Sb01g003140.1 | MLOC_76878.1 |
| IUIVLUD01BQU2O | Bradi4g29630.1 | Os09t0394100-01 | Sb02g024040.1 | MLOC_62375.5 |
| IUIVLUD02FV24Q | Bradi4g29630.1 | Os09t0394100-01 | Sb02g024040.1 | MLOC_62375.5 |
| IVLNWEC01BBPW7 | Bradi4g29630.1 | Os09t0394100-01 | Sb02g024040.1 | MLOC_62375.5 |
| IV9RU5F01DXNA6 | Bradi1g04520.1 | Os03t0809300-01 | Sb01g004030.1 | AK359885 |
| IV9RU5F02G2MTJ | Bradi1g04520.1 | Os03t0809300-01 | Sb01g004030.1 | AK359885 |
| IV9RU5F02H4DNK | Bradi1g04520.1 | Os03t0809300-01 | Sb01g004030.1 | AK359885 |
| IV9RU5F02I4HMW | Bradi4g43230.1 | Os11t0141000-01 | Sb05g002450.1 | AK248835.1 |
| IVLNWEC02J2Y2P | Bradi4g42836.1 | Os11t0148300-01 | Sb05g002890.1 | MLOC_20904.1 |
| IUIVLUD01CP7RT | Bradi4g38130.1 | Os09t0563700-01 | Sb02g011270.1 | MLOC_61306.1 |
| IV9RU5F02F98HG | Bradi1g13470.1 | Os03t0651300-01 | Sb01g013160.1 | MLOC_78591.5 |
| IUIVLUD01B7A65 | Bradi2g32930.1 | Os11t0525800-02 | Sb05g019630.1 | AK249200.1 |
| IV9RU5F02J1Z5R | Bradi4g30040.1 | Os09t0411650-01 | Sb02g024510.1 | AK358982 |
| IVLNWEC01EZYXY | Bradi4g30040.1 | Os09t0411650-01 | Sb02g024510.1 | AK358982 |
| IVLNWEC02JH84S | Bradi4g04560.1 | Os12t0563200-01 | Sb08g018320.1 | MLOC_68577.1 |
| IVLNWEC01D1J8V | Bradi4g45030.1 | Os12t0175500-01 | Sb05g000420.1 | AK252532.1 |
| IUIVLUD01BSSL5 | Bradi4g30210.1 | Os09t0413700-01 | Sb02g024630.1 | AK354786 |
| IUIVLUD01ENDWC | Bradi4g30210.1 | Os09t0413700-01 | Sb02g024630.1 | AK354786 |
| IVLNWEC02IR6I4 | Bradi4g30210.1 | Os09t0413700-01 | Sb02g024630.1 | AK354786 |
| IV9RU5F02I18K1 | Bradi4g30210.1 | Os09t0413700-02 | Sb02g024630.1 | AK354786 |
| IV9RU5F01DF2C2 | Bradi4g08120.1 | Os01t0507300-01 | Sb02g035910.1 | AK368037 |
| IV9RU5F01CY5UK | Bradi4g39210.1 | Os12t0271600-01 | Sb0011s005460.1 | MLOC_74657.2 |
| IVLNWEC01DBE60 | Bradi1g03910.1 | Os03t0818200-01 | Sb01g003480.1 | MLOC_3543.1 |
| IUIVLUD01EB7IR | Bradi3g56710.1 | Os02t0468200-01 | Sb02g031040.1 | MLOC_3981.2 |
| IV9RU5F02HV7XX | Bradi4g30600.1 | Os09t0423600-01 | Sb02g025070.1 | AK366952 |
| IVLNWEC01EW5QB | Bradi1g13470.1 | Os03t0651300-01 | Sb01g013160.1 | MLOC_78591.5 |
| IV9RU5F01D1VEO | Bradi4g30897.1 | Os09t0431600-00 | Sb02g025370.1 | MLOC_23179.3 |
| IUIVLUD01ASEDQ | Bradi4g27550.1 | Os08t0344600-01 | Sb02g020360.1 | AK367025 |
| IVLNWEC02H8SFC | Bradi1g67510.1 | Os03t0260100-01 | Sb01g040470.1 | MLOC_52306.1 |
| IV9RU5F02HZQ2N | Bradi4g32070.1 | Os09t0455400-01 | Sb02g026540.1 | AK357695 |

| | | | | |
|---|---|---|---|---|
| IUIVLUD02GJVJY | Bradi4g35540.2 | Os09t0521500-01 | Sb02g030280.1 | AK363819 |
| IVLNWEC02GJERH | Bradi4g35540.2 | Os09t0521500-01 | Sb02g030280.1 | AK363819 |
| IVLNWEC01D4UAT | Bradi1g10270.1 | Os03t0724100-01 | Sb01g009680.1 | MLOC_64122.1 |
| IVLNWEC02GU6HN | Bradi1g03170.1 | Os03t0827600-02 | Sb01g002680.1 | MLOC_71913.1 |
| IV9RU5F01DP8HW | Bradi4g25980.1 | Os01t0897000-01 | Sb08g002240.1 | MLOC_6120.2 |
| IVLNWEC02IQSXY | Bradi4g00497.1 | Os12t0638800-00 | Sb08g023080.1 | MLOC_61023.1 |
| IUIVLUD01BODEL | Bradi4g31960.1 | Os09t0453800-01 | Sb02g026430.1 | AK251848.1 |
| IVLNWEC02FP7GM | Bradi4g31960.1 | Os09t0453800-01 | Sb02g026430.1 | AK251848.1 |
| IV9RU5F02F2RZQ | Bradi3g08080.1 | Os02t0217500-01 | Sb04g007560.1 | AK369673 |
| IV9RU5F02J19E0 | Bradi1g71000.1 | Os03t0202300-01 | Sb01g043670.1 | MLOC_20830.1 |
| IVLNWEC01CZ5YO | Bradi1g71000.1 | Os03t0202300-01 | Sb01g043670.1 | MLOC_20830.1 |
| IUIVLUD02IBHFR | Bradi4g35257.1 | Os09t0516500-00 | Sb02g029890.1 | AK361767 |
| IV9RU5F01C606M | Bradi1g71160.1 | Os12t0406100-01 | Sb01g043810.1 | MLOC_10095.1 |
| IVLNWEC02JFV4K | Bradi4g23700.1 | Os11t0182400-01 | Sb05g005350.1 | AK362688 |
| IVLNWEC01CFJ1S | Bradi4g37197.1 | Os09t0550000-01 | Sb02g032020.1 | AK376014 |
| IUIVLUD01CYWGY | Bradi3g32960.1 | Os10t0552900-01 | Sb01g029600.1 | MLOC_71540.1 |
| IVLNWEC02GK10S | Bradi3g59726.1 | Os02t0740300-01 | Sb04g028420.1 | MLOC_4872.1 |
| IV9RU5F01A8IKE | Bradi3g07580.1 | Os02t0203401-00 | Sb04g007100.1 | MLOC_3451.1 |
| IV9RU5F01EONC2 | Bradi1g03570.1 | Os03t0822900-01 | Sb01g003040.1 | AK373346 |
| IV9RU5F02F0PGJ | Bradi4g08152.1 | Os12t0291000-00 | Sb08g011950.1 | MLOC_75102.1 |
| IV9RU5F02ILXI9 | Bradi4g08152.1 | Os12t0291000-00 | Sb08g011950.1 | MLOC_75102.1 |
| IUIVLUD01C7AL5 | Bradi3g53310.1 | Os02t0715200-01 | Sb04g029950.1 | AK362483 |
| IVLNWEC01BRA5B | Bradi4g01090.1 | Os12t0626300-01 | Sb03g010430.1 | MLOC_60893.1 |
| IV9RU5F02HWZI5 | Bradi2g25410.1 | Os05t0411200-01 | Sb09g020310.1 | AK357469 |
| IV9RU5F01A6XEN | Bradi3g53740.1 | Os02t0788800-02 | Sb04g035500.1 | MLOC_20163.2 |
| IVLNWEC01ASORV | Bradi4g01810.1 | Os03t0625900-01 | Sb01g013700.1 | AK372511 |
| IVLNWEC02IC3MC | Bradi4g01810.1 | Os03t0625900-01 | Sb01g013700.1 | AK372511 |
| IVLNWEC02I1NC7 | Bradi4g24280.1 | Os11t0169800-01 | Sb05g004500.1 | MLOC_11136.1 |
| IVLNWEC01E4DQ1 | Bradi1g64220.1 | Os03t0313300-01 | Sb01g037200.1 | MLOC_5906.1 |
| IV9RU5F01CLA4O | Bradi4g34650.1 | Os09t0506800-01 | Sb02g029270.1 | MLOC_12052.2 |
| IV9RU5F01AOGEQ | Bradi3g23020.1 | Os10t0198600-01 | Sb06g032780.1 | MLOC_66481.1 |
| IVLNWEC01A9S06 | Bradi3g23020.1 | Os10t0198600-01 | Sb06g032780.1 | MLOC_66481.1 |
| IUIVLUD02JGZI4 | Bradi4g24960.2 | Os11t0157400-01 | Sb05g003650.1 | AK365240 |
| IV9RU5F02ICAQB | Bradi1g52210.1 | Os02t0106100-01 | Sb04g000620.1 | MLOC_67531.6 |
| IUIVLUD02HLNGG | Bradi4g37910.1 | Os09t0560000-01 | Sb02g032725.1 | MLOC_53466.1 |
| IV9RU5F02IWLQ6 | Bradi1g67570.2 | Os03t0259300-01 | Sb01g040530.1 | MLOC_10898.2 |
| IVLNWEC01ATWDE | Bradi1g67570.2 | Os03t0259300-01 | Sb01g040530.1 | MLOC_10898.2 |
| IV9RU5F01DANWI | Bradi1g01007.1 | Os03t0856200-03 | Sb01g000620.1 | MLOC_49689.2 |
| IVLNWEC01DV3TC | Bradi1g01007.1 | Os03t0856200-04 | Sb01g000620.1 | MLOC_49689.2 |
| IVLNWEC02GXXCB | Bradi1g01007.1 | Os03t0856200-04 | Sb01g000620.1 | MLOC_49689.2 |
| IVLNWEC01C0V2L | Bradi2g19570.1 | Os05t0519400-01 | Sb01g014140.1 | AK365368 |
| IUIVLUD01A6DZ4 | Bradi4g04730.1 | Os12t0560700-01 | Sb08g018160.1 | MLOC_36301.1 |
| IUIVLUD02GTNEJ | Bradi4g04730.1 | Os12t0560700-01 | Sb08g018160.1 | MLOC_36301.1 |
| IVLNWEC01D24OH | Bradi1g02800.1 | Os03t0832900-01 | Sb01g002500.1 | AK370148 |
| IVLNWEC01CJ1NM | Bradi1g71190.1 | Os03t0200600-00 | Sb01g043840.1 | AK250731.1 |
| IVLNWEC01D52UG | Bradi1g71190.1 | Os03t0200600-00 | Sb01g043840.1 | AK250731.1 |
| IVLNWEC01DJ3BY | Bradi1g71190.1 | Os03t0200600-00 | Sb01g043840.1 | AK250731.1 |

| | | | | |
|---|---|---|---|---|
| IV9RU5F01CH606 | Bradi1g69520.1 | Os03t0223700-01 | Sb01g042440.1 | MLOC_10228.2 |
| IUIVLUD01C0IPN | Bradi1g74120.1 | Os03t0165000-00 | Sb01g046210.1 | MLOC_53883.5 |
| IV9RU5F01CFL5R | Bradi1g74120.1 | Os03t0165000-00 | Sb01g046210.1 | MLOC_53883.5 |
| IVLNWEC01AWT5C | Bradi1g64770.1 | Os11t0672400-00 | Sb01g037790.1 | MLOC_63204.1 |
| IUIVLUD02JNE9C | Bradi2g26600.1 | Os05t0391500-01 | Sb09g019350.1 | MLOC_58956.3 |
| IVLNWEC02GEZ4N | Bradi2g26600.1 | Os05t0391500-01 | Sb09g019350.1 | MLOC_58956.3 |
| IVLNWEC02I0693 | Bradi2g26600.1 | Os05t0391500-01 | Sb09g019350.1 | MLOC_58956.3 |
| IVLNWEC02IQ42V | Bradi2g26600.1 | Os05t0391500-01 | Sb09g019350.1 | MLOC_58956.3 |
| IUIVLUD02HI4ZK | Bradi1g12340.2 | Os03t0683866-00 | Sb01g011845.1 | MLOC_12803.1 |
| IUIVLUD01C2M2I | Bradi3g15520.1 | Os08t0178100-01 | Sb07g004940.1 | AK371182 |
| IUIVLUD02JFA2V | Bradi4g41777.1 | Os12t0165000-01 | Sb08g004050.2 | AK366349 |
| IUIVLUD02I2YJ3 | Bradi1g65290.1 | Os03t0295700-01 | Sb01g038270.1 | MLOC_52516.2 |
| IVLNWEC01CZ1YO | Bradi1g65290.1 | Os03t0295700-01 | Sb01g038270.1 | MLOC_52516.2 |
| IVLNWEC01DZ7ID | Bradi1g04980.1 | Os03t0803700-01 | Sb01g004640.1 | MLOC_37586.1 |
| IV9RU5F02ICK5Y | Bradi3g28787.1 | Os10t0477800-01 | Sb01g019310.1 | MLOC_64732.5 |
| IV9RU5F01BNOE5 | Bradi1g00887.1 | Os03t0858100-01 | Sb01g000490.1 | MLOC_81454.2 |
| IVLNWEC01CV4KT | Bradi1g00887.1 | Os03t0858100-01 | Sb01g000490.1 | MLOC_81454.2 |
| IVLNWEC02G225Z | Bradi1g07717.1 | Os03t0192900-01 | Sb08g014960.1 | MLOC_72276.1 |
| IVLNWEC02JMJMD | Bradi1g07717.1 | Os03t0192900-01 | Sb08g014960.1 | MLOC_72276.1 |
| IV9RU5F02F5OC2 | Bradi5g22700.1 | Os04t0628600-01 | Sb06g029400.1 | MLOC_65568.1 |
| IVLNWEC02H7TPJ | Bradi1g51890.1 | Os09t0407950-01 | Sb01g014880.1 | AK371801 |
| IVLNWEC02JOUGI | Bradi1g51890.1 | Os09t0407950-01 | Sb01g014880.1 | AK371801 |
| IVLNWEC01DG7SU | Bradi4g27157.1 | Os09t0281900-01 | Sb02g018910.1 | MLOC_63338.1 |
| IUIVLUD02JCC67 | Bradi1g10447.1 | Os03t0721200-01 | Sb01g009860.1 | MLOC_13554.5 |
| IVLNWEC01EH7OY | Bradi3g60720.1 | Os02t0831800-01 | Sb04g038440.1 | AK370298 |
| IUIVLUD01C1EY8 | Bradi3g25380.1 | Os10t0346200-01 | Sb0112s002010.1 | AK368868 |
| IUIVLUD02JF83S | Bradi4g24480.1 | Os11t0175500-01 | Sb05g004790.1 | MLOC_60365.2 |
| IVLNWEC02F672H | Bradi1g06960.1 | Os03t0777100-01 | Sb01g006400.1 | MLOC_4953.1 |
| IV9RU5F02FYEAX | Bradi4g28490.1 | Os09t0347800-01 | Sb02g022640.1 | MLOC_70141.1 |
| IVLNWEC02IBZLI | Bradi4g28490.1 | Os09t0347800-01 | Sb02g022640.1 | MLOC_70141.1 |
| IVLNWEC02JN7ZW | Bradi4g28490.1 | Os09t0347800-01 | Sb02g022640.1 | MLOC_70141.1 |
| IVLNWEC02H5J6Z | Bradi1g11960.1 | Os05t0220900-01 | Sb01g011410.1 | MLOC_63149.1 |
| IV9RU5F01CECFJ | Bradi1g08977.1 | Os03t0744300-01 | Sb01g008430.1 | MLOC_4611.1 |
| IV9RU5F01D0RWJ | Bradi1g02910.1 | Os03t0832500-01 | Sb01g002470.1 | AK360179 |
| IV9RU5F02IOU5Q | Bradi1g02910.1 | Os03t0832500-01 | Sb01g002470.1 | AK360179 |
| IVLNWEC01A2NHJ | Bradi4g35440.1 | Os09t0518700-01 | Sb02g030090.1 | MLOC_65109.6 |
| IVLNWEC02HK8G4 | Bradi2g49050.1 | Os09t0456100-01 | Sb02g026560.2 | MLOC_39928.2 |
| IVLNWEC01DK5AU | Bradi3g18970.1 | Os08t0245400-01 | Sb01g043690.1 | AK376028 |
| IV9RU5F01B7FXK | Bradi4g40560.1 | Os12t0194800-01 | Sb08g005650.1 | MLOC_67682.1 |
| IUIVLUD02H7SPW | Bradi1g11490.1 | Os03t0713000-00 | Sb01g010250.1 | MLOC_10389.1 |
| IUIVLUD02I67YP | Bradi1g70000.1 | Os03t0213900-01 | Sb01g042920.2 | AK360808 |
| IV9RU5F02HTSUS | Bradi1g70000.1 | Os03t0213900-02 | Sb01g042920.2 | AK360808 |
| IV9RU5F02IUJIG | Bradi3g41660.1 | Os08t0538800-01 | Sb07g025740.1 | AK364458 |
| IV9RU5F02IWI2U | Bradi1g69300.1 | Os03t0233500-01 | Sb01g042240.1 | AK248532.1 |
| IVLNWEC02HY5JB | Bradi1g74940.1 | Os03t0151700-01 | Sb01g046850.1 | MLOC_11400.1 |
| IVLNWEC02IEWXA | Bradi1g07390.1 | Os03t0769000-00 | Sb01g006930.1 | AK248308.1 |
| IV9RU5F01BK4C6 | Bradi1g00760.1 | Os03t0859500-01 | Sb01g000400.1 | MLOC_69405.3 |

| | | | | |
|---|---|---|---|---|
| IVLNWEC02IZNND | Bradi1g62330.1 | Os03t0352400-01 | Sb01g035280.1 | MLOC_54468.1 |
| IVLNWEC01DSK36 | Bradi1g55250.1 | Os07t0184900-01 | Sb02g000480.1 | MLOC_62977.1 |
| IVLNWEC01BY9CX | Bradi4g41970.2 | Os12t0160400-01 | Sb08g003770.1 | MLOC_63606.1 |
| IVLNWEC01AL3TG | Bradi1g67050.2 | Os03t0266800-01 | Sb01g040010.1 | MLOC_4483.1 |
| IV9RU5F01D7W15 | Bradi1g59910.2 | Os03t0417900-01 | Sb01g032420.1 | AK361277 |
| IUIVLUD01CYRK5 | Bradi3g44197.1 | Os02t0511901-00 | Sb04g020730.1 | MLOC_56305.2 |
| IVLNWEC02IJWE8 | Bradi3g44347.2 | Os02t0516600-01 | Sb04g021110.1 | AK369767 |
| IV9RU5F01CQGC5 | Bradi1g09530.1 | Os03t0735100-01 | Sb01g008930.1 | AK353866 |
| IVLNWEC02GKAEV | Bradi4g02100.1 | Os12t0609800-01 | Sb08g020980.1 | AK366127 |
| IVLNWEC02FR4WA | Bradi5g22790.1 | Os04t0630000-01 | Sb06g029510.1 | MLOC_52352.2 |
| IV9RU5F01DOITC | Bradi4g08080.1 | Os12t0407500-01 | Sb02g000980.1 | AK249633.1 |
| IV9RU5F02JUQE6 | Bradi4g08080.1 | Os12t0407500-01 | Sb02g000980.1 | AK249633.1 |
| IVLNWEC02FQJPK | Bradi4g40960.1 | Os12t0182700-01 | Sb03g038950.1 | AK370542 |
| IVLNWEC02JX5S9 | Bradi4g39550.1 | Os12t0238100-01 | Sb08g008650.1 | MLOC_82054.2 |
| IV9RU5F01EQZ2Z | Bradi4g23380.1 | Os11t0195100-00 | Sb05g005960.1 | AK368758 |
| IV9RU5F01CYHWA | Bradi2g62760.1 | Os01t0977600-02 | Sb05g002400.1 | MLOC_55536.1 |
| IUIVLUD01BUKCW | Bradi4g41970.2 | Os11t0158500-01 | Sb05g003740.1 | MLOC_60274.1 |
| IV9RU5F01AJKI6 | Bradi1g70850.1 | Os03t0205400-00 | Sb01g043560.1 | MLOC_1607.2 |
| IV9RU5F01BT97Q | Bradi1g06380.1 | Os03t0786900-01 | Sb01g005910.1 | MLOC_12376.1 |
| IV9RU5F01DD2CY | Bradi1g74450.1 | Os03t0160400-01 | Sb01g046430.1 | MLOC_18281.2 |
| IV9RU5F02INIJV | Bradi1g12140.1 | Os03t0689100-01 | Sb01g011620.1 | AK354857 |
| IVLNWEC02ICPYT | Bradi1g52530.1 | Os07t0413700-01 | Sb02g009895.1 | MLOC_33179.1 |
| IVLNWEC01CI2FS | Bradi4g08907.3 | Os09t0266400-01 | Sb02g015540.1 | MLOC_4863.2 |
| IV9RU5F02JXWNG | Bradi3g12240.1 | Os07t0476200-02 | Sb06g025910.1 | AK248246.1 |
| IUIVLUD01BRBPT | Bradi4g23600.2 | Os11t0186400-00 | Sb04g004200.1 | MLOC_64602.2 |
| IUIVLUD02JPZCL | Bradi2g34820.1 | Os05t0159000-01 | Sb09g004530.1 | AK248980.1 |
| IVLNWEC01ANWTL | Bradi2g34820.1 | Os05t0159000-01 | Sb09g004530.1 | AK248980.1 |
| IVLNWEC02GROF6 | Bradi2g34820.1 | Os05t0159000-01 | Sb09g004530.1 | AK248980.1 |
| IVLNWEC01CYKYR | Bradi3g53160.1 | Os02t0712000-01 | Sb04g030100.1 | AK248880.1 |
| IV9RU5F01CQ11Q | Bradi3g59870.1 | Os02t0736600-01 | Sb04g028590.1 | MLOC_10268.4 |
| IV9RU5F02JW1K2 | Bradi3g59870.1 | Os02t0736600-01 | Sb04g028590.1 | MLOC_10268.4 |
| IVLNWEC02HVHDK | Bradi3g29287.1 | Os10t0485600-00 | Sb01g018890.1 | MLOC_63339.1 |
| IVLNWEC02IISD1 | Bradi3g29287.1 | Os10t0485600-00 | Sb01g018890.1 | MLOC_63339.1 |
| IV9RU5F02GQG9Z | Bradi3g28760.1 | Os10t0477000-01 | Sb01g019330.1 | MLOC_71676.1 |
| IVLNWEC01C9Q6Y | Bradi3g16250.1 | Os08t0162100-01 | Sb07g004180.1 | AK360311 |
| IUIVLUD02JK8P2 | Bradi4g05110.2 | Os12t0554400-01 | Sb08g017700.1 | AK252415.1 |
| IV9RU5F02HI7ND | Bradi3g07490.1 | Os02t0202250-01 | Sb04g007000.1 | MLOC_63473.2 |
| IV9RU5F01BD62V | Bradi1g70057.1 | Os03t0213400-01 | Sb01g042980.1 | MLOC_5747.2 |
| IV9RU5F02IZW4B | Bradi1g70057.1 | Os03t0213400-01 | Sb01g042980.1 | MLOC_5747.2 |
| IV9RU5F02IVWRD | Bradi1g60890.1 | Os03t0387000-01 | Sb01g033760.1 | MLOC_57506.1 |
| IVLNWEC02H6DGG | Bradi1g11317.2 | Os03t0711200-01 | Sb01g010410.1 | AK360859 |
| IV9RU5F01DSDF8 | Bradi2g43110.1 | Os01t0607900-01 | Sb03g027400.1 | AK359073 |
| IVLNWEC02FXAUC | Bradi4g27820.1 | Os09t0309100-01 | Sb01g001310.1 | MLOC_26274.1 |
| IVLNWEC01BJ828 | Bradi4g24237.1 | Os11t0169100-01 | Sb09g030030.1 | MLOC_14088.3 |
| IVLNWEC02IXKD1 | Bradi1g01730.3 | Os03t0848600-01 | Sb01g001280.2 | MLOC_4983.1 |
| IV9RU5F01A7L6Q | Bradi3g54100.1 | Os02t0796400-00 | Sb04g035970.1 | AK370152 |
| IVLNWEC01BOI2K | Bradi3g31377.1 | Os10t0520700-01 | Sb01g031320.1 | MLOC_12086.1 |

| | | | | |
|---|---|---|---|---|
| IV9RU5F02FUDI4 | Bradi4g36976.1 | Os05t0479800-01 | Sb02g021250.1 | AK368548 |
| IVLNWEC02IFDKZ | Bradi1g10470.1 | Os03t0720800-01 | Sb01g009870.1 | MLOC_78895.1 |
| IVLNWEC01B5WDV | Bradi3g27970.1 | Os10t0457700-01 | Sb01g020140.1 | MLOC_10621.1 |
| IUIVLUD01DGKKU | Bradi4g38200.1 | Os03t0670200-01 | Sb02g011300.1 | MLOC_67950.1 |
| IV9RU5F01EM1QC | Bradi4g04030.1 | Os12t0566900-01 | Sb08g018540.1 | AK367062 |
| IVLNWEC01DV1YX | Bradi4g04030.1 | Os12t0566900-01 | Sb08g018540.1 | AK367062 |
| IVLNWEC01DIG3D | Bradi4g44660.1 | Os11t0110100-01 | Sb05g000870.1 | MLOC_38307.2 |
| IV9RU5F01D0OML | Bradi4g27660.1 | Os09t0299400-00 | Sb02g020740.1 | AK375335 |
| IV9RU5F01DLN3W | Bradi4g27660.1 | Os09t0299400-00 | Sb02g020740.1 | AK375335 |
| IV9RU5F02GCN8V | Bradi1g03260.1 | Os03t0826500-01 | Sb01g002760.1 | MLOC_10450.1 |
| IUIVLUD02J1E02 | Bradi5g02740.1 | Os01t0589900-00 | Sb09g020050.1 | MLOC_51122.1 |
| IV9RU5F01DXGHR | Bradi4g02910.1 | Os12t0594200-00 | Sb08g020025.1 | MLOC_36971.7 |
| IVLNWEC01CNGI5 | Bradi4g02910.1 | Os12t0594200-00 | Sb08g020025.1 | MLOC_36971.7 |
| IVLNWEC02IG3FR | Bradi1g05180.1 | Os03t0800000-02 | Sb01g004940.1 | AK355525 |
| IUIVLUD02JE0HK | Bradi3g24750.1 | Os10t0407000-01 | Sb01g022290.1 | MLOC_64460.1 |
| IUIVLUD02HG5OJ | Bradi1g53590.1 | Os07t0240200-01 | Sb02g007050.1 | MLOC_16913.1 |
| IV9RU5F02IY4VN | Bradi4g33540.1 | Os09t0484800-01 | Sb02g028180.1 | MLOC_65370.3 |
| IUIVLUD01AQMM5 | Bradi3g53770.1 | Os09t0504400-01 | Sb02g029140.1 | AK373501 |
| IV9RU5F01EX98S | Bradi3g53770.1 | Os09t0504400-01 | Sb02g029140.1 | AK373501 |
| IV9RU5F01ARB4L | Bradi1g09530.1 | Os03t0735100-01 | Sb01g008930.1 | AK353866 |
| IVLNWEC01E2T2U | Bradi4g23520.1 | Os11t0187100-00 | Sb05g005700.1 | MLOC_54710.1 |
| IV9RU5F01AHLH1 | Bradi1g04590.2 | Os07t0212300-01 | Sb01g003940.1 | MLOC_64862.1 |
| IV9RU5F02HUDVH | Bradi4g02910.1 | Os12t0594200-00 | Sb08g020025.1 | MLOC_36971.7 |
| IV9RU5F02HFJ67 | Bradi4g37147.1 | Os09t0549450-00 | Sb02g031970.1 | MLOC_9982.2 |
| IVLNWEC02JN2UG | Bradi5g26017.1 | Os04t0675101-00 | Sb06g032770.1 | MLOC_14650.1 |
| IUIVLUD01C54FE | Bradi1g03670.1 | Os03t0821700-01 | Sb01g003150.1 | MLOC_4490.1 |
| IVLNWEC02IMJTX | Bradi4g37917.1 | Os09t0560100-00 | Sb02g032730.1 | MLOC_71830.3 |
| IV9RU5F02H5GIC | Bradi4g05317.1 | Os12t0548100-01 | Sb08g017580.1 | MLOC_19166.3 |
| IV9RU5F01C119K | Bradi3g33980.1 | Os10t0572100-01 | Sb10g026610.1 | MLOC_2499.3 |
| IV9RU5F02HFSG8 | Bradi4g07790.1 | Os12t0456100-00 | Sb04g029550.1 | MLOC_65209.1 |
| IV9RU5F02JKODP | Bradi4g42160.1 | Os12t0156900-00 | Sb08g003540.1 | MLOC_56529.1 |
| IV9RU5F01CIF0I | Bradi1g68550.1 | Os03t0240400-01 | Sb01g041470.1 | MLOC_59553.1 |
| IUIVLUD01DFESB | Bradi1g14200.1 | Os03t0634400-01 | Sb01g014090.1 | MLOC_69381.1 |
| IUIVLUD01DXR01 | Bradi1g63330.1 | Os03t0333100-01 | Sb01g036270.1 | MLOC_60118.1 |
| IVLNWEC02H081V | Bradi2g39900.1 | Os05t0103500-01 | Sb09g000380.1 | MLOC_57152.1 |
| IUIVLUD01CXT05 | Bradi3g49830.1 | Os02t0639000-01 | Sb04g027200.1 | AK362381 |
| IVLNWEC01C93O4 | Bradi4g38130.1 | Os09t0563700-01 | Sb02g011270.1 | MLOC_61306.1 |
| IV9RU5F01CD2IS | Bradi1g02150.1 | Os03t0841900-00 | Sb01g001750.1 | AK357034 |
| IVLNWEC02H62ZN | Bradi1g02150.1 | Os03t0841900-00 | Sb01g001750.1 | AK357034 |
| IV9RU5F01B15W7 | Bradi4g38357.1 | Os09t0565450-00 | Sb02g011430.1 | MLOC_11872.2 |
| IV9RU5F01DR2RE | Bradi1g74430.1 | Os03t0161100-00 | Sb01g021680.1 | MLOC_17519.2 |
| IVLNWEC01BTIOA | Bradi1g72100.1 | Os03t0187000-01 | Sb01g044760.1 | MLOC_13505.1 |
| IV9RU5F02I8M5V | Bradi4g28877.1 | Os09t0364000-01 | Sb02g023040.1 | MLOC_54108.2 |
| IUIVLUD02GZ6YY | Bradi4g19975.1 | Os11t0311300-01 | Sb07g026060.1 | AK355627 |
| IV9RU5F01AOTFZ | Bradi4g19975.1 | Os11t0311300-01 | Sb07g026060.1 | AK355627 |
| IV9RU5F01CZ9YR | Bradi2g00627.1 | Os01t0106700-00 | Sb03g008680.1 | MLOC_9291.1 |
| IVLNWEC01BLXL1 | Bradi1g13470.1 | Os03t0651300-01 | Sb01g013160.1 | MLOC_78591.5 |

| | | | | |
|---|---|---|---|---|
| IUIVLUD01D2WSS | Bradi3g54430.1 | Os02t0803250-00 | Sb04g036270.1 | MLOC_53953.3 |
| IV9RU5F02FUC45 | Bradi3g39360.1 | Os08t0499100-01 | Sb07g028470.1 | AK375510 |
| IVLNWEC02G607I | Bradi4g35382.1 | Os09t0520200-00 | Sb02g030150.1 | MLOC_24486.1 |
| IVLNWEC02H1D9K | Bradi1g52170.2 | Os06t0104000-01 | Sb10g000445.1 | MLOC_53087.2 |
| IUIVLUD01D26KU | Bradi4g00640.1 | Os06t0498900-01 | Sb05g001920.1 | AK354479 |
| IUIVLUD01A2JZU | Bradi1g63160.1 | Os03t0336300-01 | Sb01g036110.1 | AK364634 |
| IV9RU5F02HD74I | Bradi3g44197.1 | Os02t0511900-00 | Sb04g020730.1 | MLOC_63002.1 |
| IV9RU5F01BE8CQ | Bradi1g06380.1 | Os03t0786900-01 | Sb01g005910.1 | MLOC_12376.1 |
| IVLNWEC01CLAAG | Bradi1g06380.1 | Os03t0786900-01 | Sb01g005910.1 | MLOC_12376.1 |
| IVLNWEC01B864Z | Bradi4g29310.3 | Os09t0376000-01 | Sb02g023560.1 | MLOC_67515.1 |
| IUIVLUD02FQYTU | Bradi1g33920.1 | Os04t0465700-01 | Sb06g019330.1 | AK354076 |
| IVLNWEC01DRK7N | Bradi3g08110.1 | Os10t0378400-01 | Sb05g000500.1 | MLOC_44285.1 |
| IV9RU5F01A88NQ | Bradi1g25780.1 | Os07t0531700-01 | Sb02g035270.1 | MLOC_54015.2 |
| IV9RU5F02G9SJ3 | Bradi1g17060.1 | Os12t0433500-01 | Sb03g045640.1 | MLOC_34499.1 |
| IVLNWEC01EZ5IP | Bradi1g17060.1 | Os12t0433500-01 | Sb03g045640.1 | MLOC_34499.1 |
| IUIVLUD02GLE5T | Bradi4g08907.3 | Os09t0266400-01 | Sb02g015540.1 | MLOC_4863.2 |
| IVLNWEC02IS7FT | Bradi4g08907.3 | Os09t0266400-01 | Sb02g015540.1 | MLOC_4863.2 |
| IVLNWEC01DNNL9 | Bradi1g08400.1 | Os03t0309200-01 | Sb01g007850.1 | MLOC_824.2 |
| IV9RU5F01BH95G | Bradi1g05630.1 | Os03t0795000-01 | Sb01g005330.1 | AK368582 |
| IV9RU5F01BN48A | Bradi1g70330.1 | Os03t0211600-01 | Sb01g043190.1 | MLOC_36338.1 |
| IVLNWEC02HZ2Z7 | Bradi1g01280.1 | Os03t0852000-01 | Sb01g000920.1 | MLOC_57150.1 |
| IV9RU5F02F3063 | Bradi4g07717.1 | Os06t0102800-01 | Sb10g000300.1 | MLOC_19401.1 |
| IV9RU5F02JGJAR | Bradi1g72380.1 | Os06t0314100-01 | Sb01g044940.1 | MLOC_69835.1 |
| IUIVLUD01A1J58 | Bradi3g56550.1 | Os04t0169100-01 | Sb02g035430.1 | MLOC_15819.2 |
| IV9RU5F01DQBFO | Bradi4g01080.1 | Os12t0163200-01 | Sb05g002340.1 | AK248684.1 |
| IUIVLUD02JTLGU | Bradi2g21470.1 | Os05t0491000-01 | Sb09g024030.1 | MLOC_36992.1 |
| IUIVLUD02FM9SE | Bradi5g20587.1 | Os09t0290900-01 | Sb04g012680.1 | MLOC_51418.2 |
| IUIVLUD02HTJ4Q | Bradi4g08080.1 | Os12t0407500-01 | Sb02g000980.1 | AK249633.1 |
| IVLNWEC02I43TH | Bradi2g24670.1 | Os05t0428100-00 | Sb09g021140.1 | AK363509 |
| IUIVLUD01EXQQ7 | Bradi2g25410.1 | Os05t0411200-01 | Sb09g020310.1 | AK357469 |
| IVLNWEC02IS5BN | Bradi2g61560.1 | Os01t0962600-01 | Sb03g046750.1 | MLOC_21004.1 |
| IVLNWEC02IW7ME | Bradi2g36937.1 | Os07t0227700-01 | Sb02g005340.1 | AK372407 |
| IUIVLUD01B2WJS | Bradi2g36930.1 | Os05t0277300-01 | Sb09g002890.1 | MLOC_73506.2 |
| IVLNWEC02HXF93 | Bradi1g77560.1 | Os03t0115000-01 | Sb01g049630.1 | MLOC_17803.1 |
| IV9RU5F02GXHWN | Bradi3g33810.3 | Os10t0567000-01 | Sb01g028580.1 | AK370998 |
| IVLNWEC01CY2H6 | Bradi1g48670.1 | Os06t0158900-00 | Sb07g027770.1 | MLOC_13688.1 |
| IV9RU5F01DSZBV | Bradi4g23380.1 | Os11t0195100-00 | Sb05g005960.1 | AK368758 |
| IV9RU5F02HRPGX | Bradi1g52210.1 | Os02t0106100-01 | Sb04g000620.1 | MLOC_67531.6 |
| IV9RU5F02JAPWZ | Bradi1g50010.1 | Os06t0131100-02 | Sb10g002050.1 | MLOC_58850.2 |
| IVLNWEC02HWW9A | Bradi1g12050.1 | Os02t0523500-01 | Sb04g021280.1 | MLOC_36424.1 |
| IV9RU5F02H53JC | Bradi2g51807.1 | Os01t0781200-01 | Sb03g036450.1 | MLOC_10643.2 |
| IV9RU5F02FVH6K | Bradi1g12750.1 | Os03t0672300-01 | Sb01g012240.1 | AK375629 |
| IV9RU5F01AITQE | Bradi1g14360.1 | Os03t0626600-01 | Sb01g014220.1 | MLOC_37534.8 |
| IVLNWEC01EEZB7 | Bradi1g62340.1 | Os03t0352200-01 | Sb01g035300.1 | AK374778 |
| IUIVLUD02GB56T | Bradi1g54960.1 | Os07t0188266-00 | Sb02g005170.1 | MLOC_12137.1 |
| IV9RU5F02F25MX | Bradi1g63320.1 | Os01t0769700-01 | Sb01g036260.1 | MLOC_34836.3 |
| IUIVLUD01CKMQI | Bradi1g64210.1 | Os03t0313600-01 | Sb06g023390.1 | MLOC_53997.2 |

| | | | | |
|---|---|---|---|---|
| IUIVLUD02F9FPG | Bradi1g14970.1 | Os11t0607100-01 | Sb01g014995.1 | MLOC_63226.1 |
| IVLNWEC01C7TQ0 | Bradi4g35140.1 | Os09t0514900-01 | Sb02g029820.1 | MLOC_4408.1 |
| IVLNWEC01EACTL | Bradi1g08847.1 | Os03t0746500-01 | Sb01g008310.1 | MLOC_66914.1 |
| IUIVLUD02G23FY | Bradi2g03070.1 | Os01t0514000-01 | Sb09g028470.1 | MLOC_68535.2 |
| IVLNWEC02GW33X | Bradi4g00367.1 | Os12t0640500-01 | Sb08g023210.1 | AK375316 |
| IV9RU5F01EONWS | Bradi4g04030.1 | Os12t0566800-01 | Sb08g018540.1 | AK367062 |
| IV9RU5F01EMULN | Bradi1g05610.1 | Os03t0795200-01 | Sb01g005310.1 | AK357244 |
| IV9RU5F02G433L | Bradi1g05390.1 | Os03t0798200-01 | Sb01g005090.1 | MLOC_62283.1 |
| IVLNWEC02H1CTM | Bradi4g30850.1 | Os09t0431200-01 | Sb02g025340.1 | AK365357 |
| IVLNWEC01BFE3R | Bradi5g10450.1 | Os05t0569500-01 | Sb04g031810.1 | MLOC_62259.1 |
| IVLNWEC02HDU6E | Bradi1g13787.1 | Os03t0645000-00 | Sb01g013550.1 | MLOC_53948.1 |
| IV9RU5F01AMNFL | Bradi4g29830.1 | Os09t0401300-01 | Sb02g024270.1 | AK250045.1 |
| IVLNWEC01DNXZX | Bradi1g03570.1 | Os03t0822900-01 | Sb01g003040.1 | AK373346 |
| IUIVLUD02JOUH8 | Bradi4g35376.1 | Os09t0520300-00 | Sb02g030160.1 | MLOC_11733.1 |
| IVLNWEC02H2M4S | Bradi4g31450.1 | Os09t0442400-01 | Sb02g025930.1 | AK249378.1 |
| IV9RU5F01D5X3G | Bradi4g27157.1 | Os09t0281900-01 | Sb02g018910.1 | MLOC_63338.1 |
| IV9RU5F01EQKCZ | Bradi2g26910.1 | Os03t0853700-01 | Sb01g000820.1 | MLOC_57217.1 |
| IV9RU5F02H5WX2 | Bradi2g26910.1 | Os03t0853700-01 | Sb01g000820.1 | MLOC_57217.1 |
| IV9RU5F02HY5XS | Bradi4g30500.1 | Os09t0421300-01 | Sb02g024910.1 | MLOC_54279.2 |
| IVLNWEC02HYQ5R | Bradi2g32290.1 | Os05t0212100-01 | Sb09g006640.1 | MLOC_36835.1 |
| IV9RU5F02GJRXL | Bradi1g07577.1 | Os03t0765100-01 | Sb01g007090.1 | MLOC_63880.1 |
| IV9RU5F02J2N1F | Bradi1g07577.1 | Os03t0765100-01 | Sb01g007090.1 | MLOC_63880.1 |
| IV9RU5F01E2LR5 | Bradi3g28570.1 | Os01t0511200-01 | Sb04g001080.1 | MLOC_75183.1 |
| IVLNWEC01ANGI2 | Bradi4g44720.1 | Os03t0849700-01 | Sb10g002910.1 | MLOC_15553.1 |
| IVLNWEC01BCZQT | Bradi1g05500.1 | Os03t0796600-01 | Sb01g005190.1 | MLOC_14311.2 |
| IV9RU5F02FQB9V | Bradi4g44680.1 | Os11t0109800-00 | Sb08g000870.1 | MLOC_15454.1 |
| IV9RU5F01DS9KS | Bradi4g30500.1 | Os09t0421300-01 | Sb02g024910.1 | MLOC_54279.2 |
| IV9RU5F01B4ZT6 | Bradi4g07717.1 | Os06t0102800-01 | Sb10g000300.1 | MLOC_19401.1 |
| IV9RU5F01C9YLC | Bradi4g07717.1 | Os06t0102800-01 | Sb10g000300.1 | MLOC_19401.1 |
| IVLNWEC01A2S6M | Bradi1g06810.1 | Os03t0780500-01 | Sb01g006320.1 | MLOC_10567.1 |
| IV9RU5F02I57FY | Bradi4g08527.1 | Os09t0121050-00 | Sb02g019360.1 | AK355795 |
| IVLNWEC01AKXXD | Bradi1g07380.1 | Os03t0769050-00 | Sb01g006920.1 | MLOC_44070.6 |
| IVLNWEC01ATZK5 | Bradi1g07380.1 | Os03t0769050-00 | Sb01g006920.1 | MLOC_44070.6 |
| IV9RU5F01D4W4G | Bradi4g31300.1 | Os09t0440200-01 | Sb02g025780.1 | AK362794 |
| IV9RU5F01ES176 | Bradi2g62760.1 | Os01t0977600-02 | Sb05g002400.1 | MLOC_55536.1 |
| IVLNWEC01EEVWD | Bradi2g62760.1 | Os01t0977600-02 | Sb05g002400.1 | MLOC_55536.1 |
| IVLNWEC01D83JA | Bradi4g31820.1 | Os09t0451000-01 | Sb02g026280.1 | AK359587 |
| IV9RU5F02JC8BB | Bradi4g35490.1 | Os09t0520700-01 | Sb02g030210.1 | MLOC_12901.2 |
| IVLNWEC01BTK6H | Bradi4g35490.1 | Os09t0520700-01 | Sb02g030210.1 | MLOC_12901.2 |
| IUIVLUD01EY8Q2 | Bradi4g36200.1 | Os09t0533100-01 | Sb02g030920.1 | AK251984.1 |
| IV9RU5F02HLRLL | Bradi1g02030.1 | Os03t0843400-01 | Sb01g001650.1 | MLOC_79276.1 |
| IV9RU5F02JXJ1U | Bradi1g08840.1 | Os03t0746400-01 | Sb01g008320.1 | MLOC_43312.1 |
| IUIVLUD01D7UIG | Bradi1g00760.1 | Os03t0859500-01 | Sb01g000400.1 | MLOC_69405.3 |
| IV9RU5F01DQHUR | Bradi4g27600.1 | Os09t0298700-01 | Sb02g020460.1 | AK359869 |
| IV9RU5F02GLYT0 | Bradi1g03030.1 | Os03t0831100-01 | Sb01g002280.1 | MLOC_63484.2 |
| IV9RU5F01CK09M | Bradi2g62740.1 | Os01t0977400-01 | Sb01g012973.1 | AK373944 |
| IVLNWEC01BGSIN | Bradi2g62740.1 | Os01t0977400-01 | Sb01g012973.1 | AK373944 |

| | | | | |
|---|---|---|---|---|
| IVLNWEC01ANAKF | Bradi4g31257.1 | Os09t0439500-01 | Sb02g025740.2 | MLOC_18354.1 |
| IV9RU5F02GCCA0 | Bradi4g30850.1 | Os09t0431200-01 | Sb02g025340.1 | MLOC_44275.1 |
| IVLNWEC02HHRSX | Bradi4g30850.1 | Os09t0431200-01 | Sb02g025340.1 | MLOC_44275.1 |
| IVLNWEC01DSI9V | Bradi4g37110.1 | Os09t0548400-01 | Sb02g031940.1 | MLOC_11896.2 |
| IVLNWEC02GC4PQ | Bradi4g37110.1 | Os09t0548400-01 | Sb02g031940.1 | MLOC_11896.2 |
| IV9RU5F02H09MC | Bradi4g35730.1 | Os09t0525700-01 | Sb02g030440.1 | MLOC_6781.1 |
| IV9RU5F02FLVN3 | Bradi4g34460.1 | Os09t0504800-01 | Sb03g003060.1 | MLOC_59.4 |
| IVLNWEC01DLLP4 | Bradi4g43870.1 | Os11t0129800-01 | Sb05g001800.1 | AK359497 |
| IUIVLUD02IAUBH | Bradi1g02230.1 | Os01t0959600-00 | Sb07g025050.1 | MLOC_75184.1 |
| IV9RU5F02JC3ZN | Bradi4g02117.1 | Os12t0289800-00 | Sb08g020970.1 | MLOC_52525.1 |
| IVLNWEC01D4K3S | Bradi4g01620.1 | Os12t0615600-00 | Sb08g021630.1 | MLOC_69487.3 |
| IV9RU5F02HXWJ0 | Bradi3g20530.1 | Os03t0644200-01 | Sb01g027350.1 | MLOC_63968.2 |
| IV9RU5F02IQ9R6 | Bradi3g20530.1 | Os03t0644200-01 | Sb01g027350.1 | MLOC_63968.2 |
| IV9RU5F01EKXF1 | Bradi4g39550.1 | Os12t0238100-01 | Sb08g008650.1 | MLOC_82054.2 |
| IV9RU5F02IC91K | Bradi4g06130.1 | Os12t0527900-01 | Sb08g016710.1 | MLOC_58573.1 |
| IVLNWEC01C9P6R | Bradi4g03590.1 | Os12t0580500-01 | Sb08g019340.1 | MLOC_23236.1 |
| IVLNWEC01CCFX9 | Bradi4g03590.1 | Os12t0580500-01 | Sb08g019340.1 | MLOC_23236.1 |
| IUIVLUD02FU94Y | Bradi3g10980.1 | Os02t0317800-01 | Sb07g009530.1 | MLOC_15210.1 |
| IVLNWEC02H45RO | Bradi4g16885.1 | Os11t0544000-01 | Sb05g020820.1 | AK362199 |
| IVLNWEC02IE1LN | Bradi4g19560.1 | Os11t0425300-01 | Sb01g003450.1 | MLOC_4066.1 |
| IV9RU5F02JWFEW | Bradi1g21280.1 | Os07t0621500-01 | Sb02g039580.1 | MLOC_62356.1 |
| IV9RU5F02JFWQ3 | Bradi4g29500.1 | Os09t0383300-01 | Sb02g023830.1 | MLOC_24477.2 |
| IVLNWEC01D68DK | Bradi1g07942.1 | Os03t0759600-00 | Sb01g007450.1 | MLOC_10633.1 |
| IVLNWEC02F01IU | Bradi1g07942.1 | Os03t0759600-00 | Sb01g007450.1 | MLOC_10633.1 |
| IUIVLUD01DWJCK | Bradi4g38270.1 | Os09t0565400-01 | Sb02g011350.1 | AK367105 |
| IV9RU5F01DFX7O | Bradi2g03070.1 | Os01t0514000-01 | Sb09g028470.1 | MLOC_68535.2 |
| IV9RU5F01DNQNE | Bradi2g03070.1 | Os01t0514000-01 | Sb09g028470.1 | MLOC_68535.2 |
| IVLNWEC01DGC0M | Bradi4g02910.1 | Os12t0594200-00 | Sb08g020025.1 | MLOC_36971.7 |
| IUIVLUD01B7V66 | Bradi4g16885.1 | Os11t0544000-01 | Sb05g020820.2 | AK362199 |
| IV9RU5F01DXW9Z | Bradi3g48510.1 | Os02t0611400-01 | Sb04g025740.1 | AK370682 |
| IUIVLUD01BOLY9 | Bradi3g53740.1 | Os02t0788800-02 | Sb04g035500.1 | MLOC_20163.2 |
| IVLNWEC02G81Z1 | Bradi3g59726.1 | Os02t0740300-01 | Sb04g028420.1 | MLOC_4872.1 |
| IUIVLUD02HK454 | Bradi1g48670.1 | Os06t0158900-00 | Sb07g027770.1 | MLOC_44081.2 |
| IVLNWEC01AGAUK | Bradi1g51230.1 | Os06t0144600-01 | Sb10g003060.1 | AK371533 |
| IUIVLUD01DDHE7 | Bradi3g41670.1 | Os08t0539100-01 | Sb07g025730.1 | MLOC_66731.2 |
| IVLNWEC01AJDN0 | Bradi1g49750.1 | Os04t0320200-01 | Sb10g001830.1 | MLOC_6485.2 |
| IVLNWEC02IA6XE | Bradi4g30730.1 | Os07t0124500-01 | Sb02g028310.1 | MLOC_58589.2 |
| IVLNWEC02HMKS2 | Bradi1g60740.1 | Os03t0389900-01 | Sb01g033580.1 | MLOC_57937.2 |
| IUIVLUD01BPIGN | Bradi4g37410.1 | Os09t0553900-01 | Sb02g032300.1 | MLOC_67933.1 |
| IV9RU5F01ELCHY | Bradi1g69690.1 | Os03t0219100-01 | Sb01g042670.1 | MLOC_18330.1 |
| IV9RU5F02HI4UH | Bradi1g04930.1 | Os03t0804200-01 | Sb01g004610.1 | MLOC_56327.1 |
| IVLNWEC02GHT2M | Bradi4g07290.1 | Os12t0485600-01 | Sb08g014900.1 | MLOC_6161.1 |
| IV9RU5F02JIBC6 | Bradi4g05317.1 | Os12t0548100-01 | Sb08g017580.1 | MLOC_19166.3 |
| IV9RU5F02G1YK3 | Bradi1g63330.1 | Os03t0333100-01 | Sb01g036270.1 | MLOC_60118.1 |
| IUIVLUD01AIR43 | Bradi1g56800.2 | Os07t0595800-01 | Sb02g038240.1 | MLOC_7288.1 |
| IVLNWEC02JIS38 | Bradi4g11510.3 | Os05t0188500-02 | Sb05g025690.1 | AK369248 |
| IVLNWEC02HWLGL | Bradi4g27334.1 | Os09t0286400-01 | Sb02g019500.2 | MLOC_4504.5 |

| | | | | |
|---|---|---|---|---|
| IVLNWEC02GPN4D | Bradi4g04800.1 | Os12t0560600-01 | Sb02g037450.1 | AK375918 |
| IV9RU5F02IZVQD | Bradi1g69360.1 | Os03t0225500-01 | Sb01g042300.1 | MLOC_69036.1 |
| IVLNWEC01CKYR2 | Bradi1g64580.1 | Os03t0305700-01 | Sb01g037550.1 | MLOC_54120.2 |
| IVLNWEC01BTFCB | Bradi4g14080.1 | Os03t0597200-01 | Sb01g016560.1 | MLOC_81731.1 |
| IV9RU5F02JJG8G | Bradi4g25490.1 | Os11t0153600-01 | Sb05g003290.1 | AK354532 |
| IVLNWEC01APFST | Bradi3g20070.1 | Os08t0300300-01 | Sb04g006250.1 | MLOC_6563.1 |
| IUIVLUD01AN9UC | Bradi1g04330.2 | Os03t0810600-01 | Sb01g003900.2 | MLOC_61507.1 |
| IVLNWEC02F5EDW | Bradi4g28430.1 | Os09t0346700-01 | Sb02g022610.1 | AK369117 |
| IV9RU5F02GLSJF | Bradi2g11107.1 | Os01t0286600-01 | Sb03g011670.1 | AK370908 |
| IV9RU5F01DZ2BD | Bradi1g67210.1 | Os03t0264800-00 | Sb01g040155.1 | AK374520 |
| IV9RU5F01DV2YW | Bradi1g67210.1 | Os03t0264800-00 | Sb01g040155.1 | AK374520 |
| IUIVLUD02F2TMK | Bradi1g09340.3 | Os03t0737800-01 | Sb01g008740.1 | AK367045 |
| IVLNWEC02JMA61 | Bradi1g01740.1 | Os03t0849600-01 | Sb01g001270.1 | AK370312 |
| IV9RU5F01BIRQ1 | Bradi3g54700.1 | Os02t0805900-01 | Sb10g028040.1 | MLOC_70182.1 |
| IVLNWEC01BXC5I | Bradi1g67740.1 | Os03t0255400-01 | Sb01g040730.1 | MLOC_51314.1 |
| IV9RU5F01DA7IZ | Bradi4g06370.1 | Os12t0514500-01 | Sb08g016560.1 | AK370395 |
| IV9RU5F02IOJ72 | Bradi4g32530.1 | Os08t0478800-01 | Sb02g027000.1 | MLOC_1497.2 |
| IV9RU5F01BJVQK | Bradi4g07790.1 | Os12t0456100-00 | Sb04g029550.1 | MLOC_65209.1 |
| IVLNWEC02ITMTV | Bradi3g48850.1 | Os02t0617500-01 | Sb04g026070.1 | MLOC_79740.2 |
| IVLNWEC01BGNDS | Bradi4g11330.1 | Os11t0655800-01 | Sb05g025890.1 | AK250343.1 |
| IVLNWEC02HOJ68 | Bradi1g02400.1 | Os03t0838900-01 | Sb01g001940.1 | AK368962 |
| IV9RU5F01DKVR5 | Bradi4g04650.1 | Os12t0564600-01 | Sb08g018400.1 | MLOC_12013.1 |
| IVLNWEC01C8ZK9 | Bradi1g65910.1 | Os03t0284100-01 | Sb01g038820.1 | MLOC_12732.2 |
| IV9RU5F02GGASU | Bradi4g40990.1 | Os12t0182300-01 | Sb08g005060.1 | MLOC_60385.1 |
| IVLNWEC02GG884 | Bradi4g40990.1 | Os12t0182300-01 | Sb08g005060.1 | MLOC_60385.1 |
| IV9RU5F01CW40N | Bradi4g38350.1 | Os03t0428800-01 | Sb02g011390.1 | MLOC_69252.1 |
| IVLNWEC01DKKRE | Bradi4g02810.1 | Os10t0493800-01 | Sb08g020140.1 | AK357861 |
| IUIVLUD02HPZWH | Bradi3g20530.1 | Os03t0644200-01 | Sb01g027350.1 | MLOC_63968.2 |
| IUIVLUD01DY0N6 | Bradi1g70937.1 | Os03t0203100-01 | Sb01g043640.1 | MLOC_4969.1 |
| IVLNWEC01ESCZV | Bradi4g15010.1 | Os11t0586300-01 | Sb05g022830.1 | MLOC_9792.1 |
| IVLNWEC01APQYZ | Bradi2g26170.1 | Os05t0393700-01 | Sb09g019480.1 | AK371091 |
| IV9RU5F02HYXU0 | Bradi4g35140.1 | Os09t0514900-01 | Sb02g029820.1 | MLOC_4408.1 |
| IV9RU5F02IKJJB | Bradi4g04470.1 | Os12t0562500-00 | Sb08g018240.1 | AK370509 |
| IV9RU5F02HAZ60 | Bradi2g19610.3 | Os06t0229300-01 | Sb03g034510.1 | AK368628 |
| IV9RU5F02GBI05 | Bradi3g57220.1 | Os02t0778200-01 | Sb04g034870.1 | AK371866 |
| IV9RU5F02H9244 | Bradi1g08400.1 | Os03t0752100-01 | Sb01g007850.1 | MLOC_824.2 |
| IVLNWEC02ICR02 | Bradi2g28007.1 | Os05t0235300-01 | Sb09g017790.1 | MLOC_13770.2 |
| IV9RU5F01BGEWX | Bradi4g30160.1 | Os09t0413100-01 | Sb02g024580.1 | MLOC_3999.2 |
| IVLNWEC02H1F2M | Bradi4g30160.1 | Os09t0413100-01 | Sb02g024580.1 | MLOC_3999.2 |
| IUIVLUD02HG5O6 | Bradi2g26910.1 | Os03t0853700-01 | Sb01g000820.1 | MLOC_57217.1 |
| IVLNWEC02JLQKV | Bradi2g26910.1 | Os03t0853700-01 | Sb01g000820.1 | MLOC_57217.1 |
| IVLNWEC02HXDJK | Bradi2g57720.1 | Os01t0513800-01 | Sb09g024910.1 | MLOC_67376.2 |
| IVLNWEC01DLXEJ | Bradi4g29570.1 | Os09t0386600-01 | Sb02g023980.1 | MLOC_11861.2 |
| IV9RU5F02GG4ZU | Bradi3g06727.1 | Os02t0192400-01 | Sb04g006240.1 | MLOC_6946.1 |
| IV9RU5F02HN1S6 | Bradi4g08510.1 | Os09t0134500-01 | Sb02g016800.1 | AK362848 |
| IV9RU5F02IWRP8 | Bradi4g08510.1 | Os09t0134500-01 | Sb02g016800.1 | AK362848 |
| IV9RU5F01CAETB | Bradi1g72086.1 | Os03t0187300-01 | Sb01g044740.1 | AK370665 |

| | | | | |
|---|---|---|---|---|
| IUIVLUD02FMLJO | Bradi4g05110.2 | Os12t0554400-01 | Sb08g017700.1 | AK252415.1 |
| IV9RU5F01BPN6O | Bradi4g05110.2 | Os12t0554400-01 | Sb08g017700.1 | AK252415.1 |
| IV9RU5F01DH22F | Bradi2g39790.1 | Os05t0105000-01 | Sb09g000490.1 | MLOC_55801.2 |
| IVLNWEC02HP09A | Bradi4g01930.1 | Os12t0612300-01 | Sb08g021320.1 | AK367104 |
| IVLNWEC01D8XK7 | Bradi1g02710.1 | Os03t0833900-02 | Sb02g007460.1 | AK367079 |
| IUIVLUD02HW1KO | Bradi4g08917.1 | Os09t0266600-01 | Sb02g014490.1 | MLOC_51364.3 |
| IV9RU5F01CM4T6 | Bradi4g07717.1 | Os06t0102800-01 | Sb10g000300.1 | MLOC_19401.1 |
| IVLNWEC02I6IAM | Bradi2g09177.1 | Os01t0256800-01 | Sb03g009880.1 | MLOC_12700.1 |
| IV9RU5F01AWC16 | Bradi2g39790.1 | Os05t0105000-01 | Sb09g000490.1 | MLOC_56508.1 |
| IV9RU5F01BJOGH | Bradi2g39790.1 | Os05t0105000-01 | Sb09g000490.1 | MLOC_56508.1 |
| IVLNWEC01ALAO7 | Bradi4g44720.1 | Os03t0849700-01 | Sb10g002910.1 | MLOC_15553.1 |
| IVLNWEC01CYQBP | Bradi3g05370.1 | Os02t0252600-01 | Sb08g000310.1 | MLOC_24495.4 |
| IVLNWEC01BFJ6J | Bradi4g33010.1 | Os09t0476000-01 | Sb02g027490.1 | MLOC_71276.1 |
| IV9RU5F02FUU9Z | Bradi1g04520.1 | Os03t0809300-01 | Sb01g004030.1 | AK359885 |
| IV9RU5F01DBZD5 | Bradi3g21590.1 | Os04t0218600-02 | Sb01g027630.1 | MLOC_64873.3 |
| IV9RU5F02HHVZZ | Bradi4g30600.1 | Os09t0423600-01 | Sb02g025070.1 | AK366952 |
| IV9RU5F02IHF58 | Bradi1g08060.1 | Os03t0758300-01 | Sb01g007550.1 | AK367927 |
| IVLNWEC02H5GJH | Bradi4g30897.1 | Os09t0431600-00 | Sb02g025370.1 | MLOC_23179.3 |
| IVLNWEC02HUKVE | Bradi4g35540.2 | Os09t0521500-01 | Sb02g030280.1 | AK363819 |
| IV9RU5F01CLZRN | Bradi5g08810.1 | Os04t0403400-01 | Sb06g015125.1 | MLOC_63780.6 |
| IVLNWEC02GQLWP | Bradi3g08080.1 | Os02t0217500-01 | Sb04g007560.1 | AK369673 |
| IV9RU5F02HQFH6 | Bradi1g77440.1 | Os03t0116500-01 | Sb01g049510.1 | MLOC_69599.1 |
| IV9RU5F01DL0D9 | Bradi3g54430.1 | Os02t0803250-00 | Sb04g036270.1 | MLOC_53953.3 |
| IVLNWEC02IFIZO | Bradi3g30810.3 | Os10t0508400-01 | Sb01g017550.3 | AK252323.1 |
| IVLNWEC01BMNHZ | Bradi3g32960.1 | Os10t0552900-01 | Sb01g029600.1 | MLOC_71540.1 |
| IVLNWEC01EON7B | Bradi3g08910.1 | Os02t0232400-01 | Sb04g008330.1 | MLOC_10622.1 |
| IV9RU5F01B88ME | Bradi4g01080.1 | Os12t0163200-01 | Sb05g002340.1 | AK248684.1 |
| IVLNWEC01BLAJ2 | Bradi2g25410.1 | Os05t0411200-01 | Sb09g020310.1 | AK357469 |
| IVLNWEC01BL7OW | Bradi1g08350.1 | Os03t0752700-01 | Sb01g007800.1 | MLOC_53865.1 |
| IV9RU5F02HOXKM | Bradi4g34650.1 | Os09t0506800-01 | Sb02g029270.1 | MLOC_12052.2 |
| IV9RU5F01EQJ1V | Bradi4g29410.1 | Os09t0380000-02 | Sb02g023690.1 | MLOC_13051.1 |
| IV9RU5F01DUC7N | Bradi4g24767.1 | Os11t0162000-01 | Sb05g003870.1 | MLOC_59247.2 |
| IV9RU5F01CAMMV | Bradi4g44750.1 | Os11t0109000-01 | Sb05g000750.1 | MLOC_75827.1 |
| IVLNWEC01A6VSW | Bradi1g02597.1 | Os03t0835600-01 | Sb01g002200.1 | AK355984 |
| IV9RU5F01A6L4F | Bradi2g19570.1 | Os05t0519400-01 | Sb01g014140.1 | AK365368 |
| IV9RU5F01CR5CW | Bradi1g71190.1 | Os03t0200600-00 | Sb01g043840.1 | AK250731.1 |
| IVLNWEC01EPMUM | Bradi1g69520.1 | Os03t0223700-01 | Sb01g042440.1 | MLOC_10228.2 |
| IVLNWEC01D3EIU | Bradi4g29617.1 | Os09t0393200-01 | Sb02g024010.1 | AK373293 |
| IUIVLUD01B8TO5 | Bradi4g07430.1 | Os12t0480200-01 | Sb08g014350.1 | MLOC_5095.1 |
| IUIVLUD02HZH8K | Bradi4g09997.1 | Os11t0677500-00 | Sb02g043630.1 | AK373109 |
| IV9RU5F01EOAE7 | Bradi1g65290.1 | Os03t0295700-01 | Sb01g038270.1 | MLOC_52516.2 |
| IV9RU5F01C67KQ | Bradi4g24170.1 | Os11t0176200-01 | Sb03g003740.1 | MLOC_11045.3 |
| IV9RU5F01BFRKA | Bradi1g04980.1 | Os03t0803700-01 | Sb01g004640.1 | MLOC_37586.1 |
| IV9RU5F01D9FDW | Bradi1g00887.1 | Os03t0858100-01 | Sb01g000490.1 | MLOC_81454.2 |
| IV9RU5F02HOC8L | Bradi1g00887.1 | Os03t0858100-01 | Sb01g000490.1 | MLOC_81454.2 |
| IV9RU5F02GMWOI | Bradi1g63160.1 | Os03t0336300-01 | Sb01g036110.1 | AK364634 |
| IUIVLUD02GZ9P7 | Bradi1g63160.1 | Os03t0336300-01 | Sb01g036110.1 | AK364634 |

| | | | | |
|---|---|---|---|---|
| IV9RU5F02GSMHQ | Bradi3g03317.1 | Os02t0137400-01 | Sb04g003000.1 | MLOC_78250.1 |
| IVLNWEC01A7HXA | Bradi2g52830.1 | Os12t0189900-01 | Sb03g037530.1 | MLOC_59223.1 |
| IUIVLUD01BTQKP | Bradi4g19650.1 | Os11t0414000-01 | Sb0014s003010.1 | AK357819 |
| IV9RU5F01BLLUJ | Bradi1g66850.3 | Os01t0103800-01 | Sb09g028270.1 | AK354358 |
| IVLNWEC02H6VMY | Bradi3g51720.1 | Os02t0290900-00 | Sb04g011320.1 | MLOC_10139.2 |
| IVLNWEC01BNPVR | Bradi2g36230.1 | Os05t0270200-01 | Sb04g005160.1 | MLOC_74926.1 |
| IV9RU5F02FURCE | Bradi4g05317.1 | Os12t0548100-01 | Sb08g017580.1 | MLOC_19166.3 |
| IUIVLUD01CEPSP | Bradi4g34290.1 | Os09t0500600-01 | Sb02g029000.1 | MLOC_17646.5 |
| IV9RU5F01B8XYK | Bradi4g34290.1 | Os09t0500600-01 | Sb02g029000.1 | MLOC_17646.5 |
| IVLNWEC01DIU4Z | Bradi4g31227.1 | Os09t0439000-00 | Sb02g025710.1 | MLOC_54452.3 |
| IUIVLUD01BIS9C | Bradi4g31227.1 | Os09t0439000-00 | Sb02g025710.1 | MLOC_54452.3 |
| IV9RU5F02IHC02 | Bradi1g15330.1 | Os03t0587200-01 | Sb01g015490.1 | AK369530 |
| IV9RU5F02GIIGA | Bradi1g67050.2 | Os03t0266800-01 | Sb01g040010.1 | MLOC_4483.1 |
| IUIVLUD01C956W | Bradi4g40960.1 | Os12t0182700-01 | Sb03g038950.1 | AK370542 |
| IV9RU5F01DAXBV | Bradi4g40960.1 | Os12t0182700-01 | Sb03g038950.1 | AK370542 |
| IV9RU5F01CD5BW | Bradi4g24237.1 | Os11t0169100-01 | Sb09g030030.1 | MLOC_14088.3 |
| IVLNWEC01DQ966 | Bradi1g08240.1 | Os03t0756200-01 | Sb01g007680.1 | MLOC_57253.1 |
| IVLNWEC02H2DGI | Bradi4g41970.2 | Os11t0158500-01 | Sb05g003740.1 | MLOC_60274.1 |
| IVLNWEC01CNWPA | Bradi1g06380.1 | Os03t0786900-01 | Sb01g005910.1 | MLOC_12376.1 |
| IV9RU5F02H0J3C | Bradi1g13710.1 | Os12t0617900-01 | Sb01g013460.1 | MLOC_37399.1 |
| IVLNWEC02G6YXF | Bradi1g13710.1 | Os12t0617900-01 | Sb01g013460.1 | MLOC_37399.1 |
| IV9RU5F01DWDAI | Bradi1g63500.1 | Os03t0330300-02 | Sb01g036410.1 | MLOC_10915.1 |
| IVLNWEC01EIMXM | Bradi3g59870.1 | Os02t0736600-01 | Sb04g028590.1 | MLOC_10268.4 |
| IV9RU5F02JB8IJ | Bradi4g08420.1 | Os09t0114500-01 | Sb02g013180.1 | MLOC_61378.1 |
| IV9RU5F01EI24P | Bradi1g60890.1 | Os03t0387000-01 | Sb01g033760.1 | MLOC_57506.1 |
| IVLNWEC01A2NGC | Bradi1g51757.1 | Os06t0113150-00 | Sb01g050480.1 | MLOC_54721.2 |
| IVLNWEC01A37GR | Bradi2g20250.1 | Os05t0511700-01 | Sb09g025160.1 | MLOC_22624.2 |
| IVLNWEC02G3P1O | Bradi1g08510.1 | Os03t0750100-00 | Sb01g007980.1 | MLOC_59424.2 |
| IUIVLUD01AHS1J | Bradi2g02790.1 | Os01t0595300-01 | Sb03g002010.1 | MLOC_22173.1 |
| IUIVLUD02F1UPT | Bradi4g03390.1 | Os12t0583300-01 | Sb08g019550.1 | AK369809 |
| IV9RU5F01C19AI | Bradi1g09250.1 | Os03t0738700-01 | Sb01g008680.1 | MLOC_17465.1 |
| IVLNWEC01A53XT | Bradi4g36580.1 | Os09t0540600-01 | Sb02g031330.1 | AK353826 |
| IVLNWEC01CB3XD | Bradi4g08917.1 | Os09t0266600-01 | Sb02g014490.1 | MLOC_51364.3 |
| IVLNWEC01BMG7E | Bradi4g43137.1 | Os12t0138800-01 | Sb05g002470.1 | AK373675 |
| IUIVLUD01EBUUJ | Bradi4g02910.1 | Os12t0594200-00 | Sb08g020025.1 | MLOC_36971.7 |
| IUIVLUD01EF5ZA | Bradi4g37147.1 | Os09t0549450-00 | Sb02g031970.1 | MLOC_9982.2 |
| IVLNWEC02FRK7O | Bradi2g37030.1 | Os03t0668400-01 | Sb01g012310.1 | MLOC_54332.1 |
| IUIVLUD02JBY7I | Bradi1g63050.1 | Os03t0338600-01 | Sb01g035980.1 | AK249109.1 |
| IVLNWEC01BA4VU | Bradi4g27370.1 | Os09t0287300-01 | Sb02g019520.1 | AK361521 |
| IVLNWEC02HGPFR | Bradi4g29630.1 | Os09t0394100-01 | Sb02g024040.1 | MLOC_62375.5 |
| IV9RU5F02JBPHL | Bradi1g02030.1 | Os03t0843400-01 | Sb01g001650.1 | MLOC_79276.1 |
| IV9RU5F01DJQPL | Bradi3g45620.1 | Os02t0552100-01 | Sb04g022510.1 | MLOC_6928.1 |
| IV9RU5F02HHF0V | - | Os12t0621300-02 | - | - |
| IV9RU5F01CCLJE | - | Os12t0617900-01 | - | - |
| IVLNWEC02GLXVE | - | Os12t0615000-00 | - | - |
| IV9RU5F01BMFW8 | - | Os12t0594300-00 | - | - |
| IVLNWEC01DBP27 | - | Os12t0577600-01 | - | - |

| | | | | |
|---|---|---|---|---|
| IVLNWEC01BPMYP | - | Os12t0549800-00 | - | - |
| IVLNWEC02HN4A4 | - | Os12t0549800-00 | - | - |
| IUIVLUD02HUQUI | - | Os12t0512800-01 | - | - |
| IVLNWEC02HIX4R | - | Os12t0512800-01 | - | - |
| IV9RU5F01EOK9T | - | Os12t0448900-01 | - | - |
| IVLNWEC01DMEUT | - | Os12t0448900-01 | - | - |
| IVLNWEC02GSQ8Z | - | Os12t0448900-01 | - | - |
| IVLNWEC02HK2ZI | - | Os12t0448900-01 | - | - |
| IVLNWEC02IXLQY | - | Os12t0448900-01 | - | - |
| IV9RU5F02JC1U5 | - | Os12t0431700-00 | - | - |
| IV9RU5F02F4POS | - | Os12t0431300-01 | - | - |
| IVLNWEC01DS3BG | - | Os12t0431300-01 | - | - |
| IVLNWEC02G4Q6C | - | Os12t0291000-00 | - | - |
| IVLNWEC01EAGOJ | - | Os12t0256800-00 | - | - |
| IVLNWEC02HDFWC | - | Os12t0256800-00 | - | - |
| IVLNWEC01C31SZ | - | Os12t0236100-00 | - | - |
| IV9RU5F02I0BJ6 | - | Os12t0209300-00 | - | - |
| IVLNWEC01C39J1 | - | Os11t0704100-00 | - | - |
| IV9RU5F01DU8Y9 | - | Os11t0682501-00 | - | - |
| IV9RU5F01EJ2TY | - | Os11t0682501-00 | - | - |
| IUIVLUD01CLXH7 | - | Os11t0681300-00 | - | - |
| IUIVLUD01DT9QF | - | Os11t0681300-00 | - | - |
| IVLNWEC01D4MN5 | - | Os11t0681300-00 | - | - |
| IVLNWEC02IEFO7 | - | Os11t0681300-00 | - | - |
| IV9RU5F01BFQ5J | - | Os11t0672400-00 | - | - |
| IUIVLUD02HKHBA | - | Os11t0657900-00 | - | - |
| IUIVLUD02FM3K4 | - | Os11t0609700-00 | - | - |
| IVLNWEC02H9FTV | - | Os11t0609700-00 | - | - |
| IV9RU5F01DNTXE | - | Os11t0555300-00 | - | - |
| IV9RU5F02HDVEP | - | Os11t0555300-00 | - | - |
| IVLNWEC02GAIW7 | - | Os11t0555300-00 | - | - |
| IVLNWEC02H5LXW | - | Os11t0555300-00 | - | - |
| IV9RU5F02G67J6 | - | Os11t0479100-00 | - | - |
| IV9RU5F02JH7KL | - | Os11t0479100-00 | - | - |
| IV9RU5F02JOY5W | - | Os11t0479100-00 | - | - |
| IVLNWEC02H8L99 | - | Os11t0438400-01 | - | - |
| IV9RU5F02IY9UN | - | Os11t0224800-00 | - | - |
| IVLNWEC02GD55Z | - | Os11t0224800-00 | - | - |
| IVLNWEC02GSSAP | - | Os11t0224800-00 | - | - |
| IUIVLUD01CBVZA | - | Os11t0213700-00 | - | - |
| IV9RU5F01ELWAP | - | Os11t0213700-00 | - | - |
| IV9RU5F01DL6NK | - | Os11t0211300-01 | - | - |
| IV9RU5F01CL5OX | - | Os11t0210300-01 | - | - |
| IUIVLUD01CNEHR | - | Os11t0171500-01 | - | - |
| IVLNWEC02F7F0O | - | Os11t0171500-01 | - | - |
| IV9RU5F02JMC4X | - | Os11t0148700-01 | - | - |
| IV9RU5F02GBMXF | - | Os11t0135300-00 | - | - |

| | | | | |
|---|---|---|---|---|
| IVLNWEC01E1O2S | - | Os11t0107400-01 | - | - |
| IVLNWEC02HW0T2 | - | Os10t0482700-00 | - | - |
| IV9RU5F02I3YL2 | - | Os10t0388600-00 | - | - |
| IUIVLUD01B7MQB | - | Os10t0381800-02 | - | - |
| IV9RU5F01A2ZV4 | - | Os10t0381800-02 | - | - |
| IVLNWEC02GMQRV | - | Os10t0346300-01 | - | - |
| IUIVLUD01BICRM | - | Os10t0339600-01 | - | - |
| IUIVLUD01AE8T3 | - | Os10t0147250-00 | - | - |
| IV9RU5F01CG89A | - | Os10t0147250-00 | - | - |
| IV9RU5F02H1342 | - | Os10t0147250-00 | - | - |
| IVLNWEC01DJ42U | - | Os10t0147250-00 | - | - |
| IV9RU5F01EOH10 | - | Os10t0134500-00 | - | - |
| IVLNWEC01B4BUH | - | Os10t0134500-00 | - | - |
| IVLNWEC02IZ7V9 | - | Os10t0134033-00 | - | - |
| IUIVLUD01D90PR | - | Os09t0569400-01 | - | - |
| IUIVLUD02IR816 | - | Os09t0554300-01 | - | - |
| IV9RU5F01AEZY7 | - | Os09t0554300-01 | - | - |
| IV9RU5F01BZXQF | - | Os09t0554300-01 | - | - |
| IVLNWEC01EWSA0 | - | Os09t0553900-01 | - | - |
| IUIVLUD01EXW7L | - | Os09t0551400-00 | - | - |
| IVLNWEC01ARGG3 | - | Os09t0551400-00 | - | - |
| IV9RU5F01D05YF | - | Os09t0546100-01 | - | - |
| IVLNWEC01EVPEQ | - | Os09t0545500-00 | - | - |
| IUIVLUD02FJ9TP | - | Os09t0545250-00 | - | - |
| IUIVLUD02HPPDG | - | Os09t0545250-00 | - | - |
| IVLNWEC02INSIF | - | Os09t0545250-00 | - | - |
| IUIVLUD01B6RQH | - | Os09t0479100-01 | - | - |
| IVLNWEC01ER803 | - | Os09t0479000-01 | - | - |
| IVLNWEC01D4L6Y | - | Os09t0469900-01 | - | - |
| IUIVLUD02I3TKM | - | Os09t0453500-01 | - | - |
| IVLNWEC01ARYPS | - | Os09t0453500-01 | - | - |
| IVLNWEC02I1L4M | - | Os09t0453500-01 | - | - |
| IV9RU5F01CII3B | - | Os09t0442400-01 | - | - |
| IV9RU5F01DVTOL | - | Os09t0442400-01 | - | - |
| IV9RU5F02G419X | - | Os09t0436400-01 | - | - |
| IVLNWEC01DX3LS | - | Os09t0436300-01 | - | - |
| IV9RU5F02GK7H9 | - | Os09t0428300-00 | - | - |
| IVLNWEC01ENP58 | - | Os09t0384900-01 | - | - |
| IVLNWEC02JJYKS | - | Os09t0384900-01 | - | - |
| IVLNWEC01AY12P | - | Os09t0376700-00 | - | - |
| IUIVLUD01AKY1Y | - | Os09t0362500-01 | - | - |
| IVLNWEC01EPKW3 | - | Os09t0362500-01 | - | - |
| IUIVLUD01A3MCA | - | Os09t0322500-01 | - | - |
| IUIVLUD02GE7GR | - | Os09t0322500-01 | - | - |
| IVLNWEC01DPL3O | - | Os09t0263474-00 | - | - |
| IVLNWEC01EGXTQ | - | Os09t0263474-00 | - | - |
| IVLNWEC01B3HJ6 | - | Os08t0564800-01 | - | - |

| | | | | |
|---|---|---|---|---|
| IV9RU5F01A2U63 | - | Os08t0543050-00 | - | - |
| IUIVLUD01AHERB | - | Os08t0542200-01 | - | - |
| IUIVLUD01C6HO4 | - | Os08t0539400-01 | - | - |
| IV9RU5F02GDC97 | - | Os08t0539400-01 | - | - |
| IV9RU5F02GH3SK | - | Os08t0539400-01 | - | - |
| IVLNWEC01A20VF | - | Os08t0531000-02 | - | - |
| IVLNWEC02GLBP3 | - | Os08t0480200-01 | - | - |
| IV9RU5F01C124U | - | Os08t0409900-01 | - | - |
| IVLNWEC02GYUR4 | - | Os08t0409900-01 | - | - |
| IV9RU5F02FT51U | - | Os08t0398300-01 | - | - |
| IUIVLUD02H0CU1 | - | Os08t0246300-01 | - | - |
| IV9RU5F02INM3L | - | Os08t0246300-01 | - | - |
| IVLNWEC01EVBEV | - | Os08t0246300-01 | - | - |
| IVLNWEC02ITVZW | - | Os08t0246300-01 | - | - |
| IV9RU5F01BPQP6 | - | Os08t0202400-01 | - | - |
| IV9RU5F02FKX06 | - | Os08t0158500-01 | - | - |
| IV9RU5F01DT4CY | - | Os08t0106900-00 | - | - |
| IUIVLUD01B8V8T | - | Os07t0695100-02 | - | - |
| IV9RU5F01DWAXT | - | Os07t0695100-02 | - | - |
| IV9RU5F02G4KR5 | - | Os07t0685600-01 | - | - |
| IVLNWEC01DEKN0 | - | Os07t0685600-01 | - | - |
| IV9RU5F01C7RAT | - | Os07t0645900-00 | - | - |
| IV9RU5F01ES0SR | - | Os07t0645900-00 | - | - |
| IVLNWEC01EI98U | - | Os07t0645900-00 | - | - |
| IV9RU5F01BQ5OJ | - | Os07t0554100-00 | - | - |
| IVLNWEC01C1JYP | - | Os07t0554100-00 | - | - |
| IUIVLUD01AJ2VJ | - | Os07t0422700-00 | - | - |
| IUIVLUD02I8PMC | - | Os07t0422700-00 | - | - |
| IV9RU5F02G1L4B | - | Os07t0422700-00 | - | - |
| IUIVLUD02HP6C3 | - | Os07t0421600-00 | - | - |
| IUIVLUD02I20FO | - | Os07t0277500-00 | - | - |
| IV9RU5F01BVOLG | - | Os07t0277500-00 | - | - |
| IV9RU5F02GAN7Z | - | Os07t0277500-00 | - | - |
| IVLNWEC01AM3SM | - | Os07t0277500-00 | - | - |
| IV9RU5F01CB4W3 | - | Os07t0270800-00 | - | - |
| IVLNWEC01CIFB2 | - | Os07t0270800-00 | - | - |
| IVLNWEC02IE0X4 | - | Os07t0141500-01 | - | - |
| IV9RU5F02I3MDM | - | Os06t0670633-00 | - | - |
| IVLNWEC01BNFFP | - | Os06t0670633-00 | - | - |
| IVLNWEC02I95V1 | - | Os06t0670633-00 | - | - |
| IUIVLUD02J1R2K | - | Os06t0623700-00 | - | - |
| IV9RU5F01EF5KD | - | Os06t0623700-00 | - | - |
| IV9RU5F02GBZCZ | - | Os06t0623700-00 | - | - |
| IUIVLUD02JN2SO | - | Os06t0556300-01 | - | - |
| IVLNWEC01B0KWV | - | Os06t0556300-01 | - | - |
| IV9RU5F01DSZ5R | - | Os06t0484800-00 | - | - |
| IV9RU5F01EGTJQ | - | Os06t0484800-00 | - | - |

| | | | | |
|---|---|---|---|---|
| IV9RU5F02GRXQE | - | Os06t0484800-00 | - | - |
| IV9RU5F02H3ESP | - | Os06t0484800-00 | - | - |
| IVLNWEC01CAWZ7 | - | Os06t0484800-00 | - | - |
| IVLNWEC01EXNMD | - | Os06t0484800-00 | - | - |
| IVLNWEC02FYENW | - | Os06t0484800-00 | - | - |
| IVLNWEC02IYO6H | - | Os06t0484800-00 | - | - |
| IVLNWEC02JBO4J | - | Os06t0484800-00 | - | - |
| IV9RU5F02GDPH0 | - | Os06t0286500-01 | - | - |
| IVLNWEC01A3KS1 | - | Os06t0286500-01 | - | - |
| IVLNWEC02ITAVD | - | Os06t0286500-01 | - | - |
| IVLNWEC01C5QLE | - | Os06t0279900-00 | - | - |
| IUIVLUD01C2K1B | - | Os05t0535500-01 | - | - |
| IUIVLUD02GIRFX | - | Os05t0535500-01 | - | - |
| IVLNWEC02HEUIR | - | Os05t0524500-01 | - | - |
| IVLNWEC02JAO2N | - | Os05t0524500-01 | - | - |
| IVLNWEC02JYIOH | - | Os05t0524500-01 | - | - |
| IVLNWEC01EOWWY | - | Os05t0523100-01 | - | - |
| IVLNWEC01CQZB4 | - | Os05t0508500-01 | - | - |
| IUIVLUD01EGYG5 | - | Os05t0357100-01 | - | - |
| IUIVLUD01BEGH5 | - | Os05t0352800-01 | - | - |
| IUIVLUD02HAEOE | - | Os05t0250700-00 | - | - |
| IVLNWEC01DZBGP | - | Os05t0250700-00 | - | - |
| IVLNWEC02FYZDR | - | Os05t0250700-00 | - | - |
| IV9RU5F01CLNGI | - | Os05t0223100-00 | - | - |
| IV9RU5F02I99TE | - | Os05t0223100-00 | - | - |
| IV9RU5F02F73K6 | - | Os05t0144100-01 | - | - |
| IV9RU5F01DM8NH | - | Os05t0128400-01 | - | - |
| IUIVLUD02IELSA | - | Os05t0102200-01 | - | - |
| IV9RU5F01CIUB6 | - | Os05t0102200-01 | - | - |
| IUIVLUD02H8LZ5 | - | Os04t0626500-01 | - | - |
| IV9RU5F01DTAZS | - | Os04t0614650-00 | - | - |
| IVLNWEC02GY01P | - | Os04t0614650-00 | - | - |
| IVLNWEC02JI68M | - | Os04t0540300-00 | - | - |
| IUIVLUD01EDFN7 | - | Os04t0495300-01 | - | - |
| IUIVLUD02G0YNT | - | Os04t0488400-01 | - | - |
| IVLNWEC01B9O4X | - | Os04t0378575-00 | - | - |
| IV9RU5F01CLFDM | - | Os04t0373400-01 | - | - |
| IV9RU5F01DJ6YI | - | Os04t0373400-01 | - | - |
| IVLNWEC01D43AV | - | Os04t0373400-01 | - | - |
| IV9RU5F02IU9KY | - | Os04t0369066-00 | - | - |
| IVLNWEC01C0TME | - | Os04t0364800-00 | - | - |
| IVLNWEC01AZSWY | - | Os04t0357300-01 | - | - |
| IVLNWEC01DZHIN | - | Os04t0357300-01 | - | - |
| IV9RU5F01CCIO5 | - | Os04t0356600-01 | - | - |
| IVLNWEC02FW7DV | - | Os04t0354200-00 | - | - |
| IUIVLUD02GZ202 | - | Os04t0351333-00 | - | - |
| IV9RU5F01BD62Z | - | Os04t0350000-01 | - | - |

| | | | | |
|---|---|---|---|---|
| IVLNWEC01EVEFL | - | Os04t0219600-01 | - | - |
| IUIVLUD02J1W7J | - | Os03t0850200-00 | - | - |
| IVLNWEC01CY3LO | - | Os03t0850200-00 | - | - |
| IUIVLUD02FTEN7 | - | Os03t0848700-01 | - | - |
| IVLNWEC01B612H | - | Os03t0848700-01 | - | - |
| IV9RU5F01EL44A | - | Os03t0804400-01 | - | - |
| IVLNWEC02HGUIV | - | Os03t0777700-01 | - | - |
| IV9RU5F02JSLLM | - | Os03t0777600-01 | - | - |
| IV9RU5F01DPTH2 | - | Os03t0750100-00 | - | - |
| IUIVLUD01ARQIS | - | Os03t0747300-01 | - | - |
| IVLNWEC02JAZ2L | - | Os03t0747300-01 | - | - |
| IVLNWEC01ECRKY | - | Os03t0730400-01 | - | - |
| IVLNWEC02G2SPK | - | Os03t0710100-01 | - | - |
| IVLNWEC01A35B3 | - | Os03t0699700-01 | - | - |
| IUIVLUD01EGQMT | - | Os03t0581100-01 | - | - |
| IUIVLUD02IEJBI | - | Os03t0581100-01 | - | - |
| IVLNWEC01CEPKT | - | Os03t0581100-01 | - | - |
| IVLNWEC02IB3GA | - | Os03t0581100-01 | - | - |
| IUIVLUD02F6WAH | - | Os03t0417900-01 | - | - |
| IVLNWEC02G5SRX | - | Os03t0407000-01 | - | - |
| IVLNWEC01CMEC7 | - | Os03t0386700-00 | - | - |
| IVLNWEC02GKE5G | - | Os03t0386700-00 | - | - |
| IVLNWEC01EQ5XE | - | Os03t0318500-01 | - | - |
| IV9RU5F02I1PC1 | - | Os03t0311300-03 | - | - |
| IV9RU5F02GPP81 | - | Os03t0294900-01 | - | - |
| IVLNWEC02GNZHA | - | Os03t0281600-01 | - | - |
| IVLNWEC02HCOCY | - | Os03t0281600-01 | - | - |
| IVLNWEC02GR7BG | - | Os03t0236200-01 | - | - |
| IUIVLUD02GTQ8Q | - | Os03t0138200-01 | - | - |
| IVLNWEC01A7L76 | - | Os03t0138200-01 | - | - |
| IV9RU5F02I4KXO | - | Os03t0110800-01 | - | - |
| IVLNWEC01BW2FH | - | Os03t0101600-01 | - | - |
| IVLNWEC02IK5SS | - | Os03t0101600-01 | - | - |
| IVLNWEC01DCQGO | - | Os03t0101100-01 | - | - |
| IUIVLUD01BZXGO | - | Os02t0818500-03 | - | - |
| IV9RU5F01AZLZ8 | - | Os02t0818500-03 | - | - |
| IV9RU5F02JV507 | - | Os02t0818500-03 | - | - |
| IUIVLUD01B9O4P | - | Os02t0810500-01 | - | - |
| IUIVLUD02FNARU | - | Os02t0699700-02 | - | - |
| IV9RU5F01DBY1A | - | Os02t0650800-00 | - | - |
| IVLNWEC01BGFR8 | - | Os02t0650800-00 | - | - |
| IVLNWEC01CYECT | - | Os02t0650800-00 | - | - |
| IV9RU5F02I9V5N | - | Os02t0537400-01 | - | - |
| IVLNWEC01DFZUL | - | Os02t0537400-01 | - | - |
| IVLNWEC01DIM7P | - | Os02t0537400-01 | - | - |
| IVLNWEC02H9OE7 | - | Os02t0537400-01 | - | - |
| IUIVLUD02JQO43 | - | Os02t0513700-00 | - | - |

| | | | | |
|---|---|---|---|---|
| IV9RU5F02FXJW2 | - | Os02t0491900-00 | - | - |
| IUIVLUD01D5NKF | - | Os02t0323600-01 | - | - |
| IV9RU5F02HXZ16 | - | Os02t0323600-01 | - | - |
| IV9RU5F01CTL9A | - | Os02t0284500-01 | - | - |
| IV9RU5F02FV43D | - | Os02t0284500-01 | - | - |
| IUIVLUD01B9OKC | - | Os02t0280700-01 | - | - |
| IV9RU5F01EHOJR | - | Os02t0280700-01 | - | - |
| IUIVLUD01D5P8Z | - | Os02t0277700-01 | - | - |
| IV9RU5F02GNRFQ | - | Os02t0277700-01 | - | - |
| IVLNWEC02ITKP0 | - | Os02t0272200-00 | - | - |
| IV9RU5F01EEDT3 | - | Os02t0263432-00 | - | - |
| IVLNWEC01CT9TY | - | Os02t0263432-00 | - | - |
| IV9RU5F01BXJOQ | - | Os02t0200000-01 | - | - |
| IVLNWEC01B3P0B | - | Os02t0139700-01 | - | - |
| IVLNWEC01DX707 | - | Os02t0139700-01 | - | - |
| IVLNWEC02HB8C0 | - | Os02t0100700-01 | - | - |
| IVLNWEC02GHFBW | - | Os01t0967700-01 | - | - |
| IUIVLUD02HL88C | - | Os01t0937400-02 | - | - |
| IV9RU5F02G8WR4 | - | Os01t0937400-02 | - | - |
| IV9RU5F02IQ152 | - | Os01t0937400-02 | - | - |
| IUIVLUD02HM8EZ | - | Os01t0937300-01 | - | - |
| IV9RU5F01DB979 | - | Os01t0937300-01 | - | - |
| IVLNWEC01AQYA3 | - | Os01t0937300-01 | - | - |
| IVLNWEC02HTQ95 | - | Os01t0937300-01 | - | - |
| IV9RU5F02H6YMC | - | Os01t0852650-00 | - | - |
| IVLNWEC02HTFRQ | - | Os01t0852650-00 | - | - |
| IUIVLUD02JE2LU | - | Os01t0841500-01 | - | - |
| IVLNWEC02GUL33 | - | Os01t0841500-01 | - | - |
| IV9RU5F01DZEF2 | - | Os01t0781200-01 | - | - |
| IV9RU5F02ISEMA | - | Os01t0781200-01 | - | - |
| IV9RU5F02JKL2W | - | Os01t0781200-01 | - | - |
| IUIVLUD02IQCX3 | - | Os01t0781100-01 | - | - |
| IUIVLUD02JNGGH | - | Os01t0759900-01 | - | - |
| IUIVLUD01D1GRM | - | Os01t0601625-00 | - | - |
| IV9RU5F01ECGA0 | - | Os01t0601625-00 | - | - |
| IV9RU5F02GS9XJ | - | Os01t0601625-00 | - | - |
| IV9RU5F02HIM96 | - | Os01t0601625-00 | - | - |
| IVLNWEC01BYZ32 | - | Os01t0601625-00 | - | - |
| IVLNWEC02FNL7I | - | Os01t0601625-00 | - | - |
| IVLNWEC02I0FVG | - | Os01t0601625-00 | - | - |
| IVLNWEC02IEUXT | - | Os01t0601625-00 | - | - |
| IV9RU5F01DN38E | - | Os01t0533000-00 | - | - |
| IVLNWEC02GH0LI | - | Os01t0533000-00 | - | - |
| IVLNWEC02J10QJ | - | Os01t0533000-00 | - | - |
| IV9RU5F01A54B1 | - | Os01t0509800-00 | - | - |
| IV9RU5F01B8X3X | - | Os01t0377000-01 | - | - |
| IV9RU5F02H8BVF | - | Os01t0370200-00 | - | - |

| | | | | |
|---|---|---|---|---|
| IV9RU5F02GCNMF | - | Os01t0358700-01 | - | - |
| IV9RU5F01A75E6 | - | Os01t0304100-00 | - | - |
| IV9RU5F01EZIP6 | - | Os01t0281400-00 | - | - |
| IUIVLUD01ANTVQ | - | Os01t0212400-01 | - | - |
| IVLNWEC02I8YWD | - | Os01t0205700-01 | - | - |
| IUIVLUD01DL77S | - | Os01t0201700-01 | - | - |
| IV9RU5F01D2V1K | - | Os01t0166100-01 | - | - |
| IV9RU5F02FSGXO | - | Os01t0166100-01 | - | - |
| IVLNWEC01DTFND | - | Os01t0166100-01 | - | - |
| IVLNWEC01EUT7P | - | Os01t0152950-00 | - | - |
| IVLNWEC02GFKBR | - | Os01t0152950-00 | - | - |
| IUIVLUD02GMK0N | - | Os03t0610650-01 | - | - |
| IVLNWEC01C64UA | - | Os03t0757200-01 | - | - |
| IUIVLUD01DW4MY | - | Os06t0111100-01 | - | - |
| IV9RU5F01DMK3A | - | Os03t0850200-00 | - | - |
| IVLNWEC01BU4ID | - | Os02t0191700-00 | - | - |
| IVLNWEC02IL0KW | - | Os07t0160000-00 | - | - |
| IUIVLUD01EFGSC | - | Os09t0286400-01 | - | - |
| IVLNWEC01BZBV6 | - | Os11t0121400-01 | - | - |
| IVLNWEC02H5QSD | - | Os11t0121400-01 | - | - |
| IVLNWEC01DM2OP | - | Os11t0213700-00 | - | - |
| IVLNWEC02HDO79 | - | Os11t0438400-01 | - | - |
| IV9RU5F01DI0DN | - | Os02t0272200-00 | - | - |
| IVLNWEC01BQY5A | - | Os08t0202400-01 | - | - |
| IV9RU5F01D80KD | - | Os03t0738400-01 | - | - |
| IVLNWEC01EKYG2 | - | Os03t0738400-01 | - | - |
| IVLNWEC02GZOX9 | - | Os03t0738400-01 | - | - |
| IV9RU5F01DMC42 | - | Os08t0531000-02 | - | - |
| IUIVLUD01BVTAN | - | Os12t0273900-00 | - | - |
| IV9RU5F01CD2FF | - | Os12t0273900-00 | - | - |
| IV9RU5F02IFAWH | - | Os01t0304300-01 | - | - |
| IVLNWEC01EOIAN | - | Os03t0110800-01 | - | - |
| IUIVLUD02IEMRB | - | Os09t0330700-00 | - | - |
| IVLNWEC01B381V | - | Os04t0354200-00 | - | - |
| IV9RU5F02IT6HM | - | Os08t0371608-00 | - | - |
| IV9RU5F02I39NK | - | Os06t0148250-00 | - | - |
| IVLNWEC01CYSIZ | - | Os06t0148250-00 | - | - |
| IV9RU5F02HVNQB | - | Os03t0719100-01 | - | - |
| IVLNWEC02HR78F | - | Os06t0168500-01 | - | - |
| IUIVLUD02JKCX3 | - | Os01t0574400-01 | - | - |
| IUIVLUD01ARUUJ | - | Os03t0311300-03 | - | - |
| IUIVLUD02G2Z8N | - | Os05t0144100-01 | - | - |
| IVLNWEC01EA3VP | - | Os11t0167500-00 | - | - |
| IVLNWEC02GHS3S | - | Os11t0167500-00 | - | - |
| IVLNWEC01A1Y30 | - | Os01t0702400-01 | - | - |
| IV9RU5F01A4F02 | - | Os02t0826000-00 | - | - |
| IV9RU5F01DN5D2 | - | Os03t0344700-01 | - | - |

| | | | | |
|---|---|---|---|---|
| IV9RU5F02I0KVP | - | Os03t0191000-01 | - | - |
| IV9RU5F01ERDY3 | - | Os01t0187400-01 | - | - |
| IUIVLUD02JCIT9 | - | Os03t0356700-01 | - | - |
| IVLNWEC02FYLBD | - | Os03t0356700-01 | - | - |
| IVLNWEC02HOKHE | - | Os01t0211800-01 | - | - |
| IV9RU5F02GNCPH | - | Os04t0177000-00 | - | - |
| IVLNWEC01AZRY1 | - | Os02t0826000-00 | - | - |
| IV9RU5F02I6C6Q | - | Os01t0841500-01 | - | - |
| IV9RU5F01CB18O | - | Os05t0535500-01 | - | - |
| IV9RU5F01ALXH4 | - | Os09t0436300-01 | - | - |
| IV9RU5F01BT2FF | - | Os01t0574400-01 | - | - |
| IV9RU5F02F9K7N | - | Os03t0358000-00 | - | - |
| IVLNWEC02J19LF | - | Os01t0359800-00 | - | - |
| IV9RU5F01ET4WG | - | Os09t0409950-01 | - | - |
| IV9RU5F02I57UR | - | Os09t0409950-01 | - | - |
| IVLNWEC02HDZDM | - | Os09t0409950-01 | - | - |
| IUIVLUD01EVBD9 | - | Os05t0535500-01 | - | - |
| IVLNWEC01B7Z7C | - | Os04t0373400-01 | - | - |
| IVLNWEC02JOE2A | - | Os01t0201700-01 | - | - |
| IV9RU5F01CXJPP | - | Os05t0398000-01 | - | - |
| IV9RU5F01EB090 | - | Os09t0394900-01 | - | - |
| IV9RU5F01B348L | - | Os02t0832400-01 | - | - |
| IV9RU5F02G93H9 | - | Os02t0254700-01 | - | - |
| IV9RU5F01DF961 | - | Os12t0112200-01 | - | - |
| IUIVLUD01CXD8N | - | Os01t0789000-00 | - | - |
| IUIVLUD02JJU5O | - | Os01t0789000-00 | - | - |
| IVLNWEC02G43ZP | - | Os01t0509800-00 | - | - |
| IUIVLUD01CBQNO | - | Os03t0671800-01 | - | - |
| IVLNWEC01CA0CM | - | Os03t0271400-01 | - | - |
| IV9RU5F01ASUJ7 | - | Os11t0213800-01 | - | - |
| IVLNWEC02IXBMH | - | Os09t0448900-01 | - | - |
| IV9RU5F02IW91E | - | Os09t0436400-01 | - | - |
| IVLNWEC01BOKC4 | - | Os10t0346300-01 | - | - |
| IUIVLUD02FRG84 | - | Os03t0110800-01 | - | - |
| IV9RU5F01C8LFI | - | Os03t0151800-02 | - | - |
| IV9RU5F02H8U0A | - | Os03t0151800-02 | - | - |
| IV9RU5F02FT7DO | - | Os03t0221300-01 | - | - |
| IUIVLUD01A0BT2 | - | Os03t0294900-01 | - | - |
| IV9RU5F01B7985 | - | Os11t0265400-01 | - | - |
| IUIVLUD01CJK4T | - | Os01t0205700-01 | - | - |
| IV9RU5F02HXRFJ | - | Os03t0358000-00 | - | - |
| IUIVLUD02GBMQL | - | Os05t0304400-01 | - | - |
| IVLNWEC02HSXNI | - | Os05t0304400-01 | - | - |
| IV9RU5F02FZZ1E | - | Os02t0832400-01 | - | - |
| IUIVLUD02F2KCJ | - | Os03t0278200-01 | - | - |
| IVLNWEC02G5C6Q | - | Os03t0278200-01 | - | - |
| IVLNWEC02HCG52 | - | Os03t0278200-01 | - | - |

| | | | | |
|---|---|---|---|---|
| IVLNWEC01C8GXF | - | Os03t0307100-01 | - | - |
| IVLNWEC01BL0ZK | - | Os05t0398000-01 | - | - |
| IVLNWEC02G00EY | - | Os03t0790700-01 | - | - |
| IV9RU5F01EGGKJ | - | Os12t0190000-01 | - | - |
| IUIVLUD01B7B6T | - | Os01t0183100-01 | - | - |
| IVLNWEC02GN5VS | - | Os09t0546100-01 | - | - |
| IV9RU5F01CQTQO | - | Os03t0138200-01 | - | - |
| IV9RU5F01B8CKX | - | Os02t0491900-00 | - | - |
| IVLNWEC02IZZQP | - | Os01t0166100-01 | - | - |
| IVLNWEC01EU7XK | - | Os02t0102800-01 | - | - |
| IUIVLUD01AJ4XW | - | Os03t0797000-01 | - | - |
| IVLNWEC01DW5HZ | - | Os04t0492300-01 | - | - |
| IV9RU5F01DZJSN | - | Os08t0539400-01 | - | - |
| IUIVLUD02GZ824 | - | Os02t0578300-01 | - | - |
| IVLNWEC01EZJA9 | - | Os02t0798800-00 | - | - |
| IUIVLUD01EGG4O | - | Os01t0533000-00 | - | - |
| IV9RU5F02JGOQ7 | - | Os11t0104400-01 | - | - |
| IVLNWEC02GIDFB | - | Os03t0713100-01 | - | - |
| IV9RU5F02I21H2 | - | Os03t0786700-01 | - | - |
| IUIVLUD01BTK9K | - | Os01t0254100-01 | - | - |
| IV9RU5F01CYXT5 | - | Os01t0254100-01 | - | - |
| IVLNWEC02GRBH3 | - | Os03t0790700-01 | - | - |
| IV9RU5F02FWL3L | - | Os09t0436400-01 | - | - |
| IVLNWEC02IEQVC | - | Os04t0691800-01 | - | - |
| IVLNWEC02FS6QH | - | Os09t0479000-01 | - | - |
| IUIVLUD01EEQCJ | - | Os03t0795000-01 | - | - |
| IV9RU5F02J33OO | - | Os04t0373400-01 | - | - |
| IVLNWEC01C5W1F | - | Os02t0280700-01 | - | - |
| IV9RU5F02I29D5 | - | Os05t0508500-01 | - | - |
| IV9RU5F02HLUQC | - | Os02t0807800-01 | - | - |
| IVLNWEC01B74M8 | - | Os02t0807800-01 | - | - |
| IVLNWEC02J0C2S | - | Os02t0807800-01 | - | - |
| IUIVLUD02I3CPD | - | Os08t0114300-01 | - | - |
| IV9RU5F01EBK1N | - | Os01t0567500-01 | - | - |
| IV9RU5F02H7DOK | - | Os01t0567500-01 | - | - |
| IV9RU5F02JDOS4 | - | Os08t0398300-01 | - | - |
| IVLNWEC02FNI3O | - | Os04t0364800-00 | - | - |
| IVLNWEC02GD8VL | - | Os02t0121700-01 | - | - |
| IUIVLUD01DOJ25 | - | Os04t0166000-01 | - | - |
| IVLNWEC01AFLFR | - | Os01t0937300-01 | - | - |
| IV9RU5F02IBU51 | - | Os03t0207300-01 | - | - |
| IV9RU5F02F2Y6I | - | Os09t0456200-01 | - | - |
| IV9RU5F02H9ZBK | - | Os04t0397100-01 | - | - |
| IUIVLUD01CQ8C2 | - | Os02t0200000-01 | - | - |
| IUIVLUD01AOHGQ | - | Os04t0488400-01 | - | - |
| IV9RU5F01A3HVH | - | Os04t0488400-01 | - | - |
| IV9RU5F01C3AEJ | - | Os02t0795900-01 | - | - |

| | | | | |
|---|---|---|---|---|
| IV9RU5F01AL2R4 | - | Os05t0416400-00 | - | - |
| IUIVLUD01DSE77 | - | Os05t0416400-00 | - | - |
| IUIVLUD01DOSWH | - | Os03t0598200-01 | - | - |
| IUIVLUD02JIJBQ | - | Os05t0128400-01 | - | - |
| IV9RU5F01B0TSJ | - | Os03t0152900-01 | - | - |
| IV9RU5F02GTV07 | - | Os04t0373400-01 | - | - |
| IVLNWEC02GO1MG | - | Os03t0207300-01 | - | - |
| IV9RU5F02ILWQW | - | Os01t0574400-01 | - | - |
| IV9RU5F01A5WSM | - | Os02t0102800-01 | - | - |
| IV9RU5F01DPB8T | - | Os03t0294900-01 | - | - |
| IV9RU5F02H2QZA | - | Os09t0479100-01 | - | - |
| IV9RU5F01AW49D | - | Os12t0190000-01 | - | - |
| IUIVLUD02FNO1H | - | Os03t0682200-00 | - | - |
| IV9RU5F02J307I | - | Os03t0682200-00 | - | - |
| IUIVLUD02F2CWN | - | Os11t0210300-01 | - | AK251236.1 |
| IUIVLUD02JC8OE | - | Os12t0448900-01 | - | AK251358.1 |
| IVLNWEC02IYSD2 | - | Os05t0100600-02 | - | AK355206 |
| IVLNWEC01AHUKD | - | Os01t0851700-01 | - | AK355790 |
| IVLNWEC02GKOLQ | - | Os03t0790700-01 | - | AK358358 |
| IVLNWEC02JV99O | - | Os05t0498700-01 | - | AK359220 |
| IV9RU5F01CU6FW | - | Os12t0199800-01 | - | AK360305 |
| IV9RU5F01EC66Y | - | Os12t0199800-01 | - | AK360305 |
| IVLNWEC02GKRJF | - | Os08t0265300-00 | - | AK361251 |
| IV9RU5F02HK0LJ | - | Os09t0321900-01 | - | AK362384 |
| IVLNWEC02JQ5I4 | - | Os03t0649000-01 | - | AK363790 |
| IV9RU5F02JPEMA | - | Os01t0759900-01 | - | AK364444 |
| IUIVLUD01C96RI | - | Os06t0473200-02 | - | AK364882 |
| IV9RU5F02HGQWQ | - | Os03t0803900-01 | - | AK365364 |
| IVLNWEC02H14GM | - | Os03t0281600-01 | - | AK365503 |
| IV9RU5F01CW85H | - | Os09t0394600-01 | - | AK365907 |
| IV9RU5F01CD6VE | - | Os01t0547000-01 | - | AK365963 |
| IUIVLUD02FZUEE | - | Os09t0568000-01 | - | AK366111 |
| IVLNWEC02HFVND | - | Os09t0568000-01 | - | AK366111 |
| IV9RU5F02GH8T5 | - | Os03t0802700-01 | - | AK368023 |
| IV9RU5F02GDSF2 | - | Os03t0795000-01 | - | AK368582 |
| IUIVLUD02FNHCH | - | Os03t0777600-01 | - | AK368680 |
| IV9RU5F02F5PMT | - | Os02t0100700-01 | - | AK370019 |
| IVLNWEC01BMCNZ | - | Os07t0447800-01 | - | AK372310 |
| IV9RU5F01CKQ4N | - | Os03t0151800-02 | - | AK373447 |
| IV9RU5F02JQELM | - | Os01t0663051-00 | - | AK373937 |
| IV9RU5F02I6U5X | - | Os09t0563700-01 | - | AK374134 |
| IV9RU5F01B5HAB | - | Os03t0369100-01 | - | AK374452 |
| IV9RU5F02JOMNQ | - | Os03t0369100-01 | - | AK374452 |
| IV9RU5F01BXF69 | - | Os12t0236100-00 | - | AK376057 |
| IV9RU5F02IPXD9 | - | Os12t0236100-00 | - | AK376057 |
| IVLNWEC01CEJ8A | - | Os09t0395300-01 | - | AK377103 |
| IUIVLUD02I6HPK | - | Os03t0610650-01 | - | MLOC_10176.1 |

| | | | | |
|---|---|---|---|---|
| IUIVLUD01DVO1L | - | Os08t0542200-01 | - | MLOC_10452.1 |
| IUIVLUD01DFE2U | - | Os03t0798300-01 | - | MLOC_10988.2 |
| IV9RU5F01DSGHW | - | Os05t0317200-01 | - | MLOC_11136.1 |
| IV9RU5F02FZ5OJ | - | Os04t0373400-01 | - | MLOC_11412.2 |
| IVLNWEC01CZPL2 | - | Os04t0373400-01 | - | MLOC_11412.2 |
| IVLNWEC01DGLPH | - | Os01t0295700-01 | - | MLOC_12326.1 |
| IVLNWEC01BZSL2 | - | Os11t0704100-00 | - | MLOC_12720.2 |
| IV9RU5F02HOAL9 | - | Os04t0540300-00 | - | MLOC_13091.1 |
| IVLNWEC01AQTUE | - | Os12t0112200-01 | - | MLOC_17314.1 |
| IV9RU5F01A5B31 | - | Os06t0279900-00 | - | MLOC_18184.1 |
| IUIVLUD01BI58T | - | Os08t0140300-01 | - | MLOC_18474.1 |
| IUIVLUD01DDFYK | - | Os08t0140300-01 | - | MLOC_18474.1 |
| IV9RU5F01EX9WM | - | Os08t0140300-01 | - | MLOC_18474.1 |
| IUIVLUD01C58O6 | - | Os02t0121700-01 | - | MLOC_18811.2 |
| IVLNWEC01DLX69 | - | Os06t0102900-01 | - | MLOC_19401.1 |
| IV9RU5F01DXD52 | - | Os08t0106900-00 | - | MLOC_20548.2 |
| IVLNWEC01ENM6A | - | Os12t0431700-00 | - | MLOC_20578.1 |
| IV9RU5F01DP0B9 | - | Os01t0212400-01 | - | MLOC_21289.1 |
| IVLNWEC01C6L5R | - | Os06t0286500-01 | - | MLOC_30912.2 |
| IV9RU5F02I4AE1 | - | Os05t0421300-01 | - | MLOC_34880.1 |
| IVLNWEC01D4B0V | - | Os05t0421300-01 | - | MLOC_34880.1 |
| IVLNWEC02INFOD | - | Os05t0421300-01 | - | MLOC_34880.1 |
| IUIVLUD02IZ218 | - | Os12t0512700-01 | - | MLOC_37495.1 |
| IVLNWEC02HYMZW | - | Os12t0512700-01 | - | MLOC_37495.1 |
| IUIVLUD02H14L9 | - | Os09t0431200-01 | - | MLOC_44275.1 |
| IVLNWEC02H752R | - | Os09t0554300-01 | - | MLOC_50968.1 |
| IVLNWEC02GTMME | - | Os04t0677700-00 | - | MLOC_51259.2 |
| IUIVLUD02IN1AN | - | Os03t0699700-01 | - | MLOC_54031.1 |
| IVLNWEC01CT5D1 | - | Os03t0710100-01 | - | MLOC_54142.1 |
| IVLNWEC01EH98L | - | Os03t0738200-00 | - | MLOC_55034.1 |
| IUIVLUD02GD3F0 | - | Os02t0254700-01 | - | MLOC_56270.1 |
| IVLNWEC01BXBO0 | - | Os01t0364400-00 | - | MLOC_56410.1 |
| IV9RU5F01D3C02 | - | Os04t0626500-01 | - | MLOC_57116.2 |
| IV9RU5F02IJV6D | - | Os01t0305900-01 | - | MLOC_57220.1 |
| IVLNWEC01A4H84 | - | Os01t0305900-01 | - | MLOC_57220.1 |
| IVLNWEC02JNJSL | - | Os01t0305900-01 | - | MLOC_57220.1 |
| IVLNWEC02I8DE9 | - | Os03t0300400-01 | - | MLOC_57345.1 |
| IVLNWEC02I5JAN | - | Os10t0134033-00 | - | MLOC_58037.1 |
| IVLNWEC01AQRHM | - | Os01t0896700-00 | - | MLOC_58960.1 |
| IUIVLUD01EQDZI | - | Os01t0708600-01 | - | MLOC_59846.2 |
| IUIVLUD02GWXVP | - | Os01t0708600-01 | - | MLOC_59846.2 |
| IV9RU5F02HUSZU | - | Os01t0601625-00 | - | MLOC_60328.1 |
| IVLNWEC02I4S75 | - | Os04t0691800-01 | - | MLOC_60852.1 |
| IVLNWEC01C419N | - | Os03t0803900-01 | - | MLOC_61925.4 |
| IUIVLUD01EMSK1 | - | Os09t0513000-01 | - | MLOC_62437.1 |
| IUIVLUD01E4KAG | - | Os03t0236200-01 | - | MLOC_63275.3 |
| IVLNWEC01CDXVE | - | Os03t0808000-00 | - | MLOC_6444.1 |

| | | | | |
|---|---|---|---|---|
| IVLNWEC02HTYTT | - | Os03t0808000-00 | - | MLOC_6444.1 |
| IUIVLUD01DWBST | - | Os01t0603100-01 | - | MLOC_64444.1 |
| IVLNWEC01AK4Y2 | - | Os01t0603100-01 | - | MLOC_64444.1 |
| IUIVLUD01CGTKS | - | Os10t0346300-01 | - | MLOC_65135.1 |
| IVLNWEC01CYI2A | - | Os08t0265500-01 | - | MLOC_65827.2 |
| IVLNWEC01ASIF1 | - | Os09t0553900-01 | - | MLOC_67933.1 |
| IV9RU5F01C9RQD | - | Os02t0818500-03 | - | MLOC_68230.1 |
| IV9RU5F01BALSW | - | Os05t0535500-01 | - | MLOC_68656.2 |
| IVLNWEC01CGPK9 | - | Os05t0535500-01 | - | MLOC_68656.2 |
| IVLNWEC01DWIY5 | - | Os03t0671800-01 | - | MLOC_68896.2 |
| IUIVLUD01D00A8 | - | Os09t0559800-01 | - | MLOC_70271.1 |
| IV9RU5F02HVQP6 | - | Os09t0559800-01 | - | MLOC_70271.1 |
| IVLNWEC01BT2GM | - | Os09t0559800-01 | - | MLOC_70271.1 |
| IV9RU5F01BVZL5 | - | Os02t0146400-01 | - | MLOC_70787.2 |
| IV9RU5F02GV6MH | - | Os07t0421600-00 | - | MLOC_72201.1 |
| IVLNWEC01C8EB7 | - | Os03t0710100-01 | - | MLOC_73572.1 |
| IVLNWEC02FR605 | - | Os08t0360400-00 | - | MLOC_74917.2 |
| IVLNWEC02HT8RJ | - | Os03t0251600-00 | - | MLOC_75159.1 |
| IV9RU5F01D7NWN | - | Os03t0638200-02 | - | MLOC_76072.2 |
| IUIVLUD02FWT7Y | - | Os09t0321900-01 | - | MLOC_76409.1 |
| IV9RU5F02I3CQ3 | - | Os09t0321900-01 | - | MLOC_76409.1 |
| IV9RU5F01ER6CN | - | Os08t0543050-00 | - | MLOC_78491.4 |
| IV9RU5F02GLTTI | - | Os06t0623700-00 | - | MLOC_80395.1 |
| IV9RU5F01DIPMY | - | Os06t0265000-01 | - | MLOC_63089.10 |
| IVLNWEC01ECTKT | - | Os11t0135300-00 | - | AK251637.1 |
| IUIVLUD02F3VU4 | - | Os02t0184400-01 | - | AK366557 |
| IVLNWEC02HS5PK | - | Os02t0184400-01 | - | AK366557 |
| IV9RU5F01BGTGJ | - | Os09t0363700-01 | - | AK368885 |
| IVLNWEC02IR9CX | - | Os09t0363700-01 | - | AK368885 |
| IV9RU5F01C7EM3 | - | Os08t0265500-01 | - | MLOC_65827.2 |
| IV9RU5F01BSEAJ | - | Os02t0191700-00 | - | MLOC_4612.1 |
| IV9RU5F01D0JEE | - | Os11t0672400-00 | - | MLOC_4990.2 |
| IUIVLUD01A6HVJ | - | Os11t0148700-01 | - | MLOC_54689.2 |
| IVLNWEC01CGWKB | - | Os11t0107400-01 | - | MLOC_57653.5 |
| IV9RU5F02HXY0M | - | Os02t0224200-01 | - | MLOC_55635.2 |
| IVLNWEC01ASF5H | - | Os12t0182300-01 | - | MLOC_60385.1 |
| IV9RU5F02F8YNA | - | Os09t0469400-01 | - | MLOC_10776.1 |
| IV9RU5F02G4MEY | - | Os09t0469400-01 | - | MLOC_10776.1 |
| IUIVLUD02J15O5 | - | Os12t0594300-00 | - | MLOC_38229.4 |
| IVLNWEC02HS2LD | - | Os06t0473200-02 | - | AK364882 |
| IV9RU5F02IK09M | - | Os04t0373400-01 | - | MLOC_11412.2 |
| IUIVLUD02FRNFP | - | Os07t0164100-01 | - | MLOC_37038.2 |
| IV9RU5F01A4WLS | - | Os11t0276000-01 | - | MLOC_11154.1 |
| IVLNWEC02FXA3I | - | Os03t0638200-02 | - | MLOC_76072.2 |
| IV9RU5F01EDDGV | - | Os01t0533000-00 | - | MLOC_8120.2 |
| IVLNWEC02JA7OY | - | Os03t0669800-01 | - | MLOC_4537.1 |
| IUIVLUD01EDTPL | - | Os02t0816900-01 | - | MLOC_4461.5 |

| | | | | |
|---|---|---|---|---|
| IV9RU5F02FYE95 | - | Os03t0417900-01 | - | AK361277 |
| IUIVLUD02GZM5Y | - | Os02t0326600-01 | - | MLOC_15812.1 |
| IVLNWEC01A0BIK | - | Os02t0326600-01 | - | MLOC_15812.1 |
| IVLNWEC02IKZI6 | - | Os05t0498700-01 | - | AK359220 |
| IVLNWEC02I6Z3O | - | Os08t0305300-01 | - | MLOC_52014.2 |
| IVLNWEC02I7R5Q | - | Os01t0211800-01 | - | MLOC_39203.1 |
| IV9RU5F01B9J6E | - | Os03t0143700-01 | - | AK364175 |
| IVLNWEC02JBHWI | - | Os09t0395300-01 | - | AK377103 |
| IV9RU5F01BA2PU | - | Os08t0539400-01 | - | MLOC_59797.4 |
| IVLNWEC02HNS1F | - | Os03t0203000-01 | - | MLOC_55401.4 |
| IV9RU5F02H1RPU | - | Os03t0784000-02 | - | MLOC_75599.1 |
| IV9RU5F01C61QU | - | Os06t0473200-02 | - | AK364882 |
| IV9RU5F02GKN6I | - | Os03t0707900-01 | - | AK366523 |
| IUIVLUD01DOWR6 | - | Os03t0786700-01 | - | AK249886.1 |
| IV9RU5F02HPJHF | - | Os03t0669800-01 | - | MLOC_4537.1 |
| IVLNWEC01EXJ4X | - | Os03t0210800-01 | - | AK357041 |
| IUIVLUD01C26T8 | - | Os07t0160000-00 | - | MLOC_44802.1 |
| IV9RU5F01BUOJS | - | Os12t0236100-00 | - | AK376057 |
| IVLNWEC01C7WXN | - | Os12t0236100-00 | - | AK376057 |
| IUIVLUD02GF9KU | - | Os02t0816900-01 | - | MLOC_55557.3 |
| IUIVLUD02HSPZ5 | - | Os02t0301500-01 | - | MLOC_36653.1 |
| IV9RU5F02HFDI3 | - | Os03t0318500-01 | - | MLOC_79070.2 |
| IV9RU5F02JHXSG | - | Os03t0648400-01 | - | AK250598.1 |
| IUIVLUD02GFAWB | - | Os12t0567300-00 | - | MLOC_52439.6 |
| IV9RU5F02FV1RM | - | Os12t0567300-00 | - | MLOC_52439.6 |
| IV9RU5F02FXJCG | - | Os03t0278200-01 | - | AK366489 |
| IV9RU5F02GBHF4 | - | Os03t0278200-01 | - | AK366489 |
| IV9RU5F02HFIY4 | - | Os09t0436700-01 | - | MLOC_52482.1 |
| IV9RU5F01CA5ZN | - | Os11t0264200-01 | - | MLOC_19192.2 |
| IUIVLUD01BZLXU | - | Os02t0119400-01 | - | MLOC_11180.1 |
| IUIVLUD02HO74J | - | Os02t0119400-01 | - | MLOC_11180.1 |
| IUIVLUD02FUL38 | - | Os02t0284500-01 | - | MLOC_53882.4 |
| IVLNWEC02IEKEH | - | Os11t0276000-01 | - | MLOC_11154.1 |
| IV9RU5F01D0DFS | - | Os06t0158900-00 | - | MLOC_76366.3 |
| IV9RU5F01B60FO | - | Os02t0146400-01 | - | MLOC_70787.2 |
| IVLNWEC01BRXPD | - | Os02t0146400-01 | - | MLOC_70787.2 |
| IV9RU5F02F18XB | - | Os03t0251600-00 | - | MLOC_75159.1 |
| IV9RU5F01D3EUS | - | Os01t0187400-01 | - | MLOC_54010.6 |
| IUIVLUD01EW1V6 | - | Os02t0816900-01 | - | MLOC_4461.5 |
| IVLNWEC01B6F75 | - | Os03t0417900-01 | - | AK361277 |
| IVLNWEC01DRESD | - | Os12t0582800-01 | - | AK355948 |
| IV9RU5F01EO8W6 | - | Os12t0291000-00 | - | MLOC_75102.1 |
| IV9RU5F01CKL91 | - | Os08t0398300-01 | - | MLOC_58493.2 |
| IV9RU5F02FXAFD | - | Os03t0151800-02 | - | AK373447 |
| IV9RU5F01DKYSS | - | Os08t0140300-01 | - | MLOC_18474.1 |
| IV9RU5F02JRJ9P | - | Os03t0151800-02 | - | AK373447 |
| IUIVLUD02HE7SG | - | Os03t0300400-01 | - | MLOC_57345.1 |

| | | | | |
|---|---|---|---|---|
| IUIVLUD01B6FJU | - | Os08t0496700-00 | - | MLOC_33543.1 |
| IV9RU5F02F8CDC | - | Os11t0180600-01 | - | MLOC_69650.8 |
| IV9RU5F01DA48O | - | Os11t0695900-01 | - | AK369923 |
| IV9RU5F01D8HV1 | - | Os03t0143700-01 | - | AK364175 |
| IV9RU5F01EBWMC | - | Os09t0563700-01 | - | AK374134 |
| IV9RU5F02HYUI0 | - | Os03t0300500-01 | - | MLOC_57346.1 |
| IUIVLUD01AJQWN | - | Os03t0271400-01 | - | AK251471.1 |
| IV9RU5F02GTOLH | - | Os02t0516600-01 | - | AK369767 |
| IV9RU5F02JZZYM | - | Os02t0578300-01 | - | AK358124 |
| IV9RU5F01BZG82 | - | Os08t0338700-01 | - | AK377032 |
| IUIVLUD02ILVSY | - | Os03t0236200-01 | - | MLOC_63275.3 |
| IVLNWEC01DPK3H | - | Os09t0428300-00 | - | AK371221 |
| IVLNWEC01CW4EZ | - | Os03t0191000-01 | - | MLOC_63242.2 |
| IV9RU5F01ARGQM | - | Os01t0364100-00 | - | MLOC_56410.1 |
| IV9RU5F01BFGPL | - | Os01t0364100-00 | - | MLOC_56410.1 |
| IVLNWEC01AKFEM | - | Os07t0164100-01 | - | MLOC_37038.2 |
| IV9RU5F02GOLW5 | - | Os03t0581400-00 | - | AK369964 |
| IUIVLUD02FY94I | - | Os02t0818500-03 | - | MLOC_59797.4 |
| IUIVLUD01D0GU8 | - | Os12t0431700-00 | - | MLOC_20578.1 |
| IVLNWEC02ID428 | - | Os08t0265300-00 | - | AK361251 |
| IUIVLUD01BGP8H | - | Os02t0652800-01 | - | MLOC_81672.2 |
| IVLNWEC01B6LXX | - | Os04t0166000-01 | - | AK373095 |
| IUIVLUD01BXUC9 | - | Os11t0180600-01 | - | MLOC_69650.8 |
| IV9RU5F01DZJLU | - | Os12t0582800-01 | - | AK355948 |
| IVLNWEC01CG723 | - | Os01t0851700-01 | - | MLOC_71287.2 |
| IUIVLUD01D2FG7 | - | Os11t0104400-01 | - | AK357949 |
| IV9RU5F02IUZGW | - | Os10t0578500-00 | - | MLOC_37122.1 |
| IUIVLUD02F02V2 | - | Os07t0164100-01 | - | MLOC_37038.2 |
| IV9RU5F01DD47J | - | Os06t0168500-01 | - | MLOC_38005.1 |
| IV9RU5F02JPGDR | - | Os09t0556700-01 | - | MLOC_54619.1 |
| IVLNWEC02GT715 | - | Os01t0702400-01 | - | AK363157 |
| IV9RU5F01EREDF | - | Os05t0317200-01 | - | AK370654 |
| IVLNWEC01DSWZJ | - | Os08t0564800-01 | - | MLOC_62049.1 |
| IV9RU5F02GY7RS | - | Os08t0305300-01 | - | MLOC_52014.2 |
| IV9RU5F01BEYOD | - | Os07t0447000-00 | - | MLOC_59556.1 |
| IVLNWEC02HFR83 | - | Os03t0191000-01 | - | AK365210 |
| IV9RU5F02FVJLX | - | Os09t0544900-01 | - | AK252578.1 |
| IV9RU5F02HY6UA | - | Os09t0436700-01 | - | MLOC_52482.1 |
| IVLNWEC02JLTV2 | - | Os09t0436700-01 | - | MLOC_52482.1 |
| IV9RU5F01EJB7B | - | Os05t0100600-02 | - | AK355206 |
| IV9RU5F01DCPG7 | - | Os06t0265000-01 | - | MLOC_63089.10 |
| IUIVLUD01BOVVJ | - | Os01t0304100-00 | - | MLOC_64607.5 |
| IV9RU5F02ITA06 | - | Os09t0395300-01 | - | AK377103 |
| IVLNWEC02HYFF1 | - | Os04t0204200-01 | Sb0015s007030.1 | - |
| IV9RU5F01DS9YP | - | Os02t0816900-01 | Sb01g000330.1 | - |
| IV9RU5F02JWVFT | - | Os02t0816900-01 | Sb01g000330.1 | - |
| IV9RU5F02F5PXB | - | Os03t0859800-01 | Sb01g000370.1 | - |

| | | | | |
|---|---|---|---|---|
| IV9RU5F02HZVJV | - | Os12t0512800-01 | Sb01g001180.1 | - |
| IVLNWEC02INWXO | - | Os09t0569400-01 | Sb01g001930.1 | - |
| IV9RU5F02H28WA | - | Os03t0833800-01 | Sb01g002550.1 | - |
| IVLNWEC01AX1DJ | - | Os10t0388600-00 | Sb01g002723.1 | - |
| IUIVLUD02HWLZ3 | - | Os03t0822900-01 | Sb01g003040.1 | - |
| IVLNWEC01EACX7 | - | Os03t0822900-01 | Sb01g003040.1 | - |
| IVLNWEC02FLLA4 | - | Os03t0805100-01 | Sb01g004550.1 | - |
| IVLNWEC01BAZS8 | - | Os03t0804400-01 | Sb01g004600.1 | - |
| IVLNWEC01BEL5B | - | Os04t0558200-00 | Sb01g004930.1 | - |
| IVLNWEC02FS3DM | - | Os04t0558200-00 | Sb01g004930.1 | - |
| IVLNWEC02IB8YB | - | Os04t0558200-00 | Sb01g004930.1 | - |
| IUIVLUD02J2VK1 | - | Os03t0797000-01 | Sb01g005140.1 | - |
| IV9RU5F02I8174 | - | Os03t0790700-01 | Sb01g005650.1 | - |
| IVLNWEC01CPDZN | - | Os06t0484800-00 | Sb01g005660.1 | - |
| IUIVLUD02JCOJ5 | - | Os03t0777700-01 | Sb01g006380.1 | - |
| IVLNWEC02GBP7R | - | Os03t0777700-01 | Sb01g006380.1 | - |
| IV9RU5F01AWL67 | - | Os03t0767500-01 | Sb01g006990.1 | - |
| IVLNWEC02I6WUK | - | Os03t0767500-01 | Sb01g006990.1 | - |
| IUIVLUD02I0R7K | - | Os03t0757200-01 | Sb01g007640.1 | - |
| IVLNWEC02IV3WL | - | Os03t0738900-00 | Sb01g008670.1 | - |
| IUIVLUD01CRJY2 | - | Os08t0360400-00 | Sb01g009780.1 | - |
| IVLNWEC01CV18U | - | Os03t0711200-01 | Sb01g010410.1 | - |
| IUIVLUD01DAJXI | - | Os03t0738600-01 | Sb01g011050.1 | - |
| IUIVLUD02HGT88 | - | Os03t0738600-01 | Sb01g011050.1 | - |
| IVLNWEC01DQEIW | - | Os03t0664700-01 | Sb01g012610.1 | - |
| IUIVLUD02FO7WY | - | Os03t0164400-01 | Sb01g014270.1 | - |
| IVLNWEC01A68FP | - | Os03t0164400-01 | Sb01g014270.1 | - |
| IVLNWEC01CM3S5 | - | Os03t0598200-01 | Sb01g015150.1 | - |
| IVLNWEC01BLIZX | - | Os05t0523100-01 | Sb01g020130.1 | - |
| IV9RU5F01AHC4U | - | Os10t0147250-00 | Sb01g026260.1 | - |
| IUIVLUD02JEZ2O | - | Os07t0421600-00 | Sb01g026760.1 | - |
| IUIVLUD01BY8OS | - | Os02t0513700-00 | Sb01g026820.1 | - |
| IV9RU5F01C5Z3P | - | Os02t0513700-00 | Sb01g026820.1 | - |
| IV9RU5F02GOTCY | - | Os02t0513700-00 | Sb01g026820.1 | - |
| IUIVLUD01EHCQZ | - | Os03t0101100-01 | Sb01g028940.1 | - |
| IV9RU5F01C84G2 | - | Os03t0101100-01 | Sb01g028940.1 | - |
| IUIVLUD01D38RO | - | Os06t0484800-00 | Sb01g031070.1 | - |
| IVLNWEC01AQAF9 | - | Os01t0364400-00 | Sb01g032320.1 | - |
| IUIVLUD01DKRJB | - | Os04t0204200-01 | Sb01g033680.1 | - |
| IUIVLUD01BJS8U | - | Os03t0356638-00 | Sb01g034990.1 | - |
| IV9RU5F01DDZ6A | - | Os03t0344700-01 | Sb01g035620.1 | - |
| IV9RU5F01A4XLY | - | Os03t0289300-00 | Sb01g038540.1 | - |
| IVLNWEC02JEW5A | - | Os03t0289300-00 | Sb01g038540.1 | - |
| IVLNWEC02IA1WN | - | Os03t0110800-01 | Sb01g043010.1 | - |
| IV9RU5F02I9276 | - | Os03t0193000-01 | Sb01g044280.1 | - |
| IV9RU5F02FXUUX | - | Os12t0640600-01 | Sb01g047540.1 | - |
| IV9RU5F02IVIN9 | - | Os12t0640600-01 | Sb01g047540.1 | - |

| | | | | |
|---|---|---|---|---|
| IUIVLUD01BNQAH | - | Os07t0107800-01 | Sb02g000750.1 | - |
| IV9RU5F02ICGXP | - | Os07t0107800-01 | Sb02g000750.1 | - |
| IUIVLUD01CW7R6 | - | Os07t0141500-01 | Sb02g002703.1 | - |
| IVLNWEC01AMWTP | - | Os08t0539400-01 | Sb02g006030.1 | - |
| IVLNWEC02GWYN2 | - | Os07t0447800-01 | Sb02g010840.2 | - |
| IVLNWEC01DLSU8 | - | Os11t0657900-00 | Sb02g011040.1 | - |
| IV9RU5F02HKNHF | - | Os09t0286400-01 | Sb02g019500.2 | - |
| IUIVLUD01D8F6I | - | Os09t0322500-01 | Sb02g021110.1 | - |
| IV9RU5F02GYYC5 | - | Os08t0421800-01 | Sb02g023830.1 | - |
| IV9RU5F02I6KD9 | - | Os09t0469900-01 | Sb02g025090.1 | - |
| IUIVLUD02IPNJ6 | - | Os09t0429000-00 | Sb02g025280.1 | - |
| IV9RU5F02HR7C7 | - | Os02t0237100-01 | Sb02g025980.1 | - |
| IVLNWEC01ARWAI | - | Os02t0237100-01 | Sb02g025980.1 | - |
| IVLNWEC01BXVDD | - | Os09t0456200-01 | Sb02g026570.1 | - |
| IUIVLUD01EZASV | - | Os07t0124500-01 | Sb02g028310.1 | - |
| IUIVLUD02GMX6W | - | Os07t0124500-01 | Sb02g028310.1 | - |
| IV9RU5F02JISYN | - | Os07t0124500-01 | Sb02g028310.1 | - |
| IV9RU5F02F17AZ | - | Os01t0774200-01 | Sb02g028980.1 | - |
| IVLNWEC02JJEHS | - | Os01t0852650-00 | Sb02g030865.1 | - |
| IV9RU5F01BPRWK | - | Os09t0533300-01 | Sb02g030940.1 | - |
| IV9RU5F02I6BKD | - | Os09t0533300-01 | Sb02g030940.1 | - |
| IUIVLUD01AYCQG | - | Os09t0544900-01 | Sb02g031670.1 | - |
| IV9RU5F02G4B52 | - | Os09t0544900-01 | Sb02g031670.1 | - |
| IV9RU5F02ID2NU | - | Os09t0544900-01 | Sb02g031670.1 | - |
| IV9RU5F02FQZ09 | - | Os07t0470800-00 | Sb02g032950.1 | - |
| IV9RU5F02JDCUI | - | Os07t0554100-00 | Sb02g036090.1 | - |
| IV9RU5F02F584D | - | Os01t0967700-01 | Sb02g042875.1 | - |
| IUIVLUD01D561W | - | Os10t0388600-00 | Sb03g002795.1 | - |
| IV9RU5F01BZIJD | - | Os01t0140100-01 | Sb03g006630.1 | - |
| IVLNWEC01EPZJK | - | Os01t0140100-01 | Sb03g006630.1 | - |
| IV9RU5F02JZQM6 | - | Os02t0674800-01 | Sb03g008090.1 | - |
| IV9RU5F02FUVOE | - | Os08t0543500-01 | Sb03g010030.1 | - |
| IVLNWEC01AV00T | - | Os01t0281400-00 | Sb03g011460.1 | - |
| IUIVLUD02HZSFI | - | Os08t0501000-00 | Sb03g022080.1 | - |
| IV9RU5F01D9PFW | - | Os08t0501000-00 | Sb03g022080.1 | - |
| IV9RU5F01EZMEE | - | Os01t0603500-00 | Sb03g025650.1 | - |
| IVLNWEC02HUPIQ | - | Os01t0603500-00 | Sb03g025650.1 | - |
| IVLNWEC02J1TIA | - | Os01t0574400-01 | Sb03g025820.1 | - |
| IVLNWEC02J2GAB | - | Os01t0601675-01 | Sb03g027070.1 | - |
| IVLNWEC02GPXER | - | Os12t0209300-00 | Sb03g036320.1 | - |
| IVLNWEC01E0NPY | - | Os11t0555300-00 | Sb03g036450.1 | - |
| IVLNWEC01CNDCO | - | Os01t0781100-01 | Sb03g036450.1 | - |
| IUIVLUD01D6MPM | - | Os02t0121700-01 | Sb04g001780.1 | - |
| IVLNWEC01EVNGU | - | Os02t0121700-01 | Sb04g001780.1 | - |
| IVLNWEC02JHTHV | - | Os03t0822100-01 | Sb04g002725.1 | - |
| IVLNWEC02F0M11 | - | Os02t0138100-00 | Sb04g003100.1 | - |
| IVLNWEC02IPM8B | - | Os02t0138100-00 | Sb04g003100.1 | - |

| | | | | |
|---|---|---|---|---|
| IVLNWEC01EQ4FS | - | Os01t0377000-01 | Sb04g005740.1 | - |
| IVLNWEC01DNLJU | - | Os06t0484800-00 | Sb04g014351.1 | - |
| IV9RU5F02H4NVD | - | Os04t0378575-00 | Sb04g020480.1 | - |
| IVLNWEC02I9N5A | - | Os02t0537400-01 | Sb04g021960.1 | - |
| IV9RU5F02F1DR6 | - | Os01t0370200-00 | Sb04g022250.1 | - |
| IVLNWEC01C81WC | - | Os02t0615500-01 | Sb04g026010.1 | - |
| IVLNWEC01DV3J6 | - | Os02t0615500-01 | Sb04g026010.1 | - |
| IV9RU5F02JMGJT | - | Os02t0699700-02 | Sb04g030880.1 | - |
| IVLNWEC01DP13V | - | Os02t0699700-02 | Sb04g030880.1 | - |
| IVLNWEC01DBJX9 | - | Os02t0699700-01 | Sb04g030880.1 | - |
| IV9RU5F01EAH9Y | - | Os02t0676000-01 | Sb04g031990.1 | - |
| IVLNWEC01DBXXN | - | Os02t0676000-01 | Sb04g031990.1 | - |
| IVLNWEC02JGZWL | - | Os05t0392400-01 | Sb04g033300.1 | - |
| IUIVLUD01EH9EV | - | Os02t0650800-00 | Sb04g033300.1 | - |
| IUIVLUD02IH9TH | - | Os02t0650800-00 | Sb04g033300.1 | - |
| IV9RU5F01DRYXF | - | Os02t0650800-00 | Sb04g033300.1 | - |
| IV9RU5F02HPVUP | - | Os02t0650800-00 | Sb04g033300.1 | - |
| IVLNWEC02HPA93 | - | Os02t0650800-00 | Sb04g033300.1 | - |
| IV9RU5F01CQTOR | - | Os02t0795900-01 | Sb04g035895.1 | - |
| IUIVLUD01AVD0Q | - | Os02t0816900-01 | Sb04g037210.1 | - |
| IVLNWEC02F141E | - | Os09t0376700-00 | Sb04g037520.1 | - |
| IVLNWEC01EO24E | - | Os11t0107400-01 | Sb05g000610.1 | - |
| IUIVLUD01DSSUO | - | Os06t0111100-01 | Sb05g001110.1 | - |
| IV9RU5F01A4968 | - | Os06t0111100-01 | Sb05g001110.1 | - |
| IUIVLUD01AP6YG | - | Os12t0134200-01 | Sb05g002180.1 | - |
| IV9RU5F02F4CV6 | - | Os06t0286500-01 | Sb05g003920.1 | - |
| IV9RU5F01A27EE | - | Os05t0250700-00 | Sb05g004370.1 | - |
| IVLNWEC01DKZOY | - | Os03t0822100-01 | Sb05g006263.1 | - |
| IVLNWEC02HS8WT | - | Os11t0264200-01 | Sb05g006530.1 | - |
| IVLNWEC01EF59Q | - | Os04t0219600-01 | Sb05g008140.1 | - |
| IV9RU5F02F89V0 | - | Os08t0246300-01 | Sb05g008310.1 | - |
| IV9RU5F02JUF0N | - | Os11t0213800-01 | Sb05g009130.1 | - |
| IV9RU5F02I8WP6 | - | Os02t0139700-01 | Sb05g011010.1 | - |
| IV9RU5F01BZDSX | - | Os02t0818500-03 | Sb05g022790.1 | - |
| IVLNWEC02I0V67 | - | Os11t0211300-01 | Sb05g025450.1 | - |
| IV9RU5F02HZ2VD | - | Os09t0330700-00 | Sb05g027773.1 | - |
| IUIVLUD01CO3M1 | - | Os09t0322500-01 | Sb05g027773.1 | - |
| IV9RU5F02HYIZL | - | Os04t0166000-01 | Sb06g000310.1 | - |
| IVLNWEC02G4EJ3 | - | Os06t0484800-00 | Sb06g016600.1 | - |
| IUIVLUD01CEGMS | - | Os12t0621300-02 | Sb06g021090.1 | - |
| IUIVLUD01D2RWD | - | Os04t0495300-01 | Sb06g021330.1 | - |
| IUIVLUD01DBC3D | - | Os02t0652800-01 | Sb06g024830.1 | - |
| IV9RU5F02IWQLW | - | Os02t0699000-01 | Sb06g027310.1 | - |
| IVLNWEC02HQAAH | - | Os02t0699000-01 | Sb06g027310.1 | - |
| IVLNWEC02IIYSL | - | Os02t0112900-00 | Sb06g028440.1 | - |
| IVLNWEC02JLU1V | - | Os02t0112900-00 | Sb06g028440.1 | - |
| IV9RU5F02HTPY0 | - | Os08t0137100-01 | Sb07g002810.1 | - |

| | | | | |
|---|---|---|---|---|
| IVLNWEC02FNLWG | - | Os08t0158500-01 | Sb07g003970.1 | - |
| IVLNWEC01DCQ9Y | - | Os11t0213700-00 | Sb07g005710.1 | - |
| IUIVLUD02IV18T | - | Os11t0211300-01 | Sb07g005710.1 | - |
| IV9RU5F01AEB6X | - | Os11t0213800-01 | Sb07g005720.1 | - |
| IV9RU5F01B6E9R | - | Os11t0213800-01 | Sb07g005720.1 | - |
| IVLNWEC02HHL0G | - | Os11t0213800-01 | Sb07g005720.1 | - |
| IUIVLUD02GY7DU | - | Os12t0564800-01 | Sb07g022230.1 | - |
| IVLNWEC01CHSPC | - | Os08t0564800-01 | Sb07g023602.1 | - |
| IVLNWEC02F2TYG | - | Os08t0506000-01 | Sb07g027850.1 | - |
| IV9RU5F01CYTW7 | - | Os11t0608300-01 | Sb08g001900.1 | - |
| IV9RU5F02JBS85 | - | Os11t0555300-00 | Sb08g002410.1 | - |
| IVLNWEC01EKAYY | - | Os01t0359800-00 | Sb08g002710.1 | - |
| IV9RU5F02F86QE | - | Os11t0619500-00 | Sb08g003810.1 | - |
| IVLNWEC02II8DW | - | Os11t0619500-00 | Sb08g003810.1 | - |
| IVLNWEC01A8ASS | - | Os11t0555300-00 | Sb08g005463.1 | - |
| IVLNWEC02GTVP6 | - | Os12t0621300-02 | Sb08g007745.1 | - |
| IVLNWEC01ASNF8 | - | Os12t0574000-01 | Sb08g019030.1 | - |
| IV9RU5F02H30KD | - | Os04t0350000-01 | Sb08g021140.1 | - |
| IVLNWEC01DHD43 | - | Os12t0615000-00 | Sb08g021540.1 | - |
| IVLNWEC01A4UKE | - | Os12t0621700-01 | Sb08g022070.1 | - |
| IV9RU5F02I8ZP4 | - | Os12t0641100-03 | Sb08g023290.1 | - |
| IV9RU5F02IS9IV | - | Os12t0641100-03 | Sb08g023290.1 | - |
| IV9RU5F02H8BP1 | - | Os05t0105000-01 | Sb09g000490.1 | - |
| IUIVLUD01DGLLG | - | Os05t0157100-01 | Sb09g004460.1 | - |
| IV9RU5F02HTQS2 | - | Os05t0392400-01 | Sb09g019410.1 | - |
| IVLNWEC02F99KQ | - | Os05t0535500-01 | Sb09g026710.1 | - |
| IV9RU5F02GS0CZ | - | Os06t0104000-01 | Sb10g000445.1 | - |
| IVLNWEC02HOX5E | - | Os06t0104000-01 | Sb10g000445.1 | - |
| IV9RU5F01APML3 | - | Os06t0129900-01 | Sb10g001880.1 | - |
| IV9RU5F02FTV4B | - | Os06t0129900-01 | Sb10g001880.1 | - |
| IV9RU5F02I1VCR | - | Os06t0133900-01 | Sb10g002230.1 | - |
| IV9RU5F01C0XZV | - | Os06t0225100-01 | Sb10g007930.1 | - |
| IV9RU5F02FQ8Y6 | - | Os06t0225100-01 | Sb10g007930.1 | - |
| IV9RU5F02IBGL3 | - | Os06t0225100-01 | Sb10g007930.1 | - |
| IV9RU5F01CHRPZ | - | Os08t0106900-00 | Sb10g009420.1 | - |
| IUIVLUD02JL1XN | - | Os06t0484800-00 | Sb10g022040.1 | - |
| IV9RU5F02G71JO | - | Os06t0484800-00 | Sb10g022040.1 | - |
| IVLNWEC01EJBSU | - | Os06t0484800-00 | Sb10g022040.1 | - |
| IV9RU5F01B9MLE | - | Os03t0757200-01 | Sb01g007630.1 | - |
| IVLNWEC02GGV3K | - | Os08t0532300-01 | Sb07g026170.1 | - |
| IVLNWEC01DDOBB | - | Os12t0582800-01 | Sb08g019525.1 | - |
| IVLNWEC01EVF7J | - | Os12t0582800-01 | Sb08g019525.1 | - |
| IUIVLUD01E19XR | - | Os09t0559800-01 | Sb02g032700.1 | - |
| IVLNWEC02G8UJ0 | - | Os07t0485100-01 | Sb02g033430.1 | - |
| IV9RU5F02IAKAD | - | Os12t0512700-01 | Sb01g016775.1 | - |
| IVLNWEC02FJ6SI | - | Os12t0512700-01 | Sb01g016775.1 | - |
| IUIVLUD01D3H8H | - | Os03t0182400-01 | Sb01g045110.1 | - |

| | | | | |
|---|---|---|---|---|
| IV9RU5F01C81Z1 | - | Os03t0315800-01 | Sb01g037100.1 | - |
| IVLNWEC01AS8M3 | - | Os03t0315800-01 | Sb01g037100.1 | - |
| IVLNWEC01EI3NR | - | Os02t0798800-00 | Sb04g036130.1 | - |
| IVLNWEC01ELUR9 | - | Os09t0556700-01 | Sb02g032470.1 | - |
| IVLNWEC01AV4VL | - | Os06t0498400-01 | Sb10g017820.1 | - |
| IVLNWEC01BUN89 | - | Os03t0598200-01 | Sb01g015150.1 | - |
| IVLNWEC01CU9LT | - | Os03t0193000-01 | Sb01g044280.1 | - |
| IV9RU5F01B5W7D | - | Os11t0696200-01 | Sb05g027180.1 | - |
| IVLNWEC01CW72J | - | Os11t0696200-01 | Sb05g027180.1 | - |
| IV9RU5F02GTXJ9 | - | Os02t0523500-01 | Sb04g021280.1 | - |
| IV9RU5F01CROHK | - | Os05t0105000-01 | Sb09g000490.1 | - |
| IUIVLUD01BUNEV | - | Os01t0774200-01 | Sb06g030710.1 | - |
| IV9RU5F02I9ODS | - | Os04t0356600-01 | Sb09g001750.1 | - |
| IV9RU5F01EWW73 | - | Os03t0358000-00 | Sb01g034875.1 | - |
| IUIVLUD01BGSIL | - | Os01t0601675-01 | Sb03g027070.1 | - |
| IV9RU5F01DA5F6 | - | Os01t0528800-01 | Sb04g011550.1 | - |
| IVLNWEC01C7FKB | - | Os01t0528800-01 | Sb04g011550.1 | - |
| IV9RU5F02GXP8S | - | Os09t0296400-01 | Sb02g019440.1 | - |
| IVLNWEC02HRFF2 | - | Os09t0567700-01 | Sb01g006440.1 | - |
| IV9RU5F02JOOO5 | - | Os03t0784000-02 | Sb01g006160.1 | - |
| IVLNWEC01BFVLF | - | Os09t0448900-01 | Sb02g026190.1 | - |
| IVLNWEC02JGJ9C | - | Os03t0707900-01 | Sb01g010650.1 | - |
| IVLNWEC01E099D | - | Os09t0436400-01 | Sb04g025590.1 | - |
| IVLNWEC01CEU23 | - | Os01t0183100-01 | Sb04g021020.1 | - |
| IVLNWEC02J3W23 | - | Os03t0786700-01 | Sb01g039720.1 | - |
| IV9RU5F02HJEV0 | - | Os01t0574400-01 | Sb03g025820.1 | - |
| IUIVLUD02HYBQG | - | Os03t0415200-01 | Sb01g032510.1 | - |
| IVLNWEC01BP17P | - | Os03t0151800-02 | Sb01g020910.1 | - |
| IV9RU5F01DSTGT | - | Os08t0178100-01 | Sb07g004940.1 | - |
| IV9RU5F02FULXY | - | Os08t0178100-01 | Sb07g004940.1 | - |
| IUIVLUD01BMXKH | - | Os12t0564800-01 | Sb08g018410.1 | - |
| IVLNWEC01AHLSF | - | Os03t0805100-01 | Sb01g004550.1 | - |
| IV9RU5F01APIWH | - | Os03t0358000-00 | Sb01g034875.1 | - |
| IVLNWEC01D7XHM | - | Os08t0427300-01 | Sb07g020830.1 | - |
| IVLNWEC01AMNEV | - | Os03t0852900-01 | Sb01g000850.1 | - |
| IV9RU5F02GNF8U | - | Os03t0330000-01 | Sb01g036430.1 | - |
| IVLNWEC01EUXC5 | - | Os11t0213700-00 | Sb07g005710.1 | - |
| IV9RU5F01E2EPI | - | Os11t0695900-01 | Sb05g027171.1 | - |
| IVLNWEC01ANI45 | - | Os03t0307100-01 | Sb01g037483.1 | - |
| IVLNWEC02HZGD9 | - | Os03t0307100-01 | Sb01g037483.1 | - |
| IV9RU5F01BXKAE | - | Os12t0527800-01 | Sb08g016700.1 | - |
| IUIVLUD01AI4MD | - | Os12t0640800-01 | Sb08g023260.1 | - |
| IV9RU5F01A9QVG | - | Os12t0134200-01 | Sb05g002180.1 | - |
| IV9RU5F01C72QJ | - | Os03t0711200-01 | Sb01g010410.1 | - |
| IV9RU5F02I553R | - | Os03t0332400-01 | Sb01g036340.1 | - |
| IV9RU5F02F2MCQ | - | Os02t0200000-01 | Sb10g023760.1 | - |
| IVLNWEC01DGDTQ | - | Os12t0574000-01 | Sb08g019030.1 | - |

| | | | | |
|---|---|---|---|---|
| IVLNWEC02GYSVL | - | Os07t0470800-00 | Sb02g032950.1 | - |
| IVLNWEC02JCZXW | - | Os03t0730400-01 | Sb06g018070.1 | - |
| IV9RU5F01AKRQ9 | - | Os01t0603500-00 | Sb03g025650.1 | - |
| IUIVLUD02G0VYI | - | Os02t0537400-01 | Sb06g016690.1 | - |
| IVLNWEC01B8PSR | - | Os09t0533300-01 | Sb02g030940.1 | - |
| IV9RU5F02G3BNK | - | Os02t0674800-01 | Sb02g030470.1 | - |
| IV9RU5F01A0QPQ | - | Os01t0601675-01 | Sb03g027070.1 | - |
| IVLNWEC01CUB02 | - | Os03t0193000-01 | Sb01g044280.1 | - |
| IUIVLUD01A9Y5R | - | Os08t0114300-01 | Sb07g001500.1 | - |
| IV9RU5F02G9SGI | - | Os08t0480200-01 | Sb07g028260.1 | - |
| IUIVLUD02FLMCG | - | Os07t0447000-00 | Sb08g015650.1 | - |
| IUIVLUD02II0ZD | - | Os06t0158900-00 | Sb07g027770.1 | - |
| IV9RU5F02HC04A | - | Os05t0188500-02 | Sb05g025690.1 | - |
| IUIVLUD01DH380 | - | Os11t0153700-01 | Sb05g003290.1 | - |
| IV9RU5F02JM5I6 | - | Os03t0852900-01 | Sb01g000850.1 | - |
| IUIVLUD02HYXU2 | - | Os03t0294900-01 | Sb01g038300.1 | - |
| IVLNWEC01APOJ7 | - | Os01t0574400-01 | Sb09g030660.1 | - |
| IVLNWEC01CZ5RB | - | Os06t0225100-01 | Sb10g007930.1 | - |
| IUIVLUD02H77LC | - | Os10t0346300-01 | Sb05g026630.1 | - |
| IV9RU5F01ASA2I | - | Os03t0294900-01 | Sb01g038300.1 | - |
| IV9RU5F02H3CT8 | - | Os03t0294900-01 | Sb01g038300.1 | - |
| IV9RU5F02HHLC5 | - | Os10t0392700-02 | Sb01g023260.3 | - |
| IV9RU5F02JTRGR | - | Os10t0392700-02 | Sb01g023260.3 | - |
| IUIVLUD01BF13N | - | Os07t0645900-00 | Sb01g039150.1 | - |
| IVLNWEC02JPCCK | - | Os03t0648400-01 | Sb01g013390.1 | - |
| IV9RU5F01D1JD6 | - | Os03t0713100-01 | Sb01g010240.2 | - |
| IVLNWEC01CSBHU | - | Os03t0307100-01 | Sb01g037483.1 | - |
| IUIVLUD01CXBU6 | - | Os02t0290600-01 | Sb04g011310.1 | AK248453.1 |
| IVLNWEC02JUDGA | - | Os09t0394900-01 | Sb02g024090.1 | AK249691.1 |
| IVLNWEC01CMMAN | - | Os03t0664700-01 | Sb01g012610.1 | AK359028 |
| IV9RU5F02IIJL6 | - | Os02t0284500-01 | Sb06g002515.1 | AK360162 |
| IVLNWEC02HDD3G | - | Os02t0139700-01 | Sb04g001400.1 | AK360753 |
| IVLNWEC01BZX3C | - | Os03t0711200-01 | Sb01g010410.1 | AK360859 |
| IVLNWEC02HDOSC | - | Os08t0265300-00 | Sb07g005720.1 | AK361251 |
| IV9RU5F01AGROS | - | Os03t0649000-01 | Sb08g022010.1 | AK363790 |
| IUIVLUD01CCO0F | - | Os03t0407000-01 | Sb01g032840.1 | AK363897 |
| IUIVLUD01DNG4N | - | Os03t0407000-01 | Sb01g032840.1 | AK363897 |
| IUIVLUD01D8ZFY | - | Os09t0510700-01 | Sb02g029540.1 | AK363932 |
| IUIVLUD01EFQCN | - | Os01t0547000-01 | Sb01g028490.1 | AK365963 |
| IUIVLUD01ECCW3 | - | Os03t0707900-01 | Sb01g010650.1 | AK366523 |
| IV9RU5F01DF1J6 | - | Os03t0707900-01 | Sb01g010650.1 | AK366523 |
| IV9RU5F01E3RW1 | - | Os03t0707900-01 | Sb01g010650.1 | AK366523 |
| IUIVLUD01EVFLD | - | Os01t0304300-01 | Sb09g025850.1 | AK367106 |
| IV9RU5F01BVGZ0 | - | Os09t0431200-01 | Sb02g025340.1 | AK369503 |
| IV9RU5F02I8765 | - | Os02t0615500-01 | Sb04g026010.1 | AK369808 |
| IVLNWEC02GOVR8 | - | Os03t0581400-00 | Sb01g015850.1 | AK369964 |
| IV9RU5F01ENEFD | - | Os09t0420900-01 | Sb02g024890.1 | AK371245 |

| | | | | |
|---|---|---|---|---|
| IV9RU5F02ISK1W | - | Os08t0137100-01 | Sb07g002810.1 | AK371754 |
| IV9RU5F02F1C7D | - | Os03t0859800-01 | Sb01g000370.1 | AK371781 |
| IUIVLUD02H4DBY | - | Os01t0358700-01 | Sb03g014390.1 | AK371992 |
| IV9RU5F01CBPY7 | - | Os07t0447800-01 | Sb02g010840.2 | AK372310 |
| IV9RU5F01CVDDS | - | Os07t0447800-01 | Sb02g010840.2 | AK372310 |
| IV9RU5F02F4297 | - | Os07t0447800-01 | Sb02g010840.2 | AK372310 |
| IVLNWEC01E2EFE | - | Os12t0621700-01 | Sb08g022070.1 | AK373015 |
| IVLNWEC02I9NFL | - | Os09t0279300-00 | Sb02g018630.1 | AK373149 |
| IVLNWEC02H7DQJ | - | Os08t0338700-01 | Sb02g019590.1 | AK377032 |
| IUIVLUD02HMAXK | - | Os06t0133900-01 | Sb10g002230.1 | AK377052 |
| IUIVLUD01BIML7 | - | Os05t0383100-01 | Sb04g001630.1 | MLOC_10171.2 |
| IUIVLUD01BVHIP | - | Os02t0497500-02 | Sb04g020090.1 | MLOC_10497.1 |
| IVLNWEC01A8SQ4 | - | Os02t0497500-02 | Sb04g020090.1 | MLOC_10497.1 |
| IUIVLUD02I77XL | - | Os03t0798300-01 | Sb01g005082.1 | MLOC_10988.2 |
| IV9RU5F02JF5LX | - | Os05t0157100-01 | Sb09g004460.1 | MLOC_11220.1 |
| IV9RU5F02GWJW0 | - | Os04t0177000-00 | Sb06g002970.1 | MLOC_12543.1 |
| IUIVLUD01CPMTJ | - | Os09t0448900-01 | Sb02g026190.1 | MLOC_13048.8 |
| IUIVLUD01EXWLK | - | Os12t0430000-00 | Sb05g016000.1 | MLOC_14105.3 |
| IUIVLUD02IYMI0 | - | Os11t0264200-01 | Sb05g006530.1 | MLOC_19192.2 |
| IV9RU5F02I38TP | - | Os11t0264200-01 | Sb05g006530.1 | MLOC_19192.2 |
| IUIVLUD02JLEHS | - | Os06t0102900-01 | Sb10g000310.1 | MLOC_19401.1 |
| IV9RU5F01AUQHV | - | Os03t0808000-00 | Sb01g004190.1 | MLOC_22135.1 |
| IUIVLUD02F6IAS | - | Os08t0496700-00 | Sb02g004170.1 | MLOC_33543.1 |
| IVLNWEC01AHJNS | - | Os10t0134033-00 | Sb01g026660.1 | MLOC_34476.1 |
| IUIVLUD01B4M0Q | - | Os05t0421300-01 | Sb04g024620.1 | MLOC_34880.1 |
| IUIVLUD01CXOSN | - | Os05t0421300-01 | Sb04g024620.1 | MLOC_34880.1 |
| IVLNWEC01BECKD | - | Os03t0743800-00 | Sb01g002670.1 | MLOC_36327.1 |
| IV9RU5F01DG5N3 | - | Os02t0523500-01 | Sb04g021280.1 | MLOC_36424.1 |
| IVLNWEC01BWLIF | - | Os12t0641100-03 | Sb08g023290.1 | MLOC_36509.1 |
| IV9RU5F01CNS45 | - | Os10t0578500-00 | Sb01g027870.1 | MLOC_37122.1 |
| IVLNWEC01B45KZ | - | Os10t0578500-00 | Sb01g027870.1 | MLOC_37122.1 |
| IVLNWEC02F95ZE | - | Os02t0699700-01 | Sb04g030880.1 | MLOC_38181.1 |
| IVLNWEC01COB21 | - | Os11t0265200-00 | Sb05g009970.1 | MLOC_43948.2 |
| IVLNWEC02HDWEW | - | Os11t0265200-00 | Sb05g009970.1 | MLOC_43948.2 |
| IV9RU5F01B1VG2 | - | Os10t0577700-01 | Sb01g027930.1 | MLOC_44161.3 |
| IV9RU5F01EGCUT | - | Os10t0577700-01 | Sb01g027930.1 | MLOC_44161.3 |
| IUIVLUD01CTAKP | - | Os03t0669800-01 | Sb04g024360.1 | MLOC_4537.1 |
| IUIVLUD01DSA08 | - | Os03t0669800-01 | Sb04g024360.1 | MLOC_4537.1 |
| IVLNWEC01EWL09 | - | Os11t0608300-01 | Sb04g003073.1 | MLOC_4798.7 |
| IUIVLUD01DVD7S | - | Os11t0124900-01 | Sb08g002010.1 | MLOC_4908.1 |
| IVLNWEC02FTF42 | - | Os11t0124900-01 | Sb08g002010.1 | MLOC_4908.1 |
| IVLNWEC02I12CP | - | Os11t0124900-01 | Sb08g002010.1 | MLOC_4908.1 |
| IVLNWEC01EU6WQ | - | Os08t0543500-01 | Sb07g025850.1 | MLOC_52055.2 |
| IV9RU5F01CSK80 | - | Os09t0296400-01 | Sb02g019440.1 | MLOC_53292.7 |
| IVLNWEC01CANRU | - | Os08t0506000-01 | Sb07g027850.1 | MLOC_54525.2 |
| IUIVLUD02F7RAR | - | Os11t0148700-01 | Sb05g002930.1 | MLOC_54689.2 |
| IVLNWEC02JQUSL | - | Os10t0147250-00 | Sb01g026260.1 | MLOC_54855.1 |

| | | | | |
|---|---|---|---|---|
| IVLNWEC01EGZ7C | - | Os03t0738200-00 | Sb01g008695.1 | MLOC_55034.1 |
| IVLNWEC01AH206 | - | Os02t0773300-01 | Sb04g034640.1 | MLOC_55975.1 |
| IVLNWEC01ARNF0 | - | Os03t0747300-01 | Sb01g008220.2 | MLOC_56037.3 |
| IV9RU5F02J5WQ2 | - | Os11t0153700-01 | Sb05g003290.1 | MLOC_5656.1 |
| IV9RU5F01BSNBV | - | Os12t0640600-01 | Sb01g047540.1 | MLOC_57533.7 |
| IV9RU5F02GWPC9 | - | Os12t0640600-01 | Sb01g047540.1 | MLOC_57533.7 |
| IVLNWEC01A6M1S | - | Os12t0640600-01 | Sb01g047540.1 | MLOC_57533.7 |
| IVLNWEC01D3PJ1 | - | Os12t0640600-01 | Sb01g047540.1 | MLOC_57533.7 |
| IVLNWEC01EOBDH | - | Os12t0640600-01 | Sb01g047540.1 | MLOC_57533.7 |
| IVLNWEC01B1K9V | - | Os10t0482700-00 | Sb01g019060.1 | MLOC_57694.1 |
| IUIVLUD01C2Z6P | - | Os07t0435400-01 | Sb03g013640.1 | MLOC_58976.1 |
| IVLNWEC02GODZY | - | Os04t0351333-00 | Sb05g022840.1 | MLOC_59638.1 |
| IV9RU5F02G2FIX | - | Os03t0719100-01 | Sb05g000360.1 | MLOC_60545.2 |
| IVLNWEC01C2KDM | - | Os03t0719100-01 | Sb09g002225.1 | MLOC_60545.2 |
| IVLNWEC01EN93G | - | Os08t0371608-00 | Sb02g021480.1 | MLOC_60975.2 |
| IV9RU5F01CDJFO | - | Os03t0143851-01 | Sb01g047340.1 | MLOC_64091.1 |
| IV9RU5F02F37ZL | - | Os03t0143851-01 | Sb01g047340.1 | MLOC_64091.1 |
| IUIVLUD02G9BTI | - | Os01t0781200-01 | Sb08g002350.1 | MLOC_67378.2 |
| IVLNWEC01AZUOR | - | Os09t0553900-01 | Sb02g032300.1 | MLOC_67933.1 |
| IVLNWEC01DEBAX | - | Os03t0852900-01 | Sb01g000850.1 | MLOC_71240.1 |
| IUIVLUD01A7BBG | - | Os12t0577600-01 | Sb08g019210.1 | MLOC_72552.2 |
| IV9RU5F01BIJ7Z | - | Os03t0330000-01 | Sb01g036430.1 | MLOC_73012.1 |
| IVLNWEC01EALPI | - | Os04t0397100-01 | Sb06g014590.1 | MLOC_73713.1 |
| IVLNWEC01B3UNV | - | Os06t0498400-01 | Sb10g017820.1 | MLOC_73846.5 |
| IUIVLUD01D3FTK | - | Os03t0321500-01 | Sb01g036880.1 | MLOC_74241.2 |
| IV9RU5F02ICATR | - | Os01t0663051-00 | Sb09g028790.1 | MLOC_75951.1 |
| IVLNWEC01CAM0T | - | Os04t0166000-01 | Sb02g004690.1 | MLOC_81389.2 |
| IV9RU5F01DD48S | - | Os11t0264200-01 | Sb05g006530.1 | MLOC_19192.2 |
| IVLNWEC01CR8E1 | - | Os02t0773300-01 | Sb04g034640.1 | MLOC_55975.1 |
| IUIVLUD01CMKYE | - | Os02t0224200-01 | Sb04g007940.2 | MLOC_55635.2 |
| IV9RU5F02FYLT3 | - | Os03t0251500-01 | Sb01g040970.1 | MLOC_58358.1 |
| IUIVLUD02JDA73 | - | Os02t0184400-01 | Sb10g025240.1 | AK366557 |
| IUIVLUD02ILQTM | - | Os05t0387200-01 | Sb09g019100.1 | AK249402.1 |
| IV9RU5F01A9STG | - | Os05t0387200-01 | Sb09g019100.1 | AK249402.1 |
| IV9RU5F01AKC6W | - | Os11t0310800-01 | Sb10g005130.1 | AK375706 |
| IVLNWEC02HRQCO | - | Os11t0310800-01 | Sb10g005130.1 | AK375706 |
| IV9RU5F02FV138 | - | Os05t0188500-02 | Sb05g025690.1 | AK375660 |
| IVLNWEC01COTGD | - | Os09t0510700-01 | Sb02g029540.1 | AK363932 |
| IUIVLUD01DXKOI | - | Os12t0594300-00 | Sb02g000740.1 | AK371555 |
| IUIVLUD01EBAQY | - | Os12t0582800-01 | Sb08g019525.1 | AK355948 |
| IVLNWEC01CYDAO | - | Os05t0150400-01 | Sb01g019110.1 | MLOC_16126.1 |
| IVLNWEC02IS28J | - | Os05t0150400-01 | Sb09g003860.1 | MLOC_16126.1 |
| IV9RU5F01DL784 | - | Os11t0558300-01 | Sb05g021840.1 | MLOC_66827.1 |
| IV9RU5F02F6CYI | - | Os02t0290600-01 | Sb04g011310.1 | AK248453.1 |
| IV9RU5F01A8F3E | - | Os07t0485100-01 | Sb02g033430.1 | MLOC_73067.1 |
| IV9RU5F01C36EY | - | Os12t0182300-01 | Sb08g005060.1 | MLOC_60385.1 |
| IVLNWEC01DFVNR | - | Os03t0182400-01 | Sb01g045110.1 | AK248477.1 |

| | | | | |
|---|---|---|---|---|
| IVLNWEC01BUMZJ | - | Os03t0315800-01 | Sb01g037100.1 | AK249382.1 |
| IVLNWEC01C133Z | - | Os04t0369066-00 | Sb08g014920.1 | MLOC_15351.6 |
| IV9RU5F02F3IG9 | - | Os06t0225100-01 | Sb10g007930.1 | AK356239 |
| IV9RU5F02ILQ1E | - | Os03t0803700-01 | Sb01g004640.1 | MLOC_10144.1 |
| IV9RU5F02JNSI8 | - | Os03t0152900-01 | Sb01g046750.1 | MLOC_54161.7 |
| IUIVLUD01DOE3V | - | Os05t0352800-01 | Sb06g026730.1 | MLOC_12605.2 |
| IVLNWEC01C683U | - | Os05t0383100-01 | Sb04g001630.1 | MLOC_10171.2 |
| IV9RU5F02H13U2 | - | Os09t0556700-01 | Sb02g032470.1 | MLOC_54619.1 |
| IV9RU5F01AOY8U | - | Os05t0357100-01 | Sb09g017250.1 | AK364665 |
| IV9RU5F01CNBLO | - | Os02t0816900-01 | Sb04g037210.1 | MLOC_4461.5 |
| IUIVLUD02IB660 | - | Os03t0743800-00 | Sb02g040700.1 | MLOC_46083.1 |
| IVLNWEC02I16FZ | - | Os12t0617900-01 | Sb08g021850.1 | AK375108 |
| IUIVLUD01DFU3V | - | Os02t0523500-01 | Sb04g021280.1 | MLOC_36424.1 |
| IVLNWEC01AYO2K | - | Os11t0176200-01 | Sb03g003740.1 | MLOC_11045.3 |
| IVLNWEC02IIYDX | - | Os03t0415200-01 | Sb01g032510.1 | MLOC_71887.2 |
| IV9RU5F01C92XN | - | Os08t0539400-01 | Sb07g025620.1 | MLOC_59797.4 |
| IV9RU5F02HVMT6 | - | Os04t0350000-01 | Sb05g022840.1 | MLOC_59638.1 |
| IVLNWEC01BG6OK | - | Os01t0896700-00 | Sb01g048270.1 | MLOC_58960.1 |
| IUIVLUD02GQQBB | - | Os01t0295700-01 | Sb03g012020.1 | MLOC_12326.1 |
| IV9RU5F02HICXD | - | Os01t0528800-01 | Sb04g011550.1 | AK252044.1 |
| IVLNWEC02IHAJE | - | Os01t0528800-01 | Sb04g011550.1 | AK252044.1 |
| IV9RU5F01D1PFU | - | Os09t0296400-01 | Sb02g019440.1 | MLOC_53292.7 |
| IV9RU5F01D8D40 | - | Os09t0394900-01 | Sb02g024090.1 | AK249691.1 |
| IUIVLUD02H1GQP | - | Os09t0513000-01 | Sb02g029680.1 | MLOC_62437.1 |
| IVLNWEC01EAZZ6 | - | Os09t0513000-01 | Sb02g029680.1 | MLOC_62437.1 |
| IUIVLUD01AJ1H5 | - | Os12t0577600-01 | Sb08g019210.1 | MLOC_72552.2 |
| IUIVLUD01BPIHB | - | Os09t0567700-01 | Sb01g006440.1 | AK365828 |
| IUIVLUD01DBF63 | - | Os03t0738900-00 | Sb01g008670.1 | AK375511 |
| IUIVLUD01BU1RN | - | Os12t0271600-01 | Sb0011s005460.1 | MLOC_74657.2 |
| IUIVLUD02H4XAL | - | Os12t0271600-01 | Sb0011s005460.1 | MLOC_74657.2 |
| IVLNWEC02F09HI | - | Os12t0271600-01 | Sb0011s005460.1 | MLOC_74657.2 |
| IV9RU5F02IYA5P | - | Os06t0670000-01 | Sb10g026910.1 | MLOC_2170.2 |
| IV9RU5F01AG5BR | - | Os03t0707900-01 | Sb01g010650.1 | AK366523 |
| IV9RU5F01BBSYW | - | Os03t0707900-01 | Sb01g010650.1 | AK366523 |
| IV9RU5F01DUOYQ | - | Os03t0707900-01 | Sb01g010650.1 | AK366523 |
| IV9RU5F01EFARV | - | Os03t0707900-01 | Sb01g010650.1 | AK366523 |
| IV9RU5F01A3OBZ | - | Os02t0810500-01 | Sb01g000210.1 | AK368335 |
| IV9RU5F02GSDQ4 | - | Os03t0669800-01 | Sb03g046860.1 | MLOC_4537.1 |
| IVLNWEC01DTI8O | - | Os03t0777700-01 | Sb01g006380.1 | AK360042 |
| IVLNWEC02H77W7 | - | Os07t0141500-01 | Sb02g002703.1 | MLOC_30557.1 |
| IUIVLUD01D0TZ4 | - | Os12t0430000-00 | Sb05g016000.1 | MLOC_14105.3 |
| IVLNWEC01EWR2Q | - | Os03t0221300-01 | Sb01g042570.1 | AK372460 |
| IVLNWEC02IJI5X | - | Os03t0294900-01 | Sb01g038300.1 | MLOC_34537.1 |
| IUIVLUD02G6KTF | - | Os10t0512800-01 | Sb01g017240.2 | MLOC_10504.1 |
| IV9RU5F01BFZEA | - | Os10t0512800-01 | Sb01g017240.2 | MLOC_10504.1 |
| IUIVLUD01C5BGJ | - | Os02t0301500-01 | Sb01g044300.1 | MLOC_36653.1 |
| IV9RU5F02I7H8M | - | Os11t0265400-01 | Sb05g009990.3 | MLOC_5651.1 |

| | | | | |
|---|---|---|---|---|
| IV9RU5F01BY486 | - | Os02t0802400-01 | Sb04g036210.1 | MLOC_61171.1 |
| IV9RU5F01DYVPF | - | Os02t0802400-01 | Sb04g036210.1 | MLOC_61171.1 |
| IUIVLUD02I0TMC | - | Os03t0802700-01 | Sb01g004730.1 | AK368023 |
| IVLNWEC01CR0XC | - | Os03t0802700-01 | Sb01g004730.1 | AK368023 |
| IUIVLUD02GFB7M | - | Os08t0421800-01 | Sb02g023830.1 | MLOC_24477.2 |
| IVLNWEC02IH8WU | - | Os03t0330000-01 | Sb01g036430.1 | MLOC_73012.1 |
| IV9RU5F02I2U38 | - | Os05t0421300-01 | Sb04g024620.1 | MLOC_34880.1 |
| IV9RU5F02F7000 | - | Os11t0558300-01 | Sb05g021840.1 | MLOC_66827.1 |
| IUIVLUD01D1R3Y | - | Os04t0492300-01 | Sb06g021120.1 | MLOC_36681.1 |
| IV9RU5F02FRD5P | - | Os02t0516600-01 | Sb04g021110.1 | AK369767 |
| IV9RU5F02JTNSQ | - | Os02t0516600-01 | Sb04g021110.1 | AK369767 |
| IVLNWEC02I4X2Q | - | Os07t0435400-01 | Sb03g013640.1 | MLOC_58976.1 |
| IVLNWEC01BO81J | - | Os09t0394600-01 | Sb02g024070.1 | AK365907 |
| IVLNWEC02F5VRJ | - | Os03t0321500-01 | Sb01g036880.1 | MLOC_74241.2 |
| IV9RU5F01ESFEZ | - | Os03t0356638-00 | Sb01g034990.1 | MLOC_18152.1 |
| IV9RU5F01CIDOT | - | Os12t0527800-01 | Sb08g016700.1 | AK361476 |
| IV9RU5F01DEBGO | - | Os02t0497500-02 | Sb04g020090.1 | MLOC_10497.1 |
| IV9RU5F02F8IMV | - | Os02t0497500-02 | Sb04g020090.1 | MLOC_10497.1 |
| IV9RU5F02H3CIY | - | Os12t0640800-01 | Sb08g023260.1 | MLOC_73572.1 |
| IUIVLUD02GA72T | - | Os03t0711200-01 | Sb01g010410.1 | AK360859 |
| IVLNWEC01AW2VS | - | Os03t0711200-01 | Sb01g010410.1 | AK360859 |
| IVLNWEC02I692Z | - | Os03t0711200-01 | Sb01g010410.1 | AK360859 |
| IVLNWEC02HIL27 | - | Os03t0750100-00 | Sb01g007980.1 | MLOC_59424.2 |
| IV9RU5F02G7DO3 | - | Os03t0332400-01 | Sb01g036340.1 | MLOC_12784.1 |
| IUIVLUD02FOK5Q | - | Os03t0300500-01 | Sb01g037900.1 | MLOC_57346.1 |
| IVLNWEC02FSPXW | - | Os11t0176200-01 | Sb03g003740.1 | MLOC_11045.3 |
| IV9RU5F02JTXZM | - | Os01t0309800-01 | Sb03g012690.1 | MLOC_74403.1 |
| IV9RU5F02JW7XL | - | Os01t0309800-01 | Sb03g012690.1 | MLOC_74403.1 |
| IVLNWEC01B17M3 | - | Os12t0640800-01 | Sb08g023260.1 | MLOC_54142.1 |
| IVLNWEC02I26B3 | - | Os03t0664700-01 | Sb01g012610.1 | AK359028 |
| IV9RU5F01CQ8OH | - | Os06t0286500-01 | Sb02g001150.1 | MLOC_15443.2 |
| IUIVLUD01A4MRH | - | Os05t0383100-01 | Sb04g001630.1 | MLOC_10171.2 |
| IUIVLUD01CZ49A | - | Os04t0677700-00 | Sb06g032970.1 | MLOC_51259.2 |
| IVLNWEC02IAO8L | - | Os03t0251500-01 | Sb01g040970.1 | MLOC_58358.1 |
| IUIVLUD01D7Z29 | - | Os03t0344700-01 | Sb01g035620.1 | AK362285 |
| IV9RU5F02JNQIB | - | Os01t0304300-01 | Sb09g025850.1 | AK367106 |
| IVLNWEC01A07LU | - | Os09t0545500-00 | Sb09g025180.1 | MLOC_58508.9 |
| IV9RU5F02IM26H | - | Os09t0429000-00 | Sb02g025270.1 | AK371221 |
| IV9RU5F01B1P8O | - | Os03t0804400-01 | Sb01g004600.1 | AK372303 |
| IVLNWEC01DINUQ | - | Os09t0420900-01 | Sb02g024890.1 | AK371245 |
| IVLNWEC01DEQ2W | - | Os03t0191000-01 | Sb01g044480.1 | MLOC_63242.2 |
| IVLNWEC01BAY0X | - | Os07t0485100-01 | Sb02g033430.1 | MLOC_73067.1 |
| IVLNWEC02IKD8J | - | Os09t0553900-01 | Sb02g032300.1 | MLOC_67933.1 |
| IVLNWEC01DC3UB | - | Os03t0407000-01 | Sb01g032840.1 | AK363897 |
| IV9RU5F01APQNW | - | Os03t0581400-00 | Sb01g015850.1 | AK369964 |
| IVLNWEC01BTJTE | - | Os08t0265300-00 | Sb05g025470.1 | AK361251 |
| IUIVLUD02JL271 | - | Os08t0532300-01 | Sb07g026170.1 | MLOC_57422.1 |

| | | | | |
|---|---|---|---|---|
| IVLNWEC02JFCUY | - | Os08t0532300-01 | Sb07g026170.1 | MLOC_57422.1 |
| IV9RU5F01D8TO4 | - | Os03t0737800-01 | Sb01g008740.1 | AK367045 |
| IVLNWEC02FVVX5 | - | Os03t0737800-01 | Sb01g008740.1 | AK367045 |
| IVLNWEC01EXQDO | - | Os02t0795900-01 | Sb04g035895.1 | AK374032 |
| IVLNWEC02FO2GL | - | Os07t0141500-01 | Sb02g002703.1 | MLOC_30557.1 |
| IVLNWEC01B8PPI | - | Os03t0315800-01 | Sb01g037100.1 | AK249382.1 |
| IVLNWEC02ISSKR | - | Os03t0803700-01 | Sb01g004640.1 | MLOC_10144.1 |
| IV9RU5F01E0EIT | - | Os05t0357100-01 | Sb09g017250.1 | AK364665 |
| IV9RU5F02F7UYD | - | Os03t0833800-01 | Sb01g002550.1 | MLOC_13213.1 |
| IV9RU5F02JYZV4 | - | Os03t0833800-01 | Sb01g002550.1 | MLOC_13213.1 |
| IV9RU5F01AJ6WM | - | Os09t0279300-00 | Sb02g018630.1 | AK373149 |
| IVLNWEC01ENUAD | - | Os10t0577700-01 | Sb01g027930.1 | MLOC_44161.3 |
| IV9RU5F01A9PO2 | - | Os08t0272200-00 | Sb07g011860.1 | MLOC_29481.1 |
| IV9RU5F02GFS68 | - | Os08t0272200-00 | Sb07g011860.1 | MLOC_29481.1 |
| IV9RU5F02JFJRY | - | Os03t0738900-00 | Sb01g008670.1 | AK375511 |
| IUIVLUD02JKMIK | - | Os03t0203000-01 | Sb01g043650.1 | MLOC_55401.4 |
| IV9RU5F02IXT11 | - | Os06t0670000-01 | Sb10g026910.1 | MLOC_2170.2 |
| IV9RU5F02IPRYV | - | Os03t0707900-01 | Sb01g010650.1 | AK366523 |
| IV9RU5F02FOG2Q | - | Os03t0210800-01 | Sb01g043240.1 | AK357041 |
| IVLNWEC02J3CEF | - | Os10t0339600-01 | Sb02g007180.1 | AK354222 |
| IVLNWEC01DCPOE | - | Os02t0699700-01 | Sb04g030880.1 | MLOC_38181.1 |
| IVLNWEC01CHSL4 | - | Os12t0564800-01 | Sb08g018410.1 | MLOC_1443.2 |
| IVLNWEC02H6SN2 | - | Os12t0564800-01 | Sb08g018410.1 | MLOC_1443.2 |
| IVLNWEC01DZNLB | - | Os03t0407000-01 | Sb01g032840.1 | AK363897 |
| IVLNWEC01C9P1B | - | Os08t0427300-01 | Sb07g020830.1 | MLOC_5116.4 |
| IVLNWEC02HE751 | - | Os08t0427300-01 | Sb07g020830.1 | MLOC_5116.4 |
| IV9RU5F01DXFIX | - | Os03t0802700-01 | Sb01g004730.1 | AK368023 |
| IVLNWEC02FOUJH | - | Os03t0417900-01 | Sb01g032420.1 | AK361277 |
| IUIVLUD01ASBP9 | - | Os07t0435400-01 | Sb03g013640.1 | MLOC_58976.1 |
| IV9RU5F01DGZFW | - | Os11t0124900-01 | Sb08g002010.1 | MLOC_4908.1 |
| IV9RU5F02GNTLV | - | Os11t0124900-01 | Sb08g002010.1 | MLOC_4908.1 |
| IVLNWEC01A73S8 | - | - | Sb02g029920.1 | MLOC_9917.2 |
| IUIVLUD02F7VVO | - | - | Sb02g023010.1 | MLOC_9891.3 |
| IVLNWEC01COJJ9 | - | - | Sb02g023010.1 | MLOC_9891.3 |
| IVLNWEC02JJ035 | - | - | Sb02g023010.1 | MLOC_9891.3 |
| IV9RU5F02GRKBL | - | - | Sb10g001950.1 | MLOC_81095.1 |
| IUIVLUD02GSSLV | - | - | Sb08g015230.1 | MLOC_7548.1 |
| IVLNWEC02H96WR | - | - | Sb01g033770.1 | MLOC_75024.4 |
| IUIVLUD01BN2RO | - | - | Sb01g002960.1 | MLOC_72726.2 |
| IVLNWEC01EAID6 | - | - | Sb03g015070.1 | MLOC_70842.1 |
| IVLNWEC01EBVSY | - | - | Sb03g015070.1 | MLOC_70842.1 |
| IUIVLUD02GZR0S | - | - | Sb01g011950.1 | MLOC_69563.2 |
| IV9RU5F01DB6PH | - | - | Sb01g011950.1 | MLOC_69563.2 |
| IV9RU5F01A9H8Q | - | - | Sb01g043970.1 | MLOC_6856.2 |
| IV9RU5F01BZOC0 | - | - | Sb01g043970.1 | MLOC_6856.2 |
| IVLNWEC01C4PIS | - | - | Sb02g032640.1 | MLOC_68056.2 |
| IVLNWEC01CU8IM | - | - | Sb02g032640.1 | MLOC_68056.2 |

| | | | | |
|---|---|---|---|---|
| IVLNWEC02H6KI4 | - | - | Sb02g026460.1 | MLOC_68051.1 |
| IVLNWEC01CFX59 | - | - | Sb02g030090.1 | MLOC_65109.6 |
| IVLNWEC02HG3HR | - | - | Sb01g011950.1 | MLOC_64087.2 |
| IVLNWEC01CKQE4 | - | - | Sb02g001020.1 | MLOC_62464.1 |
| IVLNWEC01BUR3K | - | - | Sb10g000660.1 | MLOC_61812.1 |
| IUIVLUD01EB79F | - | - | Sb02g023130.1 | MLOC_60983.1 |
| IV9RU5F02F3YE6 | - | - | Sb02g029600.1 | MLOC_60708.1 |
| IV9RU5F01A85DP | - | - | Sb03g025820.1 | MLOC_60551.2 |
| IVLNWEC02IDPL2 | - | - | Sb01g028683.1 | MLOC_60060.1 |
| IV9RU5F01CZDXQ | - | - | Sb01g036250.1 | MLOC_5958.2 |
| IUIVLUD02GGFJK | - | - | Sb04g009890.1 | MLOC_59368.2 |
| IV9RU5F02H5C5X | - | - | Sb01g003750.1 | MLOC_59130.2 |
| IV9RU5F01EP77K | - | - | Sb01g013710.1 | MLOC_58644.1 |
| IV9RU5F01EER8V | - | - | Sb01g000550.1 | MLOC_58215.3 |
| IV9RU5F01A6786 | - | - | Sb07g025645.1 | MLOC_58144.1 |
| IVLNWEC01CCGCE | - | - | Sb02g006030.1 | MLOC_58144.1 |
| IV9RU5F02HVF9P | - | - | Sb06g000340.1 | MLOC_57493.1 |
| IVLNWEC02FPWA5 | - | - | Sb06g000340.1 | MLOC_57493.1 |
| IV9RU5F01B8ECT | - | - | Sb01g015930.1 | MLOC_57169.1 |
| IV9RU5F01DRE32 | - | - | Sb01g015930.1 | MLOC_57169.1 |
| IV9RU5F01EKMSI | - | - | Sb02g004300.2 | MLOC_56946.2 |
| IV9RU5F02JXT6P | - | - | Sb07g004380.1 | MLOC_56869.1 |
| IV9RU5F02JLCPY | - | - | Sb01g030680.1 | MLOC_5653.2 |
| IVLNWEC01E2HLG | - | - | Sb01g011590.1 | MLOC_56050.2 |
| IUIVLUD02JQU95 | - | - | Sb01g008700.1 | MLOC_55034.1 |
| IVLNWEC02G1WZM | - | - | Sb01g012810.1 | MLOC_54949.1 |
| IVLNWEC02FKDHQ | - | - | Sb02g023480.1 | MLOC_54750.3 |
| IVLNWEC01BSJEM | - | - | Sb02g032480.1 | MLOC_54621.1 |
| IVLNWEC02G4308 | - | - | Sb04g006240.1 | MLOC_54213.1 |
| IVLNWEC02HQDNG | - | - | Sb04g006240.1 | MLOC_54213.1 |
| IVLNWEC02IV18L | - | - | Sb04g002063.1 | MLOC_54128.1 |
| IUIVLUD02JXY7Z | - | - | Sb08g022550.1 | MLOC_53665.2 |
| IV9RU5F01BH5LE | - | - | Sb01g035620.1 | MLOC_53018.1 |
| IUIVLUD01BLP31 | - | - | Sb05g002190.1 | MLOC_52548.1 |
| IVLNWEC01EZG2Q | - | - | Sb02g022610.1 | MLOC_51494.1 |
| IUIVLUD01BY3ZU | - | - | Sb04g002063.1 | MLOC_51090.1 |
| IV9RU5F01A48E1 | - | - | Sb07g006490.1 | MLOC_50726.1 |
| IV9RU5F01DT7PV | - | - | Sb02g024760.1 | MLOC_4945.1 |
| IVLNWEC01CJJJC | - | - | Sb02g025340.1 | MLOC_44275.1 |
| IV9RU5F01DU5FX | - | - | Sb09g027223.1 | MLOC_4294.1 |
| IVLNWEC01DRYWD | - | - | Sb09g027223.1 | MLOC_4294.1 |
| IVLNWEC01E09LH | - | - | Sb09g027223.1 | MLOC_4294.1 |
| IV9RU5F01DGCYN | - | - | Sb04g008547.1 | MLOC_3994.1 |
| IVLNWEC01AI6P0 | - | - | Sb02g023510.1 | MLOC_39028.1 |
| IVLNWEC02GY717 | - | - | Sb08g002290.1 | MLOC_38445.1 |
| IVLNWEC01C0WEG | - | - | Sb01g000280.1 | MLOC_38189.2 |
| IUIVLUD01AP804 | - | - | Sb07g025620.1 | MLOC_38153.1 |

| | | | | |
|---|---|---|---|---|
| IV9RU5F02F0F66 | - | - | Sb01g004640.1 | MLOC_37586.1 |
| IVLNWEC01DTYOS | - | - | Sb01g004640.1 | MLOC_37586.1 |
| IV9RU5F01DQ7YE | - | - | Sb08g002900.1 | MLOC_37447.2 |
| IVLNWEC01C97IP | - | - | Sb09g029740.1 | MLOC_37060.1 |
| IVLNWEC02H7HM9 | - | - | Sb04g024620.1 | MLOC_34880.1 |
| IUIVLUD02IN635 | - | - | Sb04g001610.1 | MLOC_2935.1 |
| IVLNWEC01EFE1N | - | - | Sb08g018220.1 | MLOC_29137.2 |
| IUIVLUD01A0QLF | - | - | Sb05g008890.1 | MLOC_24226.1 |
| IUIVLUD01ASM6A | - | - | Sb07g004420.1 | MLOC_21642.1 |
| IV9RU5F02I1RLK | - | - | Sb05g005390.1 | MLOC_20977.1 |
| IUIVLUD01D9SMN | - | - | Sb02g025730.1 | MLOC_18353.1 |
| IVLNWEC02IJII1 | - | - | Sb02g025730.1 | MLOC_18353.1 |
| IV9RU5F02FLGHR | - | - | Sb09g015250.1 | MLOC_18184.1 |
| IUIVLUD02GPF12 | - | - | Sb01g031710.1 | MLOC_17054.1 |
| IV9RU5F01DMJPX | - | - | Sb01g031710.1 | MLOC_17054.1 |
| IUIVLUD01ANA37 | - | - | Sb06g021020.1 | MLOC_14298.1 |
| IUIVLUD02I6CAM | - | - | Sb02g007180.1 | MLOC_13350.1 |
| IV9RU5F01CKMF6 | - | - | Sb04g031600.1 | MLOC_12637.2 |
| IVLNWEC02HEZOM | - | - | Sb04g031600.1 | MLOC_12637.2 |
| IV9RU5F01EC6QH | - | - | Sb09g024270.1 | MLOC_11971.1 |
| IVLNWEC01EATHB | - | - | Sb01g005082.1 | MLOC_10988.2 |
| IV9RU5F01AX46O | - | - | Sb04g009720.1 | MLOC_10500.1 |
| IV9RU5F01DCQMZ | - | - | Sb04g009720.1 | MLOC_10500.1 |
| IV9RU5F01CEUED | - | - | Sb03g014780.1 | MLOC_10329.1 |
| IV9RU5F02JCF5J | - | - | Sb08g015630.2 | AK377105 |
| IVLNWEC01CJE2Y | - | - | Sb08g015630.2 | AK377105 |
| IVLNWEC01CMPNY | - | - | Sb08g015630.2 | AK377105 |
| IVLNWEC02GHOQK | - | - | Sb08g015630.2 | AK377105 |
| IV9RU5F01CZ8NJ | - | - | Sb01g000430.1 | AK374818 |
| IUIVLUD01CU936 | - | - | Sb01g004920.1 | AK374742 |
| IV9RU5F02F2PV1 | - | - | Sb01g004920.1 | AK374742 |
| IUIVLUD02GBNGV | - | - | Sb01g005920.1 | AK371672 |
| IVLNWEC01BI4CP | - | - | Sb03g042460.1 | AK370620 |
| IV9RU5F01EMXXW | - | - | Sb05g007160.1 | AK370601 |
| IV9RU5F02HU0B6 | - | - | Sb02g028600.1 | AK370330 |
| IVLNWEC01D7E5U | - | - | Sb01g001770.1 | AK369519 |
| IVLNWEC01A9W7E | - | - | Sb02g025340.1 | AK369503 |
| IV9RU5F02FQ1FP | - | - | Sb07g001500.1 | AK367303 |
| IVLNWEC01EZ9JZ | - | - | Sb01g010650.1 | AK366523 |
| IUIVLUD01EFF7Z | - | - | Sb07g009340.1 | AK366261 |
| IVLNWEC02G4MCL | - | - | Sb03g001240.1 | AK365985 |
| IV9RU5F01EAEOY | - | - | Sb02g024050.1 | AK364936 |
| IV9RU5F02IJTA1 | - | - | Sb02g024050.1 | AK364936 |
| IVLNWEC01ED987 | - | - | Sb09g000680.1 | AK364743 |
| IVLNWEC02FVS8W | - | - | Sb03g003450.1 | AK361745 |
| IUIVLUD01CQNOJ | - | - | Sb02g022933.1 | AK358827 |
| IVLNWEC02GL8DG | - | - | Sb04g035070.1 | AK355417 |

| | | | | |
|---|---|---|---|---|
| IVLNWEC01C2OAF | - | - | Sb09g000210.1 | AK355206 |
| IVLNWEC01D4S49 | - | - | Sb09g000210.1 | AK355206 |
| IV9RU5F01B6BTN | - | - | Sb01g000730.1 | AK354917 |
| IV9RU5F01CDTXW | - | - | Sb01g000730.1 | AK354917 |
| IVLNWEC01C4LOH | - | - | Sb01g000730.1 | AK354917 |
| IVLNWEC01CWJ88 | - | - | Sb01g000730.1 | AK354917 |
| IUIVLUD01CDXAV | - | - | Sb06g019330.1 | AK354076 |
| IUIVLUD01CH2WK | - | - | Sb02g027460.1 | AK353724 |
| IUIVLUD01AML0B | - | - | Sb02g030550.1 | AK252321.1 |
| IVLNWEC01BWE2I | - | - | Sb02g030550.1 | AK252321.1 |
| IVLNWEC02HFL36 | - | - | Sb02g024090.1 | AK249691.1 |
| IV9RU5F02F6S71 | - | - | Sb02g023430.2 | AK249324.1 |
| IVLNWEC01EVHMR | - | - | Sb03g001240.1 | AK248364.1 |
| IV9RU5F02IEPLQ | - | - | Sb04g030390.1 | AK248234.1 |
| IV9RU5F02IOKEF | - | - | Sb04g030390.1 | AK248234.1 |
| IV9RU5F01DVKTN | - | - | Sb01g000550.1 | MLOC_58215.3 |
| IV9RU5F02FXFS5 | - | - | Sb07g008300.1 | AK354830 |
| IUIVLUD01A0NR2 | - | - | Sb01g000430.1 | AK374818 |
| IV9RU5F01C0810 | - | - | Sb01g006420.1 | MLOC_7003.1 |
| IVLNWEC02GF3CU | - | - | Sb01g036540.1 | MLOC_56359.2 |
| IUIVLUD01AMPWP | - | - | Sb02g019450.1 | AK376220 |
| IVLNWEC01CQW2T | - | - | Sb02g019450.1 | AK376220 |
| IVLNWEC01DF8CI | - | - | Sb02g019450.1 | AK376220 |
| IVLNWEC01EYAAI | - | - | Sb02g019450.1 | AK376220 |
| IV9RU5F02I87IM | - | - | Sb10g001950.1 | MLOC_81095.1 |
| IVLNWEC01DU6J3 | - | - | Sb10g001950.1 | MLOC_81095.1 |
| IUIVLUD01D3KT1 | - | - | Sb02g026170.1 | MLOC_53246.1 |
| IUIVLUD01DPSAC | - | - | Sb05g002380.1 | MLOC_20538.1 |
| IV9RU5F02I1UD8 | - | - | Sb05g002380.1 | MLOC_20538.1 |
| IVLNWEC01CGFN3 | - | - | Sb02g039520.1 | MLOC_60620.1 |
| IVLNWEC01DPTRC | - | - | Sb02g039520.1 | MLOC_60620.1 |
| IVLNWEC02HW56L | - | - | Sb02g039520.1 | MLOC_60620.1 |
| IUIVLUD01B5M7F | - | - | Sb02g022610.1 | AK369117 |
| IUIVLUD02H8RKZ | - | - | Sb02g022610.1 | AK369117 |
| IVLNWEC01B4URG | - | - | Sb02g022610.1 | AK369117 |
| IVLNWEC02HY1PR | - | - | Sb02g022610.1 | AK369117 |
| IV9RU5F02HBWEC | - | - | Sb02g026910.1 | AK374423 |
| IV9RU5F02JUIAD | - | - | Sb02g026910.1 | AK374423 |
| IV9RU5F01CYGAJ | - | - | Sb02g030920.1 | AK251984.1 |
| IV9RU5F02I1RF3 | - | - | Sb02g021090.1 | MLOC_10405.1 |
| IVLNWEC01DK7P0 | - | - | Sb02g021090.1 | MLOC_10405.1 |
| IVLNWEC02IM6SA | - | - | Sb02g021090.1 | MLOC_10405.1 |
| IUIVLUD01DRK6E | - | - | Sb08g012560.1 | MLOC_54346.5 |
| IV9RU5F01AZ3K6 | - | - | Sb08g012560.1 | MLOC_54346.5 |
| IVLNWEC02HNY0P | - | - | Sb08g012560.1 | MLOC_54346.5 |
| IVLNWEC01A4L1G | - | - | Sb06g021020.1 | MLOC_14298.1 |
| IV9RU5F01EJPLB | - | - | Sb02g030550.1 | AK252321.1 |

| | | | | |
|---|---|---|---|---|
| IV9RU5F01AU12J | - | - | Sb01g030430.1 | MLOC_53885.1 |
| IVLNWEC01APUZI | - | - | Sb01g030430.1 | MLOC_53885.1 |
| IUIVLUD02JU6MU | - | - | Sb03g015000.1 | MLOC_76365.1 |
| IV9RU5F02FMUKY | - | - | Sb02g020475.1 | AK364488 |
| IV9RU5F02HVFB2 | - | - | Sb02g020475.1 | AK364488 |
| IV9RU5F01ERD7G | - | - | Sb01g008700.1 | MLOC_55034.1 |
| IVLNWEC01DSX30 | - | - | Sb01g008700.1 | MLOC_55034.1 |
| IVLNWEC02HCGW2 | - | - | Sb01g011950.1 | MLOC_69563.2 |
| IVLNWEC01AVSVW | - | - | Sb01g011950.1 | MLOC_69563.2 |
| IV9RU5F02H77FY | - | - | Sb01g017540.1 | MLOC_36476.1 |
| IVLNWEC01BY50R | - | - | Sb08g004470.1 | AK365785 |
| IVLNWEC02G3S7X | - | - | Sb02g014480.1 | MLOC_34478.1 |
| IVLNWEC02JRQ9D | - | - | Sb02g014480.1 | MLOC_34478.1 |
| IV9RU5F02FO7D3 | - | - | Sb08g012360.1 | AK370517 |
| IV9RU5F01D7ZDP | - | - | Sb02g001020.1 | MLOC_62464.1 |
| IVLNWEC02FRBLR | - | - | Sb02g001020.1 | MLOC_62464.1 |
| IVLNWEC02GZQ0X | - | - | Sb02g001020.1 | MLOC_62464.1 |
| IUIVLUD01BVQ35 | - | - | Sb08g018220.1 | MLOC_29137.2 |
| IV9RU5F02F3BRF | - | - | Sb08g018220.1 | MLOC_29137.2 |
| IVLNWEC01BRN7W | - | - | Sb08g018220.1 | MLOC_29137.2 |
| IVLNWEC01DHQTG | - | - | Sb08g018220.1 | MLOC_29137.2 |
| IVLNWEC02HV0A0 | - | - | Sb08g018220.1 | MLOC_29137.2 |
| IVLNWEC02HTFYD | - | - | Sb09g027223.1 | MLOC_4294.1 |
| IUIVLUD01BGC7Q | - | - | Sb05g005390.1 | MLOC_20977.1 |
| IUIVLUD01B5FDN | - | - | Sb02g020320.1 | MLOC_59580.1 |
| IVLNWEC02IE0GB | - | - | Sb02g020320.1 | MLOC_59580.1 |
| IV9RU5F01EXEBC | - | - | Sb01g011950.1 | MLOC_69563.2 |
| IUIVLUD02HKUD7 | - | - | Sb01g013460.1 | MLOC_37399.1 |
| IV9RU5F01AF5EM | - | - | Sb07g004420.1 | MLOC_21642.1 |
| IVLNWEC01BW3OW | - | - | Sb01g014620.1 | MLOC_34557.1 |
| IUIVLUD01BLGH3 | - | - | Sb02g028990.1 | MLOC_53232.1 |
| IV9RU5F01CRDSG | - | - | Sb02g028990.1 | MLOC_53232.1 |
| IV9RU5F02HNIER | - | - | Sb08g001480.1 | MLOC_59721.1 |
| IV9RU5F02JL1AZ | - | - | Sb08g001480.1 | MLOC_59721.1 |
| IVLNWEC01D0ERW | - | - | Sb08g001480.1 | MLOC_59721.1 |
| IV9RU5F01A2WAL | - | - | Sb01g011590.1 | MLOC_56050.2 |
| IV9RU5F01DFEOD | - | - | Sb04g030880.1 | MLOC_38181.1 |
| IVLNWEC01CZE2M | - | - | Sb02g026460.1 | MLOC_68051.1 |
| IVLNWEC02IQ8E7 | - | - | Sb04g030390.1 | AK248234.1 |
| IUIVLUD02J4MI4 | - | - | Sb02g028490.1 | AK376822 |
| IV9RU5F02I3EAL | - | - | Sb01g008700.1 | MLOC_55034.1 |
| IVLNWEC02H59TL | - | - | Sb02g024520.1 | AK252367.1 |
| IVLNWEC02HKF43 | - | - | Sb01g027630.1 | MLOC_64873.3 |
| IV9RU5F01EC2ZU | - | - | Sb01g036250.1 | MLOC_5958.2 |
| IVLNWEC01DG620 | - | - | Sb02g023710.1 | MLOC_14475.1 |
| IUIVLUD01BAN96 | - | - | Sb01g019140.1 | AK370794 |
| IUIVLUD02JU124 | - | - | Sb02g024090.1 | AK249691.1 |

| | | | | |
|---|---|---|---|---|
| IVLNWEC01CWAN8 | - | - | Sb04g035070.1 | AK355417 |
| IV9RU5F01BUVRE | - | - | Sb04g009890.1 | MLOC_59368.2 |
| IV9RU5F02GBBLO | - | - | Sb08g003250.1 | MLOC_59472.1 |
| IUIVLUD01AI0EK | - | - | Sb03g042460.1 | AK370620 |
| IV9RU5F01AE2BC | - | - | Sb03g042460.1 | AK370620 |
| IVLNWEC01C8E3W | - | - | Sb03g042460.1 | AK370620 |
| IUIVLUD01BGNEY | - | - | Sb03g047410.1 | AK354583 |
| IV9RU5F01BD8B0 | - | - | Sb02g025730.1 | MLOC_18353.1 |
| IV9RU5F01EXI7P | - | - | Sb01g030680.1 | MLOC_5653.2 |
| IUIVLUD01DY8G3 | - | - | Sb01g010650.1 | AK366523 |
| IUIVLUD01ETWUW | - | - | Sb01g000210.1 | AK368335 |
| IVLNWEC01EMKKI | - | - | Sb07g001930.1 | MLOC_53998.2 |
| IVLNWEC01DJRV2 | - | - | Sb08g004950.1 | AK354521 |
| IVLNWEC02HVWIM | - | - | Sb08g004950.1 | AK354521 |
| IVLNWEC02JZ8Z1 | - | - | Sb01g008700.1 | MLOC_55034.1 |
| IV9RU5F01DYG5Z | - | - | Sb02g043940.1 | MLOC_50786.3 |
| IV9RU5F01ADZO2 | - | - | Sb02g007180.1 | AK354222 |
| IVLNWEC01DLZYZ | - | - | Sb01g019140.1 | AK370794 |
| IVLNWEC02GWXLO | - | - | Sb01g019140.1 | AK370794 |
| IUIVLUD02GYL48 | - | - | Sb04g036000.1 | AK354681 |
| IUIVLUD02HQX4A | - | - | Sb04g036000.1 | AK354681 |
| IV9RU5F01B0VNX | - | - | Sb01g026590.1 | AK252025.1 |
| IV9RU5F02F634C | - | - | Sb01g026590.1 | AK252025.1 |
| IUIVLUD01CN7Y6 | - | - | Sb08g004470.1 | MLOC_53965.1 |
| IV9RU5F01CI70V | - | - | Sb02g021790.1 | AK372591 |
| IV9RU5F02JRPM2 | - | - | Sb02g021790.1 | AK372591 |
| IUIVLUD02FQ6BL | - | - | Sb02g027310.1 | AK376040 |
| IUIVLUD02JNGIT | - | - | Sb02g023480.1 | MLOC_54750.3 |
| IV9RU5F01C0PUE | - | - | Sb02g023480.1 | MLOC_54750.3 |
| IVLNWEC02IKWXZ | - | - | Sb02g023480.1 | MLOC_54750.3 |
| IUIVLUD01E1LMN | - | - | Sb04g024620.1 | MLOC_34880.1 |
| IUIVLUD01B6XLP | - | - | Sb01g042400.1 | AK366898 |
| IVLNWEC02FM66A | - | - | Sb01g042400.1 | AK366898 |
| IV9RU5F01DTDS1 | - | - | Sb02g027460.1 | AK353724 |
| IUIVLUD01C5ZXG | - | - | Sb05g008040.1 | MLOC_64280.1 |
| IUIVLUD02J3FTD | - | - | Sb01g010410.1 | MLOC_5817.1 |
| IV9RU5F01DJ24R | - | - | Sb01g010410.1 | MLOC_5817.1 |
| IV9RU5F02HIBAS | - | - | Sb01g010410.1 | MLOC_5817.1 |
| IV9RU5F02G7O2L | - | - | Sb01g010410.1 | AK360859 |
| IVLNWEC01B35VI | - | - | Sb01g048270.1 | MLOC_58960.1 |
| IVLNWEC02GA6MF | - | - | Sb02g011240.1 | MLOC_13650.1 |
| IV9RU5F01BGA0F | - | - | Sb01g033770.1 | MLOC_75024.4 |
| IUIVLUD02I9J1P | - | - | Sb01g017540.1 | MLOC_36476.1 |
| IUIVLUD02JL6FI | - | - | Sb02g031260.1 | AK375087 |
| IV9RU5F01DGUW8 | - | - | Sb07g008300.1 | AK354830 |
| IV9RU5F01BVYE9 | - | - | Sb01g028683.1 | MLOC_60060.1 |
| IV9RU5F01BHPQL | - | - | Sb01g028683.1 | MLOC_38198.2 |

| | | | | |
|---|---|---|---|---|
| IVLNWEC02FKP8F | - | - | Sb09g016865.1 | MLOC_5527.1 |
| IV9RU5F01C3PR9 | - | - | Sb02g031600.1 | AK370769 |
| IVLNWEC01B1TYZ | - | - | Sb02g031600.1 | AK370769 |
| IVLNWEC02F67S0 | - | - | Sb01g008700.1 | MLOC_55034.1 |
| IVLNWEC02GVR88 | - | - | Sb01g008700.1 | MLOC_55034.1 |
| IVLNWEC01DXV2W | - | - | Sb02g020410.1 | MLOC_57328.1 |
| IV9RU5F02JDWRD | - | - | Sb05g007160.1 | AK370601 |
| IV9RU5F01D079A | - | - | Sb04g002063.1 | MLOC_54128.1 |
| IVLNWEC01C34OE | - | - | Sb01g043970.1 | MLOC_6856.2 |
| IVLNWEC02IJOR5 | - | - | Sb06g029220.1 | MLOC_10090.4 |
| IV9RU5F02JXCSZ | - | - | Sb04g003810.1 | MLOC_71409.1 |
| IVLNWEC02HFL5R | - | - | Sb02g029260.1 | MLOC_56084.1 |
| IUIVLUD02H1076 | - | - | Sb02g014910.1 | MLOC_58800.2 |
| IVLNWEC02JEH08 | - | - | Sb03g015070.1 | MLOC_70842.1 |
| IUIVLUD01BQEH6 | - | - | Sb09g020070.2 | AK366232 |
| IV9RU5F02F3TBI | - | - | Sb01g028683.1 | MLOC_60060.1 |
| IV9RU5F01ENBL8 | - | - | Sb01g028683.1 | MLOC_38198.2 |
| IV9RU5F01BCVTL | - | - | Sb01g017540.1 | MLOC_36476.1 |
| IUIVLUD01B4PG8 | - | - | Sb02g001045.1 | AK373752 |
| IV9RU5F01B5Q05 | - | - | Sb02g001045.1 | AK373752 |
| IUIVLUD01A2PF5 | - | - | Sb10g000660.1 | MLOC_61812.1 |
| IVLNWEC02H7MD6 | - | - | Sb04g006240.1 | MLOC_54213.1 |
| IUIVLUD02GBV8R | - | - | Sb05g024490.1 | MLOC_4685.6 |
| IV9RU5F01DMPTY | - | - | Sb08g001480.1 | MLOC_59721.1 |
| IVLNWEC02F46KQ | - | - | Sb08g001480.1 | MLOC_59721.1 |
| IV9RU5F02HJCZ4 | - | - | Sb01g011950.1 | MLOC_69563.2 |
| IV9RU5F01D2OBW | - | - | Sb01g011950.1 | MLOC_64087.2 |
| IV9RU5F02GDIEN | - | - | Sb01g011310.1 | AK353726 |
| IUIVLUD02GXQT2 | - | - | Sb02g022630.1 | AK368497 |
| IV9RU5F01EOMEU | - | - | Sb04g006240.1 | MLOC_54213.1 |
| IVLNWEC01A9GPU | - | - | Sb04g006240.1 | MLOC_54213.1 |
| IV9RU5F01E3FGL | - | - | Sb02g024900.1 | AK370249 |
| IV9RU5F01DUMAE | - | - | Sb01g000220.1 | MLOC_66589.2 |
| IV9RU5F02GCB64 | - | - | Sb01g000220.1 | MLOC_66589.2 |
| IVLNWEC01A6MIB | - | - | Sb07g004380.1 | MLOC_56869.1 |
| IVLNWEC01EUYDQ | - | - | Sb07g004380.1 | MLOC_56869.1 |
| IV9RU5F02GT2ZY | - | - | Sb04g024620.1 | MLOC_34880.1 |
| IVLNWEC02I50EA | - | - | Sb04g024620.1 | MLOC_34880.1 |
| IVLNWEC02HQYMZ | - | - | Sb01g019210.1 | MLOC_3076.3 |
| IVLNWEC02G8F54 | - | - | Sb02g027310.1 | AK376040 |
| IVLNWEC02HLJZN | - | - | Sb01g034706.1 | AK365864 |
| IVLNWEC01BHOEW | - | - | Sb01g032840.1 | AK363897 |
| IV9RU5F02HSGS2 | - | - | Sb01g013300.1 | AK363790 |
| IV9RU5F01C0LKV | - | - | Sb01g037140.1 | MLOC_19278.1 |
| IVLNWEC01C0NSC | - | - | Sb02g006040.1 | MLOC_59797.4 |
| IV9RU5F02IHU36 | - | - | Sb02g014910.1 | MLOC_29114.2 |
| IVLNWEC01D620Z | - | - | Sb01g016770.1 | MLOC_37447.2 |

| | | | | |
|---|---|---|---|---|
| IVLNWEC02IN4D3 | - | - | Sb04g028630.1 | MLOC_75294.1 |
| IVLNWEC01D1YJV | - | - | Sb03g008740.1 | MLOC_75236.1 |
| IUIVLUD02F4UDZ | - | - | Sb01g043970.1 | MLOC_6856.2 |
| IUIVLUD01EFWXT | - | - | Sb04g035070.1 | AK371721 |
| IVLNWEC02JG13B | - | - | Sb02g022630.1 | AK368497 |
| IV9RU5F01BU2N0 | - | - | Sb02g029260.1 | MLOC_56084.1 |
| IVLNWEC02G7V58 | - | - | Sb01g013300.1 | AK363790 |
| IVLNWEC01CIIF8 | - | - | Sb02g022610.1 | AK369117 |
| IV9RU5F02JNWAP | - | - | Sb03g029130.1 | AK248775.1 |
| IV9RU5F02G8NF6 | - | - | Sb03g029130.1 | AK248775.1 |
| IVLNWEC01C998T | - | - | Sb03g029130.1 | AK248775.1 |
| IVLNWEC02IMZ8N | - | - | Sb01g000220.1 | MLOC_66589.2 |
| IUIVLUD02GNUUT | - | - | Sb03g015000.1 | MLOC_76365.1 |
| IV9RU5F01BGJ51 | - | - | Sb02g020475.1 | AK364488 |
| IV9RU5F02F3GJG | - | - | Sb02g020475.1 | AK364488 |
| IV9RU5F02JI7WU | - | - | Sb01g011950.1 | MLOC_69563.2 |
| IV9RU5F02FYXPC | - | - | Sb02g022936.1 | MLOC_59632.2 |
| IVLNWEC02GEFJ7 | - | - | Sb02g022936.1 | MLOC_59632.2 |
| IVLNWEC02GO6II | - | - | Sb02g022936.1 | MLOC_59632.2 |
| IUIVLUD01B3H17 | - | - | Sb02g029500.1 | MLOC_55886.1 |
| IV9RU5F02HZUTK | - | - | Sb01g033770.1 | MLOC_75024.4 |
| IUIVLUD01CCRIO | - | - | Sb01g003750.1 | MLOC_59130.2 |
| IV9RU5F02H6MGZ | - | - | Sb01g005920.1 | AK371672 |
| IV9RU5F02IPH16 | - | - | Sb09g030646.1 | MLOC_37459.1 |
| IUIVLUD01ESS6D | - | - | Sb02g028990.1 | MLOC_53232.1 |
| IVLNWEC01DPH3S | - | - | Sb02g031260.1 | AK375087 |
| IV9RU5F01C0EX2 | - | - | Sb02g022933.1 | AK358827 |
| IVLNWEC01A3QTP | - | - | Sb01g019000.1 | MLOC_52357.1 |
| IV9RU5F01CYC95 | - | - | Sb04g024620.1 | MLOC_34880.1 |
| IVLNWEC01D3IPE | - | - | Sb01g020130.1 | MLOC_55657.5 |
| IUIVLUD02GBX1S | - | - | Sb02g024090.1 | AK249691.1 |
| IV9RU5F02HOBCP | - | - | Sb04g035070.1 | AK355417 |
| IV9RU5F02JU2KN | - | - | Sb03g042460.1 | AK370620 |
| IV9RU5F02HIO5D | - | - | Sb01g031410.1 | MLOC_76739.1 |
| IUIVLUD02H6D5L | - | - | Sb03g047410.1 | AK354583 |
| IV9RU5F01EQ950 | - | - | Sb01g000210.1 | AK368335 |
| IVLNWEC02GMN9M | - | - | Sb09g000210.1 | AK355206 |
| IV9RU5F01D0O4O | - | - | Sb02g019580.1 | AK374259 |
| IVLNWEC02J0SE5 | - | - | Sb01g042710.1 | AK365524 |
| IV9RU5F02JIJNQ | - | - | Sb01g042710.1 | AK365524 |
| IVLNWEC01DFIGZ | - | - | Sb01g027630.1 | MLOC_64873.3 |
| IUIVLUD01ENTEY | - | - | Sb02g030090.1 | MLOC_65109.6 |
| IV9RU5F01BGB1Y | - | - | Sb02g027310.1 | AK376040 |
| IVLNWEC01EPXVP | - | - | Sb07g020830.1 | MLOC_5116.4 |
| IVLNWEC02JM6IM | - | - | Sb02g023010.1 | MLOC_9891.3 |
| IVLNWEC01B1MF9 | - | - | Sb01g015490.1 | AK369530 |
| IVLNWEC02HCTDC | - | - | Sb01g015490.1 | AK369530 |

| | | | | |
|---|---|---|---|---|
| IV9RU5F02G59H4 | - | - | Sb05g008040.1 | MLOC_64280.1 |
| IUIVLUD01EPK71 | - | - | Sb01g013710.1 | MLOC_58644.1 |
| IVLNWEC01CASOR | - | - | Sb01g010410.1 | MLOC_5817.1 |
| IVLNWEC02FV48X | - | - | Sb01g010410.1 | MLOC_5817.1 |
| IV9RU5F01C1FGP | - | - | Sb01g048270.1 | MLOC_58960.1 |
| IUIVLUD02F9INR | - | - | Sb02g031260.1 | AK375087 |
| IVLNWEC01DT6N5 | - | - | Sb02g031970.1 | MLOC_9982.2 |
| IVLNWEC02J0OMR | - | - | Sb04g003073.1 | MLOC_4798.7 |
| IUIVLUD01EPYUP | - | - | Sb01g028683.1 | MLOC_38198.2 |
| IVLNWEC02GY3QR | - | - | Sb09g016865.1 | MLOC_5527.1 |
| IV9RU5F02H9PG9 | - | - | Sb02g023510.1 | MLOC_39028.1 |
| IV9RU5F02JWELL | - | - | Sb10g025160.1 | - |
| IVLNWEC02GLPEN | - | - | Sb10g025160.1 | - |
| IUIVLUD02JNWNI | - | - | Sb10g023155.1 | - |
| IVLNWEC02F82IU | - | - | Sb10g023155.1 | - |
| IUIVLUD01CTPIB | - | - | Sb10g022050.1 | - |
| IVLNWEC01B3N3K | - | - | Sb10g022050.1 | - |
| IVLNWEC01BIDN1 | - | - | Sb10g021034.1 | - |
| IVLNWEC01DHT15 | - | - | Sb10g021034.1 | - |
| IV9RU5F01B62VM | - | - | Sb10g020790.1 | - |
| IV9RU5F02F9E46 | - | - | Sb10g020790.1 | - |
| IVLNWEC01BBDFX | - | - | Sb10g020790.1 | - |
| IVLNWEC01EI2L6 | - | - | Sb10g003360.1 | - |
| IUIVLUD02FZICK | - | - | Sb10g000970.1 | - |
| IV9RU5F01B328A | - | - | Sb10g000970.1 | - |
| IUIVLUD01AI46A | - | - | Sb10g000400.1 | - |
| IUIVLUD01BHTGR | - | - | Sb10g000400.1 | - |
| IV9RU5F01EFC2D | - | - | Sb09g030660.1 | - |
| IUIVLUD02H7L9X | - | - | Sb09g030570.1 | - |
| IVLNWEC01C67JJ | - | - | Sb09g030570.1 | - |
| IUIVLUD01B1PH5 | - | - | Sb09g027720.1 | - |
| IUIVLUD01BMK65 | - | - | Sb09g027720.1 | - |
| IV9RU5F01AJ23C | - | - | Sb09g027720.1 | - |
| IVLNWEC02G1LX6 | - | - | Sb09g024270.1 | - |
| IV9RU5F01CQBV2 | - | - | Sb09g020070.2 | - |
| IV9RU5F02GBTRX | - | - | Sb09g016865.1 | - |
| IV9RU5F02HFT4B | - | - | Sb09g015250.1 | - |
| IUIVLUD01A4N4M | - | - | Sb09g007400.1 | - |
| IVLNWEC01C9U4B | - | - | Sb09g007400.1 | - |
| IV9RU5F01CO0CJ | - | - | Sb09g002195.1 | - |
| IV9RU5F02JT13S | - | - | Sb09g002195.1 | - |
| IVLNWEC01AI367 | - | - | Sb09g002195.1 | - |
| IV9RU5F02JD7NL | - | - | Sb09g000680.1 | - |
| IV9RU5F02IKAAK | - | - | Sb08g022840.1 | - |
| IV9RU5F02FRRAW | - | - | Sb08g022550.1 | - |
| IV9RU5F02HJ3E0 | - | - | Sb08g022550.1 | - |
| IUIVLUD01D2G3P | - | - | Sb08g020692.1 | - |

| | | | | |
|---|---|---|---|---|
| IUIVLUD01DAIA3 | - | - | Sb08g020692.1 | - |
| IVLNWEC01ALIB0 | - | - | Sb08g020692.1 | - |
| IVLNWEC02H7X5C | - | - | Sb08g020460.1 | - |
| IUIVLUD02JAOOJ | - | - | Sb08g019880.1 | - |
| IV9RU5F01CT5I9 | - | - | Sb08g019880.1 | - |
| IV9RU5F02JDYYE | - | - | Sb08g015230.1 | - |
| IV9RU5F01AJVVF | - | - | Sb08g007680.1 | - |
| IV9RU5F01DY882 | - | - | Sb08g007680.1 | - |
| IV9RU5F02IUTWY | - | - | Sb08g007680.1 | - |
| IVLNWEC01BQFVF | - | - | Sb08g005340.1 | - |
| IVLNWEC02GF3BS | - | - | Sb08g005340.1 | - |
| IUIVLUD01AXKB1 | - | - | Sb08g003810.1 | - |
| IV9RU5F01A654I | - | - | Sb08g003810.1 | - |
| IV9RU5F02FPNUB | - | - | Sb08g003810.1 | - |
| IVLNWEC01DPF6Q | - | - | Sb08g003810.1 | - |
| IVLNWEC01ES5XJ | - | - | Sb08g003810.1 | - |
| IVLNWEC02F6M1P | - | - | Sb08g003810.1 | - |
| IVLNWEC02FYMKW | - | - | Sb08g003810.1 | - |
| IVLNWEC02IO5SI | - | - | Sb08g003810.1 | - |
| IV9RU5F01CKJXG | - | - | Sb08g003800.1 | - |
| IVLNWEC02I99HF | - | - | Sb08g002910.1 | - |
| IVLNWEC02FRT67 | - | - | Sb08g002900.1 | - |
| IVLNWEC01DMQPA | - | - | Sb08g002290.1 | - |
| IV9RU5F01CM4P8 | - | - | Sb08g001855.1 | - |
| IV9RU5F02I6DTF | - | - | Sb08g001500.1 | - |
| IV9RU5F02IPR2W | - | - | Sb08g001500.1 | - |
| IUIVLUD02GR87W | - | - | Sb08g001480.1 | - |
| IUIVLUD01D73Q1 | - | - | Sb07g028525.1 | - |
| IV9RU5F01A5R6V | - | - | Sb07g028525.1 | - |
| IV9RU5F01CVJMT | - | - | Sb07g028525.1 | - |
| IV9RU5F02I4DQW | - | - | Sb07g028525.1 | - |
| IV9RU5F02IZTS6 | - | - | Sb07g028525.1 | - |
| IV9RU5F02JJOHF | - | - | Sb07g028525.1 | - |
| IUIVLUD01EBRR7 | - | - | Sb07g027090.1 | - |
| IVLNWEC02JODDH | - | - | Sb07g027090.1 | - |
| IV9RU5F01BIK8O | - | - | Sb07g025890.1 | - |
| IV9RU5F01D865D | - | - | Sb07g025890.1 | - |
| IUIVLUD01A2LYT | - | - | Sb07g025850.1 | - |
| IUIVLUD01CS6SR | - | - | Sb07g025850.1 | - |
| IVLNWEC02F7MHX | - | - | Sb07g025780.1 | - |
| IVLNWEC02JYH0K | - | - | Sb07g025780.1 | - |
| IVLNWEC01B36Z8 | - | - | Sb07g025620.1 | - |
| IV9RU5F01AFWO8 | - | - | Sb07g022230.1 | - |
| IV9RU5F01CMFFU | - | - | Sb07g022230.1 | - |
| IV9RU5F01D72EL | - | - | Sb07g022230.1 | - |
| IV9RU5F02HQ842 | - | - | Sb07g022230.1 | - |
| IVLNWEC01DWDIA | - | - | Sb07g022230.1 | - |

| | | | | |
|---|---|---|---|---|
| IVLNWEC02G46GS | - | - | Sb07g022230.1 | - |
| IVLNWEC02ICH4M | - | - | Sb07g009340.1 | - |
| IUIVLUD01D745O | - | - | Sb07g008300.1 | - |
| IVLNWEC02G6O9Y | - | - | Sb07g008300.1 | - |
| IUIVLUD01CQ8IP | - | - | Sb07g007680.1 | - |
| IVLNWEC01ARDZX | - | - | Sb07g007680.1 | - |
| IUIVLUD02H0SYB | - | - | Sb07g006490.1 | - |
| IV9RU5F01CN0DH | - | - | Sb07g005710.1 | - |
| IV9RU5F01CUMCL | - | - | Sb07g005710.1 | - |
| IUIVLUD02H2KQ7 | - | - | Sb07g003376.1 | - |
| IUIVLUD02IEMX8 | - | - | Sb07g003376.1 | - |
| IV9RU5F01DNLVZ | - | - | Sb07g003376.1 | - |
| IV9RU5F01EXX3S | - | - | Sb07g003376.1 | - |
| IVLNWEC01AZRS6 | - | - | Sb07g003376.1 | - |
| IVLNWEC01CS1RM | - | - | Sb07g003376.1 | - |
| IVLNWEC01DWQB9 | - | - | Sb06g029220.1 | - |
| IUIVLUD02FYKE4 | - | - | Sb06g022980.1 | - |
| IV9RU5F02HR50J | - | - | Sb06g022980.1 | - |
| IVLNWEC01AE5I1 | - | - | Sb06g021020.1 | - |
| IUIVLUD02IAJOK | - | - | Sb06g019330.1 | - |
| IV9RU5F02JBAJ8 | - | - | Sb06g019330.1 | - |
| IVLNWEC02ISDSQ | - | - | Sb06g019330.1 | - |
| IV9RU5F01AE04R | - | - | Sb06g019043.1 | - |
| IV9RU5F02JR6N9 | - | - | Sb06g019043.1 | - |
| IUIVLUD01AJIC2 | - | - | Sb06g017960.1 | - |
| IUIVLUD02JC3AV | - | - | Sb06g017960.1 | - |
| IV9RU5F02IMNUP | - | - | Sb06g017960.1 | - |
| IVLNWEC01DAEQK | - | - | Sb06g017960.1 | - |
| IUIVLUD01BG4Z1 | - | - | Sb06g016240.1 | - |
| IUIVLUD02JFR6M | - | - | Sb06g016240.1 | - |
| IVLNWEC02HDT4K | - | - | Sb06g016240.1 | - |
| IV9RU5F01DVCB8 | - | - | Sb06g004070.1 | - |
| IV9RU5F02IDFD3 | - | - | Sb06g004070.1 | - |
| IV9RU5F01E02CT | - | - | Sb06g002540.1 | - |
| IVLNWEC01D3YKG | - | - | Sb06g002540.1 | - |
| IUIVLUD01EZ454 | - | - | Sb06g001180.1 | - |
| IUIVLUD02JIIHY | - | - | Sb06g001180.1 | - |
| IV9RU5F01ALEE7 | - | - | Sb06g000577.1 | - |
| IVLNWEC01DQW72 | - | - | Sb06g000577.1 | - |
| IVLNWEC02JBRNV | - | - | Sb06g000577.1 | - |
| IUIVLUD02HCV86 | - | - | Sb05g025450.1 | - |
| IVLNWEC01D0W9A | - | - | Sb05g025450.1 | - |
| IUIVLUD02HEBJM | - | - | Sb05g024590.1 | - |
| IVLNWEC02HA74R | - | - | Sb05g024590.1 | - |
| IUIVLUD01D31PG | - | - | Sb05g024303.1 | - |
| IUIVLUD02I7JFJ | - | - | Sb05g024303.1 | - |
| IVLNWEC02FUM0S | - | - | Sb05g022840.1 | - |

| | | | | |
|---|---|---|---|---|
| IV9RU5F01DS4LF | - | - | Sb05g019570.1 | - |
| IVLNWEC02IS10Z | - | - | Sb05g019570.1 | - |
| IUIVLUD01D9A4K | - | - | Sb05g010901.1 | - |
| IUIVLUD02G8BX7 | - | - | Sb05g010901.1 | - |
| IV9RU5F02HIOJ0 | - | - | Sb05g010901.1 | - |
| IUIVLUD02FTXOG | - | - | Sb05g010321.1 | - |
| IUIVLUD01DMREB | - | - | Sb05g009430.1 | - |
| IUIVLUD01E1B70 | - | - | Sb05g009430.1 | - |
| IV9RU5F01EDNSV | - | - | Sb05g008895.1 | - |
| IVLNWEC01CUJDF | - | - | Sb05g008895.1 | - |
| IUIVLUD01C5XAJ | - | - | Sb05g008890.1 | - |
| IVLNWEC01CDDF6 | - | - | Sb05g008890.1 | - |
| IV9RU5F02HVWZX | - | - | Sb05g008360.1 | - |
| IVLNWEC02HAEVV | - | - | Sb05g008360.1 | - |
| IV9RU5F01EH2L4 | - | - | Sb05g008255.1 | - |
| IV9RU5F02JPV5C | - | - | Sb05g008255.1 | - |
| IV9RU5F02HMEB6 | - | - | Sb05g008040.1 | - |
| IUIVLUD02GQPJU | - | - | Sb05g007980.1 | - |
| IUIVLUD02JGQRV | - | - | Sb05g007980.1 | - |
| IVLNWEC01CXCCJ | - | - | Sb05g007980.1 | - |
| IUIVLUD01A650A | - | - | Sb05g005345.1 | - |
| IUIVLUD01CLN6Y | - | - | Sb05g005345.1 | - |
| IV9RU5F01DAQ58 | - | - | Sb05g005345.1 | - |
| IV9RU5F01DJH0H | - | - | Sb05g005345.1 | - |
| IUIVLUD01C8D1S | - | - | Sb05g004300.1 | - |
| IUIVLUD02HSJ08 | - | - | Sb05g004300.1 | - |
| IVLNWEC01DD6HD | - | - | Sb05g002940.1 | - |
| IV9RU5F02FTE6Y | - | - | Sb05g002190.1 | - |
| IUIVLUD01B7WWK | - | - | Sb05g001950.1 | - |
| IUIVLUD02HNNMN | - | - | Sb05g001950.1 | - |
| IVLNWEC02H61LH | - | - | Sb05g001950.1 | - |
| IVLNWEC02JMH50 | - | - | Sb05g001950.1 | - |
| IV9RU5F01AZ2BY | - | - | Sb05g001620.1 | - |
| IV9RU5F01DDQN0 | - | - | Sb05g001620.1 | - |
| IV9RU5F02GAV2G | - | - | Sb05g001620.1 | - |
| IV9RU5F02HNEBI | - | - | Sb05g001570.1 | - |
| IVLNWEC02JDCW6 | - | - | Sb05g001570.1 | - |
| IVLNWEC01DOPU4 | - | - | Sb05g001160.1 | - |
| IVLNWEC02HDXQV | - | - | Sb05g001160.1 | - |
| IVLNWEC01CEAAO | - | - | Sb05g001140.1 | - |
| IVLNWEC01EA2Q2 | - | - | Sb05g001140.1 | - |
| IV9RU5F01DCSP3 | - | - | Sb05g001110.1 | - |
| IVLNWEC01A05GC | - | - | Sb05g001110.1 | - |
| IVLNWEC01D6SVZ | - | - | Sb05g001110.1 | - |
| IUIVLUD02G3QI3 | - | - | Sb05g000800.1 | - |
| IUIVLUD02H3U9A | - | - | Sb05g000800.1 | - |
| IV9RU5F01DC22T | - | - | Sb04g037710.1 | - |

| | | | | |
|---|---|---|---|---|
| IV9RU5F02IGO1K | - | - | Sb04g037710.1 | - |
| IUIVLUD02F1K7D | - | - | Sb04g035070.1 | - |
| IV9RU5F01D2KCH | - | - | Sb04g034505.1 | - |
| IVLNWEC02IO151 | - | - | Sb04g034505.1 | - |
| IUIVLUD01A04Y6 | - | - | Sb04g030880.1 | - |
| IUIVLUD01BHI9X | - | - | Sb04g028482.1 | - |
| IUIVLUD01CL67G | - | - | Sb04g028482.1 | - |
| IUIVLUD02JUHXE | - | - | Sb04g028482.1 | - |
| IUIVLUD01CR43Y | - | - | Sb04g021960.1 | - |
| IV9RU5F02G0ZU6 | - | - | Sb04g021960.1 | - |
| IVLNWEC01CB5K3 | - | - | Sb04g021960.1 | - |
| IUIVLUD02J3CYB | - | - | Sb04g020125.1 | - |
| IV9RU5F02JGETP | - | - | Sb04g020125.1 | - |
| IVLNWEC01CFNY6 | - | - | Sb04g020110.1 | - |
| IVLNWEC02GU90T | - | - | Sb04g020110.1 | - |
| IVLNWEC02JN4ZN | - | - | Sb04g020110.1 | - |
| IV9RU5F01BU8LN | - | - | Sb04g018873.1 | - |
| IVLNWEC02JEJ2O | - | - | Sb04g018873.1 | - |
| IV9RU5F01CO6CO | - | - | Sb04g017416.1 | - |
| IV9RU5F01ES5BG | - | - | Sb04g017416.1 | - |
| IV9RU5F01A022D | - | - | Sb04g012680.1 | - |
| IV9RU5F01EBM84 | - | - | Sb04g012680.1 | - |
| IV9RU5F02GPBHS | - | - | Sb04g012680.1 | - |
| IV9RU5F02HOEZN | - | - | Sb04g012680.1 | - |
| IVLNWEC01EDRYM | - | - | Sb04g012680.1 | - |
| IVLNWEC01ETIV2 | - | - | Sb04g012680.1 | - |
| IVLNWEC02GCK8D | - | - | Sb04g012680.1 | - |
| IVLNWEC02H5P49 | - | - | Sb04g012680.1 | - |
| IVLNWEC02JNPOZ | - | - | Sb04g012680.1 | - |
| IUIVLUD01EJAID | - | - | Sb04g011140.1 | - |
| IV9RU5F01BKFVR | - | - | Sb04g011140.1 | - |
| IVLNWEC01DBVOE | - | - | Sb04g011140.1 | - |
| IUIVLUD01C6AT0 | - | - | Sb04g009720.1 | - |
| IUIVLUD01EPTBR | - | - | Sb04g009720.1 | - |
| IVLNWEC01ARVZM | - | - | Sb04g009720.1 | - |
| IVLNWEC01AVTDT | - | - | Sb04g009720.1 | - |
| IVLNWEC01C0MGX | - | - | Sb04g009720.1 | - |
| IVLNWEC01D04A5 | - | - | Sb04g009720.1 | - |
| IVLNWEC01DWX2K | - | - | Sb04g009720.1 | - |
| IVLNWEC01EJFSH | - | - | Sb04g009720.1 | - |
| IVLNWEC01EUR1S | - | - | Sb04g009720.1 | - |
| IUIVLUD01EN0QK | - | - | Sb04g008547.1 | - |
| IV9RU5F01B6UK3 | - | - | Sb04g008547.1 | - |
| IVLNWEC02HPS6J | - | - | Sb04g008547.1 | - |
| IUIVLUD01B2WJG | - | - | Sb04g006240.1 | - |
| IVLNWEC01AGZ03 | - | - | Sb04g006240.1 | - |
| IVLNWEC01DE71W | - | - | Sb04g006240.1 | - |

| | | | | |
|---|---|---|---|---|
| IV9RU5F02H739U | - | - | Sb04g003810.1 | - |
| IUIVLUD01B1M6I | - | - | Sb04g003073.1 | - |
| IV9RU5F01B1LTO | - | - | Sb04g003073.1 | - |
| IUIVLUD02GSAVD | - | - | Sb04g002063.1 | - |
| IUIVLUD02GU1YA | - | - | Sb04g001610.1 | - |
| IUIVLUD02JYGEY | - | - | Sb04g001610.1 | - |
| IV9RU5F01DTU7K | - | - | Sb04g001610.1 | - |
| IV9RU5F01EE5DI | - | - | Sb04g000875.1 | - |
| IV9RU5F02F3O46 | - | - | Sb04g000875.1 | - |
| IV9RU5F02HKO2W | - | - | Sb04g000875.1 | - |
| IUIVLUD01AHA8O | - | - | Sb03g039660.1 | - |
| IUIVLUD01BBG6V | - | - | Sb03g036763.1 | - |
| IUIVLUD01CVZS8 | - | - | Sb03g036763.1 | - |
| IUIVLUD01DJ2E5 | - | - | Sb03g036763.1 | - |
| IV9RU5F02FXU58 | - | - | Sb03g036763.1 | - |
| IVLNWEC01CGQV3 | - | - | Sb03g036763.1 | - |
| IV9RU5F01BGX58 | - | - | Sb03g036450.1 | - |
| IV9RU5F01CZAP6 | - | - | Sb03g036450.1 | - |
| IVLNWEC01AT6Z9 | - | - | Sb03g036450.1 | - |
| IV9RU5F02HNVPL | - | - | Sb03g033855.1 | - |
| IVLNWEC01B62K4 | - | - | Sb03g033855.1 | - |
| IUIVLUD01BJKDD | - | - | Sb03g029010.1 | - |
| IUIVLUD02HQPLM | - | - | Sb03g029010.1 | - |
| IV9RU5F01ECZKQ | - | - | Sb03g028280.1 | - |
| IV9RU5F01EBLPX | - | - | Sb03g027070.1 | - |
| IV9RU5F02J0HTK | - | - | Sb03g027070.1 | - |
| IUIVLUD01ER41B | - | - | Sb03g026060.1 | - |
| IUIVLUD02HWLS9 | - | - | Sb03g026060.1 | - |
| IV9RU5F01D16IX | - | - | Sb03g026060.1 | - |
| IUIVLUD01C4GSG | - | - | Sb03g025820.1 | - |
| IVLNWEC01DI43C | - | - | Sb03g025820.1 | - |
| IV9RU5F01ASST5 | - | - | Sb03g024493.1 | - |
| IV9RU5F01B86VF | - | - | Sb03g024493.1 | - |
| IVLNWEC01BO196 | - | - | Sb03g024493.1 | - |
| IUIVLUD01D8VY0 | - | - | Sb03g018250.1 | - |
| IUIVLUD02HM7ZK | - | - | Sb03g018250.1 | - |
| IUIVLUD01AWAUJ | - | - | Sb03g015536.1 | - |
| IV9RU5F02HDQXM | - | - | Sb03g015536.1 | - |
| IUIVLUD01BMI2T | - | - | Sb03g014780.1 | - |
| IVLNWEC02II28M | - | - | Sb03g011460.1 | - |
| IV9RU5F01ENCZO | - | - | Sb03g011330.1 | - |
| IV9RU5F01B08PL | - | - | Sb03g011210.1 | - |
| IV9RU5F02F6V3E | - | - | Sb03g008740.1 | - |
| IV9RU5F02JL2HB | - | - | Sb03g007636.1 | - |
| IVLNWEC02IBW0W | - | - | Sb03g007636.1 | - |
| IV9RU5F01DU9V6 | - | - | Sb03g001780.1 | - |
| IV9RU5F02GS0GC | - | - | Sb03g001780.1 | - |

| | | | | |
|---|---|---|---|---|
| IV9RU5F02H56A5 | - | - | Sb03g001780.1 | - |
| IVLNWEC02FQF91 | - | - | Sb02g043940.1 | - |
| IV9RU5F02FVX8P | - | - | Sb02g039620.1 | - |
| IVLNWEC02HCA5P | - | - | Sb02g039620.1 | - |
| IUIVLUD01DAEJX | - | - | Sb02g038980.1 | - |
| IUIVLUD01CZROC | - | - | Sb02g036555.1 | - |
| IUIVLUD01E3J5T | - | - | Sb02g036555.1 | - |
| IUIVLUD02JSX5C | - | - | Sb02g034490.3 | - |
| IVLNWEC01E2E9N | - | - | Sb02g034490.3 | - |
| IUIVLUD01DM9ZE | - | - | Sb02g032980.1 | - |
| IUIVLUD02HXURS | - | - | Sb02g032980.1 | - |
| IUIVLUD02F93Z9 | - | - | Sb02g032480.1 | - |
| IV9RU5F01BPXL7 | - | - | Sb02g031970.1 | - |
| IVLNWEC02HAUJH | - | - | Sb02g029920.1 | - |
| IVLNWEC02JQ2IW | - | - | Sb02g029920.1 | - |
| IV9RU5F01CPE8Y | - | - | Sb02g029880.1 | - |
| IVLNWEC01D0TXH | - | - | Sb02g029880.1 | - |
| IV9RU5F01DU6YD | - | - | Sb02g029620.1 | - |
| IVLNWEC01BZHPF | - | - | Sb02g029600.1 | - |
| IVLNWEC02JR9O4 | - | - | Sb02g028950.1 | - |
| IUIVLUD02I2QYH | - | - | Sb02g028810.1 | - |
| IV9RU5F02FZHDJ | - | - | Sb02g028810.1 | - |
| IVLNWEC02ID0T1 | - | - | Sb02g028600.1 | - |
| IUIVLUD02I6HRH | - | - | Sb02g028490.1 | - |
| IV9RU5F01CYZMV | - | - | Sb02g026280.1 | - |
| IVLNWEC02F6LYV | - | - | Sb02g026170.1 | - |
| IV9RU5F01DWHRP | - | - | Sb02g025566.1 | - |
| IVLNWEC01DROVY | - | - | Sb02g024900.1 | - |
| IV9RU5F01CJORI | - | - | Sb02g024760.1 | - |
| IV9RU5F02HXVWB | - | - | Sb02g024520.1 | - |
| IVLNWEC01BAZ7P | - | - | Sb02g023710.1 | - |
| IV9RU5F01CKP5W | - | - | Sb02g023480.1 | - |
| IUIVLUD02ITEUD | - | - | Sb02g023130.1 | - |
| IVLNWEC01C20AE | - | - | Sb02g022933.1 | - |
| IV9RU5F01BHHBF | - | - | Sb02g022610.1 | - |
| IUIVLUD01A03ST | - | - | Sb02g021820.1 | - |
| IV9RU5F02J2FTX | - | - | Sb02g021820.1 | - |
| IUIVLUD01BUBMN | - | - | Sb02g021110.1 | - |
| IVLNWEC01BY6HY | - | - | Sb02g021110.1 | - |
| IV9RU5F01CBJO9 | - | - | Sb02g020425.1 | - |
| IV9RU5F01DVAPA | - | - | Sb02g020425.1 | - |
| IVLNWEC01BVGVC | - | - | Sb02g020410.1 | - |
| IUIVLUD01D4Z2C | - | - | Sb02g015861.1 | - |
| IUIVLUD02JVPJ3 | - | - | Sb02g015861.1 | - |
| IV9RU5F01AOBKZ | - | - | Sb02g015861.1 | - |
| IV9RU5F01EGM0H | - | - | Sb02g015861.1 | - |
| IVLNWEC01BWXN8 | - | - | Sb02g015861.1 | - |

| | | | | |
|---|---|---|---|---|
| IVLNWEC02GUNDJ | - | - | Sb02g015861.1 | - |
| IVLNWEC02GJ0YF | - | - | Sb02g014480.1 | - |
| IV9RU5F01AP4H4 | - | - | Sb02g012480.1 | - |
| IV9RU5F01BJF1Q | - | - | Sb02g012480.1 | - |
| IUIVLUD01DJHBL | - | - | Sb02g011993.1 | - |
| IVLNWEC02FVVJH | - | - | Sb02g011993.1 | - |
| IUIVLUD01C9ODZ | - | - | Sb02g011240.1 | - |
| IV9RU5F01BEC5J | - | - | Sb02g011240.1 | - |
| IVLNWEC01B7OKU | - | - | Sb02g011240.1 | - |
| IUIVLUD01EHY09 | - | - | Sb02g008710.1 | - |
| IUIVLUD01ELA4G | - | - | Sb02g008710.1 | - |
| IUIVLUD02HLV30 | - | - | Sb02g008710.1 | - |
| IUIVLUD01CDYPT | - | - | Sb02g008430.1 | - |
| IVLNWEC01COLPV | - | - | Sb02g008430.1 | - |
| IVLNWEC02HD6BJ | - | - | Sb02g008430.1 | - |
| IUIVLUD01DX3HR | - | - | Sb02g008050.1 | - |
| IVLNWEC02H7EOJ | - | - | Sb02g008050.1 | - |
| IV9RU5F01CDWSE | - | - | Sb02g006040.1 | - |
| IVLNWEC01EK6KK | - | - | Sb02g006030.1 | - |
| IV9RU5F01EER5U | - | - | Sb02g005970.1 | - |
| IVLNWEC02IJLG5 | - | - | Sb02g005970.1 | - |
| IVLNWEC01EKD4V | - | - | Sb02g004300.2 | - |
| IVLNWEC01BDIUU | - | - | Sb02g001020.1 | - |
| IUIVLUD01C0K0L | - | - | Sb01g048580.1 | - |
| IUIVLUD02H3UFA | - | - | Sb01g048580.1 | - |
| IUIVLUD02IHXD1 | - | - | Sb01g048580.1 | - |
| IV9RU5F01BFHA2 | - | - | Sb01g048580.1 | - |
| IV9RU5F01DC4BL | - | - | Sb01g048270.1 | - |
| IVLNWEC02IFXBR | - | - | Sb01g048270.1 | - |
| IV9RU5F01AN4YW | - | - | Sb01g044380.1 | - |
| IVLNWEC02HUKNZ | - | - | Sb01g042488.1 | - |
| IUIVLUD01CITCW | - | - | Sb01g040560.1 | - |
| IVLNWEC01DUHQJ | - | - | Sb01g040560.1 | - |
| IVLNWEC01DINIL | - | - | Sb01g040440.1 | - |
| IVLNWEC02IVLYH | - | - | Sb01g040440.1 | - |
| IV9RU5F01CAJ0R | - | - | Sb01g040330.1 | - |
| IVLNWEC01DSEZ5 | - | - | Sb01g040330.1 | - |
| IV9RU5F02HIQV5 | - | - | Sb01g039390.1 | - |
| IUIVLUD01EVC6A | - | - | Sb01g036680.1 | - |
| IVLNWEC01DBI9W | - | - | Sb01g036680.1 | - |
| IUIVLUD01EK3ES | - | - | Sb01g036540.1 | - |
| IUIVLUD01EJ171 | - | - | Sb01g036355.1 | - |
| IV9RU5F02HPCN7 | - | - | Sb01g035620.1 | - |
| IUIVLUD02JVH60 | - | - | Sb01g035565.1 | - |
| IV9RU5F02GF31P | - | - | Sb01g035565.1 | - |
| IVLNWEC02GG9CU | - | - | Sb01g035565.1 | - |
| IUIVLUD02GHEP4 | - | - | Sb01g034805.1 | - |

| | | | | |
|---|---|---|---|---|
| IUIVLUD02H1C1G | - | - | Sb01g034805.1 | - |
| IV9RU5F02JR1SF | - | - | Sb01g034805.1 | - |
| IVLNWEC02F11CE | - | - | Sb01g034805.1 | - |
| IV9RU5F02HDMXW | - | - | Sb01g034706.1 | - |
| IVLNWEC01D2D7Y | - | - | Sb01g034706.1 | - |
| IUIVLUD01EUWG7 | - | - | Sb01g032900.1 | - |
| IVLNWEC02IBQ3J | - | - | Sb01g032900.1 | - |
| IVLNWEC01AS1ED | - | - | Sb01g032840.1 | - |
| IVLNWEC01CMP3O | - | - | Sb01g031410.1 | - |
| IV9RU5F01CMDJ9 | - | - | Sb01g031070.1 | - |
| IV9RU5F02GPE4E | - | - | Sb01g031070.1 | - |
| IV9RU5F02GX98U | - | - | Sb01g031070.1 | - |
| IVLNWEC01A065I | - | - | Sb01g031070.1 | - |
| IVLNWEC01C1QOZ | - | - | Sb01g031070.1 | - |
| IVLNWEC02G0GY6 | - | - | Sb01g031070.1 | - |
| IVLNWEC02HGOWQ | - | - | Sb01g031070.1 | - |
| IVLNWEC02IXABR | - | - | Sb01g031070.1 | - |
| IVLNWEC01ETDKQ | - | - | Sb01g029385.1 | - |
| IVLNWEC02G73Q9 | - | - | Sb01g029385.1 | - |
| IVLNWEC02ICONM | - | - | Sb01g029385.1 | - |
| IV9RU5F02IFW66 | - | - | Sb01g027360.1 | - |
| IVLNWEC02JRVXK | - | - | Sb01g027360.1 | - |
| IUIVLUD01CC0H7 | - | - | Sb01g027030.1 | - |
| IV9RU5F01DE093 | - | - | Sb01g027030.1 | - |
| IUIVLUD02HO4MP | - | - | Sb01g026845.1 | - |
| IV9RU5F02GK3LQ | - | - | Sb01g026845.1 | - |
| IVLNWEC01CY0OF | - | - | Sb01g026845.1 | - |
| IUIVLUD02IJQDU | - | - | Sb01g023851.1 | - |
| IVLNWEC02GVP9Z | - | - | Sb01g023851.1 | - |
| IVLNWEC02GVWFC | - | - | Sb01g020751.1 | - |
| IVLNWEC02HGDQI | - | - | Sb01g020751.1 | - |
| IUIVLUD01DGCR2 | - | - | Sb01g020130.1 | - |
| IUIVLUD02GVUX7 | - | - | Sb01g020130.1 | - |
| IV9RU5F01C1SQO | - | - | Sb01g020130.1 | - |
| IV9RU5F01CG5SW | - | - | Sb01g020130.1 | - |
| IV9RU5F01DE9XN | - | - | Sb01g020130.1 | - |
| IV9RU5F02IBDMZ | - | - | Sb01g020130.1 | - |
| IV9RU5F02JP6QW | - | - | Sb01g020130.1 | - |
| IVLNWEC01BL5YZ | - | - | Sb01g020130.1 | - |
| IVLNWEC01D0RLG | - | - | Sb01g020130.1 | - |
| IVLNWEC01DY02P | - | - | Sb01g020130.1 | - |
| IVLNWEC02JFFND | - | - | Sb01g020130.1 | - |
| IV9RU5F01C2N9X | - | - | Sb01g019210.1 | - |
| IV9RU5F02JVF4L | - | - | Sb01g019210.1 | - |
| IVLNWEC02HE3CN | - | - | Sb01g019210.1 | - |
| IV9RU5F02IWZ00 | - | - | Sb01g019065.1 | - |
| IVLNWEC02H1UYA | - | - | Sb01g019065.1 | - |

| | | | | |
|---|---|---|---|---|
| IV9RU5F01CVD3L | - | - | Sb01g019060.1 | - |
| IVLNWEC02H5RLO | - | - | Sb01g019060.1 | - |
| IUIVLUD01E0ZSK | - | - | Sb01g018510.1 | - |
| IUIVLUD02IRAN8 | - | - | Sb01g018510.1 | - |
| IV9RU5F01BRD2C | - | - | Sb01g018510.1 | - |
| IVLNWEC02GBA7Q | - | - | Sb01g018510.1 | - |
| IUIVLUD02GXGGA | - | - | Sb01g016800.1 | - |
| IV9RU5F01C0H3S | - | - | Sb01g016800.1 | - |
| IVLNWEC02JPWGF | - | - | Sb01g016800.1 | - |
| IV9RU5F02IKZ5O | - | - | Sb01g016775.1 | - |
| IV9RU5F01BR01R | - | - | Sb01g015810.1 | - |
| IVLNWEC02IK55Q | - | - | Sb01g015810.1 | - |
| IUIVLUD01CFPH0 | - | - | Sb01g015550.1 | - |
| IUIVLUD01CP5Q2 | - | - | Sb01g015550.1 | - |
| IUIVLUD01EOWX6 | - | - | Sb01g015550.1 | - |
| IV9RU5F01EK4Z4 | - | - | Sb01g015550.1 | - |
| IVLNWEC02H8GQI | - | - | Sb01g015550.1 | - |
| IV9RU5F02JDRZH | - | - | Sb01g015490.1 | - |
| IV9RU5F02F4PUN | - | - | Sb01g014620.1 | - |
| IV9RU5F01BTARL | - | - | Sb01g014270.1 | - |
| IV9RU5F02GXP73 | - | - | Sb01g014270.1 | - |
| IVLNWEC01EDLWE | - | - | Sb01g014270.1 | - |
| IUIVLUD02I7M4A | - | - | Sb01g014260.1 | - |
| IV9RU5F02GXFVN | - | - | Sb01g014260.1 | - |
| IVLNWEC01D4P4M | - | - | Sb01g012810.1 | - |
| IUIVLUD01C1FQC | - | - | Sb01g012410.1 | - |
| IV9RU5F01AVRWX | - | - | Sb01g012410.1 | - |
| IUIVLUD01EVKMR | - | - | Sb01g012030.1 | - |
| IV9RU5F01A92PC | - | - | Sb01g012030.1 | - |
| IVLNWEC01DSCBG | - | - | Sb01g011310.1 | - |
| IV9RU5F01CFBYK | - | - | Sb01g011050.1 | - |
| IV9RU5F01EFW5R | - | - | Sb01g011050.1 | - |
| IUIVLUD02FTCLA | - | - | Sb01g010930.1 | - |
| IV9RU5F02I3B4E | - | - | Sb01g010930.1 | - |
| IVLNWEC01AJGGI | - | - | Sb01g010930.1 | - |
| IVLNWEC01CZ96W | - | - | Sb01g010930.1 | - |
| IUIVLUD01C7K5F | - | - | Sb01g010025.1 | - |
| IV9RU5F01C35ZV | - | - | Sb01g010025.1 | - |
| IV9RU5F02GNOJV | - | - | Sb01g009615.1 | - |
| IV9RU5F02GRL6T | - | - | Sb01g009615.1 | - |
| IVLNWEC02G8J5A | - | - | Sb01g008700.1 | - |
| IV9RU5F02GZYZ2 | - | - | Sb01g006420.1 | - |
| IVLNWEC01DOOXF | - | - | Sb01g006175.1 | - |
| IVLNWEC01DXOVS | - | - | Sb01g006175.1 | - |
| IVLNWEC02JPI3J | - | - | Sb01g005920.1 | - |
| IVLNWEC01CFAM4 | - | - | Sb01g005660.1 | - |
| IVLNWEC02JOYWG | - | - | Sb01g005660.1 | - |

| | | | | |
|---|---|---|---|---|
| IV9RU5F02F340L | - | - | Sb01g005140.1 | - |
| IV9RU5F02IBRCH | - | - | Sb01g005140.1 | - |
| IV9RU5F02IRH4W | - | - | Sb01g005140.1 | - |
| IV9RU5F02GA0AD | - | - | Sb01g005082.1 | - |
| IUIVLUD01D1M8M | - | - | Sb01g004920.1 | - |
| IV9RU5F01DISWH | - | - | Sb01g004640.1 | - |
| IUIVLUD01ESA8S | - | - | Sb01g003590.1 | - |
| IUIVLUD02GAHOP | - | - | Sb01g003590.1 | - |
| IV9RU5F01D3WFB | - | - | Sb01g002960.1 | - |
| IVLNWEC01DJRQ1 | - | - | Sb01g001770.1 | - |
| IV9RU5F02IUR12 | - | - | Sb01g001540.1 | - |
| IVLNWEC01EHEPI | - | - | Sb01g001540.1 | - |
| IUIVLUD01DS9HE | - | - | Sb01g001200.1 | - |
| IVLNWEC01DGRV9 | - | - | Sb01g001200.1 | - |
| IUIVLUD01DQHLU | - | - | Sb01g001020.1 | - |
| IV9RU5F01A17LN | - | - | Sb01g000430.1 | - |
| IVLNWEC01CJYZX | - | - | Sb01g000330.1 | - |
| IVLNWEC01DBQG8 | - | - | Sb01g000330.1 | - |
| IUIVLUD01D97BO | - | - | Sb01g000280.1 | - |
| IUIVLUD01BQ0AO | - | - | Sb01g000220.1 | - |
| IUIVLUD02FZQ6L | - | - | Sb01g000220.1 | - |
| IUIVLUD02GVVGC | - | - | Sb01g000220.1 | - |
| IVLNWEC01ENA2B | - | - | Sb01g000220.1 | - |
| IVLNWEC02IATTJ | - | - | Sb01g000220.1 | - |
| IUIVLUD02GH76R | - | - | Sb0010s007690.1 | - |
| IV9RU5F02I9WEU | - | - | Sb0010s007690.1 | - |
| IUIVLUD02G79JR | - | - | Sb0010s007680.1 | - |
| IV9RU5F02FRSTS | - | - | Sb0010s007680.1 | - |
| IV9RU5F01BTO91 | - | - | Sb05g024490.1 | - |
| IVLNWEC01DBFI4 | - | - | Sb05g001110.1 | - |
| IUIVLUD02G0GRI | - | - | Sb05g003480.1 | - |
| IV9RU5F01DPODS | - | - | Sb05g003480.1 | - |
| IVLNWEC02HD72Z | - | - | Sb05g003480.1 | - |
| IV9RU5F01A7E27 | - | - | Sb02g024930.1 | - |
| IV9RU5F01CEI4Q | - | - | Sb01g001200.1 | - |
| IV9RU5F02H2TUT | - | - | Sb01g000220.1 | - |
| IV9RU5F01BH4XM | - | - | Sb09g029740.1 | - |
| IUIVLUD02H3Q37 | - | - | Sb02g030920.1 | - |
| IVLNWEC01C16I3 | - | - | Sb05g010321.1 | - |
| IV9RU5F02HYBNO | - | - | Sb04g012680.1 | - |
| IV9RU5F02J1G1A | - | - | Sb08g020692.1 | - |
| IVLNWEC01CBFJX | - | - | Sb03g028280.1 | - |
| IV9RU5F02F1ERR | - | - | Sb01g016775.1 | - |
| IUIVLUD02IFKCT | - | - | Sb01g016770.1 | - |
| IV9RU5F02HW8BF | - | - | Sb01g016770.1 | - |
| IVLNWEC01ETPLU | - | - | Sb08g002910.1 | - |
| IV9RU5F01AIJKL | - | - | Sb02g029620.1 | - |

| | | | | |
|---|---|---|---|---|
| IVLNWEC02HNQVB | - | - | Sb02g033400.1 | - |
| IV9RU5F01DNY4P | - | - | Sb04g017843.1 | - |
| IV9RU5F02GNBVD | - | - | Sb04g017843.1 | - |
| IUIVLUD02GUZ5Z | - | - | Sb01g020130.1 | - |
| IVLNWEC01A1XO2 | - | - | Sb01g004640.1 | - |
| IVLNWEC01BURKR | - | - | Sb02g032640.1 | - |
| IVLNWEC01DMMRZ | - | - | Sb02g020320.1 | - |
| IVLNWEC02HR83U | - | - | Sb02g020320.1 | - |
| IUIVLUD01D2NX1 | - | - | Sb09g030646.1 | - |
| IUIVLUD01AIBNO | - | - | Sb02g028990.1 | - |
| IV9RU5F02HP7QQ | - | - | Sb02g031260.1 | - |
| IV9RU5F01DPTMT | - | - | Sb02g032530.1 | - |
| IV9RU5F02GOIY0 | - | - | Sb02g032530.1 | - |
| IV9RU5F02GL1MR | - | - | Sb09g005570.1 | - |
| IUIVLUD02ICPPS | - | - | Sb08g012560.1 | - |
| IV9RU5F01D2ZMW | - | - | Sb01g019000.1 | - |
| IV9RU5F01CAUS8 | - | - | Sb04g012680.1 | - |
| IV9RU5F01DE89L | - | - | Sb01g020130.1 | - |
| IV9RU5F02IBOXR | - | - | Sb08g002290.1 | - |
| IV9RU5F01ALX9U | - | - | Sb01g001020.1 | - |
| IVLNWEC01DZTUT | - | - | Sb03g011330.1 | - |
| IVLNWEC02IG7K6 | - | - | Sb05g009140.1 | - |
| IV9RU5F01ELDJS | - | - | Sb02g038980.1 | - |
| IV9RU5F01EQVR9 | - | - | Sb01g042488.1 | - |
| IUIVLUD02IPGVM | - | - | Sb02g023710.1 | - |
| IV9RU5F01EUFG4 | - | - | Sb04g009890.1 | - |
| IVLNWEC01BFKAX | - | - | Sb09g006480.1 | - |
| IVLNWEC01BG3W3 | - | - | Sb09g006480.1 | - |
| IUIVLUD01ESE00 | - | - | Sb02g034490.3 | - |
| IVLNWEC01EDN5M | - | - | Sb08g003250.1 | - |
| IV9RU5F02JCJBF | - | - | Sb01g020130.1 | - |
| IVLNWEC01DC1VN | - | - | Sb02g023430.2 | - |
| IUIVLUD01EJE2S | - | - | Sb02g020360.1 | - |
| IVLNWEC02IJG0U | - | - | Sb02g020360.1 | - |
| IV9RU5F01D67CW | - | - | Sb01g039285.1 | - |
| IV9RU5F02G7S1R | - | - | Sb01g039285.1 | - |
| IUIVLUD02HK7MC | - | - | Sb01g020130.1 | - |
| IUIVLUD02GJRDT | - | - | Sb07g025645.1 | - |
| IUIVLUD02I8V6S | - | - | Sb01g042020.1 | - |
| IUIVLUD01BMS5A | - | - | Sb09g030660.1 | - |
| IV9RU5F01AYM0O | - | - | Sb02g043940.1 | - |
| IV9RU5F01ASBW9 | - | - | Sb01g036170.1 | - |
| IV9RU5F01CU1PH | - | - | Sb01g036170.1 | - |
| IV9RU5F01BH3G1 | - | - | Sb04g028630.1 | - |
| IV9RU5F02I87SE | - | - | Sb04g028630.1 | - |
| IV9RU5F02G2H5X | - | - | Sb02g019580.1 | - |
| IUIVLUD01BQNGO | - | - | Sb02g007180.1 | - |

| | | | | |
|---|---|---|---|---|
| IUIVLUD01B42DJ | - | - | Sb07g001930.1 | - |
| IV9RU5F01EFP7A | - | - | Sb01g013460.1 | - |
| IUIVLUD02H403U | - | - | Sb01g028683.1 | - |
| IV9RU5F01BJ2BZ | - | - | Sb01g026590.1 | - |
| IV9RU5F02ICQS7 | - | - | Sb07g020830.1 | - |
| IV9RU5F02IEFUU | - | - | Sb07g020830.1 | - |
| IUIVLUD01CH2XC | - | - | Sb01g036355.1 | - |
| IUIVLUD01BFA64 | - | - | Sb01g019370.1 | - |
| IV9RU5F01A54J7 | - | - | Sb01g019370.1 | - |
| IV9RU5F02GKMIZ | - | - | Sb01g044380.1 | - |
| IVLNWEC01B7WW4 | - | - | Sb01g044380.1 | - |
| IVLNWEC02FWTO2 | - | - | Sb02g021090.1 | - |
| IUIVLUD02F129V | - | - | Sb01g034706.1 | - |
| IVLNWEC01ERXNR | - | - | Sb01g034706.1 | - |
| IV9RU5F02H2PVM | - | - | Sb05g002190.1 | - |
| IVLNWEC02I7NI3 | - | - | Sb01g020130.1 | - |
| IUIVLUD02GOEQ6 | - | - | Sb01g010930.1 | - |
| IUIVLUD02HJZ4V | - | - | Sb01g036355.1 | - |
| IVLNWEC01D12ZS | - | - | Sb05g001950.1 | - |
| IVLNWEC01B2RFM | - | - | Sb03g039660.1 | - |
| IV9RU5F02FWM26 | - | - | Sb09g016865.1 | - |
| IV9RU5F02JZKK5 | - | - | Sb01g034300.1 | - |
| IUIVLUD01CYIOM | - | - | Sb02g031600.1 | - |
| IVLNWEC02G8FW6 | - | - | Sb04g037710.1 | - |
| IUIVLUD01A31IA | - | - | Sb05g009140.1 | - |
| IVLNWEC01CXYJX | - | - | Sb03g013450.1 | - |
| IVLNWEC01EGRHN | - | - | Sb03g013450.1 | - |
| IVLNWEC02HWHW9 | - | - | Sb02g024930.1 | - |
| IV9RU5F01DOERA | - | - | Sb03g036450.1 | - |
| IUIVLUD01DMCDA | - | - | Sb03g036450.1 | - |
| IV9RU5F01DIRZW | - | - | Sb05g002940.1 | - |
| IUIVLUD01CKB0R | - | - | Sb01g004640.1 | - |
| IVLNWEC01EAW6V | - | - | Sb02g024090.1 | - |
| IV9RU5F01DZODN | - | - | Sb05g001110.1 | - |
| IVLNWEC01ATJI5 | - | - | Sb02g034490.3 | - |
| IUIVLUD02JQF1F | - | - | Sb02g028950.1 | - |
| IVLNWEC01DE5U1 | - | - | Sb01g006990.2 | - |
| IUIVLUD02GRJAC | - | - | Sb08g001855.1 | - |
| IV9RU5F02G258A | - | - | Sb02g014910.1 | - |
| IVLNWEC02IDYL0 | - | - | Sb01g020130.1 | - |
| IV9RU5F02IEI6P | - | - | Sb03g003450.1 | - |
| IVLNWEC01AMYNK | - | - | Sb01g020130.1 | - |
| IVLNWEC01AT55S | - | - | Sb01g020130.1 | - |
| IV9RU5F02F77RB | - | - | Sb02g006040.1 | - |
| IVLNWEC02HWZ71 | - | - | Sb10g000400.1 | - |
| IV9RU5F01EM1HJ | - | - | Sb07g022230.1 | - |
| IV9RU5F01EP5YV | - | - | Sb08g001480.1 | - |

| | | | | |
|---|---|---|---|---|
| IUIVLUD01DPZMH | - | - | Sb08g003800.1 | - |
| IV9RU5F02HUR8P | - | - | Sb10g003360.1 | - |
| IV9RU5F02HNA4Z | - | - | Sb08g001480.1 | - |
| IVLNWEC01EI53F | - | - | Sb07g001500.1 | - |
| IUIVLUD02J4OJE | - | - | Sb01g034706.1 | - |
| IV9RU5F02IFOPQ | - | - | Sb01g004920.1 | - |
| IUIVLUD01A1WMC | - | - | Sb05g001570.1 | - |
| IV9RU5F02FX0PD | - | - | Sb05g001570.1 | - |
| IVLNWEC02II8Q9 | - | - | Sb01g019060.1 | - |
| IV9RU5F01AXKLJ | - | - | Sb02g026170.1 | - |
| IVLNWEC01EYZ2L | - | - | Sb02g026170.1 | - |
| IV9RU5F02HT62Y | - | - | Sb03g011210.1 | - |
| IV9RU5F01DFHBY | - | - | Sb01g001200.1 | - |
| IVLNWEC01A4MWW | - | - | Sb01g001200.1 | - |
| IVLNWEC01BA1K8 | - | - | Sb02g029500.1 | - |
| IV9RU5F01DOMS9 | - | - | Sb02g026280.1 | - |
| IV9RU5F02GVZ71 | - | - | Sb02g026280.1 | - |
| IVLNWEC01AELOT | - | - | Sb01g034300.1 | - |
| IUIVLUD02GCAVI | - | - | Sb01g019210.1 | - |
| IV9RU5F02H000M | - | - | Sb02g033400.1 | - |
| IV9RU5F02FSAPY | - | - | Sb03g011460.1 | - |
| IV9RU5F01B73Q9 | - | - | Sb08g012360.1 | - |
| IVLNWEC01BYKZL | - | - | Sb02g025566.1 | - |
| IUIVLUD02JQELI | - | - | Sb02g021110.1 | - |
| IV9RU5F01CR87L | - | - | Sb01g037140.1 | - |
| IVLNWEC02F4332 | - | - | Sb04g021960.1 | - |
| IV9RU5F02GLSA1 | - | - | Sb06g002540.1 | - |
| IUIVLUD02GCZ27 | - | - | Sb02g004300.2 | - |
| IV9RU5F02GHZG9 | - | - | Sb10g007930.1 | - |
| IV9RU5F01A335Q | - | - | Sb03g036450.1 | - |
| IV9RU5F01CTJM0 | - | - | Sb05g005345.1 | - |
| IUIVLUD02G93C9 | - | - | Sb08g022840.1 | - |
| IV9RU5F01E1RB8 | - | - | Sb02g014480.1 | - |
| IUIVLUD01BDK67 | - | - | Sb03g011330.1 | - |
| IVLNWEC02JR9K1 | - | - | Sb09g005570.1 | - |
| IV9RU5F01CVJEW | - | - | Sb01g042020.1 | - |
| IVLNWEC01EWPPI | - | - | Sb10g007930.1 | - |
| IV9RU5F01AZ2AV | - | - | Sb02g024760.1 | - |
| IUIVLUD02H6STG | - | - | Sb01g008370.1 | - |
| IV9RU5F02GPAIW | - | - | Sb01g008370.1 | - |
| IV9RU5F02J2KDY | - | - | Sb01g008370.1 | - |
| IUIVLUD02JGS7R | - | - | Sb01g039390.1 | - |
| IV9RU5F02IQNED | - | - | Sb01g006990.2 | - |
| IV9RU5F01C03E9 | - | - | Sb08g020460.1 | - |
| IV9RU5F02JO41S | - | - | Sb01g042020.1 | - |
| IV9RU5F02JR4WF | - | - | Sb04g028630.1 | - |
| IVLNWEC02FXTCC | - | - | Sb05g022840.1 | - |

| | | | | |
|---|---|---|---|---|
| IUIVLUD01DCSQO | - | - | Sb01g042400.1 | - |
| IVLNWEC02H8YSQ | - | - | Sb01g044380.1 | - |
| IV9RU5F01C205K | - | - | Sb01g012810.1 | - |
| IV9RU5F01AXNE1 | - | - | Sb01g020130.1 | - |
| IUIVLUD02IBAA5 | - | - | - | MLOC_77773.1 |
| IUIVLUD01D530N | - | - | - | AK354084 |
| IVLNWEC01BK77I | - | - | - | MLOC_43786.1 |
| IVLNWEC01CA0D3 | - | - | - | MLOC_43786.1 |
| IUIVLUD02JR3SI | - | - | - | MLOC_51107.1 |
| IVLNWEC01BWHAX | - | - | - | MLOC_7410.1 |
| IVLNWEC01EYRN1 | - | - | - | MLOC_73874.1 |
| IVLNWEC01BVVGV | - | - | - | MLOC_74974.5 |
| IVLNWEC02GMN1Y | - | - | - | MLOC_67514.1 |
| IVLNWEC02JSJRG | - | - | - | MLOC_67514.1 |
| IV9RU5F01CP61K | - | - | - | MLOC_38229.4 |
| IVLNWEC02HE9RN | - | - | - | MLOC_19278.1 |
| IVLNWEC01BO8TX | - | - | - | MLOC_69825.1 |
| IV9RU5F02F5TFE | - | - | - | MLOC_10435.2 |
| IUIVLUD01BGVD6 | - | - | - | MLOC_9984.5 |
| IV9RU5F01ANBXY | - | - | - | MLOC_54452.3 |
| IVLNWEC02FP259 | - | - | - | AK367480 |
| IVLNWEC02HSFXH | - | - | - | AK367480 |
| IUIVLUD02GYFM7 | - | - | - | AK374742 |
| IVLNWEC02G93SZ | - | - | - | AK356799 |
| IV9RU5F02IKKAE | - | - | - | MLOC_59323.1 |
| IV9RU5F01DHOXF | - | - | - | MLOC_53405.1 |
| IVLNWEC01DVHR6 | - | - | - | MLOC_53405.1 |
| IVLNWEC02FRUOP | - | - | - | MLOC_19401.1 |
| IV9RU5F02GH2XK | - | - | - | MLOC_28332.1 |
| IV9RU5F01CJ5I5 | - | - | - | AK358997 |
| IVLNWEC01DMV8F | - | - | - | AK358997 |
| IUIVLUD01ASJ7H | - | - | - | MLOC_2429.1 |
| IUIVLUD01B2FWJ | - | - | - | MLOC_2429.1 |
| IV9RU5F01E3OSW | - | - | - | MLOC_2429.1 |
| IVLNWEC01BL07T | - | - | - | MLOC_2429.1 |
| IVLNWEC02HCXXC | - | - | - | MLOC_2429.1 |
| IUIVLUD02I1E7E | - | - | - | MLOC_10243.2 |
| IV9RU5F02F1A1P | - | - | - | AK372273 |
| IV9RU5F01DZZ2I | - | - | - | AK376038 |
| IV9RU5F01EJPJG | - | - | - | MLOC_61642.4 |
| IUIVLUD02HFHG8 | - | - | - | MLOC_56792.1 |
| IVLNWEC02HN9PB | - | - | - | MLOC_56792.1 |
| IV9RU5F02G00BX | - | - | - | MLOC_77713.2 |
| IVLNWEC02FYGV6 | - | - | - | MLOC_75155.1 |
| IV9RU5F01CYGY3 | - | - | - | MLOC_11707.1 |
| IVLNWEC01DKH1J | - | - | - | MLOC_35082.1 |
| IV9RU5F01D6CXV | - | - | - | MLOC_64296.4 |

| | | | | |
|---|---|---|---|---|
| IV9RU5F02GQIBZ | - | - | - | MLOC_64296.4 |
| IVLNWEC01CQJA0 | - | - | - | MLOC_58630.2 |
| IVLNWEC02JYFNE | - | - | - | AK359447 |
| IVLNWEC01BHHRW | - | - | - | MLOC_54836.1 |
| IVLNWEC01CODMM | - | - | - | AK362289 |
| IVLNWEC01DSAIJ | - | - | - | AK362289 |
| IV9RU5F01D6TWX | - | - | - | MLOC_57426.2 |
| IV9RU5F02IAEM7 | - | - | - | AK371412 |
| IV9RU5F01CFL09 | - | - | - | MLOC_56692.1 |
| IUIVLUD02F6J2N | - | - | - | MLOC_22590.1 |
| IUIVLUD02JYAYC | - | - | - | MLOC_22590.1 |
| IV9RU5F02JSGGG | - | - | - | MLOC_22590.1 |
| IV9RU5F02G6E3Q | - | - | - | AK363711 |
| IV9RU5F02HYGLM | - | - | - | AK353660 |
| IV9RU5F02JF5JE | - | - | - | AK353660 |
| IVLNWEC02HQF8F | - | - | - | AK353660 |
| IUIVLUD01C3M34 | - | - | - | MLOC_58508.9 |
| IUIVLUD02FPJMS | - | - | - | MLOC_10070.1 |
| IV9RU5F02H4O93 | - | - | - | MLOC_10070.1 |
| IVLNWEC02H1TM3 | - | - | - | MLOC_618.2 |
| IVLNWEC02HUWHI | - | - | - | MLOC_68431.4 |
| IVLNWEC02GLCN4 | - | - | - | AK372882 |
| IUIVLUD02GN44U | - | - | - | MLOC_9982.2 |
| IUIVLUD01BKHZ1 | - | - | - | AK376547 |
| IV9RU5F02F2K80 | - | - | - | MLOC_52425.9 |
| IV9RU5F02JIN7H | - | - | - | MLOC_52425.9 |
| IVLNWEC01DZW3A | - | - | - | MLOC_52425.9 |
| IVLNWEC02IHC03 | - | - | - | MLOC_60693.1 |
| IVLNWEC01CO2AA | - | - | - | AK376476 |
| IVLNWEC01DIBE7 | - | - | - | AK376476 |
| IV9RU5F02IN76A | - | - | - | MLOC_37686.4 |
| IVLNWEC01CNW1F | - | - | - | MLOC_37686.4 |
| IV9RU5F01C2H03 | - | - | - | MLOC_20538.1 |
| IVLNWEC01D4CRB | - | - | - | MLOC_20538.1 |
| IVLNWEC01DB0AU | - | - | - | MLOC_72578.1 |
| IVLNWEC02GMHP3 | - | - | - | MLOC_72578.1 |
| IUIVLUD01A781P | - | - | - | MLOC_4614.1 |
| IV9RU5F02F06MV | - | - | - | MLOC_4614.1 |
| IV9RU5F02H8USP | - | - | - | MLOC_4614.1 |
| IVLNWEC02F4EOJ | - | - | - | MLOC_58508.9 |
| IV9RU5F02J1LMJ | - | - | - | MLOC_57426.2 |
| IV9RU5F02JCWKV | - | - | - | MLOC_57426.2 |
| IVLNWEC02H11J1 | - | - | - | MLOC_12105.1 |
| IV9RU5F02JPQ1T | - | - | - | MLOC_67515.1 |
| IVLNWEC02I3CLY | - | - | - | AK248871.1 |
| IV9RU5F02HDLJR | - | - | - | MLOC_18165.1 |
| IVLNWEC02G8EN6 | - | - | - | AK362554 |

| | | | | |
|---|---|---|---|---|
| IV9RU5F02HXXGX | - | - | - | MLOC_71365.2 |
| IUIVLUD01AW3TU | - | - | - | MLOC_64709.2 |
| IV9RU5F01D31AJ | - | - | - | MLOC_11404.1 |
| IV9RU5F02GKE3K | - | - | - | MLOC_4685.6 |
| IVLNWEC01BZOB8 | - | - | - | AK362554 |
| IVLNWEC02ISW3X | - | - | - | AK250586.1 |
| IVLNWEC01A33Q3 | - | - | - | MLOC_78400.1 |
| IVLNWEC02GBIPS | - | - | - | MLOC_28332.1 |
| IV9RU5F01EOXI8 | - | - | - | MLOC_55574.1 |
| IVLNWEC01CCIAQ | - | - | - | AK248596.1 |
| IV9RU5F01BOZUP | - | - | - | MLOC_76365.1 |
| IVLNWEC01CEWGI | - | - | - | MLOC_76365.1 |
| IV9RU5F02JZGLA | - | - | - | AK354330 |
| IV9RU5F02I2M7O | - | - | - | MLOC_36971.7 |
| IV9RU5F01BTKV7 | - | - | - | MLOC_9984.5 |
| IVLNWEC01C3BF7 | - | - | - | MLOC_9984.5 |
| IUIVLUD02FVMVD | - | - | - | MLOC_8296.1 |
| IV9RU5F01ANHYU | - | - | - | MLOC_6055.1 |
| IVLNWEC02IKKRR | - | - | - | MLOC_56876.1 |
| IVLNWEC01BRAQV | - | - | - | MLOC_3052.1 |
| IUIVLUD02IBFXX | - | - | - | MLOC_31206.1 |
| IV9RU5F01DXEE6 | - | - | - | MLOC_67515.1 |
| IV9RU5F01AMCN1 | - | - | - | AK372303 |
| IV9RU5F02JZVZH | - | - | - | MLOC_13941.2 |
| IUIVLUD01D6DNN | - | - | - | AK359097 |
| IVLNWEC01AF0L2 | - | - | - | MLOC_15823.1 |
| IV9RU5F02GPPZ2 | - | - | - | AK355984 |
| IVLNWEC01CBXS3 | - | - | - | AK355984 |
| IV9RU5F02JIIDG | - | - | - | MLOC_15017.1 |
| IVLNWEC01D9T96 | - | - | - | AK371412 |
| IV9RU5F01BVK7J | - | - | - | MLOC_55526.1 |
| IVLNWEC02F7Y4O | - | - | - | MLOC_58044.1 |
| IUIVLUD01AQBDY | - | - | - | MLOC_65338.4 |
| IV9RU5F01AFZ30 | - | - | - | MLOC_7601.1 |
| IVLNWEC02GDACU | - | - | - | MLOC_7601.1 |
| IV9RU5F02GB7CD | - | - | - | MLOC_6016.1 |
| IVLNWEC02F1LJX | - | - | - | MLOC_15443.2 |
| IV9RU5F02JTAT9 | - | - | - | AK376249 |
| IVLNWEC02F8CZZ | - | - | - | AK376249 |
| IV9RU5F01DLWWA | - | - | - | MLOC_60708.1 |
| IUIVLUD01DS5RG | - | - | - | MLOC_81021.1 |
| IV9RU5F01BS6C7 | - | - | - | AK250598.1 |
| IV9RU5F02GXI7W | - | - | - | AK371555 |
| IV9RU5F02JLJZW | - | - | - | MLOC_54267.1 |
| IV9RU5F01CTUXP | - | - | - | MLOC_16126.1 |
| IV9RU5F02G02TC | - | - | - | MLOC_16126.1 |
| IV9RU5F01A0WQV | - | - | - | AK370395 |

| | | | | |
|---|---|---|---|---|
| IVLNWEC01DB2UM | - | - | - | MLOC_44276.3 |
| IV9RU5F01BSRVO | - | - | - | MLOC_13941.2 |
| IV9RU5F02JW0GK | - | - | - | MLOC_76365.1 |
| IVLNWEC02HJFS4 | - | - | - | MLOC_76365.1 |
| IV9RU5F01DRAJY | - | - | - | AK364488 |
| IV9RU5F01AXUJZ | - | - | - | MLOC_15242.1 |
| IVLNWEC02F7GR1 | - | - | - | AK376038 |
| IV9RU5F01EID5Y | - | - | - | MLOC_81247.1 |
| IV9RU5F02GQ8IM | - | - | - | MLOC_81247.1 |
| IUIVLUD01BZ4AD | - | - | - | MLOC_62275.3 |
| IV9RU5F01BT2UZ | - | - | - | AK362566 |
| IV9RU5F01DVIZQ | - | - | - | MLOC_48910.4 |
| IUIVLUD02H9JZA | - | - | - | MLOC_18975.1 |
| IV9RU5F02FP2LG | - | - | - | MLOC_18975.1 |
| IV9RU5F01BMQ4L | - | - | - | MLOC_61121.1 |
| IV9RU5F01AFKYL | - | - | - | MLOC_12555.1 |
| IVLNWEC02H9LSW | - | - | - | MLOC_12555.1 |
| IV9RU5F02JYBZN | - | - | - | AK252929.1 |
| IUIVLUD02HPDWG | - | - | - | MLOC_53882.4 |
| IV9RU5F02GUYT6 | - | - | - | MLOC_15455.2 |
| IVLNWEC02H735Y | - | - | - | MLOC_59288.1 |
| IVLNWEC02ICQ6N | - | - | - | MLOC_59288.1 |
| IVLNWEC02G2OR3 | - | - | - | MLOC_44387.6 |
| IVLNWEC01A5SG9 | - | - | - | MLOC_62596.1 |
| IVLNWEC02IKSEG | - | - | - | MLOC_62596.1 |
| IUIVLUD02GWYIC | - | - | - | MLOC_52019.1 |
| IV9RU5F01EAOE8 | - | - | - | MLOC_34557.1 |
| IV9RU5F01CKVQM | - | - | - | MLOC_61019.2 |
| IUIVLUD01DVITE | - | - | - | MLOC_52014.2 |
| IV9RU5F02G0PFF | - | - | - | MLOC_52014.2 |
| IV9RU5F02HUVZK | - | - | - | MLOC_52014.2 |
| IUIVLUD02IL222 | - | - | - | MLOC_6833.1 |
| IVLNWEC01CKYSJ | - | - | - | MLOC_6833.1 |
| IUIVLUD02IX7OZ | - | - | - | MLOC_81247.1 |
| IVLNWEC02H0A75 | - | - | - | AK369576 |
| IV9RU5F02H0ZRN | - | - | - | MLOC_10511.4 |
| IVLNWEC01BAOOM | - | - | - | MLOC_19401.1 |
| IVLNWEC02IW2OJ | - | - | - | AK373499 |
| IV9RU5F02GK9LC | - | - | - | MLOC_10511.4 |
| IV9RU5F01ESEP2 | - | - | - | MLOC_75827.1 |
| IV9RU5F02I4ZM3 | - | - | - | MLOC_58443.1 |
| IV9RU5F01A1D0Z | - | - | - | MLOC_54883.1 |
| IVLNWEC02JIF20 | - | - | - | AK359447 |
| IVLNWEC01CERNH | - | - | - | AK359447 |
| IUIVLUD01B9ISS | - | - | - | MLOC_59845.1 |
| IVLNWEC01EZK07 | - | - | - | MLOC_80410.1 |
| IUIVLUD02F50AW | - | - | - | MLOC_43699.1 |

| | | | | |
|---|---|---|---|---|
| IUIVLUD02FV438 | - | - | - | AK355984 |
| IVLNWEC01B3Q1X | - | - | - | AK355984 |
| IVLNWEC02IPVQR | - | - | - | AK372882 |
| IUIVLUD02HH7GW | - | - | - | AK360968 |
| IV9RU5F02F5S9X | - | - | - | AK373293 |
| IV9RU5F01AUO5U | - | - | - | AK365190 |
| IUIVLUD01ELF3S | - | - | - | MLOC_69909.1 |
| IUIVLUD01AG3AV | - | - | - | MLOC_38229.4 |
| IV9RU5F02HDO3T | - | - | - | MLOC_11707.1 |
| IVLNWEC01BR7VQ | - | - | - | AK248596.1 |
| IUIVLUD01BDATJ | - | - | - | MLOC_74794.1 |
| IV9RU5F01CVY8V | - | - | - | MLOC_74794.1 |
| IV9RU5F02G4CB1 | - | - | - | MLOC_70194.5 |
| IVLNWEC01AVESD | - | - | - | MLOC_6887.1 |
| IV9RU5F02IEY4S | - | - | - | MLOC_16408.1 |
| IV9RU5F01C8VFZ | - | - | - | AK361911 |
| IV9RU5F02JIE0F | - | - | - | MLOC_50957.2 |
| IVLNWEC01BUWQ7 | - | - | - | MLOC_59824.1 |
| IVLNWEC02IHUOD | - | - | - | MLOC_54452.3 |
| IUIVLUD01A0NLM | - | - | - | MLOC_2655.1 |
| IVLNWEC02F23MP | - | - | - | MLOC_2655.1 |
| IVLNWEC02I0YIH | - | - | - | AK364487 |
| IVLNWEC02HE0UP | - | - | - | AK364487 |
| IVLNWEC02IGGFQ | - | - | - | MLOC_63089.10 |
| IV9RU5F02HZUH8 | - | - | - | AK358997 |
| IV9RU5F02IC8SN | - | - | - | MLOC_73230.1 |
| IV9RU5F02GP038 | - | - | - | MLOC_4908.1 |
| IV9RU5F01AHK8D | - | - | - | AK354330 |
| IV9RU5F02FTTNA | - | - | - | MLOC_60274.1 |
| IV9RU5F01CPHTZ | - | - | - | MLOC_58644.1 |
| IV9RU5F01D3B3E | - | - | - | MLOC_73572.1 |
| IUIVLUD02HX52V | - | - | - | MLOC_21560.3 |
| IV9RU5F01AH6WY | - | - | - | MLOC_4008.2 |
| IV9RU5F01CEOGB | - | - | - | AK359097 |
| IVLNWEC01BDO1H | - | - | - | MLOC_38307.2 |
| IV9RU5F02IU8WO | - | - | - | AK373279 |
| IV9RU5F02IXPDR | - | - | - | MLOC_68431.4 |
| IVLNWEC01ALJ6W | - | - | - | MLOC_78400.1 |
| IVLNWEC02H4ILG | - | - | - | MLOC_35082.1 |
| IVLNWEC01C4MVN | - | - | - | MLOC_59797.4 |
| IUIVLUD01DYM5Z | - | - | - | MLOC_9984.5 |
| IUIVLUD02FO5RK | - | - | - | MLOC_9984.5 |
| IV9RU5F02GVL32 | - | - | - | MLOC_9984.5 |
| IVLNWEC02JL1VQ | - | - | - | MLOC_9984.5 |
| IUIVLUD02G49CY | - | - | - | MLOC_9982.2 |
| IV9RU5F01BB0GW | - | - | - | MLOC_9956.1 |
| IVLNWEC02IZ9G8 | - | - | - | MLOC_9956.1 |

| | | | | |
|---|---|---|---|---|
| IVLNWEC02F7WY2 | - | - | - | MLOC_81998.2 |
| IVLNWEC01DWLQS | - | - | - | MLOC_81021.1 |
| IUIVLUD02FS8FT | - | - | - | MLOC_80687.1 |
| IV9RU5F02I6FDC | - | - | - | MLOC_80591.1 |
| IVLNWEC01CTWHW | - | - | - | MLOC_80410.1 |
| IVLNWEC02HDX1W | - | - | - | MLOC_78400.1 |
| IVLNWEC01C2OWI | - | - | - | MLOC_7791.1 |
| IUIVLUD02HG8LS | - | - | - | MLOC_77773.1 |
| IV9RU5F02FZGY0 | - | - | - | MLOC_77773.1 |
| IUIVLUD01CKOZA | - | - | - | MLOC_77713.2 |
| IUIVLUD02HNI9E | - | - | - | MLOC_77713.2 |
| IUIVLUD01AVRTY | - | - | - | MLOC_77043.1 |
| IVLNWEC01DQMDI | - | - | - | MLOC_77043.1 |
| IV9RU5F01AGCOQ | - | - | - | MLOC_76493.1 |
| IUIVLUD01AL135 | - | - | - | MLOC_76365.1 |
| IV9RU5F02JDGTQ | - | - | - | MLOC_76365.1 |
| IUIVLUD01ASWZW | - | - | - | MLOC_7601.1 |
| IUIVLUD01BCQVL | - | - | - | MLOC_7601.1 |
| IVLNWEC01CVC0W | - | - | - | MLOC_74974.5 |
| IV9RU5F02GKFEJ | - | - | - | MLOC_74632.1 |
| IUIVLUD01DNREY | - | - | - | MLOC_7410.1 |
| IV9RU5F01AHIPL | - | - | - | MLOC_7410.1 |
| IUIVLUD01A9DN6 | - | - | - | MLOC_74098.1 |
| IV9RU5F01A2COF | - | - | - | MLOC_74098.1 |
| IV9RU5F01DTHKZ | - | - | - | MLOC_74098.1 |
| IVLNWEC01EGT29 | - | - | - | MLOC_73874.1 |
| IUIVLUD01D7U9V | - | - | - | MLOC_73803.1 |
| IVLNWEC01BIIX6 | - | - | - | MLOC_73803.1 |
| IVLNWEC01DNTSE | - | - | - | MLOC_73803.1 |
| IV9RU5F02J1RV2 | - | - | - | MLOC_73230.1 |
| IUIVLUD02IV91D | - | - | - | MLOC_72805.2 |
| IVLNWEC02IT1SC | - | - | - | MLOC_72805.2 |
| IUIVLUD01C7PZI | - | - | - | MLOC_72572.1 |
| IV9RU5F02IRDJF | - | - | - | MLOC_72572.1 |
| IVLNWEC01DN89B | - | - | - | MLOC_72572.1 |
| IV9RU5F01EK5I4 | - | - | - | MLOC_72148.1 |
| IV9RU5F02GMTJL | - | - | - | MLOC_72148.1 |
| IV9RU5F02GOBFB | - | - | - | MLOC_72148.1 |
| IVLNWEC02GHALC | - | - | - | MLOC_72148.1 |
| IVLNWEC02HDAZE | - | - | - | MLOC_71747.1 |
| IVLNWEC02IAZIL | - | - | - | MLOC_71747.1 |
| IVLNWEC02G3MY7 | - | - | - | MLOC_71301.2 |
| IV9RU5F01BDJUT | - | - | - | MLOC_70419.2 |
| IVLNWEC01DA6RV | - | - | - | MLOC_70419.2 |
| IVLNWEC01DDRXT | - | - | - | MLOC_70419.2 |
| IUIVLUD02INW8X | - | - | - | MLOC_70194.5 |
| IUIVLUD01EE78R | - | - | - | MLOC_6995.1 |

| | | | | |
|---|---|---|---|---|
| IV9RU5F02JD1PK | - | - | - | MLOC_6995.1 |
| IVLNWEC01CAIDF | - | - | - | MLOC_69825.1 |
| IVLNWEC01BYFM7 | - | - | - | MLOC_69031.1 |
| IV9RU5F02FXPQR | - | - | - | MLOC_68905.1 |
| IVLNWEC02JHMNQ | - | - | - | MLOC_68905.1 |
| IUIVLUD02JZLA9 | - | - | - | MLOC_68734.1 |
| IUIVLUD01DR5FX | - | - | - | MLOC_68535.2 |
| IVLNWEC01BI0TT | - | - | - | MLOC_68535.2 |
| IVLNWEC01A98ZF | - | - | - | MLOC_67639.2 |
| IV9RU5F02JT5FE | - | - | - | MLOC_67515.1 |
| IVLNWEC01BKCKL | - | - | - | MLOC_67149.2 |
| IVLNWEC02ITHG8 | - | - | - | MLOC_67149.2 |
| IUIVLUD01BHLCP | - | - | - | MLOC_66827.1 |
| IV9RU5F01ARS3E | - | - | - | MLOC_66723.1 |
| IVLNWEC01B2TMW | - | - | - | MLOC_66723.1 |
| IV9RU5F01CW9Q2 | - | - | - | MLOC_6651.2 |
| IVLNWEC01DZW0Y | - | - | - | MLOC_6651.2 |
| IV9RU5F01AK7VC | - | - | - | MLOC_65917.1 |
| IVLNWEC01CQVT9 | - | - | - | MLOC_65917.1 |
| IVLNWEC02JZZ3W | - | - | - | MLOC_65917.1 |
| IUIVLUD02IRKX8 | - | - | - | MLOC_65827.2 |
| IVLNWEC01ATAO3 | - | - | - | MLOC_65827.2 |
| IV9RU5F01EV57B | - | - | - | MLOC_65819.2 |
| IVLNWEC02J15QU | - | - | - | MLOC_65819.2 |
| IUIVLUD02HR7IW | - | - | - | MLOC_65562.1 |
| IUIVLUD02IBM55 | - | - | - | MLOC_65358.1 |
| IV9RU5F02JJ6N9 | - | - | - | MLOC_65338.4 |
| IVLNWEC01DG49Z | - | - | - | MLOC_64971.1 |
| IUIVLUD01BIKWL | - | - | - | MLOC_64898.1 |
| IV9RU5F02JFL0M | - | - | - | MLOC_64898.1 |
| IV9RU5F01C8O0P | - | - | - | MLOC_64815.2 |
| IVLNWEC02GZZ4U | - | - | - | MLOC_64815.2 |
| IVLNWEC01AUOOB | - | - | - | MLOC_64709.2 |
| IUIVLUD01C4RRH | - | - | - | MLOC_64045.1 |
| IUIVLUD02JG2D3 | - | - | - | MLOC_64045.1 |
| IV9RU5F01DZPZV | - | - | - | MLOC_63760.1 |
| IV9RU5F01BSOA2 | - | - | - | MLOC_63625.1 |
| IVLNWEC02JUJHQ | - | - | - | MLOC_63625.1 |
| IUIVLUD02JT4KG | - | - | - | MLOC_62137.1 |
| IUIVLUD01AZTG6 | - | - | - | MLOC_62016.2 |
| IV9RU5F02GQNVS | - | - | - | MLOC_62016.2 |
| IVLNWEC02IFSTY | - | - | - | MLOC_618.2 |
| IV9RU5F02F7G2E | - | - | - | MLOC_61121.1 |
| IUIVLUD02IR0FC | - | - | - | MLOC_6081.1 |
| IV9RU5F01DY79M | - | - | - | MLOC_6081.1 |
| IVLNWEC02HISVU | - | - | - | MLOC_6081.1 |
| IV9RU5F02HLGOR | - | - | - | MLOC_60708.1 |

| | | | | |
|---|---|---|---|---|
| IV9RU5F02GNDT9 | - | - | - | MLOC_60693.1 |
| IVLNWEC01CSZEF | - | - | - | MLOC_6055.1 |
| IUIVLUD02FSD6Z | - | - | - | MLOC_60545.2 |
| IVLNWEC01C0D46 | - | - | - | MLOC_60545.2 |
| IVLNWEC02HLSCP | - | - | - | MLOC_6016.1 |
| IV9RU5F01CW7N8 | - | - | - | MLOC_60056.5 |
| IV9RU5F02GC94Y | - | - | - | MLOC_60035.1 |
| IV9RU5F01BDXYF | - | - | - | MLOC_59921.1 |
| IV9RU5F02IXNUT | - | - | - | MLOC_59845.1 |
| IUIVLUD01COP24 | - | - | - | MLOC_59824.1 |
| IV9RU5F01BR890 | - | - | - | MLOC_59824.1 |
| IUIVLUD02I8C3W | - | - | - | MLOC_59797.4 |
| IVLNWEC02IKSEP | - | - | - | MLOC_59797.4 |
| IV9RU5F01CAKAQ | - | - | - | MLOC_59792.1 |
| IVLNWEC02I9HWN | - | - | - | MLOC_59792.1 |
| IV9RU5F01DHM2F | - | - | - | MLOC_59699.2 |
| IVLNWEC01DQU71 | - | - | - | MLOC_59699.2 |
| IUIVLUD01BPEM5 | - | - | - | MLOC_59638.1 |
| IVLNWEC01AO7C4 | - | - | - | MLOC_59638.1 |
| IVLNWEC01AQ024 | - | - | - | MLOC_59638.1 |
| IVLNWEC02G9SNP | - | - | - | MLOC_59323.1 |
| IUIVLUD02J4B00 | - | - | - | MLOC_58630.2 |
| IVLNWEC01ANWMF | - | - | - | MLOC_58443.1 |
| IVLNWEC01ENFWY | - | - | - | MLOC_58443.1 |
| IUIVLUD02JQSYC | - | - | - | MLOC_58221.2 |
| IVLNWEC01ED63S | - | - | - | MLOC_58221.2 |
| IVLNWEC01CEYAP | - | - | - | MLOC_58044.1 |
| IUIVLUD02GIKJL | - | - | - | MLOC_57923.1 |
| IUIVLUD02IWJY5 | - | - | - | MLOC_57923.1 |
| IV9RU5F01CNQT6 | - | - | - | MLOC_57923.1 |
| IV9RU5F01DOJG6 | - | - | - | MLOC_57923.1 |
| IV9RU5F02IVZ0O | - | - | - | MLOC_57923.1 |
| IVLNWEC02GM9TI | - | - | - | MLOC_5767.2 |
| IV9RU5F01AN3QZ | - | - | - | MLOC_57619.3 |
| IVLNWEC02IL2PH | - | - | - | MLOC_57619.3 |
| IUIVLUD01ANH3O | - | - | - | MLOC_57426.2 |
| IUIVLUD01C0AQU | - | - | - | MLOC_57426.2 |
| IV9RU5F02H47RB | - | - | - | MLOC_57328.1 |
| IVLNWEC02IGRUU | - | - | - | MLOC_57328.1 |
| IUIVLUD01AXUE4 | - | - | - | MLOC_56876.1 |
| IV9RU5F01C5SA5 | - | - | - | MLOC_56792.1 |
| IVLNWEC01BSGV0 | - | - | - | MLOC_56792.1 |
| IVLNWEC02FVPCJ | - | - | - | MLOC_56792.1 |
| IUIVLUD02FZTXT | - | - | - | MLOC_56733.2 |
| IUIVLUD02HVSES | - | - | - | MLOC_56719.2 |
| IVLNWEC02HMBG6 | - | - | - | MLOC_56692.1 |
| IVLNWEC01BIZ7O | - | - | - | MLOC_55791.2 |

| | | | | |
|---|---|---|---|---|
| IV9RU5F02F89UH | - | - | - | MLOC_55586.4 |
| IVLNWEC01EJJ0I | - | - | - | MLOC_55586.4 |
| IV9RU5F02GW8RW | - | - | - | MLOC_55207.1 |
| IVLNWEC01DD986 | - | - | - | MLOC_55207.1 |
| IV9RU5F02GLZRV | - | - | - | MLOC_55034.1 |
| IV9RU5F02G6F43 | - | - | - | MLOC_54883.1 |
| IUIVLUD01EWXYE | - | - | - | MLOC_54836.1 |
| IUIVLUD02HHRRD | - | - | - | MLOC_54836.1 |
| IVLNWEC02GQLVE | - | - | - | MLOC_54836.1 |
| IUIVLUD01DAO5D | - | - | - | MLOC_54721.2 |
| IV9RU5F01DXX24 | - | - | - | MLOC_54721.2 |
| IVLNWEC01AHJCP | - | - | - | MLOC_54721.2 |
| IV9RU5F02GJ4FG | - | - | - | MLOC_54619.1 |
| IVLNWEC01BWSXV | - | - | - | MLOC_54243.2 |
| IVLNWEC02IIXR1 | - | - | - | MLOC_54243.2 |
| IUIVLUD01BQ3SN | - | - | - | MLOC_54213.1 |
| IV9RU5F02HS182 | - | - | - | MLOC_54213.1 |
| IV9RU5F02JXDON | - | - | - | MLOC_5414.1 |
| IVLNWEC02G89E9 | - | - | - | MLOC_5414.1 |
| IV9RU5F01B53TJ | - | - | - | MLOC_53882.4 |
| IVLNWEC01BHKWU | - | - | - | MLOC_53882.4 |
| IUIVLUD02GO4HB | - | - | - | MLOC_53405.1 |
| IV9RU5F02H1SQQ | - | - | - | MLOC_53405.1 |
| IVLNWEC01CMJJ2 | - | - | - | MLOC_52604.2 |
| IV9RU5F02GXVHX | - | - | - | MLOC_52055.2 |
| IVLNWEC01AY5ND | - | - | - | MLOC_52055.2 |
| IUIVLUD01AF25J | - | - | - | MLOC_51302.1 |
| IV9RU5F02FK6T0 | - | - | - | MLOC_51302.1 |
| IV9RU5F01DGP9G | - | - | - | MLOC_51107.1 |
| IV9RU5F02I3JS7 | - | - | - | MLOC_51107.1 |
| IUIVLUD01A9LQD | - | - | - | MLOC_51096.1 |
| IUIVLUD01CZ3IH | - | - | - | MLOC_51096.1 |
| IV9RU5F02G1AOI | - | - | - | MLOC_50957.2 |
| IVLNWEC02JWZA1 | - | - | - | MLOC_5095.1 |
| IV9RU5F01CW14S | - | - | - | MLOC_4908.1 |
| IUIVLUD01C9NQ5 | - | - | - | MLOC_4685.6 |
| IUIVLUD02GRRRX | - | - | - | MLOC_4685.6 |
| IUIVLUD01AMFRB | - | - | - | MLOC_44334.1 |
| IV9RU5F01DS5XR | - | - | - | MLOC_44334.1 |
| IV9RU5F01A9ACC | - | - | - | MLOC_44276.3 |
| IUIVLUD02G20AS | - | - | - | MLOC_4142.1 |
| IV9RU5F01C4BQ4 | - | - | - | MLOC_4142.1 |
| IVLNWEC01DFU9S | - | - | - | MLOC_4142.1 |
| IVLNWEC02HIFXX | - | - | - | MLOC_4142.1 |
| IVLNWEC01ETLNA | - | - | - | MLOC_40499.1 |
| IUIVLUD02GJPX4 | - | - | - | MLOC_4008.2 |
| IUIVLUD01BSH2G | - | - | - | MLOC_3871.2 |

| | | | | |
|---|---|---|---|---|
| IVLNWEC02JJMY3 | - | - | - | MLOC_38307.2 |
| IV9RU5F01C57K7 | - | - | - | MLOC_38270.1 |
| IVLNWEC02JAF61 | - | - | - | MLOC_38270.1 |
| IUIVLUD02FL2V7 | - | - | - | MLOC_37984.1 |
| IV9RU5F01CJVUV | - | - | - | MLOC_37984.1 |
| IVLNWEC02GN4MC | - | - | - | MLOC_37984.1 |
| IV9RU5F02JHKX9 | - | - | - | MLOC_37402.8 |
| IVLNWEC02I1FMP | - | - | - | MLOC_37402.8 |
| IUIVLUD01DEZVM | - | - | - | MLOC_36971.7 |
| IV9RU5F01A5TEF | - | - | - | MLOC_36868.7 |
| IVLNWEC02F1RF6 | - | - | - | MLOC_36868.7 |
| IUIVLUD02IAUEL | - | - | - | MLOC_36835.1 |
| IV9RU5F02IJB5H | - | - | - | MLOC_36835.1 |
| IVLNWEC01D9UNA | - | - | - | MLOC_36835.1 |
| IVLNWEC01DX3IR | - | - | - | MLOC_36835.1 |
| IVLNWEC02GT1YH | - | - | - | MLOC_36835.1 |
| IUIVLUD01EKJIH | - | - | - | MLOC_35625.1 |
| IVLNWEC01CE0XY | - | - | - | MLOC_35625.1 |
| IVLNWEC02FYLTM | - | - | - | MLOC_35082.1 |
| IUIVLUD01BD3ZJ | - | - | - | MLOC_34557.1 |
| IUIVLUD01ERL90 | - | - | - | MLOC_34557.1 |
| IUIVLUD01EMIKR | - | - | - | MLOC_3328.1 |
| IUIVLUD02GH0TP | - | - | - | MLOC_3328.1 |
| IUIVLUD01DAGQO | - | - | - | MLOC_31922.1 |
| IV9RU5F01BOHCZ | - | - | - | MLOC_31922.1 |
| IVLNWEC02ISZ97 | - | - | - | MLOC_31206.1 |
| IV9RU5F01AH58F | - | - | - | MLOC_30912.2 |
| IV9RU5F02FUZ7U | - | - | - | MLOC_30912.2 |
| IV9RU5F02IJW1W | - | - | - | MLOC_30912.2 |
| IV9RU5F02JZYUQ | - | - | - | MLOC_3076.3 |
| IVLNWEC02G474V | - | - | - | MLOC_3076.3 |
| IV9RU5F02JH34C | - | - | - | MLOC_3052.1 |
| IVLNWEC02I1EC0 | - | - | - | MLOC_3052.1 |
| IUIVLUD01D8CTM | - | - | - | MLOC_30512.1 |
| IVLNWEC01DRV23 | - | - | - | MLOC_30512.1 |
| IVLNWEC02I8H9R | - | - | - | MLOC_30512.1 |
| IV9RU5F01AFBC2 | - | - | - | MLOC_3013.1 |
| IVLNWEC02GTN2A | - | - | - | MLOC_3013.1 |
| IV9RU5F02G01Q0 | - | - | - | MLOC_29817.1 |
| IV9RU5F02JJRPF | - | - | - | MLOC_29817.1 |
| IUIVLUD02IOMGP | - | - | - | MLOC_28332.1 |
| IVLNWEC02F4P33 | - | - | - | MLOC_27616.1 |
| IVLNWEC02FR982 | - | - | - | MLOC_27616.1 |
| IV9RU5F01ANQ1A | - | - | - | MLOC_26232.1 |
| IV9RU5F01EBWAM | - | - | - | MLOC_26232.1 |
| IVLNWEC02IYHEN | - | - | - | MLOC_26232.1 |
| IUIVLUD01B9743 | - | - | - | MLOC_2594.1 |

| | | | | |
|---|---|---|---|---|
| IUIVLUD01EC8NG | - | - | - | MLOC_2594.1 |
| IV9RU5F02J4O0R | - | - | - | MLOC_2594.1 |
| IVLNWEC02GKZSO | - | - | - | MLOC_25835.1 |
| IVLNWEC02H3Y7M | - | - | - | MLOC_25835.1 |
| IV9RU5F01EFOFF | - | - | - | MLOC_25258.1 |
| IVLNWEC02HHO7D | - | - | - | MLOC_25258.1 |
| IV9RU5F02J2DAI | - | - | - | MLOC_2429.1 |
| IV9RU5F01CJYS3 | - | - | - | MLOC_22956.1 |
| IVLNWEC01A924J | - | - | - | MLOC_22956.1 |
| IUIVLUD01BD5W4 | - | - | - | MLOC_21137.1 |
| IUIVLUD01EWEB7 | - | - | - | MLOC_21137.1 |
| IVLNWEC01EXMHS | - | - | - | MLOC_20538.1 |
| IV9RU5F01CZNFX | - | - | - | MLOC_20159.1 |
| IVLNWEC02HFO1R | - | - | - | MLOC_20159.1 |
| IUIVLUD01A2Z1O | - | - | - | MLOC_20132.1 |
| IV9RU5F02FILUR | - | - | - | MLOC_20132.1 |
| IVLNWEC02IFQLO | - | - | - | MLOC_20132.1 |
| IV9RU5F02FU3CP | - | - | - | MLOC_19786.1 |
| IVLNWEC02G438P | - | - | - | MLOC_19786.1 |
| IVLNWEC01C8W6O | - | - | - | MLOC_18448.1 |
| IVLNWEC02HMBNO | - | - | - | MLOC_18448.1 |
| IUIVLUD01A2NLJ | - | - | - | MLOC_18265.2 |
| IVLNWEC01EGPPG | - | - | - | MLOC_18265.2 |
| IV9RU5F01CXQ70 | - | - | - | MLOC_18165.1 |
| IUIVLUD02JC58D | - | - | - | MLOC_16614.1 |
| IV9RU5F01BLZRM | - | - | - | MLOC_16614.1 |
| IV9RU5F02FVWI4 | - | - | - | MLOC_16614.1 |
| IV9RU5F02G3VDV | - | - | - | MLOC_16576.1 |
| IVLNWEC02F8EWX | - | - | - | MLOC_16576.1 |
| IV9RU5F02F58F0 | - | - | - | MLOC_16408.1 |
| IV9RU5F02HM0JH | - | - | - | MLOC_15477.1 |
| IVLNWEC02HGK8K | - | - | - | MLOC_15477.1 |
| IV9RU5F02IPV90 | - | - | - | MLOC_15443.2 |
| IV9RU5F02HIF80 | - | - | - | MLOC_15351.6 |
| IVLNWEC01C1SLF | - | - | - | MLOC_15351.6 |
| IUIVLUD02H1VMK | - | - | - | MLOC_15335.1 |
| IVLNWEC01DS8SY | - | - | - | MLOC_15335.1 |
| IV9RU5F01DHNUT | - | - | - | MLOC_15017.1 |
| IVLNWEC02JF9YI | - | - | - | MLOC_14312.4 |
| IV9RU5F02G9VHW | - | - | - | MLOC_13941.2 |
| IVLNWEC01ETWZZ | - | - | - | MLOC_13941.2 |
| IVLNWEC01DY54A | - | - | - | MLOC_12945.1 |
| IV9RU5F02GDC16 | - | - | - | MLOC_12720.2 |
| IVLNWEC01B9TH2 | - | - | - | MLOC_12720.2 |
| IV9RU5F01BC7AE | - | - | - | MLOC_12105.1 |
| IVLNWEC01ETPZ9 | - | - | - | MLOC_12105.1 |
| IVLNWEC01D13T5 | - | - | - | MLOC_11796.1 |

| | | | | |
|---|---|---|---|---|
| IV9RU5F02HAE3V | - | - | - | MLOC_11707.1 |
| IV9RU5F02I8X4F | - | - | - | MLOC_11707.1 |
| IVLNWEC02IFXAU | - | - | - | MLOC_11707.1 |
| IV9RU5F01DSN6R | - | - | - | MLOC_11404.1 |
| IV9RU5F02FUGJ2 | - | - | - | MLOC_11404.1 |
| IVLNWEC01DYLBZ | - | - | - | MLOC_11136.1 |
| IV9RU5F01DBY6P | - | - | - | MLOC_10892.1 |
| IV9RU5F02FUJ12 | - | - | - | MLOC_10793.6 |
| IVLNWEC02GNC4V | - | - | - | MLOC_10793.6 |
| IVLNWEC01B294I | - | - | - | MLOC_10497.1 |
| IVLNWEC01DK9KS | - | - | - | MLOC_10435.2 |
| IUIVLUD01C7K1Y | - | - | - | MLOC_10070.1 |
| IUIVLUD02G2KZE | - | - | - | MLOC_10070.1 |
| IV9RU5F02I4ROZ | - | - | - | MLOC_10070.1 |
| IV9RU5F02I8DXE | - | - | - | MLOC_10070.1 |
| IV9RU5F02IQKMW | - | - | - | AK376759 |
| IV9RU5F02JU336 | - | - | - | AK376759 |
| IUIVLUD02GGLMC | - | - | - | AK376547 |
| IUIVLUD01CNTK6 | - | - | - | AK376471 |
| IV9RU5F02JRBHE | - | - | - | AK376471 |
| IVLNWEC01BFCP9 | - | - | - | AK374742 |
| IVLNWEC01DD9C8 | - | - | - | AK374713 |
| IVLNWEC01BSOQM | - | - | - | AK373499 |
| IUIVLUD01CKX7P | - | - | - | AK373279 |
| IV9RU5F01D2QFL | - | - | - | AK373279 |
| IVLNWEC01AV62N | - | - | - | AK373279 |
| IVLNWEC01ETEQ2 | - | - | - | AK372303 |
| IV9RU5F01DP2OA | - | - | - | AK372273 |
| IVLNWEC01AKYW7 | - | - | - | AK371454 |
| IVLNWEC01BUNHA | - | - | - | AK371454 |
| IV9RU5F01CB9KT | - | - | - | AK371221 |
| IVLNWEC02H88JK | - | - | - | AK370751 |
| IUIVLUD01A5DD3 | - | - | - | AK370749 |
| IVLNWEC01DFPTE | - | - | - | AK370749 |
| IUIVLUD02GGOLE | - | - | - | AK370601 |
| IV9RU5F01CFTCH | - | - | - | AK370601 |
| IVLNWEC01AO7DQ | - | - | - | AK370601 |
| IV9RU5F01B7JD7 | - | - | - | AK370395 |
| IV9RU5F01D2GYP | - | - | - | AK370395 |
| IV9RU5F02HG1B4 | - | - | - | AK370395 |
| IVLNWEC01A2AMB | - | - | - | AK370334 |
| IV9RU5F01DBOEL | - | - | - | AK368828 |
| IV9RU5F02G7SRA | - | - | - | AK368828 |
| IUIVLUD01CP8MZ | - | - | - | AK368140 |
| IUIVLUD02HGLUZ | - | - | - | AK368140 |
| IV9RU5F01DTDNV | - | - | - | AK366132 |
| IV9RU5F02H1X6R | - | - | - | AK366132 |

| | | | | |
|---|---|---|---|---|
| IUIVLUD02ILFO3 | - | - | - | AK365926 |
| IV9RU5F01DLFWT | - | - | - | AK365926 |
| IUIVLUD02HH1P3 | - | - | - | AK365436 |
| IV9RU5F02HRVHQ | - | - | - | AK365230 |
| IUIVLUD01EB05P | - | - | - | AK365190 |
| IV9RU5F01CJTHJ | - | - | - | AK364839 |
| IVLNWEC01A2LBG | - | - | - | AK364839 |
| IVLNWEC02FR28B | - | - | - | AK364839 |
| IUIVLUD02JOFJY | - | - | - | AK364579 |
| IV9RU5F01BH53B | - | - | - | AK364579 |
| IVLNWEC02FRYBB | - | - | - | AK364579 |
| IUIVLUD02HFRLK | - | - | - | AK363730 |
| IVLNWEC02GXAW8 | - | - | - | AK363730 |
| IUIVLUD01CPMGU | - | - | - | AK362566 |
| IUIVLUD01C11CM | - | - | - | AK362522 |
| IV9RU5F01D87UT | - | - | - | AK362522 |
| IVLNWEC01DCOZ5 | - | - | - | AK362522 |
| IVLNWEC02H48QS | - | - | - | AK362522 |
| IVLNWEC02IG099 | - | - | - | AK362522 |
| IVLNWEC02IN9F6 | - | - | - | AK362522 |
| IUIVLUD02HK1FH | - | - | - | AK361911 |
| IV9RU5F02G79UD | - | - | - | AK361316 |
| IV9RU5F02G98DE | - | - | - | AK361316 |
| IUIVLUD02GMSK6 | - | - | - | AK360968 |
| IV9RU5F01CZC11 | - | - | - | AK360968 |
| IV9RU5F02I3TJF | - | - | - | AK360968 |
| IV9RU5F01C319T | - | - | - | AK359823 |
| IVLNWEC02HZMCN | - | - | - | AK359823 |
| IV9RU5F01A0YKK | - | - | - | AK358997 |
| IUIVLUD01E0P91 | - | - | - | AK358827 |
| IVLNWEC02G2S2W | - | - | - | AK356799 |
| IUIVLUD01EBOYP | - | - | - | AK355134 |
| IUIVLUD02IXPLQ | - | - | - | AK355134 |
| IV9RU5F02GASCW | - | - | - | AK353909 |
| IV9RU5F02HM3MI | - | - | - | AK353909 |
| IVLNWEC02ILLZG | - | - | - | AK252929.1 |
| IUIVLUD01EPQDP | - | - | - | AK252312.1 |
| IV9RU5F01B4ICV | - | - | - | AK252312.1 |
| IV9RU5F02JXJ26 | - | - | - | AK252312.1 |
| IVLNWEC01BZ9Z1 | - | - | - | AK252312.1 |
| IV9RU5F01DV6D6 | - | - | - | AK251051.1 |
| IUIVLUD02GC03X | - | - | - | AK250598.1 |
| IV9RU5F02H0OVC | - | - | - | AK250598.1 |
| IV9RU5F01CQH5I | - | - | - | AK250586.1 |
| IVLNWEC02GVVB4 | - | - | - | AK250586.1 |
| IV9RU5F01EK430 | - | - | - | AK250482.1 |
| IV9RU5F02ISFIE | - | - | - | AK250482.1 |

| | | | | |
|---|---|---|---|---|
| IV9RU5F01EZVNA | - | - | - | AK248871.1 |
| IV9RU5F02JIA2J | - | - | - | AK354084 |
| IV9RU5F01D385P | - | - | - | MLOC_18265.2 |
| IVLNWEC02IDTFM | - | - | - | MLOC_11796.1 |
| IVLNWEC02FOQV3 | - | - | - | MLOC_76365.1 |
| IVLNWEC01EKG8H | - | - | - | AK371221 |
| IV9RU5F01CYUZI | - | - | - | MLOC_57328.1 |
| IV9RU5F01D0BMN | - | - | - | MLOC_18448.1 |
| IVLNWEC01AHC0I | - | - | - | MLOC_81998.2 |
| IV9RU5F01CLUIZ | - | - | - | AK371555 |
| IVLNWEC02FO5A0 | - | - | - | MLOC_4380.1 |
| IUIVLUD02F82KE | - | - | - | MLOC_54267.1 |
| IV9RU5F01CRFGN | - | - | - | AK369576 |
| IVLNWEC02GB5R1 | - | - | - | MLOC_56733.2 |
| IV9RU5F01DR0DD | - | - | - | MLOC_62137.1 |
| IV9RU5F01E4EAD | - | - | - | MLOC_52604.2 |
| IVLNWEC01BTBNP | - | - | - | MLOC_52604.2 |
| IV9RU5F02JG9U2 | - | - | - | MLOC_66827.1 |
| IVLNWEC01CB1QB | - | - | - | AK370395 |
| IV9RU5F01CY40N | - | - | - | AK252129.1 |
| IV9RU5F02GW7B1 | - | - | - | AK252129.1 |
| IV9RU5F01D02XO | - | - | - | MLOC_8296.1 |
| IUIVLUD01CJ2W7 | - | - | - | MLOC_55574.1 |
| IUIVLUD01B2XJR | - | - | - | MLOC_29817.1 |
| IV9RU5F02HP53I | - | - | - | MLOC_69031.1 |
| IUIVLUD02F1U98 | - | - | - | MLOC_57851.1 |
| IV9RU5F02HBIZ2 | - | - | - | AK369637 |
| IVLNWEC01D000H | - | - | - | MLOC_57653.5 |
| IVLNWEC02FLCM3 | - | - | - | MLOC_57653.5 |
| IV9RU5F01DVOX2 | - | - | - | MLOC_20132.1 |
| IVLNWEC01DKLWY | - | - | - | MLOC_40499.1 |
| IV9RU5F01EIFJ1 | - | - | - | AK364488 |
| IVLNWEC01AR58N | - | - | - | MLOC_15351.6 |
| IVLNWEC01CF276 | - | - | - | MLOC_15351.6 |
| IVLNWEC02IZ34C | - | - | - | AK370751 |
| IVLNWEC01C7QA6 | - | - | - | MLOC_15242.1 |
| IVLNWEC01DRV83 | - | - | - | MLOC_15242.1 |
| IVLNWEC02FK9AP | - | - | - | MLOC_5767.2 |
| IV9RU5F02GSOG4 | - | - | - | MLOC_5767.2 |
| IVLNWEC01BJGB4 | - | - | - | MLOC_5767.2 |
| IV9RU5F01BL5QF | - | - | - | AK374103 |
| IUIVLUD02HQDQ4 | - | - | - | AK374103 |
| IV9RU5F02HI0CK | - | - | - | MLOC_38229.4 |
| IUIVLUD02GPAV5 | - | - | - | AK365436 |
| IV9RU5F02JN7O0 | - | - | - | MLOC_55791.2 |
| IV9RU5F02HW241 | - | - | - | MLOC_12945.1 |
| IVLNWEC01EH38A | - | - | - | AK363711 |

| | | | | |
|---|---|---|---|---|
| IVLNWEC02JJLFE | - | - | - | MLOC_74632.1 |
| IV9RU5F02GBNDY | - | - | - | MLOC_62275.3 |
| IVLNWEC01A70BS | - | - | - | MLOC_73803.1 |
| IV9RU5F01BJG0A | - | - | - | MLOC_9891.3 |
| IVLNWEC02H8ZVA | - | - | - | MLOC_9891.3 |
| IV9RU5F01DJ73P | - | - | - | MLOC_71365.2 |
| IVLNWEC02HBDA6 | - | - | - | MLOC_69563.2 |
| IV9RU5F01BTFVJ | - | - | - | AK362566 |
| IV9RU5F02FSS7N | - | - | - | MLOC_18265.2 |
| IV9RU5F02H30LU | - | - | - | MLOC_48910.4 |
| IVLNWEC02G4YOZ | - | - | - | MLOC_61121.1 |
| IVLNWEC02GVXCB | - | - | - | MLOC_60056.5 |
| IVLNWEC02HMNLZ | - | - | - | MLOC_57619.3 |
| IV9RU5F02JZ89K | - | - | - | MLOC_54619.1 |
| IV9RU5F02I4F8Z | - | - | - | AK369637 |
| IVLNWEC01B0XZU | - | - | - | AK369637 |
| IUIVLUD01DOK4M | - | - | - | AK252929.1 |
| IV9RU5F02JI5QV | - | - | - | AK252929.1 |
| IV9RU5F02IOEMC | - | - | - | MLOC_38988.1 |
| IVLNWEC02FYP0D | - | - | - | MLOC_38988.1 |
| IUIVLUD02HRMWY | - | - | - | MLOC_15027.2 |
| IV9RU5F02G667B | - | - | - | MLOC_15027.2 |
| IV9RU5F02FJ1EW | - | - | - | MLOC_58443.1 |
| IVLNWEC02FOYPZ | - | - | - | MLOC_15455.2 |
| IVLNWEC02GG4HK | - | - | - | MLOC_15455.2 |
| IUIVLUD02JY587 | - | - | - | MLOC_59288.1 |
| IUIVLUD02IYYX5 | - | - | - | MLOC_4380.1 |
| IV9RU5F01E1JB3 | - | - | - | MLOC_4380.1 |
| IVLNWEC02JN12S | - | - | - | MLOC_71747.1 |
| IV9RU5F01AZD8N | - | - | - | MLOC_44387.6 |
| IV9RU5F02ITHQQ | - | - | - | MLOC_60035.1 |
| IUIVLUD02JGVGH | - | - | - | MLOC_64891.2 |
| IVLNWEC02J4KDJ | - | - | - | MLOC_64891.2 |
| IVLNWEC01B26WN | - | - | - | MLOC_10892.1 |
| IV9RU5F01B2TSN | - | - | - | MLOC_69563.2 |
| IV9RU5F02FMUTU | - | - | - | MLOC_7791.1 |
| IVLNWEC02F5NH5 | - | - | - | MLOC_7791.1 |
| IV9RU5F01BQABN | - | - | - | MLOC_55526.1 |
| IVLNWEC02GGWF0 | - | - | - | MLOC_55526.1 |
| IVLNWEC01BJ139 | - | - | - | MLOC_62327.1 |
| IVLNWEC02FXDWD | - | - | - | MLOC_62327.1 |
| IVLNWEC02HYJWQ | - | - | - | MLOC_62327.1 |
| IUIVLUD01A5GOA | - | - | - | MLOC_55791.2 |
| IV9RU5F02JP7YD | - | - | - | MLOC_52019.1 |
| IVLNWEC01EI0NS | - | - | - | MLOC_68734.1 |
| IV9RU5F01BF4OU | - | - | - | MLOC_34557.1 |
| IV9RU5F01DPHLX | - | - | - | MLOC_34557.1 |

| | | | | |
|---|---|---|---|---|
| IV9RU5F01ET8R2 | - | - | - | MLOC_34557.1 |
| IV9RU5F02GEW2W | - | - | - | MLOC_34557.1 |
| IV9RU5F02IYXZJ | - | - | - | MLOC_34557.1 |
| IVLNWEC02G9EUM | - | - | - | MLOC_34557.1 |
| IVLNWEC02HUZC0 | - | - | - | MLOC_34557.1 |
| IVLNWEC02IA0IX | - | - | - | MLOC_34557.1 |
| IV9RU5F01B2C0T | - | - | - | MLOC_61019.2 |
| IUIVLUD01D1P23 | - | - | - | MLOC_52014.2 |
| IUIVLUD02G02FG | - | - | - | MLOC_52014.2 |
| IUIVLUD02HPHO1 | - | - | - | MLOC_52014.2 |
| IV9RU5F02IY379 | - | - | - | MLOC_52014.2 |
| IVLNWEC01CCKDA | - | - | - | MLOC_52014.2 |
| IVLNWEC02J0FWM | - | - | - | AK358827 |
| IUIVLUD01BAU4S | - | - | - | AK353756 |
| IVLNWEC01C2N9H | - | - | - | AK353756 |
| IV9RU5F01ELZSL | - | - | - | AK374713 |
| IV9RU5F02GLHYD | - | - | - | MLOC_37207.1 |
| IV9RU5F01AZW64 | - | - | - | MLOC_37207.1 |
| IV9RU5F01BBQ0B | - | - | - | MLOC_37207.1 |
| IV9RU5F02GSURT | - | - | - | MLOC_80591.1 |
| IVLNWEC02JFMMZ | - | - | - | MLOC_29446.1 |
| IUIVLUD01A8R3U | - | - | - | MLOC_71301.2 |
| IV9RU5F02IYFPT | - | - | - | MLOC_65358.1 |
| IVLNWEC02JC62Z | - | - | - | MLOC_71365.2 |
| IUIVLUD01B7G1Y | - | - | - | AK365240 |
| IV9RU5F02GQE82 | - | - | - | AK365240 |
| IVLNWEC02HPZNZ | - | - | - | MLOC_2655.1 |
| IUIVLUD02ITY7W | - | - | - | MLOC_53402.1 |
| IVLNWEC01BR3GB | - | - | - | MLOC_53402.1 |
| IVLNWEC01AN72Y | - | - | - | MLOC_75827.1 |
| IV9RU5F01EZUCV | - | - | - | MLOC_6016.1 |
| IV9RU5F02GB1RU | - | - | - | MLOC_61642.4 |
| IVLNWEC01BWNF7 | - | - | - | MLOC_58443.1 |
| IVLNWEC02JEFCO | - | - | - | MLOC_57851.1 |
| IVLNWEC01ELO8I | - | - | - | MLOC_54883.1 |
| IVLNWEC01BNUVN | - | - | - | MLOC_13610.1 |
| IVLNWEC02F73MP | - | - | - | MLOC_13610.1 |
| IUIVLUD02JDYGT | - | - | - | MLOC_3871.2 |
| IV9RU5F01D18XA | - | - | - | MLOC_3871.2 |
| IV9RU5F02GRJ9E | - | - | - | MLOC_3871.2 |
| IV9RU5F02IKZFR | - | - | - | MLOC_3871.2 |
| IVLNWEC01CDABX | - | - | - | MLOC_3871.2 |
| IVLNWEC02JFE98 | - | - | - | MLOC_3871.2 |
| IVLNWEC01DOD8C | - | - | - | MLOC_15823.1 |
| IV9RU5F02FQMO8 | - | - | - | MLOC_65562.1 |
| IVLNWEC02JKP8G | - | - | - | MLOC_65562.1 |
| IUIVLUD01DKZ9J | - | - | - | MLOC_18165.1 |

| | | | | |
|---|---|---|---|---|
| IV9RU5F01EF6WJ | - | - | - | MLOC_18165.1 |
| IVLNWEC02JCXN2 | - | - | - | MLOC_59921.1 |
| IV9RU5F01AN29O | - | - | - | AK365230 |
| IVLNWEC01ANNKM | - | - | - | AK365230 |
| IUIVLUD02IRKTS | - | - | - | MLOC_57642.1 |
| IVLNWEC01C8BJ3 | - | - | - | MLOC_57642.1 |
| IVLNWEC02HW4M9 | - | - | - | MLOC_43699.1 |
| IV9RU5F01DI0DV | - | - | - | MLOC_11136.1 |
| IV9RU5F02GEIYH | - | - | - | MLOC_60285.4 |
| IVLNWEC01BDF7H | - | - | - | MLOC_60285.4 |
| IUIVLUD02F0IJ6 | - | - | - | MLOC_19387.1 |
| IVLNWEC01EU2X6 | - | - | - | MLOC_19387.1 |
| IV9RU5F02GKSF0 | - | - | - | AK360968 |
| IVLNWEC01DA9ED | - | - | - | AK360968 |
| IV9RU5F02HVXOX | - | - | - | MLOC_76493.1 |
| IV9RU5F02J5QT1 | - | - | - | AK370148 |
| IVLNWEC02F979E | - | - | - | AK370148 |
| IV9RU5F02JQEPC | - | - | - | AK373293 |
| IUIVLUD02FXELD | - | - | - | MLOC_5095.1 |
| IVLNWEC01AY5LY | - | - | - | MLOC_63760.1 |
| IV9RU5F01CJJQ1 | - | - | - | AK250482.1 |
| IV9RU5F01ERRPJ | - | - | - | MLOC_64971.1 |
| IVLNWEC02G1L0C | - | - | - | MLOC_69909.1 |
| IV9RU5F02IRHPY | - | - | - | MLOC_14312.4 |
| IUIVLUD01BO3PS | - | - | - | MLOC_57973.1 |
| IVLNWEC02III58 | - | - | - | MLOC_57973.1 |
| IV9RU5F02IFYTD | - | - | - | MLOC_6887.1 |
| IV9RU5F01CRYPK | - | - | - | AK365206 |
| IVLNWEC01DUDPP | - | - | - | AK365206 |
| IV9RU5F02ICG8E | - | - | - | MLOC_50957.2 |
| IUIVLUD02GSSD3 | - | - | - | MLOC_80687.1 |
| IUIVLUD01CIB9D | - | - | - | MLOC_59288.1 |
| IUIVLUD01EUKSX | - | - | - | MLOC_59824.1 |
| IUIVLUD01DAY6M | - | - | - | AK250598.1 |
| IV9RU5F02GB5XJ | - | - | - | AK250598.1 |
| IV9RU5F01AJAK3 | - | - | - | MLOC_66827.1 |
| IV9RU5F01CK97V | - | - | - | MLOC_66827.1 |
| IVLNWEC01EP1WC | - | - | - | MLOC_66827.1 |
| IVLNWEC02GY9MZ | - | - | - | MLOC_66827.1 |
| IUIVLUD02HT79I | - | - | - | MLOC_56719.2 |
| IVLNWEC01BRYE1 | - | - | - | MLOC_56719.2 |
| IVLNWEC02H7ZP5 | - | - | - | MLOC_30512.1 |
| IVLNWEC02FPJS2 | - | - | - | AK364487 |
| IVLNWEC01A0Z9H | - | - | - | MLOC_63089.10 |
| IV9RU5F02G7Z6V | - | - | - | MLOC_75155.1 |
| IVLNWEC01ETDKT | - | - | - | MLOC_75155.1 |
| IV9RU5F01A0UG5 | - | - | - | MLOC_53364.1 |

| | | | | |
|---|---|---|---|---|
| IV9RU5F01BX3PX | - | - | - | MLOC_53364.1 |
| IVLNWEC01ETDBI | - | - | - | MLOC_53364.1 |
| IV9RU5F02JBD38 | - | - | - | MLOC_67639.2 |
| IV9RU5F02H50HI | - | - | - | AK365907 |
| IV9RU5F01DKYHB | - | - | - | AK372273 |
| IV9RU5F01DCRAR | - | - | - | AK354330 |
| IV9RU5F01D9NJC | - | - | - | MLOC_60274.1 |
| IVLNWEC02JG1HI | - | - | - | MLOC_60274.1 |
| IV9RU5F01DV2HE | - | - | - | MLOC_58644.1 |
| IVLNWEC01BUG9P | - | - | - | MLOC_58644.1 |
| IVLNWEC02G7OAV | - | - | - | AK362289 |
| IV9RU5F01EVN15 | - | - | - | MLOC_26505.2 |
| IVLNWEC02G5WP9 | - | - | - | MLOC_26505.2 |
| IV9RU5F02GSGHV | - | - | - | AK368140 |
| IVLNWEC01BFSPP | - | - | - | AK368140 |
| IUIVLUD02I38DF | - | - | - | MLOC_10497.1 |
| IVLNWEC02JT4LF | - | - | - | MLOC_73572.1 |
| IV9RU5F02JQALB | - | - | - | MLOC_10243.2 |
| IV9RU5F01BQEWF | - | - | - | AK251051.1 |
| IV9RU5F01EUCT5 | - | - | - | AK370334 |
| IVLNWEC01EVB85 | - | - | - | MLOC_21560.3 |
| IUIVLUD02HQE4E | - | - | - | MLOC_64580.5 |
| IVLNWEC02H1BZV | - | - | - | MLOC_64580.5 |
| IV9RU5F01EKYOP | - | - | - | AK365907 |
| IVLNWEC01DS8KN | - | - | - | MLOC_29446.1 |
| IV9RU5F01AKU8A | - | - | - | MLOC_11180.1 |
| IV9RU5F01B4AMW | - | - | - | MLOC_11180.1 |
| IV9RU5F01CGAU5 | - | - | - | AK354830 |
| IVLNWEC02IWH5F | - | - | - | AK354830 |
| IV9RU5F02G7B5V | - | - | - | MLOC_62016.2 |
| IUIVLUD02H31MS | - | - | - | MLOC_55034.1 |
| IVLNWEC02JIOXE | - | - | - | MLOC_73803.1 |
| IUIVLUD01EBPHM | - | - | - | MLOC_81614.1 |
| IV9RU5F02HQE6M | - | - | - | MLOC_81614.1 |
| IVLNWEC01B7K4D | - | - | - | MLOC_81614.1 |
| IVLNWEC02J0KUC | - | - | - | MLOC_2429.1 |
| IV9RU5F01CIISD | - | - | - | MLOC_73803.1 |
| IUIVLUD02IAB9Q | - | - | - | MLOC_59845.1 |
| IV9RU5F02F5GEG | - | - | - | MLOC_12945.1 |
| IVLNWEC01AVW9A | - | - | - | MLOC_12945.1 |
| IV9RU5F02HSN86 | - | - | - | MLOC_19278.1 |
| IUIVLUD01B3SLB | - | - | - | MLOC_62741.2 |
| IV9RU5F01BQK5F | - | - | - | MLOC_62741.2 |
| contig08517 | Bradi5g15797.1 | - | - | - |
| contig06254 | Bradi5g15240.1 | - | - | - |
| contig14493 | Bradi5g14130.1 | - | - | - |
| contig10858 | Bradi5g12900.1 | - | - | - |

| | | | | |
|---|---|---|---|---|
| contig17201 | Bradi5g12900.1 | - | - | - |
| contig25571 | Bradi5g12900.1 | - | - | - |
| contig03871 | Bradi5g09702.1 | - | - | - |
| contig18180 | Bradi5g03890.1 | - | - | - |
| contig01278 | Bradi5g02420.1 | - | - | - |
| contig17765 | Bradi5g02420.1 | - | - | - |
| contig23208 | Bradi5g02420.1 | - | - | - |
| contig11263 | Bradi5g02185.1 | - | - | - |
| contig05996 | Bradi5g01462.1 | - | - | - |
| contig18790 | Bradi5g01462.1 | - | - | - |
| contig18260 | Bradi5g00847.1 | - | - | - |
| contig09184 | Bradi4g45320.1 | - | - | - |
| contig06695 | Bradi4g42810.1 | - | - | - |
| contig00281 | Bradi4g42780.1 | - | - | - |
| contig10822 | Bradi4g41790.1 | - | - | - |
| contig02674 | Bradi4g41200.1 | - | - | - |
| contig09512 | Bradi4g39380.1 | - | - | - |
| contig06545 | Bradi4g38700.1 | - | - | - |
| contig02009 | Bradi4g38357.1 | - | - | - |
| contig17040 | Bradi4g38187.1 | - | - | - |
| contig09093 | Bradi4g38080.1 | - | - | - |
| contig08191 | Bradi4g37230.1 | - | - | - |
| contig21250 | Bradi4g37110.1 | - | - | - |
| contig11006 | Bradi4g34760.1 | - | - | - |
| contig07007 | Bradi4g34460.1 | - | - | - |
| contig13271 | Bradi4g34207.1 | - | - | - |
| contig17704 | Bradi4g34207.1 | - | - | - |
| contig07547 | Bradi4g34090.1 | - | - | - |
| contig02023 | Bradi4g33990.1 | - | - | - |
| contig24780 | Bradi4g33467.1 | - | - | - |
| contig05216 | Bradi4g32870.1 | - | - | - |
| contig01268 | Bradi4g32000.2 | - | - | - |
| contig09285 | Bradi4g31990.1 | - | - | - |
| contig01548 | Bradi4g31490.1 | - | - | - |
| contig00739 | Bradi4g31470.1 | - | - | - |
| contig15281 | Bradi4g30520.1 | - | - | - |
| contig20890 | Bradi4g30520.1 | - | - | - |
| contig03656 | Bradi4g30370.1 | - | - | - |
| contig24477 | Bradi4g30370.1 | - | - | - |
| contig03094 | Bradi4g30150.4 | - | - | - |
| contig15453 | Bradi4g28727.1 | - | - | - |
| contig19562 | Bradi4g28727.1 | - | - | - |
| contig19702 | Bradi4g26627.1 | - | - | - |
| contig14078 | Bradi4g25570.1 | - | - | - |
| contig19288 | Bradi4g25570.1 | - | - | - |
| contig12663 | Bradi4g24020.1 | - | - | - |
| contig04024 | Bradi4g23480.1 | - | - | - |

| | | | | |
|---|---|---|---|---|
| contig06377 | Bradi4g23480.1 | - | - | - |
| contig13938 | Bradi4g22780.1 | - | - | - |
| contig02661 | Bradi4g22000.1 | - | - | - |
| contig13713 | Bradi4g22000.1 | - | - | - |
| contig04425 | Bradi4g21870.1 | - | - | - |
| contig00319 | Bradi4g21860.1 | - | - | - |
| contig13839 | Bradi4g21363.1 | - | - | - |
| contig18923 | Bradi4g21363.1 | - | - | - |
| contig06620 | Bradi4g18800.2 | - | - | - |
| contig03736 | Bradi4g18290.1 | - | - | - |
| contig00894 | Bradi4g15790.1 | - | - | - |
| contig23220 | Bradi4g13500.1 | - | - | - |
| contig08549 | Bradi4g12210.1 | - | - | - |
| contig23139 | Bradi4g12010.1 | - | - | - |
| contig00134 | Bradi4g11635.1 | - | - | - |
| contig05730 | Bradi4g11635.1 | - | - | - |
| contig15002 | Bradi4g11120.1 | - | - | - |
| contig15877 | Bradi4g11010.1 | - | - | - |
| contig04417 | Bradi4g10987.1 | - | - | - |
| contig11144 | Bradi4g10670.1 | - | - | - |
| contig04535 | Bradi4g10164.1 | - | - | - |
| contig14997 | Bradi4g10164.1 | - | - | - |
| contig19629 | Bradi4g08720.2 | - | - | - |
| contig16431 | Bradi4g08380.1 | - | - | - |
| contig06800 | Bradi4g07770.1 | - | - | - |
| contig09450 | Bradi4g07090.1 | - | - | - |
| contig02623 | Bradi4g06880.1 | - | - | - |
| contig06633 | Bradi4g04780.1 | - | - | - |
| contig00977 | Bradi4g03490.1 | - | - | - |
| contig06938 | Bradi4g03107.1 | - | - | - |
| contig05683 | Bradi4g03040.1 | - | - | - |
| contig11011 | Bradi4g03040.1 | - | - | - |
| contig16530 | Bradi4g03040.1 | - | - | - |
| contig01234 | Bradi4g03000.1 | - | - | - |
| contig15920 | Bradi4g02647.1 | - | - | - |
| contig00552 | Bradi4g01035.1 | - | - | - |
| contig12419 | Bradi4g00860.1 | - | - | - |
| contig09477 | Bradi4g00747.1 | - | - | - |
| contig12584 | Bradi4g00747.1 | - | - | - |
| contig01239 | Bradi3g58230.1 | - | - | - |
| contig04759 | Bradi3g53510.1 | - | - | - |
| contig06022 | Bradi3g51110.1 | - | - | - |
| contig13382 | Bradi3g51077.2 | - | - | - |
| contig14314 | Bradi3g48540.1 | - | - | - |
| contig13598 | Bradi3g47640.1 | - | - | - |
| contig01160 | Bradi3g45880.1 | - | - | - |
| contig08485 | Bradi3g44980.1 | - | - | - |

| | | | | |
|---|---|---|---|---|
| contig11588 | Bradi3g43260.1 | - | - | - |
| contig08144 | Bradi3g41987.1 | - | - | - |
| contig11937 | Bradi3g39590.1 | - | - | - |
| contig21715 | Bradi3g39590.1 | - | - | - |
| contig01877 | Bradi3g36780.1 | - | - | - |
| contig08953 | Bradi3g34670.1 | - | - | - |
| contig20746 | Bradi3g31660.1 | - | - | - |
| contig14684 | Bradi3g29700.1 | - | - | - |
| contig19287 | Bradi3g28720.1 | - | - | - |
| contig18805 | Bradi3g27912.1 | - | - | - |
| contig21017 | Bradi3g27912.1 | - | - | - |
| contig00919 | Bradi3g27560.1 | - | - | - |
| contig03562 | Bradi3g21170.1 | - | - | - |
| contig18099 | Bradi3g19890.1 | - | - | - |
| contig23836 | Bradi3g19890.1 | - | - | - |
| contig25611 | Bradi3g18400.1 | - | - | - |
| contig17406 | Bradi3g17440.1 | - | - | - |
| contig02690 | Bradi3g17240.1 | - | - | - |
| contig03999 | Bradi3g17240.1 | - | - | - |
| contig04412 | Bradi3g17240.1 | - | - | - |
| contig10652 | Bradi3g17240.1 | - | - | - |
| contig13716 | Bradi3g17240.1 | - | - | - |
| contig20489 | Bradi3g17240.1 | - | - | - |
| contig13093 | Bradi3g16890.2 | - | - | - |
| contig01363 | Bradi3g14710.1 | - | - | - |
| contig13545 | Bradi3g14710.1 | - | - | - |
| contig17971 | Bradi3g14710.1 | - | - | - |
| contig14825 | Bradi3g11810.1 | - | - | - |
| contig14230 | Bradi3g10680.1 | - | - | - |
| contig00799 | Bradi3g10370.1 | - | - | - |
| contig12511 | Bradi3g10370.1 | - | - | - |
| contig24662 | Bradi3g09535.1 | - | - | - |
| contig02442 | Bradi3g09317.1 | - | - | - |
| contig06709 | Bradi3g06727.1 | - | - | - |
| contig12229 | Bradi3g06727.1 | - | - | - |
| contig05588 | Bradi3g02380.1 | - | - | - |
| contig08162 | Bradi3g02380.1 | - | - | - |
| contig16027 | Bradi3g02380.1 | - | - | - |
| contig22734 | Bradi3g02380.1 | - | - | - |
| contig14425 | Bradi3g00920.1 | - | - | - |
| contig09060 | Bradi2g55330.1 | - | - | - |
| contig24433 | Bradi2g54280.1 | - | - | - |
| contig06080 | Bradi2g52417.1 | - | - | - |
| contig11303 | Bradi2g51807.1 | - | - | - |
| contig13786 | Bradi2g51807.1 | - | - | - |
| contig14094 | Bradi2g48530.1 | - | - | - |
| contig05461 | Bradi2g43500.1 | - | - | - |

| | | | | |
|---|---|---|---|---|
| contig23615 | Bradi2g43500.1 | - | - | - |
| contig18546 | Bradi2g42890.1 | - | - | - |
| contig19279 | Bradi2g42890.1 | - | - | - |
| contig08111 | Bradi2g42660.1 | - | - | - |
| contig20293 | Bradi2g41860.1 | - | - | - |
| contig04283 | Bradi2g41080.1 | - | - | - |
| contig17401 | Bradi2g40415.1 | - | - | - |
| contig03209 | Bradi2g38850.1 | - | - | - |
| contig21769 | Bradi2g37795.1 | - | - | - |
| contig03925 | Bradi2g36525.1 | - | - | - |
| contig02213 | Bradi2g35940.1 | - | - | - |
| contig02689 | Bradi2g35660.1 | - | - | - |
| contig17059 | Bradi2g35627.1 | - | - | - |
| contig04674 | Bradi2g31530.1 | - | - | - |
| contig03008 | Bradi2g30425.1 | - | - | - |
| contig10941 | Bradi2g29227.1 | - | - | - |
| contig23478 | Bradi2g26580.1 | - | - | - |
| contig13811 | Bradi2g26500.1 | - | - | - |
| contig17979 | Bradi2g23410.1 | - | - | - |
| contig05924 | Bradi2g22907.1 | - | - | - |
| contig22521 | Bradi2g22710.1 | - | - | - |
| contig04953 | Bradi2g20200.2 | - | - | - |
| contig13825 | Bradi2g17926.1 | - | - | - |
| contig16343 | Bradi2g17890.3 | - | - | - |
| contig02965 | Bradi2g15620.1 | - | - | - |
| contig04467 | Bradi2g14450.1 | - | - | - |
| contig02682 | Bradi2g12497.1 | - | - | - |
| contig00191 | Bradi2g11590.1 | - | - | - |
| contig01186 | Bradi2g11590.1 | - | - | - |
| contig02444 | Bradi2g11590.1 | - | - | - |
| contig04629 | Bradi2g11590.1 | - | - | - |
| contig15134 | Bradi2g11590.1 | - | - | - |
| contig08426 | Bradi2g10617.1 | - | - | - |
| contig11287 | Bradi2g05030.1 | - | - | - |
| contig18440 | Bradi2g04240.1 | - | - | - |
| contig13810 | Bradi2g03530.1 | - | - | - |
| contig12194 | Bradi2g01260.1 | - | - | - |
| contig10270 | Bradi1g77310.1 | - | - | - |
| contig11882 | Bradi1g75540.1 | - | - | - |
| contig02145 | Bradi1g75240.1 | - | - | - |
| contig00158 | Bradi1g72960.1 | - | - | - |
| contig22198 | Bradi1g72720.1 | - | - | - |
| contig13324 | Bradi1g70980.1 | - | - | - |
| contig07533 | Bradi1g70970.1 | - | - | - |
| contig11961 | Bradi1g70970.1 | - | - | - |
| contig05255 | Bradi1g70510.1 | - | - | - |
| contig07828 | Bradi1g70316.1 | - | - | - |

| | | | | |
|---|---|---|---|---|
| contig01071 | Bradi1g69050.1 | - | - | - |
| contig03310 | Bradi1g67610.1 | - | - | - |
| contig16867 | Bradi1g61140.1 | - | - | - |
| contig20076 | Bradi1g61140.1 | - | - | - |
| contig24784 | Bradi1g61140.1 | - | - | - |
| contig21306 | Bradi1g59950.1 | - | - | - |
| contig06668 | Bradi1g59360.1 | - | - | - |
| contig09910 | Bradi1g58871.1 | - | - | - |
| contig05744 | Bradi1g57975.1 | - | - | - |
| contig02352 | Bradi1g56840.1 | - | - | - |
| contig20212 | Bradi1g56107.1 | - | - | - |
| contig00591 | Bradi1g53947.1 | - | - | - |
| contig00524 | Bradi1g52220.1 | - | - | - |
| contig03091 | Bradi1g51620.1 | - | - | - |
| contig03637 | Bradi1g49230.2 | - | - | - |
| contig10804 | Bradi1g48090.3 | - | - | - |
| contig06694 | Bradi1g47780.1 | - | - | - |
| contig21443 | Bradi1g46380.1 | - | - | - |
| contig11129 | Bradi1g44450.1 | - | - | - |
| contig19476 | Bradi1g43906.1 | - | - | - |
| contig12478 | Bradi1g42250.1 | - | - | - |
| contig09956 | Bradi1g41650.1 | - | - | - |
| contig23022 | Bradi1g38220.1 | - | - | - |
| contig23103 | Bradi1g32780.1 | - | - | - |
| contig19297 | Bradi1g31970.1 | - | - | - |
| contig25654 | Bradi1g31350.1 | - | - | - |
| contig03538 | Bradi1g30672.1 | - | - | - |
| contig11674 | Bradi1g29810.1 | - | - | - |
| contig26252 | Bradi1g29810.1 | - | - | - |
| contig12279 | Bradi1g27020.1 | - | - | - |
| contig18910 | Bradi1g26270.1 | - | - | - |
| contig14950 | Bradi1g22780.1 | - | - | - |
| contig10838 | Bradi1g22400.1 | - | - | - |
| contig19951 | Bradi1g21270.1 | - | - | - |
| contig06845 | Bradi1g20830.2 | - | - | - |
| contig03688 | Bradi1g20240.1 | - | - | - |
| contig06070 | Bradi1g20050.1 | - | - | - |
| contig18989 | Bradi1g19327.1 | - | - | - |
| contig10253 | Bradi1g16250.1 | - | - | - |
| contig20921 | Bradi1g16220.1 | - | - | - |
| contig03265 | Bradi1g14590.1 | - | - | - |
| contig00001 | Bradi1g12760.1 | - | - | - |
| contig07943 | Bradi1g10750.1 | - | - | - |
| contig19876 | Bradi1g10750.1 | - | - | - |
| contig19964 | Bradi1g10750.1 | - | - | - |
| contig22200 | Bradi1g10140.2 | - | - | - |
| contig00236 | Bradi1g09500.1 | - | - | - |

| | | | | |
|---|---|---|---|---|
| contig02082 | Bradi1g09140.1 | - | - | - |
| contig00195 | Bradi1g05890.1 | - | - | - |
| contig14991 | Bradi1g05890.1 | - | - | - |
| contig03319 | Bradi1g05380.1 | - | - | - |
| contig12029 | Bradi1g05380.1 | - | - | - |
| contig14046 | Bradi1g05380.1 | - | - | - |
| contig21299 | Bradi1g05380.1 | - | - | - |
| contig16827 | Bradi1g04677.1 | - | - | - |
| contig21843 | Bradi1g04220.2 | - | - | - |
| contig10073 | Bradi1g02880.1 | - | - | - |
| contig13517 | Bradi1g02830.1 | - | - | - |
| contig12440 | Bradi1g02690.1 | - | - | - |
| contig05777 | Bradi1g02597.1 | - | - | - |
| contig02872 | Bradi1g02505.1 | - | - | - |
| contig08266 | Bradi1g02360.1 | - | - | - |
| contig01662 | Bradi1g02290.1 | - | - | - |
| contig14912 | Bradi1g02247.1 | - | - | - |
| contig25199 | Bradi1g02240.1 | - | - | - |
| contig08133 | Bradi1g01840.1 | - | - | - |
| contig12066 | Bradi1g01450.1 | - | - | - |
| contig18715 | Bradi1g00960.1 | - | - | - |
| contig06516 | Bradi1g00280.1 | - | - | - |
| contig20235 | Bradi1g00227.1 | - | - | - |
| contig06432 | Bradi0023s00220.2 | - | - | - |
| contig08071 | Bradi3g43260.1 | - | - | - |
| contig01633 | Bradi1g62650.1 | - | - | - |
| contig00467 | Bradi3g09450.2 | - | - | - |
| contig23892 | Bradi2g12520.1 | - | - | - |
| contig20048 | Bradi4g32250.1 | - | - | - |
| contig10241 | Bradi4g06257.1 | - | - | - |
| contig22539 | Bradi1g05037.1 | - | - | - |
| contig14218 | Bradi4g37147.1 | - | - | - |
| contig03746 | Bradi4g45227.1 | - | - | - |
| contig03465 | Bradi4g07770.1 | - | - | - |
| contig05307 | Bradi4g36920.5 | - | - | - |
| contig01444 | Bradi4g34927.1 | - | - | - |
| contig03120 | Bradi2g42890.1 | - | - | - |
| contig19703 | Bradi2g42890.1 | - | - | - |
| contig15090 | Bradi2g19250.1 | - | - | - |
| contig07783 | Bradi4g04110.1 | - | - | - |
| contig01013 | Bradi4g20547.1 | - | - | - |
| contig04146 | Bradi4g04650.1 | - | - | - |
| contig01535 | Bradi3g10370.1 | - | - | - |
| contig00389 | Bradi1g35630.1 | - | - | - |
| contig08207 | Bradi4g01500.1 | - | - | - |
| contig04182 | Bradi1g03867.1 | - | - | - |
| contig00764 | Bradi1g57710.1 | - | - | - |

| | | | | |
|---|---|---|---|---|
| contig03777 | Bradi4g34207.1 | - | - | - |
| contig07799 | Bradi3g61040.1 | - | - | - |
| contig25393 | Bradi5g20075.1 | - | - | - |
| contig08823 | Bradi4g30520.1 | - | - | - |
| contig05617 | Bradi1g02190.1 | - | - | - |
| contig10400 | Bradi4g32250.1 | - | - | - |
| contig00436 | Bradi1g35630.1 | - | - | - |
| contig00632 | Bradi4g17317.1 | - | - | - |
| contig09245 | Bradi1g53020.1 | - | - | - |
| contig02066 | Bradi1g13880.1 | - | - | - |
| contig16528 | Bradi4g35830.1 | - | - | - |
| contig00651 | Bradi5g02170.1 | - | - | - |
| contig02695 | Bradi4g34230.2 | - | - | - |
| contig02639 | Bradi1g03700.1 | - | - | - |
| contig21597 | Bradi4g05100.1 | - | - | - |
| contig15222 | Bradi4g06970.1 | - | - | - |
| contig08215 | Bradi4g05870.1 | - | - | - |
| contig04835 | Bradi2g18110.1 | - | - | - |
| contig00781 | Bradi2g31530.1 | - | - | - |
| contig15135 | Bradi1g08287.1 | - | - | - |
| contig00317 | Bradi2g41080.1 | - | - | - |
| contig11443 | Bradi2g60950.2 | - | - | - |
| contig07872 | Bradi1g33347.1 | - | - | - |
| contig16132 | Bradi4g02950.1 | - | - | - |
| contig14706 | Bradi2g37592.1 | - | - | - |
| contig14088 | Bradi2g13535.1 | - | - | - |
| contig00765 | Bradi1g41807.1 | - | - | - |
| contig02656 | Bradi1g01936.1 | - | - | - |
| contig08525 | Bradi4g29440.1 | - | - | - |
| contig18044 | Bradi2g59730.1 | - | - | - |
| contig14961 | Bradi1g14500.1 | - | - | - |
| contig05022 | Bradi1g07987.1 | - | - | - |
| contig02464 | Bradi2g21510.1 | - | - | - |
| contig06497 | Bradi1g15080.1 | - | - | - |
| contig19670 | Bradi4g02620.1 | - | - | - |
| contig11210 | Bradi1g00410.1 | - | - | - |
| contig15749 | Bradi1g07620.1 | - | - | - |
| contig16206 | Bradi2g19950.1 | - | - | - |
| contig16518 | Bradi1g36907.1 | - | - | - |
| contig10891 | Bradi4g00200.1 | - | - | - |
| contig07108 | Bradi1g06530.1 | - | - | - |
| contig01960 | Bradi3g40640.1 | - | - | - |
| contig11923 | Bradi1g02620.1 | - | - | - |
| contig15146 | Bradi1g05162.1 | - | - | - |
| contig22011 | Bradi2g09434.1 | - | - | - |
| contig00615 | Bradi4g29947.1 | - | - | - |
| contig04751 | Bradi1g32190.1 | - | - | - |

| | | | | |
|---|---|---|---|---|
| contig06174 | Bradi1g03030.1 | - | - | - |
| contig18083 | Bradi1g00847.1 | - | - | - |
| contig14448 | Bradi1g61057.1 | - | - | - |
| contig09134 | Bradi1g68220.1 | - | - | - |
| contig00345 | Bradi2g17890.3 | - | - | - |
| contig06387 | Bradi3g45660.1 | - | - | - |
| contig23205 | Bradi4g05360.1 | - | - | - |
| contig08176 | Bradi1g21270.1 | - | - | - |
| contig10627 | Bradi5g22790.1 | - | - | - |
| contig19545 | Bradi2g09434.1 | - | - | - |
| contig02604 | Bradi1g69500.3 | - | - | - |
| contig01654 | Bradi1g32960.1 | - | - | - |
| contig09909 | Bradi4g03050.1 | - | - | - |
| contig04327 | Bradi1g04677.1 | - | - | - |
| contig18620 | Bradi3g47060.3 | - | - | - |
| contig21703 | Bradi3g08550.1 | - | - | - |
| contig13253 | Bradi3g32865.1 | - | - | - |
| contig11624 | Bradi4g33960.1 | - | - | - |
| contig19531 | Bradi4g33960.1 | - | - | - |
| contig18450 | Bradi2g17077.1 | - | - | - |
| contig09874 | Bradi4g21230.1 | - | - | - |
| contig01724 | Bradi4g36890.1 | - | - | - |
| contig14030 | Bradi2g18150.1 | - | - | - |
| contig23134 | Bradi1g33347.1 | - | - | - |
| contig07546 | Bradi1g21130.1 | - | - | - |
| contig07484 | Bradi1g27070.1 | - | - | - |
| contig25421 | Bradi3g19460.1 | - | - | - |
| contig11819 | Bradi1g00607.1 | - | - | - |
| contig15523 | Bradi2g41080.1 | - | - | - |
| contig00721 | Bradi4g29910.1 | - | - | - |
| contig18490 | Bradi1g04780.1 | - | - | - |
| contig10089 | Bradi1g05380.1 | - | - | - |
| contig24563 | Bradi2g57800.1 | - | - | - |
| contig15152 | Bradi1g05380.1 | - | - | - |
| contig15876 | Bradi4g37025.1 | - | - | - |
| contig09277 | Bradi4g33060.1 | - | - | - |
| contig01044 | Bradi3g40640.1 | - | - | - |
| contig04178 | Bradi3g09630.1 | - | - | - |
| contig04382 | Bradi1g00960.1 | - | - | - |
| contig17236 | Bradi1g09820.1 | - | - | - |
| contig04353 | Bradi1g04830.1 | - | - | - |
| contig13112 | Bradi4g07717.1 | - | - | - |
| contig01165 | Bradi1g05810.1 | - | - | - |
| contig04480 | Bradi2g38577.1 | - | - | - |
| contig25304 | Bradi1g70980.1 | - | - | - |
| contig22257 | Bradi3g29320.1 | - | - | - |
| contig08639 | Bradi2g49067.1 | - | - | - |

| | | | | |
|---|---|---|---|---|
| contig02118 | Bradi1g04292.1 | - | - | - |
| contig08582 | Bradi2g27230.1 | - | - | - |
| contig00473 | Bradi4g42950.1 | - | - | - |
| contig02902 | Bradi1g67740.1 | - | - | - |
| contig02702 | Bradi1g08727.1 | - | - | - |
| contig00531 | Bradi4g05100.1 | - | - | - |
| contig08894 | Bradi1g61340.1 | - | - | - |
| contig26398 | Bradi1g01250.1 | - | - | - |
| contig12402 | Bradi1g02180.1 | - | - | - |
| contig02136 | Bradi1g69564.1 | - | - | - |
| contig16977 | Bradi2g12930.1 | - | - | - |
| contig03186 | Bradi1g28160.1 | - | - | - |
| contig07877 | Bradi1g14410.1 | - | - | - |
| contig01188 | Bradi1g41860.1 | - | - | - |
| contig23716 | Bradi1g53980.1 | - | - | - |
| contig05086 | Bradi4g45080.1 | - | - | - |
| contig05766 | Bradi4g00747.1 | - | - | - |
| contig00807 | Bradi1g61380.2 | - | - | - |
| contig16766 | Bradi4g39580.1 | - | - | - |
| contig01892 | Bradi4g01513.1 | - | - | - |
| contig11824 | Bradi4g22780.1 | - | - | - |
| contig04877 | Bradi4g34840.2 | - | - | - |
| contig07020 | Bradi4g37070.1 | - | - | - |
| contig18306 | Bradi4g26760.1 | - | - | - |
| contig04975 | Bradi1g02180.1 | - | - | - |
| contig01521 | Bradi2g34780.1 | - | - | - |
| contig15311 | Bradi1g21860.1 | - | - | - |
| contig14924 | Bradi1g50220.1 | - | - | AK248864.1 |
| contig06367 | Bradi4g32830.1 | - | - | AK252312.1 |
| contig04249 | Bradi1g06530.1 | - | - | AK252610.1 |
| contig10710 | Bradi3g57750.4 | - | - | AK355291 |
| contig20742 | Bradi4g45310.1 | - | - | AK355534 |
| contig19579 | Bradi4g08527.1 | - | - | AK355795 |
| contig03544 | Bradi1g33347.1 | - | - | AK358556 |
| contig05219 | Bradi4g27600.1 | - | - | AK359869 |
| contig10391 | Bradi3g14710.1 | - | - | AK361404 |
| contig04348 | Bradi4g30850.1 | - | - | AK365357 |
| contig01041 | Bradi1g01436.1 | - | - | AK368910 |
| contig09965 | Bradi1g15920.1 | - | - | AK371709 |
| contig03450 | Bradi4g29190.1 | - | - | AK372711 |
| contig08161 | Bradi2g51807.1 | - | - | MLOC_10643.2 |
| contig23859 | Bradi2g51807.1 | - | - | MLOC_10643.2 |
| contig00851 | Bradi1g05380.1 | - | - | MLOC_10988.2 |
| contig23395 | Bradi3g61040.1 | - | - | MLOC_14667.2 |
| contig11054 | Bradi4g32520.1 | - | - | MLOC_1496.4 |
| contig09623 | Bradi4g33960.1 | - | - | MLOC_21667.1 |
| contig26082 | Bradi1g10750.1 | - | - | MLOC_28634.1 |

| | | | | |
|---|---|---|---|---|
| contig23825 | Bradi3g32276.1 | - | - | MLOC_2935.1 |
| contig02014 | Bradi2g23410.1 | - | - | MLOC_32116.1 |
| contig15289 | Bradi2g44280.1 | - | - | MLOC_32522.1 |
| contig05186 | Bradi4g11010.1 | - | - | MLOC_36995.1 |
| contig11602 | Bradi1g56740.6 | - | - | MLOC_37038.2 |
| contig17750 | Bradi1g14550.1 | - | - | MLOC_37768.2 |
| contig00643 | Bradi4g30120.1 | - | - | MLOC_40225.2 |
| contig12822 | Bradi1g32950.1 | - | - | MLOC_43923.3 |
| contig06730 | Bradi1g00580.1 | - | - | MLOC_44779.2 |
| contig18574 | Bradi1g70980.1 | - | - | MLOC_47578.1 |
| contig26236 | Bradi1g43070.1 | - | - | MLOC_4768.1 |
| contig14507 | Bradi1g69590.1 | - | - | MLOC_51331.2 |
| contig01756 | Bradi1g09210.1 | - | - | MLOC_51599.3 |
| contig16743 | Bradi2g24160.2 | - | - | MLOC_52790.1 |
| contig00835 | Bradi4g34090.1 | - | - | MLOC_53400.2 |
| contig04121 | Bradi4g34090.1 | - | - | MLOC_53400.2 |
| contig01495 | Bradi2g10370.1 | - | - | MLOC_54580.10 |
| contig13537 | Bradi1g08430.1 | - | - | MLOC_54983.5 |
| contig04769 | Bradi1g04740.1 | - | - | MLOC_57565.1 |
| contig08165 | Bradi4g44838.1 | - | - | MLOC_57653.5 |
| contig19080 | Bradi4g44838.1 | - | - | MLOC_57653.5 |
| contig05765 | Bradi4g26877.1 | - | - | MLOC_57760.2 |
| contig00939 | Bradi3g14710.1 | - | - | MLOC_59480.2 |
| contig25595 | Bradi4g03890.1 | - | - | MLOC_59658.3 |
| contig15485 | Bradi4g42217.1 | - | - | MLOC_61166.1 |
| contig17178 | Bradi1g08800.1 | - | - | MLOC_61175.1 |
| contig07215 | Bradi4g38130.1 | - | - | MLOC_61306.1 |
| contig04197 | Bradi1g51817.1 | - | - | MLOC_61419.1 |
| contig23313 | Bradi1g01820.1 | - | - | MLOC_62354.2 |
| contig15030 | Bradi1g21270.1 | - | - | MLOC_62356.1 |
| contig07065 | Bradi4g43690.1 | - | - | MLOC_63679.1 |
| contig25292 | Bradi4g22780.1 | - | - | MLOC_63776.1 |
| contig00490 | Bradi4g02060.1 | - | - | MLOC_64240.2 |
| contig03118 | Bradi1g06900.1 | - | - | MLOC_66071.3 |
| contig01014 | Bradi3g43630.1 | - | - | MLOC_66893.1 |
| contig04186 | Bradi4g44342.1 | - | - | MLOC_68369.1 |
| contig21119 | Bradi3g27912.1 | - | - | MLOC_68408.1 |
| contig24577 | Bradi3g27912.1 | - | - | MLOC_68408.1 |
| contig04926 | Bradi4g37990.1 | - | - | MLOC_73340.1 |
| contig22185 | Bradi3g25900.1 | - | - | MLOC_79632.1 |
| contig04484 | Bradi3g45100.1 | - | - | MLOC_80411.1 |
| contig05104 | Bradi3g43260.1 | - | - | AK365473 |
| contig03473 | Bradi4g08720.2 | - | - | AK251150.1 |
| contig24714 | Bradi4g08720.2 | - | - | AK251150.1 |
| contig15047 | Bradi2g22040.1 | - | - | AK371721 |
| contig03162 | Bradi4g44390.1 | - | - | MLOC_5324.1 |
| contig19372 | Bradi4g45310.1 | - | - | AK355534 |

| | | | | |
|---|---|---|---|---|
| contig12343 | Bradi1g08070.1 | - | - | MLOC_71287.2 |
| contig26136 | Bradi4g08887.1 | - | - | MLOC_56805.1 |
| contig10590 | Bradi4g02390.1 | - | - | AK354434 |
| contig16742 | Bradi4g02390.1 | - | - | AK354434 |
| contig05866 | Bradi1g07190.1 | - | - | AK249476.1 |
| contig00914 | Bradi4g27100.1 | - | - | AK252400.1 |
| contig00465 | Bradi3g16120.1 | - | - | MLOC_56869.1 |
| contig20851 | Bradi4g33080.1 | - | - | AK365747 |
| contig14678 | Bradi4g27730.1 | - | - | MLOC_77630.1 |
| contig01370 | Bradi3g36190.1 | - | - | MLOC_67418.1 |
| contig07387 | Bradi4g08750.1 | - | - | MLOC_34478.1 |
| contig01232 | Bradi4g27940.1 | - | - | MLOC_55839.4 |
| contig00026 | Bradi1g47510.1 | - | - | MLOC_4749.2 |
| contig08361 | Bradi4g44667.2 | - | - | MLOC_43149.1 |
| contig13208 | Bradi4g32250.1 | - | - | AK363561 |
| contig25205 | Bradi4g32250.1 | - | - | AK363561 |
| contig05496 | Bradi4g08527.1 | - | - | AK355795 |
| contig01335 | Bradi2g35627.1 | - | - | MLOC_6725.2 |
| contig20217 | Bradi1g08500.1 | - | - | MLOC_58630.2 |
| contig06777 | Bradi2g18110.1 | - | - | MLOC_34935.1 |
| contig00274 | Bradi5g20420.1 | - | - | MLOC_55154.1 |
| contig12872 | Bradi4g38187.1 | - | - | MLOC_65161.2 |
| contig19361 | Bradi4g33467.1 | - | - | AK368907 |
| contig05208 | Bradi4g05020.1 | - | - | AK365960 |
| contig09198 | Bradi4g29887.1 | - | - | MLOC_81241.5 |
| contig11190 | Bradi1g02540.1 | - | - | AK249699.1 |
| contig04225 | Bradi4g02260.1 | - | - | MLOC_6566.2 |
| contig00228 | Bradi4g00747.1 | - | - | AK365959 |
| contig19212 | Bradi4g30550.1 | - | - | MLOC_10572.1 |
| contig03283 | Bradi4g42817.1 | - | - | MLOC_69000.1 |
| contig05212 | Bradi1g06907.2 | - | - | AK360042 |
| contig11044 | Bradi4g27290.1 | - | - | AK374259 |
| contig21138 | Bradi4g27290.1 | - | - | AK374259 |
| contig02899 | Bradi1g02620.1 | - | - | MLOC_66040.1 |
| contig09012 | Bradi4g04400.1 | - | - | MLOC_5444.3 |
| contig01859 | Bradi4g26877.1 | - | - | MLOC_57760.2 |
| contig06158 | Bradi4g08870.1 | - | - | MLOC_11707.1 |
| contig09032 | Bradi4g08870.1 | - | - | MLOC_11707.1 |
| contig07127 | Bradi1g02610.1 | - | - | AK374311 |
| contig03343 | Bradi1g15120.1 | - | - | AK249984.1 |
| contig14921 | Bradi4g01180.1 | - | - | MLOC_70352.1 |
| contig14984 | Bradi3g07210.1 | - | - | MLOC_53066.1 |
| contig01697 | Bradi1g55250.1 | - | - | MLOC_62977.1 |
| contig04727 | Bradi4g05360.1 | - | - | MLOC_65527.4 |
| contig07244 | Bradi1g58087.1 | - | - | MLOC_5739.1 |
| contig15301 | Bradi1g58087.1 | - | - | MLOC_5739.1 |
| contig03878 | Bradi3g60530.2 | - | - | MLOC_71322.3 |

| | | | | |
|---|---|---|---|---|
| contig02219 | Bradi1g51757.1 | - | - | MLOC_54721.2 |
| contig00163 | Bradi4g34090.1 | - | - | MLOC_53400.2 |
| contig01082 | Bradi3g22460.1 | - | - | MLOC_54069.1 |
| contig06528 | Bradi4g02910.1 | - | - | MLOC_36925.1 |
| contig05525 | Bradi4g00540.1 | - | - | AK366806 |
| contig03737 | Bradi4g36945.1 | - | - | MLOC_65368.1 |
| contig01043 | Bradi1g29622.1 | - | - | MLOC_36683.1 |
| contig18975 | Bradi1g06100.1 | - | - | MLOC_15202.1 |
| contig19811 | Bradi2g27097.1 | - | - | AK374248 |
| contig09974 | Bradi4g34090.1 | - | - | MLOC_53400.2 |
| contig06252 | Bradi4g34090.1 | - | - | MLOC_53400.2 |
| contig08216 | Bradi4g31060.1 | - | - | MLOC_43403.1 |
| contig15452 | Bradi4g45310.1 | - | - | AK355534 |
| contig04405 | Bradi4g43790.1 | - | - | MLOC_20230.1 |
| contig10075 | Bradi4g43790.1 | - | - | MLOC_20230.1 |
| contig03523 | Bradi3g20420.1 | - | - | MLOC_74301.1 |
| contig16089 | Bradi3g09340.1 | - | - | MLOC_39203.1 |
| contig07847 | Bradi4g14620.1 | - | - | MLOC_72722.1 |
| contig08401 | Bradi1g05380.1 | - | - | MLOC_10988.2 |
| contig08659 | Bradi1g08450.1 | - | - | MLOC_5464.1 |
| contig04025 | Bradi4g28810.1 | - | - | MLOC_17216.3 |
| contig16318 | Bradi1g02180.1 | - | - | MLOC_60056.5 |
| contig09173 | Bradi4g35977.1 | - | - | MLOC_4008.2 |
| contig18853 | Bradi4g08380.1 | - | - | MLOC_74303.1 |
| contig25133 | Bradi4g04400.1 | - | - | MLOC_5444.3 |
| contig00298 | Bradi1g31350.1 | - | - | MLOC_66828.2 |
| contig24896 | Bradi4g02767.1 | - | - | MLOC_59476.2 |
| contig08120 | Bradi3g36960.1 | - | - | MLOC_38205.5 |
| contig03425 | Bradi4g27720.1 | - | - | MLOC_71104.2 |
| contig06028 | Bradi4g32860.1 | - | - | MLOC_17530.1 |
| contig00202 | Bradi1g07150.1 | - | - | MLOC_65920.1 |
| contig21687 | Bradi4g36690.1 | - | - | AK371711 |
| contig06871 | Bradi4g45320.1 | - | - | MLOC_54712.1 |
| contig14796 | Bradi4g43650.1 | - | - | MLOC_12145.8 |
| contig05181 | Bradi4g30410.1 | - | - | AK376142 |
| contig07152 | Bradi1g06810.1 | - | - | MLOC_10567.1 |
| contig03790 | Bradi4g08887.1 | - | - | MLOC_56805.1 |
| contig22205 | Bradi4g34880.2 | - | - | AK363932 |
| contig02323 | Bradi4g39630.1 | - | - | AK366562 |
| contig02728 | Bradi4g07460.1 | - | - | MLOC_48942.1 |
| contig16911 | Bradi4g07770.1 | - | - | MLOC_67020.1 |
| contig05198 | Bradi1g01800.1 | - | - | MLOC_38862.3 |
| contig05507 | Bradi4g33060.1 | - | - | MLOC_62296.1 |
| contig16752 | Bradi4g08587.1 | - | - | MLOC_36722.6 |
| contig05210 | Bradi4g30665.1 | - | - | MLOC_19512.1 |
| contig15557 | Bradi4g04360.1 | - | - | MLOC_11295.1 |
| contig04594 | Bradi4g05940.1 | - | - | MLOC_38005.1 |

| | | | | |
|---|---|---|---|---|
| contig00060 | Bradi1g08710.1 | - | - | MLOC_55557.3 |
| contig00383 | Bradi4g03630.1 | - | - | MLOC_75497.1 |
| contig10905 | Bradi1g08430.1 | - | - | MLOC_54983.5 |
| contig19582 | Bradi1g06630.2 | - | - | MLOC_58395.2 |
| contig17265 | Bradi4g30897.1 | - | - | MLOC_69213.1 |
| contig26266 | Bradi1g05162.1 | - | - | AK374742 |
| contig23062 | Bradi1g02620.1 | - | - | MLOC_66040.1 |
| contig05379 | Bradi3g30620.1 | - | - | MLOC_4008.2 |
| contig00159 | Bradi1g00887.1 | - | - | MLOC_81454.2 |
| contig04451 | Bradi4g38357.1 | - | - | MLOC_11872.2 |
| contig13276 | Bradi4g04400.1 | - | - | MLOC_5444.3 |
| contig08947 | Bradi4g08870.1 | - | - | MLOC_11707.1 |
| contig21451 | Bradi1g06945.1 | - | - | MLOC_17956.6 |
| contig01485 | Bradi1g02610.1 | - | - | AK374311 |
| contig13323 | Bradi4g27940.1 | - | - | MLOC_55839.4 |
| contig15198 | Bradi4g03420.1 | - | - | MLOC_59549.3 |
| contig05454 | Bradi4g29000.1 | - | - | MLOC_74405.2 |
| contig00323 | Bradi4g33460.3 | - | - | MLOC_31948.1 |
| contig06388 | Bradi4g24887.1 | - | - | MLOC_74641.2 |
| contig21756 | Bradi4g29887.1 | - | - | MLOC_81241.5 |
| contig04749 | Bradi4g18220.1 | - | - | MLOC_52024.5 |
| contig01592 | Bradi4g03827.1 | - | - | MLOC_81375.2 |
| contig03279 | Bradi1g01720.1 | - | Sb01g001290.1 | - |
| contig09994 | Bradi1g01720.1 | - | Sb01g001290.1 | - |
| contig06760 | Bradi1g02160.1 | - | Sb01g001760.1 | - |
| contig07824 | Bradi1g04300.2 | - | Sb01g003860.1 | - |
| contig00432 | Bradi1g05380.1 | - | Sb01g005084.1 | - |
| contig00640 | Bradi1g05380.1 | - | Sb01g005084.1 | - |
| contig02586 | Bradi1g05380.1 | - | Sb01g005084.1 | - |
| contig02588 | Bradi1g05380.1 | - | Sb01g005084.1 | - |
| contig12565 | Bradi1g05380.1 | - | Sb01g005084.1 | - |
| contig16828 | Bradi1g05380.1 | - | Sb01g005084.1 | - |
| contig23509 | Bradi1g05380.1 | - | Sb01g005084.1 | - |
| contig11480 | Bradi1g07070.1 | - | Sb01g006540.1 | - |
| contig06210 | Bradi1g07440.1 | - | Sb01g006960.1 | - |
| contig09614 | Bradi1g07870.1 | - | Sb01g007340.1 | - |
| contig08192 | Bradi1g08780.1 | - | Sb01g008230.1 | - |
| contig00180 | Bradi1g08917.1 | - | Sb01g008410.1 | - |
| contig10133 | Bradi1g09040.1 | - | Sb01g008460.1 | - |
| contig20991 | Bradi4g10017.1 | - | Sb01g026710.1 | - |
| contig15318 | Bradi1g24780.1 | - | Sb01g027310.1 | - |
| contig18261 | Bradi3g21530.1 | - | Sb01g027470.1 | - |
| contig00143 | Bradi4g24340.1 | - | Sb01g031070.1 | - |
| contig04884 | Bradi1g15920.1 | - | Sb01g031710.1 | - |
| contig00561 | Bradi1g71160.1 | - | Sb01g043810.1 | - |
| contig23620 | Bradi1g72500.1 | - | Sb01g045120.1 | - |
| contig20047 | Bradi1g01250.1 | - | Sb02g005230.1 | - |

| | | | | |
|---|---|---|---|---|
| contig09591 | Bradi1g01250.1 | - | Sb02g005235.1 | - |
| contig18827 | Bradi2g38790.1 | - | Sb02g006030.1 | - |
| contig10331 | Bradi3g06253.1 | - | Sb02g012480.1 | - |
| contig24510 | Bradi1g22640.1 | - | Sb02g020753.1 | - |
| contig14301 | Bradi4g45160.1 | - | Sb02g021110.1 | - |
| contig00070 | Bradi3g36180.1 | - | Sb02g024010.1 | - |
| contig05898 | Bradi4g30320.1 | - | Sb02g024720.1 | - |
| contig08770 | Bradi4g32250.1 | - | Sb02g026720.1 | - |
| contig14699 | Bradi4g34846.1 | - | Sb02g029500.1 | - |
| contig09555 | Bradi3g19890.1 | - | Sb02g030530.1 | - |
| contig00711 | Bradi4g15270.1 | - | Sb02g032145.1 | - |
| contig05486 | Bradi2g60990.1 | - | Sb02g034200.1 | - |
| contig12156 | Bradi4g02587.1 | - | Sb02g036590.1 | - |
| contig13274 | Bradi1g23550.1 | - | Sb02g037500.1 | - |
| contig14647 | Bradi2g07677.1 | - | Sb03g000930.1 | - |
| contig08089 | Bradi5g20075.1 | - | Sb03g004200.1 | - |
| contig12017 | Bradi1g10750.1 | - | Sb03g026060.1 | - |
| contig10883 | Bradi2g48140.1 | - | Sb03g032820.1 | - |
| contig15794 | Bradi3g32276.1 | - | Sb04g001610.1 | - |
| contig17324 | Bradi3g32276.1 | - | Sb04g001610.1 | - |
| contig17287 | Bradi3g06727.1 | - | Sb04g006240.1 | - |
| contig02021 | Bradi3g08810.1 | - | Sb04g008260.1 | - |
| contig11284 | Bradi1g22780.1 | - | Sb04g011140.1 | - |
| contig04794 | Bradi3g27560.1 | - | Sb04g017846.1 | - |
| contig00912 | Bradi2g13535.1 | - | Sb04g019780.1 | - |
| contig02230 | Bradi3g48540.1 | - | Sb04g025770.1 | - |
| contig12701 | Bradi1g24560.1 | - | Sb04g028210.1 | - |
| contig16955 | Bradi3g58300.1 | - | Sb04g033870.1 | - |
| contig10513 | Bradi4g37560.1 | - | Sb04g037070.1 | - |
| contig05243 | Bradi3g60400.1 | - | Sb04g038180.1 | - |
| contig13792 | Bradi4g18330.1 | - | Sb05g003395.1 | - |
| contig14397 | Bradi4g24852.1 | - | Sb05g003830.1 | - |
| contig08472 | Bradi4g24020.1 | - | Sb05g005185.1 | - |
| contig13856 | Bradi4g09800.1 | - | Sb06g001980.1 | - |
| contig15833 | Bradi2g31530.1 | - | Sb06g016240.1 | - |
| contig20694 | Bradi1g03680.1 | - | Sb06g021060.1 | - |
| contig01733 | Bradi5g20070.1 | - | Sb06g027130.1 | - |
| contig05610 | Bradi5g21540.1 | - | Sb06g028230.1 | - |
| contig04166 | Bradi5g24930.3 | - | Sb06g031680.1 | - |
| contig01157 | Bradi3g14710.1 | - | Sb07g002990.1 | - |
| contig13584 | Bradi3g16980.1 | - | Sb07g003550.1 | - |
| contig14931 | Bradi2g36180.1 | - | Sb07g005720.1 | - |
| contig00229 | Bradi1g30790.1 | - | Sb07g006560.1 | - |
| contig25082 | Bradi4g29810.1 | - | Sb07g009430.1 | - |
| contig19458 | Bradi4g03470.1 | - | Sb08g019410.1 | - |
| contig08540 | Bradi4g02910.1 | - | Sb08g020025.1 | - |
| contig07154 | Bradi4g01430.1 | - | Sb08g021840.1 | - |

| | | | | |
|---|---|---|---|---|
| contig23754 | Bradi4g01230.2 | - | Sb08g022140.1 | - |
| contig03082 | Bradi4g00850.1 | - | Sb08g022850.1 | - |
| contig02104 | Bradi4g00747.1 | - | Sb08g022950.1 | - |
| contig10993 | Bradi2g34580.1 | - | Sb09g004780.1 | - |
| contig11999 | Bradi2g34580.1 | - | Sb09g004780.1 | - |
| contig13940 | Bradi2g34580.1 | - | Sb09g004780.1 | - |
| contig03614 | Bradi2g25550.1 | - | Sb09g020140.1 | - |
| contig01156 | Bradi2g14450.1 | - | Sb09g030570.1 | - |
| contig08792 | Bradi2g14450.1 | - | Sb09g030570.1 | - |
| contig23771 | Bradi2g14450.1 | - | Sb09g030570.1 | - |
| contig06068 | Bradi1g48264.1 | - | Sb10g004682.1 | - |
| contig09260 | Bradi1g30648.1 | - | Sb10g025390.1 | - |
| contig05383 | Bradi1g21975.1 | - | Sb10g029405.1 | - |
| contig09142 | Bradi3g50550.1 | - | Sb02g029780.1 | - |
| contig00988 | Bradi1g13960.1 | - | Sb01g013810.1 | - |
| contig05528 | Bradi2g11337.1 | - | Sb02g028600.1 | - |
| contig02347 | Bradi4g34880.1 | - | Sb02g029540.1 | - |
| contig22399 | Bradi1g13690.1 | - | Sb08g021890.1 | - |
| contig00485 | Bradi1g08607.1 | - | Sb01g008080.1 | - |
| contig03677 | Bradi1g08607.1 | - | Sb01g008080.1 | - |
| contig14957 | Bradi2g61830.1 | - | Sb03g047060.1 | - |
| contig00816 | Bradi1g00990.1 | - | Sb01g000600.1 | - |
| contig15787 | Bradi1g00990.1 | - | Sb01g000600.1 | - |
| contig07064 | Bradi1g15920.1 | - | Sb01g031710.1 | - |
| contig03148 | Bradi4g08420.1 | - | Sb02g013180.1 | - |
| contig14439 | Bradi3g19100.1 | - | Sb07g007870.1 | - |
| contig24500 | Bradi4g36220.3 | - | Sb02g030890.3 | - |
| contig04604 | Bradi1g03920.1 | - | Sb01g003490.1 | - |
| contig13945 | Bradi4g31490.1 | - | Sb02g025966.1 | - |
| contig11194 | Bradi4g33080.1 | - | Sb02g027570.1 | - |
| contig03430 | Bradi4g12877.1 | - | Sb05g025190.1 | - |
| contig01395 | Bradi3g10630.2 | - | Sb04g011310.1 | - |
| contig02341 | Bradi4g25040.1 | - | Sb08g019990.1 | - |
| contig00902 | Bradi4g02910.1 | - | Sb08g020025.1 | - |
| contig17164 | Bradi4g02910.1 | - | Sb08g020025.1 | - |
| contig07614 | Bradi1g09210.1 | - | Sb01g008630.1 | - |
| contig04913 | Bradi1g14250.1 | - | Sb01g014150.1 | - |
| contig01202 | Bradi2g11290.1 | - | Sb02g036770.1 | - |
| contig02218 | Bradi1g08917.1 | - | Sb01g008410.1 | - |
| contig13720 | Bradi1g08917.1 | - | Sb01g008410.1 | - |
| contig04020 | Bradi4g44667.2 | - | Sb05g000820.1 | - |
| contig06680 | Bradi4g32250.1 | - | Sb02g026720.1 | - |
| contig03341 | Bradi4g29570.1 | - | Sb02g023980.1 | - |
| contig08398 | Bradi4g04370.1 | - | Sb08g018490.1 | - |
| contig02843 | Bradi3g28605.1 | - | Sb04g036540.1 | - |
| contig18039 | Bradi1g08430.1 | - | Sb01g007920.1 | - |
| contig01702 | Bradi3g24417.1 | - | Sb08g016820.1 | - |

| | | | | |
|---|---|---|---|---|
| contig04215 | Bradi2g39547.1 | - | Sb07g025630.1 | - |
| contig17119 | Bradi2g15520.2 | - | Sb02g034640.2 | - |
| contig03731 | Bradi1g08326.1 | - | Sb01g007780.1 | - |
| contig00440 | Bradi4g24960.2 | - | Sb05g003650.1 | - |
| contig08902 | Bradi4g21990.3 | - | Sb05g008235.1 | - |
| contig10796 | Bradi3g08410.1 | - | Sb04g007950.1 | - |
| contig10171 | Bradi1g05380.1 | - | Sb01g005084.1 | - |
| contig00751 | Bradi1g07620.1 | - | Sb01g007120.1 | - |
| contig15220 | Bradi4g35301.1 | - | Sb02g029920.1 | - |
| contig00397 | Bradi1g02160.1 | - | Sb01g001760.1 | - |
| contig07241 | Bradi1g67090.1 | - | Sb01g040040.1 | - |
| contig21101 | Bradi1g05162.1 | - | Sb01g004920.1 | - |
| contig19028 | Bradi4g41717.1 | - | Sb08g004180.1 | - |
| contig01405 | Bradi4g29410.1 | - | Sb02g023690.1 | - |
| contig00713 | Bradi4g07950.1 | - | Sb10g001106.1 | - |
| contig01686 | Bradi1g00730.1 | - | Sb01g000380.1 | - |
| contig00976 | Bradi1g05122.1 | - | Sb01g004840.1 | - |
| contig23396 | Bradi1g05122.1 | - | Sb01g004840.1 | - |
| contig00788 | Bradi1g13720.1 | - | Sb01g013470.1 | - |
| contig16409 | Bradi4g33920.2 | - | Sb02g028640.1 | - |
| contig02927 | Bradi4g38357.1 | - | Sb02g011430.1 | - |
| contig26092 | Bradi4g01513.1 | - | Sb02g027700.1 | - |
| contig18441 | Bradi2g16500.1 | - | Sb03g005190.1 | - |
| contig21157 | Bradi2g16500.1 | - | Sb03g005190.1 | - |
| contig08416 | Bradi1g01250.1 | - | Sb06g000340.1 | - |
| contig09697 | Bradi1g61680.1 | - | Sb01g034550.1 | - |
| contig22725 | Bradi1g05170.1 | - | Sb01g004930.1 | - |
| contig04560 | Bradi4g36000.1 | - | Sb02g030760.1 | - |
| contig06290 | Bradi4g37700.1 | - | Sb02g032540.1 | - |
| contig11245 | Bradi4g28400.1 | - | Sb02g022580.1 | - |
| contig20156 | Bradi4g33440.1 | - | Sb02g028070.1 | - |
| contig10515 | Bradi1g00607.1 | - | Sb01g000330.1 | - |
| contig05430 | Bradi2g34780.1 | - | Sb02g009510.1 | - |
| contig08432 | Bradi4g32830.1 | - | Sb02g002100.1 | - |
| contig06489 | Bradi4g03827.1 | - | Sb08g019070.1 | - |
| contig09264 | Bradi1g27807.1 | - | Sb02g033310.1 | - |
| contig05668 | Bradi2g57500.1 | - | Sb03g042460.1 | - |
| contig15379 | Bradi4g33420.1 | - | Sb02g028040.1 | - |
| contig08174 | Bradi1g04830.1 | - | Sb01g004510.1 | - |
| contig19479 | Bradi1g14530.1 | - | Sb01g014460.1 | - |
| contig10212 | Bradi1g08540.1 | - | Sb01g008020.1 | - |
| contig15259 | Bradi1g56290.1 | - | Sb10g003610.1 | - |
| contig08413 | Bradi1g49060.1 | - | Sb10g003850.1 | - |
| contig25241 | Bradi4g42950.1 | - | Sb05g002770.1 | - |
| contig03249 | Bradi1g05380.1 | - | Sb01g005084.1 | - |
| contig00505 | Bradi3g06727.1 | - | Sb04g006240.1 | - |
| contig09025 | Bradi4g02100.1 | - | Sb08g020980.1 | - |

| | | | | |
|---|---|---|---|---|
| contig21883 | Bradi4g01230.2 | - | Sb08g022140.1 | - |
| contig10809 | Bradi4g39210.1 | - | Sb0011s005460.1 | - |
| contig08576 | Bradi4g02910.1 | - | Sb08g020025.1 | - |
| contig07144 | Bradi3g54980.1 | - | Sb04g036790.1 | - |
| contig02255 | Bradi4g38147.1 | - | Sb02g011290.1 | - |
| contig06356 | Bradi2g11360.3 | - | Sb03g012030.1 | - |
| contig07765 | Bradi1g07810.1 | - | Sb01g007270.1 | - |
| contig05804 | Bradi3g12520.3 | - | Sb07g023850.1 | - |
| contig10538 | Bradi1g61750.1 | - | Sb01g034610.1 | - |
| contig14848 | Bradi1g08917.1 | - | Sb01g008410.1 | - |
| contig16695 | Bradi1g08917.1 | - | Sb01g008410.1 | - |
| contig02911 | Bradi2g31550.1 | - | Sb01g044380.1 | - |
| contig01772 | Bradi1g48264.1 | - | Sb10g004680.1 | - |
| contig13222 | Bradi1g01630.1 | - | Sb01g001120.1 | - |
| contig00384 | Bradi4g29310.3 | - | Sb02g023560.1 | - |
| contig06170 | Bradi1g20527.1 | - | Sb02g020844.1 | - |
| contig16014 | Bradi1g14410.1 | - | Sb01g043660.1 | - |
| contig15608 | Bradi1g05162.1 | - | Sb01g004920.1 | - |
| contig11453 | Bradi4g41717.1 | - | Sb08g004180.1 | - |
| contig02179 | Bradi1g02760.1 | - | Sb01g002530.1 | - |
| contig22870 | Bradi1g07830.1 | - | Sb01g007290.1 | - |
| contig08050 | Bradi2g51807.1 | - | Sb03g036450.1 | - |
| contig01205 | Bradi4g30665.1 | - | Sb02g025140.1 | - |
| contig05918 | Bradi1g01720.1 | - | Sb01g001290.1 | AK248772.1 |
| contig14695 | Bradi1g00600.1 | - | Sb02g010080.1 | AK249250.1 |
| contig00563 | Bradi1g08380.3 | - | Sb01g007830.1 | AK250552.1 |
| contig23465 | Bradi4g32830.1 | - | Sb02g028550.1 | AK252312.1 |
| contig23632 | Bradi1g06530.1 | - | Sb06g028320.1 | AK252610.1 |
| contig19141 | Bradi4g45310.1 | - | Sb08g000260.1 | AK355534 |
| contig10817 | Bradi4g01490.3 | - | Sb08g021740.1 | AK359188 |
| contig01958 | Bradi1g09537.2 | - | Sb01g008940.1 | AK359692 |
| contig08103 | Bradi1g02450.2 | - | Sb01g001990.1 | AK362468 |
| contig16954 | Bradi1g67140.1 | - | Sb01g040090.1 | AK362963 |
| contig18289 | Bradi4g32250.1 | - | Sb02g026720.1 | AK363561 |
| contig04974 | Bradi1g53217.1 | - | Sb02g007630.1 | AK364827 |
| contig10274 | Bradi3g24740.1 | - | Sb01g022250.1 | AK367195 |
| contig07939 | Bradi1g19010.1 | - | Sb02g041820.2 | AK369111 |
| contig16544 | Bradi3g18120.1 | - | Sb07g002352.1 | AK369747 |
| contig09520 | Bradi1g01900.1 | - | Sb01g001520.1 | AK370512 |
| contig13641 | Bradi4g36690.1 | - | Sb02g031440.1 | AK371711 |
| contig01203 | Bradi4g29190.2 | - | Sb02g023400.1 | AK372711 |
| contig12709 | Bradi1g69380.1 | - | Sb01g042340.1 | AK373335 |
| contig22338 | Bradi1g03180.1 | - | Sb01g002660.2 | AK374037 |
| contig08763 | Bradi4g30410.1 | - | Sb02g024810.1 | AK376142 |
| contig06660 | Bradi1g03270.2 | - | Sb01g002770.1 | MLOC_10449.1 |
| contig21240 | Bradi1g06810.1 | - | Sb01g006320.1 | MLOC_10567.1 |
| contig00098 | Bradi1g05380.1 | - | Sb01g005084.1 | MLOC_10988.2 |

| | | | | |
|---|---|---|---|---|
| contig04836 | Bradi1g05380.1 | - | Sb01g005084.1 | MLOC_10988.2 |
| contig02930 | Bradi4g38357.1 | - | Sb02g011430.1 | MLOC_11872.2 |
| contig14343 | Bradi4g31680.1 | - | Sb02g026140.1 | MLOC_12614.1 |
| contig24905 | Bradi1g06100.1 | - | Sb01g005560.1 | MLOC_15202.1 |
| contig00924 | Bradi1g47050.1 | - | Sb10g005910.1 | MLOC_18813.1 |
| contig09202 | Bradi1g05170.1 | - | Sb01g004930.1 | MLOC_21567.1 |
| contig02147 | Bradi4g45050.1 | - | Sb08g000410.1 | MLOC_37146.1 |
| contig04187 | Bradi1g78750.1 | - | Sb08g002900.1 | MLOC_37495.1 |
| contig20131 | Bradi4g26390.1 | - | Sb05g002020.1 | MLOC_4314.2 |
| contig04274 | Bradi4g26620.1 | - | Sb02g001412.1 | MLOC_4798.7 |
| contig09220 | Bradi4g18220.1 | - | Sb05g018860.1 | MLOC_52024.5 |
| contig25392 | Bradi4g18220.1 | - | Sb05g018860.1 | MLOC_52024.5 |
| contig12789 | Bradi1g39937.1 | - | Sb10g019740.1 | MLOC_52267.1 |
| contig18922 | Bradi2g16800.1 | - | Sb09g028280.1 | MLOC_53471.2 |
| contig04889 | Bradi4g06100.1 | - | Sb08g016740.1 | MLOC_54446.1 |
| contig01393 | Bradi4g00980.1 | - | Sb08g022690.1 | MLOC_54463.1 |
| contig16495 | Bradi2g62760.1 | - | Sb05g002400.1 | MLOC_55536.1 |
| contig08590 | Bradi3g17630.1 | - | Sb07g005630.1 | MLOC_55540.1 |
| contig16946 | Bradi1g52650.2 | - | Sb02g009590.1 | MLOC_59466.2 |
| contig04658 | Bradi2g01550.1 | - | Sb03g003250.1 | MLOC_62056.2 |
| contig17847 | Bradi4g32840.1 | - | Sb02g027340.1 | MLOC_63540.1 |
| contig11668 | Bradi1g10360.1 | - | Sb01g009790.1 | MLOC_64588.1 |
| contig14539 | Bradi4g05360.1 | - | Sb08g017560.1 | MLOC_65527.4 |
| contig17839 | Bradi1g72500.1 | - | Sb01g045120.1 | MLOC_6748.1 |
| contig03080 | Bradi2g55320.1 | - | Sb03g040310.1 | MLOC_71329.3 |
| contig25069 | Bradi1g53085.1 | - | Sb02g008130.1 | MLOC_77526.5 |
| contig05641 | Bradi4g33310.1 | - | Sb03g029110.1 | MLOC_78764.1 |
| contig21568 | Bradi1g04750.1 | - | Sb02g006080.1 | MLOC_79669.1 |
| contig17028 | Bradi4g37147.1 | - | Sb01g048290.1 | MLOC_9982.2 |
| contig08685 | Bradi1g03730.1 | - | Sb01g003230.1 | MLOC_51706.2 |
| contig18714 | Bradi1g01630.1 | - | Sb01g001120.1 | MLOC_880.1 |
| contig09602 | Bradi1g08070.1 | - | Sb01g007560.2 | MLOC_71287.2 |
| contig22272 | Bradi1g08070.1 | - | Sb01g007560.2 | MLOC_71287.2 |
| contig12609 | Bradi1g13570.1 | - | Sb01g013270.1 | MLOC_70676.2 |
| contig01077 | Bradi1g13590.1 | - | Sb01g013310.1 | AK360806 |
| contig02049 | Bradi4g02380.2 | - | Sb08g020810.1 | AK372643 |
| contig12313 | Bradi4g08887.1 | - | Sb07g013790.1 | MLOC_56805.1 |
| contig03287 | Bradi4g33370.1 | - | Sb02g027950.1 | MLOC_58019.2 |
| contig07674 | Bradi4g33520.1 | - | Sb02g028170.1 | AK370417 |
| contig10651 | Bradi1g03180.1 | - | Sb01g002660.2 | AK374037 |
| contig13722 | Bradi1g04120.2 | - | Sb01g003680.1 | AK365033 |
| contig17237 | Bradi1g04120.2 | - | Sb01g003680.1 | AK365033 |
| contig02167 | Bradi3g58120.1 | - | Sb04g034030.1 | MLOC_64085.2 |
| contig04549 | Bradi4g27747.1 | - | Sb02g020790.1 | AK252911.1 |
| contig08960 | Bradi1g01800.1 | - | Sb01g001140.1 | MLOC_38862.3 |
| contig10342 | Bradi1g04330.1 | - | Sb01g003900.2 | MLOC_61507.1 |
| contig04696 | Bradi1g01810.1 | - | Sb01g001360.1 | MLOC_62353.2 |

| | | | | |
|---|---|---|---|---|
| contig17799 | Bradi1g09100.1 | - | Sb01g008490.1 | AK364599 |
| contig01316 | Bradi4g27820.1 | - | Sb01g001310.1 | MLOC_26274.1 |
| contig01489 | Bradi4g36430.2 | - | Sb02g031150.1 | MLOC_50821.1 |
| contig17991 | Bradi4g04650.1 | - | Sb08g018400.1 | MLOC_12013.1 |
| contig09143 | Bradi1g15060.1 | - | Sb01g015100.1 | MLOC_75498.4 |
| contig00479 | Bradi1g14030.2 | - | Sb01g013910.1 | MLOC_71738.1 |
| contig17306 | Bradi1g04540.1 | - | Sb02g006370.1 | MLOC_62782.5 |
| contig22456 | Bradi1g08710.1 | - | Sb01g008180.1 | MLOC_55557.3 |
| contig05587 | Bradi4g06700.1 | - | Sb08g016190.1 | MLOC_14982.1 |
| contig13818 | Bradi4g40440.1 | - | Sb08g006380.1 | MLOC_72040.1 |
| contig02127 | Bradi4g44320.1 | - | Sb08g002310.1 | MLOC_65070.2 |
| contig06032 | Bradi4g42860.1 | - | Sb05g002870.1 | AK367874 |
| contig00862 | Bradi1g02000.1 | - | Sb01g001620.1 | MLOC_11570.6 |
| contig07187 | Bradi4g07610.1 | - | Sb0011s011830.1 | AK370873 |
| contig14946 | Bradi4g07610.1 | - | Sb0011s011830.1 | AK370873 |
| contig16026 | Bradi1g06387.1 | - | Sb01g005920.1 | AK371672 |
| contig08731 | Bradi1g03550.1 | - | Sb02g001020.1 | MLOC_62464.1 |
| contig00681 | Bradi4g42217.1 | - | Sb03g028750.1 | MLOC_61166.1 |
| contig09420 | Bradi4g22180.1 | - | Sb05g008740.1 | MLOC_65936.3 |
| contig14133 | Bradi4g32250.1 | - | Sb02g026720.1 | AK363561 |
| contig00120 | Bradi1g53060.2 | - | Sb02g008660.1 | AK369373 |
| contig12541 | Bradi4g02020.1 | - | Sb08g021170.1 | MLOC_54218.3 |
| contig00538 | Bradi4g08510.1 | - | Sb02g016800.1 | AK362848 |
| contig10495 | Bradi3g22480.1 | - | Sb02g018570.1 | MLOC_68440.1 |
| contig02419 | Bradi1g08470.1 | - | Sb01g007950.1 | MLOC_34938.1 |
| contig04125 | Bradi4g33310.1 | - | Sb03g029110.1 | MLOC_78764.1 |
| contig20496 | Bradi4g33310.1 | - | Sb03g029110.1 | MLOC_78764.1 |
| contig10852 | Bradi1g48264.1 | - | Sb10g004680.1 | MLOC_39065.1 |
| contig00476 | Bradi4g08567.1 | - | Sb07g010440.1 | MLOC_72899.1 |
| contig00544 | Bradi4g08567.1 | - | Sb07g010440.1 | MLOC_72899.1 |
| contig21405 | Bradi4g08567.1 | - | Sb07g010440.1 | MLOC_72899.1 |
| contig25708 | Bradi1g02510.1 | - | Sb01g002050.1 | AK366571 |
| contig10955 | Bradi1g17710.1 | - | Sb04g004540.1 | MLOC_67779.1 |
| contig03767 | Bradi1g04760.1 | - | Sb01g004400.1 | MLOC_34631.1 |
| contig03393 | Bradi4g29310.3 | - | Sb02g023560.1 | MLOC_67515.1 |
| contig14953 | Bradi4g45245.1 | - | Sb07g006230.1 | MLOC_10686.1 |
| contig04012 | Bradi4g07040.1 | - | Sb08g015410.1 | AK358846 |
| contig06614 | Bradi4g29887.1 | - | Sb02g024370.1 | MLOC_81241.5 |
| contig01215 | Bradi4g38080.1 | - | Sb02g011250.1 | AK364816 |
| contig04924 | Bradi4g34095.1 | - | Sb02g028800.1 | MLOC_53156.1 |
| contig05569 | Bradi4g30850.1 | - | Sb02g025340.1 | AK369503 |
| contig03137 | Bradi4g37327.1 | - | Sb02g032230.1 | MLOC_37844.1 |
| contig10106 | Bradi1g05660.1 | - | Sb01g005340.1 | AK354390 |
| contig09039 | Bradi1g01710.1 | - | Sb01g001300.1 | MLOC_12588.1 |
| contig10566 | Bradi4g41520.1 | - | Sb08g004540.1 | AK358200 |
| contig13084 | Bradi4g43780.1 | - | Sb08g001600.1 | MLOC_34755.2 |
| contig00808 | Bradi4g43990.1 | - | Sb05g001600.1 | MLOC_11550.1 |

| | | | | |
|---|---|---|---|---|
| contig03741 | Bradi4g29887.1 | - | Sb02g024370.1 | MLOC_81241.5 |
| contig00518 | Bradi4g34980.1 | - | Sb02g029670.1 | AK365822 |
| contig02437 | Bradi4g34005.1 | - | Sb02g028690.1 | MLOC_52057.3 |
| contig13438 | Bradi4g05840.1 | - | Sb08g017010.1 | AK365226 |
| contig01685 | Bradi1g00730.1 | - | Sb01g000380.1 | MLOC_65787.1 |
| contig01029 | Bradi1g02760.1 | - | Sb01g002530.1 | AK369869 |
| contig17519 | Bradi1g35630.1 | - | Sb08g018670.1 | MLOC_74748.1 |
| contig07630 | Bradi1g05162.1 | - | Sb01g004920.1 | AK374742 |
| contig03654 | Bradi1g13720.1 | - | Sb01g013470.1 | MLOC_25582.1 |
| contig10589 | Bradi4g38357.1 | - | Sb02g011430.1 | MLOC_11872.2 |
| contig22092 | Bradi4g38357.1 | - | Sb02g011430.1 | MLOC_11872.2 |
| contig00031 | Bradi5g22017.1 | - | Sb03g005120.1 | AK373995 |
| contig00669 | Bradi5g22017.1 | - | Sb03g005130.1 | MLOC_14592.1 |
| contig00878 | Bradi4g27157.1 | - | Sb02g018910.1 | MLOC_16526.1 |
| contig08428 | Bradi4g27157.1 | - | Sb02g018910.1 | MLOC_63338.1 |
| contig09188 | Bradi1g04070.1 | - | Sb01g003630.1 | MLOC_69944.1 |
| contig03334 | Bradi2g62760.1 | - | Sb05g002400.1 | MLOC_55536.1 |
| contig06970 | Bradi1g05170.1 | - | Sb01g004930.1 | MLOC_21567.1 |
| contig10371 | Bradi1g05170.1 | - | Sb01g004930.1 | MLOC_21567.1 |
| contig18602 | Bradi1g05170.1 | - | Sb01g004930.1 | MLOC_21567.1 |
| contig11805 | Bradi2g03680.1 | - | Sb08g018630.1 | MLOC_63808.1 |
| contig11207 | Bradi4g41390.1 | - | Sb08g004630.1 | MLOC_53531.2 |
| contig11409 | Bradi4g41390.1 | - | Sb08g004630.1 | MLOC_53531.2 |
| contig10060 | Bradi4g08080.1 | - | Sb02g000980.1 | AK365736 |
| contig00295 | Bradi1g58087.1 | - | Sb02g002670.1 | MLOC_5739.1 |
| contig09946 | Bradi4g38140.1 | - | Sb02g011280.1 | MLOC_21732.1 |
| contig01760 | Bradi1g06540.1 | - | Sb01g006100.1 | AK362698 |
| contig07809 | Bradi1g13710.1 | - | Sb01g013460.1 | MLOC_37399.1 |
| contig12755 | Bradi1g05660.1 | - | Sb01g005340.1 | AK354390 |
| contig08906 | Bradi4g32450.1 | - | Sb02g026900.1 | MLOC_67669.1 |
| contig02081 | Bradi4g44780.1 | - | Sb08g000780.1 | MLOC_45555.1 |
| contig11499 | Bradi1g53085.1 | - | Sb02g008130.1 | MLOC_77526.5 |
| contig17383 | Bradi5g20420.1 | - | Sb06g027382.1 | MLOC_12950.1 |
| contig02922 | Bradi1g14770.1 | - | Sb01g014760.1 | MLOC_11221.1 |
| contig03719 | Bradi4g02910.1 | - | Sb08g020025.1 | MLOC_36925.1 |
| contig03948 | Bradi4g02910.1 | - | Sb08g020025.1 | MLOC_36925.1 |
| contig11312 | Bradi4g05570.1 | - | Sb08g017220.1 | AK361994 |
| contig02130 | Bradi4g38187.1 | - | Sb07g024470.1 | MLOC_65161.2 |
| contig03847 | Bradi1g04140.1 | - | Sb01g003700.1 | AK250029.1 |
| contig15984 | Bradi4g43110.1 | - | Sb05g002580.1 | MLOC_10658.1 |
| contig10578 | Bradi1g04980.1 | - | Sb01g004640.1 | MLOC_37586.1 |
| contig18706 | Bradi1g02710.1 | - | Sb02g007460.1 | AK367079 |
| contig12230 | Bradi4g29310.3 | - | Sb02g023560.1 | MLOC_67515.1 |
| contig12874 | Bradi4g40020.1 | - | Sb08g001435.1 | MLOC_38921.3 |
| contig03565 | Bradi4g05720.2 | - | Sb08g017070.1 | MLOC_7790.1 |
| contig03105 | Bradi4g40020.1 | - | Sb08g001435.1 | MLOC_38921.3 |
| contig12092 | Bradi4g03800.1 | - | Sb08g019100.1 | MLOC_20816.3 |

| | | | | |
|---|---|---|---|---|
| contig12347 | Bradi1g07140.1 | - | Sb01g006630.1 | MLOC_39143.3 |
| contig03028 | Bradi4g03700.1 | - | Sb07g025280.1 | MLOC_11796.1 |
| contig01187 | Bradi4g31680.1 | - | Sb02g026140.1 | MLOC_12614.1 |
| contig21381 | Bradi1g72007.1 | - | Sb01g044660.1 | MLOC_62172.2 |
| contig02217 | Bradi1g27890.1 | - | Sb01g032070.1 | MLOC_6913.1 |
| contig21814 | Bradi1g02830.1 | - | Sb04g009720.1 | MLOC_10223.2 |
| contig19755 | Bradi1g05260.1 | - | Sb01g004970.1 | AK370319 |
| contig12598 | Bradi2g34580.1 | - | Sb09g004780.1 | AK373093 |
| contig07759 | Bradi4g41650.1 | - | Sb08g004240.1 | AK367116 |
| contig13155 | Bradi1g05380.1 | - | Sb01g005084.1 | MLOC_10988.2 |
| contig07016 | Bradi1g08430.1 | - | Sb01g007920.1 | MLOC_54983.5 |
| contig19270 | Bradi4g35280.1 | - | Sb02g029910.1 | AK377101 |
| contig01539 | Bradi4g27100.1 | - | Sb02g018870.1 | AK252400.1 |
| contig23129 | Bradi4g41717.1 | - | Sb08g004180.1 | MLOC_54805.1 |
| contig07703 | Bradi4g08567.1 | - | Sb07g010440.1 | MLOC_72899.1 |
| contig05073 | Bradi1g04620.1 | - | Sb01g004240.1 | MLOC_68131.1 |
| contig10562 | Bradi1g02440.1 | - | Sb01g001980.1 | AK361988 |
| contig00997 | Bradi4g07970.1 | - | Sb05g016000.1 | MLOC_14105.3 |
| contig00341 | Bradi4g07610.1 | - | Sb0011s011820.1 | AK370873 |
| contig19965 | Bradi4g40020.1 | - | Sb08g001435.1 | MLOC_38921.3 |
| contig10477 | Bradi4g01480.1 | - | Sb08g021750.1 | AK376083 |
| contig01200 | Bradi4g02050.1 | - | Sb08g021090.1 | MLOC_64241.1 |
| contig06515 | Bradi1g30430.1 | - | Sb10g025200.1 | MLOC_57384.1 |
| contig21317 | Bradi4g44290.1 | - | Sb09g007930.2 | MLOC_52171.1 |
| contig01333 | Bradi2g21010.1 | - | Sb06g001690.1 | MLOC_16950.1 |
| contig00556 | Bradi4g38710.1 | - | Sb02g012400.1 | MLOC_71298.1 |
| contig17023 | Bradi4g33750.1 | - | Sb02g028390.1 | MLOC_10823.1 |
| contig06072 | Bradi1g07190.1 | - | Sb01g006700.1 | AK249476.1 |
| contig07653 | Bradi1g09100.1 | - | Sb01g008490.1 | AK364599 |
| contig09576 | Bradi4g04640.1 | - | Sb08g018390.1 | AK252670.1 |
| contig00018 | Bradi4g04360.1 | - | Sb08g018493.1 | MLOC_11295.1 |
| contig00745 | Bradi4g05600.1 | - | Sb08g017190.1 | MLOC_64519.1 |
| contig00906 | Bradi2g60817.1 | - | Sb03g045910.1 | AK370672 |
| contig21853 | Bradi4g05157.1 | - | Sb08g017670.1 | AK364318 |
| contig12526 | Bradi4g08567.1 | - | Sb07g010440.1 | MLOC_72899.1 |
| contig19692 | Bradi4g08567.1 | - | Sb07g010440.1 | MLOC_72899.1 |
| contig04203 | Bradi4g08567.1 | - | Sb07g010440.1 | MLOC_72899.1 |
| contig15055 | Bradi4g08567.1 | - | Sb07g010440.1 | MLOC_72899.1 |
| contig17136 | Bradi1g05610.1 | - | Sb01g005310.1 | AK357244 |
| contig03694 | Bradi3g22387.2 | - | Sb02g019450.1 | AK376220 |
| contig17812 | Bradi4g31060.1 | - | Sb02g025575.1 | MLOC_43403.1 |
| contig18904 | Bradi4g43760.1 | - | Sb05g001860.1 | MLOC_20868.2 |
| contig00951 | Bradi1g02780.1 | - | Sb01g002520.1 | AK357591 |
| contig13295 | Bradi1g04030.1 | - | Sb01g003600.1 | MLOC_71880.1 |
| contig16259 | Bradi4g29887.1 | - | Sb02g024370.1 | MLOC_81241.5 |
| contig05154 | Bradi4g28670.1 | - | Sb02g022920.1 | MLOC_64156.7 |
| contig00328 | Bradi1g07620.1 | - | Sb01g007120.1 | AK374298 |

| | | | | |
|---|---|---|---|---|
| contig09203 | Bradi1g13480.1 | - | Sb01g013170.1 | MLOC_62824.1 |
| contig09775 | Bradi4g41470.1 | - | Sb08g004570.2 | AK353972 |
| contig07248 | Bradi3g17630.1 | - | Sb07g005630.1 | MLOC_55540.1 |
| contig15128 | Bradi1g04980.1 | - | Sb01g004640.1 | MLOC_37586.1 |
| contig00123 | Bradi4g39540.1 | - | Sb08g008680.1 | MLOC_32443.2 |
| contig09160 | Bradi1g08280.1 | - | Sb01g007710.1 | MLOC_10398.1 |
| contig06525 | Bradi1g10550.1 | - | Sb01g009960.1 | MLOC_57872.1 |
| contig05690 | Bradi4g28177.1 | - | Sb02g022180.1 | MLOC_91.1 |
| contig01374 | Bradi4g01790.1 | - | Sb08g002530.1 | MLOC_10435.2 |
| contig17210 | Bradi2g62760.1 | - | Sb05g002400.1 | MLOC_55536.1 |
| contig00094 | Bradi1g08000.1 | - | Sb01g007520.1 | MLOC_58291.1 |
| contig00340 | Bradi4g32987.1 | - | Sb02g027475.1 | MLOC_3587.2 |
| contig15043 | Bradi4g30410.1 | - | Sb02g024800.1 | AK376142 |
| contig07686 | Bradi1g01800.1 | - | Sb01g001140.1 | MLOC_38862.3 |
| contig16175 | Bradi4g29580.1 | - | Sb02g023990.1 | MLOC_58590.1 |
| contig04592 | Bradi4g08567.1 | - | Sb07g010440.1 | MLOC_72899.1 |
| contig15653 | Bradi3g21530.1 | - | Sb01g027470.1 | AK377004 |
| contig05409 | Bradi4g02910.1 | - | Sb08g020025.1 | MLOC_36925.1 |
| contig02503 | Bradi4g02910.1 | - | Sb08g020025.1 | MLOC_36925.1 |
| contig19164 | Bradi1g04980.1 | - | Sb01g004640.1 | MLOC_37586.1 |
| contig02932 | Bradi2g03400.2 | Os01t0151700-01 | - | - |
| contig05841 | Bradi2g10180.1 | Os01t0271200-01 | - | - |
| contig13599 | Bradi2g10180.1 | Os01t0271200-01 | - | - |
| contig01494 | Bradi2g10800.2 | Os01t0279400-01 | - | - |
| contig05421 | Bradi2g35987.1 | Os01t0372700-01 | - | - |
| contig06726 | Bradi4g16767.1 | Os01t0519700-00 | - | - |
| contig09036 | Bradi2g51807.1 | Os01t0781200-01 | - | - |
| contig12197 | Bradi2g60250.1 | Os01t0937400-02 | - | - |
| contig04911 | Bradi1g29634.1 | Os02t0263432-00 | - | - |
| contig16788 | Bradi1g47067.1 | Os02t0793800-00 | - | - |
| contig08530 | Bradi1g61680.1 | Os03t0364500-01 | - | - |
| contig09075 | Bradi1g61290.1 | Os03t0375900-01 | - | - |
| contig26230 | Bradi2g30425.1 | Os03t0608700-02 | - | - |
| contig21834 | Bradi1g14530.1 | Os03t0619600-01 | - | - |
| contig01963 | Bradi1g13220.1 | Os03t0657000-01 | - | - |
| contig00828 | Bradi1g16020.1 | Os03t0693000-01 | - | - |
| contig24908 | Bradi1g09140.1 | Os03t0741700-00 | - | - |
| contig11349 | Bradi1g07987.1 | Os03t0758700-01 | - | - |
| contig05090 | Bradi1g06900.1 | Os03t0778000-01 | - | - |
| contig07894 | Bradi1g02210.1 | Os03t0841100-01 | - | - |
| contig14790 | Bradi1g01847.1 | Os03t0845500-01 | - | - |
| contig11726 | Bradi5g01050.1 | Os04t0152400-00 | - | - |
| contig05136 | Bradi5g15797.1 | Os04t0307500-01 | - | - |
| contig21962 | Bradi5g22150.1 | Os04t0375300-00 | - | - |
| contig01667 | Bradi5g24370.1 | Os04t0653400-01 | - | - |
| contig15943 | Bradi2g40030.1 | Os05t0180600-01 | - | - |
| contig18680 | Bradi1g00237.1 | Os05t0250700-00 | - | - |

| | | | | |
|---|---|---|---|---|
| contig14585 | Bradi2g14450.1 | Os05t0592300-01 | - | - |
| contig15809 | Bradi1g44137.1 | Os06t0256300-01 | - | - |
| contig09569 | Bradi1g42000.1 | Os06t0483200-02 | - | - |
| contig08452 | Bradi2g11590.1 | Os06t0484800-00 | - | - |
| contig13912 | Bradi1g35840.1 | Os06t0620600-01 | - | - |
| contig12695 | Bradi1g59360.1 | Os07t0107800-01 | - | - |
| contig08367 | Bradi1g74730.1 | Os07t0180300-01 | - | - |
| contig02948 | Bradi1g25780.1 | Os07t0531700-01 | - | - |
| contig01723 | Bradi1g02180.1 | Os07t0645701-00 | - | - |
| contig18005 | Bradi3g14710.1 | Os08t0139700-01 | - | - |
| contig02493 | Bradi2g36180.1 | Os08t0265300-00 | - | - |
| contig17664 | Bradi3g22437.1 | Os08t0338600-01 | - | - |
| contig19711 | Bradi4g28160.1 | Os09t0327400-01 | - | - |
| contig10287 | Bradi4g30520.1 | Os09t0421600-00 | - | - |
| contig15811 | Bradi1g31350.1 | Os09t0429200-00 | - | - |
| contig12360 | Bradi4g33010.2 | Os09t0476000-01 | - | - |
| contig08946 | Bradi4g33430.1 | Os09t0482700-00 | - | - |
| contig02322 | Bradi4g34460.1 | Os09t0504800-01 | - | - |
| contig04873 | Bradi3g42240.1 | Os09t0545300-01 | - | - |
| contig09690 | Bradi4g38527.1 | Os09t0568200-01 | - | - |
| contig05997 | Bradi4g38560.1 | Os09t0568700-01 | - | - |
| contig06023 | Bradi3g10600.1 | Os10t0120300-01 | - | - |
| contig23347 | Bradi2g03850.1 | Os10t0151100-00 | - | - |
| contig14333 | Bradi4g23600.2 | Os11t0186400-00 | - | - |
| contig08003 | Bradi2g42290.1 | Os11t0199900-00 | - | - |
| contig11684 | Bradi1g01430.1 | Os11t0208900-01 | - | - |
| contig04630 | Bradi2g36180.1 | Os11t0211650-00 | - | - |
| contig21394 | Bradi2g38510.1 | Os11t0212300-00 | - | - |
| contig11065 | Bradi4g07541.1 | Os11t0514100-00 | - | - |
| contig03510 | Bradi1g56990.1 | Os11t0572500-01 | - | - |
| contig23769 | Bradi4g13950.1 | Os11t0610700-01 | - | - |
| contig11274 | Bradi2g36810.1 | Os11t0681300-00 | - | - |
| contig03611 | Bradi4g09597.1 | Os11t0689100-02 | - | - |
| contig00355 | Bradi4g36507.1 | Os09t0539100-01 | - | - |
| contig23699 | Bradi2g38577.1 | Os05t0133900-01 | - | - |
| contig09094 | Bradi3g28477.1 | Os10t0471350-01 | - | - |
| contig15458 | Bradi4g02647.1 | Os12t0600200-02 | - | - |
| contig21672 | Bradi4g07770.1 | Os03t0421000-01 | - | - |
| contig20723 | Bradi4g36220.3 | Os09t0532700-01 | - | - |
| contig18024 | Bradi1g33150.1 | Os06t0685300-00 | - | - |
| contig25171 | Bradi1g69880.1 | Os10t0400100-01 | - | - |
| contig12752 | Bradi4g37110.1 | Os09t0548400-01 | - | - |
| contig09117 | Bradi4g45160.1 | Os09t0343200-01 | - | - |
| contig16404 | Bradi3g03497.1 | Os02t0139700-01 | - | - |
| contig20543 | Bradi4g03890.1 | Os12t0572800-00 | - | - |
| contig02039 | Bradi2g40030.1 | Os05t0180600-01 | - | - |
| contig05222 | Bradi2g40030.1 | Os05t0180600-02 | - | - |

| | | | | |
|---|---|---|---|---|
| contig07490 | Bradi1g05130.1 | Os03t0800800-01 | - | - |
| contig22806 | Bradi1g05130.1 | Os03t0800800-01 | - | - |
| contig05874 | Bradi4g05157.1 | Os12t0552700-01 | - | - |
| contig00254 | Bradi4g35830.1 | Os09t0529700-01 | - | - |
| contig02607 | Bradi3g18250.1 | Os08t0227400-00 | - | - |
| contig19666 | Bradi1g47626.1 | Os08t0494400-01 | - | - |
| contig01430 | Bradi1g33097.2 | Os06t0684000-02 | - | - |
| contig03674 | Bradi1g08390.1 | Os03t0752200-01 | - | - |
| contig00675 | Bradi1g15970.1 | Os05t0220100-01 | - | - |
| contig03716 | Bradi1g22147.1 | Os01t0546800-01 | - | - |
| contig05793 | Bradi1g06900.1 | Os03t0778000-01 | - | - |
| contig04468 | Bradi4g13770.1 | Os11t0616200-01 | - | - |
| contig17848 | Bradi1g69090.1 | Os03t0228500-01 | - | - |
| contig01309 | Bradi2g36840.1 | Os01t0776800-02 | - | - |
| contig00164 | Bradi4g05610.1 | Os12t0538800-01 | - | - |
| contig03248 | Bradi1g01847.1 | Os03t0845500-01 | - | - |
| contig04297 | Bradi1g21030.1 | Os03t0583900-01 | - | - |
| contig04888 | Bradi4g24400.1 | Os11t0171700-01 | - | - |
| contig04699 | Bradi4g28000.1 | Os09t0322000-01 | - | - |
| contig17840 | Bradi4g34290.1 | Os09t0500600-01 | - | - |
| contig14614 | Bradi1g04397.1 | Os01t0513900-01 | - | - |
| contig18716 | Bradi4g34840.1 | Os09t0509400-01 | - | - |
| contig13318 | Bradi4g27190.1 | Os08t0337300-02 | - | - |
| contig00686 | Bradi1g05590.1 | Os03t0795400-00 | - | - |
| contig09709 | Bradi4g36972.1 | Os09t0546900-01 | - | - |
| contig00255 | Bradi5g25610.1 | Os04t0669900-00 | - | - |
| contig08860 | Bradi2g31720.1 | Os05t0251900-00 | - | - |
| contig01749 | Bradi1g13870.2 | Os03t0641700-01 | - | - |
| contig17540 | Bradi1g01847.1 | Os03t0845500-01 | - | - |
| contig09654 | Bradi2g20430.1 | Os05t0508500-01 | - | - |
| contig01016 | Bradi4g34460.1 | Os09t0504800-01 | - | - |
| contig07341 | Bradi4g06970.1 | Os08t0261000-01 | - | - |
| contig24967 | Bradi4g06970.1 | Os08t0261000-01 | - | - |
| contig02052 | Bradi4g07541.1 | Os11t0514100-00 | - | - |
| contig10222 | Bradi1g01870.1 | Os03t0845000-01 | - | - |
| contig03306 | Bradi3g09630.1 | Os02t0256800-00 | - | - |
| contig13777 | Bradi4g30810.1 | Os09t0428300-00 | - | - |
| contig18064 | Bradi5g03890.1 | Os04t0570500-01 | - | - |
| contig25162 | Bradi1g12430.1 | Os03t0682600-00 | - | - |
| contig03863 | Bradi2g38300.1 | Os09t0561450-00 | - | - |
| contig00789 | Bradi1g19470.1 | Os07t0290200-01 | - | - |
| contig06311 | Bradi4g45200.1 | Os11t0103400-00 | - | - |
| contig25992 | Bradi1g36720.1 | Os03t0422800-01 | - | - |
| contig15541 | Bradi4g03890.1 | Os12t0572800-00 | - | - |
| contig03078 | Bradi1g02990.1 | Os03t0831500-01 | - | - |
| contig00114 | Bradi1g73010.1 | Os03t0174200-01 | - | - |
| contig01305 | Bradi1g14730.1 | Os03t0610650-01 | - | - |

| | | | | |
|---|---|---|---|---|
| contig13378 | Bradi4g43257.1 | Os12t0137200-01 | - | - |
| contig05490 | Bradi4g30130.1 | Os09t0412700-01 | - | - |
| contig06139 | Bradi5g03890.1 | Os04t0570500-01 | - | - |
| contig00644 | Bradi3g45580.1 | Os11t0574725-00 | - | - |
| contig01502 | Bradi2g59730.1 | Os08t0150800-01 | - | - |
| contig11993 | Bradi1g03170.3 | Os03t0827600-02 | - | - |
| contig06332 | Bradi4g26590.1 | Os12t0143400-00 | - | - |
| contig21920 | Bradi2g09434.1 | Os10t0370100-01 | - | - |
| contig22661 | Bradi4g06970.1 | Os08t0261000-01 | - | - |
| contig01833 | Bradi2g10800.2 | Os01t0279400-01 | - | AK249459.1 |
| contig06413 | Bradi4g27100.1 | Os09t0280500-01 | - | AK252400.1 |
| contig12022 | Bradi4g01360.2 | Os12t0619000-01 | - | AK353868 |
| contig00459 | Bradi1g03800.1 | Os03t0820100-01 | - | AK354375 |
| contig14487 | Bradi4g08527.1 | Os09t0121050-00 | - | AK355795 |
| contig12032 | Bradi1g60207.1 | Os03t0407000-01 | - | AK363897 |
| contig04823 | Bradi3g43260.1 | Os02t0469200-02 | - | AK365473 |
| contig07040 | Bradi3g43260.1 | Os02t0469200-02 | - | AK365473 |
| contig09378 | Bradi3g43260.1 | Os02t0469200-02 | - | AK365473 |
| contig08749 | Bradi1g00677.1 | Os03t0860000-01 | - | AK367590 |
| contig04711 | Bradi2g18330.2 | Os05t0539700-01 | - | AK367925 |
| contig13480 | Bradi5g04567.1 | Os04t0611800-00 | - | AK369309 |
| contig18351 | Bradi4g33067.1 | Os09t0477900-01 | - | AK374386 |
| contig01637 | Bradi4g04360.1 | Os12t0566500-01 | - | MLOC_11295.1 |
| contig20299 | Bradi1g06380.1 | Os03t0786900-01 | - | MLOC_14680.1 |
| contig09754 | Bradi4g07541.1 | Os11t0514100-00 | - | MLOC_24388.12 |
| contig17998 | Bradi4g06330.1 | Os11t0637800-01 | - | MLOC_3216.2 |
| contig02652 | Bradi2g06100.1 | Os08t0496700-00 | - | MLOC_33543.1 |
| contig05150 | Bradi4g37320.1 | Os09t0552800-00 | - | MLOC_37843.1 |
| contig04350 | Bradi4g30520.1 | Os09t0421700-01 | - | MLOC_38979.4 |
| contig13928 | Bradi3g07210.1 | Os08t0323000-01 | - | MLOC_53066.1 |
| contig11636 | Bradi1g06650.1 | Os03t0782900-01 | - | MLOC_60261.1 |
| contig23504 | Bradi1g49960.1 | Os06t0130200-01 | - | MLOC_62663.2 |
| contig25372 | Bradi1g14530.1 | Os03t0620500-01 | - | MLOC_68151.2 |
| contig19185 | Bradi1g12480.1 | Os03t0681400-01 | - | AK252722.1 |
| contig10220 | Bradi1g33347.1 | Os06t0690700-01 | - | AK358556 |
| contig01961 | Bradi4g00640.1 | Os06t0498900-01 | - | AK354479 |
| contig03903 | Bradi1g62590.1 | Os03t0347200-01 | - | AK373558 |
| contig13038 | Bradi1g08780.1 | Os03t0747200-01 | - | MLOC_52950.1 |
| contig21021 | Bradi1g07760.1 | Os03t0762900-01 | - | AK374671 |
| contig00817 | Bradi2g27230.1 | Os01t0355600-01 | - | MLOC_54934.2 |
| contig00797 | Bradi4g26970.1 | Os09t0267500-01 | - | MLOC_1337.1 |
| contig03646 | Bradi2g15260.1 | Os05t0581800-01 | - | AK363158 |
| contig18164 | Bradi4g03050.1 | Os12t0590500-00 | - | MLOC_75843.3 |
| contig05224 | Bradi1g27807.1 | Os07t0481000-01 | - | AK376614 |
| contig05570 | Bradi1g14030.2 | Os03t0638200-02 | - | MLOC_76072.2 |
| contig20517 | Bradi1g01040.1 | Os02t0537400-01 | - | MLOC_31208.2 |
| contig04920 | Bradi1g08294.1 | Os03t0754900-01 | - | MLOC_54479.1 |

| | | | | |
|---|---|---|---|---|
| contig18885 | Bradi1g22000.1 | Os03t0415200-01 | - | MLOC_71887.2 |
| contig20594 | Bradi3g17620.6 | Os04t0619400-01 | - | MLOC_39114.2 |
| contig13535 | Bradi3g07210.1 | Os08t0323000-01 | - | MLOC_53066.1 |
| contig19008 | Bradi4g45240.1 | Os12t0102400-01 | - | MLOC_55862.2 |
| contig06227 | Bradi4g42217.1 | Os12t0155300-01 | - | MLOC_61166.1 |
| contig00064 | Bradi1g06480.1 | Os03t0785300-01 | - | MLOC_55658.4 |
| contig20441 | Bradi1g64100.1 | Os03t0317000-01 | - | MLOC_59288.1 |
| contig00623 | Bradi4g08510.1 | Os09t0134500-01 | - | AK362848 |
| contig15942 | Bradi4g05157.1 | Os12t0552700-01 | - | AK364318 |
| contig07142 | Bradi1g00580.1 | Os07t0438500-01 | - | MLOC_44779.2 |
| contig04737 | Bradi4g06280.1 | Os12t0515600-01 | - | AK363437 |
| contig00974 | Bradi3g19890.1 | Os06t0255700-01 | - | MLOC_70680.1 |
| contig02775 | Bradi1g01847.1 | Os03t0845500-01 | - | MLOC_17240.4 |
| contig14193 | Bradi4g04840.1 | Os12t0557800-01 | - | MLOC_37703.1 |
| contig05235 | Bradi1g00940.1 | Os03t0857400-01 | - | MLOC_7826.2 |
| contig07731 | Bradi2g13450.1 | Os01t0528800-01 | - | AK252044.1 |
| contig02756 | Bradi2g14400.1 | Os05t0593100-01 | - | AK249839.1 |
| contig11488 | Bradi1g03910.1 | Os03t0818200-01 | - | MLOC_3543.1 |
| contig15538 | Bradi3g54980.2 | Os02t0812400-01 | - | MLOC_77371.1 |
| contig13004 | Bradi1g08270.1 | Os03t0755600-00 | - | AK372882 |
| contig00678 | Bradi1g02980.1 | Os03t0831700-01 | - | MLOC_63062.2 |
| contig23788 | Bradi4g39130.1 | Os12t0279100-01 | - | MLOC_55909.3 |
| contig07696 | Bradi1g36840.1 | Os02t0205400-01 | - | MLOC_60900.1 |
| contig14485 | Bradi1g61380.2 | Os03t0372700-01 | - | MLOC_58241.2 |
| contig13664 | Bradi1g02630.1 | Os03t0835400-01 | - | AK251444.1 |
| contig03207 | Bradi3g07210.1 | Os08t0323000-01 | - | MLOC_53066.1 |
| contig06257 | Bradi3g07210.1 | Os08t0323000-01 | - | MLOC_53066.1 |
| contig05276 | Bradi1g55250.1 | Os07t0184900-01 | - | MLOC_62977.1 |
| contig08765 | Bradi1g55250.1 | Os07t0184900-01 | - | MLOC_62977.1 |
| contig03829 | Bradi1g05660.1 | Os03t0794800-01 | - | AK354390 |
| contig04780 | Bradi1g70057.1 | Os03t0743800-00 | - | MLOC_5747.2 |
| contig08601 | Bradi3g18990.1 | Os08t0248800-01 | - | AK249523.1 |
| contig06903 | Bradi4g08490.1 | Os09t0123100-01 | - | AK366742 |
| contig06181 | Bradi1g02290.1 | Os12t0614851-00 | - | MLOC_54841.1 |
| contig10957 | Bradi4g43650.1 | Os12t0131200-00 | - | MLOC_12145.8 |
| contig03114 | Bradi1g03800.1 | Os03t0820100-01 | - | AK354375 |
| contig16397 | Bradi4g35830.1 | Os09t0529700-01 | - | MLOC_70100.1 |
| contig23383 | Bradi4g07541.1 | Os11t0514100-00 | - | MLOC_24388.12 |
| contig14783 | Bradi3g16660.1 | Os05t0151000-00 | - | MLOC_5852.1 |
| contig13953 | Bradi1g07260.1 | Os03t0771100-01 | - | MLOC_52896.3 |
| contig12559 | Bradi5g20420.1 | Os04t0599650-00 | - | MLOC_55154.1 |
| contig08657 | Bradi1g03380.1 | Os03t0825700-01 | - | MLOC_55007.1 |
| contig24968 | Bradi4g33310.1 | Os12t0233600-01 | - | MLOC_78764.1 |
| contig13501 | Bradi1g33347.1 | Os06t0690700-01 | - | AK358556 |
| contig02852 | Bradi4g03050.1 | Os12t0590500-00 | - | MLOC_12612.3 |
| contig22973 | Bradi1g00940.1 | Os03t0857400-01 | - | MLOC_7826.2 |
| contig15236 | Bradi4g06380.1 | Os04t0636700-01 | - | MLOC_64489.3 |

| | | | | |
|---|---|---|---|---|
| contig00662 | Bradi1g04292.1 | Os03t0811200-01 | - | MLOC_73693.1 |
| contig01825 | Bradi2g27230.1 | Os01t0355600-01 | - | MLOC_54934.2 |
| contig08034 | Bradi2g27230.1 | Os01t0355600-01 | - | MLOC_54934.2 |
| contig06898 | Bradi1g01847.1 | Os03t0845500-01 | - | MLOC_4674.2 |
| contig26323 | Bradi4g04580.1 | Os12t0563500-01 | - | AK250022.1 |
| contig00606 | Bradi4g08510.1 | Os09t0134500-01 | - | AK362848 |
| contig16956 | Bradi3g16660.1 | Os05t0151000-00 | - | MLOC_5852.1 |
| contig07388 | Bradi4g03050.1 | Os12t0590500-00 | - | MLOC_12612.3 |
| contig00535 | Bradi1g29030.1 | Os04t0354200-00 | - | MLOC_29241.1 |
| contig02163 | Bradi4g30797.1 | Os09t0428900-01 | - | MLOC_74779.1 |
| contig08299 | Bradi4g03827.1 | Os12t0574900-01 | - | MLOC_81375.2 |
| contig00879 | Bradi4g44844.1 | Os11t0106800-00 | - | MLOC_65713.1 |
| contig02028 | Bradi1g06630.2 | Os03t0783700-00 | - | MLOC_58395.2 |
| contig04473 | Bradi1g47780.1 | Os02t0280700-01 | - | MLOC_59071.1 |
| contig01364 | Bradi1g41860.1 | Os06t0473200-02 | - | AK364882 |
| contig02878 | Bradi5g00430.1 | Os09t0101800-01 | - | MLOC_57244.2 |
| contig06479 | Bradi3g43260.1 | Os02t0469200-02 | - | AK365473 |
| contig03782 | Bradi4g28810.1 | Os09t0363400-00 | - | MLOC_17216.3 |
| contig19984 | Bradi1g09327.1 | Os03t0737900-01 | - | MLOC_12819.1 |
| contig01946 | Bradi1g00567.1 | Os03t0860900-01 | - | AK251043.1 |
| contig01271 | Bradi4g30490.1 | Os09t0421200-01 | - | AK370249 |
| contig03630 | Bradi4g03890.1 | Os12t0572800-00 | - | MLOC_59658.3 |
| contig15430 | Bradi4g29887.1 | Os09t0407600-01 | - | MLOC_81241.5 |
| contig17522 | Bradi1g00607.1 | Os02t0816900-01 | Sb01g000330.1 | - |
| contig20379 | Bradi1g00910.1 | Os03t0857750-00 | Sb01g000520.1 | - |
| contig06612 | Bradi1g01530.1 | Os02t0234800-01 | Sb01g001040.1 | - |
| contig03723 | Bradi1g01570.1 | Os03t0851200-01 | Sb01g001070.1 | - |
| contig16101 | Bradi1g01870.1 | Os03t0845000-01 | Sb01g001470.1 | - |
| contig03894 | Bradi1g01920.1 | Os03t0844600-01 | Sb01g001540.1 | - |
| contig07964 | Bradi1g03030.1 | Os03t0831100-01 | Sb01g002280.1 | - |
| contig21434 | Bradi1g03380.1 | Os03t0825700-01 | Sb01g002810.1 | - |
| contig17225 | Bradi1g05490.1 | Os03t0796800-00 | Sb01g005180.1 | - |
| contig08402 | Bradi1g07060.1 | Os03t0775700-01 | Sb01g006530.1 | - |
| contig15964 | Bradi1g07080.1 | Os03t0775500-01 | Sb01g006550.1 | - |
| contig15476 | Bradi1g08230.1 | Os03t0756300-01 | Sb01g007670.1 | - |
| contig06588 | Bradi1g08350.1 | Os03t0752700-01 | Sb01g007800.1 | - |
| contig09162 | Bradi1g08440.2 | Os03t0751000-01 | Sb01g007930.1 | - |
| contig12015 | Bradi1g08727.1 | Os03t0747600-02 | Sb01g008195.1 | - |
| contig13592 | Bradi1g11490.1 | Os03t0713000-00 | Sb01g010250.1 | - |
| contig04137 | Bradi1g13220.1 | Os03t0657000-01 | Sb01g012880.1 | - |
| contig08937 | Bradi1g13807.1 | Os03t0643700-01 | Sb01g013590.1 | - |
| contig01130 | Bradi4g36972.1 | Os09t0546900-01 | Sb01g016035.1 | - |
| contig06744 | Bradi3g29690.1 | Os10t0490666-00 | Sb01g018510.1 | - |
| contig08489 | Bradi1g02770.1 | Os09t0250700-01 | Sb01g027550.1 | - |
| contig19658 | Bradi1g02770.1 | Os09t0250700-01 | Sb01g027550.1 | - |
| contig21956 | Bradi1g02770.1 | Os09t0250700-01 | Sb01g027550.1 | - |
| contig11772 | Bradi3g08327.1 | Os12t0624400-01 | Sb01g043990.1 | - |

| | | | | |
|---|---|---|---|---|
| contig01523 | Bradi1g72490.1 | Os03t0182400-01 | Sb01g045110.1 | - |
| contig00547 | Bradi4g27590.1 | Os09t0298500-01 | Sb02g020450.1 | - |
| contig06107 | Bradi3g03106.1 | Os09t0306800-01 | Sb02g020800.1 | - |
| contig10535 | Bradi4g28160.1 | Os09t0327400-01 | Sb02g022170.1 | - |
| contig13956 | Bradi4g29347.1 | Os09t0376900-01 | Sb02g023620.1 | - |
| contig21767 | Bradi4g32870.1 | Os09t0473300-01 | Sb02g027370.1 | - |
| contig09155 | Bradi4g33080.1 | Os09t0478300-01 | Sb02g027565.1 | - |
| contig16119 | Bradi4g34660.1 | Os09t0506900-02 | Sb02g029290.1 | - |
| contig06566 | Bradi4g35450.1 | Os09t0518600-00 | Sb02g030080.1 | - |
| contig01757 | Bradi4g35382.1 | Os09t0520200-00 | Sb02g030150.1 | - |
| contig02760 | Bradi4g37110.1 | Os09t0548400-01 | Sb02g031940.1 | - |
| contig04986 | Bradi1g21280.1 | Os07t0621500-01 | Sb02g039580.1 | - |
| contig06336 | Bradi2g05420.1 | Os01t0184800-01 | Sb03g003460.1 | - |
| contig09547 | Bradi2g03530.1 | Os01t0103600-02 | Sb03g008930.1 | - |
| contig11523 | Bradi2g12497.1 | Os01t0335700-01 | Sb03g013510.1 | - |
| contig09462 | Bradi1g55350.1 | Os01t0527600-01 | Sb03g022880.1 | - |
| contig04219 | Bradi2g41400.1 | Os01t0566900-01 | Sb03g025580.1 | - |
| contig16245 | Bradi1g31970.1 | Os01t0601675-01 | Sb03g027070.1 | - |
| contig18082 | Bradi4g29760.1 | Os09t0397900-01 | Sb03g028040.1 | - |
| contig18950 | Bradi2g54640.1 | Os01t0841500-01 | Sb03g039470.1 | - |
| contig08228 | Bradi2g59137.1 | Os01t0923600-01 | Sb03g044220.1 | - |
| contig00578 | Bradi3g10370.1 | Os02t0281200-01 | Sb04g001410.1 | - |
| contig00716 | Bradi3g10640.1 | Os02t0291000-00 | Sb04g011330.1 | - |
| contig02945 | Bradi3g44940.1 | Os02t0533300-01 | Sb04g021740.1 | - |
| contig14566 | Bradi3g59980.1 | Os02t0735100-01 | Sb04g028700.1 | - |
| contig23717 | Bradi3g52470.2 | Os02t0699700-01 | Sb04g030880.1 | - |
| contig14531 | Bradi1g22170.1 | Os02t0688900-01 | Sb04g031420.1 | - |
| contig02636 | Bradi1g30610.1 | Os06t0645400-01 | Sb04g035150.1 | - |
| contig02227 | Bradi3g20710.1 | Os08t0320200-02 | Sb04g038240.1 | - |
| contig26002 | Bradi4g26877.1 | Os11t0104900-00 | Sb05g000450.1 | - |
| contig19607 | Bradi4g24852.1 | Os11t0160900-00 | Sb05g003830.1 | - |
| contig14609 | Bradi4g18300.1 | Os11t0514100-00 | Sb05g018376.1 | - |
| contig02279 | Bradi2g36180.1 | Os11t0213800-01 | Sb05g025470.1 | - |
| contig05538 | Bradi2g36430.1 | Os11t0433200-02 | Sb06g020770.1 | - |
| contig00523 | Bradi5g21937.1 | Os04t0618500-01 | Sb06g028740.1 | - |
| contig15164 | Bradi5g25620.1 | Os04t0670000-01 | Sb06g032290.1 | - |
| contig08597 | Bradi2g04960.1 | Os03t0335600-01 | Sb07g004420.1 | - |
| contig04307 | Bradi2g04960.1 | Os03t0694900-01 | Sb07g004420.1 | - |
| contig22898 | Bradi2g36180.1 | Os11t0211650-00 | Sb07g005710.1 | - |
| contig19373 | Bradi3g41960.1 | Os08t0542200-01 | Sb07g025360.1 | - |
| contig17149 | Bradi3g40520.1 | Os08t0519800-01 | Sb07g026995.1 | - |
| contig21514 | Bradi4g26390.1 | Os11t0134500-01 | Sb08g001420.1 | - |
| contig12488 | Bradi2g05445.2 | Os05t0295100-01 | Sb08g002833.1 | - |
| contig03506 | Bradi3g22760.1 | Os10t0182000-01 | Sb08g008230.1 | - |
| contig08896 | Bradi4g03050.1 | Os12t0590500-00 | Sb08g019800.1 | - |
| contig09567 | Bradi4g02030.1 | Os12t0615800-01 | Sb08g021110.1 | - |
| contig01145 | Bradi2g32570.1 | Os05t0209100-01 | Sb09g006500.1 | - |

| | | | | |
|---|---|---|---|---|
| contig02361 | Bradi2g26500.1 | Os05t0392400-01 | Sb09g019410.1 | - |
| contig13359 | Bradi2g26100.1 | Os05t0397900-01 | Sb09g019590.1 | - |
| contig18161 | Bradi2g25800.1 | Os05t0404400-01 | Sb09g019840.1 | - |
| contig07345 | Bradi2g24300.1 | Os01t0871300-01 | Sb09g021360.1 | - |
| contig04398 | Bradi1g51817.1 | Os05t0447500-00 | Sb10g000410.1 | - |
| contig00306 | Bradi1g51740.1 | Os06t0112400-01 | Sb10g000940.1 | - |
| contig16054 | Bradi1g45150.1 | Os06t0229200-01 | Sb10g008170.1 | - |
| contig00654 | Bradi2g62760.1 | Os01t0977600-01 | Sb05g002400.1 | - |
| contig02966 | Bradi3g00290.1 | Os02t0101100-01 | Sb08g022730.1 | - |
| contig03895 | Bradi4g04540.1 | Os12t0562900-01 | Sb08g018270.1 | - |
| contig03350 | Bradi2g61830.1 | Os01t0966700-01 | Sb03g047060.1 | - |
| contig03640 | Bradi1g13920.1 | Os03t0640400-01 | Sb01g013720.1 | - |
| contig00183 | Bradi4g33067.1 | Os09t0477900-01 | Sb02g027560.1 | - |
| contig22177 | Bradi4g33067.1 | Os09t0477900-01 | Sb02g027560.1 | - |
| contig24071 | Bradi4g00980.1 | Os12t0631200-01 | Sb08g022690.1 | - |
| contig01075 | Bradi3g23380.1 | Os10t0375700-01 | Sb0012s009230.1 | - |
| contig00768 | Bradi1g69880.1 | Os10t0400100-01 | Sb01g042830.1 | - |
| contig10129 | Bradi1g69880.1 | Os10t0400100-01 | Sb01g042830.1 | - |
| contig16990 | Bradi1g02697.1 | Os03t0834000-01 | Sb01g002580.1 | - |
| contig04278 | Bradi1g03920.1 | Os03t0818100-01 | Sb01g003490.1 | - |
| contig03533 | Bradi2g61850.1 | Os02t0107200-01 | Sb04g035950.1 | - |
| contig04416 | Bradi1g08847.1 | Os03t0746500-01 | Sb01g008310.1 | - |
| contig02152 | Bradi2g20490.1 | Os08t0320000-01 | Sb06g018696.1 | - |
| contig07513 | Bradi5g15070.1 | Os04t0506100-01 | Sb06g021910.1 | - |
| contig02206 | Bradi3g40520.1 | Os08t0519800-01 | Sb07g026995.2 | - |
| contig01027 | Bradi4g04650.1 | Os12t0564600-01 | Sb08g018400.1 | - |
| contig13288 | Bradi4g04650.1 | Os12t0564600-01 | Sb08g018400.1 | - |
| contig07933 | Bradi4g37110.1 | Os09t0548400-01 | Sb02g031940.1 | - |
| contig17037 | Bradi3g60140.1 | Os02t0511500-01 | Sb02g040460.1 | - |
| contig02181 | Bradi1g07830.1 | Os03t0761700-01 | Sb01g007290.1 | - |
| contig09821 | Bradi5g03890.1 | Os04t0570500-01 | Sb05g004900.2 | - |
| contig08364 | Bradi1g32610.1 | Os07t0270950-01 | Sb02g008800.1 | - |
| contig00059 | Bradi3g06930.1 | Os02t0194100-01 | Sb04g006440.1 | - |
| contig21090 | Bradi1g02770.1 | Os09t0250700-01 | Sb01g027550.1 | - |
| contig06649 | Bradi4g06310.1 | Os05t0558900-01 | Sb09g003950.1 | - |
| contig03955 | Bradi4g07990.1 | Os12t0429200-01 | Sb08g004410.1 | - |
| contig00293 | Bradi4g45240.1 | Os12t0102400-01 | Sb05g000270.1 | - |
| contig01279 | Bradi1g07520.1 | Os03t0766000-01 | Sb01g007040.1 | - |
| contig02436 | Bradi1g17710.1 | Os02t0167700-01 | Sb04g004540.1 | - |
| contig10596 | Bradi1g17710.1 | Os02t0167700-01 | Sb04g004540.1 | - |
| contig02243 | Bradi1g55350.1 | Os01t0527600-01 | Sb03g022880.1 | - |
| contig06065 | Bradi4g38090.1 | Os09t0563300-02 | Sb02g011260.1 | - |
| contig03655 | Bradi2g31490.1 | Os04t0586500-01 | Sb06g029710.1 | - |
| contig01802 | Bradi5g20420.1 | Os04t0599650-00 | Sb06g027382.1 | - |
| contig03571 | Bradi1g02190.1 | Os02t0664100-00 | Sb04g036540.1 | - |
| contig06826 | Bradi1g07590.1 | Os03t0765000-01 | Sb01g007100.1 | - |
| contig12410 | Bradi4g28380.1 | Os09t0345700-01 | Sb02g022560.1 | - |

| | | | | |
|---|---|---|---|---|
| contig10196 | Bradi1g01710.1 | Os03t0848300-01 | Sb01g001300.1 | - |
| contig06874 | Bradi4g27590.1 | Os09t0298500-01 | Sb02g020450.1 | - |
| contig16180 | Bradi4g30040.1 | Os09t0411650-01 | Sb02g024510.1 | - |
| contig01738 | Bradi4g29650.1 | Os09t0394500-01 | Sb02g024060.1 | - |
| contig02416 | Bradi4g29650.1 | Os09t0394500-01 | Sb02g024060.1 | - |
| contig00273 | Bradi4g34470.1 | Os09t0504900-01 | Sb02g029160.1 | - |
| contig02281 | Bradi2g05460.1 | Os01t0185200-03 | Sb03g003420.1 | - |
| contig02424 | Bradi1g08597.1 | Os03t0748900-01 | Sb01g008070.1 | - |
| contig03007 | Bradi1g05320.1 | Os03t0799100-01 | Sb01g005000.1 | - |
| contig01269 | Bradi2g40860.1 | Os09t0261300-01 | Sb01g000720.1 | - |
| contig02898 | Bradi4g34220.3 | Os09t0498600-01 | Sb02g028920.1 | - |
| contig16090 | Bradi4g01080.1 | Os12t0163200-01 | Sb05g002340.1 | - |
| contig09342 | Bradi4g02700.2 | Os12t0597500-01 | Sb08g020250.1 | - |
| contig02015 | Bradi1g02597.1 | Os03t0835600-01 | Sb01g002200.1 | - |
| contig24658 | Bradi3g54980.2 | Os02t0812400-01 | Sb04g036790.1 | - |
| contig19546 | Bradi3g29390.1 | Os10t0487300-01 | Sb01g018820.1 | - |
| contig02654 | Bradi3g37010.1 | Os08t0439100-01 | Sb07g021510.1 | - |
| contig04978 | Bradi1g69520.1 | Os03t0223700-01 | Sb01g042440.1 | - |
| contig02155 | Bradi1g05122.1 | Os03t0801600-01 | Sb01g004840.1 | - |
| contig04334 | Bradi5g22017.1 | Os03t0306200-01 | Sb03g005120.1 | - |
| contig00784 | Bradi5g22017.1 | Os03t0306302-00 | Sb03g005130.1 | - |
| contig10223 | Bradi4g28150.1 | Os09t0327300-01 | Sb02g022160.1 | - |
| contig02430 | Bradi4g39580.1 | Os12t0236900-01 | Sb03g029140.1 | - |
| contig19530 | Bradi1g33150.1 | Os06t0685300-00 | Sb10g027770.1 | - |
| contig04548 | Bradi1g08280.1 | Os03t0755100-01 | Sb01g007710.1 | - |
| contig06051 | Bradi1g08280.1 | Os03t0755100-01 | Sb01g007710.1 | - |
| contig05519 | Bradi1g59910.2 | Os03t0417900-01 | Sb01g032420.1 | - |
| contig12248 | Bradi3g03106.1 | Os09t0306800-01 | Sb01g007878.1 | - |
| contig05268 | Bradi4g02100.1 | Os12t0609800-01 | Sb08g020980.1 | - |
| contig19746 | Bradi1g05660.1 | Os03t0794800-01 | Sb01g005340.1 | - |
| contig01432 | Bradi4g08907.3 | Os09t0266400-01 | Sb02g015550.1 | - |
| contig06499 | Bradi1g70057.1 | Os03t0213400-01 | Sb01g042980.1 | - |
| contig01487 | Bradi4g37680.1 | Os09t0557700-00 | Sb02g032520.1 | - |
| contig02942 | Bradi1g05490.1 | Os03t0796800-00 | Sb01g005180.1 | - |
| contig04826 | Bradi1g53160.1 | Os07t0257200-01 | Sb02g007870.1 | - |
| contig21318 | Bradi1g59360.1 | Os07t0107800-01 | Sb02g000750.1 | - |
| contig02837 | Bradi4g29760.1 | Os09t0397900-01 | Sb03g028040.1 | - |
| contig04037 | Bradi1g01570.1 | Os03t0851200-01 | Sb01g001070.1 | - |
| contig03967 | Bradi2g31720.1 | Os05t0251900-00 | Sb09g009580.1 | - |
| contig16191 | Bradi1g03180.1 | Os03t0827700-01 | Sb01g002660.2 | - |
| contig00322 | Bradi1g17710.1 | Os02t0167700-01 | Sb04g004540.1 | - |
| contig06774 | Bradi4g27590.1 | Os09t0298500-01 | Sb02g020450.1 | - |
| contig11393 | Bradi2g61850.1 | Os02t0109100-01 | Sb04g035950.1 | - |
| contig15237 | Bradi4g26877.1 | Os11t0104900-00 | Sb05g000450.1 | - |
| contig04938 | Bradi4g32987.1 | Os09t0475700-01 | Sb02g027475.1 | - |
| contig17358 | Bradi4g13950.1 | Os11t0610700-01 | Sb05g024260.1 | - |
| contig10261 | Bradi1g17710.1 | Os02t0167700-01 | Sb04g004540.1 | - |

| | | | | |
|---|---|---|---|---|
| contig09236 | Bradi2g19800.1 | Os03t0824400-01 | Sb01g002910.1 | - |
| contig00509 | Bradi2g61830.1 | Os01t0966700-01 | Sb03g047060.1 | - |
| contig04685 | Bradi1g48264.1 | Os05t0163250-01 | Sb10g004685.1 | - |
| contig09301 | Bradi1g03987.1 | Os02t0511500-01 | Sb02g040460.1 | - |
| contig16392 | Bradi1g02770.1 | Os09t0250700-01 | Sb01g027550.1 | - |
| contig06558 | Bradi4g41717.1 | Os12t0166500-01 | Sb08g004180.1 | - |
| contig13285 | Bradi4g30730.1 | Os07t0124500-01 | Sb02g028310.1 | - |
| contig00731 | Bradi2g04960.1 | Os03t0694900-01 | Sb07g004420.1 | - |
| contig03549 | Bradi4g04460.1 | Os12t0562300-01 | Sb08g018230.1 | - |
| contig03197 | Bradi4g38690.1 | Os09t0569800-00 | Sb02g012380.1 | - |
| contig09305 | Bradi1g01487.2 | Os03t0851600-01 | Sb01g001010.1 | - |
| contig01357 | Bradi1g07340.1 | Os03t0769600-01 | Sb01g006880.1 | - |
| contig18612 | Bradi4g43257.1 | Os12t0137200-01 | Sb05g002420.1 | - |
| contig13969 | Bradi1g08917.1 | Os02t0162733-00 | Sb01g008410.1 | - |
| contig06888 | Bradi1g08917.1 | Os03t0744650-00 | Sb01g008410.1 | - |
| contig22457 | Bradi1g00480.2 | Os03t0862100-01 | Sb01g000240.1 | - |
| contig12432 | Bradi4g02950.1 | Os12t0592300-00 | Sb08g019900.1 | - |
| contig16740 | Bradi4g38187.1 | Os08t0556500-00 | Sb07g024470.1 | - |
| contig11086 | Bradi4g01747.1 | Os12t0613600-01 | Sb08g021450.1 | - |
| contig08301 | Bradi3g56710.1 | Os02t0468200-01 | Sb02g031040.1 | - |
| contig06288 | Bradi4g04662.1 | Os12t0564800-01 | Sb08g018420.1 | - |
| contig16216 | Bradi1g00887.1 | Os03t0858100-01 | Sb01g000490.1 | - |
| contig13577 | Bradi4g11487.1 | Os11t0648100-01 | Sb05g025710.1 | - |
| contig03361 | Bradi1g13710.1 | Os03t0646900-01 | Sb01g013460.1 | - |
| contig02409 | Bradi4g31060.1 | Os09t0436800-01 | Sb02g025575.1 | - |
| contig01982 | Bradi1g01880.1 | Os03t0844900-01 | Sb01g001480.1 | - |
| contig08613 | Bradi3g37010.1 | Os08t0439100-01 | Sb07g021510.1 | AK248279.1 |
| contig20192 | Bradi1g05150.2 | Os03t0800700-01 | Sb01g004860.1 | AK249522.1 |
| contig15814 | Bradi1g04140.1 | Os03t0815200-01 | Sb01g003700.1 | AK250029.1 |
| contig06946 | Bradi2g41610.1 | Os01t0574500-01 | Sb03g025820.1 | AK250178.1 |
| contig26327 | Bradi1g08380.2 | Os03t0752300-01 | Sb01g007830.1 | AK250552.1 |
| contig17394 | Bradi2g13450.1 | Os01t0528800-01 | Sb04g011550.1 | AK252044.1 |
| contig26205 | Bradi4g32830.1 | Os09t0471200-00 | Sb02g028550.1 | AK252312.1 |
| contig14371 | Bradi1g61290.1 | Os03t0375900-01 | Sb01g034150.1 | AK353905 |
| contig23311 | Bradi1g61290.1 | Os03t0375900-01 | Sb01g034150.1 | AK353905 |
| contig12111 | Bradi1g07520.1 | Os03t0766000-01 | Sb01g007040.1 | AK354633 |
| contig13806 | Bradi4g19590.1 | Os11t0447300-01 | Sb01g021800.1 | AK356819 |
| contig01587 | Bradi5g16350.1 | Os04t0527800-01 | Sb06g023220.1 | AK358224 |
| contig12162 | Bradi5g26890.1 | Os03t0655700-01 | Sb06g033720.1 | AK358941 |
| contig08149 | Bradi4g30040.1 | Os09t0411650-01 | Sb02g024510.1 | AK358982 |
| contig04944 | Bradi4g28860.1 | Os09t0363800-00 | Sb02g023000.1 | AK361228 |
| contig06075 | Bradi4g28860.1 | Os09t0363800-00 | Sb02g023000.1 | AK361228 |
| contig14122 | Bradi4g05590.1 | Os12t0540700-01 | Sb08g017200.1 | AK362557 |
| contig06859 | Bradi4g08510.1 | Os09t0134500-02 | Sb02g016820.1 | AK362848 |
| contig15174 | Bradi4g41527.1 | Os12t0169400-01 | Sb08g004470.1 | AK365785 |
| contig20410 | Bradi1g07820.1 | Os03t0761900-01 | Sb01g007280.1 | AK367064 |
| contig13654 | Bradi3g55920.1 | Os02t0824000-00 | Sb04g037860.1 | AK368575 |

| | | | | |
|---|---|---|---|---|
| contig12953 | Bradi1g06930.1 | Os03t0777500-01 | Sb01g006390.1 | AK368629 |
| contig10134 | Bradi1g72927.1 | Os03t0175600-01 | Sb01g045480.1 | AK369212 |
| contig03557 | Bradi4g28320.1 | Os09t0338500-01 | Sb02g022420.1 | AK369379 |
| contig20984 | Bradi4g30660.1 | Os09t0424600-01 | Sb02g025130.1 | AK369445 |
| contig12422 | Bradi3g00210.1 | Os02t0100100-01 | Sb04g000230.1 | AK370108 |
| contig06386 | Bradi1g08917.1 | Os03t0744675-00 | Sb01g008410.1 | AK370634 |
| contig11311 | Bradi1g08217.1 | Os03t0756400-01 | Sb01g007660.1 | AK370781 |
| contig02769 | Bradi3g15520.1 | Os08t0178100-01 | Sb07g004940.1 | AK371182 |
| contig02846 | Bradi4g26750.1 | Os12t0104300-01 | Sb08g000390.1 | AK372211 |
| contig17147 | Bradi1g08620.1 | Os03t0748700-01 | Sb01g008100.1 | AK372289 |
| contig03071 | Bradi1g09187.1 | Os03t0740800-01 | Sb01g008590.1 | AK372381 |
| contig22637 | Bradi4g06310.1 | Os05t0558900-01 | Sb07g027440.1 | AK373215 |
| contig01100 | Bradi2g11360.1 | Os12t0485000-02 | Sb03g012030.1 | AK373528 |
| contig03868 | Bradi4g08030.1 | Os12t0420200-01 | Sb08g005500.1 | AK373820 |
| contig06792 | Bradi2g27097.1 | Os05t0376600-01 | Sb09g018780.1 | AK374248 |
| contig16390 | Bradi4g33067.1 | Os09t0477900-01 | Sb02g027560.1 | AK374386 |
| contig14546 | Bradi3g59847.1 | Os10t0405500-01 | Sb04g005260.1 | AK375886 |
| contig04244 | Bradi1g15260.1 | Os05t0274200-01 | Sb02g038230.1 | AK375935 |
| contig20528 | Bradi3g41987.1 | Os08t0544400-01 | Sb02g021200.1 | MLOC_11283.1 |
| contig12089 | Bradi4g05550.1 | Os10t0170300-01 | Sb03g009620.1 | MLOC_11318.2 |
| contig03985 | Bradi4g37110.1 | Os09t0548400-01 | Sb02g031940.1 | MLOC_11896.2 |
| contig20687 | Bradi4g37110.1 | Os09t0548400-01 | Sb02g031940.1 | MLOC_11896.2 |
| contig12669 | Bradi4g43650.1 | Os11t0133800-00 | Sb08g001470.1 | MLOC_12145.8 |
| contig18675 | Bradi1g06380.1 | Os03t0786900-01 | Sb01g005910.1 | MLOC_12376.1 |
| contig16310 | Bradi4g31680.1 | Os09t0446800-01 | Sb02g026140.1 | MLOC_12614.1 |
| contig07331 | Bradi3g03060.1 | Os02t0134000-00 | Sb04g002790.1 | MLOC_13704.1 |
| contig19062 | Bradi1g03450.1 | Os03t0824100-01 | Sb01g002930.1 | MLOC_15169.1 |
| contig09128 | Bradi4g13770.1 | Os11t0616200-01 | Sb05g024570.1 | MLOC_21268.1 |
| contig11461 | Bradi1g05600.1 | Os03t0795300-01 | Sb01g005300.1 | MLOC_2682.1 |
| contig02353 | Bradi2g18270.1 | Os09t0241100-01 | Sb10g019730.1 | MLOC_36749.1 |
| contig05146 | Bradi1g74185.1 | Os03t0162900-01 | Sb01g046280.1 | MLOC_40201.1 |
| contig14715 | Bradi4g40176.2 | Os12t0210200-01 | Sb08g006680.1 | MLOC_4105.3 |
| contig02284 | Bradi4g29300.1 | Os09t0375900-01 | Sb02g023550.1 | MLOC_44224.1 |
| contig10591 | Bradi1g55350.1 | Os01t0527600-01 | Sb03g022880.1 | MLOC_51409.1 |
| contig00343 | Bradi4g27376.1 | Os02t0233333-00 | Sb02g019530.1 | MLOC_51410.1 |
| contig00329 | Bradi4g02070.1 | Os12t0610500-01 | Sb08g021070.1 | MLOC_52221.3 |
| contig07626 | Bradi1g52170.2 | Os06t0104000-01 | Sb10g000445.1 | MLOC_53087.2 |
| contig08258 | Bradi4g38357.1 | Os09t0565500-01 | Sb02g011420.1 | MLOC_53435.4 |
| contig25910 | Bradi3g54980.2 | Os02t0812400-01 | Sb04g036790.1 | MLOC_53556.2 |
| contig04786 | Bradi4g01035.1 | Os12t0630700-01 | Sb08g022550.1 | MLOC_53665.2 |
| contig14322 | Bradi1g25780.1 | Os07t0531700-01 | Sb02g035270.1 | MLOC_54015.2 |
| contig01955 | Bradi1g08294.1 | Os03t0754900-02 | Sb01g007730.1 | MLOC_54479.1 |
| contig08314 | Bradi2g25500.1 | Os05t0409400-01 | Sb09g020180.1 | MLOC_54602.1 |
| contig16836 | Bradi1g04790.1 | Os03t0806500-01 | Sb01g004420.1 | MLOC_54623.1 |
| contig02351 | Bradi4g39460.2 | Os12t0244200-01 | Sb02g040360.1 | MLOC_54904.2 |
| contig19497 | Bradi1g15180.1 | Os03t0595200-00 | Sb01g015280.1 | MLOC_56336.1 |
| contig02139 | Bradi2g61830.1 | Os01t0966700-01 | Sb03g047060.1 | MLOC_57254.8 |

| | | | | |
|---|---|---|---|---|
| contig22832 | Bradi1g58087.1 | Os07t0136800-01 | Sb02g002670.1 | MLOC_5739.1 |
| contig23185 | Bradi1g05850.1 | Os03t0793800-01 | Sb01g005430.1 | MLOC_57612.1 |
| contig03352 | Bradi3g58130.1 | Os10t0400800-01 | Sb04g034020.1 | MLOC_58108.2 |
| contig01237 | Bradi4g41377.1 | Os12t0174100-01 | Sb08g004650.1 | MLOC_58443.1 |
| contig06707 | Bradi4g03890.1 | Os12t0572800-00 | Sb07g023743.1 | MLOC_59658.3 |
| contig05455 | Bradi4g40300.1 | Os12t0169800-00 | Sb08g004510.1 | MLOC_59921.1 |
| contig07544 | Bradi4g01980.1 | Os12t0611900-01 | Sb08g021190.1 | MLOC_60533.1 |
| contig23043 | Bradi4g30290.1 | Os09t0416200-01 | Sb02g024690.1 | MLOC_60555.1 |
| contig06410 | Bradi4g01090.1 | Os12t0626300-01 | Sb03g010430.1 | MLOC_60893.1 |
| contig00695 | Bradi4g37530.1 | Os09t0555600-01 | Sb02g032380.1 | MLOC_61115.1 |
| contig04245 | Bradi1g02810.1 | Os03t0832600-01 | Sb01g002480.1 | MLOC_61231.1 |
| contig05468 | Bradi4g23737.1 | Os02t0309200-00 | Sb01g015810.1 | MLOC_64045.1 |
| contig25207 | Bradi4g27470.1 | Os09t0296700-01 | Sb02g019400.1 | MLOC_65221.1 |
| contig07001 | Bradi1g00460.1 | Os07t0522500-01 | Sb01g000220.1 | MLOC_66589.2 |
| contig11015 | Bradi4g13990.1 | Os11t0685600-00 | Sb08g021870.1 | MLOC_67608.3 |
| contig16476 | Bradi4g37080.1 | Os04t0563000-01 | Sb02g031900.1 | MLOC_68176.2 |
| contig06982 | Bradi4g02747.1 | Os12t0596900-01 | Sb08g020180.1 | MLOC_68708.1 |
| contig00057 | Bradi3g06727.1 | Os02t0192400-01 | Sb04g006240.1 | MLOC_6946.1 |
| contig12533 | Bradi1g00607.1 | Os03t0860700-00 | Sb02g010040.1 | MLOC_72134.2 |
| contig08705 | Bradi1g15060.1 | Os03t0599600-01 | Sb01g015100.1 | MLOC_75498.4 |
| contig20211 | Bradi4g03050.1 | Os12t0590500-00 | Sb08g019800.1 | MLOC_75843.3 |
| contig06285 | Bradi1g06780.1 | Os03t0780900-01 | Sb01g006290.1 | MLOC_77253.1 |
| contig01344 | Bradi4g28177.1 | Os09t0327600-01 | Sb02g022180.1 | MLOC_91.1 |
| contig01741 | Bradi1g08280.1 | Os03t0755100-01 | Sb01g007710.1 | AK370611 |
| contig00446 | Bradi4g37930.1 | Os09t0560300-01 | Sb02g032740.1 | MLOC_66231.1 |
| contig02421 | Bradi4g30730.1 | Os07t0124500-01 | Sb02g028310.1 | MLOC_69668.2 |
| contig09383 | Bradi1g50380.1 | Os09t0123300-01 | Sb01g027530.1 | MLOC_64891.2 |
| contig08221 | Bradi1g10710.1 | Os03t0715400-01 | Sb01g010136.1 | AK358428 |
| contig09630 | Bradi4g36380.1 | Os09t0536000-01 | Sb02g031100.1 | AK251535.1 |
| contig14685 | Bradi4g33067.1 | Os09t0477900-01 | Sb02g027560.1 | AK374386 |
| contig00303 | Bradi1g05110.1 | Os03t0801800-01 | Sb01g004800.1 | AK365509 |
| contig01545 | Bradi1g05110.1 | Os03t0801800-01 | Sb01g004800.1 | AK365509 |
| contig17827 | Bradi1g14420.1 | Os04t0394500-00 | Sb06g014360.1 | MLOC_52622.1 |
| contig17882 | Bradi1g07760.1 | Os03t0762900-01 | Sb01g007200.1 | AK374671 |
| contig00740 | Bradi1g04120.2 | Os03t0815700-01 | Sb01g003680.1 | AK365033 |
| contig10066 | Bradi3g36960.1 | Os08t0440200-01 | Sb07g021600.1 | MLOC_38205.5 |
| contig08124 | Bradi1g03530.1 | Os03t0823100-01 | Sb01g003010.1 | MLOC_65314.2 |
| contig23698 | Bradi4g14080.1 | Os03t0597200-01 | Sb01g016560.1 | MLOC_81731.1 |
| contig00062 | Bradi4g27747.1 | Os09t0306700-01 | Sb02g020790.2 | AK252911.1 |
| contig10126 | Bradi1g69880.1 | Os10t0400100-01 | Sb01g042830.1 | MLOC_68522.2 |
| contig13542 | Bradi1g69880.1 | Os10t0400100-01 | Sb01g042830.1 | MLOC_68522.2 |
| contig01373 | Bradi4g08420.1 | Os09t0114500-01 | Sb02g013180.1 | MLOC_61378.1 |
| contig03482 | Bradi1g08900.1 | Os03t0744800-01 | Sb01g008390.1 | AK366333 |
| contig04551 | Bradi1g03440.1 | Os03t0824200-01 | Sb01g002920.1 | AK364984 |
| contig00403 | Bradi4g03800.1 | Os12t0576300-01 | Sb08g019100.1 | MLOC_20816.3 |
| contig14936 | Bradi4g02930.1 | Os12t0593000-01 | Sb08g019970.1 | AK355185 |
| contig03983 | Bradi2g05445.2 | Os05t0295100-01 | Sb08g002830.1 | AK375730 |

| | | | | |
|---|---|---|---|---|
| contig09026 | Bradi1g03730.1 | Os03t0820900-01 | Sb01g003230.1 | MLOC_51706.2 |
| contig04718 | Bradi2g61320.1 | Os01t0958100-01 | Sb03g046390.1 | AK358057 |
| contig19390 | Bradi1g12820.1 | Os03t0669200-01 | Sb01g012370.1 | AK251844.1 |
| contig22379 | Bradi1g12820.1 | Os03t0669200-01 | Sb01g012370.1 | AK251844.1 |
| contig08325 | Bradi4g04650.1 | Os12t0564600-01 | Sb08g018400.1 | MLOC_1444.1 |
| contig01642 | Bradi1g04140.1 | Os03t0815200-01 | Sb01g003700.1 | AK250029.1 |
| contig00496 | Bradi1g01530.1 | Os02t0234800-01 | Sb01g001040.1 | AK368609 |
| contig00684 | Bradi4g27880.1 | Os09t0314400-01 | Sb02g021050.1 | AK362216 |
| contig12395 | Bradi1g08294.1 | Os03t0754900-02 | Sb01g007730.1 | MLOC_54479.1 |
| contig03417 | Bradi1g04980.1 | Os03t0803800-01 | Sb01g004640.1 | MLOC_37586.1 |
| contig03606 | Bradi4g32520.1 | Os09t0465500-01 | Sb02g026990.1 | MLOC_1496.4 |
| contig09037 | Bradi3g34646.1 | Os02t0296800-01 | Sb04g012200.1 | MLOC_6069.1 |
| contig00842 | Bradi1g08500.1 | Os12t0590500-00 | Sb01g007970.1 | MLOC_58630.2 |
| contig10429 | Bradi4g01980.1 | Os12t0611900-01 | Sb08g021190.1 | MLOC_60533.1 |
| contig08138 | Bradi3g36190.1 | Os01t0896300-00 | Sb03g042580.1 | MLOC_67418.1 |
| contig07595 | Bradi4g31640.1 | Os09t0446200-01 | Sb02g026125.1 | MLOC_45041.3 |
| contig10521 | Bradi4g37627.2 | Os09t0556700-01 | Sb02g032470.1 | MLOC_54619.1 |
| contig07347 | Bradi4g04580.1 | Os12t0563500-01 | Sb08g018340.1 | AK250022.1 |
| contig17931 | Bradi4g07990.2 | Os12t0429200-01 | Sb08g004410.1 | AK252929.1 |
| contig02460 | Bradi4g45240.1 | Os11t0102800-00 | Sb05g000270.1 | MLOC_55862.2 |
| contig04893 | Bradi4g44667.2 | Os12t0110000-00 | Sb05g000820.1 | MLOC_43149.1 |
| contig00356 | Bradi4g03737.1 | Os12t0577100-01 | Sb08g019140.1 | AK250638.1 |
| contig13793 | Bradi2g60817.1 | Os01t0951000-01 | Sb03g045910.1 | AK370672 |
| contig00186 | Bradi2g60817.1 | Os01t0951000-02 | Sb03g045910.1 | AK370672 |
| contig14435 | Bradi4g27580.2 | Os09t0298400-01 | Sb02g020440.1 | AK251945.1 |
| contig19385 | Bradi2g18270.1 | Os09t0241100-01 | Sb10g019730.1 | MLOC_36749.1 |
| contig03206 | Bradi1g07540.1 | Os03t0765800-00 | Sb01g007060.1 | AK367148 |
| contig03714 | Bradi1g07520.1 | Os03t0766000-01 | Sb01g007040.1 | AK354633 |
| contig05302 | Bradi4g35150.1 | Os09t0515100-02 | Sb02g029830.1 | MLOC_16969.1 |
| contig21297 | Bradi1g00580.1 | Os07t0438500-01 | Sb01g000310.1 | MLOC_44779.2 |
| contig17641 | Bradi1g00480.2 | Os03t0862100-01 | Sb01g000240.1 | MLOC_21595.1 |
| contig22538 | Bradi1g00480.2 | Os03t0862100-01 | Sb01g000240.1 | MLOC_21595.1 |
| contig22592 | Bradi1g00480.2 | Os03t0862100-01 | Sb01g000240.1 | MLOC_21595.1 |
| contig13645 | Bradi1g08727.1 | Os03t0747600-02 | Sb01g008195.1 | AK374936 |
| contig06268 | Bradi1g03380.1 | Os03t0825700-01 | Sb01g002810.1 | MLOC_55007.1 |
| contig09730 | Bradi1g03380.1 | Os03t0825700-01 | Sb01g002810.1 | MLOC_55007.1 |
| contig05050 | Bradi1g48264.1 | Os06t0167000-02 | Sb10g004680.1 | MLOC_39065.1 |
| contig03262 | Bradi4g08567.1 | Os09t0247700-01 | Sb07g010440.1 | MLOC_72899.1 |
| contig02071 | Bradi1g51817.1 | Os05t0447500-00 | Sb10g000410.1 | MLOC_14130.2 |
| contig00007 | Bradi1g17710.1 | Os02t0167700-01 | Sb04g004540.1 | MLOC_67779.1 |
| contig14524 | Bradi1g02710.1 | Os03t0833900-02 | Sb02g007460.1 | AK367079 |
| contig14401 | Bradi2g49650.1 | Os02t0816500-01 | Sb04g037170.1 | MLOC_74122.3 |
| contig04746 | Bradi4g30450.1 | Os09t0420600-01 | Sb02g024860.1 | AK251930.1 |
| contig04538 | Bradi4g29887.1 | Os09t0407600-01 | Sb02g024370.1 | MLOC_81241.5 |
| contig02372 | Bradi4g04370.1 | Os12t0566400-01 | Sb08g018490.1 | AK369426 |
| contig05343 | Bradi4g28137.1 | Os09t0327200-00 | Sb02g022150.1 | MLOC_12838.1 |
| contig11516 | Bradi4g35000.1 | Os09t0513100-02 | Sb02g029710.1 | AK369723 |

| | | | | |
|---|---|---|---|---|
| contig02701 | Bradi1g08470.1 | Os03t0750800-01 | Sb01g007950.1 | MLOC_34938.1 |
| contig02536 | Bradi4g29480.1 | Os09t0382400-01 | Sb02g023810.1 | AK355874 |
| contig06884 | Bradi4g00930.1 | Os02t0327100-01 | Sb01g012290.1 | AK354972 |
| contig10798 | Bradi4g05917.1 | Os12t0533800-01 | Sb08g016960.1 | MLOC_12056.9 |
| contig02156 | Bradi2g39790.1 | Os05t0105000-01 | Sb09g000490.1 | MLOC_56508.1 |
| contig03208 | Bradi4g06340.1 | Os12t0514900-01 | Sb08g016580.1 | MLOC_58476.1 |
| contig17080 | Bradi1g67110.1 | Os03t0266000-01 | Sb01g040060.1 | MLOC_13648.1 |
| contig04811 | Bradi4g02070.1 | Os12t0610500-01 | Sb08g021070.1 | MLOC_52221.3 |
| contig00812 | Bradi4g19590.1 | Os11t0447300-01 | Sb01g021800.1 | AK356819 |
| contig00889 | Bradi4g19590.1 | Os11t0447300-01 | Sb01g021800.1 | AK356819 |
| contig05344 | Bradi4g41520.1 | Os12t0170600-01 | Sb08g004540.1 | AK358200 |
| contig14582 | Bradi1g14220.1 | Os03t0633900-01 | Sb01g014110.1 | MLOC_58813.1 |
| contig07126 | Bradi3g36960.1 | Os08t0440200-01 | Sb07g021600.1 | MLOC_38205.5 |
| contig01976 | Bradi3g13670.1 | Os08t0114100-01 | Sb07g001450.1 | MLOC_18553.1 |
| contig01851 | Bradi4g43780.1 | Os06t0715000-01 | Sb08g001600.1 | MLOC_34755.2 |
| contig04005 | Bradi4g35087.1 | Os09t0514100-02 | Sb02g029750.1 | AK360105 |
| contig16864 | Bradi1g08217.1 | Os03t0756400-01 | Sb01g007660.1 | AK370781 |
| contig12024 | Bradi2g59260.1 | Os01t0911000-01 | Sb03g043440.1 | MLOC_62438.1 |
| contig04018 | Bradi1g08040.1 | Os03t0758400-01 | Sb01g007540.1 | MLOC_37035.1 |
| contig05664 | Bradi4g30210.1 | Os09t0413700-01 | Sb02g024630.1 | AK354786 |
| contig02283 | Bradi4g02260.1 | Os04t0606800-01 | Sb06g027850.1 | MLOC_6566.2 |
| contig06673 | Bradi1g04060.1 | Os03t0816400-02 | Sb01g003620.1 | MLOC_17833.1 |
| contig07407 | Bradi1g07640.1 | Os03t0764450-00 | Sb01g007140.1 | AK249847.1 |
| contig10150 | Bradi4g30897.1 | Os09t0431600-00 | Sb02g025370.1 | MLOC_69213.1 |
| contig07210 | Bradi1g06640.1 | Os03t0783000-01 | Sb01g020520.1 | AK371401 |
| contig21163 | Bradi4g27620.1 | Os09t0299200-01 | Sb02g020700.1 | MLOC_60074.6 |
| contig08028 | Bradi4g35540.2 | Os09t0521500-01 | Sb02g030280.1 | AK363819 |
| contig00148 | Bradi4g19560.1 | Os11t0425300-01 | Sb01g003450.1 | MLOC_4066.1 |
| contig20883 | Bradi1g03170.2 | Os03t0827600-02 | Sb01g002680.1 | MLOC_71913.1 |
| contig21300 | Bradi1g03170.2 | Os03t0827600-02 | Sb01g002680.1 | MLOC_71913.1 |
| contig13188 | Bradi1g13930.1 | Os03t0640100-01 | Sb01g013730.1 | AK374173 |
| contig22099 | Bradi1g71000.1 | Os03t0202300-01 | Sb01g043670.1 | MLOC_20830.1 |
| contig21967 | Bradi1g02810.1 | Os03t0832600-01 | Sb01g002480.1 | MLOC_61231.1 |
| contig00290 | Bradi4g01080.1 | Os12t0163200-01 | Sb05g002340.1 | AK248684.1 |
| contig01651 | Bradi4g41717.1 | Os12t0166500-01 | Sb08g004180.1 | MLOC_54805.1 |
| contig06654 | Bradi4g01810.1 | Os03t0625900-01 | Sb01g013700.1 | AK372511 |
| contig05806 | Bradi4g39110.1 | Os12t0283800-01 | Sb08g011730.1 | AK248573.1 |
| contig10780 | Bradi4g41470.1 | Os12t0171200-01 | Sb08g004570.2 | AK353972 |
| contig10903 | Bradi3g17630.1 | Os08t0200400-01 | Sb07g005630.1 | MLOC_55540.1 |
| contig25395 | Bradi3g54980.2 | Os02t0812400-01 | Sb04g036790.1 | MLOC_53556.2 |
| contig04942 | Bradi4g01090.1 | Os12t0626300-01 | Sb03g010430.1 | MLOC_60893.1 |
| contig02408 | Bradi4g03050.1 | Os12t0590500-00 | Sb08g019800.1 | MLOC_75843.3 |
| contig00042 | Bradi4g27057.1 | Os09t0279600-01 | Sb02g018660.1 | MLOC_36393.1 |
| contig00920 | Bradi4g27057.1 | Os09t0279600-01 | Sb02g018660.1 | MLOC_36393.1 |
| contig00800 | Bradi3g37010.1 | Os08t0439100-01 | Sb07g021510.1 | AK248279.1 |
| contig01721 | Bradi4g34290.1 | Os09t0500600-01 | Sb02g029000.1 | MLOC_17646.5 |
| contig06412 | Bradi1g09200.1 | Os03t0740700-01 | Sb01g008600.1 | AK369711 |

| | | | | |
|---|---|---|---|---|
| contig07611 | Bradi1g71190.1 | Os03t0200600-00 | Sb01g043840.1 | AK250731.1 |
| contig01053 | Bradi4g30660.1 | Os09t0424600-01 | Sb02g025130.1 | AK369445 |
| contig05141 | Bradi4g30660.1 | Os09t0424600-01 | Sb02g025130.1 | AK369445 |
| contig07826 | Bradi4g30660.1 | Os09t0424600-01 | Sb02g025130.1 | AK369445 |
| contig17054 | Bradi4g30660.1 | Os09t0424600-01 | Sb02g025130.1 | AK369445 |
| contig23394 | Bradi4g30660.1 | Os09t0424600-01 | Sb02g025130.1 | AK369445 |
| contig02306 | Bradi1g15440.1 | Os03t0583900-01 | Sb01g015670.1 | MLOC_53494.2 |
| contig04183 | Bradi1g64770.1 | Os11t0672400-00 | Sb01g037790.1 | MLOC_65526.2 |
| contig10143 | Bradi3g15520.1 | Os08t0178100-01 | Sb07g004940.1 | AK371182 |
| contig03898 | Bradi4g41777.1 | Os12t0165000-01 | Sb08g004050.2 | AK366349 |
| contig04196 | Bradi1g64420.1 | Os03t0308200-01 | Sb01g037420.1 | MLOC_12000.2 |
| contig03157 | Bradi3g32780.1 | Os10t0548200-01 | Sb01g029925.1 | MLOC_38151.4 |
| contig03691 | Bradi1g00887.1 | Os03t0858100-01 | Sb01g000490.1 | MLOC_81454.2 |
| contig09304 | Bradi1g63160.1 | Os03t0336300-01 | Sb01g036110.1 | AK364634 |
| contig07762 | Bradi4g38357.1 | Os09t0565500-01 | Sb02g011430.1 | MLOC_53435.4 |
| contig13418 | Bradi3g53420.1 | Os02t0717400-01 | Sb04g029850.1 | AK363507 |
| contig08643 | Bradi4g29947.1 | Os09t0410300-01 | Sb02g024440.1 | MLOC_68788.2 |
| contig12262 | Bradi4g26877.1 | Os11t0104900-00 | Sb05g000450.1 | MLOC_57760.2 |
| contig19455 | Bradi4g37530.1 | Os09t0555600-01 | Sb02g032380.1 | MLOC_61195.1 |
| contig05295 | Bradi4g21680.2 | Os11t0240900-01 | Sb05g007260.1 | AK375138 |
| contig02556 | Bradi1g36840.1 | Os02t0205400-01 | Sb04g007180.1 | MLOC_60900.1 |
| contig14809 | Bradi4g06087.1 | Os11t0642800-01 | Sb05g025630.1 | AK354495 |
| contig04812 | Bradi4g40560.1 | Os12t0194800-01 | Sb08g005650.1 | MLOC_67682.1 |
| contig04713 | Bradi1g02630.1 | Os03t0835400-01 | Sb01g002210.1 | AK251444.1 |
| contig09888 | Bradi1g02490.1 | Os03t0837300-02 | Sb01g002020.1 | MLOC_72184.1 |
| contig10110 | Bradi1g00760.1 | Os03t0859500-01 | Sb01g000400.1 | MLOC_69405.3 |
| contig04286 | Bradi4g34290.1 | Os09t0500600-01 | Sb02g029000.1 | MLOC_17646.5 |
| contig20962 | Bradi4g34290.1 | Os09t0500600-01 | Sb02g029000.1 | MLOC_17646.5 |
| contig07249 | Bradi1g05170.1 | Os03t0800100-01 | Sb01g004930.1 | MLOC_21567.1 |
| contig19758 | Bradi1g05170.1 | Os03t0800100-01 | Sb01g004930.1 | MLOC_21567.1 |
| contig12255 | Bradi1g05170.1 | Os04t0558200-00 | Sb01g004930.1 | MLOC_21567.1 |
| contig05544 | Bradi4g05710.1 | Os12t0538300-01 | Sb01g044460.1 | MLOC_11333.2 |
| contig00469 | Bradi1g08280.1 | Os03t0755100-01 | Sb01g007710.1 | MLOC_10398.1 |
| contig01397 | Bradi4g36000.1 | Os02t0271000-01 | Sb02g030760.1 | MLOC_55045.1 |
| contig02471 | Bradi1g15360.1 | Os03t0586900-01 | Sb01g015520.1 | AK364297 |
| contig06286 | Bradi4g05550.1 | Os10t0170300-01 | Sb03g009620.1 | MLOC_11318.2 |
| contig01327 | Bradi3g39580.2 | Os07t0435400-01 | Sb03g013640.1 | MLOC_58976.1 |
| contig23014 | Bradi3g39580.2 | Os07t0435400-01 | Sb03g013640.1 | MLOC_58976.1 |
| contig01705 | Bradi4g30490.1 | Os09t0421200-01 | Sb02g024900.1 | AK370249 |
| contig06653 | Bradi4g38140.1 | Os07t0134500-01 | Sb02g011280.1 | MLOC_21732.1 |
| contig11541 | Bradi1g06380.1 | Os03t0786900-01 | Sb01g005910.1 | MLOC_14680.1 |
| contig25501 | Bradi1g05660.1 | Os03t0794800-01 | Sb01g005340.1 | AK354390 |
| contig01764 | Bradi4g08907.3 | Os09t0266400-01 | Sb02g015540.1 | MLOC_4863.2 |
| contig04522 | Bradi4g08907.3 | Os09t0266400-01 | Sb02g015540.1 | MLOC_4863.2 |
| contig08945 | Bradi4g08907.3 | Os09t0266400-01 | Sb02g015540.1 | MLOC_4863.2 |
| contig00430 | Bradi4g08907.2 | Os09t0266400-01 | Sb02g015550.1 | MLOC_54822.1 |
| contig07015 | Bradi1g70057.1 | Os03t0213400-01 | Sb01g042980.1 | MLOC_5747.2 |

| | | | | |
|---|---|---|---|---|
| contig20297 | Bradi1g70057.1 | Os03t0213400-01 | Sb01g042980.1 | MLOC_5747.2 |
| contig08417 | Bradi1g60890.1 | Os03t0387000-01 | Sb01g033760.1 | MLOC_57506.1 |
| contig06747 | Bradi4g43517.3 | Os12t0134200-01 | Sb05g002180.1 | MLOC_38053.1 |
| contig08491 | Bradi1g64940.2 | Os03t0300200-01 | Sb01g037990.1 | MLOC_6079.1 |
| contig02817 | Bradi4g31947.1 | Os09t0453700-01 | Sb02g026420.1 | MLOC_26365.1 |
| contig14499 | Bradi1g64810.1 | Os03t0301400-01 | Sb01g037840.1 | AK376401 |
| contig02356 | Bradi4g28160.1 | Os09t0327400-01 | Sb02g022170.1 | MLOC_65636.1 |
| contig24015 | Bradi4g28160.1 | Os09t0327400-01 | Sb02g022170.1 | MLOC_65636.1 |
| contig12431 | Bradi4g03890.1 | Os12t0572800-00 | Sb08g018890.1 | MLOC_59658.3 |
| contig00179 | Bradi1g55300.1 | Os01t0376700-01 | Sb04g020180.1 | MLOC_6173.2 |
| contig00972 | Bradi4g37610.1 | Os09t0556400-01 | Sb02g032440.1 | AK373795 |
| contig00706 | Bradi4g31830.1 | Os09t0451500-01 | Sb02g026300.1 | MLOC_34487.1 |
| contig07356 | Bradi4g02970.1 | Os04t0619500-01 | Sb01g004350.1 | MLOC_34492.1 |
| contig15492 | Bradi1g53085.1 | Os07t0260400-01 | Sb02g008130.1 | MLOC_77526.5 |
| contig11661 | Bradi4g29760.1 | Os09t0397900-01 | Sb03g028040.1 | MLOC_55056.1 |
| contig04284 | Bradi4g05570.1 | Os12t0540900-01 | Sb08g017220.1 | AK361994 |
| contig02453 | Bradi1g08917.1 | Os03t0744675-00 | Sb01g008410.1 | AK370634 |
| contig06302 | Bradi1g08917.1 | Os03t0744675-00 | Sb01g008410.1 | AK370634 |
| contig02466 | Bradi2g27440.1 | Os05t0370000-01 | Sb09g018390.1 | MLOC_4382.1 |
| contig10051 | Bradi4g01035.1 | Os12t0630700-01 | Sb08g022550.1 | MLOC_53665.2 |
| contig20652 | Bradi4g30040.1 | Os09t0411650-01 | Sb02g024510.1 | AK358982 |
| contig13837 | Bradi1g49860.1 | Os06t0111100-01 | Sb05g001110.1 | MLOC_76661.2 |
| contig02063 | Bradi4g07990.2 | Os12t0429200-01 | Sb08g004410.1 | AK252929.1 |
| contig23226 | Bradi1g01140.1 | Os03t0852900-01 | Sb01g000850.1 | MLOC_71240.1 |
| contig01198 | Bradi4g30030.1 | Os09t0417500-01 | Sb02g024500.1 | MLOC_68565.1 |
| contig15873 | Bradi1g09250.1 | Os03t0738700-01 | Sb01g008680.1 | MLOC_17465.1 |
| contig03493 | Bradi4g37530.1 | Os09t0555600-01 | Sb02g032380.1 | MLOC_61195.1 |
| contig07389 | Bradi4g28170.1 | Os09t0327550-00 | Sb02g022180.1 | MLOC_65634.1 |
| contig22070 | Bradi4g27470.1 | Os09t0296700-01 | Sb02g019400.1 | MLOC_65221.1 |
| contig09029 | Bradi4g26390.1 | Os11t0134500-01 | Sb08g001420.1 | MLOC_4314.2 |
| contig05735 | Bradi2g15260.1 | Os05t0581800-01 | Sb09g029670.1 | AK363158 |
| contig20087 | Bradi1g05162.1 | Os03t0789466-00 | Sb01g004920.1 | AK374742 |
| contig07498 | Bradi1g01990.2 | Os03t0843700-01 | Sb01g001615.1 | MLOC_4235.4 |
| contig14629 | Bradi3g58130.1 | Os10t0400800-01 | Sb04g034020.1 | MLOC_64084.2 |
| contig02645 | Bradi1g64800.1 | Os03t0301500-00 | Sb01g037830.1 | MLOC_6489.1 |
| contig09976 | Bradi4g27070.3 | Os09t0280300-01 | Sb02g018670.1 | AK249769.1 |
| contig01468 | Bradi1g29690.1 | Os06t0728600-00 | Sb10g030940.1 | AK376429 |
| contig11562 | Bradi4g33067.1 | Os09t0477900-01 | Sb02g027560.1 | AK374386 |
| contig05897 | Bradi1g07550.1 | Os03t0765400-02 | Sb01g007070.1 | MLOC_56550.1 |
| contig01948 | Bradi4g03380.2 | Os12t0583000-01 | Sb08g019540.1 | MLOC_7254.4 |
| contig09850 | Bradi1g15920.1 | Os03t0432900-01 | Sb01g031710.1 | AK371709 |
| contig16260 | Bradi4g03050.1 | Os12t0590500-00 | Sb08g019800.1 | MLOC_75843.3 |
| contig17878 | Bradi4g03050.1 | Os12t0590500-00 | Sb08g019800.1 | MLOC_75843.3 |
| contig01596 | Bradi4g03050.1 | Os12t0590500-00 | Sb08g019810.1 | MLOC_75843.3 |
| contig16905 | Bradi3g55920.1 | Os02t0824000-00 | Sb04g037860.1 | AK368575 |
| contig26219 | Bradi2g05460.1 | Os01t0185200-03 | Sb03g003420.1 | MLOC_8121.1 |
| contig00049 | Bradi3g43700.1 | Os02t0495900-01 | Sb04g020040.1 | MLOC_22146.1 |

| | | | | |
|---|---|---|---|---|
| contig00438 | Bradi2g19800.1 | Os03t0824400-01 | Sb01g002910.1 | AK248785.1 |
| contig00604 | Bradi4g08520.1 | Os09t0240500-01 | Sb02g013390.1 | AK250156.1 |
| contig19468 | Bradi4g36160.2 | Os09t0533400-01 | Sb02g030960.1 | MLOC_71112.1 |
| contig13601 | Bradi1g08780.1 | Os03t0747200-01 | Sb01g008230.1 | MLOC_52950.1 |
| contig00756 | Bradi1g05110.1 | Os03t0801800-01 | Sb01g004800.1 | AK365509 |
| contig00182 | Bradi1g04292.1 | Os03t0811200-01 | Sb01g003850.1 | MLOC_73693.1 |
| contig11631 | Bradi4g01035.1 | Os12t0630700-01 | Sb08g022550.1 | MLOC_53665.2 |
| contig12933 | Bradi4g30730.1 | Os07t0124500-01 | Sb02g028310.1 | MLOC_69668.2 |
| contig14665 | Bradi4g02390.1 | Os12t0605500-01 | Sb08g020800.1 | AK354434 |
| contig13212 | Bradi1g07190.1 | Os03t0773600-01 | Sb01g006700.1 | AK249476.1 |
| contig11747 | Bradi4g03380.2 | Os12t0583000-01 | Sb08g019540.1 | MLOC_7254.4 |
| contig18302 | Bradi4g06800.1 | Os12t0502500-01 | Sb08g015600.1 | AK250553.1 |
| contig01002 | Bradi1g01847.1 | Os03t0845500-01 | Sb01g001450.1 | MLOC_17240.4 |
| contig22949 | Bradi4g43257.1 | Os11t0140700-02 | Sb05g002420.1 | MLOC_53116.5 |
| contig02657 | Bradi1g01530.1 | Os02t0234800-01 | Sb01g001040.1 | AK368609 |
| contig00299 | Bradi4g02607.1 | Os12t0601200-01 | Sb08g020570.1 | MLOC_73035.1 |
| contig02329 | Bradi4g04580.1 | Os12t0563500-01 | Sb08g018340.1 | AK250022.1 |
| contig02017 | Bradi4g05600.1 | Os12t0538900-01 | Sb08g017190.1 | MLOC_64519.1 |
| contig01473 | Bradi4g27580.2 | Os09t0298400-01 | Sb02g020440.1 | AK251945.1 |
| contig13783 | Bradi1g15110.1 | Os03t0598200-01 | Sb01g015150.1 | MLOC_55271.1 |
| contig03239 | Bradi1g15110.1 | Os03t0598200-01 | Sb01g015150.1 | MLOC_55271.1 |
| contig10633 | Bradi1g08727.1 | Os03t0747600-02 | Sb01g008195.1 | AK374936 |
| contig10968 | Bradi1g08727.1 | Os03t0747600-02 | Sb01g008195.1 | AK374936 |
| contig04379 | Bradi1g48264.1 | Os06t0167000-02 | Sb10g004680.1 | MLOC_44147.1 |
| contig18943 | Bradi4g08527.1 | Os09t0121050-00 | Sb02g019360.1 | MLOC_11195.1 |
| contig06637 | Bradi4g27480.3 | Os09t0296800-01 | Sb02g020290.1 | AK368229 |
| contig01453 | Bradi1g01290.1 | Os03t0852200-01 | Sb01g000910.2 | MLOC_57149.1 |
| contig05363 | Bradi4g00980.1 | Os12t0631200-01 | Sb08g022690.1 | MLOC_54463.1 |
| contig18244 | Bradi1g64810.1 | Os03t0301400-01 | Sb01g037840.1 | AK376401 |
| contig03083 | Bradi3g35517.1 | Os08t0407000-01 | Sb07g020060.1 | MLOC_28103.2 |
| contig09432 | Bradi4g44844.1 | Os11t0106800-00 | Sb05g000540.1 | MLOC_65713.1 |
| contig11672 | Bradi4g02747.1 | Os12t0596900-01 | Sb08g020180.1 | MLOC_68708.1 |
| contig00611 | Bradi1g07820.1 | Os03t0761900-01 | Sb01g007280.1 | AK367064 |
| contig03729 | Bradi4g41650.1 | Os12t0168000-01 | Sb08g004240.1 | AK367116 |
| contig09889 | Bradi1g04097.1 | Os03t0815900-01 | Sb01g003660.1 | AK361961 |
| contig04147 | Bradi1g03170.2 | Os03t0827600-02 | Sb01g002680.1 | MLOC_71913.1 |
| contig22537 | Bradi1g77830.1 | Os09t0314300-02 | Sb02g021030.1 | AK375914 |
| contig00646 | Bradi1g13930.1 | Os03t0640100-01 | Sb01g013730.1 | AK374173 |
| contig05070 | Bradi1g13930.1 | Os03t0640100-01 | Sb01g013730.1 | AK374173 |
| contig13941 | Bradi4g35257.1 | Os09t0516500-00 | Sb02g029890.1 | AK361767 |
| contig02360 | Bradi4g08860.1 | Os09t0251100-00 | Sb03g013430.1 | MLOC_76583.1 |
| contig04151 | Bradi1g01990.2 | Os03t0843700-01 | Sb01g001615.1 | MLOC_4235.4 |
| contig01336 | Bradi4g42817.1 | Os11t0148400-01 | Sb08g002880.1 | MLOC_69000.1 |
| contig02836 | Bradi3g37010.1 | Os08t0439100-01 | Sb07g021510.1 | AK248279.1 |
| contig09990 | Bradi4g34290.1 | Os09t0500600-01 | Sb02g029000.1 | MLOC_17646.5 |
| contig21115 | Bradi1g01007.1 | Os03t0856200-03 | Sb01g000620.1 | MLOC_49689.2 |
| contig05270 | Bradi1g01007.1 | Os03t0856200-04 | Sb01g000620.1 | MLOC_49689.2 |

| | | | | |
|---|---|---|---|---|
| contig00530 | Bradi4g07110.1 | Os04t0348300-01 | Sb03g044450.1 | MLOC_68734.1 |
| contig20899 | Bradi4g41960.1 | Os12t0160900-01 | Sb08g003780.1 | AK358485 |
| contig09421 | Bradi3g15520.1 | Os08t0178100-01 | Sb07g004940.1 | AK371182 |
| contig08264 | Bradi1g00887.1 | Os03t0858100-01 | Sb01g000490.1 | MLOC_81454.2 |
| contig06133 | Bradi4g38357.1 | Os09t0565500-01 | Sb02g011420.1 | MLOC_53435.4 |
| contig16726 | Bradi1g34537.1 | Os06t0704900-01 | Sb04g036220.1 | MLOC_6404.1 |
| contig11852 | Bradi1g08900.1 | Os03t0744800-01 | Sb01g008390.1 | AK366333 |
| contig25689 | Bradi1g00847.1 | Os03t0858400-01 | Sb01g000460.1 | AK366863 |
| contig09689 | Bradi1g00847.1 | Os03t0858400-01 | Sb01g000460.1 | AK366863 |
| contig04374 | Bradi4g32790.1 | Os09t0470900-01 | Sb02g027310.1 | AK376040 |
| contig05462 | Bradi1g02490.1 | Os03t0837300-02 | Sb01g002020.1 | MLOC_72184.1 |
| contig11197 | Bradi3g28720.1 | Os10t0476300-02 | Sb01g019390.1 | MLOC_72258.1 |
| contig04834 | Bradi1g62330.1 | Os03t0352400-01 | Sb01g035280.1 | MLOC_54468.1 |
| contig04977 | Bradi4g31227.1 | Os09t0439000-00 | Sb02g025710.1 | MLOC_54452.3 |
| contig02641 | Bradi3g45660.1 | Os12t0562500-00 | Sb05g026530.1 | MLOC_63199.6 |
| contig11376 | Bradi4g36050.1 | Os09t0531900-01 | Sb02g030820.1 | AK355001 |
| contig13989 | Bradi4g28177.1 | Os09t0327575-00 | Sb02g022180.1 | MLOC_91.1 |
| contig02161 | Bradi4g05550.1 | Os10t0170300-01 | Sb03g009620.1 | MLOC_11318.2 |
| contig07196 | Bradi4g39550.1 | Os12t0238100-01 | Sb08g008650.1 | MLOC_82054.2 |
| contig00832 | Bradi4g08907.3 | Os09t0266400-01 | Sb02g015545.1 | MLOC_4863.2 |
| contig15563 | Bradi4g08907.3 | Os09t0266400-01 | Sb02g015540.1 | MLOC_4863.2 |
| contig08828 | Bradi4g05110.2 | Os12t0554400-01 | Sb08g017700.1 | AK252415.1 |
| contig02512 | Bradi4g31060.1 | Os09t0436800-01 | Sb02g025575.1 | MLOC_38528.2 |
| contig08427 | Bradi2g39790.1 | Os05t0105000-01 | Sb09g000490.1 | MLOC_56508.1 |
| contig10518 | Bradi1g04397.1 | Os01t0513900-01 | Sb10g001010.1 | MLOC_38381.1 |
| contig06293 | Bradi4g02910.1 | Os12t0594200-00 | Sb08g020025.1 | MLOC_36925.1 |
| contig09570 | Bradi1g53590.1 | Os07t0240200-01 | Sb02g007050.1 | MLOC_16913.1 |
| contig09580 | - | Os12t0613600-01 | - | - |
| contig18057 | - | Os12t0613600-01 | - | - |
| contig01282 | - | Os12t0612700-01 | - | - |
| contig23909 | - | Os12t0573900-01 | - | - |
| contig00794 | - | Os12t0564800-01 | - | - |
| contig04214 | - | Os12t0539700-01 | - | - |
| contig16371 | - | Os12t0533600-01 | - | - |
| contig06699 | - | Os12t0431300-01 | - | - |
| contig04647 | - | Os12t0279000-01 | - | - |
| contig07763 | - | Os12t0166500-01 | - | - |
| contig21646 | - | Os11t0681300-00 | - | - |
| contig15558 | - | Os11t0673900-00 | - | - |
| contig10700 | - | Os11t0637300-00 | - | - |
| contig11498 | - | Os11t0593500-01 | - | - |
| contig01526 | - | Os11t0557400-01 | - | - |
| contig16738 | - | Os11t0213800-01 | - | - |
| contig26512 | - | Os11t0213800-01 | - | - |
| contig04184 | - | Os11t0213700-00 | - | - |
| contig06409 | - | Os11t0212300-00 | - | - |
| contig11719 | - | Os11t0199600-02 | - | - |

| | | | | |
|---|---|---|---|---|
| contig02565 | - | Os10t0490666-00 | - | - |
| contig05220 | - | Os10t0351414-00 | - | - |
| contig21684 | - | Os10t0136400-00 | - | - |
| contig01183 | - | Os10t0129100-01 | - | - |
| contig03799 | - | Os09t0565300-01 | - | - |
| contig19857 | - | Os09t0561600-01 | - | - |
| contig16588 | - | Os09t0551900-01 | - | - |
| contig08831 | - | Os09t0540300-00 | - | - |
| contig12700 | - | Os09t0511900-01 | - | - |
| contig09088 | - | Os09t0494800-00 | - | - |
| contig14751 | - | Os09t0442400-01 | - | - |
| contig15290 | - | Os09t0433900-01 | - | - |
| contig20923 | - | Os09t0428900-01 | - | - |
| contig03474 | - | Os09t0428300-00 | - | - |
| contig15340 | - | Os09t0343200-01 | - | - |
| contig23047 | - | Os09t0268600-01 | - | - |
| contig11005 | - | Os09t0246700-01 | - | - |
| contig07529 | - | Os09t0124800-01 | - | - |
| contig12607 | - | Os08t0542200-01 | - | - |
| contig08605 | - | Os08t0528900-01 | - | - |
| contig02921 | - | Os08t0508800-01 | - | - |
| contig09868 | - | Os08t0494400-01 | - | - |
| contig08675 | - | Os08t0324300-01 | - | - |
| contig01607 | - | Os08t0320200-02 | - | - |
| contig13845 | - | Os08t0234050-00 | - | - |
| contig03927 | - | Os07t0569800-01 | - | - |
| contig15545 | - | Os07t0466500-00 | - | - |
| contig06029 | - | Os07t0422700-00 | - | - |
| contig00978 | - | Os07t0421000-01 | - | - |
| contig00418 | - | Os07t0238800-01 | - | - |
| contig05058 | - | Os07t0238800-01 | - | - |
| contig07855 | - | Os07t0107800-01 | - | - |
| contig00415 | - | Os07t0103000-01 | - | - |
| contig18974 | - | Os06t0662000-01 | - | - |
| contig19747 | - | Os06t0662000-01 | - | - |
| contig05006 | - | Os06t0622300-03 | - | - |
| contig06983 | - | Os06t0484800-00 | - | - |
| contig07683 | - | Os06t0484800-00 | - | - |
| contig09542 | - | Os06t0484800-00 | - | - |
| contig12252 | - | Os06t0484800-00 | - | - |
| contig15834 | - | Os06t0484800-00 | - | - |
| contig21003 | - | Os06t0484800-00 | - | - |
| contig21272 | - | Os06t0484800-00 | - | - |
| contig16939 | - | Os06t0301000-01 | - | - |
| contig07049 | - | Os06t0255700-01 | - | - |
| contig12357 | - | Os06t0205337-00 | - | - |
| contig16566 | - | Os06t0148200-01 | - | - |

| | | | | |
|---|---|---|---|---|
| contig20975 | - | Os06t0143900-01 | - | - |
| contig24730 | - | Os06t0143900-01 | - | - |
| contig02427 | - | Os06t0102900-01 | - | - |
| contig15596 | - | Os05t0345900-00 | - | - |
| contig06770 | - | Os05t0326675-00 | - | - |
| contig02851 | - | Os05t0276100-01 | - | - |
| contig18432 | - | Os05t0250700-00 | - | - |
| contig00660 | - | Os04t0632600-00 | - | - |
| contig06094 | - | Os04t0620950-01 | - | - |
| contig01360 | - | Os04t0618700-01 | - | - |
| contig13859 | - | Os04t0566100-00 | - | - |
| contig14295 | - | Os04t0543200-01 | - | - |
| contig12926 | - | Os04t0452300-00 | - | - |
| contig01065 | - | Os04t0204200-01 | - | - |
| contig04011 | - | Os04t0152975-00 | - | - |
| contig10375 | - | Os04t0121300-00 | - | - |
| contig00520 | - | Os04t0115650-00 | - | - |
| contig04248 | - | Os04t0113000-01 | - | - |
| contig06351 | - | Os03t0831100-01 | - | - |
| contig15729 | - | Os03t0756000-01 | - | - |
| contig14801 | - | Os03t0652400-00 | - | - |
| contig04868 | - | Os03t0639700-01 | - | - |
| contig08469 | - | Os03t0636800-02 | - | - |
| contig00501 | - | Os03t0583900-01 | - | - |
| contig20185 | - | Os03t0421000-01 | - | - |
| contig09154 | - | Os03t0259900-01 | - | - |
| contig12871 | - | Os03t0191000-01 | - | - |
| contig06609 | - | Os03t0130350-00 | - | - |
| contig20034 | - | Os03t0109600-02 | - | - |
| contig13711 | - | Os02t0825500-01 | - | - |
| contig02815 | - | Os02t0807200-00 | - | - |
| contig14504 | - | Os02t0672200-01 | - | - |
| contig02371 | - | Os02t0650800-00 | - | - |
| contig04393 | - | Os02t0650800-00 | - | - |
| contig02896 | - | Os02t0615800-01 | - | - |
| contig09827 | - | Os02t0527950-00 | - | - |
| contig12563 | - | Os02t0527950-00 | - | - |
| contig00206 | - | Os02t0469600-01 | - | - |
| contig07417 | - | Os02t0457100-00 | - | - |
| contig03822 | - | Os02t0307075-00 | - | - |
| contig01732 | - | Os02t0286850-00 | - | - |
| contig01039 | - | Os02t0263432-00 | - | - |
| contig06393 | - | Os02t0176400-01 | - | - |
| contig02610 | - | Os02t0175200-00 | - | - |
| contig07875 | - | Os02t0175200-00 | - | - |
| contig07014 | - | Os02t0139700-01 | - | - |
| contig04149 | - | Os02t0138200-01 | - | - |

| | | | | |
|---|---|---|---|---|
| contig01506 | - | Os01t0971900-01 | - | - |
| contig02297 | - | Os01t0937300-01 | - | - |
| contig05545 | - | Os01t0937300-01 | - | - |
| contig07489 | - | Os01t0896300-00 | - | - |
| contig13876 | - | Os01t0894100-01 | - | - |
| contig10475 | - | Os01t0852650-00 | - | - |
| contig04401 | - | Os01t0841500-01 | - | - |
| contig04536 | - | Os01t0758400-01 | - | - |
| contig05966 | - | Os01t0660800-01 | - | - |
| contig12275 | - | Os01t0609900-02 | - | - |
| contig16959 | - | Os01t0601675-01 | - | - |
| contig03382 | - | Os01t0601625-00 | - | - |
| contig22831 | - | Os01t0364100-00 | - | - |
| contig11803 | - | Os01t0279400-01 | - | - |
| contig01028 | - | Os01t0153900-00 | - | - |
| contig03502 | - | Os01t0152500-00 | - | - |
| contig18875 | - | Os01t0152500-00 | - | - |
| contig10431 | - | Os09t0249700-01 | - | - |
| contig00414 | - | Os12t0556300-01 | - | - |
| contig02054 | - | Os09t0529300-01 | - | - |
| contig20776 | - | Os07t0107800-01 | - | - |
| contig03123 | - | Os05t0146900-00 | - | - |
| contig09952 | - | Os04t0304400-01 | - | - |
| contig22464 | - | Os03t0239400-01 | - | - |
| contig01189 | - | Os12t0172500-01 | - | - |
| contig18176 | - | Os01t0772600-03 | - | - |
| contig00856 | - | Os03t0638300-01 | - | - |
| contig18052 | - | Os09t0532700-01 | - | - |
| contig01158 | - | Os03t0221100-00 | - | - |
| contig14161 | - | Os09t0428300-00 | - | - |
| contig16417 | - | Os06t0619000-01 | - | - |
| contig23307 | - | Os02t0282500-00 | - | - |
| contig01527 | - | Os01t0609200-00 | - | - |
| contig25885 | - | Os12t0169400-01 | - | - |
| contig04819 | - | Os03t0730400-01 | - | - |
| contig10723 | - | Os11t0109933-01 | - | - |
| contig02274 | - | Os02t0290300-01 | - | - |
| contig26152 | - | Os12t0578500-01 | - | - |
| contig02517 | - | Os08t0398700-02 | - | - |
| contig15052 | - | Os11t0593700-01 | - | - |
| contig04752 | - | Os06t0619000-01 | - | - |
| contig01824 | - | Os02t0494000-00 | - | - |
| contig12552 | - | Os03t0230500-01 | - | - |
| contig01944 | - | Os04t0488400-01 | - | - |
| contig23016 | - | Os02t0684150-00 | - | - |
| contig00579 | - | Os01t0559600-02 | - | - |
| contig02490 | - | Os03t0639600-01 | - | - |

| | | | | |
|---|---|---|---|---|
| contig12249 | - | Os04t0599650-00 | - | - |
| contig13413 | - | Os04t0373400-01 | - | - |
| contig01666 | - | Os12t0562600-01 | - | - |
| contig00466 | - | Os12t0502300-01 | - | - |
| contig00631 | - | Os11t0518900-01 | - | - |
| contig06972 | - | Os02t0329300-01 | - | - |
| contig09455 | - | Os12t0190000-01 | - | - |
| contig02660 | - | Os01t0897000-01 | - | - |
| contig05649 | - | Os01t0304100-00 | - | - |
| contig09748 | - | Os01t0771400-00 | - | - |
| contig21726 | - | Os02t0494000-00 | - | - |
| contig02988 | - | Os04t0510300-01 | - | - |
| contig01461 | - | Os02t0220700-01 | - | - |
| contig12174 | - | Os09t0348600-00 | - | - |
| contig19375 | - | Os02t0678400-01 | - | - |
| contig16183 | - | Os03t0813200-00 | - | - |
| contig14085 | - | Os03t0643050-00 | - | - |
| contig02115 | - | Os01t0946850-00 | - | - |
| contig12992 | - | Os11t0103800-01 | - | - |
| contig00743 | - | Os12t0131701-00 | - | - |
| contig02508 | - | Os01t0382000-01 | - | - |
| contig03116 | - | Os03t0576700-01 | - | - |
| contig07371 | - | Os01t0515300-01 | - | - |
| contig03812 | - | Os05t0151000-00 | - | - |
| contig03813 | - | Os05t0151000-00 | - | - |
| contig02355 | - | Os06t0619000-01 | - | - |
| contig00557 | - | Os03t0804100-00 | - | - |
| contig10747 | - | Os02t0616300-00 | - | - |
| contig13649 | - | Os01t0126600-01 | - | - |
| contig21188 | - | Os12t0530100-01 | - | - |
| contig19652 | - | Os02t0537400-01 | - | - |
| contig07934 | - | Os09t0249700-01 | - | - |
| contig03122 | - | Os09t0460300-01 | - | - |
| contig00381 | - | Os08t0105700-01 | - | - |
| contig02967 | - | Os06t0102900-01 | - | - |
| contig12961 | - | Os11t0147000-01 | - | - |
| contig10807 | - | Os03t0767500-01 | - | - |
| contig15155 | - | Os03t0797000-01 | - | - |
| contig02580 | - | Os09t0489500-01 | - | - |
| contig01871 | - | Os08t0416400-02 | - | - |
| contig03964 | - | Os05t0117798-01 | - | - |
| contig06050 | - | Os09t0260000-01 | - | - |
| contig06513 | - | Os11t0107450-01 | - | - |
| contig08388 | - | Os02t0687900-01 | - | AK248999.1 |
| contig24633 | - | Os03t0681400-01 | - | AK252722.1 |
| contig24501 | - | Os09t0497700-01 | - | AK362409 |
| contig16477 | - | Os01t0764300-01 | - | AK370211 |

| | | | | |
|---|---|---|---|---|
| contig03225 | - | Os03t0174200-01 | - | AK373739 |
| contig01727 | - | Os01t0972900-01 | - | AK374885 |
| contig04922 | - | Os02t0728300-00 | - | MLOC_11026.1 |
| contig24270 | - | Os03t0430400-01 | - | MLOC_12624.1 |
| contig17165 | - | Os06t0484800-00 | - | MLOC_21373.1 |
| contig08404 | - | Os03t0639600-01 | - | MLOC_2356.1 |
| contig00989 | - | Os11t0514100-00 | - | MLOC_24388.12 |
| contig01055 | - | Os11t0514100-00 | - | MLOC_24388.12 |
| contig15965 | - | Os11t0514100-00 | - | MLOC_24388.12 |
| contig21559 | - | Os11t0514100-00 | - | MLOC_24388.12 |
| contig23149 | - | Os03t0643050-00 | - | MLOC_46266.1 |
| contig04531 | - | Os09t0525400-01 | - | MLOC_52707.1 |
| contig05612 | - | Os01t0708600-01 | - | MLOC_59846.2 |
| contig03956 | - | Os01t0753150-00 | - | MLOC_6122.1 |
| contig09627 | - | Os03t0236200-01 | - | MLOC_63275.3 |
| contig04385 | - | Os04t0659400-01 | - | MLOC_64592.5 |
| contig00575 | - | Os08t0226800-00 | - | MLOC_65358.1 |
| contig10450 | - | Os09t0428300-00 | - | MLOC_66828.2 |
| contig16076 | - | Os08t0289400-00 | - | MLOC_70680.1 |
| contig02102 | - | Os12t0590500-00 | - | MLOC_75843.3 |
| contig09058 | - | Os09t0249700-01 | - | AK251150.1 |
| contig03312 | - | Os09t0363700-01 | - | AK368885 |
| contig25843 | - | Os02t0537400-01 | - | MLOC_31208.2 |
| contig01835 | - | Os03t0744700-01 | - | MLOC_52486.1 |
| contig00366 | - | Os09t0469400-01 | - | MLOC_10776.1 |
| contig03671 | - | Os11t0637800-01 | - | MLOC_3216.2 |
| contig16976 | - | Os07t0164100-01 | - | MLOC_37038.2 |
| contig00275 | - | Os03t0800800-01 | - | AK366549 |
| contig02596 | - | Os07t0106000-01 | - | MLOC_21732.1 |
| contig17973 | - | Os05t0117798-01 | - | MLOC_54712.1 |
| contig01576 | - | Os09t0241100-01 | - | MLOC_36749.1 |
| contig03616 | - | Os02t0290300-01 | - | MLOC_63349.2 |
| contig00225 | - | Os05t0317200-01 | - | AK370654 |
| contig23579 | - | Os09t0505100-01 | - | MLOC_43219.1 |
| contig02428 | - | Os01t0673600-01 | - | MLOC_10404.1 |
| contig23552 | - | Os09t0252800-01 | - | MLOC_54346.5 |
| contig24255 | - | Os09t0412200-01 | - | MLOC_44757.2 |
| contig18344 | - | Os03t0769400-01 | - | MLOC_59476.2 |
| contig18175 | - | Os11t0462500-00 | - | MLOC_59845.1 |
| contig05332 | - | Os03t0700800-01 | - | MLOC_74303.1 |
| contig10198 | - | Os09t0569400-01 | - | AK354753 |
| contig03090 | - | Os12t0165000-01 | - | AK366349 |
| contig00247 | - | Os08t0428900-01 | - | MLOC_34200.3 |
| contig23965 | - | Os12t0239000-01 | - | MLOC_32443.2 |
| contig06071 | - | Os09t0410125-00 | - | AK374504 |
| contig04927 | - | Os11t0104866-00 | - | MLOC_57760.2 |
| contig02349 | - | Os05t0445900-01 | - | MLOC_11707.1 |

| | | | | |
|---|---|---|---|---|
| contig12282 | - | Os12t0556300-01 | - | MLOC_55001.1 |
| contig12461 | - | Os03t0352400-01 | - | MLOC_54468.1 |
| contig13152 | - | Os11t0148700-01 | - | MLOC_54689.2 |
| contig00708 | - | Os02t0497500-01 | - | MLOC_61890.1 |
| contig04195 | - | Os09t0286400-01 | - | AK374624 |
| contig00826 | - | Os09t0556400-01 | - | AK373795 |
| contig05026 | - | Os11t0516100-01 | - | AK249319.1 |
| contig17791 | - | Os11t0587600-01 | - | MLOC_54794.6 |
| contig05922 | - | Os03t0833800-01 | - | MLOC_13213.1 |
| contig14726 | - | Os08t0487000-01 | - | AK249956.1 |
| contig25230 | - | Os11t0636900-01 | - | MLOC_17069.1 |
| contig03620 | - | Os09t0530500-01 | - | MLOC_4008.2 |
| contig13591 | - | Os03t0757200-01 | - | MLOC_64192.1 |
| contig06986 | - | Os09t0240200-01 | - | MLOC_75092.2 |
| contig17949 | - | Os09t0316000-01 | - | MLOC_55837.1 |
| contig03429 | - | Os12t0590500-00 | - | MLOC_12612.3 |
| contig05342 | - | Os05t0363100-01 | - | MLOC_65248.1 |
| contig03322 | - | Os12t0168400-01 | - | MLOC_68899.1 |
| contig01748 | - | Os09t0478400-01 | - | AK354986 |
| contig17681 | - | Os09t0313600-00 | - | MLOC_57426.2 |
| contig02764 | - | Os03t0230500-01 | - | MLOC_4801.4 |
| contig05356 | - | Os03t0417900-01 | - | AK361277 |
| contig03273 | - | Os02t0294600-01 | - | MLOC_59257.1 |
| contig00380 | - | Os04t0490800-01 | - | AK252460.1 |
| contig00008 | - | Os11t0292050-00 | - | MLOC_18662.1 |
| contig06167 | - | Os03t0669800-01 | - | MLOC_4537.1 |
| contig01817 | - | Os05t0151000-00 | - | MLOC_66040.1 |
| contig01491 | - | Os09t0530500-01 | - | MLOC_4008.2 |
| contig03392 | - | Os02t0169400-01 | - | MLOC_39997.1 |
| contig00194 | - | Os11t0104900-00 | - | MLOC_57760.2 |
| contig16239 | - | Os12t0547600-02 | - | MLOC_65527.4 |
| contig18870 | - | Os03t0861100-01 | - | AK248140.1 |
| contig06751 | - | Os02t0497500-02 | - | MLOC_61890.1 |
| contig20017 | - | Os02t0497500-01 | - | MLOC_61890.1 |
| contig11366 | - | Os12t0207300-01 | - | AK370183 |
| contig20044 | - | Os09t0569400-01 | Sb01g001930.1 | - |
| contig13111 | - | Os03t0820100-01 | Sb01g003290.2 | - |
| contig00596 | - | Os03t0800700-01 | Sb01g004860.1 | - |
| contig00102 | - | Os03t0758900-01 | Sb01g007490.1 | - |
| contig07150 | - | Os11t0211600-00 | Sb01g015550.1 | - |
| contig06889 | - | Os10t0490666-00 | Sb01g018510.1 | - |
| contig17232 | - | Os10t0141900-01 | Sb01g026340.1 | - |
| contig00526 | - | Os03t0281600-01 | Sb01g038990.1 | - |
| contig06844 | - | Os01t0852650-00 | Sb01g045140.1 | - |
| contig11780 | - | Os03t0145500-01 | Sb01g047200.1 | - |
| contig03260 | - | Os03t0112101-00 | Sb01g049780.1 | - |
| contig00854 | - | Os08t0137200-01 | Sb01g049880.1 | - |

| | | | | | |
|---|---|---|---|---|---|
| contig25908 | - | Os06t0125000-00 | Sb02g001150.1 | - |
| contig16992 | - | Os07t0250900-01 | Sb02g007670.1 | - |
| contig24829 | - | Os07t0276000-01 | Sb02g008410.1 | - |
| contig05547 | - | Os09t0322200-01 | Sb02g021770.1 | - |
| contig00871 | - | Os09t0327300-01 | Sb02g022160.1 | - |
| contig00077 | - | Os09t0442800-01 | Sb02g025950.1 | - |
| contig03992 | - | Os09t0517800-01 | Sb02g027700.1 | - |
| contig08835 | - | Os03t0217200-01 | Sb02g028980.1 | - |
| contig12106 | - | Os09t0551800-00 | Sb02g032150.1 | - |
| contig14327 | - | Os01t0367900-01 | Sb03g014780.1 | - |
| contig18030 | - | Os03t0643050-00 | Sb03g028285.1 | - |
| contig10040 | - | Os01t0690800-01 | Sb03g031680.1 | - |
| contig06722 | - | Os11t0286800-03 | Sb04g001396.1 | - |
| contig13534 | - | Os02t0139500-02 | Sb04g003190.1 | - |
| contig03121 | - | Os02t0260700-01 | Sb04g009720.1 | - |
| contig08085 | - | Os02t0465900-01 | Sb04g017430.2 | - |
| contig15630 | - | Os02t0614966-00 | Sb04g026010.1 | - |
| contig07309 | - | Os02t0699700-01 | Sb04g030880.1 | - |
| contig01658 | - | Os02t0650800-00 | Sb04g033300.1 | - |
| contig02622 | - | Os02t0650800-00 | Sb04g033300.1 | - |
| contig02889 | - | Os02t0650800-00 | Sb04g033300.1 | - |
| contig18023 | - | Os02t0650800-00 | Sb04g033300.1 | - |
| contig21800 | - | Os02t0650800-00 | Sb04g033300.1 | - |
| contig00521 | - | Os03t0669900-00 | Sb05g002880.1 | - |
| contig05318 | - | Os11t0157400-01 | Sb05g003650.1 | - |
| contig14383 | - | Os11t0157400-01 | Sb05g003650.1 | - |
| contig02187 | - | Os11t0211300-01 | Sb05g009130.1 | - |
| contig09756 | - | Os11t0264500-00 | Sb05g009720.1 | - |
| contig07252 | - | Os11t0482100-01 | Sb05g016000.1 | - |
| contig00325 | - | Os12t0559600-00 | Sb05g022785.1 | - |
| contig13204 | - | Os11t0213700-00 | Sb07g005710.1 | - |
| contig03657 | - | Os10t0131000-00 | Sb07g022230.1 | - |
| contig05537 | - | Os08t0557700-01 | Sb07g024310.1 | - |
| contig18163 | - | Os05t0510200-01 | Sb09g025100.1 | - |
| contig21696 | - | Os06t0286500-01 | Sb10g010680.1 | - |
| contig04764 | - | Os05t0370600-01 | Sb10g024200.1 | - |
| contig01369 | - | Os09t0382500-01 | Sb02g023820.1 | - |
| contig03363 | - | Os11t0120600-00 | Sb08g002160.1 | - |
| contig13736 | - | Os08t0102100-01 | Sb07g000320.1 | - |
| contig00033 | - | Os02t0556100-01 | Sb03g016080.1 | - |
| contig01386 | - | Os11t0147000-01 | Sb05g002770.1 | - |
| contig04950 | - | Os07t0134800-01 | Sb01g006490.1 | - |
| contig00185 | - | Os01t0965000-00 | Sb03g046922.1 | - |
| contig10623 | - | Os03t0858400-01 | Sb01g000460.1 | - |
| contig04801 | - | Os09t0394100-01 | Sb02g024040.1 | - |
| contig11132 | - | Os10t0147250-00 | Sb01g026260.1 | - |
| contig17234 | - | Os11t0608700-00 | Sb05g005345.1 | - |

| | | | | |
|---|---|---|---|---|
| contig12068 | - | Os12t0177500-01 | Sb08g004800.1 | - |
| contig02058 | - | Os07t0270950-01 | Sb02g008800.1 | - |
| contig01984 | - | Os12t0429200-01 | Sb08g004410.1 | - |
| contig17772 | - | Os02t0581300-01 | Sb02g023420.1 | - |
| contig21849 | - | Os03t0852900-01 | Sb01g000850.1 | - |
| contig25197 | - | Os03t0852900-01 | Sb01g000850.1 | - |
| contig23420 | - | Os03t0852900-01 | Sb01g000850.1 | - |
| contig24259 | - | Os09t0505100-01 | Sb02g029180.1 | - |
| contig00004 | - | Os09t0393200-01 | Sb02g024010.1 | - |
| contig01431 | - | Os12t0616200-01 | Sb08g021680.1 | - |
| contig03058 | - | Os05t0585500-01 | Sb09g029950.1 | - |
| contig14291 | - | Os01t0857700-01 | Sb03g040410.1 | - |
| contig02721 | - | Os06t0186600-02 | Sb10g005890.1 | - |
| contig20954 | - | Os03t0833700-01 | Sb02g027580.1 | - |
| contig02363 | - | Os11t0148300-01 | Sb05g002890.1 | - |
| contig11664 | - | Os02t0254700-01 | Sb02g009730.2 | - |
| contig02696 | - | Os01t0513900-01 | Sb10g001010.1 | - |
| contig01891 | - | Os08t0536000-01 | Sb02g029470.1 | - |
| contig06346 | - | Os09t0569400-01 | Sb01g001930.1 | - |
| contig00249 | - | Os12t0285600-01 | Sb08g011100.1 | - |
| contig23210 | - | Os12t0239200-01 | Sb08g008680.1 | - |
| contig00116 | - | Os04t0599650-00 | Sb06g027382.1 | - |
| contig03048 | - | Os03t0805100-01 | Sb01g004550.1 | - |
| contig23718 | - | Os09t0479000-01 | Sb02g027700.1 | - |
| contig25797 | - | Os09t0517800-01 | Sb02g027690.1 | - |
| contig03359 | - | Os09t0462400-00 | Sb02g026818.1 | - |
| contig09497 | - | Os01t0513900-01 | Sb10g001010.1 | - |
| contig05267 | - | Os02t0497500-02 | Sb04g020090.1 | - |
| contig08219 | - | Os03t0743800-00 | Sb01g042980.1 | - |
| contig13761 | - | Os12t0134200-01 | Sb05g002180.1 | - |
| contig23334 | - | Os12t0134200-01 | Sb05g002180.1 | - |
| contig01034 | - | Os11t0137300-01 | Sb05g002180.1 | - |
| contig02795 | - | Os03t0586800-02 | Sb01g015530.1 | - |
| contig10065 | - | Os09t0323700-00 | Sb01g028610.1 | - |
| contig01635 | - | Os06t0151600-01 | Sb10g003620.1 | - |
| contig07364 | - | Os09t0517800-01 | Sb02g027690.1 | - |
| contig12142 | - | Os09t0544400-00 | Sb02g031610.1 | - |
| contig02334 | - | Os09t0545500-00 | Sb02g031740.1 | - |
| contig10312 | - | Os08t0505200-01 | Sb02g028255.1 | - |
| contig01500 | - | Os06t0549600-01 | Sb10g021666.1 | - |
| contig09053 | - | Os09t0401100-00 | Sb07g009430.1 | - |
| contig03760 | - | Os03t0861100-01 | Sb01g000280.1 | - |
| contig17253 | - | Os03t0852900-01 | Sb01g000850.1 | - |
| contig19486 | - | Os02t0798900-01 | Sb04g036130.1 | - |
| contig16211 | - | Os10t0162836-01 | Sb07g021550.1 | - |
| contig14254 | - | Os03t0610650-01 | Sb01g014740.1 | - |
| contig09716 | - | Os08t0536000-01 | Sb02g029470.1 | - |

| | | | | |
|---|---|---|---|---|
| contig26453 | - | Os10t0450400-01 | Sb08g003810.1 | - |
| contig11551 | - | Os07t0231400-01 | Sb02g006930.1 | - |
| contig12149 | - | Os06t0148200-01 | Sb10g003340.1 | - |
| contig01501 | - | Os03t0854800-03 | Sb01g000700.1 | - |
| contig25675 | - | Os03t0353600-01 | Sb01g011950.1 | - |
| contig09901 | - | Os07t0665300-00 | Sb01g044520.1 | - |
| contig03763 | - | Os03t0861100-01 | Sb01g000280.1 | - |
| contig02949 | - | Os03t0211400-01 | Sb01g043200.1 | - |
| contig03890 | - | Os01t0655800-01 | Sb03g029880.1 | - |
| contig00400 | - | Os09t0513500-00 | Sb02g029725.1 | - |
| contig13244 | - | Os08t0549200-01 | Sb02g031260.1 | - |
| contig07173 | - | Os06t0301000-01 | Sb05g003390.1 | AK249972.1 |
| contig08173 | - | Os01t0510100-01 | Sb03g033190.1 | AK250233.1 |
| contig12510 | - | Os08t0532700-01 | Sb01g007240.1 | AK354347 |
| contig05694 | - | Os03t0820100-01 | Sb01g003290.2 | AK354375 |
| contig04982 | - | Os11t0284032-00 | Sb05g010060.1 | AK357471 |
| contig20916 | - | Os09t0497600-01 | Sb02g028850.1 | AK358569 |
| contig18169 | - | Os12t0530100-01 | Sb08g016840.1 | AK359280 |
| contig18743 | - | Os03t0417900-01 | Sb01g032420.1 | AK361277 |
| contig13450 | - | Os03t0627500-01 | Sb01g014200.1 | AK362627 |
| contig02781 | - | Os11t0148500-02 | Sb05g002900.1 | AK363464 |
| contig07343 | - | Os03t0650900-01 | Sb01g013190.1 | AK365855 |
| contig12988 | - | Os09t0544300-01 | Sb02g031600.1 | AK370769 |
| contig12568 | - | Os03t0804100-00 | Sb01g004620.1 | AK371707 |
| contig07330 | - | Os07t0122400-01 | Sb02g001710.1 | AK373847 |
| contig02950 | - | Os01t0673600-01 | Sb02g021080.1 | MLOC_10404.1 |
| contig10343 | - | Os08t0474700-01 | Sb02g026780.1 | MLOC_10655.1 |
| contig00790 | - | Os05t0305100-01 | Sb09g027090.1 | MLOC_12242.1 |
| contig01054 | - | Os11t0514100-00 | Sb05g018376.1 | MLOC_24388.12 |
| contig05733 | - | Os08t0532500-01 | Sb07g026135.1 | MLOC_39248.1 |
| contig04369 | - | Os03t0817100-00 | Sb01g003560.1 | MLOC_54075.1 |
| contig02630 | - | Os12t0428600-01 | Sb08g012560.1 | MLOC_54346.5 |
| contig23467 | - | Os03t0352400-01 | Sb01g035280.1 | MLOC_54468.1 |
| contig10325 | - | Os05t0447580-00 | Sb10g000410.1 | MLOC_55288.2 |
| contig16138 | - | Os01t0361700-01 | Sb02g029500.1 | MLOC_55886.1 |
| contig13291 | - | Os03t0389900-01 | Sb01g033580.1 | MLOC_57937.2 |
| contig18225 | - | Os03t0833700-01 | Sb02g027580.1 | MLOC_61805.1 |
| contig16585 | - | Os05t0510200-01 | Sb09g025100.1 | MLOC_62654.4 |
| contig09263 | - | Os01t0510600-01 | Sb02g020260.1 | MLOC_6715.1 |
| contig10204 | - | Os01t0510600-01 | Sb02g020260.1 | MLOC_6715.1 |
| contig15078 | - | Os09t0509300-01 | Sb02g029480.1 | AK355067 |
| contig06706 | - | Os12t0534100-01 | Sb08g016990.1 | MLOC_37208.2 |
| contig14190 | - | Os03t0851600-02 | Sb01g001010.1 | AK356315 |
| contig04943 | - | Os07t0477000-01 | Sb02g033110.1 | AK376987 |
| contig09682 | - | Os03t0811100-01 | Sb01g003860.1 | AK355646 |
| contig00771 | - | Os12t0485000-02 | Sb03g012030.1 | AK373528 |
| contig24274 | - | Os07t0124500-01 | Sb02g028310.1 | MLOC_69668.2 |

| | | | | |
|---|---|---|---|---|
| contig10069 | - | Os09t0114500-01 | Sb02g013180.1 | MLOC_61378.1 |
| contig06402 | - | Os10t0486900-01 | Sb03g043226.1 | AK250340.1 |
| contig13322 | - | Os07t0110300-01 | Sb10g006400.1 | AK371804 |
| contig03034 | - | Os03t0803700-01 | Sb01g004640.1 | MLOC_10144.1 |
| contig13460 | - | Os03t0650900-01 | Sb01g013190.1 | AK365855 |
| contig10717 | - | Os03t0812000-01 | Sb01g003750.1 | MLOC_59130.2 |
| contig15085 | - | Os03t0812000-01 | Sb01g003750.1 | MLOC_59130.2 |
| contig22782 | - | Os03t0812000-01 | Sb01g003750.1 | MLOC_59130.2 |
| contig21903 | - | Os03t0812000-01 | Sb01g003750.1 | MLOC_72617.1 |
| contig06208 | - | Os07t0164100-01 | Sb02g003690.1 | MLOC_37038.2 |
| contig23844 | - | Os03t0627500-01 | Sb01g014200.1 | AK362627 |
| contig07089 | - | Os09t0454200-01 | Sb02g026455.1 | MLOC_9954.3 |
| contig05556 | - | Os04t0643000-01 | Sb01g005920.1 | AK371672 |
| contig14360 | - | Os03t0638200-02 | Sb01g013910.1 | MLOC_76072.2 |
| contig03453 | - | Os03t0638200-01 | Sb01g013910.1 | MLOC_76072.2 |
| contig01448 | - | Os07t0263400-00 | Sb02g008660.1 | AK369373 |
| contig00377 | - | Os02t0581300-01 | Sb02g023420.1 | MLOC_11026.1 |
| contig15444 | - | Os11t0130100-01 | Sb05g001810.1 | AK376476 |
| contig05600 | - | Os03t0852900-01 | Sb01g000850.1 | MLOC_71240.1 |
| contig01800 | - | Os12t0617900-01 | Sb01g013460.1 | AK375108 |
| contig07057 | - | Os09t0505100-01 | Sb02g029180.1 | MLOC_43219.1 |
| contig07497 | - | Os12t0430000-00 | Sb05g016000.1 | MLOC_14105.3 |
| contig07967 | - | Os12t0430000-00 | Sb05g016000.1 | MLOC_14105.3 |
| contig25145 | - | Os03t0861100-01 | Sb01g000280.1 | MLOC_19700.3 |
| contig05052 | - | Os12t0131100-01 | Sb08g001480.1 | MLOC_59721.1 |
| contig04290 | - | Os03t0815400-01 | Sb01g003690.1 | MLOC_57719.1 |
| contig06251 | - | Os04t0599650-00 | Sb06g027382.1 | MLOC_55154.1 |
| contig01689 | - | Os03t0415200-01 | Sb01g032510.1 | MLOC_71887.2 |
| contig01812 | - | Os02t0305600-01 | Sb04g016070.2 | AK370689 |
| contig06723 | - | Os03t0833700-01 | Sb02g027580.1 | MLOC_61805.1 |
| contig04597 | - | Os01t0295700-01 | Sb03g012020.1 | MLOC_12326.1 |
| contig04591 | - | Os01t0528800-01 | Sb04g011550.1 | AK252044.1 |
| contig06037 | - | Os12t0595000-01 | Sb08g020055.1 | MLOC_63764.1 |
| contig09839 | - | Os11t0123500-01 | Sb05g001480.1 | AK366149 |
| contig15328 | - | Os09t0370200-01 | Sb02g023290.1 | MLOC_58166.2 |
| contig25064 | - | Os09t0370200-01 | Sb02g023290.1 | MLOC_58166.2 |
| contig04418 | - | Os03t0861100-01 | Sb01g000280.1 | MLOC_19700.3 |
| contig06452 | - | Os07t0301200-01 | Sb02g009590.1 | MLOC_59466.2 |
| contig00801 | - | Os03t0742400-01 | Sb01g008500.1 | MLOC_51776.1 |
| contig05552 | - | Os08t0416400-01 | Sb07g020335.1 | MLOC_59304.8 |
| contig20194 | - | Os07t0155600-01 | Sb01g011025.1 | MLOC_74303.1 |
| contig21265 | - | Os03t0415200-01 | Sb01g032510.1 | MLOC_71887.2 |
| contig21939 | - | Os09t0569400-01 | Sb01g001930.1 | AK354753 |
| contig20587 | - | Os03t0389900-01 | Sb01g033580.1 | MLOC_57937.2 |
| contig25669 | - | Os03t0389900-01 | Sb01g033580.1 | MLOC_57937.2 |
| contig01433 | - | Os03t0719100-01 | Sb08g000380.1 | MLOC_60545.2 |
| contig22581 | - | Os03t0719100-01 | Sb08g000380.1 | MLOC_60545.2 |

| | | | | |
|---|---|---|---|---|
| contig00266 | - | Os12t0279100-01 | Sb03g043210.1 | MLOC_55909.3 |
| contig12813 | - | Os03t0352400-01 | Sb01g035280.1 | MLOC_54468.1 |
| contig00639 | - | Os03t0364500-01 | Sb01g034550.1 | MLOC_15954.2 |
| contig14408 | - | Os10t0170300-01 | Sb03g009620.1 | MLOC_11318.2 |
| contig02016 | - | Os07t0435400-01 | Sb03g013640.1 | MLOC_58976.1 |
| contig06579 | - | Os08t0337300-03 | Sb02g019320.1 | MLOC_59895.3 |
| contig16975 | - | Os02t0497500-02 | Sb04g020090.1 | MLOC_10497.1 |
| contig02083 | - | Os02t0497500-02 | Sb04g020090.1 | MLOC_61890.1 |
| contig22768 | - | Os12t0134200-01 | Sb05g002180.1 | MLOC_38053.1 |
| contig10988 | - | Os11t0137300-01 | Sb05g002180.1 | MLOC_68195.2 |
| contig09991 | - | Os01t0965000-00 | Sb04g003755.1 | MLOC_62016.2 |
| contig04362 | - | Os12t0533500-01 | Sb08g016860.1 | MLOC_6879.1 |
| contig20289 | - | Os04t0582900-01 | Sb01g008150.1 | MLOC_53400.2 |
| contig06771 | - | Os09t0279300-00 | Sb02g018630.1 | AK373149 |
| contig00339 | - | Os03t0861100-01 | Sb01g000280.1 | MLOC_38189.2 |
| contig21494 | - | Os11t0514100-00 | Sb05g018376.1 | MLOC_24388.12 |
| contig00203 | - | Os05t0447580-00 | Sb10g000410.1 | MLOC_55288.2 |
| contig02321 | - | Os09t0439800-01 | Sb02g025775.1 | MLOC_3895.5 |
| contig21666 | - | Os03t0389900-01 | Sb01g033580.1 | MLOC_57937.2 |
| contig05919 | - | Os12t0507600-01 | Sb08g016270.1 | MLOC_14254.1 |
| contig21567 | - | Os12t0600200-03 | Sb08g020500.1 | MLOC_67910.1 |
| contig19402 | - | Os08t0542200-01 | Sb07g025850.1 | MLOC_10452.1 |
| contig23259 | - | Os03t0833700-01 | Sb02g027580.1 | MLOC_61805.1 |
| contig13520 | - | Os03t0638000-00 | Sb01g013930.1 | MLOC_54940.1 |
| contig03420 | - | Os11t0636900-01 | Sb05g025260.2 | MLOC_17069.1 |
| contig06328 | - | Os11t0113200-01 | Sb08g002760.1 | AK248269.1 |
| contig09749 | - | Os09t0316000-01 | Sb02g021133.1 | MLOC_55837.1 |
| contig00106 | - | Os09t0280300-01 | Sb02g018670.1 | AK249769.1 |
| contig00546 | - | Os11t0140800-01 | Sb05g002430.1 | AK364131 |
| contig00109 | - | Os03t0648300-01 | Sb01g013400.1 | MLOC_13673.1 |
| contig02413 | - | Os03t0812000-01 | Sb01g003760.1 | MLOC_72617.1 |
| contig03507 | - | Os03t0637900-01 | Sb01g013930.1 | MLOC_54940.1 |
| contig16267 | - | Os03t0862100-01 | Sb01g000240.1 | MLOC_21595.1 |
| contig04571 | - | Os12t0428600-01 | Sb08g012560.1 | MLOC_54346.5 |
| contig02727 | - | Os12t0618600-01 | Sb08g021910.1 | MLOC_76757.2 |
| contig05269 | - | Os03t0826700-02 | Sb01g002710.1 | MLOC_62810.2 |
| contig03864 | - | Os09t0258000-01 | Sb02g016600.1 | MLOC_53875.1 |
| contig01836 | - | Os09t0438400-00 | Sb02g025680.1 | MLOC_3963.3 |
| contig05752 | - | Os09t0370200-01 | Sb02g023290.1 | MLOC_58166.2 |
| contig11983 | - | Os09t0498800-01 | Sb02g028940.1 | MLOC_51273.3 |
| contig22050 | - | Os03t0389900-01 | Sb01g033580.1 | MLOC_57937.2 |
| contig03009 | - | Os11t0484000-01 | Sb0019s004510.1 | AK355291 |
| contig01195 | - | Os09t0509300-01 | Sb02g029480.1 | AK355067 |
| contig00014 | - | Os09t0306800-01 | Sb02g020800.1 | MLOC_64864.1 |
| contig09687 | - | Os03t0134900-01 | Sb02g031610.1 | MLOC_19592.1 |
| contig04985 | - | - | Sb10g005050.1 | MLOC_938.1 |
| contig06014 | - | - | Sb02g040840.1 | MLOC_74495.1 |

| | | | | |
|---|---|---|---|---|
| contig01111 | - | - | Sb01g011025.1 | MLOC_74303.1 |
| contig09312 | - | - | Sb02g019340.1 | MLOC_73772.2 |
| contig11215 | - | - | Sb09g030660.1 | MLOC_72099.3 |
| contig01694 | - | - | Sb02g022160.1 | MLOC_65637.1 |
| contig03755 | - | - | Sb02g032680.1 | MLOC_65340.2 |
| contig03318 | - | - | Sb01g028470.1 | MLOC_61818.1 |
| contig04477 | - | - | Sb02g021800.2 | MLOC_57064.4 |
| contig16159 | - | - | Sb07g004380.1 | MLOC_56869.1 |
| contig21537 | - | - | Sb02g021133.1 | MLOC_55837.1 |
| contig12947 | - | - | Sb03g017630.1 | MLOC_54708.1 |
| contig02173 | - | - | Sb05g001320.1 | MLOC_52170.14 |
| contig18078 | - | - | Sb01g042540.1 | MLOC_51331.2 |
| contig08083 | - | - | Sb04g006240.1 | MLOC_48470.1 |
| contig09150 | - | - | Sb04g024360.1 | MLOC_4537.1 |
| contig17144 | - | - | Sb02g025575.1 | MLOC_43403.1 |
| contig04168 | - | - | Sb02g002150.1 | MLOC_43156.1 |
| contig05189 | - | - | Sb10g005170.1 | MLOC_36995.1 |
| contig09009 | - | - | Sb04g009750.1 | MLOC_34954.1 |
| contig00173 | - | - | Sb07g007710.1 | MLOC_31886.1 |
| contig16355 | - | - | Sb05g013420.1 | MLOC_3045.1 |
| contig06095 | - | - | Sb01g000330.1 | MLOC_19838.1 |
| contig07479 | - | - | Sb01g018400.1 | MLOC_1587.2 |
| contig03383 | - | - | Sb05g021414.1 | AK375855 |
| contig06604 | - | - | Sb05g007160.1 | AK370601 |
| contig17105 | - | - | Sb06g001580.1 | AK368079 |
| contig17254 | - | - | Sb02g027570.1 | AK364579 |
| contig09161 | - | - | Sb05g019600.1 | AK364106 |
| contig05700 | - | - | Sb02g031720.1 | AK361070 |
| contig07450 | - | - | Sb02g028850.1 | AK358569 |
| contig01624 | - | - | Sb01g004750.1 | AK358368 |
| contig15650 | - | - | Sb10g007930.1 | AK356188 |
| contig24841 | - | - | Sb05g000845.1 | AK355785 |
| contig19978 | - | - | Sb02g028255.1 | AK353941 |
| contig12348 | - | - | Sb02g018870.1 | AK252400.1 |
| contig11778 | - | - | Sb06g030015.1 | AK249099.1 |
| contig00823 | - | - | Sb01g013930.1 | MLOC_54940.1 |
| contig10038 | - | - | Sb02g029060.1 | MLOC_619.1 |
| contig05120 | - | - | Sb01g006960.1 | AK358227 |
| contig04372 | - | - | Sb02g029690.1 | MLOC_69075.2 |
| contig13129 | - | - | Sb05g008010.1 | MLOC_11269.2 |
| contig21972 | - | - | Sb02g029540.1 | AK363932 |
| contig16305 | - | - | Sb02g019450.1 | AK376220 |
| contig04387 | - | - | Sb02g026780.1 | MLOC_10655.1 |
| contig04572 | - | - | Sb03g016080.1 | AK365251 |
| contig11180 | - | - | Sb08g002980.1 | AK367504 |
| contig14805 | - | - | Sb01g020570.1 | AK248434.1 |
| contig04891 | - | - | Sb02g025515.1 | AK353915 |

| | | | | |
|---|---|---|---|---|
| contig14176 | - | - | Sb05g019600.1 | AK364106 |
| contig17573 | - | - | Sb08g002920.1 | MLOC_54689.2 |
| contig03623 | - | - | Sb07g021390.1 | AK252960.1 |
| contig03104 | - | - | Sb08g001435.1 | MLOC_38921.3 |
| contig00766 | - | - | Sb08g012560.1 | MLOC_54346.5 |
| contig13618 | - | - | Sb08g012560.1 | MLOC_54346.5 |
| contig22123 | - | - | Sb01g032150.1 | MLOC_50612.1 |
| contig03619 | - | - | Sb02g023010.1 | MLOC_9891.3 |
| contig08141 | - | - | Sb07g019250.1 | MLOC_67265.2 |
| contig21875 | - | - | Sb07g019250.1 | MLOC_67265.2 |
| contig06060 | - | - | Sb01g035670.1 | AK368525 |
| contig06662 | - | - | Sb01g003750.1 | MLOC_59130.2 |
| contig05716 | - | - | Sb01g002550.1 | MLOC_13213.1 |
| contig08436 | - | - | Sb01g005920.1 | AK371672 |
| contig21751 | - | - | Sb01g005920.1 | AK371672 |
| contig02853 | - | - | Sb02g036770.1 | MLOC_37372.1 |
| contig01585 | - | - | Sb05g000280.1 | MLOC_3548.1 |
| contig09980 | - | - | Sb06g009200.1 | MLOC_63349.2 |
| contig12554 | - | - | Sb01g011950.1 | MLOC_69563.2 |
| contig07554 | - | - | Sb02g028990.1 | MLOC_53232.1 |
| contig21957 | - | - | Sb02g031260.1 | AK375087 |
| contig22031 | - | - | Sb02g025790.1 | MLOC_72011.2 |
| contig00608 | - | - | Sb02g022933.1 | AK358827 |
| contig01219 | - | - | Sb08g012560.1 | MLOC_54346.5 |
| contig05002 | - | - | Sb02g020290.1 | AK368229 |
| contig03874 | - | - | Sb01g048270.1 | MLOC_58960.1 |
| contig11894 | - | - | Sb01g002080.1 | AK377038 |
| contig07782 | - | - | Sb01g001560.1 | AK364505 |
| contig00663 | - | - | Sb02g024425.1 | MLOC_52254.1 |
| contig00540 | - | - | Sb05g000250.1 | MLOC_10688.1 |
| contig14648 | - | - | Sb05g001470.1 | AK354845 |
| contig11981 | - | - | Sb01g042488.1 | MLOC_67507.3 |
| contig03054 | - | - | Sb05g001480.1 | AK366149 |
| contig04744 | - | - | Sb02g022870.1 | MLOC_55872.12 |
| contig08230 | - | - | Sb08g020250.1 | AK365230 |
| contig02269 | - | - | Sb01g013400.1 | AK353868 |
| contig13063 | - | - | Sb03g001240.1 | AK248364.1 |
| contig00321 | - | - | Sb02g040630.1 | MLOC_75843.3 |
| contig00664 | - | - | Sb02g028935.1 | MLOC_51273.3 |
| contig08786 | - | - | Sb02g019580.1 | AK374259 |
| contig00658 | - | - | Sb08g000380.1 | MLOC_60545.2 |
| contig15077 | - | - | Sb02g019450.1 | AK376220 |
| contig20175 | - | - | Sb02g022540.1 | AK251773.1 |
| contig14832 | - | - | Sb08g018580.1 | MLOC_52439.6 |
| contig01452 | - | - | Sb02g022940.1 | MLOC_33897.1 |
| contig19092 | - | - | Sb02g027340.1 | MLOC_63540.1 |
| contig00280 | - | - | Sb05g002930.1 | MLOC_54689.2 |

| | | | | |
|---|---|---|---|---|
| contig03125 | - | - | Sb02g021090.1 | MLOC_10405.1 |
| contig15282 | - | - | Sb02g021090.1 | MLOC_10405.1 |
| contig01509 | - | - | Sb01g034706.1 | AK365864 |
| contig04499 | - | - | Sb02g019320.1 | MLOC_59895.3 |
| contig17266 | - | - | Sb01g002100.1 | AK249699.1 |
| contig01902 | - | - | Sb02g030140.1 | AK369180 |
| contig16051 | - | - | Sb07g024470.1 | MLOC_65161.2 |
| contig05151 | - | - | Sb07g024470.1 | MLOC_65161.2 |
| contig04332 | - | - | Sb08g022030.1 | MLOC_66260.1 |
| contig07954 | - | - | Sb05g002410.1 | AK368065 |
| contig18934 | - | - | Sb05g002410.1 | AK368065 |
| contig17099 | - | - | Sb01g011950.1 | MLOC_69563.2 |
| contig04860 | - | - | Sb01g006380.1 | AK360042 |
| contig08948 | - | - | Sb03g001240.1 | AK248364.1 |
| contig06567 | - | - | Sb02g023430.2 | AK249324.1 |
| contig02445 | - | - | Sb05g001900.2 | MLOC_56099.3 |
| contig20482 | - | - | Sb02g029910.1 | AK377101 |
| contig17300 | - | - | Sb02g022190.1 | MLOC_2429.1 |
| contig24850 | - | - | Sb02g024300.1 | MLOC_34200.3 |
| contig04091 | - | - | Sb02g025270.1 | MLOC_66828.2 |
| contig10829 | - | - | Sb0011s005460.1 | MLOC_74657.2 |
| contig06074 | - | - | Sb07g003770.1 | MLOC_6549.1 |
| contig04099 | - | - | Sb08g009580.1 | AK364560 |
| contig21292 | - | - | Sb02g028820.1 | AK360950 |
| contig03196 | - | - | Sb01g031705.1 | MLOC_52287.2 |
| contig00234 | - | - | Sb01g026550.1 | AK362344 |
| contig05450 | - | - | Sb02g031150.1 | MLOC_50821.1 |
| contig10117 | - | - | Sb08g012560.1 | MLOC_54346.5 |
| contig00567 | - | - | Sb07g002945.1 | MLOC_70680.1 |
| contig02377 | - | - | Sb08g004010.1 | AK376201 |
| contig03285 | - | - | Sb01g003750.1 | MLOC_59130.2 |
| contig09201 | - | - | Sb08g018340.1 | AK250022.1 |
| contig18223 | - | - | Sb02g008660.1 | AK369373 |
| contig05681 | - | - | Sb01g012050.1 | MLOC_43949.1 |
| contig01640 | - | - | Sb08g012560.1 | MLOC_54346.5 |
| contig02382 | - | - | Sb08g012560.1 | MLOC_54346.5 |
| contig02779 | - | - | Sb01g016035.1 | MLOC_58506.12 |
| contig03337 | - | - | Sb05g001050.1 | AK368749 |
| contig16472 | - | - | Sb04g021110.1 | AK369767 |
| contig15613 | - | - | Sb02g029490.1 | MLOC_75155.1 |
| contig03478 | - | - | Sb08g002530.1 | MLOC_10435.2 |
| contig16733 | - | - | Sb02g027340.1 | MLOC_63540.1 |
| contig21362 | - | - | Sb02g027340.1 | MLOC_63540.1 |
| contig00395 | - | - | Sb02g032160.1 | MLOC_52425.9 |
| contig01926 | - | - | Sb10g027695.1 | - |
| contig03692 | - | - | Sb10g023535.1 | - |
| contig20739 | - | - | Sb10g022050.1 | - |

| | | | | |
|---|---|---|---|---|
| contig12903 | - | - | Sb10g021034.1 | - |
| contig07305 | - | - | Sb10g008600.1 | - |
| contig05508 | - | - | Sb10g008300.1 | - |
| contig02239 | - | - | Sb10g005050.1 | - |
| contig23277 | - | - | Sb10g000860.1 | - |
| contig07365 | - | - | Sb09g029220.1 | - |
| contig02434 | - | - | Sb09g019410.1 | - |
| contig13766 | - | - | Sb09g015250.1 | - |
| contig25941 | - | - | Sb09g007400.1 | - |
| contig00376 | - | - | Sb08g022550.1 | - |
| contig12769 | - | - | Sb08g020692.1 | - |
| contig05299 | - | - | Sb08g019010.1 | - |
| contig07300 | - | - | Sb08g016470.1 | - |
| contig03683 | - | - | Sb08g014910.1 | - |
| contig06380 | - | - | Sb08g007670.1 | - |
| contig12186 | - | - | Sb08g002650.1 | - |
| contig06474 | - | - | Sb07g027090.1 | - |
| contig04010 | - | - | Sb07g025890.1 | - |
| contig06264 | - | - | Sb07g025890.1 | - |
| contig20843 | - | - | Sb07g020830.1 | - |
| contig05434 | - | - | Sb07g020145.1 | - |
| contig03141 | - | - | Sb07g014850.1 | - |
| contig06578 | - | - | Sb07g006490.1 | - |
| contig03702 | - | - | Sb07g005720.1 | - |
| contig14519 | - | - | Sb07g004470.1 | - |
| contig09685 | - | - | Sb07g003376.1 | - |
| contig11249 | - | - | Sb07g002945.1 | - |
| contig12885 | - | - | Sb06g030360.1 | - |
| contig14164 | - | - | Sb06g030210.1 | - |
| contig11877 | - | - | Sb06g030015.1 | - |
| contig07605 | - | - | Sb06g027160.1 | - |
| contig15643 | - | - | Sb06g019610.1 | - |
| contig18483 | - | - | Sb06g017960.1 | - |
| contig00754 | - | - | Sb06g016980.1 | - |
| contig01798 | - | - | Sb06g016585.1 | - |
| contig08602 | - | - | Sb06g016585.1 | - |
| contig21440 | - | - | Sb06g000920.1 | - |
| contig03599 | - | - | Sb05g026940.1 | - |
| contig05504 | - | - | Sb05g023960.1 | - |
| contig00761 | - | - | Sb05g021443.1 | - |
| contig00416 | - | - | Sb05g019635.1 | - |
| contig01299 | - | - | Sb05g019635.1 | - |
| contig05089 | - | - | Sb05g019635.1 | - |
| contig12411 | - | - | Sb05g019635.1 | - |
| contig06455 | - | - | Sb05g006263.1 | - |
| contig10304 | - | - | Sb05g004925.1 | - |
| contig04824 | - | - | Sb05g004922.1 | - |

| | | | | |
|---|---|---|---|---|
| contig23486 | - | - | Sb05g004480.1 | - |
| contig09751 | - | - | Sb05g003830.1 | - |
| contig09307 | - | - | Sb05g002240.1 | - |
| contig05441 | - | - | Sb05g001110.1 | - |
| contig06149 | - | - | Sb05g001110.1 | - |
| contig10845 | - | - | Sb05g001110.1 | - |
| contig18332 | - | - | Sb05g000845.1 | - |
| contig18568 | - | - | Sb04g038510.1 | - |
| contig04126 | - | - | Sb04g033300.1 | - |
| contig15217 | - | - | Sb04g029303.1 | - |
| contig05350 | - | - | Sb04g025280.1 | - |
| contig08949 | - | - | Sb04g020125.1 | - |
| contig11577 | - | - | Sb04g019560.1 | - |
| contig03764 | - | - | Sb04g012680.1 | - |
| contig25633 | - | - | Sb04g012680.1 | - |
| contig21610 | - | - | Sb04g011140.1 | - |
| contig01227 | - | - | Sb04g009720.1 | - |
| contig01381 | - | - | Sb04g007290.1 | - |
| contig09324 | - | - | Sb04g006240.1 | - |
| contig12007 | - | - | Sb04g006240.1 | - |
| contig17863 | - | - | Sb04g006240.1 | - |
| contig14191 | - | - | Sb04g003710.1 | - |
| contig20649 | - | - | Sb04g003200.1 | - |
| contig20804 | - | - | Sb04g001610.1 | - |
| contig10093 | - | - | Sb04g000460.1 | - |
| contig08078 | - | - | Sb03g046767.1 | - |
| contig01940 | - | - | Sb03g041880.1 | - |
| contig05377 | - | - | Sb03g040410.1 | - |
| contig12976 | - | - | Sb03g040355.1 | - |
| contig05317 | - | - | Sb03g036763.1 | - |
| contig06682 | - | - | Sb03g036763.1 | - |
| contig23725 | - | - | Sb03g036763.1 | - |
| contig00457 | - | - | Sb03g028545.1 | - |
| contig09209 | - | - | Sb03g025650.1 | - |
| contig03015 | - | - | Sb03g020182.1 | - |
| contig04728 | - | - | Sb03g018255.1 | - |
| contig11347 | - | - | Sb03g018250.1 | - |
| contig08577 | - | - | Sb03g016090.1 | - |
| contig09670 | - | - | Sb03g015070.1 | - |
| contig07785 | - | - | Sb03g014780.1 | - |
| contig19314 | - | - | Sb03g013920.1 | - |
| contig16461 | - | - | Sb03g011415.1 | - |
| contig17555 | - | - | Sb03g005190.1 | - |
| contig17855 | - | - | Sb03g004250.1 | - |
| contig05330 | - | - | Sb03g002795.1 | - |
| contig08710 | - | - | Sb02g039030.3 | - |
| contig16479 | - | - | Sb02g036420.1 | - |

| | | | | |
|---|---|---|---|---|
| contig11837 | - | - | Sb02g032980.1 | - |
| contig04994 | - | - | Sb02g032690.1 | - |
| contig01818 | - | - | Sb02g032135.1 | - |
| contig02401 | - | - | Sb02g032130.1 | - |
| contig17111 | - | - | Sb02g032070.1 | - |
| contig04081 | - | - | Sb02g031670.1 | - |
| contig06999 | - | - | Sb02g031030.1 | - |
| contig11384 | - | - | Sb02g030865.1 | - |
| contig11234 | - | - | Sb02g029840.1 | - |
| contig02124 | - | - | Sb02g028040.1 | - |
| contig15565 | - | - | Sb02g027570.1 | - |
| contig11079 | - | - | Sb02g027450.1 | - |
| contig10251 | - | - | Sb02g025870.1 | - |
| contig19360 | - | - | Sb02g025870.1 | - |
| contig04069 | - | - | Sb02g025270.1 | - |
| contig14651 | - | - | Sb02g025090.1 | - |
| contig15147 | - | - | Sb02g025090.1 | - |
| contig20094 | - | - | Sb02g024900.1 | - |
| contig15122 | - | - | Sb02g023430.2 | - |
| contig12433 | - | - | Sb02g021800.2 | - |
| contig11153 | - | - | Sb02g021790.1 | - |
| contig06544 | - | - | Sb02g021220.1 | - |
| contig18196 | - | - | Sb02g021220.1 | - |
| contig21523 | - | - | Sb02g021090.1 | - |
| contig10746 | - | - | Sb02g021070.1 | - |
| contig11813 | - | - | Sb02g020425.1 | - |
| contig06156 | - | - | Sb02g018880.1 | - |
| contig13237 | - | - | Sb02g018590.1 | - |
| contig04066 | - | - | Sb02g015861.1 | - |
| contig19156 | - | - | Sb02g011250.1 | - |
| contig17938 | - | - | Sb02g009100.1 | - |
| contig08171 | - | - | Sb02g006050.1 | - |
| contig01445 | - | - | Sb02g005970.1 | - |
| contig08384 | - | - | Sb02g005320.1 | - |
| contig25479 | - | - | Sb02g004690.1 | - |
| contig23361 | - | - | Sb02g004390.1 | - |
| contig15199 | - | - | Sb02g003860.1 | - |
| contig09406 | - | - | Sb02g003700.1 | - |
| contig24232 | - | - | Sb02g002820.1 | - |
| contig00066 | - | - | Sb02g002150.1 | - |
| contig11242 | - | - | Sb02g002097.1 | - |
| contig04354 | - | - | Sb01g045978.1 | - |
| contig00728 | - | - | Sb01g044520.1 | - |
| contig03179 | - | - | Sb01g038787.1 | - |
| contig06820 | - | - | Sb01g033810.1 | - |
| contig02192 | - | - | Sb01g033010.1 | - |
| contig00506 | - | - | Sb01g033000.1 | - |

| | | | | |
|---|---|---|---|---|
| contig08837 | - | - | Sb01g031070.1 | - |
| contig12984 | - | - | Sb01g029380.1 | - |
| contig05907 | - | - | Sb01g026845.1 | - |
| contig09526 | - | - | Sb01g026845.1 | - |
| contig20226 | - | - | Sb01g022750.1 | - |
| contig13485 | - | - | Sb01g020130.1 | - |
| contig17667 | - | - | Sb01g020130.1 | - |
| contig19184 | - | - | Sb01g020130.1 | - |
| contig24335 | - | - | Sb01g020130.1 | - |
| contig00442 | - | - | Sb01g018510.1 | - |
| contig01979 | - | - | Sb01g018510.1 | - |
| contig12846 | - | - | Sb01g017436.1 | - |
| contig14551 | - | - | Sb01g017200.1 | - |
| contig24408 | - | - | Sb01g017050.1 | - |
| contig26007 | - | - | Sb01g016800.1 | - |
| contig00656 | - | - | Sb01g015550.1 | - |
| contig13669 | - | - | Sb01g010930.1 | - |
| contig04453 | - | - | Sb01g010025.1 | - |
| contig25965 | - | - | Sb01g010025.1 | - |
| contig08839 | - | - | Sb01g008520.1 | - |
| contig06480 | - | - | Sb01g005084.1 | - |
| contig18001 | - | - | Sb01g005084.1 | - |
| contig06217 | - | - | Sb01g004720.1 | - |
| contig03255 | - | - | Sb01g003290.2 | - |
| contig14977 | - | - | Sb01g002280.1 | - |
| contig00700 | - | - | Sb01g000430.1 | - |
| contig25546 | - | - | Sb01g000280.1 | - |
| contig02685 | - | - | Sb01g020130.1 | - |
| contig10162 | - | - | Sb01g020130.1 | - |
| contig02134 | - | - | Sb02g006490.1 | - |
| contig08247 | - | - | Sb04g018990.1 | - |
| contig05206 | - | - | Sb02g011340.1 | - |
| contig11492 | - | - | Sb04g027250.1 | - |
| contig00093 | - | - | Sb02g022770.1 | - |
| contig00205 | - | - | Sb04g019783.1 | - |
| contig16605 | - | - | Sb01g003680.1 | - |
| contig01740 | - | - | Sb02g026780.1 | - |
| contig10975 | - | - | Sb02g029240.1 | - |
| contig13230 | - | - | Sb02g029240.1 | - |
| contig16774 | - | - | Sb01g000600.1 | - |
| contig06271 | - | - | Sb02g011250.1 | - |
| contig00171 | - | - | Sb02g039520.1 | - |
| contig05101 | - | - | Sb05g002770.1 | - |
| contig01088 | - | - | Sb04g035713.1 | - |
| contig13139 | - | - | Sb02g027570.1 | - |
| contig25232 | - | - | Sb02g027570.1 | - |
| contig07013 | - | - | Sb08g021070.1 | - |

| | | | | |
|---|---|---|---|---|
| contig00034 | - | - | Sb01g028770.1 | - |
| contig03127 | - | - | Sb02g022933.1 | - |
| contig17053 | - | - | Sb07g025650.1 | - |
| contig03290 | - | - | Sb06g029725.1 | - |
| contig20118 | - | - | Sb02g021800.2 | - |
| contig00279 | - | - | Sb02g024000.1 | - |
| contig01009 | - | - | Sb02g022870.1 | - |
| contig01378 | - | - | Sb08g012360.1 | - |
| contig01004 | - | - | Sb08g021310.3 | - |
| contig15989 | - | - | Sb03g028755.1 | - |
| contig02778 | - | - | Sb08g003830.1 | - |
| contig05011 | - | - | Sb04g019560.1 | - |
| contig05038 | - | - | Sb02g010500.1 | - |
| contig10347 | - | - | Sb02g022270.1 | - |
| contig00482 | - | - | Sb01g026850.1 | - |
| contig01678 | - | - | Sb05g004922.1 | - |
| contig00474 | - | - | Sb10g000410.1 | - |
| contig09020 | - | - | Sb01g044520.1 | - |
| contig24074 | - | - | Sb01g044520.1 | - |
| contig12918 | - | - | Sb01g013430.1 | - |
| contig12323 | - | - | Sb01g010790.1 | - |
| contig01854 | - | - | Sb01g010790.1 | - |
| contig00515 | - | - | Sb01g016035.1 | - |
| contig09764 | - | - | Sb02g036420.1 | - |
| contig14375 | - | - | Sb01g012000.1 | - |
| contig00925 | - | - | Sb02g003030.1 | - |
| contig10478 | - | - | Sb02g025575.1 | - |
| contig07511 | - | - | Sb08g003930.1 | - |
| contig04212 | - | - | Sb04g035110.1 | - |
| contig00154 | - | - | Sb07g024310.1 | - |
| contig00964 | - | - | Sb05g001470.1 | - |
| contig14178 | - | - | Sb05g001470.1 | - |
| contig15678 | - | - | Sb02g032150.1 | - |
| contig10925 | - | - | Sb01g002240.1 | - |
| contig04584 | - | - | Sb05g026150.1 | - |
| contig02539 | - | - | Sb09g027226.1 | - |
| contig06178 | - | - | Sb01g004920.1 | - |
| contig05428 | - | - | Sb02g009800.1 | - |
| contig00168 | - | - | Sb01g003390.1 | - |
| contig00357 | - | - | Sb01g013400.1 | - |
| contig01957 | - | - | Sb02g021790.1 | - |
| contig09981 | - | - | Sb02g027700.1 | - |
| contig10130 | - | - | Sb02g027700.1 | - |
| contig07623 | - | - | Sb01g019390.1 | - |
| contig16927 | - | - | Sb03g009620.1 | - |
| contig12264 | - | - | Sb01g000280.1 | - |
| contig07420 | - | - | Sb05g016000.1 | - |

| | | | | |
|---|---|---|---|---|
| contig05816 | - | - | Sb02g024000.1 | - |
| contig13565 | - | - | Sb05g002180.1 | - |
| contig10865 | - | - | Sb01g000330.1 | - |
| contig01078 | - | - | Sb02g011240.1 | - |
| contig12289 | - | - | Sb02g024070.1 | - |
| contig05583 | - | - | Sb03g028040.1 | - |
| contig04644 | - | - | Sb01g000255.1 | - |
| contig05902 | - | - | Sb05g013840.1 | - |
| contig02956 | - | - | Sb02g029720.1 | - |
| contig10399 | - | - | Sb03g036450.1 | - |
| contig08151 | - | - | Sb01g014270.1 | - |
| contig04114 | - | - | Sb03g036450.1 | - |
| contig01829 | - | - | Sb01g000700.1 | - |
| contig13141 | - | - | Sb02g029600.1 | - |
| contig07677 | - | - | Sb10g006400.1 | - |
| contig24921 | - | - | Sb07g005720.1 | - |
| contig07246 | - | - | Sb05g026175.1 | - |
| contig03726 | - | - | Sb04g033600.1 | - |
| contig02600 | - | - | Sb04g006240.1 | - |
| contig15878 | - | - | Sb03g036450.1 | - |
| contig12577 | - | - | Sb01g044520.1 | - |
| contig12127 | - | - | Sb04g005150.1 | - |
| contig08888 | - | - | Sb08g003720.1 | - |
| contig15605 | - | - | Sb02g027690.1 | - |
| contig00212 | - | - | Sb02g025270.1 | - |
| contig04720 | - | - | Sb07g028350.1 | - |
| contig07597 | - | - | Sb10g001970.1 | - |
| contig04071 | - | - | Sb09g003670.1 | - |
| contig21992 | - | - | Sb04g036130.1 | - |
| contig14138 | - | - | Sb10g020790.1 | - |
| contig19217 | - | - | Sb02g025910.1 | - |
| contig01178 | - | - | Sb02g029540.1 | - |
| contig12244 | - | - | Sb02g029540.1 | - |
| contig21323 | - | - | Sb01g014480.1 | - |
| contig00358 | - | - | Sb01g011610.1 | - |
| contig13715 | - | - | Sb01g005460.1 | - |
| contig21378 | - | - | Sb02g029440.1 | - |
| contig20050 | - | - | Sb02g029440.1 | - |
| contig00013 | - | - | Sb04g006240.1 | - |
| contig06786 | - | - | Sb07g002945.1 | - |
| contig17457 | - | - | Sb04g008350.1 | - |
| contig01761 | - | - | Sb04g009720.1 | - |
| contig01015 | - | - | Sb03g039390.1 | - |
| contig17788 | - | - | Sb08g022950.1 | - |
| contig03617 | - | - | Sb02g022480.1 | - |
| contig05131 | - | - | Sb03g007656.1 | - |
| contig13249 | - | - | Sb01g015666.1 | - |

| | | | | |
|---|---|---|---|---|
| contig02544 | - | - | Sb03g046613.1 | - |
| contig00021 | - | - | Sb02g024760.1 | - |
| contig06761 | - | - | Sb01g005120.1 | - |
| contig19960 | - | - | Sb02g031260.1 | - |
| contig01645 | - | - | Sb05g003583.1 | - |
| contig05782 | - | - | - | MLOC_70680.1 |
| contig25643 | - | - | - | MLOC_72134.2 |
| contig00489 | - | - | - | AK354787 |
| contig05171 | - | - | - | MLOC_12838.1 |
| contig08666 | - | - | - | AK357513 |
| contig06895 | - | - | - | MLOC_30715.1 |
| contig16453 | - | - | - | MLOC_38229.4 |
| contig00393 | - | - | - | MLOC_51003.1 |
| contig03060 | - | - | - | MLOC_55862.2 |
| contig09062 | - | - | - | AK369331 |
| contig00508 | - | - | - | MLOC_4491.1 |
| contig20722 | - | - | - | MLOC_64192.1 |
| contig17039 | - | - | - | MLOC_60056.5 |
| contig21570 | - | - | - | MLOC_12234.1 |
| contig02541 | - | - | - | AK366549 |
| contig02731 | - | - | - | MLOC_62296.1 |
| contig04119 | - | - | - | MLOC_6270.1 |
| contig20545 | - | - | - | MLOC_78997.1 |
| contig15652 | - | - | - | MLOC_60694.3 |
| contig15378 | - | - | - | AK361070 |
| contig00960 | - | - | - | MLOC_34787.1 |
| contig03515 | - | - | - | MLOC_74301.1 |
| contig01266 | - | - | - | MLOC_61924.1 |
| contig18060 | - | - | - | MLOC_66533.1 |
| contig23616 | - | - | - | MLOC_52425.9 |
| contig10057 | - | - | - | MLOC_77079.2 |
| contig10426 | - | - | - | MLOC_53400.2 |
| contig14599 | - | - | - | MLOC_67418.1 |
| contig19500 | - | - | - | AK365357 |
| contig11256 | - | - | - | MLOC_60235.1 |
| contig02302 | - | - | - | AK250899.1 |
| contig04165 | - | - | - | AK358153 |
| contig18582 | - | - | - | MLOC_26193.1 |
| contig00196 | - | - | - | MLOC_45552.1 |
| contig02637 | - | - | - | MLOC_61347.1 |
| contig09189 | - | - | - | MLOC_64240.2 |
| contig00645 | - | - | - | MLOC_26926.1 |
| contig24096 | - | - | - | MLOC_15549.4 |
| contig10149 | - | - | - | MLOC_70419.2 |
| contig15056 | - | - | - | AK370645 |
| contig06198 | - | - | - | MLOC_5847.1 |
| contig10908 | - | - | - | AK358227 |

| | | | | |
|---|---|---|---|---|
| contig07247 | - | - | - | AK361278 |
| contig12851 | - | - | - | MLOC_58443.1 |
| contig00791 | - | - | - | AK369723 |
| contig07291 | - | - | - | MLOC_52950.1 |
| contig06674 | - | - | - | AK365747 |
| contig08854 | - | - | - | MLOC_56805.1 |
| contig04255 | - | - | - | MLOC_4917.1 |
| contig02993 | - | - | - | MLOC_11293.1 |
| contig11956 | - | - | - | MLOC_58205.1 |
| contig04237 | - | - | - | AK361882 |
| contig03785 | - | - | - | MLOC_61507.1 |
| contig20302 | - | - | - | MLOC_38969.11 |
| contig08931 | - | - | - | AK358964 |
| contig09310 | - | - | - | AK252129.1 |
| contig00600 | - | - | - | MLOC_4554.1 |
| contig12324 | - | - | - | MLOC_7225.1 |
| contig06179 | - | - | - | AK248352.1 |
| contig07234 | - | - | - | MLOC_52936.1 |
| contig08154 | - | - | - | MLOC_18265.2 |
| contig09904 | - | - | - | MLOC_15402.1 |
| contig17623 | - | - | - | MLOC_53882.4 |
| contig08454 | - | - | - | MLOC_37447.2 |
| contig10135 | - | - | - | MLOC_43219.1 |
| contig10665 | - | - | - | AK366097 |
| contig00848 | - | - | - | MLOC_81681.1 |
| contig00398 | - | - | - | MLOC_36354.1 |
| contig07566 | - | - | - | AK364168 |
| contig05414 | - | - | - | MLOC_46067.3 |
| contig09880 | - | - | - | AK364742 |
| contig10158 | - | - | - | AK366160 |
| contig00967 | - | - | - | MLOC_56703.1 |
| contig26172 | - | - | - | MLOC_60959.2 |
| contig01754 | - | - | - | MLOC_59921.1 |
| contig12955 | - | - | - | MLOC_59072.3 |
| contig18718 | - | - | - | MLOC_4537.1 |
| contig24698 | - | - | - | MLOC_4537.1 |
| contig00067 | - | - | - | MLOC_36722.6 |
| contig17076 | - | - | - | MLOC_38229.4 |
| contig12345 | - | - | - | AK373030 |
| contig11040 | - | - | - | MLOC_5116.4 |
| contig00076 | - | - | - | MLOC_5414.1 |
| contig05072 | - | - | - | MLOC_1068.2 |
| contig00522 | - | - | - | AK356433 |
| contig04193 | - | - | - | MLOC_37495.1 |
| contig08532 | - | - | - | AK368217 |
| contig00382 | - | - | - | MLOC_4742.4 |
| contig01147 | - | - | - | MLOC_59098.6 |

| | | | | |
|---|---|---|---|---|
| contig19580 | - | - | - | MLOC_50612.1 |
| contig02133 | - | - | - | MLOC_19557.7 |
| contig12587 | - | - | - | MLOC_52425.9 |
| contig12263 | - | - | - | MLOC_68602.3 |
| contig08430 | - | - | - | MLOC_12945.1 |
| contig07012 | - | - | - | AK249059.1 |
| contig00161 | - | - | - | MLOC_4105.3 |
| contig20074 | - | - | - | MLOC_9897.3 |
| contig21810 | - | - | - | MLOC_9897.3 |
| contig19442 | - | - | - | MLOC_91.1 |
| contig23295 | - | - | - | MLOC_8686.1 |
| contig03513 | - | - | - | MLOC_81246.1 |
| contig08957 | - | - | - | MLOC_76364.1 |
| contig17705 | - | - | - | MLOC_74415.1 |
| contig03339 | - | - | - | MLOC_72721.1 |
| contig16086 | - | - | - | MLOC_71628.2 |
| contig01018 | - | - | - | MLOC_70680.1 |
| contig11984 | - | - | - | MLOC_70680.1 |
| contig05371 | - | - | - | MLOC_70419.2 |
| contig07923 | - | - | - | MLOC_70313.1 |
| contig19114 | - | - | - | MLOC_698.2 |
| contig24514 | - | - | - | MLOC_69107.4 |
| contig08885 | - | - | - | MLOC_6791.2 |
| contig01629 | - | - | - | MLOC_6781.1 |
| contig03951 | - | - | - | MLOC_6725.2 |
| contig14107 | - | - | - | MLOC_6725.2 |
| contig17104 | - | - | - | MLOC_6651.2 |
| contig23560 | - | - | - | MLOC_65358.1 |
| contig26235 | - | - | - | MLOC_65183.1 |
| contig08719 | - | - | - | MLOC_65154.1 |
| contig05977 | - | - | - | MLOC_64898.1 |
| contig01301 | - | - | - | MLOC_64671.1 |
| contig13910 | - | - | - | MLOC_64592.5 |
| contig22978 | - | - | - | MLOC_64508.1 |
| contig20320 | - | - | - | MLOC_6390.1 |
| contig25409 | - | - | - | MLOC_6390.1 |
| contig11644 | - | - | - | MLOC_63679.1 |
| contig18305 | - | - | - | MLOC_63679.1 |
| contig06517 | - | - | - | MLOC_63540.1 |
| contig14713 | - | - | - | MLOC_62611.2 |
| contig00392 | - | - | - | MLOC_62296.1 |
| contig08281 | - | - | - | MLOC_61306.1 |
| contig18837 | - | - | - | MLOC_60894.1 |
| contig17890 | - | - | - | MLOC_60633.2 |
| contig17477 | - | - | - | MLOC_59476.2 |
| contig24313 | - | - | - | MLOC_59476.2 |
| contig12534 | - | - | - | MLOC_58507.1 |

| | | | | |
|---|---|---|---|---|
| contig14617 | - | - | - | MLOC_58150.4 |
| contig02391 | - | - | - | MLOC_57923.1 |
| contig12276 | - | - | - | MLOC_57715.1 |
| contig19841 | - | - | - | MLOC_5747.2 |
| contig09711 | - | - | - | MLOC_57426.2 |
| contig10814 | - | - | - | MLOC_57426.2 |
| contig22826 | - | - | - | MLOC_56946.2 |
| contig25549 | - | - | - | MLOC_56946.2 |
| contig24593 | - | - | - | MLOC_56869.1 |
| contig01876 | - | - | - | MLOC_56530.1 |
| contig03700 | - | - | - | MLOC_56502.1 |
| contig16033 | - | - | - | MLOC_56421.5 |
| contig08383 | - | - | - | MLOC_55837.1 |
| contig01840 | - | - | - | MLOC_55587.3 |
| contig03855 | - | - | - | MLOC_54855.1 |
| contig00327 | - | - | - | MLOC_54794.6 |
| contig17027 | - | - | - | MLOC_54712.1 |
| contig15224 | - | - | - | MLOC_54619.1 |
| contig00199 | - | - | - | MLOC_54506.1 |
| contig16232 | - | - | - | MLOC_54243.2 |
| contig06317 | - | - | - | MLOC_54213.1 |
| contig18346 | - | - | - | MLOC_53882.4 |
| contig02659 | - | - | - | MLOC_52181.1 |
| contig11838 | - | - | - | MLOC_4969.1 |
| contig15694 | - | - | - | MLOC_4969.1 |
| contig17947 | - | - | - | MLOC_4969.1 |
| contig19171 | - | - | - | MLOC_4969.1 |
| contig24031 | - | - | - | MLOC_4969.1 |
| contig20219 | - | - | - | MLOC_48470.1 |
| contig05747 | - | - | - | MLOC_48429.1 |
| contig07456 | - | - | - | MLOC_46852.1 |
| contig12667 | - | - | - | MLOC_46852.1 |
| contig20522 | - | - | - | MLOC_46348.1 |
| contig08403 | - | - | - | MLOC_44991.1 |
| contig09898 | - | - | - | MLOC_44991.1 |
| contig15731 | - | - | - | MLOC_44877.1 |
| contig17415 | - | - | - | MLOC_4294.1 |
| contig10001 | - | - | - | MLOC_40529.1 |
| contig01603 | - | - | - | MLOC_39471.1 |
| contig15232 | - | - | - | MLOC_3932.1 |
| contig06833 | - | - | - | MLOC_3895.5 |
| contig19311 | - | - | - | MLOC_38921.3 |
| contig09894 | - | - | - | MLOC_38445.1 |
| contig22387 | - | - | - | MLOC_38445.1 |
| contig14872 | - | - | - | MLOC_38418.1 |
| contig04998 | - | - | - | MLOC_36533.4 |
| contig16065 | - | - | - | MLOC_35082.1 |

| | | | | |
|---|---|---|---|---|
| contig00840 | - | - | - | MLOC_34598.1 |
| contig01096 | - | - | - | MLOC_34598.1 |
| contig21817 | - | - | - | MLOC_34598.1 |
| contig01669 | - | - | - | MLOC_33609.1 |
| contig14600 | - | - | - | MLOC_33609.1 |
| contig04546 | - | - | - | MLOC_33258.3 |
| contig05416 | - | - | - | MLOC_30912.2 |
| contig16047 | - | - | - | MLOC_30741.1 |
| contig06675 | - | - | - | MLOC_28316.1 |
| contig04967 | - | - | - | MLOC_28219.1 |
| contig04268 | - | - | - | MLOC_28102.1 |
| contig20002 | - | - | - | MLOC_24602.2 |
| contig20521 | - | - | - | MLOC_24602.2 |
| contig01057 | - | - | - | MLOC_24388.12 |
| contig23207 | - | - | - | MLOC_22809.1 |
| contig01665 | - | - | - | MLOC_22248.7 |
| contig17545 | - | - | - | MLOC_19954.2 |
| contig12999 | - | - | - | MLOC_17956.6 |
| contig16456 | - | - | - | MLOC_17896.1 |
| contig12043 | - | - | - | MLOC_17150.2 |
| contig19954 | - | - | - | MLOC_17150.2 |
| contig06657 | - | - | - | MLOC_17022.2 |
| contig10464 | - | - | - | MLOC_16408.1 |
| contig20013 | - | - | - | MLOC_16268.1 |
| contig05443 | - | - | - | MLOC_15992.6 |
| contig04916 | - | - | - | MLOC_15798.1 |
| contig13719 | - | - | - | MLOC_14667.2 |
| contig00300 | - | - | - | MLOC_14403.1 |
| contig20879 | - | - | - | MLOC_12555.1 |
| contig09413 | - | - | - | MLOC_12242.1 |
| contig16815 | - | - | - | MLOC_11283.1 |
| contig12630 | - | - | - | MLOC_11135.1 |
| contig16128 | - | - | - | MLOC_11135.1 |
| contig00002 | - | - | - | MLOC_10988.2 |
| contig02765 | - | - | - | MLOC_10971.1 |
| contig09559 | - | - | - | MLOC_10781.1 |
| contig22074 | - | - | - | MLOC_10405.1 |
| contig02086 | - | - | - | MLOC_10067.3 |
| contig04476 | - | - | - | AK376394 |
| contig15612 | - | - | - | AK374936 |
| contig00725 | - | - | - | AK374328 |
| contig07313 | - | - | - | AK374103 |
| contig16691 | - | - | - | AK374103 |
| contig19180 | - | - | - | AK374103 |
| contig04931 | - | - | - | AK373661 |
| contig21374 | - | - | - | AK373095 |
| contig24899 | - | - | - | AK372882 |

| | | | | |
|---|---|---|---|---|
| contig18123 | - | - | - | AK371991 |
| contig02318 | - | - | - | AK371706 |
| contig05672 | - | - | - | AK371555 |
| contig10759 | - | - | - | AK371221 |
| contig17517 | - | - | - | AK371221 |
| contig00142 | - | - | - | AK370749 |
| contig10019 | - | - | - | AK369519 |
| contig02199 | - | - | - | AK369331 |
| contig14571 | - | - | - | AK368910 |
| contig13329 | - | - | - | AK367438 |
| contig02555 | - | - | - | AK365058 |
| contig06426 | - | - | - | AK364579 |
| contig07632 | - | - | - | AK363790 |
| contig15343 | - | - | - | AK363464 |
| contig25928 | - | - | - | AK363464 |
| contig11878 | - | - | - | AK361404 |
| contig03602 | - | - | - | AK360753 |
| contig18473 | - | - | - | AK357711 |
| contig24410 | - | - | - | AK357647 |
| contig06134 | - | - | - | AK355408 |
| contig02469 | - | - | - | AK354908 |
| contig14202 | - | - | - | AK354908 |
| contig18760 | - | - | - | AK354908 |
| contig09372 | - | - | - | AK353827 |
| contig13955 | - | - | - | AK252937.1 |
| contig19038 | - | - | - | AK251773.1 |
| contig20130 | - | - | - | AK249518.1 |
| contig01844 | - | - | - | AK249459.1 |
| contig17010 | - | - | - | AK249161.1 |
| contig03810 | - | - | - | AK248434.1 |
| contig22488 | - | - | - | AK248269.1 |
| contig06670 | - | - | - | MLOC_6081.1 |
| contig15303 | - | - | - | MLOC_37338.2 |
| contig10316 | - | - | - | MLOC_14667.2 |
| contig10910 | - | - | - | MLOC_14667.2 |
| contig10533 | - | - | - | MLOC_13146.1 |
| contig01593 | - | - | - | MLOC_6518.1 |
| contig01008 | - | - | - | AK360968 |
| contig01799 | - | - | - | AK370127 |
| contig01630 | - | - | - | MLOC_18448.1 |
| contig09747 | - | - | - | MLOC_11269.2 |
| contig12547 | - | - | - | MLOC_59098.6 |
| contig04046 | - | - | - | AK356991 |
| contig06087 | - | - | - | AK365509 |
| contig00005 | - | - | - | AK251570.1 |
| contig11145 | - | - | - | MLOC_10658.1 |
| contig13772 | - | - | - | MLOC_64085.2 |

| | | | | |
|---|---|---|---|---|
| contig00147 | - | - | - | AK355948 |
| contig04264 | - | - | - | MLOC_55283.1 |
| contig05386 | - | - | - | AK366243 |
| contig16993 | - | - | - | MLOC_5225.1 |
| contig14342 | - | - | - | MLOC_61507.1 |
| contig01087 | - | - | - | MLOC_65248.1 |
| contig00338 | - | - | - | MLOC_57103.8 |
| contig14970 | - | - | - | AK355185 |
| contig07149 | - | - | - | AK367438 |
| contig11663 | - | - | - | MLOC_15242.1 |
| contig02572 | - | - | - | MLOC_11145.1 |
| contig15440 | - | - | - | MLOC_11145.1 |
| contig08834 | - | - | - | MLOC_64508.1 |
| contig06818 | - | - | - | MLOC_52708.1 |
| contig02291 | - | - | - | AK364742 |
| contig05246 | - | - | - | AK364579 |
| contig00056 | - | - | - | MLOC_71298.1 |
| contig08410 | - | - | - | MLOC_3013.1 |
| contig08279 | - | - | - | MLOC_78080.1 |
| contig01119 | - | - | - | MLOC_828.1 |
| contig14771 | - | - | - | MLOC_11896.2 |
| contig11985 | - | - | - | MLOC_10776.1 |
| contig13699 | - | - | - | MLOC_10776.1 |
| contig09470 | - | - | - | MLOC_59895.3 |
| contig16149 | - | - | - | MLOC_9982.2 |
| contig16520 | - | - | - | AK356191 |
| contig19389 | - | - | - | MLOC_39114.2 |
| contig18475 | - | - | - | MLOC_17896.1 |
| contig01671 | - | - | - | MLOC_15351.6 |
| contig11843 | - | - | - | AK369105 |
| contig00796 | - | - | - | MLOC_37447.2 |
| contig09507 | - | - | - | AK364579 |
| contig03500 | - | - | - | AK364444 |
| contig00227 | - | - | - | AK251945.1 |
| contig12799 | - | - | - | MLOC_79808.1 |
| contig07228 | - | - | - | AK364579 |
| contig02308 | - | - | - | MLOC_22184.1 |
| contig03445 | - | - | - | MLOC_22184.1 |
| contig00052 | - | - | - | MLOC_54346.5 |
| contig01042 | - | - | - | MLOC_55154.1 |
| contig22864 | - | - | - | AK248486.1 |
| contig13104 | - | - | - | MLOC_59172.1 |
| contig01953 | - | - | - | AK370749 |
| contig07357 | - | - | - | AK373703 |
| contig03804 | - | - | - | MLOC_17870.1 |
| contig15100 | - | - | - | MLOC_59699.2 |
| contig08754 | - | - | - | MLOC_72770.1 |

| | | | | |
|---|---|---|---|---|
| contig04969 | - | - | - | AB447465 |
| contig19869 | - | - | - | MLOC_64045.1 |
| contig02338 | - | - | - | AK365960 |
| contig05280 | - | - | - | AK376415 |
| contig02552 | - | - | - | MLOC_53416.1 |
| contig13356 | - | - | - | MLOC_14236.4 |
| contig06021 | - | - | - | MLOC_16711.1 |
| contig06635 | - | - | - | MLOC_56703.1 |
| contig21518 | - | - | - | AK373392 |
| contig02772 | - | - | - | AK365959 |
| contig03497 | - | - | - | MLOC_70419.2 |
| contig03005 | - | - | - | AK374953 |
| contig19273 | - | - | - | MLOC_66533.1 |
| contig00464 | - | - | - | MLOC_60959.2 |
| contig01272 | - | - | - | AK364742 |
| contig02348 | - | - | - | MLOC_59921.1 |
| contig24004 | - | - | - | MLOC_72199.1 |
| contig00822 | - | - | - | MLOC_36718.3 |
| contig14130 | - | - | - | MLOC_52019.1 |
| contig10751 | - | - | - | MLOC_19387.1 |
| contig07896 | - | - | - | MLOC_72552.2 |
| contig09092 | - | - | - | AK355342 |
| contig05900 | - | - | - | MLOC_70505.1 |
| contig00461 | - | - | - | AK373293 |
| contig17872 | - | - | - | AK363908 |
| contig00404 | - | - | - | MLOC_51003.1 |
| contig18337 | - | - | - | MLOC_51003.1 |
| contig00405 | - | - | - | AK370619 |
| contig19340 | - | - | - | AK369869 |
| contig01864 | - | - | - | MLOC_34200.3 |
| contig21165 | - | - | - | MLOC_32443.2 |
| contig26366 | - | - | - | MLOC_5444.3 |
| contig02368 | - | - | - | MLOC_11707.1 |
| contig10243 | - | - | - | MLOC_46067.3 |
| contig01134 | - | - | - | AK365959 |
| contig23075 | - | - | - | MLOC_57493.1 |
| contig01038 | - | - | - | MLOC_52482.1 |
| contig00310 | - | - | - | MLOC_58377.1 |
| contig14004 | - | - | - | MLOC_58377.1 |
| contig08661 | - | - | - | MLOC_5128.1 |
| contig24583 | - | - | - | AK251773.1 |
| contig00193 | - | - | - | AK364313 |
| contig05167 | - | - | - | AK353724 |
| contig18820 | - | - | - | MLOC_53797.6 |
| contig11743 | - | - | - | MLOC_10405.1 |
| contig04321 | - | - | - | MLOC_54712.1 |
| contig04359 | - | - | - | MLOC_59895.3 |

| | | | | |
|---|---|---|---|---|
| contig06104 | - | - | - | AK371791 |
| contig07949 | - | - | - | MLOC_64140.1 |
| contig02893 | - | - | - | AK370183 |
| contig18372 | - | - | - | MLOC_60555.1 |
| contig04915 | - | - | - | MLOC_53465.1 |
| contig00863 | - | - | - | MLOC_16558.1 |
| contig04337 | - | - | - | AK250340.1 |
| contig05327 | - | - | - | AK373945 |

**wheat ancestor *Triticum dicoccoides***

ıet Budak[1,3]*

| UniProt | UniGene |
|---|---|
| - | - |
| - | - |
| - | - |
| - | - |
| - | - |
| - | - |
| - | - |
| - | - |
| - | - |
| - | - |
| - | - |
| - | - |
| - | - |
| - | - |
| - | - |
| - | - |
| - | - |
| - | - |
| - | - |
| - | - |
| - | - |
| - | - |
| - | - |
| - | - |
| - | - |
| - | - |
| - | - |
| - | - |
| - | - |
| - | - |
| - | - |
| - | - |
| - | - |
| - | - |
| - | - |
| - | - |
| - | - |
| - | - |
| - | - |
| - | - |

-
-
-
-
-
-
-
-
-
-
-
-
-
-
-
-
-
-
-
-
-
-
-
-
-
-
-
-
-
-
-
-
-
-
-
-
-
-
-
-
-
-
-
-
-
-
-
-
-
-
-
-
-
-
-
-
-
-
-
-
-
-
-
-
-
-
-
-
-
-
-
-
-
-
-
-
-
-
-
-
-
-
-
-
-
-
-
-
-
-
-
-
-
-

| | |
|---|---|
| - | - |
| - | - |
| - | - |
| - | - |
| - | - |
| - | - |
| - | - |
| - | - |
| - | - |
| - | - |
| - | - |
| - | - |
| - | - |
| - | - |
| - | - |
| - | - |
| - | - |
| - | - |
| - | - |
| - | - |
| - | - |
| - | - |
| - | - |
| - | - |
| - | - |
| - | - |
| - | - |
| - | - |
| - | - |
| - | - |
| - | - |
| - | - |
| - | - |
| - | - |
| - | - |
| - | - |
| - | - |
| - | - |
| - | - |
| - | - |
| - | - |
| - | - |
| - | - |
| - | - |
| - | - |
| - | - |
| - | - |

| | |
|---|---|
| - | - |
| - | - |
| - | - |
| - | - |
| - | - |
| - | - |
| - | - |
| - | - |
| - | - |
| - | - |
| - | - |
| - | - |
| - | - |
| - | - |
| - | - |
| - | - |
| - | - |
| - | - |
| - | - |
| - | - |
| - | - |
| - | - |
| - | - |
| - | - |
| - | - |
| - | - |
| - | - |
| - | - |
| - | - |
| - | - |
| - | - |
| - | - |
| - | - |
| - | - |
| - | - |
| - | - |
| - | - |
| - | - |
| - | - |
| - | - |
| - | - |
| - | - |
| - | - |
| - | - |
| - | - |
| - | - |
| - | - |

| | |
|---|---|
| - | - |
| - | - |
| - | - |
| - | - |
| - | - |
| - | - |
| - | - |
| - | - |
| - | - |
| - | - |
| - | - |
| - | - |
| - | - |
| - | - |
| - | - |
| - | - |
| - | - |
| - | - |
| - | - |
| - | - |
| - | - |
| - | - |
| - | - |
| - | - |
| - | - |
| - | - |
| - | - |
| - | - |
| - | - |
| - | - |
| - | - |
| - | - |
| - | - |
| - | - |
| - | - |
| - | - |
| - | - |
| - | - |
| - | - |
| - | - |
| - | - |
| - | - |
| - | - |
| - | - |
| - | - |
| - | - |
| - | - |

| | |
|---|---|
| - | - |
| - | - |
| - | - |
| - | - |
| - | - |
| - | - |
| - | - |
| - | - |
| - | - |
| - | - |
| - | - |
| - | - |
| - | - |
| - | - |
| - | - |
| - | - |
| - | - |
| - | - |
| - | - |
| - | - |
| - | - |
| - | - |
| - | - |
| - | - |
| - | - |
| - | - |
| - | - |
| - | - |
| - | - |
| - | - |
| - | - |
| - | - |
| - | - |
| - | - |
| - | - |
| - | - |
| - | - |
| - | - |
| - | - |
| - | - |
| - | - |
| - | - |
| - | - |
| - | - |
| - | - |
| - | - |
| - | - |

| | |
|---|---|
| - | - |
| - | - |
| - | - |
| - | - |
| - | - |
| - | - |
| - | - |
| - | - |
| - | - |
| - | - |
| - | - |
| - | - |
| - | - |
| - | - |
| - | - |
| - | - |
| - | - |
| - | - |
| - | - |
| - | - |
| - | - |
| - | - |
| - | - |
| - | - |
| - | - |
| - | - |
| - | - |
| - | - |
| - | - |
| - | - |
| - | - |
| - | - |
| - | - |
| - | - |
| - | - |
| - | - |
| - | - |
| - | - |
| - | - |
| - | - |
| - | - |
| - | - |
| - | - |
| - | - |
| - | - |
| - | - |
| - | - |

| | |
|---|---|
| - | - |
| - | - |
| - | - |
| - | - |
| - | - |
| - | - |
| - | - |
| - | - |
| - | - |
| - | - |
| - | - |
| - | - |
| - | - |
| - | - |
| - | - |
| - | - |
| - | - |
| - | - |
| - | - |
| - | - |
| - | - |
| - | - |
| - | - |
| - | - |
| - | - |
| - | - |
| - | - |
| - | - |
| - | - |
| - | - |
| - | - |
| - | - |
| - | - |
| - | - |
| - | - |
| - | - |
| - | - |
| - | - |
| - | - |
| - | - |
| - | - |
| - | - |
| - | - |
| - | - |
| - | - |
| - | - |
| - | - |

| | |
|---|---|
| - | - |
| - | - |
| - | - |
| - | - |
| - | - |
| - | - |
| - | - |
| - | - |
| - | - |
| - | - |
| - | - |
| - | - |
| - | - |
| - | - |
| - | - |
| - | - |
| - | - |
| - | - |
| - | - |
| - | - |
| - | - |
| - | - |
| - | - |
| - | - |
| - | - |
| - | - |
| - | - |
| - | - |
| - | - |
| - | - |
| - | - |
| - | - |
| - | - |
| - | - |
| - | - |
| - | - |
| - | - |
| - | - |
| - | - |
| - | - |
| - | - |
| - | - |
| - | - |
| - | - |
| - | - |
| - | - |
| - | - |

| | |
|---|---|
| - | - |
| - | - |
| - | - |
| - | - |
| - | - |
| - | - |
| - | - |
| - | - |
| - | - |
| - | - |
| - | - |
| - | - |
| - | - |
| - | - |
| - | - |
| - | - |
| - | - |
| - | - |
| - | - |
| - | - |
| - | - |
| - | - |
| - | - |
| - | - |
| - | - |
| - | - |
| - | - |
| - | - |
| - | - |
| - | - |
| - | - |
| - | - |
| - | - |
| - | - |
| - | - |
| - | - |
| - | - |
| - | - |
| - | - |
| - | - |
| - | - |
| - | - |
| - | - |
| - | - |
| - | - |
| - | - |
| - | - |

| | |
|---|---|
| - | - |
| - | - |
| - | - |
| - | - |
| - | - |
| - | - |
| - | - |
| - | - |
| - | - |
| - | - |
| - | - |
| - | - |
| - | - |
| - | - |
| - | - |
| - | - |
| - | - |
| - | - |
| - | - |
| - | - |
| - | - |
| - | - |
| - | - |
| - | - |
| - | - |
| - | - |
| - | - |
| - | - |
| - | - |
| - | - |
| - | - |
| - | - |
| - | - |
| - | - |
| - | - |
| - | - |
| - | - |
| - | - |
| - | - |
| - | - |
| - | - |
| - | - |
| - | - |
| - | - |
| - | - |
| - | - |
| - | - |

| | |
|---|---|
| - | - |
| - | - |
| - | - |
| - | - |
| - | - |
| - | - |
| - | - |
| - | - |
| - | - |
| - | - |
| - | - |
| - | - |
| - | - |
| - | - |
| - | - |
| - | - |
| - | - |
| - | - |
| - | - |
| - | - |
| - | - |
| - | - |
| - | - |
| - | - |
| - | - |
| - | - |
| - | - |
| - | - |
| - | - |
| - | - |
| - | - |
| - | - |
| - | - |
| - | - |
| - | - |
| - | - |
| - | - |
| - | - |
| - | - |
| - | - |
| - | - |
| - | - |
| - | - |
| - | - |
| - | - |
| - | - |
| - | - |

| | |
|---|---|
| - | - |
| - | - |
| - | - |
| - | - |
| - | - |
| - | - |
| - | - |
| - | - |
| - | - |
| - | - |
| - | - |
| - | - |
| - | - |
| - | - |
| - | - |
| - | - |
| - | - |
| - | - |
| - | - |
| - | - |
| - | - |
| - | - |
| - | - |
| - | - |
| - | - |
| - | - |
| - | - |
| - | - |
| - | - |
| - | - |
| - | - |
| - | - |
| - | - |
| - | - |
| - | - |
| - | - |
| - | - |
| - | - |
| - | - |
| - | - |
| - | - |
| - | - |
| - | - |
| - | - |
| - | - |
| - | - |
| - | - |

| | |
|---|---|
| - | - |
| - | - |
| - | - |
| - | - |
| - | - |
| - | - |
| - | - |
| - | - |
| - | - |
| - | - |
| - | - |
| - | - |
| - | - |
| - | - |
| - | - |
| - | - |
| - | - |
| - | - |
| - | - |
| - | - |
| - | - |
| - | - |
| - | - |
| - | - |
| - | - |
| - | - |
| - | - |
| - | - |
| - | - |
| - | - |
| - | - |
| - | - |
| - | - |
| - | - |
| - | - |
| - | - |
| - | - |
| - | - |
| - | - |
| - | - |
| - | - |
| - | - |
| - | - |
| - | - |
| - | - |
| - | - |
| - | - |

| | |
|---|---|
| - | - |
| - | - |
| - | - |
| - | - |
| - | - |
| - | - |
| - | - |
| - | - |
| - | - |
| - | - |
| - | - |
| - | - |
| - | - |
| - | - |
| - | - |
| - | - |
| - | - |
| - | - |
| - | - |
| - | - |
| - | - |
| - | - |
| - | - |
| - | - |
| - | - |
| - | - |
| - | - |
| - | - |
| - | - |
| - | - |
| - | - |
| - | - |
| - | - |
| - | - |
| - | - |
| - | - |
| - | - |
| - | - |
| - | - |
| - | - |
| - | - |
| - | - |
| - | - |
| - | - |
| - | - |
| - | - |
| - | - |

| | |
|---|---|
| - | - |
| - | - |
| - | - |
| - | - |
| - | - |
| - | - |
| - | - |
| - | - |
| - | - |
| - | - |
| - | - |
| - | - |
| - | - |
| - | - |
| - | - |
| - | - |
| - | - |
| - | - |
| - | - |
| - | - |
| - | - |
| - | - |
| - | - |
| - | - |
| - | - |
| - | - |
| - | - |
| - | - |
| - | - |
| - | - |
| - | - |
| - | - |
| - | - |
| - | - |
| - | - |
| - | - |
| - | - |
| - | - |
| - | - |
| - | - |
| - | - |
| - | - |
| - | - |
| - | - |
| - | - |
| - | - |
| - | - |

| | |
|---|---|
| - | - |
| - | - |
| - | - |
| - | - |
| - | - |
| - | - |
| - | - |
| - | - |
| - | - |
| - | - |
| - | - |
| - | - |
| - | - |
| - | - |
| - | - |
| - | - |
| - | - |
| - | - |
| - | - |
| - | - |
| - | - |
| - | - |
| - | - |
| - | - |
| - | - |
| - | - |
| - | - |
| - | - |
| - | - |
| - | - |
| - | - |
| - | - |
| - | - |
| - | - |
| - | - |
| - | - |
| - | - |
| - | - |
| - | - |
| - | - |
| - | - |
| - | - |
| - | - |
| - | - |
| - | - |
| - | - |
| - | - |

| | |
|---|---|
| - | - |
| - | - |
| - | - |
| - | - |
| - | - |
| - | - |
| - | - |
| - | - |
| - | - |
| - | - |
| - | - |
| - | - |
| - | - |
| - | - |
| - | - |
| - | - |
| - | - |
| - | - |
| - | - |
| - | - |
| - | - |
| - | - |
| - | - |
| - | - |
| - | - |
| - | - |
| - | - |
| - | - |
| - | - |
| - | - |
| - | - |
| - | - |
| - | - |
| - | - |
| - | - |
| - | - |
| - | - |
| - | - |
| - | - |
| - | - |
| - | - |
| - | - |
| - | - |
| - | - |
| - | - |
| - | - |
| - | - |

| | |
|---|---|
| - | - |
| - | - |
| - | - |
| - | - |
| - | - |
| - | - |
| - | - |
| - | - |
| - | - |
| - | - |
| - | - |
| - | - |
| - | - |
| - | - |
| - | - |
| - | - |
| - | - |
| - | - |
| - | - |
| - | - |
| - | - |
| - | - |
| - | - |
| - | - |
| - | - |
| - | - |
| - | - |
| - | - |
| - | - |
| - | - |
| - | - |
| - | - |
| - | - |
| - | - |
| - | - |
| - | - |
| - | - |
| - | - |
| - | - |
| - | - |
| - | - |
| - | - |
| - | - |
| - | - |
| - | - |
| - | - |
| - | - |

| | |
|---|---|
| - | - |
| - | - |
| - | - |
| - | - |
| - | - |
| - | - |
| - | - |
| - | - |
| - | - |
| - | - |
| - | - |
| - | - |
| - | - |
| - | - |
| - | - |
| - | - |
| - | - |
| - | - |
| - | - |
| - | - |
| - | - |
| - | - |
| - | - |
| - | - |
| - | - |
| - | - |
| - | - |
| - | - |
| - | - |
| - | - |
| - | - |
| - | - |
| - | - |
| - | - |
| - | - |
| - | - |
| - | - |
| - | - |
| - | - |
| - | - |
| - | - |
| - | - |
| - | - |
| - | - |
| - | - |
| - | - |
| - | - |

| | |
|---|---|
| - | - |
| - | - |
| - | - |
| - | - |
| - | - |
| - | - |
| - | - |
| - | - |
| - | - |
| - | - |
| - | - |
| - | - |
| - | - |
| - | - |
| - | - |
| - | - |
| - | - |
| - | - |
| - | - |
| - | - |
| - | - |
| - | - |
| - | - |
| - | - |
| - | - |
| - | - |
| - | - |
| - | - |
| - | - |
| - | - |
| - | - |
| - | - |
| - | - |
| - | - |
| - | - |
| - | - |
| - | - |
| - | - |
| - | - |
| - | - |
| - | - |
| - | - |
| - | - |
| - | - |
| - | - |
| - | - |
| - | - |

| | |
|---|---|
| - | - |
| - | - |
| - | - |
| - | - |
| - | - |
| - | - |
| - | - |
| - | - |
| - | - |
| - | - |
| - | - |
| - | - |
| - | - |
| - | - |
| - | - |
| - | - |
| - | - |
| - | - |
| - | - |
| - | - |
| - | - |
| - | - |
| - | - |
| - | - |
| - | - |
| - | - |
| - | - |
| - | - |
| - | - |
| - | - |
| - | - |
| - | - |
| - | - |
| - | - |
| - | - |
| - | - |
| - | - |
| - | - |
| - | - |
| - | - |
| - | - |
| - | - |
| - | - |
| - | - |
| - | - |
| - | - |
| - | - |

| | |
|---|---|
| - | - |
| - | - |
| - | - |
| - | - |
| - | - |
| - | - |
| - | - |
| - | - |
| - | - |
| - | - |
| - | - |
| - | - |
| - | - |
| - | - |
| - | - |
| - | - |
| - | - |
| - | - |
| - | - |
| - | - |
| - | - |
| - | - |
| - | - |
| - | - |
| - | - |
| - | - |
| - | - |
| - | - |
| - | - |
| - | - |
| - | - |
| - | - |
| - | - |
| - | - |
| - | - |
| - | - |
| - | - |
| - | - |
| - | - |
| - | - |
| - | - |
| - | - |
| - | - |
| - | - |
| - | - |
| - | - |
| - | - |

| | |
|---|---|
| - | - |
| - | - |
| - | - |
| - | - |
| - | - |
| - | - |
| - | - |
| - | - |
| - | - |
| - | - |
| - | - |
| - | - |
| - | - |
| - | - |
| - | - |
| - | - |
| - | - |
| - | - |
| - | - |
| - | - |
| - | - |
| - | - |
| - | - |
| - | - |
| - | - |
| - | - |
| - | - |
| - | - |
| - | - |
| - | - |
| - | - |
| - | - |
| - | - |
| - | - |
| - | - |
| - | - |
| - | - |
| - | - |
| - | - |
| - | - |
| - | - |
| - | - |
| - | - |
| - | - |
| - | - |
| - | - |
| - | - |

| | |
|---|---|
| - | - |
| - | - |
| - | - |
| - | - |
| - | - |
| - | - |
| - | - |
| - | - |
| - | - |
| - | - |
| - | - |
| - | - |
| - | - |
| - | - |
| - | - |
| - | - |
| - | - |
| - | - |
| - | - |
| - | - |
| - | - |
| - | - |
| - | - |
| - | - |
| - | - |
| - | - |
| - | - |
| - | - |
| - | - |
| - | - |
| - | - |
| - | - |
| - | - |
| - | - |
| - | - |
| - | - |
| - | - |
| - | - |
| - | - |
| - | - |
| - | - |
| - | - |
| - | - |
| - | - |
| - | - |
| - | - |
| - | - |

| | |
|---|---|
| - | - |
| - | - |
| - | - |
| - | - |
| - | - |
| - | - |
| - | - |
| - | - |
| - | - |
| - | - |
| - | - |
| - | - |
| - | - |
| - | - |
| - | - |
| - | - |
| - | - |
| - | - |
| - | - |
| - | - |
| - | - |
| - | - |
| - | - |
| - | - |
| - | - |
| - | - |
| - | - |
| - | - |
| - | - |
| - | - |
| - | - |
| - | - |
| - | - |
| - | - |
| - | - |
| - | - |
| - | - |
| - | - |
| - | - |
| - | - |
| - | - |
| - | - |
| - | - |
| - | - |
| - | - |
| - | - |
| - | - |

| | |
|---|---|
| - | - |
| - | - |
| - | - |
| - | - |
| - | - |
| - | - |
| - | - |
| - | - |
| - | - |
| - | - |
| - | - |
| - | - |
| - | gnl\|UG\|Ta#S12966400 |
| - | gnl\|UG\|Ta#S18006225 |
| - | gnl\|UG\|Ta#S18012796 |
| - | gnl\|UG\|Ta#S18592052 |
| - | gnl\|UG\|Ta#S18592052 |
| - | gnl\|UG\|Ta#S18592052 |
| - | gnl\|UG\|Ta#S22368494 |
| - | gnl\|UG\|Ta#S32429129 |
| - | gnl\|UG\|Ta#S32535778 |
| - | gnl\|UG\|Ta#S32592792 |
| - | gnl\|UG\|Ta#S32592792 |
| - | gnl\|UG\|Ta#S32645457 |
| - | gnl\|UG\|Ta#S32701197 |
| - | gnl\|UG\|Ta#S50377770 |
| - | gnl\|UG\|Ta#S52541717 |
| - | gnl\|UG\|Ta#S52542520 |
| - | gnl\|UG\|Ta#S52543143 |
| - | gnl\|UG\|Ta#S52543777 |
| - | gnl\|UG\|Ta#S52544414 |
| - | gnl\|UG\|Ta#S52544428 |
| - | gnl\|UG\|Ta#S52544428 |
| - | gnl\|UG\|Ta#S52544726 |
| - | gnl\|UG\|Ta#S52544726 |
| - | gnl\|UG\|Ta#S52544726 |
| - | gnl\|UG\|Ta#S52544726 |
| - | gnl\|UG\|Ta#S52544726 |
| - | gnl\|UG\|Ta#S52545009 |
| - | gnl\|UG\|Ta#S52545451 |
| - | gnl\|UG\|Ta#S52545451 |
| - | gnl\|UG\|Ta#S52546505 |
| - | gnl\|UG\|Ta#S58842066 |
| - | gnl\|UG\|Ta#S58853951 |
| - | gnl\|UG\|Ta#S58854033 |
| - | gnl\|UG\|Ta#S58863943 |
| - | gnl\|UG\|Ta#S58867418 |

| | |
|---|---|
| - | gnl\|UG\|Ta#S58867418 |
| - | gnl\|UG\|Ta#S58869266 |
| - | gnl\|UG\|Ta#S58882509 |
| - | gnl\|UG\|Ta#S58890612 |
| - | gnl\|UG\|Ta#S58894281 |
| - | gnl\|UG\|Ta#S58895827 |
| - | gnl\|UG\|Ta#S58898783 |
| - | gnl\|UG\|Ta#S58906537 |
| - | gnl\|UG\|Ta#S58906817 |
| - | gnl\|UG\|Ta#S58908974 |
| - | gnl\|UG\|Ta#S58908974 |
| - | gnl\|UG\|Ta#S58909907 |
| - | gnl\|UG\|Ta#S58910656 |
| - | gnl\|UG\|Ta#S61518685 |
| - | gnl\|UG\|Ta#S61522520 |
| - | gnl\|UG\|Ta#S61539190 |
| - | gnl\|UG\|Ta#S61540249 |
| - | gnl\|UG\|Ta#S61541845 |
| - | gnl\|UG\|Ta#S61543811 |
| - | gnl\|UG\|Ta#S61547588 |
| - | gnl\|UG\|Ta#S61550178 |
| - | gnl\|UG\|Ta#S61554479 |
| - | gnl\|UG\|Ta#S61554479 |
| - | gnl\|UG\|Ta#S61554479 |
| - | gnl\|UG\|Ta#S61554479 |
| - | gnl\|UG\|Ta#S61554693 |
| - | gnl\|UG\|Ta#S61555488 |
| - | gnl\|UG\|Ta#S61556921 |
| - | gnl\|UG\|Ta#S61560977 |
| - | gnl\|UG\|Ta#S61561960 |
| - | gnl\|UG\|Ta#S61566619 |
| - | gnl\|UG\|Ta#S61571756 |
| - | gnl\|UG\|Ta#S61586218 |
| - | gnl\|UG\|Ta#S61593796 |
| - | gnl\|UG\|Ta#S61595167 |
| - | gnl\|UG\|Ta#S61595167 |
| - | gnl\|UG\|Ta#S61596675 |
| - | gnl\|UG\|Ta#S61604614 |
| - | gnl\|UG\|Ta#S61604614 |
| - | gnl\|UG\|Ta#S61606656 |
| - | gnl\|UG\|Ta#S61607697 |
| - | gnl\|UG\|Ta#S61609706 |
| - | gnl\|UG\|Ta#S61610970 |
| - | gnl\|UG\|Ta#S61611174 |
| - | gnl\|UG\|Ta#S61611875 |
| - | gnl\|UG\|Ta#S61613302 |
| - | gnl\|UG\|Ta#S61616216 |

| | |
|---|---|
| - | gnl\|UG\|Ta#S61621799 |
| - | gnl\|UG\|Ta#S61621799 |
| - | gnl\|UG\|Ta#S61623512 |
| - | gnl\|UG\|Ta#S61624126 |
| - | gnl\|UG\|Ta#S61624593 |
| - | gnl\|UG\|Ta#S61624959 |
| - | gnl\|UG\|Ta#S61626430 |
| - | gnl\|UG\|Ta#S61628091 |
| - | gnl\|UG\|Ta#S61632658 |
| - | gnl\|UG\|Ta#S61632666 |
| - | gnl\|UG\|Ta#S61632666 |
| - | gnl\|UG\|Ta#S61634666 |
| - | gnl\|UG\|Ta#S61637542 |
| - | gnl\|UG\|Ta#S61637542 |
| - | gnl\|UG\|Ta#S61639865 |
| - | gnl\|UG\|Ta#S61641041 |
| - | gnl\|UG\|Ta#S61641041 |
| - | gnl\|UG\|Ta#S61648388 |
| - | gnl\|UG\|Ta#S61668957 |
| - | gnl\|UG\|Ta#S61669118 |
| - | gnl\|UG\|Ta#S61669234 |
| - | gnl\|UG\|Ta#S61670310 |
| - | gnl\|UG\|Ta#S61672248 |
| - | gnl\|UG\|Ta#S61713711 |
| - | gnl\|UG\|Ta#S61720553 |
| - | gnl\|UG\|Ta#S61741897 |
| - | gnl\|UG\|Ta#S61745390 |
| - | gnl\|UG\|Ta#S61747072 |
| - | gnl\|UG\|Ta#S61750186 |
| - | gnl\|UG\|Ta#S61762911 |
| - | gnl\|UG\|Ta#S61765457 |
| - | gnl\|UG\|Ta#S61768358 |
| - | gnl\|UG\|Ta#S61768782 |
| - | gnl\|UG\|Ta#S61768893 |
| - | gnl\|UG\|Ta#S61779089 |
| - | gnl\|UG\|Ta#S61780455 |
| - | gnl\|UG\|Ta#S61781642 |
| - | gnl\|UG\|Ta#S61782156 |
| - | gnl\|UG\|Ta#S61782235 |
| - | gnl\|UG\|Ta#S61789413 |
| - | gnl\|UG\|Ta#S61791678 |
| - | gnl\|UG\|Ta#S61792222 |
| - | gnl\|UG\|Ta#S61798955 |
| - | gnl\|UG\|Ta#S61800316 |
| - | gnl\|UG\|Ta#S61800370 |
| - | gnl\|UG\|Ta#S61800370 |
| - | gnl\|UG\|Ta#S61801545 |

| | |
|---|---|
| - | gnl\|UG\|Ta#S61801545 |
| - | gnl\|UG\|Ta#S61801792 |
| - | gnl\|UG\|Ta#S61802506 |
| - | gnl\|UG\|Ta#S61802506 |
| - | gnl\|UG\|Ta#S61804187 |
| - | gnl\|UG\|Ta#S61808044 |
| - | gnl\|UG\|Ta#S61808053 |
| - | gnl\|UG\|Ta#S61808406 |
| - | gnl\|UG\|Ta#S61808903 |
| - | gnl\|UG\|Ta#S61808980 |
| - | gnl\|UG\|Ta#S61809643 |
| - | gnl\|UG\|Ta#S61809879 |
| - | gnl\|UG\|Ta#S61810100 |
| - | gnl\|UG\|Ta#S61810214 |
| - | gnl\|UG\|Ta#S61810261 |
| - | gnl\|UG\|Ta#S61810309 |
| - | gnl\|UG\|Ta#S61810811 |
| - | gnl\|UG\|Ta#S61811208 |
| - | gnl\|UG\|Ta#S61811229 |
| - | gnl\|UG\|Ta#S61811320 |
| - | gnl\|UG\|Ta#S61811332 |
| - | gnl\|UG\|Ta#S61811352 |
| - | gnl\|UG\|Ta#S61811409 |
| - | gnl\|UG\|Ta#S61811681 |
| - | gnl\|UG\|Ta#S61811827 |
| - | gnl\|UG\|Ta#S61811995 |
| - | gnl\|UG\|Ta#S61812068 |
| - | gnl\|UG\|Ta#S61812141 |
| - | gnl\|UG\|Ta#S61812141 |
| - | gnl\|UG\|Ta#S61812151 |
| - | gnl\|UG\|Ta#S61812220 |
| - | gnl\|UG\|Ta#S61812304 |
| - | gnl\|UG\|Ta#S61812317 |
| - | gnl\|UG\|Ta#S61812334 |
| - | gnl\|UG\|Ta#S61812410 |
| - | gnl\|UG\|Ta#S61812410 |
| - | gnl\|UG\|Ta#S61826588 |
| - | gnl\|UG\|Ta#S61826588 |
| - | gnl\|UG\|Ta#S61828378 |
| - | gnl\|UG\|Ta#S61828405 |
| - | gnl\|UG\|Ta#S61829174 |
| - | gnl\|UG\|Ta#S61829302 |
| - | gnl\|UG\|Ta#S61829846 |
| - | gnl\|UG\|Ta#S61829846 |
| - | gnl\|UG\|Ta#S61830858 |
| - | gnl\|UG\|Ta#S61830867 |
| - | gnl\|UG\|Ta#S61831155 |

| | |
|---|---|
| - | gnl\|UG\|Ta#S61831300 |
| - | gnl\|UG\|Ta#S61831342 |
| - | gnl\|UG\|Ta#S61831573 |
| - | gnl\|UG\|Ta#S61831713 |
| - | gnl\|UG\|Ta#S61831808 |
| - | gnl\|UG\|Ta#S61831953 |
| - | gnl\|UG\|Ta#S61832045 |
| - | gnl\|UG\|Ta#S61832130 |
| - | gnl\|UG\|Ta#S61832363 |
| - | gnl\|UG\|Ta#S61832430 |
| - | gnl\|UG\|Ta#S61832485 |
| - | gnl\|UG\|Ta#S61836589 |
| - | gnl\|UG\|Ta#S61838221 |
| - | gnl\|UG\|Ta#S61844164 |
| - | gnl\|UG\|Ta#S61863723 |
| - | gnl\|UG\|Ta#S61863892 |
| - | gnl\|UG\|Ta#S61865335 |
| - | gnl\|UG\|Ta#S61867260 |
| - | gnl\|UG\|Ta#S65589166 |
| - | gnl\|UG\|Ta#S65591060 |
| - | gnl\|UG\|Ta#S65594105 |
| - | gnl\|UG\|Ta#S65605718 |
| - | gnl\|UG\|Ta#S65606325 |
| - | gnl\|UG\|Ta#S65612899 |
| - | gnl\|UG\|Ta#S65613264 |
| - | gnl\|UG\|Ta#S65622077 |
| - | gnl\|UG\|Ta#S65629152 |
| - | gnl\|UG\|Ta#S65630248 |
| - | gnl\|UG\|Ta#S65657692 |
| - | gnl\|UG\|Ta#S65661152 |
| - | gnl\|UG\|Ta#S65668365 |
| - | gnl\|UG\|Ta#S65679950 |
| - | gnl\|UG\|Ta#S65681223 |
| - | gnl\|UG\|Ta#S65685179 |
| - | gnl\|UG\|Ta#S65690790 |
| - | gnl\|UG\|Ta#S65697370 |
| - | gnl\|UG\|Ta#S65701009 |
| - | gnl\|UG\|Ta#S65708961 |
| - | gnl\|UG\|Ta#S65710379 |
| - | gnl\|UG\|Ta#S65711079 |
| - | gnl\|UG\|Ta#S65711215 |
| - | gnl\|UG\|Ta#S65717825 |
| - | gnl\|UG\|Ta#S65723103 |
| tr\|M7YS46\|M7YS46_TRIUA | - |
| tr\|M7Z136\|M7Z136_TRIUA | - |
| tr\|M7Z1U6\|M7Z1U6_TRIUA | - |
| tr\|M7Z1U6\|M7Z1U6_TRIUA | - |

| | |
|---|---|
| tr\|M7Z2I1\|M7Z2I1_TRIUA | - |
| tr\|M7ZB18\|M7ZB18_TRIUA | - |
| tr\|M7ZNZ3\|M7ZNZ3_TRIUA | - |
| tr\|M7ZRD7\|M7ZRD7_TRIUA | - |
| tr\|M8A5L2\|M8A5L2_TRIUA | - |
| tr\|M8A811\|M8A811_TRIUA | - |
| tr\|M8AES1\|M8AES1_TRIUA | - |
| tr\|M8AH84\|M8AH84_TRIUA | - |
| tr\|M8ANN4\|M8ANN4_TRIUA | - |
| tr\|M8AZ82\|M8AZ82_TRIUA | - |
| tr\|Q5NKQ7\|Q5NKQ7_TRIMO | - |
| tr\|T1LCC9\|T1LCC9_TRIUA | - |
| tr\|T1LDK4\|T1LDK4_TRIUA | - |
| tr\|T1LL73\|T1LL73_TRIUA | - |
| tr\|T1LYG0\|T1LYG0_TRIUA | - |
| tr\|T1MF56\|T1MF56_TRIUA | - |
| tr\|T1MUC6\|T1MUC6_TRIUA | - |
| tr\|T1MUC6\|T1MUC6_TRIUA | - |
| tr\|T1N0K9\|T1N0K9_TRIUA | - |
| tr\|T1N5N7\|T1N5N7_TRIUA | - |
| tr\|T1N893\|T1N893_TRIUA | - |
| tr\|T1NCM2\|T1NCM2_TRIUA | - |
| tr\|T1NCM2\|T1NCM2_TRIUA | - |
| tr\|T1NLP0\|T1NLP0_TRIUA | - |
| tr\|W4ZZZ6\|W4ZZZ6_WHEAT | - |
| tr\|W5A312\|W5A312_WHEAT | - |
| tr\|W5A7E6\|W5A7E6_WHEAT | - |
| tr\|W5AAH5\|W5AAH5_WHEAT | - |
| tr\|W5AAI2\|W5AAI2_WHEAT | - |
| tr\|W5AAZ3\|W5AAZ3_WHEAT | - |
| tr\|W5B2F4\|W5B2F4_WHEAT | - |
| tr\|W5B7G1\|W5B7G1_WHEAT | - |
| tr\|W5BU86\|W5BU86_WHEAT | - |
| tr\|W5CJC7\|W5CJC7_WHEAT | - |
| tr\|W5DLD0\|W5DLD0_WHEAT | - |
| tr\|W5DM91\|W5DM91_WHEAT | - |
| tr\|W5DM91\|W5DM91_WHEAT | - |
| tr\|W5DMG5\|W5DMG5_WHEAT | - |
| tr\|W5DMK9\|W5DMK9_WHEAT | - |
| tr\|W5DMY3\|W5DMY3_WHEAT | - |
| tr\|W5DNU8\|W5DNU8_WHEAT | - |
| tr\|W5DTQ5\|W5DTQ5_WHEAT | - |
| tr\|W5DU55\|W5DU55_WHEAT | - |
| tr\|W5DU56\|W5DU56_WHEAT | - |
| tr\|W5DWR6\|W5DWR6_WHEAT | - |
| tr\|W5ETR8\|W5ETR8_WHEAT | - |
| tr\|W5EUT2\|W5EUT2_WHEAT | - |

| | |
|---|---|
| tr\|W5F4C3\|W5F4C3_WHEAT | - |
| tr\|W5F7G2\|W5F7G2_WHEAT | - |
| tr\|W5F833\|W5F833_WHEAT | - |
| tr\|W5F833\|W5F833_WHEAT | - |
| tr\|W5F892\|W5F892_WHEAT | - |
| tr\|W5F892\|W5F892_WHEAT | - |
| tr\|W5F8Q1\|W5F8Q1_WHEAT | - |
| tr\|W5F8T4\|W5F8T4_WHEAT | - |
| tr\|W5F8W2\|W5F8W2_WHEAT | - |
| tr\|W5F8W2\|W5F8W2_WHEAT | - |
| tr\|W5F977\|W5F977_WHEAT | - |
| tr\|W5F9G1\|W5F9G1_WHEAT | - |
| tr\|W5F9K0\|W5F9K0_WHEAT | - |
| tr\|W5FA11\|W5FA11_WHEAT | - |
| tr\|W5FAM8\|W5FAM8_WHEAT | - |
| tr\|W5FAP2\|W5FAP2_WHEAT | - |
| tr\|W5FBE2\|W5FBE2_WHEAT | - |
| tr\|W5FBW0\|W5FBW0_WHEAT | - |
| tr\|W5FC33\|W5FC33_WHEAT | - |
| tr\|W5FC36\|W5FC36_WHEAT | - |
| tr\|W5FCD1\|W5FCD1_WHEAT | - |
| tr\|W5FCZ4\|W5FCZ4_WHEAT | - |
| tr\|W5FD08\|W5FD08_WHEAT | - |
| tr\|W5FD67\|W5FD67_WHEAT | - |
| tr\|W5FDP3\|W5FDP3_WHEAT | - |
| tr\|W5FDY1\|W5FDY1_WHEAT | - |
| tr\|W5FEY2\|W5FEY2_WHEAT | - |
| tr\|W5FEZ1\|W5FEZ1_WHEAT | - |
| tr\|W5FF28\|W5FF28_WHEAT | - |
| tr\|W5FG72\|W5FG72_WHEAT | - |
| tr\|W5FGU1\|W5FGU1_WHEAT | - |
| tr\|W5FGW0\|W5FGW0_WHEAT | - |
| tr\|W5FGW0\|W5FGW0_WHEAT | - |
| tr\|W5FGW0\|W5FGW0_WHEAT | - |
| tr\|W5FH68\|W5FH68_WHEAT | - |
| tr\|W5FHF8\|W5FHF8_WHEAT | - |
| tr\|W5FHH6\|W5FHH6_WHEAT | - |
| tr\|W5FHR4\|W5FHR4_WHEAT | - |
| tr\|W5FI59\|W5FI59_WHEAT | - |
| tr\|W5FIH3\|W5FIH3_WHEAT | - |
| tr\|W5FIH6\|W5FIH6_WHEAT | - |
| tr\|W5FJ96\|W5FJ96_WHEAT | - |
| tr\|W5FJC1\|W5FJC1_WHEAT | - |
| tr\|W5FJX4\|W5FJX4_WHEAT | - |
| tr\|W5FJX8\|W5FJX8_WHEAT | - |
| tr\|W5FKS0\|W5FKS0_WHEAT | - |
| tr\|W5FKT3\|W5FKT3_WHEAT | - |

| | |
|---|---|
| tr\|W5FLX7\|W5FLX7_WHEAT | - |
| tr\|W5FMS8\|W5FMS8_WHEAT | - |
| tr\|W5FMV5\|W5FMV5_WHEAT | - |
| tr\|W5FP99\|W5FP99_WHEAT | - |
| tr\|W5FRJ0\|W5FRJ0_WHEAT | - |
| tr\|W5FS73\|W5FS73_WHEAT | - |
| tr\|W5FSB6\|W5FSB6_WHEAT | - |
| tr\|W5G168\|W5G168_WHEAT | - |
| tr\|W5G2P6\|W5G2P6_WHEAT | - |
| tr\|W5G333\|W5G333_WHEAT | - |
| tr\|W5H1L1\|W5H1L1_WHEAT | - |
| tr\|W5HEJ3\|W5HEJ3_WHEAT | - |
| tr\|W5HNS1\|W5HNS1_WHEAT | - |
| tr\|W5HQ54\|W5HQ54_WHEAT | - |
| tr\|W5HT23\|W5HT23_WHEAT | - |
| tr\|W5I2Z1\|W5I2Z1_WHEAT | - |
| tr\|W5I3B8\|W5I3B8_WHEAT | - |
| tr\|W5I5Z7\|W5I5Z7_WHEAT | - |
| tr\|W5IB38\|W5IB38_WHEAT | - |
| tr\|M7YH83\|M7YH83_TRIUA | gnl\|UG\|Ta#S12966400 |
| tr\|W5A726\|W5A726_WHEAT | gnl\|UG\|Ta#S20999693 |
| tr\|W5HNN6\|W5HNN6_WHEAT | gnl\|UG\|Ta#S22373418 |
| tr\|W5F6Y1\|W5F6Y1_WHEAT | gnl\|UG\|Ta#S37894959 |
| tr\|T1LY61\|T1LY61_TRIUA | gnl\|UG\|Ta#S52544588 |
| tr\|W5FMU8\|W5FMU8_WHEAT | gnl\|UG\|Ta#S52546959 |
| tr\|W5FM44\|W5FM44_WHEAT | gnl\|UG\|Ta#S52547038 |
| tr\|T1MVV5\|T1MVV5_TRIUA | gnl\|UG\|Ta#S61550178 |
| tr\|W5DQB7\|W5DQB7_WHEAT | gnl\|UG\|Ta#S61554472 |
| tr\|W5DR72\|W5DR72_WHEAT | gnl\|UG\|Ta#S61556412 |
| tr\|T1N9M9\|T1N9M9_TRIUA | gnl\|UG\|Ta#S61583960 |
| tr\|W5FHP8\|W5FHP8_WHEAT | gnl\|UG\|Ta#S61584946 |
| tr\|M7Z8C6\|M7Z8C6_TRIUA | gnl\|UG\|Ta#S61611875 |
| tr\|W5A1K7\|W5A1K7_WHEAT | gnl\|UG\|Ta#S61616676 |
| tr\|W5GNU6\|W5GNU6_WHEAT | gnl\|UG\|Ta#S61628091 |
| tr\|W5FBU4\|W5FBU4_WHEAT | gnl\|UG\|Ta#S61632658 |
| tr\|W5FIH3\|W5FIH3_WHEAT | gnl\|UG\|Ta#S61658050 |
| tr\|W5DMM4\|W5DMM4_WHEAT | gnl\|UG\|Ta#S61663461 |
| tr\|W5FGI1\|W5FGI1_WHEAT | gnl\|UG\|Ta#S61675718 |
| tr\|T1LBP7\|T1LBP7_TRIUA | gnl\|UG\|Ta#S61771967 |
| tr\|W5F842\|W5F842_WHEAT | gnl\|UG\|Ta#S61778646 |
| tr\|M8AUU4\|M8AUU4_TRIUA | gnl\|UG\|Ta#S61782097 |
| tr\|W5FGI8\|W5FGI8_WHEAT | gnl\|UG\|Ta#S61791678 |
| tr\|W5DHB1\|W5DHB1_WHEAT | gnl\|UG\|Ta#S61792422 |
| tr\|W5FIN8\|W5FIN8_WHEAT | gnl\|UG\|Ta#S61795840 |
| tr\|W5FES2\|W5FES2_WHEAT | gnl\|UG\|Ta#S61802148 |
| tr\|W5FTJ9\|W5FTJ9_WHEAT | gnl\|UG\|Ta#S61808011 |
| tr\|W5FMX8\|W5FMX8_WHEAT | gnl\|UG\|Ta#S61808406 |

| | |
|---|---|
| tr\|W5FMX8\|W5FMX8_WHEAT | gnl\|UG\|Ta#S61808406 |
| tr\|W5FNV7\|W5FNV7_WHEAT | gnl\|UG\|Ta#S61810907 |
| tr\|W5BSC9\|W5BSC9_WHEAT | gnl\|UG\|Ta#S61810970 |
| tr\|W5HPI5\|W5HPI5_WHEAT | gnl\|UG\|Ta#S61811603 |
| tr\|W5FH13\|W5FH13_WHEAT | gnl\|UG\|Ta#S61812310 |
| tr\|Q2QKB7\|Q2QKB7_WHEAT | gnl\|UG\|Ta#S61812336 |
| tr\|W5A6X9\|W5A6X9_WHEAT | gnl\|UG\|Ta#S61812508 |
| tr\|W5F8V2\|W5F8V2_WHEAT | gnl\|UG\|Ta#S61828504 |
| tr\|W5DVV9\|W5DVV9_WHEAT | gnl\|UG\|Ta#S61829384 |
| tr\|W5FNW0\|W5FNW0_WHEAT | gnl\|UG\|Ta#S61829605 |
| tr\|W5FG67\|W5FG67_WHEAT | gnl\|UG\|Ta#S61829705 |
| tr\|W5FLA2\|W5FLA2_WHEAT | gnl\|UG\|Ta#S61831245 |
| tr\|M8AFL2\|M8AFL2_TRIUA | gnl\|UG\|Ta#S61832061 |
| tr\|W5HT23\|W5HT23_WHEAT | gnl\|UG\|Ta#S61832293 |
| tr\|W5FEK5\|W5FEK5_WHEAT | gnl\|UG\|Ta#S61832353 |
| tr\|W5FEK5\|W5FEK5_WHEAT | gnl\|UG\|Ta#S61832353 |
| tr\|W5BEK5\|W5BEK5_WHEAT | gnl\|UG\|Ta#S61832442 |
| tr\|M7YFG7\|M7YFG7_TRIUA | gnl\|UG\|Ta#S65597914 |
| tr\|W5FB21\|W5FB21_WHEAT | gnl\|UG\|Ta#S65599324 |
| tr\|W5FHF8\|W5FHF8_WHEAT | gnl\|UG\|Ta#S65613601 |
| tr\|W5DTN1\|W5DTN1_WHEAT | gnl\|UG\|Ta#S65624501 |
| tr\|M7Z406\|M7Z406_TRIUA | gnl\|UG\|Ta#S65631148 |
| tr\|W5AB81\|W5AB81_WHEAT | gnl\|UG\|Ta#S65679382 |
| tr\|W5DWR6\|W5DWR6_WHEAT | gnl\|UG\|Ta#S65711079 |
| - | - |
| - | - |
| - | - |
| - | - |
| - | - |
| - | - |
| - | - |
| - | - |
| - | - |
| - | - |
| - | - |
| - | - |
| - | - |
| - | - |
| - | - |
| - | - |
| - | - |
| - | - |
| - | - |
| - | - |
| - | - |
| - | - |
| - | - |

| | |
|---|---|
| - | - |
| - | - |
| - | - |
| - | - |
| - | - |
| - | - |
| - | - |
| - | - |
| - | - |
| - | - |
| - | - |
| - | - |
| - | - |
| - | - |
| - | - |
| - | - |
| - | - |
| - | - |
| - | - |
| - | - |
| - | - |
| - | - |
| - | - |
| - | - |
| - | - |
| - | - |
| - | - |
| - | - |
| - | - |
| - | - |
| - | - |
| - | - |
| - | - |
| - | - |
| - | - |
| - | - |
| - | - |
| - | - |
| - | - |
| - | - |
| - | - |
| - | - |
| - | - |
| - | - |
| - | - |
| - | - |
| - | - |

| | |
|---|---|
| - | - |
| - | - |
| - | - |
| - | - |
| - | - |
| - | - |
| - | - |
| - | - |
| - | - |
| - | - |
| - | - |
| - | - |
| - | - |
| - | - |
| - | - |
| - | - |
| - | - |
| - | - |
| - | - |
| - | - |
| - | - |
| - | - |
| - | - |
| - | - |
| - | - |
| - | - |
| - | - |
| - | - |
| - | - |
| - | - |
| - | - |
| - | - |
| - | - |
| - | - |
| - | - |
| - | - |
| - | - |
| - | - |
| - | - |
| - | - |
| - | - |
| - | - |
| - | - |
| - | - |
| - | - |
| - | - |
| - | - |

| | |
|---|---|
| - | - |
| - | - |
| - | - |
| - | - |
| - | - |
| - | - |
| - | - |
| - | - |
| - | - |
| - | - |
| - | - |
| - | - |
| - | - |
| - | - |
| - | - |
| - | - |
| - | - |
| - | - |
| - | - |
| - | - |
| - | - |
| - | - |
| - | - |
| - | - |
| - | - |
| - | - |
| - | - |
| - | - |
| - | - |
| - | - |
| - | - |
| - | - |
| - | - |
| - | - |
| - | - |
| - | - |
| - | - |
| - | - |
| - | - |
| - | - |
| - | - |
| - | - |
| - | - |
| - | - |
| - | - |
| - | - |
| - | - |

| | |
|---|---|
| - | - |
| - | - |
| - | - |
| - | - |
| - | - |
| - | - |
| - | - |
| - | - |
| - | - |
| - | - |
| - | - |
| - | - |
| - | - |
| - | - |
| - | - |
| - | - |
| - | - |
| - | - |
| - | - |
| - | - |
| - | - |
| - | - |
| - | - |
| - | - |
| - | - |
| - | - |
| - | - |
| - | - |
| - | - |
| - | - |
| - | - |
| - | - |
| - | - |
| - | - |
| - | - |
| - | - |
| - | - |
| - | - |
| - | - |
| - | - |
| - | - |
| - | - |
| - | - |
| - | - |
| - | - |
| - | - |
| - | - |

| | |
|---|---|
| - | - |
| - | - |
| - | - |
| - | - |
| - | - |
| - | - |
| - | - |
| - | - |
| - | - |
| - | - |
| - | - |
| - | - |
| - | - |
| - | - |
| - | - |
| - | - |
| - | - |
| - | - |
| - | - |
| - | - |
| - | - |
| - | - |
| - | - |
| - | - |
| - | - |
| - | - |
| - | - |
| - | - |
| - | - |
| - | - |
| - | - |
| - | - |
| - | - |
| - | - |
| - | - |
| - | - |
| - | - |
| - | - |
| - | - |
| - | - |
| - | - |
| - | - |
| - | - |
| - | - |
| - | - |
| - | - |
| - | - |

| | |
|---|---|
| - | - |
| - | - |
| - | - |
| - | - |
| - | - |
| - | - |
| - | - |
| - | - |
| - | - |
| - | gnl\|UG\|Ta#S12923244 |
| - | gnl\|UG\|Ta#S16222886 |
| - | gnl\|UG\|Ta#S16242525 |
| - | gnl\|UG\|Ta#S17888282 |
| - | gnl\|UG\|Ta#S17984361 |
| - | gnl\|UG\|Ta#S18011728 |
| - | gnl\|UG\|Ta#S26022980 |
| - | gnl\|UG\|Ta#S32429129 |
| - | gnl\|UG\|Ta#S32664616 |
| - | gnl\|UG\|Ta#S43837843 |
| - | gnl\|UG\|Ta#S43837843 |
| - | gnl\|UG\|Ta#S52541003 |
| - | gnl\|UG\|Ta#S52542426 |
| - | gnl\|UG\|Ta#S52543605 |
| - | gnl\|UG\|Ta#S52543733 |
| - | gnl\|UG\|Ta#S52544187 |
| - | gnl\|UG\|Ta#S52544187 |
| - | gnl\|UG\|Ta#S52544390 |
| - | gnl\|UG\|Ta#S52544390 |
| - | gnl\|UG\|Ta#S52544516 |
| - | gnl\|UG\|Ta#S52544885 |
| - | gnl\|UG\|Ta#S52545921 |
| - | gnl\|UG\|Ta#S52546599 |
| - | gnl\|UG\|Ta#S53250795 |
| - | gnl\|UG\|Ta#S58882509 |
| - | gnl\|UG\|Ta#S58887057 |
| - | gnl\|UG\|Ta#S58891782 |
| - | gnl\|UG\|Ta#S58896896 |
| - | gnl\|UG\|Ta#S58899964 |
| - | gnl\|UG\|Ta#S58899964 |
| - | gnl\|UG\|Ta#S58901900 |
| - | gnl\|UG\|Ta#S58901900 |
| - | gnl\|UG\|Ta#S58903936 |
| - | gnl\|UG\|Ta#S58903936 |
| - | gnl\|UG\|Ta#S58905866 |
| - | gnl\|UG\|Ta#S58909995 |
| - | gnl\|UG\|Ta#S61567316 |
| - | gnl\|UG\|Ta#S61570178 |

| | |
|---|---|
| - | gnl\|UG\|Ta#S61577520 |
| - | gnl\|UG\|Ta#S61585469 |
| - | gnl\|UG\|Ta#S61585826 |
| - | gnl\|UG\|Ta#S61585826 |
| - | gnl\|UG\|Ta#S61586386 |
| - | gnl\|UG\|Ta#S61589678 |
| - | gnl\|UG\|Ta#S61596978 |
| - | gnl\|UG\|Ta#S61598568 |
| - | gnl\|UG\|Ta#S61600255 |
| - | gnl\|UG\|Ta#S61600597 |
| - | gnl\|UG\|Ta#S61603086 |
| - | gnl\|UG\|Ta#S61605385 |
| - | gnl\|UG\|Ta#S61611875 |
| - | gnl\|UG\|Ta#S61611922 |
| - | gnl\|UG\|Ta#S61613274 |
| - | gnl\|UG\|Ta#S61615120 |
| - | gnl\|UG\|Ta#S61615120 |
| - | gnl\|UG\|Ta#S61616716 |
| - | gnl\|UG\|Ta#S61618986 |
| - | gnl\|UG\|Ta#S61619919 |
| - | gnl\|UG\|Ta#S61620929 |
| - | gnl\|UG\|Ta#S61624959 |
| - | gnl\|UG\|Ta#S61630267 |
| - | gnl\|UG\|Ta#S61634783 |
| - | gnl\|UG\|Ta#S61636951 |
| - | gnl\|UG\|Ta#S61641004 |
| - | gnl\|UG\|Ta#S61641004 |
| - | gnl\|UG\|Ta#S61641041 |
| - | gnl\|UG\|Ta#S61641637 |
| - | gnl\|UG\|Ta#S61648543 |
| - | gnl\|UG\|Ta#S61650526 |
| - | gnl\|UG\|Ta#S61652423 |
| - | gnl\|UG\|Ta#S61655748 |
| - | gnl\|UG\|Ta#S61655748 |
| - | gnl\|UG\|Ta#S61655748 |
| - | gnl\|UG\|Ta#S61670511 |
| - | gnl\|UG\|Ta#S61689950 |
| - | gnl\|UG\|Ta#S61689950 |
| - | gnl\|UG\|Ta#S61715924 |
| - | gnl\|UG\|Ta#S61730173 |
| - | gnl\|UG\|Ta#S61734658 |
| - | gnl\|UG\|Ta#S61750186 |
| - | gnl\|UG\|Ta#S61755827 |
| - | gnl\|UG\|Ta#S61768358 |
| - | gnl\|UG\|Ta#S61775473 |
| - | gnl\|UG\|Ta#S61779146 |
| - | gnl\|UG\|Ta#S61779146 |

| | |
|---|---|
| - | gnl\|UG\|Ta#S61782156 |
| - | gnl\|UG\|Ta#S61785630 |
| - | gnl\|UG\|Ta#S61789535 |
| - | gnl\|UG\|Ta#S61798628 |
| - | gnl\|UG\|Ta#S61800359 |
| - | gnl\|UG\|Ta#S61801083 |
| - | gnl\|UG\|Ta#S61801359 |
| - | gnl\|UG\|Ta#S61801554 |
| - | gnl\|UG\|Ta#S61801701 |
| - | gnl\|UG\|Ta#S61802191 |
| - | gnl\|UG\|Ta#S61802366 |
| - | gnl\|UG\|Ta#S61808592 |
| - | gnl\|UG\|Ta#S61808805 |
| - | gnl\|UG\|Ta#S61808903 |
| - | gnl\|UG\|Ta#S61808949 |
| - | gnl\|UG\|Ta#S61810403 |
| - | gnl\|UG\|Ta#S61810474 |
| - | gnl\|UG\|Ta#S61810549 |
| - | gnl\|UG\|Ta#S61810549 |
| - | gnl\|UG\|Ta#S61810638 |
| - | gnl\|UG\|Ta#S61811044 |
| - | gnl\|UG\|Ta#S61811318 |
| - | gnl\|UG\|Ta#S61811318 |
| - | gnl\|UG\|Ta#S61811352 |
| - | gnl\|UG\|Ta#S61811407 |
| - | gnl\|UG\|Ta#S61811479 |
| - | gnl\|UG\|Ta#S61811480 |
| - | gnl\|UG\|Ta#S61811576 |
| - | gnl\|UG\|Ta#S61811660 |
| - | gnl\|UG\|Ta#S61811660 |
| - | gnl\|UG\|Ta#S61811730 |
| - | gnl\|UG\|Ta#S61811893 |
| - | gnl\|UG\|Ta#S61811893 |
| - | gnl\|UG\|Ta#S61811944 |
| - | gnl\|UG\|Ta#S61812220 |
| - | gnl\|UG\|Ta#S61812265 |
| - | gnl\|UG\|Ta#S61812265 |
| - | gnl\|UG\|Ta#S61812304 |
| - | gnl\|UG\|Ta#S61812340 |
| - | gnl\|UG\|Ta#S61812340 |
| - | gnl\|UG\|Ta#S61812340 |
| - | gnl\|UG\|Ta#S61812340 |
| - | gnl\|UG\|Ta#S61812379 |
| - | gnl\|UG\|Ta#S61812410 |
| - | gnl\|UG\|Ta#S61812415 |
| - | gnl\|UG\|Ta#S61812511 |
| - | gnl\|UG\|Ta#S61812519 |

| | |
|---|---|
| - | gnl\|UG\|Ta#S61812519 |
| - | gnl\|UG\|Ta#S61812545 |
| - | gnl\|UG\|Ta#S61812547 |
| - | gnl\|UG\|Ta#S61826759 |
| - | gnl\|UG\|Ta#S61827822 |
| - | gnl\|UG\|Ta#S61828378 |
| - | gnl\|UG\|Ta#S61828421 |
| - | gnl\|UG\|Ta#S61828421 |
| - | gnl\|UG\|Ta#S61828524 |
| - | gnl\|UG\|Ta#S61828896 |
| - | gnl\|UG\|Ta#S61828974 |
| - | gnl\|UG\|Ta#S61829043 |
| - | gnl\|UG\|Ta#S61829198 |
| - | gnl\|UG\|Ta#S61829605 |
| - | gnl\|UG\|Ta#S61829981 |
| - | gnl\|UG\|Ta#S61830461 |
| - | gnl\|UG\|Ta#S61830511 |
| - | gnl\|UG\|Ta#S61830616 |
| - | gnl\|UG\|Ta#S61830616 |
| - | gnl\|UG\|Ta#S61831026 |
| - | gnl\|UG\|Ta#S61831155 |
| - | gnl\|UG\|Ta#S61831155 |
| - | gnl\|UG\|Ta#S61831232 |
| - | gnl\|UG\|Ta#S61831238 |
| - | gnl\|UG\|Ta#S61831245 |
| - | gnl\|UG\|Ta#S61831245 |
| - | gnl\|UG\|Ta#S61831376 |
| - | gnl\|UG\|Ta#S61831573 |
| - | gnl\|UG\|Ta#S61831762 |
| - | gnl\|UG\|Ta#S61831868 |
| - | gnl\|UG\|Ta#S61831915 |
| - | gnl\|UG\|Ta#S61831915 |
| - | gnl\|UG\|Ta#S61831928 |
| - | gnl\|UG\|Ta#S61832174 |
| - | gnl\|UG\|Ta#S61832238 |
| - | gnl\|UG\|Ta#S61832238 |
| - | gnl\|UG\|Ta#S61832329 |
| - | gnl\|UG\|Ta#S61832342 |
| - | gnl\|UG\|Ta#S61832342 |
| - | gnl\|UG\|Ta#S61832438 |
| - | gnl\|UG\|Ta#S61832492 |
| - | gnl\|UG\|Ta#S61832495 |
| - | gnl\|UG\|Ta#S61832495 |
| - | gnl\|UG\|Ta#S61832540 |
| - | gnl\|UG\|Ta#S61832541 |
| - | gnl\|UG\|Ta#S61832541 |
| - | gnl\|UG\|Ta#S61845762 |

| | |
|---|---|
| - | gnl\|UG\|Ta#S61859993 |
| - | gnl\|UG\|Ta#S61865642 |
| - | gnl\|UG\|Ta#S65589166 |
| - | gnl\|UG\|Ta#S65607555 |
| - | gnl\|UG\|Ta#S65614014 |
| - | gnl\|UG\|Ta#S65615828 |
| - | gnl\|UG\|Ta#S65626522 |
| - | gnl\|UG\|Ta#S65634660 |
| - | gnl\|UG\|Ta#S65667218 |
| - | gnl\|UG\|Ta#S65667218 |
| - | gnl\|UG\|Ta#S65673975 |
| - | gnl\|UG\|Ta#S65681351 |
| - | gnl\|UG\|Ta#S65712998 |
| - | gnl\|UG\|Ta#S65713877 |
| tr\|M7Y5N7\|M7Y5N7_TRIUA | - |
| tr\|M7Y5N7\|M7Y5N7_TRIUA | - |
| tr\|M7YI51\|M7YI51_TRIUA | - |
| tr\|M7Z6Y8\|M7Z6Y8_TRIUA | - |
| tr\|M7Z8R8\|M7Z8R8_TRIUA | - |
| tr\|M7ZA24\|M7ZA24_TRIUA | - |
| tr\|M7ZFK4\|M7ZFK4_TRIUA | - |
| tr\|M7ZHC3\|M7ZHC3_TRIUA | - |
| tr\|M7ZKD4\|M7ZKD4_TRIUA | - |
| tr\|M7ZN21\|M7ZN21_TRIUA | - |
| tr\|M7ZZX9\|M7ZZX9_TRIUA | - |
| tr\|M8A453\|M8A453_TRIUA | - |
| tr\|M8AJ32\|M8AJ32_TRIUA | - |
| tr\|M8AKT3\|M8AKT3_TRIUA | - |
| tr\|Q9XH54\|Q9XH54_WHEAT | - |
| tr\|T1LHL5\|T1LHL5_TRIUA | - |
| tr\|T1LHL5\|T1LHL5_TRIUA | - |
| tr\|T1LJY3\|T1LJY3_TRIUA | - |
| tr\|T1MFL6\|T1MFL6_TRIUA | - |
| tr\|W4ZWW3\|W4ZWW3_WHEAT | - |
| tr\|W5A2C1\|W5A2C1_WHEAT | - |
| tr\|W5A2L8\|W5A2L8_WHEAT | - |
| tr\|W5A2S7\|W5A2S7_WHEAT | - |
| tr\|W5A5D6\|W5A5D6_WHEAT | - |
| tr\|W5A5H7\|W5A5H7_WHEAT | - |
| tr\|W5AA30\|W5AA30_WHEAT | - |
| tr\|W5AC22\|W5AC22_WHEAT | - |
| tr\|W5ANL4\|W5ANL4_WHEAT | - |
| tr\|W5AU69\|W5AU69_WHEAT | - |
| tr\|W5DM71\|W5DM71_WHEAT | - |
| tr\|W5DM90\|W5DM90_WHEAT | - |
| tr\|W5DMU9\|W5DMU9_WHEAT | - |
| tr\|W5DN03\|W5DN03_WHEAT | - |

| | |
|---|---|
| tr\|W5DNA0\|W5DNA0_WHEAT | - |
| tr\|W5DNH2\|W5DNH2_WHEAT | - |
| tr\|W5DNY3\|W5DNY3_WHEAT | - |
| tr\|W5DP85\|W5DP85_WHEAT | - |
| tr\|W5DQM6\|W5DQM6_WHEAT | - |
| tr\|W5DRD2\|W5DRD2_WHEAT | - |
| tr\|W5DSK9\|W5DSK9_WHEAT | - |
| tr\|W5DSK9\|W5DSK9_WHEAT | - |
| tr\|W5DTJ3\|W5DTJ3_WHEAT | - |
| tr\|W5DTJ3\|W5DTJ3_WHEAT | - |
| tr\|W5DUD0\|W5DUD0_WHEAT | - |
| tr\|W5DUD0\|W5DUD0_WHEAT | - |
| tr\|W5DUD0\|W5DUD0_WHEAT | - |
| tr\|W5DYA3\|W5DYA3_WHEAT | - |
| tr\|W5DZJ3\|W5DZJ3_WHEAT | - |
| tr\|W5F016\|W5F016_WHEAT | - |
| tr\|W5F087\|W5F087_WHEAT | - |
| tr\|W5F2X4\|W5F2X4_WHEAT | - |
| tr\|W5F373\|W5F373_WHEAT | - |
| tr\|W5F6P3\|W5F6P3_WHEAT | - |
| tr\|W5F6P7\|W5F6P7_WHEAT | - |
| tr\|W5F718\|W5F718_WHEAT | - |
| tr\|W5F722\|W5F722_WHEAT | - |
| tr\|W5F892\|W5F892_WHEAT | - |
| tr\|W5F8B5\|W5F8B5_WHEAT | - |
| tr\|W5F8W4\|W5F8W4_WHEAT | - |
| tr\|W5F8W4\|W5F8W4_WHEAT | - |
| tr\|W5F9G1\|W5F9G1_WHEAT | - |
| tr\|W5FA82\|W5FA82_WHEAT | - |
| tr\|W5FAB4\|W5FAB4_WHEAT | - |
| tr\|W5FAB6\|W5FAB6_WHEAT | - |
| tr\|W5FAD0\|W5FAD0_WHEAT | - |
| tr\|W5FAD7\|W5FAD7_WHEAT | - |
| tr\|W5FAF9\|W5FAF9_WHEAT | - |
| tr\|W5FAF9\|W5FAF9_WHEAT | - |
| tr\|W5FB85\|W5FB85_WHEAT | - |
| tr\|W5FBX2\|W5FBX2_WHEAT | - |
| tr\|W5FCD1\|W5FCD1_WHEAT | - |
| tr\|W5FDI2\|W5FDI2_WHEAT | - |
| tr\|W5FE25\|W5FE25_WHEAT | - |
| tr\|W5FE27\|W5FE27_WHEAT | - |
| tr\|W5FE68\|W5FE68_WHEAT | - |
| tr\|W5FE80\|W5FE80_WHEAT | - |
| tr\|W5FE80\|W5FE80_WHEAT | - |
| tr\|W5FE80\|W5FE80_WHEAT | - |
| tr\|W5FED4\|W5FED4_WHEAT | - |
| tr\|W5FEM4\|W5FEM4_WHEAT | - |

| | |
|---|---|
| tr\|W5FEN4\|W5FEN4_WHEAT | - |
| tr\|W5FF50\|W5FF50_WHEAT | - |
| tr\|W5FH13\|W5FH13_WHEAT | - |
| tr\|W5FHJ0\|W5FHJ0_WHEAT | - |
| tr\|W5FHX5\|W5FHX5_WHEAT | - |
| tr\|W5FIJ0\|W5FIJ0_WHEAT | - |
| tr\|W5FIN8\|W5FIN8_WHEAT | - |
| tr\|W5FJD8\|W5FJD8_WHEAT | - |
| tr\|W5FJD9\|W5FJD9_WHEAT | - |
| tr\|W5FJS3\|W5FJS3_WHEAT | - |
| tr\|W5FJS3\|W5FJS3_WHEAT | - |
| tr\|W5FJW4\|W5FJW4_WHEAT | - |
| tr\|W5FJX8\|W5FJX8_WHEAT | - |
| tr\|W5FKM8\|W5FKM8_WHEAT | - |
| tr\|W5FL66\|W5FL66_WHEAT | - |
| tr\|W5FL66\|W5FL66_WHEAT | - |
| tr\|W5FL71\|W5FL71_WHEAT | - |
| tr\|W5FLD8\|W5FLD8_WHEAT | - |
| tr\|W5FLQ4\|W5FLQ4_WHEAT | - |
| tr\|W5FM16\|W5FM16_WHEAT | - |
| tr\|W5FM16\|W5FM16_WHEAT | - |
| tr\|W5FMB3\|W5FMB3_WHEAT | - |
| tr\|W5FNE6\|W5FNE6_WHEAT | - |
| tr\|W5FNG5\|W5FNG5_WHEAT | - |
| tr\|W5FNU4\|W5FNU4_WHEAT | - |
| tr\|W5FPG1\|W5FPG1_WHEAT | - |
| tr\|W5FQN4\|W5FQN4_WHEAT | - |
| tr\|W5FQN4\|W5FQN4_WHEAT | - |
| tr\|W5FSP0\|W5FSP0_WHEAT | - |
| tr\|W5HNL6\|W5HNL6_WHEAT | - |
| tr\|W5I590\|W5I590_WHEAT | - |
| tr\|W4ZZB3\|W4ZZB3_WHEAT | gnl\|UG\|Ta#S16978157 |
| tr\|M7Z2W6\|M7Z2W6_TRIUA | gnl\|UG\|Ta#S50378343 |
| tr\|W5FDI2\|W5FDI2_WHEAT | gnl\|UG\|Ta#S52542175 |
| tr\|W5FKJ5\|W5FKJ5_WHEAT | gnl\|UG\|Ta#S52542379 |
| tr\|W5HV50\|W5HV50_WHEAT | gnl\|UG\|Ta#S52543081 |
| tr\|W5FMV5\|W5FMV5_WHEAT | gnl\|UG\|Ta#S52543733 |
| tr\|M7ZNW8\|M7ZNW8_TRIUA | gnl\|UG\|Ta#S52544273 |
| tr\|M7ZC23\|M7ZC23_TRIUA | gnl\|UG\|Ta#S52544360 |
| tr\|W5EHM7\|W5EHM7_WHEAT | gnl\|UG\|Ta#S52544390 |
| tr\|W5FGV5\|W5FGV5_WHEAT | gnl\|UG\|Ta#S52544911 |
| tr\|W5HW67\|W5HW67_WHEAT | gnl\|UG\|Ta#S52545194 |
| tr\|W5A5A1\|W5A5A1_WHEAT | gnl\|UG\|Ta#S58840580 |
| tr\|W5DM92\|W5DM92_WHEAT | gnl\|UG\|Ta#S58849355 |
| tr\|W5F718\|W5F718_WHEAT | gnl\|UG\|Ta#S58857354 |
| tr\|M7YI51\|M7YI51_TRIUA | gnl\|UG\|Ta#S58869079 |
| tr\|M7Y892\|M7Y892_TRIUA | gnl\|UG\|Ta#S61526827 |

| | |
|---|---|
| tr\|W5FH68\|W5FH68_WHEAT | gnl\|UG\|Ta#S61544072 |
| tr\|W5GKY9\|W5GKY9_WHEAT | gnl\|UG\|Ta#S61558917 |
| tr\|M8A4J9\|M8A4J9_TRIUA | gnl\|UG\|Ta#S61561773 |
| tr\|M7Y912\|M7Y912_TRIUA | gnl\|UG\|Ta#S61583448 |
| tr\|W5DXE8\|W5DXE8_WHEAT | gnl\|UG\|Ta#S61585826 |
| tr\|M7Z5K9\|M7Z5K9_TRIUA | gnl\|UG\|Ta#S61589705 |
| tr\|W5FM36\|W5FM36_WHEAT | gnl\|UG\|Ta#S61592013 |
| tr\|W5E2L5\|W5E2L5_WHEAT | gnl\|UG\|Ta#S61596487 |
| tr\|W5A0H1\|W5A0H1_WHEAT | gnl\|UG\|Ta#S61600351 |
| tr\|M7YUY4\|M7YUY4_TRIUA | gnl\|UG\|Ta#S61602189 |
| tr\|M7ZWW6\|M7ZWW6_TRIUA | gnl\|UG\|Ta#S61602733 |
| tr\|M8B4H5\|M8B4H5_TRIUA | gnl\|UG\|Ta#S61605385 |
| tr\|W5FPH8\|W5FPH8_WHEAT | gnl\|UG\|Ta#S61609459 |
| tr\|W5FPH8\|W5FPH8_WHEAT | gnl\|UG\|Ta#S61609459 |
| tr\|W5DVC7\|W5DVC7_WHEAT | gnl\|UG\|Ta#S61609706 |
| tr\|W5DNH2\|W5DNH2_WHEAT | gnl\|UG\|Ta#S61610829 |
| tr\|W5FAQ0\|W5FAQ0_WHEAT | gnl\|UG\|Ta#S61612433 |
| tr\|W5FAQ0\|W5FAQ0_WHEAT | gnl\|UG\|Ta#S61612433 |
| tr\|M7YX46\|M7YX46_TRIUA | gnl\|UG\|Ta#S61614770 |
| tr\|G9DR92\|G9DR92_WHEAT | gnl\|UG\|Ta#S61615120 |
| tr\|W5HUH8\|W5HUH8_WHEAT | gnl\|UG\|Ta#S61616203 |
| tr\|T1LIH7\|T1LIH7_TRIUA | gnl\|UG\|Ta#S61623512 |
| tr\|W5A398\|W5A398_WHEAT | gnl\|UG\|Ta#S61635700 |
| tr\|W5A398\|W5A398_WHEAT | gnl\|UG\|Ta#S61635700 |
| tr\|W5A398\|W5A398_WHEAT | gnl\|UG\|Ta#S61635700 |
| tr\|T1M301\|T1M301_TRIUA | gnl\|UG\|Ta#S61644961 |
| tr\|W5FBP2\|W5FBP2_WHEAT | gnl\|UG\|Ta#S61708094 |
| tr\|W5FF94\|W5FF94_WHEAT | gnl\|UG\|Ta#S61710263 |
| tr\|W5FF94\|W5FF94_WHEAT | gnl\|UG\|Ta#S61710263 |
| tr\|W5FF94\|W5FF94_WHEAT | gnl\|UG\|Ta#S61710263 |
| tr\|W5FF94\|W5FF94_WHEAT | gnl\|UG\|Ta#S61710263 |
| tr\|W5FF94\|W5FF94_WHEAT | gnl\|UG\|Ta#S61710263 |
| tr\|W5DLX4\|W5DLX4_WHEAT | gnl\|UG\|Ta#S61738160 |
| tr\|M8A6U0\|M8A6U0_TRIUA | gnl\|UG\|Ta#S61749997 |
| tr\|W5GIK8\|W5GIK8_WHEAT | gnl\|UG\|Ta#S61771990 |
| tr\|W5DLW7\|W5DLW7_WHEAT | gnl\|UG\|Ta#S61779272 |
| tr\|M7ZWL8\|M7ZWL8_TRIUA | gnl\|UG\|Ta#S61788332 |
| tr\|M7Y9N5\|M7Y9N5_TRIUA | gnl\|UG\|Ta#S61789122 |
| tr\|W5H7C9\|W5H7C9_WHEAT | gnl\|UG\|Ta#S61790154 |
| tr\|W5FET2\|W5FET2_WHEAT | gnl\|UG\|Ta#S61791669 |
| tr\|M7ZKF1\|M7ZKF1_TRIUA | gnl\|UG\|Ta#S61800359 |
| tr\|M7ZIL1\|M7ZIL1_TRIUA | gnl\|UG\|Ta#S61808053 |
| tr\|W5FPZ3\|W5FPZ3_WHEAT | gnl\|UG\|Ta#S61809051 |
| tr\|W5FG98\|W5FG98_WHEAT | gnl\|UG\|Ta#S61809398 |
| tr\|M7ZE79\|M7ZE79_TRIUA | gnl\|UG\|Ta#S61810283 |
| tr\|W5FHD1\|W5FHD1_WHEAT | gnl\|UG\|Ta#S61811208 |
| tr\|W5FHD1\|W5FHD1_WHEAT | gnl\|UG\|Ta#S61811208 |

| | |
|---|---|
| tr\|W5FP83\|W5FP83_WHEAT | gnl\|UG\|Ta#S61811469 |
| tr\|W5I5K8\|W5I5K8_WHEAT | gnl\|UG\|Ta#S61811469 |
| tr\|M7ZG18\|M7ZG18_TRIUA | gnl\|UG\|Ta#S61811893 |
| tr\|W5GM70\|W5GM70_WHEAT | gnl\|UG\|Ta#S61811910 |
| tr\|M7ZT67\|M7ZT67_TRIUA | gnl\|UG\|Ta#S61812114 |
| tr\|M7YTB8\|M7YTB8_TRIUA | gnl\|UG\|Ta#S61812287 |
| tr\|W5DR26\|W5DR26_WHEAT | gnl\|UG\|Ta#S61812304 |
| tr\|W5FXY4\|W5FXY4_WHEAT | gnl\|UG\|Ta#S61812410 |
| tr\|M7ZUJ1\|M7ZUJ1_TRIUA | gnl\|UG\|Ta#S61812544 |
| tr\|W5FES1\|W5FES1_WHEAT | gnl\|UG\|Ta#S61812547 |
| tr\|W5FES1\|W5FES1_WHEAT | gnl\|UG\|Ta#S61812547 |
| tr\|W5DTA4\|W5DTA4_WHEAT | gnl\|UG\|Ta#S61827953 |
| tr\|W5FNW0\|W5FNW0_WHEAT | gnl\|UG\|Ta#S61829605 |
| tr\|W5G2L3\|W5G2L3_WHEAT | gnl\|UG\|Ta#S61829605 |
| tr\|M7Z1H7\|M7Z1H7_TRIUA | gnl\|UG\|Ta#S61830511 |
| tr\|W5FLA2\|W5FLA2_WHEAT | gnl\|UG\|Ta#S61831245 |
| tr\|M7ZP17\|M7ZP17_TRIUA | gnl\|UG\|Ta#S61831573 |
| tr\|W5F7Q1\|W5F7Q1_WHEAT | gnl\|UG\|Ta#S61831710 |
| tr\|W5EIT0\|W5EIT0_WHEAT | gnl\|UG\|Ta#S61832342 |
| tr\|M7ZWH0\|M7ZWH0_TRIUA | gnl\|UG\|Ta#S61832406 |
| tr\|W5A8H4\|W5A8H4_WHEAT | gnl\|UG\|Ta#S61832430 |
| tr\|W5FI15\|W5FI15_WHEAT | gnl\|UG\|Ta#S61839459 |
| tr\|W5FM85\|W5FM85_WHEAT | gnl\|UG\|Ta#S61863723 |
| tr\|W5FMI9\|W5FMI9_WHEAT | gnl\|UG\|Ta#S65598501 |
| tr\|M7ZL95\|M7ZL95_TRIUA | gnl\|UG\|Ta#S65611246 |
| tr\|W5GKB0\|W5GKB0_WHEAT | gnl\|UG\|Ta#S65632102 |
| tr\|W5EVZ3\|W5EVZ3_WHEAT | gnl\|UG\|Ta#S65639709 |
| tr\|W5FE25\|W5FE25_WHEAT | gnl\|UG\|Ta#S65649808 |
| tr\|W5FIG9\|W5FIG9_WHEAT | gnl\|UG\|Ta#S65662944 |
| tr\|W5F8U2\|W5F8U2_WHEAT | gnl\|UG\|Ta#S65674235 |
| tr\|W5A5H7\|W5A5H7_WHEAT | gnl\|UG\|Ta#S65717825 |
| - | - |
| - | - |
| - | - |
| - | - |
| - | - |
| - | - |
| - | - |
| - | - |
| - | - |
| - | - |
| - | - |
| - | - |
| - | - |
| - | - |
| - | - |
| - | - |

| | |
|---|---|
| - | - |
| - | - |
| - | - |
| - | - |
| - | - |
| - | - |
| - | - |
| - | - |
| - | - |
| - | - |
| - | - |
| - | - |
| - | - |
| - | - |
| - | - |
| - | - |
| - | - |
| - | - |
| - | - |
| - | - |
| - | - |
| - | - |
| - | - |
| - | - |
| - | - |
| - | - |
| - | - |
| - | - |
| - | - |
| - | - |
| - | - |
| - | - |
| - | - |
| - | - |
| - | - |
| - | - |
| - | - |
| - | - |
| - | - |
| - | - |
| - | - |
| - | - |
| - | - |
| - | - |
| - | - |
| - | - |
| - | - |

| | |
|---|---|
| - | - |
| - | - |
| - | - |
| - | - |
| - | - |
| - | - |
| - | - |
| - | - |
| - | - |
| - | - |
| - | - |
| - | - |
| - | - |
| - | - |
| - | - |
| - | - |
| - | - |
| - | - |
| - | - |
| - | - |
| - | - |
| - | - |
| - | - |
| - | - |
| - | - |
| - | - |
| - | - |
| - | - |
| - | - |
| - | - |
| - | - |
| - | - |
| - | - |
| - | - |
| - | - |
| - | - |
| - | - |
| - | - |
| - | - |
| - | - |
| - | - |
| - | - |
| - | - |
| - | - |
| - | - |
| - | - |
| - | - |

| | |
|---|---|
| - | - |
| - | - |
| - | - |
| - | - |
| - | - |
| - | - |
| - | - |
| - | - |
| - | - |
| - | - |
| - | - |
| - | - |
| - | - |
| - | - |
| - | - |
| - | - |
| - | - |
| - | - |
| - | - |
| - | - |
| - | - |
| - | - |
| - | - |
| - | - |
| - | - |
| - | - |
| - | - |
| - | - |
| - | - |
| - | - |
| - | - |
| - | - |
| - | - |
| - | - |
| - | - |
| - | - |
| - | - |
| - | - |
| - | - |
| - | - |
| - | - |
| - | - |
| - | - |
| - | - |
| - | - |
| - | - |
| - | - |

| | |
|---|---|
| - | - |
| - | - |
| - | - |
| - | - |
| - | - |
| - | - |
| - | - |
| - | - |
| - | - |
| - | - |
| - | - |
| - | - |
| - | - |
| - | - |
| - | - |
| - | - |
| - | - |
| - | - |
| - | - |
| - | - |
| - | - |
| - | - |
| - | - |
| - | - |
| - | - |
| - | - |
| - | - |
| - | - |
| - | - |
| - | - |
| - | - |
| - | - |
| - | - |
| - | - |
| - | - |
| - | - |
| - | - |
| - | - |
| - | - |
| - | - |
| - | - |
| - | - |
| - | - |
| - | - |
| - | - |
| - | - |
| - | - |

| | |
|---|---|
| - | - |
| - | - |
| - | - |
| - | - |
| - | - |
| - | - |
| - | - |
| - | - |
| - | - |
| - | - |
| - | - |
| - | - |
| - | - |
| - | - |
| - | - |
| - | - |
| - | - |
| - | - |
| - | - |
| - | - |
| - | - |
| - | - |
| - | - |
| - | - |
| - | - |
| - | - |
| - | - |
| - | - |
| - | - |
| - | - |
| - | - |
| - | - |
| - | - |
| - | - |
| - | - |
| - | - |
| - | - |
| - | - |
| - | - |
| - | - |
| - | - |
| - | - |
| - | - |
| - | - |
| - | - |
| - | - |
| - | - |

| | |
|---|---|
| - | - |
| - | - |
| - | - |
| - | - |
| - | - |
| - | - |
| - | - |
| - | - |
| - | - |
| - | - |
| - | - |
| - | - |
| - | - |
| - | - |
| - | - |
| - | - |
| - | - |
| - | - |
| - | - |
| - | - |
| - | - |
| - | - |
| - | - |
| - | - |
| - | - |
| - | - |
| - | - |
| - | - |
| - | - |
| - | - |
| - | - |
| - | - |
| - | - |
| - | - |
| - | - |
| - | - |
| - | - |
| - | - |
| - | - |
| - | - |
| - | - |
| - | - |
| - | - |
| - | - |
| - | - |
| - | - |
| - | - |

| | |
|---|---|
| - | - |
| - | - |
| - | - |
| - | - |
| - | - |
| - | - |
| - | - |
| - | - |
| - | - |
| - | - |
| - | - |
| - | - |
| - | - |
| - | - |
| - | - |
| - | - |
| - | - |
| - | - |
| - | - |
| - | - |
| - | - |
| - | - |
| - | - |
| - | - |
| - | - |
| - | - |
| - | - |
| - | - |
| - | - |
| - | - |
| - | - |
| - | - |
| - | - |
| - | - |
| - | - |
| - | - |
| - | - |
| - | - |
| - | - |
| - | - |
| - | - |
| - | - |
| - | - |
| - | - |
| - | - |
| - | - |
| - | - |

| | |
|---|---|
| - | - |
| - | - |
| - | - |
| - | - |
| - | - |
| - | - |
| - | - |
| - | - |
| - | - |
| - | - |
| - | - |
| - | - |
| - | - |
| - | - |
| - | - |
| - | - |
| - | - |
| - | - |
| - | - |
| - | - |
| - | - |
| - | - |
| - | - |
| - | - |
| - | - |
| - | - |
| - | - |
| - | - |
| - | - |
| - | - |
| - | - |
| - | - |
| - | - |
| - | - |
| - | - |
| - | - |
| - | - |
| - | - |
| - | - |
| - | - |
| - | - |
| - | - |
| - | - |
| - | - |
| - | - |
| - | - |
| - | - |

-
-
-
-
-
-
-
-
-
-
-
-
-
-
-
-
-
-
-
-
-
-
-
-
-
-
-
-
-
-
-
-
-
-
-
-
-
-
-
-
-
-
-
-
-
-
-
-
-
-
-
-
-
-
-
-
-
-
-
-
-
-
-
-
-
-
-
-
-
-
-
-
-
-
-
-
-
-
-
-
-
-
-
-
-
-
-
-
-
-
-
-
-
-

| | |
|---|---|
| - | - |
| - | - |
| - | - |
| - | - |
| - | - |
| - | - |
| - | - |
| - | - |
| - | gnl\|UG\|Ta#S25040821 |
| - | gnl\|UG\|Ta#S27052963 |
| - | gnl\|UG\|Ta#S32422896 |
| - | gnl\|UG\|Ta#S37806908 |
| - | gnl\|UG\|Ta#S37875237 |
| - | gnl\|UG\|Ta#S52541041 |
| - | gnl\|UG\|Ta#S52541041 |
| - | gnl\|UG\|Ta#S52541041 |
| - | gnl\|UG\|Ta#S52542456 |
| - | gnl\|UG\|Ta#S52543703 |
| - | gnl\|UG\|Ta#S52543703 |
| - | gnl\|UG\|Ta#S52543703 |
| - | gnl\|UG\|Ta#S52543823 |
| - | gnl\|UG\|Ta#S52543823 |
| - | gnl\|UG\|Ta#S52544270 |
| - | gnl\|UG\|Ta#S52544506 |
| - | gnl\|UG\|Ta#S52544933 |
| - | gnl\|UG\|Ta#S52546011 |
| - | gnl\|UG\|Ta#S52546152 |
| - | gnl\|UG\|Ta#S52546323 |
| - | gnl\|UG\|Ta#S52546432 |
| - | gnl\|UG\|Ta#S52546628 |
| - | gnl\|UG\|Ta#S52546775 |
| - | gnl\|UG\|Ta#S52546805 |
| - | gnl\|UG\|Ta#S52547038 |
| - | gnl\|UG\|Ta#S52547083 |
| - | gnl\|UG\|Ta#S58849997 |
| - | gnl\|UG\|Ta#S58856577 |
| - | gnl\|UG\|Ta#S58857059 |
| - | gnl\|UG\|Ta#S58858754 |
| - | gnl\|UG\|Ta#S58859053 |
| - | gnl\|UG\|Ta#S58889605 |
| - | gnl\|UG\|Ta#S58896173 |
| - | gnl\|UG\|Ta#S58899264 |
| - | gnl\|UG\|Ta#S58902813 |
| - | gnl\|UG\|Ta#S58904510 |
| - | gnl\|UG\|Ta#S61524771 |
| - | gnl\|UG\|Ta#S61543268 |
| - | gnl\|UG\|Ta#S61547756 |

| | |
|---|---|
| - | gnl\|UG\|Ta#S61553749 |
| - | gnl\|UG\|Ta#S61553749 |
| - | gnl\|UG\|Ta#S61561960 |
| - | gnl\|UG\|Ta#S61566362 |
| - | gnl\|UG\|Ta#S61592867 |
| - | gnl\|UG\|Ta#S61596466 |
| - | gnl\|UG\|Ta#S61607104 |
| - | gnl\|UG\|Ta#S61607697 |
| - | gnl\|UG\|Ta#S61613267 |
| - | gnl\|UG\|Ta#S61616281 |
| - | gnl\|UG\|Ta#S61616281 |
| - | gnl\|UG\|Ta#S61618093 |
| - | gnl\|UG\|Ta#S61621189 |
| - | gnl\|UG\|Ta#S61632658 |
| - | gnl\|UG\|Ta#S61639512 |
| - | gnl\|UG\|Ta#S61650526 |
| - | gnl\|UG\|Ta#S61679872 |
| - | gnl\|UG\|Ta#S61682734 |
| - | gnl\|UG\|Ta#S61686166 |
| - | gnl\|UG\|Ta#S61708991 |
| - | gnl\|UG\|Ta#S61734649 |
| - | gnl\|UG\|Ta#S61736462 |
| - | gnl\|UG\|Ta#S61753558 |
| - | gnl\|UG\|Ta#S61767590 |
| - | gnl\|UG\|Ta#S61768369 |
| - | gnl\|UG\|Ta#S61770378 |
| - | gnl\|UG\|Ta#S61771177 |
| - | gnl\|UG\|Ta#S61772234 |
| - | gnl\|UG\|Ta#S61778432 |
| - | gnl\|UG\|Ta#S61781683 |
| - | gnl\|UG\|Ta#S61781683 |
| - | gnl\|UG\|Ta#S61785667 |
| - | gnl\|UG\|Ta#S61788836 |
| - | gnl\|UG\|Ta#S61792433 |
| - | gnl\|UG\|Ta#S61799385 |
| - | gnl\|UG\|Ta#S61800416 |
| - | gnl\|UG\|Ta#S61801701 |
| - | gnl\|UG\|Ta#S61801701 |
| - | gnl\|UG\|Ta#S61802148 |
| - | gnl\|UG\|Ta#S61802531 |
| - | gnl\|UG\|Ta#S61807673 |
| - | gnl\|UG\|Ta#S61808538 |
| - | gnl\|UG\|Ta#S61808538 |
| - | gnl\|UG\|Ta#S61808539 |
| - | gnl\|UG\|Ta#S61808856 |
| - | gnl\|UG\|Ta#S61809131 |
| - | gnl\|UG\|Ta#S61809392 |

| | |
|---|---|
| - | gnl|UG|Ta#S61809392 |
| - | gnl|UG|Ta#S61809392 |
| - | gnl|UG|Ta#S61809487 |
| - | gnl|UG|Ta#S61809734 |
| - | gnl|UG|Ta#S61810290 |
| - | gnl|UG|Ta#S61811494 |
| - | gnl|UG|Ta#S61811584 |
| - | gnl|UG|Ta#S61811584 |
| - | gnl|UG|Ta#S61811681 |
| - | gnl|UG|Ta#S61811731 |
| - | gnl|UG|Ta#S61811900 |
| - | gnl|UG|Ta#S61812080 |
| - | gnl|UG|Ta#S61812124 |
| - | gnl|UG|Ta#S61812260 |
| - | gnl|UG|Ta#S61812317 |
| - | gnl|UG|Ta#S61812415 |
| - | gnl|UG|Ta#S61812434 |
| - | gnl|UG|Ta#S61812441 |
| - | gnl|UG|Ta#S61812461 |
| - | gnl|UG|Ta#S61812538 |
| - | gnl|UG|Ta#S61812545 |
| - | gnl|UG|Ta#S61827924 |
| - | gnl|UG|Ta#S61828378 |
| - | gnl|UG|Ta#S61828504 |
| - | gnl|UG|Ta#S61829393 |
| - | gnl|UG|Ta#S61829393 |
| - | gnl|UG|Ta#S61829803 |
| - | gnl|UG|Ta#S61829981 |
| - | gnl|UG|Ta#S61830461 |
| - | gnl|UG|Ta#S61831211 |
| - | gnl|UG|Ta#S61831342 |
| - | gnl|UG|Ta#S61831367 |
| - | gnl|UG|Ta#S61831367 |
| - | gnl|UG|Ta#S61831561 |
| - | gnl|UG|Ta#S61831762 |
| - | gnl|UG|Ta#S61831868 |
| - | gnl|UG|Ta#S61831945 |
| - | gnl|UG|Ta#S61832168 |
| - | gnl|UG|Ta#S61832174 |
| - | gnl|UG|Ta#S61832174 |
| - | gnl|UG|Ta#S61832209 |
| - | gnl|UG|Ta#S61832271 |
| - | gnl|UG|Ta#S61832283 |
| - | gnl|UG|Ta#S61832283 |
| - | gnl|UG|Ta#S61832389 |
| - | gnl|UG|Ta#S61832419 |
| - | gnl|UG|Ta#S61832430 |

| | |
|---|---|
| - | gnl\|UG\|Ta#S61832525 |
| - | gnl\|UG\|Ta#S61832525 |
| - | gnl\|UG\|Ta#S61832525 |
| - | gnl\|UG\|Ta#S61832533 |
| - | gnl\|UG\|Ta#S61832533 |
| - | gnl\|UG\|Ta#S61833899 |
| - | gnl\|UG\|Ta#S61847383 |
| - | gnl\|UG\|Ta#S65594222 |
| - | gnl\|UG\|Ta#S65594222 |
| - | gnl\|UG\|Ta#S65600442 |
| - | gnl\|UG\|Ta#S65602357 |
| - | gnl\|UG\|Ta#S65607015 |
| - | gnl\|UG\|Ta#S65611074 |
| - | gnl\|UG\|Ta#S65616934 |
| - | gnl\|UG\|Ta#S65618144 |
| - | gnl\|UG\|Ta#S65627198 |
| - | gnl\|UG\|Ta#S65676358 |
| - | gnl\|UG\|Ta#S65710267 |
| - | gnl\|UG\|Ta#S65710267 |
| - | gnl\|UG\|Ta#S65711079 |
| - | gnl\|UG\|Ta#S65712674 |
| - | gnl\|UG\|Ta#S65714355 |
| tr\|A8BCW8\|A8BCW8_WHEAT | - |
| tr\|M7YDL5\|M7YDL5_TRIUA | - |
| tr\|M7YY51\|M7YY51_TRIUA | - |
| tr\|M7ZF26\|M7ZF26_TRIUA | - |
| tr\|M7ZIC0\|M7ZIC0_TRIUA | - |
| tr\|M7ZMZ3\|M7ZMZ3_TRIUA | - |
| tr\|M7ZNY9\|M7ZNY9_TRIUA | - |
| tr\|M7ZQ19\|M7ZQ19_TRIUA | - |
| tr\|M8A0A1\|M8A0A1_TRIUA | - |
| tr\|M8A0I9\|M8A0I9_TRIUA | - |
| tr\|M8AF88\|M8AF88_TRIUA | - |
| tr\|Q6UJY6\|Q6UJY6_TRITU | - |
| tr\|T1LK04\|T1LK04_TRIUA | - |
| tr\|W4ZVK2\|W4ZVK2_WHEAT | - |
| tr\|W5A579\|W5A579_WHEAT | - |
| tr\|W5A6D2\|W5A6D2_WHEAT | - |
| tr\|W5A746\|W5A746_WHEAT | - |
| tr\|W5AB36\|W5AB36_WHEAT | - |
| tr\|W5AC22\|W5AC22_WHEAT | - |
| tr\|W5ARH4\|W5ARH4_WHEAT | - |
| tr\|W5B7G1\|W5B7G1_WHEAT | - |
| tr\|W5DN51\|W5DN51_WHEAT | - |
| tr\|W5DUD0\|W5DUD0_WHEAT | - |
| tr\|W5DVX4\|W5DVX4_WHEAT | - |
| tr\|W5EIU2\|W5EIU2_WHEAT | - |

| | |
|---|---|
| tr\|W5EVD7\|W5EVD7_WHEAT | - |
| tr\|W5F4Z5\|W5F4Z5_WHEAT | - |
| tr\|W5F8N5\|W5F8N5_WHEAT | - |
| tr\|W5F9L2\|W5F9L2_WHEAT | - |
| tr\|W5F9R7\|W5F9R7_WHEAT | - |
| tr\|W5F9R8\|W5F9R8_WHEAT | - |
| tr\|W5FAJ4\|W5FAJ4_WHEAT | - |
| tr\|W5FB77\|W5FB77_WHEAT | - |
| tr\|W5FCJ3\|W5FCJ3_WHEAT | - |
| tr\|W5FEE3\|W5FEE3_WHEAT | - |
| tr\|W5FFY9\|W5FFY9_WHEAT | - |
| tr\|W5FH27\|W5FH27_WHEAT | - |
| tr\|W5FH68\|W5FH68_WHEAT | - |
| tr\|W5FHM6\|W5FHM6_WHEAT | - |
| tr\|W5FJ26\|W5FJ26_WHEAT | - |
| tr\|W5FJA7\|W5FJA7_WHEAT | - |
| tr\|W5FLX7\|W5FLX7_WHEAT | - |
| tr\|W5FMS8\|W5FMS8_WHEAT | - |
| tr\|W5FMV0\|W5FMV0_WHEAT | - |
| tr\|W5G0V3\|W5G0V3_WHEAT | - |
| tr\|W5G4T6\|W5G4T6_WHEAT | - |
| tr\|W5GTY4\|W5GTY4_WHEAT | - |
| tr\|W5H7S3\|W5H7S3_WHEAT | - |
| tr\|W5HPK2\|W5HPK2_WHEAT | - |
| tr\|W5HU48\|W5HU48_WHEAT | - |
| tr\|M7ZJN4\|M7ZJN4_TRIUA | gnl\|UG\|Ta#S52541041 |
| tr\|W5F8R3\|W5F8R3_WHEAT | gnl\|UG\|Ta#S52541580 |
| tr\|W5FPB7\|W5FPB7_WHEAT | gnl\|UG\|Ta#S52544097 |
| tr\|W5FH27\|W5FH27_WHEAT | gnl\|UG\|Ta#S52545304 |
| tr\|W5FVA2\|W5FVA2_WHEAT | gnl\|UG\|Ta#S52546584 |
| tr\|W5F7X4\|W5F7X4_WHEAT | gnl\|UG\|Ta#S52546628 |
| tr\|W5FQ89\|W5FQ89_WHEAT | gnl\|UG\|Ta#S58858754 |
| tr\|W5F375\|W5F375_WHEAT | gnl\|UG\|Ta#S58899204 |
| tr\|M8A956\|M8A956_TRIUA | gnl\|UG\|Ta#S61538487 |
| tr\|M8A7U2\|M8A7U2_TRIUA | gnl\|UG\|Ta#S61554693 |
| tr\|W5FI95\|W5FI95_WHEAT | gnl\|UG\|Ta#S61586211 |
| tr\|M7Z8C6\|M7Z8C6_TRIUA | gnl\|UG\|Ta#S61611875 |
| tr\|M7Z8C6\|M7Z8C6_TRIUA | gnl\|UG\|Ta#S61611875 |
| tr\|W5ELV1\|W5ELV1_WHEAT | gnl\|UG\|Ta#S61616260 |
| tr\|W5F832\|W5F832_WHEAT | gnl\|UG\|Ta#S61617424 |
| tr\|M7ZZV9\|M7ZZV9_TRIUA | gnl\|UG\|Ta#S61637542 |
| tr\|M7ZBZ4\|M7ZBZ4_TRIUA | gnl\|UG\|Ta#S61641004 |
| tr\|M7YGE1\|M7YGE1_TRIUA | gnl\|UG\|Ta#S61669611 |
| tr\|M7ZQI9\|M7ZQI9_TRIUA | gnl\|UG\|Ta#S61671779 |
| tr\|W5FB43\|W5FB43_WHEAT | gnl\|UG\|Ta#S61781384 |
| tr\|M7YSF3\|M7YSF3_TRIUA | gnl\|UG\|Ta#S61810290 |
| tr\|M8B371\|M8B371_TRIUA | gnl\|UG\|Ta#S61810654 |

| | |
|---|---|
| tr|W5E751|W5E751_WHEAT | gnl|UG|Ta#S61811584 |
| tr|M7ZH74|M7ZH74_TRIUA | gnl|UG|Ta#S61812530 |
| tr|T1LZE7|T1LZE7_TRIUA | gnl|UG|Ta#S61829883 |
| tr|W5A0L9|W5A0L9_WHEAT | gnl|UG|Ta#S61830810 |
| tr|W5FGW4|W5FGW4_WHEAT | gnl|UG|Ta#S61831109 |
| tr|W5F6A9|W5F6A9_WHEAT | gnl|UG|Ta#S61831808 |
| tr|W5HRF6|W5HRF6_WHEAT | gnl|UG|Ta#S61832093 |
| tr|T1L839|T1L839_TRIUA | gnl|UG|Ta#S65684804 |
| tr|W5DT88|W5DT88_WHEAT | gnl|UG|Ta#S65722137 |
| - | - |
| - | - |
| - | - |
| - | - |
| - | - |
| - | - |
| - | - |
| - | - |
| - | - |
| - | - |
| - | - |
| - | - |
| - | - |
| - | - |
| - | - |
| - | - |
| - | - |
| - | - |
| - | - |
| - | - |
| - | - |
| - | - |
| - | - |
| - | - |
| - | - |
| - | - |
| - | - |
| - | - |
| - | - |
| - | - |
| - | - |
| - | - |
| - | - |
| - | - |
| - | - |
| - | - |
| - | - |
| - | - |

| | |
|---|---|
| - | - |
| - | - |
| - | - |
| - | - |
| - | - |
| - | - |
| - | - |
| - | - |
| - | - |
| - | - |
| - | - |
| - | - |
| - | - |
| - | - |
| - | - |
| - | - |
| - | - |
| - | - |
| - | - |
| - | - |
| - | - |
| - | - |
| - | - |
| - | - |
| - | - |
| - | - |
| - | - |
| - | - |
| - | - |
| - | - |
| - | - |
| - | - |
| - | - |
| - | - |
| - | - |
| - | - |
| - | - |
| - | - |
| - | - |
| - | - |
| - | - |
| - | - |
| - | - |
| - | - |
| - | - |
| - | - |
| - | - |

| | |
|---|---|
| - | - |
| - | - |
| - | - |
| - | - |
| - | - |
| - | - |
| - | - |
| - | - |
| - | - |
| - | - |
| - | - |
| - | - |
| - | - |
| - | - |
| - | - |
| - | - |
| - | - |
| - | - |
| - | - |
| - | - |
| - | - |
| - | - |
| - | - |
| - | - |
| - | - |
| - | - |
| - | - |
| - | - |
| - | - |
| - | - |
| - | - |
| - | - |
| - | - |
| - | - |
| - | - |
| - | - |
| - | - |
| - | - |
| - | - |
| - | - |
| - | - |
| - | - |
| - | - |
| - | - |
| - | - |
| - | - |
| - | - |

| | |
|---|---|
| - | - |
| - | - |
| - | - |
| - | - |
| - | - |
| - | - |
| - | - |
| - | - |
| - | - |
| - | - |
| - | - |
| - | - |
| - | - |
| - | - |
| - | - |
| - | - |
| - | - |
| - | - |
| - | - |
| - | - |
| - | - |
| - | - |
| - | - |
| - | - |
| - | - |
| - | - |
| - | - |
| - | - |
| - | - |
| - | - |
| - | - |
| - | - |
| - | - |
| - | - |
| - | - |
| - | - |
| - | - |
| - | - |
| - | - |
| - | - |
| - | - |
| - | - |
| - | - |
| - | - |
| - | - |
| - | - |
| - | - |

| | |
|---|---|
| - | - |
| - | - |
| - | - |
| - | - |
| - | - |
| - | - |
| - | - |
| - | - |
| - | - |
| - | - |
| - | - |
| - | - |
| - | - |
| - | - |
| - | - |
| - | - |
| - | - |
| - | - |
| - | - |
| - | - |
| - | - |
| - | - |
| - | - |
| - | - |
| - | - |
| - | - |
| - | - |
| - | - |
| - | - |
| - | - |
| - | - |
| - | - |
| - | - |
| - | - |
| - | - |
| - | - |
| - | - |
| - | - |
| - | - |
| - | - |
| - | - |
| - | - |
| - | - |
| - | - |
| - | - |
| - | - |
| - | - |

| | |
|---|---|
| - | - |
| - | - |
| - | - |
| - | - |
| - | - |
| - | - |
| - | - |
| - | - |
| - | - |
| - | - |
| - | - |
| - | - |
| - | - |
| - | - |
| - | - |
| - | - |
| - | - |
| - | - |
| - | - |
| - | - |
| - | - |
| - | - |
| - | - |
| - | - |
| - | - |
| - | - |
| - | - |
| - | - |
| - | - |
| - | - |
| - | - |
| - | - |
| - | - |
| - | - |
| - | - |
| - | - |
| - | - |
| - | - |
| - | - |
| - | - |
| - | gnl\|UG\|Ta#S13037845 |
| - | gnl\|UG\|Ta#S17893257 |
| - | gnl\|UG\|Ta#S22381286 |
| - | gnl\|UG\|Ta#S22386058 |
| - | gnl\|UG\|Ta#S25040821 |
| - | gnl\|UG\|Ta#S26027113 |
| - | gnl\|UG\|Ta#S26029071 |

| | |
|---|---|
| - | gnl\|UG\|Ta#S32564699 |
| - | gnl\|UG\|Ta#S32630393 |
| - | gnl\|UG\|Ta#S37930561 |
| - | gnl\|UG\|Ta#S37936528 |
| - | gnl\|UG\|Ta#S43837843 |
| - | gnl\|UG\|Ta#S43837843 |
| - | gnl\|UG\|Ta#S52541003 |
| - | gnl\|UG\|Ta#S52541068 |
| - | gnl\|UG\|Ta#S52541068 |
| - | gnl\|UG\|Ta#S52541102 |
| - | gnl\|UG\|Ta#S52541562 |
| - | gnl\|UG\|Ta#S52541562 |
| - | gnl\|UG\|Ta#S52541580 |
| - | gnl\|UG\|Ta#S52541717 |
| - | gnl\|UG\|Ta#S52541717 |
| - | gnl\|UG\|Ta#S52542140 |
| - | gnl\|UG\|Ta#S52542154 |
| - | gnl\|UG\|Ta#S52542310 |
| - | gnl\|UG\|Ta#S52542310 |
| - | gnl\|UG\|Ta#S52542608 |
| - | gnl\|UG\|Ta#S52542744 |
| - | gnl\|UG\|Ta#S52542770 |
| - | gnl\|UG\|Ta#S52542912 |
| - | gnl\|UG\|Ta#S52543078 |
| - | gnl\|UG\|Ta#S52543475 |
| - | gnl\|UG\|Ta#S52543556 |
| - | gnl\|UG\|Ta#S52543768 |
| - | gnl\|UG\|Ta#S52543805 |
| - | gnl\|UG\|Ta#S52544519 |
| - | gnl\|UG\|Ta#S52544697 |
| - | gnl\|UG\|Ta#S52544707 |
| - | gnl\|UG\|Ta#S52544721 |
| - | gnl\|UG\|Ta#S52544721 |
| - | gnl\|UG\|Ta#S52544785 |
| - | gnl\|UG\|Ta#S52544878 |
| - | gnl\|UG\|Ta#S52544904 |
| - | gnl\|UG\|Ta#S52545009 |
| - | gnl\|UG\|Ta#S52545072 |
| - | gnl\|UG\|Ta#S52545072 |
| - | gnl\|UG\|Ta#S52545072 |
| - | gnl\|UG\|Ta#S52545072 |
| - | gnl\|UG\|Ta#S52545175 |
| - | gnl\|UG\|Ta#S52545434 |
| - | gnl\|UG\|Ta#S52545489 |
| - | gnl\|UG\|Ta#S52545582 |
| - | gnl\|UG\|Ta#S52545587 |
| - | gnl\|UG\|Ta#S52545621 |

| | |
|---|---|
| - | gnl\|UG\|Ta#S52545621 |
| - | gnl\|UG\|Ta#S52545748 |
| - | gnl\|UG\|Ta#S52545806 |
| - | gnl\|UG\|Ta#S52545921 |
| - | gnl\|UG\|Ta#S52545989 |
| - | gnl\|UG\|Ta#S52546054 |
| - | gnl\|UG\|Ta#S52546099 |
| - | gnl\|UG\|Ta#S52546099 |
| - | gnl\|UG\|Ta#S52546225 |
| - | gnl\|UG\|Ta#S52546249 |
| - | gnl\|UG\|Ta#S52546323 |
| - | gnl\|UG\|Ta#S52546362 |
| - | gnl\|UG\|Ta#S52546432 |
| - | gnl\|UG\|Ta#S52546628 |
| - | gnl\|UG\|Ta#S52546666 |
| - | gnl\|UG\|Ta#S52546779 |
| - | gnl\|UG\|Ta#S52546837 |
| - | gnl\|UG\|Ta#S52547068 |
| - | gnl\|UG\|Ta#S52547138 |
| - | gnl\|UG\|Ta#S52547138 |
| - | gnl\|UG\|Ta#S58853822 |
| - | gnl\|UG\|Ta#S58853822 |
| - | gnl\|UG\|Ta#S58853822 |
| - | gnl\|UG\|Ta#S58853822 |
| - | gnl\|UG\|Ta#S58857354 |
| - | gnl\|UG\|Ta#S58864265 |
| - | gnl\|UG\|Ta#S58867524 |
| - | gnl\|UG\|Ta#S58889253 |
| - | gnl\|UG\|Ta#S58890447 |
| - | gnl\|UG\|Ta#S58902813 |
| - | gnl\|UG\|Ta#S58908211 |
| - | gnl\|UG\|Ta#S58909613 |
| - | gnl\|UG\|Ta#S61516973 |
| - | gnl\|UG\|Ta#S61528543 |
| - | gnl\|UG\|Ta#S61533972 |
| - | gnl\|UG\|Ta#S61533972 |
| - | gnl\|UG\|Ta#S61536175 |
| - | gnl\|UG\|Ta#S61538090 |
| - | gnl\|UG\|Ta#S61542167 |
| - | gnl\|UG\|Ta#S61543965 |
| - | gnl\|UG\|Ta#S61543979 |
| - | gnl\|UG\|Ta#S61543979 |
| - | gnl\|UG\|Ta#S61545032 |
| - | gnl\|UG\|Ta#S61545032 |
| - | gnl\|UG\|Ta#S61545032 |
| - | gnl\|UG\|Ta#S61547588 |
| - | gnl\|UG\|Ta#S61550563 |

| | |
|---|---|
| - | gnl\|UG\|Ta#S61550563 |
| - | gnl\|UG\|Ta#S61550563 |
| - | gnl\|UG\|Ta#S61555814 |
| - | gnl\|UG\|Ta#S61572925 |
| - | gnl\|UG\|Ta#S61574079 |
| - | gnl\|UG\|Ta#S61581344 |
| - | gnl\|UG\|Ta#S61581344 |
| - | gnl\|UG\|Ta#S61581344 |
| - | gnl\|UG\|Ta#S61586386 |
| - | gnl\|UG\|Ta#S61591343 |
| - | gnl\|UG\|Ta#S61595340 |
| - | gnl\|UG\|Ta#S61596487 |
| - | gnl\|UG\|Ta#S61597002 |
| - | gnl\|UG\|Ta#S61600255 |
| - | gnl\|UG\|Ta#S61600636 |
| - | gnl\|UG\|Ta#S61601256 |
| - | gnl\|UG\|Ta#S61603098 |
| - | gnl\|UG\|Ta#S61605980 |
| - | gnl\|UG\|Ta#S61606387 |
| - | gnl\|UG\|Ta#S61609706 |
| - | gnl\|UG\|Ta#S61609706 |
| - | gnl\|UG\|Ta#S61610829 |
| - | gnl\|UG\|Ta#S61613302 |
| - | gnl\|UG\|Ta#S61615120 |
| - | gnl\|UG\|Ta#S61616260 |
| - | gnl\|UG\|Ta#S61616715 |
| - | gnl\|UG\|Ta#S61617166 |
| - | gnl\|UG\|Ta#S61621189 |
| - | gnl\|UG\|Ta#S61621189 |
| - | gnl\|UG\|Ta#S61621189 |
| - | gnl\|UG\|Ta#S61623782 |
| - | gnl\|UG\|Ta#S61623782 |
| - | gnl\|UG\|Ta#S61623782 |
| - | gnl\|UG\|Ta#S61624593 |
| - | gnl\|UG\|Ta#S61624593 |
| - | gnl\|UG\|Ta#S61626294 |
| - | gnl\|UG\|Ta#S61628441 |
| - | gnl\|UG\|Ta#S61632027 |
| - | gnl\|UG\|Ta#S61632218 |
| - | gnl\|UG\|Ta#S61632658 |
| - | gnl\|UG\|Ta#S61634015 |
| - | gnl\|UG\|Ta#S61634666 |
| - | gnl\|UG\|Ta#S61635401 |
| - | gnl\|UG\|Ta#S61635784 |
| - | gnl\|UG\|Ta#S61636247 |
| - | gnl\|UG\|Ta#S61639183 |
| - | gnl\|UG\|Ta#S61639183 |

| | |
|---|---|
| - | gnl\|UG\|Ta#S61639512 |
| - | gnl\|UG\|Ta#S61644963 |
| - | gnl\|UG\|Ta#S61652518 |
| - | gnl\|UG\|Ta#S61669075 |
| - | gnl\|UG\|Ta#S61669075 |
| - | gnl\|UG\|Ta#S61670511 |
| - | gnl\|UG\|Ta#S61670511 |
| - | gnl\|UG\|Ta#S61672259 |
| - | gnl\|UG\|Ta#S61672552 |
| - | gnl\|UG\|Ta#S61688932 |
| - | gnl\|UG\|Ta#S61696062 |
| - | gnl\|UG\|Ta#S61710756 |
| - | gnl\|UG\|Ta#S61720848 |
| - | gnl\|UG\|Ta#S61734961 |
| - | gnl\|UG\|Ta#S61745617 |
| - | gnl\|UG\|Ta#S61746792 |
| - | gnl\|UG\|Ta#S61747542 |
| - | gnl\|UG\|Ta#S61768369 |
| - | gnl\|UG\|Ta#S61768369 |
| - | gnl\|UG\|Ta#S61777641 |
| - | gnl\|UG\|Ta#S61779252 |
| - | gnl\|UG\|Ta#S61781683 |
| - | gnl\|UG\|Ta#S61782156 |
| - | gnl\|UG\|Ta#S61782281 |
| - | gnl\|UG\|Ta#S61782348 |
| - | gnl\|UG\|Ta#S61785667 |
| - | gnl\|UG\|Ta#S61790274 |
| - | gnl\|UG\|Ta#S61791016 |
| - | gnl\|UG\|Ta#S61791016 |
| - | gnl\|UG\|Ta#S61791016 |
| - | gnl\|UG\|Ta#S61791519 |
| - | gnl\|UG\|Ta#S61791923 |
| - | gnl\|UG\|Ta#S61792113 |
| - | gnl\|UG\|Ta#S61792479 |
| - | gnl\|UG\|Ta#S61792479 |
| - | gnl\|UG\|Ta#S61792483 |
| - | gnl\|UG\|Ta#S61793652 |
| - | gnl\|UG\|Ta#S61797550 |
| - | gnl\|UG\|Ta#S61797743 |
| - | gnl\|UG\|Ta#S61798628 |
| - | gnl\|UG\|Ta#S61799482 |
| - | gnl\|UG\|Ta#S61799525 |
| - | gnl\|UG\|Ta#S61799525 |
| - | gnl\|UG\|Ta#S61800172 |
| - | gnl\|UG\|Ta#S61800686 |
| - | gnl\|UG\|Ta#S61800686 |
| - | gnl\|UG\|Ta#S61800991 |

| | |
|---|---|
| - | gnl\|UG\|Ta#S61801005 |
| - | gnl\|UG\|Ta#S61801265 |
| - | gnl\|UG\|Ta#S61801463 |
| - | gnl\|UG\|Ta#S61802033 |
| - | gnl\|UG\|Ta#S61802494 |
| - | gnl\|UG\|Ta#S61802531 |
| - | gnl\|UG\|Ta#S61807708 |
| - | gnl\|UG\|Ta#S61808208 |
| - | gnl\|UG\|Ta#S61808226 |
| - | gnl\|UG\|Ta#S61808413 |
| - | gnl\|UG\|Ta#S61808562 |
| - | gnl\|UG\|Ta#S61808949 |
| - | gnl\|UG\|Ta#S61809253 |
| - | gnl\|UG\|Ta#S61809487 |
| - | gnl\|UG\|Ta#S61809696 |
| - | gnl\|UG\|Ta#S61809922 |
| - | gnl\|UG\|Ta#S61809922 |
| - | gnl\|UG\|Ta#S61810443 |
| - | gnl\|UG\|Ta#S61810474 |
| - | gnl\|UG\|Ta#S61810549 |
| - | gnl\|UG\|Ta#S61810638 |
| - | gnl\|UG\|Ta#S61810654 |
| - | gnl\|UG\|Ta#S61810843 |
| - | gnl\|UG\|Ta#S61811352 |
| - | gnl\|UG\|Ta#S61811407 |
| - | gnl\|UG\|Ta#S61811455 |
| - | gnl\|UG\|Ta#S61811468 |
| - | gnl\|UG\|Ta#S61811585 |
| - | gnl\|UG\|Ta#S61811830 |
| - | gnl\|UG\|Ta#S61811841 |
| - | gnl\|UG\|Ta#S61811944 |
| - | gnl\|UG\|Ta#S61811982 |
| - | gnl\|UG\|Ta#S61812070 |
| - | gnl\|UG\|Ta#S61812080 |
| - | gnl\|UG\|Ta#S61812211 |
| - | gnl\|UG\|Ta#S61812265 |
| - | gnl\|UG\|Ta#S61812266 |
| - | gnl\|UG\|Ta#S61812304 |
| - | gnl\|UG\|Ta#S61812354 |
| - | gnl\|UG\|Ta#S61812354 |
| - | gnl\|UG\|Ta#S61812365 |
| - | gnl\|UG\|Ta#S61812365 |
| - | gnl\|UG\|Ta#S61812441 |
| - | gnl\|UG\|Ta#S61812441 |
| - | gnl\|UG\|Ta#S61812441 |
| - | gnl\|UG\|Ta#S61812442 |
| - | gnl\|UG\|Ta#S61812442 |

| | |
|---|---|
| - | gnl\|UG\|Ta#S61812442 |
| - | gnl\|UG\|Ta#S61812508 |
| - | gnl\|UG\|Ta#S61812515 |
| - | gnl\|UG\|Ta#S61812515 |
| - | gnl\|UG\|Ta#S61812530 |
| - | gnl\|UG\|Ta#S61812545 |
| - | gnl\|UG\|Ta#S61812545 |
| - | gnl\|UG\|Ta#S61814826 |
| - | gnl\|UG\|Ta#S61827822 |
| - | gnl\|UG\|Ta#S61827822 |
| - | gnl\|UG\|Ta#S61827822 |
| - | gnl\|UG\|Ta#S61827924 |
| - | gnl\|UG\|Ta#S61828213 |
| - | gnl\|UG\|Ta#S61828213 |
| - | gnl\|UG\|Ta#S61828421 |
| - | gnl\|UG\|Ta#S61828595 |
| - | gnl\|UG\|Ta#S61828625 |
| - | gnl\|UG\|Ta#S61828913 |
| - | gnl\|UG\|Ta#S61828974 |
| - | gnl\|UG\|Ta#S61829883 |
| - | gnl\|UG\|Ta#S61830340 |
| - | gnl\|UG\|Ta#S61830340 |
| - | gnl\|UG\|Ta#S61830461 |
| - | gnl\|UG\|Ta#S61830508 |
| - | gnl\|UG\|Ta#S61830508 |
| - | gnl\|UG\|Ta#S61830508 |
| - | gnl\|UG\|Ta#S61830550 |
| - | gnl\|UG\|Ta#S61830554 |
| - | gnl\|UG\|Ta#S61830582 |
| - | gnl\|UG\|Ta#S61830677 |
| - | gnl\|UG\|Ta#S61830858 |
| - | gnl\|UG\|Ta#S61831164 |
| - | gnl\|UG\|Ta#S61831376 |
| - | gnl\|UG\|Ta#S61831376 |
| - | gnl\|UG\|Ta#S61831376 |
| - | gnl\|UG\|Ta#S61831862 |
| - | gnl\|UG\|Ta#S61831934 |
| - | gnl\|UG\|Ta#S61831934 |
| - | gnl\|UG\|Ta#S61831953 |
| - | gnl\|UG\|Ta#S61831995 |
| - | gnl\|UG\|Ta#S61832009 |
| - | gnl\|UG\|Ta#S61832061 |
| - | gnl\|UG\|Ta#S61832183 |
| - | gnl\|UG\|Ta#S61832215 |
| - | gnl\|UG\|Ta#S61832271 |
| - | gnl\|UG\|Ta#S61832283 |
| - | gnl\|UG\|Ta#S61832283 |

| | |
|---|---|
| - | gnl\|UG\|Ta#S61832283 |
| - | gnl\|UG\|Ta#S61832283 |
| - | gnl\|UG\|Ta#S61832283 |
| - | gnl\|UG\|Ta#S61832294 |
| - | gnl\|UG\|Ta#S61832329 |
| - | gnl\|UG\|Ta#S61832342 |
| - | gnl\|UG\|Ta#S61832418 |
| - | gnl\|UG\|Ta#S61832439 |
| - | gnl\|UG\|Ta#S61832442 |
| - | gnl\|UG\|Ta#S61832461 |
| - | gnl\|UG\|Ta#S61832485 |
| - | gnl\|UG\|Ta#S61832485 |
| - | gnl\|UG\|Ta#S61832522 |
| - | gnl\|UG\|Ta#S61832522 |
| - | gnl\|UG\|Ta#S61832533 |
| - | gnl\|UG\|Ta#S61832533 |
| - | gnl\|UG\|Ta#S61832538 |
| - | gnl\|UG\|Ta#S61832547 |
| - | gnl\|UG\|Ta#S61863176 |
| - | gnl\|UG\|Ta#S65588655 |
| - | gnl\|UG\|Ta#S65588655 |
| - | gnl\|UG\|Ta#S65591131 |
| - | gnl\|UG\|Ta#S65592665 |
| - | gnl\|UG\|Ta#S65594222 |
| - | gnl\|UG\|Ta#S65594222 |
| - | gnl\|UG\|Ta#S65594474 |
| - | gnl\|UG\|Ta#S65600758 |
| - | gnl\|UG\|Ta#S65600758 |
| - | gnl\|UG\|Ta#S65611074 |
| - | gnl\|UG\|Ta#S65611212 |
| - | gnl\|UG\|Ta#S65613695 |
| - | gnl\|UG\|Ta#S65620334 |
| - | gnl\|UG\|Ta#S65621396 |
| - | gnl\|UG\|Ta#S65622800 |
| - | gnl\|UG\|Ta#S65622800 |
| - | gnl\|UG\|Ta#S65623103 |
| - | gnl\|UG\|Ta#S65623103 |
| - | gnl\|UG\|Ta#S65625570 |
| - | gnl\|UG\|Ta#S65625570 |
| - | gnl\|UG\|Ta#S65625570 |
| - | gnl\|UG\|Ta#S65625570 |
| - | gnl\|UG\|Ta#S65625570 |
| - | gnl\|UG\|Ta#S65625570 |
| - | gnl\|UG\|Ta#S65625570 |
| - | gnl\|UG\|Ta#S65625570 |
| - | gnl\|UG\|Ta#S65625570 |
| - | gnl\|UG\|Ta#S65625570 |

| | |
|---|---|
| - | gnl\|UG\|Ta#S65625570 |
| - | gnl\|UG\|Ta#S65629152 |
| - | gnl\|UG\|Ta#S65634660 |
| - | gnl\|UG\|Ta#S65660438 |
| - | gnl\|UG\|Ta#S65689665 |
| - | gnl\|UG\|Ta#S65695729 |
| - | gnl\|UG\|Ta#S65706626 |
| - | gnl\|UG\|Ta#S65711079 |
| - | gnl\|UG\|Ta#S65711079 |
| - | gnl\|UG\|Ta#S65714355 |
| - | gnl\|UG\|Ta#S65715611 |
| - | gnl\|UG\|Ta#S65722902 |
| tr\|M7Y700\|M7Y700_TRIUA | - |
| tr\|M7Y700\|M7Y700_TRIUA | - |
| tr\|M7Y912\|M7Y912_TRIUA | - |
| tr\|M7YAL6\|M7YAL6_TRIUA | - |
| tr\|M7YG48\|M7YG48_TRIUA | - |
| tr\|M7YGQ6\|M7YGQ6_TRIUA | - |
| tr\|M7YIM0\|M7YIM0_TRIUA | - |
| tr\|M7YKY4\|M7YKY4_TRIUA | - |
| tr\|M7YN69\|M7YN69_TRIUA | - |
| tr\|M7YUP6\|M7YUP6_TRIUA | - |
| tr\|M7ZSS4\|M7ZSS4_TRIUA | - |
| tr\|M7ZSW1\|M7ZSW1_TRIUA | - |
| tr\|M7ZWY9\|M7ZWY9_TRIUA | - |
| tr\|M7ZX87\|M7ZX87_TRIUA | - |
| tr\|M8A2N2\|M8A2N2_TRIUA | - |
| tr\|M8A8B6\|M8A8B6_TRIUA | - |
| tr\|M8AEE9\|M8AEE9_TRIUA | - |
| tr\|M8AG18\|M8AG18_TRIUA | - |
| tr\|M8B4X3\|M8B4X3_TRIUA | - |
| tr\|Q4AEC1\|Q4AEC1_WHEAT | - |
| tr\|T1LTA5\|T1LTA5_TRIUA | - |
| tr\|T1MM86\|T1MM86_TRIUA | - |
| tr\|T1MZT0\|T1MZT0_TRIUA | - |
| tr\|T1NC62\|T1NC62_TRIUA | - |
| tr\|W5A3I4\|W5A3I4_WHEAT | - |
| tr\|W5A5F2\|W5A5F2_WHEAT | - |
| tr\|W5A5F2\|W5A5F2_WHEAT | - |
| tr\|W5ABX9\|W5ABX9_WHEAT | - |
| tr\|W5ABX9\|W5ABX9_WHEAT | - |
| tr\|W5ADP6\|W5ADP6_WHEAT | - |
| tr\|W5AGI2\|W5AGI2_WHEAT | - |
| tr\|W5DLW7\|W5DLW7_WHEAT | - |
| tr\|W5DNZ1\|W5DNZ1_WHEAT | - |
| tr\|W5DQP5\|W5DQP5_WHEAT | - |
| tr\|W5DTG5\|W5DTG5_WHEAT | - |

| | |
|---|---|
| tr\|W5DUG1\|W5DUG1_WHEAT | - |
| tr\|W5DVD0\|W5DVD0_WHEAT | - |
| tr\|W5DVJ7\|W5DVJ7_WHEAT | - |
| tr\|W5DXD3\|W5DXD3_WHEAT | - |
| tr\|W5DXD3\|W5DXD3_WHEAT | - |
| tr\|W5E047\|W5E047_WHEAT | - |
| tr\|W5E0B5\|W5E0B5_WHEAT | - |
| tr\|W5EAK1\|W5EAK1_WHEAT | - |
| tr\|W5EE02\|W5EE02_WHEAT | - |
| tr\|W5EP02\|W5EP02_WHEAT | - |
| tr\|W5EU71\|W5EU71_WHEAT | - |
| tr\|W5EX99\|W5EX99_WHEAT | - |
| tr\|W5F4E3\|W5F4E3_WHEAT | - |
| tr\|W5F5M8\|W5F5M8_WHEAT | - |
| tr\|W5F5P3\|W5F5P3_WHEAT | - |
| tr\|W5F773\|W5F773_WHEAT | - |
| tr\|W5F7X4\|W5F7X4_WHEAT | - |
| tr\|W5F7X4\|W5F7X4_WHEAT | - |
| tr\|W5F822\|W5F822_WHEAT | - |
| tr\|W5F822\|W5F822_WHEAT | - |
| tr\|W5F8H8\|W5F8H8_WHEAT | - |
| tr\|W5F8S9\|W5F8S9_WHEAT | - |
| tr\|W5F975\|W5F975_WHEAT | - |
| tr\|W5F9R5\|W5F9R5_WHEAT | - |
| tr\|W5F9R5\|W5F9R5_WHEAT | - |
| tr\|W5FAB4\|W5FAB4_WHEAT | - |
| tr\|W5FAB4\|W5FAB4_WHEAT | - |
| tr\|W5FAJ9\|W5FAJ9_WHEAT | - |
| tr\|W5FAJ9\|W5FAJ9_WHEAT | - |
| tr\|W5FD77\|W5FD77_WHEAT | - |
| tr\|W5FDE8\|W5FDE8_WHEAT | - |
| tr\|W5FEC9\|W5FEC9_WHEAT | - |
| tr\|W5FF50\|W5FF50_WHEAT | - |
| tr\|W5FFD5\|W5FFD5_WHEAT | - |
| tr\|W5FG87\|W5FG87_WHEAT | - |
| tr\|W5FGL3\|W5FGL3_WHEAT | - |
| tr\|W5FHA0\|W5FHA0_WHEAT | - |
| tr\|W5FHA0\|W5FHA0_WHEAT | - |
| tr\|W5FHH3\|W5FHH3_WHEAT | - |
| tr\|W5FJ74\|W5FJ74_WHEAT | - |
| tr\|W5FJ74\|W5FJ74_WHEAT | - |
| tr\|W5FJ74\|W5FJ74_WHEAT | - |
| tr\|W5FJK9\|W5FJK9_WHEAT | - |
| tr\|W5FK41\|W5FK41_WHEAT | - |
| tr\|W5FK61\|W5FK61_WHEAT | - |
| tr\|W5FKM7\|W5FKM7_WHEAT | - |
| tr\|W5FKX8\|W5FKX8_WHEAT | - |

| | |
|---|---|
| tr|W5FLQ4|W5FLQ4_WHEAT | - |
| tr|W5FLS1|W5FLS1_WHEAT | - |
| tr|W5FM18|W5FM18_WHEAT | - |
| tr|W5FMG3|W5FMG3_WHEAT | - |
| tr|W5FMI8|W5FMI8_WHEAT | - |
| tr|W5FMW6|W5FMW6_WHEAT | - |
| tr|W5FN02|W5FN02_WHEAT | - |
| tr|W5FN02|W5FN02_WHEAT | - |
| tr|W5FQW9|W5FQW9_WHEAT | - |
| tr|W5FWF6|W5FWF6_WHEAT | - |
| tr|W5GPG1|W5GPG1_WHEAT | - |
| tr|W5HRW8|W5HRW8_WHEAT | - |
| tr|W5I680|W5I680_WHEAT | - |
| sp|O04707|GAO1A_WHEAT | gnl|UG|Ta#S12923038 |
| tr|M7ZQG1|M7ZQG1_TRIUA | gnl|UG|Ta#S16058169 |
| tr|M7ZQG1|M7ZQG1_TRIUA | gnl|UG|Ta#S16058169 |
| tr|W5FHL1|W5FHL1_WHEAT | gnl|UG|Ta#S22379969 |
| tr|W5F4S3|W5F4S3_WHEAT | gnl|UG|Ta#S32692189 |
| tr|W5FLK3|W5FLK3_WHEAT | gnl|UG|Ta#S50388888 |
| tr|W5FL10|W5FL10_WHEAT | gnl|UG|Ta#S52541003 |
| tr|W5DWS5|W5DWS5_WHEAT | gnl|UG|Ta#S52541102 |
| tr|M8AGK4|M8AGK4_TRIUA | gnl|UG|Ta#S52541562 |
| tr|M7ZTR3|M7ZTR3_TRIUA | gnl|UG|Ta#S52542310 |
| tr|W5FLC4|W5FLC4_WHEAT | gnl|UG|Ta#S52542608 |
| tr|W5FS65|W5FS65_WHEAT | gnl|UG|Ta#S52542901 |
| tr|W5E024|W5E024_WHEAT | gnl|UG|Ta#S52543038 |
| tr|W5E024|W5E024_WHEAT | gnl|UG|Ta#S52543038 |
| tr|W5FNN1|W5FNN1_WHEAT | gnl|UG|Ta#S52543615 |
| tr|M7YS08|M7YS08_TRIUA | gnl|UG|Ta#S52543768 |
| tr|W5DLD0|W5DLD0_WHEAT | gnl|UG|Ta#S52543805 |
| tr|M7ZHW5|M7ZHW5_TRIUA | gnl|UG|Ta#S52544206 |
| tr|M7ZHW5|M7ZHW5_TRIUA | gnl|UG|Ta#S52544206 |
| tr|T1MQH3|T1MQH3_TRIUA | gnl|UG|Ta#S52544270 |
| tr|M8ABT8|M8ABT8_TRIUA | gnl|UG|Ta#S52544574 |
| tr|T1M058|T1M058_TRIUA | gnl|UG|Ta#S52544697 |
| tr|W5EJ23|W5EJ23_WHEAT | gnl|UG|Ta#S52544707 |
| tr|W5EXZ9|W5EXZ9_WHEAT | gnl|UG|Ta#S52544892 |
| tr|W5FPD5|W5FPD5_WHEAT | gnl|UG|Ta#S52544966 |
| tr|M7ZU33|M7ZU33_TRIUA | gnl|UG|Ta#S52545621 |
| tr|W5DWT9|W5DWT9_WHEAT | gnl|UG|Ta#S52545806 |
| tr|M7YAL6|M7YAL6_TRIUA | gnl|UG|Ta#S52546225 |
| tr|W5F606|W5F606_WHEAT | gnl|UG|Ta#S52546514 |
| tr|W5G472|W5G472_WHEAT | gnl|UG|Ta#S52546514 |
| tr|W5FEF7|W5FEF7_WHEAT | gnl|UG|Ta#S52546584 |
| tr|W5FG87|W5FG87_WHEAT | gnl|UG|Ta#S52546837 |
| tr|M8B4X3|M8B4X3_TRIUA | gnl|UG|Ta#S58897580 |
| tr|W5DUQ7|W5DUQ7_WHEAT | gnl|UG|Ta#S58897671 |

| | |
|---|---|
| tr\|M7ZQG1\|M7ZQG1_TRIUA | gnl\|UG\|Ta#S58899264 |
| tr\|M7ZQG1\|M7ZQG1_TRIUA | gnl\|UG\|Ta#S58899264 |
| tr\|W5F822\|W5F822_WHEAT | gnl\|UG\|Ta#S58902813 |
| tr\|M7ZN71\|M7ZN71_TRIUA | gnl\|UG\|Ta#S58903936 |
| tr\|W5DMX2\|W5DMX2_WHEAT | gnl\|UG\|Ta#S61522762 |
| tr\|W5DS21\|W5DS21_WHEAT | gnl\|UG\|Ta#S61528543 |
| tr\|W5FA95\|W5FA95_WHEAT | gnl\|UG\|Ta#S61543599 |
| tr\|M7ZZP3\|M7ZZP3_TRIUA | gnl\|UG\|Ta#S61545032 |
| tr\|M7ZZP3\|M7ZZP3_TRIUA | gnl\|UG\|Ta#S61545032 |
| tr\|W5FHZ3\|W5FHZ3_WHEAT | gnl\|UG\|Ta#S61550563 |
| tr\|W5FHZ3\|W5FHZ3_WHEAT | gnl\|UG\|Ta#S61550563 |
| tr\|W5FLK3\|W5FLK3_WHEAT | gnl\|UG\|Ta#S61568791 |
| tr\|M7YGZ9\|M7YGZ9_TRIUA | gnl\|UG\|Ta#S61572925 |
| tr\|W5FPF0\|W5FPF0_WHEAT | gnl\|UG\|Ta#S61574012 |
| tr\|W5F408\|W5F408_WHEAT | gnl\|UG\|Ta#S61582279 |
| tr\|W5F8G8\|W5F8G8_WHEAT | gnl\|UG\|Ta#S61585469 |
| tr\|W5DXE8\|W5DXE8_WHEAT | gnl\|UG\|Ta#S61585826 |
| tr\|W5A153\|W5A153_WHEAT | gnl\|UG\|Ta#S61591476 |
| tr\|W5ACQ5\|W5ACQ5_WHEAT | gnl\|UG\|Ta#S61596690 |
| tr\|Q66NG6\|Q66NG6_WHEAT | gnl\|UG\|Ta#S61600255 |
| tr\|M8A8B6\|M8A8B6_TRIUA | gnl\|UG\|Ta#S61613274 |
| tr\|W5FIM4\|W5FIM4_WHEAT | gnl\|UG\|Ta#S61614134 |
| tr\|M8A984\|M8A984_TRIUA | gnl\|UG\|Ta#S61614770 |
| tr\|W5A2X0\|W5A2X0_WHEAT | gnl\|UG\|Ta#S61632027 |
| tr\|W5A1E3\|W5A1E3_WHEAT | gnl\|UG\|Ta#S61634666 |
| tr\|W5A1E3\|W5A1E3_WHEAT | gnl\|UG\|Ta#S61634666 |
| tr\|W5G3N1\|W5G3N1_WHEAT | gnl\|UG\|Ta#S61635401 |
| tr\|M8B2F0\|M8B2F0_TRIUA | gnl\|UG\|Ta#S61670310 |
| tr\|W5FK55\|W5FK55_WHEAT | gnl\|UG\|Ta#S61677225 |
| tr\|W5GLB0\|W5GLB0_WHEAT | gnl\|UG\|Ta#S61711094 |
| tr\|W5F5K3\|W5F5K3_WHEAT | gnl\|UG\|Ta#S61724966 |
| tr\|T1N3S2\|T1N3S2_TRIUA | gnl\|UG\|Ta#S61726686 |
| tr\|W5FA64\|W5FA64_WHEAT | gnl\|UG\|Ta#S61733563 |
| tr\|W5DZ65\|W5DZ65_WHEAT | gnl\|UG\|Ta#S61746204 |
| tr\|W5FI85\|W5FI85_WHEAT | gnl\|UG\|Ta#S61782156 |
| tr\|W5F3F2\|W5F3F2_WHEAT | gnl\|UG\|Ta#S61782348 |
| tr\|M7ZWL8\|M7ZWL8_TRIUA | gnl\|UG\|Ta#S61788332 |
| tr\|W5FNE3\|W5FNE3_WHEAT | gnl\|UG\|Ta#S61791016 |
| tr\|W5HI93\|W5HI93_WHEAT | gnl\|UG\|Ta#S61792169 |
| tr\|W5DP85\|W5DP85_WHEAT | gnl\|UG\|Ta#S61792400 |
| tr\|M7ZPV1\|M7ZPV1_TRIUA | gnl\|UG\|Ta#S61797718 |
| tr\|W5H5T3\|W5H5T3_WHEAT | gnl\|UG\|Ta#S61798594 |
| tr\|W5F8W6\|W5F8W6_WHEAT | gnl\|UG\|Ta#S61798628 |
| tr\|M8AF88\|M8AF88_TRIUA | gnl\|UG\|Ta#S61800074 |
| tr\|W5FP84\|W5FP84_WHEAT | gnl\|UG\|Ta#S61800991 |
| tr\|W5FDE8\|W5FDE8_WHEAT | gnl\|UG\|Ta#S61801144 |
| tr\|W5G148\|W5G148_WHEAT | gnl\|UG\|Ta#S61801463 |

| | |
|---|---|
| tr\|M7ZWI2\|M7ZWI2_TRIUA | gnl\|UG\|Ta#S61802494 |
| tr\|W5GPV5\|W5GPV5_WHEAT | gnl\|UG\|Ta#S61808376 |
| tr\|M7ZQZ2\|M7ZQZ2_TRIUA | gnl\|UG\|Ta#S61808949 |
| tr\|M7Z177\|M7Z177_TRIUA | gnl\|UG\|Ta#S61809487 |
| tr\|M8A6X4\|M8A6X4_TRIUA | gnl\|UG\|Ta#S61809696 |
| tr\|W5FMF3\|W5FMF3_WHEAT | gnl\|UG\|Ta#S61809922 |
| tr\|W5G3S4\|W5G3S4_WHEAT | gnl\|UG\|Ta#S61809978 |
| tr\|W5FNN2\|W5FNN2_WHEAT | gnl\|UG\|Ta#S61809996 |
| tr\|M7ZP22\|M7ZP22_TRIUA | gnl\|UG\|Ta#S61810999 |
| tr\|W5G1Q6\|W5G1Q6_WHEAT | gnl\|UG\|Ta#S61811291 |
| tr\|W5DWR4\|W5DWR4_WHEAT | gnl\|UG\|Ta#S61811900 |
| tr\|W5GM70\|W5GM70_WHEAT | gnl\|UG\|Ta#S61811910 |
| tr\|W5GM70\|W5GM70_WHEAT | gnl\|UG\|Ta#S61811910 |
| tr\|W5FMK4\|W5FMK4_WHEAT | gnl\|UG\|Ta#S61811944 |
| tr\|W5DMW9\|W5DMW9_WHEAT | gnl\|UG\|Ta#S61812078 |
| tr\|W5F8X4\|W5F8X4_WHEAT | gnl\|UG\|Ta#S61812080 |
| tr\|W5DQA1\|W5DQA1_WHEAT | gnl\|UG\|Ta#S61812266 |
| tr\|W5DQA1\|W5DQA1_WHEAT | gnl\|UG\|Ta#S61812266 |
| tr\|W5FH13\|W5FH13_WHEAT | gnl\|UG\|Ta#S61812310 |
| tr\|M7Z842\|M7Z842_TRIUA | gnl\|UG\|Ta#S61812515 |
| tr\|M7ZH74\|M7ZH74_TRIUA | gnl\|UG\|Ta#S61812530 |
| tr\|M7ZH74\|M7ZH74_TRIUA | gnl\|UG\|Ta#S61812530 |
| tr\|W5F8Y2\|W5F8Y2_WHEAT | gnl\|UG\|Ta#S61812530 |
| tr\|W5GLR8\|W5GLR8_WHEAT | gnl\|UG\|Ta#S61812544 |
| tr\|W5GM24\|W5GM24_WHEAT | gnl\|UG\|Ta#S61812544 |
| tr\|M8A4U4\|M8A4U4_TRIUA | gnl\|UG\|Ta#S61815543 |
| tr\|M8ADX5\|M8ADX5_TRIUA | gnl\|UG\|Ta#S61827822 |
| tr\|M8ADX5\|M8ADX5_TRIUA | gnl\|UG\|Ta#S61827822 |
| tr\|W5FM42\|W5FM42_WHEAT | gnl\|UG\|Ta#S61827924 |
| tr\|W5F8P4\|W5F8P4_WHEAT | gnl\|UG\|Ta#S61830076 |
| tr\|W5EWB0\|W5EWB0_WHEAT | gnl\|UG\|Ta#S61830308 |
| tr\|W5FJN8\|W5FJN8_WHEAT | gnl\|UG\|Ta#S61830554 |
| tr\|W5F6Z0\|W5F6Z0_WHEAT | gnl\|UG\|Ta#S61831232 |
| tr\|W5EJ84\|W5EJ84_WHEAT | gnl\|UG\|Ta#S61831439 |
| tr\|M8AMD9\|M8AMD9_TRIUA | gnl\|UG\|Ta#S61831824 |
| tr\|M8A9E0\|M8A9E0_TRIUA | gnl\|UG\|Ta#S61831934 |
| tr\|M8A3B4\|M8A3B4_TRIUA | gnl\|UG\|Ta#S61832183 |
| tr\|W5DZW9\|W5DZW9_WHEAT | gnl\|UG\|Ta#S61832193 |
| tr\|W5DP30\|W5DP30_WHEAT | gnl\|UG\|Ta#S61832271 |
| tr\|W5FPG1\|W5FPG1_WHEAT | gnl\|UG\|Ta#S61832276 |
| tr\|M8A2S6\|M8A2S6_TRIUA | gnl\|UG\|Ta#S61832283 |
| tr\|M8A2S6\|M8A2S6_TRIUA | gnl\|UG\|Ta#S61832283 |
| tr\|W5EGT4\|W5EGT4_WHEAT | gnl\|UG\|Ta#S61832342 |
| tr\|W5A5W4\|W5A5W4_WHEAT | gnl\|UG\|Ta#S61832438 |
| tr\|W5DYT4\|W5DYT4_WHEAT | gnl\|UG\|Ta#S61832440 |
| tr\|W5HNI3\|W5HNI3_WHEAT | gnl\|UG\|Ta#S61832547 |
| tr\|W5FJR9\|W5FJR9_WHEAT | gnl\|UG\|Ta#S65596596 |

| | |
|---|---|
| tr|W5FPL8|W5FPL8_WHEAT | gnl|UG|Ta#S65601858 |
| tr|M7YYL7|M7YYL7_TRIUA | gnl|UG|Ta#S65615254 |
| tr|W5FHZ3|W5FHZ3_WHEAT | gnl|UG|Ta#S65625570 |
| tr|W5DRU5|W5DRU5_WHEAT | gnl|UG|Ta#S65652338 |
| tr|W5DW67|W5DW67_WHEAT | gnl|UG|Ta#S65667530 |
| tr|W5FCU1|W5FCU1_WHEAT | gnl|UG|Ta#S65679950 |
| tr|W5G4M0|W5G4M0_WHEAT | gnl|UG|Ta#S65689665 |
| tr|M7YG49|M7YG49_TRIUA | gnl|UG|Ta#S65698377 |
| tr|M7YBQ4|M7YBQ4_TRIUA | gnl|UG|Ta#S65699612 |
| tr|T1LYV5|T1LYV5_TRIUA | gnl|UG|Ta#S65706626 |
| tr|W5FEQ3|W5FEQ3_WHEAT | gnl|UG|Ta#S65719718 |
| - | - |
| - | - |
| - | - |
| - | - |
| - | - |
| - | - |
| - | - |
| - | - |
| - | - |
| - | - |
| - | - |
| - | - |
| - | - |
| - | - |
| - | - |
| - | - |
| - | - |
| - | - |
| - | - |
| - | - |
| - | - |
| - | - |
| - | - |
| - | - |
| - | - |
| - | - |
| - | - |
| - | - |
| - | - |
| - | - |
| - | - |
| - | - |
| - | - |
| - | - |
| - | - |
| - | - |

| | |
|---|---|
| - | - |
| - | - |
| - | - |
| - | - |
| - | - |
| - | - |
| - | - |
| - | - |
| - | - |
| - | - |
| - | - |
| - | - |
| - | - |
| - | - |
| - | - |
| - | - |
| - | - |
| - | - |
| - | - |
| - | - |
| - | - |
| - | - |
| - | - |
| - | - |
| - | - |
| - | - |
| - | - |
| - | - |
| - | - |
| - | - |
| - | - |
| - | - |
| - | - |
| - | - |
| - | - |
| - | - |
| - | - |
| - | - |
| - | - |
| - | - |
| - | - |
| - | - |
| - | - |
| - | - |
| - | - |
| - | - |
| - | - |

| | |
|---|---|
| - | - |
| - | - |
| - | - |
| - | - |
| - | - |
| - | - |
| - | - |
| - | - |
| - | - |
| - | - |
| - | - |
| - | - |
| - | - |
| - | - |
| - | - |
| - | - |
| - | - |
| - | - |
| - | - |
| - | - |
| - | - |
| - | - |
| - | - |
| - | - |
| - | - |
| - | - |
| - | - |
| - | - |
| - | - |
| - | - |
| - | - |
| - | - |
| - | - |
| - | - |
| - | - |
| - | - |
| - | - |
| - | - |
| - | - |
| - | - |
| - | - |
| - | - |
| - | - |
| - | - |
| - | - |
| - | - |
| - | - |

| | |
|---|---|
| - | - |
| - | - |
| - | - |
| - | - |
| - | - |
| - | - |
| - | - |
| - | - |
| - | - |
| - | - |
| - | - |
| - | - |
| - | - |
| - | - |
| - | - |
| - | - |
| - | - |
| - | - |
| - | - |
| - | - |
| - | - |
| - | - |
| - | - |
| - | - |
| - | - |
| - | - |
| - | - |
| - | - |
| - | - |
| - | - |
| - | - |
| - | - |
| - | - |
| - | - |
| - | - |
| - | - |
| - | - |
| - | - |
| - | - |
| - | - |
| - | - |
| - | - |
| - | - |
| - | - |
| - | - |
| - | - |
| - | - |

| | |
|---|---|
| - | - |
| - | - |
| - | - |
| - | - |
| - | - |
| - | - |
| - | - |
| - | - |
| - | - |
| - | - |
| - | - |
| - | - |
| - | - |
| - | - |
| - | - |
| - | - |
| - | - |
| - | - |
| - | - |
| - | - |
| - | - |
| - | - |
| - | - |
| - | - |
| - | - |
| - | - |
| - | - |
| - | - |
| - | - |
| - | - |
| - | - |
| - | - |
| - | - |
| - | - |
| - | - |
| - | - |
| - | - |
| - | - |
| - | - |
| - | - |
| - | - |
| - | - |
| - | - |
| - | - |
| - | - |
| - | - |
| - | - |

| | |
|---|---|
| - | - |
| - | - |
| - | - |
| - | - |
| - | - |
| - | - |
| - | - |
| - | - |
| - | - |
| - | - |
| - | - |
| - | - |
| - | - |
| - | - |
| - | - |
| - | - |
| - | - |
| - | - |
| - | - |
| - | - |
| - | - |
| - | - |
| - | - |
| - | - |
| - | - |
| - | - |
| - | - |
| - | - |
| - | - |
| - | - |
| - | - |
| - | - |
| - | - |
| - | - |
| - | - |
| - | - |
| - | - |
| - | - |
| - | - |
| - | - |
| - | - |
| - | - |
| - | - |
| - | - |
| - | - |
| - | - |
| - | - |

| | |
|---|---|
| - | - |
| - | - |
| - | - |
| - | - |
| - | - |
| - | - |
| - | - |
| - | - |
| - | - |
| - | - |
| - | - |
| - | - |
| - | - |
| - | - |
| - | - |
| - | - |
| - | - |
| - | - |
| - | - |
| - | gnl\|UG\|Ta#S12945079 |
| - | gnl\|UG\|Ta#S16058215 |
| - | gnl\|UG\|Ta#S32557620 |
| - | gnl\|UG\|Ta#S37948509 |
| - | gnl\|UG\|Ta#S37948509 |
| - | gnl\|UG\|Ta#S50378517 |
| - | gnl\|UG\|Ta#S52541021 |
| - | gnl\|UG\|Ta#S52543615 |
| - | gnl\|UG\|Ta#S52544904 |
| - | gnl\|UG\|Ta#S52545434 |
| - | gnl\|UG\|Ta#S52546782 |
| - | gnl\|UG\|Ta#S52546886 |
| - | gnl\|UG\|Ta#S52546905 |
| - | gnl\|UG\|Ta#S58857032 |
| - | gnl\|UG\|Ta#S58861289 |
| - | gnl\|UG\|Ta#S58895933 |
| - | gnl\|UG\|Ta#S58912125 |
| - | gnl\|UG\|Ta#S58912126 |
| - | gnl\|UG\|Ta#S61517640 |
| - | gnl\|UG\|Ta#S61544078 |
| - | gnl\|UG\|Ta#S61553615 |
| - | gnl\|UG\|Ta#S61603101 |
| - | gnl\|UG\|Ta#S61612325 |
| - | gnl\|UG\|Ta#S61612325 |
| - | gnl\|UG\|Ta#S61612970 |
| - | gnl\|UG\|Ta#S61641041 |
| - | gnl\|UG\|Ta#S61646225 |
| - | gnl\|UG\|Ta#S61650526 |

| | |
|---|---|
| - | gnl\|UG\|Ta#S61689923 |
| - | gnl\|UG\|Ta#S61690023 |
| - | gnl\|UG\|Ta#S61697425 |
| - | gnl\|UG\|Ta#S61720116 |
| - | gnl\|UG\|Ta#S61720553 |
| - | gnl\|UG\|Ta#S61722210 |
| - | gnl\|UG\|Ta#S61768919 |
| - | gnl\|UG\|Ta#S61772254 |
| - | gnl\|UG\|Ta#S61778976 |
| - | gnl\|UG\|Ta#S61779567 |
| - | gnl\|UG\|Ta#S61788689 |
| - | gnl\|UG\|Ta#S61801893 |
| - | gnl\|UG\|Ta#S61802148 |
| - | gnl\|UG\|Ta#S61802318 |
| - | gnl\|UG\|Ta#S61808011 |
| - | gnl\|UG\|Ta#S61808208 |
| - | gnl\|UG\|Ta#S61809596 |
| - | gnl\|UG\|Ta#S61809952 |
| - | gnl\|UG\|Ta#S61811886 |
| - | gnl\|UG\|Ta#S61812107 |
| - | gnl\|UG\|Ta#S61812141 |
| - | gnl\|UG\|Ta#S61812141 |
| - | gnl\|UG\|Ta#S61812141 |
| - | gnl\|UG\|Ta#S61812151 |
| - | gnl\|UG\|Ta#S61812223 |
| - | gnl\|UG\|Ta#S61812334 |
| - | gnl\|UG\|Ta#S61812461 |
| - | gnl\|UG\|Ta#S61824142 |
| - | gnl\|UG\|Ta#S61826588 |
| - | gnl\|UG\|Ta#S61828531 |
| - | gnl\|UG\|Ta#S61828896 |
| - | gnl\|UG\|Ta#S61829846 |
| - | gnl\|UG\|Ta#S61830746 |
| - | gnl\|UG\|Ta#S61830765 |
| - | gnl\|UG\|Ta#S61831320 |
| - | gnl\|UG\|Ta#S61831654 |
| - | gnl\|UG\|Ta#S61831713 |
| - | gnl\|UG\|Ta#S61831808 |
| - | gnl\|UG\|Ta#S61832081 |
| - | gnl\|UG\|Ta#S61832081 |
| - | gnl\|UG\|Ta#S61832084 |
| - | gnl\|UG\|Ta#S61832276 |
| - | gnl\|UG\|Ta#S61832389 |
| - | gnl\|UG\|Ta#S61834547 |
| - | gnl\|UG\|Ta#S65596953 |
| - | gnl\|UG\|Ta#S65622991 |
| - | gnl\|UG\|Ta#S65629152 |

| | |
|---|---|
| - | gnl\|UG\|Ta#S65634825 |
| - | gnl\|UG\|Ta#S65717627 |
| - | gnl\|UG\|Ta#S65723103 |
| tr\|M7YDU3\|M7YDU3_TRIUA | - |
| tr\|M7YEV7\|M7YEV7_TRIUA | - |
| tr\|M7YPG2\|M7YPG2_TRIUA | - |
| tr\|M7Z141\|M7Z141_TRIUA | - |
| tr\|M7Z562\|M7Z562_TRIUA | - |
| tr\|M7ZGC7\|M7ZGC7_TRIUA | - |
| tr\|M7ZM62\|M7ZM62_TRIUA | - |
| tr\|M7ZRB9\|M7ZRB9_TRIUA | - |
| tr\|M8AW18\|M8AW18_TRIUA | - |
| tr\|T1L8N0\|T1L8N0_TRIUA | - |
| tr\|T1LLK2\|T1LLK2_TRIUA | - |
| tr\|T1LTD1\|T1LTD1_TRIUA | - |
| tr\|T1MHD6\|T1MHD6_TRIUA | - |
| tr\|W5A0B2\|W5A0B2_WHEAT | - |
| tr\|W5A6K2\|W5A6K2_WHEAT | - |
| tr\|W5A8P3\|W5A8P3_WHEAT | - |
| tr\|W5D2N5\|W5D2N5_WHEAT | - |
| tr\|W5D5A0\|W5D5A0_WHEAT | - |
| tr\|W5DLJ8\|W5DLJ8_WHEAT | - |
| tr\|W5DLJ8\|W5DLJ8_WHEAT | - |
| tr\|W5DP23\|W5DP23_WHEAT | - |
| tr\|W5DS67\|W5DS67_WHEAT | - |
| tr\|W5DSL1\|W5DSL1_WHEAT | - |
| tr\|W5DSN5\|W5DSN5_WHEAT | - |
| tr\|W5DXZ3\|W5DXZ3_WHEAT | - |
| tr\|W5DZJ3\|W5DZJ3_WHEAT | - |
| tr\|W5F4Z5\|W5F4Z5_WHEAT | - |
| tr\|W5F787\|W5F787_WHEAT | - |
| tr\|W5F7K2\|W5F7K2_WHEAT | - |
| tr\|W5F833\|W5F833_WHEAT | - |
| tr\|W5F8P4\|W5F8P4_WHEAT | - |
| tr\|W5F921\|W5F921_WHEAT | - |
| tr\|W5FA11\|W5FA11_WHEAT | - |
| tr\|W5FB25\|W5FB25_WHEAT | - |
| tr\|W5FBV9\|W5FBV9_WHEAT | - |
| tr\|W5FCL5\|W5FCL5_WHEAT | - |
| tr\|W5FDQ7\|W5FDQ7_WHEAT | - |
| tr\|W5FDT0\|W5FDT0_WHEAT | - |
| tr\|W5FDX8\|W5FDX8_WHEAT | - |
| tr\|W5FER0\|W5FER0_WHEAT | - |
| tr\|W5FIZ1\|W5FIZ1_WHEAT | - |
| tr\|W5FJ06\|W5FJ06_WHEAT | - |
| tr\|W5FJC1\|W5FJC1_WHEAT | - |
| tr\|W5FMS8\|W5FMS8_WHEAT | - |

| | |
|---|---|
| tr\|W5FNX4\|W5FNX4_WHEAT | - |
| tr\|W5FP61\|W5FP61_WHEAT | - |
| tr\|W5FYD0\|W5FYD0_WHEAT | - |
| tr\|W5FYD0\|W5FYD0_WHEAT | - |
| tr\|W5G3N1\|W5G3N1_WHEAT | - |
| tr\|W5GIJ1\|W5GIJ1_WHEAT | - |
| tr\|W5HUR7\|W5HUR7_WHEAT | - |
| tr\|W5FED0\|W5FED0_WHEAT | gnl\|UG\|Ta#S18011728 |
| tr\|W5AAZ3\|W5AAZ3_WHEAT | gnl\|UG\|Ta#S32621472 |
| tr\|M7ZWZ8\|M7ZWZ8_TRIUA | gnl\|UG\|Ta#S52546782 |
| tr\|W5DUY1\|W5DUY1_WHEAT | gnl\|UG\|Ta#S52546905 |
| tr\|M7Z7B7\|M7Z7B7_TRIUA | gnl\|UG\|Ta#S58860731 |
| tr\|W4ZTL5\|W4ZTL5_WHEAT | gnl\|UG\|Ta#S61526416 |
| tr\|W5DNS0\|W5DNS0_WHEAT | gnl\|UG\|Ta#S61554479 |
| tr\|W5FE64\|W5FE64_WHEAT | gnl\|UG\|Ta#S61647901 |
| tr\|M7ZGC7\|M7ZGC7_TRIUA | gnl\|UG\|Ta#S61767574 |
| tr\|W5F0V2\|W5F0V2_WHEAT | gnl\|UG\|Ta#S61778432 |
| tr\|T1LPQ7\|T1LPQ7_TRIUA | gnl\|UG\|Ta#S61779567 |
| tr\|W5F9C5\|W5F9C5_WHEAT | gnl\|UG\|Ta#S61780251 |
| tr\|W5F9C5\|W5F9C5_WHEAT | gnl\|UG\|Ta#S61780251 |
| tr\|W5FHF6\|W5FHF6_WHEAT | gnl\|UG\|Ta#S61782336 |
| tr\|W5FIW6\|W5FIW6_WHEAT | gnl\|UG\|Ta#S61810348 |
| tr\|W5FLR8\|W5FLR8_WHEAT | gnl\|UG\|Ta#S61810653 |
| tr\|W5A0W6\|W5A0W6_WHEAT | gnl\|UG\|Ta#S61831184 |
| tr\|W5GPJ5\|W5GPJ5_WHEAT | gnl\|UG\|Ta#S61832499 |
| tr\|W5FLK4\|W5FLK4_WHEAT | gnl\|UG\|Ta#S65622991 |
| tr\|Q2L3W7\|Q2L3W7_WHEAT | gnl\|UG\|Ta#S65663931 |
| tr\|W5FD08\|W5FD08_WHEAT | gnl\|UG\|Ta#S65680582 |
| tr\|W5FN96\|W5FN96_WHEAT | gnl\|UG\|Ta#S65714383 |
| - | - |
| - | - |
| - | - |
| - | - |
| - | - |
| - | - |
| - | - |
| - | - |
| - | - |
| - | - |
| - | - |
| - | - |
| - | - |
| - | - |
| - | - |
| - | - |
| - | - |
| - | - |

| | |
|---|---|
| - | - |
| - | - |
| - | - |
| - | - |
| - | - |
| - | - |
| - | - |
| - | - |
| - | - |
| - | - |
| - | - |
| - | - |
| - | - |
| - | - |
| - | - |
| - | - |
| - | - |
| - | - |
| - | - |
| - | - |
| - | - |
| - | - |
| - | - |
| - | - |
| - | - |
| - | - |
| - | - |
| - | - |
| - | - |
| - | - |
| - | - |
| - | - |
| - | - |
| - | - |
| - | - |
| - | - |
| - | - |
| - | - |
| - | - |
| - | - |
| - | - |
| - | - |
| - | - |
| - | - |
| - | - |
| - | - |
| - | - |

-
-
-
-
-
-
-
-
-
-
-
-
-
-
-
-
-
-
-
-
-
-
-
-
-
-
-
-
-
-
-
-
-
-
-
-
-
-
-
-
-
-
-
-
-
-
-

-
-
-
-
-
-
-
-
-
-
-
-
-
-
-
-
-
-
-
-
-
-
-
-
-
-
-
-
-
-
-
-
-
-
-
-
-
-
-
-
-
-
-
-
-
-
-

| | |
|---|---|
| - | - |
| - | - |
| - | - |
| - | - |
| - | - |
| - | - |
| - | - |
| - | - |
| - | - |
| - | - |
| - | - |
| - | - |
| - | - |
| - | - |
| - | - |
| - | - |
| - | - |
| - | - |
| - | - |
| - | - |
| - | - |
| - | - |
| - | - |
| - | - |
| - | - |
| - | - |
| - | - |
| - | - |
| - | - |
| - | - |
| - | gnl\|UG\|Ta#S12985063 |
| - | gnl\|UG\|Ta#S12985063 |
| - | gnl\|UG\|Ta#S17890456 |
| - | gnl\|UG\|Ta#S22379618 |
| - | gnl\|UG\|Ta#S22388788 |
| - | gnl\|UG\|Ta#S26026092 |
| - | gnl\|UG\|Ta#S52541021 |
| - | gnl\|UG\|Ta#S52541022 |
| - | gnl\|UG\|Ta#S52542253 |
| - | gnl\|UG\|Ta#S52542253 |
| - | gnl\|UG\|Ta#S52543379 |
| - | gnl\|UG\|Ta#S52543379 |
| - | gnl\|UG\|Ta#S52543556 |
| - | gnl\|UG\|Ta#S52543615 |
| - | gnl\|UG\|Ta#S52543615 |
| - | gnl\|UG\|Ta#S52543615 |
| - | gnl\|UG\|Ta#S52543615 |

| | |
|---|---|
| - | gnl\|UG\|Ta#S52543615 |
| - | gnl\|UG\|Ta#S52543615 |
| - | gnl\|UG\|Ta#S52544273 |
| - | gnl\|UG\|Ta#S52544273 |
| - | gnl\|UG\|Ta#S52544726 |
| - | gnl\|UG\|Ta#S52544726 |
| - | gnl\|UG\|Ta#S52544726 |
| - | gnl\|UG\|Ta#S52544726 |
| - | gnl\|UG\|Ta#S52544770 |
| - | gnl\|UG\|Ta#S52544785 |
| - | gnl\|UG\|Ta#S52544877 |
| - | gnl\|UG\|Ta#S52544904 |
| - | gnl\|UG\|Ta#S58853985 |
| - | gnl\|UG\|Ta#S58865993 |
| - | gnl\|UG\|Ta#S58899046 |
| - | gnl\|UG\|Ta#S58902006 |
| - | gnl\|UG\|Ta#S61516468 |
| - | gnl\|UG\|Ta#S61516468 |
| - | gnl\|UG\|Ta#S61516468 |
| - | gnl\|UG\|Ta#S61531827 |
| - | gnl\|UG\|Ta#S61531827 |
| - | gnl\|UG\|Ta#S61536175 |
| - | gnl\|UG\|Ta#S61554197 |
| - | gnl\|UG\|Ta#S61587548 |
| - | gnl\|UG\|Ta#S61588706 |
| - | gnl\|UG\|Ta#S61596978 |
| - | gnl\|UG\|Ta#S61598568 |
| - | gnl\|UG\|Ta#S61603229 |
| - | gnl\|UG\|Ta#S61605086 |
| - | gnl\|UG\|Ta#S61607104 |
| - | gnl\|UG\|Ta#S61619919 |
| - | gnl\|UG\|Ta#S61620929 |
| - | gnl\|UG\|Ta#S61620929 |
| - | gnl\|UG\|Ta#S61626193 |
| - | gnl\|UG\|Ta#S61634015 |
| - | gnl\|UG\|Ta#S61635116 |
| - | gnl\|UG\|Ta#S61635700 |
| - | gnl\|UG\|Ta#S61636247 |
| - | gnl\|UG\|Ta#S61641083 |
| - | gnl\|UG\|Ta#S61668558 |
| - | gnl\|UG\|Ta#S61705706 |
| - | gnl\|UG\|Ta#S61745685 |
| - | gnl\|UG\|Ta#S61768919 |
| - | gnl\|UG\|Ta#S61770427 |
| - | gnl\|UG\|Ta#S61780822 |
| - | gnl\|UG\|Ta#S61780822 |
| - | gnl\|UG\|Ta#S61780860 |

| | |
|---|---|
| - | gnl\|UG\|Ta#S61780873 |
| - | gnl\|UG\|Ta#S61781218 |
| - | gnl\|UG\|Ta#S61782437 |
| - | gnl\|UG\|Ta#S61789945 |
| - | gnl\|UG\|Ta#S61791033 |
| - | gnl\|UG\|Ta#S61798594 |
| - | gnl\|UG\|Ta#S61799763 |
| - | gnl\|UG\|Ta#S61799763 |
| - | gnl\|UG\|Ta#S61801008 |
| - | gnl\|UG\|Ta#S61801144 |
| - | gnl\|UG\|Ta#S61802318 |
| - | gnl\|UG\|Ta#S61802539 |
| - | gnl\|UG\|Ta#S61802539 |
| - | gnl\|UG\|Ta#S61808592 |
| - | gnl\|UG\|Ta#S61809572 |
| - | gnl\|UG\|Ta#S61810100 |
| - | gnl\|UG\|Ta#S61810261 |
| - | gnl\|UG\|Ta#S61810544 |
| - | gnl\|UG\|Ta#S61810904 |
| - | gnl\|UG\|Ta#S61810904 |
| - | gnl\|UG\|Ta#S61811404 |
| - | gnl\|UG\|Ta#S61811903 |
| - | gnl\|UG\|Ta#S61812022 |
| - | gnl\|UG\|Ta#S61812022 |
| - | gnl\|UG\|Ta#S61812408 |
| - | gnl\|UG\|Ta#S61812408 |
| - | gnl\|UG\|Ta#S61827768 |
| - | gnl\|UG\|Ta#S61827768 |
| - | gnl\|UG\|Ta#S61828421 |
| - | gnl\|UG\|Ta#S61828421 |
| - | gnl\|UG\|Ta#S61829252 |
| - | gnl\|UG\|Ta#S61830003 |
| - | gnl\|UG\|Ta#S61830511 |
| - | gnl\|UG\|Ta#S61830572 |
| - | gnl\|UG\|Ta#S61831450 |
| - | gnl\|UG\|Ta#S61831450 |
| - | gnl\|UG\|Ta#S61831654 |
| - | gnl\|UG\|Ta#S61831654 |
| - | gnl\|UG\|Ta#S61831654 |
| - | gnl\|UG\|Ta#S61831654 |
| - | gnl\|UG\|Ta#S61831808 |
| - | gnl\|UG\|Ta#S61831824 |
| - | gnl\|UG\|Ta#S61831854 |
| - | gnl\|UG\|Ta#S61832040 |
| - | gnl\|UG\|Ta#S61832215 |
| - | gnl\|UG\|Ta#S61832492 |
| - | gnl\|UG\|Ta#S61832492 |

| | |
|---|---|
| - | gnl\|UG\|Ta#S65590008 |
| - | gnl\|UG\|Ta#S65596551 |
| - | gnl\|UG\|Ta#S65596953 |
| - | gnl\|UG\|Ta#S65600561 |
| - | gnl\|UG\|Ta#S65633207 |
| - | gnl\|UG\|Ta#S65687854 |
| - | gnl\|UG\|Ta#S65697070 |
| - | gnl\|UG\|Ta#S65707827 |
| tr\|M7Z8R8\|M7Z8R8_TRIUA | - |
| tr\|M7ZGA3\|M7ZGA3_TRIUA | - |
| tr\|M7ZHM6\|M7ZHM6_TRIUA | - |
| tr\|M7ZKU8\|M7ZKU8_TRIUA | - |
| tr\|M8ACG1\|M8ACG1_TRIUA | - |
| tr\|M8AX58\|M8AX58_TRIUA | - |
| tr\|W5A431\|W5A431_WHEAT | - |
| tr\|W5A5H7\|W5A5H7_WHEAT | - |
| tr\|W5A9U9\|W5A9U9_WHEAT | - |
| tr\|W5DLC6\|W5DLC6_WHEAT | - |
| tr\|W5DU30\|W5DU30_WHEAT | - |
| tr\|W5DW61\|W5DW61_WHEAT | - |
| tr\|W5DZV8\|W5DZV8_WHEAT | - |
| tr\|W5F7C1\|W5F7C1_WHEAT | - |
| tr\|W5F7F6\|W5F7F6_WHEAT | - |
| tr\|W5F894\|W5F894_WHEAT | - |
| tr\|W5F894\|W5F894_WHEAT | - |
| tr\|W5F922\|W5F922_WHEAT | - |
| tr\|W5F935\|W5F935_WHEAT | - |
| tr\|W5F935\|W5F935_WHEAT | - |
| tr\|W5F938\|W5F938_WHEAT | - |
| tr\|W5F938\|W5F938_WHEAT | - |
| tr\|W5F9G1\|W5F9G1_WHEAT | - |
| tr\|W5F9M5\|W5F9M5_WHEAT | - |
| tr\|W5FBJ3\|W5FBJ3_WHEAT | - |
| tr\|W5FC15\|W5FC15_WHEAT | - |
| tr\|W5FCM6\|W5FCM6_WHEAT | - |
| tr\|W5FE68\|W5FE68_WHEAT | - |
| tr\|W5FE68\|W5FE68_WHEAT | - |
| tr\|W5FFB5\|W5FFB5_WHEAT | - |
| tr\|W5FGW6\|W5FGW6_WHEAT | - |
| tr\|W5FH13\|W5FH13_WHEAT | - |
| tr\|W5FH56\|W5FH56_WHEAT | - |
| tr\|W5FH56\|W5FH56_WHEAT | - |
| tr\|W5FHQ4\|W5FHQ4_WHEAT | - |
| tr\|W5FHW3\|W5FHW3_WHEAT | - |
| tr\|W5FI44\|W5FI44_WHEAT | - |
| tr\|W5FI44\|W5FI44_WHEAT | - |
| tr\|W5FJP0\|W5FJP0_WHEAT | - |

| | |
|---|---|
| tr\|W5FJX8\|W5FJX8_WHEAT | - |
| tr\|W5FKX8\|W5FKX8_WHEAT | - |
| tr\|W5FL03\|W5FL03_WHEAT | - |
| tr\|W5FL03\|W5FL03_WHEAT | - |
| tr\|W5FL03\|W5FL03_WHEAT | - |
| tr\|W5FLW6\|W5FLW6_WHEAT | - |
| tr\|W5FM16\|W5FM16_WHEAT | - |
| tr\|W5FM16\|W5FM16_WHEAT | - |
| tr\|W5FM19\|W5FM19_WHEAT | - |
| tr\|W5FP70\|W5FP70_WHEAT | - |
| tr\|W5FPE6\|W5FPE6_WHEAT | - |
| tr\|W5FQN4\|W5FQN4_WHEAT | - |
| tr\|W5FZP8\|W5FZP8_WHEAT | - |
| tr\|W5GIJ5\|W5GIJ5_WHEAT | - |
| tr\|W5GIJ5\|W5GIJ5_WHEAT | - |
| tr\|W5GJW3\|W5GJW3_WHEAT | - |
| tr\|M7ZRB9\|M7ZRB9_TRIUA | gnl\|UG\|Ta#S13028222 |
| tr\|W5FDJ9\|W5FDJ9_WHEAT | gnl\|UG\|Ta#S24623136 |
| tr\|M7YEV7\|M7YEV7_TRIUA | gnl\|UG\|Ta#S37948509 |
| tr\|W5FBH7\|W5FBH7_WHEAT | gnl\|UG\|Ta#S52542744 |
| tr\|W5HTG7\|W5HTG7_WHEAT | gnl\|UG\|Ta#S52542840 |
| tr\|M8A008\|M8A008_TRIUA | gnl\|UG\|Ta#S52543379 |
| tr\|M7ZJR9\|M7ZJR9_TRIUA | gnl\|UG\|Ta#S52543417 |
| tr\|T1LC05\|T1LC05_TRIUA | gnl\|UG\|Ta#S52543615 |
| tr\|W5FPB7\|W5FPB7_WHEAT | gnl\|UG\|Ta#S52544097 |
| tr\|M7ZC23\|M7ZC23_TRIUA | gnl\|UG\|Ta#S52544360 |
| tr\|W5DZE5\|W5DZE5_WHEAT | gnl\|UG\|Ta#S52544390 |
| tr\|W5FQC4\|W5FQC4_WHEAT | gnl\|UG\|Ta#S52544726 |
| tr\|W5DQ61\|W5DQ61_WHEAT | gnl\|UG\|Ta#S58844003 |
| tr\|W5FE48\|W5FE48_WHEAT | gnl\|UG\|Ta#S58860858 |
| tr\|W5FE48\|W5FE48_WHEAT | gnl\|UG\|Ta#S58860858 |
| tr\|W5DUQ7\|W5DUQ7_WHEAT | gnl\|UG\|Ta#S58897671 |
| tr\|Q7XA60\|Q7XA60_WHEAT | gnl\|UG\|Ta#S61561773 |
| tr\|W5FHZ7\|W5FHZ7_WHEAT | gnl\|UG\|Ta#S61587548 |
| tr\|T1NQF4\|T1NQF4_TRIUA | gnl\|UG\|Ta#S61600597 |
| tr\|W5FGL3\|W5FGL3_WHEAT | gnl\|UG\|Ta#S61614622 |
| tr\|W5A398\|W5A398_WHEAT | gnl\|UG\|Ta#S61635700 |
| tr\|W5FBJ0\|W5FBJ0_WHEAT | gnl\|UG\|Ta#S61650526 |
| tr\|W5FBE2\|W5FBE2_WHEAT | gnl\|UG\|Ta#S61668558 |
| tr\|W5FHA8\|W5FHA8_WHEAT | gnl\|UG\|Ta#S61708006 |
| tr\|W5FHA8\|W5FHA8_WHEAT | gnl\|UG\|Ta#S61708006 |
| tr\|M7ZKB7\|M7ZKB7_TRIUA | gnl\|UG\|Ta#S61780643 |
| tr\|W5FDK2\|W5FDK2_WHEAT | gnl\|UG\|Ta#S61780643 |
| tr\|W5FDJ7\|W5FDJ7_WHEAT | gnl\|UG\|Ta#S61780822 |
| tr\|M7ZSG8\|M7ZSG8_TRIUA | gnl\|UG\|Ta#S61780860 |
| tr\|M7ZKF1\|M7ZKF1_TRIUA | gnl\|UG\|Ta#S61800359 |
| tr\|W5EMD7\|W5EMD7_WHEAT | gnl\|UG\|Ta#S61802191 |

| | |
|---|---|
| tr\|M7ZP83\|M7ZP83_TRIUA | gnl\|UG\|Ta#S61809228 |
| tr\|W5FK41\|W5FK41_WHEAT | gnl\|UG\|Ta#S61810680 |
| tr\|W5FHF6\|W5FHF6_WHEAT | gnl\|UG\|Ta#S61810891 |
| tr\|W5DU62\|W5DU62_WHEAT | gnl\|UG\|Ta#S61810928 |
| tr\|W5DUI6\|W5DUI6_WHEAT | gnl\|UG\|Ta#S61811479 |
| tr\|W5A0C9\|W5A0C9_WHEAT | gnl\|UG\|Ta#S61811886 |
| tr\|M7ZZP7\|M7ZZP7_TRIUA | gnl\|UG\|Ta#S61812304 |
| tr\|W5FDR2\|W5FDR2_WHEAT | gnl\|UG\|Ta#S61812461 |
| tr\|W5FDR2\|W5FDR2_WHEAT | gnl\|UG\|Ta#S61812461 |
| tr\|T1MFJ9\|T1MFJ9_TRIUA | gnl\|UG\|Ta#S61828784 |
| tr\|M8AJE6\|M8AJE6_TRIUA | gnl\|UG\|Ta#S61829369 |
| tr\|W5DXX3\|W5DXX3_WHEAT | gnl\|UG\|Ta#S61829384 |
| tr\|W5DXX3\|W5DXX3_WHEAT | gnl\|UG\|Ta#S61829384 |
| tr\|W5HTG7\|W5HTG7_WHEAT | gnl\|UG\|Ta#S61830316 |
| tr\|M8A9B1\|M8A9B1_TRIUA | gnl\|UG\|Ta#S61830616 |
| tr\|T1MNP6\|T1MNP6_TRIUA | gnl\|UG\|Ta#S61832130 |
| tr\|W5F7G6\|W5F7G6_WHEAT | gnl\|UG\|Ta#S65597520 |
| tr\|W5FD75\|W5FD75_WHEAT | gnl\|UG\|Ta#S65622983 |
| tr\|W5FM19\|W5FM19_WHEAT | gnl\|UG\|Ta#S65644008 |
| tr\|M7YIY1\|M7YIY1_TRIUA | gnl\|UG\|Ta#S65674801 |
| tr\|M7ZVY6\|M7ZVY6_TRIUA | gnl\|UG\|Ta#S65697070 |
| - | - |
| - | - |
| - | - |
| - | - |
| - | - |
| - | - |
| - | - |
| - | - |
| - | - |
| - | - |
| - | - |
| - | - |
| - | - |
| - | - |
| - | - |
| - | - |
| - | - |
| - | - |
| - | - |
| - | - |
| - | - |
| - | - |
| - | - |
| - | - |
| - | - |
| - | - |

| | |
|---|---|
| - | - |
| - | - |
| - | - |
| - | - |
| - | - |
| - | - |
| - | - |
| - | - |
| - | - |
| - | - |
| - | - |
| - | - |
| - | - |
| - | - |
| - | - |
| - | - |
| - | - |
| - | - |
| - | - |
| - | - |
| - | - |
| - | - |
| - | - |
| - | - |
| - | - |
| - | - |
| - | - |
| - | - |
| - | - |
| - | - |
| - | - |
| - | - |
| - | - |
| - | - |
| - | - |
| - | - |
| - | - |
| - | - |
| - | - |
| - | - |
| - | - |
| - | - |
| - | - |
| - | - |
| - | - |
| - | - |
| - | - |

| | |
|---|---|
| - | - |
| - | - |
| - | - |
| - | - |
| - | - |
| - | - |
| - | - |
| - | - |
| - | - |
| - | - |
| - | - |
| - | - |
| - | - |
| - | - |
| - | - |
| - | - |
| - | - |
| - | - |
| - | - |
| - | - |
| - | - |
| - | - |
| - | - |
| - | - |
| - | - |
| - | - |
| - | - |
| - | - |
| - | - |
| - | - |
| - | - |
| - | - |
| - | - |
| - | - |
| - | - |
| - | - |
| - | - |
| - | - |
| - | - |
| - | - |
| - | - |
| - | - |
| - | - |
| - | - |
| - | - |
| - | - |
| - | - |

| | |
|---|---|
| - | - |
| - | - |
| - | - |
| - | - |
| - | - |
| - | - |
| - | - |
| - | - |
| - | - |
| - | - |
| - | - |
| - | - |
| - | - |
| - | - |
| - | - |
| - | - |
| - | - |
| - | - |
| - | - |
| - | - |
| - | - |
| - | - |
| - | - |
| - | - |
| - | - |
| - | - |
| - | - |
| - | - |
| - | - |
| - | - |
| - | - |
| - | - |
| - | - |
| - | - |
| - | - |
| - | - |
| - | - |
| - | - |
| - | - |
| - | - |
| - | - |
| - | - |
| - | - |
| - | - |
| - | - |
| - | - |
| - | - |

| | |
|---|---|
| - | - |
| - | - |
| - | - |
| - | - |
| - | - |
| - | - |
| - | - |
| - | - |
| - | - |
| - | - |
| - | - |
| - | - |
| - | - |
| - | - |
| - | - |
| - | - |
| - | - |
| - | - |
| - | - |
| - | - |
| - | - |
| - | - |
| - | - |
| - | - |
| - | - |
| - | - |
| - | - |
| - | - |
| - | - |
| - | - |
| - | - |
| - | - |
| - | - |
| - | - |
| - | - |
| - | - |
| - | - |
| - | - |
| - | - |
| - | - |
| - | - |
| - | - |
| - | - |
| - | - |
| - | - |
| - | - |
| - | - |

| | |
|---|---|
| - | - |
| - | - |
| - | - |
| - | - |
| - | - |
| - | - |
| - | - |
| - | - |
| - | - |
| - | - |
| - | - |
| - | - |
| - | - |
| - | - |
| - | - |
| - | - |
| - | - |
| - | - |
| - | - |
| - | - |
| - | - |
| - | - |
| - | - |
| - | - |
| - | - |
| - | - |
| - | - |
| - | - |
| - | - |
| - | - |
| - | - |
| - | - |
| - | - |
| - | - |
| - | - |
| - | - |
| - | - |
| - | - |
| - | - |
| - | - |
| - | - |
| - | - |
| - | - |
| - | - |
| - | - |
| - | - |
| - | - |

| | |
|---|---|
| - | - |
| - | - |
| - | - |
| - | - |
| - | - |
| - | - |
| - | - |
| - | - |
| - | - |
| - | - |
| - | - |
| - | - |
| - | - |
| - | - |
| - | - |
| - | - |
| - | - |
| - | - |
| - | - |
| - | - |
| - | - |
| - | - |
| - | - |
| - | - |
| - | - |
| - | - |
| - | - |
| - | - |
| - | - |
| - | - |
| - | - |
| - | - |
| - | - |
| - | - |
| - | - |
| - | - |
| - | - |
| - | - |
| - | - |
| - | - |
| - | - |
| - | - |
| - | - |
| - | - |
| - | - |
| - | - |
| - | - |

| | |
|---|---|
| - | - |
| - | - |
| - | - |
| - | - |
| - | - |
| - | - |
| - | - |
| - | - |
| - | - |
| - | - |
| - | - |
| - | - |
| - | - |
| - | - |
| - | - |
| - | - |
| - | - |
| - | - |
| - | - |
| - | - |
| - | - |
| - | - |
| - | - |
| - | - |
| - | - |
| - | - |
| - | - |
| - | - |
| - | - |
| - | - |
| - | - |
| - | - |
| - | - |
| - | - |
| - | - |
| - | - |
| - | - |
| - | - |
| - | - |
| - | - |
| - | - |
| - | - |
| - | - |
| - | - |
| - | - |
| - | - |
| - | - |

| | |
|---|---|
| - | - |
| - | - |
| - | - |
| - | - |
| - | - |
| - | - |
| - | - |
| - | - |
| - | - |
| - | - |
| - | - |
| - | - |
| - | - |
| - | - |
| - | - |
| - | - |
| - | - |
| - | - |
| - | - |
| - | - |
| - | - |
| - | - |
| - | - |
| - | - |
| - | - |
| - | - |
| - | - |
| - | - |
| - | - |
| - | - |
| - | - |
| - | - |
| - | - |
| - | - |
| - | - |
| - | - |
| - | - |
| - | - |
| - | - |
| - | - |
| - | gnl\|UG\|Ta#S12950024 |
| - | gnl\|UG\|Ta#S16058472 |
| - | gnl\|UG\|Ta#S18006781 |
| - | gnl\|UG\|Ta#S27052963 |
| - | gnl\|UG\|Ta#S37781531 |
| - | gnl\|UG\|Ta#S37917887 |
| - | gnl\|UG\|Ta#S52541041 |

| | |
|---|---|
| - | gnl\|UG\|Ta#S52541068 |
| - | gnl\|UG\|Ta#S52541068 |
| - | gnl\|UG\|Ta#S52541068 |
| - | gnl\|UG\|Ta#S52541580 |
| - | gnl\|UG\|Ta#S52542126 |
| - | gnl\|UG\|Ta#S52542426 |
| - | gnl\|UG\|Ta#S52542434 |
| - | gnl\|UG\|Ta#S52542515 |
| - | gnl\|UG\|Ta#S52542515 |
| - | gnl\|UG\|Ta#S52543038 |
| - | gnl\|UG\|Ta#S52543220 |
| - | gnl\|UG\|Ta#S52543220 |
| - | gnl\|UG\|Ta#S52543445 |
| - | gnl\|UG\|Ta#S52543456 |
| - | gnl\|UG\|Ta#S52543703 |
| - | gnl\|UG\|Ta#S52543703 |
| - | gnl\|UG\|Ta#S52544160 |
| - | gnl\|UG\|Ta#S52544177 |
| - | gnl\|UG\|Ta#S52544770 |
| - | gnl\|UG\|Ta#S52544770 |
| - | gnl\|UG\|Ta#S52544785 |
| - | gnl\|UG\|Ta#S52544832 |
| - | gnl\|UG\|Ta#S52544878 |
| - | gnl\|UG\|Ta#S52544878 |
| - | gnl\|UG\|Ta#S52544878 |
| - | gnl\|UG\|Ta#S52545265 |
| - | gnl\|UG\|Ta#S52545489 |
| - | gnl\|UG\|Ta#S52545541 |
| - | gnl\|UG\|Ta#S52545972 |
| - | gnl\|UG\|Ta#S52545972 |
| - | gnl\|UG\|Ta#S52545989 |
| - | gnl\|UG\|Ta#S52546028 |
| - | gnl\|UG\|Ta#S52546078 |
| - | gnl\|UG\|Ta#S52546332 |
| - | gnl\|UG\|Ta#S52546439 |
| - | gnl\|UG\|Ta#S52546475 |
| - | gnl\|UG\|Ta#S52546538 |
| - | gnl\|UG\|Ta#S52546569 |
| - | gnl\|UG\|Ta#S58852454 |
| - | gnl\|UG\|Ta#S58853311 |
| - | gnl\|UG\|Ta#S58858754 |
| - | gnl\|UG\|Ta#S58861596 |
| - | gnl\|UG\|Ta#S58861596 |
| - | gnl\|UG\|Ta#S58861596 |
| - | gnl\|UG\|Ta#S58886335 |
| - | gnl\|UG\|Ta#S58892130 |
| - | gnl\|UG\|Ta#S58892130 |

| | |
|---|---|
| - | gnl\|UG\|Ta#S58893586 |
| - | gnl\|UG\|Ta#S58900458 |
| - | gnl\|UG\|Ta#S58902006 |
| - | gnl\|UG\|Ta#S58906069 |
| - | gnl\|UG\|Ta#S58907652 |
| - | gnl\|UG\|Ta#S59657928 |
| - | gnl\|UG\|Ta#S61566367 |
| - | gnl\|UG\|Ta#S61566367 |
| - | gnl\|UG\|Ta#S61586218 |
| - | gnl\|UG\|Ta#S61595333 |
| - | gnl\|UG\|Ta#S61597517 |
| - | gnl\|UG\|Ta#S61599608 |
| - | gnl\|UG\|Ta#S61600255 |
| - | gnl\|UG\|Ta#S61604010 |
| - | gnl\|UG\|Ta#S61608238 |
| - | gnl\|UG\|Ta#S61617166 |
| - | gnl\|UG\|Ta#S61617418 |
| - | gnl\|UG\|Ta#S61624959 |
| - | gnl\|UG\|Ta#S61625144 |
| - | gnl\|UG\|Ta#S61625144 |
| - | gnl\|UG\|Ta#S61625144 |
| - | gnl\|UG\|Ta#S61625144 |
| - | gnl\|UG\|Ta#S61635292 |
| - | gnl\|UG\|Ta#S61635401 |
| - | gnl\|UG\|Ta#S61635401 |
| - | gnl\|UG\|Ta#S61641133 |
| - | gnl\|UG\|Ta#S61643350 |
| - | gnl\|UG\|Ta#S61643350 |
| - | gnl\|UG\|Ta#S61647925 |
| - | gnl\|UG\|Ta#S61648840 |
| - | gnl\|UG\|Ta#S61657223 |
| - | gnl\|UG\|Ta#S61665118 |
| - | gnl\|UG\|Ta#S61672080 |
| - | gnl\|UG\|Ta#S61679690 |
| - | gnl\|UG\|Ta#S61681911 |
| - | gnl\|UG\|Ta#S61700560 |
| - | gnl\|UG\|Ta#S61740718 |
| - | gnl\|UG\|Ta#S61763747 |
| - | gnl\|UG\|Ta#S61768511 |
| - | gnl\|UG\|Ta#S61768919 |
| - | gnl\|UG\|Ta#S61770963 |
| - | gnl\|UG\|Ta#S61771853 |
| - | gnl\|UG\|Ta#S61779146 |
| - | gnl\|UG\|Ta#S61779567 |
| - | gnl\|UG\|Ta#S61779820 |
| - | gnl\|UG\|Ta#S61780981 |
| - | gnl\|UG\|Ta#S61781861 |

| | |
|---|---|
| - | gnl\|UG\|Ta#S61788689 |
| - | gnl\|UG\|Ta#S61790402 |
| - | gnl\|UG\|Ta#S61797743 |
| - | gnl\|UG\|Ta#S61797786 |
| - | gnl\|UG\|Ta#S61800091 |
| - | gnl\|UG\|Ta#S61800416 |
| - | gnl\|UG\|Ta#S61802310 |
| - | gnl\|UG\|Ta#S61807729 |
| - | gnl\|UG\|Ta#S61808538 |
| - | gnl\|UG\|Ta#S61809106 |
| - | gnl\|UG\|Ta#S61810290 |
| - | gnl\|UG\|Ta#S61810588 |
| - | gnl\|UG\|Ta#S61810928 |
| - | gnl\|UG\|Ta#S61810983 |
| - | gnl\|UG\|Ta#S61811483 |
| - | gnl\|UG\|Ta#S61811518 |
| - | gnl\|UG\|Ta#S61811692 |
| - | gnl\|UG\|Ta#S61811730 |
| - | gnl\|UG\|Ta#S61811946 |
| - | gnl\|UG\|Ta#S61812124 |
| - | gnl\|UG\|Ta#S61812220 |
| - | gnl\|UG\|Ta#S61812271 |
| - | gnl\|UG\|Ta#S61812365 |
| - | gnl\|UG\|Ta#S61812379 |
| - | gnl\|UG\|Ta#S61812515 |
| - | gnl\|UG\|Ta#S61816230 |
| - | gnl\|UG\|Ta#S61825841 |
| - | gnl\|UG\|Ta#S61827925 |
| - | gnl\|UG\|Ta#S61827953 |
| - | gnl\|UG\|Ta#S61828595 |
| - | gnl\|UG\|Ta#S61828974 |
| - | gnl\|UG\|Ta#S61829756 |
| - | gnl\|UG\|Ta#S61829756 |
| - | gnl\|UG\|Ta#S61829756 |
| - | gnl\|UG\|Ta#S61829760 |
| - | gnl\|UG\|Ta#S61829803 |
| - | gnl\|UG\|Ta#S61829803 |
| - | gnl\|UG\|Ta#S61829883 |
| - | gnl\|UG\|Ta#S61829981 |
| - | gnl\|UG\|Ta#S61830550 |
| - | gnl\|UG\|Ta#S61830912 |
| - | gnl\|UG\|Ta#S61832035 |
| - | gnl\|UG\|Ta#S61832084 |
| - | gnl\|UG\|Ta#S61832174 |
| - | gnl\|UG\|Ta#S61832238 |
| - | gnl\|UG\|Ta#S61832272 |
| - | gnl\|UG\|Ta#S61832321 |

| | |
|---|---|
| - | gnl\|UG\|Ta#S61832329 |
| - | gnl\|UG\|Ta#S61832389 |
| - | gnl\|UG\|Ta#S61832406 |
| - | gnl\|UG\|Ta#S61832406 |
| - | gnl\|UG\|Ta#S61832461 |
| - | gnl\|UG\|Ta#S61832524 |
| - | gnl\|UG\|Ta#S61832525 |
| - | gnl\|UG\|Ta#S61832532 |
| - | gnl\|UG\|Ta#S61832532 |
| - | gnl\|UG\|Ta#S61832538 |
| - | gnl\|UG\|Ta#S65589436 |
| - | gnl\|UG\|Ta#S65607523 |
| - | gnl\|UG\|Ta#S65617689 |
| - | gnl\|UG\|Ta#S65623049 |
| - | gnl\|UG\|Ta#S65627072 |
| - | gnl\|UG\|Ta#S65631341 |
| - | gnl\|UG\|Ta#S65658529 |
| - | gnl\|UG\|Ta#S65660171 |
| - | gnl\|UG\|Ta#S65674801 |
| tr\|M5FMS1\|M5FMS1_WHEAT | - |
| tr\|M7YMP6\|M7YMP6_TRIUA | - |
| tr\|M7YQT2\|M7YQT2_TRIUA | - |
| tr\|M7Z1U6\|M7Z1U6_TRIUA | - |
| tr\|M7Z4D6\|M7Z4D6_TRIUA | - |
| tr\|M7ZBN2\|M7ZBN2_TRIUA | - |
| tr\|M7ZKB8\|M7ZKB8_TRIUA | - |
| tr\|M7ZS67\|M7ZS67_TRIUA | - |
| tr\|M7ZVY5\|M7ZVY5_TRIUA | - |
| tr\|M8AAP7\|M8AAP7_TRIUA | - |
| tr\|M8B4L4\|M8B4L4_TRIUA | - |
| tr\|T1MLI5\|T1MLI5_TRIUA | - |
| tr\|T1MW81\|T1MW81_TRIUA | - |
| tr\|T1MW81\|T1MW81_TRIUA | - |
| tr\|W4ZZN0\|W4ZZN0_WHEAT | - |
| tr\|W5ABL2\|W5ABL2_WHEAT | - |
| tr\|W5ABV0\|W5ABV0_WHEAT | - |
| tr\|W5B185\|W5B185_WHEAT | - |
| tr\|W5DLJ5\|W5DLJ5_WHEAT | - |
| tr\|W5DMD8\|W5DMD8_WHEAT | - |
| tr\|W5DMS8\|W5DMS8_WHEAT | - |
| tr\|W5DQ60\|W5DQ60_WHEAT | - |
| tr\|W5DUB2\|W5DUB2_WHEAT | - |
| tr\|W5E041\|W5E041_WHEAT | - |
| tr\|W5EDY9\|W5EDY9_WHEAT | - |
| tr\|W5F762\|W5F762_WHEAT | - |
| tr\|W5F7E4\|W5F7E4_WHEAT | - |
| tr\|W5F7K2\|W5F7K2_WHEAT | - |

| | |
|---|---|
| tr\|W5F9R8\|W5F9R8_WHEAT | - |
| tr\|W5FB21\|W5FB21_WHEAT | - |
| tr\|W5FBS8\|W5FBS8_WHEAT | - |
| tr\|W5FCJ5\|W5FCJ5_WHEAT | - |
| tr\|W5FD08\|W5FD08_WHEAT | - |
| tr\|W5FFF3\|W5FFF3_WHEAT | - |
| tr\|W5FFX5\|W5FFX5_WHEAT | - |
| tr\|W5FHQ0\|W5FHQ0_WHEAT | - |
| tr\|W5FI42\|W5FI42_WHEAT | - |
| tr\|W5FI63\|W5FI63_WHEAT | - |
| tr\|W5FKJ1\|W5FKJ1_WHEAT | - |
| tr\|W5FKJ1\|W5FKJ1_WHEAT | - |
| tr\|W5FL67\|W5FL67_WHEAT | - |
| tr\|W5FMS1\|W5FMS1_WHEAT | - |
| tr\|W5FMS1\|W5FMS1_WHEAT | - |
| tr\|W5FMS1\|W5FMS1_WHEAT | - |
| tr\|W5FN68\|W5FN68_WHEAT | - |
| tr\|W5FPE6\|W5FPE6_WHEAT | - |
| tr\|W5G107\|W5G107_WHEAT | - |
| tr\|W5HT35\|W5HT35_WHEAT | - |
| tr\|W5FI26\|W5FI26_WHEAT | gnl\|UG\|Ta#S12890084 |
| tr\|W5FSY5\|W5FSY5_WHEAT | gnl\|UG\|Ta#S12972187 |
| tr\|M7ZP51\|M7ZP51_TRIUA | gnl\|UG\|Ta#S24513530 |
| tr\|M7ZXC1\|M7ZXC1_TRIUA | gnl\|UG\|Ta#S43837843 |
| tr\|W5EKB0\|W5EKB0_WHEAT | gnl\|UG\|Ta#S52541068 |
| tr\|W5A593\|W5A593_WHEAT | gnl\|UG\|Ta#S52543821 |
| tr\|W5FM60\|W5FM60_WHEAT | gnl\|UG\|Ta#S58910675 |
| tr\|W5FM60\|W5FM60_WHEAT | gnl\|UG\|Ta#S58910675 |
| tr\|M7YRA1\|M7YRA1_TRIUA | gnl\|UG\|Ta#S59657928 |
| tr\|M7ZTZ8\|M7ZTZ8_TRIUA | gnl\|UG\|Ta#S61549792 |
| tr\|W5FHP8\|W5FHP8_WHEAT | gnl\|UG\|Ta#S61584946 |
| tr\|W5FLF6\|W5FLF6_WHEAT | gnl\|UG\|Ta#S61590227 |
| tr\|M8AKB3\|M8AKB3_TRIUA | gnl\|UG\|Ta#S61595333 |
| tr\|M8AKB3\|M8AKB3_TRIUA | gnl\|UG\|Ta#S61595333 |
| tr\|W5FPW5\|W5FPW5_WHEAT | gnl\|UG\|Ta#S61599608 |
| tr\|W5A6K2\|W5A6K2_WHEAT | gnl\|UG\|Ta#S61612221 |
| tr\|W5FRS0\|W5FRS0_WHEAT | gnl\|UG\|Ta#S61613001 |
| tr\|W5GIJ5\|W5GIJ5_WHEAT | gnl\|UG\|Ta#S61614134 |
| tr\|M7ZRG4\|M7ZRG4_TRIUA | gnl\|UG\|Ta#S61616337 |
| tr\|M7YXF4\|M7YXF4_TRIUA | gnl\|UG\|Ta#S61625144 |
| tr\|W5FM87\|W5FM87_WHEAT | gnl\|UG\|Ta#S61634783 |
| tr\|W5DQ60\|W5DQ60_WHEAT | gnl\|UG\|Ta#S61637542 |
| tr\|W5AAI8\|W5AAI8_WHEAT | gnl\|UG\|Ta#S61682908 |
| tr\|W5FDY4\|W5FDY4_WHEAT | gnl\|UG\|Ta#S61717475 |
| tr\|W5FK91\|W5FK91_WHEAT | gnl\|UG\|Ta#S61733056 |
| tr\|W5FXB4\|W5FXB4_WHEAT | gnl\|UG\|Ta#S61737398 |
| tr\|W5BCW0\|W5BCW0_WHEAT | gnl\|UG\|Ta#S61770035 |

| | |
|---|---|
| tr\|W5FMP9\|W5FMP9_WHEAT | gnl\|UG\|Ta#S61809952 |
| tr\|W5DWK2\|W5DWK2_WHEAT | gnl\|UG\|Ta#S61810958 |
| tr\|M7ZUY3\|M7ZUY3_TRIUA | gnl\|UG\|Ta#S61812124 |
| tr\|W5E2V3\|W5E2V3_WHEAT | gnl\|UG\|Ta#S61812271 |
| tr\|M7YN69\|M7YN69_TRIUA | gnl\|UG\|Ta#S61812461 |
| tr\|M8A056\|M8A056_TRIUA | gnl\|UG\|Ta#S61812527 |
| tr\|M7Z684\|M7Z684_TRIUA | gnl\|UG\|Ta#S61828531 |
| tr\|W5FN95\|W5FN95_WHEAT | gnl\|UG\|Ta#S61830249 |
| tr\|W5FN95\|W5FN95_WHEAT | gnl\|UG\|Ta#S61830249 |
| tr\|W5FCY5\|W5FCY5_WHEAT | gnl\|UG\|Ta#S61830572 |
| tr\|W5FA92\|W5FA92_WHEAT | gnl\|UG\|Ta#S61830728 |
| tr\|W5FK91\|W5FK91_WHEAT | gnl\|UG\|Ta#S61830923 |
| tr\|M8AMD9\|M8AMD9_TRIUA | gnl\|UG\|Ta#S61831824 |
| tr\|M8AS09\|M8AS09_TRIUA | gnl\|UG\|Ta#S61831950 |
| tr\|W5A5U6\|W5A5U6_WHEAT | gnl\|UG\|Ta#S61832081 |
| tr\|W5A7W7\|W5A7W7_WHEAT | gnl\|UG\|Ta#S61832321 |
| tr\|M8AS20\|M8AS20_TRIUA | gnl\|UG\|Ta#S61832327 |
| tr\|M7Z657\|M7Z657_TRIUA | gnl\|UG\|Ta#S61832455 |
| tr\|M7Z657\|M7Z657_TRIUA | gnl\|UG\|Ta#S61832455 |
| tr\|M7Z657\|M7Z657_TRIUA | gnl\|UG\|Ta#S61832455 |
| tr\|W5FH94\|W5FH94_WHEAT | gnl\|UG\|Ta#S61845168 |
| tr\|W5DZ14\|W5DZ14_WHEAT | gnl\|UG\|Ta#S65623049 |
| tr\|W5FLH5\|W5FLH5_WHEAT | gnl\|UG\|Ta#S65623496 |
| tr\|M7YJI1\|M7YJI1_TRIUA | gnl\|UG\|Ta#S65627198 |
| tr\|W5FCJ3\|W5FCJ3_WHEAT | gnl\|UG\|Ta#S65655685 |
| tr\|Q2L3W7\|Q2L3W7_WHEAT | gnl\|UG\|Ta#S65663931 |
| tr\|W5A349\|W5A349_WHEAT | gnl\|UG\|Ta#S65688621 |
| tr\|W5AH58\|W5AH58_WHEAT | gnl\|UG\|Ta#S65688621 |
| - | - |
| - | - |
| - | - |
| - | - |
| - | - |
| - | - |
| - | - |
| - | - |
| - | - |
| - | - |
| - | - |
| - | - |
| - | - |
| - | - |
| - | - |
| - | - |
| - | - |
| - | - |
| - | - |

| | |
|---|---|
| - | - |
| - | - |
| - | - |
| - | - |
| - | - |
| - | - |
| - | - |
| - | - |
| - | - |
| - | - |
| - | - |
| - | - |
| - | - |
| - | - |
| - | - |
| - | - |
| - | - |
| - | - |
| - | - |
| - | - |
| - | - |
| - | - |
| - | - |
| - | - |
| - | - |
| - | - |
| - | - |
| - | - |
| - | - |
| - | - |
| - | - |
| - | - |
| - | - |
| - | - |
| - | - |
| - | - |
| - | - |
| - | - |
| - | - |
| - | - |
| - | - |
| - | - |
| - | - |
| - | - |
| - | - |
| - | - |
| - | - |

| | |
|---|---|
| - | - |
| - | - |
| - | - |
| - | - |
| - | - |
| - | - |
| - | - |
| - | - |
| - | - |
| - | - |
| - | - |
| - | - |
| - | - |
| - | - |
| - | - |
| - | - |
| - | - |
| - | - |
| - | - |
| - | - |
| - | - |
| - | - |
| - | - |
| - | - |
| - | - |
| - | - |
| - | - |
| - | - |
| - | - |
| - | - |
| - | - |
| - | - |
| - | - |
| - | - |
| - | - |
| - | - |
| - | - |
| - | - |
| - | - |
| - | - |
| - | - |
| - | - |
| - | - |
| - | - |
| - | - |
| - | - |
| - | - |

| | |
|---|---|
| - | - |
| - | - |
| - | - |
| - | - |
| - | - |
| - | - |
| - | - |
| - | - |
| - | - |
| - | - |
| - | - |
| - | - |
| - | - |
| - | - |
| - | - |
| - | - |
| - | - |
| - | - |
| - | - |
| - | - |
| - | - |
| - | - |
| - | - |
| - | - |
| - | - |
| - | - |
| - | - |
| - | - |
| - | - |
| - | - |
| - | - |
| - | - |
| - | - |
| - | - |
| - | - |
| - | - |
| - | - |
| - | - |
| - | - |
| - | - |
| - | - |
| - | - |
| - | - |
| - | - |
| - | - |
| - | - |
| - | - |

| | |
|---|---|
| - | - |
| - | - |
| - | - |
| - | - |
| - | - |
| - | - |
| - | - |
| - | - |
| - | - |
| - | - |
| - | - |
| - | - |
| - | - |
| - | - |
| - | - |
| - | - |
| - | - |
| - | - |
| - | - |
| - | - |
| - | - |
| - | - |
| - | - |
| - | - |
| - | - |
| - | - |
| - | - |
| - | - |
| - | - |
| - | - |
| - | - |
| - | - |
| - | - |
| - | - |
| - | - |
| - | - |
| - | - |
| - | - |
| - | - |
| - | - |
| - | - |
| - | - |
| - | - |
| - | - |
| - | - |
| - | - |
| - | - |

| | |
|---|---|
| - | - |
| - | - |
| - | - |
| - | - |
| - | - |
| - | - |
| - | - |
| - | - |
| - | - |
| - | - |
| - | - |
| - | - |
| - | - |
| - | - |
| - | - |
| - | - |
| - | - |
| - | - |
| - | - |
| - | - |
| - | - |
| - | - |
| - | - |
| - | - |
| - | - |
| - | - |
| - | - |
| - | - |
| - | - |
| - | - |
| - | - |
| - | - |
| - | - |
| - | - |
| - | - |
| - | - |
| - | - |
| - | - |
| - | - |
| - | - |
| - | - |
| - | - |
| - | - |
| - | - |
| - | - |
| - | - |
| - | - |

| | |
|---|---|
| - | - |
| - | - |
| - | - |
| - | - |
| - | - |
| - | - |
| - | - |
| - | - |
| - | - |
| - | - |
| - | - |
| - | - |
| - | - |
| - | - |
| - | - |
| - | - |
| - | - |
| - | - |
| - | - |
| - | - |
| - | - |
| - | - |
| - | - |
| - | - |
| - | - |
| - | - |
| - | - |
| - | - |
| - | - |
| - | - |
| - | - |
| - | - |
| - | - |
| - | - |
| - | - |
| - | - |
| - | - |
| - | - |
| - | - |
| - | - |
| - | - |
| - | - |
| - | - |
| - | - |
| - | - |
| - | - |
| - | - |

| | |
|---|---|
| - | - |
| - | - |
| - | - |
| - | - |
| - | - |
| - | - |
| - | - |
| - | - |
| - | - |
| - | - |
| - | - |
| - | - |
| - | - |
| - | - |
| - | - |
| - | - |
| - | - |
| - | - |
| - | - |
| - | - |
| - | - |
| - | - |
| - | - |
| - | - |
| - | - |
| - | - |
| - | - |
| - | - |
| - | - |
| - | - |
| - | - |
| - | - |
| - | - |
| - | - |
| - | - |
| - | - |
| - | - |
| - | - |
| - | - |
| - | - |
| - | - |
| - | - |
| - | - |
| - | - |
| - | - |
| - | - |
| - | - |

| | |
|---|---|
| - | - |
| - | gnl\|UG\|Ta#S13011468 |
| - | gnl\|UG\|Ta#S13049762 |
| - | gnl\|UG\|Ta#S13118843 |
| - | gnl\|UG\|Ta#S16058102 |
| - | gnl\|UG\|Ta#S16058472 |
| - | gnl\|UG\|Ta#S16228124 |
| - | gnl\|UG\|Ta#S16235077 |
| - | gnl\|UG\|Ta#S22379618 |
| - | gnl\|UG\|Ta#S22379618 |
| - | gnl\|UG\|Ta#S22389895 |
| - | gnl\|UG\|Ta#S32514261 |
| - | gnl\|UG\|Ta#S32560716 |
| - | gnl\|UG\|Ta#S37917887 |
| - | gnl\|UG\|Ta#S50385579 |
| - | gnl\|UG\|Ta#S52541021 |
| - | gnl\|UG\|Ta#S52541041 |
| - | gnl\|UG\|Ta#S52541041 |
| - | gnl\|UG\|Ta#S52541495 |
| - | gnl\|UG\|Ta#S52542126 |
| - | gnl\|UG\|Ta#S52542126 |
| - | gnl\|UG\|Ta#S52542196 |
| - | gnl\|UG\|Ta#S52542310 |
| - | gnl\|UG\|Ta#S52542310 |
| - | gnl\|UG\|Ta#S52542434 |
| - | gnl\|UG\|Ta#S52542563 |
| - | gnl\|UG\|Ta#S52542901 |
| - | gnl\|UG\|Ta#S52542903 |
| - | gnl\|UG\|Ta#S52542917 |
| - | gnl\|UG\|Ta#S52543220 |
| - | gnl\|UG\|Ta#S52543556 |
| - | gnl\|UG\|Ta#S52543821 |
| - | gnl\|UG\|Ta#S52544160 |
| - | gnl\|UG\|Ta#S52544160 |
| - | gnl\|UG\|Ta#S52544160 |
| - | gnl\|UG\|Ta#S52544177 |
| - | gnl\|UG\|Ta#S52544198 |
| - | gnl\|UG\|Ta#S52544198 |
| - | gnl\|UG\|Ta#S52544198 |
| - | gnl\|UG\|Ta#S52544203 |
| - | gnl\|UG\|Ta#S52544203 |
| - | gnl\|UG\|Ta#S52544203 |
| - | gnl\|UG\|Ta#S52544270 |
| - | gnl\|UG\|Ta#S52544273 |
| - | gnl\|UG\|Ta#S52544574 |
| - | gnl\|UG\|Ta#S52544574 |
| - | gnl\|UG\|Ta#S52544574 |

| | |
|---|---|
| - | gnl\|UG\|Ta#S52544639 |
| - | gnl\|UG\|Ta#S52544785 |
| - | gnl\|UG\|Ta#S52544785 |
| - | gnl\|UG\|Ta#S52544911 |
| - | gnl\|UG\|Ta#S52544911 |
| - | gnl\|UG\|Ta#S52544933 |
| - | gnl\|UG\|Ta#S52544966 |
| - | gnl\|UG\|Ta#S52545129 |
| - | gnl\|UG\|Ta#S52545442 |
| - | gnl\|UG\|Ta#S52545582 |
| - | gnl\|UG\|Ta#S52545621 |
| - | gnl\|UG\|Ta#S52545764 |
| - | gnl\|UG\|Ta#S52545989 |
| - | gnl\|UG\|Ta#S52545989 |
| - | gnl\|UG\|Ta#S52545998 |
| - | gnl\|UG\|Ta#S52545998 |
| - | gnl\|UG\|Ta#S52546305 |
| - | gnl\|UG\|Ta#S52546444 |
| - | gnl\|UG\|Ta#S52546700 |
| - | gnl\|UG\|Ta#S58850698 |
| - | gnl\|UG\|Ta#S58851816 |
| - | gnl\|UG\|Ta#S58855071 |
| - | gnl\|UG\|Ta#S58856030 |
| - | gnl\|UG\|Ta#S58856030 |
| - | gnl\|UG\|Ta#S58856030 |
| - | gnl\|UG\|Ta#S58863788 |
| - | gnl\|UG\|Ta#S58865512 |
| - | gnl\|UG\|Ta#S58866344 |
| - | gnl\|UG\|Ta#S58866695 |
| - | gnl\|UG\|Ta#S58870090 |
| - | gnl\|UG\|Ta#S58870090 |
| - | gnl\|UG\|Ta#S58886335 |
| - | gnl\|UG\|Ta#S58890447 |
| - | gnl\|UG\|Ta#S58893285 |
| - | gnl\|UG\|Ta#S58893586 |
| - | gnl\|UG\|Ta#S58893586 |
| - | gnl\|UG\|Ta#S58895933 |
| - | gnl\|UG\|Ta#S58899219 |
| - | gnl\|UG\|Ta#S58899219 |
| - | gnl\|UG\|Ta#S58900941 |
| - | gnl\|UG\|Ta#S58901079 |
| - | gnl\|UG\|Ta#S58901079 |
| - | gnl\|UG\|Ta#S58901079 |
| - | gnl\|UG\|Ta#S58901079 |
| - | gnl\|UG\|Ta#S58902006 |
| - | gnl\|UG\|Ta#S58902006 |
| - | gnl\|UG\|Ta#S58907530 |

| | |
|---|---|
| - | gnl\|UG\|Ta#S58907530 |
| - | gnl\|UG\|Ta#S58908761 |
| - | gnl\|UG\|Ta#S58908769 |
| - | gnl\|UG\|Ta#S58909613 |
| - | gnl\|UG\|Ta#S61516476 |
| - | gnl\|UG\|Ta#S61516476 |
| - | gnl\|UG\|Ta#S61516973 |
| - | gnl\|UG\|Ta#S61529336 |
| - | gnl\|UG\|Ta#S61537276 |
| - | gnl\|UG\|Ta#S61545000 |
| - | gnl\|UG\|Ta#S61545000 |
| - | gnl\|UG\|Ta#S61547588 |
| - | gnl\|UG\|Ta#S61548524 |
| - | gnl\|UG\|Ta#S61549412 |
| - | gnl\|UG\|Ta#S61553833 |
| - | gnl\|UG\|Ta#S61556373 |
| - | gnl\|UG\|Ta#S61556373 |
| - | gnl\|UG\|Ta#S61556921 |
| - | gnl\|UG\|Ta#S61558097 |
| - | gnl\|UG\|Ta#S61558387 |
| - | gnl\|UG\|Ta#S61558649 |
| - | gnl\|UG\|Ta#S61558792 |
| - | gnl\|UG\|Ta#S61560148 |
| - | gnl\|UG\|Ta#S61560730 |
| - | gnl\|UG\|Ta#S61560730 |
| - | gnl\|UG\|Ta#S61563987 |
| - | gnl\|UG\|Ta#S61569683 |
| - | gnl\|UG\|Ta#S61572348 |
| - | gnl\|UG\|Ta#S61579489 |
| - | gnl\|UG\|Ta#S61579489 |
| - | gnl\|UG\|Ta#S61581471 |
| - | gnl\|UG\|Ta#S61583802 |
| - | gnl\|UG\|Ta#S61586167 |
| - | gnl\|UG\|Ta#S61590227 |
| - | gnl\|UG\|Ta#S61590842 |
| - | gnl\|UG\|Ta#S61597330 |
| - | gnl\|UG\|Ta#S61600255 |
| - | gnl\|UG\|Ta#S61601256 |
| - | gnl\|UG\|Ta#S61601256 |
| - | gnl\|UG\|Ta#S61601256 |
| - | gnl\|UG\|Ta#S61602189 |
| - | gnl\|UG\|Ta#S61603101 |
| - | gnl\|UG\|Ta#S61604237 |
| - | gnl\|UG\|Ta#S61604336 |
| - | gnl\|UG\|Ta#S61604614 |
| - | gnl\|UG\|Ta#S61604614 |
| - | gnl\|UG\|Ta#S61604666 |

| | |
|---|---|
| - | gnl\|UG\|Ta#S61607242 |
| - | gnl\|UG\|Ta#S61607436 |
| - | gnl\|UG\|Ta#S61607436 |
| - | gnl\|UG\|Ta#S61607436 |
| - | gnl\|UG\|Ta#S61607814 |
| - | gnl\|UG\|Ta#S61608869 |
| - | gnl\|UG\|Ta#S61613004 |
| - | gnl\|UG\|Ta#S61614452 |
| - | gnl\|UG\|Ta#S61614472 |
| - | gnl\|UG\|Ta#S61614825 |
| - | gnl\|UG\|Ta#S61614825 |
| - | gnl\|UG\|Ta#S61614825 |
| - | gnl\|UG\|Ta#S61615643 |
| - | gnl\|UG\|Ta#S61616260 |
| - | gnl\|UG\|Ta#S61616337 |
| - | gnl\|UG\|Ta#S61616337 |
| - | gnl\|UG\|Ta#S61616616 |
| - | gnl\|UG\|Ta#S61617166 |
| - | gnl\|UG\|Ta#S61617286 |
| - | gnl\|UG\|Ta#S61617950 |
| - | gnl\|UG\|Ta#S61621189 |
| - | gnl\|UG\|Ta#S61622478 |
| - | gnl\|UG\|Ta#S61624083 |
| - | gnl\|UG\|Ta#S61624593 |
| - | gnl\|UG\|Ta#S61625153 |
| - | gnl\|UG\|Ta#S61625946 |
| - | gnl\|UG\|Ta#S61626291 |
| - | gnl\|UG\|Ta#S61632658 |
| - | gnl\|UG\|Ta#S61634666 |
| - | gnl\|UG\|Ta#S61634666 |
| - | gnl\|UG\|Ta#S61634666 |
| - | gnl\|UG\|Ta#S61634783 |
| - | gnl\|UG\|Ta#S61635292 |
| - | gnl\|UG\|Ta#S61635292 |
| - | gnl\|UG\|Ta#S61636951 |
| - | gnl\|UG\|Ta#S61638248 |
| - | gnl\|UG\|Ta#S61638248 |
| - | gnl\|UG\|Ta#S61638483 |
| - | gnl\|UG\|Ta#S61638483 |
| - | gnl\|UG\|Ta#S61641130 |
| - | gnl\|UG\|Ta#S61641147 |
| - | gnl\|UG\|Ta#S61644963 |
| - | gnl\|UG\|Ta#S61645525 |
| - | gnl\|UG\|Ta#S61650526 |
| - | gnl\|UG\|Ta#S61654089 |
| - | gnl\|UG\|Ta#S61669075 |
| - | gnl\|UG\|Ta#S61671486 |

| | |
|---|---|
| - | gnl\|UG\|Ta#S61708006 |
| - | gnl\|UG\|Ta#S61715160 |
| - | gnl\|UG\|Ta#S61721586 |
| - | gnl\|UG\|Ta#S61732882 |
| - | gnl\|UG\|Ta#S61736668 |
| - | gnl\|UG\|Ta#S61743196 |
| - | gnl\|UG\|Ta#S61768724 |
| - | gnl\|UG\|Ta#S61771853 |
| - | gnl\|UG\|Ta#S61771853 |
| - | gnl\|UG\|Ta#S61776127 |
| - | gnl\|UG\|Ta#S61776127 |
| - | gnl\|UG\|Ta#S61777575 |
| - | gnl\|UG\|Ta#S61777575 |
| - | gnl\|UG\|Ta#S61777884 |
| - | gnl\|UG\|Ta#S61779114 |
| - | gnl\|UG\|Ta#S61779114 |
| - | gnl\|UG\|Ta#S61779447 |
| - | gnl\|UG\|Ta#S61780178 |
| - | gnl\|UG\|Ta#S61780509 |
| - | gnl\|UG\|Ta#S61780509 |
| - | gnl\|UG\|Ta#S61780509 |
| - | gnl\|UG\|Ta#S61781566 |
| - | gnl\|UG\|Ta#S61781566 |
| - | gnl\|UG\|Ta#S61781566 |
| - | gnl\|UG\|Ta#S61782281 |
| - | gnl\|UG\|Ta#S61782348 |
| - | gnl\|UG\|Ta#S61784527 |
| - | gnl\|UG\|Ta#S61784823 |
| - | gnl\|UG\|Ta#S61788457 |
| - | gnl\|UG\|Ta#S61789523 |
| - | gnl\|UG\|Ta#S61789523 |
| - | gnl\|UG\|Ta#S61789535 |
| - | gnl\|UG\|Ta#S61791033 |
| - | gnl\|UG\|Ta#S61791923 |
| - | gnl\|UG\|Ta#S61791923 |
| - | gnl\|UG\|Ta#S61791923 |
| - | gnl\|UG\|Ta#S61791923 |
| - | gnl\|UG\|Ta#S61792113 |
| - | gnl\|UG\|Ta#S61792297 |
| - | gnl\|UG\|Ta#S61792483 |
| - | gnl\|UG\|Ta#S61797632 |
| - | gnl\|UG\|Ta#S61797786 |
| - | gnl\|UG\|Ta#S61798599 |
| - | gnl\|UG\|Ta#S61799525 |
| - | gnl\|UG\|Ta#S61800172 |
| - | gnl\|UG\|Ta#S61800316 |
| - | gnl\|UG\|Ta#S61800686 |

| | |
|---|---|
| - | gnl\|UG\|Ta#S61800732 |
| - | gnl\|UG\|Ta#S61800732 |
| - | gnl\|UG\|Ta#S61801005 |
| - | gnl\|UG\|Ta#S61801554 |
| - | gnl\|UG\|Ta#S61801571 |
| - | gnl\|UG\|Ta#S61801686 |
| - | gnl\|UG\|Ta#S61801808 |
| - | gnl\|UG\|Ta#S61801808 |
| - | gnl\|UG\|Ta#S61802145 |
| - | gnl\|UG\|Ta#S61802177 |
| - | gnl\|UG\|Ta#S61802177 |
| - | gnl\|UG\|Ta#S61807729 |
| - | gnl\|UG\|Ta#S61807810 |
| - | gnl\|UG\|Ta#S61807861 |
| - | gnl\|UG\|Ta#S61808053 |
| - | gnl\|UG\|Ta#S61808199 |
| - | gnl\|UG\|Ta#S61808243 |
| - | gnl\|UG\|Ta#S61808376 |
| - | gnl\|UG\|Ta#S61808571 |
| - | gnl\|UG\|Ta#S61808689 |
| - | gnl\|UG\|Ta#S61808689 |
| - | gnl\|UG\|Ta#S61809104 |
| - | gnl\|UG\|Ta#S61809106 |
| - | gnl\|UG\|Ta#S61809592 |
| - | gnl\|UG\|Ta#S61809707 |
| - | gnl\|UG\|Ta#S61809750 |
| - | gnl\|UG\|Ta#S61809750 |
| - | gnl\|UG\|Ta#S61809818 |
| - | gnl\|UG\|Ta#S61810100 |
| - | gnl\|UG\|Ta#S61810474 |
| - | gnl\|UG\|Ta#S61810636 |
| - | gnl\|UG\|Ta#S61810636 |
| - | gnl\|UG\|Ta#S61810697 |
| - | gnl\|UG\|Ta#S61810843 |
| - | gnl\|UG\|Ta#S61810976 |
| - | gnl\|UG\|Ta#S61811367 |
| - | gnl\|UG\|Ta#S61811367 |
| - | gnl\|UG\|Ta#S61811404 |
| - | gnl\|UG\|Ta#S61811404 |
| - | gnl\|UG\|Ta#S61811404 |
| - | gnl\|UG\|Ta#S61811490 |
| - | gnl\|UG\|Ta#S61811521 |
| - | gnl\|UG\|Ta#S61811521 |
| - | gnl\|UG\|Ta#S61811660 |
| - | gnl\|UG\|Ta#S61811666 |
| - | gnl\|UG\|Ta#S61811666 |
| - | gnl\|UG\|Ta#S61811666 |

| | |
|---|---|
| - | gnl\|UG\|Ta#S61811692 |
| - | gnl\|UG\|Ta#S61811774 |
| - | gnl\|UG\|Ta#S61811774 |
| - | gnl\|UG\|Ta#S61811900 |
| - | gnl\|UG\|Ta#S61812070 |
| - | gnl\|UG\|Ta#S61812070 |
| - | gnl\|UG\|Ta#S61812070 |
| - | gnl\|UG\|Ta#S61812070 |
| - | gnl\|UG\|Ta#S61812114 |
| - | gnl\|UG\|Ta#S61812124 |
| - | gnl\|UG\|Ta#S61812150 |
| - | gnl\|UG\|Ta#S61812151 |
| - | gnl\|UG\|Ta#S61812151 |
| - | gnl\|UG\|Ta#S61812317 |
| - | gnl\|UG\|Ta#S61812336 |
| - | gnl\|UG\|Ta#S61812377 |
| - | gnl\|UG\|Ta#S61812377 |
| - | gnl\|UG\|Ta#S61812415 |
| - | gnl\|UG\|Ta#S61812415 |
| - | gnl\|UG\|Ta#S61812421 |
| - | gnl\|UG\|Ta#S61812516 |
| - | gnl\|UG\|Ta#S61812516 |
| - | gnl\|UG\|Ta#S61812530 |
| - | gnl\|UG\|Ta#S61812545 |
| - | gnl\|UG\|Ta#S61817694 |
| - | gnl\|UG\|Ta#S61827768 |
| - | gnl\|UG\|Ta#S61827953 |
| - | gnl\|UG\|Ta#S61827978 |
| - | gnl\|UG\|Ta#S61828099 |
| - | gnl\|UG\|Ta#S61828099 |
| - | gnl\|UG\|Ta#S61828099 |
| - | gnl\|UG\|Ta#S61828101 |
| - | gnl\|UG\|Ta#S61828182 |
| - | gnl\|UG\|Ta#S61828213 |
| - | gnl\|UG\|Ta#S61828213 |
| - | gnl\|UG\|Ta#S61828405 |
| - | gnl\|UG\|Ta#S61828504 |
| - | gnl\|UG\|Ta#S61828524 |
| - | gnl\|UG\|Ta#S61829104 |
| - | gnl\|UG\|Ta#S61829167 |
| - | gnl\|UG\|Ta#S61829384 |
| - | gnl\|UG\|Ta#S61829384 |
| - | gnl\|UG\|Ta#S61829619 |
| - | gnl\|UG\|Ta#S61829866 |
| - | gnl\|UG\|Ta#S61830019 |
| - | gnl\|UG\|Ta#S61830096 |
| - | gnl\|UG\|Ta#S61830226 |

| | |
|---|---|
| - | gnl\|UG\|Ta#S61830249 |
| - | gnl\|UG\|Ta#S61831026 |
| - | gnl\|UG\|Ta#S61831155 |
| - | gnl\|UG\|Ta#S61831238 |
| - | gnl\|UG\|Ta#S61831280 |
| - | gnl\|UG\|Ta#S61831402 |
| - | gnl\|UG\|Ta#S61831441 |
| - | gnl\|UG\|Ta#S61831458 |
| - | gnl\|UG\|Ta#S61831561 |
| - | gnl\|UG\|Ta#S61831573 |
| - | gnl\|UG\|Ta#S61831623 |
| - | gnl\|UG\|Ta#S61831623 |
| - | gnl\|UG\|Ta#S61831696 |
| - | gnl\|UG\|Ta#S61831808 |
| - | gnl\|UG\|Ta#S61831927 |
| - | gnl\|UG\|Ta#S61831928 |
| - | gnl\|UG\|Ta#S61832035 |
| - | gnl\|UG\|Ta#S61832069 |
| - | gnl\|UG\|Ta#S61832106 |
| - | gnl\|UG\|Ta#S61832262 |
| - | gnl\|UG\|Ta#S61832271 |
| - | gnl\|UG\|Ta#S61832272 |
| - | gnl\|UG\|Ta#S61832297 |
| - | gnl\|UG\|Ta#S61832327 |
| - | gnl\|UG\|Ta#S61832335 |
| - | gnl\|UG\|Ta#S61832389 |
| - | gnl\|UG\|Ta#S61832389 |
| - | gnl\|UG\|Ta#S61832389 |
| - | gnl\|UG\|Ta#S61832394 |
| - | gnl\|UG\|Ta#S61832400 |
| - | gnl\|UG\|Ta#S61832400 |
| - | gnl\|UG\|Ta#S61832418 |
| - | gnl\|UG\|Ta#S61832418 |
| - | gnl\|UG\|Ta#S61832439 |
| - | gnl\|UG\|Ta#S61832441 |
| - | gnl\|UG\|Ta#S61832461 |
| - | gnl\|UG\|Ta#S61832473 |
| - | gnl\|UG\|Ta#S61832522 |
| - | gnl\|UG\|Ta#S61832522 |
| - | gnl\|UG\|Ta#S61832524 |
| - | gnl\|UG\|Ta#S61832533 |
| - | gnl\|UG\|Ta#S61834147 |
| - | gnl\|UG\|Ta#S61841454 |
| - | gnl\|UG\|Ta#S61854177 |
| - | gnl\|UG\|Ta#S61862121 |
| - | gnl\|UG\|Ta#S61863261 |
| - | gnl\|UG\|Ta#S65589186 |

| | |
|---|---|
| - | gnl\|UG\|Ta#S65590008 |
| - | gnl\|UG\|Ta#S65591015 |
| - | gnl\|UG\|Ta#S65591060 |
| - | gnl\|UG\|Ta#S65594331 |
| - | gnl\|UG\|Ta#S65601858 |
| - | gnl\|UG\|Ta#S65601858 |
| - | gnl\|UG\|Ta#S65607523 |
| - | gnl\|UG\|Ta#S65608434 |
| - | gnl\|UG\|Ta#S65608434 |
| - | gnl\|UG\|Ta#S65609111 |
| - | gnl\|UG\|Ta#S65611212 |
| - | gnl\|UG\|Ta#S65613284 |
| - | gnl\|UG\|Ta#S65613284 |
| - | gnl\|UG\|Ta#S65614522 |
| - | gnl\|UG\|Ta#S65617689 |
| - | gnl\|UG\|Ta#S65618599 |
| - | gnl\|UG\|Ta#S65620191 |
| - | gnl\|UG\|Ta#S65621388 |
| - | gnl\|UG\|Ta#S65621388 |
| - | gnl\|UG\|Ta#S65623583 |
| - | gnl\|UG\|Ta#S65624415 |
| - | gnl\|UG\|Ta#S65628900 |
| - | gnl\|UG\|Ta#S65633872 |
| - | gnl\|UG\|Ta#S65639709 |
| - | gnl\|UG\|Ta#S65658546 |
| - | gnl\|UG\|Ta#S65661119 |
| - | gnl\|UG\|Ta#S65679156 |
| - | gnl\|UG\|Ta#S65685611 |
| - | gnl\|UG\|Ta#S65688621 |
| - | gnl\|UG\|Ta#S65703817 |
| - | gnl\|UG\|Ta#S65706298 |
| - | gnl\|UG\|Ta#S65711079 |
| - | gnl\|UG\|Ta#S65720200 |
| tr\|M7YDD6\|M7YDD6_TRIUA | - |
| tr\|M7YF72\|M7YF72_TRIUA | - |
| tr\|M7YF74\|M7YF74_TRIUA | - |
| tr\|M7YFM2\|M7YFM2_TRIUA | - |
| tr\|M7YKD6\|M7YKD6_TRIUA | - |
| tr\|M7YKD6\|M7YKD6_TRIUA | - |
| tr\|M7YT40\|M7YT40_TRIUA | - |
| tr\|M7YTB8\|M7YTB8_TRIUA | - |
| tr\|M7YW50\|M7YW50_TRIUA | - |
| tr\|M7YZC0\|M7YZC0_TRIUA | - |
| tr\|M7Z0R0\|M7Z0R0_TRIUA | - |
| tr\|M7Z0R0\|M7Z0R0_TRIUA | - |
| tr\|M7Z657\|M7Z657_TRIUA | - |
| tr\|M7ZE42\|M7ZE42_TRIUA | - |

| | |
|---|---|
| tr\|M7ZM78\|M7ZM78_TRIUA | - |
| tr\|M7ZMX7\|M7ZMX7_TRIUA | - |
| tr\|M7ZRG4\|M7ZRG4_TRIUA | - |
| tr\|M7ZS76\|M7ZS76_TRIUA | - |
| tr\|M7ZSP4\|M7ZSP4_TRIUA | - |
| tr\|M7ZTQ2\|M7ZTQ2_TRIUA | - |
| tr\|M7ZY48\|M7ZY48_TRIUA | - |
| tr\|M8A7W2\|M8A7W2_TRIUA | - |
| tr\|M8A7W2\|M8A7W2_TRIUA | - |
| tr\|M8A8X2\|M8A8X2_TRIUA | - |
| tr\|M8AI48\|M8AI48_TRIUA | - |
| tr\|M8AJE6\|M8AJE6_TRIUA | - |
| tr\|M8APC2\|M8APC2_TRIUA | - |
| tr\|M8AQT1\|M8AQT1_TRIUA | - |
| tr\|M8AQT1\|M8AQT1_TRIUA | - |
| tr\|M8AQT4\|M8AQT4_TRIUA | - |
| tr\|M8AQT4\|M8AQT4_TRIUA | - |
| tr\|Q66NG8\|Q66NG8_WHEAT | - |
| tr\|T1LLS5\|T1LLS5_TRIUA | - |
| tr\|T1LVF9\|T1LVF9_TRIUA | - |
| tr\|T1LY63\|T1LY63_TRIUA | - |
| tr\|T1N0K9\|T1N0K9_TRIUA | - |
| tr\|T1N4L7\|T1N4L7_TRIUA | - |
| tr\|T1N6U6\|T1N6U6_TRIUA | - |
| tr\|T1NRR3\|T1NRR3_TRIUA | - |
| tr\|W4ZP47\|W4ZP47_WHEAT | - |
| tr\|W4ZWX4\|W4ZWX4_WHEAT | - |
| tr\|W4ZXR7\|W4ZXR7_WHEAT | - |
| tr\|W4ZZA4\|W4ZZA4_WHEAT | - |
| tr\|W5A617\|W5A617_WHEAT | - |
| tr\|W5A8K2\|W5A8K2_WHEAT | - |
| tr\|W5A925\|W5A925_WHEAT | - |
| tr\|W5A9F3\|W5A9F3_WHEAT | - |
| tr\|W5AC56\|W5AC56_WHEAT | - |
| tr\|W5AF40\|W5AF40_WHEAT | - |
| tr\|W5BNM4\|W5BNM4_WHEAT | - |
| tr\|W5DLZ1\|W5DLZ1_WHEAT | - |
| tr\|W5DNJ2\|W5DNJ2_WHEAT | - |
| tr\|W5DNQ6\|W5DNQ6_WHEAT | - |
| tr\|W5DPQ7\|W5DPQ7_WHEAT | - |
| tr\|W5DTA2\|W5DTA2_WHEAT | - |
| tr\|W5DV05\|W5DV05_WHEAT | - |
| tr\|W5DVP8\|W5DVP8_WHEAT | - |
| tr\|W5DW13\|W5DW13_WHEAT | - |
| tr\|W5DXI7\|W5DXI7_WHEAT | - |
| tr\|W5DZC6\|W5DZC6_WHEAT | - |
| tr\|W5E6K5\|W5E6K5_WHEAT | - |

| | |
|---|---|
| tr\|W5EAM7\|W5EAM7_WHEAT | - |
| tr\|W5EVX6\|W5EVX6_WHEAT | - |
| tr\|W5EYE0\|W5EYE0_WHEAT | - |
| tr\|W5F4D5\|W5F4D5_WHEAT | - |
| tr\|W5F5S5\|W5F5S5_WHEAT | - |
| tr\|W5F685\|W5F685_WHEAT | - |
| tr\|W5F6P6\|W5F6P6_WHEAT | - |
| tr\|W5F755\|W5F755_WHEAT | - |
| tr\|W5F7E4\|W5F7E4_WHEAT | - |
| tr\|W5F823\|W5F823_WHEAT | - |
| tr\|W5F880\|W5F880_WHEAT | - |
| tr\|W5F8G0\|W5F8G0_WHEAT | - |
| tr\|W5F8H4\|W5F8H4_WHEAT | - |
| tr\|W5F8N5\|W5F8N5_WHEAT | - |
| tr\|W5F8W4\|W5F8W4_WHEAT | - |
| tr\|W5F8Y2\|W5F8Y2_WHEAT | - |
| tr\|W5F922\|W5F922_WHEAT | - |
| tr\|W5F922\|W5F922_WHEAT | - |
| tr\|W5F9I5\|W5F9I5_WHEAT | - |
| tr\|W5F9M5\|W5F9M5_WHEAT | - |
| tr\|W5F9P3\|W5F9P3_WHEAT | - |
| tr\|W5F9P3\|W5F9P3_WHEAT | - |
| tr\|W5F9Z6\|W5F9Z6_WHEAT | - |
| tr\|W5FA75\|W5FA75_WHEAT | - |
| tr\|W5FAJ8\|W5FAJ8_WHEAT | - |
| tr\|W5FAK7\|W5FAK7_WHEAT | - |
| tr\|W5FAL9\|W5FAL9_WHEAT | - |
| tr\|W5FBP2\|W5FBP2_WHEAT | - |
| tr\|W5FBP2\|W5FBP2_WHEAT | - |
| tr\|W5FCC0\|W5FCC0_WHEAT | - |
| tr\|W5FCI4\|W5FCI4_WHEAT | - |
| tr\|W5FDG3\|W5FDG3_WHEAT | - |
| tr\|W5FDG3\|W5FDG3_WHEAT | - |
| tr\|W5FE52\|W5FE52_WHEAT | - |
| tr\|W5FE68\|W5FE68_WHEAT | - |
| tr\|W5FE68\|W5FE68_WHEAT | - |
| tr\|W5FEE3\|W5FEE3_WHEAT | - |
| tr\|W5FEU6\|W5FEU6_WHEAT | - |
| tr\|W5FEU6\|W5FEU6_WHEAT | - |
| tr\|W5FF04\|W5FF04_WHEAT | - |
| tr\|W5FH73\|W5FH73_WHEAT | - |
| tr\|W5FH94\|W5FH94_WHEAT | - |
| tr\|W5FI20\|W5FI20_WHEAT | - |
| tr\|W5FI44\|W5FI44_WHEAT | - |
| tr\|W5FIA8\|W5FIA8_WHEAT | - |
| tr\|W5FIA9\|W5FIA9_WHEAT | - |
| tr\|W5FIA9\|W5FIA9_WHEAT | - |

| | |
|---|---|
| tr\|W5FJ96\|W5FJ96_WHEAT | - |
| tr\|W5FJA7\|W5FJA7_WHEAT | - |
| tr\|W5FJA7\|W5FJA7_WHEAT | - |
| tr\|W5FKD6\|W5FKD6_WHEAT | - |
| tr\|W5FKD6\|W5FKD6_WHEAT | - |
| tr\|W5FKJ5\|W5FKJ5_WHEAT | - |
| tr\|W5FKL7\|W5FKL7_WHEAT | - |
| tr\|W5FKS9\|W5FKS9_WHEAT | - |
| tr\|W5FKX2\|W5FKX2_WHEAT | - |
| tr\|W5FLD8\|W5FLD8_WHEAT | - |
| tr\|W5FLI3\|W5FLI3_WHEAT | - |
| tr\|W5FM06\|W5FM06_WHEAT | - |
| tr\|W5FM06\|W5FM06_WHEAT | - |
| tr\|W5FMU3\|W5FMU3_WHEAT | - |
| tr\|W5FN72\|W5FN72_WHEAT | - |
| tr\|W5FNE2\|W5FNE2_WHEAT | - |
| tr\|W5FNE2\|W5FNE2_WHEAT | - |
| tr\|W5FNU6\|W5FNU6_WHEAT | - |
| tr\|W5FP53\|W5FP53_WHEAT | - |
| tr\|W5FP84\|W5FP84_WHEAT | - |
| tr\|W5FPD4\|W5FPD4_WHEAT | - |
| tr\|W5FRB4\|W5FRB4_WHEAT | - |
| tr\|W5FYG1\|W5FYG1_WHEAT | - |
| tr\|W5FYG1\|W5FYG1_WHEAT | - |
| tr\|W5FZ85\|W5FZ85_WHEAT | - |
| tr\|W5G1A0\|W5G1A0_WHEAT | - |
| tr\|W5G1A0\|W5G1A0_WHEAT | - |
| tr\|W5G1L1\|W5G1L1_WHEAT | - |
| tr\|W5G3Q5\|W5G3Q5_WHEAT | - |
| tr\|W5GHV6\|W5GHV6_WHEAT | - |
| tr\|W5GJD4\|W5GJD4_WHEAT | - |
| tr\|W5GLC7\|W5GLC7_WHEAT | - |
| tr\|W5HDJ2\|W5HDJ2_WHEAT | - |
| tr\|W5HI93\|W5HI93_WHEAT | - |
| tr\|W5HTH6\|W5HTH6_WHEAT | - |
| tr\|W5I5A6\|W5I5A6_WHEAT | - |
| tr\|W5FI26\|W5FI26_WHEAT | gnl\|UG\|Ta#S12890084 |
| tr\|T1NS24\|T1NS24_TRIUA | gnl\|UG\|Ta#S13045650 |
| tr\|M8AXI3\|M8AXI3_TRIUA | gnl\|UG\|Ta#S13211046 |
| tr\|W5E1Z7\|W5E1Z7_WHEAT | gnl\|UG\|Ta#S16228124 |
| tr\|M8ASU2\|M8ASU2_TRIUA | gnl\|UG\|Ta#S22369533 |
| tr\|M7ZPE1\|M7ZPE1_TRIUA | gnl\|UG\|Ta#S32692189 |
| tr\|W5FEE0\|W5FEE0_WHEAT | gnl\|UG\|Ta#S37810183 |
| tr\|W5EET5\|W5EET5_WHEAT | gnl\|UG\|Ta#S52541068 |
| tr\|W5FGW6\|W5FGW6_WHEAT | gnl\|UG\|Ta#S52541732 |
| tr\|M7YNW6\|M7YNW6_TRIUA | gnl\|UG\|Ta#S52542426 |
| tr\|W5FZ77\|W5FZ77_WHEAT | gnl\|UG\|Ta#S52542520 |

| | |
|---|---|
| tr\|W5FP12\|W5FP12_WHEAT | gnl\|UG\|Ta#S52542917 |
| tr\|W5E024\|W5E024_WHEAT | gnl\|UG\|Ta#S52543038 |
| tr\|M8A008\|M8A008_TRIUA | gnl\|UG\|Ta#S52543379 |
| tr\|W5FFX0\|W5FFX0_WHEAT | gnl\|UG\|Ta#S52544203 |
| tr\|M7ZF91\|M7ZF91_TRIUA | gnl\|UG\|Ta#S52545260 |
| tr\|W5FH27\|W5FH27_WHEAT | gnl\|UG\|Ta#S52545304 |
| tr\|W5FDZ3\|W5FDZ3_WHEAT | gnl\|UG\|Ta#S52545541 |
| tr\|W5F731\|W5F731_WHEAT | gnl\|UG\|Ta#S52545582 |
| tr\|M8AGH9\|M8AGH9_TRIUA | gnl\|UG\|Ta#S52545989 |
| tr\|M7ZR95\|M7ZR95_TRIUA | gnl\|UG\|Ta#S52546277 |
| tr\|W5DX39\|W5DX39_WHEAT | gnl\|UG\|Ta#S52546277 |
| tr\|W5E9U6\|W5E9U6_WHEAT | gnl\|UG\|Ta#S52546444 |
| tr\|M7ZES3\|M7ZES3_TRIUA | gnl\|UG\|Ta#S52546475 |
| tr\|W5G942\|W5G942_WHEAT | gnl\|UG\|Ta#S52546616 |
| tr\|M7ZWZ8\|M7ZWZ8_TRIUA | gnl\|UG\|Ta#S52546782 |
| tr\|M7ZXC5\|M7ZXC5_TRIUA | gnl\|UG\|Ta#S52547038 |
| tr\|W5FY62\|W5FY62_WHEAT | gnl\|UG\|Ta#S58840547 |
| tr\|W5DQ61\|W5DQ61_WHEAT | gnl\|UG\|Ta#S58844003 |
| tr\|W5DYW2\|W5DYW2_WHEAT | gnl\|UG\|Ta#S58844736 |
| tr\|M7Z7B7\|M7Z7B7_TRIUA | gnl\|UG\|Ta#S58860731 |
| tr\|W5FG40\|W5FG40_WHEAT | gnl\|UG\|Ta#S58882566 |
| tr\|W4ZV02\|W4ZV02_WHEAT | gnl\|UG\|Ta#S58895933 |
| tr\|W5DZB7\|W5DZB7_WHEAT | gnl\|UG\|Ta#S58897580 |
| tr\|W5FXD7\|W5FXD7_WHEAT | gnl\|UG\|Ta#S58899219 |
| tr\|W5FXD7\|W5FXD7_WHEAT | gnl\|UG\|Ta#S58899219 |
| tr\|W5F814\|W5F814_WHEAT | gnl\|UG\|Ta#S58901079 |
| tr\|M8B3Z7\|M8B3Z7_TRIUA | gnl\|UG\|Ta#S61545000 |
| tr\|W5FM06\|W5FM06_WHEAT | gnl\|UG\|Ta#S61548524 |
| tr\|M7Y7R5\|M7Y7R5_TRIUA | gnl\|UG\|Ta#S61560148 |
| tr\|M7ZUS6\|M7ZUS6_TRIUA | gnl\|UG\|Ta#S61560647 |
| tr\|W5A5D8\|W5A5D8_WHEAT | gnl\|UG\|Ta#S61579408 |
| tr\|W5FKY3\|W5FKY3_WHEAT | gnl\|UG\|Ta#S61590842 |
| tr\|W5FM36\|W5FM36_WHEAT | gnl\|UG\|Ta#S61592013 |
| tr\|W5A1K7\|W5A1K7_WHEAT | gnl\|UG\|Ta#S61596675 |
| tr\|W5C0C6\|W5C0C6_WHEAT | gnl\|UG\|Ta#S61597330 |
| tr\|Q66NG6\|Q66NG6_WHEAT | gnl\|UG\|Ta#S61600255 |
| tr\|M8A398\|M8A398_TRIUA | gnl\|UG\|Ta#S61601256 |
| tr\|W5FFF6\|W5FFF6_WHEAT | gnl\|UG\|Ta#S61604010 |
| tr\|W5FFF6\|W5FFF6_WHEAT | gnl\|UG\|Ta#S61604010 |
| tr\|W5F922\|W5F922_WHEAT | gnl\|UG\|Ta#S61604237 |
| tr\|W5F922\|W5F922_WHEAT | gnl\|UG\|Ta#S61604237 |
| tr\|M8A4B0\|M8A4B0_TRIUA | gnl\|UG\|Ta#S61604267 |
| tr\|M7Z068\|M7Z068_TRIUA | gnl\|UG\|Ta#S61607436 |
| tr\|T1N0L0\|T1N0L0_TRIUA | gnl\|UG\|Ta#S61607817 |
| tr\|W5FAV9\|W5FAV9_WHEAT | gnl\|UG\|Ta#S61611803 |
| tr\|W5FAV9\|W5FAV9_WHEAT | gnl\|UG\|Ta#S61611803 |
| tr\|M7ZG18\|M7ZG18_TRIUA | gnl\|UG\|Ta#S61614123 |

| | |
|---|---|
| tr\|M7ZRN3\|M7ZRN3_TRIUA | gnl\|UG\|Ta#S61635292 |
| tr\|M7ZRN3\|M7ZRN3_TRIUA | gnl\|UG\|Ta#S61635292 |
| tr\|T1NMD0\|T1NMD0_TRIUA | gnl\|UG\|Ta#S61654089 |
| tr\|W5FPC1\|W5FPC1_WHEAT | gnl\|UG\|Ta#S61669301 |
| tr\|M7ZQI9\|M7ZQI9_TRIUA | gnl\|UG\|Ta#S61671779 |
| tr\|T1MIU8\|T1MIU8_TRIUA | gnl\|UG\|Ta#S61736756 |
| tr\|W5F6Y8\|W5F6Y8_WHEAT | gnl\|UG\|Ta#S61744367 |
| tr\|T1NJX7\|T1NJX7_TRIUA | gnl\|UG\|Ta#S61746969 |
| tr\|W5FIU1\|W5FIU1_WHEAT | gnl\|UG\|Ta#S61765200 |
| tr\|W5FIU1\|W5FIU1_WHEAT | gnl\|UG\|Ta#S61765200 |
| tr\|W5EVB4\|W5EVB4_WHEAT | gnl\|UG\|Ta#S61767438 |
| tr\|W5FKE6\|W5FKE6_WHEAT | gnl\|UG\|Ta#S61768369 |
| tr\|M8A062\|M8A062_TRIUA | gnl\|UG\|Ta#S61771853 |
| tr\|W5FGL8\|W5FGL8_WHEAT | gnl\|UG\|Ta#S61781566 |
| tr\|W5A095\|W5A095_WHEAT | gnl\|UG\|Ta#S61792338 |
| tr\|M7ZDB8\|M7ZDB8_TRIUA | gnl\|UG\|Ta#S61797786 |
| tr\|W5F1B9\|W5F1B9_WHEAT | gnl\|UG\|Ta#S61798563 |
| tr\|W5FH72\|W5FH72_WHEAT | gnl\|UG\|Ta#S61799525 |
| tr\|W5EY15\|W5EY15_WHEAT | gnl\|UG\|Ta#S61800732 |
| tr\|W5AYP1\|W5AYP1_WHEAT | gnl\|UG\|Ta#S61802033 |
| tr\|W5GQ60\|W5GQ60_WHEAT | gnl\|UG\|Ta#S61802145 |
| tr\|W5A561\|W5A561_WHEAT | gnl\|UG\|Ta#S61802146 |
| tr\|W5GJS2\|W5GJS2_WHEAT | gnl\|UG\|Ta#S61802210 |
| tr\|W4ZU18\|W4ZU18_WHEAT | gnl\|UG\|Ta#S61802297 |
| tr\|M7ZQJ0\|M7ZQJ0_TRIUA | gnl\|UG\|Ta#S61808199 |
| tr\|W5FJF2\|W5FJF2_WHEAT | gnl\|UG\|Ta#S61808226 |
| tr\|W5FPD8\|W5FPD8_WHEAT | gnl\|UG\|Ta#S61808403 |
| tr\|W5A617\|W5A617_WHEAT | gnl\|UG\|Ta#S61809592 |
| tr\|W5FHW3\|W5FHW3_WHEAT | gnl\|UG\|Ta#S61809989 |
| tr\|T1M7T6\|T1M7T6_TRIUA | gnl\|UG\|Ta#S61810474 |
| tr\|W5FK41\|W5FK41_WHEAT | gnl\|UG\|Ta#S61810680 |
| tr\|M7ZUB2\|M7ZUB2_TRIUA | gnl\|UG\|Ta#S61810714 |
| tr\|W5FHF6\|W5FHF6_WHEAT | gnl\|UG\|Ta#S61810891 |
| tr\|M7ZP22\|M7ZP22_TRIUA | gnl\|UG\|Ta#S61810999 |
| tr\|W4ZZC1\|W4ZZC1_WHEAT | gnl\|UG\|Ta#S61811490 |
| tr\|M7ZU63\|M7ZU63_TRIUA | gnl\|UG\|Ta#S61811666 |
| tr\|M7Z2I9\|M7Z2I9_TRIUA | gnl\|UG\|Ta#S61811692 |
| tr\|W5FJ90\|W5FJ90_WHEAT | gnl\|UG\|Ta#S61811730 |
| tr\|M8A087\|M8A087_TRIUA | gnl\|UG\|Ta#S61811944 |
| tr\|M7YBF3\|M7YBF3_TRIUA | gnl\|UG\|Ta#S61811995 |
| tr\|W5GFN6\|W5GFN6_WHEAT | gnl\|UG\|Ta#S61812151 |
| tr\|M7Y8E5\|M7Y8E5_TRIUA | gnl\|UG\|Ta#S61812260 |
| tr\|W5FEQ3\|W5FEQ3_WHEAT | gnl\|UG\|Ta#S61812317 |
| tr\|M7ZL45\|M7ZL45_TRIUA | gnl\|UG\|Ta#S61812377 |
| tr\|M7ZL45\|M7ZL45_TRIUA | gnl\|UG\|Ta#S61812377 |
| tr\|M7ZTQ2\|M7ZTQ2_TRIUA | gnl\|UG\|Ta#S61812379 |
| tr\|W5DW38\|W5DW38_WHEAT | gnl\|UG\|Ta#S61812379 |

| | |
|---|---|
| tr\|W5GRS1\|W5GRS1_WHEAT | gnl\|UG\|Ta#S61812432 |
| tr\|W5CHN0\|W5CHN0_WHEAT | gnl\|UG\|Ta#S61828326 |
| tr\|M7YXC5\|M7YXC5_TRIUA | gnl\|UG\|Ta#S61828595 |
| tr\|T1MFJ9\|T1MFJ9_TRIUA | gnl\|UG\|Ta#S61828784 |
| tr\|T1N3P8\|T1N3P8_TRIUA | gnl\|UG\|Ta#S61829781 |
| tr\|W5A9N2\|W5A9N2_WHEAT | gnl\|UG\|Ta#S61830003 |
| tr\|M7ZXH6\|M7ZXH6_TRIUA | gnl\|UG\|Ta#S61830189 |
| tr\|W5FIW1\|W5FIW1_WHEAT | gnl\|UG\|Ta#S61830418 |
| tr\|W5FIW1\|W5FIW1_WHEAT | gnl\|UG\|Ta#S61830418 |
| tr\|W5FKM0\|W5FKM0_WHEAT | gnl\|UG\|Ta#S61830614 |
| tr\|W5FU73\|W5FU73_WHEAT | gnl\|UG\|Ta#S61830614 |
| tr\|W5F3I7\|W5F3I7_WHEAT | gnl\|UG\|Ta#S61831164 |
| tr\|W5DZ19\|W5DZ19_WHEAT | gnl\|UG\|Ta#S61831238 |
| tr\|W5FKB7\|W5FKB7_WHEAT | gnl\|UG\|Ta#S61831696 |
| tr\|W5FKB7\|W5FKB7_WHEAT | gnl\|UG\|Ta#S61831696 |
| tr\|W5DLR5\|W5DLR5_WHEAT | gnl\|UG\|Ta#S61831738 |
| tr\|W5FI93\|W5FI93_WHEAT | gnl\|UG\|Ta#S61831913 |
| tr\|T1MGT9\|T1MGT9_TRIUA | gnl\|UG\|Ta#S61832035 |
| tr\|M8A7W2\|M8A7W2_TRIUA | gnl\|UG\|Ta#S61832106 |
| tr\|W5F1J0\|W5F1J0_WHEAT | gnl\|UG\|Ta#S61832238 |
| tr\|W5F1J0\|W5F1J0_WHEAT | gnl\|UG\|Ta#S61832238 |
| tr\|M8A5Z5\|M8A5Z5_TRIUA | gnl\|UG\|Ta#S61832378 |
| tr\|M7YHV8\|M7YHV8_TRIUA | gnl\|UG\|Ta#S61832400 |
| tr\|W5F7J3\|W5F7J3_WHEAT | gnl\|UG\|Ta#S61832449 |
| tr\|M8A2T8\|M8A2T8_TRIUA | gnl\|UG\|Ta#S61832524 |
| tr\|W5IA51\|W5IA51_WHEAT | gnl\|UG\|Ta#S61832540 |
| tr\|W5A2Q9\|W5A2Q9_WHEAT | gnl\|UG\|Ta#S61856355 |
| tr\|W5FK80\|W5FK80_WHEAT | gnl\|UG\|Ta#S65594281 |
| tr\|W5DNB8\|W5DNB8_WHEAT | gnl\|UG\|Ta#S65596852 |
| tr\|W5FNE6\|W5FNE6_WHEAT | gnl\|UG\|Ta#S65600879 |
| tr\|W5FBS8\|W5FBS8_WHEAT | gnl\|UG\|Ta#S65611327 |
| tr\|W5EYR4\|W5EYR4_WHEAT | gnl\|UG\|Ta#S65621180 |
| tr\|W5F8X7\|W5F8X7_WHEAT | gnl\|UG\|Ta#S65624890 |
| tr\|M7YXX3\|M7YXX3_TRIUA | gnl\|UG\|Ta#S65626522 |
| tr\|W4ZYP3\|W4ZYP3_WHEAT | gnl\|UG\|Ta#S65633872 |
| tr\|W5EVZ3\|W5EVZ3_WHEAT | gnl\|UG\|Ta#S65639709 |
| tr\|W5DPK8\|W5DPK8_WHEAT | gnl\|UG\|Ta#S65642019 |
| tr\|W5DWH8\|W5DWH8_WHEAT | gnl\|UG\|Ta#S65654829 |
| tr\|M7ZB78\|M7ZB78_TRIUA | gnl\|UG\|Ta#S65660171 |
| tr\|W5F862\|W5F862_WHEAT | gnl\|UG\|Ta#S65662808 |
| tr\|M7YI57\|M7YI57_TRIUA | gnl\|UG\|Ta#S65673537 |
| tr\|W5FA60\|W5FA60_WHEAT | gnl\|UG\|Ta#S65696835 |
| - | - |
| - | - |
| - | - |
| - | - |
| - | - |

| | |
|---|---|
| - | - |
| - | - |
| - | - |
| - | - |
| - | - |
| - | - |
| - | - |
| - | - |
| - | - |
| - | - |
| - | - |
| - | - |
| - | - |
| - | - |
| - | - |
| - | - |
| - | - |
| - | - |
| - | - |
| - | - |
| - | - |
| - | - |
| - | - |
| - | - |
| - | - |
| - | - |
| - | - |
| - | - |
| - | - |
| - | - |
| - | - |
| - | - |
| - | - |
| - | - |
| - | - |
| - | - |
| - | - |
| - | - |
| - | - |
| - | - |
| - | - |
| - | - |
| - | - |
| - | - |
| - | - |
| - | - |
| - | - |

| | |
|---|---|
| - | - |
| - | - |
| - | - |
| - | - |
| - | - |
| - | - |
| - | - |
| - | - |
| - | - |
| - | - |
| - | - |
| - | - |
| - | - |
| - | - |
| - | - |
| - | - |
| - | - |
| - | - |
| - | - |
| - | - |
| - | - |
| - | - |
| - | - |
| - | - |
| - | - |
| - | - |
| - | - |
| - | - |
| - | - |
| - | - |
| - | - |
| - | - |
| - | - |
| - | - |
| - | - |
| - | - |
| - | - |
| - | - |
| - | - |
| - | - |
| - | - |
| - | - |
| - | - |
| - | - |
| - | - |
| - | - |
| - | - |

| | |
|---|---|
| - | - |
| - | - |
| - | - |
| - | - |
| - | - |
| - | - |
| - | - |
| - | - |
| - | - |
| - | - |
| - | - |
| - | - |
| - | - |
| - | - |
| - | - |
| - | - |
| - | - |
| - | - |
| - | - |
| - | - |
| - | - |
| - | - |
| - | - |
| - | - |
| - | - |
| - | - |
| - | - |
| - | - |
| - | - |
| - | - |
| - | - |
| - | - |
| - | - |
| - | - |
| - | - |
| - | - |
| - | - |
| - | - |
| - | - |
| - | - |
| - | - |
| - | - |
| - | - |
| - | - |
| - | - |
| - | - |
| - | - |

| | |
|---|---|
| - | - |
| - | - |
| - | - |
| - | - |
| - | - |
| - | - |
| - | - |
| - | - |
| - | - |
| - | - |
| - | - |
| - | - |
| - | - |
| - | - |
| - | - |
| - | - |
| - | - |
| - | - |
| - | - |
| - | - |
| - | - |
| - | - |
| - | - |
| - | - |
| - | - |
| - | - |
| - | - |
| - | - |
| - | - |
| - | - |
| - | - |
| - | - |
| - | - |
| - | - |
| - | - |
| - | - |
| - | - |
| - | - |
| - | - |
| - | - |
| - | - |
| - | - |
| - | - |
| - | - |
| - | - |
| - | - |
| - | - |

-
-
-
-
-
-
-
-
-
-
-
-
-
-
-
-
-
-
-
-
-
-
-
-
-
-
-
-
-
-
-
-
-
-
-
-
-
-
-
-
-
-
-
-
-
-
-
-
-
-
-
-
-
-
-
-
-
-
-
-
-
-
-
-
-
-
-
-
-
-
-
-
-
-
-
-
-
-
-
-
-
-
-
-
-
-
-
-
-
-
-
-
-
-

| | |
|---|---|
| - | - |
| - | - |
| - | - |
| - | - |
| - | - |
| - | - |
| - | - |
| - | - |
| - | - |
| - | - |
| - | - |
| - | - |
| - | - |
| - | - |
| - | - |
| - | - |
| - | - |
| - | - |
| - | - |
| - | - |
| - | - |
| - | - |
| - | - |
| - | - |
| - | - |
| - | - |
| - | - |
| - | - |
| - | - |
| - | - |
| - | - |
| - | - |
| - | - |
| - | - |
| - | - |
| - | - |
| - | - |
| - | - |
| - | - |
| - | - |
| - | - |
| - | - |
| - | - |
| - | - |
| - | - |
| - | - |
| - | - |

| | |
|---|---|
| - | - |
| - | - |
| - | - |
| - | - |
| - | - |
| - | - |
| - | - |
| - | - |
| - | - |
| - | - |
| - | - |
| - | gnl\|UG\|Ta#S12923035 |
| - | gnl\|UG\|Ta#S12928189 |
| - | gnl\|UG\|Ta#S13022491 |
| - | gnl\|UG\|Ta#S32565736 |
| - | gnl\|UG\|Ta#S52541399 |
| - | gnl\|UG\|Ta#S52541749 |
| - | gnl\|UG\|Ta#S52542520 |
| - | gnl\|UG\|Ta#S52545531 |
| - | gnl\|UG\|Ta#S52545531 |
| - | gnl\|UG\|Ta#S52897632 |
| - | gnl\|UG\|Ta#S58846782 |
| - | gnl\|UG\|Ta#S58863943 |
| - | gnl\|UG\|Ta#S58868221 |
| - | gnl\|UG\|Ta#S58907813 |
| - | gnl\|UG\|Ta#S58907813 |
| - | gnl\|UG\|Ta#S58907813 |
| - | gnl\|UG\|Ta#S58910045 |
| - | gnl\|UG\|Ta#S58910562 |
| - | gnl\|UG\|Ta#S58910571 |
| - | gnl\|UG\|Ta#S61516857 |
| - | gnl\|UG\|Ta#S61517413 |
| - | gnl\|UG\|Ta#S61541540 |
| - | gnl\|UG\|Ta#S61561554 |
| - | gnl\|UG\|Ta#S61566353 |
| - | gnl\|UG\|Ta#S61571600 |
| - | gnl\|UG\|Ta#S61571600 |
| - | gnl\|UG\|Ta#S61586128 |
| - | gnl\|UG\|Ta#S61589523 |
| - | gnl\|UG\|Ta#S61592818 |
| - | gnl\|UG\|Ta#S61597368 |
| - | gnl\|UG\|Ta#S61601283 |
| - | gnl\|UG\|Ta#S61612325 |
| - | gnl\|UG\|Ta#S61612325 |
| - | gnl\|UG\|Ta#S61614597 |
| - | gnl\|UG\|Ta#S61623539 |
| - | gnl\|UG\|Ta#S61623782 |

| | |
|---|---|
| - | gnl\|UG\|Ta#S61625946 |
| - | gnl\|UG\|Ta#S61636223 |
| - | gnl\|UG\|Ta#S61641041 |
| - | gnl\|UG\|Ta#S61641041 |
| - | gnl\|UG\|Ta#S61647475 |
| - | gnl\|UG\|Ta#S61651987 |
| - | gnl\|UG\|Ta#S61657494 |
| - | gnl\|UG\|Ta#S61660221 |
| - | gnl\|UG\|Ta#S61674044 |
| - | gnl\|UG\|Ta#S61691843 |
| - | gnl\|UG\|Ta#S61713711 |
| - | gnl\|UG\|Ta#S61734561 |
| - | gnl\|UG\|Ta#S61747003 |
| - | gnl\|UG\|Ta#S61771674 |
| - | gnl\|UG\|Ta#S61771674 |
| - | gnl\|UG\|Ta#S61771674 |
| - | gnl\|UG\|Ta#S61771693 |
| - | gnl\|UG\|Ta#S61778187 |
| - | gnl\|UG\|Ta#S61778326 |
| - | gnl\|UG\|Ta#S61779157 |
| - | gnl\|UG\|Ta#S61781034 |
| - | gnl\|UG\|Ta#S61781943 |
| - | gnl\|UG\|Ta#S61782499 |
| - | gnl\|UG\|Ta#S61792469 |
| - | gnl\|UG\|Ta#S61792500 |
| - | gnl\|UG\|Ta#S61792500 |
| - | gnl\|UG\|Ta#S61797973 |
| - | gnl\|UG\|Ta#S61798955 |
| - | gnl\|UG\|Ta#S61802381 |
| - | gnl\|UG\|Ta#S61807747 |
| - | gnl\|UG\|Ta#S61807830 |
| - | gnl\|UG\|Ta#S61809733 |
| - | gnl\|UG\|Ta#S61810344 |
| - | gnl\|UG\|Ta#S61811032 |
| - | gnl\|UG\|Ta#S61811785 |
| - | gnl\|UG\|Ta#S61811785 |
| - | gnl\|UG\|Ta#S61811957 |
| - | gnl\|UG\|Ta#S61812138 |
| - | gnl\|UG\|Ta#S61828292 |
| - | gnl\|UG\|Ta#S61828901 |
| - | gnl\|UG\|Ta#S61829162 |
| - | gnl\|UG\|Ta#S61829821 |
| - | gnl\|UG\|Ta#S61829821 |
| - | gnl\|UG\|Ta#S61830303 |
| - | gnl\|UG\|Ta#S61831515 |
| - | gnl\|UG\|Ta#S61831515 |
| - | gnl\|UG\|Ta#S61831515 |

| | |
|---|---|
| - | gnl\|UG\|Ta#S61832258 |
| - | gnl\|UG\|Ta#S61844158 |
| - | gnl\|UG\|Ta#S65620340 |
| - | gnl\|UG\|Ta#S65621366 |
| - | gnl\|UG\|Ta#S65633582 |
| - | gnl\|UG\|Ta#S65656136 |
| tr\|M7Z762\|M7Z762_TRIUA | - |
| tr\|M7Z9Q7\|M7Z9Q7_TRIUA | - |
| tr\|M7ZXZ1\|M7ZXZ1_TRIUA | - |
| tr\|M8ASB1\|M8ASB1_TRIUA | - |
| tr\|T1M3U0\|T1M3U0_TRIUA | - |
| tr\|W4ZVV9\|W4ZVV9_WHEAT | - |
| tr\|W5A9M9\|W5A9M9_WHEAT | - |
| tr\|W5BKR1\|W5BKR1_WHEAT | - |
| tr\|W5DC31\|W5DC31_WHEAT | - |
| tr\|W5DJZ1\|W5DJZ1_WHEAT | - |
| tr\|W5DVJ0\|W5DVJ0_WHEAT | - |
| tr\|W5EEM1\|W5EEM1_WHEAT | - |
| tr\|W5EFV2\|W5EFV2_WHEAT | - |
| tr\|W5EXL5\|W5EXL5_WHEAT | - |
| tr\|W5EXL5\|W5EXL5_WHEAT | - |
| tr\|W5F9K4\|W5F9K4_WHEAT | - |
| tr\|W5FB11\|W5FB11_WHEAT | - |
| tr\|W5FBK8\|W5FBK8_WHEAT | - |
| tr\|W5FBW0\|W5FBW0_WHEAT | - |
| tr\|W5FDU5\|W5FDU5_WHEAT | - |
| tr\|W5FF90\|W5FF90_WHEAT | - |
| tr\|W5FJS5\|W5FJS5_WHEAT | - |
| tr\|W5FJX4\|W5FJX4_WHEAT | - |
| tr\|W5FKW4\|W5FKW4_WHEAT | - |
| tr\|W5FKW4\|W5FKW4_WHEAT | - |
| tr\|W5FKW4\|W5FKW4_WHEAT | - |
| tr\|W5FMI7\|W5FMI7_WHEAT | - |
| tr\|W5FMN3\|W5FMN3_WHEAT | - |
| tr\|W5FMN3\|W5FMN3_WHEAT | - |
| tr\|W5FNZ2\|W5FNZ2_WHEAT | - |
| tr\|W5GQN8\|W5GQN8_WHEAT | - |
| tr\|W5GT12\|W5GT12_WHEAT | - |
| tr\|W5HIY6\|W5HIY6_WHEAT | - |
| tr\|W5HSN8\|W5HSN8_WHEAT | - |
| tr\|M7ZKV8\|M7ZKV8_TRIUA | gnl\|UG\|Ta#S17990041 |
| tr\|B1Q3K4\|B1Q3K4_WHEAT | gnl\|UG\|Ta#S43309413 |
| tr\|W5FLU4\|W5FLU4_WHEAT | gnl\|UG\|Ta#S52545226 |
| tr\|W5GSC5\|W5GSC5_WHEAT | gnl\|UG\|Ta#S52545526 |
| tr\|M7ZLH2\|M7ZLH2_TRIUA | gnl\|UG\|Ta#S58846130 |
| tr\|M7ZLH2\|M7ZLH2_TRIUA | gnl\|UG\|Ta#S58846130 |
| tr\|W5DC83\|W5DC83_WHEAT | gnl\|UG\|Ta#S58857458 |

| | |
|---|---|
| tr\|W5FL45\|W5FL45_WHEAT | gnl\|UG\|Ta#S61537826 |
| tr\|W5FL05\|W5FL05_WHEAT | gnl\|UG\|Ta#S61570289 |
| tr\|W5F716\|W5F716_WHEAT | gnl\|UG\|Ta#S61619919 |
| tr\|W5A9Y6\|W5A9Y6_WHEAT | gnl\|UG\|Ta#S61620969 |
| tr\|M7YSI3\|M7YSI3_TRIUA | gnl\|UG\|Ta#S61769042 |
| tr\|W5EWW6\|W5EWW6_WHEAT | gnl\|UG\|Ta#S61778187 |
| tr\|W5DZQ7\|W5DZQ7_WHEAT | gnl\|UG\|Ta#S61797618 |
| tr\|W5FW00\|W5FW00_WHEAT | gnl\|UG\|Ta#S61808539 |
| tr\|M8ASB1\|M8ASB1_TRIUA | gnl\|UG\|Ta#S61811254 |
| tr\|W5DVX4\|W5DVX4_WHEAT | gnl\|UG\|Ta#S61812138 |
| tr\|Q2L3U4\|Q2L3U4_WHEAT | gnl\|UG\|Ta#S65603192 |
| tr\|W5FM50\|W5FM50_WHEAT | gnl\|UG\|Ta#S65621366 |
| tr\|W5FKF0\|W5FKF0_WHEAT | gnl\|UG\|Ta#S65683947 |
| tr\|W5FKF0\|W5FKF0_WHEAT | gnl\|UG\|Ta#S65683947 |
| - | - |
| - | - |
| - | - |
| - | - |
| - | - |
| - | - |
| - | - |
| - | - |
| - | - |
| - | - |
| - | - |
| - | - |
| - | - |
| - | - |
| - | - |
| - | - |
| - | - |
| - | - |
| - | - |
| - | - |
| - | - |
| - | - |
| - | - |
| - | - |
| - | - |
| - | - |
| - | - |
| - | - |
| - | - |
| - | - |
| - | - |
| - | - |
| - | - |

| | |
|---|---|
| - | - |
| - | - |
| - | - |
| - | - |
| - | - |
| - | - |
| - | - |
| - | - |
| - | - |
| - | - |
| - | - |
| - | - |
| - | - |
| - | - |
| - | - |
| - | - |
| - | - |
| - | - |
| - | - |
| - | - |
| - | - |
| - | - |
| - | - |
| - | - |
| - | - |
| - | - |
| - | - |
| - | - |
| - | - |
| - | - |
| - | - |
| - | - |
| - | - |
| - | - |
| - | - |
| - | - |
| - | - |
| - | - |
| - | - |
| - | - |
| - | - |
| - | - |
| - | - |
| - | - |
| - | - |
| - | - |
| - | - |

| | |
|---|---|
| - | - |
| - | - |
| - | - |
| - | - |
| - | - |
| - | - |
| - | - |
| - | - |
| - | - |
| - | - |
| - | - |
| - | - |
| - | - |
| - | - |
| - | - |
| - | - |
| - | - |
| - | - |
| - | - |
| - | - |
| - | - |
| - | - |
| - | - |
| - | gnl\|UG\|Ta#S13058199 |
| - | gnl\|UG\|Ta#S16208040 |
| - | gnl\|UG\|Ta#S21311119 |
| - | gnl\|UG\|Ta#S21311119 |
| - | gnl\|UG\|Ta#S22386197 |
| - | gnl\|UG\|Ta#S22386197 |
| - | gnl\|UG\|Ta#S52541326 |
| - | gnl\|UG\|Ta#S52541399 |
| - | gnl\|UG\|Ta#S52542903 |
| - | gnl\|UG\|Ta#S58854033 |
| - | gnl\|UG\|Ta#S58891791 |
| - | gnl\|UG\|Ta#S58892728 |
| - | gnl\|UG\|Ta#S58899219 |
| - | gnl\|UG\|Ta#S59489969 |
| - | gnl\|UG\|Ta#S59489969 |
| - | gnl\|UG\|Ta#S61525665 |
| - | gnl\|UG\|Ta#S61559072 |
| - | gnl\|UG\|Ta#S61562335 |
| - | gnl\|UG\|Ta#S61586334 |
| - | gnl\|UG\|Ta#S61587619 |
| - | gnl\|UG\|Ta#S61598401 |
| - | gnl\|UG\|Ta#S61611130 |
| - | gnl\|UG\|Ta#S61612568 |
| - | gnl\|UG\|Ta#S61616960 |

| | |
|---|---|
| - | gnl\|UG\|Ta#S61617950 |
| - | gnl\|UG\|Ta#S61617961 |
| - | gnl\|UG\|Ta#S61617961 |
| - | gnl\|UG\|Ta#S61632330 |
| - | gnl\|UG\|Ta#S61639637 |
| - | gnl\|UG\|Ta#S61647475 |
| - | gnl\|UG\|Ta#S61650547 |
| - | gnl\|UG\|Ta#S61659361 |
| - | gnl\|UG\|Ta#S61709758 |
| - | gnl\|UG\|Ta#S61789781 |
| - | gnl\|UG\|Ta#S61798764 |
| - | gnl\|UG\|Ta#S61801610 |
| - | gnl\|UG\|Ta#S61809367 |
| - | gnl\|UG\|Ta#S61810666 |
| - | gnl\|UG\|Ta#S61811185 |
| - | gnl\|UG\|Ta#S61811667 |
| - | gnl\|UG\|Ta#S61811832 |
| - | gnl\|UG\|Ta#S61812111 |
| - | gnl\|UG\|Ta#S61812111 |
| - | gnl\|UG\|Ta#S61812350 |
| - | gnl\|UG\|Ta#S61812436 |
| - | gnl\|UG\|Ta#S61829102 |
| - | gnl\|UG\|Ta#S61830284 |
| - | gnl\|UG\|Ta#S61831444 |
| - | gnl\|UG\|Ta#S61831444 |
| - | gnl\|UG\|Ta#S61831515 |
| - | gnl\|UG\|Ta#S61831515 |
| - | gnl\|UG\|Ta#S61832122 |
| - | gnl\|UG\|Ta#S65604935 |
| - | gnl\|UG\|Ta#S65629286 |
| - | gnl\|UG\|Ta#S65629286 |
| - | gnl\|UG\|Ta#S65631066 |
| tr\|M7ZGS4\|M7ZGS4_TRIUA | - |
| tr\|M7ZT74\|M7ZT74_TRIUA | - |
| tr\|M7ZUW4\|M7ZUW4_TRIUA | - |
| tr\|M7ZUW4\|M7ZUW4_TRIUA | - |
| tr\|M7ZYQ5\|M7ZYQ5_TRIUA | - |
| tr\|M8A271\|M8A271_TRIUA | - |
| tr\|M8A7Q7\|M8A7Q7_TRIUA | - |
| tr\|M8AE21\|M8AE21_TRIUA | - |
| tr\|T1M9D9\|T1M9D9_TRIUA | - |
| tr\|W5A467\|W5A467_WHEAT | - |
| tr\|W5A4J9\|W5A4J9_WHEAT | - |
| tr\|W5A5Z7\|W5A5Z7_WHEAT | - |
| tr\|W5A817\|W5A817_WHEAT | - |
| tr\|W5AJR7\|W5AJR7_WHEAT | - |
| tr\|W5AUH8\|W5AUH8_WHEAT | - |

| | |
|---|---|
| tr\|W5CGP8\|W5CGP8_WHEAT | - |
| tr\|W5DN61\|W5DN61_WHEAT | - |
| tr\|W5DPW4\|W5DPW4_WHEAT | - |
| tr\|W5DU77\|W5DU77_WHEAT | - |
| tr\|W5DXU6\|W5DXU6_WHEAT | - |
| tr\|W5DZI5\|W5DZI5_WHEAT | - |
| tr\|W5E040\|W5E040_WHEAT | - |
| tr\|W5EZE1\|W5EZE1_WHEAT | - |
| tr\|W5F775\|W5F775_WHEAT | - |
| tr\|W5F7M9\|W5F7M9_WHEAT | - |
| tr\|W5FBA0\|W5FBA0_WHEAT | - |
| tr\|W5FCD5\|W5FCD5_WHEAT | - |
| tr\|W5FEZ5\|W5FEZ5_WHEAT | - |
| tr\|W5FHL6\|W5FHL6_WHEAT | - |
| tr\|W5FHL6\|W5FHL6_WHEAT | - |
| tr\|W5FL30\|W5FL30_WHEAT | - |
| tr\|W5FMC6\|W5FMC6_WHEAT | - |
| tr\|W5FNF4\|W5FNF4_WHEAT | - |
| tr\|W5FPL6\|W5FPL6_WHEAT | - |
| tr\|W5GJW9\|W5GJW9_WHEAT | - |
| tr\|W5GNG0\|W5GNG0_WHEAT | - |
| tr\|W5HIY6\|W5HIY6_WHEAT | - |
| tr\|W5DN61\|W5DN61_WHEAT | gnl\|UG\|Ta#S32660053 |
| tr\|T1M9D9\|T1M9D9_TRIUA | gnl\|UG\|Ta#S52543675 |
| tr\|W5D4S2\|W5D4S2_WHEAT | gnl\|UG\|Ta#S52545062 |
| tr\|W5FD11\|W5FD11_WHEAT | gnl\|UG\|Ta#S52546007 |
| tr\|W5A792\|W5A792_WHEAT | gnl\|UG\|Ta#S58850511 |
| tr\|W5FL30\|W5FL30_WHEAT | gnl\|UG\|Ta#S61586334 |
| tr\|W5FM14\|W5FM14_WHEAT | gnl\|UG\|Ta#S61589523 |
| tr\|W5FDX4\|W5FDX4_WHEAT | gnl\|UG\|Ta#S61590455 |
| tr\|W5GLK9\|W5GLK9_WHEAT | gnl\|UG\|Ta#S61614597 |
| tr\|W5F6U6\|W5F6U6_WHEAT | gnl\|UG\|Ta#S61626193 |
| tr\|M7Z562\|M7Z562_TRIUA | gnl\|UG\|Ta#S61628477 |
| tr\|W5FGC4\|W5FGC4_WHEAT | gnl\|UG\|Ta#S61639637 |
| tr\|W5F7T7\|W5F7T7_WHEAT | gnl\|UG\|Ta#S61684096 |
| tr\|W5ELF9\|W5ELF9_WHEAT | gnl\|UG\|Ta#S61768679 |
| tr\|W5FQ06\|W5FQ06_WHEAT | gnl\|UG\|Ta#S61772041 |
| tr\|W5FE22\|W5FE22_WHEAT | gnl\|UG\|Ta#S61797504 |
| tr\|W5FE22\|W5FE22_WHEAT | gnl\|UG\|Ta#S61797504 |
| tr\|W5AAN7\|W5AAN7_WHEAT | gnl\|UG\|Ta#S61810345 |
| tr\|T1M333\|T1M333_TRIUA | gnl\|UG\|Ta#S61831498 |
| tr\|M7YB54\|M7YB54_TRIUA | gnl\|UG\|Ta#S61831528 |
| tr\|W5FMU2\|W5FMU2_WHEAT | gnl\|UG\|Ta#S61848977 |
| - | - |
| - | - |
| - | - |
| - | - |

| | |
|---|---|
| - | - |
| - | - |
| - | - |
| - | - |
| - | - |
| - | - |
| - | - |
| - | - |
| - | - |
| - | - |
| - | - |
| - | - |
| - | - |
| - | - |
| - | - |
| - | - |
| - | - |
| - | - |
| - | - |
| - | - |
| - | - |
| - | - |
| - | - |
| - | - |
| - | - |
| - | - |
| - | - |
| - | - |
| - | - |
| - | - |
| - | - |
| - | - |
| - | - |
| - | - |
| - | - |
| - | - |
| - | - |
| - | - |
| - | - |
| - | - |
| - | - |
| - | - |
| - | - |
| - | - |
| - | - |
| - | - |
| - | - |

| | |
|---|---|
| - | - |
| - | - |
| - | - |
| - | - |
| - | - |
| - | - |
| - | - |
| - | - |
| - | - |
| - | - |
| - | - |
| - | - |
| - | - |
| - | - |
| - | - |
| - | - |
| - | - |
| - | - |
| - | - |
| - | - |
| - | - |
| - | - |
| - | - |
| - | - |
| - | - |
| - | - |
| - | - |
| - | - |
| - | - |
| - | - |
| - | - |
| - | - |
| - | - |
| - | - |
| - | - |
| - | - |
| - | - |
| - | - |
| - | - |
| - | - |
| - | - |
| - | - |
| - | - |
| - | - |
| - | - |
| - | - |
| - | - |

| | |
|---|---|
| - | - |
| - | - |
| - | - |
| - | - |
| - | - |
| - | - |
| - | - |
| - | - |
| - | - |
| - | - |
| - | - |
| - | - |
| - | - |
| - | - |
| - | - |
| - | - |
| - | - |
| - | - |
| - | - |
| - | - |
| - | - |
| - | - |
| - | - |
| - | - |
| - | - |
| - | - |
| - | - |
| - | - |
| - | - |
| - | - |
| - | - |
| - | - |
| - | - |
| - | - |
| - | - |
| - | - |
| - | - |
| - | - |
| - | - |
| - | - |
| - | - |
| - | - |
| - | - |
| - | - |
| - | - |
| - | - |
| - | - |

| | |
|---|---|
| - | - |
| - | - |
| - | - |
| - | - |
| - | - |
| - | - |
| - | - |
| - | - |
| - | - |
| - | - |
| - | - |
| - | - |
| - | - |
| - | - |
| - | - |
| - | - |
| - | - |
| - | - |
| - | - |
| - | - |
| - | - |
| - | - |
| - | - |
| - | - |
| - | - |
| - | - |
| - | - |
| - | - |
| - | - |
| - | - |
| - | - |
| - | - |
| - | - |
| - | - |
| - | - |
| - | - |
| - | - |
| - | - |
| - | gnl\|UG\|Ta#S12928189 |
| - | gnl\|UG\|Ta#S17878740 |
| - | gnl\|UG\|Ta#S52543675 |
| - | gnl\|UG\|Ta#S52543675 |
| - | gnl\|UG\|Ta#S52544430 |
| - | gnl\|UG\|Ta#S58856030 |
| - | gnl\|UG\|Ta#S58891736 |
| - | gnl\|UG\|Ta#S58891736 |
| - | gnl\|UG\|Ta#S58900458 |

| | |
|---|---|
| - | gnl\|UG\|Ta#S58900763 |
| - | gnl\|UG\|Ta#S58900763 |
| - | gnl\|UG\|Ta#S61547109 |
| - | gnl\|UG\|Ta#S61590455 |
| - | gnl\|UG\|Ta#S61614066 |
| - | gnl\|UG\|Ta#S61619919 |
| - | gnl\|UG\|Ta#S61622005 |
| - | gnl\|UG\|Ta#S61626936 |
| - | gnl\|UG\|Ta#S61626936 |
| - | gnl\|UG\|Ta#S61641147 |
| - | gnl\|UG\|Ta#S61654089 |
| - | gnl\|UG\|Ta#S61675135 |
| - | gnl\|UG\|Ta#S61706998 |
| - | gnl\|UG\|Ta#S61734561 |
| - | gnl\|UG\|Ta#S61762911 |
| - | gnl\|UG\|Ta#S61780623 |
| - | gnl\|UG\|Ta#S61780623 |
| - | gnl\|UG\|Ta#S61780859 |
| - | gnl\|UG\|Ta#S61791887 |
| - | gnl\|UG\|Ta#S61798764 |
| - | gnl\|UG\|Ta#S61807830 |
| - | gnl\|UG\|Ta#S61809367 |
| - | gnl\|UG\|Ta#S61809388 |
| - | gnl\|UG\|Ta#S61810271 |
| - | gnl\|UG\|Ta#S61810666 |
| - | gnl\|UG\|Ta#S61811229 |
| - | gnl\|UG\|Ta#S61811297 |
| - | gnl\|UG\|Ta#S61811785 |
| - | gnl\|UG\|Ta#S61812124 |
| - | gnl\|UG\|Ta#S61812124 |
| - | gnl\|UG\|Ta#S61827779 |
| - | gnl\|UG\|Ta#S61829050 |
| - | gnl\|UG\|Ta#S61829162 |
| - | gnl\|UG\|Ta#S61830054 |
| - | gnl\|UG\|Ta#S61830125 |
| - | gnl\|UG\|Ta#S61830800 |
| - | gnl\|UG\|Ta#S61831622 |
| - | gnl\|UG\|Ta#S61832222 |
| - | gnl\|UG\|Ta#S61832258 |
| - | gnl\|UG\|Ta#S61832258 |
| - | gnl\|UG\|Ta#S61832276 |
| - | gnl\|UG\|Ta#S61832521 |
| - | gnl\|UG\|Ta#S61832532 |
| - | gnl\|UG\|Ta#S61832533 |
| - | gnl\|UG\|Ta#S65603368 |
| - | gnl\|UG\|Ta#S65622149 |
| - | gnl\|UG\|Ta#S65658707 |

| | |
|---|---|
| - | gnl\|UG\|Ta#S65665101 |
| - | gnl\|UG\|Ta#S65714482 |
| tr\|M7Z2B8\|M7Z2B8_TRIUA | - |
| tr\|W5DQ82\|W5DQ82_WHEAT | - |
| tr\|W5F078\|W5F078_WHEAT | - |
| tr\|W5FAM6\|W5FAM6_WHEAT | - |
| tr\|W5FBH6\|W5FBH6_WHEAT | - |
| tr\|W5FD41\|W5FD41_WHEAT | - |
| tr\|W5FMI7\|W5FMI7_WHEAT | - |
| tr\|W5FND7\|W5FND7_WHEAT | - |
| tr\|W5H2B3\|W5H2B3_WHEAT | - |
| tr\|W5HT35\|W5HT35_WHEAT | - |
| tr\|M7YNW6\|M7YNW6_TRIUA | gnl\|UG\|Ta#S52542426 |
| tr\|M7ZF91\|M7ZF91_TRIUA | gnl\|UG\|Ta#S52545260 |
| tr\|W5FG18\|W5FG18_WHEAT | gnl\|UG\|Ta#S61613001 |
| tr\|M7YXF4\|M7YXF4_TRIUA | gnl\|UG\|Ta#S61625144 |
| tr\|W5FJE2\|W5FJE2_WHEAT | gnl\|UG\|Ta#S61799964 |
| tr\|W5DS50\|W5DS50_WHEAT | gnl\|UG\|Ta#S61807002 |
| tr\|W5DRK5\|W5DRK5_WHEAT | gnl\|UG\|Ta#S61810344 |
| tr\|M7YXF4\|M7YXF4_TRIUA | gnl\|UG\|Ta#S61812138 |
| tr\|M7YXF4\|M7YXF4_TRIUA | gnl\|UG\|Ta#S61812138 |
| tr\|W4ZNM6\|W4ZNM6_WHEAT | gnl\|UG\|Ta#S61828738 |
| tr\|W4ZNM6\|W4ZNM6_WHEAT | gnl\|UG\|Ta#S61828738 |
| tr\|W5FEN3\|W5FEN3_WHEAT | gnl\|UG\|Ta#S61829091 |
| tr\|W5FGF9\|W5FGF9_WHEAT | gnl\|UG\|Ta#S61830284 |
| tr\|W5EEM1\|W5EEM1_WHEAT | gnl\|UG\|Ta#S61830584 |
| tr\|M7ZI26\|M7ZI26_TRIUA | gnl\|UG\|Ta#S61832258 |
| - | - |
| - | - |
| - | - |
| - | - |
| - | - |
| - | - |
| - | - |
| - | - |
| - | - |
| - | - |
| - | - |
| - | - |
| - | - |
| - | - |
| - | - |
| - | - |
| - | - |
| - | - |
| - | - |
| - | - |

| | |
|---|---|
| - | - |
| - | - |
| - | - |
| - | - |
| - | - |
| - | - |
| - | - |
| - | - |
| - | - |
| - | - |
| - | - |
| - | - |
| - | - |
| - | - |
| - | - |
| - | - |
| - | - |
| - | - |
| - | - |
| - | - |
| - | - |
| - | - |
| - | - |
| - | - |
| - | - |
| - | - |
| - | - |
| - | - |
| - | - |
| - | - |
| - | - |
| - | - |
| - | - |
| - | - |
| - | - |
| - | - |
| - | - |
| - | - |
| - | - |
| - | - |
| - | - |
| - | - |
| - | - |
| - | - |
| - | - |
| - | - |
| - | - |

| | |
|---|---|
| - | - |
| - | - |
| - | - |
| - | - |
| - | - |
| - | - |
| - | - |
| - | - |
| - | - |
| - | - |
| - | - |
| - | - |
| - | - |
| - | - |
| - | - |
| - | - |
| - | - |
| - | - |
| - | - |
| - | - |
| - | - |
| - | - |
| - | - |
| - | - |
| - | - |
| - | - |
| - | - |
| - | gnl\|UG\|Ta#S12945984 |
| - | gnl\|UG\|Ta#S12979669 |
| - | gnl\|UG\|Ta#S16058010 |
| - | gnl\|UG\|Ta#S17978335 |
| - | gnl\|UG\|Ta#S21311119 |
| - | gnl\|UG\|Ta#S52541775 |
| - | gnl\|UG\|Ta#S52541775 |
| - | gnl\|UG\|Ta#S52542109 |
| - | gnl\|UG\|Ta#S52542109 |
| - | gnl\|UG\|Ta#S52542426 |
| - | gnl\|UG\|Ta#S52542813 |
| - | gnl\|UG\|Ta#S52543105 |
| - | gnl\|UG\|Ta#S52543675 |
| - | gnl\|UG\|Ta#S52544279 |
| - | gnl\|UG\|Ta#S52544279 |
| - | gnl\|UG\|Ta#S52546164 |
| - | gnl\|UG\|Ta#S52547138 |
| - | gnl\|UG\|Ta#S58856030 |
| - | gnl\|UG\|Ta#S58899219 |
| - | gnl\|UG\|Ta#S58900458 |

| | |
|---|---|
| - | gnl\|UG\|Ta#S58900763 |
| - | gnl\|UG\|Ta#S58910733 |
| - | gnl\|UG\|Ta#S61541016 |
| - | gnl\|UG\|Ta#S61544072 |
| - | gnl\|UG\|Ta#S61563083 |
| - | gnl\|UG\|Ta#S61574978 |
| - | gnl\|UG\|Ta#S61578155 |
| - | gnl\|UG\|Ta#S61590455 |
| - | gnl\|UG\|Ta#S61595768 |
| - | gnl\|UG\|Ta#S61616960 |
| - | gnl\|UG\|Ta#S61623197 |
| - | gnl\|UG\|Ta#S61624959 |
| - | gnl\|UG\|Ta#S61641147 |
| - | gnl\|UG\|Ta#S61649462 |
| - | gnl\|UG\|Ta#S61688546 |
| - | gnl\|UG\|Ta#S61709758 |
| - | gnl\|UG\|Ta#S61730725 |
| - | gnl\|UG\|Ta#S61749100 |
| - | gnl\|UG\|Ta#S61780527 |
| - | gnl\|UG\|Ta#S61780623 |
| - | gnl\|UG\|Ta#S61780623 |
| - | gnl\|UG\|Ta#S61780859 |
| - | gnl\|UG\|Ta#S61781034 |
| - | gnl\|UG\|Ta#S61781642 |
| - | gnl\|UG\|Ta#S61781642 |
| - | gnl\|UG\|Ta#S61788363 |
| - | gnl\|UG\|Ta#S61791887 |
| - | gnl\|UG\|Ta#S61792200 |
| - | gnl\|UG\|Ta#S61792297 |
| - | gnl\|UG\|Ta#S61792297 |
| - | gnl\|UG\|Ta#S61792297 |
| - | gnl\|UG\|Ta#S61802244 |
| - | gnl\|UG\|Ta#S61809367 |
| - | gnl\|UG\|Ta#S61809367 |
| - | gnl\|UG\|Ta#S61809367 |
| - | gnl\|UG\|Ta#S61809367 |
| - | gnl\|UG\|Ta#S61809764 |
| - | gnl\|UG\|Ta#S61811185 |
| - | gnl\|UG\|Ta#S61811474 |
| - | gnl\|UG\|Ta#S61811827 |
| - | gnl\|UG\|Ta#S61811931 |
| - | gnl\|UG\|Ta#S61811957 |
| - | gnl\|UG\|Ta#S61812138 |
| - | gnl\|UG\|Ta#S61812179 |
| - | gnl\|UG\|Ta#S61812179 |
| - | gnl\|UG\|Ta#S61812436 |
| - | gnl\|UG\|Ta#S61828292 |

| | |
|---|---|
| - | gnl\|UG\|Ta#S61830014 |
| - | gnl\|UG\|Ta#S61830014 |
| - | gnl\|UG\|Ta#S61830269 |
| - | gnl\|UG\|Ta#S61830269 |
| - | gnl\|UG\|Ta#S61830728 |
| - | gnl\|UG\|Ta#S61830800 |
| - | gnl\|UG\|Ta#S61831313 |
| - | gnl\|UG\|Ta#S61831329 |
| - | gnl\|UG\|Ta#S61831404 |
| - | gnl\|UG\|Ta#S61831441 |
| - | gnl\|UG\|Ta#S61831441 |
| - | gnl\|UG\|Ta#S61831543 |
| - | gnl\|UG\|Ta#S61831754 |
| - | gnl\|UG\|Ta#S61831757 |
| - | gnl\|UG\|Ta#S61832138 |
| - | gnl\|UG\|Ta#S61832276 |
| - | gnl\|UG\|Ta#S61832496 |
| - | gnl\|UG\|Ta#S61832496 |
| - | gnl\|UG\|Ta#S61832521 |
| - | gnl\|UG\|Ta#S61832533 |
| - | gnl\|UG\|Ta#S61832533 |
| - | gnl\|UG\|Ta#S61832533 |
| - | gnl\|UG\|Ta#S65594281 |
| - | gnl\|UG\|Ta#S65603368 |
| - | gnl\|UG\|Ta#S65667672 |
| tr\|M7Y8E5\|M7Y8E5_TRIUA | - |
| tr\|M7YIT5\|M7YIT5_TRIUA | - |
| tr\|M7YIT5\|M7YIT5_TRIUA | - |
| tr\|M7YNQ7\|M7YNQ7_TRIUA | - |
| tr\|M7YY31\|M7YY31_TRIUA | - |
| tr\|M8AQJ5\|M8AQJ5_TRIUA | - |
| tr\|W4ZUG7\|W4ZUG7_WHEAT | - |
| tr\|W5AYF7\|W5AYF7_WHEAT | - |
| tr\|W5BWR1\|W5BWR1_WHEAT | - |
| tr\|W5ELA0\|W5ELA0_WHEAT | - |
| tr\|W5FBB9\|W5FBB9_WHEAT | - |
| tr\|W5FBZ3\|W5FBZ3_WHEAT | - |
| tr\|W5FCD5\|W5FCD5_WHEAT | - |
| tr\|W5FEI4\|W5FEI4_WHEAT | - |
| tr\|W5FEM1\|W5FEM1_WHEAT | - |
| tr\|W5FEZ5\|W5FEZ5_WHEAT | - |
| tr\|W5FG88\|W5FG88_WHEAT | - |
| tr\|W5FHY4\|W5FHY4_WHEAT | - |
| tr\|W5FLP6\|W5FLP6_WHEAT | - |
| tr\|W5FMC6\|W5FMC6_WHEAT | - |
| tr\|W5GNB8\|W5GNB8_WHEAT | - |
| tr\|W5F8I3\|W5F8I3_WHEAT | gnl\|UG\|Ta#S17878740 |

| | |
|---|---|
| tr\|W5F8I3\|W5F8I3_WHEAT | gnl\|UG\|Ta#S17878740 |
| tr\|M7YNU9\|M7YNU9_TRIUA | gnl\|UG\|Ta#S52546444 |
| tr\|M7YNU9\|M7YNU9_TRIUA | gnl\|UG\|Ta#S52546444 |
| tr\|W5GM47\|W5GM47_WHEAT | gnl\|UG\|Ta#S58857458 |
| tr\|W4ZZ07\|W4ZZ07_WHEAT | gnl\|UG\|Ta#S58861289 |
| tr\|W5EHR4\|W5EHR4_WHEAT | gnl\|UG\|Ta#S58900763 |
| tr\|W5FH68\|W5FH68_WHEAT | gnl\|UG\|Ta#S61544072 |
| tr\|W5A1P0\|W5A1P0_WHEAT | gnl\|UG\|Ta#S61595768 |
| tr\|W5FDD2\|W5FDD2_WHEAT | gnl\|UG\|Ta#S61608057 |
| tr\|W5FDD2\|W5FDD2_WHEAT | gnl\|UG\|Ta#S61608057 |
| tr\|W5F800\|W5F800_WHEAT | gnl\|UG\|Ta#S61661947 |
| tr\|W5BHH6\|W5BHH6_WHEAT | gnl\|UG\|Ta#S61778056 |
| tr\|W5A3H4\|W5A3H4_WHEAT | gnl\|UG\|Ta#S61790071 |
| tr\|W5A3H4\|W5A3H4_WHEAT | gnl\|UG\|Ta#S61790071 |
| tr\|W5FJC7\|W5FJC7_WHEAT | gnl\|UG\|Ta#S61792200 |
| tr\|M7ZD90\|M7ZD90_TRIUA | gnl\|UG\|Ta#S61800036 |
| tr\|W5HBT1\|W5HBT1_WHEAT | gnl\|UG\|Ta#S61802244 |
| tr\|W5EAH0\|W5EAH0_WHEAT | gnl\|UG\|Ta#S61809367 |
| tr\|W5DXM9\|W5DXM9_WHEAT | gnl\|UG\|Ta#S61811667 |
| tr\|M7Z785\|M7Z785_TRIUA | gnl\|UG\|Ta#S61811772 |
| tr\|M7Z036\|M7Z036_TRIUA | gnl\|UG\|Ta#S61812497 |
| tr\|W5FNQ1\|W5FNQ1_WHEAT | gnl\|UG\|Ta#S61827779 |
| tr\|W5FNQ1\|W5FNQ1_WHEAT | gnl\|UG\|Ta#S61827779 |
| tr\|W5FLP6\|W5FLP6_WHEAT | gnl\|UG\|Ta#S61828526 |
| tr\|W5FP33\|W5FP33_WHEAT | gnl\|UG\|Ta#S61830054 |
| tr\|W5FP33\|W5FP33_WHEAT | gnl\|UG\|Ta#S61830054 |
| tr\|W5FT97\|W5FT97_WHEAT | gnl\|UG\|Ta#S61830269 |
| tr\|W5F8D7\|W5F8D7_WHEAT | gnl\|UG\|Ta#S61831280 |
| tr\|M8ACH0\|M8ACH0_TRIUA | gnl\|UG\|Ta#S61831543 |
| tr\|W5F8R6\|W5F8R6_WHEAT | gnl\|UG\|Ta#S61831867 |
| tr\|W5F8R6\|W5F8R6_WHEAT | gnl\|UG\|Ta#S61831867 |
| - | - |
| - | - |
| - | - |
| - | - |
| - | - |
| - | - |
| - | - |
| - | - |
| - | - |
| - | - |
| - | - |
| - | - |
| - | - |
| - | - |
| - | - |
| - | - |

| | |
|---|---|
| - | - |
| - | - |
| - | - |
| - | - |
| - | - |
| - | - |
| - | - |
| - | - |
| - | - |
| - | - |
| - | - |
| - | - |
| - | - |
| - | - |
| - | - |
| - | - |
| - | - |
| - | - |
| - | - |
| - | - |
| - | - |
| - | - |
| - | - |
| - | - |
| - | - |
| - | - |
| - | - |
| - | - |
| - | - |
| - | - |
| - | - |
| - | - |
| - | - |
| - | - |
| - | - |
| - | - |
| - | - |
| - | - |
| - | - |
| - | - |
| - | - |
| - | - |
| - | - |
| - | - |
| - | - |
| - | - |
| - | - |

| | |
|---|---|
| - | - |
| - | - |
| - | - |
| - | - |
| - | - |
| - | - |
| - | - |
| - | - |
| - | - |
| - | - |
| - | - |
| - | - |
| - | - |
| - | - |
| - | - |
| - | - |
| - | - |
| - | - |
| - | - |
| - | - |
| - | - |
| - | - |
| - | - |
| - | - |
| - | - |
| - | - |
| - | - |
| - | - |
| - | - |
| - | - |
| - | - |
| - | - |
| - | - |
| - | - |
| - | - |
| - | - |
| - | - |
| - | - |
| - | - |
| - | - |
| - | - |
| - | - |
| - | - |
| - | - |
| - | - |
| - | - |
| - | - |

| | |
|---|---|
| - | - |
| - | - |
| - | - |
| - | - |
| - | - |
| - | - |
| - | - |
| - | - |
| - | - |
| - | - |
| - | - |
| - | - |
| - | - |
| - | - |
| - | - |
| - | gnl\|UG\|Ta#S12917219 |
| - | gnl\|UG\|Ta#S13043069 |
| - | gnl\|UG\|Ta#S26024008 |
| - | gnl\|UG\|Ta#S37785040 |
| - | gnl\|UG\|Ta#S52542854 |
| - | gnl\|UG\|Ta#S52542915 |
| - | gnl\|UG\|Ta#S52542915 |
| - | gnl\|UG\|Ta#S52542915 |
| - | gnl\|UG\|Ta#S52542915 |
| - | gnl\|UG\|Ta#S52543447 |
| - | gnl\|UG\|Ta#S52543447 |
| - | gnl\|UG\|Ta#S52543933 |
| - | gnl\|UG\|Ta#S52544949 |
| - | gnl\|UG\|Ta#S52544949 |
| - | gnl\|UG\|Ta#S52545265 |
| - | gnl\|UG\|Ta#S52545265 |
| - | gnl\|UG\|Ta#S52545265 |
| - | gnl\|UG\|Ta#S52545582 |
| - | gnl\|UG\|Ta#S52545582 |
| - | gnl\|UG\|Ta#S52545582 |
| - | gnl\|UG\|Ta#S52545582 |
| - | gnl\|UG\|Ta#S52545598 |
| - | gnl\|UG\|Ta#S52545598 |
| - | gnl\|UG\|Ta#S52545972 |
| - | gnl\|UG\|Ta#S58857610 |
| - | gnl\|UG\|Ta#S58857610 |
| - | gnl\|UG\|Ta#S58857610 |
| - | gnl\|UG\|Ta#S58883774 |
| - | gnl\|UG\|Ta#S58883774 |
| - | gnl\|UG\|Ta#S58883774 |
| - | gnl\|UG\|Ta#S58885569 |
| - | gnl\|UG\|Ta#S58894891 |

| | |
|---|---|
| - | gnl\|UG\|Ta#S58899651 |
| - | gnl\|UG\|Ta#S58899651 |
| - | gnl\|UG\|Ta#S58902464 |
| - | gnl\|UG\|Ta#S58909708 |
| - | gnl\|UG\|Ta#S58909708 |
| - | gnl\|UG\|Ta#S61520434 |
| - | gnl\|UG\|Ta#S61520434 |
| - | gnl\|UG\|Ta#S61532565 |
| - | gnl\|UG\|Ta#S61533995 |
| - | gnl\|UG\|Ta#S61566327 |
| - | gnl\|UG\|Ta#S61582158 |
| - | gnl\|UG\|Ta#S61582693 |
| - | gnl\|UG\|Ta#S61582693 |
| - | gnl\|UG\|Ta#S61593101 |
| - | gnl\|UG\|Ta#S61596468 |
| - | gnl\|UG\|Ta#S61596468 |
| - | gnl\|UG\|Ta#S61596468 |
| - | gnl\|UG\|Ta#S61608995 |
| - | gnl\|UG\|Ta#S61608995 |
| - | gnl\|UG\|Ta#S61608995 |
| - | gnl\|UG\|Ta#S61608995 |
| - | gnl\|UG\|Ta#S61608995 |
| - | gnl\|UG\|Ta#S61611107 |
| - | gnl\|UG\|Ta#S61614472 |
| - | gnl\|UG\|Ta#S61615691 |
| - | gnl\|UG\|Ta#S61615691 |
| - | gnl\|UG\|Ta#S61618594 |
| - | gnl\|UG\|Ta#S61624959 |
| - | gnl\|UG\|Ta#S61626294 |
| - | gnl\|UG\|Ta#S61626639 |
| - | gnl\|UG\|Ta#S61626908 |
| - | gnl\|UG\|Ta#S61626908 |
| - | gnl\|UG\|Ta#S61628266 |
| - | gnl\|UG\|Ta#S61628266 |
| - | gnl\|UG\|Ta#S61628266 |
| - | gnl\|UG\|Ta#S61630030 |
| - | gnl\|UG\|Ta#S61639458 |
| - | gnl\|UG\|Ta#S61666206 |
| - | gnl\|UG\|Ta#S61685330 |
| - | gnl\|UG\|Ta#S61709984 |
| - | gnl\|UG\|Ta#S61737633 |
| - | gnl\|UG\|Ta#S61754351 |
| - | gnl\|UG\|Ta#S61756150 |
| - | gnl\|UG\|Ta#S61771035 |
| - | gnl\|UG\|Ta#S61778289 |
| - | gnl\|UG\|Ta#S61778874 |
| - | gnl\|UG\|Ta#S61781034 |

| | |
|---|---|
| - | gnl\|UG\|Ta#S61781183 |
| - | gnl\|UG\|Ta#S61781478 |
| - | gnl\|UG\|Ta#S61782530 |
| - | gnl\|UG\|Ta#S61798276 |
| - | gnl\|UG\|Ta#S61798276 |
| - | gnl\|UG\|Ta#S61798276 |
| - | gnl\|UG\|Ta#S61799833 |
| - | gnl\|UG\|Ta#S61800808 |
| - | gnl\|UG\|Ta#S61802338 |
| - | gnl\|UG\|Ta#S61809367 |
| - | gnl\|UG\|Ta#S61809764 |
| - | gnl\|UG\|Ta#S61810904 |
| - | gnl\|UG\|Ta#S61811255 |
| - | gnl\|UG\|Ta#S61811255 |
| - | gnl\|UG\|Ta#S61811396 |
| - | gnl\|UG\|Ta#S61811421 |
| - | gnl\|UG\|Ta#S61811772 |
| - | gnl\|UG\|Ta#S61811801 |
| - | gnl\|UG\|Ta#S61811801 |
| - | gnl\|UG\|Ta#S61812404 |
| - | gnl\|UG\|Ta#S61812404 |
| - | gnl\|UG\|Ta#S61827982 |
| - | gnl\|UG\|Ta#S61827982 |
| - | gnl\|UG\|Ta#S61828477 |
| - | gnl\|UG\|Ta#S61829341 |
| - | gnl\|UG\|Ta#S61829341 |
| - | gnl\|UG\|Ta#S61829853 |
| - | gnl\|UG\|Ta#S61831268 |
| - | gnl\|UG\|Ta#S61831268 |
| - | gnl\|UG\|Ta#S61831268 |
| - | gnl\|UG\|Ta#S61831313 |
| - | gnl\|UG\|Ta#S61831474 |
| - | gnl\|UG\|Ta#S61831474 |
| - | gnl\|UG\|Ta#S61831551 |
| - | gnl\|UG\|Ta#S61831921 |
| - | gnl\|UG\|Ta#S61832533 |
| - | gnl\|UG\|Ta#S61832533 |
| - | gnl\|UG\|Ta#S61832533 |
| - | gnl\|UG\|Ta#S61832533 |
| - | gnl\|UG\|Ta#S61840519 |
| - | gnl\|UG\|Ta#S65594406 |
| - | gnl\|UG\|Ta#S65597071 |
| - | gnl\|UG\|Ta#S65622922 |
| - | gnl\|UG\|Ta#S65625818 |
| - | gnl\|UG\|Ta#S65631084 |
| - | gnl\|UG\|Ta#S65653203 |
| - | gnl\|UG\|Ta#S65653370 |

| | |
|---|---|
| - | gnl\|UG\|Ta#S65665693 |
| - | gnl\|UG\|Ta#S65688304 |
| - | gnl\|UG\|Ta#S65688304 |
| - | gnl\|UG\|Ta#S65690620 |
| - | gnl\|UG\|Ta#S65701551 |
| tr\|A3FM72\|A3FM72_WHEAT | - |
| tr\|M7YG48\|M7YG48_TRIUA | - |
| tr\|M7YJV4\|M7YJV4_TRIUA | - |
| tr\|M7YMN5\|M7YMN5_TRIUA | - |
| tr\|M7ZKY6\|M7ZKY6_TRIUA | - |
| tr\|T1LX57\|T1LX57_TRIUA | - |
| tr\|T1MKQ3\|T1MKQ3_TRIUA | - |
| tr\|T1NTP9\|T1NTP9_TRIUA | - |
| tr\|T1NV33\|T1NV33_TRIUA | - |
| tr\|W4ZZV5\|W4ZZV5_WHEAT | - |
| tr\|W5A092\|W5A092_WHEAT | - |
| tr\|W5A092\|W5A092_WHEAT | - |
| tr\|W5A5T4\|W5A5T4_WHEAT | - |
| tr\|W5DPV2\|W5DPV2_WHEAT | - |
| tr\|W5DPV2\|W5DPV2_WHEAT | - |
| tr\|W5DPY9\|W5DPY9_WHEAT | - |
| tr\|W5DS94\|W5DS94_WHEAT | - |
| tr\|W5DSK9\|W5DSK9_WHEAT | - |
| tr\|W5EE02\|W5EE02_WHEAT | - |
| tr\|W5EE02\|W5EE02_WHEAT | - |
| tr\|W5ENY3\|W5ENY3_WHEAT | - |
| tr\|W5ERJ5\|W5ERJ5_WHEAT | - |
| tr\|W5FB92\|W5FB92_WHEAT | - |
| tr\|W5FCW3\|W5FCW3_WHEAT | - |
| tr\|W5FFE3\|W5FFE3_WHEAT | - |
| tr\|W5FFE3\|W5FFE3_WHEAT | - |
| tr\|W5FG86\|W5FG86_WHEAT | - |
| tr\|W5FG99\|W5FG99_WHEAT | - |
| tr\|W5FG99\|W5FG99_WHEAT | - |
| tr\|W5FI47\|W5FI47_WHEAT | - |
| tr\|W5FI47\|W5FI47_WHEAT | - |
| tr\|W5FIF8\|W5FIF8_WHEAT | - |
| tr\|W5FIF8\|W5FIF8_WHEAT | - |
| tr\|W5FJI6\|W5FJI6_WHEAT | - |
| tr\|W5FJV7\|W5FJV7_WHEAT | - |
| tr\|W5FLM0\|W5FLM0_WHEAT | - |
| tr\|W5FLP6\|W5FLP6_WHEAT | - |
| tr\|W5FLW6\|W5FLW6_WHEAT | - |
| tr\|W5FMP8\|W5FMP8_WHEAT | - |
| tr\|W5FNF4\|W5FNF4_WHEAT | - |
| tr\|W5FNP6\|W5FNP6_WHEAT | - |
| tr\|W5FU43\|W5FU43_WHEAT | - |

| | |
|---|---|
| tr\|W5GKG5\|W5GKG5_WHEAT | - |
| tr\|W5GSC2\|W5GSC2_WHEAT | - |
| tr\|M7YMN5\|M7YMN5_TRIUA | gnl\|UG\|Ta#S13050103 |
| tr\|M7YUW0\|M7YUW0_TRIUA | gnl\|UG\|Ta#S16058184 |
| tr\|W5FCW3\|W5FCW3_WHEAT | gnl\|UG\|Ta#S17976579 |
| tr\|T1MKQ3\|T1MKQ3_TRIUA | gnl\|UG\|Ta#S26028387 |
| tr\|W5FLW6\|W5FLW6_WHEAT | gnl\|UG\|Ta#S52544883 |
| tr\|W5F731\|W5F731_WHEAT | gnl\|UG\|Ta#S52545582 |
| tr\|M7Z8B9\|M7Z8B9_TRIUA | gnl\|UG\|Ta#S58883216 |
| tr\|W5FK83\|W5FK83_WHEAT | gnl\|UG\|Ta#S58883216 |
| tr\|W5FK83\|W5FK83_WHEAT | gnl\|UG\|Ta#S58883216 |
| tr\|W5FG99\|W5FG99_WHEAT | gnl\|UG\|Ta#S58896173 |
| tr\|M7YQS9\|M7YQS9_TRIUA | gnl\|UG\|Ta#S58902464 |
| tr\|W5FIE0\|W5FIE0_WHEAT | gnl\|UG\|Ta#S58909708 |
| tr\|W5FIE0\|W5FIE0_WHEAT | gnl\|UG\|Ta#S58909708 |
| tr\|W5DNC6\|W5DNC6_WHEAT | gnl\|UG\|Ta#S61532565 |
| tr\|W5EX82\|W5EX82_WHEAT | gnl\|UG\|Ta#S61549911 |
| tr\|W5EX82\|W5EX82_WHEAT | gnl\|UG\|Ta#S61549911 |
| tr\|W5FA86\|W5FA86_WHEAT | gnl\|UG\|Ta#S61549911 |
| tr\|W5FDR3\|W5FDR3_WHEAT | gnl\|UG\|Ta#S61565580 |
| tr\|M8A6S6\|M8A6S6_TRIUA | gnl\|UG\|Ta#S61567032 |
| tr\|W5FUY6\|W5FUY6_WHEAT | gnl\|UG\|Ta#S61586121 |
| tr\|W5FCW0\|W5FCW0_WHEAT | gnl\|UG\|Ta#S61593513 |
| tr\|T1NC83\|T1NC83_TRIUA | gnl\|UG\|Ta#S61618986 |
| tr\|W5EXE7\|W5EXE7_WHEAT | gnl\|UG\|Ta#S61626908 |
| tr\|W5FF97\|W5FF97_WHEAT | gnl\|UG\|Ta#S61627164 |
| tr\|W5FX13\|W5FX13_WHEAT | gnl\|UG\|Ta#S61640151 |
| tr\|W5A1H0\|W5A1H0_WHEAT | gnl\|UG\|Ta#S61645240 |
| tr\|W5FIF8\|W5FIF8_WHEAT | gnl\|UG\|Ta#S61704290 |
| tr\|W5A6X7\|W5A6X7_WHEAT | gnl\|UG\|Ta#S61738379 |
| tr\|M7ZL05\|M7ZL05_TRIUA | gnl\|UG\|Ta#S61781034 |
| tr\|W5G906\|W5G906_WHEAT | gnl\|UG\|Ta#S61781183 |
| tr\|W5FC92\|W5FC92_WHEAT | gnl\|UG\|Ta#S61798276 |
| tr\|W5A0R2\|W5A0R2_WHEAT | gnl\|UG\|Ta#S61798733 |
| tr\|W5FM63\|W5FM63_WHEAT | gnl\|UG\|Ta#S61799833 |
| tr\|T1NUT3\|T1NUT3_TRIUA | gnl\|UG\|Ta#S61809764 |
| tr\|W5EYC8\|W5EYC8_WHEAT | gnl\|UG\|Ta#S61810345 |
| tr\|W5FCY4\|W5FCY4_WHEAT | gnl\|UG\|Ta#S61811749 |
| tr\|M8ARP3\|M8ARP3_TRIUA | gnl\|UG\|Ta#S61812016 |
| tr\|W5E379\|W5E379_WHEAT | gnl\|UG\|Ta#S61812016 |
| tr\|W5A095\|W5A095_WHEAT | gnl\|UG\|Ta#S61812329 |
| tr\|W5FV98\|W5FV98_WHEAT | gnl\|UG\|Ta#S61828405 |
| tr\|W5FJV7\|W5FJV7_WHEAT | gnl\|UG\|Ta#S61829853 |
| tr\|W5FPG0\|W5FPG0_WHEAT | gnl\|UG\|Ta#S61830054 |
| tr\|W5FQT1\|W5FQT1_WHEAT | gnl\|UG\|Ta#S61830694 |
| tr\|W5FNY5\|W5FNY5_WHEAT | gnl\|UG\|Ta#S61831164 |
| tr\|W5FNY5\|W5FNY5_WHEAT | gnl\|UG\|Ta#S61831164 |

| | |
|---|---|
| tr\|T1MXY6\|T1MXY6_TRIUA | gnl\|UG\|Ta#S61831921 |
| tr\|M7ZDQ6\|M7ZDQ6_TRIUA | gnl\|UG\|Ta#S61832044 |
| tr\|M8AGB5\|M8AGB5_TRIUA | gnl\|UG\|Ta#S61832533 |
| tr\|M8AGB5\|M8AGB5_TRIUA | gnl\|UG\|Ta#S61832533 |
| tr\|W5HHQ5\|W5HHQ5_WHEAT | gnl\|UG\|Ta#S61840519 |
| tr\|W5FF97\|W5FF97_WHEAT | gnl\|UG\|Ta#S65625818 |
| tr\|M7ZYP5\|M7ZYP5_TRIUA | gnl\|UG\|Ta#S65639709 |
| tr\|W5F7E5\|W5F7E5_WHEAT | gnl\|UG\|Ta#S65651826 |
| tr\|W5A092\|W5A092_WHEAT | gnl\|UG\|Ta#S65653370 |
| tr\|W5FBV8\|W5FBV8_WHEAT | gnl\|UG\|Ta#S65665693 |
| tr\|W5FKK7\|W5FKK7_WHEAT | gnl\|UG\|Ta#S65716912 |
| - | - |
| - | - |
| - | - |
| - | - |
| - | - |
| - | - |
| - | - |
| - | - |
| - | - |
| - | - |
| - | - |
| - | - |
| - | - |
| - | - |
| - | - |
| - | - |
| - | - |
| - | - |
| - | - |
| - | - |
| - | - |
| - | - |
| - | - |
| - | - |
| - | - |
| - | - |
| - | - |
| - | - |
| - | - |
| - | - |
| - | - |
| - | - |
| - | - |
| - | - |
| - | - |

| | |
|---|---|
| - | - |
| - | - |
| - | - |
| - | - |
| - | - |
| - | - |
| - | - |
| - | - |
| - | - |
| - | - |
| - | - |
| - | - |
| - | - |
| - | - |
| - | - |
| - | - |
| - | - |
| - | - |
| - | - |
| - | - |
| - | - |
| - | - |
| - | - |
| - | - |
| - | - |
| - | - |
| - | - |
| - | - |
| - | - |
| - | - |
| - | - |
| - | - |
| - | - |
| - | - |
| - | - |
| - | - |
| - | - |
| - | - |
| - | - |
| - | - |
| - | - |
| - | - |
| - | - |
| - | - |
| - | - |
| - | - |
| - | - |

| | |
|---|---|
| - | - |
| - | - |
| - | - |
| - | - |
| - | - |
| - | - |
| - | - |
| - | - |
| - | - |
| - | - |
| - | - |
| - | - |
| - | - |
| - | - |
| - | - |
| - | - |
| - | - |
| - | - |
| - | - |
| - | - |
| - | - |
| - | - |
| - | - |
| - | - |
| - | - |
| - | - |
| - | - |
| - | - |
| - | - |
| - | - |
| - | - |
| - | - |
| - | - |
| - | - |
| - | - |
| - | - |
| - | - |
| - | - |
| - | - |
| - | - |
| - | - |
| - | - |
| - | - |
| - | - |
| - | - |
| - | - |
| - | - |

-
-
-
-
-
-
-
-
-
-
-
-
-
-
-
-
-
-
-
-
-
-
-
-
-
-
-
-
-
-
-
-
-
-
-
-
-
-
-
-
-
-
-
-
-
-
-
-
-
-
-
-
-
-
-
-
-
-
-
-
-
-
-
-
-
-
-
-
-
-
-
-
-
-
-
-
-
-
-
-
-
-
-
-
-
-
-
-
-
-
-
-
-
-

| | |
|---|---|
| - | - |
| - | - |
| - | - |
| - | - |
| - | - |
| - | - |
| - | - |
| - | - |
| - | - |
| - | - |
| - | - |
| - | - |
| - | - |
| - | - |
| - | - |
| - | - |
| - | - |
| - | - |
| - | - |
| - | - |
| - | - |
| - | - |
| - | - |
| - | - |
| - | - |
| - | - |
| - | - |
| - | - |
| - | - |
| - | - |
| - | - |
| - | - |
| - | - |
| - | - |
| - | - |
| - | - |
| - | - |
| - | - |
| - | - |
| - | - |
| - | - |
| - | - |
| - | - |
| - | - |
| - | - |
| - | - |
| - | - |

| | |
|---|---|
| - | - |
| - | - |
| - | - |
| - | - |
| - | - |
| - | - |
| - | - |
| - | - |
| - | - |
| - | - |
| - | - |
| - | - |
| - | - |
| - | - |
| - | - |
| - | - |
| - | - |
| - | - |
| - | - |
| - | - |
| - | - |
| - | - |
| - | - |
| - | - |
| - | - |
| - | - |
| - | - |
| - | - |
| - | - |
| - | - |
| - | - |
| - | - |
| - | - |
| - | - |
| - | - |
| - | - |
| - | - |
| - | - |
| - | - |
| - | - |
| - | - |
| - | - |
| - | - |
| - | - |
| - | - |
| - | - |
| - | - |

| | |
|---|---|
| - | - |
| - | - |
| - | - |
| - | - |
| - | - |
| - | - |
| - | - |
| - | - |
| - | - |
| - | - |
| - | - |
| - | - |
| - | - |
| - | - |
| - | - |
| - | - |
| - | - |
| - | - |
| - | - |
| - | - |
| - | - |
| - | - |
| - | - |
| - | - |
| - | - |
| - | - |
| - | - |
| - | - |
| - | - |
| - | - |
| - | - |
| - | - |
| - | - |
| - | - |
| - | - |
| - | - |
| - | - |
| - | - |
| - | - |
| - | - |
| - | - |
| - | - |
| - | - |
| - | - |
| - | - |
| - | - |
| - | - |
| - | - |

-
-
-
-
-
-
-
-
-
-
-
-
-
-
-
-
-
-
-
-
-
-
-
-
-
-
-
-
-
-
-
-
-
-
-
-
-
-
-
-
-
-
-
-
-
-
-
-
-
-
-
-
-
-
-
-
-
-
-
-
-
-
-
-
-
-
-
-
-
-
-
-
-
-
-
-
-
-
-
-
-
-
-
-
-
-
-
-
-
-
-
-
-
-

-
-
-
-
-
-
-
-
-
-
-
-
-
-
-
-
-
-
-
-
-
-
-
-
-
-
-
-
-
-
-
-
-
-
-
-
-
-
-
-
-
-
-
-
-
-
-
-
-
-
-
-
-
-
-
-
-
-
-
-
-
-
-
-
-
-
-
-
-
-
-
-
-
-
-
-
-
-
-
-
-
-
-
-
-
-
-
-
-
-
-
-
-
-

| | |
|---|---|
| - | - |
| - | - |
| - | - |
| - | - |
| - | - |
| - | - |
| - | - |
| - | - |
| - | - |
| - | - |
| - | - |
| - | - |
| - | - |
| - | - |
| - | - |
| - | - |
| - | - |
| - | - |
| - | - |
| - | - |
| - | - |
| - | - |
| - | - |
| - | - |
| - | - |
| - | - |
| - | - |
| - | - |
| - | - |
| - | - |
| - | - |
| - | - |
| - | - |
| - | - |
| - | - |
| - | - |
| - | - |
| - | - |
| - | - |
| - | - |
| - | - |
| - | - |
| - | - |
| - | - |
| - | - |
| - | - |
| - | - |

| | |
|---|---|
| - | - |
| - | - |
| - | - |
| - | - |
| - | - |
| - | - |
| - | - |
| - | - |
| - | - |
| - | - |
| - | - |
| - | - |
| - | - |
| - | - |
| - | - |
| - | - |
| - | - |
| - | - |
| - | - |
| - | - |
| - | - |
| - | - |
| - | - |
| - | - |
| - | - |
| - | - |
| - | - |
| - | - |
| - | gnl\|UG\|Ta#S12913303 |
| - | gnl\|UG\|Ta#S13022491 |
| - | gnl\|UG\|Ta#S26028042 |
| - | gnl\|UG\|Ta#S26028042 |
| - | gnl\|UG\|Ta#S26028042 |
| - | gnl\|UG\|Ta#S32507624 |
| - | gnl\|UG\|Ta#S37806908 |
| - | gnl\|UG\|Ta#S50371846 |
| - | gnl\|UG\|Ta#S52541805 |
| - | gnl\|UG\|Ta#S52545972 |
| - | gnl\|UG\|Ta#S58849005 |
| - | gnl\|UG\|Ta#S58850375 |
| - | gnl\|UG\|Ta#S58882065 |
| - | gnl\|UG\|Ta#S58887568 |
| - | gnl\|UG\|Ta#S58891736 |
| - | gnl\|UG\|Ta#S58891736 |
| - | gnl\|UG\|Ta#S58891736 |
| - | gnl\|UG\|Ta#S58907016 |
| - | gnl\|UG\|Ta#S58908970 |

| | |
|---|---|
| - | gnl\|UG\|Ta#S58910045 |
| - | gnl\|UG\|Ta#S61532710 |
| - | gnl\|UG\|Ta#S61532710 |
| - | gnl\|UG\|Ta#S61533547 |
| - | gnl\|UG\|Ta#S61544072 |
| - | gnl\|UG\|Ta#S61607701 |
| - | gnl\|UG\|Ta#S61615691 |
| - | gnl\|UG\|Ta#S61615691 |
| - | gnl\|UG\|Ta#S61618986 |
| - | gnl\|UG\|Ta#S61626908 |
| - | gnl\|UG\|Ta#S61627159 |
| - | gnl\|UG\|Ta#S61628150 |
| - | gnl\|UG\|Ta#S61628150 |
| - | gnl\|UG\|Ta#S61630388 |
| - | gnl\|UG\|Ta#S61641599 |
| - | gnl\|UG\|Ta#S61645240 |
| - | gnl\|UG\|Ta#S61647802 |
| - | gnl\|UG\|Ta#S61664714 |
| - | gnl\|UG\|Ta#S61688848 |
| - | gnl\|UG\|Ta#S61736816 |
| - | gnl\|UG\|Ta#S61739216 |
| - | gnl\|UG\|Ta#S61767553 |
| - | gnl\|UG\|Ta#S61772499 |
| - | gnl\|UG\|Ta#S61777018 |
| - | gnl\|UG\|Ta#S61778289 |
| - | gnl\|UG\|Ta#S61781478 |
| - | gnl\|UG\|Ta#S61781575 |
| - | gnl\|UG\|Ta#S61781575 |
| - | gnl\|UG\|Ta#S61781770 |
| - | gnl\|UG\|Ta#S61782530 |
| - | gnl\|UG\|Ta#S61798464 |
| - | gnl\|UG\|Ta#S61799194 |
| - | gnl\|UG\|Ta#S61800172 |
| - | gnl\|UG\|Ta#S61800172 |
| - | gnl\|UG\|Ta#S61800847 |
| - | gnl\|UG\|Ta#S61800847 |
| - | gnl\|UG\|Ta#S61807738 |
| - | gnl\|UG\|Ta#S61810508 |
| - | gnl\|UG\|Ta#S61810979 |
| - | gnl\|UG\|Ta#S61811229 |
| - | gnl\|UG\|Ta#S61811421 |
| - | gnl\|UG\|Ta#S61811584 |
| - | gnl\|UG\|Ta#S61811584 |
| - | gnl\|UG\|Ta#S61811585 |
| - | gnl\|UG\|Ta#S61811585 |
| - | gnl\|UG\|Ta#S61811749 |
| - | gnl\|UG\|Ta#S61811772 |

| | |
|---|---|
| - | gnl\|UG\|Ta#S61811887 |
| - | gnl\|UG\|Ta#S61812223 |
| - | gnl\|UG\|Ta#S61812528 |
| - | gnl\|UG\|Ta#S61827982 |
| - | gnl\|UG\|Ta#S61830054 |
| - | gnl\|UG\|Ta#S61830054 |
| - | gnl\|UG\|Ta#S61831783 |
| - | gnl\|UG\|Ta#S61831830 |
| - | gnl\|UG\|Ta#S61831830 |
| - | gnl\|UG\|Ta#S61831945 |
| - | gnl\|UG\|Ta#S61831945 |
| - | gnl\|UG\|Ta#S61832211 |
| - | gnl\|UG\|Ta#S61832401 |
| - | gnl\|UG\|Ta#S61832401 |
| - | gnl\|UG\|Ta#S61832532 |
| - | gnl\|UG\|Ta#S61845194 |
| - | gnl\|UG\|Ta#S65600758 |
| - | gnl\|UG\|Ta#S65620995 |
| - | gnl\|UG\|Ta#S65630248 |
| - | gnl\|UG\|Ta#S65651273 |
| - | gnl\|UG\|Ta#S65665693 |
| - | gnl\|UG\|Ta#S65675666 |
| - | gnl\|UG\|Ta#S65688304 |
| - | gnl\|UG\|Ta#S65688817 |
| - | gnl\|UG\|Ta#S65688873 |
| - | gnl\|UG\|Ta#S65689408 |
| - | gnl\|UG\|Ta#S65689408 |
| - | gnl\|UG\|Ta#S65701113 |
| - | gnl\|UG\|Ta#S65705450 |
| tr\|E0Y9Z4\|E0Y9Z4_WHEAT | - |
| tr\|M7YML0\|M7YML0_TRIUA | - |
| tr\|M7Z8L2\|M7Z8L2_TRIUA | - |
| tr\|M7ZL05\|M7ZL05_TRIUA | - |
| tr\|M7ZNL3\|M7ZNL3_TRIUA | - |
| tr\|M8A614\|M8A614_TRIUA | - |
| tr\|T1MIA9\|T1MIA9_TRIUA | - |
| tr\|T1N3G0\|T1N3G0_TRIUA | - |
| tr\|T1N7X5\|T1N7X5_TRIUA | - |
| tr\|T1NTP9\|T1NTP9_TRIUA | - |
| tr\|W5A666\|W5A666_WHEAT | - |
| tr\|W5A850\|W5A850_WHEAT | - |
| tr\|W5A991\|W5A991_WHEAT | - |
| tr\|W5A9K3\|W5A9K3_WHEAT | - |
| tr\|W5A9M9\|W5A9M9_WHEAT | - |
| tr\|W5DNL8\|W5DNL8_WHEAT | - |
| tr\|W5DNN8\|W5DNN8_WHEAT | - |
| tr\|W5DP60\|W5DP60_WHEAT | - |

| | |
|---|---|
| tr\|W5DUZ7\|W5DUZ7_WHEAT | - |
| tr\|W5EEY9\|W5EEY9_WHEAT | - |
| tr\|W5ENL1\|W5ENL1_WHEAT | - |
| tr\|W5F3J5\|W5F3J5_WHEAT | - |
| tr\|W5F3Y7\|W5F3Y7_WHEAT | - |
| tr\|W5F7J8\|W5F7J8_WHEAT | - |
| tr\|W5F8K6\|W5F8K6_WHEAT | - |
| tr\|W5F8K6\|W5F8K6_WHEAT | - |
| tr\|W5FAM9\|W5FAM9_WHEAT | - |
| tr\|W5FBI9\|W5FBI9_WHEAT | - |
| tr\|W5FBI9\|W5FBI9_WHEAT | - |
| tr\|W5FCP3\|W5FCP3_WHEAT | - |
| tr\|W5FCX1\|W5FCX1_WHEAT | - |
| tr\|W5FCX1\|W5FCX1_WHEAT | - |
| tr\|W5FDR3\|W5FDR3_WHEAT | - |
| tr\|W5FEE3\|W5FEE3_WHEAT | - |
| tr\|W5FEE3\|W5FEE3_WHEAT | - |
| tr\|W5FI59\|W5FI59_WHEAT | - |
| tr\|W5FJI6\|W5FJI6_WHEAT | - |
| tr\|W5FLX6\|W5FLX6_WHEAT | - |
| tr\|W5FP03\|W5FP03_WHEAT | - |
| tr\|W5FP85\|W5FP85_WHEAT | - |
| tr\|W5FTT6\|W5FTT6_WHEAT | - |
| tr\|W5GAJ1\|W5GAJ1_WHEAT | - |
| tr\|W5GRD8\|W5GRD8_WHEAT | - |
| tr\|W5HEW8\|W5HEW8_WHEAT | - |
| tr\|W5HJK9\|W5HJK9_WHEAT | - |
| tr\|W5F9R7\|W5F9R7_WHEAT | gnl\|UG\|Ta#S13039846 |
| tr\|W5FUU8\|W5FUU8_WHEAT | gnl\|UG\|Ta#S50378190 |
| tr\|W5DS84\|W5DS84_WHEAT | gnl\|UG\|Ta#S52543489 |
| tr\|W5A917\|W5A917_WHEAT | gnl\|UG\|Ta#S52544108 |
| tr\|M7ZTZ8\|M7ZTZ8_TRIUA | gnl\|UG\|Ta#S61569793 |
| tr\|T1MWF2\|T1MWF2_TRIUA | gnl\|UG\|Ta#S61582693 |
| tr\|W5FFR0\|W5FFR0_WHEAT | gnl\|UG\|Ta#S61583960 |
| tr\|W5AMP4\|W5AMP4_WHEAT | gnl\|UG\|Ta#S61630388 |
| tr\|M7YBJ7\|M7YBJ7_TRIUA | gnl\|UG\|Ta#S61638433 |
| tr\|W5FAX8\|W5FAX8_WHEAT | gnl\|UG\|Ta#S61669959 |
| tr\|W5EY91\|W5EY91_WHEAT | gnl\|UG\|Ta#S61713102 |
| tr\|W5FBE8\|W5FBE8_WHEAT | gnl\|UG\|Ta#S61747568 |
| tr\|W5FBE8\|W5FBE8_WHEAT | gnl\|UG\|Ta#S61747568 |
| tr\|W5FBE8\|W5FBE8_WHEAT | gnl\|UG\|Ta#S61747568 |
| tr\|W5DVU9\|W5DVU9_WHEAT | gnl\|UG\|Ta#S61752688 |
| tr\|T1N3G0\|T1N3G0_TRIUA | gnl\|UG\|Ta#S61771929 |
| tr\|M7ZAG1\|M7ZAG1_TRIUA | gnl\|UG\|Ta#S61797051 |
| tr\|M8AWX6\|M8AWX6_TRIUA | gnl\|UG\|Ta#S61810718 |
| tr\|W5GHT9\|W5GHT9_WHEAT | gnl\|UG\|Ta#S61811585 |
| tr\|W5AAZ8\|W5AAZ8_WHEAT | gnl\|UG\|Ta#S61815016 |

| | |
|---|---|
| tr\|M7YZZ2\|M7YZZ2_TRIUA | gnl\|UG\|Ta#S61831474 |
| tr\|M7ZMJ2\|M7ZMJ2_TRIUA | gnl\|UG\|Ta#S61831945 |
| tr\|M8A3H6\|M8A3H6_TRIUA | gnl\|UG\|Ta#S61856569 |
| tr\|T1LQ40\|T1LQ40_TRIUA | gnl\|UG\|Ta#S65626964 |
| tr\|W5IB38\|W5IB38_WHEAT | - |
| tr\|W5HUN9\|W5HUN9_WHEAT | - |
| tr\|W5G852\|W5G852_WHEAT | - |
| tr\|W5G852\|W5G852_WHEAT | - |
| tr\|W5FX78\|W5FX78_WHEAT | - |
| tr\|W5FSE8\|W5FSE8_WHEAT | - |
| tr\|W5FRJ8\|W5FRJ8_WHEAT | - |
| tr\|W5FQQ6\|W5FQQ6_WHEAT | - |
| tr\|W5FNU4\|W5FNU4_WHEAT | - |
| tr\|W5FNU4\|W5FNU4_WHEAT | - |
| tr\|W5FMT7\|W5FMT7_WHEAT | - |
| tr\|W5FMP8\|W5FMP8_WHEAT | - |
| tr\|W5FMI7\|W5FMI7_WHEAT | - |
| tr\|W5FLW7\|W5FLW7_WHEAT | - |
| tr\|W5FL04\|W5FL04_WHEAT | - |
| tr\|W5FKM0\|W5FKM0_WHEAT | - |
| tr\|W5FKG5\|W5FKG5_WHEAT | - |
| tr\|W5FKG5\|W5FKG5_WHEAT | - |
| tr\|W5FKF0\|W5FKF0_WHEAT | - |
| tr\|W5FK01\|W5FK01_WHEAT | - |
| tr\|W5FJT1\|W5FJT1_WHEAT | - |
| tr\|W5FJF8\|W5FJF8_WHEAT | - |
| tr\|W5FJF8\|W5FJF8_WHEAT | - |
| tr\|W5FJB9\|W5FJB9_WHEAT | - |
| tr\|W5FJ86\|W5FJ86_WHEAT | - |
| tr\|W5FIU6\|W5FIU6_WHEAT | - |
| tr\|W5FIU6\|W5FIU6_WHEAT | - |
| tr\|W5FIT3\|W5FIT3_WHEAT | - |
| tr\|W5FIT3\|W5FIT3_WHEAT | - |
| tr\|W5FIT3\|W5FIT3_WHEAT | - |
| tr\|W5FIT3\|W5FIT3_WHEAT | - |
| tr\|W5FIT3\|W5FIT3_WHEAT | - |
| tr\|W5FIJ4\|W5FIJ4_WHEAT | - |
| tr\|W5FHN9\|W5FHN9_WHEAT | - |
| tr\|W5FGS8\|W5FGS8_WHEAT | - |
| tr\|W5FFX7\|W5FFX7_WHEAT | - |
| tr\|W5FFA6\|W5FFA6_WHEAT | - |
| tr\|W5FFA6\|W5FFA6_WHEAT | - |
| tr\|W5FF74\|W5FF74_WHEAT | - |
| tr\|W5FES8\|W5FES8_WHEAT | - |
| tr\|W5FES1\|W5FES1_WHEAT | - |
| tr\|W5FEK3\|W5FEK3_WHEAT | - |
| tr\|W5FE25\|W5FE25_WHEAT | - |

| | |
|---|---|
| tr\|W5FE25\|W5FE25_WHEAT | - |
| tr\|W5FDR2\|W5FDR2_WHEAT | - |
| tr\|W5FDN5\|W5FDN5_WHEAT | - |
| tr\|W5FDI3\|W5FDI3_WHEAT | - |
| tr\|W5FDC4\|W5FDC4_WHEAT | - |
| tr\|W5FDC4\|W5FDC4_WHEAT | - |
| tr\|W5FDB5\|W5FDB5_WHEAT | - |
| tr\|W5FD66\|W5FD66_WHEAT | - |
| tr\|W5FD13\|W5FD13_WHEAT | - |
| tr\|W5FCW4\|W5FCW4_WHEAT | - |
| tr\|W5FCW4\|W5FCW4_WHEAT | - |
| tr\|W5FCW4\|W5FCW4_WHEAT | - |
| tr\|W5FCU5\|W5FCU5_WHEAT | - |
| tr\|W5FCR3\|W5FCR3_WHEAT | - |
| tr\|W5FCR3\|W5FCR3_WHEAT | - |
| tr\|W5FCR3\|W5FCR3_WHEAT | - |
| tr\|W5FBZ3\|W5FBZ3_WHEAT | - |
| tr\|W5FBV9\|W5FBV9_WHEAT | - |
| tr\|W5FBV9\|W5FBV9_WHEAT | - |
| tr\|W5FBL9\|W5FBL9_WHEAT | - |
| tr\|W5FBC4\|W5FBC4_WHEAT | - |
| tr\|W5FAZ0\|W5FAZ0_WHEAT | - |
| tr\|W5FA95\|W5FA95_WHEAT | - |
| tr\|W5F9Y0\|W5F9Y0_WHEAT | - |
| tr\|W5F9U9\|W5F9U9_WHEAT | - |
| tr\|W5F9U9\|W5F9U9_WHEAT | - |
| tr\|W5F9U9\|W5F9U9_WHEAT | - |
| tr\|W5F9I6\|W5F9I6_WHEAT | - |
| tr\|W5F992\|W5F992_WHEAT | - |
| tr\|W5F992\|W5F992_WHEAT | - |
| tr\|W5F8X6\|W5F8X6_WHEAT | - |
| tr\|W5F8X6\|W5F8X6_WHEAT | - |
| tr\|W5F8J0\|W5F8J0_WHEAT | - |
| tr\|W5F8J0\|W5F8J0_WHEAT | - |
| tr\|W5F894\|W5F894_WHEAT | - |
| tr\|W5F894\|W5F894_WHEAT | - |
| tr\|W5F858\|W5F858_WHEAT | - |
| tr\|W5F858\|W5F858_WHEAT | - |
| tr\|W5F858\|W5F858_WHEAT | - |
| tr\|W5F7T2\|W5F7T2_WHEAT | - |
| tr\|W5F7F8\|W5F7F8_WHEAT | - |
| tr\|W5F7F8\|W5F7F8_WHEAT | - |
| tr\|W5F782\|W5F782_WHEAT | - |
| tr\|W5F722\|W5F722_WHEAT | - |
| tr\|W5F4B7\|W5F4B7_WHEAT | - |
| tr\|W5EW92\|W5EW92_WHEAT | - |
| tr\|W5EFX2\|W5EFX2_WHEAT | - |

| | |
|---|---|
| tr\|W5DY85\|W5DY85_WHEAT | - |
| tr\|W5DUT8\|W5DUT8_WHEAT | - |
| tr\|W5DU18\|W5DU18_WHEAT | - |
| tr\|W5DSK9\|W5DSK9_WHEAT | - |
| tr\|W5DQR8\|W5DQR8_WHEAT | - |
| tr\|W5DL66\|W5DL66_WHEAT | - |
| tr\|W5D010\|W5D010_WHEAT | - |
| tr\|W5CD44\|W5CD44_WHEAT | - |
| tr\|W5AKY3\|W5AKY3_WHEAT | - |
| tr\|W5AC22\|W5AC22_WHEAT | - |
| tr\|W5A8Y0\|W5A8Y0_WHEAT | - |
| tr\|W5A8Y0\|W5A8Y0_WHEAT | - |
| tr\|W5A5C0\|W5A5C0_WHEAT | - |
| tr\|W4ZYP3\|W4ZYP3_WHEAT | - |
| tr\|W4ZXM2\|W4ZXM2_WHEAT | - |
| tr\|W4ZXM2\|W4ZXM2_WHEAT | - |
| tr\|W4ZMG2\|W4ZMG2_WHEAT | - |
| tr\|T1NWZ5\|T1NWZ5_TRIUA | - |
| tr\|T1MZJ1\|T1MZJ1_TRIUA | - |
| tr\|T1MQ65\|T1MQ65_TRIUA | - |
| tr\|T1M4K0\|T1M4K0_TRIUA | - |
| tr\|M8A8X2\|M8A8X2_TRIUA | - |
| tr\|M7ZYU3\|M7ZYU3_TRIUA | - |
| tr\|M7ZY99\|M7ZY99_TRIUA | - |
| tr\|M7ZVF3\|M7ZVF3_TRIUA | - |
| tr\|M7ZUS3\|M7ZUS3_TRIUA | - |
| tr\|M7ZP22\|M7ZP22_TRIUA | - |
| tr\|M7ZP22\|M7ZP22_TRIUA | - |
| tr\|M7ZLR3\|M7ZLR3_TRIUA | - |
| tr\|M7ZGX5\|M7ZGX5_TRIUA | - |
| tr\|M7ZEY6\|M7ZEY6_TRIUA | - |
| tr\|M7Z7W8\|M7Z7W8_TRIUA | - |
| tr\|M7Z762\|M7Z762_TRIUA | - |
| tr\|M7Z4E2\|M7Z4E2_TRIUA | - |
| tr\|M7Z4E2\|M7Z4E2_TRIUA | - |
| tr\|M7YVQ9\|M7YVQ9_TRIUA | - |
| tr\|M7YPF1\|M7YPF1_TRIUA | - |
| tr\|M7YEN5\|M7YEN5_TRIUA | - |
| tr\|M7YEN5\|M7YEN5_TRIUA | - |
| tr\|T1MTN4\|T1MTN4_TRIUA | gnl\|UG\|Ta#S13037380 |
| tr\|W5GRD1\|W5GRD1_WHEAT | gnl\|UG\|Ta#S17988998 |
| tr\|T1LZI1\|T1LZI1_TRIUA | gnl\|UG\|Ta#S24512966 |
| tr\|W5FG90\|W5FG90_WHEAT | gnl\|UG\|Ta#S52543105 |
| tr\|M8B5B4\|M8B5B4_TRIUA | gnl\|UG\|Ta#S52544097 |
| tr\|W5AAQ9\|W5AAQ9_WHEAT | gnl\|UG\|Ta#S52544279 |
| tr\|W5AAQ9\|W5AAQ9_WHEAT | gnl\|UG\|Ta#S52544279 |
| tr\|W5FM44\|W5FM44_WHEAT | gnl\|UG\|Ta#S52547038 |

| | |
|---|---|
| tr\|M7Z112\|M7Z112_TRIUA | gnl\|UG\|Ta#S56541630 |
| tr\|M7ZY99\|M7ZY99_TRIUA | gnl\|UG\|Ta#S58856760 |
| tr\|M7YQS9\|M7YQS9_TRIUA | gnl\|UG\|Ta#S58902464 |
| tr\|M7YQS9\|M7YQS9_TRIUA | gnl\|UG\|Ta#S58902464 |
| tr\|W5F8S5\|W5F8S5_WHEAT | gnl\|UG\|Ta#S58909708 |
| tr\|M7ZPD6\|M7ZPD6_TRIUA | gnl\|UG\|Ta#S59657911 |
| tr\|W5FGS8\|W5FGS8_WHEAT | gnl\|UG\|Ta#S61522423 |
| tr\|W5F7T9\|W5F7T9_WHEAT | gnl\|UG\|Ta#S61533004 |
| tr\|W5F7T9\|W5F7T9_WHEAT | gnl\|UG\|Ta#S61533004 |
| tr\|W5AC12\|W5AC12_WHEAT | gnl\|UG\|Ta#S61554556 |
| tr\|M7YJR2\|M7YJR2_TRIUA | gnl\|UG\|Ta#S61569415 |
| tr\|W5FF65\|W5FF65_WHEAT | gnl\|UG\|Ta#S61572242 |
| tr\|W5FCE5\|W5FCE5_WHEAT | gnl\|UG\|Ta#S61573690 |
| tr\|W5FCE5\|W5FCE5_WHEAT | gnl\|UG\|Ta#S61573690 |
| tr\|W5DQ37\|W5DQ37_WHEAT | gnl\|UG\|Ta#S61575017 |
| tr\|M7ZT06\|M7ZT06_TRIUA | gnl\|UG\|Ta#S61596134 |
| tr\|M7ZT06\|M7ZT06_TRIUA | gnl\|UG\|Ta#S61596134 |
| tr\|W5F7J1\|W5F7J1_WHEAT | gnl\|UG\|Ta#S61597651 |
| tr\|W5FP14\|W5FP14_WHEAT | gnl\|UG\|Ta#S61604115 |
| tr\|W5FUF0\|W5FUF0_WHEAT | gnl\|UG\|Ta#S61607405 |
| tr\|M7ZQE0\|M7ZQE0_TRIUA | gnl\|UG\|Ta#S61608592 |
| tr\|M7ZQE0\|M7ZQE0_TRIUA | gnl\|UG\|Ta#S61608592 |
| tr\|M7YUC2\|M7YUC2_TRIUA | gnl\|UG\|Ta#S61611320 |
| tr\|M7Z147\|M7Z147_TRIUA | gnl\|UG\|Ta#S61620601 |
| tr\|M7Z147\|M7Z147_TRIUA | gnl\|UG\|Ta#S61620601 |
| tr\|M7ZP05\|M7ZP05_TRIUA | gnl\|UG\|Ta#S61626236 |
| tr\|M8A7X8\|M8A7X8_TRIUA | gnl\|UG\|Ta#S61626639 |
| tr\|W5A2I9\|W5A2I9_WHEAT | gnl\|UG\|Ta#S61634446 |
| tr\|W5FGC4\|W5FGC4_WHEAT | gnl\|UG\|Ta#S61639637 |
| tr\|W5FGC4\|W5FGC4_WHEAT | gnl\|UG\|Ta#S61639637 |
| tr\|W5FGC4\|W5FGC4_WHEAT | gnl\|UG\|Ta#S61639637 |
| tr\|W4ZZQ3\|W4ZZQ3_WHEAT | gnl\|UG\|Ta#S61650254 |
| tr\|W4ZZQ3\|W4ZZQ3_WHEAT | gnl\|UG\|Ta#S61650254 |
| tr\|W5F7T9\|W5F7T9_WHEAT | gnl\|UG\|Ta#S61653897 |
| tr\|W5FNX1\|W5FNX1_WHEAT | gnl\|UG\|Ta#S61663004 |
| tr\|W5FE50\|W5FE50_WHEAT | gnl\|UG\|Ta#S61680594 |
| tr\|W5FBP2\|W5FBP2_WHEAT | gnl\|UG\|Ta#S61708094 |
| tr\|W5FAG2\|W5FAG2_WHEAT | gnl\|UG\|Ta#S61766447 |
| tr\|W5FE50\|W5FE50_WHEAT | gnl\|UG\|Ta#S61768893 |
| tr\|W5FHF6\|W5FHF6_WHEAT | gnl\|UG\|Ta#S61782336 |
| tr\|W5F5C4\|W5F5C4_WHEAT | gnl\|UG\|Ta#S61800777 |
| tr\|W5FSZ4\|W5FSZ4_WHEAT | gnl\|UG\|Ta#S61801496 |
| tr\|W5FDN5\|W5FDN5_WHEAT | gnl\|UG\|Ta#S61801504 |
| tr\|M7ZB80\|M7ZB80_TRIUA | gnl\|UG\|Ta#S61801504 |
| tr\|W5G157\|W5G157_WHEAT | gnl\|UG\|Ta#S61801671 |
| tr\|M7ZQZ2\|M7ZQZ2_TRIUA | gnl\|UG\|Ta#S61808949 |
| tr\|W5FJC3\|W5FJC3_WHEAT | gnl\|UG\|Ta#S61809545 |

| | |
|---|---|
| tr\|W4ZXV6\|W4ZXV6_WHEAT | gnl\|UG\|Ta#S61810441 |
| tr\|M7ZP22\|M7ZP22_TRIUA | gnl\|UG\|Ta#S61810999 |
| tr\|W5FAZ0\|W5FAZ0_WHEAT | gnl\|UG\|Ta#S61811416 |
| tr\|M8AHZ2\|M8AHZ2_TRIUA | gnl\|UG\|Ta#S61811466 |
| tr\|W5F9X4\|W5F9X4_WHEAT | gnl\|UG\|Ta#S61811730 |
| tr\|W5A2P7\|W5A2P7_WHEAT | gnl\|UG\|Ta#S61812194 |
| tr\|M7YX52\|M7YX52_TRIUA | gnl\|UG\|Ta#S61812316 |
| tr\|W5FMT7\|W5FMT7_WHEAT | gnl\|UG\|Ta#S61812326 |
| tr\|W5FES1\|W5FES1_WHEAT | gnl\|UG\|Ta#S61812547 |
| tr\|W5AC22\|W5AC22_WHEAT | gnl\|UG\|Ta#S61822675 |
| tr\|W5FPC7\|W5FPC7_WHEAT | gnl\|UG\|Ta#S61828078 |
| tr\|W5FPC7\|W5FPC7_WHEAT | gnl\|UG\|Ta#S61828078 |
| tr\|M8ARH4\|M8ARH4_TRIUA | gnl\|UG\|Ta#S61828331 |
| tr\|T1NRX1\|T1NRX1_TRIUA | gnl\|UG\|Ta#S61828338 |
| tr\|W5FTB0\|W5FTB0_WHEAT | gnl\|UG\|Ta#S61828346 |
| tr\|W5FNW0\|W5FNW0_WHEAT | gnl\|UG\|Ta#S61829605 |
| tr\|W5FG42\|W5FG42_WHEAT | gnl\|UG\|Ta#S61829846 |
| tr\|M8A0F5\|M8A0F5_TRIUA | gnl\|UG\|Ta#S61830224 |
| tr\|W5FKM0\|W5FKM0_WHEAT | gnl\|UG\|Ta#S61830614 |
| tr\|W5DPJ8\|W5DPJ8_WHEAT | gnl\|UG\|Ta#S61830811 |
| tr\|W5DLQ6\|W5DLQ6_WHEAT | gnl\|UG\|Ta#S61830811 |
| tr\|W5DTQ0\|W5DTQ0_WHEAT | gnl\|UG\|Ta#S61831433 |
| tr\|M7ZMD4\|M7ZMD4_TRIUA | gnl\|UG\|Ta#S61831433 |
| tr\|T1M333\|T1M333_TRIUA | gnl\|UG\|Ta#S61831498 |
| tr\|W5HVC5\|W5HVC5_WHEAT | gnl\|UG\|Ta#S61831753 |
| tr\|W5GMV1\|W5GMV1_WHEAT | gnl\|UG\|Ta#S61831823 |
| tr\|W5F8R6\|W5F8R6_WHEAT | gnl\|UG\|Ta#S61831867 |
| tr\|M7Y901\|M7Y901_TRIUA | gnl\|UG\|Ta#S61831966 |
| tr\|T1MGT9\|T1MGT9_TRIUA | gnl\|UG\|Ta#S61832035 |
| tr\|W5FLQ0\|W5FLQ0_WHEAT | gnl\|UG\|Ta#S61832044 |
| tr\|W5FNN9\|W5FNN9_WHEAT | gnl\|UG\|Ta#S61832521 |
| tr\|M7YQX8\|M7YQX8_TRIUA | gnl\|UG\|Ta#S65592825 |
| tr\|W5FH13\|W5FH13_WHEAT | gnl\|UG\|Ta#S65599697 |
| tr\|M7ZVF3\|M7ZVF3_TRIUA | gnl\|UG\|Ta#S65604932 |
| tr\|W5FKB0\|W5FKB0_WHEAT | gnl\|UG\|Ta#S65607523 |
| tr\|W5GKB0\|W5GKB0_WHEAT | gnl\|UG\|Ta#S65632102 |
| tr\|W5FBC4\|W5FBC4_WHEAT | gnl\|UG\|Ta#S65638971 |
| tr\|W5D010\|W5D010_WHEAT | gnl\|UG\|Ta#S65662190 |
| tr\|W5FEK3\|W5FEK3_WHEAT | gnl\|UG\|Ta#S65717910 |
| tr\|W5FNF4\|W5FNF4_WHEAT | gnl\|UG\|Ta#S65718984 |
| - | - |
| - | - |
| - | - |
| - | - |
| - | - |
| - | - |
| - | - |

| | |
|---|---|
| - | - |
| - | - |
| - | - |
| - | - |
| - | - |
| - | - |
| - | - |
| - | - |
| - | - |
| - | - |
| - | - |
| - | - |
| - | - |
| - | - |
| - | - |
| - | - |
| - | - |
| - | - |
| - | - |
| - | - |
| - | - |
| - | - |
| - | - |
| - | - |
| - | - |
| - | - |
| - | - |
| - | - |
| - | - |
| - | - |
| - | - |
| - | - |
| - | - |
| - | - |
| - | - |
| - | - |
| - | - |
| - | - |
| - | - |
| - | - |
| - | - |
| - | - |
| - | - |
| - | - |
| - | - |
| - | - |
| - | - |

| | |
|---|---|
| - | - |
| - | - |
| - | - |
| - | - |
| - | - |
| - | - |
| - | - |
| - | - |
| - | - |
| - | - |
| - | - |
| - | - |
| - | - |
| - | - |
| - | - |
| - | - |
| - | - |
| - | - |
| - | - |
| - | - |
| - | - |
| - | - |
| - | - |
| - | - |
| - | - |
| - | - |
| - | - |
| - | - |
| - | - |
| - | - |
| - | - |
| - | - |
| - | - |
| - | - |
| - | - |
| - | - |
| - | - |
| - | - |
| - | - |
| - | - |
| - | - |
| - | - |
| - | - |
| - | - |
| - | - |
| - | - |
| - | - |

| | |
|---|---|
| - | - |
| - | - |
| - | - |
| - | - |
| - | - |
| - | - |
| - | - |
| - | - |
| - | - |
| - | - |
| - | - |
| - | - |
| - | - |
| - | - |
| - | - |
| - | - |
| - | - |
| - | - |
| - | - |
| - | - |
| - | - |
| - | - |
| - | - |
| - | - |
| - | - |
| - | - |
| - | - |
| - | - |
| - | - |
| - | - |
| - | - |
| - | - |
| - | - |
| - | - |
| - | - |
| - | - |
| - | - |
| - | - |
| - | - |
| - | - |
| - | - |
| - | - |
| - | - |
| - | - |
| - | - |
| - | - |
| - | - |

| | |
|---|---|
| - | - |
| - | - |
| - | - |
| - | - |
| - | - |
| - | - |
| - | - |
| - | - |
| - | - |
| - | - |
| - | - |
| - | - |
| - | - |
| - | - |
| - | - |
| - | - |
| - | - |
| - | - |
| - | - |
| - | - |
| - | - |
| - | - |
| - | - |
| - | - |
| - | - |
| - | - |
| - | - |
| - | - |
| - | - |
| - | - |
| - | - |
| - | - |
| - | - |
| - | - |
| - | - |
| - | - |
| - | - |
| - | - |
| - | - |
| - | - |
| - | - |
| - | - |
| - | - |
| - | - |
| - | - |
| - | - |
| - | - |

| | |
|---|---|
| - | - |
| - | - |
| - | - |
| - | - |
| - | - |
| - | - |
| - | - |
| - | - |
| - | - |
| - | - |
| - | - |
| - | - |
| - | - |
| - | - |
| - | - |
| - | - |
| - | - |
| - | - |
| - | - |
| - | - |
| - | - |
| - | - |
| - | - |
| - | - |
| - | - |
| - | - |
| - | - |
| - | - |
| - | - |
| - | - |
| - | - |
| - | - |
| - | - |
| - | - |
| - | - |
| - | - |
| - | - |
| - | - |
| - | - |
| - | - |
| - | - |
| - | - |
| - | - |
| - | - |
| - | - |
| - | - |
| - | - |

| | |
|---|---|
| - | - |
| - | - |
| - | - |
| - | - |
| - | - |
| - | - |
| - | - |
| - | - |
| - | - |
| - | - |
| - | - |
| - | - |
| - | - |
| - | - |
| - | - |
| - | - |
| - | - |
| - | - |
| - | - |
| - | - |
| - | - |
| - | - |
| - | - |
| - | - |
| - | - |
| - | - |
| - | - |
| - | - |
| - | - |
| - | - |
| - | - |
| - | - |
| - | - |
| - | - |
| - | - |
| - | - |
| - | - |
| - | - |
| - | - |
| - | - |
| - | - |
| - | - |
| - | - |
| - | - |
| - | - |
| - | - |
| - | - |

| | |
|---|---|
| - | - |
| - | - |
| - | - |
| - | - |
| - | - |
| - | - |
| - | - |
| - | - |
| - | - |
| - | - |
| - | - |
| - | - |
| - | - |
| - | - |
| - | - |
| - | - |
| - | - |
| - | - |
| - | - |
| - | - |
| - | - |
| - | - |
| - | - |
| - | - |
| - | - |
| - | - |
| - | - |
| - | - |
| - | - |
| - | - |
| - | - |
| - | - |
| - | - |
| - | - |
| - | - |
| - | - |
| - | - |
| - | - |
| - | - |
| - | - |
| - | - |
| - | - |
| - | - |
| - | - |
| - | - |
| - | - |
| - | - |

| | |
|---|---|
| - | - |
| - | - |
| - | - |
| - | - |
| - | - |
| - | - |
| - | - |
| - | - |
| - | - |
| - | - |
| - | - |
| - | - |
| - | - |
| - | - |
| - | - |
| - | - |
| - | - |
| - | - |
| - | - |
| - | - |
| - | - |
| - | - |
| - | - |
| - | - |
| - | - |
| - | - |
| - | - |
| - | - |
| - | - |
| - | - |
| - | - |
| - | - |
| - | - |
| - | - |
| - | - |
| - | - |
| - | - |
| - | - |
| - | - |
| - | - |
| - | - |
| - | - |
| - | - |
| - | - |
| - | - |
| - | - |
| - | - |

| | |
|---|---|
| - | - |
| - | gnl\|UG\|Ta#S12913984 |
| - | gnl\|UG\|Ta#S16222886 |
| - | gnl\|UG\|Ta#S17978550 |
| - | gnl\|UG\|Ta#S17987023 |
| - | gnl\|UG\|Ta#S18011728 |
| - | gnl\|UG\|Ta#S22119324 |
| - | gnl\|UG\|Ta#S32668000 |
| - | gnl\|UG\|Ta#S52542257 |
| - | gnl\|UG\|Ta#S52543105 |
| - | gnl\|UG\|Ta#S52543693 |
| - | gnl\|UG\|Ta#S52544097 |
| - | gnl\|UG\|Ta#S52544206 |
| - | gnl\|UG\|Ta#S52544330 |
| - | gnl\|UG\|Ta#S52544336 |
| - | gnl\|UG\|Ta#S52544728 |
| - | gnl\|UG\|Ta#S52544728 |
| - | gnl\|UG\|Ta#S52546164 |
| - | gnl\|UG\|Ta#S52547038 |
| - | gnl\|UG\|Ta#S52547045 |
| - | gnl\|UG\|Ta#S52547045 |
| - | gnl\|UG\|Ta#S53281794 |
| - | gnl\|UG\|Ta#S58844166 |
| - | gnl\|UG\|Ta#S58851961 |
| - | gnl\|UG\|Ta#S58855941 |
| - | gnl\|UG\|Ta#S58884639 |
| - | gnl\|UG\|Ta#S58888502 |
| - | gnl\|UG\|Ta#S58891791 |
| - | gnl\|UG\|Ta#S58891791 |
| - | gnl\|UG\|Ta#S58895187 |
| - | gnl\|UG\|Ta#S58906886 |
| - | gnl\|UG\|Ta#S58909708 |
| - | gnl\|UG\|Ta#S58910733 |
| - | gnl\|UG\|Ta#S58910733 |
| - | gnl\|UG\|Ta#S58910733 |
| - | gnl\|UG\|Ta#S59657911 |
| - | gnl\|UG\|Ta#S59657911 |
| - | gnl\|UG\|Ta#S61518391 |
| - | gnl\|UG\|Ta#S61518725 |
| - | gnl\|UG\|Ta#S61518725 |
| - | gnl\|UG\|Ta#S61523950 |
| - | gnl\|UG\|Ta#S61524771 |
| - | gnl\|UG\|Ta#S61525665 |
| - | gnl\|UG\|Ta#S61527255 |
| - | gnl\|UG\|Ta#S61529393 |
| - | gnl\|UG\|Ta#S61534361 |
| - | gnl\|UG\|Ta#S61548588 |

| | |
|---|---|
| - | gnl\|UG\|Ta#S61550178 |
| - | gnl\|UG\|Ta#S61554555 |
| - | gnl\|UG\|Ta#S61559464 |
| - | gnl\|UG\|Ta#S61561718 |
| - | gnl\|UG\|Ta#S61561718 |
| - | gnl\|UG\|Ta#S61562186 |
| - | gnl\|UG\|Ta#S61565233 |
| - | gnl\|UG\|Ta#S61569415 |
| - | gnl\|UG\|Ta#S61570827 |
| - | gnl\|UG\|Ta#S61572242 |
| - | gnl\|UG\|Ta#S61575017 |
| - | gnl\|UG\|Ta#S61577520 |
| - | gnl\|UG\|Ta#S61588679 |
| - | gnl\|UG\|Ta#S61590455 |
| - | gnl\|UG\|Ta#S61594093 |
| - | gnl\|UG\|Ta#S61594093 |
| - | gnl\|UG\|Ta#S61597651 |
| - | gnl\|UG\|Ta#S61597651 |
| - | gnl\|UG\|Ta#S61598188 |
| - | gnl\|UG\|Ta#S61598188 |
| - | gnl\|UG\|Ta#S61600740 |
| - | gnl\|UG\|Ta#S61600740 |
| - | gnl\|UG\|Ta#S61601905 |
| - | gnl\|UG\|Ta#S61607405 |
| - | gnl\|UG\|Ta#S61607405 |
| - | gnl\|UG\|Ta#S61608592 |
| - | gnl\|UG\|Ta#S61608846 |
| - | gnl\|UG\|Ta#S61608846 |
| - | gnl\|UG\|Ta#S61608900 |
| - | gnl\|UG\|Ta#S61611320 |
| - | gnl\|UG\|Ta#S61614770 |
| - | gnl\|UG\|Ta#S61616616 |
| - | gnl\|UG\|Ta#S61616616 |
| - | gnl\|UG\|Ta#S61616670 |
| - | gnl\|UG\|Ta#S61618594 |
| - | gnl\|UG\|Ta#S61619055 |
| - | gnl\|UG\|Ta#S61619055 |
| - | gnl\|UG\|Ta#S61620966 |
| - | gnl\|UG\|Ta#S61620966 |
| - | gnl\|UG\|Ta#S61621852 |
| - | gnl\|UG\|Ta#S61621852 |
| - | gnl\|UG\|Ta#S61621852 |
| - | gnl\|UG\|Ta#S61623166 |
| - | gnl\|UG\|Ta#S61626236 |
| - | gnl\|UG\|Ta#S61626430 |
| - | gnl\|UG\|Ta#S61626639 |
| - | gnl\|UG\|Ta#S61626639 |

| | |
|---|---|
| - | gnl\|UG\|Ta#S61626639 |
| - | gnl\|UG\|Ta#S61626639 |
| - | gnl\|UG\|Ta#S61626639 |
| - | gnl\|UG\|Ta#S61626639 |
| - | gnl\|UG\|Ta#S61626639 |
| - | gnl\|UG\|Ta#S61626639 |
| - | gnl\|UG\|Ta#S61634446 |
| - | gnl\|UG\|Ta#S61639637 |
| - | gnl\|UG\|Ta#S61639637 |
| - | gnl\|UG\|Ta#S61639637 |
| - | gnl\|UG\|Ta#S61639637 |
| - | gnl\|UG\|Ta#S61639637 |
| - | gnl\|UG\|Ta#S61640151 |
| - | gnl\|UG\|Ta#S61649366 |
| - | gnl\|UG\|Ta#S61649366 |
| - | gnl\|UG\|Ta#S61653730 |
| - | gnl\|UG\|Ta#S61665534 |
| - | gnl\|UG\|Ta#S61678210 |
| - | gnl\|UG\|Ta#S61678210 |
| - | gnl\|UG\|Ta#S61689116 |
| - | gnl\|UG\|Ta#S61692732 |
| - | gnl\|UG\|Ta#S61717259 |
| - | gnl\|UG\|Ta#S61719053 |
| - | gnl\|UG\|Ta#S61752704 |
| - | gnl\|UG\|Ta#S61772135 |
| - | gnl\|UG\|Ta#S61772135 |
| - | gnl\|UG\|Ta#S61779097 |
| - | gnl\|UG\|Ta#S61781343 |
| - | gnl\|UG\|Ta#S61781343 |
| - | gnl\|UG\|Ta#S61782336 |
| - | gnl\|UG\|Ta#S61788199 |
| - | gnl\|UG\|Ta#S61792126 |
| - | gnl\|UG\|Ta#S61800777 |
| - | gnl\|UG\|Ta#S61801359 |
| - | gnl\|UG\|Ta#S61801496 |
| - | gnl\|UG\|Ta#S61802160 |
| - | gnl\|UG\|Ta#S61802160 |
| - | gnl\|UG\|Ta#S61802494 |
| - | gnl\|UG\|Ta#S61802494 |
| - | gnl\|UG\|Ta#S61802494 |
| - | gnl\|UG\|Ta#S61802494 |
| - | gnl\|UG\|Ta#S61802494 |
| - | gnl\|UG\|Ta#S61802494 |
| - | gnl\|UG\|Ta#S61807852 |
| - | gnl\|UG\|Ta#S61808043 |
| - | gnl\|UG\|Ta#S61808043 |
| - | gnl\|UG\|Ta#S61808112 |

| | |
|---|---|
| - | gnl\|UG\|Ta#S61808112 |
| - | gnl\|UG\|Ta#S61808173 |
| - | gnl\|UG\|Ta#S61808827 |
| - | gnl\|UG\|Ta#S61808827 |
| - | gnl\|UG\|Ta#S61809221 |
| - | gnl\|UG\|Ta#S61809221 |
| - | gnl\|UG\|Ta#S61809545 |
| - | gnl\|UG\|Ta#S61809818 |
| - | gnl\|UG\|Ta#S61810305 |
| - | gnl\|UG\|Ta#S61810305 |
| - | gnl\|UG\|Ta#S61810381 |
| - | gnl\|UG\|Ta#S61810381 |
| - | gnl\|UG\|Ta#S61811466 |
| - | gnl\|UG\|Ta#S61811466 |
| - | gnl\|UG\|Ta#S61811480 |
| - | gnl\|UG\|Ta#S61811660 |
| - | gnl\|UG\|Ta#S61811660 |
| - | gnl\|UG\|Ta#S61811730 |
| - | gnl\|UG\|Ta#S61811944 |
| - | gnl\|UG\|Ta#S61812040 |
| - | gnl\|UG\|Ta#S61812219 |
| - | gnl\|UG\|Ta#S61812291 |
| - | gnl\|UG\|Ta#S61812316 |
| - | gnl\|UG\|Ta#S61812529 |
| - | gnl\|UG\|Ta#S61821912 |
| - | gnl\|UG\|Ta#S61821912 |
| - | gnl\|UG\|Ta#S61828338 |
| - | gnl\|UG\|Ta#S61829185 |
| - | gnl\|UG\|Ta#S61829185 |
| - | gnl\|UG\|Ta#S61829846 |
| - | gnl\|UG\|Ta#S61829995 |
| - | gnl\|UG\|Ta#S61830022 |
| - | gnl\|UG\|Ta#S61830224 |
| - | gnl\|UG\|Ta#S61830284 |
| - | gnl\|UG\|Ta#S61830284 |
| - | gnl\|UG\|Ta#S61831329 |
| - | gnl\|UG\|Ta#S61831329 |
| - | gnl\|UG\|Ta#S61831329 |
| - | gnl\|UG\|Ta#S61831329 |
| - | gnl\|UG\|Ta#S61831376 |
| - | gnl\|UG\|Ta#S61831376 |
| - | gnl\|UG\|Ta#S61831396 |
| - | gnl\|UG\|Ta#S61831433 |
| - | gnl\|UG\|Ta#S61831498 |
| - | gnl\|UG\|Ta#S61831531 |
| - | gnl\|UG\|Ta#S61831531 |
| - | gnl\|UG\|Ta#S61831588 |

| | |
|---|---|
| - | gnl\|UG\|Ta#S61831588 |
| - | gnl\|UG\|Ta#S61831588 |
| - | gnl\|UG\|Ta#S61831645 |
| - | gnl\|UG\|Ta#S61831754 |
| - | gnl\|UG\|Ta#S61831880 |
| - | gnl\|UG\|Ta#S61831966 |
| - | gnl\|UG\|Ta#S61832035 |
| - | gnl\|UG\|Ta#S61832035 |
| - | gnl\|UG\|Ta#S61832044 |
| - | gnl\|UG\|Ta#S61832044 |
| - | gnl\|UG\|Ta#S61832083 |
| - | gnl\|UG\|Ta#S61832086 |
| - | gnl\|UG\|Ta#S61832086 |
| - | gnl\|UG\|Ta#S61832310 |
| - | gnl\|UG\|Ta#S61832310 |
| - | gnl\|UG\|Ta#S61832496 |
| - | gnl\|UG\|Ta#S61832521 |
| - | gnl\|UG\|Ta#S61835256 |
| - | gnl\|UG\|Ta#S61846510 |
| - | gnl\|UG\|Ta#S61860902 |
| - | gnl\|UG\|Ta#S65592825 |
| - | gnl\|UG\|Ta#S65607281 |
| - | gnl\|UG\|Ta#S65607281 |
| - | gnl\|UG\|Ta#S65613174 |
| - | gnl\|UG\|Ta#S65616150 |
| - | gnl\|UG\|Ta#S65629286 |
| - | gnl\|UG\|Ta#S65629286 |
| - | gnl\|UG\|Ta#S65631084 |
| - | gnl\|UG\|Ta#S65631084 |
| - | gnl\|UG\|Ta#S65646581 |
| - | gnl\|UG\|Ta#S65662275 |
| - | gnl\|UG\|Ta#S65662659 |
| - | gnl\|UG\|Ta#S65692176 |
| - | gnl\|UG\|Ta#S65692176 |
| - | gnl\|UG\|Ta#S65692176 |
| - | gnl\|UG\|Ta#S65699362 |
| - | gnl\|UG\|Ta#S65704386 |
| - | gnl\|UG\|Ta#S65705477 |
| - | gnl\|UG\|Ta#S65706073 |
| - | gnl\|UG\|Ta#S65706073 |
| - | gnl\|UG\|Ta#S65709702 |
| - | gnl\|UG\|Ta#S65717536 |
| - | gnl\|UG\|Ta#S65717536 |
| - | - |
| - | - |
| - | - |
| - | - |

| | |
|---|---|
| - | - |
| - | - |
| - | - |
| - | - |
| - | - |
| - | - |
| - | - |
| - | - |
| - | - |
| - | - |
| - | - |
| - | - |
| - | - |
| - | - |
| - | - |
| - | - |
| - | - |
| - | - |
| - | - |
| - | - |
| - | - |
| - | - |
| - | - |
| - | - |
| - | - |
| - | - |
| - | - |
| - | - |
| - | - |
| - | - |
| - | - |
| - | - |
| - | - |
| - | - |
| - | - |
| - | - |
| - | - |
| - | - |
| - | - |
| - | - |
| - | - |
| - | - |
| - | - |
| - | - |
| - | - |
| - | - |
| - | - |

| | |
|---|---|
| - | - |
| - | - |
| - | - |
| - | - |
| - | - |
| - | - |
| - | - |
| - | - |
| - | - |
| - | - |
| - | - |
| - | - |
| - | - |
| - | - |
| - | - |
| - | - |
| - | - |
| - | - |
| - | - |
| - | - |
| - | - |
| - | - |
| - | - |
| - | - |
| - | - |
| - | - |
| - | - |
| - | - |
| - | - |
| - | - |
| - | - |
| - | - |
| - | - |
| - | - |
| - | - |
| - | - |
| - | - |
| - | - |
| - | - |
| - | - |
| - | - |
| - | - |
| - | - |
| - | - |
| - | - |
| - | - |
| - | - |

| | |
|---|---|
| - | - |
| - | - |
| - | - |
| - | - |
| - | - |
| - | - |
| - | - |
| - | - |
| - | - |
| - | - |
| - | - |
| - | - |
| - | - |
| - | - |
| - | - |
| - | - |
| - | - |
| - | - |
| - | - |
| - | - |
| - | - |
| - | - |
| - | - |
| - | - |
| - | - |
| - | - |
| - | - |
| - | - |
| - | - |
| - | - |
| - | - |
| - | - |
| - | - |
| - | - |
| - | - |
| - | - |
| - | - |
| - | - |
| - | - |
| - | - |
| - | - |
| - | - |
| - | - |
| - | - |
| - | - |
| - | - |
| - | - |

| | |
|---|---|
| - | - |
| - | - |
| - | - |
| - | - |
| - | - |
| - | - |
| - | - |
| - | - |
| - | - |
| - | - |
| - | - |
| - | - |
| - | - |
| - | - |
| - | - |
| - | - |
| - | - |
| - | - |
| - | - |
| - | - |
| - | - |
| - | - |
| - | - |
| - | - |
| - | - |
| - | - |
| - | - |
| - | - |
| - | - |
| - | - |
| - | - |
| - | - |
| - | - |
| - | - |
| - | - |
| - | - |
| - | - |
| - | - |
| - | - |
| - | - |
| - | - |
| - | - |
| - | - |
| - | - |
| - | - |
| - | - |
| - | - |

| | |
|---|---|
| - | - |
| - | - |
| - | - |
| - | - |
| - | - |
| - | - |
| - | - |
| - | - |
| - | - |
| - | - |
| - | - |
| - | - |
| - | - |
| - | - |
| - | - |
| - | - |
| - | - |
| - | - |
| - | - |
| - | - |
| - | - |
| - | - |
| - | - |
| - | - |
| - | gnl\|UG\|Ta#S12864179 |
| - | gnl\|UG\|Ta#S12989535 |
| - | gnl\|UG\|Ta#S13168511 |
| - | gnl\|UG\|Ta#S16058021 |
| - | gnl\|UG\|Ta#S17972207 |
| - | gnl\|UG\|Ta#S37842046 |
| - | gnl\|UG\|Ta#S52541121 |
| - | gnl\|UG\|Ta#S52542770 |
| - | gnl\|UG\|Ta#S52544003 |
| - | gnl\|UG\|Ta#S52544173 |
| - | gnl\|UG\|Ta#S52544642 |
| - | gnl\|UG\|Ta#S58849737 |
| - | gnl\|UG\|Ta#S58882570 |
| - | gnl\|UG\|Ta#S58882570 |
| - | gnl\|UG\|Ta#S58886514 |
| - | gnl\|UG\|Ta#S58893514 |
| - | gnl\|UG\|Ta#S58893575 |
| - | gnl\|UG\|Ta#S58895933 |
| - | gnl\|UG\|Ta#S58912125 |
| - | gnl\|UG\|Ta#S61516556 |
| - | gnl\|UG\|Ta#S61519802 |
| - | gnl\|UG\|Ta#S61520913 |
| - | gnl\|UG\|Ta#S61526987 |

| | |
|---|---|
| - | gnl\|UG\|Ta#S61533425 |
| - | gnl\|UG\|Ta#S61537445 |
| - | gnl\|UG\|Ta#S61550910 |
| - | gnl\|UG\|Ta#S61569276 |
| - | gnl\|UG\|Ta#S61577520 |
| - | gnl\|UG\|Ta#S61600810 |
| - | gnl\|UG\|Ta#S61624030 |
| - | gnl\|UG\|Ta#S61626670 |
| - | gnl\|UG\|Ta#S61644576 |
| - | gnl\|UG\|Ta#S61648747 |
| - | gnl\|UG\|Ta#S61649374 |
| - | gnl\|UG\|Ta#S61652846 |
| - | gnl\|UG\|Ta#S61653626 |
| - | gnl\|UG\|Ta#S61657691 |
| - | gnl\|UG\|Ta#S61669373 |
| - | gnl\|UG\|Ta#S61674062 |
| - | gnl\|UG\|Ta#S61684064 |
| - | gnl\|UG\|Ta#S61687310 |
| - | gnl\|UG\|Ta#S61691487 |
| - | gnl\|UG\|Ta#S61695531 |
| - | gnl\|UG\|Ta#S61705196 |
| - | gnl\|UG\|Ta#S61733365 |
| - | gnl\|UG\|Ta#S61746469 |
| - | gnl\|UG\|Ta#S61754155 |
| - | gnl\|UG\|Ta#S61780856 |
| - | gnl\|UG\|Ta#S61780998 |
| - | gnl\|UG\|Ta#S61781231 |
| - | gnl\|UG\|Ta#S61781775 |
| - | gnl\|UG\|Ta#S61781838 |
| - | gnl\|UG\|Ta#S61782182 |
| - | gnl\|UG\|Ta#S61784126 |
| - | gnl\|UG\|Ta#S61791388 |
| - | gnl\|UG\|Ta#S61792222 |
| - | gnl\|UG\|Ta#S61800560 |
| - | gnl\|UG\|Ta#S61801987 |
| - | gnl\|UG\|Ta#S61807819 |
| - | gnl\|UG\|Ta#S61807929 |
| - | gnl\|UG\|Ta#S61808827 |
| - | gnl\|UG\|Ta#S61809253 |
| - | gnl\|UG\|Ta#S61809277 |
| - | gnl\|UG\|Ta#S61809364 |
| - | gnl\|UG\|Ta#S61812083 |
| - | gnl\|UG\|Ta#S61812279 |
| - | gnl\|UG\|Ta#S61812354 |
| - | gnl\|UG\|Ta#S61812410 |
| - | gnl\|UG\|Ta#S61812511 |
| - | gnl\|UG\|Ta#S61825075 |

| | |
|---|---|
| - | gnl\|UG\|Ta#S61827599 |
| - | gnl\|UG\|Ta#S61828864 |
| - | gnl\|UG\|Ta#S61828936 |
| - | gnl\|UG\|Ta#S61830032 |
| - | gnl\|UG\|Ta#S61830078 |
| - | gnl\|UG\|Ta#S61830923 |
| - | gnl\|UG\|Ta#S61831079 |
| - | gnl\|UG\|Ta#S61831090 |
| - | gnl\|UG\|Ta#S61831573 |
| - | gnl\|UG\|Ta#S61835950 |
| - | gnl\|UG\|Ta#S61838399 |
| - | gnl\|UG\|Ta#S61840817 |
| - | gnl\|UG\|Ta#S61850076 |
| - | gnl\|UG\|Ta#S61851226 |
| - | gnl\|UG\|Ta#S61862542 |
| - | gnl\|UG\|Ta#S65592792 |
| - | gnl\|UG\|Ta#S65593731 |
| - | gnl\|UG\|Ta#S65630994 |
| - | gnl\|UG\|Ta#S65663454 |
| - | gnl\|UG\|Ta#S65674860 |
| - | gnl\|UG\|Ta#S65687679 |
| - | gnl\|UG\|Ta#S65704608 |
| tr\|M7YVE5\|M7YVE5_TRIUA | - |
| tr\|M7Z7S5\|M7Z7S5_TRIUA | - |
| tr\|T1LCQ7\|T1LCQ7_TRIUA | - |
| tr\|T1MIW2\|T1MIW2_TRIUA | - |
| tr\|W4ZTX2\|W4ZTX2_WHEAT | - |
| tr\|W5DUD0\|W5DUD0_WHEAT | - |
| tr\|W5F4A5\|W5F4A5_WHEAT | - |
| tr\|W5F9X3\|W5F9X3_WHEAT | - |
| tr\|W5FBH1\|W5FBH1_WHEAT | - |
| tr\|W5FBN8\|W5FBN8_WHEAT | - |
| tr\|W5FCP9\|W5FCP9_WHEAT | - |
| tr\|W5FF85\|W5FF85_WHEAT | - |
| tr\|W5FFP8\|W5FFP8_WHEAT | - |
| tr\|W5FG37\|W5FG37_WHEAT | - |
| tr\|W5FGQ6\|W5FGQ6_WHEAT | - |
| tr\|W5FH11\|W5FH11_WHEAT | - |
| tr\|W5FHH6\|W5FHH6_WHEAT | - |
| tr\|W5FIL0\|W5FIL0_WHEAT | - |
| tr\|W5FJ03\|W5FJ03_WHEAT | - |
| tr\|W5FJB9\|W5FJB9_WHEAT | - |
| tr\|W5FJD9\|W5FJD9_WHEAT | - |
| tr\|W5FMN6\|W5FMN6_WHEAT | - |
| tr\|W5G2P6\|W5G2P6_WHEAT | - |
| tr\|W5FBV3\|W5FBV3_WHEAT | gnl\|UG\|Ta#S12902004 |
| tr\|W5FP26\|W5FP26_WHEAT | gnl\|UG\|Ta#S37755546 |

| | |
|---|---|
| tr\|W5FAR1\|W5FAR1_WHEAT | gnl\|UG\|Ta#S52542530 |
| tr\|T1LC05\|T1LC05_TRIUA | gnl\|UG\|Ta#S52543615 |
| tr\|W5FA91\|W5FA91_WHEAT | gnl\|UG\|Ta#S52545646 |
| tr\|M7ZWZ8\|M7ZWZ8_TRIUA | gnl\|UG\|Ta#S52546782 |
| tr\|W5FHA3\|W5FHA3_WHEAT | gnl\|UG\|Ta#S61622478 |
| tr\|W5FL94\|W5FL94_WHEAT | gnl\|UG\|Ta#S61669373 |
| tr\|W5FEB2\|W5FEB2_WHEAT | gnl\|UG\|Ta#S61697984 |
| tr\|M8A2Y2\|M8A2Y2_TRIUA | gnl\|UG\|Ta#S61711035 |
| tr\|W5FEE9\|W5FEE9_WHEAT | gnl\|UG\|Ta#S61766989 |
| tr\|W5DZN5\|W5DZN5_WHEAT | gnl\|UG\|Ta#S61791038 |
| tr\|M7Z407\|M7Z407_TRIUA | gnl\|UG\|Ta#S61791928 |
| tr\|W5FPY0\|W5FPY0_WHEAT | gnl\|UG\|Ta#S61792090 |
| tr\|M8A948\|M8A948_TRIUA | gnl\|UG\|Ta#S61808980 |
| tr\|W5DP41\|W5DP41_WHEAT | gnl\|UG\|Ta#S61809596 |
| tr\|W5FUI9\|W5FUI9_WHEAT | gnl\|UG\|Ta#S61810015 |
| tr\|W5F785\|W5F785_WHEAT | gnl\|UG\|Ta#S61811923 |
| tr\|W5FMI3\|W5FMI3_WHEAT | gnl\|UG\|Ta#S61828625 |
| tr\|W5F1B2\|W5F1B2_WHEAT | gnl\|UG\|Ta#S61828978 |
| tr\|W5FMX9\|W5FMX9_WHEAT | gnl\|UG\|Ta#S61829756 |
| tr\|W5FBM8\|W5FBM8_WHEAT | gnl\|UG\|Ta#S61830138 |
| tr\|W5FKD4\|W5FKD4_WHEAT | gnl\|UG\|Ta#S61832175 |
| tr\|W5FES8\|W5FES8_WHEAT | gnl\|UG\|Ta#S61868261 |
| tr\|W5F9F6\|W5F9F6_WHEAT | gnl\|UG\|Ta#S65606997 |
| tr\|W5FAD5\|W5FAD5_WHEAT | gnl\|UG\|Ta#S65616923 |
| tr\|W5FB66\|W5FB66_WHEAT | gnl\|UG\|Ta#S65674715 |
| tr\|W5F7U1\|W5F7U1_WHEAT | gnl\|UG\|Ta#S65687779 |
| tr\|W5G4J4\|W5G4J4_WHEAT | gnl\|UG\|Ta#S65715434 |
| - | - |
| - | - |
| - | - |
| - | - |
| - | - |
| - | - |
| - | - |
| - | - |
| - | - |
| - | - |
| - | - |
| - | - |
| - | - |
| - | - |
| - | - |
| - | - |
| - | - |
| - | - |
| - | - |
| - | - |

| | |
|---|---|
| - | - |
| - | - |
| - | - |
| - | - |
| - | - |
| - | - |
| - | - |
| - | - |
| - | - |
| - | - |
| - | - |
| - | - |
| - | - |
| - | - |
| - | - |
| - | - |
| - | - |
| - | - |
| - | - |
| - | - |
| - | - |
| - | - |
| - | - |
| - | - |
| - | - |
| - | - |
| - | - |
| - | - |
| - | - |
| - | - |
| - | - |
| - | - |
| - | - |
| - | - |
| - | - |
| - | - |
| - | - |
| - | - |
| - | - |
| - | - |
| - | - |
| - | gnl\|UG\|Ta#S12864179 |
| - | gnl\|UG\|Ta#S16057969 |
| - | gnl\|UG\|Ta#S16057969 |
| - | gnl\|UG\|Ta#S16058184 |
| - | gnl\|UG\|Ta#S22378223 |
| - | gnl\|UG\|Ta#S22378671 |

| | |
|---|---|
| - | gnl\|UG\|Ta#S43837843 |
| - | gnl\|UG\|Ta#S52542198 |
| - | gnl\|UG\|Ta#S52545434 |
| - | gnl\|UG\|Ta#S52545434 |
| - | gnl\|UG\|Ta#S52545998 |
| - | gnl\|UG\|Ta#S58856884 |
| - | gnl\|UG\|Ta#S58864467 |
| - | gnl\|UG\|Ta#S58868541 |
| - | gnl\|UG\|Ta#S61524036 |
| - | gnl\|UG\|Ta#S61578820 |
| - | gnl\|UG\|Ta#S61582693 |
| - | gnl\|UG\|Ta#S61585194 |
| - | gnl\|UG\|Ta#S61594577 |
| - | gnl\|UG\|Ta#S61599754 |
| - | gnl\|UG\|Ta#S61600810 |
| - | gnl\|UG\|Ta#S61600810 |
| - | gnl\|UG\|Ta#S61636247 |
| - | gnl\|UG\|Ta#S61650367 |
| - | gnl\|UG\|Ta#S61671450 |
| - | gnl\|UG\|Ta#S61687310 |
| - | gnl\|UG\|Ta#S61711681 |
| - | gnl\|UG\|Ta#S61713164 |
| - | gnl\|UG\|Ta#S61747729 |
| - | gnl\|UG\|Ta#S61771319 |
| - | gnl\|UG\|Ta#S61781904 |
| - | gnl\|UG\|Ta#S61791731 |
| - | gnl\|UG\|Ta#S61798193 |
| - | gnl\|UG\|Ta#S61802086 |
| - | gnl\|UG\|Ta#S61802539 |
| - | gnl\|UG\|Ta#S61811195 |
| - | gnl\|UG\|Ta#S61811474 |
| - | gnl\|UG\|Ta#S61811749 |
| - | gnl\|UG\|Ta#S61811749 |
| - | gnl\|UG\|Ta#S61812279 |
| - | gnl\|UG\|Ta#S61812519 |
| - | gnl\|UG\|Ta#S61812538 |
| - | gnl\|UG\|Ta#S61812547 |
| - | gnl\|UG\|Ta#S61812547 |
| - | gnl\|UG\|Ta#S61828896 |
| - | gnl\|UG\|Ta#S61829445 |
| - | gnl\|UG\|Ta#S61829623 |
| - | gnl\|UG\|Ta#S61830735 |
| - | gnl\|UG\|Ta#S61831026 |
| - | gnl\|UG\|Ta#S61831079 |
| - | gnl\|UG\|Ta#S61831734 |
| - | gnl\|UG\|Ta#S61831734 |
| - | gnl\|UG\|Ta#S61832375 |

| | |
|---|---|
| - | gnl\|UG\|Ta#S61832540 |
| - | gnl\|UG\|Ta#S65633020 |
| - | gnl\|UG\|Ta#S65635614 |
| - | gnl\|UG\|Ta#S65642458 |
| - | gnl\|UG\|Ta#S65646387 |
| - | gnl\|UG\|Ta#S65702526 |
| tr\|M7YEC0\|M7YEC0_TRIUA | - |
| tr\|T1LUB7\|T1LUB7_TRIUA | - |
| tr\|W5A0T4\|W5A0T4_WHEAT | - |
| tr\|W5EU63\|W5EU63_WHEAT | - |
| tr\|W5EVZ7\|W5EVZ7_WHEAT | - |
| tr\|W5F6R8\|W5F6R8_WHEAT | - |
| tr\|W5F8X1\|W5F8X1_WHEAT | - |
| tr\|W5FA37\|W5FA37_WHEAT | - |
| tr\|W5FA37\|W5FA37_WHEAT | - |
| tr\|W5FB26\|W5FB26_WHEAT | - |
| tr\|W5FBP1\|W5FBP1_WHEAT | - |
| tr\|W5FF61\|W5FF61_WHEAT | - |
| tr\|W5FF85\|W5FF85_WHEAT | - |
| tr\|W5FFK3\|W5FFK3_WHEAT | - |
| tr\|W5FG11\|W5FG11_WHEAT | - |
| tr\|W5FGS4\|W5FGS4_WHEAT | - |
| tr\|W5FH13\|W5FH13_WHEAT | - |
| tr\|W5FHD1\|W5FHD1_WHEAT | - |
| tr\|W5FLE4\|W5FLE4_WHEAT | - |
| tr\|W5FLU0\|W5FLU0_WHEAT | - |
| tr\|W5FN26\|W5FN26_WHEAT | - |
| tr\|W5FNJ1\|W5FNJ1_WHEAT | - |
| tr\|W5FU92\|W5FU92_WHEAT | - |
| tr\|W5FWQ0\|W5FWQ0_WHEAT | - |
| tr\|M7Z1G7\|M7Z1G7_TRIUA | gnl\|UG\|Ta#S18006914 |
| tr\|W5FIJ1\|W5FIJ1_WHEAT | gnl\|UG\|Ta#S32652920 |
| tr\|W5FIC9\|W5FIC9_WHEAT | gnl\|UG\|Ta#S37753025 |
| tr\|T1MF73\|T1MF73_TRIUA | gnl\|UG\|Ta#S37875728 |
| tr\|W5F949\|W5F949_WHEAT | gnl\|UG\|Ta#S50389132 |
| tr\|M8AM83\|M8AM83_TRIUA | gnl\|UG\|Ta#S52541311 |
| tr\|W5FFH5\|W5FFH5_WHEAT | gnl\|UG\|Ta#S52542198 |
| tr\|W5F790\|W5F790_WHEAT | gnl\|UG\|Ta#S52542813 |
| tr\|B6E107\|B6E107_WHEAT | gnl\|UG\|Ta#S52542998 |
| tr\|W5FL74\|W5FL74_WHEAT | gnl\|UG\|Ta#S52543666 |
| tr\|W5FJB2\|W5FJB2_WHEAT | gnl\|UG\|Ta#S52544173 |
| tr\|M7ZTC5\|M7ZTC5_TRIUA | gnl\|UG\|Ta#S52544506 |
| tr\|W5FG37\|W5FG37_WHEAT | gnl\|UG\|Ta#S61541888 |
| tr\|W5FU87\|W5FU87_WHEAT | gnl\|UG\|Ta#S61545696 |
| tr\|W5FFB4\|W5FFB4_WHEAT | gnl\|UG\|Ta#S61549834 |
| tr\|W5FPJ8\|W5FPJ8_WHEAT | gnl\|UG\|Ta#S61583467 |
| tr\|T1M4G9\|T1M4G9_TRIUA | gnl\|UG\|Ta#S61589523 |

| | |
|---|---|
| tr\|W5FCE1\|W5FCE1_WHEAT | gnl\|UG\|Ta#S61605334 |
| tr\|W5FNX5\|W5FNX5_WHEAT | gnl\|UG\|Ta#S61674448 |
| tr\|W5FFD5\|W5FFD5_WHEAT | gnl\|UG\|Ta#S61711910 |
| tr\|W5F216\|W5F216_WHEAT | gnl\|UG\|Ta#S61790900 |
| tr\|W5FH72\|W5FH72_WHEAT | gnl\|UG\|Ta#S61799525 |
| tr\|M7ZIC8\|M7ZIC8_TRIUA | gnl\|UG\|Ta#S61809253 |
| tr\|W5FAD6\|W5FAD6_WHEAT | gnl\|UG\|Ta#S61812279 |
| tr\|W5EXU0\|W5EXU0_WHEAT | gnl\|UG\|Ta#S61812310 |
| tr\|W5FAL1\|W5FAL1_WHEAT | gnl\|UG\|Ta#S61812377 |
| tr\|W5FEI6\|W5FEI6_WHEAT | gnl\|UG\|Ta#S61812472 |
| tr\|W5FLE4\|W5FLE4_WHEAT | gnl\|UG\|Ta#S61812519 |
| tr\|W5FES1\|W5FES1_WHEAT | gnl\|UG\|Ta#S61812547 |
| tr\|W5FIN8\|W5FIN8_WHEAT | gnl\|UG\|Ta#S61827822 |
| tr\|W5FD03\|W5FD03_WHEAT | gnl\|UG\|Ta#S61828896 |
| tr\|W5FIP2\|W5FIP2_WHEAT | gnl\|UG\|Ta#S61829892 |
| tr\|M8A1P7\|M8A1P7_TRIUA | gnl\|UG\|Ta#S61831397 |
| tr\|W5F6W3\|W5F6W3_WHEAT | gnl\|UG\|Ta#S61843910 |
| tr\|W5FHT1\|W5FHT1_WHEAT | gnl\|UG\|Ta#S65596618 |
| tr\|W5FIH3\|W5FIH3_WHEAT | gnl\|UG\|Ta#S65610149 |
| tr\|M7Z9L5\|M7Z9L5_TRIUA | gnl\|UG\|Ta#S65622650 |
| tr\|W5FT51\|W5FT51_WHEAT | gnl\|UG\|Ta#S65627072 |
| tr\|M7ZXG9\|M7ZXG9_TRIUA | gnl\|UG\|Ta#S65701597 |
| - | - |
| - | - |
| - | - |
| - | - |
| - | - |
| - | - |
| - | - |
| - | - |
| - | - |
| - | - |
| - | - |
| - | - |
| - | - |
| - | - |
| - | - |
| - | - |
| - | - |
| - | - |
| - | - |
| - | - |
| - | - |
| - | - |
| - | - |
| - | - |
| - | - |

| | |
|---|---|
| - | - |
| - | - |
| - | - |
| - | - |
| - | - |
| - | - |
| - | - |
| - | - |
| - | - |
| - | - |
| - | - |
| - | - |
| - | - |
| - | - |
| - | - |
| - | - |
| - | - |
| - | - |
| - | - |
| - | - |
| - | - |
| - | - |
| - | - |
| - | - |
| - | - |
| - | - |
| - | - |
| - | - |
| - | - |
| - | - |
| - | - |
| - | - |
| - | - |
| - | - |
| - | - |
| - | - |
| - | - |
| - | - |
| - | - |
| - | - |
| - | - |
| - | - |
| - | - |
| - | - |
| - | - |
| - | - |
| - | - |

| | |
|---|---|
| - | - |
| - | - |
| - | - |
| - | - |
| - | - |
| - | - |
| - | - |
| - | - |
| - | - |
| - | - |
| - | - |
| - | - |
| - | - |
| - | gnl\|UG\|Ta#S16057917 |
| - | gnl\|UG\|Ta#S26021345 |
| - | gnl\|UG\|Ta#S32511849 |
| - | gnl\|UG\|Ta#S52542813 |
| - | gnl\|UG\|Ta#S52543318 |
| - | gnl\|UG\|Ta#S52543703 |
| - | gnl\|UG\|Ta#S52543703 |
| - | gnl\|UG\|Ta#S52543760 |
| - | gnl\|UG\|Ta#S52544735 |
| - | gnl\|UG\|Ta#S52544735 |
| - | gnl\|UG\|Ta#S52545421 |
| - | gnl\|UG\|Ta#S52545489 |
| - | gnl\|UG\|Ta#S52545921 |
| - | gnl\|UG\|Ta#S52545940 |
| - | gnl\|UG\|Ta#S52546432 |
| - | gnl\|UG\|Ta#S58852847 |
| - | gnl\|UG\|Ta#S58868541 |
| - | gnl\|UG\|Ta#S61519787 |
| - | gnl\|UG\|Ta#S61532276 |
| - | gnl\|UG\|Ta#S61533505 |
| - | gnl\|UG\|Ta#S61538348 |
| - | gnl\|UG\|Ta#S61538348 |
| - | gnl\|UG\|Ta#S61543979 |
| - | gnl\|UG\|Ta#S61558759 |
| - | gnl\|UG\|Ta#S61595665 |
| - | gnl\|UG\|Ta#S61599608 |
| - | gnl\|UG\|Ta#S61599608 |
| - | gnl\|UG\|Ta#S61599754 |
| - | gnl\|UG\|Ta#S61600810 |
| - | gnl\|UG\|Ta#S61607436 |
| - | gnl\|UG\|Ta#S61671309 |
| - | gnl\|UG\|Ta#S61671376 |
| - | gnl\|UG\|Ta#S61671805 |
| - | gnl\|UG\|Ta#S61718012 |

| | |
|---|---|
| - | gnl\|UG\|Ta#S61723667 |
| - | gnl\|UG\|Ta#S61755040 |
| - | gnl\|UG\|Ta#S61771988 |
| - | gnl\|UG\|Ta#S61772135 |
| - | gnl\|UG\|Ta#S61772547 |
| - | gnl\|UG\|Ta#S61773954 |
| - | gnl\|UG\|Ta#S61779647 |
| - | gnl\|UG\|Ta#S61807929 |
| - | gnl\|UG\|Ta#S61808856 |
| - | gnl\|UG\|Ta#S61809205 |
| - | gnl\|UG\|Ta#S61809250 |
| - | gnl\|UG\|Ta#S61809253 |
| - | gnl\|UG\|Ta#S61809487 |
| - | gnl\|UG\|Ta#S61810680 |
| - | gnl\|UG\|Ta#S61810728 |
| - | gnl\|UG\|Ta#S61810841 |
| - | gnl\|UG\|Ta#S61812018 |
| - | gnl\|UG\|Ta#S61812018 |
| - | gnl\|UG\|Ta#S61812441 |
| - | gnl\|UG\|Ta#S61812469 |
| - | gnl\|UG\|Ta#S61812472 |
| - | gnl\|UG\|Ta#S61829426 |
| - | gnl\|UG\|Ta#S61829760 |
| - | gnl\|UG\|Ta#S61829760 |
| - | gnl\|UG\|Ta#S61829968 |
| - | gnl\|UG\|Ta#S61830425 |
| - | gnl\|UG\|Ta#S61830468 |
| - | gnl\|UG\|Ta#S61830814 |
| - | gnl\|UG\|Ta#S61830827 |
| - | gnl\|UG\|Ta#S61831342 |
| - | gnl\|UG\|Ta#S65601476 |
| - | gnl\|UG\|Ta#S65605638 |
| - | gnl\|UG\|Ta#S65687779 |
| - | gnl\|UG\|Ta#S65693870 |
| - | gnl\|UG\|Ta#S65701597 |
| tr\|M7YVW9\|M7YVW9_TRIUA | - |
| tr\|M7ZXH3\|M7ZXH3_TRIUA | - |
| tr\|M8A7I6\|M8A7I6_TRIUA | - |
| tr\|W5DMZ4\|W5DMZ4_WHEAT | - |
| tr\|W5DUC1\|W5DUC1_WHEAT | - |
| tr\|W5F119\|W5F119_WHEAT | - |
| tr\|W5F7J5\|W5F7J5_WHEAT | - |
| tr\|W5F8P8\|W5F8P8_WHEAT | - |
| tr\|W5F8Q9\|W5F8Q9_WHEAT | - |
| tr\|W5FF85\|W5FF85_WHEAT | - |
| tr\|W5FKR7\|W5FKR7_WHEAT | - |
| tr\|W5FMF5\|W5FMF5_WHEAT | - |

| | |
|---|---|
| tr\|W5FMG2\|W5FMG2_WHEAT | - |
| tr\|W5FNZ9\|W5FNZ9_WHEAT | - |
| tr\|W5G4H6\|W5G4H6_WHEAT | - |
| tr\|W5G4W2\|W5G4W2_WHEAT | - |
| tr\|W5F0P2\|W5F0P2_WHEAT | gnl\|UG\|Ta#S37747465 |
| tr\|W5G4E6\|W5G4E6_WHEAT | gnl\|UG\|Ta#S52542447 |
| tr\|M7YN39\|M7YN39_TRIUA | gnl\|UG\|Ta#S58902682 |
| tr\|W5EWR6\|W5EWR6_WHEAT | gnl\|UG\|Ta#S58904400 |
| tr\|W5FGG3\|W5FGG3_WHEAT | gnl\|UG\|Ta#S61573692 |
| tr\|W5FHY7\|W5FHY7_WHEAT | gnl\|UG\|Ta#S61599608 |
| tr\|W5FHY7\|W5FHY7_WHEAT | gnl\|UG\|Ta#S61599608 |
| tr\|W5FD21\|W5FD21_WHEAT | gnl\|UG\|Ta#S61608154 |
| tr\|W5FBU4\|W5FBU4_WHEAT | gnl\|UG\|Ta#S61632658 |
| tr\|W5FET3\|W5FET3_WHEAT | gnl\|UG\|Ta#S61652381 |
| tr\|W5FHW8\|W5FHW8_WHEAT | gnl\|UG\|Ta#S61709136 |
| tr\|M7YHM3\|M7YHM3_TRIUA | gnl\|UG\|Ta#S61792126 |
| tr\|M8A948\|M8A948_TRIUA | gnl\|UG\|Ta#S61808980 |
| tr\|M7YST5\|M7YST5_TRIUA | gnl\|UG\|Ta#S61809253 |
| tr\|M7Z177\|M7Z177_TRIUA | gnl\|UG\|Ta#S61809487 |
| tr\|W5F9M2\|W5F9M2_WHEAT | gnl\|UG\|Ta#S61812117 |
| tr\|W5G082\|W5G082_WHEAT | gnl\|UG\|Ta#S61819423 |
| tr\|E0Y9Z4\|E0Y9Z4_WHEAT | gnl\|UG\|Ta#S65662144 |
| tr\|W5FFB4\|W5FFB4_WHEAT | gnl\|UG\|Ta#S65674801 |
| - | - |
| - | - |
| - | - |
| - | - |
| - | - |
| - | - |
| - | - |
| - | - |
| - | - |
| - | - |
| - | - |
| - | - |
| - | - |
| - | - |
| - | - |
| - | - |
| - | - |
| - | - |
| - | - |
| - | - |
| - | - |
| - | - |
| - | - |
| - | - |

| | |
|---|---|
| - | - |
| - | - |
| - | - |
| - | - |
| - | - |
| - | - |
| - | - |
| - | - |
| - | - |
| - | - |
| - | - |
| - | - |
| - | - |
| - | - |
| - | - |
| - | - |
| - | - |
| - | - |
| - | - |
| - | - |
| - | - |
| - | - |
| - | - |
| - | - |
| - | - |
| - | - |
| - | - |
| - | - |
| - | gnl\|UG\|Ta#S21008213 |
| - | gnl\|UG\|Ta#S37877799 |
| - | gnl\|UG\|Ta#S43837843 |
| - | gnl\|UG\|Ta#S43837843 |
| - | gnl\|UG\|Ta#S46933443 |
| - | gnl\|UG\|Ta#S52541593 |
| - | gnl\|UG\|Ta#S52542140 |
| - | gnl\|UG\|Ta#S52542198 |
| - | gnl\|UG\|Ta#S52542527 |
| - | gnl\|UG\|Ta#S52542912 |
| - | gnl\|UG\|Ta#S52543067 |
| - | gnl\|UG\|Ta#S52543364 |
| - | gnl\|UG\|Ta#S52543364 |
| - | gnl\|UG\|Ta#S52543475 |
| - | gnl\|UG\|Ta#S52544471 |
| - | gnl\|UG\|Ta#S52544506 |
| - | gnl\|UG\|Ta#S52545541 |
| - | gnl\|UG\|Ta#S58850121 |

| | |
|---|---|
| - | gnl\|UG\|Ta#S58853822 |
| - | gnl\|UG\|Ta#S58855217 |
| - | gnl\|UG\|Ta#S58857532 |
| - | gnl\|UG\|Ta#S58895933 |
| - | gnl\|UG\|Ta#S58897472 |
| - | gnl\|UG\|Ta#S58903613 |
| - | gnl\|UG\|Ta#S61561773 |
| - | gnl\|UG\|Ta#S61563963 |
| - | gnl\|UG\|Ta#S61568375 |
| - | gnl\|UG\|Ta#S61568791 |
| - | gnl\|UG\|Ta#S61569683 |
| - | gnl\|UG\|Ta#S61572680 |
| - | gnl\|UG\|Ta#S61581344 |
| - | gnl\|UG\|Ta#S61585301 |
| - | gnl\|UG\|Ta#S61585301 |
| - | gnl\|UG\|Ta#S61593513 |
| - | gnl\|UG\|Ta#S61596468 |
| - | gnl\|UG\|Ta#S61598372 |
| - | gnl\|UG\|Ta#S61600597 |
| - | gnl\|UG\|Ta#S61600810 |
| - | gnl\|UG\|Ta#S61604779 |
| - | gnl\|UG\|Ta#S61609459 |
| - | gnl\|UG\|Ta#S61611803 |
| - | gnl\|UG\|Ta#S61612725 |
| - | gnl\|UG\|Ta#S61614098 |
| - | gnl\|UG\|Ta#S61614134 |
| - | gnl\|UG\|Ta#S61614134 |
| - | gnl\|UG\|Ta#S61632658 |
| - | gnl\|UG\|Ta#S61634748 |
| - | gnl\|UG\|Ta#S61634748 |
| - | gnl\|UG\|Ta#S61634748 |
| - | gnl\|UG\|Ta#S61639865 |
| - | gnl\|UG\|Ta#S61641130 |
| - | gnl\|UG\|Ta#S61658231 |
| - | gnl\|UG\|Ta#S61659119 |
| - | gnl\|UG\|Ta#S61670340 |
| - | gnl\|UG\|Ta#S61688932 |
| - | gnl\|UG\|Ta#S61696975 |
| - | gnl\|UG\|Ta#S61718247 |
| - | gnl\|UG\|Ta#S61730251 |
| - | gnl\|UG\|Ta#S61730589 |
| - | gnl\|UG\|Ta#S61737240 |
| - | gnl\|UG\|Ta#S61757407 |
| - | gnl\|UG\|Ta#S61770308 |
| - | gnl\|UG\|Ta#S61778485 |
| - | gnl\|UG\|Ta#S61780151 |
| - | gnl\|UG\|Ta#S61780360 |

| | |
|---|---|
| - | gnl\|UG\|Ta#S61781904 |
| - | gnl\|UG\|Ta#S61800433 |
| - | gnl\|UG\|Ta#S61807921 |
| - | gnl\|UG\|Ta#S61809996 |
| - | gnl\|UG\|Ta#S61810841 |
| - | gnl\|UG\|Ta#S61812117 |
| - | gnl\|UG\|Ta#S61812265 |
| - | gnl\|UG\|Ta#S61812354 |
| - | gnl\|UG\|Ta#S61812441 |
| - | gnl\|UG\|Ta#S61812472 |
| - | gnl\|UG\|Ta#S61812472 |
| - | gnl\|UG\|Ta#S61812527 |
| - | gnl\|UG\|Ta#S61812527 |
| - | gnl\|UG\|Ta#S61812530 |
| - | gnl\|UG\|Ta#S61812530 |
| - | gnl\|UG\|Ta#S61817837 |
| - | gnl\|UG\|Ta#S61827942 |
| - | gnl\|UG\|Ta#S61830468 |
| - | gnl\|UG\|Ta#S61830468 |
| - | gnl\|UG\|Ta#S61830468 |
| - | gnl\|UG\|Ta#S61830490 |
| - | gnl\|UG\|Ta#S61831570 |
| - | gnl\|UG\|Ta#S61831570 |
| - | gnl\|UG\|Ta#S61831623 |
| - | gnl\|UG\|Ta#S61831734 |
| - | gnl\|UG\|Ta#S61831857 |
| - | gnl\|UG\|Ta#S61831934 |
| - | gnl\|UG\|Ta#S61832238 |
| - | gnl\|UG\|Ta#S61832278 |
| - | gnl\|UG\|Ta#S65591986 |
| - | gnl\|UG\|Ta#S65592629 |
| - | gnl\|UG\|Ta#S65608357 |
| - | gnl\|UG\|Ta#S65610767 |
| - | gnl\|UG\|Ta#S65628183 |
| - | gnl\|UG\|Ta#S65630293 |
| - | gnl\|UG\|Ta#S65630293 |
| - | gnl\|UG\|Ta#S65634660 |
| - | gnl\|UG\|Ta#S65648968 |
| - | gnl\|UG\|Ta#S65662200 |
| - | gnl\|UG\|Ta#S65716526 |
| - | gnl\|UG\|Ta#S65719718 |
| tr\|M7Z3S1\|M7Z3S1_TRIUA | - |
| tr\|M8A8X2\|M8A8X2_TRIUA | - |
| tr\|T1MII9\|T1MII9_TRIUA | - |
| tr\|T1MRZ1\|T1MRZ1_TRIUA | - |
| tr\|W4ZVY9\|W4ZVY9_WHEAT | - |
| tr\|W4ZWV7\|W4ZWV7_WHEAT | - |

| | |
|---|---|
| tr\|W5F0Q6\|W5F0Q6_WHEAT | - |
| tr\|W5F5A5\|W5F5A5_WHEAT | - |
| tr\|W5F6Z0\|W5F6Z0_WHEAT | - |
| tr\|W5F8N2\|W5F8N2_WHEAT | - |
| tr\|W5F9L9\|W5F9L9_WHEAT | - |
| tr\|W5FAL2\|W5FAL2_WHEAT | - |
| tr\|W5FAV6\|W5FAV6_WHEAT | - |
| tr\|W5FC10\|W5FC10_WHEAT | - |
| tr\|W5FDK3\|W5FDK3_WHEAT | - |
| tr\|W5FF85\|W5FF85_WHEAT | - |
| tr\|W5FFD5\|W5FFD5_WHEAT | - |
| tr\|W5FFU8\|W5FFU8_WHEAT | - |
| tr\|W5FFZ6\|W5FFZ6_WHEAT | - |
| tr\|W5FGR4\|W5FGR4_WHEAT | - |
| tr\|W5FHZ3\|W5FHZ3_WHEAT | - |
| tr\|W5FIG6\|W5FIG6_WHEAT | - |
| tr\|W5FK57\|W5FK57_WHEAT | - |
| tr\|W5FL32\|W5FL32_WHEAT | - |
| tr\|W5FMQ2\|W5FMQ2_WHEAT | - |
| tr\|W5FN43\|W5FN43_WHEAT | - |
| tr\|W5FNU8\|W5FNU8_WHEAT | - |
| tr\|W5FP67\|W5FP67_WHEAT | - |
| tr\|W5HZD7\|W5HZD7_WHEAT | - |
| tr\|W5F8W0\|W5F8W0_WHEAT | gnl\|UG\|Ta#S12906881 |
| tr\|W5FN04\|W5FN04_WHEAT | gnl\|UG\|Ta#S24623068 |
| tr\|W5F8M3\|W5F8M3_WHEAT | gnl\|UG\|Ta#S37780295 |
| tr\|W5FGX1\|W5FGX1_WHEAT | gnl\|UG\|Ta#S52543344 |
| tr\|M8AIH1\|M8AIH1_TRIUA | gnl\|UG\|Ta#S52545998 |
| tr\|W5FIZ0\|W5FIZ0_WHEAT | gnl\|UG\|Ta#S58853822 |
| tr\|M8ANT8\|M8ANT8_TRIUA | gnl\|UG\|Ta#S58895933 |
| tr\|W5FPJ8\|W5FPJ8_WHEAT | gnl\|UG\|Ta#S61583467 |
| tr\|W5FN83\|W5FN83_WHEAT | gnl\|UG\|Ta#S61601664 |
| tr\|W5FQ82\|W5FQ82_WHEAT | gnl\|UG\|Ta#S61607314 |
| tr\|M7YXS5\|M7YXS5_TRIUA | gnl\|UG\|Ta#S61613729 |
| tr\|M8AK09\|M8AK09_TRIUA | gnl\|UG\|Ta#S61634748 |
| tr\|M8AK09\|M8AK09_TRIUA | gnl\|UG\|Ta#S61634748 |
| tr\|W5FEV6\|W5FEV6_WHEAT | gnl\|UG\|Ta#S61634748 |
| tr\|W5FEV6\|W5FEV6_WHEAT | gnl\|UG\|Ta#S61634748 |
| tr\|W5FVA5\|W5FVA5_WHEAT | gnl\|UG\|Ta#S61649849 |
| tr\|M7ZVP6\|M7ZVP6_TRIUA | gnl\|UG\|Ta#S61711885 |
| tr\|W5FCG4\|W5FCG4_WHEAT | gnl\|UG\|Ta#S61730879 |
| tr\|W5F3G2\|W5F3G2_WHEAT | gnl\|UG\|Ta#S61759667 |
| tr\|W5FBE4\|W5FBE4_WHEAT | gnl\|UG\|Ta#S61781024 |
| tr\|W5F799\|W5F799_WHEAT | gnl\|UG\|Ta#S61781428 |
| tr\|W5FG14\|W5FG14_WHEAT | gnl\|UG\|Ta#S61781904 |
| tr\|M7ZFX1\|M7ZFX1_TRIUA | gnl\|UG\|Ta#S61800556 |
| tr\|M7YFL2\|M7YFL2_TRIUA | gnl\|UG\|Ta#S61807929 |

| | |
|---|---|
| tr\|W5FEN6\|W5FEN6_WHEAT | gnl\|UG\|Ta#S61808180 |
| tr\|W5FIW6\|W5FIW6_WHEAT | gnl\|UG\|Ta#S61810348 |
| tr\|W5FJE0\|W5FJE0_WHEAT | gnl\|UG\|Ta#S61810530 |
| tr\|W5FEQ3\|W5FEQ3_WHEAT | gnl\|UG\|Ta#S61812317 |
| tr\|W5FNL9\|W5FNL9_WHEAT | gnl\|UG\|Ta#S61812476 |
| tr\|M7YJF8\|M7YJF8_TRIUA | gnl\|UG\|Ta#S61830662 |
| tr\|M7ZCX5\|M7ZCX5_TRIUA | gnl\|UG\|Ta#S61830834 |
| tr\|W5FH03\|W5FH03_WHEAT | gnl\|UG\|Ta#S61831249 |
| tr\|W5FLW7\|W5FLW7_WHEAT | gnl\|UG\|Ta#S61831644 |
| tr\|W5FE68\|W5FE68_WHEAT | gnl\|UG\|Ta#S61831928 |
| tr\|W5FA80\|W5FA80_WHEAT | gnl\|UG\|Ta#S61832265 |
| tr\|M8A6D0\|M8A6D0_TRIUA | gnl\|UG\|Ta#S61839105 |
| tr\|W5F949\|W5F949_WHEAT | gnl\|UG\|Ta#S61856293 |
| tr\|W5DL93\|W5DL93_WHEAT | gnl\|UG\|Ta#S61862782 |
| tr\|W5FJU8\|W5FJU8_WHEAT | gnl\|UG\|Ta#S65592692 |
| tr\|W5FHZ3\|W5FHZ3_WHEAT | gnl\|UG\|Ta#S65625570 |
| tr\|W5FBZ9\|W5FBZ9_WHEAT | gnl\|UG\|Ta#S65645571 |
| tr\|M7ZPX9\|M7ZPX9_TRIUA | gnl\|UG\|Ta#S65674281 |
| tr\|W5FM30\|W5FM30_WHEAT | gnl\|UG\|Ta#S65674281 |
| tr\|M7YKY4\|M7YKY4_TRIUA | gnl\|UG\|Ta#S65719718 |
| - | - |
| - | - |
| - | - |
| - | - |
| - | - |
| - | - |
| - | - |
| - | - |
| - | - |
| - | - |
| - | - |
| - | - |
| - | - |
| - | - |
| - | - |
| - | - |
| - | - |
| - | - |
| - | - |
| - | - |
| - | - |
| - | - |
| - | - |
| - | - |
| - | - |
| - | - |
| - | - |

| | |
|---|---|
| - | - |
| - | - |
| - | - |
| - | - |
| - | - |
| - | - |
| - | - |
| - | - |
| - | - |
| - | - |
| - | - |
| - | - |
| - | - |
| - | - |
| - | - |
| - | - |
| - | - |
| - | - |
| - | - |
| - | - |
| - | - |
| - | - |
| - | - |
| - | - |
| - | - |
| - | - |
| - | - |
| - | - |
| - | - |
| - | - |
| - | - |
| - | - |
| - | - |
| - | gnl\|UG\|Ta#S22388699 |
| - | gnl\|UG\|Ta#S52543417 |
| - | gnl\|UG\|Ta#S52543703 |
| - | gnl\|UG\|Ta#S52543920 |
| - | gnl\|UG\|Ta#S52544173 |
| - | gnl\|UG\|Ta#S52545940 |
| - | gnl\|UG\|Ta#S58854185 |
| - | gnl\|UG\|Ta#S58900038 |
| - | gnl\|UG\|Ta#S58908769 |
| - | gnl\|UG\|Ta#S61541540 |
| - | gnl\|UG\|Ta#S61554479 |
| - | gnl\|UG\|Ta#S61556371 |
| - | gnl\|UG\|Ta#S61594194 |
| - | gnl\|UG\|Ta#S61594194 |

| | |
|---|---|
| - | gnl\|UG\|Ta#S61597200 |
| - | gnl\|UG\|Ta#S61597200 |
| - | gnl\|UG\|Ta#S61613729 |
| - | gnl\|UG\|Ta#S61685283 |
| - | gnl\|UG\|Ta#S61694125 |
| - | gnl\|UG\|Ta#S61712033 |
| - | gnl\|UG\|Ta#S61731636 |
| - | gnl\|UG\|Ta#S61732880 |
| - | gnl\|UG\|Ta#S61734074 |
| - | gnl\|UG\|Ta#S61753636 |
| - | gnl\|UG\|Ta#S61789625 |
| - | gnl\|UG\|Ta#S61790707 |
| - | gnl\|UG\|Ta#S61800680 |
| - | gnl\|UG\|Ta#S61807819 |
| - | gnl\|UG\|Ta#S61808504 |
| - | gnl\|UG\|Ta#S61811479 |
| - | gnl\|UG\|Ta#S61811841 |
| - | gnl\|UG\|Ta#S61812316 |
| - | gnl\|UG\|Ta#S61830245 |
| - | gnl\|UG\|Ta#S61830418 |
| - | gnl\|UG\|Ta#S61831496 |
| - | gnl\|UG\|Ta#S61831531 |
| - | gnl\|UG\|Ta#S61832481 |
| - | gnl\|UG\|Ta#S61836423 |
| - | gnl\|UG\|Ta#S61846588 |
| - | gnl\|UG\|Ta#S61866621 |
| - | gnl\|UG\|Ta#S65599663 |
| - | gnl\|UG\|Ta#S65606779 |
| - | gnl\|UG\|Ta#S65624025 |
| tr\|W5DSN5\|W5DSN5_WHEAT | - |
| tr\|W5FCL5\|W5FCL5_WHEAT | - |
| tr\|W5FD08\|W5FD08_WHEAT | - |
| tr\|W5FD08\|W5FD08_WHEAT | - |
| tr\|W5FH29\|W5FH29_WHEAT | - |
| tr\|W5FH49\|W5FH49_WHEAT | - |
| tr\|W5FHP1\|W5FHP1_WHEAT | - |
| tr\|W5FHX1\|W5FHX1_WHEAT | - |
| tr\|W5FK94\|W5FK94_WHEAT | - |
| tr\|W5FKF0\|W5FKF0_WHEAT | - |
| tr\|W5FLE6\|W5FLE6_WHEAT | - |
| tr\|W5FNA2\|W5FNA2_WHEAT | - |
| tr\|W5FPV1\|W5FPV1_WHEAT | - |
| tr\|W5FW29\|W5FW29_WHEAT | - |
| tr\|W5G263\|W5G263_WHEAT | - |
| tr\|W5G4E4\|W5G4E4_WHEAT | - |
| tr\|W5FLB8\|W5FLB8_WHEAT | gnl\|UG\|Ta#S58848062 |
| sp\|Q9ST57\|SPZ2A_WHEAT | gnl\|UG\|Ta#S58869591 |

| | |
|---|---|
| tr\|W5FBM0\|W5FBM0_WHEAT | gnl\|UG\|Ta#S58897382 |
| tr\|W5FIJ6\|W5FIJ6_WHEAT | gnl\|UG\|Ta#S58902390 |
| tr\|W5F7J2\|W5F7J2_WHEAT | gnl\|UG\|Ta#S61564953 |
| tr\|M7ZRB8\|M7ZRB8_TRIUA | gnl\|UG\|Ta#S61696460 |
| tr\|W5EY59\|W5EY59_WHEAT | gnl\|UG\|Ta#S61782182 |
| tr\|M8A6V9\|M8A6V9_TRIUA | gnl\|UG\|Ta#S61801554 |
| tr\|W5DV32\|W5DV32_WHEAT | gnl\|UG\|Ta#S61811335 |
| tr\|W5FXY4\|W5FXY4_WHEAT | gnl\|UG\|Ta#S61812410 |
| tr\|W5FD08\|W5FD08_WHEAT | gnl\|UG\|Ta#S65685399 |
| - | - |
| - | - |
| - | - |
| - | - |
| - | - |
| - | - |
| - | - |
| - | - |
| - | - |
| - | - |
| - | - |
| - | - |
| - | - |
| - | - |
| - | - |
| - | - |
| - | - |
| - | - |
| - | - |
| - | - |
| - | - |
| - | - |
| - | - |
| - | - |
| - | gnl\|UG\|Ta#S17988750 |
| - | gnl\|UG\|Ta#S26024214 |
| - | gnl\|UG\|Ta#S37929544 |
| - | gnl\|UG\|Ta#S52541102 |
| - | gnl\|UG\|Ta#S52541772 |
| - | gnl\|UG\|Ta#S52542879 |
| - | gnl\|UG\|Ta#S52543615 |
| - | gnl\|UG\|Ta#S52544726 |
| - | gnl\|UG\|Ta#S52545000 |
| - | gnl\|UG\|Ta#S58853985 |
| - | gnl\|UG\|Ta#S58854874 |
| - | gnl\|UG\|Ta#S58903613 |
| - | gnl\|UG\|Ta#S61536339 |
| - | gnl\|UG\|Ta#S61548506 |

| | |
|---|---|
| - | gnl\|UG\|Ta#S61562659 |
| - | gnl\|UG\|Ta#S61568505 |
| - | gnl\|UG\|Ta#S61594912 |
| - | gnl\|UG\|Ta#S61597657 |
| - | gnl\|UG\|Ta#S61598372 |
| - | gnl\|UG\|Ta#S61600577 |
| - | gnl\|UG\|Ta#S61608592 |
| - | gnl\|UG\|Ta#S61611803 |
| - | gnl\|UG\|Ta#S61613729 |
| - | gnl\|UG\|Ta#S61616281 |
| - | gnl\|UG\|Ta#S61619410 |
| - | gnl\|UG\|Ta#S61633115 |
| - | gnl\|UG\|Ta#S61767278 |
| - | gnl\|UG\|Ta#S61771778 |
| - | gnl\|UG\|Ta#S61780591 |
| - | gnl\|UG\|Ta#S61780623 |
| - | gnl\|UG\|Ta#S61781350 |
| - | gnl\|UG\|Ta#S61792483 |
| - | gnl\|UG\|Ta#S61810690 |
| - | gnl\|UG\|Ta#S61811416 |
| - | gnl\|UG\|Ta#S61811928 |
| - | gnl\|UG\|Ta#S61812262 |
| - | gnl\|UG\|Ta#S61828446 |
| - | gnl\|UG\|Ta#S61828978 |
| - | gnl\|UG\|Ta#S61829435 |
| - | gnl\|UG\|Ta#S61830735 |
| - | gnl\|UG\|Ta#S61830735 |
| - | gnl\|UG\|Ta#S61831026 |
| - | gnl\|UG\|Ta#S61831026 |
| - | gnl\|UG\|Ta#S61832278 |
| - | gnl\|UG\|Ta#S61832522 |
| - | gnl\|UG\|Ta#S65604950 |
| tr\|M8A5M0\|M8A5M0_TRIUA | - |
| tr\|W5DUB8\|W5DUB8_WHEAT | - |
| tr\|W5F752\|W5F752_WHEAT | - |
| tr\|W5FCJ4\|W5FCJ4_WHEAT | - |
| tr\|W5FCU6\|W5FCU6_WHEAT | - |
| tr\|W5FEB3\|W5FEB3_WHEAT | - |
| tr\|W5FEX5\|W5FEX5_WHEAT | - |
| tr\|W5FG58\|W5FG58_WHEAT | - |
| tr\|W5FHW1\|W5FHW1_WHEAT | - |
| tr\|W5FI40\|W5FI40_WHEAT | - |
| tr\|W5FIM4\|W5FIM4_WHEAT | - |
| tr\|W5FKL2\|W5FKL2_WHEAT | - |
| tr\|W5FMW4\|W5FMW4_WHEAT | - |
| tr\|W5FVP1\|W5FVP1_WHEAT | - |
| tr\|W5FM77\|W5FM77_WHEAT | gnl\|UG\|Ta#S17985102 |

| | |
|---|---|
| tr|W5FAR1|W5FAR1_WHEAT | gnl|UG|Ta#S52542530 |
| tr|T1LC05|T1LC05_TRIUA | gnl|UG|Ta#S52543615 |
| tr|T1LC05|T1LC05_TRIUA | gnl|UG|Ta#S52543615 |
| tr|W5FJE5|W5FJE5_WHEAT | gnl|UG|Ta#S58894194 |
| tr|W5FNQ7|W5FNQ7_WHEAT | gnl|UG|Ta#S61592450 |
| tr|W5FAV9|W5FAV9_WHEAT | gnl|UG|Ta#S61611803 |
| tr|W5F7K2|W5F7K2_WHEAT | gnl|UG|Ta#S61664144 |
| tr|W5FMW4|W5FMW4_WHEAT | gnl|UG|Ta#S61704562 |
| tr|W5FHL8|W5FHL8_WHEAT | gnl|UG|Ta#S61749357 |
| tr|W5F7A9|W5F7A9_WHEAT | gnl|UG|Ta#S61765457 |
| tr|W5FPL2|W5FPL2_WHEAT | gnl|UG|Ta#S61782414 |
| tr|W5FAX1|W5FAX1_WHEAT | gnl|UG|Ta#S61790194 |
| tr|M8AE98|M8AE98_TRIUA | gnl|UG|Ta#S61790900 |
| tr|W5F7N6|W5F7N6_WHEAT | gnl|UG|Ta#S61799527 |
| tr|W5FHY6|W5FHY6_WHEAT | gnl|UG|Ta#S61809596 |
| tr|W5FUX6|W5FUX6_WHEAT | gnl|UG|Ta#S61811576 |
| tr|M7ZHC3|M7ZHC3_TRIUA | gnl|UG|Ta#S61812334 |
| tr|W5FG11|W5FG11_WHEAT | gnl|UG|Ta#S61827891 |
| tr|M7YMS0|M7YMS0_TRIUA | gnl|UG|Ta#S61828810 |
| tr|W5FAQ9|W5FAQ9_WHEAT | gnl|UG|Ta#S61830550 |
| tr|W5FI11|W5FI11_WHEAT | gnl|UG|Ta#S61831698 |
| tr|M7ZF26|M7ZF26_TRIUA | gnl|UG|Ta#S65600911 |
| tr|W5FJI4|W5FJI4_WHEAT | gnl|UG|Ta#S65622650 |
| - | - |
| - | - |
| - | - |
| - | - |
| - | - |
| - | - |
| - | - |
| - | - |
| - | - |
| - | - |
| - | - |
| - | - |
| - | - |
| - | - |
| - | - |
| - | - |
| - | - |
| - | - |
| - | - |
| - | - |
| - | - |
| - | - |
| - | - |
| - | - |

-
-
-
-
-
-
-
-
-
-
-
-
-
-
-
-
-
-
-
-
-
-
-
-
-
-
-
-
-
-
-
-
-
-
-
-
-
-
-
-
-
-
-
-
-
-
-
-
-
-
-
-
-
-
-
-
-
-
-
-
-
-
-
-
-
-
-
-
-
-
-
-
-
-
-
-
-
-
-
-
-
-
-
-
-
-
-
-
-
-
-
-
-
-
-
-
-
-
-
-

| | |
|---|---|
| - | - |
| - | - |
| - | - |
| - | - |
| - | - |
| - | - |
| - | - |
| - | gnl\|UG\|Ta#S12896958 |
| - | gnl\|UG\|Ta#S13244586 |
| - | gnl\|UG\|Ta#S17984525 |
| - | gnl\|UG\|Ta#S25040821 |
| - | gnl\|UG\|Ta#S26026576 |
| - | gnl\|UG\|Ta#S52542196 |
| - | gnl\|UG\|Ta#S52542196 |
| - | gnl\|UG\|Ta#S52543119 |
| - | gnl\|UG\|Ta#S52544473 |
| - | gnl\|UG\|Ta#S52544877 |
| - | gnl\|UG\|Ta#S52544877 |
| - | gnl\|UG\|Ta#S52546144 |
| - | gnl\|UG\|Ta#S52546432 |
| - | gnl\|UG\|Ta#S52546847 |
| - | gnl\|UG\|Ta#S52547115 |
| - | gnl\|UG\|Ta#S58845066 |
| - | gnl\|UG\|Ta#S58853705 |
| - | gnl\|UG\|Ta#S58861359 |
| - | gnl\|UG\|Ta#S58895933 |
| - | gnl\|UG\|Ta#S58895933 |
| - | gnl\|UG\|Ta#S58908761 |
| - | gnl\|UG\|Ta#S61519792 |
| - | gnl\|UG\|Ta#S61557707 |
| - | gnl\|UG\|Ta#S61564953 |
| - | gnl\|UG\|Ta#S61583826 |
| - | gnl\|UG\|Ta#S61586888 |
| - | gnl\|UG\|Ta#S61594002 |
| - | gnl\|UG\|Ta#S61594384 |
| - | gnl\|UG\|Ta#S61597651 |
| - | gnl\|UG\|Ta#S61597657 |
| - | gnl\|UG\|Ta#S61612863 |
| - | gnl\|UG\|Ta#S61641130 |
| - | gnl\|UG\|Ta#S61641130 |
| - | gnl\|UG\|Ta#S61717137 |
| - | gnl\|UG\|Ta#S61738842 |
| - | gnl\|UG\|Ta#S61746292 |
| - | gnl\|UG\|Ta#S61751871 |
| - | gnl\|UG\|Ta#S61751918 |
| - | gnl\|UG\|Ta#S61753558 |
| - | gnl\|UG\|Ta#S61767862 |

| | |
|---|---|
| - | gnl\|UG\|Ta#S61768587 |
| - | gnl\|UG\|Ta#S61780972 |
| - | gnl\|UG\|Ta#S61789523 |
| - | gnl\|UG\|Ta#S61790554 |
| - | gnl\|UG\|Ta#S61790554 |
| - | gnl\|UG\|Ta#S61790644 |
| - | gnl\|UG\|Ta#S61796936 |
| - | gnl\|UG\|Ta#S61799676 |
| - | gnl\|UG\|Ta#S61802351 |
| - | gnl\|UG\|Ta#S61804016 |
| - | gnl\|UG\|Ta#S61808011 |
| - | gnl\|UG\|Ta#S61808403 |
| - | gnl\|UG\|Ta#S61808827 |
| - | gnl\|UG\|Ta#S61810441 |
| - | gnl\|UG\|Ta#S61810690 |
| - | gnl\|UG\|Ta#S61811006 |
| - | gnl\|UG\|Ta#S61811332 |
| - | gnl\|UG\|Ta#S61811692 |
| - | gnl\|UG\|Ta#S61812018 |
| - | gnl\|UG\|Ta#S61812527 |
| - | gnl\|UG\|Ta#S61812527 |
| - | gnl\|UG\|Ta#S61829726 |
| - | gnl\|UG\|Ta#S61829756 |
| - | gnl\|UG\|Ta#S61830374 |
| - | gnl\|UG\|Ta#S61830662 |
| - | gnl\|UG\|Ta#S61830662 |
| - | gnl\|UG\|Ta#S61831280 |
| - | gnl\|UG\|Ta#S61831312 |
| - | gnl\|UG\|Ta#S61831561 |
| - | gnl\|UG\|Ta#S61832278 |
| - | gnl\|UG\|Ta#S61832297 |
| - | gnl\|UG\|Ta#S61832522 |
| - | gnl\|UG\|Ta#S65596793 |
| - | gnl\|UG\|Ta#S65602331 |
| - | gnl\|UG\|Ta#S65602357 |
| - | gnl\|UG\|Ta#S65619890 |
| - | gnl\|UG\|Ta#S65625001 |
| - | gnl\|UG\|Ta#S65664629 |
| - | gnl\|UG\|Ta#S65668335 |
| tr\|M7YY67\|M7YY67_TRIUA | - |
| tr\|M7Z6K8\|M7Z6K8_TRIUA | - |
| tr\|M7Z8W5\|M7Z8W5_TRIUA | - |
| tr\|M7ZIE0\|M7ZIE0_TRIUA | - |
| tr\|M7ZXA3\|M7ZXA3_TRIUA | - |
| tr\|M8A6D0\|M8A6D0_TRIUA | - |
| tr\|W5DRV1\|W5DRV1_WHEAT | - |
| tr\|W5F663\|W5F663_WHEAT | - |

| | |
|---|---|
| tr\|W5F6T5\|W5F6T5_WHEAT | - |
| tr\|W5F7I0\|W5F7I0_WHEAT | - |
| tr\|W5FBU4\|W5FBU4_WHEAT | - |
| tr\|W5FDV4\|W5FDV4_WHEAT | - |
| tr\|W5FED9\|W5FED9_WHEAT | - |
| tr\|W5FGR4\|W5FGR4_WHEAT | - |
| tr\|W5FI26\|W5FI26_WHEAT | - |
| tr\|W5FN47\|W5FN47_WHEAT | - |
| tr\|W5FPD5\|W5FPD5_WHEAT | - |
| tr\|W5FH55\|W5FH55_WHEAT | gnl\|UG\|Ta#S32611508 |
| tr\|W5DSC7\|W5DSC7_WHEAT | gnl\|UG\|Ta#S52541512 |
| tr\|W5F9B2\|W5F9B2_WHEAT | gnl\|UG\|Ta#S52543456 |
| tr\|W5FBM0\|W5FBM0_WHEAT | gnl\|UG\|Ta#S58897382 |
| tr\|M7YH76\|M7YH76_TRIUA | gnl\|UG\|Ta#S61599608 |
| tr\|M7YH76\|M7YH76_TRIUA | gnl\|UG\|Ta#S61599608 |
| tr\|W5FJW3\|W5FJW3_WHEAT | gnl\|UG\|Ta#S61617395 |
| tr\|T1MHM8\|T1MHM8_TRIUA | gnl\|UG\|Ta#S61685993 |
| tr\|W5F8F9\|W5F8F9_WHEAT | gnl\|UG\|Ta#S61718443 |
| tr\|W5FL37\|W5FL37_WHEAT | gnl\|UG\|Ta#S61759540 |
| tr\|W5GVP2\|W5GVP2_WHEAT | gnl\|UG\|Ta#S61797632 |
| tr\|W5FLV2\|W5FLV2_WHEAT | gnl\|UG\|Ta#S61798263 |
| tr\|M7ZL45\|M7ZL45_TRIUA | gnl\|UG\|Ta#S61812377 |
| tr\|W5EQL7\|W5EQL7_WHEAT | gnl\|UG\|Ta#S61831627 |
| tr\|W5F7E3\|W5F7E3_WHEAT | gnl\|UG\|Ta#S61832238 |
| tr\|W5F6R8\|W5F6R8_WHEAT | gnl\|UG\|Ta#S65589875 |
| tr\|M8ASZ6\|M8ASZ6_TRIUA | gnl\|UG\|Ta#S65595762 |
| - | - |
| - | - |
| - | - |
| - | - |
| - | - |
| - | - |
| - | - |
| - | - |
| - | - |
| - | - |
| - | - |
| - | - |
| - | - |
| - | - |
| - | - |
| - | - |
| - | - |
| - | - |
| - | - |
| - | - |
| - | - |

| | |
|---|---|
| - | - |
| - | - |
| - | - |
| - | - |
| - | - |
| - | - |
| - | - |
| - | - |
| - | - |
| - | - |
| - | - |
| - | - |
| - | - |
| - | - |
| - | - |
| - | - |
| - | - |
| - | - |
| - | - |
| - | - |
| - | - |
| - | - |
| - | - |
| - | - |
| - | - |
| - | - |
| - | - |
| - | - |
| - | - |
| - | - |
| - | - |
| - | - |
| - | - |
| - | - |
| - | - |
| - | - |
| - | - |
| - | - |
| - | - |
| - | - |
| - | - |
| - | - |
| - | - |
| - | - |
| - | - |
| - | - |
| - | - |

| | |
|---|---|
| - | - |
| - | - |
| - | - |
| - | - |
| - | - |
| - | - |
| - | - |
| - | - |
| - | - |
| - | - |
| - | - |
| - | - |
| - | - |
| - | - |
| - | - |
| - | - |
| - | - |
| - | - |
| - | - |
| - | - |
| - | - |
| - | - |
| - | - |
| - | gnl\|UG\|Ta#S16198784 |
| - | gnl\|UG\|Ta#S17879993 |
| - | gnl\|UG\|Ta#S22379570 |
| - | gnl\|UG\|Ta#S32559241 |
| - | gnl\|UG\|Ta#S37768439 |
| - | gnl\|UG\|Ta#S52542126 |
| - | gnl\|UG\|Ta#S52542196 |
| - | gnl\|UG\|Ta#S52542496 |
| - | gnl\|UG\|Ta#S52542496 |
| - | gnl\|UG\|Ta#S52542579 |
| - | gnl\|UG\|Ta#S52542879 |
| - | gnl\|UG\|Ta#S52543364 |
| - | gnl\|UG\|Ta#S52543648 |
| - | gnl\|UG\|Ta#S52543692 |
| - | gnl\|UG\|Ta#S52544203 |
| - | gnl\|UG\|Ta#S52544471 |
| - | gnl\|UG\|Ta#S52544877 |
| - | gnl\|UG\|Ta#S52544877 |
| - | gnl\|UG\|Ta#S52545489 |
| - | gnl\|UG\|Ta#S52546323 |
| - | gnl\|UG\|Ta#S52546354 |
| - | gnl\|UG\|Ta#S52546463 |
| - | gnl\|UG\|Ta#S52546514 |
| - | gnl\|UG\|Ta#S52546693 |

| | |
|---|---|
| - | gnl\|UG\|Ta#S58863788 |
| - | gnl\|UG\|Ta#S58865847 |
| - | gnl\|UG\|Ta#S58870090 |
| - | gnl\|UG\|Ta#S58870090 |
| - | gnl\|UG\|Ta#S58895933 |
| - | gnl\|UG\|Ta#S58900016 |
| - | gnl\|UG\|Ta#S58900941 |
| - | gnl\|UG\|Ta#S61541853 |
| - | gnl\|UG\|Ta#S61548506 |
| - | gnl\|UG\|Ta#S61549788 |
| - | gnl\|UG\|Ta#S61551520 |
| - | gnl\|UG\|Ta#S61559658 |
| - | gnl\|UG\|Ta#S61570667 |
| - | gnl\|UG\|Ta#S61572072 |
| - | gnl\|UG\|Ta#S61578820 |
| - | gnl\|UG\|Ta#S61585202 |
| - | gnl\|UG\|Ta#S61590455 |
| - | gnl\|UG\|Ta#S61592450 |
| - | gnl\|UG\|Ta#S61597651 |
| - | gnl\|UG\|Ta#S61597657 |
| - | gnl\|UG\|Ta#S61599754 |
| - | gnl\|UG\|Ta#S61606492 |
| - | gnl\|UG\|Ta#S61607314 |
| - | gnl\|UG\|Ta#S61607314 |
| - | gnl\|UG\|Ta#S61608332 |
| - | gnl\|UG\|Ta#S61608440 |
| - | gnl\|UG\|Ta#S61609250 |
| - | gnl\|UG\|Ta#S61612863 |
| - | gnl\|UG\|Ta#S61613302 |
| - | gnl\|UG\|Ta#S61616281 |
| - | gnl\|UG\|Ta#S61617395 |
| - | gnl\|UG\|Ta#S61617395 |
| - | gnl\|UG\|Ta#S61617395 |
| - | gnl\|UG\|Ta#S61622478 |
| - | gnl\|UG\|Ta#S61625153 |
| - | gnl\|UG\|Ta#S61625153 |
| - | gnl\|UG\|Ta#S61632658 |
| - | gnl\|UG\|Ta#S61634748 |
| - | gnl\|UG\|Ta#S61635864 |
| - | gnl\|UG\|Ta#S61641133 |
| - | gnl\|UG\|Ta#S61643444 |
| - | gnl\|UG\|Ta#S61667980 |
| - | gnl\|UG\|Ta#S61670440 |
| - | gnl\|UG\|Ta#S61676289 |
| - | gnl\|UG\|Ta#S61678668 |
| - | gnl\|UG\|Ta#S61712168 |
| - | gnl\|UG\|Ta#S61718281 |

| | |
|---|---|
| - | gnl\|UG\|Ta#S61740494 |
| - | gnl\|UG\|Ta#S61753658 |
| - | gnl\|UG\|Ta#S61755046 |
| - | gnl\|UG\|Ta#S61757524 |
| - | gnl\|UG\|Ta#S61765200 |
| - | gnl\|UG\|Ta#S61768004 |
| - | gnl\|UG\|Ta#S61769206 |
| - | gnl\|UG\|Ta#S61772528 |
| - | gnl\|UG\|Ta#S61778311 |
| - | gnl\|UG\|Ta#S61778311 |
| - | gnl\|UG\|Ta#S61778485 |
| - | gnl\|UG\|Ta#S61778714 |
| - | gnl\|UG\|Ta#S61778880 |
| - | gnl\|UG\|Ta#S61778931 |
| - | gnl\|UG\|Ta#S61780151 |
| - | gnl\|UG\|Ta#S61781964 |
| - | gnl\|UG\|Ta#S61787820 |
| - | gnl\|UG\|Ta#S61791293 |
| - | gnl\|UG\|Ta#S61791477 |
| - | gnl\|UG\|Ta#S61791923 |
| - | gnl\|UG\|Ta#S61798193 |
| - | gnl\|UG\|Ta#S61798305 |
| - | gnl\|UG\|Ta#S61798508 |
| - | gnl\|UG\|Ta#S61799525 |
| - | gnl\|UG\|Ta#S61800374 |
| - | gnl\|UG\|Ta#S61800699 |
| - | gnl\|UG\|Ta#S61800732 |
| - | gnl\|UG\|Ta#S61800991 |
| - | gnl\|UG\|Ta#S61801554 |
| - | gnl\|UG\|Ta#S61801554 |
| - | gnl\|UG\|Ta#S61801823 |
| - | gnl\|UG\|Ta#S61802177 |
| - | gnl\|UG\|Ta#S61807831 |
| - | gnl\|UG\|Ta#S61808403 |
| - | gnl\|UG\|Ta#S61809487 |
| - | gnl\|UG\|Ta#S61809750 |
| - | gnl\|UG\|Ta#S61810061 |
| - | gnl\|UG\|Ta#S61810348 |
| - | gnl\|UG\|Ta#S61810530 |
| - | gnl\|UG\|Ta#S61810690 |
| - | gnl\|UG\|Ta#S61810983 |
| - | gnl\|UG\|Ta#S61811168 |
| - | gnl\|UG\|Ta#S61811325 |
| - | gnl\|UG\|Ta#S61811325 |
| - | gnl\|UG\|Ta#S61811332 |
| - | gnl\|UG\|Ta#S61811389 |
| - | gnl\|UG\|Ta#S61811455 |

| | |
|---|---|
| - | gnl\|UG\|Ta#S61811666 |
| - | gnl\|UG\|Ta#S61811802 |
| - | gnl\|UG\|Ta#S61811802 |
| - | gnl\|UG\|Ta#S61811802 |
| - | gnl\|UG\|Ta#S61811802 |
| - | gnl\|UG\|Ta#S61811802 |
| - | gnl\|UG\|Ta#S61811841 |
| - | gnl\|UG\|Ta#S61812107 |
| - | gnl\|UG\|Ta#S61812124 |
| - | gnl\|UG\|Ta#S61812150 |
| - | gnl\|UG\|Ta#S61812338 |
| - | gnl\|UG\|Ta#S61812365 |
| - | gnl\|UG\|Ta#S61812377 |
| - | gnl\|UG\|Ta#S61812379 |
| - | gnl\|UG\|Ta#S61812472 |
| - | gnl\|UG\|Ta#S61812483 |
| - | gnl\|UG\|Ta#S61812511 |
| - | gnl\|UG\|Ta#S61812538 |
| - | gnl\|UG\|Ta#S61818862 |
| - | gnl\|UG\|Ta#S61828043 |
| - | gnl\|UG\|Ta#S61828446 |
| - | gnl\|UG\|Ta#S61829043 |
| - | gnl\|UG\|Ta#S61829104 |
| - | gnl\|UG\|Ta#S61829435 |
| - | gnl\|UG\|Ta#S61829858 |
| - | gnl\|UG\|Ta#S61830226 |
| - | gnl\|UG\|Ta#S61830418 |
| - | gnl\|UG\|Ta#S61830418 |
| - | gnl\|UG\|Ta#S61830468 |
| - | gnl\|UG\|Ta#S61830468 |
| - | gnl\|UG\|Ta#S61830468 |
| - | gnl\|UG\|Ta#S61830525 |
| - | gnl\|UG\|Ta#S61830662 |
| - | gnl\|UG\|Ta#S61830814 |
| - | gnl\|UG\|Ta#S61830893 |
| - | gnl\|UG\|Ta#S61831360 |
| - | gnl\|UG\|Ta#S61831543 |
| - | gnl\|UG\|Ta#S61831543 |
| - | gnl\|UG\|Ta#S61831698 |
| - | gnl\|UG\|Ta#S61831857 |
| - | gnl\|UG\|Ta#S61832106 |
| - | gnl\|UG\|Ta#S61832278 |
| - | gnl\|UG\|Ta#S61832297 |
| - | gnl\|UG\|Ta#S61832297 |
| - | gnl\|UG\|Ta#S61832297 |
| - | gnl\|UG\|Ta#S61832297 |
| - | gnl\|UG\|Ta#S61832522 |

| | |
|---|---|
| - | gnl\|UG\|Ta#S61832522 |
| - | gnl\|UG\|Ta#S61832524 |
| - | gnl\|UG\|Ta#S61832532 |
| - | gnl\|UG\|Ta#S61834825 |
| - | gnl\|UG\|Ta#S61838698 |
| - | gnl\|UG\|Ta#S65592024 |
| - | gnl\|UG\|Ta#S65596811 |
| - | gnl\|UG\|Ta#S65596811 |
| - | gnl\|UG\|Ta#S65600911 |
| - | gnl\|UG\|Ta#S65601371 |
| - | gnl\|UG\|Ta#S65609318 |
| - | gnl\|UG\|Ta#S65611246 |
| - | gnl\|UG\|Ta#S65621128 |
| - | gnl\|UG\|Ta#S65622077 |
| - | gnl\|UG\|Ta#S65625001 |
| - | gnl\|UG\|Ta#S65634660 |
| - | gnl\|UG\|Ta#S65677103 |
| - | gnl\|UG\|Ta#S65677103 |
| - | gnl\|UG\|Ta#S65692893 |
| tr\|M7Y8U0\|M7Y8U0_TRIUA | - |
| tr\|M7ZTE5\|M7ZTE5_TRIUA | - |
| tr\|W5DTW7\|W5DTW7_WHEAT | - |
| tr\|W5F7J1\|W5F7J1_WHEAT | - |
| tr\|W5F9H4\|W5F9H4_WHEAT | - |
| tr\|W5FA38\|W5FA38_WHEAT | - |
| tr\|W5FBS8\|W5FBS8_WHEAT | - |
| tr\|W5FC38\|W5FC38_WHEAT | - |
| tr\|W5FCD7\|W5FCD7_WHEAT | - |
| tr\|W5FCJ9\|W5FCJ9_WHEAT | - |
| tr\|W5FFH0\|W5FFH0_WHEAT | - |
| tr\|W5FFS7\|W5FFS7_WHEAT | - |
| tr\|W5FGZ5\|W5FGZ5_WHEAT | - |
| tr\|W5FHA1\|W5FHA1_WHEAT | - |
| tr\|W5FI19\|W5FI19_WHEAT | - |
| tr\|W5FIF4\|W5FIF4_WHEAT | - |
| tr\|W5FJF3\|W5FJF3_WHEAT | - |
| tr\|W5FJM3\|W5FJM3_WHEAT | - |
| tr\|W5FJW8\|W5FJW8_WHEAT | - |
| tr\|W5FKL4\|W5FKL4_WHEAT | - |
| tr\|W5FL84\|W5FL84_WHEAT | - |
| tr\|W5FM11\|W5FM11_WHEAT | - |
| tr\|W5FM16\|W5FM16_WHEAT | - |
| tr\|W5FM16\|W5FM16_WHEAT | - |
| tr\|W5FM16\|W5FM16_WHEAT | - |
| tr\|W5FMS1\|W5FMS1_WHEAT | - |
| tr\|W5FPE6\|W5FPE6_WHEAT | - |
| tr\|W5FTM9\|W5FTM9_WHEAT | - |

| | |
|---|---|
| tr\|M7ZNA6\|M7ZNA6_TRIUA | gnl\|UG\|Ta#S12928726 |
| tr\|W5FIX0\|W5FIX0_WHEAT | gnl\|UG\|Ta#S16058102 |
| tr\|M7ZJN4\|M7ZJN4_TRIUA | gnl\|UG\|Ta#S52541041 |
| tr\|W5FIZ3\|W5FIZ3_WHEAT | gnl\|UG\|Ta#S52541772 |
| tr\|M7Y6A9\|M7Y6A9_TRIUA | gnl\|UG\|Ta#S52542496 |
| tr\|W5FAR1\|W5FAR1_WHEAT | gnl\|UG\|Ta#S52542530 |
| tr\|W5F3U3\|W5F3U3_WHEAT | gnl\|UG\|Ta#S52543048 |
| tr\|M7YEY7\|M7YEY7_TRIUA | gnl\|UG\|Ta#S52544898 |
| tr\|W5FPM7\|W5FPM7_WHEAT | gnl\|UG\|Ta#S52545434 |
| tr\|W5FEY1\|W5FEY1_WHEAT | gnl\|UG\|Ta#S52545998 |
| tr\|M8AI25\|M8AI25_TRIUA | gnl\|UG\|Ta#S52546659 |
| tr\|W5FNR3\|W5FNR3_WHEAT | gnl\|UG\|Ta#S58890907 |
| tr\|W5FEN7\|W5FEN7_WHEAT | gnl\|UG\|Ta#S58894194 |
| tr\|W5FQM0\|W5FQM0_WHEAT | gnl\|UG\|Ta#S58897382 |
| tr\|M7ZS79\|M7ZS79_TRIUA | gnl\|UG\|Ta#S58900941 |
| tr\|W5G3I8\|W5G3I8_WHEAT | gnl\|UG\|Ta#S58901401 |
| tr\|W5FNQ7\|W5FNQ7_WHEAT | gnl\|UG\|Ta#S61592450 |
| tr\|M8AD50\|M8AD50_TRIUA | gnl\|UG\|Ta#S61601664 |
| tr\|M7ZHV2\|M7ZHV2_TRIUA | gnl\|UG\|Ta#S61608332 |
| tr\|W5F716\|W5F716_WHEAT | gnl\|UG\|Ta#S61619919 |
| tr\|W5FGQ3\|W5FGQ3_WHEAT | gnl\|UG\|Ta#S61619919 |
| tr\|W5FHA3\|W5FHA3_WHEAT | gnl\|UG\|Ta#S61622478 |
| tr\|W5FHA3\|W5FHA3_WHEAT | gnl\|UG\|Ta#S61622478 |
| tr\|W5FZQ9\|W5FZQ9_WHEAT | gnl\|UG\|Ta#S61632658 |
| tr\|W5FJY0\|W5FJY0_WHEAT | gnl\|UG\|Ta#S61636247 |
| tr\|W5FVA0\|W5FVA0_WHEAT | gnl\|UG\|Ta#S61645444 |
| tr\|M8A0B9\|M8A0B9_TRIUA | gnl\|UG\|Ta#S61652612 |
| tr\|W5F557\|W5F557_WHEAT | gnl\|UG\|Ta#S61664076 |
| tr\|W5E4S5\|W5E4S5_WHEAT | gnl\|UG\|Ta#S61779775 |
| tr\|W5FNS2\|W5FNS2_WHEAT | gnl\|UG\|Ta#S61781861 |
| tr\|W5FAX1\|W5FAX1_WHEAT | gnl\|UG\|Ta#S61790194 |
| tr\|W5FLH1\|W5FLH1_WHEAT | gnl\|UG\|Ta#S61791877 |
| tr\|W5FIV3\|W5FIV3_WHEAT | gnl\|UG\|Ta#S61798354 |
| tr\|W5FDK3\|W5FDK3_WHEAT | gnl\|UG\|Ta#S61800074 |
| tr\|W5FBS1\|W5FBS1_WHEAT | gnl\|UG\|Ta#S61801370 |
| tr\|W5F7Q7\|W5F7Q7_WHEAT | gnl\|UG\|Ta#S61801554 |
| tr\|W5FMV7\|W5FMV7_WHEAT | gnl\|UG\|Ta#S61801555 |
| tr\|M8A6M4\|M8A6M4_TRIUA | gnl\|UG\|Ta#S61801823 |
| tr\|W5FY36\|W5FY36_WHEAT | gnl\|UG\|Ta#S61801823 |
| tr\|W5FGN1\|W5FGN1_WHEAT | gnl\|UG\|Ta#S61807729 |
| tr\|W5FFR8\|W5FFR8_WHEAT | gnl\|UG\|Ta#S61808691 |
| tr\|W5FHA1\|W5FHA1_WHEAT | gnl\|UG\|Ta#S61809301 |
| tr\|W5FDF7\|W5FDF7_WHEAT | gnl\|UG\|Ta#S61811195 |
| tr\|M7ZF28\|M7ZF28_TRIUA | gnl\|UG\|Ta#S61811332 |
| tr\|M7YPL3\|M7YPL3_TRIUA | gnl\|UG\|Ta#S61811389 |
| tr\|M7Z1J9\|M7Z1J9_TRIUA | gnl\|UG\|Ta#S61811404 |
| tr\|W5F6Y4\|W5F6Y4_WHEAT | gnl\|UG\|Ta#S61811404 |

| | |
|---|---|
| tr\|M7YJP4\|M7YJP4_TRIUA | gnl\|UG\|Ta#S61811409 |
| tr\|W5FLD5\|W5FLD5_WHEAT | gnl\|UG\|Ta#S61811773 |
| tr\|M7ZUY3\|M7ZUY3_TRIUA | gnl\|UG\|Ta#S61812124 |
| tr\|M7ZL45\|M7ZL45_TRIUA | gnl\|UG\|Ta#S61812377 |
| tr\|W5FEI6\|W5FEI6_WHEAT | gnl\|UG\|Ta#S61812472 |
| tr\|W5FK46\|W5FK46_WHEAT | gnl\|UG\|Ta#S61814735 |
| tr\|M7ZWM7\|M7ZWM7_TRIUA | gnl\|UG\|Ta#S61822723 |
| tr\|W5DTS6\|W5DTS6_WHEAT | gnl\|UG\|Ta#S61828864 |
| tr\|W5FHS3\|W5FHS3_WHEAT | gnl\|UG\|Ta#S61828864 |
| tr\|W5FJV7\|W5FJV7_WHEAT | gnl\|UG\|Ta#S61829853 |
| tr\|M7Z3R5\|M7Z3R5_TRIUA | gnl\|UG\|Ta#S61829858 |
| tr\|W5FAI5\|W5FAI5_WHEAT | gnl\|UG\|Ta#S61830212 |
| tr\|M8AJ70\|M8AJ70_TRIUA | gnl\|UG\|Ta#S61830249 |
| tr\|W5FKM0\|W5FKM0_WHEAT | gnl\|UG\|Ta#S61830614 |
| tr\|M7YZC5\|M7YZC5_TRIUA | gnl\|UG\|Ta#S61830923 |
| tr\|W5FWL5\|W5FWL5_WHEAT | gnl\|UG\|Ta#S61831136 |
| tr\|W5FUI0\|W5FUI0_WHEAT | gnl\|UG\|Ta#S61831249 |
| tr\|M8A687\|M8A687_TRIUA | gnl\|UG\|Ta#S61831360 |
| tr\|W5F527\|W5F527_WHEAT | gnl\|UG\|Ta#S61831808 |
| tr\|M8A652\|M8A652_TRIUA | gnl\|UG\|Ta#S61832297 |
| tr\|W5F9Q6\|W5F9Q6_WHEAT | gnl\|UG\|Ta#S61832297 |
| tr\|W5FMP3\|W5FMP3_WHEAT | gnl\|UG\|Ta#S61832461 |
| tr\|T1M4N8\|T1M4N8_TRIUA | gnl\|UG\|Ta#S61847633 |
| tr\|W5FIU1\|W5FIU1_WHEAT | gnl\|UG\|Ta#S61854246 |
| tr\|W5FAG5\|W5FAG5_WHEAT | gnl\|UG\|Ta#S61861586 |
| tr\|W5FM30\|W5FM30_WHEAT | gnl\|UG\|Ta#S65602960 |
| tr\|W5FGI0\|W5FGI0_WHEAT | gnl\|UG\|Ta#S65618599 |
| - | - |
| - | - |
| - | - |
| - | - |
| - | - |
| - | - |
| - | - |
| - | - |
| - | - |
| - | - |
| - | - |
| - | - |
| - | - |
| - | - |
| - | - |
| - | - |
| - | - |
| - | - |
| - | - |
| - | - |

| | |
|---|---|
| - | - |
| - | - |
| - | - |
| - | - |
| - | - |
| - | - |
| - | - |
| - | - |
| - | - |
| - | - |
| - | - |
| - | - |
| - | - |
| - | - |
| - | - |
| - | - |
| - | - |
| - | - |
| - | - |
| - | - |
| - | - |
| - | - |
| - | - |
| - | - |
| - | - |
| - | - |
| - | - |
| - | - |
| - | - |
| - | - |
| - | - |
| - | - |
| - | - |
| - | - |
| - | - |
| - | - |
| - | - |
| - | - |
| - | - |
| - | - |
| - | - |
| - | - |
| - | - |
| - | - |
| - | - |
| - | - |
| - | - |

| | |
|---|---|
| - | - |
| - | - |
| - | - |
| - | - |
| - | - |
| - | - |
| - | - |
| - | - |
| - | - |
| - | - |
| - | - |
| - | - |
| - | - |
| - | - |
| - | - |
| - | - |
| - | - |
| - | - |
| - | - |
| - | - |
| - | - |
| - | - |
| - | - |
| - | - |
| - | - |
| - | - |
| - | - |
| - | - |
| - | - |
| - | - |
| - | - |
| - | - |
| - | - |
| - | - |
| - | - |
| - | - |
| - | - |
| - | - |
| - | - |
| - | - |
| - | - |
| - | - |
| - | - |
| - | - |
| - | - |
| - | - |
| - | - |

| | |
|---|---|
| - | - |
| - | - |
| - | - |
| - | - |
| - | - |
| - | - |
| - | - |
| - | - |
| - | - |
| - | - |
| - | - |
| - | - |
| - | - |
| - | - |
| - | - |
| - | - |
| - | - |
| - | gnl\|UG\|Ta#S16057969 |
| - | gnl\|UG\|Ta#S16193032 |
| - | gnl\|UG\|Ta#S17879385 |
| - | gnl\|UG\|Ta#S22387302 |
| - | gnl\|UG\|Ta#S32654061 |
| - | gnl\|UG\|Ta#S41658045 |
| - | gnl\|UG\|Ta#S52542998 |
| - | gnl\|UG\|Ta#S52543168 |
| - | gnl\|UG\|Ta#S52544885 |
| - | gnl\|UG\|Ta#S52545434 |
| - | gnl\|UG\|Ta#S52545940 |
| - | gnl\|UG\|Ta#S58847700 |
| - | gnl\|UG\|Ta#S58887057 |
| - | gnl\|UG\|Ta#S58890333 |
| - | gnl\|UG\|Ta#S58912126 |
| - | gnl\|UG\|Ta#S61560305 |
| - | gnl\|UG\|Ta#S61582158 |
| - | gnl\|UG\|Ta#S61594415 |
| - | gnl\|UG\|Ta#S61599754 |
| - | gnl\|UG\|Ta#S61615413 |
| - | gnl\|UG\|Ta#S61636976 |
| - | gnl\|UG\|Ta#S61640151 |
| - | gnl\|UG\|Ta#S61652182 |
| - | gnl\|UG\|Ta#S61675100 |
| - | gnl\|UG\|Ta#S61692999 |
| - | gnl\|UG\|Ta#S61694931 |
| - | gnl\|UG\|Ta#S61713696 |
| - | gnl\|UG\|Ta#S61722333 |
| - | gnl\|UG\|Ta#S61770361 |
| - | gnl\|UG\|Ta#S61771750 |

| | |
|---|---|
| - | gnl\|UG\|Ta#S61777267 |
| - | gnl\|UG\|Ta#S61778187 |
| - | gnl\|UG\|Ta#S61806240 |
| - | gnl\|UG\|Ta#S61809879 |
| - | gnl\|UG\|Ta#S61809980 |
| - | gnl\|UG\|Ta#S61811456 |
| - | gnl\|UG\|Ta#S61811579 |
| - | gnl\|UG\|Ta#S61811665 |
| - | gnl\|UG\|Ta#S61812141 |
| - | gnl\|UG\|Ta#S61812454 |
| - | gnl\|UG\|Ta#S61828512 |
| - | gnl\|UG\|Ta#S61830550 |
| - | gnl\|UG\|Ta#S61830923 |
| - | gnl\|UG\|Ta#S61831029 |
| - | gnl\|UG\|Ta#S61831515 |
| - | gnl\|UG\|Ta#S61831832 |
| - | gnl\|UG\|Ta#S61831960 |
| - | gnl\|UG\|Ta#S65687206 |
| - | gnl\|UG\|Ta#S65692178 |
| - | gnl\|UG\|Ta#S65716793 |
| tr\|F8SXZ9\|F8SXZ9_WHEAT | - |
| tr\|M8AFT7\|M8AFT7_TRIUA | - |
| tr\|W5F2Q8\|W5F2Q8_WHEAT | - |
| tr\|W5FD31\|W5FD31_WHEAT | - |
| tr\|W5FD31\|W5FD31_WHEAT | - |
| tr\|W5FIK0\|W5FIK0_WHEAT | - |
| tr\|W5FJG0\|W5FJG0_WHEAT | - |
| tr\|W5FKF0\|W5FKF0_WHEAT | - |
| tr\|W5FMD8\|W5FMD8_WHEAT | - |
| tr\|W5FNN5\|W5FNN5_WHEAT | - |
| tr\|W5FUK1\|W5FUK1_WHEAT | - |
| tr\|F6LAX4\|F6LAX4_WHEAT | gnl\|UG\|Ta#S16057969 |
| tr\|W5FDB6\|W5FDB6_WHEAT | gnl\|UG\|Ta#S16229452 |
| tr\|Q58A32\|Q58A32_WHEAT | gnl\|UG\|Ta#S24318187 |
| tr\|W5FM09\|W5FM09_WHEAT | gnl\|UG\|Ta#S52542905 |
| tr\|W5F8Q9\|W5F8Q9_WHEAT | gnl\|UG\|Ta#S52545646 |
| tr\|T1N3G0\|T1N3G0_TRIUA | gnl\|UG\|Ta#S61571973 |
| tr\|W5EW52\|W5EW52_WHEAT | gnl\|UG\|Ta#S61770110 |
| tr\|W5FHI1\|W5FHI1_WHEAT | gnl\|UG\|Ta#S61807730 |
| tr\|W5FG21\|W5FG21_WHEAT | gnl\|UG\|Ta#S61811130 |
| tr\|W5FIC9\|W5FIC9_WHEAT | gnl\|UG\|Ta#S61832348 |
| tr\|W5FCZ1\|W5FCZ1_WHEAT | gnl\|UG\|Ta#S61848513 |
| tr\|W5F879\|W5F879_WHEAT | gnl\|UG\|Ta#S65647798 |
| - | - |
| - | - |
| - | - |
| - | - |

| | |
|---|---|
| - | - |
| - | - |
| - | - |
| - | - |
| - | - |
| - | - |
| - | - |
| - | - |
| - | - |
| - | - |
| - | - |
| - | - |
| - | - |
| - | - |
| - | - |
| - | - |
| - | - |
| - | - |
| - | - |
| - | - |
| - | gnl\|UG\|Ta#S16057969 |
| - | gnl\|UG\|Ta#S22386197 |
| - | gnl\|UG\|Ta#S58857201 |
| - | gnl\|UG\|Ta#S58893674 |
| - | gnl\|UG\|Ta#S59489969 |
| - | gnl\|UG\|Ta#S61516913 |
| - | gnl\|UG\|Ta#S61586334 |
| - | gnl\|UG\|Ta#S61597200 |
| - | gnl\|UG\|Ta#S61605469 |
| - | gnl\|UG\|Ta#S61606996 |
| - | gnl\|UG\|Ta#S61608440 |
| - | gnl\|UG\|Ta#S61615413 |
| - | gnl\|UG\|Ta#S61626193 |
| - | gnl\|UG\|Ta#S61626869 |
| - | gnl\|UG\|Ta#S61638387 |
| - | gnl\|UG\|Ta#S61641599 |
| - | gnl\|UG\|Ta#S61756836 |
| - | gnl\|UG\|Ta#S61782544 |
| - | gnl\|UG\|Ta#S61801671 |
| - | gnl\|UG\|Ta#S61811208 |
| - | gnl\|UG\|Ta#S61811497 |
| - | gnl\|UG\|Ta#S61812150 |
| - | gnl\|UG\|Ta#S61812450 |
| - | gnl\|UG\|Ta#S61812476 |
| - | gnl\|UG\|Ta#S61812511 |
| - | gnl\|UG\|Ta#S61812538 |
| - | gnl\|UG\|Ta#S61812547 |

| | |
|---|---|
| - | gnl\|UG\|Ta#S61830166 |
| - | gnl\|UG\|Ta#S61830249 |
| - | gnl\|UG\|Ta#S61832098 |
| - | gnl\|UG\|Ta#S61832496 |
| - | gnl\|UG\|Ta#S65592843 |
| - | gnl\|UG\|Ta#S65609318 |
| tr\|M7ZWK9\|M7ZWK9_TRIUA | - |
| tr\|W5FA01\|W5FA01_WHEAT | - |
| tr\|W5FDD2\|W5FDD2_WHEAT | - |
| tr\|W5FEH7\|W5FEH7_WHEAT | - |
| tr\|W5FGU2\|W5FGU2_WHEAT | - |
| tr\|W5FH13\|W5FH13_WHEAT | - |
| tr\|W5FHH1\|W5FHH1_WHEAT | - |
| tr\|W5FIY4\|W5FIY4_WHEAT | - |
| tr\|W5FKX7\|W5FKX7_WHEAT | - |
| tr\|W5FMW4\|W5FMW4_WHEAT | - |
| tr\|W5HUL5\|W5HUL5_WHEAT | - |
| tr\|M7Z540\|M7Z540_TRIUA | gnl\|UG\|Ta#S52542329 |
| tr\|W5F7P9\|W5F7P9_WHEAT | gnl\|UG\|Ta#S52542529 |
| tr\|W5FDB5\|W5FDB5_WHEAT | gnl\|UG\|Ta#S61521837 |
| tr\|M7ZFQ9\|M7ZFQ9_TRIUA | gnl\|UG\|Ta#S61560162 |
| tr\|M8AE21\|M8AE21_TRIUA | gnl\|UG\|Ta#S61617950 |
| tr\|W5FMH0\|W5FMH0_WHEAT | gnl\|UG\|Ta#S61620929 |
| tr\|W5F0P2\|W5F0P2_WHEAT | gnl\|UG\|Ta#S61631969 |
| tr\|W5G0Q7\|W5G0Q7_WHEAT | gnl\|UG\|Ta#S61810728 |
| tr\|W5FFK0\|W5FFK0_WHEAT | gnl\|UG\|Ta#S61811185 |
| tr\|W5FAD6\|W5FAD6_WHEAT | gnl\|UG\|Ta#S61812279 |
| tr\|W5FH13\|W5FH13_WHEAT | gnl\|UG\|Ta#S61812310 |
| tr\|W5AFE3\|W5AFE3_WHEAT | gnl\|UG\|Ta#S61812363 |
| tr\|M8A710\|M8A710_TRIUA | gnl\|UG\|Ta#S61812538 |
| tr\|W5FP19\|W5FP19_WHEAT | gnl\|UG\|Ta#S61831079 |
| tr\|W5FKM5\|W5FKM5_WHEAT | gnl\|UG\|Ta#S61831408 |
| tr\|W5F6C2\|W5F6C2_WHEAT | gnl\|UG\|Ta#S61832496 |
| tr\|W5FLM8\|W5FLM8_WHEAT | gnl\|UG\|Ta#S61832496 |
| tr\|M7ZVV5\|M7ZVV5_TRIUA | gnl\|UG\|Ta#S65609370 |
| - | - |
| - | - |
| - | - |
| - | - |
| - | - |
| - | - |
| - | - |
| - | - |
| - | - |
| - | - |
| - | - |
| - | - |

| | |
|---|---|
| - | - |
| - | - |
| - | - |
| - | - |
| - | - |
| - | - |
| - | - |
| - | - |
| - | - |
| - | - |
| - | - |
| - | - |
| - | - |
| - | - |
| - | - |
| - | - |
| - | - |
| - | - |
| - | - |
| - | - |
| - | - |
| - | - |
| - | - |
| - | - |
| - | - |
| - | - |
| - | - |
| - | - |
| - | - |
| - | - |
| - | - |
| - | - |
| - | - |
| - | - |
| - | - |
| - | - |
| - | gnl\|UG\|Ta#S18006252 |
| - | gnl\|UG\|Ta#S32643400 |
| - | gnl\|UG\|Ta#S32684428 |
| - | gnl\|UG\|Ta#S52544781 |
| - | gnl\|UG\|Ta#S52545646 |
| - | gnl\|UG\|Ta#S52546243 |
| - | gnl\|UG\|Ta#S58849757 |
| - | gnl\|UG\|Ta#S58865836 |
| - | gnl\|UG\|Ta#S58899829 |
| - | gnl\|UG\|Ta#S61517974 |
| - | gnl\|UG\|Ta#S61537246 |

| | |
|---|---|
| - | gnl\|UG\|Ta#S61580653 |
| - | gnl\|UG\|Ta#S61583826 |
| - | gnl\|UG\|Ta#S61597651 |
| - | gnl\|UG\|Ta#S61608652 |
| - | gnl\|UG\|Ta#S61613001 |
| - | gnl\|UG\|Ta#S61613001 |
| - | gnl\|UG\|Ta#S61613004 |
| - | gnl\|UG\|Ta#S61626869 |
| - | gnl\|UG\|Ta#S61661526 |
| - | gnl\|UG\|Ta#S61669085 |
| - | gnl\|UG\|Ta#S61743683 |
| - | gnl\|UG\|Ta#S61746307 |
| - | gnl\|UG\|Ta#S61764679 |
| - | gnl\|UG\|Ta#S61771991 |
| - | gnl\|UG\|Ta#S61782348 |
| - | gnl\|UG\|Ta#S61782499 |
| - | gnl\|UG\|Ta#S61787890 |
| - | gnl\|UG\|Ta#S61807722 |
| - | gnl\|UG\|Ta#S61811497 |
| - | gnl\|UG\|Ta#S61811543 |
| - | gnl\|UG\|Ta#S61812476 |
| - | gnl\|UG\|Ta#S61828393 |
| - | gnl\|UG\|Ta#S61829050 |
| - | gnl\|UG\|Ta#S61829473 |
| - | gnl\|UG\|Ta#S61830085 |
| - | gnl\|UG\|Ta#S61831144 |
| - | gnl\|UG\|Ta#S61831496 |
| - | gnl\|UG\|Ta#S61832496 |
| - | gnl\|UG\|Ta#S61832522 |
| - | gnl\|UG\|Ta#S61832532 |
| - | gnl\|UG\|Ta#S61832532 |
| - | gnl\|UG\|Ta#S61832532 |
| tr\|W5EDA7\|W5EDA7_WHEAT | - |
| tr\|W5F7F1\|W5F7F1_WHEAT | - |
| tr\|W5F8K2\|W5F8K2_WHEAT | - |
| tr\|W5F8Y7\|W5F8Y7_WHEAT | - |
| tr\|W5F9F8\|W5F9F8_WHEAT | - |
| tr\|W5F9Z8\|W5F9Z8_WHEAT | - |
| tr\|W5FAV4\|W5FAV4_WHEAT | - |
| tr\|W5FFD8\|W5FFD8_WHEAT | - |
| tr\|W5FJJ5\|W5FJJ5_WHEAT | - |
| tr\|W5FKM5\|W5FKM5_WHEAT | - |
| tr\|W5FKM6\|W5FKM6_WHEAT | - |
| tr\|W5FME1\|W5FME1_WHEAT | - |
| tr\|W5FNT5\|W5FNT5_WHEAT | - |
| sp\|Q41593\|SPZ1A_WHEAT | gnl\|UG\|Ta#S12923035 |
| tr\|M7ZKH8\|M7ZKH8_TRIUA | gnl\|UG\|Ta#S22366685 |

| | |
|---|---|
| tr\|T1LB35\|T1LB35_TRIUA | gnl\|UG\|Ta#S61522516 |
| tr\|M8A135\|M8A135_TRIUA | gnl\|UG\|Ta#S61543858 |
| tr\|W5DLZ6\|W5DLZ6_WHEAT | gnl\|UG\|Ta#S61571600 |
| tr\|W5FDK6\|W5FDK6_WHEAT | gnl\|UG\|Ta#S61612946 |
| tr\|W5ERJ5\|W5ERJ5_WHEAT | gnl\|UG\|Ta#S61618594 |
| tr\|W5F8T3\|W5F8T3_WHEAT | gnl\|UG\|Ta#S61828711 |
| tr\|W5FQA9\|W5FQA9_WHEAT | gnl\|UG\|Ta#S61831408 |
| tr\|M7YWE3\|M7YWE3_TRIUA | gnl\|UG\|Ta#S61831852 |
| tr\|M7Z1Y2\|M7Z1Y2_TRIUA | gnl\|UG\|Ta#S61832384 |
| tr\|W5FBE6\|W5FBE6_WHEAT | gnl\|UG\|Ta#S65600487 |
| tr\|W5FF97\|W5FF97_WHEAT | gnl\|UG\|Ta#S65625818 |
| - | - |
| - | - |
| - | - |
| - | - |
| - | - |
| - | - |
| - | - |
| - | - |
| - | - |
| - | - |
| - | - |
| - | - |
| - | - |
| - | - |
| - | - |
| - | - |
| - | - |
| - | - |
| - | - |
| - | - |
| - | - |
| - | - |
| - | - |
| - | - |
| - | - |
| - | - |
| - | - |
| - | - |
| - | gnl\|UG\|Ta#S17987607 |
| - | gnl\|UG\|Ta#S37929428 |
| - | gnl\|UG\|Ta#S52541512 |
| - | gnl\|UG\|Ta#S52541957 |
| - | gnl\|UG\|Ta#S52542434 |
| - | gnl\|UG\|Ta#S52542447 |
| - | gnl\|UG\|Ta#S52544898 |

| | |
|---|---|
| - | gnl\|UG\|Ta#S52545489 |
| - | gnl\|UG\|Ta#S58841169 |
| - | gnl\|UG\|Ta#S58889665 |
| - | gnl\|UG\|Ta#S61544072 |
| - | gnl\|UG\|Ta#S61577778 |
| - | gnl\|UG\|Ta#S61586121 |
| - | gnl\|UG\|Ta#S61586121 |
| - | gnl\|UG\|Ta#S61586121 |
| - | gnl\|UG\|Ta#S61586121 |
| - | gnl\|UG\|Ta#S61586334 |
| - | gnl\|UG\|Ta#S61587768 |
| - | gnl\|UG\|Ta#S61588059 |
| - | gnl\|UG\|Ta#S61593513 |
| - | gnl\|UG\|Ta#S61598401 |
| - | gnl\|UG\|Ta#S61598401 |
| - | gnl\|UG\|Ta#S61604779 |
| - | gnl\|UG\|Ta#S61608648 |
| - | gnl\|UG\|Ta#S61608962 |
| - | gnl\|UG\|Ta#S61613004 |
| - | gnl\|UG\|Ta#S61624959 |
| - | gnl\|UG\|Ta#S61626869 |
| - | gnl\|UG\|Ta#S61628441 |
| - | gnl\|UG\|Ta#S61628441 |
| - | gnl\|UG\|Ta#S61637393 |
| - | gnl\|UG\|Ta#S61639627 |
| - | gnl\|UG\|Ta#S61648502 |
| - | gnl\|UG\|Ta#S61678329 |
| - | gnl\|UG\|Ta#S61688546 |
| - | gnl\|UG\|Ta#S61770534 |
| - | gnl\|UG\|Ta#S61771991 |
| - | gnl\|UG\|Ta#S61780527 |
| - | gnl\|UG\|Ta#S61780608 |
| - | gnl\|UG\|Ta#S61781223 |
| - | gnl\|UG\|Ta#S61792106 |
| - | gnl\|UG\|Ta#S61799492 |
| - | gnl\|UG\|Ta#S61799492 |
| - | gnl\|UG\|Ta#S61801911 |
| - | gnl\|UG\|Ta#S61809521 |
| - | gnl\|UG\|Ta#S61809678 |
| - | gnl\|UG\|Ta#S61811130 |
| - | gnl\|UG\|Ta#S61811208 |
| - | gnl\|UG\|Ta#S61811297 |
| - | gnl\|UG\|Ta#S61811497 |
| - | gnl\|UG\|Ta#S61811734 |
| - | gnl\|UG\|Ta#S61811734 |
| - | gnl\|UG\|Ta#S61811923 |
| - | gnl\|UG\|Ta#S61811923 |

| | |
|---|---|
| - | gnl\|UG\|Ta#S61812262 |
| - | gnl\|UG\|Ta#S61830249 |
| - | gnl\|UG\|Ta#S61830425 |
| - | gnl\|UG\|Ta#S61831360 |
| - | gnl\|UG\|Ta#S61831543 |
| - | gnl\|UG\|Ta#S61832481 |
| - | gnl\|UG\|Ta#S61832496 |
| - | gnl\|UG\|Ta#S61832496 |
| - | gnl\|UG\|Ta#S61832532 |
| - | gnl\|UG\|Ta#S61832532 |
| - | gnl\|UG\|Ta#S65722944 |
| tr\|A8BCW8\|A8BCW8_WHEAT | - |
| tr\|W5F7P5\|W5F7P5_WHEAT | - |
| tr\|W5F800\|W5F800_WHEAT | - |
| tr\|W5FBP0\|W5FBP0_WHEAT | - |
| tr\|W5FEB3\|W5FEB3_WHEAT | - |
| tr\|W5FFL6\|W5FFL6_WHEAT | - |
| tr\|W5FHF3\|W5FHF3_WHEAT | - |
| tr\|W5FIA1\|W5FIA1_WHEAT | - |
| tr\|W5FMN1\|W5FMN1_WHEAT | - |
| tr\|W5FNJ9\|W5FNJ9_WHEAT | - |
| tr\|W5FP89\|W5FP89_WHEAT | - |
| tr\|W5G492\|W5G492_WHEAT | - |
| tr\|W5FB15\|W5FB15_WHEAT | gnl\|UG\|Ta#S18349078 |
| tr\|W5FGU2\|W5FGU2_WHEAT | gnl\|UG\|Ta#S30065901 |
| tr\|M8B3L5\|M8B3L5_TRIUA | gnl\|UG\|Ta#S52543649 |
| tr\|W5FKX7\|W5FKX7_WHEAT | gnl\|UG\|Ta#S58866707 |
| tr\|W5FJF3\|W5FJF3_WHEAT | gnl\|UG\|Ta#S58866975 |
| tr\|W5EU75\|W5EU75_WHEAT | gnl\|UG\|Ta#S58892014 |
| tr\|W5FD53\|W5FD53_WHEAT | gnl\|UG\|Ta#S61580864 |
| tr\|W5FFQ8\|W5FFQ8_WHEAT | gnl\|UG\|Ta#S61586121 |
| tr\|W5FB15\|W5FB15_WHEAT | gnl\|UG\|Ta#S61601968 |
| tr\|W5FJW3\|W5FJW3_WHEAT | gnl\|UG\|Ta#S61617395 |
| tr\|W5FP08\|W5FP08_WHEAT | gnl\|UG\|Ta#S61641599 |
| tr\|W5FPG7\|W5FPG7_WHEAT | gnl\|UG\|Ta#S61645368 |
| tr\|W5F9B9\|W5F9B9_WHEAT | gnl\|UG\|Ta#S61648428 |
| tr\|W5F9W5\|W5F9W5_WHEAT | gnl\|UG\|Ta#S61719330 |
| tr\|W5EZC2\|W5EZC2_WHEAT | gnl\|UG\|Ta#S61781559 |
| tr\|W5FD12\|W5FD12_WHEAT | gnl\|UG\|Ta#S61799492 |
| tr\|W5F8L3\|W5F8L3_WHEAT | gnl\|UG\|Ta#S61811265 |
| tr\|W5FIA1\|W5FIA1_WHEAT | gnl\|UG\|Ta#S61811734 |
| tr\|W5FLK0\|W5FLK0_WHEAT | gnl\|UG\|Ta#S61829888 |
| tr\|W5FEJ2\|W5FEJ2_WHEAT | gnl\|UG\|Ta#S61830338 |
| tr\|M8AL24\|M8AL24_TRIUA | gnl\|UG\|Ta#S61831312 |
| tr\|W5FJQ8\|W5FJQ8_WHEAT | gnl\|UG\|Ta#S65663510 |
| - | - |
| - | - |

| | |
|---|---|
| - | - |
| - | - |
| - | - |
| - | - |
| - | - |
| - | - |
| - | - |
| - | - |
| - | - |
| - | - |
| - | - |
| - | - |
| - | - |
| - | - |
| - | - |
| - | - |
| - | - |
| - | - |
| - | - |
| - | - |
| - | - |
| - | - |
| - | - |
| - | - |
| - | - |
| - | - |
| - | - |
| - | - |
| - | - |
| - | - |
| - | - |
| - | - |
| - | - |
| - | - |
| - | - |
| - | gnl\|UG\|Ta#S13008921 |
| - | gnl\|UG\|Ta#S18006218 |
| - | gnl\|UG\|Ta#S18012388 |
| - | gnl\|UG\|Ta#S32551013 |
| - | gnl\|UG\|Ta#S32718008 |
| - | gnl\|UG\|Ta#S52542813 |
| - | gnl\|UG\|Ta#S52542915 |
| - | gnl\|UG\|Ta#S52543438 |
| - | gnl\|UG\|Ta#S52544781 |
| - | gnl\|UG\|Ta#S52544914 |
| - | gnl\|UG\|Ta#S52545145 |
| - | gnl\|UG\|Ta#S52545772 |

| | |
|---|---|
| - | gnl\|UG\|Ta#S58848148 |
| - | gnl\|UG\|Ta#S58854033 |
| - | gnl\|UG\|Ta#S58856841 |
| - | gnl\|UG\|Ta#S58857094 |
| - | gnl\|UG\|Ta#S58883774 |
| - | gnl\|UG\|Ta#S58883774 |
| - | gnl\|UG\|Ta#S61520307 |
| - | gnl\|UG\|Ta#S61561718 |
| - | gnl\|UG\|Ta#S61578524 |
| - | gnl\|UG\|Ta#S61578524 |
| - | gnl\|UG\|Ta#S61583448 |
| - | gnl\|UG\|Ta#S61586121 |
| - | gnl\|UG\|Ta#S61588056 |
| - | gnl\|UG\|Ta#S61593513 |
| - | gnl\|UG\|Ta#S61593513 |
| - | gnl\|UG\|Ta#S61595665 |
| - | gnl\|UG\|Ta#S61596978 |
| - | gnl\|UG\|Ta#S61615413 |
| - | gnl\|UG\|Ta#S61618594 |
| - | gnl\|UG\|Ta#S61626908 |
| - | gnl\|UG\|Ta#S61627164 |
| - | gnl\|UG\|Ta#S61639979 |
| - | gnl\|UG\|Ta#S61640151 |
| - | gnl\|UG\|Ta#S61641599 |
| - | gnl\|UG\|Ta#S61645444 |
| - | gnl\|UG\|Ta#S61653521 |
| - | gnl\|UG\|Ta#S61766570 |
| - | gnl\|UG\|Ta#S61770378 |
| - | gnl\|UG\|Ta#S61771674 |
| - | gnl\|UG\|Ta#S61772466 |
| - | gnl\|UG\|Ta#S61780811 |
| - | gnl\|UG\|Ta#S61791038 |
| - | gnl\|UG\|Ta#S61792106 |
| - | gnl\|UG\|Ta#S61797600 |
| - | gnl\|UG\|Ta#S61808827 |
| - | gnl\|UG\|Ta#S61809802 |
| - | gnl\|UG\|Ta#S61810481 |
| - | gnl\|UG\|Ta#S61811168 |
| - | gnl\|UG\|Ta#S61811265 |
| - | gnl\|UG\|Ta#S61811749 |
| - | gnl\|UG\|Ta#S61811923 |
| - | gnl\|UG\|Ta#S61813942 |
| - | gnl\|UG\|Ta#S61831039 |
| - | gnl\|UG\|Ta#S61831444 |
| - | gnl\|UG\|Ta#S61831871 |
| - | gnl\|UG\|Ta#S61832070 |
| - | gnl\|UG\|Ta#S61832098 |

| | |
|---|---|
| - | gnl\|UG\|Ta#S61832211 |
| - | gnl\|UG\|Ta#S61832211 |
| - | gnl\|UG\|Ta#S61832401 |
| - | gnl\|UG\|Ta#S61832481 |
| - | gnl\|UG\|Ta#S61845320 |
| - | gnl\|UG\|Ta#S65600846 |
| - | gnl\|UG\|Ta#S65648968 |
| - | gnl\|UG\|Ta#S65679421 |
| - | gnl\|UG\|Ta#S65681709 |
| tr\|M7Z605\|M7Z605_TRIUA | - |
| tr\|M7Z605\|M7Z605_TRIUA | - |
| tr\|W5DNC6\|W5DNC6_WHEAT | - |
| tr\|W5EVT9\|W5EVT9_WHEAT | - |
| tr\|W5F6Q2\|W5F6Q2_WHEAT | - |
| tr\|W5FAJ1\|W5FAJ1_WHEAT | - |
| tr\|W5FBJ2\|W5FBJ2_WHEAT | - |
| tr\|W5FFU8\|W5FFU8_WHEAT | - |
| tr\|W5FIT3\|W5FIT3_WHEAT | - |
| tr\|W5FIY7\|W5FIY7_WHEAT | - |
| tr\|W5FLU0\|W5FLU0_WHEAT | - |
| tr\|W5FNZ9\|W5FNZ9_WHEAT | - |
| tr\|W5EWD3\|W5EWD3_WHEAT | gnl\|UG\|Ta#S17894269 |
| tr\|W5FLE0\|W5FLE0_WHEAT | gnl\|UG\|Ta#S52540999 |
| tr\|W5FXQ7\|W5FXQ7_WHEAT | gnl\|UG\|Ta#S52544563 |
| tr\|T1MKN7\|T1MKN7_TRIUA | gnl\|UG\|Ta#S52545421 |
| tr\|W5F7D2\|W5F7D2_WHEAT | gnl\|UG\|Ta#S52546262 |
| tr\|M7YLP2\|M7YLP2_TRIUA | gnl\|UG\|Ta#S58857532 |
| tr\|W5FP08\|W5FP08_WHEAT | gnl\|UG\|Ta#S58883774 |
| tr\|M8AK74\|M8AK74_TRIUA | gnl\|UG\|Ta#S61529834 |
| tr\|W5FC57\|W5FC57_WHEAT | gnl\|UG\|Ta#S61574510 |
| tr\|W5FBG1\|W5FBG1_WHEAT | gnl\|UG\|Ta#S61586121 |
| tr\|W5FNQ7\|W5FNQ7_WHEAT | gnl\|UG\|Ta#S61592450 |
| tr\|W5FDW0\|W5FDW0_WHEAT | gnl\|UG\|Ta#S61604779 |
| tr\|M7YHQ6\|M7YHQ6_TRIUA | gnl\|UG\|Ta#S61640873 |
| tr\|W5FP08\|W5FP08_WHEAT | gnl\|UG\|Ta#S61641599 |
| tr\|W5FP08\|W5FP08_WHEAT | gnl\|UG\|Ta#S61641599 |
| tr\|W5F766\|W5F766_WHEAT | gnl\|UG\|Ta#S61807462 |
| tr\|W5F7R8\|W5F7R8_WHEAT | gnl\|UG\|Ta#S61809617 |
| tr\|W5FCB9\|W5FCB9_WHEAT | gnl\|UG\|Ta#S61831441 |
| tr\|M7YJK5\|M7YJK5_TRIUA | gnl\|UG\|Ta#S61831531 |
| tr\|W5FLW7\|W5FLW7_WHEAT | gnl\|UG\|Ta#S61831644 |
| tr\|W5FKH8\|W5FKH8_WHEAT | gnl\|UG\|Ta#S61832070 |
| tr\|W5FKH8\|W5FKH8_WHEAT | gnl\|UG\|Ta#S61832070 |
| tr\|W5F9U9\|W5F9U9_WHEAT | gnl\|UG\|Ta#S65654907 |
| - | - |
| - | - |
| - | - |

| | |
|---|---|
| - | - |
| - | - |
| - | - |
| - | - |
| - | - |
| - | - |
| - | - |
| - | - |
| - | - |
| - | - |
| - | - |
| - | - |
| - | - |
| - | - |
| - | - |
| - | - |
| - | - |
| - | - |
| - | - |
| - | - |
| - | - |
| - | - |
| - | - |
| - | - |
| - | - |
| - | - |
| - | - |
| - | - |
| - | - |
| - | - |
| - | - |
| - | - |
| - | - |
| - | - |
| - | - |
| - | - |
| - | - |
| - | - |
| - | - |
| - | - |
| - | - |
| - | - |
| - | - |
| - | - |
| - | - |
| - | - |
| - | - |

| | |
|---|---|
| - | - |
| - | - |
| - | - |
| - | - |
| - | - |
| - | - |
| - | - |
| - | - |
| - | - |
| - | - |
| - | - |
| - | - |
| - | - |
| - | - |
| - | - |
| - | - |
| - | - |
| - | - |
| - | - |
| - | - |
| - | - |
| - | - |
| - | - |
| - | - |
| - | - |
| - | - |
| - | - |
| - | - |
| - | - |
| - | - |
| - | - |
| - | - |
| - | - |
| - | - |
| - | - |
| - | - |
| - | - |
| - | - |
| - | - |
| - | - |
| - | - |
| - | - |
| - | - |
| - | - |
| - | - |
| - | - |
| - | - |

| | |
|---|---|
| - | - |
| - | - |
| - | - |
| - | - |
| - | - |
| - | - |
| - | - |
| - | - |
| - | - |
| - | - |
| - | - |
| - | - |
| - | - |
| - | - |
| - | - |
| - | - |
| - | - |
| - | - |
| - | - |
| - | - |
| - | - |
| - | - |
| - | - |
| - | - |
| - | - |
| - | - |
| - | - |
| - | gnl\|UG\|Ta#S12861187 |
| - | gnl\|UG\|Ta#S12861187 |
| - | gnl\|UG\|Ta#S12883260 |
| - | gnl\|UG\|Ta#S13208132 |
| - | gnl\|UG\|Ta#S16209972 |
| - | gnl\|UG\|Ta#S16239455 |
| - | gnl\|UG\|Ta#S17890499 |
| - | gnl\|UG\|Ta#S52542804 |
| - | gnl\|UG\|Ta#S52543364 |
| - | gnl\|UG\|Ta#S52543438 |
| - | gnl\|UG\|Ta#S52543537 |
| - | gnl\|UG\|Ta#S52543537 |
| - | gnl\|UG\|Ta#S52544735 |
| - | gnl\|UG\|Ta#S52545130 |
| - | gnl\|UG\|Ta#S52545265 |
| - | gnl\|UG\|Ta#S52545646 |
| - | gnl\|UG\|Ta#S58855885 |
| - | gnl\|UG\|Ta#S58868541 |
| - | gnl\|UG\|Ta#S58868541 |
| - | gnl\|UG\|Ta#S61520070 |

| | |
|---|---|
| - | gnl\|UG\|Ta#S61539367 |
| - | gnl\|UG\|Ta#S61543175 |
| - | gnl\|UG\|Ta#S61547270 |
| - | gnl\|UG\|Ta#S61567142 |
| - | gnl\|UG\|Ta#S61572386 |
| - | gnl\|UG\|Ta#S61581995 |
| - | gnl\|UG\|Ta#S61586606 |
| - | gnl\|UG\|Ta#S61593101 |
| - | gnl\|UG\|Ta#S61597217 |
| - | gnl\|UG\|Ta#S61598375 |
| - | gnl\|UG\|Ta#S61608360 |
| - | gnl\|UG\|Ta#S61613697 |
| - | gnl\|UG\|Ta#S61614413 |
| - | gnl\|UG\|Ta#S61616923 |
| - | gnl\|UG\|Ta#S61622594 |
| - | gnl\|UG\|Ta#S61634954 |
| - | gnl\|UG\|Ta#S61635864 |
| - | gnl\|UG\|Ta#S61640184 |
| - | gnl\|UG\|Ta#S61640184 |
| - | gnl\|UG\|Ta#S61645368 |
| - | gnl\|UG\|Ta#S61650699 |
| - | gnl\|UG\|Ta#S61682250 |
| - | gnl\|UG\|Ta#S61697351 |
| - | gnl\|UG\|Ta#S61702322 |
| - | gnl\|UG\|Ta#S61707123 |
| - | gnl\|UG\|Ta#S61729734 |
| - | gnl\|UG\|Ta#S61730879 |
| - | gnl\|UG\|Ta#S61731931 |
| - | gnl\|UG\|Ta#S61736096 |
| - | gnl\|UG\|Ta#S61758531 |
| - | gnl\|UG\|Ta#S61780811 |
| - | gnl\|UG\|Ta#S61780811 |
| - | gnl\|UG\|Ta#S61781954 |
| - | gnl\|UG\|Ta#S61811928 |
| - | gnl\|UG\|Ta#S61812003 |
| - | gnl\|UG\|Ta#S61812279 |
| - | gnl\|UG\|Ta#S61812354 |
| - | gnl\|UG\|Ta#S61812377 |
| - | gnl\|UG\|Ta#S61812973 |
| - | gnl\|UG\|Ta#S61828228 |
| - | gnl\|UG\|Ta#S61829341 |
| - | gnl\|UG\|Ta#S61829426 |
| - | gnl\|UG\|Ta#S61829473 |
| - | gnl\|UG\|Ta#S61830212 |
| - | gnl\|UG\|Ta#S61831360 |
| - | gnl\|UG\|Ta#S61831408 |
| - | gnl\|UG\|Ta#S61831900 |

| | |
|---|---|
| - | gnl\|UG\|Ta#S61832213 |
| - | gnl\|UG\|Ta#S61832532 |
| - | gnl\|UG\|Ta#S65605638 |
| - | gnl\|UG\|Ta#S65610505 |
| - | gnl\|UG\|Ta#S65613174 |
| - | gnl\|UG\|Ta#S65625001 |
| - | gnl\|UG\|Ta#S65655562 |
| - | gnl\|UG\|Ta#S65671336 |
| - | gnl\|UG\|Ta#S65688639 |
| - | gnl\|UG\|Ta#S65722398 |
| - | gnl\|UG\|Ta#S65723085 |
| tr\|E0Y9Z4\|E0Y9Z4_WHEAT | - |
| tr\|G1FI00\|G1FI00_9POAL | - |
| tr\|M7YIH3\|M7YIH3_TRIUA | - |
| tr\|M7ZSC6\|M7ZSC6_TRIUA | - |
| tr\|M7ZSN9\|M7ZSN9_TRIUA | - |
| tr\|M8AJN0\|M8AJN0_TRIUA | - |
| tr\|Q2L3S0\|Q2L3S0_WHEAT | - |
| tr\|W5BQ01\|W5BQ01_WHEAT | - |
| tr\|W5DQ53\|W5DQ53_WHEAT | - |
| tr\|W5EY54\|W5EY54_WHEAT | - |
| tr\|W5F7J6\|W5F7J6_WHEAT | - |
| tr\|W5F8P3\|W5F8P3_WHEAT | - |
| tr\|W5FAJ6\|W5FAJ6_WHEAT | - |
| tr\|W5FBE3\|W5FBE3_WHEAT | - |
| tr\|W5FCD8\|W5FCD8_WHEAT | - |
| tr\|W5FDR6\|W5FDR6_WHEAT | - |
| tr\|W5FME1\|W5FME1_WHEAT | - |
| tr\|W5FME1\|W5FME1_WHEAT | - |
| tr\|W5FPH1\|W5FPH1_WHEAT | - |
| tr\|W5F9G0\|W5F9G0_WHEAT | gnl\|UG\|Ta#S32589786 |
| tr\|M7YJZ3\|M7YJZ3_TRIUA | gnl\|UG\|Ta#S52542813 |
| tr\|W5F790\|W5F790_WHEAT | gnl\|UG\|Ta#S52542813 |
| tr\|W5FM09\|W5FM09_WHEAT | gnl\|UG\|Ta#S52542905 |
| tr\|M7ZZD2\|M7ZZD2_TRIUA | gnl\|UG\|Ta#S52543897 |
| tr\|W5F2U2\|W5F2U2_WHEAT | gnl\|UG\|Ta#S52544885 |
| tr\|T1M9J8\|T1M9J8_TRIUA | gnl\|UG\|Ta#S52545231 |
| tr\|W5F781\|W5F781_WHEAT | gnl\|UG\|Ta#S52545231 |
| tr\|W5FFN4\|W5FFN4_WHEAT | gnl\|UG\|Ta#S58859259 |
| tr\|W5FSU9\|W5FSU9_WHEAT | gnl\|UG\|Ta#S61529834 |
| tr\|W5FK65\|W5FK65_WHEAT | gnl\|UG\|Ta#S61573314 |
| tr\|W5FAL2\|W5FAL2_WHEAT | gnl\|UG\|Ta#S61653260 |
| tr\|W5FGX8\|W5FGX8_WHEAT | gnl\|UG\|Ta#S61788783 |
| tr\|M8AKV7\|M8AKV7_TRIUA | gnl\|UG\|Ta#S61807673 |
| tr\|M8A520\|M8A520_TRIUA | gnl\|UG\|Ta#S61807761 |
| tr\|M7ZMW0\|M7ZMW0_TRIUA | gnl\|UG\|Ta#S61811064 |
| tr\|M8A6X3\|M8A6X3_TRIUA | gnl\|UG\|Ta#S61811841 |

| | |
|---|---|
| tr\|W5GII3\|W5GII3_WHEAT | gnl\|UG\|Ta#S61812160 |
| tr\|W5H4L2\|W5H4L2_WHEAT | gnl\|UG\|Ta#S61832252 |
| tr\|W5FIG7\|W5FIG7_WHEAT | gnl\|UG\|Ta#S65590756 |
| tr\|W5FF97\|W5FF97_WHEAT | gnl\|UG\|Ta#S65625818 |
| tr\|W5FJD5\|W5FJD5_WHEAT | gnl\|UG\|Ta#S65705921 |
| tr\|W5FZ86\|W5FZ86_WHEAT | - |
| tr\|W5FV45\|W5FV45_WHEAT | - |
| tr\|W5FTS3\|W5FTS3_WHEAT | - |
| tr\|W5FTK4\|W5FTK4_WHEAT | - |
| tr\|W5FP28\|W5FP28_WHEAT | - |
| tr\|W5FNV4\|W5FNV4_WHEAT | - |
| tr\|W5FMT7\|W5FMT7_WHEAT | - |
| tr\|W5FM29\|W5FM29_WHEAT | - |
| tr\|W5FJP5\|W5FJP5_WHEAT | - |
| tr\|W5FJ31\|W5FJ31_WHEAT | - |
| tr\|W5FHQ2\|W5FHQ2_WHEAT | - |
| tr\|W5FHH1\|W5FHH1_WHEAT | - |
| tr\|W5FGS4\|W5FGS4_WHEAT | - |
| tr\|W5FGS0\|W5FGS0_WHEAT | - |
| tr\|W5FGD9\|W5FGD9_WHEAT | - |
| tr\|W5FG37\|W5FG37_WHEAT | - |
| tr\|W5FFP0\|W5FFP0_WHEAT | - |
| tr\|W5FER7\|W5FER7_WHEAT | - |
| tr\|W5FDZ7\|W5FDZ7_WHEAT | - |
| tr\|W5FDA1\|W5FDA1_WHEAT | - |
| tr\|W5FD61\|W5FD61_WHEAT | - |
| tr\|W5FB26\|W5FB26_WHEAT | - |
| tr\|W5FB23\|W5FB23_WHEAT | - |
| tr\|W5FAJ3\|W5FAJ3_WHEAT | - |
| tr\|W5F9U9\|W5F9U9_WHEAT | - |
| tr\|W5F8W2\|W5F8W2_WHEAT | - |
| tr\|W5F7P5\|W5F7P5_WHEAT | - |
| tr\|W5F7L0\|W5F7L0_WHEAT | - |
| tr\|W5F7E4\|W5F7E4_WHEAT | - |
| tr\|W5F1A1\|W5F1A1_WHEAT | - |
| tr\|W5EWG6\|W5EWG6_WHEAT | - |
| tr\|W5E5L3\|W5E5L3_WHEAT | - |
| tr\|W5E0Q6\|W5E0Q6_WHEAT | - |
| tr\|W5DS15\|W5DS15_WHEAT | - |
| tr\|W5DRM7\|W5DRM7_WHEAT | - |
| tr\|W5C578\|W5C578_WHEAT | - |
| tr\|W5AFG7\|W5AFG7_WHEAT | - |
| tr\|W5ABK1\|W5ABK1_WHEAT | - |
| tr\|M7ZC62\|M7ZC62_TRIUA | - |
| tr\|M7Z6V5\|M7Z6V5_TRIUA | - |
| tr\|W5FJ16\|W5FJ16_WHEAT | gnl\|UG\|Ta#S17984949 |
| tr\|Q93ZK5\|Q93ZK5_WHEAT | gnl\|UG\|Ta#S22391606 |

| | |
|---|---|
| tr\|W5HGF6\|W5HGF6_WHEAT | gnl\|UG\|Ta#S26025003 |
| tr\|W5DMX5\|W5DMX5_WHEAT | gnl\|UG\|Ta#S52541234 |
| tr\|W5F9T6\|W5F9T6_WHEAT | gnl\|UG\|Ta#S52541503 |
| tr\|M7ZSZ3\|M7ZSZ3_TRIUA | gnl\|UG\|Ta#S52541772 |
| tr\|W5FG75\|W5FG75_WHEAT | gnl\|UG\|Ta#S52542196 |
| tr\|W5FFH5\|W5FFH5_WHEAT | gnl\|UG\|Ta#S52542198 |
| tr\|M7ZS53\|M7ZS53_TRIUA | gnl\|UG\|Ta#S52542653 |
| tr\|W5FBH7\|W5FBH7_WHEAT | gnl\|UG\|Ta#S52542744 |
| tr\|M7ZC23\|M7ZC23_TRIUA | gnl\|UG\|Ta#S52544360 |
| tr\|W5F9J4\|W5F9J4_WHEAT | gnl\|UG\|Ta#S52544885 |
| tr\|W5FDZ3\|W5FDZ3_WHEAT | gnl\|UG\|Ta#S52545541 |
| tr\|M8AIW9\|M8AIW9_TRIUA | gnl\|UG\|Ta#S52545940 |
| tr\|W5FLH7\|W5FLH7_WHEAT | gnl\|UG\|Ta#S52546421 |
| tr\|W5G4N2\|W5G4N2_WHEAT | gnl\|UG\|Ta#S52547045 |
| tr\|W5EX56\|W5EX56_WHEAT | gnl\|UG\|Ta#S58890727 |
| tr\|T1LAS1\|T1LAS1_TRIUA | gnl\|UG\|Ta#S58898085 |
| tr\|W5F7E5\|W5F7E5_WHEAT | gnl\|UG\|Ta#S61519406 |
| tr\|W5A8R9\|W5A8R9_WHEAT | gnl\|UG\|Ta#S61525508 |
| tr\|W5G031\|W5G031_WHEAT | gnl\|UG\|Ta#S61570827 |
| tr\|W5FPL5\|W5FPL5_WHEAT | gnl\|UG\|Ta#S61597217 |
| tr\|W5FP14\|W5FP14_WHEAT | gnl\|UG\|Ta#S61604116 |
| tr\|W5FSW2\|W5FSW2_WHEAT | gnl\|UG\|Ta#S61615168 |
| tr\|W5FJ78\|W5FJ78_WHEAT | gnl\|UG\|Ta#S61626869 |
| tr\|W5FNZ7\|W5FNZ7_WHEAT | gnl\|UG\|Ta#S61636976 |
| tr\|M8AQT0\|M8AQT0_TRIUA | gnl\|UG\|Ta#S61651398 |
| tr\|W5F8F9\|W5F8F9_WHEAT | gnl\|UG\|Ta#S61653789 |
| tr\|W5FLZ4\|W5FLZ4_WHEAT | gnl\|UG\|Ta#S61654041 |
| tr\|W5HR48\|W5HR48_WHEAT | gnl\|UG\|Ta#S61655796 |
| tr\|W5FK49\|W5FK49_WHEAT | gnl\|UG\|Ta#S61717026 |
| tr\|W5FM46\|W5FM46_WHEAT | gnl\|UG\|Ta#S61770036 |
| tr\|W5FHI0\|W5FHI0_WHEAT | gnl\|UG\|Ta#S61781265 |
| tr\|M7YUI7\|M7YUI7_TRIUA | gnl\|UG\|Ta#S61807761 |
| tr\|W5FM27\|W5FM27_WHEAT | gnl\|UG\|Ta#S61808173 |
| tr\|W5FTL3\|W5FTL3_WHEAT | gnl\|UG\|Ta#S61809207 |
| tr\|W5FFK0\|W5FFK0_WHEAT | gnl\|UG\|Ta#S61811185 |
| tr\|W5FFK0\|W5FFK0_WHEAT | gnl\|UG\|Ta#S61811185 |
| tr\|W5FU87\|W5FU87_WHEAT | gnl\|UG\|Ta#S61811608 |
| tr\|W5FMT7\|W5FMT7_WHEAT | gnl\|UG\|Ta#S61812326 |
| tr\|W5FLJ9\|W5FLJ9_WHEAT | gnl\|UG\|Ta#S61813843 |
| tr\|W5FP33\|W5FP33_WHEAT | gnl\|UG\|Ta#S61830054 |
| tr\|W5FLD4\|W5FLD4_WHEAT | gnl\|UG\|Ta#S61832192 |
| tr\|W5FM19\|W5FM19_WHEAT | gnl\|UG\|Ta#S61855581 |
| tr\|M7ZCT2\|M7ZCT2_TRIUA | gnl\|UG\|Ta#S61862638 |
| tr\|M7Z7U3\|M7Z7U3_TRIUA | gnl\|UG\|Ta#S65588561 |
| tr\|W5F9T4\|W5F9T4_WHEAT | gnl\|UG\|Ta#S65594346 |
| tr\|W5G3A3\|W5G3A3_WHEAT | gnl\|UG\|Ta#S65614553 |
| tr\|W5FD07\|W5FD07_WHEAT | gnl\|UG\|Ta#S65628405 |

| | |
|---|---|
| tr\|M7ZA47\|M7ZA47_TRIUA | gnl\|UG\|Ta#S65633749 |
| tr\|W5FAQ4\|W5FAQ4_WHEAT | gnl\|UG\|Ta#S65652227 |
| tr\|W5F9U9\|W5F9U9_WHEAT | gnl\|UG\|Ta#S65654907 |
| tr\|W5FH95\|W5FH95_WHEAT | gnl\|UG\|Ta#S65667196 |
| tr\|M7YRY2\|M7YRY2_TRIUA | gnl\|UG\|Ta#S65672481 |
| tr\|W5EYR2\|W5EYR2_WHEAT | gnl\|UG\|Ta#S65682185 |
| sp\|O04437\|GSTZ_WHEAT | gnl\|UG\|Ta#S65708882 |
| - | - |
| - | - |
| - | - |
| - | - |
| - | - |
| - | - |
| - | - |
| - | - |
| - | - |
| - | - |
| - | - |
| - | - |
| - | - |
| - | - |
| - | - |
| - | - |
| - | - |
| - | - |
| - | - |
| - | - |
| - | - |
| - | - |
| - | - |
| - | - |
| - | - |
| - | - |
| - | - |
| - | - |
| - | - |
| - | - |
| - | - |
| - | - |
| - | - |
| - | - |
| - | - |
| - | - |
| - | - |
| - | - |
| - | - |
| - | - |

| | |
|---|---|
| - | - |
| - | - |
| - | - |
| - | - |
| - | - |
| - | - |
| - | - |
| - | - |
| - | - |
| - | - |
| - | - |
| - | - |
| - | - |
| - | - |
| - | - |
| - | - |
| - | - |
| - | - |
| - | - |
| - | - |
| - | - |
| - | - |
| - | - |
| - | - |
| - | - |
| - | - |
| - | - |
| - | - |
| - | - |
| - | - |
| - | - |
| - | - |
| - | - |
| - | - |
| - | - |
| - | - |
| - | - |
| - | - |
| - | - |
| - | - |
| - | - |
| - | - |
| - | - |
| - | - |
| - | - |
| - | - |
| - | - |

| | |
|---|---|
| - | - |
| - | - |
| - | - |
| - | - |
| - | - |
| - | - |
| - | - |
| - | - |
| - | - |
| - | - |
| - | - |
| - | - |
| - | - |
| - | - |
| - | - |
| - | - |
| - | - |
| - | - |
| - | - |
| - | - |
| - | - |
| - | - |
| - | - |
| - | - |
| - | - |
| - | - |
| - | - |
| - | - |
| - | - |
| - | - |
| - | - |
| - | - |
| - | - |
| - | - |
| - | - |
| - | - |
| - | - |
| - | - |
| - | - |
| - | - |
| - | - |
| - | - |
| - | - |
| - | - |
| - | - |
| - | - |
| - | - |

| | |
|---|---|
| - | - |
| - | - |
| - | - |
| - | - |
| - | - |
| - | - |
| - | - |
| - | - |
| - | - |
| - | - |
| - | - |
| - | - |
| - | - |
| - | - |
| - | - |
| - | - |
| - | - |
| - | - |
| - | - |
| - | - |
| - | - |
| - | - |
| - | - |
| - | - |
| - | - |
| - | - |
| - | - |
| - | - |
| - | - |
| - | - |
| - | - |
| - | gnl\|UG\|Ta#S12891400 |
| - | gnl\|UG\|Ta#S16058021 |
| - | gnl\|UG\|Ta#S17889042 |
| - | gnl\|UG\|Ta#S17889042 |
| - | gnl\|UG\|Ta#S17889572 |
| - | gnl\|UG\|Ta#S17893541 |
| - | gnl\|UG\|Ta#S32428706 |
| - | gnl\|UG\|Ta#S32429159 |
| - | gnl\|UG\|Ta#S32668000 |
| - | gnl\|UG\|Ta#S32718008 |
| - | gnl\|UG\|Ta#S37916533 |
| - | gnl\|UG\|Ta#S37930664 |
| - | gnl\|UG\|Ta#S52542496 |
| - | gnl\|UG\|Ta#S52543168 |
| - | gnl\|UG\|Ta#S52543419 |
| - | gnl\|UG\|Ta#S52543475 |

| | |
|---|---|
| - | gnl\|UG\|Ta#S52543675 |
| - | gnl\|UG\|Ta#S52543901 |
| - | gnl\|UG\|Ta#S52544716 |
| - | gnl\|UG\|Ta#S52544911 |
| - | gnl\|UG\|Ta#S52545541 |
| - | gnl\|UG\|Ta#S52545885 |
| - | gnl\|UG\|Ta#S52545973 |
| - | gnl\|UG\|Ta#S52546514 |
| - | gnl\|UG\|Ta#S52547034 |
| - | gnl\|UG\|Ta#S52547046 |
| - | gnl\|UG\|Ta#S58854401 |
| - | gnl\|UG\|Ta#S58854401 |
| - | gnl\|UG\|Ta#S58860703 |
| - | gnl\|UG\|Ta#S58862493 |
| - | gnl\|UG\|Ta#S58865880 |
| - | gnl\|UG\|Ta#S58868541 |
| - | gnl\|UG\|Ta#S58891237 |
| - | gnl\|UG\|Ta#S58893656 |
| - | gnl\|UG\|Ta#S58901294 |
| - | gnl\|UG\|Ta#S58903046 |
| - | gnl\|UG\|Ta#S58908769 |
| - | gnl\|UG\|Ta#S59489969 |
| - | gnl\|UG\|Ta#S59489969 |
| - | gnl\|UG\|Ta#S61525011 |
| - | gnl\|UG\|Ta#S61543599 |
| - | gnl\|UG\|Ta#S61549792 |
| - | gnl\|UG\|Ta#S61568505 |
| - | gnl\|UG\|Ta#S61572386 |
| - | gnl\|UG\|Ta#S61581803 |
| - | gnl\|UG\|Ta#S61594391 |
| - | gnl\|UG\|Ta#S61595161 |
| - | gnl\|UG\|Ta#S61600690 |
| - | gnl\|UG\|Ta#S61607976 |
| - | gnl\|UG\|Ta#S61608332 |
| - | gnl\|UG\|Ta#S61608846 |
| - | gnl\|UG\|Ta#S61620693 |
| - | gnl\|UG\|Ta#S61640166 |
| - | gnl\|UG\|Ta#S61640166 |
| - | gnl\|UG\|Ta#S61641599 |
| - | gnl\|UG\|Ta#S61647697 |
| - | gnl\|UG\|Ta#S61650699 |
| - | gnl\|UG\|Ta#S61653442 |
| - | gnl\|UG\|Ta#S61665149 |
| - | gnl\|UG\|Ta#S61725672 |
| - | gnl\|UG\|Ta#S61748714 |
| - | gnl\|UG\|Ta#S61752565 |
| - | gnl\|UG\|Ta#S61755040 |

| | |
|---|---|
| - | gnl\|UG\|Ta#S61755442 |
| - | gnl\|UG\|Ta#S61761485 |
| - | gnl\|UG\|Ta#S61771319 |
| - | gnl\|UG\|Ta#S61774462 |
| - | gnl\|UG\|Ta#S61780711 |
| - | gnl\|UG\|Ta#S61781176 |
| - | gnl\|UG\|Ta#S61781210 |
| - | gnl\|UG\|Ta#S61781265 |
| - | gnl\|UG\|Ta#S61782020 |
| - | gnl\|UG\|Ta#S61782097 |
| - | gnl\|UG\|Ta#S61791857 |
| - | gnl\|UG\|Ta#S61800629 |
| - | gnl\|UG\|Ta#S61802459 |
| - | gnl\|UG\|Ta#S61807761 |
| - | gnl\|UG\|Ta#S61807839 |
| - | gnl\|UG\|Ta#S61808173 |
| - | gnl\|UG\|Ta#S61808316 |
| - | gnl\|UG\|Ta#S61808380 |
| - | gnl\|UG\|Ta#S61809617 |
| - | gnl\|UG\|Ta#S61810381 |
| - | gnl\|UG\|Ta#S61811044 |
| - | gnl\|UG\|Ta#S61811579 |
| - | gnl\|UG\|Ta#S61811672 |
| - | gnl\|UG\|Ta#S61811730 |
| - | gnl\|UG\|Ta#S61811832 |
| - | gnl\|UG\|Ta#S61811851 |
| - | gnl\|UG\|Ta#S61811851 |
| - | gnl\|UG\|Ta#S61812028 |
| - | gnl\|UG\|Ta#S61812117 |
| - | gnl\|UG\|Ta#S61812450 |
| - | gnl\|UG\|Ta#S61812476 |
| - | gnl\|UG\|Ta#S61812519 |
| - | gnl\|UG\|Ta#S61812547 |
| - | gnl\|UG\|Ta#S61812904 |
| - | gnl\|UG\|Ta#S61828625 |
| - | gnl\|UG\|Ta#S61829968 |
| - | gnl\|UG\|Ta#S61830164 |
| - | gnl\|UG\|Ta#S61830358 |
| - | gnl\|UG\|Ta#S61830358 |
| - | gnl\|UG\|Ta#S61830374 |
| - | gnl\|UG\|Ta#S61831039 |
| - | gnl\|UG\|Ta#S61831142 |
| - | gnl\|UG\|Ta#S61831551 |
| - | gnl\|UG\|Ta#S61831676 |
| - | gnl\|UG\|Ta#S61832211 |
| - | gnl\|UG\|Ta#S61832348 |
| - | gnl\|UG\|Ta#S61832481 |

| | |
|---|---|
| - | gnl\|UG\|Ta#S61867721 |
| - | gnl\|UG\|Ta#S65595031 |
| - | gnl\|UG\|Ta#S65609370 |
| - | gnl\|UG\|Ta#S65622829 |
| - | gnl\|UG\|Ta#S65639365 |
| - | gnl\|UG\|Ta#S65659322 |
| - | gnl\|UG\|Ta#S65678974 |
| - | gnl\|UG\|Ta#S65709965 |

| Contig/read name | B | R | S | B | UniProt | UniGene |
|---|---|---|---|---|---|---|
| contig14591 | - | - | - | - | tr\|W5HJ75\|W5HJ75_WHEAT | - |
| contig12447 | - | - | - | - | tr\|W5G2V9\|W5G2V9_WHEAT | - |
| contig06595 | - | - | - | - | tr\|W5FW55\|W5FW55_WHEAT | - |
| contig05076 | - | - | - | - | tr\|W5FVU4\|W5FVU4_WHEAT | - |
| contig09757 | - | - | - | - | tr\|W5FTE4\|W5FTE4_WHEAT | - |
| contig22022 | - | - | - | - | tr\|W5FS10\|W5FS10_WHEAT | - |
| contig11166 | - | - | - | - | tr\|W5FQ61\|W5FQ61_WHEAT | - |
| contig20627 | - | - | - | - | tr\|W5FPR6\|W5FPR6_WHEAT | - |
| contig06184 | - | - | - | - | tr\|W5FP89\|W5FP89_WHEAT | - |
| contig09095 | - | - | - | - | tr\|W5FP66\|W5FP66_WHEAT | - |
| contig19143 | - | - | - | - | tr\|W5FP64\|W5FP64_WHEAT | - |
| contig08468 | - | - | - | - | tr\|W5FP42\|W5FP42_WHEAT | - |
| contig04409 | - | - | - | - | tr\|W5FNV4\|W5FNV4_WHEAT | - |
| contig05890 | - | - | - | - | tr\|W5FNQ3\|W5FNQ3_WHEAT | - |
| contig04710 | - | - | - | - | tr\|W5FNL1\|W5FNL1_WHEAT | - |
| contig00231 | - | - | - | - | tr\|W5FNH1\|W5FNH1_WHEAT | - |
| contig15251 | - | - | - | - | tr\|W5FM09\|W5FM09_WHEAT | - |
| contig01923 | - | - | - | - | tr\|W5FLZ9\|W5FLZ9_WHEAT | - |
| contig12124 | - | - | - | - | tr\|W5FLX4\|W5FLX4_WHEAT | - |
| contig17505 | - | - | - | - | tr\|W5FLW2\|W5FLW2_WHEAT | - |
| contig16241 | - | - | - | - | tr\|W5FLU0\|W5FLU0_WHEAT | - |
| contig19052 | - | - | - | - | tr\|W5FLM3\|W5FLM3_WHEAT | - |
| contig01260 | - | - | - | - | tr\|W5FLI0\|W5FLI0_WHEAT | - |
| contig03559 | - | - | - | - | tr\|W5FLH6\|W5FLH6_WHEAT | - |
| contig19193 | - | - | - | - | tr\|W5FLC5\|W5FLC5_WHEAT | - |
| contig01565 | - | - | - | - | tr\|W5FL25\|W5FL25_WHEAT | - |
| contig20620 | - | - | - | - | tr\|W5FL20\|W5FL20_WHEAT | - |
| contig02112 | - | - | - | - | tr\|W5FJZ4\|W5FJZ4_WHEAT | - |
| contig06598 | - | - | - | - | tr\|W5FJT5\|W5FJT5_WHEAT | - |
| contig00259 | - | - | - | - | tr\|W5FJS3\|W5FJS3_WHEAT | - |
| contig08851 | - | - | - | - | tr\|W5FJS3\|W5FJS3_WHEAT | - |
| contig04478 | - | - | - | - | tr\|W5FJR5\|W5FJR5_WHEAT | - |
| contig06253 | - | - | - | - | tr\|W5FJ43\|W5FJ43_WHEAT | - |
| contig16055 | - | - | - | - | tr\|W5FIT3\|W5FIT3_WHEAT | - |
| contig23397 | - | - | - | - | tr\|W5FIL6\|W5FIL6_WHEAT | - |
| contig07794 | - | - | - | - | tr\|W5FIA6\|W5FIA6_WHEAT | - |
| contig02328 | - | - | - | - | tr\|W5FI83\|W5FI83_WHEAT | - |
| contig24808 | - | - | - | - | tr\|W5FI83\|W5FI83_WHEAT | - |
| contig14934 | - | - | - | - | tr\|W5FI80\|W5FI80_WHEAT | - |
| contig20307 | - | - | - | - | tr\|W5FHX1\|W5FHX1_WHEAT | - |
| contig02158 | - | - | - | - | tr\|W5FHV7\|W5FHV7_WHEAT | - |
| contig11336 | - | - | - | - | tr\|W5FHV7\|W5FHV7_WHEAT | - |
| contig18640 | - | - | - | - | tr\|W5FHQ4\|W5FHQ4_WHEAT | - |
| contig24105 | - | - | - | - | tr\|W5FHQ4\|W5FHQ4_WHEAT | - |
| contig22045 | - | - | - | - | tr\|W5FHK6\|W5FHK6_WHEAT | - |
| contig12726 | - | - | - | - | tr\|W5FHJ1\|W5FHJ1_WHEAT | - |
| contig24299 | - | - | - | - | tr\|W5FHB7\|W5FHB7_WHEAT | - |
| contig10147 | - | - | - | - | tr\|W5FH82\|W5FH82_WHEAT | - |
| contig10730 | - | - | - | - | tr\|W5FH26\|W5FH26_WHEAT | - |

| | | | | | | | |
|---|---|---|---|---|---|---|---|
| contig13874 | - | - | - | - | tr\|W5FGM7\|W5FGM7_WHEAT | - |
| contig00166 | - | - | - | - | tr\|W5FGD2\|W5FGD2_WHEAT | - |
| contig02470 | - | - | - | - | tr\|W5FFE0\|W5FFE0_WHEAT | - |
| contig26302 | - | - | - | - | tr\|W5FFC8\|W5FFC8_WHEAT | - |
| contig13991 | - | - | - | - | tr\|W5FEX5\|W5FEX5_WHEAT | - |
| contig16655 | - | - | - | - | tr\|W5FEX3\|W5FEX3_WHEAT | - |
| contig25239 | - | - | - | - | tr\|W5FEW6\|W5FEW6_WHEAT | - |
| contig15521 | - | - | - | - | tr\|W5FES0\|W5FES0_WHEAT | - |
| contig00244 | - | - | - | - | tr\|W5FEG3\|W5FEG3_WHEAT | - |
| contig14733 | - | - | - | - | tr\|W5FEG3\|W5FEG3_WHEAT | - |
| contig24406 | - | - | - | - | tr\|W5FEB3\|W5FEB3_WHEAT | - |
| contig23493 | - | - | - | - | tr\|W5FE05\|W5FE05_WHEAT | - |
| contig01116 | - | - | - | - | tr\|W5FDW0\|W5FDW0_WHEAT | - |
| contig11283 | - | - | - | - | tr\|W5FDS6\|W5FDS6_WHEAT | - |
| contig03200 | - | - | - | - | tr\|W5FDN9\|W5FDN9_WHEAT | - |
| contig14568 | - | - | - | - | tr\|W5FDM9\|W5FDM9_WHEAT | - |
| contig08175 | - | - | - | - | tr\|W5FDA0\|W5FDA0_WHEAT | - |
| contig11137 | - | - | - | - | tr\|W5FCP7\|W5FCP7_WHEAT | - |
| contig16603 | - | - | - | - | tr\|W5FCN2\|W5FCN2_WHEAT | - |
| contig09227 | - | - | - | - | tr\|W5FCB7\|W5FCB7_WHEAT | - |
| contig08889 | - | - | - | - | tr\|W5FBR4\|W5FBR4_WHEAT | - |
| contig10049 | - | - | - | - | tr\|W5FBL9\|W5FBL9_WHEAT | - |
| contig15941 | - | - | - | - | tr\|W5FBF3\|W5FBF3_WHEAT | - |
| contig15189 | - | - | - | - | tr\|W5FB94\|W5FB94_WHEAT | - |
| contig17596 | - | - | - | - | tr\|W5FB40\|W5FB40_WHEAT | - |
| contig07699 | - | - | - | - | tr\|W5FB33\|W5FB33_WHEAT | - |
| contig09947 | - | - | - | - | tr\|W5FAY1\|W5FAY1_WHEAT | - |
| contig14881 | - | - | - | - | tr\|W5FAY1\|W5FAY1_WHEAT | - |
| contig04690 | - | - | - | - | tr\|W5FA90\|W5FA90_WHEAT | - |
| contig25716 | - | - | - | - | tr\|W5FA19\|W5FA19_WHEAT | - |
| contig18813 | - | - | - | - | tr\|W5F9T6\|W5F9T6_WHEAT | - |
| contig07648 | - | - | - | - | tr\|W5F9T4\|W5F9T4_WHEAT | - |
| contig06144 | - | - | - | - | tr\|W5F9R7\|W5F9R7_WHEAT | - |
| contig20306 | - | - | - | - | tr\|W5F9K1\|W5F9K1_WHEAT | - |
| contig21144 | - | - | - | - | tr\|W5F9G3\|W5F9G3_WHEAT | - |
| contig08616 | - | - | - | - | tr\|W5F970\|W5F970_WHEAT | - |
| contig03331 | - | - | - | - | tr\|W5F8Q1\|W5F8Q1_WHEAT | - |
| contig08886 | - | - | - | - | tr\|W5F8P3\|W5F8P3_WHEAT | - |
| contig18016 | - | - | - | - | tr\|W5F8C5\|W5F8C5_WHEAT | - |
| contig09787 | - | - | - | - | tr\|W5F8A6\|W5F8A6_WHEAT | - |
| contig07608 | - | - | - | - | tr\|W5F7X7\|W5F7X7_WHEAT | - |
| contig18231 | - | - | - | - | tr\|W5F7S1\|W5F7S1_WHEAT | - |
| contig23093 | - | - | - | - | tr\|W5F7J5\|W5F7J5_WHEAT | - |
| contig12137 | - | - | - | - | tr\|W5F7D6\|W5F7D6_WHEAT | - |
| contig10573 | - | - | - | - | tr\|W5F7A3\|W5F7A3_WHEAT | - |
| contig04753 | - | - | - | - | tr\|W5F785\|W5F785_WHEAT | - |
| contig25371 | - | - | - | - | tr\|W5F785\|W5F785_WHEAT | - |
| contig08806 | - | - | - | - | tr\|W5F733\|W5F733_WHEAT | - |
| contig00668 | - | - | - | - | tr\|W5F6Y8\|W5F6Y8_WHEAT | - |
| contig13511 | - | - | - | - | tr\|W5F6Y7\|W5F6Y7_WHEAT | - |

| | | | |
|---|---|---|---|
| contig04318 | - - - - | tr|W5F6X6|W5F6X6_WHEAT | - |
| contig03933 | - - - - | tr|W5F6U5|W5F6U5_WHEAT | - |
| contig17385 | - - - - | tr|W5F6P8|W5F6P8_WHEAT | - |
| contig21377 | - - - - | tr|W5F352|W5F352_WHEAT | - |
| contig03503 | - - - - | tr|W5F2K9|W5F2K9_WHEAT | - |
| contig00560 | - - - - | tr|W5F2G2|W5F2G2_WHEAT | - |
| contig24695 | - - - - | tr|W5EZA5|W5EZA5_WHEAT | - |
| contig07151 | - - - - | tr|W5EZ42|W5EZ42_WHEAT | - |
| contig14143 | - - - - | tr|W5EWP9|W5EWP9_WHEAT | - |
| contig22497 | - - - - | tr|W5EWD6|W5EWD6_WHEAT | - |
| contig09204 | - - - - | tr|W5EW23|W5EW23_WHEAT | - |
| contig02390 | - - - - | tr|W5EAB0|W5EAB0_WHEAT | - |
| contig14060 | - - - - | tr|W5DX61|W5DX61_WHEAT | - |
| contig24557 | - - - - | tr|W5DUZ7|W5DUZ7_WHEAT | - |
| contig23178 | - - - - | tr|W5DP52|W5DP52_WHEAT | - |
| contig05567 | - - - - | tr|W5DMB2|W5DMB2_WHEAT | - |
| contig22564 | - - - - | tr|W5DM06|W5DM06_WHEAT | - |
| contig03852 | - - - - | tr|W5CTC7|W5CTC7_WHEAT | - |
| contig24992 | - - - - | tr|W5CRT1|W5CRT1_WHEAT | - |
| contig09459 | - - - - | tr|W5BXF4|W5BXF4_WHEAT | - |
| contig00441 | - - - - | tr|W5BJF4|W5BJF4_WHEAT | - |
| contig01763 | - - - - | tr|W5BEX9|W5BEX9_WHEAT | - |
| contig10505 | - - - - | tr|W5B4F3|W5B4F3_WHEAT | - |
| contig16156 | - - - - | tr|T1NPW0|T1NPW0_TRIUA | - |
| contig07808 | - - - - | tr|T1NJ26|T1NJ26_TRIUA | - |
| contig15107 | - - - - | tr|T1N922|T1N922_TRIUA | - |
| contig04164 | - - - - | tr|T1MVS7|T1MVS7_TRIUA | - |
| contig02754 | - - - - | tr|T1LSA1|T1LSA1_TRIUA | - |
| contig05169 | - - - - | tr|Q8LK33|Q8LK33_WHEAT | - |
| contig09411 | - - - - | tr|Q58A29|Q58A29_WHEAT | - |
| contig03972 | - - - - | tr|M8B2H0|M8B2H0_TRIUA | - |
| contig08924 | - - - - | tr|M8AQT0|M8AQT0_TRIUA | - |
| contig11017 | - - - - | tr|M8A9I1|M8A9I1_TRIUA | - |
| contig24394 | - - - - | tr|M8A883|M8A883_TRIUA | - |
| contig00517 | - - - - | tr|M8A571|M8A571_TRIUA | - |
| contig05394 | - - - - | tr|M8A175|M8A175_TRIUA | - |
| contig07070 | - - - - | tr|M7ZXT0|M7ZXT0_TRIUA | - |
| contig05203 | - - - - | tr|M7ZWH8|M7ZWH8_TRIUA | - |
| contig04084 | - - - - | tr|M7ZSX6|M7ZSX6_TRIUA | - |
| contig18719 | - - - - | tr|M7ZPU2|M7ZPU2_TRIUA | - |
| contig02855 | - - - - | tr|M7ZNY9|M7ZNY9_TRIUA | - |
| contig21698 | - - - - | tr|M7ZL38|M7ZL38_TRIUA | - |
| contig06355 | - - - - | tr|M7ZGU5|M7ZGU5_TRIUA | - |
| contig05984 | - - - - | tr|M7Z7H2|M7Z7H2_TRIUA | - |
| contig04740 | - - - - | tr|M7Z4A3|M7Z4A3_TRIUA | - |
| contig10683 | - - - - | tr|M7YWV0|M7YWV0_TRIUA | - |
| contig09076 | - - - - | tr|M7YT37|M7YT37_TRIUA | - |
| contig17042 | - - - - | tr|M7YS28|M7YS28_TRIUA | - |
| contig15123 | - - - - | tr|M7YLT7|M7YLT7_TRIUA | - |
| contig13580 | - - - - | tr|M7YJR9|M7YJR9_TRIUA | - |

| | | | | | | | |
|---|---|---|---|---|---|---|---|
| contig17066 | - | - | - | - | tr\|M7YFQ4\|M7YFQ4_TRIUA | - |
| contig04818 | - | - | - | - | tr\|M7YEA6\|M7YEA6_TRIUA | - |
| contig20282 | - | - | - | - | tr\|M7YCC0\|M7YCC0_TRIUA | - |
| contig25253 | - | - | - | - | tr\|W5DLG3\|W5DLG3_WHEAT | gnl\|UG\|Ta#S12945079 |
| contig24919 | - | - | - | - | tr\|W5FJ40\|W5FJ40_WHEAT | gnl\|UG\|Ta#S12984322 |
| contig07153 | - | - | - | - | tr\|W5DUP9\|W5DUP9_WHEAT | gnl\|UG\|Ta#S19222463 |
| contig00529 | - | - | - | - | tr\|T1LXS0\|T1LXS0_TRIUA | gnl\|UG\|Ta#S32585126 |
| contig02055 | - | - | - | - | tr\|M7YRL4\|M7YRL4_TRIUA | gnl\|UG\|Ta#S32654061 |
| contig01476 | - | - | - | - | tr\|W5FHX4\|W5FHX4_WHEAT | gnl\|UG\|Ta#S32669625 |
| contig15515 | - | - | - | - | tr\|M7Z5M6\|M7Z5M6_TRIUA | gnl\|UG\|Ta#S37871927 |
| contig08737 | - | - | - | - | tr\|W5FAK4\|W5FAK4_WHEAT | gnl\|UG\|Ta#S52542126 |
| contig03284 | - | - | - | - | tr\|W5FCA8\|W5FCA8_WHEAT | gnl\|UG\|Ta#S52542233 |
| contig19778 | - | - | - | - | tr\|T1MS69\|T1MS69_TRIUA | gnl\|UG\|Ta#S52542905 |
| contig04009 | - | - | - | - | tr\|W4ZPM4\|W4ZPM4_WHEAT | gnl\|UG\|Ta#S52543192 |
| contig14993 | - | - | - | - | tr\|W5FPG6\|W5FPG6_WHEAT | gnl\|UG\|Ta#S52543321 |
| contig01163 | - | - | - | - | tr\|W5FAT0\|W5FAT0_WHEAT | gnl\|UG\|Ta#S52543760 |
| contig17874 | - | - | - | - | tr\|D0QMR4\|D0QMR4_WHEAT | gnl\|UG\|Ta#S52543897 |
| contig19563 | - | - | - | - | tr\|M7ZLA3\|M7ZLA3_TRIUA | gnl\|UG\|Ta#S52543920 |
| contig13331 | - | - | - | - | tr\|W5FG63\|W5FG63_WHEAT | gnl\|UG\|Ta#S52543944 |
| contig01941 | - | - | - | - | tr\|W5FID0\|W5FID0_WHEAT | gnl\|UG\|Ta#S52544387 |
| contig02431 | - | - | - | - | tr\|W5FN67\|W5FN67_WHEAT | gnl\|UG\|Ta#S52544411 |
| contig24453 | - | - | - | - | tr\|W5FN67\|W5FN67_WHEAT | gnl\|UG\|Ta#S52544411 |
| contig01736 | - | - | - | - | tr\|W5FFS3\|W5FFS3_WHEAT | gnl\|UG\|Ta#S52544546 |
| contig02871 | - | - | - | - | tr\|W5A1F5\|W5A1F5_WHEAT | gnl\|UG\|Ta#S52546102 |
| contig04636 | - | - | - | - | tr\|W5F884\|W5F884_WHEAT | gnl\|UG\|Ta#S52546198 |
| contig02398 | - | - | - | - | tr\|W5FG44\|W5FG44_WHEAT | gnl\|UG\|Ta#S52546554 |
| contig05236 | - | - | - | - | tr\|W5FKN6\|W5FKN6_WHEAT | gnl\|UG\|Ta#S52546693 |
| contig15903 | - | - | - | - | tr\|W5FNP7\|W5FNP7_WHEAT | gnl\|UG\|Ta#S52547034 |
| contig20266 | - | - | - | - | tr\|T1MAS9\|T1MAS9_TRIUA | gnl\|UG\|Ta#S58838319 |
| contig04384 | - | - | - | - | tr\|W5FIF0\|W5FIF0_WHEAT | gnl\|UG\|Ta#S58854401 |
| contig02819 | - | - | - | - | tr\|W5FBM4\|W5FBM4_WHEAT | gnl\|UG\|Ta#S58859076 |
| contig12180 | - | - | - | - | tr\|W5F8J0\|W5F8J0_WHEAT | gnl\|UG\|Ta#S58860002 |
| contig06195 | - | - | - | - | tr\|W5F7P1\|W5F7P1_WHEAT | gnl\|UG\|Ta#S58867418 |
| contig03707 | - | - | - | - | tr\|M7Y728\|M7Y728_TRIUA | gnl\|UG\|Ta#S58883091 |
| contig00748 | - | - | - | - | tr\|W5BIQ1\|W5BIQ1_WHEAT | gnl\|UG\|Ta#S58885231 |
| contig08699 | - | - | - | - | tr\|W5FHS7\|W5FHS7_WHEAT | gnl\|UG\|Ta#S58887006 |
| contig15466 | - | - | - | - | tr\|W5FHS7\|W5FHS7_WHEAT | gnl\|UG\|Ta#S58887006 |
| contig09298 | - | - | - | - | tr\|S4SPB8\|S4SPB8_TRITD | gnl\|UG\|Ta#S58893674 |
| contig01328 | - | - | - | - | tr\|W5F7E5\|W5F7E5_WHEAT | gnl\|UG\|Ta#S61519406 |
| contig04962 | - | - | - | - | tr\|W5FFF7\|W5FFF7_WHEAT | gnl\|UG\|Ta#S61521559 |
| contig00803 | - | - | - | - | tr\|M8APT0\|M8APT0_TRIUA | gnl\|UG\|Ta#S61523988 |
| contig05955 | - | - | - | - | tr\|W5F9P9\|W5F9P9_WHEAT | gnl\|UG\|Ta#S61524128 |
| contig11595 | - | - | - | - | tr\|W5F9P9\|W5F9P9_WHEAT | gnl\|UG\|Ta#S61524128 |
| contig18958 | - | - | - | - | tr\|M8A7Q7\|M8A7Q7_TRIUA | gnl\|UG\|Ta#S61528219 |
| contig06429 | - | - | - | - | tr\|W5FP99\|W5FP99_WHEAT | gnl\|UG\|Ta#S61540607 |
| contig14887 | - | - | - | - | tr\|M7Z0Q3\|M7Z0Q3_TRIUA | gnl\|UG\|Ta#S61543396 |
| contig03218 | - | - | - | - | tr\|W5FJQ8\|W5FJQ8_WHEAT | gnl\|UG\|Ta#S61548818 |
| contig07017 | - | - | - | - | tr\|W5FJQ8\|W5FJQ8_WHEAT | gnl\|UG\|Ta#S61548818 |
| contig22413 | - | - | - | - | tr\|W5FCD1\|W5FCD1_WHEAT | gnl\|UG\|Ta#S61556387 |
| contig06035 | - | - | - | - | tr\|W5FD46\|W5FD46_WHEAT | gnl\|UG\|Ta#S61564953 |

| | | | |
|---|---|---|---|
| contig17328 | - - - - | tr\|T1MDI6\|T1MDI6_TRIUA | gnl\|UG\|Ta#S61567142 |
| contig03017 | - - - - | tr\|M8ACM1\|M8ACM1_TRIUA | gnl\|UG\|Ta#S61568522 |
| contig16918 | - - - - | tr\|M7ZRK7\|M7ZRK7_TRIUA | gnl\|UG\|Ta#S61582158 |
| contig01676 | - - - - | tr\|M7Z476\|M7Z476_TRIUA | gnl\|UG\|Ta#S61586606 |
| contig06568 | - - - - | tr\|W5F8L8\|W5F8L8_WHEAT | gnl\|UG\|Ta#S61597200 |
| contig20604 | - - - - | tr\|W5F8L8\|W5F8L8_WHEAT | gnl\|UG\|Ta#S61597200 |
| contig10964 | - - - - | tr\|W5FLY4\|W5FLY4_WHEAT | gnl\|UG\|Ta#S61609447 |
| contig14672 | - - - - | tr\|W5AFE3\|W5AFE3_WHEAT | gnl\|UG\|Ta#S61611243 |
| contig19128 | - - - - | tr\|W5AFE3\|W5AFE3_WHEAT | gnl\|UG\|Ta#S61611243 |
| contig06589 | - - - - | tr\|M8A0J4\|M8A0J4_TRIUA | gnl\|UG\|Ta#S61611980 |
| contig07105 | - - - - | tr\|W5F7T1\|W5F7T1_WHEAT | gnl\|UG\|Ta#S61616923 |
| contig00248 | - - - - | tr\|W5F7E5\|W5F7E5_WHEAT | gnl\|UG\|Ta#S61625946 |
| contig10689 | - - - - | tr\|W5FS98\|W5FS98_WHEAT | gnl\|UG\|Ta#S61637380 |
| contig17911 | - - - - | tr\|T1N0K9\|T1N0K9_TRIUA | gnl\|UG\|Ta#S61638683 |
| contig25030 | - - - - | tr\|W5G0K2\|W5G0K2_WHEAT | gnl\|UG\|Ta#S61640184 |
| contig05884 | - - - - | tr\|W5F6Q4\|W5F6Q4_WHEAT | gnl\|UG\|Ta#S61640184 |
| contig11945 | - - - - | tr\|W5FTU8\|W5FTU8_WHEAT | gnl\|UG\|Ta#S61645325 |
| contig23115 | - - - - | tr\|W5F494\|W5F494_WHEAT | gnl\|UG\|Ta#S61645368 |
| contig12146 | - - - - | tr\|G9DR93\|G9DR93_WHEAT | gnl\|UG\|Ta#S61648747 |
| contig17535 | - - - - | tr\|W5FK07\|W5FK07_WHEAT | gnl\|UG\|Ta#S61657691 |
| contig12453 | - - - - | tr\|W5FDB3\|W5FDB3_WHEAT | gnl\|UG\|Ta#S61658716 |
| contig03135 | - - - - | tr\|W5F587\|W5F587_WHEAT | gnl\|UG\|Ta#S61668110 |
| contig18303 | - - - - | tr\|W5FKB9\|W5FKB9_WHEAT | gnl\|UG\|Ta#S61687893 |
| contig13561 | - - - - | tr\|W5F892\|W5F892_WHEAT | gnl\|UG\|Ta#S61701300 |
| contig18352 | - - - - | tr\|W5F9N6\|W5F9N6_WHEAT | gnl\|UG\|Ta#S61702796 |
| contig02196 | - - - - | tr\|M7YFE5\|M7YFE5_TRIUA | gnl\|UG\|Ta#S61706043 |
| contig08118 | - - - - | tr\|W5FJS3\|W5FJS3_WHEAT | gnl\|UG\|Ta#S61706631 |
| contig08040 | - - - - | tr\|W5FHW8\|W5FHW8_WHEAT | gnl\|UG\|Ta#S61709136 |
| contig08347 | - - - - | tr\|W5FDM9\|W5FDM9_WHEAT | gnl\|UG\|Ta#S61716690 |
| contig04573 | - - - - | tr\|W5F899\|W5F899_WHEAT | gnl\|UG\|Ta#S61726409 |
| contig07290 | - - - - | tr\|M8A0J7\|M8A0J7_TRIUA | gnl\|UG\|Ta#S61760859 |
| contig01577 | - - - - | tr\|W5FT79\|W5FT79_WHEAT | gnl\|UG\|Ta#S61768083 |
| contig22781 | - - - - | tr\|W5FKS7\|W5FKS7_WHEAT | gnl\|UG\|Ta#S61769430 |
| contig04176 | - - - - | tr\|W5G3K0\|W5G3K0_WHEAT | gnl\|UG\|Ta#S61770099 |
| contig06692 | - - - - | tr\|W5FPZ6\|W5FPZ6_WHEAT | gnl\|UG\|Ta#S61770928 |
| contig00307 | - - - - | tr\|W5FJJ3\|W5FJJ3_WHEAT | gnl\|UG\|Ta#S61774937 |
| contig01664 | - - - - | tr\|W5FHW1\|W5FHW1_WHEAT | gnl\|UG\|Ta#S61775859 |
| contig18591 | - - - - | tr\|W5FFU4\|W5FFU4_WHEAT | gnl\|UG\|Ta#S61780998 |
| contig24637 | - - - - | tr\|M7ZNJ3\|M7ZNJ3_TRIUA | gnl\|UG\|Ta#S61782182 |
| contig10233 | - - - - | tr\|W5FP96\|W5FP96_WHEAT | gnl\|UG\|Ta#S61790111 |
| contig00352 | - - - - | tr\|W5F1R9\|W5F1R9_WHEAT | gnl\|UG\|Ta#S61792090 |
| contig05061 | - - - - | tr\|M7ZB00\|M7ZB00_TRIUA | gnl\|UG\|Ta#S61798436 |
| contig02007 | - - - - | tr\|W5FGM7\|W5FGM7_WHEAT | gnl\|UG\|Ta#S61799499 |
| contig07200 | - - - - | tr\|W5FBP0\|W5FBP0_WHEAT | gnl\|UG\|Ta#S61801911 |
| contig06442 | - - - - | tr\|W5F8X6\|W5F8X6_WHEAT | gnl\|UG\|Ta#S61802353 |
| contig03597 | - - - - | tr\|W5F888\|W5F888_WHEAT | gnl\|UG\|Ta#S61803294 |
| contig01204 | - - - - | tr\|W5FIY3\|W5FIY3_WHEAT | gnl\|UG\|Ta#S61807673 |
| contig00063 | - - - - | tr\|W5FN82\|W5FN82_WHEAT | gnl\|UG\|Ta#S61808516 |
| contig01498 | - - - - | tr\|W5F9S7\|W5F9S7_WHEAT | gnl\|UG\|Ta#S61808693 |
| contig13881 | - - - - | tr\|W5FPN2\|W5FPN2_WHEAT | gnl\|UG\|Ta#S61809250 |

| | | | |
|---|---|---|---|
| contig16617 | - - - - | tr\|W5FL65\|W5FL65_WHEAT | gnl\|UG\|Ta#S61809735 |
| contig01402 | - - - - | tr\|W5FD79\|W5FD79_WHEAT | gnl\|UG\|Ta#S61810015 |
| contig10163 | - - - - | tr\|W5FTF3\|W5FTF3_WHEAT | gnl\|UG\|Ta#S61810348 |
| contig11213 | - - - - | tr\|M7ZU43\|M7ZU43_TRIUA | gnl\|UG\|Ta#S61810351 |
| contig03492 | - - - - | tr\|W5FNT4\|W5FNT4_WHEAT | gnl\|UG\|Ta#S61810441 |
| contig08625 | - - - - | tr\|M7ZMW0\|M7ZMW0_TRIUA | gnl\|UG\|Ta#S61811064 |
| contig24576 | - - - - | tr\|M7ZMW0\|M7ZMW0_TRIUA | gnl\|UG\|Ta#S61811064 |
| contig03728 | - - - - | tr\|W5FG28\|W5FG28_WHEAT | gnl\|UG\|Ta#S61812028 |
| contig01567 | - - - - | tr\|W5FA17\|W5FA17_WHEAT | gnl\|UG\|Ta#S61812269 |
| contig02784 | - - - - | tr\|W5FA17\|W5FA17_WHEAT | gnl\|UG\|Ta#S61812269 |
| contig03182 | - - - - | tr\|W5F9D2\|W5F9D2_WHEAT | gnl\|UG\|Ta#S61812354 |
| contig06453 | - - - - | tr\|M8A440\|M8A440_TRIUA | gnl\|UG\|Ta#S61812354 |
| contig09323 | - - - - | tr\|W5FAE0\|W5FAE0_WHEAT | gnl\|UG\|Ta#S61828316 |
| contig12678 | - - - - | tr\|W5FCS6\|W5FCS6_WHEAT | gnl\|UG\|Ta#S61829325 |
| contig18755 | - - - - | tr\|W5FCS6\|W5FCS6_WHEAT | gnl\|UG\|Ta#S61829325 |
| contig00153 | - - - - | tr\|W5FDH0\|W5FDH0_WHEAT | gnl\|UG\|Ta#S61829645 |
| contig14310 | - - - - | tr\|W5FJ00\|W5FJ00_WHEAT | gnl\|UG\|Ta#S61829895 |
| contig03412 | - - - - | tr\|W5F9M4\|W5F9M4_WHEAT | gnl\|UG\|Ta#S61830032 |
| contig06655 | - - - - | tr\|W5F968\|W5F968_WHEAT | gnl\|UG\|Ta#S61830280 |
| contig06223 | - - - - | tr\|W5FC05\|W5FC05_WHEAT | gnl\|UG\|Ta#S61830716 |
| contig19473 | - - - - | tr\|W5FK65\|W5FK65_WHEAT | gnl\|UG\|Ta#S61831039 |
| contig12303 | - - - - | tr\|W5CSL0\|W5CSL0_WHEAT | gnl\|UG\|Ta#S61831312 |
| contig13749 | - - - - | tr\|W5FK39\|W5FK39_WHEAT | gnl\|UG\|Ta#S61831531 |
| contig00905 | - - - - | tr\|W5FJF8\|W5FJF8_WHEAT | gnl\|UG\|Ta#S61831770 |
| contig07685 | - - - - | tr\|W5FDM9\|W5FDM9_WHEAT | gnl\|UG\|Ta#S61832213 |
| contig09731 | - - - - | tr\|W5AXZ8\|W5AXZ8_WHEAT | gnl\|UG\|Ta#S61832213 |
| contig11276 | - - - - | tr\|W5ELJ5\|W5ELJ5_WHEAT | gnl\|UG\|Ta#S61832522 |
| contig07542 | - - - - | tr\|W5FC46\|W5FC46_WHEAT | gnl\|UG\|Ta#S61840963 |
| contig03395 | - - - - | tr\|W5FC32\|W5FC32_WHEAT | gnl\|UG\|Ta#S61846433 |
| contig04191 | - - - - | tr\|M7ZIP2\|M7ZIP2_TRIUA | gnl\|UG\|Ta#S65588561 |
| contig01434 | - - - - | tr\|M7YYZ4\|M7YYZ4_TRIUA | gnl\|UG\|Ta#S65589155 |
| contig00368 | - - - - | tr\|W5FDZ5\|W5FDZ5_WHEAT | gnl\|UG\|Ta#S65594860 |
| contig05374 | - - - - | tr\|W5FDN9\|W5FDN9_WHEAT | gnl\|UG\|Ta#S65603192 |
| contig01660 | - - - - | tr\|M8A2N0\|M8A2N0_TRIUA | gnl\|UG\|Ta#S65609345 |
| contig00717 | - - - - | tr\|W5FN33\|W5FN33_WHEAT | gnl\|UG\|Ta#S65610213 |
| contig11864 | - - - - | tr\|W5FJQ8\|W5FJQ8_WHEAT | gnl\|UG\|Ta#S65620757 |
| contig17379 | - - - - | tr\|W5FTA0\|W5FTA0_WHEAT | gnl\|UG\|Ta#S65671356 |
| contig03792 | - - - - | tr\|W5FXW5\|W5FXW5_WHEAT | gnl\|UG\|Ta#S65685225 |
| contig00103 | - - - - | tr\|M7ZSK7\|M7ZSK7_TRIUA | gnl\|UG\|Ta#S65689303 |
| contig17097 | - - - - - | | gnl\|UG\|Ta#S12860450 |
| contig02365 | - - - - - | | gnl\|UG\|Ta#S12861188 |
| contig09530 | - - - - - | | gnl\|UG\|Ta#S12863726 |
| contig17723 | - - - - - | | gnl\|UG\|Ta#S12872236 |
| contig03504 | - - - - - | | gnl\|UG\|Ta#S12877383 |
| contig01897 | - - - - - | | gnl\|UG\|Ta#S12883375 |
| contig16488 | - - - - - | | gnl\|UG\|Ta#S12902607 |
| contig04615 | - - - - - | | gnl\|UG\|Ta#S12902946 |
| contig07879 | - - - - - | | gnl\|UG\|Ta#S12907462 |
| contig20999 | - - - - - | | gnl\|UG\|Ta#S12907462 |
| contig13853 | - - - - - | | gnl\|UG\|Ta#S12922861 |

| | | |
|---|---|---|
| contig14679 | - - - - - | gnl\|UG\|Ta#S12922861 |
| contig21482 | - - - - - | gnl\|UG\|Ta#S12923035 |
| contig01486 | - - - - - | gnl\|UG\|Ta#S12923172 |
| contig04896 | - - - - - | gnl\|UG\|Ta#S12926520 |
| contig02925 | - - - - - | gnl\|UG\|Ta#S12935253 |
| contig01886 | - - - - - | gnl\|UG\|Ta#S12949482 |
| contig05023 | - - - - - | gnl\|UG\|Ta#S12958442 |
| contig12297 | - - - - - | gnl\|UG\|Ta#S12958937 |
| contig07815 | - - - - - | gnl\|UG\|Ta#S12961618 |
| contig20377 | - - - - - | gnl\|UG\|Ta#S12961618 |
| contig14683 | - - - - - | gnl\|UG\|Ta#S12986866 |
| contig06154 | - - - - - | gnl\|UG\|Ta#S12986971 |
| contig04507 | - - - - - | gnl\|UG\|Ta#S12990701 |
| contig04482 | - - - - - | gnl\|UG\|Ta#S12992137 |
| contig13079 | - - - - - | gnl\|UG\|Ta#S13002311 |
| contig08268 | - - - - - | gnl\|UG\|Ta#S13008383 |
| contig11715 | - - - - - | gnl\|UG\|Ta#S13022279 |
| contig13875 | - - - - - | gnl\|UG\|Ta#S13022279 |
| contig01942 | - - - - - | gnl\|UG\|Ta#S13022371 |
| contig15505 | - - - - - | gnl\|UG\|Ta#S13025892 |
| contig23381 | - - - - - | gnl\|UG\|Ta#S13046896 |
| contig10122 | - - - - - | gnl\|UG\|Ta#S13046971 |
| contig17758 | - - - - - | gnl\|UG\|Ta#S13048599 |
| contig26142 | - - - - - | gnl\|UG\|Ta#S13048599 |
| contig04402 | - - - - - | gnl\|UG\|Ta#S13057312 |
| contig03830 | - - - - - | gnl\|UG\|Ta#S13109255 |
| contig14754 | - - - - - | gnl\|UG\|Ta#S13115498 |
| contig25919 | - - - - - | gnl\|UG\|Ta#S13115498 |
| contig17175 | - - - - - | gnl\|UG\|Ta#S13119743 |
| contig05155 | - - - - - | gnl\|UG\|Ta#S13122260 |
| contig00500 | - - - - - | gnl\|UG\|Ta#S13126589 |
| contig25432 | - - - - - | gnl\|UG\|Ta#S13140806 |
| contig18931 | - - - - - | gnl\|UG\|Ta#S13179656 |
| contig10077 | - - - - - | gnl\|UG\|Ta#S13216390 |
| contig06096 | - - - - - | gnl\|UG\|Ta#S13267325 |
| contig18458 | - - - - - | gnl\|UG\|Ta#S13267931 |
| contig19256 | - - - - - | gnl\|UG\|Ta#S13268792 |
| contig11090 | - - - - - | gnl\|UG\|Ta#S15824301 |
| contig00921 | - - - - - | gnl\|UG\|Ta#S15902889 |
| contig22893 | - - - - - | gnl\|UG\|Ta#S15902889 |
| contig25996 | - - - - - | gnl\|UG\|Ta#S16057969 |
| contig04360 | - - - - - | gnl\|UG\|Ta#S16058102 |
| contig12315 | - - - - - | gnl\|UG\|Ta#S16058102 |
| contig23600 | - - - - - | gnl\|UG\|Ta#S16058102 |
| contig09832 | - - - - - | gnl\|UG\|Ta#S16204548 |
| contig19189 | - - - - - | gnl\|UG\|Ta#S16206438 |
| contig00875 | - - - - - | gnl\|UG\|Ta#S16207418 |
| contig17155 | - - - - - | gnl\|UG\|Ta#S16207418 |
| contig05957 | - - - - - | gnl\|UG\|Ta#S16215150 |
| contig00750 | - - - - - | gnl\|UG\|Ta#S16217371 |

| | | |
|---|---|---|
| contig08619 | - - - - - | gnl\|UG\|Ta#S16226011 |
| contig01746 | - - - - - | gnl\|UG\|Ta#S16230781 |
| contig02282 | - - - - - | gnl\|UG\|Ta#S16232262 |
| contig19054 | - - - - - | gnl\|UG\|Ta#S16232464 |
| contig04613 | - - - - - | gnl\|UG\|Ta#S16233104 |
| contig18093 | - - - - - | gnl\|UG\|Ta#S16235625 |
| contig23555 | - - - - - | gnl\|UG\|Ta#S16235625 |
| contig18183 | - - - - - | gnl\|UG\|Ta#S16237852 |
| contig20397 | - - - - - | gnl\|UG\|Ta#S16237852 |
| contig19363 | - - - - - | gnl\|UG\|Ta#S16239451 |
| contig21912 | - - - - - | gnl\|UG\|Ta#S16241672 |
| contig10213 | - - - - - | gnl\|UG\|Ta#S16246576 |
| contig01141 | - - - - - | gnl\|UG\|Ta#S16247043 |
| contig19842 | - - - - - | gnl\|UG\|Ta#S16252280 |
| contig09111 | - - - - - | gnl\|UG\|Ta#S16259681 |
| contig01003 | - - - - - | gnl\|UG\|Ta#S16977773 |
| contig20132 | - - - - - | gnl\|UG\|Ta#S17879835 |
| contig07628 | - - - - - | gnl\|UG\|Ta#S17879993 |
| contig09982 | - - - - - | gnl\|UG\|Ta#S17884166 |
| contig24336 | - - - - - | gnl\|UG\|Ta#S17884330 |
| contig03138 | - - - - - | gnl\|UG\|Ta#S17890647 |
| contig12027 | - - - - - | gnl\|UG\|Ta#S17973628 |
| contig16811 | - - - - - | gnl\|UG\|Ta#S17984361 |
| contig12221 | - - - - - | gnl\|UG\|Ta#S17985102 |
| contig26157 | - - - - - | gnl\|UG\|Ta#S17986624 |
| contig03195 | - - - - - | gnl\|UG\|Ta#S17987472 |
| contig08238 | - - - - - | gnl\|UG\|Ta#S17987472 |
| contig20277 | - - - - - | gnl\|UG\|Ta#S17987643 |
| contig19868 | - - - - - | gnl\|UG\|Ta#S17987953 |
| contig11707 | - - - - - | gnl\|UG\|Ta#S17988113 |
| contig03881 | - - - - - | gnl\|UG\|Ta#S17988574 |
| contig09781 | - - - - - | gnl\|UG\|Ta#S17989154 |
| contig19686 | - - - - - | gnl\|UG\|Ta#S18007330 |
| contig15638 | - - - - - | gnl\|UG\|Ta#S18011450 |
| contig07848 | - - - - - | gnl\|UG\|Ta#S18592052 |
| contig06819 | - - - - - | gnl\|UG\|Ta#S21627511 |
| contig04798 | - - - - - | gnl\|UG\|Ta#S22119324 |
| contig14776 | - - - - - | gnl\|UG\|Ta#S22370655 |
| contig24639 | - - - - - | gnl\|UG\|Ta#S22379653 |
| contig08275 | - - - - - | gnl\|UG\|Ta#S22380229 |
| contig07155 | - - - - - | gnl\|UG\|Ta#S22383267 |
| contig12906 | - - - - - | gnl\|UG\|Ta#S22385777 |
| contig21508 | - - - - - | gnl\|UG\|Ta#S26021019 |
| contig20520 | - - - - - | gnl\|UG\|Ta#S26027000 |
| contig00710 | - - - - - | gnl\|UG\|Ta#S32217406 |
| contig11620 | - - - - - | gnl\|UG\|Ta#S32217406 |
| contig02740 | - - - - - | gnl\|UG\|Ta#S32424550 |
| contig02362 | - - - - - | gnl\|UG\|Ta#S32432921 |
| contig01589 | - - - - - | gnl\|UG\|Ta#S32433882 |
| contig05007 | - - - - - | gnl\|UG\|Ta#S32505676 |

| | | |
|---|---|---|
| contig15772 | - - - - - | gnl\|UG\|Ta#S32510096 |
| contig07048 | - - - - - | gnl\|UG\|Ta#S32516478 |
| contig07997 | - - - - - | gnl\|UG\|Ta#S32516478 |
| contig00895 | - - - - - | gnl\|UG\|Ta#S32532291 |
| contig11350 | - - - - - | gnl\|UG\|Ta#S32532291 |
| contig00177 | - - - - - | gnl\|UG\|Ta#S32558954 |
| contig09517 | - - - - - | gnl\|UG\|Ta#S32571152 |
| contig00838 | - - - - - | gnl\|UG\|Ta#S32588503 |
| contig19025 | - - - - - | gnl\|UG\|Ta#S32601075 |
| contig11301 | - - - - - | gnl\|UG\|Ta#S32605660 |
| contig19616 | - - - - - | gnl\|UG\|Ta#S32605660 |
| contig20139 | - - - - - | gnl\|UG\|Ta#S32607037 |
| contig24758 | - - - - - | gnl\|UG\|Ta#S32607037 |
| contig18254 | - - - - - | gnl\|UG\|Ta#S32610994 |
| contig04937 | - - - - - | gnl\|UG\|Ta#S32612672 |
| contig14061 | - - - - - | gnl\|UG\|Ta#S32620739 |
| contig05166 | - - - - - | gnl\|UG\|Ta#S32637836 |
| contig07103 | - - - - - | gnl\|UG\|Ta#S32654061 |
| contig01019 | - - - - - | gnl\|UG\|Ta#S32665150 |
| contig03900 | - - - - - | gnl\|UG\|Ta#S32665150 |
| contig26491 | - - - - - | gnl\|UG\|Ta#S32665150 |
| contig00780 | - - - - - | gnl\|UG\|Ta#S32668034 |
| contig18907 | - - - - - | gnl\|UG\|Ta#S32668645 |
| contig14132 | - - - - - | gnl\|UG\|Ta#S32686734 |
| contig11164 | - - - - - | gnl\|UG\|Ta#S32688910 |
| contig03595 | - - - - - | gnl\|UG\|Ta#S32692064 |
| contig20750 | - - - - - | gnl\|UG\|Ta#S32693695 |
| contig02381 | - - - - - | gnl\|UG\|Ta#S32725041 |
| contig00592 | - - - - - | gnl\|UG\|Ta#S37748597 |
| contig06608 | - - - - - | gnl\|UG\|Ta#S37758607 |
| contig14488 | - - - - - | gnl\|UG\|Ta#S37763965 |
| contig17602 | - - - - - | gnl\|UG\|Ta#S37763965 |
| contig04490 | - - - - - | gnl\|UG\|Ta#S37768276 |
| contig04470 | - - - - - | gnl\|UG\|Ta#S37779133 |
| contig17907 | - - - - - | gnl\|UG\|Ta#S37787950 |
| contig18752 | - - - - - | gnl\|UG\|Ta#S37787950 |
| contig00433 | - - - - - | gnl\|UG\|Ta#S37807398 |
| contig04929 | - - - - - | gnl\|UG\|Ta#S37818091 |
| contig24238 | - - - - - | gnl\|UG\|Ta#S37818091 |
| contig13368 | - - - - - | gnl\|UG\|Ta#S37821256 |
| contig06835 | - - - - - | gnl\|UG\|Ta#S37828354 |
| contig25617 | - - - - - | gnl\|UG\|Ta#S37856486 |
| contig16654 | - - - - - | gnl\|UG\|Ta#S37866174 |
| contig21160 | - - - - - | gnl\|UG\|Ta#S37875112 |
| contig06988 | - - - - - | gnl\|UG\|Ta#S37876769 |
| contig13110 | - - - - - | gnl\|UG\|Ta#S37903265 |
| contig00235 | - - - - - | gnl\|UG\|Ta#S37916533 |
| contig02410 | - - - - - | gnl\|UG\|Ta#S37919319 |
| contig20105 | - - - - - | gnl\|UG\|Ta#S37928418 |
| contig01943 | - - - - - | gnl\|UG\|Ta#S41658045 |

| | | |
|---|---|---|
| contig04291 | - - - - - | gnl\|UG\|Ta#S43837843 |
| contig17884 | - - - - - | gnl\|UG\|Ta#S50383792 |
| contig05285 | - - - - - | gnl\|UG\|Ta#S50384352 |
| contig07276 | - - - - - | gnl\|UG\|Ta#S50385251 |
| contig03398 | - - - - - | gnl\|UG\|Ta#S50388970 |
| contig06031 | - - - - - | gnl\|UG\|Ta#S52541041 |
| contig26274 | - - - - - | gnl\|UG\|Ta#S52541340 |
| contig24724 | - - - - - | gnl\|UG\|Ta#S52541952 |
| contig01348 | - - - - - | gnl\|UG\|Ta#S52542072 |
| contig11462 | - - - - - | gnl\|UG\|Ta#S52542124 |
| contig24939 | - - - - - | gnl\|UG\|Ta#S52542124 |
| contig06147 | - - - - - | gnl\|UG\|Ta#S52542126 |
| contig02768 | - - - - - | gnl\|UG\|Ta#S52542196 |
| contig17432 | - - - - - | gnl\|UG\|Ta#S52542196 |
| contig08126 | - - - - - | gnl\|UG\|Ta#S52542198 |
| contig05708 | - - - - - | gnl\|UG\|Ta#S52542227 |
| contig15968 | - - - - - | gnl\|UG\|Ta#S52542227 |
| contig04750 | - - - - - | gnl\|UG\|Ta#S52542804 |
| contig10960 | - - - - - | gnl\|UG\|Ta#S52542804 |
| contig07996 | - - - - - | gnl\|UG\|Ta#S52542915 |
| contig10646 | - - - - - | gnl\|UG\|Ta#S52542915 |
| contig05079 | - - - - - | gnl\|UG\|Ta#S52542998 |
| contig14810 | - - - - - | gnl\|UG\|Ta#S52543227 |
| contig02122 | - - - - - | gnl\|UG\|Ta#S52543321 |
| contig04063 | - - - - - | gnl\|UG\|Ta#S52543321 |
| contig06584 | - - - - - | gnl\|UG\|Ta#S52543321 |
| contig09870 | - - - - - | gnl\|UG\|Ta#S52543321 |
| contig17235 | - - - - - | gnl\|UG\|Ta#S52543321 |
| contig17024 | - - - - - | gnl\|UG\|Ta#S52543344 |
| contig11722 | - - - - - | gnl\|UG\|Ta#S52543364 |
| contig15409 | - - - - - | gnl\|UG\|Ta#S52543419 |
| contig05415 | - - - - - | gnl\|UG\|Ta#S52543438 |
| contig08179 | - - - - - | gnl\|UG\|Ta#S52543488 |
| contig19231 | - - - - - | gnl\|UG\|Ta#S52543513 |
| contig09735 | - - - - - | gnl\|UG\|Ta#S52543560 |
| contig08331 | - - - - - | gnl\|UG\|Ta#S52543616 |
| contig08332 | - - - - - | gnl\|UG\|Ta#S52543616 |
| contig08334 | - - - - - | gnl\|UG\|Ta#S52543616 |
| contig23060 | - - - - - | gnl\|UG\|Ta#S52543616 |
| contig25411 | - - - - - | gnl\|UG\|Ta#S52543616 |
| contig08305 | - - - - - | gnl\|UG\|Ta#S52543760 |
| contig15350 | - - - - - | gnl\|UG\|Ta#S52543897 |
| contig05174 | - - - - - | gnl\|UG\|Ta#S52544003 |
| contig03467 | - - - - - | gnl\|UG\|Ta#S52544173 |
| contig17619 | - - - - - | gnl\|UG\|Ta#S52544173 |
| contig00204 | - - - - - | gnl\|UG\|Ta#S52544221 |
| contig01072 | - - - - - | gnl\|UG\|Ta#S52544221 |
| contig11154 | - - - - - | gnl\|UG\|Ta#S52544221 |
| contig11736 | - - - - - | gnl\|UG\|Ta#S52544221 |
| contig04988 | - - - - - | gnl\|UG\|Ta#S52544280 |

| | | |
|---|---|---|
| contig18778 | - - - - - | gnl\|UG\|Ta#S52544280 |
| contig25072 | - - - - - | gnl\|UG\|Ta#S52544280 |
| contig03783 | - - - - - | gnl\|UG\|Ta#S52544411 |
| contig09422 | - - - - - | gnl\|UG\|Ta#S52544411 |
| contig00486 | - - - - - | gnl\|UG\|Ta#S52544716 |
| contig17609 | - - - - - | gnl\|UG\|Ta#S52544760 |
| contig04376 | - - - - - | gnl\|UG\|Ta#S52544885 |
| contig18090 | - - - - - | gnl\|UG\|Ta#S52545007 |
| contig19971 | - - - - - | gnl\|UG\|Ta#S52545007 |
| contig19119 | - - - - - | gnl\|UG\|Ta#S52545130 |
| contig09120 | - - - - - | gnl\|UG\|Ta#S52545221 |
| contig15713 | - - - - - | gnl\|UG\|Ta#S52545293 |
| contig02030 | - - - - - | gnl\|UG\|Ta#S52545434 |
| contig15253 | - - - - - | gnl\|UG\|Ta#S52545598 |
| contig06189 | - - - - - | gnl\|UG\|Ta#S52545646 |
| contig22213 | - - - - - | gnl\|UG\|Ta#S52545812 |
| contig10393 | - - - - - | gnl\|UG\|Ta#S52545886 |
| contig03721 | - - - - - | gnl\|UG\|Ta#S52546007 |
| contig00990 | - - - - - | gnl\|UG\|Ta#S52546127 |
| contig15760 | - - - - - | gnl\|UG\|Ta#S52546144 |
| contig08618 | - - - - - | gnl\|UG\|Ta#S52546152 |
| contig18772 | - - - - - | gnl\|UG\|Ta#S52546219 |
| contig04578 | - - - - - | gnl\|UG\|Ta#S52546243 |
| contig00624 | - - - - - | gnl\|UG\|Ta#S52546414 |
| contig02880 | - - - - - | gnl\|UG\|Ta#S52546414 |
| contig19063 | - - - - - | gnl\|UG\|Ta#S52546414 |
| contig23006 | - - - - - | gnl\|UG\|Ta#S52546414 |
| contig18009 | - - - - - | gnl\|UG\|Ta#S52546439 |
| contig03168 | - - - - - | gnl\|UG\|Ta#S52546475 |
| contig12484 | - - - - - | gnl\|UG\|Ta#S52546513 |
| contig08734 | - - - - - | gnl\|UG\|Ta#S52546514 |
| contig07097 | - - - - - | gnl\|UG\|Ta#S52546847 |
| contig06596 | - - - - - | gnl\|UG\|Ta#S52546959 |
| contig08067 | - - - - - | gnl\|UG\|Ta#S52547034 |
| contig10136 | - - - - - | gnl\|UG\|Ta#S52547034 |
| contig03165 | - - - - - | gnl\|UG\|Ta#S58844132 |
| contig10428 | - - - - - | gnl\|UG\|Ta#S58844132 |
| contig14910 | - - - - - | gnl\|UG\|Ta#S58844146 |
| contig08842 | - - - - - | gnl\|UG\|Ta#S58844172 |
| contig17476 | - - - - - | gnl\|UG\|Ta#S58844378 |
| contig06510 | - - - - - | gnl\|UG\|Ta#S58844407 |
| contig00346 | - - - - - | gnl\|UG\|Ta#S58844684 |
| contig04082 | - - - - - | gnl\|UG\|Ta#S58844870 |
| contig23096 | - - - - - | gnl\|UG\|Ta#S58845005 |
| contig25507 | - - - - - | gnl\|UG\|Ta#S58845005 |
| contig02048 | - - - - - | gnl\|UG\|Ta#S58845185 |
| contig03717 | - - - - - | gnl\|UG\|Ta#S58845739 |
| contig09444 | - - - - - | gnl\|UG\|Ta#S58846030 |
| contig13159 | - - - - - | gnl\|UG\|Ta#S58846447 |
| contig24674 | - - - - - | gnl\|UG\|Ta#S58846447 |

| | | |
|---|---|---|
| contig04171 | - - - - - | gnl\|UG\|Ta#S58846587 |
| contig15829 | - - - - - | gnl\|UG\|Ta#S58846645 |
| contig01572 | - - - - - | gnl\|UG\|Ta#S58846652 |
| contig00936 | - - - - - | gnl\|UG\|Ta#S58847107 |
| contig16283 | - - - - - | gnl\|UG\|Ta#S58847226 |
| contig07881 | - - - - - | gnl\|UG\|Ta#S58847493 |
| contig13920 | - - - - - | gnl\|UG\|Ta#S58847497 |
| contig26344 | - - - - - | gnl\|UG\|Ta#S58847497 |
| contig03440 | - - - - - | gnl\|UG\|Ta#S58847577 |
| contig05483 | - - - - - | gnl\|UG\|Ta#S58847594 |
| contig05017 | - - - - - | gnl\|UG\|Ta#S58848148 |
| contig09596 | - - - - - | gnl\|UG\|Ta#S58848504 |
| contig12060 | - - - - - | gnl\|UG\|Ta#S58849115 |
| contig04705 | - - - - - | gnl\|UG\|Ta#S58849170 |
| contig02468 | - - - - - | gnl\|UG\|Ta#S58849221 |
| contig05783 | - - - - - | gnl\|UG\|Ta#S58849405 |
| contig14863 | - - - - - | gnl\|UG\|Ta#S58849525 |
| contig08751 | - - - - - | gnl\|UG\|Ta#S58849526 |
| contig02261 | - - - - - | gnl\|UG\|Ta#S58849603 |
| contig25854 | - - - - - | gnl\|UG\|Ta#S58850025 |
| contig07615 | - - - - - | gnl\|UG\|Ta#S58850281 |
| contig02725 | - - - - - | gnl\|UG\|Ta#S58850292 |
| contig15555 | - - - - - | gnl\|UG\|Ta#S58851243 |
| contig00222 | - - - - - | gnl\|UG\|Ta#S58851286 |
| contig10031 | - - - - - | gnl\|UG\|Ta#S58851930 |
| contig13500 | - - - - - | gnl\|UG\|Ta#S58852044 |
| contig03066 | - - - - - | gnl\|UG\|Ta#S58852411 |
| contig13122 | - - - - - | gnl\|UG\|Ta#S58852577 |
| contig02686 | - - - - - | gnl\|UG\|Ta#S58852661 |
| contig08573 | - - - - - | gnl\|UG\|Ta#S58852661 |
| contig01708 | - - - - - | gnl\|UG\|Ta#S58852757 |
| contig18148 | - - - - - | gnl\|UG\|Ta#S58853233 |
| contig00256 | - - - - - | gnl\|UG\|Ta#S58853329 |
| contig07046 | - - - - - | gnl\|UG\|Ta#S58853833 |
| contig19024 | - - - - - | gnl\|UG\|Ta#S58853833 |
| contig22232 | - - - - - | gnl\|UG\|Ta#S58853833 |
| contig14093 | - - - - - | gnl\|UG\|Ta#S58853985 |
| contig18801 | - - - - - | gnl\|UG\|Ta#S58854185 |
| contig20253 | - - - - - | gnl\|UG\|Ta#S58854185 |
| contig02168 | - - - - - | gnl\|UG\|Ta#S58854401 |
| contig06085 | - - - - - | gnl\|UG\|Ta#S58854401 |
| contig12464 | - - - - - | gnl\|UG\|Ta#S58855030 |
| contig01362 | - - - - - | gnl\|UG\|Ta#S58855240 |
| contig04530 | - - - - - | gnl\|UG\|Ta#S58855240 |
| contig06113 | - - - - - | gnl\|UG\|Ta#S58856178 |
| contig03771 | - - - - - | gnl\|UG\|Ta#S58856307 |
| contig16554 | - - - - - | gnl\|UG\|Ta#S58856433 |
| contig20754 | - - - - - | gnl\|UG\|Ta#S58856533 |
| contig24703 | - - - - - | gnl\|UG\|Ta#S58856533 |
| contig05497 | - - - - - | gnl\|UG\|Ta#S58856730 |

| | | |
|---|---|---|
| contig06625 | - - - - - | gnl\|UG\|Ta#S58856743 |
| contig07672 | - - - - - | gnl\|UG\|Ta#S58856743 |
| contig00119 | - - - - - | gnl\|UG\|Ta#S58857133 |
| contig10551 | - - - - - | gnl\|UG\|Ta#S58857201 |
| contig14849 | - - - - - | gnl\|UG\|Ta#S58857201 |
| contig07989 | - - - - - | gnl\|UG\|Ta#S58857909 |
| contig01292 | - - - - - | gnl\|UG\|Ta#S58858414 |
| contig01294 | - - - - - | gnl\|UG\|Ta#S58858414 |
| contig00839 | - - - - - | gnl\|UG\|Ta#S58858820 |
| contig01751 | - - - - - | gnl\|UG\|Ta#S58858891 |
| contig19536 | - - - - - | gnl\|UG\|Ta#S58859076 |
| contig00351 | - - - - - | gnl\|UG\|Ta#S58859222 |
| contig01417 | - - - - - | gnl\|UG\|Ta#S58859222 |
| contig02036 | - - - - - | gnl\|UG\|Ta#S58859222 |
| contig13829 | - - - - - | gnl\|UG\|Ta#S58859222 |
| contig17126 | - - - - - | gnl\|UG\|Ta#S58859755 |
| contig13335 | - - - - - | gnl\|UG\|Ta#S58860258 |
| contig22554 | - - - - - | gnl\|UG\|Ta#S58860384 |
| contig08958 | - - - - - | gnl\|UG\|Ta#S58861899 |
| contig15702 | - - - - - | gnl\|UG\|Ta#S58861899 |
| contig18650 | - - - - - | gnl\|UG\|Ta#S58862383 |
| contig23751 | - - - - - | gnl\|UG\|Ta#S58862383 |
| contig19243 | - - - - - | gnl\|UG\|Ta#S58862432 |
| contig02266 | - - - - - | gnl\|UG\|Ta#S58863246 |
| contig07090 | - - - - - | gnl\|UG\|Ta#S58864225 |
| contig01973 | - - - - - | gnl\|UG\|Ta#S58864639 |
| contig18839 | - - - - - | gnl\|UG\|Ta#S58865169 |
| contig12734 | - - - - - | gnl\|UG\|Ta#S58865324 |
| contig05906 | - - - - - | gnl\|UG\|Ta#S58865776 |
| contig15933 | - - - - - | gnl\|UG\|Ta#S58867116 |
| contig03697 | - - - - - | gnl\|UG\|Ta#S58867418 |
| contig12826 | - - - - - | gnl\|UG\|Ta#S58867701 |
| contig10792 | - - - - - | gnl\|UG\|Ta#S58868578 |
| contig07288 | - - - - - | gnl\|UG\|Ta#S58868579 |
| contig05226 | - - - - - | gnl\|UG\|Ta#S58868954 |
| contig05529 | - - - - - | gnl\|UG\|Ta#S58869637 |
| contig09439 | - - - - - | gnl\|UG\|Ta#S58882078 |
| contig00650 | - - - - - | gnl\|UG\|Ta#S58882412 |
| contig00885 | - - - - - | gnl\|UG\|Ta#S58882412 |
| contig02568 | - - - - - | gnl\|UG\|Ta#S58882412 |
| contig17161 | - - - - - | gnl\|UG\|Ta#S58882412 |
| contig04934 | - - - - - | gnl\|UG\|Ta#S58883316 |
| contig21754 | - - - - - | gnl\|UG\|Ta#S58883554 |
| contig16171 | - - - - - | gnl\|UG\|Ta#S58884345 |
| contig17933 | - - - - - | gnl\|UG\|Ta#S58884345 |
| contig25982 | - - - - - | gnl\|UG\|Ta#S58884345 |
| contig01828 | - - - - - | gnl\|UG\|Ta#S58884553 |
| contig02680 | - - - - - | gnl\|UG\|Ta#S58885231 |
| contig04461 | - - - - - | gnl\|UG\|Ta#S58885558 |
| contig00720 | - - - - - | gnl\|UG\|Ta#S58887006 |

| | | |
|---|---|---|
| contig03023 | - - - - - | gnl\|UG\|Ta#S58887662 |
| contig16302 | - - - - - | gnl\|UG\|Ta#S58889717 |
| contig09086 | - - - - - | gnl\|UG\|Ta#S58890094 |
| contig11827 | - - - - - | gnl\|UG\|Ta#S58890170 |
| contig23078 | - - - - - | gnl\|UG\|Ta#S58890447 |
| contig06450 | - - - - - | gnl\|UG\|Ta#S58890570 |
| contig06698 | - - - - - | gnl\|UG\|Ta#S58890612 |
| contig00621 | - - - - - | gnl\|UG\|Ta#S58890856 |
| contig12868 | - - - - - | gnl\|UG\|Ta#S58891026 |
| contig17585 | - - - - - | gnl\|UG\|Ta#S58891540 |
| contig03328 | - - - - - | gnl\|UG\|Ta#S58892018 |
| contig06899 | - - - - - | gnl\|UG\|Ta#S58893980 |
| contig22157 | - - - - - | gnl\|UG\|Ta#S58893980 |
| contig11291 | - - - - - | gnl\|UG\|Ta#S58895652 |
| contig20817 | - - - - - | gnl\|UG\|Ta#S58897392 |
| contig20612 | - - - - - | gnl\|UG\|Ta#S58897472 |
| contig05417 | - - - - - | gnl\|UG\|Ta#S58900941 |
| contig01340 | - - - - - | gnl\|UG\|Ta#S58901623 |
| contig15900 | - - - - - | gnl\|UG\|Ta#S58902005 |
| contig06109 | - - - - - | gnl\|UG\|Ta#S58902682 |
| contig03386 | - - - - - | gnl\|UG\|Ta#S58904088 |
| contig21943 | - - - - - | gnl\|UG\|Ta#S58904773 |
| contig11085 | - - - - - | gnl\|UG\|Ta#S58905261 |
| contig13637 | - - - - - | gnl\|UG\|Ta#S58905261 |
| contig00149 | - - - - - | gnl\|UG\|Ta#S58905356 |
| contig12739 | - - - - - | gnl\|UG\|Ta#S58906817 |
| contig02366 | - - - - - | gnl\|UG\|Ta#S58908769 |
| contig06444 | - - - - - | gnl\|UG\|Ta#S58910657 |
| contig15009 | - - - - - | gnl\|UG\|Ta#S58912130 |
| contig02037 | - - - - - | gnl\|UG\|Ta#S58912267 |
| contig00884 | - - - - - | gnl\|UG\|Ta#S58912275 |
| contig26145 | - - - - - | gnl\|UG\|Ta#S61516913 |
| contig09318 | - - - - - | gnl\|UG\|Ta#S61517321 |
| contig02483 | - - - - - | gnl\|UG\|Ta#S61518394 |
| contig03875 | - - - - - | gnl\|UG\|Ta#S61518654 |
| contig04886 | - - - - - | gnl\|UG\|Ta#S61519052 |
| contig08285 | - - - - - | gnl\|UG\|Ta#S61519406 |
| contig17970 | - - - - - | gnl\|UG\|Ta#S61519792 |
| contig13939 | - - - - - | gnl\|UG\|Ta#S61520159 |
| contig19425 | - - - - - | gnl\|UG\|Ta#S61520159 |
| contig15997 | - - - - - | gnl\|UG\|Ta#S61520307 |
| contig15685 | - - - - - | gnl\|UG\|Ta#S61521618 |
| contig03276 | - - - - - | gnl\|UG\|Ta#S61522300 |
| contig04179 | - - - - - | gnl\|UG\|Ta#S61522534 |
| contig04921 | - - - - - | gnl\|UG\|Ta#S61522534 |
| contig03839 | - - - - - | gnl\|UG\|Ta#S61523553 |
| contig02567 | - - - - - | gnl\|UG\|Ta#S61523722 |
| contig06727 | - - - - - | gnl\|UG\|Ta#S61523988 |
| contig04185 | - - - - - | gnl\|UG\|Ta#S61524170 |
| contig19301 | - - - - - | gnl\|UG\|Ta#S61524804 |

| | | |
|---|---|---|
| contig21511 | - - - - - | gnl\|UG\|Ta#S61526033 |
| contig02773 | - - - - - | gnl\|UG\|Ta#S61526177 |
| contig13964 | - - - - - | gnl\|UG\|Ta#S61526177 |
| contig11370 | - - - - - | gnl\|UG\|Ta#S61527944 |
| contig10672 | - - - - - | gnl\|UG\|Ta#S61528219 |
| contig02755 | - - - - - | gnl\|UG\|Ta#S61529447 |
| contig25007 | - - - - - | gnl\|UG\|Ta#S61529834 |
| contig10802 | - - - - - | gnl\|UG\|Ta#S61530063 |
| contig18539 | - - - - - | gnl\|UG\|Ta#S61531271 |
| contig20300 | - - - - - | gnl\|UG\|Ta#S61531271 |
| contig08985 | - - - - - | gnl\|UG\|Ta#S61532255 |
| contig04346 | - - - - - | gnl\|UG\|Ta#S61532379 |
| contig02510 | - - - - - | gnl\|UG\|Ta#S61532799 |
| contig01132 | - - - - - | gnl\|UG\|Ta#S61533262 |
| contig07004 | - - - - - | gnl\|UG\|Ta#S61533487 |
| contig09151 | - - - - - | gnl\|UG\|Ta#S61533604 |
| contig01241 | - - - - - | gnl\|UG\|Ta#S61533903 |
| contig04002 | - - - - - | gnl\|UG\|Ta#S61533903 |
| contig00815 | - - - - - | gnl\|UG\|Ta#S61534114 |
| contig15234 | - - - - - | gnl\|UG\|Ta#S61534114 |
| contig04427 | - - - - - | gnl\|UG\|Ta#S61534264 |
| contig07780 | - - - - - | gnl\|UG\|Ta#S61534461 |
| contig00687 | - - - - - | gnl\|UG\|Ta#S61535205 |
| contig01808 | - - - - - | gnl\|UG\|Ta#S61535454 |
| contig05637 | - - - - - | gnl\|UG\|Ta#S61536377 |
| contig05734 | - - - - - | gnl\|UG\|Ta#S61536377 |
| contig00793 | - - - - - | gnl\|UG\|Ta#S61537246 |
| contig08481 | - - - - - | gnl\|UG\|Ta#S61537464 |
| contig03738 | - - - - - | gnl\|UG\|Ta#S61538117 |
| contig15793 | - - - - - | gnl\|UG\|Ta#S61539654 |
| contig24715 | - - - - - | gnl\|UG\|Ta#S61539654 |
| contig06092 | - - - - - | gnl\|UG\|Ta#S61540097 |
| contig00192 | - - - - - | gnl\|UG\|Ta#S61540223 |
| contig06169 | - - - - - | gnl\|UG\|Ta#S61541684 |
| contig02991 | - - - - - | gnl\|UG\|Ta#S61542287 |
| contig00313 | - - - - - | gnl\|UG\|Ta#S61542440 |
| contig19855 | - - - - - | gnl\|UG\|Ta#S61542440 |
| contig10410 | - - - - - | gnl\|UG\|Ta#S61542808 |
| contig25094 | - - - - - | gnl\|UG\|Ta#S61542808 |
| contig04436 | - - - - - | gnl\|UG\|Ta#S61543175 |
| contig11580 | - - - - - | gnl\|UG\|Ta#S61543396 |
| contig05581 | - - - - - | gnl\|UG\|Ta#S61543729 |
| contig16833 | - - - - - | gnl\|UG\|Ta#S61543858 |
| contig01387 | - - - - - | gnl\|UG\|Ta#S61544532 |
| contig18982 | - - - - - | gnl\|UG\|Ta#S61545857 |
| contig23297 | - - - - - | gnl\|UG\|Ta#S61545857 |
| contig01252 | - - - - - | gnl\|UG\|Ta#S61547142 |
| contig03509 | - - - - - | gnl\|UG\|Ta#S61547272 |
| contig18167 | - - - - - | gnl\|UG\|Ta#S61548588 |
| contig24120 | - - - - - | gnl\|UG\|Ta#S61548588 |

| | | |
|---|---|---|
| contig08104 | - - - - - | gnl\|UG\|Ta#S61548897 |
| contig00553 | - - - - - | gnl\|UG\|Ta#S61550003 |
| contig17272 | - - - - - | gnl\|UG\|Ta#S61550448 |
| contig21310 | - - - - - | gnl\|UG\|Ta#S61551520 |
| contig10898 | - - - - - | gnl\|UG\|Ta#S61552385 |
| contig09930 | - - - - - | gnl\|UG\|Ta#S61553212 |
| contig23891 | - - - - - | gnl\|UG\|Ta#S61553313 |
| contig09052 | - - - - - | gnl\|UG\|Ta#S61553692 |
| contig11608 | - - - - - | gnl\|UG\|Ta#S61554086 |
| contig07681 | - - - - - | gnl\|UG\|Ta#S61554479 |
| contig02211 | - - - - - | gnl\|UG\|Ta#S61554500 |
| contig00040 | - - - - - | gnl\|UG\|Ta#S61555302 |
| contig03715 | - - - - - | gnl\|UG\|Ta#S61557317 |
| contig02583 | - - - - - | gnl\|UG\|Ta#S61558722 |
| contig15636 | - - - - - | gnl\|UG\|Ta#S61558723 |
| contig16771 | - - - - - | gnl\|UG\|Ta#S61558723 |
| contig07842 | - - - - - | gnl\|UG\|Ta#S61559658 |
| contig18566 | - - - - - | gnl\|UG\|Ta#S61559658 |
| contig12304 | - - - - - | gnl\|UG\|Ta#S61560264 |
| contig10720 | - - - - - | gnl\|UG\|Ta#S61561462 |
| contig18055 | - - - - - | gnl\|UG\|Ta#S61562696 |
| contig20903 | - - - - - | gnl\|UG\|Ta#S61562696 |
| contig01428 | - - - - - | gnl\|UG\|Ta#S61563822 |
| contig01118 | - - - - - | gnl\|UG\|Ta#S61563963 |
| contig09818 | - - - - - | gnl\|UG\|Ta#S61564953 |
| contig07262 | - - - - - | gnl\|UG\|Ta#S61564964 |
| contig05736 | - - - - - | gnl\|UG\|Ta#S61565779 |
| contig20630 | - - - - - | gnl\|UG\|Ta#S61565857 |
| contig09973 | - - - - - | gnl\|UG\|Ta#S61567142 |
| contig18075 | - - - - - | gnl\|UG\|Ta#S61567675 |
| contig25318 | - - - - - | gnl\|UG\|Ta#S61567675 |
| contig07329 | - - - - - | gnl\|UG\|Ta#S61569415 |
| contig16075 | - - - - - | gnl\|UG\|Ta#S61569415 |
| contig11362 | - - - - - | gnl\|UG\|Ta#S61572015 |
| contig20784 | - - - - - | gnl\|UG\|Ta#S61573813 |
| contig14800 | - - - - - | gnl\|UG\|Ta#S61574623 |
| contig07188 | - - - - - | gnl\|UG\|Ta#S61576919 |
| contig02383 | - - - - - | gnl\|UG\|Ta#S61577181 |
| contig08970 | - - - - - | gnl\|UG\|Ta#S61578612 |
| contig02749 | - - - - - | gnl\|UG\|Ta#S61579027 |
| contig00289 | - - - - - | gnl\|UG\|Ta#S61581029 |
| contig08194 | - - - - - | gnl\|UG\|Ta#S61582033 |
| contig25930 | - - - - - | gnl\|UG\|Ta#S61582279 |
| contig24887 | - - - - - | gnl\|UG\|Ta#S61582693 |
| contig13417 | - - - - - | gnl\|UG\|Ta#S61583267 |
| contig01954 | - - - - - | gnl\|UG\|Ta#S61584185 |
| contig01247 | - - - - - | gnl\|UG\|Ta#S61584997 |
| contig05944 | - - - - - | gnl\|UG\|Ta#S61585301 |
| contig10182 | - - - - - | gnl\|UG\|Ta#S61585532 |
| contig03561 | - - - - - | gnl\|UG\|Ta#S61585609 |

| | | |
|---|---|---|
| contig03662 | - - - - - | gnl\|UG\|Ta#S61587631 |
| contig20259 | - - - - - | gnl\|UG\|Ta#S61587768 |
| contig04628 | - - - - - | gnl\|UG\|Ta#S61589286 |
| contig08237 | - - - - - | gnl\|UG\|Ta#S61589288 |
| contig02114 | - - - - - | gnl\|UG\|Ta#S61589567 |
| contig07316 | - - - - - | gnl\|UG\|Ta#S61590455 |
| contig09320 | - - - - - | gnl\|UG\|Ta#S61590455 |
| contig05207 | - - - - - | gnl\|UG\|Ta#S61592415 |
| contig08529 | - - - - - | gnl\|UG\|Ta#S61592415 |
| contig07186 | - - - - - | gnl\|UG\|Ta#S61592450 |
| contig20239 | - - - - - | gnl\|UG\|Ta#S61592450 |
| contig04723 | - - - - - | gnl\|UG\|Ta#S61592901 |
| contig05623 | - - - - - | gnl\|UG\|Ta#S61592901 |
| contig11857 | - - - - - | gnl\|UG\|Ta#S61593513 |
| contig23835 | - - - - - | gnl\|UG\|Ta#S61593664 |
| contig24574 | - - - - - | gnl\|UG\|Ta#S61593664 |
| contig00296 | - - - - - | gnl\|UG\|Ta#S61594002 |
| contig19289 | - - - - - | gnl\|UG\|Ta#S61594002 |
| contig02790 | - - - - - | gnl\|UG\|Ta#S61594093 |
| contig12796 | - - - - - | gnl\|UG\|Ta#S61594093 |
| contig04352 | - - - - - | gnl\|UG\|Ta#S61594194 |
| contig00785 | - - - - - | gnl\|UG\|Ta#S61594384 |
| contig05514 | - - - - - | gnl\|UG\|Ta#S61594391 |
| contig13951 | - - - - - | gnl\|UG\|Ta#S61596319 |
| contig09534 | - - - - - | gnl\|UG\|Ta#S61596468 |
| contig26475 | - - - - - | gnl\|UG\|Ta#S61597200 |
| contig20597 | - - - - - | gnl\|UG\|Ta#S61597217 |
| contig05008 | - - - - - | gnl\|UG\|Ta#S61598009 |
| contig18590 | - - - - - | gnl\|UG\|Ta#S61598375 |
| contig09252 | - - - - - | gnl\|UG\|Ta#S61599176 |
| contig03160 | - - - - - | gnl\|UG\|Ta#S61599518 |
| contig10050 | - - - - - | gnl\|UG\|Ta#S61599754 |
| contig10430 | - - - - - | gnl\|UG\|Ta#S61602972 |
| contig05145 | - - - - - | gnl\|UG\|Ta#S61603632 |
| contig15075 | - - - - - | gnl\|UG\|Ta#S61603873 |
| contig12629 | - - - - - | gnl\|UG\|Ta#S61604115 |
| contig11055 | - - - - - | gnl\|UG\|Ta#S61605469 |
| contig07466 | - - - - - | gnl\|UG\|Ta#S61606492 |
| contig02003 | - - - - - | gnl\|UG\|Ta#S61607976 |
| contig10605 | - - - - - | gnl\|UG\|Ta#S61608332 |
| contig07243 | - - - - - | gnl\|UG\|Ta#S61608686 |
| contig16876 | - - - - - | gnl\|UG\|Ta#S61609459 |
| contig22942 | - - - - - | gnl\|UG\|Ta#S61611243 |
| contig23605 | - - - - - | gnl\|UG\|Ta#S61611243 |
| contig24794 | - - - - - | gnl\|UG\|Ta#S61611243 |
| contig12995 | - - - - - | gnl\|UG\|Ta#S61612725 |
| contig04054 | - - - - - | gnl\|UG\|Ta#S61613893 |
| contig09238 | - - - - - | gnl\|UG\|Ta#S61615413 |
| contig09273 | - - - - - | gnl\|UG\|Ta#S61616716 |
| contig14334 | - - - - - | gnl\|UG\|Ta#S61616923 |

| | | |
|---|---|---|
| contig04907 | - - - - - | gnl\|UG\|Ta#S61617846 |
| contig01793 | - - - - - | gnl\|UG\|Ta#S61618924 |
| contig19705 | - - - - - | gnl\|UG\|Ta#S61618924 |
| contig00246 | - - - - - | gnl\|UG\|Ta#S61619734 |
| contig22374 | - - - - - | gnl\|UG\|Ta#S61624307 |
| contig06948 | - - - - - | gnl\|UG\|Ta#S61628441 |
| contig02373 | - - - - - | gnl\|UG\|Ta#S61629513 |
| contig16313 | - - - - - | gnl\|UG\|Ta#S61630219 |
| contig17475 | - - - - - | gnl\|UG\|Ta#S61630686 |
| contig22925 | - - - - - | gnl\|UG\|Ta#S61630846 |
| contig00144 | - - - - - | gnl\|UG\|Ta#S61631969 |
| contig23114 | - - - - - | gnl\|UG\|Ta#S61631969 |
| contig23729 | - - - - - | gnl\|UG\|Ta#S61633729 |
| contig07579 | - - - - - | gnl\|UG\|Ta#S61634380 |
| contig11760 | - - - - - | gnl\|UG\|Ta#S61634380 |
| contig19147 | - - - - - | gnl\|UG\|Ta#S61634380 |
| contig02669 | - - - - - | gnl\|UG\|Ta#S61634954 |
| contig10506 | - - - - - | gnl\|UG\|Ta#S61634954 |
| contig20386 | - - - - - | gnl\|UG\|Ta#S61634954 |
| contig00152 | - - - - - | gnl\|UG\|Ta#S61636851 |
| contig06488 | - - - - - | gnl\|UG\|Ta#S61638387 |
| contig24357 | - - - - - | gnl\|UG\|Ta#S61639812 |
| contig16997 | - - - - - | gnl\|UG\|Ta#S61639943 |
| contig01634 | - - - - - | gnl\|UG\|Ta#S61639979 |
| contig16809 | - - - - - | gnl\|UG\|Ta#S61639979 |
| contig01562 | - - - - - | gnl\|UG\|Ta#S61640166 |
| contig03069 | - - - - - | gnl\|UG\|Ta#S61640184 |
| contig17668 | - - - - - | gnl\|UG\|Ta#S61640930 |
| contig17677 | - - - - - | gnl\|UG\|Ta#S61641796 |
| contig21141 | - - - - - | gnl\|UG\|Ta#S61641796 |
| contig14214 | - - - - - | gnl\|UG\|Ta#S61642322 |
| contig12772 | - - - - - | gnl\|UG\|Ta#S61642413 |
| contig25321 | - - - - - | gnl\|UG\|Ta#S61642748 |
| contig02761 | - - - - - | gnl\|UG\|Ta#S61643669 |
| contig08690 | - - - - - | gnl\|UG\|Ta#S61644213 |
| contig13282 | - - - - - | gnl\|UG\|Ta#S61644576 |
| contig19417 | - - - - - | gnl\|UG\|Ta#S61645325 |
| contig26249 | - - - - - | gnl\|UG\|Ta#S61645325 |
| contig17202 | - - - - - | gnl\|UG\|Ta#S61645470 |
| contig08991 | - - - - - | gnl\|UG\|Ta#S61646722 |
| contig04016 | - - - - - | gnl\|UG\|Ta#S61646767 |
| contig12097 | - - - - - | gnl\|UG\|Ta#S61646767 |
| contig06131 | - - - - - | gnl\|UG\|Ta#S61646819 |
| contig04954 | - - - - - | gnl\|UG\|Ta#S61647946 |
| contig05541 | - - - - - | gnl\|UG\|Ta#S61647946 |
| contig07035 | - - - - - | gnl\|UG\|Ta#S61647946 |
| contig01081 | - - - - - | gnl\|UG\|Ta#S61648042 |
| contig22739 | - - - - - | gnl\|UG\|Ta#S61648391 |
| contig09394 | - - - - - | gnl\|UG\|Ta#S61648627 |
| contig01171 | - - - - - | gnl\|UG\|Ta#S61649454 |

| | | |
|---|---|---|
| contig04511 | - - - - - | gnl\|UG\|Ta#S61649517 |
| contig19432 | - - - - - | gnl\|UG\|Ta#S61649625 |
| contig18394 | - - - - - | gnl\|UG\|Ta#S61649849 |
| contig12545 | - - - - - | gnl\|UG\|Ta#S61649924 |
| contig02919 | - - - - - | gnl\|UG\|Ta#S61650101 |
| contig13573 | - - - - - | gnl\|UG\|Ta#S61650163 |
| contig14205 | - - - - - | gnl\|UG\|Ta#S61650163 |
| contig16594 | - - - - - | gnl\|UG\|Ta#S61650283 |
| contig19665 | - - - - - | gnl\|UG\|Ta#S61650545 |
| contig07892 | - - - - - | gnl\|UG\|Ta#S61650626 |
| contig08310 | - - - - - | gnl\|UG\|Ta#S61650910 |
| contig13066 | - - - - - | gnl\|UG\|Ta#S61650910 |
| contig22709 | - - - - - | gnl\|UG\|Ta#S61650910 |
| contig14403 | - - - - - | gnl\|UG\|Ta#S61651108 |
| contig19030 | - - - - - | gnl\|UG\|Ta#S61651481 |
| contig08312 | - - - - - | gnl\|UG\|Ta#S61651706 |
| contig00221 | - - - - - | gnl\|UG\|Ta#S61652093 |
| contig00727 | - - - - - | gnl\|UG\|Ta#S61652175 |
| contig00388 | - - - - - | gnl\|UG\|Ta#S61652198 |
| contig00847 | - - - - - | gnl\|UG\|Ta#S61652218 |
| contig04627 | - - - - - | gnl\|UG\|Ta#S61652595 |
| contig12537 | - - - - - | gnl\|UG\|Ta#S61652598 |
| contig13988 | - - - - - | gnl\|UG\|Ta#S61652654 |
| contig12161 | - - - - - | gnl\|UG\|Ta#S61652676 |
| contig03682 | - - - - - | gnl\|UG\|Ta#S61653059 |
| contig18276 | - - - - - | gnl\|UG\|Ta#S61653393 |
| contig09761 | - - - - - | gnl\|UG\|Ta#S61653653 |
| contig03022 | - - - - - | gnl\|UG\|Ta#S61653984 |
| contig05282 | - - - - - | gnl\|UG\|Ta#S61654208 |
| contig17255 | - - - - - | gnl\|UG\|Ta#S61654311 |
| contig06864 | - - - - - | gnl\|UG\|Ta#S61654389 |
| contig05175 | - - - - - | gnl\|UG\|Ta#S61654399 |
| contig03846 | - - - - - | gnl\|UG\|Ta#S61654472 |
| contig04465 | - - - - - | gnl\|UG\|Ta#S61654484 |
| contig08460 | - - - - - | gnl\|UG\|Ta#S61654742 |
| contig00983 | - - - - - | gnl\|UG\|Ta#S61655353 |
| contig11220 | - - - - - | gnl\|UG\|Ta#S61655497 |
| contig10281 | - - - - - | gnl\|UG\|Ta#S61655515 |
| contig05253 | - - - - - | gnl\|UG\|Ta#S61655686 |
| contig04901 | - - - - - | gnl\|UG\|Ta#S61655691 |
| contig16412 | - - - - - | gnl\|UG\|Ta#S61655756 |
| contig03311 | - - - - - | gnl\|UG\|Ta#S61655830 |
| contig19506 | - - - - - | gnl\|UG\|Ta#S61656083 |
| contig14798 | - - - - - | gnl\|UG\|Ta#S61656185 |
| contig21043 | - - - - - | gnl\|UG\|Ta#S61656335 |
| contig05223 | - - - - - | gnl\|UG\|Ta#S61656396 |
| contig02578 | - - - - - | gnl\|UG\|Ta#S61656596 |
| contig04570 | - - - - - | gnl\|UG\|Ta#S61657228 |
| contig06125 | - - - - - | gnl\|UG\|Ta#S61657566 |
| contig20527 | - - - - - | gnl\|UG\|Ta#S61657727 |

| | | |
|---|---|---|
| contig14640 | - - - - - | gnl\|UG\|Ta#S61657733 |
| contig01903 | - - - - - | gnl\|UG\|Ta#S61657764 |
| contig06823 | - - - - - | gnl\|UG\|Ta#S61657790 |
| contig11201 | - - - - - | gnl\|UG\|Ta#S61658362 |
| contig02258 | - - - - - | gnl\|UG\|Ta#S61658388 |
| contig24437 | - - - - - | gnl\|UG\|Ta#S61658461 |
| contig06763 | - - - - - | gnl\|UG\|Ta#S61658515 |
| contig09027 | - - - - - | gnl\|UG\|Ta#S61658587 |
| contig08658 | - - - - - | gnl\|UG\|Ta#S61658625 |
| contig06305 | - - - - - | gnl\|UG\|Ta#S61658699 |
| contig13655 | - - - - - | gnl\|UG\|Ta#S61658740 |
| contig18786 | - - - - - | gnl\|UG\|Ta#S61658906 |
| contig05336 | - - - - - | gnl\|UG\|Ta#S61658989 |
| contig11066 | - - - - - | gnl\|UG\|Ta#S61659137 |
| contig26114 | - - - - - | gnl\|UG\|Ta#S61659137 |
| contig01173 | - - - - - | gnl\|UG\|Ta#S61659275 |
| contig10484 | - - - - - | gnl\|UG\|Ta#S61659680 |
| contig01010 | - - - - - | gnl\|UG\|Ta#S61659882 |
| contig07437 | - - - - - | gnl\|UG\|Ta#S61659944 |
| contig06746 | - - - - - | gnl\|UG\|Ta#S61659949 |
| contig05980 | - - - - - | gnl\|UG\|Ta#S61660169 |
| contig09403 | - - - - - | gnl\|UG\|Ta#S61660708 |
| contig02367 | - - - - - | gnl\|UG\|Ta#S61660729 |
| contig08654 | - - - - - | gnl\|UG\|Ta#S61660908 |
| contig13950 | - - - - - | gnl\|UG\|Ta#S61661139 |
| contig01280 | - - - - - | gnl\|UG\|Ta#S61661359 |
| contig02996 | - - - - - | gnl\|UG\|Ta#S61661411 |
| contig01591 | - - - - - | gnl\|UG\|Ta#S61661717 |
| contig06565 | - - - - - | gnl\|UG\|Ta#S61661854 |
| contig02006 | - - - - - | gnl\|UG\|Ta#S61662031 |
| contig06532 | - - - - - | gnl\|UG\|Ta#S61662156 |
| contig19935 | - - - - - | gnl\|UG\|Ta#S61662253 |
| contig23644 | - - - - - | gnl\|UG\|Ta#S61662253 |
| contig07470 | - - - - - | gnl\|UG\|Ta#S61662446 |
| contig15053 | - - - - - | gnl\|UG\|Ta#S61662558 |
| contig03475 | - - - - - | gnl\|UG\|Ta#S61662582 |
| contig07383 | - - - - - | gnl\|UG\|Ta#S61662589 |
| contig11369 | - - - - - | gnl\|UG\|Ta#S61662730 |
| contig17901 | - - - - - | gnl\|UG\|Ta#S61662993 |
| contig00447 | - - - - - | gnl\|UG\|Ta#S61663093 |
| contig17378 | - - - - - | gnl\|UG\|Ta#S61663527 |
| contig03720 | - - - - - | gnl\|UG\|Ta#S61663648 |
| contig07045 | - - - - - | gnl\|UG\|Ta#S61663664 |
| contig11572 | - - - - - | gnl\|UG\|Ta#S61664072 |
| contig18844 | - - - - - | gnl\|UG\|Ta#S61664122 |
| contig00472 | - - - - - | gnl\|UG\|Ta#S61664368 |
| contig00110 | - - - - - | gnl\|UG\|Ta#S61664461 |
| contig00073 | - - - - - | gnl\|UG\|Ta#S61664734 |
| contig04483 | - - - - - | gnl\|UG\|Ta#S61665185 |
| contig02482 | - - - - - | gnl\|UG\|Ta#S61665230 |

| | | |
|---|---|---|
| contig22254 | - - - - - | gnl\|UG\|Ta#S61665569 |
| contig11001 | - - - - - | gnl\|UG\|Ta#S61667808 |
| contig19496 | - - - - - | gnl\|UG\|Ta#S61667808 |
| contig21488 | - - - - - | gnl\|UG\|Ta#S61667808 |
| contig05217 | - - - - - | gnl\|UG\|Ta#S61667969 |
| contig07424 | - - - - - | gnl\|UG\|Ta#S61668138 |
| contig05655 | - - - - - | gnl\|UG\|Ta#S61668279 |
| contig00399 | - - - - - | gnl\|UG\|Ta#S61668832 |
| contig22124 | - - - - - | gnl\|UG\|Ta#S61669680 |
| contig02675 | - - - - - | gnl\|UG\|Ta#S61669890 |
| contig04458 | - - - - - | gnl\|UG\|Ta#S61670005 |
| contig07160 | - - - - - | gnl\|UG\|Ta#S61670322 |
| contig11278 | - - - - - | gnl\|UG\|Ta#S61670648 |
| contig05328 | - - - - - | gnl\|UG\|Ta#S61670881 |
| contig18012 | - - - - - | gnl\|UG\|Ta#S61670881 |
| contig07380 | - - - - - | gnl\|UG\|Ta#S61670972 |
| contig01934 | - - - - - | gnl\|UG\|Ta#S61671043 |
| contig21475 | - - - - - | gnl\|UG\|Ta#S61671123 |
| contig09424 | - - - - - | gnl\|UG\|Ta#S61671153 |
| contig06520 | - - - - - | gnl\|UG\|Ta#S61671541 |
| contig19370 | - - - - - | gnl\|UG\|Ta#S61671568 |
| contig07043 | - - - - - | gnl\|UG\|Ta#S61672024 |
| contig07831 | - - - - - | gnl\|UG\|Ta#S61672216 |
| contig20365 | - - - - - | gnl\|UG\|Ta#S61672258 |
| contig14719 | - - - - - | gnl\|UG\|Ta#S61672668 |
| contig09769 | - - - - - | gnl\|UG\|Ta#S61672680 |
| contig04019 | - - - - - | gnl\|UG\|Ta#S61672910 |
| contig01035 | - - - - - | gnl\|UG\|Ta#S61673237 |
| contig20438 | - - - - - | gnl\|UG\|Ta#S61673394 |
| contig01855 | - - - - - | gnl\|UG\|Ta#S61673843 |
| contig13319 | - - - - - | gnl\|UG\|Ta#S61674604 |
| contig13415 | - - - - - | gnl\|UG\|Ta#S61674888 |
| contig02008 | - - - - - | gnl\|UG\|Ta#S61674987 |
| contig08473 | - - - - - | gnl\|UG\|Ta#S61675120 |
| contig00752 | - - - - - | gnl\|UG\|Ta#S61675266 |
| contig21162 | - - - - - | gnl\|UG\|Ta#S61675269 |
| contig14413 | - - - - - | gnl\|UG\|Ta#S61675631 |
| contig14736 | - - - - - | gnl\|UG\|Ta#S61675732 |
| contig02933 | - - - - - | gnl\|UG\|Ta#S61676178 |
| contig10369 | - - - - - | gnl\|UG\|Ta#S61676279 |
| contig13822 | - - - - - | gnl\|UG\|Ta#S61676279 |
| contig04851 | - - - - - | gnl\|UG\|Ta#S61676602 |
| contig05686 | - - - - - | gnl\|UG\|Ta#S61676729 |
| contig17939 | - - - - - | gnl\|UG\|Ta#S61676876 |
| contig14270 | - - - - - | gnl\|UG\|Ta#S61676962 |
| contig06918 | - - - - - | gnl\|UG\|Ta#S61677511 |
| contig15518 | - - - - - | gnl\|UG\|Ta#S61677612 |
| contig08406 | - - - - - | gnl\|UG\|Ta#S61677702 |
| contig04524 | - - - - - | gnl\|UG\|Ta#S61678180 |
| contig05471 | - - - - - | gnl\|UG\|Ta#S61678290 |

| | | |
|---|---|---|
| contig18296 | - - - - - | gnl\|UG\|Ta#S61678574 |
| contig16286 | - - - - - | gnl\|UG\|Ta#S61678614 |
| contig09604 | - - - - - | gnl\|UG\|Ta#S61678765 |
| contig14742 | - - - - - | gnl\|UG\|Ta#S61678905 |
| contig00122 | - - - - - | gnl\|UG\|Ta#S61679124 |
| contig07457 | - - - - - | gnl\|UG\|Ta#S61679623 |
| contig00429 | - - - - - | gnl\|UG\|Ta#S61679789 |
| contig01427 | - - - - - | gnl\|UG\|Ta#S61679876 |
| contig12210 | - - - - - | gnl\|UG\|Ta#S61680114 |
| contig01098 | - - - - - | gnl\|UG\|Ta#S61680194 |
| contig09083 | - - - - - | gnl\|UG\|Ta#S61680506 |
| contig01915 | - - - - - | gnl\|UG\|Ta#S61680627 |
| contig07353 | - - - - - | gnl\|UG\|Ta#S61680835 |
| contig02476 | - - - - - | gnl\|UG\|Ta#S61680928 |
| contig02198 | - - - - - | gnl\|UG\|Ta#S61681050 |
| contig13303 | - - - - - | gnl\|UG\|Ta#S61681121 |
| contig04523 | - - - - - | gnl\|UG\|Ta#S61681364 |
| contig01460 | - - - - - | gnl\|UG\|Ta#S61681543 |
| contig15817 | - - - - - | gnl\|UG\|Ta#S61682087 |
| contig18563 | - - - - - | gnl\|UG\|Ta#S61682389 |
| contig00637 | - - - - - | gnl\|UG\|Ta#S61682530 |
| contig11385 | - - - - - | gnl\|UG\|Ta#S61682699 |
| contig01999 | - - - - - | gnl\|UG\|Ta#S61682739 |
| contig19982 | - - - - - | gnl\|UG\|Ta#S61682912 |
| contig20808 | - - - - - | gnl\|UG\|Ta#S61683096 |
| contig17655 | - - - - - | gnl\|UG\|Ta#S61683218 |
| contig20902 | - - - - - | gnl\|UG\|Ta#S61683218 |
| contig05515 | - - - - - | gnl\|UG\|Ta#S61683440 |
| contig14909 | - - - - - | gnl\|UG\|Ta#S61683481 |
| contig12188 | - - - - - | gnl\|UG\|Ta#S61684102 |
| contig04634 | - - - - - | gnl\|UG\|Ta#S61685093 |
| contig14212 | - - - - - | gnl\|UG\|Ta#S61685814 |
| contig06243 | - - - - - | gnl\|UG\|Ta#S61685952 |
| contig08172 | - - - - - | gnl\|UG\|Ta#S61685952 |
| contig20799 | - - - - - | gnl\|UG\|Ta#S61686054 |
| contig04702 | - - - - - | gnl\|UG\|Ta#S61686143 |
| contig13254 | - - - - - | gnl\|UG\|Ta#S61686195 |
| contig04095 | - - - - - | gnl\|UG\|Ta#S61686226 |
| contig03867 | - - - - - | gnl\|UG\|Ta#S61686329 |
| contig08482 | - - - - - | gnl\|UG\|Ta#S61687148 |
| contig07850 | - - - - - | gnl\|UG\|Ta#S61687462 |
| contig11822 | - - - - - | gnl\|UG\|Ta#S61687893 |
| contig00111 | - - - - - | gnl\|UG\|Ta#S61687950 |
| contig09815 | - - - - - | gnl\|UG\|Ta#S61688568 |
| contig00570 | - - - - - | gnl\|UG\|Ta#S61689137 |
| contig14277 | - - - - - | gnl\|UG\|Ta#S61689518 |
| contig01985 | - - - - - | gnl\|UG\|Ta#S61689606 |
| contig02001 | - - - - - | gnl\|UG\|Ta#S61689836 |
| contig15882 | - - - - - | gnl\|UG\|Ta#S61690476 |
| contig11371 | - - - - - | gnl\|UG\|Ta#S61690609 |

| | | |
|---|---|---|
| contig09727 | - - - - - | gnl\|UG\|Ta#S61690627 |
| contig21091 | - - - - - | gnl\|UG\|Ta#S61690639 |
| contig15203 | - - - - - | gnl\|UG\|Ta#S61690821 |
| contig01451 | - - - - - | gnl\|UG\|Ta#S61690851 |
| contig05663 | - - - - - | gnl\|UG\|Ta#S61690851 |
| contig03861 | - - - - - | gnl\|UG\|Ta#S61691110 |
| contig10711 | - - - - - | gnl\|UG\|Ta#S61691228 |
| contig22513 | - - - - - | gnl\|UG\|Ta#S61691739 |
| contig04602 | - - - - - | gnl\|UG\|Ta#S61691848 |
| contig02418 | - - - - - | gnl\|UG\|Ta#S61691952 |
| contig06715 | - - - - - | gnl\|UG\|Ta#S61691971 |
| contig15668 | - - - - - | gnl\|UG\|Ta#S61692080 |
| contig06564 | - - - - - | gnl\|UG\|Ta#S61692323 |
| contig00024 | - - - - - | gnl\|UG\|Ta#S61692356 |
| contig19581 | - - - - - | gnl\|UG\|Ta#S61692368 |
| contig22798 | - - - - - | gnl\|UG\|Ta#S61692368 |
| contig01161 | - - - - - | gnl\|UG\|Ta#S61692757 |
| contig08026 | - - - - - | gnl\|UG\|Ta#S61692869 |
| contig14174 | - - - - - | gnl\|UG\|Ta#S61693126 |
| contig01544 | - - - - - | gnl\|UG\|Ta#S61693825 |
| contig25137 | - - - - - | gnl\|UG\|Ta#S61693945 |
| contig02425 | - - - - - | gnl\|UG\|Ta#S61694458 |
| contig11490 | - - - - - | gnl\|UG\|Ta#S61694555 |
| contig06212 | - - - - - | gnl\|UG\|Ta#S61694816 |
| contig14114 | - - - - - | gnl\|UG\|Ta#S61694968 |
| contig18255 | - - - - - | gnl\|UG\|Ta#S61695410 |
| contig08684 | - - - - - | gnl\|UG\|Ta#S61695525 |
| contig16240 | - - - - - | gnl\|UG\|Ta#S61695796 |
| contig04335 | - - - - - | gnl\|UG\|Ta#S61695826 |
| contig02570 | - - - - - | gnl\|UG\|Ta#S61695876 |
| contig02267 | - - - - - | gnl\|UG\|Ta#S61695905 |
| contig00597 | - - - - - | gnl\|UG\|Ta#S61695961 |
| contig20629 | - - - - - | gnl\|UG\|Ta#S61695985 |
| contig00497 | - - - - - | gnl\|UG\|Ta#S61696046 |
| contig07396 | - - - - - | gnl\|UG\|Ta#S61696092 |
| contig01283 | - - - - - | gnl\|UG\|Ta#S61696257 |
| contig07334 | - - - - - | gnl\|UG\|Ta#S61697068 |
| contig01343 | - - - - - | gnl\|UG\|Ta#S61697127 |
| contig02888 | - - - - - | gnl\|UG\|Ta#S61697171 |
| contig01614 | - - - - - | gnl\|UG\|Ta#S61697534 |
| contig00577 | - - - - - | gnl\|UG\|Ta#S61697656 |
| contig08930 | - - - - - | gnl\|UG\|Ta#S61697699 |
| contig19041 | - - - - - | gnl\|UG\|Ta#S61697921 |
| contig00010 | - - - - - | gnl\|UG\|Ta#S61697968 |
| contig05516 | - - - - - | gnl\|UG\|Ta#S61698172 |
| contig00417 | - - - - - | gnl\|UG\|Ta#S61698213 |
| contig03329 | - - - - - | gnl\|UG\|Ta#S61698325 |
| contig01668 | - - - - - | gnl\|UG\|Ta#S61698413 |
| contig00263 | - - - - - | gnl\|UG\|Ta#S61698458 |
| contig06783 | - - - - - | gnl\|UG\|Ta#S61698547 |

| | | |
|---|---|---|
| contig01939 | - - - - - | gnl\|UG\|Ta#S61699379 |
| contig08680 | - - - - - | gnl\|UG\|Ta#S61700039 |
| contig17499 | - - - - - | gnl\|UG\|Ta#S61700108 |
| contig06710 | - - - - - | gnl\|UG\|Ta#S61700143 |
| contig07663 | - - - - - | gnl\|UG\|Ta#S61700466 |
| contig08832 | - - - - - | gnl\|UG\|Ta#S61700528 |
| contig02280 | - - - - - | gnl\|UG\|Ta#S61700682 |
| contig04383 | - - - - - | gnl\|UG\|Ta#S61700694 |
| contig07251 | - - - - - | gnl\|UG\|Ta#S61701175 |
| contig01083 | - - - - - | gnl\|UG\|Ta#S61701202 |
| contig03841 | - - - - - | gnl\|UG\|Ta#S61701499 |
| contig17130 | - - - - - | gnl\|UG\|Ta#S61701649 |
| contig02895 | - - - - - | gnl\|UG\|Ta#S61701704 |
| contig06108 | - - - - - | gnl\|UG\|Ta#S61701777 |
| contig12079 | - - - - - | gnl\|UG\|Ta#S61702033 |
| contig24256 | - - - - - | gnl\|UG\|Ta#S61702823 |
| contig00097 | - - - - - | gnl\|UG\|Ta#S61703481 |
| contig16898 | - - - - - | gnl\|UG\|Ta#S61703597 |
| contig00573 | - - - - - | gnl\|UG\|Ta#S61703651 |
| contig11889 | - - - - - | gnl\|UG\|Ta#S61703653 |
| contig03422 | - - - - - | gnl\|UG\|Ta#S61703903 |
| contig07311 | - - - - - | gnl\|UG\|Ta#S61703968 |
| contig08358 | - - - - - | gnl\|UG\|Ta#S61704026 |
| contig08867 | - - - - - | gnl\|UG\|Ta#S61704317 |
| contig19974 | - - - - - | gnl\|UG\|Ta#S61704331 |
| contig03827 | - - - - - | gnl\|UG\|Ta#S61704688 |
| contig23689 | - - - - - | gnl\|UG\|Ta#S61704688 |
| contig25335 | - - - - - | gnl\|UG\|Ta#S61704752 |
| contig08752 | - - - - - | gnl\|UG\|Ta#S61704775 |
| contig18677 | - - - - - | gnl\|UG\|Ta#S61704973 |
| contig12098 | - - - - - | gnl\|UG\|Ta#S61705185 |
| contig13393 | - - - - - | gnl\|UG\|Ta#S61705185 |
| contig07771 | - - - - - | gnl\|UG\|Ta#S61705555 |
| contig01994 | - - - - - | gnl\|UG\|Ta#S61705966 |
| contig16433 | - - - - - | gnl\|UG\|Ta#S61706043 |
| contig24920 | - - - - - | gnl\|UG\|Ta#S61706459 |
| contig07415 | - - - - - | gnl\|UG\|Ta#S61706535 |
| contig06628 | - - - - - | gnl\|UG\|Ta#S61706689 |
| contig06485 | - - - - - | gnl\|UG\|Ta#S61706773 |
| contig00982 | - - - - - | gnl\|UG\|Ta#S61706798 |
| contig03672 | - - - - - | gnl\|UG\|Ta#S61706798 |
| contig08871 | - - - - - | gnl\|UG\|Ta#S61707062 |
| contig03282 | - - - - - | gnl\|UG\|Ta#S61707123 |
| contig12890 | - - - - - | gnl\|UG\|Ta#S61707342 |
| contig06959 | - - - - - | gnl\|UG\|Ta#S61707580 |
| contig24812 | - - - - - | gnl\|UG\|Ta#S61707595 |
| contig05505 | - - - - - | gnl\|UG\|Ta#S61708764 |
| contig12783 | - - - - - | gnl\|UG\|Ta#S61708945 |
| contig14225 | - - - - - | gnl\|UG\|Ta#S61708945 |
| contig22645 | - - - - - | gnl\|UG\|Ta#S61708945 |

| | | |
|---|---|---|
| contig02787 | - - - - - | gnl\|UG\|Ta#S61709074 |
| contig07031 | - - - - - | gnl\|UG\|Ta#S61709289 |
| contig07460 | - - - - - | gnl\|UG\|Ta#S61709595 |
| contig01911 | - - - - - | gnl\|UG\|Ta#S61709641 |
| contig14394 | - - - - - | gnl\|UG\|Ta#S61710168 |
| contig24511 | - - - - - | gnl\|UG\|Ta#S61710168 |
| contig10636 | - - - - - | gnl\|UG\|Ta#S61710828 |
| contig13284 | - - - - - | gnl\|UG\|Ta#S61711057 |
| contig16205 | - - - - - | gnl\|UG\|Ta#S61711329 |
| contig24561 | - - - - - | gnl\|UG\|Ta#S61711886 |
| contig03776 | - - - - - | gnl\|UG\|Ta#S61712362 |
| contig10390 | - - - - - | gnl\|UG\|Ta#S61712460 |
| contig02494 | - - - - - | gnl\|UG\|Ta#S61712728 |
| contig10416 | - - - - - | gnl\|UG\|Ta#S61712857 |
| contig10317 | - - - - - | gnl\|UG\|Ta#S61713041 |
| contig09688 | - - - - - | gnl\|UG\|Ta#S61713096 |
| contig06955 | - - - - - | gnl\|UG\|Ta#S61713400 |
| contig23303 | - - - - - | gnl\|UG\|Ta#S61713416 |
| contig26310 | - - - - - | gnl\|UG\|Ta#S61713416 |
| contig00741 | - - - - - | gnl\|UG\|Ta#S61713420 |
| contig00115 | - - - - - | gnl\|UG\|Ta#S61713662 |
| contig02535 | - - - - - | gnl\|UG\|Ta#S61713992 |
| contig05633 | - - - - - | gnl\|UG\|Ta#S61714053 |
| contig00680 | - - - - - | gnl\|UG\|Ta#S61714245 |
| contig06437 | - - - - - | gnl\|UG\|Ta#S61714365 |
| contig00187 | - - - - - | gnl\|UG\|Ta#S61714551 |
| contig13557 | - - - - - | gnl\|UG\|Ta#S61714956 |
| contig06321 | - - - - - | gnl\|UG\|Ta#S61715093 |
| contig13407 | - - - - - | gnl\|UG\|Ta#S61715334 |
| contig16068 | - - - - - | gnl\|UG\|Ta#S61716237 |
| contig19831 | - - - - - | gnl\|UG\|Ta#S61716278 |
| contig10187 | - - - - - | gnl\|UG\|Ta#S61716364 |
| contig03613 | - - - - - | gnl\|UG\|Ta#S61716409 |
| contig16372 | - - - - - | gnl\|UG\|Ta#S61716903 |
| contig00550 | - - - - - | gnl\|UG\|Ta#S61716939 |
| contig01033 | - - - - - | gnl\|UG\|Ta#S61717159 |
| contig07225 | - - - - - | gnl\|UG\|Ta#S61717342 |
| contig19226 | - - - - - | gnl\|UG\|Ta#S61718281 |
| contig14303 | - - - - - | gnl\|UG\|Ta#S61718422 |
| contig06436 | - - - - - | gnl\|UG\|Ta#S61718503 |
| contig08033 | - - - - - | gnl\|UG\|Ta#S61718698 |
| contig19248 | - - - - - | gnl\|UG\|Ta#S61719052 |
| contig23962 | - - - - - | gnl\|UG\|Ta#S61719312 |
| contig25726 | - - - - - | gnl\|UG\|Ta#S61719312 |
| contig23559 | - - - - - | gnl\|UG\|Ta#S61719716 |
| contig03254 | - - - - - | gnl\|UG\|Ta#S61719827 |
| contig22172 | - - - - - | gnl\|UG\|Ta#S61720101 |
| contig01514 | - - - - - | gnl\|UG\|Ta#S61720234 |
| contig07805 | - - - - - | gnl\|UG\|Ta#S61720343 |
| contig09096 | - - - - - | gnl\|UG\|Ta#S61720820 |

| | | |
|---|---|---|
| contig09886 | - - - - - | gnl\|UG\|Ta#S61720865 |
| contig17980 | - - - - - | gnl\|UG\|Ta#S61721262 |
| contig18112 | - - - - - | gnl\|UG\|Ta#S61721553 |
| contig00304 | - - - - - | gnl\|UG\|Ta#S61721627 |
| contig04526 | - - - - - | gnl\|UG\|Ta#S61721836 |
| contig01728 | - - - - - | gnl\|UG\|Ta#S61722288 |
| contig20247 | - - - - - | gnl\|UG\|Ta#S61722378 |
| contig05124 | - - - - - | gnl\|UG\|Ta#S61722481 |
| contig01899 | - - - - - | gnl\|UG\|Ta#S61722522 |
| contig00003 | - - - - - | gnl\|UG\|Ta#S61722553 |
| contig10338 | - - - - - | gnl\|UG\|Ta#S61722932 |
| contig09741 | - - - - - | gnl\|UG\|Ta#S61723047 |
| contig14692 | - - - - - | gnl\|UG\|Ta#S61723843 |
| contig11885 | - - - - - | gnl\|UG\|Ta#S61724008 |
| contig01403 | - - - - - | gnl\|UG\|Ta#S61724855 |
| contig22187 | - - - - - | gnl\|UG\|Ta#S61725091 |
| contig07578 | - - - - - | gnl\|UG\|Ta#S61725523 |
| contig26404 | - - - - - | gnl\|UG\|Ta#S61725791 |
| contig00200 | - - - - - | gnl\|UG\|Ta#S61726064 |
| contig12201 | - - - - - | gnl\|UG\|Ta#S61726165 |
| contig19033 | - - - - - | gnl\|UG\|Ta#S61726421 |
| contig14960 | - - - - - | gnl\|UG\|Ta#S61726448 |
| contig10666 | - - - - - | gnl\|UG\|Ta#S61726608 |
| contig25693 | - - - - - | gnl\|UG\|Ta#S61726608 |
| contig13002 | - - - - - | gnl\|UG\|Ta#S61726693 |
| contig09881 | - - - - - | gnl\|UG\|Ta#S61726880 |
| contig08809 | - - - - - | gnl\|UG\|Ta#S61726904 |
| contig21345 | - - - - - | gnl\|UG\|Ta#S61727235 |
| contig02858 | - - - - - | gnl\|UG\|Ta#S61727857 |
| contig07603 | - - - - - | gnl\|UG\|Ta#S61727879 |
| contig01058 | - - - - - | gnl\|UG\|Ta#S61727958 |
| contig12521 | - - - - - | gnl\|UG\|Ta#S61728021 |
| contig12013 | - - - - - | gnl\|UG\|Ta#S61728125 |
| contig15276 | - - - - - | gnl\|UG\|Ta#S61728371 |
| contig03215 | - - - - - | gnl\|UG\|Ta#S61728384 |
| contig01617 | - - - - - | gnl\|UG\|Ta#S61728730 |
| contig04033 | - - - - - | gnl\|UG\|Ta#S61728820 |
| contig03242 | - - - - - | gnl\|UG\|Ta#S61728841 |
| contig16902 | - - - - - | gnl\|UG\|Ta#S61729263 |
| contig25026 | - - - - - | gnl\|UG\|Ta#S61729263 |
| contig12278 | - - - - - | gnl\|UG\|Ta#S61729599 |
| contig25088 | - - - - - | gnl\|UG\|Ta#S61729730 |
| contig19196 | - - - - - | gnl\|UG\|Ta#S61730399 |
| contig10618 | - - - - - | gnl\|UG\|Ta#S61730422 |
| contig00198 | - - - - - | gnl\|UG\|Ta#S61730624 |
| contig06333 | - - - - - | gnl\|UG\|Ta#S61730724 |
| contig21177 | - - - - - | gnl\|UG\|Ta#S61730791 |
| contig22016 | - - - - - | gnl\|UG\|Ta#S61730791 |
| contig17392 | - - - - - | gnl\|UG\|Ta#S61730977 |
| contig18733 | - - - - - | gnl\|UG\|Ta#S61730977 |

| | | |
|---|---|---|
| contig04240 | - - - - - | gnl\|UG\|Ta#S61731589 |
| contig13215 | - - - - - | gnl\|UG\|Ta#S61731692 |
| contig15268 | - - - - - | gnl\|UG\|Ta#S61731692 |
| contig13796 | - - - - - | gnl\|UG\|Ta#S61731858 |
| contig18400 | - - - - - | gnl\|UG\|Ta#S61731933 |
| contig15240 | - - - - - | gnl\|UG\|Ta#S61732284 |
| contig07438 | - - - - - | gnl\|UG\|Ta#S61732314 |
| contig19489 | - - - - - | gnl\|UG\|Ta#S61732397 |
| contig23050 | - - - - - | gnl\|UG\|Ta#S61732427 |
| contig25118 | - - - - - | gnl\|UG\|Ta#S61732427 |
| contig01201 | - - - - - | gnl\|UG\|Ta#S61733006 |
| contig01358 | - - - - - | gnl\|UG\|Ta#S61733006 |
| contig01611 | - - - - - | gnl\|UG\|Ta#S61733052 |
| contig03288 | - - - - - | gnl\|UG\|Ta#S61734114 |
| contig00511 | - - - - - | gnl\|UG\|Ta#S61734150 |
| contig13607 | - - - - - | gnl\|UG\|Ta#S61734915 |
| contig25868 | - - - - - | gnl\|UG\|Ta#S61734915 |
| contig05710 | - - - - - | gnl\|UG\|Ta#S61735101 |
| contig03644 | - - - - - | gnl\|UG\|Ta#S61735319 |
| contig06945 | - - - - - | gnl\|UG\|Ta#S61735358 |
| contig11239 | - - - - - | gnl\|UG\|Ta#S61735464 |
| contig01267 | - - - - - | gnl\|UG\|Ta#S61735526 |
| contig12834 | - - - - - | gnl\|UG\|Ta#S61735529 |
| contig01909 | - - - - - | gnl\|UG\|Ta#S61735643 |
| contig15777 | - - - - - | gnl\|UG\|Ta#S61735856 |
| contig17886 | - - - - - | gnl\|UG\|Ta#S61735856 |
| contig23500 | - - - - - | gnl\|UG\|Ta#S61735863 |
| contig04304 | - - - - - | gnl\|UG\|Ta#S61736650 |
| contig00342 | - - - - - | gnl\|UG\|Ta#S61736651 |
| contig01080 | - - - - - | gnl\|UG\|Ta#S61736944 |
| contig07884 | - - - - - | gnl\|UG\|Ta#S61737257 |
| contig02151 | - - - - - | gnl\|UG\|Ta#S61737939 |
| contig09063 | - - - - - | gnl\|UG\|Ta#S61738166 |
| contig06089 | - - - - - | gnl\|UG\|Ta#S61738372 |
| contig09016 | - - - - - | gnl\|UG\|Ta#S61738437 |
| contig06417 | - - - - - | gnl\|UG\|Ta#S61738462 |
| contig06422 | - - - - - | gnl\|UG\|Ta#S61738830 |
| contig19082 | - - - - - | gnl\|UG\|Ta#S61739065 |
| contig04174 | - - - - - | gnl\|UG\|Ta#S61739518 |
| contig05399 | - - - - - | gnl\|UG\|Ta#S61739534 |
| contig13604 | - - - - - | gnl\|UG\|Ta#S61739845 |
| contig17043 | - - - - - | gnl\|UG\|Ta#S61739845 |
| contig02251 | - - - - - | gnl\|UG\|Ta#S61739958 |
| contig00252 | - - - - - | gnl\|UG\|Ta#S61740174 |
| contig12334 | - - - - - | gnl\|UG\|Ta#S61740693 |
| contig05953 | - - - - - | gnl\|UG\|Ta#S61740943 |
| contig15700 | - - - - - | gnl\|UG\|Ta#S61741593 |
| contig06347 | - - - - - | gnl\|UG\|Ta#S61742022 |
| contig20405 | - - - - - | gnl\|UG\|Ta#S61742022 |
| contig13784 | - - - - - | gnl\|UG\|Ta#S61742279 |

| | | |
|---|---|---|
| contig02782 | - - - - - | gnl\|UG\|Ta#S61742322 |
| contig14737 | - - - - - | gnl\|UG\|Ta#S61742336 |
| contig10247 | - - - - - | gnl\|UG\|Ta#S61742407 |
| contig04620 | - - - - - | gnl\|UG\|Ta#S61742532 |
| contig05142 | - - - - - | gnl\|UG\|Ta#S61742609 |
| contig03912 | - - - - - | gnl\|UG\|Ta#S61742750 |
| contig06324 | - - - - - | gnl\|UG\|Ta#S61742857 |
| contig11731 | - - - - - | gnl\|UG\|Ta#S61742857 |
| contig02762 | - - - - - | gnl\|UG\|Ta#S61742943 |
| contig07779 | - - - - - | gnl\|UG\|Ta#S61742943 |
| contig08554 | - - - - - | gnl\|UG\|Ta#S61743070 |
| contig15333 | - - - - - | gnl\|UG\|Ta#S61743150 |
| contig22478 | - - - - - | gnl\|UG\|Ta#S61743358 |
| contig04958 | - - - - - | gnl\|UG\|Ta#S61743611 |
| contig14898 | - - - - - | gnl\|UG\|Ta#S61744325 |
| contig15335 | - - - - - | gnl\|UG\|Ta#S61744325 |
| contig18991 | - - - - - | gnl\|UG\|Ta#S61744591 |
| contig02592 | - - - - - | gnl\|UG\|Ta#S61744831 |
| contig00519 | - - - - - | gnl\|UG\|Ta#S61745050 |
| contig09212 | - - - - - | gnl\|UG\|Ta#S61745163 |
| contig11161 | - - - - - | gnl\|UG\|Ta#S61745389 |
| contig18243 | - - - - - | gnl\|UG\|Ta#S61745389 |
| contig04903 | - - - - - | gnl\|UG\|Ta#S61745422 |
| contig06142 | - - - - - | gnl\|UG\|Ta#S61746353 |
| contig07493 | - - - - - | gnl\|UG\|Ta#S61746353 |
| contig01388 | - - - - - | gnl\|UG\|Ta#S61746469 |
| contig19018 | - - - - - | gnl\|UG\|Ta#S61746835 |
| contig13461 | - - - - - | gnl\|UG\|Ta#S61746860 |
| contig07778 | - - - - - | gnl\|UG\|Ta#S61747079 |
| contig05769 | - - - - - | gnl\|UG\|Ta#S61747323 |
| contig15028 | - - - - - | gnl\|UG\|Ta#S61747360 |
| contig00846 | - - - - - | gnl\|UG\|Ta#S61747401 |
| contig15455 | - - - - - | gnl\|UG\|Ta#S61747844 |
| contig02240 | - - - - - | gnl\|UG\|Ta#S61747855 |
| contig17803 | - - - - - | gnl\|UG\|Ta#S61747998 |
| contig15472 | - - - - - | gnl\|UG\|Ta#S61748107 |
| contig09404 | - - - - - | gnl\|UG\|Ta#S61748178 |
| contig02032 | - - - - - | gnl\|UG\|Ta#S61748368 |
| contig12915 | - - - - - | gnl\|UG\|Ta#S61748368 |
| contig00655 | - - - - - | gnl\|UG\|Ta#S61748416 |
| contig14570 | - - - - - | gnl\|UG\|Ta#S61748425 |
| contig17249 | - - - - - | gnl\|UG\|Ta#S61748425 |
| contig10235 | - - - - - | gnl\|UG\|Ta#S61748663 |
| contig02672 | - - - - - | gnl\|UG\|Ta#S61748798 |
| contig19815 | - - - - - | gnl\|UG\|Ta#S61748868 |
| contig25758 | - - - - - | gnl\|UG\|Ta#S61749199 |
| contig05448 | - - - - - | gnl\|UG\|Ta#S61749663 |
| contig14577 | - - - - - | gnl\|UG\|Ta#S61749663 |
| contig15908 | - - - - - | gnl\|UG\|Ta#S61750798 |
| contig03270 | - - - - - | gnl\|UG\|Ta#S61750828 |

| | | |
|---|---|---|
| contig00948 | - - - - - | gnl\|UG\|Ta#S61750853 |
| contig00770 | - - - - - | gnl\|UG\|Ta#S61751147 |
| contig06292 | - - - - - | gnl\|UG\|Ta#S61751451 |
| contig02160 | - - - - - | gnl\|UG\|Ta#S61751453 |
| contig10438 | - - - - - | gnl\|UG\|Ta#S61752297 |
| contig10356 | - - - - - | gnl\|UG\|Ta#S61752305 |
| contig16801 | - - - - - | gnl\|UG\|Ta#S61752305 |
| contig04622 | - - - - - | gnl\|UG\|Ta#S61752461 |
| contig05886 | - - - - - | gnl\|UG\|Ta#S61752643 |
| contig24144 | - - - - - | gnl\|UG\|Ta#S61752643 |
| contig02542 | - - - - - | gnl\|UG\|Ta#S61753603 |
| contig09905 | - - - - - | gnl\|UG\|Ta#S61754497 |
| contig17001 | - - - - - | gnl\|UG\|Ta#S61754497 |
| contig14920 | - - - - - | gnl\|UG\|Ta#S61754520 |
| contig26006 | - - - - - | gnl\|UG\|Ta#S61754692 |
| contig08389 | - - - - - | gnl\|UG\|Ta#S61754712 |
| contig18200 | - - - - - | gnl\|UG\|Ta#S61754788 |
| contig22180 | - - - - - | gnl\|UG\|Ta#S61755040 |
| contig04672 | - - - - - | gnl\|UG\|Ta#S61755157 |
| contig04083 | - - - - - | gnl\|UG\|Ta#S61755404 |
| contig04603 | - - - - - | gnl\|UG\|Ta#S61756718 |
| contig04796 | - - - - - | gnl\|UG\|Ta#S61756770 |
| contig13563 | - - - - - | gnl\|UG\|Ta#S61756805 |
| contig02808 | - - - - - | gnl\|UG\|Ta#S61756836 |
| contig11454 | - - - - - | gnl\|UG\|Ta#S61757407 |
| contig00475 | - - - - - | gnl\|UG\|Ta#S61758303 |
| contig17844 | - - - - - | gnl\|UG\|Ta#S61758662 |
| contig02780 | - - - - - | gnl\|UG\|Ta#S61758856 |
| contig23745 | - - - - - | gnl\|UG\|Ta#S61758998 |
| contig07932 | - - - - - | gnl\|UG\|Ta#S61759041 |
| contig17536 | - - - - - | gnl\|UG\|Ta#S61759332 |
| contig06626 | - - - - - | gnl\|UG\|Ta#S61759441 |
| contig14302 | - - - - - | gnl\|UG\|Ta#S61759441 |
| contig06960 | - - - - - | gnl\|UG\|Ta#S61759474 |
| contig25707 | - - - - - | gnl\|UG\|Ta#S61759803 |
| contig23917 | - - - - - | gnl\|UG\|Ta#S61759887 |
| contig11578 | - - - - - | gnl\|UG\|Ta#S61760063 |
| contig05756 | - - - - - | gnl\|UG\|Ta#S61760468 |
| contig03696 | - - - - - | gnl\|UG\|Ta#S61760492 |
| contig09533 | - - - - - | gnl\|UG\|Ta#S61760744 |
| contig08206 | - - - - - | gnl\|UG\|Ta#S61760859 |
| contig03250 | - - - - - | gnl\|UG\|Ta#S61760867 |
| contig00320 | - - - - - | gnl\|UG\|Ta#S61760950 |
| contig01302 | - - - - - | gnl\|UG\|Ta#S61761309 |
| contig07619 | - - - - - | gnl\|UG\|Ta#S61761611 |
| contig23571 | - - - - - | gnl\|UG\|Ta#S61761827 |
| contig06542 | - - - - - | gnl\|UG\|Ta#S61762239 |
| contig00019 | - - - - - | gnl\|UG\|Ta#S61762425 |
| contig00703 | - - - - - | gnl\|UG\|Ta#S61763340 |
| contig01959 | - - - - - | gnl\|UG\|Ta#S61763340 |

| | | |
|---|---|---|
| contig10537 | - - - - - | gnl\|UG\|Ta#S61763413 |
| contig00132 | - - - - - | gnl\|UG\|Ta#S61763502 |
| contig14607 | - - - - - | gnl\|UG\|Ta#S61763550 |
| contig02591 | - - - - - | gnl\|UG\|Ta#S61763608 |
| contig09442 | - - - - - | gnl\|UG\|Ta#S61763769 |
| contig00445 | - - - - - | gnl\|UG\|Ta#S61764016 |
| contig03695 | - - - - - | gnl\|UG\|Ta#S61764048 |
| contig25923 | - - - - - | gnl\|UG\|Ta#S61764327 |
| contig13474 | - - - - - | gnl\|UG\|Ta#S61764583 |
| contig20262 | - - - - - | gnl\|UG\|Ta#S61764761 |
| contig25097 | - - - - - | gnl\|UG\|Ta#S61765522 |
| contig03621 | - - - - - | gnl\|UG\|Ta#S61766004 |
| contig05060 | - - - - - | gnl\|UG\|Ta#S61766406 |
| contig15634 | - - - - - | gnl\|UG\|Ta#S61766556 |
| contig06498 | - - - - - | gnl\|UG\|Ta#S61766695 |
| contig04029 | - - - - - | gnl\|UG\|Ta#S61766718 |
| contig19450 | - - - - - | gnl\|UG\|Ta#S61766741 |
| contig24657 | - - - - - | gnl\|UG\|Ta#S61766741 |
| contig12319 | - - - - - | gnl\|UG\|Ta#S61766742 |
| contig12239 | - - - - - | gnl\|UG\|Ta#S61766907 |
| contig18997 | - - - - - | gnl\|UG\|Ta#S61767267 |
| contig08509 | - - - - - | gnl\|UG\|Ta#S61767563 |
| contig05165 | - - - - - | gnl\|UG\|Ta#S61767590 |
| contig05319 | - - - - - | gnl\|UG\|Ta#S61767891 |
| contig15160 | - - - - - | gnl\|UG\|Ta#S61767891 |
| contig00086 | - - - - - | gnl\|UG\|Ta#S61768045 |
| contig05780 | - - - - - | gnl\|UG\|Ta#S61768045 |
| contig03870 | - - - - - | gnl\|UG\|Ta#S61768351 |
| contig01492 | - - - - - | gnl\|UG\|Ta#S61768561 |
| contig02395 | - - - - - | gnl\|UG\|Ta#S61768609 |
| contig12019 | - - - - - | gnl\|UG\|Ta#S61768784 |
| contig12670 | - - - - - | gnl\|UG\|Ta#S61769430 |
| contig23471 | - - - - - | gnl\|UG\|Ta#S61769430 |
| contig05975 | - - - - - | gnl\|UG\|Ta#S61769719 |
| contig00291 | - - - - - | gnl\|UG\|Ta#S61769731 |
| contig07100 | - - - - - | gnl\|UG\|Ta#S61769966 |
| contig00676 | - - - - - | gnl\|UG\|Ta#S61770299 |
| contig17913 | - - - - - | gnl\|UG\|Ta#S61770299 |
| contig10783 | - - - - - | gnl\|UG\|Ta#S61770410 |
| contig15494 | - - - - - | gnl\|UG\|Ta#S61770410 |
| contig17711 | - - - - - | gnl\|UG\|Ta#S61770504 |
| contig14163 | - - - - - | gnl\|UG\|Ta#S61770752 |
| contig08594 | - - - - - | gnl\|UG\|Ta#S61770906 |
| contig09116 | - - - - - | gnl\|UG\|Ta#S61770928 |
| contig10005 | - - - - - | gnl\|UG\|Ta#S61770928 |
| contig05391 | - - - - - | gnl\|UG\|Ta#S61771075 |
| contig23219 | - - - - - | gnl\|UG\|Ta#S61771075 |
| contig04080 | - - - - - | gnl\|UG\|Ta#S61771186 |
| contig05629 | - - - - - | gnl\|UG\|Ta#S61771468 |
| contig08696 | - - - - - | gnl\|UG\|Ta#S61771690 |

| | | |
|---|---|---|
| contig15243 | - - - - - | gnl\|UG\|Ta#S61771690 |
| contig16527 | - - - - - | gnl\|UG\|Ta#S61771690 |
| contig00882 | - - - - - | gnl\|UG\|Ta#S61771929 |
| contig07594 | - - - - - | gnl\|UG\|Ta#S61771939 |
| contig09418 | - - - - - | gnl\|UG\|Ta#S61772110 |
| contig11886 | - - - - - | gnl\|UG\|Ta#S61772466 |
| contig02566 | - - - - - | gnl\|UG\|Ta#S61772476 |
| contig02881 | - - - - - | gnl\|UG\|Ta#S61772476 |
| contig09743 | - - - - - | gnl\|UG\|Ta#S61772539 |
| contig16510 | - - - - - | gnl\|UG\|Ta#S61772839 |
| contig17003 | - - - - - | gnl\|UG\|Ta#S61772949 |
| contig20571 | - - - - - | gnl\|UG\|Ta#S61772949 |
| contig04589 | - - - - - | gnl\|UG\|Ta#S61773121 |
| contig00379 | - - - - - | gnl\|UG\|Ta#S61773239 |
| contig22986 | - - - - - | gnl\|UG\|Ta#S61773340 |
| contig18645 | - - - - - | gnl\|UG\|Ta#S61773446 |
| contig11659 | - - - - - | gnl\|UG\|Ta#S61773506 |
| contig15322 | - - - - - | gnl\|UG\|Ta#S61773698 |
| contig01869 | - - - - - | gnl\|UG\|Ta#S61773771 |
| contig08419 | - - - - - | gnl\|UG\|Ta#S61773803 |
| contig22676 | - - - - - | gnl\|UG\|Ta#S61773876 |
| contig01249 | - - - - - | gnl\|UG\|Ta#S61773954 |
| contig18290 | - - - - - | gnl\|UG\|Ta#S61773976 |
| contig18919 | - - - - - | gnl\|UG\|Ta#S61773976 |
| contig12939 | - - - - - | gnl\|UG\|Ta#S61774288 |
| contig04300 | - - - - - | gnl\|UG\|Ta#S61775144 |
| contig15547 | - - - - - | gnl\|UG\|Ta#S61775278 |
| contig24250 | - - - - - | gnl\|UG\|Ta#S61775656 |
| contig00787 | - - - - - | gnl\|UG\|Ta#S61775758 |
| contig10380 | - - - - - | gnl\|UG\|Ta#S61775938 |
| contig01620 | - - - - - | gnl\|UG\|Ta#S61776063 |
| contig02516 | - - - - - | gnl\|UG\|Ta#S61776233 |
| contig20945 | - - - - - | gnl\|UG\|Ta#S61776656 |
| contig22058 | - - - - - | gnl\|UG\|Ta#S61776656 |
| contig10098 | - - - - - | gnl\|UG\|Ta#S61776721 |
| contig13754 | - - - - - | gnl\|UG\|Ta#S61776733 |
| contig16287 | - - - - - | gnl\|UG\|Ta#S61777267 |
| contig03920 | - - - - - | gnl\|UG\|Ta#S61777321 |
| contig10782 | - - - - - | gnl\|UG\|Ta#S61777391 |
| contig00050 | - - - - - | gnl\|UG\|Ta#S61777608 |
| contig05444 | - - - - - | gnl\|UG\|Ta#S61777608 |
| contig00284 | - - - - - | gnl\|UG\|Ta#S61777870 |
| contig02952 | - - - - - | gnl\|UG\|Ta#S61777877 |
| contig03910 | - - - - - | gnl\|UG\|Ta#S61777952 |
| contig16737 | - - - - - | gnl\|UG\|Ta#S61777952 |
| contig12445 | - - - - - | gnl\|UG\|Ta#S61778147 |
| contig05398 | - - - - - | gnl\|UG\|Ta#S61778298 |
| contig03735 | - - - - - | gnl\|UG\|Ta#S61778432 |
| contig02511 | - - - - - | gnl\|UG\|Ta#S61778714 |
| contig00354 | - - - - - | gnl\|UG\|Ta#S61778880 |

| | | |
|---|---|---|
| contig01154 | - - - - - | gnl\|UG\|Ta#S61778880 |
| contig04822 | - - - - - | gnl\|UG\|Ta#S61778880 |
| contig08049 | - - - - - | gnl\|UG\|Ta#S61778880 |
| contig11912 | - - - - - | gnl\|UG\|Ta#S61778880 |
| contig08069 | - - - - - | gnl\|UG\|Ta#S61778893 |
| contig06811 | - - - - - | gnl\|UG\|Ta#S61778931 |
| contig03668 | - - - - - | gnl\|UG\|Ta#S61779038 |
| contig05926 | - - - - - | gnl\|UG\|Ta#S61779038 |
| contig17510 | - - - - - | gnl\|UG\|Ta#S61779038 |
| contig12719 | - - - - - | gnl\|UG\|Ta#S61779114 |
| contig18056 | - - - - - | gnl\|UG\|Ta#S61779219 |
| contig08155 | - - - - - | gnl\|UG\|Ta#S61779390 |
| contig01209 | - - - - - | gnl\|UG\|Ta#S61779586 |
| contig02585 | - - - - - | gnl\|UG\|Ta#S61779647 |
| contig05867 | - - - - - | gnl\|UG\|Ta#S61780083 |
| contig09579 | - - - - - | gnl\|UG\|Ta#S61780360 |
| contig11903 | - - - - - | gnl\|UG\|Ta#S61780360 |
| contig24678 | - - - - - | gnl\|UG\|Ta#S61780360 |
| contig18802 | - - - - - | gnl\|UG\|Ta#S61780513 |
| contig12916 | - - - - - | gnl\|UG\|Ta#S61780669 |
| contig00065 | - - - - - | gnl\|UG\|Ta#S61780671 |
| contig01418 | - - - - - | gnl\|UG\|Ta#S61780711 |
| contig06548 | - - - - - | gnl\|UG\|Ta#S61780711 |
| contig04632 | - - - - - | gnl\|UG\|Ta#S61780856 |
| contig08025 | - - - - - | gnl\|UG\|Ta#S61780998 |
| contig13009 | - - - - - | gnl\|UG\|Ta#S61780998 |
| contig20027 | - - - - - | gnl\|UG\|Ta#S61780998 |
| contig20270 | - - - - - | gnl\|UG\|Ta#S61781084 |
| contig09926 | - - - - - | gnl\|UG\|Ta#S61781176 |
| contig07714 | - - - - - | gnl\|UG\|Ta#S61781210 |
| contig07859 | - - - - - | gnl\|UG\|Ta#S61781428 |
| contig01217 | - - - - - | gnl\|UG\|Ta#S61781580 |
| contig06213 | - - - - - | gnl\|UG\|Ta#S61781580 |
| contig06221 | - - - - - | gnl\|UG\|Ta#S61781636 |
| contig19724 | - - - - - | gnl\|UG\|Ta#S61781678 |
| contig20494 | - - - - - | gnl\|UG\|Ta#S61781678 |
| contig21210 | - - - - - | gnl\|UG\|Ta#S61781678 |
| contig23151 | - - - - - | gnl\|UG\|Ta#S61781678 |
| contig11978 | - - - - - | gnl\|UG\|Ta#S61781779 |
| contig11534 | - - - - - | gnl\|UG\|Ta#S61781838 |
| contig12240 | - - - - - | gnl\|UG\|Ta#S61781838 |
| contig20993 | - - - - - | gnl\|UG\|Ta#S61781838 |
| contig07908 | - - - - - | gnl\|UG\|Ta#S61782043 |
| contig13963 | - - - - - | gnl\|UG\|Ta#S61782048 |
| contig08941 | - - - - - | gnl\|UG\|Ta#S61782182 |
| contig07983 | - - - - - | gnl\|UG\|Ta#S61782183 |
| contig00251 | - - - - - | gnl\|UG\|Ta#S61782298 |
| contig10022 | - - - - - | gnl\|UG\|Ta#S61782298 |
| contig22300 | - - - - - | gnl\|UG\|Ta#S61782529 |
| contig21836 | - - - - - | gnl\|UG\|Ta#S61782575 |

| | | |
|---|---|---|
| contig21296 | - - - - - | gnl\|UG\|Ta#S61783271 |
| contig09563 | - - - - - | gnl\|UG\|Ta#S61783442 |
| contig08628 | - - - - - | gnl\|UG\|Ta#S61783557 |
| contig18353 | - - - - - | gnl\|UG\|Ta#S61783557 |
| contig09366 | - - - - - | gnl\|UG\|Ta#S61783680 |
| contig03821 | - - - - - | gnl\|UG\|Ta#S61783729 |
| contig02831 | - - - - - | gnl\|UG\|Ta#S61783958 |
| contig00883 | - - - - - | gnl\|UG\|Ta#S61784008 |
| contig15629 | - - - - - | gnl\|UG\|Ta#S61784290 |
| contig01641 | - - - - - | gnl\|UG\|Ta#S61784402 |
| contig19085 | - - - - - | gnl\|UG\|Ta#S61784516 |
| contig01291 | - - - - - | gnl\|UG\|Ta#S61784716 |
| contig00043 | - - - - - | gnl\|UG\|Ta#S61785080 |
| contig01175 | - - - - - | gnl\|UG\|Ta#S61785331 |
| contig25957 | - - - - - | gnl\|UG\|Ta#S61785331 |
| contig03397 | - - - - - | gnl\|UG\|Ta#S61785568 |
| contig10565 | - - - - - | gnl\|UG\|Ta#S61785740 |
| contig12344 | - - - - - | gnl\|UG\|Ta#S61785909 |
| contig03264 | - - - - - | gnl\|UG\|Ta#S61786182 |
| contig03216 | - - - - - | gnl\|UG\|Ta#S61786204 |
| contig02799 | - - - - - | gnl\|UG\|Ta#S61786374 |
| contig16933 | - - - - - | gnl\|UG\|Ta#S61786451 |
| contig09395 | - - - - - | gnl\|UG\|Ta#S61786503 |
| contig00652 | - - - - - | gnl\|UG\|Ta#S61786906 |
| contig03358 | - - - - - | gnl\|UG\|Ta#S61787067 |
| contig00677 | - - - - - | gnl\|UG\|Ta#S61787266 |
| contig11943 | - - - - - | gnl\|UG\|Ta#S61787309 |
| contig06776 | - - - - - | gnl\|UG\|Ta#S61787330 |
| contig17377 | - - - - - | gnl\|UG\|Ta#S61787452 |
| contig10572 | - - - - - | gnl\|UG\|Ta#S61787592 |
| contig01410 | - - - - - | gnl\|UG\|Ta#S61788465 |
| contig01597 | - - - - - | gnl\|UG\|Ta#S61788465 |
| contig02985 | - - - - - | gnl\|UG\|Ta#S61788529 |
| contig10244 | - - - - - | gnl\|UG\|Ta#S61788604 |
| contig02758 | - - - - - | gnl\|UG\|Ta#S61788946 |
| contig00499 | - - - - - | gnl\|UG\|Ta#S61789083 |
| contig08234 | - - - - - | gnl\|UG\|Ta#S61789588 |
| contig10541 | - - - - - | gnl\|UG\|Ta#S61789625 |
| contig16732 | - - - - - | gnl\|UG\|Ta#S61789659 |
| contig11884 | - - - - - | gnl\|UG\|Ta#S61790323 |
| contig12627 | - - - - - | gnl\|UG\|Ta#S61790707 |
| contig08956 | - - - - - | gnl\|UG\|Ta#S61790966 |
| contig10271 | - - - - - | gnl\|UG\|Ta#S61791087 |
| contig06686 | - - - - - | gnl\|UG\|Ta#S61791165 |
| contig09170 | - - - - - | gnl\|UG\|Ta#S61791165 |
| contig01537 | - - - - - | gnl\|UG\|Ta#S61791277 |
| contig15604 | - - - - - | gnl\|UG\|Ta#S61791293 |
| contig23912 | - - - - - | gnl\|UG\|Ta#S61791342 |
| contig01571 | - - - - - | gnl\|UG\|Ta#S61791574 |
| contig10847 | - - - - - | gnl\|UG\|Ta#S61791708 |

| | | |
|---|---|---|
| contig23353 | - - - - - | gnl\|UG\|Ta#S61791731 |
| contig00777 | - - - - - | gnl\|UG\|Ta#S61791741 |
| contig16464 | - - - - - | gnl\|UG\|Ta#S61791780 |
| contig14175 | - - - - - | gnl\|UG\|Ta#S61792066 |
| contig03133 | - - - - - | gnl\|UG\|Ta#S61792074 |
| contig22659 | - - - - - | gnl\|UG\|Ta#S61792359 |
| contig18447 | - - - - - | gnl\|UG\|Ta#S61792684 |
| contig22814 | - - - - - | gnl\|UG\|Ta#S61792684 |
| contig15775 | - - - - - | gnl\|UG\|Ta#S61792687 |
| contig14020 | - - - - - | gnl\|UG\|Ta#S61792793 |
| contig01884 | - - - - - | gnl\|UG\|Ta#S61792935 |
| contig09456 | - - - - - | gnl\|UG\|Ta#S61793367 |
| contig09619 | - - - - - | gnl\|UG\|Ta#S61793457 |
| contig16682 | - - - - - | gnl\|UG\|Ta#S61793560 |
| contig14752 | - - - - - | gnl\|UG\|Ta#S61793669 |
| contig16473 | - - - - - | gnl\|UG\|Ta#S61794044 |
| contig02022 | - - - - - | gnl\|UG\|Ta#S61794284 |
| contig21748 | - - - - - | gnl\|UG\|Ta#S61794453 |
| contig07720 | - - - - - | gnl\|UG\|Ta#S61794538 |
| contig12850 | - - - - - | gnl\|UG\|Ta#S61794752 |
| contig22377 | - - - - - | gnl\|UG\|Ta#S61794831 |
| contig08766 | - - - - - | gnl\|UG\|Ta#S61795072 |
| contig02254 | - - - - - | gnl\|UG\|Ta#S61795521 |
| contig23608 | - - - - - | gnl\|UG\|Ta#S61796276 |
| contig21589 | - - - - - | gnl\|UG\|Ta#S61796658 |
| contig14266 | - - - - - | gnl\|UG\|Ta#S61796969 |
| contig14458 | - - - - - | gnl\|UG\|Ta#S61797134 |
| contig03314 | - - - - - | gnl\|UG\|Ta#S61797210 |
| contig14730 | - - - - - | gnl\|UG\|Ta#S61797332 |
| contig11790 | - - - - - | gnl\|UG\|Ta#S61797695 |
| contig12391 | - - - - - | gnl\|UG\|Ta#S61797754 |
| contig04996 | - - - - - | gnl\|UG\|Ta#S61798464 |
| contig18470 | - - - - - | gnl\|UG\|Ta#S61798464 |
| contig10959 | - - - - - | gnl\|UG\|Ta#S61798508 |
| contig12837 | - - - - - | gnl\|UG\|Ta#S61799097 |
| contig04344 | - - - - - | gnl\|UG\|Ta#S61799246 |
| contig05102 | - - - - - | gnl\|UG\|Ta#S61799472 |
| contig04806 | - - - - - | gnl\|UG\|Ta#S61799488 |
| contig12654 | - - - - - | gnl\|UG\|Ta#S61799570 |
| contig05970 | - - - - - | gnl\|UG\|Ta#S61799676 |
| contig08311 | - - - - - | gnl\|UG\|Ta#S61799763 |
| contig08368 | - - - - - | gnl\|UG\|Ta#S61799987 |
| contig04651 | - - - - - | gnl\|UG\|Ta#S61800284 |
| contig18149 | - - - - - | gnl\|UG\|Ta#S61800389 |
| contig09069 | - - - - - | gnl\|UG\|Ta#S61800470 |
| contig06531 | - - - - - | gnl\|UG\|Ta#S61800687 |
| contig09426 | - - - - - | gnl\|UG\|Ta#S61801009 |
| contig18315 | - - - - - | gnl\|UG\|Ta#S61801072 |
| contig21706 | - - - - - | gnl\|UG\|Ta#S61801555 |
| contig07324 | - - - - - | gnl\|UG\|Ta#S61801610 |

| | | |
|---|---|---|
| contig21595 | - - - - - | gnl\|UG\|Ta#S61801610 |
| contig11233 | - - - - - | gnl\|UG\|Ta#S61801701 |
| contig12861 | - - - - - | gnl\|UG\|Ta#S61801714 |
| contig08543 | - - - - - | gnl\|UG\|Ta#S61801767 |
| contig03512 | - - - - - | gnl\|UG\|Ta#S61801987 |
| contig04805 | - - - - - | gnl\|UG\|Ta#S61802148 |
| contig07486 | - - - - - | gnl\|UG\|Ta#S61802351 |
| contig15743 | - - - - - | gnl\|UG\|Ta#S61802351 |
| contig09087 | - - - - - | gnl\|UG\|Ta#S61802841 |
| contig01900 | - - - - - | gnl\|UG\|Ta#S61802855 |
| contig03800 | - - - - - | gnl\|UG\|Ta#S61803323 |
| contig25370 | - - - - - | gnl\|UG\|Ta#S61803483 |
| contig15802 | - - - - - | gnl\|UG\|Ta#S61804015 |
| contig19895 | - - - - - | gnl\|UG\|Ta#S61804032 |
| contig08407 | - - - - - | gnl\|UG\|Ta#S61804100 |
| contig11000 | - - - - - | gnl\|UG\|Ta#S61804303 |
| contig09723 | - - - - - | gnl\|UG\|Ta#S61804892 |
| contig21991 | - - - - - | gnl\|UG\|Ta#S61804952 |
| contig02943 | - - - - - | gnl\|UG\|Ta#S61805409 |
| contig03576 | - - - - - | gnl\|UG\|Ta#S61805679 |
| contig13103 | - - - - - | gnl\|UG\|Ta#S61805961 |
| contig18561 | - - - - - | gnl\|UG\|Ta#S61805961 |
| contig14198 | - - - - - | gnl\|UG\|Ta#S61806042 |
| contig10138 | - - - - - | gnl\|UG\|Ta#S61806308 |
| contig03893 | - - - - - | gnl\|UG\|Ta#S61806370 |
| contig01089 | - - - - - | gnl\|UG\|Ta#S61806690 |
| contig05842 | - - - - - | gnl\|UG\|Ta#S61806726 |
| contig13510 | - - - - - | gnl\|UG\|Ta#S61807003 |
| contig14481 | - - - - - | gnl\|UG\|Ta#S61807003 |
| contig07977 | - - - - - | gnl\|UG\|Ta#S61807260 |
| contig00502 | - - - - - | gnl\|UG\|Ta#S61807632 |
| contig13191 | - - - - - | gnl\|UG\|Ta#S61807632 |
| contig10825 | - - - - - | gnl\|UG\|Ta#S61807772 |
| contig20354 | - - - - - | gnl\|UG\|Ta#S61807831 |
| contig07268 | - - - - - | gnl\|UG\|Ta#S61807910 |
| contig09476 | - - - - - | gnl\|UG\|Ta#S61807910 |
| contig02811 | - - - - - | gnl\|UG\|Ta#S61807944 |
| contig14709 | - - - - - | gnl\|UG\|Ta#S61808180 |
| contig01262 | - - - - - | gnl\|UG\|Ta#S61808380 |
| contig15298 | - - - - - | gnl\|UG\|Ta#S61808609 |
| contig06772 | - - - - - | gnl\|UG\|Ta#S61808661 |
| contig00170 | - - - - - | gnl\|UG\|Ta#S61808817 |
| contig05536 | - - - - - | gnl\|UG\|Ta#S61809026 |
| contig18426 | - - - - - | gnl\|UG\|Ta#S61809026 |
| contig09939 | - - - - - | gnl\|UG\|Ta#S61809138 |
| contig20004 | - - - - - | gnl\|UG\|Ta#S61809138 |
| contig13023 | - - - - - | gnl\|UG\|Ta#S61809250 |
| contig23547 | - - - - - | gnl\|UG\|Ta#S61809250 |
| contig15033 | - - - - - | gnl\|UG\|Ta#S61809253 |
| contig07124 | - - - - - | gnl\|UG\|Ta#S61809427 |

| | | |
|---|---|---|
| contig22946 | - - - - - | gnl\|UG\|Ta#S61809430 |
| contig17751 | - - - - - | gnl\|UG\|Ta#S61809434 |
| contig16095 | - - - - - | gnl\|UG\|Ta#S61809487 |
| contig11281 | - - - - - | gnl\|UG\|Ta#S61809492 |
| contig08634 | - - - - - | gnl\|UG\|Ta#S61809493 |
| contig00426 | - - - - - | gnl\|UG\|Ta#S61809521 |
| contig11244 | - - - - - | gnl\|UG\|Ta#S61809521 |
| contig16460 | - - - - - | gnl\|UG\|Ta#S61809521 |
| contig00769 | - - - - - | gnl\|UG\|Ta#S61809687 |
| contig08353 | - - - - - | gnl\|UG\|Ta#S61809733 |
| contig10395 | - - - - - | gnl\|UG\|Ta#S61809735 |
| contig00927 | - - - - - | gnl\|UG\|Ta#S61809750 |
| contig14201 | - - - - - | gnl\|UG\|Ta#S61809866 |
| contig08371 | - - - - - | gnl\|UG\|Ta#S61809869 |
| contig05338 | - - - - - | gnl\|UG\|Ta#S61809879 |
| contig06483 | - - - - - | gnl\|UG\|Ta#S61809980 |
| contig10208 | - - - - - | gnl\|UG\|Ta#S61809980 |
| contig12380 | - - - - - | gnl\|UG\|Ta#S61809980 |
| contig15118 | - - - - - | gnl\|UG\|Ta#S61809980 |
| contig09441 | - - - - - | gnl\|UG\|Ta#S61809981 |
| contig05266 | - - - - - | gnl\|UG\|Ta#S61810015 |
| contig09996 | - - - - - | gnl\|UG\|Ta#S61810048 |
| contig19542 | - - - - - | gnl\|UG\|Ta#S61810048 |
| contig25350 | - - - - - | gnl\|UG\|Ta#S61810061 |
| contig02293 | - - - - - | gnl\|UG\|Ta#S61810082 |
| contig11694 | - - - - - | gnl\|UG\|Ta#S61810082 |
| contig02106 | - - - - - | gnl\|UG\|Ta#S61810145 |
| contig20309 | - - - - - | gnl\|UG\|Ta#S61810145 |
| contig00100 | - - - - - | gnl\|UG\|Ta#S61810160 |
| contig05928 | - - - - - | gnl\|UG\|Ta#S61810177 |
| contig15382 | - - - - - | gnl\|UG\|Ta#S61810177 |
| contig15715 | - - - - - | gnl\|UG\|Ta#S61810177 |
| contig16853 | - - - - - | gnl\|UG\|Ta#S61810177 |
| contig13054 | - - - - - | gnl\|UG\|Ta#S61810351 |
| contig18690 | - - - - - | gnl\|UG\|Ta#S61810351 |
| contig00032 | - - - - - | gnl\|UG\|Ta#S61810436 |
| contig07093 | - - - - - | gnl\|UG\|Ta#S61810664 |
| contig07041 | - - - - - | gnl\|UG\|Ta#S61810690 |
| contig19000 | - - - - - | gnl\|UG\|Ta#S61810752 |
| contig25862 | - - - - - | gnl\|UG\|Ta#S61810752 |
| contig05691 | - - - - - | gnl\|UG\|Ta#S61810907 |
| contig08098 | - - - - - | gnl\|UG\|Ta#S61810913 |
| contig13787 | - - - - - | gnl\|UG\|Ta#S61810913 |
| contig01243 | - - - - - | gnl\|UG\|Ta#S61810977 |
| contig01225 | - - - - - | gnl\|UG\|Ta#S61810998 |
| contig13098 | - - - - - | gnl\|UG\|Ta#S61810998 |
| contig06569 | - - - - - | gnl\|UG\|Ta#S61811064 |
| contig10929 | - - - - - | gnl\|UG\|Ta#S61811064 |
| contig13386 | - - - - - | gnl\|UG\|Ta#S61811064 |
| contig00468 | - - - - - | gnl\|UG\|Ta#S61811127 |

| | | |
|---|---|---|
| contig00272 | - - - - - | gnl\|UG\|Ta#S61811181 |
| contig02961 | - - - - - | gnl\|UG\|Ta#S61811181 |
| contig11115 | - - - - - | gnl\|UG\|Ta#S61811249 |
| contig20330 | - - - - - | gnl\|UG\|Ta#S61811249 |
| contig05644 | - - - - - | gnl\|UG\|Ta#S61811259 |
| contig18499 | - - - - - | gnl\|UG\|Ta#S61811297 |
| contig11200 | - - - - - | gnl\|UG\|Ta#S61811378 |
| contig08007 | - - - - - | gnl\|UG\|Ta#S61811404 |
| contig21135 | - - - - - | gnl\|UG\|Ta#S61811475 |
| contig24018 | - - - - - | gnl\|UG\|Ta#S61811475 |
| contig11026 | - - - - - | gnl\|UG\|Ta#S61811479 |
| contig14220 | - - - - - | gnl\|UG\|Ta#S61811479 |
| contig05368 | - - - - - | gnl\|UG\|Ta#S61811511 |
| contig10527 | - - - - - | gnl\|UG\|Ta#S61811579 |
| contig02333 | - - - - - | gnl\|UG\|Ta#S61811733 |
| contig04076 | - - - - - | gnl\|UG\|Ta#S61811733 |
| contig04077 | - - - - - | gnl\|UG\|Ta#S61811733 |
| contig04078 | - - - - - | gnl\|UG\|Ta#S61811733 |
| contig08220 | - - - - - | gnl\|UG\|Ta#S61811733 |
| contig03550 | - - - - - | gnl\|UG\|Ta#S61811749 |
| contig00015 | - - - - - | gnl\|UG\|Ta#S61811751 |
| contig17422 | - - - - - | gnl\|UG\|Ta#S61811827 |
| contig06842 | - - - - - | gnl\|UG\|Ta#S61811832 |
| contig09914 | - - - - - | gnl\|UG\|Ta#S61811841 |
| contig14995 | - - - - - | gnl\|UG\|Ta#S61811841 |
| contig19709 | - - - - - | gnl\|UG\|Ta#S61811841 |
| contig06225 | - - - - - | gnl\|UG\|Ta#S61811866 |
| contig05533 | - - - - - | gnl\|UG\|Ta#S61811923 |
| contig01447 | - - - - - | gnl\|UG\|Ta#S61811958 |
| contig03303 | - - - - - | gnl\|UG\|Ta#S61811958 |
| contig13843 | - - - - - | gnl\|UG\|Ta#S61811960 |
| contig17071 | - - - - - | gnl\|UG\|Ta#S61811960 |
| contig20710 | - - - - - | gnl\|UG\|Ta#S61811960 |
| contig08847 | - - - - - | gnl\|UG\|Ta#S61811976 |
| contig00613 | - - - - - | gnl\|UG\|Ta#S61812107 |
| contig18577 | - - - - - | gnl\|UG\|Ta#S61812107 |
| contig14596 | - - - - - | gnl\|UG\|Ta#S61812111 |
| contig18816 | - - - - - | gnl\|UG\|Ta#S61812150 |
| contig06720 | - - - - - | gnl\|UG\|Ta#S61812160 |
| contig00582 | - - - - - | gnl\|UG\|Ta#S61812168 |
| contig06651 | - - - - - | gnl\|UG\|Ta#S61812224 |
| contig06849 | - - - - - | gnl\|UG\|Ta#S61812224 |
| contig03932 | - - - - - | gnl\|UG\|Ta#S61812279 |
| contig11587 | - - - - - | gnl\|UG\|Ta#S61812279 |
| contig11788 | - - - - - | gnl\|UG\|Ta#S61812279 |
| contig01264 | - - - - - | gnl\|UG\|Ta#S61812322 |
| contig06319 | - - - - - | gnl\|UG\|Ta#S61812326 |
| contig10096 | - - - - - | gnl\|UG\|Ta#S61812343 |
| contig01712 | - - - - - | gnl\|UG\|Ta#S61812354 |
| contig06773 | - - - - - | gnl\|UG\|Ta#S61812354 |

| | | |
|---|---|---|
| contig15127 | - - - - - | gnl\|UG\|Ta#S61812356 |
| contig03494 | - - - - - | gnl\|UG\|Ta#S61812363 |
| contig05660 | - - - - - | gnl\|UG\|Ta#S61812375 |
| contig06233 | - - - - - | gnl\|UG\|Ta#S61812403 |
| contig09715 | - - - - - | gnl\|UG\|Ta#S61812403 |
| contig00331 | - - - - - | gnl\|UG\|Ta#S61812435 |
| contig00968 | - - - - - | gnl\|UG\|Ta#S61812435 |
| contig01323 | - - - - - | gnl\|UG\|Ta#S61812435 |
| contig02827 | - - - - - | gnl\|UG\|Ta#S61812435 |
| contig03918 | - - - - - | gnl\|UG\|Ta#S61812454 |
| contig06056 | - - - - - | gnl\|UG\|Ta#S61812454 |
| contig00841 | - - - - - | gnl\|UG\|Ta#S61812464 |
| contig03073 | - - - - - | gnl\|UG\|Ta#S61812464 |
| contig08761 | - - - - - | gnl\|UG\|Ta#S61812464 |
| contig04726 | - - - - - | gnl\|UG\|Ta#S61812469 |
| contig02277 | - - - - - | gnl\|UG\|Ta#S61812476 |
| contig04855 | - - - - - | gnl\|UG\|Ta#S61812511 |
| contig21791 | - - - - - | gnl\|UG\|Ta#S61812519 |
| contig14223 | - - - - - | gnl\|UG\|Ta#S61812527 |
| contig05323 | - - - - - | gnl\|UG\|Ta#S61812538 |
| contig23308 | - - - - - | gnl\|UG\|Ta#S61813000 |
| contig17885 | - - - - - | gnl\|UG\|Ta#S61813179 |
| contig10761 | - - - - - | gnl\|UG\|Ta#S61813268 |
| contig13047 | - - - - - | gnl\|UG\|Ta#S61813275 |
| contig05296 | - - - - - | gnl\|UG\|Ta#S61813319 |
| contig16284 | - - - - - | gnl\|UG\|Ta#S61813427 |
| contig18758 | - - - - - | gnl\|UG\|Ta#S61813958 |
| contig26364 | - - - - - | gnl\|UG\|Ta#S61813958 |
| contig10497 | - - - - - | gnl\|UG\|Ta#S61814224 |
| contig21125 | - - - - - | gnl\|UG\|Ta#S61814543 |
| contig12328 | - - - - - | gnl\|UG\|Ta#S61814552 |
| contig15808 | - - - - - | gnl\|UG\|Ta#S61814596 |
| contig03089 | - - - - - | gnl\|UG\|Ta#S61814650 |
| contig05066 | - - - - - | gnl\|UG\|Ta#S61815327 |
| contig01690 | - - - - - | gnl\|UG\|Ta#S61815647 |
| contig23477 | - - - - - | gnl\|UG\|Ta#S61816008 |
| contig16158 | - - - - - | gnl\|UG\|Ta#S61816219 |
| contig13926 | - - - - - | gnl\|UG\|Ta#S61816373 |
| contig10594 | - - - - - | gnl\|UG\|Ta#S61816475 |
| contig14036 | - - - - - | gnl\|UG\|Ta#S61816910 |
| contig03699 | - - - - - | gnl\|UG\|Ta#S61816970 |
| contig03415 | - - - - - | gnl\|UG\|Ta#S61817170 |
| contig12942 | - - - - - | gnl\|UG\|Ta#S61817185 |
| contig08853 | - - - - - | gnl\|UG\|Ta#S61817837 |
| contig19225 | - - - - - | gnl\|UG\|Ta#S61818209 |
| contig19828 | - - - - - | gnl\|UG\|Ta#S61818209 |
| contig13216 | - - - - - | gnl\|UG\|Ta#S61818394 |
| contig22891 | - - - - - | gnl\|UG\|Ta#S61818394 |
| contig15765 | - - - - - | gnl\|UG\|Ta#S61818395 |
| contig09817 | - - - - - | gnl\|UG\|Ta#S61818489 |

| | | |
|---|---|---|
| contig01244 | - - - - - | gnl\|UG\|Ta#S61818754 |
| contig15708 | - - - - - | gnl\|UG\|Ta#S61818899 |
| contig22836 | - - - - - | gnl\|UG\|Ta#S61819078 |
| contig17715 | - - - - - | gnl\|UG\|Ta#S61819290 |
| contig03454 | - - - - - | gnl\|UG\|Ta#S61819335 |
| contig15627 | - - - - - | gnl\|UG\|Ta#S61819664 |
| contig22629 | - - - - - | gnl\|UG\|Ta#S61819664 |
| contig23686 | - - - - - | gnl\|UG\|Ta#S61819664 |
| contig03586 | - - - - - | gnl\|UG\|Ta#S61820101 |
| contig10192 | - - - - - | gnl\|UG\|Ta#S61820395 |
| contig14747 | - - - - - | gnl\|UG\|Ta#S61820934 |
| contig26346 | - - - - - | gnl\|UG\|Ta#S61820970 |
| contig06581 | - - - - - | gnl\|UG\|Ta#S61821290 |
| contig01766 | - - - - - | gnl\|UG\|Ta#S61823246 |
| contig05638 | - - - - - | gnl\|UG\|Ta#S61823321 |
| contig16060 | - - - - - | gnl\|UG\|Ta#S61823538 |
| contig04766 | - - - - - | gnl\|UG\|Ta#S61823742 |
| contig20884 | - - - - - | gnl\|UG\|Ta#S61824063 |
| contig03865 | - - - - - | gnl\|UG\|Ta#S61824643 |
| contig24246 | - - - - - | gnl\|UG\|Ta#S61825026 |
| contig08780 | - - - - - | gnl\|UG\|Ta#S61825123 |
| contig16519 | - - - - - | gnl\|UG\|Ta#S61825191 |
| contig05720 | - - - - - | gnl\|UG\|Ta#S61825264 |
| contig06259 | - - - - - | gnl\|UG\|Ta#S61825264 |
| contig05791 | - - - - - | gnl\|UG\|Ta#S61825764 |
| contig20625 | - - - - - | gnl\|UG\|Ta#S61826220 |
| contig02527 | - - - - - | gnl\|UG\|Ta#S61826272 |
| contig16499 | - - - - - | gnl\|UG\|Ta#S61826559 |
| contig01318 | - - - - - | gnl\|UG\|Ta#S61826931 |
| contig14897 | - - - - - | gnl\|UG\|Ta#S61827269 |
| contig03097 | - - - - - | gnl\|UG\|Ta#S61827822 |
| contig02551 | - - - - - | gnl\|UG\|Ta#S61827964 |
| contig06977 | - - - - - | gnl\|UG\|Ta#S61828030 |
| contig17013 | - - - - - | gnl\|UG\|Ta#S61828232 |
| contig20352 | - - - - - | gnl\|UG\|Ta#S61828232 |
| contig18011 | - - - - - | gnl\|UG\|Ta#S61828349 |
| contig25844 | - - - - - | gnl\|UG\|Ta#S61828349 |
| contig07568 | - - - - - | gnl\|UG\|Ta#S61828378 |
| contig15334 | - - - - - | gnl\|UG\|Ta#S61828386 |
| contig01406 | - - - - - | gnl\|UG\|Ta#S61828497 |
| contig03447 | - - - - - | gnl\|UG\|Ta#S61828497 |
| contig05476 | - - - - - | gnl\|UG\|Ta#S61828497 |
| contig02046 | - - - - - | gnl\|UG\|Ta#S61828538 |
| contig14862 | - - - - - | gnl\|UG\|Ta#S61828538 |
| contig20942 | - - - - - | gnl\|UG\|Ta#S61828583 |
| contig04432 | - - - - - | gnl\|UG\|Ta#S61828711 |
| contig17873 | - - - - - | gnl\|UG\|Ta#S61828711 |
| contig18672 | - - - - - | gnl\|UG\|Ta#S61828730 |
| contig20560 | - - - - - | gnl\|UG\|Ta#S61828730 |
| contig14855 | - - - - - | gnl\|UG\|Ta#S61828797 |

| | | |
|---|---|---|
| contig24923 | - - - - - | gnl\|UG\|Ta#S61828810 |
| contig07909 | - - - - - | gnl\|UG\|Ta#S61829043 |
| contig15216 | - - - - - | gnl\|UG\|Ta#S61829050 |
| contig10962 | - - - - - | gnl\|UG\|Ta#S61829091 |
| contig01870 | - - - - - | gnl\|UG\|Ta#S61829143 |
| contig19712 | - - - - - | gnl\|UG\|Ta#S61829313 |
| contig05385 | - - - - - | gnl\|UG\|Ta#S61829325 |
| contig22683 | - - - - - | gnl\|UG\|Ta#S61829341 |
| contig03977 | - - - - - | gnl\|UG\|Ta#S61829426 |
| contig09122 | - - - - - | gnl\|UG\|Ta#S61829473 |
| contig16903 | - - - - - | gnl\|UG\|Ta#S61829473 |
| contig01037 | - - - - - | gnl\|UG\|Ta#S61829519 |
| contig01480 | - - - - - | gnl\|UG\|Ta#S61829519 |
| contig11098 | - - - - - | gnl\|UG\|Ta#S61829645 |
| contig06168 | - - - - - | gnl\|UG\|Ta#S61829668 |
| contig10230 | - - - - - | gnl\|UG\|Ta#S61829810 |
| contig23743 | - - - - - | gnl\|UG\|Ta#S61829810 |
| contig06522 | - - - - - | gnl\|UG\|Ta#S61829853 |
| contig15859 | - - - - - | gnl\|UG\|Ta#S61829853 |
| contig06462 | - - - - - | gnl\|UG\|Ta#S61829895 |
| contig16405 | - - - - - | gnl\|UG\|Ta#S61829895 |
| contig02530 | - - - - - | gnl\|UG\|Ta#S61830085 |
| contig05333 | - - - - - | gnl\|UG\|Ta#S61830085 |
| contig09258 | - - - - - | gnl\|UG\|Ta#S61830085 |
| contig16551 | - - - - - | gnl\|UG\|Ta#S61830226 |
| contig03548 | - - - - - | gnl\|UG\|Ta#S61830280 |
| contig05459 | - - - - - | gnl\|UG\|Ta#S61830280 |
| contig06685 | - - - - - | gnl\|UG\|Ta#S61830280 |
| contig15261 | - - - - - | gnl\|UG\|Ta#S61830280 |
| contig05676 | - - - - - | gnl\|UG\|Ta#S61830344 |
| contig03994 | - - - - - | gnl\|UG\|Ta#S61830374 |
| contig15457 | - - - - - | gnl\|UG\|Ta#S61830374 |
| contig02522 | - - - - - | gnl\|UG\|Ta#S61830408 |
| contig17898 | - - - - - | gnl\|UG\|Ta#S61830408 |
| contig01573 | - - - - - | gnl\|UG\|Ta#S61830468 |
| contig07158 | - - - - - | gnl\|UG\|Ta#S61830468 |
| contig22335 | - - - - - | gnl\|UG\|Ta#S61830468 |
| contig01613 | - - - - - | gnl\|UG\|Ta#S61830492 |
| contig08641 | - - - - - | gnl\|UG\|Ta#S61830492 |
| contig00620 | - - - - - | gnl\|UG\|Ta#S61830559 |
| contig08708 | - - - - - | gnl\|UG\|Ta#S61830565 |
| contig13924 | - - - - - | gnl\|UG\|Ta#S61830565 |
| contig13277 | - - - - - | gnl\|UG\|Ta#S61830605 |
| contig01718 | - - - - - | gnl\|UG\|Ta#S61830611 |
| contig03882 | - - - - - | gnl\|UG\|Ta#S61830659 |
| contig00315 | - - - - - | gnl\|UG\|Ta#S61830717 |
| contig19102 | - - - - - | gnl\|UG\|Ta#S61830729 |
| contig21990 | - - - - - | gnl\|UG\|Ta#S61830729 |
| contig23267 | - - - - - | gnl\|UG\|Ta#S61830754 |
| contig24110 | - - - - - | gnl\|UG\|Ta#S61830754 |

| | | |
|---|---|---|
| contig11545 | - - - - - | gnl\|UG\|Ta#S61830759 |
| contig06205 | - - - - - | gnl\|UG\|Ta#S61830812 |
| contig06954 | - - - - - | gnl\|UG\|Ta#S61830879 |
| contig17841 | - - - - - | gnl\|UG\|Ta#S61831029 |
| contig08789 | - - - - - | gnl\|UG\|Ta#S61831079 |
| contig12592 | - - - - - | gnl\|UG\|Ta#S61831079 |
| contig11364 | - - - - - | gnl\|UG\|Ta#S61831090 |
| contig12208 | - - - - - | gnl\|UG\|Ta#S61831136 |
| contig13872 | - - - - - | gnl\|UG\|Ta#S61831136 |
| contig07320 | - - - - - | gnl\|UG\|Ta#S61831280 |
| contig17779 | - - - - - | gnl\|UG\|Ta#S61831285 |
| contig26490 | - - - - - | gnl\|UG\|Ta#S61831285 |
| contig00534 | - - - - - | gnl\|UG\|Ta#S61831314 |
| contig16819 | - - - - - | gnl\|UG\|Ta#S61831376 |
| contig02062 | - - - - - | gnl\|UG\|Ta#S61831397 |
| contig04935 | - - - - - | gnl\|UG\|Ta#S61831397 |
| contig01307 | - - - - - | gnl\|UG\|Ta#S61831483 |
| contig13901 | - - - - - | gnl\|UG\|Ta#S61831498 |
| contig18181 | - - - - - | gnl\|UG\|Ta#S61831515 |
| contig25512 | - - - - - | gnl\|UG\|Ta#S61831515 |
| contig09013 | - - - - - | gnl\|UG\|Ta#S61831557 |
| contig14082 | - - - - - | gnl\|UG\|Ta#S61831622 |
| contig03563 | - - - - - | gnl\|UG\|Ta#S61831644 |
| contig00987 | - - - - - | gnl\|UG\|Ta#S61831696 |
| contig03289 | - - - - - | gnl\|UG\|Ta#S61831698 |
| contig12931 | - - - - - | gnl\|UG\|Ta#S61831862 |
| contig23238 | - - - - - | gnl\|UG\|Ta#S61831917 |
| contig03247 | - - - - - | gnl\|UG\|Ta#S61831960 |
| contig18726 | - - - - - | gnl\|UG\|Ta#S61832007 |
| contig25509 | - - - - - | gnl\|UG\|Ta#S61832007 |
| contig07664 | - - - - - | gnl\|UG\|Ta#S61832033 |
| contig05292 | - - - - - | gnl\|UG\|Ta#S61832073 |
| contig18103 | - - - - - | gnl\|UG\|Ta#S61832073 |
| contig21365 | - - - - - | gnl\|UG\|Ta#S61832095 |
| contig00283 | - - - - - | gnl\|UG\|Ta#S61832098 |
| contig09635 | - - - - - | gnl\|UG\|Ta#S61832140 |
| contig12550 | - - - - - | gnl\|UG\|Ta#S61832140 |
| contig00804 | - - - - - | gnl\|UG\|Ta#S61832174 |
| contig19805 | - - - - - | gnl\|UG\|Ta#S61832186 |
| contig08425 | - - - - - | gnl\|UG\|Ta#S61832208 |
| contig03479 | - - - - - | gnl\|UG\|Ta#S61832213 |
| contig06711 | - - - - - | gnl\|UG\|Ta#S61832213 |
| contig11860 | - - - - - | gnl\|UG\|Ta#S61832265 |
| contig14038 | - - - - - | gnl\|UG\|Ta#S61832278 |
| contig16758 | - - - - - | gnl\|UG\|Ta#S61832278 |
| contig13697 | - - - - - | gnl\|UG\|Ta#S61832340 |
| contig17586 | - - - - - | gnl\|UG\|Ta#S61832348 |
| contig02590 | - - - - - | gnl\|UG\|Ta#S61832353 |
| contig03797 | - - - - - | gnl\|UG\|Ta#S61832353 |
| contig16677 | - - - - - | gnl\|UG\|Ta#S61832353 |

| | | |
|---|---|---|
| contig23528 | - - - - - | gnl\|UG\|Ta#S61832353 |
| contig00201 | - - - - - | gnl\|UG\|Ta#S61832375 |
| contig02141 | - - - - - | gnl\|UG\|Ta#S61832401 |
| contig10542 | - - - - - | gnl\|UG\|Ta#S61832401 |
| contig11334 | - - - - - | gnl\|UG\|Ta#S61832408 |
| contig14411 | - - - - - | gnl\|UG\|Ta#S61832436 |
| contig07891 | - - - - - | gnl\|UG\|Ta#S61832481 |
| contig04876 | - - - - - | gnl\|UG\|Ta#S61832496 |
| contig15752 | - - - - - | gnl\|UG\|Ta#S61832496 |
| contig00213 | - - - - - | gnl\|UG\|Ta#S61832505 |
| contig01246 | - - - - - | gnl\|UG\|Ta#S61832505 |
| contig02070 | - - - - - | gnl\|UG\|Ta#S61832505 |
| contig04843 | - - - - - | gnl\|UG\|Ta#S61832521 |
| contig12398 | - - - - - | gnl\|UG\|Ta#S61832521 |
| contig05946 | - - - - - | gnl\|UG\|Ta#S61832528 |
| contig24605 | - - - - - | gnl\|UG\|Ta#S61832532 |
| contig02605 | - - - - - | gnl\|UG\|Ta#S61832550 |
| contig03490 | - - - - - | gnl\|UG\|Ta#S61832550 |
| contig06615 | - - - - - | gnl\|UG\|Ta#S61832550 |
| contig09587 | - - - - - | gnl\|UG\|Ta#S61832550 |
| contig15082 | - - - - - | gnl\|UG\|Ta#S61832718 |
| contig13830 | - - - - - | gnl\|UG\|Ta#S61832999 |
| contig24252 | - - - - - | gnl\|UG\|Ta#S61833428 |
| contig20432 | - - - - - | gnl\|UG\|Ta#S61833544 |
| contig02537 | - - - - - | gnl\|UG\|Ta#S61833825 |
| contig13022 | - - - - - | gnl\|UG\|Ta#S61833871 |
| contig08596 | - - - - - | gnl\|UG\|Ta#S61834287 |
| contig08106 | - - - - - | gnl\|UG\|Ta#S61834497 |
| contig11029 | - - - - - | gnl\|UG\|Ta#S61834920 |
| contig22436 | - - - - - | gnl\|UG\|Ta#S61835074 |
| contig19640 | - - - - - | gnl\|UG\|Ta#S61835217 |
| contig25781 | - - - - - | gnl\|UG\|Ta#S61835253 |
| contig10376 | - - - - - | gnl\|UG\|Ta#S61835725 |
| contig07955 | - - - - - | gnl\|UG\|Ta#S61835858 |
| contig15756 | - - - - - | gnl\|UG\|Ta#S61836099 |
| contig24075 | - - - - - | gnl\|UG\|Ta#S61836297 |
| contig01477 | - - - - - | gnl\|UG\|Ta#S61836500 |
| contig15540 | - - - - - | gnl\|UG\|Ta#S61836739 |
| contig16851 | - - - - - | gnl\|UG\|Ta#S61836810 |
| contig06202 | - - - - - | gnl\|UG\|Ta#S61837111 |
| contig19026 | - - - - - | gnl\|UG\|Ta#S61837340 |
| contig14374 | - - - - - | gnl\|UG\|Ta#S61837825 |
| contig07485 | - - - - - | gnl\|UG\|Ta#S61837900 |
| contig14288 | - - - - - | gnl\|UG\|Ta#S61837948 |
| contig04265 | - - - - - | gnl\|UG\|Ta#S61838693 |
| contig10239 | - - - - - | gnl\|UG\|Ta#S61838753 |
| contig09662 | - - - - - | gnl\|UG\|Ta#S61839338 |
| contig00047 | - - - - - | gnl\|UG\|Ta#S61839596 |
| contig10221 | - - - - - | gnl\|UG\|Ta#S61840030 |
| contig04716 | - - - - - | gnl\|UG\|Ta#S61840152 |

| | | |
|---|---|---|
| contig08791 | - - - - - | gnl\|UG\|Ta#S61840152 |
| contig13399 | - - - - - | gnl\|UG\|Ta#S61840318 |
| contig07695 | - - - - - | gnl\|UG\|Ta#S61840727 |
| contig21126 | - - - - - | gnl\|UG\|Ta#S61840963 |
| contig22586 | - - - - - | gnl\|UG\|Ta#S61841018 |
| contig00679 | - - - - - | gnl\|UG\|Ta#S61841581 |
| contig07537 | - - - - - | gnl\|UG\|Ta#S61842708 |
| contig07116 | - - - - - | gnl\|UG\|Ta#S61842791 |
| contig01929 | - - - - - | gnl\|UG\|Ta#S61842856 |
| contig01931 | - - - - - | gnl\|UG\|Ta#S61842856 |
| contig15143 | - - - - - | gnl\|UG\|Ta#S61842856 |
| contig13707 | - - - - - | gnl\|UG\|Ta#S61842953 |
| contig15697 | - - - - - | gnl\|UG\|Ta#S61843319 |
| contig00510 | - - - - - | gnl\|UG\|Ta#S61843588 |
| contig05961 | - - - - - | gnl\|UG\|Ta#S61843725 |
| contig05594 | - - - - - | gnl\|UG\|Ta#S61844275 |
| contig07165 | - - - - - | gnl\|UG\|Ta#S61844275 |
| contig03238 | - - - - - | gnl\|UG\|Ta#S61844630 |
| contig13614 | - - - - - | gnl\|UG\|Ta#S61845019 |
| contig17774 | - - - - - | gnl\|UG\|Ta#S61845148 |
| contig16280 | - - - - - | gnl\|UG\|Ta#S61845157 |
| contig17665 | - - - - - | gnl\|UG\|Ta#S61845320 |
| contig00373 | - - - - - | gnl\|UG\|Ta#S61845772 |
| contig18471 | - - - - - | gnl\|UG\|Ta#S61846022 |
| contig05635 | - - - - - | gnl\|UG\|Ta#S61846641 |
| contig14700 | - - - - - | gnl\|UG\|Ta#S61846641 |
| contig11318 | - - - - - | gnl\|UG\|Ta#S61846731 |
| contig17308 | - - - - - | gnl\|UG\|Ta#S61846796 |
| contig05252 | - - - - - | gnl\|UG\|Ta#S61846830 |
| contig15821 | - - - - - | gnl\|UG\|Ta#S61846990 |
| contig06804 | - - - - - | gnl\|UG\|Ta#S61847226 |
| contig04447 | - - - - - | gnl\|UG\|Ta#S61847599 |
| contig23638 | - - - - - | gnl\|UG\|Ta#S61847965 |
| contig19838 | - - - - - | gnl\|UG\|Ta#S61848058 |
| contig05301 | - - - - - | gnl\|UG\|Ta#S61848248 |
| contig11043 | - - - - - | gnl\|UG\|Ta#S61848853 |
| contig00424 | - - - - - | gnl\|UG\|Ta#S61848875 |
| contig05839 | - - - - - | gnl\|UG\|Ta#S61848919 |
| contig15384 | - - - - - | gnl\|UG\|Ta#S61849076 |
| contig06600 | - - - - - | gnl\|UG\|Ta#S61849889 |
| contig03485 | - - - - - | gnl\|UG\|Ta#S61850035 |
| contig04550 | - - - - - | gnl\|UG\|Ta#S61850239 |
| contig16874 | - - - - - | gnl\|UG\|Ta#S61850335 |
| contig20618 | - - - - - | gnl\|UG\|Ta#S61850396 |
| contig13202 | - - - - - | gnl\|UG\|Ta#S61850503 |
| contig12388 | - - - - - | gnl\|UG\|Ta#S61850906 |
| contig04650 | - - - - - | gnl\|UG\|Ta#S61851657 |
| contig01536 | - - - - - | gnl\|UG\|Ta#S61851930 |
| contig01113 | - - - - - | gnl\|UG\|Ta#S61852224 |
| contig00209 | - - - - - | gnl\|UG\|Ta#S61852309 |

| | | |
|---|---|---|
| contig11798 | - - - - - | gnl\|UG\|Ta#S61852689 |
| contig07822 | - - - - - | gnl\|UG\|Ta#S61852835 |
| contig18418 | - - - - - | gnl\|UG\|Ta#S61852943 |
| contig16974 | - - - - - | gnl\|UG\|Ta#S61853198 |
| contig11712 | - - - - - | gnl\|UG\|Ta#S61853319 |
| contig07698 | - - - - - | gnl\|UG\|Ta#S61853552 |
| contig00129 | - - - - - | gnl\|UG\|Ta#S61853560 |
| contig15762 | - - - - - | gnl\|UG\|Ta#S61854010 |
| contig16449 | - - - - - | gnl\|UG\|Ta#S61854286 |
| contig16547 | - - - - - | gnl\|UG\|Ta#S61854286 |
| contig09718 | - - - - - | gnl\|UG\|Ta#S61854525 |
| contig10983 | - - - - - | gnl\|UG\|Ta#S61854903 |
| contig03997 | - - - - - | gnl\|UG\|Ta#S61854936 |
| contig19364 | - - - - - | gnl\|UG\|Ta#S61854949 |
| contig00776 | - - - - - | gnl\|UG\|Ta#S61855196 |
| contig05412 | - - - - - | gnl\|UG\|Ta#S61855571 |
| contig04600 | - - - - - | gnl\|UG\|Ta#S61855590 |
| contig06371 | - - - - - | gnl\|UG\|Ta#S61855617 |
| contig03371 | - - - - - | gnl\|UG\|Ta#S61855685 |
| contig00907 | - - - - - | gnl\|UG\|Ta#S61856250 |
| contig04833 | - - - - - | gnl\|UG\|Ta#S61856259 |
| contig20864 | - - - - - | gnl\|UG\|Ta#S61856722 |
| contig10749 | - - - - - | gnl\|UG\|Ta#S61857192 |
| contig16590 | - - - - - | gnl\|UG\|Ta#S61857421 |
| contig16064 | - - - - - | gnl\|UG\|Ta#S61857610 |
| contig00245 | - - - - - | gnl\|UG\|Ta#S61858182 |
| contig22362 | - - - - - | gnl\|UG\|Ta#S61858227 |
| contig01538 | - - - - - | gnl\|UG\|Ta#S61858525 |
| contig00599 | - - - - - | gnl\|UG\|Ta#S61858598 |
| contig10061 | - - - - - | gnl\|UG\|Ta#S61858641 |
| contig02757 | - - - - - | gnl\|UG\|Ta#S61858810 |
| contig05840 | - - - - - | gnl\|UG\|Ta#S61858904 |
| contig05572 | - - - - - | gnl\|UG\|Ta#S61858946 |
| contig06458 | - - - - - | gnl\|UG\|Ta#S61858995 |
| contig02753 | - - - - - | gnl\|UG\|Ta#S61859373 |
| contig15618 | - - - - - | gnl\|UG\|Ta#S61859407 |
| contig21655 | - - - - - | gnl\|UG\|Ta#S61860428 |
| contig16279 | - - - - - | gnl\|UG\|Ta#S61860972 |
| contig07436 | - - - - - | gnl\|UG\|Ta#S61861061 |
| contig06617 | - - - - - | gnl\|UG\|Ta#S61861972 |
| contig12476 | - - - - - | gnl\|UG\|Ta#S61862296 |
| contig14770 | - - - - - | gnl\|UG\|Ta#S61862562 |
| contig11842 | - - - - - | gnl\|UG\|Ta#S61862584 |
| contig03428 | - - - - - | gnl\|UG\|Ta#S61862589 |
| contig03913 | - - - - - | gnl\|UG\|Ta#S61862686 |
| contig20837 | - - - - - | gnl\|UG\|Ta#S61863012 |
| contig14779 | - - - - - | gnl\|UG\|Ta#S61863347 |
| contig20241 | - - - - - | gnl\|UG\|Ta#S61863347 |
| contig08196 | - - - - - | gnl\|UG\|Ta#S61864300 |
| contig05215 | - - - - - | gnl\|UG\|Ta#S61864881 |

| | | |
|---|---|---|
| contig02977 | - - - - - | gnl\|UG\|Ta#S61865109 |
| contig03128 | - - - - - | gnl\|UG\|Ta#S61865201 |
| contig00937 | - - - - - | gnl\|UG\|Ta#S61865389 |
| contig00208 | - - - - - | gnl\|UG\|Ta#S61865553 |
| contig03934 | - - - - - | gnl\|UG\|Ta#S61865556 |
| contig15355 | - - - - - | gnl\|UG\|Ta#S61865607 |
| contig01632 | - - - - - | gnl\|UG\|Ta#S61865689 |
| contig03604 | - - - - - | gnl\|UG\|Ta#S61865882 |
| contig04414 | - - - - - | gnl\|UG\|Ta#S61866068 |
| contig22087 | - - - - - | gnl\|UG\|Ta#S61866536 |
| contig16565 | - - - - - | gnl\|UG\|Ta#S61866892 |
| contig16061 | - - - - - | gnl\|UG\|Ta#S61867132 |
| contig20973 | - - - - - | gnl\|UG\|Ta#S61867420 |
| contig23357 | - - - - - | gnl\|UG\|Ta#S61867453 |
| contig18409 | - - - - - | gnl\|UG\|Ta#S61867558 |
| contig26183 | - - - - - | gnl\|UG\|Ta#S61867558 |
| contig00649 | - - - - - | gnl\|UG\|Ta#S61868063 |
| contig07719 | - - - - - | gnl\|UG\|Ta#S65589245 |
| contig09953 | - - - - - | gnl\|UG\|Ta#S65589322 |
| contig20775 | - - - - - | gnl\|UG\|Ta#S65590809 |
| contig23227 | - - - - - | gnl\|UG\|Ta#S65591783 |
| contig02125 | - - - - - | gnl\|UG\|Ta#S65592843 |
| contig01846 | - - - - - | gnl\|UG\|Ta#S65593544 |
| contig02019 | - - - - - | gnl\|UG\|Ta#S65593892 |
| contig08372 | - - - - - | gnl\|UG\|Ta#S65593892 |
| contig26000 | - - - - - | gnl\|UG\|Ta#S65595231 |
| contig01714 | - - - - - | gnl\|UG\|Ta#S65596618 |
| contig01905 | - - - - - | gnl\|UG\|Ta#S65597629 |
| contig08869 | - - - - - | gnl\|UG\|Ta#S65600237 |
| contig07338 | - - - - - | gnl\|UG\|Ta#S65601521 |
| contig00092 | - - - - - | gnl\|UG\|Ta#S65602695 |
| contig01935 | - - - - - | gnl\|UG\|Ta#S65602706 |
| contig06081 | - - - - - | gnl\|UG\|Ta#S65602736 |
| contig04771 | - - - - - | gnl\|UG\|Ta#S65602862 |
| contig13342 | - - - - - | gnl\|UG\|Ta#S65604939 |
| contig17554 | - - - - - | gnl\|UG\|Ta#S65604983 |
| contig25955 | - - - - - | gnl\|UG\|Ta#S65604983 |
| contig02465 | - - - - - | gnl\|UG\|Ta#S65605334 |
| contig10427 | - - - - - | gnl\|UG\|Ta#S65606779 |
| contig09231 | - - - - - | gnl\|UG\|Ta#S65607281 |
| contig02640 | - - - - - | gnl\|UG\|Ta#S65607669 |
| contig08309 | - - - - - | gnl\|UG\|Ta#S65607669 |
| contig04505 | - - - - - | gnl\|UG\|Ta#S65609318 |
| contig00188 | - - - - - | gnl\|UG\|Ta#S65609374 |
| contig10581 | - - - - - | gnl\|UG\|Ta#S65609732 |
| contig21376 | - - - - - | gnl\|UG\|Ta#S65611027 |
| contig02415 | - - - - - | gnl\|UG\|Ta#S65611045 |
| contig09443 | - - - - - | gnl\|UG\|Ta#S65611327 |
| contig04694 | - - - - - | gnl\|UG\|Ta#S65611328 |
| contig00718 | - - - - - | gnl\|UG\|Ta#S65611784 |

| | | |
|---|---|---|
| contig18251 | - - - - - | gnl\|UG\|Ta#S65612876 |
| contig16995 | - - - - - | gnl\|UG\|Ta#S65613150 |
| contig14789 | - - - - - | gnl\|UG\|Ta#S65613700 |
| contig10659 | - - - - - | gnl\|UG\|Ta#S65613925 |
| contig19133 | - - - - - | gnl\|UG\|Ta#S65614187 |
| contig11927 | - - - - - | gnl\|UG\|Ta#S65615778 |
| contig20661 | - - - - - | gnl\|UG\|Ta#S65615947 |
| contig08336 | - - - - - | gnl\|UG\|Ta#S65616823 |
| contig26513 | - - - - - | gnl\|UG\|Ta#S65616923 |
| contig04892 | - - - - - | gnl\|UG\|Ta#S65616934 |
| contig13024 | - - - - - | gnl\|UG\|Ta#S65617051 |
| contig13569 | - - - - - | gnl\|UG\|Ta#S65617362 |
| contig03134 | - - - - - | gnl\|UG\|Ta#S65618651 |
| contig07435 | - - - - - | gnl\|UG\|Ta#S65620303 |
| contig11275 | - - - - - | gnl\|UG\|Ta#S65620379 |
| contig01908 | - - - - - | gnl\|UG\|Ta#S65620757 |
| contig15897 | - - - - - | gnl\|UG\|Ta#S65621114 |
| contig18916 | - - - - - | gnl\|UG\|Ta#S65621114 |
| contig20353 | - - - - - | gnl\|UG\|Ta#S65621114 |
| contig00910 | - - - - - | gnl\|UG\|Ta#S65621442 |
| contig23028 | - - - - - | gnl\|UG\|Ta#S65624625 |
| contig05940 | - - - - - | gnl\|UG\|Ta#S65625323 |
| contig07267 | - - - - - | gnl\|UG\|Ta#S65626395 |
| contig24051 | - - - - - | gnl\|UG\|Ta#S65626736 |
| contig12800 | - - - - - | gnl\|UG\|Ta#S65627795 |
| contig23409 | - - - - - | gnl\|UG\|Ta#S65627795 |
| contig18120 | - - - - - | gnl\|UG\|Ta#S65628017 |
| contig12451 | - - - - - | gnl\|UG\|Ta#S65628416 |
| contig19328 | - - - - - | gnl\|UG\|Ta#S65628416 |
| contig11227 | - - - - - | gnl\|UG\|Ta#S65629443 |
| contig04756 | - - - - - | gnl\|UG\|Ta#S65630280 |
| contig09944 | - - - - - | gnl\|UG\|Ta#S65630676 |
| contig12317 | - - - - - | gnl\|UG\|Ta#S65630945 |
| contig23563 | - - - - - | gnl\|UG\|Ta#S65631674 |
| contig07836 | - - - - - | gnl\|UG\|Ta#S65633086 |
| contig03584 | - - - - - | gnl\|UG\|Ta#S65636477 |
| contig03947 | - - - - - | gnl\|UG\|Ta#S65637061 |
| contig24942 | - - - - - | gnl\|UG\|Ta#S65637222 |
| contig10924 | - - - - - | gnl\|UG\|Ta#S65638773 |
| contig17405 | - - - - - | gnl\|UG\|Ta#S65639206 |
| contig26525 | - - - - - | gnl\|UG\|Ta#S65639206 |
| contig03151 | - - - - - | gnl\|UG\|Ta#S65642631 |
| contig04038 | - - - - - | gnl\|UG\|Ta#S65642768 |
| contig16537 | - - - - - | gnl\|UG\|Ta#S65642845 |
| contig00324 | - - - - - | gnl\|UG\|Ta#S65643043 |
| contig04598 | - - - - - | gnl\|UG\|Ta#S65643114 |
| contig12825 | - - - - - | gnl\|UG\|Ta#S65643133 |
| contig16842 | - - - - - | gnl\|UG\|Ta#S65645098 |
| contig01516 | - - - - - | gnl\|UG\|Ta#S65646022 |
| contig05214 | - - - - - | gnl\|UG\|Ta#S65646022 |

| | | |
|---|---|---|
| contig17527 | - - - - - | gnl\|UG\|Ta#S65646147 |
| contig19318 | - - - - - | gnl\|UG\|Ta#S65646147 |
| contig05436 | - - - - - | gnl\|UG\|Ta#S65646672 |
| contig16021 | - - - - - | gnl\|UG\|Ta#S65646683 |
| contig20810 | - - - - - | gnl\|UG\|Ta#S65647058 |
| contig04090 | - - - - - | gnl\|UG\|Ta#S65647608 |
| contig09742 | - - - - - | gnl\|UG\|Ta#S65647972 |
| contig13376 | - - - - - | gnl\|UG\|Ta#S65648878 |
| contig16985 | - - - - - | gnl\|UG\|Ta#S65648951 |
| contig07708 | - - - - - | gnl\|UG\|Ta#S65649973 |
| contig04345 | - - - - - | gnl\|UG\|Ta#S65651712 |
| contig06491 | - - - - - | gnl\|UG\|Ta#S65651803 |
| contig12034 | - - - - - | gnl\|UG\|Ta#S65651967 |
| contig04881 | - - - - - | gnl\|UG\|Ta#S65652260 |
| contig07523 | - - - - - | gnl\|UG\|Ta#S65652321 |
| contig05993 | - - - - - | gnl\|UG\|Ta#S65652416 |
| contig00084 | - - - - - | gnl\|UG\|Ta#S65653906 |
| contig18915 | - - - - - | gnl\|UG\|Ta#S65655210 |
| contig11600 | - - - - - | gnl\|UG\|Ta#S65655427 |
| contig04709 | - - - - - | gnl\|UG\|Ta#S65655762 |
| contig01788 | - - - - - | gnl\|UG\|Ta#S65656136 |
| contig14354 | - - - - - | gnl\|UG\|Ta#S65656709 |
| contig11316 | - - - - - | gnl\|UG\|Ta#S65657241 |
| contig15465 | - - - - - | gnl\|UG\|Ta#S65658121 |
| contig14877 | - - - - - | gnl\|UG\|Ta#S65658843 |
| contig21776 | - - - - - | gnl\|UG\|Ta#S65659313 |
| contig03989 | - - - - - | gnl\|UG\|Ta#S65659531 |
| contig00525 | - - - - - | gnl\|UG\|Ta#S65659670 |
| contig12233 | - - - - - | gnl\|UG\|Ta#S65662347 |
| contig22744 | - - - - - | gnl\|UG\|Ta#S65662566 |
| contig15788 | - - - - - | gnl\|UG\|Ta#S65663026 |
| contig11294 | - - - - - | gnl\|UG\|Ta#S65663550 |
| contig08783 | - - - - - | gnl\|UG\|Ta#S65665406 |
| contig04838 | - - - - - | gnl\|UG\|Ta#S65665450 |
| contig00211 | - - - - - | gnl\|UG\|Ta#S65665681 |
| contig23258 | - - - - - | gnl\|UG\|Ta#S65666303 |
| contig04322 | - - - - - | gnl\|UG\|Ta#S65666464 |
| contig15549 | - - - - - | gnl\|UG\|Ta#S65666994 |
| contig19106 | - - - - - | gnl\|UG\|Ta#S65667196 |
| contig06415 | - - - - - | gnl\|UG\|Ta#S65668113 |
| contig01206 | - - - - - | gnl\|UG\|Ta#S65668498 |
| contig21226 | - - - - - | gnl\|UG\|Ta#S65669119 |
| contig06472 | - - - - - | gnl\|UG\|Ta#S65669154 |
| contig16498 | - - - - - | gnl\|UG\|Ta#S65669221 |
| contig06132 | - - - - - | gnl\|UG\|Ta#S65669635 |
| contig01803 | - - - - - | gnl\|UG\|Ta#S65670585 |
| contig01354 | - - - - - | gnl\|UG\|Ta#S65670642 |
| contig21235 | - - - - - | gnl\|UG\|Ta#S65670730 |
| contig08379 | - - - - - | gnl\|UG\|Ta#S65670790 |
| contig10812 | - - - - - | gnl\|UG\|Ta#S65670916 |

| | | |
|---|---|---|
| contig07607 | - - - - - | gnl\|UG\|Ta#S65671024 |
| contig17251 | - - - - - | gnl\|UG\|Ta#S65671356 |
| contig18463 | - - - - - | gnl\|UG\|Ta#S65671361 |
| contig03883 | - - - - - | gnl\|UG\|Ta#S65671567 |
| contig26311 | - - - - - | gnl\|UG\|Ta#S65671795 |
| contig13760 | - - - - - | gnl\|UG\|Ta#S65672197 |
| contig24446 | - - - - - | gnl\|UG\|Ta#S65672351 |
| contig14237 | - - - - - | gnl\|UG\|Ta#S65672523 |
| contig10836 | - - - - - | gnl\|UG\|Ta#S65674860 |
| contig17860 | - - - - - | gnl\|UG\|Ta#S65675634 |
| contig19863 | - - - - - | gnl\|UG\|Ta#S65675749 |
| contig10637 | - - - - - | gnl\|UG\|Ta#S65677447 |
| contig17169 | - - - - - | gnl\|UG\|Ta#S65677452 |
| contig04695 | - - - - - | gnl\|UG\|Ta#S65677605 |
| contig14927 | - - - - - | gnl\|UG\|Ta#S65678830 |
| contig09492 | - - - - - | gnl\|UG\|Ta#S65678919 |
| contig14808 | - - - - - | gnl\|UG\|Ta#S65678919 |
| contig17041 | - - - - - | gnl\|UG\|Ta#S65678919 |
| contig15436 | - - - - - | gnl\|UG\|Ta#S65679153 |
| contig00666 | - - - - - | gnl\|UG\|Ta#S65679314 |
| contig06349 | - - - - - | gnl\|UG\|Ta#S65680152 |
| contig04396 | - - - - - | gnl\|UG\|Ta#S65680760 |
| contig19626 | - - - - - | gnl\|UG\|Ta#S65680814 |
| contig16016 | - - - - - | gnl\|UG\|Ta#S65680959 |
| contig08240 | - - - - - | gnl\|UG\|Ta#S65680960 |
| contig18420 | - - - - - | gnl\|UG\|Ta#S65681014 |
| contig08665 | - - - - - | gnl\|UG\|Ta#S65681108 |
| contig12738 | - - - - - | gnl\|UG\|Ta#S65681111 |
| contig19182 | - - - - - | gnl\|UG\|Ta#S65681877 |
| contig05390 | - - - - - | gnl\|UG\|Ta#S65682388 |
| contig13384 | - - - - - | gnl\|UG\|Ta#S65682388 |
| contig18025 | - - - - - | gnl\|UG\|Ta#S65684148 |
| contig07161 | - - - - - | gnl\|UG\|Ta#S65684280 |
| contig04803 | - - - - - | gnl\|UG\|Ta#S65684327 |
| contig07006 | - - - - - | gnl\|UG\|Ta#S65684574 |
| contig02143 | - - - - - | gnl\|UG\|Ta#S65685251 |
| contig03418 | - - - - - | gnl\|UG\|Ta#S65685813 |
| contig14297 | - - - - - | gnl\|UG\|Ta#S65686094 |
| contig16036 | - - - - - | gnl\|UG\|Ta#S65686825 |
| contig23489 | - - - - - | gnl\|UG\|Ta#S65686825 |
| contig05445 | - - - - - | gnl\|UG\|Ta#S65687028 |
| contig07995 | - - - - - | gnl\|UG\|Ta#S65687336 |
| contig10713 | - - - - - | gnl\|UG\|Ta#S65687579 |
| contig14735 | - - - - - | gnl\|UG\|Ta#S65688639 |
| contig05920 | - - - - - | gnl\|UG\|Ta#S65688955 |
| contig12689 | - - - - - | gnl\|UG\|Ta#S65689169 |
| contig13264 | - - - - - | gnl\|UG\|Ta#S65689169 |
| contig26389 | - - - - - | gnl\|UG\|Ta#S65689196 |
| contig12975 | - - - - - | gnl\|UG\|Ta#S65689755 |
| contig21917 | - - - - - | gnl\|UG\|Ta#S65689755 |

| | | |
|---|---|---|
| contig01166 | - - - - - | gnl\|UG\|Ta#S65689864 |
| contig06445 | - - - - - | gnl\|UG\|Ta#S65690564 |
| contig11353 | - - - - - | gnl\|UG\|Ta#S65690732 |
| contig06378 | - - - - - | gnl\|UG\|Ta#S65690740 |
| contig15957 | - - - - - | gnl\|UG\|Ta#S65690740 |
| contig07266 | - - - - - | gnl\|UG\|Ta#S65691132 |
| contig03012 | - - - - - | gnl\|UG\|Ta#S65691610 |
| contig17776 | - - - - - | gnl\|UG\|Ta#S65691704 |
| contig10826 | - - - - - | gnl\|UG\|Ta#S65692254 |
| contig01550 | - - - - - | gnl\|UG\|Ta#S65692419 |
| contig02336 | - - - - - | gnl\|UG\|Ta#S65692837 |
| contig00082 | - - - - - | gnl\|UG\|Ta#S65693552 |
| contig19992 | - - - - - | gnl\|UG\|Ta#S65694302 |
| contig08897 | - - - - - | gnl\|UG\|Ta#S65695265 |
| contig12878 | - - - - - | gnl\|UG\|Ta#S65695265 |
| contig09622 | - - - - - | gnl\|UG\|Ta#S65695901 |
| contig17846 | - - - - - | gnl\|UG\|Ta#S65695963 |
| contig02300 | - - - - - | gnl\|UG\|Ta#S65696625 |
| contig10083 | - - - - - | gnl\|UG\|Ta#S65696944 |
| contig23873 | - - - - - | gnl\|UG\|Ta#S65696988 |
| contig15938 | - - - - - | gnl\|UG\|Ta#S65699142 |
| contig18628 | - - - - - | gnl\|UG\|Ta#S65699884 |
| contig03307 | - - - - - | gnl\|UG\|Ta#S65699995 |
| contig06354 | - - - - - | gnl\|UG\|Ta#S65699995 |
| contig11563 | - - - - - | gnl\|UG\|Ta#S65700390 |
| contig10002 | - - - - - | gnl\|UG\|Ta#S65701911 |
| contig24226 | - - - - - | gnl\|UG\|Ta#S65702316 |
| contig09214 | - - - - - | gnl\|UG\|Ta#S65702702 |
| contig09527 | - - - - - | gnl\|UG\|Ta#S65703386 |
| contig15445 | - - - - - | gnl\|UG\|Ta#S65703413 |
| contig16429 | - - - - - | gnl\|UG\|Ta#S65703413 |
| contig05852 | - - - - - | gnl\|UG\|Ta#S65703515 |
| contig11168 | - - - - - | gnl\|UG\|Ta#S65703688 |
| contig04528 | - - - - - | gnl\|UG\|Ta#S65704056 |
| contig14418 | - - - - - | gnl\|UG\|Ta#S65704608 |
| contig15730 | - - - - - | gnl\|UG\|Ta#S65705298 |
| contig02005 | - - - - - | gnl\|UG\|Ta#S65705372 |
| contig13850 | - - - - - | gnl\|UG\|Ta#S65706187 |
| contig22392 | - - - - - | gnl\|UG\|Ta#S65706287 |
| contig09970 | - - - - - | gnl\|UG\|Ta#S65706351 |
| contig06932 | - - - - - | gnl\|UG\|Ta#S65708577 |
| contig17397 | - - - - - | gnl\|UG\|Ta#S65708622 |
| contig02917 | - - - - - | gnl\|UG\|Ta#S65709170 |
| contig17930 | - - - - - | gnl\|UG\|Ta#S65709195 |
| contig22663 | - - - - - | gnl\|UG\|Ta#S65709195 |
| contig04583 | - - - - - | gnl\|UG\|Ta#S65709333 |
| contig01916 | - - - - - | gnl\|UG\|Ta#S65709604 |
| contig02891 | - - - - - | gnl\|UG\|Ta#S65709965 |
| contig01361 | - - - - - | gnl\|UG\|Ta#S65710740 |
| contig06856 | - - - - - | gnl\|UG\|Ta#S65710822 |

| | | | |
|---|---|---|---|
| contig06729 | - - - - - | | gnl\|UG\|Ta#S65711066 |
| contig14208 | - - - - - | | gnl\|UG\|Ta#S65712348 |
| contig14125 | - - - - - | | gnl\|UG\|Ta#S65712383 |
| contig15406 | - - - - - | | gnl\|UG\|Ta#S65712532 |
| contig12660 | - - - - - | | gnl\|UG\|Ta#S65713287 |
| contig22089 | - - - - - | | gnl\|UG\|Ta#S65713287 |
| contig02480 | - - - - - | | gnl\|UG\|Ta#S65713363 |
| contig10045 | - - - - - | | gnl\|UG\|Ta#S65713577 |
| contig26030 | - - - - - | | gnl\|UG\|Ta#S65714092 |
| contig15830 | - - - - - | | gnl\|UG\|Ta#S65715517 |
| contig11088 | - - - - - | | gnl\|UG\|Ta#S65716038 |
| contig00992 | - - - - - | | gnl\|UG\|Ta#S65716773 |
| contig05143 | - - - - - | | gnl\|UG\|Ta#S65717180 |
| contig04250 | - - - - - | | gnl\|UG\|Ta#S65717832 |
| contig06315 | - - - - - | | gnl\|UG\|Ta#S65717910 |
| contig04093 | - - - - - | | gnl\|UG\|Ta#S65718881 |
| contig23595 | - - - - - | | gnl\|UG\|Ta#S65719308 |
| contig13447 | - - - - - | | gnl\|UG\|Ta#S65719406 |
| contig15363 | - - - - - | | gnl\|UG\|Ta#S65721136 |
| contig00401 | - - - - - | | gnl\|UG\|Ta#S65722211 |
| contig09097 | - - - - - | | gnl\|UG\|Ta#S65722343 |
| contig06993 | - - - - - | | gnl\|UG\|Ta#S65722373 |
| contig06994 | - - - - - | | gnl\|UG\|Ta#S65722373 |
| contig17871 | - - - - - | | gnl\|UG\|Ta#S65722373 |
| contig00932 | - - - - - | | gnl\|UG\|Ta#S65723020 |
| IVLNWEC02GOD9Y | - - - - | tr\|W5HVX3\|W5HVX3_WHEAT | - |
| IVLNWEC02HSP8T | - - - - | tr\|W5HVX3\|W5HVX3_WHEAT | - |
| IV9RU5F01E2N0I | - - - - | tr\|W5GGJ4\|W5GGJ4_WHEAT | - |
| IV9RU5F02HWI7H | - - - - | tr\|W5GGJ4\|W5GGJ4_WHEAT | - |
| IUIVLUD01CSGQM | - - - - | tr\|W5G0B8\|W5G0B8_WHEAT | - |
| IVLNWEC02F58VF | - - - - | tr\|W5G0B8\|W5G0B8_WHEAT | - |
| IV9RU5F02HMCW8 | - - - - | tr\|W5FWJ3\|W5FWJ3_WHEAT | - |
| IVLNWEC01DBDNL | - - - - | tr\|W5FWJ3\|W5FWJ3_WHEAT | - |
| IV9RU5F01CECHT | - - - - | tr\|W5FS07\|W5FS07_WHEAT | - |
| IV9RU5F02HBU9X | - - - - | tr\|W5FS07\|W5FS07_WHEAT | - |
| IUIVLUD02FQGEJ | - - - - | tr\|W5FPN5\|W5FPN5_WHEAT | - |
| IUIVLUD01ANKWA | - - - - | tr\|W5FPJ4\|W5FPJ4_WHEAT | - |
| IV9RU5F01B8D3B | - - - - | tr\|W5FPJ4\|W5FPJ4_WHEAT | - |
| IVLNWEC01DGTT5 | - - - - | tr\|W5FPJ3\|W5FPJ3_WHEAT | - |
| IV9RU5F01BYD31 | - - - - | tr\|W5FP98\|W5FP98_WHEAT | - |
| IV9RU5F01CIBCF | - - - - | tr\|W5FP03\|W5FP03_WHEAT | - |
| IV9RU5F01ECZ6U | - - - - | tr\|W5FP03\|W5FP03_WHEAT | - |
| IV9RU5F01DN0S0 | - - - - | tr\|W5FNY3\|W5FNY3_WHEAT | - |
| IVLNWEC02JI5SZ | - - - - | tr\|W5FNG1\|W5FNG1_WHEAT | - |
| IUIVLUD02G2W58 | - - - - | tr\|W5FND7\|W5FND7_WHEAT | - |
| IVLNWEC02HUNHC | - - - - | tr\|W5FND7\|W5FND7_WHEAT | - |
| IUIVLUD02GHS73 | - - - - | tr\|W5FN33\|W5FN33_WHEAT | - |
| IVLNWEC01CZRUK | - - - - | tr\|W5FM44\|W5FM44_WHEAT | - |
| IVLNWEC02HJXQ9 | - - - - | tr\|W5FM44\|W5FM44_WHEAT | - |
| IUIVLUD02JJ75C | - - - - | tr\|W5FL76\|W5FL76_WHEAT | - |

| | | | |
|---|---|---|---|
| IV9RU5F01CV9IK | - - - - | tr\|W5FK17\|W5FK17_WHEAT | - |
| IVLNWEC02G6H83 | - - - - | tr\|W5FJY4\|W5FJY4_WHEAT | - |
| IVLNWEC02JLU1A | - - - - | tr\|W5FJY4\|W5FJY4_WHEAT | - |
| IVLNWEC02FZ88P | - - - - | tr\|W5FJR5\|W5FJR5_WHEAT | - |
| IVLNWEC02JJ02P | - - - - | tr\|W5FJR5\|W5FJR5_WHEAT | - |
| IUIVLUD02IYDXD | - - - - | tr\|W5FJF8\|W5FJF8_WHEAT | - |
| IV9RU5F01BC18T | - - - - | tr\|W5FJF8\|W5FJF8_WHEAT | - |
| IVLNWEC02G3OO9 | - - - - | tr\|W5FJF8\|W5FJF8_WHEAT | - |
| IVLNWEC01AXA0V | - - - - | tr\|W5FJ86\|W5FJ86_WHEAT | - |
| IVLNWEC02HIZ5V | - - - - | tr\|W5FJ86\|W5FJ86_WHEAT | - |
| IV9RU5F01B77HY | - - - - | tr\|W5FJ67\|W5FJ67_WHEAT | - |
| IVLNWEC01CZIA2 | - - - - | tr\|W5FJ67\|W5FJ67_WHEAT | - |
| IV9RU5F02HDIBZ | - - - - | tr\|W5FIA8\|W5FIA8_WHEAT | - |
| IVLNWEC01DLJO0 | - - - - | tr\|W5FIA8\|W5FIA8_WHEAT | - |
| IUIVLUD01BIX4A | - - - - | tr\|W5FI66\|W5FI66_WHEAT | - |
| IV9RU5F02G5R8E | - - - - | tr\|W5FI66\|W5FI66_WHEAT | - |
| IUIVLUD02F5RIB | - - - - | tr\|W5FHY8\|W5FHY8_WHEAT | - |
| IUIVLUD02HP6T6 | - - - - | tr\|W5FHY8\|W5FHY8_WHEAT | - |
| IVLNWEC01ALGWE | - - - - | tr\|W5FHP8\|W5FHP8_WHEAT | - |
| IVLNWEC01A3BQ8 | - - - - | tr\|W5FH75\|W5FH75_WHEAT | - |
| IVLNWEC01B2E5H | - - - - | tr\|W5FH75\|W5FH75_WHEAT | - |
| IV9RU5F02F1UES | - - - - | tr\|W5FH74\|W5FH74_WHEAT | - |
| IVLNWEC01ENRAJ | - - - - | tr\|W5FH74\|W5FH74_WHEAT | - |
| IV9RU5F01EL4EQ | - - - - | tr\|W5FGM7\|W5FGM7_WHEAT | - |
| IVLNWEC02IQ15K | - - - - | tr\|W5FGM7\|W5FGM7_WHEAT | - |
| IV9RU5F01CS3YL | - - - - | tr\|W5FEI4\|W5FEI4_WHEAT | - |
| IVLNWEC02IFQC5 | - - - - | tr\|W5FEI4\|W5FEI4_WHEAT | - |
| IVLNWEC01DS87Z | - - - - | tr\|W5FDY0\|W5FDY0_WHEAT | - |
| IUIVLUD02GF5YJ | - - - - | tr\|W5FDR1\|W5FDR1_WHEAT | - |
| IV9RU5F02IYI4P | - - - - | tr\|W5FDR1\|W5FDR1_WHEAT | - |
| IV9RU5F01D9NN4 | - - - - | tr\|W5FCN8\|W5FCN8_WHEAT | - |
| IV9RU5F02HMH0J | - - - - | tr\|W5FCJ0\|W5FCJ0_WHEAT | - |
| IV9RU5F01EDVUD | - - - - | tr\|W5FC68\|W5FC68_WHEAT | - |
| IVLNWEC02HWUEC | - - - - | tr\|W5FC68\|W5FC68_WHEAT | - |
| IUIVLUD01D10LZ | - - - - | tr\|W5FBN4\|W5FBN4_WHEAT | - |
| IV9RU5F01A38OL | - - - - | tr\|W5FBN4\|W5FBN4_WHEAT | - |
| IV9RU5F02HM25T | - - - - | tr\|W5FBN4\|W5FBN4_WHEAT | - |
| IV9RU5F01CEA35 | - - - - | tr\|W5FBA5\|W5FBA5_WHEAT | - |
| IV9RU5F02JZ3RP | - - - - | tr\|W5FBA5\|W5FBA5_WHEAT | - |
| IV9RU5F02FSV8C | - - - - | tr\|W5FBA1\|W5FBA1_WHEAT | - |
| IVLNWEC01A3WDO | - - - - | tr\|W5FBA1\|W5FBA1_WHEAT | - |
| IVLNWEC02JEBWI | - - - - | tr\|W5FBA1\|W5FBA1_WHEAT | - |
| IVLNWEC01DW5U0 | - - - - | tr\|W5FB25\|W5FB25_WHEAT | - |
| IUIVLUD01BWXO1 | - - - - | tr\|W5FAH4\|W5FAH4_WHEAT | - |
| IV9RU5F01A0M41 | - - - - | tr\|W5FAH4\|W5FAH4_WHEAT | - |
| IVLNWEC02FSAIG | - - - - | tr\|W5FA38\|W5FA38_WHEAT | - |
| IVLNWEC02JXX7S | - - - - | tr\|W5FA38\|W5FA38_WHEAT | - |
| IV9RU5F01CMC37 | - - - - | tr\|W5F925\|W5F925_WHEAT | - |
| IV9RU5F02H2XAL | - - - - | tr\|W5F8X6\|W5F8X6_WHEAT | - |
| IVLNWEC01DB8BC | - - - - | tr\|W5F8X6\|W5F8X6_WHEAT | - |

| | | | | | | |
|---|---|---|---|---|---|---|
| IUIVLUD01COF7I | - | - | - | - | tr\|W5F8Q1\|W5F8Q1_WHEAT | - |
| IUIVLUD02HD9ID | - | - | - | - | tr\|W5F8Q1\|W5F8Q1_WHEAT | - |
| IVLNWEC01DJCN5 | - | - | - | - | tr\|W5F8Q1\|W5F8Q1_WHEAT | - |
| IV9RU5F01DT8GG | - | - | - | - | tr\|W5F8P4\|W5F8P4_WHEAT | - |
| IV9RU5F02F5BAC | - | - | - | - | tr\|W5F8J9\|W5F8J9_WHEAT | - |
| IVLNWEC02GG5XX | - | - | - | - | tr\|W5F8J9\|W5F8J9_WHEAT | - |
| IUIVLUD01DH7GJ | - | - | - | - | tr\|W5F8C1\|W5F8C1_WHEAT | - |
| IVLNWEC02G1UHO | - | - | - | - | tr\|W5F8C1\|W5F8C1_WHEAT | - |
| IVLNWEC02HTILS | - | - | - | - | tr\|W5F8C1\|W5F8C1_WHEAT | - |
| IVLNWEC02I3OXB | - | - | - | - | tr\|W5F8C1\|W5F8C1_WHEAT | - |
| IV9RU5F01D6BVB | - | - | - | - | tr\|W5F8A7\|W5F8A7_WHEAT | - |
| IVLNWEC01EF303 | - | - | - | - | tr\|W5F8A7\|W5F8A7_WHEAT | - |
| IV9RU5F01CI0HH | - | - | - | - | tr\|W5F888\|W5F888_WHEAT | - |
| IV9RU5F02JSA1N | - | - | - | - | tr\|W5F888\|W5F888_WHEAT | - |
| IVLNWEC01BEGLH | - | - | - | - | tr\|W5F888\|W5F888_WHEAT | - |
| IUIVLUD01DHKD7 | - | - | - | - | tr\|W5F884\|W5F884_WHEAT | - |
| IVLNWEC02IR5IL | - | - | - | - | tr\|W5F884\|W5F884_WHEAT | - |
| IV9RU5F01CW5W8 | - | - | - | - | tr\|W5F7N3\|W5F7N3_WHEAT | - |
| IVLNWEC01D3MIU | - | - | - | - | tr\|W5F7N3\|W5F7N3_WHEAT | - |
| IUIVLUD01CCSPU | - | - | - | - | tr\|W5F6S7\|W5F6S7_WHEAT | - |
| IV9RU5F01CJGGU | - | - | - | - | tr\|W5F6S7\|W5F6S7_WHEAT | - |
| IVLNWEC01BKAWB | - | - | - | - | tr\|W5DXT7\|W5DXT7_WHEAT | - |
| IV9RU5F01EZ51E | - | - | - | - | tr\|W5DUY0\|W5DUY0_WHEAT | - |
| IV9RU5F02I0PWB | - | - | - | - | tr\|W5DUY0\|W5DUY0_WHEAT | - |
| IV9RU5F02JH0GD | - | - | - | - | tr\|W5DUY0\|W5DUY0_WHEAT | - |
| IVLNWEC01DOL30 | - | - | - | - | tr\|W5DUY0\|W5DUY0_WHEAT | - |
| IVLNWEC01EPD94 | - | - | - | - | tr\|W5DUY0\|W5DUY0_WHEAT | - |
| IUIVLUD02IOB0B | - | - | - | - | tr\|W5DTE1\|W5DTE1_WHEAT | - |
| IV9RU5F02GR2RY | - | - | - | - | tr\|W5DTE1\|W5DTE1_WHEAT | - |
| IVLNWEC02FSG88 | - | - | - | - | tr\|W5DSW0\|W5DSW0_WHEAT | - |
| IV9RU5F01BEIFI | - | - | - | - | tr\|W5DS97\|W5DS97_WHEAT | - |
| IV9RU5F02HZQ4I | - | - | - | - | tr\|W5DS97\|W5DS97_WHEAT | - |
| IV9RU5F02HA48G | - | - | - | - | tr\|W5DPY6\|W5DPY6_WHEAT | - |
| IV9RU5F02HV28K | - | - | - | - | tr\|W5DPY6\|W5DPY6_WHEAT | - |
| IUIVLUD01ECOSL | - | - | - | - | tr\|W5DNN8\|W5DNN8_WHEAT | - |
| IV9RU5F01A2P2P | - | - | - | - | tr\|W5DNN8\|W5DNN8_WHEAT | - |
| IV9RU5F01A4JLG | - | - | - | - | tr\|W5DNN8\|W5DNN8_WHEAT | - |
| IVLNWEC02FTSR0 | - | - | - | - | tr\|W5DND9\|W5DND9_WHEAT | - |
| IVLNWEC02JKWO7 | - | - | - | - | tr\|W5DND9\|W5DND9_WHEAT | - |
| IUIVLUD01ET4B4 | - | - | - | - | tr\|W5DL47\|W5DL47_WHEAT | - |
| IUIVLUD02GAFP9 | - | - | - | - | tr\|W5DL47\|W5DL47_WHEAT | - |
| IV9RU5F01B43NA | - | - | - | - | tr\|W5BKM9\|W5BKM9_WHEAT | - |
| IV9RU5F02G9RQ2 | - | - | - | - | tr\|W5BKM9\|W5BKM9_WHEAT | - |
| IUIVLUD01EBDTL | - | - | - | - | tr\|W5ABY4\|W5ABY4_WHEAT | - |
| IV9RU5F01BCSA3 | - | - | - | - | tr\|W5ABN6\|W5ABN6_WHEAT | - |
| IV9RU5F01CNIAX | - | - | - | - | tr\|W5AB10\|W5AB10_WHEAT | - |
| IV9RU5F01DYSA4 | - | - | - | - | tr\|W5AB10\|W5AB10_WHEAT | - |
| IUIVLUD02HRF6E | - | - | - | - | tr\|W5A9M0\|W5A9M0_WHEAT | - |
| IVLNWEC02HT9BW | - | - | - | - | tr\|W5A9M0\|W5A9M0_WHEAT | - |
| IUIVLUD01EHGA1 | - | - | - | - | tr\|W5A7T8\|W5A7T8_WHEAT | - |

| | | | |
|---|---|---|---|
| IVLNWEC02I0HRD | - - - - | tr\|W5A7T8\|W5A7T8_WHEAT | - |
| IV9RU5F02JVOF7 | - - - - | tr\|W5A7E9\|W5A7E9_WHEAT | - |
| IVLNWEC02G3YMU | - - - - | tr\|W5A7E9\|W5A7E9_WHEAT | - |
| IV9RU5F01CCU4Y | - - - - | tr\|W5A768\|W5A768_WHEAT | - |
| IV9RU5F02IFY71 | - - - - | tr\|W5A768\|W5A768_WHEAT | - |
| IUIVLUD02I2WKI | - - - - | tr\|T1NP75\|T1NP75_TRIUA | - |
| IV9RU5F01AYSS2 | - - - - | tr\|T1NP75\|T1NP75_TRIUA | - |
| IV9RU5F02F6GIU | - - - - | tr\|T1NP75\|T1NP75_TRIUA | - |
| IV9RU5F01BEWX9 | - - - - | tr\|T1NJ26\|T1NJ26_TRIUA | - |
| IVLNWEC02IP67T | - - - - | tr\|T1NJ26\|T1NJ26_TRIUA | - |
| IV9RU5F02G0GQQ | - - - - | tr\|T1MXL5\|T1MXL5_TRIUA | - |
| IVLNWEC02H83BB | - - - - | tr\|T1MXL5\|T1MXL5_TRIUA | - |
| IV9RU5F02F6FE0 | - - - - | tr\|T1MCE4\|T1MCE4_TRIUA | - |
| IV9RU5F02H10US | - - - - | tr\|T1MCE4\|T1MCE4_TRIUA | - |
| IV9RU5F02J1NSL | - - - - | tr\|T1M6T6\|T1M6T6_TRIUA | - |
| IV9RU5F02JNDEM | - - - - | tr\|T1M6T6\|T1M6T6_TRIUA | - |
| IVLNWEC01AM9R3 | - - - - | tr\|T1M1V2\|T1M1V2_TRIUA | - |
| IVLNWEC01B7G6O | - - - - | tr\|T1M1V2\|T1M1V2_TRIUA | - |
| IV9RU5F01DVYSM | - - - - | tr\|M8B257\|M8B257_TRIUA | - |
| IVLNWEC02IK0ZG | - - - - | tr\|M8B257\|M8B257_TRIUA | - |
| IV9RU5F02GCQWA | - - - - | tr\|M8AKH3\|M8AKH3_TRIUA | - |
| IVLNWEC02I6UXA | - - - - | tr\|M8AKH3\|M8AKH3_TRIUA | - |
| IV9RU5F01B4HC7 | - - - - | tr\|M8A7V6\|M8A7V6_TRIUA | - |
| IVLNWEC02JP76O | - - - - | tr\|M8A7V6\|M8A7V6_TRIUA | - |
| IUIVLUD02JDEQF | - - - - | tr\|M8A6U9\|M8A6U9_TRIUA | - |
| IV9RU5F01AYQLW | - - - - | tr\|M8A4I1\|M8A4I1_TRIUA | - |
| IVLNWEC02IM8RW | - - - - | tr\|M8A4I1\|M8A4I1_TRIUA | - |
| IV9RU5F02HI5ZE | - - - - | tr\|M8A3Q6\|M8A3Q6_TRIUA | - |
| IV9RU5F01DGRCE | - - - - | tr\|M8A1C3\|M8A1C3_TRIUA | - |
| IVLNWEC01COMR4 | - - - - | tr\|M8A1C3\|M8A1C3_TRIUA | - |
| IVLNWEC02FWG75 | - - - - | tr\|M8A0M4\|M8A0M4_TRIUA | - |
| IV9RU5F02H3VVH | - - - - | tr\|M7ZRN5\|M7ZRN5_TRIUA | - |
| IVLNWEC02JSG7P | - - - - | tr\|M7ZRN5\|M7ZRN5_TRIUA | - |
| IV9RU5F01EJ02T | - - - - | tr\|M7ZJC8\|M7ZJC8_TRIUA | - |
| IV9RU5F02JK83Q | - - - - | tr\|M7ZJC8\|M7ZJC8_TRIUA | - |
| IV9RU5F01BOVCS | - - - - | tr\|M7ZJ83\|M7ZJ83_TRIUA | - |
| IVLNWEC02I4PV3 | - - - - | tr\|M7ZJ83\|M7ZJ83_TRIUA | - |
| IUIVLUD01ER779 | - - - - | tr\|M7ZD83\|M7ZD83_TRIUA | - |
| IV9RU5F02FLVFD | - - - - | tr\|M7Z7U3\|M7Z7U3_TRIUA | - |
| IVLNWEC02F9T7O | - - - - | tr\|M7Z7U3\|M7Z7U3_TRIUA | - |
| IV9RU5F01ET5M8 | - - - - | tr\|M7Z7E7\|M7Z7E7_TRIUA | - |
| IVLNWEC02JDVBA | - - - - | tr\|M7Z5P2\|M7Z5P2_TRIUA | - |
| IUIVLUD01EVDAO | - - - - | tr\|M7Z1W7\|M7Z1W7_TRIUA | - |
| IUIVLUD02G9Z5R | - - - - | tr\|M7Z1W7\|M7Z1W7_TRIUA | - |
| IV9RU5F02GDNX9 | - - - - | tr\|M7YWE3\|M7YWE3_TRIUA | - |
| IV9RU5F01DEH93 | - - - - | tr\|M7YUW0\|M7YUW0_TRIUA | - |
| IV9RU5F02G20A9 | - - - - | tr\|M7YNW6\|M7YNW6_TRIUA | - |
| IVLNWEC02G2TEI | - - - - | tr\|M7YNW6\|M7YNW6_TRIUA | - |
| IUIVLUD02IXRJ1 | - - - - | tr\|M7YID7\|M7YID7_TRIUA | - |
| IV9RU5F02II9X0 | - - - - | tr\|M7YID7\|M7YID7_TRIUA | - |

| | | | |
|---|---|---|---|
| IV9RU5F02JM16U | - - - - | tr\|M7YBW1\|M7YBW1_TRIUA | - |
| IVLNWEC02G7GAO | - - - - | tr\|M7YBW1\|M7YBW1_TRIUA | - |
| IVLNWEC01D35NI | - - - - | tr\|W5FB42\|W5FB42_WHEAT | gnl\|UG\|Ta#S12881932 |
| IVLNWEC01EUT61 | - - - - | tr\|W5FNX3\|W5FNX3_WHEAT | gnl\|UG\|Ta#S12902190 |
| IV9RU5F02HWMKX | - - - - | tr\|M7YUW0\|M7YUW0_TRIUA | gnl\|UG\|Ta#S16058184 |
| IUIVLUD01B2OBY | - - - - | tr\|M8ARL7\|M8ARL7_TRIUA | gnl\|UG\|Ta#S16252219 |
| IV9RU5F02I44NO | - - - - | tr\|M7ZYM4\|M7ZYM4_TRIUA | gnl\|UG\|Ta#S22368494 |
| IV9RU5F02FU2JX | - - - - | tr\|A0MPL3\|A0MPL3_WHEAT | gnl\|UG\|Ta#S35718008 |
| IUIVLUD01ERXB6 | - - - - | tr\|W5GC63\|W5GC63_WHEAT | gnl\|UG\|Ta#S50377770 |
| IVLNWEC02HE3LM | - - - - | tr\|W5GC63\|W5GC63_WHEAT | gnl\|UG\|Ta#S50377770 |
| IUIVLUD02GT7K1 | - - - - | tr\|W5FMB0\|W5FMB0_WHEAT | gnl\|UG\|Ta#S52541509 |
| IVLNWEC02GW4N3 | - - - - | tr\|W5FMB0\|W5FMB0_WHEAT | gnl\|UG\|Ta#S52541509 |
| IV9RU5F01AK40D | - - - - | tr\|W5BUI2\|W5BUI2_WHEAT | gnl\|UG\|Ta#S52542198 |
| IVLNWEC02IBAF8 | - - - - | tr\|W5DTU5\|W5DTU5_WHEAT | gnl\|UG\|Ta#S52542257 |
| IV9RU5F02F1XND | - - - - | tr\|Q2L3S4\|Q2L3S4_WHEAT | gnl\|UG\|Ta#S52542527 |
| IV9RU5F02JEE47 | - - - - | tr\|W5FBC1\|W5FBC1_WHEAT | gnl\|UG\|Ta#S52542599 |
| IV9RU5F01BXGWN | - - - - | tr\|W5FM09\|W5FM09_WHEAT | gnl\|UG\|Ta#S52542905 |
| IV9RU5F02HFA08 | - - - - | tr\|W5FM09\|W5FM09_WHEAT | gnl\|UG\|Ta#S52542905 |
| IV9RU5F02HZEPG | - - - - | tr\|W5FM09\|W5FM09_WHEAT | gnl\|UG\|Ta#S52542905 |
| IV9RU5F02I2NS6 | - - - - | tr\|W5FM09\|W5FM09_WHEAT | gnl\|UG\|Ta#S52542905 |
| IVLNWEC01EWL1H | - - - - | tr\|W5FM09\|W5FM09_WHEAT | gnl\|UG\|Ta#S52542905 |
| IUIVLUD02GEND3 | - - - - | tr\|W5FNG7\|W5FNG7_WHEAT | gnl\|UG\|Ta#S52543160 |
| IVLNWEC01DQBN2 | - - - - | tr\|W5FNG7\|W5FNG7_WHEAT | gnl\|UG\|Ta#S52543160 |
| IUIVLUD01EV1LZ | - - - - | tr\|W5F473\|W5F473_WHEAT | gnl\|UG\|Ta#S52543417 |
| IVLNWEC01CNA7J | - - - - | tr\|W5FN58\|W5FN58_WHEAT | gnl\|UG\|Ta#S52543560 |
| IUIVLUD02IY56D | - - - - | tr\|W5FGW0\|W5FGW0_WHEAT | gnl\|UG\|Ta#S52543566 |
| IV9RU5F01A430K | - - - - | tr\|W5FMJ4\|W5FMJ4_WHEAT | gnl\|UG\|Ta#S52543616 |
| IUIVLUD01CJ5SL | - - - - | tr\|W5FMA6\|W5FMA6_WHEAT | gnl\|UG\|Ta#S52543846 |
| IV9RU5F02JESIO | - - - - | tr\|W5FMR2\|W5FMR2_WHEAT | gnl\|UG\|Ta#S52543897 |
| IUIVLUD02JQ6OM | - - - - | tr\|W5GLW9\|W5GLW9_WHEAT | gnl\|UG\|Ta#S52544548 |
| IV9RU5F01EJXCX | - - - - | tr\|M8APG1\|M8APG1_TRIUA | gnl\|UG\|Ta#S52544548 |
| IUIVLUD01EAQBI | - - - - | tr\|W5DSI8\|W5DSI8_WHEAT | gnl\|UG\|Ta#S52544843 |
| IV9RU5F02GDGL5 | - - - - | tr\|W5F2U2\|W5F2U2_WHEAT | gnl\|UG\|Ta#S52544885 |
| IV9RU5F01CVKJ4 | - - - - | tr\|M8B1S8\|M8B1S8_TRIUA | gnl\|UG\|Ta#S52545072 |
| IVLNWEC01ET3C8 | - - - - | tr\|W5EQK8\|W5EQK8_WHEAT | gnl\|UG\|Ta#S52545515 |
| IUIVLUD01EB2I6 | - - - - | tr\|W5FA91\|W5FA91_WHEAT | gnl\|UG\|Ta#S52545646 |
| IVLNWEC01EX6GL | - - - - | tr\|W5FD11\|W5FD11_WHEAT | gnl\|UG\|Ta#S52546007 |
| IUIVLUD01C4082 | - - - - | tr\|W5FXJ9\|W5FXJ9_WHEAT | gnl\|UG\|Ta#S52546065 |
| IV9RU5F02JIO4H | - - - - | tr\|W5F7U3\|W5F7U3_WHEAT | gnl\|UG\|Ta#S52546065 |
| IVLNWEC02FLVCD | - - - - | tr\|W5FCA3\|W5FCA3_WHEAT | gnl\|UG\|Ta#S52546513 |
| IVLNWEC02H4GRG | - - - - | tr\|W5FP82\|W5FP82_WHEAT | gnl\|UG\|Ta#S52547045 |
| IV9RU5F01ASM67 | - - - - | tr\|W5FEN4\|W5FEN4_WHEAT | gnl\|UG\|Ta#S58849420 |
| IVLNWEC01CVUFC | - - - - | tr\|W5FEN4\|W5FEN4_WHEAT | gnl\|UG\|Ta#S58849420 |
| IVLNWEC02F2EBY | - - - - | tr\|W5FAJ7\|W5FAJ7_WHEAT | gnl\|UG\|Ta#S58867212 |
| IVLNWEC02F9TTH | - - - - | tr\|W5FAJ7\|W5FAJ7_WHEAT | gnl\|UG\|Ta#S58867212 |
| IVLNWEC02GSSRV | - - - - | tr\|W5FAJ7\|W5FAJ7_WHEAT | gnl\|UG\|Ta#S58867212 |
| IUIVLUD01BRA0E | - - - - | tr\|W5DPP3\|W5DPP3_WHEAT | gnl\|UG\|Ta#S58869837 |
| IV9RU5F02JPDKW | - - - - | tr\|W5FDT1\|W5FDT1_WHEAT | gnl\|UG\|Ta#S58890062 |
| IV9RU5F01BY6IZ | - - - - | tr\|W5FL26\|W5FL26_WHEAT | gnl\|UG\|Ta#S58894753 |
| IUIVLUD01EJVOX | - - - - | tr\|W5FLX6\|W5FLX6_WHEAT | gnl\|UG\|Ta#S58910045 |

| | | | |
|---|---|---|---|
| IVLNWEC01CFRAS | - - - - | tr\|W5GNH7\|W5GNH7_WHEAT | gnl\|UG\|Ta#S58911197 |
| IV9RU5F02FW0WI | - - - - | tr\|W5AFN3\|W5AFN3_WHEAT | gnl\|UG\|Ta#S61516800 |
| IVLNWEC01CJIO0 | - - - - | tr\|M7Z1W7\|M7Z1W7_TRIUA | gnl\|UG\|Ta#S61517413 |
| IUIVLUD01AVI9I | - - - - | tr\|T1LGV4\|T1LGV4_TRIUA | gnl\|UG\|Ta#S61523950 |
| IVLNWEC01AR5F7 | - - - - | tr\|T1LGV4\|T1LGV4_TRIUA | gnl\|UG\|Ta#S61523950 |
| IUIVLUD02G7YZJ | - - - - | tr\|W5FP99\|W5FP99_WHEAT | gnl\|UG\|Ta#S61527490 |
| IV9RU5F01CSB4M | - - - - | tr\|W5FP99\|W5FP99_WHEAT | gnl\|UG\|Ta#S61527490 |
| IV9RU5F02HF80U | - - - - | tr\|W5FVH1\|W5FVH1_WHEAT | gnl\|UG\|Ta#S61529185 |
| IVLNWEC02JAPJR | - - - - | tr\|W5FMI1\|W5FMI1_WHEAT | gnl\|UG\|Ta#S61543175 |
| IV9RU5F02J0LN4 | - - - - | tr\|W5FL76\|W5FL76_WHEAT | gnl\|UG\|Ta#S61548004 |
| IV9RU5F02HTKEV | - - - - | tr\|M8AJL3\|M8AJL3_TRIUA | gnl\|UG\|Ta#S61561507 |
| IUIVLUD01COT02 | - - - - | tr\|W5DW44\|W5DW44_WHEAT | gnl\|UG\|Ta#S61562186 |
| IVLNWEC02H73CB | - - - - | tr\|W5ABY4\|W5ABY4_WHEAT | gnl\|UG\|Ta#S61562955 |
| IUIVLUD02IIRU1 | - - - - | tr\|W5FH40\|W5FH40_WHEAT | gnl\|UG\|Ta#S61571703 |
| IUIVLUD02FNHAL | - - - - | tr\|W5FPF0\|W5FPF0_WHEAT | gnl\|UG\|Ta#S61574012 |
| IVLNWEC01D7W2V | - - - - | tr\|W5AXZ8\|W5AXZ8_WHEAT | gnl\|UG\|Ta#S61580410 |
| IVLNWEC01BBCA6 | - - - - | tr\|W5FHP8\|W5FHP8_WHEAT | gnl\|UG\|Ta#S61584946 |
| IUIVLUD02FUNS0 | - - - - | tr\|W5FL30\|W5FL30_WHEAT | gnl\|UG\|Ta#S61586334 |
| IUIVLUD01BQP05 | - - - - | tr\|M7ZM62\|M7ZM62_TRIUA | gnl\|UG\|Ta#S61587421 |
| IVLNWEC02I8RS4 | - - - - | tr\|M8B3P4\|M8B3P4_TRIUA | gnl\|UG\|Ta#S61590135 |
| IV9RU5F01BIIZE | - - - - | tr\|W5EVX6\|W5EVX6_WHEAT | gnl\|UG\|Ta#S61590842 |
| IV9RU5F01B3EQS | - - - - | tr\|W5FIV1\|W5FIV1_WHEAT | gnl\|UG\|Ta#S61596319 |
| IV9RU5F01CJNCJ | - - - - | tr\|W5FA98\|W5FA98_WHEAT | gnl\|UG\|Ta#S61596468 |
| IUIVLUD02JXF8O | - - - - | tr\|M7ZHP6\|M7ZHP6_TRIUA | gnl\|UG\|Ta#S61598188 |
| IV9RU5F02F1XF1 | - - - - | tr\|W5FFN8\|W5FFN8_WHEAT | gnl\|UG\|Ta#S61599083 |
| IV9RU5F02GSNBU | - - - - | tr\|W5FCW3\|W5FCW3_WHEAT | gnl\|UG\|Ta#S61604819 |
| IV9RU5F02IHXI2 | - - - - | tr\|W5A3G8\|W5A3G8_WHEAT | gnl\|UG\|Ta#S61615491 |
| IVLNWEC01A0FCR | - - - - | tr\|W5FJW3\|W5FJW3_WHEAT | gnl\|UG\|Ta#S61617394 |
| IVLNWEC01DQGIW | - - - - | tr\|W5FJW3\|W5FJW3_WHEAT | gnl\|UG\|Ta#S61617394 |
| IVLNWEC01ENF16 | - - - - | tr\|W5FCJ0\|W5FCJ0_WHEAT | gnl\|UG\|Ta#S61620966 |
| IUIVLUD01CREZ9 | - - - - | tr\|M8A7X8\|M8A7X8_TRIUA | gnl\|UG\|Ta#S61626639 |
| IVLNWEC01EH1CR | - - - - | tr\|M7Z7E7\|M7Z7E7_TRIUA | gnl\|UG\|Ta#S61629986 |
| IV9RU5F01A0X2S | - - - - | tr\|W5HFT1\|W5HFT1_WHEAT | gnl\|UG\|Ta#S61637542 |
| IVLNWEC02HHGLZ | - - - - | tr\|M7ZBZ4\|M7ZBZ4_TRIUA | gnl\|UG\|Ta#S61641004 |
| IV9RU5F01B5KC7 | - - - - | tr\|W5FBN4\|W5FBN4_WHEAT | gnl\|UG\|Ta#S61654356 |
| IV9RU5F01D41F7 | - - - - | tr\|W5FHC4\|W5FHC4_WHEAT | gnl\|UG\|Ta#S61658911 |
| IV9RU5F02H7R5E | - - - - | tr\|W5FJE2\|W5FJE2_WHEAT | gnl\|UG\|Ta#S61668167 |
| IUIVLUD02GEN8F | - - - - | tr\|W5FK55\|W5FK55_WHEAT | gnl\|UG\|Ta#S61677225 |
| IV9RU5F01B1T1G | - - - - | tr\|M7Z1M8\|M7Z1M8_TRIUA | gnl\|UG\|Ta#S61679002 |
| IV9RU5F02H56QQ | - - - - | tr\|M7ZN22\|M7ZN22_TRIUA | gnl\|UG\|Ta#S61688930 |
| IV9RU5F02IA5H5 | - - - - | tr\|M7ZN22\|M7ZN22_TRIUA | gnl\|UG\|Ta#S61688930 |
| IUIVLUD02I99L5 | - - - - | tr\|W5FP17\|W5FP17_WHEAT | gnl\|UG\|Ta#S61692560 |
| IV9RU5F02HMG1A | - - - - | tr\|W5FP17\|W5FP17_WHEAT | gnl\|UG\|Ta#S61692560 |
| IVLNWEC02GCMLK | - - - - | tr\|W5FP17\|W5FP17_WHEAT | gnl\|UG\|Ta#S61692560 |
| IVLNWEC02GPZCG | - - - - | tr\|W5GHT3\|W5GHT3_WHEAT | gnl\|UG\|Ta#S61695205 |
| IV9RU5F02JO7FX | - - - - | tr\|W5DU74\|W5DU74_WHEAT | gnl\|UG\|Ta#S61698687 |
| IVLNWEC02HUSMJ | - - - - | tr\|W5DU74\|W5DU74_WHEAT | gnl\|UG\|Ta#S61698687 |
| IVLNWEC01DY19B | - - - - | tr\|W5FCN8\|W5FCN8_WHEAT | gnl\|UG\|Ta#S61702871 |
| IUIVLUD02G0XDJ | - - - - | tr\|W5AA67\|W5AA67_WHEAT | gnl\|UG\|Ta#S61706984 |
| IVLNWEC02GJ051 | - - - - | tr\|W5FHC4\|W5FHC4_WHEAT | gnl\|UG\|Ta#S61706998 |

| | | | |
|---|---|---|---|
| IV9RU5F01AE2AK | - - - - | tr\|W5FB25\|W5FB25_WHEAT | gnl\|UG\|Ta#S61731436 |
| IVLNWEC02HH0M8 | - - - - | tr\|W5FB25\|W5FB25_WHEAT | gnl\|UG\|Ta#S61731436 |
| IV9RU5F01D7GWN | - - - - | tr\|W5FPF8\|W5FPF8_WHEAT | gnl\|UG\|Ta#S61733447 |
| IV9RU5F01DSD4J | - - - - | tr\|M8A748\|M8A748_TRIUA | gnl\|UG\|Ta#S61735705 |
| IVLNWEC01EBSHW | - - - - | tr\|W5FLT0\|W5FLT0_WHEAT | gnl\|UG\|Ta#S61737376 |
| IV9RU5F01D9FT5 | - - - - | tr\|T1MM15\|T1MM15_TRIUA | gnl\|UG\|Ta#S61741123 |
| IVLNWEC02HP9V8 | - - - - | tr\|T1MM15\|T1MM15_TRIUA | gnl\|UG\|Ta#S61741123 |
| IV9RU5F01ALGAJ | - - - - | tr\|M8B574\|M8B574_TRIUA | gnl\|UG\|Ta#S61741924 |
| IVLNWEC01BSKNQ | - - - - | tr\|W5FIR3\|W5FIR3_WHEAT | gnl\|UG\|Ta#S61744003 |
| IVLNWEC02HVF7I | - - - - | tr\|W5FBN5\|W5FBN5_WHEAT | gnl\|UG\|Ta#S61749856 |
| IVLNWEC02IDOZO | - - - - | tr\|W5FDY0\|W5FDY0_WHEAT | gnl\|UG\|Ta#S61756089 |
| IV9RU5F01AFPQA | - - - - | tr\|W5ABN6\|W5ABN6_WHEAT | gnl\|UG\|Ta#S61759141 |
| IVLNWEC02I45TI | - - - - | tr\|W5FA58\|W5FA58_WHEAT | gnl\|UG\|Ta#S61770339 |
| IV9RU5F01DXDPD | - - - - | tr\|M8A0M4\|M8A0M4_TRIUA | gnl\|UG\|Ta#S61771589 |
| IV9RU5F01DQ0NF | - - - - | tr\|W5FJY9\|W5FJY9_WHEAT | gnl\|UG\|Ta#S61771869 |
| IV9RU5F01BYF1V | - - - - | tr\|W5F842\|W5F842_WHEAT | gnl\|UG\|Ta#S61778646 |
| IV9RU5F01BJ4XU | - - - - | tr\|W5AXZ8\|W5AXZ8_WHEAT | gnl\|UG\|Ta#S61778880 |
| IV9RU5F02G08XR | - - - - | tr\|W5AXZ8\|W5AXZ8_WHEAT | gnl\|UG\|Ta#S61778880 |
| IVLNWEC01EXWJ9 | - - - - | tr\|W5AXZ8\|W5AXZ8_WHEAT | gnl\|UG\|Ta#S61778880 |
| IVLNWEC02GEG04 | - - - - | tr\|W5AXZ8\|W5AXZ8_WHEAT | gnl\|UG\|Ta#S61778880 |
| IV9RU5F02GBPZB | - - - - | tr\|W5FK17\|W5FK17_WHEAT | gnl\|UG\|Ta#S61780998 |
| IVLNWEC01CHR40 | - - - - | tr\|W5FK17\|W5FK17_WHEAT | gnl\|UG\|Ta#S61780998 |
| IVLNWEC01BWXZD | - - - - | tr\|M8A0W8\|M8A0W8_TRIUA | gnl\|UG\|Ta#S61781770 |
| IV9RU5F01EQ42N | - - - - | tr\|M8A8A5\|M8A8A5_TRIUA | gnl\|UG\|Ta#S61781966 |
| IV9RU5F01CNPTS | - - - - | tr\|W5F6H0\|W5F6H0_WHEAT | gnl\|UG\|Ta#S61782350 |
| IV9RU5F02H9KSY | - - - - | tr\|W5FBN5\|W5FBN5_WHEAT | gnl\|UG\|Ta#S61784647 |
| IV9RU5F02HH059 | - - - - | tr\|W5FBN5\|W5FBN5_WHEAT | gnl\|UG\|Ta#S61784647 |
| IV9RU5F02H5CFX | - - - - | tr\|W5AXZ8\|W5AXZ8_WHEAT | gnl\|UG\|Ta#S61787776 |
| IV9RU5F01AYRY2 | - - - - | tr\|W5FDL9\|W5FDL9_WHEAT | gnl\|UG\|Ta#S61791554 |
| IV9RU5F02HCXWX | - - - - | tr\|W5FDL9\|W5FDL9_WHEAT | gnl\|UG\|Ta#S61791554 |
| IVLNWEC01CSLLC | - - - - | tr\|W5FDL9\|W5FDL9_WHEAT | gnl\|UG\|Ta#S61791554 |
| IV9RU5F02JM0XS | - - - - | tr\|W5FI08\|W5FI08_WHEAT | gnl\|UG\|Ta#S61791639 |
| IVLNWEC01BHH98 | - - - - | tr\|W5FGI8\|W5FGI8_WHEAT | gnl\|UG\|Ta#S61791678 |
| IV9RU5F01AUA9O | - - - - | tr\|W5FIK1\|W5FIK1_WHEAT | gnl\|UG\|Ta#S61791780 |
| IVLNWEC01BT7X4 | - - - - | tr\|W5FIK1\|W5FIK1_WHEAT | gnl\|UG\|Ta#S61791780 |
| IUIVLUD02GDHFE | - - - - | tr\|W5F889\|W5F889_WHEAT | gnl\|UG\|Ta#S61794409 |
| IV9RU5F02GZHKI | - - - - | tr\|W5G072\|W5G072_WHEAT | gnl\|UG\|Ta#S61797061 |
| IVLNWEC01EEUYM | - - - - | tr\|W5FES7\|W5FES7_WHEAT | gnl\|UG\|Ta#S61798170 |
| IVLNWEC02JA474 | - - - - | tr\|W5FES7\|W5FES7_WHEAT | gnl\|UG\|Ta#S61798170 |
| IUIVLUD01EFYG7 | - - - - | tr\|M7Z1X0\|M7Z1X0_TRIUA | gnl\|UG\|Ta#S61799069 |
| IVLNWEC02ITPQQ | - - - - | tr\|M7Z1X0\|M7Z1X0_TRIUA | gnl\|UG\|Ta#S61799069 |
| IUIVLUD01BVRYW | - - - - | tr\|W5DPY0\|W5DPY0_WHEAT | gnl\|UG\|Ta#S61800168 |
| IV9RU5F02GWIMK | - - - - | tr\|M7ZFX1\|M7ZFX1_TRIUA | gnl\|UG\|Ta#S61800556 |
| IUIVLUD01DLT25 | - - - - | tr\|M8AL97\|M8AL97_TRIUA | gnl\|UG\|Ta#S61800870 |
| IV9RU5F02FTR20 | - - - - | tr\|W5GID1\|W5GID1_WHEAT | gnl\|UG\|Ta#S61801964 |
| IV9RU5F02HH378 | - - - - | tr\|W5GID1\|W5GID1_WHEAT | gnl\|UG\|Ta#S61801964 |
| IVLNWEC02F67YX | - - - - | tr\|W5DUG6\|W5DUG6_WHEAT | gnl\|UG\|Ta#S61808212 |
| IV9RU5F01DMPQ2 | - - - - | tr\|W5FNG1\|W5FNG1_WHEAT | gnl\|UG\|Ta#S61808263 |
| IVLNWEC01EF00M | - - - - | tr\|W5FNG1\|W5FNG1_WHEAT | gnl\|UG\|Ta#S61808263 |
| IV9RU5F01CUBR7 | - - - - | tr\|W5FKY9\|W5FKY9_WHEAT | gnl\|UG\|Ta#S61808686 |

| | | | |
|---|---|---|---|
| IV9RU5F02JR01R | - - - - | tr\|W5FKY9\|W5FKY9_WHEAT | gnl\|UG\|Ta#S61808686 |
| IVLNWEC01CI5H0 | - - - - | tr\|W5FKY9\|W5FKY9_WHEAT | gnl\|UG\|Ta#S61808686 |
| IVLNWEC01CIZXQ | - - - - | tr\|M8B3F4\|M8B3F4_TRIUA | gnl\|UG\|Ta#S61808780 |
| IUIVLUD01AT23P | - - - - | tr\|W5FC73\|W5FC73_WHEAT | gnl\|UG\|Ta#S61809603 |
| IUIVLUD02G9B1W | - - - - | tr\|W5FC73\|W5FC73_WHEAT | gnl\|UG\|Ta#S61809603 |
| IV9RU5F01BGJLR | - - - - | tr\|W5FC73\|W5FC73_WHEAT | gnl\|UG\|Ta#S61809603 |
| IV9RU5F02HVYZS | - - - - | tr\|W5FC73\|W5FC73_WHEAT | gnl\|UG\|Ta#S61809603 |
| IUIVLUD01BEII0 | - - - - | tr\|M7Z5P2\|M7Z5P2_TRIUA | gnl\|UG\|Ta#S61810040 |
| IV9RU5F02F7WCW | - - - - | tr\|W5F444\|W5F444_WHEAT | gnl\|UG\|Ta#S61810381 |
| IUIVLUD02JV4CI | - - - - | tr\|W5FTG6\|W5FTG6_WHEAT | gnl\|UG\|Ta#S61810463 |
| IVLNWEC01E5ZC3 | - - - - | tr\|W5E0N0\|W5E0N0_WHEAT | gnl\|UG\|Ta#S61810883 |
| IUIVLUD01A13CV | - - - - | tr\|W5FCN4\|W5FCN4_WHEAT | gnl\|UG\|Ta#S61811546 |
| IVLNWEC01DH8AD | - - - - | tr\|W5FPZ3\|W5FPZ3_WHEAT | gnl\|UG\|Ta#S61811660 |
| IV9RU5F02IVLQE | - - - - | tr\|W5FNF7\|W5FNF7_WHEAT | gnl\|UG\|Ta#S61811866 |
| IUIVLUD01CWZFY | - - - - | tr\|W5FA17\|W5FA17_WHEAT | gnl\|UG\|Ta#S61812269 |
| IUIVLUD01DFP4J | - - - - | tr\|W5FA17\|W5FA17_WHEAT | gnl\|UG\|Ta#S61812269 |
| IVLNWEC02GS95W | - - - - | tr\|W5FA17\|W5FA17_WHEAT | gnl\|UG\|Ta#S61812269 |
| IV9RU5F02F8BNS | - - - - | tr\|W5DQV7\|W5DQV7_WHEAT | gnl\|UG\|Ta#S61812473 |
| IV9RU5F02HYWEP | - - - - | tr\|W5DL32\|W5DL32_WHEAT | gnl\|UG\|Ta#S61827824 |
| IV9RU5F01DIXAO | - - - - | tr\|W5FG11\|W5FG11_WHEAT | gnl\|UG\|Ta#S61827891 |
| IUIVLUD01D0PFB | - - - - | tr\|W5F8V9\|W5F8V9_WHEAT | gnl\|UG\|Ta#S61828328 |
| IV9RU5F02IOQKL | - - - - | tr\|W5FV98\|W5FV98_WHEAT | gnl\|UG\|Ta#S61828405 |
| IV9RU5F01BSR4G | - - - - | tr\|W5FP98\|W5FP98_WHEAT | gnl\|UG\|Ta#S61829049 |
| IVLNWEC01A7I9I | - - - - | tr\|T1N0B3\|T1N0B3_TRIUA | gnl\|UG\|Ta#S61829049 |
| IVLNWEC02HCWTB | - - - - | tr\|T1N0B3\|T1N0B3_TRIUA | gnl\|UG\|Ta#S61829049 |
| IUIVLUD01CPXL2 | - - - - | tr\|W5FEN3\|W5FEN3_WHEAT | gnl\|UG\|Ta#S61829091 |
| IVLNWEC02GCTA3 | - - - - | tr\|W5FEN3\|W5FEN3_WHEAT | gnl\|UG\|Ta#S61829091 |
| IVLNWEC02GU0PL | - - - - | tr\|T1LM33\|T1LM33_TRIUA | gnl\|UG\|Ta#S61829407 |
| IVLNWEC01CYNE5 | - - - - | tr\|W5FG67\|W5FG67_WHEAT | gnl\|UG\|Ta#S61829705 |
| IVLNWEC02GXF2E | - - - - | tr\|W5FG67\|W5FG67_WHEAT | gnl\|UG\|Ta#S61829705 |
| IUIVLUD02G6OEY | - - - - | tr\|M7YLW1\|M7YLW1_TRIUA | gnl\|UG\|Ta#S61829803 |
| IV9RU5F02JCHWH | - - - - | tr\|W5DSA0\|W5DSA0_WHEAT | gnl\|UG\|Ta#S61829858 |
| IV9RU5F02GAH5Z | - - - - | tr\|M7Z3R5\|M7Z3R5_TRIUA | gnl\|UG\|Ta#S61829858 |
| IVLNWEC02IK0N0 | - - - - | tr\|W5F8P4\|W5F8P4_WHEAT | gnl\|UG\|Ta#S61830076 |
| IVLNWEC01DJ5Q0 | - - - - | tr\|W5FDS0\|W5FDS0_WHEAT | gnl\|UG\|Ta#S61830217 |
| IVLNWEC02HO7J7 | - - - - | tr\|W5FDS0\|W5FDS0_WHEAT | gnl\|UG\|Ta#S61830217 |
| IVLNWEC02JVU4Y | - - - - | tr\|W5FDS0\|W5FDS0_WHEAT | gnl\|UG\|Ta#S61830217 |
| IVLNWEC02IQJ28 | - - - - | tr\|M8A5L2\|M8A5L2_TRIUA | gnl\|UG\|Ta#S61830765 |
| IV9RU5F02GDG8O | - - - - | tr\|W5FHN0\|W5FHN0_WHEAT | gnl\|UG\|Ta#S61830867 |
| IVLNWEC02IVU6T | - - - - | tr\|W5FHN0\|W5FHN0_WHEAT | gnl\|UG\|Ta#S61830867 |
| IV9RU5F01EXRCT | - - - - | tr\|W5DSW0\|W5DSW0_WHEAT | gnl\|UG\|Ta#S61830879 |
| IV9RU5F02I2MRK | - - - - | tr\|W5DSW0\|W5DSW0_WHEAT | gnl\|UG\|Ta#S61830879 |
| IV9RU5F02G8NBC | - - - - | tr\|W5FBV6\|W5FBV6_WHEAT | gnl\|UG\|Ta#S61830996 |
| IV9RU5F01C2D8C | - - - - | tr\|M7YRM6\|M7YRM6_TRIUA | gnl\|UG\|Ta#S61831107 |
| IV9RU5F01CMI70 | - - - - | tr\|W5A0W6\|W5A0W6_WHEAT | gnl\|UG\|Ta#S61831184 |
| IV9RU5F01APEHP | - - - - | tr\|W5FLB3\|W5FLB3_WHEAT | gnl\|UG\|Ta#S61831397 |
| IV9RU5F02HTHM5 | - - - - | tr\|W5FPN5\|W5FPN5_WHEAT | gnl\|UG\|Ta#S61831483 |
| IV9RU5F02HHV04 | - - - - | tr\|W5GHD5\|W5GHD5_WHEAT | gnl\|UG\|Ta#S61831533 |
| IVLNWEC01DDOB9 | - - - - | tr\|W5FPV1\|W5FPV1_WHEAT | gnl\|UG\|Ta#S61831651 |
| IV9RU5F02G2P01 | - - - - | tr\|W5FCU2\|W5FCU2_WHEAT | gnl\|UG\|Ta#S61831651 |

| | | | |
|---|---|---|---|
| IVLNWEC02GTVBC | - - - - | tr\|W5FJF8\|W5FJF8_WHEAT | gnl\|UG\|Ta#S61831770 |
| IUIVLUD02G5F4H | - - - - | tr\|M7YWE3\|M7YWE3_TRIUA | gnl\|UG\|Ta#S61831852 |
| IUIVLUD02ICKXE | - - - - | tr\|M7YWE3\|M7YWE3_TRIUA | gnl\|UG\|Ta#S61831852 |
| IVLNWEC01B5HFF | - - - - | tr\|M7YWE3\|M7YWE3_TRIUA | gnl\|UG\|Ta#S61831852 |
| IUIVLUD01AKERN | - - - - | tr\|M8A3Q6\|M8A3Q6_TRIUA | gnl\|UG\|Ta#S61831856 |
| IV9RU5F02G3MFU | - - - - | tr\|M8A3Q6\|M8A3Q6_TRIUA | gnl\|UG\|Ta#S61831856 |
| IV9RU5F02HUM1M | - - - - | tr\|W5FBL0\|W5FBL0_WHEAT | gnl\|UG\|Ta#S61832122 |
| IV9RU5F02I8PO3 | - - - - | tr\|W5BUI2\|W5BUI2_WHEAT | gnl\|UG\|Ta#S61832213 |
| IUIVLUD01B19GQ | - - - - | tr\|W5AXZ8\|W5AXZ8_WHEAT | gnl\|UG\|Ta#S61832213 |
| IV9RU5F01BBMNZ | - - - - | tr\|W5AXZ8\|W5AXZ8_WHEAT | gnl\|UG\|Ta#S61832213 |
| IUIVLUD02IM2UB | - - - - | tr\|W5FC49\|W5FC49_WHEAT | gnl\|UG\|Ta#S61832375 |
| IUIVLUD02JNB7P | - - - - | tr\|W5FC49\|W5FC49_WHEAT | gnl\|UG\|Ta#S61832375 |
| IV9RU5F02FMAXA | - - - - | tr\|W5FC49\|W5FC49_WHEAT | gnl\|UG\|Ta#S61832375 |
| IVLNWEC01DK819 | - - - - | tr\|W5FC49\|W5FC49_WHEAT | gnl\|UG\|Ta#S61832375 |
| IUIVLUD01DS7QS | - - - - | tr\|M7Z082\|M7Z082_TRIUA | gnl\|UG\|Ta#S61832404 |
| IV9RU5F02F67HF | - - - - | tr\|M7YVX6\|M7YVX6_TRIUA | gnl\|UG\|Ta#S61832521 |
| IUIVLUD02HLY1H | - - - - | tr\|W5DXT7\|W5DXT7_WHEAT | gnl\|UG\|Ta#S61832541 |
| IV9RU5F02G2AU3 | - - - - | tr\|W5FB95\|W5FB95_WHEAT | gnl\|UG\|Ta#S61858914 |
| IV9RU5F02JK7PV | - - - - | tr\|W5FB95\|W5FB95_WHEAT | gnl\|UG\|Ta#S61858914 |
| IUIVLUD01EFQ0R | - - - - | tr\|W5FJR5\|W5FJR5_WHEAT | gnl\|UG\|Ta#S61861606 |
| IUIVLUD01DC9CL | - - - - | tr\|W5FKX8\|W5FKX8_WHEAT | gnl\|UG\|Ta#S65594474 |
| IVLNWEC02ITT6Y | - - - - | tr\|W5FNY3\|W5FNY3_WHEAT | gnl\|UG\|Ta#S65596334 |
| IV9RU5F01EYW42 | - - - - | tr\|W5FJF8\|W5FJF8_WHEAT | gnl\|UG\|Ta#S65608944 |
| IV9RU5F01BA7UR | - - - - | tr\|M8A2N0\|M8A2N0_TRIUA | gnl\|UG\|Ta#S65609345 |
| IVLNWEC01A1XHV | - - - - | tr\|W5FCU1\|W5FCU1_WHEAT | gnl\|UG\|Ta#S65612613 |
| IV9RU5F02HHZB1 | - - - - | tr\|W5FC36\|W5FC36_WHEAT | gnl\|UG\|Ta#S65624906 |
| IV9RU5F02IEUMM | - - - - | tr\|W5FPD7\|W5FPD7_WHEAT | gnl\|UG\|Ta#S65628416 |
| IUIVLUD01D5Y8U | - - - - | tr\|W5FN93\|W5FN93_WHEAT | gnl\|UG\|Ta#S65632646 |
| IVLNWEC02II5H4 | - - - - | tr\|W5FN93\|W5FN93_WHEAT | gnl\|UG\|Ta#S65632646 |
| IV9RU5F01CPHG9 | - - - - | tr\|W5F925\|W5F925_WHEAT | gnl\|UG\|Ta#S65634736 |
| IVLNWEC01DEYV2 | - - - - | tr\|W5F925\|W5F925_WHEAT | gnl\|UG\|Ta#S65634736 |
| IVLNWEC01EYICX | - - - - | tr\|W5FJQ8\|W5FJQ8_WHEAT | gnl\|UG\|Ta#S65645505 |
| IVLNWEC01DBEYU | - - - - | tr\|W5FN33\|W5FN33_WHEAT | gnl\|UG\|Ta#S65648649 |
| IV9RU5F01CVD1H | - - - - | tr\|W5FCU1\|W5FCU1_WHEAT | gnl\|UG\|Ta#S65652253 |
| IV9RU5F01A8MNO | - - - - | tr\|W5F8E5\|W5F8E5_WHEAT | gnl\|UG\|Ta#S65654883 |
| IVLNWEC02HCQYG | - - - - | tr\|W5DQW7\|W5DQW7_WHEAT | gnl\|UG\|Ta#S65657897 |
| IV9RU5F02GY7FU | - - - - | tr\|W5FLG5\|W5FLG5_WHEAT | gnl\|UG\|Ta#S65658707 |
| IV9RU5F02I0RHO | - - - - | tr\|W5FLG5\|W5FLG5_WHEAT | gnl\|UG\|Ta#S65658707 |
| IV9RU5F02H0R6C | - - - - | tr\|W5FH67\|W5FH67_WHEAT | gnl\|UG\|Ta#S65658717 |
| IV9RU5F02HTXTV | - - - - | tr\|W5FH67\|W5FH67_WHEAT | gnl\|UG\|Ta#S65658717 |
| IV9RU5F02JJ8JK | - - - - | tr\|W5FH67\|W5FH67_WHEAT | gnl\|UG\|Ta#S65658717 |
| IUIVLUD02HT5KJ | - - - - | tr\|M7ZD83\|M7ZD83_TRIUA | gnl\|UG\|Ta#S65660946 |
| IVLNWEC01C12WB | - - - - | tr\|M8A6U9\|M8A6U9_TRIUA | gnl\|UG\|Ta#S65678892 |
| IV9RU5F01ALBK9 | - - - - | tr\|W5FPJ3\|W5FPJ3_WHEAT | gnl\|UG\|Ta#S65685260 |
| IV9RU5F02IY35Q | - - - - | tr\|W5FFQ9\|W5FFQ9_WHEAT | gnl\|UG\|Ta#S65697722 |
| IUIVLUD01BHGG6 | - - - - | tr\|W5F6Y8\|W5F6Y8_WHEAT | gnl\|UG\|Ta#S65698248 |
| IV9RU5F01CR5N9 | - - - - | tr\|M7YLK0\|M7YLK0_TRIUA | gnl\|UG\|Ta#S65702597 |
| IV9RU5F02J3FN4 | - - - - | tr\|M8A6G1\|M8A6G1_TRIUA | gnl\|UG\|Ta#S65722581 |
| IV9RU5F02ISLBJ | - - - - - | | gnl\|UG\|Ta#S12877179 |
| IVLNWEC02F9GYE | - - - - - | | gnl\|UG\|Ta#S12877179 |

| | | |
|---|---|---|
| IVLNWEC02I38FE | - - - - - | gnl\|UG\|Ta#S12877179 |
| IV9RU5F01C0ZTZ | - - - - - | gnl\|UG\|Ta#S12881932 |
| IVLNWEC01DMBRH | - - - - - | gnl\|UG\|Ta#S12886824 |
| IVLNWEC01EHGYK | - - - - - | gnl\|UG\|Ta#S12886824 |
| IVLNWEC01B64JE | - - - - - | gnl\|UG\|Ta#S12887722 |
| IVLNWEC02GV5TM | - - - - - | gnl\|UG\|Ta#S12887722 |
| IVLNWEC02JB62J | - - - - - | gnl\|UG\|Ta#S12887722 |
| IUIVLUD01B7QPU | - - - - - | gnl\|UG\|Ta#S12895341 |
| IV9RU5F02GH9PZ | - - - - - | gnl\|UG\|Ta#S12895341 |
| IV9RU5F01DEFJX | - - - - - | gnl\|UG\|Ta#S12895411 |
| IV9RU5F02HD876 | - - - - - | gnl\|UG\|Ta#S12895411 |
| IVLNWEC02HUEQU | - - - - - | gnl\|UG\|Ta#S12902190 |
| IV9RU5F02GA6B4 | - - - - - | gnl\|UG\|Ta#S12917219 |
| IVLNWEC01CWCAD | - - - - - | gnl\|UG\|Ta#S12917219 |
| IVLNWEC02FUOIY | - - - - - | gnl\|UG\|Ta#S12918303 |
| IVLNWEC02FVMJY | - - - - - | gnl\|UG\|Ta#S12918303 |
| IV9RU5F01BRITU | - - - - - | gnl\|UG\|Ta#S12922596 |
| IVLNWEC02HCPJ7 | - - - - - | gnl\|UG\|Ta#S12922596 |
| IV9RU5F01B1CRZ | - - - - - | gnl\|UG\|Ta#S12922878 |
| IV9RU5F01B9UFL | - - - - - | gnl\|UG\|Ta#S12922878 |
| IV9RU5F02JMOX3 | - - - - - | gnl\|UG\|Ta#S12922878 |
| IVLNWEC01EPEWK | - - - - - | gnl\|UG\|Ta#S12922878 |
| IV9RU5F01EEUJM | - - - - - | gnl\|UG\|Ta#S12922917 |
| IV9RU5F02G263A | - - - - - | gnl\|UG\|Ta#S12922917 |
| IUIVLUD01CJK4G | - - - - - | gnl\|UG\|Ta#S12923506 |
| IVLNWEC02G0UCX | - - - - - | gnl\|UG\|Ta#S12923506 |
| IUIVLUD01DEI5A | - - - - - | gnl\|UG\|Ta#S12932393 |
| IV9RU5F02ICQ0V | - - - - - | gnl\|UG\|Ta#S12932393 |
| IV9RU5F01C4HJZ | - - - - - | gnl\|UG\|Ta#S12936810 |
| IV9RU5F01C7KOC | - - - - - | gnl\|UG\|Ta#S12936810 |
| IV9RU5F01COF8P | - - - - - | gnl\|UG\|Ta#S12936810 |
| IVLNWEC01CC62H | - - - - - | gnl\|UG\|Ta#S12936810 |
| IV9RU5F01D2PHJ | - - - - - | gnl\|UG\|Ta#S12936833 |
| IVLNWEC02F0OZG | - - - - - | gnl\|UG\|Ta#S12936833 |
| IUIVLUD01ECB2Q | - - - - - | gnl\|UG\|Ta#S12940550 |
| IV9RU5F01A23Q8 | - - - - - | gnl\|UG\|Ta#S12940550 |
| IUIVLUD02HYGJ5 | - - - - - | gnl\|UG\|Ta#S12947479 |
| IV9RU5F01EDVG3 | - - - - - | gnl\|UG\|Ta#S12947479 |
| IVLNWEC02J4NJH | - - - - - | gnl\|UG\|Ta#S12947479 |
| IVLNWEC02JUA2R | - - - - - | gnl\|UG\|Ta#S12947479 |
| IV9RU5F01DWNCI | - - - - - | gnl\|UG\|Ta#S12955148 |
| IVLNWEC02JA6D5 | - - - - - | gnl\|UG\|Ta#S12955148 |
| IV9RU5F02IS872 | - - - - - | gnl\|UG\|Ta#S12956749 |
| IVLNWEC01CRQK3 | - - - - - | gnl\|UG\|Ta#S12956749 |
| IVLNWEC02G6C5P | - - - - - | gnl\|UG\|Ta#S12956749 |
| IVLNWEC01D1X7U | - - - - - | gnl\|UG\|Ta#S12958442 |
| IVLNWEC01E5I7Q | - - - - - | gnl\|UG\|Ta#S12958442 |
| IUIVLUD01B32R8 | - - - - - | gnl\|UG\|Ta#S12962699 |
| IVLNWEC02HPTVS | - - - - - | gnl\|UG\|Ta#S12962699 |
| IVLNWEC02I66EV | - - - - - | gnl\|UG\|Ta#S12962699 |

| | | |
|---|---|---|
| IV9RU5F02I37CP | - - - - - | gnl\|UG\|Ta#S12976533 |
| IVLNWEC01BL3JR | - - - - - | gnl\|UG\|Ta#S12976533 |
| IVLNWEC02HH1IA | - - - - - | gnl\|UG\|Ta#S12976533 |
| IVLNWEC02IRYQJ | - - - - - | gnl\|UG\|Ta#S12976533 |
| IV9RU5F01CJW4G | - - - - - | gnl\|UG\|Ta#S12978092 |
| IVLNWEC01C9V9F | - - - - - | gnl\|UG\|Ta#S12978092 |
| IVLNWEC02HWTSD | - - - - - | gnl\|UG\|Ta#S12982430 |
| IVLNWEC02IF00Z | - - - - - | gnl\|UG\|Ta#S12982430 |
| IUIVLUD01A9E7U | - - - - - | gnl\|UG\|Ta#S12984322 |
| IV9RU5F02GV9T8 | - - - - - | gnl\|UG\|Ta#S12984322 |
| IUIVLUD02HTCV1 | - - - - - | gnl\|UG\|Ta#S12985063 |
| IVLNWEC01A2ESX | - - - - - | gnl\|UG\|Ta#S12985063 |
| IUIVLUD01DEA60 | - - - - - | gnl\|UG\|Ta#S12989160 |
| IV9RU5F01BTM9S | - - - - - | gnl\|UG\|Ta#S12989160 |
| IV9RU5F02JDLDJ | - - - - - | gnl\|UG\|Ta#S12989160 |
| IVLNWEC01DGYXF | - - - - - | gnl\|UG\|Ta#S12989160 |
| IV9RU5F01CFWA2 | - - - - - | gnl\|UG\|Ta#S12989737 |
| IV9RU5F02FRBA4 | - - - - - | gnl\|UG\|Ta#S12989737 |
| IVLNWEC02J21LG | - - - - - | gnl\|UG\|Ta#S12989737 |
| IV9RU5F01AQDHA | - - - - - | gnl\|UG\|Ta#S13002311 |
| IVLNWEC01EH6FN | - - - - - | gnl\|UG\|Ta#S13002311 |
| IVLNWEC01C4EYD | - - - - - | gnl\|UG\|Ta#S13022323 |
| IVLNWEC02JYAUG | - - - - - | gnl\|UG\|Ta#S13022323 |
| IUIVLUD01CGAR8 | - - - - - | gnl\|UG\|Ta#S13045734 |
| IV9RU5F01A8XNX | - - - - - | gnl\|UG\|Ta#S13045734 |
| IUIVLUD01DCZVB | - - - - - | gnl\|UG\|Ta#S13047059 |
| IVLNWEC01ES98L | - - - - - | gnl\|UG\|Ta#S13047059 |
| IUIVLUD01B9F6F | - - - - - | gnl\|UG\|Ta#S13119141 |
| IUIVLUD02HZRU9 | - - - - - | gnl\|UG\|Ta#S13119141 |
| IV9RU5F02IBWF3 | - - - - - | gnl\|UG\|Ta#S13119141 |
| IV9RU5F01EM4OZ | - - - - - | gnl\|UG\|Ta#S13123626 |
| IV9RU5F02HA6LN | - - - - - | gnl\|UG\|Ta#S13123626 |
| IVLNWEC01C64ZA | - - - - - | gnl\|UG\|Ta#S13123626 |
| IUIVLUD02JLEV9 | - - - - - | gnl\|UG\|Ta#S13136564 |
| IV9RU5F02H80T8 | - - - - - | gnl\|UG\|Ta#S13136564 |
| IVLNWEC02G3WJF | - - - - - | gnl\|UG\|Ta#S13136564 |
| IV9RU5F02G57ZH | - - - - - | gnl\|UG\|Ta#S13202564 |
| IVLNWEC01EQ5HH | - - - - - | gnl\|UG\|Ta#S13202564 |
| IV9RU5F01BEKS4 | - - - - - | gnl\|UG\|Ta#S13212207 |
| IV9RU5F01C6O3A | - - - - - | gnl\|UG\|Ta#S13212207 |
| IUIVLUD02H47XS | - - - - - | gnl\|UG\|Ta#S13234487 |
| IV9RU5F01BQJQ2 | - - - - - | gnl\|UG\|Ta#S13234487 |
| IV9RU5F01AFSX4 | - - - - - | gnl\|UG\|Ta#S13234815 |
| IVLNWEC02JOJ6Y | - - - - - | gnl\|UG\|Ta#S13234815 |
| IVLNWEC01EJYH7 | - - - - - | gnl\|UG\|Ta#S15824131 |
| IVLNWEC02IUO54 | - - - - - | gnl\|UG\|Ta#S15824131 |
| IUIVLUD01DVFTP | - - - - - | gnl\|UG\|Ta#S16057890 |
| IV9RU5F01CWZKH | - - - - - | gnl\|UG\|Ta#S16057890 |
| IUIVLUD02G0D2I | - - - - - | gnl\|UG\|Ta#S16058005 |
| IVLNWEC01CJWIN | - - - - - | gnl\|UG\|Ta#S16058005 |

| | | |
|---|---|---|
| IUIVLUD02J24WS | - - - - - | gnl\|UG\|Ta#S16058457 |
| IV9RU5F01AY43D | - - - - - | gnl\|UG\|Ta#S16058457 |
| IV9RU5F01CZQ4K | - - - - - | gnl\|UG\|Ta#S16058457 |
| IV9RU5F02GTWZX | - - - - - | gnl\|UG\|Ta#S16193357 |
| IVLNWEC02JK0C1 | - - - - - | gnl\|UG\|Ta#S16193357 |
| IV9RU5F01A7NMR | - - - - - | gnl\|UG\|Ta#S16194371 |
| IV9RU5F02I174Z | - - - - - | gnl\|UG\|Ta#S16194371 |
| IVLNWEC01A44IM | - - - - - | gnl\|UG\|Ta#S16202871 |
| IVLNWEC01EPP2K | - - - - - | gnl\|UG\|Ta#S16202871 |
| IUIVLUD01DTBRE | - - - - - | gnl\|UG\|Ta#S16207819 |
| IV9RU5F02FUDPK | - - - - - | gnl\|UG\|Ta#S16207819 |
| IUIVLUD01ATJTK | - - - - - | gnl\|UG\|Ta#S16228689 |
| IV9RU5F02JYYSY | - - - - - | gnl\|UG\|Ta#S16228689 |
| IVLNWEC01C1FED | - - - - - | gnl\|UG\|Ta#S16230571 |
| IVLNWEC01EOSD6 | - - - - - | gnl\|UG\|Ta#S16230571 |
| IV9RU5F01CD2E2 | - - - - - | gnl\|UG\|Ta#S16235973 |
| IV9RU5F02FP5KU | - - - - - | gnl\|UG\|Ta#S16235973 |
| IV9RU5F02GCK3J | - - - - - | gnl\|UG\|Ta#S16235973 |
| IV9RU5F02GMYHI | - - - - - | gnl\|UG\|Ta#S16235973 |
| IVLNWEC01BFDQN | - - - - - | gnl\|UG\|Ta#S16242865 |
| IVLNWEC02H31KN | - - - - - | gnl\|UG\|Ta#S16242865 |
| IVLNWEC02HGLWX | - - - - - | gnl\|UG\|Ta#S16242865 |
| IUIVLUD02FO0PX | - - - - - | gnl\|UG\|Ta#S16243622 |
| IUIVLUD02IPZ2R | - - - - - | gnl\|UG\|Ta#S16243622 |
| IV9RU5F01BIKZ8 | - - - - - | gnl\|UG\|Ta#S16243622 |
| IVLNWEC02J30PC | - - - - - | gnl\|UG\|Ta#S16243622 |
| IUIVLUD02JKIRP | - - - - - | gnl\|UG\|Ta#S16244091 |
| IV9RU5F01EWH3H | - - - - - | gnl\|UG\|Ta#S16244091 |
| IVLNWEC01EP36X | - - - - - | gnl\|UG\|Ta#S16244091 |
| IVLNWEC02GBU60 | - - - - - | gnl\|UG\|Ta#S16244091 |
| IV9RU5F02FQOWB | - - - - - | gnl\|UG\|Ta#S16246750 |
| IV9RU5F02GFQ6L | - - - - - | gnl\|UG\|Ta#S16246750 |
| IVLNWEC02F03ME | - - - - - | gnl\|UG\|Ta#S16246750 |
| IV9RU5F02GXSRI | - - - - - | gnl\|UG\|Ta#S16247431 |
| IVLNWEC01DINI0 | - - - - - | gnl\|UG\|Ta#S16247431 |
| IV9RU5F02GN4SN | - - - - - | gnl\|UG\|Ta#S16252219 |
| IV9RU5F02HL67V | - - - - - | gnl\|UG\|Ta#S16258338 |
| IV9RU5F02IWDTN | - - - - - | gnl\|UG\|Ta#S16258338 |
| IVLNWEC01EX5Z9 | - - - - - | gnl\|UG\|Ta#S16258338 |
| IVLNWEC02F67KK | - - - - - | gnl\|UG\|Ta#S16258338 |
| IUIVLUD01CYS8N | - - - - - | gnl\|UG\|Ta#S16264474 |
| IVLNWEC01DCRJC | - - - - - | gnl\|UG\|Ta#S16264474 |
| IUIVLUD01B2JU9 | - - - - - | gnl\|UG\|Ta#S16266881 |
| IV9RU5F01B74Y2 | - - - - - | gnl\|UG\|Ta#S16266881 |
| IVLNWEC01A0ZH6 | - - - - - | gnl\|UG\|Ta#S16266881 |
| IUIVLUD01B7PZ3 | - - - - - | gnl\|UG\|Ta#S17878983 |
| IV9RU5F01E1UA4 | - - - - - | gnl\|UG\|Ta#S17878983 |
| IVLNWEC01CGJ4B | - - - - - | gnl\|UG\|Ta#S17885596 |
| IVLNWEC02FSEE3 | - - - - - | gnl\|UG\|Ta#S17885596 |
| IVLNWEC01A8GM5 | - - - - - | gnl\|UG\|Ta#S17888401 |

| | | |
|---|---|---|
| IVLNWEC01AJMJQ | - - - - - | gnl\|UG\|Ta#S17888401 |
| IVLNWEC01CJKGS | - - - - - | gnl\|UG\|Ta#S17888401 |
| IUIVLUD02JNEUM | - - - - - | gnl\|UG\|Ta#S17890031 |
| IV9RU5F01EWN48 | - - - - - | gnl\|UG\|Ta#S17890031 |
| IV9RU5F01B5F7N | - - - - - | gnl\|UG\|Ta#S17894007 |
| IV9RU5F02I4B7S | - - - - - | gnl\|UG\|Ta#S17894007 |
| IV9RU5F01CUQXA | - - - - - | gnl\|UG\|Ta#S17898862 |
| IV9RU5F02G875F | - - - - - | gnl\|UG\|Ta#S17898862 |
| IV9RU5F02HSEJO | - - - - - | gnl\|UG\|Ta#S17898862 |
| IUIVLUD02IXTZD | - - - - - | gnl\|UG\|Ta#S17976579 |
| IVLNWEC01BMGS5 | - - - - - | gnl\|UG\|Ta#S17976579 |
| IVLNWEC01ELZ59 | - - - - - | gnl\|UG\|Ta#S17986352 |
| IVLNWEC02GOGKN | - - - - - | gnl\|UG\|Ta#S17986352 |
| IVLNWEC01BMG9J | - - - - - | gnl\|UG\|Ta#S17986613 |
| IVLNWEC01ETI2Z | - - - - - | gnl\|UG\|Ta#S17986613 |
| IUIVLUD01DR9SC | - - - - - | gnl\|UG\|Ta#S17987326 |
| IVLNWEC01BLAC8 | - - - - - | gnl\|UG\|Ta#S17987326 |
| IUIVLUD01CK34J | - - - - - | gnl\|UG\|Ta#S17987515 |
| IVLNWEC01BMVFV | - - - - - | gnl\|UG\|Ta#S17987515 |
| IVLNWEC01BWIIC | - - - - - | gnl\|UG\|Ta#S17987515 |
| IUIVLUD01AG3P9 | - - - - - | gnl\|UG\|Ta#S17987930 |
| IV9RU5F01CZATG | - - - - - | gnl\|UG\|Ta#S17987930 |
| IUIVLUD01CHINZ | - - - - - | gnl\|UG\|Ta#S17988079 |
| IVLNWEC01BFZCO | - - - - - | gnl\|UG\|Ta#S17988079 |
| IVLNWEC02JPABI | - - - - - | gnl\|UG\|Ta#S17988079 |
| IV9RU5F01AVFQB | - - - - - | gnl\|UG\|Ta#S17988389 |
| IVLNWEC02F70ZC | - - - - - | gnl\|UG\|Ta#S17988389 |
| IV9RU5F01DPRZ0 | - - - - - | gnl\|UG\|Ta#S18006638 |
| IV9RU5F02JS5MJ | - - - - - | gnl\|UG\|Ta#S18006638 |
| IV9RU5F02FUU05 | - - - - - | gnl\|UG\|Ta#S18008200 |
| IVLNWEC01DD811 | - - - - - | gnl\|UG\|Ta#S18008200 |
| IV9RU5F01BSQEX | - - - - - | gnl\|UG\|Ta#S18012331 |
| IV9RU5F01EGLK3 | - - - - - | gnl\|UG\|Ta#S18012331 |
| IVLNWEC02JFNCB | - - - - - | gnl\|UG\|Ta#S18012331 |
| IV9RU5F02HAM54 | - - - - - | gnl\|UG\|Ta#S19220548 |
| IVLNWEC01ER6JS | - - - - - | gnl\|UG\|Ta#S19220548 |
| IVLNWEC01BRJ5X | - - - - - | gnl\|UG\|Ta#S19223067 |
| IVLNWEC01EBWJJ | - - - - - | gnl\|UG\|Ta#S19223067 |
| IUIVLUD02IADRD | - - - - - | gnl\|UG\|Ta#S19225630 |
| IVLNWEC02IQEUL | - - - - - | gnl\|UG\|Ta#S19225630 |
| IUIVLUD01CPJMX | - - - - - | gnl\|UG\|Ta#S21001678 |
| IUIVLUD02J0BCK | - - - - - | gnl\|UG\|Ta#S21001678 |
| IUIVLUD02HFWZR | - - - - - | gnl\|UG\|Ta#S21611203 |
| IV9RU5F01BBNDN | - - - - - | gnl\|UG\|Ta#S21611203 |
| IVLNWEC01AYA9E | - - - - - | gnl\|UG\|Ta#S21611203 |
| IUIVLUD01B0RZX | - - - - - | gnl\|UG\|Ta#S21611285 |
| IVLNWEC02H78PC | - - - - - | gnl\|UG\|Ta#S21611285 |
| IVLNWEC02GXAJB | - - - - - | gnl\|UG\|Ta#S22368494 |
| IV9RU5F01EATSI | - - - - - | gnl\|UG\|Ta#S22368785 |
| IVLNWEC01ENQSM | - - - - - | gnl\|UG\|Ta#S22368785 |

| | | |
|---|---|---|
| IUIVLUD02HUBXA | - - - - - | gnl\|UG\|Ta#S22370445 |
| IVLNWEC01AZJ3V | - - - - - | gnl\|UG\|Ta#S22370445 |
| IV9RU5F01CEY1H | - - - - - | gnl\|UG\|Ta#S22371501 |
| IV9RU5F02I4BCR | - - - - - | gnl\|UG\|Ta#S22371501 |
| IVLNWEC02HWCYM | - - - - - | gnl\|UG\|Ta#S22371501 |
| IUIVLUD01DCZRH | - - - - - | gnl\|UG\|Ta#S22378041 |
| IUIVLUD01EHVH5 | - - - - - | gnl\|UG\|Ta#S22378041 |
| IVLNWEC01C3RFL | - - - - - | gnl\|UG\|Ta#S22378041 |
| IV9RU5F01C3A8Y | - - - - - | gnl\|UG\|Ta#S22383102 |
| IV9RU5F02JU8EQ | - - - - - | gnl\|UG\|Ta#S22383102 |
| IV9RU5F01CIADX | - - - - - | gnl\|UG\|Ta#S22385670 |
| IVLNWEC01ETPWM | - - - - - | gnl\|UG\|Ta#S22385670 |
| IV9RU5F02GC0X2 | - - - - - | gnl\|UG\|Ta#S22389895 |
| IV9RU5F02GPVRN | - - - - - | gnl\|UG\|Ta#S22389895 |
| IV9RU5F02HW89F | - - - - - | gnl\|UG\|Ta#S22389895 |
| IUIVLUD02HG2N1 | - - - - - | gnl\|UG\|Ta#S24318185 |
| IUIVLUD02IIPIW | - - - - - | gnl\|UG\|Ta#S24318185 |
| IV9RU5F01BY4EL | - - - - - | gnl\|UG\|Ta#S24318185 |
| IV9RU5F01D9JST | - - - - - | gnl\|UG\|Ta#S24318185 |
| IVLNWEC02F4W9S | - - - - - | gnl\|UG\|Ta#S24318185 |
| IVLNWEC02H9QTA | - - - - - | gnl\|UG\|Ta#S24318185 |
| IV9RU5F01AY1CK | - - - - - | gnl\|UG\|Ta#S24512966 |
| IVLNWEC02GLXMA | - - - - - | gnl\|UG\|Ta#S24512966 |
| IUIVLUD02HL50S | - - - - - | gnl\|UG\|Ta#S24513540 |
| IVLNWEC02ILTHS | - - - - - | gnl\|UG\|Ta#S24513540 |
| IV9RU5F02IHL39 | - - - - - | gnl\|UG\|Ta#S25040821 |
| IVLNWEC01EFEQY | - - - - - | gnl\|UG\|Ta#S25040821 |
| IV9RU5F01EL01X | - - - - - | gnl\|UG\|Ta#S26022044 |
| IV9RU5F02FPZUU | - - - - - | gnl\|UG\|Ta#S26022044 |
| IUIVLUD01EPZUT | - - - - - | gnl\|UG\|Ta#S26022980 |
| IVLNWEC02JCQUF | - - - - - | gnl\|UG\|Ta#S26022980 |
| IUIVLUD01A474T | - - - - - | gnl\|UG\|Ta#S26023479 |
| IUIVLUD02I8WMS | - - - - - | gnl\|UG\|Ta#S26023479 |
| IVLNWEC02HM0K3 | - - - - - | gnl\|UG\|Ta#S26023479 |
| IV9RU5F01C1LXF | - - - - - | gnl\|UG\|Ta#S26026001 |
| IVLNWEC01EVDY0 | - - - - - | gnl\|UG\|Ta#S26026001 |
| IV9RU5F02F9DT1 | - - - - - | gnl\|UG\|Ta#S26026431 |
| IVLNWEC01BMORQ | - - - - - | gnl\|UG\|Ta#S26026431 |
| IUIVLUD02JPHSV | - - - - - | gnl\|UG\|Ta#S26027518 |
| IV9RU5F01A6WVS | - - - - - | gnl\|UG\|Ta#S26027518 |
| IV9RU5F01A74K2 | - - - - - | gnl\|UG\|Ta#S26027518 |
| IVLNWEC02HBUJS | - - - - - | gnl\|UG\|Ta#S26027518 |
| IV9RU5F01A8J1J | - - - - - | gnl\|UG\|Ta#S26027727 |
| IV9RU5F01BULWY | - - - - - | gnl\|UG\|Ta#S26027727 |
| IV9RU5F01ARMSB | - - - - - | gnl\|UG\|Ta#S27052963 |
| IV9RU5F01D4YPM | - - - - - | gnl\|UG\|Ta#S27052963 |
| IV9RU5F01ET0TR | - - - - - | gnl\|UG\|Ta#S27052963 |
| IV9RU5F02IQAG8 | - - - - - | gnl\|UG\|Ta#S32429957 |
| IVLNWEC02GPRAW | - - - - - | gnl\|UG\|Ta#S32429957 |
| IV9RU5F01E2DIN | - - - - - | gnl\|UG\|Ta#S32501549 |

| | | |
|---|---|---|
| IV9RU5F02GBOLW | - - - - - | gnl\|UG\|Ta#S32501549 |
| IUIVLUD01CZNNI | - - - - - | gnl\|UG\|Ta#S32505676 |
| IV9RU5F02FYCZ5 | - - - - - | gnl\|UG\|Ta#S32505676 |
| IVLNWEC02H76D8 | - - - - - | gnl\|UG\|Ta#S32505676 |
| IUIVLUD01AZ1F9 | - - - - - | gnl\|UG\|Ta#S32510096 |
| IV9RU5F02FRGFQ | - - - - - | gnl\|UG\|Ta#S32510096 |
| IV9RU5F02IGU3O | - - - - - | gnl\|UG\|Ta#S32510096 |
| IVLNWEC01DCPPW | - - - - - | gnl\|UG\|Ta#S32510096 |
| IV9RU5F01C6VAP | - - - - - | gnl\|UG\|Ta#S32518132 |
| IVLNWEC01EF4G1 | - - - - - | gnl\|UG\|Ta#S32518132 |
| IV9RU5F01CWVW6 | - - - - - | gnl\|UG\|Ta#S32521499 |
| IV9RU5F02IGZYG | - - - - - | gnl\|UG\|Ta#S32521499 |
| IUIVLUD01DCBK5 | - - - - - | gnl\|UG\|Ta#S32525808 |
| IUIVLUD02FP0RJ | - - - - - | gnl\|UG\|Ta#S32525808 |
| IUIVLUD01EYUS3 | - - - - - | gnl\|UG\|Ta#S32554261 |
| IV9RU5F02FN3SM | - - - - - | gnl\|UG\|Ta#S32554261 |
| IV9RU5F02G3QWY | - - - - - | gnl\|UG\|Ta#S32559869 |
| IVLNWEC02IP096 | - - - - - | gnl\|UG\|Ta#S32559869 |
| IUIVLUD01AZIQN | - - - - - | gnl\|UG\|Ta#S32561250 |
| IVLNWEC01D8OSE | - - - - - | gnl\|UG\|Ta#S32561250 |
| IVLNWEC02FWL5R | - - - - - | gnl\|UG\|Ta#S32561250 |
| IUIVLUD02HHC1T | - - - - - | gnl\|UG\|Ta#S32561686 |
| IUIVLUD02JMNMO | - - - - - | gnl\|UG\|Ta#S32561686 |
| IV9RU5F01CC8UQ | - - - - - | gnl\|UG\|Ta#S32566905 |
| IVLNWEC01ENKIZ | - - - - - | gnl\|UG\|Ta#S32566905 |
| IV9RU5F01BDJID | - - - - - | gnl\|UG\|Ta#S32600725 |
| IV9RU5F01DM7BY | - - - - - | gnl\|UG\|Ta#S32600725 |
| IV9RU5F02JUZ1I | - - - - - | gnl\|UG\|Ta#S32600725 |
| IV9RU5F02J2IIX | - - - - - | gnl\|UG\|Ta#S32605616 |
| IVLNWEC01C1ZTP | - - - - - | gnl\|UG\|Ta#S32605616 |
| IUIVLUD02H0O7A | - - - - - | gnl\|UG\|Ta#S32620479 |
| IV9RU5F01APKI1 | - - - - - | gnl\|UG\|Ta#S32620479 |
| IUIVLUD02FX21F | - - - - - | gnl\|UG\|Ta#S32621754 |
| IV9RU5F02GHYXG | - - - - - | gnl\|UG\|Ta#S32621754 |
| IV9RU5F02IJF44 | - - - - - | gnl\|UG\|Ta#S32631250 |
| IVLNWEC02HSG88 | - - - - - | gnl\|UG\|Ta#S32631250 |
| IUIVLUD01DF43V | - - - - - | gnl\|UG\|Ta#S32639344 |
| IVLNWEC01BTA8G | - - - - - | gnl\|UG\|Ta#S32639344 |
| IV9RU5F02INDQT | - - - - - | gnl\|UG\|Ta#S32645457 |
| IVLNWEC01ANZ0O | - - - - - | gnl\|UG\|Ta#S32645457 |
| IVLNWEC02G7MX5 | - - - - - | gnl\|UG\|Ta#S32645457 |
| IV9RU5F02FUW3M | - - - - - | gnl\|UG\|Ta#S32645894 |
| IVLNWEC01A356L | - - - - - | gnl\|UG\|Ta#S32645894 |
| IUIVLUD02HGXGK | - - - - - | gnl\|UG\|Ta#S32662163 |
| IV9RU5F02HLI2F | - - - - - | gnl\|UG\|Ta#S32662163 |
| IUIVLUD01EI51S | - - - - - | gnl\|UG\|Ta#S32664990 |
| IUIVLUD02HAK4V | - - - - - | gnl\|UG\|Ta#S32664990 |
| IVLNWEC01EIEW4 | - - - - - | gnl\|UG\|Ta#S32664990 |
| IV9RU5F01AMI9X | - - - - - | gnl\|UG\|Ta#S32670052 |
| IV9RU5F01AP5KC | - - - - - | gnl\|UG\|Ta#S32670052 |

| | | |
|---|---|---|
| IV9RU5F01EOXMR | - - - - - | gnl\|UG\|Ta#S32673587 |
| IV9RU5F02GJ1A7 | - - - - - | gnl\|UG\|Ta#S32673587 |
| IVLNWEC01C8SVE | - - - - - | gnl\|UG\|Ta#S32678877 |
| IVLNWEC02F5Z5V | - - - - - | gnl\|UG\|Ta#S32678877 |
| IUIVLUD01CX7OI | - - - - - | gnl\|UG\|Ta#S32680503 |
| IUIVLUD02FVMU9 | - - - - - | gnl\|UG\|Ta#S32680503 |
| IV9RU5F01ERUDU | - - - - - | gnl\|UG\|Ta#S32680503 |
| IUIVLUD01DJUN7 | - - - - - | gnl\|UG\|Ta#S32696806 |
| IV9RU5F02GIHIM | - - - - - | gnl\|UG\|Ta#S32696806 |
| IVLNWEC01EG0WF | - - - - - | gnl\|UG\|Ta#S32696806 |
| IUIVLUD01ER4HP | - - - - - | gnl\|UG\|Ta#S32701197 |
| IV9RU5F01D8157 | - - - - - | gnl\|UG\|Ta#S32701197 |
| IUIVLUD01C7HER | - - - - - | gnl\|UG\|Ta#S32739578 |
| IV9RU5F01CZFNY | - - - - - | gnl\|UG\|Ta#S32739578 |
| IV9RU5F01C0Y4W | - - - - - | gnl\|UG\|Ta#S35718008 |
| IUIVLUD01EW36B | - - - - - | gnl\|UG\|Ta#S37758652 |
| IVLNWEC01ARDRU | - - - - - | gnl\|UG\|Ta#S37758652 |
| IUIVLUD01AOZHE | - - - - - | gnl\|UG\|Ta#S37768276 |
| IUIVLUD01BRAVC | - - - - - | gnl\|UG\|Ta#S37768276 |
| IV9RU5F01EDQ4U | - - - - - | gnl\|UG\|Ta#S37768276 |
| IVLNWEC01CKRPK | - - - - - | gnl\|UG\|Ta#S37768276 |
| IVLNWEC01E1R4C | - - - - - | gnl\|UG\|Ta#S37768276 |
| IUIVLUD02H5YN2 | - - - - - | gnl\|UG\|Ta#S37770341 |
| IUIVLUD02IUNJM | - - - - - | gnl\|UG\|Ta#S37770341 |
| IVLNWEC01BBYMW | - - - - - | gnl\|UG\|Ta#S37775314 |
| IVLNWEC02GZ0K5 | - - - - - | gnl\|UG\|Ta#S37775314 |
| IV9RU5F02IAP38 | - - - - - | gnl\|UG\|Ta#S37803527 |
| IVLNWEC02GDPV4 | - - - - - | gnl\|UG\|Ta#S37803527 |
| IV9RU5F02H5FL5 | - - - - - | gnl\|UG\|Ta#S37806699 |
| IVLNWEC01CATOU | - - - - - | gnl\|UG\|Ta#S37806699 |
| IUIVLUD01BQFWF | - - - - - | gnl\|UG\|Ta#S37816802 |
| IV9RU5F01EAXRR | - - - - - | gnl\|UG\|Ta#S37816802 |
| IUIVLUD01DHXDF | - - - - - | gnl\|UG\|Ta#S37816897 |
| IV9RU5F01A0JJS | - - - - - | gnl\|UG\|Ta#S37816897 |
| IUIVLUD02H4QMS | - - - - - | gnl\|UG\|Ta#S37822452 |
| IV9RU5F02GC5ZX | - - - - - | gnl\|UG\|Ta#S37822452 |
| IV9RU5F02F4KTG | - - - - - | gnl\|UG\|Ta#S37839212 |
| IVLNWEC01C962T | - - - - - | gnl\|UG\|Ta#S37839212 |
| IUIVLUD01D3V1E | - - - - - | gnl\|UG\|Ta#S37843084 |
| IVLNWEC02GUIAK | - - - - - | gnl\|UG\|Ta#S37843084 |
| IVLNWEC02G4DLJ | - - - - - | gnl\|UG\|Ta#S37850812 |
| IVLNWEC02H5SPJ | - - - - - | gnl\|UG\|Ta#S37850812 |
| IUIVLUD01A6VNU | - - - - - | gnl\|UG\|Ta#S37853394 |
| IUIVLUD02FL2DK | - - - - - | gnl\|UG\|Ta#S37853394 |
| IV9RU5F02F6YKY | - - - - - | gnl\|UG\|Ta#S37861880 |
| IVLNWEC02FTIJE | - - - - - | gnl\|UG\|Ta#S37861880 |
| IUIVLUD02H62DZ | - - - - - | gnl\|UG\|Ta#S37921485 |
| IVLNWEC02GDM7W | - - - - - | gnl\|UG\|Ta#S37921485 |
| IV9RU5F01AEO0I | - - - - - | gnl\|UG\|Ta#S37928831 |
| IV9RU5F01C5VU2 | - - - - - | gnl\|UG\|Ta#S37928831 |

| | | |
|---|---|---|
| IUIVLUD02FPJM7 | - - - - - | gnl\|UG\|Ta#S37929428 |
| IVLNWEC01CUUAA | - - - - - | gnl\|UG\|Ta#S37929428 |
| IV9RU5F02HVSV1 | - - - - - | gnl\|UG\|Ta#S37930875 |
| IV9RU5F02IKG3I | - - - - - | gnl\|UG\|Ta#S37930875 |
| IUIVLUD02FSVUF | - - - - - | gnl\|UG\|Ta#S41658072 |
| IV9RU5F01E1IWR | - - - - - | gnl\|UG\|Ta#S41658072 |
| IVLNWEC01C6VEG | - - - - - | gnl\|UG\|Ta#S50384149 |
| IVLNWEC02IJ4H2 | - - - - - | gnl\|UG\|Ta#S50384149 |
| IV9RU5F01A0H9W | - - - - - | gnl\|UG\|Ta#S50385433 |
| IV9RU5F01BNBA9 | - - - - - | gnl\|UG\|Ta#S50385433 |
| IVLNWEC01C2NUA | - - - - - | gnl\|UG\|Ta#S50388970 |
| IVLNWEC01CUFP6 | - - - - - | gnl\|UG\|Ta#S50388970 |
| IVLNWEC02FQYP5 | - - - - - | gnl\|UG\|Ta#S50388970 |
| IV9RU5F01BUA9W | - - - - - | gnl\|UG\|Ta#S52541026 |
| IV9RU5F02GNF0O | - - - - - | gnl\|UG\|Ta#S52541026 |
| IV9RU5F01DKFG6 | - - - - - | gnl\|UG\|Ta#S52541041 |
| IV9RU5F02FYOR8 | - - - - - | gnl\|UG\|Ta#S52541041 |
| IVLNWEC01DL9AK | - - - - - | gnl\|UG\|Ta#S52541041 |
| IVLNWEC02FV84G | - - - - - | gnl\|UG\|Ta#S52541041 |
| IVLNWEC02GY0OW | - - - - - | gnl\|UG\|Ta#S52541041 |
| IUIVLUD02JDMJC | - - - - - | gnl\|UG\|Ta#S52541068 |
| IV9RU5F02IZF72 | - - - - - | gnl\|UG\|Ta#S52541068 |
| IV9RU5F01D4W7O | - - - - - | gnl\|UG\|Ta#S52541173 |
| IV9RU5F01D5MIR | - - - - - | gnl\|UG\|Ta#S52541173 |
| IV9RU5F02FV88J | - - - - - | gnl\|UG\|Ta#S52541302 |
| IVLNWEC02FNFOE | - - - - - | gnl\|UG\|Ta#S52541302 |
| IVLNWEC01B47QB | - - - - - | gnl\|UG\|Ta#S52541509 |
| IV9RU5F01B5CYC | - - - - - | gnl\|UG\|Ta#S52541593 |
| IVLNWEC01CWDF5 | - - - - - | gnl\|UG\|Ta#S52541593 |
| IVLNWEC02GZDOC | - - - - - | gnl\|UG\|Ta#S52541749 |
| IVLNWEC02H31Y4 | - - - - - | gnl\|UG\|Ta#S52541749 |
| IUIVLUD01AMC2C | - - - - - | gnl\|UG\|Ta#S52541789 |
| IVLNWEC01CY1RF | - - - - - | gnl\|UG\|Ta#S52541789 |
| IUIVLUD02JHG01 | - - - - - | gnl\|UG\|Ta#S52541805 |
| IV9RU5F02FUB2K | - - - - - | gnl\|UG\|Ta#S52541805 |
| IUIVLUD01CZQ18 | - - - - - | gnl\|UG\|Ta#S52542124 |
| IUIVLUD02F5K59 | - - - - - | gnl\|UG\|Ta#S52542124 |
| IUIVLUD02JDR7D | - - - - - | gnl\|UG\|Ta#S52542124 |
| IV9RU5F02HGJVO | - - - - - | gnl\|UG\|Ta#S52542124 |
| IVLNWEC01CJMRT | - - - - - | gnl\|UG\|Ta#S52542124 |
| IVLNWEC01DMNQN | - - - - - | gnl\|UG\|Ta#S52542124 |
| IV9RU5F01BEQD3 | - - - - - | gnl\|UG\|Ta#S52542149 |
| IV9RU5F01DBD12 | - - - - - | gnl\|UG\|Ta#S52542149 |
| IV9RU5F02HC23K | - - - - - | gnl\|UG\|Ta#S52542149 |
| IVLNWEC01DMYWG | - - - - - | gnl\|UG\|Ta#S52542149 |
| IUIVLUD01B0UII | - - - - - | gnl\|UG\|Ta#S52542198 |
| IUIVLUD01C784P | - - - - - | gnl\|UG\|Ta#S52542198 |
| IV9RU5F01CYDLI | - - - - - | gnl\|UG\|Ta#S52542198 |
| IV9RU5F01ELM2Q | - - - - - | gnl\|UG\|Ta#S52542198 |
| IV9RU5F02GNDNB | - - - - - | gnl\|UG\|Ta#S52542198 |

| | | |
|---|---|---|
| IVLNWEC01ANWKX | - - - - - | gnl\|UG\|Ta#S52542198 |
| IVLNWEC01EFZ5F | - - - - - | gnl\|UG\|Ta#S52542227 |
| IVLNWEC02HGR8D | - - - - - | gnl\|UG\|Ta#S52542227 |
| IVLNWEC01AO6EJ | - - - - - | gnl\|UG\|Ta#S52542257 |
| IVLNWEC02GUI74 | - - - - - | gnl\|UG\|Ta#S52542257 |
| IV9RU5F01BVC35 | - - - - - | gnl\|UG\|Ta#S52542329 |
| IV9RU5F02GH4UG | - - - - - | gnl\|UG\|Ta#S52542329 |
| IVLNWEC01BJV17 | - - - - - | gnl\|UG\|Ta#S52542329 |
| IVLNWEC01BZY64 | - - - - - | gnl\|UG\|Ta#S52542329 |
| IUIVLUD01A8R2M | - - - - - | gnl\|UG\|Ta#S52542334 |
| IUIVLUD01AR5J4 | - - - - - | gnl\|UG\|Ta#S52542334 |
| IV9RU5F02I5HX4 | - - - - - | gnl\|UG\|Ta#S52542334 |
| IV9RU5F02JHWBM | - - - - - | gnl\|UG\|Ta#S52542334 |
| IVLNWEC02GH6AB | - - - - - | gnl\|UG\|Ta#S52542334 |
| IUIVLUD02IC116 | - - - - - | gnl\|UG\|Ta#S52542426 |
| IVLNWEC02HKBQV | - - - - - | gnl\|UG\|Ta#S52542426 |
| IV9RU5F02HZKGV | - - - - - | gnl\|UG\|Ta#S52542430 |
| IV9RU5F02IJCAC | - - - - - | gnl\|UG\|Ta#S52542430 |
| IV9RU5F02JT6JW | - - - - - | gnl\|UG\|Ta#S52542430 |
| IVLNWEC02H8MQG | - - - - - | gnl\|UG\|Ta#S52542430 |
| IV9RU5F02ID6TP | - - - - - | gnl\|UG\|Ta#S52542527 |
| IVLNWEC02G6VSH | - - - - - | gnl\|UG\|Ta#S52542527 |
| IVLNWEC01C2NWC | - - - - - | gnl\|UG\|Ta#S52542599 |
| IVLNWEC02JPQYA | - - - - - | gnl\|UG\|Ta#S52542599 |
| IUIVLUD02JEXZ0 | - - - - - | gnl\|UG\|Ta#S52542855 |
| IV9RU5F02HBBK6 | - - - - - | gnl\|UG\|Ta#S52542855 |
| IVLNWEC01CVF4I | - - - - - | gnl\|UG\|Ta#S52542855 |
| IVLNWEC01DYZ5Y | - - - - - | gnl\|UG\|Ta#S52542855 |
| IVLNWEC01D76EU | - - - - - | gnl\|UG\|Ta#S52542905 |
| IUIVLUD01BQKSU | - - - - - | gnl\|UG\|Ta#S52543010 |
| IV9RU5F02HV3IN | - - - - - | gnl\|UG\|Ta#S52543010 |
| IV9RU5F01D3W1B | - - - - - | gnl\|UG\|Ta#S52543105 |
| IV9RU5F01D5G1U | - - - - - | gnl\|UG\|Ta#S52543105 |
| IVLNWEC01CV5XF | - - - - - | gnl\|UG\|Ta#S52543105 |
| IVLNWEC01EEXC0 | - - - - - | gnl\|UG\|Ta#S52543105 |
| IUIVLUD02F1WVN | - - - - - | gnl\|UG\|Ta#S52543129 |
| IV9RU5F01A1WRQ | - - - - - | gnl\|UG\|Ta#S52543129 |
| IV9RU5F01DU6QR | - - - - - | gnl\|UG\|Ta#S52543129 |
| IUIVLUD02HPXY5 | - - - - - | gnl\|UG\|Ta#S52543160 |
| IVLNWEC01DE2BJ | - - - - - | gnl\|UG\|Ta#S52543160 |
| IVLNWEC02GOX11 | - - - - - | gnl\|UG\|Ta#S52543160 |
| IV9RU5F02IVWC7 | - - - - - | gnl\|UG\|Ta#S52543203 |
| IVLNWEC02G3LRD | - - - - - | gnl\|UG\|Ta#S52543203 |
| IV9RU5F01AUWCT | - - - - - | gnl\|UG\|Ta#S52543328 |
| IV9RU5F02GR6IT | - - - - - | gnl\|UG\|Ta#S52543328 |
| IVLNWEC02JJGMF | - - - - - | gnl\|UG\|Ta#S52543328 |
| IUIVLUD01DY6B6 | - - - - - | gnl\|UG\|Ta#S52543344 |
| IVLNWEC01D5FXY | - - - - - | gnl\|UG\|Ta#S52543344 |
| IUIVLUD01BU28X | - - - - - | gnl\|UG\|Ta#S52543372 |
| IVLNWEC01D2YLL | - - - - - | gnl\|UG\|Ta#S52543372 |

| | | |
|---|---|---|
| IV9RU5F01CUSFO | - - - - - | gnl\|UG\|Ta#S52543397 |
| IVLNWEC02G5OZK | - - - - - | gnl\|UG\|Ta#S52543397 |
| IUIVLUD02FPEDB | - - - - - | gnl\|UG\|Ta#S52543411 |
| IVLNWEC02GRTFU | - - - - - | gnl\|UG\|Ta#S52543411 |
| IUIVLUD02F80FP | - - - - - | gnl\|UG\|Ta#S52543417 |
| IUIVLUD01C8H5T | - - - - - | gnl\|UG\|Ta#S52543462 |
| IV9RU5F01AWFZD | - - - - - | gnl\|UG\|Ta#S52543462 |
| IV9RU5F01A5QV9 | - - - - - | gnl\|UG\|Ta#S52543525 |
| IVLNWEC02FUE6P | - - - - - | gnl\|UG\|Ta#S52543525 |
| IV9RU5F02GCNPB | - - - - - | gnl\|UG\|Ta#S52543528 |
| IV9RU5F02IXHD3 | - - - - - | gnl\|UG\|Ta#S52543528 |
| IV9RU5F01EYPTI | - - - - - | gnl\|UG\|Ta#S52543560 |
| IUIVLUD01DI5KS | - - - - - | gnl\|UG\|Ta#S52543566 |
| IUIVLUD01B8Z1S | - - - - - | gnl\|UG\|Ta#S52543616 |
| IUIVLUD02JC2M3 | - - - - - | gnl\|UG\|Ta#S52543616 |
| IV9RU5F01AUOJU | - - - - - | gnl\|UG\|Ta#S52543616 |
| IV9RU5F02HFBUP | - - - - - | gnl\|UG\|Ta#S52543616 |
| IVLNWEC02F0GGD | - - - - - | gnl\|UG\|Ta#S52543616 |
| IUIVLUD01DRC4V | - - - - - | gnl\|UG\|Ta#S52543703 |
| IVLNWEC02IJ5EF | - - - - - | gnl\|UG\|Ta#S52543703 |
| IUIVLUD01EG4NS | - - - - - | gnl\|UG\|Ta#S52543777 |
| IV9RU5F01BRNLI | - - - - - | gnl\|UG\|Ta#S52543777 |
| IV9RU5F01AMT0V | - - - - - | gnl\|UG\|Ta#S52543846 |
| IV9RU5F01DM1WM | - - - - - | gnl\|UG\|Ta#S52543846 |
| IV9RU5F02GEAMR | - - - - - | gnl\|UG\|Ta#S52543897 |
| IVLNWEC02HCLHC | - - - - - | gnl\|UG\|Ta#S52543897 |
| IUIVLUD02GEW99 | - - - - - | gnl\|UG\|Ta#S52543916 |
| IV9RU5F01ASHB1 | - - - - - | gnl\|UG\|Ta#S52543916 |
| IVLNWEC02GBU4A | - - - - - | gnl\|UG\|Ta#S52543944 |
| IVLNWEC02GPXWP | - - - - - | gnl\|UG\|Ta#S52543944 |
| IUIVLUD01D945E | - - - - - | gnl\|UG\|Ta#S52543992 |
| IV9RU5F02GXCY7 | - - - - - | gnl\|UG\|Ta#S52543992 |
| IV9RU5F01B5284 | - - - - - | gnl\|UG\|Ta#S52544116 |
| IVLNWEC01ANM2Y | - - - - - | gnl\|UG\|Ta#S52544116 |
| IVLNWEC01EEOVJ | - - - - - | gnl\|UG\|Ta#S52544116 |
| IVLNWEC01BCP71 | - - - - - | gnl\|UG\|Ta#S52544123 |
| IVLNWEC01EOZYH | - - - - - | gnl\|UG\|Ta#S52544123 |
| IV9RU5F01DE3W9 | - - - - - | gnl\|UG\|Ta#S52544173 |
| IVLNWEC01EEH3O | - - - - - | gnl\|UG\|Ta#S52544173 |
| IV9RU5F02JFHU5 | - - - - - | gnl\|UG\|Ta#S52544203 |
| IVLNWEC01BGYJ6 | - - - - - | gnl\|UG\|Ta#S52544203 |
| IV9RU5F02HVFQI | - - - - - | gnl\|UG\|Ta#S52544227 |
| IVLNWEC01BW0EB | - - - - - | gnl\|UG\|Ta#S52544227 |
| IVLNWEC01CB6OS | - - - - - | gnl\|UG\|Ta#S52544227 |
| IVLNWEC02H5I9F | - - - - - | gnl\|UG\|Ta#S52544227 |
| IVLNWEC02I9ODT | - - - - - | gnl\|UG\|Ta#S52544227 |
| IV9RU5F02GY9P3 | - - - - - | gnl\|UG\|Ta#S52544360 |
| IV9RU5F02IQACV | - - - - - | gnl\|UG\|Ta#S52544360 |
| IUIVLUD02J1UYM | - - - - - | gnl\|UG\|Ta#S52544506 |
| IV9RU5F02GDB32 | - - - - - | gnl\|UG\|Ta#S52544506 |

| | | |
|---|---|---|
| IVLNWEC02J2QHU | - - - - - | gnl\|UG\|Ta#S52544506 |
| IV9RU5F01BZ4O3 | - - - - - | gnl\|UG\|Ta#S52544639 |
| IV9RU5F02GD6Q9 | - - - - - | gnl\|UG\|Ta#S52544639 |
| IV9RU5F01CIW40 | - - - - - | gnl\|UG\|Ta#S52544654 |
| IV9RU5F02IZ2X4 | - - - - - | gnl\|UG\|Ta#S52544654 |
| IV9RU5F01CH2EE | - - - - - | gnl\|UG\|Ta#S52544728 |
| IVLNWEC01D9I22 | - - - - - | gnl\|UG\|Ta#S52544728 |
| IUIVLUD02H9Y6H | - - - - - | gnl\|UG\|Ta#S52544730 |
| IV9RU5F01AR1NA | - - - - - | gnl\|UG\|Ta#S52544730 |
| IV9RU5F01CE0DI | - - - - - | gnl\|UG\|Ta#S52544735 |
| IVLNWEC02I6FNY | - - - - - | gnl\|UG\|Ta#S52544735 |
| IV9RU5F02IB3YD | - - - - - | gnl\|UG\|Ta#S52544785 |
| IVLNWEC01BLQDS | - - - - - | gnl\|UG\|Ta#S52544785 |
| IUIVLUD01DMSPH | - - - - - | gnl\|UG\|Ta#S52544809 |
| IUIVLUD02GW0QM | - - - - - | gnl\|UG\|Ta#S52544809 |
| IV9RU5F02HAFZI | - - - - - | gnl\|UG\|Ta#S52544832 |
| IVLNWEC01C8TWP | - - - - - | gnl\|UG\|Ta#S52544832 |
| IVLNWEC02H3SN3 | - - - - - | gnl\|UG\|Ta#S52544832 |
| IVLNWEC02JIMF0 | - - - - - | gnl\|UG\|Ta#S52544832 |
| IV9RU5F01BYL0B | - - - - - | gnl\|UG\|Ta#S52544843 |
| IVLNWEC02F7BHG | - - - - - | gnl\|UG\|Ta#S52544843 |
| IV9RU5F02HO0ME | - - - - - | gnl\|UG\|Ta#S52544885 |
| IVLNWEC01C9R4N | - - - - - | gnl\|UG\|Ta#S52544885 |
| IV9RU5F02I6SQ6 | - - - - - | gnl\|UG\|Ta#S52545011 |
| IVLNWEC01BG8Q4 | - - - - - | gnl\|UG\|Ta#S52545011 |
| IV9RU5F02F4I8Z | - - - - - | gnl\|UG\|Ta#S52545072 |
| IV9RU5F01BZ34T | - - - - - | gnl\|UG\|Ta#S52545153 |
| IV9RU5F01C446K | - - - - - | gnl\|UG\|Ta#S52545153 |
| IUIVLUD02HIWM8 | - - - - - | gnl\|UG\|Ta#S52545260 |
| IV9RU5F02JWM9J | - - - - - | gnl\|UG\|Ta#S52545260 |
| IUIVLUD01CND92 | - - - - - | gnl\|UG\|Ta#S52545265 |
| IUIVLUD01EGO2S | - - - - - | gnl\|UG\|Ta#S52545265 |
| IVLNWEC02H5Y5K | - - - - - | gnl\|UG\|Ta#S52545265 |
| IV9RU5F01DOYOA | - - - - - | gnl\|UG\|Ta#S52545294 |
| IVLNWEC01AJ291 | - - - - - | gnl\|UG\|Ta#S52545294 |
| IV9RU5F01CHVBT | - - - - - | gnl\|UG\|Ta#S52545485 |
| IV9RU5F02IKVMO | - - - - - | gnl\|UG\|Ta#S52545485 |
| IV9RU5F01DXTT0 | - - - - - | gnl\|UG\|Ta#S52545515 |
| IUIVLUD01DV0Z4 | - - - - - | gnl\|UG\|Ta#S52545531 |
| IV9RU5F01BO688 | - - - - - | gnl\|UG\|Ta#S52545531 |
| IVLNWEC01ASA5P | - - - - - | gnl\|UG\|Ta#S52545531 |
| IV9RU5F01A0C0J | - - - - - | gnl\|UG\|Ta#S52545532 |
| IVLNWEC02FZ5T0 | - - - - - | gnl\|UG\|Ta#S52545532 |
| IV9RU5F01EOQJM | - - - - - | gnl\|UG\|Ta#S52545646 |
| IUIVLUD01CCDUM | - - - - - | gnl\|UG\|Ta#S52545772 |
| IV9RU5F01C813U | - - - - - | gnl\|UG\|Ta#S52545772 |
| IV9RU5F01BGD85 | - - - - - | gnl\|UG\|Ta#S52545892 |
| IV9RU5F01ESRTE | - - - - - | gnl\|UG\|Ta#S52545892 |
| IV9RU5F02I4YLF | - - - - - | gnl\|UG\|Ta#S52545940 |
| IV9RU5F02JOYBL | - - - - - | gnl\|UG\|Ta#S52545940 |

| | | |
|---|---|---|
| IUIVLUD02HQZR2 | - - - - - | gnl\|UG\|Ta#S52545958 |
| IVLNWEC02IURUH | - - - - - | gnl\|UG\|Ta#S52545958 |
| IV9RU5F01AILQ4 | - - - - - | gnl\|UG\|Ta#S52545986 |
| IVLNWEC01AR3W2 | - - - - - | gnl\|UG\|Ta#S52545986 |
| IVLNWEC01CEU88 | - - - - - | gnl\|UG\|Ta#S52545989 |
| IVLNWEC02IHPNI | - - - - - | gnl\|UG\|Ta#S52545989 |
| IV9RU5F02IN8ZP | - - - - - | gnl\|UG\|Ta#S52545995 |
| IVLNWEC01ENX4O | - - - - - | gnl\|UG\|Ta#S52545995 |
| IVLNWEC02GK58A | - - - - - | gnl\|UG\|Ta#S52545995 |
| IUIVLUD02GU7I3 | - - - - - | gnl\|UG\|Ta#S52546007 |
| IUIVLUD01B18UF | - - - - - | gnl\|UG\|Ta#S52546144 |
| IVLNWEC01CKSS6 | - - - - - | gnl\|UG\|Ta#S52546144 |
| IV9RU5F02HVGZM | - - - - - | gnl\|UG\|Ta#S52546249 |
| IVLNWEC01DTH5B | - - - - - | gnl\|UG\|Ta#S52546249 |
| IUIVLUD01BM444 | - - - - - | gnl\|UG\|Ta#S52546277 |
| IV9RU5F01DNMUR | - - - - - | gnl\|UG\|Ta#S52546277 |
| IV9RU5F02JD08J | - - - - - | gnl\|UG\|Ta#S52546277 |
| IV9RU5F02JHRK5 | - - - - - | gnl\|UG\|Ta#S52546277 |
| IV9RU5F01C7TFF | - - - - - | gnl\|UG\|Ta#S52546414 |
| IV9RU5F01E0XHT | - - - - - | gnl\|UG\|Ta#S52546414 |
| IVLNWEC01EKANU | - - - - - | gnl\|UG\|Ta#S52546414 |
| IUIVLUD01AKFN4 | - - - - - | gnl\|UG\|Ta#S52546513 |
| IV9RU5F02IRKSO | - - - - - | gnl\|UG\|Ta#S52546513 |
| IV9RU5F02JYH8V | - - - - - | gnl\|UG\|Ta#S52546513 |
| IVLNWEC02JZH4D | - - - - - | gnl\|UG\|Ta#S52546513 |
| IV9RU5F01CTKBZ | - - - - - | gnl\|UG\|Ta#S52546584 |
| IV9RU5F01DHQP9 | - - - - - | gnl\|UG\|Ta#S52546584 |
| IV9RU5F02GEBA3 | - - - - - | gnl\|UG\|Ta#S52546584 |
| IV9RU5F02I1HXS | - - - - - | gnl\|UG\|Ta#S52546584 |
| IV9RU5F02GZWS8 | - - - - - | gnl\|UG\|Ta#S52546669 |
| IVLNWEC01B4OS6 | - - - - - | gnl\|UG\|Ta#S52546669 |
| IVLNWEC01CMJQG | - - - - - | gnl\|UG\|Ta#S52546669 |
| IV9RU5F02JSAXE | - - - - - | gnl\|UG\|Ta#S52546693 |
| IVLNWEC01D4MDM | - - - - - | gnl\|UG\|Ta#S52546693 |
| IVLNWEC01B376V | - - - - - | gnl\|UG\|Ta#S52546780 |
| IVLNWEC01DTA3R | - - - - - | gnl\|UG\|Ta#S52546780 |
| IUIVLUD02H3MHD | - - - - - | gnl\|UG\|Ta#S52546782 |
| IVLNWEC02H2TAM | - - - - - | gnl\|UG\|Ta#S52546782 |
| IUIVLUD02HTD66 | - - - - - | gnl\|UG\|Ta#S52546786 |
| IUIVLUD02JZ5RT | - - - - - | gnl\|UG\|Ta#S52546786 |
| IUIVLUD01D1PJA | - - - - - | gnl\|UG\|Ta#S52546835 |
| IV9RU5F01AWWWB | - - - - - | gnl\|UG\|Ta#S52546835 |
| IV9RU5F01BWPUS | - - - - - | gnl\|UG\|Ta#S52546835 |
| IVLNWEC02GMHAE | - - - - - | gnl\|UG\|Ta#S52546835 |
| IV9RU5F02I4OXR | - - - - - | gnl\|UG\|Ta#S52546850 |
| IVLNWEC01BI5MU | - - - - - | gnl\|UG\|Ta#S52546850 |
| IV9RU5F02IXCVF | - - - - - | gnl\|UG\|Ta#S52547045 |
| IUIVLUD02IJ62Q | - - - - - | gnl\|UG\|Ta#S52897515 |
| IV9RU5F01B8TE0 | - - - - - | gnl\|UG\|Ta#S52897515 |
| IV9RU5F01CQSIA | - - - - - | gnl\|UG\|Ta#S52897632 |

| | | |
|---|---|---|
| IV9RU5F01D5LNP | - - - - - | gnl\|UG\|Ta#S52897632 |
| IUIVLUD01EAYXI | - - - - - | gnl\|UG\|Ta#S52898600 |
| IVLNWEC01COWIW | - - - - - | gnl\|UG\|Ta#S52898600 |
| IV9RU5F02FXDMN | - - - - - | gnl\|UG\|Ta#S56344616 |
| IVLNWEC01C70K4 | - - - - - | gnl\|UG\|Ta#S56344616 |
| IVLNWEC01DOGWE | - - - - - | gnl\|UG\|Ta#S56344616 |
| IUIVLUD02GKTX8 | - - - - - | gnl\|UG\|Ta#S58841349 |
| IV9RU5F02F3ZWW | - - - - - | gnl\|UG\|Ta#S58841349 |
| IVLNWEC02GTSS5 | - - - - - | gnl\|UG\|Ta#S58841349 |
| IUIVLUD02F7JPP | - - - - - | gnl\|UG\|Ta#S58842750 |
| IV9RU5F02FW0W0 | - - - - - | gnl\|UG\|Ta#S58842750 |
| IUIVLUD02JHALS | - - - - - | gnl\|UG\|Ta#S58843205 |
| IV9RU5F01EUXOU | - - - - - | gnl\|UG\|Ta#S58843205 |
| IV9RU5F02JX5SM | - - - - - | gnl\|UG\|Ta#S58843205 |
| IV9RU5F02HKWJC | - - - - - | gnl\|UG\|Ta#S58846030 |
| IV9RU5F02ITP82 | - - - - - | gnl\|UG\|Ta#S58846030 |
| IVLNWEC01B404S | - - - - - | gnl\|UG\|Ta#S58846030 |
| IV9RU5F01C33QA | - - - - - | gnl\|UG\|Ta#S58846157 |
| IV9RU5F02IWX4A | - - - - - | gnl\|UG\|Ta#S58846157 |
| IVLNWEC01BV7N4 | - - - - - | gnl\|UG\|Ta#S58846157 |
| IV9RU5F01B2SLS | - - - - - | gnl\|UG\|Ta#S58846645 |
| IV9RU5F02IHTRH | - - - - - | gnl\|UG\|Ta#S58846645 |
| IVLNWEC02HGWSG | - - - - - | gnl\|UG\|Ta#S58846645 |
| IVLNWEC02I5GKE | - - - - - | gnl\|UG\|Ta#S58846645 |
| IVLNWEC02H3GB3 | - - - - - | gnl\|UG\|Ta#S58847350 |
| IVLNWEC02IW5VP | - - - - - | gnl\|UG\|Ta#S58847350 |
| IV9RU5F02JNXUO | - - - - - | gnl\|UG\|Ta#S58848081 |
| IVLNWEC02IIW0B | - - - - - | gnl\|UG\|Ta#S58848081 |
| IV9RU5F02G3KR0 | - - - - - | gnl\|UG\|Ta#S58848290 |
| IV9RU5F02JIJD4 | - - - - - | gnl\|UG\|Ta#S58848290 |
| IV9RU5F01BA04O | - - - - - | gnl\|UG\|Ta#S58848764 |
| IV9RU5F01D25TS | - - - - - | gnl\|UG\|Ta#S58848764 |
| IVLNWEC02F313A | - - - - - | gnl\|UG\|Ta#S58848764 |
| IVLNWEC02HP81S | - - - - - | gnl\|UG\|Ta#S58848764 |
| IV9RU5F01AL899 | - - - - - | gnl\|UG\|Ta#S58849345 |
| IV9RU5F02II9FM | - - - - - | gnl\|UG\|Ta#S58849345 |
| IUIVLUD02GN0UO | - - - - - | gnl\|UG\|Ta#S58849420 |
| IV9RU5F01B3B8N | - - - - - | gnl\|UG\|Ta#S58849533 |
| IVLNWEC01CC076 | - - - - - | gnl\|UG\|Ta#S58849533 |
| IUIVLUD02IBDZX | - - - - - | gnl\|UG\|Ta#S58850770 |
| IV9RU5F01D3OST | - - - - - | gnl\|UG\|Ta#S58850770 |
| IUIVLUD01DWP9M | - - - - - | gnl\|UG\|Ta#S58851525 |
| IVLNWEC01EVWAM | - - - - - | gnl\|UG\|Ta#S58851525 |
| IV9RU5F01DDUTM | - - - - - | gnl\|UG\|Ta#S58851775 |
| IVLNWEC02ISCU3 | - - - - - | gnl\|UG\|Ta#S58851775 |
| IV9RU5F01AIV67 | - - - - - | gnl\|UG\|Ta#S58852789 |
| IV9RU5F01DLNS3 | - - - - - | gnl\|UG\|Ta#S58852789 |
| IVLNWEC02I04KI | - - - - - | gnl\|UG\|Ta#S58852789 |
| IUIVLUD01BM606 | - - - - - | gnl\|UG\|Ta#S58852862 |
| IVLNWEC01B00PR | - - - - - | gnl\|UG\|Ta#S58852862 |

| | | |
|---|---|---|
| IUIVLUD01DN7PN | - - - - - | gnl\|UG\|Ta#S58853888 |
| IUIVLUD02ITIAX | - - - - - | gnl\|UG\|Ta#S58853888 |
| IVLNWEC01E0J1G | - - - - - | gnl\|UG\|Ta#S58853888 |
| IVLNWEC01CPX90 | - - - - - | gnl\|UG\|Ta#S58854020 |
| IVLNWEC02FS2AT | - - - - - | gnl\|UG\|Ta#S58854020 |
| IVLNWEC02IW75A | - - - - - | gnl\|UG\|Ta#S58854020 |
| IV9RU5F01AWYJ8 | - - - - - | gnl\|UG\|Ta#S58854033 |
| IVLNWEC01BX8L6 | - - - - - | gnl\|UG\|Ta#S58854033 |
| IV9RU5F02GN84Z | - - - - - | gnl\|UG\|Ta#S58854185 |
| IV9RU5F02JSD5Z | - - - - - | gnl\|UG\|Ta#S58854185 |
| IV9RU5F02JP6QD | - - - - - | gnl\|UG\|Ta#S58854539 |
| IVLNWEC01EOP1C | - - - - - | gnl\|UG\|Ta#S58854539 |
| IUIVLUD02IEFWN | - - - - - | gnl\|UG\|Ta#S58854824 |
| IV9RU5F01C2KTR | - - - - - | gnl\|UG\|Ta#S58854824 |
| IUIVLUD01BWACQ | - - - - - | gnl\|UG\|Ta#S58857465 |
| IVLNWEC02JG7NW | - - - - - | gnl\|UG\|Ta#S58857465 |
| IV9RU5F01CLT98 | - - - - - | gnl\|UG\|Ta#S58857944 |
| IVLNWEC01EYZGH | - - - - - | gnl\|UG\|Ta#S58857944 |
| IUIVLUD01CGP7P | - - - - - | gnl\|UG\|Ta#S58860377 |
| IVLNWEC01C0LEO | - - - - - | gnl\|UG\|Ta#S58860377 |
| IV9RU5F01BC8XG | - - - - - | gnl\|UG\|Ta#S58860858 |
| IVLNWEC01D1P9V | - - - - - | gnl\|UG\|Ta#S58860858 |
| IV9RU5F02IZ9SL | - - - - - | gnl\|UG\|Ta#S58861596 |
| IVLNWEC02GAAZA | - - - - - | gnl\|UG\|Ta#S58861596 |
| IVLNWEC01AIRUQ | - - - - - | gnl\|UG\|Ta#S58862431 |
| IVLNWEC01CKBMS | - - - - - | gnl\|UG\|Ta#S58862431 |
| IV9RU5F02G697F | - - - - - | gnl\|UG\|Ta#S58862432 |
| IVLNWEC02IM9TZ | - - - - - | gnl\|UG\|Ta#S58862432 |
| IUIVLUD02IA3S9 | - - - - - | gnl\|UG\|Ta#S58863852 |
| IV9RU5F01BQERF | - - - - - | gnl\|UG\|Ta#S58863852 |
| IV9RU5F02F5T1U | - - - - - | gnl\|UG\|Ta#S58863852 |
| IV9RU5F02HRKRU | - - - - - | gnl\|UG\|Ta#S58864470 |
| IVLNWEC01C52OW | - - - - - | gnl\|UG\|Ta#S58864470 |
| IUIVLUD01BF097 | - - - - - | gnl\|UG\|Ta#S58864636 |
| IV9RU5F02GV50W | - - - - - | gnl\|UG\|Ta#S58864636 |
| IVLNWEC02HV76V | - - - - - | gnl\|UG\|Ta#S58864636 |
| IV9RU5F02GSUXY | - - - - - | gnl\|UG\|Ta#S58864639 |
| IVLNWEC01DWTNI | - - - - - | gnl\|UG\|Ta#S58864639 |
| IUIVLUD01CPW4X | - - - - - | gnl\|UG\|Ta#S58865323 |
| IUIVLUD02JX9SK | - - - - - | gnl\|UG\|Ta#S58865323 |
| IV9RU5F02GIU7D | - - - - - | gnl\|UG\|Ta#S58866344 |
| IV9RU5F02GVWQF | - - - - - | gnl\|UG\|Ta#S58866344 |
| IUIVLUD02ICZTR | - - - - - | gnl\|UG\|Ta#S58866699 |
| IV9RU5F01A61NB | - - - - - | gnl\|UG\|Ta#S58866699 |
| IV9RU5F02HC8Z2 | - - - - - | gnl\|UG\|Ta#S58866699 |
| IVLNWEC01BYBKJ | - - - - - | gnl\|UG\|Ta#S58866699 |
| IV9RU5F02H0IM0 | - - - - - | gnl\|UG\|Ta#S58867212 |
| IV9RU5F02IFO92 | - - - - - | gnl\|UG\|Ta#S58867212 |
| IVLNWEC01D2CLC | - - - - - | gnl\|UG\|Ta#S58867212 |
| IV9RU5F01DI2OE | - - - - - | gnl\|UG\|Ta#S58867904 |

| | | |
|---|---|---|
| IVLNWEC01BRKDT | - - - - - | gnl\|UG\|Ta#S58867904 |
| IVLNWEC01BUC71 | - - - - - | gnl\|UG\|Ta#S58869079 |
| IVLNWEC01EUU13 | - - - - - | gnl\|UG\|Ta#S58869079 |
| IV9RU5F01DLTX0 | - - - - - | gnl\|UG\|Ta#S58869342 |
| IV9RU5F02GZIKO | - - - - - | gnl\|UG\|Ta#S58869342 |
| IV9RU5F02I48GI | - - - - - | gnl\|UG\|Ta#S58869342 |
| IVLNWEC02H8BM0 | - - - - - | gnl\|UG\|Ta#S58869837 |
| IV9RU5F01C14AG | - - - - - | gnl\|UG\|Ta#S58881890 |
| IVLNWEC01DCNG1 | - - - - - | gnl\|UG\|Ta#S58881890 |
| IVLNWEC02ITVWM | - - - - - | gnl\|UG\|Ta#S58882108 |
| IVLNWEC02JZV9I | - - - - - | gnl\|UG\|Ta#S58882108 |
| IV9RU5F01CPKT6 | - - - - - | gnl\|UG\|Ta#S58882412 |
| IV9RU5F01ELMFP | - - - - - | gnl\|UG\|Ta#S58882412 |
| IVLNWEC01BT3O6 | - - - - - | gnl\|UG\|Ta#S58882523 |
| IVLNWEC01BXET5 | - - - - - | gnl\|UG\|Ta#S58882523 |
| IV9RU5F01BNUJD | - - - - - | gnl\|UG\|Ta#S58882943 |
| IVLNWEC02IH8CZ | - - - - - | gnl\|UG\|Ta#S58882943 |
| IV9RU5F01CFXN5 | - - - - - | gnl\|UG\|Ta#S58883121 |
| IVLNWEC01EKIIG | - - - - - | gnl\|UG\|Ta#S58883121 |
| IV9RU5F02GS72T | - - - - - | gnl\|UG\|Ta#S58883797 |
| IVLNWEC02HJ377 | - - - - - | gnl\|UG\|Ta#S58883797 |
| IUIVLUD02FZFK0 | - - - - - | gnl\|UG\|Ta#S58884789 |
| IV9RU5F01A28JD | - - - - - | gnl\|UG\|Ta#S58884789 |
| IUIVLUD01ETMRF | - - - - - | gnl\|UG\|Ta#S58885081 |
| IV9RU5F02HGSCV | - - - - - | gnl\|UG\|Ta#S58885081 |
| IUIVLUD01CNIJL | - - - - - | gnl\|UG\|Ta#S58886623 |
| IVLNWEC01CFNWQ | - - - - - | gnl\|UG\|Ta#S58886623 |
| IUIVLUD01CDGY5 | - - - - - | gnl\|UG\|Ta#S58887172 |
| IUIVLUD02JNPA5 | - - - - - | gnl\|UG\|Ta#S58887172 |
| IUIVLUD02I3R8G | - - - - - | gnl\|UG\|Ta#S58887568 |
| IVLNWEC02HW2U0 | - - - - - | gnl\|UG\|Ta#S58887568 |
| IV9RU5F02F8WFY | - - - - - | gnl\|UG\|Ta#S58888193 |
| IVLNWEC02HG5D1 | - - - - - | gnl\|UG\|Ta#S58888193 |
| IVLNWEC02JIX2D | - - - - - | gnl\|UG\|Ta#S58888193 |
| IUIVLUD01DL5E4 | - - - - - | gnl\|UG\|Ta#S58889097 |
| IV9RU5F01BUJAL | - - - - - | gnl\|UG\|Ta#S58889097 |
| IVLNWEC01EEH03 | - - - - - | gnl\|UG\|Ta#S58889097 |
| IUIVLUD01BXZWI | - - - - - | gnl\|UG\|Ta#S58889970 |
| IV9RU5F01DARVA | - - - - - | gnl\|UG\|Ta#S58889970 |
| IVLNWEC02I3ZG0 | - - - - - | gnl\|UG\|Ta#S58890062 |
| IV9RU5F02FRNW3 | - - - - - | gnl\|UG\|Ta#S58891101 |
| IVLNWEC02IU14L | - - - - - | gnl\|UG\|Ta#S58891101 |
| IVLNWEC01EFMHF | - - - - - | gnl\|UG\|Ta#S58891385 |
| IVLNWEC02HCXEQ | - - - - - | gnl\|UG\|Ta#S58891385 |
| IUIVLUD02HISU1 | - - - - - | gnl\|UG\|Ta#S58891736 |
| IUIVLUD02I98KI | - - - - - | gnl\|UG\|Ta#S58891736 |
| IV9RU5F02HQK23 | - - - - - | gnl\|UG\|Ta#S58891736 |
| IVLNWEC02FNCYI | - - - - - | gnl\|UG\|Ta#S58891736 |
| IV9RU5F01B9QAB | - - - - - | gnl\|UG\|Ta#S58892193 |
| IVLNWEC02GJCO7 | - - - - - | gnl\|UG\|Ta#S58892193 |

| | | |
|---|---|---|
| IV9RU5F01DWK2O | - - - - - | gnl\|UG\|Ta#S58892333 |
| IVLNWEC02G3QQ3 | - - - - - | gnl\|UG\|Ta#S58892333 |
| IV9RU5F01E3091 | - - - - - | gnl\|UG\|Ta#S58892780 |
| IVLNWEC01DA88C | - - - - - | gnl\|UG\|Ta#S58892780 |
| IUIVLUD01A1NVM | - - - - - | gnl\|UG\|Ta#S58893514 |
| IV9RU5F02FPAEO | - - - - - | gnl\|UG\|Ta#S58893514 |
| IV9RU5F01EIE2X | - - - - - | gnl\|UG\|Ta#S58893820 |
| IV9RU5F02F41GY | - - - - - | gnl\|UG\|Ta#S58893820 |
| IVLNWEC01DJUGJ | - - - - - | gnl\|UG\|Ta#S58893820 |
| IV9RU5F02I03QF | - - - - - | gnl\|UG\|Ta#S58894380 |
| IVLNWEC01BBNKM | - - - - - | gnl\|UG\|Ta#S58894380 |
| IUIVLUD01DT5U4 | - - - - - | gnl\|UG\|Ta#S58894753 |
| IV9RU5F01AZU3U | - - - - - | gnl\|UG\|Ta#S58894753 |
| IV9RU5F01CCLYH | - - - - - | gnl\|UG\|Ta#S58894753 |
| IV9RU5F02GSOOE | - - - - - | gnl\|UG\|Ta#S58894753 |
| IV9RU5F02I0TYK | - - - - - | gnl\|UG\|Ta#S58895350 |
| IVLNWEC01D6EKD | - - - - - | gnl\|UG\|Ta#S58895350 |
| IV9RU5F01CU979 | - - - - - | gnl\|UG\|Ta#S58895933 |
| IV9RU5F02JE1E8 | - - - - - | gnl\|UG\|Ta#S58895933 |
| IUIVLUD02FSO4Z | - - - - - | gnl\|UG\|Ta#S58901254 |
| IV9RU5F02HHXQE | - - - - - | gnl\|UG\|Ta#S58901254 |
| IVLNWEC01BLT2Z | - - - - - | gnl\|UG\|Ta#S58904400 |
| IVLNWEC01C0J17 | - - - - - | gnl\|UG\|Ta#S58904400 |
| IUIVLUD02F6LY8 | - - - - - | gnl\|UG\|Ta#S58905261 |
| IV9RU5F02I91UF | - - - - - | gnl\|UG\|Ta#S58905261 |
| IVLNWEC02GFXVZ | - - - - - | gnl\|UG\|Ta#S58905261 |
| IUIVLUD01DZYS0 | - - - - - | gnl\|UG\|Ta#S58905476 |
| IVLNWEC02IVB4Y | - - - - - | gnl\|UG\|Ta#S58905476 |
| IV9RU5F01DBDEI | - - - - - | gnl\|UG\|Ta#S58906504 |
| IV9RU5F01E0FRK | - - - - - | gnl\|UG\|Ta#S58906504 |
| IV9RU5F02H0BFH | - - - - - | gnl\|UG\|Ta#S58906504 |
| IVLNWEC01BYLGB | - - - - - | gnl\|UG\|Ta#S58906504 |
| IVLNWEC01ENG6C | - - - - - | gnl\|UG\|Ta#S58906504 |
| IVLNWEC01EXAEA | - - - - - | gnl\|UG\|Ta#S58906504 |
| IVLNWEC02FQ4KD | - - - - - | gnl\|UG\|Ta#S58906504 |
| IVLNWEC02IGSDW | - - - - - | gnl\|UG\|Ta#S58906504 |
| IVLNWEC02IMNFG | - - - - - | gnl\|UG\|Ta#S58906504 |
| IVLNWEC02JF4LI | - - - - - | gnl\|UG\|Ta#S58906504 |
| IUIVLUD01AN0NY | - - - - - | gnl\|UG\|Ta#S58906839 |
| IV9RU5F01BGKWB | - - - - - | gnl\|UG\|Ta#S58906839 |
| IV9RU5F01A7OLG | - - - - - | gnl\|UG\|Ta#S58906990 |
| IVLNWEC01ENWO8 | - - - - - | gnl\|UG\|Ta#S58906990 |
| IUIVLUD01E0QYL | - - - - - | gnl\|UG\|Ta#S58907785 |
| IV9RU5F02J3USG | - - - - - | gnl\|UG\|Ta#S58907785 |
| IUIVLUD01B11ET | - - - - - | gnl\|UG\|Ta#S58910045 |
| IV9RU5F02JU95H | - - - - - | gnl\|UG\|Ta#S58910045 |
| IUIVLUD02HXTX8 | - - - - - | gnl\|UG\|Ta#S58910574 |
| IV9RU5F02HA08P | - - - - - | gnl\|UG\|Ta#S58910574 |
| IV9RU5F01EBJ7K | - - - - - | gnl\|UG\|Ta#S58911197 |
| IUIVLUD01EOGJ6 | - - - - - | gnl\|UG\|Ta#S61516645 |

| | | |
|---|---|---|
| IUIVLUD02H12VO | - - - - - | gnl\|UG\|Ta#S61516645 |
| IUIVLUD01BURNO | - - - - - | gnl\|UG\|Ta#S61516800 |
| IV9RU5F01BP6EE | - - - - - | gnl\|UG\|Ta#S61517675 |
| IV9RU5F02GTL7H | - - - - - | gnl\|UG\|Ta#S61517675 |
| IV9RU5F02HMX3Y | - - - - - | gnl\|UG\|Ta#S61517786 |
| IVLNWEC02G316Q | - - - - - | gnl\|UG\|Ta#S61517786 |
| IV9RU5F02F5T4B | - - - - - | gnl\|UG\|Ta#S61518397 |
| IVLNWEC01ATJ15 | - - - - - | gnl\|UG\|Ta#S61518397 |
| IV9RU5F01BPZ5N | - - - - - | gnl\|UG\|Ta#S61518685 |
| IVLNWEC02ILIHV | - - - - - | gnl\|UG\|Ta#S61518685 |
| IV9RU5F02GDP2J | - - - - - | gnl\|UG\|Ta#S61519093 |
| IVLNWEC01CBG54 | - - - - - | gnl\|UG\|Ta#S61519093 |
| IVLNWEC01ESQNV | - - - - - | gnl\|UG\|Ta#S61519424 |
| IVLNWEC02IVV2Q | - - - - - | gnl\|UG\|Ta#S61519424 |
| IUIVLUD01BJRHG | - - - - - | gnl\|UG\|Ta#S61521127 |
| IV9RU5F02F9H51 | - - - - - | gnl\|UG\|Ta#S61521127 |
| IUIVLUD01BO8HY | - - - - - | gnl\|UG\|Ta#S61521798 |
| IUIVLUD02J0YS9 | - - - - - | gnl\|UG\|Ta#S61521798 |
| IUIVLUD02FKTV1 | - - - - - | gnl\|UG\|Ta#S61523651 |
| IVLNWEC02JAGSX | - - - - - | gnl\|UG\|Ta#S61523651 |
| IVLNWEC02F4Q50 | - - - - - | gnl\|UG\|Ta#S61523950 |
| IUIVLUD01B0ZIV | - - - - - | gnl\|UG\|Ta#S61524170 |
| IUIVLUD02ILM79 | - - - - - | gnl\|UG\|Ta#S61524170 |
| IVLNWEC01AI3FW | - - - - - | gnl\|UG\|Ta#S61524170 |
| IUIVLUD01EPVAL | - - - - - | gnl\|UG\|Ta#S61524327 |
| IV9RU5F01B9D7X | - - - - - | gnl\|UG\|Ta#S61524327 |
| IUIVLUD01CE1HA | - - - - - | gnl\|UG\|Ta#S61525033 |
| IVLNWEC01D0XHQ | - - - - - | gnl\|UG\|Ta#S61525033 |
| IVLNWEC02IMH0C | - - - - - | gnl\|UG\|Ta#S61525033 |
| IUIVLUD01DGYNY | - - - - - | gnl\|UG\|Ta#S61525035 |
| IVLNWEC02IMTVG | - - - - - | gnl\|UG\|Ta#S61525035 |
| IUIVLUD01C76DK | - - - - - | gnl\|UG\|Ta#S61525041 |
| IVLNWEC01DR5RH | - - - - - | gnl\|UG\|Ta#S61525041 |
| IV9RU5F01AWN3Q | - - - - - | gnl\|UG\|Ta#S61525166 |
| IV9RU5F01DLEIP | - - - - - | gnl\|UG\|Ta#S61525166 |
| IV9RU5F01BOS25 | - - - - - | gnl\|UG\|Ta#S61526624 |
| IV9RU5F02HM9EH | - - - - - | gnl\|UG\|Ta#S61526624 |
| IVLNWEC01CC4SU | - - - - - | gnl\|UG\|Ta#S61526624 |
| IVLNWEC02ICLKG | - - - - - | gnl\|UG\|Ta#S61526624 |
| IV9RU5F02JMTX5 | - - - - - | gnl\|UG\|Ta#S61526808 |
| IVLNWEC01E1BTW | - - - - - | gnl\|UG\|Ta#S61526808 |
| IUIVLUD02F8O95 | - - - - - | gnl\|UG\|Ta#S61526999 |
| IVLNWEC02JFITH | - - - - - | gnl\|UG\|Ta#S61526999 |
| IVLNWEC01DTIXF | - - - - - | gnl\|UG\|Ta#S61528389 |
| IVLNWEC02G6NGX | - - - - - | gnl\|UG\|Ta#S61528389 |
| IVLNWEC01EYX1E | - - - - - | gnl\|UG\|Ta#S61529185 |
| IVLNWEC02G9RR5 | - - - - - | gnl\|UG\|Ta#S61529185 |
| IV9RU5F01ASB6Z | - - - - - | gnl\|UG\|Ta#S61529250 |
| IV9RU5F02F8KZ5 | - - - - - | gnl\|UG\|Ta#S61529250 |
| IV9RU5F02JD9RO | - - - - - | gnl\|UG\|Ta#S61529250 |

| | | |
|---|---|---|
| IV9RU5F01BAQ8J | - - - - - | gnl\|UG\|Ta#S61529393 |
| IVLNWEC02I6QVO | - - - - - | gnl\|UG\|Ta#S61529393 |
| IV9RU5F01CD8EJ | - - - - - | gnl\|UG\|Ta#S61529652 |
| IVLNWEC01C88FF | - - - - - | gnl\|UG\|Ta#S61529652 |
| IV9RU5F02HYUML | - - - - - | gnl\|UG\|Ta#S61531271 |
| IVLNWEC02I16N4 | - - - - - | gnl\|UG\|Ta#S61531271 |
| IVLNWEC01AY1ZG | - - - - - | gnl\|UG\|Ta#S61532521 |
| IVLNWEC01D0KZE | - - - - - | gnl\|UG\|Ta#S61532521 |
| IVLNWEC01B3DU5 | - - - - - | gnl\|UG\|Ta#S61532975 |
| IVLNWEC02HP2BL | - - - - - | gnl\|UG\|Ta#S61532975 |
| IVLNWEC02IQMU8 | - - - - - | gnl\|UG\|Ta#S61532975 |
| IUIVLUD02G593Y | - - - - - | gnl\|UG\|Ta#S61532997 |
| IV9RU5F02F7S47 | - - - - - | gnl\|UG\|Ta#S61532997 |
| IV9RU5F02JNL9T | - - - - - | gnl\|UG\|Ta#S61533995 |
| IVLNWEC01A3H9L | - - - - - | gnl\|UG\|Ta#S61533995 |
| IUIVLUD01D2JOZ | - - - - - | gnl\|UG\|Ta#S61536424 |
| IUIVLUD02FTF4Z | - - - - - | gnl\|UG\|Ta#S61536424 |
| IV9RU5F02GW1JT | - - - - - | gnl\|UG\|Ta#S61536424 |
| IV9RU5F02JZSL5 | - - - - - | gnl\|UG\|Ta#S61536424 |
| IVLNWEC01E1IK3 | - - - - - | gnl\|UG\|Ta#S61536544 |
| IVLNWEC01ECABR | - - - - - | gnl\|UG\|Ta#S61536544 |
| IVLNWEC02I1963 | - - - - - | gnl\|UG\|Ta#S61536544 |
| IUIVLUD01BAF6Y | - - - - - | gnl\|UG\|Ta#S61537228 |
| IV9RU5F01BNY6P | - - - - - | gnl\|UG\|Ta#S61537228 |
| IV9RU5F02H6XEI | - - - - - | gnl\|UG\|Ta#S61537228 |
| IUIVLUD02F5ZJK | - - - - - | gnl\|UG\|Ta#S61539919 |
| IV9RU5F02JK2NA | - - - - - | gnl\|UG\|Ta#S61539919 |
| IV9RU5F02IBDKD | - - - - - | gnl\|UG\|Ta#S61540318 |
| IVLNWEC01E0A3M | - - - - - | gnl\|UG\|Ta#S61540318 |
| IVLNWEC02HAREP | - - - - - | gnl\|UG\|Ta#S61541173 |
| IVLNWEC02HU2ZF | - - - - - | gnl\|UG\|Ta#S61541173 |
| IV9RU5F01DHLVP | - - - - - | gnl\|UG\|Ta#S61541540 |
| IV9RU5F01EAXEV | - - - - - | gnl\|UG\|Ta#S61541540 |
| IV9RU5F01BRXWP | - - - - - | gnl\|UG\|Ta#S61541830 |
| IV9RU5F01DUCK3 | - - - - - | gnl\|UG\|Ta#S61541830 |
| IVLNWEC02HZ6PA | - - - - - | gnl\|UG\|Ta#S61541830 |
| IV9RU5F01APJ2M | - - - - - | gnl\|UG\|Ta#S61542500 |
| IVLNWEC01CHQA9 | - - - - - | gnl\|UG\|Ta#S61542500 |
| IV9RU5F01CIP2W | - - - - - | gnl\|UG\|Ta#S61542662 |
| IVLNWEC01B7EKK | - - - - - | gnl\|UG\|Ta#S61542662 |
| IVLNWEC02HPJPG | - - - - - | gnl\|UG\|Ta#S61542662 |
| IUIVLUD01CVENB | - - - - - | gnl\|UG\|Ta#S61543175 |
| IUIVLUD02F95OC | - - - - - | gnl\|UG\|Ta#S61543175 |
| IV9RU5F01EKUHB | - - - - - | gnl\|UG\|Ta#S61543175 |
| IV9RU5F02GKIIM | - - - - - | gnl\|UG\|Ta#S61543839 |
| IV9RU5F02GM70A | - - - - - | gnl\|UG\|Ta#S61543839 |
| IV9RU5F02HIP6B | - - - - - | gnl\|UG\|Ta#S61543839 |
| IUIVLUD01AYF9J | - - - - - | gnl\|UG\|Ta#S61544374 |
| IV9RU5F01BR313 | - - - - - | gnl\|UG\|Ta#S61544374 |
| IV9RU5F01DJWWW | - - - - - | gnl\|UG\|Ta#S61544374 |

| | | |
|---|---|---|
| IVLNWEC01CQJJC | - - - - - | gnl\|UG\|Ta#S61544374 |
| IUIVLUD01EC6G4 | - - - - - | gnl\|UG\|Ta#S61548557 |
| IV9RU5F01BW2ED | - - - - - | gnl\|UG\|Ta#S61548557 |
| IVLNWEC01DGK30 | - - - - - | gnl\|UG\|Ta#S61548557 |
| IV9RU5F01BQ5PW | - - - - - | gnl\|UG\|Ta#S61548897 |
| IVLNWEC01E09PG | - - - - - | gnl\|UG\|Ta#S61548897 |
| IV9RU5F02FMGSW | - - - - - | gnl\|UG\|Ta#S61549422 |
| IVLNWEC02H77C6 | - - - - - | gnl\|UG\|Ta#S61549422 |
| IV9RU5F02JLCMD | - - - - - | gnl\|UG\|Ta#S61550178 |
| IVLNWEC01C9JTG | - - - - - | gnl\|UG\|Ta#S61550178 |
| IVLNWEC01EC5D8 | - - - - - | gnl\|UG\|Ta#S61550178 |
| IV9RU5F01B4RAA | - - - - - | gnl\|UG\|Ta#S61553220 |
| IV9RU5F02IJKR4 | - - - - - | gnl\|UG\|Ta#S61553220 |
| IV9RU5F02IRDS0 | - - - - - | gnl\|UG\|Ta#S61553220 |
| IUIVLUD02JQP6Z | - - - - - | gnl\|UG\|Ta#S61553236 |
| IVLNWEC01DGGP9 | - - - - - | gnl\|UG\|Ta#S61553236 |
| IV9RU5F02FR2ER | - - - - - | gnl\|UG\|Ta#S61554355 |
| IVLNWEC02G3URT | - - - - - | gnl\|UG\|Ta#S61554355 |
| IV9RU5F01BZPAD | - - - - - | gnl\|UG\|Ta#S61554479 |
| IV9RU5F01D5744 | - - - - - | gnl\|UG\|Ta#S61554479 |
| IVLNWEC01AMUTV | - - - - - | gnl\|UG\|Ta#S61554479 |
| IV9RU5F01DYBFP | - - - - - | gnl\|UG\|Ta#S61554680 |
| IV9RU5F02GU346 | - - - - - | gnl\|UG\|Ta#S61554680 |
| IV9RU5F02FVHL7 | - - - - - | gnl\|UG\|Ta#S61556703 |
| IVLNWEC02JBSSV | - - - - - | gnl\|UG\|Ta#S61556703 |
| IV9RU5F01BQHZT | - - - - - | gnl\|UG\|Ta#S61556794 |
| IV9RU5F01DVMBF | - - - - - | gnl\|UG\|Ta#S61556794 |
| IVLNWEC01ERBEI | - - - - - | gnl\|UG\|Ta#S61557267 |
| IVLNWEC02H0YPX | - - - - - | gnl\|UG\|Ta#S61557267 |
| IV9RU5F01AQWVZ | - - - - - | gnl\|UG\|Ta#S61558235 |
| IV9RU5F01CGI6U | - - - - - | gnl\|UG\|Ta#S61558235 |
| IVLNWEC01BGRHU | - - - - - | gnl\|UG\|Ta#S61558490 |
| IVLNWEC02F2U65 | - - - - - | gnl\|UG\|Ta#S61558490 |
| IUIVLUD02HMYJU | - - - - - | gnl\|UG\|Ta#S61559464 |
| IV9RU5F01EB2YK | - - - - - | gnl\|UG\|Ta#S61559464 |
| IV9RU5F01BZG66 | - - - - - | gnl\|UG\|Ta#S61559793 |
| IVLNWEC01EQKKO | - - - - - | gnl\|UG\|Ta#S61559793 |
| IUIVLUD01EBS09 | - - - - - | gnl\|UG\|Ta#S61560522 |
| IUIVLUD02H8VIJ | - - - - - | gnl\|UG\|Ta#S61560522 |
| IV9RU5F01EVDVT | - - - - - | gnl\|UG\|Ta#S61561507 |
| IVLNWEC01EGTXY | - - - - - | gnl\|UG\|Ta#S61561507 |
| IVLNWEC02IK7XM | - - - - - | gnl\|UG\|Ta#S61561507 |
| IUIVLUD01E0CF4 | - - - - - | gnl\|UG\|Ta#S61562186 |
| IVLNWEC01A8909 | - - - - - | gnl\|UG\|Ta#S61562186 |
| IV9RU5F01DUQ5Q | - - - - - | gnl\|UG\|Ta#S61562714 |
| IVLNWEC01A7G81 | - - - - - | gnl\|UG\|Ta#S61562714 |
| IVLNWEC01CYW2O | - - - - - | gnl\|UG\|Ta#S61562714 |
| IVLNWEC01EU1PU | - - - - - | gnl\|UG\|Ta#S61562714 |
| IVLNWEC02HP3U3 | - - - - - | gnl\|UG\|Ta#S61562807 |
| IVLNWEC02J2PL9 | - - - - - | gnl\|UG\|Ta#S61562807 |

| | | |
|---|---|---|
| IUIVLUD02JVZVY | - - - - - | gnl\|UG\|Ta#S61563584 |
| IV9RU5F02HCK5X | - - - - - | gnl\|UG\|Ta#S61563584 |
| IV9RU5F02JQ71M | - - - - - | gnl\|UG\|Ta#S61564794 |
| IVLNWEC01EY0XZ | - - - - - | gnl\|UG\|Ta#S61564794 |
| IUIVLUD01C2YMT | - - - - - | gnl\|UG\|Ta#S61565848 |
| IV9RU5F01ALQXU | - - - - - | gnl\|UG\|Ta#S61565848 |
| IVLNWEC02HF9QS | - - - - - | gnl\|UG\|Ta#S61565848 |
| IUIVLUD01A2DTI | - - - - - | gnl\|UG\|Ta#S61566619 |
| IVLNWEC01BBRPM | - - - - - | gnl\|UG\|Ta#S61566619 |
| IV9RU5F01CI08G | - - - - - | gnl\|UG\|Ta#S61568667 |
| IV9RU5F02INAG6 | - - - - - | gnl\|UG\|Ta#S61568667 |
| IVLNWEC01CES0I | - - - - - | gnl\|UG\|Ta#S61568667 |
| IV9RU5F01A9RBW | - - - - - | gnl\|UG\|Ta#S61569037 |
| IV9RU5F02HZGAY | - - - - - | gnl\|UG\|Ta#S61569037 |
| IUIVLUD01A3AZB | - - - - - | gnl\|UG\|Ta#S61570773 |
| IVLNWEC02GSGCH | - - - - - | gnl\|UG\|Ta#S61570773 |
| IUIVLUD01EBK60 | - - - - - | gnl\|UG\|Ta#S61570777 |
| IV9RU5F01DYGIX | - - - - - | gnl\|UG\|Ta#S61570777 |
| IUIVLUD01BU6UH | - - - - - | gnl\|UG\|Ta#S61571703 |
| IUIVLUD02HJWPO | - - - - - | gnl\|UG\|Ta#S61571703 |
| IUIVLUD02JXRMG | - - - - - | gnl\|UG\|Ta#S61572016 |
| IV9RU5F01EYN7B | - - - - - | gnl\|UG\|Ta#S61572016 |
| IUIVLUD02JHJML | - - - - - | gnl\|UG\|Ta#S61573236 |
| IV9RU5F01EOE19 | - - - - - | gnl\|UG\|Ta#S61573236 |
| IVLNWEC02H35I4 | - - - - - | gnl\|UG\|Ta#S61574012 |
| IVLNWEC01AI6HU | - - - - - | gnl\|UG\|Ta#S61574566 |
| IVLNWEC02I3W6M | - - - - - | gnl\|UG\|Ta#S61574566 |
| IVLNWEC01BIEXE | - - - - - | gnl\|UG\|Ta#S61576919 |
| IVLNWEC02HKJ1A | - - - - - | gnl\|UG\|Ta#S61576919 |
| IV9RU5F01BFR02 | - - - - - | gnl\|UG\|Ta#S61577686 |
| IVLNWEC01DQ37G | - - - - - | gnl\|UG\|Ta#S61577686 |
| IUIVLUD02IQOCD | - - - - - | gnl\|UG\|Ta#S61578399 |
| IV9RU5F01CQJLF | - - - - - | gnl\|UG\|Ta#S61578399 |
| IVLNWEC01EE0GU | - - - - - | gnl\|UG\|Ta#S61578399 |
| IUIVLUD01BPU10 | - - - - - | gnl\|UG\|Ta#S61579516 |
| IUIVLUD02JO441 | - - - - - | gnl\|UG\|Ta#S61579516 |
| IVLNWEC02FUHUQ | - - - - - | gnl\|UG\|Ta#S61579516 |
| IVLNWEC02HI9Q2 | - - - - - | gnl\|UG\|Ta#S61579516 |
| IUIVLUD01DX1B7 | - - - - - | gnl\|UG\|Ta#S61579721 |
| IV9RU5F02IYZEF | - - - - - | gnl\|UG\|Ta#S61579721 |
| IVLNWEC01BFW5W | - - - - - | gnl\|UG\|Ta#S61579721 |
| IVLNWEC01BQAK8 | - - - - - | gnl\|UG\|Ta#S61579721 |
| IVLNWEC02I8IEJ | - - - - - | gnl\|UG\|Ta#S61579721 |
| IUIVLUD02G176V | - - - - - | gnl\|UG\|Ta#S61580360 |
| IV9RU5F02FIFKB | - - - - - | gnl\|UG\|Ta#S61580360 |
| IV9RU5F01DSTB6 | - - - - - | gnl\|UG\|Ta#S61581379 |
| IV9RU5F02JQ0PJ | - - - - - | gnl\|UG\|Ta#S61581379 |
| IUIVLUD01B4197 | - - - - - | gnl\|UG\|Ta#S61581462 |
| IVLNWEC02GZZVP | - - - - - | gnl\|UG\|Ta#S61581462 |
| IUIVLUD01D4SDZ | - - - - - | gnl\|UG\|Ta#S61582037 |

| | | |
|---|---|---|
| IV9RU5F01C49YX | - - - - - | gnl\|UG\|Ta#S61582037 |
| IVLNWEC01ERIOF | - - - - - | gnl\|UG\|Ta#S61582037 |
| IV9RU5F01B46Z5 | - - - - - | gnl\|UG\|Ta#S61582158 |
| IV9RU5F02G7EJA | - - - - - | gnl\|UG\|Ta#S61582158 |
| IVLNWEC01B187B | - - - - - | gnl\|UG\|Ta#S61582158 |
| IVLNWEC01COWDL | - - - - - | gnl\|UG\|Ta#S61583803 |
| IVLNWEC02FNCY6 | - - - - - | gnl\|UG\|Ta#S61583803 |
| IVLNWEC01B3VCO | - - - - - | gnl\|UG\|Ta#S61585469 |
| IVLNWEC02JXZR2 | - - - - - | gnl\|UG\|Ta#S61585469 |
| IUIVLUD01D2IKX | - - - - - | gnl\|UG\|Ta#S61585532 |
| IUIVLUD01DEWG9 | - - - - - | gnl\|UG\|Ta#S61585532 |
| IV9RU5F02JUFXO | - - - - - | gnl\|UG\|Ta#S61585532 |
| IUIVLUD01CHVEF | - - - - - | gnl\|UG\|Ta#S61585609 |
| IV9RU5F02JWGD4 | - - - - - | gnl\|UG\|Ta#S61585609 |
| IV9RU5F01BPZLS | - - - - - | gnl\|UG\|Ta#S61586121 |
| IV9RU5F02FWYBC | - - - - - | gnl\|UG\|Ta#S61586121 |
| IVLNWEC02IKHMM | - - - - - | gnl\|UG\|Ta#S61586334 |
| IV9RU5F01EJ45G | - - - - - | gnl\|UG\|Ta#S61587421 |
| IVLNWEC01BLMK4 | - - - - - | gnl\|UG\|Ta#S61587421 |
| IVLNWEC01E2GK3 | - - - - - | gnl\|UG\|Ta#S61587421 |
| IV9RU5F01BV84E | - - - - - | gnl\|UG\|Ta#S61587437 |
| IV9RU5F02FRTXB | - - - - - | gnl\|UG\|Ta#S61587437 |
| IV9RU5F02IN2MP | - - - - - | gnl\|UG\|Ta#S61587437 |
| IUIVLUD02GA46M | - - - - - | gnl\|UG\|Ta#S61588059 |
| IVLNWEC02GU5J1 | - - - - - | gnl\|UG\|Ta#S61588059 |
| IV9RU5F01EIV4Y | - - - - - | gnl\|UG\|Ta#S61588131 |
| IVLNWEC01C968M | - - - - - | gnl\|UG\|Ta#S61588131 |
| IUIVLUD01C5NRJ | - - - - - | gnl\|UG\|Ta#S61588855 |
| IV9RU5F02GZ104 | - - - - - | gnl\|UG\|Ta#S61588855 |
| IUIVLUD01CN45T | - - - - - | gnl\|UG\|Ta#S61589293 |
| IVLNWEC01D7BSI | - - - - - | gnl\|UG\|Ta#S61589293 |
| IVLNWEC01EPSGM | - - - - - | gnl\|UG\|Ta#S61589293 |
| IV9RU5F02GTLHO | - - - - - | gnl\|UG\|Ta#S61589418 |
| IV9RU5F02IF43V | - - - - - | gnl\|UG\|Ta#S61589418 |
| IVLNWEC02H7H2Z | - - - - - | gnl\|UG\|Ta#S61589418 |
| IUIVLUD02HZFVJ | - - - - - | gnl\|UG\|Ta#S61589523 |
| IV9RU5F01A6MUM | - - - - - | gnl\|UG\|Ta#S61589523 |
| IVLNWEC01BDMF9 | - - - - - | gnl\|UG\|Ta#S61589523 |
| IUIVLUD01B00F0 | - - - - - | gnl\|UG\|Ta#S61589844 |
| IVLNWEC01BGJYC | - - - - - | gnl\|UG\|Ta#S61589844 |
| IVLNWEC02IQO2P | - - - - - | gnl\|UG\|Ta#S61590135 |
| IVLNWEC02JHTI3 | - - - - - | gnl\|UG\|Ta#S61590135 |
| IUIVLUD01D9GRV | - - - - - | gnl\|UG\|Ta#S61590227 |
| IV9RU5F02IAINR | - - - - - | gnl\|UG\|Ta#S61590227 |
| IV9RU5F01CXT35 | - - - - - | gnl\|UG\|Ta#S61590346 |
| IVLNWEC01CUKYY | - - - - - | gnl\|UG\|Ta#S61590346 |
| IV9RU5F01EIWOA | - - - - - | gnl\|UG\|Ta#S61590842 |
| IUIVLUD02I0CSO | - - - - - | gnl\|UG\|Ta#S61590861 |
| IV9RU5F02I0UYH | - - - - - | gnl\|UG\|Ta#S61590861 |
| IUIVLUD02HQ5PY | - - - - - | gnl\|UG\|Ta#S61591024 |

| | | |
|---|---|---|
| IV9RU5F01AX87N | - - - - - | gnl\|UG\|Ta#S61591024 |
| IVLNWEC01DEXSC | - - - - - | gnl\|UG\|Ta#S61591024 |
| IV9RU5F01BCL72 | - - - - - | gnl\|UG\|Ta#S61591343 |
| IV9RU5F01BWLW9 | - - - - - | gnl\|UG\|Ta#S61591343 |
| IUIVLUD01EWB3G | - - - - - | gnl\|UG\|Ta#S61591757 |
| IV9RU5F01APDX2 | - - - - - | gnl\|UG\|Ta#S61591757 |
| IVLNWEC02FXHYL | - - - - - | gnl\|UG\|Ta#S61591757 |
| IVLNWEC02JSI3J | - - - - - | gnl\|UG\|Ta#S61591757 |
| IV9RU5F02IX3JR | - - - - - | gnl\|UG\|Ta#S61592450 |
| IVLNWEC01CP4J8 | - - - - - | gnl\|UG\|Ta#S61592450 |
| IVLNWEC02HAIYH | - - - - - | gnl\|UG\|Ta#S61593039 |
| IVLNWEC02JMLFM | - - - - - | gnl\|UG\|Ta#S61593039 |
| IV9RU5F01AG03K | - - - - - | gnl\|UG\|Ta#S61593215 |
| IVLNWEC01CUHJO | - - - - - | gnl\|UG\|Ta#S61593215 |
| IVLNWEC01EKGT9 | - - - - - | gnl\|UG\|Ta#S61593215 |
| IUIVLUD01CR1MX | - - - - - | gnl\|UG\|Ta#S61594577 |
| IV9RU5F01D57XY | - - - - - | gnl\|UG\|Ta#S61594577 |
| IV9RU5F01EMKUY | - - - - - | gnl\|UG\|Ta#S61594577 |
| IVLNWEC01BJFJA | - - - - - | gnl\|UG\|Ta#S61594577 |
| IVLNWEC02IW3JO | - - - - - | gnl\|UG\|Ta#S61594577 |
| IV9RU5F01AUZRR | - - - - - | gnl\|UG\|Ta#S61595161 |
| IV9RU5F01EYEQ9 | - - - - - | gnl\|UG\|Ta#S61595161 |
| IV9RU5F01DIWSM | - - - - - | gnl\|UG\|Ta#S61596069 |
| IVLNWEC02FPQFN | - - - - - | gnl\|UG\|Ta#S61596069 |
| IVLNWEC02H5C6W | - - - - - | gnl\|UG\|Ta#S61596069 |
| IV9RU5F01D7159 | - - - - - | gnl\|UG\|Ta#S61596134 |
| IVLNWEC01B9FKB | - - - - - | gnl\|UG\|Ta#S61596134 |
| IV9RU5F02G7UPE | - - - - - | gnl\|UG\|Ta#S61596319 |
| IVLNWEC02J0UBB | - - - - - | gnl\|UG\|Ta#S61596468 |
| IV9RU5F02FJ7OB | - - - - - | gnl\|UG\|Ta#S61596831 |
| IVLNWEC01CGE7R | - - - - - | gnl\|UG\|Ta#S61596831 |
| IUIVLUD02JN1ZV | - - - - - | gnl\|UG\|Ta#S61598188 |
| IVLNWEC02GEOSX | - - - - - | gnl\|UG\|Ta#S61598188 |
| IUIVLUD01C53JF | - - - - - | gnl\|UG\|Ta#S61598803 |
| IV9RU5F01A4O7C | - - - - - | gnl\|UG\|Ta#S61598803 |
| IV9RU5F01AS9RN | - - - - - | gnl\|UG\|Ta#S61599047 |
| IV9RU5F02G00M3 | - - - - - | gnl\|UG\|Ta#S61599047 |
| IV9RU5F02H8TOE | - - - - - | gnl\|UG\|Ta#S61599047 |
| IVLNWEC01DXLGX | - - - - - | gnl\|UG\|Ta#S61599047 |
| IUIVLUD01EYHZV | - - - - - | gnl\|UG\|Ta#S61599083 |
| IV9RU5F01DQHTK | - - - - - | gnl\|UG\|Ta#S61599083 |
| IV9RU5F02JOAJN | - - - - - | gnl\|UG\|Ta#S61599083 |
| IVLNWEC01EK2GP | - - - - - | gnl\|UG\|Ta#S61599083 |
| IUIVLUD01DD82C | - - - - - | gnl\|UG\|Ta#S61599435 |
| IV9RU5F01EE7IU | - - - - - | gnl\|UG\|Ta#S61599435 |
| IUIVLUD01AZRJ1 | - - - - - | gnl\|UG\|Ta#S61600082 |
| IVLNWEC02INRS9 | - - - - - | gnl\|UG\|Ta#S61600082 |
| IVLNWEC01B2D0T | - - - - - | gnl\|UG\|Ta#S61600477 |
| IVLNWEC01CU0EC | - - - - - | gnl\|UG\|Ta#S61600477 |
| IUIVLUD01DYV7R | - - - - - | gnl\|UG\|Ta#S61600740 |

| | | |
|---|---|---|
| IUIVLUD02GD9Y4 | - - - - - | gnl\|UG\|Ta#S61600740 |
| IV9RU5F02HU9TE | - - - - - | gnl\|UG\|Ta#S61601435 |
| IV9RU5F02HZIZO | - - - - - | gnl\|UG\|Ta#S61601435 |
| IUIVLUD01BF3BU | - - - - - | gnl\|UG\|Ta#S61601831 |
| IV9RU5F01DEST7 | - - - - - | gnl\|UG\|Ta#S61601831 |
| IV9RU5F02I26KH | - - - - - | gnl\|UG\|Ta#S61602892 |
| IV9RU5F02IBYGB | - - - - - | gnl\|UG\|Ta#S61602892 |
| IV9RU5F01BETG9 | - - - - - | gnl\|UG\|Ta#S61602960 |
| IV9RU5F01ETVF4 | - - - - - | gnl\|UG\|Ta#S61602960 |
| IUIVLUD01A83QP | - - - - - | gnl\|UG\|Ta#S61603521 |
| IV9RU5F01EWY0X | - - - - - | gnl\|UG\|Ta#S61603521 |
| IV9RU5F02I592F | - - - - - | gnl\|UG\|Ta#S61604819 |
| IVLNWEC01BJQUQ | - - - - - | gnl\|UG\|Ta#S61604819 |
| IV9RU5F01EIS6B | - - - - - | gnl\|UG\|Ta#S61607104 |
| IV9RU5F02GPA4B | - - - - - | gnl\|UG\|Ta#S61607104 |
| IV9RU5F01C3KER | - - - - - | gnl\|UG\|Ta#S61607593 |
| IVLNWEC02HGIAK | - - - - - | gnl\|UG\|Ta#S61607593 |
| IV9RU5F01CJYO3 | - - - - - | gnl\|UG\|Ta#S61607643 |
| IVLNWEC02GWSSF | - - - - - | gnl\|UG\|Ta#S61607643 |
| IUIVLUD01ALCOW | - - - - - | gnl\|UG\|Ta#S61608681 |
| IV9RU5F01AUYB9 | - - - - - | gnl\|UG\|Ta#S61608681 |
| IV9RU5F01AVKU1 | - - - - - | gnl\|UG\|Ta#S61608900 |
| IV9RU5F01D53LF | - - - - - | gnl\|UG\|Ta#S61608900 |
| IV9RU5F02IKN4M | - - - - - | gnl\|UG\|Ta#S61608995 |
| IV9RU5F02JJVFW | - - - - - | gnl\|UG\|Ta#S61608995 |
| IV9RU5F01A2CQE | - - - - - | gnl\|UG\|Ta#S61609018 |
| IVLNWEC02JSO1P | - - - - - | gnl\|UG\|Ta#S61609018 |
| IV9RU5F01AIQ5N | - - - - - | gnl\|UG\|Ta#S61609445 |
| IV9RU5F02FS89Y | - - - - - | gnl\|UG\|Ta#S61609445 |
| IUIVLUD02F1LQ3 | - - - - - | gnl\|UG\|Ta#S61609564 |
| IV9RU5F02HO9V6 | - - - - - | gnl\|UG\|Ta#S61609564 |
| IV9RU5F02FVW9V | - - - - - | gnl\|UG\|Ta#S61609706 |
| IVLNWEC02HM0AI | - - - - - | gnl\|UG\|Ta#S61609706 |
| IVLNWEC02J4XQ1 | - - - - - | gnl\|UG\|Ta#S61609706 |
| IV9RU5F01CP5TU | - - - - - | gnl\|UG\|Ta#S61609979 |
| IVLNWEC01EMSZ3 | - - - - - | gnl\|UG\|Ta#S61609979 |
| IVLNWEC01CN0FF | - - - - - | gnl\|UG\|Ta#S61611320 |
| IVLNWEC02GEPBM | - - - - - | gnl\|UG\|Ta#S61611320 |
| IUIVLUD01D3XY5 | - - - - - | gnl\|UG\|Ta#S61611922 |
| IUIVLUD02HR1X9 | - - - - - | gnl\|UG\|Ta#S61611922 |
| IV9RU5F01DQ8Q2 | - - - - - | gnl\|UG\|Ta#S61612360 |
| IVLNWEC02HQO5Y | - - - - - | gnl\|UG\|Ta#S61612360 |
| IUIVLUD01EB894 | - - - - - | gnl\|UG\|Ta#S61614123 |
| IV9RU5F02FX5A2 | - - - - - | gnl\|UG\|Ta#S61614123 |
| IV9RU5F02HWZWU | - - - - - | gnl\|UG\|Ta#S61614123 |
| IUIVLUD02F9U6W | - - - - - | gnl\|UG\|Ta#S61614799 |
| IV9RU5F01CO0JK | - - - - - | gnl\|UG\|Ta#S61614799 |
| IVLNWEC02JHHL1 | - - - - - | gnl\|UG\|Ta#S61614799 |
| IV9RU5F02IASWM | - - - - - | gnl\|UG\|Ta#S61614825 |
| IVLNWEC01COTHA | - - - - - | gnl\|UG\|Ta#S61614825 |

| | | |
|---|---|---|
| IVLNWEC02FV9F2 | - - - - - | gnl\|UG\|Ta#S61614825 |
| IUIVLUD02G52LI | - - - - - | gnl\|UG\|Ta#S61615491 |
| IV9RU5F02FRNYV | - - - - - | gnl\|UG\|Ta#S61616159 |
| IV9RU5F02JOQP5 | - - - - - | gnl\|UG\|Ta#S61616159 |
| IV9RU5F01BYYYM | - - - - - | gnl\|UG\|Ta#S61616216 |
| IV9RU5F02G0FIX | - - - - - | gnl\|UG\|Ta#S61616216 |
| IVLNWEC02F7S95 | - - - - - | gnl\|UG\|Ta#S61616216 |
| IUIVLUD01B58MR | - - - - - | gnl\|UG\|Ta#S61616676 |
| IVLNWEC01D998J | - - - - - | gnl\|UG\|Ta#S61616676 |
| IUIVLUD01C04FY | - - - - - | gnl\|UG\|Ta#S61616822 |
| IVLNWEC02FXGBU | - - - - - | gnl\|UG\|Ta#S61616822 |
| IVLNWEC01BWC0N | - - - - - | gnl\|UG\|Ta#S61617394 |
| IV9RU5F01E3RO0 | - - - - - | gnl\|UG\|Ta#S61617846 |
| IVLNWEC02HUOS1 | - - - - - | gnl\|UG\|Ta#S61617846 |
| IV9RU5F02HYIG6 | - - - - - | gnl\|UG\|Ta#S61618918 |
| IV9RU5F02I4TJ2 | - - - - - | gnl\|UG\|Ta#S61618918 |
| IV9RU5F02IE220 | - - - - - | gnl\|UG\|Ta#S61618918 |
| IV9RU5F01DLNAS | - - - - - | gnl\|UG\|Ta#S61618924 |
| IVLNWEC02IT9YD | - - - - - | gnl\|UG\|Ta#S61618924 |
| IV9RU5F01CEPV9 | - - - - - | gnl\|UG\|Ta#S61619055 |
| IVLNWEC01BB2MA | - - - - - | gnl\|UG\|Ta#S61619055 |
| IV9RU5F01AHFJN | - - - - - | gnl\|UG\|Ta#S61619919 |
| IVLNWEC01CAE6L | - - - - - | gnl\|UG\|Ta#S61619919 |
| IVLNWEC02GIF8P | - - - - - | gnl\|UG\|Ta#S61619919 |
| IV9RU5F02G6711 | - - - - - | gnl\|UG\|Ta#S61620231 |
| IV9RU5F02H95Z2 | - - - - - | gnl\|UG\|Ta#S61620231 |
| IV9RU5F01DQY39 | - - - - - | gnl\|UG\|Ta#S61620617 |
| IVLNWEC02G5430 | - - - - - | gnl\|UG\|Ta#S61620617 |
| IUIVLUD02H4LY1 | - - - - - | gnl\|UG\|Ta#S61620966 |
| IV9RU5F01AZOY1 | - - - - - | gnl\|UG\|Ta#S61620966 |
| IV9RU5F01E027J | - - - - - | gnl\|UG\|Ta#S61620966 |
| IVLNWEC02I00S6 | - - - - - | gnl\|UG\|Ta#S61620966 |
| IUIVLUD02J16UM | - - - - - | gnl\|UG\|Ta#S61623782 |
| IVLNWEC02I1W63 | - - - - - | gnl\|UG\|Ta#S61623782 |
| IV9RU5F01BZEHR | - - - - - | gnl\|UG\|Ta#S61623891 |
| IV9RU5F02G9D2U | - - - - - | gnl\|UG\|Ta#S61623891 |
| IV9RU5F02JZNCR | - - - - - | gnl\|UG\|Ta#S61624083 |
| IVLNWEC01CPHPI | - - - - - | gnl\|UG\|Ta#S61624083 |
| IVLNWEC02INNG9 | - - - - - | gnl\|UG\|Ta#S61624083 |
| IVLNWEC02JBEP2 | - - - - - | gnl\|UG\|Ta#S61624083 |
| IUIVLUD01C28OD | - - - - - | gnl\|UG\|Ta#S61625893 |
| IV9RU5F01BDCRD | - - - - - | gnl\|UG\|Ta#S61625893 |
| IVLNWEC02H6L64 | - - - - - | gnl\|UG\|Ta#S61625893 |
| IVLNWEC01A49ZS | - - - - - | gnl\|UG\|Ta#S61625946 |
| IVLNWEC02JWKAY | - - - - - | gnl\|UG\|Ta#S61625946 |
| IUIVLUD02JHTSJ | - - - - - | gnl\|UG\|Ta#S61626171 |
| IV9RU5F01BK41Z | - - - - - | gnl\|UG\|Ta#S61626171 |
| IV9RU5F01D5VYL | - - - - - | gnl\|UG\|Ta#S61626171 |
| IV9RU5F02HOH6C | - - - - - | gnl\|UG\|Ta#S61626171 |
| IUIVLUD02IJTS2 | - - - - - | gnl\|UG\|Ta#S61626193 |

| | | |
|---|---|---|
| IV9RU5F01BZZBD | - - - - - | gnl\|UG\|Ta#S61626193 |
| IV9RU5F01DJPJE | - - - - - | gnl\|UG\|Ta#S61626339 |
| IVLNWEC01A1EFD | - - - - - | gnl\|UG\|Ta#S61626339 |
| IUIVLUD01CIFZ3 | - - - - - | gnl\|UG\|Ta#S61626430 |
| IV9RU5F02IB2OM | - - - - - | gnl\|UG\|Ta#S61626430 |
| IVLNWEC02FV82X | - - - - - | gnl\|UG\|Ta#S61626639 |
| IVLNWEC01CGA74 | - - - - - | gnl\|UG\|Ta#S61626908 |
| IVLNWEC01D6N32 | - - - - - | gnl\|UG\|Ta#S61626908 |
| IVLNWEC01EXNZW | - - - - - | gnl\|UG\|Ta#S61626908 |
| IUIVLUD02I4RYU | - - - - - | gnl\|UG\|Ta#S61630362 |
| IVLNWEC01AJ8SI | - - - - - | gnl\|UG\|Ta#S61630362 |
| IUIVLUD01CW89M | - - - - - | gnl\|UG\|Ta#S61632218 |
| IV9RU5F02JLKW8 | - - - - - | gnl\|UG\|Ta#S61632218 |
| IV9RU5F02JH5KV | - - - - - | gnl\|UG\|Ta#S61634061 |
| IVLNWEC02JD9Z5 | - - - - - | gnl\|UG\|Ta#S61634061 |
| IUIVLUD02HMWVO | - - - - - | gnl\|UG\|Ta#S61634783 |
| IVLNWEC02IMHI5 | - - - - - | gnl\|UG\|Ta#S61634783 |
| IUIVLUD02FTQ53 | - - - - - | gnl\|UG\|Ta#S61636247 |
| IV9RU5F02FVGZK | - - - - - | gnl\|UG\|Ta#S61636247 |
| IV9RU5F01EIDMM | - - - - - | gnl\|UG\|Ta#S61636383 |
| IVLNWEC01BZAQZ | - - - - - | gnl\|UG\|Ta#S61636383 |
| IV9RU5F01CDN44 | - - - - - | gnl\|UG\|Ta#S61637542 |
| IV9RU5F01CZE1O | - - - - - | gnl\|UG\|Ta#S61637542 |
| IV9RU5F02H1H07 | - - - - - | gnl\|UG\|Ta#S61637542 |
| IV9RU5F02JRLRS | - - - - - | gnl\|UG\|Ta#S61638483 |
| IVLNWEC01C46XU | - - - - - | gnl\|UG\|Ta#S61638483 |
| IV9RU5F02FWSTS | - - - - - | gnl\|UG\|Ta#S61638496 |
| IVLNWEC02G8GCY | - - - - - | gnl\|UG\|Ta#S61638496 |
| IV9RU5F01AP7G9 | - - - - - | gnl\|UG\|Ta#S61639637 |
| IV9RU5F01DD1QO | - - - - - | gnl\|UG\|Ta#S61639637 |
| IVLNWEC02JP3XF | - - - - - | gnl\|UG\|Ta#S61641004 |
| IVLNWEC01D5ZN1 | - - - - - | gnl\|UG\|Ta#S61641224 |
| IVLNWEC01ELFGE | - - - - - | gnl\|UG\|Ta#S61641224 |
| IUIVLUD01DH5P9 | - - - - - | gnl\|UG\|Ta#S61641353 |
| IUIVLUD01EMBBV | - - - - - | gnl\|UG\|Ta#S61641353 |
| IVLNWEC02HHAAR | - - - - - | gnl\|UG\|Ta#S61641353 |
| IV9RU5F01E2RQ1 | - - - - - | gnl\|UG\|Ta#S61641412 |
| IVLNWEC01ECPC6 | - - - - - | gnl\|UG\|Ta#S61641412 |
| IV9RU5F01A930P | - - - - - | gnl\|UG\|Ta#S61642221 |
| IVLNWEC02IH7QA | - - - - - | gnl\|UG\|Ta#S61642221 |
| IUIVLUD01DSLHD | - - - - - | gnl\|UG\|Ta#S61642322 |
| IV9RU5F02HU4BZ | - - - - - | gnl\|UG\|Ta#S61642322 |
| IV9RU5F01D8GHM | - - - - - | gnl\|UG\|Ta#S61643292 |
| IVLNWEC02HIL7U | - - - - - | gnl\|UG\|Ta#S61643292 |
| IVLNWEC02FUZTM | - - - - - | gnl\|UG\|Ta#S61643530 |
| IVLNWEC02JIJAA | - - - - - | gnl\|UG\|Ta#S61643530 |
| IUIVLUD01EZUP4 | - - - - - | gnl\|UG\|Ta#S61643669 |
| IUIVLUD02GWRHP | - - - - - | gnl\|UG\|Ta#S61643669 |
| IUIVLUD02HKWBK | - - - - - | gnl\|UG\|Ta#S61643669 |
| IVLNWEC01BNYA4 | - - - - - | gnl\|UG\|Ta#S61643669 |

| | | |
|---|---|---|
| IV9RU5F02F4AL7 | - - - - - | gnl\|UG\|Ta#S61644576 |
| IVLNWEC01DDAVL | - - - - - | gnl\|UG\|Ta#S61644576 |
| IVLNWEC02IWJQ9 | - - - - - | gnl\|UG\|Ta#S61644576 |
| IUIVLUD02HWNYZ | - - - - - | gnl\|UG\|Ta#S61644963 |
| IV9RU5F02GGJNC | - - - - - | gnl\|UG\|Ta#S61644963 |
| IV9RU5F02ITYUK | - - - - - | gnl\|UG\|Ta#S61644963 |
| IVLNWEC01CC990 | - - - - - | gnl\|UG\|Ta#S61644963 |
| IUIVLUD01BZ5M4 | - - - - - | gnl\|UG\|Ta#S61645271 |
| IVLNWEC02HK3QH | - - - - - | gnl\|UG\|Ta#S61645271 |
| IV9RU5F01CJPA4 | - - - - - | gnl\|UG\|Ta#S61645832 |
| IVLNWEC01B0UQG | - - - - - | gnl\|UG\|Ta#S61645832 |
| IVLNWEC02GAAWU | - - - - - | gnl\|UG\|Ta#S61645832 |
| IVLNWEC02J452L | - - - - - | gnl\|UG\|Ta#S61645832 |
| IV9RU5F01BTMHI | - - - - - | gnl\|UG\|Ta#S61646117 |
| IV9RU5F02G6KAT | - - - - - | gnl\|UG\|Ta#S61646117 |
| IV9RU5F02GTLC8 | - - - - - | gnl\|UG\|Ta#S61646117 |
| IV9RU5F01CICQ2 | - - - - - | gnl\|UG\|Ta#S61646194 |
| IV9RU5F01E0VSJ | - - - - - | gnl\|UG\|Ta#S61646194 |
| IV9RU5F02G6DGI | - - - - - | gnl\|UG\|Ta#S61646225 |
| IVLNWEC02G2V3K | - - - - - | gnl\|UG\|Ta#S61646225 |
| IV9RU5F02HJ7MS | - - - - - | gnl\|UG\|Ta#S61646502 |
| IVLNWEC01C001D | - - - - - | gnl\|UG\|Ta#S61646502 |
| IUIVLUD02IX891 | - - - - - | gnl\|UG\|Ta#S61646539 |
| IV9RU5F02HMI0C | - - - - - | gnl\|UG\|Ta#S61646539 |
| IVLNWEC01D1FYQ | - - - - - | gnl\|UG\|Ta#S61646539 |
| IV9RU5F01AI822 | - - - - - | gnl\|UG\|Ta#S61646849 |
| IV9RU5F01CCON7 | - - - - - | gnl\|UG\|Ta#S61646849 |
| IUIVLUD02F6KNF | - - - - - | gnl\|UG\|Ta#S61646897 |
| IV9RU5F02GC2P5 | - - - - - | gnl\|UG\|Ta#S61646897 |
| IV9RU5F01CALX5 | - - - - - | gnl\|UG\|Ta#S61647133 |
| IV9RU5F02JJCCJ | - - - - - | gnl\|UG\|Ta#S61647133 |
| IVLNWEC02H7S5I | - - - - - | gnl\|UG\|Ta#S61647133 |
| IV9RU5F02ILSVQ | - - - - - | gnl\|UG\|Ta#S61647611 |
| IV9RU5F02JHAQ5 | - - - - - | gnl\|UG\|Ta#S61647611 |
| IUIVLUD01DG31D | - - - - - | gnl\|UG\|Ta#S61647852 |
| IUIVLUD02GM1EF | - - - - - | gnl\|UG\|Ta#S61647852 |
| IUIVLUD02HH2TK | - - - - - | gnl\|UG\|Ta#S61647852 |
| IV9RU5F01CO5D3 | - - - - - | gnl\|UG\|Ta#S61647852 |
| IVLNWEC02GZ953 | - - - - - | gnl\|UG\|Ta#S61647873 |
| IVLNWEC02INK8R | - - - - - | gnl\|UG\|Ta#S61647873 |
| IVLNWEC02GOOLU | - - - - - | gnl\|UG\|Ta#S61648313 |
| IVLNWEC02J21WK | - - - - - | gnl\|UG\|Ta#S61648313 |
| IV9RU5F01E1PQ6 | - - - - - | gnl\|UG\|Ta#S61648528 |
| IV9RU5F02F936N | - - - - - | gnl\|UG\|Ta#S61648528 |
| IUIVLUD02INGIF | - - - - - | gnl\|UG\|Ta#S61648710 |
| IUIVLUD02JO4JU | - - - - - | gnl\|UG\|Ta#S61648710 |
| IV9RU5F02GL6HW | - - - - - | gnl\|UG\|Ta#S61648710 |
| IVLNWEC02HAIXP | - - - - - | gnl\|UG\|Ta#S61648710 |
| IVLNWEC02IM934 | - - - - - | gnl\|UG\|Ta#S61648710 |
| IUIVLUD02FX2AG | - - - - - | gnl\|UG\|Ta#S61648718 |

| | | |
|---|---|---|
| IVLNWEC02I6647 | - - - - - | gnl\|UG\|Ta#S61648718 |
| IV9RU5F01BRGQP | - - - - - | gnl\|UG\|Ta#S61649188 |
| IVLNWEC01DTC2E | - - - - - | gnl\|UG\|Ta#S61649188 |
| IV9RU5F01CMAX7 | - - - - - | gnl\|UG\|Ta#S61649256 |
| IV9RU5F02ILT5E | - - - - - | gnl\|UG\|Ta#S61649256 |
| IUIVLUD01DDXD4 | - - - - - | gnl\|UG\|Ta#S61649366 |
| IVLNWEC01EFKQA | - - - - - | gnl\|UG\|Ta#S61649366 |
| IV9RU5F02IMNAT | - - - - - | gnl\|UG\|Ta#S61649934 |
| IVLNWEC02H154Z | - - - - - | gnl\|UG\|Ta#S61649934 |
| IV9RU5F02J25CI | - - - - - | gnl\|UG\|Ta#S61649953 |
| IVLNWEC02JFJI0 | - - - - - | gnl\|UG\|Ta#S61649953 |
| IV9RU5F01BMXO2 | - - - - - | gnl\|UG\|Ta#S61650328 |
| IV9RU5F01BWM6E | - - - - - | gnl\|UG\|Ta#S61650328 |
| IVLNWEC01CLAGD | - - - - - | gnl\|UG\|Ta#S61650705 |
| IVLNWEC02FQDCD | - - - - - | gnl\|UG\|Ta#S61650705 |
| IVLNWEC02I3LRV | - - - - - | gnl\|UG\|Ta#S61650705 |
| IV9RU5F02JJP5P | - - - - - | gnl\|UG\|Ta#S61650975 |
| IVLNWEC01AZWET | - - - - - | gnl\|UG\|Ta#S61650975 |
| IV9RU5F01B2PVA | - - - - - | gnl\|UG\|Ta#S61651901 |
| IVLNWEC02IDY1Z | - - - - - | gnl\|UG\|Ta#S61651901 |
| IUIVLUD02JJZNS | - - - - - | gnl\|UG\|Ta#S61652031 |
| IV9RU5F02IA84S | - - - - - | gnl\|UG\|Ta#S61652031 |
| IVLNWEC02HBOMS | - - - - - | gnl\|UG\|Ta#S61652637 |
| IVLNWEC02I2RYJ | - - - - - | gnl\|UG\|Ta#S61652637 |
| IV9RU5F01BVD2Q | - - - - - | gnl\|UG\|Ta#S61652790 |
| IV9RU5F01EQ11E | - - - - - | gnl\|UG\|Ta#S61652790 |
| IVLNWEC01CY9MM | - - - - - | gnl\|UG\|Ta#S61652831 |
| IVLNWEC02F111K | - - - - - | gnl\|UG\|Ta#S61652831 |
| IV9RU5F01AROOS | - - - - - | gnl\|UG\|Ta#S61652897 |
| IVLNWEC02H30JG | - - - - - | gnl\|UG\|Ta#S61652897 |
| IV9RU5F02HB7F9 | - - - - - | gnl\|UG\|Ta#S61653056 |
| IVLNWEC01EVYYJ | - - - - - | gnl\|UG\|Ta#S61653056 |
| IVLNWEC02F6BM2 | - - - - - | gnl\|UG\|Ta#S61653056 |
| IUIVLUD01DNG0T | - - - - - | gnl\|UG\|Ta#S61653390 |
| IVLNWEC01BC7D6 | - - - - - | gnl\|UG\|Ta#S61653390 |
| IV9RU5F02IFJO2 | - - - - - | gnl\|UG\|Ta#S61653654 |
| IVLNWEC01ETRDI | - - - - - | gnl\|UG\|Ta#S61653654 |
| IVLNWEC02GEY14 | - - - - - | gnl\|UG\|Ta#S61653934 |
| IVLNWEC02H57RY | - - - - - | gnl\|UG\|Ta#S61653934 |
| IVLNWEC02HIRZJ | - - - - - | gnl\|UG\|Ta#S61653934 |
| IVLNWEC02JZ6QT | - - - - - | gnl\|UG\|Ta#S61653934 |
| IUIVLUD01D9LZ1 | - - - - - | gnl\|UG\|Ta#S61654160 |
| IVLNWEC01AZ05X | - - - - - | gnl\|UG\|Ta#S61654160 |
| IVLNWEC01DON8K | - - - - - | gnl\|UG\|Ta#S61654830 |
| IVLNWEC02GZRZ6 | - - - - - | gnl\|UG\|Ta#S61654830 |
| IVLNWEC02IZTFP | - - - - - | gnl\|UG\|Ta#S61654830 |
| IV9RU5F02JF88D | - - - - - | gnl\|UG\|Ta#S61655052 |
| IVLNWEC02G4SR5 | - - - - - | gnl\|UG\|Ta#S61655052 |
| IUIVLUD01ELMLK | - - - - - | gnl\|UG\|Ta#S61655075 |
| IV9RU5F01E10WQ | - - - - - | gnl\|UG\|Ta#S61655075 |

| | | |
|---|---|---|
| IV9RU5F01A8WGD | - - - - - | gnl\|UG\|Ta#S61655186 |
| IVLNWEC02FSDSQ | - - - - - | gnl\|UG\|Ta#S61655186 |
| IUIVLUD02F9M27 | - - - - - | gnl\|UG\|Ta#S61655313 |
| IV9RU5F02FZ3H6 | - - - - - | gnl\|UG\|Ta#S61655313 |
| IVLNWEC01CFGFN | - - - - - | gnl\|UG\|Ta#S61655313 |
| IVLNWEC01D139U | - - - - - | gnl\|UG\|Ta#S61655313 |
| IV9RU5F01C7K75 | - - - - - | gnl\|UG\|Ta#S61656177 |
| IV9RU5F02GIFGU | - - - - - | gnl\|UG\|Ta#S61656177 |
| IUIVLUD01D7P9Y | - - - - - | gnl\|UG\|Ta#S61656572 |
| IV9RU5F01EIJM8 | - - - - - | gnl\|UG\|Ta#S61656572 |
| IV9RU5F01D0AU4 | - - - - - | gnl\|UG\|Ta#S61656647 |
| IV9RU5F02GZJEY | - - - - - | gnl\|UG\|Ta#S61656647 |
| IV9RU5F02JJHDI | - - - - - | gnl\|UG\|Ta#S61656647 |
| IVLNWEC01CHJOS | - - - - - | gnl\|UG\|Ta#S61656647 |
| IUIVLUD02GPUKB | - - - - - | gnl\|UG\|Ta#S61657261 |
| IV9RU5F01BNFYT | - - - - - | gnl\|UG\|Ta#S61657261 |
| IVLNWEC01DC9GL | - - - - - | gnl\|UG\|Ta#S61657261 |
| IUIVLUD02FUT08 | - - - - - | gnl\|UG\|Ta#S61657829 |
| IVLNWEC02JIXIO | - - - - - | gnl\|UG\|Ta#S61657829 |
| IV9RU5F01CCYEN | - - - - - | gnl\|UG\|Ta#S61657942 |
| IV9RU5F02G9J5G | - - - - - | gnl\|UG\|Ta#S61657942 |
| IV9RU5F01BFMI6 | - - - - - | gnl\|UG\|Ta#S61658152 |
| IV9RU5F01CHKGW | - - - - - | gnl\|UG\|Ta#S61658152 |
| IVLNWEC01BAI0A | - - - - - | gnl\|UG\|Ta#S61658152 |
| IVLNWEC01EF3CG | - - - - - | gnl\|UG\|Ta#S61658461 |
| IVLNWEC02JY6L7 | - - - - - | gnl\|UG\|Ta#S61658461 |
| IUIVLUD01ESDDX | - - - - - | gnl\|UG\|Ta#S61658775 |
| IVLNWEC02JXCFL | - - - - - | gnl\|UG\|Ta#S61658775 |
| IUIVLUD01BETTA | - - - - - | gnl\|UG\|Ta#S61659259 |
| IV9RU5F01DT4K9 | - - - - - | gnl\|UG\|Ta#S61659259 |
| IUIVLUD02IH8OY | - - - - - | gnl\|UG\|Ta#S61659566 |
| IUIVLUD02IKEXH | - - - - - | gnl\|UG\|Ta#S61659566 |
| IVLNWEC01BLA13 | - - - - - | gnl\|UG\|Ta#S61659566 |
| IUIVLUD02I8D7G | - - - - - | gnl\|UG\|Ta#S61659888 |
| IVLNWEC01AO5TT | - - - - - | gnl\|UG\|Ta#S61659888 |
| IVLNWEC01BB8V7 | - - - - - | gnl\|UG\|Ta#S61660473 |
| IVLNWEC01CYCQ4 | - - - - - | gnl\|UG\|Ta#S61660473 |
| IVLNWEC01CZAAR | - - - - - | gnl\|UG\|Ta#S61660473 |
| IVLNWEC02HDG2G | - - - - - | gnl\|UG\|Ta#S61660473 |
| IUIVLUD02JDRGU | - - - - - | gnl\|UG\|Ta#S61660954 |
| IV9RU5F01EP3KH | - - - - - | gnl\|UG\|Ta#S61660954 |
| IV9RU5F01A3WJE | - - - - - | gnl\|UG\|Ta#S61661314 |
| IV9RU5F01DJYKA | - - - - - | gnl\|UG\|Ta#S61661314 |
| IV9RU5F01EGGR7 | - - - - - | gnl\|UG\|Ta#S61661314 |
| IUIVLUD01DW7NV | - - - - - | gnl\|UG\|Ta#S61661415 |
| IVLNWEC02GNF7X | - - - - - | gnl\|UG\|Ta#S61661415 |
| IUIVLUD02HA6F8 | - - - - - | gnl\|UG\|Ta#S61661909 |
| IV9RU5F02G406X | - - - - - | gnl\|UG\|Ta#S61661909 |
| IVLNWEC02JI0XS | - - - - - | gnl\|UG\|Ta#S61661909 |
| IUIVLUD01CRXX6 | - - - - - | gnl\|UG\|Ta#S61662140 |

| | | |
|---|---|---|
| IV9RU5F02GMKVX | - - - - - | gnl|UG|Ta#S61662140 |
| IV9RU5F02IYSDP | - - - - - | gnl|UG|Ta#S61662572 |
| IVLNWEC02JBTXQ | - - - - - | gnl|UG|Ta#S61662572 |
| IUIVLUD01DX634 | - - - - - | gnl|UG|Ta#S61662769 |
| IUIVLUD02H6VWK | - - - - - | gnl|UG|Ta#S61662769 |
| IV9RU5F01BHCW9 | - - - - - | gnl|UG|Ta#S61662871 |
| IV9RU5F02IL7AC | - - - - - | gnl|UG|Ta#S61662871 |
| IVLNWEC02FO1Q9 | - - - - - | gnl|UG|Ta#S61662876 |
| IVLNWEC02HKZKA | - - - - - | gnl|UG|Ta#S61662876 |
| IUIVLUD01DME5N | - - - - - | gnl|UG|Ta#S61662902 |
| IV9RU5F01CECU4 | - - - - - | gnl|UG|Ta#S61662902 |
| IVLNWEC02IZOMT | - - - - - | gnl|UG|Ta#S61662902 |
| IUIVLUD02G5Q9H | - - - - - | gnl|UG|Ta#S61663107 |
| IUIVLUD02IOK8L | - - - - - | gnl|UG|Ta#S61663107 |
| IV9RU5F02JTO83 | - - - - - | gnl|UG|Ta#S61663107 |
| IUIVLUD02JG7FF | - - - - - | gnl|UG|Ta#S61663163 |
| IV9RU5F01CTMMZ | - - - - - | gnl|UG|Ta#S61663163 |
| IVLNWEC01C7U8M | - - - - - | gnl|UG|Ta#S61663250 |
| IVLNWEC02JTD4W | - - - - - | gnl|UG|Ta#S61663250 |
| IV9RU5F02IP4ZN | - - - - - | gnl|UG|Ta#S61664388 |
| IVLNWEC02H59HT | - - - - - | gnl|UG|Ta#S61664388 |
| IV9RU5F01BBE02 | - - - - - | gnl|UG|Ta#S61665352 |
| IV9RU5F01BINNO | - - - - - | gnl|UG|Ta#S61665352 |
| IV9RU5F01D0JDS | - - - - - | gnl|UG|Ta#S61665352 |
| IV9RU5F01EGV15 | - - - - - | gnl|UG|Ta#S61665352 |
| IV9RU5F02HIBVZ | - - - - - | gnl|UG|Ta#S61665352 |
| IV9RU5F02HX5BD | - - - - - | gnl|UG|Ta#S61665470 |
| IVLNWEC01EEYB4 | - - - - - | gnl|UG|Ta#S61665470 |
| IV9RU5F01DB5TI | - - - - - | gnl|UG|Ta#S61665554 |
| IV9RU5F02JELEM | - - - - - | gnl|UG|Ta#S61665554 |
| IVLNWEC01AKIYX | - - - - - | gnl|UG|Ta#S61665742 |
| IVLNWEC02IG1Z6 | - - - - - | gnl|UG|Ta#S61665742 |
| IV9RU5F02FM28D | - - - - - | gnl|UG|Ta#S61665956 |
| IVLNWEC01DZSQ8 | - - - - - | gnl|UG|Ta#S61665956 |
| IV9RU5F01DHYPH | - - - - - | gnl|UG|Ta#S61666086 |
| IVLNWEC01A99AN | - - - - - | gnl|UG|Ta#S61666086 |
| IVLNWEC01E22G0 | - - - - - | gnl|UG|Ta#S61666086 |
| IUIVLUD01CC5K9 | - - - - - | gnl|UG|Ta#S61666655 |
| IV9RU5F02G9Q2C | - - - - - | gnl|UG|Ta#S61666655 |
| IVLNWEC02H21VP | - - - - - | gnl|UG|Ta#S61666655 |
| IVLNWEC02I28FW | - - - - - | gnl|UG|Ta#S61666655 |
| IV9RU5F01BW33F | - - - - - | gnl|UG|Ta#S61667059 |
| IVLNWEC01E5PLA | - - - - - | gnl|UG|Ta#S61667059 |
| IVLNWEC02H0GF5 | - - - - - | gnl|UG|Ta#S61667059 |
| IUIVLUD01E1DJ6 | - - - - - | gnl|UG|Ta#S61667275 |
| IUIVLUD02F58LZ | - - - - - | gnl|UG|Ta#S61667275 |
| IV9RU5F02GOXBD | - - - - - | gnl|UG|Ta#S61667275 |
| IVLNWEC02GU1EL | - - - - - | gnl|UG|Ta#S61667275 |
| IV9RU5F01D0B4X | - - - - - | gnl|UG|Ta#S61667499 |
| IV9RU5F02F5O6F | - - - - - | gnl|UG|Ta#S61667499 |

| | | |
|---|---|---|
| IUIVLUD01B8CTC | - - - - - | gnl\|UG\|Ta#S61667647 |
| IV9RU5F01DKVS2 | - - - - - | gnl\|UG\|Ta#S61667647 |
| IV9RU5F02H1XTI | - - - - - | gnl\|UG\|Ta#S61667808 |
| IVLNWEC02ISM63 | - - - - - | gnl\|UG\|Ta#S61667808 |
| IUIVLUD02HNRBN | - - - - - | gnl\|UG\|Ta#S61668167 |
| IUIVLUD01A5V9Z | - - - - - | gnl\|UG\|Ta#S61668230 |
| IUIVLUD02JSCC9 | - - - - - | gnl\|UG\|Ta#S61668230 |
| IV9RU5F02JL1SJ | - - - - - | gnl\|UG\|Ta#S61668230 |
| IV9RU5F02HGK74 | - - - - - | gnl\|UG\|Ta#S61668772 |
| IVLNWEC02GMLVI | - - - - - | gnl\|UG\|Ta#S61668772 |
| IUIVLUD01BSBLU | - - - - - | gnl\|UG\|Ta#S61669680 |
| IUIVLUD02HUAYO | - - - - - | gnl\|UG\|Ta#S61669680 |
| IUIVLUD02JU8I2 | - - - - - | gnl\|UG\|Ta#S61669680 |
| IVLNWEC02G0PMU | - - - - - | gnl\|UG\|Ta#S61669680 |
| IVLNWEC02HC18K | - - - - - | gnl\|UG\|Ta#S61669680 |
| IV9RU5F01DJCUB | - - - - - | gnl\|UG\|Ta#S61669687 |
| IV9RU5F01ERU8C | - - - - - | gnl\|UG\|Ta#S61669687 |
| IV9RU5F02HX4NO | - - - - - | gnl\|UG\|Ta#S61669687 |
| IV9RU5F02JYCZY | - - - - - | gnl\|UG\|Ta#S61669687 |
| IV9RU5F02F3IIU | - - - - - | gnl\|UG\|Ta#S61669714 |
| IVLNWEC01CBFUN | - - - - - | gnl\|UG\|Ta#S61669714 |
| IUIVLUD01A2L34 | - - - - - | gnl\|UG\|Ta#S61669723 |
| IVLNWEC02FOXU1 | - - - - - | gnl\|UG\|Ta#S61669723 |
| IVLNWEC01D6YMT | - - - - - | gnl\|UG\|Ta#S61669796 |
| IVLNWEC02IHCWC | - - - - - | gnl\|UG\|Ta#S61669796 |
| IV9RU5F02GNH12 | - - - - - | gnl\|UG\|Ta#S61670391 |
| IV9RU5F02H3VCC | - - - - - | gnl\|UG\|Ta#S61670391 |
| IV9RU5F02J3B4M | - - - - - | gnl\|UG\|Ta#S61670391 |
| IVLNWEC01EQWRT | - - - - - | gnl\|UG\|Ta#S61670391 |
| IV9RU5F01AXUXH | - - - - - | gnl\|UG\|Ta#S61670433 |
| IVLNWEC01C8YYF | - - - - - | gnl\|UG\|Ta#S61670433 |
| IV9RU5F01EF7UW | - - - - - | gnl\|UG\|Ta#S61670511 |
| IV9RU5F01ESK5H | - - - - - | gnl\|UG\|Ta#S61670511 |
| IV9RU5F02GKAV7 | - - - - - | gnl\|UG\|Ta#S61670767 |
| IV9RU5F02JPKDY | - - - - - | gnl\|UG\|Ta#S61670767 |
| IUIVLUD01EXAIY | - - - - - | gnl\|UG\|Ta#S61670881 |
| IVLNWEC01AGG6H | - - - - - | gnl\|UG\|Ta#S61670881 |
| IV9RU5F01BV6HJ | - - - - - | gnl\|UG\|Ta#S61670940 |
| IV9RU5F01DHWKP | - - - - - | gnl\|UG\|Ta#S61670940 |
| IVLNWEC01D0JUJ | - - - - - | gnl\|UG\|Ta#S61670940 |
| IV9RU5F02HEMIQ | - - - - - | gnl\|UG\|Ta#S61671550 |
| IVLNWEC01EGKLB | - - - - - | gnl\|UG\|Ta#S61671550 |
| IUIVLUD01CPOO5 | - - - - - | gnl\|UG\|Ta#S61671954 |
| IV9RU5F01AYVIC | - - - - - | gnl\|UG\|Ta#S61671954 |
| IVLNWEC01D0W3G | - - - - - | gnl\|UG\|Ta#S61672248 |
| IVLNWEC02IL66M | - - - - - | gnl\|UG\|Ta#S61672248 |
| IVLNWEC01B97PQ | - - - - - | gnl\|UG\|Ta#S61672342 |
| IVLNWEC02IHKS7 | - - - - - | gnl\|UG\|Ta#S61672342 |
| IUIVLUD02GWY00 | - - - - - | gnl\|UG\|Ta#S61672567 |
| IUIVLUD02H6CMC | - - - - - | gnl\|UG\|Ta#S61672567 |

| | | |
|---|---|---|
| IVLNWEC02G1EUK | - - - - - | gnl\|UG\|Ta#S61672567 |
| IUIVLUD02IVG51 | - - - - - | gnl\|UG\|Ta#S61673890 |
| IVLNWEC01EF3SG | - - - - - | gnl\|UG\|Ta#S61673890 |
| IV9RU5F01DOKKD | - - - - - | gnl\|UG\|Ta#S61674351 |
| IV9RU5F02GPJQT | - - - - - | gnl\|UG\|Ta#S61674351 |
| IV9RU5F01C6CLJ | - - - - - | gnl\|UG\|Ta#S61674576 |
| IV9RU5F01EV406 | - - - - - | gnl\|UG\|Ta#S61674576 |
| IUIVLUD01CUMAW | - - - - - | gnl\|UG\|Ta#S61674728 |
| IUIVLUD01DVE0Y | - - - - - | gnl\|UG\|Ta#S61674728 |
| IV9RU5F01B2P97 | - - - - - | gnl\|UG\|Ta#S61674728 |
| IV9RU5F01EQQOH | - - - - - | gnl\|UG\|Ta#S61674728 |
| IUIVLUD02ISARH | - - - - - | gnl\|UG\|Ta#S61674940 |
| IV9RU5F01DSH0I | - - - - - | gnl\|UG\|Ta#S61674940 |
| IVLNWEC01CWO1J | - - - - - | gnl\|UG\|Ta#S61674940 |
| IVLNWEC02H8AZK | - - - - - | gnl\|UG\|Ta#S61674940 |
| IV9RU5F01CR7BD | - - - - - | gnl\|UG\|Ta#S61675187 |
| IVLNWEC01D69N5 | - - - - - | gnl\|UG\|Ta#S61675187 |
| IUIVLUD01C7ZQ8 | - - - - - | gnl\|UG\|Ta#S61676232 |
| IV9RU5F02HWA23 | - - - - - | gnl\|UG\|Ta#S61676232 |
| IV9RU5F02JK7AR | - - - - - | gnl\|UG\|Ta#S61676756 |
| IVLNWEC01B09AH | - - - - - | gnl\|UG\|Ta#S61676756 |
| IUIVLUD02J2NII | - - - - - | gnl\|UG\|Ta#S61677217 |
| IV9RU5F02GW6W2 | - - - - - | gnl\|UG\|Ta#S61677217 |
| IUIVLUD02J46V5 | - - - - - | gnl\|UG\|Ta#S61677225 |
| IV9RU5F01CYJIH | - - - - - | gnl\|UG\|Ta#S61678138 |
| IVLNWEC01BPKGO | - - - - - | gnl\|UG\|Ta#S61678138 |
| IVLNWEC01A5CFF | - - - - - | gnl\|UG\|Ta#S61678589 |
| IVLNWEC01C39LL | - - - - - | gnl\|UG\|Ta#S61678589 |
| IVLNWEC02H4UKO | - - - - - | gnl\|UG\|Ta#S61678589 |
| IV9RU5F01BLSAS | - - - - - | gnl\|UG\|Ta#S61678849 |
| IV9RU5F02IQ4G3 | - - - - - | gnl\|UG\|Ta#S61678849 |
| IUIVLUD01EAOY2 | - - - - - | gnl\|UG\|Ta#S61678958 |
| IUIVLUD02GYLPI | - - - - - | gnl\|UG\|Ta#S61678958 |
| IV9RU5F01B3NUU | - - - - - | gnl\|UG\|Ta#S61678998 |
| IV9RU5F02F7UTG | - - - - - | gnl\|UG\|Ta#S61678998 |
| IUIVLUD02G7FH8 | - - - - - | gnl\|UG\|Ta#S61679002 |
| IUIVLUD02GJK78 | - - - - - | gnl\|UG\|Ta#S61679250 |
| IV9RU5F02G0C7X | - - - - - | gnl\|UG\|Ta#S61679250 |
| IVLNWEC02IT412 | - - - - - | gnl\|UG\|Ta#S61679250 |
| IUIVLUD01BWVT6 | - - - - - | gnl\|UG\|Ta#S61679536 |
| IVLNWEC02F4263 | - - - - - | gnl\|UG\|Ta#S61679536 |
| IV9RU5F01DR075 | - - - - - | gnl\|UG\|Ta#S61679828 |
| IV9RU5F02GK5UY | - - - - - | gnl\|UG\|Ta#S61679828 |
| IV9RU5F01AY3SM | - - - - - | gnl\|UG\|Ta#S61680115 |
| IV9RU5F01DFO92 | - - - - - | gnl\|UG\|Ta#S61680115 |
| IV9RU5F02HDEH7 | - - - - - | gnl\|UG\|Ta#S61680225 |
| IVLNWEC02GAPEO | - - - - - | gnl\|UG\|Ta#S61680225 |
| IV9RU5F01A0Y5L | - - - - - | gnl\|UG\|Ta#S61681393 |
| IV9RU5F01C18NY | - - - - - | gnl\|UG\|Ta#S61681393 |
| IV9RU5F01CB6AZ | - - - - - | gnl\|UG\|Ta#S61681393 |

| | | |
|---|---|---|
| IV9RU5F01D1EYB | - - - - - | gnl\|UG\|Ta#S61681393 |
| IVLNWEC01D3VPS | - - - - - | gnl\|UG\|Ta#S61681393 |
| IUIVLUD02J3A4M | - - - - - | gnl\|UG\|Ta#S61681435 |
| IV9RU5F01D1QPX | - - - - - | gnl\|UG\|Ta#S61681435 |
| IV9RU5F02JO1PB | - - - - - | gnl\|UG\|Ta#S61681435 |
| IVLNWEC02JZQWF | - - - - - | gnl\|UG\|Ta#S61681435 |
| IVLNWEC01ASXOE | - - - - - | gnl\|UG\|Ta#S61681952 |
| IVLNWEC01BD301 | - - - - - | gnl\|UG\|Ta#S61681952 |
| IV9RU5F01CA6MS | - - - - - | gnl\|UG\|Ta#S61682092 |
| IV9RU5F02H36A3 | - - - - - | gnl\|UG\|Ta#S61682092 |
| IV9RU5F01BAOYU | - - - - - | gnl\|UG\|Ta#S61682592 |
| IV9RU5F02IGEB4 | - - - - - | gnl\|UG\|Ta#S61682592 |
| IV9RU5F01CKGY1 | - - - - - | gnl\|UG\|Ta#S61682704 |
| IVLNWEC02IB1VO | - - - - - | gnl\|UG\|Ta#S61682704 |
| IV9RU5F02HZZVW | - - - - - | gnl\|UG\|Ta#S61682797 |
| IVLNWEC02GSY97 | - - - - - | gnl\|UG\|Ta#S61682797 |
| IUIVLUD01AU9Q4 | - - - - - | gnl\|UG\|Ta#S61683295 |
| IVLNWEC01AVWC3 | - - - - - | gnl\|UG\|Ta#S61683295 |
| IVLNWEC02JEKBY | - - - - - | gnl\|UG\|Ta#S61683295 |
| IUIVLUD02HKYRR | - - - - - | gnl\|UG\|Ta#S61683444 |
| IV9RU5F01DOY3D | - - - - - | gnl\|UG\|Ta#S61683444 |
| IV9RU5F01CG0BG | - - - - - | gnl\|UG\|Ta#S61683963 |
| IV9RU5F01EGOY5 | - - - - - | gnl\|UG\|Ta#S61683963 |
| IV9RU5F01B1ZQL | - - - - - | gnl\|UG\|Ta#S61684027 |
| IVLNWEC01B1JJY | - - - - - | gnl\|UG\|Ta#S61684027 |
| IV9RU5F01EYIFY | - - - - - | gnl\|UG\|Ta#S61684709 |
| IVLNWEC01BW4P9 | - - - - - | gnl\|UG\|Ta#S61684709 |
| IV9RU5F01BQAL9 | - - - - - | gnl\|UG\|Ta#S61685330 |
| IVLNWEC01C6UYB | - - - - - | gnl\|UG\|Ta#S61685330 |
| IVLNWEC01AJLRQ | - - - - - | gnl\|UG\|Ta#S61685482 |
| IVLNWEC02GNFQL | - - - - - | gnl\|UG\|Ta#S61685482 |
| IUIVLUD01BYH15 | - - - - - | gnl\|UG\|Ta#S61685696 |
| IV9RU5F01DPH25 | - - - - - | gnl\|UG\|Ta#S61685696 |
| IV9RU5F02HF17O | - - - - - | gnl\|UG\|Ta#S61685954 |
| IVLNWEC02F1LJ4 | - - - - - | gnl\|UG\|Ta#S61685954 |
| IUIVLUD02GY9W5 | - - - - - | gnl\|UG\|Ta#S61686259 |
| IVLNWEC01AUAID | - - - - - | gnl\|UG\|Ta#S61686259 |
| IVLNWEC02I5RX4 | - - - - - | gnl\|UG\|Ta#S61686259 |
| IV9RU5F01DKWAF | - - - - - | gnl\|UG\|Ta#S61686380 |
| IVLNWEC01ETI0S | - - - - - | gnl\|UG\|Ta#S61686380 |
| IVLNWEC02FR4AN | - - - - - | gnl\|UG\|Ta#S61686380 |
| IUIVLUD01ECQGN | - - - - - | gnl\|UG\|Ta#S61687843 |
| IUIVLUD02J092Z | - - - - - | gnl\|UG\|Ta#S61687843 |
| IV9RU5F01CUJO8 | - - - - - | gnl\|UG\|Ta#S61687843 |
| IUIVLUD02HYVA0 | - - - - - | gnl\|UG\|Ta#S61687941 |
| IV9RU5F02HPJ3Y | - - - - - | gnl\|UG\|Ta#S61687941 |
| IVLNWEC02F013S | - - - - - | gnl\|UG\|Ta#S61688278 |
| IVLNWEC02GXGAE | - - - - - | gnl\|UG\|Ta#S61688278 |
| IVLNWEC01BL6RW | - - - - - | gnl\|UG\|Ta#S61688568 |
| IVLNWEC02GE41T | - - - - - | gnl\|UG\|Ta#S61688568 |

| | | |
|---|---|---|
| IUIVLUD01EMVJM | - - - - - | gnl\|UG\|Ta#S61689113 |
| IUIVLUD02FKNF7 | - - - - - | gnl\|UG\|Ta#S61689113 |
| IV9RU5F01B5AZU | - - - - - | gnl\|UG\|Ta#S61689113 |
| IV9RU5F01CUJFA | - - - - - | gnl\|UG\|Ta#S61689113 |
| IVLNWEC02IKMVN | - - - - - | gnl\|UG\|Ta#S61689113 |
| IV9RU5F02H1LH8 | - - - - - | gnl\|UG\|Ta#S61689399 |
| IVLNWEC01BPIMX | - - - - - | gnl\|UG\|Ta#S61689399 |
| IVLNWEC02J0JRG | - - - - - | gnl\|UG\|Ta#S61689399 |
| IV9RU5F02H7MB2 | - - - - - | gnl\|UG\|Ta#S61689538 |
| IV9RU5F02INWFZ | - - - - - | gnl\|UG\|Ta#S61689538 |
| IVLNWEC01EJWTO | - - - - - | gnl\|UG\|Ta#S61689538 |
| IV9RU5F02I14IN | - - - - - | gnl\|UG\|Ta#S61689541 |
| IVLNWEC01BZ2AJ | - - - - - | gnl\|UG\|Ta#S61689541 |
| IV9RU5F01APPGK | - - - - - | gnl\|UG\|Ta#S61689652 |
| IV9RU5F01EEUAB | - - - - - | gnl\|UG\|Ta#S61689652 |
| IV9RU5F01DHWHA | - - - - - | gnl\|UG\|Ta#S61690656 |
| IV9RU5F02HXGI4 | - - - - - | gnl\|UG\|Ta#S61690656 |
| IVLNWEC02H20PP | - - - - - | gnl\|UG\|Ta#S61690656 |
| IUIVLUD02IAFE5 | - - - - - | gnl\|UG\|Ta#S61691581 |
| IVLNWEC01B60WN | - - - - - | gnl\|UG\|Ta#S61691581 |
| IUIVLUD02J1GT9 | - - - - - | gnl\|UG\|Ta#S61692150 |
| IV9RU5F02G1OHQ | - - - - - | gnl\|UG\|Ta#S61692150 |
| IV9RU5F02GYE46 | - - - - - | gnl\|UG\|Ta#S61692150 |
| IUIVLUD01E37ZL | - - - - - | gnl\|UG\|Ta#S61692428 |
| IUIVLUD02JDXSV | - - - - - | gnl\|UG\|Ta#S61692428 |
| IUIVLUD02IK7E3 | - - - - - | gnl\|UG\|Ta#S61692516 |
| IV9RU5F01BGJUB | - - - - - | gnl\|UG\|Ta#S61692516 |
| IUIVLUD01BOW2X | - - - - - | gnl\|UG\|Ta#S61692560 |
| IVLNWEC01DJXZ8 | - - - - - | gnl\|UG\|Ta#S61692573 |
| IVLNWEC02HC7M5 | - - - - - | gnl\|UG\|Ta#S61692573 |
| IV9RU5F02JOQLP | - - - - - | gnl\|UG\|Ta#S61692834 |
| IVLNWEC02HJKB8 | - - - - - | gnl\|UG\|Ta#S61692834 |
| IV9RU5F02H2NS0 | - - - - - | gnl\|UG\|Ta#S61692906 |
| IVLNWEC02GOK0Z | - - - - - | gnl\|UG\|Ta#S61692906 |
| IUIVLUD01A1F1J | - - - - - | gnl\|UG\|Ta#S61693191 |
| IUIVLUD02G4QZQ | - - - - - | gnl\|UG\|Ta#S61693191 |
| IUIVLUD02H6VV6 | - - - - - | gnl\|UG\|Ta#S61693438 |
| IVLNWEC01EZMCF | - - - - - | gnl\|UG\|Ta#S61693438 |
| IUIVLUD02HHFTV | - - - - - | gnl\|UG\|Ta#S61693943 |
| IVLNWEC02I3RRM | - - - - - | gnl\|UG\|Ta#S61693943 |
| IUIVLUD02GX9N6 | - - - - - | gnl\|UG\|Ta#S61694182 |
| IV9RU5F02G5SBI | - - - - - | gnl\|UG\|Ta#S61694182 |
| IUIVLUD01A0RGJ | - - - - - | gnl\|UG\|Ta#S61694624 |
| IV9RU5F02JKGYG | - - - - - | gnl\|UG\|Ta#S61694624 |
| IVLNWEC01DYZHH | - - - - - | gnl\|UG\|Ta#S61694624 |
| IUIVLUD01CBJ21 | - - - - - | gnl\|UG\|Ta#S61694796 |
| IUIVLUD02GS2JB | - - - - - | gnl\|UG\|Ta#S61694796 |
| IV9RU5F01DVFIH | - - - - - | gnl\|UG\|Ta#S61694796 |
| IUIVLUD01BEVQ0 | - - - - - | gnl\|UG\|Ta#S61694847 |
| IVLNWEC01BE83U | - - - - - | gnl\|UG\|Ta#S61694847 |

| | | |
|---|---|---|
| IV9RU5F01AXELX | - - - - - | gnl\|UG\|Ta#S61694952 |
| IVLNWEC01AV1I6 | - - - - - | gnl\|UG\|Ta#S61694952 |
| IVLNWEC02J1KD1 | - - - - - | gnl\|UG\|Ta#S61694952 |
| IVLNWEC02GGC30 | - - - - - | gnl\|UG\|Ta#S61694990 |
| IVLNWEC02HW38F | - - - - - | gnl\|UG\|Ta#S61694990 |
| IUIVLUD02JYTXK | - - - - - | gnl\|UG\|Ta#S61695167 |
| IUIVLUD02JZQ8A | - - - - - | gnl\|UG\|Ta#S61695167 |
| IVLNWEC01B2K2V | - - - - - | gnl\|UG\|Ta#S61695204 |
| IVLNWEC01EUCPQ | - - - - - | gnl\|UG\|Ta#S61695204 |
| IUIVLUD01BSA99 | - - - - - | gnl\|UG\|Ta#S61695205 |
| IV9RU5F01DGVF8 | - - - - - | gnl\|UG\|Ta#S61695332 |
| IV9RU5F01EI3Z2 | - - - - - | gnl\|UG\|Ta#S61695332 |
| IV9RU5F02G91B8 | - - - - - | gnl\|UG\|Ta#S61695332 |
| IVLNWEC02I5RW0 | - - - - - | gnl\|UG\|Ta#S61695332 |
| IV9RU5F01DUBH9 | - - - - - | gnl\|UG\|Ta#S61696645 |
| IV9RU5F02JPIW9 | - - - - - | gnl\|UG\|Ta#S61696645 |
| IUIVLUD02H952N | - - - - - | gnl\|UG\|Ta#S61696718 |
| IVLNWEC02JBI83 | - - - - - | gnl\|UG\|Ta#S61696718 |
| IV9RU5F02IK8HY | - - - - - | gnl\|UG\|Ta#S61697029 |
| IVLNWEC02HPPFD | - - - - - | gnl\|UG\|Ta#S61697029 |
| IV9RU5F02F25RA | - - - - - | gnl\|UG\|Ta#S61697219 |
| IV9RU5F02IHDWZ | - - - - - | gnl\|UG\|Ta#S61697219 |
| IV9RU5F01CN2AA | - - - - - | gnl\|UG\|Ta#S61697370 |
| IVLNWEC01CDN3W | - - - - - | gnl\|UG\|Ta#S61697370 |
| IUIVLUD02GNKA4 | - - - - - | gnl\|UG\|Ta#S61697522 |
| IV9RU5F01CMVFY | - - - - - | gnl\|UG\|Ta#S61697522 |
| IV9RU5F01B6AC2 | - - - - - | gnl\|UG\|Ta#S61697712 |
| IV9RU5F01BXOHV | - - - - - | gnl\|UG\|Ta#S61697712 |
| IVLNWEC01CXDZ7 | - - - - - | gnl\|UG\|Ta#S61697768 |
| IVLNWEC01D6YSJ | - - - - - | gnl\|UG\|Ta#S61697768 |
| IV9RU5F02IAP66 | - - - - - | gnl\|UG\|Ta#S61697865 |
| IVLNWEC02JKAY5 | - - - - - | gnl\|UG\|Ta#S61697865 |
| IUIVLUD01B3QJ2 | - - - - - | gnl\|UG\|Ta#S61698444 |
| IUIVLUD02GXC6D | - - - - - | gnl\|UG\|Ta#S61698444 |
| IUIVLUD02FOSDR | - - - - - | gnl\|UG\|Ta#S61699659 |
| IVLNWEC01A8RUY | - - - - - | gnl\|UG\|Ta#S61699659 |
| IV9RU5F02GBWAV | - - - - - | gnl\|UG\|Ta#S61699675 |
| IVLNWEC01CWJ12 | - - - - - | gnl\|UG\|Ta#S61699675 |
| IVLNWEC01BOVJ0 | - - - - - | gnl\|UG\|Ta#S61700523 |
| IVLNWEC02HCC6I | - - - - - | gnl\|UG\|Ta#S61700523 |
| IV9RU5F01BOOKP | - - - - - | gnl\|UG\|Ta#S61701778 |
| IV9RU5F02HE8XY | - - - - - | gnl\|UG\|Ta#S61701778 |
| IVLNWEC02F46TJ | - - - - - | gnl\|UG\|Ta#S61701778 |
| IVLNWEC02JN8XN | - - - - - | gnl\|UG\|Ta#S61701778 |
| IUIVLUD01EB36T | - - - - - | gnl\|UG\|Ta#S61702095 |
| IVLNWEC01EIXQ6 | - - - - - | gnl\|UG\|Ta#S61702095 |
| IUIVLUD01CVQGM | - - - - - | gnl\|UG\|Ta#S61702475 |
| IV9RU5F01DQ7QP | - - - - - | gnl\|UG\|Ta#S61702475 |
| IVLNWEC01ETJWN | - - - - - | gnl\|UG\|Ta#S61702475 |
| IVLNWEC02GJFKQ | - - - - - | gnl\|UG\|Ta#S61702475 |

| | | |
|---|---|---|
| IV9RU5F02HTYG7 | - - - - - | gnl\|UG\|Ta#S61704142 |
| IV9RU5F02J3X7T | - - - - - | gnl\|UG\|Ta#S61704142 |
| IUIVLUD01BU4T8 | - - - - - | gnl\|UG\|Ta#S61704224 |
| IVLNWEC01A8593 | - - - - - | gnl\|UG\|Ta#S61704224 |
| IVLNWEC01BJ47V | - - - - - | gnl\|UG\|Ta#S61704224 |
| IV9RU5F01DGTI2 | - - - - - | gnl\|UG\|Ta#S61704288 |
| IVLNWEC01ATBJV | - - - - - | gnl\|UG\|Ta#S61704288 |
| IVLNWEC01C888C | - - - - - | gnl\|UG\|Ta#S61704288 |
| IVLNWEC01EWQV0 | - - - - - | gnl\|UG\|Ta#S61704288 |
| IV9RU5F02GLTSD | - - - - - | gnl\|UG\|Ta#S61704289 |
| IV9RU5F02I404A | - - - - - | gnl\|UG\|Ta#S61704289 |
| IUIVLUD02GKNJ4 | - - - - - | gnl\|UG\|Ta#S61705055 |
| IV9RU5F01BY03F | - - - - - | gnl\|UG\|Ta#S61705055 |
| IV9RU5F02GL813 | - - - - - | gnl\|UG\|Ta#S61705055 |
| IV9RU5F02GPE0W | - - - - - | gnl\|UG\|Ta#S61705055 |
| IUIVLUD02GU4O7 | - - - - - | gnl\|UG\|Ta#S61705059 |
| IV9RU5F01D47B0 | - - - - - | gnl\|UG\|Ta#S61705059 |
| IV9RU5F02INO2K | - - - - - | gnl\|UG\|Ta#S61705923 |
| IVLNWEC02IBWIH | - - - - - | gnl\|UG\|Ta#S61705923 |
| IUIVLUD02ITNNK | - - - - - | gnl\|UG\|Ta#S61706062 |
| IV9RU5F01CYGYH | - - - - - | gnl\|UG\|Ta#S61706062 |
| IV9RU5F02FMPIQ | - - - - - | gnl\|UG\|Ta#S61706067 |
| IVLNWEC01ERSE2 | - - - - - | gnl\|UG\|Ta#S61706067 |
| IUIVLUD01AI6OP | - - - - - | gnl\|UG\|Ta#S61706276 |
| IVLNWEC02HVFAK | - - - - - | gnl\|UG\|Ta#S61706276 |
| IV9RU5F01EDHVK | - - - - - | gnl\|UG\|Ta#S61706599 |
| IVLNWEC01AOR5V | - - - - - | gnl\|UG\|Ta#S61706599 |
| IV9RU5F01BJCBH | - - - - - | gnl\|UG\|Ta#S61706858 |
| IV9RU5F02GVHCM | - - - - - | gnl\|UG\|Ta#S61706858 |
| IV9RU5F01CN6I6 | - - - - - | gnl\|UG\|Ta#S61706959 |
| IV9RU5F02HP9KR | - - - - - | gnl\|UG\|Ta#S61706959 |
| IVLNWEC01CBL5Z | - - - - - | gnl\|UG\|Ta#S61706984 |
| IUIVLUD02FYJ73 | - - - - - | gnl\|UG\|Ta#S61707040 |
| IV9RU5F01EC1LE | - - - - - | gnl\|UG\|Ta#S61707040 |
| IV9RU5F01ES5OB | - - - - - | gnl\|UG\|Ta#S61707040 |
| IVLNWEC01BB3UJ | - - - - - | gnl\|UG\|Ta#S61707040 |
| IVLNWEC02HQYUI | - - - - - | gnl\|UG\|Ta#S61707040 |
| IUIVLUD02JK48C | - - - - - | gnl\|UG\|Ta#S61707818 |
| IV9RU5F01EWLQX | - - - - - | gnl\|UG\|Ta#S61707818 |
| IUIVLUD02FKZF8 | - - - - - | gnl\|UG\|Ta#S61708896 |
| IV9RU5F02HEJB5 | - - - - - | gnl\|UG\|Ta#S61708896 |
| IVLNWEC02I5IZS | - - - - - | gnl\|UG\|Ta#S61708896 |
| IUIVLUD02H0J3A | - - - - - | gnl\|UG\|Ta#S61709160 |
| IV9RU5F02GE7X8 | - - - - - | gnl\|UG\|Ta#S61709160 |
| IVLNWEC01CLJSG | - - - - - | gnl\|UG\|Ta#S61709657 |
| IVLNWEC02I4U14 | - - - - - | gnl\|UG\|Ta#S61709657 |
| IUIVLUD02FXJEY | - - - - - | gnl\|UG\|Ta#S61709999 |
| IV9RU5F01EX82C | - - - - - | gnl\|UG\|Ta#S61709999 |
| IV9RU5F01AW7QP | - - - - - | gnl\|UG\|Ta#S61710893 |
| IVLNWEC01BKX9U | - - - - - | gnl\|UG\|Ta#S61710893 |

| | | |
|---|---|---|
| IUIVLUD01B4648 | - - - - - | gnl\|UG\|Ta#S61711424 |
| IVLNWEC01B6WEO | - - - - - | gnl\|UG\|Ta#S61711424 |
| IUIVLUD02H6OSX | - - - - - | gnl\|UG\|Ta#S61711777 |
| IV9RU5F02FYBWA | - - - - - | gnl\|UG\|Ta#S61711777 |
| IVLNWEC01EIV3X | - - - - - | gnl\|UG\|Ta#S61711777 |
| IV9RU5F01CRI4T | - - - - - | gnl\|UG\|Ta#S61711891 |
| IVLNWEC01D360J | - - - - - | gnl\|UG\|Ta#S61711891 |
| IV9RU5F01EBCAW | - - - - - | gnl\|UG\|Ta#S61712297 |
| IV9RU5F02JPAGZ | - - - - - | gnl\|UG\|Ta#S61712297 |
| IUIVLUD02H66EH | - - - - - | gnl\|UG\|Ta#S61712525 |
| IV9RU5F01CWO75 | - - - - - | gnl\|UG\|Ta#S61712525 |
| IV9RU5F01AXRO9 | - - - - - | gnl\|UG\|Ta#S61712788 |
| IV9RU5F02G1XU2 | - - - - - | gnl\|UG\|Ta#S61712788 |
| IV9RU5F02F3588 | - - - - - | gnl\|UG\|Ta#S61712997 |
| IVLNWEC02JFG1M | - - - - - | gnl\|UG\|Ta#S61712997 |
| IVLNWEC01ED48M | - - - - - | gnl\|UG\|Ta#S61713062 |
| IVLNWEC02IAB92 | - - - - - | gnl\|UG\|Ta#S61713062 |
| IV9RU5F01BZNV4 | - - - - - | gnl\|UG\|Ta#S61713216 |
| IV9RU5F01CTU7D | - - - - - | gnl\|UG\|Ta#S61713216 |
| IUIVLUD01BQQTI | - - - - - | gnl\|UG\|Ta#S61713922 |
| IV9RU5F02JT3RP | - - - - - | gnl\|UG\|Ta#S61713922 |
| IV9RU5F01EFG57 | - - - - - | gnl\|UG\|Ta#S61714554 |
| IV9RU5F02GE2ZB | - - - - - | gnl\|UG\|Ta#S61714554 |
| IUIVLUD01BP7EM | - - - - - | gnl\|UG\|Ta#S61714631 |
| IUIVLUD02GFMYK | - - - - - | gnl\|UG\|Ta#S61714631 |
| IVLNWEC02G5VIB | - - - - - | gnl\|UG\|Ta#S61714631 |
| IUIVLUD02GFDW9 | - - - - - | gnl\|UG\|Ta#S61714726 |
| IV9RU5F02GKUM1 | - - - - - | gnl\|UG\|Ta#S61714726 |
| IUIVLUD02GSXUD | - - - - - | gnl\|UG\|Ta#S61714968 |
| IVLNWEC02INAEL | - - - - - | gnl\|UG\|Ta#S61714968 |
| IVLNWEC02G9782 | - - - - - | gnl\|UG\|Ta#S61715047 |
| IVLNWEC02H3V10 | - - - - - | gnl\|UG\|Ta#S61715047 |
| IUIVLUD02ICVBT | - - - - - | gnl\|UG\|Ta#S61715311 |
| IVLNWEC01CA13R | - - - - - | gnl\|UG\|Ta#S61715311 |
| IV9RU5F01ETRQU | - - - - - | gnl\|UG\|Ta#S61715447 |
| IVLNWEC01A1OO9 | - - - - - | gnl\|UG\|Ta#S61715447 |
| IUIVLUD02G5AO0 | - - - - - | gnl\|UG\|Ta#S61716337 |
| IVLNWEC02JM8VG | - - - - - | gnl\|UG\|Ta#S61716337 |
| IV9RU5F01CIP48 | - - - - - | gnl\|UG\|Ta#S61716523 |
| IVLNWEC01BZ7XM | - - - - - | gnl\|UG\|Ta#S61716523 |
| IVLNWEC01CO8JY | - - - - - | gnl\|UG\|Ta#S61716523 |
| IV9RU5F01A4JG7 | - - - - - | gnl\|UG\|Ta#S61716657 |
| IV9RU5F02IM3SI | - - - - - | gnl\|UG\|Ta#S61716657 |
| IUIVLUD02GQTO5 | - - - - - | gnl\|UG\|Ta#S61716699 |
| IV9RU5F01EO497 | - - - - - | gnl\|UG\|Ta#S61716699 |
| IV9RU5F02HEXPS | - - - - - | gnl\|UG\|Ta#S61717807 |
| IV9RU5F02HXMJH | - - - - - | gnl\|UG\|Ta#S61717807 |
| IV9RU5F02GUGUQ | - - - - - | gnl\|UG\|Ta#S61717910 |
| IVLNWEC02G2978 | - - - - - | gnl\|UG\|Ta#S61717910 |
| IUIVLUD01A10J2 | - - - - - | gnl\|UG\|Ta#S61718129 |

| | | |
|---|---|---|
| IUIVLUD02HPY3W | - - - - - | gnl\|UG\|Ta#S61718129 |
| IVLNWEC02GGE1S | - - - - - | gnl\|UG\|Ta#S61718129 |
| IV9RU5F02JU30L | - - - - - | gnl\|UG\|Ta#S61719140 |
| IVLNWEC01DZA2T | - - - - - | gnl\|UG\|Ta#S61719140 |
| IVLNWEC02GBB0K | - - - - - | gnl\|UG\|Ta#S61719140 |
| IV9RU5F02HRI46 | - - - - - | gnl\|UG\|Ta#S61720532 |
| IVLNWEC02JR4JG | - - - - - | gnl\|UG\|Ta#S61720532 |
| IUIVLUD02IEXP7 | - - - - - | gnl\|UG\|Ta#S61720728 |
| IV9RU5F01BGSH4 | - - - - - | gnl\|UG\|Ta#S61720728 |
| IVLNWEC02IUTD3 | - - - - - | gnl\|UG\|Ta#S61720975 |
| IVLNWEC02JR62A | - - - - - | gnl\|UG\|Ta#S61720975 |
| IV9RU5F02GS967 | - - - - - | gnl\|UG\|Ta#S61721260 |
| IV9RU5F02JHX7R | - - - - - | gnl\|UG\|Ta#S61721260 |
| IUIVLUD01CSQG3 | - - - - - | gnl\|UG\|Ta#S61721268 |
| IUIVLUD02JEIM6 | - - - - - | gnl\|UG\|Ta#S61721268 |
| IV9RU5F02ID4I2 | - - - - - | gnl\|UG\|Ta#S61721268 |
| IV9RU5F01DG5N6 | - - - - - | gnl\|UG\|Ta#S61721888 |
| IVLNWEC01CZ8K6 | - - - - - | gnl\|UG\|Ta#S61721888 |
| IV9RU5F01BQ8BC | - - - - - | gnl\|UG\|Ta#S61722101 |
| IVLNWEC01C2YFC | - - - - - | gnl\|UG\|Ta#S61722101 |
| IUIVLUD01C3G15 | - - - - - | gnl\|UG\|Ta#S61722457 |
| IVLNWEC01ALTB5 | - - - - - | gnl\|UG\|Ta#S61722457 |
| IUIVLUD02G6RUI | - - - - - | gnl\|UG\|Ta#S61722484 |
| IV9RU5F02GGU8B | - - - - - | gnl\|UG\|Ta#S61722484 |
| IV9RU5F01CHMXQ | - - - - - | gnl\|UG\|Ta#S61722530 |
| IV9RU5F01CU3UB | - - - - - | gnl\|UG\|Ta#S61722530 |
| IV9RU5F02JZ9E0 | - - - - - | gnl\|UG\|Ta#S61722530 |
| IVLNWEC01A2QNX | - - - - - | gnl\|UG\|Ta#S61722753 |
| IVLNWEC01BM960 | - - - - - | gnl\|UG\|Ta#S61722753 |
| IUIVLUD01A9XIG | - - - - - | gnl\|UG\|Ta#S61722801 |
| IVLNWEC02IKQPW | - - - - - | gnl\|UG\|Ta#S61722801 |
| IV9RU5F01C3PKL | - - - - - | gnl\|UG\|Ta#S61723533 |
| IVLNWEC02HMNN8 | - - - - - | gnl\|UG\|Ta#S61723533 |
| IV9RU5F01DT20N | - - - - - | gnl\|UG\|Ta#S61723769 |
| IV9RU5F02FQ736 | - - - - - | gnl\|UG\|Ta#S61723769 |
| IUIVLUD01C8LCQ | - - - - - | gnl\|UG\|Ta#S61723917 |
| IVLNWEC02HWVGD | - - - - - | gnl\|UG\|Ta#S61723917 |
| IVLNWEC01A5OZN | - - - - - | gnl\|UG\|Ta#S61724286 |
| IVLNWEC01DH2HU | - - - - - | gnl\|UG\|Ta#S61724286 |
| IVLNWEC02I3JAX | - - - - - | gnl\|UG\|Ta#S61724286 |
| IVLNWEC02GHM7Q | - - - - - | gnl\|UG\|Ta#S61724620 |
| IVLNWEC02HNDW7 | - - - - - | gnl\|UG\|Ta#S61724620 |
| IVLNWEC02I9RUO | - - - - - | gnl\|UG\|Ta#S61724620 |
| IV9RU5F01B66RJ | - - - - - | gnl\|UG\|Ta#S61724726 |
| IV9RU5F01D14LX | - - - - - | gnl\|UG\|Ta#S61724726 |
| IVLNWEC02GKVU7 | - - - - - | gnl\|UG\|Ta#S61724726 |
| IV9RU5F02IE78T | - - - - - | gnl\|UG\|Ta#S61725399 |
| IVLNWEC01DBPTR | - - - - - | gnl\|UG\|Ta#S61725399 |
| IVLNWEC01BNWYP | - - - - - | gnl\|UG\|Ta#S61725846 |
| IVLNWEC01DD84H | - - - - - | gnl\|UG\|Ta#S61725846 |

| | | |
|---|---|---|
| IV9RU5F02FWAVE | - - - - - | gnl\|UG\|Ta#S61725996 |
| IV9RU5F02JVHFW | - - - - - | gnl\|UG\|Ta#S61725996 |
| IVLNWEC01B0LZ7 | - - - - - | gnl\|UG\|Ta#S61726565 |
| IVLNWEC01ETZYT | - - - - - | gnl\|UG\|Ta#S61726565 |
| IVLNWEC02IUWQZ | - - - - - | gnl\|UG\|Ta#S61726633 |
| IVLNWEC02JEJ28 | - - - - - | gnl\|UG\|Ta#S61726633 |
| IV9RU5F02G9B3J | - - - - - | gnl\|UG\|Ta#S61727617 |
| IVLNWEC02GP952 | - - - - - | gnl\|UG\|Ta#S61727617 |
| IUIVLUD01DDFDK | - - - - - | gnl\|UG\|Ta#S61728221 |
| IVLNWEC01DK4JU | - - - - - | gnl\|UG\|Ta#S61728221 |
| IV9RU5F02G1IPM | - - - - - | gnl\|UG\|Ta#S61728265 |
| IV9RU5F02HTQND | - - - - - | gnl\|UG\|Ta#S61728265 |
| IV9RU5F02J0C28 | - - - - - | gnl\|UG\|Ta#S61728265 |
| IV9RU5F01AR71K | - - - - - | gnl\|UG\|Ta#S61728427 |
| IVLNWEC02JCK6R | - - - - - | gnl\|UG\|Ta#S61728427 |
| IV9RU5F01BB5EF | - - - - - | gnl\|UG\|Ta#S61728528 |
| IV9RU5F02JBFUM | - - - - - | gnl\|UG\|Ta#S61728528 |
| IUIVLUD02GOHZ2 | - - - - - | gnl\|UG\|Ta#S61728696 |
| IVLNWEC01EG4LL | - - - - - | gnl\|UG\|Ta#S61728696 |
| IUIVLUD01A83BW | - - - - - | gnl\|UG\|Ta#S61728805 |
| IV9RU5F01AOL4T | - - - - - | gnl\|UG\|Ta#S61728805 |
| IV9RU5F02FYHRB | - - - - - | gnl\|UG\|Ta#S61728831 |
| IV9RU5F02JLZRZ | - - - - - | gnl\|UG\|Ta#S61728831 |
| IVLNWEC01C5U46 | - - - - - | gnl\|UG\|Ta#S61729006 |
| IVLNWEC02FQC7E | - - - - - | gnl\|UG\|Ta#S61729006 |
| IVLNWEC01BVQCK | - - - - - | gnl\|UG\|Ta#S61729337 |
| IVLNWEC01CW24J | - - - - - | gnl\|UG\|Ta#S61729337 |
| IVLNWEC01EYVGG | - - - - - | gnl\|UG\|Ta#S61729337 |
| IVLNWEC01CI0Y1 | - - - - - | gnl\|UG\|Ta#S61729424 |
| IVLNWEC02IA9B0 | - - - - - | gnl\|UG\|Ta#S61729424 |
| IUIVLUD01A9T4G | - - - - - | gnl\|UG\|Ta#S61729835 |
| IUIVLUD01C88EG | - - - - - | gnl\|UG\|Ta#S61729835 |
| IV9RU5F02GHJX8 | - - - - - | gnl\|UG\|Ta#S61729869 |
| IV9RU5F02JNGAF | - - - - - | gnl\|UG\|Ta#S61729869 |
| IVLNWEC01BKNS5 | - - - - - | gnl\|UG\|Ta#S61729869 |
| IUIVLUD02HGMW6 | - - - - - | gnl\|UG\|Ta#S61730255 |
| IVLNWEC02J02MH | - - - - - | gnl\|UG\|Ta#S61730255 |
| IUIVLUD02I0ATS | - - - - - | gnl\|UG\|Ta#S61730349 |
| IV9RU5F01BKPMJ | - - - - - | gnl\|UG\|Ta#S61730349 |
| IVLNWEC02IQ8AH | - - - - - | gnl\|UG\|Ta#S61730349 |
| IV9RU5F01D7ACU | - - - - - | gnl\|UG\|Ta#S61730394 |
| IVLNWEC01CJ7BG | - - - - - | gnl\|UG\|Ta#S61730394 |
| IVLNWEC01ETG5K | - - - - - | gnl\|UG\|Ta#S61730394 |
| IV9RU5F01ER4YB | - - - - - | gnl\|UG\|Ta#S61730503 |
| IV9RU5F02JO577 | - - - - - | gnl\|UG\|Ta#S61730503 |
| IV9RU5F02GWTZD | - - - - - | gnl\|UG\|Ta#S61730928 |
| IVLNWEC01C52J2 | - - - - - | gnl\|UG\|Ta#S61730928 |
| IV9RU5F02IEWJB | - - - - - | gnl\|UG\|Ta#S61731086 |
| IVLNWEC02GY7V1 | - - - - - | gnl\|UG\|Ta#S61731086 |
| IVLNWEC01EU2PQ | - - - - - | gnl\|UG\|Ta#S61731436 |

| | | |
|---|---|---|
| IUIVLUD02FNCXR | - - - - - | gnl\|UG\|Ta#S61731600 |
| IUIVLUD02GRP6C | - - - - - | gnl\|UG\|Ta#S61731600 |
| IV9RU5F02FLZX0 | - - - - - | gnl\|UG\|Ta#S61731600 |
| IVLNWEC01AXY1U | - - - - - | gnl\|UG\|Ta#S61731600 |
| IV9RU5F02HFKCV | - - - - - | gnl\|UG\|Ta#S61731705 |
| IVLNWEC01A3QZV | - - - - - | gnl\|UG\|Ta#S61731705 |
| IUIVLUD01CTIP3 | - - - - - | gnl\|UG\|Ta#S61731758 |
| IUIVLUD02IAJNZ | - - - - - | gnl\|UG\|Ta#S61731758 |
| IV9RU5F01BDD7M | - - - - - | gnl\|UG\|Ta#S61731758 |
| IVLNWEC01DUZYC | - - - - - | gnl\|UG\|Ta#S61731758 |
| IV9RU5F02J329V | - - - - - | gnl\|UG\|Ta#S61731802 |
| IVLNWEC01ASOXP | - - - - - | gnl\|UG\|Ta#S61731802 |
| IUIVLUD01EKD7J | - - - - - | gnl\|UG\|Ta#S61731856 |
| IV9RU5F02H5PP6 | - - - - - | gnl\|UG\|Ta#S61731856 |
| IV9RU5F02FK2OE | - - - - - | gnl\|UG\|Ta#S61732061 |
| IV9RU5F02IGKO9 | - - - - - | gnl\|UG\|Ta#S61732061 |
| IVLNWEC02HBRDT | - - - - - | gnl\|UG\|Ta#S61732061 |
| IV9RU5F02F7618 | - - - - - | gnl\|UG\|Ta#S61732480 |
| IV9RU5F02HYFTZ | - - - - - | gnl\|UG\|Ta#S61732480 |
| IVLNWEC01D12XK | - - - - - | gnl\|UG\|Ta#S61733052 |
| IVLNWEC02GGPO9 | - - - - - | gnl\|UG\|Ta#S61733052 |
| IVLNWEC02JFTCK | - - - - - | gnl\|UG\|Ta#S61733447 |
| IUIVLUD01CWJ3W | - - - - - | gnl\|UG\|Ta#S61733563 |
| IVLNWEC01CN3QE | - - - - - | gnl\|UG\|Ta#S61733563 |
| IVLNWEC02GGSTJ | - - - - - | gnl\|UG\|Ta#S61733563 |
| IV9RU5F01CUIIO | - - - - - | gnl\|UG\|Ta#S61734094 |
| IV9RU5F02HQ0RE | - - - - - | gnl\|UG\|Ta#S61734094 |
| IUIVLUD02JQS83 | - - - - - | gnl\|UG\|Ta#S61734102 |
| IVLNWEC02JMKK7 | - - - - - | gnl\|UG\|Ta#S61734102 |
| IV9RU5F01C7UTB | - - - - - | gnl\|UG\|Ta#S61734120 |
| IVLNWEC01D62RR | - - - - - | gnl\|UG\|Ta#S61734120 |
| IVLNWEC01DVCQC | - - - - - | gnl\|UG\|Ta#S61734447 |
| IVLNWEC02HQ8N2 | - - - - - | gnl\|UG\|Ta#S61734447 |
| IUIVLUD01DQZCX | - - - - - | gnl\|UG\|Ta#S61734648 |
| IV9RU5F01ATHIC | - - - - - | gnl\|UG\|Ta#S61734648 |
| IV9RU5F02GNC0V | - - - - - | gnl\|UG\|Ta#S61734649 |
| IVLNWEC02GUP62 | - - - - - | gnl\|UG\|Ta#S61734649 |
| IUIVLUD02JEHKA | - - - - - | gnl\|UG\|Ta#S61734787 |
| IV9RU5F01D9RS7 | - - - - - | gnl\|UG\|Ta#S61734787 |
| IV9RU5F01EXT2A | - - - - - | gnl\|UG\|Ta#S61734787 |
| IV9RU5F02IUKT7 | - - - - - | gnl\|UG\|Ta#S61734787 |
| IVLNWEC01ENJZR | - - - - - | gnl\|UG\|Ta#S61734787 |
| IV9RU5F01D4RI9 | - - - - - | gnl\|UG\|Ta#S61735075 |
| IVLNWEC02GWQ25 | - - - - - | gnl\|UG\|Ta#S61735075 |
| IUIVLUD01EPFD7 | - - - - - | gnl\|UG\|Ta#S61735236 |
| IUIVLUD02GJUCP | - - - - - | gnl\|UG\|Ta#S61735236 |
| IUIVLUD01A0PSH | - - - - - | gnl\|UG\|Ta#S61735424 |
| IV9RU5F02G0CAY | - - - - - | gnl\|UG\|Ta#S61735424 |
| IVLNWEC02GDI7Q | - - - - - | gnl\|UG\|Ta#S61735424 |
| IVLNWEC02IRCCM | - - - - - | gnl\|UG\|Ta#S61735424 |

| | | |
|---|---|---|
| IUIVLUD02GIBFS | - - - - - | gnl\|UG\|Ta#S61735705 |
| IV9RU5F01AI8YW | - - - - - | gnl\|UG\|Ta#S61735705 |
| IV9RU5F02F4K8F | - - - - - | gnl\|UG\|Ta#S61735705 |
| IUIVLUD01CZ2DB | - - - - - | gnl\|UG\|Ta#S61735863 |
| IV9RU5F02JHPPR | - - - - - | gnl\|UG\|Ta#S61735863 |
| IVLNWEC02IHV0X | - - - - - | gnl\|UG\|Ta#S61735863 |
| IV9RU5F01DT7R7 | - - - - - | gnl\|UG\|Ta#S61736278 |
| IV9RU5F02JD6AB | - - - - - | gnl\|UG\|Ta#S61736278 |
| IVLNWEC01EEIUH | - - - - - | gnl\|UG\|Ta#S61736278 |
| IV9RU5F02IJ05O | - - - - - | gnl\|UG\|Ta#S61736408 |
| IVLNWEC01APZHV | - - - - - | gnl\|UG\|Ta#S61736408 |
| IVLNWEC01A6J6X | - - - - - | gnl\|UG\|Ta#S61736419 |
| IVLNWEC02GFHJC | - - - - - | gnl\|UG\|Ta#S61736419 |
| IVLNWEC02H4E8L | - - - - - | gnl\|UG\|Ta#S61736419 |
| IV9RU5F02HXNAQ | - - - - - | gnl\|UG\|Ta#S61736684 |
| IVLNWEC02FO1ME | - - - - - | gnl\|UG\|Ta#S61736684 |
| IVLNWEC02HCAJG | - - - - - | gnl\|UG\|Ta#S61736684 |
| IUIVLUD02J2QGD | - - - - - | gnl\|UG\|Ta#S61736840 |
| IV9RU5F01D6O66 | - - - - - | gnl\|UG\|Ta#S61736840 |
| IV9RU5F01ECDMW | - - - - - | gnl\|UG\|Ta#S61736840 |
| IVLNWEC01AMPGC | - - - - - | gnl\|UG\|Ta#S61736905 |
| IVLNWEC01DRCZE | - - - - - | gnl\|UG\|Ta#S61736905 |
| IVLNWEC02G2JYR | - - - - - | gnl\|UG\|Ta#S61736905 |
| IVLNWEC02H4ESP | - - - - - | gnl\|UG\|Ta#S61736905 |
| IV9RU5F01CE1AF | - - - - - | gnl\|UG\|Ta#S61736944 |
| IV9RU5F02G7WU8 | - - - - - | gnl\|UG\|Ta#S61736944 |
| IVLNWEC02GX3XF | - - - - - | gnl\|UG\|Ta#S61736944 |
| IVLNWEC01CB1P0 | - - - - - | gnl\|UG\|Ta#S61737137 |
| IVLNWEC02G388J | - - - - - | gnl\|UG\|Ta#S61737137 |
| IVLNWEC02GXYKJ | - - - - - | gnl\|UG\|Ta#S61737376 |
| IUIVLUD02IFR9H | - - - - - | gnl\|UG\|Ta#S61738063 |
| IV9RU5F01DOCIN | - - - - - | gnl\|UG\|Ta#S61738063 |
| IV9RU5F01ENDP2 | - - - - - | gnl\|UG\|Ta#S61738868 |
| IV9RU5F02H7IFP | - - - - - | gnl\|UG\|Ta#S61738868 |
| IV9RU5F02JW4OF | - - - - - | gnl\|UG\|Ta#S61738868 |
| IV9RU5F01BXP8W | - - - - - | gnl\|UG\|Ta#S61738919 |
| IV9RU5F02HG0DH | - - - - - | gnl\|UG\|Ta#S61738919 |
| IVLNWEC01CLFZX | - - - - - | gnl\|UG\|Ta#S61738919 |
| IVLNWEC02FS8V3 | - - - - - | gnl\|UG\|Ta#S61738919 |
| IUIVLUD01CH7BV | - - - - - | gnl\|UG\|Ta#S61739379 |
| IUIVLUD01EA6SA | - - - - - | gnl\|UG\|Ta#S61739379 |
| IVLNWEC02IME2C | - - - - - | gnl\|UG\|Ta#S61739379 |
| IV9RU5F01BJ6CG | - - - - - | gnl\|UG\|Ta#S61739408 |
| IVLNWEC01A4XV5 | - - - - - | gnl\|UG\|Ta#S61739408 |
| IUIVLUD01DS5IF | - - - - - | gnl\|UG\|Ta#S61739485 |
| IV9RU5F02HC1HE | - - - - - | gnl\|UG\|Ta#S61739485 |
| IVLNWEC01DETMP | - - - - - | gnl\|UG\|Ta#S61739485 |
| IV9RU5F01DOOGV | - - - - - | gnl\|UG\|Ta#S61739564 |
| IV9RU5F02G8W3T | - - - - - | gnl\|UG\|Ta#S61739564 |
| IVLNWEC01B5V9K | - - - - - | gnl\|UG\|Ta#S61739883 |

| | | |
|---|---|---|
| IVLNWEC01CHB9U | - - - - - | gnl\|UG\|Ta#S61739883 |
| IV9RU5F01CFXTL | - - - - - | gnl\|UG\|Ta#S61740026 |
| IVLNWEC01CMOPL | - - - - - | gnl\|UG\|Ta#S61740026 |
| IV9RU5F01CFX3U | - - - - - | gnl\|UG\|Ta#S61740088 |
| IV9RU5F02F6XEL | - - - - - | gnl\|UG\|Ta#S61740088 |
| IV9RU5F02IT5S8 | - - - - - | gnl\|UG\|Ta#S61740093 |
| IVLNWEC02IRNHS | - - - - - | gnl\|UG\|Ta#S61740093 |
| IUIVLUD02JW1SO | - - - - - | gnl\|UG\|Ta#S61740490 |
| IV9RU5F01DMYZM | - - - - - | gnl\|UG\|Ta#S61740490 |
| IV9RU5F01DUG15 | - - - - - | gnl\|UG\|Ta#S61740490 |
| IV9RU5F01C305F | - - - - - | gnl\|UG\|Ta#S61741013 |
| IVLNWEC02I2W0C | - - - - - | gnl\|UG\|Ta#S61741013 |
| IVLNWEC01DNPKH | - - - - - | gnl\|UG\|Ta#S61741568 |
| IVLNWEC02I2UB1 | - - - - - | gnl\|UG\|Ta#S61741568 |
| IV9RU5F01D99M4 | - - - - - | gnl\|UG\|Ta#S61741598 |
| IVLNWEC02HKHJK | - - - - - | gnl\|UG\|Ta#S61741598 |
| IUIVLUD01DKXUZ | - - - - - | gnl\|UG\|Ta#S61741686 |
| IVLNWEC02HVW2I | - - - - - | gnl\|UG\|Ta#S61741686 |
| IVLNWEC01DPX65 | - - - - - | gnl\|UG\|Ta#S61741924 |
| IV9RU5F02HNGOA | - - - - - | gnl\|UG\|Ta#S61742323 |
| IVLNWEC02G463A | - - - - - | gnl\|UG\|Ta#S61742323 |
| IV9RU5F01CHV8Y | - - - - - | gnl\|UG\|Ta#S61743054 |
| IV9RU5F02FR3PF | - - - - - | gnl\|UG\|Ta#S61743054 |
| IV9RU5F02GB9JF | - - - - - | gnl\|UG\|Ta#S61743054 |
| IV9RU5F01D9Q62 | - - - - - | gnl\|UG\|Ta#S61743940 |
| IV9RU5F02HCRKD | - - - - - | gnl\|UG\|Ta#S61743940 |
| IV9RU5F01CCLIM | - - - - - | gnl\|UG\|Ta#S61744003 |
| IVLNWEC01BBF8E | - - - - - | gnl\|UG\|Ta#S61744026 |
| IVLNWEC01DUCMS | - - - - - | gnl\|UG\|Ta#S61744026 |
| IVLNWEC02JLKR2 | - - - - - | gnl\|UG\|Ta#S61744026 |
| IV9RU5F01A6SVQ | - - - - - | gnl\|UG\|Ta#S61744044 |
| IV9RU5F01B49C3 | - - - - - | gnl\|UG\|Ta#S61744044 |
| IV9RU5F02J4I4K | - - - - - | gnl\|UG\|Ta#S61744373 |
| IVLNWEC02IF2ET | - - - - - | gnl\|UG\|Ta#S61744373 |
| IVLNWEC01CSFHP | - - - - - | gnl\|UG\|Ta#S61744605 |
| IVLNWEC02HEXB3 | - - - - - | gnl\|UG\|Ta#S61744605 |
| IV9RU5F01EG3BY | - - - - - | gnl\|UG\|Ta#S61744728 |
| IV9RU5F02IFFNG | - - - - - | gnl\|UG\|Ta#S61744728 |
| IV9RU5F02JS8LL | - - - - - | gnl\|UG\|Ta#S61744728 |
| IV9RU5F02ISUFL | - - - - - | gnl\|UG\|Ta#S61745536 |
| IV9RU5F02IWS75 | - - - - - | gnl\|UG\|Ta#S61745536 |
| IVLNWEC02H77PV | - - - - - | gnl\|UG\|Ta#S61745536 |
| IUIVLUD02HWFOT | - - - - - | gnl\|UG\|Ta#S61745588 |
| IV9RU5F02HNHGM | - - - - - | gnl\|UG\|Ta#S61745588 |
| IUIVLUD01DG8UF | - - - - - | gnl\|UG\|Ta#S61745760 |
| IVLNWEC01BO6WN | - - - - - | gnl\|UG\|Ta#S61745760 |
| IV9RU5F01DZAXA | - - - - - | gnl\|UG\|Ta#S61745996 |
| IV9RU5F02FWU2M | - - - - - | gnl\|UG\|Ta#S61745996 |
| IUIVLUD02GETU4 | - - - - - | gnl\|UG\|Ta#S61746739 |
| IV9RU5F01B86MD | - - - - - | gnl\|UG\|Ta#S61746739 |

| | | |
|---|---|---|
| IVLNWEC02HQK7N | - - - - - | gnl\|UG\|Ta#S61746739 |
| IV9RU5F01BBMPH | - - - - - | gnl\|UG\|Ta#S61746841 |
| IVLNWEC02JS8N6 | - - - - - | gnl\|UG\|Ta#S61746841 |
| IV9RU5F01CPF4S | - - - - - | gnl\|UG\|Ta#S61747051 |
| IV9RU5F02FOE9P | - - - - - | gnl\|UG\|Ta#S61747051 |
| IV9RU5F02HC1CV | - - - - - | gnl\|UG\|Ta#S61747051 |
| IUIVLUD01A4O4N | - - - - - | gnl\|UG\|Ta#S61747170 |
| IVLNWEC02I5LYJ | - - - - - | gnl\|UG\|Ta#S61747170 |
| IV9RU5F01DXVQ3 | - - - - - | gnl\|UG\|Ta#S61747185 |
| IVLNWEC01CDU8U | - - - - - | gnl\|UG\|Ta#S61747185 |
| IVLNWEC01DC6BW | - - - - - | gnl\|UG\|Ta#S61747459 |
| IVLNWEC02IG1N6 | - - - - - | gnl\|UG\|Ta#S61747459 |
| IV9RU5F01AVXYA | - - - - - | gnl\|UG\|Ta#S61747637 |
| IV9RU5F02IXM1O | - - - - - | gnl\|UG\|Ta#S61747637 |
| IV9RU5F01DM5RF | - - - - - | gnl\|UG\|Ta#S61747818 |
| IVLNWEC01B6KEM | - - - - - | gnl\|UG\|Ta#S61747818 |
| IV9RU5F01ANDDX | - - - - - | gnl\|UG\|Ta#S61747902 |
| IV9RU5F02IWKEE | - - - - - | gnl\|UG\|Ta#S61747902 |
| IUIVLUD01ATW1U | - - - - - | gnl\|UG\|Ta#S61747920 |
| IV9RU5F01CIRQH | - - - - - | gnl\|UG\|Ta#S61747920 |
| IUIVLUD02FMPBG | - - - - - | gnl\|UG\|Ta#S61748477 |
| IVLNWEC01BZYLE | - - - - - | gnl\|UG\|Ta#S61748477 |
| IV9RU5F01BZFKF | - - - - - | gnl\|UG\|Ta#S61748755 |
| IV9RU5F01D0PWR | - - - - - | gnl\|UG\|Ta#S61748755 |
| IV9RU5F01A9N0E | - - - - - | gnl\|UG\|Ta#S61749326 |
| IV9RU5F01BRLW1 | - - - - - | gnl\|UG\|Ta#S61749326 |
| IV9RU5F01C2JJC | - - - - - | gnl\|UG\|Ta#S61749787 |
| IV9RU5F02F470H | - - - - - | gnl\|UG\|Ta#S61749787 |
| IVLNWEC02IFR5Z | - - - - - | gnl\|UG\|Ta#S61749787 |
| IVLNWEC01COEX4 | - - - - - | gnl\|UG\|Ta#S61750186 |
| IVLNWEC01DDIWH | - - - - - | gnl\|UG\|Ta#S61750186 |
| IV9RU5F02FWH0L | - - - - - | gnl\|UG\|Ta#S61750566 |
| IVLNWEC01EA5DL | - - - - - | gnl\|UG\|Ta#S61750566 |
| IUIVLUD01BP0ZC | - - - - - | gnl\|UG\|Ta#S61750655 |
| IV9RU5F01BH0GD | - - - - - | gnl\|UG\|Ta#S61750655 |
| IUIVLUD02GAB4R | - - - - - | gnl\|UG\|Ta#S61751547 |
| IVLNWEC01EDJKQ | - - - - - | gnl\|UG\|Ta#S61751547 |
| IV9RU5F01A3HNE | - - - - - | gnl\|UG\|Ta#S61751666 |
| IV9RU5F01D9W6R | - - - - - | gnl\|UG\|Ta#S61751666 |
| IV9RU5F01EOIR6 | - - - - - | gnl\|UG\|Ta#S61751666 |
| IUIVLUD01AUZ6R | - - - - - | gnl\|UG\|Ta#S61752109 |
| IVLNWEC02GDNL4 | - - - - - | gnl\|UG\|Ta#S61752109 |
| IVLNWEC01EQN2J | - - - - - | gnl\|UG\|Ta#S61752903 |
| IVLNWEC02GR6Q4 | - - - - - | gnl\|UG\|Ta#S61752903 |
| IUIVLUD02J083P | - - - - - | gnl\|UG\|Ta#S61753324 |
| IV9RU5F02JH61A | - - - - - | gnl\|UG\|Ta#S61753324 |
| IUIVLUD02HOF8F | - - - - - | gnl\|UG\|Ta#S61753631 |
| IV9RU5F02IJ13P | - - - - - | gnl\|UG\|Ta#S61753631 |
| IVLNWEC01D13AM | - - - - - | gnl\|UG\|Ta#S61753631 |
| IVLNWEC02HR6H8 | - - - - - | gnl\|UG\|Ta#S61753631 |

| | | |
|---|---|---|
| IUIVLUD02FLGHB | - - - - - | gnl\|UG\|Ta#S61753636 |
| IV9RU5F02F5QPP | - - - - - | gnl\|UG\|Ta#S61753636 |
| IUIVLUD01AL6M6 | - - - - - | gnl\|UG\|Ta#S61754131 |
| IUIVLUD01AUHR8 | - - - - - | gnl\|UG\|Ta#S61754131 |
| IV9RU5F01A07BI | - - - - - | gnl\|UG\|Ta#S61754131 |
| IV9RU5F01ESC6B | - - - - - | gnl\|UG\|Ta#S61754131 |
| IV9RU5F02G6I5Z | - - - - - | gnl\|UG\|Ta#S61754131 |
| IVLNWEC02FMLFU | - - - - - | gnl\|UG\|Ta#S61754131 |
| IVLNWEC02IAQ6H | - - - - - | gnl\|UG\|Ta#S61754131 |
| IVLNWEC02JB49T | - - - - - | gnl\|UG\|Ta#S61754131 |
| IUIVLUD01CW014 | - - - - - | gnl\|UG\|Ta#S61754245 |
| IV9RU5F01C55OA | - - - - - | gnl\|UG\|Ta#S61754245 |
| IV9RU5F01A0TGD | - - - - - | gnl\|UG\|Ta#S61754403 |
| IVLNWEC01ECOQ5 | - - - - - | gnl\|UG\|Ta#S61754403 |
| IVLNWEC01EIKT3 | - - - - - | gnl\|UG\|Ta#S61754403 |
| IV9RU5F02IMGE8 | - - - - - | gnl\|UG\|Ta#S61754498 |
| IVLNWEC02H5IPB | - - - - - | gnl\|UG\|Ta#S61754498 |
| IUIVLUD02F4TVZ | - - - - - | gnl\|UG\|Ta#S61754625 |
| IVLNWEC02FRHLR | - - - - - | gnl\|UG\|Ta#S61754625 |
| IUIVLUD02IKVO6 | - - - - - | gnl\|UG\|Ta#S61754728 |
| IVLNWEC01E1HK7 | - - - - - | gnl\|UG\|Ta#S61754728 |
| IUIVLUD01AVSSO | - - - - - | gnl\|UG\|Ta#S61755021 |
| IUIVLUD02F3GNA | - - - - - | gnl\|UG\|Ta#S61755021 |
| IVLNWEC01DEJ6U | - - - - - | gnl\|UG\|Ta#S61755021 |
| IV9RU5F01EWHI5 | - - - - - | gnl\|UG\|Ta#S61755122 |
| IVLNWEC01AQ95N | - - - - - | gnl\|UG\|Ta#S61755122 |
| IUIVLUD01BX6MU | - - - - - | gnl\|UG\|Ta#S61755827 |
| IV9RU5F02HY404 | - - - - - | gnl\|UG\|Ta#S61755827 |
| IV9RU5F02HIDGC | - - - - - | gnl\|UG\|Ta#S61756089 |
| IV9RU5F02JYK86 | - - - - - | gnl\|UG\|Ta#S61756089 |
| IVLNWEC02JI02B | - - - - - | gnl\|UG\|Ta#S61756089 |
| IV9RU5F01EKRVJ | - - - - - | gnl\|UG\|Ta#S61756472 |
| IVLNWEC02IN7CA | - - - - - | gnl\|UG\|Ta#S61756472 |
| IUIVLUD02FKNJY | - - - - - | gnl\|UG\|Ta#S61756577 |
| IV9RU5F01EWXQR | - - - - - | gnl\|UG\|Ta#S61756577 |
| IV9RU5F01B52YV | - - - - - | gnl\|UG\|Ta#S61756718 |
| IVLNWEC02GQNOD | - - - - - | gnl\|UG\|Ta#S61756718 |
| IV9RU5F01B03B4 | - - - - - | gnl\|UG\|Ta#S61757326 |
| IVLNWEC01CEFJB | - - - - - | gnl\|UG\|Ta#S61757326 |
| IV9RU5F02FORG1 | - - - - - | gnl\|UG\|Ta#S61757493 |
| IVLNWEC01CW9K3 | - - - - - | gnl\|UG\|Ta#S61757493 |
| IV9RU5F01D8QEN | - - - - - | gnl\|UG\|Ta#S61757570 |
| IV9RU5F02G0NXO | - - - - - | gnl\|UG\|Ta#S61757570 |
| IVLNWEC01A8DPM | - - - - - | gnl\|UG\|Ta#S61757570 |
| IV9RU5F02HOR2B | - - - - - | gnl\|UG\|Ta#S61757894 |
| IVLNWEC02JJLWE | - - - - - | gnl\|UG\|Ta#S61757894 |
| IUIVLUD02FRY9A | - - - - - | gnl\|UG\|Ta#S61757987 |
| IV9RU5F02JDFW9 | - - - - - | gnl\|UG\|Ta#S61757987 |
| IVLNWEC01AO4GM | - - - - - | gnl\|UG\|Ta#S61758083 |
| IVLNWEC02HQZU8 | - - - - - | gnl\|UG\|Ta#S61758083 |

| | | |
|---|---|---|
| IV9RU5F02GPK3V | - - - - - | gnl\|UG\|Ta#S61758237 |
| IV9RU5F02JKQLH | - - - - - | gnl\|UG\|Ta#S61758237 |
| IVLNWEC01CASRK | - - - - - | gnl\|UG\|Ta#S61758302 |
| IVLNWEC02G6CPD | - - - - - | gnl\|UG\|Ta#S61758302 |
| IV9RU5F02GYRIN | - - - - - | gnl\|UG\|Ta#S61758391 |
| IVLNWEC02IY162 | - - - - - | gnl\|UG\|Ta#S61758391 |
| IV9RU5F02I7ERE | - - - - - | gnl\|UG\|Ta#S61758704 |
| IVLNWEC01D0P9A | - - - - - | gnl\|UG\|Ta#S61758704 |
| IUIVLUD01DFLOD | - - - - - | gnl\|UG\|Ta#S61759296 |
| IVLNWEC01BGQYM | - - - - - | gnl\|UG\|Ta#S61759296 |
| IUIVLUD02JGTQQ | - - - - - | gnl\|UG\|Ta#S61759462 |
| IVLNWEC02IV5QB | - - - - - | gnl\|UG\|Ta#S61759462 |
| IVLNWEC02IZ78X | - - - - - | gnl\|UG\|Ta#S61759462 |
| IUIVLUD01D9YGY | - - - - - | gnl\|UG\|Ta#S61760368 |
| IUIVLUD02GN6Y0 | - - - - - | gnl\|UG\|Ta#S61760368 |
| IVLNWEC02ILGLO | - - - - - | gnl\|UG\|Ta#S61760368 |
| IV9RU5F01A0ZQI | - - - - - | gnl\|UG\|Ta#S61760656 |
| IVLNWEC01CMUVK | - - - - - | gnl\|UG\|Ta#S61760656 |
| IV9RU5F02G5Y1F | - - - - - | gnl\|UG\|Ta#S61760666 |
| IVLNWEC02JY3PR | - - - - - | gnl\|UG\|Ta#S61760666 |
| IUIVLUD02GYOKD | - - - - - | gnl\|UG\|Ta#S61760738 |
| IVLNWEC01BI6BB | - - - - - | gnl\|UG\|Ta#S61760738 |
| IV9RU5F01D8S62 | - - - - - | gnl\|UG\|Ta#S61760869 |
| IVLNWEC02JRHME | - - - - - | gnl\|UG\|Ta#S61760869 |
| IUIVLUD01CCPF6 | - - - - - | gnl\|UG\|Ta#S61761158 |
| IVLNWEC01DM7DC | - - - - - | gnl\|UG\|Ta#S61761158 |
| IUIVLUD01BX8LU | - - - - - | gnl\|UG\|Ta#S61761468 |
| IVLNWEC02F06CR | - - - - - | gnl\|UG\|Ta#S61761468 |
| IV9RU5F01CBQ54 | - - - - - | gnl\|UG\|Ta#S61762283 |
| IVLNWEC01DCNOB | - - - - - | gnl\|UG\|Ta#S61762283 |
| IVLNWEC01A8MTZ | - - - - - | gnl\|UG\|Ta#S61763016 |
| IVLNWEC01E2NPC | - - - - - | gnl\|UG\|Ta#S61763016 |
| IV9RU5F01A6JZV | - - - - - | gnl\|UG\|Ta#S61763133 |
| IV9RU5F02IZP5E | - - - - - | gnl\|UG\|Ta#S61763133 |
| IVLNWEC01DDLAP | - - - - - | gnl\|UG\|Ta#S61763188 |
| IVLNWEC02I1NS8 | - - - - - | gnl\|UG\|Ta#S61763188 |
| IUIVLUD01C1814 | - - - - - | gnl\|UG\|Ta#S61763379 |
| IUIVLUD01DAI7O | - - - - - | gnl\|UG\|Ta#S61763379 |
| IVLNWEC01DCLRH | - - - - - | gnl\|UG\|Ta#S61763379 |
| IVLNWEC02F6KKO | - - - - - | gnl\|UG\|Ta#S61764679 |
| IVLNWEC02JOCCK | - - - - - | gnl\|UG\|Ta#S61764679 |
| IV9RU5F02F5H29 | - - - - - | gnl\|UG\|Ta#S61764849 |
| IVLNWEC01CGHYZ | - - - - - | gnl\|UG\|Ta#S61764849 |
| IUIVLUD01B0MO0 | - - - - - | gnl\|UG\|Ta#S61764987 |
| IV9RU5F01DK3CS | - - - - - | gnl\|UG\|Ta#S61764987 |
| IV9RU5F02FS3H8 | - - - - - | gnl\|UG\|Ta#S61764987 |
| IV9RU5F02I4KF3 | - - - - - | gnl\|UG\|Ta#S61764987 |
| IUIVLUD02HFROI | - - - - - | gnl\|UG\|Ta#S61765353 |
| IV9RU5F02JK9EU | - - - - - | gnl\|UG\|Ta#S61765353 |
| IV9RU5F01EPDBG | - - - - - | gnl\|UG\|Ta#S61765607 |

| | | |
|---|---|---|
| IVLNWEC01APEC6 | - - - - - | gnl\|UG\|Ta#S61765607 |
| IUIVLUD01A0S4N | - - - - - | gnl\|UG\|Ta#S61765662 |
| IUIVLUD02G1PZ0 | - - - - - | gnl\|UG\|Ta#S61765662 |
| IV9RU5F02F102N | - - - - - | gnl\|UG\|Ta#S61765662 |
| IVLNWEC02GPZN6 | - - - - - | gnl\|UG\|Ta#S61765662 |
| IUIVLUD02FK5JH | - - - - - | gnl\|UG\|Ta#S61765870 |
| IV9RU5F01CYID6 | - - - - - | gnl\|UG\|Ta#S61765870 |
| IVLNWEC01BD20E | - - - - - | gnl\|UG\|Ta#S61765870 |
| IV9RU5F01DAQRB | - - - - - | gnl\|UG\|Ta#S61765990 |
| IV9RU5F02F2GB1 | - - - - - | gnl\|UG\|Ta#S61765990 |
| IVLNWEC02HB0LK | - - - - - | gnl\|UG\|Ta#S61765990 |
| IVLNWEC01CCHIM | - - - - - | gnl\|UG\|Ta#S61766288 |
| IVLNWEC01DC5IU | - - - - - | gnl\|UG\|Ta#S61766288 |
| IUIVLUD02JC44Q | - - - - - | gnl\|UG\|Ta#S61766595 |
| IV9RU5F02IMNGF | - - - - - | gnl\|UG\|Ta#S61766595 |
| IV9RU5F01AY93W | - - - - - | gnl\|UG\|Ta#S61766612 |
| IVLNWEC02I1U2D | - - - - - | gnl\|UG\|Ta#S61766612 |
| IV9RU5F01C51SU | - - - - - | gnl\|UG\|Ta#S61767256 |
| IVLNWEC01DTDJK | - - - - - | gnl\|UG\|Ta#S61767256 |
| IV9RU5F02JQ1I2 | - - - - - | gnl\|UG\|Ta#S61767333 |
| IVLNWEC01CLPBB | - - - - - | gnl\|UG\|Ta#S61767333 |
| IVLNWEC02IWQAE | - - - - - | gnl\|UG\|Ta#S61767333 |
| IUIVLUD02GS2NJ | - - - - - | gnl\|UG\|Ta#S61767553 |
| IV9RU5F01CCQ8U | - - - - - | gnl\|UG\|Ta#S61767553 |
| IVLNWEC02H6RJT | - - - - - | gnl\|UG\|Ta#S61767553 |
| IUIVLUD02GDCQW | - - - - - | gnl\|UG\|Ta#S61767808 |
| IV9RU5F01AF8GA | - - - - - | gnl\|UG\|Ta#S61767808 |
| IVLNWEC02HIID9 | - - - - - | gnl\|UG\|Ta#S61767808 |
| IVLNWEC02I4RNQ | - - - - - | gnl\|UG\|Ta#S61767808 |
| IV9RU5F02I52HB | - - - - - | gnl\|UG\|Ta#S61767813 |
| IV9RU5F02JQ48F | - - - - - | gnl\|UG\|Ta#S61767813 |
| IV9RU5F02F67SA | - - - - - | gnl\|UG\|Ta#S61767835 |
| IV9RU5F02GAT9G | - - - - - | gnl\|UG\|Ta#S61767835 |
| IV9RU5F02GJKI9 | - - - - - | gnl\|UG\|Ta#S61767835 |
| IV9RU5F02JUPW7 | - - - - - | gnl\|UG\|Ta#S61767835 |
| IV9RU5F02G07SE | - - - - - | gnl\|UG\|Ta#S61767866 |
| IVLNWEC02IHZPE | - - - - - | gnl\|UG\|Ta#S61767866 |
| IVLNWEC02JAXD6 | - - - - - | gnl\|UG\|Ta#S61767866 |
| IV9RU5F02IUBX5 | - - - - - | gnl\|UG\|Ta#S61768045 |
| IV9RU5F02JEZ6U | - - - - - | gnl\|UG\|Ta#S61768045 |
| IUIVLUD02G0PWK | - - - - - | gnl\|UG\|Ta#S61768072 |
| IV9RU5F01B09CT | - - - - - | gnl\|UG\|Ta#S61768072 |
| IVLNWEC02HQRPO | - - - - - | gnl\|UG\|Ta#S61768072 |
| IV9RU5F01A309A | - - - - - | gnl\|UG\|Ta#S61768369 |
| IVLNWEC01BG8EQ | - - - - - | gnl\|UG\|Ta#S61768369 |
| IVLNWEC01BLM5U | - - - - - | gnl\|UG\|Ta#S61768369 |
| IVLNWEC02GJ78L | - - - - - | gnl\|UG\|Ta#S61768369 |
| IV9RU5F01DNRMR | - - - - - | gnl\|UG\|Ta#S61768492 |
| IV9RU5F01E2MJU | - - - - - | gnl\|UG\|Ta#S61768492 |
| IUIVLUD01EWSMK | - - - - - | gnl\|UG\|Ta#S61768651 |

| | | |
|---|---|---|
| IVLNWEC01DT785 | - - - - - | gnl\|UG\|Ta#S61768651 |
| IV9RU5F01A4L4K | - - - - - | gnl\|UG\|Ta#S61768894 |
| IV9RU5F01AV1Q4 | - - - - - | gnl\|UG\|Ta#S61768894 |
| IV9RU5F01B8NX4 | - - - - - | gnl\|UG\|Ta#S61768894 |
| IUIVLUD02HEJC8 | - - - - - | gnl\|UG\|Ta#S61769431 |
| IV9RU5F02I80BW | - - - - - | gnl\|UG\|Ta#S61769431 |
| IV9RU5F02J0VO4 | - - - - - | gnl\|UG\|Ta#S61769431 |
| IVLNWEC01CUV61 | - - - - - | gnl\|UG\|Ta#S61769431 |
| IVLNWEC01DM7A2 | - - - - - | gnl\|UG\|Ta#S61769431 |
| IUIVLUD01A2XNN | - - - - - | gnl\|UG\|Ta#S61769697 |
| IV9RU5F02IJLWE | - - - - - | gnl\|UG\|Ta#S61769697 |
| IVLNWEC01EDD2J | - - - - - | gnl\|UG\|Ta#S61769697 |
| IUIVLUD02HXL1L | - - - - - | gnl\|UG\|Ta#S61769723 |
| IV9RU5F01AKI7X | - - - - - | gnl\|UG\|Ta#S61769723 |
| IV9RU5F01DEN4U | - - - - - | gnl\|UG\|Ta#S61769723 |
| IVLNWEC01BNU14 | - - - - - | gnl\|UG\|Ta#S61769723 |
| IVLNWEC02HDY6L | - - - - - | gnl\|UG\|Ta#S61769723 |
| IUIVLUD02GNEYP | - - - - - | gnl\|UG\|Ta#S61770046 |
| IVLNWEC01D5QTJ | - - - - - | gnl\|UG\|Ta#S61770046 |
| IVLNWEC01EEH0Z | - - - - - | gnl\|UG\|Ta#S61770046 |
| IUIVLUD01B3H8W | - - - - - | gnl\|UG\|Ta#S61770099 |
| IUIVLUD01BZKVM | - - - - - | gnl\|UG\|Ta#S61770099 |
| IUIVLUD01CG6JX | - - - - - | gnl\|UG\|Ta#S61770296 |
| IUIVLUD02INOU6 | - - - - - | gnl\|UG\|Ta#S61770296 |
| IV9RU5F02I7CBK | - - - - - | gnl\|UG\|Ta#S61770296 |
| IVLNWEC01A67TK | - - - - - | gnl\|UG\|Ta#S61770339 |
| IV9RU5F01B9MRL | - - - - - | gnl\|UG\|Ta#S61770548 |
| IVLNWEC02G8MS4 | - - - - - | gnl\|UG\|Ta#S61770548 |
| IV9RU5F02JBEZ6 | - - - - - | gnl\|UG\|Ta#S61770575 |
| IVLNWEC01DQIQL | - - - - - | gnl\|UG\|Ta#S61770575 |
| IUIVLUD01AI4JU | - - - - - | gnl\|UG\|Ta#S61770680 |
| IUIVLUD02GJ3AC | - - - - - | gnl\|UG\|Ta#S61770680 |
| IV9RU5F01ATG5E | - - - - - | gnl\|UG\|Ta#S61770680 |
| IUIVLUD01BEIEK | - - - - - | gnl\|UG\|Ta#S61770752 |
| IV9RU5F02GPDI1 | - - - - - | gnl\|UG\|Ta#S61770752 |
| IUIVLUD02JNOZN | - - - - - | gnl\|UG\|Ta#S61770839 |
| IV9RU5F01B8IQG | - - - - - | gnl\|UG\|Ta#S61770839 |
| IVLNWEC02GQ0UB | - - - - - | gnl\|UG\|Ta#S61771055 |
| IVLNWEC02HD2OL | - - - - - | gnl\|UG\|Ta#S61771055 |
| IUIVLUD01B0C9T | - - - - - | gnl\|UG\|Ta#S61771468 |
| IV9RU5F01BIAYI | - - - - - | gnl\|UG\|Ta#S61771468 |
| IV9RU5F01CFREM | - - - - - | gnl\|UG\|Ta#S61771586 |
| IVLNWEC02FOITV | - - - - - | gnl\|UG\|Ta#S61771586 |
| IV9RU5F01DQ8VR | - - - - - | gnl\|UG\|Ta#S61771589 |
| IV9RU5F01CVOHV | - - - - - | gnl\|UG\|Ta#S61771869 |
| IV9RU5F01ARWU6 | - - - - - | gnl\|UG\|Ta#S61771929 |
| IV9RU5F02FSAFC | - - - - - | gnl\|UG\|Ta#S61771929 |
| IVLNWEC02H3JX4 | - - - - - | gnl\|UG\|Ta#S61772110 |
| IVLNWEC02JBW5B | - - - - - | gnl\|UG\|Ta#S61772110 |
| IVLNWEC01C6VPI | - - - - - | gnl\|UG\|Ta#S61772148 |

| | | |
|---|---|---|
| IVLNWEC01CA72Q | - - - - - | gnl\|UG\|Ta#S61772148 |
| IUIVLUD01AXH9I | - - - - - | gnl\|UG\|Ta#S61772268 |
| IUIVLUD01EHC55 | - - - - - | gnl\|UG\|Ta#S61772268 |
| IV9RU5F01CUH3I | - - - - - | gnl\|UG\|Ta#S61772268 |
| IV9RU5F02HZBP3 | - - - - - | gnl\|UG\|Ta#S61772539 |
| IV9RU5F02JE2ZZ | - - - - - | gnl\|UG\|Ta#S61772539 |
| IVLNWEC02G5O55 | - - - - - | gnl\|UG\|Ta#S61772539 |
| IVLNWEC02H9RLY | - - - - - | gnl\|UG\|Ta#S61772539 |
| IVLNWEC02GQ3FH | - - - - - | gnl\|UG\|Ta#S61772543 |
| IVLNWEC02GRVCP | - - - - - | gnl\|UG\|Ta#S61772543 |
| IV9RU5F01ESIXV | - - - - - | gnl\|UG\|Ta#S61772559 |
| IVLNWEC01CGRIT | - - - - - | gnl\|UG\|Ta#S61772559 |
| IUIVLUD01CH7J5 | - - - - - | gnl\|UG\|Ta#S61772589 |
| IV9RU5F01A8H2Z | - - - - - | gnl\|UG\|Ta#S61772589 |
| IV9RU5F02H57J8 | - - - - - | gnl\|UG\|Ta#S61772589 |
| IUIVLUD01AT5QH | - - - - - | gnl\|UG\|Ta#S61772694 |
| IUIVLUD01DH56U | - - - - - | gnl\|UG\|Ta#S61772694 |
| IUIVLUD02F2OR0 | - - - - - | gnl\|UG\|Ta#S61772694 |
| IV9RU5F01E3L0B | - - - - - | gnl\|UG\|Ta#S61773255 |
| IVLNWEC02F53KC | - - - - - | gnl\|UG\|Ta#S61773255 |
| IVLNWEC02J041J | - - - - - | gnl\|UG\|Ta#S61773255 |
| IV9RU5F01ANBYL | - - - - - | gnl\|UG\|Ta#S61774705 |
| IVLNWEC02G2J62 | - - - - - | gnl\|UG\|Ta#S61774705 |
| IVLNWEC02H3SDI | - - - - - | gnl\|UG\|Ta#S61774705 |
| IUIVLUD02GP8EP | - - - - - | gnl\|UG\|Ta#S61774709 |
| IVLNWEC01DM4K8 | - - - - - | gnl\|UG\|Ta#S61774709 |
| IV9RU5F01CUA69 | - - - - - | gnl\|UG\|Ta#S61774746 |
| IV9RU5F02HOCQ7 | - - - - - | gnl\|UG\|Ta#S61774746 |
| IVLNWEC01BYN83 | - - - - - | gnl\|UG\|Ta#S61774746 |
| IVLNWEC02HKO1X | - - - - - | gnl\|UG\|Ta#S61774746 |
| IV9RU5F01BTLLQ | - - - - - | gnl\|UG\|Ta#S61775282 |
| IV9RU5F01DYPB9 | - - - - - | gnl\|UG\|Ta#S61775282 |
| IV9RU5F02GHUSW | - - - - - | gnl\|UG\|Ta#S61776204 |
| IV9RU5F02GT2JY | - - - - - | gnl\|UG\|Ta#S61776204 |
| IUIVLUD01ARP87 | - - - - - | gnl\|UG\|Ta#S61776327 |
| IVLNWEC01B63AK | - - - - - | gnl\|UG\|Ta#S61776327 |
| IVLNWEC01DE1X5 | - - - - - | gnl\|UG\|Ta#S61776573 |
| IVLNWEC02H5NZD | - - - - - | gnl\|UG\|Ta#S61776573 |
| IUIVLUD02JMIMK | - - - - - | gnl\|UG\|Ta#S61777030 |
| IVLNWEC02GSWJS | - - - - - | gnl\|UG\|Ta#S61777030 |
| IV9RU5F02G06BG | - - - - - | gnl\|UG\|Ta#S61777600 |
| IVLNWEC01BNIN0 | - - - - - | gnl\|UG\|Ta#S61777600 |
| IV9RU5F02F25TH | - - - - - | gnl\|UG\|Ta#S61777774 |
| IV9RU5F02H4MVM | - - - - - | gnl\|UG\|Ta#S61777774 |
| IV9RU5F02JOSI4 | - - - - - | gnl\|UG\|Ta#S61777774 |
| IUIVLUD02I25E7 | - - - - - | gnl\|UG\|Ta#S61777809 |
| IV9RU5F01BQNVI | - - - - - | gnl\|UG\|Ta#S61777809 |
| IV9RU5F01AQ5CH | - - - - - | gnl\|UG\|Ta#S61777877 |
| IVLNWEC02GM15B | - - - - - | gnl\|UG\|Ta#S61777877 |
| IVLNWEC02GDFGV | - - - - - | gnl\|UG\|Ta#S61778115 |

| | | |
|---|---|---|
| IVLNWEC02H2THN | - - - - - | gnl\|UG\|Ta#S61778115 |
| IV9RU5F01AZT9V | - - - - - | gnl\|UG\|Ta#S61778223 |
| IV9RU5F02GD49W | - - - - - | gnl\|UG\|Ta#S61778223 |
| IVLNWEC02ILFKU | - - - - - | gnl\|UG\|Ta#S61778223 |
| IV9RU5F02HNJAG | - - - - - | gnl\|UG\|Ta#S61778241 |
| IVLNWEC02GYB4M | - - - - - | gnl\|UG\|Ta#S61778241 |
| IUIVLUD02GBD7R | - - - - - | gnl\|UG\|Ta#S61778375 |
| IV9RU5F02IE2QZ | - - - - - | gnl\|UG\|Ta#S61778375 |
| IV9RU5F01D51M5 | - - - - - | gnl\|UG\|Ta#S61778436 |
| IVLNWEC01EJ9H0 | - - - - - | gnl\|UG\|Ta#S61778436 |
| IV9RU5F01APXIP | - - - - - | gnl\|UG\|Ta#S61778646 |
| IUIVLUD01EAOQ2 | - - - - - | gnl\|UG\|Ta#S61778828 |
| IVLNWEC02H9UKW | - - - - - | gnl\|UG\|Ta#S61778828 |
| IUIVLUD02G8HHV | - - - - - | gnl\|UG\|Ta#S61778880 |
| IUIVLUD02IBGIC | - - - - - | gnl\|UG\|Ta#S61778880 |
| IUIVLUD02JUE51 | - - - - - | gnl\|UG\|Ta#S61778880 |
| IV9RU5F02G4VA8 | - - - - - | gnl\|UG\|Ta#S61778880 |
| IVLNWEC01A4RFU | - - - - - | gnl\|UG\|Ta#S61778880 |
| IVLNWEC02HOE7E | - - - - - | gnl\|UG\|Ta#S61778880 |
| IUIVLUD01A9I19 | - - - - - | gnl\|UG\|Ta#S61778893 |
| IV9RU5F01DBV36 | - - - - - | gnl\|UG\|Ta#S61778893 |
| IV9RU5F02H1G19 | - - - - - | gnl\|UG\|Ta#S61778893 |
| IV9RU5F02HEKAT | - - - - - | gnl\|UG\|Ta#S61778893 |
| IV9RU5F02JWX49 | - - - - - | gnl\|UG\|Ta#S61778931 |
| IVLNWEC01CHYGK | - - - - - | gnl\|UG\|Ta#S61778931 |
| IVLNWEC02HUD6U | - - - - - | gnl\|UG\|Ta#S61778931 |
| IV9RU5F02GN7BP | - - - - - | gnl\|UG\|Ta#S61779001 |
| IV9RU5F02HSZLN | - - - - - | gnl\|UG\|Ta#S61779001 |
| IV9RU5F02IKHKI | - - - - - | gnl\|UG\|Ta#S61779001 |
| IVLNWEC01AN5SR | - - - - - | gnl\|UG\|Ta#S61779001 |
| IV9RU5F01DOYK4 | - - - - - | gnl\|UG\|Ta#S61779197 |
| IVLNWEC01BU380 | - - - - - | gnl\|UG\|Ta#S61779197 |
| IUIVLUD01DS5MU | - - - - - | gnl\|UG\|Ta#S61779221 |
| IVLNWEC01A9YH8 | - - - - - | gnl\|UG\|Ta#S61779221 |
| IUIVLUD01CUABD | - - - - - | gnl\|UG\|Ta#S61779390 |
| IV9RU5F02INJPN | - - - - - | gnl\|UG\|Ta#S61779390 |
| IV9RU5F01CXQ7R | - - - - - | gnl\|UG\|Ta#S61779560 |
| IVLNWEC01BBJAY | - - - - - | gnl\|UG\|Ta#S61779560 |
| IV9RU5F01D74BQ | - - - - - | gnl\|UG\|Ta#S61779586 |
| IV9RU5F02I5ZVJ | - - - - - | gnl\|UG\|Ta#S61779586 |
| IUIVLUD02GCP6V | - - - - - | gnl\|UG\|Ta#S61779645 |
| IVLNWEC01B5THK | - - - - - | gnl\|UG\|Ta#S61779645 |
| IV9RU5F01B8NPB | - - - - - | gnl\|UG\|Ta#S61779790 |
| IV9RU5F02GG7IT | - - - - - | gnl\|UG\|Ta#S61779790 |
| IV9RU5F02GNKBO | - - - - - | gnl\|UG\|Ta#S61779790 |
| IVLNWEC01B8S8W | - - - - - | gnl\|UG\|Ta#S61779790 |
| IVLNWEC01BB0W3 | - - - - - | gnl\|UG\|Ta#S61779790 |
| IVLNWEC01C7UQE | - - - - - | gnl\|UG\|Ta#S61779790 |
| IVLNWEC02GQ2QS | - - - - - | gnl\|UG\|Ta#S61779790 |
| IVLNWEC01D6SX9 | - - - - - | gnl\|UG\|Ta#S61780083 |

| | | |
|---|---|---|
| IVLNWEC02HOJKP | - - - - - | gnl\|UG\|Ta#S61780083 |
| IUIVLUD01EI20D | - - - - - | gnl\|UG\|Ta#S61780368 |
| IUIVLUD02HE7FB | - - - - - | gnl\|UG\|Ta#S61780368 |
| IV9RU5F01A8IGV | - - - - - | gnl\|UG\|Ta#S61780368 |
| IV9RU5F01CEH6O | - - - - - | gnl\|UG\|Ta#S61780368 |
| IUIVLUD01ELPXK | - - - - - | gnl\|UG\|Ta#S61780449 |
| IV9RU5F01EC4SR | - - - - - | gnl\|UG\|Ta#S61780449 |
| IV9RU5F02GE97F | - - - - - | gnl\|UG\|Ta#S61780487 |
| IV9RU5F02IA7U1 | - - - - - | gnl\|UG\|Ta#S61780487 |
| IV9RU5F02IE8J1 | - - - - - | gnl\|UG\|Ta#S61780487 |
| IV9RU5F01CE2SN | - - - - - | gnl\|UG\|Ta#S61780491 |
| IVLNWEC02IH00V | - - - - - | gnl\|UG\|Ta#S61780491 |
| IUIVLUD01BM3Q9 | - - - - - | gnl\|UG\|Ta#S61780503 |
| IV9RU5F02G5J2V | - - - - - | gnl\|UG\|Ta#S61780503 |
| IVLNWEC02JYK24 | - - - - - | gnl\|UG\|Ta#S61780503 |
| IV9RU5F01A7V9C | - - - - - | gnl\|UG\|Ta#S61780623 |
| IVLNWEC01EREC2 | - - - - - | gnl\|UG\|Ta#S61780623 |
| IVLNWEC01BZO29 | - - - - - | gnl\|UG\|Ta#S61780766 |
| IVLNWEC02GYHY4 | - - - - - | gnl\|UG\|Ta#S61780766 |
| IUIVLUD02GEEPJ | - - - - - | gnl\|UG\|Ta#S61780844 |
| IV9RU5F02JEUNI | - - - - - | gnl\|UG\|Ta#S61780844 |
| IUIVLUD02H5UX3 | - - - - - | gnl\|UG\|Ta#S61780856 |
| IV9RU5F01DILHL | - - - - - | gnl\|UG\|Ta#S61780856 |
| IVLNWEC02HWVXM | - - - - - | gnl\|UG\|Ta#S61780856 |
| IVLNWEC02JOZ2W | - - - - - | gnl\|UG\|Ta#S61780856 |
| IVLNWEC01C19GO | - - - - - | gnl\|UG\|Ta#S61780859 |
| IVLNWEC02FK0DK | - - - - - | gnl\|UG\|Ta#S61780859 |
| IUIVLUD02I0NNW | - - - - - | gnl\|UG\|Ta#S61780923 |
| IV9RU5F01BJFDJ | - - - - - | gnl\|UG\|Ta#S61780923 |
| IV9RU5F01ECU7X | - - - - - | gnl\|UG\|Ta#S61780923 |
| IV9RU5F02GPNFY | - - - - - | gnl\|UG\|Ta#S61780923 |
| IVLNWEC02H58PG | - - - - - | gnl\|UG\|Ta#S61780923 |
| IVLNWEC01AY8MM | - - - - - | gnl\|UG\|Ta#S61780998 |
| IV9RU5F02HWLMP | - - - - - | gnl\|UG\|Ta#S61781034 |
| IVLNWEC02IGVAQ | - - - - - | gnl\|UG\|Ta#S61781034 |
| IV9RU5F02F2TCU | - - - - - | gnl\|UG\|Ta#S61781084 |
| IVLNWEC02JSZBZ | - - - - - | gnl\|UG\|Ta#S61781084 |
| IVLNWEC01D581T | - - - - - | gnl\|UG\|Ta#S61781119 |
| IVLNWEC02H0VZI | - - - - - | gnl\|UG\|Ta#S61781119 |
| IV9RU5F01E0EF8 | - - - - - | gnl\|UG\|Ta#S61781176 |
| IVLNWEC01DWZZ8 | - - - - - | gnl\|UG\|Ta#S61781176 |
| IUIVLUD02JZJRE | - - - - - | gnl\|UG\|Ta#S61781216 |
| IV9RU5F02GJSEA | - - - - - | gnl\|UG\|Ta#S61781216 |
| IV9RU5F02INXU3 | - - - - - | gnl\|UG\|Ta#S61781268 |
| IVLNWEC01C3QZX | - - - - - | gnl\|UG\|Ta#S61781268 |
| IVLNWEC02IKQHV | - - - - - | gnl\|UG\|Ta#S61781268 |
| IV9RU5F01BZ3LY | - - - - - | gnl\|UG\|Ta#S61781441 |
| IVLNWEC02HCV8L | - - - - - | gnl\|UG\|Ta#S61781441 |
| IUIVLUD01BO8S7 | - - - - - | gnl\|UG\|Ta#S61781494 |
| IV9RU5F01DR9QX | - - - - - | gnl\|UG\|Ta#S61781494 |

| | | |
|---|---|---|
| IV9RU5F02I4UAF | - - - - - | gnl\|UG\|Ta#S61781494 |
| IVLNWEC01CA4VI | - - - - - | gnl\|UG\|Ta#S61781494 |
| IVLNWEC01DLVO1 | - - - - - | gnl\|UG\|Ta#S61781659 |
| IVLNWEC01DSI98 | - - - - - | gnl\|UG\|Ta#S61781659 |
| IVLNWEC02IW9QY | - - - - - | gnl\|UG\|Ta#S61781659 |
| IV9RU5F01DDZGG | - - - - - | gnl\|UG\|Ta#S61781770 |
| IV9RU5F01DRRNO | - - - - - | gnl\|UG\|Ta#S61781770 |
| IVLNWEC02FUVF2 | - - - - - | gnl\|UG\|Ta#S61781770 |
| IUIVLUD01AM4PO | - - - - - | gnl\|UG\|Ta#S61781808 |
| IVLNWEC01BAI29 | - - - - - | gnl\|UG\|Ta#S61781808 |
| IV9RU5F01ED5AZ | - - - - - | gnl\|UG\|Ta#S61781849 |
| IV9RU5F02JYRYE | - - - - - | gnl\|UG\|Ta#S61781849 |
| IVLNWEC01ERMPO | - - - - - | gnl\|UG\|Ta#S61781849 |
| IUIVLUD02HXSV0 | - - - - - | gnl\|UG\|Ta#S61781947 |
| IV9RU5F01BUEYI | - - - - - | gnl\|UG\|Ta#S61781947 |
| IV9RU5F02ISQWL | - - - - - | gnl\|UG\|Ta#S61781947 |
| IUIVLUD01B9Q5D | - - - - - | gnl\|UG\|Ta#S61781966 |
| IV9RU5F02I1A1H | - - - - - | gnl\|UG\|Ta#S61781966 |
| IVLNWEC02G2WVK | - - - - - | gnl\|UG\|Ta#S61781966 |
| IV9RU5F01A2I6Y | - - - - - | gnl\|UG\|Ta#S61782304 |
| IV9RU5F01EGIR2 | - - - - - | gnl\|UG\|Ta#S61782304 |
| IV9RU5F01A27XZ | - - - - - | gnl\|UG\|Ta#S61782350 |
| IV9RU5F02FK8YB | - - - - - | gnl\|UG\|Ta#S61782350 |
| IVLNWEC02GWRWI | - - - - - | gnl\|UG\|Ta#S61782350 |
| IUIVLUD01DPJZX | - - - - - | gnl\|UG\|Ta#S61782457 |
| IV9RU5F01BHNI4 | - - - - - | gnl\|UG\|Ta#S61782457 |
| IV9RU5F02IES9G | - - - - - | gnl\|UG\|Ta#S61782457 |
| IVLNWEC01A4JV0 | - - - - - | gnl\|UG\|Ta#S61782457 |
| IVLNWEC01AHYV6 | - - - - - | gnl\|UG\|Ta#S61782457 |
| IVLNWEC01C85MY | - - - - - | gnl\|UG\|Ta#S61782770 |
| IVLNWEC01DERBO | - - - - - | gnl\|UG\|Ta#S61782770 |
| IV9RU5F01AN7N2 | - - - - - | gnl\|UG\|Ta#S61782826 |
| IV9RU5F01DPGQ3 | - - - - - | gnl\|UG\|Ta#S61782826 |
| IVLNWEC01A4UHW | - - - - - | gnl\|UG\|Ta#S61783180 |
| IVLNWEC01BXSTE | - - - - - | gnl\|UG\|Ta#S61783180 |
| IV9RU5F02JOYDJ | - - - - - | gnl\|UG\|Ta#S61783333 |
| IVLNWEC02JYA4N | - - - - - | gnl\|UG\|Ta#S61783333 |
| IUIVLUD01CV7XT | - - - - - | gnl\|UG\|Ta#S61783512 |
| IV9RU5F02IDX7P | - - - - - | gnl\|UG\|Ta#S61783512 |
| IUIVLUD01AHUZ4 | - - - - - | gnl\|UG\|Ta#S61783737 |
| IV9RU5F02I1EN2 | - - - - - | gnl\|UG\|Ta#S61783737 |
| IV9RU5F02FI18I | - - - - - | gnl\|UG\|Ta#S61783931 |
| IVLNWEC01EFS1K | - - - - - | gnl\|UG\|Ta#S61783931 |
| IVLNWEC01AI41X | - - - - - | gnl\|UG\|Ta#S61783956 |
| IVLNWEC01DHMV0 | - - - - - | gnl\|UG\|Ta#S61783956 |
| IV9RU5F02JSVUL | - - - - - | gnl\|UG\|Ta#S61784126 |
| IVLNWEC01B8BX6 | - - - - - | gnl\|UG\|Ta#S61784126 |
| IV9RU5F02HXX90 | - - - - - | gnl\|UG\|Ta#S61784208 |
| IVLNWEC02G6FX7 | - - - - - | gnl\|UG\|Ta#S61784208 |
| IUIVLUD01ES92N | - - - - - | gnl\|UG\|Ta#S61784369 |

| | | |
|---|---|---|
| IV9RU5F01BC8F3 | - - - - - | gnl\|UG\|Ta#S61784369 |
| IV9RU5F01DXT3L | - - - - - | gnl\|UG\|Ta#S61784369 |
| IV9RU5F02HTD2Q | - - - - - | gnl\|UG\|Ta#S61784369 |
| IVLNWEC01CSCCR | - - - - - | gnl\|UG\|Ta#S61784369 |
| IV9RU5F01EDVKJ | - - - - - | gnl\|UG\|Ta#S61784556 |
| IVLNWEC02IB5AG | - - - - - | gnl\|UG\|Ta#S61784556 |
| IUIVLUD01B8NRT | - - - - - | gnl\|UG\|Ta#S61784611 |
| IVLNWEC01AJ7PA | - - - - - | gnl\|UG\|Ta#S61784611 |
| IV9RU5F01C7C4C | - - - - - | gnl\|UG\|Ta#S61784834 |
| IV9RU5F02IEZ3E | - - - - - | gnl\|UG\|Ta#S61784834 |
| IV9RU5F01DPUOY | - - - - - | gnl\|UG\|Ta#S61785065 |
| IVLNWEC02F2P8Y | - - - - - | gnl\|UG\|Ta#S61785065 |
| IUIVLUD01BRTGT | - - - - - | gnl\|UG\|Ta#S61785074 |
| IVLNWEC01B1B9T | - - - - - | gnl\|UG\|Ta#S61785074 |
| IUIVLUD02JRT2Y | - - - - - | gnl\|UG\|Ta#S61785325 |
| IVLNWEC02HDOZZ | - - - - - | gnl\|UG\|Ta#S61785325 |
| IV9RU5F01CGDYP | - - - - - | gnl\|UG\|Ta#S61785364 |
| IV9RU5F02GLGRH | - - - - - | gnl\|UG\|Ta#S61785364 |
| IUIVLUD01APMKB | - - - - - | gnl\|UG\|Ta#S61785667 |
| IUIVLUD02JYVJG | - - - - - | gnl\|UG\|Ta#S61785667 |
| IV9RU5F01BBJUG | - - - - - | gnl\|UG\|Ta#S61785667 |
| IV9RU5F02FX0CI | - - - - - | gnl\|UG\|Ta#S61785709 |
| IV9RU5F02GKOGH | - - - - - | gnl\|UG\|Ta#S61785709 |
| IUIVLUD01A7R73 | - - - - - | gnl\|UG\|Ta#S61786013 |
| IVLNWEC01BB775 | - - - - - | gnl\|UG\|Ta#S61786013 |
| IVLNWEC02JZ16Z | - - - - - | gnl\|UG\|Ta#S61786013 |
| IVLNWEC01ALAZD | - - - - - | gnl\|UG\|Ta#S61786146 |
| IVLNWEC02HQCJJ | - - - - - | gnl\|UG\|Ta#S61786146 |
| IV9RU5F02HL04F | - - - - - | gnl\|UG\|Ta#S61786234 |
| IVLNWEC01B162U | - - - - - | gnl\|UG\|Ta#S61786234 |
| IVLNWEC02GYKQH | - - - - - | gnl\|UG\|Ta#S61786234 |
| IVLNWEC02IZZYM | - - - - - | gnl\|UG\|Ta#S61786234 |
| IUIVLUD01BKVQX | - - - - - | gnl\|UG\|Ta#S61786366 |
| IUIVLUD02JB2ZV | - - - - - | gnl\|UG\|Ta#S61786366 |
| IV9RU5F01E0VL3 | - - - - - | gnl\|UG\|Ta#S61786366 |
| IV9RU5F02G9CAV | - - - - - | gnl\|UG\|Ta#S61786366 |
| IVLNWEC01CGC4F | - - - - - | gnl\|UG\|Ta#S61786366 |
| IUIVLUD01BJBWN | - - - - - | gnl\|UG\|Ta#S61786459 |
| IUIVLUD01EF5VM | - - - - - | gnl\|UG\|Ta#S61786459 |
| IVLNWEC01AYOLU | - - - - - | gnl\|UG\|Ta#S61786459 |
| IV9RU5F01EPA56 | - - - - - | gnl\|UG\|Ta#S61786994 |
| IV9RU5F02IEFWX | - - - - - | gnl\|UG\|Ta#S61786994 |
| IV9RU5F01CUMS7 | - - - - - | gnl\|UG\|Ta#S61787452 |
| IV9RU5F02H4TKF | - - - - - | gnl\|UG\|Ta#S61787452 |
| IV9RU5F02JZADS | - - - - - | gnl\|UG\|Ta#S61787452 |
| IVLNWEC01EPHXS | - - - - - | gnl\|UG\|Ta#S61787486 |
| IVLNWEC02JP9BO | - - - - - | gnl\|UG\|Ta#S61787486 |
| IUIVLUD01AZ8KT | - - - - - | gnl\|UG\|Ta#S61787690 |
| IUIVLUD02I2ZNH | - - - - - | gnl\|UG\|Ta#S61787690 |
| IUIVLUD02H6IC5 | - - - - - | gnl\|UG\|Ta#S61787776 |

| | | |
|---|---|---|
| IV9RU5F02J4IGN | - - - - - | gnl\|UG\|Ta#S61788015 |
| IVLNWEC01EX7IV | - - - - - | gnl\|UG\|Ta#S61788015 |
| IUIVLUD01EIF0I | - - - - - | gnl\|UG\|Ta#S61788126 |
| IV9RU5F01BEC1C | - - - - - | gnl\|UG\|Ta#S61788126 |
| IVLNWEC02GZA04 | - - - - - | gnl\|UG\|Ta#S61788337 |
| IVLNWEC02HHSDU | - - - - - | gnl\|UG\|Ta#S61788337 |
| IUIVLUD01CIJQP | - - - - - | gnl\|UG\|Ta#S61788725 |
| IV9RU5F01B87P5 | - - - - - | gnl\|UG\|Ta#S61788725 |
| IV9RU5F02J1IYV | - - - - - | gnl\|UG\|Ta#S61788725 |
| IVLNWEC01ALI7I | - - - - - | gnl\|UG\|Ta#S61789388 |
| IVLNWEC02IMBNR | - - - - - | gnl\|UG\|Ta#S61789388 |
| IUIVLUD02F041M | - - - - - | gnl\|UG\|Ta#S61789588 |
| IUIVLUD02IDJ2T | - - - - - | gnl\|UG\|Ta#S61789588 |
| IV9RU5F02GRV5J | - - - - - | gnl\|UG\|Ta#S61789588 |
| IV9RU5F02IHSLS | - - - - - | gnl\|UG\|Ta#S61789588 |
| IV9RU5F02IZZJL | - - - - - | gnl\|UG\|Ta#S61789588 |
| IVLNWEC01CER27 | - - - - - | gnl\|UG\|Ta#S61789588 |
| IVLNWEC02IKD9I | - - - - - | gnl\|UG\|Ta#S61789588 |
| IUIVLUD01BS9WQ | - - - - - | gnl\|UG\|Ta#S61789758 |
| IUIVLUD01DFGPA | - - - - - | gnl\|UG\|Ta#S61789758 |
| IUIVLUD02GDE74 | - - - - - | gnl\|UG\|Ta#S61789758 |
| IVLNWEC02H5G9Q | - - - - - | gnl\|UG\|Ta#S61789758 |
| IUIVLUD01D7S9X | - - - - - | gnl\|UG\|Ta#S61790008 |
| IUIVLUD02F4H6O | - - - - - | gnl\|UG\|Ta#S61790008 |
| IV9RU5F01BBPM1 | - - - - - | gnl\|UG\|Ta#S61790008 |
| IV9RU5F01EZHD9 | - - - - - | gnl\|UG\|Ta#S61790019 |
| IV9RU5F02I4H2D | - - - - - | gnl\|UG\|Ta#S61790019 |
| IUIVLUD01C2VZD | - - - - - | gnl\|UG\|Ta#S61790111 |
| IV9RU5F01E2GNW | - - - - - | gnl\|UG\|Ta#S61790111 |
| IV9RU5F01ANK61 | - - - - - | gnl\|UG\|Ta#S61790480 |
| IV9RU5F01D3MQI | - - - - - | gnl\|UG\|Ta#S61790480 |
| IV9RU5F02JATXJ | - - - - - | gnl\|UG\|Ta#S61790685 |
| IV9RU5F02JFC34 | - - - - - | gnl\|UG\|Ta#S61790685 |
| IUIVLUD01DUTTB | - - - - - | gnl\|UG\|Ta#S61790984 |
| IV9RU5F01B9YB0 | - - - - - | gnl\|UG\|Ta#S61790984 |
| IUIVLUD01A8OPL | - - - - - | gnl\|UG\|Ta#S61791061 |
| IV9RU5F01DBHJS | - - - - - | gnl\|UG\|Ta#S61791061 |
| IVLNWEC02G1PQ0 | - - - - - | gnl\|UG\|Ta#S61791061 |
| IUIVLUD01EGH2Q | - - - - - | gnl\|UG\|Ta#S61791071 |
| IVLNWEC01DCZXN | - - - - - | gnl\|UG\|Ta#S61791071 |
| IV9RU5F01CF1D4 | - - - - - | gnl\|UG\|Ta#S61791087 |
| IVLNWEC01ADTSY | - - - - - | gnl\|UG\|Ta#S61791087 |
| IV9RU5F01BS7ZH | - - - - - | gnl\|UG\|Ta#S61791127 |
| IV9RU5F01EWSL6 | - - - - - | gnl\|UG\|Ta#S61791127 |
| IVLNWEC01BVPKC | - - - - - | gnl\|UG\|Ta#S61791127 |
| IVLNWEC02GQJW2 | - - - - - | gnl\|UG\|Ta#S61791127 |
| IVLNWEC02H7121 | - - - - - | gnl\|UG\|Ta#S61791234 |
| IVLNWEC02JXPMD | - - - - - | gnl\|UG\|Ta#S61791234 |
| IV9RU5F02FWNGS | - - - - - | gnl\|UG\|Ta#S61791545 |
| IVLNWEC01CM1MM | - - - - - | gnl\|UG\|Ta#S61791545 |

| | | |
|---|---|---|
| IVLNWEC02IBO12 | - - - - - | gnl\|UG\|Ta#S61791545 |
| IV9RU5F01AP5TU | - - - - - | gnl\|UG\|Ta#S61791554 |
| IVLNWEC02GJ7DB | - - - - - | gnl\|UG\|Ta#S61791639 |
| IVLNWEC02JQRYK | - - - - - | gnl\|UG\|Ta#S61791639 |
| IVLNWEC02GTEH0 | - - - - - | gnl\|UG\|Ta#S61791678 |
| IUIVLUD01DS1EY | - - - - - | gnl\|UG\|Ta#S61791774 |
| IV9RU5F02GTHZ5 | - - - - - | gnl\|UG\|Ta#S61791774 |
| IV9RU5F02JCH45 | - - - - - | gnl\|UG\|Ta#S61791780 |
| IV9RU5F01A9SM7 | - - - - - | gnl\|UG\|Ta#S61791903 |
| IVLNWEC02GERHB | - - - - - | gnl\|UG\|Ta#S61791903 |
| IUIVLUD02J3H0B | - - - - - | gnl\|UG\|Ta#S61792066 |
| IV9RU5F01BL4UE | - - - - - | gnl\|UG\|Ta#S61792066 |
| IV9RU5F02GCRQ7 | - - - - - | gnl\|UG\|Ta#S61792066 |
| IUIVLUD01BMYDZ | - - - - - | gnl\|UG\|Ta#S61792167 |
| IVLNWEC02IQJZE | - - - - - | gnl\|UG\|Ta#S61792167 |
| IUIVLUD02JJSFT | - - - - - | gnl\|UG\|Ta#S61792233 |
| IV9RU5F02J2R12 | - - - - - | gnl\|UG\|Ta#S61792233 |
| IVLNWEC01CWV1K | - - - - - | gnl\|UG\|Ta#S61792233 |
| IVLNWEC01DKR9P | - - - - - | gnl\|UG\|Ta#S61792233 |
| IUIVLUD02FSWCJ | - - - - - | gnl\|UG\|Ta#S61792469 |
| IUIVLUD02IR7SU | - - - - - | gnl\|UG\|Ta#S61792469 |
| IVLNWEC02IJE6Y | - - - - - | gnl\|UG\|Ta#S61792469 |
| IV9RU5F02FN0JQ | - - - - - | gnl\|UG\|Ta#S61792500 |
| IVLNWEC01CN640 | - - - - - | gnl\|UG\|Ta#S61792500 |
| IVLNWEC02GZ05B | - - - - - | gnl\|UG\|Ta#S61792500 |
| IV9RU5F01BB7XU | - - - - - | gnl\|UG\|Ta#S61792814 |
| IV9RU5F01BYQUB | - - - - - | gnl\|UG\|Ta#S61792814 |
| IVLNWEC01DPTNW | - - - - - | gnl\|UG\|Ta#S61792814 |
| IV9RU5F02FW0UT | - - - - - | gnl\|UG\|Ta#S61792884 |
| IVLNWEC01EHL3F | - - - - - | gnl\|UG\|Ta#S61792884 |
| IV9RU5F02GEKXY | - - - - - | gnl\|UG\|Ta#S61792985 |
| IV9RU5F02IESOY | - - - - - | gnl\|UG\|Ta#S61792985 |
| IUIVLUD01BBA5O | - - - - - | gnl\|UG\|Ta#S61793063 |
| IVLNWEC02HNP3G | - - - - - | gnl\|UG\|Ta#S61793063 |
| IUIVLUD01BDK9B | - - - - - | gnl\|UG\|Ta#S61793066 |
| IVLNWEC02G49JU | - - - - - | gnl\|UG\|Ta#S61793066 |
| IV9RU5F02JLG8E | - - - - - | gnl\|UG\|Ta#S61793091 |
| IVLNWEC02H5EKK | - - - - - | gnl\|UG\|Ta#S61793091 |
| IV9RU5F02GNUQU | - - - - - | gnl\|UG\|Ta#S61793709 |
| IV9RU5F02IY50P | - - - - - | gnl\|UG\|Ta#S61793709 |
| IVLNWEC02HKZTF | - - - - - | gnl\|UG\|Ta#S61793709 |
| IV9RU5F01CCO8R | - - - - - | gnl\|UG\|Ta#S61793939 |
| IVLNWEC02FQM2A | - - - - - | gnl\|UG\|Ta#S61793939 |
| IUIVLUD02FXQUZ | - - - - - | gnl\|UG\|Ta#S61794409 |
| IV9RU5F02HVPBF | - - - - - | gnl\|UG\|Ta#S61794421 |
| IVLNWEC01CO29Y | - - - - - | gnl\|UG\|Ta#S61794421 |
| IVLNWEC01COQ08 | - - - - - | gnl\|UG\|Ta#S61794421 |
| IV9RU5F02JFWTE | - - - - - | gnl\|UG\|Ta#S61794486 |
| IVLNWEC01B30QG | - - - - - | gnl\|UG\|Ta#S61794486 |
| IVLNWEC01CNBQ6 | - - - - - | gnl\|UG\|Ta#S61794575 |

| | | |
|---|---|---|
| IVLNWEC02IO1ZG | - - - - - | gnl\|UG\|Ta#S61794575 |
| IUIVLUD02IFHPV | - - - - - | gnl\|UG\|Ta#S61794643 |
| IUIVLUD02J2NO9 | - - - - - | gnl\|UG\|Ta#S61794643 |
| IV9RU5F02FSTPE | - - - - - | gnl\|UG\|Ta#S61794643 |
| IUIVLUD02GQH4F | - - - - - | gnl\|UG\|Ta#S61794907 |
| IV9RU5F02FTUBB | - - - - - | gnl\|UG\|Ta#S61794907 |
| IV9RU5F02F2DZ1 | - - - - - | gnl\|UG\|Ta#S61795015 |
| IV9RU5F02GBLMJ | - - - - - | gnl\|UG\|Ta#S61795015 |
| IVLNWEC02G9S67 | - - - - - | gnl\|UG\|Ta#S61795554 |
| IVLNWEC02GOVPQ | - - - - - | gnl\|UG\|Ta#S61795554 |
| IV9RU5F01CDMPU | - - - - - | gnl\|UG\|Ta#S61795782 |
| IV9RU5F01EBOBB | - - - - - | gnl\|UG\|Ta#S61795782 |
| IV9RU5F02H3CDX | - - - - - | gnl\|UG\|Ta#S61795782 |
| IUIVLUD01AQR8D | - - - - - | gnl\|UG\|Ta#S61795898 |
| IV9RU5F01DOEYX | - - - - - | gnl\|UG\|Ta#S61795898 |
| IUIVLUD01AQUF6 | - - - - - | gnl\|UG\|Ta#S61795964 |
| IUIVLUD02GI1UO | - - - - - | gnl\|UG\|Ta#S61795964 |
| IV9RU5F02IQ1PM | - - - - - | gnl\|UG\|Ta#S61796276 |
| IVLNWEC01ETK46 | - - - - - | gnl\|UG\|Ta#S61796276 |
| IUIVLUD02GJ5HV | - - - - - | gnl\|UG\|Ta#S61796463 |
| IVLNWEC01DHZAD | - - - - - | gnl\|UG\|Ta#S61796463 |
| IUIVLUD01CS7YZ | - - - - - | gnl\|UG\|Ta#S61797061 |
| IV9RU5F02IWT1Y | - - - - - | gnl\|UG\|Ta#S61797061 |
| IV9RU5F02FR1SS | - - - - - | gnl\|UG\|Ta#S61797270 |
| IVLNWEC01CIG0K | - - - - - | gnl\|UG\|Ta#S61797270 |
| IVLNWEC01E4BLO | - - - - - | gnl\|UG\|Ta#S61797270 |
| IV9RU5F01A2QWT | - - - - - | gnl\|UG\|Ta#S61797618 |
| IVLNWEC01C61CC | - - - - - | gnl\|UG\|Ta#S61797618 |
| IVLNWEC02G02NP | - - - - - | gnl\|UG\|Ta#S61797618 |
| IV9RU5F02GKXK3 | - - - - - | gnl\|UG\|Ta#S61798048 |
| IVLNWEC02I5RU3 | - - - - - | gnl\|UG\|Ta#S61798048 |
| IUIVLUD01DVG3R | - - - - - | gnl\|UG\|Ta#S61798386 |
| IVLNWEC01ASC7G | - - - - - | gnl\|UG\|Ta#S61798386 |
| IUIVLUD01DFJVB | - - - - - | gnl\|UG\|Ta#S61798395 |
| IV9RU5F01DK5NN | - - - - - | gnl\|UG\|Ta#S61798395 |
| IUIVLUD01ESQTB | - - - - - | gnl\|UG\|Ta#S61798464 |
| IV9RU5F01CXYIZ | - - - - - | gnl\|UG\|Ta#S61798464 |
| IV9RU5F01AGM8V | - - - - - | gnl\|UG\|Ta#S61798520 |
| IV9RU5F01B5N7Y | - - - - - | gnl\|UG\|Ta#S61798520 |
| IV9RU5F01EATF7 | - - - - - | gnl\|UG\|Ta#S61798520 |
| IV9RU5F02FKWBD | - - - - - | gnl\|UG\|Ta#S61798520 |
| IV9RU5F02H0T35 | - - - - - | gnl\|UG\|Ta#S61798520 |
| IV9RU5F02JAOOH | - - - - - | gnl\|UG\|Ta#S61798520 |
| IV9RU5F01CSELH | - - - - - | gnl\|UG\|Ta#S61798598 |
| IV9RU5F01DWXOU | - - - - - | gnl\|UG\|Ta#S61798598 |
| IV9RU5F02HFXFF | - - - - - | gnl\|UG\|Ta#S61798598 |
| IV9RU5F01AKXZW | - - - - - | gnl\|UG\|Ta#S61798863 |
| IV9RU5F01BO2ES | - - - - - | gnl\|UG\|Ta#S61798863 |
| IUIVLUD02GMMVO | - - - - - | gnl\|UG\|Ta#S61799140 |
| IV9RU5F01AD8MJ | - - - - - | gnl\|UG\|Ta#S61799140 |

| | | |
|---|---|---|
| IV9RU5F01EEECA | - - - - - | gnl\|UG\|Ta#S61799140 |
| IV9RU5F02ISFKS | - - - - - | gnl\|UG\|Ta#S61799140 |
| IUIVLUD01AOUGW | - - - - - | gnl\|UG\|Ta#S61799246 |
| IUIVLUD01ASIBD | - - - - - | gnl\|UG\|Ta#S61799246 |
| IVLNWEC02G43SC | - - - - - | gnl\|UG\|Ta#S61799246 |
| IUIVLUD02HAQJK | - - - - - | gnl\|UG\|Ta#S61799492 |
| IVLNWEC01BGYS8 | - - - - - | gnl\|UG\|Ta#S61799492 |
| IV9RU5F01DWAKS | - - - - - | gnl\|UG\|Ta#S61799688 |
| IV9RU5F02F2YFD | - - - - - | gnl\|UG\|Ta#S61799688 |
| IV9RU5F01BJ23U | - - - - - | gnl\|UG\|Ta#S61799987 |
| IVLNWEC01DZP0D | - - - - - | gnl\|UG\|Ta#S61799987 |
| IVLNWEC01EELH2 | - - - - - | gnl\|UG\|Ta#S61800156 |
| IVLNWEC02I0GL5 | - - - - - | gnl\|UG\|Ta#S61800156 |
| IV9RU5F01AQ72A | - - - - - | gnl\|UG\|Ta#S61800168 |
| IV9RU5F02HLYXA | - - - - - | gnl\|UG\|Ta#S61800172 |
| IVLNWEC01EJWRO | - - - - - | gnl\|UG\|Ta#S61800172 |
| IV9RU5F02JNX9G | - - - - - | gnl\|UG\|Ta#S61800284 |
| IVLNWEC02G52LX | - - - - - | gnl\|UG\|Ta#S61800284 |
| IV9RU5F01D0DB9 | - - - - - | gnl\|UG\|Ta#S61800416 |
| IVLNWEC01CJ5HX | - - - - - | gnl\|UG\|Ta#S61800416 |
| IVLNWEC02HQTN8 | - - - - - | gnl\|UG\|Ta#S61800550 |
| IVLNWEC02JPVT5 | - - - - - | gnl\|UG\|Ta#S61800550 |
| IVLNWEC01EUL4Z | - - - - - | gnl\|UG\|Ta#S61800556 |
| IV9RU5F02HRXI2 | - - - - - | gnl\|UG\|Ta#S61800772 |
| IVLNWEC02GMOV5 | - - - - - | gnl\|UG\|Ta#S61800772 |
| IV9RU5F02FOU8M | - - - - - | gnl\|UG\|Ta#S61800847 |
| IV9RU5F02GLSEY | - - - - - | gnl\|UG\|Ta#S61800847 |
| IV9RU5F02I661R | - - - - - | gnl\|UG\|Ta#S61800847 |
| IVLNWEC02JNAWG | - - - - - | gnl\|UG\|Ta#S61800847 |
| IVLNWEC01AIHF3 | - - - - - | gnl\|UG\|Ta#S61800870 |
| IV9RU5F02J16QL | - - - - - | gnl\|UG\|Ta#S61800964 |
| IV9RU5F02JPYIV | - - - - - | gnl\|UG\|Ta#S61800964 |
| IVLNWEC01B78NT | - - - - - | gnl\|UG\|Ta#S61800964 |
| IVLNWEC02G7XSG | - - - - - | gnl\|UG\|Ta#S61800964 |
| IVLNWEC02J26BQ | - - - - - | gnl\|UG\|Ta#S61800964 |
| IV9RU5F01ELFOA | - - - - - | gnl\|UG\|Ta#S61800984 |
| IVLNWEC01BBIZH | - - - - - | gnl\|UG\|Ta#S61800984 |
| IVLNWEC01DWBNY | - - - - - | gnl\|UG\|Ta#S61800984 |
| IUIVLUD02F3GIV | - - - - - | gnl\|UG\|Ta#S61801009 |
| IV9RU5F01A44MT | - - - - - | gnl\|UG\|Ta#S61801009 |
| IV9RU5F01BHN43 | - - - - - | gnl\|UG\|Ta#S61801144 |
| IV9RU5F01DETQS | - - - - - | gnl\|UG\|Ta#S61801144 |
| IVLNWEC01AV6BX | - - - - - | gnl\|UG\|Ta#S61801144 |
| IVLNWEC01CO1AY | - - - - - | gnl\|UG\|Ta#S61801144 |
| IUIVLUD01CHMDX | - - - - - | gnl\|UG\|Ta#S61801265 |
| IUIVLUD01DUFCW | - - - - - | gnl\|UG\|Ta#S61801265 |
| IV9RU5F01BYU9D | - - - - - | gnl\|UG\|Ta#S61801686 |
| IVLNWEC02H35RW | - - - - - | gnl\|UG\|Ta#S61801686 |
| IVLNWEC02FQ4K2 | - - - - - | gnl\|UG\|Ta#S61801714 |
| IVLNWEC02G5U05 | - - - - - | gnl\|UG\|Ta#S61801714 |

| | | |
|---|---|---|
| IV9RU5F01DUI7C | - - - - - | gnl\|UG\|Ta#S61801827 |
| IV9RU5F02F21AA | - - - - - | gnl\|UG\|Ta#S61801827 |
| IV9RU5F02HDKBO | - - - - - | gnl\|UG\|Ta#S61801827 |
| IUIVLUD02J21CQ | - - - - - | gnl\|UG\|Ta#S61802021 |
| IVLNWEC01D9MLH | - - - - - | gnl\|UG\|Ta#S61802021 |
| IUIVLUD01EW979 | - - - - - | gnl\|UG\|Ta#S61802148 |
| IV9RU5F01BUCC8 | - - - - - | gnl\|UG\|Ta#S61802148 |
| IV9RU5F02I3K3D | - - - - - | gnl\|UG\|Ta#S61802148 |
| IV9RU5F01D40ZB | - - - - - | gnl\|UG\|Ta#S61802287 |
| IVLNWEC02GS2X6 | - - - - - | gnl\|UG\|Ta#S61802287 |
| IVLNWEC02GDJ0M | - - - - - | gnl\|UG\|Ta#S61802309 |
| IVLNWEC02JTCA6 | - - - - - | gnl\|UG\|Ta#S61802309 |
| IV9RU5F01BL6L4 | - - - - - | gnl\|UG\|Ta#S61802456 |
| IVLNWEC01BDKJN | - - - - - | gnl\|UG\|Ta#S61802456 |
| IUIVLUD01BN7Y5 | - - - - - | gnl\|UG\|Ta#S61802533 |
| IVLNWEC01E2NJI | - - - - - | gnl\|UG\|Ta#S61802533 |
| IVLNWEC02I1LMG | - - - - - | gnl\|UG\|Ta#S61802533 |
| IVLNWEC01CS89M | - - - - - | gnl\|UG\|Ta#S61802550 |
| IVLNWEC01DPR1S | - - - - - | gnl\|UG\|Ta#S61802550 |
| IV9RU5F01C6G0T | - - - - - | gnl\|UG\|Ta#S61802925 |
| IV9RU5F02FLP9W | - - - - - | gnl\|UG\|Ta#S61802925 |
| IV9RU5F02INH2D | - - - - - | gnl\|UG\|Ta#S61802925 |
| IUIVLUD01A78GV | - - - - - | gnl\|UG\|Ta#S61803735 |
| IUIVLUD01ALFBX | - - - - - | gnl\|UG\|Ta#S61803735 |
| IV9RU5F01A3QSL | - - - - - | gnl\|UG\|Ta#S61803735 |
| IUIVLUD01B9OL5 | - - - - - | gnl\|UG\|Ta#S61804018 |
| IV9RU5F01BKFVV | - - - - - | gnl\|UG\|Ta#S61804018 |
| IUIVLUD02GBSXL | - - - - - | gnl\|UG\|Ta#S61804460 |
| IVLNWEC01BBEYL | - - - - - | gnl\|UG\|Ta#S61804460 |
| IV9RU5F01CUK32 | - - - - - | gnl\|UG\|Ta#S61804476 |
| IVLNWEC01BAJCU | - - - - - | gnl\|UG\|Ta#S61804476 |
| IV9RU5F01EIMKY | - - - - - | gnl\|UG\|Ta#S61804817 |
| IV9RU5F01ES9ER | - - - - - | gnl\|UG\|Ta#S61804817 |
| IUIVLUD01B0L0R | - - - - - | gnl\|UG\|Ta#S61806121 |
| IVLNWEC02GKG8X | - - - - - | gnl\|UG\|Ta#S61806121 |
| IUIVLUD01C9HSG | - - - - - | gnl\|UG\|Ta#S61806362 |
| IVLNWEC01EIJUM | - - - - - | gnl\|UG\|Ta#S61806362 |
| IV9RU5F01A81CM | - - - - - | gnl\|UG\|Ta#S61806802 |
| IV9RU5F01AF6W4 | - - - - - | gnl\|UG\|Ta#S61806802 |
| IUIVLUD02GZVJ8 | - - - - - | gnl\|UG\|Ta#S61806808 |
| IVLNWEC01A1XFM | - - - - - | gnl\|UG\|Ta#S61806808 |
| IV9RU5F02FVD81 | - - - - - | gnl\|UG\|Ta#S61806811 |
| IV9RU5F02I5AZH | - - - - - | gnl\|UG\|Ta#S61806811 |
| IUIVLUD02H0EJJ | - - - - - | gnl\|UG\|Ta#S61807475 |
| IUIVLUD02HFYK7 | - - - - - | gnl\|UG\|Ta#S61807475 |
| IV9RU5F01C93RX | - - - - - | gnl\|UG\|Ta#S61807475 |
| IVLNWEC01E23UZ | - - - - - | gnl\|UG\|Ta#S61807475 |
| IV9RU5F02H8B8P | - - - - - | gnl\|UG\|Ta#S61807584 |
| IV9RU5F02IETKF | - - - - - | gnl\|UG\|Ta#S61807584 |
| IVLNWEC01B8RKN | - - - - - | gnl\|UG\|Ta#S61807632 |

| | | |
|---|---|---|
| IVLNWEC01BMSLG | - - - - - | gnl\|UG\|Ta#S61807632 |
| IVLNWEC02G9XML | - - - - - | gnl\|UG\|Ta#S61807632 |
| IUIVLUD01AUUOW | - - - - - | gnl\|UG\|Ta#S61807868 |
| IV9RU5F02IT4KI | - - - - - | gnl\|UG\|Ta#S61807868 |
| IVLNWEC01AMS2C | - - - - - | gnl\|UG\|Ta#S61808053 |
| IVLNWEC02HEBFF | - - - - - | gnl\|UG\|Ta#S61808053 |
| IV9RU5F01EXA6C | - - - - - | gnl\|UG\|Ta#S61808068 |
| IVLNWEC01D9Y8Z | - - - - - | gnl\|UG\|Ta#S61808068 |
| IVLNWEC02J3IJA | - - - - - | gnl\|UG\|Ta#S61808068 |
| IUIVLUD01C7NML | - - - - - | gnl\|UG\|Ta#S61808126 |
| IUIVLUD02HDEFH | - - - - - | gnl\|UG\|Ta#S61808126 |
| IV9RU5F01AFGA9 | - - - - - | gnl\|UG\|Ta#S61808126 |
| IV9RU5F02ILZ28 | - - - - - | gnl\|UG\|Ta#S61808126 |
| IV9RU5F01CRFDV | - - - - - | gnl\|UG\|Ta#S61808200 |
| IV9RU5F02H4KCS | - - - - - | gnl\|UG\|Ta#S61808200 |
| IVLNWEC02GLJWE | - - - - - | gnl\|UG\|Ta#S61808212 |
| IV9RU5F01CEC36 | - - - - - | gnl\|UG\|Ta#S61808227 |
| IV9RU5F02JHGKW | - - - - - | gnl\|UG\|Ta#S61808227 |
| IVLNWEC02JRUV8 | - - - - - | gnl\|UG\|Ta#S61808263 |
| IUIVLUD01CIMH8 | - - - - - | gnl\|UG\|Ta#S61808539 |
| IUIVLUD02F1BSV | - - - - - | gnl\|UG\|Ta#S61808539 |
| IV9RU5F01BF5QY | - - - - - | gnl\|UG\|Ta#S61808566 |
| IVLNWEC01C2EMB | - - - - - | gnl\|UG\|Ta#S61808566 |
| IVLNWEC02FNNGW | - - - - - | gnl\|UG\|Ta#S61808566 |
| IVLNWEC02HOU5Z | - - - - - | gnl\|UG\|Ta#S61808566 |
| IV9RU5F01C0U99 | - - - - - | gnl\|UG\|Ta#S61808575 |
| IVLNWEC01C4RMX | - - - - - | gnl\|UG\|Ta#S61808575 |
| IUIVLUD02JMMB2 | - - - - - | gnl\|UG\|Ta#S61808576 |
| IVLNWEC02JV0EU | - - - - - | gnl\|UG\|Ta#S61808576 |
| IV9RU5F01D5ER5 | - - - - - | gnl\|UG\|Ta#S61808621 |
| IVLNWEC02G3E5E | - - - - - | gnl\|UG\|Ta#S61808621 |
| IV9RU5F01BXV4N | - - - - - | gnl\|UG\|Ta#S61808645 |
| IV9RU5F01CCH38 | - - - - - | gnl\|UG\|Ta#S61808645 |
| IVLNWEC02JI71U | - - - - - | gnl\|UG\|Ta#S61808645 |
| IVLNWEC02HVRYA | - - - - - | gnl\|UG\|Ta#S61808686 |
| IVLNWEC02JVL0V | - - - - - | gnl\|UG\|Ta#S61808686 |
| IUIVLUD01B470K | - - - - - | gnl\|UG\|Ta#S61808688 |
| IUIVLUD02IXTGI | - - - - - | gnl\|UG\|Ta#S61808688 |
| IVLNWEC01BSIHC | - - - - - | gnl\|UG\|Ta#S61808775 |
| IVLNWEC02IS3RO | - - - - - | gnl\|UG\|Ta#S61808775 |
| IVLNWEC01COIF2 | - - - - - | gnl\|UG\|Ta#S61808780 |
| IUIVLUD01EAA0E | - - - - - | gnl\|UG\|Ta#S61808803 |
| IV9RU5F01A1M6X | - - - - - | gnl\|UG\|Ta#S61808803 |
| IV9RU5F02G7EBY | - - - - - | gnl\|UG\|Ta#S61808803 |
| IUIVLUD02IUUT5 | - - - - - | gnl\|UG\|Ta#S61808883 |
| IV9RU5F02GO42O | - - - - - | gnl\|UG\|Ta#S61808883 |
| IV9RU5F01DB0AY | - - - - - | gnl\|UG\|Ta#S61808922 |
| IVLNWEC01CJIRG | - - - - - | gnl\|UG\|Ta#S61808922 |
| IV9RU5F02HIJWJ | - - - - - | gnl\|UG\|Ta#S61808949 |
| IV9RU5F02IPW95 | - - - - - | gnl\|UG\|Ta#S61808949 |

| | | |
|---|---|---|
| IV9RU5F01DWP1G | - - - - - | gnl\|UG\|Ta#S61808970 |
| IV9RU5F02HPDBZ | - - - - - | gnl\|UG\|Ta#S61808970 |
| IV9RU5F02F0N55 | - - - - - | gnl\|UG\|Ta#S61809015 |
| IVLNWEC01CHAIU | - - - - - | gnl\|UG\|Ta#S61809015 |
| IUIVLUD01D679A | - - - - - | gnl\|UG\|Ta#S61809026 |
| IUIVLUD02IPMTA | - - - - - | gnl\|UG\|Ta#S61809026 |
| IV9RU5F02IZKHP | - - - - - | gnl\|UG\|Ta#S61809026 |
| IVLNWEC01C9SHH | - - - - - | gnl\|UG\|Ta#S61809026 |
| IUIVLUD02GREZI | - - - - - | gnl\|UG\|Ta#S61809051 |
| IUIVLUD02HBEDG | - - - - - | gnl\|UG\|Ta#S61809051 |
| IVLNWEC02I10SA | - - - - - | gnl\|UG\|Ta#S61809051 |
| IVLNWEC01CLA4W | - - - - - | gnl\|UG\|Ta#S61809138 |
| IVLNWEC01EV0CE | - - - - - | gnl\|UG\|Ta#S61809138 |
| IV9RU5F02G7QUM | - - - - - | gnl\|UG\|Ta#S61809217 |
| IV9RU5F02GP20V | - - - - - | gnl\|UG\|Ta#S61809217 |
| IUIVLUD01ATVG8 | - - - - - | gnl\|UG\|Ta#S61809293 |
| IUIVLUD01ERVP8 | - - - - - | gnl\|UG\|Ta#S61809293 |
| IV9RU5F01ESBQO | - - - - - | gnl\|UG\|Ta#S61809293 |
| IUIVLUD02HU3IQ | - - - - - | gnl\|UG\|Ta#S61809301 |
| IV9RU5F02JZZ2D | - - - - - | gnl\|UG\|Ta#S61809301 |
| IUIVLUD02I6VDP | - - - - - | gnl\|UG\|Ta#S61809398 |
| IV9RU5F01BESFT | - - - - - | gnl\|UG\|Ta#S61809398 |
| IV9RU5F01DURUB | - - - - - | gnl\|UG\|Ta#S61809427 |
| IVLNWEC01D9A5Z | - - - - - | gnl\|UG\|Ta#S61809427 |
| IUIVLUD01CNU8L | - - - - - | gnl\|UG\|Ta#S61809574 |
| IV9RU5F01BPI4I | - - - - - | gnl\|UG\|Ta#S61809574 |
| IVLNWEC01BONGJ | - - - - - | gnl\|UG\|Ta#S61809574 |
| IVLNWEC02I5VJG | - - - - - | gnl\|UG\|Ta#S61809574 |
| IUIVLUD01A9K7U | - - - - - | gnl\|UG\|Ta#S61809643 |
| IVLNWEC02I928S | - - - - - | gnl\|UG\|Ta#S61809643 |
| IUIVLUD02H1YNF | - - - - - | gnl\|UG\|Ta#S61809731 |
| IV9RU5F02HM3SC | - - - - - | gnl\|UG\|Ta#S61809731 |
| IUIVLUD02GM0WI | - - - - - | gnl\|UG\|Ta#S61809733 |
| IV9RU5F01BL3H0 | - - - - - | gnl\|UG\|Ta#S61809733 |
| IVLNWEC01A9EYI | - - - - - | gnl\|UG\|Ta#S61809733 |
| IVLNWEC02FN2E6 | - - - - - | gnl\|UG\|Ta#S61809733 |
| IVLNWEC01E0XGX | - - - - - | gnl\|UG\|Ta#S61809869 |
| IVLNWEC02GOJH2 | - - - - - | gnl\|UG\|Ta#S61809869 |
| IV9RU5F01DERU7 | - - - - - | gnl\|UG\|Ta#S61810048 |
| IVLNWEC02ICTG9 | - - - - - | gnl\|UG\|Ta#S61810048 |
| IV9RU5F02IJMDY | - - - - - | gnl\|UG\|Ta#S61810100 |
| IVLNWEC01BWBXU | - - - - - | gnl\|UG\|Ta#S61810100 |
| IVLNWEC01BIG69 | - - - - - | gnl\|UG\|Ta#S61810145 |
| IVLNWEC02I0VPP | - - - - - | gnl\|UG\|Ta#S61810145 |
| IUIVLUD01EDO25 | - - - - - | gnl\|UG\|Ta#S61810160 |
| IUIVLUD02GDIRL | - - - - - | gnl\|UG\|Ta#S61810160 |
| IV9RU5F01AVLMW | - - - - - | gnl\|UG\|Ta#S61810160 |
| IV9RU5F02JC0O4 | - - - - - | gnl\|UG\|Ta#S61810160 |
| IVLNWEC02F2LAQ | - - - - - | gnl\|UG\|Ta#S61810160 |
| IUIVLUD01EE7OX | - - - - - | gnl\|UG\|Ta#S61810261 |

| | | |
|---|---|---|
| IV9RU5F01BF4K7 | - - - - - | gnl\|UG\|Ta#S61810261 |
| IVLNWEC01AR1TC | - - - - - | gnl\|UG\|Ta#S61810261 |
| IV9RU5F01E1JMA | - - - - - | gnl\|UG\|Ta#S61810379 |
| IV9RU5F02I9T8T | - - - - - | gnl\|UG\|Ta#S61810379 |
| IVLNWEC02F7HWR | - - - - - | gnl\|UG\|Ta#S61810379 |
| IVLNWEC01E2KYJ | - - - - - | gnl\|UG\|Ta#S61810381 |
| IUIVLUD01CXR0V | - - - - - | gnl\|UG\|Ta#S61810412 |
| IVLNWEC02FMTKN | - - - - - | gnl\|UG\|Ta#S61810412 |
| IUIVLUD02JKHMW | - - - - - | gnl\|UG\|Ta#S61810463 |
| IV9RU5F01CDJ1D | - - - - - | gnl\|UG\|Ta#S61810463 |
| IV9RU5F02JS1JW | - - - - - | gnl\|UG\|Ta#S61810463 |
| IVLNWEC01BWVYU | - - - - - | gnl\|UG\|Ta#S61810463 |
| IV9RU5F01B8N1K | - - - - - | gnl\|UG\|Ta#S61810486 |
| IV9RU5F02IPLW2 | - - - - - | gnl\|UG\|Ta#S61810486 |
| IVLNWEC01AQ8Z9 | - - - - - | gnl\|UG\|Ta#S61810486 |
| IVLNWEC01EFTL2 | - - - - - | gnl\|UG\|Ta#S61810486 |
| IUIVLUD01E1PRM | - - - - - | gnl\|UG\|Ta#S61810519 |
| IV9RU5F01CXL02 | - - - - - | gnl\|UG\|Ta#S61810519 |
| IV9RU5F01EO9Z9 | - - - - - | gnl\|UG\|Ta#S61810519 |
| IVLNWEC02G1XFW | - - - - - | gnl\|UG\|Ta#S61810519 |
| IUIVLUD01B408R | - - - - - | gnl\|UG\|Ta#S61810530 |
| IV9RU5F01D8NAA | - - - - - | gnl\|UG\|Ta#S61810530 |
| IV9RU5F02IBOWU | - - - - - | gnl\|UG\|Ta#S61810549 |
| IV9RU5F02J0EMK | - - - - - | gnl\|UG\|Ta#S61810549 |
| IV9RU5F02JG6OZ | - - - - - | gnl\|UG\|Ta#S61810549 |
| IUIVLUD01C3NS9 | - - - - - | gnl\|UG\|Ta#S61810642 |
| IUIVLUD02HN7Q9 | - - - - - | gnl\|UG\|Ta#S61810642 |
| IV9RU5F01AYQ6C | - - - - - | gnl\|UG\|Ta#S61810642 |
| IVLNWEC01AW1WQ | - - - - - | gnl\|UG\|Ta#S61810642 |
| IVLNWEC01BSR4P | - - - - - | gnl\|UG\|Ta#S61810642 |
| IVLNWEC02IKAF2 | - - - - - | gnl\|UG\|Ta#S61810642 |
| IV9RU5F02JWWA3 | - - - - - | gnl\|UG\|Ta#S61810725 |
| IVLNWEC01EAMX6 | - - - - - | gnl\|UG\|Ta#S61810725 |
| IV9RU5F02FTI5R | - - - - - | gnl\|UG\|Ta#S61810873 |
| IV9RU5F02JSXYX | - - - - - | gnl\|UG\|Ta#S61810873 |
| IVLNWEC01CDPUX | - - - - - | gnl\|UG\|Ta#S61810873 |
| IUIVLUD01AR56K | - - - - - | gnl\|UG\|Ta#S61810883 |
| IV9RU5F02H8L8Z | - - - - - | gnl\|UG\|Ta#S61810883 |
| IVLNWEC01D3892 | - - - - - | gnl\|UG\|Ta#S61810883 |
| IUIVLUD01APJGA | - - - - - | gnl\|UG\|Ta#S61810913 |
| IVLNWEC02IBCX4 | - - - - - | gnl\|UG\|Ta#S61810913 |
| IVLNWEC02JUJGG | - - - - - | gnl\|UG\|Ta#S61810913 |
| IUIVLUD01COZDN | - - - - - | gnl\|UG\|Ta#S61810977 |
| IV9RU5F02G1LUV | - - - - - | gnl\|UG\|Ta#S61810977 |
| IV9RU5F02HP0H1 | - - - - - | gnl\|UG\|Ta#S61810977 |
| IVLNWEC01B0NE3 | - - - - - | gnl\|UG\|Ta#S61810977 |
| IVLNWEC01BSGZL | - - - - - | gnl\|UG\|Ta#S61810977 |
| IUIVLUD02FNJ5E | - - - - - | gnl\|UG\|Ta#S61811024 |
| IV9RU5F01ECORC | - - - - - | gnl\|UG\|Ta#S61811024 |
| IVLNWEC01DPTU8 | - - - - - | gnl\|UG\|Ta#S61811024 |

| | | |
|---|---|---|
| IVLNWEC01BMGFF | - - - - - | gnl\|UG\|Ta#S61811032 |
| IVLNWEC02JJH2F | - - - - - | gnl\|UG\|Ta#S61811032 |
| IUIVLUD01BQ8NK | - - - - - | gnl\|UG\|Ta#S61811066 |
| IV9RU5F02F8QZF | - - - - - | gnl\|UG\|Ta#S61811066 |
| IUIVLUD02HKJE5 | - - - - - | gnl\|UG\|Ta#S61811101 |
| IVLNWEC01DRVYT | - - - - - | gnl\|UG\|Ta#S61811101 |
| IVLNWEC02H6Q9A | - - - - - | gnl\|UG\|Ta#S61811101 |
| IUIVLUD02G0IHS | - - - - - | gnl\|UG\|Ta#S61811229 |
| IV9RU5F01AX0BA | - - - - - | gnl\|UG\|Ta#S61811229 |
| IVLNWEC01ANGD6 | - - - - - | gnl\|UG\|Ta#S61811309 |
| IVLNWEC02H81XW | - - - - - | gnl\|UG\|Ta#S61811309 |
| IUIVLUD01CVSWD | - - - - - | gnl\|UG\|Ta#S61811324 |
| IV9RU5F01BN8W4 | - - - - - | gnl\|UG\|Ta#S61811324 |
| IV9RU5F02HW617 | - - - - - | gnl\|UG\|Ta#S61811324 |
| IVLNWEC01C9993 | - - - - - | gnl\|UG\|Ta#S61811324 |
| IUIVLUD02GGDAF | - - - - - | gnl\|UG\|Ta#S61811378 |
| IVLNWEC01AVH25 | - - - - - | gnl\|UG\|Ta#S61811378 |
| IUIVLUD01EDSZB | - - - - - | gnl\|UG\|Ta#S61811416 |
| IV9RU5F02JXXJP | - - - - - | gnl\|UG\|Ta#S61811416 |
| IV9RU5F01D7KXM | - - - - - | gnl\|UG\|Ta#S61811462 |
| IVLNWEC01EJSMF | - - - - - | gnl\|UG\|Ta#S61811462 |
| IUIVLUD01APPHS | - - - - - | gnl\|UG\|Ta#S61811474 |
| IUIVLUD02JGF4L | - - - - - | gnl\|UG\|Ta#S61811474 |
| IV9RU5F01EZTYG | - - - - - | gnl\|UG\|Ta#S61811497 |
| IVLNWEC02ITD40 | - - - - - | gnl\|UG\|Ta#S61811497 |
| IV9RU5F02F791R | - - - - - | gnl\|UG\|Ta#S61811511 |
| IVLNWEC01BR2M8 | - - - - - | gnl\|UG\|Ta#S61811511 |
| IVLNWEC02JJMDL | - - - - - | gnl\|UG\|Ta#S61811511 |
| IVLNWEC01A4OB3 | - - - - - | gnl\|UG\|Ta#S61811546 |
| IVLNWEC01CYAQL | - - - - - | gnl\|UG\|Ta#S61811546 |
| IVLNWEC01BS6M0 | - - - - - | gnl\|UG\|Ta#S61811585 |
| IVLNWEC01ENCH0 | - - - - - | gnl\|UG\|Ta#S61811585 |
| IV9RU5F02GTYST | - - - - - | gnl\|UG\|Ta#S61811621 |
| IVLNWEC01C8PNA | - - - - - | gnl\|UG\|Ta#S61811621 |
| IV9RU5F02FW8DM | - - - - - | gnl\|UG\|Ta#S61811660 |
| IV9RU5F02GRLE7 | - - - - - | gnl\|UG\|Ta#S61811660 |
| IV9RU5F02IDWM4 | - - - - - | gnl\|UG\|Ta#S61811660 |
| IUIVLUD02IOBI8 | - - - - - | gnl\|UG\|Ta#S61811672 |
| IV9RU5F01DKF4B | - - - - - | gnl\|UG\|Ta#S61811672 |
| IV9RU5F02F8Y7M | - - - - - | gnl\|UG\|Ta#S61811672 |
| IV9RU5F02GHWLK | - - - - - | gnl\|UG\|Ta#S61811672 |
| IVLNWEC01E2MNW | - - - - - | gnl\|UG\|Ta#S61811672 |
| IUIVLUD02GSHEI | - - - - - | gnl\|UG\|Ta#S61811773 |
| IVLNWEC02IQBV9 | - - - - - | gnl\|UG\|Ta#S61811773 |
| IV9RU5F01BKIZ4 | - - - - - | gnl\|UG\|Ta#S61811830 |
| IVLNWEC02F6OPE | - - - - - | gnl\|UG\|Ta#S61811830 |
| IUIVLUD02FOC4M | - - - - - | gnl\|UG\|Ta#S61811866 |
| IUIVLUD02JWX7S | - - - - - | gnl\|UG\|Ta#S61811866 |
| IVLNWEC01AIACT | - - - - - | gnl\|UG\|Ta#S61811866 |
| IUIVLUD02IWP0O | - - - - - | gnl\|UG\|Ta#S61811889 |

| | | |
|---|---|---|
| IV9RU5F01AOXE6 | - - - - - | gnl\|UG\|Ta#S61811889 |
| IV9RU5F01CLMVJ | - - - - - | gnl\|UG\|Ta#S61811938 |
| IV9RU5F01DU3PI | - - - - - | gnl\|UG\|Ta#S61811938 |
| IVLNWEC01A5Y1J | - - - - - | gnl\|UG\|Ta#S61811957 |
| IVLNWEC01C3797 | - - - - - | gnl\|UG\|Ta#S61811957 |
| IVLNWEC02I4PZ5 | - - - - - | gnl\|UG\|Ta#S61811957 |
| IV9RU5F02FSEAS | - - - - - | gnl\|UG\|Ta#S61811960 |
| IV9RU5F02IZR5F | - - - - - | gnl\|UG\|Ta#S61811960 |
| IV9RU5F01DWQWG | - - - - - | gnl\|UG\|Ta#S61811976 |
| IV9RU5F02GS93G | - - - - - | gnl\|UG\|Ta#S61811976 |
| IVLNWEC02HNAQM | - - - - - | gnl\|UG\|Ta#S61811976 |
| IUIVLUD02IG73O | - - - - - | gnl\|UG\|Ta#S61812003 |
| IV9RU5F01ECD0C | - - - - - | gnl\|UG\|Ta#S61812003 |
| IV9RU5F02IPW5M | - - - - - | gnl\|UG\|Ta#S61812003 |
| IV9RU5F01BLV64 | - - - - - | gnl\|UG\|Ta#S61812022 |
| IVLNWEC01BEKBV | - - - - - | gnl\|UG\|Ta#S61812022 |
| IV9RU5F01EW032 | - - - - - | gnl\|UG\|Ta#S61812100 |
| IVLNWEC02F28F7 | - - - - - | gnl\|UG\|Ta#S61812100 |
| IVLNWEC02F8G46 | - - - - - | gnl\|UG\|Ta#S61812118 |
| IVLNWEC02GQFUQ | - - - - - | gnl\|UG\|Ta#S61812118 |
| IUIVLUD01B2WF8 | - - - - - | gnl\|UG\|Ta#S61812134 |
| IV9RU5F02JMDJ3 | - - - - - | gnl\|UG\|Ta#S61812134 |
| IVLNWEC01CXZO7 | - - - - - | gnl\|UG\|Ta#S61812134 |
| IV9RU5F01CJSNV | - - - - - | gnl\|UG\|Ta#S61812141 |
| IVLNWEC01EPX7C | - - - - - | gnl\|UG\|Ta#S61812141 |
| IV9RU5F01ATTEX | - - - - - | gnl\|UG\|Ta#S61812151 |
| IV9RU5F01DJRA5 | - - - - - | gnl\|UG\|Ta#S61812151 |
| IVLNWEC01B2QWF | - - - - - | gnl\|UG\|Ta#S61812160 |
| IVLNWEC01EXT3V | - - - - - | gnl\|UG\|Ta#S61812160 |
| IV9RU5F01EMXFM | - - - - - | gnl\|UG\|Ta#S61812219 |
| IV9RU5F02GTMG1 | - - - - - | gnl\|UG\|Ta#S61812219 |
| IVLNWEC01A6C1A | - - - - - | gnl\|UG\|Ta#S61812219 |
| IVLNWEC02F236F | - - - - - | gnl\|UG\|Ta#S61812244 |
| IVLNWEC02IHAM2 | - - - - - | gnl\|UG\|Ta#S61812244 |
| IV9RU5F01EP7HL | - - - - - | gnl\|UG\|Ta#S61812260 |
| IV9RU5F01EU3BW | - - - - - | gnl\|UG\|Ta#S61812260 |
| IVLNWEC02F0V3R | - - - - - | gnl\|UG\|Ta#S61812260 |
| IV9RU5F02H6NV1 | - - - - - | gnl\|UG\|Ta#S61812308 |
| IV9RU5F02H7M55 | - - - - - | gnl\|UG\|Ta#S61812308 |
| IVLNWEC02IOHEV | - - - - - | gnl\|UG\|Ta#S61812308 |
| IVLNWEC02JFPEN | - - - - - | gnl\|UG\|Ta#S61812308 |
| IV9RU5F01AY9HU | - - - - - | gnl\|UG\|Ta#S61812334 |
| IVLNWEC02HZKL0 | - - - - - | gnl\|UG\|Ta#S61812334 |
| IV9RU5F01CAIWR | - - - - - | gnl\|UG\|Ta#S61812356 |
| IVLNWEC01DDYUD | - - - - - | gnl\|UG\|Ta#S61812356 |
| IUIVLUD01C96TR | - - - - - | gnl\|UG\|Ta#S61812405 |
| IVLNWEC02GYAAV | - - - - - | gnl\|UG\|Ta#S61812405 |
| IUIVLUD02HY1QB | - - - - - | gnl\|UG\|Ta#S61812411 |
| IVLNWEC02GRQKY | - - - - - | gnl\|UG\|Ta#S61812411 |
| IUIVLUD02J0XNS | - - - - - | gnl\|UG\|Ta#S61812415 |

| | | |
|---|---|---|
| IV9RU5F01EK4P2 | - - - - - | gnl\|UG\|Ta#S61812415 |
| IUIVLUD01BQUK6 | - - - - - | gnl\|UG\|Ta#S61812435 |
| IUIVLUD01DOT0B | - - - - - | gnl\|UG\|Ta#S61812435 |
| IV9RU5F02GN357 | - - - - - | gnl\|UG\|Ta#S61812435 |
| IVLNWEC01AKK3N | - - - - - | gnl\|UG\|Ta#S61812435 |
| IVLNWEC01AV605 | - - - - - | gnl\|UG\|Ta#S61812435 |
| IVLNWEC01COL03 | - - - - - | gnl\|UG\|Ta#S61812435 |
| IVLNWEC02FYSPL | - - - - - | gnl\|UG\|Ta#S61812435 |
| IV9RU5F02F7CA2 | - - - - - | gnl\|UG\|Ta#S61812442 |
| IVLNWEC02GF4YB | - - - - - | gnl\|UG\|Ta#S61812442 |
| IV9RU5F01B85Y3 | - - - - - | gnl\|UG\|Ta#S61812454 |
| IV9RU5F01DP82M | - - - - - | gnl\|UG\|Ta#S61812454 |
| IV9RU5F02H1CY5 | - - - - - | gnl\|UG\|Ta#S61812454 |
| IV9RU5F02HQP9Q | - - - - - | gnl\|UG\|Ta#S61812454 |
| IVLNWEC01DYMCW | - - - - - | gnl\|UG\|Ta#S61812454 |
| IV9RU5F01BK48D | - - - - - | gnl\|UG\|Ta#S61812461 |
| IV9RU5F01CPA11 | - - - - - | gnl\|UG\|Ta#S61812461 |
| IUIVLUD01EU0C1 | - - - - - | gnl\|UG\|Ta#S61812473 |
| IV9RU5F01BPP1G | - - - - - | gnl\|UG\|Ta#S61812473 |
| IV9RU5F01DR5P0 | - - - - - | gnl\|UG\|Ta#S61812473 |
| IUIVLUD02F4703 | - - - - - | gnl\|UG\|Ta#S61812477 |
| IV9RU5F01DJES8 | - - - - - | gnl\|UG\|Ta#S61812477 |
| IV9RU5F02JD5OU | - - - - - | gnl\|UG\|Ta#S61812503 |
| IVLNWEC01DAXNX | - - - - - | gnl\|UG\|Ta#S61812503 |
| IV9RU5F02FYZSB | - - - - - | gnl\|UG\|Ta#S61812507 |
| IVLNWEC02I9SH6 | - - - - - | gnl\|UG\|Ta#S61812507 |
| IV9RU5F02JYIJ7 | - - - - - | gnl\|UG\|Ta#S61812509 |
| IVLNWEC01CCYQT | - - - - - | gnl\|UG\|Ta#S61812509 |
| IV9RU5F01EAMV5 | - - - - - | gnl\|UG\|Ta#S61812515 |
| IV9RU5F01ETO1N | - - - - - | gnl\|UG\|Ta#S61812515 |
| IVLNWEC02HPSHT | - - - - - | gnl\|UG\|Ta#S61812515 |
| IUIVLUD01CPVK6 | - - - - - | gnl\|UG\|Ta#S61812516 |
| IV9RU5F02H629C | - - - - - | gnl\|UG\|Ta#S61812516 |
| IV9RU5F01C38RJ | - - - - - | gnl\|UG\|Ta#S61812528 |
| IVLNWEC01B5UDA | - - - - - | gnl\|UG\|Ta#S61812528 |
| IV9RU5F01AI9NN | - - - - - | gnl\|UG\|Ta#S61812530 |
| IV9RU5F01C9TP8 | - - - - - | gnl\|UG\|Ta#S61812530 |
| IUIVLUD01C39WY | - - - - - | gnl\|UG\|Ta#S61812545 |
| IV9RU5F01CHRCW | - - - - - | gnl\|UG\|Ta#S61812545 |
| IV9RU5F01D2LYK | - - - - - | gnl\|UG\|Ta#S61812545 |
| IV9RU5F01C6RPG | - - - - - | gnl\|UG\|Ta#S61812547 |
| IV9RU5F01CXC10 | - - - - - | gnl\|UG\|Ta#S61812547 |
| IV9RU5F02HAYLE | - - - - - | gnl\|UG\|Ta#S61812547 |
| IVLNWEC02GO9RD | - - - - - | gnl\|UG\|Ta#S61812547 |
| IV9RU5F02JXBI1 | - - - - - | gnl\|UG\|Ta#S61812606 |
| IVLNWEC01CHNA4 | - - - - - | gnl\|UG\|Ta#S61812606 |
| IV9RU5F02G4L84 | - - - - - | gnl\|UG\|Ta#S61813059 |
| IV9RU5F02G83JI | - - - - - | gnl\|UG\|Ta#S61813059 |
| IUIVLUD01EXYCU | - - - - - | gnl\|UG\|Ta#S61813087 |
| IVLNWEC02GL9HB | - - - - - | gnl\|UG\|Ta#S61813087 |

| | | |
|---|---|---|
| IV9RU5F01CJPNO | - - - - - | gnl\|UG\|Ta#S61813552 |
| IVLNWEC01CC73Q | - - - - - | gnl\|UG\|Ta#S61813552 |
| IVLNWEC01CCT8W | - - - - - | gnl\|UG\|Ta#S61815225 |
| IVLNWEC01DRGLO | - - - - - | gnl\|UG\|Ta#S61815225 |
| IUIVLUD02GCIA0 | - - - - - | gnl\|UG\|Ta#S61815421 |
| IV9RU5F02GGIRA | - - - - - | gnl\|UG\|Ta#S61815421 |
| IVLNWEC01E1NYX | - - - - - | gnl\|UG\|Ta#S61815421 |
| IVLNWEC01DUK9Z | - - - - - | gnl\|UG\|Ta#S61815682 |
| IVLNWEC02F1HAI | - - - - - | gnl\|UG\|Ta#S61815682 |
| IVLNWEC02GMJ1L | - - - - - | gnl\|UG\|Ta#S61815682 |
| IVLNWEC02GQRC7 | - - - - - | gnl\|UG\|Ta#S61815682 |
| IVLNWEC01EJJDI | - - - - - | gnl\|UG\|Ta#S61815754 |
| IVLNWEC02JYB8Z | - - - - - | gnl\|UG\|Ta#S61815754 |
| IVLNWEC01CBV9V | - - - - - | gnl\|UG\|Ta#S61815902 |
| IVLNWEC02GNW8U | - - - - - | gnl\|UG\|Ta#S61815902 |
| IUIVLUD02H599L | - - - - - | gnl\|UG\|Ta#S61816422 |
| IV9RU5F01CARTC | - - - - - | gnl\|UG\|Ta#S61816422 |
| IV9RU5F01EO22X | - - - - - | gnl\|UG\|Ta#S61816514 |
| IVLNWEC01CE2DO | - - - - - | gnl\|UG\|Ta#S61816514 |
| IVLNWEC01B6R6G | - - - - - | gnl\|UG\|Ta#S61817364 |
| IVLNWEC02GLY5H | - - - - - | gnl\|UG\|Ta#S61817364 |
| IV9RU5F01CSBXX | - - - - - | gnl\|UG\|Ta#S61817422 |
| IVLNWEC01ERKZZ | - - - - - | gnl\|UG\|Ta#S61817422 |
| IUIVLUD01CAIMZ | - - - - - | gnl\|UG\|Ta#S61817547 |
| IVLNWEC01C9EEW | - - - - - | gnl\|UG\|Ta#S61817547 |
| IUIVLUD01C1I0X | - - - - - | gnl\|UG\|Ta#S61817583 |
| IV9RU5F01C86OX | - - - - - | gnl\|UG\|Ta#S61817583 |
| IV9RU5F02FNF66 | - - - - - | gnl\|UG\|Ta#S61817583 |
| IV9RU5F02H4COU | - - - - - | gnl\|UG\|Ta#S61817583 |
| IUIVLUD02JZTMG | - - - - - | gnl\|UG\|Ta#S61817628 |
| IVLNWEC02JBKNT | - - - - - | gnl\|UG\|Ta#S61817628 |
| IV9RU5F01A58TU | - - - - - | gnl\|UG\|Ta#S61817989 |
| IV9RU5F01ELIZY | - - - - - | gnl\|UG\|Ta#S61817989 |
| IVLNWEC01E1GLN | - - - - - | gnl\|UG\|Ta#S61817989 |
| IUIVLUD01DR2NE | - - - - - | gnl\|UG\|Ta#S61819893 |
| IVLNWEC02I55QT | - - - - - | gnl\|UG\|Ta#S61819893 |
| IV9RU5F01CTAIC | - - - - - | gnl\|UG\|Ta#S61820665 |
| IVLNWEC01B0RJK | - - - - - | gnl\|UG\|Ta#S61820665 |
| IV9RU5F02G8UKT | - - - - - | gnl\|UG\|Ta#S61821134 |
| IV9RU5F02GINC5 | - - - - - | gnl\|UG\|Ta#S61821134 |
| IV9RU5F02HVKQH | - - - - - | gnl\|UG\|Ta#S61821134 |
| IV9RU5F01C2PHY | - - - - - | gnl\|UG\|Ta#S61821312 |
| IV9RU5F02G4TQR | - - - - - | gnl\|UG\|Ta#S61821312 |
| IV9RU5F02GENFT | - - - - - | gnl\|UG\|Ta#S61821349 |
| IVLNWEC01AY9BV | - - - - - | gnl\|UG\|Ta#S61821349 |
| IVLNWEC02I1N3B | - - - - - | gnl\|UG\|Ta#S61821349 |
| IUIVLUD01BIGJR | - - - - - | gnl\|UG\|Ta#S61821482 |
| IUIVLUD02H3WCP | - - - - - | gnl\|UG\|Ta#S61821482 |
| IV9RU5F01DNAWW | - - - - - | gnl\|UG\|Ta#S61821912 |
| IVLNWEC02FZ05Z | - - - - - | gnl\|UG\|Ta#S61821912 |

| | | |
|---|---|---|
| IVLNWEC02I555Y | - - - - - | gnl\|UG\|Ta#S61821912 |
| IV9RU5F01EQ1MI | - - - - - | gnl\|UG\|Ta#S61822116 |
| IV9RU5F02FS667 | - - - - - | gnl\|UG\|Ta#S61822116 |
| IV9RU5F02F27AF | - - - - - | gnl\|UG\|Ta#S61823057 |
| IV9RU5F02HCN6K | - - - - - | gnl\|UG\|Ta#S61823057 |
| IV9RU5F02I9EPP | - - - - - | gnl\|UG\|Ta#S61823057 |
| IV9RU5F02GAYOG | - - - - - | gnl\|UG\|Ta#S61823447 |
| IVLNWEC01DH66J | - - - - - | gnl\|UG\|Ta#S61823447 |
| IUIVLUD01BGRPU | - - - - - | gnl\|UG\|Ta#S61823574 |
| IVLNWEC02JMK2L | - - - - - | gnl\|UG\|Ta#S61823574 |
| IUIVLUD02JOSFQ | - - - - - | gnl\|UG\|Ta#S61824003 |
| IV9RU5F01EVNM0 | - - - - - | gnl\|UG\|Ta#S61824003 |
| IV9RU5F02HEWYW | - - - - - | gnl\|UG\|Ta#S61824094 |
| IVLNWEC02HVC95 | - - - - - | gnl\|UG\|Ta#S61824094 |
| IUIVLUD02GD4KK | - - - - - | gnl\|UG\|Ta#S61825019 |
| IV9RU5F01EI199 | - - - - - | gnl\|UG\|Ta#S61825019 |
| IV9RU5F01CD6R5 | - - - - - | gnl\|UG\|Ta#S61825214 |
| IV9RU5F01EA0S3 | - - - - - | gnl\|UG\|Ta#S61825214 |
| IUIVLUD02HZ3Q7 | - - - - - | gnl\|UG\|Ta#S61825445 |
| IV9RU5F01BJW7E | - - - - - | gnl\|UG\|Ta#S61825445 |
| IV9RU5F01DOA0T | - - - - - | gnl\|UG\|Ta#S61825445 |
| IUIVLUD02IHMD3 | - - - - - | gnl\|UG\|Ta#S61825720 |
| IVLNWEC01AJ5QO | - - - - - | gnl\|UG\|Ta#S61825720 |
| IVLNWEC01DZ14G | - - - - - | gnl\|UG\|Ta#S61825720 |
| IV9RU5F01DLGKY | - - - - - | gnl\|UG\|Ta#S61826190 |
| IV9RU5F02FVA7J | - - - - - | gnl\|UG\|Ta#S61826190 |
| IVLNWEC01DK92J | - - - - - | gnl\|UG\|Ta#S61826190 |
| IV9RU5F01BM5DO | - - - - - | gnl\|UG\|Ta#S61826485 |
| IVLNWEC01CNRP4 | - - - - - | gnl\|UG\|Ta#S61826485 |
| IV9RU5F01AY42M | - - - - - | gnl\|UG\|Ta#S61827082 |
| IVLNWEC02GNSV4 | - - - - - | gnl\|UG\|Ta#S61827082 |
| IVLNWEC02I8JNJ | - - - - - | gnl\|UG\|Ta#S61827082 |
| IV9RU5F02JOXJF | - - - - - | gnl\|UG\|Ta#S61827083 |
| IVLNWEC02IW5S6 | - - - - - | gnl\|UG\|Ta#S61827083 |
| IUIVLUD01EMI1H | - - - - - | gnl\|UG\|Ta#S61827089 |
| IVLNWEC02HCYEW | - - - - - | gnl\|UG\|Ta#S61827089 |
| IUIVLUD02G3WM2 | - - - - - | gnl\|UG\|Ta#S61827136 |
| IV9RU5F02JK6AQ | - - - - - | gnl\|UG\|Ta#S61827136 |
| IVLNWEC02J4P8H | - - - - - | gnl\|UG\|Ta#S61827136 |
| IV9RU5F02IW41S | - - - - - | gnl\|UG\|Ta#S61827454 |
| IVLNWEC01AKX5X | - - - - - | gnl\|UG\|Ta#S61827454 |
| IVLNWEC02H7KVQ | - - - - - | gnl\|UG\|Ta#S61827454 |
| IVLNWEC02HQYGV | - - - - - | gnl\|UG\|Ta#S61827454 |
| IV9RU5F02IZTON | - - - - - | gnl\|UG\|Ta#S61827656 |
| IVLNWEC01CM65J | - - - - - | gnl\|UG\|Ta#S61827656 |
| IV9RU5F01C0RD7 | - - - - - | gnl\|UG\|Ta#S61827676 |
| IVLNWEC02ISDLN | - - - - - | gnl\|UG\|Ta#S61827676 |
| IUIVLUD02HVDNZ | - - - - - | gnl\|UG\|Ta#S61827747 |
| IV9RU5F01AQS9U | - - - - - | gnl\|UG\|Ta#S61827747 |
| IV9RU5F01B2TP0 | - - - - - | gnl\|UG\|Ta#S61827747 |

| | | |
|---|---|---|
| IVLNWEC01EZHE3 | - - - - - | gnl\|UG\|Ta#S61827753 |
| IVLNWEC02G04D6 | - - - - - | gnl\|UG\|Ta#S61827753 |
| IUIVLUD02HZDI6 | - - - - - | gnl\|UG\|Ta#S61827822 |
| IVLNWEC02H50WR | - - - - - | gnl\|UG\|Ta#S61827822 |
| IVLNWEC01A1GCR | - - - - - | gnl\|UG\|Ta#S61827824 |
| IV9RU5F01BW74F | - - - - - | gnl\|UG\|Ta#S61827891 |
| IV9RU5F02G8LWZ | - - - - - | gnl\|UG\|Ta#S61827891 |
| IVLNWEC02JN03M | - - - - - | gnl\|UG\|Ta#S61827891 |
| IV9RU5F01DRBH3 | - - - - - | gnl\|UG\|Ta#S61827953 |
| IVLNWEC02F5JZ3 | - - - - - | gnl\|UG\|Ta#S61827953 |
| IUIVLUD02F6S51 | - - - - - | gnl\|UG\|Ta#S61828147 |
| IUIVLUD02IEQ3L | - - - - - | gnl\|UG\|Ta#S61828147 |
| IV9RU5F02I89FJ | - - - - - | gnl\|UG\|Ta#S61828163 |
| IVLNWEC01ARNHM | - - - - - | gnl\|UG\|Ta#S61828163 |
| IVLNWEC02HLMNP | - - - - - | gnl\|UG\|Ta#S61828163 |
| IV9RU5F01CLAXW | - - - - - | gnl\|UG\|Ta#S61828232 |
| IV9RU5F02I9595 | - - - - - | gnl\|UG\|Ta#S61828232 |
| IVLNWEC01AP013 | - - - - - | gnl\|UG\|Ta#S61828232 |
| IUIVLUD02I33IE | - - - - - | gnl\|UG\|Ta#S61828264 |
| IV9RU5F02GXE80 | - - - - - | gnl\|UG\|Ta#S61828264 |
| IVLNWEC01CB3II | - - - - - | gnl\|UG\|Ta#S61828264 |
| IUIVLUD01DLY4L | - - - - - | gnl\|UG\|Ta#S61828271 |
| IUIVLUD02HQ1ZE | - - - - - | gnl\|UG\|Ta#S61828271 |
| IV9RU5F01CZU8L | - - - - - | gnl\|UG\|Ta#S61828271 |
| IVLNWEC01ECXH8 | - - - - - | gnl\|UG\|Ta#S61828271 |
| IVLNWEC02JFPEA | - - - - - | gnl\|UG\|Ta#S61828271 |
| IV9RU5F01EIVGU | - - - - - | gnl\|UG\|Ta#S61828316 |
| IV9RU5F01EJ17F | - - - - - | gnl\|UG\|Ta#S61828316 |
| IV9RU5F02HZGDG | - - - - - | gnl\|UG\|Ta#S61828316 |
| IV9RU5F02JZ7DB | - - - - - | gnl\|UG\|Ta#S61828316 |
| IVLNWEC01BK55B | - - - - - | gnl\|UG\|Ta#S61828316 |
| IV9RU5F01ANSGE | - - - - - | gnl\|UG\|Ta#S61828328 |
| IUIVLUD02F3RCJ | - - - - - | gnl\|UG\|Ta#S61828331 |
| IVLNWEC02FXWUL | - - - - - | gnl\|UG\|Ta#S61828331 |
| IV9RU5F02IOXQF | - - - - - | gnl\|UG\|Ta#S61828378 |
| IVLNWEC02JX8SX | - - - - - | gnl\|UG\|Ta#S61828378 |
| IUIVLUD02GC8QL | - - - - - | gnl\|UG\|Ta#S61828383 |
| IUIVLUD02GXHX4 | - - - - - | gnl\|UG\|Ta#S61828383 |
| IVLNWEC01A1793 | - - - - - | gnl\|UG\|Ta#S61828383 |
| IVLNWEC02ITUZR | - - - - - | gnl\|UG\|Ta#S61828383 |
| IV9RU5F01CPDWS | - - - - - | gnl\|UG\|Ta#S61828386 |
| IV9RU5F02ILNNI | - - - - - | gnl\|UG\|Ta#S61828386 |
| IUIVLUD01BV4A7 | - - - - - | gnl\|UG\|Ta#S61828405 |
| IV9RU5F02IYR7S | - - - - - | gnl\|UG\|Ta#S61828405 |
| IUIVLUD01CIP6X | - - - - - | gnl\|UG\|Ta#S61828497 |
| IV9RU5F01DKB2F | - - - - - | gnl\|UG\|Ta#S61828497 |
| IVLNWEC01BS72U | - - - - - | gnl\|UG\|Ta#S61828497 |
| IV9RU5F01ECPPL | - - - - - | gnl\|UG\|Ta#S61828538 |
| IV9RU5F02JB9O2 | - - - - - | gnl\|UG\|Ta#S61828538 |
| IVLNWEC01A64OD | - - - - - | gnl\|UG\|Ta#S61828538 |

| | | |
|---|---|---|
| IV9RU5F02G3LI8 | - - - - - | gnl\|UG\|Ta#S61828638 |
| IV9RU5F02HZJH6 | - - - - - | gnl\|UG\|Ta#S61828638 |
| IUIVLUD02J25NY | - - - - - | gnl\|UG\|Ta#S61828711 |
| IV9RU5F02IPQ0F | - - - - - | gnl\|UG\|Ta#S61828711 |
| IVLNWEC02J4MWT | - - - - - | gnl\|UG\|Ta#S61828711 |
| IV9RU5F02IRQUH | - - - - - | gnl\|UG\|Ta#S61828896 |
| IVLNWEC02IYXT8 | - - - - - | gnl\|UG\|Ta#S61828896 |
| IV9RU5F01BRLJ7 | - - - - - | gnl\|UG\|Ta#S61829091 |
| IV9RU5F01DLBYK | - - - - - | gnl\|UG\|Ta#S61829091 |
| IVLNWEC02IU7A1 | - - - - - | gnl\|UG\|Ta#S61829091 |
| IUIVLUD02GFPKL | - - - - - | gnl\|UG\|Ta#S61829094 |
| IV9RU5F02FWXQZ | - - - - - | gnl\|UG\|Ta#S61829094 |
| IUIVLUD01ELHJZ | - - - - - | gnl\|UG\|Ta#S61829198 |
| IV9RU5F01AJQ43 | - - - - - | gnl\|UG\|Ta#S61829198 |
| IV9RU5F02HRJHG | - - - - - | gnl\|UG\|Ta#S61829198 |
| IUIVLUD02HHLR3 | - - - - - | gnl\|UG\|Ta#S61829202 |
| IVLNWEC01DDT3T | - - - - - | gnl\|UG\|Ta#S61829202 |
| IV9RU5F01A2S0H | - - - - - | gnl\|UG\|Ta#S61829205 |
| IV9RU5F02IDF45 | - - - - - | gnl\|UG\|Ta#S61829205 |
| IV9RU5F02JWXP0 | - - - - - | gnl\|UG\|Ta#S61829205 |
| IVLNWEC01BW3DC | - - - - - | gnl\|UG\|Ta#S61829205 |
| IVLNWEC01C7NLF | - - - - - | gnl\|UG\|Ta#S61829205 |
| IVLNWEC01CKRQZ | - - - - - | gnl\|UG\|Ta#S61829205 |
| IVLNWEC01DH0HQ | - - - - - | gnl\|UG\|Ta#S61829205 |
| IVLNWEC02HUW8D | - - - - - | gnl\|UG\|Ta#S61829205 |
| IUIVLUD02J2B70 | - - - - - | gnl\|UG\|Ta#S61829216 |
| IVLNWEC02I7JAR | - - - - - | gnl\|UG\|Ta#S61829216 |
| IV9RU5F01E146M | - - - - - | gnl\|UG\|Ta#S61829341 |
| IVLNWEC02JPKSX | - - - - - | gnl\|UG\|Ta#S61829341 |
| IV9RU5F01DDY7C | - - - - - | gnl\|UG\|Ta#S61829407 |
| IVLNWEC01DWR3H | - - - - - | gnl\|UG\|Ta#S61829407 |
| IUIVLUD02JK3B5 | - - - - - | gnl\|UG\|Ta#S61829435 |
| IV9RU5F02IFTQB | - - - - - | gnl\|UG\|Ta#S61829435 |
| IUIVLUD02JRKMC | - - - - - | gnl\|UG\|Ta#S61829543 |
| IV9RU5F02GWULL | - - - - - | gnl\|UG\|Ta#S61829543 |
| IVLNWEC01DVWGX | - - - - - | gnl\|UG\|Ta#S61829543 |
| IVLNWEC02FT4FJ | - - - - - | gnl\|UG\|Ta#S61829543 |
| IVLNWEC02I47KR | - - - - - | gnl\|UG\|Ta#S61829608 |
| IVLNWEC02JUK0V | - - - - - | gnl\|UG\|Ta#S61829608 |
| IUIVLUD01DP5W6 | - - - - - | gnl\|UG\|Ta#S61829668 |
| IUIVLUD02FLR50 | - - - - - | gnl\|UG\|Ta#S61829668 |
| IVLNWEC02I8I7X | - - - - - | gnl\|UG\|Ta#S61829668 |
| IVLNWEC02ICCRJ | - - - - - | gnl\|UG\|Ta#S61829668 |
| IVLNWEC01AWR0Z | - - - - - | gnl\|UG\|Ta#S61829669 |
| IVLNWEC01EVJMK | - - - - - | gnl\|UG\|Ta#S61829669 |
| IV9RU5F02IVELV | - - - - - | gnl\|UG\|Ta#S61829726 |
| IVLNWEC01D9T3R | - - - - - | gnl\|UG\|Ta#S61829726 |
| IUIVLUD02GBBTK | - - - - - | gnl\|UG\|Ta#S61829744 |
| IVLNWEC01BF8DH | - - - - - | gnl\|UG\|Ta#S61829744 |
| IUIVLUD01EUFHZ | - - - - - | gnl\|UG\|Ta#S61829756 |

| | | |
|---|---|---|
| IV9RU5F01CBVRF | - - - - - | gnl\|UG\|Ta#S61829756 |
| IVLNWEC01CXZ6J | - - - - - | gnl\|UG\|Ta#S61829756 |
| IUIVLUD02JX2Z1 | - - - - - | gnl\|UG\|Ta#S61829803 |
| IV9RU5F01BK1FU | - - - - - | gnl\|UG\|Ta#S61829803 |
| IUIVLUD01AJ7UC | - - - - - | gnl\|UG\|Ta#S61829810 |
| IV9RU5F01B9R1N | - - - - - | gnl\|UG\|Ta#S61829810 |
| IVLNWEC01E25L6 | - - - - - | gnl\|UG\|Ta#S61829810 |
| IUIVLUD01AILRH | - - - - - | gnl\|UG\|Ta#S61829826 |
| IVLNWEC02FW1LB | - - - - - | gnl\|UG\|Ta#S61829826 |
| IV9RU5F02GLIOC | - - - - - | gnl\|UG\|Ta#S61829883 |
| IVLNWEC02JDVWR | - - - - - | gnl\|UG\|Ta#S61829883 |
| IV9RU5F02FLFON | - - - - - | gnl\|UG\|Ta#S61830096 |
| IVLNWEC01EX8XO | - - - - - | gnl\|UG\|Ta#S61830096 |
| IVLNWEC02I05AO | - - - - - | gnl\|UG\|Ta#S61830096 |
| IUIVLUD01AH8GY | - - - - - | gnl\|UG\|Ta#S61830166 |
| IUIVLUD02G79BY | - - - - - | gnl\|UG\|Ta#S61830166 |
| IV9RU5F01ADNCH | - - - - - | gnl\|UG\|Ta#S61830166 |
| IV9RU5F02HSPS0 | - - - - - | gnl\|UG\|Ta#S61830166 |
| IVLNWEC01B8QQQ | - - - - - | gnl\|UG\|Ta#S61830166 |
| IVLNWEC01DJKYX | - - - - - | gnl\|UG\|Ta#S61830166 |
| IV9RU5F01CD2D7 | - - - - - | gnl\|UG\|Ta#S61830171 |
| IVLNWEC02H431X | - - - - - | gnl\|UG\|Ta#S61830171 |
| IVLNWEC01EEFAY | - - - - - | gnl\|UG\|Ta#S61830217 |
| IVLNWEC02F4KUN | - - - - - | gnl\|UG\|Ta#S61830217 |
| IUIVLUD01B5TFE | - - - - - | gnl\|UG\|Ta#S61830245 |
| IUIVLUD02G4GWT | - - - - - | gnl\|UG\|Ta#S61830245 |
| IV9RU5F01D6XLN | - - - - - | gnl\|UG\|Ta#S61830280 |
| IVLNWEC02HF5YK | - - - - - | gnl\|UG\|Ta#S61830280 |
| IUIVLUD01E1BEM | - - - - - | gnl\|UG\|Ta#S61830344 |
| IVLNWEC01B7EUS | - - - - - | gnl\|UG\|Ta#S61830344 |
| IV9RU5F01C3SHJ | - - - - - | gnl\|UG\|Ta#S61830668 |
| IVLNWEC02GQF0N | - - - - - | gnl\|UG\|Ta#S61830668 |
| IV9RU5F01AWN00 | - - - - - | gnl\|UG\|Ta#S61830683 |
| IV9RU5F01CEJVK | - - - - - | gnl\|UG\|Ta#S61830683 |
| IV9RU5F01AM1V2 | - - - - - | gnl\|UG\|Ta#S61830716 |
| IV9RU5F01CX335 | - - - - - | gnl\|UG\|Ta#S61830716 |
| IVLNWEC02GG4ZQ | - - - - - | gnl\|UG\|Ta#S61830716 |
| IVLNWEC02GORYA | - - - - - | gnl\|UG\|Ta#S61830716 |
| IUIVLUD01BXYYS | - - - - - | gnl\|UG\|Ta#S61830731 |
| IV9RU5F01AFA1L | - - - - - | gnl\|UG\|Ta#S61830731 |
| IUIVLUD01BWCK2 | - - - - - | gnl\|UG\|Ta#S61830765 |
| IV9RU5F01EORK9 | - - - - - | gnl\|UG\|Ta#S61830765 |
| IV9RU5F02HEXPI | - - - - - | gnl\|UG\|Ta#S61830765 |
| IVLNWEC02HNEAU | - - - - - | gnl\|UG\|Ta#S61830765 |
| IV9RU5F01DVZDB | - - - - - | gnl\|UG\|Ta#S61830834 |
| IV9RU5F02IB03Y | - - - - - | gnl\|UG\|Ta#S61830834 |
| IV9RU5F02GGNNN | - - - - - | gnl\|UG\|Ta#S61830879 |
| IV9RU5F01B8K6N | - - - - - | gnl\|UG\|Ta#S61830920 |
| IVLNWEC01ES2ZV | - - - - - | gnl\|UG\|Ta#S61830920 |
| IV9RU5F01BTRVS | - - - - - | gnl\|UG\|Ta#S61830940 |

| | | |
|---|---|---|
| IV9RU5F01ERXGF | - - - - - | gnl\|UG\|Ta#S61830940 |
| IVLNWEC01BYJBU | - - - - - | gnl\|UG\|Ta#S61830940 |
| IUIVLUD02GJ9MU | - - - - - | gnl\|UG\|Ta#S61830982 |
| IV9RU5F01BQLUM | - - - - - | gnl\|UG\|Ta#S61830982 |
| IUIVLUD01AZG3W | - - - - - | gnl\|UG\|Ta#S61830996 |
| IV9RU5F02HL6TP | - - - - - | gnl\|UG\|Ta#S61830996 |
| IVLNWEC02JOIPH | - - - - - | gnl\|UG\|Ta#S61830996 |
| IUIVLUD02IVYOQ | - - - - - | gnl\|UG\|Ta#S61831036 |
| IUIVLUD02JLZ35 | - - - - - | gnl\|UG\|Ta#S61831036 |
| IUIVLUD01EAFZ6 | - - - - - | gnl\|UG\|Ta#S61831043 |
| IUIVLUD02JY79L | - - - - - | gnl\|UG\|Ta#S61831043 |
| IV9RU5F02IUZ59 | - - - - - | gnl\|UG\|Ta#S61831043 |
| IVLNWEC02F89PQ | - - - - - | gnl\|UG\|Ta#S61831043 |
| IV9RU5F02JAJLR | - - - - - | gnl\|UG\|Ta#S61831059 |
| IVLNWEC02JANN5 | - - - - - | gnl\|UG\|Ta#S61831059 |
| IUIVLUD02GFZVQ | - - - - - | gnl\|UG\|Ta#S61831107 |
| IV9RU5F01BVCND | - - - - - | gnl\|UG\|Ta#S61831107 |
| IV9RU5F02FVXJ1 | - - - - - | gnl\|UG\|Ta#S61831107 |
| IVLNWEC02JVTI9 | - - - - - | gnl\|UG\|Ta#S61831107 |
| IV9RU5F01C7U40 | - - - - - | gnl\|UG\|Ta#S61831109 |
| IV9RU5F01DCEPF | - - - - - | gnl\|UG\|Ta#S61831109 |
| IUIVLUD01BKT86 | - - - - - | gnl\|UG\|Ta#S61831144 |
| IVLNWEC01D3GZ9 | - - - - - | gnl\|UG\|Ta#S61831144 |
| IVLNWEC02I5K6M | - - - - - | gnl\|UG\|Ta#S61831144 |
| IV9RU5F01CWKD5 | - - - - - | gnl\|UG\|Ta#S61831149 |
| IVLNWEC02F11JB | - - - - - | gnl\|UG\|Ta#S61831149 |
| IUIVLUD02JEGS1 | - - - - - | gnl\|UG\|Ta#S61831155 |
| IV9RU5F01ATIRA | - - - - - | gnl\|UG\|Ta#S61831155 |
| IV9RU5F02IJ1YU | - - - - - | gnl\|UG\|Ta#S61831155 |
| IVLNWEC01E0R7W | - - - - - | gnl\|UG\|Ta#S61831155 |
| IUIVLUD01DP2ZO | - - - - - | gnl\|UG\|Ta#S61831164 |
| IVLNWEC01DR87K | - - - - - | gnl\|UG\|Ta#S61831164 |
| IVLNWEC01DY1L1 | - - - - - | gnl\|UG\|Ta#S61831164 |
| IVLNWEC02H6UW7 | - - - - - | gnl\|UG\|Ta#S61831164 |
| IVLNWEC01C097L | - - - - - | gnl\|UG\|Ta#S61831184 |
| IV9RU5F01DVZIR | - - - - - | gnl\|UG\|Ta#S61831211 |
| IVLNWEC01CX68W | - - - - - | gnl\|UG\|Ta#S61831211 |
| IVLNWEC01DPSB4 | - - - - - | gnl\|UG\|Ta#S61831211 |
| IVLNWEC02HCMT8 | - - - - - | gnl\|UG\|Ta#S61831211 |
| IUIVLUD02GW9XW | - - - - - | gnl\|UG\|Ta#S61831245 |
| IUIVLUD02HY55Z | - - - - - | gnl\|UG\|Ta#S61831245 |
| IV9RU5F01BUZ7O | - - - - - | gnl\|UG\|Ta#S61831263 |
| IV9RU5F01BWCFV | - - - - - | gnl\|UG\|Ta#S61831263 |
| IV9RU5F02FP63D | - - - - - | gnl\|UG\|Ta#S61831268 |
| IVLNWEC02H4KNU | - - - - - | gnl\|UG\|Ta#S61831268 |
| IUIVLUD02HDVG2 | - - - - - | gnl\|UG\|Ta#S61831285 |
| IVLNWEC01B136T | - - - - - | gnl\|UG\|Ta#S61831285 |
| IV9RU5F01EYB7K | - - - - - | gnl\|UG\|Ta#S61831297 |
| IVLNWEC01E3MAN | - - - - - | gnl\|UG\|Ta#S61831297 |
| IV9RU5F02G1E3G | - - - - - | gnl\|UG\|Ta#S61831341 |

| | | |
|---|---|---|
| IVLNWEC02H956U | - - - - - | gnl\|UG\|Ta#S61831341 |
| IVLNWEC01A23JR | - - - - - | gnl\|UG\|Ta#S61831367 |
| IVLNWEC02ID6M7 | - - - - - | gnl\|UG\|Ta#S61831367 |
| IUIVLUD01DIWY5 | - - - - - | gnl\|UG\|Ta#S61831396 |
| IVLNWEC02JQUXY | - - - - - | gnl\|UG\|Ta#S61831396 |
| IV9RU5F02HNN0Z | - - - - - | gnl\|UG\|Ta#S61831397 |
| IUIVLUD01EYX4C | - - - - - | gnl\|UG\|Ta#S61831444 |
| IVLNWEC01B9VIE | - - - - - | gnl\|UG\|Ta#S61831444 |
| IVLNWEC02G6T4W | - - - - - | gnl\|UG\|Ta#S61831444 |
| IUIVLUD01CQN5C | - - - - - | gnl\|UG\|Ta#S61831463 |
| IVLNWEC01CT5HP | - - - - - | gnl\|UG\|Ta#S61831463 |
| IUIVLUD01BZ1LG | - - - - - | gnl\|UG\|Ta#S61831483 |
| IV9RU5F01BDCGI | - - - - - | gnl\|UG\|Ta#S61831483 |
| IV9RU5F01EPRQA | - - - - - | gnl\|UG\|Ta#S61831483 |
| IV9RU5F02JAC48 | - - - - - | gnl\|UG\|Ta#S61831483 |
| IUIVLUD01AY1PL | - - - - - | gnl\|UG\|Ta#S61831498 |
| IUIVLUD01COAFG | - - - - - | gnl\|UG\|Ta#S61831498 |
| IV9RU5F01C2TC4 | - - - - - | gnl\|UG\|Ta#S61831533 |
| IUIVLUD01DDJKF | - - - - - | gnl\|UG\|Ta#S61831543 |
| IV9RU5F01EPFQE | - - - - - | gnl\|UG\|Ta#S61831543 |
| IVLNWEC01AF3ZQ | - - - - - | gnl\|UG\|Ta#S61831543 |
| IVLNWEC02HBCOV | - - - - - | gnl\|UG\|Ta#S61831543 |
| IV9RU5F02GKVJ3 | - - - - - | gnl\|UG\|Ta#S61831570 |
| IV9RU5F02JSZ5S | - - - - - | gnl\|UG\|Ta#S61831570 |
| IVLNWEC02GRNSX | - - - - - | gnl\|UG\|Ta#S61831622 |
| IVLNWEC02JEGOC | - - - - - | gnl\|UG\|Ta#S61831622 |
| IV9RU5F02GMHLE | - - - - - | gnl\|UG\|Ta#S61831644 |
| IVLNWEC01EBH0M | - - - - - | gnl\|UG\|Ta#S61831644 |
| IVLNWEC02GUGVI | - - - - - | gnl\|UG\|Ta#S61831644 |
| IUIVLUD01ER7VI | - - - - - | gnl\|UG\|Ta#S61831651 |
| IV9RU5F02HP8V9 | - - - - - | gnl\|UG\|Ta#S61831651 |
| IUIVLUD02FO167 | - - - - - | gnl\|UG\|Ta#S61831713 |
| IV9RU5F01CRZ3G | - - - - - | gnl\|UG\|Ta#S61831713 |
| IUIVLUD02FXFFR | - - - - - | gnl\|UG\|Ta#S61831797 |
| IV9RU5F02GU2OE | - - - - - | gnl\|UG\|Ta#S61831797 |
| IUIVLUD01B1J9C | - - - - - | gnl\|UG\|Ta#S61831803 |
| IV9RU5F02GLV22 | - - - - - | gnl\|UG\|Ta#S61831803 |
| IV9RU5F01ARJSU | - - - - - | gnl\|UG\|Ta#S61831808 |
| IV9RU5F01AWIUI | - - - - - | gnl\|UG\|Ta#S61831808 |
| IV9RU5F02J3QP3 | - - - - - | gnl\|UG\|Ta#S61831808 |
| IUIVLUD02HKRSN | - - - - - | gnl\|UG\|Ta#S61831833 |
| IUIVLUD02IGAPA | - - - - - | gnl\|UG\|Ta#S61831833 |
| IVLNWEC01B6RBY | - - - - - | gnl\|UG\|Ta#S61831833 |
| IVLNWEC02JAFNB | - - - - - | gnl\|UG\|Ta#S61831833 |
| IVLNWEC02GQYX8 | - - - - - | gnl\|UG\|Ta#S61831836 |
| IVLNWEC02IEXV2 | - - - - - | gnl\|UG\|Ta#S61831836 |
| IUIVLUD01BF8OW | - - - - - | gnl\|UG\|Ta#S61831852 |
| IVLNWEC02GD1Z2 | - - - - - | gnl\|UG\|Ta#S61831852 |
| IUIVLUD02GZAQV | - - - - - | gnl\|UG\|Ta#S61831856 |
| IV9RU5F01AGPW9 | - - - - - | gnl\|UG\|Ta#S61831856 |

| | | |
|---|---|---|
| IV9RU5F01EE25T | - - - - - | gnl\|UG\|Ta#S61831856 |
| IUIVLUD02GNT8O | - - - - - | gnl\|UG\|Ta#S61831862 |
| IVLNWEC02FJ7EV | - - - - - | gnl\|UG\|Ta#S61831862 |
| IUIVLUD02FM1L3 | - - - - - | gnl\|UG\|Ta#S61831870 |
| IV9RU5F02I9W6K | - - - - - | gnl\|UG\|Ta#S61831870 |
| IVLNWEC02H624H | - - - - - | gnl\|UG\|Ta#S61831870 |
| IV9RU5F01AXSN9 | - - - - - | gnl\|UG\|Ta#S61831930 |
| IVLNWEC02IR8Z4 | - - - - - | gnl\|UG\|Ta#S61831930 |
| IVLNWEC02JWS5L | - - - - - | gnl\|UG\|Ta#S61831930 |
| IV9RU5F01CGOVL | - - - - - | gnl\|UG\|Ta#S61831942 |
| IVLNWEC01DIGOT | - - - - - | gnl\|UG\|Ta#S61831942 |
| IVLNWEC01CW5BS | - - - - - | gnl\|UG\|Ta#S61831956 |
| IVLNWEC02GILZ3 | - - - - - | gnl\|UG\|Ta#S61831956 |
| IV9RU5F02G5O7H | - - - - - | gnl\|UG\|Ta#S61831960 |
| IV9RU5F02IB2ER | - - - - - | gnl\|UG\|Ta#S61831960 |
| IVLNWEC01BTCJ6 | - - - - - | gnl\|UG\|Ta#S61831960 |
| IVLNWEC01D8HHP | - - - - - | gnl\|UG\|Ta#S61831960 |
| IUIVLUD02FHWLJ | - - - - - | gnl\|UG\|Ta#S61831975 |
| IV9RU5F02FY6F2 | - - - - - | gnl\|UG\|Ta#S61831975 |
| IV9RU5F01AU259 | - - - - - | gnl\|UG\|Ta#S61832028 |
| IVLNWEC02IBREE | - - - - - | gnl\|UG\|Ta#S61832028 |
| IUIVLUD02G7AD8 | - - - - - | gnl\|UG\|Ta#S61832033 |
| IV9RU5F02JZQEK | - - - - - | gnl\|UG\|Ta#S61832033 |
| IUIVLUD01DMLB4 | - - - - - | gnl\|UG\|Ta#S61832044 |
| IUIVLUD01EWBBF | - - - - - | gnl\|UG\|Ta#S61832044 |
| IV9RU5F02HB7KI | - - - - - | gnl\|UG\|Ta#S61832044 |
| IV9RU5F01C2TDW | - - - - - | gnl\|UG\|Ta#S61832092 |
| IVLNWEC01AT0J4 | - - - - - | gnl\|UG\|Ta#S61832092 |
| IV9RU5F01EZKSA | - - - - - | gnl\|UG\|Ta#S61832122 |
| IUIVLUD01EDWK7 | - - - - - | gnl\|UG\|Ta#S61832174 |
| IUIVLUD02JA0PD | - - - - - | gnl\|UG\|Ta#S61832174 |
| IV9RU5F02GHH0K | - - - - - | gnl\|UG\|Ta#S61832174 |
| IVLNWEC02HCUW4 | - - - - - | gnl\|UG\|Ta#S61832174 |
| IUIVLUD02H6KBD | - - - - - | gnl\|UG\|Ta#S61832196 |
| IVLNWEC02G5UAI | - - - - - | gnl\|UG\|Ta#S61832196 |
| IUIVLUD02F6WWU | - - - - - | gnl\|UG\|Ta#S61832213 |
| IVLNWEC02HAQZ2 | - - - - - | gnl\|UG\|Ta#S61832213 |
| IVLNWEC02IIZ6E | - - - - - | gnl\|UG\|Ta#S61832213 |
| IUIVLUD01BGNNI | - - - - - | gnl\|UG\|Ta#S61832215 |
| IVLNWEC01CKFY7 | - - - - - | gnl\|UG\|Ta#S61832215 |
| IVLNWEC01DZ4FG | - - - - - | gnl\|UG\|Ta#S61832215 |
| IUIVLUD02HHSRN | - - - - - | gnl\|UG\|Ta#S61832225 |
| IVLNWEC02G4O4L | - - - - - | gnl\|UG\|Ta#S61832225 |
| IUIVLUD01D12H7 | - - - - - | gnl\|UG\|Ta#S61832238 |
| IVLNWEC01DZD1Q | - - - - - | gnl\|UG\|Ta#S61832238 |
| IUIVLUD01DQ6SK | - - - - - | gnl\|UG\|Ta#S61832252 |
| IV9RU5F01C5KAO | - - - - - | gnl\|UG\|Ta#S61832252 |
| IVLNWEC02GSU6C | - - - - - | gnl\|UG\|Ta#S61832252 |
| IUIVLUD01ERZ2D | - - - - - | gnl\|UG\|Ta#S61832276 |
| IV9RU5F02H2OSA | - - - - - | gnl\|UG\|Ta#S61832276 |

| | | |
|---|---|---|
| IV9RU5F02H2T8C | - - - - - | gnl\|UG\|Ta#S61832329 |
| IVLNWEC02HLTOE | - - - - - | gnl\|UG\|Ta#S61832329 |
| IUIVLUD02IQ4R1 | - - - - - | gnl\|UG\|Ta#S61832335 |
| IV9RU5F01D8PS8 | - - - - - | gnl\|UG\|Ta#S61832335 |
| IUIVLUD02HPEWP | - - - - - | gnl\|UG\|Ta#S61832342 |
| IV9RU5F01BAGH3 | - - - - - | gnl\|UG\|Ta#S61832342 |
| IV9RU5F01EGF05 | - - - - - | gnl\|UG\|Ta#S61832342 |
| IVLNWEC02FON3T | - - - - - | gnl\|UG\|Ta#S61832342 |
| IVLNWEC02HDX3A | - - - - - | gnl\|UG\|Ta#S61832342 |
| IUIVLUD02HS8FR | - - - - - | gnl\|UG\|Ta#S61832375 |
| IVLNWEC01BAGRV | - - - - - | gnl\|UG\|Ta#S61832375 |
| IVLNWEC02HW0M7 | - - - - - | gnl\|UG\|Ta#S61832375 |
| IVLNWEC02I4EJS | - - - - - | gnl\|UG\|Ta#S61832375 |
| IV9RU5F01BT8ZG | - - - - - | gnl\|UG\|Ta#S61832380 |
| IV9RU5F01EN2RQ | - - - - - | gnl\|UG\|Ta#S61832380 |
| IUIVLUD01CJXHV | - - - - - | gnl\|UG\|Ta#S61832389 |
| IV9RU5F01CS090 | - - - - - | gnl\|UG\|Ta#S61832389 |
| IVLNWEC01AXU4G | - - - - - | gnl\|UG\|Ta#S61832389 |
| IVLNWEC02HP5FA | - - - - - | gnl\|UG\|Ta#S61832389 |
| IUIVLUD01C92CZ | - - - - - | gnl\|UG\|Ta#S61832404 |
| IV9RU5F01CI258 | - - - - - | gnl\|UG\|Ta#S61832404 |
| IV9RU5F01AIQI8 | - - - - - | gnl\|UG\|Ta#S61832410 |
| IV9RU5F01BBDOB | - - - - - | gnl\|UG\|Ta#S61832410 |
| IVLNWEC01ETX83 | - - - - - | gnl\|UG\|Ta#S61832441 |
| IVLNWEC02J25XM | - - - - - | gnl\|UG\|Ta#S61832441 |
| IUIVLUD02H4VTX | - - - - - | gnl\|UG\|Ta#S61832443 |
| IVLNWEC01CVBI0 | - - - - - | gnl\|UG\|Ta#S61832443 |
| IUIVLUD01A2BTT | - - - - - | gnl\|UG\|Ta#S61832446 |
| IV9RU5F02I0WVW | - - - - - | gnl\|UG\|Ta#S61832446 |
| IUIVLUD02GUVUZ | - - - - - | gnl\|UG\|Ta#S61832476 |
| IV9RU5F02IGI6V | - - - - - | gnl\|UG\|Ta#S61832476 |
| IV9RU5F01BTV9H | - - - - - | gnl\|UG\|Ta#S61832496 |
| IV9RU5F01C4NKM | - - - - - | gnl\|UG\|Ta#S61832496 |
| IV9RU5F02IQNLK | - - - - - | gnl\|UG\|Ta#S61832496 |
| IVLNWEC01CW0TQ | - - - - - | gnl\|UG\|Ta#S61832496 |
| IVLNWEC02IGPXX | - - - - - | gnl\|UG\|Ta#S61832496 |
| IVLNWEC02JNJO3 | - - - - - | gnl\|UG\|Ta#S61832496 |
| IUIVLUD01BMPU6 | - - - - - | gnl\|UG\|Ta#S61832521 |
| IUIVLUD02GY8ZE | - - - - - | gnl\|UG\|Ta#S61832521 |
| IUIVLUD02I0VVI | - - - - - | gnl\|UG\|Ta#S61832521 |
| IV9RU5F01BTQV9 | - - - - - | gnl\|UG\|Ta#S61832521 |
| IV9RU5F02I1OFX | - - - - - | gnl\|UG\|Ta#S61832521 |
| IVLNWEC01BDBME | - - - - - | gnl\|UG\|Ta#S61832521 |
| IVLNWEC01ERTS6 | - - - - - | gnl\|UG\|Ta#S61832521 |
| IUIVLUD01CMG4G | - - - - - | gnl\|UG\|Ta#S61832522 |
| IV9RU5F02GO835 | - - - - - | gnl\|UG\|Ta#S61832522 |
| IVLNWEC02HGDSG | - - - - - | gnl\|UG\|Ta#S61832522 |
| IV9RU5F01B56CT | - - - - - | gnl\|UG\|Ta#S61832533 |
| IV9RU5F02F94MF | - - - - - | gnl\|UG\|Ta#S61832533 |
| IV9RU5F01BUDDC | - - - - - | gnl\|UG\|Ta#S61832534 |

| | | |
|---|---|---|
| IV9RU5F02HAPJ8 | - - - - - | gnl\|UG\|Ta#S61832534 |
| IVLNWEC01B8XBX | - - - - - | gnl\|UG\|Ta#S61832534 |
| IVLNWEC01DM3D5 | - - - - - | gnl\|UG\|Ta#S61832534 |
| IUIVLUD02JM7I3 | - - - - - | gnl\|UG\|Ta#S61832538 |
| IV9RU5F02HU0KG | - - - - - | gnl\|UG\|Ta#S61832538 |
| IV9RU5F01AP5PE | - - - - - | gnl\|UG\|Ta#S61832540 |
| IVLNWEC02JIGUN | - - - - - | gnl\|UG\|Ta#S61832540 |
| IVLNWEC02ICK9O | - - - - - | gnl\|UG\|Ta#S61832541 |
| IUIVLUD01DXI22 | - - - - - | gnl\|UG\|Ta#S61832550 |
| IUIVLUD02GT7ZP | - - - - - | gnl\|UG\|Ta#S61832550 |
| IUIVLUD02HH253 | - - - - - | gnl\|UG\|Ta#S61832550 |
| IV9RU5F01A0L6O | - - - - - | gnl\|UG\|Ta#S61832550 |
| IV9RU5F01AT6SF | - - - - - | gnl\|UG\|Ta#S61832550 |
| IV9RU5F01BJU9H | - - - - - | gnl\|UG\|Ta#S61832550 |
| IV9RU5F01BQVN3 | - - - - - | gnl\|UG\|Ta#S61832550 |
| IV9RU5F01CCQML | - - - - - | gnl\|UG\|Ta#S61832550 |
| IV9RU5F01DQPHM | - - - - - | gnl\|UG\|Ta#S61832550 |
| IV9RU5F01EJ812 | - - - - - | gnl\|UG\|Ta#S61832550 |
| IV9RU5F02GBGI4 | - - - - - | gnl\|UG\|Ta#S61832550 |
| IV9RU5F02GMODY | - - - - - | gnl\|UG\|Ta#S61832550 |
| IV9RU5F02JW3SV | - - - - - | gnl\|UG\|Ta#S61832550 |
| IVLNWEC01AT7BM | - - - - - | gnl\|UG\|Ta#S61832550 |
| IVLNWEC02IL6SK | - - - - - | gnl\|UG\|Ta#S61832550 |
| IUIVLUD02H79GD | - - - - - | gnl\|UG\|Ta#S61832872 |
| IV9RU5F01CA8WG | - - - - - | gnl\|UG\|Ta#S61832872 |
| IUIVLUD02GMX9J | - - - - - | gnl\|UG\|Ta#S61833488 |
| IUIVLUD02IK2U0 | - - - - - | gnl\|UG\|Ta#S61833488 |
| IVLNWEC02HEE7A | - - - - - | gnl\|UG\|Ta#S61833488 |
| IV9RU5F01BJT4T | - - - - - | gnl\|UG\|Ta#S61834246 |
| IV9RU5F01DK8YD | - - - - - | gnl\|UG\|Ta#S61834246 |
| IVLNWEC02G9EK2 | - - - - - | gnl\|UG\|Ta#S61834246 |
| IUIVLUD02HQ10C | - - - - - | gnl\|UG\|Ta#S61834710 |
| IVLNWEC02IYMCN | - - - - - | gnl\|UG\|Ta#S61834710 |
| IV9RU5F02I4ID1 | - - - - - | gnl\|UG\|Ta#S61834714 |
| IVLNWEC01DTVWY | - - - - - | gnl\|UG\|Ta#S61834714 |
| IV9RU5F01D2NN9 | - - - - - | gnl\|UG\|Ta#S61834723 |
| IVLNWEC01B0MCS | - - - - - | gnl\|UG\|Ta#S61834723 |
| IUIVLUD02IS8XC | - - - - - | gnl\|UG\|Ta#S61834986 |
| IV9RU5F02HTS7A | - - - - - | gnl\|UG\|Ta#S61834986 |
| IV9RU5F02HNGT7 | - - - - - | gnl\|UG\|Ta#S61835368 |
| IVLNWEC02HPE5V | - - - - - | gnl\|UG\|Ta#S61835368 |
| IUIVLUD01EPU1Y | - - - - - | gnl\|UG\|Ta#S61835420 |
| IUIVLUD02HFGJK | - - - - - | gnl\|UG\|Ta#S61835420 |
| IV9RU5F02IOAZN | - - - - - | gnl\|UG\|Ta#S61836068 |
| IVLNWEC01DTU9L | - - - - - | gnl\|UG\|Ta#S61836068 |
| IV9RU5F02J0RV3 | - - - - - | gnl\|UG\|Ta#S61837151 |
| IVLNWEC01BKXQ5 | - - - - - | gnl\|UG\|Ta#S61837151 |
| IUIVLUD01AZ6DV | - - - - - | gnl\|UG\|Ta#S61837870 |
| IV9RU5F01CMGWI | - - - - - | gnl\|UG\|Ta#S61837870 |
| IVLNWEC02HPP7Z | - - - - - | gnl\|UG\|Ta#S61837870 |

| | | |
|---|---|---|
| IUIVLUD01BFTFS | - - - - - | gnl\|UG\|Ta#S61837875 |
| IV9RU5F01CQKU9 | - - - - - | gnl\|UG\|Ta#S61837875 |
| IV9RU5F02IOXL1 | - - - - - | gnl\|UG\|Ta#S61837875 |
| IVLNWEC02HE94Q | - - - - - | gnl\|UG\|Ta#S61837875 |
| IUIVLUD01BPI6G | - - - - - | gnl\|UG\|Ta#S61838231 |
| IV9RU5F01BVXPV | - - - - - | gnl\|UG\|Ta#S61838231 |
| IVLNWEC01DVH22 | - - - - - | gnl\|UG\|Ta#S61838231 |
| IV9RU5F02JKEIG | - - - - - | gnl\|UG\|Ta#S61838652 |
| IVLNWEC02GWW0N | - - - - - | gnl\|UG\|Ta#S61838652 |
| IVLNWEC02I5PPJ | - - - - - | gnl\|UG\|Ta#S61838652 |
| IVLNWEC02IV6Y8 | - - - - - | gnl\|UG\|Ta#S61838698 |
| IVLNWEC02JUVB5 | - - - - - | gnl\|UG\|Ta#S61838698 |
| IUIVLUD02I5JIN | - - - - - | gnl\|UG\|Ta#S61839271 |
| IV9RU5F01DXLB5 | - - - - - | gnl\|UG\|Ta#S61839271 |
| IVLNWEC02G4WO1 | - - - - - | gnl\|UG\|Ta#S61839271 |
| IV9RU5F02HT1I6 | - - - - - | gnl\|UG\|Ta#S61839341 |
| IVLNWEC01DSIIF | - - - - - | gnl\|UG\|Ta#S61839341 |
| IVLNWEC01C0DM8 | - - - - - | gnl\|UG\|Ta#S61839582 |
| IVLNWEC02ICSOJ | - - - - - | gnl\|UG\|Ta#S61839582 |
| IV9RU5F02F33QY | - - - - - | gnl\|UG\|Ta#S61839829 |
| IV9RU5F02JJ8PP | - - - - - | gnl\|UG\|Ta#S61839829 |
| IVLNWEC02HY642 | - - - - - | gnl\|UG\|Ta#S61839829 |
| IVLNWEC02IP0GA | - - - - - | gnl\|UG\|Ta#S61839829 |
| IVLNWEC01A1RNS | - - - - - | gnl\|UG\|Ta#S61840090 |
| IVLNWEC01BTHNZ | - - - - - | gnl\|UG\|Ta#S61840090 |
| IV9RU5F02G6ILR | - - - - - | gnl\|UG\|Ta#S61841003 |
| IVLNWEC01EHKL5 | - - - - - | gnl\|UG\|Ta#S61841003 |
| IVLNWEC02GIL4V | - - - - - | gnl\|UG\|Ta#S61841003 |
| IUIVLUD01DH1RQ | - - - - - | gnl\|UG\|Ta#S61841823 |
| IV9RU5F01B3C0O | - - - - - | gnl\|UG\|Ta#S61841823 |
| IVLNWEC01C7WLV | - - - - - | gnl\|UG\|Ta#S61841823 |
| IV9RU5F01CH1A5 | - - - - - | gnl\|UG\|Ta#S61841904 |
| IV9RU5F02H09QF | - - - - - | gnl\|UG\|Ta#S61841904 |
| IVLNWEC02H6VQV | - - - - - | gnl\|UG\|Ta#S61841904 |
| IV9RU5F02G402W | - - - - - | gnl\|UG\|Ta#S61842067 |
| IVLNWEC01B64EG | - - - - - | gnl\|UG\|Ta#S61842067 |
| IV9RU5F01DIQXX | - - - - - | gnl\|UG\|Ta#S61842198 |
| IV9RU5F01EAKJQ | - - - - - | gnl\|UG\|Ta#S61842198 |
| IV9RU5F02IPW3C | - - - - - | gnl\|UG\|Ta#S61842198 |
| IVLNWEC02FRG6G | - - - - - | gnl\|UG\|Ta#S61842411 |
| IVLNWEC02HKEIS | - - - - - | gnl\|UG\|Ta#S61842411 |
| IVLNWEC02JGQ83 | - - - - - | gnl\|UG\|Ta#S61842411 |
| IV9RU5F02H5E78 | - - - - - | gnl\|UG\|Ta#S61842660 |
| IV9RU5F02IC5QS | - - - - - | gnl\|UG\|Ta#S61842660 |
| IUIVLUD01EIH20 | - - - - - | gnl\|UG\|Ta#S61842853 |
| IVLNWEC01BY2M3 | - - - - - | gnl\|UG\|Ta#S61842853 |
| IVLNWEC02HQXRP | - - - - - | gnl\|UG\|Ta#S61842853 |
| IUIVLUD01BE677 | - - - - - | gnl\|UG\|Ta#S61844165 |
| IV9RU5F01CE4SN | - - - - - | gnl\|UG\|Ta#S61844165 |
| IUIVLUD01BITFI | - - - - - | gnl\|UG\|Ta#S61844307 |

| | | |
|---|---|---|
| IV9RU5F02H4D5T | - - - - - | gnl\|UG\|Ta#S61844307 |
| IV9RU5F02G76RV | - - - - - | gnl\|UG\|Ta#S61844509 |
| IVLNWEC02HU88K | - - - - - | gnl\|UG\|Ta#S61844509 |
| IUIVLUD01B85Q6 | - - - - - | gnl\|UG\|Ta#S61844608 |
| IVLNWEC01EJM92 | - - - - - | gnl\|UG\|Ta#S61844608 |
| IUIVLUD02GD5OC | - - - - - | gnl\|UG\|Ta#S61844630 |
| IV9RU5F02INXKL | - - - - - | gnl\|UG\|Ta#S61844630 |
| IUIVLUD01ED2L2 | - - - - - | gnl\|UG\|Ta#S61844688 |
| IUIVLUD01ELURF | - - - - - | gnl\|UG\|Ta#S61844688 |
| IUIVLUD01EGPLC | - - - - - | gnl\|UG\|Ta#S61845135 |
| IV9RU5F01ANXOC | - - - - - | gnl\|UG\|Ta#S61845135 |
| IV9RU5F01BIH6U | - - - - - | gnl\|UG\|Ta#S61845135 |
| IV9RU5F02G2H4T | - - - - - | gnl\|UG\|Ta#S61845135 |
| IVLNWEC01APIAC | - - - - - | gnl\|UG\|Ta#S61845163 |
| IVLNWEC01AS7ES | - - - - - | gnl\|UG\|Ta#S61845163 |
| IVLNWEC01BAZZJ | - - - - - | gnl\|UG\|Ta#S61845163 |
| IUIVLUD01D9LWV | - - - - - | gnl\|UG\|Ta#S61845190 |
| IV9RU5F02JX5HC | - - - - - | gnl\|UG\|Ta#S61845190 |
| IV9RU5F02I3WB9 | - - - - - | gnl\|UG\|Ta#S61845304 |
| IV9RU5F02IX545 | - - - - - | gnl\|UG\|Ta#S61845304 |
| IVLNWEC01A89TW | - - - - - | gnl\|UG\|Ta#S61846291 |
| IVLNWEC02GYMQB | - - - - - | gnl\|UG\|Ta#S61846291 |
| IUIVLUD02I45L6 | - - - - - | gnl\|UG\|Ta#S61847267 |
| IV9RU5F02JYZCC | - - - - - | gnl\|UG\|Ta#S61847267 |
| IV9RU5F02GKITB | - - - - - | gnl\|UG\|Ta#S61847527 |
| IV9RU5F02JAJO1 | - - - - - | gnl\|UG\|Ta#S61847527 |
| IV9RU5F02GQ3OK | - - - - - | gnl\|UG\|Ta#S61847638 |
| IVLNWEC01C2FR5 | - - - - - | gnl\|UG\|Ta#S61847638 |
| IUIVLUD02GIZ2J | - - - - - | gnl\|UG\|Ta#S61849128 |
| IUIVLUD02HVHVZ | - - - - - | gnl\|UG\|Ta#S61849128 |
| IVLNWEC01APX6T | - - - - - | gnl\|UG\|Ta#S61849128 |
| IUIVLUD01BOIX5 | - - - - - | gnl\|UG\|Ta#S61849157 |
| IUIVLUD02IIQ1B | - - - - - | gnl\|UG\|Ta#S61849157 |
| IV9RU5F02IAXP4 | - - - - - | gnl\|UG\|Ta#S61850003 |
| IVLNWEC02FS5AL | - - - - - | gnl\|UG\|Ta#S61850003 |
| IUIVLUD02I3YV0 | - - - - - | gnl\|UG\|Ta#S61850108 |
| IUIVLUD02JBDXH | - - - - - | gnl\|UG\|Ta#S61850108 |
| IUIVLUD01CX91E | - - - - - | gnl\|UG\|Ta#S61850270 |
| IV9RU5F02I3F1E | - - - - - | gnl\|UG\|Ta#S61850270 |
| IVLNWEC01CX6BN | - - - - - | gnl\|UG\|Ta#S61850309 |
| IVLNWEC02G1PPP | - - - - - | gnl\|UG\|Ta#S61850309 |
| IV9RU5F01BCXG3 | - - - - - | gnl\|UG\|Ta#S61850774 |
| IV9RU5F02H6EEK | - - - - - | gnl\|UG\|Ta#S61850774 |
| IVLNWEC01DLI0N | - - - - - | gnl\|UG\|Ta#S61850774 |
| IVLNWEC02IM28J | - - - - - | gnl\|UG\|Ta#S61850774 |
| IVLNWEC01DXR37 | - - - - - | gnl\|UG\|Ta#S61851171 |
| IVLNWEC02FYRRV | - - - - - | gnl\|UG\|Ta#S61851171 |
| IVLNWEC02I8OKY | - - - - - | gnl\|UG\|Ta#S61851171 |
| IVLNWEC01A8BTJ | - - - - - | gnl\|UG\|Ta#S61851455 |
| IVLNWEC01CJNHI | - - - - - | gnl\|UG\|Ta#S61851455 |

| | | |
|---|---|---|
| IVLNWEC01AJ9XK | - - - - - | gnl\|UG\|Ta#S61851553 |
| IVLNWEC01EC3CS | - - - - - | gnl\|UG\|Ta#S61851553 |
| IV9RU5F02HE6AZ | - - - - - | gnl\|UG\|Ta#S61851602 |
| IVLNWEC01CWV6Y | - - - - - | gnl\|UG\|Ta#S61851602 |
| IVLNWEC01DIZW1 | - - - - - | gnl\|UG\|Ta#S61851602 |
| IUIVLUD01EHXC7 | - - - - - | gnl\|UG\|Ta#S61851625 |
| IV9RU5F01DOLEP | - - - - - | gnl\|UG\|Ta#S61851625 |
| IV9RU5F01EEUUL | - - - - - | gnl\|UG\|Ta#S61852161 |
| IV9RU5F02HPPX6 | - - - - - | gnl\|UG\|Ta#S61852161 |
| IVLNWEC01CDH7C | - - - - - | gnl\|UG\|Ta#S61852270 |
| IVLNWEC01CWFTU | - - - - - | gnl\|UG\|Ta#S61852270 |
| IV9RU5F01D6C2U | - - - - - | gnl\|UG\|Ta#S61852351 |
| IVLNWEC02JBCBZ | - - - - - | gnl\|UG\|Ta#S61852351 |
| IUIVLUD02H6LZJ | - - - - - | gnl\|UG\|Ta#S61853573 |
| IVLNWEC02I519A | - - - - - | gnl\|UG\|Ta#S61853573 |
| IV9RU5F01AXVFR | - - - - - | gnl\|UG\|Ta#S61853644 |
| IVLNWEC01B1RVU | - - - - - | gnl\|UG\|Ta#S61853644 |
| IUIVLUD02GUA6W | - - - - - | gnl\|UG\|Ta#S61854318 |
| IV9RU5F02JO0F8 | - - - - - | gnl\|UG\|Ta#S61854318 |
| IVLNWEC01CCN1N | - - - - - | gnl\|UG\|Ta#S61854672 |
| IVLNWEC02H15SQ | - - - - - | gnl\|UG\|Ta#S61854672 |
| IV9RU5F01A9R6B | - - - - - | gnl\|UG\|Ta#S61855423 |
| IV9RU5F02G4ZLS | - - - - - | gnl\|UG\|Ta#S61855423 |
| IUIVLUD01CSHGU | - - - - - | gnl\|UG\|Ta#S61855464 |
| IVLNWEC01BI7BS | - - - - - | gnl\|UG\|Ta#S61855464 |
| IVLNWEC02G8TMW | - - - - - | gnl\|UG\|Ta#S61855464 |
| IVLNWEC02HLK3V | - - - - - | gnl\|UG\|Ta#S61855464 |
| IV9RU5F01ANBSS | - - - - - | gnl\|UG\|Ta#S61855471 |
| IVLNWEC01A8UCT | - - - - - | gnl\|UG\|Ta#S61855471 |
| IUIVLUD02GW7U7 | - - - - - | gnl\|UG\|Ta#S61855554 |
| IVLNWEC01AUA2I | - - - - - | gnl\|UG\|Ta#S61855554 |
| IV9RU5F02JOTJW | - - - - - | gnl\|UG\|Ta#S61856617 |
| IVLNWEC01BGSR9 | - - - - - | gnl\|UG\|Ta#S61856617 |
| IV9RU5F01EY6BQ | - - - - - | gnl\|UG\|Ta#S61856726 |
| IV9RU5F02HSS59 | - - - - - | gnl\|UG\|Ta#S61856726 |
| IVLNWEC01DT28V | - - - - - | gnl\|UG\|Ta#S61856726 |
| IV9RU5F01A20T0 | - - - - - | gnl\|UG\|Ta#S61856736 |
| IVLNWEC01DAELW | - - - - - | gnl\|UG\|Ta#S61856736 |
| IV9RU5F02H3P9W | - - - - - | gnl\|UG\|Ta#S61857554 |
| IVLNWEC01D558Z | - - - - - | gnl\|UG\|Ta#S61857554 |
| IVLNWEC02FXL4I | - - - - - | gnl\|UG\|Ta#S61857554 |
| IUIVLUD01CEKA0 | - - - - - | gnl\|UG\|Ta#S61857826 |
| IV9RU5F01EKL1M | - - - - - | gnl\|UG\|Ta#S61857826 |
| IV9RU5F01CURF3 | - - - - - | gnl\|UG\|Ta#S61858199 |
| IVLNWEC01EKX1C | - - - - - | gnl\|UG\|Ta#S61858199 |
| IVLNWEC02IT8JP | - - - - - | gnl\|UG\|Ta#S61858199 |
| IV9RU5F01DJ75N | - - - - - | gnl\|UG\|Ta#S61858299 |
| IV9RU5F02GPXYL | - - - - - | gnl\|UG\|Ta#S61858299 |
| IUIVLUD01BOKGQ | - - - - - | gnl\|UG\|Ta#S61860068 |
| IUIVLUD01D7W7A | - - - - - | gnl\|UG\|Ta#S61860068 |

| | | |
|---|---|---|
| IUIVLUD01D9YLJ | - - - - - | gnl\|UG\|Ta#S61860767 |
| IVLNWEC02F300P | - - - - - | gnl\|UG\|Ta#S61860767 |
| IVLNWEC02IMG4A | - - - - - | gnl\|UG\|Ta#S61860767 |
| IV9RU5F01AR7DR | - - - - - | gnl\|UG\|Ta#S61860797 |
| IV9RU5F01EM22N | - - - - - | gnl\|UG\|Ta#S61860797 |
| IV9RU5F02IBE12 | - - - - - | gnl\|UG\|Ta#S61860808 |
| IVLNWEC02INQ40 | - - - - - | gnl\|UG\|Ta#S61860808 |
| IV9RU5F01D5BX2 | - - - - - | gnl\|UG\|Ta#S61861746 |
| IVLNWEC02FQORN | - - - - - | gnl\|UG\|Ta#S61861746 |
| IUIVLUD01BWS7E | - - - - - | gnl\|UG\|Ta#S61862170 |
| IUIVLUD02G4DMG | - - - - - | gnl\|UG\|Ta#S61862170 |
| IVLNWEC01D1S0W | - - - - - | gnl\|UG\|Ta#S61862170 |
| IVLNWEC01ED66J | - - - - - | gnl\|UG\|Ta#S61862170 |
| IUIVLUD02J0FRW | - - - - - | gnl\|UG\|Ta#S61862496 |
| IUIVLUD02JV0XQ | - - - - - | gnl\|UG\|Ta#S61862496 |
| IV9RU5F01CZD9G | - - - - - | gnl\|UG\|Ta#S61862918 |
| IVLNWEC01AH169 | - - - - - | gnl\|UG\|Ta#S61862918 |
| IUIVLUD01ARHEL | - - - - - | gnl\|UG\|Ta#S61863335 |
| IUIVLUD02HGT26 | - - - - - | gnl\|UG\|Ta#S61863335 |
| IV9RU5F01CANO1 | - - - - - | gnl\|UG\|Ta#S61864012 |
| IVLNWEC01DU9SB | - - - - - | gnl\|UG\|Ta#S61864012 |
| IVLNWEC02IHMPF | - - - - - | gnl\|UG\|Ta#S61864012 |
| IV9RU5F02IXAS5 | - - - - - | gnl\|UG\|Ta#S61864143 |
| IV9RU5F02JS7DG | - - - - - | gnl\|UG\|Ta#S61864143 |
| IVLNWEC02GV0DC | - - - - - | gnl\|UG\|Ta#S61864143 |
| IV9RU5F01B4863 | - - - - - | gnl\|UG\|Ta#S61864688 |
| IVLNWEC02FU6S9 | - - - - - | gnl\|UG\|Ta#S61864688 |
| IV9RU5F01DTG57 | - - - - - | gnl\|UG\|Ta#S61864975 |
| IVLNWEC01EJKVV | - - - - - | gnl\|UG\|Ta#S61864975 |
| IVLNWEC01AXBG8 | - - - - - | gnl\|UG\|Ta#S61865335 |
| IVLNWEC01C7WNJ | - - - - - | gnl\|UG\|Ta#S61865335 |
| IV9RU5F01DERRO | - - - - - | gnl\|UG\|Ta#S61865460 |
| IVLNWEC02INKQU | - - - - - | gnl\|UG\|Ta#S61865460 |
| IUIVLUD01A1HCU | - - - - - | gnl\|UG\|Ta#S61865986 |
| IVLNWEC01DE16W | - - - - - | gnl\|UG\|Ta#S61865986 |
| IVLNWEC01BKUDI | - - - - - | gnl\|UG\|Ta#S61866415 |
| IVLNWEC02GZZYW | - - - - - | gnl\|UG\|Ta#S61866415 |
| IV9RU5F01C1M58 | - - - - - | gnl\|UG\|Ta#S61866690 |
| IV9RU5F02GV7GM | - - - - - | gnl\|UG\|Ta#S61866690 |
| IVLNWEC01EXGI4 | - - - - - | gnl\|UG\|Ta#S61866890 |
| IVLNWEC02FV5A2 | - - - - - | gnl\|UG\|Ta#S61866890 |
| IV9RU5F02I0AVT | - - - - - | gnl\|UG\|Ta#S61867532 |
| IVLNWEC01D9G7L | - - - - - | gnl\|UG\|Ta#S61867532 |
| IUIVLUD02IQVTL | - - - - - | gnl\|UG\|Ta#S61867552 |
| IVLNWEC01EDJWR | - - - - - | gnl\|UG\|Ta#S61867552 |
| IVLNWEC01CHL71 | - - - - - | gnl\|UG\|Ta#S61867557 |
| IVLNWEC02FQVZ0 | - - - - - | gnl\|UG\|Ta#S61867557 |
| IUIVLUD02GA17R | - - - - - | gnl\|UG\|Ta#S61867876 |
| IVLNWEC02ICHRJ | - - - - - | gnl\|UG\|Ta#S61867876 |
| IUIVLUD01CG2N5 | - - - - - | gnl\|UG\|Ta#S61868063 |

| | | |
|---|---|---|
| IVLNWEC02JYWN9 | - - - - - | gnl\|UG\|Ta#S61868063 |
| IV9RU5F02H4YRQ | - - - - - | gnl\|UG\|Ta#S65588252 |
| IVLNWEC01CLB0M | - - - - - | gnl\|UG\|Ta#S65588252 |
| IUIVLUD01AZG8K | - - - - - | gnl\|UG\|Ta#S65589712 |
| IV9RU5F02G97NG | - - - - - | gnl\|UG\|Ta#S65589712 |
| IVLNWEC01C57DK | - - - - - | gnl\|UG\|Ta#S65589712 |
| IV9RU5F02ITUFK | - - - - - | gnl\|UG\|Ta#S65591551 |
| IVLNWEC02HADBR | - - - - - | gnl\|UG\|Ta#S65591551 |
| IUIVLUD02HX3BZ | - - - - - | gnl\|UG\|Ta#S65591587 |
| IVLNWEC02I404D | - - - - - | gnl\|UG\|Ta#S65591587 |
| IV9RU5F02GK9ZV | - - - - - | gnl\|UG\|Ta#S65591867 |
| IVLNWEC01D6GAW | - - - - - | gnl\|UG\|Ta#S65591867 |
| IV9RU5F01C490Z | - - - - - | gnl\|UG\|Ta#S65592304 |
| IVLNWEC02HEQUH | - - - - - | gnl\|UG\|Ta#S65592304 |
| IUIVLUD02FWUXF | - - - - - | gnl\|UG\|Ta#S65592718 |
| IVLNWEC01EKEW1 | - - - - - | gnl\|UG\|Ta#S65592718 |
| IV9RU5F01A6TG1 | - - - - - | gnl\|UG\|Ta#S65592786 |
| IV9RU5F01EK5OR | - - - - - | gnl\|UG\|Ta#S65592786 |
| IUIVLUD02I6CBE | - - - - - | gnl\|UG\|Ta#S65592792 |
| IV9RU5F01DGG55 | - - - - - | gnl\|UG\|Ta#S65592792 |
| IV9RU5F01EMSVW | - - - - - | gnl\|UG\|Ta#S65592792 |
| IUIVLUD02IKBVX | - - - - - | gnl\|UG\|Ta#S65592887 |
| IV9RU5F02JBMON | - - - - - | gnl\|UG\|Ta#S65592887 |
| IV9RU5F01CWSRW | - - - - - | gnl\|UG\|Ta#S65593892 |
| IV9RU5F02F4MCK | - - - - - | gnl\|UG\|Ta#S65593892 |
| IVLNWEC01BW34J | - - - - - | gnl\|UG\|Ta#S65593892 |
| IVLNWEC01CYA0B | - - - - - | gnl\|UG\|Ta#S65593892 |
| IVLNWEC02G0VK0 | - - - - - | gnl\|UG\|Ta#S65593892 |
| IVLNWEC02GH9P2 | - - - - - | gnl\|UG\|Ta#S65593892 |
| IV9RU5F01DBAGH | - - - - - | gnl\|UG\|Ta#S65594474 |
| IV9RU5F02HX3JH | - - - - - | gnl\|UG\|Ta#S65594474 |
| IV9RU5F01B1WGE | - - - - - | gnl\|UG\|Ta#S65595137 |
| IV9RU5F01BA6DW | - - - - - | gnl\|UG\|Ta#S65595137 |
| IVLNWEC01BLPZ7 | - - - - - | gnl\|UG\|Ta#S65595137 |
| IVLNWEC01CWPT8 | - - - - - | gnl\|UG\|Ta#S65595137 |
| IV9RU5F01BW27P | - - - - - | gnl\|UG\|Ta#S65597716 |
| IVLNWEC01C0EMH | - - - - - | gnl\|UG\|Ta#S65597716 |
| IV9RU5F01BHOQW | - - - - - | gnl\|UG\|Ta#S65598185 |
| IVLNWEC01BY05P | - - - - - | gnl\|UG\|Ta#S65598185 |
| IUIVLUD02GNXQH | - - - - - | gnl\|UG\|Ta#S65599619 |
| IV9RU5F01AK6YC | - - - - - | gnl\|UG\|Ta#S65599619 |
| IVLNWEC02HE50Z | - - - - - | gnl\|UG\|Ta#S65599619 |
| IV9RU5F02HKM5U | - - - - - | gnl\|UG\|Ta#S65600237 |
| IV9RU5F02IO8GY | - - - - - | gnl\|UG\|Ta#S65600237 |
| IVLNWEC01DQTP2 | - - - - - | gnl\|UG\|Ta#S65600237 |
| IVLNWEC02H8JQA | - - - - - | gnl\|UG\|Ta#S65600237 |
| IUIVLUD01AUCKZ | - - - - - | gnl\|UG\|Ta#S65600756 |
| IVLNWEC01CD8K0 | - - - - - | gnl\|UG\|Ta#S65600756 |
| IV9RU5F01BOK3L | - - - - - | gnl\|UG\|Ta#S65600876 |
| IVLNWEC02J425K | - - - - - | gnl\|UG\|Ta#S65600876 |

| | | |
|---|---|---|
| IUIVLUD01BYH8C | - - - - - | gnl\|UG\|Ta#S65601384 |
| IUIVLUD02GVBHR | - - - - - | gnl\|UG\|Ta#S65601384 |
| IV9RU5F01DWYR1 | - - - - - | gnl\|UG\|Ta#S65601384 |
| IUIVLUD02ICZ4S | - - - - - | gnl\|UG\|Ta#S65603396 |
| IV9RU5F02GAH76 | - - - - - | gnl\|UG\|Ta#S65603396 |
| IVLNWEC01APQTC | - - - - - | gnl\|UG\|Ta#S65603396 |
| IVLNWEC02F173Y | - - - - - | gnl\|UG\|Ta#S65603396 |
| IUIVLUD01DRD9J | - - - - - | gnl\|UG\|Ta#S65604838 |
| IV9RU5F02IC8TB | - - - - - | gnl\|UG\|Ta#S65604838 |
| IUIVLUD02HA649 | - - - - - | gnl\|UG\|Ta#S65607767 |
| IVLNWEC02JVV0N | - - - - - | gnl\|UG\|Ta#S65607767 |
| IUIVLUD01APV4X | - - - - - | gnl\|UG\|Ta#S65609345 |
| IUIVLUD02JLH0R | - - - - - | gnl\|UG\|Ta#S65609370 |
| IVLNWEC01BL93H | - - - - - | gnl\|UG\|Ta#S65609370 |
| IVLNWEC02F1YBH | - - - - - | gnl\|UG\|Ta#S65609576 |
| IVLNWEC02GKG05 | - - - - - | gnl\|UG\|Ta#S65609576 |
| IVLNWEC02GZTIG | - - - - - | gnl\|UG\|Ta#S65609576 |
| IVLNWEC02IQAEJ | - - - - - | gnl\|UG\|Ta#S65609576 |
| IUIVLUD01CIKHA | - - - - - | gnl\|UG\|Ta#S65610129 |
| IVLNWEC02G3447 | - - - - - | gnl\|UG\|Ta#S65610129 |
| IVLNWEC02G6KX5 | - - - - - | gnl\|UG\|Ta#S65610129 |
| IVLNWEC02JIL76 | - - - - - | gnl\|UG\|Ta#S65610129 |
| IUIVLUD01A4RAN | - - - - - | gnl\|UG\|Ta#S65611045 |
| IV9RU5F02IM627 | - - - - - | gnl\|UG\|Ta#S65611045 |
| IUIVLUD02GKVBH | - - - - - | gnl\|UG\|Ta#S65611246 |
| IUIVLUD02HZ79R | - - - - - | gnl\|UG\|Ta#S65611246 |
| IV9RU5F02FLD0M | - - - - - | gnl\|UG\|Ta#S65611246 |
| IV9RU5F02GOYQO | - - - - - | gnl\|UG\|Ta#S65611246 |
| IV9RU5F02I6XJJ | - - - - - | gnl\|UG\|Ta#S65611246 |
| IVLNWEC02IMYKX | - - - - - | gnl\|UG\|Ta#S65611246 |
| IUIVLUD02II3KZ | - - - - - | gnl\|UG\|Ta#S65611597 |
| IVLNWEC01ESX06 | - - - - - | gnl\|UG\|Ta#S65611597 |
| IVLNWEC02H9BVE | - - - - - | gnl\|UG\|Ta#S65611597 |
| IV9RU5F02H1AJ7 | - - - - - | gnl\|UG\|Ta#S65612613 |
| IUIVLUD02GZLUF | - - - - - | gnl\|UG\|Ta#S65613477 |
| IUIVLUD02JTWQJ | - - - - - | gnl\|UG\|Ta#S65613477 |
| IVLNWEC02IH0RP | - - - - - | gnl\|UG\|Ta#S65613477 |
| IVLNWEC01D8J0E | - - - - - | gnl\|UG\|Ta#S65613520 |
| IVLNWEC02FWICK | - - - - - | gnl\|UG\|Ta#S65613520 |
| IV9RU5F01BZGDR | - - - - - | gnl\|UG\|Ta#S65614553 |
| IV9RU5F01CWFMH | - - - - - | gnl\|UG\|Ta#S65614553 |
| IV9RU5F02GPEFH | - - - - - | gnl\|UG\|Ta#S65614553 |
| IV9RU5F01A0V33 | - - - - - | gnl\|UG\|Ta#S65615453 |
| IV9RU5F02GWKST | - - - - - | gnl\|UG\|Ta#S65615453 |
| IV9RU5F02H8X6X | - - - - - | gnl\|UG\|Ta#S65615453 |
| IUIVLUD02HY943 | - - - - - | gnl\|UG\|Ta#S65616563 |
| IV9RU5F01EEGRW | - - - - - | gnl\|UG\|Ta#S65616563 |
| IVLNWEC02F0YKX | - - - - - | gnl\|UG\|Ta#S65616563 |
| IVLNWEC02HEPXU | - - - - - | gnl\|UG\|Ta#S65616563 |
| IUIVLUD02GBVJ2 | - - - - - | gnl\|UG\|Ta#S65617835 |

| | | |
|---|---|---|
| IV9RU5F01ECI8N | - - - - - | gnl\|UG\|Ta#S65617835 |
| IV9RU5F02HS6NQ | - - - - - | gnl\|UG\|Ta#S65619509 |
| IVLNWEC02GDTPY | - - - - - | gnl\|UG\|Ta#S65619509 |
| IUIVLUD01AU1KW | - - - - - | gnl\|UG\|Ta#S65620303 |
| IV9RU5F02F20J0 | - - - - - | gnl\|UG\|Ta#S65620303 |
| IV9RU5F01BZP8L | - - - - - | gnl\|UG\|Ta#S65622087 |
| IV9RU5F01ENCIC | - - - - - | gnl\|UG\|Ta#S65622087 |
| IUIVLUD02IO31J | - - - - - | gnl\|UG\|Ta#S65622982 |
| IV9RU5F02JLQ1O | - - - - - | gnl\|UG\|Ta#S65622982 |
| IUIVLUD01EXSZU | - - - - - | gnl\|UG\|Ta#S65623679 |
| IV9RU5F02F1UA1 | - - - - - | gnl\|UG\|Ta#S65623679 |
| IV9RU5F02F145B | - - - - - | gnl\|UG\|Ta#S65624099 |
| IV9RU5F02IAK27 | - - - - - | gnl\|UG\|Ta#S65624099 |
| IVLNWEC01BAZLC | - - - - - | gnl\|UG\|Ta#S65624099 |
| IVLNWEC02H7ZY8 | - - - - - | gnl\|UG\|Ta#S65624099 |
| IUIVLUD02JAVTR | - - - - - | gnl\|UG\|Ta#S65624179 |
| IV9RU5F01CCRTH | - - - - - | gnl\|UG\|Ta#S65624179 |
| IUIVLUD01BSCBT | - - - - - | gnl\|UG\|Ta#S65624542 |
| IUIVLUD01BUJZ3 | - - - - - | gnl\|UG\|Ta#S65624542 |
| IVLNWEC02H0FLM | - - - - - | gnl\|UG\|Ta#S65624906 |
| IUIVLUD01CCP0S | - - - - - | gnl\|UG\|Ta#S65625809 |
| IUIVLUD01DEPOH | - - - - - | gnl\|UG\|Ta#S65625809 |
| IVLNWEC02J2PVP | - - - - - | gnl\|UG\|Ta#S65625809 |
| IV9RU5F01DIWJC | - - - - - | gnl\|UG\|Ta#S65626607 |
| IV9RU5F02HEIZW | - - - - - | gnl\|UG\|Ta#S65626607 |
| IVLNWEC02I60WE | - - - - - | gnl\|UG\|Ta#S65626607 |
| IV9RU5F01CBZW1 | - - - - - | gnl\|UG\|Ta#S65627316 |
| IV9RU5F02FLWGH | - - - - - | gnl\|UG\|Ta#S65627316 |
| IVLNWEC01BK5OV | - - - - - | gnl\|UG\|Ta#S65628416 |
| IUIVLUD02F1E3U | - - - - - | gnl\|UG\|Ta#S65629546 |
| IUIVLUD02GB5TQ | - - - - - | gnl\|UG\|Ta#S65629546 |
| IV9RU5F01AQLRH | - - - - - | gnl\|UG\|Ta#S65635286 |
| IV9RU5F01BA90W | - - - - - | gnl\|UG\|Ta#S65635286 |
| IVLNWEC01C028A | - - - - - | gnl\|UG\|Ta#S65636386 |
| IVLNWEC02HMQO8 | - - - - - | gnl\|UG\|Ta#S65636386 |
| IUIVLUD01EHB5N | - - - - - | gnl\|UG\|Ta#S65636977 |
| IUIVLUD01EHHJ9 | - - - - - | gnl\|UG\|Ta#S65636977 |
| IV9RU5F02GSVWB | - - - - - | gnl\|UG\|Ta#S65636977 |
| IV9RU5F01CZAA2 | - - - - - | gnl\|UG\|Ta#S65637309 |
| IVLNWEC01AUC5I | - - - - - | gnl\|UG\|Ta#S65637309 |
| IUIVLUD01DP4PT | - - - - - | gnl\|UG\|Ta#S65637516 |
| IUIVLUD01DX2JB | - - - - - | gnl\|UG\|Ta#S65637516 |
| IV9RU5F02HLG2M | - - - - - | gnl\|UG\|Ta#S65637516 |
| IV9RU5F01DBOR8 | - - - - - | gnl\|UG\|Ta#S65639158 |
| IVLNWEC02H03RQ | - - - - - | gnl\|UG\|Ta#S65639158 |
| IVLNWEC02J4QDC | - - - - - | gnl\|UG\|Ta#S65639158 |
| IV9RU5F02IOGKR | - - - - - | gnl\|UG\|Ta#S65641058 |
| IVLNWEC02JAH6N | - - - - - | gnl\|UG\|Ta#S65641058 |
| IV9RU5F02HR859 | - - - - - | gnl\|UG\|Ta#S65641107 |
| IVLNWEC01CEI42 | - - - - - | gnl\|UG\|Ta#S65641107 |

| | | |
|---|---|---|
| IV9RU5F01DDDKH | - - - - - | gnl\|UG\|Ta#S65642631 |
| IV9RU5F02IKWX7 | - - - - - | gnl\|UG\|Ta#S65642631 |
| IVLNWEC02FMAGH | - - - - - | gnl\|UG\|Ta#S65643051 |
| IVLNWEC02H0Y28 | - - - - - | gnl\|UG\|Ta#S65643051 |
| IVLNWEC02HUFQP | - - - - - | gnl\|UG\|Ta#S65643051 |
| IVLNWEC01ATXYN | - - - - - | gnl\|UG\|Ta#S65643228 |
| IVLNWEC02HS5P1 | - - - - - | gnl\|UG\|Ta#S65643228 |
| IV9RU5F01CR1F3 | - - - - - | gnl\|UG\|Ta#S65644308 |
| IV9RU5F02IAYXU | - - - - - | gnl\|UG\|Ta#S65644308 |
| IV9RU5F02H792X | - - - - - | gnl\|UG\|Ta#S65645136 |
| IVLNWEC01C1ATK | - - - - - | gnl\|UG\|Ta#S65645136 |
| IVLNWEC01CF6BU | - - - - - | gnl\|UG\|Ta#S65645136 |
| IV9RU5F02G7I6S | - - - - - | gnl\|UG\|Ta#S65645505 |
| IV9RU5F02HMEEC | - - - - - | gnl\|UG\|Ta#S65645505 |
| IV9RU5F02IH0DZ | - - - - - | gnl\|UG\|Ta#S65645505 |
| IVLNWEC02G8QFJ | - - - - - | gnl\|UG\|Ta#S65645505 |
| IUIVLUD02HVWAP | - - - - - | gnl\|UG\|Ta#S65645935 |
| IV9RU5F02JEPGA | - - - - - | gnl\|UG\|Ta#S65645935 |
| IV9RU5F01BD4T4 | - - - - - | gnl\|UG\|Ta#S65646090 |
| IV9RU5F02ILLNH | - - - - - | gnl\|UG\|Ta#S65646090 |
| IV9RU5F01DGSQ1 | - - - - - | gnl\|UG\|Ta#S65646444 |
| IV9RU5F02IH1EI | - - - - - | gnl\|UG\|Ta#S65646444 |
| IV9RU5F01A5XL7 | - - - - - | gnl\|UG\|Ta#S65648146 |
| IV9RU5F02GJW6K | - - - - - | gnl\|UG\|Ta#S65648146 |
| IVLNWEC02IW7ZD | - - - - - | gnl\|UG\|Ta#S65648146 |
| IVLNWEC01CD72O | - - - - - | gnl\|UG\|Ta#S65648831 |
| IVLNWEC02G6EPI | - - - - - | gnl\|UG\|Ta#S65648831 |
| IV9RU5F01AR1NH | - - - - - | gnl\|UG\|Ta#S65649197 |
| IV9RU5F01CBYOH | - - - - - | gnl\|UG\|Ta#S65649197 |
| IUIVLUD02ITDXZ | - - - - - | gnl\|UG\|Ta#S65649231 |
| IVLNWEC02ID4BY | - - - - - | gnl\|UG\|Ta#S65649231 |
| IV9RU5F01BMVZH | - - - - - | gnl\|UG\|Ta#S65650265 |
| IVLNWEC01DIXSG | - - - - - | gnl\|UG\|Ta#S65650265 |
| IVLNWEC01EIMQT | - - - - - | gnl\|UG\|Ta#S65651610 |
| IVLNWEC02FY0RY | - - - - - | gnl\|UG\|Ta#S65651610 |
| IV9RU5F01C8E28 | - - - - - | gnl\|UG\|Ta#S65651826 |
| IV9RU5F01DBEYK | - - - - - | gnl\|UG\|Ta#S65651826 |
| IV9RU5F02HSRE0 | - - - - - | gnl\|UG\|Ta#S65652043 |
| IVLNWEC01DS0RF | - - - - - | gnl\|UG\|Ta#S65652043 |
| IUIVLUD02F0VRB | - - - - - | gnl\|UG\|Ta#S65652085 |
| IV9RU5F01CEEVW | - - - - - | gnl\|UG\|Ta#S65652085 |
| IV9RU5F01BISPG | - - - - - | gnl\|UG\|Ta#S65652305 |
| IV9RU5F01CWKQC | - - - - - | gnl\|UG\|Ta#S65652305 |
| IVLNWEC01DB7X9 | - - - - - | gnl\|UG\|Ta#S65652305 |
| IVLNWEC01EWP66 | - - - - - | gnl\|UG\|Ta#S65652305 |
| IUIVLUD01CIQ1O | - - - - - | gnl\|UG\|Ta#S65652995 |
| IV9RU5F02HIR9X | - - - - - | gnl\|UG\|Ta#S65652995 |
| IV9RU5F02IF91Y | - - - - - | gnl\|UG\|Ta#S65654883 |
| IV9RU5F02JNFT4 | - - - - - | gnl\|UG\|Ta#S65656489 |
| IVLNWEC01CIG8V | - - - - - | gnl\|UG\|Ta#S65656489 |

| | | |
|---|---|---|
| IUIVLUD01CF1AN | - - - - - | gnl\|UG\|Ta#S65656709 |
| IVLNWEC01CU15R | - - - - - | gnl\|UG\|Ta#S65656709 |
| IUIVLUD01B0PX5 | - - - - - | gnl\|UG\|Ta#S65657760 |
| IV9RU5F02HINKX | - - - - - | gnl\|UG\|Ta#S65657760 |
| IV9RU5F01CACEB | - - - - - | gnl\|UG\|Ta#S65657771 |
| IV9RU5F02HTGWP | - - - - - | gnl\|UG\|Ta#S65657771 |
| IV9RU5F01CASEN | - - - - - | gnl\|UG\|Ta#S65657897 |
| IUIVLUD01BP9DJ | - - - - - | gnl\|UG\|Ta#S65658145 |
| IV9RU5F02IYDGL | - - - - - | gnl\|UG\|Ta#S65658145 |
| IVLNWEC01CXYHN | - - - - - | gnl\|UG\|Ta#S65658145 |
| IV9RU5F01D0GQO | - - - - - | gnl\|UG\|Ta#S65662140 |
| IV9RU5F02I2WE3 | - - - - - | gnl\|UG\|Ta#S65662140 |
| IUIVLUD02F6EF8 | - - - - - | gnl\|UG\|Ta#S65662160 |
| IUIVLUD02IN6LN | - - - - - | gnl\|UG\|Ta#S65662160 |
| IV9RU5F01DNQNS | - - - - - | gnl\|UG\|Ta#S65662160 |
| IVLNWEC02H9XYE | - - - - - | gnl\|UG\|Ta#S65662659 |
| IVLNWEC02HYKVO | - - - - - | gnl\|UG\|Ta#S65662659 |
| IVLNWEC01BWGQR | - - - - - | gnl\|UG\|Ta#S65662856 |
| IVLNWEC01DF9BH | - - - - - | gnl\|UG\|Ta#S65662856 |
| IV9RU5F01BP2WS | - - - - - | gnl\|UG\|Ta#S65662978 |
| IV9RU5F01BTDL7 | - - - - - | gnl\|UG\|Ta#S65662978 |
| IV9RU5F02HUD3C | - - - - - | gnl\|UG\|Ta#S65662978 |
| IV9RU5F01BGU8V | - - - - - | gnl\|UG\|Ta#S65663271 |
| IVLNWEC01ETO98 | - - - - - | gnl\|UG\|Ta#S65663271 |
| IV9RU5F01CTNJH | - - - - - | gnl\|UG\|Ta#S65663945 |
| IV9RU5F02JAVES | - - - - - | gnl\|UG\|Ta#S65663945 |
| IUIVLUD02HFP4G | - - - - - | gnl\|UG\|Ta#S65664904 |
| IVLNWEC01E52AJ | - - - - - | gnl\|UG\|Ta#S65664904 |
| IV9RU5F01BFXNL | - - - - - | gnl\|UG\|Ta#S65665911 |
| IV9RU5F01DYSIM | - - - - - | gnl\|UG\|Ta#S65665911 |
| IV9RU5F02FH6PO | - - - - - | gnl\|UG\|Ta#S65665911 |
| IVLNWEC01AU39J | - - - - - | gnl\|UG\|Ta#S65667988 |
| IVLNWEC02HZT7X | - - - - - | gnl\|UG\|Ta#S65667988 |
| IV9RU5F02GXPYH | - - - - - | gnl\|UG\|Ta#S65668151 |
| IVLNWEC02I9BGO | - - - - - | gnl\|UG\|Ta#S65668151 |
| IVLNWEC02JZ58H | - - - - - | gnl\|UG\|Ta#S65668151 |
| IUIVLUD02GDNRN | - - - - - | gnl\|UG\|Ta#S65669231 |
| IVLNWEC02ID8NV | - - - - - | gnl\|UG\|Ta#S65669231 |
| IUIVLUD02ISG5V | - - - - - | gnl\|UG\|Ta#S65670027 |
| IVLNWEC02G3980 | - - - - - | gnl\|UG\|Ta#S65670027 |
| IUIVLUD01DKAY6 | - - - - - | gnl\|UG\|Ta#S65670594 |
| IV9RU5F01D2GLK | - - - - - | gnl\|UG\|Ta#S65670594 |
| IV9RU5F01ET8ND | - - - - - | gnl\|UG\|Ta#S65671292 |
| IV9RU5F02IYBRE | - - - - - | gnl\|UG\|Ta#S65671292 |
| IVLNWEC02F3PW5 | - - - - - | gnl\|UG\|Ta#S65675843 |
| IVLNWEC02F4ADG | - - - - - | gnl\|UG\|Ta#S65675843 |
| IUIVLUD01DDXGR | - - - - - | gnl\|UG\|Ta#S65676031 |
| IV9RU5F02HV74O | - - - - - | gnl\|UG\|Ta#S65676031 |
| IUIVLUD01C1JYE | - - - - - | gnl\|UG\|Ta#S65676358 |
| IVLNWEC01DS19I | - - - - - | gnl\|UG\|Ta#S65676358 |

| | | |
|---|---|---|
| IV9RU5F01CC6GB | - - - - - | gnl\|UG\|Ta#S65677532 |
| IVLNWEC02GEQBE | - - - - - | gnl\|UG\|Ta#S65677532 |
| IVLNWEC02IAJ5Y | - - - - - | gnl\|UG\|Ta#S65677532 |
| IV9RU5F02FUJF6 | - - - - - | gnl\|UG\|Ta#S65678337 |
| IVLNWEC01EBWZJ | - - - - - | gnl\|UG\|Ta#S65678337 |
| IVLNWEC01CBOQ4 | - - - - - | gnl\|UG\|Ta#S65678892 |
| IUIVLUD01DQA7D | - - - - - | gnl\|UG\|Ta#S65679314 |
| IVLNWEC02HSTZ6 | - - - - - | gnl\|UG\|Ta#S65679314 |
| IUIVLUD02IA1WO | - - - - - | gnl\|UG\|Ta#S65679830 |
| IUIVLUD02IAEZK | - - - - - | gnl\|UG\|Ta#S65679830 |
| IV9RU5F02H6ZYS | - - - - - | gnl\|UG\|Ta#S65679830 |
| IUIVLUD01B96JP | - - - - - | gnl\|UG\|Ta#S65680194 |
| IUIVLUD01CVM6U | - - - - - | gnl\|UG\|Ta#S65680194 |
| IUIVLUD02F5KBC | - - - - - | gnl\|UG\|Ta#S65681223 |
| IV9RU5F02GJN61 | - - - - - | gnl\|UG\|Ta#S65681223 |
| IUIVLUD02JX2W1 | - - - - - | gnl\|UG\|Ta#S65682641 |
| IVLNWEC01A28LW | - - - - - | gnl\|UG\|Ta#S65682641 |
| IUIVLUD01CMX2G | - - - - - | gnl\|UG\|Ta#S65683539 |
| IUIVLUD01ERJSO | - - - - - | gnl\|UG\|Ta#S65683539 |
| IUIVLUD02GA290 | - - - - - | gnl\|UG\|Ta#S65683539 |
| IVLNWEC01AJLI3 | - - - - - | gnl\|UG\|Ta#S65683541 |
| IVLNWEC01EXG0K | - - - - - | gnl\|UG\|Ta#S65683541 |
| IV9RU5F02IHI70 | - - - - - | gnl\|UG\|Ta#S65684074 |
| IVLNWEC01A8DLF | - - - - - | gnl\|UG\|Ta#S65684074 |
| IVLNWEC02GOO4M | - - - - - | gnl\|UG\|Ta#S65684573 |
| IVLNWEC02JUG3Q | - - - - - | gnl\|UG\|Ta#S65684573 |
| IUIVLUD02I0SC0 | - - - - - | gnl\|UG\|Ta#S65685101 |
| IVLNWEC02IHC5N | - - - - - | gnl\|UG\|Ta#S65685101 |
| IUIVLUD01AOBON | - - - - - | gnl\|UG\|Ta#S65685543 |
| IUIVLUD02F7TYQ | - - - - - | gnl\|UG\|Ta#S65685543 |
| IVLNWEC01A09GB | - - - - - | gnl\|UG\|Ta#S65685543 |
| IUIVLUD01EIHG3 | - - - - - | gnl\|UG\|Ta#S65685635 |
| IVLNWEC01CL6BF | - - - - - | gnl\|UG\|Ta#S65685635 |
| IV9RU5F02IM9R6 | - - - - - | gnl\|UG\|Ta#S65685749 |
| IVLNWEC01BP77E | - - - - - | gnl\|UG\|Ta#S65685749 |
| IVLNWEC02I132W | - - - - - | gnl\|UG\|Ta#S65685749 |
| IVLNWEC01COF6M | - - - - - | gnl\|UG\|Ta#S65686296 |
| IVLNWEC02HUFCE | - - - - - | gnl\|UG\|Ta#S65686296 |
| IV9RU5F01BS602 | - - - - - | gnl\|UG\|Ta#S65686867 |
| IV9RU5F02ISDUH | - - - - - | gnl\|UG\|Ta#S65686867 |
| IUIVLUD01CB5RZ | - - - - - | gnl\|UG\|Ta#S65687194 |
| IUIVLUD02JSFSZ | - - - - - | gnl\|UG\|Ta#S65687194 |
| IV9RU5F01CJEP8 | - - - - - | gnl\|UG\|Ta#S65687194 |
| IVLNWEC01DNOWL | - - - - - | gnl\|UG\|Ta#S65687390 |
| IVLNWEC01DSNL1 | - - - - - | gnl\|UG\|Ta#S65687390 |
| IV9RU5F01DCMYD | - - - - - | gnl\|UG\|Ta#S65687484 |
| IV9RU5F02JMVYL | - - - - - | gnl\|UG\|Ta#S65687484 |
| IUIVLUD01DOZVR | - - - - - | gnl\|UG\|Ta#S65688304 |
| IUIVLUD01DSV55 | - - - - - | gnl\|UG\|Ta#S65688304 |
| IV9RU5F02F41E0 | - - - - - | gnl\|UG\|Ta#S65688304 |

| | | |
|---|---|---|
| IV9RU5F01EF049 | - - - - - | gnl\|UG\|Ta#S65688993 |
| IV9RU5F01EQW9Q | - - - - - | gnl\|UG\|Ta#S65688993 |
| IUIVLUD02JCXNA | - - - - - | gnl\|UG\|Ta#S65689062 |
| IV9RU5F01AFB3G | - - - - - | gnl\|UG\|Ta#S65689062 |
| IUIVLUD01B6EBH | - - - - - | gnl\|UG\|Ta#S65689488 |
| IVLNWEC02JFZZB | - - - - - | gnl\|UG\|Ta#S65689488 |
| IV9RU5F01ES6KK | - - - - - | gnl\|UG\|Ta#S65689573 |
| IV9RU5F02HLKRW | - - - - - | gnl\|UG\|Ta#S65689573 |
| IV9RU5F01BDDJN | - - - - - | gnl\|UG\|Ta#S65690617 |
| IVLNWEC01B0II5 | - - - - - | gnl\|UG\|Ta#S65690617 |
| IVLNWEC01BRST7 | - - - - - | gnl\|UG\|Ta#S65690732 |
| IVLNWEC02F6390 | - - - - - | gnl\|UG\|Ta#S65690732 |
| IV9RU5F02GP4XT | - - - - - | gnl\|UG\|Ta#S65690943 |
| IVLNWEC02JZPDP | - - - - - | gnl\|UG\|Ta#S65690943 |
| IUIVLUD01AUH20 | - - - - - | gnl\|UG\|Ta#S65692855 |
| IV9RU5F01D1XOF | - - - - - | gnl\|UG\|Ta#S65692855 |
| IV9RU5F02IDLKK | - - - - - | gnl\|UG\|Ta#S65692855 |
| IUIVLUD01C983B | - - - - - | gnl\|UG\|Ta#S65693141 |
| IVLNWEC01EVVIU | - - - - - | gnl\|UG\|Ta#S65693141 |
| IVLNWEC02JEKNR | - - - - - | gnl\|UG\|Ta#S65693141 |
| IV9RU5F02HW1BH | - - - - - | gnl\|UG\|Ta#S65694312 |
| IVLNWEC01COGW0 | - - - - - | gnl\|UG\|Ta#S65694312 |
| IUIVLUD02IXFRH | - - - - - | gnl\|UG\|Ta#S65695265 |
| IVLNWEC01BHUGT | - - - - - | gnl\|UG\|Ta#S65695265 |
| IV9RU5F01C72UI | - - - - - | gnl\|UG\|Ta#S65695949 |
| IVLNWEC01BYVQS | - - - - - | gnl\|UG\|Ta#S65695949 |
| IUIVLUD01EGYWA | - - - - - | gnl\|UG\|Ta#S65696620 |
| IV9RU5F02H82M3 | - - - - - | gnl\|UG\|Ta#S65696620 |
| IUIVLUD01A3GHI | - - - - - | gnl\|UG\|Ta#S65696703 |
| IUIVLUD02JW4RJ | - - - - - | gnl\|UG\|Ta#S65696703 |
| IV9RU5F02JKJ4C | - - - - - | gnl\|UG\|Ta#S65696703 |
| IV9RU5F01CUSQH | - - - - - | gnl\|UG\|Ta#S65696909 |
| IV9RU5F02H4HMS | - - - - - | gnl\|UG\|Ta#S65696909 |
| IVLNWEC01C77IG | - - - - - | gnl\|UG\|Ta#S65696909 |
| IV9RU5F01AG2UK | - - - - - | gnl\|UG\|Ta#S65696938 |
| IV9RU5F01AW614 | - - - - - | gnl\|UG\|Ta#S65696938 |
| IVLNWEC01BA5D7 | - - - - - | gnl\|UG\|Ta#S65696938 |
| IVLNWEC01EPQOE | - - - - - | gnl\|UG\|Ta#S65696938 |
| IUIVLUD02FWOL9 | - - - - - | gnl\|UG\|Ta#S65697404 |
| IVLNWEC02I1B6U | - - - - - | gnl\|UG\|Ta#S65697404 |
| IUIVLUD01D6JU3 | - - - - - | gnl\|UG\|Ta#S65697637 |
| IV9RU5F01C41QP | - - - - - | gnl\|UG\|Ta#S65697637 |
| IV9RU5F02F2QFN | - - - - - | gnl\|UG\|Ta#S65697637 |
| IVLNWEC01EJ0R7 | - - - - - | gnl\|UG\|Ta#S65697722 |
| IUIVLUD02HWX49 | - - - - - | gnl\|UG\|Ta#S65698248 |
| IV9RU5F01C9QUC | - - - - - | gnl\|UG\|Ta#S65698723 |
| IV9RU5F02GU3VG | - - - - - | gnl\|UG\|Ta#S65698723 |
| IUIVLUD02IJBPN | - - - - - | gnl\|UG\|Ta#S65699551 |
| IVLNWEC01C6IH6 | - - - - - | gnl\|UG\|Ta#S65699551 |
| IVLNWEC02FVNDG | - - - - - | gnl\|UG\|Ta#S65699551 |

| | | |
|---|---|---|
| IUIVLUD02JBMN4 | - - - - - | gnl\|UG\|Ta#S65700333 |
| IV9RU5F02HTB8M | - - - - - | gnl\|UG\|Ta#S65700333 |
| IV9RU5F01ESK88 | - - - - - | gnl\|UG\|Ta#S65701100 |
| IV9RU5F01ESUBN | - - - - - | gnl\|UG\|Ta#S65701100 |
| IV9RU5F02G512O | - - - - - | gnl\|UG\|Ta#S65701100 |
| IV9RU5F02FM4FK | - - - - - | gnl\|UG\|Ta#S65701169 |
| IVLNWEC01API3U | - - - - - | gnl\|UG\|Ta#S65701169 |
| IVLNWEC01BFNQC | - - - - - | gnl\|UG\|Ta#S65701169 |
| IV9RU5F02H8AMB | - - - - - | gnl\|UG\|Ta#S65701237 |
| IV9RU5F02HNSVV | - - - - - | gnl\|UG\|Ta#S65701237 |
| IVLNWEC01CIKVK | - - - - - | gnl\|UG\|Ta#S65701342 |
| IVLNWEC01DI0OU | - - - - - | gnl\|UG\|Ta#S65701342 |
| IVLNWEC02FRNZM | - - - - - | gnl\|UG\|Ta#S65702597 |
| IUIVLUD02JTQZL | - - - - - | gnl\|UG\|Ta#S65703463 |
| IVLNWEC02I4EBG | - - - - - | gnl\|UG\|Ta#S65703463 |
| IUIVLUD02G4ND8 | - - - - - | gnl\|UG\|Ta#S65703473 |
| IVLNWEC02IIH14 | - - - - - | gnl\|UG\|Ta#S65703473 |
| IV9RU5F01BEX3X | - - - - - | gnl\|UG\|Ta#S65703499 |
| IVLNWEC02HB4ZJ | - - - - - | gnl\|UG\|Ta#S65703499 |
| IV9RU5F01AS9FT | - - - - - | gnl\|UG\|Ta#S65705349 |
| IVLNWEC01BY3ZB | - - - - - | gnl\|UG\|Ta#S65705349 |
| IUIVLUD02IKL5J | - - - - - | gnl\|UG\|Ta#S65705543 |
| IV9RU5F01AUYKC | - - - - - | gnl\|UG\|Ta#S65705543 |
| IVLNWEC01BYAB1 | - - - - - | gnl\|UG\|Ta#S65706341 |
| IVLNWEC01C2RHB | - - - - - | gnl\|UG\|Ta#S65706341 |
| IVLNWEC01D87UU | - - - - - | gnl\|UG\|Ta#S65706341 |
| IUIVLUD01CYPQZ | - - - - - | gnl\|UG\|Ta#S65706483 |
| IV9RU5F01AFQQ2 | - - - - - | gnl\|UG\|Ta#S65706483 |
| IV9RU5F01D4WMO | - - - - - | gnl\|UG\|Ta#S65706483 |
| IV9RU5F01DM27D | - - - - - | gnl\|UG\|Ta#S65707710 |
| IVLNWEC01DPDWV | - - - - - | gnl\|UG\|Ta#S65707710 |
| IV9RU5F02FOV78 | - - - - - | gnl\|UG\|Ta#S65709229 |
| IV9RU5F02GC4XL | - - - - - | gnl\|UG\|Ta#S65709229 |
| IV9RU5F01CBDTI | - - - - - | gnl\|UG\|Ta#S65710348 |
| IV9RU5F02HL0X6 | - - - - - | gnl\|UG\|Ta#S65710348 |
| IVLNWEC02H7TM5 | - - - - - | gnl\|UG\|Ta#S65710379 |
| IVLNWEC02HFTOF | - - - - - | gnl\|UG\|Ta#S65710379 |
| IV9RU5F01CHO7A | - - - - - | gnl\|UG\|Ta#S65712204 |
| IVLNWEC01EN8K8 | - - - - - | gnl\|UG\|Ta#S65712204 |
| IVLNWEC01EVP7M | - - - - - | gnl\|UG\|Ta#S65712204 |
| IV9RU5F01DPZVA | - - - - - | gnl\|UG\|Ta#S65712501 |
| IV9RU5F02FI13V | - - - - - | gnl\|UG\|Ta#S65712501 |
| IVLNWEC01A0BAW | - - - - - | gnl\|UG\|Ta#S65712755 |
| IVLNWEC02IQID2 | - - - - - | gnl\|UG\|Ta#S65712755 |
| IV9RU5F01C2VYV | - - - - - | gnl\|UG\|Ta#S65713201 |
| IVLNWEC02GHKRY | - - - - - | gnl\|UG\|Ta#S65713201 |
| IUIVLUD02I9C35 | - - - - - | gnl\|UG\|Ta#S65714391 |
| IVLNWEC01DPR8T | - - - - - | gnl\|UG\|Ta#S65714391 |
| IV9RU5F02JSDZY | - - - - - | gnl\|UG\|Ta#S65716794 |
| IVLNWEC01BUGA5 | - - - - - | gnl\|UG\|Ta#S65716794 |

| | | |
|---|---|---|
| IUIVLUD01BQMKG | - - - - - | gnl\|UG\|Ta#S65718785 |
| IVLNWEC02I7TIR | - - - - - | gnl\|UG\|Ta#S65718785 |
| IVLNWEC01D9F14 | - - - - - | gnl\|UG\|Ta#S65718823 |
| IVLNWEC02GIQKA | - - - - - | gnl\|UG\|Ta#S65718823 |
| IUIVLUD01BYGEN | - - - - - | gnl\|UG\|Ta#S65719201 |
| IUIVLUD02HC4WQ | - - - - - | gnl\|UG\|Ta#S65719201 |
| IV9RU5F01DN4TZ | - - - - - | gnl\|UG\|Ta#S65719336 |
| IV9RU5F01DR7PW | - - - - - | gnl\|UG\|Ta#S65719336 |
| IVLNWEC02JDJUL | - - - - - | gnl\|UG\|Ta#S65719336 |
| IV9RU5F02ICOSY | - - - - - | gnl\|UG\|Ta#S65721035 |
| IV9RU5F02IZOME | - - - - - | gnl\|UG\|Ta#S65721035 |
| IV9RU5F01EAFJB | - - - - - | gnl\|UG\|Ta#S65721058 |
| IVLNWEC01AMBY1 | - - - - - | gnl\|UG\|Ta#S65721058 |
| IVLNWEC01BMXNB | - - - - - | gnl\|UG\|Ta#S65721179 |
| IVLNWEC02H02MR | - - - - - | gnl\|UG\|Ta#S65721179 |
| IV9RU5F01BMUFQ | - - - - - | gnl\|UG\|Ta#S65721336 |
| IV9RU5F01D525S | - - - - - | gnl\|UG\|Ta#S65721336 |
| IV9RU5F02F1FE5 | - - - - - | gnl\|UG\|Ta#S65722270 |
| IV9RU5F02F1L24 | - - - - - | gnl\|UG\|Ta#S65722270 |
| IUIVLUD01EJQC4 | - - - - - | gnl\|UG\|Ta#S65722581 |

| Seq. Name |
|---|
| contig00049 |
| contig00057 |
| contig00059 |
| contig00186 |
| contig00509 |
| contig00523 |
| contig00578 |
| contig00716 |
| contig00800 |
| contig01145 |
| contig01587 |
| contig01802 |
| contig02139 |
| contig02156 |
| contig02206 |
| contig02227 |
| contig02281 |
| contig02361 |
| contig02466 |
| contig02654 |
| contig02769 |
| contig02836 |
| contig02945 |
| contig03083 |
| contig03350 |
| contig03352 |
| contig03967 |
| contig04219 |
| contig04718 |
| contig05735 |
| contig06336 |
| contig06792 |
| contig06946 |
| contig07126 |
| contig07331 |
| contig07345 |
| contig07513 |
| contig08314 |
| contig08427 |
| contig08613 |
| contig09037 |
| contig09421 |
| contig09547 |
| contig10066 |
| contig10143 |
| contig10903 |

contig11523
contig12024
contig13359
contig13418
contig13654
contig13793
contig14546
contig14566
contig14629
contig15164
contig16905
contig17149
contig18950
contig19373
contig23717
contig24658
contig25395
contig25910
IUIVLUD01CPRZD
IUIVLUD01D3I7B
IUIVLUD01DSHQ7
IUIVLUD01CA2MO
IUIVLUD01COVHD
IUIVLUD01CYRK5
IUIVLUD01CZLP5
IUIVLUD01BD1V5
IUIVLUD01CXT05
IUIVLUD01AHS1J
IUIVLUD01D2WSS
IUIVLUD01BOLY9
IUIVLUD01ED174
IUIVLUD01CDXRL
IUIVLUD01EUAQ0
IUIVLUD01BM1GQ
IUIVLUD01DDHE7
IUIVLUD01A2CE8
IUIVLUD01ASY1O
IUIVLUD02IRAJV
IUIVLUD02JNE9C
IUIVLUD02HPHYQ
IUIVLUD02JC5VM
IUIVLUD02GU0L3
IUIVLUD02IX0AT
IUIVLUD02H0X44
IUIVLUD02ICLXK
IUIVLUD02FU94Y
IUIVLUD02JSHYK

IUIVLUD02JWMTT
IUIVLUD02JPZCL
IUIVLUD02JFRAB
IUIVLUD02I6O98
IUIVLUD02JTLGU
IUIVLUD02GJ2I7
IUIVLUD02H5S26
IUIVLUD02G23FY
IUIVLUD02GLS5P
IUIVLUD02IDGZZ
IVLNWEC01EYZEC
IVLNWEC01BTTOW
IVLNWEC01ASDMG
IVLNWEC01DR35L
IVLNWEC01CYKYR
IVLNWEC01D4E0H
IVLNWEC01EIMXM
IVLNWEC01BIULG
IVLNWEC01CIX2C
IVLNWEC01DW9BR
IVLNWEC01BPC6J
IVLNWEC01CAVNO
IVLNWEC01BLAJ2
IVLNWEC01ECFGC
IVLNWEC01D2OAF
IVLNWEC01C9XCY
IVLNWEC01CDH0V
IVLNWEC01A3FWG
IVLNWEC01AZ16F
IVLNWEC01A26IU
IVLNWEC01ANWTL
IVLNWEC01CT6ZL
IVLNWEC01A15FQ
IVLNWEC01BG7GD
IVLNWEC01DPFR4
IVLNWEC01CPVEU
IVLNWEC01EXM7A
IVLNWEC01D2H11
IVLNWEC01CEJRZ
IVLNWEC01A0KVJ
IVLNWEC01A39TO
IVLNWEC01EECM7
IVLNWEC01DH1FI
IVLNWEC01A8JDT
IVLNWEC01CK51S
IVLNWEC01BU4L4
IVLNWEC01CEEZG

IVLNWEC02J37D1
IVLNWEC02FTRC4
IVLNWEC02FQ1AY
IVLNWEC02GEZ4N
IVLNWEC02IQ5RR
IVLNWEC02H2UHF
IVLNWEC02GOIM6
IVLNWEC02G81Z1
IVLNWEC02IIP75
IVLNWEC02ILRH3
IVLNWEC02HQINX
IVLNWEC02IS5BN
IVLNWEC02JHQQ8
IVLNWEC02JN2UG
IVLNWEC02JNRSM
IVLNWEC02I6IAM
IVLNWEC02FUBHX
IVLNWEC02H6CZ5
IVLNWEC02F7THL
IVLNWEC02GQLWP
IVLNWEC02GROF6
IVLNWEC02JZCQH
IVLNWEC02F7TMR
IVLNWEC02F1XI5
IVLNWEC02H081V
IVLNWEC02GK10S
IVLNWEC02I43TH
IVLNWEC02IJWE8
IVLNWEC02ICR02
IVLNWEC02H6VMY
IVLNWEC02JJK28
IVLNWEC02HYQ5R
IVLNWEC02I29CH
IVLNWEC02IUQ2Q
IVLNWEC02GHKAJ
IVLNWEC02ITMTV
IVLNWEC02HXDJK
IVLNWEC02I0693
IVLNWEC02HPXQC
IVLNWEC02IQ42V
IVLNWEC02HF9K5
IV9RU5F01COBQT
IV9RU5F01CRKXQ
IV9RU5F01BGZ1X
IV9RU5F01DSDF8
IV9RU5F01AMBXW
IV9RU5F01DH22F

IV9RU5F01EQCFD
IV9RU5F01CQ11Q
IV9RU5F01EWRJX
IV9RU5F01DZ544
IV9RU5F01DBLFE
IV9RU5F01DA6MZ
IV9RU5F01BB7DS
IV9RU5F01CX1QD
IV9RU5F01EHDUX
IV9RU5F01BVBFA
IV9RU5F01A6XEN
IV9RU5F01CZ9YR
IV9RU5F01DVTBD
IV9RU5F01DL0D9
IV9RU5F01EY1PF
IV9RU5F01EL5DL
IV9RU5F01ECUSE
IV9RU5F01DPSVL
IV9RU5F01B7YKX
IV9RU5F01A9MRR
IV9RU5F01AUSLI
IV9RU5F01BJOGH
IV9RU5F01AWC16
IV9RU5F01ENOBQ
IV9RU5F01A7L6Q
IV9RU5F01CXBH0
IV9RU5F01DGSOK
IV9RU5F01DWPIO
IV9RU5F01CKMJM
IV9RU5F01DXW9Z
IV9RU5F01DNQNE
IV9RU5F01AMZ4Y
IV9RU5F01C3N3T
IV9RU5F01DFX7O
IV9RU5F02GSMHQ
IV9RU5F02IQUQ6
IV9RU5F02IADLX
IV9RU5F02JOFGA
IV9RU5F02GLSJF
IV9RU5F02IJUKJ
IV9RU5F02H53JC
IV9RU5F02HI7ND
IV9RU5F02HTXVZ
IV9RU5F02J3N61
IV9RU5F02HD74I
IV9RU5F02I9R7M
IV9RU5F02I7JRZ

IV9RU5F02GQ4N4
IV9RU5F02GSSAB
IV9RU5F02JFG5S
IV9RU5F02JW1K2
IV9RU5F02HWZI5
IV9RU5F02JWEDW
IV9RU5F02FUC45
IV9RU5F02F2RZQ
IV9RU5F02J087N
IV9RU5F02GBI05
IV9RU5F02J34OY
IV9RU5F02IKXV0
IV9RU5F02JAG2A
IV9RU5F02HQSPB
IV9RU5F02J5TRW
IV9RU5F02JX6K1
IV9RU5F02HQ8OX
IV9RU5F02GFQVS
IV9RU5F02GG4ZU
IV9RU5F02IUJIG
IV9RU5F02J3HJL
IVLNWEC01CYB15
IV9RU5F01EY5XH
IVLNWEC01BVY0W
IV9RU5F02II6H0
IUIVLUD01EXPFT
IVLNWEC02JZA4G
IVLNWEC01CH1U0
IVLNWEC02GT55G
IV9RU5F01EOJUE
IV9RU5F02FY2Q2
IVLNWEC02I16R4
IVLNWEC02GZELA
IVLNWEC01EUJ78
IUIVLUD01C2M2I
IUIVLUD01EXQQ7
contig08228
contig18161
contig12422
contig01976
contig26219

| Seq. Description | Seq. Lengtl |
|---|---|
| hypothetical protein F775_32519 | 4413 |
| lysine-specific demethylase 5b | 4220 |
| cop9 signalosome complex subunit 4 | 4186 |
| hypothetical protein F775_15038 | 3101 |
| beta- insoluble isoenzyme 4 | 1734 |
| gamma-soluble nsf attachment protein | 2367 |
| predicted protein | 2301 |
| hypothetical protein OsI_06812 | 2162 |
| hypothetical protein TRIUR3_09049 | 2095 |
| endoplasmic reticulum mannosyl-oligosaccharide -alpha-mannosidase | 1886 |
| hypothetical protein TRIUR3_11621 | 1694 |
| lupus brain antigen 1-like protein | 1627 |
| predicted protein | 1533 |
| methionine s-methyltransferase | 1529 |
| pumilio-like protein 5 | 1515 |
| oligopeptide transporter 5 | 1511 |
| glutaminyl-trna synthetase | 1499 |
| transcription termination factor 2 | 1478 |
| hypothetical protein TRIUR3_15376 | 1459 |
| hypothetical protein TRIUR3_09049 | 1425 |
| vacuolar sorting-associated protein 18-like protein | 1407 |
| hypothetical protein TRIUR3_09049 | 1392 |
| bifunctional monodehydroascorbate reductase and carbonic anhydrase nectarin-3 | 1375 |
| trna modification gtpase mnme | 1352 |
| fructan exohydrolase | 1298 |
| phenylalanyl-trna synthetase alpha chain | 1310 |
| metal-nicotianamine transporter ysl3 | 1228 |
| predicted protein | 1183 |
| cell division protein chloroplastic-like isoform x1 | 1139 |
| predicted protein | 1028 |
| hypothetical protein F775_28676 | 981 |
| pax-interacting protein 1 | 950 |
| cell division protease ftsh-like mitochondrial | 937 |
| poly -specific ribonuclease parn-like protein | 930 |
| hypothetical protein F775_03927 | 911 |
| aspartate aminotransferase | 912 |
| probable leucine-rich repeat receptor-like protein kinase at5g63930-like | 896 |
| hypothetical protein TRIUR3_20200 | 844 |
| hypothetical protein OsI_18110 | 836 |
| hypothetical protein F775_06862 | 827 |
| sirohydrochlorin ferrochelatase | 802 |
| predicted protein | 778 |
| 3-beta-hydroxysteroid-delta -isomerase | 770 |
| poly -specific ribonuclease parn-like protein | 735 |
| predicted protein | 736 |
| predicted protein | 694 |

| | |
|---|---|
| disease resistance protein rpm1 | 663 |
| predicted protein | 636 |
| Kinesin-4 | 571 |
| hypothetical protein F775_27532 | 568 |
| predicted protein | 558 |
| hypothetical protein F775_15038 | 551 |
| hypothetical protein F775_18198 | 518 |
| hypothetical protein F775_02171 | 519 |
| predicted protein | 513 |
| predicted protein | 491 |
| pentatricopeptide repeat-containing protein mitochondrial-like | 418 |
| pumilio-like protein 5 | 409 |
| cop9 signalosome complex subunit 4 | 339 |
| disease resistance protein rga1-like | 323 |
| dna topoisomerase 2 | 180 |
| predicted protein | 152 |
| translation initiation factor eif-2b subunit epsilon | 131 |
| predicted protein | 116 |
| af414176_1nbs-lrr-like protein | 344 |
| dna damage-binding protein 1 | 399 |
| hypothetical protein ZEAMMB73_042343 | 494 |
| lysine-specific demethylase 5b | 445 |
| proliferation-associated protein 2g4 | 359 |
| dna polymerase epsilon catalytic subunit a | 416 |
| disease resistance rpp13-like protein 1 | 306 |
| hypothetical protein F775_05149 | 365 |
| rna polymerase ii c-terminal domain phosphatase-like 1 | 354 |
| hypothetical protein F775_17292 | 327 |
| hypothetical protein F775_02375 | 452 |
| hypothetical protein TRIUR3_33326 | 377 |
| hypothetical protein F775_27913 | 464 |
| lysine-specific demethylase 5b | 326 |
| predicted protein | 346 |
| hypothetical protein OsI_14700 | 379 |
| probable s-acyltransferase at3g26935-like | 193 |
| unknown | 221 |
| gdsl esterase lipase 7 | 430 |
| hypothetical protein F775_27323 | 140 |
| predicted protein | 312 |
| Separin | 366 |
| hypothetical protein TRIUR3_27519 | 329 |
| hypothetical protein F775_06876 | 431 |
| lysine-specific demethylase 5b | 321 |
| pentatricopeptide repeat-containing protein mitochondrial-like | 257 |
| probable atp-dependent dna helicase hfm1-like | 322 |
| hypothetical protein F775_29226 | 401 |
| cation h(+) antiporter 15-like | 362 |

| | |
|---|---|
| predicted protein | 289 |
| e3 ubiquitin-protein ligase upl6 | 256 |
| hypothetical protein F775_14546 | 272 |
| hypothetical protein ZEAMMB73_019909, partial | 304 |
| unknown | 363 |
| hypothetical protein OsJ_01351 | 355 |
| cation h(+) antiporter 15 | 294 |
| predicted protein | 327 |
| ring finger protein 10 | 217 |
| lrr receptor-like serine threonine-protein kinase gso1 | 203 |
| hypothetical protein TRIUR3_12465 | 403 |
| methionine s-methyltransferase-like | 376 |
| 3-beta-hydroxysteroid-delta -isomerase | 456 |
| isoleucyl-trna synthetase | 419 |
| uncharacterized protein LOC100382830 | 296 |
| methyl- -binding domain-containing protein 9 | 345 |
| atp-dependent rna helicase kurz | 425 |
| hypothetical protein ZEAMMB73_296869 | 428 |
| hypothetical protein OsI_02280 | 447 |
| nuclear pore complex protein nup205-like | 378 |
| hypothetical protein F775_43843 | 394 |
| hypothetical protein SORBIDRAFT_07g004940 | 453 |
| hypothetical protein F775_10025 | 441 |
| target of rapamycin | 409 |
| serine threonine-protein kinase atm | 482 |
| unknown protein | 430 |
| hypothetical protein OsI_01450 | 415 |
| hypothetical protein F775_07946 | 386 |
| glutaminyl-trna synthetase | 416 |
| phospholipase a2-1-like protein | 494 |
| e3 ubiquitin-protein ligase upl6 | 361 |
| pentatricopeptide repeat-containing protein mitochondrial-like | 321 |
| hypothetical protein F775_43843 | 424 |
| hypothetical protein TRIUR3_08334 | 449 |
| pentatricopeptide repeat-containing protein mitochondrial-like | 433 |
| hypothetical protein F775_18698 | 482 |
| PREDICTED: uncharacterized protein LOC100842951 | 410 |
| hypothetical protein F775_21121 | 379 |
| hypothetical protein SORBIDRAFT_06g015320 | 422 |
| low quality protein: probable trna modification gtpase mnme-like | 407 |
| Putative S-acyltransferase | 402 |
| protease do-like 7-like | 393 |
| Separin | 418 |
| serine threonine-protein kinase atm | 288 |
| hypothetical protein SORBIDRAFT_04g035685 | 435 |
| predicted protein | 386 |
| predicted protein | 409 |

| | |
|---|---|
| disease resistance rpp13-like protein 1 | 334 |
| 40s ribosomal protein s10-like | 420 |
| pentatricopeptide repeat-containing protein mitochondrial-like | 378 |
| mannosylglycoprotein endo-beta-mannosidase | 376 |
| n-alpha-acetyltransferase auxiliary subunit-like | 275 |
| predicted protein | 334 |
| mannosylglycoprotein endo-beta-mannosidase | 418 |
| predicted protein | 413 |
| udp-glucose:sterol glucosyltransferase | 381 |
| glutaminyl-trna synthetase | 356 |
| zinc finger ccch domain-containing protein 4-like | 343 |
| 40s ribosomal protein s10-like | 398 |
| predicted protein | 427 |
| Sacsin | 409 |
| lrr receptor-like serine threonine-protein kinase gso1 | 457 |
| hypothetical protein OsJ_01156 | 415 |
| hypothetical protein F775_29226 | 419 |
| hypothetical protein OsJ_27256 | 347 |
| beta-galactosidase 7 | 432 |
| hypothetical protein TRIUR3_09742 | 418 |
| e3 ubiquitin-protein ligase upl6 | 380 |
| hypothetical protein F775_26089 | 370 |
| pentatricopeptide repeat-containing protein mitochondrial-like | 395 |
| af414171_1nbs-lrr-like protein | 348 |
| hypothetical protein TRIUR3_08334 | 326 |
| protein cdch | 405 |
| beta-galactosidase 7 | 416 |
| ring finger protein 10 | 390 |
| serine threonine-protein kinase tor | 217 |
| gdsl esterase lipase 7-like | 395 |
| hypothetical protein F775_27323 | 378 |
| hypothetical protein F775_05716 | 324 |
| phospholipase a2-1-like protein | 253 |
| nudix hydrolase 3 | 417 |
| vacuolar sorting-associated protein 18-like protein | 314 |
| atp-dependent dna helicase hfm1 | 260 |
| hypothetical protein TRIUR3_26118 | 355 |
| predicted protein | 373 |
| 2-aminoethanethiol dioxygenase | 309 |
| mannosylglycoprotein endo-beta-mannosidase | 193 |
| TPA: hypothetical protein ZEAMMB73_029969 | 262 |
| rna polymerase ii c-terminal domain phosphatase-like 1 | 324 |
| predicted protein | 473 |
| trna modification gtpase mnme | 462 |
| predicted protein | 474 |
| hypothetical protein F775_06876 | 474 |
| methionine s-methyltransferase-like | 512 |

| | |
|---|---|
| lysine-specific demethylase 5b | 485 |
| hypothetical protein F775_11969 | 513 |
| phospholipase a2-like protein 1 | 503 |
| hypothetical protein TRIUR3_20200 | 495 |
| hypothetical protein SORBIDRAFT_06g015320 | 481 |
| predicted protein | 513 |
| hypothetical protein F775_05716 | 405 |
| vacuolar sorting-associated protein 18-like protein | 463 |
| beta- insoluble isoenzyme 4 | 522 |
| 12-oxophytodienoate reductase 3 | 499 |
| hypothetical protein TRIUR3_33326 | 514 |
| serine threonine-protein kinase atm | 486 |
| hypothetical protein OsI_21216 | 495 |
| hypothetical protein F775_02375 | 497 |
| hypothetical protein OsJ_19154 | 501 |
| hypothetical protein F775_08290 | 429 |
| hypothetical protein F775_27913 | 476 |
| lysine-specific demethylase 5b | 425 |
| beta- insoluble isoenzyme 4 | 411 |
| lrr receptor-like serine threonine-protein kinase gso1 | 428 |
| trna modification gtpase mnme | 488 |
| methionine s-methyltransferase-like isoform x2 | 462 |
| methionine s-methyltransferase-like isoform x2 | 470 |
| target of rapamycin | 397 |
| hypothetical protein F775_14546 | 413 |
| hypothetical protein F775_08546 | 453 |
| methionine s-methyltransferase-like isoform x2 | 363 |
| predicted protein | 425 |
| hypothetical protein F775_29226 | 372 |
| hypothetical protein F775_05149 | 377 |
| predicted protein | 359 |
| uncharacterized protein LOC100272422 | 414 |
| fructan exohydrolase | 413 |
| predicted protein | 339 |
| predicted protein | 373 |
| glutaminyl-trna synthetase | 478 |
| 2-aminoethanethiol dioxygenase | 352 |
| hypothetical protein F775_27913 | 352 |
| protoporphyrinogen chloroplastic | 368 |
| bifunctional monodehydroascorbate reductase and carbonic anhydrase nectarin-3 | 464 |
| disease resistance protein | 466 |
| hypothetical protein TRIUR3_13415 | 518 |
| flavin-containing monooxygenase yucca11 | 448 |
| hypothetical protein OsI_02280 | 425 |
| dna polymerase epsilon catalytic subunit a | 491 |
| unknown | 436 |
| hypothetical protein F775_04815 | 516 |

| | |
|---|---|
| atp-dependent helicase | 495 |
| predicted protein | 534 |
| hypothetical protein OsJ_27256 | 463 |
| probable atp-dependent rna helicase dhx37-like | 491 |
| predicted protein | 250 |
| probable atp-dependent dna helicase hfm1-like | 354 |
| predicted protein | 506 |
| hypothetical protein TRIUR3_09742 | 474 |
| PREDICTED: uncharacterized protein LOC101772169 | 444 |
| isoleucyl-trna synthetase | 486 |
| glutaminyl-trna synthetase | 351 |
| protein bonzai 3 | 490 |
| exonuclease 1 | 358 |
| PREDICTED: uncharacterized protein LOC101772169 | 443 |
| predicted protein | 468 |
| pentatricopeptide repeat-containing protein mitochondrial-like | 410 |
| hypothetical protein F775_52156 | 454 |
| e3 ubiquitin-protein ligase upl6 | 221 |
| hypothetical protein TRIUR3_28170 | 511 |
| predicted protein | 442 |
| 2-aminoethanethiol dioxygenase | 359 |
| --None-- | |
| --None-- | |
| --None-- | |
| --None-- | |
| --None-- | |
| --None-- | |
| --None-- | |
| --None-- | |
| --None-- | |
| --None-- | |
| --None-- | |
| --None-- | |
| --None-- | |
| --None-- | |
| --None-- | |
| --None-- | |
| --None-- | |
| --None-- | |
| --None-- | |
| --None-- | |

| #Hits | min. eValu | mean Simil | #GOs | GOs |
|---|---|---|---|---|
| 1 | 1,25E-26 | 100,00% | 0 | - |
| 1 | 2,17E-130 | 97,00% | 4 | C:nucleus; I |
| 1 | 1,15E-54 | 89,00% | 5 | F:DNA bind |
| 1 | 1,44E-52 | 97,00% | 0 | - |
| 1 | 5,60E-177 | 91,00% | 2 | F:hydrolase |
| 1 | 8,67E-10 | 87,00% | 2 | C:plasma m |
| 1 | 0 | 68,00% | 1 | F:ADP bind |
| 1 | 2,57E-11 | 84,00% | 4 | F:calcium ic |
| 1 | 8,25E-27 | 98,00% | 2 | C:plasma m |
| 1 | 0 | 88,00% | 7 | C:endosom |
| 1 | 1,74E-16 | 95,00% | 2 | C:chloropla |
| 1 | 1,22E-155 | 97,00% | 1 | F:ATP bindi |
| 1 | 2,09E-52 | 76,00% | 2 | F:hydrolase |
| 1 | 4,47E-74 | 67,00% | 2 | F:transfera: |
| 1 | 8,69E-55 | 80,00% | 1 | F:RNA bind |
| 1 | 0 | 85,00% | 3 | F:oligopept |
| 1 | 6,59E-22 | 89,00% | 4 | C:cytoplasr |
| 1 | 3,66E-38 | 85,00% | 4 | F:nucleic ac |
| 1 | 0 | 95,00% | 0 | - |
| 1 | 3,33E-28 | 85,00% | 2 | C:plasma m |
| 1 | 3,36E-70 | 74,00% | 1 | P:transport |
| 1 | 9,25E-24 | 96,00% | 2 | C:plasma m |
| 1 | 3,57E-40 | 98,00% | 4 | C:chloropla |
| 1 | 1,63E-26 | 86,00% | 1 | F:GTP bindi |
| 1 | 0 | 99,00% | 2 | F:hydrolase |
| 1 | 1,90E-27 | 100,00% | 5 | C:cytoplasr |
| 1 | 1,79E-128 | 81,00% | 1 | P:transmer |
| 1 | 6,28E-23 | 83,00% | 3 | F:RNA bind |
| 1 | 2,61E-23 | 94,00% | 0 | - |
| 1 | 3,40E-77 | 96,00% | 0 | - |
| 1 | 7,41E-11 | 82,00% | 0 | - |
| 1 | 4,73E-30 | 100,00% | 0 | - |
| 1 | 1,72E-111 | 96,00% | 6 | C:membrar |
| 1 | 1,88E-48 | 95,00% | 2 | C:nucleus; I |
| 1 | 1,82E-16 | 65,00% | 0 | - |
| 1 | 5,44E-09 | 85,00% | 5 | C:chloropla |
| 1 | 1,62E-24 | 87,00% | 4 | C:integral c |
| 1 | 5,91E-45 | 91,00% | 3 | F:microtub |
| 1 | 2,66E-88 | 83,00% | 7 | C:cytosol; F |
| 1 | 1,53E-09 | 94,00% | 1 | C:plasma m |
| 1 | 1,28E-16 | 100,00% | 3 | F:lyase acti |
| 1 | 3,25E-23 | 95,00% | 3 | F:zinc ion b |
| 1 | 1,32E-45 | 82,00% | 4 | C:endoplas |
| 1 | 1,31E-38 | 96,00% | 2 | C:nucleus; I |
| 1 | 1,06E-27 | 100,00% | 3 | F:zinc ion b |
| 1 | 3,90E-68 | 97,00% | 1 | F:RNA bind |

| | | | | |
|---|---|---|---|---|
| 1 | 4,40E-120 | 95,00% | 5 | F:ATP bindi |
| 1 | 8,26E-41 | 98,00% | 0 | - |
| 1 | 3,93E-15 | 88,00% | 6 | C:kinesin cc |
| 1 | 2,33E-22 | 98,00% | 1 | C:cytoplasr |
| 1 | 3,27E-92 | 95,00% | 0 | - |
| 1 | 3,71E-12 | 97,00% | 0 | - |
| 1 | 3,85E-41 | 100,00% | 0 | - |
| 1 | 9,80E-27 | 91,00% | 3 | C:membrar |
| 1 | 1,91E-26 | 98,00% | 5 | C:cytoplasr |
| 1 | 1,17E-16 | 87,00% | 1 | C:integral c |
| 1 | 5,87E-73 | 94,00% | 0 | - |
| 1 | 2,85E-11 | 89,00% | 1 | F:RNA bind |
| 1 | 1,36E-29 | 73,00% | 4 | F:binding; F |
| 1 | 1,42E-47 | 87,00% | 0 | - |
| 1 | 2,22E-24 | 88,00% | 5 | F:DNA bind |
| 1 | 4,08E-22 | 100,00% | 2 | F:nucleotid |
| 1 | 1,02E-19 | 100,00% | 2 | F:translatio |
| 1 | 2,86E-11 | 94,00% | 2 | F:nucleotid |
| 1 | 5,04E-26 | 90,00% | 2 | F:ADP bind |
| 1 | 3,86E-51 | 96,00% | 7 | C:nucleus; ( |
| 1 | 1,44E-09 | 94,00% | 0 | - |
| 1 | 2,66E-33 | 85,00% | 4 | C:nucleus; I |
| 1 | 2,99E-32 | 97,00% | 5 | C:nucleolus |
| 1 | 2,94E-32 | 100,00% | 6 | C:epsilon D |
| 1 | 8,00E-49 | 91,00% | 2 | F:ADP bind |
| 1 | 1,18E-51 | 96,00% | 0 | - |
| 1 | 5,77E-22 | 100,00% | 5 | F:phosphos |
| 1 | 7,93E-53 | 98,00% | 1 | F:zinc ion b |
| 1 | 2,12E-44 | 100,00% | 0 | - |
| 1 | 8,32E-29 | 97,00% | 1 | C:integral c |
| 1 | 8,91E-46 | 97,00% | 0 | - |
| 1 | 3,01E-25 | 84,00% | 4 | C:nucleus; I |
| 1 | 9,38E-15 | 93,00% | 0 | - |
| 1 | 4,29E-11 | 77,00% | 2 | P:metaboli |
| 1 | 3,90E-20 | 95,00% | 4 | C:integral c |
| 1 | 1,98E-34 | 98,00% | 1 | F:calcium ic |
| 1 | 5,38E-20 | 94,00% | 2 | F:hydrolase |
| 1 | 6,83E-19 | 95,00% | 0 | - |
| 1 | 3,09E-66 | 99,00% | 3 | C:vacuole; |
| 1 | 2,40E-63 | 100,00% | 8 | C:nucleus; I |
| 1 | 3,50E-16 | 100,00% | 1 | C:nucleus |
| 1 | 4,48E-73 | 95,00% | 1 | C:chloropla |
| 1 | 8,14E-34 | 87,00% | 4 | C:nucleus; I |
| 1 | 2,86E-32 | 86,00% | 0 | - |
| 1 | 2,86E-29 | 90,00% | 0 | - |
| 1 | 1,08E-16 | 94,00% | 0 | - |
| 1 | 1,52E-23 | 86,00% | 3 | C:integral c |

| | | | | |
|---|---|---|---|---|
| 1 | 2,21E-33 | 89,00% | 0 | - |
| 1 | 4,79E-26 | 100,00% | 3 | F:ubiquitin- |
| 1 | 4,55E-42 | 91,00% | 0 | - |
| 1 | 7,77E-15 | 100,00% | 0 | - |
| 1 | 1,01E-29 | 98,00% | 1 | F:calcium io |
| 1 | 4,23E-13 | 92,00% | 4 | C:plastid; F |
| 1 | 2,02E-34 | 97,00% | 3 | C:integral c |
| 1 | 1,38E-32 | 100,00% | 2 | C:cytosolic |
| 1 | 6,47E-25 | 81,00% | 1 | F:zinc ion b |
| 1 | 2,04E-29 | 81,00% | 2 | F:kinase ac |
| 1 | 1,19E-21 | 100,00% | 0 | - |
| 1 | 6,30E-50 | 87,00% | 0 | - |
| 1 | 4,72E-47 | 92,00% | 7 | C:endoplas |
| 1 | 4,80E-45 | 100,00% | 7 | C:chloropla |
| 1 | 2,49E-30 | 96,00% | 0 | - |
| 1 | 6,64E-16 | 82,00% | 2 | C:nucleus; |
| 1 | 1,70E-61 | 100,00% | 4 | F:nucleic ac |
| 1 | 2,23E-14 | 100,00% | 3 | C:vacuole; |
| 1 | 4,31E-23 | 88,00% | 3 | C:1,3-beta- |
| 1 | 1,88E-15 | 87,00% | 2 | C:nuclear p |
| 1 | 3,44E-17 | 81,00% | 0 | - |
| 1 | 2,12E-47 | 94,00% | 3 | F:zinc ion b |
| 1 | 4,20E-57 | 99,00% | 4 | C:chloropla |
| 1 | 1,32E-49 | 98,00% | 15 | F:regulator |
| 1 | 3,18E-34 | 100,00% | 4 | F:ATP bindi |
| 1 | 1,13E-30 | 96,00% | 14 | F:regulator |
| 1 | 8,95E-35 | 95,00% | 5 | C:chloropla |
| 1 | 2,19E-20 | 100,00% | 0 | - |
| 1 | 3,32E-30 | 86,00% | 4 | C:cytoplasr |
| 1 | 7,82E-11 | 80,00% | 6 | F:phosphol |
| 1 | 3,19E-30 | 100,00% | 3 | F:ubiquitin- |
| 1 | 1,15E-55 | 96,00% | 0 | - |
| 1 | 3,21E-12 | 75,00% | 0 | - |
| 1 | 9,69E-48 | 96,00% | 1 | F:copper io |
| 1 | 1,70E-75 | 95,00% | 0 | - |
| 1 | 4,57E-16 | 89,00% | 0 | - |
| 1 | 1,12E-31 | 73,00% | 0 | - |
| 1 | 3,88E-18 | 70,00% | 4 | F:ATP bindi |
| 1 | 8,26E-48 | 79,00% | 10 | C:mitochor |
| 1 | 4,92E-36 | 97,00% | 0 | - |
| 1 | 3,08E-61 | 98,00% | 6 | C:integral c |
| 1 | 2,17E-09 | 84,00% | 0 | - |
| 1 | 1,57E-22 | 92,00% | 3 | C:nucleus; |
| 1 | 1,50E-23 | 100,00% | 4 | F:ATP bindi |
| 1 | 3,07E-28 | 92,00% | 3 | C:vacuole; |
| 1 | 3,04E-24 | 98,00% | 5 | C:chloropla |
| 1 | 6,80E-26 | 81,00% | 1 | F:ATP bindi |

| | | | | |
|---|---|---|---|---|
| 1 | 5,33E-42 | 88,00% | 2 | F:ADP bind |
| 1 | 4,76E-37 | 84,00% | 0 | - |
| 1 | 4,19E-52 | 93,00% | 0 | - |
| 1 | 5,14E-17 | 76,00% | 1 | F:hydrolase |
| 1 | 2,05E-18 | 100,00% | 2 | C:cytosol; C |
| 1 | 5,20E-11 | 91,00% | 0 | - |
| 1 | 2,10E-62 | 97,00% | 3 | C:vacuole; |
| 1 | 2,86E-20 | 100,00% | 3 | F:ATP bindi |
| 1 | 6,96E-20 | 87,00% | 2 | F:sterol 3-b |
| 1 | 5,98E-26 | 97,00% | 4 | C:cytoplasn |
| 1 | 1,98E-19 | 90,00% | 0 | - |
| 1 | 5,79E-36 | 96,00% | 0 | - |
| 1 | 1,19E-12 | 89,00% | 2 | F:hydrolase |
| 1 | 1,04E-55 | 100,00% | 2 | C:plasmode |
| 1 | 2,22E-39 | 98,00% | 2 | F:kinase ac |
| 1 | 6,04E-40 | 85,00% | 6 | C:chloropla |
| 1 | 7,95E-35 | 100,00% | 0 | - |
| 1 | 7,29E-11 | 91,00% | 5 | C:intracellu |
| 1 | 9,80E-33 | 100,00% | 3 | F:beta-gala |
| 1 | 4,44E-25 | 98,00% | 5 | C:mRNA cle |
| 1 | 1,57E-21 | 100,00% | 3 | F:ubiquitin- |
| 1 | 3,72E-13 | 97,00% | 0 | - |
| 1 | 3,69E-16 | 93,00% | 0 | - |
| 1 | 2,42E-45 | 86,00% | 2 | F:ADP bind |
| 1 | 1,83E-49 | 100,00% | 1 | F:copper io |
| 1 | 2,58E-20 | 100,00% | 1 | F:ATP bindi |
| 1 | 2,54E-23 | 93,00% | 3 | F:beta-gala |
| 1 | 2,39E-64 | 100,00% | 1 | F:zinc ion b |
| 1 | 1,58E-18 | 100,00% | 15 | F:regulator |
| 1 | 1,33E-54 | 100,00% | 2 | F:hydrolase |
| 1 | 2,76E-18 | 88,00% | 0 | - |
| 1 | 1,23E-50 | 98,00% | 1 | P:embryo c |
| 1 | 1,69E-12 | 79,00% | 6 | F:phosphol |
| 1 | 4,84E-17 | 97,00% | 3 | C:vacuole; |
| 1 | 2,48E-23 | 92,00% | 3 | F:zinc ion b |
| 1 | 1,91E-30 | 98,00% | 4 | F:nucleic ac |
| 1 | 5,20E-47 | 100,00% | 0 | - |
| 1 | 8,57E-32 | 92,00% | 3 | C:vacuole; |
| 1 | 1,22E-09 | 82,00% | 2 | F:cysteamir |
| 1 | 1,48E-36 | 100,00% | 3 | C:vacuole; |
| 1 | 1,30E-13 | 81,00% | 0 | - |
| 1 | 1,34E-36 | 98,00% | 5 | F:phosphos |
| 1 | 7,83E-15 | 100,00% | 5 | C:nucleus; I |
| 1 | 4,80E-23 | 97,00% | 1 | F:GTP bindi |
| 1 | 1,84E-88 | 97,00% | 4 | C:integral c |
| 1 | 1,89E-52 | 95,00% | 1 | C:chloropla |
| 1 | 3,79E-33 | 80,00% | 0 | - |

| | | | | |
|---|---|---|---|---|
| 1 | 5,08E-28 | 73,00% | 1 | F:metal ion |
| 1 | 5,71E-51 | 92,00% | 0 | - |
| 1 | 1,01E-07 | 82,00% | 0 | - |
| 1 | 3,42E-17 | 89,00% | 3 | F:microtub |
| 1 | 6,34E-57 | 83,00% | 10 | C:mitochor |
| 1 | 1,63E-34 | 79,00% | 4 | C:integral c |
| 1 | 2,33E-59 | 89,00% | 1 | P:embryo c |
| 1 | 3,79E-12 | 81,00% | 3 | F:zinc ion b |
| 1 | 8,40E-95 | 97,00% | 2 | F:hydrolase |
| 1 | 1,30E-17 | 87,00% | 6 | F:FMN binc |
| 1 | 1,90E-29 | 78,00% | 1 | C:membrar |
| 1 | 1,88E-38 | 100,00% | 4 | F:ATP bindi |
| 1 | 2,32E-15 | 100,00% | 7 | C:nucleus; |
| 1 | 8,87E-21 | 100,00% | 0 | - |
| 1 | 3,60E-23 | 79,00% | 1 | C:outer me |
| 1 | 6,78E-28 | 82,00% | 0 | - |
| 1 | 4,11E-45 | 97,00% | 0 | - |
| 1 | 2,75E-27 | 98,00% | 4 | C:nucleus; |
| 1 | 9,79E-38 | 66,00% | 1 | F:hydrolase |
| 1 | 1,02E-69 | 90,00% | 2 | F:kinase ac |
| 1 | 4,24E-33 | 98,00% | 1 | F:GTP bindi |
| 1 | 2,15E-48 | 92,00% | 7 | C:cytosol; F |
| 1 | 4,95E-41 | 90,00% | 7 | C:cytosol; F |
| 1 | 1,23E-20 | 100,00% | 15 | F:regulator |
| 1 | 7,84E-50 | 96,00% | 0 | - |
| 1 | 6,40E-14 | 100,00% | 0 | - |
| 1 | 3,85E-37 | 95,00% | 7 | C:cytosol; F |
| 1 | 1,09E-28 | 94,00% | 2 | C:nucleus; |
| 1 | 1,28E-26 | 90,00% | 0 | - |
| 1 | 1,05E-32 | 95,00% | 0 | - |
| 1 | 5,53E-38 | 97,00% | 2 | C:cytosolic |
| 1 | 1,65E-64 | 100,00% | 2 | C:nucleus; |
| 1 | 7,63E-55 | 100,00% | 2 | F:hydrolase |
| 1 | 1,00E-33 | 97,00% | 2 | C:cytosolic |
| 1 | 3,53E-69 | 99,00% | 7 | C:nucleus; |
| 1 | 9,00E-54 | 100,00% | 4 | C:cytoplasr |
| 1 | 1,26E-14 | 68,00% | 3 | P:oxidation |
| 1 | 5,71E-47 | 95,00% | 0 | - |
| 1 | 1,72E-29 | 100,00% | 5 | C:chloropla |
| 1 | 9,26E-20 | 92,00% | 4 | C:chloropla |
| 1 | 1,37E-10 | 97,00% | 2 | F:ADP bind |
| 1 | 5,08E-34 | 97,00% | 1 | C:nuclear p |
| 1 | 4,85E-31 | 100,00% | 4 | F:N,N-dime |
| 1 | 5,58E-29 | 81,00% | 3 | C:1,3-beta- |
| 1 | 2,57E-15 | 100,00% | 6 | C:nucleus; |
| 1 | 5,51E-67 | 81,00% | 3 | F:FMN binc |
| 1 | 3,05E-17 | 100,00% | 1 | C:nucleus |

| | | | | |
|---|---|---|---|---|
| 1 | 1,05E-79 | 92,00% | 3 | F:helicase a |
| 1 | 7,83E-22 | 97,00% | 2 | C:nucleus; |
| 1 | 3,24E-12 | 100,00% | 5 | C:intracellu |
| 1 | 1,24E-22 | 92,00% | 0 | - |
| 1 | 2,31E-21 | 100,00% | 4 | C:chloropla |
| 1 | 8,90E-24 | 91,00% | 0 | - |
| 1 | 6,08E-96 | 96,00% | 5 | F:ATP bindi |
| 1 | 7,00E-30 | 100,00% | 5 | C:mRNA cle |
| 1 | 2,63E-23 | 92,00% | 2 | F:nucleic ac |
| 1 | 9,72E-27 | 100,00% | 6 | C:cytoplasr |
| 1 | 1,28E-19 | 92,00% | 4 | C:cytoplasr |
| 1 | 1,03E-38 | 76,00% | 1 | P:negative |
| 1 | 1,48E-15 | 90,00% | 5 | C:nucleus; |
| 1 | 7,20E-33 | 91,00% | 2 | F:nucleic ac |
| 1 | 1,27E-48 | 98,00% | 5 | F:ATP bindi |
| 1 | 1,41E-55 | 90,00% | 0 | - |
| 1 | 3,39E-29 | 100,00% | 3 | C:vacuole; |
| 1 | 1,68E-15 | 85,00% | 3 | F:ubiquitin- |
| 1 | 3,07E-30 | 95,00% | 0 | - |
| 1 | 2,30E-17 | 93,00% | 0 | - |
| 1 | 1,85E-17 | 91,00% | 2 | F:cysteamir |

# Molecular organization and comparative analysis of chromosome 5B of the

Bala Ani Akpinar[1], Meral Yuce[1], Stuart Lucas[1], Jan Vrána[2], Veronika Burešová[2], Jaroslav Doležel

**Supplementary Table 3. Predicted Tdic5B miRNAs and their respective targets.**

| Unique Hit ID | New miRNA Id | New miRNA sequence |
|---|---|---|
| 11_IV9RU5F01BFHPW | candidate-miR169b-5p | UAGUCAAGGAUGACUUGCCUAUG |
| 14_contig00597 | candidate-miR5062b-3p | UGAACCUUAGGGAACAGCCGCAU |
| 15_contig04934 | candidate-miR167b-5p | UGAAGCUGCCAGCAUGAUCUGA |
| 16_contig06207 | candidate-miR167b-5p | UGAAGCUGCCAGCAUGAUCUAU |
| 17_contig02570 | candidate-miR167b-5p | UGAAGCUGCCAGCAUGAUCUAG |
| 26_IVLNWEC01EUHN7 | candidate-miR2118d-3p | UUCCUGAUGCCUCCCAUGCCUA |
| 27_IVLNWEC01D12C9 | candidate-miR2118d-3p | UUCCUGAUGCCUCCCAUGCCUA |
| 28_IVLNWEC01C1E8V | candidate-miR2118d-3p | UUCCUGAUGCCUCCCAUGCCUA |
| 29_IVLNWEC01B0RJK | candidate-miR2118d-3p | UUCCUGAUGCCUCCCAUGCCUA |
| 30_IUIVLUD02JNJRF | candidate-miR2118d-3p | UUCCUGAUGCCUCCCAUGCCUA |
| 31_IUIVLUD02HV36R | candidate-miR2118d-3p | UUCCUGAUGCCUCCCAUGCCUA |
| 32_IUIVLUD02H2QF2 | candidate-miR2118d-3p | UUCCUGAUGCCUCCCAUGCCUA |
| 33_IUIVLUD01DO4ZV | candidate-miR2118d-3p | UUCCUGAUGCCUCCCAUGCCUA |
| 36_contig15634 | candidate-miR2118d-3p | UUCCUGAUGCCUCCCAUGCCUA |
| 37_contig12404 | candidate-miR2118d-3p | UUCCUGAUGCCUCCCAUGCCUA |
| 38_contig12404 | candidate-miR2118d-3p | UUCCCAAUGCCUCCCAUGCCUA |
| 39_contig03699 | candidate-miR2118d-3p | UUCCUGAUGCCUCCCAUGCCUA |
| 44_contig03108 | candidate-miR2118d-3p | UUCCUGAUGCCUCCCAUGCCUA |
| 45_contig02267 | candidate-miR2118d-3p | UUCCUGAUGCCUCCCAUGCCUA |
| 48_contig01460 | candidate-miR2118d-3p | UUCCUGAUGCCUCCCAUGCCUA |
| 50_contig00983 | candidate-miR2118d-3p | UUCCUGAUGCCUCCCAUGCCUA |
| 51_contig00113 | candidate-miR2118d-3p | UUCCUGAUGCCUCCCAUGCCUA |
| 54_IV9RU5F02HWNI4 | candidate-miR2118d-3p | UUCCUGAUGCCUCCCAUUCCUA |
| 55_IV9RU5F02JPIW9 | candidate-miR2118d-3p | UUCCUAAUGCCUCCCAUGCCUA |
| 58_IV9RU5F01BTDDP | candidate-miR2118d-3p | UUCCNGAUGCCUCCCAUGCCUA |
| 60_IV9RU5F01DUBH9 | candidate-miR2118d-3p | UUCCUAAUGCCUCCCAUGCCUA |
| 61_IV9RU5F01DUBH9 | candidate-miR2118d-3p | UUCCCGAUGCCUCCCUUGCCUA |
| 64_IVLNWEC01C7L9O | candidate-miR2118d-3p | UUCCUGAUGCCUCCCAUUCCUA |
| 65_IUIVLUD02GOBIZ | candidate-miR2118d-3p | UUCCCGAUGCCUCCCAUGCCUA |
| 67_contig15489 | candidate-miR2118d-3p | UUCCUGAUGCCUCUCAUGCCUA |
| 69_contig03963 | candidate-miR2118d-3p | UUCCUCAUGCCUCCCAUGCCUA |
| 70_contig02414 | candidate-miR2118d-3p | UUCCCGAUGCCUCCCAUGCCUA |
| 76_IV9RU5F02GNYTM | candidate-miR2118d-3p | UUCCUGAUGCCUCCCAUUUCUA |
| 78_IV9RU5F02HIF1A | candidate-miR2118d-3p | UUUCUGAUGCCUCCCAUUCCUA |
| 94_IVLNWEC01DHZAD | candidate-miR2118d-3p | UUUCUGAUGCCUCCCAUUCCUA |
| 95_IVLNWEC01B0CK4 | candidate-miR2118d-3p | UUCCCGAUGCCUCCCUUGCCUA |
| 106_contig19478 | candidate-miR2118d-3p | UUCCCGAUGCCUCUCAUGCCUA |
| 109_contig12973 | candidate-miR2118d-3p | UUCCUGAUGCCUCUCAUUCCUA |
| 111_contig10421 | candidate-miR2118d-3p | UUCCCAAUGCCUCCCAUGCCUA |
| 114_contig00982 | candidate-miR2118d-3p | UUCCUGAUGCCUCUCAUGCCAA |

| | | |
|---|---|---|
| 115_contig00821 | candidate-miR2118d-3p | UUUCUGAUGCCUCCCAUUCCUA |
| 160_contig16902 | candidate-miR2118d-3p | UUUCUGAUGCCUCCCAUUUCUA |
| 177_contig03266 | candidate-miR2118d-3p | UUCCUGAUGCCUCAUAUUCCUA |
| 186_contig00047 | candidate-miR2118d-3p | UUCUCGAUGCCUCUCAUGCCUA |
| 216_IV9RU5F02GFRM4 | candidate-miR9863b-5p | UGUUAUGAUUUGCUUCUCAUC |
| 250_IV9RU5F02IVBDY | candidate-miR2275c-3p | AUUGGUUUACUCGAAUAUCUCA |
| 275_IUIVLUD02FMRPQ | candidate-miR169g-5p | AAGCCAAGGAUCAUUUGCCGA |
| 279_IV9RU5F01BFHPW | candidate-miR169i-5p | UAGUCAAGGAUGACUUGCCUA |
| 280_IVLNWEC01B62WO | candidate-miR169i-5p | UAGCCAAGGAUGAAUUGCCAG |
| 281_IV9RU5F01BFHPW | candidate-miR169c-3p | GGCAGUCACCUUGGCUAGC |
| 306_contig06207 | candidate-miR167c-3p | GAUCAUGACUGACAGCCUCAUU |
| 309_IV9RU5F02GNYTM | candidate-miR2118a-3p | GGGAAUGGGAACAUGGAGGAA |
| 311_contig12973 | candidate-miR2118a-3p | GGGAAUGGGAACAUGGAGGAA |
| 317_IV9RU5F02JPIW9 | candidate-miR2118a-3p | GGGCAUGGGAACAUGGAGGAA |
| 320_IV9RU5F01DUBH9 | candidate-miR2118a-3p | GGGCAUGGGAACAUGGAGGAA |
| 321_IV9RU5F01DUBH9 | candidate-miR2118a-3p | GGGCAUGGGAACAUGGAGGAA |
| 322_IVLNWEC01AFOQC | candidate-miR2118a-3p | GGGCAUGGGAACAUGGAGGAA |
| 323_IVLNWEC01EUHN7 | candidate-miR2118a-3p | GGGCAUGGGAACAUGGAGGAA |
| 325_IVLNWEC01B0CK4 | candidate-miR2118a-3p | GGGCAUGGGAACAUGGAGGAA |
| 326_IVLNWEC01C1E8V | candidate-miR2118a-3p | GGGCAUGGGAACAUGGAGGAA |
| 327_IVLNWEC01B0RJK | candidate-miR2118a-3p | GGGCAUGGGAACAUGGAGGAA |
| 329_IUIVLUD02JNJRF | candidate-miR2118a-3p | GGGCAUGGGAACAUGGAGGAA |
| 330_IUIVLUD02GOBIZ | candidate-miR2118a-3p | GGGCAUGGGAACAUGGAGGAA |
| 331_IUIVLUD02H2QF2 | candidate-miR2118a-3p | GGGCAUGGGAACAUGGAGGAA |
| 332_IUIVLUD01CISTK | candidate-miR2118a-3p | GGGGAUGGGAACAUGGAGGAA |
| 334_IUIVLUD01DO4ZV | candidate-miR2118a-3p | GGGCAUGGGAACAUGGAGGAA |
| 337_contig19478 | candidate-miR2118a-3p | GGGCAUGGGAACAUGGAGGAA |
| 338_contig15634 | candidate-miR2118a-3p | GGGCAUGGGAACAUGGAGGAA |
| 340_contig12404 | candidate-miR2118a-3p | GGGCAUGGGAACAUGGAGGAA |
| 341_contig12404 | candidate-miR2118a-3p | GGGCAUGGGAACAUGGAGGAA |
| 342_contig10421 | candidate-miR2118a-3p | GGGCAUGGGAACAUGGAGGAA |
| 343_contig09442 | candidate-miR2118a-3p | GGGCAUGGGAACAUGGAGGAA |
| 346_contig03108 | candidate-miR2118a-3p | GGGUAUGGGAACAUGGAGGAA |
| 348_contig02414 | candidate-miR2118a-3p | GGGCAUGGGAACAUGGAGGAA |
| 349_contig02267 | candidate-miR2118a-3p | GGGCAUGGGAACAUGGAGGAA |
| 351_contig01460 | candidate-miR2118a-3p | GGGCAUGGGAACAUGGAGGAA |
| 352_contig01460 | candidate-miR2118a-3p | GGGCAUGGGAACAUGGAGGAA |
| 353_contig00983 | candidate-miR2118a-3p | GGGCAUGGGAACAUGGAGGAA |
| 355_contig00113 | candidate-miR2118a-3p | GGGCAUGGGAACAUGGAGGAA |
| 356_contig00113 | candidate-miR2118a-3p | GGGCAUGGGAACAUGGAGGAA |
| 358_IV9RU5F02HWNI4 | candidate-miR2118a-3p | AGGAAUGGGAACAUAGAGGAA |
| 359_IVLNWEC01C7L9O | candidate-miR2118a-3p | AGGAAUGGGAACAUAGAGGAA |
| 361_contig20101 | candidate-miR2118a-3p | AGGAAUGGGAACAUUGAGGAA |
| 367_IVLNWEC02JYWN9 | candidate-miR2118a-3p | AUGAAUGGGAACAUCGAGGAA |
| 371_IUIVLUD02HV36R | candidate-miR2118a-3p | GGGCAUGGGAACAUGCAGGAA |
| 376_contig15489 | candidate-miR2118a-3p | AGGCAUGGGAACAUUGAGGAA |
| 379_contig05202 | candidate-miR2118a-3p | AUGAAUGGGAACAUCGAGGAA |

| | | |
|---|---|---|
| 384_contig00241 | candidate-miR2118a-3p | AUGAAUGGGAACAUCGAGGAA |
| 386_IV9RU5F02HIF1A | candidate-miR2118a-3p | AGGAAUGGGAACAUGAAGAAA |
| 389_IVLNWEC01DHZAD | candidate-miR2118a-3p | AGGAAUGGGAACAUGAAGAAA |
| 393_contig16902 | candidate-miR2118a-3p | AGGAAUGGGAACAUGAAGAAA |
| 394_contig00821 | candidate-miR2118a-3p | AGGAAUGGGAACAUGAAGAAA |
| 403_IV9RU5F02HSXSH | candidate-miR399a-5p | GGGCGCUUCUCCAUUGGCACGG |
| 404_IVLNWEC02I6NFF | candidate-miR399a-5p | GGGCGCUUCUCCAUUGGCACGG |
| 460_IUIVLUD01CISTK | candidate-miR2118a-5p | UUCCCGAUGUCUCCUAUUCCUA |
| 520_IV9RU5F02HSXSH | candidate-miR399a-3p | UGCCAAAGGAGAAUUGCCCUG |
| 522_IVLNWEC02I6NFF | candidate-miR399a-3p | UGCCAAAGGAGAAUUGCCCUG |
| 689_IUIVLUD01DQSUI | candidate-miR5084-5p | AUUCUCUGCAGUACUGUACGGUGC |
| 708_IVLNWEC02I55SE | candidate-miR398f-3p | UGUGUUCUCAGGUCACCCCUU |
| 710_IVLNWEC01AVIEM | candidate-miR9772b-5p | UGAGAUGAGAUUACCCCAUAC |
| 711_IVLNWEC01AVIEM | candidate-miR9772b-5p | UGAGGUAAGAUUACCCCAUAC |
| 713_IVLNWEC02GO0BP | candidate-miR9772b-5p | UGAGGUAAGAUUACCCCAUAC |
| 724_contig06207 | candidate-miR167e-5p | UGAAGCUGCCAGCAUGAUCUA |
| 725_contig02570 | candidate-miR167e-5p | UGAAGCUGCCAGCAUGAUCUA |
| 726_contig04934 | candidate-miR167e-5p | UGAAGCUGCCAGCAUGAUCUG |
| 740_IV9RU5F02HSXSH | candidate-miR399b-5p | CAGGGCGCUUCUCCAUUGGCA |
| 741_IV9RU5F02HSXSH | candidate-miR399b-5p | CAGGGCAAUUCUCCUUUGGCA |
| 742_IVLNWEC02I6NFF | candidate-miR399b-5p | CAGGGCGCUUCUCCAUUGGCA |
| 743_IVLNWEC02I6NFF | candidate-miR399b-5p | CAGGGCAAUUCUCCUUUGGCA |
| 758_contig02570 | candidate-miR167e-3p | GGUCAUGCUGCGGCAGCCUCACU |
| 769_IUIVLUD01DQSUI | candidate-miR5084-3p | ACCAUACGGUACUGCAGAGGAUC |
| 773_IV9RU5F02FKAON | candidate-miR5084-3p | ACCGUAUAGUACUGCAGAGGAUC |
| 774_IV9RU5F02G6NHP | candidate-miR5084-3p | ACCGUAUAGUACUGCAGAGGAUC |
| 1101_IV9RU5F01DOCIN | candidate-miR2118b | UUCCUGGUGCCUCAUAUUCCUA |
| 1129_IUIVLUD02IFR9H | candidate-miR2118b | UUCCUGGUGCCUCAUAUUCCUA |
| 1143_contig12508 | candidate-miR2118b | UUCCUGAUGCCUCAUAUUUCUA |
| 1222_IV9RU5F01BFHPW | candidate-miR169m | UAGUCAAGGAUGACUUGCCU |
| 1223_IVLNWEC01B62WO | candidate-miR169m | UAGCCAAGGAUGAAUUGCCA |
| 1267_IVLNWEC02FQ1MJ | candidate-miR5180a | UAGGUGUCACAGUUUUGAACG |
| 1325_IVLNWEC02ILW4A | candidate-miR7731-3p | AGGUUUGCUCAGGAGUUUGGAAUC |
| 1558_IVLNWEC02GWESA | candidate-miR5175b | UCUCUGUACCUAAAUACUUGU |
| 1651_IUIVLUD01AJRY0 | candidate-miR393b-3p | UCAGUGCGAUCCCUUUGGAUG |
| 1709_IVLNWEC02GWESA | candidate-miR5049a | ACCUAAAUACUUGUAGUUGGG |
| 1712_IVLNWEC02F0WQR | candidate-miR5049a | ACCAAAAUACUUGUAGUUGGG |
| 1843_IVLNWEC02GWESA | candidate-miR5049b | AAUAUUUAGGUACAGAGGGAG |
| 1855_contig02733 | candidate-miR5049b | AGUAUUGUGGUACAGAGGGAG |
| 1859_IVLNWEC02GWESA | candidate-miR5049e | AAAUAUUUAGGUACAGAGGGA |
| 2168_IUIVLUD02I7XZ2 | candidate-miR2118e | UUCCCCAUGCCUCUCAUGCUUA |
| 2201_contig00047 | candidate-miR2118e | UUCCCCAUGCCUCUCAUGCUUA |
| 2412_contig03699 | candidate-miR2118o | UUCCUGAUGCCUCCCAUGCCUA |
| 2416_contig03108 | candidate-miR2118o | UUCCUGAUGCCUCCCAUGCCUA |
| 2419_contig01460 | candidate-miR2118o | UUCCUGAUGCCUCCCAUGCCUA |
| 2420_contig00983 | candidate-miR2118o | UUCCUGAUGCCUCCCAUGCCUA |
| 2421_contig00113 | candidate-miR2118o | UUCCUGAUGCCUCCCAUGCCUA |

| | | |
|---|---|---|
| 2448_contig03963 | candidate-miR2118o | UUCCUCAUGCCUCCCAUGCCUA |
| 2767_contig05202 | candidate-miR5522 | AACAAUAGGAAUGGGAGGCAU |
| 2819_contig00113 | candidate-miR5522 | AACAUUAGGCAUGGGAGGCAU |
| 2845_contig02414 | candidate-miR5522 | AACAUUAGGCAUGGGAGGCAU |
| 2847_contig01449 | candidate-miR5522 | AACAGUAGUAAUGGGAGGCAU |
| 2896_contig06207 | candidate-miR167a-3p | AUCAUGACUGACAGCCUCAUUU |
| 3513_IV9RU5F01BFHPW | candidate-miR169d-3p | GGGCAGUCACCUUGGCUAGC |
| 3598_IV9RU5F02JPIW9 | candidate-miR2118-5p | GGCAUGGGAACAUGGAGGAAAG |
| 3599_IV9RU5F01DUBH9 | candidate-miR2118-5p | GGCAUGGGAACAUGGAGGAAGG |
| 3600_IV9RU5F01DUBH9 | candidate-miR2118-5p | GGCAUGGGAACAUGGAGGAAAG |
| 3603_IVLNWEC01B0CK4 | candidate-miR2118-5p | GGCAUGGGAACAUGGAGGAAGG |
| 3604_IUIVLUD02GOBIZ | candidate-miR2118-5p | GGCAUGGGAACAUGGAGGAAGG |
| 3605_IUIVLUD02HV36R | candidate-miR2118-5p | GGCAUGGGAACAUGCAGGAAGG |
| 3606_contig12404 | candidate-miR2118-5p | GGCAUGGGAACAUGGAGGAAGG |
| 3607_contig12404 | candidate-miR2118-5p | GGCAUGGGAACAUGGAGGAAAG |
| 3608_contig10421 | candidate-miR2118-5p | GGCAUGGGAACAUGGAGGAAGG |
| 3611_contig02267 | candidate-miR2118-5p | GGCAUGGGAACAUGGAGGAAAG |
| 3613_contig01460 | candidate-miR2118-5p | GGCAUGGGAACAUGGAGGAAAG |
| 3617_contig02414 | candidate-miR2118-5p | GGCAUGGGAACAUGGAGGAAGU |
| 3618_contig00113 | candidate-miR2118-5p | GGCAUGGGAACAUGGAGGAAGU |
| 3619_contig00113 | candidate-miR2118-5p | GGCAUGGGAACAUGGAGGAAAG |
| 3624_IVLNWEC02FZKCU | candidate-miR2118-5p | GACAUGGGAACAUGGAGGAAGG |
| 3627_IVLNWEC01EUHN7 | candidate-miR2118-5p | GGCAUGGGAACAUGGAGGAAAG |
| 3628_IVLNWEC01D12C9 | candidate-miR2118-5p | GGCAUGGGAACAUGGAGGAAAG |
| 3629_IVLNWEC01C1E8V | candidate-miR2118-5p | GGCAUGGGAACAUGGAGGAAAG |
| 3630_IVLNWEC01B0RJK | candidate-miR2118-5p | GGCAUGGGAACAUGGAGGAAAG |
| 3632_IUIVLUD02JNJRF | candidate-miR2118-5p | GGCAUGGGAACAUGGAGGAAAG |
| 3633_IUIVLUD02H2QF2 | candidate-miR2118-5p | GGCAUGGGAACAUGGAGGAAAG |
| 3636_IUIVLUD01DO4ZV | candidate-miR2118-5p | GGCAUGGGAACAUGGAGGAAAG |
| 3640_contig19478 | candidate-miR2118-5p | GGCAUGGGAACAUGGAGGAAAG |
| 3641_contig15634 | candidate-miR2118-5p | GGCAUGGGAACAUGGAGGAAAG |
| 3642_contig15489 | candidate-miR2118-5p | GGCAUGGGAACAUUGAGGAAGG |
| 3646_contig00983 | candidate-miR2118-5p | GGCAUGGGAACAUGGAGGAAAG |
| 3647_contig00982 | candidate-miR2118-5p | GGCAUGGGAACAUGGAGGAAAG |
| 3769_IV9RU5F02JDX8X | candidate-miR1127b-3p | ACAAGUAUUUCGGUACGGAGU |
| 3799_IVLNWEC02JKYI7 | candidate-miR9663-5p | AAGCGUAGUCGAACGAAUCUG |
| 3812_IV9RU5F02IFUNJ | candidate-miR1133 | CAUAUACUCCCUCCGUCCGAAA |
| 3931_IV9RU5F02IFUNJ | candidate-miR1128 | AUAUACUCCCUCCGUCCGAAA |
| 3932_IVLNWEC01EACXK | candidate-miR1128 | AUAUACUCCCUCCGUCCGAAA |
| 4083_IV9RU5F02HSXSH | candidate-miR399 | UGCCAAAGGAGAAUUGCCC |
| 4085_IVLNWEC02I6NFF | candidate-miR399 | UGCCAAAGGAGAAUUGCCC |
| 4087_IV9RU5F01DVYJC | candidate-miR399 | UGCCAAAGGAGACUUGCCC |
| 4446_IV9RU5F01BFHPW | candidate-miR169i-3p | GGCAGUCACCUUGGCUAG |
| 4451_IV9RU5F02JBCAV | candidate-miR399f-5p | GGGCAGGGUCUCCUUUGGCAGA |
| 4654_IUIVLUD01AJRY0 | candidate-miR393a-3p | AUCAGUGCGAUCCCUUUGGAUG |
| 4750_contig05069 | candidate-miR159a | UUGGAAUGAAAGGAGCUCCA |
| 4779_IV9RU5F02HRFA2 | candidate-miR1223h | AUGUAGAGUCACGCUCGUCUG |

| | | |
|---|---|---|
| 4884_contig06207 | candidate-miR167 | UGAAGCUGCCAGCAUGAUCUA |
| 4885_contig04934 | candidate-miR167 | UGAAGCUGCCAGCAUGAUCUG |
| 5363_IV9RU5F02HSXSH | candidate-miR399b-5p | GGGCGCUUCUCCAUUGGCACG |
| 5364_IVLNWEC02I6NFF | candidate-miR399b-5p | GGGCGCUUCUCCAUUGGCACG |
| 5461_contig06207 | candidate-miR167c-5p | GAAGCUGCCAGCAUGAUCUAU |
| 5462_contig04934 | candidate-miR167c-5p | GAAGCUGCCAGCAUGAUCUGA |
| 5463_contig02570 | candidate-miR167c-5p | GAAGCUGCCAGCAUGAUCUAG |
| 5949_IV9RU5F01BFHPW | candidate-miR169l | UAGUCAAGGAUGACUUGCCUAU |
| 5950_IVLNWEC01B62WO | candidate-miR169l | UAGCCAAGGAUGAAUUGCCAGC |
| 5951_IUIVLUD01AXDSA | candidate-miR169l | UAGCCAAGGAUGAAUUGGCAGC |
| 5983_contig06207 | candidate-miR167d | UGAAGCUGCCAGCAUGAUCU |
| 5984_contig04934 | candidate-miR167d | UGAAGCUGCCAGCAUGAUCU |
| 5985_contig02570 | candidate-miR167d | UGAAGCUGCCAGCAUGAUCU |
| 6135_contig06207 | candidate-miR167e | UGAAGCUGCCAGCAUGAUCUA |
| 6137_contig04934 | candidate-miR167e | UGAAGCUGCCAGCAUGAUCUG |
| 6270_IVLNWEC02I55SE | candidate-miR398 | UGUGUUCUCAGGUCACCCCU |
| 6272_IVLNWEC01D0RL9 | candidate-miR398 | UGUUUUCCCAGGUCACCCCU |
| 6273_IVLNWEC01BV0LE | candidate-miR398 | UGUUUUCCCAGGUCACCCCU |
| 6442_IV9RU5F01BUPRF | candidate-miR1533 | AUAAUAAAAAUAAGAAUGA |
| 6515_IV9RU5F01BFHPW | candidate-miR169e | AGUCAAGGAUGACUUGCCUA |
| 6516_IVLNWEC01B62WO | candidate-miR169e | AGCCAAGGAUGAAUUGCCAG |
| 6661_contig05069 | candidate-miR319c | UUGGAAUGAAAGGAGCUCCA |
| 7315_IUIVLUD02IVG51 | candidate-miR482 | UUUUUCCAAUGCCUCCCAUUCC |
| 7620_IVLNWEC01B62WO | candidate-miR169d | GUAGCCAAGGAUGAAUUGCCA |
| 7647_IV9RU5F02HSXSH | candidate-miR399b | CGUGCCAAAGGAGAAUUGCCC |
| 7649_IVLNWEC02I6NFF | candidate-miR399b | CGUGCCAAAGGAGAAUUGCCC |
| 7651_IV9RU5F01DVYJC | candidate-miR399b | ACUGCCAAAGGAGACUUGCCC |
| 8036_contig04934 | candidate-miR167b-5p | UGAAGCUGCCAGCAUGAUCUGA |
| 1_IV9RU5F02GGKXZ | candidate-miR9672-5p | CUUAAUGACAGUCGUGGUGUC |
| 2_IVLNWEC02GFHJC | candidate-miR9672-5p | CUUAAUGACAGUCGUGGUGUC |
| 301_contig04934 | candidate-miR167b-3p | AGGUCAUGCUGGAGUUUCAUC |
| 778_IVLNWEC02GFHJC | candidate-miR9672-3p | UACCACGACUGUCAUUAAGCA |
| 1710_IVLNWEC02GWESA | candidate-miR5049a | ACCUAAAUAUUUGUAGUUGGG |
| 4077_IVLNWEC02GFHJC | candidate-miR9672a-3p | CCACGACUGUCAUUAAGCAUC |
| 4447_contig04934 | candidate-miR167g-3p | GGUCAUGCUGGAGUUUCAUC |
| 5386_IV9RU5F02H1CWJ | candidate-miR834-3p | UGGGAGUAGUAGCGGUGGUAG |

**wild wheat ancestor *Triticum dicoccoides***

l[2], Hikmet Budak[1,3]*

| New miRNA length | Conserved miRNA ID | Conserved miRNA sequence | Mismatch |
|---|---|---|---|
| 23 | ata-miR169b-5p | UAGCCAAGGAUGAUUUGCCUGUG | 3 |
| 23 | ata-miR5062b-3p | UGAACCUUAGGGAAAAGCCGCAU | 1 |
| 22 | ata-miR167b-5p | UGAAGCUGCCAGCAUGAUCUGA | 0 |
| 22 | ata-miR167b-5p | UGAAGCUGCCAGCAUGAUCUGA | 2 |
| 22 | ata-miR167b-5p | UGAAGCUGCCAGCAUGAUCUGA | 2 |
| 22 | ata-miR2118d-3p | UUCCUGAUGCCUCCCAUGCCUA | 0 |
| 22 | ata-miR2118d-3p | UUCCUGAUGCCUCCCAUGCCUA | 0 |
| 22 | ata-miR2118d-3p | UUCCUGAUGCCUCCCAUGCCUA | 0 |
| 22 | ata-miR2118d-3p | UUCCUGAUGCCUCCCAUGCCUA | 0 |
| 22 | ata-miR2118d-3p | UUCCUGAUGCCUCCCAUGCCUA | 0 |
| 22 | ata-miR2118d-3p | UUCCUGAUGCCUCCCAUGCCUA | 0 |
| 22 | ata-miR2118d-3p | UUCCUGAUGCCUCCCAUGCCUA | 0 |
| 22 | ata-miR2118d-3p | UUCCUGAUGCCUCCCAUGCCUA | 0 |
| 22 | ata-miR2118d-3p | UUCCUGAUGCCUCCCAUGCCUA | 0 |
| 22 | ata-miR2118d-3p | UUCCUGAUGCCUCCCAUGCCUA | 0 |
| 22 | ata-miR2118d-3p | UUCCUGAUGCCUCCCAUGCCUA | 2 |
| 22 | ata-miR2118d-3p | UUCCUGAUGCCUCCCAUGCCUA | 0 |
| 22 | ata-miR2118d-3p | UUCCUGAUGCCUCCCAUGCCUA | 0 |
| 22 | ata-miR2118d-3p | UUCCUGAUGCCUCCCAUGCCUA | 0 |
| 22 | ata-miR2118d-3p | UUCCUGAUGCCUCCCAUGCCUA | 0 |
| 22 | ata-miR2118d-3p | UUCCUGAUGCCUCCCAUGCCUA | 0 |
| 22 | ata-miR2118d-3p | UUCCUGAUGCCUCCCAUGCCUA | 0 |
| 22 | ata-miR2118d-3p | UUCCUGAUGCCUCCCAUGCCUA | 1 |
| 22 | ata-miR2118d-3p | UUCCUGAUGCCUCCCAUGCCUA | 1 |
| 22 | ata-miR2118d-3p | UUCCUGAUGCCUCCCAUGCCUA | 1 |
| 22 | ata-miR2118d-3p | UUCCUGAUGCCUCCCAUGCCUA | 1 |
| 22 | ata-miR2118d-3p | UUCCUGAUGCCUCCCAUGCCUA | 2 |
| 22 | ata-miR2118d-3p | UUCCUGAUGCCUCCCAUGCCUA | 1 |
| 22 | ata-miR2118d-3p | UUCCUGAUGCCUCCCAUGCCUA | 1 |
| 22 | ata-miR2118d-3p | UUCCUGAUGCCUCCCAUGCCUA | 1 |
| 22 | ata-miR2118d-3p | UUCCUGAUGCCUCCCAUGCCUA | 1 |
| 22 | ata-miR2118d-3p | UUCCUGAUGCCUCCCAUGCCUA | 1 |
| 22 | ata-miR2118d-3p | UUCCUGAUGCCUCCCAUGCCUA | 2 |
| 22 | ata-miR2118d-3p | UUCCUGAUGCCUCCCAUGCCUA | 2 |
| 22 | ata-miR2118d-3p | UUCCUGAUGCCUCCCAUGCCUA | 2 |
| 22 | ata-miR2118d-3p | UUCCUGAUGCCUCCCAUGCCUA | 2 |
| 22 | ata-miR2118d-3p | UUCCUGAUGCCUCCCAUGCCUA | 2 |
| 22 | ata-miR2118d-3p | UUCCUGAUGCCUCCCAUGCCUA | 2 |
| 22 | ata-miR2118d-3p | UUCCUGAUGCCUCCCAUGCCUA | 2 |
| 22 | ata-miR2118d-3p | UUCCUGAUGCCUCCCAUGCCUA | 2 |

| | | | |
|---|---|---|---|
| 22 | ata-miR2118d-3p | UUCCUGAUGCCUCCCAUGCCUA | 2 |
| 22 | ata-miR2118d-3p | UUCCUGAUGCCUCCCAUGCCUA | 3 |
| 22 | ata-miR2118d-3p | UUCCUGAUGCCUCCCAUGCCUA | 3 |
| 22 | ata-miR2118d-3p | UUCCUGAUGCCUCCCAUGCCUA | 3 |
| 21 | ata-miR9863b-5p | UGUUAUGAUCUGCUUCUCAUC | 1 |
| 22 | ata-miR2275c-3p | UUUGGUUUCCUCCAAUAUCUCA | 3 |
| 21 | ata-miR169g-5p | CAGCCAAGGAUGACUUGCCGA | 3 |
| 21 | ata-miR169i-5p | UAGCCAAGGAUGACUUGCCUG | 2 |
| 21 | ata-miR169i-5p | UAGCCAAGGAUGACUUGCCUG | 2 |
| 19 | ata-miR169c-3p | GGCGGUCACCUUGGCUAGC | 1 |
| 22 | ata-miR167c-3p | GAUCAUGACUGACAGCCUCAUU | 0 |
| 21 | ata-miR2118a-3p | GGGAAUGGGAACAUGGAGGAA | 0 |
| 21 | ata-miR2118a-3p | GGGAAUGGGAACAUGGAGGAA | 0 |
| 21 | ata-miR2118a-3p | GGGAAUGGGAACAUGGAGGAA | 1 |
| 21 | ata-miR2118a-3p | GGGAAUGGGAACAUGGAGGAA | 1 |
| 21 | ata-miR2118a-3p | GGGAAUGGGAACAUGGAGGAA | 1 |
| 21 | ata-miR2118a-3p | GGGAAUGGGAACAUGGAGGAA | 1 |
| 21 | ata-miR2118a-3p | GGGAAUGGGAACAUGGAGGAA | 1 |
| 21 | ata-miR2118a-3p | GGGAAUGGGAACAUGGAGGAA | 1 |
| 21 | ata-miR2118a-3p | GGGAAUGGGAACAUGGAGGAA | 1 |
| 21 | ata-miR2118a-3p | GGGAAUGGGAACAUGGAGGAA | 1 |
| 21 | ata-miR2118a-3p | GGGAAUGGGAACAUGGAGGAA | 1 |
| 21 | ata-miR2118a-3p | GGGAAUGGGAACAUGGAGGAA | 1 |
| 21 | ata-miR2118a-3p | GGGAAUGGGAACAUGGAGGAA | 1 |
| 21 | ata-miR2118a-3p | GGGAAUGGGAACAUGGAGGAA | 1 |
| 21 | ata-miR2118a-3p | GGGAAUGGGAACAUGGAGGAA | 1 |
| 21 | ata-miR2118a-3p | GGGAAUGGGAACAUGGAGGAA | 1 |
| 21 | ata-miR2118a-3p | GGGAAUGGGAACAUGGAGGAA | 1 |
| 21 | ata-miR2118a-3p | GGGAAUGGGAACAUGGAGGAA | 1 |
| 21 | ata-miR2118a-3p | GGGAAUGGGAACAUGGAGGAA | 1 |
| 21 | ata-miR2118a-3p | GGGAAUGGGAACAUGGAGGAA | 1 |
| 21 | ata-miR2118a-3p | GGGAAUGGGAACAUGGAGGAA | 1 |
| 21 | ata-miR2118a-3p | GGGAAUGGGAACAUGGAGGAA | 1 |
| 21 | ata-miR2118a-3p | GGGAAUGGGAACAUGGAGGAA | 1 |
| 21 | ata-miR2118a-3p | GGGAAUGGGAACAUGGAGGAA | 1 |
| 21 | ata-miR2118a-3p | GGGAAUGGGAACAUGGAGGAA | 1 |
| 21 | ata-miR2118a-3p | GGGAAUGGGAACAUGGAGGAA | 1 |
| 21 | ata-miR2118a-3p | GGGAAUGGGAACAUGGAGGAA | 1 |
| 21 | ata-miR2118a-3p | GGGAAUGGGAACAUGGAGGAA | 1 |
| 21 | ata-miR2118a-3p | GGGAAUGGGAACAUGGAGGAA | 1 |
| 21 | ata-miR2118a-3p | GGGAAUGGGAACAUGGAGGAA | 2 |
| 21 | ata-miR2118a-3p | GGGAAUGGGAACAUGGAGGAA | 2 |
| 21 | ata-miR2118a-3p | GGGAAUGGGAACAUGGAGGAA | 2 |
| 21 | ata-miR2118a-3p | GGGAAUGGGAACAUGGAGGAA | 3 |
| 21 | ata-miR2118a-3p | GGGAAUGGGAACAUGGAGGAA | 2 |
| 21 | ata-miR2118a-3p | GGGAAUGGGAACAUGGAGGAA | 3 |
| 21 | ata-miR2118a-3p | GGGAAUGGGAACAUGGAGGAA | 3 |

| | | | |
|---|---|---|---|
| 21 | ata-miR2118a-3p | GGGAAUGGGAACAUGGAGGAA | 3 |
| 21 | ata-miR2118a-3p | GGGAAUGGGAACAUGGAGGAA | 3 |
| 21 | ata-miR2118a-3p | GGGAAUGGGAACAUGGAGGAA | 3 |
| 21 | ata-miR2118a-3p | GGGAAUGGGAACAUGGAGGAA | 3 |
| 21 | ata-miR2118a-3p | GGGAAUGGGAACAUGGAGGAA | 3 |
| 22 | ata-miR399a-5p | GGGCGCUUCUCCAUUGGCACGG | 0 |
| 22 | ata-miR399a-5p | GGGCGCUUCUCCAUUGGCACGG | 0 |
| 22 | ata-miR2118a-5p | UUCCUAAUGUCUCCCAUUCCUA | 3 |
| 21 | ata-miR399a-3p | UGCCAAAGGAGAAUUGCCCUG | 0 |
| 21 | ata-miR399a-3p | UGCCAAAGGAGAAUUGCCCUG | 0 |
| 24 | ata-miR5084-5p | AUCCUCUACAGUACUGUACGGUGC | 2 |
| 21 | ata-miR398f-3p | UGUGUUCUCAGGUCGCCCCCG | 3 |
| 21 | ata-miR9772b-5p | UGAGAUGAGAUUACCCCAUAC | 0 |
| 21 | ata-miR9772b-5p | UGAGAUGAGAUUACCCCAUAC | 2 |
| 21 | ata-miR9772b-5p | UGAGAUGAGAUUACCCCAUAC | 2 |
| 21 | ata-miR167e-5p | UGAAGCUGCCAGCAUGAUCUA | 0 |
| 21 | ata-miR167e-5p | UGAAGCUGCCAGCAUGAUCUA | 0 |
| 21 | ata-miR167e-5p | UGAAGCUGCCAGCAUGAUCUA | 1 |
| 21 | ata-miR399b-5p | CAGGGCGCUUCUCCUUUGGCA | 1 |
| 21 | ata-miR399b-5p | CAGGGCGCUUCUCCUUUGGCA | 2 |
| 21 | ata-miR399b-5p | CAGGGCGCUUCUCCUUUGGCA | 1 |
| 21 | ata-miR399b-5p | CAGGGCGCUUCUCCUUUGGCA | 2 |
| 23 | ata-miR167e-3p | GGUCAUGCUGCGGCAGCCUCACU | 0 |
| 23 | ata-miR5084-3p | ACCAUACGGUACUGCAGAGGAUC | 0 |
| 23 | ata-miR5084-3p | ACCAUACGGUACUGCAGAGGAUC | 3 |
| 23 | ata-miR5084-3p | ACCAUACGGUACUGCAGAGGAUC | 3 |
| 22 | bdi-miR2118b | UUCCUGAUGCCUCCCAUUCCUA | 3 |
| 22 | bdi-miR2118b | UUCCUGAUGCCUCCCAUUCCUA | 3 |
| 22 | bdi-miR2118b | UUCCUGAUGCCUCCCAUUCCUA | 3 |
| 20 | bdi-miR169m | UAGCCAAGGAUGACUUGCCG | 2 |
| 20 | bdi-miR169m | UAGCCAAGGAUGACUUGCCG | 2 |
| 21 | bdi-miR5180a | UAAGUGUCUCAGUUUUGAACU | 3 |
| 24 | bdi-miR7731-3p | AGGUUUGCUCUGGACUUUGGAAUC | 2 |
| 21 | bdi-miR5175b | CCUCUGUUCCUAAAUUCUUGU | 3 |
| 21 | bdi-miR393b-3p | UCAGUGCAAUCCCUUUGGAAU | 3 |
| 21 | hvu-miR5049a | UCCUAAAUACUUGUUGUUGGG | 2 |
| 21 | hvu-miR5049a | UCCUAAAUACUUGUUGUUGGG | 3 |
| 21 | hvu-miR5049b | AGUAUUUAGGUACAGAGGGAG | 1 |
| 21 | hvu-miR5049b | AGUAUUUAGGUACAGAGGGAG | 2 |
| 21 | hvu-miR5049e | AAUUAUUUAGGUACAGAGGGA | 1 |
| 22 | osa-miR2118e | UUCCCAAUGCCUCCCAUGCCUA | 3 |
| 22 | osa-miR2118e | UUCCCAAUGCCUCCCAUGCCUA | 3 |
| 22 | osa-miR2118o | CUCCUGAUGCCUCCCAAGCCUA | 2 |
| 22 | osa-miR2118o | CUCCUGAUGCCUCCCAAGCCUA | 2 |
| 22 | osa-miR2118o | CUCCUGAUGCCUCCCAAGCCUA | 2 |
| 22 | osa-miR2118o | CUCCUGAUGCCUCCCAAGCCUA | 2 |
| 22 | osa-miR2118o | CUCCUGAUGCCUCCCAAGCCUA | 2 |

| | | | |
|---|---|---|---|
| 22 | osa-miR2118o | CUCCUGAUGCCUCCCAAGCCUA | 3 |
| 21 | osa-miR5522 | AACAAUAGGAAUGGGAGGCAU | 0 |
| 21 | osa-miR5522 | AACAAUAGGAAUGGGAGGCAU | 2 |
| 21 | osa-miR5522 | AACAAUAGGAAUGGGAGGCAU | 2 |
| 21 | osa-miR5522 | AACAAUAGGAAUGGGAGGCAU | 2 |
| 22 | osa-miR167a-3p | AUCAUGCAUGACAGCCUCAUUU | 2 |
| 20 | sbi-miR169d-3p | GGGCGGUCACCUUGGCUAGC | 1 |
| 22 | sbi-miR2118-5p | GGCAUGGGAACAUGUAGGAAGG | 2 |
| 22 | sbi-miR2118-5p | GGCAUGGGAACAUGUAGGAAGG | 1 |
| 22 | sbi-miR2118-5p | GGCAUGGGAACAUGUAGGAAGG | 2 |
| 22 | sbi-miR2118-5p | GGCAUGGGAACAUGUAGGAAGG | 1 |
| 22 | sbi-miR2118-5p | GGCAUGGGAACAUGUAGGAAGG | 1 |
| 22 | sbi-miR2118-5p | GGCAUGGGAACAUGUAGGAAGG | 1 |
| 22 | sbi-miR2118-5p | GGCAUGGGAACAUGUAGGAAGG | 1 |
| 22 | sbi-miR2118-5p | GGCAUGGGAACAUGUAGGAAGG | 2 |
| 22 | sbi-miR2118-5p | GGCAUGGGAACAUGUAGGAAGG | 1 |
| 22 | sbi-miR2118-5p | GGCAUGGGAACAUGUAGGAAGG | 2 |
| 22 | sbi-miR2118-5p | GGCAUGGGAACAUGUAGGAAGG | 2 |
| 22 | sbi-miR2118-5p | GGCAUGGGAACAUGUAGGAAGG | 2 |
| 22 | sbi-miR2118-5p | GGCAUGGGAACAUGUAGGAAGG | 2 |
| 22 | sbi-miR2118-5p | GGCAUGGGAACAUGUAGGAAGG | 2 |
| 22 | sbi-miR2118-5p | GGCAUGGGAACAUGUAGGAAGG | 2 |
| 22 | sbi-miR2118-5p | GGCAUGGGAACAUGUAGGAAGG | 2 |
| 22 | sbi-miR2118-5p | GGCAUGGGAACAUGUAGGAAGG | 2 |
| 22 | sbi-miR2118-5p | GGCAUGGGAACAUGUAGGAAGG | 2 |
| 22 | sbi-miR2118-5p | GGCAUGGGAACAUGUAGGAAGG | 2 |
| 22 | sbi-miR2118-5p | GGCAUGGGAACAUGUAGGAAGG | 2 |
| 22 | sbi-miR2118-5p | GGCAUGGGAACAUGUAGGAAGG | 2 |
| 22 | sbi-miR2118-5p | GGCAUGGGAACAUGUAGGAAGG | 2 |
| 22 | sbi-miR2118-5p | GGCAUGGGAACAUGUAGGAAGG | 2 |
| 22 | sbi-miR2118-5p | GGCAUGGGAACAUGUAGGAAGG | 2 |
| 22 | sbi-miR2118-5p | GGCAUGGGAACAUGUAGGAAGG | 2 |
| 22 | sbi-miR2118-5p | GGCAUGGGAACAUGUAGGAAGG | 2 |
| 22 | sbi-miR2118-5p | GGCAUGGGAACAUGUAGGAAGG | 2 |
| 22 | sbi-miR2118-5p | GGCAUGGGAACAUGUAGGAAGG | 2 |
| 21 | tae-miR1127b-3p | ACAAGUAUUUCUGGACGGAGG | 3 |
| 21 | tae-miR9663-5p | AAGCGUAGUCGAACGAAUCUG | 0 |
| 22 | tae-miR1133 | CAUAUACUCCCUCCGUCCGAAA | 0 |
| 21 | tae-miR1128 | UACUACUCCCUCCGUCCGAAA | 3 |
| 21 | tae-miR1128 | UACUACUCCCUCCGUCCGAAA | 3 |
| 19 | tae-miR399 | UGCCAAAGGAGAAUUGCCC | 0 |
| 19 | tae-miR399 | UGCCAAAGGAGAAUUGCCC | 0 |
| 19 | tae-miR399 | UGCCAAAGGAGAAUUGCCC | 1 |
| 18 | zma-miR169i-3p | GGCAGUCUCCUUGGCUAG | 1 |
| 22 | zma-miR399f-5p | GGGCAACUUCUCCUUUGGCAGA | 3 |
| 22 | zma-miR393a-3p | AUCAGUGCAAUCCCUUUGGAAU | 3 |
| 20 | pta-miR159a | UUGGAUUGAAGGGAGCUCCA | 2 |
| 21 | ppt-miR1223h | UUGUAGAGUCACGCACCUCUG | 3 |

| | | | |
|---|---|---|---|
| 21 | ppt-miR167 | GGAAGCUGCCAGCAUGAUCCU | 3 |
| 21 | ppt-miR167 | GGAAGCUGCCAGCAUGAUCCU | 3 |
| 21 | aly-miR399b-5p | GGGCGCCUCUCCAUUGGCAGG | 2 |
| 21 | aly-miR399b-5p | GGGCGCCUCUCCAUUGGCAGG | 2 |
| 21 | aly-miR167c-5p | UAAGCUGCCAGCAUGAUCUUG | 3 |
| 21 | aly-miR167c-5p | UAAGCUGCCAGCAUGAUCUUG | 3 |
| 21 | aly-miR167c-5p | UAAGCUGCCAGCAUGAUCUUG | 2 |
| 22 | bna-miR169l | UAGCCAAGGAUGACUUGCCUGC | 3 |
| 22 | bna-miR169l | UAGCCAAGGAUGACUUGCCUGC | 2 |
| 22 | bna-miR169l | UAGCCAAGGAUGACUUGCCUGC | 3 |
| 20 | bna-miR167d | UGAAGCUGCCAGCAUGAUCU | 0 |
| 20 | bna-miR167d | UGAAGCUGCCAGCAUGAUCU | 0 |
| 20 | bna-miR167d | UGAAGCUGCCAGCAUGAUCU | 0 |
| 21 | cme-miR167e | UCAAGCUGCCAGCAUGAUCUA | 1 |
| 21 | cme-miR167e | UCAAGCUGCCAGCAUGAUCUA | 2 |
| 20 | ahy-miR398 | UGUGUUCUCAGGUCACCCCU | 0 |
| 20 | ahy-miR398 | UGUGUUCUCAGGUCACCCCU | 2 |
| 20 | ahy-miR398 | UGUGUUCUCAGGUCACCCCU | 2 |
| 19 | gma-miR1533 | AUAAUAAAAAUAAUAAUGA | 1 |
| 20 | gma-miR169e | AGCCAAGGAUGACUUGCCGG | 3 |
| 20 | gma-miR169e | AGCCAAGGAUGACUUGCCGG | 2 |
| 20 | gma-miR319c | UUGGACUGAAAGGAGCUCCU | 2 |
| 22 | gra-miR482 | UCUUUCCAAUUCCUCCCAUUCC | 2 |
| 21 | ppe-miR169d | UGAGCCAAGGAUGACUUGCCA | 3 |
| 21 | ppe-miR399b | UCUGCCAAAGGAGAAUUGCCC | 2 |
| 21 | ppe-miR399b | UCUGCCAAAGGAGAAUUGCCC | 2 |
| 21 | ppe-miR399b | UCUGCCAAAGGAGAAUUGCCC | 2 |
| 22 | sly-miR167b-5p | UAAAGCUGCCAGCAUGAUCUGG | 2 |
| 21 | ata-miR9672-5p | CUUAAUGACAGUCGUGGUGUC | 0 |
| 21 | ata-miR9672-5p | CUUAAUGACAGUCGUGGUGUC | 0 |
| 21 | ata-miR167b-3p | AGGUCAUGCUGGAGUUUCAUC | 0 |
| 21 | ata-miR9672-3p | UACCACGACUGUCAUUAAGCA | 0 |
| 21 | hvu-miR5049a | UCCUAAAUACUUGUUGUUGGG | 3 |
| 21 | tae-miR9672a-3p | CCACGACUGUCAUUAAGCAUC | 0 |
| 20 | zma-miR167g-3p | GGUCAUGCUGUAGUUUCAUC | 1 |
| 21 | aly-miR834-3p | UGGUAGCAGUAGCGGUGGUAA | 3 |

| miRNA start | miRNA end | miRNA* start | miRNA* end | miRNA* sequence |
|---|---|---|---|---|
| 21 | 43 | 90 | 110 | UGGGCAGUCACCUUGGCUAGC |
| 21 | 43 | 137 | 161 | GCGGAUUUUUCACCAAAGGUUCAAG |
| 21 | 42 | 77 | 97 | AGGUCAUGCUGGAGUUUCAUC |
| 21 | 42 | 75 | 97 | Not defined |
| 21 | 42 | 102 | 125 | AGGUCAUGCUGCGGCAGCCUCACU |
| 91 | 112 | 21 | 42 | GGCAUGGGAACAUGGAGGAAAG |
| 91 | 112 | 19 | 42 | Not defined |
| 92 | 113 | 21 | 42 | GGCAUGGGAACAUGGAGGAAAG |
| 91 | 112 | 21 | 42 | GGCAUGGGAACAUGGAGGAAAG |
| 92 | 113 | 21 | 41 | GGCAUGGGAACAUGGAGGAAA |
| 21 | 42 | 177 | 198 | GGCAUGGGAACAUGCAGGAAGG |
| 91 | 112 | 21 | 42 | GGCAUGGGAACAUGGAGGAAAG |
| 91 | 112 | 21 | 42 | GGCAUGGGAACAUGGAGGAAAG |
| 90 | 111 | 21 | 42 | GGCAUGGGAACAUGGAGGAAAG |
| 91 | 112 | 21 | 42 | GGCAUGGGAACAUGGAGGAAAG |
| 94 | 115 | 21 | 41 | GGCAUGGGAACAUGGAGGAAG |
| 91 | 112 | 21 | 42 | GGCAUGGGAACCUCGAGGAAAG |
| 91 | 112 | 21 | 42 | GGUAUGGGAACAUGGAGGAAAG |
| 91 | 112 | 21 | 42 | GGCAUGGGAACAUGGAGGAAAG |
| 91 | 112 | 21 | 42 | GGCAUGGGAACAUGGAGGAAAG |
| 91 | 112 | 21 | 42 | GGCAUGGGAACAUGGAGGAAAG |
| 91 | 112 | 21 | 42 | GGCAUGGGAACAUGGAGGAAAG |
| 100 | 121 | 21 | 42 | GGAAUGGGAACAUAGAGGAAAG |
| 91 | 112 | 21 | 42 | GGCAUGGGAACAUGGAGGAAAG |
| 93 | 114 | 21 | 42 | GGUAUGGGAACAUGGAGGAAAG |
| 91 | 112 | 21 | 42 | GGCAUGGGAACAUGGAGGAAAG |
| 94 | 115 | 21 | 41 | GGCAUGGGAACAUGGAGGAAG |
| 100 | 121 | 21 | 42 | GGAAUGGGAACAUAGAGGAAAG |
| 95 | 116 | 21 | 41 | GGCAUGGGAACAUGGAGGAAG |
| 95 | 116 | 21 | 42 | GGCAUGGGAACAUUGAGGAAGG |
| 91 | 112 | 21 | 42 | GGCAUGGAAACAUGGAGGAAAG |
| 95 | 116 | 21 | 41 | GGCAUGGGAACAUGGAGGAAG |
| 101 | 122 | 21 | 42 | GGAAUGGGAACAUGGAGGAAAG |
| 90 | 111 | 21 | 42 | GGAAUGGGAACAUGAAGAAAAU |
| 90 | 111 | 21 | 42 | GGAAUGGGAACAUGAAGAAAAU |
| 94 | 115 | 21 | 41 | GGCAUGGGAACAUGGAGGAAG |
| 91 | 112 | 21 | 41 | GGCAUGGGAACAUGGAGGAAA |
| 97 | 118 | 21 | 42 | GGGAUGAGAGCAUAGAGGAAAG |
| 94 | 115 | 21 | 41 | GGCAUGGGAACAUGGAGGAAG |
| 91 | 112 | 21 | 42 | GGCAUGGGAACAUGGAGGAAAG |

| | | | | |
|---|---|---|---|---|
| 90 | 111 | 21 | 42 | GGAAUGGGAACAUGAAGAAAAU |
| 90 | 111 | 21 | 42 | GGAAUGGGAACAUGAAGAAAAU |
| 95 | 116 | 21 | 42 | GGAAGAGGAUCAUAGAGGAAAG |
| 95 | 116 | 21 | 41 | GACAUGGGGACAUGGAGGAAG |
| 21 | 41 | 80 | 103 | AGUUUGAGAGAGAUCAUAAACAAG |
| 54 | 75 | 21 | 44 | AGAAAUUCGGUAAACGCCGAAUGU |
| 86 | 106 | 21 | 41 | GGCAAAUGAUCCUUGACUUUA |
| 21 | 41 | 92 | 110 | GGCAGUCACCUUGGCUAGC |
| 21 | 41 | 78 | 98 | GGCAAGUCAUUACUGGCUACG |
[truncated: 680,652 more chars]
